# Integrating mRNA and miRNA Weighted Gene Co-Expression Networks with eQTLs in the Nucleus Accumbens of Subjects with Alcohol Dependence

**DOI:** 10.1371/journal.pone.0137671

**Published:** 2015-09-18

**Authors:** Mohammed Mamdani, Vernell Williamson, Gowon O. McMichael, Tana Blevins, Fazil Aliev, Amy Adkins, Laura Hack, Tim Bigdeli, Andrew D. van der Vaart, Bradley Todd Web, Silviu-Alin Bacanu, Gursharan Kalsi, COGA Consortium, Kenneth S. Kendler, Michael F. Miles, Danielle Dick, Brien P. Riley, Catherine Dumur, Vladimir I. Vladimirov

**Affiliations:** 1 Virginia Institute for Psychiatric and Behavioral Genetics, Virginia Commonwealth University, Richmond, VA, United States of America; 2 Department of Pathology, Virginia Commonwealth University, Richmond, VA, United States of America; 3 Department of Psychiatry, Virginia Commonwealth University, Richmond, VA, United States of America; 4 Department of Human & Molecular Genetics, Virginia Commonwealth University, Richmond, VA, United States of America; 5 Department of Pharmacology & Toxicology, Virginia Commonwealth University, Richmond, VA, United States of America; 6 Department of Psychology, Virginia Commonwealth University, Richmond, VA, United States of America; 7 Center for Biomarker Research and Personalized Medicine, Virginia Commonwealth University, Richmond, VA, United States of America; 8 Department of Social, Genetic and Developmental Psychiatry, Institute of Psychiatry, London SE5 8AF, United Kingdom; 9 Lieber Institute for Brain Development, Johns Hopkins University, Baltimore, MD, United States of America; IRCCS-Policlinico San Donato, ITALY

## Abstract

Alcohol consumption is known to lead to gene expression changes in the brain. After performing weighted gene co-expression network analyses (WGCNA) on genome-wide mRNA and microRNA (miRNA) expression in Nucleus Accumbens (NAc) of subjects with alcohol dependence (AD; N = 18) and of matched controls (N = 18), six mRNA and three miRNA modules significantly correlated with AD were identified (Bonferoni-adj. p≤ 0.05). Cell-type-specific transcriptome analyses revealed two of the mRNA modules to be enriched for neuronal specific marker genes and downregulated in AD, whereas the remaining four mRNA modules were enriched for astrocyte and microglial specific marker genes and upregulated in AD. Gene set enrichment analysis demonstrated that neuronal specific modules were enriched for genes involved in oxidative phosphorylation, mitochondrial dysfunction and MAPK signaling. Glial-specific modules were predominantly enriched for genes involved in processes related to immune functions, i.e. cytokine signaling (all adj. p≤ 0.05). In mRNA and miRNA modules, 461 and 25 candidate hub genes were identified, respectively. In contrast to the expected biological functions of miRNAs, correlation analyses between mRNA and miRNA hub genes revealed a higher number of positive than negative correlations (χ^2^ test p≤ 0.0001). Integration of hub gene expression with genome-wide genotypic data resulted in 591 mRNA cis-eQTLs and 62 miRNA cis-eQTLs. mRNA cis-eQTLs were significantly enriched for AD diagnosis and AD symptom counts (adj. p = 0.014 and p = 0.024, respectively) in AD GWAS signals in a large, independent genetic sample from the Collaborative Study on Genetics of Alcohol (COGA). In conclusion, our study identified putative gene network hubs coordinating mRNA and miRNA co-expression changes in the NAc of AD subjects, and our genetic (cis-eQTL) analysis provides novel insights into the etiological mechanisms of AD.

## Introduction

Alcohol dependence (AD) is a chronic, debilitating substance use disorder. Over the past few decades, research has unveiled the vast complexity of the genetic architecture underlying AD and alcohol-related phenotypes (ARP). Family, twin and adoption studies currently estimate the heritability of AD to be 50–60% [1, 2]. Both animal and human postmortem brain studies reveal that chronic alcohol consumption leads to broad transcriptional changes in brain regions not known to previously play a role in AD [3]. Early postmortem human brain expression studies focused on the prefrontal cortex (PFC), where genes related to GABA_A_ receptor subunits and mitochondrial function were found to be differentially expressed in chronic alcoholics [4, 5]. Similarly, genome-wide expression studies in PFC implicated variation in the expression of genes related to processes such as myelination, cell cycling, oxidative stress, and transcription [6–11]. Research into other brain regions, such as nucleus accumbens (NAc) or the ventral tegmental area (VTA), revealed differential expression of genes related to cell architecture, cell signaling, vesicle formation, and synaptic transmission [8]. These findings suggest that there are brain region-specific susceptibilities and adaptations to chronic alcohol consumption that likely have a distinct effect on the behavioral phenotypes comprising AD [6, 8, 12].

Evaluation of the regulatory mechanisms underlying genetic differentiation is necessary to better understand the neurobiology of AD [13]. Transcriptional and translation regulation by microRNAs (miRNAs) in substance use disorders and AD is a growing field of interest in recent years [14]. MiRNAs are small, non-coding, regulatory RNA molecules that function primarily to repress translation of an estimated 30–50% of all protein-coding genes by downregulation of mRNA [15]. MiRNA play a pivotal role in regulation of the central nervous system (CNS), where approximately 70% of known miRNAs are expressed. Similarly, mRNA in the CNS have longer 3’ untranslated regions (3’ UTRs), which represent a large number of potential miRNA target sites [16, 17]. The cooperative and combinatorial targeting ability of miRNA allow precise and robust gene regulation at both the single-gene and the gene-network level [18]. To date, there have been limited studies in rodent and cell-based models, and even fewer studies of genome-wide miRNA expression in AD postmortem brain tissue [19–23]. One such study on the PFC of AD subjects identified 35 upregulated miRNAs, which are known to target mRNAs that function in apoptosis, cell adhesion, cell cycling, signaling, and neuronal development [20]. Another recent study profiled mRNAs and miRNAs in the PFC of rats following chronic exposure to alcohol, where mRNAs with functions in neurotransmission, axonal guidance, neuroadaptation, and neurotransmitter signaling were found to be differentially expressed [24]. While the specific relationship between miRNA and mRNA associated with AD is difficult to ascertain, due to the complexity of the transcriptome in AD, several miRNA:mRNA interactions have been experimentally validated [25–28]. Combined genomic profiling of miRNA and mRNA in human NAc has not been conducted, despite the well-established role of NAc in the mesocorticolimbic pathway central to the rewarding properties of alcohol and other drugs of abuse.

Individual assessment of gene expression cannot alone explain the complex etiology of AD; thus, an integrative approach to assessing gene expression in a network framework is necessary to unravel the molecular underpinnings of AD. Weighted gene co-expression network analysis (WGCNA) is a tool that has been used to successfully build and identify gene networks involved in various disorders including schizophrenia, major depression, AD, and ARPs [29, 30]. Integrating dysregulated gene networks in AD, identified by WGCNA, with genetic data, identified by GWAS, provides an invaluable tool to further discern the genetic basis of AD susceptibility. This approach, termed ‘genetical genomics’, classifies associations between genetic variants and gene expression as quantitative trait loci (eQTLs), which are then modeled as quantitative traits [31–33]. As the majority of genetic variants are located outside of protein-coding regions, their influence on cell function likely involves subtle modification of gene transcription and translation [34]. The connection between genetic variation and gene expression may identify functional loci not previously associated with AD, as well as offer specific, testable hypotheses for polymorphisms associated with AD [34–36]. Recent studies in rodent models have identified hub genes which play a role in the behavioral responses to alcohol by a coordinate analysis of acute alcohol-responsive gene networks, linked genetic intervals, and alcohol behavioral responses [37]. Linkage disequilibrium (LD) of eQTLs with genetic variants implicated in AD and ARPs can provide a biological mechanism for disease-associated variants with no otherwise apparent functions as there is empirical evidence suggesting that eQTLs are over-represented among GWAS signals [38, 39].

In this study we evaluated mRNA and miRNA expression patterns in the NAc of 18 AD cases and 18 matched controls and perform gene co-expression network analysis to identify gene networks associated with AD. We then integrated gene expression with genotypic data to identify eQTLs that impact the expression of network hub genes in NAc, and provide evidence that these eQTLs are enriched for AD GWAS signals in the Collaborative Study on the Genetics of Alcoholism (COGA) sample.

## Results

Based on our univariate analysis, we identified systemic changes in mRNA and miRNA expression levels in the NAc between subjects with AD and healthy controls. Specifically, at a nominal p≤ 0.05 we identified 4,571 (25%) differentially expressed mRNA transcripts and 240 (14%) differentially expressed miRNAs, which are statistically much greater than these expected by chance (hypergeometric p = 5x 10^−5^ and p = 9x10^-9^, respectively). Our results are also in agreement with results from previous postmortem brain expression studies, which have shown similar widespread changes in gene expression in PFC and VTA [3, 40]. The univariate analyses results for the mRNA and miRNA transcripts are provided in S1 Table, respectively.

### Identification of mRNA co-expression modules

**Fig 1:**
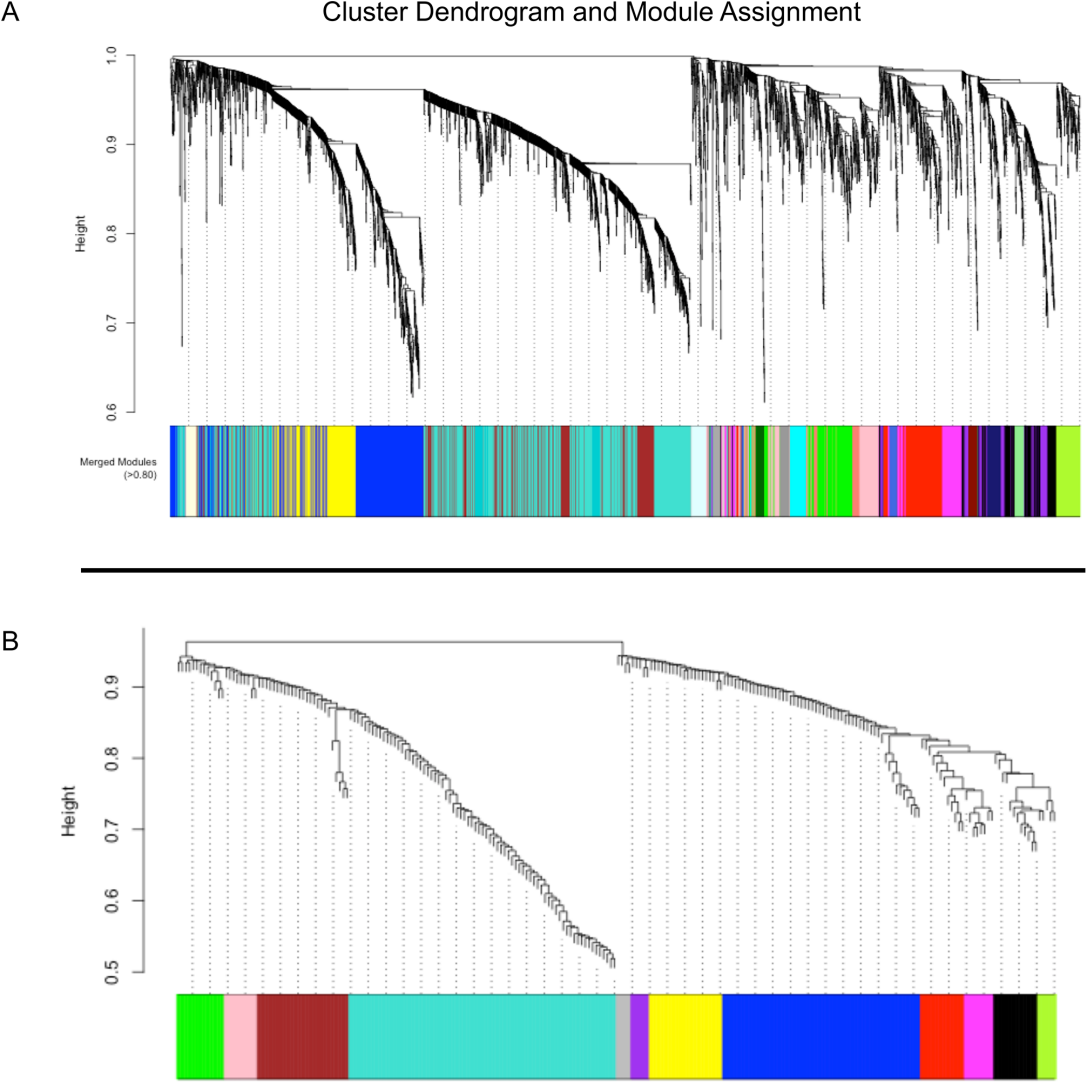
(A) Cluster dendrogram and module assignment for mRNA modules from WGCNA. Topological overlap dissimilarity measure is clustered by average linkage hierarchical clustering and module assignments (dynamic hybrid algorithm) are denoted in the color bar (bottom). 4571 transcripts were assigned to one of 24 modules including Mgrey. (B). Following the same outline, 240 miRNAs are assigned to one of 12 modules indicated by color (including Mgrey).

**Fig 2:**
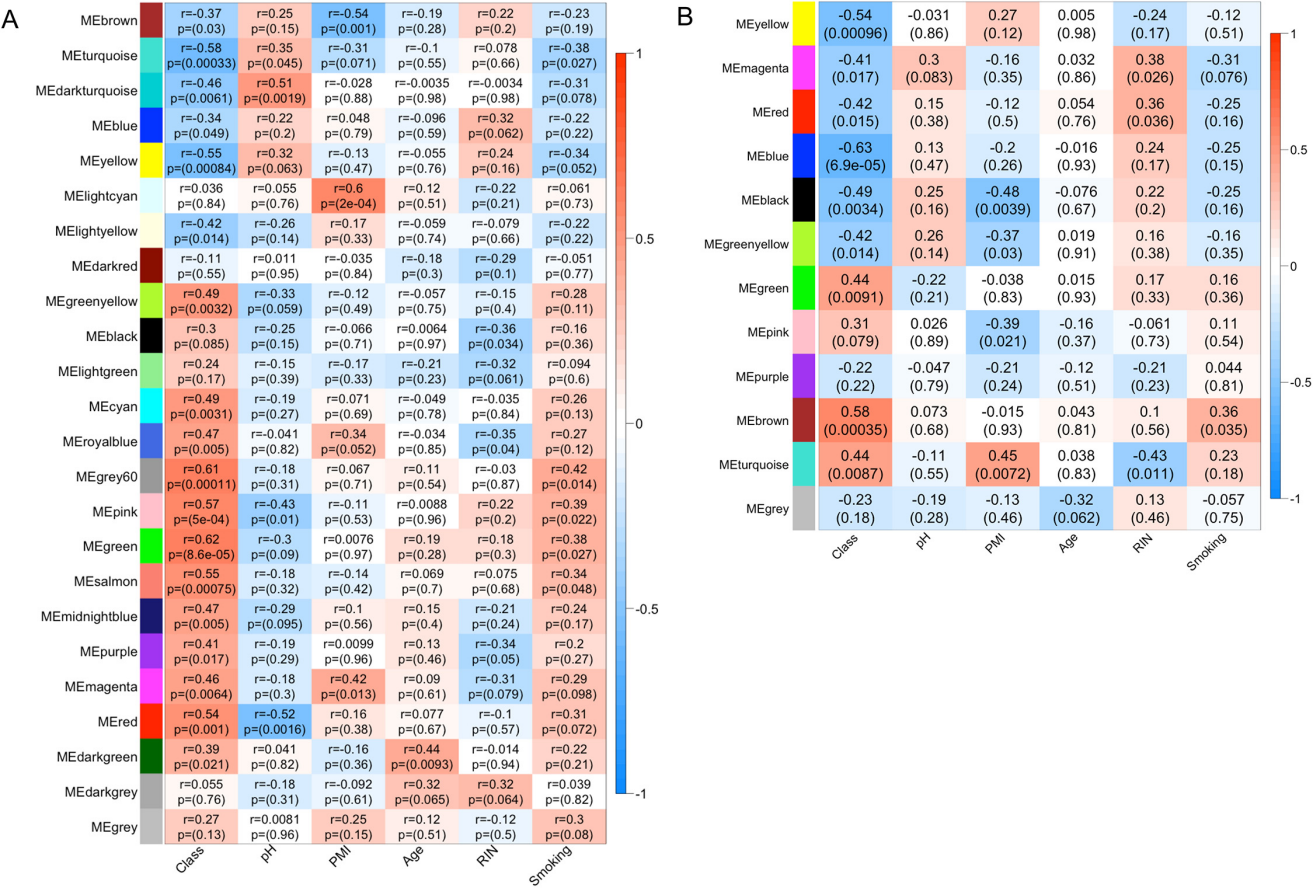
Module-trait relationships. (**A**) mRNA module MEs are correlated (Pearson) to AD case-status (Class), brain pH, PMI, Age, RIN and subject smoking status to assess for confounding. P-values shown are unadjusted for multiple testing. After adjusting for number of modules tested, ME_*turquoise*_, ME_*yellow*_, ME_*grey60*_, ME_*pink*_, ME_*green*_ and ME_*salmon*_ are significantly correlated with AD case-status (Class). (**B**). Similarly, after adjusting p-values for number of modules tested, miRNAs ME_*yellow*_, ME_*blue*_ and ME_*brown*_ modules are significantly correlated with AD case-status (Class).

The major limitation of most genomic studies is the focus on individual genes with the highest statistical significance. In this study we utilized a network based approach, to establish a better functional understanding of changes occurring in the NAc transcriptome of subjects with AD. All nominally significant transcripts identified in the univariate analyses (p≤ 0.05) were used to generate AD-relevant gene co-expression networks. This significance threshold was chosen to allow the inclusion of true positive signals with smaller effect size (which would otherwise be excluded with more stringent statistical criteria) and to retain a sufficient number of genes with biological importance in AD in the building of gene co-expression networks. A total of 24 modules were identified, including the *grey* module (*M*_*grey*_), which contains 13 transcripts unassigned to any of the other 23 modules [41]. The module sizes varied from 1106 transcripts in *M*_*turquoise*_ to 35 transcripts in *M*_*darkgrey*_ (Fig 1A). Next, to assess the quality of sample matching and to detect any confounded modules, the module eigengenes (MEs), which represent the sum of gene expression profiles of each module, were correlated to four matching demographics (age, pH, Postmortem Interval (PMI) and RNA integrity Number (RIN)) and to smoking status. Four modules were significantly correlated (adj. p≤ 0.05) with brain pH and PMI, and were removed from all subsequent analyses. Of the remaining 20 modules, six were significantly correlated with AD case-status (Fig 2A). *M*_*turquoise*_ and *M*_*yellow*_ were downregulated in AD cases, while *M*_*grey60*_, *M*_*pink*_, *M*_*green*_ and *M*_*salmon*_ were upregulated, and these six modules contained 45% of the 4571 transcripts. A full table containing module size, correlations and p-values for all mRNA modules is provided in S2 Table.

### Identification of miRNA co-expression modules

The differentially expressed miRNAs clustered into 12 modules, including *M*_*grey*_ (Fig 1B). Module sizes varied from 73 transcripts (*M*_*turquoise*_) to five transcripts (*M*_*greenyellow*_ and *M*_*purple*_), with *M*_*grey*_ containing four otherwise unassigned transcripts. After correlation to matching demographics and smoking status, only one module, *M*_*black*_, was significantly correlated with PMI (subsequently removed from analysis). Of the remaining modules, three (*M*_*blue*_, *M*_*yellow*_ and *M*_*brown*_) were significantly correlated with AD status (adj. p≤ 0.05) (Fig 2B). These three modules contained 41% of the 240 differentially expressed miRNAs analyzed by WGCNA. A full table containing module size, correlations, and p-values for all modules is available in S2 Table.

### Detection of network hub genes

**Fig 3:**
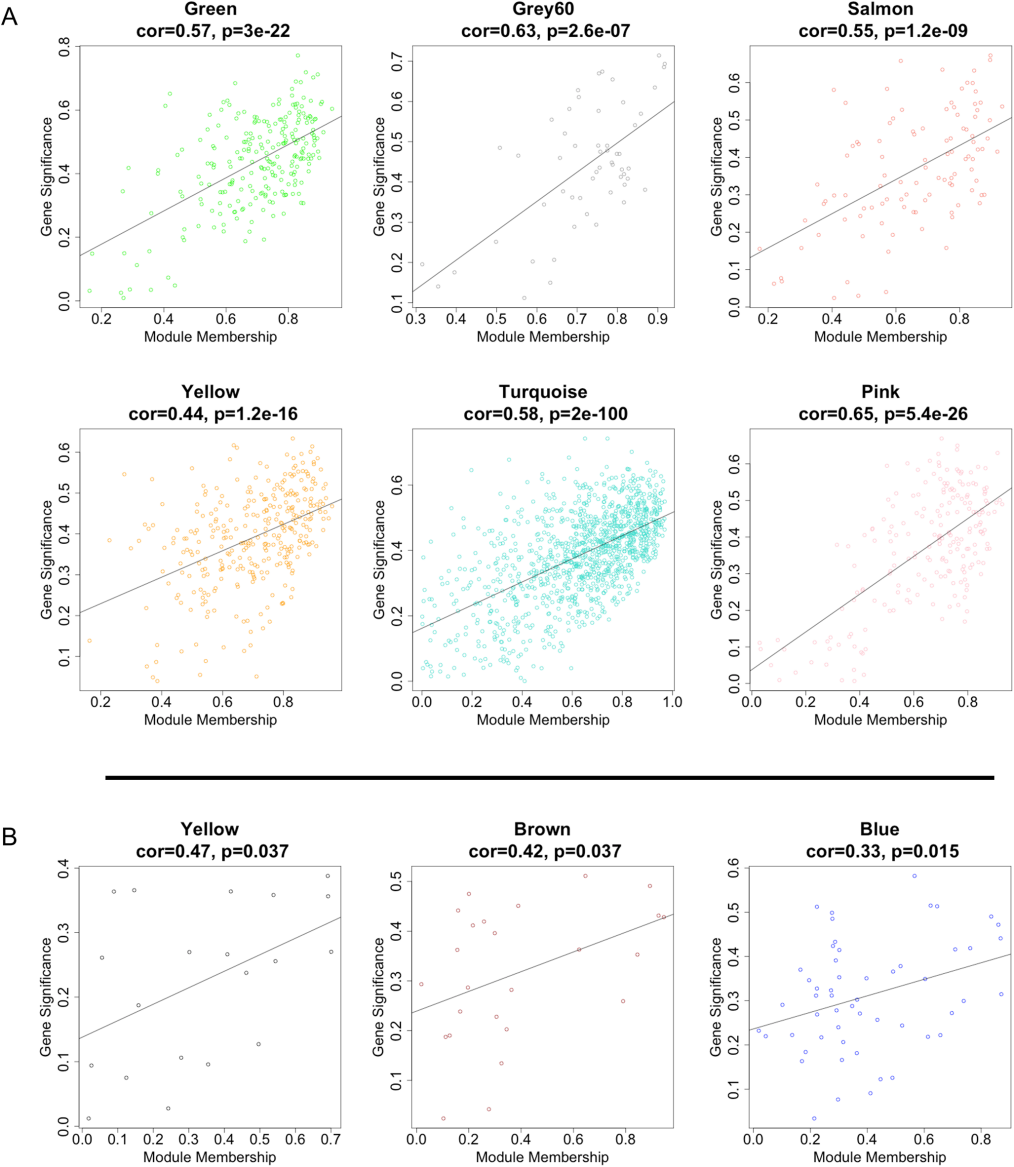
(A). mRNA modules significantly correlated with AD case-status. Each point represents an individual transcript within each module, which are plotted by the absolute value of their expression correlation to AD case-status (Gene Significance) on the y-axis and module eigengene (Module Membership) on the x-axis. The regression line, correlation value and p-value is shown for each plot, which indicates that GS of transcripts increases with increasing intramodular connectivity (MM). (B). miRNA modules significantly correlated with AD case-status. Each point represents an individual miRNA transcript within each module, which are plotted by the absolute value of their expression correlation to AD case-status (Gene Significance) on the y-axis and module eigengene (Module Membership) on the x-axis. Similarly to mRNA modules, GS of miRNA transcripts increases with increasing intramodular connectivity (MM).

The six mRNA modules significantly correlated with AD were explored to identify hub genes. In scale-free network topology, ‘hubs’ are the most highly connected genes (of which there are relatively few among all the nodes within a network). A highly significant positive correlation between module membership ((MM), correlation of individual gene expression with ME of its respective module) and gene significance ((GS), correlation of individual gene expression with AD case-status) for NAc was observed, supporting previous observations in PFC, where genes significantly correlated with AD were also the most important (or central) elements of the module for AD (Fig 3A) [42]. Of the 2034 transcripts clustered in the six modules, 518 transcripts were located in the top quartile of MM and were selected as candidate hub transcripts (see Material and Methods). After collapsing their transcript IDs to unique Hugo Gene Nomenclature (HGNC) symbols, 461 unique genes were identified. Among these, three genes, guanylate cyclase activator 1A (GUCA1A), polypyrimidine tract binding protein 1 (PTBP1), and transgelin 2 (TAGLN2) were shared as hub genes in more than one module. Full transcript, GS, MM, and gene symbol annotation for candidate hub transcripts are available in S3 Table.

From the three significant miRNA modules, 26 miRNA hub genes in the top quartile of MM were identified (Fig 3B); however, one miRNA, hsa-miR-3676, was removed from analysis as it was reported as a tRNA fragment and not processed as a miRNA according to miRBase (www.mirbase.org). Many of the miRNA hub genes also belonged to the same miRNA gene family (<10 kb genomic distance apart). For example, hsa-miR-377-5p, -134-5p, and -382-5p from *M*_*blue*_ are located in the same genomic cluster of chromosome 14q; this region was previously reported to contain overexpressed miRNAs in the PFC of AD cases [21]. In addition, miR-382 overexpression in the NAc was shown to attenuate voluntary alcohol intake in a two-bottle choice rat model [26]. Hsa-miR-132-3p and hsa-miR-212-3p, also in *M*_*blue*_, have been previously associated with schizophrenia/bipolar disorder and cocaine dependence [43–46]. Both hsa-miR-132-3p and hsa-miR-212-3p are in the same miRNA family and are important for neuronal function and long-term potentiation, as well as for neuronal survival in Alzheimer’s disease. *M*_*brown*_ included miRNA from the hsa-miR-34b and hsa-miR-34c family, which have been implicated in neurodegenerative disorders including Huntington’s, Alzheimer’s and Parkinson’s disorders [47, 48]. More importantly, in agreement with our data from NAc, hsa-miR-34c-5p was recently reported to be upregulated in the PFC of human AD subjects [20]. A full table of the significant miRNA modules with GS and MM information is available in S3 Table.

### Detection of cell specific co-expression modules in NAc

The mRNA co-expression modules significantly correlated with AD were assessed for enrichment of cell-type specific marker genes. All six modules showed cell-specific enrichment after correction for multiple testing (Table 1). Similar to other alcohol related studies in Prefrontal Cortex and Ventral Tegmental Area, our analysis revealed that alcohol has discrete effects on different cell types [40]. For example, the neuronal expression-associated modules, *M*_*yellow*_ and *M*_*turquoise*_, were negatively correlated with AD status, while the glial and microglial-associated modules, *M*_*green*_, *M*_*grey60*_, and *M*_*salmon*_, were positively correlated with AD. *M*_*pink*_ was also positively correlated with AD; however, this module was significantly enriched for astrocyte cell specific gene expression only.

**Table 1:** Brain list enrichment for cell type specific modules.

Module	Gene sets	p-values	Adj. p-values	Enriched Genes
**Green**	Microglia (M8)	6.37E-48	3.82E-47	49
**Green**	Microglia (M10)	2.34E-08	1.41E-07	13
**Green**	Astrocytes (M3)	1.61E-07	9.63E-07	30
**Grey60**	Astrocytes (M3)	1.84E-04	1.11E-03	9
**Grey60**	Microglia (M8)	8.62E-04	5.17E-03	5
**Pink**	Astrocytes (M3)	1.27E-39	7.60E-39	64
**Salmon**	Microglia (M10)	1.86E-06	1.12E-05	8
**Salmon**	Astrocytes (M3)	9.14E-04	5.49E-03	13
**Salmon**	Microglia (M8)	7.39E-03	4.44E-02	6
**Turquoise**	Neuron (M11)	3.02E-18	1.81E-17	86
**Yellow**	Neuron (M13)	4.05E-03	2.43E-02	10

### Gene set enrichment analysis

WGCNA allows for the generation of gene modules that are related by their co-expression patterns. Studies employing WGCNA have found that modules organized by co-expression patterns are enriched for biologically relevant functions [49–51]. Thus, the mRNA modules significantly correlated with AD were assessed for enrichment of cellular process and biological functional categories using the gene set enrichment analysis (GSEA) software. Gene lists for each module were generated by ranking all 4571 differentially expressed transcripts according to their MM in each of the six significant mRNA modules, which was achieved by correlating individual gene expression values to the ME of each of the six significant modules (as previously described) [52]. To assess for enrichment of cellular processes and functional categories, all transcripts from the mRNA modules were collapsed into unique, functionally annotated genes by GSEA, resulting in 3742 unique gene symbols. Using the default parameters in GSEA, we identified 364 *a priori* gene sets significantly enriched (FDR≤ 0.10) within the six MM ranked gene lists. Of these, 117 *a priori* gene sets were enriched in multiple (≥2) modules. Interestingly, no *a priori* gene sets were shared between glial cell-associated modules, *M*_*green*_, *M*_*grey60*_, *M*_*pink*_ and *M*_*salmon*_, and the neuron-associated modules, *M*_*turquoise*_ and *M*_*yellow*_.

Sixty-eight *a priori* gene sets shared enrichment in *M*_*turquoise*_ and *M*_*yellow*_, with genes predominantly involved in the ‘neuronal system’ and neurotransmission, neurodegenerative disorders (Huntington’s, Parkinson’s and Alzheimer’s), protein metabolism, DNA repair and replication, transcription and mRNA processing, cell cycling, oxidative phosphorylation, glucose metabolism, mitochondrial function, and MAPK cell signaling pathways. Both *M*_*turquoise*_ and *M*_*yellow*_ were negatively correlated with AD, suggesting decreased activity/function of these pathways and processes. The full list of shared enriched gene sets between the neuronal modules is available in S4 Table. Shared *a priori* gene set enrichment between the glial cell-associated modules was predominantly between *M*_*green*_, *M*_*pink*_ and *M*_*salmon*_, totaling 18 gene sets, with genes involved in cytokine/immune signaling, cell surface interactions, and cell signaling pathways (JAK/STAT, RhoA and TGF-β), which were upregulated in AD cases. The full list of shared enriched gene sets between the glial enriched modules is available in S4 Table.

While overlapping *a priori* gene sets between modules suggests shared functionality between these gene co-expression networks, we also identified *a priori* gene sets that were unique to each of the significant mRNA modules. Specifically, 51 *a priori* gene sets were unique among five of the six modules, with no unique gene sets enriched in *M*_*grey60*_. Of these, 24 gene sets were uniquely enriched in M_turquoise_, a neuron-associated module, with genes involved in mRNA processing and degradation, DNA repair, protein modification, and glucose and carbohydrate metabolism. In *M*_*yellow*_, also a neuron-associated module, genes involved in neurotransmitter binding/transmission and opioid signaling, long-term potentiation, calcium signaling and translation regulation pathways were enriched among 9 gene sets. Of the glial cell-associated modules, *M*_*green*_ exhibited enrichment with 12 gene sets related to immune function and signaling, apoptosis, and actin cytoskeleton regulation. *M*_*pink*_ and *M*_*salmon*_ were enriched for gene sets with genes involved in glycosaminoglycan metabolism, Notch signaling, and lysosomal activity, respectively. The full list of unique enriched gene sets is given in S5 Table.

### Assessment of mRNA:miRNA module interactions in AD

**Fig 4:**
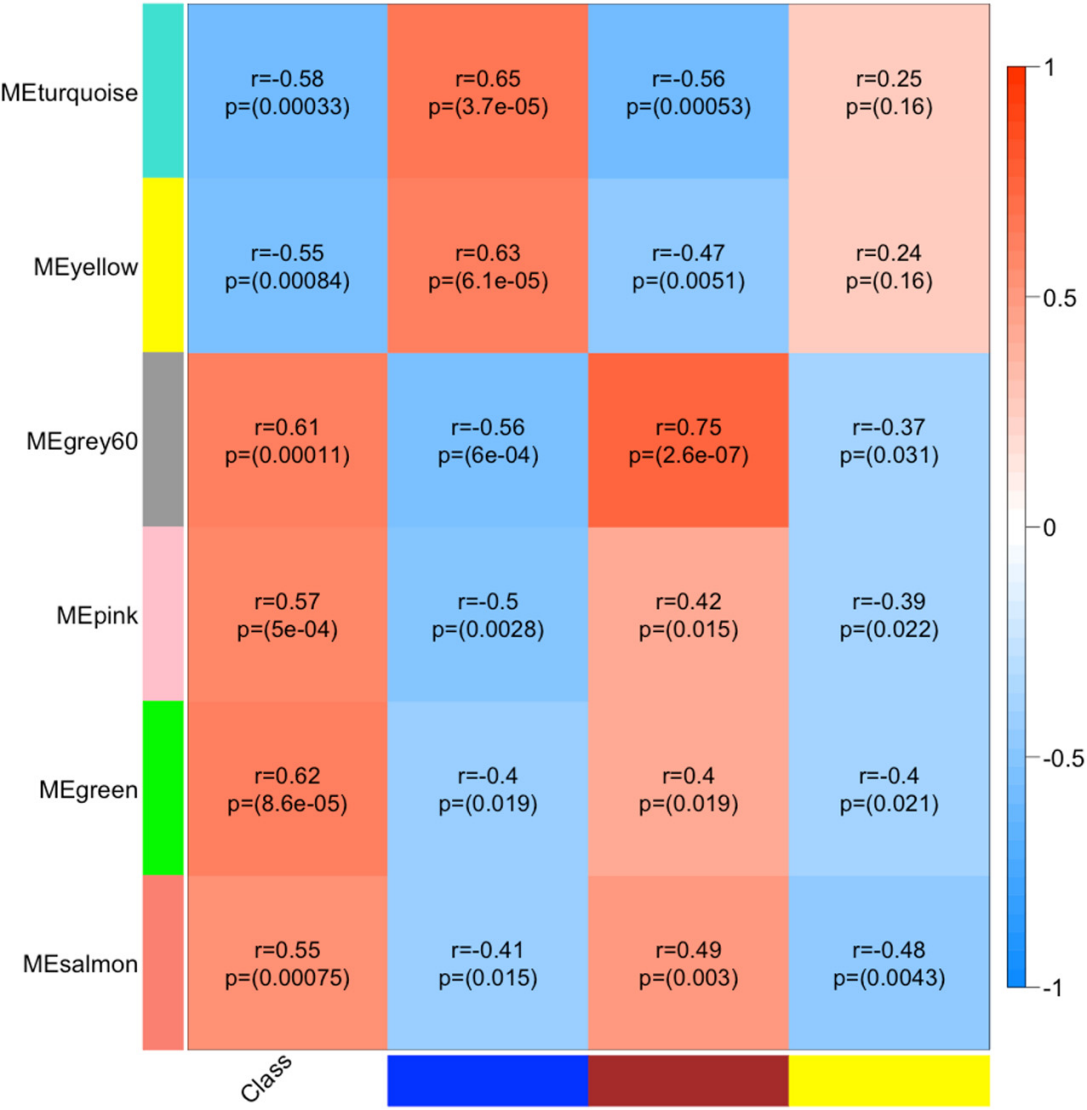
miRNA:mRNA Module relationships: Significant mRNA module MEs (rows) are correlated (Pearson) to significant miRNA MEs for blue, brown and yellow modules (columns). Strongest correlations is positive between miRNA MEbrown and mRNA MEgrey60 (r = 0.75) and strongest negative correlation is between miRNA MEbrown and mRNA MEturquoise (r = -0.56) and miRNA MEblue and mRNA MEgrey60 (r = -0.56). miRNA MEblue and MEyellow are negatively correlated with glial cell-associated mRNA modules and miRNA MEbrown is negatively with neuron-associated mRNA modules.

The purpose of correlating mRNA and miRNA MEs was to investigate whether general correlation patterns could be detected at the gene network level. As MEs are representative of module-wide gene expression profiles, this analysis gives a global perspective of miRNA:mRNA interactions in our AD sample. Although the strongest correlations between mRNA and miRNA MEs were positive (ranging between r = 0.63 and 0.75), negative correlations were also prominent (ranging between r = -0.37 and -0.56) (Fig 4). After assessing miRNA:mRNA ME correlations, our next step was to examine the individual miRNA:mRNA hub gene interactions driving the module correlations. The Pearson correlations between 518 transcripts, representing the 461 mRNA hubs and 25 miRNA hubs, resulted in 12,950 correlations (S6 Table), of which a higher number of positive versus negative correlations were observed (59% vs. 41%; χ^2^ p≤ 0.0001). Interestingly, Nunez, *et al*. (2013) reported similar findings in a mouse model of AD where miRNA:mRNA positive correlations were more abundant than negative correlations [22]. It is unclear, however, whether these positive correlations represent direct miRNA upregulation of target mRNAs or secondary effects of the miRNA targeting mediating molecules. Therefore, we elected to focus on negative correlations, as the predominant (and canonical) effect of miRNAs on gene expression is through mRNA downregulation, which then manifests as negative miRNA:mRNA expression correlations [53].

Next, the significant negative miRNA:mRNA correlations were intersected with bioinformatic predictions to identify the most likely direct miRNA:mRNA target interactions. At FDR <0.10, 2445 significant negative correlations were retained. The intersection resulted in the identification of 481 miRNA:mRNA targeting pairs between 25 hub miRNAs and 244 hub mRNAs. Similar to the ME network-level correlations, a clear separation of module miRNAs:mRNA interactions was also detected. For example, miRNAs from the *M*_*blue*_ and *M*_*yellow*_ modules targeted only the glial cell-associated mRNA modules, *M*_*green*_, *M*_*grey60*_, *M*_*pink*_ and *M*_*salmon*_, whereas miRNAs from the *M*_*brown*_ targeted only the neuron-associated mRNA modules, *M*_*turquoise*_ and *M*_*yellow*_. Within each mRNA module we identified several instances of cooperative miRNA targeting, i.e. multiple miRNAs targeting a single mRNA gene. It has previously been shown that cooperative miRNA regulation of mRNAs leads to an enhanced repressive effect and greater specificity of target regulation [54]. The most prominent case of such cooperative targeting by miRNAs was for AF4/FMR2 family, member 1 (AFF1), which was targeted by 8 miRNAs from miRNA: *M*_*blue*_ and *M*_*yellow*_. The full list of the 481 significant negatively correlated and bioinformatically predicted miRNA:mRNA targeting pairs is given in S7 Table.

### Identifying eQTLs for candidate mRNA and miRNA hub genes

**Fig 5:**
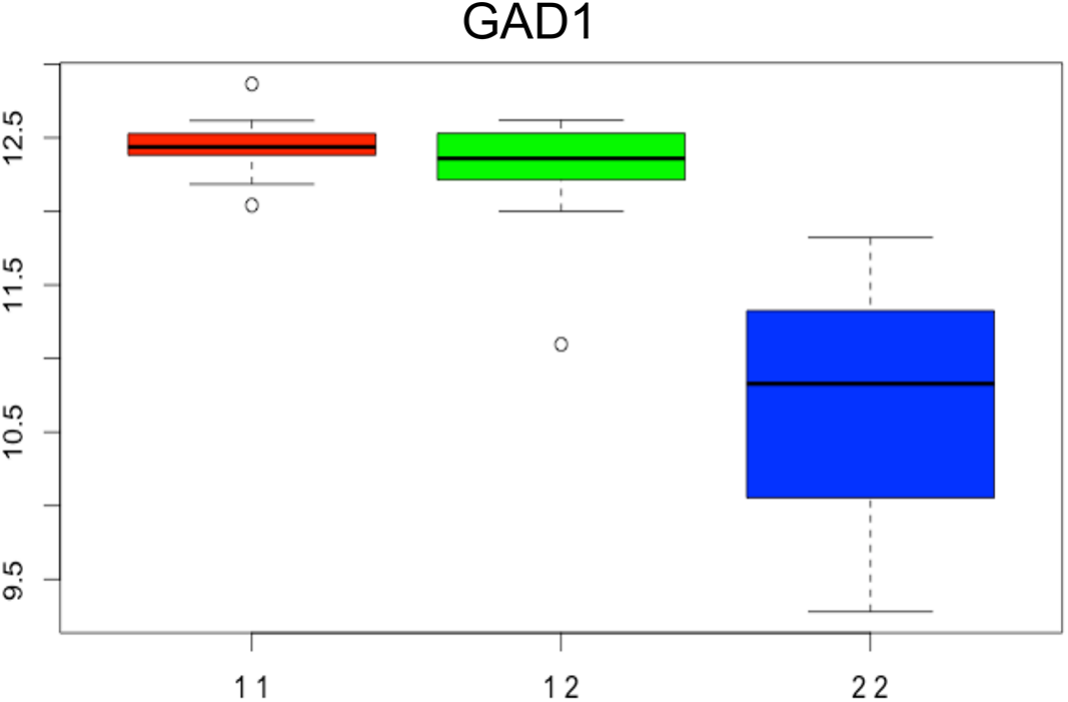
eQTL (chr2:170783092:D) effect on the expression of the glutamate decarboxylase (GAD1) gene. The bar plot depicts the differential expression of GAD1 among homozygote for the major (11, red), heterozygote (12, green) and homozygote for the minor alleles (22, blue) subjects.

To better understand the underlying genetic mechanisms of AD in NAc, we integrated expression data with previously collected genotypic data to identify eQTLs affecting the expression of hub mRNA and miRNA genes. Only cis-eQTLs (defined within 1 megabases) were considered in our analysis, as our study was underpowered to identify trans-eQTLs. Five hundred and ninety-one significant mRNA hub cis-eQTLs were identified (FDR≤ 0.10). In some cases, a single gene was associated with multiple eQTLs. Thus, the total number of mRNAs for which eQTLs were detected was 383. The full list of the identified mRNA eQTLs is given in S8 Table. The most significant mRNA cis-eQTL signal we detected was for glutamate decarboxylase (GAD1; p = 5.5x10^-7^), the rate-limiting enzyme in gamma-aminobutyric acid (GABA) biosynthesis (Fig 5). Interestingly, variants in the GAD1 gene have been previously associated with AD in Han Taiwanese men [55].

**Fig 6:**
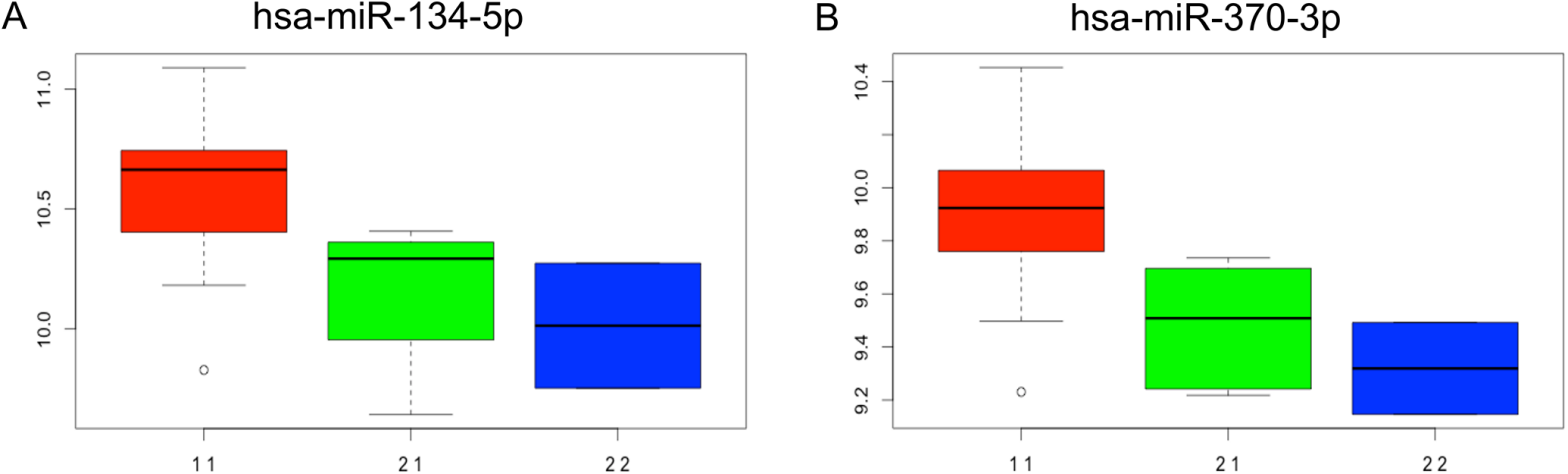
(A). rs11626307 effect on the hsa-miR-134-5p (A) and hsa-miR-370-3p (B) expressions.

At an FDR≤ 0.10, 68 miRNA cis-eQTLs were detected; the most significant cis-eQTLs were detected for hsa-miR-134-5p (p = 7.1x10^-5^) and -370-3p (p = 8.2x10^-5^) (Fig 6A and 6B). We also identified many instances of single variants associated with the expression of multiple miRNAs. This is not surprising in that many miRNAs are clustered genomically (within 10kb), and being expressed on the same primary transcript, will show similar regulatory patterns [56]. The full list of the identified eQTLs for the miRNA hub genes is given in S8 Table.

### eQTLs in NAc are enriched in alcohol related GWAS

All significant eQTLs for the mRNA and miRNA hubs were queried against GWAS case/ control data from a large, independent genetic sample, the Collaborative Studies On Genetics of Alcohol (COGA) (N = 1399), to assess whether our eQTLs were associated with risk for AD or alcohol related phenotypes (ARPs) [57]. Since *a priori* molecular evidence increases the likelihood of true association, we queried our eQTLs against GWAS loci associated with AD and other ARPs using a more liberal threshold of p≤10^−3^. In addition to providing explanatory power to the GWAS signals, this analysis could also provide candidates for functional studies to investigate the molecular mechanism(s) through which eQTLs confer risk for developing AD.

We first determined whether there was enrichment of AD association signals from the COGA sample within our significant eQTLs. To that end, we tested the European Americans in the COGA sample (for a set-based enrichment of AD and ARP, i.e. AD case/ status, AD symptom count and maximum number of drinks in 24 hours) with association signals within our eQTLs. Based on this analysis, we observed a significant set-based enrichment for AD diagnosis and AD symptom counts, which also passed the Simes’ correction for multiple testing (adj. p = 0.014 and p = 0.024, respectively). Among the enriched eQTL sets, the most significant GWAS signals in COGA at p≤10^−3^ were: 1) rs1780705 for neuronatin (NNAT), a mRNA hub gene in *M*_*yellow*_, associated with AD at p = 2.2x10^-4^, 2) rs13392737 for a long non-coding RNA (PKI55), a mRNA hub gene in *M*_*turquoise*_, associated with AD at p = 1.6x10^-4^ and 3) rs4243820 for replication protein A2, 32kDa (RPA2), a mRNA hub gene in *M*_*turquoise*_, associated with an AD at p = 4.1x10^-4^[58, 59].

## Discussion

The purpose of this study was to perform a transcriptome analysis of mRNA and miRNA expression to identify gene co-expression modules correlated with excessive alcohol consumption in human NAc. The NAc is a central component of the mesocorticolimbic system (MCL) and has been shown to be involved in addictive behaviors. A current theory on the mechanism by which the NAc modulates addiction is that the NAc integrates signals from other MCL regions on the single-neuron level in order to modulate goal- and motivation-directed behavior [60–62]. We assembled our expression data with previously generated genome-wide genotype data to identify NAc-specific cis-eQTLs affecting the expression of mRNA and miRNA network hub genes. We then tested whether the eQTLs were enriched for AD GWAS signals. This is the first study to show that genetic variants (previously shown to be associated with AD or ARP) are eQTLs, which affect the differential expression of genes between AD cases and controls in NAc.

Cell type-specific profiling of our co-expression networks revealed significant enrichment of neuronal or glial marker genes within specific modules. The genes in the neuronal and glial specific modules showed interesting and opposing patterns of expression. Genes from the neuronal expression associated modules were downregulated in AD, while those from the glial modules were upregulated in AD. We speculate that the opposing patterns of expression reflect cytological changes occurring in the brains of AD as a result of prolonged alcohol consumption, which is known to have strong neurotoxic effects [63]. We could not differentiate whether these changes are the result of a global downregulation of expression in neurons or the result of a progressive loss of neuronal cell mass. A previous study on the amygdala of chronic alcoholics reported downregulation of neuronal gene expression, coupled with the upregulation of glial cell expression[40]. We would like to point out that using cell deconvolution to estimate cell-specific gene expression relies on computational algorithms; thus, it is conceivable that these approaches will be inherently noisier than a direct estimation of cell proportions.

In an effort to better understand the biological processes influencing the organization of our co-expressed gene networks in NAc, we performed GSEA, utilizing a well-curated collection of gene sets ascertained from both physiological and pathological cellular states and functions. This analysis revealed that neuron-specific modules were downregulated for gene sets enriched with: 1) brain related functions such as neuronal signaling, neurotransmission, long-term potentiation, and constitutive cell maintenance, 2) growth functions such as glucose metabolism, oxidative phosphorylation, mitochondrial function, and MAPK signaling, and 3) involvement in the etiology of neurodegenerative disorders such as Alzheimer’s, Parkinson’s, and Huntington’s. In support of our findings, alcohol has been shown to modulate MAPK signaling cascades depending on cell type, brain region, and ethanol treatment paradigm [13, 64, 65]. However, our results contrast those of an earlier study assessing gene expression in the PFC, VTA and NAc of chronic alcoholics [8]. The authors reported that genes involved in oxidative phosphorylation and energy production were only differentially expressed in PFC, whereas we observed these functional pathways to be differentially expressed in the NAc. Potential reasons for this discrepancy could be either the small sample size of the previous study or technical, platform-dependent differences.

The negative impact of alcohol intake on mitochondrial function has been well documented in several animal models, and excessive alcohol consumption in mice has been linked to degradation of mitochondrial DNA [66–69]. As AD is a risk factor for dementia, prolonged alcohol consumption has been shown to have a toxic effect on amyloid precursor protein. This leads to an accumulation of beta-amyloid in neurons from alcohol dependent rats, perhaps mediated through increased production of reactive oxygen species and mitochondrial dysfunction [70]. Results from human studies, however, have been more equivocal. While a postmortem brain analysis of chronic alcoholics did not detect greater incidence of neuropathological lesions compared to control subjects, a Swedish national cohort study concluded that history of alcohol dependence conferred a greater risk for Parkinson’s disease diagnosis [71, 72].

Astrocyte and microglial associated modules exhibited increased expression in AD subjects and were predominantly enriched with immune related processes. The immune signaling processes resulting from repeated alcohol abuse arise from activation of astrocytes and microglia; when coupled with alcohol-induced loss of neurogenesis, are thought to enhance the negative emotional states that lead to addiction [73]. In this study, *M*_*green*_ was the module most significantly associated with these immune related processes, i.e. Toll-like receptor 4 (TLR4) signaling and inflammatory cytokine pathways. Injection of lipopolysaccharides (LPS) in mice has been shown to induce the expression of innate immune genes through activation of TLR4 in microglia and astrocytes, leading to depression-like behaviors [73]. LPS infusions in human studies were also reported to reduce reward responses and increase depressed mood [74]. Furthermore, induction of innate immune genes in mice resulted in increased ethanol consumption, whereas inactivation of such genes reduced drinking behavior [75, 76]. These observations highlight the role of immune signaling in the neurobiology of addiction and support our GSEA findings in astrocyte and microglial associated modules.

By integrating miRNA and mRNA co-expression modules, we were able to examine the regulatory roles that miRNA have on their gene targets (as both mRNA and miRNA data was generated from the same subjects). Performing a module eigengene (ME) correlation analysis, we found that the significant miRNA and mRNA AD modules were also negatively correlated with each other,i.e.miRNA modules upregulated in chronic alcoholics were negatively correlated with the downregulated mRNA modules and *vice versa—*suggesting that the neuropathology of AD is at least partially modulated by specific miRNA:mRNA interactions. Interestingly, we also observed positively correlated miRNA and mRNA modules that contained miRNA:mRNA targeting pairs. Although these interactions were not further assessed, they may be important for disease development. Considering the canonical role of miRNA in negatively modulating gene expression, these results are surprising. Similar observations were also reported in an animal based study, where highly significant positive miRNA:mRNA correlations were detected in the PFC of mice undergoing acute alcohol exposure [22]. One potential explanation is that the positive miRNA:mRNA expression correlations observed in the animal model are the result of an uncompensated miRNA response to the increase in gene expression following the alcohol consumption. Considering that our postmortem sample consists of chronic alcoholics with drinking histories of several decades, attributing our positive miRNA:mRNA module correlations to a temporal artifact seems unlikely. A more plausible explanation, which stems directly from the negative regulatory capacity of miRNA function, could be that the observed positive correlations reflect secondary miRNA targets, as the expression of such secondary miRNA targets are expected to be positively correlated with miRNA expression [77]. Regardless of the mechanism by which these positive correlations are occurring, we are the first to report that such positive correlations appear to be preserved in chronic alcoholic subjects and that these may reflect either an adaptive or decompensated state resulting from excessive alcohol consumption.

By exploring miRNA:mRNA interactions at the single hub level, we observed that many of the mRNA hub genes were predicted to be targeted by multiple miRNAs, thus highlighting the cooperative capacity of these molecules. Cooperative targeting by miRNAs produces synergistic mRNA target repression and enhances the ‘fine-tuning’ capacity of these molecules [54]. In addition, cooperative targeting is thought to increase the cell’s ability to buffer induction of gene expression by external stimuli. We speculate that the cooperative regulation of a single mRNA hub gene by multiple miRNAs provides redundancy within the system to respond to the loss of a critical miRNA:mRNA interaction. In such a case, the presence of additional miRNA target sites ensures that repression of gene expression will be maintained.

Another observation from our results is that miRNA belonging to a miRNA family act cooperatively to control the expression of a single mRNA target. For example, members of the hsa-miR-34 family (hsa-miR-34b and hsa-miR-34c) clustered into miRNA module *M*_*brown*_, and were shown to target numerous hub genes in the neuronal expression associated mRNA modules, *M*_*turquoise*_ and *M*_*yellow*_. This miRNA family has been shown to play role in a wide range of human disease phenotypes including neuropsychiatric and neurodegenerative disorders, and is also reported to be upregulated in the PFC of human chronic alcoholics [20, 47, 48]. In addition, we identified that hsa-miR-34c-5p and hsa-miR-34b-5p cooperatively target neuronatin (NNAT; rs1780705) and proteosome subunit beta, type 5 (PSMB5; rs10137082)–two mRNA hub genes with significant eQTLs that were also associated with AD (p< 10^−3^) in the COGA sample. Our most significant eQTL signal was associated with glutatamate decarboxylase (GAD1) expression, the rate-limiting enzyme in GABA biosynthesis and previously implicated in AD. GAD1 was also cooperatively targeted by the hsa-miR-34 family of miRNAs [55, 78]. Thus, our observations here corroborate *a priori* evidence implicating the involvement of the hsa-miR-34 family in alcohol addiction phenotypes and reinforce its significance.

There is empirical evidence that eQTLs are over-represented in GWAS signals, and several reports have linked AD association signals with eQTLs [31, 38, 39, 79, 80]. Our study adds to this growing body of evidence, as we find that AD GWAS signals are significantly over-represented in our eQTLs, thus strengthening the significance of these genetic associations and providing potentially causal mechanisms of action for our eQTL findings. For example, in addition to NNAT and PSMB5, eQTLs for a long non-coding RNA (PKI55; rs13392737), adaptor related protein complex 1, sigma 1 subunit (AP1S1; rs10279545) and translocation associated membrane protein 1 (TRAM1; rs2959574) were also associated with AD at p = p = 9.1x10^-4^ and p = 6.2x10^-3^, respectively. In particular, NNAT and AP1S1 have prior evidence of involvement with AD [81]. NNAT, an imprinted gene expressed early in brain development, was shown to regulate dendritic calcium levels in hippocampal neurons and was differentially expressed in the NAc, PFC, and VTA of an acute ethanol response mouse model [82, 83]. AP1S1, a clathrin-related protein involved in membrane trafficking and endocytosis, as well as the causal gene for MENDIK (mental retardation, enteropathy, deafness, neuropathy, ichthyosis and keratoderma) syndrome, was differentially expressed in the PFC of chronic alcoholics [9, 84]. Although we did not observe significant enrichment of AD GWAS signals among our significant eQTLs for miRNA hubs, NNAT and PSMB5 from neuronal expression-associated mRNA modules, *M*_*yellow*_ and *M*_*turquoise*_, respectively, were both cooperatively targeted by hsa-miR-34 family miRNAs. It is possible that hsa-miR-34 family miRNAs exhibit trans-eQTL effects with variants associated with NNAT and PSMB5; however, as this analysis requires much larger samples, we did not have the power test this hypothesis here.

## Conclusions

In this study, we identified mRNA and miRNA co-expression modules differentially expressed in a matched AD case-control postmortem sample, including cell type-specific associations and the differential enrichment of biological processes therein. Our results corroborate previously reported findings in the literature; however, we are first to identify the dysregulation of these processes in the NAc of human alcoholics. While central to the MCL system, the NAc also acts in conjunction with other critical brain regions, and we believe our study advances our knowledge of the effect of alcohol on this prominent reward/reinforcement circuit.

Our most important finding is the identification of the effects that alcohol relevant eQTLs have on gene expression in the brain. To our knowledge, this is the first study to perform such analyses in human postmortem brains of subjects with AD.

While novel in its approach to integrating genetic and molecular data in postmortem alcohol research, our study is not without limitations. First, postmortem brain studies are observational, as the manipulation of the brain of living human subjects is not possible. Although the cross-sectional nature of these studies limits causal inferences, we believe our eQTL analysis, and subsequent integration with GWAS data, are major steps toward clarifying the directionality of these observations. Secondly, although our sample size (N = 36) is the largest postmortem alcohol study in the NAc to date, the sample size is still small. In an attempt to reduce the experimental variance and allow for increased power, we performed stringent case/ control matching for factors known to systemically impact gene expression levels, an approach that has been successfully applied before [85–88]. We believe that by using this experimental design and implementing integrative multivariate approaches, we can greatly further our understanding of the alcohol addiction processes and translate these advances into effective therapeutic strategies for patients suffering from substance use disorders.

## Methods and Materials

### Postmortem tissue

Tissues from 41 AD cases and 41 controls were received from the Australian Brain Donor Program, New South Wales Tissue Resource Centre, which is supported by The University of Sydney, National Health and Medical Research Council of Australia, Schizophrenia Research Institute, National Institute of Alcohol Abuse and Alcoholism, and the New South Wales Department of Health (http://sydney.edu.au/medicine/pathology/trc/). Cases were excluded if there was: 1) a history of infectious disease (i.e. HIV/AIDS, hepatitis B or C, or Creutzfeldt-Jakob disease), 2) an unsatisfactory agonal status (determined from the circumstances surrounding the death), 3) a post-mortem interval >48 hours, or 4) significant head injury. In addition to case status, age, sex, ethnicity, brain weight, brain pH, post-mortem interval (PMI), tissue hemisphere, clinical cause of death, blood toxicology at time of death, smoking status, neuropathology, and liver pathology were provided for each subject (S9 Table).

### RNA isolation and sample selection

Total RNA containing the small RNA fraction was isolated from 100mg of frozen tissue from the nucleus accumbens (NAc) using the mirVana-PARIS kit (Life Technologies, Carlsbad, CA), following manufacturer's protocols. RNA concentration was measured using the Quant-iT Broad Range RNA Assay kit (Life Technologies), and the RNA Integrity Number (RIN) was measured on the Agilent 2100 Bioanalyzer (Agilent Technologies, Inc., Santa Clara, CA). All subjects were initially included for matching based on age, sex, ethnicity, brain pH, PMI and RIN, and this yielded 18 appropriately matched case-control pairs with RINs ≥6 (N = 36).

### mRNA expression microarrays

The Affymetrix® protocol (Affymetrix, Santa Clara, CA) has been previously described [89]. Briefly, starting from 300ng of total RNA, cDNA synthesis and cRNA labeling were performed using the GeneChip^®^ 3' IVT Express Kit (Affymetrix). Ten μg of fragmented cRNA were hybridized on the Affymetrix GeneChip^®^ Human Genome U133A 2.0 (HG-U133A 2.0). This array provides comprehensive coverage of the transcribed human genome using 22,214 probesets, and captures the expression of ≈18,400 human transcripts. Each array was scanned on the Affymetrix GeneChip^®^ Scanner 3000 7G (Affymetrix), and raw probe intensities stored in.CEL files by the GeneChip^®^ Operating Software (GCOS v1.4). Array quality was assessed by monitoring the 3′/5′ ratios of GAPDH, and the percentage of “Present” genes (%P) and array exhibiting GAPDH 3′/5′ < 3.0 and %P > 40% were considered of good quality. Based on these metrics no arrays were excluded.

### miRNA expression microarrays

The Affymetrix GeneChip miRNA 3.0 microarray contains probesets to measure the expression level of 1733 human mature miRNAs from miRBase v.17 (www.mirbase.org), the primary repository for annotated miRNAs [90]. Total RNA (500ng) from each specimen was labeled using the FlashTag™ Biotin HSR RNA labeling kit (Affymetrix). Each RNA sample was spiked with five different oligonucleotides (as positive endogenous controls) to assess the efficiency of the labeling reaction. The RNA samples were subjected to a brief Poly(A) tailing reaction followed by ligation of a biotinylated signal molecule. Each labeled sample was then hybridized onto a GeneChip^®^ miRNA 3.0 Array, and scanned on a GeneChip^®^ Scanner 3000 7G as described above. The microarray data was submitted to the NCBI GEO archive and are available under *GSE62699*.

### Microarray normalization

Expression values were calculated following the pre-processing procedure: 1) GCRMA background correction, 2) log_2_ transformation, 3) quantile normalization, and 4) median-polish probeset summarization using Partek Genomics Suite v6.23 (PGS; Partek Inc., St. Louis, MO) [91, 92]. The batch effect removal option in PGS was used to control for batch effect. mRNA and miRNA microarray quality was assessed by principal component analysis (PCA) of the expression values for both the miRNA and mRNA arrays. Samples were plotted along the first three principle components (PCs) to identify potential microarray outliers. Of the 36 HG-U133A 2.0 microarrays, one AD sample did not load onto two of the first three PCs and was removed from subsequent analysis (n = 35). No samples were removed from miRNA microarray analysis.

### Microarray analyses

Single gene analysis for differential expression of mRNA and miRNA transcripts was performed in the Number Cruncher Statistical Software (NCSS) v9, using a robust multiple regression model. Prior to the main analyses, a step-wise regression analysis was performed to assess the impact of smoking and liver and brain pathology on expression (as these covariates could not be effectively matched). Only measures significant at a nominal p<0.05 were included as covariates in the regression model to evaluate dependence of gene expression on AD case-status. Smoking was the only covariate with a systemic effect on mRNA expression, and was included as covariate in the robust multiple regression analysis.

To evaluate the reliability of the expression microarrays, expression levels of five genes were validated by quantitative real-time PCR using a Taqman approach (Applied Biosystems, Foster City, CA): CD63 (catalog# Hs01041237_g1), LPPR1 (catalog# Hs00214827_m1), PLK2 (catalog# Hs01573405_g), AKR1A1 (catalog #Hs00895477_m1) and MAPT (catalog #Hs00902194_m1). The expression of these genes was normalized using two reference genes as endogenous controls: POLR2A (catalog #Hs00172187) and RPS17 (Hs #00734303-g). The efficiencies of the target and reference genes expression were assessed by LinRegPCR software (http://www.gene-quantification.com/LinRegPCR_help_manual_v11.0.pdf). The expression levels of the five genes measured by the two platforms were well correlated (S1 Fig). The individual expression values reported in S10 Table also demonstrate an agreement in magnitude and direction of expression between the microarray and PCR based results.

### WGCNA–construction of mRNA modules

The method for constructing scale-free networks by WGCNA has been described in previous studies [41, 93]. The gene co-expression networks were constructed by using the WGCNA v1.36 package in R environment (v3.02). In order to construct gene modules, pair-wise Pearson correlation coefficients were first calculated between all differentially expressed transcripts to generate a signed similarity matrix selecting for positive correlations only. To emphasize (weight) stronger correlations at the expense of weaker correlations, the signed similarity matrix was then raised to the lowest power, β = 14, that approximated a scale-free network topology (R^2^>0.80), to generate an adjacency matrix. Following this, a topological overlap measure (TOM) was calculated, which assessed transcript interconnectedness. A dissimilarity measure was calculated from the TOM and was subsequently used for average linkage hierarchical clustering. Module definition parameters included a minimum module size of 35 genes and a minimum module merge height of 0.8 (default parameter).

Following module definition, the first principal component of each module–the module eigengene (ME)–was calculated as a synthetic gene representing the expression profile of all genes within a given module. Modules were named by a conventional color scheme and then correlated to AD case-status, matching demographics and relevant covariates. Statistical significance was assessed at Bonferoroni-adj. p≤ 0.05 (corrected for number of tested modules).

### Construction of miRNA modules

The steps for constructing miRNA co-expression modules were as described above (with a few differences). After generating the signed similarity matrix, a β = 5 was chosen to generate the adjacency matrix. A TOM and dissimilarity measure was calculated as previously described and a minimum module size of five miRNA genes was chosen. Five was chosen as the minimum module size for the miRNA genes due to the smaller size of the miRNA transcriptome relative to the mRNA transcriptome. The default minimum module merge height of 0.8 was retained. After modules were defined, MEs were calculated and correlated to AD case-status and demographics, and all confounded modules were removed from subsequent analysis.

### Brain list enrichment

The mRNA modules significantly associated with AD case-status were assessed for enrichment of brain cell-type specific co-expression genes using the ‘*UserListEnrichment’* option in WGCNA, which allows for detection of statistical enrichment of external *a priori* gene sets associated with several phenotypes and tissues. Statistical significance of enrichment of genes from cell-type specific gene sets within our mRNA co-expression modules was assessed by one tail hypergeometric test (adj. p≤ 0.05) [94].

### Gene set enrichment analysis

Gene set enrichment analysis (GSEA) was used for detection of known biological processes and pathways enriched within the mRNA modules using GSEA v2.0.14 software from the Broad Institute, as previously described [95, 96]. Individual gene lists for each of the mRNA modules significantly correlated with AD case-status were generated by rank-ordering all differentially expressed mRNA transcripts by their module membership (MM) to each of the AD-associated modules.

In GSEA software, the Affymetrix HG-U133A 2.0 transcript IDs of all nominally differentially expressed (p≤ 0.05) mRNA transcripts were converted to HUGO Gene Nomenclature Committee (HGNC) gene symbols and in cases of multiple transcripts representing a single gene, the probeset with the highest MM was retained [97]. *A priori* gene sets were obtained from the Molecular Signatures Database v4.0 (MSigDB; http://www.broadinstitute.org/gsea/msigdb) from the Broad Institute. A total of 1320 gene sets from the Canonical Pathways subset of the C2: Curated Pathways collection of MSigDB was assessed. Default parameters were then applied to give a minimum and maximum *a priori* gene set size between 15 and 500 genes, respectively. Of the 1320 a priori gene sets used for GSEA from the C2: Curated Pathways collection from MSigDB, 929 gene sets were excluded due to gene set size parameters (i.e. gene sets smaller than 15 genes) and 391 a priori gene sets were used for final GSEA analysis.

In order to identify module-specific *a priori* gene sets, the leading edge analysis (LEA) tool within GSEA software was applied. The leading edge genes are the subset of genes in the gene list preceding the point of maximum ES, i.e. the genes that contributed to the net increases in ES and constituted the significant enrichment of the *a priori* gene set. After identifying the LE genes for each ranked gene list, we selected those *a priori* gene sets containing at least one module member gene to be module-specific gene sets.

### Candidate gene prioritization

Hub genes comprise the highly interconnected nodes within gene co-expression modules and have been shown to be functionally significant [98]. In our study, candidate hub genes were defined by their intramodular connectivity, the strength of which is measured by the absolute value of the Pearson correlation (r≥ 0.8) between individual gene expression and ME, referred also as a module membership (MM) [99, 100]. The upper quartile of transcripts with the highest MM in each AD-significant module (employed for both mRNA and miRNA modules) was chosen as criterion for selection of candidate hub genes.

### miRNA:mRNA Analysis

The miRNA and mRNA hub genes from the modules significantly correlated with AD were correlated (Pearson product moment) with each other. Since one sample was removed from the mRNA arrays, for the correlation analysis the same sample was removed from the miRNA arrays as well. In the correlation procedure, we focused only on the negative miRNA:mRNA MEs correlations, as these represent the direct, gene silencing effects of miRNAs on their targets, and the significant negative correlations were adjusted at a FDR ≤0.10.

Next, the significant negative correlations were intersected with miRNA:mRNA interactions that were predicted computationally by miRanda software using the default parameters [101]. All Affymetrix candidate hub mRNA transcript IDs were converted to HGNC gene symbols with the Affymetrix HG-U133A 2.0 annotation file, version 34.

The miRanda software (August 2010 version release) was run locally to predict putative targets for hub genes as previously described [102]. The intersection between miRanda predictions and expression correlations was shown to give a set of miRNA:mRNA targeting pairs that had fewer false positives compared to predictions alone [103].

### eQTL detection

Genotype calls for the postmortem AD sample were generated as part of a larger GWAS and integrated with miRNA and mRNA hub genes to identify expression quantitative trait loci (eQTLs). The genotype data used to generate the eQTLs for the mRNA and miRNA genes are provided in S11 Table, respectively. Briefly, all samples were genotyped on the Affymetrix Genome-Wide Human SNP Array 6.0; imputed genotype probabilities greater than 98% were converted into hard call genotypes of 0,1, or 2 using GTOOL software and filtered to eliminate ambiguity, i.e. no “unknown” calls [104]. Genetic variants were then filtered with Plink v1.07 to exclude variants in LD (R^2^≥ 0.8) [105]. Based on a sample size of n = 34 (one individual was removed from the sample due to genotype missingness), for a reliable estimation of the eQTL effects on gene expression a minor allele frequency ≥24% was required.

eQTLs were detected by MatrixEQTL software package in R within a linear regression framework using an additive model, accounting for the potential effects of smoking and AD case-status [106]. All significant results were adjusted for multiple testing at FDR≤ 0.10.

### Test for GWAS association signals enrichment

The enrichment analysis was performed by assessing the association between AD and whole gene eQTLs SNP-set in the R package gskat. Gskat performs a set-based test for the effects of a SNP set in association studies for both quantitative and discrete phenotypes using the generalized estimation equation approach. Specifically, a Kernel Machine (KM) estimating based statistic was constructed to test for the association between an AD and other alcohol related phenotype and a SNP set.

Specifically in the COGA European American (EA) sample, we tested for the following phenotypes: alcohol diagnosis (AD_DX), alcohol symptoms count (AD_SX), and maximum drinks in 24 hour (MAX24), using mRNA (591 SNPs) and miRNA(68 SNPs) eQTL sets. Correction for multiple testing was assessed using the Simes test; Simes is modification of Bonferroni test and performs better for small number of multiple tests [107]. MAX24 phenotype didn’t show significance in any set based analysis. None of the three phenotypes show set-based signal with miRNA set, most likely due to lack of power. Additional pertinent information for the enriched eQTLs, such as chromosome position and minor allele frequency is provided in S12 Table.

### COGA Sample

The test of enrichment for AD association signals was performed in a case-control sample of 1399 phenotyped subjects of European descent only who were selected from the COGA sample. Cases had a lifetime diagnosis of AD by DSM-IV criteria. Controls reported consuming alcohol but did not have a diagnosis of AD or alcohol abuse by any of the diagnostic criteria assessed by SSAGA, and did not meet diagnostic criteria for dependence on cocaine, marijuana, opioids, sedatives or stimulants. Controls could not share a known common ancestor with a case and were preferentially selected to be above the age of 25 years. Genotyping was completed using the Illumina Human 1 M DNA Analysis BeadChip at the Center for Inherited Disease Research. Additional details on the COGA GWAS sample can be found in Edenberg et al. (2010) and Yan et al. (2014) [108, 109].

The Collaborative Study on the Genetic of Alcoholism was approved under VCU IRB protocol (# HM20000289); informed written consent, has been obtained from all participants.

## Supporting Information

S1 Fig Validation of the microarray expression data using quantitative PCR. Expression levels of five genes measured by the expression array-based approach were validated using quantitative PCR in all 36 postmortem Nucleus Accumbens (NAc) RNA samples. The correlation coefficients were calculated using the Pearson product moment: The correlation coefficients for CD63, LPPR1, PLK2, TPD52L1 and MAPT was 0.891, 0.926, 0.907, 0.845 and 0.628, respectively.
(TIF)

S1 Table (A) Results from the univariate mRNA analysis. The analysis was performed using multiple regression model implemented in the Number Cruncher Statistical Software (NCSSv.9). (B). Results from the univariate miRNA analysis. The analysis was performed using multiple regression model implemented in the Number Cruncher Statistical Software (NCSSv.9). (DOCX)

S2 Table (A) MRNA modules. MRNA module size and ME correlation to AD case-status (Class) and sample demographics with un-adjusted p-values. (B) MiRNA modules. MiRNA module size and ME correlation to AD case-status (Class) and sample demographics with un-adjusted p-values. (DOCX)

S3 Table (A) Candidate hub mRNA transcripts from top quartile of MM from 6 mRNA modules significantly correlated with AD case-status. P-values are unadjusted. (B) Candidate hub miRNA transcripts from top quartile of MM from 3 miRNA modules significantly correlated with AD case-status. P-values are unadjusted. (DOCX)

S4 Table (A) Shared gene sets enriched between significant modules associated with neuronal cell expression. (B) Shared gene sets enriched between significant modules associated with glial cell expression. (DOCX)

S5 Table Unique significant enriched a priori gene sets for the significant modules. (DOCX)

S6 Table The entire list of Pearson correlation coefficients resulting from the correlations of 461 mRNA hubs and 25 miRNA hubs. (DOCX)

S7 Table Significant negatively correlated miRNAs: mRNAs with bioinformatic support. (DOCX)

S8 Table (A) Table of the significant cis-eQTLs for the mRNA hubs at FDR ≤0.1. (B) Table of the significant cis-eQTLs for the miRNA hubs at FDR ≤0.1. (DOCX)

S9 Table Brain Sample Demographics. The lines in bold represent the matched case-control samples (N = 36) used in this study.
(DOCX)

S10 Table The table reports the individual expression values of the validated genes between the microarray and the PCR platforms. The letters behind each ID number reflects the disease status of the subject (i.e., A = Alcoholics and C = Controls).
(DOCX)

S11 Table (A) Genotype calls for the mRNA hub genes. In the GEN file used by Gtool to convert between PED and GEN files, the genotypes are expressed as pairs of 1,2,0 where 1 corresponds to allele A from the GEN file and 2 corresponds to allele B. If none of the probabilities are over the calling threshold then the pair is unknown, 0 0. This should allow the conversion of indels and other biallelic structural variants from the 1000 Genomes. (B) Genotype calls for the miRNA hub genes. (XLSX)

S12 Table (A) List of the mRNA eQTLs identified in the Australian postmortem sample and tested for enrichment in COGA EA sample; Table abbreviations: chromosome (CHR), base pair position (BP), minor allele frequency (MAF) and Australian (AUS). (B) List of the miRNA eQTLs identified in the Australian postmortem sample and tested for enrichment in COGA EA sample. Table abbreviations: chromosome (CHR), base pair position (BP), minor allele frequency (MAF) and Australian (AUS). (DOCX)
