# Supplementary figures and images for: Integrating mRNA and miRNA Weighted Gene Co-Expression Networks with eQTLs in the Nucleus Accumbens of Subjects with Alcohol Dependence
Source: PLoS One. 2015 Sep 18;10(9):e0137671. doi: 10.1371/journal.pone.0137671 (PMC4575063; doi:10.1371/journal.pone.0137671)

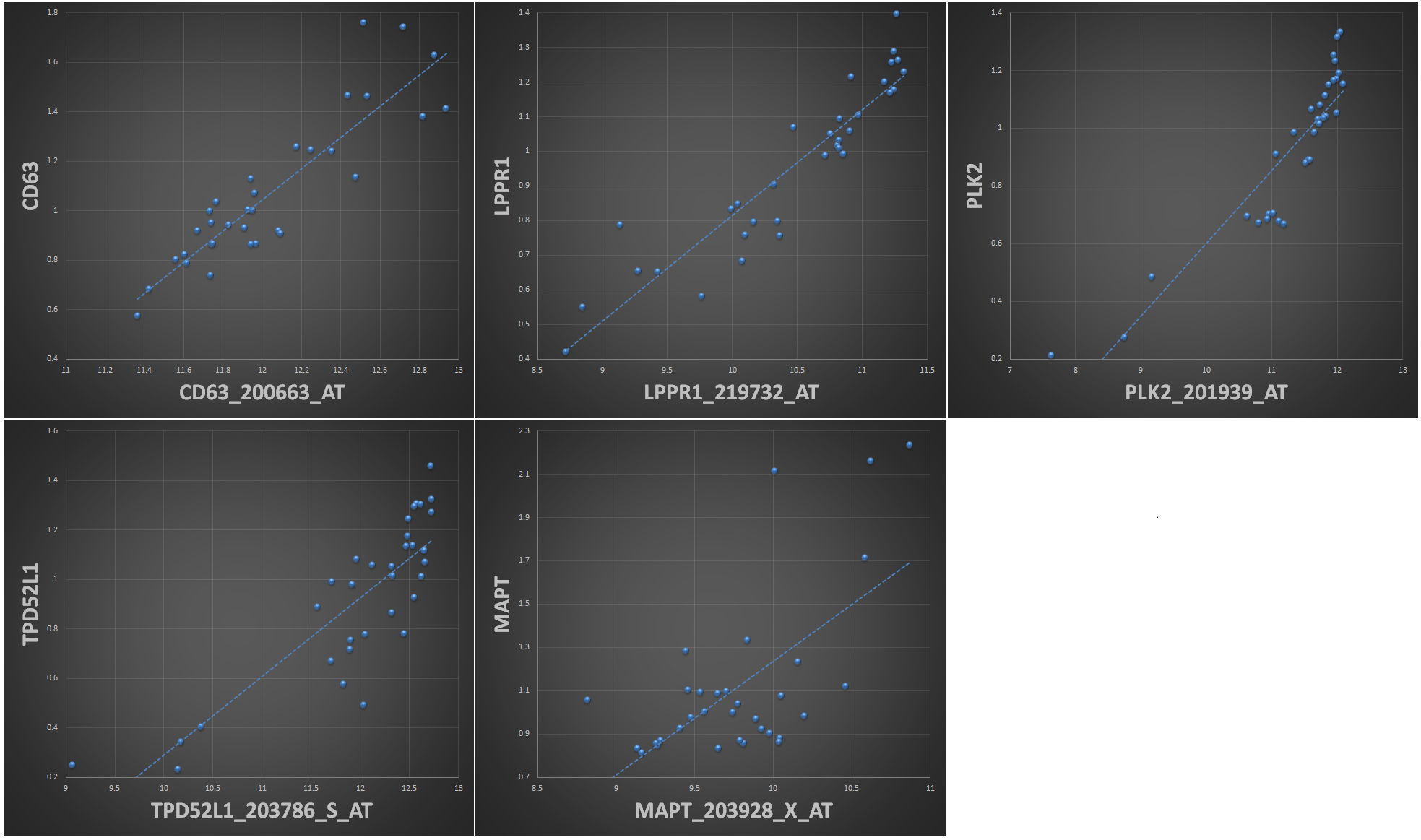

Supplement: S1 Fig — Expression levels of five genes measured by the expression array-based approach were validated using quantitative PCR in all 36 postmortem Nucleus Accumbens (NAc) RNA samples. The correlation coefficients were calculated using the Pearson product moment: The correlation coefficients for CD63, LPPR1, PLK2, TPD52L1 and MAPT was 0.891, 0.926, 0.907, 0.845 and 0.628, respectively. (TIF) [file pone.0137671.s001.tif]
