# Supplementary material for: Integrating mRNA and miRNA Weighted Gene Co-Expression Networks with eQTLs in the Nucleus Accumbens of Subjects with Alcohol Dependence
Source: PLoS One. 2015 Sep 18;10(9):e0137671. doi: 10.1371/journal.pone.0137671 (PMC4575063; doi:10.1371/journal.pone.0137671)
Supplement: S2 Table — (DOCX) [file pone.0137671.s003.docx]

(A).

|  |  | **Class** | | **pH** | | **PMI** | |
| --- | --- | --- | --- | --- | --- | --- | --- |
| **Module** | **Size** | **Corr.** | **P-value** | **Corr.** | **P-value** | **Corr.** | **P-value** |
| **MEblack** | 210 | 0.300 | 8.51E-02 | -0.252 | 1.50E-01 | -0.066 | 7.11E-01 |
| **MEblue** | 556 | -0.341 | 4.86E-02 | 0.223 | 2.04E-01 | 0.048 | 7.88E-01 |
| **MEbrown** | 416 | -0.372 | 3.04E-02 | 0.254 | 1.46E-01 | -0.538 | 1.03E-03 |
| **MEcyan** | 98 | 0.493 | 3.08E-03 | -0.195 | 2.70E-01 | 0.071 | 6.90E-01 |
| **MEdarkgreen** | 41 | 0.394 | 2.12E-02 | 0.041 | 8.18E-01 | -0.164 | 3.55E-01 |
| **MEdarkgrey** | 35 | 0.055 | 7.59E-01 | -0.181 | 3.06E-01 | -0.092 | 6.05E-01 |
| **MEdarkred** | 44 | -0.107 | 5.48E-01 | 0.011 | 9.49E-01 | -0.035 | 8.43E-01 |
| **MEdarkturquoise** | 157 | -0.461 | 6.12E-03 | 0.513 | 1.90E-03 | -0.028 | 8.76E-01 |
| **MEgreen** | 242 | 0.622 | 8.56E-05 | -0.295 | 9.01E-02 | 0.008 | 9.66E-01 |
| **MEgreenyellow** | 117 | 0.491 | 3.21E-03 | -0.327 | 5.92E-02 | -0.122 | 4.92E-01 |
| **MEgrey** | 13 | 0.265 | 1.30E-01 | 0.008 | 9.64E-01 | 0.253 | 1.48E-01 |
| **MEgrey60** | 55 | 0.614 | 1.14E-04 | -0.178 | 3.14E-01 | 0.067 | 7.06E-01 |
| **MElightcyan** | 75 | 0.036 | 8.40E-01 | 0.055 | 7.59E-01 | 0.596 | 1.98E-04 |
| **MElightgreen** | 52 | 0.241 | 1.70E-01 | -0.151 | 3.93E-01 | -0.173 | 3.29E-01 |
| **MElightyellow** | 52 | -0.418 | 1.40E-02 | -0.257 | 1.43E-01 | 0.173 | 3.28E-01 |
| **MEmagenta** | 167 | 0.463 | 5.87E-03 | -0.183 | 3.01E-01 | 0.423 | 1.26E-02 |
| **MEmidnightblue** | 81 | 0.470 | 5.04E-03 | -0.291 | 9.46E-02 | 0.105 | 5.56E-01 |
| **MEpink** | 205 | 0.565 | 4.98E-04 | -0.434 | 1.03E-02 | -0.113 | 5.26E-01 |
| **MEpurple** | 144 | 0.407 | 1.68E-02 | -0.187 | 2.89E-01 | 0.010 | 9.56E-01 |
| **MEred** | 231 | 0.537 | 1.07E-03 | -0.523 | 1.52E-03 | 0.154 | 3.84E-01 |
| **MEroyalblue** | 48 | 0.470 | 4.99E-03 | -0.041 | 8.20E-01 | 0.336 | 5.23E-02 |
| **MEsalmon** | 105 | 0.550 | 7.51E-04 | -0.177 | 3.18E-01 | -0.142 | 4.23E-01 |
| **MEturquoise** | 1106 | -0.580 | 3.28E-04 | 0.347 | 4.46E-02 | -0.314 | 7.10E-02 |
| **MEyellow** | 321 | -0.546 | 8.40E-04 | 0.323 | 6.25E-02 | -0.127 | 4.73E-01 |

|  |  | **Age** | | **RIN** | | **Smoking** | |
| --- | --- | --- | --- | --- | --- | --- | --- |
| **Module** | **Size** | **Corr.** | **P-value** | **Corr.** | **P-value** | **Corr.** | **P-value** |
| **MEblack** | 210 | 0.006 | 9.71E-01 | -0.364 | 3.44E-02 | 0.162 | 3.61E-01 |
| **MEblue** | 556 | -0.096 | 5.89E-01 | 0.324 | 6.18E-02 | -0.217 | 2.18E-01 |
| **MEbrown** | 416 | -0.191 | 2.78E-01 | 0.225 | 2.02E-01 | -0.231 | 1.89E-01 |
| **MEcyan** | 98 | -0.049 | 7.82E-01 | -0.035 | 8.42E-01 | 0.264 | 1.31E-01 |
| **MEdarkgreen** | 41 | 0.439 | 9.32E-03 | -0.014 | 9.35E-01 | 0.223 | 2.05E-01 |
| **MEdarkgrey** | 35 | 0.320 | 6.52E-02 | 0.321 | 6.42E-02 | 0.039 | 8.25E-01 |
| **MEdarkred** | 44 | -0.184 | 2.96E-01 | -0.286 | 1.01E-01 | -0.051 | 7.74E-01 |
| **MEdarkturquoise** | 157 | -0.004 | 9.84E-01 | -0.003 | 9.85E-01 | -0.307 | 7.76E-02 |
| **MEgreen** | 242 | 0.190 | 2.81E-01 | 0.183 | 3.00E-01 | 0.379 | 2.69E-02 |
| **MEgreenyellow** | 117 | -0.057 | 7.50E-01 | -0.149 | 3.99E-01 | 0.277 | 1.12E-01 |
| **MEgrey** | 13 | 0.117 | 5.11E-01 | -0.119 | 5.01E-01 | 0.304 | 8.03E-02 |
| **MEgrey60** | 55 | 0.110 | 5.37E-01 | -0.030 | 8.66E-01 | 0.417 | 1.43E-02 |
| **MElightcyan** | 75 | 0.118 | 5.06E-01 | -0.219 | 2.12E-01 | 0.061 | 7.31E-01 |
| **MElightgreen** | 52 | -0.211 | 2.31E-01 | -0.324 | 6.13E-02 | 0.094 | 5.95E-01 |
| **MElightyellow** | 52 | -0.059 | 7.40E-01 | -0.079 | 6.58E-01 | -0.218 | 2.15E-01 |
| **MEmagenta** | 167 | 0.083 | 6.42E-01 | -0.298 | 8.71E-02 | 0.291 | 9.44E-02 |
| **MEmidnightblue** | 81 | 0.149 | 3.99E-01 | -0.208 | 2.38E-01 | 0.243 | 1.65E-01 |
| **MEpink** | 205 | 0.009 | 9.61E-01 | 0.225 | 2.02E-01 | 0.393 | 2.15E-02 |
| **MEpurple** | 144 | 0.132 | 4.57E-01 | -0.338 | 5.04E-02 | 0.196 | 2.67E-01 |
| **MEred** | 231 | 0.080 | 6.52E-01 | -0.103 | 5.62E-01 | 0.311 | 7.34E-02 |
| **MEroyalblue** | 48 | -0.034 | 8.49E-01 | -0.354 | 3.98E-02 | 0.275 | 1.16E-01 |
| **MEsalmon** | 105 | 0.069 | 6.99E-01 | 0.075 | 6.75E-01 | 0.341 | 4.81E-02 |
| **MEturquoise** | 1106 | -0.105 | 5.55E-01 | 0.078 | 6.60E-01 | -0.379 | 2.71E-02 |
| **MEyellow** | 321 | -0.055 | 7.59E-01 | 0.245 | 1.63E-01 | -0.336 | 5.24E-02 |

(B)

|  |  | **Class** | | **pH** | | **PMI** | |
| --- | --- | --- | --- | --- | --- | --- | --- |
| **Module** | **Size** | **Corr.** | **P-value** | **Corr.** | **P-value** | **Corr.** | **P-value** |
| **MEblack** | 12 | -0.488 | 3.38E-03 | 0.247 | 1.59E-01 | -0.482 | 3.91E-03 |
| **MEblue** | 54 | -0.629 | 6.87E-05 | 0.129 | 4.66E-01 | -0.199 | 2.59E-01 |
| **MEbrown** | 25 | 0.577 | 3.54E-04 | 0.073 | 6.82E-01 | -0.015 | 9.35E-01 |
| **MEgreen** | 13 | 0.441 | 9.10E-03 | -0.221 | 2.09E-01 | -0.038 | 8.32E-01 |
| **MEgreenyellow** | 5 | -0.419 | 1.36E-02 | 0.259 | 1.39E-01 | -0.373 | 2.96E-02 |
| **MEgrey** | 4 | -0.235 | 1.81E-01 | -0.191 | 2.79E-01 | -0.132 | 4.55E-01 |
| **MEmagenta** | 8 | -0.406 | 1.73E-02 | 0.301 | 8.33E-02 | -0.164 | 3.53E-01 |
| **MEpink** | 9 | 0.306 | 7.86E-02 | 0.026 | 8.85E-01 | -0.393 | 2.14E-02 |
| **MEpurple** | 5 | -0.217 | 2.18E-01 | -0.047 | 7.91E-01 | -0.207 | 2.40E-01 |
| **MEred** | 12 | -0.415 | 1.46E-02 | 0.154 | 3.84E-01 | -0.121 | 4.96E-01 |
| **MEturquoise** | 73 | 0.443 | 8.69E-03 | -0.106 | 5.51E-01 | 0.453 | 7.17E-03 |
| **MEyellow** | 20 | -0.541 | 9.65E-04 | -0.031 | 8.61E-01 | 0.273 | 1.19E-01 |
|  |  |  |  |  |  |  |  |
|  |  | **Age** | | **RIN** | | **Smoking** | |
| **Module** | **Size** | **Corr.** | **P-value** | **Corr.** | **P-value** | **Corr.** | **P-value** |
| **MEblack** | 12 | -0.076 | 6.67E-01 | 0.225 | 2.01E-01 | -0.246 | 1.61E-01 |
| **MEblue** | 54 | -0.016 | 9.28E-01 | 0.242 | 1.68E-01 | -0.253 | 1.49E-01 |
| **MEbrown** | 25 | 0.043 | 8.10E-01 | 0.105 | 5.56E-01 | 0.362 | 3.54E-02 |
| **MEgreen** | 13 | 0.015 | 9.33E-01 | 0.171 | 3.35E-01 | 0.161 | 3.62E-01 |
| **MEgreenyellow** | 5 | 0.019 | 9.13E-01 | 0.156 | 3.78E-01 | -0.164 | 3.54E-01 |
| **MEgrey** | 4 | -0.324 | 6.18E-02 | 0.132 | 4.57E-01 | -0.057 | 7.50E-01 |
| **MEmagenta** | 8 | 0.032 | 8.58E-01 | 0.381 | 2.63E-02 | -0.308 | 7.64E-02 |
| **MEpink** | 9 | -0.159 | 3.71E-01 | -0.061 | 7.32E-01 | 0.109 | 5.41E-01 |
| **MEpurple** | 5 | -0.116 | 5.14E-01 | -0.211 | 2.30E-01 | 0.044 | 8.05E-01 |
| **MEred** | 12 | 0.054 | 7.61E-01 | 0.362 | 3.55E-02 | -0.247 | 1.59E-01 |
| **MEturquoise** | 73 | 0.038 | 8.30E-01 | -0.430 | 1.11E-02 | 0.235 | 1.82E-01 |
| **MEyellow** | 20 | 0.005 | 9.78E-01 | -0.240 | 1.71E-01 | -0.117 | 5.10E-01 |
