# Supplementary material for: Integrating mRNA and miRNA Weighted Gene Co-Expression Networks with eQTLs in the Nucleus Accumbens of Subjects with Alcohol Dependence
Source: PLoS One. 2015 Sep 18;10(9):e0137671. doi: 10.1371/journal.pone.0137671 (PMC4575063; doi:10.1371/journal.pone.0137671)
Supplement: S3 Table — (DOCX) [file pone.0137671.s004.docx]

**Table S3**. (A)

| **Probeset ID** | **Gene** | **Module** | **GS** | **p** | **MM** | **p** |
| --- | --- | --- | --- | --- | --- | --- |
| 208659_at | CLIC1 | salmon | 0.536 | 1.08E-03 | 0.933 | 9.80E-16 |
| 208998_at | UCP2 | salmon | 0.415 | 1.46E-02 | 0.918 | 2.25E-14 |
| 200916_at | TAGLN2 | salmon | 0.673 | 1.31E-05 | 0.896 | 8.00E-13 |
| 201999_s_at | DYNLT1 | salmon | 0.661 | 2.12E-05 | 0.895 | 9.35E-13 |
| 200663_at | CD63 | salmon | 0.500 | 2.63E-03 | 0.894 | 1.05E-12 |
| 211271_x_at | PTBP1 | salmon | 0.451 | 7.47E-03 | 0.887 | 2.77E-12 |
| 200701_at | NPC2 | salmon | 0.300 | 8.47E-02 | 0.877 | 1.02E-11 |
| 217746_s_at | PDCD6IP | salmon | 0.422 | 1.28E-02 | 0.871 | 2.16E-11 |
| 204194_at | BACH1 | salmon | 0.299 | 8.56E-02 | 0.868 | 2.87E-11 |
| 212063_at | CD44 | salmon | 0.547 | 8.25E-04 | 0.865 | 4.00E-11 |
| 201887_at | IL13RA1 | salmon | 0.278 | 1.12E-01 | 0.863 | 5.42E-11 |
| 200967_at | PPIB | salmon | 0.421 | 1.31E-02 | 0.857 | 1.03E-10 |
| 202269_x_at | GBP1 | salmon | 0.526 | 1.38E-03 | 0.855 | 1.24E-10 |
| 206989_s_at | SCAF11 | salmon | 0.338 | 5.08E-02 | 0.852 | 1.61E-10 |
| 201029_s_at | CD99 | salmon | 0.576 | 3.59E-04 | 0.850 | 2.02E-10 |
| 202133_at | WWTR1 | salmon | 0.509 | 2.14E-03 | 0.848 | 2.40E-10 |
| 221667_s_at | HSPB8 | salmon | 0.543 | 9.04E-04 | 0.848 | 2.49E-10 |
| 202376_at | SERPINA3 | salmon | 0.632 | 6.03E-05 | 0.845 | 3.26E-10 |
| 202096_s_at | TSPO | salmon | 0.441 | 8.97E-03 | 0.843 | 3.99E-10 |
| 200600_at | MSN | salmon | 0.396 | 2.06E-02 | 0.841 | 4.85E-10 |
| 200905_x_at | HLA-E | salmon | 0.599 | 1.82E-04 | 0.840 | 5.03E-10 |
| 204070_at | RARRES3 | salmon | 0.457 | 6.58E-03 | 0.839 | 5.49E-10 |
| 217947_at | CMTM6 | salmon | 0.312 | 7.23E-02 | 0.838 | 6.15E-10 |
| 48531_at | TNIP2 | salmon | 0.368 | 3.24E-02 | 0.837 | 6.86E-10 |
| 203411_s_at | LMNA | salmon | 0.403 | 1.82E-02 | 0.827 | 1.63E-09 |
| 213293_s_at | TRIM22 | salmon | 0.600 | 1.78E-04 | 0.824 | 2.21E-09 |
| 201160_s_at | YBX3 | green | 0.604 | 1.56E-04 | 0.940 | 1.61E-16 |
| 204326_x_at | MT1X | green | 0.530 | 1.26E-03 | 0.912 | 5.95E-14 |
| 209122_at | PLIN2 | green | 0.626 | 7.59E-05 | 0.908 | 1.31E-13 |
| 208690_s_at | PDLIM1 | green | 0.619 | 9.49E-05 | 0.904 | 2.22E-13 |
| 202430_s_at | PLSCR1 | green | 0.449 | 7.66E-03 | 0.899 | 5.14E-13 |
| 203645_s_at | CD163 | green | 0.469 | 5.09E-03 | 0.895 | 9.10E-13 |
| 212203_x_at | IFITM3 | green | 0.712 | 2.32E-06 | 0.894 | 1.06E-12 |
| 218854_at | DSE | green | 0.511 | 2.01E-03 | 0.893 | 1.30E-12 |
| 207643_s_at | TNFRSF1A | green | 0.572 | 4.06E-04 | 0.887 | 2.84E-12 |
| 217730_at | TMBIM1 | green | 0.504 | 2.37E-03 | 0.884 | 4.17E-12 |
| 205097_at | SLC26A2 | green | 0.571 | 4.26E-04 | 0.884 | 4.23E-12 |
| 217995_at | SQRDL | green | 0.512 | 1.94E-03 | 0.883 | 4.94E-12 |
| 210592_s_at | SAT1 | green | 0.489 | 3.32E-03 | 0.882 | 5.40E-12 |
| 202180_s_at | MVP | green | 0.521 | 1.57E-03 | 0.881 | 6.28E-12 |
| 201315_x_at | IFITM2 | green | 0.703 | 3.61E-06 | 0.880 | 7.47E-12 |
| 201761_at | MTHFD2 | green | 0.538 | 1.04E-03 | 0.880 | 7.54E-12 |
| 201319_at | MYL12A | green | 0.459 | 6.37E-03 | 0.879 | 7.56E-12 |
| 214829_at | AASS | green | 0.563 | 5.24E-04 | 0.877 | 1.09E-11 |
| 221009_s_at | ANGPTL4 | green | 0.526 | 1.39E-03 | 0.876 | 1.14E-11 |
| 204787_at | VSIG4 | green | 0.447 | 7.98E-03 | 0.873 | 1.67E-11 |
| 219911_s_at | SLCO4A1 | green | 0.629 | 6.72E-05 | 0.872 | 1.90E-11 |
| 212067_s_at | C1R | green | 0.579 | 3.38E-04 | 0.871 | 2.15E-11 |
| 203973_s_at | CEBPD | green | 0.581 | 3.13E-04 | 0.868 | 2.86E-11 |
| 212110_at | SLC39A14 | green | 0.431 | 1.10E-02 | 0.866 | 3.64E-11 |
| 201161_s_at | YBX3 | green | 0.460 | 6.19E-03 | 0.865 | 4.02E-11 |
| 212687_at | LIMS1 | green | 0.407 | 1.68E-02 | 0.865 | 4.20E-11 |
| 203455_s_at | SAT1 | green | 0.447 | 8.09E-03 | 0.864 | 4.87E-11 |
| 208981_at | PECAM1 | green | 0.574 | 3.90E-04 | 0.863 | 5.06E-11 |
| 212501_at | CEBPB | green | 0.560 | 5.73E-04 | 0.859 | 7.73E-11 |
| 210978_s_at | TAGLN2 | green | 0.495 | 2.90E-03 | 0.857 | 9.73E-11 |
| 200986_at | SERPING1 | green | 0.719 | 1.68E-06 | 0.856 | 1.10E-10 |
| 208991_at | STAT3 | green | 0.577 | 3.57E-04 | 0.855 | 1.24E-10 |
| 201601_x_at | IFITM1 | green | 0.639 | 4.77E-05 | 0.849 | 2.26E-10 |
| 201859_at | SRGN | green | 0.447 | 8.00E-03 | 0.849 | 2.28E-10 |
| 218322_s_at | ACSL5 | green | 0.590 | 2.42E-04 | 0.848 | 2.38E-10 |
| 217546_at | MT1M | green | 0.307 | 7.72E-02 | 0.848 | 2.48E-10 |
| 203854_at | CFI | green | 0.480 | 4.11E-03 | 0.848 | 2.50E-10 |
| 200797_s_at | MCL1 | green | 0.511 | 2.02E-03 | 0.847 | 2.59E-10 |
| 202864_s_at | SP100 | green | 0.465 | 5.56E-03 | 0.845 | 3.17E-10 |
| 214022_s_at | IFITM1 | green | 0.612 | 1.21E-04 | 0.843 | 3.99E-10 |
| 212099_at | RHOB | green | 0.469 | 5.19E-03 | 0.842 | 4.19E-10 |
| 212460_at | SPTSSA | green | 0.372 | 3.04E-02 | 0.842 | 4.40E-10 |
| 207198_s_at | LIMS1 | green | 0.449 | 7.74E-03 | 0.842 | 4.49E-10 |
| 211999_at | H3F3A | green | 0.308 | 7.61E-02 | 0.841 | 4.76E-10 |
| 201666_at | TIMP1 | green | 0.688 | 6.99E-06 | 0.840 | 5.42E-10 |
| 213572_s_at | SERPINB1 | green | 0.446 | 8.23E-03 | 0.839 | 5.67E-10 |
| 200798_x_at | MCL1 | green | 0.595 | 2.06E-04 | 0.834 | 8.70E-10 |
| 201324_at | EMP1 | green | 0.570 | 4.37E-04 | 0.834 | 9.31E-10 |
| 205119_s_at | FPR1 | green | 0.337 | 5.16E-02 | 0.834 | 9.35E-10 |
| 218559_s_at | MAFB | green | 0.584 | 2.89E-04 | 0.833 | 1.01E-09 |
| 202948_at | IL1R1 | green | 0.772 | 9.12E-08 | 0.830 | 1.24E-09 |
| 209183_s_at | C10orf10 | green | 0.560 | 5.78E-04 | 0.830 | 1.26E-09 |
| 218507_at | HILPDA | green | 0.506 | 2.25E-03 | 0.830 | 1.28E-09 |
| 36711_at | MAFF | green | 0.620 | 9.13E-05 | 0.828 | 1.51E-09 |
| 202863_at | SP100 | green | 0.422 | 1.30E-02 | 0.828 | 1.55E-09 |
| 208581_x_at | MT1X | green | 0.453 | 7.08E-03 | 0.826 | 1.75E-09 |
| 209732_at | CLEC2B | green | 0.496 | 2.87E-03 | 0.824 | 2.10E-09 |
| 219582_at | OGFRL1 | green | 0.608 | 1.39E-04 | 0.824 | 2.15E-09 |
| 205856_at | SLC14A1 | green | 0.690 | 6.39E-06 | 0.824 | 2.16E-09 |
| 221741_s_at | YTHDF1 | green | 0.538 | 1.04E-03 | 0.823 | 2.30E-09 |
| 200989_at | HIF1A | green | 0.378 | 2.77E-02 | 0.822 | 2.49E-09 |
| 200677_at | PTTG1IP | pink | 0.490 | 3.29E-03 | 0.927 | 3.49E-15 |
| 201412_at | LRP10 | pink | 0.501 | 2.55E-03 | 0.918 | 2.00E-14 |
| 208782_at | FSTL1 | pink | 0.630 | 6.62E-05 | 0.910 | 9.09E-14 |
| 222043_at | CLU | pink | 0.494 | 2.98E-03 | 0.906 | 1.81E-13 |
| 201146_at | NFE2L2 | pink | 0.469 | 5.18E-03 | 0.899 | 5.40E-13 |
| 201753_s_at | ADD3 | pink | 0.368 | 3.20E-02 | 0.880 | 7.16E-12 |
| 217936_at | ARHGAP5 | pink | 0.464 | 5.71E-03 | 0.876 | 1.13E-11 |
| 209513_s_at | HSDL2 | pink | 0.382 | 2.60E-02 | 0.873 | 1.74E-11 |
| 210817_s_at | CALCOCO2 | pink | 0.492 | 3.13E-03 | 0.872 | 1.81E-11 |
| 214150_x_at | ATP6V0E1 | pink | 0.368 | 3.24E-02 | 0.871 | 2.10E-11 |
| 202370_s_at | CBFB | pink | 0.535 | 1.11E-03 | 0.869 | 2.51E-11 |
| 221958_s_at | WLS | pink | 0.499 | 2.65E-03 | 0.867 | 3.35E-11 |
| 218656_s_at | LHFP | pink | 0.588 | 2.55E-04 | 0.865 | 4.07E-11 |
| 201924_at | AFF1 | pink | 0.524 | 1.47E-03 | 0.864 | 4.43E-11 |
| 212195_at | IL6ST | pink | 0.419 | 1.36E-02 | 0.862 | 5.62E-11 |
| 202834_at | AGT | pink | 0.518 | 1.70E-03 | 0.862 | 5.82E-11 |
| 210906_x_at | AQP4 | pink | 0.531 | 1.22E-03 | 0.861 | 6.51E-11 |
| 221796_at | NTRK2 | pink | 0.329 | 5.74E-02 | 0.857 | 9.71E-11 |
| 210068_s_at | AQP4 | pink | 0.409 | 1.64E-02 | 0.855 | 1.17E-10 |
| 202149_at | NEDD9 | pink | 0.468 | 5.27E-03 | 0.855 | 1.27E-10 |
| 208818_s_at | COMT | pink | 0.474 | 4.61E-03 | 0.851 | 1.75E-10 |
| 201398_s_at | TRAM1 | pink | 0.414 | 1.49E-02 | 0.849 | 2.20E-10 |
| 207761_s_at | METTL7A | pink | 0.299 | 8.61E-02 | 0.848 | 2.34E-10 |
| 203704_s_at | RREB1 | pink | 0.440 | 9.22E-03 | 0.847 | 2.70E-10 |
| 200804_at | TMBIM6 | pink | 0.415 | 1.46E-02 | 0.847 | 2.71E-10 |
| 210105_s_at | FYN | pink | 0.434 | 1.04E-02 | 0.844 | 3.76E-10 |
| 212977_at | CXCR7 | pink | 0.461 | 6.06E-03 | 0.842 | 4.23E-10 |
| 204554_at | PPP1R3D | pink | 0.331 | 5.56E-02 | 0.839 | 5.54E-10 |
| 200906_s_at | PALLD | pink | 0.592 | 2.28E-04 | 0.838 | 6.38E-10 |
| 218285_s_at | BDH2 | pink | 0.468 | 5.27E-03 | 0.831 | 1.17E-09 |
| 213217_at | ADCY2 | pink | 0.544 | 8.81E-04 | 0.830 | 1.30E-09 |
| 201180_s_at | GNAI3 | pink | 0.430 | 1.12E-02 | 0.829 | 1.34E-09 |
| 201172_x_at | ATP6V0E1 | pink | 0.355 | 3.93E-02 | 0.827 | 1.67E-09 |
| 202936_s_at | SOX9 | pink | 0.379 | 2.71E-02 | 0.826 | 1.87E-09 |
| 210946_at | PPAP2A | pink | 0.468 | 5.21E-03 | 0.821 | 2.69E-09 |
| 202071_at | SDC4 | pink | 0.547 | 8.09E-04 | 0.820 | 2.93E-09 |
| 200673_at | LAPTM4A | pink | 0.325 | 6.07E-02 | 0.816 | 4.02E-09 |
| 211962_s_at | ZFP36L1 | pink | 0.577 | 3.57E-04 | 0.811 | 5.92E-09 |
| 209476_at | TMX1 | pink | 0.316 | 6.85E-02 | 0.809 | 7.07E-09 |
| 201656_at | ITGA6 | pink | 0.494 | 3.02E-03 | 0.808 | 7.48E-09 |
| 202543_s_at | GMFB | pink | 0.423 | 1.26E-02 | 0.807 | 7.89E-09 |
| 213005_s_at | KANK1 | pink | 0.413 | 1.51E-02 | 0.806 | 8.80E-09 |
| 218706_s_at | GRAMD3 | pink | 0.477 | 4.31E-03 | 0.805 | 9.67E-09 |
| 203313_s_at | TGIF1 | pink | 0.544 | 8.80E-04 | 0.802 | 1.18E-08 |
| 204068_at | STK3 | pink | 0.368 | 3.22E-02 | 0.802 | 1.18E-08 |
| 202975_s_at | RHOBTB3 | pink | 0.515 | 1.84E-03 | 0.799 | 1.50E-08 |
| 218005_at | ZNF22 | pink | 0.268 | 1.25E-01 | 0.797 | 1.68E-08 |
| 212321_at | SGPL1 | pink | 0.197 | 2.64E-01 | 0.797 | 1.71E-08 |
| 212015_x_at | PTBP1 | pink | 0.368 | 3.24E-02 | 0.797 | 1.76E-08 |
| 208809_s_at | C6orf62 | pink | 0.510 | 2.08E-03 | 0.796 | 1.80E-08 |
| 203685_at | BCL2 | pink | 0.255 | 1.45E-01 | 0.795 | 1.92E-08 |
| 203120_at | TP53BP2 | pink | 0.284 | 1.04E-01 | 0.789 | 2.88E-08 |
| 201590_x_at | ANXA2 | grey60 | 0.693 | 5.49E-06 | 0.917 | 2.38E-14 |
| 210427_x_at | ANXA2 | grey60 | 0.685 | 7.78E-06 | 0.916 | 3.36E-14 |
| 213503_x_at | ANXA2 | grey60 | 0.714 | 2.10E-06 | 0.903 | 2.77E-13 |
| 201012_at | ANXA1 | grey60 | 0.635 | 5.57E-05 | 0.893 | 1.28E-12 |
| 214428_x_at | C4B | grey60 | 0.381 | 2.64E-02 | 0.869 | 2.79E-11 |
| 202252_at | RAB13 | grey60 | 0.570 | 4.39E-04 | 0.858 | 8.77E-11 |
| 203723_at | ITPKB | grey60 | 0.541 | 9.54E-04 | 0.843 | 3.79E-10 |
| 1007_s_at | DDR1 | grey60 | 0.433 | 1.05E-02 | 0.828 | 1.57E-09 |
| 217820_s_at | ENAH | grey60 | 0.408 | 1.65E-02 | 0.826 | 1.88E-09 |
| 211270_x_at | PTBP1 | grey60 | 0.393 | 2.14E-02 | 0.818 | 3.54E-09 |
| 202587_s_at | AK1 | grey60 | 0.419 | 1.35E-02 | 0.816 | 4.03E-09 |
| 208451_s_at | C4A | grey60 | 0.349 | 4.29E-02 | 0.816 | 4.08E-09 |
| 209108_at | TSPAN6 | grey60 | 0.598 | 1.89E-04 | 0.814 | 4.71E-09 |
| 213592_at | APLNR | grey60 | 0.431 | 1.10E-02 | 0.811 | 6.04E-09 |
| 219714_s_at | CACNA2D3 | yellow | -0.468 | 5.29E-03 | 0.959 | 3.62E-19 |
| 201725_at | CDC123 | yellow | -0.438 | 9.56E-03 | 0.948 | 1.59E-17 |
| 203156_at | AKAP11 | yellow | -0.487 | 3.46E-03 | 0.940 | 1.90E-16 |
| 207054_at | IMPG1 | yellow | -0.522 | 1.55E-03 | 0.938 | 2.58E-16 |
| 208679_s_at | ARPC2 | yellow | -0.453 | 7.08E-03 | 0.937 | 3.20E-16 |
| 207717_s_at | PKP2 | yellow | -0.511 | 2.00E-03 | 0.931 | 1.37E-15 |
| 213904_at | RP11-526J3.3 | yellow | -0.437 | 9.77E-03 | 0.931 | 1.42E-15 |
| 212271_at | MAPK1 | yellow | -0.409 | 1.62E-02 | 0.930 | 1.73E-15 |
| 205324_s_at | FTSJ1 | yellow | -0.473 | 4.70E-03 | 0.930 | 2.00E-15 |
| 206803_at | PDYN | yellow | -0.558 | 6.13E-04 | 0.923 | 8.58E-15 |
| 205489_at | CRYM | yellow | -0.479 | 4.12E-03 | 0.921 | 1.13E-14 |
| 210972_x_at | TRAC | yellow | -0.454 | 7.03E-03 | 0.921 | 1.30E-14 |
| 202564_x_at | ARL2 | yellow | -0.464 | 5.76E-03 | 0.919 | 1.89E-14 |
| 204072_s_at | FRY | yellow | -0.585 | 2.77E-04 | 0.912 | 6.04E-14 |
| 218201_at | NDUFB2 | yellow | -0.472 | 4.82E-03 | 0.910 | 8.32E-14 |
| 217837_s_at | CHMP3 | yellow | -0.370 | 3.13E-02 | 0.908 | 1.34E-13 |
| 203150_at | RABEPK | yellow | -0.523 | 1.52E-03 | 0.904 | 2.23E-13 |
| 204239_s_at | NNAT | yellow | -0.571 | 4.26E-04 | 0.900 | 4.79E-13 |
| 215522_at | SORCS3 | yellow | -0.576 | 3.69E-04 | 0.899 | 5.01E-13 |
| 209671_x_at | TRAC | yellow | -0.469 | 5.13E-03 | 0.899 | 5.26E-13 |
| 206935_at | PCDH8 | yellow | -0.535 | 1.10E-03 | 0.898 | 5.86E-13 |
| 218200_s_at | NDUFB2 | yellow | -0.528 | 1.33E-03 | 0.895 | 8.85E-13 |
| 201145_at | HAX1 | yellow | -0.379 | 2.70E-02 | 0.895 | 9.72E-13 |
| 207400_at | NPY5R | yellow | -0.614 | 1.13E-04 | 0.894 | 1.03E-12 |
| 206671_at | SAG | yellow | -0.510 | 2.07E-03 | 0.893 | 1.33E-12 |
| 221696_s_at | STYK1 | yellow | -0.448 | 7.89E-03 | 0.892 | 1.52E-12 |
| 200739_s_at | SUMO3 | yellow | -0.486 | 3.55E-03 | 0.891 | 1.76E-12 |
| 213911_s_at | H2AFZ | yellow | -0.438 | 9.65E-03 | 0.890 | 1.92E-12 |
| 213272_s_at | TMEM159 | yellow | -0.469 | 5.18E-03 | 0.889 | 2.24E-12 |
| 218432_at | FBXO3 | yellow | -0.601 | 1.73E-04 | 0.889 | 2.29E-12 |
| 219326_s_at | B3GNT2 | yellow | -0.478 | 4.25E-03 | 0.888 | 2.48E-12 |
| 218160_at | NDUFA8 | yellow | -0.409 | 1.62E-02 | 0.887 | 2.94E-12 |
| 213552_at | GLCE | yellow | -0.436 | 1.00E-02 | 0.886 | 3.33E-12 |
| 209265_s_at | METTL3 | yellow | -0.375 | 2.91E-02 | 0.885 | 3.70E-12 |
| 218292_s_at | PRKAG2 | yellow | -0.350 | 4.25E-02 | 0.885 | 3.85E-12 |
| 202475_at | TMEM147 | yellow | -0.453 | 7.07E-03 | 0.881 | 6.04E-12 |
| 200853_at | H2AFZ | yellow | -0.374 | 2.95E-02 | 0.881 | 6.27E-12 |
| 215884_s_at | UBQLN2 | yellow | -0.541 | 9.52E-04 | 0.881 | 6.33E-12 |
| 221488_s_at | CUTA | yellow | -0.547 | 8.07E-04 | 0.880 | 7.41E-12 |
| 202471_s_at | IDH3G | yellow | -0.417 | 1.42E-02 | 0.876 | 1.20E-11 |
| 206099_at | PRKCH | yellow | -0.553 | 7.02E-04 | 0.874 | 1.43E-11 |
| 219683_at | FZD3 | yellow | -0.394 | 2.11E-02 | 0.874 | 1.43E-11 |
| 210434_x_at | JTB | yellow | -0.379 | 2.70E-02 | 0.874 | 1.44E-11 |
| 210962_s_at | AKAP9 | yellow | -0.508 | 2.16E-03 | 0.874 | 1.53E-11 |
| 207988_s_at | ARPC2 | yellow | -0.436 | 9.86E-03 | 0.872 | 1.87E-11 |
| 221874_at | KIAA1324 | yellow | -0.485 | 3.69E-03 | 0.870 | 2.34E-11 |
| 215307_at | ZNF529 | yellow | -0.511 | 2.00E-03 | 0.868 | 2.88E-11 |
| 201512_s_at | TOMM70A | yellow | -0.377 | 2.78E-02 | 0.867 | 3.28E-11 |
| 205413_at | MPPED2 | yellow | -0.422 | 1.29E-02 | 0.865 | 4.11E-11 |
| 201592_at | EIF3H | yellow | -0.554 | 6.79E-04 | 0.863 | 4.97E-11 |
| 212310_at | MIA3 | yellow | -0.574 | 3.85E-04 | 0.863 | 5.00E-11 |
| 204002_s_at | ICA1 | yellow | -0.453 | 7.14E-03 | 0.862 | 5.61E-11 |
| 219297_at | WDR44 | yellow | -0.596 | 2.02E-04 | 0.859 | 8.20E-11 |
| 1255_g_at | GUCA1A | yellow | -0.532 | 1.22E-03 | 0.859 | 8.36E-11 |
| 205196_s_at | AP1S1 | yellow | -0.395 | 2.07E-02 | 0.857 | 1.02E-10 |
| 210448_s_at | P2RX5 | yellow | -0.383 | 2.54E-02 | 0.854 | 1.34E-10 |
| 217997_at | PHLDA1 | yellow | -0.383 | 2.53E-02 | 0.853 | 1.48E-10 |
| 218384_at | CARHSP1 | yellow | -0.441 | 9.02E-03 | 0.853 | 1.51E-10 |
| 212727_at | DLG3 | yellow | -0.366 | 3.32E-02 | 0.852 | 1.68E-10 |
| 218882_s_at | WDR3 | yellow | -0.552 | 7.08E-04 | 0.848 | 2.40E-10 |
| 206875_s_at | SLK | yellow | -0.482 | 3.91E-03 | 0.848 | 2.48E-10 |
| 210927_x_at | JTB | yellow | -0.344 | 4.66E-02 | 0.846 | 3.01E-10 |
| 202121_s_at | CHMP2A | yellow | -0.498 | 2.75E-03 | 0.844 | 3.78E-10 |
| 206356_s_at | GNAL | yellow | -0.444 | 8.51E-03 | 0.842 | 4.48E-10 |
| 222360_at | DPH5 | yellow | -0.427 | 1.19E-02 | 0.841 | 4.87E-10 |
| 203781_at | MRPL33 | yellow | -0.567 | 4.77E-04 | 0.839 | 5.73E-10 |
| 205758_at | CD8A | yellow | -0.495 | 2.92E-03 | 0.838 | 6.09E-10 |
| 211902_x_at | YME1L1 | yellow | -0.403 | 1.81E-02 | 0.837 | 6.82E-10 |
| 207830_s_at | PPP1R8 | yellow | -0.545 | 8.60E-04 | 0.834 | 8.92E-10 |
| 218526_s_at | RANGRF | yellow | -0.447 | 8.11E-03 | 0.834 | 9.28E-10 |
| 211376_s_at | NSMCE4A | yellow | -0.561 | 5.55E-04 | 0.834 | 9.42E-10 |
| 220251_at | DIEXF | yellow | -0.585 | 2.75E-04 | 0.833 | 9.84E-10 |
| 218048_at | COMMD3 | yellow | -0.434 | 1.03E-02 | 0.832 | 1.09E-09 |
| 208697_s_at | EIF3E | yellow | -0.499 | 2.66E-03 | 0.831 | 1.15E-09 |
| 218970_s_at | CUTC | yellow | -0.633 | 5.82E-05 | 0.830 | 1.24E-09 |
| 218283_at | SS18L2 | yellow | -0.310 | 7.44E-02 | 0.830 | 1.28E-09 |
| 204766_s_at | NUDT1 | yellow | -0.466 | 5.53E-03 | 0.830 | 1.30E-09 |
| 208457_at | GABRD | yellow | -0.265 | 1.30E-01 | 0.829 | 1.34E-09 |
| 206805_at | SEMA3A | yellow | -0.457 | 6.61E-03 | 0.828 | 1.48E-09 |
| 210872_x_at | GAS7 | yellow | -0.541 | 9.54E-04 | 0.828 | 1.49E-09 |
| 213496_at | LPPR4 | yellow | -0.304 | 8.08E-02 | 0.828 | 1.51E-09 |
| 208857_s_at | PCMT1 | turquoise | -0.550 | 7.62E-04 | 0.969 | 5.01E-21 |
| 209228_x_at | TUSC3 | turquoise | -0.464 | 5.75E-03 | 0.969 | 6.26E-21 |
| 210156_s_at | PCMT1 | turquoise | -0.497 | 2.82E-03 | 0.968 | 9.01E-21 |
| 205202_at | PCMT1 | turquoise | -0.612 | 1.22E-04 | 0.963 | 7.87E-20 |
| 203079_s_at | CUL2 | turquoise | -0.491 | 3.23E-03 | 0.962 | 1.10E-19 |
| 201054_at | HNRNPA0 | turquoise | -0.586 | 2.69E-04 | 0.961 | 2.15E-19 |
| 204744_s_at | IARS | turquoise | -0.445 | 8.40E-03 | 0.960 | 2.93E-19 |
| 210149_s_at | ATP5H | turquoise | -0.546 | 8.40E-04 | 0.957 | 8.71E-19 |
| 212600_s_at | UQCRC2 | turquoise | -0.478 | 4.25E-03 | 0.956 | 1.13E-18 |
| 202395_at | NSF | turquoise | -0.471 | 4.92E-03 | 0.955 | 2.00E-18 |
| 211855_s_at | SLC25A14 | turquoise | -0.627 | 7.29E-05 | 0.954 | 2.52E-18 |
| 203889_at | SCG5 | turquoise | -0.574 | 3.87E-04 | 0.954 | 3.00E-18 |
| 203816_at | DGUOK | turquoise | -0.575 | 3.80E-04 | 0.952 | 5.60E-18 |
| 200883_at | UQCRC2 | turquoise | -0.523 | 1.49E-03 | 0.950 | 8.75E-18 |
| 202077_at | NDUFAB1 | turquoise | -0.529 | 1.28E-03 | 0.950 | 1.06E-17 |
| 206042_x_at | PAR-SN | turquoise | -0.477 | 4.32E-03 | 0.949 | 1.42E-17 |
| 200978_at | MDH1 | turquoise | -0.533 | 1.18E-03 | 0.948 | 1.78E-17 |
| 218866_s_at | POLR3K | turquoise | -0.485 | 3.64E-03 | 0.946 | 2.92E-17 |
| 218667_at | PJA1 | turquoise | -0.493 | 3.05E-03 | 0.946 | 2.95E-17 |
| 209755_at | NMNAT2 | turquoise | -0.554 | 6.82E-04 | 0.946 | 3.27E-17 |
| 202670_at | MAP2K1 | turquoise | -0.481 | 4.00E-03 | 0.946 | 3.30E-17 |
| 205279_s_at | GLRB | turquoise | -0.448 | 7.91E-03 | 0.945 | 4.56E-17 |
| 213423_x_at | TUSC3 | turquoise | -0.415 | 1.46E-02 | 0.940 | 1.74E-16 |
| 213738_s_at | ATP5A1 | turquoise | -0.544 | 8.93E-04 | 0.938 | 2.59E-16 |
| 218654_s_at | MRPS33 | turquoise | -0.511 | 2.00E-03 | 0.938 | 2.64E-16 |
| 203817_at | GUCY1B3 | turquoise | -0.516 | 1.79E-03 | 0.938 | 2.70E-16 |
| 208838_at | CAND1 | turquoise | -0.559 | 5.97E-04 | 0.936 | 4.18E-16 |
| 203094_at | MAD2L1BP | turquoise | -0.510 | 2.05E-03 | 0.936 | 4.21E-16 |
| 211951_at | NOLC1 | turquoise | -0.600 | 1.77E-04 | 0.936 | 4.40E-16 |
| 201966_at | NDUFS2 | turquoise | -0.450 | 7.58E-03 | 0.936 | 4.42E-16 |
| 202825_at | SLC25A4 | turquoise | -0.479 | 4.14E-03 | 0.936 | 4.50E-16 |
| 200816_s_at | PAFAH1B1 | turquoise | -0.492 | 3.15E-03 | 0.934 | 7.04E-16 |
| 208909_at | UQCRFS1 | turquoise | -0.462 | 6.00E-03 | 0.934 | 7.81E-16 |
| 208121_s_at | PTPRO | turquoise | -0.470 | 5.00E-03 | 0.934 | 8.03E-16 |
| 208813_at | GOT1 | turquoise | -0.388 | 2.33E-02 | 0.933 | 9.30E-16 |
| 212038_s_at | VDAC1 | turquoise | -0.397 | 2.00E-02 | 0.933 | 9.84E-16 |
| 200662_s_at | TOMM20 | turquoise | -0.522 | 1.54E-03 | 0.933 | 9.89E-16 |
| 205031_at | EFNB3 | turquoise | -0.485 | 3.61E-03 | 0.932 | 1.17E-15 |
| 216218_s_at | PLCL2 | turquoise | -0.641 | 4.48E-05 | 0.932 | 1.18E-15 |
| 217957_at | C16orf80 | turquoise | -0.525 | 1.43E-03 | 0.931 | 1.44E-15 |
| 222230_s_at | ACTR10 | turquoise | -0.485 | 3.66E-03 | 0.931 | 1.49E-15 |
| 200820_at | PSMD8 | turquoise | -0.474 | 4.60E-03 | 0.931 | 1.49E-15 |
| 209507_at | RPA3 | turquoise | -0.569 | 4.49E-04 | 0.931 | 1.64E-15 |
| 205550_s_at | BRE | turquoise | -0.457 | 6.53E-03 | 0.930 | 1.65E-15 |
| 218597_s_at | CISD1 | turquoise | -0.519 | 1.66E-03 | 0.930 | 1.76E-15 |
| 210418_s_at | IDH3B | turquoise | -0.545 | 8.52E-04 | 0.930 | 1.77E-15 |
| 209569_x_at | NSG1 | turquoise | -0.563 | 5.34E-04 | 0.930 | 1.96E-15 |
| 205775_at | FAM50B | turquoise | -0.582 | 3.02E-04 | 0.930 | 2.01E-15 |
| 221449_s_at | ITFG1 | turquoise | -0.378 | 2.73E-02 | 0.929 | 2.17E-15 |
| 215952_s_at | OAZ1 | turquoise | -0.403 | 1.80E-02 | 0.927 | 3.28E-15 |
| 207507_s_at | ATP5G3 | turquoise | -0.449 | 7.75E-03 | 0.927 | 3.41E-15 |
| 200614_at | CLTC | turquoise | -0.436 | 9.99E-03 | 0.926 | 4.02E-15 |
| 202614_at | SLC30A9 | turquoise | -0.529 | 1.30E-03 | 0.925 | 5.00E-15 |
| 206544_x_at | SMARCA2 | turquoise | -0.418 | 1.38E-02 | 0.925 | 5.76E-15 |
| 209227_at | TUSC3 | turquoise | -0.487 | 3.48E-03 | 0.925 | 5.82E-15 |
| 218788_s_at | SMYD3 | turquoise | -0.549 | 7.83E-04 | 0.925 | 5.94E-15 |
| 208826_x_at | HINT1 | turquoise | -0.494 | 2.99E-03 | 0.924 | 6.35E-15 |
| 217882_at | EMC3 | turquoise | -0.424 | 1.24E-02 | 0.924 | 6.51E-15 |
| 211615_s_at | LRPPRC | turquoise | -0.629 | 6.84E-05 | 0.923 | 7.36E-15 |
| 221688_s_at | IMP3 | turquoise | -0.463 | 5.88E-03 | 0.923 | 8.20E-15 |
| 218224_at | PNMA1 | turquoise | -0.392 | 2.17E-02 | 0.922 | 9.64E-15 |
| 209248_at | GHITM | turquoise | -0.568 | 4.59E-04 | 0.920 | 1.55E-14 |
| 205705_at | ANKRD26 | turquoise | -0.516 | 1.79E-03 | 0.918 | 2.24E-14 |
| 202373_s_at | AURKAPS1 | turquoise | -0.512 | 1.98E-03 | 0.916 | 3.07E-14 |
| 210534_s_at | B9D1 | turquoise | -0.496 | 2.86E-03 | 0.915 | 4.02E-14 |
| 200641_s_at | YWHAZ | turquoise | -0.377 | 2.81E-02 | 0.913 | 4.90E-14 |
| 205280_at | GLRB | turquoise | -0.436 | 9.88E-03 | 0.911 | 6.89E-14 |
| 204587_at | SLC25A14 | turquoise | -0.644 | 3.91E-05 | 0.911 | 7.66E-14 |
| 202591_s_at | SSBP1 | turquoise | -0.595 | 2.06E-04 | 0.911 | 7.79E-14 |
| 205110_s_at | FGF13 | turquoise | -0.573 | 4.02E-04 | 0.909 | 1.03E-13 |
| 208868_s_at | GABARAPL1 | turquoise | -0.420 | 1.33E-02 | 0.909 | 1.12E-13 |
| 201256_at | COX7A2L | turquoise | -0.600 | 1.79E-04 | 0.908 | 1.21E-13 |
| 211595_s_at | MRPS11 | turquoise | -0.371 | 3.06E-02 | 0.907 | 1.43E-13 |
| 212887_at | SEC23A | turquoise | -0.455 | 6.86E-03 | 0.907 | 1.53E-13 |
| 207776_s_at | CACNB2 | turquoise | -0.616 | 1.05E-04 | 0.906 | 1.67E-13 |
| 201272_at | AKR1B1 | turquoise | -0.561 | 5.64E-04 | 0.906 | 1.71E-13 |
| 203157_s_at | GLS | turquoise | -0.464 | 5.68E-03 | 0.905 | 2.08E-13 |
| 200812_at | CCT7 | turquoise | -0.446 | 8.17E-03 | 0.903 | 2.89E-13 |
| 209157_at | DNAJA2 | turquoise | -0.367 | 3.29E-02 | 0.901 | 3.86E-13 |
| 201198_s_at | PSMD1 | turquoise | -0.410 | 1.60E-02 | 0.900 | 4.46E-13 |
| 200638_s_at | YWHAZ | turquoise | -0.380 | 2.64E-02 | 0.900 | 4.59E-13 |
| 206984_s_at | RIT2 | turquoise | -0.607 | 1.42E-04 | 0.900 | 4.59E-13 |
| 213011_s_at | TPI1 | turquoise | -0.449 | 7.75E-03 | 0.899 | 4.98E-13 |
| 212961_x_at | CXorf40B | turquoise | -0.474 | 4.62E-03 | 0.899 | 5.06E-13 |
| 213714_at | CACNB2 | turquoise | -0.668 | 1.59E-05 | 0.899 | 5.52E-13 |
| 208678_at | ATP6V1E1 | turquoise | -0.518 | 1.69E-03 | 0.899 | 5.55E-13 |
| 209075_s_at | ISCU | turquoise | -0.482 | 3.85E-03 | 0.898 | 6.09E-13 |
| 210968_s_at | RTN4 | turquoise | -0.436 | 9.97E-03 | 0.898 | 6.22E-13 |
| 209550_at | NDN | turquoise | -0.526 | 1.38E-03 | 0.898 | 6.34E-13 |
| 211763_s_at | UBE2B | turquoise | -0.469 | 5.19E-03 | 0.897 | 6.65E-13 |
| 206542_s_at | SMARCA2 | turquoise | -0.457 | 6.56E-03 | 0.897 | 6.90E-13 |
| 215527_at | KHDRBS2 | turquoise | -0.385 | 2.45E-02 | 0.897 | 7.14E-13 |
| 208839_s_at | CAND1 | turquoise | -0.481 | 4.02E-03 | 0.896 | 8.07E-13 |
| 219421_at | TTC33 | turquoise | -0.525 | 1.42E-03 | 0.896 | 8.21E-13 |
| 211558_s_at | DHPS | turquoise | -0.528 | 1.31E-03 | 0.896 | 8.38E-13 |
| 209303_at | NDUFS4 | turquoise | -0.400 | 1.91E-02 | 0.895 | 8.81E-13 |
| 221515_s_at | LCMT1 | turquoise | -0.522 | 1.55E-03 | 0.895 | 8.90E-13 |
| 200626_s_at | MATR3 | turquoise | -0.397 | 2.00E-02 | 0.895 | 8.98E-13 |
| 212217_at | PREPL | turquoise | -0.403 | 1.82E-02 | 0.895 | 9.32E-13 |
| 203404_at | ARMCX2 | turquoise | -0.431 | 1.09E-02 | 0.895 | 9.33E-13 |
| 222216_s_at | MRPL17 | turquoise | -0.422 | 1.29E-02 | 0.895 | 9.48E-13 |
| 201628_s_at | RRAGA | turquoise | -0.504 | 2.39E-03 | 0.895 | 9.69E-13 |
| 202078_at | COPS3 | turquoise | -0.413 | 1.52E-02 | 0.894 | 1.03E-12 |
| 200030_s_at | SLC25A3 | turquoise | -0.508 | 2.17E-03 | 0.894 | 1.07E-12 |
| 203613_s_at | NDUFB6 | turquoise | -0.495 | 2.89E-03 | 0.894 | 1.16E-12 |
| 200786_at | PSMB7 | turquoise | -0.461 | 6.10E-03 | 0.893 | 1.21E-12 |
| 219263_at | RNF128 | turquoise | -0.523 | 1.51E-03 | 0.892 | 1.35E-12 |
| 218488_at | EIF2B3 | turquoise | -0.445 | 8.33E-03 | 0.891 | 1.56E-12 |
| 208827_at | PSMB6 | turquoise | -0.534 | 1.14E-03 | 0.890 | 1.81E-12 |
| 207508_at | ATP5G3 | turquoise | -0.444 | 8.47E-03 | 0.890 | 1.88E-12 |
| 202507_s_at | SNAP25 | turquoise | -0.400 | 1.89E-02 | 0.889 | 2.23E-12 |
| 213533_at | NSG1 | turquoise | -0.523 | 1.52E-03 | 0.888 | 2.56E-12 |
| 203189_s_at | NDUFS8 | turquoise | -0.547 | 8.24E-04 | 0.887 | 2.81E-12 |
| 218120_s_at | HMOX2 | turquoise | -0.450 | 7.53E-03 | 0.887 | 2.83E-12 |
| 218491_s_at | THYN1 | turquoise | -0.551 | 7.36E-04 | 0.887 | 2.88E-12 |
| 213887_s_at | POLR2E | turquoise | -0.478 | 4.23E-03 | 0.887 | 2.90E-12 |
| 217923_at | PEF1 | turquoise | -0.572 | 4.06E-04 | 0.886 | 3.24E-12 |
| 218163_at | MCTS1 | turquoise | -0.368 | 3.25E-02 | 0.886 | 3.44E-12 |
| 200818_at | ATP5O | turquoise | -0.432 | 1.07E-02 | 0.885 | 3.76E-12 |
| 205633_s_at | ALAS1 | turquoise | -0.335 | 5.30E-02 | 0.885 | 3.78E-12 |
| 208745_at | ATP5L | turquoise | -0.451 | 7.46E-03 | 0.884 | 4.18E-12 |
| 201322_at | ATP5B | turquoise | -0.383 | 2.52E-02 | 0.884 | 4.41E-12 |
| 210453_x_at | ATP5L | turquoise | -0.511 | 2.02E-03 | 0.884 | 4.53E-12 |
| 203663_s_at | COX5A | turquoise | -0.407 | 1.68E-02 | 0.883 | 4.81E-12 |
| 218133_s_at | NIF3L1 | turquoise | -0.501 | 2.56E-03 | 0.883 | 5.03E-12 |
| 202325_s_at | ATP5J | turquoise | -0.380 | 2.67E-02 | 0.882 | 5.24E-12 |
| 221531_at | WDR61 | turquoise | -0.544 | 8.71E-04 | 0.882 | 5.26E-12 |
| 202233_s_at | UQCRH | turquoise | -0.388 | 2.33E-02 | 0.881 | 5.88E-12 |
| 209733_at | MID2 | turquoise | -0.619 | 9.45E-05 | 0.881 | 6.15E-12 |
| 215416_s_at | STOML2 | turquoise | -0.460 | 6.24E-03 | 0.881 | 6.37E-12 |
| 205005_s_at | NMT2 | turquoise | -0.525 | 1.43E-03 | 0.880 | 6.99E-12 |
| 202736_s_at | LSM4 | turquoise | -0.383 | 2.53E-02 | 0.880 | 7.41E-12 |
| 217906_at | KLHDC2 | turquoise | -0.494 | 2.95E-03 | 0.880 | 7.42E-12 |
| 205963_s_at | DNAJA3 | turquoise | -0.400 | 1.91E-02 | 0.880 | 7.49E-12 |
| 201443_s_at | ATP6AP2 | turquoise | -0.474 | 4.60E-03 | 0.879 | 8.55E-12 |
| 219481_at | TTC13 | turquoise | -0.561 | 5.65E-04 | 0.878 | 8.85E-12 |
| 217960_s_at | TOMM22 | turquoise | -0.463 | 5.88E-03 | 0.877 | 9.78E-12 |
| 221699_s_at | DDX50 | turquoise | -0.430 | 1.10E-02 | 0.877 | 1.09E-11 |
| 201988_s_at | CREBL2 | turquoise | -0.442 | 8.91E-03 | 0.876 | 1.11E-11 |
| 207573_x_at | ATP5L | turquoise | -0.483 | 3.79E-03 | 0.876 | 1.18E-11 |
| 212053_at | PDXDC1 | turquoise | -0.392 | 2.18E-02 | 0.875 | 1.30E-11 |
| 208911_s_at | PDHB | turquoise | -0.509 | 2.10E-03 | 0.875 | 1.36E-11 |
| 203362_s_at | MAD2L1 | turquoise | -0.621 | 8.87E-05 | 0.875 | 1.36E-11 |
| 208860_s_at | ATRX | turquoise | -0.421 | 1.31E-02 | 0.875 | 1.39E-11 |
| 213227_at | PGRMC2 | turquoise | -0.478 | 4.29E-03 | 0.874 | 1.41E-11 |
| 211479_s_at | HTR2C | turquoise | -0.452 | 7.31E-03 | 0.874 | 1.41E-11 |
| 209046_s_at | GABARAPL2 | turquoise | -0.485 | 3.66E-03 | 0.874 | 1.49E-11 |
| 201112_s_at | CSE1L | turquoise | -0.574 | 3.82E-04 | 0.873 | 1.66E-11 |
| 214629_x_at | RTN4 | turquoise | -0.475 | 4.57E-03 | 0.873 | 1.70E-11 |
| 202594_at | LEPROTL1 | turquoise | -0.414 | 1.50E-02 | 0.873 | 1.75E-11 |
| 209104_s_at | NHP2 | turquoise | -0.465 | 5.58E-03 | 0.872 | 1.79E-11 |
| 200822_x_at | TPI1 | turquoise | -0.436 | 9.96E-03 | 0.872 | 1.80E-11 |
| 211297_s_at | CDK7 | turquoise | -0.482 | 3.89E-03 | 0.870 | 2.27E-11 |
| 212820_at | DMXL2 | turquoise | -0.554 | 6.77E-04 | 0.868 | 2.90E-11 |
| 219355_at | CXorf57 | turquoise | -0.581 | 3.12E-04 | 0.868 | 3.09E-11 |
| 218976_at | DNAJC12 | turquoise | -0.525 | 1.42E-03 | 0.867 | 3.43E-11 |
| 206062_at | GUCA1A | turquoise | -0.597 | 1.91E-04 | 0.866 | 3.88E-11 |
| 203033_x_at | FH | turquoise | -0.496 | 2.83E-03 | 0.865 | 4.03E-11 |
| 208869_s_at | GABARAPL1 | turquoise | -0.383 | 2.52E-02 | 0.865 | 4.35E-11 |
| 201410_at | PLEKHB2 | turquoise | -0.544 | 8.79E-04 | 0.864 | 4.67E-11 |
| 202741_at | PRKACB | turquoise | -0.530 | 1.26E-03 | 0.864 | 4.73E-11 |
| 44669_at | SDHAF1 | turquoise | -0.541 | 9.54E-04 | 0.863 | 5.03E-11 |
| 205217_at | TIMM8A | turquoise | -0.538 | 1.02E-03 | 0.862 | 5.64E-11 |
| 207812_s_at | GORASP2 | turquoise | -0.582 | 3.04E-04 | 0.862 | 5.71E-11 |
| 202868_s_at | POP4 | turquoise | -0.298 | 8.68E-02 | 0.862 | 5.80E-11 |
| 212157_at | SDC2 | turquoise | -0.618 | 9.72E-05 | 0.862 | 5.93E-11 |
| 217780_at | WDR83OS | turquoise | -0.485 | 3.68E-03 | 0.861 | 6.39E-11 |
| 204119_s_at | ADK | turquoise | -0.515 | 1.85E-03 | 0.861 | 6.51E-11 |
| 210406_s_at | RAB6A | turquoise | -0.525 | 1.43E-03 | 0.859 | 7.68E-11 |
| 201106_at | GPX4 | turquoise | -0.545 | 8.65E-04 | 0.859 | 7.79E-11 |
| 201570_at | SAMM50 | turquoise | -0.448 | 7.84E-03 | 0.859 | 7.84E-11 |
| 201066_at | CYC1 | turquoise | -0.374 | 2.94E-02 | 0.859 | 8.36E-11 |
| 202658_at | PEX11B | turquoise | -0.396 | 2.05E-02 | 0.858 | 8.59E-11 |
| 200903_s_at | AHCY | turquoise | -0.476 | 4.40E-03 | 0.858 | 8.65E-11 |
| 201484_at | SUPT4H1 | turquoise | -0.276 | 1.14E-01 | 0.858 | 9.01E-11 |
| 201086_x_at | SON | turquoise | -0.571 | 4.18E-04 | 0.857 | 9.80E-11 |
| 210240_s_at | CDKN2D | turquoise | -0.378 | 2.75E-02 | 0.857 | 1.02E-10 |
| 201756_at | RPA2 | turquoise | -0.510 | 2.06E-03 | 0.857 | 1.03E-10 |
| 209549_s_at | DGUOK | turquoise | -0.590 | 2.38E-04 | 0.856 | 1.11E-10 |
| 211475_s_at | BAG1 | turquoise | -0.498 | 2.72E-03 | 0.856 | 1.13E-10 |
| 215058_at | DENND5B | turquoise | -0.570 | 4.27E-04 | 0.855 | 1.25E-10 |
| 208731_at | RAB2A | turquoise | -0.450 | 7.56E-03 | 0.854 | 1.40E-10 |
| 212407_at | METTL13 | turquoise | -0.534 | 1.14E-03 | 0.853 | 1.43E-10 |
| 211971_s_at | LRPPRC | turquoise | -0.370 | 3.10E-02 | 0.853 | 1.53E-10 |
| 207120_at | ZNF667 | turquoise | -0.456 | 6.76E-03 | 0.853 | 1.56E-10 |
| 203621_at | NDUFB5 | turquoise | -0.396 | 2.06E-02 | 0.852 | 1.69E-10 |
| 210014_x_at | IDH3B | turquoise | -0.481 | 3.96E-03 | 0.852 | 1.72E-10 |
| 201989_s_at | CREBL2 | turquoise | -0.614 | 1.13E-04 | 0.850 | 2.11E-10 |
| 218982_s_at | MRPS17 | turquoise | -0.400 | 1.89E-02 | 0.850 | 2.11E-10 |
| 212990_at | SYNJ1 | turquoise | -0.522 | 1.54E-03 | 0.850 | 2.11E-10 |
| 200079_s_at | KARS | turquoise | -0.457 | 6.57E-03 | 0.849 | 2.27E-10 |
| 202920_at | ANK2 | turquoise | -0.408 | 1.67E-02 | 0.848 | 2.44E-10 |
| 203302_at | DCK | turquoise | -0.503 | 2.45E-03 | 0.847 | 2.61E-10 |
| 208832_at | ATXN10 | turquoise | -0.454 | 7.04E-03 | 0.846 | 2.98E-10 |
| 211318_s_at | RAE1 | turquoise | -0.431 | 1.10E-02 | 0.846 | 3.04E-10 |
| 201527_at | ATP6V1F | turquoise | -0.475 | 4.52E-03 | 0.846 | 3.07E-10 |
| 205278_at | GAD1 | turquoise | -0.452 | 7.26E-03 | 0.845 | 3.17E-10 |
| 219628_at | ZMAT3 | turquoise | -0.434 | 1.04E-02 | 0.845 | 3.20E-10 |
| 220329_s_at | RMND1 | turquoise | -0.605 | 1.49E-04 | 0.844 | 3.53E-10 |
| 203560_at | GGH | turquoise | -0.451 | 7.41E-03 | 0.844 | 3.54E-10 |
| 203159_at | GLS | turquoise | -0.427 | 1.18E-02 | 0.844 | 3.55E-10 |
| 204957_at | ORC5 | turquoise | -0.630 | 6.51E-05 | 0.844 | 3.62E-10 |
| 218557_at | NIT2 | turquoise | -0.561 | 5.53E-04 | 0.843 | 3.90E-10 |
| 203137_at | WTAP | turquoise | -0.380 | 2.68E-02 | 0.843 | 4.08E-10 |
| 202232_s_at | EIF3M | turquoise | -0.589 | 2.48E-04 | 0.842 | 4.24E-10 |
| 221263_s_at | SF3B5 | turquoise | -0.387 | 2.36E-02 | 0.842 | 4.43E-10 |
| 202641_at | ARL3 | turquoise | -0.619 | 9.55E-05 | 0.842 | 4.51E-10 |
| 211672_s_at | ARPC4 | turquoise | -0.443 | 8.66E-03 | 0.842 | 4.54E-10 |
| 201313_at | ENO2 | turquoise | -0.377 | 2.79E-02 | 0.841 | 4.60E-10 |
| 208946_s_at | BECN1 | turquoise | -0.408 | 1.65E-02 | 0.841 | 4.68E-10 |
| 204992_s_at | PFN2 | turquoise | -0.399 | 1.93E-02 | 0.841 | 4.71E-10 |
| 207922_s_at | MAEA | turquoise | -0.397 | 2.01E-02 | 0.840 | 5.04E-10 |
| 218694_at | ARMCX1 | turquoise | -0.468 | 5.21E-03 | 0.839 | 5.52E-10 |
| 200077_s_at | OAZ1 | turquoise | -0.317 | 6.73E-02 | 0.838 | 6.08E-10 |
| 200097_s_at | HNRNPK | turquoise | -0.453 | 7.18E-03 | 0.838 | 6.34E-10 |
| 210501_x_at | EIF3K | turquoise | -0.447 | 8.04E-03 | 0.838 | 6.41E-10 |
| 201900_s_at | AKR1A1 | turquoise | -0.702 | 3.79E-06 | 0.837 | 6.75E-10 |
| 200862_at | DHCR24 | turquoise | -0.531 | 1.24E-03 | 0.836 | 7.40E-10 |
| 211769_x_at | SERINC3 | turquoise | -0.329 | 5.77E-02 | 0.836 | 7.45E-10 |
| 205512_s_at | AIFM1 | turquoise | -0.340 | 4.90E-02 | 0.836 | 7.55E-10 |
| 200708_at | GOT2 | turquoise | -0.418 | 1.38E-02 | 0.836 | 7.74E-10 |
| 214717_at | PKI55 | turquoise | -0.583 | 2.99E-04 | 0.835 | 8.33E-10 |
| 218316_at | TIMM9 | turquoise | -0.561 | 5.55E-04 | 0.835 | 8.54E-10 |
| 207079_s_at | MED6 | turquoise | -0.470 | 5.00E-03 | 0.834 | 8.76E-10 |
| 217968_at | TSSC1 | turquoise | -0.544 | 8.90E-04 | 0.834 | 9.13E-10 |
| 212716_s_at | EIF3K | turquoise | -0.544 | 8.72E-04 | 0.834 | 9.22E-10 |
| 205531_s_at | GLS2 | turquoise | -0.612 | 1.19E-04 | 0.832 | 1.04E-09 |
| 212092_at | PEG10 | turquoise | -0.490 | 3.28E-03 | 0.832 | 1.05E-09 |
| 206015_s_at | FOXJ3 | turquoise | -0.432 | 1.07E-02 | 0.832 | 1.06E-09 |
| 202427_s_at | MPC2 | turquoise | -0.398 | 1.99E-02 | 0.832 | 1.07E-09 |
| 219760_at | LIN7B | turquoise | -0.438 | 9.58E-03 | 0.832 | 1.08E-09 |
| 202382_s_at | GNPDA1 | turquoise | -0.446 | 8.13E-03 | 0.832 | 1.10E-09 |
| 201274_at | PSMA5 | turquoise | -0.332 | 5.48E-02 | 0.832 | 1.11E-09 |
| 208761_s_at | SUMO1 | turquoise | -0.417 | 1.42E-02 | 0.831 | 1.17E-09 |
| 202779_s_at | UBE2S | turquoise | -0.282 | 1.06E-01 | 0.831 | 1.17E-09 |
| 204245_s_at | RPP14 | turquoise | -0.455 | 6.89E-03 | 0.831 | 1.22E-09 |
| 207088_s_at | SLC25A11 | turquoise | -0.355 | 3.93E-02 | 0.830 | 1.30E-09 |
| 203721_s_at | UTP18 | turquoise | -0.426 | 1.21E-02 | 0.830 | 1.30E-09 |
| 208675_s_at | DDOST | turquoise | -0.356 | 3.88E-02 | 0.830 | 1.30E-09 |
| 203983_at | TSNAX | turquoise | -0.521 | 1.57E-03 | 0.830 | 1.30E-09 |
| 201411_s_at | PLEKHB2 | turquoise | -0.392 | 2.20E-02 | 0.830 | 1.33E-09 |
| 215171_s_at | TIMM17A | turquoise | -0.395 | 2.07E-02 | 0.829 | 1.39E-09 |
| 211963_s_at | ARPC5 | turquoise | -0.512 | 1.98E-03 | 0.829 | 1.39E-09 |
| 212645_x_at | BRE | turquoise | -0.548 | 7.84E-04 | 0.828 | 1.50E-09 |
| 208799_at | PSMB5 | turquoise | -0.422 | 1.29E-02 | 0.828 | 1.57E-09 |
| 202712_s_at | CKMT1A | turquoise | -0.553 | 6.87E-04 | 0.828 | 1.57E-09 |
| 212215_at | PREPL | turquoise | -0.648 | 3.44E-05 | 0.827 | 1.65E-09 |
| 202930_s_at | SUCLA2 | turquoise | -0.451 | 7.49E-03 | 0.827 | 1.69E-09 |
| 219960_s_at | UCHL5 | turquoise | -0.443 | 8.76E-03 | 0.827 | 1.70E-09 |
| 210278_s_at | AP4S1 | turquoise | -0.402 | 1.84E-02 | 0.827 | 1.72E-09 |
| 200053_at | SPAG7 | turquoise | -0.596 | 1.98E-04 | 0.826 | 1.75E-09 |
| 209243_s_at | PEG3 | turquoise | -0.415 | 1.46E-02 | 0.826 | 1.76E-09 |
| 215506_s_at | DIRAS3 | turquoise | -0.544 | 8.76E-04 | 0.826 | 1.81E-09 |
| 202802_at | DHPS | turquoise | -0.455 | 6.92E-03 | 0.826 | 1.85E-09 |
| 211566_x_at | BRE | turquoise | -0.488 | 3.38E-03 | 0.825 | 1.90E-09 |
| 218214_at | C12orf44 | turquoise | -0.292 | 9.40E-02 | 0.825 | 1.92E-09 |
| 213710_s_at | CALM1 | turquoise | -0.505 | 2.32E-03 | 0.825 | 1.93E-09 |
| 211404_s_at | APLP2 | turquoise | -0.269 | 1.23E-01 | 0.825 | 1.97E-09 |
| 211658_at | PRDX2 | turquoise | -0.483 | 3.80E-03 | 0.824 | 2.19E-09 |
| 217286_s_at | NDRG3 | turquoise | -0.513 | 1.91E-03 | 0.823 | 2.27E-09 |
| 207831_x_at | DHPS | turquoise | -0.505 | 2.32E-03 | 0.823 | 2.30E-09 |
| 204125_at | NDUFAF1 | turquoise | -0.450 | 7.65E-03 | 0.823 | 2.40E-09 |
| 200720_s_at | ACTR1A | turquoise | -0.648 | 3.42E-05 | 0.822 | 2.52E-09 |
| 206849_at | GABRG2 | turquoise | -0.356 | 3.91E-02 | 0.822 | 2.56E-09 |
| 203944_x_at | BTN2A1 | turquoise | -0.503 | 2.42E-03 | 0.821 | 2.76E-09 |
| 200843_s_at | EPRS | turquoise | -0.650 | 3.21E-05 | 0.820 | 2.95E-09 |
| 203415_at | PDCD6 | turquoise | -0.267 | 1.27E-01 | 0.820 | 3.00E-09 |
| 209570_s_at | NSG1 | turquoise | -0.564 | 5.20E-04 | 0.820 | 3.06E-09 |
| 218226_s_at | NDUFB4 | turquoise | -0.411 | 1.58E-02 | 0.819 | 3.25E-09 |
| 200040_at | KHDRBS1 | turquoise | -0.592 | 2.29E-04 | 0.819 | 3.31E-09 |
| 203893_at | TAF9 | turquoise | -0.422 | 1.28E-02 | 0.817 | 3.73E-09 |
| 217860_at | NDUFA10 | turquoise | -0.335 | 5.26E-02 | 0.817 | 3.80E-09 |
| 217959_s_at | TRAPPC4 | turquoise | -0.442 | 8.80E-03 | 0.815 | 4.35E-09 |
| 202613_at | CTPS1 | turquoise | -0.477 | 4.38E-03 | 0.814 | 4.83E-09 |
| 209598_at | PNMA2 | turquoise | -0.534 | 1.14E-03 | 0.814 | 4.83E-09 |
| 209840_s_at | LRRN3 | turquoise | -0.516 | 1.79E-03 | 0.814 | 4.96E-09 |
| 202929_s_at | DDT | turquoise | -0.313 | 7.19E-02 | 0.813 | 5.03E-09 |

(B)

| **miRBase ID** | **Probe.Set.ID** | **Family** | **Module** | **GS** | **p-value** | **MM** | **p-value** |
| --- | --- | --- | --- | --- | --- | --- | --- |
| hsa-miR-34b-5p | hsa-miR-34b-star_st | mir-34 | brown | 0.428 | 1.15E-02 | 0.946 | 3.12E-17 |
| hsa-miR-34c-5p | hsa-miR-34c-5p_st | mir-34 | brown | 0.431 | 1.09E-02 | 0.926 | 4.57E-15 |
| hsa-miR-34c-3p | hsa-miR-34c-3p_st | mir-34 | brown | 0.491 | 3.21E-03 | 0.893 | 1.30E-12 |
| hsa-miR-375 | hsa-miR-375_st | mir-375 | brown | 0.353 | 4.07E-02 | 0.845 | 3.33E-10 |
| hsa-miR-34b-3p | hsa-miR-34b_st | mir-34 | brown | 0.259 | 1.39E-01 | 0.790 | 2.74E-08 |
| hsa-miR-4652-3p | hsa-miR-4652-3p_st |  | brown | 0.511 | 2.01E-03 | 0.646 | 3.68E-05 |
| hsa-miR-4423-3p | hsa-miR-4423-3p_st |  | brown | 0.363 | 3.49E-02 | 0.622 | 8.60E-05 |
| hsa-miR-383-5p | hsa-miR-383_st | mir-383 | blue | -0.315 | 6.99E-02 | 0.871 | 2.20E-11 |
| hsa-miR-212-3p | hsa-miR-212_st | mir-132 | blue | -0.441 | 9.06E-03 | 0.868 | 2.91E-11 |
| hsa-miR-377-5p | hsa-miR-377-star_st | mir-134 | blue | -0.472 | 4.85E-03 | 0.860 | 6.90E-11 |
| hsa-miR-132-3p | hsa-miR-132_st | mir-132 | blue | -0.490 | 3.25E-03 | 0.835 | 8.09E-10 |
| hsa-miR-1912 | hsa-miR-1912_st | mir-1912 | blue | -0.419 | 1.37E-02 | 0.761 | 1.70E-07 |
| hsa-miR-1180-3p | hsa-miR-1180_st | mir-1180 | blue | -0.299 | 8.54E-02 | 0.739 | 6.11E-07 |
| hsa-miR-382-5p | hsa-miR-382_st | mir-134 | blue | -0.416 | 1.44E-02 | 0.709 | 2.70E-06 |
| hsa-miR-370-3p | hsa-miR-370_st | mir-370 | blue | -0.272 | 1.20E-01 | 0.697 | 4.66E-06 |
| hsa-miR-361-5p | hsa-miR-361-5p_st | mir-361 | blue | -0.222 | 2.07E-01 | 0.657 | 2.47E-05 |
| hsa-miR-4760-3p | hsa-miR-4760-3p_st |  | blue | -0.513 | 1.90E-03 | 0.645 | 3.78E-05 |
| hsa-miR-3189-5p | hsa-miR-3189-5p_st |  | blue | -0.515 | 1.84E-03 | 0.622 | 8.48E-05 |
| hsa-miR-134-5p | hsa-miR-134_st | mir-134 | blue | -0.218 | 2.14E-01 | 0.613 | 1.16E-04 |
| hsa-miR-523-3p | hsa-miR-523_st | mir-515 | blue | -0.349 | 4.30E-02 | 0.603 | 1.59E-04 |
| hsa-miR-4720-3p | hsa-miR-4720-3p_st |  | blue | -0.582 | 3.06E-04 | 0.566 | 4.83E-04 |
| hsa-miR-4633-5p | hsa-miR-4633-5p_st |  | yellow | -0.270 | 1.22E-01 | 0.701 | 3.91E-06 |
| hsa-miR-4762-5p | hsa-miR-4762-5p_st |  | yellow | -0.356 | 3.86E-02 | 0.692 | 5.80E-06 |
| hsa-miR-4311 | hsa-miR-4311_st |  | yellow | -0.388 | 2.34E-02 | 0.691 | 6.02E-06 |
| hsa-miR-555 | hsa-miR-555_st | mir-555 | yellow | -0.256 | 1.44E-01 | 0.544 | 8.85E-04 |
| Removed | hsa-miR-3676_st |  | yellow | -0.358 | 3.75E-02 | 0.538 | 1.02E-03 |
