# Supplementary material for: Integrating mRNA and miRNA Weighted Gene Co-Expression Networks with eQTLs in the Nucleus Accumbens of Subjects with Alcohol Dependence
Source: PLoS One. 2015 Sep 18;10(9):e0137671. doi: 10.1371/journal.pone.0137671 (PMC4575063; doi:10.1371/journal.pone.0137671)
Supplement: S4 Table — (DOCX) [file pone.0137671.s005.docx]

**Table S4.** (A)

| **Enriched gene sets** | **Turquoise** | **Yellow** | **Total** |
| --- | --- | --- | --- |
| Reactome: METABOLISM OF PROTEINS | + | + | 52 |
| Reactome: CELL CYCLE | + | + | 48 |
| Reactome: TCA CYCLE AND RESPIRATORY ELECTRON TRANSPORT | + | + | 44 |
| Reactome: CELL CYCLE MITOTIC | + | + | 44 |
| Reactome: HIV INFECTION | + | + | 43 |
| Reactome: CLASS I MHC MEDIATED ANTIGEN PROCESSING PRESENTATION | + | + | 43 |
| Reactome: ANTIGEN PROCESSING UBIQUITINATION PROTEASOME DEGRADATION | + | + | 40 |
| KEGG: HUNTINGTONS DISEASE | + | + | 40 |
| Reactome: METABOLISM OF RNA | + | + | 38 |
| KEGG: PARKINSONS DISEASE | + | + | 37 |
| KEGG: ALZHEIMERS DISEASE | + | + | 36 |
| Reactome: RESPIRATORY ELECTRON TRANSPORT ATP SYNTHESIS BY CHEMIOSMOTIC COUPLING AND HEAT PRODUCTION BY UNCOUPLING PROTEINS | + | + | 35 |
| KEGG: OXIDATIVE PHOSPHORYLATION | + | + | 34 |
| Reactome: DNA REPLICATION | + | + | 33 |
| Reactome: MITOTIC M M G1 PHASES | + | + | 31 |
| Reactome: RESPIRATORY ELECTRON TRANSPORT | + | + | 27 |
| Reactome: HOST INTERACTIONS OF HIV FACTORS | + | + | 27 |
| Reactome: NEURONAL SYSTEM | + | + | 26 |
| Reactome: CELL CYCLE CHECKPOINTS | + | + | 26 |
| Reactome: MITOTIC G1 G1 S PHASES | + | + | 25 |
| Reactome: MRNA PROCESSING | + | + | 25 |
| Reactome: METABOLISM OF AMINO ACIDS AND DERIVATIVES | + | + | 25 |
| Reactome: S PHASE | + | + | 24 |
| Reactome: G1 S TRANSITION | + | + | 24 |
| Reactome: SYNTHESIS OF DNA | + | + | 22 |
| Reactome: TRANSMISSION ACROSS CHEMICAL SYNAPSES | + | + | 21 |
| Reactome: M G1 TRANSITION | + | + | 20 |
| KEGG: UBIQUITIN MEDIATED PROTEOLYSIS | + | + | 19 |
| Reactome: HIV LIFE CYCLE | + | + | 19 |
| Reactome: TRANSCRIPTION | + | + | 18 |
| Reactome: REGULATION OF MITOTIC CELL CYCLE | + | + | 18 |
| Reactome: ORC1 REMOVAL FROM CHROMATIN | + | + | 18 |
| Reactome: APC C CDC20 MEDIATED DEGRADATION OF MITOTIC PROTEINS | + | + | 18 |
| Reactome: ASSEMBLY OF THE PRE REPLICATIVE COMPLEX | + | + | 18 |
| Reactome: DNA REPAIR | + | + | 17 |
| Reactome: SIGNALING BY WNT | + | + | 17 |
| Reactome: MITOCHONDRIAL PROTEIN IMPORT | + | + | 17 |
| Reactome: CDT1 ASSOCIATION WITH THE CDC6 ORC ORIGIN COMPLEX | + | + | 16 |
| Reactome: LATE PHASE OF HIV LIFE CYCLE | + | + | 16 |
| Reactome: ANTIGEN PROCESSING CROSS PRESENTATION | + | + | 16 |
| Reactome: VIF MEDIATED DEGRADATION OF APOBEC3G | + | + | 16 |
| Reactome: AUTODEGRADATION OF CDH1 BY CDH1 APC C | + | + | 16 |
| Reactome: APC C CDH1 MEDIATED DEGRADATION OF CDC20 AND OTHER APC C CDH1 TARGETED PROTEINS IN LATE MITOSIS EARLY G1 | + | + | 16 |
| Reactome: REGULATION OF ORNITHINE DECARBOXYLASE ODC | + | + | 16 |
| Reactome: ER PHAGOSOME PATHWAY | + | + | 16 |
| Reactome: CYCLIN E ASSOCIATED EVENTS DURING G1 S TRANSITION | + | + | 16 |
| Reactome: DESTABILIZATION OF MRNA BY AUF1 HNRNP D0 | + | + | 15 |
| Reactome: DOWNSTREAM SIGNALING EVENTS OF B CELL RECEPTOR BCR | + | + | 15 |
| Reactome: ACTIVATION OF NF KAPPAB IN B CELLS | + | + | 15 |
| Reactome: SCFSKP2 MEDIATED DEGRADATION OF P27 P21 | + | + | 15 |
| KEGG: CARDIAC MUSCLE CONTRACTION | + | + | 15 |
| Reactome: CDK MEDIATED PHOSPHORYLATION AND REMOVAL OF CDC6 | + | + | 14 |
| Reactome: SCF BETA TRCP MEDIATED DEGRADATION OF EMI1 | + | + | 14 |
| Reactome: P53 DEPENDENT G1 DNA DAMAGE RESPONSE | + | + | 14 |
| KEGG: PROTEASOME | + | + | 14 |
| Reactome: CROSS PRESENTATION OF SOLUBLE EXOGENOUS ANTIGENS ENDOSOMES | + | + | 14 |
| Reactome: P53 INDEPENDENT G1 S DNA DAMAGE CHECKPOINT | + | + | 14 |
| Reactome: REGULATION OF APOPTOSIS | + | + | 14 |
| Reactome: AUTODEGRADATION OF THE E3 UBIQUITIN LIGASE COP1 | + | + | 14 |
| Reactome: ACTIVATION OF THE MRNA UPON BINDING OF THE CAP BINDING COMPLEX AND EIFS AND SUBSEQUENT BINDING TO 43S | + | + | 12 |
| KEGG: OOCYTE MEIOSIS | + | + | 12 |
| KEGG: CITRATE CYCLE TCA CYCLE | + | + | 9 |
| Reactome: PYRUVATE METABOLISM AND CITRIC ACID TCA CYCLE | + | + | 9 |
| KEGG: GLYCOLYSIS GLUCONEOGENESIS | + | + | 9 |
| Reactome: G2 M CHECKPOINTS | + | + | 9 |
| Reactome: CHROMOSOME MAINTENANCE | + | + | 8 |
| KEGG: AMINO SUGAR AND NUCLEOTIDE SUGAR METABOLISM | + | + | 5 |
| Reactome: ACTIVATION OF NMDA RECEPTOR UPON GLUTAMATE BINDING AND POSTSYNAPTIC EVENTS | + | + | 5 |

(B)

| **Enriched gene sets** | **Green** | **Grey60** | **Pink** | **Salmon** | **Total** |
| --- | --- | --- | --- | --- | --- |
| KEGG: CYTOKINE CYTOKINE RECEPTOR INTERACTION | + | + | + | + | 12 |
| KEGG: CELL ADHESION MOLECULES CAMS | + | + | + | + | 12 |
| PID: ANGIOPOIETINRECEPTOR PATHWAY | + | + | + | + | 7 |
| Reactome: CYTOKINE SIGNALING IN IMMUNE SYSTEM | + |  | + | + | 26 |
| KEGG: FOCAL ADHESION | + |  | + | + | 20 |
| KEGG: PATHWAYS IN CANCER | + |  | + | + | 18 |
| KEGG: LEUKOCYTE TRANSENDOTHELIAL MIGRATION | + |  | + | + | 14 |
| PID: CXCR4 PATHWAY | + |  | + | + | 11 |
| Reactome: RESPONSE TO ELEVATED PLATELET CYTOSOLIC CA2 | + |  | + | + | 10 |
| Reactome: INTEGRIN CELL SURFACE INTERACTIONS | + |  | + | + | 9 |
| Reactome: SIGNALING BY ILS | + |  | + | + | 9 |
| PID: INTEGRIN1 PATHWAY | + |  | + | + | 8 |
| KEGG: ECM RECEPTOR INTERACTION | + |  | + | + | 8 |
| PID: IL6 7PATHWAY | + |  | + | + | 8 |
| PID: INTEGRIN3 PATHWAY | + |  | + | + | 8 |
| KEGG: SMALL CELL LUNG CANCER | + |  | + | + | 7 |
| PID: LYSOPHOSPHOLIPID: PATHWAY | + |  | + | + | 7 |
| KEGG: JAK STAT SIGNALING PATHWAY | + |  | + | + | 7 |
| Reactome: EXTRACELLULAR MATRIX ORGANIZATION | + |  | + | + | 6 |
| PID: RHOA PATHWAY | + |  | + | + | 6 |
| KEGG: TGF BETA SIGNALING PATHWAY | + |  | + | + | 3 |
| Reactome: PLATELET ACTIVATION SIGNALING AND AGGREGATION | + | + |  | + | 11 |
| Reactome: TCR SIGNALING | + | + |  | + | 10 |
| Reactome: CELL SURFACE INTERACTIONS AT THE VASCULAR WALL | + | + | + |  | 11 |
| Reactome: IMMUNE SYSTEM | + |  |  | + | 41 |
| Reactome: HEMOSTASIS | + |  |  | + | 17 |
| Reactome: INNATE IMMUNE SYSTEM | + |  |  | + | 13 |
| Reactome: INTERFERON SIGNALING | + |  |  | + | 13 |
| Reactome: SLC MEDIATED TRANSMEMBRANE TRANSPORT | + |  |  | + | 11 |
| Reactome: INTERFERON GAMMA SIGNALING | + |  |  | + | 8 |
| PID: FCER1PATHWAY | + |  |  | + | 4 |
| Reactome: SEMAPHORIN INTERACTIONS | + |  |  | + | 4 |
| PID: TGFBRPATHWAY | + |  |  | + | 4 |
| PID: AP1 PATHWAY | + |  | + |  | 11 |
| Reactome: CELL CELL COMMUNICATION | + |  | + |  | 10 |
| Reactome: TRANSPORT OF GLUCOSE AND OTHER SUGARS BILE SALTS AND ORGANIC ACIDS METAL IONS AND AMINE COMPOUNDS | + |  | + |  | 9 |
| PID: IL2 1PATHWAY | + |  | + |  | 7 |
| PID: VEGFR1 2 PATHWAY | + |  | + |  | 7 |
| PID: MYC REPRESSPATHWAY | + |  | + |  | 7 |
| Reactome: CELL JUNCTION ORGANIZATION | + |  | + |  | 7 |
| KEGG: ADIPOCYTOKINE SIGNALING PATHWAY | + |  | + |  | 6 |
| PID: A6B1 A6B4 INTEGRIN PATHWAY | + |  | + |  | 5 |
| PID: FAK PATHWAY | + |  | + |  | 5 |
| KEGG: PPAR SIGNALING PATHWAY | + |  | + |  | 5 |
| PID: TAP63PATHWAY | + |  | + |  | 4 |
| PID: ERBB1 RECEPTOR PROXIMAL PATHWAY | + |  | + |  | 4 |
| PID: PTP1BPATHWAY | + |  | + |  | 3 |
| KEGG: ANTIGEN PROCESSING AND PRESENTATION | + |  |  | + | 3 |
| PID: ILK PATHWAY |  |  | + | + | 5 |
