# Supplementary material for: Integrating mRNA and miRNA Weighted Gene Co-Expression Networks with eQTLs in the Nucleus Accumbens of Subjects with Alcohol Dependence
Source: PLoS One. 2015 Sep 18;10(9):e0137671. doi: 10.1371/journal.pone.0137671 (PMC4575063; doi:10.1371/journal.pone.0137671)
Supplement: S5 Table — (DOCX) [file pone.0137671.s006.docx]

**Table S5**.

| **Module** | **Enriched Gene set** | **Total Genes** |
| --- | --- | --- |
| Green | KEGG: APOPTOSIS | 7 |
|  | Reactome: ACTIVATED TLR4 SIGNALLING | 7 |
|  | Reactome: TOLL RECEPTOR CASCADES | 7 |
|  | KEGG: REGULATION OF ACTIN CYTOSKELETON | 6 |
|  | KEGG: LEISHMANIA INFECTION | 5 |
|  | BIOCARTA HIVNEF PATHWAY | 4 |
|  | KEGG: NATURAL KILLER CELL MEDIATED CYTOTOXICITY | 3 |
|  | PID: IL4 2PATHWAY | 3 |
|  | PID: P38 ALPHABETA DOWNSTREAM PATHWAY | 3 |
|  | ST T CELL SIGNAL TRANSDUCTION | 3 |
|  | PID: SMAD2 3NUCLEARPATHWAY | 2 |
|  | Reactome: PI3K EVENTS IN ERBB2 SIGNALING | 1 |
| Pink | KEGG: NOTCH SIGNALING PATHWAY | 4 |
|  | Reactome: ACTIVATION OF CHAPERONE GENES BY XBP1S | 3 |
|  | Reactome: CHONDROITIN SULFATE DERMATAN SULFATE METABOLISM | 3 |
|  | PID: NCADHERINPATHWAY | 1 |
| Salmon | KEGG: LYSOSOME | 5 |
|  | PID: SHP2 PATHWAY | 1 |
| Turquoise | Reactome: METABOLISM OF MRNA | 24 |
|  | Reactome: PROCESSING OF CAPPED INTRON CONTAINING PRE MRNA | 17 |
|  | KEGG: SPLICEOSOME | 16 |
|  | Reactome: POST TRANSLATIONAL PROTEIN MODIFICATION | 16 |
|  | Reactome: REGULATION OF MRNA STABILITY BY PROTEINS THAT BIND AU RICH ELEMENTS | 16 |
|  | Reactome: METABOLISM OF CARBOHYDRATES | 15 |
|  | Reactome: SIGNALING BY THE B CELL RECEPTOR BCR | 15 |
|  | Reactome: RNA POL II TRANSCRIPTION | 13 |
|  | BIOCARTA PROTEASOME PATHWAY | 11 |
|  | Reactome: GLUCOSE METABOLISM | 11 |
|  | PID: MYC ACTIVPATHWAY | 10 |
|  | KEGG: PYRIMIDINE METABOLISM | 9 |
|  | Reactome: GLUCONEOGENESIS | 9 |
|  | Reactome: RNA POL II PRE TRANSCRIPTION EVENTS | 9 |
|  | KEGG: RNA DEGRADATION | 7 |
|  | Reactome: FORMATION OF RNA POL II ELONGATION COMPLEX | 7 |
|  | BIOCARTA MPR PATHWAY | 6 |
|  | KEGG: NUCLEOTIDE EXCISION REPAIR | 6 |
|  | KEGG: PYRUVATE METABOLISM | 6 |
|  | Reactome: NUCLEOTIDE EXCISION REPAIR | 6 |
|  | Reactome: TRANSCRIPTION COUPLED NER TC NER | 6 |
|  | Reactome: TRANSPORT OF MATURE TRANSCRIPT TO CYTOPLASM | 6 |
|  | Reactome: PROTEIN FOLDING | 5 |
|  | KEGG: VIBRIO CHOLERAE INFECTION | 4 |
| Yellow | Reactome: NEUROTRANSMITTER RECEPTOR BINDING AND DOWNSTREAM TRANSMISSION IN THE POSTSYNAPTIC CELL | 6 |
|  | Reactome: 3 UTR MEDIATED TRANSLATIONAL REGULATION | 4 |
|  | Reactome: FORMATION OF THE TERNARY COMPLEX AND SUBSEQUENTLY THE 43S COMPLEX | 4 |
|  | Reactome: OPIOID SIGNALLING | 4 |
|  | KEGG: CALCIUM SIGNALING PATHWAY | 2 |
|  | KEGG: LONG TERM POTENTIATION | 2 |
|  | PID: CD8TCRDOWNSTREAMPATHWAY | 2 |
|  | BIOCARTA NFAT PATHWAY | 1 |
|  | KEGG: PURINE METABOLISM | 1 |
