# Supplementary material for: Integrating mRNA and miRNA Weighted Gene Co-Expression Networks with eQTLs in the Nucleus Accumbens of Subjects with Alcohol Dependence
Source: PLoS One. 2015 Sep 18;10(9):e0137671. doi: 10.1371/journal.pone.0137671 (PMC4575063; doi:10.1371/journal.pone.0137671)
Supplement: S6 Table — (DOCX) [file pone.0137671.s007.docx]

Table S6.

| mRNA | miRNA | correlation | p-value |
| --- | --- | --- | --- |
| 211951_at | hsa-miR-34c-3p_st | **-0.7503437739** | **0.0000012898** |
| 211566_x_at | hsa-miR-34c-3p_st | **-0.7133689840** | **0.0000046293** |
| 211951_at | hsa-miR-34c-5p_st | **-0.7115355233** | **0.0000049634** |
| 205550_s_at | hsa-miR-34c-3p_st | **-0.7100076394** | **0.0000052610** |
| 202975_s_at | hsa-miR-382_st | **-0.7070058849** | **0.0000059003** |
| 203889_at | hsa-miR-34c-3p_st | **-0.6999236058** | **0.0000077387** |
| 208946_s_at | hsa-miR-34c-5p_st | **-0.6919786096** | **0.0000104808** |
| 208678_at | hsa-miR-34c-3p_st | **-0.6916730328** | **0.0000106032** |
| 208911_s_at | hsa-miR-34c-3p_st | **-0.6892284186** | **0.0000116336** |
| 213503_x_at | hsa-miR-4720-3p_st | **-0.6873949580** | **0.0000124688** |
| 204125_at | hsa-miR-34c-3p_st | **-0.6785332315** | **0.0000173737** |
| 211763_s_at | hsa-miR-34c-5p_st | **-0.6782276547** | **0.0000175716** |
| 211763_s_at | hsa-miR-34c-3p_st | **-0.6779220779** | **0.0000177716** |
| 202252_at | hsa-miR-377-star_st | **-0.6776165011** | **0.0000179738** |
| 202370_s_at | hsa-miR-377-star_st | **-0.6748663102** | **0.0000198926** |
| 213714_at | hsa-miR-34c-3p_st | **-0.6736440031** | **0.0000208054** |
| 211566_x_at | hsa-miR-34c-5p_st | **-0.6702826585** | **0.0000235202** |
| 200786_at | hsa-miR-34c-3p_st | **-0.6699770817** | **0.0000237827** |
| 208911_s_at | hsa-miR-34c-5p_st | **-0.6693659282** | **0.0000243157** |
| 201590_x_at | hsa-miR-4720-3p_st | **-0.6675324675** | **0.0000259819** |
| 217882_at | hsa-miR-34c-3p_st | **-0.6649858679** | **0.0000284715** |
| 201180_s_at | hsa-miR-4760-3p_st | **-0.6647822765** | **0.0000286798** |
| 209755_at | hsa-miR-34c-3p_st | **-0.6644766998** | **0.0000289949** |
| 205550_s_at | hsa-miR-34c-5p_st | **-0.6638655462** | **0.0000296349** |
| 208782_at | hsa-miR-1912_st | **-0.6638655462** | **0.0000296349** |
| 202395_at | hsa-miR-34c-3p_st | **-0.6632543927** | **0.0000302878** |
| 201756_at | hsa-miR-34c-3p_st | **-0.6629488159** | **0.0000306192** |
| 218133_s_at | hsa-miR-34c-3p_st | **-0.6614209320** | **0.0000323267** |
| 207812_s_at | hsa-miR-34c-3p_st | **-0.6608097785** | **0.0000330338** |
| 218788_s_at | hsa-miR-34c-3p_st | **-0.6608097785** | **0.0000330338** |
| 200662_s_at | hsa-miR-34c-3p_st | **-0.6605042017** | **0.0000333927** |
| 208946_s_at | hsa-miR-34b-star_st | **-0.6605042017** | **0.0000333927** |
| 210427_x_at | hsa-miR-4720-3p_st | **-0.6589763178** | **0.0000352412** |
| 212645_x_at | hsa-miR-34c-3p_st | **-0.6583651642** | **0.0000360065** |
| 205005_s_at | hsa-miR-34c-5p_st | **-0.6580595875** | **0.0000363949** |
| 214829_at | hsa-miR-1912_st | **-0.6580595875** | **0.0000363949** |
| 217286_s_at | hsa-miR-34b-star_st | **-0.6580595875** | **0.0000363949** |
| 218316_at | hsa-miR-34c-3p_st | **-0.6577540107** | **0.0000367870** |
| 207776_s_at | hsa-miR-34c-3p_st | **-0.6559205500** | **0.0000392223** |
| 206805_at | hsa-miR-34c-5p_st | **-0.6556149733** | **0.0000396422** |
| 204125_at | hsa-miR-34c-5p_st | **-0.6550038197** | **0.0000404943** |
| 210817_s_at | hsa-miR-377-star_st | **-0.6546982429** | **0.0000409266** |
| 208946_s_at | hsa-miR-34c-3p_st | **-0.6543926662** | **0.0000413631** |
| 217286_s_at | hsa-miR-34c-5p_st | **-0.6537815126** | **0.0000422490** |
| 205856_at | hsa-miR-4720-3p_st | **-0.6501145913** | **0.0000479369** |
| 217286_s_at | hsa-miR-34c-3p_st | **-0.6498090145** | **0.0000484410** |
| 36711_at | hsa-miR-4720-3p_st | **-0.6482811306** | **0.0000510346** |
| 200786_at | hsa-miR-34c-5p_st | **-0.6479755539** | **0.0000515683** |
| 202252_at | hsa-miR-1912_st | **-0.6470588235** | **0.0000531997** |
| 205856_at | hsa-miR-1912_st | **-0.6461420932** | **0.0000548778** |
| 200916_at | hsa-miR-377-star_st | **-0.6455309396** | **0.0000560231** |
| 201198_s_at | hsa-miR-34c-3p_st | **-0.6452253629** | **0.0000566038** |
| 201924_at | hsa-miR-377-star_st | **-0.6452253629** | **0.0000566038** |
| 205196_s_at | hsa-miR-34c-3p_st | **-0.6449197861** | **0.0000571900** |
| 205005_s_at | hsa-miR-34c-3p_st | **-0.6443086325** | **0.0000583789** |
| 206805_at | hsa-miR-34c-3p_st | **-0.6436974790** | **0.0000595902** |
| 217923_at | hsa-miR-34c-3p_st | **-0.6436974790** | **0.0000595902** |
| 203150_at | hsa-miR-34c-3p_st | **-0.6418640183** | **0.0000633619** |
| 211763_s_at | hsa-miR-34b-star_st | **-0.6412528648** | **0.0000646664** |
| 203033_x_at | hsa-miR-34c-3p_st | **-0.6409472880** | **0.0000653277** |
| 211951_at | hsa-miR-34b-star_st | **-0.6394194041** | **0.0000687269** |
| 217882_at | hsa-miR-34c-5p_st | **-0.6383986572** | **0.0000710861** |
| 217968_at | hsa-miR-34c-3p_st | **-0.6375859435** | **0.0000730165** |
| 218316_at | hsa-miR-34c-5p_st | **-0.6375859435** | **0.0000730165** |
| 212645_x_at | hsa-miR-34c-5p_st | **-0.6366692131** | **0.0000752507** |
| 209248_at | hsa-miR-34c-3p_st | **-0.6364122564** | **0.0000758880** |
| 208911_s_at | hsa-miR-34b-star_st | **-0.6363636364** | **0.0000760091** |
| 208826_x_at | hsa-miR-34c-3p_st | **-0.6360580596** | **0.0000767743** |
| 200662_s_at | hsa-miR-34c-5p_st | **-0.6351413293** | **0.0000791120** |
| 203094_at | hsa-miR-34c-3p_st | **-0.6348357525** | **0.0000799054** |
| 206935_at | hsa-miR-34c-3p_st | **-0.6348357525** | **0.0000799054** |
| 208678_at | hsa-miR-34c-5p_st | **-0.6330022918** | **0.0000848182** |
| 200916_at | hsa-miR-1912_st | **-0.6326967150** | **0.0000856630** |
| 205202_at | hsa-miR-34c-3p_st | **-0.6323911383** | **0.0000865154** |
| 204744_s_at | hsa-miR-34c-3p_st | **-0.6320855615** | **0.0000873754** |
| 208991_at | hsa-miR-4760-3p_st | **-0.6320855615** | **0.0000873754** |
| 201180_s_at | hsa-miR-377-star_st | **-0.6317799847** | **0.0000882431** |
| 203889_at | hsa-miR-34c-5p_st | **-0.6314744079** | **0.0000891185** |
| 202370_s_at | hsa-miR-132_st | **-0.6308632544** | **0.0000908929** |
| 203157_s_at | hsa-miR-34c-3p_st | **-0.6287242170** | **0.0000973568** |
| 48531_at | hsa-miR-377-star_st | **-0.6287242170** | **0.0000973568** |
| 205196_s_at | hsa-miR-34c-5p_st | **-0.6259740260** | **0.0001062743** |
| 218133_s_at | hsa-miR-34c-5p_st | **-0.6250572956** | **0.0001094060** |
| 202370_s_at | hsa-miR-1912_st | **-0.6244461421** | **0.0001115397** |
| 203816_at | hsa-miR-34c-3p_st | **-0.6238349885** | **0.0001137107** |
| 205633_s_at | hsa-miR-34c-3p_st | **-0.6235294118** | **0.0001148104** |
| 200816_s_at | hsa-miR-34c-3p_st | **-0.6229182582** | **0.0001170385** |
| 206042_x_at | hsa-miR-34c-3p_st | **-0.6226126814** | **0.0001181670** |
| 207507_s_at | hsa-miR-34c-3p_st | **-0.6216959511** | **0.0001216115** |
| 202641_at | hsa-miR-34c-3p_st | **-0.6213903743** | **0.0001227795** |
| 201272_at | hsa-miR-34c-3p_st | **-0.6210847976** | **0.0001239576** |
| 201756_at | hsa-miR-34c-5p_st | **-0.6210847976** | **0.0001239576** |
| 202507_s_at | hsa-miR-34c-3p_st | **-0.6207792208** | **0.0001251458** |
| 208991_at | hsa-miR-377-star_st | **-0.6207792208** | **0.0001251458** |
| 1007_s_at | hsa-miR-1912_st | **-0.6201680672** | **0.0001275530** |
| 213738_s_at | hsa-miR-34c-3p_st | **-0.6201680672** | **0.0001275530** |
| 212038_s_at | hsa-miR-34c-3p_st | **-0.6198624905** | **0.0001287721** |
| 204992_s_at | hsa-miR-34c-3p_st | **-0.6195569137** | **0.0001300017** |
| 215416_s_at | hsa-miR-34c-3p_st | **-0.6195569137** | **0.0001300017** |
| 204587_at | hsa-miR-34c-3p_st | **-0.6192513369** | **0.0001312417** |
| 208838_at | hsa-miR-34c-3p_st | **-0.6186401833** | **0.0001337538** |
| 207830_s_at | hsa-miR-34c-3p_st | **-0.6177234530** | **0.0001376028** |
| 201484_at | hsa-miR-34c-3p_st | **-0.6161955691** | **0.0001442390** |
| 203159_at | hsa-miR-34c-3p_st | **-0.6161955691** | **0.0001442390** |
| 201999_s_at | hsa-miR-1912_st | **-0.6158899924** | **0.0001456002** |
| 212727_at | hsa-miR-34c-3p_st | **-0.6155844156** | **0.0001469728** |
| 201315_x_at | hsa-miR-1912_st | **-0.6149732620** | **0.0001497530** |
| 201753_s_at | hsa-miR-382_st | **-0.6142562475** | **0.0001530748** |
| 203560_at | hsa-miR-34c-3p_st | **-0.6137509549** | **0.0001554552** |
| 201256_at | hsa-miR-34c-3p_st | **-0.6134453782** | **0.0001569108** |
| 209755_at | hsa-miR-34c-5p_st | **-0.6134453782** | **0.0001569108** |
| 200916_at | hsa-miR-4720-3p_st | **-0.6125286478** | **0.0001613511** |
| 205110_s_at | hsa-miR-34c-3p_st | **-0.6125286478** | **0.0001613511** |
| 200986_at | hsa-miR-1912_st | **-0.6119174943** | **0.0001643735** |
| 211566_x_at | hsa-miR-34b-star_st | **-0.6116119175** | **0.0001659036** |
| 218976_at | hsa-miR-34c-3p_st | **-0.6113063407** | **0.0001674465** |
| 202395_at | hsa-miR-34c-5p_st | **-0.6110007639** | **0.0001690022** |
| 205633_s_at | hsa-miR-34c-5p_st | **-0.6097784568** | **0.0001753553** |
| 203313_s_at | hsa-miR-4720-3p_st | **-0.6091673033** | **0.0001786112** |
| 205550_s_at | hsa-miR-34b-star_st | **-0.6085561497** | **0.0001819212** |
| 211615_s_at | hsa-miR-34c-3p_st | **-0.6085561497** | **0.0001819212** |
| 212645_x_at | hsa-miR-34b-star_st | **-0.6085561497** | **0.0001819212** |
| 217936_at | hsa-miR-1912_st | **-0.6085561497** | **0.0001819212** |
| 219481_at | hsa-miR-34c-3p_st | **-0.6085561497** | **0.0001819212** |
| 200818_at | hsa-miR-34c-3p_st | **-0.6082505730** | **0.0001835967** |
| 210156_s_at | hsa-miR-34c-3p_st | **-0.6082505730** | **0.0001835967** |
| 214717_at | hsa-miR-34c-3p_st | **-0.6082505730** | **0.0001835967** |
| 204068_at | hsa-miR-377-star_st | **-0.6079449962** | **0.0001852859** |
| 210117_at | hsa-miR-4720-3p_st | **-0.6079449962** | **0.0001852859** |
| 212961_x_at | hsa-miR-34c-3p_st | **-0.6076394194** | **0.0001869891** |
| 202507_s_at | hsa-miR-34c-5p_st | **-0.6073338426** | **0.0001887062** |
| 203983_at | hsa-miR-34c-3p_st | **-0.6067226891** | **0.0001921829** |
| 205775_at | hsa-miR-34c-3p_st | **-0.6067226891** | **0.0001921829** |
| 203150_at | hsa-miR-34c-5p_st | **-0.6064171123** | **0.0001939426** |
| 218788_s_at | hsa-miR-34c-5p_st | **-0.6064171123** | **0.0001939426** |
| 201966_at | hsa-miR-34c-3p_st | **-0.6055003820** | **0.0001993086** |
| 201999_s_at | hsa-miR-4760-3p_st | **-0.6055003820** | **0.0001993086** |
| 204070_at | hsa-miR-1912_st | **-0.6051948052** | **0.0002011265** |
| 201924_at | hsa-miR-1912_st | **-0.6045836516** | **0.0002048069** |
| 212990_at | hsa-miR-34c-3p_st | **-0.6039724981** | **0.0002085473** |
| 210240_s_at | hsa-miR-34c-3p_st | **-0.6033613445** | **0.0002123487** |
| 208857_s_at | hsa-miR-34c-3p_st | **-0.6030557678** | **0.0002142725** |
| 212961_x_at | hsa-miR-34c-5p_st | **-0.6027501910** | **0.0002162119** |
| 218283_at | hsa-miR-34c-5p_st | **-0.6026434428** | **0.0002168931** |
| 201054_at | hsa-miR-34c-3p_st | **-0.6024446142** | **0.0002181669** |
| 202430_s_at | hsa-miR-4311_st | **-0.6021850426** | **0.0002198400** |
| 203033_x_at | hsa-miR-34c-5p_st | **-0.6021390374** | **0.0002201377** |
| 203094_at | hsa-miR-34c-5p_st | **-0.6021390374** | **0.0002201377** |
| 203973_s_at | hsa-miR-4720-3p_st | **-0.6021390374** | **0.0002201377** |
| 205279_s_at | hsa-miR-34c-3p_st | **-0.6021390374** | **0.0002201377** |
| 207812_s_at | hsa-miR-34c-5p_st | **-0.6021390374** | **0.0002201377** |
| 212038_s_at | hsa-miR-34c-5p_st | **-0.6021390374** | **0.0002201377** |
| 212203_x_at | hsa-miR-1912_st | **-0.6015278839** | **0.0002241272** |
| 203033_x_at | hsa-miR-34b-star_st | **-0.6006111536** | **0.0002302324** |
| 207830_s_at | hsa-miR-34c-5p_st | **-0.6006111536** | **0.0002302324** |
| 221699_s_at | hsa-miR-34c-3p_st | **-0.6003055768** | **0.0002323003** |
| 205856_at | hsa-miR-377-star_st | **-0.6000000000** | **0.0002343847** |
| 208826_x_at | hsa-miR-34c-5p_st | **-0.6000000000** | **0.0002343847** |
| 212271_at | hsa-miR-34c-3p_st | **-0.6000000000** | **0.0002343847** |
| 218283_at | hsa-miR-34b-star_st | **-0.5997402416** | **0.0002361696** |
| 204119_s_at | hsa-miR-34c-3p_st | **-0.5996944232** | **0.0002364858** |
| 206875_s_at | hsa-miR-34b-star_st | **-0.5996944232** | **0.0002364858** |
| 208909_at | hsa-miR-34c-3p_st | **-0.5993888464** | **0.0002386037** |
| 201198_s_at | hsa-miR-34c-5p_st | **-0.5987776929** | **0.0002428903** |
| 210749_x_at | hsa-miR-1912_st | **-0.5984721161** | **0.0002450593** |
| 213714_at | hsa-miR-34c-5p_st | **-0.5981665393** | **0.0002472456** |
| 204744_s_at | hsa-miR-34c-5p_st | **-0.5978609626** | **0.0002494493** |
| 200662_s_at | hsa-miR-34b-star_st | **-0.5975553858** | **0.0002516704** |
| 217820_s_at | hsa-miR-1912_st | **-0.5975553858** | **0.0002516704** |
| 203156_at | hsa-miR-34c-3p_st | **-0.5969442322** | **0.0002561658** |
| 208745_at | hsa-miR-34c-3p_st | **-0.5969442322** | **0.0002561658** |
| 215416_s_at | hsa-miR-34c-5p_st | **-0.5966386555** | **0.0002584403** |
| 202948_at | hsa-miR-4720-3p_st | **-0.5951107716** | **0.0002700850** |
| 206935_at | hsa-miR-34c-5p_st | **-0.5948051948** | **0.0002724694** |
| 203983_at | hsa-miR-34c-5p_st | **-0.5941940413** | **0.0002772944** |
| 200079_s_at | hsa-miR-34c-3p_st | **-0.5938884645** | **0.0002797354** |
| 209122_at | hsa-miR-377-star_st | **-0.5935828877** | **0.0002821955** |
| 202543_s_at | hsa-miR-377-star_st | **-0.5929717341** | **0.0002871738** |
| 210534_s_at | hsa-miR-34c-3p_st | **-0.5928642389** | **0.0002880574** |
| 218316_at | hsa-miR-34b-star_st | **-0.5926661574** | **0.0002896921** |
| 208839_s_at | hsa-miR-34c-3p_st | **-0.5923605806** | **0.0002922301** |
| 200916_at | hsa-miR-4760-3p_st | **-0.5920550038** | **0.0002947879** |
| 207776_s_at | hsa-miR-34c-5p_st | **-0.5917494270** | **0.0002973657** |
| 216218_s_at | hsa-miR-34c-3p_st | **-0.5917494270** | **0.0002973657** |
| 217906_at | hsa-miR-34c-3p_st | **-0.5917494270** | **0.0002973657** |
| 200786_at | hsa-miR-34b-star_st | **-0.5914438503** | **0.0002999635** |
| 203157_s_at | hsa-miR-34c-5p_st | **-0.5914438503** | **0.0002999635** |
| 207643_s_at | hsa-miR-1912_st | **-0.5911382735** | **0.0003025816** |
| 209549_s_at | hsa-miR-34c-3p_st | **-0.5911382735** | **0.0003025816** |
| 218283_at | hsa-miR-34c-3p_st | **-0.5910306381** | **0.0003035086** |
| 205278_at | hsa-miR-34c-3p_st | **-0.5908326967** | **0.0003052200** |
| 201272_at | hsa-miR-34c-5p_st | **-0.5902215432** | **0.0003105583** |
| 212053_at | hsa-miR-34c-3p_st | **-0.5896103896** | **0.0003159797** |
| 202376_at | hsa-miR-1912_st | **-0.5889992361** | **0.0003214853** |
| 204125_at | hsa-miR-34b-star_st | **-0.5883880825** | **0.0003270761** |
| 221449_s_at | hsa-miR-34c-3p_st | **-0.5880825057** | **0.0003299039** |
| 203723_at | hsa-miR-1912_st | **-0.5878218368** | **0.0003323333** |
| 202670_at | hsa-miR-34c-3p_st | **-0.5874713522** | **0.0003356248** |
| 200720_s_at | hsa-miR-34c-3p_st | **-0.5871657754** | **0.0003385183** |
| 205531_s_at | hsa-miR-34c-3p_st | **-0.5871657754** | **0.0003385183** |
| 208832_at | hsa-miR-34c-3p_st | **-0.5871657754** | **0.0003385183** |
| 207507_s_at | hsa-miR-34c-5p_st | **-0.5868601986** | **0.0003414340** |
| 200663_at | hsa-miR-1912_st | **-0.5862490451** | **0.0003473325** |
| 205005_s_at | hsa-miR-34b-star_st | **-0.5862490451** | **0.0003473325** |
| 205031_at | hsa-miR-34c-3p_st | **-0.5862490451** | **0.0003473325** |
| 217968_at | hsa-miR-34c-5p_st | **-0.5862490451** | **0.0003473325** |
| 200978_at | hsa-miR-34c-3p_st | **-0.5856378915** | **0.0003533215** |
| 205279_s_at | hsa-miR-34c-5p_st | **-0.5850267380** | **0.0003594024** |
| 211297_s_at | hsa-miR-34c-3p_st | **-0.5847211612** | **0.0003624776** |
| 209248_at | hsa-miR-34c-5p_st | **-0.5844602355** | **0.0003651219** |
| 205489_at | hsa-miR-34c-3p_st | **-0.5841100076** | **0.0003686983** |
| 209228_x_at | hsa-miR-34c-3p_st | **-0.5841100076** | **0.0003686983** |
| 206875_s_at | hsa-miR-34c-5p_st | **-0.5834988541** | **0.0003750139** |
| 222230_s_at | hsa-miR-34c-3p_st | **-0.5834988541** | **0.0003750139** |
| 205202_at | hsa-miR-34c-5p_st | **-0.5831932773** | **0.0003782076** |
| 210418_s_at | hsa-miR-34c-3p_st | **-0.5828877005** | **0.0003814256** |
| 218597_s_at | hsa-miR-34c-3p_st | **-0.5825821238** | **0.0003846678** |
| 213293_s_at | hsa-miR-1912_st | **-0.5822765470** | **0.0003879346** |
| 202077_at | hsa-miR-34c-3p_st | **-0.5819709702** | **0.0003912260** |
| 203079_s_at | hsa-miR-34c-3p_st | **-0.5816653934** | **0.0003945423** |
| 217286_s_at | hsa-miR-375_st | **-0.5816653934** | **0.0003945423** |
| 201590_x_at | hsa-miR-1912_st | **-0.5807486631** | **0.0004046415** |
| 201592_at | hsa-miR-34c-3p_st | **-0.5807486631** | **0.0004046415** |
| 202825_at | hsa-miR-34c-3p_st | **-0.5807486631** | **0.0004046415** |
| 208678_at | hsa-miR-34b-star_st | **-0.5807486631** | **0.0004046415** |
| 210240_s_at | hsa-miR-34c-5p_st | **-0.5804430863** | **0.0004080587** |
| 202948_at | hsa-miR-377-star_st | **-0.5798319328** | **0.0004149701** |
| 203983_at | hsa-miR-34b-star_st | **-0.5795263560** | **0.0004184646** |
| 201989_s_at | hsa-miR-34c-3p_st | **-0.5792207792** | **0.0004219853** |
| 219481_at | hsa-miR-34c-5p_st | **-0.5789152024** | **0.0004255324** |
| 200843_s_at | hsa-miR-34c-3p_st | **-0.5786096257** | **0.0004291059** |
| 208946_s_at | hsa-miR-34b_st | **-0.5786096257** | **0.0004291059** |
| 206062_at | hsa-miR-34c-3p_st | **-0.5783040489** | **0.0004327060** |
| 217882_at | hsa-miR-34b-star_st | **-0.5777370328** | **0.0004394576** |
| 202975_s_at | hsa-miR-1912_st | **-0.5776928953** | **0.0004399870** |
| 203817_at | hsa-miR-34c-3p_st | **-0.5776928953** | **0.0004399870** |
| 218654_s_at | hsa-miR-34c-3p_st | **-0.5776928953** | **0.0004399870** |
| 201527_at | hsa-miR-34c-3p_st | **-0.5773873186** | **0.0004436682** |
| 212271_at | hsa-miR-34c-5p_st | **-0.5773873186** | **0.0004436682** |
| 220251_at | hsa-miR-34c-3p_st | **-0.5773873186** | **0.0004436682** |
| 205196_s_at | hsa-miR-34b-star_st | **-0.5770817418** | **0.0004473767** |
| 206015_s_at | hsa-miR-34c-3p_st | **-0.5770817418** | **0.0004473767** |
| 201756_at | hsa-miR-34b-star_st | **-0.5764705882** | **0.0004548765** |
| 219714_s_at | hsa-miR-34c-3p_st | **-0.5764705882** | **0.0004548765** |
| 200818_at | hsa-miR-34c-5p_st | **-0.5761650115** | **0.0004586681** |
| 201966_at | hsa-miR-34c-5p_st | **-0.5761650115** | **0.0004586681** |
| 204119_s_at | hsa-miR-34c-5p_st | **-0.5761650115** | **0.0004586681** |
| 205110_s_at | hsa-miR-34c-5p_st | **-0.5758594347** | **0.0004624877** |
| 200677_at | hsa-miR-377-star_st | **-0.5755538579** | **0.0004663356** |
| 201628_s_at | hsa-miR-34c-3p_st | **-0.5752482811** | **0.0004702120** |
| 211658_at | hsa-miR-34c-3p_st | **-0.5752482811** | **0.0004702120** |
| 217906_at | hsa-miR-34c-5p_st | **-0.5749427044** | **0.0004741169** |
| 201999_s_at | hsa-miR-377-star_st | **-0.5743315508** | **0.0004820133** |
| 202078_at | hsa-miR-34c-3p_st | **-0.5743315508** | **0.0004820133** |
| 206805_at | hsa-miR-34b-star_st | **-0.5743315508** | **0.0004820133** |
| 208121_s_at | hsa-miR-34c-3p_st | **-0.5743315508** | **0.0004820133** |
| 203362_s_at | hsa-miR-34c-3p_st | **-0.5731092437** | **0.0004981573** |
| 212215_at | hsa-miR-34c-3p_st | **-0.5731092437** | **0.0004981573** |
| 200820_at | hsa-miR-34c-3p_st | **-0.5728036669** | **0.0005022676** |
| 200883_at | hsa-miR-34c-3p_st | **-0.5728036669** | **0.0005022676** |
| 201484_at | hsa-miR-34c-5p_st | **-0.5728036669** | **0.0005022676** |
| 202370_s_at | hsa-miR-4760-3p_st | **-0.5728036669** | **0.0005022676** |
| 218866_s_at | hsa-miR-34c-3p_st | **-0.5728036669** | **0.0005022676** |
| 202269_x_at | hsa-miR-1912_st | **-0.5724980901** | **0.0005064080** |
| 215307_at | hsa-miR-34c-3p_st | **-0.5721925134** | **0.0005105787** |
| 204245_s_at | hsa-miR-34c-5p_st | **-0.5712757830** | **0.0005232742** |
| 215884_s_at | hsa-miR-34c-3p_st | **-0.5712757830** | **0.0005232742** |
| 218667_at | hsa-miR-34c-3p_st | **-0.5712757830** | **0.0005232742** |
| 218854_at | hsa-miR-4311_st | **-0.5710138301** | **0.0005269531** |
| 203156_at | hsa-miR-34c-5p_st | **-0.5709702063** | **0.0005275679** |
| 201012_at | hsa-miR-1912_st | **-0.5706646295** | **0.0005318929** |
| 209122_at | hsa-miR-382_st | **-0.5705554299** | **0.0005334460** |
| 208779_x_at | hsa-miR-1912_st | **-0.5700534759** | **0.0005406373** |
| 211566_x_at | hsa-miR-375_st | **-0.5700534759** | **0.0005406373** |
| 212727_at | hsa-miR-34c-5p_st | **-0.5700534759** | **0.0005406373** |
| 200816_s_at | hsa-miR-34c-5p_st | **-0.5697478992** | **0.0005450571** |
| 202741_at | hsa-miR-34c-3p_st | **-0.5697478992** | **0.0005450571** |
| 205856_at | hsa-miR-382_st | **-0.5696386295** | **0.0005466454** |
| 207830_s_at | hsa-miR-34b-star_st | **-0.5694423224** | **0.0005495090** |
| 210427_x_at | hsa-miR-1912_st | **-0.5694423224** | **0.0005495090** |
| 221741_s_at | hsa-miR-4311_st | **-0.5693330294** | **0.0005511091** |
| 210156_s_at | hsa-miR-34c-5p_st | **-0.5691367456** | **0.0005539932** |
| 201112_s_at | hsa-miR-34c-3p_st | **-0.5688311688** | **0.0005585097** |
| 218160_at | hsa-miR-34c-3p_st | **-0.5688311688** | **0.0005585097** |
| 212687_at | hsa-miR-4311_st | **-0.5687218291** | **0.0005601338** |
| 200677_at | hsa-miR-1912_st | **-0.5685255921** | **0.0005630590** |
| 200822_x_at | hsa-miR-34c-3p_st | **-0.5685255921** | **0.0005630590** |
| 203816_at | hsa-miR-34c-5p_st | **-0.5685255921** | **0.0005630590** |
| 218976_at | hsa-miR-34c-5p_st | **-0.5685255921** | **0.0005630590** |
| 204125_at | hsa-miR-34b_st | **-0.5682200153** | **0.0005676410** |
| 218133_s_at | hsa-miR-34b-star_st | **-0.5682200153** | **0.0005676410** |
| 200614_at | hsa-miR-34c-3p_st | **-0.5679578288** | **0.0005715988** |
| 217820_s_at | hsa-miR-377-star_st | **-0.5676088617** | **0.0005769045** |
| 216218_s_at | hsa-miR-34c-5p_st | **-0.5673032850** | **0.0005815864** |
| 218292_s_at | hsa-miR-34c-5p_st | **-0.5673032850** | **0.0005815864** |
| 209733_at | hsa-miR-34c-3p_st | **-0.5666921314** | **0.0005910513** |
| 203944_x_at | hsa-miR-34c-3p_st | **-0.5663865546** | **0.0005958348** |
| 205775_at | hsa-miR-34c-5p_st | **-0.5663865546** | **0.0005958348** |
| 209075_s_at | hsa-miR-34c-3p_st | **-0.5663865546** | **0.0005958348** |
| 218656_s_at | hsa-miR-1912_st | **-0.5663865546** | **0.0005958348** |
| 201753_s_at | hsa-miR-1912_st | **-0.5654698243** | **0.0006103919** |
| 202133_at | hsa-miR-4720-3p_st | **-0.5648586707** | **0.0006202710** |
| 208868_s_at | hsa-miR-34c-3p_st | **-0.5645530940** | **0.0006252635** |
| 221449_s_at | hsa-miR-34c-5p_st | **-0.5642475172** | **0.0006302917** |
| 201443_s_at | hsa-miR-34c-3p_st | **-0.5636363636** | **0.0006404555** |
| 212217_at | hsa-miR-34c-3p_st | **-0.5636363636** | **0.0006404555** |
| 208827_at | hsa-miR-34c-3p_st | **-0.5633307869** | **0.0006455917** |
| 211855_s_at | hsa-miR-34c-3p_st | **-0.5633307869** | **0.0006455917** |
| 200905_x_at | hsa-miR-132_st | **-0.5630252101** | **0.0006507644** |
| 202180_s_at | hsa-miR-4760-3p_st | **-0.5630252101** | **0.0006507644** |
| 202594_at | hsa-miR-34c-3p_st | **-0.5627196333** | **0.0006559738** |
| 210149_s_at | hsa-miR-34c-3p_st | **-0.5624140565** | **0.0006612201** |
| 217923_at | hsa-miR-34c-5p_st | **-0.5624140565** | **0.0006612201** |
| 206875_s_at | hsa-miR-34c-3p_st | **-0.5621084798** | **0.0006665035** |
| 203889_at | hsa-miR-34b-star_st | **-0.5618029030** | **0.0006718244** |
| 210817_s_at | hsa-miR-132_st | **-0.5614973262** | **0.0006771828** |
| 208909_at | hsa-miR-34c-5p_st | **-0.5611917494** | **0.0006825791** |
| 201054_at | hsa-miR-34c-5p_st | **-0.5605805959** | **0.0006934860** |
| 201315_x_at | hsa-miR-4720-3p_st | **-0.5605805959** | **0.0006934860** |
| 202948_at | hsa-miR-1912_st | **-0.5605805959** | **0.0006934860** |
| 204957_at | hsa-miR-34c-3p_st | **-0.5605805959** | **0.0006934860** |
| 207643_s_at | hsa-miR-212_st | **-0.5605805959** | **0.0006934860** |
| 200641_s_at | hsa-miR-34c-3p_st | **-0.5602750191** | **0.0006989972** |
| 201274_at | hsa-miR-34c-5p_st | **-0.5602750191** | **0.0006989972** |
| 201443_s_at | hsa-miR-34c-5p_st | **-0.5602750191** | **0.0006989972** |
| 217968_at | hsa-miR-34b-star_st | **-0.5599694423** | **0.0007045471** |
| 218788_s_at | hsa-miR-34b-star_st | **-0.5599694423** | **0.0007045471** |
| 206042_x_at | hsa-miR-34c-5p_st | **-0.5596638655** | **0.0007101360** |
| 202868_s_at | hsa-miR-34c-3p_st | **-0.5593582888** | **0.0007157641** |
| 212038_s_at | hsa-miR-34b-star_st | **-0.5593582888** | **0.0007157641** |
| 218292_s_at | hsa-miR-34c-3p_st | **-0.5590527120** | **0.0007214318** |
| 202373_s_at | hsa-miR-34c-3p_st | **-0.5587471352** | **0.0007271391** |
| 201256_at | hsa-miR-34c-5p_st | **-0.5584415584** | **0.0007328863** |
| 201924_at | hsa-miR-132_st | **-0.5584415584** | **0.0007328863** |
| 215416_s_at | hsa-miR-34b-star_st | **-0.5584415584** | **0.0007328863** |
| 215522_at | hsa-miR-34c-3p_st | **-0.5584415584** | **0.0007328863** |
| 204245_s_at | hsa-miR-34c-3p_st | **-0.5581359817** | **0.0007386738** |
| 211558_s_at | hsa-miR-34c-3p_st | **-0.5581359817** | **0.0007386738** |
| 209248_at | hsa-miR-34b-star_st | **-0.5580258248** | **0.0007407700** |
| 202149_at | hsa-miR-377-star_st | **-0.5578304049** | **0.0007445017** |
| 204002_s_at | hsa-miR-34c-3p_st | **-0.5575248281** | **0.0007503702** |
| 213503_x_at | hsa-miR-4760-3p_st | **-0.5572192513** | **0.0007562797** |
| 217960_s_at | hsa-miR-34c-3p_st | **-0.5572192513** | **0.0007562797** |
| 206805_at | hsa-miR-375_st | **-0.5569136746** | **0.0007622303** |
| 213503_x_at | hsa-miR-1912_st | **-0.5566080978** | **0.0007682223** |
| 201315_x_at | hsa-miR-132_st | **-0.5559969442** | **0.0007803316** |
| 205280_at | hsa-miR-34c-3p_st | **-0.5559969442** | **0.0007803316** |
| 218322_s_at | hsa-miR-377-star_st | **-0.5559969442** | **0.0007803316** |
| 200663_at | hsa-miR-377-star_st | **-0.5556913675** | **0.0007864494** |
| 203560_at | hsa-miR-34c-5p_st | **-0.5556913675** | **0.0007864494** |
| 202252_at | hsa-miR-132_st | **-0.5550802139** | **0.0007988124** |
| 204070_at | hsa-miR-132_st | **-0.5550802139** | **0.0007988124** |
| 202868_s_at | hsa-miR-34c-5p_st | **-0.5547746371** | **0.0008050582** |
| 209507_at | hsa-miR-34c-3p_st | **-0.5547746371** | **0.0008050582** |
| 215527_at | hsa-miR-34c-3p_st | **-0.5547746371** | **0.0008050582** |
| 210978_s_at | hsa-miR-4720-3p_st | **-0.5544690604** | **0.0008113471** |
| 212820_at | hsa-miR-34c-3p_st | **-0.5544690604** | **0.0008113471** |
| 218656_s_at | hsa-miR-377-star_st | **-0.5544690604** | **0.0008113471** |
| 202975_s_at | hsa-miR-132_st | **-0.5541634836** | **0.0008176794** |
| 208745_at | hsa-miR-34c-5p_st | **-0.5541634836** | **0.0008176794** |
| 211769_x_at | hsa-miR-34c-5p_st | **-0.5541634836** | **0.0008176794** |
| 206544_x_at | hsa-miR-34c-3p_st | **-0.5538579068** | **0.0008240554** |
| 218656_s_at | hsa-miR-4720-3p_st | **-0.5535523300** | **0.0008304754** |
| 48531_at | hsa-miR-1912_st | **-0.5535523300** | **0.0008304754** |
| 212321_at | hsa-miR-382_st | **-0.5534418230** | **0.0008328080** |
| 203094_at | hsa-miR-34b-star_st | **-0.5529411765** | **0.0008434482** |
| 211318_s_at | hsa-miR-34c-3p_st | **-0.5529411765** | **0.0008434482** |
| 219481_at | hsa-miR-34b-star_st | **-0.5529411765** | **0.0008434482** |
| 202078_at | hsa-miR-34c-5p_st | **-0.5526355997** | **0.0008500016** |
| 202252_at | hsa-miR-212_st | **-0.5526355997** | **0.0008500016** |
| 209183_s_at | hsa-miR-4720-3p_st | **-0.5526355997** | **0.0008500016** |
| 212407_at | hsa-miR-34c-3p_st | **-0.5526355997** | **0.0008500016** |
| 200804_at | hsa-miR-370_st | **-0.5523300229** | **0.0008566000** |
| 206875_s_at | hsa-miR-375_st | **-0.5523300229** | **0.0008566000** |
| 202543_s_at | hsa-miR-4760-3p_st | **-0.5520244461** | **0.0008632436** |
| 204245_s_at | hsa-miR-34b-star_st | **-0.5517188694** | **0.0008699327** |
| 222230_s_at | hsa-miR-34c-5p_st | **-0.5517188694** | **0.0008699327** |
| 212203_x_at | hsa-miR-132_st | **-0.5514132926** | **0.0008766677** |
| 213738_s_at | hsa-miR-34c-5p_st | **-0.5514132926** | **0.0008766677** |
| 215884_s_at | hsa-miR-34c-5p_st | **-0.5514132926** | **0.0008766677** |
| 202670_at | hsa-miR-34c-5p_st | **-0.5511077158** | **0.0008834487** |
| 211615_s_at | hsa-miR-34c-5p_st | **-0.5511077158** | **0.0008834487** |
| 212203_x_at | hsa-miR-4720-3p_st | **-0.5511077158** | **0.0008834487** |
| 205705_at | hsa-miR-34c-3p_st | **-0.5504965623** | **0.0008971500** |
| 205512_s_at | hsa-miR-34c-3p_st | **-0.5501909855** | **0.0009040708** |
| 211318_s_at | hsa-miR-34c-5p_st | **-0.5501909855** | **0.0009040708** |
| 213592_at | hsa-miR-1912_st | **-0.5501909855** | **0.0009040708** |
| 212310_at | hsa-miR-34c-3p_st | **-0.5498854087** | **0.0009110388** |
| 208690_s_at | hsa-miR-4720-3p_st | **-0.5495798319** | **0.0009180542** |
| 208838_at | hsa-miR-34c-5p_st | **-0.5495798319** | **0.0009180542** |
| 210534_s_at | hsa-miR-34c-5p_st | **-0.5494690214** | **0.0009206099** |
| 201274_at | hsa-miR-34c-3p_st | **-0.5492742552** | **0.0009251173** |
| 201398_s_at | hsa-miR-377-star_st | **-0.5492742552** | **0.0009251173** |
| 202370_s_at | hsa-miR-383_st | **-0.5492742552** | **0.0009251173** |
| 203159_at | hsa-miR-34c-5p_st | **-0.5492742552** | **0.0009251173** |
| 208946_s_at | hsa-miR-375_st | **-0.5492742552** | **0.0009251173** |
| 202641_at | hsa-miR-34c-5p_st | **-0.5486631016** | **0.0009393877** |
| 202658_at | hsa-miR-34c-3p_st | **-0.5486631016** | **0.0009393877** |
| 205633_s_at | hsa-miR-34b-star_st | **-0.5486631016** | **0.0009393877** |
| 201924_at | hsa-miR-4720-3p_st | **-0.5483575248** | **0.0009465956** |
| 206062_at | hsa-miR-34c-5p_st | **-0.5483575248** | **0.0009465956** |
| 207643_s_at | hsa-miR-4720-3p_st | **-0.5483575248** | **0.0009465956** |
| 200905_x_at | hsa-miR-4760-3p_st | **-0.5480519481** | **0.0009538524** |
| 207507_s_at | hsa-miR-34b-star_st | **-0.5477463713** | **0.0009611582** |
| 211479_s_at | hsa-miR-34c-3p_st | **-0.5477463713** | **0.0009611582** |
| 202395_at | hsa-miR-34b-star_st | **-0.5471352177** | **0.0009759183** |
| 200862_at | hsa-miR-34c-3p_st | **-0.5462184874** | **0.0009984341** |
| 201012_at | hsa-miR-4720-3p_st | **-0.5462184874** | **0.0009984341** |
| 221688_s_at | hsa-miR-34c-3p_st | **-0.5462184874** | **0.0009984341** |
| 209108_at | hsa-miR-4720-3p_st | **-0.5459129106** | **0.0010060406** |
| 213217_at | hsa-miR-1912_st | **-0.5459129106** | **0.0010060406** |
| 201029_s_at | hsa-miR-1912_st | **-0.5456073338** | **0.0010136983** |
| 201988_s_at | hsa-miR-34c-3p_st | **-0.5456073338** | **0.0010136983** |
| 212727_at | hsa-miR-34b-star_st | **-0.5456073338** | **0.0010136983** |
| 212990_at | hsa-miR-34c-5p_st | **-0.5453017571** | **0.0010214074** |
| 201601_x_at | hsa-miR-4311_st | **-0.5451906197** | **0.0010242240** |
| 208857_s_at | hsa-miR-34c-5p_st | **-0.5449961803** | **0.0010291682** |
| 208826_x_at | hsa-miR-34b-star_st | **-0.5446906035** | **0.0010369811** |
| 218120_s_at | hsa-miR-34c-3p_st | **-0.5446906035** | **0.0010369811** |
| 201999_s_at | hsa-miR-132_st | **-0.5443850267** | **0.0010448463** |
| 202252_at | hsa-miR-4760-3p_st | **-0.5437738732** | **0.0010607348** |
| 208659_at | hsa-miR-1912_st | **-0.5437738732** | **0.0010607348** |
| 200677_at | hsa-miR-132_st | **-0.5434682964** | **0.0010687587** |
| 208679_s_at | hsa-miR-34c-3p_st | **-0.5434682964** | **0.0010687587** |
| 219714_s_at | hsa-miR-34c-5p_st | **-0.5434682964** | **0.0010687587** |
| 202825_at | hsa-miR-34c-5p_st | **-0.5431627196** | **0.0010768362** |
| 1007_s_at | hsa-miR-377-star_st | **-0.5428571429** | **0.0010849675** |
| 205489_at | hsa-miR-34c-5p_st | **-0.5425515661** | **0.0010931530** |
| 208799_at | hsa-miR-34c-3p_st | **-0.5425515661** | **0.0010931530** |
| 208813_at | hsa-miR-34c-3p_st | **-0.5425515661** | **0.0010931530** |
| 204992_s_at | hsa-miR-34c-5p_st | **-0.5422459893** | **0.0011013929** |
| 200812_at | hsa-miR-34c-3p_st | **-0.5419404125** | **0.0011096876** |
| 217837_s_at | hsa-miR-34c-5p_st | **-0.5419404125** | **0.0011096876** |
| 213496_at | hsa-miR-34c-3p_st | **-0.5413292590** | **0.0011264425** |
| 205005_s_at | hsa-miR-375_st | **-0.5410236822** | **0.0011349033** |
| 205278_at | hsa-miR-34c-5p_st | **-0.5410236822** | **0.0011349033** |
| 212321_at | hsa-miR-1912_st | **-0.5410236822** | **0.0011349033** |
| 221515_s_at | hsa-miR-34c-3p_st | **-0.5410236822** | **0.0011349033** |
| 202252_at | hsa-miR-4720-3p_st | **-0.5407181054** | **0.0011434202** |
| 208659_at | hsa-miR-377-star_st | **-0.5407181054** | **0.0011434202** |
| 214829_at | hsa-miR-377-star_st | **-0.5407181054** | **0.0011434202** |
| 201590_x_at | hsa-miR-377-star_st | **-0.5404125286** | **0.0011519934** |
| 201315_x_at | hsa-miR-377-star_st | **-0.5401069519** | **0.0011606233** |
| 208818_s_at | hsa-miR-132_st | **-0.5401069519** | **0.0011606233** |
| 210501_x_at | hsa-miR-34c-3p_st | **-0.5401069519** | **0.0011606233** |
| 213714_at | hsa-miR-34b-star_st | **-0.5401069519** | **0.0011606233** |
| 200816_s_at | hsa-miR-34b-star_st | **-0.5398013751** | **0.0011693101** |
| 201160_s_at | hsa-miR-4720-3p_st | **-0.5398013751** | **0.0011693101** |
| 210978_s_at | hsa-miR-4760-3p_st | **-0.5398013751** | **0.0011693101** |
| 214022_s_at | hsa-miR-377-star_st | **-0.5398013751** | **0.0011693101** |
| 48531_at | hsa-miR-132_st | **-0.5398013751** | **0.0011693101** |
| 209549_s_at | hsa-miR-34c-5p_st | **-0.5394957983** | **0.0011780542** |
| 211479_s_at | hsa-miR-34c-5p_st | **-0.5394957983** | **0.0011780542** |
| 218654_s_at | hsa-miR-34c-5p_st | **-0.5391902215** | **0.0011868560** |
| 200804_at | hsa-miR-4760-3p_st | **-0.5388846448** | **0.0011957156** |
| 202614_at | hsa-miR-34c-3p_st | **-0.5388846448** | **0.0011957156** |
| 207812_s_at | hsa-miR-34b-star_st | **-0.5388846448** | **0.0011957156** |
| 201590_x_at | hsa-miR-4760-3p_st | **-0.5385790680** | **0.0012046336** |
| 202591_s_at | hsa-miR-34c-3p_st | **-0.5385790680** | **0.0012046336** |
| 205856_at | hsa-miR-132_st | **-0.5385790680** | **0.0012046336** |
| 215307_at | hsa-miR-34c-5p_st | **-0.5385790680** | **0.0012046336** |
| 204587_at | hsa-miR-34c-5p_st | **-0.5379679144** | **0.0012226456** |
| 208991_at | hsa-miR-132_st | **-0.5373567609** | **0.0012408947** |
| 221958_s_at | hsa-miR-4720-3p_st | **-0.5367456073** | **0.0012593835** |
| 201272_at | hsa-miR-34b-star_st | **-0.5364400306** | **0.0012687186** |
| 208832_at | hsa-miR-34c-5p_st | **-0.5364400306** | **0.0012687186** |
| 211270_x_at | hsa-miR-1912_st | **-0.5364400306** | **0.0012687186** |
| 201256_at | hsa-miR-34b-star_st | **-0.5361344538** | **0.0012781146** |
| 201601_x_at | hsa-miR-1912_st | **-0.5361344538** | **0.0012781146** |
| 205512_s_at | hsa-miR-34b_st | **-0.5361344538** | **0.0012781146** |
| 221699_s_at | hsa-miR-34c-5p_st | **-0.5361344538** | **0.0012781146** |
| 205324_s_at | hsa-miR-34c-3p_st | **-0.5358288770** | **0.0012875719** |
| 206935_at | hsa-miR-34b-star_st | **-0.5355233002** | **0.0012970908** |
| 217820_s_at | hsa-miR-4720-3p_st | **-0.5355233002** | **0.0012970908** |
| 201411_s_at | hsa-miR-34c-5p_st | **-0.5352177235** | **0.0013066717** |
| 201411_s_at | hsa-miR-34c-3p_st | **-0.5352177235** | **0.0013066717** |
| 201966_at | hsa-miR-34b-star_st | **-0.5352177235** | **0.0013066717** |
| 201761_at | hsa-miR-377-star_st | **-0.5349121467** | **0.0013163149** |
| 201160_s_at | hsa-miR-377-star_st | **-0.5346065699** | **0.0013260207** |
| 208782_at | hsa-miR-377-star_st | **-0.5346065699** | **0.0013260207** |
| 214022_s_at | hsa-miR-1912_st | **-0.5346065699** | **0.0013260207** |
| 201412_at | hsa-miR-1912_st | **-0.5343009931** | **0.0013357895** |
| 208991_at | hsa-miR-1912_st | **-0.5339954163** | **0.0013456217** |
| 209755_at | hsa-miR-34b-star_st | **-0.5339954163** | **0.0013456217** |
| 208675_s_at | hsa-miR-34c-3p_st | **-0.5333842628** | **0.0013654775** |
| 200053_at | hsa-miR-34c-3p_st | **-0.5330786860** | **0.0013755018** |
| 205031_at | hsa-miR-34c-5p_st | **-0.5330786860** | **0.0013755018** |
| 202507_s_at | hsa-miR-34b-star_st | **-0.5324675325** | **0.0013957450** |
| 208782_at | hsa-miR-4760-3p_st | **-0.5324675325** | **0.0013957450** |
| 221696_s_at | hsa-miR-34c-3p_st | **-0.5324675325** | **0.0013957450** |
| 201666_at | hsa-miR-4762-5p_st | **-0.5321619557** | **0.0014059646** |
| 208911_s_at | hsa-miR-34b_st | **-0.5321619557** | **0.0014059646** |
| 215884_s_at | hsa-miR-34b-star_st | **-0.5321619557** | **0.0014059646** |
| 203079_s_at | hsa-miR-34c-5p_st | **-0.5318563789** | **0.0014162501** |
| 203189_s_at | hsa-miR-34c-3p_st | **-0.5318563789** | **0.0014162501** |
| 203362_s_at | hsa-miR-34c-5p_st | **-0.5318563789** | **0.0014162501** |
| 204070_at | hsa-miR-377-star_st | **-0.5318563789** | **0.0014162501** |
| 208869_s_at | hsa-miR-34c-3p_st | **-0.5318563789** | **0.0014162501** |
| 215307_at | hsa-miR-34b-star_st | **-0.5315508021** | **0.0014266017** |
| 201180_s_at | hsa-miR-132_st | **-0.5312452254** | **0.0014370199** |
| 203817_at | hsa-miR-34c-5p_st | **-0.5312452254** | **0.0014370199** |
| 210817_s_at | hsa-miR-1912_st | **-0.5312452254** | **0.0014370199** |
| 208827_at | hsa-miR-34c-5p_st | **-0.5309396486** | **0.0014475050** |
| 211769_x_at | hsa-miR-34c-3p_st | **-0.5309396486** | **0.0014475050** |
| 213887_s_at | hsa-miR-34c-3p_st | **-0.5309396486** | **0.0014475050** |
| 204326_x_at | hsa-miR-382_st | **-0.5306746138** | **0.0014566535** |
| 218160_at | hsa-miR-34c-5p_st | **-0.5303284950** | **0.0014686775** |
| 221488_s_at | hsa-miR-34c-3p_st | **-0.5303284950** | **0.0014686775** |
| 203723_at | hsa-miR-132_st | **-0.5300634136** | **0.0014779453** |
| 217837_s_at | hsa-miR-34c-3p_st | **-0.5300229183** | **0.0014793656** |
| 36711_at | hsa-miR-377-star_st | **-0.5300229183** | **0.0014793656** |
| 208659_at | hsa-miR-132_st | **-0.5297173415** | **0.0014901220** |
| 208839_s_at | hsa-miR-34c-5p_st | **-0.5297173415** | **0.0014901220** |
| 213503_x_at | hsa-miR-377-star_st | **-0.5297173415** | **0.0014901220** |
| 217936_at | hsa-miR-383_st | **-0.5297173415** | **0.0014901220** |
| 203560_at | hsa-miR-34b-star_st | **-0.5294117647** | **0.0015009473** |
| 222043_at | hsa-miR-1912_st | **-0.5294117647** | **0.0015009473** |
| 201410_at | hsa-miR-34c-3p_st | **-0.5291061879** | **0.0015118417** |
| 205110_s_at | hsa-miR-34b-star_st | **-0.5291061879** | **0.0015118417** |
| 213293_s_at | hsa-miR-377-star_st | **-0.5291061879** | **0.0015118417** |
| 218120_s_at | hsa-miR-34c-5p_st | **-0.5291061879** | **0.0015118417** |
| 212015_x_at | hsa-miR-1912_st | **-0.5288006112** | **0.0015228055** |
| 218224_at | hsa-miR-34c-3p_st | **-0.5288006112** | **0.0015228055** |
| 202370_s_at | hsa-miR-212_st | **-0.5284950344** | **0.0015338393** |
| 203411_s_at | hsa-miR-132_st | **-0.5284950344** | **0.0015338393** |
| 212887_at | hsa-miR-34c-3p_st | **-0.5284950344** | **0.0015338393** |
| 200641_s_at | hsa-miR-34c-5p_st | **-0.5278838808** | **0.0015561181** |
| 200720_s_at | hsa-miR-34c-5p_st | **-0.5278838808** | **0.0015561181** |
| 200818_at | hsa-miR-34b-star_st | **-0.5278838808** | **0.0015561181** |
| 210453_x_at | hsa-miR-34c-3p_st | **-0.5275783040** | **0.0015673638** |
| 201198_s_at | hsa-miR-34b-star_st | **-0.5272727273** | **0.0015786810** |
| 201725_at | hsa-miR-34c-3p_st | **-0.5272727273** | **0.0015786810** |
| 201180_s_at | hsa-miR-1912_st | **-0.5266615737** | **0.0016015311** |
| 201999_s_at | hsa-miR-1180_st | **-0.5263559969** | **0.0016130649** |
| 208911_s_at | hsa-miR-375_st | **-0.5263559969** | **0.0016130649** |
| 217906_at | hsa-miR-34b-star_st | **-0.5263559969** | **0.0016130649** |
| 217286_s_at | hsa-miR-34b_st | **-0.5257448434** | **0.0016363517** |
| 218133_s_at | hsa-miR-4652-3p_st | **-0.5257448434** | **0.0016363517** |
| 200820_at | hsa-miR-34c-5p_st | **-0.5254392666** | **0.0016481055** |
| 201761_at | hsa-miR-4720-3p_st | **-0.5254392666** | **0.0016481055** |
| 202269_x_at | hsa-miR-377-star_st | **-0.5254392666** | **0.0016481055** |
| 203621_at | hsa-miR-34c-3p_st | **-0.5254392666** | **0.0016481055** |
| 218667_at | hsa-miR-34c-5p_st | **-0.5254392666** | **0.0016481055** |
| 200862_at | hsa-miR-34c-5p_st | **-0.5251336898** | **0.0016599335** |
| 200903_s_at | hsa-miR-34c-3p_st | **-0.5251336898** | **0.0016599335** |
| 200986_at | hsa-miR-4720-3p_st | **-0.5251336898** | **0.0016599335** |
| 212600_s_at | hsa-miR-34c-3p_st | **-0.5251336898** | **0.0016599335** |
| 201628_s_at | hsa-miR-34c-5p_st | **-0.5248281131** | **0.0016718361** |
| 205202_at | hsa-miR-34b-star_st | **-0.5248281131** | **0.0016718361** |
| 208451_s_at | hsa-miR-4720-3p_st | **-0.5248281131** | **0.0016718361** |
| 217960_s_at | hsa-miR-34c-5p_st | **-0.5248281131** | **0.0016718361** |
| 201989_s_at | hsa-miR-34c-5p_st | **-0.5245225363** | **0.0016838136** |
| 204072_s_at | hsa-miR-34c-3p_st | **-0.5245225363** | **0.0016838136** |
| 208745_at | hsa-miR-34b-star_st | **-0.5245225363** | **0.0016838136** |
| 210014_x_at | hsa-miR-34c-3p_st | **-0.5245225363** | **0.0016838136** |
| 212053_at | hsa-miR-34c-5p_st | **-0.5245225363** | **0.0016838136** |
| 214717_at | hsa-miR-34c-5p_st | **-0.5245225363** | **0.0016838136** |
| 218316_at | hsa-miR-34b_st | **-0.5245225363** | **0.0016838136** |
| 200614_at | hsa-miR-34c-5p_st | **-0.5244098113** | **0.0016882510** |
| 201592_at | hsa-miR-34c-5p_st | **-0.5242169595** | **0.0016958665** |
| 212961_x_at | hsa-miR-34b_st | **-0.5242169595** | **0.0016958665** |
| 218597_s_at | hsa-miR-34c-5p_st | **-0.5242169595** | **0.0016958665** |
| 200916_at | hsa-miR-132_st | **-0.5239113827** | **0.0017079951** |
| 201106_at | hsa-miR-34c-3p_st | **-0.5239113827** | **0.0017079951** |
| 202180_s_at | hsa-miR-377-star_st | **-0.5239113827** | **0.0017079951** |
| 208679_s_at | hsa-miR-34c-5p_st | **-0.5239113827** | **0.0017079951** |
| 217936_at | hsa-miR-377-star_st | **-0.5239113827** | **0.0017079951** |
| 207831_x_at | hsa-miR-34c-3p_st | **-0.5236058060** | **0.0017201999** |
| 217820_s_at | hsa-miR-383_st | **-0.5233002292** | **0.0017324812** |
| 201398_s_at | hsa-miR-1912_st | **-0.5229946524** | **0.0017448396** |
| 203816_at | hsa-miR-34b-star_st | **-0.5226890756** | **0.0017572753** |
| 205279_s_at | hsa-miR-34b-star_st | **-0.5226890756** | **0.0017572753** |
| 213710_s_at | hsa-miR-34c-3p_st | **-0.5226890756** | **0.0017572753** |
| 209122_at | hsa-miR-132_st | **-0.5223834989** | **0.0017697888** |
| 44669_at | hsa-miR-34c-3p_st | **-0.5223834989** | **0.0017697888** |
| 218557_at | hsa-miR-34c-3p_st | **-0.5217723453** | **0.0017950508** |
| 203411_s_at | hsa-miR-4760-3p_st | **-0.5214667685** | **0.0018078002** |
| 205324_s_at | hsa-miR-34c-5p_st | **-0.5214667685** | **0.0018078002** |
| 209046_s_at | hsa-miR-34c-3p_st | **-0.5214667685** | **0.0018078002** |
| 209513_s_at | hsa-miR-377-star_st | **-0.5214667685** | **0.0018078002** |
| 210946_at | hsa-miR-377-star_st | **-0.5214667685** | **0.0018078002** |
| 213904_at | hsa-miR-34c-3p_st | **-0.5214667685** | **0.0018078002** |
| 201324_at | hsa-miR-4720-3p_st | **-0.5208556150** | **0.0018335378** |
| 203613_s_at | hsa-miR-34c-3p_st | **-0.5207426098** | **0.0018383319** |
| 207079_s_at | hsa-miR-34c-3p_st | **-0.5202444614** | **0.0018595966** |
| 202834_at | hsa-miR-1912_st | **-0.5199388846** | **0.0018727475** |
| 203973_s_at | hsa-miR-377-star_st | **-0.5199388846** | **0.0018727475** |
| 211962_s_at | hsa-miR-4720-3p_st | **-0.5199388846** | **0.0018727475** |
| 217923_at | hsa-miR-34b-star_st | **-0.5199388846** | **0.0018727475** |
| 200638_s_at | hsa-miR-34c-3p_st | **-0.5196333079** | **0.0018859800** |
| 200978_at | hsa-miR-34c-5p_st | **-0.5196333079** | **0.0018859800** |
| 202975_s_at | hsa-miR-377-star_st | **-0.5196333079** | **0.0018859800** |
| 203150_at | hsa-miR-34b-star_st | **-0.5196333079** | **0.0018859800** |
| 204587_at | hsa-miR-34b-star_st | **-0.5193277311** | **0.0018992945** |
| 209549_s_at | hsa-miR-34b-star_st | **-0.5193277311** | **0.0018992945** |
| 211297_s_at | hsa-miR-34c-5p_st | **-0.5193277311** | **0.0018992945** |
| 201112_s_at | hsa-miR-34c-5p_st | **-0.5190221543** | **0.0019126914** |
| 200883_at | hsa-miR-34c-5p_st | **-0.5187165775** | **0.0019261712** |
| 203313_s_at | hsa-miR-4760-3p_st | **-0.5187165775** | **0.0019261712** |
| 218292_s_at | hsa-miR-34b-star_st | **-0.5187165775** | **0.0019261712** |
| 203313_s_at | hsa-miR-1912_st | **-0.5184110008** | **0.0019397343** |
| 208121_s_at | hsa-miR-34c-5p_st | **-0.5184110008** | **0.0019397343** |
| 208659_at | hsa-miR-4720-3p_st | **-0.5184110008** | **0.0019397343** |
| 210278_s_at | hsa-miR-34c-3p_st | **-0.5184110008** | **0.0019397343** |
| 219760_at | hsa-miR-34c-3p_st | **-0.5184110008** | **0.0019397343** |
| 208868_s_at | hsa-miR-34c-5p_st | **-0.5181054240** | **0.0019533812** |
| 221688_s_at | hsa-miR-34c-5p_st | **-0.5181054240** | **0.0019533812** |
| 200079_s_at | hsa-miR-34c-5p_st | **-0.5177998472** | **0.0019671122** |
| 204119_s_at | hsa-miR-34b-star_st | **-0.5177998472** | **0.0019671122** |
| 214829_at | hsa-miR-382_st | **-0.5176866086** | **0.0019722220** |
| 202376_at | hsa-miR-377-star_st | **-0.5174942704** | **0.0019809278** |
| 215416_s_at | hsa-miR-34b_st | **-0.5174942704** | **0.0019809278** |
| 218882_s_at | hsa-miR-34c-3p_st | **-0.5174942704** | **0.0019809278** |
| 212990_at | hsa-miR-34b-star_st | **-0.5171886937** | **0.0019948285** |
| 200843_s_at | hsa-miR-34c-5p_st | **-0.5168831169** | **0.0020088148** |
| 201988_s_at | hsa-miR-34c-5p_st | **-0.5168831169** | **0.0020088148** |
| 202252_at | hsa-miR-383_st | **-0.5168831169** | **0.0020088148** |
| 208659_at | hsa-miR-1180_st | **-0.5165775401** | **0.0020228869** |
| 221741_s_at | hsa-miR-382_st | **-0.5163114080** | **0.0020352130** |
| 217959_s_at | hsa-miR-34c-3p_st | **-0.5162719633** | **0.0020370455** |
| 200626_s_at | hsa-miR-34c-3p_st | **-0.5159663866** | **0.0020512909** |
| 200916_at | hsa-miR-1180_st | **-0.5159663866** | **0.0020512909** |
| 202382_s_at | hsa-miR-34c-3p_st | **-0.5159663866** | **0.0020512909** |
| 206015_s_at | hsa-miR-34c-5p_st | **-0.5159663866** | **0.0020512909** |
| 211962_s_at | hsa-miR-1912_st | **-0.5159663866** | **0.0020512909** |
| 209108_at | hsa-miR-1912_st | **-0.5156608098** | **0.0020656236** |
| 210427_x_at | hsa-miR-4760-3p_st | **-0.5153552330** | **0.0020800440** |
| 212961_x_at | hsa-miR-34b-star_st | **-0.5153552330** | **0.0020800440** |
| 218866_s_at | hsa-miR-34c-5p_st | **-0.5153552330** | **0.0020800440** |
| 203721_s_at | hsa-miR-34c-3p_st | **-0.5150496562** | **0.0020945527** |
| 205280_at | hsa-miR-34c-5p_st | **-0.5150496562** | **0.0020945527** |
| 201160_s_at | hsa-miR-4311_st | **-0.5147834074** | **0.0021072663** |
| 207776_s_at | hsa-miR-34b-star_st | **-0.5147440794** | **0.0021091500** |
| 207054_at | hsa-miR-34c-3p_st | **-0.5144385027** | **0.0021238365** |
| 211763_s_at | hsa-miR-375_st | **-0.5144385027** | **0.0021238365** |
| 212203_x_at | hsa-miR-382_st | **-0.5143250072** | **0.0021293140** |
| 202373_s_at | hsa-miR-34c-5p_st | **-0.5141329259** | **0.0021386125** |
| 203415_at | hsa-miR-34c-3p_st | **-0.5141329259** | **0.0021386125** |
| 203944_x_at | hsa-miR-34c-5p_st | **-0.5141329259** | **0.0021386125** |
| 209157_at | hsa-miR-34c-3p_st | **-0.5141329259** | **0.0021386125** |
| 209227_at | hsa-miR-34c-3p_st | **-0.5141329259** | **0.0021386125** |
| 209228_x_at | hsa-miR-34c-5p_st | **-0.5141329259** | **0.0021386125** |
| 218970_s_at | hsa-miR-34c-3p_st | **-0.5141329259** | **0.0021386125** |
| 200798_x_at | hsa-miR-4720-3p_st | **-0.5138273491** | **0.0021534785** |
| 206671_at | hsa-miR-34c-3p_st | **-0.5138273491** | **0.0021534785** |
| 213496_at | hsa-miR-34c-5p_st | **-0.5138273491** | **0.0021534785** |
| 203302_at | hsa-miR-34c-3p_st | **-0.5135217723** | **0.0021684351** |
| 203415_at | hsa-miR-34c-5p_st | **-0.5135217723** | **0.0021684351** |
| 210427_x_at | hsa-miR-377-star_st | **-0.5135217723** | **0.0021684351** |
| 217820_s_at | hsa-miR-4760-3p_st | **-0.5135217723** | **0.0021684351** |
| 219628_at | hsa-miR-34c-3p_st | **-0.5135217723** | **0.0021684351** |
| 200040_at | hsa-miR-34c-3p_st | **-0.5132161956** | **0.0021834826** |
| 208675_s_at | hsa-miR-34c-5p_st | **-0.5132161956** | **0.0021834826** |
| 212217_at | hsa-miR-34c-5p_st | **-0.5129106188** | **0.0021986216** |
| 213227_at | hsa-miR-34c-3p_st | **-0.5129106188** | **0.0021986216** |
| 215527_at | hsa-miR-34c-5p_st | **-0.5129106188** | **0.0021986216** |
| 217957_at | hsa-miR-34c-3p_st | **-0.5129106188** | **0.0021986216** |
| 202975_s_at | hsa-miR-370_st | **-0.5126050420** | **0.0022138524** |
| 213738_s_at | hsa-miR-34b-star_st | **-0.5126050420** | **0.0022138524** |
| 218163_at | hsa-miR-34c-3p_st | **-0.5126050420** | **0.0022138524** |
| 218200_s_at | hsa-miR-34c-3p_st | **-0.5126050420** | **0.0022138524** |
| 200903_s_at | hsa-miR-34c-5p_st | **-0.5122994652** | **0.0022291757** |
| 201753_s_at | hsa-miR-370_st | **-0.5122994652** | **0.0022291757** |
| 221488_s_at | hsa-miR-34c-5p_st | **-0.5122994652** | **0.0022291757** |
| 208782_at | hsa-miR-132_st | **-0.5119938885** | **0.0022445918** |
| 213272_s_at | hsa-miR-34c-3p_st | **-0.5119938885** | **0.0022445918** |
| 201315_x_at | hsa-miR-382_st | **-0.5118802062** | **0.0022503508** |
| 205856_at | hsa-miR-4311_st | **-0.5117274062** | **0.0022581118** |
| 214022_s_at | hsa-miR-132_st | **-0.5116883117** | **0.0022601012** |
| 202825_at | hsa-miR-34b-star_st | **-0.5113827349** | **0.0022757045** |
| 207988_s_at | hsa-miR-34c-3p_st | **-0.5113827349** | **0.0022757045** |
| 209733_at | hsa-miR-34c-5p_st | **-0.5113827349** | **0.0022757045** |
| 219263_at | hsa-miR-34c-3p_st | **-0.5113827349** | **0.0022757045** |
| 208909_at | hsa-miR-34b-star_st | **-0.5110771581** | **0.0022914020** |
| 212063_at | hsa-miR-1912_st | **-0.5110771581** | **0.0022914020** |
| 202475_at | hsa-miR-34c-3p_st | **-0.5107715814** | **0.0023071943** |
| 212407_at | hsa-miR-34c-5p_st | **-0.5107715814** | **0.0023071943** |
| 217960_s_at | hsa-miR-34b-star_st | **-0.5107715814** | **0.0023071943** |
| 202370_s_at | hsa-miR-382_st | **-0.5106578058** | **0.0023130985** |
| 221874_at | hsa-miR-34c-3p_st | **-0.5104660046** | **0.0023230818** |
| 204744_s_at | hsa-miR-34b-star_st | **-0.5101604278** | **0.0023390651** |
| 210501_x_at | hsa-miR-34c-5p_st | **-0.5101604278** | **0.0023390651** |
| 213423_x_at | hsa-miR-34c-3p_st | **-0.5101604278** | **0.0023390651** |
| 219911_s_at | hsa-miR-377-star_st | **-0.5101604278** | **0.0023390651** |
| 207643_s_at | hsa-miR-132_st | **-0.5098548510** | **0.0023551446** |
| 208679_s_at | hsa-miR-34b-star_st | **-0.5098548510** | **0.0023551446** |
| 222230_s_at | hsa-miR-34b-star_st | **-0.5098548510** | **0.0023551446** |
| 211951_at | hsa-miR-4652-3p_st | **-0.5095492743** | **0.0023713208** |
| 219481_at | hsa-miR-375_st | **-0.5092436975** | **0.0023875941** |
| 202741_at | hsa-miR-34c-5p_st | **-0.5089381207** | **0.0024039652** |
| 208813_at | hsa-miR-34c-5p_st | **-0.5089381207** | **0.0024039652** |
| 202077_at | hsa-miR-34c-5p_st | **-0.5083269672** | **0.0024370024** |
| 203313_s_at | hsa-miR-377-star_st | **-0.5080213904** | **0.0024536696** |
| 202864_s_at | hsa-miR-377-star_st | **-0.5077158136** | **0.0024704364** |
| 203540_at | hsa-miR-1912_st | **-0.5077158136** | **0.0024704364** |
| 209569_x_at | hsa-miR-34c-3p_st | **-0.5077158136** | **0.0024704364** |
| 202834_at | hsa-miR-377-star_st | **-0.5074102368** | **0.0024873034** |
| 204002_s_at | hsa-miR-34c-5p_st | **-0.5074102368** | **0.0024873034** |
| 206544_x_at | hsa-miR-34c-5p_st | **-0.5074102368** | **0.0024873034** |
| 206671_at | hsa-miR-34c-5p_st | **-0.5074102368** | **0.0024873034** |
| 216218_s_at | hsa-miR-34b-star_st | **-0.5074102368** | **0.0024873034** |
| 210418_s_at | hsa-miR-34c-5p_st | **-0.5071046600** | **0.0025042711** |
| 201256_at | hsa-miR-375_st | **-0.5067990833** | **0.0025213399** |
| 210749_x_at | hsa-miR-377-star_st | **-0.5067990833** | **0.0025213399** |
| 202233_s_at | hsa-miR-34c-3p_st | **-0.5064935065** | **0.0025385105** |
| 205489_at | hsa-miR-34b-star_st | **-0.5064935065** | **0.0025385105** |
| 210817_s_at | hsa-miR-4760-3p_st | **-0.5064935065** | **0.0025385105** |
| 212271_at | hsa-miR-34b-star_st | **-0.5064935065** | **0.0025385105** |
| 218292_s_at | hsa-miR-375_st | **-0.5064935065** | **0.0025385105** |
| 202948_at | hsa-miR-132_st | **-0.5061879297** | **0.0025557833** |
| 207054_at | hsa-miR-34c-5p_st | **-0.5061879297** | **0.0025557833** |
| 202180_s_at | hsa-miR-1912_st | **-0.5058823529** | **0.0025731588** |
| 212157_at | hsa-miR-34c-3p_st | **-0.5058823529** | **0.0025731588** |
| 201066_at | hsa-miR-34c-3p_st | **-0.5055767762** | **0.0025906376** |
| 201443_s_at | hsa-miR-34b-star_st | **-0.5055767762** | **0.0025906376** |
| 202471_s_at | hsa-miR-34c-3p_st | **-0.5055767762** | **0.0025906376** |
| 218488_at | hsa-miR-34c-3p_st | **-0.5055767762** | **0.0025906376** |
| 201054_at | hsa-miR-34b-star_st | **-0.5052711994** | **0.0026082201** |
| 202591_s_at | hsa-miR-34c-5p_st | **-0.5052711994** | **0.0026082201** |
| 208799_at | hsa-miR-34c-5p_st | **-0.5052711994** | **0.0026082201** |
| 208818_s_at | hsa-miR-1912_st | **-0.5052711994** | **0.0026082201** |
| 215952_s_at | hsa-miR-34c-3p_st | **-0.5046600458** | **0.0026436984** |
| 201398_s_at | hsa-miR-4760-3p_st | **-0.5043544691** | **0.0026615953** |
| 203156_at | hsa-miR-34b-star_st | **-0.5043544691** | **0.0026615953** |
| 208827_at | hsa-miR-34b-star_st | **-0.5043544691** | **0.0026615953** |
| 44669_at | hsa-miR-34c-5p_st | **-0.5043544691** | **0.0026615953** |
| 201086_x_at | hsa-miR-34c-3p_st | **-0.5040488923** | **0.0026795981** |
| 207400_at | hsa-miR-34c-3p_st | **-0.5040488923** | **0.0026795981** |
| 209840_s_at | hsa-miR-34c-3p_st | **-0.5040488923** | **0.0026795981** |
| 203157_s_at | hsa-miR-34b-star_st | **-0.5037433155** | **0.0026977072** |
| 213533_at | hsa-miR-34c-3p_st | **-0.5037433155** | **0.0026977072** |
| 212203_x_at | hsa-miR-4311_st | **-0.5036290029** | **0.0027045090** |
| 200916_at | hsa-miR-212_st | **-0.5034377387** | **0.0027159232** |
| 202868_s_at | hsa-miR-34b-star_st | **-0.5034377387** | **0.0027159232** |
| 209507_at | hsa-miR-34c-5p_st | **-0.5034377387** | **0.0027159232** |
| 201315_x_at | hsa-miR-4311_st | **-0.5033234028** | **0.0027227666** |
| 202133_at | hsa-miR-4311_st | **-0.5033234028** | **0.0027227666** |
| 202180_s_at | hsa-miR-4720-3p_st | **-0.5031321620** | **0.0027342466** |
| 207717_s_at | hsa-miR-34c-3p_st | **-0.5031321620** | **0.0027342466** |
| 207988_s_at | hsa-miR-34c-5p_st | **-0.5031321620** | **0.0027342466** |
| 209550_at | hsa-miR-34c-3p_st | **-0.5031321620** | **0.0027342466** |
| 209598_at | hsa-miR-34c-3p_st | **-0.5028265852** | **0.0027526780** |
| 202121_s_at | hsa-miR-34c-3p_st | **-0.5025210084** | **0.0027712179** |
| 218970_s_at | hsa-miR-34b-star_st | **-0.5025210084** | **0.0027712179** |
| 208818_s_at | hsa-miR-377-star_st | **-0.5022154316** | **0.0027898668** |
| 200626_s_at | hsa-miR-34c-5p_st | **-0.5019098549** | **0.0028086253** |
| 214829_at | hsa-miR-132_st | **-0.5019098549** | **0.0028086253** |
| 214022_s_at | hsa-miR-4311_st | **-0.5017954022** | **0.0028156796** |
| 202180_s_at | hsa-miR-4311_st | **-0.5013370020** | **0.0028440883** |
| 202543_s_at | hsa-miR-383_st | **-0.5012987013** | **0.0028464732** |
| 205512_s_at | hsa-miR-34c-5p_st | **-0.5012987013** | **0.0028464732** |
| 200986_at | hsa-miR-132_st | **-0.5009931245** | **0.0028655636** |
| 202180_s_at | hsa-miR-1180_st | **-0.5009931245** | **0.0028655636** |
| 206099_at | hsa-miR-34c-3p_st | **-0.5009931245** | **0.0028655636** |
| 218656_s_at | hsa-miR-132_st | **-0.5006875477** | **0.0028847659** |
| 201527_at | hsa-miR-34c-5p_st | **-0.5003819710** | **0.0029040803** |
| 210156_s_at | hsa-miR-34b-star_st | **-0.5003819710** | **0.0029040803** |
| 214629_x_at | hsa-miR-34c-5p_st | **-0.5003819710** | **0.0029040803** |
| 202779_s_at | hsa-miR-34c-3p_st | **-0.5000763942** | **0.0029235077** |
| 211270_x_at | hsa-miR-4760-3p_st | **-0.5000763942** | **0.0029235077** |
| 212215_at | hsa-miR-34c-5p_st | **-0.5000763942** | **0.0029235077** |
| 209513_s_at | hsa-miR-1912_st | **-0.4997708174** | **0.0029430484** |
| 214629_x_at | hsa-miR-34c-3p_st | **-0.4997708174** | **0.0029430484** |
| 218322_s_at | hsa-miR-4311_st | **-0.4995034013** | **0.0029602424** |
| 206805_at | hsa-miR-34b_st | **-0.4994652406** | **0.0029627031** |
| 214150_x_at | hsa-miR-377-star_st | **-0.4994652406** | **0.0029627031** |
| 207643_s_at | hsa-miR-3189-5p_st | **-0.4991596639** | **0.0029824723** |
| 212645_x_at | hsa-miR-375_st | **-0.4991596639** | **0.0029824723** |
| 212501_at | hsa-miR-382_st | **-0.4988922010** | **0.0029998702** |
| 201753_s_at | hsa-miR-132_st | **-0.4985485103** | **0.0030223566** |
| 201999_s_at | hsa-miR-383_st | **-0.4985485103** | **0.0030223566** |
| 208839_s_at | hsa-miR-34b-star_st | **-0.4985485103** | **0.0030223566** |
| 212887_at | hsa-miR-34c-5p_st | **-0.4985485103** | **0.0030223566** |
| 210749_x_at | hsa-miR-132_st | **-0.4982429335** | **0.0030424727** |
| 211318_s_at | hsa-miR-34b-star_st | **-0.4982429335** | **0.0030424727** |
| 208838_at | hsa-miR-34b-star_st | **-0.4979373568** | **0.0030627056** |
| 200638_s_at | hsa-miR-34c-5p_st | **-0.4976317800** | **0.0030830558** |
| 211855_s_at | hsa-miR-34c-5p_st | **-0.4976317800** | **0.0030830558** |
| 218656_s_at | hsa-miR-382_st | **-0.4975170005** | **0.0030907301** |
| 211658_at | hsa-miR-34c-5p_st | **-0.4973262032** | **0.0031035239** |
| 211963_s_at | hsa-miR-34c-3p_st | **-0.4973262032** | **0.0031035239** |
| 212820_at | hsa-miR-34c-5p_st | **-0.4973262032** | **0.0031035239** |
| 200662_s_at | hsa-miR-375_st | **-0.4967150497** | **0.0031448161** |
| 202658_at | hsa-miR-34c-5p_st | **-0.4967150497** | **0.0031448161** |
| 209075_s_at | hsa-miR-34c-5p_st | **-0.4967150497** | **0.0031448161** |
| 1255_g_at | hsa-miR-34c-3p_st | **-0.4964094729** | **0.0031656414** |
| 202376_at | hsa-miR-4720-3p_st | **-0.4964094729** | **0.0031656414** |
| 207120_at | hsa-miR-34c-3p_st | **-0.4964094729** | **0.0031656414** |
| 210406_s_at | hsa-miR-34c-3p_st | **-0.4964094729** | **0.0031656414** |
| 201601_x_at | hsa-miR-132_st | **-0.4961038961** | **0.0031865869** |
| 207573_x_at | hsa-miR-34c-3p_st | **-0.4961038961** | **0.0031865869** |
| 203893_at | hsa-miR-34c-3p_st | **-0.4957983193** | **0.0032076532** |
| 206671_at | hsa-miR-34b-star_st | **-0.4957983193** | **0.0032076532** |
| 200812_at | hsa-miR-34c-5p_st | **-0.4954927426** | **0.0032288408** |
| 200853_at | hsa-miR-34c-5p_st | **-0.4954927426** | **0.0032288408** |
| 218976_at | hsa-miR-34b-star_st | **-0.4954927426** | **0.0032288408** |
| 221696_s_at | hsa-miR-34c-5p_st | **-0.4954927426** | **0.0032288408** |
| 219326_s_at | hsa-miR-34c-5p_st | **-0.4948815890** | **0.0032715825** |
| 202121_s_at | hsa-miR-34c-5p_st | **-0.4945760122** | **0.0032931378** |
| 211479_s_at | hsa-miR-34b-star_st | **-0.4945760122** | **0.0032931378** |
| 213011_s_at | hsa-miR-34c-3p_st | **-0.4945760122** | **0.0032931378** |
| 218160_at | hsa-miR-34b-star_st | **-0.4945760122** | **0.0032931378** |
| 218970_s_at | hsa-miR-34c-5p_st | **-0.4945760122** | **0.0032931378** |
| 203415_at | hsa-miR-34b-star_st | **-0.4939648587** | **0.0033366201** |
| 202370_s_at | hsa-miR-1180_st | **-0.4936592819** | **0.0033585483** |
| 203721_s_at | hsa-miR-34c-5p_st | **-0.4936592819** | **0.0033585483** |
| 205278_at | hsa-miR-34b-star_st | **-0.4936592819** | **0.0033585483** |
| 1007_s_at | hsa-miR-132_st | **-0.4933537051** | **0.0033806020** |
| 208998_at | hsa-miR-4760-3p_st | **-0.4933537051** | **0.0033806020** |
| 211271_x_at | hsa-miR-4760-3p_st | **-0.4933537051** | **0.0033806020** |
| 218854_at | hsa-miR-382_st | **-0.4932385987** | **0.0033889420** |
| 208782_at | hsa-miR-382_st | **-0.4930857987** | **0.0034000408** |
| 205531_s_at | hsa-miR-34c-5p_st | **-0.4930481283** | **0.0034027819** |
| 207508_at | hsa-miR-34c-3p_st | **-0.4930481283** | **0.0034027819** |
| 219326_s_at | hsa-miR-34c-3p_st | **-0.4930481283** | **0.0034027819** |
| 204068_at | hsa-miR-4311_st | **-0.4929329986** | **0.0034111712** |
| 201666_at | hsa-miR-3189-5p_st | **-0.4927425516** | **0.0034250885** |
| 202180_s_at | hsa-miR-132_st | **-0.4927425516** | **0.0034250885** |
| 202594_at | hsa-miR-34c-5p_st | **-0.4927425516** | **0.0034250885** |
| 204070_at | hsa-miR-383_st | **-0.4927425516** | **0.0034250885** |
| 209122_at | hsa-miR-4760-3p_st | **-0.4927425516** | **0.0034250885** |
| 213293_s_at | hsa-miR-1180_st | **-0.4927425516** | **0.0034250885** |
| 217820_s_at | hsa-miR-132_st | **-0.4927425516** | **0.0034250885** |
| 218654_s_at | hsa-miR-34b-star_st | **-0.4927425516** | **0.0034250885** |
| 200905_x_at | hsa-miR-1180_st | **-0.4924369748** | **0.0034475223** |
| 202370_s_at | hsa-miR-370_st | **-0.4924369748** | **0.0034475223** |
| 212645_x_at | hsa-miR-34b_st | **-0.4924369748** | **0.0034475223** |
| 218526_s_at | hsa-miR-34c-3p_st | **-0.4924369748** | **0.0034475223** |
| 222043_at | hsa-miR-377-star_st | **-0.4924369748** | **0.0034475223** |
| 201412_at | hsa-miR-382_st | **-0.4923217984** | **0.0034560112** |
| 201989_s_at | hsa-miR-34b-star_st | **-0.4921313980** | **0.0034700842** |
| 202641_at | hsa-miR-34b-star_st | **-0.4921313980** | **0.0034700842** |
| 201398_s_at | hsa-miR-132_st | **-0.4918258212** | **0.0034927745** |
| 201725_at | hsa-miR-34c-5p_st | **-0.4918258212** | **0.0034927745** |
| 203983_at | hsa-miR-4652-3p_st | **-0.4918258212** | **0.0034927745** |
| 203150_at | hsa-miR-375_st | **-0.4915202445** | **0.0035155940** |
| 217837_s_at | hsa-miR-34b-star_st | **-0.4912146677** | **0.0035385433** |
| 222216_s_at | hsa-miR-34c-3p_st | **-0.4912146677** | **0.0035385433** |
| 201410_at | hsa-miR-34c-5p_st | **-0.4909090909** | **0.0035616229** |
| 205758_at | hsa-miR-34c-3p_st | **-0.4909090909** | **0.0035616229** |
| 206356_s_at | hsa-miR-375_st | **-0.4909090909** | **0.0035616229** |
| 210240_s_at | hsa-miR-34b-star_st | **-0.4909090909** | **0.0035616229** |
| 211270_x_at | hsa-miR-377-star_st | **-0.4909090909** | **0.0035616229** |
| 201753_s_at | hsa-miR-377-star_st | **-0.4906035141** | **0.0035848336** |
| 205775_at | hsa-miR-34b-star_st | **-0.4906035141** | **0.0035848336** |
| 206042_x_at | hsa-miR-34b-star_st | **-0.4906035141** | **0.0035848336** |
| 200030_s_at | hsa-miR-34c-3p_st | **-0.4902979374** | **0.0036081759** |
| 200853_at | hsa-miR-34c-3p_st | **-0.4902979374** | **0.0036081759** |
| 208782_at | hsa-miR-212_st | **-0.4902979374** | **0.0036081759** |
| 215522_at | hsa-miR-34c-5p_st | **-0.4902979374** | **0.0036081759** |
| 218488_at | hsa-miR-34c-5p_st | **-0.4902979374** | **0.0036081759** |
| 201999_s_at | hsa-miR-4720-3p_st | **-0.4899923606** | **0.0036316504** |
| 214829_at | hsa-miR-212_st | **-0.4899923606** | **0.0036316504** |
| 203613_s_at | hsa-miR-34c-5p_st | **-0.4897241973** | **0.0036523602** |
| 203621_at | hsa-miR-34c-5p_st | **-0.4896867838** | **0.0036552578** |
| 202078_at | hsa-miR-34b-star_st | **-0.4893812070** | **0.0036789986** |
| 200906_s_at | hsa-miR-4720-3p_st | **-0.4890756303** | **0.0037028736** |
| 201601_x_at | hsa-miR-377-star_st | **-0.4890756303** | **0.0037028736** |
| 218005_at | hsa-miR-377-star_st | **-0.4890756303** | **0.0037028736** |
| 207830_s_at | hsa-miR-4652-3p_st | **-0.4887700535** | **0.0037268834** |
| 212203_x_at | hsa-miR-377-star_st | **-0.4887700535** | **0.0037268834** |
| 200905_x_at | hsa-miR-1912_st | **-0.4884644767** | **0.0037510285** |
| 218214_at | hsa-miR-34c-5p_st | **-0.4884644767** | **0.0037510285** |
| 218432_at | hsa-miR-34c-3p_st | **-0.4884644767** | **0.0037510285** |
| 203302_at | hsa-miR-34c-5p_st | **-0.4881588999** | **0.0037753097** |
| 205196_s_at | hsa-miR-34b_st | **-0.4881588999** | **0.0037753097** |
| 211999_at | hsa-miR-4311_st | **-0.4880806846** | **0.0037815466** |
| 201924_at | hsa-miR-382_st | **-0.4880433966** | **0.0037845232** |
| 202975_s_at | hsa-miR-212_st | **-0.4878533231** | **0.0037997275** |
| 218283_at | hsa-miR-34b_st | **-0.4877377965** | **0.0038089947** |
| 201859_at | hsa-miR-4311_st | **-0.4875849965** | **0.0038212820** |
| 208581_x_at | hsa-miR-1912_st | **-0.4875477464** | **0.0038242827** |
| 219714_s_at | hsa-miR-34b-star_st | **-0.4875477464** | **0.0038242827** |
| 201411_s_at | hsa-miR-34b-star_st | **-0.4872421696** | **0.0038489758** |
| 202779_s_at | hsa-miR-34c-5p_st | **-0.4872421696** | **0.0038489758** |
| 203411_s_at | hsa-miR-377-star_st | **-0.4872421696** | **0.0038489758** |
| 48531_at | hsa-miR-212_st | **-0.4869365928** | **0.0038738075** |
| 202149_at | hsa-miR-382_st | **-0.4866681961** | **0.0038957327** |
| 200641_s_at | hsa-miR-34b-star_st | **-0.4866310160** | **0.0038987784** |
| 201628_s_at | hsa-miR-34b-star_st | **-0.4866310160** | **0.0038987784** |
| 204992_s_at | hsa-miR-34b-star_st | **-0.4866310160** | **0.0038987784** |
| 218557_at | hsa-miR-34c-5p_st | **-0.4866310160** | **0.0038987784** |
| 209227_at | hsa-miR-34c-5p_st | **-0.4863254393** | **0.0039238892** |
| 220329_s_at | hsa-miR-34c-3p_st | **-0.4863254393** | **0.0039238892** |
| 200822_x_at | hsa-miR-34c-5p_st | **-0.4860198625** | **0.0039491406** |
| 200986_at | hsa-miR-377-star_st | **-0.4860198625** | **0.0039491406** |
| 202802_at | hsa-miR-34c-3p_st | **-0.4860198625** | **0.0039491406** |
| 204957_at | hsa-miR-34c-5p_st | **-0.4860198625** | **0.0039491406** |
| 207812_s_at | hsa-miR-4652-3p_st | **-0.4860198625** | **0.0039491406** |
| 211615_s_at | hsa-miR-34b-star_st | **-0.4860198625** | **0.0039491406** |
| 218316_at | hsa-miR-375_st | **-0.4860198625** | **0.0039491406** |
| 214022_s_at | hsa-miR-382_st | **-0.4857513957** | **0.0039714419** |
| 1007_s_at | hsa-miR-4760-3p_st | **-0.4857142857** | **0.0039745332** |
| 210149_s_at | hsa-miR-34c-5p_st | **-0.4857142857** | **0.0039745332** |
| 218597_s_at | hsa-miR-34b-star_st | **-0.4857142857** | **0.0039745332** |
| 218283_at | hsa-miR-375_st | **-0.4855985957** | **0.0039841837** |
| 204068_at | hsa-miR-4760-3p_st | **-0.4854087089** | **0.0040000676** |
| 205413_at | hsa-miR-34c-3p_st | **-0.4854087089** | **0.0040000676** |
| 213272_s_at | hsa-miR-34c-5p_st | **-0.4854087089** | **0.0040000676** |
| 210946_at | hsa-miR-382_st | **-0.4851401955** | **0.0040226226** |
| 201988_s_at | hsa-miR-34b-star_st | **-0.4851031322** | **0.0040257445** |
| 209157_at | hsa-miR-34c-5p_st | **-0.4851031322** | **0.0040257445** |
| 211376_s_at | hsa-miR-34c-5p_st | **-0.4851031322** | **0.0040257445** |
| 212321_at | hsa-miR-377-star_st | **-0.4851031322** | **0.0040257445** |
| 220251_at | hsa-miR-34c-5p_st | **-0.4851031322** | **0.0040257445** |
| 205550_s_at | hsa-miR-375_st | **-0.4847975554** | **0.0040515647** |
| 215058_at | hsa-miR-34c-3p_st | **-0.4847975554** | **0.0040515647** |
| 200708_at | hsa-miR-34c-3p_st | **-0.4841864018** | **0.0041036371** |
| 202614_at | hsa-miR-34c-5p_st | **-0.4841864018** | **0.0041036371** |
| 210749_x_at | hsa-miR-4760-3p_st | **-0.4841864018** | **0.0041036371** |
| 213592_at | hsa-miR-377-star_st | **-0.4841864018** | **0.0041036371** |
| 201601_x_at | hsa-miR-382_st | **-0.4839177950** | **0.0041267068** |
| 1007_s_at | hsa-miR-383_st | **-0.4838808251** | **0.0041298908** |
| 201512_s_at | hsa-miR-34c-5p_st | **-0.4838808251** | **0.0041298908** |
| 212015_x_at | hsa-miR-4760-3p_st | **-0.4838808251** | **0.0041298908** |
| 217882_at | hsa-miR-375_st | **-0.4837649949** | **0.0041398804** |
| 202475_at | hsa-miR-34c-5p_st | **-0.4835752483** | **0.0041562903** |
| 211270_x_at | hsa-miR-4720-3p_st | **-0.4835752483** | **0.0041562903** |
| 211558_s_at | hsa-miR-34c-5p_st | **-0.4835752483** | **0.0041562903** |
| 203817_at | hsa-miR-34b-star_st | **-0.4832696715** | **0.0041828363** |
| 205705_at | hsa-miR-34c-5p_st | **-0.4832696715** | **0.0041828363** |
| 206984_s_at | hsa-miR-34c-3p_st | **-0.4832696715** | **0.0041828363** |
| 200906_s_at | hsa-miR-377-star_st | **-0.4829640947** | **0.0042095295** |
| 201924_at | hsa-miR-4760-3p_st | **-0.4829640947** | **0.0042095295** |
| 202712_s_at | hsa-miR-34c-3p_st | **-0.4829640947** | **0.0042095295** |
| 204245_s_at | hsa-miR-375_st | **-0.4829640947** | **0.0042095295** |
| 205217_at | hsa-miR-34c-3p_st | **-0.4829640947** | **0.0042095295** |
| 203723_at | hsa-miR-377-star_st | **-0.4828481945** | **0.0042196925** |
| 204070_at | hsa-miR-4720-3p_st | **-0.4826585180** | **0.0042363707** |
| 208991_at | hsa-miR-4720-3p_st | **-0.4826585180** | **0.0042363707** |
| 210872_x_at | hsa-miR-34c-5p_st | **-0.4826585180** | **0.0042363707** |
| 214717_at | hsa-miR-34b-star_st | **-0.4826585180** | **0.0042363707** |
| 202929_s_at | hsa-miR-34c-3p_st | **-0.4820473644** | **0.0042904992** |
| 212321_at | hsa-miR-132_st | **-0.4820473644** | **0.0042904992** |
| 205963_s_at | hsa-miR-34c-3p_st | **-0.4817417876** | **0.0043177880** |
| 208679_s_at | hsa-miR-375_st | **-0.4817417876** | **0.0043177880** |
| 208779_x_at | hsa-miR-377-star_st | **-0.4817417876** | **0.0043177880** |
| 206356_s_at | hsa-miR-34c-5p_st | **-0.4814362108** | **0.0043452275** |
| 213904_at | hsa-miR-34c-5p_st | **-0.4814362108** | **0.0043452275** |
| 201160_s_at | hsa-miR-4760-3p_st | **-0.4811306341** | **0.0043728182** |
| 202325_s_at | hsa-miR-34c-5p_st | **-0.4811306341** | **0.0043728182** |
| 217936_at | hsa-miR-212_st | **-0.4811306341** | **0.0043728182** |
| 208581_x_at | hsa-miR-382_st | **-0.4810145938** | **0.0043833354** |
| 201484_at | hsa-miR-34b-star_st | **-0.4808250573** | **0.0044005609** |
| 203723_at | hsa-miR-4720-3p_st | **-0.4807089937** | **0.0044111381** |
| 207508_at | hsa-miR-34c-5p_st | **-0.4805194805** | **0.0044284564** |
| 218491_s_at | hsa-miR-34c-3p_st | **-0.4805194805** | **0.0044284564** |
| 202613_at | hsa-miR-34c-3p_st | **-0.4802139037** | **0.0044565052** |
| 203362_s_at | hsa-miR-34b-star_st | **-0.4799083270** | **0.0044847081** |
| 201666_at | hsa-miR-132_st | **-0.4796027502** | **0.0045130658** |
| 211376_s_at | hsa-miR-34c-3p_st | **-0.4792971734** | **0.0045415789** |
| 218706_s_at | hsa-miR-382_st | **-0.4790281930** | **0.0045668066** |
| 201160_s_at | hsa-miR-1912_st | **-0.4789915966** | **0.0045702483** |
| 203159_at | hsa-miR-34b-star_st | **-0.4789915966** | **0.0045702483** |
| 208782_at | hsa-miR-383_st | **-0.4786860199** | **0.0045990746** |
| 1007_s_at | hsa-miR-212_st | **-0.4783804431** | **0.0046280585** |
| 203189_s_at | hsa-miR-34c-5p_st | **-0.4783804431** | **0.0046280585** |
| 204326_x_at | hsa-miR-1912_st | **-0.4783804431** | **0.0046280585** |
| 219582_at | hsa-miR-377-star_st | **-0.4783804431** | **0.0046280585** |
| 200600_at | hsa-miR-4762-5p_st | **-0.4780748663** | **0.0046572007** |
| 201012_at | hsa-miR-377-star_st | **-0.4780748663** | **0.0046572007** |
| 211963_s_at | hsa-miR-34c-5p_st | **-0.4780748663** | **0.0046572007** |
| 221958_s_at | hsa-miR-1912_st | **-0.4780748663** | **0.0046572007** |
| 213552_at | hsa-miR-34c-3p_st | **-0.4777692895** | **0.0046865020** |
| 202920_at | hsa-miR-34c-3p_st | **-0.4771581360** | **0.0047455846** |
| 210240_s_at | hsa-miR-375_st | **-0.4771581360** | **0.0047455846** |
| 211479_s_at | hsa-miR-375_st | **-0.4771581360** | **0.0047455846** |
| 218214_at | hsa-miR-34c-3p_st | **-0.4771581360** | **0.0047455846** |
| 219911_s_at | hsa-miR-4720-3p_st | **-0.4771581360** | **0.0047455846** |
| 202948_at | hsa-miR-382_st | **-0.4768889921** | **0.0047718079** |
| 201180_s_at | hsa-miR-1180_st | **-0.4768525592** | **0.0047753673** |
| 208869_s_at | hsa-miR-34c-5p_st | **-0.4768525592** | **0.0047753673** |
| 211595_s_at | hsa-miR-34c-3p_st | **-0.4768525592** | **0.0047753673** |
| 202670_at | hsa-miR-34b-star_st | **-0.4765469824** | **0.0048053120** |
| 205609_at | hsa-miR-1912_st | **-0.4765469824** | **0.0048053120** |
| 210872_x_at | hsa-miR-34c-3p_st | **-0.4765469824** | **0.0048053120** |
| 211271_x_at | hsa-miR-1912_st | **-0.4765469824** | **0.0048053120** |
| 208659_at | hsa-miR-4760-3p_st | **-0.4762414057** | **0.0048354193** |
| 218559_s_at | hsa-miR-1912_st | **-0.4762414057** | **0.0048354193** |
| 221449_s_at | hsa-miR-34b-star_st | **-0.4759358289** | **0.0048656901** |
| 200739_s_at | hsa-miR-34c-3p_st | **-0.4753246753** | **0.0049267247** |
| 206062_at | hsa-miR-34b-star_st | **-0.4753246753** | **0.0049267247** |
| 201512_s_at | hsa-miR-34c-3p_st | **-0.4750190985** | **0.0049574900** |
| 208857_s_at | hsa-miR-34b-star_st | **-0.4750190985** | **0.0049574900** |
| 212600_s_at | hsa-miR-34c-5p_st | **-0.4750190985** | **0.0049574900** |
| 202269_x_at | hsa-miR-4311_st | **-0.4749025913** | **0.0049692637** |
| 201924_at | hsa-miR-212_st | **-0.4747135218** | **0.0049884217** |
| 201999_s_at | hsa-miR-212_st | **-0.4747135218** | **0.0049884217** |
| 208731_at | hsa-miR-34c-3p_st | **-0.4747135218** | **0.0049884217** |
| 210406_s_at | hsa-miR-34c-5p_st | **-0.4747135218** | **0.0049884217** |
| 217936_at | hsa-miR-4760-3p_st | **-0.4747135218** | **0.0049884217** |
| 222360_at | hsa-miR-34c-3p_st | **-0.4747135218** | **0.0049884217** |
| 200614_at | hsa-miR-34b-star_st | **-0.4745969912** | **0.0050002614** |
| 206935_at | hsa-miR-375_st | **-0.4744079450** | **0.0050195205** |
| 218882_s_at | hsa-miR-34c-5p_st | **-0.4744079450** | **0.0050195205** |
| 204070_at | hsa-miR-1180_st | **-0.4741023682** | **0.0050507871** |
| 213710_s_at | hsa-miR-4652-3p_st | **-0.4741023682** | **0.0050507871** |
| 201112_s_at | hsa-miR-34b-star_st | **-0.4734912147** | **0.0051138268** |
| 211763_s_at | hsa-miR-4652-3p_st | **-0.4734912147** | **0.0051138268** |
| 1007_s_at | hsa-miR-1180_st | **-0.4731856379** | **0.0051456014** |
| 202133_at | hsa-miR-377-star_st | **-0.4731856379** | **0.0051456014** |
| 205856_at | hsa-miR-212_st | **-0.4731856379** | **0.0051456014** |
| 219911_s_at | hsa-miR-4760-3p_st | **-0.4731856379** | **0.0051456014** |
| 202868_s_at | hsa-miR-34b_st | **-0.4728800611** | **0.0051775469** |
| 218163_at | hsa-miR-34c-5p_st | **-0.4728800611** | **0.0051775469** |
| 200978_at | hsa-miR-34b-star_st | **-0.4725744843** | **0.0052096639** |
| 211376_s_at | hsa-miR-375_st | **-0.4725744843** | **0.0052096639** |
| 215416_s_at | hsa-miR-375_st | **-0.4725744843** | **0.0052096639** |
| 201601_x_at | hsa-miR-1180_st | **-0.4722689076** | **0.0052419533** |
| 206542_s_at | hsa-miR-34c-3p_st | **-0.4722689076** | **0.0052419533** |
| 210946_at | hsa-miR-132_st | **-0.4722689076** | **0.0052419533** |
| 201411_s_at | hsa-miR-375_st | **-0.4719633308** | **0.0052744159** |
| 213496_at | hsa-miR-34b-star_st | **-0.4719633308** | **0.0052744159** |
| 213005_s_at | hsa-miR-382_st | **-0.4716937901** | **0.0053031945** |
| 202376_at | hsa-miR-132_st | **-0.4716577540** | **0.0053070523** |
| 212727_at | hsa-miR-4652-3p_st | **-0.4716577540** | **0.0053070523** |
| 210534_s_at | hsa-miR-34b-star_st | **-0.4713881899** | **0.0053359874** |
| 201274_at | hsa-miR-34b-star_st | **-0.4710466005** | **0.0053728499** |
| 201512_s_at | hsa-miR-375_st | **-0.4710466005** | **0.0053728499** |
| 210453_x_at | hsa-miR-34c-5p_st | **-0.4710466005** | **0.0053728499** |
| 211763_s_at | hsa-miR-34b_st | **-0.4710466005** | **0.0053728499** |
| 213217_at | hsa-miR-377-star_st | **-0.4710466005** | **0.0053728499** |
| 217923_at | hsa-miR-375_st | **-0.4710466005** | **0.0053728499** |
| 200905_x_at | hsa-miR-377-star_st | **-0.4707410237** | **0.0054060126** |
| 200916_at | hsa-miR-383_st | **-0.4707410237** | **0.0054060126** |
| 206803_at | hsa-miR-34c-3p_st | **-0.4707410237** | **0.0054060126** |
| 212063_at | hsa-miR-377-star_st | **-0.4707410237** | **0.0054060126** |
| 217968_at | hsa-miR-375_st | **-0.4707410237** | **0.0054060126** |
| 218982_s_at | hsa-miR-34c-3p_st | **-0.4707410237** | **0.0054060126** |
| 213005_s_at | hsa-miR-523_st | **-0.4704354469** | **0.0054393522** |
| 213710_s_at | hsa-miR-34c-5p_st | **-0.4704354469** | **0.0054393522** |
| 205097_at | hsa-miR-4311_st | **-0.4703185895** | **0.0054521488** |
| 202736_s_at | hsa-miR-34c-3p_st | **-0.4701298701** | **0.0054728697** |
| 206356_s_at | hsa-miR-34c-3p_st | **-0.4701298701** | **0.0054728697** |
| 201412_at | hsa-miR-132_st | **-0.4698242934** | **0.0055065656** |
| 212053_at | hsa-miR-34b-star_st | **-0.4698242934** | **0.0055065656** |
| 202948_at | hsa-miR-4311_st | **-0.4695545892** | **0.0055364548** |
| 209046_s_at | hsa-miR-34c-5p_st | **-0.4695187166** | **0.0055404409** |
| 205324_s_at | hsa-miR-34b-star_st | **-0.4692131398** | **0.0055744963** |
| 212310_at | hsa-miR-34c-5p_st | **-0.4692131398** | **0.0055744963** |
| 203540_at | hsa-miR-4720-3p_st | **-0.4689075630** | **0.0056087325** |
| 214150_x_at | hsa-miR-4311_st | **-0.4687905889** | **0.0056218861** |
| 201601_x_at | hsa-miR-4762-5p_st | **-0.4686019862** | **0.0056431505** |
| 201666_at | hsa-miR-1912_st | **-0.4682964095** | **0.0056777509** |
| 202507_s_at | hsa-miR-34b_st | **-0.4682964095** | **0.0056777509** |
| 211475_s_at | hsa-miR-34c-3p_st | **-0.4682964095** | **0.0056777509** |
| 218224_at | hsa-miR-34c-5p_st | **-0.4682964095** | **0.0056777509** |
| 200986_at | hsa-miR-212_st | **-0.4679908327** | **0.0057125346** |
| 201145_at | hsa-miR-34c-3p_st | **-0.4679908327** | **0.0057125346** |
| 201322_at | hsa-miR-34c-3p_st | **-0.4679908327** | **0.0057125346** |
| 201900_s_at | hsa-miR-34c-3p_st | **-0.4679908327** | **0.0057125346** |
| 201966_at | hsa-miR-375_st | **-0.4679908327** | **0.0057125346** |
| 203079_s_at | hsa-miR-34b-star_st | **-0.4679908327** | **0.0057125346** |
| 205280_at | hsa-miR-34b-star_st | **-0.4679908327** | **0.0057125346** |
| 205856_at | hsa-miR-383_st | **-0.4679908327** | **0.0057125346** |
| 211855_s_at | hsa-miR-34b-star_st | **-0.4679908327** | **0.0057125346** |
| 212977_at | hsa-miR-1912_st | **-0.4679908327** | **0.0057125346** |
| 217820_s_at | hsa-miR-1180_st | **-0.4676852559** | **0.0057475023** |
| 217860_at | hsa-miR-34c-3p_st | **-0.4676852559** | **0.0057475023** |
| 218982_s_at | hsa-miR-34c-5p_st | **-0.4676852559** | **0.0057475023** |
| 208818_s_at | hsa-miR-382_st | **-0.4674153883** | **0.0057785375** |
| 218283_at | hsa-miR-4652-3p_st | **-0.4674153883** | **0.0057785375** |
| 203560_at | hsa-miR-4652-3p_st | **-0.4673796791** | **0.0057826549** |
| 206099_at | hsa-miR-34c-5p_st | **-0.4673796791** | **0.0057826549** |
| 207831_x_at | hsa-miR-34c-5p_st | **-0.4673796791** | **0.0057826549** |
| 218507_at | hsa-miR-4720-3p_st | **-0.4673796791** | **0.0057826549** |
| 200967_at | hsa-miR-4760-3p_st | **-0.4670741024** | **0.0058179932** |
| 201180_s_at | hsa-miR-383_st | **-0.4667685256** | **0.0058535180** |
| 211769_x_at | hsa-miR-34b-star_st | **-0.4667685256** | **0.0058535180** |
| 213217_at | hsa-miR-4720-3p_st | **-0.4667685256** | **0.0058535180** |
| 221263_s_at | hsa-miR-34c-3p_st | **-0.4667685256** | **0.0058535180** |
| 221696_s_at | hsa-miR-34b-star_st | **-0.4667685256** | **0.0058535180** |
| 201256_at | hsa-miR-4652-3p_st | **-0.4664629488** | **0.0058892300** |
| 204119_s_at | hsa-miR-375_st | **-0.4664629488** | **0.0058892300** |
| 208998_at | hsa-miR-377-star_st | **-0.4664629488** | **0.0058892300** |
| 201146_at | hsa-miR-382_st | **-0.4663457879** | **0.0059029722** |
| 1255_g_at | hsa-miR-34c-5p_st | **-0.4661573720** | **0.0059251302** |
| 202077_at | hsa-miR-34b-star_st | **-0.4661573720** | **0.0059251302** |
| 202929_s_at | hsa-miR-34c-5p_st | **-0.4661573720** | **0.0059251302** |
| 203663_s_at | hsa-miR-34c-3p_st | **-0.4661573720** | **0.0059251302** |
| 204072_s_at | hsa-miR-34c-5p_st | **-0.4661573720** | **0.0059251302** |
| 205005_s_at | hsa-miR-34b_st | **-0.4661573720** | **0.0059251302** |
| 210972_x_at | hsa-miR-34c-3p_st | **-0.4661573720** | **0.0059251302** |
| 217730_at | hsa-miR-132_st | **-0.4661573720** | **0.0059251302** |
| 217936_at | hsa-miR-132_st | **-0.4661573720** | **0.0059251302** |
| 219760_at | hsa-miR-34c-5p_st | **-0.4661573720** | **0.0059251302** |
| 203889_at | hsa-miR-375_st | **-0.4658517953** | **0.0059612192** |
| 203973_s_at | hsa-miR-1912_st | **-0.4658517953** | **0.0059612192** |
| 208782_at | hsa-miR-4720-3p_st | **-0.4658517953** | **0.0059612192** |
| 218200_s_at | hsa-miR-34c-5p_st | **-0.4658517953** | **0.0059612192** |
| 218866_s_at | hsa-miR-34b-star_st | **-0.4658517953** | **0.0059612192** |
| 201656_at | hsa-miR-1912_st | **-0.4655462185** | **0.0059974980** |
| 203893_at | hsa-miR-34c-5p_st | **-0.4655462185** | **0.0059974980** |
| 207400_at | hsa-miR-34c-5p_st | **-0.4655462185** | **0.0059974980** |
| 215527_at | hsa-miR-34b-star_st | **-0.4655462185** | **0.0059974980** |
| 203645_s_at | hsa-miR-4311_st | **-0.4654289875** | **0.0060114665** |
| 205633_s_at | hsa-miR-34b_st | **-0.4652406417** | **0.0060339673** |
| 210278_s_at | hsa-miR-34c-5p_st | **-0.4652406417** | **0.0060339673** |
| 221488_s_at | hsa-miR-34b-star_st | **-0.4652406417** | **0.0060339673** |
| 201924_at | hsa-miR-383_st | **-0.4649350649** | **0.0060706280** |
| 207717_s_at | hsa-miR-34c-5p_st | **-0.4649350649** | **0.0060706280** |
| 201146_at | hsa-miR-4311_st | **-0.4648177873** | **0.0060847491** |
| 205489_at | hsa-miR-375_st | **-0.4646294882** | **0.0061074809** |
| 200040_at | hsa-miR-34b-star_st | **-0.4643239114** | **0.0061445268** |
| 219421_at | hsa-miR-34c-3p_st | **-0.4643239114** | **0.0061445268** |
| 201066_at | hsa-miR-34c-5p_st | **-0.4640183346** | **0.0061817666** |
| 201315_x_at | hsa-miR-212_st | **-0.4640183346** | **0.0061817666** |
| 202543_s_at | hsa-miR-1912_st | **-0.4640183346** | **0.0061817666** |
| 204002_s_at | hsa-miR-34b-star_st | **-0.4640183346** | **0.0061817666** |
| 211672_s_at | hsa-miR-34c-3p_st | **-0.4640183346** | **0.0061817666** |
| 213887_s_at | hsa-miR-34c-5p_st | **-0.4640183346** | **0.0061817666** |
| 215058_at | hsa-miR-34c-5p_st | **-0.4640183346** | **0.0061817666** |
| 212038_s_at | hsa-miR-375_st | **-0.4637127578** | **0.0062192010** |
| 203723_at | hsa-miR-212_st | **-0.4635953868** | **0.0062336313** |
| 201313_at | hsa-miR-34c-3p_st | **-0.4634071811** | **0.0062568309** |
| 213503_x_at | hsa-miR-132_st | **-0.4634071811** | **0.0062568309** |
| 219628_at | hsa-miR-34c-5p_st | **-0.4634071811** | **0.0062568309** |
| 200720_s_at | hsa-miR-34b-star_st | **-0.4631016043** | **0.0062946572** |
| 201590_x_at | hsa-miR-212_st | **-0.4627960275** | **0.0063326807** |
| 202149_at | hsa-miR-132_st | **-0.4627960275** | **0.0063326807** |
| 214022_s_at | hsa-miR-1180_st | **-0.4627960275** | **0.0063326807** |
| 211971_s_at | hsa-miR-34c-3p_st | **-0.4626785864** | **0.0063473467** |
| 200663_at | hsa-miR-132_st | **-0.4624904507** | **0.0063709022** |
| 201160_s_at | hsa-miR-132_st | **-0.4624904507** | **0.0063709022** |
| 210434_x_at | hsa-miR-34c-3p_st | **-0.4624904507** | **0.0063709022** |
| 212067_s_at | hsa-miR-1912_st | **-0.4624904507** | **0.0063709022** |
| 212203_x_at | hsa-miR-4762-5p_st | **-0.4624904507** | **0.0063709022** |
| 212820_at | hsa-miR-34b-star_st | **-0.4624904507** | **0.0063709022** |
| 214150_x_at | hsa-miR-4760-3p_st | **-0.4624904507** | **0.0063709022** |
| 201988_s_at | hsa-miR-375_st | **-0.4615737204** | **0.0064867633** |
| 217820_s_at | hsa-miR-212_st | **-0.4615737204** | **0.0064867633** |
| 203723_at | hsa-miR-383_st | **-0.4614561859** | **0.0065017485** |
| 201315_x_at | hsa-miR-4762-5p_st | **-0.4612681436** | **0.0065257854** |
| 201315_x_at | hsa-miR-383_st | **-0.4612681436** | **0.0065257854** |
| 213293_s_at | hsa-miR-132_st | **-0.4612681436** | **0.0065257854** |
| 202834_at | hsa-miR-4762-5p_st | **-0.4609625668** | **0.0065650098** |
| 218854_at | hsa-miR-4762-5p_st | **-0.4609625668** | **0.0065650098** |
| 200906_s_at | hsa-miR-4760-3p_st | **-0.4606569901** | **0.0066044373** |
| 202613_at | hsa-miR-34c-5p_st | **-0.4606569901** | **0.0066044373** |
| 207988_s_at | hsa-miR-34b-star_st | **-0.4606569901** | **0.0066044373** |
| 213710_s_at | hsa-miR-34b-star_st | **-0.4606569901** | **0.0066044373** |
| 200804_at | hsa-miR-1912_st | **-0.4603514133** | **0.0066440688** |
| 209513_s_at | hsa-miR-383_st | **-0.4603514133** | **0.0066440688** |
| 201592_at | hsa-miR-34b-star_st | **-0.4600458365** | **0.0066839051** |
| 201753_s_at | hsa-miR-212_st | **-0.4600458365** | **0.0066839051** |
| 201086_x_at | hsa-miR-34c-5p_st | **-0.4597402597** | **0.0067239472** |
| 202269_x_at | hsa-miR-4720-3p_st | **-0.4597402597** | **0.0067239472** |
| 218160_at | hsa-miR-375_st | **-0.4591291062** | **0.0068046520** |
| 208991_at | hsa-miR-4311_st | **-0.4590113849** | **0.0068202930** |
| 202543_s_at | hsa-miR-4311_st | **-0.4588585849** | **0.0068406409** |
| 201180_s_at | hsa-miR-4720-3p_st | **-0.4588235294** | **0.0068453165** |
| 210749_x_at | hsa-miR-4720-3p_st | **-0.4588235294** | **0.0068453165** |
| 221874_at | hsa-miR-34c-5p_st | **-0.4588235294** | **0.0068453165** |
| 212501_at | hsa-miR-4311_st | **-0.4587057848** | **0.0068610411** |
| 215952_s_at | hsa-miR-34c-5p_st | **-0.4585179526** | **0.0068861902** |
| 200677_at | hsa-miR-382_st | **-0.4582473846** | **0.0069225565** |
| 200638_s_at | hsa-miR-34b-star_st | **-0.4582123759** | **0.0069272740** |
| 221688_s_at | hsa-miR-34b-star_st | **-0.4582123759** | **0.0069272740** |
| 221874_at | hsa-miR-375_st | **-0.4582123759** | **0.0069272740** |
| 213217_at | hsa-miR-212_st | **-0.4579067991** | **0.0069685688** |
| 213227_at | hsa-miR-34c-5p_st | **-0.4579067991** | **0.0069685688** |
| 200040_at | hsa-miR-34c-5p_st | **-0.4576012223** | **0.0070100754** |
| 201590_x_at | hsa-miR-132_st | **-0.4576012223** | **0.0070100754** |
| 203781_at | hsa-miR-34c-3p_st | **-0.4576012223** | **0.0070100754** |
| 206671_at | hsa-miR-375_st | **-0.4576012223** | **0.0070100754** |
| 207079_s_at | hsa-miR-34c-5p_st | **-0.4572956455** | **0.0070517948** |
| 207088_s_at | hsa-miR-34c-3p_st | **-0.4572956455** | **0.0070517948** |
| 212977_at | hsa-miR-4720-3p_st | **-0.4572956455** | **0.0070517948** |
| 201570_at | hsa-miR-34c-3p_st | **-0.4569900688** | **0.0070937278** |
| 205413_at | hsa-miR-34c-5p_st | **-0.4566844920** | **0.0071358753** |
| 217957_at | hsa-miR-34c-5p_st | **-0.4566844920** | **0.0071358753** |
| 217959_s_at | hsa-miR-34c-5p_st | **-0.4566844920** | **0.0071358753** |
| 202149_at | hsa-miR-1912_st | **-0.4563789152** | **0.0071782383** |
| 208799_at | hsa-miR-34b-star_st | **-0.4563789152** | **0.0071782383** |
| 212727_at | hsa-miR-375_st | **-0.4563789152** | **0.0071782383** |
| 218133_s_at | hsa-miR-375_st | **-0.4563789152** | **0.0071782383** |
| 200820_at | hsa-miR-34b-star_st | **-0.4560733384** | **0.0072208175** |
| 211404_s_at | hsa-miR-34c-5p_st | **-0.4560733384** | **0.0072208175** |
| 217882_at | hsa-miR-34b_st | **-0.4559553837** | **0.0072373115** |
| 200843_s_at | hsa-miR-34b-star_st | **-0.4557677617** | **0.0072636140** |
| 205279_s_at | hsa-miR-34b_st | **-0.4557677617** | **0.0072636140** |
| 200053_at | hsa-miR-34c-5p_st | **-0.4554621849** | **0.0073066286** |
| 202096_s_at | hsa-miR-1912_st | **-0.4554621849** | **0.0073066286** |
| 205031_at | hsa-miR-34b-star_st | **-0.4554621849** | **0.0073066286** |
| 213738_s_at | hsa-miR-375_st | **-0.4554621849** | **0.0073066286** |
| 209733_at | hsa-miR-34b-star_st | **-0.4551566081** | **0.0073498623** |
| 201172_x_at | hsa-miR-555_st | **-0.4550385833** | **0.0073666195** |
| 200786_at | hsa-miR-375_st | **-0.4548510313** | **0.0073933159** |
| 202543_s_at | hsa-miR-1180_st | **-0.4548510313** | **0.0073933159** |
| 205196_s_at | hsa-miR-375_st | **-0.4548510313** | **0.0073933159** |
| 219683_at | hsa-miR-34c-3p_st | **-0.4545454545** | **0.0074369903** |
| 204587_at | hsa-miR-375_st | **-0.4542398778** | **0.0074808865** |
| 210033_s_at | hsa-miR-4720-3p_st | **-0.4542398778** | **0.0074808865** |
| 213005_s_at | hsa-miR-377-star_st | **-0.4542398778** | **0.0074808865** |
| 215058_at | hsa-miR-375_st | **-0.4542398778** | **0.0074808865** |
| 219960_s_at | hsa-miR-34c-3p_st | **-0.4542398778** | **0.0074808865** |
| 202834_at | hsa-miR-382_st | **-0.4539689829** | **0.0075199868** |
| 202471_s_at | hsa-miR-34c-5p_st | **-0.4539343010** | **0.0075250053** |
| 201656_at | hsa-miR-4720-3p_st | **-0.4536287242** | **0.0075693478** |
| 210427_x_at | hsa-miR-132_st | **-0.4536287242** | **0.0075693478** |
| 217820_s_at | hsa-miR-523_st | **-0.4536287242** | **0.0075693478** |
| 209248_at | hsa-miR-375_st | **-0.4535105827** | **0.0075865515** |
| 200906_s_at | hsa-miR-1912_st | **-0.4533231474** | **0.0076139148** |
| 201272_at | hsa-miR-375_st | **-0.4533231474** | **0.0076139148** |
| 217936_at | hsa-miR-370_st | **-0.4533231474** | **0.0076139148** |
| 201753_s_at | hsa-miR-383_st | **-0.4530175707** | **0.0076587072** |
| 208659_at | hsa-miR-383_st | **-0.4530175707** | **0.0076587072** |
| 213272_s_at | hsa-miR-34b-star_st | **-0.4530175707** | **0.0076587072** |
| 216218_s_at | hsa-miR-34b_st | **-0.4530175707** | **0.0076587072** |
| 201315_x_at | hsa-miR-1180_st | **-0.4527119939** | **0.0077037260** |
| 202948_at | hsa-miR-212_st | **-0.4527119939** | **0.0077037260** |
| 210434_x_at | hsa-miR-34c-5p_st | **-0.4527119939** | **0.0077037260** |
| 213592_at | hsa-miR-4720-3p_st | **-0.4527119939** | **0.0077037260** |
| 217820_s_at | hsa-miR-4311_st | **-0.4525937824** | **0.0077212023** |
| 219297_at | hsa-miR-34c-3p_st | **-0.4521008403** | **0.0077944464** |
| 200883_at | hsa-miR-34b-star_st | **-0.4517952636** | **0.0078401498** |
| 209507_at | hsa-miR-34b-star_st | **-0.4517952636** | **0.0078401498** |
| 202325_s_at | hsa-miR-34c-3p_st | **-0.4514896868** | **0.0078860834** |
| 202373_s_at | hsa-miR-34b-star_st | **-0.4514896868** | **0.0078860834** |
| 210014_x_at | hsa-miR-34c-5p_st | **-0.4511841100** | **0.0079322480** |
| 214629_x_at | hsa-miR-34b-star_st | **-0.4511841100** | **0.0079322480** |
| 221263_s_at | hsa-miR-34c-5p_st | **-0.4511841100** | **0.0079322480** |
| 203889_at | hsa-miR-4652-3p_st | **-0.4508785332** | **0.0079786446** |
| 208832_at | hsa-miR-34b-star_st | **-0.4508785332** | **0.0079786446** |
| 36711_at | hsa-miR-1912_st | **-0.4508785332** | **0.0079786446** |
| 202741_at | hsa-miR-34b-star_st | **-0.4505729565** | **0.0080252742** |
| 209108_at | hsa-miR-377-star_st | **-0.4505729565** | **0.0080252742** |
| 221531_at | hsa-miR-34c-3p_st | **-0.4505729565** | **0.0080252742** |
| 200812_at | hsa-miR-34b-star_st | **-0.4502673797** | **0.0080721376** |
| 202975_s_at | hsa-miR-134_st | **-0.4502673797** | **0.0080721376** |
| 208981_at | hsa-miR-4720-3p_st | **-0.4502673797** | **0.0080721376** |
| 200797_s_at | hsa-miR-4720-3p_st | **-0.4499618029** | **0.0081192359** |
| 200804_at | hsa-miR-212_st | **-0.4499618029** | **0.0081192359** |
| 201756_at | hsa-miR-375_st | **-0.4499618029** | **0.0081192359** |
| 208991_at | hsa-miR-212_st | **-0.4499618029** | **0.0081192359** |
| 200663_at | hsa-miR-383_st | **-0.4493506494** | **0.0082141407** |
| 205097_at | hsa-miR-1912_st | **-0.4493506494** | **0.0082141407** |
| 210749_x_at | hsa-miR-383_st | **-0.4493506494** | **0.0082141407** |
| 210749_x_at | hsa-miR-212_st | **-0.4493506494** | **0.0082141407** |
| 211951_at | hsa-miR-375_st | **-0.4490450726** | **0.0082619492** |
| 213227_at | hsa-miR-34b-star_st | **-0.4490450726** | **0.0082619492** |
| 214829_at | hsa-miR-4720-3p_st | **-0.4490450726** | **0.0082619492** |
| 215506_s_at | hsa-miR-34c-3p_st | **-0.4490450726** | **0.0082619492** |
| 221741_s_at | hsa-miR-4762-5p_st | **-0.4490450726** | **0.0082619492** |
| 200853_at | hsa-miR-34b-star_st | **-0.4487394958** | **0.0083099963** |
| 204002_s_at | hsa-miR-375_st | **-0.4487394958** | **0.0083099963** |
| 205097_at | hsa-miR-4720-3p_st | **-0.4487394958** | **0.0083099963** |
| 213714_at | hsa-miR-375_st | **-0.4487394958** | **0.0083099963** |
| 201601_x_at | hsa-miR-4720-3p_st | **-0.4484339190** | **0.0083582831** |
| 211297_s_at | hsa-miR-34b-star_st | **-0.4484339190** | **0.0083582831** |
| 212321_at | hsa-miR-4760-3p_st | **-0.4484339190** | **0.0083582831** |
| 212501_at | hsa-miR-4720-3p_st | **-0.4484339190** | **0.0083582831** |
| 213423_x_at | hsa-miR-34c-5p_st | **-0.4484339190** | **0.0083582831** |
| 215058_at | hsa-miR-34b-star_st | **-0.4484339190** | **0.0083582831** |
| 200739_s_at | hsa-miR-34c-5p_st | **-0.4481283422** | **0.0084068105** |
| 201756_at | hsa-miR-34b_st | **-0.4481283422** | **0.0084068105** |
| 202591_s_at | hsa-miR-34b-star_st | **-0.4481283422** | **0.0084068105** |
| 203854_at | hsa-miR-4720-3p_st | **-0.4481283422** | **0.0084068105** |
| 212157_at | hsa-miR-34c-5p_st | **-0.4481283422** | **0.0084068105** |
| 218120_s_at | hsa-miR-34b-star_st | **-0.4481283422** | **0.0084068105** |
| 211971_s_at | hsa-miR-34c-5p_st | **-0.4480097805** | **0.0084257038** |
| 207643_s_at | hsa-miR-383_st | **-0.4478227655** | **0.0084555795** |
| 200663_at | hsa-miR-4760-3p_st | **-0.4475171887** | **0.0085045910** |
| 209122_at | hsa-miR-1912_st | **-0.4475171887** | **0.0085045910** |
| 212887_at | hsa-miR-34b-star_st | **-0.4475171887** | **0.0085045910** |
| 218656_s_at | hsa-miR-383_st | **-0.4475171887** | **0.0085045910** |
| 218667_at | hsa-miR-34b-star_st | **-0.4475171887** | **0.0085045910** |
| 219714_s_at | hsa-miR-375_st | **-0.4475171887** | **0.0085045910** |
| 48531_at | hsa-miR-4760-3p_st | **-0.4475171887** | **0.0085045910** |
| 207573_x_at | hsa-miR-34c-5p_st | **-0.4472116119** | **0.0085538460** |
| 208121_s_at | hsa-miR-34b-star_st | **-0.4472116119** | **0.0085538460** |
| 221667_s_at | hsa-miR-377-star_st | **-0.4472116119** | **0.0085538460** |
| 203411_s_at | hsa-miR-1912_st | **-0.4469060351** | **0.0086033455** |
| 213533_at | hsa-miR-34c-5p_st | **-0.4469060351** | **0.0086033455** |
| 36711_at | hsa-miR-4311_st | **-0.4466345800** | **0.0086475236** |
| 202149_at | hsa-miR-4720-3p_st | **-0.4466004584** | **0.0086530905** |
| 210453_x_at | hsa-miR-34b-star_st | **-0.4466004584** | **0.0086530905** |
| 206849_at | hsa-miR-34c-5p_st | **-0.4462948816** | **0.0087030819** |
| 208991_at | hsa-miR-4762-5p_st | **-0.4462948816** | **0.0087030819** |
| 211376_s_at | hsa-miR-34b-star_st | **-0.4462948816** | **0.0087030819** |
| 218788_s_at | hsa-miR-375_st | **-0.4462948816** | **0.0087030819** |
| 200079_s_at | hsa-miR-34b-star_st | **-0.4459893048** | **0.0087533208** |
| 203944_x_at | hsa-miR-34b-star_st | **-0.4459893048** | **0.0087533208** |
| 205280_at | hsa-miR-34b_st | **-0.4459893048** | **0.0087533208** |
| 205512_s_at | hsa-miR-34b-star_st | **-0.4459893048** | **0.0087533208** |
| 208581_x_at | hsa-miR-370_st | **-0.4459893048** | **0.0087533208** |
| 210427_x_at | hsa-miR-212_st | **-0.4459893048** | **0.0087533208** |
| 213293_s_at | hsa-miR-370_st | **-0.4459893048** | **0.0087533208** |
| 217995_at | hsa-miR-4762-5p_st | **-0.4459893048** | **0.0087533208** |
| 203613_s_at | hsa-miR-34b-star_st | **-0.4457177796** | **0.0087981699** |
| 203302_at | hsa-miR-34b-star_st | **-0.4453781513** | **0.0088545450** |
| 205110_s_at | hsa-miR-375_st | **-0.4453781513** | **0.0088545450** |
| 208827_at | hsa-miR-375_st | **-0.4453781513** | **0.0088545450** |
| 212203_x_at | hsa-miR-212_st | **-0.4453781513** | **0.0088545450** |
| 201146_at | hsa-miR-377-star_st | **-0.4450725745** | **0.0089055322** |
| 213005_s_at | hsa-miR-132_st | **-0.4450725745** | **0.0089055322** |
| 218048_at | hsa-miR-34c-5p_st | **-0.4450725745** | **0.0089055322** |
| 218292_s_at | hsa-miR-34b_st | **-0.4450725745** | **0.0089055322** |
| 201412_at | hsa-miR-370_st | **-0.4447669977** | **0.0089567708** |
| 202587_s_at | hsa-miR-4720-3p_st | **-0.4447669977** | **0.0089567708** |
| 203663_s_at | hsa-miR-34c-5p_st | **-0.4447669977** | **0.0089567708** |
| 207508_at | hsa-miR-34b-star_st | **-0.4447669977** | **0.0089567708** |
| 209075_s_at | hsa-miR-34b-star_st | **-0.4447669977** | **0.0089567708** |
| 205856_at | hsa-miR-370_st | **-0.4444614209** | **0.0090082618** |
| 213503_x_at | hsa-miR-212_st | **-0.4444614209** | **0.0090082618** |
| 210427_x_at | hsa-miR-383_st | **-0.4441558442** | **0.0090600063** |
| 213005_s_at | hsa-miR-1912_st | **-0.4441558442** | **0.0090600063** |
| 200677_at | hsa-miR-4760-3p_st | **-0.4438502674** | **0.0091120052** |
| 201180_s_at | hsa-miR-212_st | **-0.4438502674** | **0.0091120052** |
| 202802_at | hsa-miR-34c-5p_st | **-0.4438502674** | **0.0091120052** |
| 207079_s_at | hsa-miR-34b-star_st | **-0.4438502674** | **0.0091120052** |
| 211951_at | hsa-miR-34b_st | **-0.4438502674** | **0.0091120052** |
| 211962_s_at | hsa-miR-377-star_st | **-0.4438502674** | **0.0091120052** |
| 212310_at | hsa-miR-34b-star_st | **-0.4438502674** | **0.0091120052** |
| 200798_x_at | hsa-miR-4311_st | **-0.4435785787** | **0.0091584520** |
| 203404_at | hsa-miR-34c-3p_st | **-0.4435446906** | **0.0091642596** |
| 206849_at | hsa-miR-34c-3p_st | **-0.4435446906** | **0.0091642596** |
| 209550_at | hsa-miR-34c-5p_st | **-0.4435446906** | **0.0091642596** |
| 217936_at | hsa-miR-382_st | **-0.4434257787** | **0.0091846631** |
| 210427_x_at | hsa-miR-1180_st | **-0.4432391138** | **0.0092167704** |
| 207830_s_at | hsa-miR-375_st | **-0.4429335371** | **0.0092695387** |
| 208818_s_at | hsa-miR-523_st | **-0.4429335371** | **0.0092695387** |
| 218970_s_at | hsa-miR-375_st | **-0.4429335371** | **0.0092695387** |
| 200804_at | hsa-miR-1180_st | **-0.4426279603** | **0.0093225655** |
| 204070_at | hsa-miR-212_st | **-0.4426279603** | **0.0093225655** |
| 208678_at | hsa-miR-375_st | **-0.4426279603** | **0.0093225655** |
| 217780_at | hsa-miR-34c-3p_st | **-0.4426279603** | **0.0093225655** |
| 221667_s_at | hsa-miR-1912_st | **-0.4426279603** | **0.0093225655** |
| 221874_at | hsa-miR-34b-star_st | **-0.4426279603** | **0.0093225655** |
| 201161_s_at | hsa-miR-4720-3p_st | **-0.4423223835** | **0.0093758518** |
| 201315_x_at | hsa-miR-523_st | **-0.4423223835** | **0.0093758518** |
| 202096_s_at | hsa-miR-132_st | **-0.4423223835** | **0.0093758518** |
| 204068_at | hsa-miR-132_st | **-0.4423223835** | **0.0093758518** |
| 202587_s_at | hsa-miR-1912_st | **-0.4420168067** | **0.0094293986** |
| 203704_s_at | hsa-miR-1912_st | **-0.4420168067** | **0.0094293986** |
| 209569_x_at | hsa-miR-34c-5p_st | **-0.4420168067** | **0.0094293986** |
| 217997_at | hsa-miR-34c-3p_st | **-0.4420168067** | **0.0094293986** |
| 218694_at | hsa-miR-34c-3p_st | **-0.4420168067** | **0.0094293986** |
| 221009_s_at | hsa-miR-1912_st | **-0.4420168067** | **0.0094293986** |
| 202395_at | hsa-miR-375_st | **-0.4414056532** | **0.0095372779** |
| 208991_at | hsa-miR-1180_st | **-0.4414056532** | **0.0095372779** |
| 214829_at | hsa-miR-523_st | **-0.4414056532** | **0.0095372779** |
| 202121_s_at | hsa-miR-34b-star_st | **-0.4411000764** | **0.0095916125** |
| 207120_at | hsa-miR-34c-5p_st | **-0.4411000764** | **0.0095916125** |
| 213911_s_at | hsa-miR-34c-3p_st | **-0.4411000764** | **0.0095916125** |
| 218322_s_at | hsa-miR-4720-3p_st | **-0.4411000764** | **0.0095916125** |
| 221699_s_at | hsa-miR-34b-star_st | **-0.4411000764** | **0.0095916125** |
| 200862_at | hsa-miR-34b-star_st | **-0.4407944996** | **0.0096462117** |
| 213293_s_at | hsa-miR-383_st | **-0.4407944996** | **0.0096462117** |
| 200662_s_at | hsa-miR-34b_st | **-0.4404889228** | **0.0097010766** |
| 203781_at | hsa-miR-34c-5p_st | **-0.4404889228** | **0.0097010766** |
| 212015_x_at | hsa-miR-377-star_st | **-0.4404889228** | **0.0097010766** |
| 221741_s_at | hsa-miR-377-star_st | **-0.4404889228** | **0.0097010766** |
| 202834_at | hsa-miR-4311_st | **-0.4403697774** | **0.0097225409** |
| 209598_at | hsa-miR-34c-5p_st | **-0.4401833461** | **0.0097562083** |
| 218384_at | hsa-miR-34c-3p_st | **-0.4401833461** | **0.0097562083** |
| 201198_s_at | hsa-miR-34b_st | **-0.4398777693** | **0.0098116077** |
| 203150_at | hsa-miR-4652-3p_st | **-0.4398777693** | **0.0098116077** |
| 208731_at | hsa-miR-34c-5p_st | **-0.4398777693** | **0.0098116077** |
| 213272_s_at | hsa-miR-375_st | **-0.4398777693** | **0.0098116077** |
| 218224_at | hsa-miR-34b-star_st | **-0.4398777693** | **0.0098116077** |
| 201999_s_at | hsa-miR-370_st | **-0.4395721925** | **0.0098672759** |
| 209671_x_at | hsa-miR-34c-3p_st | **-0.4395721925** | **0.0098672759** |
| 210817_s_at | hsa-miR-212_st | **-0.4395721925** | **0.0098672759** |
| 211672_s_at | hsa-miR-34c-5p_st | **-0.4395721925** | **0.0098672759** |
| 220251_at | hsa-miR-34b-star_st | **-0.4395721925** | **0.0098672759** |
| 202252_at | hsa-miR-1180_st | **-0.4392666157** | **0.0099232140** |
| 205413_at | hsa-miR-34b-star_st | **-0.4392666157** | **0.0099232140** |
| 204239_s_at | hsa-miR-34c-3p_st | **-0.4389610390** | **0.0099794230** |
| 210817_s_at | hsa-miR-383_st | **-0.4389610390** | **0.0099794230** |
| 212110_at | hsa-miR-4720-3p_st | **-0.4389610390** | **0.0099794230** |
| 207054_at | hsa-miR-34b-star_st | **-0.4386554622** | **0.0100359040** |
| 213293_s_at | hsa-miR-4720-3p_st | **-0.4386554622** | **0.0100359040** |
| 210817_s_at | hsa-miR-382_st | **-0.4383833766** | **0.0100864244** |
| 218322_s_at | hsa-miR-132_st | **-0.4383498854** | **0.0100926580** |
| 202376_at | hsa-miR-4762-5p_st | **-0.4380443086** | **0.0101496861** |
| 208991_at | hsa-miR-3676_st | **-0.4380443086** | **0.0101496861** |
| 212203_x_at | hsa-miR-1180_st | **-0.4380443086** | **0.0101496861** |
| 214022_s_at | hsa-miR-4760-3p_st | **-0.4380443086** | **0.0101496861** |
| 217995_at | hsa-miR-4760-3p_st | **-0.4380443086** | **0.0101496861** |
| 218322_s_at | hsa-miR-4760-3p_st | **-0.4380443086** | **0.0101496861** |
| 210448_s_at | hsa-miR-34c-3p_st | **-0.4377387319** | **0.0102069893** |
| 210978_s_at | hsa-miR-1912_st | **-0.4377387319** | **0.0102069893** |
| 212038_s_at | hsa-miR-34b_st | **-0.4377387319** | **0.0102069893** |
| 201410_at | hsa-miR-34b-star_st | **-0.4374331551** | **0.0102645688** |
| 202233_s_at | hsa-miR-34c-5p_st | **-0.4374331551** | **0.0102645688** |
| 202736_s_at | hsa-miR-34c-5p_st | **-0.4374331551** | **0.0102645688** |
| 203973_s_at | hsa-miR-4311_st | **-0.4373137762** | **0.0102871384** |
| 200906_s_at | hsa-miR-382_st | **-0.4371609761** | **0.0103160885** |
| 208813_at | hsa-miR-34b-star_st | **-0.4371275783** | **0.0103224255** |
| 206015_s_at | hsa-miR-34b-star_st | **-0.4365164248** | **0.0104389750** |
| 208779_x_at | hsa-miR-212_st | **-0.4365164248** | **0.0104389750** |
| 202594_at | hsa-miR-34b-star_st | **-0.4362108480** | **0.0104976700** |
| 209228_x_at | hsa-miR-34b-star_st | **-0.4362108480** | **0.0104976700** |
| 213572_s_at | hsa-miR-377-star_st | **-0.4362108480** | **0.0104976700** |
| 200677_at | hsa-miR-212_st | **-0.4359052712** | **0.0105566466** |
| 201887_at | hsa-miR-377-star_st | **-0.4359052712** | **0.0105566466** |
| 202370_s_at | hsa-miR-4720-3p_st | **-0.4359052712** | **0.0105566466** |
| 211595_s_at | hsa-miR-34c-5p_st | **-0.4359052712** | **0.0105566466** |
| 201761_at | hsa-miR-4311_st | **-0.4356329755** | **0.0106094380** |
| 205324_s_at | hsa-miR-375_st | **-0.4355996944** | **0.0106159058** |
| 215522_at | hsa-miR-34b-star_st | **-0.4355996944** | **0.0106159058** |
| 208998_at | hsa-miR-1912_st | **-0.4352941176** | **0.0106754487** |
| 209840_s_at | hsa-miR-34c-5p_st | **-0.4352941176** | **0.0106754487** |
| 218322_s_at | hsa-miR-1912_st | **-0.4352941176** | **0.0106754487** |
| 205609_at | hsa-miR-377-star_st | **-0.4349885409** | **0.0107352765** |
| 214428_x_at | hsa-miR-4720-3p_st | **-0.4349885409** | **0.0107352765** |
| 219263_at | hsa-miR-34c-5p_st | **-0.4343773873** | **0.0108557909** |
| 219326_s_at | hsa-miR-375_st | **-0.4343773873** | **0.0108557909** |
| 212203_x_at | hsa-miR-383_st | **-0.4340718105** | **0.0109164797** |
| 210749_x_at | hsa-miR-4762-5p_st | **-0.4337662338** | **0.0109774578** |
| 213503_x_at | hsa-miR-1180_st | **-0.4337662338** | **0.0109774578** |
| 201512_s_at | hsa-miR-34b-star_st | **-0.4334606570** | **0.0110387261** |
| 201666_at | hsa-miR-4760-3p_st | **-0.4334606570** | **0.0110387261** |
| 202543_s_at | hsa-miR-132_st | **-0.4334606570** | **0.0110387261** |
| 209755_at | hsa-miR-375_st | **-0.4334606570** | **0.0110387261** |
| 212015_x_at | hsa-miR-4720-3p_st | **-0.4334606570** | **0.0110387261** |
| 211999_at | hsa-miR-382_st | **-0.4332212714** | **0.0110869266** |
| 203621_at | hsa-miR-34b-star_st | **-0.4331550802** | **0.0111002858** |
| 205110_s_at | hsa-miR-4652-3p_st | **-0.4331550802** | **0.0111002858** |
| 210149_s_at | hsa-miR-34b-star_st | **-0.4331550802** | **0.0111002858** |
| 200097_s_at | hsa-miR-34c-3p_st | **-0.4328495034** | **0.0111621381** |
| 200626_s_at | hsa-miR-34b-star_st | **-0.4328495034** | **0.0111621381** |
| 218133_s_at | hsa-miR-34b_st | **-0.4328495034** | **0.0111621381** |
| 219326_s_at | hsa-miR-34b-star_st | **-0.4328495034** | **0.0111621381** |
| 219355_at | hsa-miR-34c-3p_st | **-0.4328495034** | **0.0111621381** |
| 200677_at | hsa-miR-383_st | **-0.4325439267** | **0.0112242840** |
| 205489_at | hsa-miR-4652-3p_st | **-0.4325439267** | **0.0112242840** |
| 212203_x_at | hsa-miR-3189-5p_st | **-0.4325439267** | **0.0112242840** |
| 215884_s_at | hsa-miR-375_st | **-0.4322383499** | **0.0112867246** |
| 201319_at | hsa-miR-4311_st | **-0.4319657740** | **0.0113426716** |
| 201590_x_at | hsa-miR-383_st | **-0.4319327731** | **0.0113494611** |
| 203645_s_at | hsa-miR-4762-5p_st | **-0.4319327731** | **0.0113494611** |
| 201029_s_at | hsa-miR-377-star_st | **-0.4316271963** | **0.0114124946** |
| 203854_at | hsa-miR-3189-5p_st | **-0.4316271963** | **0.0114124946** |
| 202834_at | hsa-miR-4720-3p_st | **-0.4313216196** | **0.0114758261** |
| 203721_s_at | hsa-miR-34b-star_st | **-0.4313216196** | **0.0114758261** |
| 208818_s_at | hsa-miR-383_st | **-0.4313216196** | **0.0114758261** |
| 208868_s_at | hsa-miR-34b-star_st | **-0.4313216196** | **0.0114758261** |
| 210972_x_at | hsa-miR-34c-5p_st | **-0.4313216196** | **0.0114758261** |
| 218557_at | hsa-miR-34b-star_st | **-0.4313216196** | **0.0114758261** |
| 208779_x_at | hsa-miR-132_st | **-0.4310160428** | **0.0115394569** |
| 211404_s_at | hsa-miR-34c-3p_st | **-0.4310160428** | **0.0115394569** |
| 221667_s_at | hsa-miR-4720-3p_st | **-0.4310160428** | **0.0115394569** |
| 201106_at | hsa-miR-34c-5p_st | **-0.4307104660** | **0.0116033880** |
| 202149_at | hsa-miR-212_st | **-0.4307104660** | **0.0116033880** |
| 208981_at | hsa-miR-4311_st | **-0.4304377734** | **0.0116606938** |
| 206935_at | hsa-miR-4652-3p_st | **-0.4304048892** | **0.0116676206** |
| 207717_s_at | hsa-miR-34b-star_st | **-0.4304048892** | **0.0116676206** |
| 214022_s_at | hsa-miR-212_st | **-0.4304048892** | **0.0116676206** |
| 205278_at | hsa-miR-4652-3p_st | **-0.4300993125** | **0.0117321558** |
| 206542_s_at | hsa-miR-34c-5p_st | **-0.4300993125** | **0.0117321558** |
| 213911_s_at | hsa-miR-34c-5p_st | **-0.4300993125** | **0.0117321558** |
| 201666_at | hsa-miR-212_st | **-0.4297937357** | **0.0117969948** |
| 201761_at | hsa-miR-1912_st | **-0.4297937357** | **0.0117969948** |
| 204070_at | hsa-miR-370_st | **-0.4297937357** | **0.0117969948** |
| 212092_at | hsa-miR-34c-3p_st | **-0.4297937357** | **0.0117969948** |
| 218706_s_at | hsa-miR-1912_st | **-0.4297937357** | **0.0117969948** |
| 221741_s_at | hsa-miR-132_st | **-0.4297937357** | **0.0117969948** |
| 203156_at | hsa-miR-375_st | **-0.4294881589** | **0.0118621386** |
| 219297_at | hsa-miR-34c-5p_st | **-0.4294881589** | **0.0118621386** |
| 200906_s_at | hsa-miR-132_st | **-0.4291825821** | **0.0119275884** |
| 201859_at | hsa-miR-4720-3p_st | **-0.4288770053** | **0.0119933454** |
| 202325_s_at | hsa-miR-34b-star_st | **-0.4288770053** | **0.0119933454** |
| 202948_at | hsa-miR-383_st | **-0.4288770053** | **0.0119933454** |
| 208809_s_at | hsa-miR-4720-3p_st | **-0.4288770053** | **0.0119933454** |
| 209227_at | hsa-miR-34b-star_st | **-0.4288770053** | **0.0119933454** |
| 210117_at | hsa-miR-377-star_st | **-0.4288770053** | **0.0119933454** |
| 217997_at | hsa-miR-34c-5p_st | **-0.4288770053** | **0.0119933454** |
| 217995_at | hsa-miR-1912_st | **-0.4285714286** | **0.0120594107** |
| 221741_s_at | hsa-miR-1912_st | **-0.4285714286** | **0.0120594107** |
| 202096_s_at | hsa-miR-1180_st | **-0.4282658518** | **0.0121257855** |
| 208998_at | hsa-miR-132_st | **-0.4282658518** | **0.0121257855** |
| 211297_s_at | hsa-miR-375_st | **-0.4282658518** | **0.0121257855** |
| 212460_at | hsa-miR-377-star_st | **-0.4282658518** | **0.0121257855** |
| 202834_at | hsa-miR-4760-3p_st | **-0.4279602750** | **0.0121924709** |
| 212407_at | hsa-miR-34b-star_st | **-0.4279602750** | **0.0121924709** |
| 202252_at | hsa-miR-382_st | **-0.4278401724** | **0.0122187659** |
| 222043_at | hsa-miR-382_st | **-0.4278401724** | **0.0122187659** |
| 202269_x_at | hsa-miR-4633-5p_st | **-0.4276546982** | **0.0122594681** |
| 210240_s_at | hsa-miR-34b_st | **-0.4276546982** | **0.0122594681** |
| 202180_s_at | hsa-miR-383_st | **-0.4273491215** | **0.0123267781** |
| 218656_s_at | hsa-miR-4633-5p_st | **-0.4273491215** | **0.0123267781** |
| 221515_s_at | hsa-miR-34c-5p_st | **-0.4273491215** | **0.0123267781** |
| 1007_s_at | hsa-miR-4720-3p_st | **-0.4270435447** | **0.0123944023** |
| 201527_at | hsa-miR-34b-star_st | **-0.4270435447** | **0.0123944023** |
| 200077_s_at | hsa-miR-34c-3p_st | **-0.4267379679** | **0.0124623417** |
| 203816_at | hsa-miR-375_st | **-0.4267379679** | **0.0124623417** |
| 217860_at | hsa-miR-34c-5p_st | **-0.4267379679** | **0.0124623417** |
| 211475_s_at | hsa-miR-34c-5p_st | **-0.4264323911** | **0.0125305976** |
| 212321_at | hsa-miR-212_st | **-0.4264323911** | **0.0125305976** |
| 213904_at | hsa-miR-34b-star_st | **-0.4264323911** | **0.0125305976** |
| 203723_at | hsa-miR-523_st | **-0.4261593717** | **0.0125918497** |
| 200030_s_at | hsa-miR-34c-5p_st | **-0.4261268144** | **0.0125991710** |
| 200798_x_at | hsa-miR-3676_st | **-0.4261268144** | **0.0125991710** |
| 201411_s_at | hsa-miR-34b_st | **-0.4261268144** | **0.0125991710** |
| 203893_at | hsa-miR-34b-star_st | **-0.4261268144** | **0.0125991710** |
| 206062_at | hsa-miR-375_st | **-0.4261268144** | **0.0125991710** |
| 207120_at | hsa-miR-34b-star_st | **-0.4261268144** | **0.0125991710** |
| 210501_x_at | hsa-miR-34b-star_st | **-0.4261268144** | **0.0125991710** |
| 218163_at | hsa-miR-34b_st | **-0.4261268144** | **0.0125991710** |
| 218654_s_at | hsa-miR-4652-3p_st | **-0.4261268144** | **0.0125991710** |
| 219714_s_at | hsa-miR-4652-3p_st | **-0.4261268144** | **0.0125991710** |
| 48531_at | hsa-miR-383_st | **-0.4261268144** | **0.0125991710** |
| 206803_at | hsa-miR-34c-5p_st | **-0.4258212376** | **0.0126680631** |
| 208818_s_at | hsa-miR-370_st | **-0.4258212376** | **0.0126680631** |
| 209598_at | hsa-miR-34b-star_st | **-0.4258212376** | **0.0126680631** |
| 217837_s_at | hsa-miR-375_st | **-0.4258212376** | **0.0126680631** |
| 217906_at | hsa-miR-34b_st | **-0.4258212376** | **0.0126680631** |
| 201322_at | hsa-miR-34c-5p_st | **-0.4255156608** | **0.0127372752** |
| 203094_at | hsa-miR-375_st | **-0.4255156608** | **0.0127372752** |
| 210418_s_at | hsa-miR-34b-star_st | **-0.4252100840** | **0.0128068084** |
| 212053_at | hsa-miR-375_st | **-0.4252100840** | **0.0128068084** |
| 44669_at | hsa-miR-34b-star_st | **-0.4252100840** | **0.0128068084** |
| 202430_s_at | hsa-miR-4720-3p_st | **-0.4249045073** | **0.0128766639** |
| 212407_at | hsa-miR-34b_st | **-0.4245989305** | **0.0129468429** |
| 213552_at | hsa-miR-34c-5p_st | **-0.4245989305** | **0.0129468429** |
| 204070_at | hsa-miR-4760-3p_st | **-0.4242933537** | **0.0130173465** |
| 218163_at | hsa-miR-34b-star_st | **-0.4242933537** | **0.0130173465** |
| 218706_s_at | hsa-miR-370_st | **-0.4242933537** | **0.0130173465** |
| 202232_s_at | hsa-miR-34c-3p_st | **-0.4239877769** | **0.0130881760** |
| 208675_s_at | hsa-miR-34b-star_st | **-0.4239877769** | **0.0130881760** |
| 200720_s_at | hsa-miR-4652-3p_st | **-0.4236822002** | **0.0131593325** |
| 203663_s_at | hsa-miR-34b-star_st | **-0.4236822002** | **0.0131593325** |
| 217820_s_at | hsa-miR-4762-5p_st | **-0.4236822002** | **0.0131593325** |
| 218048_at | hsa-miR-34c-3p_st | **-0.4236822002** | **0.0131593325** |
| 219960_s_at | hsa-miR-34c-5p_st | **-0.4236822002** | **0.0131593325** |
| 201315_x_at | hsa-miR-370_st | **-0.4233766234** | **0.0132308172** |
| 203415_at | hsa-miR-375_st | **-0.4233766234** | **0.0132308172** |
| 203983_at | hsa-miR-375_st | **-0.4233766234** | **0.0132308172** |
| 219628_at | hsa-miR-375_st | **-0.4233766234** | **0.0132308172** |
| 208779_x_at | hsa-miR-383_st | **-0.4230710466** | **0.0133026314** |
| 221488_s_at | hsa-miR-375_st | **-0.4230710466** | **0.0133026314** |
| 213217_at | hsa-miR-132_st | **-0.4227654698** | **0.0133747761** |
| 210156_s_at | hsa-miR-34b_st | **-0.4224598930** | **0.0134472528** |
| 212271_at | hsa-miR-375_st | **-0.4224598930** | **0.0134472528** |
| 212321_at | hsa-miR-383_st | **-0.4224598930** | **0.0134472528** |
| 213572_s_at | hsa-miR-4760-3p_st | **-0.4224598930** | **0.0134472528** |
| 221531_at | hsa-miR-34c-5p_st | **-0.4224598930** | **0.0134472528** |
| 208779_x_at | hsa-miR-4760-3p_st | **-0.4221543163** | **0.0135200624** |
| 210968_s_at | hsa-miR-34c-3p_st | **-0.4221543163** | **0.0135200624** |
| 205097_at | hsa-miR-382_st | **-0.4220337700** | **0.0135488768** |
| 200862_at | hsa-miR-375_st | **-0.4218487395** | **0.0135932063** |
| 207079_s_at | hsa-miR-34b_st | **-0.4218487395** | **0.0135932063** |
| 208998_at | hsa-miR-4720-3p_st | **-0.4218487395** | **0.0135932063** |
| 218226_s_at | hsa-miR-34c-3p_st | **-0.4218487395** | **0.0135932063** |
| 208451_s_at | hsa-miR-1912_st | **-0.4215431627** | **0.0136666857** |
| 209570_s_at | hsa-miR-34c-3p_st | **-0.4215431627** | **0.0136666857** |
| 217997_at | hsa-miR-375_st | **-0.4215431627** | **0.0136666857** |
| 218982_s_at | hsa-miR-34b_st | **-0.4215431627** | **0.0136666857** |
| 205550_s_at | hsa-miR-34b_st | **-0.4212375859** | **0.0137405017** |
| 205705_at | hsa-miR-34b-star_st | **-0.4212375859** | **0.0137405017** |
| 217936_at | hsa-miR-4720-3p_st | **-0.4212375859** | **0.0137405017** |
| 218982_s_at | hsa-miR-34b-star_st | **-0.4212375859** | **0.0137405017** |
| 219421_at | hsa-miR-34c-5p_st | **-0.4212375859** | **0.0137405017** |
| 201590_x_at | hsa-miR-1180_st | **-0.4209320092** | **0.0138146557** |
| 201601_x_at | hsa-miR-370_st | **-0.4209320092** | **0.0138146557** |
| 205097_at | hsa-miR-377-star_st | **-0.4209320092** | **0.0138146557** |
| 211658_at | hsa-miR-34b-star_st | **-0.4209320092** | **0.0138146557** |
| 212977_at | hsa-miR-377-star_st | **-0.4209320092** | **0.0138146557** |
| 222360_at | hsa-miR-34c-5p_st | **-0.4209320092** | **0.0138146557** |
| 202180_s_at | hsa-miR-555_st | **-0.4206585695** | **0.0138812984** |
| 200906_s_at | hsa-miR-4762-5p_st | **-0.4206264324** | **0.0138891487** |
| 200967_at | hsa-miR-132_st | **-0.4206264324** | **0.0138891487** |
| 201756_at | hsa-miR-4652-3p_st | **-0.4206264324** | **0.0138891487** |
| 212203_x_at | hsa-miR-370_st | **-0.4206264324** | **0.0138891487** |
| 213011_s_at | hsa-miR-34c-5p_st | **-0.4206264324** | **0.0138891487** |
| 217286_s_at | hsa-miR-4652-3p_st | **-0.4206264324** | **0.0138891487** |
| 217546_at | hsa-miR-370_st | **-0.4206264324** | **0.0138891487** |
| 209303_at | hsa-miR-34c-5p_st | **-0.4203529694** | **0.0139561016** |
| 200097_s_at | hsa-miR-34c-5p_st | **-0.4203208556** | **0.0139639821** |
| 203362_s_at | hsa-miR-375_st | **-0.4203208556** | **0.0139639821** |
| 204245_s_at | hsa-miR-34b_st | **-0.4203208556** | **0.0139639821** |
| 210872_x_at | hsa-miR-375_st | **-0.4203208556** | **0.0139639821** |
| 212203_x_at | hsa-miR-4760-3p_st | **-0.4203208556** | **0.0139639821** |
| 218432_at | hsa-miR-34c-5p_st | **-0.4203208556** | **0.0139639821** |
| 209476_at | hsa-miR-4311_st | **-0.4202001693** | **0.0139936312** |
| 202149_at | hsa-miR-370_st | **-0.4200152788** | **0.0140391570** |
| 206356_s_at | hsa-miR-34b-star_st | **-0.4200152788** | **0.0140391570** |
| 210968_s_at | hsa-miR-34c-5p_st | **-0.4200152788** | **0.0140391570** |
| 213714_at | hsa-miR-34b_st | **-0.4200152788** | **0.0140391570** |
| 218120_s_at | hsa-miR-375_st | **-0.4200152788** | **0.0140391570** |
| 200804_at | hsa-miR-132_st | **-0.4197097021** | **0.0141146747** |
| 202920_at | hsa-miR-34c-5p_st | **-0.4197097021** | **0.0141146747** |
| 203685_at | hsa-miR-377-star_st | **-0.4197097021** | **0.0141146747** |
| 218526_s_at | hsa-miR-34c-5p_st | **-0.4197097021** | **0.0141146747** |
| 201145_at | hsa-miR-34c-5p_st | **-0.4194041253** | **0.0141905364** |
| 206989_s_at | hsa-miR-4720-3p_st | **-0.4194041253** | **0.0141905364** |
| 208731_at | hsa-miR-34b-star_st | **-0.4194041253** | **0.0141905364** |
| 208659_at | hsa-miR-4311_st | **-0.4192833689** | **0.0142206102** |
| 214150_x_at | hsa-miR-555_st | **-0.4191305689** | **0.0142587417** |
| 218384_at | hsa-miR-34c-5p_st | **-0.4190985485** | **0.0142667434** |
| 201315_x_at | hsa-miR-3189-5p_st | **-0.4187929717** | **0.0143432968** |
| 202382_s_at | hsa-miR-34c-5p_st | **-0.4187929717** | **0.0143432968** |
| 205963_s_at | hsa-miR-34c-5p_st | **-0.4187929717** | **0.0143432968** |
| 210434_x_at | hsa-miR-34b-star_st | **-0.4187929717** | **0.0143432968** |
| 212215_at | hsa-miR-34b-star_st | **-0.4187929717** | **0.0143432968** |
| 209303_at | hsa-miR-34c-3p_st | **-0.4186721687** | **0.0143736564** |
| 209122_at | hsa-miR-4720-3p_st | **-0.4184873950** | **0.0144201980** |
| 200978_at | hsa-miR-4652-3p_st | **-0.4181818182** | **0.0144974482** |
| 203781_at | hsa-miR-34b-star_st | **-0.4181818182** | **0.0144974482** |
| 211270_x_at | hsa-miR-132_st | **-0.4181818182** | **0.0144974482** |
| 218882_s_at | hsa-miR-4652-3p_st | **-0.4181818182** | **0.0144974482** |
| 200739_s_at | hsa-miR-375_st | **-0.4178762414** | **0.0145750485** |
| 201988_s_at | hsa-miR-34b_st | **-0.4178762414** | **0.0145750485** |
| 208860_s_at | hsa-miR-34c-3p_st | **-0.4178762414** | **0.0145750485** |
| 219297_at | hsa-miR-34b-star_st | **-0.4178762414** | **0.0145750485** |
| 211999_at | hsa-miR-377-star_st | **-0.4177553683** | **0.0146058409** |
| 206544_x_at | hsa-miR-34b-star_st | **-0.4175706646** | **0.0146530004** |
| 209733_at | hsa-miR-375_st | **-0.4175706646** | **0.0146530004** |
| 203987_at | hsa-miR-4720-3p_st | **-0.4172650879** | **0.0147313049** |
| 211963_s_at | hsa-miR-34b-star_st | **-0.4172650879** | **0.0147313049** |
| 213293_s_at | hsa-miR-4311_st | **-0.4171441681** | **0.0147623885** |
| 201086_x_at | hsa-miR-34b-star_st | **-0.4169595111** | **0.0148099634** |
| 202252_at | hsa-miR-523_st | **-0.4169595111** | **0.0148099634** |
| 213503_x_at | hsa-miR-383_st | **-0.4169595111** | **0.0148099634** |
| 221009_s_at | hsa-miR-382_st | **-0.4168385680** | **0.0148411935** |
| 201666_at | hsa-miR-4720-3p_st | **-0.4166539343** | **0.0148889772** |
| 207643_s_at | hsa-miR-377-star_st | **-0.4166539343** | **0.0148889772** |
| 212063_at | hsa-miR-4720-3p_st | **-0.4166539343** | **0.0148889772** |
| 215171_s_at | hsa-miR-34c-3p_st | **-0.4166539343** | **0.0148889772** |
| 200600_at | hsa-miR-3676_st | **-0.4163483575** | **0.0149683474** |
| 200708_at | hsa-miR-34c-5p_st | **-0.4163483575** | **0.0149683474** |
| 201172_x_at | hsa-miR-377-star_st | **-0.4160427807** | **0.0150480754** |
| 201324_at | hsa-miR-1912_st | **-0.4160427807** | **0.0150480754** |
| 202658_at | hsa-miR-34b-star_st | **-0.4160427807** | **0.0150480754** |
| 218488_at | hsa-miR-34b-star_st | **-0.4160427807** | **0.0150480754** |
| 202613_at | hsa-miR-34b-star_st | **-0.4157372040** | **0.0151281623** |
| 202975_s_at | hsa-miR-383_st | **-0.4157372040** | **0.0151281623** |
| 204766_s_at | hsa-miR-34c-5p_st | **-0.4157372040** | **0.0151281623** |
| 212067_s_at | hsa-miR-132_st | **-0.4154316272** | **0.0152086096** |
| 205775_at | hsa-miR-375_st | **-0.4151260504** | **0.0152894184** |
| 201172_x_at | hsa-miR-4311_st | **-0.4146993671** | **0.0154028607** |
| 201398_s_at | hsa-miR-383_st | **-0.4145148969** | **0.0154521257** |
| 201989_s_at | hsa-miR-375_st | **-0.4145148969** | **0.0154521257** |
| 207573_x_at | hsa-miR-34b-star_st | **-0.4145148969** | **0.0154521257** |
| 217780_at | hsa-miR-34c-5p_st | **-0.4145148969** | **0.0154521257** |
| 207400_at | hsa-miR-34b-star_st | **-0.4142093201** | **0.0155340268** |
| 210872_x_at | hsa-miR-34b-star_st | **-0.4142093201** | **0.0155340268** |
| 217746_s_at | hsa-miR-377-star_st | **-0.4142093201** | **0.0155340268** |
| 222216_s_at | hsa-miR-34c-5p_st | **-0.4142093201** | **0.0155340268** |
| 213293_s_at | hsa-miR-382_st | **-0.4140881668** | **0.0155665999** |
| 201725_at | hsa-miR-34b-star_st | **-0.4135981665** | **0.0156989303** |
| 203781_at | hsa-miR-4652-3p_st | **-0.4135981665** | **0.0156989303** |
| 201590_x_at | hsa-miR-523_st | **-0.4132925898** | **0.0157819352** |
| 201900_s_at | hsa-miR-34c-5p_st | **-0.4132925898** | **0.0157819352** |
| 202252_at | hsa-miR-3189-5p_st | **-0.4132925898** | **0.0157819352** |
| 219582_at | hsa-miR-1912_st | **-0.4132925898** | **0.0157819352** |
| 201966_at | hsa-miR-34b_st | **-0.4129870130** | **0.0158653107** |
| 203987_at | hsa-miR-1912_st | **-0.4129870130** | **0.0158653107** |
| 217730_at | hsa-miR-1912_st | **-0.4129870130** | **0.0158653107** |
| 217995_at | hsa-miR-3676_st | **-0.4129870130** | **0.0158653107** |
| 202564_x_at | hsa-miR-34c-3p_st | **-0.4126814362** | **0.0159490579** |
| 202825_at | hsa-miR-34b_st | **-0.4126814362** | **0.0159490579** |
| 218322_s_at | hsa-miR-382_st | **-0.4125601662** | **0.0159823969** |
| 202975_s_at | hsa-miR-523_st | **-0.4123758594** | **0.0160331783** |
| 208782_at | hsa-miR-370_st | **-0.4123758594** | **0.0160331783** |
| 202864_s_at | hsa-miR-4311_st | **-0.4121017660** | **0.0161089503** |
| 203685_at | hsa-miR-1912_st | **-0.4120702827** | **0.0161176731** |
| 203983_at | hsa-miR-34b_st | **-0.4120702827** | **0.0161176731** |
| 206984_s_at | hsa-miR-34c-5p_st | **-0.4120702827** | **0.0161176731** |
| 203411_s_at | hsa-miR-383_st | **-0.4117647059** | **0.0162025436** |
| 210278_s_at | hsa-miR-34b-star_st | **-0.4117647059** | **0.0162025436** |
| 219628_at | hsa-miR-34b-star_st | **-0.4117647059** | **0.0162025436** |
| 203411_s_at | hsa-miR-555_st | **-0.4116433659** | **0.0162363489** |
| 200797_s_at | hsa-miR-377-star_st | **-0.4114591291** | **0.0162877911** |
| 201656_at | hsa-miR-383_st | **-0.4114591291** | **0.0162877911** |
| 207054_at | hsa-miR-4652-3p_st | **-0.4114591291** | **0.0162877911** |
| 208457_at | hsa-miR-34c-3p_st | **-0.4114591291** | **0.0162877911** |
| 208678_at | hsa-miR-4652-3p_st | **-0.4114591291** | **0.0162877911** |
| 210068_s_at | hsa-miR-4762-5p_st | **-0.4114591291** | **0.0162877911** |
| 210946_at | hsa-miR-1912_st | **-0.4114591291** | **0.0162877911** |
| 213217_at | hsa-miR-383_st | **-0.4114591291** | **0.0162877911** |
| 201656_at | hsa-miR-132_st | **-0.4111535523** | **0.0163734169** |
| 202121_s_at | hsa-miR-375_st | **-0.4111535523** | **0.0163734169** |
| 211769_x_at | hsa-miR-34b_st | **-0.4111535523** | **0.0163734169** |
| 212990_at | hsa-miR-4652-3p_st | **-0.4111535523** | **0.0163734169** |
| 217546_at | hsa-miR-382_st | **-0.4108793656** | **0.0164505700** |
| 202741_at | hsa-miR-4652-3p_st | **-0.4108479756** | **0.0164594224** |
| 203189_s_at | hsa-miR-375_st | **-0.4108479756** | **0.0164594224** |
| 204070_at | hsa-miR-523_st | **-0.4108479756** | **0.0164594224** |
| 215307_at | hsa-miR-375_st | **-0.4108479756** | **0.0164594224** |
| 205856_at | hsa-miR-4760-3p_st | **-0.4105423988** | **0.0165458088** |
| 208745_at | hsa-miR-34b_st | **-0.4105423988** | **0.0165458088** |
| 202133_at | hsa-miR-4760-3p_st | **-0.4102368220** | **0.0166325774** |
| 214829_at | hsa-miR-370_st | **-0.4102368220** | **0.0166325774** |
| 201753_s_at | hsa-miR-523_st | **-0.4099312452** | **0.0167197297** |
| 203944_x_at | hsa-miR-34b_st | **-0.4099312452** | **0.0167197297** |
| 218788_s_at | hsa-miR-34b_st | **-0.4099312452** | **0.0167197297** |
| 200720_s_at | hsa-miR-34b_st | **-0.4096256684** | **0.0168072668** |
| 221696_s_at | hsa-miR-375_st | **-0.4096256684** | **0.0168072668** |
| 208832_at | hsa-miR-4652-3p_st | **-0.4093200917** | **0.0168951902** |
| 209513_s_at | hsa-miR-4760-3p_st | **-0.4093200917** | **0.0168951902** |
| 209550_at | hsa-miR-34b-star_st | **-0.4093200917** | **0.0168951902** |
| 217959_s_at | hsa-miR-34b-star_st | **-0.4093200917** | **0.0168951902** |
| 200816_s_at | hsa-miR-34b_st | **-0.4090145149** | **0.0169835011** |
| 201412_at | hsa-miR-383_st | **-0.4090145149** | **0.0169835011** |
| 202929_s_at | hsa-miR-34b-star_st | **-0.4090145149** | **0.0169835011** |
| 203157_s_at | hsa-miR-34b_st | **-0.4090145149** | **0.0169835011** |
| 203189_s_at | hsa-miR-34b-star_st | **-0.4090145149** | **0.0169835011** |
| 210749_x_at | hsa-miR-1180_st | **-0.4090145149** | **0.0169835011** |
| 200843_s_at | hsa-miR-4652-3p_st | **-0.4087089381** | **0.0170722008** |
| 205758_at | hsa-miR-34c-5p_st | **-0.4087089381** | **0.0170722008** |
| 201601_x_at | hsa-miR-212_st | **-0.4084033613** | **0.0171612908** |
| 202825_at | hsa-miR-375_st | **-0.4084033613** | **0.0171612908** |
| 205217_at | hsa-miR-34c-5p_st | **-0.4084033613** | **0.0171612908** |
| 207988_s_at | hsa-miR-375_st | **-0.4084033613** | **0.0171612908** |
| 208911_s_at | hsa-miR-4652-3p_st | **-0.4084033613** | **0.0171612908** |
| 219582_at | hsa-miR-4720-3p_st | **-0.4084033613** | **0.0171612908** |
| 200677_at | hsa-miR-370_st | **-0.4080977846** | **0.0172507724** |
| 201601_x_at | hsa-miR-4760-3p_st | **-0.4080977846** | **0.0172507724** |
| 1255_g_at | hsa-miR-34b-star_st | **-0.4077922078** | **0.0173406468** |
| 216218_s_at | hsa-miR-375_st | **-0.4077922078** | **0.0173406468** |
| 217730_at | hsa-miR-523_st | **-0.4077922078** | **0.0173406468** |
| 218491_s_at | hsa-miR-34c-5p_st | **-0.4077922078** | **0.0173406468** |
| 219263_at | hsa-miR-34b-star_st | **-0.4077922078** | **0.0173406468** |
| 221009_s_at | hsa-miR-212_st | **-0.4077922078** | **0.0173406468** |
| 201315_x_at | hsa-miR-4760-3p_st | **-0.4074866310** | **0.0174309154** |
| 207922_s_at | hsa-miR-34c-3p_st | **-0.4074866310** | **0.0174309154** |
| 211672_s_at | hsa-miR-34b-star_st | **-0.4074866310** | **0.0174309154** |
| 212203_x_at | hsa-miR-555_st | **-0.4073649641** | **0.0174669663** |
| 217960_s_at | hsa-miR-34b_st | **-0.4071810542** | **0.0175215796** |
| 200663_at | hsa-miR-1180_st | **-0.4068754775** | **0.0176126407** |
| 202373_s_at | hsa-miR-34b_st | **-0.4068754775** | **0.0176126407** |
| 209549_s_at | hsa-miR-375_st | **-0.4068754775** | **0.0176126407** |
| 214428_x_at | hsa-miR-1912_st | **-0.4068754775** | **0.0176126407** |
| 218559_s_at | hsa-miR-382_st | **-0.4066009638** | **0.0176947846** |
| 200905_x_at | hsa-miR-4720-3p_st | **-0.4065699007** | **0.0177041001** |
| 203415_at | hsa-miR-34b_st | **-0.4065699007** | **0.0177041001** |
| 213217_at | hsa-miR-4760-3p_st | **-0.4065699007** | **0.0177041001** |
| 213503_x_at | hsa-miR-3189-5p_st | **-0.4062643239** | **0.0177959591** |
| 200903_s_at | hsa-miR-34b-star_st | **-0.4059587471** | **0.0178882190** |
| 202376_at | hsa-miR-212_st | **-0.4059587471** | **0.0178882190** |
| 203854_at | hsa-miR-555_st | **-0.4056841635** | **0.0179714646** |
| 212716_s_at | hsa-miR-34c-3p_st | **-0.4056531704** | **0.0179808813** |
| 36711_at | hsa-miR-132_st | **-0.4056531704** | **0.0179808813** |
| 210427_x_at | hsa-miR-523_st | **-0.4053475936** | **0.0180739472** |
| 218201_at | hsa-miR-34c-3p_st | **-0.4053475936** | **0.0180739472** |
| 200905_x_at | hsa-miR-212_st | **-0.4050420168** | **0.0181674182** |
| 201412_at | hsa-miR-377-star_st | **-0.4050420168** | **0.0181674182** |
| 201570_at | hsa-miR-34c-5p_st | **-0.4050420168** | **0.0181674182** |
| 203540_at | hsa-miR-377-star_st | **-0.4050420168** | **0.0181674182** |
| 210105_s_at | hsa-miR-377-star_st | **-0.4050420168** | **0.0181674182** |
| 212600_s_at | hsa-miR-34b-star_st | **-0.4050420168** | **0.0181674182** |
| 203973_s_at | hsa-miR-132_st | **-0.4047364400** | **0.0182612956** |
| 207120_at | hsa-miR-375_st | **-0.4047364400** | **0.0182612956** |
| 208981_at | hsa-miR-132_st | **-0.4047364400** | **0.0182612956** |
| 212195_at | hsa-miR-4311_st | **-0.4046145630** | **0.0182988516** |
| 200816_s_at | hsa-miR-4652-3p_st | **-0.4044308633** | **0.0183555808** |
| 200822_x_at | hsa-miR-34b-star_st | **-0.4044308633** | **0.0183555808** |
| 206099_at | hsa-miR-34b-star_st | **-0.4044308633** | **0.0183555808** |
| 206099_at | hsa-miR-375_st | **-0.4044308633** | **0.0183555808** |
| 209122_at | hsa-miR-4311_st | **-0.4041561628** | **0.0184406883** |
| 204587_at | hsa-miR-4652-3p_st | **-0.4041252865** | **0.0184502751** |
| 205633_s_at | hsa-miR-375_st | **-0.4038197097** | **0.0185453800** |
| 210978_s_at | hsa-miR-377-star_st | **-0.4038197097** | **0.0185453800** |
| 217997_at | hsa-miR-34b-star_st | **-0.4038197097** | **0.0185453800** |
| 207198_s_at | hsa-miR-4311_st | **-0.4036977627** | **0.0185834485** |
| 209248_at | hsa-miR-4652-3p_st | **-0.4036977627** | **0.0185834485** |
| 209157_at | hsa-miR-34b-star_st | **-0.4035141329** | **0.0186408967** |
| 210406_s_at | hsa-miR-34b-star_st | **-0.4035141329** | **0.0186408967** |
| 1007_s_at | hsa-miR-370_st | **-0.4032085561** | **0.0187368267** |
| 202614_at | hsa-miR-34b-star_st | **-0.4032085561** | **0.0187368267** |
| 208779_x_at | hsa-miR-523_st | **-0.4032085561** | **0.0187368267** |
| 213904_at | hsa-miR-375_st | **-0.4032085561** | **0.0187368267** |
| 220329_s_at | hsa-miR-34c-5p_st | **-0.4032085561** | **0.0187368267** |
| 205413_at | hsa-miR-4652-3p_st | **-0.4029029794** | **0.0188331714** |
| 212501_at | hsa-miR-1912_st | **-0.4029029794** | **0.0188331714** |
| 218005_at | hsa-miR-1912_st | **-0.4029029794** | **0.0188331714** |
| 202779_s_at | hsa-miR-34b-star_st | **-0.4025974026** | **0.0189299321** |
| 208690_s_at | hsa-miR-3189-5p_st | **-0.4025974026** | **0.0189299321** |
| 218200_s_at | hsa-miR-34b-star_st | **-0.4025974026** | **0.0189299321** |
| 218432_at | hsa-miR-34b-star_st | **-0.4025974026** | **0.0189299321** |
| 200600_at | hsa-miR-4311_st | **-0.4023225621** | **0.0190173167** |
| 203723_at | hsa-miR-1180_st | **-0.4023225621** | **0.0190173167** |
| 204072_s_at | hsa-miR-34b-star_st | **-0.4022918258** | **0.0190271102** |
| 208745_at | hsa-miR-4652-3p_st | **-0.4022918258** | **0.0190271102** |
| 202269_x_at | hsa-miR-4762-5p_st | **-0.4019862490** | **0.0191247072** |
| 207831_x_at | hsa-miR-34b-star_st | **-0.4019862490** | **0.0191247072** |
| 209122_at | hsa-miR-212_st | **-0.4019862490** | **0.0191247072** |
| 210014_x_at | hsa-miR-34b-star_st | **-0.4019862490** | **0.0191247072** |
| 215952_s_at | hsa-miR-34b-star_st | **-0.4019862490** | **0.0191247072** |
| 217957_at | hsa-miR-34b-star_st | **-0.4019862490** | **0.0191247072** |
| 208998_at | hsa-miR-1180_st | **-0.4016806723** | **0.0192227244** |
| 212310_at | hsa-miR-4652-3p_st | **-0.4016806723** | **0.0192227244** |
| 209513_s_at | hsa-miR-212_st | **-0.4013750955** | **0.0193211632** |
| 211270_x_at | hsa-miR-212_st | **-0.4013750955** | **0.0193211632** |
| 203645_s_at | hsa-miR-382_st | **-0.4012529617** | **0.0193606257** |
| 201999_s_at | hsa-miR-3189-5p_st | **-0.4010695187** | **0.0194200249** |
| 202587_s_at | hsa-miR-377-star_st | **-0.4010695187** | **0.0194200249** |
| 202975_s_at | hsa-miR-4760-3p_st | **-0.4010695187** | **0.0194200249** |
| 204957_at | hsa-miR-34b-star_st | **-0.4010695187** | **0.0194200249** |
| 205775_at | hsa-miR-4652-3p_st | **-0.4010695187** | **0.0194200249** |
| 208782_at | hsa-miR-1180_st | **-0.4010695187** | **0.0194200249** |
| 211962_s_at | hsa-miR-132_st | **-0.4010695187** | **0.0194200249** |
| 214829_at | hsa-miR-383_st | **-0.4010695187** | **0.0194200249** |
| 207507_s_at | hsa-miR-34b_st | **-0.4007639419** | **0.0195193111** |
| 202507_s_at | hsa-miR-375_st | **-0.4004583652** | **0.0196190231** |
| 210534_s_at | hsa-miR-375_st | **-0.4000305612** | **0.0197593368** |
| 200663_at | hsa-miR-4720-3p_st | **-0.3998472116** | **0.0198197301** |
| 207776_s_at | hsa-miR-375_st | **-0.3998472116** | **0.0198197301** |
| 213592_at | hsa-miR-132_st | **-0.3998472116** | **0.0198197301** |
| 203781_at | hsa-miR-375_st | **-0.3995416348** | **0.0199207280** |
| 205531_s_at | hsa-miR-34b-star_st | **-0.3995416348** | **0.0199207280** |
| 212099_at | hsa-miR-4720-3p_st | **-0.3995416348** | **0.0199207280** |
| 214022_s_at | hsa-miR-4762-5p_st | **-0.3995416348** | **0.0199207280** |
| 214717_at | hsa-miR-4652-3p_st | **-0.3995416348** | **0.0199207280** |
| 209046_s_at | hsa-miR-34b-star_st | **-0.3992360581** | **0.0200221573** |
| 218970_s_at | hsa-miR-4652-3p_st | **-0.3992360581** | **0.0200221573** |
| 202376_at | hsa-miR-4311_st | **-0.3989609607** | **0.0201138399** |
| 208659_at | hsa-miR-212_st | **-0.3989304813** | **0.0201240195** |
| 201272_at | hsa-miR-4652-3p_st | **-0.3986249045** | **0.0202263159** |
| 203411_s_at | hsa-miR-4720-3p_st | **-0.3986249045** | **0.0202263159** |
| 204125_at | hsa-miR-4652-3p_st | **-0.3986249045** | **0.0202263159** |
| 207776_s_at | hsa-miR-34b_st | **-0.3986249045** | **0.0202263159** |
| 208981_at | hsa-miR-4760-3p_st | **-0.3986249045** | **0.0202263159** |
| 218048_at | hsa-miR-34b-star_st | **-0.3986249045** | **0.0202263159** |
| 201999_s_at | hsa-miR-382_st | **-0.3985025606** | **0.0202673945** |
| 203120_at | hsa-miR-382_st | **-0.3985025606** | **0.0202673945** |
| 201398_s_at | hsa-miR-212_st | **-0.3983193277** | **0.0203290481** |
| 205278_at | hsa-miR-375_st | **-0.3983193277** | **0.0203290481** |
| 207643_s_at | hsa-miR-1180_st | **-0.3983193277** | **0.0203290481** |
| 202864_s_at | hsa-miR-382_st | **-0.3980441604** | **0.0204219309** |
| 200798_x_at | hsa-miR-4760-3p_st | **-0.3980137510** | **0.0204322174** |
| 209840_s_at | hsa-miR-34b-star_st | **-0.3980137510** | **0.0204322174** |
| 213911_s_at | hsa-miR-34b-star_st | **-0.3980137510** | **0.0204322174** |
| 221449_s_at | hsa-miR-34b_st | **-0.3980137510** | **0.0204322174** |
| 222043_at | hsa-miR-383_st | **-0.3980137510** | **0.0204322174** |
| 205856_at | hsa-miR-3189-5p_st | **-0.3977081742** | **0.0205358252** |
| 208779_x_at | hsa-miR-4720-3p_st | **-0.3977081742** | **0.0205358252** |
| 209840_s_at | hsa-miR-4652-3p_st | **-0.3977081742** | **0.0205358252** |
| 218005_at | hsa-miR-4760-3p_st | **-0.3977081742** | **0.0205358252** |
| 208809_s_at | hsa-miR-377-star_st | **-0.3974025974** | **0.0206398731** |
| 213503_x_at | hsa-miR-4762-5p_st | **-0.3974025974** | **0.0206398731** |
| 218507_at | hsa-miR-377-star_st | **-0.3974025974** | **0.0206398731** |
| 200989_at | hsa-miR-4720-3p_st | **-0.3970970206** | **0.0207443623** |
| 202180_s_at | hsa-miR-3189-5p_st | **-0.3970970206** | **0.0207443623** |
| 212015_x_at | hsa-miR-212_st | **-0.3970970206** | **0.0207443623** |
| 203621_at | hsa-miR-4652-3p_st | **-0.3967914439** | **0.0208492944** |
| 217960_s_at | hsa-miR-375_st | **-0.3967914439** | **0.0208492944** |
| 218882_s_at | hsa-miR-34b-star_st | **-0.3967914439** | **0.0208492944** |
| 200040_at | hsa-miR-375_st | **-0.3964858671** | **0.0209546709** |
| 200818_at | hsa-miR-375_st | **-0.3961802903** | **0.0210604930** |
| 201313_at | hsa-miR-34c-5p_st | **-0.3958747135** | **0.0211667623** |
| 202269_x_at | hsa-miR-132_st | **-0.3958747135** | **0.0211667623** |
| 203721_s_at | hsa-miR-375_st | **-0.3958747135** | **0.0211667623** |
| 205413_at | hsa-miR-375_st | **-0.3958747135** | **0.0211667623** |
| 210906_x_at | hsa-miR-1912_st | **-0.3958747135** | **0.0211667623** |
| 213572_s_at | hsa-miR-132_st | **-0.3958747135** | **0.0211667623** |
| 219911_s_at | hsa-miR-212_st | **-0.3955691367** | **0.0212734803** |
| 200916_at | hsa-miR-3189-5p_st | **-0.3952635600** | **0.0213806483** |
| 202834_at | hsa-miR-132_st | **-0.3952635600** | **0.0213806483** |
| 209513_s_at | hsa-miR-132_st | **-0.3952635600** | **0.0213806483** |
| 211271_x_at | hsa-miR-212_st | **-0.3952635600** | **0.0213806483** |
| 213227_at | hsa-miR-34b_st | **-0.3952635600** | **0.0213806483** |
| 214022_s_at | hsa-miR-4720-3p_st | **-0.3952635600** | **0.0213806483** |
| 219911_s_at | hsa-miR-1912_st | **-0.3952635600** | **0.0213806483** |
| 203150_at | hsa-miR-34b_st | **-0.3949579832** | **0.0214882679** |
| 208659_at | hsa-miR-3189-5p_st | **-0.3949579832** | **0.0214882679** |
| 211271_x_at | hsa-miR-132_st | **-0.3949579832** | **0.0214882679** |
| 211318_s_at | hsa-miR-34b_st | **-0.3949579832** | **0.0214882679** |
| 200053_at | hsa-miR-34b-star_st | **-0.3946524064** | **0.0215963404** |
| 200905_x_at | hsa-miR-383_st | **-0.3946524064** | **0.0215963404** |
| 201054_at | hsa-miR-375_st | **-0.3946524064** | **0.0215963404** |
| 210434_x_at | hsa-miR-375_st | **-0.3946524064** | **0.0215963404** |
| 200986_at | hsa-miR-4762-5p_st | **-0.3943468296** | **0.0217048674** |
| 201161_s_at | hsa-miR-4762-5p_st | **-0.3943468296** | **0.0217048674** |
| 203663_s_at | hsa-miR-4652-3p_st | **-0.3943468296** | **0.0217048674** |
| 203816_at | hsa-miR-34b_st | **-0.3943468296** | **0.0217048674** |
| 203816_at | hsa-miR-4652-3p_st | **-0.3943468296** | **0.0217048674** |
| 208857_s_at | hsa-miR-34b_st | **-0.3943468296** | **0.0217048674** |
| 213533_at | hsa-miR-34b-star_st | **-0.3943468296** | **0.0217048674** |
| 218694_at | hsa-miR-34c-5p_st | **-0.3943468296** | **0.0217048674** |
| 201054_at | hsa-miR-4652-3p_st | **-0.3940412529** | **0.0218138502** |
| 202180_s_at | hsa-miR-212_st | **-0.3940412529** | **0.0218138502** |
| 207761_s_at | hsa-miR-382_st | **-0.3937657587** | **0.0219124963** |
| 201319_at | hsa-miR-4633-5p_st | **-0.3937356761** | **0.0219232905** |
| 211404_s_at | hsa-miR-34b-star_st | **-0.3937356761** | **0.0219232905** |
| 212053_at | hsa-miR-4652-3p_st | **-0.3937356761** | **0.0219232905** |
| 212217_at | hsa-miR-34b-star_st | **-0.3937356761** | **0.0219232905** |
| 213592_at | hsa-miR-383_st | **-0.3937356761** | **0.0219232905** |
| 218432_at | hsa-miR-375_st | **-0.3937356761** | **0.0219232905** |
| 200614_at | hsa-miR-34b_st | **-0.3936129586** | **0.0219673699** |
| 201666_at | hsa-miR-1180_st | **-0.3934300993** | **0.0220331895** |
| 201924_at | hsa-miR-370_st | **-0.3934300993** | **0.0220331895** |
| 203094_at | hsa-miR-4652-3p_st | **-0.3934300993** | **0.0220331895** |
| 204072_s_at | hsa-miR-4652-3p_st | **-0.3934300993** | **0.0220331895** |
| 212099_at | hsa-miR-377-star_st | **-0.3934300993** | **0.0220331895** |
| 213592_at | hsa-miR-1180_st | **-0.3934300993** | **0.0220331895** |
| 200916_at | hsa-miR-4311_st | **-0.3933073585** | **0.0220774621** |
| 219582_at | hsa-miR-4311_st | **-0.3933073585** | **0.0220774621** |
| 200986_at | hsa-miR-523_st | **-0.3931245225** | **0.0221435488** |
| 201512_s_at | hsa-miR-34b_st | **-0.3931245225** | **0.0221435488** |
| 201590_x_at | hsa-miR-4762-5p_st | **-0.3931245225** | **0.0221435488** |
| 219421_at | hsa-miR-34b-star_st | **-0.3931245225** | **0.0221435488** |
| 218507_at | hsa-miR-4311_st | **-0.3930017583** | **0.0221880152** |
| 201146_at | hsa-miR-4760-3p_st | **-0.3928189458** | **0.0222543699** |
| 205202_at | hsa-miR-375_st | **-0.3928189458** | **0.0222543699** |
| 212887_at | hsa-miR-34b_st | **-0.3928189458** | **0.0222543699** |
| 219421_at | hsa-miR-4652-3p_st | **-0.3928189458** | **0.0222543699** |
| 210427_x_at | hsa-miR-4762-5p_st | **-0.3925133690** | **0.0223656543** |
| 215506_s_at | hsa-miR-34c-5p_st | **-0.3925133690** | **0.0223656543** |
| 218488_at | hsa-miR-34b_st | **-0.3922077922** | **0.0224774033** |
| 211999_at | hsa-miR-4762-5p_st | **-0.3919321579** | **0.0225786023** |
| 203033_x_at | hsa-miR-34b_st | **-0.3915966387** | **0.0227023014** |
| 203313_s_at | hsa-miR-3676_st | **-0.3915966387** | **0.0227023014** |
| 205512_s_at | hsa-miR-375_st | **-0.3915966387** | **0.0227023014** |
| 208860_s_at | hsa-miR-34c-5p_st | **-0.3912910619** | **0.0228154534** |
| 209476_at | hsa-miR-4762-5p_st | **-0.3912910619** | **0.0228154534** |
| 222230_s_at | hsa-miR-4652-3p_st | **-0.3912910619** | **0.0228154534** |
| 200798_x_at | hsa-miR-377-star_st | **-0.3909854851** | **0.0229290761** |
| 217730_at | hsa-miR-4760-3p_st | **-0.3909854851** | **0.0229290761** |
| 202376_at | hsa-miR-382_st | **-0.3907097574** | **0.0230320051** |
| 202149_at | hsa-miR-383_st | **-0.3906799083** | **0.0230431709** |
| 202641_at | hsa-miR-375_st | **-0.3906799083** | **0.0230431709** |
| 207508_at | hsa-miR-34b_st | **-0.3906799083** | **0.0230431709** |
| 210156_s_at | hsa-miR-375_st | **-0.3906799083** | **0.0230431709** |
| 203404_at | hsa-miR-34c-5p_st | **-0.3903743316** | **0.0231577393** |
| 218976_at | hsa-miR-4652-3p_st | **-0.3903743316** | **0.0231577393** |
| 201106_at | hsa-miR-34b-star_st | **-0.3900687548** | **0.0232727828** |
| 201753_s_at | hsa-miR-134_st | **-0.3900687548** | **0.0232727828** |
| 210448_s_at | hsa-miR-34c-5p_st | **-0.3900687548** | **0.0232727828** |
| 211297_s_at | hsa-miR-34b_st | **-0.3900687548** | **0.0232727828** |
| 201398_s_at | hsa-miR-1180_st | **-0.3897631780** | **0.0233883029** |
| 212157_at | hsa-miR-34b-star_st | **-0.3897631780** | **0.0233883029** |
| 215527_at | hsa-miR-375_st | **-0.3897631780** | **0.0233883029** |
| 208981_at | hsa-miR-1912_st | **-0.3894576012** | **0.0235043011** |
| 200600_at | hsa-miR-4760-3p_st | **-0.3891520244** | **0.0236207789** |
| 205202_at | hsa-miR-4652-3p_st | **-0.3891520244** | **0.0236207789** |
| 201146_at | hsa-miR-555_st | **-0.3890289567** | **0.0236678249** |
| 200967_at | hsa-miR-1912_st | **-0.3888464477** | **0.0237377378** |
| 201761_at | hsa-miR-4760-3p_st | **-0.3888464477** | **0.0237377378** |
| 206875_s_at | hsa-miR-4652-3p_st | **-0.3888464477** | **0.0237377378** |
| 211566_x_at | hsa-miR-34b_st | **-0.3888464477** | **0.0237377378** |
| 218005_at | hsa-miR-132_st | **-0.3888464477** | **0.0237377378** |
| 48531_at | hsa-miR-1180_st | **-0.3888464477** | **0.0237377378** |
| 202430_s_at | hsa-miR-555_st | **-0.3887233566** | **0.0237849870** |
| 201322_at | hsa-miR-34b-star_st | **-0.3885408709** | **0.0238551794** |
| 207831_x_at | hsa-miR-375_st | **-0.3885408709** | **0.0238551794** |
| 203854_at | hsa-miR-4633-5p_st | **-0.3882352941** | **0.0239731050** |
| 218694_at | hsa-miR-34b-star_st | **-0.3882352941** | **0.0239731050** |
| 200641_s_at | hsa-miR-375_st | **-0.3879297173** | **0.0240915163** |
| 200986_at | hsa-miR-383_st | **-0.3879297173** | **0.0240915163** |
| 202077_at | hsa-miR-4652-3p_st | **-0.3879297173** | **0.0240915163** |
| 204070_at | hsa-miR-4762-5p_st | **-0.3879297173** | **0.0240915163** |
| 204239_s_at | hsa-miR-34c-5p_st | **-0.3879297173** | **0.0240915163** |
| 208991_at | hsa-miR-383_st | **-0.3879297173** | **0.0240915163** |
| 213293_s_at | hsa-miR-4762-5p_st | **-0.3879297173** | **0.0240915163** |
| 213293_s_at | hsa-miR-4760-3p_st | **-0.3879297173** | **0.0240915163** |
| 204072_s_at | hsa-miR-375_st | **-0.3876241406** | **0.0242104147** |
| 210033_s_at | hsa-miR-1912_st | **-0.3876241406** | **0.0242104147** |
| 215171_s_at | hsa-miR-34c-5p_st | **-0.3876241406** | **0.0242104147** |
| 219683_at | hsa-miR-34c-5p_st | **-0.3876241406** | **0.0242104147** |
| 208809_s_at | hsa-miR-1912_st | **-0.3873185638** | **0.0243298017** |
| 212990_at | hsa-miR-34b_st | **-0.3873185638** | **0.0243298017** |
| 203156_at | hsa-miR-4652-3p_st | **-0.3870129870** | **0.0244496790** |
| 205217_at | hsa-miR-34b-star_st | **-0.3870129870** | **0.0244496790** |
| 200641_s_at | hsa-miR-34b_st | **-0.3867074102** | **0.0245700479** |
| 202376_at | hsa-miR-383_st | **-0.3867074102** | **0.0245700479** |
| 202475_at | hsa-miR-34b-star_st | **-0.3867074102** | **0.0245700479** |
| 202670_at | hsa-miR-375_st | **-0.3867074102** | **0.0245700479** |
| 203704_s_at | hsa-miR-4720-3p_st | **-0.3867074102** | **0.0245700479** |
| 204766_s_at | hsa-miR-34c-3p_st | **-0.3867074102** | **0.0245700479** |
| 205856_at | hsa-miR-1180_st | **-0.3867074102** | **0.0245700479** |
| 208869_s_at | hsa-miR-34b-star_st | **-0.3867074102** | **0.0245700479** |
| 218005_at | hsa-miR-4720-3p_st | **-0.3867074102** | **0.0245700479** |
| 201315_x_at | hsa-miR-555_st | **-0.3865841558** | **0.0246187383** |
| 218656_s_at | hsa-miR-4311_st | **-0.3865841558** | **0.0246187383** |
| 200663_at | hsa-miR-212_st | **-0.3864018335** | **0.0246909101** |
| 208818_s_at | hsa-miR-212_st | **-0.3864018335** | **0.0246909101** |
| 209513_s_at | hsa-miR-4720-3p_st | **-0.3864018335** | **0.0246909101** |
| 211558_s_at | hsa-miR-34b-star_st | **-0.3864018335** | **0.0246909101** |
| 212687_at | hsa-miR-4762-5p_st | **-0.3864018335** | **0.0246909101** |
| 203613_s_at | hsa-miR-4652-3p_st | **-0.3862785556** | **0.0247398091** |
| 1007_s_at | hsa-miR-4762-5p_st | **-0.3860962567** | **0.0248122670** |
| 201859_at | hsa-miR-377-star_st | **-0.3860962567** | **0.0248122670** |
| 205279_s_at | hsa-miR-375_st | **-0.3860962567** | **0.0248122670** |
| 211271_x_at | hsa-miR-4762-5p_st | **-0.3860962567** | **0.0248122670** |
| 200097_s_at | hsa-miR-34b-star_st | **-0.3857906799** | **0.0249341202** |
| 201666_at | hsa-miR-3676_st | **-0.3857906799** | **0.0249341202** |
| 202712_s_at | hsa-miR-34c-5p_st | **-0.3857906799** | **0.0249341202** |
| 202948_at | hsa-miR-3189-5p_st | **-0.3857906799** | **0.0249341202** |
| 203540_at | hsa-miR-132_st | **-0.3857906799** | **0.0249341202** |
| 207507_s_at | hsa-miR-375_st | **-0.3857906799** | **0.0249341202** |
| 217730_at | hsa-miR-377-star_st | **-0.3857906799** | **0.0249341202** |
| 207812_s_at | hsa-miR-375_st | **-0.3854851031** | **0.0250564712** |
| 213423_x_at | hsa-miR-34b-star_st | **-0.3854851031** | **0.0250564712** |
| 218201_at | hsa-miR-34c-5p_st | **-0.3854851031** | **0.0250564712** |
| 218559_s_at | hsa-miR-4720-3p_st | **-0.3854851031** | **0.0250564712** |
| 206875_s_at | hsa-miR-34b_st | **-0.3848739496** | **0.0253026728** |
| 211855_s_at | hsa-miR-375_st | **-0.3848739496** | **0.0253026728** |
| 213887_s_at | hsa-miR-34b-star_st | **-0.3848739496** | **0.0253026728** |
| 202948_at | hsa-miR-4760-3p_st | **-0.3845683728** | **0.0254265265** |
| 212063_at | hsa-miR-4311_st | **-0.3844449549** | **0.0254766919** |
| 209671_x_at | hsa-miR-34c-5p_st | **-0.3842627960** | **0.0255508841** |
| 221741_s_at | hsa-miR-212_st | **-0.3842627960** | **0.0255508841** |
| 211999_at | hsa-miR-1912_st | **-0.3839865547** | **0.0256637383** |
| 201274_at | hsa-miR-34b_st | **-0.3839572193** | **0.0256757472** |
| 221958_s_at | hsa-miR-377-star_st | **-0.3839572193** | **0.0256757472** |
| 201160_s_at | hsa-miR-383_st | **-0.3836516425** | **0.0258011173** |
| 201180_s_at | hsa-miR-4762-5p_st | **-0.3836516425** | **0.0258011173** |
| 201412_at | hsa-miR-212_st | **-0.3836516425** | **0.0258011173** |
| 206062_at | hsa-miR-4652-3p_st | **-0.3836516425** | **0.0258011173** |
| 208731_at | hsa-miR-4652-3p_st | **-0.3836516425** | **0.0258011173** |
| 210117_at | hsa-miR-1912_st | **-0.3836516425** | **0.0258011173** |
| 218322_s_at | hsa-miR-1180_st | **-0.3836516425** | **0.0258011173** |
| 200030_s_at | hsa-miR-34b-star_st | **-0.3833460657** | **0.0259269961** |
| 201029_s_at | hsa-miR-4720-3p_st | **-0.3833460657** | **0.0259269961** |
| 201592_at | hsa-miR-4652-3p_st | **-0.3833460657** | **0.0259269961** |
| 202071_at | hsa-miR-1912_st | **-0.3833460657** | **0.0259269961** |
| 202543_s_at | hsa-miR-370_st | **-0.3833460657** | **0.0259269961** |
| 213714_at | hsa-miR-4652-3p_st | **-0.3833460657** | **0.0259269961** |
| 214022_s_at | hsa-miR-523_st | **-0.3833460657** | **0.0259269961** |
| 218854_at | hsa-miR-4720-3p_st | **-0.3833460657** | **0.0259269961** |
| 203120_at | hsa-miR-370_st | **-0.3830404889** | **0.0260533850** |
| 208826_x_at | hsa-miR-4652-3p_st | **-0.3830404889** | **0.0260533850** |
| 210427_x_at | hsa-miR-3189-5p_st | **-0.3830404889** | **0.0260533850** |
| 218322_s_at | hsa-miR-4633-5p_st | **-0.3830404889** | **0.0260533850** |
| 201601_x_at | hsa-miR-383_st | **-0.3827349121** | **0.0261802856** |
| 218322_s_at | hsa-miR-555_st | **-0.3826113542** | **0.0262317427** |
| 200989_at | hsa-miR-4311_st | **-0.3824585541** | **0.0262954941** |
| 202096_s_at | hsa-miR-377-star_st | **-0.3824293354** | **0.0263076995** |
| 203560_at | hsa-miR-375_st | **-0.3824293354** | **0.0263076995** |
| 207400_at | hsa-miR-375_st | **-0.3824293354** | **0.0263076995** |
| 210817_s_at | hsa-miR-1180_st | **-0.3824293354** | **0.0263076995** |
| 212063_at | hsa-miR-1180_st | **-0.3824293354** | **0.0263076995** |
| 200967_at | hsa-miR-377-star_st | **-0.3821237586** | **0.0264356281** |
| 206542_s_at | hsa-miR-34b-star_st | **-0.3821237586** | **0.0264356281** |
| 212820_at | hsa-miR-4652-3p_st | **-0.3821237586** | **0.0264356281** |
| 213496_at | hsa-miR-375_st | **-0.3821237586** | **0.0264356281** |
| 201412_at | hsa-miR-3189-5p_st | **-0.3818181818** | **0.0265640732** |
| 202975_s_at | hsa-miR-4762-5p_st | **-0.3818181818** | **0.0265640732** |
| 209104_s_at | hsa-miR-34c-3p_st | **-0.3818181818** | **0.0265640732** |
| 201666_at | hsa-miR-383_st | **-0.3815126050** | **0.0266930362** |
| 204326_x_at | hsa-miR-370_st | **-0.3815126050** | **0.0266930362** |
| 211902_x_at | hsa-miR-34c-3p_st | **-0.3815126050** | **0.0266930362** |
| 212067_s_at | hsa-miR-4720-3p_st | **-0.3815126050** | **0.0266930362** |
| 221958_s_at | hsa-miR-3676_st | **-0.3815126050** | **0.0266930362** |
| 201656_at | hsa-miR-377-star_st | **-0.3812070283** | **0.0268225187** |
| 219760_at | hsa-miR-34b-star_st | **-0.3812070283** | **0.0268225187** |
| 212015_x_at | hsa-miR-382_st | **-0.3809305535** | **0.0269401187** |
| 202736_s_at | hsa-miR-34b-star_st | **-0.3809014515** | **0.0269525223** |
| 208675_s_at | hsa-miR-375_st | **-0.3809014515** | **0.0269525223** |
| 208826_x_at | hsa-miR-34b_st | **-0.3809014515** | **0.0269525223** |
| 208981_at | hsa-miR-377-star_st | **-0.3809014515** | **0.0269525223** |
| 210406_s_at | hsa-miR-34b_st | **-0.3809014515** | **0.0269525223** |
| 212092_at | hsa-miR-34c-5p_st | **-0.3809014515** | **0.0269525223** |
| 217995_at | hsa-miR-377-star_st | **-0.3809014515** | **0.0269525223** |
| 222360_at | hsa-miR-34b-star_st | **-0.3809014515** | **0.0269525223** |
| 201410_at | hsa-miR-4652-3p_st | **-0.3805958747** | **0.0270830485** |
| 202232_s_at | hsa-miR-34c-5p_st | **-0.3805958747** | **0.0270830485** |
| 212195_at | hsa-miR-377-star_st | **-0.3805958747** | **0.0270830485** |
| 214022_s_at | hsa-miR-383_st | **-0.3805958747** | **0.0270830485** |
| 218384_at | hsa-miR-34b-star_st | **-0.3805958747** | **0.0270830485** |
| 214022_s_at | hsa-miR-370_st | **-0.3802902979** | **0.0272140989** |
| 218224_at | hsa-miR-4652-3p_st | **-0.3802902979** | **0.0272140989** |
| 201160_s_at | hsa-miR-212_st | **-0.3799847212** | **0.0273456752** |
| 208457_at | hsa-miR-34c-5p_st | **-0.3799847212** | **0.0273456752** |
| 210978_s_at | hsa-miR-212_st | **-0.3799847212** | **0.0273456752** |
| 200739_s_at | hsa-miR-34b-star_st | **-0.3796791444** | **0.0274777788** |
| 218559_s_at | hsa-miR-377-star_st | **-0.3796791444** | **0.0274777788** |
| 221449_s_at | hsa-miR-375_st | **-0.3796791444** | **0.0274777788** |
| 203685_at | hsa-miR-4720-3p_st | **-0.3793735676** | **0.0276104113** |
| 208839_s_at | hsa-miR-375_st | **-0.3793735676** | **0.0276104113** |
| 212203_x_at | hsa-miR-523_st | **-0.3790679908** | **0.0277435744** |
| 200843_s_at | hsa-miR-375_st | **-0.3787624141** | **0.0278772696** |
| 202376_at | hsa-miR-1180_st | **-0.3787624141** | **0.0278772696** |
| 217730_at | hsa-miR-382_st | **-0.3786385526** | **0.0279316131** |
| 201029_s_at | hsa-miR-4762-5p_st | **-0.3784568373** | **0.0280114985** |
| 211672_s_at | hsa-miR-34b_st | **-0.3784568373** | **0.0280114985** |
| 218854_at | hsa-miR-377-star_st | **-0.3784568373** | **0.0280114985** |
| 221699_s_at | hsa-miR-34b_st | **-0.3784568373** | **0.0280114985** |
| 203540_at | hsa-miR-212_st | **-0.3781512605** | **0.0281462627** |
| 209122_at | hsa-miR-370_st | **-0.3781512605** | **0.0281462627** |
| 211769_x_at | hsa-miR-375_st | **-0.3778456837** | **0.0282815638** |
| 213552_at | hsa-miR-34b-star_st | **-0.3778456837** | **0.0282815638** |
| 220329_s_at | hsa-miR-34b-star_st | **-0.3778456837** | **0.0282815638** |
| 44669_at | hsa-miR-375_st | **-0.3778456837** | **0.0282815638** |
| 213503_x_at | hsa-miR-523_st | **-0.3775401070** | **0.0284174034** |
| 218706_s_at | hsa-miR-134_st | **-0.3775401070** | **0.0284174034** |
| 201753_s_at | hsa-miR-4760-3p_st | **-0.3772345302** | **0.0285537830** |
| 203973_s_at | hsa-miR-382_st | **-0.3771105519** | **0.0286092692** |
| 201592_at | hsa-miR-375_st | **-0.3769289534** | **0.0286907044** |
| 209569_x_at | hsa-miR-34b-star_st | **-0.3769289534** | **0.0286907044** |
| 211318_s_at | hsa-miR-375_st | **-0.3769289534** | **0.0286907044** |
| 210749_x_at | hsa-miR-523_st | **-0.3766233766** | **0.0288281690** |
| 215884_s_at | hsa-miR-4652-3p_st | **-0.3766233766** | **0.0288281690** |
| 200797_s_at | hsa-miR-4760-3p_st | **-0.3763177998** | **0.0289661785** |
| 201725_at | hsa-miR-375_st | **-0.3763177998** | **0.0289661785** |
| 202096_s_at | hsa-miR-383_st | **-0.3763177998** | **0.0289661785** |
| 202543_s_at | hsa-miR-212_st | **-0.3763177998** | **0.0289661785** |
| 221009_s_at | hsa-miR-132_st | **-0.3763177998** | **0.0289661785** |
| 204326_x_at | hsa-miR-377-star_st | **-0.3760122231** | **0.0291047344** |
| 202149_at | hsa-miR-4311_st | **-0.3757353514** | **0.0292307480** |
| 208809_s_at | hsa-miR-132_st | **-0.3757066463** | **0.0292438385** |
| 218970_s_at | hsa-miR-34b_st | **-0.3754010695** | **0.0293834923** |
| 219297_at | hsa-miR-375_st | **-0.3754010695** | **0.0293834923** |
| 202471_s_at | hsa-miR-375_st | **-0.3750954927** | **0.0295236974** |
| 208690_s_at | hsa-miR-1912_st | **-0.3750954927** | **0.0295236974** |
| 210978_s_at | hsa-miR-132_st | **-0.3750954927** | **0.0295236974** |
| 201410_at | hsa-miR-375_st | **-0.3747899160** | **0.0296644554** |
| 206042_x_at | hsa-miR-375_st | **-0.3747899160** | **0.0296644554** |
| 210033_s_at | hsa-miR-377-star_st | **-0.3747899160** | **0.0296644554** |
| 218559_s_at | hsa-miR-132_st | **-0.3747899160** | **0.0296644554** |
| 200986_at | hsa-miR-4760-3p_st | **-0.3744843392** | **0.0298057680** |
| 212215_at | hsa-miR-4652-3p_st | **-0.3744843392** | **0.0298057680** |
| 213503_x_at | hsa-miR-3676_st | **-0.3744843392** | **0.0298057680** |
| 217968_at | hsa-miR-4652-3p_st | **-0.3744843392** | **0.0298057680** |
| 218976_at | hsa-miR-34b_st | **-0.3744843392** | **0.0298057680** |
| 201628_s_at | hsa-miR-375_st | **-0.3741787624** | **0.0299476367** |
| 204245_s_at | hsa-miR-4652-3p_st | **-0.3741787624** | **0.0299476367** |
| 212407_at | hsa-miR-375_st | **-0.3741787624** | **0.0299476367** |
| 202594_at | hsa-miR-4652-3p_st | **-0.3738731856** | **0.0300900632** |
| 208659_at | hsa-miR-4762-5p_st | **-0.3738731856** | **0.0300900632** |
| 210927_x_at | hsa-miR-34c-5p_st | **-0.3738731856** | **0.0300900632** |
| 202825_at | hsa-miR-4652-3p_st | **-0.3735676089** | **0.0302330492** |
| 202864_s_at | hsa-miR-4720-3p_st | **-0.3735676089** | **0.0302330492** |
| 208779_x_at | hsa-miR-1180_st | **-0.3735676089** | **0.0302330492** |
| 209228_x_at | hsa-miR-375_st | **-0.3735676089** | **0.0302330492** |
| 217746_s_at | hsa-miR-4760-3p_st | **-0.3735676089** | **0.0302330492** |
| 222043_at | hsa-miR-132_st | **-0.3735676089** | **0.0302330492** |
| 200816_s_at | hsa-miR-375_st | **-0.3732620321** | **0.0303765962** |
| 201198_s_at | hsa-miR-375_st | **-0.3732620321** | **0.0303765962** |
| 202149_at | hsa-miR-4760-3p_st | **-0.3732620321** | **0.0303765962** |
| 200986_at | hsa-miR-3189-5p_st | **-0.3729564553** | **0.0305207059** |
| 207830_s_at | hsa-miR-34b_st | **-0.3729564553** | **0.0305207059** |
| 213592_at | hsa-miR-212_st | **-0.3729564553** | **0.0305207059** |
| 219355_at | hsa-miR-34c-5p_st | **-0.3729564553** | **0.0305207059** |
| 214150_x_at | hsa-miR-382_st | **-0.3728321502** | **0.0305794894** |
| 203723_at | hsa-miR-4760-3p_st | **-0.3726793502** | **0.0306518762** |
| 203817_at | hsa-miR-375_st | **-0.3726508785** | **0.0306653798** |
| 204125_at | hsa-miR-375_st | **-0.3726508785** | **0.0306653798** |
| 206015_s_at | hsa-miR-375_st | **-0.3726508785** | **0.0306653798** |
| 207643_s_at | hsa-miR-4760-3p_st | **-0.3726508785** | **0.0306653798** |
| 218559_s_at | hsa-miR-4311_st | **-0.3725265501** | **0.0307244045** |
| 201029_s_at | hsa-miR-4760-3p_st | **-0.3723453018** | **0.0308106197** |
| 204068_at | hsa-miR-1912_st | **-0.3723453018** | **0.0308106197** |
| 208761_s_at | hsa-miR-34b_st | **-0.3723453018** | **0.0308106197** |
| 221531_at | hsa-miR-34b_st | **-0.3723453018** | **0.0308106197** |
| 208782_at | hsa-miR-3189-5p_st | **-0.3720397250** | **0.0309564272** |
| 203987_at | hsa-miR-4760-3p_st | **-0.3717341482** | **0.0311028039** |
| 206989_s_at | hsa-miR-377-star_st | **-0.3717341482** | **0.0311028039** |
| 208857_s_at | hsa-miR-375_st | **-0.3717341482** | **0.0311028039** |
| 208991_at | hsa-miR-370_st | **-0.3717341482** | **0.0311028039** |
| 212310_at | hsa-miR-375_st | **-0.3717341482** | **0.0311028039** |
| 200673_at | hsa-miR-4720-3p_st | **-0.3714285714** | **0.0312497514** |
| 202614_at | hsa-miR-34b_st | **-0.3714285714** | **0.0312497514** |
| 205856_at | hsa-miR-523_st | **-0.3714285714** | **0.0312497514** |
| 212716_s_at | hsa-miR-34c-5p_st | **-0.3714285714** | **0.0312497514** |
| 217906_at | hsa-miR-375_st | **-0.3714285714** | **0.0312497514** |
| 204002_s_at | hsa-miR-34b_st | **-0.3711229947** | **0.0313972715** |
| 204068_at | hsa-miR-370_st | **-0.3711229947** | **0.0313972715** |
| 205031_at | hsa-miR-4652-3p_st | **-0.3711229947** | **0.0313972715** |
| 205856_at | hsa-miR-4762-5p_st | **-0.3711229947** | **0.0313972715** |
| 222043_at | hsa-miR-212_st | **-0.3711229947** | **0.0313972715** |
| 210906_x_at | hsa-miR-4762-5p_st | **-0.3708174179** | **0.0315453656** |
| 210927_x_at | hsa-miR-34c-3p_st | **-0.3708174179** | **0.0315453656** |
| 219960_s_at | hsa-miR-34b-star_st | **-0.3708174179** | **0.0315453656** |
| 202370_s_at | hsa-miR-4311_st | **-0.3705401493** | **0.0316802388** |
| 202564_x_at | hsa-miR-34c-5p_st | **-0.3705118411** | **0.0316940356** |
| 204068_at | hsa-miR-383_st | **-0.3705118411** | **0.0316940356** |
| 208813_at | hsa-miR-34b_st | **-0.3705118411** | **0.0316940356** |
| 218656_s_at | hsa-miR-212_st | **-0.3705118411** | **0.0316940356** |
| 200079_s_at | hsa-miR-375_st | **-0.3702062643** | **0.0318432829** |
| 200853_at | hsa-miR-375_st | **-0.3702062643** | **0.0318432829** |
| 205202_at | hsa-miR-34b_st | **-0.3702062643** | **0.0318432829** |
| 206803_at | hsa-miR-34b-star_st | **-0.3702062643** | **0.0318432829** |
| 209122_at | hsa-miR-3189-5p_st | **-0.3702062643** | **0.0318432829** |
| 211475_s_at | hsa-miR-375_st | **-0.3702062643** | **0.0318432829** |
| 212321_at | hsa-miR-370_st | **-0.3702062643** | **0.0318432829** |
| 217947_at | hsa-miR-377-star_st | **-0.3702062643** | **0.0318432829** |
| 218226_s_at | hsa-miR-34c-5p_st | **-0.3702062643** | **0.0318432829** |
| 204070_at | hsa-miR-4311_st | **-0.3700817491** | **0.0319042636** |
| 200905_x_at | hsa-miR-361-5p_st | **-0.3699006875** | **0.0319931094** |
| 203033_x_at | hsa-miR-4652-3p_st | **-0.3699006875** | **0.0319931094** |
| 206849_at | hsa-miR-34b-star_st | **-0.3699006875** | **0.0319931094** |
| 209122_at | hsa-miR-383_st | **-0.3699006875** | **0.0319931094** |
| 218597_s_at | hsa-miR-375_st | **-0.3699006875** | **0.0319931094** |
| 201666_at | hsa-miR-523_st | **-0.3695951108** | **0.0321435166** |
| 202096_s_at | hsa-miR-523_st | **-0.3695951108** | **0.0321435166** |
| 209243_s_at | hsa-miR-34c-3p_st | **-0.3695951108** | **0.0321435166** |
| 221741_s_at | hsa-miR-1180_st | **-0.3695951108** | **0.0321435166** |
| 200905_x_at | hsa-miR-4311_st | **-0.3693177488** | **0.0322805404** |
| 221531_at | hsa-miR-34b-star_st | **-0.3692895340** | **0.0322945061** |
| 202133_at | hsa-miR-4762-5p_st | **-0.3689839572** | **0.0324460797** |
| 202471_s_at | hsa-miR-34b-star_st | **-0.3689839572** | **0.0324460797** |
| 203313_s_at | hsa-miR-212_st | **-0.3689839572** | **0.0324460797** |
| 207054_at | hsa-miR-375_st | **-0.3689839572** | **0.0324460797** |
| 213293_s_at | hsa-miR-212_st | **-0.3689839572** | **0.0324460797** |
| 212460_at | hsa-miR-4311_st | **-0.3688593486** | **0.0325080567** |
| 211615_s_at | hsa-miR-375_st | **-0.3686783804** | **0.0325982391** |
| 212015_x_at | hsa-miR-132_st | **-0.3686783804** | **0.0325982391** |
| 204194_at | hsa-miR-4311_st | **-0.3684009484** | **0.0327368926** |
| 201112_s_at | hsa-miR-375_st | **-0.3683728037** | **0.0327509857** |
| 219911_s_at | hsa-miR-132_st | **-0.3683728037** | **0.0327509857** |
| 217995_at | hsa-miR-4311_st | **-0.3680953483** | **0.0328901857** |
| 201012_at | hsa-miR-4760-3p_st | **-0.3680672269** | **0.0329043214** |
| 210946_at | hsa-miR-4720-3p_st | **-0.3680672269** | **0.0329043214** |
| 211270_x_at | hsa-miR-383_st | **-0.3680672269** | **0.0329043214** |
| 210117_at | hsa-miR-4311_st | **-0.3679425483** | **0.0329670537** |
| 201989_s_at | hsa-miR-4652-3p_st | **-0.3677616501** | **0.0330582479** |
| 202376_at | hsa-miR-523_st | **-0.3677616501** | **0.0330582479** |
| 202864_s_at | hsa-miR-1912_st | **-0.3677616501** | **0.0330582479** |
| 203094_at | hsa-miR-34b_st | **-0.3677616501** | **0.0330582479** |
| 209507_at | hsa-miR-4652-3p_st | **-0.3677616501** | **0.0330582479** |
| 212067_s_at | hsa-miR-4762-5p_st | **-0.3677616501** | **0.0330582479** |
| 201172_x_at | hsa-miR-382_st | **-0.3676369481** | **0.0331212334** |
| 203313_s_at | hsa-miR-132_st | **-0.3674560733** | **0.0332127666** |
| 210501_x_at | hsa-miR-375_st | **-0.3674560733** | **0.0332127666** |
| 212887_at | hsa-miR-375_st | **-0.3671504966** | **0.0333678794** |
| 215307_at | hsa-miR-4652-3p_st | **-0.3671504966** | **0.0333678794** |
| 217936_at | hsa-miR-1180_st | **-0.3671504966** | **0.0333678794** |
| 213572_s_at | hsa-miR-4311_st | **-0.3668729478** | **0.0335092812** |
| 200986_at | hsa-miR-1180_st | **-0.3668449198** | **0.0335235879** |
| 203033_x_at | hsa-miR-375_st | **-0.3668449198** | **0.0335235879** |
| 204744_s_at | hsa-miR-375_st | **-0.3668449198** | **0.0335235879** |
| 208826_x_at | hsa-miR-375_st | **-0.3668449198** | **0.0335235879** |
| 218214_at | hsa-miR-34b-star_st | **-0.3668449198** | **0.0335235879** |
| 221958_s_at | hsa-miR-4760-3p_st | **-0.3668449198** | **0.0335235879** |
| 200701_at | hsa-miR-4760-3p_st | **-0.3662337662** | **0.0338367987** |
| 201761_at | hsa-miR-132_st | **-0.3662337662** | **0.0338367987** |
| 203362_s_at | hsa-miR-4652-3p_st | **-0.3662337662** | **0.0338367987** |
| 204766_s_at | hsa-miR-34b-star_st | **-0.3662337662** | **0.0338367987** |
| 212687_at | hsa-miR-4760-3p_st | **-0.3662337662** | **0.0338367987** |
| 201315_x_at | hsa-miR-4633-5p_st | **-0.3659281895** | **0.0339943044** |
| 207079_s_at | hsa-miR-375_st | **-0.3659281895** | **0.0339943044** |
| 1255_g_at | hsa-miR-375_st | **-0.3656226127** | **0.0341524124** |
| 209108_at | hsa-miR-212_st | **-0.3656226127** | **0.0341524124** |
| 211271_x_at | hsa-miR-4720-3p_st | **-0.3656226127** | **0.0341524124** |
| 212820_at | hsa-miR-34b_st | **-0.3656226127** | **0.0341524124** |
| 218976_at | hsa-miR-375_st | **-0.3656226127** | **0.0341524124** |
| 201999_s_at | hsa-miR-4311_st | **-0.3654977473** | **0.0342171926** |
| 200638_s_at | hsa-miR-375_st | **-0.3653170359** | **0.0343111246** |
| 200786_at | hsa-miR-34b_st | **-0.3653170359** | **0.0343111246** |
| 201412_at | hsa-miR-134_st | **-0.3653170359** | **0.0343111246** |
| 202233_s_at | hsa-miR-34b-star_st | **-0.3653170359** | **0.0343111246** |
| 203817_at | hsa-miR-4652-3p_st | **-0.3653170359** | **0.0343111246** |
| 211271_x_at | hsa-miR-377-star_st | **-0.3653170359** | **0.0343111246** |
| 206989_s_at | hsa-miR-555_st | **-0.3651921471** | **0.0343761643** |
| 202395_at | hsa-miR-4652-3p_st | **-0.3650114591** | **0.0344704425** |
| 203704_s_at | hsa-miR-377-star_st | **-0.3650114591** | **0.0344704425** |
| 214629_x_at | hsa-miR-375_st | **-0.3650114591** | **0.0344704425** |
| 222043_at | hsa-miR-370_st | **-0.3650114591** | **0.0344704425** |
| 202078_at | hsa-miR-375_st | **-0.3644003056** | **0.0347909025** |
| 207088_s_at | hsa-miR-34c-5p_st | **-0.3644003056** | **0.0347909025** |
| 209732_at | hsa-miR-3189-5p_st | **-0.3644003056** | **0.0347909025** |
| 215522_at | hsa-miR-4652-3p_st | **-0.3644003056** | **0.0347909025** |
| 220251_at | hsa-miR-375_st | **-0.3644003056** | **0.0347909025** |
| 201412_at | hsa-miR-523_st | **-0.3640947288** | **0.0349520479** |
| 212990_at | hsa-miR-375_st | **-0.3640947288** | **0.0349520479** |
| 218285_s_at | hsa-miR-4762-5p_st | **-0.3640947288** | **0.0349520479** |
| 218656_s_at | hsa-miR-3189-5p_st | **-0.3640947288** | **0.0349520479** |
| 204068_at | hsa-miR-382_st | **-0.3639697467** | **0.0350181334** |
| 201160_s_at | hsa-miR-1180_st | **-0.3637891520** | **0.0351138058** |
| 217746_s_at | hsa-miR-1912_st | **-0.3637891520** | **0.0351138058** |
| 218200_s_at | hsa-miR-375_st | **-0.3637891520** | **0.0351138058** |
| 36711_at | hsa-miR-4760-3p_st | **-0.3637891520** | **0.0351138058** |
| 201590_x_at | hsa-miR-4311_st | **-0.3635113465** | **0.0352613960** |
| 200804_at | hsa-miR-383_st | **-0.3634835752** | **0.0352761780** |
| 202613_at | hsa-miR-375_st | **-0.3634835752** | **0.0352761780** |
| 205196_s_at | hsa-miR-4652-3p_st | **-0.3634835752** | **0.0352761780** |
| 210972_x_at | hsa-miR-34b-star_st | **-0.3634835752** | **0.0352761780** |
| 201180_s_at | hsa-miR-4311_st | **-0.3633585464** | **0.0353427911** |
| 209046_s_at | hsa-miR-4652-3p_st | **-0.3631779985** | **0.0354391661** |
| 218285_s_at | hsa-miR-382_st | **-0.3630529463** | **0.0355060442** |
| 203411_s_at | hsa-miR-1180_st | **-0.3628724217** | **0.0356027718** |
| 206544_x_at | hsa-miR-375_st | **-0.3628724217** | **0.0356027718** |
| 213572_s_at | hsa-miR-4762-5p_st | **-0.3628724217** | **0.0356027718** |
| 218322_s_at | hsa-miR-212_st | **-0.3628724217** | **0.0356027718** |
| 218788_s_at | hsa-miR-4652-3p_st | **-0.3628724217** | **0.0356027718** |
| 201590_x_at | hsa-miR-3189-5p_st | **-0.3625668449** | **0.0357669968** |
| 201172_x_at | hsa-miR-4760-3p_st | **-0.3622612681** | **0.0359318429** |
| 204068_at | hsa-miR-1180_st | **-0.3622612681** | **0.0359318429** |
| 215171_s_at | hsa-miR-34b_st | **-0.3622612681** | **0.0359318429** |
| 217995_at | hsa-miR-132_st | **-0.3622612681** | **0.0359318429** |
| 212099_at | hsa-miR-4311_st | **-0.3621361459** | **0.0359995208** |
| 211270_x_at | hsa-miR-4311_st | **-0.3619833458** | **0.0360823112** |
| 200677_at | hsa-miR-4720-3p_st | **-0.3619556914** | **0.0360973117** |
| 201324_at | hsa-miR-3676_st | **-0.3619556914** | **0.0360973117** |
| 203159_at | hsa-miR-34b_st | **-0.3619556914** | **0.0360973117** |
| 204068_at | hsa-miR-4720-3p_st | **-0.3619556914** | **0.0360973117** |
| 200906_s_at | hsa-miR-212_st | **-0.3616501146** | **0.0362634049** |
| 203973_s_at | hsa-miR-212_st | **-0.3616501146** | **0.0362634049** |
| 206984_s_at | hsa-miR-34b-star_st | **-0.3616501146** | **0.0362634049** |
| 210427_x_at | hsa-miR-3676_st | **-0.3616501146** | **0.0362634049** |
| 210946_at | hsa-miR-370_st | **-0.3616501146** | **0.0362634049** |
| 220251_at | hsa-miR-4652-3p_st | **-0.3616501146** | **0.0362634049** |
| 202930_s_at | hsa-miR-34c-3p_st | **-0.3613445378** | **0.0364301242** |
| 205280_at | hsa-miR-4652-3p_st | **-0.3613445378** | **0.0364301242** |
| 208761_s_at | hsa-miR-34c-3p_st | **-0.3613445378** | **0.0364301242** |
| 210978_s_at | hsa-miR-1180_st | **-0.3613445378** | **0.0364301242** |
| 221515_s_at | hsa-miR-34b-star_st | **-0.3613445378** | **0.0364301242** |
| 201322_at | hsa-miR-375_st | **-0.3610389610** | **0.0365974715** |
| 203079_s_at | hsa-miR-375_st | **-0.3610389610** | **0.0365974715** |
| 203854_at | hsa-miR-377-star_st | **-0.3610389610** | **0.0365974715** |
| 203944_x_at | hsa-miR-4652-3p_st | **-0.3610389610** | **0.0365974715** |
| 207776_s_at | hsa-miR-4652-3p_st | **-0.3610389610** | **0.0365974715** |
| 208457_at | hsa-miR-375_st | **-0.3610389610** | **0.0365974715** |
| 214629_x_at | hsa-miR-34b_st | **-0.3610389610** | **0.0365974715** |
| 222230_s_at | hsa-miR-375_st | **-0.3610389610** | **0.0365974715** |
| 209104_s_at | hsa-miR-34c-5p_st | **-0.3607333843** | **0.0367654482** |
| 202863_at | hsa-miR-4311_st | **-0.3606081453** | **0.0368344749** |
| 202078_at | hsa-miR-4652-3p_st | **-0.3604278075** | **0.0369340563** |
| 207198_s_at | hsa-miR-4720-3p_st | **-0.3604278075** | **0.0369340563** |
| 212977_at | hsa-miR-212_st | **-0.3604278075** | **0.0369340563** |
| 200798_x_at | hsa-miR-555_st | **-0.3601497451** | **0.0370880328** |
| 201924_at | hsa-miR-555_st | **-0.3601497451** | **0.0370880328** |
| 201753_s_at | hsa-miR-4762-5p_st | **-0.3601222307** | **0.0371032974** |
| 204554_at | hsa-miR-4760-3p_st | **-0.3601222307** | **0.0371032974** |
| 208909_at | hsa-miR-4652-3p_st | **-0.3601222307** | **0.0371032974** |
| 209513_s_at | hsa-miR-370_st | **-0.3601222307** | **0.0371032974** |
| 218656_s_at | hsa-miR-555_st | **-0.3598441450** | **0.0372578643** |
| 208813_at | hsa-miR-375_st | **-0.3598166539** | **0.0372731731** |
| 211475_s_at | hsa-miR-34b-star_st | **-0.3598166539** | **0.0372731731** |
| 218667_at | hsa-miR-375_st | **-0.3598166539** | **0.0372731731** |
| 219960_s_at | hsa-miR-34b_st | **-0.3598166539** | **0.0372731731** |
| 202779_s_at | hsa-miR-34b_st | **-0.3592055004** | **0.0376148357** |
| 203157_s_at | hsa-miR-375_st | **-0.3592055004** | **0.0376148357** |
| 209183_s_at | hsa-miR-3676_st | **-0.3592055004** | **0.0376148357** |
| 218654_s_at | hsa-miR-375_st | **-0.3592055004** | **0.0376148357** |
| 202543_s_at | hsa-miR-382_st | **-0.3589273446** | **0.0377711841** |
| 200903_s_at | hsa-miR-375_st | **-0.3588999236** | **0.0377866259** |
| 203302_at | hsa-miR-375_st | **-0.3588999236** | **0.0377866259** |
| 209549_s_at | hsa-miR-4652-3p_st | **-0.3588999236** | **0.0377866259** |
| 213496_at | hsa-miR-4652-3p_st | **-0.3588999236** | **0.0377866259** |
| 214629_x_at | hsa-miR-4652-3p_st | **-0.3588999236** | **0.0377866259** |
| 202252_at | hsa-miR-4311_st | **-0.3587745446** | **0.0378572975** |
| 210068_s_at | hsa-miR-382_st | **-0.3587745446** | **0.0378572975** |
| 204766_s_at | hsa-miR-375_st | **-0.3585943468** | **0.0379590577** |
| 218866_s_at | hsa-miR-375_st | **-0.3585943468** | **0.0379590577** |
| 210105_s_at | hsa-miR-382_st | **-0.3580105442** | **0.0382902777** |
| 203137_at | hsa-miR-34c-3p_st | **-0.3579831933** | **0.0383058531** |
| 200967_at | hsa-miR-3189-5p_st | **-0.3576776165** | **0.0384802200** |
| 212217_at | hsa-miR-375_st | **-0.3576776165** | **0.0384802200** |
| 213887_s_at | hsa-miR-375_st | **-0.3576776165** | **0.0384802200** |
| 213293_s_at | hsa-miR-361-5p_st | **-0.3573720397** | **0.0386552355** |
| 202641_at | hsa-miR-4652-3p_st | **-0.3570664629** | **0.0388309013** |
| 203854_at | hsa-miR-1912_st | **-0.3570664629** | **0.0388309013** |
| 206849_at | hsa-miR-375_st | **-0.3570664629** | **0.0388309013** |
| 210749_x_at | hsa-miR-370_st | **-0.3570664629** | **0.0388309013** |
| 211962_s_at | hsa-miR-3676_st | **-0.3570664629** | **0.0388309013** |
| 202864_s_at | hsa-miR-555_st | **-0.3569409438** | **0.0389032468** |
| 202864_s_at | hsa-miR-132_st | **-0.3567608862** | **0.0390072190** |
| 206042_x_at | hsa-miR-34b_st | **-0.3567608862** | **0.0390072190** |
| 208991_at | hsa-miR-382_st | **-0.3564825436** | **0.0391683915** |
| 206671_at | hsa-miR-4652-3p_st | **-0.3564553094** | **0.0391841905** |
| 202180_s_at | hsa-miR-3676_st | **-0.3558441558** | **0.0395401016** |
| 204744_s_at | hsa-miR-34b_st | **-0.3558441558** | **0.0395401016** |
| 209507_at | hsa-miR-375_st | **-0.3558441558** | **0.0395401016** |
| 210817_s_at | hsa-miR-4720-3p_st | **-0.3558441558** | **0.0395401016** |
| 210968_s_at | hsa-miR-34b-star_st | **-0.3555385791** | **0.0397190446** |
| 200978_at | hsa-miR-375_st | **-0.3552330023** | **0.0398986484** |
| 208581_x_at | hsa-miR-377-star_st | **-0.3552330023** | **0.0398986484** |
| 212067_s_at | hsa-miR-212_st | **-0.3552330023** | **0.0398986484** |
| 212215_at | hsa-miR-375_st | **-0.3552330023** | **0.0398986484** |
| 213293_s_at | hsa-miR-3189-5p_st | **-0.3552330023** | **0.0398986484** |
| 210906_x_at | hsa-miR-382_st | **-0.3551073431** | **0.0399726971** |
| 203613_s_at | hsa-miR-375_st | **-0.3549545430** | **0.0400628906** |
| 204554_at | hsa-miR-377-star_st | **-0.3549274255** | **0.0400789146** |
| 210946_at | hsa-miR-383_st | **-0.3549274255** | **0.0400789146** |
| 212063_at | hsa-miR-132_st | **-0.3549274255** | **0.0400789146** |
| 214717_at | hsa-miR-375_st | **-0.3549274255** | **0.0400789146** |
| 203854_at | hsa-miR-382_st | **-0.3546489429** | **0.0402437758** |
| 201924_at | hsa-miR-1180_st | **-0.3546218487** | **0.0402598450** |
| 209108_at | hsa-miR-4760-3p_st | **-0.3546218487** | **0.0402598450** |
| 221263_s_at | hsa-miR-34b-star_st | **-0.3546218487** | **0.0402598450** |
| 201601_x_at | hsa-miR-555_st | **-0.3544961428** | **0.0403344680** |
| 203540_at | hsa-miR-4762-5p_st | **-0.3543162720** | **0.0404414413** |
| 200916_at | hsa-miR-4762-5p_st | **-0.3537051184** | **0.0408066388** |
| 201761_at | hsa-miR-1180_st | **-0.3537051184** | **0.0408066388** |
| 210906_x_at | hsa-miR-4720-3p_st | **-0.3537051184** | **0.0408066388** |
| 213533_at | hsa-miR-375_st | **-0.3537051184** | **0.0408066388** |
| 214150_x_at | hsa-miR-4762-5p_st | **-0.3537051184** | **0.0408066388** |
| 219326_s_at | hsa-miR-34b_st | **-0.3537051184** | **0.0408066388** |
| 222230_s_at | hsa-miR-34b_st | **-0.3537051184** | **0.0408066388** |
| 202430_s_at | hsa-miR-4762-5p_st | **-0.3533995416** | **0.0409902434** |
| 217820_s_at | hsa-miR-4633-5p_st | **-0.3533995416** | **0.0409902434** |
| 201066_at | hsa-miR-34b-star_st | **-0.3530939649** | **0.0411745210** |
| 201887_at | hsa-miR-132_st | **-0.3530939649** | **0.0411745210** |
| 205031_at | hsa-miR-375_st | **-0.3530939649** | **0.0411745210** |
| 212977_at | hsa-miR-382_st | **-0.3528153422** | **0.0413431320** |
| 200673_at | hsa-miR-4762-5p_st | **-0.3527883881** | **0.0413594733** |
| 204239_s_at | hsa-miR-4652-3p_st | **-0.3527883881** | **0.0413594733** |
| 208838_at | hsa-miR-34b_st | **-0.3527883881** | **0.0413594733** |
| 210105_s_at | hsa-miR-1912_st | **-0.3527883881** | **0.0413594733** |
| 210972_x_at | hsa-miR-375_st | **-0.3527883881** | **0.0413594733** |
| 214829_at | hsa-miR-4760-3p_st | **-0.3527883881** | **0.0413594733** |
| 210427_x_at | hsa-miR-4311_st | **-0.3526625421** | **0.0414358390** |
| 200040_at | hsa-miR-4652-3p_st | **-0.3524828113** | **0.0415451021** |
| 201012_at | hsa-miR-212_st | **-0.3524828113** | **0.0415451021** |
| 202269_x_at | hsa-miR-1180_st | **-0.3524828113** | **0.0415451021** |
| 204239_s_at | hsa-miR-34b-star_st | **-0.3524828113** | **0.0415451021** |
| 204326_x_at | hsa-miR-4720-3p_st | **-0.3524828113** | **0.0415451021** |
| 208868_s_at | hsa-miR-375_st | **-0.3524828113** | **0.0415451021** |
| 221796_at | hsa-miR-1912_st | **-0.3524828113** | **0.0415451021** |
| 202736_s_at | hsa-miR-34b_st | **-0.3521772345** | **0.0417314091** |
| 208809_s_at | hsa-miR-212_st | **-0.3521772345** | **0.0417314091** |
| 212067_s_at | hsa-miR-3189-5p_st | **-0.3521772345** | **0.0417314091** |
| 200905_x_at | hsa-miR-4762-5p_st | **-0.3518716578** | **0.0419183961** |
| 203455_s_at | hsa-miR-4720-3p_st | **-0.3518716578** | **0.0419183961** |
| 203704_s_at | hsa-miR-4760-3p_st | **-0.3518716578** | **0.0419183961** |
| 204119_s_at | hsa-miR-34b_st | **-0.3518716578** | **0.0419183961** |
| 211297_s_at | hsa-miR-4652-3p_st | **-0.3518716578** | **0.0419183961** |
| 222216_s_at | hsa-miR-34b-star_st | **-0.3518716578** | **0.0419183961** |
| 219683_at | hsa-miR-34b-star_st | **-0.3515660810** | **0.0421060648** |
| 1255_g_at | hsa-miR-4652-3p_st | **-0.3512605042** | **0.0422944171** |
| 209108_at | hsa-miR-370_st | **-0.3512605042** | **0.0422944171** |
| 209227_at | hsa-miR-4652-3p_st | **-0.3512605042** | **0.0422944171** |
| 201666_at | hsa-miR-4311_st | **-0.3511345415** | **0.0423722577** |
| 201029_s_at | hsa-miR-523_st | **-0.3509549274** | **0.0424834546** |
| 208809_s_at | hsa-miR-4760-3p_st | **-0.3509549274** | **0.0424834546** |
| 200798_x_at | hsa-miR-4762-5p_st | **-0.3506493506** | **0.0426731793** |
| 205097_at | hsa-miR-132_st | **-0.3506493506** | **0.0426731793** |
| 214150_x_at | hsa-miR-132_st | **-0.3506493506** | **0.0426731793** |
| 221488_s_at | hsa-miR-4652-3p_st | **-0.3506493506** | **0.0426731793** |
| 36711_at | hsa-miR-212_st | **-0.3506493506** | **0.0426731793** |
| 202741_at | hsa-miR-34b_st | **-0.3503437739** | **0.0428635927** |
| 203685_at | hsa-miR-212_st | **-0.3503437739** | **0.0428635927** |
| 212067_s_at | hsa-miR-377-star_st | **-0.3503437739** | **0.0428635927** |
| 213572_s_at | hsa-miR-555_st | **-0.3502177411** | **0.0429423283** |
| 200077_s_at | hsa-miR-34c-5p_st | **-0.3500381971** | **0.0430546967** |
| 201601_x_at | hsa-miR-3189-5p_st | **-0.3500381971** | **0.0430546967** |
| 202180_s_at | hsa-miR-370_st | **-0.3500381971** | **0.0430546967** |
| 204239_s_at | hsa-miR-375_st | **-0.3500381971** | **0.0430546967** |
| 222043_at | hsa-miR-4720-3p_st | **-0.3500381971** | **0.0430546967** |
| 200662_s_at | hsa-miR-4652-3p_st | **-0.3497326203** | **0.0432464932** |
| 202096_s_at | hsa-miR-4720-3p_st | **-0.3497326203** | **0.0432464932** |
| 204992_s_at | hsa-miR-4652-3p_st | **-0.3497326203** | **0.0432464932** |
| 210278_s_at | hsa-miR-4652-3p_st | **-0.3497326203** | **0.0432464932** |
| 210927_x_at | hsa-miR-34b-star_st | **-0.3497326203** | **0.0432464932** |
| 211376_s_at | hsa-miR-34b_st | **-0.3497326203** | **0.0432464932** |
| 218694_at | hsa-miR-34b_st | **-0.3497326203** | **0.0432464932** |
| 202269_x_at | hsa-miR-382_st | **-0.3496065409** | **0.0433258296** |
| 1007_s_at | hsa-miR-523_st | **-0.3494270435** | **0.0434389837** |
| 202864_s_at | hsa-miR-4633-5p_st | **-0.3494270435** | **0.0434389837** |
| 209157_at | hsa-miR-375_st | **-0.3494270435** | **0.0434389837** |
| 209227_at | hsa-miR-375_st | **-0.3494270435** | **0.0434389837** |
| 211595_s_at | hsa-miR-34b-star_st | **-0.3494270435** | **0.0434389837** |
| 219582_at | hsa-miR-4760-3p_st | **-0.3494270435** | **0.0434389837** |
| 222043_at | hsa-miR-4760-3p_st | **-0.3494270435** | **0.0434389837** |
| 200677_at | hsa-miR-1180_st | **-0.3491214668** | **0.0436321702** |
| 209303_at | hsa-miR-34b-star_st | **-0.3489953406** | **0.0437121109** |
| 201601_x_at | hsa-miR-361-5p_st | **-0.3488158900** | **0.0438260544** |
| 206849_at | hsa-miR-34b_st | **-0.3488158900** | **0.0438260544** |
| 210418_s_at | hsa-miR-375_st | **-0.3488158900** | **0.0438260544** |
| 214022_s_at | hsa-miR-361-5p_st | **-0.3488158900** | **0.0438260544** |
| 215171_s_at | hsa-miR-34b-star_st | **-0.3488158900** | **0.0438260544** |
| 208799_at | hsa-miR-4652-3p_st | **-0.3485103132** | **0.0440206381** |
| 209157_at | hsa-miR-34b_st | **-0.3485103132** | **0.0440206381** |
| 217860_at | hsa-miR-34b-star_st | **-0.3485103132** | **0.0440206381** |
| 219582_at | hsa-miR-132_st | **-0.3485103132** | **0.0440206381** |
| 200701_at | hsa-miR-555_st | **-0.3482313403** | **0.0441988934** |
| 200986_at | hsa-miR-382_st | **-0.3482313403** | **0.0441988934** |
| 208909_at | hsa-miR-375_st | **-0.3482047364** | **0.0442159231** |
| 210453_x_at | hsa-miR-375_st | **-0.3482047364** | **0.0442159231** |
| 212961_x_at | hsa-miR-375_st | **-0.3482047364** | **0.0442159231** |
| 200701_at | hsa-miR-4762-5p_st | **-0.3478991597** | **0.0444119111** |
| 218316_at | hsa-miR-4652-3p_st | **-0.3478991597** | **0.0444119111** |
| 201656_at | hsa-miR-212_st | **-0.3475935829** | **0.0446086039** |
| 214428_x_at | hsa-miR-377-star_st | **-0.3475935829** | **0.0446086039** |
| 204070_at | hsa-miR-382_st | **-0.3474673400** | **0.0446900698** |
| 218882_s_at | hsa-miR-375_st | **-0.3472880061** | **0.0448060034** |
| 211999_at | hsa-miR-132_st | **-0.3471617399** | **0.0448877768** |
| 202180_s_at | hsa-miR-4633-5p_st | **-0.3469824293** | **0.0450041112** |
| 209122_at | hsa-miR-1180_st | **-0.3469824293** | **0.0450041112** |
| 209243_s_at | hsa-miR-34c-5p_st | **-0.3469824293** | **0.0450041112** |
| 211963_s_at | hsa-miR-375_st | **-0.3469824293** | **0.0450041112** |
| 221688_s_at | hsa-miR-375_st | **-0.3469824293** | **0.0450041112** |
| 202948_at | hsa-miR-1180_st | **-0.3466768526** | **0.0452029293** |
| 203987_at | hsa-miR-377-star_st | **-0.3466768526** | **0.0452029293** |
| 204068_at | hsa-miR-212_st | **-0.3466768526** | **0.0452029293** |
| 212063_at | hsa-miR-4760-3p_st | **-0.3466768526** | **0.0452029293** |
| 213005_s_at | hsa-miR-3189-5p_st | **-0.3466768526** | **0.0452029293** |
| 213738_s_at | hsa-miR-4652-3p_st | **-0.3466768526** | **0.0452029293** |
| 202180_s_at | hsa-miR-382_st | **-0.3463977396** | **0.0453851513** |
| 201029_s_at | hsa-miR-132_st | **-0.3463712758** | **0.0454024593** |
| 202252_at | hsa-miR-4762-5p_st | **-0.3463712758** | **0.0454024593** |
| 210962_s_at | hsa-miR-34c-3p_st | **-0.3463712758** | **0.0454024593** |
| 218982_s_at | hsa-miR-375_st | **-0.3463712758** | **0.0454024593** |
| 200614_at | hsa-miR-375_st | **-0.3460921394** | **0.0455853485** |
| 208690_s_at | hsa-miR-4760-3p_st | **-0.3460656990** | **0.0456027032** |
| 218005_at | hsa-miR-383_st | **-0.3460656990** | **0.0456027032** |
| 221699_s_at | hsa-miR-375_st | **-0.3460656990** | **0.0456027032** |
| 201666_at | hsa-miR-382_st | **-0.3457865393** | **0.0457862613** |
| 209303_at | hsa-miR-34b_st | **-0.3457865393** | **0.0457862613** |
| 202071_at | hsa-miR-4720-3p_st | **-0.3457601222** | **0.0458036625** |
| 209569_x_at | hsa-miR-375_st | **-0.3457601222** | **0.0458036625** |
| 218322_s_at | hsa-miR-370_st | **-0.3457601222** | **0.0458036625** |
| 201753_s_at | hsa-miR-555_st | **-0.3456337393** | **0.0458869866** |
| 211971_s_at | hsa-miR-34b-star_st | **-0.3456337393** | **0.0458869866** |
| 203079_s_at | hsa-miR-34b_st | **-0.3454545455** | **0.0460053393** |
| 217947_at | hsa-miR-4760-3p_st | **-0.3454545455** | **0.0460053393** |
| 200812_at | hsa-miR-375_st | **-0.3448433919** | **0.0464108521** |
| 203156_at | hsa-miR-34b_st | **-0.3448433919** | **0.0464108521** |
| 210592_s_at | hsa-miR-4311_st | **-0.3447169389** | **0.0464951167** |
| 211971_s_at | hsa-miR-34b_st | **-0.3447169389** | **0.0464951167** |
| 201146_at | hsa-miR-4762-5p_st | **-0.3445378151** | **0.0466146917** |
| 210105_s_at | hsa-miR-132_st | **-0.3445378151** | **0.0466146917** |
| 215506_s_at | hsa-miR-34b-star_st | **-0.3445378151** | **0.0466146917** |
| 202269_x_at | hsa-miR-383_st | **-0.3442322383** | **0.0468192559** |
| 205758_at | hsa-miR-34b-star_st | **-0.3442322383** | **0.0468192559** |
| 208818_s_at | hsa-miR-361-5p_st | **-0.3442322383** | **0.0468192559** |
| 204744_s_at | hsa-miR-4652-3p_st | **-0.3439266616** | **0.0470245464** |
| 218285_s_at | hsa-miR-1912_st | **-0.3439266616** | **0.0470245464** |
| 202180_s_at | hsa-miR-4762-5p_st | **-0.3436210848** | **0.0472305651** |
| 203663_s_at | hsa-miR-34b_st | **-0.3436210848** | **0.0472305651** |
| 207922_s_at | hsa-miR-34c-5p_st | **-0.3436210848** | **0.0472305651** |
| 212977_at | hsa-miR-132_st | **-0.3436210848** | **0.0472305651** |
| 211999_at | hsa-miR-370_st | **-0.3434945384** | **0.0473160958** |
| 201484_at | hsa-miR-34b_st | **-0.3433155080** | **0.0474373138** |
| 202802_at | hsa-miR-34b-star_st | **-0.3433155080** | **0.0474373138** |
| 205609_at | hsa-miR-523_st | **-0.3433155080** | **0.0474373138** |
| 211962_s_at | hsa-miR-4760-3p_st | **-0.3433155080** | **0.0474373138** |
| 200673_at | hsa-miR-4311_st | **-0.3431889383** | **0.0475231632** |
| 219582_at | hsa-miR-382_st | **-0.3431889383** | **0.0475231632** |
| 201753_s_at | hsa-miR-361-5p_st | **-0.3430099312** | **0.0476447942** |
| 202712_s_at | hsa-miR-34b-star_st | **-0.3430099312** | **0.0476447942** |
| 211558_s_at | hsa-miR-375_st | **-0.3430099312** | **0.0476447942** |
| 218507_at | hsa-miR-1912_st | **-0.3430099312** | **0.0476447942** |
| 200797_s_at | hsa-miR-1912_st | **-0.3427043545** | **0.0478530082** |
| 201443_s_at | hsa-miR-375_st | **-0.3427043545** | **0.0478530082** |
| 208761_s_at | hsa-miR-34c-5p_st | **-0.3427043545** | **0.0478530082** |
| 211271_x_at | hsa-miR-383_st | **-0.3427043545** | **0.0478530082** |
| 211615_s_at | hsa-miR-4652-3p_st | **-0.3423987777** | **0.0480619575** |
| 201145_at | hsa-miR-34b-star_st | **-0.3420932009** | **0.0482716441** |
| 202920_at | hsa-miR-34b-star_st | **-0.3420932009** | **0.0482716441** |
| 212195_at | hsa-miR-132_st | **-0.3420932009** | **0.0482716441** |
| 213738_s_at | hsa-miR-34b_st | **-0.3420932009** | **0.0482716441** |
| 215522_at | hsa-miR-375_st | **-0.3420932009** | **0.0482716441** |
| 217957_at | hsa-miR-375_st | **-0.3420932009** | **0.0482716441** |
| 206803_at | hsa-miR-4652-3p_st | **-0.3417876241** | **0.0484820696** |
| 202936_s_at | hsa-miR-1912_st | **-0.3414820474** | **0.0486932360** |
| 211902_x_at | hsa-miR-34c-5p_st | **-0.3414820474** | **0.0486932360** |
| 217546_at | hsa-miR-1912_st | **-0.3414820474** | **0.0486932360** |
| 218285_s_at | hsa-miR-370_st | **-0.3414820474** | **0.0486932360** |
| 221741_s_at | hsa-miR-370_st | **-0.3414820474** | **0.0486932360** |
| 203313_s_at | hsa-miR-4311_st | **-0.3413553375** | **0.0487810155** |
| 201146_at | hsa-miR-132_st | **-0.3411764706** | **0.0489051449** |
| 221009_s_at | hsa-miR-377-star_st | **-0.3411764706** | **0.0489051449** |
| 200804_at | hsa-miR-377-star_st | **-0.3408708938** | **0.0491177984** |
| 206875_s_at | hsa-miR-4423-3p_st | **-0.3408708938** | **0.0491177984** |
| 208946_s_at | hsa-miR-4652-3p_st | **-0.3408708938** | **0.0491177984** |
| 200030_s_at | hsa-miR-375_st | **-0.3405653170** | **0.0493311980** |
| 206015_s_at | hsa-miR-34b_st | **-0.3405653170** | **0.0493311980** |
| 203540_at | hsa-miR-4311_st | **-0.3404385372** | **0.0494199542** |
| 212687_at | hsa-miR-382_st | **-0.3402857371** | **0.0495270980** |
| 200626_s_at | hsa-miR-375_st | **-0.3402597403** | **0.0495453457** |
| 201410_at | hsa-miR-34b_st | **-0.3402597403** | **0.0495453457** |
| 201443_s_at | hsa-miR-4652-3p_st | **-0.3402597403** | **0.0495453457** |
| 202948_at | hsa-miR-370_st | **-0.3402597403** | **0.0495453457** |
| 209476_at | hsa-miR-377-star_st | **-0.3402597403** | **0.0495453457** |
| 208860_s_at | hsa-miR-34b-star_st | **-0.3399541635** | **0.0497602433** |
| 214150_x_at | hsa-miR-1912_st | **-0.3399541635** | **0.0497602433** |
| 218597_s_at | hsa-miR-34b_st | **-0.3399541635** | **0.0497602433** |
| 209755_at | hsa-miR-4652-3p_st | **-0.3396485867** | **0.0499758926** |
| 202395_at | hsa-miR-34b_st | -0.3393430099 | 0.0501922954 |
| 207643_s_at | hsa-miR-370_st | -0.3393430099 | 0.0501922954 |
| 218200_s_at | hsa-miR-4652-3p_st | -0.3393430099 | 0.0501922954 |
| 213293_s_at | hsa-miR-555_st | -0.3392161367 | 0.0502823661 |
| 202232_s_at | hsa-miR-34b-star_st | -0.3390374332 | 0.0504094536 |
| 212092_at | hsa-miR-34b-star_st | -0.3390374332 | 0.0504094536 |
| 221958_s_at | hsa-miR-132_st | -0.3390374332 | 0.0504094536 |
| 207643_s_at | hsa-miR-382_st | -0.3389105366 | 0.0504998551 |
| 201160_s_at | hsa-miR-382_st | -0.3387577365 | 0.0506088837 |
| 217882_at | hsa-miR-4652-3p_st | -0.3387577365 | 0.0506088837 |
| 203973_s_at | hsa-miR-370_st | -0.3387318564 | 0.0506273689 |
| 204119_s_at | hsa-miR-4652-3p_st | -0.3384262796 | 0.0508460432 |
| 205531_s_at | hsa-miR-34b_st | -0.3384262796 | 0.0508460432 |
| 205758_at | hsa-miR-375_st | -0.3384262796 | 0.0508460432 |
| 208838_at | hsa-miR-375_st | -0.3384262796 | 0.0508460432 |
| 209108_at | hsa-miR-132_st | -0.3384262796 | 0.0508460432 |
| 210149_s_at | hsa-miR-4652-3p_st | -0.3384262796 | 0.0508460432 |
| 201570_at | hsa-miR-34b-star_st | -0.3381207028 | 0.0510654783 |
| 201887_at | hsa-miR-4720-3p_st | -0.3381207028 | 0.0510654783 |
| 210906_x_at | hsa-miR-377-star_st | -0.3381207028 | 0.0510654783 |
| 212067_s_at | hsa-miR-523_st | -0.3381207028 | 0.0510654783 |
| 212195_at | hsa-miR-1912_st | -0.3381207028 | 0.0510654783 |
| 217730_at | hsa-miR-212_st | -0.3381207028 | 0.0510654783 |
| 207643_s_at | hsa-miR-523_st | -0.3378151261 | 0.0512856760 |
| 218491_s_at | hsa-miR-34b-star_st | -0.3378151261 | 0.0512856760 |
| 201012_at | hsa-miR-523_st | -0.3372039725 | 0.0517283667 |
| 207717_s_at | hsa-miR-375_st | -0.3372039725 | 0.0517283667 |
| 207761_s_at | hsa-miR-370_st | -0.3372039725 | 0.0517283667 |
| 208675_s_at | hsa-miR-34b_st | -0.3368983957 | 0.0519508633 |
| 200739_s_at | hsa-miR-4652-3p_st | -0.3365928189 | 0.0521741298 |
| 203893_at | hsa-miR-4652-3p_st | -0.3365928189 | 0.0521741298 |
| 205531_s_at | hsa-miR-375_st | -0.3365928189 | 0.0521741298 |
| 208690_s_at | hsa-miR-212_st | -0.3365928189 | 0.0521741298 |
| 212501_at | hsa-miR-4762-5p_st | -0.3365928189 | 0.0521741298 |
| 201666_at | hsa-miR-377-star_st | -0.3362872422 | 0.0523981681 |
| 203540_at | hsa-miR-383_st | -0.3362872422 | 0.0523981681 |
| 200905_x_at | hsa-miR-382_st | -0.3360073354 | 0.0526040648 |
| 200053_at | hsa-miR-4652-3p_st | -0.3359816654 | 0.0526229800 |
| 201180_s_at | hsa-miR-370_st | -0.3359816654 | 0.0526229800 |
| 202658_at | hsa-miR-375_st | -0.3359816654 | 0.0526229800 |
| 201887_at | hsa-miR-4311_st | -0.3358545353 | 0.0527167375 |
| 210534_s_at | hsa-miR-4652-3p_st | -0.3358545353 | 0.0527167375 |
| 201029_s_at | hsa-miR-212_st | -0.3356760886 | 0.0528485673 |
| 201761_at | hsa-miR-212_st | -0.3356760886 | 0.0528485673 |
| 202252_at | hsa-miR-370_st | -0.3356760886 | 0.0528485673 |
| 205005_s_at | hsa-miR-4652-3p_st | -0.3356760886 | 0.0528485673 |
| 210453_x_at | hsa-miR-4652-3p_st | -0.3356760886 | 0.0528485673 |
| 213423_x_at | hsa-miR-4652-3p_st | -0.3356760886 | 0.0528485673 |
| 218526_s_at | hsa-miR-34b-star_st | -0.3356760886 | 0.0528485673 |
| 201172_x_at | hsa-miR-4762-5p_st | -0.3353705118 | 0.0530749319 |
| 202834_at | hsa-miR-370_st | -0.3353705118 | 0.0530749319 |
| 200883_at | hsa-miR-375_st | -0.3350649351 | 0.0533020755 |
| 200967_at | hsa-miR-3676_st | -0.3350649351 | 0.0533020755 |
| 218201_at | hsa-miR-34b-star_st | -0.3350649351 | 0.0533020755 |
| 200673_at | hsa-miR-377-star_st | -0.3347593583 | 0.0535300002 |
| 208832_at | hsa-miR-34b_st | -0.3347593583 | 0.0535300002 |
| 218160_at | hsa-miR-4652-3p_st | -0.3347593583 | 0.0535300002 |
| 203137_at | hsa-miR-34c-5p_st | -0.3344537815 | 0.0537587075 |
| 203540_at | hsa-miR-1180_st | -0.3344537815 | 0.0537587075 |
| 207508_at | hsa-miR-375_st | -0.3344537815 | 0.0537587075 |
| 207831_x_at | hsa-miR-4652-3p_st | -0.3344537815 | 0.0537587075 |
| 211658_at | hsa-miR-375_st | -0.3344537815 | 0.0537587075 |
| 217730_at | hsa-miR-4720-3p_st | -0.3344537815 | 0.0537587075 |
| 201601_x_at | hsa-miR-523_st | -0.3341482047 | 0.0539881995 |
| 212501_at | hsa-miR-3189-5p_st | -0.3341482047 | 0.0539881995 |
| 202269_x_at | hsa-miR-212_st | -0.3338426280 | 0.0542184779 |
| 202587_s_at | hsa-miR-212_st | -0.3338426280 | 0.0542184779 |
| 212063_at | hsa-miR-383_st | -0.3338426280 | 0.0542184779 |
| 202373_s_at | hsa-miR-375_st | -0.3335370512 | 0.0544495446 |
| 211270_x_at | hsa-miR-4633-5p_st | -0.3335370512 | 0.0544495446 |
| 218285_s_at | hsa-miR-377-star_st | -0.3335370512 | 0.0544495446 |
| 218384_at | hsa-miR-375_st | -0.3335370512 | 0.0544495446 |
| 201398_s_at | hsa-miR-4720-3p_st | -0.3332314744 | 0.0546814015 |
| 203560_at | hsa-miR-34b_st | -0.3332314744 | 0.0546814015 |
| 205758_at | hsa-miR-4652-3p_st | -0.3332314744 | 0.0546814015 |
| 208818_s_at | hsa-miR-4760-3p_st | -0.3332314744 | 0.0546814015 |
| 208998_at | hsa-miR-4311_st | -0.3329513342 | 0.0548946541 |
| 202834_at | hsa-miR-523_st | -0.3329258976 | 0.0549140502 |
| 210946_at | hsa-miR-212_st | -0.3329258976 | 0.0549140502 |
| 202834_at | hsa-miR-212_st | -0.3326203209 | 0.0551474928 |
| 202930_s_at | hsa-miR-4652-3p_st | -0.3326203209 | 0.0551474928 |
| 208745_at | hsa-miR-375_st | -0.3326203209 | 0.0551474928 |
| 209228_x_at | hsa-miR-4652-3p_st | -0.3326203209 | 0.0551474928 |
| 213217_at | hsa-miR-523_st | -0.3326203209 | 0.0551474928 |
| 218214_at | hsa-miR-375_st | -0.3326203209 | 0.0551474928 |
| 44669_at | hsa-miR-34b_st | -0.3326203209 | 0.0551474928 |
| 209183_s_at | hsa-miR-4311_st | -0.3324929340 | 0.0552450437 |
| 201012_at | hsa-miR-132_st | -0.3323147441 | 0.0553817310 |
| 208121_s_at | hsa-miR-375_st | -0.3323147441 | 0.0553817310 |
| 212195_at | hsa-miR-4760-3p_st | -0.3323147441 | 0.0553817310 |
| 213572_s_at | hsa-miR-4720-3p_st | -0.3323147441 | 0.0553817310 |
| 36711_at | hsa-miR-383_st | -0.3323147441 | 0.0553817310 |
| 200797_s_at | hsa-miR-4311_st | -0.3317289337 | 0.0558330122 |
| 202133_at | hsa-miR-1912_st | -0.3317035905 | 0.0558526018 |
| 202370_s_at | hsa-miR-3189-5p_st | -0.3317035905 | 0.0558526018 |
| 213572_s_at | hsa-miR-1180_st | -0.3317035905 | 0.0558526018 |
| 209732_at | hsa-miR-555_st | -0.3315761336 | 0.0559512059 |
| 207198_s_at | hsa-miR-382_st | -0.3314233335 | 0.0560696000 |
| 203893_at | hsa-miR-34b_st | -0.3313980138 | 0.0560892380 |
| 205963_s_at | hsa-miR-34b-star_st | -0.3313980138 | 0.0560892380 |
| 208998_at | hsa-miR-383_st | -0.3313980138 | 0.0560892380 |
| 211962_s_at | hsa-miR-383_st | -0.3313980138 | 0.0560892380 |
| 204326_x_at | hsa-miR-4311_st | -0.3311177334 | 0.0563069908 |
| 200986_at | hsa-miR-370_st | -0.3310924370 | 0.0563266772 |
| 201180_s_at | hsa-miR-3676_st | -0.3310924370 | 0.0563266772 |
| 201656_at | hsa-miR-1180_st | -0.3310924370 | 0.0563266772 |
| 202930_s_at | hsa-miR-34c-5p_st | -0.3310924370 | 0.0563266772 |
| 203685_at | hsa-miR-4760-3p_st | -0.3310924370 | 0.0563266772 |
| 213227_at | hsa-miR-4652-3p_st | -0.3310924370 | 0.0563266772 |
| 203411_s_at | hsa-miR-212_st | -0.3307868602 | 0.0565649212 |
| 210817_s_at | hsa-miR-370_st | -0.3307868602 | 0.0565649212 |
| 212716_s_at | hsa-miR-34b-star_st | -0.3304812834 | 0.0568039720 |
| 218226_s_at | hsa-miR-4652-3p_st | -0.3304812834 | 0.0568039720 |
| 212195_at | hsa-miR-382_st | -0.3303537331 | 0.0569039929 |
| 210592_s_at | hsa-miR-382_st | -0.3302009330 | 0.0570239995 |
| 201029_s_at | hsa-miR-1180_st | -0.3301757066 | 0.0570438313 |
| 202594_at | hsa-miR-375_st | -0.3301757066 | 0.0570438313 |
| 206984_s_at | hsa-miR-4652-3p_st | -0.3301757066 | 0.0570438313 |
| 207507_s_at | hsa-miR-4652-3p_st | -0.3301757066 | 0.0570438313 |
| 208451_s_at | hsa-miR-377-star_st | -0.3301757066 | 0.0570438313 |
| 208697_s_at | hsa-miR-34c-3p_st | -0.3301757066 | 0.0570438313 |
| 212203_x_at | hsa-miR-361-5p_st | -0.3301757066 | 0.0570438313 |
| 220329_s_at | hsa-miR-34b_st | -0.3301757066 | 0.0570438313 |
| 202834_at | hsa-miR-4633-5p_st | -0.3298701299 | 0.0572845011 |
| 210033_s_at | hsa-miR-4760-3p_st | -0.3298701299 | 0.0572845011 |
| 200906_s_at | hsa-miR-4311_st | -0.3295897328 | 0.0575060540 |
| 208779_x_at | hsa-miR-370_st | -0.3295645531 | 0.0575259830 |
| 213293_s_at | hsa-miR-523_st | -0.3295645531 | 0.0575259830 |
| 200862_at | hsa-miR-34b_st | -0.3292589763 | 0.0577682791 |
| 201146_at | hsa-miR-1912_st | -0.3292589763 | 0.0577682791 |
| 202121_s_at | hsa-miR-4652-3p_st | -0.3292589763 | 0.0577682791 |
| 203404_at | hsa-miR-34b-star_st | -0.3292589763 | 0.0577682791 |
| 203944_x_at | hsa-miR-375_st | -0.3292589763 | 0.0577682791 |
| 208581_x_at | hsa-miR-212_st | -0.3292589763 | 0.0577682791 |
| 208690_s_at | hsa-miR-377-star_st | -0.3292589763 | 0.0577682791 |
| 210149_s_at | hsa-miR-375_st | -0.3292589763 | 0.0577682791 |
| 201412_at | hsa-miR-4720-3p_st | -0.3289533995 | 0.0580113911 |
| 202180_s_at | hsa-miR-361-5p_st | -0.3289533995 | 0.0580113911 |
| 203540_at | hsa-miR-523_st | -0.3289533995 | 0.0580113911 |
| 212195_at | hsa-miR-370_st | -0.3289533995 | 0.0580113911 |
| 201086_x_at | hsa-miR-4652-3p_st | -0.3286478228 | 0.0582553210 |
| 201666_at | hsa-miR-370_st | -0.3286478228 | 0.0582553210 |
| 202096_s_at | hsa-miR-370_st | -0.3286478228 | 0.0582553210 |
| 208799_at | hsa-miR-34b_st | -0.3286478228 | 0.0582553210 |
| 219582_at | hsa-miR-523_st | -0.3286478228 | 0.0582553210 |
| 209122_at | hsa-miR-555_st | -0.3283673323 | 0.0584799468 |
| 202430_s_at | hsa-miR-4633-5p_st | -0.3283422460 | 0.0585000704 |
| 210872_x_at | hsa-miR-34b_st | -0.3283422460 | 0.0585000704 |
| 213011_s_at | hsa-miR-34b-star_st | -0.3283422460 | 0.0585000704 |
| 204554_at | hsa-miR-4311_st | -0.3280617322 | 0.0587254691 |
| 202564_x_at | hsa-miR-375_st | -0.3280366692 | 0.0587456414 |
| 204957_at | hsa-miR-4652-3p_st | -0.3280366692 | 0.0587456414 |
| 210014_x_at | hsa-miR-375_st | -0.3280366692 | 0.0587456414 |
| 211672_s_at | hsa-miR-375_st | -0.3280366692 | 0.0587456414 |
| 44669_at | hsa-miR-4652-3p_st | -0.3280366692 | 0.0587456414 |
| 201161_s_at | hsa-miR-4311_st | -0.3279089321 | 0.0588485388 |
| 210946_at | hsa-miR-4311_st | -0.3279089321 | 0.0588485388 |
| 200989_at | hsa-miR-4762-5p_st | -0.3277310924 | 0.0589920357 |
| 219911_s_at | hsa-miR-3189-5p_st | -0.3277310924 | 0.0589920357 |
| 221874_at | hsa-miR-4652-3p_st | -0.3277310924 | 0.0589920357 |
| 201590_x_at | hsa-miR-3676_st | -0.3274255157 | 0.0592392552 |
| 202233_s_at | hsa-miR-34b_st | -0.3274255157 | 0.0592392552 |
| 209732_at | hsa-miR-4760-3p_st | -0.3274255157 | 0.0592392552 |
| 201999_s_at | hsa-miR-4762-5p_st | -0.3271199389 | 0.0594873018 |
| 202475_at | hsa-miR-4652-3p_st | -0.3271199389 | 0.0594873018 |
| 201160_s_at | hsa-miR-4762-5p_st | -0.3268143621 | 0.0597361773 |
| 207573_x_at | hsa-miR-375_st | -0.3268143621 | 0.0597361773 |
| 218120_s_at | hsa-miR-34b_st | -0.3268143621 | 0.0597361773 |
| 221531_at | hsa-miR-375_st | -0.3268143621 | 0.0597361773 |
| 200905_x_at | hsa-miR-555_st | -0.3265337316 | 0.0599654672 |
| 218201_at | hsa-miR-375_st | -0.3265087853 | 0.0599858836 |
| 218526_s_at | hsa-miR-4652-3p_st | -0.3262032086 | 0.0602364225 |
| 218557_at | hsa-miR-375_st | -0.3262032086 | 0.0602364225 |
| 203120_at | hsa-miR-1912_st | -0.3255920550 | 0.0607400055 |
| 210068_s_at | hsa-miR-1912_st | -0.3255920550 | 0.0607400055 |
| 208731_at | hsa-miR-375_st | -0.3252864782 | 0.0609930534 |
| 209570_s_at | hsa-miR-34c-5p_st | -0.3252864782 | 0.0609930534 |
| 210946_at | hsa-miR-3189-5p_st | -0.3252864782 | 0.0609930534 |
| 219355_at | hsa-miR-34b-star_st | -0.3252864782 | 0.0609930534 |
| 213911_s_at | hsa-miR-375_st | -0.3249809015 | 0.0612469414 |
| 209671_x_at | hsa-miR-34b-star_st | -0.3246753247 | 0.0615016712 |
| 209840_s_at | hsa-miR-375_st | -0.3246753247 | 0.0615016712 |
| 211962_s_at | hsa-miR-370_st | -0.3246753247 | 0.0615016712 |
| 217546_at | hsa-miR-377-star_st | -0.3246753247 | 0.0615016712 |
| 201900_s_at | hsa-miR-34b-star_st | -0.3243697479 | 0.0617572448 |
| 213005_s_at | hsa-miR-212_st | -0.3243697479 | 0.0617572448 |
| 36711_at | hsa-miR-4762-5p_st | -0.3243697479 | 0.0617572448 |
| 202802_at | hsa-miR-375_st | -0.3240641711 | 0.0620136640 |
| 208779_x_at | hsa-miR-4762-5p_st | -0.3240641711 | 0.0620136640 |
| 212157_at | hsa-miR-375_st | -0.3240641711 | 0.0620136640 |
| 213592_at | hsa-miR-523_st | -0.3240641711 | 0.0620136640 |
| 218322_s_at | hsa-miR-383_st | -0.3240641711 | 0.0620136640 |
| 48531_at | hsa-miR-370_st | -0.3240641711 | 0.0620136640 |
| 212321_at | hsa-miR-4311_st | -0.3239361305 | 0.0621213588 |
| 201412_at | hsa-miR-4760-3p_st | -0.3237585943 | 0.0622709307 |
| 210448_s_at | hsa-miR-34b-star_st | -0.3237585943 | 0.0622709307 |
| 209265_s_at | hsa-miR-34c-3p_st | -0.3234530176 | 0.0625290467 |
| 202543_s_at | hsa-miR-4762-5p_st | -0.3231474408 | 0.0627880140 |
| 208690_s_at | hsa-miR-132_st | -0.3231474408 | 0.0627880140 |
| 211479_s_at | hsa-miR-34b_st | -0.3231474408 | 0.0627880140 |
| 202370_s_at | hsa-miR-4762-5p_st | -0.3228418640 | 0.0630478343 |
| 203621_at | hsa-miR-375_st | -0.3228418640 | 0.0630478343 |
| 217957_at | hsa-miR-4652-3p_st | -0.3228418640 | 0.0630478343 |
| 218597_s_at | hsa-miR-4652-3p_st | -0.3228418640 | 0.0630478343 |
| 200600_at | hsa-miR-555_st | -0.3225609300 | 0.0632874560 |
| 209248_at | hsa-miR-34b_st | -0.3225609300 | 0.0632874560 |
| 201887_at | hsa-miR-1912_st | -0.3225362872 | 0.0633085095 |
| 202834_at | hsa-miR-3676_st | -0.3222307105 | 0.0635700415 |
| 213005_s_at | hsa-miR-383_st | -0.3222307105 | 0.0635700415 |
| 200820_at | hsa-miR-375_st | -0.3219251337 | 0.0638324321 |
| 201324_at | hsa-miR-4762-5p_st | -0.3219251337 | 0.0638324321 |
| 202658_at | hsa-miR-34b_st | -0.3219251337 | 0.0638324321 |
| 201172_x_at | hsa-miR-132_st | -0.3216195569 | 0.0640956832 |
| 202948_at | hsa-miR-4633-5p_st | -0.3216195569 | 0.0640956832 |
| 206989_s_at | hsa-miR-132_st | -0.3213139801 | 0.0643597967 |
| 201313_at | hsa-miR-34b-star_st | -0.3210084034 | 0.0646247744 |
| 208839_s_at | hsa-miR-34b_st | -0.3210084034 | 0.0646247744 |
| 212195_at | hsa-miR-4762-5p_st | -0.3210084034 | 0.0646247744 |
| 201443_s_at | hsa-miR-34b_st | -0.3207028266 | 0.0648906182 |
| 203189_s_at | hsa-miR-4652-3p_st | -0.3207028266 | 0.0648906182 |
| 218656_s_at | hsa-miR-370_st | -0.3207028266 | 0.0648906182 |
| 203854_at | hsa-miR-132_st | -0.3203972498 | 0.0651573299 |
| 210592_s_at | hsa-miR-4720-3p_st | -0.3203972498 | 0.0651573299 |
| 217995_at | hsa-miR-1180_st | -0.3203972498 | 0.0651573299 |
| 211404_s_at | hsa-miR-375_st | -0.3200916730 | 0.0654249115 |
| 213572_s_at | hsa-miR-1912_st | -0.3200916730 | 0.0654249115 |
| 212195_at | hsa-miR-555_st | -0.3199633289 | 0.0655375572 |
| 210968_s_at | hsa-miR-34b_st | -0.3197860963 | 0.0656933647 |
| 213911_s_at | hsa-miR-4652-3p_st | -0.3197860963 | 0.0656933647 |
| 217820_s_at | hsa-miR-370_st | -0.3197860963 | 0.0656933647 |
| 221796_at | hsa-miR-4720-3p_st | -0.3197860963 | 0.0656933647 |
| 218854_at | hsa-miR-555_st | -0.3196577288 | 0.0658063977 |
| 201086_x_at | hsa-miR-375_st | -0.3194805195 | 0.0659626915 |
| 208827_at | hsa-miR-4652-3p_st | -0.3194805195 | 0.0659626915 |
| 221667_s_at | hsa-miR-4762-5p_st | -0.3194805195 | 0.0659626915 |
| 200916_at | hsa-miR-382_st | -0.3193521287 | 0.0660761126 |
| 203723_at | hsa-miR-370_st | -0.3193521287 | 0.0660761126 |
| 209513_s_at | hsa-miR-1180_st | -0.3191749427 | 0.0662328936 |
| 210068_s_at | hsa-miR-4720-3p_st | -0.3191749427 | 0.0662328936 |
| 211376_s_at | hsa-miR-4652-3p_st | -0.3191749427 | 0.0662328936 |
| 200905_x_at | hsa-miR-370_st | -0.3188693659 | 0.0665039730 |
| 202382_s_at | hsa-miR-34b-star_st | -0.3188693659 | 0.0665039730 |
| 202929_s_at | hsa-miR-34b_st | -0.3188693659 | 0.0665039730 |
| 219714_s_at | hsa-miR-34b_st | -0.3188693659 | 0.0665039730 |
| 202370_s_at | hsa-miR-555_st | -0.3185881284 | 0.0667542378 |
| 213217_at | hsa-miR-382_st | -0.3185881284 | 0.0667542378 |
| 200053_at | hsa-miR-375_st | -0.3185637892 | 0.0667759316 |
| 202078_at | hsa-miR-34b_st | -0.3185637892 | 0.0667759316 |
| 202948_at | hsa-miR-523_st | -0.3185637892 | 0.0667759316 |
| 213904_at | hsa-miR-4652-3p_st | -0.3185637892 | 0.0667759316 |
| 221741_s_at | hsa-miR-4760-3p_st | -0.3185637892 | 0.0667759316 |
| 203411_s_at | hsa-miR-3189-5p_st | -0.3182582124 | 0.0670487711 |
| 217746_s_at | hsa-miR-4311_st | -0.3181297282 | 0.0671637540 |
| 204326_x_at | hsa-miR-132_st | -0.3179526356 | 0.0673224935 |
| 208909_at | hsa-miR-34b_st | -0.3179526356 | 0.0673224935 |
| 217995_at | hsa-miR-4720-3p_st | -0.3179526356 | 0.0673224935 |
| 218488_at | hsa-miR-375_st | -0.3179526356 | 0.0673224935 |
| 219297_at | hsa-miR-4652-3p_st | -0.3179526356 | 0.0673224935 |
| 209598_at | hsa-miR-4652-3p_st | -0.3176470588 | 0.0675971006 |
| 211962_s_at | hsa-miR-212_st | -0.3176470588 | 0.0675971006 |
| 48531_at | hsa-miR-382_st | -0.3175185279 | 0.0677128699 |
| 201887_at | hsa-miR-555_st | -0.3173657279 | 0.0678507030 |
| 201066_at | hsa-miR-375_st | -0.3173414820 | 0.0678725943 |
| 201628_s_at | hsa-miR-4652-3p_st | -0.3173414820 | 0.0678725943 |
| 208998_at | hsa-miR-212_st | -0.3173414820 | 0.0678725943 |
| 212038_s_at | hsa-miR-4652-3p_st | -0.3173414820 | 0.0678725943 |
| 201412_at | hsa-miR-4762-5p_st | -0.3170359053 | 0.0681489764 |
| 209671_x_at | hsa-miR-375_st | -0.3170359053 | 0.0681489764 |
| 202427_s_at | hsa-miR-34c-3p_st | -0.3167303285 | 0.0684262488 |
| 212645_x_at | hsa-miR-4652-3p_st | -0.3167303285 | 0.0684262488 |
| 217730_at | hsa-miR-383_st | -0.3167303285 | 0.0684262488 |
| 200804_at | hsa-miR-134_st | -0.3164247517 | 0.0687044134 |
| 201272_at | hsa-miR-34b_st | -0.3164247517 | 0.0687044134 |
| 201398_s_at | hsa-miR-370_st | -0.3164247517 | 0.0687044134 |
| 210906_x_at | hsa-miR-4760-3p_st | -0.3164247517 | 0.0687044134 |
| 221958_s_at | hsa-miR-212_st | -0.3164247517 | 0.0687044134 |
| 202975_s_at | hsa-miR-4311_st | -0.3162961274 | 0.0688217665 |
| 209549_s_at | hsa-miR-34b_st | -0.3161191749 | 0.0689834721 |
| 212687_at | hsa-miR-1912_st | -0.3161191749 | 0.0689834721 |
| 213217_at | hsa-miR-370_st | -0.3161191749 | 0.0689834721 |
| 213710_s_at | hsa-miR-375_st | -0.3161191749 | 0.0689834721 |
| 221009_s_at | hsa-miR-4720-3p_st | -0.3161191749 | 0.0689834721 |
| 202543_s_at | hsa-miR-4633-5p_st | -0.3158135982 | 0.0692634266 |
| 209108_at | hsa-miR-4762-5p_st | -0.3158135982 | 0.0692634266 |
| 209183_s_at | hsa-miR-377-star_st | -0.3158135982 | 0.0692634266 |
| 209733_at | hsa-miR-4652-3p_st | -0.3158135982 | 0.0692634266 |
| 218285_s_at | hsa-miR-4760-3p_st | -0.3158135982 | 0.0692634266 |
| 201900_s_at | hsa-miR-375_st | -0.3155080214 | 0.0695442789 |
| 203973_s_at | hsa-miR-383_st | -0.3155080214 | 0.0695442789 |
| 205531_s_at | hsa-miR-4652-3p_st | -0.3155080214 | 0.0695442789 |
| 208857_s_at | hsa-miR-4652-3p_st | -0.3155080214 | 0.0695442789 |
| 217730_at | hsa-miR-3189-5p_st | -0.3155080214 | 0.0695442789 |
| 204554_at | hsa-miR-4720-3p_st | -0.3152024446 | 0.0698260308 |
| 211270_x_at | hsa-miR-1180_st | -0.3152024446 | 0.0698260308 |
| 212600_s_at | hsa-miR-375_st | -0.3152024446 | 0.0698260308 |
| 200818_at | hsa-miR-34b_st | -0.3148968678 | 0.0701086842 |
| 201319_at | hsa-miR-377-star_st | -0.3148968678 | 0.0701086842 |
| 202269_x_at | hsa-miR-4760-3p_st | -0.3148968678 | 0.0701086842 |
| 212501_at | hsa-miR-377-star_st | -0.3148968678 | 0.0701086842 |
| 203159_at | hsa-miR-375_st | -0.3145912911 | 0.0703922410 |
| 211271_x_at | hsa-miR-523_st | -0.3145912911 | 0.0703922410 |
| 213503_x_at | hsa-miR-4311_st | -0.3143097266 | 0.0706543170 |
| 200916_at | hsa-miR-370_st | -0.3142857143 | 0.0706767030 |
| 202930_s_at | hsa-miR-34b-star_st | -0.3142857143 | 0.0706767030 |
| 207812_s_at | hsa-miR-34b_st | -0.3142857143 | 0.0706767030 |
| 208581_x_at | hsa-miR-132_st | -0.3142857143 | 0.0706767030 |
| 208981_at | hsa-miR-1180_st | -0.3142857143 | 0.0706767030 |
| 212501_at | hsa-miR-370_st | -0.3142857143 | 0.0706767030 |
| 219683_at | hsa-miR-375_st | -0.3142857143 | 0.0706767030 |
| 209755_at | hsa-miR-34b_st | -0.3139801375 | 0.0709620720 |
| 210749_x_at | hsa-miR-3189-5p_st | -0.3139801375 | 0.0709620720 |
| 201180_s_at | hsa-miR-382_st | -0.3136985264 | 0.0712258650 |
| 201887_at | hsa-miR-4760-3p_st | -0.3136745607 | 0.0712483500 |
| 202373_s_at | hsa-miR-4652-3p_st | -0.3136745607 | 0.0712483500 |
| 212203_x_at | hsa-miR-4633-5p_st | -0.3136745607 | 0.0712483500 |
| 213217_at | hsa-miR-4762-5p_st | -0.3136745607 | 0.0712483500 |
| 213423_x_at | hsa-miR-375_st | -0.3136745607 | 0.0712483500 |
| 208659_at | hsa-miR-370_st | -0.3133689840 | 0.0715355389 |
| 209075_s_at | hsa-miR-4652-3p_st | -0.3133689840 | 0.0715355389 |
| 201398_s_at | hsa-miR-382_st | -0.3130873261 | 0.0718010563 |
| 201484_at | hsa-miR-375_st | -0.3130634072 | 0.0718236403 |
| 202975_s_at | hsa-miR-361-5p_st | -0.3130634072 | 0.0718236403 |
| 204194_at | hsa-miR-4720-3p_st | -0.3130634072 | 0.0718236403 |
| 200614_at | hsa-miR-4652-3p_st | -0.3127817260 | 0.0720900228 |
| 202376_at | hsa-miR-4760-3p_st | -0.3127578304 | 0.0721126564 |
| 36711_at | hsa-miR-382_st | -0.3126289260 | 0.0722348495 |
| 200786_at | hsa-miR-4652-3p_st | -0.3124522536 | 0.0724025888 |
| 200905_x_at | hsa-miR-3189-5p_st | -0.3124522536 | 0.0724025888 |
| 202670_at | hsa-miR-4652-3p_st | -0.3124522536 | 0.0724025888 |
| 201999_s_at | hsa-miR-555_st | -0.3121705258 | 0.0726707068 |
| 202975_s_at | hsa-miR-555_st | -0.3121705258 | 0.0726707068 |
| 201160_s_at | hsa-miR-370_st | -0.3121466769 | 0.0726934395 |
| 203817_at | hsa-miR-34b_st | -0.3121466769 | 0.0726934395 |
| 202376_at | hsa-miR-370_st | -0.3118411001 | 0.0729852103 |
| 211271_x_at | hsa-miR-1180_st | -0.3118411001 | 0.0729852103 |
| 200663_at | hsa-miR-523_st | -0.3115355233 | 0.0732779030 |
| 202376_at | hsa-miR-3189-5p_st | -0.3115355233 | 0.0732779030 |
| 202543_s_at | hsa-miR-4720-3p_st | -0.3115355233 | 0.0732779030 |
| 207198_s_at | hsa-miR-4762-5p_st | -0.3115355233 | 0.0732779030 |
| 211855_s_at | hsa-miR-4652-3p_st | -0.3115355233 | 0.0732779030 |
| 211962_s_at | hsa-miR-523_st | -0.3115355233 | 0.0732779030 |
| 212687_at | hsa-miR-377-star_st | -0.3115355233 | 0.0732779030 |
| 203723_at | hsa-miR-4762-5p_st | -0.3114065255 | 0.0734017392 |
| 200967_at | hsa-miR-555_st | -0.3112537254 | 0.0735486383 |
| 213592_at | hsa-miR-4760-3p_st | -0.3112299465 | 0.0735715197 |
| 200701_at | hsa-miR-4311_st | -0.3111009253 | 0.0736957689 |
| 200626_s_at | hsa-miR-34b_st | -0.3109243697 | 0.0738660620 |
| 203411_s_at | hsa-miR-523_st | -0.3109243697 | 0.0738660620 |
| 201924_at | hsa-miR-4311_st | -0.3107953252 | 0.0739907252 |
| 208782_at | hsa-miR-4311_st | -0.3106425252 | 0.0741385514 |
| 200883_at | hsa-miR-34b_st | -0.3106187930 | 0.0741615319 |
| 202269_x_at | hsa-miR-523_st | -0.3106187930 | 0.0741615319 |
| 208761_s_at | hsa-miR-34b-star_st | -0.3106187930 | 0.0741615319 |
| 210927_x_at | hsa-miR-375_st | -0.3106187930 | 0.0741615319 |
| 212460_at | hsa-miR-4720-3p_st | -0.3106187930 | 0.0741615319 |
| 221796_at | hsa-miR-3676_st | -0.3106187930 | 0.0741615319 |
| 202071_at | hsa-miR-382_st | -0.3104897251 | 0.0742866100 |
| 200677_at | hsa-miR-4762-5p_st | -0.3103132162 | 0.0744579313 |
| 206042_x_at | hsa-miR-4652-3p_st | -0.3103132162 | 0.0744579313 |
| 208991_at | hsa-miR-3189-5p_st | -0.3103132162 | 0.0744579313 |
| 217936_at | hsa-miR-523_st | -0.3103132162 | 0.0744579313 |
| 219760_at | hsa-miR-375_st | -0.3103132162 | 0.0744579313 |
| 201761_at | hsa-miR-383_st | -0.3100076394 | 0.0747552619 |
| 210968_s_at | hsa-miR-375_st | -0.3100076394 | 0.0747552619 |
| 214829_at | hsa-miR-3189-5p_st | -0.3100076394 | 0.0747552619 |
| 211962_s_at | hsa-miR-382_st | -0.3097257248 | 0.0750303964 |
| 209122_at | hsa-miR-4762-5p_st | -0.3097020626 | 0.0750535257 |
| 209183_s_at | hsa-miR-4633-5p_st | -0.3097020626 | 0.0750535257 |
| 214022_s_at | hsa-miR-3189-5p_st | -0.3097020626 | 0.0750535257 |
| 202096_s_at | hsa-miR-212_st | -0.3093964859 | 0.0753527245 |
| 202591_s_at | hsa-miR-375_st | -0.3093964859 | 0.0753527245 |
| 212063_at | hsa-miR-523_st | -0.3093964859 | 0.0753527245 |
| 218656_s_at | hsa-miR-4762-5p_st | -0.3093964859 | 0.0753527245 |
| 219263_at | hsa-miR-375_st | -0.3093964859 | 0.0753527245 |
| 201601_x_at | hsa-miR-134_st | -0.3090909091 | 0.0756528601 |
| 205097_at | hsa-miR-3189-5p_st | -0.3090909091 | 0.0756528601 |
| 210978_s_at | hsa-miR-4762-5p_st | -0.3090909091 | 0.0756528601 |
| 200798_x_at | hsa-miR-382_st | -0.3089617245 | 0.0757800267 |
| 208981_at | hsa-miR-382_st | -0.3089617245 | 0.0757800267 |
| 213005_s_at | hsa-miR-555_st | -0.3088089244 | 0.0759306565 |
| 201146_at | hsa-miR-370_st | -0.3087853323 | 0.0759539345 |
| 201966_at | hsa-miR-4652-3p_st | -0.3087853323 | 0.0759539345 |
| 201172_x_at | hsa-miR-4720-3p_st | -0.3084797555 | 0.0762559495 |
| 201274_at | hsa-miR-4652-3p_st | -0.3084797555 | 0.0762559495 |
| 202096_s_at | hsa-miR-4760-3p_st | -0.3084797555 | 0.0762559495 |
| 217906_at | hsa-miR-4652-3p_st | -0.3081741788 | 0.0765589069 |
| 218048_at | hsa-miR-375_st | -0.3081741788 | 0.0765589069 |
| 205278_at | hsa-miR-34b_st | -0.3078686020 | 0.0768628087 |
| 218706_s_at | hsa-miR-132_st | -0.3078686020 | 0.0768628087 |
| 200798_x_at | hsa-miR-132_st | -0.3075630252 | 0.0771676566 |
| 201725_at | hsa-miR-4652-3p_st | -0.3075630252 | 0.0771676566 |
| 204070_at | hsa-miR-361-5p_st | -0.3075630252 | 0.0771676566 |
| 208868_s_at | hsa-miR-34b_st | -0.3075630252 | 0.0771676566 |
| 210946_at | hsa-miR-361-5p_st | -0.3072574484 | 0.0774734525 |
| 213272_s_at | hsa-miR-4652-3p_st | -0.3072574484 | 0.0774734525 |
| 217546_at | hsa-miR-212_st | -0.3072574484 | 0.0774734525 |
| 209513_s_at | hsa-miR-382_st | -0.3069753237 | 0.0777566228 |
| 201012_at | hsa-miR-383_st | -0.3069518717 | 0.0777801983 |
| 203685_at | hsa-miR-383_st | -0.3069518717 | 0.0777801983 |
| 209046_s_at | hsa-miR-375_st | -0.3069518717 | 0.0777801983 |
| 222360_at | hsa-miR-375_st | -0.3069518717 | 0.0777801983 |
| 203455_s_at | hsa-miR-382_st | -0.3068225236 | 0.0779103277 |
| 200986_at | hsa-miR-4311_st | -0.3066697236 | 0.0780642708 |
| 202382_s_at | hsa-miR-4652-3p_st | -0.3066462949 | 0.0780878958 |
| 204957_at | hsa-miR-34b_st | -0.3063407181 | 0.0783965469 |
| 210501_x_at | hsa-miR-34b_st | -0.3063407181 | 0.0783965469 |
| 211658_at | hsa-miR-34b_st | -0.3063407181 | 0.0783965469 |
| 218432_at | hsa-miR-4652-3p_st | -0.3063407181 | 0.0783965469 |
| 218706_s_at | hsa-miR-4762-5p_st | -0.3063407181 | 0.0783965469 |
| 202376_at | hsa-miR-4633-5p_st | -0.3060351413 | 0.0787061535 |
| 210946_at | hsa-miR-134_st | -0.3060351413 | 0.0787061535 |
| 217995_at | hsa-miR-212_st | -0.3060351413 | 0.0787061535 |
| 218559_s_at | hsa-miR-383_st | -0.3060351413 | 0.0787061535 |
| 221667_s_at | hsa-miR-523_st | -0.3060351413 | 0.0787061535 |
| 208659_at | hsa-miR-382_st | -0.3059057233 | 0.0788375667 |
| 200638_s_at | hsa-miR-34b_st | -0.3057295646 | 0.0790167174 |
| 200720_s_at | hsa-miR-375_st | -0.3057295646 | 0.0790167174 |
| 203313_s_at | hsa-miR-1180_st | -0.3057295646 | 0.0790167174 |
| 203704_s_at | hsa-miR-212_st | -0.3057295646 | 0.0790167174 |
| 203854_at | hsa-miR-523_st | -0.3057295646 | 0.0790167174 |
| 206803_at | hsa-miR-375_st | -0.3057295646 | 0.0790167174 |
| 215307_at | hsa-miR-34b_st | -0.3057295646 | 0.0790167174 |
| 201029_s_at | hsa-miR-383_st | -0.3054239878 | 0.0793282404 |
| 203704_s_at | hsa-miR-132_st | -0.3054239878 | 0.0793282404 |
| 200708_at | hsa-miR-34b-star_st | -0.3051184110 | 0.0796407244 |
| 209598_at | hsa-miR-34b_st | -0.3051184110 | 0.0796407244 |
| 212195_at | hsa-miR-3676_st | -0.3051184110 | 0.0796407244 |
| 208991_at | hsa-miR-555_st | -0.3049889229 | 0.0797734298 |
| 202741_at | hsa-miR-375_st | -0.3048128342 | 0.0799541713 |
| 210946_at | hsa-miR-4760-3p_st | -0.3048128342 | 0.0799541713 |
| 212203_x_at | hsa-miR-134_st | -0.3048128342 | 0.0799541713 |
| 200798_x_at | hsa-miR-1912_st | -0.3045072574 | 0.0802685829 |
| 202077_at | hsa-miR-375_st | -0.3045072574 | 0.0802685829 |
| 202149_at | hsa-miR-1180_st | -0.3045072574 | 0.0802685829 |
| 217746_s_at | hsa-miR-132_st | -0.3045072574 | 0.0802685829 |
| 211271_x_at | hsa-miR-4311_st | -0.3043777226 | 0.0804021544 |
| 202591_s_at | hsa-miR-4652-3p_st | -0.3042016807 | 0.0805839610 |
| 203685_at | hsa-miR-370_st | -0.3042016807 | 0.0805839610 |
| 204194_at | hsa-miR-4633-5p_st | -0.3042016807 | 0.0805839610 |
| 209243_s_at | hsa-miR-34b-star_st | -0.3042016807 | 0.0805839610 |
| 213496_at | hsa-miR-34b_st | -0.3038961039 | 0.0809003076 |
| 217746_s_at | hsa-miR-1180_st | -0.3038961039 | 0.0809003076 |
| 203411_s_at | hsa-miR-4762-5p_st | -0.3035905271 | 0.0812176244 |
| 203685_at | hsa-miR-132_st | -0.3035905271 | 0.0812176244 |
| 212063_at | hsa-miR-212_st | -0.3035905271 | 0.0812176244 |
| 212195_at | hsa-miR-383_st | -0.3035905271 | 0.0812176244 |
| 212977_at | hsa-miR-370_st | -0.3035905271 | 0.0812176244 |
| 217959_s_at | hsa-miR-34b_st | -0.3035905271 | 0.0812176244 |
| 203302_at | hsa-miR-4652-3p_st | -0.3032849503 | 0.0815359133 |
| 201412_at | hsa-miR-1180_st | -0.3029793736 | 0.0818551762 |
| 202370_s_at | hsa-miR-361-5p_st | -0.3029793736 | 0.0818551762 |
| 205280_at | hsa-miR-375_st | -0.3029793736 | 0.0818551762 |
| 208679_s_at | hsa-miR-34b_st | -0.3026737968 | 0.0821754149 |
| 208981_at | hsa-miR-4762-5p_st | -0.3026737968 | 0.0821754149 |
| 208991_at | hsa-miR-4633-5p_st | -0.3026737968 | 0.0821754149 |
| 200804_at | hsa-miR-3676_st | -0.3023682200 | 0.0824966313 |
| 200905_x_at | hsa-miR-523_st | -0.3023682200 | 0.0824966313 |
| 205550_s_at | hsa-miR-4652-3p_st | -0.3023682200 | 0.0824966313 |
| 209104_s_at | hsa-miR-34b-star_st | -0.3023682200 | 0.0824966313 |
| 209108_at | hsa-miR-383_st | -0.3023682200 | 0.0824966313 |
| 218226_s_at | hsa-miR-34b-star_st | -0.3023682200 | 0.0824966313 |
| 202543_s_at | hsa-miR-555_st | -0.3020857217 | 0.0827944592 |
| 200906_s_at | hsa-miR-383_st | -0.3020626432 | 0.0828188271 |
| 205217_at | hsa-miR-4652-3p_st | -0.3020626432 | 0.0828188271 |
| 219421_at | hsa-miR-375_st | -0.3020626432 | 0.0828188271 |
| 201656_at | hsa-miR-370_st | -0.3017570665 | 0.0831420043 |
| 202613_at | hsa-miR-4652-3p_st | -0.3017570665 | 0.0831420043 |
| 204766_s_at | hsa-miR-4652-3p_st | -0.3014514897 | 0.0834661647 |
| 206989_s_at | hsa-miR-4760-3p_st | -0.3014514897 | 0.0834661647 |
| 201319_at | hsa-miR-4720-3p_st | -0.3011459129 | 0.0837913101 |
| 218656_s_at | hsa-miR-3676_st | -0.3011459129 | 0.0837913101 |
| 218694_at | hsa-miR-4652-3p_st | -0.3011459129 | 0.0837913101 |
| 221688_s_at | hsa-miR-34b_st | -0.3011459129 | 0.0837913101 |
| 206989_s_at | hsa-miR-4311_st | -0.3010161213 | 0.0839297119 |
| 201256_at | hsa-miR-34b_st | -0.3008403361 | 0.0841174424 |
| 203159_at | hsa-miR-4652-3p_st | -0.3008403361 | 0.0841174424 |
| 208659_at | hsa-miR-523_st | -0.3008403361 | 0.0841174424 |
| 211558_s_at | hsa-miR-4652-3p_st | -0.3008403361 | 0.0841174424 |
| 212067_s_at | hsa-miR-382_st | -0.3004049210 | 0.0845838556 |
| 201999_s_at | hsa-miR-523_st | -0.3002291826 | 0.0847726750 |
| 209550_at | hsa-miR-375_st | -0.3002291826 | 0.0847726750 |
| 210817_s_at | hsa-miR-3189-5p_st | -0.3002291826 | 0.0847726750 |
| 217780_at | hsa-miR-34b-star_st | -0.3002291826 | 0.0847726750 |
| 210434_x_at | hsa-miR-4652-3p_st | -0.2999236058 | 0.0851017790 |
| 218048_at | hsa-miR-4652-3p_st | -0.2999236058 | 0.0851017790 |
| 201398_s_at | hsa-miR-555_st | -0.2993353206 | 0.0857381590 |
| 202475_at | hsa-miR-375_st | -0.2993124523 | 0.0857629715 |
| 218322_s_at | hsa-miR-3189-5p_st | -0.2993124523 | 0.0857629715 |
| 222043_at | hsa-miR-1180_st | -0.2993124523 | 0.0857629715 |
| 201180_s_at | hsa-miR-555_st | -0.2991825205 | 0.0859040556 |
| 202325_s_at | hsa-miR-34b_st | -0.2990068755 | 0.0860950638 |
| 212157_at | hsa-miR-4652-3p_st | -0.2990068755 | 0.0860950638 |
| 216218_s_at | hsa-miR-4652-3p_st | -0.2990068755 | 0.0860950638 |
| 218706_s_at | hsa-miR-377-star_st | -0.2990068755 | 0.0860950638 |
| 200701_at | hsa-miR-3676_st | -0.2987012987 | 0.0864281557 |
| 201570_at | hsa-miR-375_st | -0.2987012987 | 0.0864281557 |
| 205279_s_at | hsa-miR-4652-3p_st | -0.2987012987 | 0.0864281557 |
| 48531_at | hsa-miR-4720-3p_st | -0.2987012987 | 0.0864281557 |
| 210105_s_at | hsa-miR-4760-3p_st | -0.2983957219 | 0.0867622493 |
| 212820_at | hsa-miR-375_st | -0.2983957219 | 0.0867622493 |
| 204068_at | hsa-miR-555_st | -0.2981129201 | 0.0870723365 |
| 204957_at | hsa-miR-375_st | -0.2980901451 | 0.0870973463 |
| 208998_at | hsa-miR-361-5p_st | -0.2980901451 | 0.0870973463 |
| 210817_s_at | hsa-miR-4762-5p_st | -0.2980901451 | 0.0870973463 |
| 214150_x_at | hsa-miR-4720-3p_st | -0.2980901451 | 0.0870973463 |
| 206356_s_at | hsa-miR-34b_st | -0.2977845684 | 0.0874334485 |
| 210406_s_at | hsa-miR-375_st | -0.2977845684 | 0.0874334485 |
| 212203_x_at | hsa-miR-3676_st | -0.2977845684 | 0.0874334485 |
| 212217_at | hsa-miR-34b_st | -0.2977845684 | 0.0874334485 |
| 212687_at | hsa-miR-4720-3p_st | -0.2977845684 | 0.0874334485 |
| 212977_at | hsa-miR-4760-3p_st | -0.2977845684 | 0.0874334485 |
| 203704_s_at | hsa-miR-382_st | -0.2976545199 | 0.0875767935 |
| 203313_s_at | hsa-miR-383_st | -0.2974789916 | 0.0877705579 |
| 208981_at | hsa-miR-212_st | -0.2971734148 | 0.0881086761 |
| 218322_s_at | hsa-miR-361-5p_st | -0.2971734148 | 0.0881086761 |
| 209476_at | hsa-miR-555_st | -0.2970433197 | 0.0882529320 |
| 203540_at | hsa-miR-4760-3p_st | -0.2968678380 | 0.0884478051 |
| 208697_s_at | hsa-miR-34c-5p_st | -0.2968678380 | 0.0884478051 |
| 200600_at | hsa-miR-1912_st | -0.2965622613 | 0.0887879467 |
| 201106_at | hsa-miR-375_st | -0.2965622613 | 0.0887879467 |
| 202948_at | hsa-miR-4762-5p_st | -0.2965622613 | 0.0887879467 |
| 205110_s_at | hsa-miR-34b_st | -0.2965622613 | 0.0887879467 |
| 200797_s_at | hsa-miR-132_st | -0.2962566845 | 0.0891291027 |
| 202587_s_at | hsa-miR-523_st | -0.2962566845 | 0.0891291027 |
| 203987_at | hsa-miR-212_st | -0.2962566845 | 0.0891291027 |
| 209550_at | hsa-miR-4652-3p_st | -0.2962566845 | 0.0891291027 |
| 201753_s_at | hsa-miR-4311_st | -0.2961265193 | 0.0892747319 |
| 214022_s_at | hsa-miR-555_st | -0.2961265193 | 0.0892747319 |
| 203723_at | hsa-miR-4311_st | -0.2959963325 | 0.0894205699 |
| 202936_s_at | hsa-miR-382_st | -0.2959737193 | 0.0894459206 |
| 221688_s_at | hsa-miR-4652-3p_st | -0.2959511077 | 0.0894712749 |
| 210946_at | hsa-miR-555_st | -0.2958209192 | 0.0896173638 |
| 211999_at | hsa-miR-4760-3p_st | -0.2956681191 | 0.0897890617 |
| 200677_at | hsa-miR-523_st | -0.2956455309 | 0.0898144652 |
| 203854_at | hsa-miR-4760-3p_st | -0.2956455309 | 0.0898144652 |
| 209570_s_at | hsa-miR-34b-star_st | -0.2956455309 | 0.0898144652 |
| 218694_at | hsa-miR-375_st | -0.2956455309 | 0.0898144652 |
| 201274_at | hsa-miR-375_st | -0.2953399542 | 0.0901586753 |
| 208809_s_at | hsa-miR-3189-5p_st | -0.2953399542 | 0.0901586753 |
| 218656_s_at | hsa-miR-523_st | -0.2953399542 | 0.0901586753 |
| 221667_s_at | hsa-miR-132_st | -0.2953399542 | 0.0901586753 |
| 200797_s_at | hsa-miR-382_st | -0.2952097189 | 0.0903056863 |
| 200820_at | hsa-miR-34b_st | -0.2950343774 | 0.0905039072 |
| 200906_s_at | hsa-miR-3189-5p_st | -0.2950343774 | 0.0905039072 |
| 200989_at | hsa-miR-3676_st | -0.2950343774 | 0.0905039072 |
| 211271_x_at | hsa-miR-3189-5p_st | -0.2950343774 | 0.0905039072 |
| 212015_x_at | hsa-miR-383_st | -0.2950343774 | 0.0905039072 |
| 218005_at | hsa-miR-212_st | -0.2950343774 | 0.0905039072 |
| 201146_at | hsa-miR-4720-3p_st | -0.2947288006 | 0.0908501626 |
| 202564_x_at | hsa-miR-34b-star_st | -0.2947288006 | 0.0908501626 |
| 208860_s_at | hsa-miR-34b_st | -0.2947288006 | 0.0908501626 |
| 209732_at | hsa-miR-132_st | -0.2947288006 | 0.0908501626 |
| 208782_at | hsa-miR-3676_st | -0.2944232238 | 0.0911974433 |
| 209570_s_at | hsa-miR-375_st | -0.2944232238 | 0.0911974433 |
| 221667_s_at | hsa-miR-4311_st | -0.2941401185 | 0.0915201024 |
| 211566_x_at | hsa-miR-4652-3p_st | -0.2941176471 | 0.0915457512 |
| 213552_at | hsa-miR-4652-3p_st | -0.2941176471 | 0.0915457512 |
| 221958_s_at | hsa-miR-4762-5p_st | -0.2941176471 | 0.0915457512 |
| 203723_at | hsa-miR-4633-5p_st | -0.2938345184 | 0.0918693903 |
| 201398_s_at | hsa-miR-4762-5p_st | -0.2938120703 | 0.0918950882 |
| 201527_at | hsa-miR-375_st | -0.2938120703 | 0.0918950882 |
| 201761_at | hsa-miR-370_st | -0.2938120703 | 0.0918950882 |
| 204554_at | hsa-miR-3676_st | -0.2938120703 | 0.0918950882 |
| 210946_at | hsa-miR-523_st | -0.2938120703 | 0.0918950882 |
| 218163_at | hsa-miR-4652-3p_st | -0.2938120703 | 0.0918950882 |
| 202325_s_at | hsa-miR-375_st | -0.2935064935 | 0.0922454559 |
| 202834_at | hsa-miR-1180_st | -0.2935064935 | 0.0922454559 |
| 206935_at | hsa-miR-34b_st | -0.2935064935 | 0.0922454559 |
| 209183_s_at | hsa-miR-3189-5p_st | -0.2935064935 | 0.0922454559 |
| 218285_s_at | hsa-miR-132_st | -0.2935064935 | 0.0922454559 |
| 210534_s_at | hsa-miR-34b_st | -0.2933761182 | 0.0923952555 |
| 201924_at | hsa-miR-3189-5p_st | -0.2932009167 | 0.0925968562 |
| 204070_at | hsa-miR-3189-5p_st | -0.2932009167 | 0.0925968562 |
| 218654_s_at | hsa-miR-34b_st | -0.2932009167 | 0.0925968562 |
| 218559_s_at | hsa-miR-212_st | -0.2928953400 | 0.0929492909 |
| 211971_s_at | hsa-miR-375_st | -0.2927649180 | 0.0931000276 |
| 202587_s_at | hsa-miR-4760-3p_st | -0.2925897632 | 0.0933027620 |
| 200673_at | hsa-miR-4760-3p_st | -0.2922841864 | 0.0936572710 |
| 201656_at | hsa-miR-4633-5p_st | -0.2922841864 | 0.0936572710 |
| 203973_s_at | hsa-miR-4760-3p_st | -0.2922841864 | 0.0936572710 |
| 212215_at | hsa-miR-34b_st | -0.2922841864 | 0.0936572710 |
| 202834_at | hsa-miR-383_st | -0.2919786096 | 0.0940128200 |
| 208981_at | hsa-miR-3189-5p_st | -0.2919786096 | 0.0940128200 |
| 209183_s_at | hsa-miR-4760-3p_st | -0.2919786096 | 0.0940128200 |
| 212195_at | hsa-miR-1180_st | -0.2919786096 | 0.0940128200 |
| 200843_s_at | hsa-miR-34b_st | -0.2916730328 | 0.0943694106 |
| 202868_s_at | hsa-miR-375_st | -0.2916730328 | 0.0943694106 |
| 218285_s_at | hsa-miR-3676_st | -0.2916730328 | 0.0943694106 |
| 200077_s_at | hsa-miR-34b-star_st | -0.2913674561 | 0.0947270447 |
| 208659_at | hsa-miR-361-5p_st | -0.2913674561 | 0.0947270447 |
| 208690_s_at | hsa-miR-4762-5p_st | -0.2913674561 | 0.0947270447 |
| 208782_at | hsa-miR-4762-5p_st | -0.2910618793 | 0.0950857240 |
| 208869_s_at | hsa-miR-375_st | -0.2910618793 | 0.0950857240 |
| 212687_at | hsa-miR-4633-5p_st | -0.2910618793 | 0.0950857240 |
| 201029_s_at | hsa-miR-4311_st | -0.2909313172 | 0.0952392941 |
| 203313_s_at | hsa-miR-370_st | -0.2907563025 | 0.0954454505 |
| 207717_s_at | hsa-miR-4652-3p_st | -0.2907563025 | 0.0954454505 |
| 208457_at | hsa-miR-34b-star_st | -0.2907563025 | 0.0954454505 |
| 209570_s_at | hsa-miR-4652-3p_st | -0.2907563025 | 0.0954454505 |
| 200903_s_at | hsa-miR-4652-3p_st | -0.2904507257 | 0.0958062259 |
| 201012_at | hsa-miR-1180_st | -0.2904507257 | 0.0958062259 |
| 209243_s_at | hsa-miR-375_st | -0.2904507257 | 0.0958062259 |
| 211962_s_at | hsa-miR-4762-5p_st | -0.2904507257 | 0.0958062259 |
| 201160_s_at | hsa-miR-555_st | -0.2903201170 | 0.0959607479 |
| 219911_s_at | hsa-miR-4311_st | -0.2903201170 | 0.0959607479 |
| 202071_at | hsa-miR-4762-5p_st | -0.2901451490 | 0.0961680520 |
| 212271_at | hsa-miR-4652-3p_st | -0.2901451490 | 0.0961680520 |
| 215058_at | hsa-miR-4652-3p_st | -0.2898395722 | 0.0965309305 |
| 215952_s_at | hsa-miR-375_st | -0.2898395722 | 0.0965309305 |
| 218559_s_at | hsa-miR-370_st | -0.2898395722 | 0.0965309305 |
| 200600_at | hsa-miR-4720-3p_st | -0.2895339954 | 0.0968948634 |
| 205705_at | hsa-miR-375_st | -0.2895339954 | 0.0968948634 |
| 208981_at | hsa-miR-3676_st | -0.2895339954 | 0.0968948634 |
| 221796_at | hsa-miR-382_st | -0.2894033166 | 0.0970508203 |
| 200677_at | hsa-miR-3189-5p_st | -0.2892284186 | 0.0972598524 |
| 210592_s_at | hsa-miR-377-star_st | -0.2892284186 | 0.0972598524 |
| 213227_at | hsa-miR-375_st | -0.2889228419 | 0.0976258992 |
| 217546_at | hsa-miR-132_st | -0.2889228419 | 0.0976258992 |
| 208451_s_at | hsa-miR-4311_st | -0.2887921164 | 0.0977828173 |
| 201989_s_at | hsa-miR-34b_st | -0.2886172651 | 0.0979930057 |
| 203704_s_at | hsa-miR-3676_st | -0.2886172651 | 0.0979930057 |
| 217947_at | hsa-miR-132_st | -0.2886172651 | 0.0979930057 |
| 221696_s_at | hsa-miR-4652-3p_st | -0.2886172651 | 0.0979930057 |
| 211270_x_at | hsa-miR-382_st | -0.2883337162 | 0.0983345984 |
| 218285_s_at | hsa-miR-4311_st | -0.2883337162 | 0.0983345984 |
| 200701_at | hsa-miR-132_st | -0.2883116883 | 0.0983611738 |
| 200798_x_at | hsa-miR-3189-5p_st | -0.2883116883 | 0.0983611738 |
| 206989_s_at | hsa-miR-3189-5p_st | -0.2883116883 | 0.0983611738 |
| 212092_at | hsa-miR-375_st | -0.2883116883 | 0.0983611738 |
| 218656_s_at | hsa-miR-1180_st | -0.2883116883 | 0.0983611738 |
| 214829_at | hsa-miR-4311_st | -0.2881809161 | 0.0985190568 |
| 209075_s_at | hsa-miR-375_st | -0.2880061115 | 0.0987304050 |
| 217968_at | hsa-miR-34b_st | -0.2880061115 | 0.0987304050 |
| 218432_at | hsa-miR-34b_st | -0.2880061115 | 0.0987304050 |
| 48531_at | hsa-miR-523_st | -0.2880061115 | 0.0987304050 |
| 205097_at | hsa-miR-212_st | -0.2877005348 | 0.0991007013 |
| 219263_at | hsa-miR-4652-3p_st | -0.2877005348 | 0.0991007013 |
| 205856_at | hsa-miR-555_st | -0.2875697159 | 0.0992595530 |
| 201012_at | hsa-miR-4762-5p_st | -0.2873949580 | 0.0994720645 |
| 210448_s_at | hsa-miR-375_st | -0.2873949580 | 0.0994720645 |
| 212887_at | hsa-miR-4652-3p_st | -0.2873949580 | 0.0994720645 |
| 212977_at | hsa-miR-523_st | -0.2873949580 | 0.0994720645 |
| 218160_at | hsa-miR-34b_st | -0.2873949580 | 0.0994720645 |
| 203455_s_at | hsa-miR-4311_st | -0.2872641157 | 0.0996314019 |
| 202591_s_at | hsa-miR-34b_st | -0.2870893812 | 0.0998444963 |
| 48531_at | hsa-miR-4762-5p_st | -0.2870893812 | 0.0998444963 |
| 201484_at | hsa-miR-4652-3p_st | -0.2867838044 | 0.1002179984 |
| 203313_s_at | hsa-miR-3189-5p_st | -0.2867838044 | 0.1002179984 |
| 217995_at | hsa-miR-383_st | -0.2867838044 | 0.1002179984 |
| 218559_s_at | hsa-miR-1180_st | -0.2867838044 | 0.1002179984 |
| 202670_at | hsa-miR-34b_st | -0.2861726509 | 0.1009682212 |
| 209046_s_at | hsa-miR-34b_st | -0.2861726509 | 0.1009682212 |
| 212195_at | hsa-miR-4720-3p_st | -0.2861726509 | 0.1009682212 |
| 222043_at | hsa-miR-4311_st | -0.2860417153 | 0.1011295106 |
| 200853_at | hsa-miR-34b_st | -0.2858670741 | 0.1013449452 |
| 205097_at | hsa-miR-1180_st | -0.2858670741 | 0.1013449452 |
| 221009_s_at | hsa-miR-3189-5p_st | -0.2858670741 | 0.1013449452 |
| 204326_x_at | hsa-miR-212_st | -0.2855614973 | 0.1017227468 |
| 208818_s_at | hsa-miR-134_st | -0.2855614973 | 0.1017227468 |
| 211270_x_at | hsa-miR-4762-5p_st | -0.2855614973 | 0.1017227468 |
| 208911_s_at | hsa-miR-4423-3p_st | -0.2852559206 | 0.1021016277 |
| 209183_s_at | hsa-miR-1912_st | -0.2852559206 | 0.1021016277 |
| 218559_s_at | hsa-miR-523_st | -0.2852559206 | 0.1021016277 |
| 221515_s_at | hsa-miR-375_st | -0.2852559206 | 0.1021016277 |
| 201315_x_at | hsa-miR-361-5p_st | -0.2849503438 | 0.1024815897 |
| 208659_at | hsa-miR-555_st | -0.2848193148 | 0.1026448461 |
| 48531_at | hsa-miR-4311_st | -0.2846665147 | 0.1028354800 |
| 200663_at | hsa-miR-370_st | -0.2846447670 | 0.1028626345 |
| 201570_at | hsa-miR-34b_st | -0.2846447670 | 0.1028626345 |
| 204002_s_at | hsa-miR-4652-3p_st | -0.2846447670 | 0.1028626345 |
| 221741_s_at | hsa-miR-3189-5p_st | -0.2846447670 | 0.1028626345 |
| 202133_at | hsa-miR-382_st | -0.2845137146 | 0.1030263850 |
| 208809_s_at | hsa-miR-382_st | -0.2843609146 | 0.1032175614 |
| 202427_s_at | hsa-miR-34c-5p_st | -0.2843391902 | 0.1032447639 |
| 205097_at | hsa-miR-4633-5p_st | -0.2843391902 | 0.1032447639 |
| 208818_s_at | hsa-miR-555_st | -0.2842081145 | 0.1034090094 |
| 203411_s_at | hsa-miR-382_st | -0.2840553145 | 0.1036007292 |
| 201146_at | hsa-miR-3189-5p_st | -0.2840336134 | 0.1036279797 |
| 202834_at | hsa-miR-3189-5p_st | -0.2840336134 | 0.1036279797 |
| 203621_at | hsa-miR-34b_st | -0.2840336134 | 0.1036279797 |
| 208839_s_at | hsa-miR-4652-3p_st | -0.2840336134 | 0.1036279797 |
| 217860_at | hsa-miR-34b_st | -0.2840336134 | 0.1036279797 |
| 218970_s_at | hsa-miR-4423-3p_st | -0.2840336134 | 0.1036279797 |
| 203987_at | hsa-miR-4762-5p_st | -0.2837280367 | 0.1040122837 |
| 210978_s_at | hsa-miR-383_st | -0.2837280367 | 0.1040122837 |
| 218507_at | hsa-miR-132_st | -0.2837280367 | 0.1040122837 |
| 200677_at | hsa-miR-555_st | -0.2835969143 | 0.1041775218 |
| 207830_s_at | hsa-miR-4423-3p_st | -0.2834224599 | 0.1043976775 |
| 211962_s_at | hsa-miR-4311_st | -0.2831385141 | 0.1047567688 |
| 210418_s_at | hsa-miR-4652-3p_st | -0.2831168831 | 0.1047841630 |
| 210592_s_at | hsa-miR-1912_st | -0.2831168831 | 0.1047841630 |
| 200916_at | hsa-miR-3676_st | -0.2828113063 | 0.1051717419 |
| 200989_at | hsa-miR-4633-5p_st | -0.2828113063 | 0.1051717419 |
| 218656_s_at | hsa-miR-4760-3p_st | -0.2828113063 | 0.1051717419 |
| 214428_x_at | hsa-miR-4311_st | -0.2825273138 | 0.1055329262 |
| 200967_at | hsa-miR-4762-5p_st | -0.2825057296 | 0.1055604160 |
| 209108_at | hsa-miR-3676_st | -0.2825057296 | 0.1055604160 |
| 212501_at | hsa-miR-132_st | -0.2825057296 | 0.1055604160 |
| 213552_at | hsa-miR-375_st | -0.2825057296 | 0.1055604160 |
| 218285_s_at | hsa-miR-555_st | -0.2823745138 | 0.1057276506 |
| 217995_at | hsa-miR-382_st | -0.2822217137 | 0.1059226495 |
| 201324_at | hsa-miR-3189-5p_st | -0.2822001528 | 0.1059501870 |
| 205705_at | hsa-miR-4652-3p_st | -0.2822001528 | 0.1059501870 |
| 213005_s_at | hsa-miR-4762-5p_st | -0.2822001528 | 0.1059501870 |
| 215506_s_at | hsa-miR-375_st | -0.2822001528 | 0.1059501870 |
| 211271_x_at | hsa-miR-555_st | -0.2819161136 | 0.1063134716 |
| 201412_at | hsa-miR-3676_st | -0.2818945760 | 0.1063410568 |
| 211962_s_at | hsa-miR-3189-5p_st | -0.2818945760 | 0.1063410568 |
| 215416_s_at | hsa-miR-4652-3p_st | -0.2818945760 | 0.1063410568 |
| 219911_s_at | hsa-miR-382_st | -0.2817633135 | 0.1065092952 |
| 200822_x_at | hsa-miR-375_st | -0.2815889992 | 0.1067330269 |
| 201160_s_at | hsa-miR-3676_st | -0.2815889992 | 0.1067330269 |
| 202370_s_at | hsa-miR-523_st | -0.2815889992 | 0.1067330269 |
| 208659_at | hsa-miR-3676_st | -0.2815889992 | 0.1067330269 |
| 212716_s_at | hsa-miR-375_st | -0.2815889992 | 0.1067330269 |
| 214150_x_at | hsa-miR-370_st | -0.2815889992 | 0.1067330269 |
| 218557_at | hsa-miR-4652-3p_st | -0.2815889992 | 0.1067330269 |
| 219911_s_at | hsa-miR-370_st | -0.2815889992 | 0.1067330269 |
| 203313_s_at | hsa-miR-4762-5p_st | -0.2812834225 | 0.1071260993 |
| 210906_x_at | hsa-miR-132_st | -0.2812834225 | 0.1071260993 |
| 222043_at | hsa-miR-4762-5p_st | -0.2812834225 | 0.1071260993 |
| 201324_at | hsa-miR-377-star_st | -0.2809778457 | 0.1075202755 |
| 202133_at | hsa-miR-4633-5p_st | -0.2809778457 | 0.1075202755 |
| 203137_at | hsa-miR-34b-star_st | -0.2809778457 | 0.1075202755 |
| 209733_at | hsa-miR-34b_st | -0.2809778457 | 0.1075202755 |
| 210962_s_at | hsa-miR-34c-5p_st | -0.2809778457 | 0.1075202755 |
| 217936_at | hsa-miR-134_st | -0.2809778457 | 0.1075202755 |
| 206544_x_at | hsa-miR-34b_st | -0.2806722689 | 0.1079155575 |
| 212407_at | hsa-miR-4652-3p_st | -0.2806722689 | 0.1079155575 |
| 213005_s_at | hsa-miR-4760-3p_st | -0.2806722689 | 0.1079155575 |
| 214829_at | hsa-miR-1180_st | -0.2806722689 | 0.1079155575 |
| 219911_s_at | hsa-miR-1180_st | -0.2806722689 | 0.1079155575 |
| 202736_s_at | hsa-miR-375_st | -0.2803666921 | 0.1083119469 |
| 210278_s_at | hsa-miR-34b_st | -0.2800611154 | 0.1087094454 |
| 221699_s_at | hsa-miR-4652-3p_st | -0.2800611154 | 0.1087094454 |
| 202543_s_at | hsa-miR-361-5p_st | -0.2797555386 | 0.1091080548 |
| 204992_s_at | hsa-miR-375_st | -0.2797555386 | 0.1091080548 |
| 208731_at | hsa-miR-34b_st | -0.2797555386 | 0.1091080548 |
| 208827_at | hsa-miR-34b_st | -0.2797555386 | 0.1091080548 |
| 210972_x_at | hsa-miR-4652-3p_st | -0.2797555386 | 0.1091080548 |
| 203120_at | hsa-miR-4311_st | -0.2796241127 | 0.1092798353 |
| 201924_at | hsa-miR-523_st | -0.2794499618 | 0.1095077769 |
| 204587_at | hsa-miR-34b_st | -0.2794499618 | 0.1095077769 |
| 208679_s_at | hsa-miR-4652-3p_st | -0.2794499618 | 0.1095077769 |
| 215058_at | hsa-miR-34b_st | -0.2794499618 | 0.1095077769 |
| 1007_s_at | hsa-miR-4311_st | -0.2791657125 | 0.1098806011 |
| 202834_at | hsa-miR-555_st | -0.2791657125 | 0.1098806011 |
| 210749_x_at | hsa-miR-555_st | -0.2791657125 | 0.1098806011 |
| 200673_at | hsa-miR-3676_st | -0.2791443850 | 0.1099086133 |
| 219628_at | hsa-miR-34b_st | -0.2791443850 | 0.1099086133 |
| 202096_s_at | hsa-miR-361-5p_st | -0.2788388083 | 0.1103105659 |
| 202779_s_at | hsa-miR-375_st | -0.2788388083 | 0.1103105659 |
| 200989_at | hsa-miR-377-star_st | -0.2785332315 | 0.1107136362 |
| 210068_s_at | hsa-miR-132_st | -0.2785332315 | 0.1107136362 |
| 201887_at | hsa-miR-1180_st | -0.2782276547 | 0.1111178261 |
| 208678_at | hsa-miR-34b_st | -0.2782276547 | 0.1111178261 |
| 209265_s_at | hsa-miR-34c-5p_st | -0.2782276547 | 0.1111178261 |
| 210068_s_at | hsa-miR-4760-3p_st | -0.2782276547 | 0.1111178261 |
| 213592_at | hsa-miR-4311_st | -0.2780961121 | 0.1112921642 |
| 206984_s_at | hsa-miR-375_st | -0.2779220779 | 0.1115231373 |
| 207761_s_at | hsa-miR-1912_st | -0.2779220779 | 0.1115231373 |
| 210448_s_at | hsa-miR-4652-3p_st | -0.2779220779 | 0.1115231373 |
| 220329_s_at | hsa-miR-4652-3p_st | -0.2779220779 | 0.1115231373 |
| 209104_s_at | hsa-miR-375_st | -0.2776165011 | 0.1119295715 |
| 210817_s_at | hsa-miR-361-5p_st | -0.2776165011 | 0.1119295715 |
| 211902_x_at | hsa-miR-34b-star_st | -0.2776165011 | 0.1119295715 |
| 212063_at | hsa-miR-4633-5p_st | -0.2776165011 | 0.1119295715 |
| 213592_at | hsa-miR-4762-5p_st | -0.2776165011 | 0.1119295715 |
| 202712_s_at | hsa-miR-375_st | -0.2773109244 | 0.1123371304 |
| 210906_x_at | hsa-miR-4311_st | -0.2770265116 | 0.1127174741 |
| 200967_at | hsa-miR-212_st | -0.2770053476 | 0.1127458157 |
| 202071_at | hsa-miR-132_st | -0.2770053476 | 0.1127458157 |
| 202096_s_at | hsa-miR-3189-5p_st | -0.2770053476 | 0.1127458157 |
| 206989_s_at | hsa-miR-4633-5p_st | -0.2770053476 | 0.1127458157 |
| 207088_s_at | hsa-miR-34b-star_st | -0.2770053476 | 0.1127458157 |
| 215506_s_at | hsa-miR-4652-3p_st | -0.2770053476 | 0.1127458157 |
| 200883_at | hsa-miR-4652-3p_st | -0.2766997708 | 0.1131556292 |
| 205097_at | hsa-miR-4760-3p_st | -0.2766997708 | 0.1131556292 |
| 210149_s_at | hsa-miR-34b_st | -0.2766997708 | 0.1131556292 |
| 211963_s_at | hsa-miR-4652-3p_st | -0.2766997708 | 0.1131556292 |
| 218005_at | hsa-miR-370_st | -0.2766997708 | 0.1131556292 |
| 200967_at | hsa-miR-383_st | -0.2763941940 | 0.1135665726 |
| 201527_at | hsa-miR-4652-3p_st | -0.2763941940 | 0.1135665726 |
| 202864_s_at | hsa-miR-4760-3p_st | -0.2763941940 | 0.1135665726 |
| 200797_s_at | hsa-miR-212_st | -0.2760886173 | 0.1139786475 |
| 202614_at | hsa-miR-375_st | -0.2760886173 | 0.1139786475 |
| 207643_s_at | hsa-miR-3676_st | -0.2760886173 | 0.1139786475 |
| 209507_at | hsa-miR-34b_st | -0.2760886173 | 0.1139786475 |
| 218005_at | hsa-miR-1180_st | -0.2760886173 | 0.1139786475 |
| 210117_at | hsa-miR-4633-5p_st | -0.2757830405 | 0.1143918557 |
| 217997_at | hsa-miR-34b_st | -0.2754774637 | 0.1148061989 |
| 218706_s_at | hsa-miR-4720-3p_st | -0.2754774637 | 0.1148061989 |
| 201324_at | hsa-miR-4760-3p_st | -0.2751718869 | 0.1152216788 |
| 217837_s_at | hsa-miR-4652-3p_st | -0.2751718869 | 0.1152216788 |
| 221741_s_at | hsa-miR-383_st | -0.2751718869 | 0.1152216788 |
| 208581_x_at | hsa-miR-4311_st | -0.2748873108 | 0.1156096288 |
| 200701_at | hsa-miR-377-star_st | -0.2748663102 | 0.1156382971 |
| 201160_s_at | hsa-miR-4633-5p_st | -0.2748663102 | 0.1156382971 |
| 218854_at | hsa-miR-132_st | -0.2748663102 | 0.1156382971 |
| 219960_s_at | hsa-miR-4652-3p_st | -0.2748663102 | 0.1156382971 |
| 221696_s_at | hsa-miR-34b_st | -0.2748663102 | 0.1156382971 |
| 201315_x_at | hsa-miR-134_st | -0.2745607334 | 0.1160560555 |
| 204766_s_at | hsa-miR-34b_st | -0.2745607334 | 0.1160560555 |
| 202376_at | hsa-miR-555_st | -0.2742761105 | 0.1164461945 |
| 201887_at | hsa-miR-383_st | -0.2742551566 | 0.1164749556 |
| 208782_at | hsa-miR-134_st | -0.2742551566 | 0.1164749556 |
| 200822_x_at | hsa-miR-4652-3p_st | -0.2739495798 | 0.1168949992 |
| 201924_at | hsa-miR-4762-5p_st | -0.2739495798 | 0.1168949992 |
| 203540_at | hsa-miR-4633-5p_st | -0.2739495798 | 0.1168949992 |
| 212961_x_at | hsa-miR-4652-3p_st | -0.2739495798 | 0.1168949992 |
| 212321_at | hsa-miR-4720-3p_st | -0.2736440031 | 0.1173161880 |
| 218507_at | hsa-miR-212_st | -0.2736440031 | 0.1173161880 |
| 219481_at | hsa-miR-34b_st | -0.2736440031 | 0.1173161880 |
| 218005_at | hsa-miR-4311_st | -0.2735121102 | 0.1174983355 |
| 202868_s_at | hsa-miR-4652-3p_st | -0.2733384263 | 0.1177385237 |
| 209108_at | hsa-miR-523_st | -0.2733384263 | 0.1177385237 |
| 212015_x_at | hsa-miR-370_st | -0.2733384263 | 0.1177385237 |
| 201753_s_at | hsa-miR-1180_st | -0.2730328495 | 0.1181620078 |
| 204554_at | hsa-miR-1912_st | -0.2730328495 | 0.1181620078 |
| 218866_s_at | hsa-miR-34b_st | -0.2730328495 | 0.1181620078 |
| 200916_at | hsa-miR-555_st | -0.2727481099 | 0.1185576500 |
| 201924_at | hsa-miR-361-5p_st | -0.2727272727 | 0.1185866422 |
| 202133_at | hsa-miR-132_st | -0.2727272727 | 0.1185866422 |
| 202975_s_at | hsa-miR-1180_st | -0.2727272727 | 0.1185866422 |
| 205217_at | hsa-miR-34b_st | -0.2727272727 | 0.1185866422 |
| 218285_s_at | hsa-miR-383_st | -0.2727272727 | 0.1185866422 |
| 218285_s_at | hsa-miR-134_st | -0.2727272727 | 0.1185866422 |
| 221796_at | hsa-miR-4762-5p_st | -0.2727272727 | 0.1185866422 |
| 203302_at | hsa-miR-34b_st | -0.2724216960 | 0.1190124285 |
| 210418_s_at | hsa-miR-34b_st | -0.2724216960 | 0.1190124285 |
| 207079_s_at | hsa-miR-4652-3p_st | -0.2721161192 | 0.1194393684 |
| 208818_s_at | hsa-miR-3189-5p_st | -0.2721161192 | 0.1194393684 |
| 209108_at | hsa-miR-1180_st | -0.2721161192 | 0.1194393684 |
| 212015_x_at | hsa-miR-523_st | -0.2721161192 | 0.1194393684 |
| 218656_s_at | hsa-miR-361-5p_st | -0.2721161192 | 0.1194393684 |
| 213005_s_at | hsa-miR-4311_st | -0.2718313095 | 0.1198383333 |
| 200967_at | hsa-miR-1180_st | -0.2718105424 | 0.1198674635 |
| 202936_s_at | hsa-miR-4720-3p_st | -0.2718105424 | 0.1198674635 |
| 211270_x_at | hsa-miR-370_st | -0.2718105424 | 0.1198674635 |
| 217746_s_at | hsa-miR-555_st | -0.2715257094 | 0.1202675395 |
| 202863_at | hsa-miR-4720-3p_st | -0.2715049656 | 0.1202967155 |
| 205963_s_at | hsa-miR-34b_st | -0.2715049656 | 0.1202967155 |
| 209476_at | hsa-miR-4720-3p_st | -0.2715049656 | 0.1202967155 |
| 200862_at | hsa-miR-4652-3p_st | -0.2711993888 | 0.1207271261 |
| 202232_s_at | hsa-miR-34b_st | -0.2711993888 | 0.1207271261 |
| 205324_s_at | hsa-miR-4652-3p_st | -0.2711993888 | 0.1207271261 |
| 206542_s_at | hsa-miR-34b_st | -0.2711993888 | 0.1207271261 |
| 208998_at | hsa-miR-3189-5p_st | -0.2711993888 | 0.1207271261 |
| 217959_s_at | hsa-miR-4652-3p_st | -0.2711993888 | 0.1207271261 |
| 200097_s_at | hsa-miR-375_st | -0.2708938121 | 0.1211586970 |
| 200673_at | hsa-miR-132_st | -0.2708938121 | 0.1211586970 |
| 201656_at | hsa-miR-523_st | -0.2708938121 | 0.1211586970 |
| 202930_s_at | hsa-miR-34b_st | -0.2708938121 | 0.1211586970 |
| 208818_s_at | hsa-miR-1180_st | -0.2708938121 | 0.1211586970 |
| 210105_s_at | hsa-miR-4720-3p_st | -0.2708938121 | 0.1211586970 |
| 212600_s_at | hsa-miR-4652-3p_st | -0.2708938121 | 0.1211586970 |
| 221515_s_at | hsa-miR-4652-3p_st | -0.2708938121 | 0.1211586970 |
| 210749_x_at | hsa-miR-4311_st | -0.2707617091 | 0.1213456277 |
| 205963_s_at | hsa-miR-375_st | -0.2705882353 | 0.1215914297 |
| 218526_s_at | hsa-miR-375_st | -0.2705882353 | 0.1215914297 |
| 210978_s_at | hsa-miR-4311_st | -0.2703033089 | 0.1219959674 |
| 217860_at | hsa-miR-375_st | -0.2702826585 | 0.1220253261 |
| 200673_at | hsa-miR-555_st | -0.2701505088 | 0.1222133296 |
| 200030_s_at | hsa-miR-4652-3p_st | -0.2699770817 | 0.1224603876 |
| 203893_at | hsa-miR-375_st | -0.2699770817 | 0.1224603876 |
| 212063_at | hsa-miR-4762-5p_st | -0.2699770817 | 0.1224603876 |
| 217820_s_at | hsa-miR-3189-5p_st | -0.2699770817 | 0.1224603876 |
| 222043_at | hsa-miR-523_st | -0.2699770817 | 0.1224603876 |
| 222216_s_at | hsa-miR-375_st | -0.2699770817 | 0.1224603876 |
| 206989_s_at | hsa-miR-4762-5p_st | -0.2696715050 | 0.1228966161 |
| 207922_s_at | hsa-miR-34b-star_st | -0.2696715050 | 0.1228966161 |
| 214428_x_at | hsa-miR-4762-5p_st | -0.2696715050 | 0.1228966161 |
| 220329_s_at | hsa-miR-375_st | -0.2696715050 | 0.1228966161 |
| 209108_at | hsa-miR-382_st | -0.2695393086 | 0.1230856960 |
| 200916_at | hsa-miR-523_st | -0.2693659282 | 0.1233340131 |
| 202232_s_at | hsa-miR-375_st | -0.2693659282 | 0.1233340131 |
| 202936_s_at | hsa-miR-212_st | -0.2693659282 | 0.1233340131 |
| 204787_at | hsa-miR-4720-3p_st | -0.2693659282 | 0.1233340131 |
| 205097_at | hsa-miR-383_st | -0.2693659282 | 0.1233340131 |
| 208690_s_at | hsa-miR-3676_st | -0.2693659282 | 0.1233340131 |
| 208799_at | hsa-miR-375_st | -0.2693659282 | 0.1233340131 |
| 210068_s_at | hsa-miR-3676_st | -0.2693659282 | 0.1233340131 |
| 210749_x_at | hsa-miR-4633-5p_st | -0.2693659282 | 0.1233340131 |
| 203723_at | hsa-miR-382_st | -0.2691014670 | 0.1237135025 |
| 213005_s_at | hsa-miR-370_st | -0.2690603514 | 0.1237725803 |
| 201656_at | hsa-miR-3676_st | -0.2687547746 | 0.1242123193 |
| 202507_s_at | hsa-miR-4652-3p_st | -0.2687547746 | 0.1242123193 |
| 208782_at | hsa-miR-4633-5p_st | -0.2687547746 | 0.1242123193 |
| 208838_at | hsa-miR-4652-3p_st | -0.2687547746 | 0.1242123193 |
| 210592_s_at | hsa-miR-4762-5p_st | -0.2687547746 | 0.1242123193 |
| 217837_s_at | hsa-miR-34b_st | -0.2687547746 | 0.1242123193 |
| 207988_s_at | hsa-miR-4652-3p_st | -0.2684491979 | 0.1246532317 |
| 208451_s_at | hsa-miR-4762-5p_st | -0.2684491979 | 0.1246532317 |
| 218201_at | hsa-miR-4652-3p_st | -0.2684491979 | 0.1246532317 |
| 221009_s_at | hsa-miR-370_st | -0.2684491979 | 0.1246532317 |
| 36711_at | hsa-miR-3189-5p_st | -0.2684491979 | 0.1246532317 |
| 208998_at | hsa-miR-555_st | -0.2681641080 | 0.1250656434 |
| 201054_at | hsa-miR-34b_st | -0.2681436211 | 0.1250953193 |
| 204068_at | hsa-miR-4762-5p_st | -0.2681436211 | 0.1250953193 |
| 217936_at | hsa-miR-361-5p_st | -0.2681436211 | 0.1250953193 |
| 218214_at | hsa-miR-34b_st | -0.2681436211 | 0.1250953193 |
| 204194_at | hsa-miR-555_st | -0.2678585079 | 0.1255088627 |
| 200663_at | hsa-miR-3189-5p_st | -0.2678380443 | 0.1255385836 |
| 211270_x_at | hsa-miR-3189-5p_st | -0.2678380443 | 0.1255385836 |
| 212271_at | hsa-miR-34b_st | -0.2678380443 | 0.1255385836 |
| 218488_at | hsa-miR-4652-3p_st | -0.2678380443 | 0.1255385836 |
| 201761_at | hsa-miR-382_st | -0.2677057079 | 0.1257309141 |
| 201590_x_at | hsa-miR-4633-5p_st | -0.2675324675 | 0.1259830263 |
| 201656_at | hsa-miR-4760-3p_st | -0.2675324675 | 0.1259830263 |
| 206671_at | hsa-miR-34b_st | -0.2675324675 | 0.1259830263 |
| 213293_s_at | hsa-miR-4633-5p_st | -0.2675324675 | 0.1259830263 |
| 201412_at | hsa-miR-555_st | -0.2674001077 | 0.1261759017 |
| 200673_at | hsa-miR-1912_st | -0.2672268908 | 0.1264286491 |
| 201106_at | hsa-miR-4652-3p_st | -0.2672268908 | 0.1264286491 |
| 205856_at | hsa-miR-361-5p_st | -0.2672268908 | 0.1264286491 |
| 210749_x_at | hsa-miR-3676_st | -0.2672268908 | 0.1264286491 |
| 211962_s_at | hsa-miR-1180_st | -0.2672268908 | 0.1264286491 |
| 221667_s_at | hsa-miR-383_st | -0.2672268908 | 0.1264286491 |
| 201324_at | hsa-miR-4311_st | -0.2670945076 | 0.1266220702 |
| 212321_at | hsa-miR-555_st | -0.2669417076 | 0.1268455978 |
| 206542_s_at | hsa-miR-375_st | -0.2669213140 | 0.1268754534 |
| 207400_at | hsa-miR-34b_st | -0.2669213140 | 0.1268754534 |
| 210978_s_at | hsa-miR-3189-5p_st | -0.2669213140 | 0.1268754534 |
| 208451_s_at | hsa-miR-4633-5p_st | -0.2666157372 | 0.1273234410 |
| 211270_x_at | hsa-miR-523_st | -0.2666157372 | 0.1273234410 |
| 213005_s_at | hsa-miR-4720-3p_st | -0.2666157372 | 0.1273234410 |
| 202543_s_at | hsa-miR-3189-5p_st | -0.2663101604 | 0.1277726135 |
| 206805_at | hsa-miR-4652-3p_st | -0.2663101604 | 0.1277726135 |
| 209075_s_at | hsa-miR-34b_st | -0.2663101604 | 0.1277726135 |
| 210817_s_at | hsa-miR-523_st | -0.2663101604 | 0.1277726135 |
| 213217_at | hsa-miR-3189-5p_st | -0.2663101604 | 0.1277726135 |
| 217746_s_at | hsa-miR-4720-3p_st | -0.2663101604 | 0.1277726135 |
| 201012_at | hsa-miR-4311_st | -0.2661777072 | 0.1279676772 |
| 203613_s_at | hsa-miR-34b_st | -0.2660249072 | 0.1281929828 |
| 201628_s_at | hsa-miR-34b_st | -0.2660045837 | 0.1282229725 |
| 213533_at | hsa-miR-34b_st | -0.2660045837 | 0.1282229725 |
| 219628_at | hsa-miR-3676_st | -0.2660045837 | 0.1282229725 |
| 219911_s_at | hsa-miR-383_st | -0.2660045837 | 0.1282229725 |
| 205217_at | hsa-miR-375_st | -0.2656990069 | 0.1286745196 |
| 200677_at | hsa-miR-361-5p_st | -0.2653934301 | 0.1291272565 |
| 201012_at | hsa-miR-4633-5p_st | -0.2653934301 | 0.1291272565 |
| 201322_at | hsa-miR-34b_st | -0.2653934301 | 0.1291272565 |
| 201859_at | hsa-miR-1912_st | -0.2653934301 | 0.1291272565 |
| 202614_at | hsa-miR-4652-3p_st | -0.2653934301 | 0.1291272565 |
| 200663_at | hsa-miR-382_st | -0.2651081068 | 0.1295510616 |
| 203404_at | hsa-miR-375_st | -0.2650878533 | 0.1295811846 |
| 203411_s_at | hsa-miR-361-5p_st | -0.2650878533 | 0.1295811846 |
| 210749_x_at | hsa-miR-382_st | -0.2648025067 | 0.1300061384 |
| 218283_at | hsa-miR-4423-3p_st | -0.2648025067 | 0.1300061384 |
| 201012_at | hsa-miR-3189-5p_st | -0.2647822765 | 0.1300363057 |
| 209227_at | hsa-miR-34b_st | -0.2647822765 | 0.1300363057 |
| 212321_at | hsa-miR-3676_st | -0.2647822765 | 0.1300363057 |
| 221958_s_at | hsa-miR-4311_st | -0.2646497066 | 0.1302341247 |
| 218706_s_at | hsa-miR-4311_st | -0.2644969066 | 0.1304624098 |
| 200967_at | hsa-miR-4720-3p_st | -0.2644766998 | 0.1304926214 |
| 201900_s_at | hsa-miR-34b_st | -0.2644766998 | 0.1304926214 |
| 217820_s_at | hsa-miR-3676_st | -0.2644766998 | 0.1304926214 |
| 36711_at | hsa-miR-1180_st | -0.2644766998 | 0.1304926214 |
| 202948_at | hsa-miR-555_st | -0.2643441065 | 0.1306909940 |
| 200978_at | hsa-miR-34b_st | -0.2641711230 | 0.1309501332 |
| 208581_x_at | hsa-miR-4720-3p_st | -0.2641711230 | 0.1309501332 |
| 208809_s_at | hsa-miR-383_st | -0.2641711230 | 0.1309501332 |
| 218982_s_at | hsa-miR-4652-3p_st | -0.2641711230 | 0.1309501332 |
| 203889_at | hsa-miR-34b_st | -0.2638655462 | 0.1314088427 |
| 214022_s_at | hsa-miR-134_st | -0.2638655462 | 0.1314088427 |
| 219582_at | hsa-miR-4633-5p_st | -0.2638655462 | 0.1314088427 |
| 203411_s_at | hsa-miR-4311_st | -0.2637329063 | 0.1316083253 |
| 212460_at | hsa-miR-382_st | -0.2637329063 | 0.1316083253 |
| 203404_at | hsa-miR-4652-3p_st | -0.2632543927 | 0.1323298613 |
| 203987_at | hsa-miR-3676_st | -0.2632543927 | 0.1323298613 |
| 208782_at | hsa-miR-523_st | -0.2632543927 | 0.1323298613 |
| 212321_at | hsa-miR-1180_st | -0.2632543927 | 0.1323298613 |
| 218706_s_at | hsa-miR-212_st | -0.2632543927 | 0.1323298613 |
| 218384_at | hsa-miR-4652-3p_st | -0.2629488159 | 0.1327921736 |
| 210427_x_at | hsa-miR-370_st | -0.2626432391 | 0.1332556899 |
| 221958_s_at | hsa-miR-382_st | -0.2625105058 | 0.1334574030 |
| 203663_s_at | hsa-miR-375_st | -0.2623376623 | 0.1337204119 |
| 203973_s_at | hsa-miR-1180_st | -0.2623376623 | 0.1337204119 |
| 211763_s_at | hsa-miR-4423-3p_st | -0.2623376623 | 0.1337204119 |
| 213293_s_at | hsa-miR-3676_st | -0.2623376623 | 0.1337204119 |
| 204787_at | hsa-miR-4311_st | -0.2622049056 | 0.1339226847 |
| 210817_s_at | hsa-miR-4311_st | -0.2622049056 | 0.1339226847 |
| 208782_at | hsa-miR-361-5p_st | -0.2620320856 | 0.1341863412 |
| 210033_s_at | hsa-miR-523_st | -0.2620320856 | 0.1341863412 |
| 212321_at | hsa-miR-523_st | -0.2620320856 | 0.1341863412 |
| 200673_at | hsa-miR-370_st | -0.2617265088 | 0.1346534792 |
| 201999_s_at | hsa-miR-3676_st | -0.2617265088 | 0.1346534792 |
| 205609_at | hsa-miR-212_st | -0.2617265088 | 0.1346534792 |
| 208981_at | hsa-miR-383_st | -0.2617265088 | 0.1346534792 |
| 218285_s_at | hsa-miR-4720-3p_st | -0.2617265088 | 0.1346534792 |
| 210962_s_at | hsa-miR-4652-3p_st | -0.2614209320 | 0.1351218277 |
| 212195_at | hsa-miR-4633-5p_st | -0.2614209320 | 0.1351218277 |
| 217730_at | hsa-miR-4762-5p_st | -0.2614209320 | 0.1351218277 |
| 219760_at | hsa-miR-34b_st | -0.2614209320 | 0.1351218277 |
| 201319_at | hsa-miR-555_st | -0.2612881053 | 0.1353257850 |
| 209476_at | hsa-miR-382_st | -0.2612881053 | 0.1353257850 |
| 208690_s_at | hsa-miR-4311_st | -0.2611353052 | 0.1355606952 |
| 202233_s_at | hsa-miR-4652-3p_st | -0.2611153552 | 0.1355913880 |
| 210156_s_at | hsa-miR-4652-3p_st | -0.2611153552 | 0.1355913880 |
| 218491_s_at | hsa-miR-4652-3p_st | -0.2611153552 | 0.1355913880 |
| 222360_at | hsa-miR-4652-3p_st | -0.2611153552 | 0.1355913880 |
| 201145_at | hsa-miR-375_st | -0.2608097785 | 0.1360621619 |
| 202269_x_at | hsa-miR-3676_st | -0.2608097785 | 0.1360621619 |
| 217947_at | hsa-miR-4720-3p_st | -0.2608097785 | 0.1360621619 |
| 218316_at | hsa-miR-4423-3p_st | -0.2608097785 | 0.1360621619 |
| 201398_s_at | hsa-miR-4311_st | -0.2605241050 | 0.1365033715 |
| 221958_s_at | hsa-miR-383_st | -0.2605042017 | 0.1365341509 |
| 201161_s_at | hsa-miR-132_st | -0.2601986249 | 0.1370073564 |
| 204326_x_at | hsa-miR-4762-5p_st | -0.2601986249 | 0.1370073564 |
| 205324_s_at | hsa-miR-34b_st | -0.2601986249 | 0.1370073564 |
| 208121_s_at | hsa-miR-4652-3p_st | -0.2601986249 | 0.1370073564 |
| 211595_s_at | hsa-miR-4652-3p_st | -0.2601986249 | 0.1370073564 |
| 217286_s_at | hsa-miR-4423-3p_st | -0.2601986249 | 0.1370073564 |
| 221741_s_at | hsa-miR-4720-3p_st | -0.2601986249 | 0.1370073564 |
| 200079_s_at | hsa-miR-4652-3p_st | -0.2598930481 | 0.1374817801 |
| 203313_s_at | hsa-miR-4633-5p_st | -0.2595874714 | 0.1379574236 |
| 221796_at | hsa-miR-383_st | -0.2595874714 | 0.1379574236 |
| 203079_s_at | hsa-miR-4652-3p_st | -0.2592818946 | 0.1384342883 |
| 200853_at | hsa-miR-4652-3p_st | -0.2589763178 | 0.1389123758 |
| 208581_x_at | hsa-miR-383_st | -0.2589763178 | 0.1389123758 |
| 203723_at | hsa-miR-3189-5p_st | -0.2588433043 | 0.1391208633 |
| 200905_x_at | hsa-miR-134_st | -0.2586707410 | 0.1393916876 |
| 218854_at | hsa-miR-4760-3p_st | -0.2583651642 | 0.1398722253 |
| 208981_at | hsa-miR-555_st | -0.2582321040 | 0.1400818539 |
| 200903_s_at | hsa-miR-34b_st | -0.2580595875 | 0.1403539904 |
| 204992_s_at | hsa-miR-34b_st | -0.2580595875 | 0.1403539904 |
| 209732_at | hsa-miR-377-star_st | -0.2580595875 | 0.1403539904 |
| 214150_x_at | hsa-miR-3676_st | -0.2577540107 | 0.1408369844 |
| 201029_s_at | hsa-miR-370_st | -0.2574484339 | 0.1413212088 |
| 202071_at | hsa-miR-3676_st | -0.2574484339 | 0.1413212088 |
| 221009_s_at | hsa-miR-523_st | -0.2574484339 | 0.1413212088 |
| 202975_s_at | hsa-miR-3189-5p_st | -0.2571428571 | 0.1418066653 |
| 212099_at | hsa-miR-4633-5p_st | -0.2571428571 | 0.1418066653 |
| 212099_at | hsa-miR-1912_st | -0.2571428571 | 0.1418066653 |
| 218005_at | hsa-miR-382_st | -0.2568569035 | 0.1422620645 |
| 200812_at | hsa-miR-34b_st | -0.2568372804 | 0.1422933552 |
| 203157_s_at | hsa-miR-4652-3p_st | -0.2568372804 | 0.1422933552 |
| 209476_at | hsa-miR-4633-5p_st | -0.2568372804 | 0.1422933552 |
| 210068_s_at | hsa-miR-377-star_st | -0.2568372804 | 0.1422933552 |
| 201666_at | hsa-miR-555_st | -0.2565513034 | 0.1427499473 |
| 1007_s_at | hsa-miR-4633-5p_st | -0.2565317036 | 0.1427812800 |
| 202863_at | hsa-miR-377-star_st | -0.2565317036 | 0.1427812800 |
| 218706_s_at | hsa-miR-523_st | -0.2565317036 | 0.1427812800 |
| 210427_x_at | hsa-miR-555_st | -0.2563985033 | 0.1429943524 |
| 206356_s_at | hsa-miR-4652-3p_st | -0.2562261268 | 0.1432704414 |
| 211271_x_at | hsa-miR-3676_st | -0.2562261268 | 0.1432704414 |
| 212460_at | hsa-miR-4423-3p_st | -0.2562261268 | 0.1432704414 |
| 218292_s_at | hsa-miR-4652-3p_st | -0.2562261268 | 0.1432704414 |
| 219582_at | hsa-miR-383_st | -0.2562261268 | 0.1432704414 |
| 203973_s_at | hsa-miR-4762-5p_st | -0.2559205500 | 0.1437608408 |
| 202929_s_at | hsa-miR-375_st | -0.2556149733 | 0.1442524796 |
| 203973_s_at | hsa-miR-3189-5p_st | -0.2556149733 | 0.1442524796 |
| 217995_at | hsa-miR-370_st | -0.2556149733 | 0.1442524796 |
| 201656_at | hsa-miR-4311_st | -0.2554817029 | 0.1444672852 |
| 202269_x_at | hsa-miR-370_st | -0.2553093965 | 0.1447453595 |
| 209732_at | hsa-miR-4720-3p_st | -0.2553093965 | 0.1447453595 |
| 217936_at | hsa-miR-3189-5p_st | -0.2553093965 | 0.1447453595 |
| 217959_s_at | hsa-miR-375_st | -0.2553093965 | 0.1447453595 |
| 221449_s_at | hsa-miR-4652-3p_st | -0.2553093965 | 0.1447453595 |
| 211271_x_at | hsa-miR-382_st | -0.2551761028 | 0.1449607445 |
| 211270_x_at | hsa-miR-555_st | -0.2550233028 | 0.1452079403 |
| 211615_s_at | hsa-miR-34b_st | -0.2550038197 | 0.1452394818 |
| 212687_at | hsa-miR-3676_st | -0.2546982429 | 0.1457348481 |
| 221009_s_at | hsa-miR-4760-3p_st | -0.2546982429 | 0.1457348481 |
| 201656_at | hsa-miR-382_st | -0.2545649026 | 0.1459513945 |
| 211999_at | hsa-miR-555_st | -0.2544315403 | 0.1461682138 |
| 208981_at | hsa-miR-361-5p_st | -0.2543926662 | 0.1462314599 |
| 211962_s_at | hsa-miR-555_st | -0.2541065024 | 0.1466976529 |
| 201180_s_at | hsa-miR-3189-5p_st | -0.2540870894 | 0.1467293185 |
| 212977_at | hsa-miR-383_st | -0.2540870894 | 0.1467293185 |
| 201725_at | hsa-miR-34b_st | -0.2537815126 | 0.1472284256 |
| 210427_x_at | hsa-miR-4633-5p_st | -0.2537815126 | 0.1472284256 |
| 218559_s_at | hsa-miR-3189-5p_st | -0.2537815126 | 0.1472284256 |
| 202587_s_at | hsa-miR-132_st | -0.2534759358 | 0.1477287826 |
| 207400_at | hsa-miR-4652-3p_st | -0.2534759358 | 0.1477287826 |
| 210105_s_at | hsa-miR-4762-5p_st | -0.2534759358 | 0.1477287826 |
| 201601_x_at | hsa-miR-4633-5p_st | -0.2531703591 | 0.1482303909 |
| 208581_x_at | hsa-miR-4760-3p_st | -0.2528647823 | 0.1487332520 |
| 208809_s_at | hsa-miR-4423-3p_st | -0.2528647823 | 0.1487332520 |
| 208998_at | hsa-miR-370_st | -0.2528647823 | 0.1487332520 |
| 202269_x_at | hsa-miR-555_st | -0.2527313018 | 0.1489533027 |
| 200600_at | hsa-miR-4633-5p_st | -0.2525592055 | 0.1492373674 |
| 202325_s_at | hsa-miR-4652-3p_st | -0.2525592055 | 0.1492373674 |
| 218322_s_at | hsa-miR-4762-5p_st | -0.2525592055 | 0.1492373674 |
| 209303_at | hsa-miR-4652-3p_st | -0.2524257017 | 0.1494580050 |
| 202936_s_at | hsa-miR-370_st | -0.2522536287 | 0.1497427385 |
| 202936_s_at | hsa-miR-4760-3p_st | -0.2522536287 | 0.1497427385 |
| 203983_at | hsa-miR-4423-3p_st | -0.2522536287 | 0.1497427385 |
| 208809_s_at | hsa-miR-523_st | -0.2522536287 | 0.1497427385 |
| 219582_at | hsa-miR-3189-5p_st | -0.2522536287 | 0.1497427385 |
| 202133_at | hsa-miR-555_st | -0.2519673015 | 0.1502174150 |
| 201313_at | hsa-miR-375_st | -0.2519480519 | 0.1502493667 |
| 208998_at | hsa-miR-382_st | -0.2516617014 | 0.1507252614 |
| 200600_at | hsa-miR-377-star_st | -0.2516424752 | 0.1507572536 |
| 203455_s_at | hsa-miR-4423-3p_st | -0.2516424752 | 0.1507572536 |
| 212099_at | hsa-miR-4423-3p_st | -0.2516424752 | 0.1507572536 |
| 212110_at | hsa-miR-3676_st | -0.2516424752 | 0.1507572536 |
| 217546_at | hsa-miR-4311_st | -0.2515089013 | 0.1509796570 |
| 202071_at | hsa-miR-370_st | -0.2513368984 | 0.1512664006 |
| 209569_x_at | hsa-miR-4652-3p_st | -0.2510313216 | 0.1517768091 |
| 211271_x_at | hsa-miR-370_st | -0.2510313216 | 0.1517768091 |
| 219355_at | hsa-miR-375_st | -0.2510313216 | 0.1517768091 |
| 212015_x_at | hsa-miR-4311_st | -0.2507449010 | 0.1522563673 |
| 201753_s_at | hsa-miR-4720-3p_st | -0.2507257448 | 0.1522884805 |
| 202430_s_at | hsa-miR-3676_st | -0.2507257448 | 0.1522884805 |
| 205609_at | hsa-miR-383_st | -0.2507257448 | 0.1522884805 |
| 222360_at | hsa-miR-34b_st | -0.2507257448 | 0.1522884805 |
| 201999_s_at | hsa-miR-361-5p_st | -0.2504201681 | 0.1528014162 |
| 209122_at | hsa-miR-134_st | -0.2504201681 | 0.1528014162 |
| 212460_at | hsa-miR-4760-3p_st | -0.2504201681 | 0.1528014162 |
| 219582_at | hsa-miR-212_st | -0.2504201681 | 0.1528014162 |
| 212067_s_at | hsa-miR-4311_st | -0.2502865008 | 0.1530261855 |
| 203987_at | hsa-miR-132_st | -0.2501145913 | 0.1533156178 |
| 210105_s_at | hsa-miR-4423-3p_st | -0.2501145913 | 0.1533156178 |
| 201161_s_at | hsa-miR-382_st | -0.2498281007 | 0.1537988535 |
| 208869_s_at | hsa-miR-34b_st | -0.2498090145 | 0.1538310865 |
| 209183_s_at | hsa-miR-212_st | -0.2498090145 | 0.1538310865 |
| 218706_s_at | hsa-miR-4760-3p_st | -0.2498090145 | 0.1538310865 |
| 201398_s_at | hsa-miR-3189-5p_st | -0.2495034377 | 0.1543478239 |
| 210927_x_at | hsa-miR-4652-3p_st | -0.2495034377 | 0.1543478239 |
| 212157_at | hsa-miR-34b_st | -0.2495034377 | 0.1543478239 |
| 210406_s_at | hsa-miR-4652-3p_st | -0.2491978610 | 0.1548658314 |
| 210906_x_at | hsa-miR-3676_st | -0.2491978610 | 0.1548658314 |
| 214150_x_at | hsa-miR-212_st | -0.2491978610 | 0.1548658314 |
| 200797_s_at | hsa-miR-370_st | -0.2488922842 | 0.1553851103 |
| 202613_at | hsa-miR-34b_st | -0.2488922842 | 0.1553851103 |
| 203120_at | hsa-miR-4720-3p_st | -0.2488922842 | 0.1553851103 |
| 208860_s_at | hsa-miR-375_st | -0.2488922842 | 0.1553851103 |
| 212321_at | hsa-miR-4762-5p_st | -0.2488922842 | 0.1553851103 |
| 201324_at | hsa-miR-555_st | -0.2487585002 | 0.1556128553 |
| 204194_at | hsa-miR-4762-5p_st | -0.2485867074 | 0.1559056620 |
| 210946_at | hsa-miR-1180_st | -0.2485867074 | 0.1559056620 |
| 201198_s_at | hsa-miR-4652-3p_st | -0.2482811306 | 0.1564274881 |
| 201859_at | hsa-miR-132_st | -0.2482811306 | 0.1564274881 |
| 202641_at | hsa-miR-34b_st | -0.2482811306 | 0.1564274881 |
| 200663_at | hsa-miR-4762-5p_st | -0.2479755539 | 0.1569505897 |
| 200701_at | hsa-miR-1912_st | -0.2479755539 | 0.1569505897 |
| 36711_at | hsa-miR-3676_st | -0.2479755539 | 0.1569505897 |
| 202864_s_at | hsa-miR-212_st | -0.2476699771 | 0.1574749685 |
| 205705_at | hsa-miR-34b_st | -0.2476699771 | 0.1574749685 |
| 208675_s_at | hsa-miR-4652-3p_st | -0.2476699771 | 0.1574749685 |
| 211902_x_at | hsa-miR-4652-3p_st | -0.2476699771 | 0.1574749685 |
| 217997_at | hsa-miR-4652-3p_st | -0.2476699771 | 0.1574749685 |
| 218559_s_at | hsa-miR-4762-5p_st | -0.2476699771 | 0.1574749685 |
| 201112_s_at | hsa-miR-34b_st | -0.2473644003 | 0.1580006256 |
| 201146_at | hsa-miR-4633-5p_st | -0.2473644003 | 0.1580006256 |
| 204068_at | hsa-miR-4633-5p_st | -0.2473644003 | 0.1580006256 |
| 208818_s_at | hsa-miR-4720-3p_st | -0.2473644003 | 0.1580006256 |
| 218866_s_at | hsa-miR-4652-3p_st | -0.2473644003 | 0.1580006256 |
| 204554_at | hsa-miR-370_st | -0.2470588235 | 0.1585275626 |
| 210117_at | hsa-miR-4760-3p_st | -0.2467532468 | 0.1590557807 |
| 217923_at | hsa-miR-4652-3p_st | -0.2467532468 | 0.1590557807 |
| 219263_at | hsa-miR-34b_st | -0.2467532468 | 0.1590557807 |
| 208832_at | hsa-miR-375_st | -0.2464476700 | 0.1595852815 |
| 218559_s_at | hsa-miR-4760-3p_st | -0.2461420932 | 0.1601160661 |
| 221263_s_at | hsa-miR-375_st | -0.2461420932 | 0.1601160661 |
| 213503_x_at | hsa-miR-555_st | -0.2460080991 | 0.1603492182 |
| 210592_s_at | hsa-miR-4423-3p_st | -0.2458365164 | 0.1606481361 |
| 221796_at | hsa-miR-132_st | -0.2458365164 | 0.1606481361 |
| 201887_at | hsa-miR-523_st | -0.2455309396 | 0.1611814927 |
| 207988_s_at | hsa-miR-34b_st | -0.2455309396 | 0.1611814927 |
| 213293_s_at | hsa-miR-134_st | -0.2455309396 | 0.1611814927 |
| 201029_s_at | hsa-miR-382_st | -0.2452440988 | 0.1616833194 |
| 201029_s_at | hsa-miR-3189-5p_st | -0.2452253629 | 0.1617161374 |
| 201172_x_at | hsa-miR-4423-3p_st | -0.2452253629 | 0.1617161374 |
| 203362_s_at | hsa-miR-34b_st | -0.2452253629 | 0.1617161374 |
| 212600_s_at | hsa-miR-34b_st | -0.2452253629 | 0.1617161374 |
| 213904_at | hsa-miR-34b_st | -0.2452253629 | 0.1617161374 |
| 210068_s_at | hsa-miR-4311_st | -0.2449384987 | 0.1622192153 |
| 200818_at | hsa-miR-4652-3p_st | -0.2449197861 | 0.1622520714 |
| 204070_at | hsa-miR-4633-5p_st | -0.2449197861 | 0.1622520714 |
| 210105_s_at | hsa-miR-212_st | -0.2449197861 | 0.1622520714 |
| 210906_x_at | hsa-miR-212_st | -0.2446142093 | 0.1627892961 |
| 214150_x_at | hsa-miR-383_st | -0.2446142093 | 0.1627892961 |
| 219326_s_at | hsa-miR-4652-3p_st | -0.2446142093 | 0.1627892961 |
| 201146_at | hsa-miR-134_st | -0.2443086325 | 0.1633278129 |
| 206099_at | hsa-miR-34b_st | -0.2436974790 | 0.1644087280 |
| 211902_x_at | hsa-miR-375_st | -0.2433919022 | 0.1649511289 |
| 212195_at | hsa-miR-361-5p_st | -0.2433919022 | 0.1649511289 |
| 202658_at | hsa-miR-4652-3p_st | -0.2430863254 | 0.1654948273 |
| 201112_s_at | hsa-miR-4652-3p_st | -0.2427807487 | 0.1660398243 |
| 201315_x_at | hsa-miR-3676_st | -0.2427807487 | 0.1660398243 |
| 209598_at | hsa-miR-375_st | -0.2427807487 | 0.1660398243 |
| 212321_at | hsa-miR-3189-5p_st | -0.2427807487 | 0.1660398243 |
| 215884_s_at | hsa-miR-34b_st | -0.2427807487 | 0.1660398243 |
| 221958_s_at | hsa-miR-1180_st | -0.2427807487 | 0.1660398243 |
| 209513_s_at | hsa-miR-4311_st | -0.2426464978 | 0.1662796721 |
| 202948_at | hsa-miR-361-5p_st | -0.2424751719 | 0.1665861213 |
| 208451_s_at | hsa-miR-132_st | -0.2424751719 | 0.1665861213 |
| 213572_s_at | hsa-miR-383_st | -0.2424751719 | 0.1665861213 |
| 209732_at | hsa-miR-382_st | -0.2423408976 | 0.1668265825 |
| 211999_at | hsa-miR-383_st | -0.2423408976 | 0.1668265825 |
| 202269_x_at | hsa-miR-3189-5p_st | -0.2421695951 | 0.1671337197 |
| 219355_at | hsa-miR-4652-3p_st | -0.2421695951 | 0.1671337197 |
| 212687_at | hsa-miR-555_st | -0.2420352975 | 0.1673747950 |
| 205856_at | hsa-miR-4633-5p_st | -0.2415584416 | 0.1682328255 |
| 207761_s_at | hsa-miR-4720-3p_st | -0.2415584416 | 0.1682328255 |
| 209228_x_at | hsa-miR-34b_st | -0.2415584416 | 0.1682328255 |
| 209513_s_at | hsa-miR-4762-5p_st | -0.2412528648 | 0.1687843356 |
| 213572_s_at | hsa-miR-3676_st | -0.2412528648 | 0.1687843356 |
| 217746_s_at | hsa-miR-4633-5p_st | -0.2412528648 | 0.1687843356 |
| 200906_s_at | hsa-miR-555_st | -0.2409656971 | 0.1693038115 |
| 217730_at | hsa-miR-555_st | -0.2409656971 | 0.1693038115 |
| 203455_s_at | hsa-miR-1912_st | -0.2409472880 | 0.1693371522 |
| 206989_s_at | hsa-miR-1912_st | -0.2409472880 | 0.1693371522 |
| 218224_at | hsa-miR-375_st | -0.2409472880 | 0.1693371522 |
| 213503_x_at | hsa-miR-382_st | -0.2406600970 | 0.1698578994 |
| 201412_at | hsa-miR-361-5p_st | -0.2406417112 | 0.1698912766 |
| 202071_at | hsa-miR-212_st | -0.2406417112 | 0.1698912766 |
| 212015_x_at | hsa-miR-1180_st | -0.2406417112 | 0.1698912766 |
| 202427_s_at | hsa-miR-34b-star_st | -0.2403361345 | 0.1704467100 |
| 203540_at | hsa-miR-3189-5p_st | -0.2403361345 | 0.1704467100 |
| 204194_at | hsa-miR-377-star_st | -0.2403361345 | 0.1704467100 |
| 210906_x_at | hsa-miR-523_st | -0.2403361345 | 0.1704467100 |
| 210962_s_at | hsa-miR-34b-star_st | -0.2403361345 | 0.1704467100 |
| 212067_s_at | hsa-miR-1180_st | -0.2403361345 | 0.1704467100 |
| 218854_at | hsa-miR-4633-5p_st | -0.2403361345 | 0.1704467100 |
| 219481_at | hsa-miR-4652-3p_st | -0.2403361345 | 0.1704467100 |
| 200986_at | hsa-miR-3676_st | -0.2400305577 | 0.1710034538 |
| 201146_at | hsa-miR-383_st | -0.2400305577 | 0.1710034538 |
| 203313_s_at | hsa-miR-382_st | -0.2398960967 | 0.1712488498 |
| 204194_at | hsa-miR-3676_st | -0.2397249809 | 0.1715615092 |
| 208690_s_at | hsa-miR-523_st | -0.2397249809 | 0.1715615092 |
| 213217_at | hsa-miR-3676_st | -0.2397249809 | 0.1715615092 |
| 214150_x_at | hsa-miR-1180_st | -0.2397249809 | 0.1715615092 |
| 217746_s_at | hsa-miR-4762-5p_st | -0.2397249809 | 0.1715615092 |
| 200663_at | hsa-miR-4311_st | -0.2394376965 | 0.1720873558 |
| 210033_s_at | hsa-miR-212_st | -0.2394194041 | 0.1721208775 |
| 201029_s_at | hsa-miR-555_st | -0.2391320964 | 0.1726480024 |
| 203854_at | hsa-miR-212_st | -0.2391138273 | 0.1726815599 |
| 203987_at | hsa-miR-1180_st | -0.2391138273 | 0.1726815599 |
| 211855_s_at | hsa-miR-34b_st | -0.2391138273 | 0.1726815599 |
| 209183_s_at | hsa-miR-132_st | -0.2388082506 | 0.1732435577 |
| 202149_at | hsa-miR-3189-5p_st | -0.2385026738 | 0.1738068722 |
| 209476_at | hsa-miR-4760-3p_st | -0.2385026738 | 0.1738068722 |
| 212099_at | hsa-miR-382_st | -0.2383680960 | 0.1740553770 |
| 202594_at | hsa-miR-34b_st | -0.2381970970 | 0.1743715045 |
| 210033_s_at | hsa-miR-4633-5p_st | -0.2381970970 | 0.1743715045 |
| 201590_x_at | hsa-miR-370_st | -0.2378915202 | 0.1749374560 |
| 203455_s_at | hsa-miR-377-star_st | -0.2378915202 | 0.1749374560 |
| 207761_s_at | hsa-miR-377-star_st | -0.2378915202 | 0.1749374560 |
| 217746_s_at | hsa-miR-3189-5p_st | -0.2378915202 | 0.1749374560 |
| 217820_s_at | hsa-miR-361-5p_st | -0.2378915202 | 0.1749374560 |
| 221667_s_at | hsa-miR-4760-3p_st | -0.2378915202 | 0.1749374560 |
| 208779_x_at | hsa-miR-4311_st | -0.2376040957 | 0.1754709930 |
| 200820_at | hsa-miR-4652-3p_st | -0.2375859435 | 0.1755047278 |
| 201172_x_at | hsa-miR-1912_st | -0.2375859435 | 0.1755047278 |
| 212977_at | hsa-miR-3189-5p_st | -0.2375859435 | 0.1755047278 |
| 217947_at | hsa-miR-1180_st | -0.2375859435 | 0.1755047278 |
| 204554_at | hsa-miR-4423-3p_st | -0.2372803667 | 0.1760733212 |
| 202430_s_at | hsa-miR-382_st | -0.2369928955 | 0.1766094327 |
| 205775_at | hsa-miR-34b_st | -0.2369747899 | 0.1766432374 |
| 217546_at | hsa-miR-4762-5p_st | -0.2366692131 | 0.1772144776 |
| 221796_at | hsa-miR-370_st | -0.2366692131 | 0.1772144776 |
| 200673_at | hsa-miR-383_st | -0.2363636364 | 0.1777870431 |
| 201180_s_at | hsa-miR-4633-5p_st | -0.2363636364 | 0.1777870431 |
| 201180_s_at | hsa-miR-361-5p_st | -0.2363636364 | 0.1777870431 |
| 203704_s_at | hsa-miR-523_st | -0.2363636364 | 0.1777870431 |
| 208451_s_at | hsa-miR-212_st | -0.2363636364 | 0.1777870431 |
| 218285_s_at | hsa-miR-212_st | -0.2363636364 | 0.1777870431 |
| 221667_s_at | hsa-miR-1180_st | -0.2363636364 | 0.1777870431 |
| 210033_s_at | hsa-miR-4311_st | -0.2360760951 | 0.1783270264 |
| 221667_s_at | hsa-miR-382_st | -0.2360760951 | 0.1783270264 |
| 210117_at | hsa-miR-212_st | -0.2360580596 | 0.1783609350 |
| 201160_s_at | hsa-miR-3189-5p_st | -0.2357524828 | 0.1789361546 |
| 207761_s_at | hsa-miR-132_st | -0.2357524828 | 0.1789361546 |
| 217820_s_at | hsa-miR-382_st | -0.2354648949 | 0.1794787257 |
| 200600_at | hsa-miR-132_st | -0.2354469060 | 0.1795127031 |
| 201172_x_at | hsa-miR-370_st | -0.2354469060 | 0.1795127031 |
| 207922_s_at | hsa-miR-34b_st | -0.2354469060 | 0.1795127031 |
| 208697_s_at | hsa-miR-34b-star_st | -0.2354469060 | 0.1795127031 |
| 221667_s_at | hsa-miR-212_st | -0.2354469060 | 0.1795127031 |
| 222043_at | hsa-miR-3189-5p_st | -0.2354469060 | 0.1795127031 |
| 202370_s_at | hsa-miR-134_st | -0.2351413293 | 0.1800905816 |
| 205609_at | hsa-miR-132_st | -0.2351413293 | 0.1800905816 |
| 212015_x_at | hsa-miR-3189-5p_st | -0.2351413293 | 0.1800905816 |
| 221958_s_at | hsa-miR-3189-5p_st | -0.2351413293 | 0.1800905816 |
| 201324_at | hsa-miR-132_st | -0.2348357525 | 0.1806697913 |
| 213217_at | hsa-miR-1180_st | -0.2348357525 | 0.1806697913 |
| 204787_at | hsa-miR-382_st | -0.2347008946 | 0.1809258333 |
| 217820_s_at | hsa-miR-555_st | -0.2345480945 | 0.1812162541 |
| 202543_s_at | hsa-miR-3676_st | -0.2345301757 | 0.1812503335 |
| 221958_s_at | hsa-miR-370_st | -0.2345301757 | 0.1812503335 |
| 210014_x_at | hsa-miR-4652-3p_st | -0.2342245989 | 0.1818322092 |
| 217546_at | hsa-miR-4760-3p_st | -0.2342245989 | 0.1818322092 |
| 218226_s_at | hsa-miR-34b_st | -0.2342245989 | 0.1818322092 |
| 200905_x_at | hsa-miR-3676_st | -0.2339190222 | 0.1824154198 |
| 202712_s_at | hsa-miR-4652-3p_st | -0.2339190222 | 0.1824154198 |
| 212015_x_at | hsa-miR-555_st | -0.2336312941 | 0.1829657861 |
| 201161_s_at | hsa-miR-3189-5p_st | -0.2336134454 | 0.1829999662 |
| 202430_s_at | hsa-miR-377-star_st | -0.2336134454 | 0.1829999662 |
| 205119_s_at | hsa-miR-4423-3p_st | -0.2336134454 | 0.1829999662 |
| 215527_at | hsa-miR-4652-3p_st | -0.2336134454 | 0.1829999662 |
| 201146_at | hsa-miR-1180_st | -0.2333078686 | 0.1835858497 |
| 206989_s_at | hsa-miR-3676_st | -0.2333078686 | 0.1835858497 |
| 209569_x_at | hsa-miR-34b_st | -0.2333078686 | 0.1835858497 |
| 213592_at | hsa-miR-4633-5p_st | -0.2333078686 | 0.1835858497 |
| 201590_x_at | hsa-miR-382_st | -0.2328672938 | 0.1844329214 |
| 201999_s_at | hsa-miR-134_st | -0.2326967150 | 0.1847616327 |
| 202864_s_at | hsa-miR-4762-5p_st | -0.2326967150 | 0.1847616327 |
| 207120_at | hsa-miR-34b_st | -0.2326967150 | 0.1847616327 |
| 210278_s_at | hsa-miR-375_st | -0.2326967150 | 0.1847616327 |
| 212099_at | hsa-miR-132_st | -0.2326967150 | 0.1847616327 |
| 217936_at | hsa-miR-4762-5p_st | -0.2326967150 | 0.1847616327 |
| 201145_at | hsa-miR-4652-3p_st | -0.2323911383 | 0.1853515345 |
| 202077_at | hsa-miR-34b_st | -0.2323911383 | 0.1853515345 |
| 202936_s_at | hsa-miR-377-star_st | -0.2323911383 | 0.1853515345 |
| 214717_at | hsa-miR-34b_st | -0.2323911383 | 0.1853515345 |
| 218285_s_at | hsa-miR-4423-3p_st | -0.2323911383 | 0.1853515345 |
| 202096_s_at | hsa-miR-4311_st | -0.2321032935 | 0.1859084325 |
| 200798_x_at | hsa-miR-383_st | -0.2320855615 | 0.1859427779 |
| 208690_s_at | hsa-miR-1180_st | -0.2320855615 | 0.1859427779 |
| 217546_at | hsa-miR-4720-3p_st | -0.2320855615 | 0.1859427779 |
| 1007_s_at | hsa-miR-382_st | -0.2319504935 | 0.1862045414 |
| 200677_at | hsa-miR-4311_st | -0.2317976934 | 0.1865009861 |
| 204070_at | hsa-miR-555_st | -0.2314920933 | 0.1870948838 |
| 1007_s_at | hsa-miR-3189-5p_st | -0.2314744079 | 0.1871292943 |
| 204326_x_at | hsa-miR-383_st | -0.2314744079 | 0.1871292943 |
| 217746_s_at | hsa-miR-383_st | -0.2314744079 | 0.1871292943 |
| 201012_at | hsa-miR-555_st | -0.2311864931 | 0.1876901268 |
| 200701_at | hsa-miR-4720-3p_st | -0.2311688312 | 0.1877245696 |
| 200798_x_at | hsa-miR-370_st | -0.2311688312 | 0.1877245696 |
| 205196_s_at | hsa-miR-4423-3p_st | -0.2311688312 | 0.1877245696 |
| 205856_at | hsa-miR-134_st | -0.2311688312 | 0.1877245696 |
| 208779_x_at | hsa-miR-4633-5p_st | -0.2311688312 | 0.1877245696 |
| 212321_at | hsa-miR-4633-5p_st | -0.2311688312 | 0.1877245696 |
| 218507_at | hsa-miR-382_st | -0.2310336931 | 0.1879882530 |
| 203120_at | hsa-miR-134_st | -0.2308632544 | 0.1883211911 |
| 203987_at | hsa-miR-370_st | -0.2308632544 | 0.1883211911 |
| 212501_at | hsa-miR-212_st | -0.2308632544 | 0.1883211911 |
| 214428_x_at | hsa-miR-132_st | -0.2308632544 | 0.1883211911 |
| 201029_s_at | hsa-miR-3676_st | -0.2305576776 | 0.1889191598 |
| 212195_at | hsa-miR-3189-5p_st | -0.2305576776 | 0.1889191598 |
| 201590_x_at | hsa-miR-555_st | -0.2304224928 | 0.1891841266 |
| 200677_at | hsa-miR-3676_st | -0.2302521008 | 0.1895184770 |
| 203411_s_at | hsa-miR-3676_st | -0.2302521008 | 0.1895184770 |
| 211658_at | hsa-miR-4652-3p_st | -0.2302521008 | 0.1895184770 |
| 214150_x_at | hsa-miR-361-5p_st | -0.2302521008 | 0.1895184770 |
| 201319_at | hsa-miR-4762-5p_st | -0.2299465241 | 0.1901191436 |
| 202471_s_at | hsa-miR-4652-3p_st | -0.2299465241 | 0.1901191436 |
| 211963_s_at | hsa-miR-34b_st | -0.2299465241 | 0.1901191436 |
| 201161_s_at | hsa-miR-377-star_st | -0.2296409473 | 0.1907211609 |
| 201398_s_at | hsa-miR-361-5p_st | -0.2296409473 | 0.1907211609 |
| 204068_at | hsa-miR-361-5p_st | -0.2296409473 | 0.1907211609 |
| 205097_at | hsa-miR-4762-5p_st | -0.2296409473 | 0.1907211609 |
| 210906_x_at | hsa-miR-383_st | -0.2296409473 | 0.1907211609 |
| 200804_at | hsa-miR-382_st | -0.2293528924 | 0.1912898958 |
| 201012_at | hsa-miR-382_st | -0.2292000923 | 0.1915920719 |
| 201324_at | hsa-miR-382_st | -0.2290472923 | 0.1918945862 |
| 211404_s_at | hsa-miR-34b_st | -0.2290297937 | 0.1919292515 |
| 212321_at | hsa-miR-134_st | -0.2290297937 | 0.1919292515 |
| 212687_at | hsa-miR-370_st | -0.2290297937 | 0.1919292515 |
| 200673_at | hsa-miR-1180_st | -0.2287242170 | 0.1925353271 |
| 201859_at | hsa-miR-4633-5p_st | -0.2287242170 | 0.1925353271 |
| 202071_at | hsa-miR-523_st | -0.2287242170 | 0.1925353271 |
| 203721_s_at | hsa-miR-34b_st | -0.2287242170 | 0.1925353271 |
| 203313_s_at | hsa-miR-555_st | -0.2285888921 | 0.1928041609 |
| 200916_at | hsa-miR-4633-5p_st | -0.2284186402 | 0.1931427576 |
| 201887_at | hsa-miR-4633-5p_st | -0.2284186402 | 0.1931427576 |
| 217923_at | hsa-miR-34b_st | -0.2284186402 | 0.1931427576 |
| 205097_at | hsa-miR-555_st | -0.2282832920 | 0.1934122382 |
| 202071_at | hsa-miR-4311_st | -0.2281304919 | 0.1937167856 |
| 209108_at | hsa-miR-4311_st | -0.2281304919 | 0.1937167856 |
| 211999_at | hsa-miR-1180_st | -0.2281304919 | 0.1937167856 |
| 201656_at | hsa-miR-4762-5p_st | -0.2281130634 | 0.1937515441 |
| 217947_at | hsa-miR-1912_st | -0.2281130634 | 0.1937515441 |
| 201859_at | hsa-miR-382_st | -0.2279776919 | 0.1940216723 |
| 217936_at | hsa-miR-4311_st | -0.2279776919 | 0.1940216723 |
| 209476_at | hsa-miR-1912_st | -0.2278074866 | 0.1943616876 |
| 213503_x_at | hsa-miR-370_st | -0.2278074866 | 0.1943616876 |
| 214428_x_at | hsa-miR-212_st | -0.2278074866 | 0.1943616876 |
| 218163_at | hsa-miR-375_st | -0.2278074866 | 0.1943616876 |
| 213710_s_at | hsa-miR-34b_st | -0.2275019099 | 0.1949731893 |
| 215527_at | hsa-miR-34b_st | -0.2275019099 | 0.1949731893 |
| 218285_s_at | hsa-miR-361-5p_st | -0.2275019099 | 0.1949731893 |
| 207922_s_at | hsa-miR-375_st | -0.2271963331 | 0.1955860501 |
| 221741_s_at | hsa-miR-134_st | -0.2271963331 | 0.1955860501 |
| 202430_s_at | hsa-miR-4760-3p_st | -0.2268907563 | 0.1962002711 |
| 212460_at | hsa-miR-4762-5p_st | -0.2268907563 | 0.1962002711 |
| 214150_x_at | hsa-miR-4633-5p_st | -0.2265851795 | 0.1968158533 |
| 212460_at | hsa-miR-132_st | -0.2262796028 | 0.1974327978 |
| 215171_s_at | hsa-miR-4652-3p_st | -0.2262796028 | 0.1974327978 |
| 208581_x_at | hsa-miR-1180_st | -0.2259740260 | 0.1980511056 |
| 212460_at | hsa-miR-1912_st | -0.2259740260 | 0.1980511056 |
| 212687_at | hsa-miR-1180_st | -0.2259740260 | 0.1980511056 |
| 218559_s_at | hsa-miR-361-5p_st | -0.2259740260 | 0.1980511056 |
| 218706_s_at | hsa-miR-3676_st | -0.2259740260 | 0.1980511056 |
| 200804_at | hsa-miR-4720-3p_st | -0.2256684492 | 0.1986707777 |
| 200916_at | hsa-miR-361-5p_st | -0.2256684492 | 0.1986707777 |
| 214428_x_at | hsa-miR-4633-5p_st | -0.2256684492 | 0.1986707777 |
| 217746_s_at | hsa-miR-3676_st | -0.2256684492 | 0.1986707777 |
| 208690_s_at | hsa-miR-382_st | -0.2255328909 | 0.1989461104 |
| 201161_s_at | hsa-miR-4760-3p_st | -0.2253628724 | 0.1992918151 |
| 201172_x_at | hsa-miR-3676_st | -0.2253628724 | 0.1992918151 |
| 201412_at | hsa-miR-4633-5p_st | -0.2253628724 | 0.1992918151 |
| 203704_s_at | hsa-miR-3189-5p_st | -0.2253628724 | 0.1992918151 |
| 213011_s_at | hsa-miR-375_st | -0.2253628724 | 0.1992918151 |
| 200798_x_at | hsa-miR-1180_st | -0.2250572956 | 0.1999142188 |
| 203973_s_at | hsa-miR-3676_st | -0.2250572956 | 0.1999142188 |
| 219421_at | hsa-miR-34b_st | -0.2247517189 | 0.2005379899 |
| 200797_s_at | hsa-miR-1180_st | -0.2244461421 | 0.2011631294 |
| 218322_s_at | hsa-miR-3676_st | -0.2241405653 | 0.2017896381 |
| 201398_s_at | hsa-miR-3676_st | -0.2238349885 | 0.2024175172 |
| 207198_s_at | hsa-miR-4423-3p_st | -0.2238349885 | 0.2024175172 |
| 201601_x_at | hsa-miR-3676_st | -0.2235294118 | 0.2030467675 |
| 219960_s_at | hsa-miR-375_st | -0.2235294118 | 0.2030467675 |
| 211999_at | hsa-miR-212_st | -0.2232408899 | 0.2036421574 |
| 208690_s_at | hsa-miR-383_st | -0.2232238350 | 0.2036773901 |
| 208946_s_at | hsa-miR-4423-3p_st | -0.2232238350 | 0.2036773901 |
| 200097_s_at | hsa-miR-4652-3p_st | -0.2229182582 | 0.2043093860 |
| 218120_s_at | hsa-miR-4652-3p_st | -0.2229182582 | 0.2043093860 |
| 218667_at | hsa-miR-34b_st | -0.2229182582 | 0.2043093860 |
| 221958_s_at | hsa-miR-4633-5p_st | -0.2229182582 | 0.2043093860 |
| 207198_s_at | hsa-miR-555_st | -0.2227824898 | 0.2045906240 |
| 200030_s_at | hsa-miR-34b_st | -0.2226126814 | 0.2049427560 |
| 200673_at | hsa-miR-4633-5p_st | -0.2226126814 | 0.2049427560 |
| 202834_at | hsa-miR-134_st | -0.2226126814 | 0.2049427560 |
| 218559_s_at | hsa-miR-3676_st | -0.2226126814 | 0.2049427560 |
| 200967_at | hsa-miR-523_st | -0.2223071047 | 0.2055775011 |
| 200989_at | hsa-miR-1912_st | -0.2223071047 | 0.2055775011 |
| 206062_at | hsa-miR-34b_st | -0.2223071047 | 0.2055775011 |
| 213005_s_at | hsa-miR-134_st | -0.2223071047 | 0.2055775011 |
| 212977_at | hsa-miR-3676_st | -0.2220015279 | 0.2062136224 |
| 203723_at | hsa-miR-555_st | -0.2217298289 | 0.2067803765 |
| 210817_s_at | hsa-miR-555_st | -0.2217128893 | 0.2068157478 |
| 202920_at | hsa-miR-375_st | -0.2216959511 | 0.2068511206 |
| 203313_s_at | hsa-miR-523_st | -0.2216959511 | 0.2068511206 |
| 210592_s_at | hsa-miR-132_st | -0.2216959511 | 0.2068511206 |
| 202864_s_at | hsa-miR-383_st | -0.2213903743 | 0.2074899969 |
| 217957_at | hsa-miR-34b_st | -0.2213903743 | 0.2074899969 |
| 218507_at | hsa-miR-4760-3p_st | -0.2213903743 | 0.2074899969 |
| 201172_x_at | hsa-miR-361-5p_st | -0.2210847976 | 0.2081302520 |
| 202133_at | hsa-miR-383_st | -0.2210847976 | 0.2081302520 |
| 209732_at | hsa-miR-1912_st | -0.2210847976 | 0.2081302520 |
| 211595_s_at | hsa-miR-375_st | -0.2210847976 | 0.2081302520 |
| 200663_at | hsa-miR-361-5p_st | -0.2207792208 | 0.2087718869 |
| 203120_at | hsa-miR-4762-5p_st | -0.2207792208 | 0.2087718869 |
| 203987_at | hsa-miR-3189-5p_st | -0.2207792208 | 0.2087718869 |
| 209122_at | hsa-miR-523_st | -0.2207792208 | 0.2087718869 |
| 212977_at | hsa-miR-4762-5p_st | -0.2207792208 | 0.2087718869 |
| 201859_at | hsa-miR-4762-5p_st | -0.2204736440 | 0.2094149026 |
| 204326_x_at | hsa-miR-523_st | -0.2204736440 | 0.2094149026 |
| 208998_at | hsa-miR-3676_st | -0.2204736440 | 0.2094149026 |
| 200600_at | hsa-miR-382_st | -0.2203376888 | 0.2097014330 |
| 1007_s_at | hsa-miR-3676_st | -0.2201680672 | 0.2100592999 |
| 200708_at | hsa-miR-375_st | -0.2201680672 | 0.2100592999 |
| 200797_s_at | hsa-miR-4762-5p_st | -0.2201680672 | 0.2100592999 |
| 205856_at | hsa-miR-3676_st | -0.2201680672 | 0.2100592999 |
| 208818_s_at | hsa-miR-4762-5p_st | -0.2201680672 | 0.2100592999 |
| 203120_at | hsa-miR-4760-3p_st | -0.2198624905 | 0.2107050797 |
| 202427_s_at | hsa-miR-4652-3p_st | -0.2195569137 | 0.2113522430 |
| 211270_x_at | hsa-miR-3676_st | -0.2195569137 | 0.2113522430 |
| 212110_at | hsa-miR-4311_st | -0.2192680883 | 0.2119652020 |
| 201761_at | hsa-miR-4762-5p_st | -0.2192513369 | 0.2120007907 |
| 213572_s_at | hsa-miR-212_st | -0.2192513369 | 0.2120007907 |
| 218706_s_at | hsa-miR-361-5p_st | -0.2192513369 | 0.2120007907 |
| 210872_x_at | hsa-miR-4652-3p_st | -0.2186401833 | 0.2133020425 |
| 221667_s_at | hsa-miR-3189-5p_st | -0.2186401833 | 0.2133020425 |
| 218226_s_at | hsa-miR-375_st | -0.2183346066 | 0.2139547484 |
| 203973_s_at | hsa-miR-4633-5p_st | -0.2180290298 | 0.2146088422 |
| 207643_s_at | hsa-miR-4633-5p_st | -0.2180290298 | 0.2146088422 |
| 202948_at | hsa-miR-3676_st | -0.2177234530 | 0.2152643247 |
| 208581_x_at | hsa-miR-4762-5p_st | -0.2177234530 | 0.2152643247 |
| 218133_s_at | hsa-miR-4423-3p_st | -0.2177234530 | 0.2152643247 |
| 48531_at | hsa-miR-3189-5p_st | -0.2177234530 | 0.2152643247 |
| 212460_at | hsa-miR-555_st | -0.2174344876 | 0.2158854530 |
| 208659_at | hsa-miR-4633-5p_st | -0.2174178762 | 0.2159211967 |
| 213572_s_at | hsa-miR-370_st | -0.2174178762 | 0.2159211967 |
| 210117_at | hsa-miR-4762-5p_st | -0.2168067227 | 0.2172391129 |
| 212310_at | hsa-miR-34b_st | -0.2168067227 | 0.2172391129 |
| 217780_at | hsa-miR-375_st | -0.2168067227 | 0.2172391129 |
| 211999_at | hsa-miR-4720-3p_st | -0.2165176872 | 0.2178643396 |
| 200600_at | hsa-miR-212_st | -0.2161955691 | 0.2185625973 |
| 212501_at | hsa-miR-383_st | -0.2161955691 | 0.2185625973 |
| 212727_at | hsa-miR-34b_st | -0.2161955691 | 0.2185625973 |
| 201656_at | hsa-miR-361-5p_st | -0.2158899924 | 0.2192264297 |
| 202133_at | hsa-miR-212_st | -0.2158899924 | 0.2192264297 |
| 219582_at | hsa-miR-4762-5p_st | -0.2155844156 | 0.2198916566 |
| 202121_s_at | hsa-miR-34b_st | -0.2149732620 | 0.2212262973 |
| 210946_at | hsa-miR-4762-5p_st | -0.2149732620 | 0.2212262973 |
| 217546_at | hsa-miR-1180_st | -0.2149732620 | 0.2212262973 |
| 212063_at | hsa-miR-555_st | -0.2146840865 | 0.2218597476 |
| 201012_at | hsa-miR-3676_st | -0.2143621085 | 0.2225665258 |
| 202376_at | hsa-miR-3676_st | -0.2143621085 | 0.2225665258 |
| 202096_s_at | hsa-miR-382_st | -0.2140728863 | 0.2232027250 |
| 200701_at | hsa-miR-1180_st | -0.2140565317 | 0.2232387374 |
| 202834_at | hsa-miR-361-5p_st | -0.2140565317 | 0.2232387374 |
| 203455_s_at | hsa-miR-4762-5p_st | -0.2140565317 | 0.2232387374 |
| 210105_s_at | hsa-miR-4311_st | -0.2139200862 | 0.2235393438 |
| 200077_s_at | hsa-miR-375_st | -0.2137509549 | 0.2239123483 |
| 201887_at | hsa-miR-3189-5p_st | -0.2137509549 | 0.2239123483 |
| 203645_s_at | hsa-miR-4720-3p_st | -0.2137509549 | 0.2239123483 |
| 210068_s_at | hsa-miR-370_st | -0.2134453782 | 0.2245873593 |
| 219297_at | hsa-miR-34b_st | -0.2134453782 | 0.2245873593 |
| 213572_s_at | hsa-miR-382_st | -0.2133088859 | 0.2248893195 |
| 201412_at | hsa-miR-4311_st | -0.2131560859 | 0.2252276891 |
| 203704_s_at | hsa-miR-383_st | -0.2131398014 | 0.2252637711 |
| 218322_s_at | hsa-miR-134_st | -0.2131398014 | 0.2252637711 |
| 221796_at | hsa-miR-212_st | -0.2131398014 | 0.2252637711 |
| 209303_at | hsa-miR-375_st | -0.2130032858 | 0.2255664090 |
| 200600_at | hsa-miR-523_st | -0.2125286478 | 0.2266208002 |
| 203781_at | hsa-miR-34b_st | -0.2125286478 | 0.2266208002 |
| 207198_s_at | hsa-miR-377-star_st | -0.2125286478 | 0.2266208002 |
| 201324_at | hsa-miR-212_st | -0.2122230710 | 0.2273014190 |
| 205609_at | hsa-miR-4720-3p_st | -0.2122230710 | 0.2273014190 |
| 205963_s_at | hsa-miR-4652-3p_st | -0.2122230710 | 0.2273014190 |
| 218491_s_at | hsa-miR-34b_st | -0.2122230710 | 0.2273014190 |
| 200797_s_at | hsa-miR-555_st | -0.2120864855 | 0.2276060934 |
| 200097_s_at | hsa-miR-34b_st | -0.2119174943 | 0.2279834415 |
| 200798_x_at | hsa-miR-212_st | -0.2119174943 | 0.2279834415 |
| 201146_at | hsa-miR-212_st | -0.2119174943 | 0.2279834415 |
| 218667_at | hsa-miR-4652-3p_st | -0.2119174943 | 0.2279834415 |
| 202232_s_at | hsa-miR-4652-3p_st | -0.2116119175 | 0.2286668686 |
| 206805_at | hsa-miR-134_st | -0.2116119175 | 0.2286668686 |
| 208981_at | hsa-miR-370_st | -0.2116119175 | 0.2286668686 |
| 218854_at | hsa-miR-1912_st | -0.2116119175 | 0.2286668686 |
| 210427_x_at | hsa-miR-382_st | -0.2113224851 | 0.2293154843 |
| 202382_s_at | hsa-miR-375_st | -0.2113063407 | 0.2293517010 |
| 209108_at | hsa-miR-3189-5p_st | -0.2113063407 | 0.2293517010 |
| 213572_s_at | hsa-miR-361-5p_st | -0.2113063407 | 0.2293517010 |
| 202930_s_at | hsa-miR-375_st | -0.2110007639 | 0.2300379393 |
| 212092_at | hsa-miR-34b_st | -0.2110007639 | 0.2300379393 |
| 213572_s_at | hsa-miR-3189-5p_st | -0.2110007639 | 0.2300379393 |
| 208581_x_at | hsa-miR-523_st | -0.2106951872 | 0.2307255843 |
| 208779_x_at | hsa-miR-3189-5p_st | -0.2106951872 | 0.2307255843 |
| 201172_x_at | hsa-miR-4633-5p_st | -0.2103896104 | 0.2314146367 |
| 201859_at | hsa-miR-1180_st | -0.2103896104 | 0.2314146367 |
| 201398_s_at | hsa-miR-4633-5p_st | -0.2100840336 | 0.2321050972 |
| 210105_s_at | hsa-miR-383_st | -0.2100840336 | 0.2321050972 |
| 202071_at | hsa-miR-377-star_st | -0.2097784568 | 0.2327969664 |
| 202936_s_at | hsa-miR-132_st | -0.2097784568 | 0.2327969664 |
| 204070_at | hsa-miR-134_st | -0.2097784568 | 0.2327969664 |
| 218507_at | hsa-miR-370_st | -0.2097784568 | 0.2327969664 |
| 218706_s_at | hsa-miR-3189-5p_st | -0.2097784568 | 0.2327969664 |
| 218882_s_at | hsa-miR-34b_st | -0.2097784568 | 0.2327969664 |
| 208779_x_at | hsa-miR-382_st | -0.2094888844 | 0.2334539001 |
| 200677_at | hsa-miR-134_st | -0.2094728801 | 0.2334902450 |
| 204070_at | hsa-miR-3676_st | -0.2094728801 | 0.2334902450 |
| 212063_at | hsa-miR-3189-5p_st | -0.2094728801 | 0.2334902450 |
| 221874_at | hsa-miR-34b_st | -0.2094728801 | 0.2334902450 |
| 201319_at | hsa-miR-382_st | -0.2093360843 | 0.2338010578 |
| 207573_x_at | hsa-miR-4652-3p_st | -0.2091673033 | 0.2341849338 |
| 208690_s_at | hsa-miR-555_st | -0.2090304842 | 0.2344964312 |
| 206984_s_at | hsa-miR-34b_st | -0.2088617265 | 0.2348810333 |
| 200804_at | hsa-miR-3189-5p_st | -0.2085561497 | 0.2355785443 |
| 201924_at | hsa-miR-3676_st | -0.2085561497 | 0.2355785443 |
| 202096_s_at | hsa-miR-4762-5p_st | -0.2085561497 | 0.2355785443 |
| 209671_x_at | hsa-miR-4652-3p_st | -0.2085561497 | 0.2355785443 |
| 204554_at | hsa-miR-132_st | -0.2082505730 | 0.2362774674 |
| 200797_s_at | hsa-miR-383_st | -0.2079449962 | 0.2369778032 |
| 205413_at | hsa-miR-34b_st | -0.2076394194 | 0.2376795524 |
| 200673_at | hsa-miR-382_st | -0.2075024836 | 0.2379944807 |
| 212501_at | hsa-miR-523_st | -0.2073338426 | 0.2383827156 |
| 218706_s_at | hsa-miR-383_st | -0.2073338426 | 0.2383827156 |
| 201160_s_at | hsa-miR-523_st | -0.2070282659 | 0.2390872934 |
| 203120_at | hsa-miR-132_st | -0.2070282659 | 0.2390872934 |
| 219683_at | hsa-miR-34b_st | -0.2070282659 | 0.2390872934 |
| 207054_at | hsa-miR-34b_st | -0.2067226891 | 0.2397932865 |
| 210978_s_at | hsa-miR-3676_st | -0.2067226891 | 0.2397932865 |
| 200797_s_at | hsa-miR-3676_st | -0.2064171123 | 0.2405006954 |
| 217730_at | hsa-miR-370_st | -0.2064171123 | 0.2405006954 |
| 203455_s_at | hsa-miR-3189-5p_st | -0.2061115355 | 0.2412095208 |
| 218557_at | hsa-miR-34b_st | -0.2061115355 | 0.2412095208 |
| 202180_s_at | hsa-miR-523_st | -0.2058059587 | 0.2419197633 |
| 205609_at | hsa-miR-1180_st | -0.2058059587 | 0.2419197633 |
| 209157_at | hsa-miR-4652-3p_st | -0.2058059587 | 0.2419197633 |
| 212195_at | hsa-miR-4423-3p_st | -0.2058059587 | 0.2419197633 |
| 208121_s_at | hsa-miR-34b_st | -0.2055003820 | 0.2426314234 |
| 203854_at | hsa-miR-4311_st | -0.2052104827 | 0.2433078831 |
| 217947_at | hsa-miR-555_st | -0.2050576826 | 0.2436649451 |
| 205512_s_at | hsa-miR-4652-3p_st | -0.2048892284 | 0.2440589989 |
| 36711_at | hsa-miR-4633-5p_st | -0.2045836516 | 0.2447749154 |
| 203685_at | hsa-miR-1180_st | -0.2042780749 | 0.2454922519 |
| 205119_s_at | hsa-miR-4762-5p_st | -0.2042780749 | 0.2454922519 |
| 207717_s_at | hsa-miR-34b_st | -0.2042780749 | 0.2454922519 |
| 222216_s_at | hsa-miR-34b_st | -0.2042780749 | 0.2454922519 |
| 202233_s_at | hsa-miR-375_st | -0.2039724981 | 0.2462110089 |
| 219582_at | hsa-miR-361-5p_st | -0.2039724981 | 0.2462110089 |
| 200989_at | hsa-miR-4760-3p_st | -0.2036669213 | 0.2469311869 |
| 202149_at | hsa-miR-4762-5p_st | -0.2036669213 | 0.2469311869 |
| 207761_s_at | hsa-miR-134_st | -0.2036669213 | 0.2469311869 |
| 211475_s_at | hsa-miR-34b_st | -0.2036669213 | 0.2469311869 |
| 212321_at | hsa-miR-361-5p_st | -0.2036669213 | 0.2469311869 |
| 201161_s_at | hsa-miR-555_st | -0.2035296820 | 0.2472550926 |
| 200906_s_at | hsa-miR-523_st | -0.2030557678 | 0.2483758081 |
| 210117_at | hsa-miR-382_st | -0.2029184818 | 0.2487011020 |
| 217947_at | hsa-miR-4311_st | -0.2027656817 | 0.2490634937 |
| 203120_at | hsa-miR-383_st | -0.2027501910 | 0.2491002524 |
| 203120_at | hsa-miR-212_st | -0.2027501910 | 0.2491002524 |
| 221667_s_at | hsa-miR-4633-5p_st | -0.2027501910 | 0.2491002524 |
| 207508_at | hsa-miR-4652-3p_st | -0.2024446142 | 0.2498261199 |
| 221009_s_at | hsa-miR-383_st | -0.2024446142 | 0.2498261199 |
| 201161_s_at | hsa-miR-212_st | -0.2021390374 | 0.2505534110 |
| 202920_at | hsa-miR-34b_st | -0.2021390374 | 0.2505534110 |
| 208690_s_at | hsa-miR-4633-5p_st | -0.2021390374 | 0.2505534110 |
| 211999_at | hsa-miR-361-5p_st | -0.2020016814 | 0.2508807905 |
| 204194_at | hsa-miR-4423-3p_st | -0.2018334607 | 0.2512821263 |
| 208809_s_at | hsa-miR-3676_st | -0.2018334607 | 0.2512821263 |
| 212015_x_at | hsa-miR-4762-5p_st | -0.2018334607 | 0.2512821263 |
| 202370_s_at | hsa-miR-3676_st | -0.2015278839 | 0.2520122662 |
| 210946_at | hsa-miR-4423-3p_st | -0.2012223071 | 0.2527438313 |
| 217960_s_at | hsa-miR-4652-3p_st | -0.2009167303 | 0.2534768220 |
| 218214_at | hsa-miR-134_st | -0.2009167303 | 0.2534768220 |
| 218224_at | hsa-miR-34b_st | -0.2009167303 | 0.2534768220 |
| 203987_at | hsa-miR-383_st | -0.2006111536 | 0.2542112388 |
| 205633_s_at | hsa-miR-4652-3p_st | -0.2006111536 | 0.2542112388 |
| 212501_at | hsa-miR-4633-5p_st | -0.2006111536 | 0.2542112388 |
| 217936_at | hsa-miR-3676_st | -0.2006111536 | 0.2542112388 |
| 218005_at | hsa-miR-4423-3p_st | -0.2006111536 | 0.2542112388 |
| 210068_s_at | hsa-miR-555_st | -0.2003208807 | 0.2549101956 |
| 201146_at | hsa-miR-361-5p_st | -0.2003055768 | 0.2549470821 |
| 205119_s_at | hsa-miR-34b_st | -0.2003055768 | 0.2549470821 |
| 217780_at | hsa-miR-134_st | -0.2003055768 | 0.2549470821 |
| 202133_at | hsa-miR-3189-5p_st | -0.2000000000 | 0.2556843525 |
| 210117_at | hsa-miR-132_st | -0.2000000000 | 0.2556843525 |
| 212067_s_at | hsa-miR-4760-3p_st | -0.2000000000 | 0.2556843525 |
| 212195_at | hsa-miR-212_st | -0.2000000000 | 0.2556843525 |
| 218005_at | hsa-miR-4633-5p_st | -0.2000000000 | 0.2556843525 |
| 219582_at | hsa-miR-1180_st | -0.2000000000 | 0.2556843525 |
| 200739_s_at | hsa-miR-34b_st | -0.1996944232 | 0.2564230504 |
| 200804_at | hsa-miR-4311_st | -0.1995568804 | 0.2567560108 |
| 201859_at | hsa-miR-370_st | -0.1993888464 | 0.2571631761 |
| 212110_at | hsa-miR-377-star_st | -0.1993888464 | 0.2571631761 |
| 214428_x_at | hsa-miR-523_st | -0.1993888464 | 0.2571631761 |
| 217546_at | hsa-miR-383_st | -0.1993888464 | 0.2571631761 |
| 203973_s_at | hsa-miR-523_st | -0.1990832697 | 0.2579047303 |
| 212067_s_at | hsa-miR-383_st | -0.1990832697 | 0.2579047303 |
| 221796_at | hsa-miR-4760-3p_st | -0.1990832697 | 0.2579047303 |
| 206099_at | hsa-miR-4652-3p_st | -0.1987776929 | 0.2586477132 |
| 210014_x_at | hsa-miR-34b_st | -0.1987776929 | 0.2586477132 |
| 201274_at | hsa-miR-134_st | -0.1984721161 | 0.2593921253 |
| 217730_at | hsa-miR-1180_st | -0.1981665393 | 0.2601379670 |
| 211962_s_at | hsa-miR-4633-5p_st | -0.1978609626 | 0.2608852388 |
| 221741_s_at | hsa-miR-3676_st | -0.1978609626 | 0.2608852388 |
| 200708_at | hsa-miR-34b_st | -0.1975553858 | 0.2616339409 |
| 201029_s_at | hsa-miR-4633-5p_st | -0.1975553858 | 0.2616339409 |
| 203455_s_at | hsa-miR-523_st | -0.1975553858 | 0.2616339409 |
| 206989_s_at | hsa-miR-383_st | -0.1975553858 | 0.2616339409 |
| 209122_at | hsa-miR-361-5p_st | -0.1975553858 | 0.2616339409 |
| 213592_at | hsa-miR-370_st | -0.1975553858 | 0.2616339409 |
| 204787_at | hsa-miR-4633-5p_st | -0.1972498090 | 0.2623840739 |
| 218491_s_at | hsa-miR-375_st | -0.1972498090 | 0.2623840739 |
| 210240_s_at | hsa-miR-4652-3p_st | -0.1969442322 | 0.2631356381 |
| 210978_s_at | hsa-miR-370_st | -0.1969442322 | 0.2631356381 |
| 213887_s_at | hsa-miR-34b_st | -0.1969442322 | 0.2631356381 |
| 217746_s_at | hsa-miR-361-5p_st | -0.1969442322 | 0.2631356381 |
| 217947_at | hsa-miR-383_st | -0.1969442322 | 0.2631356381 |
| 217995_at | hsa-miR-3189-5p_st | -0.1969442322 | 0.2631356381 |
| 203854_at | hsa-miR-383_st | -0.1966386555 | 0.2638886338 |
| 209476_at | hsa-miR-132_st | -0.1966386555 | 0.2638886338 |
| 201086_x_at | hsa-miR-34b_st | -0.1963330787 | 0.2646430615 |
| 201666_at | hsa-miR-134_st | -0.1963330787 | 0.2646430615 |
| 204766_s_at | hsa-miR-134_st | -0.1963330787 | 0.2646430615 |
| 203987_at | hsa-miR-382_st | -0.1961952791 | 0.2649837387 |
| 201180_s_at | hsa-miR-523_st | -0.1957219251 | 0.2661562141 |
| 204194_at | hsa-miR-4760-3p_st | -0.1957219251 | 0.2661562141 |
| 205489_at | hsa-miR-34b_st | -0.1957219251 | 0.2661562141 |
| 200986_at | hsa-miR-4633-5p_st | -0.1951107716 | 0.2676750987 |
| 201146_at | hsa-miR-4423-3p_st | -0.1951107716 | 0.2676750987 |
| 201887_at | hsa-miR-4762-5p_st | -0.1951107716 | 0.2676750987 |
| 200804_at | hsa-miR-361-5p_st | -0.1941940413 | 0.2699641788 |
| 202133_at | hsa-miR-523_st | -0.1941940413 | 0.2699641788 |
| 207761_s_at | hsa-miR-4760-3p_st | -0.1941940413 | 0.2699641788 |
| 202864_s_at | hsa-miR-3189-5p_st | -0.1938884645 | 0.2707300743 |
| 203404_at | hsa-miR-34b_st | -0.1932773109 | 0.2722661704 |
| 201319_at | hsa-miR-4760-3p_st | -0.1929717341 | 0.2730363715 |
| 202736_s_at | hsa-miR-4652-3p_st | -0.1929717341 | 0.2730363715 |
| 202802_at | hsa-miR-4652-3p_st | -0.1929717341 | 0.2730363715 |
| 214829_at | hsa-miR-4762-5p_st | -0.1929717341 | 0.2730363715 |
| 200663_at | hsa-miR-555_st | -0.1923752775 | 0.2745438669 |
| 200798_x_at | hsa-miR-4633-5p_st | -0.1923605806 | 0.2745810812 |
| 202427_s_at | hsa-miR-34b_st | -0.1923605806 | 0.2745810812 |
| 208998_at | hsa-miR-4762-5p_st | -0.1923605806 | 0.2745810812 |
| 210592_s_at | hsa-miR-370_st | -0.1923605806 | 0.2745810812 |
| 213503_x_at | hsa-miR-4633-5p_st | -0.1923605806 | 0.2745810812 |
| 214428_x_at | hsa-miR-4760-3p_st | -0.1920550038 | 0.2753555904 |
| 222216_s_at | hsa-miR-4652-3p_st | -0.1920550038 | 0.2753555904 |
| 203854_at | hsa-miR-4762-5p_st | -0.1914438503 | 0.2769089186 |
| 208998_at | hsa-miR-4633-5p_st | -0.1914438503 | 0.2769089186 |
| 200626_s_at | hsa-miR-4652-3p_st | -0.1911382735 | 0.2776877380 |
| 203033_x_at | hsa-miR-4423-3p_st | -0.1911382735 | 0.2776877380 |
| 210453_x_at | hsa-miR-34b_st | -0.1911382735 | 0.2776877380 |
| 210592_s_at | hsa-miR-4760-3p_st | -0.1911382735 | 0.2776877380 |
| 219355_at | hsa-miR-34b_st | -0.1911382735 | 0.2776877380 |
| 202864_s_at | hsa-miR-523_st | -0.1908326967 | 0.2784679948 |
| 212063_at | hsa-miR-382_st | -0.1906944768 | 0.2788213962 |
| 205119_s_at | hsa-miR-34b-star_st | -0.1905271199 | 0.2792496890 |
| 203893_at | hsa-miR-4423-3p_st | -0.1902215432 | 0.2800328209 |
| 208761_s_at | hsa-miR-375_st | -0.1902215432 | 0.2800328209 |
| 209243_s_at | hsa-miR-34b_st | -0.1902215432 | 0.2800328209 |
| 203723_at | hsa-miR-361-5p_st | -0.1900832766 | 0.2803876428 |
| 203704_s_at | hsa-miR-370_st | -0.1899159664 | 0.2808173906 |
| 212063_at | hsa-miR-370_st | -0.1899159664 | 0.2808173906 |
| 201887_at | hsa-miR-361-5p_st | -0.1896103896 | 0.2816033985 |
| 36711_at | hsa-miR-370_st | -0.1896103896 | 0.2816033985 |
| 208782_at | hsa-miR-555_st | -0.1893192763 | 0.2823535411 |
| 201859_at | hsa-miR-3189-5p_st | -0.1893048128 | 0.2823908446 |
| 208451_s_at | hsa-miR-1180_st | -0.1893048128 | 0.2823908446 |
| 203973_s_at | hsa-miR-555_st | -0.1891664762 | 0.2827477996 |
| 202587_s_at | hsa-miR-383_st | -0.1889992361 | 0.2831797292 |
| 214022_s_at | hsa-miR-4633-5p_st | -0.1889992361 | 0.2831797292 |
| 210978_s_at | hsa-miR-382_st | -0.1888608761 | 0.2835373958 |
| 202564_x_at | hsa-miR-4652-3p_st | -0.1883880825 | 0.2847618143 |
| 204125_at | hsa-miR-4423-3p_st | -0.1883880825 | 0.2847618143 |
| 207761_s_at | hsa-miR-4762-5p_st | -0.1883880825 | 0.2847618143 |
| 209265_s_at | hsa-miR-4652-3p_st | -0.1883880825 | 0.2847618143 |
| 212067_s_at | hsa-miR-370_st | -0.1883880825 | 0.2847618143 |
| 221796_at | hsa-miR-4423-3p_st | -0.1883880825 | 0.2847618143 |
| 202475_at | hsa-miR-34b_st | -0.1880825057 | 0.2855550152 |
| 207761_s_at | hsa-miR-3676_st | -0.1880825057 | 0.2855550152 |
| 201161_s_at | hsa-miR-1912_st | -0.1877769290 | 0.2863496552 |
| 202543_s_at | hsa-miR-523_st | -0.1874713522 | 0.2871457345 |
| 209476_at | hsa-miR-370_st | -0.1874713522 | 0.2871457345 |
| 202802_at | hsa-miR-34b_st | -0.1871657754 | 0.2879432531 |
| 204554_at | hsa-miR-4762-5p_st | -0.1871657754 | 0.2879432531 |
| 204766_s_at | hsa-miR-4423-3p_st | -0.1871657754 | 0.2879432531 |
| 209265_s_at | hsa-miR-34b-star_st | -0.1871657754 | 0.2879432531 |
| 213533_at | hsa-miR-4652-3p_st | -0.1871657754 | 0.2879432531 |
| 203685_at | hsa-miR-382_st | -0.1868744753 | 0.2887048515 |
| 203663_s_at | hsa-miR-4423-3p_st | -0.1868601986 | 0.2887422112 |
| 210068_s_at | hsa-miR-212_st | -0.1868601986 | 0.2887422112 |
| 221667_s_at | hsa-miR-370_st | -0.1868601986 | 0.2887422112 |
| 1007_s_at | hsa-miR-555_st | -0.1865688752 | 0.2895052432 |
| 205609_at | hsa-miR-4760-3p_st | -0.1865546218 | 0.2895426089 |
| 206015_s_at | hsa-miR-4652-3p_st | -0.1865546218 | 0.2895426089 |
| 210962_s_at | hsa-miR-375_st | -0.1865546218 | 0.2895426089 |
| 212687_at | hsa-miR-132_st | -0.1865546218 | 0.2895426089 |
| 218005_at | hsa-miR-3676_st | -0.1865546218 | 0.2895426089 |
| 218507_at | hsa-miR-1180_st | -0.1865546218 | 0.2895426089 |
| 204554_at | hsa-miR-1180_st | -0.1862490451 | 0.2903444464 |
| 221515_s_at | hsa-miR-34b_st | -0.1862490451 | 0.2903444464 |
| 201887_at | hsa-miR-212_st | -0.1859434683 | 0.2911477236 |
| 205097_at | hsa-miR-3676_st | -0.1859434683 | 0.2911477236 |
| 208457_at | hsa-miR-34b_st | -0.1859434683 | 0.2911477236 |
| 218507_at | hsa-miR-4762-5p_st | -0.1859434683 | 0.2911477236 |
| 207761_s_at | hsa-miR-4423-3p_st | -0.1856378915 | 0.2919524408 |
| 212110_at | hsa-miR-4762-5p_st | -0.1853323147 | 0.2927585979 |
| 203721_s_at | hsa-miR-4652-3p_st | -0.1850267380 | 0.2935661951 |
| 208451_s_at | hsa-miR-383_st | -0.1850267380 | 0.2935661951 |
| 209732_at | hsa-miR-4762-5p_st | -0.1850267380 | 0.2935661951 |
| 212195_at | hsa-miR-134_st | -0.1850267380 | 0.2935661951 |
| 215952_s_at | hsa-miR-4652-3p_st | -0.1850267380 | 0.2935661951 |
| 221796_at | hsa-miR-377-star_st | -0.1850267380 | 0.2935661951 |
| 215952_s_at | hsa-miR-34b_st | -0.1847211612 | 0.2943752325 |
| 200906_s_at | hsa-miR-3676_st | -0.1844155844 | 0.2951857101 |
| 203854_at | hsa-miR-3676_st | -0.1844155844 | 0.2951857101 |
| 208761_s_at | hsa-miR-4652-3p_st | -0.1844155844 | 0.2951857101 |
| 214829_at | hsa-miR-3676_st | -0.1844155844 | 0.2951857101 |
| 207761_s_at | hsa-miR-4311_st | -0.1842768742 | 0.2955540846 |
| 204554_at | hsa-miR-212_st | -0.1841100076 | 0.2959976279 |
| 208581_x_at | hsa-miR-134_st | -0.1841100076 | 0.2959976279 |
| 212015_x_at | hsa-miR-4633-5p_st | -0.1841100076 | 0.2959976279 |
| 213005_s_at | hsa-miR-361-5p_st | -0.1841100076 | 0.2959976279 |
| 217968_at | hsa-miR-4423-3p_st | -0.1841100076 | 0.2959976279 |
| 201160_s_at | hsa-miR-361-5p_st | -0.1838044309 | 0.2968109860 |
| 202936_s_at | hsa-miR-523_st | -0.1838044309 | 0.2968109860 |
| 210592_s_at | hsa-miR-555_st | -0.1836656740 | 0.2971807926 |
| 201319_at | hsa-miR-4423-3p_st | -0.1834988541 | 0.2976257844 |
| 204554_at | hsa-miR-383_st | -0.1834988541 | 0.2976257844 |
| 202930_s_at | hsa-miR-4423-3p_st | -0.1831932773 | 0.2984420232 |
| 208457_at | hsa-miR-134_st | -0.1831932773 | 0.2984420232 |
| 208745_at | hsa-miR-4423-3p_st | -0.1831932773 | 0.2984420232 |
| 201999_s_at | hsa-miR-4633-5p_st | -0.1828877005 | 0.2992597024 |
| 202133_at | hsa-miR-1180_st | -0.1828877005 | 0.2992597024 |
| 207573_x_at | hsa-miR-34b_st | -0.1828877005 | 0.2992597024 |
| 208868_s_at | hsa-miR-4652-3p_st | -0.1828877005 | 0.2992597024 |
| 210968_s_at | hsa-miR-4652-3p_st | -0.1828877005 | 0.2992597024 |
| 201859_at | hsa-miR-383_st | -0.1825821238 | 0.3000788219 |
| 201924_at | hsa-miR-4633-5p_st | -0.1825821238 | 0.3000788219 |
| 205609_at | hsa-miR-4762-5p_st | -0.1825821238 | 0.3000788219 |
| 210906_x_at | hsa-miR-4633-5p_st | -0.1825821238 | 0.3000788219 |
| 214150_x_at | hsa-miR-4423-3p_st | -0.1825821238 | 0.3000788219 |
| 203704_s_at | hsa-miR-4633-5p_st | -0.1822765470 | 0.3008993818 |
| 204326_x_at | hsa-miR-4760-3p_st | -0.1822765470 | 0.3008993818 |
| 210068_s_at | hsa-miR-3189-5p_st | -0.1822765470 | 0.3008993818 |
| 208697_s_at | hsa-miR-4652-3p_st | -0.1816653934 | 0.3025448228 |
| 209732_at | hsa-miR-361-5p_st | -0.1816653934 | 0.3025448228 |
| 212687_at | hsa-miR-383_st | -0.1816653934 | 0.3025448228 |
| 201172_x_at | hsa-miR-383_st | -0.1810542399 | 0.3041960252 |
| 208991_at | hsa-miR-361-5p_st | -0.1810542399 | 0.3041960252 |
| 211271_x_at | hsa-miR-4633-5p_st | -0.1810542399 | 0.3041960252 |
| 205097_at | hsa-miR-370_st | -0.1807486631 | 0.3050237868 |
| 214428_x_at | hsa-miR-1180_st | -0.1807486631 | 0.3050237868 |
| 201859_at | hsa-miR-555_st | -0.1801512726 | 0.3066461879 |
| 201012_at | hsa-miR-370_st | -0.1801375095 | 0.3066836307 |
| 218656_s_at | hsa-miR-134_st | -0.1801375095 | 0.3066836307 |
| 210906_x_at | hsa-miR-3189-5p_st | -0.1798319328 | 0.3075157128 |
| 212053_at | hsa-miR-34b_st | -0.1798319328 | 0.3075157128 |
| 203645_s_at | hsa-miR-555_st | -0.1796928724 | 0.3078948494 |
| 205031_at | hsa-miR-34b_st | -0.1795263560 | 0.3083492349 |
| 209513_s_at | hsa-miR-523_st | -0.1792207792 | 0.3091841970 |
| 203540_at | hsa-miR-382_st | -0.1787760720 | 0.3104018934 |
| 203987_at | hsa-miR-555_st | -0.1787760720 | 0.3104018934 |
| 201161_s_at | hsa-miR-3676_st | -0.1786096257 | 0.3108584407 |
| 201411_s_at | hsa-miR-4652-3p_st | -0.1786096257 | 0.3108584407 |
| 210434_x_at | hsa-miR-34b_st | -0.1786096257 | 0.3108584407 |
| 213592_at | hsa-miR-382_st | -0.1783176718 | 0.3116602753 |
| 202252_at | hsa-miR-361-5p_st | -0.1783040489 | 0.3116977221 |
| 204072_s_at | hsa-miR-34b_st | -0.1783040489 | 0.3116977221 |
| 201753_s_at | hsa-miR-3189-5p_st | -0.1779984721 | 0.3125384430 |
| 203411_s_at | hsa-miR-370_st | -0.1779984721 | 0.3125384430 |
| 203645_s_at | hsa-miR-3676_st | -0.1779984721 | 0.3125384430 |
| 204068_at | hsa-miR-523_st | -0.1779984721 | 0.3125384430 |
| 208451_s_at | hsa-miR-3676_st | -0.1779984721 | 0.3125384430 |
| 210068_s_at | hsa-miR-383_st | -0.1779984721 | 0.3125384430 |
| 211558_s_at | hsa-miR-34b_st | -0.1779984721 | 0.3125384430 |
| 48531_at | hsa-miR-4633-5p_st | -0.1779984721 | 0.3125384430 |
| 200701_at | hsa-miR-361-5p_st | -0.1776928953 | 0.3133806034 |
| 207198_s_at | hsa-miR-4633-5p_st | -0.1773873186 | 0.3142242030 |
| 221796_at | hsa-miR-4633-5p_st | -0.1770817418 | 0.3150692418 |
| 200600_at | hsa-miR-3189-5p_st | -0.1767761650 | 0.3159157196 |
| 208451_s_at | hsa-miR-4760-3p_st | -0.1767761650 | 0.3159157196 |
| 218048_at | hsa-miR-34b_st | -0.1767761650 | 0.3159157196 |
| 211951_at | hsa-miR-4423-3p_st | -0.1764705882 | 0.3167636363 |
| 213272_s_at | hsa-miR-34b_st | -0.1764705882 | 0.3167636363 |
| 208779_x_at | hsa-miR-3676_st | -0.1761650115 | 0.3176129916 |
| 204068_at | hsa-miR-3189-5p_st | -0.1758594347 | 0.3184637854 |
| 212501_at | hsa-miR-134_st | -0.1758594347 | 0.3184637854 |
| 219911_s_at | hsa-miR-4633-5p_st | -0.1758594347 | 0.3184637854 |
| 200701_at | hsa-miR-4633-5p_st | -0.1755538579 | 0.3193160175 |
| 207761_s_at | hsa-miR-212_st | -0.1755538579 | 0.3193160175 |
| 208451_s_at | hsa-miR-523_st | -0.1755538579 | 0.3193160175 |
| 200673_at | hsa-miR-212_st | -0.1752482811 | 0.3201696878 |
| 200906_s_at | hsa-miR-370_st | -0.1752482811 | 0.3201696878 |
| 202149_at | hsa-miR-134_st | -0.1752482811 | 0.3201696878 |
| 210117_at | hsa-miR-3676_st | -0.1752482811 | 0.3201696878 |
| 212099_at | hsa-miR-4760-3p_st | -0.1752482811 | 0.3201696878 |
| 221796_at | hsa-miR-523_st | -0.1752482811 | 0.3201696878 |
| 205119_s_at | hsa-miR-555_st | -0.1744976703 | 0.3222727249 |
| 209732_at | hsa-miR-4423-3p_st | -0.1743315508 | 0.3227393254 |
| 212099_at | hsa-miR-370_st | -0.1743315508 | 0.3227393254 |
| 217746_s_at | hsa-miR-212_st | -0.1743315508 | 0.3227393254 |
| 201761_at | hsa-miR-3189-5p_st | -0.1740259740 | 0.3235987461 |
| 211475_s_at | hsa-miR-4652-3p_st | -0.1740259740 | 0.3235987461 |
| 219582_at | hsa-miR-4423-3p_st | -0.1740259740 | 0.3235987461 |
| 208697_s_at | hsa-miR-375_st | -0.1737203972 | 0.3244596039 |
| 210906_x_at | hsa-miR-370_st | -0.1737203972 | 0.3244596039 |
| 212099_at | hsa-miR-555_st | -0.1735808699 | 0.3248531521 |
| 213710_s_at | hsa-miR-4423-3p_st | -0.1734148205 | 0.3253218985 |
| 200600_at | hsa-miR-1180_st | -0.1731092437 | 0.3261856296 |
| 202180_s_at | hsa-miR-134_st | -0.1731092437 | 0.3261856296 |
| 200708_at | hsa-miR-4652-3p_st | -0.1724980901 | 0.3279174005 |
| 201900_s_at | hsa-miR-4652-3p_st | -0.1724980901 | 0.3279174005 |
| 48531_at | hsa-miR-555_st | -0.1723584694 | 0.3283138370 |
| 36711_at | hsa-miR-523_st | -0.1721925134 | 0.3287854397 |
| 207120_at | hsa-miR-4652-3p_st | -0.1715813598 | 0.3305258242 |
| 209183_s_at | hsa-miR-4762-5p_st | -0.1715813598 | 0.3305258242 |
| 219683_at | hsa-miR-4652-3p_st | -0.1715813598 | 0.3305258242 |
| 202252_at | hsa-miR-555_st | -0.1712888690 | 0.3313607823 |
| 201761_at | hsa-miR-3676_st | -0.1712757830 | 0.3313981689 |
| 211595_s_at | hsa-miR-34b_st | -0.1712757830 | 0.3313981689 |
| 211999_at | hsa-miR-3676_st | -0.1711360690 | 0.3317974953 |
| 204326_x_at | hsa-miR-1180_st | -0.1709702063 | 0.3322719481 |
| 202936_s_at | hsa-miR-3676_st | -0.1706646295 | 0.3331471616 |
| 209840_s_at | hsa-miR-34b_st | -0.1706646295 | 0.3331471616 |
| 211479_s_at | hsa-miR-4652-3p_st | -0.1706646295 | 0.3331471616 |
| 214829_at | hsa-miR-134_st | -0.1706646295 | 0.3331471616 |
| 215416_s_at | hsa-miR-4423-3p_st | -0.1703590527 | 0.3340238090 |
| 217947_at | hsa-miR-212_st | -0.1703590527 | 0.3340238090 |
| 221009_s_at | hsa-miR-134_st | -0.1703590527 | 0.3340238090 |
| 210978_s_at | hsa-miR-555_st | -0.1702192686 | 0.3344253036 |
| 218706_s_at | hsa-miR-1180_st | -0.1700534759 | 0.3349018900 |
| 212977_at | hsa-miR-4311_st | -0.1697608684 | 0.3357440468 |
| 201324_at | hsa-miR-523_st | -0.1697478992 | 0.3357814042 |
| 202948_at | hsa-miR-134_st | -0.1697478992 | 0.3357814042 |
| 213005_s_at | hsa-miR-4633-5p_st | -0.1697478992 | 0.3357814042 |
| 218507_at | hsa-miR-383_st | -0.1694423224 | 0.3366623512 |
| 200641_s_at | hsa-miR-4652-3p_st | -0.1691367456 | 0.3375447308 |
| 201319_at | hsa-miR-1912_st | -0.1691367456 | 0.3375447308 |
| 211999_at | hsa-miR-4633-5p_st | -0.1689968681 | 0.3379491171 |
| 202252_at | hsa-miR-4633-5p_st | -0.1688311688 | 0.3384285425 |
| 206544_x_at | hsa-miR-4652-3p_st | -0.1688311688 | 0.3384285425 |
| 210592_s_at | hsa-miR-383_st | -0.1688311688 | 0.3384285425 |
| 210592_s_at | hsa-miR-3189-5p_st | -0.1685255921 | 0.3393137859 |
| 202587_s_at | hsa-miR-1180_st | -0.1682200153 | 0.3402004607 |
| 203189_s_at | hsa-miR-34b_st | -0.1682200153 | 0.3402004607 |
| 218854_at | hsa-miR-3189-5p_st | -0.1682200153 | 0.3402004607 |
| 203137_at | hsa-miR-375_st | -0.1679144385 | 0.3410885665 |
| 203415_at | hsa-miR-4652-3p_st | -0.1679144385 | 0.3410885665 |
| 207761_s_at | hsa-miR-555_st | -0.1677744676 | 0.3414958453 |
| 218706_s_at | hsa-miR-555_st | -0.1674688675 | 0.3423861048 |
| 215171_s_at | hsa-miR-375_st | -0.1673032850 | 0.3428690692 |
| 219582_at | hsa-miR-3676_st | -0.1673032850 | 0.3428690692 |
| 220251_at | hsa-miR-34b_st | -0.1673032850 | 0.3428690692 |
| 205119_s_at | hsa-miR-3676_st | -0.1669977082 | 0.3437614654 |
| 207088_s_at | hsa-miR-3676_st | -0.1669977082 | 0.3437614654 |
| 218694_at | hsa-miR-4423-3p_st | -0.1669977082 | 0.3437614654 |
| 200040_at | hsa-miR-34b_st | -0.1663865546 | 0.3455505451 |
| 209513_s_at | hsa-miR-4633-5p_st | -0.1660809778 | 0.3464472278 |
| 210068_s_at | hsa-miR-523_st | -0.1657754011 | 0.3473453384 |
| 202936_s_at | hsa-miR-4311_st | -0.1656352667 | 0.3477576800 |
| 211999_at | hsa-miR-134_st | -0.1654824667 | 0.3482076323 |
| 201066_at | hsa-miR-34b_st | -0.1654698243 | 0.3482448764 |
| 217947_at | hsa-miR-4423-3p_st | -0.1654698243 | 0.3482448764 |
| 218201_at | hsa-miR-34b_st | -0.1654698243 | 0.3482448764 |
| 221796_at | hsa-miR-4311_st | -0.1651768666 | 0.3491086072 |
| 201761_at | hsa-miR-555_st | -0.1648712664 | 0.3500110088 |
| 217947_at | hsa-miR-3189-5p_st | -0.1645530940 | 0.3509520505 |
| 200663_at | hsa-miR-4633-5p_st | -0.1642475172 | 0.3518572935 |
| 212501_at | hsa-miR-3676_st | -0.1642475172 | 0.3518572935 |
| 203455_s_at | hsa-miR-132_st | -0.1633307869 | 0.3545815702 |
| 201656_at | hsa-miR-3189-5p_st | -0.1630252101 | 0.3554925099 |
| 212460_at | hsa-miR-4633-5p_st | -0.1630252101 | 0.3554925099 |
| 221741_s_at | hsa-miR-555_st | -0.1628848656 | 0.3559113606 |
| 200673_at | hsa-miR-3189-5p_st | -0.1627196333 | 0.3564048725 |
| 201570_at | hsa-miR-4652-3p_st | -0.1627196333 | 0.3564048725 |
| 203150_at | hsa-miR-134_st | -0.1627196333 | 0.3564048725 |
| 48531_at | hsa-miR-361-5p_st | -0.1627196333 | 0.3564048725 |
| 210448_s_at | hsa-miR-34b_st | -0.1624140565 | 0.3573186573 |
| 209476_at | hsa-miR-3676_st | -0.1618029030 | 0.3591504915 |
| 218507_at | hsa-miR-4633-5p_st | -0.1618029030 | 0.3591504915 |
| 214428_x_at | hsa-miR-383_st | -0.1614973262 | 0.3600685398 |
| 217746_s_at | hsa-miR-382_st | -0.1613568650 | 0.3604910055 |
| 203560_at | hsa-miR-4423-3p_st | -0.1608861727 | 0.3619088955 |
| 218214_at | hsa-miR-4652-3p_st | -0.1608861727 | 0.3619088955 |
| 203120_at | hsa-miR-377-star_st | -0.1605805959 | 0.3628312017 |
| 212092_at | hsa-miR-4652-3p_st | -0.1605805959 | 0.3628312017 |
| 219582_at | hsa-miR-555_st | -0.1604400646 | 0.3632558358 |
| 203120_at | hsa-miR-3676_st | -0.1602750191 | 0.3637549261 |
| 210817_s_at | hsa-miR-4423-3p_st | -0.1602750191 | 0.3637549261 |
| 212067_s_at | hsa-miR-4633-5p_st | -0.1602750191 | 0.3637549261 |
| 213217_at | hsa-miR-4633-5p_st | -0.1602750191 | 0.3637549261 |
| 218005_at | hsa-miR-3189-5p_st | -0.1602750191 | 0.3637549261 |
| 218285_s_at | hsa-miR-4633-5p_st | -0.1602750191 | 0.3637549261 |
| 204245_s_at | hsa-miR-4423-3p_st | -0.1599694423 | 0.3646800680 |
| 214829_at | hsa-miR-361-5p_st | -0.1599694423 | 0.3646800680 |
| 201527_at | hsa-miR-34b_st | -0.1596638655 | 0.3656066267 |
| 204554_at | hsa-miR-382_st | -0.1595232643 | 0.3660334285 |
| 206671_at | hsa-miR-4423-3p_st | -0.1593582888 | 0.3665346016 |
| 221958_s_at | hsa-miR-523_st | -0.1593582888 | 0.3665346016 |
| 201146_at | hsa-miR-3676_st | -0.1590527120 | 0.3674639921 |
| 201398_s_at | hsa-miR-523_st | -0.1590527120 | 0.3674639921 |
| 202376_at | hsa-miR-361-5p_st | -0.1587471352 | 0.3683947975 |
| 210978_s_at | hsa-miR-523_st | -0.1587471352 | 0.3683947975 |
| 201887_at | hsa-miR-382_st | -0.1586064639 | 0.3688237662 |
| 202133_at | hsa-miR-3676_st | -0.1581359817 | 0.3702606501 |
| 202543_s_at | hsa-miR-4423-3p_st | -0.1581359817 | 0.3702606501 |
| 203704_s_at | hsa-miR-4311_st | -0.1579952637 | 0.3706910630 |
| 201592_at | hsa-miR-34b_st | -0.1578304049 | 0.3711956960 |
| 204326_x_at | hsa-miR-134_st | -0.1578304049 | 0.3711956960 |
| 204326_x_at | hsa-miR-555_st | -0.1575368635 | 0.3720952443 |
| 200804_at | hsa-miR-4762-5p_st | -0.1575248281 | 0.3721321541 |
| 206015_s_at | hsa-miR-3676_st | -0.1575248281 | 0.3721321541 |
| 200986_at | hsa-miR-555_st | -0.1573840634 | 0.3725640107 |
| 202071_at | hsa-miR-4633-5p_st | -0.1572192513 | 0.3730700235 |
| 204554_at | hsa-miR-4633-5p_st | -0.1572192513 | 0.3730700235 |
| 201924_at | hsa-miR-134_st | -0.1569136746 | 0.3740093037 |
| 213423_x_at | hsa-miR-34b_st | -0.1566080978 | 0.3749499938 |
| 213592_at | hsa-miR-3676_st | -0.1566080978 | 0.3749499938 |
| 201172_x_at | hsa-miR-3189-5p_st | -0.1563025210 | 0.3758920932 |
| 201319_at | hsa-miR-523_st | -0.1559969442 | 0.3768356011 |
| 201988_s_at | hsa-miR-4652-3p_st | -0.1559969442 | 0.3768356011 |
| 201324_at | hsa-miR-383_st | -0.1556913675 | 0.3777805167 |
| 209108_at | hsa-miR-4633-5p_st | -0.1556913675 | 0.3777805167 |
| 205119_s_at | hsa-miR-4311_st | -0.1552448626 | 0.3791637450 |
| 201410_at | hsa-miR-4423-3p_st | -0.1550802139 | 0.3796745679 |
| 204194_at | hsa-miR-132_st | -0.1547746371 | 0.3806237021 |
| 217995_at | hsa-miR-555_st | -0.1544808622 | 0.3815375034 |
| 203704_s_at | hsa-miR-4762-5p_st | -0.1544690604 | 0.3815742409 |
| 210501_x_at | hsa-miR-4652-3p_st | -0.1544690604 | 0.3815742409 |
| 205119_s_at | hsa-miR-34c-5p_st | -0.1541634836 | 0.3825261835 |
| 209476_at | hsa-miR-383_st | -0.1541634836 | 0.3825261835 |
| 212063_at | hsa-miR-3676_st | -0.1541634836 | 0.3825261835 |
| 221488_s_at | hsa-miR-34b_st | -0.1541634836 | 0.3825261835 |
| 36711_at | hsa-miR-555_st | -0.1540224621 | 0.3829659714 |
| 200701_at | hsa-miR-383_st | -0.1538579068 | 0.3834795291 |
| 202864_s_at | hsa-miR-1180_st | -0.1535523300 | 0.3844342768 |
| 203781_at | hsa-miR-4423-3p_st | -0.1535523300 | 0.3844342768 |
| 221531_at | hsa-miR-4652-3p_st | -0.1535523300 | 0.3844342768 |
| 222043_at | hsa-miR-4633-5p_st | -0.1532467532 | 0.3853904260 |
| 209550_at | hsa-miR-34b_st | -0.1529411765 | 0.3863479756 |
| 210033_s_at | hsa-miR-4762-5p_st | -0.1529411765 | 0.3863479756 |
| 203723_at | hsa-miR-3676_st | -0.1526472615 | 0.3872703026 |
| 212067_s_at | hsa-miR-3676_st | -0.1526355997 | 0.3873069250 |
| 212110_at | hsa-miR-212_st | -0.1526355997 | 0.3873069250 |
| 206542_s_at | hsa-miR-4652-3p_st | -0.1523300229 | 0.3882672731 |
| 210117_at | hsa-miR-523_st | -0.1523300229 | 0.3882672731 |
| 206989_s_at | hsa-miR-382_st | -0.1521888613 | 0.3887113793 |
| 214829_at | hsa-miR-555_st | -0.1520360613 | 0.3891924373 |
| 209104_s_at | hsa-miR-4652-3p_st | -0.1520244461 | 0.3892290192 |
| 200967_at | hsa-miR-382_st | -0.1517304611 | 0.3901556010 |
| 202863_at | hsa-miR-4633-5p_st | -0.1517188694 | 0.3901921624 |
| 218285_s_at | hsa-miR-523_st | -0.1517188694 | 0.3901921624 |
| 200906_s_at | hsa-miR-1180_st | -0.1514132926 | 0.3911567018 |
| 211376_s_at | hsa-miR-4423-3p_st | -0.1514132926 | 0.3911567018 |
| 201256_at | hsa-miR-4423-3p_st | -0.1511077158 | 0.3921226365 |
| 209265_s_at | hsa-miR-375_st | -0.1508021390 | 0.3930899656 |
| 201666_at | hsa-miR-361-5p_st | -0.1504965623 | 0.3940586882 |
| 203120_at | hsa-miR-4423-3p_st | -0.1504965623 | 0.3940586882 |
| 211672_s_at | hsa-miR-4652-3p_st | -0.1504965623 | 0.3940586882 |
| 201274_at | hsa-miR-361-5p_st | -0.1501909855 | 0.3950288033 |
| 202863_at | hsa-miR-1912_st | -0.1495798319 | 0.3969732076 |
| 212687_at | hsa-miR-4423-3p_st | -0.1495798319 | 0.3969732076 |
| 221741_s_at | hsa-miR-4633-5p_st | -0.1495798319 | 0.3969732076 |
| 200673_at | hsa-miR-4423-3p_st | -0.1492742552 | 0.3979474949 |
| 207922_s_at | hsa-miR-3676_st | -0.1492742552 | 0.3979474949 |
| 212063_at | hsa-miR-361-5p_st | -0.1492742552 | 0.3979474949 |
| 218200_s_at | hsa-miR-34b_st | -0.1492742552 | 0.3979474949 |
| 202975_s_at | hsa-miR-4720-3p_st | -0.1489686784 | 0.3989231710 |
| 212501_at | hsa-miR-1180_st | -0.1489686784 | 0.3989231710 |
| 221667_s_at | hsa-miR-3676_st | -0.1483575248 | 0.4008786857 |
| 205609_at | hsa-miR-382_st | -0.1482160597 | 0.4013321235 |
| 209243_s_at | hsa-miR-4652-3p_st | -0.1480519481 | 0.4018585223 |
| 212217_at | hsa-miR-4652-3p_st | -0.1480519481 | 0.4018585223 |
| 211769_x_at | hsa-miR-4652-3p_st | -0.1477463713 | 0.4028397439 |
| 201161_s_at | hsa-miR-383_st | -0.1474407945 | 0.4038223493 |
| 218048_at | hsa-miR-4423-3p_st | -0.1474407945 | 0.4038223493 |
| 208813_at | hsa-miR-4652-3p_st | -0.1471352177 | 0.4048063377 |
| 205609_at | hsa-miR-3189-5p_st | -0.1468296409 | 0.4057917078 |
| 212977_at | hsa-miR-555_st | -0.1466880591 | 0.4062487242 |
| 209732_at | hsa-miR-4311_st | -0.1460768589 | 0.4082250355 |
| 200989_at | hsa-miR-382_st | -0.1459240588 | 0.4087199752 |
| 202929_s_at | hsa-miR-4652-3p_st | -0.1459129106 | 0.4087560991 |
| 206989_s_at | hsa-miR-1180_st | -0.1459129106 | 0.4087560991 |
| 210105_s_at | hsa-miR-523_st | -0.1459129106 | 0.4087560991 |
| 201656_at | hsa-miR-555_st | -0.1457712587 | 0.4092152594 |
| 208991_at | hsa-miR-134_st | -0.1456073338 | 0.4097469863 |
| 217780_at | hsa-miR-4652-3p_st | -0.1456073338 | 0.4097469863 |
| 200701_at | hsa-miR-382_st | -0.1454656586 | 0.4102068607 |
| 200797_s_at | hsa-miR-3189-5p_st | -0.1453017571 | 0.4107392502 |
| 208679_s_at | hsa-miR-4423-3p_st | -0.1453017571 | 0.4107392502 |
| 218005_at | hsa-miR-555_st | -0.1450072584 | 0.4116968429 |
| 204326_x_at | hsa-miR-3189-5p_st | -0.1449961803 | 0.4117328895 |
| 206805_at | hsa-miR-4423-3p_st | -0.1449961803 | 0.4117328895 |
| 214829_at | hsa-miR-4633-5p_st | -0.1446906035 | 0.4127279034 |
| 202920_at | hsa-miR-4652-3p_st | -0.1443850267 | 0.4137242905 |
| 209104_s_at | hsa-miR-34b_st | -0.1443850267 | 0.4137242905 |
| 221741_s_at | hsa-miR-361-5p_st | -0.1440794500 | 0.4147220500 |
| 210033_s_at | hsa-miR-383_st | -0.1437738732 | 0.4157211806 |
| 210068_s_at | hsa-miR-4633-5p_st | -0.1437738732 | 0.4157211806 |
| 213005_s_at | hsa-miR-1180_st | -0.1437738732 | 0.4157211806 |
| 202936_s_at | hsa-miR-4762-5p_st | -0.1434682964 | 0.4167216812 |
| 210592_s_at | hsa-miR-3676_st | -0.1434682964 | 0.4167216812 |
| 207198_s_at | hsa-miR-4760-3p_st | -0.1431627196 | 0.4177235507 |
| 212990_at | hsa-miR-4423-3p_st | -0.1431627196 | 0.4177235507 |
| 207643_s_at | hsa-miR-4311_st | -0.1428680576 | 0.4186909305 |
| 201859_at | hsa-miR-4760-3p_st | -0.1428571429 | 0.4187267880 |
| 212977_at | hsa-miR-1180_st | -0.1428571429 | 0.4187267880 |
| 217947_at | hsa-miR-3676_st | -0.1428571429 | 0.4187267880 |
| 201066_at | hsa-miR-4652-3p_st | -0.1425515661 | 0.4197313919 |
| 202071_at | hsa-miR-4760-3p_st | -0.1425515661 | 0.4197313919 |
| 207643_s_at | hsa-miR-134_st | -0.1425515661 | 0.4197313919 |
| 212217_at | hsa-miR-3676_st | -0.1425515661 | 0.4197313919 |
| 214022_s_at | hsa-miR-3676_st | -0.1425515661 | 0.4197313919 |
| 210906_x_at | hsa-miR-555_st | -0.1424096574 | 0.4201983905 |
| 201172_x_at | hsa-miR-212_st | -0.1422459893 | 0.4207373612 |
| 202149_at | hsa-miR-523_st | -0.1422459893 | 0.4207373612 |
| 218292_s_at | hsa-miR-4423-3p_st | -0.1422459893 | 0.4207373612 |
| 207088_s_at | hsa-miR-375_st | -0.1419404125 | 0.4217446949 |
| 218432_at | hsa-miR-4423-3p_st | -0.1419404125 | 0.4217446949 |
| 213217_at | hsa-miR-555_st | -0.1416456571 | 0.4227176476 |
| 200638_s_at | hsa-miR-4652-3p_st | -0.1416348358 | 0.4227533916 |
| 201172_x_at | hsa-miR-1180_st | -0.1416348358 | 0.4227533916 |
| 202427_s_at | hsa-miR-375_st | -0.1416348358 | 0.4227533916 |
| 217546_at | hsa-miR-3189-5p_st | -0.1416348358 | 0.4227533916 |
| 222043_at | hsa-miR-134_st | -0.1416348358 | 0.4227533916 |
| 222043_at | hsa-miR-555_st | -0.1414928570 | 0.4232225212 |
| 201656_at | hsa-miR-134_st | -0.1413292590 | 0.4237634502 |
| 218559_s_at | hsa-miR-134_st | -0.1413292590 | 0.4237634502 |
| 200812_at | hsa-miR-4652-3p_st | -0.1410236822 | 0.4247748696 |
| 211999_at | hsa-miR-523_st | -0.1408816568 | 0.4252454182 |
| 208779_x_at | hsa-miR-555_st | -0.1407288567 | 0.4257519923 |
| 200906_s_at | hsa-miR-4633-5p_st | -0.1407181054 | 0.4257876485 |
| 201859_at | hsa-miR-212_st | -0.1407181054 | 0.4257876485 |
| 203685_at | hsa-miR-3676_st | -0.1407181054 | 0.4257876485 |
| 217746_s_at | hsa-miR-370_st | -0.1404125286 | 0.4268017857 |
| 200822_x_at | hsa-miR-34b_st | -0.1401069519 | 0.4278172800 |
| 202779_s_at | hsa-miR-1180_st | -0.1398013751 | 0.4288341302 |
| 213217_at | hsa-miR-134_st | -0.1398013751 | 0.4288341302 |
| 218656_s_at | hsa-miR-4423-3p_st | -0.1398013751 | 0.4288341302 |
| 200786_at | hsa-miR-4423-3p_st | -0.1391902215 | 0.4308718931 |
| 203854_at | hsa-miR-1180_st | -0.1391902215 | 0.4308718931 |
| 208690_s_at | hsa-miR-370_st | -0.1391902215 | 0.4308718931 |
| 209183_s_at | hsa-miR-1180_st | -0.1391902215 | 0.4308718931 |
| 213011_s_at | hsa-miR-34b_st | -0.1391902215 | 0.4308718931 |
| 201161_s_at | hsa-miR-523_st | -0.1388846448 | 0.4318928033 |
| 211318_s_at | hsa-miR-4652-3p_st | -0.1388846448 | 0.4318928033 |
| 212099_at | hsa-miR-1180_st | -0.1388846448 | 0.4318928033 |
| 202071_at | hsa-miR-383_st | -0.1385790680 | 0.4329150644 |
| 212099_at | hsa-miR-212_st | -0.1385790680 | 0.4329150644 |
| 202587_s_at | hsa-miR-555_st | -0.1382840557 | 0.4339032639 |
| 202430_s_at | hsa-miR-370_st | -0.1382734912 | 0.4339386751 |
| 210033_s_at | hsa-miR-555_st | -0.1379784556 | 0.4349282546 |
| 214428_x_at | hsa-miR-3676_st | -0.1379679144 | 0.4349636340 |
| 213592_at | hsa-miR-555_st | -0.1376728555 | 0.4359545925 |
| 203645_s_at | hsa-miR-377-star_st | -0.1376623377 | 0.4359899399 |
| 218854_at | hsa-miR-212_st | -0.1376623377 | 0.4359899399 |
| 212067_s_at | hsa-miR-134_st | -0.1370511841 | 0.4380465873 |
| 202779_s_at | hsa-miR-4652-3p_st | -0.1367456073 | 0.4390769263 |
| 210427_x_at | hsa-miR-361-5p_st | -0.1364400306 | 0.4401086069 |
| 209108_at | hsa-miR-555_st | -0.1362976549 | 0.4405897498 |
| 201859_at | hsa-miR-3676_st | -0.1361344538 | 0.4411416279 |
| 203540_at | hsa-miR-3676_st | -0.1361344538 | 0.4411416279 |
| 208659_at | hsa-miR-134_st | -0.1361344538 | 0.4411416279 |
| 210117_at | hsa-miR-1180_st | -0.1361344538 | 0.4411416279 |
| 201666_at | hsa-miR-4633-5p_st | -0.1358288770 | 0.4421759880 |
| 209513_s_at | hsa-miR-4423-3p_st | -0.1358288770 | 0.4421759880 |
| 213552_at | hsa-miR-34b_st | -0.1358288770 | 0.4421759880 |
| 205097_at | hsa-miR-361-5p_st | -0.1355233002 | 0.4432116856 |
| 207988_s_at | hsa-miR-134_st | -0.1355233002 | 0.4432116856 |
| 209476_at | hsa-miR-1180_st | -0.1352177235 | 0.4442487196 |
| 213911_s_at | hsa-miR-34b_st | -0.1352177235 | 0.4442487196 |
| 207643_s_at | hsa-miR-4762-5p_st | -0.1349121467 | 0.4452870884 |
| 207761_s_at | hsa-miR-383_st | -0.1349121467 | 0.4452870884 |
| 212716_s_at | hsa-miR-34b_st | -0.1349121467 | 0.4452870884 |
| 213227_at | hsa-miR-4423-3p_st | -0.1349121467 | 0.4452870884 |
| 217947_at | hsa-miR-4762-5p_st | -0.1349121467 | 0.4452870884 |
| 200986_at | hsa-miR-134_st | -0.1346065699 | 0.4463267908 |
| 204787_at | hsa-miR-1912_st | -0.1346065699 | 0.4463267908 |
| 202149_at | hsa-miR-361-5p_st | -0.1343009931 | 0.4473678252 |
| 210117_at | hsa-miR-383_st | -0.1339954163 | 0.4484101904 |
| 202936_s_at | hsa-miR-383_st | -0.1336898396 | 0.4494538849 |
| 203540_at | hsa-miR-370_st | -0.1336898396 | 0.4494538849 |
| 209732_at | hsa-miR-523_st | -0.1336898396 | 0.4494538849 |
| 202863_at | hsa-miR-361-5p_st | -0.1333842628 | 0.4504989072 |
| 212110_at | hsa-miR-3189-5p_st | -0.1333842628 | 0.4504989072 |
| 200967_at | hsa-miR-361-5p_st | -0.1327731092 | 0.4525929297 |
| 209243_s_at | hsa-miR-3676_st | -0.1327731092 | 0.4525929297 |
| 213887_s_at | hsa-miR-4652-3p_st | -0.1327731092 | 0.4525929297 |
| 208731_at | hsa-miR-4423-3p_st | -0.1324675325 | 0.4536419270 |
| 201319_at | hsa-miR-3189-5p_st | -0.1321619557 | 0.4546922463 |
| 217730_at | hsa-miR-4311_st | -0.1320192532 | 0.4551831915 |
| 208991_at | hsa-miR-523_st | -0.1318563789 | 0.4557438863 |
| 201319_at | hsa-miR-132_st | -0.1315508021 | 0.4567968454 |
| 201753_s_at | hsa-miR-3676_st | -0.1315508021 | 0.4567968454 |
| 203455_s_at | hsa-miR-370_st | -0.1315508021 | 0.4567968454 |
| 204194_at | hsa-miR-3189-5p_st | -0.1315508021 | 0.4567968454 |
| 204787_at | hsa-miR-377-star_st | -0.1315508021 | 0.4567968454 |
| 213011_s_at | hsa-miR-4652-3p_st | -0.1315508021 | 0.4567968454 |
| 215506_s_at | hsa-miR-34b_st | -0.1315508021 | 0.4567968454 |
| 221263_s_at | hsa-miR-34b_st | -0.1315508021 | 0.4567968454 |
| 204787_at | hsa-miR-361-5p_st | -0.1312452254 | 0.4578511221 |
| 201324_at | hsa-miR-1180_st | -0.1309396486 | 0.4589067150 |
| 202430_s_at | hsa-miR-1912_st | -0.1309396486 | 0.4589067150 |
| 203854_at | hsa-miR-370_st | -0.1309396486 | 0.4589067150 |
| 212099_at | hsa-miR-3189-5p_st | -0.1309396486 | 0.4589067150 |
| 218854_at | hsa-miR-1180_st | -0.1309396486 | 0.4589067150 |
| 204787_at | hsa-miR-555_st | -0.1307968527 | 0.4594004438 |
| 203540_at | hsa-miR-555_st | -0.1306440526 | 0.4599290807 |
| 203987_at | hsa-miR-4311_st | -0.1306440526 | 0.4599290807 |
| 1007_s_at | hsa-miR-361-5p_st | -0.1306340718 | 0.4599636225 |
| 200701_at | hsa-miR-3189-5p_st | -0.1306340718 | 0.4599636225 |
| 218005_at | hsa-miR-4762-5p_st | -0.1306340718 | 0.4599636225 |
| 201756_at | hsa-miR-4423-3p_st | -0.1300229183 | 0.4620813753 |
| 206989_s_at | hsa-miR-212_st | -0.1300229183 | 0.4620813753 |
| 212099_at | hsa-miR-383_st | -0.1300229183 | 0.4620813753 |
| 218854_at | hsa-miR-383_st | -0.1300229183 | 0.4620813753 |
| 200701_at | hsa-miR-523_st | -0.1297173415 | 0.4631422175 |
| 200989_at | hsa-miR-212_st | -0.1297173415 | 0.4631422175 |
| 202269_x_at | hsa-miR-361-5p_st | -0.1297173415 | 0.4631422175 |
| 206989_s_at | hsa-miR-523_st | -0.1297173415 | 0.4631422175 |
| 201172_x_at | hsa-miR-523_st | -0.1294117647 | 0.4642043683 |
| 201398_s_at | hsa-miR-134_st | -0.1294117647 | 0.4642043683 |
| 200989_at | hsa-miR-4423-3p_st | -0.1288006112 | 0.4663325891 |
| 201029_s_at | hsa-miR-361-5p_st | -0.1288006112 | 0.4663325891 |
| 200797_s_at | hsa-miR-4633-5p_st | -0.1284950344 | 0.4673986560 |
| 202430_s_at | hsa-miR-1180_st | -0.1284950344 | 0.4673986560 |
| 212687_at | hsa-miR-361-5p_st | -0.1284950344 | 0.4673986560 |
| 1255_g_at | hsa-miR-34b_st | -0.1281894576 | 0.4684660252 |
| 202863_at | hsa-miR-1180_st | -0.1278838808 | 0.4695346950 |
| 208818_s_at | hsa-miR-4423-3p_st | -0.1278838808 | 0.4695346950 |
| 212038_s_at | hsa-miR-4423-3p_st | -0.1278838808 | 0.4695346950 |
| 200989_at | hsa-miR-132_st | -0.1275783040 | 0.4706046638 |
| 203411_s_at | hsa-miR-4633-5p_st | -0.1275783040 | 0.4706046638 |
| 207643_s_at | hsa-miR-555_st | -0.1274352513 | 0.4711060050 |
| 202096_s_at | hsa-miR-134_st | -0.1272727273 | 0.4716759301 |
| 202471_s_at | hsa-miR-34b_st | -0.1272727273 | 0.4716759301 |
| 210906_x_at | hsa-miR-1180_st | -0.1272727273 | 0.4716759301 |
| 212687_at | hsa-miR-212_st | -0.1272727273 | 0.4716759301 |
| 209732_at | hsa-miR-212_st | -0.1269671505 | 0.4727484923 |
| 212015_x_at | hsa-miR-3676_st | -0.1269671505 | 0.4727484923 |
| 218285_s_at | hsa-miR-1180_st | -0.1269671505 | 0.4727484923 |
| 210592_s_at | hsa-miR-212_st | -0.1266615737 | 0.4738223486 |
| 201761_at | hsa-miR-4633-5p_st | -0.1263559969 | 0.4748974975 |
| 212015_x_at | hsa-miR-361-5p_st | -0.1260504202 | 0.4759739373 |
| 202543_s_at | hsa-miR-134_st | -0.1257448434 | 0.4770516664 |
| 203137_at | hsa-miR-34b_st | -0.1257448434 | 0.4770516664 |
| 222043_at | hsa-miR-3676_st | -0.1257448434 | 0.4770516664 |
| 212099_at | hsa-miR-3676_st | -0.1254392666 | 0.4781306831 |
| 212501_at | hsa-miR-555_st | -0.1252960505 | 0.4786368337 |
| 201443_s_at | hsa-miR-4423-3p_st | -0.1251336898 | 0.4792109857 |
| 205413_at | hsa-miR-4423-3p_st | -0.1251336898 | 0.4792109857 |
| 200701_at | hsa-miR-370_st | -0.1248281131 | 0.4802925726 |
| 219582_at | hsa-miR-370_st | -0.1248281131 | 0.4802925726 |
| 200663_at | hsa-miR-3676_st | -0.1245225363 | 0.4813754420 |
| 218226_s_at | hsa-miR-4423-3p_st | -0.1245225363 | 0.4813754420 |
| 221263_s_at | hsa-miR-4652-3p_st | -0.1245225363 | 0.4813754420 |
| 200053_at | hsa-miR-34b_st | -0.1242169595 | 0.4824595923 |
| 208581_x_at | hsa-miR-3676_st | -0.1242169595 | 0.4824595923 |
| 217947_at | hsa-miR-34b_st | -0.1239113827 | 0.4835450217 |
| 218285_s_at | hsa-miR-3189-5p_st | -0.1239113827 | 0.4835450217 |
| 201324_at | hsa-miR-370_st | -0.1236058060 | 0.4846317286 |
| 208761_s_at | hsa-miR-4423-3p_st | -0.1236058060 | 0.4846317286 |
| 209183_s_at | hsa-miR-555_st | -0.1233096497 | 0.4856861512 |
| 214150_x_at | hsa-miR-3189-5p_st | -0.1233002292 | 0.4857197112 |
| 201106_at | hsa-miR-34b_st | -0.1229946524 | 0.4868089677 |
| 218160_at | hsa-miR-4423-3p_st | -0.1229946524 | 0.4868089677 |
| 205119_s_at | hsa-miR-4652-3p_st | -0.1226890756 | 0.4878994965 |
| 206099_at | hsa-miR-134_st | -0.1226890756 | 0.4878994965 |
| 222043_at | hsa-miR-361-5p_st | -0.1226890756 | 0.4878994965 |
| 210817_s_at | hsa-miR-134_st | -0.1223834989 | 0.4889912958 |
| 202149_at | hsa-miR-4423-3p_st | -0.1220779221 | 0.4900843638 |
| 208981_at | hsa-miR-134_st | -0.1220779221 | 0.4900843638 |
| 213533_at | hsa-miR-3676_st | -0.1220779221 | 0.4900843638 |
| 217947_at | hsa-miR-370_st | -0.1220779221 | 0.4900843638 |
| 219760_at | hsa-miR-4652-3p_st | -0.1220779221 | 0.4900843638 |
| 200701_at | hsa-miR-212_st | -0.1217723453 | 0.4911786987 |
| 209732_at | hsa-miR-3676_st | -0.1217723453 | 0.4911786987 |
| 213572_s_at | hsa-miR-523_st | -0.1217723453 | 0.4911786987 |
| 217906_at | hsa-miR-4423-3p_st | -0.1214667685 | 0.4922742987 |
| 210972_x_at | hsa-miR-34b_st | -0.1208556150 | 0.4944692872 |
| 212015_x_at | hsa-miR-134_st | -0.1208556150 | 0.4944692872 |
| 202863_at | hsa-miR-382_st | -0.1207120486 | 0.4949856443 |
| 202920_at | hsa-miR-3676_st | -0.1205500382 | 0.4955686720 |
| 202325_s_at | hsa-miR-4423-3p_st | -0.1202444614 | 0.4966693147 |
| 200677_at | hsa-miR-4633-5p_st | -0.1199388846 | 0.4977712135 |
| 210033_s_at | hsa-miR-1180_st | -0.1199388846 | 0.4977712135 |
| 211404_s_at | hsa-miR-3676_st | -0.1199388846 | 0.4977712135 |
| 202149_at | hsa-miR-555_st | -0.1197952483 | 0.4982895946 |
| 202096_s_at | hsa-miR-4633-5p_st | -0.1196333079 | 0.4988743666 |
| 207198_s_at | hsa-miR-1912_st | -0.1196333079 | 0.4988743666 |
| 208998_at | hsa-miR-134_st | -0.1196333079 | 0.4988743666 |
| 201988_s_at | hsa-miR-4423-3p_st | -0.1193277311 | 0.4999787722 |
| 208581_x_at | hsa-miR-3189-5p_st | -0.1193277311 | 0.4999787722 |
| 209183_s_at | hsa-miR-383_st | -0.1193277311 | 0.4999787722 |
| 213572_s_at | hsa-miR-4633-5p_st | -0.1193277311 | 0.4999787722 |
| 217947_at | hsa-miR-4633-5p_st | -0.1193277311 | 0.4999787722 |
| 205119_s_at | hsa-miR-375_st | -0.1190221543 | 0.5010844283 |
| 210962_s_at | hsa-miR-34b_st | -0.1190221543 | 0.5010844283 |
| 207831_x_at | hsa-miR-34b_st | -0.1187165775 | 0.5021913332 |
| 217546_at | hsa-miR-523_st | -0.1187165775 | 0.5021913332 |
| 221009_s_at | hsa-miR-1180_st | -0.1187165775 | 0.5021913332 |
| 200600_at | hsa-miR-383_st | -0.1181054240 | 0.5044088816 |
| 201146_at | hsa-miR-523_st | -0.1174942704 | 0.5066314024 |
| 201272_at | hsa-miR-4423-3p_st | -0.1174942704 | 0.5066314024 |
| 221741_s_at | hsa-miR-523_st | -0.1174942704 | 0.5066314024 |
| 211999_at | hsa-miR-3189-5p_st | -0.1171976472 | 0.5077118900 |
| 201859_at | hsa-miR-361-5p_st | -0.1171886937 | 0.5077445227 |
| 202370_s_at | hsa-miR-4633-5p_st | -0.1171886937 | 0.5077445227 |
| 209513_s_at | hsa-miR-361-5p_st | -0.1171886937 | 0.5077445227 |
| 219911_s_at | hsa-miR-555_st | -0.1170448472 | 0.5082689387 |
| 202096_s_at | hsa-miR-3676_st | -0.1168831169 | 0.5088588803 |
| 204787_at | hsa-miR-132_st | -0.1168831169 | 0.5088588803 |
| 209476_at | hsa-miR-4423-3p_st | -0.1168831169 | 0.5088588803 |
| 212977_at | hsa-miR-4633-5p_st | -0.1168831169 | 0.5088588803 |
| 212067_s_at | hsa-miR-361-5p_st | -0.1165775401 | 0.5099744733 |
| 217936_at | hsa-miR-4633-5p_st | -0.1165775401 | 0.5099744733 |
| 218854_at | hsa-miR-3676_st | -0.1165775401 | 0.5099744733 |
| 202863_at | hsa-miR-4762-5p_st | -0.1162719633 | 0.5110912999 |
| 202864_s_at | hsa-miR-4423-3p_st | -0.1162719633 | 0.5110912999 |
| 201887_at | hsa-miR-370_st | -0.1159663866 | 0.5122093580 |
| 203455_s_at | hsa-miR-383_st | -0.1159663866 | 0.5122093580 |
| 209122_at | hsa-miR-3676_st | -0.1159663866 | 0.5122093580 |
| 200798_x_at | hsa-miR-4423-3p_st | -0.1156608098 | 0.5133286458 |
| 204326_x_at | hsa-miR-3676_st | -0.1156608098 | 0.5133286458 |
| 221667_s_at | hsa-miR-555_st | -0.1155168465 | 0.5138563898 |
| 202936_s_at | hsa-miR-4423-3p_st | -0.1153552330 | 0.5144491612 |
| 210906_x_at | hsa-miR-4423-3p_st | -0.1150496562 | 0.5155709022 |
| 212407_at | hsa-miR-134_st | -0.1147440794 | 0.5166938670 |
| 221796_at | hsa-miR-3189-5p_st | -0.1147440794 | 0.5166938670 |
| 201512_s_at | hsa-miR-4652-3p_st | -0.1144385027 | 0.5178180535 |
| 207198_s_at | hsa-miR-3676_st | -0.1144385027 | 0.5178180535 |
| 210592_s_at | hsa-miR-1180_st | -0.1144385027 | 0.5178180535 |
| 206356_s_at | hsa-miR-134_st | -0.1141329259 | 0.5189434597 |
| 210749_x_at | hsa-miR-134_st | -0.1141329259 | 0.5189434597 |
| 218507_at | hsa-miR-3189-5p_st | -0.1141329259 | 0.5189434597 |
| 221796_at | hsa-miR-1180_st | -0.1141329259 | 0.5189434597 |
| 203455_s_at | hsa-miR-555_st | -0.1139888459 | 0.5194745132 |
| 208809_s_at | hsa-miR-370_st | -0.1138273491 | 0.5200700837 |
| 212110_at | hsa-miR-132_st | -0.1138273491 | 0.5200700837 |
| 203455_s_at | hsa-miR-3676_st | -0.1135217723 | 0.5211979233 |
| 204766_s_at | hsa-miR-361-5p_st | -0.1135217723 | 0.5211979233 |
| 218526_s_at | hsa-miR-34b_st | -0.1135217723 | 0.5211979233 |
| 206989_s_at | hsa-miR-4423-3p_st | -0.1129106188 | 0.5234572416 |
| 219911_s_at | hsa-miR-523_st | -0.1126050420 | 0.5245887161 |
| 202096_s_at | hsa-miR-555_st | -0.1123080453 | 0.5256895783 |
| 203704_s_at | hsa-miR-555_st | -0.1120024451 | 0.5268235187 |
| 200816_s_at | hsa-miR-4423-3p_st | -0.1119938885 | 0.5268552859 |
| 204194_at | hsa-miR-1912_st | -0.1119938885 | 0.5268552859 |
| 202825_at | hsa-miR-4423-3p_st | -0.1116883117 | 0.5279903770 |
| 210817_s_at | hsa-miR-3676_st | -0.1116883117 | 0.5279903770 |
| 210749_x_at | hsa-miR-361-5p_st | -0.1113827349 | 0.5291266694 |
| 213503_x_at | hsa-miR-361-5p_st | -0.1113827349 | 0.5291266694 |
| 218214_at | hsa-miR-361-5p_st | -0.1113827349 | 0.5291266694 |
| 217947_at | hsa-miR-382_st | -0.1112384448 | 0.5296636315 |
| 208860_s_at | hsa-miR-4652-3p_st | -0.1110771581 | 0.5302641611 |
| 210033_s_at | hsa-miR-132_st | -0.1107715814 | 0.5314028500 |
| 219628_at | hsa-miR-4652-3p_st | -0.1107715814 | 0.5314028500 |
| 202252_at | hsa-miR-3676_st | -0.1104660046 | 0.5325427339 |
| 200986_at | hsa-miR-361-5p_st | -0.1101604278 | 0.5336838108 |
| 217546_at | hsa-miR-134_st | -0.1101604278 | 0.5336838108 |
| 217947_at | hsa-miR-361-5p_st | -0.1101604278 | 0.5336838108 |
| 202863_at | hsa-miR-370_st | -0.1095492743 | 0.5359695351 |
| 212460_at | hsa-miR-370_st | -0.1095492743 | 0.5359695351 |
| 201989_s_at | hsa-miR-4423-3p_st | -0.1092436975 | 0.5371141782 |
| 208981_at | hsa-miR-523_st | -0.1092436975 | 0.5371141782 |
| 217746_s_at | hsa-miR-523_st | -0.1092436975 | 0.5371141782 |
| 209248_at | hsa-miR-4423-3p_st | -0.1090992440 | 0.5376556910 |
| 202936_s_at | hsa-miR-4633-5p_st | -0.1089381207 | 0.5382600057 |
| 210592_s_at | hsa-miR-523_st | -0.1086325439 | 0.5394070155 |
| 208451_s_at | hsa-miR-3189-5p_st | -0.1080213904 | 0.5417045733 |
| 210105_s_at | hsa-miR-555_st | -0.1078768435 | 0.5422486683 |
| 200797_s_at | hsa-miR-523_st | -0.1077158136 | 0.5428551169 |
| 203685_at | hsa-miR-4762-5p_st | -0.1077158136 | 0.5428551169 |
| 222216_s_at | hsa-miR-3676_st | -0.1074102368 | 0.5440068342 |
| 217936_at | hsa-miR-555_st | -0.1072656432 | 0.5445522151 |
| 213217_at | hsa-miR-4311_st | -0.1071128432 | 0.5451288341 |
| 203685_at | hsa-miR-4423-3p_st | -0.1071046600 | 0.5451597228 |
| 213227_at | hsa-miR-3676_st | -0.1071046600 | 0.5451597228 |
| 222043_at | hsa-miR-4423-3p_st | -0.1071046600 | 0.5451597228 |
| 200798_x_at | hsa-miR-523_st | -0.1067990833 | 0.5463137806 |
| 202376_at | hsa-miR-134_st | -0.1064935065 | 0.5474690053 |
| 203854_at | hsa-miR-134_st | -0.1064935065 | 0.5474690053 |
| 206989_s_at | hsa-miR-361-5p_st | -0.1064935065 | 0.5474690053 |
| 207079_s_at | hsa-miR-4423-3p_st | -0.1064935065 | 0.5474690053 |
| 209104_s_at | hsa-miR-134_st | -0.1064935065 | 0.5474690053 |
| 205758_at | hsa-miR-134_st | -0.1061879297 | 0.5486253948 |
| 208981_at | hsa-miR-4633-5p_st | -0.1061879297 | 0.5486253948 |
| 212099_at | hsa-miR-4762-5p_st | -0.1061879297 | 0.5486253948 |
| 213592_at | hsa-miR-361-5p_st | -0.1061879297 | 0.5486253948 |
| 203120_at | hsa-miR-3189-5p_st | -0.1058823529 | 0.5497829468 |
| 211672_s_at | hsa-miR-4423-3p_st | -0.1058823529 | 0.5497829468 |
| 218882_s_at | hsa-miR-4423-3p_st | -0.1058823529 | 0.5497829468 |
| 200077_s_at | hsa-miR-3676_st | -0.1055767762 | 0.5509416590 |
| 204068_at | hsa-miR-134_st | -0.1055767762 | 0.5509416590 |
| 203120_at | hsa-miR-1180_st | -0.1052711994 | 0.5521015293 |
| 217959_s_at | hsa-miR-4423-3p_st | -0.1052711994 | 0.5521015293 |
| 207812_s_at | hsa-miR-4423-3p_st | -0.1043544691 | 0.5555880655 |
| 203685_at | hsa-miR-4311_st | -0.1042096420 | 0.5561398236 |
| 211971_s_at | hsa-miR-3676_st | -0.1040568419 | 0.5567222364 |
| 200600_at | hsa-miR-370_st | -0.1040488923 | 0.5567525452 |
| 201322_at | hsa-miR-4652-3p_st | -0.1040488923 | 0.5567525452 |
| 219911_s_at | hsa-miR-3676_st | -0.1040488923 | 0.5567525452 |
| 200967_at | hsa-miR-4311_st | -0.1037512418 | 0.5578879220 |
| 201590_x_at | hsa-miR-361-5p_st | -0.1037433155 | 0.5579181714 |
| 212460_at | hsa-miR-383_st | -0.1037433155 | 0.5579181714 |
| 218120_s_at | hsa-miR-134_st | -0.1037433155 | 0.5579181714 |
| 208818_s_at | hsa-miR-4311_st | -0.1031400416 | 0.5602227246 |
| 210117_at | hsa-miR-370_st | -0.1031321620 | 0.5602528547 |
| 202071_at | hsa-miR-555_st | -0.1025288413 | 0.5625620870 |
| 211658_at | hsa-miR-3676_st | -0.1025210084 | 0.5625920968 |
| 209671_x_at | hsa-miR-34b_st | -0.1022154316 | 0.5637634216 |
| 204787_at | hsa-miR-3676_st | -0.1019098549 | 0.5649358791 |
| 212099_at | hsa-miR-4652-3p_st | -0.1019098549 | 0.5649358791 |
| 213011_s_at | hsa-miR-3676_st | -0.1019098549 | 0.5649358791 |
| 217730_at | hsa-miR-361-5p_st | -0.1019098549 | 0.5649358791 |
| 201761_at | hsa-miR-523_st | -0.1016042781 | 0.5661094670 |
| 203621_at | hsa-miR-4423-3p_st | -0.1016042781 | 0.5661094670 |
| 210117_at | hsa-miR-3189-5p_st | -0.1016042781 | 0.5661094670 |
| 212716_s_at | hsa-miR-4652-3p_st | -0.1016042781 | 0.5661094670 |
| 201172_x_at | hsa-miR-134_st | -0.1012987013 | 0.5672841828 |
| 208869_s_at | hsa-miR-3676_st | -0.1012987013 | 0.5672841828 |
| 212321_at | hsa-miR-4423-3p_st | -0.1012987013 | 0.5672841828 |
| 220329_s_at | hsa-miR-4423-3p_st | -0.1012987013 | 0.5672841828 |
| 1007_s_at | hsa-miR-134_st | -0.1009931245 | 0.5684600243 |
| 200804_at | hsa-miR-523_st | -0.1009931245 | 0.5684600243 |
| 202591_s_at | hsa-miR-4423-3p_st | -0.1009931245 | 0.5684600243 |
| 202863_at | hsa-miR-132_st | -0.1006875477 | 0.5696369890 |
| 208869_s_at | hsa-miR-4652-3p_st | -0.1006875477 | 0.5696369890 |
| 205633_s_at | hsa-miR-3676_st | -0.1003819710 | 0.5708150746 |
| 201753_s_at | hsa-miR-4633-5p_st | -0.1000763942 | 0.5719942786 |
| 214629_x_at | hsa-miR-4423-3p_st | -0.1000763942 | 0.5719942786 |
| 218854_at | hsa-miR-370_st | -0.1000763942 | 0.5719942786 |
| 202658_at | hsa-miR-3676_st | -0.0997708174 | 0.5731745988 |
| 215884_s_at | hsa-miR-4423-3p_st | -0.0997708174 | 0.5731745988 |
| 212110_at | hsa-miR-1912_st | -0.0994652406 | 0.5743560325 |
| 200673_at | hsa-miR-361-5p_st | -0.0988540871 | 0.5767222313 |
| 205280_at | hsa-miR-4423-3p_st | -0.0988540871 | 0.5767222313 |
| 207198_s_at | hsa-miR-132_st | -0.0988540871 | 0.5767222313 |
| 210068_s_at | hsa-miR-4423-3p_st | -0.0988540871 | 0.5767222313 |
| 210105_s_at | hsa-miR-370_st | -0.0988540871 | 0.5767222313 |
| 200662_s_at | hsa-miR-4423-3p_st | -0.0982429335 | 0.5790928556 |
| 200708_at | hsa-miR-3676_st | -0.0982429335 | 0.5790928556 |
| 201484_at | hsa-miR-4423-3p_st | -0.0979373568 | 0.5802798212 |
| 214629_x_at | hsa-miR-361-5p_st | -0.0979373568 | 0.5802798212 |
| 202564_x_at | hsa-miR-134_st | -0.0976317800 | 0.5814678858 |
| 217780_at | hsa-miR-34b_st | -0.0976317800 | 0.5814678858 |
| 200862_at | hsa-miR-361-5p_st | -0.0973262032 | 0.5826570470 |
| 201859_at | hsa-miR-4423-3p_st | -0.0973262032 | 0.5826570470 |
| 210033_s_at | hsa-miR-370_st | -0.0973262032 | 0.5826570470 |
| 211999_at | hsa-miR-4423-3p_st | -0.0965696389 | 0.5856059419 |
| 202613_at | hsa-miR-4423-3p_st | -0.0961038961 | 0.5874246074 |
| 209513_s_at | hsa-miR-3189-5p_st | -0.0961038961 | 0.5874246074 |
| 214150_x_at | hsa-miR-134_st | -0.0961038961 | 0.5874246074 |
| 213592_at | hsa-miR-3189-5p_st | -0.0957983193 | 0.5886192140 |
| 214428_x_at | hsa-miR-3189-5p_st | -0.0957983193 | 0.5886192140 |
| 211318_s_at | hsa-miR-3676_st | -0.0951871658 | 0.5910116692 |
| 208581_x_at | hsa-miR-361-5p_st | -0.0948815890 | 0.5922095129 |
| 212501_at | hsa-miR-4760-3p_st | -0.0948815890 | 0.5922095129 |
| 202471_s_at | hsa-miR-134_st | -0.0945760122 | 0.5934084305 |
| 203150_at | hsa-miR-4423-3p_st | -0.0945760122 | 0.5934084305 |
| 205119_s_at | hsa-miR-4720-3p_st | -0.0945760122 | 0.5934084305 |
| 210927_x_at | hsa-miR-34b_st | -0.0945760122 | 0.5934084305 |
| 201319_at | hsa-miR-3676_st | -0.0942704354 | 0.5946084195 |
| 215522_at | hsa-miR-34b_st | -0.0942704354 | 0.5946084195 |
| 202430_s_at | hsa-miR-3189-5p_st | -0.0939648587 | 0.5958094774 |
| 209598_at | hsa-miR-3676_st | -0.0939648587 | 0.5958094774 |
| 211971_s_at | hsa-miR-4633-5p_st | -0.0938192378 | 0.5963822100 |
| 202071_at | hsa-miR-3189-5p_st | -0.0936592819 | 0.5970116016 |
| 203645_s_at | hsa-miR-132_st | -0.0936592819 | 0.5970116016 |
| 202430_s_at | hsa-miR-132_st | -0.0933537051 | 0.5982147894 |
| 210068_s_at | hsa-miR-1180_st | -0.0933537051 | 0.5982147894 |
| 212110_at | hsa-miR-1180_st | -0.0933537051 | 0.5982147894 |
| 213496_at | hsa-miR-4423-3p_st | -0.0930481283 | 0.5994190384 |
| 218788_s_at | hsa-miR-4423-3p_st | -0.0930481283 | 0.5994190384 |
| 209513_s_at | hsa-miR-555_st | -0.0929024374 | 0.5999935652 |
| 203120_at | hsa-miR-523_st | -0.0927425516 | 0.6006243459 |
| 203455_s_at | hsa-miR-212_st | -0.0924369748 | 0.6018307093 |
| 205005_s_at | hsa-miR-4423-3p_st | -0.0924369748 | 0.6018307093 |
| 208799_at | hsa-miR-4423-3p_st | -0.0924369748 | 0.6018307093 |
| 210033_s_at | hsa-miR-3189-5p_st | -0.0924369748 | 0.6018307093 |
| 210240_s_at | hsa-miR-361-5p_st | -0.0921313980 | 0.6030381261 |
| 219297_at | hsa-miR-4423-3p_st | -0.0921313980 | 0.6030381261 |
| 200916_at | hsa-miR-134_st | -0.0918258212 | 0.6042465936 |
| 200905_x_at | hsa-miR-4633-5p_st | -0.0915202445 | 0.6054561091 |
| 218706_s_at | hsa-miR-4633-5p_st | -0.0915202445 | 0.6054561091 |
| 203156_at | hsa-miR-4423-3p_st | -0.0909090909 | 0.6078782740 |
| 204326_x_at | hsa-miR-361-5p_st | -0.0909090909 | 0.6078782740 |
| 210278_s_at | hsa-miR-4423-3p_st | -0.0909090909 | 0.6078782740 |
| 212727_at | hsa-miR-4423-3p_st | -0.0909090909 | 0.6078782740 |
| 218559_s_at | hsa-miR-4633-5p_st | -0.0909090909 | 0.6078782740 |
| 218654_s_at | hsa-miR-4423-3p_st | -0.0909090909 | 0.6078782740 |
| 218982_s_at | hsa-miR-4423-3p_st | -0.0909090909 | 0.6078782740 |
| 200989_at | hsa-miR-555_st | -0.0907632366 | 0.6084569494 |
| 203415_at | hsa-miR-4423-3p_st | -0.0906035141 | 0.6090909180 |
| 203987_at | hsa-miR-523_st | -0.0906035141 | 0.6090909180 |
| 204554_at | hsa-miR-555_st | -0.0904576364 | 0.6096701817 |
| 200853_at | hsa-miR-4423-3p_st | -0.0902979374 | 0.6103045995 |
| 201012_at | hsa-miR-361-5p_st | -0.0902979374 | 0.6103045995 |
| 212887_at | hsa-miR-4423-3p_st | -0.0902979374 | 0.6103045995 |
| 218322_s_at | hsa-miR-523_st | -0.0902979374 | 0.6103045995 |
| 221958_s_at | hsa-miR-555_st | -0.0899992363 | 0.6114919725 |
| 205609_at | hsa-miR-370_st | -0.0899923606 | 0.6115193158 |
| 203094_at | hsa-miR-4423-3p_st | -0.0893812070 | 0.6139518424 |
| 210946_at | hsa-miR-4633-5p_st | -0.0890756303 | 0.6151696472 |
| 218005_at | hsa-miR-361-5p_st | -0.0890756303 | 0.6151696472 |
| 219355_at | hsa-miR-3676_st | -0.0890756303 | 0.6151696472 |
| 201172_x_at | hsa-miR-34b_st | -0.0887700535 | 0.6163884762 |
| 201324_at | hsa-miR-4633-5p_st | -0.0887700535 | 0.6163884762 |
| 208998_at | hsa-miR-523_st | -0.0887700535 | 0.6163884762 |
| 203645_s_at | hsa-miR-3189-5p_st | -0.0884644767 | 0.6176083265 |
| 216218_s_at | hsa-miR-4423-3p_st | -0.0884644767 | 0.6176083265 |
| 207507_s_at | hsa-miR-4423-3p_st | -0.0881588999 | 0.6188291956 |
| 213217_at | hsa-miR-361-5p_st | -0.0878533231 | 0.6200510807 |
| 217960_s_at | hsa-miR-4423-3p_st | -0.0878533231 | 0.6200510807 |
| 201066_at | hsa-miR-3676_st | -0.0872421696 | 0.6224978878 |
| 202864_s_at | hsa-miR-370_st | -0.0872421696 | 0.6224978878 |
| 217730_at | hsa-miR-134_st | -0.0872421696 | 0.6224978878 |
| 213572_s_at | hsa-miR-134_st | -0.0869365928 | 0.6237228045 |
| 202868_s_at | hsa-miR-4423-3p_st | -0.0866310160 | 0.6249487262 |
| 208457_at | hsa-miR-361-5p_st | -0.0866310160 | 0.6249487262 |
| 214150_x_at | hsa-miR-523_st | -0.0866310160 | 0.6249487262 |
| 204194_at | hsa-miR-382_st | -0.0863320348 | 0.6261491578 |
| 207761_s_at | hsa-miR-361-5p_st | -0.0863254393 | 0.6261756501 |
| 207761_s_at | hsa-miR-3189-5p_st | -0.0863254393 | 0.6261756501 |
| 212110_at | hsa-miR-4760-3p_st | -0.0863254393 | 0.6261756501 |
| 222230_s_at | hsa-miR-4423-3p_st | -0.0863254393 | 0.6261756501 |
| 209303_at | hsa-miR-361-5p_st | -0.0861792347 | 0.6267630309 |
| 202587_s_at | hsa-miR-3189-5p_st | -0.0857142857 | 0.6286324938 |
| 203645_s_at | hsa-miR-1912_st | -0.0857142857 | 0.6286324938 |
| 209183_s_at | hsa-miR-382_st | -0.0855680345 | 0.6292210157 |
| 208827_at | hsa-miR-4423-3p_st | -0.0854087089 | 0.6298624080 |
| 209476_at | hsa-miR-212_st | -0.0854087089 | 0.6298624080 |
| 209513_s_at | hsa-miR-3676_st | -0.0854087089 | 0.6298624080 |
| 200677_at | hsa-miR-4423-3p_st | -0.0851031322 | 0.6310933133 |
| 218854_at | hsa-miR-523_st | -0.0851031322 | 0.6310933133 |
| 213005_s_at | hsa-miR-4423-3p_st | -0.0847975554 | 0.6323252071 |
| 214629_x_at | hsa-miR-134_st | -0.0847975554 | 0.6323252071 |
| 202507_s_at | hsa-miR-4423-3p_st | -0.0844919786 | 0.6335580864 |
| 208581_x_at | hsa-miR-555_st | -0.0843456340 | 0.6341488762 |
| 202587_s_at | hsa-miR-3676_st | -0.0841864018 | 0.6347919484 |
| 202863_at | hsa-miR-523_st | -0.0841864018 | 0.6347919484 |
| 214428_x_at | hsa-miR-382_st | -0.0838872338 | 0.6360008826 |
| 201859_at | hsa-miR-523_st | -0.0838808251 | 0.6360267905 |
| 203455_s_at | hsa-miR-4760-3p_st | -0.0838808251 | 0.6360267905 |
| 221667_s_at | hsa-miR-361-5p_st | -0.0838808251 | 0.6360267905 |
| 200967_at | hsa-miR-370_st | -0.0835752483 | 0.6372626096 |
| 209183_s_at | hsa-miR-370_st | -0.0835752483 | 0.6372626096 |
| 208809_s_at | hsa-miR-1180_st | -0.0832696715 | 0.6384994031 |
| 210972_x_at | hsa-miR-134_st | -0.0832696715 | 0.6384994031 |
| 200989_at | hsa-miR-370_st | -0.0826585180 | 0.6409759015 |
| 208826_x_at | hsa-miR-4423-3p_st | -0.0826585180 | 0.6409759015 |
| 208832_at | hsa-miR-4423-3p_st | -0.0817417876 | 0.6446978850 |
| 208697_s_at | hsa-miR-134_st | -0.0814362108 | 0.6459404642 |
| 201313_at | hsa-miR-4652-3p_st | -0.0811306341 | 0.6471839978 |
| 211962_s_at | hsa-miR-361-5p_st | -0.0811306341 | 0.6471839978 |
| 202936_s_at | hsa-miR-555_st | -0.0809840326 | 0.6477809248 |
| 202149_at | hsa-miR-4633-5p_st | -0.0808250573 | 0.6484284827 |
| 202149_at | hsa-miR-3676_st | -0.0808250573 | 0.6484284827 |
| 202587_s_at | hsa-miR-4633-5p_st | -0.0808250573 | 0.6484284827 |
| 48531_at | hsa-miR-3676_st | -0.0808250573 | 0.6484284827 |
| 200720_s_at | hsa-miR-4423-3p_st | -0.0805194805 | 0.6496739160 |
| 204787_at | hsa-miR-4762-5p_st | -0.0799083270 | 0.6521676164 |
| 209476_at | hsa-miR-3189-5p_st | -0.0799083270 | 0.6521676164 |
| 212099_at | hsa-miR-361-5p_st | -0.0799083270 | 0.6521676164 |
| 217882_at | hsa-miR-4423-3p_st | -0.0796088321 | 0.6533910244 |
| 200079_s_at | hsa-miR-34b_st | -0.0796027502 | 0.6534158777 |
| 212460_at | hsa-miR-1180_st | -0.0796027502 | 0.6534158777 |
| 212687_at | hsa-miR-134_st | -0.0796027502 | 0.6534158777 |
| 210068_s_at | hsa-miR-134_st | -0.0792971734 | 0.6546650757 |
| 208451_s_at | hsa-miR-382_st | -0.0791504319 | 0.6552652872 |
| 203120_at | hsa-miR-361-5p_st | -0.0786860199 | 0.6571662703 |
| 205005_s_at | hsa-miR-134_st | -0.0786860199 | 0.6571662703 |
| 208909_at | hsa-miR-4423-3p_st | -0.0786860199 | 0.6571662703 |
| 202382_s_at | hsa-miR-34b_st | -0.0783804431 | 0.6584182611 |
| 203645_s_at | hsa-miR-1180_st | -0.0783804431 | 0.6584182611 |
| 212460_at | hsa-miR-212_st | -0.0780748663 | 0.6596711768 |
| 219960_s_at | hsa-miR-4423-3p_st | -0.0780748663 | 0.6596711768 |
| 221796_at | hsa-miR-555_st | -0.0777752313 | 0.6609006255 |
| 207508_at | hsa-miR-4423-3p_st | -0.0777692895 | 0.6609250146 |
| 204554_at | hsa-miR-34b_st | -0.0774637128 | 0.6621797714 |
| 208451_s_at | hsa-miR-370_st | -0.0771581360 | 0.6634354443 |
| 208809_s_at | hsa-miR-4633-5p_st | -0.0771581360 | 0.6634354443 |
| 212961_x_at | hsa-miR-4423-3p_st | -0.0771581360 | 0.6634354443 |
| 210817_s_at | hsa-miR-34b_st | -0.0768525592 | 0.6646920302 |
| 203723_at | hsa-miR-134_st | -0.0767056309 | 0.6652965497 |
| 201180_s_at | hsa-miR-134_st | -0.0765469824 | 0.6659495263 |
| 200077_s_at | hsa-miR-4652-3p_st | -0.0762414057 | 0.6672079295 |
| 201161_s_at | hsa-miR-1180_st | -0.0759358289 | 0.6684672367 |
| 212110_at | hsa-miR-382_st | -0.0756360305 | 0.6697036065 |
| 200641_s_at | hsa-miR-4423-3p_st | -0.0756302521 | 0.6697274451 |
| 200989_at | hsa-miR-383_st | -0.0756302521 | 0.6697274451 |
| 202641_at | hsa-miR-4423-3p_st | -0.0756302521 | 0.6697274451 |
| 211404_s_at | hsa-miR-4652-3p_st | -0.0756302521 | 0.6697274451 |
| 213911_s_at | hsa-miR-4423-3p_st | -0.0756302521 | 0.6697274451 |
| 215171_s_at | hsa-miR-4423-3p_st | -0.0756302521 | 0.6697274451 |
| 219911_s_at | hsa-miR-4762-5p_st | -0.0756302521 | 0.6697274451 |
| 205217_at | hsa-miR-4423-3p_st | -0.0753246753 | 0.6709885515 |
| 210105_s_at | hsa-miR-4633-5p_st | -0.0753246753 | 0.6709885515 |
| 201412_at | hsa-miR-4423-3p_st | -0.0750190985 | 0.6722505528 |
| 202071_at | hsa-miR-134_st | -0.0750190985 | 0.6722505528 |
| 202121_s_at | hsa-miR-134_st | -0.0750190985 | 0.6722505528 |
| 204194_at | hsa-miR-1180_st | -0.0750190985 | 0.6722505528 |
| 211595_s_at | hsa-miR-134_st | -0.0750190985 | 0.6722505528 |
| 202864_s_at | hsa-miR-361-5p_st | -0.0747135218 | 0.6735134462 |
| 219911_s_at | hsa-miR-361-5p_st | -0.0747135218 | 0.6735134462 |
| 202121_s_at | hsa-miR-4423-3p_st | -0.0744079450 | 0.6747772285 |
| 210117_at | hsa-miR-555_st | -0.0739552298 | 0.6766511628 |
| 205758_at | hsa-miR-34b_st | -0.0734912147 | 0.6785738786 |
| 203704_s_at | hsa-miR-1180_st | -0.0731856379 | 0.6798411861 |
| 207761_s_at | hsa-miR-1180_st | -0.0731856379 | 0.6798411861 |
| 210968_s_at | hsa-miR-361-5p_st | -0.0731856379 | 0.6798411861 |
| 208451_s_at | hsa-miR-555_st | -0.0728856294 | 0.6810862505 |
| 201313_at | hsa-miR-3676_st | -0.0728800611 | 0.6811093672 |
| 201725_at | hsa-miR-134_st | -0.0725744843 | 0.6823784189 |
| 203540_at | hsa-miR-361-5p_st | -0.0725744843 | 0.6823784189 |
| 204119_s_at | hsa-miR-4423-3p_st | -0.0722689076 | 0.6836483380 |
| 207088_s_at | hsa-miR-4652-3p_st | -0.0722689076 | 0.6836483380 |
| 218854_at | hsa-miR-134_st | -0.0722689076 | 0.6836483380 |
| 203613_s_at | hsa-miR-4423-3p_st | -0.0719688290 | 0.6848962487 |
| 201066_at | hsa-miR-134_st | -0.0719633308 | 0.6849191214 |
| 201887_at | hsa-miR-3676_st | -0.0719633308 | 0.6849191214 |
| 210406_s_at | hsa-miR-4633-5p_st | -0.0716577540 | 0.6861907661 |
| 212407_at | hsa-miR-361-5p_st | -0.0716577540 | 0.6861907661 |
| 215307_at | hsa-miR-4423-3p_st | -0.0716577540 | 0.6861907661 |
| 210817_s_at | hsa-miR-4633-5p_st | -0.0713521772 | 0.6874632689 |
| 218559_s_at | hsa-miR-555_st | -0.0708992286 | 0.6893510395 |
| 202863_at | hsa-miR-4423-3p_st | -0.0707410237 | 0.6900108365 |
| 212092_at | hsa-miR-3676_st | -0.0707410237 | 0.6900108365 |
| 202133_at | hsa-miR-370_st | -0.0704354469 | 0.6912858949 |
| 209108_at | hsa-miR-134_st | -0.0701298701 | 0.6925617990 |
| 212195_at | hsa-miR-34b_st | -0.0701298701 | 0.6925617990 |
| 221009_s_at | hsa-miR-555_st | -0.0699824282 | 0.6931777290 |
| 200097_s_at | hsa-miR-4423-3p_st | -0.0698242934 | 0.6938385455 |
| 203137_at | hsa-miR-4652-3p_st | -0.0698242934 | 0.6938385455 |
| 205489_at | hsa-miR-4423-3p_st | -0.0698242934 | 0.6938385455 |
| 208809_s_at | hsa-miR-555_st | -0.0695240280 | 0.6950939176 |
| 214428_x_at | hsa-miR-555_st | -0.0695240280 | 0.6950939176 |
| 201512_s_at | hsa-miR-134_st | -0.0695187166 | 0.6951161314 |
| 201887_at | hsa-miR-4423-3p_st | -0.0695187166 | 0.6951161314 |
| 204194_at | hsa-miR-383_st | -0.0695187166 | 0.6951161314 |
| 219421_at | hsa-miR-4423-3p_st | -0.0695187166 | 0.6951161314 |
| 217820_s_at | hsa-miR-134_st | -0.0689075630 | 0.6976738084 |
| 217837_s_at | hsa-miR-4423-3p_st | -0.0689075630 | 0.6976738084 |
| 44669_at | hsa-miR-361-5p_st | -0.0689075630 | 0.6976738084 |
| 203120_at | hsa-miR-4633-5p_st | -0.0682964095 | 0.7002348047 |
| 205097_at | hsa-miR-134_st | -0.0682964095 | 0.7002348047 |
| 217780_at | hsa-miR-370_st | -0.0682964095 | 0.7002348047 |
| 218200_s_at | hsa-miR-134_st | -0.0682964095 | 0.7002348047 |
| 200818_at | hsa-miR-4423-3p_st | -0.0679908327 | 0.7015165396 |
| 205119_s_at | hsa-miR-34c-3p_st | -0.0679908327 | 0.7015165396 |
| 209476_at | hsa-miR-361-5p_st | -0.0679908327 | 0.7015165396 |
| 200614_at | hsa-miR-3676_st | -0.0678432273 | 0.7021359610 |
| 218226_s_at | hsa-miR-134_st | -0.0676852559 | 0.7027990948 |
| 207922_s_at | hsa-miR-4652-3p_st | -0.0673796791 | 0.7040824670 |
| 213738_s_at | hsa-miR-4423-3p_st | -0.0673796791 | 0.7040824670 |
| 209732_at | hsa-miR-383_st | -0.0670741024 | 0.7053666532 |
| 221696_s_at | hsa-miR-4423-3p_st | -0.0670741024 | 0.7053666532 |
| 212067_s_at | hsa-miR-555_st | -0.0669264270 | 0.7059875496 |
| 204787_at | hsa-miR-3189-5p_st | -0.0664629488 | 0.7079374541 |
| 218507_at | hsa-miR-3676_st | -0.0661573720 | 0.7092240626 |
| 221699_s_at | hsa-miR-3676_st | -0.0661573720 | 0.7092240626 |
| 201628_s_at | hsa-miR-4423-3p_st | -0.0658517953 | 0.7105114720 |
| 203704_s_at | hsa-miR-4423-3p_st | -0.0658517953 | 0.7105114720 |
| 202587_s_at | hsa-miR-370_st | -0.0655462185 | 0.7117996791 |
| 210406_s_at | hsa-miR-4423-3p_st | -0.0655462185 | 0.7117996791 |
| 211902_x_at | hsa-miR-134_st | -0.0655462185 | 0.7117996791 |
| 203302_at | hsa-miR-4423-3p_st | -0.0652406417 | 0.7130886808 |
| 203816_at | hsa-miR-4423-3p_st | -0.0652406417 | 0.7130886808 |
| 209732_at | hsa-miR-1180_st | -0.0649350649 | 0.7143784737 |
| 221009_s_at | hsa-miR-4311_st | -0.0647872261 | 0.7150027619 |
| 212977_at | hsa-miR-134_st | -0.0640183346 | 0.7182525674 |
| 217780_at | hsa-miR-1180_st | -0.0640183346 | 0.7182525674 |
| 201484_at | hsa-miR-3676_st | -0.0637127578 | 0.7195454927 |
| 206803_at | hsa-miR-34b_st | -0.0637127578 | 0.7195454927 |
| 210105_s_at | hsa-miR-3676_st | -0.0637127578 | 0.7195454927 |
| 218201_at | hsa-miR-134_st | -0.0637127578 | 0.7195454927 |
| 218854_at | hsa-miR-4423-3p_st | -0.0634071811 | 0.7208391930 |
| 208809_s_at | hsa-miR-4311_st | -0.0631064254 | 0.7221132357 |
| 200708_at | hsa-miR-1180_st | -0.0627960275 | 0.7234289051 |
| 204787_at | hsa-miR-4423-3p_st | -0.0627960275 | 0.7234289051 |
| 207988_s_at | hsa-miR-4423-3p_st | -0.0627960275 | 0.7234289051 |
| 221688_s_at | hsa-miR-4423-3p_st | -0.0627960275 | 0.7234289051 |
| 202252_at | hsa-miR-134_st | -0.0624904507 | 0.7247249103 |
| 210240_s_at | hsa-miR-134_st | -0.0624904507 | 0.7247249103 |
| 201161_s_at | hsa-miR-370_st | -0.0618792972 | 0.7273192028 |
| 203159_at | hsa-miR-3676_st | -0.0618792972 | 0.7273192028 |
| 205097_at | hsa-miR-4423-3p_st | -0.0618792972 | 0.7273192028 |
| 203854_at | hsa-miR-361-5p_st | -0.0612681436 | 0.7299165156 |
| 205202_at | hsa-miR-4423-3p_st | -0.0612681436 | 0.7299165156 |
| 200673_at | hsa-miR-34b_st | -0.0609625668 | 0.7312162964 |
| 204787_at | hsa-miR-523_st | -0.0609625668 | 0.7312162964 |
| 204194_at | hsa-miR-34b_st | -0.0606569901 | 0.7325168223 |
| 217997_at | hsa-miR-4423-3p_st | -0.0606569901 | 0.7325168223 |
| 221263_s_at | hsa-miR-134_st | -0.0603514133 | 0.7338180901 |
| 206849_at | hsa-miR-3676_st | -0.0600458365 | 0.7351200962 |
| 217995_at | hsa-miR-4633-5p_st | -0.0600458365 | 0.7351200962 |
| 221958_s_at | hsa-miR-4423-3p_st | -0.0597402597 | 0.7364228374 |
| 200822_x_at | hsa-miR-3676_st | -0.0594346830 | 0.7377263104 |
| 201054_at | hsa-miR-4423-3p_st | -0.0594346830 | 0.7377263104 |
| 209549_s_at | hsa-miR-4423-3p_st | -0.0594346830 | 0.7377263104 |
| 205110_s_at | hsa-miR-4423-3p_st | -0.0591291062 | 0.7390305118 |
| 200797_s_at | hsa-miR-4423-3p_st | -0.0582123759 | 0.7429474523 |
| 200967_at | hsa-miR-4633-5p_st | -0.0582123759 | 0.7429474523 |
| 204239_s_at | hsa-miR-34b_st | -0.0582123759 | 0.7429474523 |
| 212460_at | hsa-miR-3676_st | -0.0582123759 | 0.7429474523 |
| 204068_at | hsa-miR-4423-3p_st | -0.0579067991 | 0.7442545333 |
| 207088_s_at | hsa-miR-34b_st | -0.0579067991 | 0.7442545333 |
| 205324_s_at | hsa-miR-4423-3p_st | -0.0576012223 | 0.7455623259 |
| 218316_at | hsa-miR-3676_st | -0.0576012223 | 0.7455623259 |
| 210592_s_at | hsa-miR-4633-5p_st | -0.0572956455 | 0.7468708264 |
| 217995_at | hsa-miR-4423-3p_st | -0.0569900688 | 0.7481800317 |
| 218226_s_at | hsa-miR-4633-5p_st | -0.0569900688 | 0.7481800317 |
| 203455_s_at | hsa-miR-4633-5p_st | -0.0566844920 | 0.7494899382 |
| 210033_s_at | hsa-miR-382_st | -0.0563832227 | 0.7507820630 |
| 202475_at | hsa-miR-134_st | -0.0563789152 | 0.7508005426 |
| 215058_at | hsa-miR-4423-3p_st | -0.0560733384 | 0.7521118413 |
| 206989_s_at | hsa-miR-370_st | -0.0557677617 | 0.7534238311 |
| 212501_at | hsa-miR-361-5p_st | -0.0557677617 | 0.7534238311 |
| 200614_at | hsa-miR-4423-3p_st | -0.0554664223 | 0.7547183006 |
| 202430_s_at | hsa-miR-383_st | -0.0554621849 | 0.7547365084 |
| 203137_at | hsa-miR-3676_st | -0.0554621849 | 0.7547365084 |
| 201319_at | hsa-miR-1180_st | -0.0551566081 | 0.7560498698 |
| 205550_s_at | hsa-miR-3676_st | -0.0551566081 | 0.7560498698 |
| 207643_s_at | hsa-miR-361-5p_st | -0.0551566081 | 0.7560498698 |
| 212195_at | hsa-miR-523_st | -0.0551566081 | 0.7560498698 |
| 210149_s_at | hsa-miR-4423-3p_st | -0.0548510313 | 0.7573639119 |
| 207054_at | hsa-miR-4423-3p_st | -0.0542398778 | 0.7599940244 |
| 218163_at | hsa-miR-4423-3p_st | -0.0542398778 | 0.7599940244 |
| 218384_at | hsa-miR-34b_st | -0.0542398778 | 0.7599940244 |
| 200673_at | hsa-miR-523_st | -0.0536287242 | 0.7626268182 |
| 201398_s_at | hsa-miR-4423-3p_st | -0.0536287242 | 0.7626268182 |
| 201484_at | hsa-miR-134_st | -0.0536287242 | 0.7626268182 |
| 211963_s_at | hsa-miR-4423-3p_st | -0.0536287242 | 0.7626268182 |
| 201274_at | hsa-miR-4423-3p_st | -0.0533231474 | 0.7639442118 |
| 205119_s_at | hsa-miR-382_st | -0.0527160212 | 0.7665635913 |
| 200077_s_at | hsa-miR-34b_st | -0.0524064171 | 0.7679003385 |
| 218507_at | hsa-miR-555_st | -0.0519520209 | 0.7698634463 |
| 205609_at | hsa-miR-4633-5p_st | -0.0517952636 | 0.7705410093 |
| 208869_s_at | hsa-miR-1180_st | -0.0514896868 | 0.7718623101 |
| 202712_s_at | hsa-miR-34b_st | -0.0511841100 | 0.7731842500 |
| 209122_at | hsa-miR-4633-5p_st | -0.0511841100 | 0.7731842500 |
| 203120_at | hsa-miR-555_st | -0.0510352206 | 0.7738285833 |
| 202802_at | hsa-miR-134_st | -0.0508785332 | 0.7745068252 |
| 214428_x_at | hsa-miR-370_st | -0.0508785332 | 0.7745068252 |
| 202071_at | hsa-miR-4423-3p_st | -0.0505729565 | 0.7758300324 |
| 206542_s_at | hsa-miR-3676_st | -0.0505729565 | 0.7758300324 |
| 210448_s_at | hsa-miR-134_st | -0.0505729565 | 0.7758300324 |
| 201145_at | hsa-miR-34b_st | -0.0502673797 | 0.7771538680 |
| 209732_at | hsa-miR-134_st | -0.0502673797 | 0.7771538680 |
| 208809_s_at | hsa-miR-4762-5p_st | -0.0496562261 | 0.7798034102 |
| 210105_s_at | hsa-miR-34b_st | -0.0496562261 | 0.7798034102 |
| 217746_s_at | hsa-miR-4423-3p_st | -0.0496562261 | 0.7798034102 |
| 218224_at | hsa-miR-4423-3p_st | -0.0493506494 | 0.7811291098 |
| 206544_x_at | hsa-miR-3676_st | -0.0490450726 | 0.7824554237 |
| 210968_s_at | hsa-miR-134_st | -0.0490450726 | 0.7824554237 |
| 212310_at | hsa-miR-4423-3p_st | -0.0490450726 | 0.7824554237 |
| 219911_s_at | hsa-miR-134_st | -0.0490450726 | 0.7824554237 |
| 221488_s_at | hsa-miR-4423-3p_st | -0.0487394958 | 0.7837823483 |
| 201411_s_at | hsa-miR-4423-3p_st | -0.0481283422 | 0.7864380154 |
| 203645_s_at | hsa-miR-4633-5p_st | -0.0481283422 | 0.7864380154 |
| 208832_at | hsa-miR-3676_st | -0.0481283422 | 0.7864380154 |
| 211271_x_at | hsa-miR-361-5p_st | -0.0481283422 | 0.7864380154 |
| 206099_at | hsa-miR-555_st | -0.0479792193 | 0.7870863699 |
| 200739_s_at | hsa-miR-4423-3p_st | -0.0478227655 | 0.7877667509 |
| 211558_s_at | hsa-miR-134_st | -0.0478227655 | 0.7877667509 |
| 200843_s_at | hsa-miR-4423-3p_st | -0.0475171887 | 0.7890960828 |
| 202863_at | hsa-miR-4760-3p_st | -0.0475171887 | 0.7890960828 |
| 210927_x_at | hsa-miR-4423-3p_st | -0.0475171887 | 0.7890960828 |
| 209303_at | hsa-miR-134_st | -0.0473680191 | 0.7897452218 |
| 200989_at | hsa-miR-1180_st | -0.0472116119 | 0.7904260076 |
| 209122_at | hsa-miR-4423-3p_st | -0.0469060351 | 0.7917565218 |
| 209265_s_at | hsa-miR-34b_st | -0.0469060351 | 0.7917565218 |
| 209570_s_at | hsa-miR-34b_st | -0.0469060351 | 0.7917565218 |
| 209671_x_at | hsa-miR-134_st | -0.0469060351 | 0.7917565218 |
| 211297_s_at | hsa-miR-4423-3p_st | -0.0466004584 | 0.7930876217 |
| 201160_s_at | hsa-miR-134_st | -0.0462948816 | 0.7944193038 |
| 201319_at | hsa-miR-212_st | -0.0462948816 | 0.7944193038 |
| 206356_s_at | hsa-miR-361-5p_st | -0.0462948816 | 0.7944193038 |
| 201012_at | hsa-miR-134_st | -0.0459893048 | 0.7957515645 |
| 201656_at | hsa-miR-4423-3p_st | -0.0456837280 | 0.7970844002 |
| 221009_s_at | hsa-miR-361-5p_st | -0.0456837280 | 0.7970844002 |
| 200903_s_at | hsa-miR-134_st | -0.0453781513 | 0.7984178073 |
| 206805_at | hsa-miR-361-5p_st | -0.0450725745 | 0.7997517822 |
| 221263_s_at | hsa-miR-1180_st | -0.0447669977 | 0.8010863212 |
| 200673_at | hsa-miR-134_st | -0.0444614209 | 0.8024214208 |
| 201761_at | hsa-miR-361-5p_st | -0.0444614209 | 0.8024214208 |
| 210872_x_at | hsa-miR-4423-3p_st | -0.0441558442 | 0.8037570774 |
| 217860_at | hsa-miR-3676_st | -0.0441558442 | 0.8037570774 |
| 218976_at | hsa-miR-4423-3p_st | -0.0441558442 | 0.8037570774 |
| 219714_s_at | hsa-miR-4423-3p_st | -0.0438502674 | 0.8050932873 |
| 217546_at | hsa-miR-3676_st | -0.0432391138 | 0.8077673525 |
| 221958_s_at | hsa-miR-134_st | -0.0432391138 | 0.8077673525 |
| 202779_s_at | hsa-miR-361-5p_st | -0.0429335371 | 0.8091052006 |
| 204587_at | hsa-miR-4423-3p_st | -0.0429335371 | 0.8091052006 |
| 208675_s_at | hsa-miR-134_st | -0.0429335371 | 0.8091052006 |
| 209303_at | hsa-miR-4423-3p_st | -0.0426312172 | 0.8104293199 |
| 202936_s_at | hsa-miR-134_st | -0.0426279603 | 0.8104435875 |
| 205633_s_at | hsa-miR-4423-3p_st | -0.0426279603 | 0.8104435875 |
| 203973_s_at | hsa-miR-361-5p_st | -0.0423223835 | 0.8117825096 |
| 221488_s_at | hsa-miR-134_st | -0.0423223835 | 0.8117825096 |
| 206935_at | hsa-miR-4423-3p_st | -0.0420168067 | 0.8131219631 |
| 209476_at | hsa-miR-523_st | -0.0420168067 | 0.8131219631 |
| 202232_s_at | hsa-miR-4423-3p_st | -0.0417112299 | 0.8144619445 |
| 212687_at | hsa-miR-3189-5p_st | -0.0417112299 | 0.8144619445 |
| 211902_x_at | hsa-miR-34b_st | -0.0414056532 | 0.8158024501 |
| 203685_at | hsa-miR-4633-5p_st | -0.0411000764 | 0.8171434763 |
| 204787_at | hsa-miR-370_st | -0.0407944996 | 0.8184850193 |
| 205097_at | hsa-miR-34b_st | -0.0407944996 | 0.8184850193 |
| 202269_x_at | hsa-miR-134_st | -0.0401833461 | 0.8211696412 |
| 204326_x_at | hsa-miR-4633-5p_st | -0.0401833461 | 0.8211696412 |
| 209108_at | hsa-miR-361-5p_st | -0.0401833461 | 0.8211696412 |
| 200638_s_at | hsa-miR-3676_st | -0.0398777693 | 0.8225127128 |
| 207761_s_at | hsa-miR-523_st | -0.0398777693 | 0.8225127128 |
| 202936_s_at | hsa-miR-3189-5p_st | -0.0395721925 | 0.8238562865 |
| 210453_x_at | hsa-miR-4423-3p_st | -0.0395721925 | 0.8238562865 |
| 200967_at | hsa-miR-34b_st | -0.0389610390 | 0.8265449258 |
| 218694_at | hsa-miR-3676_st | -0.0389610390 | 0.8265449258 |
| 201512_s_at | hsa-miR-4423-3p_st | -0.0380443086 | 0.8305815586 |
| 200906_s_at | hsa-miR-4423-3p_st | -0.0377387319 | 0.8319280678 |
| 211270_x_at | hsa-miR-361-5p_st | -0.0377387319 | 0.8319280678 |
| 44669_at | hsa-miR-4423-3p_st | -0.0377387319 | 0.8319280678 |
| 203645_s_at | hsa-miR-370_st | -0.0374331551 | 0.8332750533 |
| 201313_at | hsa-miR-34b_st | -0.0371275783 | 0.8346225114 |
| 201319_at | hsa-miR-361-5p_st | -0.0371275783 | 0.8346225114 |
| 202834_at | hsa-miR-4423-3p_st | -0.0368220015 | 0.8359704383 |
| 208457_at | hsa-miR-4652-3p_st | -0.0368220015 | 0.8359704383 |
| 217546_at | hsa-miR-555_st | -0.0366720148 | 0.8366322139 |
| 208779_x_at | hsa-miR-361-5p_st | -0.0365164248 | 0.8373188303 |
| 201319_at | hsa-miR-370_st | -0.0359052712 | 0.8400169949 |
| 211658_at | hsa-miR-4423-3p_st | -0.0359052712 | 0.8400169949 |
| 217746_s_at | hsa-miR-134_st | -0.0359052712 | 0.8400169949 |
| 200663_at | hsa-miR-4423-3p_st | -0.0355996944 | 0.8413667599 |
| 202741_at | hsa-miR-4423-3p_st | -0.0355996944 | 0.8413667599 |
| 221009_s_at | hsa-miR-4762-5p_st | -0.0355996944 | 0.8413667599 |
| 209157_at | hsa-miR-3676_st | -0.0352941176 | 0.8427169752 |
| 200040_at | hsa-miR-4423-3p_st | -0.0349885409 | 0.8440676370 |
| 218507_at | hsa-miR-523_st | -0.0346829641 | 0.8454187414 |
| 202564_x_at | hsa-miR-34b_st | -0.0343773873 | 0.8467702849 |
| 204194_at | hsa-miR-361-5p_st | -0.0343773873 | 0.8467702849 |
| 210105_s_at | hsa-miR-3189-5p_st | -0.0343773873 | 0.8467702849 |
| 201988_s_at | hsa-miR-3676_st | -0.0337662338 | 0.8494746737 |
| 202078_at | hsa-miR-4423-3p_st | -0.0337662338 | 0.8494746737 |
| 202863_at | hsa-miR-3189-5p_st | -0.0337662338 | 0.8494746737 |
| 203685_at | hsa-miR-3189-5p_st | -0.0337662338 | 0.8494746737 |
| 200804_at | hsa-miR-555_st | -0.0336160135 | 0.8501396697 |
| 203455_s_at | hsa-miR-1180_st | -0.0331550802 | 0.8521807733 |
| 219760_at | hsa-miR-3676_st | -0.0331550802 | 0.8521807733 |
| 203157_s_at | hsa-miR-3676_st | -0.0328495034 | 0.8535344553 |
| 203817_at | hsa-miR-4423-3p_st | -0.0328495034 | 0.8535344553 |
| 217780_at | hsa-miR-361-5p_st | -0.0322383499 | 0.8562430648 |
| 48531_at | hsa-miR-134_st | -0.0322383499 | 0.8562430648 |
| 205705_at | hsa-miR-4423-3p_st | -0.0319327731 | 0.8575979848 |
| 209046_s_at | hsa-miR-4423-3p_st | -0.0319327731 | 0.8575979848 |
| 201753_s_at | hsa-miR-4423-3p_st | -0.0316271963 | 0.8589533099 |
| 205119_s_at | hsa-miR-3189-5p_st | -0.0316271963 | 0.8589533099 |
| 200798_x_at | hsa-miR-361-5p_st | -0.0313216196 | 0.8603090362 |
| 217995_at | hsa-miR-523_st | -0.0313216196 | 0.8603090362 |
| 208832_at | hsa-miR-134_st | -0.0310160428 | 0.8616651601 |
| 210978_s_at | hsa-miR-134_st | -0.0310160428 | 0.8616651601 |
| 201086_x_at | hsa-miR-4423-3p_st | -0.0307104660 | 0.8630216778 |
| 202975_s_at | hsa-miR-3676_st | -0.0297937357 | 0.8670935552 |
| 203645_s_at | hsa-miR-134_st | -0.0294881589 | 0.8684516099 |
| 208818_s_at | hsa-miR-3676_st | -0.0291825821 | 0.8698100393 |
| 200641_s_at | hsa-miR-3676_st | -0.0288770053 | 0.8711688397 |
| 205278_at | hsa-miR-4423-3p_st | -0.0288770053 | 0.8711688397 |
| 209157_at | hsa-miR-134_st | -0.0288770053 | 0.8711688397 |
| 218120_s_at | hsa-miR-361-5p_st | -0.0288770053 | 0.8711688397 |
| 202587_s_at | hsa-miR-382_st | -0.0285736115 | 0.8725182965 |
| 201313_at | hsa-miR-134_st | -0.0282658518 | 0.8738875381 |
| 203685_at | hsa-miR-523_st | -0.0282658518 | 0.8738875381 |
| 208731_at | hsa-miR-3676_st | -0.0279602750 | 0.8752474285 |
| 218133_s_at | hsa-miR-134_st | -0.0279602750 | 0.8752474285 |
| 201319_at | hsa-miR-383_st | -0.0276546982 | 0.8766076745 |
| 204002_s_at | hsa-miR-4423-3p_st | -0.0276546982 | 0.8766076745 |
| 208678_at | hsa-miR-4423-3p_st | -0.0276546982 | 0.8766076745 |
| 201966_at | hsa-miR-4423-3p_st | -0.0267379679 | 0.8806905087 |
| 200626_s_at | hsa-miR-3676_st | -0.0261268144 | 0.8834141064 |
| 200967_at | hsa-miR-4423-3p_st | -0.0258212376 | 0.8847764063 |
| 212820_at | hsa-miR-4423-3p_st | -0.0258212376 | 0.8847764063 |
| 204766_s_at | hsa-miR-555_st | -0.0256704103 | 0.8854489347 |
| 207761_s_at | hsa-miR-4633-5p_st | -0.0255156608 | 0.8861390351 |
| 210406_s_at | hsa-miR-3676_st | -0.0255156608 | 0.8861390351 |
| 202929_s_at | hsa-miR-4423-3p_st | -0.0252100840 | 0.8875019889 |
| 209513_s_at | hsa-miR-134_st | -0.0252100840 | 0.8875019889 |
| 210105_s_at | hsa-miR-1180_st | -0.0252100840 | 0.8875019889 |
| 210978_s_at | hsa-miR-4633-5p_st | -0.0252100840 | 0.8875019889 |
| 205609_at | hsa-miR-4311_st | -0.0250592101 | 0.8881750455 |
| 200097_s_at | hsa-miR-3676_st | -0.0249045073 | 0.8888652639 |
| 200663_at | hsa-miR-134_st | -0.0249045073 | 0.8888652639 |
| 201145_at | hsa-miR-134_st | -0.0249045073 | 0.8888652639 |
| 204002_s_at | hsa-miR-134_st | -0.0249045073 | 0.8888652639 |
| 202587_s_at | hsa-miR-4762-5p_st | -0.0245989305 | 0.8902288562 |
| 218201_at | hsa-miR-4423-3p_st | -0.0245989305 | 0.8902288562 |
| 218854_at | hsa-miR-361-5p_st | -0.0245989305 | 0.8902288562 |
| 211971_s_at | hsa-miR-4652-3p_st | -0.0244480099 | 0.8909024337 |
| 200798_x_at | hsa-miR-34b_st | -0.0242933537 | 0.8915927621 |
| 202868_s_at | hsa-miR-134_st | -0.0239877769 | 0.8929569776 |
| 210033_s_at | hsa-miR-3676_st | -0.0239877769 | 0.8929569776 |
| 208857_s_at | hsa-miR-4423-3p_st | -0.0236822002 | 0.8943214990 |
| 219683_at | hsa-miR-3676_st | -0.0236822002 | 0.8943214990 |
| 209227_at | hsa-miR-4423-3p_st | -0.0233766234 | 0.8956863222 |
| 209507_at | hsa-miR-4423-3p_st | -0.0230710466 | 0.8970514436 |
| 210501_x_at | hsa-miR-134_st | -0.0230710466 | 0.8970514436 |
| 202507_s_at | hsa-miR-361-5p_st | -0.0227654698 | 0.8984168591 |
| 210156_s_at | hsa-miR-4423-3p_st | -0.0227654698 | 0.8984168591 |
| 218882_s_at | hsa-miR-134_st | -0.0227654698 | 0.8984168591 |
| 200701_at | hsa-miR-134_st | -0.0224598930 | 0.8997825651 |
| 200843_s_at | hsa-miR-3676_st | -0.0224598930 | 0.8997825651 |
| 208946_s_at | hsa-miR-361-5p_st | -0.0224598930 | 0.8997825651 |
| 202373_s_at | hsa-miR-4423-3p_st | -0.0221543163 | 0.9011485575 |
| 205279_s_at | hsa-miR-4423-3p_st | -0.0221543163 | 0.9011485575 |
| 208860_s_at | hsa-miR-3676_st | -0.0221543163 | 0.9011485575 |
| 202507_s_at | hsa-miR-3676_st | -0.0218487395 | 0.9025148325 |
| 206849_at | hsa-miR-4652-3p_st | -0.0218487395 | 0.9025148325 |
| 212460_at | hsa-miR-3189-5p_st | -0.0215431627 | 0.9038813864 |
| 212645_x_at | hsa-miR-4423-3p_st | -0.0215431627 | 0.9038813864 |
| 219714_s_at | hsa-miR-134_st | -0.0215431627 | 0.9038813864 |
| 201924_at | hsa-miR-4423-3p_st | -0.0212375859 | 0.9052482150 |
| 200739_s_at | hsa-miR-134_st | -0.0209320092 | 0.9066153147 |
| 208697_s_at | hsa-miR-34b_st | -0.0209320092 | 0.9066153147 |
| 209075_s_at | hsa-miR-3676_st | -0.0209320092 | 0.9066153147 |
| 218706_s_at | hsa-miR-4423-3p_st | -0.0209320092 | 0.9066153147 |
| 203411_s_at | hsa-miR-134_st | -0.0206264324 | 0.9079826816 |
| 211962_s_at | hsa-miR-134_st | -0.0206264324 | 0.9079826816 |
| 219481_at | hsa-miR-4423-3p_st | -0.0200152788 | 0.9107182011 |
| 205512_s_at | hsa-miR-4423-3p_st | -0.0197097021 | 0.9120863460 |
| 213496_at | hsa-miR-134_st | -0.0197097021 | 0.9120863460 |
| 200701_at | hsa-miR-4423-3p_st | -0.0194041253 | 0.9134547425 |
| 202071_at | hsa-miR-1180_st | -0.0190985485 | 0.9148233867 |
| 212887_at | hsa-miR-4633-5p_st | -0.0190985485 | 0.9148233867 |
| 219326_s_at | hsa-miR-134_st | -0.0190985485 | 0.9148233867 |
| 203621_at | hsa-miR-134_st | -0.0187929717 | 0.9161922748 |
| 209732_at | hsa-miR-4633-5p_st | -0.0187929717 | 0.9161922748 |
| 217730_at | hsa-miR-3676_st | -0.0187929717 | 0.9161922748 |
| 221667_s_at | hsa-miR-4423-3p_st | -0.0187929717 | 0.9161922748 |
| 202864_s_at | hsa-miR-3676_st | -0.0184873950 | 0.9175614028 |
| 217936_at | hsa-miR-4423-3p_st | -0.0184873950 | 0.9175614028 |
| 208838_at | hsa-miR-4423-3p_st | -0.0181818182 | 0.9189307668 |
| 208813_at | hsa-miR-3676_st | -0.0175706646 | 0.9216701873 |
| 211404_s_at | hsa-miR-4762-5p_st | -0.0175706646 | 0.9216701873 |
| 212099_at | hsa-miR-523_st | -0.0172650879 | 0.9230402360 |
| 203645_s_at | hsa-miR-4760-3p_st | -0.0169595111 | 0.9244105052 |
| 210240_s_at | hsa-miR-3189-5p_st | -0.0169595111 | 0.9244105052 |
| 201198_s_at | hsa-miR-3676_st | -0.0163483575 | 0.9271516892 |
| 205324_s_at | hsa-miR-3676_st | -0.0163483575 | 0.9271516892 |
| 218005_at | hsa-miR-523_st | -0.0163483575 | 0.9271516892 |
| 218005_at | hsa-miR-34b_st | -0.0163483575 | 0.9271516892 |
| 206805_at | hsa-miR-555_st | -0.0160440065 | 0.9285170969 |
| 221958_s_at | hsa-miR-361-5p_st | -0.0160427807 | 0.9285225962 |
| 212961_x_at | hsa-miR-361-5p_st | -0.0157372040 | 0.9298937081 |
| 208818_s_at | hsa-miR-4633-5p_st | -0.0154316272 | 0.9312650208 |
| 211558_s_at | hsa-miR-361-5p_st | -0.0154316272 | 0.9312650208 |
| 219582_at | hsa-miR-134_st | -0.0154316272 | 0.9312650208 |
| 201410_at | hsa-miR-4633-5p_st | -0.0151260504 | 0.9326365306 |
| 201274_at | hsa-miR-370_st | -0.0148204736 | 0.9340082334 |
| 211769_x_at | hsa-miR-3676_st | -0.0148204736 | 0.9340082334 |
| 48531_at | hsa-miR-4423-3p_st | -0.0148204736 | 0.9340082334 |
| 208779_x_at | hsa-miR-134_st | -0.0145148969 | 0.9353801254 |
| 212407_at | hsa-miR-555_st | -0.0143632058 | 0.9360612141 |
| 208998_at | hsa-miR-4423-3p_st | -0.0142093201 | 0.9367522026 |
| 219326_s_at | hsa-miR-4423-3p_st | -0.0142093201 | 0.9367522026 |
| 202430_s_at | hsa-miR-212_st | -0.0139037433 | 0.9381244612 |
| 210427_x_at | hsa-miR-134_st | -0.0139037433 | 0.9381244612 |
| 213572_s_at | hsa-miR-4423-3p_st | -0.0139037433 | 0.9381244612 |
| 201411_s_at | hsa-miR-555_st | -0.0137520055 | 0.9388059386 |
| 202587_s_at | hsa-miR-4311_st | -0.0135992055 | 0.9394922307 |
| 202975_s_at | hsa-miR-4423-3p_st | -0.0135981665 | 0.9394968971 |
| 203150_at | hsa-miR-361-5p_st | -0.0132925898 | 0.9408695066 |
| 200862_at | hsa-miR-134_st | -0.0129870130 | 0.9422422856 |
| 200883_at | hsa-miR-4423-3p_st | -0.0129870130 | 0.9422422856 |
| 202975_s_at | hsa-miR-4633-5p_st | -0.0129870130 | 0.9422422856 |
| 221531_at | hsa-miR-4423-3p_st | -0.0129870130 | 0.9422422856 |
| 44669_at | hsa-miR-134_st | -0.0129870130 | 0.9422422856 |
| 202325_s_at | hsa-miR-134_st | -0.0123758594 | 0.9449883366 |
| 221874_at | hsa-miR-4423-3p_st | -0.0123758594 | 0.9449883366 |
| 218322_s_at | hsa-miR-4423-3p_st | -0.0120702827 | 0.9463616008 |
| 202427_s_at | hsa-miR-4423-3p_st | -0.0117647059 | 0.9477350188 |
| 203685_at | hsa-miR-4652-3p_st | -0.0117647059 | 0.9477350188 |
| 219960_s_at | hsa-miR-4633-5p_st | -0.0117647059 | 0.9477350188 |
| 221009_s_at | hsa-miR-4633-5p_st | -0.0117647059 | 0.9477350188 |
| 200812_at | hsa-miR-3676_st | -0.0114591291 | 0.9491085867 |
| 202936_s_at | hsa-miR-1180_st | -0.0114591291 | 0.9491085867 |
| 203944_x_at | hsa-miR-4423-3p_st | -0.0114591291 | 0.9491085867 |
| 201512_s_at | hsa-miR-555_st | -0.0111544045 | 0.9504784696 |
| 208839_s_at | hsa-miR-4423-3p_st | -0.0108479756 | 0.9518561567 |
| 210978_s_at | hsa-miR-361-5p_st | -0.0108479756 | 0.9518561567 |
| 210434_x_at | hsa-miR-4423-3p_st | -0.0105423988 | 0.9532301508 |
| 213503_x_at | hsa-miR-134_st | -0.0105423988 | 0.9532301508 |
| 213714_at | hsa-miR-4423-3p_st | -0.0105423988 | 0.9532301508 |
| 217730_at | hsa-miR-4423-3p_st | -0.0105423988 | 0.9532301508 |
| 200978_at | hsa-miR-4423-3p_st | -0.0102368220 | 0.9546042792 |
| 201029_s_at | hsa-miR-134_st | -0.0102368220 | 0.9546042792 |
| 203362_s_at | hsa-miR-4423-3p_st | -0.0102368220 | 0.9546042792 |
| 208675_s_at | hsa-miR-361-5p_st | -0.0102368220 | 0.9546042792 |
| 211271_x_at | hsa-miR-134_st | -0.0099312452 | 0.9559785378 |
| 211376_s_at | hsa-miR-134_st | -0.0099312452 | 0.9559785378 |
| 201172_x_at | hsa-miR-4652-3p_st | -0.0096256684 | 0.9573529227 |
| 202233_s_at | hsa-miR-3676_st | -0.0096256684 | 0.9573529227 |
| 202078_at | hsa-miR-3676_st | -0.0093200917 | 0.9587274300 |
| 203313_s_at | hsa-miR-134_st | -0.0093200917 | 0.9587274300 |
| 202133_at | hsa-miR-361-5p_st | -0.0090145149 | 0.9601020558 |
| 204554_at | hsa-miR-4652-3p_st | -0.0090145149 | 0.9601020558 |
| 208690_s_at | hsa-miR-361-5p_st | -0.0090145149 | 0.9601020558 |
| 211595_s_at | hsa-miR-361-5p_st | -0.0090145149 | 0.9601020558 |
| 203987_at | hsa-miR-4633-5p_st | -0.0087089381 | 0.9614767961 |
| 209570_s_at | hsa-miR-3676_st | -0.0087089381 | 0.9614767961 |
| 215416_s_at | hsa-miR-3676_st | -0.0087089381 | 0.9614767961 |
| 221741_s_at | hsa-miR-34c-5p_st | -0.0087089381 | 0.9614767961 |
| 201322_at | hsa-miR-3676_st | -0.0084033613 | 0.9628516470 |
| 203987_at | hsa-miR-134_st | -0.0084033613 | 0.9628516470 |
| 218559_s_at | hsa-miR-4423-3p_st | -0.0080977846 | 0.9642266045 |
| 200638_s_at | hsa-miR-4423-3p_st | -0.0077922078 | 0.9656016647 |
| 202382_s_at | hsa-miR-3676_st | -0.0077922078 | 0.9656016647 |
| 202936_s_at | hsa-miR-4652-3p_st | -0.0077922078 | 0.9656016647 |
| 212110_at | hsa-miR-370_st | -0.0077922078 | 0.9656016647 |
| 217995_at | hsa-miR-134_st | -0.0074866310 | 0.9669768236 |
| 207717_s_at | hsa-miR-4423-3p_st | -0.0071810542 | 0.9683520774 |
| 218866_s_at | hsa-miR-3676_st | -0.0071810542 | 0.9683520774 |
| 210534_s_at | hsa-miR-134_st | -0.0067232027 | 0.9704128152 |
| 201161_s_at | hsa-miR-4423-3p_st | -0.0065699007 | 0.9711028535 |
| 208869_s_at | hsa-miR-134_st | -0.0065699007 | 0.9711028535 |
| 212407_at | hsa-miR-4423-3p_st | -0.0065699007 | 0.9711028535 |
| 200720_s_at | hsa-miR-3676_st | -0.0062643239 | 0.9724783680 |
| 212961_x_at | hsa-miR-134_st | -0.0062643239 | 0.9724783680 |
| 221796_at | hsa-miR-134_st | -0.0059587471 | 0.9738539615 |
| 203411_s_at | hsa-miR-34b_st | -0.0056531704 | 0.9752296301 |
| 203987_at | hsa-miR-361-5p_st | -0.0053475936 | 0.9766053698 |
| 211658_at | hsa-miR-4633-5p_st | -0.0053475936 | 0.9766053698 |
| 218597_s_at | hsa-miR-4423-3p_st | -0.0053475936 | 0.9766053698 |
| 202929_s_at | hsa-miR-1180_st | -0.0044308633 | 0.9807329763 |
| 203645_s_at | hsa-miR-34b_st | -0.0044308633 | 0.9807329763 |
| 204068_at | hsa-miR-3676_st | -0.0044308633 | 0.9807329763 |
| 204194_at | hsa-miR-523_st | -0.0038197097 | 0.9834849971 |
| 219911_s_at | hsa-miR-4423-3p_st | -0.0038197097 | 0.9834849971 |
| 202077_at | hsa-miR-4423-3p_st | -0.0025974026 | 0.9889895764 |
| 220329_s_at | hsa-miR-3676_st | -0.0019862490 | 0.9917420716 |
| 202868_s_at | hsa-miR-361-5p_st | -0.0016806723 | 0.9931183568 |
| 216218_s_at | hsa-miR-134_st | -0.0016806723 | 0.9931183568 |
| 218214_at | hsa-miR-1180_st | -0.0016806723 | 0.9931183568 |
| 209569_x_at | hsa-miR-3676_st | -0.0013750955 | 0.9944946617 |
| 202233_s_at | hsa-miR-1180_st | -0.0010695187 | 0.9958709825 |
| 202233_s_at | hsa-miR-4423-3p_st | -0.0010695187 | 0.9958709825 |
| 204125_at | hsa-miR-361-5p_st | -0.0007639419 | 0.9972473150 |
| 219263_at | hsa-miR-3676_st | -0.0007639419 | 0.9972473150 |
| 208782_at | hsa-miR-4423-3p_st | -0.0004583652 | 0.9986236555 |
| 211297_s_at | hsa-miR-134_st | -0.0004583652 | 0.9986236555 |
| 201628_s_at | hsa-miR-3676_st | 0.0001527884 | 0.9993118275 |
| 204554_at | hsa-miR-361-5p_st | 0.0001527884 | 0.9993118275 |
| 208832_at | hsa-miR-370_st | 0.0001527884 | 0.9993118275 |
| 203645_s_at | hsa-miR-212_st | 0.0004583652 | 0.9979354846 |
| 207054_at | hsa-miR-134_st | 0.0004583652 | 0.9979354846 |
| 207812_s_at | hsa-miR-3676_st | 0.0004583652 | 0.9979354846 |
| 208838_at | hsa-miR-3676_st | 0.0004583652 | 0.9979354846 |
| 212321_at | hsa-miR-4652-3p_st | 0.0004583652 | 0.9979354846 |
| 202779_s_at | hsa-miR-3676_st | 0.0007639419 | 0.9965591475 |
| 218224_at | hsa-miR-3676_st | 0.0007639419 | 0.9965591475 |
| 218226_s_at | hsa-miR-3676_st | 0.0007639419 | 0.9965591475 |
| 219960_s_at | hsa-miR-3676_st | 0.0007639419 | 0.9965591475 |
| 209476_at | hsa-miR-134_st | 0.0013750955 | 0.9938065070 |
| 221696_s_at | hsa-miR-134_st | 0.0013750955 | 0.9938065070 |
| 202863_at | hsa-miR-212_st | 0.0016806723 | 0.9924302115 |
| 204194_at | hsa-miR-370_st | 0.0016806723 | 0.9924302115 |
| 207198_s_at | hsa-miR-523_st | 0.0016806723 | 0.9924302115 |
| 222360_at | hsa-miR-4423-3p_st | 0.0016806723 | 0.9924302115 |
| 213005_s_at | hsa-miR-3676_st | 0.0019862490 | 0.9910539377 |
| 200804_at | hsa-miR-4633-5p_st | 0.0022918258 | 0.9896776896 |
| 204992_s_at | hsa-miR-3676_st | 0.0022918258 | 0.9896776896 |
| 212977_at | hsa-miR-361-5p_st | 0.0022918258 | 0.9896776896 |
| 202863_at | hsa-miR-4652-3p_st | 0.0029029794 | 0.9869252863 |
| 221874_at | hsa-miR-4311_st | 0.0030560012 | 0.9862361563 |
| 203079_s_at | hsa-miR-3676_st | 0.0032085561 | 0.9855491390 |
| 203189_s_at | hsa-miR-134_st | 0.0032085561 | 0.9855491390 |
| 217957_at | hsa-miR-4423-3p_st | 0.0032085561 | 0.9855491390 |
| 212321_at | hsa-miR-34b_st | 0.0035141329 | 0.9841730332 |
| 210278_s_at | hsa-miR-134_st | 0.0038197097 | 0.9827969729 |
| 217546_at | hsa-miR-361-5p_st | 0.0038197097 | 0.9827969729 |
| 200804_at | hsa-miR-4423-3p_st | 0.0041252865 | 0.9814209620 |
| 217546_at | hsa-miR-4652-3p_st | 0.0041252865 | 0.9814209620 |
| 218160_at | hsa-miR-134_st | 0.0041252865 | 0.9814209620 |
| 218285_s_at | hsa-miR-4652-3p_st | 0.0041252865 | 0.9814209620 |
| 208457_at | hsa-miR-3189-5p_st | 0.0044308633 | 0.9800450044 |
| 207088_s_at | hsa-miR-1180_st | 0.0047364400 | 0.9786691041 |
| 201146_at | hsa-miR-4652-3p_st | 0.0050420168 | 0.9772932651 |
| 212460_at | hsa-miR-523_st | 0.0050420168 | 0.9772932651 |
| 218557_at | hsa-miR-4423-3p_st | 0.0050420168 | 0.9772932651 |
| 203313_s_at | hsa-miR-361-5p_st | 0.0053475936 | 0.9759174913 |
| 205963_s_at | hsa-miR-134_st | 0.0053475936 | 0.9759174913 |
| 210448_s_at | hsa-miR-3676_st | 0.0053475936 | 0.9759174913 |
| 202543_s_at | hsa-miR-4652-3p_st | 0.0059587471 | 0.9731661551 |
| 218005_at | hsa-miR-4652-3p_st | 0.0059587471 | 0.9731661551 |
| 203120_at | hsa-miR-4652-3p_st | 0.0062643239 | 0.9717906006 |
| 203973_s_at | hsa-miR-134_st | 0.0065699007 | 0.9704151271 |
| 217959_s_at | hsa-miR-3676_st | 0.0065699007 | 0.9704151271 |
| 207761_s_at | hsa-miR-4652-3p_st | 0.0068754775 | 0.9690397386 |
| 211376_s_at | hsa-miR-555_st | 0.0070288028 | 0.9683496606 |
| 204072_s_at | hsa-miR-134_st | 0.0071810542 | 0.9676644389 |
| 205775_at | hsa-miR-134_st | 0.0071810542 | 0.9676644389 |
| 205609_at | hsa-miR-555_st | 0.0076400031 | 0.9655990381 |
| 208690_s_at | hsa-miR-134_st | 0.0077922078 | 0.9649141220 |
| 213904_at | hsa-miR-4423-3p_st | 0.0077922078 | 0.9649141220 |
| 210501_x_at | hsa-miR-361-5p_st | 0.0084033613 | 0.9621642080 |
| 211962_s_at | hsa-miR-4423-3p_st | 0.0084033613 | 0.9621642080 |
| 218285_s_at | hsa-miR-34b_st | 0.0084033613 | 0.9621642080 |
| 208731_at | hsa-miR-4311_st | 0.0085568034 | 0.9614738550 |
| 210534_s_at | hsa-miR-4423-3p_st | 0.0088624036 | 0.9600990099 |
| 205097_at | hsa-miR-523_st | 0.0090145149 | 0.9594147284 |
| 206989_s_at | hsa-miR-34b_st | 0.0093200917 | 0.9580401613 |
| 221515_s_at | hsa-miR-3676_st | 0.0093200917 | 0.9580401613 |
| 219683_at | hsa-miR-4423-3p_st | 0.0096256684 | 0.9566657147 |
| 202325_s_at | hsa-miR-361-5p_st | 0.0099312452 | 0.9552913924 |
| 207079_s_at | hsa-miR-3676_st | 0.0099312452 | 0.9552913924 |
| 221667_s_at | hsa-miR-134_st | 0.0099312452 | 0.9552913924 |
| 221699_s_at | hsa-miR-4423-3p_st | 0.0099312452 | 0.9552913924 |
| 210592_s_at | hsa-miR-361-5p_st | 0.0105423988 | 0.9525431367 |
| 218048_at | hsa-miR-3676_st | 0.0105423988 | 0.9525431367 |
| 206042_x_at | hsa-miR-4423-3p_st | 0.0108479756 | 0.9511692112 |
| 208457_at | hsa-miR-555_st | 0.0108488044 | 0.9511654848 |
| 204125_at | hsa-miR-134_st | 0.0114591291 | 0.9484217843 |
| 207198_s_at | hsa-miR-3189-5p_st | 0.0114591291 | 0.9484217843 |
| 217860_at | hsa-miR-4652-3p_st | 0.0114591291 | 0.9484217843 |
| 221263_s_at | hsa-miR-3676_st | 0.0114591291 | 0.9484217843 |
| 211855_s_at | hsa-miR-4423-3p_st | 0.0123758594 | 0.9443017635 |
| 202430_s_at | hsa-miR-4423-3p_st | 0.0126814362 | 0.9429287375 |
| 200030_s_at | hsa-miR-4423-3p_st | 0.0129870130 | 0.9415558751 |
| 206356_s_at | hsa-miR-4423-3p_st | 0.0129870130 | 0.9415558751 |
| 201180_s_at | hsa-miR-4423-3p_st | 0.0132925898 | 0.9401831804 |
| 201590_x_at | hsa-miR-134_st | 0.0132925898 | 0.9401831804 |
| 209243_s_at | hsa-miR-4762-5p_st | 0.0135981665 | 0.9388106572 |
| 211595_s_at | hsa-miR-1180_st | 0.0135981665 | 0.9388106572 |
| 203411_s_at | hsa-miR-4423-3p_st | 0.0139037433 | 0.9374383095 |
| 204326_x_at | hsa-miR-4423-3p_st | 0.0139037433 | 0.9374383095 |
| 205196_s_at | hsa-miR-134_st | 0.0139037433 | 0.9374383095 |
| 207573_x_at | hsa-miR-4423-3p_st | 0.0139037433 | 0.9374383095 |
| 213904_at | hsa-miR-134_st | 0.0139037433 | 0.9374383095 |
| 218005_at | hsa-miR-134_st | 0.0139037433 | 0.9374383095 |
| 208745_at | hsa-miR-134_st | 0.0142093201 | 0.9360661411 |
| 200967_at | hsa-miR-134_st | 0.0145148969 | 0.9346941560 |
| 202863_at | hsa-miR-383_st | 0.0145148969 | 0.9346941560 |
| 218488_at | hsa-miR-1180_st | 0.0145148969 | 0.9346941560 |
| 205119_s_at | hsa-miR-132_st | 0.0148204736 | 0.9333223581 |
| 202614_at | hsa-miR-4423-3p_st | 0.0151260504 | 0.9319507513 |
| 210906_x_at | hsa-miR-134_st | 0.0151260504 | 0.9319507513 |
| 212215_at | hsa-miR-4633-5p_st | 0.0151260504 | 0.9319507513 |
| 207400_at | hsa-miR-4423-3p_st | 0.0154316272 | 0.9305793396 |
| 202370_s_at | hsa-miR-4423-3p_st | 0.0157372040 | 0.9292081268 |
| 203889_at | hsa-miR-4423-3p_st | 0.0157372040 | 0.9292081268 |
| 209104_s_at | hsa-miR-361-5p_st | 0.0157372040 | 0.9292081268 |
| 211270_x_at | hsa-miR-134_st | 0.0157372040 | 0.9292081268 |
| 218557_at | hsa-miR-134_st | 0.0157372040 | 0.9292081268 |
| 220251_at | hsa-miR-4423-3p_st | 0.0157372040 | 0.9292081268 |
| 202863_at | hsa-miR-555_st | 0.0158912064 | 0.9285171493 |
| 205512_s_at | hsa-miR-3676_st | 0.0160427807 | 0.9278371169 |
| 206015_s_at | hsa-miR-134_st | 0.0160427807 | 0.9278371169 |
| 207776_s_at | hsa-miR-4423-3p_st | 0.0160427807 | 0.9278371169 |
| 208868_s_at | hsa-miR-361-5p_st | 0.0163483575 | 0.9264663137 |
| 209598_at | hsa-miR-4423-3p_st | 0.0166539343 | 0.9250957212 |
| 212215_at | hsa-miR-3676_st | 0.0166539343 | 0.9250957212 |
| 210968_s_at | hsa-miR-4423-3p_st | 0.0169595111 | 0.9237253433 |
| 212217_at | hsa-miR-4633-5p_st | 0.0169595111 | 0.9237253433 |
| 206935_at | hsa-miR-134_st | 0.0172650879 | 0.9223551839 |
| 221009_s_at | hsa-miR-4423-3p_st | 0.0172650879 | 0.9223551839 |
| 218292_s_at | hsa-miR-134_st | 0.0178762414 | 0.9196155361 |
| 202149_at | hsa-miR-34b_st | 0.0181818182 | 0.9182460555 |
| 208832_at | hsa-miR-361-5p_st | 0.0184873950 | 0.9168768090 |
| 203189_s_at | hsa-miR-4423-3p_st | 0.0187929717 | 0.9155078005 |
| 206984_s_at | hsa-miR-3676_st | 0.0187929717 | 0.9155078005 |
| 221449_s_at | hsa-miR-3676_st | 0.0187929717 | 0.9155078005 |
| 208826_x_at | hsa-miR-361-5p_st | 0.0190985485 | 0.9141390339 |
| 200862_at | hsa-miR-1180_st | 0.0194041253 | 0.9127705130 |
| 201725_at | hsa-miR-4423-3p_st | 0.0197097021 | 0.9114022418 |
| 208761_s_at | hsa-miR-361-5p_st | 0.0197097021 | 0.9114022418 |
| 210105_s_at | hsa-miR-361-5p_st | 0.0197097021 | 0.9114022418 |
| 210946_at | hsa-miR-3676_st | 0.0197097021 | 0.9114022418 |
| 202382_s_at | hsa-miR-134_st | 0.0200152788 | 0.9100342242 |
| 203156_at | hsa-miR-134_st | 0.0200152788 | 0.9100342242 |
| 209265_s_at | hsa-miR-134_st | 0.0200152788 | 0.9100342242 |
| 209755_at | hsa-miR-134_st | 0.0203208556 | 0.9086664639 |
| 205775_at | hsa-miR-4423-3p_st | 0.0209320092 | 0.9059317313 |
| 210278_s_at | hsa-miR-370_st | 0.0209320092 | 0.9059317313 |
| 216218_s_at | hsa-miR-361-5p_st | 0.0209320092 | 0.9059317313 |
| 209046_s_at | hsa-miR-3676_st | 0.0212375859 | 0.9045647666 |
| 208581_x_at | hsa-miR-4423-3p_st | 0.0215431627 | 0.9031980749 |
| 212600_s_at | hsa-miR-4423-3p_st | 0.0218487395 | 0.9018316599 |
| 205550_s_at | hsa-miR-4423-3p_st | 0.0221543163 | 0.9004655257 |
| 209570_s_at | hsa-miR-4633-5p_st | 0.0221543163 | 0.9004655257 |
| 202149_at | hsa-miR-4652-3p_st | 0.0227654698 | 0.8977341148 |
| 209183_s_at | hsa-miR-523_st | 0.0227654698 | 0.8977341148 |
| 217997_at | hsa-miR-4762-5p_st | 0.0227654698 | 0.8977341148 |
| 202395_at | hsa-miR-4423-3p_st | 0.0230710466 | 0.8963688459 |
| 208826_x_at | hsa-miR-134_st | 0.0230710466 | 0.8963688459 |
| 208946_s_at | hsa-miR-134_st | 0.0230710466 | 0.8963688459 |
| 209733_at | hsa-miR-4423-3p_st | 0.0230710466 | 0.8963688459 |
| 221449_s_at | hsa-miR-361-5p_st | 0.0230710466 | 0.8963688459 |
| 200708_at | hsa-miR-361-5p_st | 0.0233766234 | 0.8950038731 |
| 201274_at | hsa-miR-1180_st | 0.0236822002 | 0.8936392003 |
| 204992_s_at | hsa-miR-4423-3p_st | 0.0236822002 | 0.8936392003 |
| 208675_s_at | hsa-miR-4423-3p_st | 0.0236822002 | 0.8936392003 |
| 210418_s_at | hsa-miR-361-5p_st | 0.0236822002 | 0.8936392003 |
| 215506_s_at | hsa-miR-3676_st | 0.0239877769 | 0.8922748314 |
| 206356_s_at | hsa-miR-3189-5p_st | 0.0242933537 | 0.8909107702 |
| 218283_at | hsa-miR-134_st | 0.0244480099 | 0.8902205207 |
| 203645_s_at | hsa-miR-361-5p_st | 0.0245989305 | 0.8895470206 |
| 208839_s_at | hsa-miR-3676_st | 0.0245989305 | 0.8895470206 |
| 218163_at | hsa-miR-3676_st | 0.0245989305 | 0.8895470206 |
| 202594_at | hsa-miR-4423-3p_st | 0.0249045073 | 0.8881835865 |
| 205705_at | hsa-miR-3676_st | 0.0249045073 | 0.8881835865 |
| 212110_at | hsa-miR-4423-3p_st | 0.0249045073 | 0.8881835865 |
| 212501_at | hsa-miR-4423-3p_st | 0.0249045073 | 0.8881835865 |
| 217837_s_at | hsa-miR-134_st | 0.0249045073 | 0.8881835865 |
| 200626_s_at | hsa-miR-4423-3p_st | 0.0252100840 | 0.8868204716 |
| 200797_s_at | hsa-miR-361-5p_st | 0.0252100840 | 0.8868204716 |
| 211404_s_at | hsa-miR-134_st | 0.0252100840 | 0.8868204716 |
| 212217_at | hsa-miR-4762-5p_st | 0.0252100840 | 0.8868204716 |
| 202507_s_at | hsa-miR-134_st | 0.0255156608 | 0.8854576798 |
| 210068_s_at | hsa-miR-361-5p_st | 0.0255156608 | 0.8854576798 |
| 201319_at | hsa-miR-134_st | 0.0258212376 | 0.8840952150 |
| 201570_at | hsa-miR-361-5p_st | 0.0258212376 | 0.8840952150 |
| 210434_x_at | hsa-miR-134_st | 0.0258212376 | 0.8840952150 |
| 205609_at | hsa-miR-361-5p_st | 0.0261268144 | 0.8827330810 |
| 210946_at | hsa-miR-4652-3p_st | 0.0264323911 | 0.8813712817 |
| 202430_s_at | hsa-miR-361-5p_st | 0.0267379679 | 0.8800098208 |
| 204787_at | hsa-miR-134_st | 0.0267379679 | 0.8800098208 |
| 205005_s_at | hsa-miR-361-5p_st | 0.0267379679 | 0.8800098208 |
| 209243_s_at | hsa-miR-4633-5p_st | 0.0267379679 | 0.8800098208 |
| 212460_at | hsa-miR-34b_st | 0.0267379679 | 0.8800098208 |
| 212887_at | hsa-miR-3676_st | 0.0267379679 | 0.8800098208 |
| 217995_at | hsa-miR-361-5p_st | 0.0267379679 | 0.8800098208 |
| 218597_s_at | hsa-miR-3676_st | 0.0267379679 | 0.8800098208 |
| 210962_s_at | hsa-miR-4423-3p_st | 0.0270435447 | 0.8786487022 |
| 211769_x_at | hsa-miR-4423-3p_st | 0.0273491215 | 0.8772879298 |
| 207508_at | hsa-miR-3676_st | 0.0276546982 | 0.8759275073 |
| 212063_at | hsa-miR-134_st | 0.0276546982 | 0.8759275073 |
| 200883_at | hsa-miR-3676_st | 0.0282658518 | 0.8732077275 |
| 201900_s_at | hsa-miR-3676_st | 0.0282658518 | 0.8732077275 |
| 206062_at | hsa-miR-4423-3p_st | 0.0282658518 | 0.8732077275 |
| 208731_at | hsa-miR-4633-5p_st | 0.0282658518 | 0.8732077275 |
| 212687_at | hsa-miR-523_st | 0.0282658518 | 0.8732077275 |
| 221263_s_at | hsa-miR-370_st | 0.0282658518 | 0.8732077275 |
| 204068_at | hsa-miR-4652-3p_st | 0.0285714286 | 0.8718483778 |
| 210014_x_at | hsa-miR-361-5p_st | 0.0285714286 | 0.8718483778 |
| 218226_s_at | hsa-miR-361-5p_st | 0.0285714286 | 0.8718483778 |
| 208868_s_at | hsa-miR-1180_st | 0.0288770053 | 0.8704893934 |
| 218214_at | hsa-miR-370_st | 0.0288770053 | 0.8704893934 |
| 200818_at | hsa-miR-134_st | 0.0291825821 | 0.8691307780 |
| 212990_at | hsa-miR-3676_st | 0.0291825821 | 0.8691307780 |
| 36711_at | hsa-miR-4423-3p_st | 0.0291825821 | 0.8691307780 |
| 203645_s_at | hsa-miR-4423-3p_st | 0.0294881589 | 0.8677725355 |
| 220251_at | hsa-miR-3676_st | 0.0294881589 | 0.8677725355 |
| 205278_at | hsa-miR-4311_st | 0.0296432119 | 0.8670834920 |
| 200097_s_at | hsa-miR-4633-5p_st | 0.0297937357 | 0.8664146696 |
| 201198_s_at | hsa-miR-4423-3p_st | 0.0297937357 | 0.8664146696 |
| 208868_s_at | hsa-miR-134_st | 0.0297937357 | 0.8664146696 |
| 200906_s_at | hsa-miR-134_st | 0.0300993125 | 0.8650571842 |
| 201761_at | hsa-miR-4423-3p_st | 0.0300993125 | 0.8650571842 |
| 202930_s_at | hsa-miR-4633-5p_st | 0.0300993125 | 0.8650571842 |
| 204787_at | hsa-miR-1180_st | 0.0300993125 | 0.8650571842 |
| 212271_at | hsa-miR-4423-3p_st | 0.0300993125 | 0.8650571842 |
| 215527_at | hsa-miR-4423-3p_st | 0.0300993125 | 0.8650571842 |
| 200600_at | hsa-miR-134_st | 0.0304048892 | 0.8637000831 |
| 201592_at | hsa-miR-4423-3p_st | 0.0304048892 | 0.8637000831 |
| 217730_at | hsa-miR-4633-5p_st | 0.0304048892 | 0.8637000831 |
| 204744_s_at | hsa-miR-4423-3p_st | 0.0307104660 | 0.8623433700 |
| 213887_s_at | hsa-miR-3676_st | 0.0307104660 | 0.8623433700 |
| 212271_at | hsa-miR-134_st | 0.0310160428 | 0.8609870487 |
| 202594_at | hsa-miR-3676_st | 0.0313216196 | 0.8596311231 |
| 203854_at | hsa-miR-4423-3p_st | 0.0313216196 | 0.8596311231 |
| 208697_s_at | hsa-miR-370_st | 0.0313216196 | 0.8596311231 |
| 200798_x_at | hsa-miR-134_st | 0.0316271963 | 0.8582755969 |
| 212157_at | hsa-miR-4423-3p_st | 0.0316271963 | 0.8582755969 |
| 218526_s_at | hsa-miR-4423-3p_st | 0.0316271963 | 0.8582755969 |
| 209243_s_at | hsa-miR-4311_st | 0.0316296127 | 0.8582648794 |
| 214717_at | hsa-miR-4423-3p_st | 0.0319327731 | 0.8569204740 |
| 212217_at | hsa-miR-134_st | 0.0322383499 | 0.8555657580 |
| 202736_s_at | hsa-miR-1180_st | 0.0325439267 | 0.8542114527 |
| 209183_s_at | hsa-miR-4423-3p_st | 0.0325439267 | 0.8542114527 |
| 206356_s_at | hsa-miR-555_st | 0.0326992132 | 0.8535233871 |
| 204194_at | hsa-miR-212_st | 0.0328495034 | 0.8528575621 |
| 201106_at | hsa-miR-4423-3p_st | 0.0331550802 | 0.8515040897 |
| 206099_at | hsa-miR-4762-5p_st | 0.0331550802 | 0.8515040897 |
| 210014_x_at | hsa-miR-1180_st | 0.0331550802 | 0.8515040897 |
| 209303_at | hsa-miR-1180_st | 0.0333104134 | 0.8508162434 |
| 36711_at | hsa-miR-361-5p_st | 0.0334606570 | 0.8501510394 |
| 210927_x_at | hsa-miR-134_st | 0.0337662338 | 0.8487984149 |
| 200906_s_at | hsa-miR-361-5p_st | 0.0340718105 | 0.8474462200 |
| 202641_at | hsa-miR-134_st | 0.0340718105 | 0.8474462200 |
| 217546_at | hsa-miR-4423-3p_st | 0.0343773873 | 0.8460944585 |
| 218488_at | hsa-miR-4423-3p_st | 0.0343773873 | 0.8460944585 |
| 218982_s_at | hsa-miR-4311_st | 0.0345328139 | 0.8454070747 |
| 217906_at | hsa-miR-3676_st | 0.0349885409 | 0.8433922505 |
| 217923_at | hsa-miR-4423-3p_st | 0.0349885409 | 0.8433922505 |
| 201322_at | hsa-miR-4423-3p_st | 0.0352941176 | 0.8420418115 |
| 208868_s_at | hsa-miR-3676_st | 0.0352941176 | 0.8420418115 |
| 203704_s_at | hsa-miR-134_st | 0.0355996944 | 0.8406918209 |
| 205031_at | hsa-miR-134_st | 0.0355996944 | 0.8406918209 |
| 206542_s_at | hsa-miR-4423-3p_st | 0.0355996944 | 0.8406918209 |
| 202929_s_at | hsa-miR-3189-5p_st | 0.0359052712 | 0.8393422823 |
| 201410_at | hsa-miR-4311_st | 0.0360608145 | 0.8386555211 |
| 211376_s_at | hsa-miR-3189-5p_st | 0.0362108480 | 0.8379931996 |
| 218488_at | hsa-miR-361-5p_st | 0.0362108480 | 0.8379931996 |
| 201160_s_at | hsa-miR-4423-3p_st | 0.0365164248 | 0.8366445764 |
| 217860_at | hsa-miR-1180_st | 0.0365164248 | 0.8366445764 |
| 200989_at | hsa-miR-4652-3p_st | 0.0368220015 | 0.8352964164 |
| 221796_at | hsa-miR-361-5p_st | 0.0368220015 | 0.8352964164 |
| 218488_at | hsa-miR-134_st | 0.0374331551 | 0.8326015013 |
| 204239_s_at | hsa-miR-4423-3p_st | 0.0380443086 | 0.8299084838 |
| 207400_at | hsa-miR-134_st | 0.0380443086 | 0.8299084838 |
| 202658_at | hsa-miR-134_st | 0.0386554622 | 0.8272173937 |
| 202395_at | hsa-miR-134_st | 0.0389610390 | 0.8258725806 |
| 203540_at | hsa-miR-134_st | 0.0389610390 | 0.8258725806 |
| 204957_at | hsa-miR-3676_st | 0.0389610390 | 0.8258725806 |
| 208869_s_at | hsa-miR-361-5p_st | 0.0389610390 | 0.8258725806 |
| 215522_at | hsa-miR-4423-3p_st | 0.0389610390 | 0.8258725806 |
| 200989_at | hsa-miR-523_st | 0.0392666157 | 0.8245282606 |
| 221741_s_at | hsa-miR-34b-star_st | 0.0392666157 | 0.8245282606 |
| 217997_at | hsa-miR-555_st | 0.0394224159 | 0.8238430419 |
| 201145_at | hsa-miR-3676_st | 0.0395721925 | 0.8231844371 |
| 203645_s_at | hsa-miR-383_st | 0.0395721925 | 0.8231844371 |
| 208809_s_at | hsa-miR-361-5p_st | 0.0395721925 | 0.8231844371 |
| 203685_at | hsa-miR-555_st | 0.0397280160 | 0.8224993704 |
| 202736_s_at | hsa-miR-4423-3p_st | 0.0398777693 | 0.8218411140 |
| 220329_s_at | hsa-miR-4633-5p_st | 0.0398777693 | 0.8218411140 |
| 208799_at | hsa-miR-1180_st | 0.0401833461 | 0.8204982949 |
| 210592_s_at | hsa-miR-134_st | 0.0401833461 | 0.8204982949 |
| 210946_at | hsa-miR-34b_st | 0.0401833461 | 0.8204982949 |
| 202670_at | hsa-miR-4423-3p_st | 0.0404889228 | 0.8191559834 |
| 207761_s_at | hsa-miR-34b_st | 0.0404889228 | 0.8191559834 |
| 203157_s_at | hsa-miR-4423-3p_st | 0.0407944996 | 0.8178141834 |
| 212460_at | hsa-miR-361-5p_st | 0.0407944996 | 0.8178141834 |
| 203663_s_at | hsa-miR-3676_st | 0.0411000764 | 0.8164728984 |
| 214150_x_at | hsa-miR-4652-3p_st | 0.0411000764 | 0.8164728984 |
| 218866_s_at | hsa-miR-4423-3p_st | 0.0411000764 | 0.8164728984 |
| 221449_s_at | hsa-miR-134_st | 0.0411000764 | 0.8164728984 |
| 44669_at | hsa-miR-3676_st | 0.0414056532 | 0.8151321320 |
| 200053_at | hsa-miR-4423-3p_st | 0.0417112299 | 0.8137918881 |
| 201512_s_at | hsa-miR-361-5p_st | 0.0417112299 | 0.8137918881 |
| 202591_s_at | hsa-miR-134_st | 0.0417112299 | 0.8137918881 |
| 211404_s_at | hsa-miR-4423-3p_st | 0.0417112299 | 0.8137918881 |
| 213272_s_at | hsa-miR-4423-3p_st | 0.0417112299 | 0.8137918881 |
| 209570_s_at | hsa-miR-4311_st | 0.0417144168 | 0.8137779134 |
| 205531_s_at | hsa-miR-361-5p_st | 0.0423223835 | 0.8111129819 |
| 218507_at | hsa-miR-361-5p_st | 0.0423223835 | 0.8111129819 |
| 201410_at | hsa-miR-3676_st | 0.0426279603 | 0.8097743269 |
| 203621_at | hsa-miR-361-5p_st | 0.0426279603 | 0.8097743269 |
| 208761_s_at | hsa-miR-3676_st | 0.0426279603 | 0.8097743269 |
| 212157_at | hsa-miR-3676_st | 0.0426279603 | 0.8097743269 |
[truncated: 516,984 more chars]
