# Supplementary material for: Integrating mRNA and miRNA Weighted Gene Co-Expression Networks with eQTLs in the Nucleus Accumbens of Subjects with Alcohol Dependence
Source: PLoS One. 2015 Sep 18;10(9):e0137671. doi: 10.1371/journal.pone.0137671 (PMC4575063; doi:10.1371/journal.pone.0137671)
Supplement: S7 Table — (DOCX) [file pone.0137671.s008.docx]

**Table S7**.

| mRNA | miRNA | miRNA module | Correlation | FDR (q-value) |
| --- | --- | --- | --- | --- |
| ACSL5 | hsa-miR-1180-3p | blue | -0.383651642 | 0.07115243 |
| ACSL5 | hsa-miR-555 | yellow | -0.382611354 | 0.07183326 |
| ACTR1A | hsa-miR-34b-5p | brown | -0.463101604 | 0.03114673 |
| ACTR1A | hsa-miR-34c-5p | brown | -0.527883881 | 0.01627305 |
| ADCY2 | hsa-miR-377-5p | blue | -0.4710466 | 0.02905436 |
| ADCY2 | hsa-miR-383-5p | blue | -0.411459129 | 0.05385778 |
| ADCY2 | hsa-miR-4720-3p | blue | -0.466768526 | 0.03018215 |
| ADD3 | hsa-miR-370-3p | blue | -0.512299465 | 0.01857199 |
| ADD3 | hsa-miR-382-5p | blue | -0.614256247 | 0.007820095 |
| ADK | hsa-miR-34c-5p | brown | -0.576165011 | 0.01027268 |
| AFF1 | hsa-miR-132-3p | blue | -0.558441558 | 0.01206645 |
| AFF1 | hsa-miR-212-3p | blue | -0.474713522 | 0.0278185 |
| AFF1 | hsa-miR-370-3p | blue | -0.393430099 | 0.06452494 |
| AFF1 | hsa-miR-377-5p | blue | -0.645225363 | 0.005783386 |
| AFF1 | hsa-miR-382-5p | blue | -0.488043397 | 0.0241198 |
| AFF1 | hsa-miR-383-5p | blue | -0.464935065 | 0.03050201 |
| AFF1 | hsa-miR-4760-3p | blue | -0.482964095 | 0.02512095 |
| AFF1 | hsa-miR-555 | yellow | -0.360149745 | 0.08881672 |
| AIFM1 | hsa-miR-34b-3p | brown | -0.536134454 | 0.01501174 |
| AIFM1 | hsa-miR-34c-5p | brown | -0.501298701 | 0.02096763 |
| AKAP11 | hsa-miR-34b-5p | brown | -0.504354469 | 0.02028092 |
| AKAP11 | hsa-miR-34c-5p | brown | -0.570970206 | 0.010778 |
| ANGPT1 | hsa-miR-1912 | blue | -0.476546982 | 0.02720879 |
| ANK2 | hsa-miR-34c-5p | brown | -0.419709702 | 0.04923227 |
| ANXA2 | hsa-miR-377-5p | blue | -0.540412529 | 0.01434285 |
| AP4S1 | hsa-miR-34b-5p | brown | -0.411764706 | 0.05385778 |
| APLNR | hsa-miR-383-5p | blue | -0.393735676 | 0.06444189 |
| APLP2 | hsa-miR-34b-5p | brown | -0.393735676 | 0.06444189 |
| APLP2 | hsa-miR-34c-5p | brown | -0.456073338 | 0.03390965 |
| AQP4 | hsa-miR-4720-3p | blue | -0.353705118 | 0.0950494 |
| AQP4 | hsa-miR-4762-5p | yellow | -0.411459129 | 0.05385778 |
| ARHGAP5 | hsa-miR-370-3p | blue | -0.453323147 | 0.0349545 |
| ARHGAP5 | hsa-miR-382-5p | blue | -0.443425779 | 0.03852127 |
| ARHGAP5 | hsa-miR-4760-3p | blue | -0.474713522 | 0.0278185 |
| ARL3 | hsa-miR-4652-3p | brown | -0.357066463 | 0.09176102 |
| ARPC2 | hsa-miR-34b-5p | brown | -0.509854851 | 0.01896457 |
| ARPC2 | hsa-miR-34c-5p | brown | -0.523911383 | 0.01666425 |
| ARPC4 | hsa-miR-34b-3p | brown | -0.378456837 | 0.07478746 |
| ATP6V0E1 | hsa-miR-4760-3p | blue | -0.462490451 | 0.03121686 |
| ATP6V0E1 | hsa-miR-555 | yellow | -0.455038583 | 0.03433205 |
| ATP6V1E1 | hsa-miR-34c-5p | brown | -0.633002292 | 0.006320558 |
| B3GNT2 | hsa-miR-34b-3p | brown | -0.353705118 | 0.0950494 |
| B9D1 | hsa-miR-34b-5p | brown | -0.47138819 | 0.02902525 |
| BAG1 | hsa-miR-34b-5p | brown | -0.359816654 | 0.08903074 |
| BAG1 | hsa-miR-34c-5p | brown | -0.426432391 | 0.04634216 |
| BCL2 | hsa-miR-212-3p | blue | -0.350343774 | 0.09802455 |
| BCL2 | hsa-miR-4720-3p | blue | -0.379373568 | 0.07400232 |
| BRE | hsa-miR-34b-5p | brown | -0.60855615 | 0.007945749 |
| BTN2A1 | hsa-miR-34b-5p | brown | -0.445989305 | 0.03756662 |
| CACNA2D3 | hsa-miR-34b-5p | brown | -0.487547746 | 0.0242336 |
| CACNA2D3 | hsa-miR-375 | brown | -0.447517189 | 0.03707439 |
| CACNB2 | hsa-miR-34b-5p | brown | -0.540106952 | 0.01434285 |
| CACNB2 | hsa-miR-34c-5p | brown | -0.591749427 | 0.008836893 |
| CALCOCO2 | hsa-miR-1912 | blue | -0.531245225 | 0.01583584 |
| CALCOCO2 | hsa-miR-377-5p | blue | -0.654698243 | 0.005311852 |
| CALCOCO2 | hsa-miR-382-5p | blue | -0.438383377 | 0.04060552 |
| CALCOCO2 | hsa-miR-4760-3p | blue | -0.506493506 | 0.01980683 |
| CALM1 | hsa-miR-34c-3p | brown | -0.522689076 | 0.01696553 |
| CAND1 | hsa-miR-34b-3p | brown | -0.352788388 | 0.09585279 |
| CAND1 | hsa-miR-34b-5p | brown | -0.497937357 | 0.02183362 |
| CAND1 | hsa-miR-34c-3p | brown | -0.618640183 | 0.007326 |
| CAND1 | hsa-miR-34c-5p | brown | -0.549579832 | 0.01292361 |
| CARHSP1 | hsa-miR-34c-5p | brown | -0.419098549 | 0.04957514 |
| CBFB | hsa-miR-132-3p | blue | -0.630863254 | 0.006364804 |
| CBFB | hsa-miR-4720-3p | blue | -0.435905271 | 0.04174365 |
| CBFB | hsa-miR-4760-3p | blue | -0.572803667 | 0.01056481 |
| CD44 | hsa-miR-1912 | blue | -0.511077158 | 0.01877931 |
| CD44 | hsa-miR-377-5p | blue | -0.470741024 | 0.02906323 |
| CD99 | hsa-miR-4760-3p | blue | -0.372345302 | 0.07968413 |
| CFI | hsa-miR-555 | yellow | -0.405684163 | 0.05729852 |
| CLTC | hsa-miR-34b-5p | brown | -0.474596991 | 0.02785644 |
| CLTC | hsa-miR-34c-5p | brown | -0.524409811 | 0.01666425 |
| CLU | hsa-miR-370-3p | blue | -0.365011459 | 0.08501582 |
| CLU | hsa-miR-377-5p | blue | -0.492436975 | 0.02300566 |
| CLU | hsa-miR-383-5p | blue | -0.398013751 | 0.06183317 |
| COMMD3 | hsa-miR-34b-5p | brown | -0.398624905 | 0.06158062 |
| COMT | hsa-miR-1912 | blue | -0.505271199 | 0.02001203 |
| COMT | hsa-miR-377-5p | blue | -0.502215432 | 0.02071617 |
| COX7A2L | hsa-miR-34b-5p | brown | -0.536134454 | 0.01501174 |
| CREBL2 | hsa-miR-34b-3p | brown | -0.417876241 | 0.05017372 |
| CUL2 | hsa-miR-34b-5p | brown | -0.467990833 | 0.02989758 |
| CUTA | hsa-miR-34b-5p | brown | -0.465240642 | 0.03037298 |
| CUTA | hsa-miR-34c-5p | brown | -0.512299465 | 0.01857199 |
| CUTC | hsa-miR-34b-3p | brown | -0.37540107 | 0.07751525 |
| DDR1 | hsa-miR-1180-3p | blue | -0.473185638 | 0.02838033 |
| DDR1 | hsa-miR-370-3p | blue | -0.403208556 | 0.05869316 |
| DDR1 | hsa-miR-377-5p | blue | -0.542857143 | 0.0140563 |
| DENND5B | hsa-miR-34c-3p | brown | -0.484797555 | 0.02471142 |
| DENND5B | hsa-miR-34c-5p | brown | -0.464018335 | 0.03075318 |
| DENND5B | hsa-miR-375 | brown | -0.454239878 | 0.03460223 |
| DHCR24 | hsa-miR-34c-5p | brown | -0.52513369 | 0.01666425 |
| DHPS | hsa-miR-34b-5p | brown | -0.401986249 | 0.05933701 |
| DHPS | hsa-miR-34c-5p | brown | -0.483575248 | 0.02501915 |
| DIEXF | hsa-miR-34b-5p | brown | -0.439572193 | 0.0399603 |
| DIEXF | hsa-miR-34c-3p | brown | -0.577387319 | 0.01022655 |
| DIEXF | hsa-miR-34c-5p | brown | -0.485103132 | 0.0246082 |
| DIEXF | hsa-miR-4652-3p | brown | -0.361650115 | 0.08771716 |
| DLG3 | hsa-miR-34b-5p | brown | -0.545607334 | 0.01354536 |
| DLG3 | hsa-miR-34c-3p | brown | -0.615584416 | 0.007670316 |
| DLG3 | hsa-miR-34c-5p | brown | -0.570053476 | 0.010778 |
| DLG3 | hsa-miR-4652-3p | brown | -0.471657754 | 0.02889627 |
| DMXL2 | hsa-miR-34b-5p | brown | -0.462490451 | 0.03121686 |
| DNAJA2 | hsa-miR-34b-3p | brown | -0.348510313 | 0.09935625 |
| DNAJA3 | hsa-miR-34c-3p | brown | -0.481741788 | 0.025492 |
| DNAJC12 | hsa-miR-34c-3p | brown | -0.611306341 | 0.007945749 |
| DSE | hsa-miR-382-5p | blue | -0.493238599 | 0.02299465 |
| DSE | hsa-miR-4720-3p | blue | -0.383346066 | 0.07121556 |
| DSE | hsa-miR-4311 | yellow | -0.57101383 | 0.010778 |
| DYNLT1 | hsa-miR-4760-3p | blue | -0.605500382 | 0.008011854 |
| DYNLT1 | hsa-miR-4311 | yellow | -0.365497747 | 0.08480713 |
| EFNB3 | hsa-miR-34b-5p | brown | -0.455462185 | 0.03410993 |
| EFNB3 | hsa-miR-34c-5p | brown | -0.533078686 | 0.0156248 |
| EIF3H | hsa-miR-34b-5p | brown | -0.460045837 | 0.03229289 |
| EIF3H | hsa-miR-34c-3p | brown | -0.580748663 | 0.009948786 |
| EIF3H | hsa-miR-4652-3p | brown | -0.383346066 | 0.07121556 |
| EMC3 | hsa-miR-34c-5p | brown | -0.638398657 | 0.006225869 |
| EMP1 | hsa-miR-1912 | blue | -0.416042781 | 0.05116531 |
| ENAH | hsa-miR-377-5p | blue | -0.567608862 | 0.01089227 |
| ENAH | hsa-miR-383-5p | blue | -0.523300229 | 0.01684374 |
| ENAH | hsa-miR-4720-3p | blue | -0.5355233 | 0.0151382 |
| ENAH | hsa-miR-4760-3p | blue | -0.513521772 | 0.0184267 |
| ENAH | hsa-miR-4311 | yellow | -0.452593782 | 0.03518426 |
| ENAH | hsa-miR-4633-5p | yellow | -0.353399542 | 0.09539673 |
| ENAH | hsa-miR-4762-5p | yellow | -0.4236822 | 0.0476735 |
| FBXO3 | hsa-miR-34c-3p | brown | -0.488464477 | 0.024017 |
| FBXO3 | hsa-miR-34c-5p | brown | -0.420320856 | 0.0490157 |
| FBXO3 | hsa-miR-375 | brown | -0.393735676 | 0.06444189 |
| FGF13 | hsa-miR-4652-3p | brown | -0.43315508 | 0.04321378 |
| FOXJ3 | hsa-miR-34b-5p | brown | -0.436516425 | 0.04148593 |
| FOXJ3 | hsa-miR-34c-3p | brown | -0.577081742 | 0.01022681 |
| FRY | hsa-miR-34c-5p | brown | -0.466157372 | 0.03018215 |
| FSTL1 | hsa-miR-132-3p | blue | -0.511993888 | 0.01864427 |
| FSTL1 | hsa-miR-1912 | blue | -0.663865546 | 0.005311852 |
| FSTL1 | hsa-miR-212-3p | blue | -0.490297937 | 0.02345526 |
| FSTL1 | hsa-miR-370-3p | blue | -0.412375859 | 0.05352779 |
| FSTL1 | hsa-miR-383-5p | blue | -0.47868602 | 0.02664092 |
| FSTL1 | hsa-miR-4760-3p | blue | -0.532467532 | 0.01573232 |
| FTSJ1 | hsa-miR-375 | brown | -0.435599694 | 0.04188815 |
| FZD3 | hsa-miR-34b-5p | brown | -0.351566081 | 0.0968527 |
| FZD3 | hsa-miR-34c-3p | brown | -0.454545455 | 0.0345436 |
| FZD6 | hsa-miR-1912 | blue | -0.412987013 | 0.05312766 |
| GABRG2 | hsa-miR-34b-3p | brown | -0.34881589 | 0.09907876 |
| GABRG2 | hsa-miR-34b-5p | brown | -0.369900688 | 0.08126074 |
| GABRG2 | hsa-miR-34c-3p | brown | -0.443544691 | 0.03846486 |
| GABRG2 | hsa-miR-34c-5p | brown | -0.446294882 | 0.03756662 |
| GAD1 | hsa-miR-34b-5p | brown | -0.493659282 | 0.02299465 |
| GAD1 | hsa-miR-34c-3p | brown | -0.590832697 | 0.008840194 |
| GAD1 | hsa-miR-4652-3p | brown | -0.430099312 | 0.04466778 |
| GBP1 | hsa-miR-382-5p | blue | -0.349606541 | 0.09848543 |
| GBP1 | hsa-miR-4311 | yellow | -0.474902591 | 0.0278185 |
| GBP1 | hsa-miR-4633-5p | yellow | -0.427654698 | 0.04579296 |
| GFAP | hsa-miR-1912 | blue | -0.507715814 | 0.01962876 |
| GFAP | hsa-miR-377-5p | blue | -0.405042017 | 0.05762738 |
| GGH | hsa-miR-4652-3p | brown | -0.467379679 | 0.03000905 |
| GHITM | hsa-miR-34b-5p | brown | -0.558025825 | 0.01208832 |
| GLCE | hsa-miR-34b-5p | brown | -0.377845684 | 0.07529048 |
| GLCE | hsa-miR-34c-5p | brown | -0.42459893 | 0.04721288 |
| GLRB | hsa-miR-34b-3p | brown | -0.455767762 | 0.03402397 |
| GLS | hsa-miR-34b-3p | brown | -0.409014515 | 0.05523382 |
| GLS | hsa-miR-34b-5p | brown | -0.503743316 | 0.02041276 |
| GLS | hsa-miR-34c-3p | brown | -0.628724217 | 0.006649109 |
| GLS | hsa-miR-34c-5p | brown | -0.59144385 | 0.008836893 |
| GLS2 | hsa-miR-34b-5p | brown | -0.399541635 | 0.06098587 |
| GLS2 | hsa-miR-34c-5p | brown | -0.493048128 | 0.02299465 |
| GMFB | hsa-miR-132-3p | blue | -0.433460657 | 0.04309544 |
| GMFB | hsa-miR-212-3p | blue | -0.3763178 | 0.07659699 |
| GMFB | hsa-miR-377-5p | blue | -0.592971734 | 0.008836893 |
| GMFB | hsa-miR-382-5p | blue | -0.358927345 | 0.08986914 |
| GMFB | hsa-miR-383-5p | blue | -0.501298701 | 0.02096763 |
| GMFB | hsa-miR-4760-3p | blue | -0.552024446 | 0.01273457 |
| GMFB | hsa-miR-4311 | yellow | -0.458858585 | 0.03284327 |
| GNAI3 | hsa-miR-4311 | yellow | -0.363358546 | 0.08639696 |
| GNAL | hsa-miR-34b-5p | brown | -0.420015279 | 0.04909268 |
| GNAL | hsa-miR-34c-5p | brown | -0.481436211 | 0.02559936 |
| GNPDA1 | hsa-miR-34c-5p | brown | -0.418792972 | 0.04968511 |
| GORASP2 | hsa-miR-34c-5p | brown | -0.602139037 | 0.008011854 |
| GORASP2 | hsa-miR-4652-3p | brown | -0.486019862 | 0.02451924 |
| GOT2 | hsa-miR-34c-5p | brown | -0.416348358 | 0.05101965 |
| GRAMD3 | hsa-miR-134-5p | blue | -0.377540107 | 0.07557936 |
| GUCY1B3 | hsa-miR-34c-5p | brown | -0.531245225 | 0.01583584 |
| H2AFZ | hsa-miR-34c-3p | brown | -0.490297937 | 0.02345526 |
| HAX1 | hsa-miR-34c-3p | brown | -0.467990833 | 0.02989758 |
| HIF1A | hsa-miR-4311 | yellow | -0.382458554 | 0.07183326 |
| HLA-E | hsa-miR-4311 | yellow | -0.369317749 | 0.08180092 |
| HNRNPK | hsa-miR-34c-3p | brown | -0.432849503 | 0.04330221 |
| HSDL2 | hsa-miR-132-3p | blue | -0.39526356 | 0.06369292 |
| HSDL2 | hsa-miR-212-3p | blue | -0.401375095 | 0.05981235 |
| HSDL2 | hsa-miR-4760-3p | blue | -0.409320092 | 0.05514112 |
| HTR2C | hsa-miR-34b-5p | brown | -0.494576012 | 0.02268697 |
| HTR2C | hsa-miR-34c-5p | brown | -0.539495798 | 0.0143863 |
| IARS | hsa-miR-34b-3p | brown | -0.355844156 | 0.09307908 |
| ICA1 | hsa-miR-34b-5p | brown | -0.464018335 | 0.03075318 |
| ICA1 | hsa-miR-34c-5p | brown | -0.507410237 | 0.01962876 |
| IDH3B | hsa-miR-34c-3p | brown | -0.582887701 | 0.009768733 |
| IL13RA1 | hsa-miR-132-3p | blue | -0.353093965 | 0.09570481 |
| IL13RA1 | hsa-miR-377-5p | blue | -0.435905271 | 0.04174365 |
| IL1R1 | hsa-miR-132-3p | blue | -0.50618793 | 0.0198855 |
| IL1R1 | hsa-miR-212-3p | blue | -0.452711994 | 0.03513356 |
| IL1R1 | hsa-miR-3189-5p | blue | -0.38579068 | 0.06948894 |
| IL1R1 | hsa-miR-377-5p | blue | -0.579831933 | 0.01011284 |
| IL6ST | hsa-miR-377-5p | blue | -0.380595875 | 0.07294227 |
| IMP3 | hsa-miR-34b-5p | brown | -0.458212376 | 0.03306443 |
| IMPG1 | hsa-miR-34b-5p | brown | -0.438655462 | 0.04043599 |
| IMPG1 | hsa-miR-34c-5p | brown | -0.50618793 | 0.0198855 |
| IMPG1 | hsa-miR-4652-3p | brown | -0.411459129 | 0.05385778 |
| ISCU | hsa-miR-34c-5p | brown | -0.49671505 | 0.02216194 |
| ITFG1 | hsa-miR-34c-3p | brown | -0.588082506 | 0.009311369 |
| ITGA6 | hsa-miR-1912 | blue | -0.465546218 | 0.03029969 |
| ITGA6 | hsa-miR-377-5p | blue | -0.381207028 | 0.07273636 |
| ITPKB | hsa-miR-383-5p | blue | -0.461456186 | 0.03177356 |
| ITPKB | hsa-miR-4760-3p | blue | -0.37267935 | 0.07949432 |
| KHDRBS1 | hsa-miR-4652-3p | brown | -0.352482811 | 0.09596138 |
| KIAA1324 | hsa-miR-34b-5p | brown | -0.44262796 | 0.03880544 |
| KIAA1324 | hsa-miR-34c-3p | brown | -0.510466005 | 0.01889895 |
| KIAA1324 | hsa-miR-34c-5p | brown | -0.458823529 | 0.03284327 |
| KLHDC2 | hsa-miR-34b-5p | brown | -0.526355997 | 0.01664827 |
| LCMT1 | hsa-miR-34b-5p | brown | -0.361344538 | 0.08792821 |
| LCMT1 | hsa-miR-34c-5p | brown | -0.427349121 | 0.0459513 |
| LEPROTL1 | hsa-miR-34b-5p | brown | -0.436210848 | 0.04162947 |
| LEPROTL1 | hsa-miR-34c-3p | brown | -0.562719633 | 0.01166832 |
| LEPROTL1 | hsa-miR-34c-5p | brown | -0.492742552 | 0.02299465 |
| LHFP | hsa-miR-4311 | yellow | -0.386584156 | 0.06920267 |
| LIMS1 | hsa-miR-4720-3p | blue | -0.360427807 | 0.08864174 |
| LIMS1 | hsa-miR-4760-3p | blue | -0.366233766 | 0.08416599 |
| LIMS1 | hsa-miR-4311 | yellow | -0.568721829 | 0.010778 |
| LMNA | hsa-miR-377-5p | blue | -0.48724217 | 0.02430655 |
| LMNA | hsa-miR-4720-3p | blue | -0.398624905 | 0.06158062 |
| LRP10 | hsa-miR-134-5p | blue | -0.365317036 | 0.08481195 |
| LRP10 | hsa-miR-1912 | blue | -0.534300993 | 0.01533109 |
| LRP10 | hsa-miR-3189-5p | blue | -0.381818182 | 0.07228355 |
| LRP10 | hsa-miR-370-3p | blue | -0.444766998 | 0.03802675 |
| LRPPRC | hsa-miR-34c-3p | brown | -0.60855615 | 0.007945749 |
| LRPPRC | hsa-miR-34c-5p | brown | -0.551107716 | 0.01276041 |
| MAD2L1 | hsa-miR-34b-5p | brown | -0.479908327 | 0.02614268 |
| MAD2L1 | hsa-miR-4652-3p | brown | -0.366233766 | 0.08416599 |
| MAFB | hsa-miR-382-5p | blue | -0.406600964 | 0.05661219 |
| MAFB | hsa-miR-4720-3p | blue | -0.385485103 | 0.06968949 |
| MAFB | hsa-miR-4311 | yellow | -0.37252655 | 0.07961003 |
| MAFF | hsa-miR-4720-3p | blue | -0.648281131 | 0.005783386 |
| MAP2K1 | hsa-miR-34b-5p | brown | -0.476546982 | 0.02720879 |
| MAP2K1 | hsa-miR-34c-3p | brown | -0.587471352 | 0.009316832 |
| MAP2K1 | hsa-miR-34c-5p | brown | -0.551107716 | 0.01276041 |
| MAPK1 | hsa-miR-34b-5p | brown | -0.506493506 | 0.01980683 |
| MAPK1 | hsa-miR-34c-5p | brown | -0.577387319 | 0.01022655 |
| MCL1 | hsa-miR-4760-3p | blue | -0.398013751 | 0.06183317 |
| MDH1 | hsa-miR-34b-5p | brown | -0.472574484 | 0.02859113 |
| METTL13 | hsa-miR-34c-5p | brown | -0.510771581 | 0.01882507 |
| MIA3 | hsa-miR-34b-5p | brown | -0.443850267 | 0.03836196 |
| MIA3 | hsa-miR-34c-5p | brown | -0.46921314 | 0.02965203 |
| MIA3 | hsa-miR-4652-3p | brown | -0.401680672 | 0.05957429 |
| MPPED2 | hsa-miR-34c-3p | brown | -0.485408709 | 0.0246082 |
| MPPED2 | hsa-miR-4652-3p | brown | -0.402902979 | 0.05889491 |
| MRPL17 | hsa-miR-34b-5p | brown | -0.351871658 | 0.09646113 |
| MRPL33 | hsa-miR-34b-5p | brown | -0.418181818 | 0.05003112 |
| MRPL33 | hsa-miR-34c-5p | brown | -0.440488923 | 0.03966471 |
| MRPS17 | hsa-miR-34b-3p | brown | -0.421543163 | 0.04877684 |
| MT1X | hsa-miR-1912 | blue | -0.487547746 | 0.0242336 |
| MTHFD2 | hsa-miR-377-5p | blue | -0.534912147 | 0.015234 |
| MTHFD2 | hsa-miR-4720-3p | blue | -0.525439267 | 0.01666425 |
| MTHFD2 | hsa-miR-4760-3p | blue | -0.388846448 | 0.06782911 |
| MTHFD2 | hsa-miR-4311 | yellow | -0.435632976 | 0.04188815 |
| MVP | hsa-miR-1180-3p | blue | -0.500993125 | 0.02102427 |
| MVP | hsa-miR-3189-5p | blue | -0.397097021 | 0.06243624 |
| MVP | hsa-miR-370-3p | blue | -0.350038197 | 0.09821797 |
| NDRG3 | hsa-miR-34b-3p | brown | -0.525744843 | 0.01666425 |
| NDUFAB1 | hsa-miR-4652-3p | brown | -0.387929717 | 0.06838085 |
| NDUFB5 | hsa-miR-34b-5p | brown | -0.43315508 | 0.04321378 |
| NDUFB5 | hsa-miR-34c-5p | brown | -0.489686784 | 0.02365016 |
| NDUFB5 | hsa-miR-4652-3p | brown | -0.396791444 | 0.06264981 |
| NDUFS2 | hsa-miR-34b-3p | brown | -0.412987013 | 0.05312766 |
| NEDD9 | hsa-miR-132-3p | blue | -0.462796028 | 0.03121686 |
| NEDD9 | hsa-miR-212-3p | blue | -0.430710466 | 0.04439138 |
| NEDD9 | hsa-miR-370-3p | blue | -0.420015279 | 0.04909268 |
| NEDD9 | hsa-miR-377-5p | blue | -0.557830405 | 0.01211348 |
| NEDD9 | hsa-miR-383-5p | blue | -0.390679908 | 0.06649704 |
| NEDD9 | hsa-miR-4720-3p | blue | -0.446600458 | 0.03748543 |
| NEDD9 | hsa-miR-4760-3p | blue | -0.373262032 | 0.07911645 |
| NFE2L2 | hsa-miR-382-5p | blue | -0.466345788 | 0.03018215 |
| NIT2 | hsa-miR-34c-3p | brown | -0.521772345 | 0.01718342 |
| NMNAT2 | hsa-miR-34b-5p | brown | -0.533995416 | 0.01538012 |
| NNAT | hsa-miR-34b-5p | brown | -0.352482811 | 0.09596138 |
| NNAT | hsa-miR-34c-5p | brown | -0.387929717 | 0.06838085 |
| NOLC1 | hsa-miR-34b-3p | brown | -0.443850267 | 0.03836196 |
| NOLC1 | hsa-miR-34b-5p | brown | -0.639419404 | 0.006225869 |
| NOLC1 | hsa-miR-375 | brown | -0.449045073 | 0.03674044 |
| NSF | hsa-miR-34b-5p | brown | -0.547135218 | 0.01329749 |
| NSF | hsa-miR-4652-3p | brown | -0.365011459 | 0.08501582 |
| NTRK2 | hsa-miR-1912 | blue | -0.352482811 | 0.09596138 |
| OGFRL1 | hsa-miR-1912 | blue | -0.41329259 | 0.05297674 |
| OGFRL1 | hsa-miR-377-5p | blue | -0.478380443 | 0.026697 |
| OGFRL1 | hsa-miR-4720-3p | blue | -0.408403361 | 0.05555077 |
| OGFRL1 | hsa-miR-4760-3p | blue | -0.349427044 | 0.09848543 |
| OGFRL1 | hsa-miR-4311 | yellow | -0.393307358 | 0.06458621 |
| ORC5 | hsa-miR-34b-5p | brown | -0.401069519 | 0.05981714 |
| ORC5 | hsa-miR-34c-5p | brown | -0.486019862 | 0.02451924 |
| PAFAH1B1 | hsa-miR-34c-3p | brown | -0.622918258 | 0.007260291 |
| PAFAH1B1 | hsa-miR-34c-5p | brown | -0.569747899 | 0.010778 |
| PAFAH1B1 | hsa-miR-375 | brown | -0.373262032 | 0.07911645 |
| PAFAH1B1 | hsa-miR-4652-3p | brown | -0.404430863 | 0.05795837 |
| PALLD | hsa-miR-382-5p | blue | -0.437160976 | 0.04108177 |
| PALLD | hsa-miR-4760-3p | blue | -0.46065699 | 0.03202081 |
| PALLD | hsa-miR-4762-5p | yellow | -0.420626432 | 0.04897054 |
| PCDH8 | hsa-miR-34b-5p | brown | -0.5355233 | 0.0151382 |
| PDCD6 | hsa-miR-34c-3p | brown | -0.514132926 | 0.01839938 |
| PDCD6 | hsa-miR-375 | brown | -0.423376623 | 0.04780724 |
| PDXDC1 | hsa-miR-34b-5p | brown | -0.469824293 | 0.02940378 |
| PDXDC1 | hsa-miR-34c-5p | brown | -0.524522536 | 0.01666425 |
| PDYN | hsa-miR-34b-5p | brown | -0.370206264 | 0.08110361 |
| PDYN | hsa-miR-34c-5p | brown | -0.425821238 | 0.04644117 |
| PECAM1 | hsa-miR-4311 | yellow | -0.430437773 | 0.04451398 |
| PEF1 | hsa-miR-34b-5p | brown | -0.519938885 | 0.0175892 |
| PEG10 | hsa-miR-34c-5p | brown | -0.380901451 | 0.07276786 |
| PEG3 | hsa-miR-34c-3p | brown | -0.369595111 | 0.0814931 |
| PEX11B | hsa-miR-34b-5p | brown | -0.416042781 | 0.05116531 |
| PKP2 | hsa-miR-34b-5p | brown | -0.430404889 | 0.04451398 |
| PKP2 | hsa-miR-34c-5p | brown | -0.464935065 | 0.03050201 |
| PLEKHB2 | hsa-miR-34b-3p | brown | -0.426126814 | 0.04634216 |
| PLEKHB2 | hsa-miR-34c-3p | brown | -0.535217723 | 0.0151541 |
| POLR2E | hsa-miR-34c-5p | brown | -0.464018335 | 0.03075318 |
| POP4 | hsa-miR-34b-5p | brown | -0.503437739 | 0.02041276 |
| POP4 | hsa-miR-34c-5p | brown | -0.554774637 | 0.0125302 |
| PPAP2A | hsa-miR-370-3p | blue | -0.361650115 | 0.08771716 |
| PPP1R3D | hsa-miR-377-5p | blue | -0.354927426 | 0.09386814 |
| PPP1R3D | hsa-miR-4760-3p | blue | -0.360122231 | 0.08881672 |
| PPP1R8 | hsa-miR-34b-5p | brown | -0.569442322 | 0.010778 |
| PPP1R8 | hsa-miR-34c-5p | brown | -0.600611154 | 0.008075552 |
| PRDX2 | hsa-miR-34b-5p | brown | -0.420932009 | 0.04895751 |
| PREPL | hsa-miR-34b-5p | brown | -0.418792972 | 0.04968511 |
| PREPL | hsa-miR-34c-5p | brown | -0.512910619 | 0.01851259 |
| PRKACB | hsa-miR-34b-3p | brown | -0.350343774 | 0.09802455 |
| PRKACB | hsa-miR-4652-3p | brown | -0.410847976 | 0.05413408 |
| PRKCH | hsa-miR-34c-5p | brown | -0.467379679 | 0.03000905 |
| PSMA5 | hsa-miR-34b-3p | brown | -0.383957219 | 0.07105464 |
| PSMA5 | hsa-miR-34c-3p | brown | -0.549274255 | 0.01292361 |
| PSMA5 | hsa-miR-34c-5p | brown | -0.560275019 | 0.01189801 |
| PSMB5 | hsa-miR-34b-5p | brown | -0.456378915 | 0.03376702 |
| PSMB5 | hsa-miR-34c-5p | brown | -0.505271199 | 0.02001203 |
| PSMB6 | hsa-miR-34c-5p | brown | -0.530939649 | 0.01585663 |
| PSMB7 | hsa-miR-34b-3p | brown | -0.365317036 | 0.08481195 |
| PTBP1 | hsa-miR-382-5p | blue | -0.380930553 | 0.07276786 |
| PTTG1IP | hsa-miR-1180-3p | blue | -0.349121467 | 0.0988829 |
| PTTG1IP | hsa-miR-1912 | blue | -0.568525592 | 0.010778 |
| PTTG1IP | hsa-miR-370-3p | blue | -0.408097785 | 0.05577514 |
| RAB13 | hsa-miR-4760-3p | blue | -0.543773873 | 0.01390518 |
| RAB2A | hsa-miR-34b-5p | brown | -0.419404125 | 0.04940343 |
| RAB2A | hsa-miR-34c-5p | brown | -0.439877769 | 0.03988083 |
| RAB2A | hsa-miR-4652-3p | brown | -0.383651642 | 0.07115243 |
| RABEPK | hsa-miR-34c-5p | brown | -0.606417112 | 0.007947337 |
| RABEPK | hsa-miR-4652-3p | brown | -0.439877769 | 0.03988083 |
| RAE1 | hsa-miR-34b-5p | brown | -0.498242934 | 0.02171737 |
| RAE1 | hsa-miR-34c-5p | brown | -0.550190985 | 0.01292331 |
| RHOB | hsa-miR-4311 | yellow | -0.362136146 | 0.08749971 |
| RHOBTB3 | hsa-miR-132-3p | blue | -0.554163484 | 0.0125302 |
| RHOBTB3 | hsa-miR-134-5p | blue | -0.45026738 | 0.03627544 |
| RHOBTB3 | hsa-miR-382-5p | blue | -0.707005885 | 0.005311852 |
| RHOBTB3 | hsa-miR-4760-3p | blue | -0.401069519 | 0.05981714 |
| RHOBTB3 | hsa-miR-4762-5p | yellow | -0.381818182 | 0.07228355 |
| RNF128 | hsa-miR-34b-5p | brown | -0.407792208 | 0.05586981 |
| RNF128 | hsa-miR-34c-3p | brown | -0.511382735 | 0.01870609 |
| RPP14 | hsa-miR-34b-3p | brown | -0.420320856 | 0.0490157 |
| RPP14 | hsa-miR-4652-3p | brown | -0.374178762 | 0.07844239 |
| RREB1 | hsa-miR-1912 | blue | -0.442016807 | 0.03895701 |
| RREB1 | hsa-miR-377-5p | blue | -0.365011459 | 0.08501582 |
| RREB1 | hsa-miR-4760-3p | blue | -0.351871658 | 0.09646113 |
| RTN4 | hsa-miR-34c-5p | brown | -0.500381971 | 0.02119442 |
| SAG | hsa-miR-375 | brown | -0.457601222 | 0.03328733 |
| SAT1 | hsa-miR-4720-3p | blue | -0.351871658 | 0.09646113 |
| SEMA3A | hsa-miR-34b-5p | brown | -0.574331551 | 0.01041601 |
| SEMA3A | hsa-miR-34c-5p | brown | -0.655614973 | 0.005311852 |
| SERINC3 | hsa-miR-34b-3p | brown | -0.411153552 | 0.05401177 |
| SERINC3 | hsa-miR-34b-5p | brown | -0.466768526 | 0.03018215 |
| SERPINA3 | hsa-miR-377-5p | blue | -0.51749427 | 0.01787682 |
| SERPINA3 | hsa-miR-383-5p | blue | -0.38670741 | 0.06913607 |
| SERPINA3 | hsa-miR-4720-3p | blue | -0.496409473 | 0.0221956 |
| SERPINB1 | hsa-miR-132-3p | blue | -0.395874714 | 0.06332857 |
| SERPINB1 | hsa-miR-377-5p | blue | -0.436210848 | 0.04162947 |
| SERPINB1 | hsa-miR-4760-3p | blue | -0.422459893 | 0.04833671 |
| SERPINB1 | hsa-miR-4311 | yellow | -0.366872948 | 0.0835748 |
| SERPING1 | hsa-miR-1912 | blue | -0.611917494 | 0.007945749 |
| SERPING1 | hsa-miR-3189-5p | blue | -0.372956455 | 0.07934236 |
| SGPL1 | hsa-miR-1912 | blue | -0.541023682 | 0.01433398 |
| SGPL1 | hsa-miR-370-3p | blue | -0.370206264 | 0.08110361 |
| SGPL1 | hsa-miR-377-5p | blue | -0.485103132 | 0.0246082 |
| SGPL1 | hsa-miR-382-5p | blue | -0.553441823 | 0.01262217 |
| SGPL1 | hsa-miR-383-5p | blue | -0.422459893 | 0.04833671 |
| SLC14A1 | hsa-miR-3189-5p | blue | -0.397708174 | 0.06201102 |
| SLC14A1 | hsa-miR-377-5p | blue | -0.6 | 0.008075552 |
| SLC14A1 | hsa-miR-4760-3p | blue | -0.410542399 | 0.05435357 |
| SLC14A1 | hsa-miR-4311 | yellow | -0.511727406 | 0.01868891 |
| SLC25A3 | hsa-miR-34b-5p | brown | -0.383346066 | 0.07121556 |
| SLC25A3 | hsa-miR-34c-5p | brown | -0.426126814 | 0.04634216 |
| SLC25A4 | hsa-miR-34c-5p | brown | -0.54316272 | 0.0139837 |
| SLC25A4 | hsa-miR-4652-3p | brown | -0.373567609 | 0.07885395 |
| SLC26A2 | hsa-miR-1912 | blue | -0.449350649 | 0.03667524 |
| SLC26A2 | hsa-miR-382-5p | blue | -0.42203377 | 0.04860725 |
| SLC26A2 | hsa-miR-4720-3p | blue | -0.448739496 | 0.03683566 |
| SLC26A2 | hsa-miR-4311 | yellow | -0.47031859 | 0.02922605 |
| SLC30A9 | hsa-miR-34b-3p | brown | -0.371428571 | 0.08040634 |
| SLC30A9 | hsa-miR-34b-5p | brown | -0.403208556 | 0.05869316 |
| SLC30A9 | hsa-miR-34c-3p | brown | -0.538884645 | 0.0144556 |
| SLC30A9 | hsa-miR-34c-5p | brown | -0.484186402 | 0.02491912 |
| SLCO4A1 | hsa-miR-212-3p | blue | -0.395569137 | 0.06361346 |
| SLK | hsa-miR-34b-3p | brown | -0.38487395 | 0.07026827 |
| SLK | hsa-miR-34b-5p | brown | -0.599694423 | 0.008075552 |
| SLK | hsa-miR-34c-3p | brown | -0.56210848 | 0.01174235 |
| SMYD3 | hsa-miR-4652-3p | brown | -0.362872422 | 0.08676411 |
| SNAP25 | hsa-miR-34b-3p | brown | -0.468296409 | 0.02989758 |
| SON | hsa-miR-34b-5p | brown | -0.416959511 | 0.05069846 |
| SON | hsa-miR-34c-3p | brown | -0.504048892 | 0.02033407 |
| SON | hsa-miR-34c-5p | brown | -0.45974026 | 0.03242971 |
| SORCS3 | hsa-miR-4652-3p | brown | -0.364400306 | 0.08561533 |
| SP100 | hsa-miR-377-5p | blue | -0.507715814 | 0.01962876 |
| SP100 | hsa-miR-382-5p | blue | -0.39804416 | 0.06183317 |
| SPAG1 | hsa-miR-4311 | yellow | -0.367942548 | 0.08289716 |
| SPTSSA | hsa-miR-377-5p | blue | -0.428265852 | 0.04547786 |
| STAT3 | hsa-miR-212-3p | blue | -0.449961803 | 0.03636892 |
| STAT3 | hsa-miR-370-3p | blue | -0.371734148 | 0.08021478 |
| STAT3 | hsa-miR-4720-3p | blue | -0.482658518 | 0.0251455 |
| STK3 | hsa-miR-382-5p | blue | -0.363969747 | 0.08598327 |
| SYNJ1 | hsa-miR-34c-5p | brown | -0.545301757 | 0.01361548 |
| TAGLN2 | hsa-miR-383-5p | blue | -0.470741024 | 0.02906323 |
| TGIF1 | hsa-miR-4760-3p | blue | -0.518716578 | 0.01773655 |
| TMBIM1 | hsa-miR-1912 | blue | -0.412987013 | 0.05312766 |
| TMBIM6 | hsa-miR-370-3p | blue | -0.552330023 | 0.01267035 |
| TNFRSF1A | hsa-miR-1180-3p | blue | -0.398319328 | 0.06172354 |
| TNFRSF1A | hsa-miR-1912 | blue | -0.591138273 | 0.008836893 |
| TNFRSF1A | hsa-miR-3189-5p | blue | -0.499159664 | 0.02148312 |
| TNFRSF1A | hsa-miR-377-5p | blue | -0.416653934 | 0.05081173 |
| TNIP2 | hsa-miR-1180-3p | blue | -0.388846448 | 0.06782911 |
| TNIP2 | hsa-miR-383-5p | blue | -0.426126814 | 0.04634216 |
| TOMM20 | hsa-miR-34b-3p | brown | -0.440488923 | 0.03966471 |
| TOMM20 | hsa-miR-34c-3p | brown | -0.660504202 | 0.005311852 |
| TOMM22 | hsa-miR-34c-5p | brown | -0.524828113 | 0.01666425 |
| TOMM70A | hsa-miR-34c-5p | brown | -0.483880825 | 0.02496891 |
| TP53BP2 | hsa-miR-370-3p | blue | -0.383040489 | 0.07142088 |
| TRAC | hsa-miR-34c-5p | brown | -0.384262796 | 0.07081538 |
| TRAPPC4 | hsa-miR-34c-5p | brown | -0.456684492 | 0.03368231 |
| TRIM22 | hsa-miR-1180-3p | blue | -0.492742552 | 0.02299465 |
| TRIM22 | hsa-miR-361-5p | blue | -0.35737204 | 0.09154142 |
| TRIM22 | hsa-miR-377-5p | blue | -0.529106188 | 0.0160528 |
| TRIM22 | hsa-miR-383-5p | blue | -0.4407945 | 0.03955733 |
| TRIM22 | hsa-miR-4760-3p | blue | -0.387929717 | 0.06838085 |
| TRIM22 | hsa-miR-4311 | yellow | -0.417144168 | 0.05062959 |
| TRIM22 | hsa-miR-4762-5p | yellow | -0.387929717 | 0.06838085 |
| TSNAX | hsa-miR-34b-3p | brown | -0.412070283 | 0.05368029 |
| TSNAX | hsa-miR-34c-3p | brown | -0.606722689 | 0.007947337 |
| TSNAX | hsa-miR-4652-3p | brown | -0.491825821 | 0.02314015 |
| TSPAN6 | hsa-miR-212-3p | blue | -0.365622613 | 0.08468451 |
| TSPAN6 | hsa-miR-370-3p | blue | -0.351260504 | 0.09716475 |
| TSPAN6 | hsa-miR-4760-3p | blue | -0.354621849 | 0.09413249 |
| TSSC1 | hsa-miR-375 | brown | -0.470741024 | 0.02906323 |
| TSSC1 | hsa-miR-4652-3p | brown | -0.374484339 | 0.07818184 |
| TUSC3 | hsa-miR-34b-5p | brown | -0.436210848 | 0.04162947 |
| TUSC3 | hsa-miR-34c-5p | brown | -0.514132926 | 0.01839938 |
| UBQLN2 | hsa-miR-375 | brown | -0.43223835 | 0.04366305 |
| UBQLN2 | hsa-miR-4652-3p | brown | -0.376623377 | 0.07641468 |
| UCHL5 | hsa-miR-34b-3p | brown | -0.359816654 | 0.08903074 |
| UCHL5 | hsa-miR-34b-5p | brown | -0.370817418 | 0.08086606 |
| UCHL5 | hsa-miR-34c-3p | brown | -0.454239878 | 0.03460223 |
| UCHL5 | hsa-miR-34c-5p | brown | -0.4236822 | 0.0476735 |
| UCP2 | hsa-miR-377-5p | blue | -0.466462949 | 0.03018215 |
| WDR3 | hsa-miR-34b-5p | brown | -0.396791444 | 0.06264981 |
| WDR3 | hsa-miR-34c-5p | brown | -0.474407945 | 0.02790753 |
| WDR3 | hsa-miR-4652-3p | brown | -0.418181818 | 0.05003112 |
| WDR44 | hsa-miR-34c-5p | brown | -0.429488159 | 0.04491537 |
| WDR61 | hsa-miR-34b-5p | brown | -0.369289534 | 0.08180092 |
| WDR61 | hsa-miR-34c-5p | brown | -0.422459893 | 0.04833671 |
| WLS | hsa-miR-1912 | blue | -0.478074866 | 0.02675351 |
| WWTR1 | hsa-miR-377-5p | blue | -0.473185638 | 0.02838033 |
| WWTR1 | hsa-miR-4720-3p | blue | -0.564858671 | 0.01136205 |
| WWTR1 | hsa-miR-4760-3p | blue | -0.410236822 | 0.05457379 |
| WWTR1 | hsa-miR-4311 | yellow | -0.503323403 | 0.02041276 |
| YBX3 | hsa-miR-4311 | yellow | -0.514783407 | 0.01839938 |
| YBX3 | hsa-miR-4762-5p | yellow | -0.39434683 | 0.06410642 |
| YWHAZ | hsa-miR-34b-3p | brown | -0.38670741 | 0.06913607 |
| YWHAZ | hsa-miR-34b-5p | brown | -0.486631016 | 0.02445356 |
| ZFP36L1 | hsa-miR-4720-3p | blue | -0.519938885 | 0.0175892 |
| ZMAT3 | hsa-miR-34b-5p | brown | -0.411764706 | 0.05385778 |
| ZMAT3 | hsa-miR-34c-5p | brown | -0.463407181 | 0.03098728 |
| ZMAT3 | hsa-miR-375 | brown | -0.423376623 | 0.04780724 |
| ZNF22 | hsa-miR-4760-3p | blue | -0.397708174 | 0.06201102 |
