# Supplementary material for: Integrating mRNA and miRNA Weighted Gene Co-Expression Networks with eQTLs in the Nucleus Accumbens of Subjects with Alcohol Dependence
Source: PLoS One. 2015 Sep 18;10(9):e0137671. doi: 10.1371/journal.pone.0137671 (PMC4575063; doi:10.1371/journal.pone.0137671)
Supplement: S8 Table — (DOCX) [file pone.0137671.s009.docx]

**Table S8.**

| **SNP ID** | **probe ID** | **Gene Symbol** | **raw p-value** | **q-value** |
| --- | --- | --- | --- | --- |
| chr2:170783092:D | 205278_at | GAD1 | 5.51E-07 | 0.003 |
| rs1205026 | 205775_at | FAM50B | 6.50E-06 | 0.008 |
| rs34044693 | 212310_at | MIA3 | 9.26E-06 | 0.008 |
| rs701743 | 204992_s_at | PFN2 | 9.50E-06 | 0.008 |
| rs10911064 | 209755_at | NMNAT2 | 9.79E-06 | 0.008 |
| rs10911070 | 209755_at | NMNAT2 | 1.00E-05 | 0.008 |
| rs1711564 | 201512_s_at | TOMM70A | 1.07E-05 | 0.008 |
| rs6503859 | 201484_at | SUPT4H1 | 1.28E-05 | 0.008 |
| rs6844456 | 203362_s_at | MAD2L1 | 1.37E-05 | 0.008 |
| rs582149 | 210068_s_at | AQP4 | 1.48E-05 | 0.008 |
| rs2681101 | 204992_s_at | PFN2 | 1.56E-05 | 0.008 |
| rs876492 | 218432_at | FBXO3 | 1.70E-05 | 0.008 |
| rs752021 | 207508_at | ATP5G3 | 1.77E-05 | 0.008 |
| rs6602633 | 201725_at | CDC123 | 2.04E-05 | 0.009 |
| rs2804263 | 213005_s_at | KANK1 | 2.41E-05 | 0.009 |
| rs2863231 | 202232_s_at | EIF3M | 2.45E-05 | 0.009 |
| rs2641988 | 213005_s_at | KANK1 | 2.75E-05 | 0.010 |
| rs701748 | 204992_s_at | PFN2 | 3.00E-05 | 0.010 |
| chr3:148952063:D | 204992_s_at | PFN2 | 3.24E-05 | 0.010 |
| rs35107119 | 202825_at | SLC25A4 | 3.41E-05 | 0.010 |
| rs2756191 | 205413_at | MPPED2 | 3.60E-05 | 0.010 |
| rs11259457 | 205005_s_at | NMT2 | 3.73E-05 | 0.010 |
| rs6744982 | 214629_x_at | RTN4 | 4.38E-05 | 0.011 |
| rs12757009 | 203120_at | TP53BP2 | 4.46E-05 | 0.011 |
| rs3102512 | 212157_at | SDC2 | 4.52E-05 | 0.011 |
| rs9532432 | 218656_s_at | LHFP | 4.75E-05 | 0.011 |
| rs2931312 | 208731_at | RAB2A | 5.53E-05 | 0.012 |
| chr8:103661359:D | 203987_at | FZD6 | 5.54E-05 | 0.012 |
| rs12049040 | 202427_s_at | BRP44 | 6.69E-05 | 0.014 |
| rs1972551 | 218292_s_at | PRKAG2 | 7.48E-05 | 0.015 |
| rs5748304 | 208818_s_at | COMT | 8.17E-05 | 0.016 |
| rs1968908 | 208731_at | RAB2A | 9.54E-05 | 0.018 |
| chr6:8041919:D | 203704_s_at | RREB1 | 9.73E-05 | 0.018 |
| rs7138718 | 201313_at | ENO2 | 0.000106034 | 0.019 |
| rs35264487 | 205705_at | ANKRD26 | 0.00011214 | 0.020 |
| rs55927952 | 218224_at | PNMA1 | 0.000117766 | 0.020 |
| chr17:8930219:D | 210872_x_at | GAS7 | 0.000132542 | 0.022 |
| rs10206142 | 205278_at | GAD1 | 0.000136234 | 0.022 |
| rs8037444 | 210427_x_at | ANXA2 | 0.000139105 | 0.022 |
| rs62140389 | 203781_at | MRPL33 | 0.000146809 | 0.023 |
| rs34215541 | 209513_s_at | HSDL2 | 0.00016691 | 0.025 |
| rs3802872 | 210453_x_at | ATP5L | 0.000179263 | 0.026 |
| rs7761672 | 218854_at | DSE | 0.000180179 | 0.026 |
| rs11191242 | 202641_at | ARL3 | 0.00018452 | 0.026 |
| rs56666273 | 212600_s_at | UQCRC2 | 0.000195283 | 0.026 |
| rs13123051 | 205279_s_at | GLRB | 0.000207753 | 0.026 |
| rs6760912 | 206671_at | SAG | 0.000207907 | 0.026 |
| rs2495987 | 221488_s_at | CUTA | 0.000211055 | 0.026 |
| rs17626344 | 212038_s_at | VDAC1 | 0.000220037 | 0.026 |
| rs2724122 | 212092_at | PEG10 | 0.000229497 | 0.026 |
| rs11958087 | 209303_at | NDUFS4 | 0.00023138 | 0.026 |
| rs3814341 | 211963_s_at | ARPC5 | 0.000243614 | 0.026 |
| rs2027270 | 218322_s_at | ACSL5 | 0.000244143 | 0.026 |
| rs13283083 | 213904_at | --- | 0.000247095 | 0.026 |
| rs2358343 | 213714_at | CACNB2 | 0.000251721 | 0.026 |
| rs76874578 | 221531_at | WDR61 | 0.000255855 | 0.026 |
| rs1580985 | 218384_at | CARHSP1 | 0.000262844 | 0.026 |
| rs67796509 | 207812_s_at | GORASP2 | 0.000274001 | 0.026 |
| rs10211223 | 218133_s_at | NIF3L1 | 0.000274171 | 0.026 |
| rs9862408 | 204245_s_at | RPP14 | 0.0002752 | 0.026 |
| rs12363217 | 200986_at | SERPING1 | 0.000276774 | 0.026 |
| rs4595717 | 209476_at | TMX1 | 0.000282642 | 0.026 |
| rs10279545 | 205196_s_at | AP1S1 | 0.000287762 | 0.026 |
| rs12122027 | 202741_at | PRKACB | 0.000293832 | 0.026 |
| rs58091773 | 202475_at | TMEM147 | 0.000293934 | 0.026 |
| rs10866849 | 209598_at | PNMA2 | 0.000294403 | 0.026 |
| rs7770335 | 203137_at | WTAP | 0.000295966 | 0.026 |
| rs10865503 | 205550_s_at | BRE | 0.000297405 | 0.026 |
| rs6073758 | 202071_at | SDC4 | 0.000298721 | 0.026 |
| rs2705579 | 214717_at | PKI55 | 0.000302596 | 0.026 |
| rs1946182 | 201272_at | AKR1B1 | 0.000304432 | 0.026 |
| rs6670279 | 201180_s_at | GNAI3 | 0.00031374 | 0.026 |
| rs10878722 | 208838_at | CAND1 | 0.000320913 | 0.026 |
| rs13200739 | 207054_at | IMPG1 | 0.000321373 | 0.026 |
| rs1241 | 218882_s_at | WDR3 | 0.000323122 | 0.026 |
| rs135014 | 202096_s_at | TSPO | 0.000328681 | 0.026 |
| chr1:43273847:D | 206015_s_at | FOXJ3 | 0.000330145 | 0.026 |
| rs681660 | 200862_at | DHCR24 | 0.000331215 | 0.026 |
| rs4813909 | 202507_s_at | SNAP25 | 0.000339824 | 0.026 |
| rs12731712 | 200798_x_at | MCL1 | 0.000344818 | 0.026 |
| rs1711564 | 218557_at | NIT2 | 0.000345202 | 0.026 |
| rs4734491 | 210117_at | SPAG1 | 0.000351464 | 0.026 |
| rs10822920 | 218976_at | DNAJC12 | 0.00035234 | 0.026 |
| rs4734491 | 200638_s_at | YWHAZ | 0.000359414 | 0.026 |
| rs752555 | 208675_s_at | DDOST | 0.000359471 | 0.026 |
| rs7249866 | 44669_at | SDHAF1 | 0.000365837 | 0.026 |
| chr12:10120983:I | 209732_at | CLEC2B | 0.000372165 | 0.026 |
| rs1771991 | 218005_at | ZNF22 | 0.000379372 | 0.026 |
| rs2269668 | 201570_at | SAMM50 | 0.000392348 | 0.027 |
| chr20:1339981:I | 206803_at | PDYN | 0.000403064 | 0.027 |
| rs2428965 | 218488_at | EIF2B3 | 0.000408 | 0.027 |
| rs9900917 | 210448_s_at | P2RX5 | 0.00042642 | 0.028 |
| rs7964018 | 201160_s_at | CSDA | 0.000428561 | 0.028 |
| rs11199316 | 209248_at | GHITM | 0.000429701 | 0.028 |
| chr20:55445889:D | 211318_s_at | RAE1 | 0.000432249 | 0.028 |
| rs4847000 | 202834_at | AGT | 0.000442211 | 0.028 |
| rs148927935 | 200708_at | GOT2 | 0.000449921 | 0.028 |
| rs12050659 | 203889_at | SCG5 | 0.000456983 | 0.028 |
| rs34299790 | 202779_s_at | UBE2S | 0.000457755 | 0.028 |
| rs2224396 | 212407_at | METTL13 | 0.000458039 | 0.028 |
| chr18:40750466:D | 206984_s_at | RIT2 | 0.000459596 | 0.028 |
| rs115696548 | 214428_x_at | C4A | 0.000467406 | 0.028 |
| rs9327415 | 218706_s_at | GRAMD3 | 0.000474701 | 0.028 |
| rs4805496 | 202868_s_at | POP4 | 0.000483751 | 0.028 |
| rs62006060 | 218224_at | PNMA1 | 0.000493603 | 0.028 |
| rs10814323 | 215416_s_at | STOML2 | 0.000493905 | 0.028 |
| rs12133213 | 221874_at | KIAA1324 | 0.000502368 | 0.028 |
| rs61261949 | 205202_at | PCMT1 | 0.000503116 | 0.028 |
| chr14:22451707:I | 201412_at | LRP10 | 0.000504047 | 0.028 |
| rs11638916 | 221688_s_at | IMP3 | 0.000506571 | 0.028 |
| rs7301213 | 201988_s_at | CREBL2 | 0.000536205 | 0.029 |
| rs921120 | 212215_at | PREPL | 0.000536573 | 0.029 |
| rs3775500 | 200853_at | H2AFZ | 0.000540997 | 0.029 |
| rs10923570 | 218882_s_at | WDR3 | 0.000544474 | 0.029 |
| rs57326741 | 218491_s_at | THYN1 | 0.000551947 | 0.029 |
| rs6518508 | 208678_at | ATP6V1E1 | 0.000566191 | 0.029 |
| rs114659906 | 200905_x_at | HLA-E | 0.000572268 | 0.029 |
| rs35417171 | 205609_at | ANGPT1 | 0.000573869 | 0.029 |
| rs1883816 | 206062_at | GUCA1A | 0.000575491 | 0.029 |
| rs13021399 | 207198_s_at | LIMS1 | 0.000587011 | 0.029 |
| rs7250630 | 205119_s_at | FPR1 | 0.000594724 | 0.029 |
| rs10180866 | 201256_at | COX7A2L | 0.000597786 | 0.029 |
| rs1039519 | 200816_s_at | PAFAH1B1 | 0.000601147 | 0.029 |
| rs7497946 | 206042_x_at | PAR-SN | 0.000607381 | 0.029 |
| rs10844054 | 207717_s_at | PKP2 | 0.000610238 | 0.029 |
| rs2398664 | 204766_s_at | NUDT1 | 0.000613039 | 0.029 |
| rs1935148 | 212321_at | SGPL1 | 0.000620072 | 0.029 |
| rs3851068 | 203079_s_at | CUL2 | 0.000626088 | 0.029 |
| rs9902000 | 208827_at | PSMB6 | 0.000632735 | 0.030 |
| rs7636418 | 202430_s_at | PLSCR1 | 0.000642021 | 0.030 |
| rs678814 | 208679_s_at | ARPC2 | 0.000650543 | 0.030 |
| rs115834361 | 208659_at | CLIC1 | 0.000653477 | 0.030 |
| rs41302651 | 202587_s_at | AK1 | 0.000671073 | 0.030 |
| rs56101391 | 210501_x_at | EIF3K | 0.000680689 | 0.030 |
| rs10954176 | 218507_at | HILPDA | 0.000686063 | 0.030 |
| rs12809946 | 210149_s_at | ATP5H | 0.000689519 | 0.030 |
| rs289020 | 210946_at | PPAP2A | 0.000693037 | 0.030 |
| rs1294488 | 213710_s_at | LOC100652987 | 0.000694514 | 0.030 |
| rs7082919 | 215522_at | SORCS3 | 0.000702878 | 0.030 |
| rs2242090 | 201066_at | CYC1 | 0.000731704 | 0.031 |
| chr2:231040102:I | 202864_s_at | SP100 | 0.000739415 | 0.031 |
| rs114583166 | 1007_s_at | DDR1 | 0.000752463 | 0.032 |
| chr17:4571289:I | 200053_at | SPAG7 | 0.000758149 | 0.032 |
| rs1042303 | 208809_s_at | C6orf62 | 0.000763603 | 0.032 |
| rs1507055 | 206356_s_at | GNAL | 0.000766698 | 0.032 |
| rs9616037 | 208832_at | ATXN10 | 0.000772334 | 0.032 |
| rs113374069 | 202149_at | NEDD9 | 0.000794473 | 0.032 |
| rs138769756 | 209228_x_at | TUSC3 | 0.000812809 | 0.033 |
| rs9718306 | 208857_s_at | PCMT1 | 0.00081772 | 0.033 |
| rs629146 | 201753_s_at | ADD3 | 0.00082721 | 0.033 |
| chr2:218989013:I | 207988_s_at | ARPC2 | 0.000828718 | 0.033 |
| rs3753078 | 212271_at | MAPK1 | 0.000849064 | 0.033 |
| rs1610180 | 217882_at | EMC3 | 0.000851097 | 0.033 |
| rs7470954 | 218160_at | NDUFA8 | 0.00086227 | 0.033 |
| rs3755459 | 201761_at | MTHFD2 | 0.000864 | 0.033 |
| rs7297997 | 215058_at | DENND5B | 0.000867537 | 0.033 |
| rs57137815 | 208826_x_at | HINT1 | 0.000875226 | 0.033 |
| rs9915014 | 202936_s_at | SOX9 | 0.000876787 | 0.033 |
| rs12767657 | 218970_s_at | CUTC | 0.000890153 | 0.033 |
| rs11122483 | 219481_at | TTC13 | 0.00089066 | 0.033 |
| chr6:151493133:D | 220329_s_at | RMND1 | 0.000908077 | 0.034 |
| chr20:42913981:I | 211769_x_at | SERINC3 | 0.000912618 | 0.034 |
| rs11140062 | 200097_s_at | HNRNPK | 0.0009161 | 0.034 |
| chr1:98991321:I | 213496_at | LPPR4 | 0.000936303 | 0.034 |
| rs2011702 | 205119_s_at | FPR1 | 0.000958935 | 0.034 |
| rs4711750 | 203094_at | MAD2L1BP | 0.000961688 | 0.034 |
| chr3:148952063:D | 202133_at | WWTR1 | 0.000968879 | 0.034 |
| rs34003734 | 213738_s_at | ATP5A1 | 0.00097178 | 0.034 |
| rs1449508 | 203156_at | AKAP11 | 0.000974008 | 0.034 |
| rs4701068 | 209104_s_at | NHP2 | 0.000983368 | 0.034 |
| rs11580613 | 210927_x_at | JTB | 0.000987929 | 0.034 |
| rs6918529 | 218285_s_at | BDH2 | 0.000990978 | 0.034 |
| rs2315484 | 203313_s_at | TGIF1 | 0.000995367 | 0.034 |
| rs641648 | 202507_s_at | SNAP25 | 0.00100341 | 0.034 |
| rs12539370 | 204957_at | ORC5 | 0.00100972 | 0.034 |
| rs7949056 | 212063_at | CD44 | 0.001010534 | 0.034 |
| rs12974285 | 207120_at | ZNF667 | 0.001015995 | 0.034 |
| rs62036940 | 218120_s_at | HMOX2 | 0.00101688 | 0.034 |
| rs10845200 | 208868_s_at | GABARAPL1 | 0.001030863 | 0.034 |
| rs1793719 | 201592_at | EIF3H | 0.001038503 | 0.034 |
| rs7130708 | 207573_x_at | ATP5L | 0.001043493 | 0.034 |
| rs2044787 | 217957_at | C16orf80 | 0.001045383 | 0.034 |
| rs2925206 | 219683_at | FZD3 | 0.001048319 | 0.034 |
| chr3:33175159:I | 217746_s_at | PDCD6IP | 0.001054146 | 0.034 |
| rs13110042 | 213533_at | D4S234E | 0.001057334 | 0.034 |
| rs4758407 | 213293_s_at | TRIM22 | 0.001073419 | 0.035 |
| rs62007276 | 221531_at | WDR61 | 0.001095106 | 0.035 |
| rs269238 | 218200_s_at | NDUFB2 | 0.001103832 | 0.035 |
| rs62044232 | 212600_s_at | UQCRC2 | 0.001107098 | 0.035 |
| chr15:51106304:I | 212820_at | DMXL2 | 0.001110738 | 0.035 |
| chr14:63209184:I | 200989_at | HIF1A | 0.001114596 | 0.035 |
| chr9:76660814:D | 201012_at | ANXA1 | 0.001132867 | 0.035 |
| rs266372 | 202670_at | MAP2K1 | 0.001143815 | 0.035 |
| rs13123051 | 203817_at | GUCY1B3 | 0.001145557 | 0.035 |
| rs73402972 | 201315_x_at | IFITM2 | 0.001151574 | 0.035 |
| rs11639660 | 221515_s_at | LCMT1 | 0.00115203 | 0.035 |
| rs4989513 | 215171_s_at | TIMM17A | 0.001176076 | 0.036 |
| rs6059839 | 200903_s_at | AHCY | 0.001186756 | 0.036 |
| rs11785301 | 213423_x_at | TUSC3 | 0.001208541 | 0.036 |
| rs206138 | 204072_s_at | FRY | 0.001217867 | 0.036 |
| rs2050047 | 205202_at | PCMT1 | 0.00121797 | 0.036 |
| rs35257551 | 212990_at | SYNJ1 | 0.001230178 | 0.036 |
| rs1834228 | 205217_at | TIMM8A | 0.001250958 | 0.036 |
| rs4073620 | 222216_s_at | MRPL17 | 0.001252099 | 0.036 |
| rs11769320 | 209507_at | RPA3 | 0.001257095 | 0.036 |
| rs142086710 | 200822_x_at | TPI1 | 0.001259116 | 0.036 |
| rs8049603 | 202077_at | NDUFAB1 | 0.0012604 | 0.036 |
| rs12339807 | 201628_s_at | RRAGA | 0.00126567 | 0.036 |
| rs2769577 | 203721_s_at | UTP18 | 0.001269653 | 0.036 |
| chr6:32514538:D | 208451_s_at | C4A | 0.001275658 | 0.036 |
| rs1014971 | 208909_at | UQCRFS1 | 0.00128086 | 0.036 |
| rs11621149 | 202543_s_at | GMFB | 0.001286286 | 0.036 |
| rs6584540 | 206875_s_at | SLK | 0.001312799 | 0.037 |
| rs12129379 | 200916_at | TAGLN2 | 0.001330895 | 0.037 |
| rs10992660 | 204744_s_at | IARS | 0.001345423 | 0.037 |
| rs1096722 | 212687_at | LIMS1 | 0.001346141 | 0.037 |
| rs369940 | 219421_at | TTC33 | 0.001354603 | 0.037 |
| rs4845039 | 220251_at | DIEXF | 0.001354912 | 0.037 |
| rs576521 | 218226_s_at | NDUFB4 | 0.001358396 | 0.037 |
| rs11102950 | 201274_at | PSMA5 | 0.001360706 | 0.037 |
| rs13243252 | 205196_s_at | AP1S1 | 0.001374057 | 0.037 |
| rs35020435 | 209569_x_at | D4S234E | 0.001396626 | 0.037 |
| rs7692006 | 207922_s_at | MAEA | 0.0014036 | 0.037 |
| rs13392737 | 214717_at | PKI55 | 0.001414921 | 0.038 |
| rs6985160 | 212110_at | SLC39A14 | 0.001438707 | 0.038 |
| chr14:50811184:I | 217906_at | KLHDC2 | 0.001444322 | 0.038 |
| rs3178327 | 200820_at | PSMD8 | 0.001452754 | 0.038 |
| chr1:227485662:D | 203723_at | ITPKB | 0.00147926 | 0.039 |
| chr10:75934398:I | 204119_s_at | ADK | 0.001521194 | 0.040 |
| rs2075467 | 205963_s_at | DNAJA3 | 0.001545045 | 0.040 |
| rs7694682 | 213911_s_at | H2AFZ | 0.001549048 | 0.040 |
| rs2169122 | 209550_at | NDN | 0.001557126 | 0.040 |
| rs7532791 | 221958_s_at | WLS | 0.001559644 | 0.040 |
| rs6546066 | 200978_at | MDH1 | 0.001565348 | 0.040 |
| rs7655694 | 207400_at | NPY5R | 0.00158373 | 0.040 |
| rs11055826 | 201324_at | EMP1 | 0.001595146 | 0.040 |
| chr8:71519924:D | 201398_s_at | TRAM1 | 0.001611001 | 0.040 |
| rs193327 | 217820_s_at | ENAH | 0.001626359 | 0.041 |
| rs1263653 | 211902_x_at | YME1L1 | 0.001645 | 0.041 |
| rs10152106 | 212460_at | SPTSSA | 0.001657876 | 0.041 |
| rs2246441 | 202180_s_at | MVP | 0.001663959 | 0.041 |
| rs2490430 | 201966_at | NDUFS2 | 0.001699076 | 0.042 |
| rs11937659 | 202614_at | SLC30A9 | 0.001704924 | 0.042 |
| rs10762317 | 221699_s_at | DDX50 | 0.001733932 | 0.042 |
| rs9645570 | 215522_at | SORCS3 | 0.001748034 | 0.042 |
| rs6596974 | 213572_s_at | SERPINB1 | 0.001750623 | 0.042 |
| rs17439519 | 203157_s_at | GLS | 0.001757264 | 0.042 |
| rs11639978 | 218866_s_at | POLR3K | 0.001769647 | 0.042 |
| rs7180378 | 209550_at | NDN | 0.001771287 | 0.042 |
| chr2:74408733:I | 209549_s_at | DGUOK | 0.00177376 | 0.042 |
| rs458461 | 204194_at | BACH1 | 0.001777335 | 0.042 |
| rs487976 | 203854_at | CFI | 0.001777765 | 0.042 |
| rs12277519 | 204070_at | RARRES3 | 0.001789871 | 0.042 |
| rs12614861 | 207507_s_at | ATP5G3 | 0.00179558 | 0.042 |
| rs10248025 | 214829_at | AASS | 0.001803306 | 0.042 |
| rs61767323 | 217923_at | PEF1 | 0.001808243 | 0.042 |
| rs1642018 | 202180_s_at | MVP | 0.001812527 | 0.042 |
| rs2044787 | 208581_x_at | MT1X | 0.00182506 | 0.042 |
| rs9788415 | 212887_at | SEC23A | 0.00182567 | 0.042 |
| chr19:57447040:D | 209243_s_at | PEG3 | 0.001830622 | 0.042 |
| rs10876947 | 201322_at | ATP5B | 0.001856128 | 0.042 |
| rs6899791 | 201999_s_at | DYNLT1 | 0.00186926 | 0.042 |
| rs17582459 | 202920_at | ANK2 | 0.001869845 | 0.042 |
| rs6751950 | 215952_s_at | OAZ1 | 0.00190155 | 0.043 |
| rs7562649 | 201656_at | ITGA6 | 0.001911504 | 0.043 |
| rs4624763 | 213217_at | ADCY2 | 0.001926536 | 0.043 |
| chr3:10282255:D | 211672_s_at | ARPC4 | 0.001942581 | 0.043 |
| rs3859580 | 221741_s_at | YTHDF1 | 0.001947156 | 0.043 |
| rs2090035 | 200673_at | LAPTM4A | 0.001963869 | 0.043 |
| rs1041045 | 215506_s_at | DIRAS3 | 0.001967095 | 0.043 |
| rs61916978 | 208121_s_at | PTPRO | 0.001972734 | 0.043 |
| rs2090690 | 203033_x_at | FH | 0.001991613 | 0.044 |
| rs2466946 | 207088_s_at | SLC25A11 | 0.002007074 | 0.044 |
| rs2695094 | 201146_at | NFE2L2 | 0.002011439 | 0.044 |
| rs12704713 | 212092_at | PEG10 | 0.002021489 | 0.044 |
| rs12582584 | 221696_s_at | STYK1 | 0.002022492 | 0.044 |
| rs10163112 | 203663_s_at | COX5A | 0.002084404 | 0.045 |
| rs7307406 | 212067_s_at | C1R | 0.002084706 | 0.045 |
| rs7589667 | 202863_at | SP100 | 0.002090718 | 0.045 |
| rs13295416 | 221796_at | NTRK2 | 0.002098707 | 0.045 |
| rs12942267 | 205031_at | EFNB3 | 0.002111802 | 0.045 |
| rs35033649 | 202948_at | IL1R1 | 0.002126973 | 0.045 |
| rs1294173 | 220329_s_at | RMND1 | 0.002149527 | 0.045 |
| rs222975 | 202325_s_at | ATP5J | 0.002156372 | 0.045 |
| rs12202149 | 210501_x_at | EIF3K | 0.002177126 | 0.046 |
| rs495406 | 211763_s_at | UBE2B | 0.002191132 | 0.046 |
| rs2050047 | 220329_s_at | RMND1 | 0.002192439 | 0.046 |
| rs61919598 | 213011_s_at | TPI1 | 0.002216232 | 0.046 |
| rs59591052 | 210240_s_at | CDKN2D | 0.002241271 | 0.046 |
| rs4878509 | 211475_s_at | BAG1 | 0.002248027 | 0.046 |
| rs2437000 | 208697_s_at | EIF3E | 0.002248118 | 0.046 |
| rs11895 | 222043_at | CLU | 0.002263429 | 0.046 |
| rs6016400 | 218559_s_at | MAFB | 0.002269615 | 0.046 |
| rs4732986 | 202594_at | LEPROTL1 | 0.00228017 | 0.046 |
| rs3826700 | 202736_s_at | LSM4 | 0.002302129 | 0.046 |
| rs168914 | 209046_s_at | GABARAPL2 | 0.002305655 | 0.046 |
| rs35247064 | 218283_at | SS18L2 | 0.002309054 | 0.046 |
| rs34279656 | 218788_s_at | SMYD3 | 0.002331772 | 0.047 |
| rs4815589 | 210014_x_at | IDH3B | 0.002355982 | 0.047 |
| rs55695577 | 207643_s_at | TNFRSF1A | 0.00237051 | 0.047 |
| rs2645477 | 200614_at | CLTC | 0.002379626 | 0.047 |
| chr14:72009084:I | 207079_s_at | MED6 | 0.002409061 | 0.048 |
| chr17:5295252:I | 208827_at | PSMB6 | 0.002440214 | 0.048 |
| rs2301733 | 209840_s_at | LRRN3 | 0.00246945 | 0.049 |
| rs9312367 | 200906_s_at | PALLD | 0.002476287 | 0.049 |
| rs17833053 | 218316_at | TIMM9 | 0.002506192 | 0.049 |
| rs1898517 | 217968_at | TSSC1 | 0.00251348 | 0.049 |
| rs6727086 | 200812_at | CCT7 | 0.002520412 | 0.049 |
| rs35774314 | 200818_at | ATP5O | 0.002524327 | 0.049 |
| rs62494668 | 209598_at | PNMA2 | 0.002529275 | 0.049 |
| rs7176730 | 201590_x_at | ANXA2 | 0.002550131 | 0.049 |
| chr2:62112221:I | 219326_s_at | B3GNT2 | 0.002551692 | 0.049 |
| chr2:203069480:D | 208761_s_at | SUMO1 | 0.002574523 | 0.049 |
| rs1712355 | 212460_at | SPTSSA | 0.002587323 | 0.049 |
| rs216312 | 213011_s_at | TPI1 | 0.002591349 | 0.049 |
| rs932554 | 211962_s_at | ZFP36L1 | 0.002619317 | 0.049 |
| rs4443097 | 217960_s_at | TOMM22 | 0.002630387 | 0.049 |
| rs4721321 | 204766_s_at | NUDT1 | 0.002641637 | 0.049 |
| rs7524452 | 218488_at | EIF2B3 | 0.002648268 | 0.049 |
| chr19:320220:I | 211270_x_at | PTBP1 | 0.002649159 | 0.049 |
| chr1:32206487:I | 200040_at | KHDRBS1 | 0.002658447 | 0.049 |
| rs6023939 | 204239_s_at | NNAT | 0.002685416 | 0.050 |
| rs10845179 | 221696_s_at | STYK1 | 0.002689344 | 0.050 |
| rs11730758 | 203302_at | DCK | 0.002697569 | 0.050 |
| rs73402972 | 212203_x_at | IFITM3 | 0.002697604 | 0.050 |
| rs6106668 | 210418_s_at | IDH3B | 0.002728261 | 0.050 |
| chr16:76192043:I | 200079_s_at | KARS | 0.002809196 | 0.051 |
| rs2419725 | 211558_s_at | DHPS | 0.002816767 | 0.051 |
| rs7305558 | 200804_at | TMBIM6 | 0.002831286 | 0.051 |
| rs2448424 | 212195_at | IL6ST | 0.002865574 | 0.051 |
| rs808213 | 222230_s_at | ACTR10 | 0.002866393 | 0.051 |
| rs650546 | 203685_at | BCL2 | 0.002868799 | 0.051 |
| rs139233474 | 203540_at | GFAP | 0.002902219 | 0.052 |
| rs9660850 | 210927_x_at | JTB | 0.002943863 | 0.052 |
| rs55865513 | 202920_at | ANK2 | 0.002947604 | 0.052 |
| rs4371315 | 212645_x_at | BRE | 0.002948468 | 0.052 |
| rs3735643 | 218507_at | HILPDA | 0.002964092 | 0.052 |
| rs808213 | 218316_at | TIMM9 | 0.002978911 | 0.052 |
| rs6967785 | 218982_s_at | MRPS17 | 0.002983936 | 0.052 |
| rs747740 | 217860_at | NDUFA10 | 0.002998933 | 0.052 |
| rs2413508 | 36711_at | MAFF | 0.003008486 | 0.052 |
| rs10269573 | 204002_s_at | ICA1 | 0.003013486 | 0.052 |
| rs7160237 | 202376_at | SERPINA3 | 0.003015042 | 0.052 |
| rs17177097 | 207079_s_at | MED6 | 0.003045035 | 0.053 |
| rs12528898 | 207054_at | IMPG1 | 0.003053699 | 0.053 |
| chr3:179577915:I | 203621_at | NDUFB5 | 0.003105312 | 0.053 |
| rs10884001 | 215522_at | SORCS3 | 0.003109757 | 0.053 |
| chr3:57568478:I | 208911_s_at | PDHB | 0.003115882 | 0.053 |
| rs2479729 | 202587_s_at | AK1 | 0.003141081 | 0.054 |
| rs11844749 | 211902_x_at | YME1L1 | 0.0031944 | 0.054 |
| chr17:69136395:I | 202936_s_at | SOX9 | 0.003249953 | 0.055 |
| rs9467703 | 203944_x_at | BTN2A1 | 0.003253878 | 0.055 |
| rs6967957 | 218654_s_at | MRPS33 | 0.003270395 | 0.055 |
| rs117303347 | 221009_s_at | ANGPTL4 | 0.003290803 | 0.055 |
| rs7927425 | 211404_s_at | APLP2 | 0.003294798 | 0.055 |
| rs17496908 | 211615_s_at | LRPPRC | 0.003295256 | 0.055 |
| rs10001332 | 48531_at | TNIP2 | 0.003313437 | 0.055 |
| rs2932983 | 203137_at | WTAP | 0.003323042 | 0.055 |
| rs223586 | 221263_s_at | SF3B5 | 0.003350806 | 0.056 |
| rs61957797 | 205531_s_at | GLS2 | 0.003395608 | 0.056 |
| rs35097149 | 201172_x_at | ATP6V0E1 | 0.003402954 | 0.056 |
| rs12979985 | 202736_s_at | LSM4 | 0.003424784 | 0.056 |
| rs11931860 | 207922_s_at | MAEA | 0.003436957 | 0.056 |
| rs1951722 | 206935_at | PCDH8 | 0.00345122 | 0.056 |
| rs9465751 | 202149_at | NEDD9 | 0.003473993 | 0.056 |
| chr14:23726371:I | 210972_x_at | TRAC | 0.003474451 | 0.056 |
| rs960633 | 201527_at | ATP6V1F | 0.003483157 | 0.056 |
| rs4293 | 208981_at | PECAM1 | 0.003551232 | 0.057 |
| rs11714155 | 219628_at | ZMAT3 | 0.003567788 | 0.057 |
| rs215377 | 217286_s_at | NDRG3 | 0.003569998 | 0.057 |
| rs2508445 | 210453_x_at | ATP5L | 0.003582349 | 0.057 |
| rs2451269 | 203137_at | WTAP | 0.003639976 | 0.058 |
| rs7504530 | 201319_at | MYL12A | 0.003645134 | 0.058 |
| rs563082 | 200967_at | PPIB | 0.003691224 | 0.058 |
| rs56017041 | 207643_s_at | TNFRSF1A | 0.003693731 | 0.058 |
| rs55988458 | 204766_s_at | NUDT1 | 0.003698862 | 0.058 |
| rs11595598 | 208813_at | GOT1 | 0.003701521 | 0.058 |
| rs62197303 | 219911_s_at | SLCO4A1 | 0.003707476 | 0.058 |
| rs6449986 | 211297_s_at | CDK7 | 0.0037137 | 0.058 |
| rs11709187 | 217947_at | CMTM6 | 0.003726469 | 0.058 |
| rs7740085 | 215527_at | KHDRBS2 | 0.00374236 | 0.058 |
| rs11858956 | 213552_at | GLCE | 0.003761318 | 0.058 |
| chr7:101007525:D | 205196_s_at | AP1S1 | 0.003767458 | 0.058 |
| rs10875869 | 217286_s_at | NDRG3 | 0.003768167 | 0.058 |
| rs3791938 | 217730_at | TMBIM1 | 0.00381182 | 0.059 |
| rs7554126 | 203033_x_at | FH | 0.003815807 | 0.059 |
| rs7111826 | 212063_at | CD44 | 0.003830564 | 0.059 |
| rs2471636 | 217997_at | PHLDA1 | 0.003837815 | 0.059 |
| rs4790213 | 208827_at | PSMB6 | 0.003837955 | 0.059 |
| rs11632235 | 211595_s_at | MRPS11 | 0.003840531 | 0.059 |
| chr1:101859018:I | 222360_at | DPH5 | 0.003846777 | 0.059 |
| rs1446558 | 208857_s_at | PCMT1 | 0.003859053 | 0.059 |
| rs1661725 | 211999_at | H3F3A | 0.003869372 | 0.059 |
| rs1492254 | 200030_s_at | SLC25A3 | 0.003883247 | 0.059 |
| rs525357 | 215171_s_at | TIMM17A | 0.00390898 | 0.059 |
| rs10281564 | 209507_at | RPA3 | 0.0039494 | 0.059 |
| rs10148691 | 212460_at | SPTSSA | 0.003972283 | 0.059 |
| chr2:132451880:I | 201411_s_at | PLEKHB2 | 0.003975508 | 0.059 |
| rs12444268 | 213272_s_at | TMEM159 | 0.003982288 | 0.059 |
| chr17:46735822:D | 210817_s_at | CALCOCO2 | 0.003984548 | 0.059 |
| rs11934833 | 205279_s_at | GLRB | 0.003995085 | 0.059 |
| rs2646255 | 212977_at | CXCR7 | 0.003997006 | 0.059 |
| rs1330865 | 215522_at | SORCS3 | 0.004007675 | 0.059 |
| rs8017061 | 208799_at | PSMB5 | 0.004032057 | 0.059 |
| rs56101391 | 205202_at | PCMT1 | 0.004041336 | 0.059 |
| chr6:76634964:D | 207054_at | IMPG1 | 0.004044843 | 0.059 |
| rs11264529 | 203411_s_at | LMNA | 0.004065993 | 0.059 |
| rs7296156 | 203645_s_at | CD163 | 0.004078397 | 0.059 |
| rs194828 | 204957_at | ORC5 | 0.004105348 | 0.059 |
| chr5:95192337:I | 202975_s_at | RHOBTB3 | 0.004108226 | 0.059 |
| rs12141090 | 202269_x_at | GBP1 | 0.004112266 | 0.059 |
| rs75772313 | 204554_at | PPP1R3D | 0.004117794 | 0.059 |
| rs8106216 | 205119_s_at | FPR1 | 0.004138613 | 0.060 |
| rs947889 | 207573_x_at | ATP5L | 0.004149677 | 0.060 |
| rs1967827 | 219960_s_at | UCHL5 | 0.004163386 | 0.060 |
| rs4503602 | 221667_s_at | HSPB8 | 0.00417351 | 0.060 |
| rs13175611 | 203893_at | TAF9 | 0.004190062 | 0.060 |
| rs12983907 | 217780_at | WDR83OS | 0.004222784 | 0.060 |
| rs117989086 | 202395_at | LOC100507699 | 0.00423643 | 0.060 |
| rs4780052 | 203889_at | SCG5 | 0.004288044 | 0.061 |
| rs115069213 | 215952_s_at | OAZ1 | 0.004304327 | 0.061 |
| rs6765381 | 219714_s_at | CACNA2D3 | 0.004317164 | 0.061 |
| rs4304175 | 220329_s_at | RMND1 | 0.004322233 | 0.061 |
| rs6075339 | 206803_at | PDYN | 0.004334781 | 0.061 |
| rs9996585 | 209570_s_at | D4S234E | 0.004355794 | 0.061 |
| rs4685318 | 216218_s_at | PLCL2 | 0.004398537 | 0.061 |
| rs254428 | 202382_s_at | GNPDA1 | 0.004401297 | 0.061 |
| rs3786047 | 208827_at | PSMB6 | 0.004405529 | 0.061 |
| rs2670288 | 208782_at | FSTL1 | 0.004422811 | 0.061 |
| rs6763776 | 216218_s_at | PLCL2 | 0.004452406 | 0.062 |
| rs7962563 | 203645_s_at | CD163 | 0.004464238 | 0.062 |
| rs11128855 | 216218_s_at | PLCL2 | 0.004489137 | 0.062 |
| rs6130371 | 204239_s_at | NNAT | 0.004503084 | 0.062 |
| rs576627 | 210978_s_at | TAGLN2 | 0.00455257 | 0.062 |
| rs182888673 | 202802_at | DHPS | 0.004578199 | 0.063 |
| rs931803 | 214150_x_at | ATP6V0E1 | 0.004580327 | 0.063 |
| rs12924648 | 204326_x_at | MT1X | 0.004635486 | 0.063 |
| rs586104 | 211615_s_at | LRPPRC | 0.004655969 | 0.063 |
| rs7863990 | 206542_s_at | SMARCA2 | 0.004658416 | 0.063 |
| rs1292131 | 208998_at | UCP2 | 0.004672777 | 0.063 |
| rs111674972 | 211376_s_at | NSMCE4A | 0.004798313 | 0.065 |
| rs2274578 | 1255_g_at | GUCA1A | 0.004801399 | 0.065 |
| rs10923360 | 218882_s_at | WDR3 | 0.004824885 | 0.065 |
| rs62044232 | 205489_at | CRYM | 0.004858739 | 0.065 |
| rs4465517 | 209476_at | TMX1 | 0.004858881 | 0.065 |
| rs4240205 | 217837_s_at | CHMP3 | 0.004862398 | 0.065 |
| rs35261662 | 207054_at | IMPG1 | 0.004892123 | 0.065 |
| rs2886722 | 217837_s_at | CHMP3 | 0.004915897 | 0.065 |
| rs6029108 | 218559_s_at | MAFB | 0.004962753 | 0.066 |
| chr18:24655122:D | 210906_x_at | AQP4 | 0.005029019 | 0.066 |
| rs59772551 | 200786_at | PSMB7 | 0.00503256 | 0.066 |
| rs6424952 | 212407_at | METTL13 | 0.005035455 | 0.066 |
| rs13297158 | 215416_s_at | STOML2 | 0.005055481 | 0.066 |
| rs2504735 | 219582_at | OGFRL1 | 0.005056993 | 0.066 |
| rs61936484 | 209075_s_at | ISCU | 0.005100072 | 0.067 |
| rs1286459 | 213710_s_at | LOC100652987 | 0.005116837 | 0.067 |
| rs7514026 | 201145_at | HAX1 | 0.005193635 | 0.068 |
| rs250854 | 212038_s_at | VDAC1 | 0.005215916 | 0.068 |
| rs4254841 | 203302_at | DCK | 0.005224503 | 0.068 |
| rs7644973 | 205633_s_at | ALAS1 | 0.005257735 | 0.068 |
| rs852347 | 201112_s_at | CSE1L | 0.005337098 | 0.069 |
| rs1147944 | 209183_s_at | C10orf10 | 0.005337256 | 0.069 |
| rs2784262 | 202373_s_at | AURKAPS1 | 0.005407801 | 0.070 |
| rs6015739 | 204554_at | PPP1R3D | 0.005530804 | 0.071 |
| rs6657810 | 218882_s_at | WDR3 | 0.005548925 | 0.071 |
| rs10495471 | 203033_x_at | FH | 0.005550578 | 0.071 |
| rs8130506 | 201086_x_at | SON | 0.005579423 | 0.071 |
| rs1780705 | 204239_s_at | NNAT | 0.005600457 | 0.071 |
| rs2488094 | 221263_s_at | SF3B5 | 0.005649877 | 0.072 |
| rs1870063 | 202736_s_at | LSM4 | 0.005664023 | 0.072 |
| rs60964576 | 212460_at | SPTSSA | 0.005678136 | 0.072 |
| rs1862849 | 217546_at | MT1M | 0.005744121 | 0.072 |
| rs34003734 | 205856_at | SLC14A1 | 0.005763559 | 0.072 |
| rs1152230 | 219760_at | LIN7B | 0.005768984 | 0.072 |
| rs663818 | 218488_at | EIF2B3 | 0.005774023 | 0.072 |
| rs340515 | 211971_s_at | LRPPRC | 0.005790432 | 0.072 |
| rs11621456 | 209671_x_at | TRAC | 0.005794391 | 0.072 |
| rs4243820 | 201756_at | RPA2 | 0.005798133 | 0.072 |
| rs4687658 | 205633_s_at | ALAS1 | 0.00580681 | 0.072 |
| rs17087144 | 221796_at | NTRK2 | 0.005817833 | 0.072 |
| rs6062234 | 219911_s_at | SLCO4A1 | 0.005842409 | 0.072 |
| rs10813878 | 203613_s_at | NDUFB6 | 0.00584925 | 0.072 |
| rs34270 | 209075_s_at | ISCU | 0.00587578 | 0.072 |
| rs7246292 | 202475_at | TMEM147 | 0.005938661 | 0.072 |
| rs2008366 | 203150_at | RABEPK | 0.005944039 | 0.072 |
| rs12532041 | 209507_at | RPA3 | 0.005955691 | 0.072 |
| rs3754176 | 202613_at | CTPS1 | 0.005959097 | 0.072 |
| rs4865825 | 209303_at | NDUFS4 | 0.005965337 | 0.072 |
| rs12444698 | 218120_s_at | HMOX2 | 0.005966675 | 0.072 |
| rs66477917 | 216218_s_at | PLCL2 | 0.005967997 | 0.072 |
| rs6585012 | 201753_s_at | ADD3 | 0.005973967 | 0.072 |
| rs12599013 | 205963_s_at | DNAJA3 | 0.006003437 | 0.072 |
| rs2192756 | 202948_at | IL1R1 | 0.006039411 | 0.073 |
| rs11096592 | 212099_at | RHOB | 0.006069742 | 0.073 |
| rs4976055 | 211297_s_at | CDK7 | 0.006075057 | 0.073 |
| rs7517707 | 203411_s_at | LMNA | 0.006091791 | 0.073 |
| rs11248945 | 218866_s_at | POLR3K | 0.006107177 | 0.073 |
| rs12649814 | 209569_x_at | D4S234E | 0.00612998 | 0.073 |
| rs6696511 | 206015_s_at | FOXJ3 | 0.006195143 | 0.074 |
| rs764946 | 202370_s_at | CBFB | 0.006222542 | 0.074 |
| rs35477883 | 205758_at | CD8A | 0.006310442 | 0.075 |
| rs2459994 | 208457_at | GABRD | 0.006315563 | 0.075 |
| rs570772 | 201601_x_at | IFITM1 | 0.006320143 | 0.075 |
| chr7:9214553:D | 204002_s_at | ICA1 | 0.006352842 | 0.075 |
| rs61166009 | 219582_at | OGFRL1 | 0.006364024 | 0.075 |
| rs9316362 | 202930_s_at | SUCLA2 | 0.006380295 | 0.075 |
| chr8:99197259:I | 204068_at | STK3 | 0.006407844 | 0.075 |
| rs4663003 | 210406_s_at | RAB6A | 0.006478055 | 0.076 |
| rs7974872 | 209075_s_at | ISCU | 0.006502361 | 0.076 |
| rs114001271 | 221488_s_at | CUTA | 0.006520765 | 0.076 |
| rs11265633 | 202252_at | RAB13 | 0.006565132 | 0.076 |
| rs1229735 | 201054_at | HNRNPA0 | 0.006582361 | 0.076 |
| rs155960 | 202975_s_at | RHOBTB3 | 0.006663942 | 0.077 |
| rs10483863 | 200701_at | NPC2 | 0.006710723 | 0.077 |
| rs1556880 | 210501_x_at | EIF3K | 0.006745711 | 0.078 |
| rs35856900 | 213227_at | LOC100652849 | 0.006766446 | 0.078 |
| rs7914700 | 218597_s_at | CISD1 | 0.006861014 | 0.079 |
| rs2999386 | 209476_at | TMX1 | 0.006897051 | 0.079 |
| rs111742659 | 202370_s_at | CBFB | 0.006911003 | 0.079 |
| rs28448715 | 210278_s_at | AP4S1 | 0.006919138 | 0.079 |
| rs4687657 | 205633_s_at | ALAS1 | 0.006959358 | 0.079 |
| rs335685 | 221688_s_at | IMP3 | 0.007046674 | 0.080 |
| rs839856 | 201274_at | PSMA5 | 0.007047316 | 0.080 |
| chr5:95253458:I | 202975_s_at | RHOBTB3 | 0.007060121 | 0.080 |
| chr17:8930219:D | 218526_s_at | RANGRF | 0.007120958 | 0.080 |
| rs10231832 | 206805_at | SEMA3A | 0.007151375 | 0.080 |
| rs12129556 | 202658_at | PEX11B | 0.007164116 | 0.080 |
| rs7615055 | 208911_s_at | PDHB | 0.007167275 | 0.080 |
| rs3790997 | 212977_at | CXCR7 | 0.007182351 | 0.080 |
| rs9324628 | 205097_at | SLC26A2 | 0.007203641 | 0.081 |
| rs2220530 | 200989_at | HIF1A | 0.007325708 | 0.082 |
| rs7564499 | 217968_at | TSSC1 | 0.007378864 | 0.082 |
| rs62176560 | 201410_at | PLEKHB2 | 0.007391495 | 0.082 |
| rs9394166 | 221488_s_at | CUTA | 0.007505617 | 0.083 |
| rs10137082 | 208799_at | PSMB5 | 0.007603623 | 0.084 |
| chr10:45079660:I | 209183_s_at | C10orf10 | 0.007629244 | 0.084 |
| rs4238500 | 206042_x_at | PAR-SN | 0.007696915 | 0.085 |
| rs6727791 | 206671_at | SAG | 0.007698259 | 0.085 |
| rs809871 | 203944_x_at | BTN2A1 | 0.007704622 | 0.085 |
| rs11778274 | 201592_at | EIF3H | 0.007780689 | 0.085 |
| rs4408643 | 221009_s_at | ANGPTL4 | 0.00780804 | 0.085 |
| rs74846680 | 213592_at | APLNR | 0.007834487 | 0.086 |
| rs10733852 | 201859_at | SRGN | 0.007887307 | 0.086 |
| rs1406050 | 209840_s_at | LRRN3 | 0.007907236 | 0.086 |
| rs1150994 | 207717_s_at | PKP2 | 0.007912902 | 0.086 |
| rs12100712 | 212887_at | SEC23A | 0.007929232 | 0.086 |
| chr14:63012781:D | 206099_at | PRKCH | 0.007946871 | 0.086 |
| rs6095888 | 212501_at | CEBPB | 0.007979135 | 0.086 |
| rs419174 | 219760_at | LIN7B | 0.0081098 | 0.087 |
| rs2184423 | 215522_at | SORCS3 | 0.008119452 | 0.087 |
| rs2959574 | 201398_s_at | TRAM1 | 0.008135767 | 0.087 |
| rs4802324 | 212716_s_at | EIF3K | 0.008149611 | 0.087 |
| chr12:98571401:I | 200030_s_at | SLC25A3 | 0.008377146 | 0.090 |
| rs9920305 | 213552_at | GLCE | 0.008405006 | 0.090 |
| rs1362765 | 207088_s_at | SLC25A11 | 0.008460184 | 0.090 |
| rs10178599 | 212977_at | CXCR7 | 0.008567954 | 0.091 |
| rs3933430 | 213887_s_at | POLR2E | 0.008572702 | 0.091 |
| rs13127918 | 203817_at | GUCY1B3 | 0.00859724 | 0.091 |
| rs11867362 | 208946_s_at | BECN1 | 0.00860694 | 0.091 |
| rs10085108 | 202975_s_at | RHOBTB3 | 0.008608462 | 0.091 |
| rs6016348 | 218559_s_at | MAFB | 0.008626178 | 0.091 |
| rs161977 | 207717_s_at | PKP2 | 0.008651089 | 0.091 |
| chr1:220550647:D | 200843_s_at | EPRS | 0.008675841 | 0.091 |
| rs10445831 | 201198_s_at | PSMD1 | 0.008690197 | 0.091 |
| rs11844114 | 202376_at | SERPINA3 | 0.008715384 | 0.091 |
| rs4296182 | 209476_at | TMX1 | 0.008779944 | 0.092 |
| rs6049210 | 210014_x_at | IDH3B | 0.008787766 | 0.092 |
| rs2099984 | 206989_s_at | SCAF11 | 0.008829307 | 0.092 |
| rs1388483 | 203302_at | DCK | 0.008852732 | 0.092 |
| rs6693877 | 210978_s_at | TAGLN2 | 0.008912902 | 0.092 |
| rs6684194 | 201180_s_at | GNAI3 | 0.0089482 | 0.093 |
| rs62044232 | 213272_s_at | TMEM159 | 0.008958173 | 0.093 |
| rs618006 | 202564_x_at | ARL2 | 0.008979711 | 0.093 |
| rs1263646 | 208799_at | PSMB5 | 0.008991925 | 0.093 |
| chr3:178991864:D | 219628_at | ZMAT3 | 0.009133431 | 0.094 |
| rs7805875 | 218654_s_at | MRPS33 | 0.00922694 | 0.095 |
| chr18:40708701:I | 206984_s_at | RIT2 | 0.009360908 | 0.096 |
| rs10819141 | 203150_at | RABEPK | 0.009570919 | 0.098 |
| rs8054024 | 218120_s_at | HMOX2 | 0.009588296 | 0.098 |
| chr3:52340636:D | 205633_s_at | ALAS1 | 0.009607231 | 0.098 |
| rs326640 | 207717_s_at | PKP2 | 0.009616113 | 0.098 |
| rs34001136 | 205609_at | ANGPT1 | 0.009669819 | 0.098 |
| rs7093411 | 208813_at | GOT1 | 0.009680376 | 0.098 |
| rs12466 | 215171_s_at | TIMM17A | 0.009709312 | 0.098 |
| rs2152786 | 200843_s_at | EPRS | 0.009790274 | 0.099 |
| rs12434436 | 209671_x_at | TRAC | 0.009804207 | 0.099 |
| rs7397265 | 207761_s_at | METTL7A | 0.00981314 | 0.099 |
| rs7537915 | 218882_s_at | WDR3 | 0.009839687 | 0.099 |
| rs748694 | 208827_at | PSMB6 | 0.00994341 | 0.100 |
| rs13277972 | 201398_s_at | TRAM1 | 0.009951172 | 0.100 |
| rs7368883 | 211971_s_at | LRPPRC | 0.00995544 | 0.100 |
| rs2595585 | 210418_s_at | IDH3B | 0.009976084 | 0.100 |

(B)

| SNP | Gene Symbol | p-value | q-value |
| --- | --- | --- | --- |
| rs11626307 | hsa-miR-134-5p | 7.11E-05 | 0.019 |
| rs11626307 | hsa-miR-370-3p | 8.15E-05 | 0.019 |
| rs4889332 | hsa-miR-4720-3p | 9.21E-05 | 0.019 |
| rs62046623 | hsa-miR-4720-3p | 9.39E-05 | 0.019 |
| rs2447472 | hsa-miR-4720-3p | 0.000185351 | 0.031 |
| rs11626307 | hsa-miR-382-5p | 0.000557512 | 0.063 |
| rs8011440 | hsa-miR-134-5p | 0.000740694 | 0.075 |
| rs12916425 | hsa-miR-4311 | 0.000866152 | 0.080 |
| rs11624974 | hsa-miR-377-5p | 0.001229057 | 0.088 |
| rs331072 | hsa-miR-4633-5p | 0.001264661 | 0.088 |
| rs13164401 | hsa-miR-4633-5p | 0.001480117 | 0.088 |
| rs11624974 | hsa-miR-382-5p | 0.001545244 | 0.088 |
| rs8011440 | hsa-miR-370-3p | 0.001796001 | 0.088 |
| rs11624974 | hsa-miR-134-5p | 0.002066849 | 0.088 |
| rs9893575 | hsa-miR-212-3p | 0.002096748 | 0.088 |
| rs28568867 | hsa-miR-4652-3p | 0.002344653 | 0.088 |
| rs11624974 | hsa-miR-370-3p | 0.002469148 | 0.088 |
| chr7:92792768:D | hsa-miR-4652-3p | 0.002471309 | 0.088 |
| rs3785965 | hsa-miR-132-3p | 0.002910124 | 0.088 |
| rs10144321 | hsa-miR-382-5p | 0.003099445 | 0.088 |
| rs11624049 | hsa-miR-382-5p | 0.003099445 | 0.088 |
| rs1212911 | hsa-miR-382-5p | 0.003099445 | 0.088 |
| rs113289613 | hsa-miR-132-3p | 0.003104862 | 0.088 |
| rs9893575 | hsa-miR-132-3p | 0.003231618 | 0.088 |
| rs74551863 | hsa-miR-383-5p | 0.003663736 | 0.088 |
| rs2119261 | hsa-miR-4311 | 0.003669425 | 0.088 |
| rs11649448 | hsa-miR-4720-3p | 0.003715492 | 0.088 |
| rs1190715 | hsa-miR-134-5p | 0.003822763 | 0.088 |
| rs42508 | hsa-miR-4652-3p | 0.003999963 | 0.088 |
| rs13243787 | hsa-miR-4652-3p | 0.003999963 | 0.088 |
| rs17711548 | hsa-miR-4652-3p | 0.003999963 | 0.088 |
| rs35504285 | hsa-miR-4652-3p | 0.003999963 | 0.088 |
| rs3802055 | hsa-miR-4652-3p | 0.003999963 | 0.088 |
| rs28597901 | hsa-miR-134-5p | 0.004132578 | 0.088 |
| rs12599630 | hsa-miR-4720-3p | 0.004213864 | 0.088 |
| rs7157984 | hsa-miR-370-3p | 0.004286764 | 0.088 |
| rs7223273 | hsa-miR-212-3p | 0.004537036 | 0.091 |
| rs9324019 | hsa-miR-377-5p | 0.004769532 | 0.091 |
| chr19:19295118:I | hsa-miR-3189-5p | 0.004904668 | 0.091 |
| rs11984874 | hsa-miR-383-5p | 0.004920015 | 0.091 |
| rs13245529 | hsa-miR-4652-3p | 0.004972065 | 0.091 |
| rs1051322 | hsa-miR-212-3p | 0.005063817 | 0.091 |
| chr15:66944217:I | hsa-miR-4311 | 0.005078322 | 0.091 |
| rs72807926 | hsa-miR-4720-3p | 0.005374218 | 0.091 |
| rs612345 | hsa-miR-34b-3p | 0.005380058 | 0.091 |
| rs10953075 | hsa-miR-4652-3p | 0.005668089 | 0.091 |
| rs552850 | hsa-miR-4633-5p | 0.005861541 | 0.091 |
| rs463035 | hsa-miR-4633-5p | 0.005912749 | 0.091 |
| rs915380 | hsa-miR-382-5p | 0.005943514 | 0.091 |
| rs11859045 | hsa-miR-4720-3p | 0.006166045 | 0.091 |
| rs6564783 | hsa-miR-4720-3p | 0.006220037 | 0.091 |
| rs12925874 | hsa-miR-4720-3p | 0.006301735 | 0.091 |
| rs11214031 | hsa-miR-34b-3p | 0.006801513 | 0.095 |
| rs7106006 | hsa-miR-34c-3p | 0.006890228 | 0.095 |
| rs56103101 | hsa-miR-4311 | 0.007042334 | 0.096 |
| rs76102375 | hsa-miR-383-5p | 0.007375484 | 0.096 |
| rs59874704 | hsa-miR-4652-3p | 0.007502934 | 0.096 |
| rs4835948 | hsa-miR-4633-5p | 0.008409463 | 0.096 |
| rs11624049 | hsa-miR-134-5p | 0.008478435 | 0.096 |
| rs10144321 | hsa-miR-134-5p | 0.008478435 | 0.096 |
| rs1212911 | hsa-miR-134-5p | 0.008478435 | 0.096 |
| rs8008884 | hsa-miR-377-5p | 0.008569557 | 0.096 |
| rs17113227 | hsa-miR-34b-3p | 0.008933436 | 0.096 |
| rs4936678 | hsa-miR-34b-3p | 0.008933436 | 0.096 |
| rs7157984 | hsa-miR-377-5p | 0.009486262 | 0.097 |
| chr19:18328289:D | hsa-miR-3189-5p | 0.009487085 | 0.097 |
| rs2580312 | hsa-miR-4720-3p | 0.009803774 | 0.099 |
| rs61811421 | hsa-miR-555 | 0.009891084 | 0.099 |
