# Supplementary material for: Integrating mRNA and miRNA Weighted Gene Co-Expression Networks with eQTLs in the Nucleus Accumbens of Subjects with Alcohol Dependence
Source: PLoS One. 2015 Sep 18;10(9):e0137671. doi: 10.1371/journal.pone.0137671 (PMC4575063; doi:10.1371/journal.pone.0137671)
Supplement: S9 Table — The lines in bold represent the matched case-control samples (N = 36) used in this study. (DOCX) [file pone.0137671.s010.docx]

**Table S9**.

| # | Age began | Alcohol mean daily gms | Total drinking Years | Age | Sex | RIN | Alcohol Status | Brain (g) | pH | PMI | Smoke |
| --- | --- | --- | --- | --- | --- | --- | --- | --- | --- | --- | --- |
| **43** | 25 | 80 | 29 | **54** | **1** | **7.8** | **Cases** | **1340** | **6.41** | **17** | **Yes** |
| **45** | 25 | 150 | 20 | **46** | **1** | **8** | **Cases** | **1200** | **6.51** | **24** | **Unknown** |
| 28 | 25 | 100 | 25 | 65 | 1 | 5.8 | Cases | 1330 | 5.66 | 32 | Unknown |
| 5 | 20 | 210 | 26 | 46 | 2 |  | Cases | 1220 | 6.5 | 27 | Yes |
| 44 |  |  |  | 37 | 1 | 3.7 | Controls | 1280 | 5.3 | 11 | Yes |
| 23 | 17 | 150 | 34 | 51 | 1 | 3.7 | Cases | 1240 | 5.58 | 27 | **Yes** |
| **59** |  | 0 | 25 | **50** | **1** | **5.3** | **Controls** | **1320** | **6.68** | **29** | Yes |
| **57** | 30 | 85 | 20 | **50** | **1** | **6.2** | **Cases** | **1420** | **6.59** | **24** | **No** |
| 32 | 25 | 90 | 54 | 79 | 1 | 6.6 | Cases | 1300 | 6.34 | 48 | Unknown |
| **48** | 25 | 203 | 48 | **73** | **1** | **8.5** | **Cases** | **1300** | **6.3** | **24** | **Yes** |
| 55 | 25 | 80 | 36 | 61 | 1 | 4.6 | Cases | 1410 | 5.29 | 28 | Unknown |
| **51** |  |  |  | **56** | **1** | **7.8** | **Controls** | **1635** | **6.53** | **24** | Yes |
| **82** |  |  |  | **59** | **1** | **5.3** | **Controls** | **1360** | **6.56** | **20** | **Unknown** |
| **46** | 25 | 80 | 14 | **39** | **1** | **7.6** | **Cases** | **1360** | **6.56** | **24** | **No** |
| **35** |  |  |  | **43** | **1** | **8.3** | **Controls** | **1500** | **6.43** | **13** | **Yes** |
| 63 | 25 | 300 | 23 | 52 | 1 | 6.4 | Cases | 1570 | 6.04 | 35 | **No** |
| **3** |  |  |  | **37** | **1** |  | **Controls** | **1520** | **6.37** | **24** | **Unknown** |
| 34 | 15 | 262 | 55 | 70 | 1 | 5.8 | Cases | 1450 | 6.24 | 33.5 | **Yes** |
| **24** | 25 | 190 | 31 | **56** | **1** | **6.4** | **Cases** | **1284** | **6.51** | **45** | Yes |
| 78 |  |  |  | 51 | 1 | 4.7 | Controls | 1530 | 5.88 | 20 | Yes |
| **27** |  |  |  | **46** | **1** | **7** | **Controls** | **1490** | **6.65** | **25** | **Yes** |
| **40** | 25 | 100 | 34 | **59** | **1** | **6.7** | **Cases** | **1520** | **6.57** | **24** | Yes |
| **42** | 25 | 80 | 31 | **56** | **1** | **8.2** | **Cases** | **1230** | **6.52** | **22** | **Yes** |
| **1** | 25 | 80 | 12 | **37** | **1** |  | **Cases** | **1500** | **6.33** | **17** | **Yes** |
| **49** |  |  |  | **58** | **1** | **4.6** | **Controls** | **1350** | **6.46** | **12** | Yes |
| 12 | 25 | 20 | 35 | 60 | 2 | 6.3 | Controls | 1200 | 6.8 | 21 | **No** |
| **54** | 25 | 9 | 25 | **50** | **1** | **7.3** | **Controls** | **1500** | **6.26** | **19** | **No** |
| **73** | 25 | 10 | 57 | **82** | **1** | **5.3** | **Controls** | **1300** | **6.24** | **36** | **Yes** |
| **60** | 18 | 167.5 | 32 | **50** | **1** | **6.3** | **Cases** | **1520** | **6.3** | **17** | Yes |
| **61** | 25 | 175 | 26 | **51** | **1** | **6.4** | **Cases** | **1460** | **6.35** | **46** | No |
| 52 | 25 | 10 | 8 | 53 | 1 | 8.6 | Controls | 1590 | 6.84 | 16 | Yes |
| 67 | 25 | 195.5 | 42 | 67 | 1 | 5.1 | Cases | 1460 | 6.4 | 48 | **Yes** |
| 16 | 25 | 154 | 33 | 58 | 2 | 4.5 | Cases | 1220 | 6.4 | 48 | **Yes** |
| **33** | 25 | 305 | 27 | **52** | **1** | **6** | **Cases** | **1380** | **6.78** | **45.5** | **Yes** |
| 79 |  |  |  | 78 | 2 | 2.8 | Controls | 1480 | 6.54 | 37 | **Yes** |
| 31 | 25 | 126 | 31 | 56 | 1 | 7 | Cases | 1140 | 6.13 | 31 | Yes |
| **13** | 25 | 268 | 36 | **61** | **1** | **6.9** | **Cases** | **1340** | **6.93** | **21** | **Yes** |
| 75 | 25 | 7 | 23 | 48 | 1 |  | Controls | 1330 | 6.73 | 24 | **Yes** |
| **70** | 25 | 488 | 28 | **53** | **1** | **5.8** | **Cases** | **1340** | **6.75** | **57** | Yes |
| 7 | 25 | 250 | 16 | 41 | 1 | 2.7 | Cases | 1580 | 6.7 | 54 | Yes |
| **18** |  |  |  | **44** | **1** | **6.9** | **Controls** | **1220** | **6.6** | **50** | Ex-smoker |
| **30** |  |  |  | **56** | **1** | **7.1** | **Controls** | **1510** | **6.76** | **37** | Yes |
| 2 |  |  |  | 43 | 1 |  | Controls | 1400 | 6.2 | 66 | **No** |
| **26** | 18 | 120 | 24 | **42** | **1** | **6.4** | **Cases** | **1400** | **6.5** | **41** | **Yes** |
| **80** | 25 | 10 | 32 | **57** | **1** | **7.7** | **Controls** | **1360** | **6.6** | **18** | **Yes** |
| 53 | 25 | 42 | 35 | 60 | 1 | 6 | Controls | 1610 | 6.7 | 25 | **Ex-smoker** |
| **11** |  |  |  | **71** | **2** | **4.1** | **Controls** | **1260** | **6.2** | **16** | **Unknown** |
| 58 |  |  |  | 60 | 1 | 7.4 | Controls | 1420 | 6.8 | 28 | Unknown |
| 39 |  |  |  | 54 | 1 | 6.2 | Controls | 1510 | 6.8 | 29 | **Unknown** |
| **15** | 42 | 112 | 28 | **75** | **2** | **4.9** | **Cases** | **1150** | **6** | **9** | **Yes** |
| **29** | 18 | 162 | 33 | **58** | **1** | **7.6** | **Cases** | **1250** | **6.64** | **20** | Unknown |
| 8 | 25 | 80 | 18 | 43 | 1 | 4.7 | Cases | 1400 | 6.29 | 29 | **Ex-smoker** |
| **25** | 25 | 20 | 38 | **63** | **1** | **7.3** | **Controls** | **1570** | **6.94** | **24** | **No** |
| 17 | 36 | 250 | 13 | 49 | 2 | 4.9 | Cases | 1284 | 6.11 | 48 | No |
| **77** | 25 | 30 | 48 | **73** | **1** | **6** | **Controls** | **1380** | **6.8** | **48** | Unknown |
| **74** | 25 | 20 | 39 | **64** | **1** | **6.5** | **Controls** | **1390** | **6.94** | **9.5** | Yes |
| 19 |  |  |  | 49 | 2 | 6.9 | Controls | 1330 | 6.93 | 15 | **Ex-smoker** |
| 36 | 25 | 170 | 32 | 57 | 1 | 5.9 | Cases | 1280 | 6.46 | 43 | **Yes** |
| **38** |  |  |  | **53** | **1** | **3.7** | **Controls** | **1450** | **6.64** | **27** | Unknown |
| 41 |  |  |  | 69 | 1 | 3.4 | Controls | 1460 | 6.34 | 19 | **Ex-smoker** |
| **22** | 25 | 200 | 35 | **60** | **1** | **6.9** | **Cases** | **1470** | **6.48** | **16.5** | No |
| 81 | 25 | 10 | 27 | 52 | 2 | 2.7 | Controls | 1200 | 6.21 | 11 | **Ex-smoker** |
| **50** | 25 | 10 | 35 | **60** | **1** | **2.4** | **Controls** | **1620** | **6.66** | **21.5** | No |
| **69** | 25 | 14 | 30 | **55** | **1** | **6.3** | **Controls** | **1560** | **6.89** | **39** | No |
| 21 | 25 | 33 | 39 | 64 | 1 | 5.7 | Controls | 1470 | 6.68 | 39.5 | **Yes** |
| 47 | 25 | 10 | 41 | 66 | 1 | 7 | Controls | 1430 | 6.74 | 23 | **Yes** |
| **20** | 25 | 10 | 37 | **62** | **1** | **8.1** | **Controls** | **1480** | **6.56** | **37.5** | **Yes** |
| **37** | 20 | 187 | 38 | **58** | **1** | **7.5** | **Cases** | **1462** | **6.65** | **21.5** | Unknown |
| 14 | 25 |  | 31 | 56 | 2 | 6.5 | Cases | 1180 | 6.54 | 38 | **Unknown** |
| **56** | 25 | 138 | 37 | **63** | **1** | **5.5** | **Cases** | **1616** | **6.21** | **25.5** | No |
| **76** | 25 | 94 | 48 | **73** | **1** | **6.8** | **Cases** | **1188** | **6.84** | **19** | Yes |
| 72 | 25 | 135 | 29 | 54 | 1 | 2.7 | Cases | 1408 | 6.16 | 27 | **No** |
| **64** | 24 | 197 | 31 | **55** | **1** | **6.9** | **Cases** | **1362** | **7.02** | **48** | No |
| 10 | 25 | 10 | 37 | 63 | 2 |  | Controls | 1346 | 7.02 | 42 | Ex-smoker |
| **66** | 25 | 15 | 22 | **47** | **1** | **6.5** | **Controls** | **1534** | **6.74** | **38** | **Unknown** |
| 6 | 25 | 18 | 20 | 52 | 2 |  | Controls | 1304 | 6.33 | 43 | Yes |
| **62** | 25 | 147 | 39 | **64** | **1** | **6.7** | **Cases** | **1370** | **6.76** | **39** | **Yes** |
| 71 | 25 | 25 | 43 | 68 | 1 | 5 | Controls | 1408 | 6.12 | 45.5 | Yes |
| 4 | 25 | 162 | 41.5 | 67 | 2 | 2.7 | Cases | 1308 | 5.89 | 18 | No |
| **68** |  |  |  | **50** | **1** | **6.7** | **Controls** | **1426** | **6.37** | **30** | **Yes** |
| 9 | 25 | 129 | 27 | 52 | 2 | 4.3 | Cases | 1456 | 6.44 | 23 | **No** |
| **65** | 25 | 20 | 30 | **55** | **1** | **6.7** | **Controls** | **1631** | **6.39** | **12** | **No** |
