# Supplementary material for: Integrating mRNA and miRNA Weighted Gene Co-Expression Networks with eQTLs in the Nucleus Accumbens of Subjects with Alcohol Dependence
Source: PLoS One. 2015 Sep 18;10(9):e0137671. doi: 10.1371/journal.pone.0137671 (PMC4575063; doi:10.1371/journal.pone.0137671)
Supplement: S10 Table — The letters behind each ID number reflects the disease status of the subject (i.e., A = Alcoholics and C = Controls). (DOCX) [file pone.0137671.s011.docx]

| Sample | TPD52L1_203786_s_at | TPD52L1 | CD63_200663_at | CD63 | PLK2_201939_at | PLK2 | MAPT_203928_x_at | MAPT | LPPR1_219732_at | LPPR1 |
| --- | --- | --- | --- | --- | --- | --- | --- | --- | --- | --- |
| 13A | 11.9023 | 0.754504478 | 11.768 | 1.035 | 11.832 | 1.041 | 10.461 | 1.120 | 10.1681 | 0.795 |
| 18C | 12.5729 | 1.305667127 | 11.745 | 0.862 | 11.968 | 1.233 | 9.976 | 0.903 | 11.2435 | 1.177 |
| 20C | 12.4896 | 1.243737632 | 11.929 | 1.004 | 11.990 | 1.053 | 9.163 | 0.813 | 10.9049 | 1.058 |
| 24A | 11.8256 | 0.574005258 | 12.537 | 1.461 | 11.105 | 0.677 | 9.702 | 1.097 | 9.76311 | 0.581 |
| 25C | 12.5375 | 1.137506811 | 11.910 | 0.929 | 11.610 | 1.064 | 10.040 | 0.880 | 10.8111 | 1.017 |
| 26A | 11.9201 | 0.977390769 | 11.963 | 1.070 | 11.343 | 0.986 | 10.156 | 1.232 | 10.4724 | 1.068 |
| 27C | 12.6485 | 1.116914644 | 11.952 | 1.001 | 11.792 | 1.034 | 9.654 | 0.832 | 11.1738 | 1.200 |
| 30C | 12.4481 | 0.779669545 | 11.969 | 0.866 | 11.561 | 0.891 | 8.820 | 1.055 | 10.8251 | 1.008 |
| 33A | 11.7137 | 0.988933185 | 12.877 | 1.627 | 9.171 | 0.483 | 9.460 | 1.102 | 9.13843 | 0.787 |
| 35C | 12.7228 | 1.27042829 | 11.743 | 0.950 | 12.048 | 1.333 | 9.743 | 0.998 | 10.9181 | 1.214 |
| 40A | 11.9616 | 1.081641795 | 12.354 | 1.240 | 11.068 | 0.909 | 9.446 | 1.283 | 10.0432 | 0.847 |
| 42A | 12.3274 | 1.014474914 | 11.426 | 0.681 | 11.648 | 0.986 | 9.777 | 1.037 | 11.2765 | 1.263 |
| 43A | 11.8928 | 0.714872754 | 12.248 | 1.245 | 10.953 | 0.703 | 10.050 | 1.076 | 10.0976 | 0.758 |
| 45A | 12.6239 | 1.010568434 | 11.747 | 0.866 | 11.517 | 0.880 | 9.812 | 0.857 | 10.9722 | 1.104 |
| 46A | 11.7028 | 0.669907458 | 12.085 | 0.917 | 11.701 | 1.029 | 10.199 | 0.984 | 10.3491 | 0.797 |
| 48A | 12.0339 | 0.489179289 | 12.478 | 1.135 | 11.184 | 0.667 | 9.790 | 0.868 | 10.0758 | 0.682 |
| 51C | 12.5518 | 0.926527999 | 11.942 | 1.129 | 11.944 | 1.254 | 9.476 | 0.975 | 10.8279 | 1.094 |
| 54C | 12.659 | 1.070128887 | 11.670 | 0.920 | 12.000 | 1.316 | 9.266 | 0.846 | 11.2692 | 1.396 |
| 57A | 10.1425 | 0.230134733 | 12.436 | 1.464 | 10.932 | 0.685 | 10.583 | 1.712 | 8.72006 | 0.420 |
| 59C | 9.06781 | 0.247697505 | 12.721 | 1.743 | 7.635 | 0.211 | 10.619 | 2.159 | 8.84514 | 0.549 |
| 60A | 10.3816 | 0.401562049 | 12.516 | 1.758 | 8.747 | 0.275 | 10.009 | 2.113 | 9.27562 | 0.654 |
| 61A | 11.5614 | 0.887859762 | 12.937 | 1.411 | 10.629 | 0.694 | 9.837 | 1.334 | 9.99323 | 0.832 |
| 64A | 12.1255 | 1.058140723 | 12.094 | 0.906 | 11.729 | 1.015 | 9.645 | 1.086 | 10.3192 | 0.904 |
| 66C | 12.7192 | 1.456480024 | 11.828 | 0.942 | 11.820 | 1.114 | 9.254 | 0.857 | 11.321 | 1.228 |
| 68C | 12.6167 | 1.301828125 | 11.737 | 0.737 | 11.875 | 1.150 | 9.409 | 0.927 | 11.2478 | 1.287 |
| 69C | 12.5488 | 1.293342815 | 11.617 | 0.788 | 11.993 | 1.171 | 10.036 | 0.863 | 11.2294 | 1.256 |
| 70A | 12.3206 | 0.864524027 | 11.559 | 0.803 | 11.736 | 1.079 | 9.537 | 1.091 | 10.7565 | 1.049 |
| 73C | 10.1703 | 0.341073306 | 12.173 | 1.258 | 10.802 | 0.673 | 10.868 | 2.234 | 9.4246 | 0.651 |
| 74C | 12.4689 | 1.133138676 | 11.735 | 0.999 | 12.031 | 1.190 | 9.284 | 0.868 | 10.7182 | 0.987 |
| 76A | 12.0463 | 0.777038772 | 12.818 | 1.381 | 11.026 | 0.704 | 9.929 | 0.923 | 10.3638 | 0.755 |
| 77C | 12.4839 | 1.174607332 | 11.364 | 0.573 | 12.088 | 1.153 | 9.891 | 0.969 | 10.8577 | 0.992 |
| 80C | 12.7273 | 1.324343687 | 11.605 | 0.822 | 11.946 | 1.165 | 9.136 | 0.832 | 11.2185 | 1.169 |
| 82C | 12.3199 | 1.050153133 | 11.945 | 0.862 | 11.582 | 0.890 | 9.564 | 1.002 | 10.8246 | 1.031 |
