# Supplementary material for: Integrating mRNA and miRNA Weighted Gene Co-Expression Networks with eQTLs in the Nucleus Accumbens of Subjects with Alcohol Dependence
Source: PLoS One. 2015 Sep 18;10(9):e0137671. doi: 10.1371/journal.pone.0137671 (PMC4575063; doi:10.1371/journal.pone.0137671)
Supplement: S12 Table — (DOCX) [file pone.0137671.s013.docx]

| SNP | CHR | BP | COGA EA MAF | AUS MAF |
| --- | --- | --- | --- | --- |
| chr10:45079660:I | 10 | 45079660 | 0.3383 | 0.34804 |
| chr10:75934398:I | 10 | 75934398 | 0.4921 | 0.49896 |
| chr1:101859018:I | 1 | 101859018 | 0.3604 | 0.40754 |
| chr12:10120983:I | 12 | 10120983 | 0.4094 | 0.48478 |
| chr1:220550647:D | 1 | 220550647 | 0.06903 | 0.09411 |
| chr1:227485662:D | 1 | 227485662 | 0.4143 | 0.48055 |
| chr12:98571401:I | 12 | 98571401 | 0.3043 | 0.32195 |
| chr1:32206487:I | 1 | 32206487 | 0.3147 | 0.39141 |
| chr14:22451707:I | 14 | 22451707 | 0.07904 | 0.12889 |
| chr14:23726371:I | 14 | 23726371 | 0.2412 | 0.34302 |
| chr1:43273847:D | 1 | 43273847 | 0.4443 | 0.4822 |
| chr14:50811184:I | 14 | 50811184 | 0.3106 | 0.28327 |
| chr14:63012781:D | 14 | 63012781 | 0.3177 | 0.28339 |
| chr14:63209184:I | 14 | 63209184 | 0.4502 | 0.4987 |
| chr14:72009084:I | 14 | 72009084 | 0.2299 | 0.24704 |
| chr15:51106304:I | 15 | 51106304 | 0.45 | 0.48835 |
| chr16:76192043:I | 16 | 76192043 | 0.4074 | 0.42019 |
| chr17:4571289:I | 17 | 4571289 | 0.3216 | 0.3716 |
| chr17:46735822:D | 17 | 46735822 | 0.3103 | 0.3499 |
| chr17:5295252:I | 17 | 5295252 | 0.3222 | 0.37786 |
| chr17:69136395:I | 17 | 69136395 | 0.4596 | 0.49394 |
| chr17:8930219:D | 17 | 8930219 | 0.2638 | 0.25342 |
| chr18:24655122:D | 18 | 24655122 | 0.4278 | 0.45231 |
| chr18:40708701:I | 18 | 40708701 | 0.3792 | 0.44238 |
| chr18:40750466:D | 18 | 40750466 | 0.3226 | 0.38927 |
| chr19:320220:I | 19 | 320220 | 0.3714 | 0.39465 |
| chr19:57447040:D | 19 | 57447040 | 0.1643 | 0.19393 |
| chr1:98991321:I | 1 | 98991321 | 0.292 | 0.38361 |
| chr20:1339981:I | 20 | 1339981 | 0.2296 | 0.30999 |
| chr20:42913981:I | 20 | 42913981 | 0.2124 | 0.24445 |
| chr20:55445889:D | 20 | 55445889 | 0.2985 | 0.36972 |
| chr2:132451880:I | 2 | 132451880 | 0.4462 | 0.49822 |
| chr2:170783092:D | 2 | 170783092 | 0.3375 | 0.3038 |
| chr2:203069480:D | 2 | 203069480 | 0.2641 | 0.26112 |
| chr2:218989013:I | 2 | 218989013 | 0.4958 | 0.47331 |
| chr2:231040102:I | 2 | 231040102 | 0.3545 | 0.32731 |
| chr2:62112221:I | 2 | 62112221 | 0.4131 | 0.37658 |
| chr2:74408733:I | 2 | 74408733 | 0.3941 | 0.37851 |
| chr3:10282255:D | 3 | 10282255 | 0.4101 | 0.46208 |
| chr3:148952063:D | 3 | 148952063 | 0.2441 | 0.26115 |
| chr3:178991864:D | 3 | 178991864 | 0.4446 | 0.42749 |
| chr3:179577915:I | 3 | 179577915 | 0.4491 | 0.49849 |
| chr3:33175159:I | 3 | 33175159 | 0.3314 | 0.32019 |
| chr3:52340636:D | 3 | 52340636 | 0.3675 | 0.44674 |
| chr3:57568478:I | 3 | 57568478 | 0.4934 | 0.49458 |
| chr5:95192337:I | 5 | 95192337 | 0.1801 | 0.19998 |
| chr5:95253458:I | 5 | 95253458 | 0.3435 | 0.31566 |
| chr6:151493133:D | 6 | 151493133 | 0.4005 | 0.40437 |
| chr6:32514538:D | 6 | 32514538 | 0.3174 | 0.29636 |
| chr6:76634964:D | 6 | 76634964 | 0.1621 | 0.1707 |
| chr6:8041919:D | 6 | 8041919 | 0.1638 | 0.27309 |
| chr7:101007525:D | 7 | 101007525 | 0.4978 | 0.48732 |
| chr7:9214553:D | 7 | 9214553 | 0.392 | 0.4056 |
| chr8:103661359:D | 8 | 103661359 | 0.4569 | 0.45381 |
| chr8:71519924:D | 8 | 71519924 | 0.2792 | 0.35969 |
| chr8:99197259:I | 8 | 99197259 | 0.2835 | 0.36059 |
| chr9:76660814:D | 9 | 76660814 | 0.3095 | 0.36322 |
| rs10001332 | 4 | 2534477 | 0.2109 | 0.25083 |
| rs10085108 | 5 | 95928738 | 0.2485 | 0.29034 |
| rs10137082 | 14 | 23840033 | 0.2459 | 0.28481 |
| rs10148691 | 14 | 35556813 | 0.2153 | 0.25255 |
| rs1014971 | 22 | 39332623 | 0.3592 | 0.35235 |
| rs10152106 | 14 | 34205643 | 0.2598 | 0.25949 |
| rs10163112 | 15 | 76015397 | 0.3113 | 0.37975 |
| rs10178599 | 2 | 238264223 | 0.2609 | 0.29114 |
| rs10180866 | 2 | 42077657 | 0.4734 | 0.44304 |
| rs10206142 | 2 | 170887333 | 0.2956 | 0.27339 |
| rs10211223 | 2 | 202621002 | 0.1582 | 0.18354 |
| rs10231832 | 7 | 83366142 | 0.3224 | 0.34767 |
| rs10248025 | 7 | 122109781 | 0.3922 | 0.43018 |
| rs10269573 | 7 | 8840179 | 0.462 | 0.40492 |
| rs10279545 | 7 | 101171028 | 0.4929 | 0.44707 |
| rs10281564 | 7 | 7894736 | 0.1968 | 0.15648 |
| rs1039519 | 17 | 3447914 | 0.4085 | 0.37342 |
| rs1041045 | 1 | 69002803 | 0.3095 | 0.2913 |
| rs1042303 | 6 | 24437458 | 0.4547 | 0.38602 |
| rs10445831 | 2 | 232264384 | 0.4559 | 0.3595 |
| rs10483863 | 14 | 75322327 | 0.4852 | 0.43671 |
| rs10495471 | 1 | 240706107 | 0.1251 | 0.13924 |
| rs10733852 | 10 | 71171968 | 0.3852 | 0.37342 |
| rs10762317 | 10 | 71607758 | 0.4985 | 0.43362 |
| rs10813878 | 9 | 32859234 | 0.4414 | 0.44304 |
| rs10814323 | 9 | 36031610 | 0.2091 | 0.15823 |
| rs10819141 | 9 | 128988326 | 0.4618 | 0.45883 |
| rs10822920 | 10 | 68572526 | 0.4288 | 0.42303 |
| rs10844054 | 12 | 32067260 | 0.4366 | 0.41483 |
| rs10845179 | 12 | 10759489 | 0.3892 | 0.34331 |
| rs10845200 | 12 | 10824855 | 0.4058 | 0.40075 |
| rs10865503 | 2 | 28817870 | 0.2806 | 0.34326 |
| rs10866849 | 8 | 26302790 | 0.3794 | 0.4154 |
| rs10875869 | 12 | 49138481 | 0.4108 | 0.40345 |
| rs10876947 | 12 | 57283232 | 0.432 | 0.42442 |
| rs10878722 | 12 | 68383829 | 0.2445 | 0.26689 |
| rs10884001 | 10 | 106191341 | 0.436 | 0.46259 |
| rs10911064 | 1 | 182324168 | 0.3186 | 0.35442 |
| rs10911070 | 1 | 182365334 | 0.3188 | 0.34809 |
| rs10923360 | 1 | 118166877 | 0.3335 | 0.36076 |
| rs10923570 | 1 | 118964561 | 0.4608 | 0.4261 |
| rs10954176 | 7 | 127907319 | 0.4337 | 0.44937 |
| rs1096722 | 2 | 108727338 | 0.4449 | 0.46204 |
| rs10992660 | 9 | 95923174 | 0.4576 | 0.39818 |
| rs11055826 | 12 | 14335596 | 0.4691 | 0.45437 |
| rs11096592 | 2 | 19680042 | 0.3452 | 0.35443 |
| rs11102950 | 1 | 109722590 | 0.3785 | 0.29228 |
| rs11122483 | 1 | 230428624 | 0.208 | 0.22152 |
| rs11128855 | 3 | 17809203 | 0.4839 | 0.49603 |
| rs11140062 | 9 | 85993689 | 0.4318 | 0.45758 |
| rs111674972 | 10 | 123960811 | 0.3992 | 0.47401 |
| rs111742659 | 16 | 67819873 | 0.061 | 0.08862 |
| rs11191242 | 10 | 103983087 | 0.4914 | 0.48721 |
| rs11199316 | 10 | 85343296 | 0.2541 | 0.31028 |
| rs11248945 | 16 | 768596 | 0.178 | 0.21569 |
| rs11259457 | 10 | 15107077 | 0.2202 | 0.23418 |
| rs11264529 | 1 | 156679345 | 0.4081 | 0.41772 |
| rs11265633 | 1 | 153646700 | 0.4599 | 0.49692 |
| rs113374069 | 6 | 10546806 | 0.3393 | 0.38195 |
| rs114001271 | 6 | 32706801 | 0.3903 | 0.35456 |
| rs1147944 | 10 | 44959750 | 0.2545 | 0.24051 |
| rs115069213 | 2 | 69676898 | 0.08352 | 0.13525 |
| rs1150994 | 12 | 32068691 | 0.4823 | 0.48154 |
| rs1152230 | 19 | 50433503 | 0.2941 | 0.30179 |
| rs115696548 | 6 | 31351764 | 0.398 | 0.41139 |
| rs11580613 | 1 | 154291559 | 0.1596 | 0.16501 |
| rs11595598 | 10 | 101662856 | 0.474 | 0.43445 |
| rs11621149 | 14 | 55162554 | 0.3212 | 0.36967 |
| rs11621456 | 14 | 22611697 | 0.46 | 0.38608 |
| rs11632235 | 15 | 88234025 | 0.2893 | 0.39031 |
| rs11638916 | 15 | 76364596 | 0.4276 | 0.48933 |
| rs11639660 | 16 | 25934664 | 0.3878 | 0.44304 |
| rs11639978 | 16 | 639042 | 0.1749 | 0.26252 |
| rs11709187 | 3 | 31820684 | 0.361 | 0.33548 |
| rs11714155 | 3 | 179005701 | 0.4415 | 0.43697 |
| rs117303347 | 19 | 8104482 | 0.1441 | 0.25347 |
| rs11730758 | 4 | 72565508 | 0.3372 | 0.29747 |
| rs11769320 | 7 | 7886002 | 0.3743 | 0.40712 |
| rs11778274 | 8 | 117547928 | 0.3389 | 0.37962 |
| rs11785301 | 8 | 15012676 | 0.297 | 0.33953 |
| rs117989086 | 17 | 44350395 | 0.1712 | 0.20976 |
| rs11844114 | 14 | 94460529 | 0.4094 | 0.44937 |
| rs11844749 | 14 | 22277136 | 0.2581 | 0.23418 |
| rs11858956 | 15 | 70261228 | 0.3463 | 0.40509 |
| rs11867362 | 17 | 41938082 | 0.1297 | 0.18354 |
| rs11895 | 8 | 28286071 | 0.1219 | 0.15823 |
| rs11931860 | 4 | 1519502 | 0.3895 | 0.32911 |
| rs11934833 | 4 | 157557583 | 0.2898 | 0.31143 |
| rs11937659 | 4 | 42210117 | 0.3158 | 0.32911 |
| rs11958087 | 5 | 52664692 | 0.279 | 0.33308 |
| rs12049040 | 1 | 168521615 | 0.2026 | 0.23418 |
| rs1205026 | 6 | 3603809 | 0.3961 | 0.4215 |
| rs12050659 | 15 | 33974316 | 0.2437 | 0.28714 |
| rs12100712 | 14 | 40172991 | 0.3442 | 0.3086 |
| rs12122027 | 1 | 84031555 | 0.4029 | 0.39241 |
| rs12129379 | 1 | 159965261 | 0.1895 | 0.23608 |
| rs12129556 | 1 | 146482133 | 0.3174 | 0.41301 |
| rs12133213 | 1 | 110191395 | 0.3249 | 0.35234 |
| rs12141090 | 1 | 88647867 | 0.4031 | 0.45506 |
| rs12202149 | 6 | 149283505 | 0.3631 | 0.36083 |
| rs12277519 | 11 | 63154309 | 0.3035 | 0.33233 |
| rs1229735 | 5 | 136680804 | 0.2852 | 0.31644 |
| rs12339807 | 9 | 18510413 | 0.4347 | 0.42018 |
| rs12363217 | 11 | 56412474 | 0.4057 | 0.4304 |
| rs1241 | 1 | 117685560 | 0.2719 | 0.31013 |
| rs12434436 | 14 | 22094954 | 0.4782 | 0.47468 |
| rs12444268 | 16 | 20342572 | 0.2584 | 0.36709 |
| rs12444698 | 16 | 5052889 | 0.3053 | 0.31013 |
| rs12466 | 1 | 201197808 | 0.4061 | 0.4557 |
| rs12528898 | 6 | 77309002 | 0.4465 | 0.49149 |
| rs12532041 | 7 | 7904969 | 0.2374 | 0.20307 |
| rs12539370 | 7 | 104005655 | 0.2698 | 0.25874 |
| rs12582584 | 12 | 9824138 | 0.4286 | 0.41023 |
| rs12599013 | 16 | 4167739 | 0.3684 | 0.40544 |
| rs12614861 | 2 | 175047184 | 0.3448 | 0.3481 |
| rs1263646 | 14 | 23017530 | 0.1738 | 0.18987 |
| rs1263653 | 14 | 23018665 | 0.4462 | 0.36076 |
| rs12649814 | 4 | 4642440 | 0.4442 | 0.48053 |
| rs12704713 | 7 | 93838454 | 0.299 | 0.31091 |
| rs12731712 | 1 | 150666316 | 0.09649 | 0.21433 |
| rs12757009 | 1 | 223646781 | 0.4235 | 0.39241 |
| rs12767657 | 10 | 100720606 | 0.2435 | 0.17279 |
| rs12809946 | 12 | 69712608 | 0.4811 | 0.4872 |
| rs1286459 | 14 | 91075150 | 0.3331 | 0.33951 |
| rs1292131 | 11 | 74064863 | 0.4521 | 0.48477 |
| rs12924648 | 16 | 57567948 | 0.3267 | 0.29737 |
| rs1294173 | 6 | 151576281 | 0.4987 | 0.44937 |
| rs12942267 | 17 | 7372637 | 0.3753 | 0.41711 |
| rs1294488 | 14 | 90948987 | 0.1965 | 0.22802 |
| rs12974285 | 19 | 57445328 | 0.2723 | 0.23418 |
| rs12979985 | 19 | 18406470 | 0.2067 | 0.27884 |
| rs12983907 | 19 | 13367703 | 0.2462 | 0.22999 |
| rs13021399 | 2 | 109006665 | 0.2537 | 0.32911 |
| rs13110042 | 4 | 4686358 | 0.2998 | 0.40571 |
| rs13123051 | 4 | 157167065 | 0.4517 | 0.49121 |
| rs13127918 | 4 | 156879479 | 0.3209 | 0.30367 |
| rs13175611 | 5 | 68242039 | 0.42 | 0.44502 |
| rs13200739 | 6 | 76635522 | 0.4725 | 0.48065 |
| rs13243252 | 7 | 101019522 | 0.4874 | 0.49382 |
| rs13277972 | 8 | 72025535 | 0.2465 | 0.1962 |
| rs13295416 | 9 | 86798665 | 0.2162 | 0.2128 |
| rs13297158 | 9 | 35969701 | 0.3133 | 0.40098 |
| rs1330865 | 10 | 106147425 | 0.4879 | 0.43037 |
| rs13392737 | 2 | 216369179 | 0.1823 | 0.19447 |
| rs135014 | 22 | 43497898 | 0.4446 | 0.47934 |
| rs1362765 | 17 | 5730117 | 0.3678 | 0.3607 |
| rs138769756 | 8 | 14664899 | 0.4953 | 0.46909 |
| rs1388483 | 4 | 72591971 | 0.4269 | 0.34793 |
| rs139233474 | 17 | 43495660 | 0.4272 | 0.41098 |
| rs1406050 | 7 | 110405196 | 0.3072 | 0.33587 |
| rs142086710 | 1 | 76352239 | 0.3818 | 0.43208 |
| rs1446558 | 6 | 149526277 | 0.348 | 0.29452 |
| rs1449508 | 13 | 42848799 | 0.496 | 0.41762 |
| rs148927935 | 16 | 59109700 | 0.2739 | 0.26215 |
| rs1492254 | 12 | 99699284 | 0.3639 | 0.3847 |
| rs1507055 | 18 | 10948635 | 0.4793 | 0.47853 |
| rs1556880 | 6 | 150615827 | 0.2933 | 0.32915 |
| rs155960 | 5 | 95799701 | 0.446 | 0.42766 |
| rs1580985 | 16 | 8026291 | 0.4136 | 0.40283 |
| rs1610180 | 3 | 10917457 | 0.4621 | 0.40535 |
| rs161977 | 12 | 32421457 | 0.4133 | 0.49368 |
| rs1642018 | 16 | 29140448 | 0.3573 | 0.36886 |
| rs1661725 | 17 | 73560134 | 0.3842 | 0.42634 |
| rs168914 | 16 | 76212736 | 0.3844 | 0.38608 |
| rs17087144 | 9 | 86964718 | 0.2582 | 0.36076 |
| rs1711564 | 3 | 99422587 | 0.4076 | 0.40506 |
| rs1712355 | 14 | 35347512 | 0.4857 | 0.47896 |
| rs17177097 | 14 | 71253069 | 0.2802 | 0.26582 |
| rs17439519 | 2 | 192130220 | 0.1058 | 0.11395 |
| rs17496908 | 2 | 44260347 | 0.1481 | 0.19959 |
| rs17582459 | 4 | 115254307 | 0.2867 | 0.33411 |
| rs17626344 | 5 | 132627902 | 0.4486 | 0.44287 |
| rs1771991 | 10 | 45267690 | 0.2799 | 0.28493 |
| rs1780705 | 20 | 35729950 | 0.1501 | 0.1519 |
| rs17833053 | 14 | 59181924 | 0.1151 | 0.12025 |
| rs1793719 | 8 | 118273125 | 0.4408 | 0.43652 |
| rs182888673 | 19 | 13712969 | 0.1147 | 0.24772 |
| rs1834228 | 2 | 163893641 | 0.2865 | 0.28482 |
| rs1862849 | 16 | 56639494 | 0.4703 | 0.4869 |
| rs1870063 | 19 | 18170962 | 0.3759 | 0.35443 |
| rs1883816 | 6 | 41644441 | 0.3227 | 0.28194 |
| rs1898517 | 2 | 4186285 | 0.37 | 0.37975 |
| rs193327 | 1 | 226095513 | 0.3338 | 0.40096 |
| rs1935148 | 10 | 72885253 | 0.4447 | 0.47706 |
| rs1946182 | 7 | 134053431 | 0.3737 | 0.33973 |
| rs194828 | 7 | 103753765 | 0.4935 | 0.49996 |
| rs1951722 | 13 | 53679747 | 0.4725 | 0.44524 |
| rs1967827 | 1 | 193643052 | 0.1421 | 0.16251 |
| rs1968908 | 8 | 62143702 | 0.4971 | 0.49999 |
| rs1972551 | 7 | 152139854 | 0.3633 | 0.34335 |
| rs2008366 | 9 | 128987552 | 0.4201 | 0.49547 |
| rs2011702 | 19 | 53022053 | 0.3812 | 0.40091 |
| rs2027270 | 10 | 115058004 | 0.127 | 0.1417 |
| rs2044787 | 16 | 57640853 | 0.3149 | 0.28712 |
| rs2050047 | 6 | 150764310 | 0.456 | 0.41153 |
| rs206138 | 13 | 32879195 | 0.3875 | 0.43039 |
| rs2075467 | 16 | 4873242 | 0.3978 | 0.32911 |
| rs2090035 | 2 | 20372270 | 0.4483 | 0.44409 |
| rs2090690 | 1 | 242217919 | 0.2335 | 0.30724 |
| rs2099984 | 12 | 46537123 | 0.2905 | 0.29747 |
| rs2152786 | 1 | 219178352 | 0.2575 | 0.27198 |
| rs215377 | 12 | 47752171 | 0.148 | 0.1962 |
| rs216312 | 12 | 6128984 | 0.4428 | 0.41139 |
| rs2169122 | 15 | 24273049 | 0.3537 | 0.31079 |
| rs2184423 | 10 | 106196151 | 0.4908 | 0.42279 |
| rs2192756 | 2 | 103180530 | 0.4417 | 0.37025 |
| rs2220530 | 14 | 63169714 | 0.3735 | 0.36076 |
| rs2224396 | 1 | 171955221 | 0.3771 | 0.31013 |
| rs222975 | 21 | 27879388 | 0.3006 | 0.27533 |
| rs223586 | 6 | 143719278 | 0.3714 | 0.42405 |
| rs2242090 | 8 | 144688736 | 0.4613 | 0.46166 |
| rs2246441 | 16 | 29114352 | 0.4976 | 0.49685 |
| rs2269668 | 22 | 43699774 | 0.153 | 0.17089 |
| rs2274578 | 6 | 41888827 | 0.4853 | 0.41758 |
| rs2301733 | 7 | 110737149 | 0.431 | 0.45717 |
| rs2315484 | 18 | 4311818 | 0.3538 | 0.37577 |
| rs2358343 | 10 | 19452483 | 0.2801 | 0.35305 |
| rs2398664 | 7 | 2367445 | 0.4528 | 0.41633 |
| rs2413508 | 22 | 38597174 | 0.2709 | 0.31101 |
| rs2419725 | 19 | 13683510 | 0.2426 | 0.28641 |
| rs2428965 | 1 | 44502909 | 0.3367 | 0.3481 |
| rs2437000 | 8 | 109045279 | 0.1938 | 0.24996 |
| rs2448424 | 5 | 55918201 | 0.4678 | 0.4104 |
| rs2451269 | 6 | 159556897 | 0.4031 | 0.39237 |
| rs2459994 | 1 | 2024064 | 0.178 | 0.12658 |
| rs2466946 | 17 | 3993715 | 0.4662 | 0.42525 |
| rs2471636 | 12 | 75500806 | 0.3034 | 0.31851 |
| rs2479729 | 9 | 129714044 | 0.2278 | 0.23445 |
| rs2488094 | 6 | 144026503 | 0.4831 | 0.47522 |
| rs2490430 | 1 | 161941306 | 0.3606 | 0.29935 |
| rs2495987 | 6 | 33955175 | 0.3714 | 0.33397 |
| rs2504735 | 6 | 71623655 | 0.2743 | 0.3481 |
| rs2508445 | 11 | 117872544 | 0.3287 | 0.34249 |
| rs250854 | 5 | 132730239 | 0.4403 | 0.46383 |
| rs254428 | 5 | 141777439 | 0.4294 | 0.3581 |
| rs2595585 | 20 | 3602060 | 0.2244 | 0.25316 |
| rs2641988 | 9 | 552783 | 0.3056 | 0.30168 |
| rs2645477 | 17 | 57845624 | 0.4703 | 0.38564 |
| rs2646255 | 2 | 238262982 | 0.3201 | 0.36749 |
| rs266372 | 15 | 67226458 | 0.3767 | 0.37342 |
| rs2670288 | 3 | 119345630 | 0.4852 | 0.49891 |
| rs2681101 | 3 | 148948734 | 0.2055 | 0.1739 |
| rs269238 | 7 | 140356141 | 0.2759 | 0.29269 |
| rs2695094 | 2 | 178760030 | 0.3963 | 0.38402 |
| rs2705579 | 2 | 217831965 | 0.3949 | 0.42405 |
| rs2724122 | 7 | 93446357 | 0.4925 | 0.47555 |
| rs2756191 | 11 | 31135882 | 0.3497 | 0.37342 |
| rs2769577 | 17 | 50358178 | 0.4899 | 0.49984 |
| rs2784262 | 1 | 221153278 | 0.3352 | 0.35417 |
| rs2804263 | 9 | 559758 | 0.296 | 0.29747 |
| rs28448715 | 14 | 31542492 | 0.35 | 0.39925 |
| rs2863231 | 11 | 31796560 | 0.3417 | 0.31013 |
| rs2886722 | 2 | 85742297 | 0.3915 | 0.44937 |
| rs289020 | 5 | 55675575 | 0.4887 | 0.49087 |
| rs2925206 | 8 | 29096520 | 0.3896 | 0.41196 |
| rs2931312 | 8 | 62100651 | 0.4942 | 0.48734 |
| rs2932983 | 6 | 159571349 | 0.376 | 0.36924 |
| rs2959574 | 8 | 70621828 | 0.3824 | 0.32854 |
| rs2999386 | 14 | 51652628 | 0.3238 | 0.34177 |
| rs3102512 | 8 | 96804668 | 0.35 | 0.4301 |
| rs3178327 | 19 | 38798086 | 0.2745 | 0.33451 |
| rs326640 | 12 | 32399049 | 0.4028 | 0.48731 |
| rs335685 | 15 | 76194286 | 0.4692 | 0.49373 |
| rs34001136 | 8 | 108522573 | 0.3788 | 0.34721 |
| rs34003734 | 18 | 43637429 | 0.5 | 0.46558 |
| rs34044693 | 1 | 223083499 | 0.3257 | 0.34209 |
| rs340515 | 2 | 45188370 | 0.3847 | 0.41144 |
| rs34215541 | 9 | 114666493 | 0.1557 | 0.18988 |
| rs34270 | 12 | 109597930 | 0.307 | 0.32564 |
| rs34279656 | 1 | 246834035 | 0.4988 | 0.49237 |
| rs34299790 | 17 | 14648148 | 0.4156 | 0.41303 |
| rs35020435 | 4 | 4076195 | 0.1537 | 0.32467 |
| rs35033649 | 2 | 102206602 | 0.2276 | 0.19724 |
| rs35097149 | 5 | 172125843 | 0.4963 | 0.48744 |
| rs35107119 | 4 | 186538431 | 0.1612 | 0.179 |
| rs35247064 | 3 | 42865270 | 0.2913 | 0.33336 |
| rs35257551 | 21 | 33352064 | 0.4402 | 0.44799 |
| rs35261662 | 6 | 76638612 | 0.3498 | 0.35443 |
| rs35264487 | 10 | 27585415 | 0.3296 | 0.22349 |
| rs35417171 | 8 | 108771065 | 0.317 | 0.30675 |
| rs35477883 | 2 | 86083571 | 0.4103 | 0.38861 |
| rs35774314 | 21 | 35295709 | 0.2276 | 0.27213 |
| rs35856900 | 4 | 128290531 | 0.3511 | 0.37719 |
| rs369940 | 5 | 40726247 | 0.3662 | 0.33808 |
| rs3735643 | 7 | 127224193 | 0.3593 | 0.30702 |
| rs3753078 | 22 | 22712742 | 0.2387 | 0.23503 |
| rs3754176 | 1 | 41235946 | 0.4206 | 0.35443 |
| rs3755459 | 2 | 75321603 | 0.3835 | 0.4557 |
| rs3775500 | 4 | 100743513 | 0.2505 | 0.17722 |
| rs3786047 | 17 | 4615098 | 0.3084 | 0.34177 |
| rs3790997 | 2 | 238260448 | 0.4332 | 0.38608 |
| rs3791938 | 2 | 218753636 | 0.3277 | 0.33392 |
| rs3802872 | 11 | 117776526 | 0.4044 | 0.43142 |
| rs3814341 | 1 | 184596806 | 0.2347 | 0.40825 |
| rs3826700 | 19 | 17426501 | 0.4407 | 0.42405 |
| rs3851068 | 10 | 34704074 | 0.287 | 0.26582 |
| rs3859580 | 20 | 61816552 | 0.3082 | 0.35994 |
| rs3933430 | 19 | 1365105 | 0.3725 | 0.33092 |
| rs4073620 | 11 | 7491018 | 0.3555 | 0.37156 |
| rs41302651 | 9 | 130167338 | 0.3752 | 0.37976 |
| rs419174 | 19 | 49755660 | 0.2734 | 0.32278 |
| rs4238500 | 15 | 24370448 | 0.3972 | 0.34759 |
| rs4240205 | 2 | 87056805 | 0.321 | 0.31646 |
| rs4243820 | 1 | 28292739 | 0.357 | 0.28403 |
| rs4254841 | 4 | 70880614 | 0.2248 | 0.26456 |
| rs4293 | 17 | 61555666 | 0.4772 | 0.46203 |
| rs4296182 | 14 | 51662167 | 0.4621 | 0.4878 |
| rs4304175 | 6 | 151923982 | 0.2827 | 0.22867 |
| rs4371315 | 2 | 28821149 | 0.2784 | 0.35443 |
| rs4408643 | 19 | 9096252 | 0.4192 | 0.41532 |
| rs4443097 | 22 | 39209839 | 0.4918 | 0.42681 |
| rs4465517 | 14 | 51673715 | 0.2974 | 0.31709 |
| rs4503602 | 12 | 120588852 | 0.222 | 0.3038 |
| rs458461 | 21 | 31129999 | 0.2641 | 0.25397 |
| rs4595717 | 14 | 51566776 | 0.3767 | 0.3672 |
| rs4624763 | 5 | 7163630 | 0.2484 | 0.18339 |
| rs4663003 | 2 | 130648707 | 0.4189 | 0.44937 |
| rs4685318 | 3 | 16207153 | 0.3664 | 0.39862 |
| rs4687657 | 3 | 52852538 | 0.2673 | 0.27851 |
| rs4687658 | 3 | 52860366 | 0.2269 | 0.25313 |
| rs4701068 | 5 | 178224133 | 0.315 | 0.35412 |
| rs4711750 | 6 | 43757082 | 0.4957 | 0.43325 |
| rs4721321 | 7 | 2068470 | 0.2641 | 0.24056 |
| rs4732986 | 8 | 29485927 | 0.4217 | 0.33458 |
| rs4734491 | 8 | 101877914 | 0.3512 | 0.36686 |
| rs4758407 | 11 | 6327406 | 0.4314 | 0.48505 |
| rs4780052 | 15 | 33154851 | 0.4843 | 0.48111 |
| rs4790213 | 17 | 4572677 | 0.2883 | 0.3231 |
| rs4802324 | 19 | 38785094 | 0.3006 | 0.37606 |
| rs4805496 | 19 | 30378841 | 0.4307 | 0.47356 |
| rs4813909 | 20 | 10023689 | 0.3159 | 0.30759 |
| rs4815589 | 20 | 3418549 | 0.2889 | 0.3163 |
| rs4845039 | 1 | 210697177 | 0.1301 | 0.17798 |
| rs4847000 | 1 | 230781741 | 0.2189 | 0.20886 |
| rs4865825 | 5 | 53723362 | 0.2102 | 0.22165 |
| rs4878509 | 9 | 32884254 | 0.3553 | 0.37483 |
| rs487976 | 4 | 111294345 | 0.307 | 0.34125 |
| rs495406 | 5 | 134444120 | 0.3688 | 0.37968 |
| rs4976055 | 5 | 68237170 | 0.241 | 0.27477 |
| rs4989513 | 1 | 202929586 | 0.3525 | 0.29747 |
| rs525357 | 1 | 202588595 | 0.4983 | 0.48216 |
| rs55695577 | 12 | 5739231 | 0.2135 | 0.29727 |
| rs55865513 | 4 | 115276501 | 0.4276 | 0.49032 |
| rs55927952 | 14 | 75021952 | 0.4742 | 0.43044 |
| rs55988458 | 7 | 2059761 | 0.1932 | 0.18385 |
| rs56017041 | 12 | 5698378 | 0.2041 | 0.28278 |
| rs56101391 | 6 | 150499049 | 0.1225 | 0.16289 |
| rs563082 | 15 | 64762593 | 0.3361 | 0.33544 |
| rs56666273 | 16 | 22728306 | 0.2637 | 0.3376 |
| rs570772 | 11 | 224048 | 0.2027 | 0.26878 |
| rs57137815 | 5 | 130517013 | 0.08831 | 0.17204 |
| rs5748304 | 22 | 19562795 | 0.4968 | 0.47507 |
| rs576521 | 3 | 120621061 | 0.4982 | 0.46275 |
| rs576627 | 1 | 160702475 | 0.167 | 0.17839 |
| rs58091773 | 19 | 35265847 | 0.3105 | 0.3346 |
| rs582149 | 18 | 25178261 | 0.312 | 0.29747 |
| rs586104 | 2 | 44893874 | 0.3579 | 0.37623 |
| rs59591052 | 19 | 10135154 | 0.2705 | 0.26695 |
| rs59772551 | 9 | 126412034 | 0.3093 | 0.37975 |
| rs6015739 | 20 | 58955530 | 0.4217 | 0.46143 |
| rs6016348 | 20 | 39038828 | 0.15 | 0.17722 |
| rs6016400 | 20 | 39280054 | 0.3037 | 0.27215 |
| rs6023939 | 20 | 36832526 | 0.4607 | 0.49278 |
| rs6029108 | 20 | 39048873 | 0.1665 | 0.1962 |
| rs6049210 | 20 | 2441687 | 0.1025 | 0.15215 |
| rs6059839 | 20 | 33029374 | 0.4502 | 0.49357 |
| rs6062234 | 20 | 61166540 | 0.4782 | 0.44937 |
| rs6073758 | 20 | 44115041 | 0.3609 | 0.35047 |
| rs6075339 | 20 | 1894315 | 0.3788 | 0.39247 |
| rs6095888 | 20 | 48981926 | 0.3648 | 0.35887 |
| rs60964576 | 14 | 34015705 | 0.4484 | 0.46166 |
| rs6106668 | 20 | 2399539 | 0.316 | 0.31975 |
| rs61166009 | 6 | 71652548 | 0.4441 | 0.40624 |
| rs61261949 | 6 | 150802258 | 0.2539 | 0.22166 |
| rs6130371 | 20 | 35655720 | 0.1488 | 0.15209 |
| rs61767323 | 1 | 31314851 | 0.2772 | 0.27779 |
| rs618006 | 11 | 64680819 | 0.2816 | 0.23823 |
| rs61916978 | 12 | 16420518 | 0.2372 | 0.24735 |
| rs61919598 | 12 | 7370116 | 0.3308 | 0.3908 |
| rs61936484 | 12 | 109805928 | 0.3494 | 0.28491 |
| rs61957797 | 12 | 55899567 | 0.2808 | 0.28879 |
| rs62006060 | 14 | 74176679 | 0.4884 | 0.46225 |
| rs62007276 | 15 | 77700304 | 0.2005 | 0.24408 |
| rs62036940 | 16 | 4006163 | 0.3313 | 0.295 |
| rs62044232 | 16 | 21714238 | 0.2363 | 0.26478 |
| rs62140389 | 2 | 28206268 | 0.2731 | 0.31563 |
| rs62176560 | 2 | 132302165 | 0.4135 | 0.34876 |
| rs62197303 | 20 | 61654430 | 0.1177 | 0.14744 |
| rs62494668 | 8 | 26573697 | 0.1231 | 0.14557 |
| rs629146 | 10 | 111598788 | 0.4982 | 0.48767 |
| rs641648 | 20 | 10023442 | 0.4289 | 0.48101 |
| rs6424952 | 1 | 172463995 | 0.2782 | 0.32199 |
| rs6449986 | 5 | 68234769 | 0.4635 | 0.41772 |
| rs6503859 | 17 | 56460852 | 0.2089 | 0.26587 |
| rs650546 | 18 | 60469666 | 0.4808 | 0.42325 |
| rs6518508 | 22 | 19004772 | 0.2916 | 0.28481 |
| rs6546066 | 2 | 64562805 | 0.255 | 0.24692 |
| rs6584540 | 10 | 104927634 | 0.4123 | 0.38908 |
| rs6585012 | 10 | 112543217 | 0.1767 | 0.18987 |
| rs6596974 | 6 | 3465833 | 0.4806 | 0.47468 |
| rs6602633 | 10 | 13176047 | 0.1864 | 0.18697 |
| rs663818 | 1 | 44578559 | 0.4682 | 0.49367 |
| rs66477917 | 3 | 16767232 | 0.1738 | 0.24051 |
| rs6657810 | 1 | 117715026 | 0.3679 | 0.38608 |
| rs6670279 | 1 | 110487907 | 0.233 | 0.26603 |
| rs6684194 | 1 | 110055211 | 0.4988 | 0.41095 |
| rs6693877 | 1 | 160146790 | 0.3288 | 0.32278 |
| rs6696511 | 1 | 42110888 | 0.3864 | 0.35447 |
| rs6727086 | 2 | 73075006 | 0.2862 | 0.29724 |
| rs6727791 | 2 | 235172781 | 0.4249 | 0.44304 |
| rs6744982 | 2 | 54659980 | 0.4176 | 0.49367 |
| rs6751950 | 2 | 70560939 | 0.1937 | 0.17722 |
| rs6760912 | 2 | 234110944 | 0.4644 | 0.44602 |
| rs6763776 | 3 | 16293151 | 0.287 | 0.24684 |
| rs6765381 | 3 | 54173258 | 0.2553 | 0.25316 |
| rs67796509 | 2 | 171746228 | 0.4945 | 0.4936 |
| rs678814 | 2 | 219359287 | 0.4207 | 0.39971 |
| rs681660 | 1 | 54471189 | 0.4266 | 0.36723 |
| rs6844456 | 4 | 121831539 | 0.1444 | 0.18092 |
| rs6899791 | 6 | 158498054 | 0.4893 | 0.44589 |
| rs6918529 | 6 | 99536858 | 0.4219 | 0.38778 |
| rs6967785 | 7 | 56802478 | 0.393 | 0.29182 |
| rs6967957 | 7 | 139878184 | 0.4446 | 0.43527 |
| rs6985160 | 8 | 23226094 | 0.3371 | 0.31529 |
| rs701743 | 3 | 148953025 | 0.2573 | 0.2222 |
| rs701748 | 3 | 148941275 | 0.2234 | 0.18987 |
| rs7082919 | 10 | 106144354 | 0.4749 | 0.45569 |
| rs7093411 | 10 | 100215967 | 0.3411 | 0.31889 |
| rs7111826 | 11 | 36176258 | 0.2291 | 0.25949 |
| rs7130708 | 11 | 117400550 | 0.2685 | 0.28974 |
| rs7138718 | 12 | 7402193 | 0.3631 | 0.36036 |
| rs7160237 | 14 | 95463806 | 0.4298 | 0.47668 |
| rs7176730 | 15 | 60577374 | 0.3838 | 0.4557 |
| rs7180378 | 15 | 24355278 | 0.2502 | 0.20266 |
| rs7246292 | 19 | 36572564 | 0.4578 | 0.43038 |
| rs7249866 | 19 | 36638037 | 0.1956 | 0.33574 |
| rs7250630 | 19 | 53100323 | 0.1794 | 0.26582 |
| rs7296156 | 12 | 7844620 | 0.4149 | 0.42813 |
| rs7297997 | 12 | 31579728 | 0.4067 | 0.46203 |
| rs7301213 | 12 | 13386887 | 0.2826 | 0.33544 |
| rs7305558 | 12 | 50473377 | 0.2419 | 0.29114 |
| rs7307406 | 12 | 7373646 | 0.3671 | 0.38577 |
| rs73402972 | 11 | 648476 | 0.1938 | 0.22375 |
| rs7368883 | 2 | 43378720 | 0.3714 | 0.3303 |
| rs7397265 | 12 | 50691167 | 0.4351 | 0.43072 |
| rs7470954 | 9 | 124958345 | 0.2139 | 0.27175 |
| rs747740 | 2 | 241073043 | 0.2106 | 0.24659 |
| rs74846680 | 11 | 57771538 | 0.3239 | 0.43143 |
| rs748694 | 17 | 4550189 | 0.4664 | 0.48734 |
| rs7497946 | 15 | 24680647 | 0.4941 | 0.48643 |
| rs7504530 | 18 | 4106560 | 0.4127 | 0.49885 |
| rs7514026 | 1 | 154330042 | 0.4053 | 0.4244 |
| rs7517707 | 1 | 156136589 | 0.3807 | 0.48984 |
| rs752021 | 2 | 175189322 | 0.4819 | 0.48759 |
| rs7524452 | 1 | 44535660 | 0.4268 | 0.41139 |
| rs752555 | 1 | 21032041 | 0.2528 | 0.29114 |
| rs7532791 | 1 | 68969879 | 0.431 | 0.45797 |
| rs7537915 | 1 | 117999364 | 0.4928 | 0.49366 |
| rs7554126 | 1 | 241887181 | 0.3964 | 0.37561 |
| rs7562649 | 2 | 174147227 | 0.3111 | 0.39819 |
| rs7564499 | 2 | 2799480 | 0.2879 | 0.24935 |
| rs75772313 | 20 | 59080716 | 0.3991 | 0.41108 |
| rs7589667 | 2 | 231013656 | 0.2896 | 0.39145 |
| rs7615055 | 3 | 58168473 | 0.3433 | 0.39241 |
| rs7636418 | 3 | 145506122 | 0.4789 | 0.41772 |
| rs7644973 | 3 | 53082937 | 0.2292 | 0.24051 |
| rs764946 | 16 | 66270344 | 0.1518 | 0.22718 |
| rs7655694 | 4 | 164519194 | 0.4443 | 0.49375 |
| rs76874578 | 15 | 78586642 | 0.009124 | 0.05765 |
| rs7692006 | 4 | 1511831 | 0.4174 | 0.36709 |
| rs7694682 | 4 | 101448667 | 0.4968 | 0.46962 |
| rs7740085 | 6 | 62652892 | 0.3796 | 0.48213 |
| rs7761672 | 6 | 116933018 | 0.3325 | 0.34004 |
| rs7770335 | 6 | 159337131 | 0.3063 | 0.28437 |
| rs7805875 | 7 | 140017013 | 0.2172 | 0.23961 |
| rs7863990 | 9 | 2210993 | 0.2167 | 0.27215 |
| rs7914700 | 10 | 60786027 | 0.436 | 0.42895 |
| rs7927425 | 11 | 130393150 | 0.2279 | 0.18209 |
| rs7949056 | 11 | 35999988 | 0.4074 | 0.42405 |
| rs7962563 | 12 | 7353514 | 0.4884 | 0.49996 |
| rs7964018 | 12 | 9866399 | 0.3812 | 0.4494 |
| rs7974872 | 12 | 109836164 | 0.3163 | 0.27119 |
| rs8017061 | 14 | 22502880 | 0.3345 | 0.41043 |
| rs8037444 | 15 | 60437458 | 0.4182 | 0.33258 |
| rs8049603 | 16 | 23067260 | 0.2261 | 0.21517 |
| rs8054024 | 16 | 5530617 | 0.3581 | 0.39241 |
| rs808213 | 14 | 58370958 | 0.3228 | 0.34813 |
| rs809871 | 6 | 26256526 | 0.4142 | 0.39873 |
| rs8106216 | 19 | 52294472 | 0.3904 | 0.30338 |
| rs8130506 | 21 | 34381769 | 0.2856 | 0.20886 |
| rs839856 | 1 | 109471469 | 0.4235 | 0.46977 |
| rs852347 | 20 | 46825424 | 0.3632 | 0.43102 |
| rs876492 | 11 | 34615897 | 0.4817 | 0.44294 |
| rs921120 | 2 | 45273041 | 0.3091 | 0.28481 |
| rs9312367 | 4 | 169718700 | 0.4156 | 0.38088 |
| rs9316362 | 13 | 48718746 | 0.2041 | 0.3038 |
| rs931803 | 5 | 172031468 | 0.3582 | 0.32911 |
| rs9324628 | 5 | 149187134 | 0.3019 | 0.27848 |
| rs932554 | 14 | 68293923 | 0.2262 | 0.25948 |
| rs9327415 | 5 | 126011535 | 0.4507 | 0.49544 |
| rs9394166 | 6 | 33751122 | 0.3898 | 0.39631 |
| rs9465751 | 6 | 10272211 | 0.4758 | 0.41139 |
| rs9467703 | 6 | 26318903 | 0.3753 | 0.32278 |
| rs947889 | 11 | 117874531 | 0.4553 | 0.44304 |
| rs9532432 | 13 | 40352602 | 0.4061 | 0.38689 |
| rs960633 | 7 | 128560761 | 0.2816 | 0.28262 |
| rs9616037 | 22 | 46920187 | 0.2197 | 0.23358 |
| rs9645570 | 10 | 106165941 | 0.4613 | 0.46155 |
| rs9660850 | 1 | 154317281 | 0.1648 | 0.16456 |
| rs9718306 | 6 | 149489178 | 0.3602 | 0.35552 |
| rs9788415 | 14 | 39958630 | 0.3695 | 0.36076 |
| rs9862408 | 3 | 57354328 | 0.2773 | 0.26582 |
| rs9900917 | 17 | 2678687 | 0.2183 | 0.23391 |
| rs9902000 | 17 | 4582426 | 0.2269 | 0.25715 |
| rs9915014 | 17 | 70412402 | 0.4227 | 0.46389 |
| rs9920305 | 15 | 69955799 | 0.3197 | 0.40697 |
| rs9996585 | 4 | 4607318 | 0.1759 | 0.23277 |

(B)

| SNP | CHR | BP | COGA EA MAF | AUS MAF |
| --- | --- | --- | --- | --- |
| chr15:66944217:I | 15 | 66944217 | 0.43 | 0.36602 |
| chr19:18328289:D | 19 | 18328289 | 0.4277 | 0.4764 |
| chr19:19295118:I | 19 | 19295118 | 0.3581 | 0.40338 |
| chr7:92792768:D | 7 | 92792768 | 0.1765 | 0.20337 |
| rs10144321 | 14 | 100882405 | 0.2538 | 0.20253 |
| rs1051322 | 17 | 1946503 | 0.1834 | 0.23418 |
| rs10953075 | 7 | 92532973 | 0.2196 | 0.25539 |
| rs11214031 | 11 | 111766039 | 0.3624 | 0.48755 |
| rs113289613 | 17 | 1420714 | 0.1286 | 0.13369 |
| rs11624049 | 14 | 100852820 | 0.2563 | 0.20253 |
| rs11624974 | 14 | 100805794 | 0.2309 | 0.20345 |
| rs11626307 | 14 | 100942065 | 0.2835 | 0.24627 |
| rs11649448 | 16 | 81815716 | 0.1724 | 0.28117 |
| rs11859045 | 16 | 80532625 | 0.4942 | 0.47502 |
| rs1190715 | 14 | 102023079 | 0.4728 | 0.438 |
| rs11984874 | 8 | 13738160 | 0.3965 | 0.39835 |
| rs1212911 | 14 | 100917372 | 0.2634 | 0.20253 |
| rs12599630 | 16 | 81662106 | 0.3877 | 0.4288 |
| rs12916425 | 15 | 66927677 | 0.2631 | 0.29354 |
| rs12925874 | 16 | 81236973 | 0.2906 | 0.35443 |
| rs13164401 | 5 | 128736512 | 0.2726 | 0.3813 |
| rs13243787 | 7 | 92851445 | 0.1707 | 0.20254 |
| rs13245529 | 7 | 92863895 | 0.1815 | 0.22784 |
| rs17113227 | 11 | 111620491 | 0.2866 | 0.24684 |
| rs17711548 | 7 | 92977414 | 0.1769 | 0.20886 |
| rs2119261 | 15 | 67011980 | 0.3588 | 0.38608 |
| rs2447472 | 16 | 81429806 | 0.4074 | 0.37636 |
| rs2580312 | 16 | 80841509 | 0.4643 | 0.49367 |
| rs28568867 | 7 | 92538421 | 0.2082 | 0.21846 |
| rs28597901 | 14 | 101701780 | 0.4924 | 0.46362 |
| rs331072 | 5 | 127722834 | 0.327 | 0.37775 |
| rs35504285 | 7 | 92883758 | 0.1724 | 0.20259 |
| rs3785965 | 17 | 1454810 | 0.1279 | 0.13237 |
| rs3802055 | 7 | 92944147 | 0.1735 | 0.20886 |
| rs42508 | 7 | 92818808 | 0.171 | 0.20257 |
| rs463035 | 5 | 127657056 | 0.3311 | 0.36381 |
| rs4835948 | 5 | 127870190 | 0.4768 | 0.4738 |
| rs4889332 | 16 | 81559706 | 0.2389 | 0.20253 |
| rs4936678 | 11 | 111625085 | 0.2573 | 0.22152 |
| rs552850 | 5 | 127700694 | 0.2527 | 0.22176 |
| rs56103101 | 15 | 65332582 | 0.2238 | 0.31863 |
| rs59874704 | 7 | 92946010 | 0.2255 | 0.25337 |
| rs612345 | 11 | 111639122 | 0.3274 | 0.45017 |
| rs61811421 | 1 | 154653951 | 0.2112 | 0.2095 |
| rs62046623 | 16 | 81573842 | 0.2104 | 0.15225 |
| rs6564783 | 16 | 80725445 | 0.2521 | 0.27581 |
| rs7106006 | 11 | 111459389 | 0.4864 | 0.39325 |
| rs7157984 | 14 | 100625456 | 0.2885 | 0.30501 |
| rs7223273 | 17 | 2746761 | 0.4551 | 0.42162 |
| rs72807926 | 16 | 80745797 | 0.362 | 0.41187 |
| rs74551863 | 8 | 14743584 | 0.2731 | 0.28726 |
| rs76102375 | 8 | 14738021 | 0.3343 | 0.32501 |
| rs8008884 | 14 | 100653787 | 0.2646 | 0.35443 |
| rs8011440 | 14 | 102025158 | 0.4092 | 0.34648 |
| rs915380 | 14 | 101689191 | 0.4843 | 0.48031 |
| rs9324019 | 14 | 100792093 | 0.3024 | 0.23237 |
| rs9893575 | 17 | 2590598 | 0.4932 | 0.47734 |
