# Supplementary material for: Integrating mRNA and miRNA Weighted Gene Co-Expression Networks with eQTLs in the Nucleus Accumbens of Subjects with Alcohol Dependence
Source: PLoS One. 2015 Sep 18;10(9):e0137671. doi: 10.1371/journal.pone.0137671 (PMC4575063; doi:10.1371/journal.pone.0137671)
Supplement: S1 Table — (DOCX) [file pone.0137671.s002.docx]

(A)

| Probe ID | b(i) | SE | T-Value | P-Value | q-value |
| --- | --- | --- | --- | --- | --- |
| X203786_s_at | -0.2942 | 0.0309 | -9.529 | 1.00E-10 | 1.11E-06 |
| X200663_at | 0.3105 | 0.0326 | 9.526 | 1.01E-10 | 1.11E-06 |
| X219732_at | -0.416 | 0.0478 | -8.707 | 7.84E-10 | 5.78E-06 |
| X216060_s_at | 0.3947 | 0.0464 | 8.514 | 1.29E-09 | 7.16E-06 |
| X201939_at | -0.3169 | 0.0388 | -8.166 | 3.19E-09 | 1.42E-05 |
| X201900_s_at | -0.2429 | 0.0301 | -8.064 | 4.18E-09 | 1.54E-05 |
| X203929_s_at | 0.1538 | 0.0192 | 8.007 | 4.85E-09 | 1.54E-05 |
| X210372_s_at | -0.4271 | 0.0551 | -7.755 | 9.47E-09 | 2.63E-05 |
| X212310_at | -0.2685 | 0.0352 | -7.638 | 1.30E-08 | 3.21E-05 |
| X200876_s_at | -0.1518 | 0.0204 | -7.425 | 2.31E-08 | 4.70E-05 |
| X218217_at | 0.3279 | 0.0442 | 7.423 | 2.33E-08 | 4.70E-05 |
| X211754_s_at | -0.0865 | 0.0119 | -7.291 | 3.32E-08 | 6.15E-05 |
| X208782_at | 0.3777 | 0.0526 | 7.186 | 4.44E-08 | 7.49E-05 |
| X208153_s_at | -0.8472 | 0.1183 | -7.164 | 4.72E-08 | 7.49E-05 |
| X213503_x_at | 0.9673 | 0.1363 | 7.096 | 5.68E-08 | 7.55E-05 |
| X203230_at | 0.1505 | 0.0212 | 7.096 | 5.69E-08 | 7.55E-05 |
| X204955_at | 0.958 | 0.1354 | 7.073 | 6.06E-08 | 7.55E-05 |
| X201590_x_at | 0.8932 | 0.1264 | 7.066 | 6.17E-08 | 7.55E-05 |
| X213955_at | -0.5589 | 0.0793 | -7.05 | 6.45E-08 | 7.55E-05 |
| X219374_s_at | -0.4922 | 0.0706 | -6.968 | 8.10E-08 | 9.00E-05 |
| X211932_at | -0.1275 | 0.0184 | -6.918 | 9.28E-08 | 9.40E-05 |
| X209421_at | -0.2521 | 0.0365 | -6.909 | 9.53E-08 | 9.40E-05 |
| X212599_at | -0.2233 | 0.0324 | -6.901 | 9.73E-08 | 9.40E-05 |
| X202278_s_at | -0.022 | 0.0032 | -6.88 | 1.03E-07 | 9.55E-05 |
| X208679_s_at | -0.148 | 0.0216 | -6.849 | 1.13E-07 | 0.0001 |
| X205549_at | -0.1796 | 0.0265 | -6.768 | 1.41E-07 | 0.000121 |
| X203189_s_at | -0.3697 | 0.0548 | -6.742 | 1.52E-07 | 0.000123 |
| X214655_at | -0.3515 | 0.0522 | -6.733 | 1.55E-07 | 0.000123 |
| X203376_at | -0.3326 | 0.0495 | -6.716 | 1.63E-07 | 0.000124 |
| X212371_at | -0.2825 | 0.0421 | -6.706 | 1.67E-07 | 0.000124 |
| X207174_at | -0.381 | 0.0569 | -6.691 | 1.75E-07 | 0.000125 |
| X212626_x_at | -0.0665 | 0.01 | -6.636 | 2.04E-07 | 0.000137 |
| X210427_x_at | 0.9237 | 0.1395 | 6.622 | 2.12E-07 | 0.000137 |
| X206414_s_at | -0.2868 | 0.0433 | -6.622 | 2.12E-07 | 0.000137 |
| X201943_s_at | -0.1021 | 0.0154 | -6.616 | 2.15E-07 | 0.000137 |
| X204283_at | -0.231 | 0.0351 | -6.587 | 2.34E-07 | 0.000143 |
| X200843_s_at | -0.1666 | 0.0253 | -6.579 | 2.39E-07 | 0.000143 |
| X202948_at | 0.8796 | 0.1339 | 6.57 | 2.45E-07 | 0.000143 |
| X204798_at | -0.4842 | 0.0741 | -6.538 | 2.68E-07 | 0.000153 |
| X212646_at | 0.8254 | 0.1268 | 6.507 | 2.92E-07 | 0.00016 |
| X202138_x_at | -0.2055 | 0.0316 | -6.503 | 2.95E-07 | 0.00016 |
| X221988_at | -0.2391 | 0.037 | -6.463 | 3.31E-07 | 0.000175 |
| X201651_s_at | 0.2426 | 0.0382 | 6.357 | 4.47E-07 | 0.000231 |
| X203367_at | -0.2853 | 0.045 | -6.333 | 4.77E-07 | 0.000241 |
| X205661_s_at | -0.1074 | 0.017 | -6.321 | 4.93E-07 | 0.000244 |
| X214553_s_at | -0.5383 | 0.0854 | -6.301 | 5.23E-07 | 0.000249 |
| X202441_at | -0.2753 | 0.0437 | -6.294 | 5.33E-07 | 0.000249 |
| X218499_at | -0.4415 | 0.0702 | -6.29 | 5.39E-07 | 0.000249 |
| X202674_s_at | -0.5175 | 0.0824 | -6.282 | 5.52E-07 | 0.00025 |
| X201112_s_at | -0.1831 | 0.0292 | -6.269 | 5.73E-07 | 0.000251 |
| X201410_at | -0.1251 | 0.02 | -6.266 | 5.76E-07 | 0.000251 |
| X201781_s_at | -0.3034 | 0.0486 | -6.249 | 6.05E-07 | 0.000259 |
| X203708_at | -0.2009 | 0.0322 | -6.231 | 6.37E-07 | 0.000266 |
| X218719_s_at | -0.1782 | 0.0286 | -6.226 | 6.47E-07 | 0.000266 |
| X218868_at | -0.3924 | 0.0631 | -6.215 | 6.67E-07 | 0.00027 |
| X200657_at | -0.1556 | 0.0251 | -6.201 | 6.93E-07 | 0.000275 |
| X219410_at | -0.361 | 0.0583 | -6.189 | 7.17E-07 | 0.000279 |
| X203843_at | -0.2361 | 0.0384 | -6.153 | 7.95E-07 | 0.000304 |
| X220038_at | -0.247 | 0.0404 | -6.111 | 8.95E-07 | 0.000337 |
| X205311_at | -0.5387 | 0.0886 | -6.082 | 9.73E-07 | 0.00036 |
| X201865_x_at | -0.1308 | 0.0215 | -6.075 | 9.92E-07 | 0.000361 |
| X32042_at | -0.2037 | 0.0337 | -6.04 | 1.10E-06 | 0.000392 |
| X218970_s_at | -0.4068 | 0.0677 | -6.011 | 1.19E-06 | 0.00042 |
| X202976_s_at | 0.2878 | 0.0481 | 5.984 | 1.28E-06 | 0.000446 |
| X201725_at | -0.2654 | 0.0444 | -5.976 | 1.31E-06 | 0.000449 |
| X206875_s_at | -0.3047 | 0.0511 | -5.966 | 1.35E-06 | 0.000454 |
| X212063_at | 1.3348 | 0.2239 | 5.961 | 1.37E-06 | 0.000454 |
| X211671_s_at | -0.1373 | 0.0232 | -5.928 | 1.51E-06 | 0.000492 |
| X205229_s_at | -0.3755 | 0.0634 | -5.923 | 1.53E-06 | 0.000492 |
| X209321_s_at | -0.4434 | 0.075 | -5.91 | 1.58E-06 | 0.000503 |
| X200958_s_at | -0.1668 | 0.0283 | -5.904 | 1.61E-06 | 0.000505 |
| X212798_s_at | -0.1911 | 0.0324 | -5.898 | 1.64E-06 | 0.000506 |
| X203339_at | 0.2542 | 0.0431 | 5.893 | 1.66E-06 | 0.000507 |
| X205774_at | -0.405 | 0.0688 | -5.887 | 1.70E-06 | 0.000509 |
| X218200_s_at | -0.233 | 0.0398 | -5.856 | 1.85E-06 | 0.000548 |
| X203862_s_at | -0.1476 | 0.0253 | -5.843 | 1.92E-06 | 0.000558 |
| X201807_at | -0.0589 | 0.0101 | -5.839 | 1.94E-06 | 0.000558 |
| X200916_at | 0.626 | 0.1073 | 5.836 | 1.96E-06 | 0.000558 |
| X204635_at | -0.1662 | 0.0285 | -5.824 | 2.03E-06 | 0.000563 |
| X220359_s_at | -0.188 | 0.0323 | -5.824 | 2.03E-06 | 0.000563 |
| X216321_s_at | -0.1459 | 0.0251 | -5.816 | 2.08E-06 | 0.000569 |
| X221653_x_at | 0.1856 | 0.0321 | 5.789 | 2.24E-06 | 0.000606 |
| X205080_at | -0.3905 | 0.0676 | -5.78 | 2.30E-06 | 0.000614 |
| X210962_s_at | -0.197 | 0.0342 | -5.754 | 2.47E-06 | 0.00065 |
| X210312_s_at | 0.2147 | 0.0373 | 5.752 | 2.49E-06 | 0.00065 |
| X213338_at | -0.4488 | 0.0781 | -5.742 | 2.56E-06 | 0.000657 |
| X215343_at | -0.5966 | 0.104 | -5.739 | 2.58E-06 | 0.000657 |
| X209080_x_at | -0.1942 | 0.0339 | -5.734 | 2.62E-06 | 0.000657 |
| X201256_at | -0.1539 | 0.0269 | -5.733 | 2.63E-06 | 0.000657 |
| X202429_s_at | -0.2863 | 0.05 | -5.725 | 2.69E-06 | 0.000663 |
| X200999_s_at | 0.4238 | 0.0743 | 5.704 | 2.85E-06 | 0.000687 |
| X201030_x_at | -0.0953 | 0.0167 | -5.704 | 2.85E-06 | 0.000687 |
| X219271_at | 0.0186 | 0.0033 | 5.701 | 2.88E-06 | 0.000687 |
| X200045_at | -0.1969 | 0.0346 | -5.698 | 2.91E-06 | 0.000687 |
| X217728_at | 0.3139 | 0.0552 | 5.683 | 3.03E-06 | 0.000708 |
| X207830_s_at | -0.1872 | 0.033 | -5.672 | 3.12E-06 | 0.000723 |
| X218270_at | -0.0963 | 0.017 | -5.668 | 3.16E-06 | 0.000724 |
| X202379_s_at | 0.2929 | 0.0517 | 5.663 | 3.21E-06 | 0.000728 |
| X218545_at | -0.1165 | 0.0206 | -5.651 | 3.33E-06 | 0.000747 |
| X215111_s_at | 0.1626 | 0.0288 | 5.636 | 3.47E-06 | 0.000771 |
| X203156_at | -0.2073 | 0.0368 | -5.63 | 3.53E-06 | 0.000777 |
| X205111_s_at | 0.6043 | 0.1076 | 5.619 | 3.65E-06 | 0.000794 |
| X218177_at | -0.5079 | 0.0906 | -5.605 | 3.79E-06 | 0.000817 |
| X209046_s_at | -0.0992 | 0.0177 | -5.587 | 3.99E-06 | 0.000853 |
| X201832_s_at | -0.1068 | 0.0191 | -5.578 | 4.09E-06 | 0.000859 |
| X202309_at | -0.3107 | 0.0557 | -5.577 | 4.10E-06 | 0.000859 |
| X202020_s_at | -0.1552 | 0.0279 | -5.558 | 4.33E-06 | 0.0009 |
| X201681_s_at | 0.3246 | 0.0585 | 5.551 | 4.42E-06 | 0.000907 |
| X209507_at | -0.2706 | 0.0488 | -5.546 | 4.48E-06 | 0.000907 |
| X221482_s_at | -0.2153 | 0.0388 | -5.546 | 4.49E-06 | 0.000907 |
| X212176_at | 0.1944 | 0.0351 | 5.536 | 4.62E-06 | 0.000917 |
| X218432_at | -0.2501 | 0.0452 | -5.536 | 4.62E-06 | 0.000917 |
| X50314_i_at | -0.5524 | 0.0998 | -5.533 | 4.66E-06 | 0.000917 |
| X201519_at | -0.343 | 0.0622 | -5.513 | 4.93E-06 | 0.000961 |
| X219617_at | -0.2306 | 0.0419 | -5.507 | 5.02E-06 | 0.00097 |
| X203704_s_at | 0.2604 | 0.0474 | 5.489 | 5.28E-06 | 0.001012 |
| X201925_s_at | 0.4791 | 0.0875 | 5.474 | 5.52E-06 | 0.00104 |
| X212977_at | 0.5116 | 0.0935 | 5.473 | 5.53E-06 | 0.00104 |
| X204872_at | -0.2254 | 0.0414 | -5.446 | 5.98E-06 | 0.001116 |
| X218133_s_at | -0.2468 | 0.0453 | -5.442 | 6.05E-06 | 0.00112 |
| X218841_at | -0.0852 | 0.0157 | -5.435 | 6.17E-06 | 0.001125 |
| X203752_s_at | 0.2268 | 0.0417 | 5.435 | 6.18E-06 | 0.001125 |
| X207307_at | -0.4896 | 0.0904 | -5.418 | 6.48E-06 | 0.001154 |
| X201012_at | 1.3941 | 0.2573 | 5.418 | 6.49E-06 | 0.001154 |
| X201836_s_at | -0.1588 | 0.0293 | -5.417 | 6.49E-06 | 0.001154 |
| X205202_at | -0.205 | 0.0379 | -5.408 | 6.67E-06 | 0.001176 |
| X210835_s_at | 0.135 | 0.025 | 5.404 | 6.74E-06 | 0.001179 |
| X208451_s_at | 1.0295 | 0.1906 | 5.4 | 6.82E-06 | 0.001183 |
| X204759_at | -0.1754 | 0.0325 | -5.396 | 6.91E-06 | 0.00119 |
| X217077_s_at | 0.613 | 0.1138 | 5.387 | 7.07E-06 | 0.001201 |
| X202759_s_at | 0.2523 | 0.0469 | 5.383 | 7.16E-06 | 0.001201 |
| X213388_at | 0.1985 | 0.0369 | 5.381 | 7.19E-06 | 0.001201 |
| X219714_s_at | -0.3801 | 0.0706 | -5.381 | 7.21E-06 | 0.001201 |
| X209318_x_at | 0.2039 | 0.0379 | 5.379 | 7.25E-06 | 0.001201 |
| X213592_at | 1.0698 | 0.1991 | 5.374 | 7.34E-06 | 0.001208 |
| X208808_s_at | 0.371 | 0.0691 | 5.367 | 7.50E-06 | 0.001225 |
| X202376_at | 2.0313 | 0.3795 | 5.353 | 7.81E-06 | 0.001259 |
| X209475_at | -0.152 | 0.0284 | -5.352 | 7.82E-06 | 0.001259 |
| X201570_at | -0.1487 | 0.0278 | -5.342 | 8.05E-06 | 0.001287 |
| X208911_s_at | -0.169 | 0.0317 | -5.337 | 8.17E-06 | 0.001296 |
| X220217_x_at | -0.0177 | 0.0033 | -5.328 | 8.37E-06 | 0.001319 |
| X216307_at | -0.4432 | 0.0833 | -5.32 | 8.57E-06 | 0.00134 |
| X206046_at | -0.4679 | 0.088 | -5.318 | 8.63E-06 | 0.001341 |
| X208712_at | -0.3411 | 0.0642 | -5.31 | 8.82E-06 | 0.001361 |
| X36711_at | 1.4715 | 0.2778 | 5.297 | 9.17E-06 | 0.001405 |
| X204072_s_at | -0.1506 | 0.0285 | -5.291 | 9.31E-06 | 0.001417 |
| X217908_s_at | -0.1271 | 0.024 | -5.288 | 9.41E-06 | 0.001422 |
| X204082_at | -0.2501 | 0.0474 | -5.276 | 9.74E-06 | 0.001461 |
| X204300_at | -0.1344 | 0.0255 | -5.272 | 9.86E-06 | 0.00147 |
| X219326_s_at | -0.7314 | 0.1392 | -5.254 | 1.04E-05 | 0.00153 |
| X201761_at | 0.663 | 0.1263 | 5.251 | 1.04E-05 | 0.00153 |
| X203540_at | 0.5764 | 0.1099 | 5.247 | 1.06E-05 | 0.00153 |
| X203087_s_at | -0.126 | 0.024 | -5.246 | 1.06E-05 | 0.00153 |
| X205626_s_at | -0.6447 | 0.1229 | -5.245 | 1.06E-05 | 0.00153 |
| X200652_at | -0.1513 | 0.0289 | -5.242 | 1.07E-05 | 0.00153 |
| X200986_at | 0.5585 | 0.1066 | 5.241 | 1.07E-05 | 0.00153 |
| X217923_at | -0.2364 | 0.0451 | -5.236 | 1.09E-05 | 0.001535 |
| X203190_at | -0.1817 | 0.0347 | -5.235 | 1.10E-05 | 0.001535 |
| X221758_at | 0.0581 | 0.0111 | 5.232 | 1.10E-05 | 0.001535 |
| X221958_s_at | 0.2984 | 0.057 | 5.231 | 1.11E-05 | 0.001535 |
| X219528_s_at | -0.2747 | 0.0525 | -5.229 | 1.11E-05 | 0.001535 |
| X205856_at | 1.2189 | 0.2333 | 5.225 | 1.13E-05 | 0.001545 |
| X205625_s_at | -0.6034 | 0.1156 | -5.22 | 1.14E-05 | 0.001559 |
| X214429_at | -0.089 | 0.0171 | -5.216 | 1.16E-05 | 0.001563 |
| X213465_s_at | -0.268 | 0.0514 | -5.215 | 1.16E-05 | 0.001563 |
| X208835_s_at | 0.2434 | 0.0467 | 5.211 | 1.17E-05 | 0.001569 |
| X208106_x_at | 0.0159 | 0.003 | 5.207 | 1.19E-05 | 0.001578 |
| X200981_x_at | 0.1835 | 0.0353 | 5.202 | 1.20E-05 | 0.001586 |
| X203362_s_at | -0.3239 | 0.0623 | -5.201 | 1.21E-05 | 0.001586 |
| X209078_s_at | -0.2234 | 0.043 | -5.193 | 1.23E-05 | 0.00161 |
| X210138_at | -0.245 | 0.0472 | -5.192 | 1.24E-05 | 0.00161 |
| X203001_s_at | 0.1537 | 0.0296 | 5.184 | 1.27E-05 | 0.001635 |
| X222077_s_at | -0.1959 | 0.0379 | -5.171 | 1.32E-05 | 0.00169 |
| X209095_at | -0.2202 | 0.0426 | -5.165 | 1.34E-05 | 0.001711 |
| X219067_s_at | -0.228 | 0.0442 | -5.162 | 1.35E-05 | 0.001711 |
| X206302_s_at | 0.3684 | 0.0714 | 5.158 | 1.36E-05 | 0.001718 |
| X218178_s_at | -0.3939 | 0.0764 | -5.153 | 1.38E-05 | 0.001718 |
| X204434_at | -0.1254 | 0.0243 | -5.152 | 1.39E-05 | 0.001718 |
| X205883_at | 0.8871 | 0.1722 | 5.152 | 1.39E-05 | 0.001718 |
| X218190_s_at | -0.0645 | 0.0125 | -5.151 | 1.39E-05 | 0.001718 |
| X203373_at | 0.3082 | 0.0599 | 5.143 | 1.43E-05 | 0.001748 |
| X205590_at | -0.3102 | 0.0603 | -5.141 | 1.43E-05 | 0.001748 |
| X213070_at | -0.3935 | 0.0767 | -5.133 | 1.47E-05 | 0.001783 |
| X212124_at | 0.173 | 0.0337 | 5.129 | 1.48E-05 | 0.001784 |
| X201666_at | 0.4117 | 0.0803 | 5.128 | 1.49E-05 | 0.001784 |
| X40562_at | 0.1664 | 0.0325 | 5.123 | 1.51E-05 | 0.0018 |
| X205613_at | 0.3464 | 0.0676 | 5.122 | 1.52E-05 | 0.0018 |
| X201214_s_at | -0.2933 | 0.0574 | -5.109 | 1.57E-05 | 0.001856 |
| X207574_s_at | 0.7796 | 0.1531 | 5.092 | 1.65E-05 | 0.00194 |
| X217872_at | -0.1296 | 0.0255 | -5.083 | 1.69E-05 | 0.001973 |
| X216336_x_at | 0.2771 | 0.0545 | 5.082 | 1.70E-05 | 0.001973 |
| X207480_s_at | -0.1865 | 0.0367 | -5.08 | 1.71E-05 | 0.001975 |
| X201029_s_at | 0.359 | 0.0708 | 5.069 | 1.76E-05 | 0.002027 |
| X205110_s_at | -0.1717 | 0.0339 | -5.064 | 1.79E-05 | 0.002045 |
| X204731_at | 0.2945 | 0.0582 | 5.061 | 1.80E-05 | 0.002056 |
| X212730_at | 0.2186 | 0.0432 | 5.055 | 1.83E-05 | 0.002077 |
| X212273_x_at | 0.1877 | 0.0372 | 5.04 | 1.92E-05 | 0.002161 |
| X208827_at | -0.1893 | 0.0376 | -5.035 | 1.94E-05 | 0.002167 |
| X210149_s_at | -0.1041 | 0.0207 | -5.035 | 1.94E-05 | 0.002167 |
| X212792_at | 0.4399 | 0.0876 | 5.024 | 2.01E-05 | 0.002221 |
| X213228_at | 0.2347 | 0.0467 | 5.023 | 2.01E-05 | 0.002221 |
| X202388_at | -0.4192 | 0.0835 | -5.022 | 2.02E-05 | 0.002221 |
| X207076_s_at | 0.1875 | 0.0374 | 5.016 | 2.05E-05 | 0.002239 |
| X210031_at | -0.0391 | 0.0078 | -5.015 | 2.06E-05 | 0.002239 |
| X218541_s_at | 0.3089 | 0.0617 | 5.009 | 2.09E-05 | 0.002251 |
| X204796_at | -0.2594 | 0.0518 | -5.009 | 2.09E-05 | 0.002251 |
| X211951_at | -0.0838 | 0.0167 | -5.008 | 2.10E-05 | 0.002251 |
| X217797_at | -0.1175 | 0.0235 | -5.007 | 2.11E-05 | 0.002251 |
| X214717_at | -0.2266 | 0.0454 | -4.996 | 2.17E-05 | 0.002307 |
| X201284_s_at | -0.3212 | 0.0643 | -4.995 | 2.18E-05 | 0.002307 |
| X208690_s_at | 0.2952 | 0.0591 | 4.992 | 2.20E-05 | 0.002315 |
| X211558_s_at | -0.206 | 0.0414 | -4.977 | 2.29E-05 | 0.002392 |
| X204156_at | -0.2072 | 0.0416 | -4.976 | 2.30E-05 | 0.002392 |
| X218882_s_at | -0.1231 | 0.0247 | -4.974 | 2.31E-05 | 0.002392 |
| X210117_at | 0.327 | 0.0657 | 4.974 | 2.32E-05 | 0.002392 |
| X208887_at | -0.1156 | 0.0233 | -4.963 | 2.39E-05 | 0.002452 |
| X206711_at | -0.608 | 0.1225 | -4.962 | 2.40E-05 | 0.002452 |
| X207400_at | -0.3789 | 0.0765 | -4.951 | 2.47E-05 | 0.002513 |
| X203313_s_at | 0.4489 | 0.0907 | 4.95 | 2.48E-05 | 0.002513 |
| X218081_at | -0.3294 | 0.0666 | -4.948 | 2.49E-05 | 0.002514 |
| X202232_s_at | -0.1193 | 0.0241 | -4.945 | 2.52E-05 | 0.002529 |
| X213791_at | -0.2811 | 0.0569 | -4.94 | 2.55E-05 | 0.002538 |
| X209304_x_at | 0.5737 | 0.1161 | 4.939 | 2.56E-05 | 0.002538 |
| X213301_x_at | -0.1129 | 0.0229 | -4.937 | 2.57E-05 | 0.002538 |
| X204599_s_at | -0.2538 | 0.0514 | -4.936 | 2.58E-05 | 0.002538 |
| X204766_s_at | -0.305 | 0.0618 | -4.936 | 2.58E-05 | 0.002538 |
| X209111_at | -0.1949 | 0.0395 | -4.931 | 2.62E-05 | 0.002559 |
| X214113_s_at | -0.1294 | 0.0263 | -4.929 | 2.63E-05 | 0.002565 |
| X212762_s_at | 0.2565 | 0.0521 | 4.927 | 2.65E-05 | 0.002569 |
| X207812_s_at | -0.0945 | 0.0192 | -4.923 | 2.68E-05 | 0.002581 |
| X200780_x_at | 0.1783 | 0.0362 | 4.922 | 2.68E-05 | 0.002581 |
| X210458_s_at | -0.2629 | 0.0534 | -4.92 | 2.70E-05 | 0.002588 |
| X201175_at | -0.0972 | 0.0198 | -4.917 | 2.73E-05 | 0.002599 |
| X218167_at | -0.104 | 0.0212 | -4.912 | 2.76E-05 | 0.0026 |
| X211943_x_at | -0.0564 | 0.0115 | -4.91 | 2.78E-05 | 0.0026 |
| X222273_at | -0.1812 | 0.0369 | -4.909 | 2.78E-05 | 0.0026 |
| X41220_at | 0.349 | 0.0711 | 4.908 | 2.79E-05 | 0.0026 |
| X214988_s_at | -0.1187 | 0.0242 | -4.908 | 2.79E-05 | 0.0026 |
| X214853_s_at | 0.3415 | 0.0696 | 4.908 | 2.80E-05 | 0.0026 |
| X215388_s_at | 0.4118 | 0.0839 | 4.905 | 2.82E-05 | 0.002606 |
| X206115_at | -0.2922 | 0.0597 | -4.897 | 2.89E-05 | 0.002621 |
| X204576_s_at | -0.2304 | 0.0471 | -4.896 | 2.89E-05 | 0.002621 |
| X210534_s_at | -0.4154 | 0.0849 | -4.894 | 2.91E-05 | 0.002621 |
| X209489_at | -0.1816 | 0.0371 | -4.894 | 2.91E-05 | 0.002621 |
| X206552_s_at | -0.1993 | 0.0407 | -4.894 | 2.91E-05 | 0.002621 |
| X214620_x_at | -0.2176 | 0.0445 | -4.894 | 2.91E-05 | 0.002621 |
| X205726_at | -0.22 | 0.045 | -4.893 | 2.91E-05 | 0.002621 |
| X205249_at | -0.5341 | 0.1092 | -4.89 | 2.95E-05 | 0.00264 |
| X209142_s_at | -0.1376 | 0.0282 | -4.886 | 2.98E-05 | 0.002658 |
| X217883_at | -0.152 | 0.0312 | -4.874 | 3.08E-05 | 0.00273 |
| X214652_at | -0.5246 | 0.1076 | -4.874 | 3.08E-05 | 0.00273 |
| X209506_s_at | 0.3942 | 0.081 | 4.866 | 3.15E-05 | 0.002769 |
| X217492_s_at | 0.1985 | 0.0408 | 4.866 | 3.15E-05 | 0.002769 |
| X213564_x_at | -0.08 | 0.0164 | -4.864 | 3.17E-05 | 0.002774 |
| X220168_at | 0.1335 | 0.0275 | 4.855 | 3.25E-05 | 0.002823 |
| X205866_at | 0.0064 | 0.0013 | 4.855 | 3.25E-05 | 0.002823 |
| X201405_s_at | -0.1767 | 0.0364 | -4.848 | 3.32E-05 | 0.002865 |
| X206805_at | -0.8014 | 0.1653 | -4.847 | 3.33E-05 | 0.002865 |
| X213052_at | 0.1617 | 0.0334 | 4.845 | 3.35E-05 | 0.002872 |
| X208463_at | -0.3746 | 0.0773 | -4.843 | 3.37E-05 | 0.002876 |
| X218570_at | -0.1539 | 0.0318 | -4.837 | 3.43E-05 | 0.002916 |
| X202184_s_at | -0.1381 | 0.0286 | -4.835 | 3.45E-05 | 0.002925 |
| X203493_s_at | -0.1447 | 0.0299 | -4.831 | 3.49E-05 | 0.002947 |
| X221538_s_at | 0.1867 | 0.0387 | 4.826 | 3.54E-05 | 0.002959 |
| X219911_s_at | 0.725 | 0.1503 | 4.825 | 3.54E-05 | 0.002959 |
| X205990_s_at | 0.301 | 0.0624 | 4.825 | 3.54E-05 | 0.002959 |
| X214548_x_at | 0.1738 | 0.036 | 4.821 | 3.59E-05 | 0.002986 |
| X201762_s_at | 0.1873 | 0.0389 | 4.812 | 3.68E-05 | 0.003052 |
| X202332_at | 0.1695 | 0.0353 | 4.808 | 3.73E-05 | 0.003076 |
| X200779_at | 0.1982 | 0.0412 | 4.806 | 3.75E-05 | 0.003081 |
| X218273_s_at | -0.4027 | 0.0839 | -4.801 | 3.79E-05 | 0.0031 |
| X213504_at | -0.1755 | 0.0366 | -4.801 | 3.80E-05 | 0.0031 |
| X213738_s_at | -0.1103 | 0.023 | -4.798 | 3.82E-05 | 0.003112 |
| X201999_s_at | 0.2283 | 0.0478 | 4.779 | 4.04E-05 | 0.003275 |
| X217997_at | -0.3111 | 0.0651 | -4.777 | 4.07E-05 | 0.003287 |
| X214825_at | 0.2748 | 0.0576 | 4.774 | 4.10E-05 | 0.003295 |
| X205559_s_at | 0.4384 | 0.0919 | 4.773 | 4.12E-05 | 0.003295 |
| X219582_at | 0.3573 | 0.0749 | 4.772 | 4.12E-05 | 0.003295 |
| X216218_s_at | -0.5 | 0.1049 | -4.766 | 4.20E-05 | 0.003343 |
| X203864_s_at | -0.5263 | 0.1105 | -4.763 | 4.23E-05 | 0.003356 |
| X203139_at | -0.178 | 0.0374 | -4.76 | 4.27E-05 | 0.003372 |
| X201549_x_at | -0.2784 | 0.0586 | -4.753 | 4.35E-05 | 0.00342 |
| X202296_s_at | -0.1666 | 0.0351 | -4.753 | 4.36E-05 | 0.00342 |
| X32099_at | 0.2024 | 0.0426 | 4.75 | 4.39E-05 | 0.003433 |
| X201430_s_at | 0.4326 | 0.0911 | 4.746 | 4.44E-05 | 0.003464 |
| X208857_s_at | -0.2388 | 0.0504 | -4.742 | 4.49E-05 | 0.003489 |
| X211858_x_at | 0.1841 | 0.0388 | 4.74 | 4.52E-05 | 0.003502 |
| X201272_at | -0.2632 | 0.0556 | -4.738 | 4.55E-05 | 0.003508 |
| X218440_at | -0.1344 | 0.0284 | -4.728 | 4.67E-05 | 0.003591 |
| X40016_g_at | 0.2416 | 0.0511 | 4.723 | 4.74E-05 | 0.003616 |
| X203674_at | 0.1633 | 0.0346 | 4.723 | 4.74E-05 | 0.003616 |
| X213965_s_at | 0.301 | 0.0637 | 4.722 | 4.75E-05 | 0.003616 |
| X213924_at | -0.2163 | 0.0458 | -4.72 | 4.78E-05 | 0.003616 |
| X64408_s_at | 0.0747 | 0.0158 | 4.72 | 4.79E-05 | 0.003616 |
| X221488_s_at | -0.166 | 0.0352 | -4.718 | 4.81E-05 | 0.00362 |
| X209183_s_at | 0.66 | 0.14 | 4.713 | 4.88E-05 | 0.003662 |
| X209122_at | 0.6173 | 0.1311 | 4.71 | 4.93E-05 | 0.003684 |
| X201325_s_at | 0.5032 | 0.1069 | 4.708 | 4.95E-05 | 0.003687 |
| X40020_at | 0.1919 | 0.0408 | 4.705 | 4.99E-05 | 0.003701 |
| X211297_s_at | -0.2609 | 0.0555 | -4.705 | 5.00E-05 | 0.003701 |
| X216520_s_at | -0.0812 | 0.0173 | -4.694 | 5.15E-05 | 0.003804 |
| X209104_s_at | -0.2007 | 0.0428 | -4.687 | 5.25E-05 | 0.003857 |
| X204846_at | 0.4032 | 0.086 | 4.687 | 5.26E-05 | 0.003857 |
| X209320_at | -0.2797 | 0.0598 | -4.68 | 5.37E-05 | 0.003921 |
| X220251_at | -0.2984 | 0.0638 | -4.677 | 5.42E-05 | 0.003945 |
| X212958_x_at | -0.1902 | 0.0407 | -4.672 | 5.49E-05 | 0.00398 |
| X221637_s_at | -0.0746 | 0.016 | -4.671 | 5.50E-05 | 0.00398 |
| X200739_s_at | -0.1183 | 0.0254 | -4.667 | 5.57E-05 | 0.004019 |
| X201740_at | -0.1776 | 0.0381 | -4.665 | 5.60E-05 | 0.004027 |
| X211964_at | 0.3716 | 0.0797 | 4.661 | 5.66E-05 | 0.004058 |
| X208116_s_at | -0.6996 | 0.1502 | -4.659 | 5.69E-05 | 0.004064 |
| X212053_at | -0.1782 | 0.0383 | -4.656 | 5.75E-05 | 0.004081 |
| X205562_at | -0.2068 | 0.0444 | -4.656 | 5.75E-05 | 0.004081 |
| X215522_at | -0.3742 | 0.0804 | -4.654 | 5.78E-05 | 0.004087 |
| X207054_at | -1.1842 | 0.2549 | -4.646 | 5.91E-05 | 0.004165 |
| X200014_s_at | -0.1071 | 0.0231 | -4.645 | 5.93E-05 | 0.004165 |
| X212859_x_at | 0.3148 | 0.0678 | 4.643 | 5.96E-05 | 0.004178 |
| X203621_at | -0.1541 | 0.0332 | -4.639 | 6.03E-05 | 0.00421 |
| X203798_s_at | 0.5804 | 0.1252 | 4.636 | 6.09E-05 | 0.004225 |
| X209532_at | -0.1162 | 0.0251 | -4.635 | 6.10E-05 | 0.004225 |
| X206335_at | 0.1765 | 0.0381 | 4.635 | 6.11E-05 | 0.004225 |
| X212203_x_at | 0.5605 | 0.1211 | 4.627 | 6.24E-05 | 0.004306 |
| X207826_s_at | 0.6537 | 0.1418 | 4.61 | 6.55E-05 | 0.004502 |
| X201267_s_at | -0.3019 | 0.0657 | -4.599 | 6.76E-05 | 0.004637 |
| X202753_at | -0.1025 | 0.0223 | -4.595 | 6.82E-05 | 0.004664 |
| X212947_at | -0.0244 | 0.0053 | -4.594 | 6.86E-05 | 0.004672 |
| X221760_at | -0.4211 | 0.0917 | -4.591 | 6.92E-05 | 0.004694 |
| X201973_s_at | 0.0832 | 0.0181 | 4.59 | 6.93E-05 | 0.004694 |
| X212518_at | 0.1184 | 0.0258 | 4.588 | 6.98E-05 | 0.00471 |
| X221696_s_at | -0.4462 | 0.0973 | -4.586 | 7.02E-05 | 0.004724 |
| X212692_s_at | -0.1861 | 0.0406 | -4.581 | 7.11E-05 | 0.004771 |
| X202811_at | -0.1204 | 0.0263 | -4.576 | 7.21E-05 | 0.004816 |
| X204326_x_at | 0.6621 | 0.1447 | 4.576 | 7.22E-05 | 0.004816 |
| X211675_s_at | 0.3953 | 0.0865 | 4.571 | 7.32E-05 | 0.004846 |
| X205646_s_at | 0.2878 | 0.063 | 4.57 | 7.34E-05 | 0.004846 |
| X211419_s_at | -0.2792 | 0.0611 | -4.57 | 7.34E-05 | 0.004846 |
| X206671_at | -0.7829 | 0.1714 | -4.569 | 7.36E-05 | 0.004846 |
| X205112_at | 0.2991 | 0.0655 | 4.568 | 7.37E-05 | 0.004846 |
| X218654_s_at | -0.1901 | 0.0416 | -4.567 | 7.40E-05 | 0.004849 |
| X212207_at | 0.2715 | 0.0595 | 4.565 | 7.44E-05 | 0.004849 |
| X201509_at | -0.1726 | 0.0378 | -4.565 | 7.44E-05 | 0.004849 |
| X202475_at | -0.1446 | 0.0317 | -4.563 | 7.49E-05 | 0.004865 |
| X219726_at | -0.1355 | 0.0297 | -4.559 | 7.56E-05 | 0.004897 |
| X205639_at | -0.5178 | 0.1136 | -4.557 | 7.62E-05 | 0.00492 |
| X212157_at | -0.386 | 0.0847 | -4.556 | 7.64E-05 | 0.00492 |
| X208581_x_at | 0.4347 | 0.0955 | 4.552 | 7.72E-05 | 0.004954 |
| X205413_at | -0.2667 | 0.0586 | -4.55 | 7.77E-05 | 0.004966 |
| X208371_s_at | -0.0978 | 0.0215 | -4.549 | 7.80E-05 | 0.004966 |
| X211855_s_at | -0.3676 | 0.0808 | -4.548 | 7.80E-05 | 0.004966 |
| X203018_s_at | -0.2692 | 0.0592 | -4.546 | 7.85E-05 | 0.004978 |
| X210021_s_at | -0.0927 | 0.0204 | -4.545 | 7.88E-05 | 0.004978 |
| X218160_at | -0.141 | 0.031 | -4.545 | 7.89E-05 | 0.004978 |
| X214839_at | 0.27 | 0.0594 | 4.543 | 7.91E-05 | 0.00498 |
| X202733_at | 0.5304 | 0.1168 | 4.541 | 7.97E-05 | 0.004991 |
| X217724_at | -0.0963 | 0.0212 | -4.541 | 7.98E-05 | 0.004991 |
| X203973_s_at | 0.969 | 0.2136 | 4.537 | 8.05E-05 | 0.005014 |
| X209295_at | 0.3104 | 0.0684 | 4.537 | 8.06E-05 | 0.005014 |
| X204391_x_at | -0.0803 | 0.0177 | -4.531 | 8.19E-05 | 0.00508 |
| X212344_at | 0.1469 | 0.0324 | 4.53 | 8.21E-05 | 0.00508 |
| X212239_at | 0.2113 | 0.0467 | 4.529 | 8.25E-05 | 0.005093 |
| X207551_s_at | -0.1451 | 0.032 | -4.527 | 8.28E-05 | 0.005095 |
| X214629_x_at | -0.0861 | 0.019 | -4.527 | 8.30E-05 | 0.005095 |
| X212585_at | -0.1848 | 0.0408 | -4.525 | 8.33E-05 | 0.005097 |
| X48825_at | -0.1027 | 0.0227 | -4.523 | 8.40E-05 | 0.005105 |
| X202193_at | 0.5804 | 0.1283 | 4.522 | 8.40E-05 | 0.005105 |
| X208858_s_at | 0.274 | 0.0606 | 4.522 | 8.41E-05 | 0.005105 |
| X213562_s_at | -0.0415 | 0.0092 | -4.519 | 8.48E-05 | 0.00513 |
| X206874_s_at | -0.2376 | 0.0526 | -4.517 | 8.54E-05 | 0.005151 |
| X219464_at | -0.2319 | 0.0513 | -4.516 | 8.56E-05 | 0.005151 |
| X202236_s_at | 0.3754 | 0.0832 | 4.514 | 8.60E-05 | 0.005166 |
| X218260_at | -0.1756 | 0.0389 | -4.512 | 8.66E-05 | 0.005185 |
| X202060_at | -0.1015 | 0.0225 | -4.506 | 8.81E-05 | 0.005261 |
| X203013_at | -0.1006 | 0.0223 | -4.504 | 8.85E-05 | 0.005261 |
| X212209_at | 0.2818 | 0.0626 | 4.504 | 8.86E-05 | 0.005261 |
| X203569_s_at | 0.2188 | 0.0486 | 4.501 | 8.93E-05 | 0.005288 |
| X204239_s_at | -0.5578 | 0.124 | -4.5 | 8.96E-05 | 0.005291 |
| X213887_s_at | -0.3299 | 0.0733 | -4.499 | 8.98E-05 | 0.005292 |
| X221939_at | 0.1652 | 0.0367 | 4.495 | 9.07E-05 | 0.005332 |
| X222236_s_at | 0.0379 | 0.0084 | 4.493 | 9.14E-05 | 0.005348 |
| X211376_s_at | -0.4257 | 0.0948 | -4.492 | 9.15E-05 | 0.005348 |
| X209515_s_at | 0.1167 | 0.026 | 4.49 | 9.20E-05 | 0.005366 |
| X209757_s_at | -0.1583 | 0.0353 | -4.489 | 9.23E-05 | 0.005366 |
| X202724_s_at | -0.2915 | 0.065 | -4.484 | 9.38E-05 | 0.00544 |
| X203377_s_at | -0.3674 | 0.082 | -4.482 | 9.41E-05 | 0.005446 |
| X218866_s_at | -0.2221 | 0.0496 | -4.481 | 9.46E-05 | 0.005456 |
| X212500_at | -0.1477 | 0.033 | -4.477 | 9.55E-05 | 0.005488 |
| X201160_s_at | 0.6117 | 0.1366 | 4.477 | 9.56E-05 | 0.005488 |
| X49306_at | 0.5535 | 0.1237 | 4.474 | 9.64E-05 | 0.005518 |
| X201515_s_at | -0.1249 | 0.0279 | -4.47 | 9.74E-05 | 0.005565 |
| X212214_at | -0.229 | 0.0512 | -4.469 | 9.77E-05 | 0.005566 |
| X210095_s_at | 1.1539 | 0.2584 | 4.465 | 9.89E-05 | 0.005617 |
| X201364_s_at | -0.2496 | 0.0559 | -4.464 | 9.93E-05 | 0.005626 |
| X203440_at | 0.117 | 0.0262 | 4.462 | 9.97E-05 | 0.005626 |
| X202834_at | 0.3753 | 0.0841 | 4.462 | 9.98E-05 | 0.005626 |
| X214091_s_at | -0.4251 | 0.0953 | -4.46 | 0.000100326 | 0.005642 |
| X218764_at | -0.5157 | 0.1157 | -4.457 | 0.000101063 | 0.005669 |
| X205711_x_at | -0.0906 | 0.0203 | -4.456 | 0.000101389 | 0.005673 |
| X203233_at | 0.017 | 0.0038 | 4.455 | 0.000101849 | 0.005685 |
| X211948_x_at | 0.1873 | 0.0421 | 4.452 | 0.000102482 | 0.005706 |
| X201861_s_at | 0.3655 | 0.0822 | 4.449 | 0.000103415 | 0.005733 |
| X205721_at | 0.02 | 0.0045 | 4.449 | 0.000103491 | 0.005733 |
| X214077_x_at | 0.2242 | 0.0504 | 4.448 | 0.00010378 | 0.005735 |
| X200609_s_at | 0.2613 | 0.0588 | 4.446 | 0.000104242 | 0.005746 |
| X218507_at | 0.4582 | 0.1031 | 4.445 | 0.000104706 | 0.005757 |
| X202591_s_at | -0.1484 | 0.0334 | -4.442 | 0.00010556 | 0.005778 |
| X208131_s_at | -0.4276 | 0.0963 | -4.442 | 0.000105608 | 0.005778 |
| X204957_at | -0.2497 | 0.0562 | -4.44 | 0.000106179 | 0.005781 |
| X202113_s_at | -0.2417 | 0.0544 | -4.44 | 0.000106187 | 0.005781 |
| X204929_s_at | 0.2099 | 0.0473 | 4.437 | 0.000107093 | 0.005817 |
| X201600_at | -0.0903 | 0.0204 | -4.424 | 0.000111148 | 0.006013 |
| X214829_at | 0.2743 | 0.062 | 4.423 | 0.000111273 | 0.006013 |
| X204721_s_at | -0.0712 | 0.0161 | -4.423 | 0.000111513 | 0.006013 |
| X206434_at | -0.224 | 0.0507 | -4.421 | 0.000112138 | 0.006032 |
| X212908_at | -0.1539 | 0.0348 | -4.418 | 0.000113061 | 0.006067 |
| X203369_x_at | -0.0118 | 0.0027 | -4.414 | 0.000114177 | 0.006112 |
| X221934_s_at | -0.0413 | 0.0094 | -4.408 | 0.000116278 | 0.006209 |
| X213307_at | 0.2067 | 0.047 | 4.4 | 0.000118904 | 0.006334 |
| X212085_at | -0.2167 | 0.0493 | -4.397 | 0.000119866 | 0.006357 |
| X204185_x_at | -0.1329 | 0.0302 | -4.396 | 0.000120197 | 0.006357 |
| X204671_s_at | 0.4102 | 0.0933 | 4.395 | 0.000120461 | 0.006357 |
| X32094_at | 0.048 | 0.0109 | 4.395 | 0.000120482 | 0.006357 |
| X212845_at | 0.2743 | 0.0625 | 4.393 | 0.000121277 | 0.006384 |
| X208992_s_at | 0.0901 | 0.0205 | 4.39 | 0.000122335 | 0.006424 |
| X218377_s_at | -0.2246 | 0.0512 | -4.389 | 0.000122729 | 0.00643 |
| X201315_x_at | 0.5613 | 0.128 | 4.387 | 0.000123456 | 0.006435 |
| X212490_at | -0.2071 | 0.0472 | -4.386 | 0.000123616 | 0.006435 |
| X212354_at | 0.3926 | 0.0895 | 4.386 | 0.0001237 | 0.006435 |
| X215527_at | -0.3696 | 0.0843 | -4.382 | 0.000125047 | 0.006472 |
| X204019_s_at | -0.1004 | 0.0229 | -4.382 | 0.000125155 | 0.006472 |
| X212199_at | -0.0885 | 0.0202 | -4.381 | 0.000125307 | 0.006472 |
| X219492_at | 0.2406 | 0.0549 | 4.38 | 0.000125631 | 0.006472 |
| X205963_s_at | -0.1663 | 0.038 | -4.38 | 0.000125869 | 0.006472 |
| X219035_s_at | -0.1527 | 0.0349 | -4.378 | 0.00012638 | 0.006475 |
| X206261_at | -0.2017 | 0.0461 | -4.378 | 0.000126503 | 0.006475 |
| X202973_x_at | 0.2019 | 0.0462 | 4.373 | 0.000128134 | 0.006538 |
| X220495_s_at | -0.1249 | 0.0286 | -4.373 | 0.000128322 | 0.006538 |
| X212491_s_at | -0.0911 | 0.0208 | -4.37 | 0.000129289 | 0.00656 |
| X214909_s_at | 0.1919 | 0.0439 | 4.37 | 0.000129355 | 0.00656 |
| X221009_s_at | 0.8176 | 0.1873 | 4.366 | 0.000130883 | 0.006619 |
| X201324_at | 0.832 | 0.1906 | 4.365 | 0.000131103 | 0.006619 |
| X221706_s_at | -0.1293 | 0.0296 | -4.364 | 0.000131756 | 0.006637 |
| X209108_at | 0.3843 | 0.0881 | 4.362 | 0.000132257 | 0.006639 |
| X207700_s_at | 0.1316 | 0.0302 | 4.361 | 0.000132643 | 0.006639 |
| X204676_at | -0.0845 | 0.0194 | -4.361 | 0.000132699 | 0.006639 |
| X201348_at | -0.4106 | 0.0942 | -4.358 | 0.0001338 | 0.006679 |
| X212750_at | -0.2163 | 0.0497 | -4.353 | 0.000135645 | 0.006756 |
| X201303_at | -0.122 | 0.0281 | -4.342 | 0.000140177 | 0.006955 |
| X208971_at | -0.1304 | 0.03 | -4.341 | 0.000140267 | 0.006955 |
| X203315_at | 0.2534 | 0.0584 | 4.339 | 0.000141358 | 0.006981 |
| X201516_at | -0.1464 | 0.0337 | -4.339 | 0.000141411 | 0.006981 |
| X211383_s_at | 0.0848 | 0.0196 | 4.332 | 0.000143928 | 0.007069 |
| X214737_x_at | -0.0701 | 0.0162 | -4.332 | 0.000144065 | 0.007069 |
| X219332_at | 0.0778 | 0.018 | 4.332 | 0.000144151 | 0.007069 |
| X201010_s_at | 0.7524 | 0.1737 | 4.33 | 0.00014478 | 0.007077 |
| X214247_s_at | -0.0869 | 0.0201 | -4.33 | 0.000144948 | 0.007077 |
| X218493_at | -0.1449 | 0.0335 | -4.326 | 0.000146405 | 0.007131 |
| X210649_s_at | -0.0288 | 0.0067 | -4.326 | 0.000146696 | 0.007131 |
| X201587_s_at | 0.1822 | 0.0422 | 4.323 | 0.000147916 | 0.007174 |
| X214104_at | 0.1769 | 0.0409 | 4.32 | 0.000149183 | 0.00722 |
| X213438_at | 0.1652 | 0.0383 | 4.318 | 0.000149944 | 0.007241 |
| X218472_s_at | -0.1527 | 0.0354 | -4.312 | 0.000152531 | 0.007341 |
| X201435_s_at | -0.2648 | 0.0614 | -4.311 | 0.000152681 | 0.007341 |
| X212872_s_at | -0.049 | 0.0114 | -4.31 | 0.000153111 | 0.007346 |
| X212501_at | 0.3173 | 0.0736 | 4.309 | 0.000153751 | 0.007361 |
| X218761_at | -0.1531 | 0.0356 | -4.307 | 0.000154616 | 0.007386 |
| X207173_x_at | -0.1512 | 0.0351 | -4.305 | 0.000155587 | 0.007407 |
| X212038_s_at | -0.1268 | 0.0294 | -4.304 | 0.000155726 | 0.007407 |
| X208991_at | 0.3012 | 0.07 | 4.303 | 0.000156124 | 0.007411 |
| X206099_at | -0.4265 | 0.0991 | -4.302 | 0.000156868 | 0.007412 |
| X200790_at | 0.2904 | 0.0675 | 4.302 | 0.000156873 | 0.007412 |
| X201086_x_at | -0.16 | 0.0372 | -4.301 | 0.000157164 | 0.007412 |
| X564_at | 0.157 | 0.0365 | 4.3 | 0.000157686 | 0.007421 |
| X209191_at | 0.8645 | 0.2011 | 4.298 | 0.000158357 | 0.007437 |
| X218201_at | -0.171 | 0.0399 | -4.286 | 0.000164106 | 0.007685 |
| X203884_s_at | -0.1759 | 0.0411 | -4.285 | 0.000164462 | 0.007685 |
| X202590_s_at | -0.0945 | 0.0221 | -4.285 | 0.000164668 | 0.007685 |
| X202374_s_at | -0.1339 | 0.0313 | -4.283 | 0.000165291 | 0.007698 |
| X201858_s_at | 0.7324 | 0.171 | 4.282 | 0.000165821 | 0.007706 |
| X205208_at | 0.3236 | 0.0756 | 4.281 | 0.000166368 | 0.007715 |
| X203889_at | -0.1753 | 0.041 | -4.279 | 0.000167126 | 0.007729 |
| X203116_s_at | -0.161 | 0.0376 | -4.279 | 0.000167358 | 0.007729 |
| X221094_s_at | -0.2587 | 0.0605 | -4.276 | 0.000168592 | 0.00777 |
| X201092_at | -0.1795 | 0.042 | -4.274 | 0.000169621 | 0.007801 |
| X202207_at | 0.4983 | 0.1167 | 4.271 | 0.000171284 | 0.007861 |
| X217849_s_at | 0.1834 | 0.043 | 4.266 | 0.000173665 | 0.007954 |
| X208659_at | 0.6091 | 0.1429 | 4.262 | 0.000175661 | 0.008029 |
| X209177_at | -0.1212 | 0.0285 | -4.26 | 0.000176611 | 0.008056 |
| X201995_at | 0.2008 | 0.0472 | 4.258 | 0.000177261 | 0.008069 |
| X209546_s_at | 0.0138 | 0.0032 | 4.257 | 0.000177966 | 0.008085 |
| X202190_at | -0.0086 | 0.002 | -4.255 | 0.000178857 | 0.008108 |
| X204350_s_at | -0.1867 | 0.0439 | -4.254 | 0.00017931 | 0.008112 |
| X203816_at | -0.2115 | 0.0497 | -4.252 | 0.00018073 | 0.00816 |
| X203086_at | -0.1984 | 0.0467 | -4.25 | 0.000181681 | 0.008186 |
| X222251_s_at | -0.0408 | 0.0096 | -4.248 | 0.000182674 | 0.008214 |
| X207776_s_at | -0.3848 | 0.0907 | -4.244 | 0.00018463 | 0.00827 |
| X203861_s_at | -0.3553 | 0.0837 | -4.244 | 0.000184649 | 0.00827 |
| X210968_s_at | -0.1455 | 0.0343 | -4.243 | 0.000185021 | 0.00827 |
| X201009_s_at | 0.8614 | 0.2031 | 4.242 | 0.000185514 | 0.008275 |
| X202942_at | -0.1511 | 0.0356 | -4.24 | 0.000186472 | 0.008301 |
| X206458_s_at | -0.5044 | 0.119 | -4.239 | 0.000187471 | 0.008329 |
| X207949_s_at | -0.236 | 0.0557 | -4.234 | 0.000190129 | 0.00843 |
| X215329_s_at | -0.0082 | 0.0019 | -4.231 | 0.000191253 | 0.008438 |
| X207560_at | 0.012 | 0.0028 | 4.231 | 0.000191717 | 0.008438 |
| X216651_s_at | -0.9967 | 0.2356 | -4.23 | 0.000192003 | 0.008438 |
| X208722_s_at | -0.1397 | 0.033 | -4.23 | 0.000192143 | 0.008438 |
| X212992_at | 0.2371 | 0.0561 | 4.23 | 0.000192207 | 0.008438 |
| X204797_s_at | -0.1762 | 0.0417 | -4.228 | 0.000193102 | 0.008461 |
| X203386_at | 0.0811 | 0.0192 | 4.227 | 0.000193744 | 0.008462 |
| X202090_s_at | -0.0832 | 0.0197 | -4.227 | 0.000193895 | 0.008462 |
| X212826_s_at | -0.1129 | 0.0267 | -4.224 | 0.00019548 | 0.008499 |
| X218010_x_at | 0.0159 | 0.0038 | 4.224 | 0.000195503 | 0.008499 |
| X210511_s_at | -0.466 | 0.1104 | -4.222 | 0.000196579 | 0.008529 |
| X218283_at | -0.0924 | 0.0219 | -4.219 | 0.000198251 | 0.008585 |
| X203234_at | 0.0563 | 0.0134 | 4.217 | 0.000199171 | 0.008608 |
| X219100_at | -0.1878 | 0.0446 | -4.216 | 0.00019991 | 0.008623 |
| X213714_at | -0.2663 | 0.0632 | -4.214 | 0.000200944 | 0.008641 |
| X201988_s_at | -0.3032 | 0.072 | -4.214 | 0.000201096 | 0.008641 |
| X212511_at | -0.1783 | 0.0423 | -4.211 | 0.000202601 | 0.008688 |
| X214030_at | -0.2802 | 0.0666 | -4.209 | 0.000203444 | 0.008693 |
| X208982_at | 0.5087 | 0.1209 | 4.209 | 0.000203661 | 0.008693 |
| X218754_at | -0.1384 | 0.0329 | -4.209 | 0.000203885 | 0.008693 |
| X202935_s_at | 0.3202 | 0.0761 | 4.207 | 0.000204691 | 0.008711 |
| X204691_x_at | -0.0198 | 0.0047 | -4.206 | 0.000205467 | 0.008727 |
| X221531_at | -0.2522 | 0.06 | -4.205 | 0.000206245 | 0.008743 |
| X209208_at | -0.1721 | 0.041 | -4.2 | 0.000208755 | 0.008833 |
| X220329_s_at | -0.1812 | 0.0432 | -4.199 | 0.000209431 | 0.008836 |
| X200030_s_at | -0.0921 | 0.0219 | -4.199 | 0.000209658 | 0.008836 |
| X219421_at | -0.2741 | 0.0653 | -4.198 | 0.000210013 | 0.008836 |
| X214265_at | 0.0244 | 0.0058 | 4.192 | 0.00021382 | 0.008956 |
| X210802_s_at | -0.2009 | 0.0479 | -4.191 | 0.000214394 | 0.008956 |
| X219297_at | -0.1965 | 0.0469 | -4.19 | 0.00021464 | 0.008956 |
| X221486_at | 0.2112 | 0.0504 | 4.19 | 0.000214973 | 0.008956 |
| X210605_s_at | -0.0388 | 0.0093 | -4.189 | 0.00021534 | 0.008956 |
| X210418_s_at | -0.1682 | 0.0402 | -4.189 | 0.000215567 | 0.008956 |
| X202206_at | 0.3849 | 0.0919 | 4.188 | 0.000215856 | 0.008956 |
| X202574_s_at | 0.1758 | 0.042 | 4.188 | 0.000216109 | 0.008956 |
| X202641_at | -0.1738 | 0.0415 | -4.187 | 0.00021665 | 0.008957 |
| X209271_at | -0.1317 | 0.0315 | -4.185 | 0.000217618 | 0.008957 |
| X202760_s_at | 0.4216 | 0.1007 | 4.185 | 0.000217903 | 0.008957 |
| X210285_x_at | -0.3245 | 0.0775 | -4.184 | 0.000218193 | 0.008957 |
| X207772_s_at | -0.5595 | 0.1337 | -4.184 | 0.000218578 | 0.008957 |
| X216836_s_at | 0.3321 | 0.0794 | 4.183 | 0.000218829 | 0.008957 |
| X211535_s_at | 0.1387 | 0.0332 | 4.183 | 0.000218955 | 0.008957 |
| X201411_s_at | -0.2542 | 0.0608 | -4.182 | 0.000219974 | 0.008979 |
| X202336_s_at | -0.1883 | 0.045 | -4.181 | 0.000220299 | 0.008979 |
| X204002_s_at | -0.3177 | 0.076 | -4.18 | 0.000220975 | 0.00899 |
| X201753_s_at | 0.0963 | 0.023 | 4.177 | 0.000222942 | 0.009054 |
| X206349_at | -0.1654 | 0.0396 | -4.174 | 0.000224993 | 0.00912 |
| X200978_at | -0.1449 | 0.0347 | -4.172 | 0.0002261 | 0.009141 |
| X202624_s_at | 0.022 | 0.0053 | 4.171 | 0.000226312 | 0.009141 |
| X218982_s_at | -0.2147 | 0.0515 | -4.171 | 0.000226841 | 0.009145 |
| X212027_at | 0.2566 | 0.0616 | 4.163 | 0.000231798 | 0.009328 |
| X204416_x_at | -0.3474 | 0.0835 | -4.161 | 0.000233031 | 0.009361 |
| X205278_at | -0.1081 | 0.026 | -4.16 | 0.000233665 | 0.009369 |
| X206445_s_at | -0.1776 | 0.0427 | -4.158 | 0.000235128 | 0.009404 |
| X213513_x_at | -0.1034 | 0.0249 | -4.157 | 0.00023537 | 0.009404 |
| X209467_s_at | 0.2228 | 0.0536 | 4.156 | 0.000236651 | 0.009438 |
| X34206_at | 0.0794 | 0.0191 | 4.154 | 0.00023744 | 0.009439 |
| X202440_s_at | 0.1254 | 0.0302 | 4.154 | 0.000237549 | 0.009439 |
| X209190_s_at | -0.2001 | 0.0482 | -4.153 | 0.000238354 | 0.009439 |
| X206356_s_at | -0.7712 | 0.1857 | -4.153 | 0.000238373 | 0.009439 |
| X208842_s_at | -0.0452 | 0.0109 | -4.152 | 0.000239265 | 0.009457 |
| X217673_x_at | 0.1201 | 0.029 | 4.149 | 0.000240782 | 0.0095 |
| X201105_at | 0.3216 | 0.0776 | 4.143 | 0.000244929 | 0.009635 |
| X204119_s_at | -0.2997 | 0.0723 | -4.143 | 0.000245057 | 0.009635 |
| X217785_s_at | -0.4685 | 0.1132 | -4.141 | 0.00024683 | 0.009681 |
| X214100_x_at | 0.1014 | 0.0245 | 4.14 | 0.000247106 | 0.009681 |
| X218269_at | -0.1797 | 0.0434 | -4.138 | 0.0002486 | 0.009723 |
| X215171_s_at | -0.1323 | 0.032 | -4.137 | 0.000249518 | 0.009724 |
| X205489_at | -0.5704 | 0.1379 | -4.136 | 0.000249726 | 0.009724 |
| X202216_x_at | -0.1217 | 0.0294 | -4.136 | 0.000250046 | 0.009724 |
| X212742_at | -0.0945 | 0.0229 | -4.135 | 0.000250476 | 0.009724 |
| X217802_s_at | 0.1115 | 0.027 | 4.135 | 0.000250819 | 0.009724 |
| X203492_x_at | -0.1593 | 0.0386 | -4.132 | 0.000252883 | 0.009768 |
| X209306_s_at | 0.3651 | 0.0884 | 4.131 | 0.000253182 | 0.009768 |
| X217968_at | -0.1767 | 0.0428 | -4.131 | 0.000253283 | 0.009768 |
| X221437_s_at | -0.1421 | 0.0344 | -4.13 | 0.0002539 | 0.009775 |
| X219378_at | 0.158 | 0.0383 | 4.129 | 0.000254618 | 0.009786 |
| X215646_s_at | 0.4111 | 0.0996 | 4.128 | 0.000255781 | 0.009813 |
| X209141_at | -0.0968 | 0.0235 | -4.126 | 0.000256993 | 0.009843 |
| X45633_at | -0.2158 | 0.0524 | -4.121 | 0.000260478 | 0.009933 |
| X220070_at | -0.0173 | 0.0042 | -4.121 | 0.000260613 | 0.009933 |
| X203856_at | -0.1766 | 0.0429 | -4.121 | 0.000260692 | 0.009933 |
| X210766_s_at | -0.1357 | 0.0329 | -4.119 | 0.000262223 | 0.009974 |
| X219162_s_at | -0.1451 | 0.0352 | -4.118 | 0.000262766 | 0.009978 |
| X218491_s_at | -0.1348 | 0.0328 | -4.114 | 0.000265648 | 0.010051 |
| X213106_at | -0.133 | 0.0323 | -4.113 | 0.000266234 | 0.010051 |
| X203640_at | -0.1578 | 0.0384 | -4.113 | 0.000266389 | 0.010051 |
| X214004_s_at | 0.1514 | 0.0368 | 4.113 | 0.000266494 | 0.010051 |
| X221742_at | -0.2489 | 0.0605 | -4.112 | 0.000267095 | 0.010056 |
| X202234_s_at | 0.4507 | 0.1097 | 4.109 | 0.000269352 | 0.010124 |
| X204249_s_at | 0.2754 | 0.0671 | 4.107 | 0.000271442 | 0.010186 |
| X219623_at | -0.2078 | 0.0506 | -4.106 | 0.00027206 | 0.010186 |
| X218002_s_at | 0.2151 | 0.0524 | 4.105 | 0.00027281 | 0.010186 |
| X200096_s_at | 0.1102 | 0.0269 | 4.105 | 0.000272839 | 0.010186 |
| X203622_s_at | -0.3102 | 0.0756 | -4.102 | 0.000274629 | 0.010231 |
| X206042_x_at | -0.2148 | 0.0524 | -4.102 | 0.000275074 | 0.010231 |
| X203221_at | -0.1377 | 0.0336 | -4.101 | 0.000275454 | 0.010231 |
| X218427_at | 0.1819 | 0.0444 | 4.101 | 0.000275868 | 0.010231 |
| X215416_s_at | -0.2363 | 0.0577 | -4.097 | 0.000278505 | 0.010311 |
| X202630_at | -0.1079 | 0.0263 | -4.095 | 0.000280059 | 0.010347 |
| X209352_s_at | 0.1365 | 0.0333 | 4.094 | 0.000280847 | 0.010347 |
| X202252_at | 0.3136 | 0.0766 | 4.094 | 0.000281291 | 0.010347 |
| X203607_at | -0.086 | 0.021 | -4.094 | 0.000281442 | 0.010347 |
| X218706_s_at | 0.2998 | 0.0732 | 4.093 | 0.000281796 | 0.010347 |
| X210600_s_at | 0.2136 | 0.0522 | 4.092 | 0.000283006 | 0.010368 |
| X203344_s_at | 0.2483 | 0.0607 | 4.091 | 0.000283562 | 0.010368 |
| X209249_s_at | -0.2167 | 0.053 | -4.091 | 0.000283765 | 0.010368 |
| X217882_at | -0.1167 | 0.0286 | -4.088 | 0.000286077 | 0.010435 |
| X215695_s_at | 0.4443 | 0.1087 | 4.086 | 0.00028728 | 0.010462 |
| X210976_s_at | -0.1175 | 0.0288 | -4.085 | 0.000288089 | 0.010474 |
| X217960_s_at | -0.2293 | 0.0562 | -4.083 | 0.000289648 | 0.010513 |
| X200791_s_at | 0.3241 | 0.0794 | 4.083 | 0.000290108 | 0.010513 |
| X202471_s_at | -0.3673 | 0.09 | -4.08 | 0.000292598 | 0.010586 |
| X210964_s_at | 0.2692 | 0.066 | 4.077 | 0.000295057 | 0.010658 |
| X210653_s_at | -0.0073 | 0.0018 | -4.073 | 0.000297914 | 0.010743 |
| X205434_s_at | 0.138 | 0.0339 | 4.066 | 0.000304045 | 0.010947 |
| X201813_s_at | 0.1093 | 0.0269 | 4.064 | 0.000305959 | 0.010991 |
| X213309_at | -0.2165 | 0.0533 | -4.063 | 0.000306269 | 0.010991 |
| X209134_s_at | 0.0707 | 0.0174 | 4.063 | 0.000306764 | 0.010991 |
| X217820_s_at | 0.1876 | 0.0462 | 4.062 | 0.000307626 | 0.010997 |
| X203753_at | 0.2381 | 0.0586 | 4.061 | 0.000308272 | 0.010997 |
| X207760_s_at | 0.1186 | 0.0292 | 4.061 | 0.000308415 | 0.010997 |
| X202564_x_at | -0.2717 | 0.067 | -4.057 | 0.000311549 | 0.01108 |
| X203467_at | -0.2157 | 0.0532 | -4.057 | 0.000311873 | 0.01108 |
| X214924_s_at | 0.1761 | 0.0434 | 4.056 | 0.000312292 | 0.01108 |
| X201054_at | -0.2036 | 0.0502 | -4.055 | 0.000313029 | 0.01108 |
| X209227_at | -0.3659 | 0.0902 | -4.055 | 0.000313489 | 0.01108 |
| X209305_s_at | 0.394 | 0.0972 | 4.055 | 0.000313736 | 0.01108 |
| X202975_s_at | 0.3159 | 0.0779 | 4.053 | 0.000315077 | 0.01111 |
| X206183_s_at | -0.2261 | 0.0558 | -4.049 | 0.000318587 | 0.011216 |
| X202208_s_at | 0.2671 | 0.066 | 4.047 | 0.000320242 | 0.011256 |
| X201512_s_at | -0.5702 | 0.1409 | -4.046 | 0.000321454 | 0.011273 |
| X219347_at | -0.1473 | 0.0364 | -4.046 | 0.000321736 | 0.011273 |
| X210471_s_at | -0.3691 | 0.0913 | -4.043 | 0.000323923 | 0.011332 |
| X214151_s_at | 0.1159 | 0.0287 | 4.041 | 0.000326224 | 0.011381 |
| X202972_s_at | 0.1054 | 0.0261 | 4.041 | 0.000326355 | 0.011381 |
| X211386_at | 0.3028 | 0.075 | 4.04 | 0.000327085 | 0.011389 |
| X211302_s_at | -0.2635 | 0.0653 | -4.035 | 0.000331414 | 0.011521 |
| X214180_at | 0.284 | 0.0704 | 4.032 | 0.000334511 | 0.011611 |
| X211073_x_at | -0.0769 | 0.0191 | -4.03 | 0.000336241 | 0.011653 |
| X206254_at | 0.016 | 0.004 | 4.027 | 0.000338438 | 0.01171 |
| X214821_at | -0.1538 | 0.0382 | -4.022 | 0.000343195 | 0.01185 |
| X207996_s_at | -0.3238 | 0.0805 | -4.022 | 0.000343537 | 0.01185 |
| X206793_at | -0.1484 | 0.0369 | -4.021 | 0.000344228 | 0.011855 |
| X210349_at | -0.2103 | 0.0523 | -4.02 | 0.000345377 | 0.011876 |
| X207661_s_at | -0.0133 | 0.0033 | -4.018 | 0.000347248 | 0.011922 |
| X200079_s_at | -0.1057 | 0.0263 | -4.017 | 0.000348017 | 0.01193 |
| X212771_at | -0.1308 | 0.0326 | -4.017 | 0.000348606 | 0.011932 |
| X210947_s_at | -0.1869 | 0.0465 | -4.015 | 0.00035027 | 0.011971 |
| X205428_s_at | 0.1772 | 0.0441 | 4.014 | 0.000351114 | 0.011972 |
| X205510_s_at | -0.1707 | 0.0425 | -4.014 | 0.00035139 | 0.011972 |
| X200624_s_at | -0.1039 | 0.0259 | -4.012 | 0.000352902 | 0.012005 |
| X200998_s_at | 0.3524 | 0.0879 | 4.011 | 0.000354724 | 0.012021 |
| X204489_s_at | 0.5374 | 0.134 | 4.01 | 0.000354919 | 0.012021 |
| X205741_s_at | 0.6401 | 0.1596 | 4.01 | 0.000355004 | 0.012021 |
| X209567_at | -0.0668 | 0.0167 | -4.007 | 0.000358067 | 0.012107 |
| X217962_at | -0.1079 | 0.0269 | -4.005 | 0.000360541 | 0.012172 |
| X202219_at | 0.2577 | 0.0644 | 4.002 | 0.00036349 | 0.012227 |
| X201301_s_at | 0.15 | 0.0375 | 4.001 | 0.000363845 | 0.012227 |
| X213245_at | 0.462 | 0.1155 | 4.001 | 0.000364137 | 0.012227 |
| X215058_at | -0.1889 | 0.0472 | -4.001 | 0.000364369 | 0.012227 |
| X200883_at | -0.1907 | 0.0477 | -4 | 0.000365718 | 0.01225 |
| X213641_at | -0.0155 | 0.0039 | -3.999 | 0.000366151 | 0.01225 |
| X208696_at | -0.2024 | 0.0506 | -3.998 | 0.000367725 | 0.012284 |
| X217152_at | 0.1712 | 0.0428 | 3.997 | 0.000368497 | 0.012291 |
| X218656_s_at | 0.2861 | 0.0716 | 3.996 | 0.000369215 | 0.012296 |
| X211962_s_at | 0.3389 | 0.0848 | 3.995 | 0.000370479 | 0.01232 |
| X201609_x_at | -0.0835 | 0.0209 | -3.994 | 0.000371448 | 0.012324 |
| X200686_s_at | 0.1167 | 0.0292 | 3.994 | 0.000371854 | 0.012324 |
| X221571_at | 0.1935 | 0.0485 | 3.993 | 0.000372444 | 0.012324 |
| X220513_at | -0.2788 | 0.0698 | -3.993 | 0.000372918 | 0.012324 |
| X201494_at | 0.1411 | 0.0353 | 3.992 | 0.000373837 | 0.012324 |
| X217957_at | -0.1491 | 0.0374 | -3.992 | 0.000373931 | 0.012324 |
| X202974_at | -0.2118 | 0.0531 | -3.987 | 0.000378514 | 0.012457 |
| X213304_at | -0.1481 | 0.0371 | -3.986 | 0.000379669 | 0.012459 |
| X201705_at | -0.1272 | 0.0319 | -3.986 | 0.000379718 | 0.012459 |
| X207149_at | -0.4959 | 0.1244 | -3.985 | 0.000380974 | 0.012472 |
| X213293_s_at | 0.4911 | 0.1233 | 3.985 | 0.000381238 | 0.012472 |
| X209403_at | 0.211 | 0.053 | 3.984 | 0.000382222 | 0.012486 |
| X215133_s_at | 0.256 | 0.0643 | 3.983 | 0.000383465 | 0.012509 |
| X201729_s_at | -0.1587 | 0.0399 | -3.98 | 0.000385653 | 0.012548 |
| X210027_s_at | -0.0956 | 0.024 | -3.98 | 0.00038581 | 0.012548 |
| X214353_at | 0.1839 | 0.0462 | 3.977 | 0.0003892 | 0.012623 |
| X203656_at | -0.2545 | 0.064 | -3.977 | 0.000389251 | 0.012623 |
| X201386_s_at | -0.0989 | 0.0249 | -3.975 | 0.000392032 | 0.012695 |
| X206062_at | -0.8855 | 0.2228 | -3.974 | 0.000393023 | 0.012708 |
| X200827_at | 0.2049 | 0.0516 | 3.973 | 0.000394067 | 0.012724 |
| X208657_s_at | 0.0479 | 0.0121 | 3.97 | 0.000397239 | 0.012807 |
| X218559_s_at | 0.4597 | 0.1158 | 3.969 | 0.000397965 | 0.012812 |
| X218322_s_at | 0.6861 | 0.173 | 3.966 | 0.000401588 | 0.012895 |
| X213041_s_at | -0.1381 | 0.0348 | -3.966 | 0.000401813 | 0.012895 |
| X208687_x_at | -0.2558 | 0.0645 | -3.965 | 0.000402317 | 0.012895 |
| X209882_at | -0.1876 | 0.0473 | -3.964 | 0.000403412 | 0.012895 |
| X202077_at | -0.0913 | 0.023 | -3.964 | 0.00040345 | 0.012895 |
| X221676_s_at | 0.2453 | 0.0619 | 3.963 | 0.000405193 | 0.012932 |
| X204987_at | -0.1129 | 0.0285 | -3.961 | 0.000406564 | 0.012952 |
| X210215_at | 0.015 | 0.0038 | 3.961 | 0.000407105 | 0.012952 |
| X205457_at | 0.2232 | 0.0564 | 3.961 | 0.000407563 | 0.012952 |
| X221806_s_at | 0.1801 | 0.0455 | 3.957 | 0.000411802 | 0.013068 |
| X212601_at | 0.056 | 0.0142 | 3.954 | 0.000415346 | 0.013162 |
| X202344_at | 0.0164 | 0.0042 | 3.946 | 0.000424162 | 0.013422 |
| X206625_at | 0.0197 | 0.005 | 3.945 | 0.000425688 | 0.013451 |
| X210978_s_at | 0.0231 | 0.0059 | 3.942 | 0.000428542 | 0.013522 |
| X206013_s_at | -0.4469 | 0.1134 | -3.942 | 0.000429166 | 0.013523 |
| X204741_at | 0.0741 | 0.0188 | 3.939 | 0.000432436 | 0.013606 |
| X219061_s_at | -0.2416 | 0.0614 | -3.936 | 0.000436701 | 0.013717 |
| X218995_s_at | 0.3898 | 0.0991 | 3.935 | 0.000437198 | 0.013717 |
| X205217_at | -0.3544 | 0.0901 | -3.934 | 0.000438829 | 0.013749 |
| X209723_at | 0.4752 | 0.1208 | 3.932 | 0.000440984 | 0.013797 |
| X204697_s_at | 0.2199 | 0.0559 | 3.931 | 0.000442404 | 0.013822 |
| X209171_at | -0.2165 | 0.0551 | -3.928 | 0.000446062 | 0.01389 |
| X206809_s_at | -0.1447 | 0.0368 | -3.928 | 0.000446126 | 0.01389 |
| X202284_s_at | 0.248 | 0.0631 | 3.927 | 0.000446967 | 0.01389 |
| X203371_s_at | -0.0873 | 0.0222 | -3.927 | 0.000447077 | 0.01389 |
| X221879_at | 0.0456 | 0.0116 | 3.926 | 0.00044815 | 0.013904 |
| X220940_at | 0.4324 | 0.1102 | 3.925 | 0.000449243 | 0.013904 |
| X202075_s_at | 0.1468 | 0.0374 | 3.925 | 0.000449392 | 0.013904 |
| X218604_at | -0.1068 | 0.0272 | -3.924 | 0.000451116 | 0.013938 |
| X206399_x_at | -0.0131 | 0.0033 | -3.923 | 0.000451887 | 0.013942 |
| X214022_s_at | 0.3463 | 0.0883 | 3.922 | 0.000453639 | 0.013977 |
| X202415_s_at | -0.3058 | 0.0781 | -3.918 | 0.00045818 | 0.014079 |
| X212886_at | 0.1847 | 0.0472 | 3.918 | 0.000458694 | 0.014079 |
| X213792_s_at | 0.2391 | 0.061 | 3.918 | 0.000458875 | 0.014079 |
| X213104_at | -0.1834 | 0.0468 | -3.917 | 0.000459486 | 0.014079 |
| X209626_s_at | 0.0344 | 0.0088 | 3.916 | 0.00046121 | 0.014112 |
| X202930_s_at | -0.1127 | 0.0288 | -3.915 | 0.000462151 | 0.014121 |
| X212321_at | 0.1411 | 0.0361 | 3.914 | 0.000463215 | 0.014134 |
| X207248_at | -0.7304 | 0.1868 | -3.91 | 0.000468651 | 0.014281 |
| X209270_at | -0.0218 | 0.0056 | -3.907 | 0.000472391 | 0.014362 |
| X201592_at | -0.1665 | 0.0426 | -3.907 | 0.000472619 | 0.014362 |
| X212926_at | 0.4157 | 0.1065 | 3.905 | 0.000474752 | 0.014404 |
| X219128_at | -0.0807 | 0.0207 | -3.905 | 0.000475462 | 0.014404 |
| X218343_s_at | -0.099 | 0.0254 | -3.904 | 0.000476417 | 0.014404 |
| X218286_s_at | -0.1236 | 0.0316 | -3.904 | 0.000476582 | 0.014404 |
| X205570_at | 0.3769 | 0.0966 | 3.903 | 0.000477801 | 0.014414 |
| X208781_x_at | -0.0799 | 0.0205 | -3.902 | 0.000479655 | 0.014414 |
| X211787_s_at | -0.083 | 0.0213 | -3.901 | 0.000479991 | 0.014414 |
| X203811_s_at | -0.4548 | 0.1166 | -3.901 | 0.000480124 | 0.014414 |
| X208860_s_at | -0.0834 | 0.0214 | -3.901 | 0.000480353 | 0.014414 |
| X204868_at | -0.0894 | 0.0229 | -3.901 | 0.000480825 | 0.014414 |
| X213272_s_at | -0.7021 | 0.1801 | -3.898 | 0.000485182 | 0.014507 |
| X218792_s_at | -0.1637 | 0.042 | -3.898 | 0.000485217 | 0.014507 |
| X201800_s_at | -0.0979 | 0.0251 | -3.896 | 0.000486792 | 0.014527 |
| X202382_s_at | -0.1717 | 0.0441 | -3.896 | 0.000487184 | 0.014527 |
| X208799_at | -0.1059 | 0.0272 | -3.894 | 0.000489417 | 0.014559 |
| X204060_s_at | 0.1224 | 0.0314 | 3.894 | 0.000489897 | 0.014559 |
| X203964_at | 0.2344 | 0.0602 | 3.894 | 0.000490224 | 0.014559 |
| X209900_s_at | 0.4058 | 0.1043 | 3.89 | 0.000494793 | 0.014667 |
| X217932_at | -0.1399 | 0.036 | -3.89 | 0.000495201 | 0.014667 |
| X213385_at | -0.3084 | 0.0793 | -3.889 | 0.000497067 | 0.014688 |
| X215005_at | -0.3187 | 0.0819 | -3.889 | 0.00049723 | 0.014688 |
| X218955_at | -0.1231 | 0.0317 | -3.886 | 0.000500474 | 0.014747 |
| X222146_s_at | 0.1881 | 0.0484 | 3.886 | 0.00050057 | 0.014747 |
| X212747_at | 0.0674 | 0.0173 | 3.886 | 0.000501222 | 0.014747 |
| X206498_at | -0.8783 | 0.2261 | -3.884 | 0.000503266 | 0.014788 |
| X218319_at | -0.1751 | 0.0451 | -3.883 | 0.00050501 | 0.014819 |
| X208747_s_at | 0.3231 | 0.0832 | 3.882 | 0.000506957 | 0.014844 |
| X208839_s_at | -0.2053 | 0.0529 | -3.881 | 0.000507852 | 0.014844 |
| X213447_at | 0.1454 | 0.0375 | 3.881 | 0.000507867 | 0.014844 |
| X207761_s_at | 0.1493 | 0.0385 | 3.879 | 0.000511145 | 0.014914 |
| X203448_s_at | 0.1138 | 0.0293 | 3.878 | 0.000511605 | 0.014914 |
| X210156_s_at | -0.3019 | 0.0779 | -3.877 | 0.000514162 | 0.014962 |
| X202214_s_at | -0.1358 | 0.035 | -3.876 | 0.000515046 | 0.014962 |
| X1255_g_at | -0.4225 | 0.109 | -3.875 | 0.000515821 | 0.014962 |
| X210906_x_at | 0.3082 | 0.0795 | 3.875 | 0.000516287 | 0.014962 |
| X221227_x_at | -0.1174 | 0.0303 | -3.875 | 0.000516597 | 0.014962 |
| X211963_s_at | -0.1915 | 0.0494 | -3.874 | 0.000518168 | 0.014974 |
| X213203_at | -0.0512 | 0.0132 | -3.874 | 0.000518352 | 0.014974 |
| X209926_at | -0.049 | 0.0126 | -3.872 | 0.000520091 | 0.015004 |
| X207616_s_at | -0.2018 | 0.0521 | -3.871 | 0.000521702 | 0.015012 |
| X214052_x_at | 0.3587 | 0.0927 | 3.871 | 0.000521812 | 0.015012 |
| X218694_at | -0.0808 | 0.0209 | -3.871 | 0.0005224 | 0.015012 |
| X202133_at | 0.3161 | 0.0817 | 3.869 | 0.000524459 | 0.015052 |
| X209248_at | -0.1132 | 0.0293 | -3.866 | 0.000528753 | 0.015156 |
| X212039_x_at | -0.0745 | 0.0193 | -3.865 | 0.000530431 | 0.015173 |
| X203408_s_at | 0.2414 | 0.0625 | 3.864 | 0.000531915 | 0.015173 |
| X207418_s_at | -0.0279 | 0.0072 | -3.864 | 0.000532182 | 0.015173 |
| X222027_at | 0.155 | 0.0401 | 3.864 | 0.000532629 | 0.015173 |
| X211933_s_at | -0.0913 | 0.0236 | -3.864 | 0.000532779 | 0.015173 |
| X201460_at | 0.1734 | 0.0449 | 3.861 | 0.000536112 | 0.015243 |
| X218888_s_at | -0.1983 | 0.0514 | -3.861 | 0.000536616 | 0.015243 |
| X213721_at | 0.1744 | 0.0452 | 3.859 | 0.000540287 | 0.015328 |
| X201268_at | -0.1017 | 0.0264 | -3.858 | 0.000541013 | 0.015329 |
| X219590_x_at | -0.1691 | 0.0438 | -3.858 | 0.000541834 | 0.015333 |
| X202001_s_at | -0.0889 | 0.0231 | -3.857 | 0.000542863 | 0.015342 |
| X200597_at | -0.0975 | 0.0253 | -3.856 | 0.000544119 | 0.015358 |
| X209385_s_at | -0.1633 | 0.0424 | -3.855 | 0.000544958 | 0.015363 |
| X206291_at | 0.874 | 0.2268 | 3.853 | 0.000548162 | 0.015413 |
| X217803_at | 0.105 | 0.0273 | 3.853 | 0.000548421 | 0.015413 |
| X202594_at | -0.128 | 0.0332 | -3.853 | 0.000548811 | 0.015413 |
| X214129_at | 0.3018 | 0.0784 | 3.85 | 0.000552992 | 0.015507 |
| X205178_s_at | 0.2117 | 0.055 | 3.849 | 0.000554305 | 0.015507 |
| X201071_x_at | 0.0872 | 0.0227 | 3.849 | 0.000554338 | 0.015507 |
| X221569_at | 0.1271 | 0.033 | 3.849 | 0.000554956 | 0.015507 |
| X202505_at | -0.0652 | 0.0169 | -3.847 | 0.000557378 | 0.015555 |
| X213435_at | 0.7543 | 0.1961 | 3.847 | 0.000558453 | 0.015565 |
| X217746_s_at | 0.1867 | 0.0486 | 3.845 | 0.000560526 | 0.015586 |
| X213453_x_at | -0.0599 | 0.0156 | -3.845 | 0.000561074 | 0.015586 |
| X59625_at | 0.2246 | 0.0584 | 3.845 | 0.000561291 | 0.015586 |
| X212149_at | 0.2594 | 0.0675 | 3.841 | 0.000566549 | 0.015702 |
| X202557_at | -0.1234 | 0.0321 | -3.841 | 0.000566906 | 0.015702 |
| X211323_s_at | -0.2746 | 0.0715 | -3.841 | 0.000567631 | 0.015703 |
| X206138_s_at | 0.0554 | 0.0144 | 3.839 | 0.000570734 | 0.015769 |
| X204587_at | -0.2371 | 0.0618 | -3.836 | 0.000575293 | 0.015875 |
| X217743_s_at | -0.1566 | 0.0408 | -3.835 | 0.000577139 | 0.015906 |
| X203133_at | -0.0842 | 0.022 | -3.834 | 0.000578489 | 0.01591 |
| X200904_at | 0.3201 | 0.0835 | 3.834 | 0.000578706 | 0.01591 |
| X215823_x_at | 0.1138 | 0.0297 | 3.832 | 0.000580891 | 0.01595 |
| X204228_at | -0.073 | 0.0191 | -3.831 | 0.000582657 | 0.015979 |
| X205234_at | 0.2354 | 0.0615 | 3.83 | 0.000583841 | 0.015992 |
| X219384_s_at | -0.1709 | 0.0446 | -3.828 | 0.000588245 | 0.016093 |
| X202300_at | -0.0976 | 0.0255 | -3.825 | 0.000592613 | 0.016179 |
| X202844_s_at | 0.2072 | 0.0542 | 3.824 | 0.000593806 | 0.016179 |
| X210972_x_at | -1.0309 | 0.2696 | -3.824 | 0.000594284 | 0.016179 |
| X201322_at | -0.1523 | 0.0398 | -3.824 | 0.00059495 | 0.016179 |
| X201798_s_at | 0.5377 | 0.1406 | 3.824 | 0.000595038 | 0.016179 |
| X200005_at | -0.0877 | 0.0229 | -3.823 | 0.000596227 | 0.016191 |
| X40255_at | -0.2414 | 0.0632 | -3.821 | 0.000599818 | 0.016252 |
| X203261_at | -0.0836 | 0.0219 | -3.821 | 0.000599905 | 0.016252 |
| X202857_at | -0.1301 | 0.0341 | -3.819 | 0.000602722 | 0.016308 |
| X203179_at | -0.2667 | 0.0699 | -3.816 | 0.000607675 | 0.016405 |
| X213804_at | 0.0465 | 0.0122 | 3.816 | 0.000607988 | 0.016405 |
| X200067_x_at | -0.0769 | 0.0201 | -3.815 | 0.00060853 | 0.016405 |
| X203079_s_at | -0.1498 | 0.0393 | -3.815 | 0.000609493 | 0.016411 |
| X212350_at | 0.1898 | 0.0498 | 3.813 | 0.00061317 | 0.01649 |
| X215157_x_at | 0.1017 | 0.0267 | 3.812 | 0.000614885 | 0.016516 |
| X202559_x_at | -0.1408 | 0.037 | -3.811 | 0.000616237 | 0.016533 |
| X211755_s_at | -0.0932 | 0.0245 | -3.81 | 0.000617794 | 0.016555 |
| X211126_s_at | 0.3832 | 0.1006 | 3.809 | 0.000618692 | 0.016557 |
| X211965_at | 0.3602 | 0.0946 | 3.809 | 0.000619384 | 0.016557 |
| X202010_s_at | 0.1049 | 0.0276 | 3.808 | 0.000620371 | 0.016564 |
| X212386_at | 0.2103 | 0.0552 | 3.807 | 0.000622937 | 0.016612 |
| X206976_s_at | -0.2596 | 0.0682 | -3.806 | 0.000624628 | 0.016637 |
| X201527_at | -0.0926 | 0.0243 | -3.802 | 0.000630722 | 0.016752 |
| X212215_at | -0.1166 | 0.0307 | -3.802 | 0.000630883 | 0.016752 |
| X202114_at | -0.085 | 0.0224 | -3.802 | 0.000631338 | 0.016752 |
| X218623_at | -0.1776 | 0.0467 | -3.801 | 0.000632257 | 0.016752 |
| X216591_s_at | -0.1249 | 0.0329 | -3.801 | 0.000632746 | 0.016752 |
| X218203_at | -0.0751 | 0.0198 | -3.801 | 0.000633466 | 0.016752 |
| X218168_s_at | -0.0977 | 0.0257 | -3.8 | 0.000634826 | 0.016756 |
| X222368_at | 0.3412 | 0.0898 | 3.8 | 0.000635113 | 0.016756 |
| X200931_s_at | 0.2188 | 0.0576 | 3.799 | 0.000637382 | 0.016796 |
| X218789_s_at | -0.2049 | 0.054 | -3.798 | 0.000639098 | 0.016821 |
| X219979_s_at | -0.1753 | 0.0462 | -3.796 | 0.000641228 | 0.016857 |
| X219292_at | -0.1533 | 0.0404 | -3.795 | 0.000644116 | 0.016902 |
| X204053_x_at | 0.1369 | 0.0361 | 3.795 | 0.000644456 | 0.016902 |
| X206290_s_at | -0.213 | 0.0562 | -3.793 | 0.000646279 | 0.016913 |
| X211509_s_at | -0.0772 | 0.0204 | -3.793 | 0.000646392 | 0.016913 |
| X217188_s_at | -0.1553 | 0.041 | -3.792 | 0.000648346 | 0.016944 |
| X91682_at | -0.2153 | 0.0568 | -3.792 | 0.000649553 | 0.016956 |
| X219345_at | -0.0756 | 0.0199 | -3.79 | 0.000652262 | 0.017006 |
| X208668_x_at | 0.1352 | 0.0357 | 3.789 | 0.000653847 | 0.017012 |
| X218421_at | 0.186 | 0.0491 | 3.789 | 0.000653996 | 0.017012 |
| X203571_s_at | 0.4014 | 0.106 | 3.788 | 0.000656375 | 0.017053 |
| X209276_s_at | 0.1322 | 0.0349 | 3.784 | 0.000663853 | 0.017228 |
| X208457_at | -0.5436 | 0.1437 | -3.783 | 0.00066543 | 0.017248 |
| X208731_at | -0.1041 | 0.0275 | -3.779 | 0.000671976 | 0.017398 |
| X201195_s_at | 0.3683 | 0.0975 | 3.778 | 0.000674468 | 0.017442 |
| X202351_at | -0.1468 | 0.0389 | -3.775 | 0.000679402 | 0.017529 |
| X205125_at | 0.2598 | 0.0688 | 3.775 | 0.00067947 | 0.017529 |
| X217140_s_at | -0.2299 | 0.0609 | -3.775 | 0.000680393 | 0.017529 |
| X203324_s_at | 0.3934 | 0.1042 | 3.774 | 0.000680987 | 0.017529 |
| X210379_s_at | -0.1451 | 0.0385 | -3.772 | 0.000684665 | 0.017603 |
| X212407_at | -0.2634 | 0.0699 | -3.771 | 0.000687883 | 0.017651 |
| X207767_s_at | -0.2463 | 0.0653 | -3.771 | 0.00068811 | 0.017651 |
| X201131_s_at | -0.1484 | 0.0394 | -3.77 | 0.000690068 | 0.017681 |
| X218751_s_at | 0.2152 | 0.0571 | 3.769 | 0.000691106 | 0.017687 |
| X209670_at | -0.4411 | 0.1171 | -3.768 | 0.000692675 | 0.017694 |
| X212185_x_at | 0.3354 | 0.089 | 3.767 | 0.000695616 | 0.017694 |
| X43427_at | 0.3381 | 0.0898 | 3.767 | 0.000695869 | 0.017694 |
| X201898_s_at | -0.2008 | 0.0533 | -3.766 | 0.000696184 | 0.017694 |
| X202167_s_at | -0.1686 | 0.0448 | -3.765 | 0.000698097 | 0.017694 |
| X217356_s_at | -0.2592 | 0.0689 | -3.765 | 0.000698751 | 0.017694 |
| X208981_at | 0.4503 | 0.1196 | 3.765 | 0.000699518 | 0.017694 |
| X205775_at | -0.2647 | 0.0703 | -3.764 | 0.000699749 | 0.017694 |
| X202922_at | -0.1347 | 0.0358 | -3.764 | 0.000700838 | 0.017694 |
| X202071_at | 0.3665 | 0.0974 | 3.764 | 0.000700906 | 0.017694 |
| X203407_at | 0.0338 | 0.009 | 3.764 | 0.000701187 | 0.017694 |
| X201186_at | -0.1941 | 0.0516 | -3.764 | 0.00070143 | 0.017694 |
| X216032_s_at | -0.1625 | 0.0432 | -3.763 | 0.000701833 | 0.017694 |
| X205219_s_at | -0.1668 | 0.0443 | -3.763 | 0.000702537 | 0.017694 |
| X204541_at | 0.1496 | 0.0398 | 3.762 | 0.000703847 | 0.017696 |
| X218297_at | -0.135 | 0.0359 | -3.762 | 0.000704416 | 0.017696 |
| X203785_s_at | -0.0795 | 0.0211 | -3.762 | 0.000704999 | 0.017696 |
| X201797_s_at | -0.1631 | 0.0434 | -3.759 | 0.000709979 | 0.017801 |
| X202654_x_at | -0.1541 | 0.041 | -3.756 | 0.000716617 | 0.017947 |
| X213011_s_at | -0.1618 | 0.0431 | -3.754 | 0.000720135 | 0.018015 |
| X201142_at | -0.0972 | 0.0259 | -3.752 | 0.000724798 | 0.018111 |
| X203710_at | -0.155 | 0.0413 | -3.75 | 0.000727246 | 0.018126 |
| X221829_s_at | 0.2472 | 0.0659 | 3.75 | 0.000727576 | 0.018126 |
| X218214_at | -0.1443 | 0.0385 | -3.75 | 0.000727833 | 0.018126 |
| X207988_s_at | -0.1329 | 0.0354 | -3.748 | 0.000731959 | 0.018208 |
| X201106_at | -0.1583 | 0.0423 | -3.747 | 0.000734523 | 0.018237 |
| X204700_x_at | -0.1016 | 0.0271 | -3.747 | 0.000734774 | 0.018237 |
| X201535_at | -0.135 | 0.0361 | -3.743 | 0.000742388 | 0.018384 |
| X211980_at | 0.2944 | 0.0786 | 3.743 | 0.000742749 | 0.018384 |
| X207630_s_at | -0.193 | 0.0516 | -3.742 | 0.000743595 | 0.018384 |
| X200996_at | -0.0894 | 0.0239 | -3.742 | 0.000743988 | 0.018384 |
| X211456_x_at | 0.278 | 0.0743 | 3.741 | 0.000745313 | 0.018396 |
| X207030_s_at | 0.3711 | 0.0993 | 3.739 | 0.000750678 | 0.018508 |
| X209818_s_at | -0.168 | 0.0449 | -3.737 | 0.000753463 | 0.018556 |
| X203414_at | -0.155 | 0.0415 | -3.736 | 0.000757335 | 0.018631 |
| X202370_s_at | 0.3823 | 0.1024 | 3.735 | 0.00075948 | 0.018663 |
| X209702_at | -0.1478 | 0.0396 | -3.732 | 0.000764536 | 0.018766 |
| X202121_s_at | -0.0996 | 0.0267 | -3.73 | 0.000768562 | 0.018844 |
| X208843_s_at | -0.0942 | 0.0253 | -3.728 | 0.000773126 | 0.018935 |
| X201008_s_at | 0.6641 | 0.1782 | 3.726 | 0.000777115 | 0.019012 |
| X207831_x_at | -0.2012 | 0.054 | -3.725 | 0.000780513 | 0.019069 |
| X217941_s_at | 0.2656 | 0.0713 | 3.724 | 0.000781162 | 0.019069 |
| X208620_at | -0.1126 | 0.0302 | -3.724 | 0.000782043 | 0.019069 |
| X202068_s_at | 0.0822 | 0.0221 | 3.722 | 0.000785339 | 0.019129 |
| X201323_at | -0.1156 | 0.0311 | -3.72 | 0.000789881 | 0.019207 |
| X200924_s_at | 0.1413 | 0.038 | 3.72 | 0.000790287 | 0.019207 |
| X201645_at | 0.6359 | 0.1711 | 3.717 | 0.000796043 | 0.019326 |
| X208816_x_at | 0.0549 | 0.0148 | 3.716 | 0.000799784 | 0.019396 |
| X203081_at | 0.1792 | 0.0483 | 3.714 | 0.000804337 | 0.019438 |
| X209671_x_at | -0.9463 | 0.2548 | -3.714 | 0.000804353 | 0.019438 |
| X200903_s_at | -0.1747 | 0.0471 | -3.713 | 0.00080462 | 0.019438 |
| X202951_at | 0.119 | 0.032 | 3.713 | 0.000805217 | 0.019438 |
| X216944_s_at | -0.264 | 0.0711 | -3.713 | 0.000805903 | 0.019438 |
| X200611_s_at | 0.2025 | 0.0546 | 3.712 | 0.000807917 | 0.019465 |
| X208756_at | -0.1064 | 0.0287 | -3.71 | 0.000811477 | 0.01953 |
| X208735_s_at | -0.0462 | 0.0125 | -3.707 | 0.000818852 | 0.019668 |
| X208739_x_at | -0.0947 | 0.0256 | -3.707 | 0.000818986 | 0.019668 |
| X201794_s_at | -0.1094 | 0.0295 | -3.705 | 0.000823842 | 0.019763 |
| X217620_s_at | 0.2781 | 0.0751 | 3.703 | 0.000827452 | 0.019828 |
| X209731_at | -0.1513 | 0.0409 | -3.703 | 0.000828528 | 0.019833 |
| X201601_x_at | 0.4812 | 0.13 | 3.701 | 0.000831241 | 0.019861 |
| X214428_x_at | 0.6924 | 0.1871 | 3.701 | 0.000832138 | 0.019861 |
| X207547_s_at | 0.3254 | 0.0879 | 3.701 | 0.000832384 | 0.019861 |
| X206754_s_at | 0.0092 | 0.0025 | 3.7 | 0.000834126 | 0.019876 |
| X211732_x_at | 0.0088 | 0.0024 | 3.7 | 0.000834807 | 0.019876 |
| X213219_at | 0.8119 | 0.2195 | 3.699 | 0.000836613 | 0.019894 |
| X211270_x_at | 0.1705 | 0.0461 | 3.699 | 0.000837728 | 0.019894 |
| X209834_at | 0.0197 | 0.0053 | 3.698 | 0.000838239 | 0.019894 |
| X202550_s_at | -0.1342 | 0.0363 | -3.698 | 0.000839195 | 0.019895 |
| X209990_s_at | 0.4052 | 0.1096 | 3.697 | 0.00084109 | 0.019919 |
| X219141_s_at | -0.0245 | 0.0066 | -3.697 | 0.000842325 | 0.019927 |
| X212581_x_at | -0.0613 | 0.0166 | -3.694 | 0.000849173 | 0.020056 |
| X219144_at | -0.0667 | 0.0181 | -3.693 | 0.000849605 | 0.020056 |
| X200905_x_at | 0.3536 | 0.0957 | 3.693 | 0.00085114 | 0.020071 |
| X37831_at | 0.0261 | 0.0071 | 3.691 | 0.000854387 | 0.020127 |
| X221689_s_at | -0.1997 | 0.0541 | -3.691 | 0.000855631 | 0.020135 |
| X201231_s_at | -0.1297 | 0.0351 | -3.69 | 0.000858643 | 0.020184 |
| X200723_s_at | -0.0785 | 0.0213 | -3.688 | 0.000862779 | 0.020241 |
| X214055_x_at | 0.1854 | 0.0503 | 3.688 | 0.000862903 | 0.020241 |
| X208770_s_at | 0.0615 | 0.0167 | 3.687 | 0.000864663 | 0.020261 |
| X206833_s_at | -0.1014 | 0.0275 | -3.683 | 0.000874838 | 0.020478 |
| X220028_at | 0.0171 | 0.0047 | 3.68 | 0.000881009 | 0.020601 |
| X201966_at | -0.2396 | 0.0651 | -3.679 | 0.000882816 | 0.020621 |
| X201859_at | 0.5468 | 0.1486 | 3.679 | 0.000883984 | 0.020627 |
| X220609_at | 0.4567 | 0.1242 | 3.678 | 0.00088651 | 0.020664 |
| X213256_at | 0.3244 | 0.0882 | 3.676 | 0.000889799 | 0.020713 |
| X209485_s_at | 0.3041 | 0.0827 | 3.676 | 0.000890659 | 0.020713 |
| X204646_at | 0.2931 | 0.0798 | 3.676 | 0.000891394 | 0.020713 |
| X205068_s_at | 0.4858 | 0.1322 | 3.674 | 0.000895003 | 0.020775 |
| X205531_s_at | -0.1788 | 0.0487 | -3.671 | 0.00090298 | 0.020938 |
| X210338_s_at | -0.2988 | 0.0814 | -3.67 | 0.000904594 | 0.020954 |
| X218459_at | -0.1402 | 0.0382 | -3.67 | 0.000906357 | 0.020955 |
| X218108_at | -0.1189 | 0.0324 | -3.67 | 0.000906528 | 0.020955 |
| X201134_x_at | -0.0601 | 0.0164 | -3.669 | 0.000907606 | 0.020958 |
| X203385_at | -0.1316 | 0.0359 | -3.667 | 0.000914162 | 0.021079 |
| X202242_at | -0.1414 | 0.0386 | -3.666 | 0.00091475 | 0.021079 |
| X200614_at | -0.091 | 0.0248 | -3.665 | 0.000916711 | 0.021102 |
| X220014_at | -0.228 | 0.0622 | -3.665 | 0.000918136 | 0.021113 |
| X213217_at | 0.2299 | 0.0628 | 3.662 | 0.000924239 | 0.021222 |
| X220030_at | -0.4427 | 0.1209 | -3.662 | 0.000924774 | 0.021222 |
| X214121_x_at | -0.0582 | 0.0159 | -3.661 | 0.000927508 | 0.021263 |
| X202350_s_at | 0.4615 | 0.1261 | 3.66 | 0.000931645 | 0.02131 |
| X215165_x_at | -0.0854 | 0.0233 | -3.659 | 0.000932094 | 0.02131 |
| X216593_s_at | -0.2489 | 0.068 | -3.659 | 0.000932425 | 0.02131 |
| X217780_at | -0.1802 | 0.0493 | -3.657 | 0.000939232 | 0.021443 |
| X211662_s_at | -0.0897 | 0.0245 | -3.654 | 0.000945617 | 0.021563 |
| X219140_s_at | -0.2628 | 0.0719 | -3.653 | 0.000947968 | 0.021563 |
| X205978_at | -0.4219 | 0.1155 | -3.653 | 0.000948406 | 0.021563 |
| X217225_x_at | -0.2034 | 0.0557 | -3.653 | 0.000949207 | 0.021563 |
| X203094_at | -0.1973 | 0.054 | -3.653 | 0.000949336 | 0.021563 |
| X212957_s_at | -0.1512 | 0.0414 | -3.651 | 0.000953838 | 0.021643 |
| X213478_at | 0.2038 | 0.0559 | 3.648 | 0.000960953 | 0.021722 |
| X202839_s_at | -0.0906 | 0.0248 | -3.648 | 0.000960972 | 0.021722 |
| X201866_s_at | -0.0145 | 0.004 | -3.648 | 0.000961026 | 0.021722 |
| X209384_at | -0.1065 | 0.0292 | -3.648 | 0.000961215 | 0.021722 |
| X204678_s_at | -0.2124 | 0.0582 | -3.646 | 0.000965328 | 0.021792 |
| X205970_at | 0.1028 | 0.0282 | 3.645 | 0.000969199 | 0.021858 |
| X219663_s_at | -0.0524 | 0.0144 | -3.645 | 0.000970349 | 0.021861 |
| X217815_at | -0.112 | 0.0307 | -3.643 | 0.000974021 | 0.021922 |
| X202024_at | -0.2289 | 0.0628 | -3.643 | 0.000975138 | 0.021925 |
| X203570_at | 0.0308 | 0.0085 | 3.641 | 0.000979855 | 0.021985 |
| X217047_s_at | 0.1308 | 0.0359 | 3.641 | 0.000979985 | 0.021985 |
| X217004_s_at | -0.2174 | 0.0597 | -3.64 | 0.000981476 | 0.021985 |
| X211479_s_at | -0.9349 | 0.2568 | -3.64 | 0.000981761 | 0.021985 |
| X201466_s_at | 0.5612 | 0.1543 | 3.638 | 0.00098723 | 0.022085 |
| X201484_at | -0.1291 | 0.0355 | -3.637 | 0.000989338 | 0.02211 |
| X219006_at | -0.1625 | 0.0447 | -3.637 | 0.000990382 | 0.022111 |
| X222129_at | -0.162 | 0.0446 | -3.636 | 0.000993073 | 0.022132 |
| X218007_s_at | 0.0791 | 0.0218 | 3.636 | 0.000994194 | 0.022132 |
| X208994_s_at | 0.1031 | 0.0283 | 3.636 | 0.000994344 | 0.022132 |
| X218938_at | -0.0517 | 0.0142 | -3.635 | 0.000995326 | 0.022132 |
| X212360_at | -0.2355 | 0.0648 | -3.633 | 0.001000727 | 0.02221 |
| X208370_s_at | -0.0993 | 0.0273 | -3.633 | 0.001000841 | 0.02221 |
| X221699_s_at | -0.1377 | 0.0379 | -3.632 | 0.001004153 | 0.022262 |
| X218512_at | -0.1214 | 0.0335 | -3.629 | 0.001012335 | 0.02241 |
| X204248_at | 0.1746 | 0.0481 | 3.628 | 0.001013839 | 0.02241 |
| X206113_s_at | -0.2012 | 0.0555 | -3.628 | 0.00101385 | 0.02241 |
| X217898_at | -0.0928 | 0.0256 | -3.627 | 0.001018736 | 0.022495 |
| X208206_s_at | -0.3643 | 0.1005 | -3.625 | 0.001024107 | 0.022591 |
| X211721_s_at | -0.1378 | 0.038 | -3.624 | 0.00102725 | 0.022638 |
| X220661_s_at | 0.1734 | 0.0479 | 3.622 | 0.001032242 | 0.022707 |
| X63009_at | -0.0805 | 0.0222 | -3.622 | 0.001032542 | 0.022707 |
| X202536_at | -0.148 | 0.0409 | -3.621 | 0.00103413 | 0.022707 |
| X213648_at | -0.024 | 0.0066 | -3.621 | 0.001034475 | 0.022707 |
| X218774_at | -0.0285 | 0.0079 | -3.62 | 0.001037117 | 0.022731 |
| X206829_x_at | -0.1845 | 0.051 | -3.62 | 0.001037599 | 0.022731 |
| X218292_s_at | -0.3226 | 0.0891 | -3.619 | 0.001040695 | 0.022776 |
| X219628_at | -0.1645 | 0.0455 | -3.618 | 0.001041909 | 0.02278 |
| X213891_s_at | 0.1803 | 0.0498 | 3.617 | 0.001044239 | 0.022809 |
| X200035_at | 0.0889 | 0.0246 | 3.616 | 0.001048064 | 0.02287 |
| X214545_s_at | -0.2637 | 0.0729 | -3.616 | 0.001049582 | 0.022875 |
| X202155_s_at | 0.0285 | 0.0079 | 3.615 | 0.001050338 | 0.022875 |
| X202926_at | -0.1138 | 0.0315 | -3.614 | 0.001052979 | 0.022905 |
| X205226_at | -0.2529 | 0.07 | -3.614 | 0.001054582 | 0.022905 |
| X218462_at | -0.3188 | 0.0882 | -3.614 | 0.00105483 | 0.022905 |
| X201078_at | -0.227 | 0.0628 | -3.611 | 0.001061341 | 0.023024 |
| X212403_at | 0.0752 | 0.0208 | 3.611 | 0.001063685 | 0.023039 |
| X221667_s_at | 0.3137 | 0.0869 | 3.611 | 0.001064122 | 0.023039 |
| X200661_at | 0.1519 | 0.0421 | 3.61 | 0.001065926 | 0.023053 |
| X203299_s_at | -0.1108 | 0.0307 | -3.609 | 0.001067721 | 0.023053 |
| X209197_at | -0.0543 | 0.0151 | -3.609 | 0.001067974 | 0.023053 |
| X217852_s_at | -0.0783 | 0.0217 | -3.609 | 0.001068903 | 0.023053 |
| X204392_at | -0.1772 | 0.0491 | -3.608 | 0.001072787 | 0.023086 |
| X207079_s_at | -0.2613 | 0.0724 | -3.607 | 0.001074054 | 0.023086 |
| X213407_at | -0.1273 | 0.0353 | -3.607 | 0.001074883 | 0.023086 |
| X216862_s_at | 0.0807 | 0.0224 | 3.607 | 0.001075371 | 0.023086 |
| X219515_at | -0.1737 | 0.0482 | -3.607 | 0.001075621 | 0.023086 |
| X36907_at | 0.0205 | 0.0057 | 3.606 | 0.001076688 | 0.023086 |
| X203817_at | -0.2009 | 0.0557 | -3.605 | 0.001078926 | 0.023112 |
| X206235_at | -0.226 | 0.0627 | -3.604 | 0.001081658 | 0.023148 |
| X209969_s_at | 0.1631 | 0.0453 | 3.603 | 0.001086787 | 0.023236 |
| X202006_at | 0.1398 | 0.0388 | 3.602 | 0.001090158 | 0.023285 |
| X214016_s_at | 0.1278 | 0.0355 | 3.601 | 0.001092756 | 0.023318 |
| X202428_x_at | 0.2127 | 0.0591 | 3.6 | 0.001095339 | 0.023338 |
| X208978_at | -0.2753 | 0.0765 | -3.6 | 0.001095752 | 0.023338 |
| X201393_s_at | 0.1716 | 0.0477 | 3.599 | 0.001098299 | 0.023369 |
| X201417_at | 0.2261 | 0.0628 | 3.598 | 0.001101017 | 0.023386 |
| X209157_at | -0.1346 | 0.0374 | -3.598 | 0.001101178 | 0.023386 |
| X201619_at | -0.1185 | 0.033 | -3.597 | 0.001104026 | 0.023424 |
| X201991_s_at | 0.1517 | 0.0422 | 3.595 | 0.001111117 | 0.023548 |
| X207619_at | -0.0048 | 0.0013 | -3.594 | 0.001111998 | 0.023548 |
| X218481_at | -0.3828 | 0.1065 | -3.593 | 0.001114425 | 0.023577 |
| X203922_s_at | 0.0116 | 0.0032 | 3.591 | 0.001121248 | 0.023677 |
| X203781_at | -0.1423 | 0.0396 | -3.591 | 0.001121283 | 0.023677 |
| X204203_at | 0.2552 | 0.0711 | 3.591 | 0.00112263 | 0.023683 |
| X208838_at | -0.1198 | 0.0334 | -3.587 | 0.001133014 | 0.023879 |
| X202572_s_at | -0.1436 | 0.04 | -3.587 | 0.001134985 | 0.023898 |
| X218241_at | -0.0745 | 0.0208 | -3.586 | 0.001136605 | 0.02391 |
| X214264_s_at | 0.2574 | 0.0718 | 3.586 | 0.001138138 | 0.023919 |
| X212368_at | 0.1491 | 0.0416 | 3.584 | 0.001141946 | 0.023976 |
| X209931_s_at | -0.5893 | 0.1644 | -3.584 | 0.001143683 | 0.023976 |
| X200830_at | -0.1034 | 0.0289 | -3.584 | 0.001144083 | 0.023976 |
| X201944_at | -0.241 | 0.0673 | -3.581 | 0.001151368 | 0.024099 |
| X200980_s_at | -0.1405 | 0.0392 | -3.581 | 0.001152121 | 0.024099 |
| X220988_s_at | -0.1934 | 0.054 | -3.581 | 0.001153224 | 0.024099 |
| X221207_s_at | -0.1712 | 0.0478 | -3.58 | 0.001154302 | 0.024099 |
| X221911_at | -0.2254 | 0.063 | -3.575 | 0.001169842 | 0.024385 |
| X209048_s_at | 0.1516 | 0.0424 | 3.575 | 0.001170405 | 0.024385 |
| X218820_at | 0.1091 | 0.0305 | 3.575 | 0.001171986 | 0.024385 |
| X212143_s_at | 0.0159 | 0.0045 | 3.575 | 0.00117236 | 0.024385 |
| X219478_at | -0.4645 | 0.13 | -3.574 | 0.001173752 | 0.024391 |
| X200670_at | -0.192 | 0.0537 | -3.573 | 0.001179036 | 0.024478 |
| X217993_s_at | -0.1078 | 0.0302 | -3.572 | 0.001182399 | 0.024489 |
| X213342_at | 0.2326 | 0.0651 | 3.571 | 0.00118267 | 0.024489 |
| X210907_s_at | -0.0958 | 0.0268 | -3.571 | 0.001182872 | 0.024489 |
| X201096_s_at | -0.1941 | 0.0544 | -3.57 | 0.001185606 | 0.024522 |
| X214703_s_at | -0.2164 | 0.0606 | -3.569 | 0.001190781 | 0.024607 |
| X206309_at | -0.1886 | 0.0529 | -3.568 | 0.001194103 | 0.024652 |
| X201532_at | -0.0945 | 0.0265 | -3.567 | 0.001195542 | 0.024659 |
| X213637_at | 0.1815 | 0.0509 | 3.565 | 0.001201726 | 0.024764 |
| X219348_at | -0.1448 | 0.0406 | -3.564 | 0.001205289 | 0.024814 |
| X203278_s_at | 0.1684 | 0.0473 | 3.564 | 0.001206387 | 0.024814 |
| X210667_s_at | -0.3652 | 0.1025 | -3.563 | 0.001208782 | 0.02484 |
| X204436_at | -0.0622 | 0.0175 | -3.563 | 0.001210443 | 0.024848 |
| X208610_s_at | 0.318 | 0.0893 | 3.563 | 0.001211428 | 0.024848 |
| X217826_s_at | 0.2421 | 0.068 | 3.562 | 0.001213411 | 0.024866 |
| X202670_at | -0.279 | 0.0783 | -3.561 | 0.00121553 | 0.024886 |
| X211152_s_at | -0.0473 | 0.0133 | -3.561 | 0.001217728 | 0.024908 |
| X211031_s_at | 0.1507 | 0.0424 | 3.559 | 0.00122439 | 0.025001 |
| X203723_at | 0.2755 | 0.0774 | 3.559 | 0.001224491 | 0.025001 |
| X219171_s_at | -0.1251 | 0.0352 | -3.558 | 0.001226907 | 0.025008 |
| X207038_at | 0.4811 | 0.1352 | 3.558 | 0.001227102 | 0.025008 |
| X222158_s_at | -0.3043 | 0.0856 | -3.556 | 0.001232933 | 0.025104 |
| X215409_at | -0.1242 | 0.0349 | -3.555 | 0.001236056 | 0.025128 |
| X210648_x_at | -0.0704 | 0.0198 | -3.555 | 0.001236398 | 0.025128 |
| X200084_at | -0.0996 | 0.028 | -3.554 | 0.001239485 | 0.025168 |
| X212480_at | 0.0797 | 0.0224 | 3.55 | 0.001251516 | 0.025388 |
| X218278_at | -0.0542 | 0.0153 | -3.55 | 0.001253518 | 0.025388 |
| X207957_s_at | -0.2791 | 0.0786 | -3.55 | 0.001253725 | 0.025388 |
| X210293_s_at | -0.1002 | 0.0283 | -3.547 | 0.00126456 | 0.025563 |
| X221840_at | -0.1652 | 0.0466 | -3.546 | 0.001265545 | 0.025563 |
| X206659_at | -0.0753 | 0.0212 | -3.546 | 0.001266679 | 0.025563 |
| X215153_at | 0.0352 | 0.0099 | 3.545 | 0.001268424 | 0.025563 |
| X218350_s_at | 0.3799 | 0.1072 | 3.545 | 0.001268869 | 0.025563 |
| X221289_at | -0.0194 | 0.0055 | -3.545 | 0.001270315 | 0.025563 |
| X204065_at | -0.1238 | 0.0349 | -3.545 | 0.001271259 | 0.025563 |
| X200688_at | -0.1587 | 0.0448 | -3.544 | 0.001272707 | 0.025563 |
| X218023_s_at | 0.0786 | 0.0222 | 3.544 | 0.001272751 | 0.025563 |
| X205521_at | 0.2336 | 0.066 | 3.541 | 0.001282382 | 0.025733 |
| X202700_s_at | -0.0305 | 0.0086 | -3.54 | 0.001285673 | 0.025776 |
| X221641_s_at | 0.0408 | 0.0115 | 3.54 | 0.001287589 | 0.025791 |
| X211715_s_at | -0.2665 | 0.0753 | -3.539 | 0.001289789 | 0.025812 |
| X219111_s_at | -0.2827 | 0.0799 | -3.539 | 0.001292297 | 0.025839 |
| X218174_s_at | -0.1448 | 0.0409 | -3.537 | 0.001295805 | 0.025881 |
| X206879_s_at | 0.0262 | 0.0074 | 3.537 | 0.00129671 | 0.025881 |
| X201989_s_at | -0.1295 | 0.0366 | -3.536 | 0.001299292 | 0.025899 |
| X210817_s_at | 0.3559 | 0.1007 | 3.536 | 0.001299953 | 0.025899 |
| X203006_at | -0.1607 | 0.0455 | -3.536 | 0.001301247 | 0.025899 |
| X203342_at | -0.1899 | 0.0537 | -3.535 | 0.001303193 | 0.025899 |
| X213904_at | -0.1751 | 0.0495 | -3.535 | 0.001303568 | 0.025899 |
| X222046_at | 0.0121 | 0.0034 | 3.535 | 0.001304637 | 0.025899 |
| X219551_at | 0.2931 | 0.0829 | 3.534 | 0.001308091 | 0.025945 |
| X202712_s_at | -0.2659 | 0.0753 | -3.532 | 0.001313867 | 0.026036 |
| X203105_s_at | -0.2253 | 0.0638 | -3.53 | 0.001322169 | 0.026177 |
| X212135_s_at | 0.317 | 0.0898 | 3.529 | 0.00132506 | 0.026211 |
| X201468_s_at | -0.2216 | 0.0628 | -3.527 | 0.001331377 | 0.026291 |
| X209307_at | 0.2838 | 0.0805 | 3.527 | 0.001331485 | 0.026291 |
| X205609_at | 0.63 | 0.1787 | 3.525 | 0.001339692 | 0.026406 |
| X214157_at | 0.2939 | 0.0834 | 3.525 | 0.001340024 | 0.026406 |
| X213298_at | 0.0425 | 0.012 | 3.525 | 0.001340842 | 0.026406 |
| X218157_x_at | 0.2688 | 0.0763 | 3.523 | 0.001345659 | 0.026477 |
| X203863_at | -0.151 | 0.0429 | -3.522 | 0.001349772 | 0.026511 |
| X205621_at | -0.114 | 0.0324 | -3.522 | 0.001349856 | 0.026511 |
| X214464_at | 0.2037 | 0.0578 | 3.522 | 0.001350974 | 0.026511 |
| X221891_x_at | -0.1874 | 0.0532 | -3.522 | 0.001352296 | 0.026514 |
| X221688_s_at | -0.1428 | 0.0406 | -3.521 | 0.001354547 | 0.026534 |
| X202956_at | -0.1281 | 0.0364 | -3.519 | 0.001360728 | 0.026615 |
| X221449_s_at | -0.2029 | 0.0577 | -3.519 | 0.001361972 | 0.026615 |
| X212082_s_at | 0.089 | 0.0253 | 3.519 | 0.001362275 | 0.026615 |
| X207717_s_at | -0.7343 | 0.2087 | -3.519 | 0.001363615 | 0.026618 |
| X202395_at | -0.1816 | 0.0516 | -3.518 | 0.001366267 | 0.026646 |
| X202000_at | -0.097 | 0.0276 | -3.517 | 0.00136838 | 0.026664 |
| X218539_at | -0.1327 | 0.0377 | -3.516 | 0.001371876 | 0.026709 |
| X213058_at | -0.1286 | 0.0366 | -3.515 | 0.001376766 | 0.026781 |
| X203983_at | -0.0862 | 0.0245 | -3.514 | 0.001379429 | 0.0268 |
| X207486_x_at | -0.1448 | 0.0412 | -3.514 | 0.001380173 | 0.0268 |
| X209821_at | -0.369 | 0.105 | -3.514 | 0.001381853 | 0.026809 |
| X205896_at | 0.3015 | 0.0858 | 3.513 | 0.001383616 | 0.02682 |
| X202269_x_at | 0.3716 | 0.1058 | 3.512 | 0.001387506 | 0.026872 |
| X209732_at | 0.6897 | 0.1964 | 3.511 | 0.001390726 | 0.026911 |
| X205330_at | -0.2268 | 0.0646 | -3.508 | 0.001402061 | 0.027095 |
| X205055_at | -0.1318 | 0.0376 | -3.508 | 0.001403741 | 0.027095 |
| X202508_s_at | -0.0849 | 0.0242 | -3.508 | 0.00140388 | 0.027095 |
| X209593_s_at | 0.0674 | 0.0192 | 3.507 | 0.001406069 | 0.027113 |
| X218685_s_at | -0.061 | 0.0174 | -3.506 | 0.001410577 | 0.027157 |
| X208530_s_at | -0.4065 | 0.116 | -3.506 | 0.001410781 | 0.027157 |
| X218513_at | -0.1929 | 0.055 | -3.505 | 0.00141523 | 0.027193 |
| X220727_at | 0.4961 | 0.1416 | 3.504 | 0.001416014 | 0.027193 |
| X202375_at | 0.2843 | 0.0811 | 3.504 | 0.001416342 | 0.027193 |
| X203106_s_at | -0.2375 | 0.0678 | -3.502 | 0.001424342 | 0.027323 |
| X202849_x_at | 0.013 | 0.0037 | 3.501 | 0.001430021 | 0.027409 |
| X208937_s_at | 0.3914 | 0.1118 | 3.5 | 0.001431299 | 0.027409 |
| X205071_x_at | -0.264 | 0.0755 | -3.496 | 0.001447679 | 0.027688 |
| X210075_at | 0.2674 | 0.0765 | 3.496 | 0.001448341 | 0.027688 |
| X209916_at | 0.0759 | 0.0217 | 3.494 | 0.001454505 | 0.027782 |
| X214577_at | 0.318 | 0.0911 | 3.492 | 0.001465985 | 0.027977 |
| X211202_s_at | -0.218 | 0.0624 | -3.491 | 0.001468302 | 0.027997 |
| X213022_s_at | -0.3392 | 0.0972 | -3.49 | 0.001472504 | 0.02802 |
| X210958_s_at | 0.0884 | 0.0253 | 3.49 | 0.001473286 | 0.02802 |
| X202774_s_at | 0.1533 | 0.0439 | 3.49 | 0.001473644 | 0.02802 |
| X210017_at | 0.1255 | 0.036 | 3.489 | 0.001474534 | 0.02802 |
| X221950_at | 0.1971 | 0.0565 | 3.488 | 0.001481796 | 0.028134 |
| X213849_s_at | -0.0973 | 0.0279 | -3.487 | 0.001483435 | 0.028141 |
| X201444_s_at | -0.2281 | 0.0654 | -3.486 | 0.001488105 | 0.028187 |
| X209074_s_at | 0.255 | 0.0731 | 3.486 | 0.001488414 | 0.028187 |
| X211228_s_at | -0.1359 | 0.039 | -3.485 | 0.001493038 | 0.028251 |
| X208785_s_at | -0.1359 | 0.039 | -3.483 | 0.00150013 | 0.028361 |
| X206984_s_at | -0.428 | 0.1229 | -3.482 | 0.001503842 | 0.028407 |
| X211475_s_at | -0.1714 | 0.0493 | -3.48 | 0.001511642 | 0.02853 |
| X209740_s_at | -0.1351 | 0.0388 | -3.478 | 0.001518021 | 0.028621 |
| X208189_s_at | 0.0116 | 0.0033 | 3.478 | 0.001519599 | 0.028621 |
| X208760_at | -0.1866 | 0.0536 | -3.478 | 0.001520357 | 0.028621 |
| X202196_s_at | -0.2175 | 0.0625 | -3.477 | 0.001522828 | 0.028644 |
| X218011_at | -0.0894 | 0.0257 | -3.476 | 0.001526621 | 0.028691 |
| X212887_at | -0.1106 | 0.0318 | -3.476 | 0.001529221 | 0.028715 |
| X219287_at | 0.303 | 0.0872 | 3.475 | 0.001532441 | 0.028751 |
| X211959_at | 0.1512 | 0.0435 | 3.473 | 0.001539537 | 0.028842 |
| X219637_at | -0.2395 | 0.069 | -3.473 | 0.001539883 | 0.028842 |
| X200052_s_at | -0.1087 | 0.0313 | -3.472 | 0.001543115 | 0.028878 |
| X204559_s_at | -0.1612 | 0.0464 | -3.472 | 0.001546036 | 0.028894 |
| X213216_at | -0.1873 | 0.054 | -3.472 | 0.001546571 | 0.028894 |
| X211944_at | 0.1 | 0.0288 | 3.469 | 0.001555392 | 0.029035 |
| X202390_s_at | 0.1422 | 0.041 | 3.469 | 0.001556686 | 0.029035 |
| X200899_s_at | 0.0914 | 0.0263 | 3.468 | 0.001559511 | 0.029058 |
| X208433_s_at | 0.1618 | 0.0467 | 3.468 | 0.001560555 | 0.029058 |
| X201365_at | -0.115 | 0.0332 | -3.467 | 0.001563543 | 0.029089 |
| X213383_at | 0.2124 | 0.0613 | 3.466 | 0.001567529 | 0.029106 |
| X217185_s_at | -0.2333 | 0.0673 | -3.466 | 0.001567771 | 0.029106 |
| X202578_s_at | -0.1118 | 0.0323 | -3.466 | 0.001568372 | 0.029106 |
| X208972_s_at | -0.1118 | 0.0323 | -3.466 | 0.001571195 | 0.029113 |
| X206857_s_at | -0.1715 | 0.0495 | -3.466 | 0.001571376 | 0.029113 |
| X201123_s_at | -0.3121 | 0.0902 | -3.462 | 0.001586459 | 0.029368 |
| X201431_s_at | 0.1861 | 0.0538 | 3.459 | 0.001598181 | 0.029539 |
| X218256_s_at | 0.102 | 0.0295 | 3.459 | 0.001599325 | 0.029539 |
| X209704_at | -0.2043 | 0.0591 | -3.459 | 0.001599674 | 0.029539 |
| X203150_at | -0.1744 | 0.0504 | -3.458 | 0.001601455 | 0.029547 |
| X201513_at | -0.09 | 0.026 | -3.458 | 0.001603646 | 0.029563 |
| X201628_s_at | -0.0921 | 0.0266 | -3.457 | 0.001609252 | 0.029642 |
| X200710_at | 0.1853 | 0.0536 | 3.455 | 0.00161707 | 0.029748 |
| X207507_s_at | -0.1111 | 0.0322 | -3.455 | 0.001617698 | 0.029748 |
| X213531_s_at | 0.0825 | 0.0239 | 3.453 | 0.001626176 | 0.029879 |
| X215548_s_at | -0.102 | 0.0296 | -3.452 | 0.001627617 | 0.029881 |
| X221708_s_at | 0.0202 | 0.0059 | 3.451 | 0.00163311 | 0.029957 |
| X218968_s_at | -0.1314 | 0.0381 | -3.451 | 0.001635751 | 0.029981 |
| X202096_s_at | 0.2345 | 0.068 | 3.45 | 0.001639036 | 0.03 |
| X213675_at | 0.1148 | 0.0333 | 3.45 | 0.001639585 | 0.03 |
| X208708_x_at | -0.1629 | 0.0472 | -3.449 | 0.00164087 | 0.03 |
| X206214_at | -0.278 | 0.0806 | -3.448 | 0.001645538 | 0.030041 |
| X201239_s_at | -0.1276 | 0.037 | -3.448 | 0.001645816 | 0.030041 |
| X200709_at | -0.2215 | 0.0642 | -3.447 | 0.001649224 | 0.030079 |
| X202069_s_at | -0.2244 | 0.0651 | -3.447 | 0.001650892 | 0.030079 |
| X214672_at | 0.1801 | 0.0522 | 3.447 | 0.001652465 | 0.030079 |
| X201097_s_at | -0.0952 | 0.0276 | -3.447 | 0.001653328 | 0.030079 |
| X202453_s_at | -0.319 | 0.0926 | -3.445 | 0.001659059 | 0.030144 |
| X202149_at | 0.4942 | 0.1434 | 3.445 | 0.001659614 | 0.030144 |
| X214293_at | -0.1551 | 0.045 | -3.445 | 0.001662207 | 0.030167 |
| X213051_at | 0.2449 | 0.0711 | 3.444 | 0.00166557 | 0.030194 |
| X217286_s_at | -0.0946 | 0.0275 | -3.444 | 0.001666543 | 0.030194 |
| X202806_at | -0.1693 | 0.0492 | -3.443 | 0.001669792 | 0.030194 |
| X201782_s_at | -0.1331 | 0.0387 | -3.442 | 0.00167155 | 0.030194 |
| X202201_at | -0.1626 | 0.0472 | -3.442 | 0.001672478 | 0.030194 |
| X205466_s_at | 0.2062 | 0.0599 | 3.442 | 0.001674385 | 0.030194 |
| X204388_s_at | 0.3301 | 0.0959 | 3.442 | 0.001674393 | 0.030194 |
| X201297_s_at | 0.1562 | 0.0454 | 3.441 | 0.001675897 | 0.030194 |
| X213451_x_at | 0.1466 | 0.0426 | 3.441 | 0.001675931 | 0.030194 |
| X202796_at | 0.1313 | 0.0382 | 3.437 | 0.001694792 | 0.030491 |
| X210543_s_at | -0.1633 | 0.0475 | -3.437 | 0.001695139 | 0.030491 |
| X221563_at | 0.3292 | 0.0958 | 3.437 | 0.001696993 | 0.030499 |
| X218511_s_at | -0.1415 | 0.0412 | -3.434 | 0.001710484 | 0.030717 |
| X206920_s_at | -0.0263 | 0.0077 | -3.432 | 0.0017173 | 0.030804 |
| X210872_x_at | -0.3397 | 0.099 | -3.432 | 0.001718127 | 0.030804 |
| X221777_at | -0.2522 | 0.0735 | -3.432 | 0.001720423 | 0.030821 |
| X202289_s_at | -0.141 | 0.0411 | -3.43 | 0.001726086 | 0.030897 |
| X47550_at | -0.2043 | 0.0596 | -3.428 | 0.001738577 | 0.03108 |
| X201839_s_at | -0.2082 | 0.0607 | -3.427 | 0.001740138 | 0.03108 |
| X203035_s_at | 0.149 | 0.0435 | 3.427 | 0.001740528 | 0.03108 |
| X213425_at | 0.4026 | 0.1175 | 3.426 | 0.001744375 | 0.031124 |
| X211559_s_at | -0.3894 | 0.1137 | -3.425 | 0.001751185 | 0.031221 |
| X207088_s_at | -0.1863 | 0.0545 | -3.419 | 0.00177703 | 0.031656 |
| X210132_at | -0.1065 | 0.0312 | -3.419 | 0.001779726 | 0.031679 |
| X201821_s_at | -0.1897 | 0.0555 | -3.417 | 0.001787386 | 0.031789 |
| X201145_at | -0.1682 | 0.0492 | -3.416 | 0.001792208 | 0.031822 |
| X206433_s_at | -0.247 | 0.0723 | -3.416 | 0.001793201 | 0.031822 |
| X203551_s_at | -0.1302 | 0.0381 | -3.416 | 0.001793502 | 0.031822 |
| X203780_at | 0.1242 | 0.0364 | 3.414 | 0.001801512 | 0.031938 |
| X212090_at | -0.1599 | 0.0468 | -3.413 | 0.001805291 | 0.031958 |
| X216135_at | -0.034 | 0.01 | -3.413 | 0.001805469 | 0.031958 |
| X207624_s_at | 0.2858 | 0.0837 | 3.413 | 0.001807996 | 0.031964 |
| X203337_x_at | -0.4777 | 0.14 | -3.413 | 0.001808734 | 0.031964 |
| X200713_s_at | 0.1442 | 0.0423 | 3.412 | 0.001811888 | 0.031995 |
| X209739_s_at | -0.1231 | 0.0361 | -3.411 | 0.001815003 | 0.032024 |
| X205318_at | 0.1566 | 0.0459 | 3.411 | 0.001817762 | 0.032032 |
| X200910_at | -0.0637 | 0.0187 | -3.411 | 0.001819549 | 0.032032 |
| X219226_at | -0.111 | 0.0326 | -3.41 | 0.001820173 | 0.032032 |
| X200820_at | -0.1248 | 0.0366 | -3.41 | 0.001821193 | 0.032032 |
| X200959_at | -0.1266 | 0.0371 | -3.409 | 0.001824739 | 0.032069 |
| X217294_s_at | -0.3246 | 0.0952 | -3.409 | 0.001827376 | 0.03209 |
| X201931_at | -0.076 | 0.0223 | -3.408 | 0.001831326 | 0.032134 |
| X201656_at | 0.196 | 0.0575 | 3.407 | 0.001835733 | 0.032185 |
| X200826_at | -0.0686 | 0.0201 | -3.407 | 0.001839102 | 0.032208 |
| X218156_s_at | -0.1225 | 0.036 | -3.406 | 0.001839912 | 0.032208 |
| X204373_s_at | 0.1574 | 0.0462 | 3.406 | 0.001842523 | 0.032215 |
| X204178_s_at | -0.0796 | 0.0234 | -3.405 | 0.001844646 | 0.032215 |
| X206557_at | -0.1339 | 0.0393 | -3.405 | 0.001845871 | 0.032215 |
| X221898_at | 0.2124 | 0.0624 | 3.405 | 0.001846243 | 0.032215 |
| X218976_at | -0.1476 | 0.0433 | -3.404 | 0.001849993 | 0.032215 |
| X201063_at | -0.1612 | 0.0474 | -3.404 | 0.001852504 | 0.032215 |
| X205051_s_at | -0.2656 | 0.078 | -3.404 | 0.00185269 | 0.032215 |
| X207643_s_at | 0.2757 | 0.081 | 3.404 | 0.00185283 | 0.032215 |
| X214597_at | -0.4374 | 0.1285 | -3.404 | 0.00185339 | 0.032215 |
| X209733_at | -0.3537 | 0.104 | -3.402 | 0.001860093 | 0.032307 |
| X209833_at | -0.1852 | 0.0544 | -3.402 | 0.001862173 | 0.032317 |
| X210014_x_at | -0.1996 | 0.0587 | -3.401 | 0.001866101 | 0.03236 |
| X212582_at | -0.1818 | 0.0535 | -3.4 | 0.001869293 | 0.03236 |
| X205005_s_at | -0.2535 | 0.0746 | -3.4 | 0.001871054 | 0.03236 |
| X205788_s_at | 0.0637 | 0.0187 | 3.4 | 0.001871159 | 0.03236 |
| X211023_at | -0.1379 | 0.0406 | -3.4 | 0.001871925 | 0.03236 |
| X220159_at | -0.1705 | 0.0502 | -3.399 | 0.001874506 | 0.03238 |
| X213122_at | -0.1327 | 0.039 | -3.399 | 0.001877426 | 0.032384 |
| X217249_x_at | -0.0947 | 0.0279 | -3.399 | 0.001877674 | 0.032384 |
| X219467_at | -0.0523 | 0.0154 | -3.398 | 0.00188207 | 0.032409 |
| X220864_s_at | -0.1166 | 0.0343 | -3.398 | 0.001883047 | 0.032409 |
| X210033_s_at | 0.9509 | 0.2799 | 3.397 | 0.001884004 | 0.032409 |
| X201926_s_at | 0.407 | 0.1198 | 3.397 | 0.00188497 | 0.032409 |
| X217959_s_at | -0.2331 | 0.0686 | -3.396 | 0.001890856 | 0.032444 |
| X212787_at | -0.0666 | 0.0196 | -3.396 | 0.00189252 | 0.032444 |
| X216212_s_at | 0.1291 | 0.038 | 3.396 | 0.001893407 | 0.032444 |
| X204985_s_at | -0.2235 | 0.0658 | -3.395 | 0.001893933 | 0.032444 |
| X212492_s_at | 0.3395 | 0.1 | 3.395 | 0.001895073 | 0.032444 |
| X212552_at | 0.345 | 0.1016 | 3.395 | 0.001897145 | 0.032444 |
| X209243_s_at | -0.2908 | 0.0856 | -3.395 | 0.001897187 | 0.032444 |
| X201530_x_at | -0.064 | 0.0188 | -3.394 | 0.001898911 | 0.032448 |
| X201066_at | -0.1744 | 0.0514 | -3.394 | 0.001901163 | 0.032462 |
| X213428_s_at | 0.2475 | 0.0729 | 3.393 | 0.001907518 | 0.032512 |
| X221547_at | -0.1479 | 0.0436 | -3.392 | 0.001908928 | 0.032512 |
| X204424_s_at | -0.1032 | 0.0304 | -3.392 | 0.001910231 | 0.032512 |
| X203167_at | 0.2007 | 0.0592 | 3.392 | 0.001910866 | 0.032512 |
| X202619_s_at | 0.3078 | 0.0907 | 3.392 | 0.001911616 | 0.032512 |
| X204633_s_at | -0.1114 | 0.0328 | -3.392 | 0.00191294 | 0.032512 |
| X221804_s_at | -0.0867 | 0.0256 | -3.391 | 0.001914421 | 0.032512 |
| X218665_at | 0.0704 | 0.0207 | 3.391 | 0.001915813 | 0.032512 |
| X211615_s_at | -0.126 | 0.0371 | -3.391 | 0.001918254 | 0.03252 |
| X204211_x_at | 0.0782 | 0.0231 | 3.39 | 0.001919208 | 0.03252 |
| X201163_s_at | 0.288 | 0.085 | 3.39 | 0.001922262 | 0.032547 |
| X204049_s_at | 0.2257 | 0.0666 | 3.388 | 0.001932438 | 0.032694 |
| X214037_s_at | -0.0285 | 0.0084 | -3.386 | 0.001942324 | 0.032833 |
| X212382_at | 0.2504 | 0.074 | 3.386 | 0.001943641 | 0.032833 |
| X204906_at | 0.2324 | 0.0687 | 3.385 | 0.001945487 | 0.03284 |
| X211902_x_at | -0.8029 | 0.2373 | -3.384 | 0.001953265 | 0.032946 |
| X204173_at | -0.1304 | 0.0385 | -3.383 | 0.001958013 | 0.033001 |
| X204482_at | 0.3046 | 0.0901 | 3.382 | 0.001964257 | 0.033081 |
| X201870_at | -0.3242 | 0.0959 | -3.381 | 0.001969171 | 0.033139 |
| X206107_at | 0.0135 | 0.004 | 3.38 | 0.001971925 | 0.03316 |
| X209228_x_at | -0.2876 | 0.0851 | -3.38 | 0.001974532 | 0.033179 |
| X211763_s_at | -0.122 | 0.0361 | -3.377 | 0.001988751 | 0.033376 |
| X219949_at | 0.1633 | 0.0484 | 3.377 | 0.001989274 | 0.033376 |
| X200862_at | -0.2232 | 0.0661 | -3.376 | 0.001992495 | 0.033405 |
| X211672_s_at | -0.2548 | 0.0755 | -3.376 | 0.001994816 | 0.033418 |
| X213897_s_at | -0.1318 | 0.0391 | -3.375 | 0.001998594 | 0.033455 |
| X219196_at | -0.1015 | 0.0301 | -3.375 | 0.002000865 | 0.033455 |
| X210466_s_at | 0.0792 | 0.0235 | 3.374 | 0.002004782 | 0.033455 |
| X220261_s_at | -0.1942 | 0.0576 | -3.374 | 0.002005892 | 0.033455 |
| X201899_s_at | -0.0941 | 0.0279 | -3.373 | 0.002009449 | 0.033455 |
| X222338_x_at | -0.1004 | 0.0298 | -3.373 | 0.002010017 | 0.033455 |
| X218109_s_at | 0.1637 | 0.0485 | 3.373 | 0.002011638 | 0.033455 |
| X218407_x_at | -0.1203 | 0.0357 | -3.373 | 0.002012136 | 0.033455 |
| X205097_at | 0.4865 | 0.1442 | 3.372 | 0.002012996 | 0.033455 |
| X211595_s_at | -0.1621 | 0.0481 | -3.372 | 0.002014407 | 0.033455 |
| X200812_at | -0.1367 | 0.0405 | -3.372 | 0.00201442 | 0.033455 |
| X203272_s_at | -0.123 | 0.0365 | -3.372 | 0.002015042 | 0.033455 |
| X212099_at | 0.3092 | 0.0918 | 3.368 | 0.002034316 | 0.033749 |
| X209782_s_at | -0.3755 | 0.1115 | -3.368 | 0.002038597 | 0.033795 |
| X206050_s_at | -0.1534 | 0.0456 | -3.366 | 0.002050084 | 0.03396 |
| X208675_s_at | -0.1593 | 0.0474 | -3.364 | 0.002059628 | 0.034093 |
| X211781_x_at | 0.0302 | 0.009 | 3.363 | 0.002061432 | 0.034097 |
| X212181_s_at | 0.1746 | 0.0519 | 3.363 | 0.002066362 | 0.034153 |
| X207103_at | -0.1644 | 0.0489 | -3.361 | 0.002073636 | 0.034228 |
| X209432_s_at | -0.1272 | 0.0378 | -3.361 | 0.002073959 | 0.034228 |
| X202897_at | 0.1297 | 0.0386 | 3.361 | 0.00207678 | 0.034249 |
| X219641_at | -0.1098 | 0.0327 | -3.36 | 0.002082349 | 0.034316 |
| X203643_at | -0.1413 | 0.0421 | -3.359 | 0.002085519 | 0.034342 |
| X221626_at | 0.0599 | 0.0178 | 3.358 | 0.002092823 | 0.034437 |
| X219056_at | -0.1685 | 0.0502 | -3.357 | 0.002095504 | 0.034447 |
| X219176_at | -0.0838 | 0.025 | -3.357 | 0.002096529 | 0.034447 |
| X210826_x_at | -0.1572 | 0.0469 | -3.354 | 0.0021121 | 0.034668 |
| X202659_at | -0.1836 | 0.0548 | -3.354 | 0.002113117 | 0.034668 |
| X213346_at | 0.0382 | 0.0114 | 3.353 | 0.002118789 | 0.034709 |
| X207541_s_at | 0.1026 | 0.0306 | 3.353 | 0.002119781 | 0.034709 |
| X204677_at | 0.1683 | 0.0502 | 3.353 | 0.002120264 | 0.034709 |
| X218666_s_at | 0.1619 | 0.0483 | 3.352 | 0.002125121 | 0.034745 |
| X218723_s_at | 0.3512 | 0.1048 | 3.352 | 0.002125629 | 0.034745 |
| X202406_s_at | 0.1783 | 0.0532 | 3.351 | 0.002129513 | 0.034772 |
| X203646_at | -0.0397 | 0.0119 | -3.351 | 0.002130373 | 0.034772 |
| X201561_s_at | 0.1362 | 0.0407 | 3.35 | 0.00213711 | 0.034813 |
| X41577_at | -0.1593 | 0.0475 | -3.35 | 0.002137276 | 0.034813 |
| X216583_x_at | -0.0949 | 0.0283 | -3.35 | 0.002137631 | 0.034813 |
| X218625_at | 0.5009 | 0.1496 | 3.349 | 0.00214093 | 0.034817 |
| X215884_s_at | -0.1076 | 0.0321 | -3.349 | 0.002141012 | 0.034817 |
| X204237_at | -0.1657 | 0.0495 | -3.348 | 0.002145816 | 0.03487 |
| X222192_s_at | -0.362 | 0.1081 | -3.348 | 0.002147411 | 0.03487 |
| X218443_s_at | 0.0906 | 0.0271 | 3.346 | 0.002159063 | 0.03501 |
| X219696_at | -0.263 | 0.0786 | -3.346 | 0.002160713 | 0.03501 |
| X213616_at | -0.1887 | 0.0564 | -3.345 | 0.002163041 | 0.03501 |
| X202393_s_at | 0.3371 | 0.1008 | 3.345 | 0.002165548 | 0.03501 |
| X208158_s_at | 0.2455 | 0.0734 | 3.345 | 0.002166549 | 0.03501 |
| X201217_x_at | -0.0749 | 0.0224 | -3.345 | 0.002166741 | 0.03501 |
| X212600_s_at | -0.1252 | 0.0374 | -3.345 | 0.002167032 | 0.03501 |
| X212896_at | -0.0896 | 0.0268 | -3.343 | 0.002176117 | 0.035131 |
| X203228_at | -0.2497 | 0.0747 | -3.342 | 0.002180697 | 0.035178 |
| X219675_s_at | -0.1498 | 0.0448 | -3.342 | 0.002182184 | 0.035178 |
| X203406_at | -0.1 | 0.0299 | -3.341 | 0.002189529 | 0.035258 |
| X217726_at | -0.0835 | 0.025 | -3.341 | 0.002190324 | 0.035258 |
| X202775_s_at | 0.1006 | 0.0301 | 3.339 | 0.002198636 | 0.035338 |
| X58994_at | 0.1701 | 0.0509 | 3.339 | 0.002199052 | 0.035338 |
| X200989_at | 0.2153 | 0.0645 | 3.339 | 0.002200059 | 0.035338 |
| X221874_at | -0.1878 | 0.0562 | -3.338 | 0.002203882 | 0.035374 |
| X221494_x_at | -0.1099 | 0.0329 | -3.337 | 0.002209425 | 0.035437 |
| X209841_s_at | -0.1844 | 0.0553 | -3.337 | 0.002212288 | 0.035457 |
| X207064_s_at | 0.0106 | 0.0032 | 3.335 | 0.002222678 | 0.035589 |
| X208899_x_at | -0.2194 | 0.0658 | -3.335 | 0.002223736 | 0.035589 |
| X210453_x_at | -0.0943 | 0.0283 | -3.333 | 0.002234947 | 0.035739 |
| X212141_at | -0.2808 | 0.0843 | -3.333 | 0.002236309 | 0.035739 |
| X208943_s_at | 0.0914 | 0.0274 | 3.332 | 0.002239617 | 0.035742 |
| X201712_s_at | -0.1206 | 0.0362 | -3.332 | 0.002239733 | 0.035742 |
| X201370_s_at | -0.1234 | 0.037 | -3.331 | 0.002247811 | 0.035798 |
| X218120_s_at | -0.2196 | 0.0659 | -3.331 | 0.002247849 | 0.035798 |
| X209592_s_at | -0.0161 | 0.0048 | -3.331 | 0.002248055 | 0.035798 |
| X202912_at | 0.4498 | 0.1351 | 3.33 | 0.002250534 | 0.035812 |
| X219703_at | 0.4779 | 0.1436 | 3.327 | 0.002269081 | 0.036081 |
| X213423_x_at | -0.215 | 0.0646 | -3.327 | 0.002272597 | 0.036087 |
| X214930_at | -0.1905 | 0.0573 | -3.327 | 0.002272705 | 0.036087 |
| X208870_x_at | -0.0611 | 0.0184 | -3.326 | 0.002274782 | 0.036094 |
| X222217_s_at | 0.0113 | 0.0034 | 3.326 | 0.002277903 | 0.036118 |
| X218563_at | -0.0911 | 0.0274 | -3.324 | 0.002285007 | 0.036205 |
| X201290_at | 0.1616 | 0.0486 | 3.324 | 0.002287834 | 0.036224 |
| X209814_at | -0.1354 | 0.0408 | -3.322 | 0.002296975 | 0.036343 |
| X210091_s_at | 0.4067 | 0.1224 | 3.322 | 0.002299852 | 0.03635 |
| X221550_at | -0.1962 | 0.0591 | -3.322 | 0.002300699 | 0.03635 |
| X219595_at | -0.1377 | 0.0415 | -3.32 | 0.002309958 | 0.03646 |
| X212271_at | -0.2207 | 0.0665 | -3.32 | 0.002310978 | 0.03646 |
| X206780_at | -0.2149 | 0.0648 | -3.317 | 0.00233085 | 0.036748 |
| X208946_s_at | -0.098 | 0.0296 | -3.317 | 0.002333229 | 0.036759 |
| X201238_s_at | -0.1506 | 0.0454 | -3.316 | 0.002335009 | 0.036761 |
| X212162_at | 0.1341 | 0.0405 | 3.316 | 0.002338296 | 0.036787 |
| X202729_s_at | 0.2223 | 0.0671 | 3.315 | 0.002344549 | 0.036859 |
| X202963_at | 0.1763 | 0.0532 | 3.313 | 0.002353525 | 0.036952 |
| X209915_s_at | -0.1645 | 0.0497 | -3.313 | 0.002353819 | 0.036952 |
| X214585_s_at | -0.0957 | 0.0289 | -3.313 | 0.002355559 | 0.036954 |
| X214210_at | -0.0318 | 0.0096 | -3.313 | 0.002357734 | 0.036962 |
| X220466_at | 0.0154 | 0.0046 | 3.312 | 0.002359445 | 0.036962 |
| X212067_s_at | 0.2854 | 0.0862 | 3.312 | 0.002361563 | 0.03697 |
| X218280_x_at | 0.2803 | 0.0846 | 3.311 | 0.002367205 | 0.037032 |
| X218184_at | -0.1022 | 0.0309 | -3.311 | 0.002370053 | 0.03705 |
| X204403_x_at | 0.0996 | 0.0301 | 3.31 | 0.002372329 | 0.03706 |
| X201575_at | -0.0404 | 0.0122 | -3.31 | 0.002375753 | 0.037087 |
| X202727_s_at | 0.2154 | 0.0651 | 3.309 | 0.002378899 | 0.03711 |
| X202228_s_at | -0.0987 | 0.0298 | -3.308 | 0.002385426 | 0.037186 |
| X202675_at | -0.1341 | 0.0406 | -3.307 | 0.002392905 | 0.037275 |
| X201317_s_at | -0.1071 | 0.0324 | -3.307 | 0.002394931 | 0.037275 |
| X209669_s_at | 0.099 | 0.0299 | 3.306 | 0.002396205 | 0.037275 |
| X201162_at | 0.3322 | 0.1005 | 3.306 | 0.002399707 | 0.037304 |
| X219167_at | 0.5261 | 0.1592 | 3.305 | 0.002406189 | 0.037378 |
| X201087_at | 0.011 | 0.0033 | 3.304 | 0.002412532 | 0.037451 |
| X209707_at | -0.1153 | 0.0349 | -3.303 | 0.002416939 | 0.037456 |
| X205156_s_at | -0.0951 | 0.0288 | -3.303 | 0.002417713 | 0.037456 |
| X202801_at | 0.0593 | 0.018 | 3.303 | 0.002417901 | 0.037456 |
| X222190_s_at | -0.0214 | 0.0065 | -3.302 | 0.002421193 | 0.03748 |
| X209242_at | -0.1316 | 0.0399 | -3.301 | 0.00242813 | 0.037562 |
| X212645_x_at | -0.1122 | 0.034 | -3.3 | 0.002435107 | 0.037643 |
| X213937_s_at | -0.0406 | 0.0123 | -3.299 | 0.00244418 | 0.037757 |
| X216119_s_at | -0.271 | 0.0822 | -3.298 | 0.002446701 | 0.037758 |
| X209727_at | -0.0112 | 0.0034 | -3.298 | 0.002448186 | 0.037758 |
| X219800_s_at | -0.1351 | 0.041 | -3.298 | 0.002449333 | 0.037758 |
| X209747_at | 0.1682 | 0.051 | 3.298 | 0.002451795 | 0.03777 |
| X204165_at | -0.1306 | 0.0396 | -3.297 | 0.002455755 | 0.037805 |
| X217995_at | 0.3218 | 0.0976 | 3.296 | 0.002462576 | 0.037883 |
| X204547_at | -0.0901 | 0.0273 | -3.296 | 0.002464573 | 0.037888 |
| X202689_at | -0.0254 | 0.0077 | -3.295 | 0.002468175 | 0.037892 |
| X213118_at | -0.1234 | 0.0374 | -3.295 | 0.002468225 | 0.037892 |
| X204218_at | -0.022 | 0.0067 | -3.292 | 0.00248647 | 0.038145 |
| X203137_at | -0.1888 | 0.0574 | -3.292 | 0.002489769 | 0.03817 |
| X203891_s_at | -0.0335 | 0.0102 | -3.291 | 0.002492982 | 0.038192 |
| X208818_s_at | 0.1475 | 0.0448 | 3.291 | 0.002497435 | 0.038234 |
| X217773_s_at | -0.0915 | 0.0278 | -3.29 | 0.002501552 | 0.038245 |
| X219170_at | 0.1769 | 0.0538 | 3.29 | 0.002501579 | 0.038245 |
| X203693_s_at | -0.129 | 0.0392 | -3.289 | 0.00250894 | 0.038331 |
| X206015_s_at | -0.1027 | 0.0312 | -3.288 | 0.002513813 | 0.038379 |
| X212070_at | 0.2807 | 0.0854 | 3.288 | 0.002516884 | 0.038391 |
| X203839_s_at | 0.1557 | 0.0474 | 3.288 | 0.002518057 | 0.038391 |
| X201424_s_at | 0.0973 | 0.0296 | 3.286 | 0.002525771 | 0.038463 |
| X218876_at | 0.5809 | 0.1768 | 3.286 | 0.002526255 | 0.038463 |
| X202250_s_at | -0.1352 | 0.0412 | -3.285 | 0.002535826 | 0.038581 |
| X201237_at | -0.169 | 0.0514 | -3.285 | 0.002537425 | 0.038581 |
| X39966_at | -0.1633 | 0.0497 | -3.284 | 0.00254174 | 0.03862 |
| X202520_s_at | -0.0931 | 0.0284 | -3.283 | 0.002547266 | 0.038655 |
| X214150_x_at | 0.1098 | 0.0335 | 3.283 | 0.002547556 | 0.038655 |
| X219220_x_at | -0.0669 | 0.0204 | -3.283 | 0.002551274 | 0.038685 |
| X215321_at | -0.1304 | 0.0397 | -3.281 | 0.002558729 | 0.038765 |
| X220364_at | -0.0838 | 0.0256 | -3.281 | 0.002559989 | 0.038765 |
| X202725_at | -0.0535 | 0.0163 | -3.281 | 0.002563444 | 0.03879 |
| X201799_s_at | -0.0185 | 0.0057 | -3.28 | 0.002568444 | 0.03884 |
| X202887_s_at | 0.4756 | 0.145 | 3.279 | 0.002573003 | 0.038882 |
| X219281_at | -0.0991 | 0.0302 | -3.278 | 0.002579997 | 0.038961 |
| X203017_s_at | -0.2052 | 0.0626 | -3.278 | 0.002582908 | 0.038979 |
| X201903_at | -0.1982 | 0.0605 | -3.276 | 0.002593639 | 0.039114 |
| X205072_s_at | -0.2514 | 0.0767 | -3.275 | 0.002600133 | 0.039164 |
| X209387_s_at | 0.3445 | 0.1052 | 3.275 | 0.00260045 | 0.039164 |
| X214778_at | -0.2395 | 0.0731 | -3.275 | 0.00260497 | 0.039202 |
| X204662_at | 0.2132 | 0.0651 | 3.274 | 0.002606516 | 0.039202 |
| X218352_at | 0.1647 | 0.0503 | 3.273 | 0.002617109 | 0.039318 |
| X39705_at | 0.0694 | 0.0212 | 3.273 | 0.002617759 | 0.039318 |
| X213627_at | 0.1104 | 0.0337 | 3.272 | 0.002622579 | 0.039363 |
| X222216_s_at | -0.1134 | 0.0346 | -3.272 | 0.00262593 | 0.039365 |
| X216326_s_at | -0.1022 | 0.0312 | -3.271 | 0.002626224 | 0.039365 |
| X214665_s_at | 0.1151 | 0.0352 | 3.271 | 0.00263218 | 0.039428 |
| X221518_s_at | 0.0995 | 0.0304 | 3.27 | 0.002634169 | 0.039431 |
| X202188_at | -0.211 | 0.0646 | -3.268 | 0.002653298 | 0.039663 |
| X206116_s_at | -0.1497 | 0.0458 | -3.268 | 0.002653373 | 0.039663 |
| X206855_s_at | 0.1307 | 0.04 | 3.267 | 0.002655041 | 0.039663 |
| X206803_at | -0.7455 | 0.2283 | -3.265 | 0.002668145 | 0.039832 |
| X216483_s_at | -0.1469 | 0.045 | -3.265 | 0.002671727 | 0.039843 |
| X200750_s_at | -0.1565 | 0.048 | -3.265 | 0.002673934 | 0.039843 |
| X203215_s_at | 0.2075 | 0.0636 | 3.264 | 0.002675663 | 0.039843 |
| X91684_g_at | -0.1489 | 0.0456 | -3.264 | 0.002676038 | 0.039843 |
| X200797_s_at | 0.2774 | 0.085 | 3.264 | 0.002678276 | 0.039849 |
| X200677_at | 0.1969 | 0.0603 | 3.264 | 0.00268166 | 0.039857 |
| X218503_at | -0.1009 | 0.0309 | -3.263 | 0.002682398 | 0.039857 |
| X204048_s_at | 0.2611 | 0.08 | 3.262 | 0.002688969 | 0.039924 |
| X218244_at | -0.0892 | 0.0273 | -3.262 | 0.00269049 | 0.039924 |
| X202515_at | -0.0884 | 0.0271 | -3.262 | 0.002692466 | 0.039927 |
| X202490_at | -0.2084 | 0.0639 | -3.26 | 0.002706778 | 0.040112 |
| X201306_s_at | 0.2441 | 0.0749 | 3.26 | 0.002709972 | 0.040133 |
| X218032_at | -0.1268 | 0.0389 | -3.258 | 0.002718561 | 0.040233 |
| X200040_at | -0.1385 | 0.0425 | -3.258 | 0.002722624 | 0.040248 |
| X203010_at | 0.0083 | 0.0025 | 3.258 | 0.002723268 | 0.040248 |
| X214507_s_at | -0.0216 | 0.0066 | -3.257 | 0.002726733 | 0.040248 |
| X203359_s_at | 0.1265 | 0.0388 | 3.257 | 0.00272682 | 0.040248 |
| X209061_at | 0.1398 | 0.0429 | 3.256 | 0.0027316 | 0.040271 |
| X208890_s_at | 0.0732 | 0.0225 | 3.256 | 0.002731994 | 0.040271 |
| X206109_at | 0.01 | 0.0031 | 3.256 | 0.002735851 | 0.040271 |
| X219033_at | 0.2576 | 0.0791 | 3.256 | 0.002736248 | 0.040271 |
| X218342_s_at | -0.1174 | 0.0361 | -3.256 | 0.002737455 | 0.040271 |
| X203063_at | 0.166 | 0.051 | 3.255 | 0.002739305 | 0.040272 |
| X202706_s_at | -0.0983 | 0.0302 | -3.254 | 0.002748818 | 0.040385 |
| X204247_s_at | -0.2037 | 0.0627 | -3.251 | 0.002772808 | 0.040711 |
| X215696_s_at | 0.0704 | 0.0217 | 3.25 | 0.002776211 | 0.040734 |
| X211616_s_at | 0.2856 | 0.0879 | 3.249 | 0.002786738 | 0.040861 |
| X53991_at | 0.1638 | 0.0504 | 3.248 | 0.002790938 | 0.040896 |
| X217651_at | -0.1428 | 0.044 | -3.247 | 0.002797786 | 0.040969 |
| X217868_s_at | -0.1017 | 0.0313 | -3.247 | 0.002800704 | 0.040985 |
| X217991_x_at | 0.0165 | 0.0051 | 3.247 | 0.002802648 | 0.040986 |
| X222034_at | 0.0248 | 0.0077 | 3.245 | 0.002816643 | 0.041164 |
| X208736_at | -0.1436 | 0.0443 | -3.244 | 0.002820361 | 0.041191 |
| X203675_at | -0.1119 | 0.0345 | -3.244 | 0.002822897 | 0.041197 |
| X203581_at | 0.1338 | 0.0413 | 3.244 | 0.002824481 | 0.041197 |
| X217456_x_at | 0.2243 | 0.0692 | 3.243 | 0.002830035 | 0.041251 |
| X201607_at | -0.146 | 0.045 | -3.242 | 0.002834802 | 0.041261 |
| X213923_at | 0.0551 | 0.017 | 3.242 | 0.002835189 | 0.041261 |
| X209569_x_at | -0.433 | 0.1336 | -3.242 | 0.002836279 | 0.041261 |
| X203746_s_at | -0.1505 | 0.0464 | -3.242 | 0.002839766 | 0.041276 |
| X214911_s_at | 0.1945 | 0.06 | 3.241 | 0.002841059 | 0.041276 |
| X201251_at | -0.1892 | 0.0584 | -3.241 | 0.002843358 | 0.041283 |
| X201684_s_at | -0.1003 | 0.0309 | -3.24 | 0.002848408 | 0.041329 |
| X215653_at | -0.492 | 0.1519 | -3.24 | 0.002851091 | 0.041341 |
| X203019_x_at | -0.1126 | 0.0348 | -3.24 | 0.002854792 | 0.041357 |
| X209124_at | 0.2978 | 0.0919 | 3.239 | 0.00285778 | 0.041357 |
| X205932_s_at | 0.1727 | 0.0533 | 3.239 | 0.002857804 | 0.041357 |
| X209806_at | -0.1772 | 0.0547 | -3.238 | 0.002863125 | 0.041407 |
| X206542_s_at | -0.1511 | 0.0467 | -3.238 | 0.002866273 | 0.041426 |
| X201111_at | -0.0841 | 0.026 | -3.238 | 0.00286933 | 0.041443 |
| X206444_at | -0.4729 | 0.1461 | -3.237 | 0.002872446 | 0.041461 |
| X205381_at | -0.1155 | 0.0357 | -3.236 | 0.002882448 | 0.041578 |
| X204501_at | -0.1665 | 0.0515 | -3.235 | 0.002886824 | 0.041614 |
| X204151_x_at | 0.3033 | 0.0938 | 3.235 | 0.002889154 | 0.041621 |
| X212268_at | 0.0103 | 0.0032 | 3.234 | 0.002894936 | 0.041658 |
| X214232_at | -0.013 | 0.004 | -3.234 | 0.002895484 | 0.041658 |
| X208908_s_at | 0.2006 | 0.0621 | 3.232 | 0.002914153 | 0.041885 |
| X204170_s_at | -0.4576 | 0.1416 | -3.232 | 0.00291506 | 0.041885 |
| X212217_at | -0.2127 | 0.0658 | -3.231 | 0.002916933 | 0.041885 |
| X213808_at | -0.1622 | 0.0502 | -3.231 | 0.002920923 | 0.041898 |
| X74694_s_at | 0.1896 | 0.0587 | 3.23 | 0.002923978 | 0.041898 |
| X214959_s_at | -0.2552 | 0.079 | -3.23 | 0.002924612 | 0.041898 |
| X209329_x_at | -0.1162 | 0.036 | -3.23 | 0.002925367 | 0.041898 |
| X211298_s_at | -0.0297 | 0.0092 | -3.229 | 0.002931624 | 0.041954 |
| X201791_s_at | 0.0628 | 0.0194 | 3.229 | 0.002933058 | 0.041954 |
| X202584_at | -0.0692 | 0.0214 | -3.228 | 0.002943961 | 0.042061 |
| X209226_s_at | 0.1381 | 0.0428 | 3.228 | 0.002945546 | 0.042061 |
| X217772_s_at | -0.1033 | 0.032 | -3.227 | 0.002948112 | 0.042061 |
| X210840_s_at | 0.2658 | 0.0824 | 3.227 | 0.002950599 | 0.042061 |
| X218315_s_at | -0.0839 | 0.026 | -3.227 | 0.002952668 | 0.042061 |
| X209265_s_at | -0.1724 | 0.0534 | -3.226 | 0.002955801 | 0.042061 |
| X213912_at | -0.1826 | 0.0566 | -3.226 | 0.002958723 | 0.042061 |
| X205796_at | -0.1808 | 0.0561 | -3.226 | 0.002960542 | 0.042061 |
| X220094_s_at | 0.2247 | 0.0697 | 3.226 | 0.002960555 | 0.042061 |
| X221712_s_at | -0.1711 | 0.0531 | -3.225 | 0.002963226 | 0.042061 |
| X217317_s_at | 0.1696 | 0.0526 | 3.225 | 0.002963882 | 0.042061 |
| X207181_s_at | 0.1529 | 0.0474 | 3.225 | 0.002964966 | 0.042061 |
| X201180_s_at | 0.1939 | 0.0601 | 3.225 | 0.002968375 | 0.042061 |
| X219356_s_at | -0.1031 | 0.032 | -3.224 | 0.002969604 | 0.042061 |
| X206896_s_at | -0.2092 | 0.0649 | -3.224 | 0.002969628 | 0.042061 |
| X211327_x_at | 0.0082 | 0.0026 | 3.224 | 0.002970791 | 0.042061 |
| X219365_s_at | -0.3005 | 0.0933 | -3.222 | 0.002986842 | 0.042244 |
| X222275_at | -0.17 | 0.0528 | -3.222 | 0.002987542 | 0.042244 |
| X209840_s_at | -0.2115 | 0.0657 | -3.221 | 0.002999419 | 0.042385 |
| X218569_s_at | -0.1465 | 0.0455 | -3.22 | 0.003007736 | 0.042475 |
| X208064_s_at | -0.3208 | 0.0997 | -3.219 | 0.003014697 | 0.042547 |
| X212453_at | -0.1129 | 0.0351 | -3.218 | 0.003021429 | 0.042615 |
| X219103_at | 0.124 | 0.0385 | 3.216 | 0.003031885 | 0.042735 |
| X209561_at | -0.0471 | 0.0146 | -3.215 | 0.003040862 | 0.042834 |
| X202325_s_at | -0.0722 | 0.0224 | -3.215 | 0.003043599 | 0.042845 |
| X218603_at | 0.1349 | 0.042 | 3.215 | 0.003045443 | 0.042845 |
| X219683_at | -0.2199 | 0.0684 | -3.214 | 0.003050741 | 0.042892 |
| X212910_at | -0.0913 | 0.0284 | -3.214 | 0.003054717 | 0.042921 |
| X203258_at | -0.0653 | 0.0203 | -3.213 | 0.003060177 | 0.04297 |
| X204021_s_at | 0.1034 | 0.0322 | 3.212 | 0.003065084 | 0.043012 |
| X206559_x_at | -0.0386 | 0.012 | -3.212 | 0.003071499 | 0.043036 |
| X201636_at | 0.0924 | 0.0288 | 3.211 | 0.003071806 | 0.043036 |
| X208763_s_at | 0.2449 | 0.0763 | 3.211 | 0.003072637 | 0.043036 |
| X200811_at | -0.0878 | 0.0274 | -3.211 | 0.003079124 | 0.043078 |
| X218006_s_at | 0.1953 | 0.0608 | 3.211 | 0.003079472 | 0.043078 |
| X200853_at | -0.1677 | 0.0523 | -3.21 | 0.003087398 | 0.043135 |
| X205584_at | -0.1909 | 0.0595 | -3.209 | 0.003088511 | 0.043135 |
| X209971_x_at | -0.062 | 0.0193 | -3.209 | 0.003089383 | 0.043135 |
| X205012_s_at | -0.116 | 0.0362 | -3.208 | 0.003096555 | 0.043183 |
| X212723_at | 0.1913 | 0.0596 | 3.208 | 0.003096713 | 0.043183 |
| X212296_at | -0.0669 | 0.0209 | -3.208 | 0.003101768 | 0.043221 |
| X202364_at | 0.1532 | 0.0478 | 3.208 | 0.003103314 | 0.043221 |
| X212756_s_at | 0.1002 | 0.0312 | 3.207 | 0.003106028 | 0.043231 |
| X213956_at | 0.232 | 0.0724 | 3.206 | 0.003117385 | 0.043362 |
| X201354_s_at | 0.0718 | 0.0224 | 3.205 | 0.003122937 | 0.043412 |
| X205550_s_at | -0.2294 | 0.0716 | -3.205 | 0.003125857 | 0.043426 |
| X208091_s_at | 0.1329 | 0.0415 | 3.204 | 0.003129354 | 0.043447 |
| X202430_s_at | 0.3183 | 0.0994 | 3.204 | 0.003134457 | 0.043491 |
| X202736_s_at | -0.1964 | 0.0613 | -3.203 | 0.003136966 | 0.043491 |
| X213572_s_at | 0.456 | 0.1424 | 3.203 | 0.003139655 | 0.043491 |
| X205824_at | -0.0181 | 0.0056 | -3.203 | 0.00314037 | 0.043491 |
| X203162_s_at | -0.116 | 0.0362 | -3.203 | 0.003143671 | 0.04351 |
| X206577_at | 0.5078 | 0.1586 | 3.202 | 0.003146907 | 0.043528 |
| X202449_s_at | 0.1233 | 0.0385 | 3.202 | 0.003152087 | 0.043572 |
| X205270_s_at | 0.0153 | 0.0048 | 3.201 | 0.003158164 | 0.04362 |
| X202102_s_at | 0.1479 | 0.0462 | 3.201 | 0.00315951 | 0.04362 |
| X205478_at | -0.384 | 0.12 | -3.199 | 0.003169675 | 0.043734 |
| X221532_s_at | -0.1132 | 0.0354 | -3.199 | 0.003175947 | 0.043793 |
| X201318_s_at | 0.0904 | 0.0283 | 3.197 | 0.003189277 | 0.04395 |
| X218183_at | 0.0886 | 0.0277 | 3.195 | 0.003203633 | 0.044103 |
| X214106_s_at | -0.1451 | 0.0454 | -3.195 | 0.003204388 | 0.044103 |
| X211686_s_at | -0.1618 | 0.0507 | -3.195 | 0.003209359 | 0.044144 |
| X209225_x_at | 0.2172 | 0.068 | 3.194 | 0.003217119 | 0.044223 |
| X201067_at | -0.1109 | 0.0347 | -3.193 | 0.003219848 | 0.044234 |
| X209953_s_at | -0.2373 | 0.0743 | -3.193 | 0.00322278 | 0.044247 |
| X212891_s_at | -0.0986 | 0.0309 | -3.192 | 0.003229504 | 0.044284 |
| X204842_x_at | 0.1522 | 0.0477 | 3.192 | 0.00322953 | 0.044284 |
| X212401_s_at | 0.1072 | 0.0336 | 3.192 | 0.00323469 | 0.044328 |
| X202530_at | -0.1003 | 0.0314 | -3.191 | 0.003237089 | 0.044333 |
| X217745_s_at | -0.1492 | 0.0468 | -3.191 | 0.00324189 | 0.044372 |
| X214490_at | -0.0062 | 0.0019 | -3.19 | 0.003249189 | 0.044422 |
| X200734_s_at | -0.149 | 0.0467 | -3.19 | 0.003249559 | 0.044422 |
| X222150_s_at | 0.4197 | 0.1316 | 3.189 | 0.003256618 | 0.044491 |
| X209076_s_at | 0.0686 | 0.0215 | 3.187 | 0.003274354 | 0.044706 |
| X205518_s_at | 0.1538 | 0.0483 | 3.186 | 0.003278969 | 0.044741 |
| X213900_at | 0.2374 | 0.0745 | 3.186 | 0.003284445 | 0.044789 |
| X205139_s_at | -0.1506 | 0.0473 | -3.185 | 0.003286479 | 0.044789 |
| X203896_s_at | 0.4007 | 0.1259 | 3.184 | 0.003297562 | 0.044912 |
| X202871_at | 0.0464 | 0.0146 | 3.183 | 0.00330986 | 0.045052 |
| X208702_x_at | -0.1658 | 0.0521 | -3.178 | 0.003346909 | 0.045529 |
| X205352_at | -0.1128 | 0.0355 | -3.178 | 0.00335189 | 0.045568 |
| X218788_s_at | -0.1084 | 0.0341 | -3.177 | 0.003355641 | 0.045588 |
| X210434_x_at | -0.0994 | 0.0313 | -3.177 | 0.003357398 | 0.045588 |
| X217824_at | -0.0817 | 0.0257 | -3.177 | 0.003359554 | 0.045589 |
| X212770_at | 0.0835 | 0.0263 | 3.177 | 0.003363409 | 0.045613 |
| X207636_at | -0.1166 | 0.0367 | -3.176 | 0.00336826 | 0.045651 |
| X213246_at | -0.1615 | 0.0509 | -3.175 | 0.003374266 | 0.045705 |
| X222369_at | 0.0724 | 0.0228 | 3.174 | 0.00338619 | 0.045819 |
| X210189_at | -0.0611 | 0.0192 | -3.174 | 0.003386821 | 0.045819 |
| X219960_s_at | -0.0906 | 0.0285 | -3.174 | 0.003389464 | 0.045827 |
| X218949_s_at | -0.3229 | 0.1018 | -3.173 | 0.003392237 | 0.045836 |
| X213489_at | -0.128 | 0.0403 | -3.172 | 0.003405268 | 0.045985 |
| X221853_s_at | -0.2389 | 0.0753 | -3.171 | 0.003410125 | 0.046022 |
| X218220_at | -0.1153 | 0.0364 | -3.171 | 0.003415558 | 0.046068 |
| X201569_s_at | -0.1226 | 0.0387 | -3.169 | 0.003432594 | 0.046269 |
| X212716_s_at | -0.1056 | 0.0333 | -3.168 | 0.003441654 | 0.046363 |
| X202291_s_at | 0.6399 | 0.202 | 3.167 | 0.003447638 | 0.046416 |
| X212681_at | 0.1557 | 0.0492 | 3.166 | 0.003456468 | 0.046506 |
| X211594_s_at | -0.1295 | 0.0409 | -3.166 | 0.003459583 | 0.046516 |
| X217822_at | -0.0941 | 0.0297 | -3.165 | 0.003461399 | 0.046516 |
| X202304_at | -0.1628 | 0.0514 | -3.165 | 0.003464351 | 0.046528 |
| X219463_at | -0.4536 | 0.1433 | -3.165 | 0.003468262 | 0.046552 |
| X49452_at | 0.4285 | 0.1355 | 3.164 | 0.003478583 | 0.046638 |
| X205638_at | -0.1398 | 0.0442 | -3.164 | 0.003478825 | 0.046638 |
| X208664_s_at | 0.3038 | 0.0961 | 3.162 | 0.003494467 | 0.046795 |
| X202864_s_at | 0.2776 | 0.0878 | 3.162 | 0.00349477 | 0.046795 |
| X214131_at | 0.137 | 0.0434 | 3.161 | 0.003502578 | 0.046823 |
| X211212_s_at | -0.2459 | 0.0778 | -3.16 | 0.003506161 | 0.046823 |
| X218783_at | -0.2862 | 0.0906 | -3.16 | 0.003508274 | 0.046823 |
| X202087_s_at | 0.0932 | 0.0295 | 3.16 | 0.0035087 | 0.046823 |
| X212008_at | 0.2174 | 0.0688 | 3.16 | 0.003509631 | 0.046823 |
| X203518_at | -0.1471 | 0.0466 | -3.16 | 0.003511346 | 0.046823 |
| X215744_at | -0.0217 | 0.0069 | -3.16 | 0.003511622 | 0.046823 |
| X203404_at | -0.254 | 0.0804 | -3.159 | 0.003515499 | 0.046847 |
| X208290_s_at | -0.2054 | 0.065 | -3.158 | 0.003529724 | 0.046989 |
| X219698_s_at | -0.1206 | 0.0382 | -3.158 | 0.003530408 | 0.046989 |
| X203428_s_at | -0.1893 | 0.06 | -3.157 | 0.003540178 | 0.047091 |
| X206355_at | -0.293 | 0.0928 | -3.156 | 0.003546559 | 0.047135 |
| X203645_s_at | 0.6442 | 0.2041 | 3.156 | 0.003547784 | 0.047135 |
| X201924_at | 0.2426 | 0.0769 | 3.155 | 0.003552737 | 0.047149 |
| X210502_s_at | -0.1518 | 0.0481 | -3.155 | 0.003553016 | 0.047149 |
| X201917_s_at | 0.167 | 0.053 | 3.154 | 0.003566549 | 0.0473 |
| X212509_s_at | -0.1491 | 0.0473 | -3.153 | 0.003572221 | 0.047347 |
| X217546_at | 0.4798 | 0.1522 | 3.153 | 0.003578103 | 0.047379 |
| X215963_x_at | -0.0693 | 0.022 | -3.153 | 0.003579414 | 0.047379 |
| X205607_s_at | 0.0452 | 0.0143 | 3.152 | 0.003581024 | 0.047379 |
| X212869_x_at | -0.0427 | 0.0136 | -3.151 | 0.003589507 | 0.047463 |
| X209768_s_at | -0.094 | 0.0298 | -3.15 | 0.003598414 | 0.047552 |
| X221504_s_at | -0.1701 | 0.054 | -3.15 | 0.003605437 | 0.047617 |
| X218935_at | 0.0713 | 0.0226 | 3.149 | 0.003608391 | 0.047627 |
| X207573_x_at | -0.1155 | 0.0367 | -3.148 | 0.003616986 | 0.047712 |
| X203895_at | 0.3711 | 0.1179 | 3.148 | 0.003621496 | 0.047743 |
| X201305_x_at | 0.2516 | 0.0799 | 3.148 | 0.003623593 | 0.047743 |
| X201502_s_at | 0.4412 | 0.1402 | 3.147 | 0.003629752 | 0.047768 |
| X221921_s_at | -0.153 | 0.0486 | -3.147 | 0.003631172 | 0.047768 |
| X209575_at | -0.1028 | 0.0327 | -3.146 | 0.003635378 | 0.047768 |
| X219053_s_at | -0.0983 | 0.0312 | -3.146 | 0.003635891 | 0.047768 |
| X221983_at | -0.1544 | 0.0491 | -3.146 | 0.00363624 | 0.047768 |
| X208787_at | -0.0831 | 0.0264 | -3.145 | 0.00365304 | 0.047949 |
| X200053_at | -0.078 | 0.0248 | -3.144 | 0.003654376 | 0.047949 |
| X203078_at | -0.1917 | 0.061 | -3.143 | 0.003665943 | 0.048073 |
| X210852_s_at | 0.1313 | 0.0418 | 3.143 | 0.003671397 | 0.048116 |
| X204226_at | -0.1458 | 0.0464 | -3.141 | 0.003683627 | 0.048204 |
| X202950_at | 0.3254 | 0.1036 | 3.141 | 0.003684794 | 0.048204 |
| X216668_at | -0.0102 | 0.0033 | -3.141 | 0.003685217 | 0.048204 |
| X201523_x_at | -0.2135 | 0.068 | -3.141 | 0.003686821 | 0.048204 |
| X204094_s_at | 0.0921 | 0.0293 | 3.141 | 0.003689205 | 0.048207 |
| X217854_s_at | -0.1581 | 0.0504 | -3.14 | 0.003701444 | 0.048339 |
| X209389_x_at | 0.2207 | 0.0703 | 3.139 | 0.003709898 | 0.04842 |
| X201392_s_at | 0.2449 | 0.0781 | 3.138 | 0.003718176 | 0.048472 |
| X212820_at | -0.1529 | 0.0487 | -3.138 | 0.003718207 | 0.048472 |
| X218049_s_at | -0.1334 | 0.0425 | -3.137 | 0.003726797 | 0.048555 |
| X218633_x_at | -0.1075 | 0.0343 | -3.136 | 0.003735084 | 0.048633 |
| X201784_s_at | -0.1249 | 0.0398 | -3.136 | 0.003737152 | 0.048633 |
| X201770_at | -0.1346 | 0.0429 | -3.135 | 0.003748992 | 0.048705 |
| X203823_at | 0.1772 | 0.0565 | 3.134 | 0.003750452 | 0.048705 |
| X215040_at | 0.0676 | 0.0216 | 3.134 | 0.003752168 | 0.048705 |
| X213786_at | -0.1409 | 0.045 | -3.134 | 0.003756683 | 0.048705 |
| X212018_s_at | -0.1246 | 0.0398 | -3.134 | 0.003757621 | 0.048705 |
| X203721_s_at | -0.1329 | 0.0424 | -3.133 | 0.003759474 | 0.048705 |
| X200805_at | -0.0949 | 0.0303 | -3.133 | 0.003761829 | 0.048705 |
| X221496_s_at | 0.4967 | 0.1585 | 3.133 | 0.003761899 | 0.048705 |
| X209101_at | 0.5785 | 0.1846 | 3.133 | 0.003762399 | 0.048705 |
| X202143_s_at | -0.1676 | 0.0535 | -3.131 | 0.003779248 | 0.048895 |
| X208625_s_at | 0.1061 | 0.0339 | 3.131 | 0.003785873 | 0.048925 |
| X209711_at | 0.1394 | 0.0445 | 3.131 | 0.003788261 | 0.048925 |
| X209363_s_at | -0.2678 | 0.0855 | -3.13 | 0.003790863 | 0.048925 |
| X219572_at | 0.299 | 0.0955 | 3.13 | 0.003794219 | 0.048925 |
| X209944_at | 0.0993 | 0.0317 | 3.13 | 0.003797722 | 0.048925 |
| X200944_s_at | 0.111 | 0.0355 | 3.129 | 0.003799157 | 0.048925 |
| X212136_at | 0.2776 | 0.0887 | 3.129 | 0.003799161 | 0.048925 |
| X204976_s_at | 0.3992 | 0.1276 | 3.129 | 0.003799197 | 0.048925 |
| X214920_at | -0.1793 | 0.0573 | -3.129 | 0.00380189 | 0.048931 |
| X206495_s_at | -0.0126 | 0.004 | -3.128 | 0.003808689 | 0.04899 |
| X216210_x_at | 0.0947 | 0.0303 | 3.128 | 0.003814076 | 0.049031 |
| X204321_at | 0.1814 | 0.058 | 3.128 | 0.003816342 | 0.049032 |
| X221255_s_at | 0.0639 | 0.0204 | 3.127 | 0.003823775 | 0.049099 |
| X219110_at | -0.0794 | 0.0254 | -3.126 | 0.003828679 | 0.049134 |
| X203944_x_at | -0.1195 | 0.0382 | -3.125 | 0.003844683 | 0.049311 |
| X211571_s_at | 0.2219 | 0.071 | 3.124 | 0.003853567 | 0.049396 |
| X218384_at | -0.4921 | 0.1576 | -3.123 | 0.003862622 | 0.049483 |
| X222239_s_at | 0.153 | 0.049 | 3.122 | 0.003868383 | 0.049529 |
| X203430_at | 0.1953 | 0.0626 | 3.122 | 0.003876416 | 0.049603 |
| X220294_at | -0.3611 | 0.1157 | -3.121 | 0.003883066 | 0.049659 |
| X211185_s_at | 0.0752 | 0.0241 | 3.12 | 0.003892158 | 0.049747 |
| X222064_s_at | -0.1598 | 0.0512 | -3.119 | 0.003901039 | 0.049832 |
| X209558_s_at | 0.1015 | 0.0325 | 3.118 | 0.003908509 | 0.049899 |
| X221276_s_at | 0.4094 | 0.1313 | 3.118 | 0.003911706 | 0.049911 |
| X204517_at | 0.1994 | 0.064 | 3.117 | 0.003925188 | 0.050054 |
| X210278_s_at | -0.3071 | 0.0986 | -3.116 | 0.003929064 | 0.050056 |
| X203427_at | -0.1606 | 0.0515 | -3.116 | 0.003930166 | 0.050056 |
| X212641_at | -0.2824 | 0.0906 | -3.116 | 0.003932096 | 0.050056 |
| X202458_at | 0.2 | 0.0642 | 3.115 | 0.003943579 | 0.050173 |
| X200914_x_at | 0.1427 | 0.0458 | 3.114 | 0.003954539 | 0.050217 |
| X210068_s_at | 0.2973 | 0.0955 | 3.114 | 0.003954813 | 0.050217 |
| X221522_at | 0.1395 | 0.0448 | 3.114 | 0.003955998 | 0.050217 |
| X202334_s_at | -0.072 | 0.0231 | -3.114 | 0.00395733 | 0.050217 |
| X220744_s_at | -0.296 | 0.0951 | -3.113 | 0.00395833 | 0.050217 |
| X37384_at | 0.061 | 0.0196 | 3.113 | 0.003962254 | 0.050238 |
| X206707_x_at | -0.012 | 0.0039 | -3.113 | 0.003966596 | 0.050265 |
| X202867_s_at | -0.0178 | 0.0057 | -3.112 | 0.003969999 | 0.050279 |
| X213134_x_at | -0.2206 | 0.0709 | -3.111 | 0.003984068 | 0.05041 |
| X214556_at | 0.0063 | 0.002 | 3.111 | 0.003985884 | 0.05041 |
| X213105_s_at | -0.0797 | 0.0256 | -3.111 | 0.003987178 | 0.05041 |
| X201952_at | -0.0994 | 0.032 | -3.11 | 0.003991801 | 0.050419 |
| X211150_s_at | -0.2412 | 0.0775 | -3.11 | 0.003992365 | 0.050419 |
| X208695_s_at | 0.0649 | 0.0209 | 3.11 | 0.003994646 | 0.050419 |
| X211612_s_at | 0.2519 | 0.081 | 3.109 | 0.003999313 | 0.050449 |
| X213078_x_at | -0.2818 | 0.0907 | -3.108 | 0.004011472 | 0.050574 |
| X215063_x_at | 0.0292 | 0.0094 | 3.108 | 0.004013984 | 0.050577 |
| X214769_at | -0.1218 | 0.0392 | -3.107 | 0.004027021 | 0.050712 |
| X209382_at | -0.112 | 0.0361 | -3.106 | 0.004030515 | 0.050727 |
| X206618_at | 0.0127 | 0.0041 | 3.106 | 0.004032906 | 0.050729 |
| X217398_x_at | -0.0512 | 0.0165 | -3.105 | 0.004048692 | 0.050868 |
| X215130_s_at | -0.1463 | 0.0471 | -3.105 | 0.004050312 | 0.050868 |
| X204176_at | -0.1381 | 0.0445 | -3.104 | 0.004051885 | 0.050868 |
| X219525_at | 0.0154 | 0.005 | 3.104 | 0.004053161 | 0.050868 |
| X203987_at | 0.2232 | 0.0719 | 3.103 | 0.004066762 | 0.05101 |
| X213750_at | -0.1376 | 0.0443 | -3.103 | 0.004070179 | 0.051011 |
| X212295_s_at | 0.1369 | 0.0441 | 3.102 | 0.004073164 | 0.051011 |
| X200606_at | -0.576 | 0.1857 | -3.102 | 0.004073739 | 0.051011 |
| X214415_at | 0.2482 | 0.08 | 3.101 | 0.004083044 | 0.051099 |
| X213911_s_at | -0.1388 | 0.0448 | -3.101 | 0.004086972 | 0.051119 |
| X202845_s_at | 0.0689 | 0.0222 | 3.101 | 0.004092254 | 0.051157 |
| X212178_s_at | 0.131 | 0.0423 | 3.1 | 0.00409728 | 0.051191 |
| X207231_at | -0.1497 | 0.0483 | -3.1 | 0.004101978 | 0.051221 |
| X200081_s_at | 0.0793 | 0.0256 | 3.099 | 0.00410547 | 0.051235 |
| X209239_at | -0.0429 | 0.0139 | -3.098 | 0.004119892 | 0.051378 |
| X217949_s_at | -0.1361 | 0.0439 | -3.098 | 0.004121518 | 0.051378 |
| X221307_at | -0.2829 | 0.0914 | -3.097 | 0.004130593 | 0.051462 |
| X212507_at | -0.111 | 0.0359 | -3.097 | 0.004134097 | 0.051464 |
| X210740_s_at | 0.1322 | 0.0427 | 3.096 | 0.004138296 | 0.051464 |
| X204705_x_at | 0.0054 | 0.0017 | 3.096 | 0.004138784 | 0.051464 |
| X206929_s_at | 0.122 | 0.0394 | 3.096 | 0.004141205 | 0.051464 |
| X213620_s_at | 0.4639 | 0.1499 | 3.096 | 0.004142338 | 0.051464 |
| X209973_at | -0.0174 | 0.0056 | -3.095 | 0.004145765 | 0.05147 |
| X209549_s_at | -0.1602 | 0.0518 | -3.095 | 0.004147436 | 0.05147 |
| X215356_at | -0.2168 | 0.0701 | -3.094 | 0.004163934 | 0.051646 |
| X206014_at | -0.2421 | 0.0783 | -3.092 | 0.004177772 | 0.051784 |
| X203140_at | 0.3224 | 0.1043 | 3.092 | 0.004179731 | 0.051784 |
| X203032_s_at | -0.3542 | 0.1146 | -3.092 | 0.004186826 | 0.051843 |
| X214044_at | -0.1284 | 0.0415 | -3.091 | 0.004190717 | 0.051851 |
| X212351_at | -0.1284 | 0.0415 | -3.091 | 0.004192144 | 0.051851 |
| X218449_at | -0.1691 | 0.0547 | -3.091 | 0.004196965 | 0.051882 |
| X219575_s_at | -0.1229 | 0.0398 | -3.089 | 0.004217095 | 0.052102 |
| X218382_s_at | -0.0082 | 0.0026 | -3.088 | 0.004223811 | 0.052156 |
| X222036_s_at | -0.1259 | 0.0408 | -3.087 | 0.004237995 | 0.052302 |
| X212170_at | -0.141 | 0.0457 | -3.086 | 0.00424323 | 0.052337 |
| X211986_at | 0.2537 | 0.0822 | 3.086 | 0.004248525 | 0.052373 |
| X207657_x_at | 0.1898 | 0.0615 | 3.085 | 0.004254379 | 0.052416 |
| X215169_at | 0.5158 | 0.1672 | 3.084 | 0.004264934 | 0.052495 |
| X210406_s_at | -0.068 | 0.0221 | -3.084 | 0.004265458 | 0.052495 |
| X215307_at | -0.2338 | 0.0758 | -3.084 | 0.00426797 | 0.052497 |
| X201506_at | 0.0918 | 0.0298 | 3.082 | 0.004292637 | 0.052771 |
| X212234_at | 0.0363 | 0.0118 | 3.081 | 0.004300369 | 0.052837 |
| X213571_s_at | 0.1042 | 0.0338 | 3.081 | 0.004305837 | 0.052849 |
| X207120_at | -0.2697 | 0.0875 | -3.081 | 0.004306173 | 0.052849 |
| X202038_at | -0.067 | 0.0217 | -3.079 | 0.004323821 | 0.053022 |
| X205412_at | -0.0696 | 0.0226 | -3.079 | 0.004325037 | 0.053022 |
| X206806_at | -0.301 | 0.0978 | -3.079 | 0.004329927 | 0.05304 |
| X214011_s_at | -0.098 | 0.0318 | -3.078 | 0.004331285 | 0.05304 |
| X207196_s_at | 0.0839 | 0.0273 | 3.078 | 0.004334397 | 0.053049 |
| X206584_at | 0.392 | 0.1274 | 3.077 | 0.004349143 | 0.053174 |
| X209755_at | -0.2139 | 0.0695 | -3.077 | 0.004349349 | 0.053174 |
| X212991_at | -0.1902 | 0.0618 | -3.076 | 0.004355155 | 0.053189 |
| X201400_at | -0.0898 | 0.0292 | -3.076 | 0.004355425 | 0.053189 |
| X208832_at | -0.1912 | 0.0622 | -3.076 | 0.004361486 | 0.053234 |
| X203257_s_at | -0.0646 | 0.021 | -3.075 | 0.004368305 | 0.053279 |
| X200048_s_at | -0.105 | 0.0341 | -3.075 | 0.004369931 | 0.053279 |
| X201372_s_at | -0.2466 | 0.0802 | -3.074 | 0.004382861 | 0.053393 |
| X219754_at | 0.1761 | 0.0573 | 3.074 | 0.004384083 | 0.053393 |
| X211931_s_at | 0.0851 | 0.0277 | 3.073 | 0.004392454 | 0.053465 |
| X202907_s_at | -0.0915 | 0.0298 | -3.072 | 0.004397953 | 0.053494 |
| X218989_x_at | -0.1465 | 0.0477 | -3.072 | 0.004400698 | 0.053494 |
| X202277_at | -0.0868 | 0.0282 | -3.072 | 0.004402024 | 0.053494 |
| X202825_at | -0.1362 | 0.0443 | -3.072 | 0.004404441 | 0.053494 |
| X201594_s_at | 0.1413 | 0.046 | 3.072 | 0.004408213 | 0.053506 |
| X221483_s_at | -0.1473 | 0.0479 | -3.071 | 0.004410257 | 0.053506 |
| X209785_s_at | -0.184 | 0.0599 | -3.071 | 0.004416551 | 0.053518 |
| X202414_at | 0.147 | 0.0479 | 3.071 | 0.004416623 | 0.053518 |
| X204423_at | 0.0419 | 0.0136 | 3.071 | 0.004418484 | 0.053518 |
| X215566_x_at | -0.0277 | 0.009 | -3.07 | 0.004421963 | 0.053531 |
| X202743_at | 0.1536 | 0.05 | 3.069 | 0.004434783 | 0.053657 |
| X208056_s_at | -0.0545 | 0.0178 | -3.068 | 0.004444038 | 0.05374 |
| X1007_s_at | 0.195 | 0.0636 | 3.068 | 0.004453195 | 0.053792 |
| X211711_s_at | 0.1554 | 0.0507 | 3.068 | 0.004453199 | 0.053792 |
| X207768_at | -0.2515 | 0.082 | -3.066 | 0.004467276 | 0.053933 |
| X214608_s_at | -0.5545 | 0.1809 | -3.065 | 0.004477216 | 0.054012 |
| X213047_x_at | -0.1219 | 0.0398 | -3.065 | 0.004480106 | 0.054012 |
| X202588_at | 0.0486 | 0.0159 | 3.065 | 0.004483366 | 0.054012 |
| X214527_s_at | -0.1817 | 0.0593 | -3.065 | 0.004485034 | 0.054012 |
| X201309_x_at | 0.0143 | 0.0047 | 3.065 | 0.004485983 | 0.054012 |
| X203412_at | 0.0483 | 0.0158 | 3.064 | 0.004488817 | 0.054017 |
| X201219_at | 0.2368 | 0.0773 | 3.064 | 0.004491773 | 0.054018 |
| X209184_s_at | 0.1281 | 0.0418 | 3.064 | 0.004493813 | 0.054018 |
| X221622_s_at | -0.1003 | 0.0328 | -3.063 | 0.004508837 | 0.054169 |
| X213918_s_at | 0.1942 | 0.0634 | 3.061 | 0.004524544 | 0.054329 |
| X212644_s_at | -0.0619 | 0.0202 | -3.059 | 0.004551793 | 0.054626 |
| X211752_s_at | -0.1517 | 0.0496 | -3.058 | 0.00456128 | 0.054695 |
| X208628_s_at | 0.1367 | 0.0447 | 3.058 | 0.004562847 | 0.054695 |
| X204357_s_at | 0.0179 | 0.0059 | 3.058 | 0.004564903 | 0.054695 |
| X214373_at | 0.0336 | 0.011 | 3.057 | 0.004574105 | 0.054776 |
| X212710_at | -0.1297 | 0.0425 | -3.055 | 0.004596788 | 0.055018 |
| X221865_at | -0.145 | 0.0475 | -3.055 | 0.004599974 | 0.055026 |
| X208104_s_at | 0.1665 | 0.0545 | 3.055 | 0.004603459 | 0.055038 |
| X202969_at | -0.1568 | 0.0513 | -3.054 | 0.004608544 | 0.05507 |
| X202633_at | -0.0948 | 0.031 | -3.053 | 0.004625977 | 0.055248 |
| X200822_x_at | -0.149 | 0.0488 | -3.052 | 0.004630768 | 0.055276 |
| X209991_x_at | 0.3572 | 0.117 | 3.051 | 0.004639751 | 0.055353 |
| X213226_at | 0.0928 | 0.0304 | 3.051 | 0.004645295 | 0.055368 |
| X218234_at | -0.0613 | 0.0201 | -3.051 | 0.004645976 | 0.055368 |
| X213145_at | -0.101 | 0.0331 | -3.051 | 0.004649508 | 0.055374 |
| X201398_s_at | 0.1971 | 0.0646 | 3.05 | 0.004651503 | 0.055374 |
| X201330_at | -0.0634 | 0.0208 | -3.05 | 0.004659104 | 0.055435 |
| X201119_s_at | -0.1101 | 0.0361 | -3.049 | 0.004665304 | 0.055479 |
| X219892_at | 0.3491 | 0.1145 | 3.049 | 0.004672227 | 0.055532 |
| X202145_at | -0.0195 | 0.0064 | -3.048 | 0.00467732 | 0.055563 |
| X203945_at | 0.0255 | 0.0084 | 3.048 | 0.004682514 | 0.055586 |
| X201756_at | -0.1922 | 0.0631 | -3.048 | 0.004684272 | 0.055586 |
| X205799_s_at | -0.2278 | 0.0748 | -3.047 | 0.00469358 | 0.055666 |
| X212900_at | 0.1319 | 0.0433 | 3.047 | 0.004696759 | 0.055674 |
| X210927_x_at | -0.0888 | 0.0292 | -3.046 | 0.004707185 | 0.055768 |
| X204744_s_at | -0.1123 | 0.0369 | -3.045 | 0.004712448 | 0.055801 |
| X200738_s_at | -0.0814 | 0.0267 | -3.044 | 0.004733576 | 0.056018 |
| X213154_s_at | 0.1151 | 0.0378 | 3.043 | 0.004738387 | 0.056018 |
| X217770_at | 0.0452 | 0.0149 | 3.043 | 0.004740146 | 0.056018 |
| X204687_at | -0.1853 | 0.0609 | -3.043 | 0.004740916 | 0.056018 |
| X208909_at | -0.1199 | 0.0394 | -3.043 | 0.004746972 | 0.056044 |
| X222313_at | 0.2689 | 0.0884 | 3.042 | 0.004748165 | 0.056044 |
| X218116_at | -0.0702 | 0.0231 | -3.042 | 0.004758864 | 0.056141 |
| X213553_x_at | -0.206 | 0.0677 | -3.041 | 0.004766811 | 0.056196 |
| X204794_at | -0.0519 | 0.0171 | -3.041 | 0.004770262 | 0.056196 |
| X210990_s_at | -0.0087 | 0.0029 | -3.041 | 0.004771151 | 0.056196 |
| X211337_s_at | -0.2749 | 0.0904 | -3.04 | 0.004777157 | 0.056237 |
| X212406_s_at | -0.1159 | 0.0381 | -3.039 | 0.004791369 | 0.05635 |
| X210647_x_at | -0.032 | 0.0105 | -3.039 | 0.004792576 | 0.05635 |
| X218442_at | -0.0546 | 0.018 | -3.039 | 0.004794382 | 0.05635 |
| X65133_i_at | -0.0076 | 0.0025 | -3.038 | 0.004798247 | 0.056363 |
| X206411_s_at | -0.0116 | 0.0038 | -3.038 | 0.004804551 | 0.056363 |
| X218316_at | -0.0832 | 0.0274 | -3.038 | 0.004804722 | 0.056363 |
| X203152_at | -0.0859 | 0.0283 | -3.038 | 0.004805562 | 0.056363 |
| X201216_at | -0.138 | 0.0454 | -3.037 | 0.00481892 | 0.056418 |
| X206106_at | -0.108 | 0.0356 | -3.037 | 0.004819204 | 0.056418 |
| X220325_at | -0.0559 | 0.0184 | -3.037 | 0.004819423 | 0.056418 |
| X213725_x_at | 0.1656 | 0.0545 | 3.036 | 0.004820746 | 0.056418 |
| X218104_at | -0.1066 | 0.0351 | -3.036 | 0.004823086 | 0.056418 |
| X219459_at | -0.1802 | 0.0594 | -3.036 | 0.00482551 | 0.056418 |
| X201978_s_at | 0.0779 | 0.0257 | 3.035 | 0.004835384 | 0.056504 |
| X51176_at | -0.1 | 0.033 | -3.035 | 0.004842527 | 0.056557 |
| X209120_at | 0.3146 | 0.1037 | 3.034 | 0.004852222 | 0.056625 |
| X202658_at | -0.0824 | 0.0272 | -3.034 | 0.00485346 | 0.056625 |
| X212435_at | -0.0893 | 0.0294 | -3.033 | 0.004858025 | 0.056649 |
| X201001_s_at | 0.1586 | 0.0523 | 3.031 | 0.004890499 | 0.056998 |
| X201224_s_at | 0.2876 | 0.0949 | 3.031 | 0.004894377 | 0.057013 |
| X202936_s_at | 0.257 | 0.0848 | 3.03 | 0.004904632 | 0.057102 |
| X221434_s_at | -0.1019 | 0.0336 | -3.029 | 0.00491288 | 0.057169 |
| X37425_g_at | -0.0809 | 0.0267 | -3.029 | 0.004918883 | 0.057208 |
| X205586_x_at | -0.0422 | 0.0139 | -3.028 | 0.004928298 | 0.057264 |
| X201213_at | -0.1179 | 0.0389 | -3.028 | 0.004928775 | 0.057264 |
| X220435_at | -0.1017 | 0.0336 | -3.027 | 0.004944147 | 0.057412 |
| X217763_s_at | 0.1464 | 0.0484 | 3.026 | 0.004956788 | 0.057525 |
| X215948_x_at | 0.1495 | 0.0494 | 3.025 | 0.004959152 | 0.057525 |
| X210240_s_at | -0.2396 | 0.0792 | -3.025 | 0.004961647 | 0.057525 |
| X202917_s_at | 1.2806 | 0.4234 | 3.024 | 0.004969873 | 0.05759 |
| X212069_s_at | 0.1647 | 0.0545 | 3.023 | 0.004990734 | 0.057793 |
| X201521_s_at | -0.0991 | 0.0328 | -3.022 | 0.004998709 | 0.057793 |
| X204672_s_at | 0.2355 | 0.0779 | 3.022 | 0.004999997 | 0.057793 |
| X202676_x_at | 0.1099 | 0.0364 | 3.022 | 0.005002047 | 0.057793 |
| X200011_s_at | -0.147 | 0.0487 | -3.022 | 0.005003262 | 0.057793 |
| X203919_at | -0.1552 | 0.0514 | -3.022 | 0.005004665 | 0.057793 |
| X208771_s_at | -0.0868 | 0.0287 | -3.022 | 0.00500561 | 0.057793 |
| X212448_at | -0.1874 | 0.062 | -3.021 | 0.005016095 | 0.057855 |
| X209496_at | 0.1841 | 0.061 | 3.021 | 0.005016111 | 0.057855 |
| X220044_x_at | 0.0856 | 0.0283 | 3.02 | 0.00502258 | 0.057857 |
| X205324_s_at | -0.2204 | 0.073 | -3.02 | 0.005025418 | 0.057857 |
| X200923_at | 0.1117 | 0.037 | 3.02 | 0.005025578 | 0.057857 |
| X209511_at | -0.0286 | 0.0095 | -3.02 | 0.005026767 | 0.057857 |
| X209174_s_at | -0.068 | 0.0225 | -3.02 | 0.005031517 | 0.057882 |
| X215448_at | 0.2268 | 0.0751 | 3.019 | 0.005043888 | 0.057994 |
| X219512_at | -0.1408 | 0.0467 | -3.018 | 0.00505744 | 0.058113 |
| X218048_at | -0.0876 | 0.029 | -3.017 | 0.00505942 | 0.058113 |
| X221423_s_at | -0.2348 | 0.0778 | -3.017 | 0.005062478 | 0.058118 |
| X213254_at | 0.0962 | 0.0319 | 3.016 | 0.005078644 | 0.058273 |
| X215273_s_at | -0.1454 | 0.0482 | -3.016 | 0.005083093 | 0.058283 |
| X200049_at | -0.1166 | 0.0387 | -3.015 | 0.005086897 | 0.058283 |
| X205060_at | -0.1241 | 0.0412 | -3.015 | 0.005087385 | 0.058283 |
| X209109_s_at | 0.3946 | 0.1309 | 3.015 | 0.005092807 | 0.058315 |
| X205652_s_at | -0.0474 | 0.0157 | -3.014 | 0.005102566 | 0.058368 |
| X201749_at | 0.1252 | 0.0415 | 3.014 | 0.005102648 | 0.058368 |
| X210518_at | -0.3308 | 0.1098 | -3.014 | 0.005105504 | 0.05837 |
| X218431_at | -0.0522 | 0.0173 | -3.014 | 0.005108414 | 0.058374 |
| X202384_s_at | 0.1272 | 0.0422 | 3.013 | 0.005117488 | 0.058447 |
| X221971_x_at | 0.1276 | 0.0423 | 3.013 | 0.005123221 | 0.058483 |
| X218613_at | -0.1243 | 0.0413 | -3.011 | 0.005139392 | 0.058637 |
| X216858_x_at | 0.1758 | 0.0584 | 3.01 | 0.005150118 | 0.058719 |
| X215141_at | -0.0087 | 0.0029 | -3.01 | 0.005151884 | 0.058719 |
| X212556_at | 0.0773 | 0.0257 | 3.009 | 0.005166459 | 0.058855 |
| X215990_s_at | 0.0165 | 0.0055 | 3.009 | 0.005170546 | 0.058855 |
| X204477_at | -0.0384 | 0.0128 | -3.009 | 0.005171903 | 0.058855 |
| X201416_at | 0.0591 | 0.0197 | 3.009 | 0.005175122 | 0.058855 |
| X71933_at | -0.1082 | 0.036 | -3.008 | 0.005177614 | 0.058855 |
| X205204_at | 0.0874 | 0.0291 | 3.008 | 0.005179657 | 0.058855 |
| X202357_s_at | 0.0157 | 0.0052 | 3.008 | 0.005183889 | 0.058873 |
| X208813_at | -0.1877 | 0.0624 | -3.007 | 0.005195019 | 0.058941 |
| X206935_at | -0.2567 | 0.0854 | -3.007 | 0.005195198 | 0.058941 |
| X217860_at | -0.1459 | 0.0485 | -3.005 | 0.005218796 | 0.059178 |
| X208579_x_at | -0.3573 | 0.119 | -3.003 | 0.005244282 | 0.059437 |
| X206005_s_at | 0.0851 | 0.0283 | 3.002 | 0.005257785 | 0.05956 |
| X209224_s_at | -0.0632 | 0.021 | -3.002 | 0.005261271 | 0.059569 |
| X218357_s_at | -0.0659 | 0.0219 | -3.002 | 0.005266112 | 0.059593 |
| X213252_at | 0.0288 | 0.0096 | 3.001 | 0.005273153 | 0.059642 |
| X206956_at | -0.0777 | 0.0259 | -3 | 0.005282276 | 0.059715 |
| X213545_x_at | -0.0973 | 0.0324 | -3 | 0.005287312 | 0.059742 |
| X204205_at | 0.1005 | 0.0335 | 3 | 0.005289977 | 0.059742 |
| X207508_at | -0.0809 | 0.027 | -3 | 0.005295445 | 0.059773 |
| X204168_at | 0.3494 | 0.1165 | 2.999 | 0.005306025 | 0.059862 |
| X220676_at | -0.0274 | 0.0091 | -2.998 | 0.005311401 | 0.059864 |
| X209106_at | -0.0759 | 0.0253 | -2.998 | 0.005311629 | 0.059864 |
| X210104_at | -0.1826 | 0.0609 | -2.998 | 0.005319757 | 0.059926 |
| X213142_x_at | 0.368 | 0.1228 | 2.997 | 0.005322601 | 0.059927 |
| X202698_x_at | -0.068 | 0.0227 | -2.997 | 0.005333841 | 0.060023 |
| X212240_s_at | 0.2473 | 0.0825 | 2.996 | 0.005337578 | 0.060035 |
| X203302_at | -0.1717 | 0.0573 | -2.996 | 0.005340941 | 0.060042 |
| X214713_at | -0.1602 | 0.0535 | -2.995 | 0.005360887 | 0.060216 |
| X202866_at | -0.0667 | 0.0223 | -2.995 | 0.005361825 | 0.060216 |
| X212687_at | 0.2417 | 0.0807 | 2.994 | 0.005367488 | 0.060249 |
| X213028_at | -0.2373 | 0.0793 | -2.994 | 0.005371831 | 0.060268 |
| X206801_at | -0.4969 | 0.166 | -2.993 | 0.005383975 | 0.060373 |
| X201032_at | -0.1788 | 0.0598 | -2.991 | 0.00541086 | 0.060644 |
| X219428_s_at | -0.0361 | 0.0121 | -2.99 | 0.005427993 | 0.060787 |
| X202112_at | 0.448 | 0.1499 | 2.99 | 0.00542907 | 0.060787 |
| X201772_at | 0.1663 | 0.0556 | 2.989 | 0.005433297 | 0.060804 |
| X201099_at | -0.0676 | 0.0226 | -2.989 | 0.005438833 | 0.06083 |
| X205875_s_at | 0.0078 | 0.0026 | 2.989 | 0.005441136 | 0.06083 |
| X209129_at | 0.1555 | 0.052 | 2.988 | 0.005447391 | 0.060858 |
| X204140_at | 0.1721 | 0.0576 | 2.988 | 0.005449113 | 0.060858 |
| X206098_at | -0.0508 | 0.017 | -2.988 | 0.005453662 | 0.060875 |
| X218238_at | -0.1096 | 0.0367 | -2.988 | 0.005456137 | 0.060875 |
| X203549_s_at | -0.323 | 0.1081 | -2.987 | 0.005464146 | 0.060904 |
| X209024_s_at | 0.1387 | 0.0464 | 2.987 | 0.00546439 | 0.060904 |
| X200654_at | 0.0497 | 0.0166 | 2.987 | 0.005466946 | 0.060904 |
| X212637_s_at | -0.1942 | 0.0651 | -2.985 | 0.005488886 | 0.061097 |
| X201252_at | -0.0361 | 0.0121 | -2.985 | 0.005489776 | 0.061097 |
| X213689_x_at | -0.1279 | 0.0428 | -2.985 | 0.005492714 | 0.061099 |
| X203044_at | 0.2562 | 0.0859 | 2.985 | 0.005500166 | 0.061152 |
| X202126_at | -0.0834 | 0.0279 | -2.984 | 0.005508408 | 0.061212 |
| X212496_s_at | 0.1463 | 0.049 | 2.984 | 0.00551185 | 0.06122 |
| X202373_s_at | -0.1441 | 0.0483 | -2.983 | 0.005516793 | 0.06123 |
| X206215_at | 0.3917 | 0.1313 | 2.983 | 0.005518231 | 0.06123 |
| X200892_s_at | -0.1035 | 0.0347 | -2.982 | 0.005531442 | 0.061336 |
| X205280_at | -0.1237 | 0.0415 | -2.982 | 0.005533369 | 0.061336 |
| X214288_s_at | -0.0521 | 0.0175 | -2.981 | 0.005550051 | 0.061461 |
| X221952_x_at | 0.1035 | 0.0347 | 2.981 | 0.005550104 | 0.061461 |
| X202224_at | 0.1227 | 0.0412 | 2.98 | 0.005565393 | 0.061599 |
| X201412_at | 0.1884 | 0.0632 | 2.979 | 0.005570762 | 0.061628 |
| X220643_s_at | 0.1132 | 0.038 | 2.978 | 0.005587035 | 0.061777 |
| X203531_at | -0.1016 | 0.0341 | -2.978 | 0.005598093 | 0.061865 |
| X202860_at | 0.074 | 0.0249 | 2.977 | 0.005601519 | 0.061865 |
| X200600_at | 0.1213 | 0.0407 | 2.977 | 0.005603364 | 0.061865 |
| X200027_at | -0.0597 | 0.02 | -2.977 | 0.00560952 | 0.061903 |
| X209230_s_at | 0.2647 | 0.0889 | 2.976 | 0.00561307 | 0.061911 |
| X206461_x_at | 0.2773 | 0.0932 | 2.976 | 0.005623478 | 0.061995 |
| X213581_at | -0.1064 | 0.0358 | -2.975 | 0.005630474 | 0.062041 |
| X201938_at | 0.1594 | 0.0536 | 2.975 | 0.005638388 | 0.062098 |
| X210256_s_at | 0.0124 | 0.0042 | 2.974 | 0.005649597 | 0.062154 |
| X200877_at | -0.1477 | 0.0497 | -2.974 | 0.005651207 | 0.062154 |
| X211074_at | 0.2351 | 0.0791 | 2.974 | 0.005651927 | 0.062154 |
| X204630_s_at | 0.0806 | 0.0271 | 2.973 | 0.005657789 | 0.062188 |
| X202549_at | 0.2705 | 0.091 | 2.973 | 0.005667563 | 0.062265 |
| X202748_at | 0.079 | 0.0266 | 2.972 | 0.005678731 | 0.062337 |
| X215903_s_at | 0.077 | 0.0259 | 2.972 | 0.005679738 | 0.062337 |
| X201356_at | -0.0609 | 0.0205 | -2.97 | 0.005701225 | 0.062542 |
| X201985_at | -0.0723 | 0.0243 | -2.97 | 0.005707696 | 0.062582 |
| X204120_s_at | -0.1854 | 0.0624 | -2.969 | 0.005722275 | 0.062672 |
| X210241_s_at | -0.0601 | 0.0202 | -2.969 | 0.005722699 | 0.062672 |
| X221333_at | -0.0102 | 0.0034 | -2.969 | 0.005724405 | 0.062672 |
| X209796_s_at | -0.0866 | 0.0292 | -2.968 | 0.00574025 | 0.062815 |
| X200861_at | -0.0319 | 0.0107 | -2.967 | 0.005752766 | 0.062921 |
| X213484_at | -0.1308 | 0.0441 | -2.966 | 0.005761167 | 0.062951 |
| X218471_s_at | -0.0782 | 0.0264 | -2.966 | 0.00576118 | 0.062951 |
| X212366_at | -0.1088 | 0.0367 | -2.966 | 0.005767214 | 0.062986 |
| X213817_at | 0.2284 | 0.077 | 2.965 | 0.005774037 | 0.063029 |
| X212400_at | -0.1484 | 0.0501 | -2.965 | 0.005777134 | 0.063032 |
| X207628_s_at | -0.1236 | 0.0417 | -2.965 | 0.005782276 | 0.063051 |
| X205316_at | -0.3137 | 0.1058 | -2.965 | 0.005784581 | 0.063051 |
| X218336_at | 0.0451 | 0.0152 | 2.964 | 0.005788024 | 0.063058 |
| X220072_at | 0.1964 | 0.0663 | 2.964 | 0.00579126 | 0.063062 |
| X205923_at | -0.2021 | 0.0682 | -2.963 | 0.00580758 | 0.063199 |
| X202187_s_at | -0.1196 | 0.0404 | -2.963 | 0.005809501 | 0.063199 |
| X213546_at | 0.0711 | 0.024 | 2.962 | 0.00581484 | 0.06321 |
| X212629_s_at | 0.1332 | 0.045 | 2.962 | 0.005816232 | 0.06321 |
| X202191_s_at | -0.1151 | 0.0389 | -2.961 | 0.005830836 | 0.063338 |
| X206675_s_at | -0.0173 | 0.0059 | -2.961 | 0.005843765 | 0.063405 |
| X200075_s_at | -0.1425 | 0.0481 | -2.96 | 0.00584447 | 0.063405 |
| X209595_at | -0.1247 | 0.0421 | -2.96 | 0.005845574 | 0.063405 |
| X204238_s_at | -0.2038 | 0.0689 | -2.96 | 0.00585447 | 0.063471 |
| X212013_at | 0.0254 | 0.0086 | 2.959 | 0.005868268 | 0.063589 |
| X210243_s_at | -0.2116 | 0.0715 | -2.959 | 0.005872227 | 0.063601 |
| X208640_at | -0.04 | 0.0135 | -2.958 | 0.005881185 | 0.063667 |
| X205279_s_at | -0.2335 | 0.0789 | -2.958 | 0.005887651 | 0.063706 |
| X218020_s_at | 0.1005 | 0.034 | 2.957 | 0.005891363 | 0.063715 |
| X202877_s_at | 0.0102 | 0.0035 | 2.957 | 0.005901599 | 0.063778 |
| X200708_at | -0.0758 | 0.0257 | -2.957 | 0.005902939 | 0.063778 |
| X214306_at | -0.1025 | 0.0347 | -2.956 | 0.005913905 | 0.063837 |
| X208791_at | 0.1522 | 0.0515 | 2.956 | 0.005916993 | 0.063837 |
| X209609_s_at | -0.0652 | 0.0221 | -2.956 | 0.005917022 | 0.063837 |
| X219049_at | 0.1925 | 0.0652 | 2.954 | 0.005943208 | 0.06408 |
| X218634_at | 0.0575 | 0.0195 | 2.954 | 0.005945302 | 0.06408 |
| X203529_at | 0.114 | 0.0386 | 2.952 | 0.005965533 | 0.064267 |
| X208961_s_at | 0.2937 | 0.0995 | 2.952 | 0.005972262 | 0.064308 |
| X221831_at | -0.0974 | 0.033 | -2.951 | 0.005979236 | 0.064352 |
| X201792_at | 0.4141 | 0.1403 | 2.951 | 0.005985447 | 0.064388 |
| X214913_at | -0.3918 | 0.1328 | -2.951 | 0.005990301 | 0.064409 |
| X212312_at | 0.0197 | 0.0067 | 2.95 | 0.005997954 | 0.06446 |
| X207797_s_at | 0.1347 | 0.0457 | 2.949 | 0.006009792 | 0.064556 |
| X211060_x_at | -0.1493 | 0.0506 | -2.949 | 0.006021477 | 0.064649 |
| X52078_at | 0.0393 | 0.0133 | 2.948 | 0.006026454 | 0.064649 |
| X205975_s_at | -0.0234 | 0.0079 | -2.948 | 0.006027235 | 0.064649 |
| X211815_s_at | -0.0055 | 0.0019 | -2.947 | 0.006040116 | 0.064756 |
| X203166_at | 0.0689 | 0.0234 | 2.947 | 0.006046864 | 0.064797 |
| X219146_at | -0.1445 | 0.0491 | -2.946 | 0.006062043 | 0.064929 |
| X200594_x_at | -0.0588 | 0.02 | -2.945 | 0.006074249 | 0.065028 |
| X218441_s_at | 0.035 | 0.0119 | 2.944 | 0.006089469 | 0.06516 |
| X222170_at | -0.0273 | 0.0093 | -2.944 | 0.006097124 | 0.065188 |
| X215228_at | 0.2731 | 0.0928 | 2.944 | 0.006098529 | 0.065188 |
| X215952_s_at | -0.1828 | 0.0621 | -2.943 | 0.006101455 | 0.065188 |
| X201241_at | -0.0804 | 0.0273 | -2.943 | 0.006103889 | 0.065188 |
| X213410_at | -0.0734 | 0.025 | -2.942 | 0.006125443 | 0.065358 |
| X218281_at | -0.1085 | 0.0369 | -2.942 | 0.006125637 | 0.065358 |
| X219898_at | -0.2398 | 0.0815 | -2.941 | 0.006136717 | 0.065445 |
| X216338_s_at | -0.0386 | 0.0131 | -2.94 | 0.006151614 | 0.065571 |
| X218306_s_at | 0.1107 | 0.0377 | 2.94 | 0.006154518 | 0.065571 |
| X203910_at | 0.321 | 0.1092 | 2.94 | 0.006157553 | 0.065572 |
| X217191_x_at | -0.0249 | 0.0085 | -2.939 | 0.006166427 | 0.065621 |
| X200755_s_at | -0.1405 | 0.0478 | -2.939 | 0.006168009 | 0.065621 |
| X219244_s_at | -0.2091 | 0.0712 | -2.938 | 0.006180898 | 0.065707 |
| X201563_at | 0.1848 | 0.0629 | 2.938 | 0.006184134 | 0.065707 |
| X212276_at | -0.0981 | 0.0334 | -2.938 | 0.00618495 | 0.065707 |
| X200639_s_at | -0.0956 | 0.0325 | -2.938 | 0.006191551 | 0.065745 |
| X217707_x_at | -0.0978 | 0.0333 | -2.937 | 0.006203739 | 0.065843 |
| X212177_at | 0.2633 | 0.0897 | 2.936 | 0.006213887 | 0.065845 |
| X218785_s_at | -0.1685 | 0.0574 | -2.936 | 0.006214452 | 0.065845 |
| X211769_x_at | -0.1855 | 0.0632 | -2.936 | 0.006215965 | 0.065845 |
| X201038_s_at | -0.0972 | 0.0331 | -2.936 | 0.006217415 | 0.065845 |
| X217617_at | 0.313 | 0.1066 | 2.936 | 0.006218749 | 0.065845 |
| X220432_s_at | 0.0886 | 0.0302 | 2.935 | 0.006238141 | 0.066019 |
| X208926_at | -0.0967 | 0.033 | -2.934 | 0.006245944 | 0.06607 |
| X221428_s_at | -0.1569 | 0.0535 | -2.933 | 0.006255659 | 0.066141 |
| X208977_x_at | 0.1033 | 0.0352 | 2.933 | 0.00625908 | 0.066146 |
| X205358_at | -0.1556 | 0.0531 | -2.933 | 0.006263098 | 0.066157 |
| X221190_s_at | 0.0552 | 0.0188 | 2.933 | 0.006266936 | 0.066166 |
| X221582_at | -0.259 | 0.0883 | -2.932 | 0.006282129 | 0.066295 |
| X201990_s_at | -0.1046 | 0.0357 | -2.931 | 0.006296146 | 0.066411 |
| X203720_s_at | -0.0598 | 0.0204 | -2.93 | 0.006307219 | 0.066497 |
| X204133_at | -0.0095 | 0.0033 | -2.929 | 0.006330752 | 0.066713 |
| X205031_at | -0.3167 | 0.1081 | -2.928 | 0.006338844 | 0.066761 |
| X214363_s_at | -0.0514 | 0.0176 | -2.928 | 0.006341327 | 0.066761 |
| X201464_x_at | 0.4137 | 0.1414 | 2.927 | 0.006363893 | 0.066967 |
| X210731_s_at | -0.2289 | 0.0782 | -2.925 | 0.0063842 | 0.067149 |
| X203533_s_at | 0.082 | 0.028 | 2.925 | 0.006388071 | 0.067158 |
| X217809_at | -0.1363 | 0.0466 | -2.924 | 0.006403011 | 0.067247 |
| X213812_s_at | -0.329 | 0.1125 | -2.924 | 0.006403046 | 0.067247 |
| X204620_s_at | 0.2799 | 0.0957 | 2.924 | 0.006406168 | 0.067247 |
| X212852_s_at | 0.1588 | 0.0543 | 2.924 | 0.006408656 | 0.067247 |
| X205953_at | -0.0163 | 0.0056 | -2.923 | 0.006414727 | 0.067279 |
| X204787_at | 0.0863 | 0.0295 | 2.923 | 0.006424587 | 0.067351 |
| X202271_at | -0.0768 | 0.0263 | -2.922 | 0.006430478 | 0.06738 |
| X203323_at | 0.3761 | 0.1287 | 2.922 | 0.00643407 | 0.067386 |
| X213698_at | 0.2107 | 0.0721 | 2.922 | 0.006439546 | 0.06739 |
| X212982_at | -0.0546 | 0.0187 | -2.922 | 0.006440492 | 0.06739 |
| X208761_s_at | -0.079 | 0.027 | -2.921 | 0.006451695 | 0.067476 |
| X219511_s_at | -0.2496 | 0.0854 | -2.921 | 0.006455612 | 0.067485 |
| X220992_s_at | -0.141 | 0.0483 | -2.92 | 0.006470132 | 0.067605 |
| X206846_s_at | 0.1318 | 0.0451 | 2.92 | 0.006477585 | 0.067651 |
| X218667_at | -0.2088 | 0.0715 | -2.919 | 0.006488497 | 0.067733 |
| X200919_at | 0.1187 | 0.0407 | 2.919 | 0.006493818 | 0.067757 |
| X219065_s_at | 0.0682 | 0.0234 | 2.918 | 0.006509223 | 0.067885 |
| X220551_at | 0.0865 | 0.0296 | 2.916 | 0.006531364 | 0.068076 |
| X206752_s_at | 0.0875 | 0.03 | 2.916 | 0.006533639 | 0.068076 |
| X55081_at | 0.0389 | 0.0133 | 2.916 | 0.006536731 | 0.068076 |
| X213929_at | 0.4836 | 0.1658 | 2.916 | 0.006540398 | 0.068083 |
| X216044_x_at | -0.1682 | 0.0577 | -2.915 | 0.006548028 | 0.068084 |
| X207941_s_at | -0.0546 | 0.0187 | -2.915 | 0.006549254 | 0.068084 |
| X218385_at | -0.0636 | 0.0218 | -2.915 | 0.006549744 | 0.068084 |
| X201441_at | -0.085 | 0.0292 | -2.915 | 0.006557591 | 0.068134 |
| X213670_x_at | 0.0729 | 0.025 | 2.914 | 0.006562041 | 0.068148 |
| X206376_at | 0.2303 | 0.079 | 2.914 | 0.006566728 | 0.068165 |
| X210749_x_at | 0.2052 | 0.0704 | 2.913 | 0.006579456 | 0.068265 |
| X204224_s_at | -0.1809 | 0.0621 | -2.912 | 0.006600732 | 0.068384 |
| X217985_s_at | 0.2756 | 0.0946 | 2.912 | 0.006601118 | 0.068384 |
| X213676_at | 0.2331 | 0.08 | 2.912 | 0.006601328 | 0.068384 |
| X211929_at | -0.1241 | 0.0426 | -2.912 | 0.006603184 | 0.068384 |
| X208942_s_at | 0.0766 | 0.0263 | 2.911 | 0.006609827 | 0.068421 |
| X207124_s_at | -0.2093 | 0.0719 | -2.911 | 0.006619946 | 0.068493 |
| X213605_s_at | 0.202 | 0.0694 | 2.91 | 0.006628507 | 0.06855 |
| X203631_s_at | -0.0085 | 0.0029 | -2.91 | 0.006633759 | 0.068573 |
| X201024_x_at | 0.1481 | 0.0509 | 2.91 | 0.00663895 | 0.068594 |
| X209524_at | 0.1097 | 0.0377 | 2.909 | 0.006652682 | 0.068704 |
| X202495_at | -0.0935 | 0.0321 | -2.909 | 0.006656077 | 0.068707 |
| X202327_s_at | -0.0058 | 0.002 | -2.908 | 0.006661243 | 0.068726 |
| X219900_s_at | 0.0082 | 0.0028 | 2.908 | 0.006664039 | 0.068726 |
| X200006_at | -0.0667 | 0.0229 | -2.907 | 0.006681362 | 0.068847 |
| X213837_at | 0.0316 | 0.0109 | 2.907 | 0.006681995 | 0.068847 |
| X218083_at | -0.0891 | 0.0307 | -2.905 | 0.006710274 | 0.069103 |
| X209369_at | 0.4362 | 0.1502 | 2.905 | 0.006713074 | 0.069103 |
| X212674_s_at | -0.0854 | 0.0294 | -2.904 | 0.006736215 | 0.069309 |
| X200846_s_at | -0.1767 | 0.0609 | -2.903 | 0.006745035 | 0.069368 |
| X206544_x_at | -0.2372 | 0.0817 | -2.903 | 0.006748218 | 0.069368 |
| X218210_at | -0.0973 | 0.0335 | -2.903 | 0.0067562 | 0.069418 |
| X213500_at | -0.1133 | 0.039 | -2.902 | 0.006771714 | 0.069545 |
| X203893_at | -0.0811 | 0.028 | -2.9 | 0.006798824 | 0.06977 |
| X218839_at | -0.1341 | 0.0462 | -2.9 | 0.006799885 | 0.06977 |
| X219134_at | 0.4255 | 0.1467 | 2.9 | 0.006808697 | 0.069823 |
| X207121_s_at | -0.0896 | 0.0309 | -2.899 | 0.006811313 | 0.069823 |
| X219355_at | -0.5331 | 0.1839 | -2.899 | 0.006818263 | 0.069862 |
| X219540_at | -0.1494 | 0.0515 | -2.898 | 0.006828526 | 0.069935 |
| X211703_s_at | -0.1559 | 0.0538 | -2.898 | 0.006839941 | 0.07002 |
| X218134_s_at | -0.0683 | 0.0236 | -2.898 | 0.006845211 | 0.070031 |
| X204303_s_at | 0.1316 | 0.0454 | 2.897 | 0.006847383 | 0.070031 |
| X211484_s_at | 0.1108 | 0.0383 | 2.897 | 0.006860889 | 0.070137 |
| X212213_x_at | -0.1216 | 0.042 | -2.894 | 0.00689986 | 0.070503 |
| X204589_at | 0.1752 | 0.0606 | 2.892 | 0.006937096 | 0.070822 |
| X208113_x_at | 0.0934 | 0.0323 | 2.892 | 0.006939182 | 0.070822 |
| X217761_at | 0.1939 | 0.067 | 2.892 | 0.00694065 | 0.070822 |
| X217672_x_at | -0.0187 | 0.0065 | -2.891 | 0.00696506 | 0.071039 |
| X210611_s_at | 0.3962 | 0.1371 | 2.89 | 0.006973688 | 0.071094 |
| X213448_at | -0.1357 | 0.047 | -2.888 | 0.00700469 | 0.071377 |
| X207981_s_at | -0.1784 | 0.0618 | -2.888 | 0.007011615 | 0.071415 |
| X209576_at | 0.1265 | 0.0438 | 2.886 | 0.007037718 | 0.071626 |
| X206481_s_at | -0.212 | 0.0735 | -2.886 | 0.007038768 | 0.071626 |
| X218009_s_at | -0.0234 | 0.0081 | -2.886 | 0.007045247 | 0.071659 |
| X204257_at | 0.1446 | 0.0501 | 2.885 | 0.007055833 | 0.071734 |
| X204610_s_at | -0.2275 | 0.0788 | -2.885 | 0.00706014 | 0.071745 |
| X203432_at | 0.2056 | 0.0713 | 2.884 | 0.007071919 | 0.071832 |
| X221541_at | 0.5065 | 0.1756 | 2.884 | 0.007076779 | 0.071841 |
| X200623_s_at | -0.1289 | 0.0447 | -2.884 | 0.00707989 | 0.071841 |
| X206078_at | -0.0521 | 0.0181 | -2.884 | 0.007082579 | 0.071841 |
| X212284_x_at | -0.0448 | 0.0155 | -2.884 | 0.007085863 | 0.071842 |
| X218488_at | -0.1366 | 0.0474 | -2.883 | 0.007106177 | 0.072015 |
| X205290_s_at | 0.0076 | 0.0026 | 2.882 | 0.00711458 | 0.072067 |
| X40472_at | -0.2316 | 0.0804 | -2.882 | 0.007120697 | 0.072096 |
| X204863_s_at | 0.3491 | 0.1212 | 2.881 | 0.007131252 | 0.07217 |
| X219201_s_at | 0.0485 | 0.0168 | 2.881 | 0.00713601 | 0.072185 |
| X215034_s_at | 0.5654 | 0.1963 | 2.88 | 0.007144529 | 0.072239 |
| X213518_at | -0.113 | 0.0392 | -2.879 | 0.007167078 | 0.072434 |
| X222113_s_at | 0.1897 | 0.0659 | 2.878 | 0.007186048 | 0.072592 |
| X204738_s_at | -0.0119 | 0.0042 | -2.878 | 0.007194863 | 0.072648 |
| X201250_s_at | 0.3122 | 0.1086 | 2.876 | 0.00721651 | 0.072834 |
| X202459_s_at | -0.1253 | 0.0436 | -2.876 | 0.007220868 | 0.072845 |
| X212092_at | -0.8522 | 0.2963 | -2.876 | 0.00723074 | 0.072911 |
| X200976_s_at | -0.0595 | 0.0207 | -2.875 | 0.007235846 | 0.07293 |
| X214487_s_at | -0.1159 | 0.0403 | -2.875 | 0.007248323 | 0.072983 |
| X219913_s_at | -0.1868 | 0.065 | -2.875 | 0.007249013 | 0.072983 |
| X202927_at | -0.1553 | 0.054 | -2.874 | 0.007250989 | 0.072983 |
| X206315_at | -0.5174 | 0.18 | -2.874 | 0.00726575 | 0.073098 |
| X217972_at | -0.0813 | 0.0283 | -2.872 | 0.007287431 | 0.073253 |
| X200733_s_at | 0.1338 | 0.0466 | 2.872 | 0.00728767 | 0.073253 |
| X200088_x_at | 0.1017 | 0.0354 | 2.872 | 0.007291321 | 0.073256 |
| X218396_at | 0.1388 | 0.0483 | 2.871 | 0.007303989 | 0.07335 |
| X222068_s_at | 0.3068 | 0.1069 | 2.87 | 0.007326194 | 0.07354 |
| X218894_s_at | -0.1087 | 0.0379 | -2.869 | 0.007344802 | 0.07368 |
| X217897_at | 0.3162 | 0.1102 | 2.869 | 0.007346773 | 0.07368 |
| X204932_at | 0.0968 | 0.0337 | 2.869 | 0.007350178 | 0.073681 |
| X204334_at | 0.1347 | 0.047 | 2.868 | 0.007373212 | 0.073849 |
| X200816_s_at | -0.1026 | 0.0358 | -2.868 | 0.007373615 | 0.073849 |
| X210009_s_at | -0.2056 | 0.0717 | -2.867 | 0.007385905 | 0.073939 |
| X206233_at | -0.1049 | 0.0366 | -2.865 | 0.007415226 | 0.074158 |
| X202066_at | 0.1084 | 0.0378 | 2.865 | 0.007416465 | 0.074158 |
| X201583_s_at | -0.1297 | 0.0453 | -2.865 | 0.007417774 | 0.074158 |
| X214257_s_at | -0.1192 | 0.0416 | -2.865 | 0.007427721 | 0.074224 |
| X208962_s_at | 0.1861 | 0.065 | 2.864 | 0.007442891 | 0.074342 |
| X204875_s_at | -0.0639 | 0.0223 | -2.863 | 0.007458393 | 0.074434 |
| X212195_at | 0.1216 | 0.0425 | 2.863 | 0.007458854 | 0.074434 |
| X211033_s_at | -0.1237 | 0.0432 | -2.863 | 0.007463117 | 0.074444 |
| X214333_x_at | -0.1007 | 0.0352 | -2.862 | 0.00746912 | 0.074469 |
| X203455_s_at | 0.1714 | 0.0599 | 2.862 | 0.007472375 | 0.074469 |
| X48531_at | 0.1723 | 0.0602 | 2.862 | 0.007475991 | 0.074472 |
| X213464_at | 0.1422 | 0.0497 | 2.862 | 0.00748089 | 0.074487 |
| X221249_s_at | -0.1748 | 0.0611 | -2.861 | 0.007490618 | 0.074521 |
| X213326_at | 0.272 | 0.0951 | 2.861 | 0.007492626 | 0.074521 |
| X208995_s_at | 0.201 | 0.0703 | 2.861 | 0.007497754 | 0.074521 |
| X213452_at | -0.2367 | 0.0827 | -2.861 | 0.00749777 | 0.074521 |
| X207891_s_at | -0.1732 | 0.0606 | -2.858 | 0.007560099 | 0.075075 |
| X219028_at | 0.3839 | 0.1343 | 2.858 | 0.007560659 | 0.075075 |
| X204644_at | 0.0194 | 0.0068 | 2.857 | 0.007563609 | 0.075075 |
| X202450_s_at | 0.0346 | 0.0121 | 2.857 | 0.007573962 | 0.07508 |
| X219034_at | 0.0074 | 0.0026 | 2.857 | 0.007574903 | 0.07508 |
| X207801_s_at | 0.103 | 0.0361 | 2.857 | 0.007577534 | 0.07508 |
| X202742_s_at | -0.2367 | 0.0829 | -2.857 | 0.007577577 | 0.07508 |
| X208758_at | -0.0878 | 0.0308 | -2.853 | 0.007639797 | 0.075662 |
| X203560_at | -0.1604 | 0.0562 | -2.852 | 0.007672015 | 0.075916 |
| X217837_s_at | -0.2125 | 0.0745 | -2.852 | 0.007672209 | 0.075916 |
| X213947_s_at | 0.0064 | 0.0022 | 2.851 | 0.007676466 | 0.075924 |
| X213601_at | 0.2159 | 0.0758 | 2.85 | 0.007709807 | 0.07622 |
| X215794_x_at | -0.2199 | 0.0772 | -2.85 | 0.0077135 | 0.076222 |
| X219009_at | 0.0216 | 0.0076 | 2.849 | 0.007732802 | 0.076379 |
| X212815_at | -0.1816 | 0.0638 | -2.848 | 0.007748621 | 0.076501 |
| X202329_at | 0.0414 | 0.0145 | 2.847 | 0.007758982 | 0.076565 |
| X200818_at | -0.0804 | 0.0282 | -2.847 | 0.007761965 | 0.076565 |
| X206249_at | -0.1501 | 0.0527 | -2.846 | 0.007776326 | 0.076673 |
| X204497_at | -0.2247 | 0.079 | -2.845 | 0.007793187 | 0.076774 |
| X210554_s_at | 0.085 | 0.0299 | 2.845 | 0.007794213 | 0.076774 |
| X217499_x_at | 0.2211 | 0.0777 | 2.845 | 0.007798232 | 0.076774 |
| X204871_at | -0.0353 | 0.0124 | -2.845 | 0.007800459 | 0.076774 |
| X208927_at | 0.0957 | 0.0337 | 2.844 | 0.007812578 | 0.076859 |
| X208685_x_at | 0.1705 | 0.06 | 2.844 | 0.007827356 | 0.076971 |
| X215170_s_at | -0.018 | 0.0063 | -2.843 | 0.007840839 | 0.077039 |
| X203905_at | -0.1023 | 0.036 | -2.843 | 0.007841227 | 0.077039 |
| X219481_at | -0.1651 | 0.0581 | -2.842 | 0.007849315 | 0.077084 |
| X201023_at | -0.088 | 0.031 | -2.841 | 0.007880042 | 0.077352 |
| X205871_at | 0.2284 | 0.0804 | 2.841 | 0.007887976 | 0.077394 |
| X222360_at | -0.1793 | 0.0631 | -2.84 | 0.007897126 | 0.077394 |
| X221970_s_at | -0.0758 | 0.0267 | -2.84 | 0.007897799 | 0.077394 |
| X213281_at | 0.0861 | 0.0303 | 2.84 | 0.007900992 | 0.077394 |
| X203352_at | -0.2476 | 0.0872 | -2.84 | 0.007901747 | 0.077394 |
| X78047_s_at | -0.0406 | 0.0143 | -2.84 | 0.007905692 | 0.077396 |
| X221743_at | -0.1015 | 0.0358 | -2.839 | 0.007914349 | 0.077396 |
| X41644_at | 0.1292 | 0.0455 | 2.839 | 0.00792067 | 0.077396 |
| X212317_at | 0.0689 | 0.0243 | 2.839 | 0.007921029 | 0.077396 |
| X207515_s_at | -0.0538 | 0.019 | -2.839 | 0.007923315 | 0.077396 |
| X217553_at | -0.0153 | 0.0054 | -2.839 | 0.007924356 | 0.077396 |
| X202891_at | -0.1117 | 0.0394 | -2.839 | 0.007926342 | 0.077396 |
| X208146_s_at | -0.3986 | 0.1405 | -2.838 | 0.007935164 | 0.077448 |
| X221796_at | 0.1452 | 0.0512 | 2.838 | 0.007942388 | 0.077485 |
| X214710_s_at | -0.0729 | 0.0257 | -2.837 | 0.007959259 | 0.077615 |
| X203282_at | 0.1843 | 0.065 | 2.837 | 0.007966395 | 0.077651 |
| X209291_at | 0.1935 | 0.0682 | 2.836 | 0.00797145 | 0.077654 |
| X212795_at | -0.0969 | 0.0342 | -2.836 | 0.007980774 | 0.077654 |
| X212482_at | -0.1273 | 0.0449 | -2.836 | 0.007981473 | 0.077654 |
| X64418_at | -0.0591 | 0.0209 | -2.836 | 0.007983709 | 0.077654 |
| X204786_s_at | 0.1537 | 0.0542 | 2.835 | 0.0079877 | 0.077654 |
| X222001_x_at | 0.0911 | 0.0321 | 2.835 | 0.007987747 | 0.077654 |
| X202348_s_at | -0.1242 | 0.0438 | -2.835 | 0.007991418 | 0.077656 |
| X207729_at | -0.2566 | 0.0905 | -2.835 | 0.00800131 | 0.077718 |
| X210592_s_at | 0.1625 | 0.0574 | 2.833 | 0.008034505 | 0.078006 |
| X219854_at | -0.1116 | 0.0394 | -2.833 | 0.008043516 | 0.078057 |
| X201465_s_at | 0.8138 | 0.2873 | 2.832 | 0.008046747 | 0.078057 |
| X203403_s_at | -0.1109 | 0.0392 | -2.832 | 0.008061919 | 0.07817 |
| X206762_at | -0.2254 | 0.0796 | -2.832 | 0.00806597 | 0.078175 |
| X212160_at | 0.0789 | 0.0279 | 2.831 | 0.008080181 | 0.078279 |
| X219289_at | -0.1326 | 0.0468 | -2.83 | 0.008095867 | 0.078376 |
| X206051_at | 0.5579 | 0.1971 | 2.83 | 0.008097229 | 0.078376 |
| X200912_s_at | -0.0465 | 0.0164 | -2.83 | 0.008102587 | 0.078393 |
| X203355_s_at | -0.1191 | 0.0421 | -2.829 | 0.008107146 | 0.078403 |
| X220235_s_at | -0.1606 | 0.0568 | -2.829 | 0.008115591 | 0.078451 |
| X219036_at | -0.0713 | 0.0252 | -2.829 | 0.00812463 | 0.078467 |
| X202795_x_at | 0.0708 | 0.025 | 2.828 | 0.008129342 | 0.078467 |
| X202254_at | -0.1383 | 0.0489 | -2.828 | 0.008133386 | 0.078467 |
| X203285_s_at | -0.1638 | 0.0579 | -2.828 | 0.008134119 | 0.078467 |
| X204472_at | 0.5453 | 0.1928 | 2.828 | 0.008134951 | 0.078467 |
| X217329_x_at | -0.0398 | 0.0141 | -2.828 | 0.008141794 | 0.078476 |
| X210813_s_at | -0.286 | 0.1012 | -2.828 | 0.008142935 | 0.078476 |
| X219406_at | -0.1672 | 0.0591 | -2.827 | 0.008152119 | 0.078506 |
| X209362_at | -0.1643 | 0.0581 | -2.827 | 0.008155364 | 0.078506 |
| X214177_s_at | 0.1326 | 0.0469 | 2.827 | 0.008156671 | 0.078506 |
| X212568_s_at | -0.1828 | 0.0647 | -2.827 | 0.008162326 | 0.078527 |
| X216242_x_at | -0.1722 | 0.0609 | -2.826 | 0.008176444 | 0.078628 |
| X202614_at | -0.0982 | 0.0347 | -2.826 | 0.00818211 | 0.078634 |
| X211568_at | 0.3288 | 0.1164 | 2.825 | 0.008187261 | 0.078634 |
| X204924_at | 0.01 | 0.0035 | 2.825 | 0.008187658 | 0.078634 |
| X201014_s_at | 0.1093 | 0.0387 | 2.825 | 0.008195585 | 0.078676 |
| X204230_s_at | 0.5225 | 0.1851 | 2.823 | 0.008244541 | 0.079051 |
| X219217_at | -0.1517 | 0.0537 | -2.823 | 0.008245058 | 0.079051 |
| X202243_s_at | -0.0692 | 0.0245 | -2.823 | 0.008245251 | 0.079051 |
| X214057_at | 0.0182 | 0.0064 | 2.822 | 0.008249256 | 0.079055 |
| X215293_s_at | -0.0509 | 0.018 | -2.822 | 0.008264323 | 0.079165 |
| X215983_s_at | -0.093 | 0.033 | -2.821 | 0.008279802 | 0.079279 |
| X211070_x_at | 0.2011 | 0.0713 | 2.821 | 0.008286528 | 0.079309 |
| X206494_s_at | 0.0063 | 0.0022 | 2.82 | 0.008294863 | 0.079355 |
| X202723_s_at | -0.1096 | 0.0389 | -2.82 | 0.008304914 | 0.079417 |
| X204879_at | 0.0117 | 0.0041 | 2.819 | 0.008311093 | 0.079442 |
| X204935_at | -0.1665 | 0.0591 | -2.819 | 0.008324006 | 0.079489 |
| X201953_at | 0.1529 | 0.0543 | 2.819 | 0.008324809 | 0.079489 |
| X209520_s_at | -0.2338 | 0.0829 | -2.819 | 0.00832681 | 0.079489 |
| X208714_at | -0.1854 | 0.0658 | -2.818 | 0.008343033 | 0.07961 |
| X221692_s_at | -0.1279 | 0.0454 | -2.815 | 0.00839508 | 0.080072 |
| X219957_at | 0.2758 | 0.098 | 2.815 | 0.008399768 | 0.080083 |
| X209284_s_at | -0.1128 | 0.0401 | -2.814 | 0.008421701 | 0.080257 |
| X211025_x_at | -0.0609 | 0.0216 | -2.814 | 0.008430172 | 0.080279 |
| X203304_at | 0.2725 | 0.0968 | 2.814 | 0.008431188 | 0.080279 |
| X216197_at | -0.0328 | 0.0117 | -2.813 | 0.008449757 | 0.080421 |
| X210735_s_at | -0.3909 | 0.139 | -2.812 | 0.008462851 | 0.080478 |
| X204068_at | 0.2368 | 0.0842 | 2.812 | 0.008463029 | 0.080478 |
| X209283_at | 0.3031 | 0.1078 | 2.812 | 0.008470749 | 0.080517 |
| X212007_at | 0.1724 | 0.0613 | 2.811 | 0.008484791 | 0.080584 |
| X218026_at | -0.0642 | 0.0228 | -2.811 | 0.00849086 | 0.080584 |
| X208792_s_at | 0.1439 | 0.0512 | 2.811 | 0.008491769 | 0.080584 |
| X218112_at | -0.1594 | 0.0567 | -2.811 | 0.008492294 | 0.080584 |
| X219437_s_at | 0.0887 | 0.0316 | 2.81 | 0.008499538 | 0.080619 |
| X201042_at | 0.3164 | 0.1127 | 2.809 | 0.008536374 | 0.080933 |
| X213713_s_at | 0.011 | 0.0039 | 2.808 | 0.008543237 | 0.080964 |
| X207169_x_at | 0.2347 | 0.0836 | 2.807 | 0.008565144 | 0.081137 |
| X208678_at | -0.1145 | 0.0408 | -2.807 | 0.008573791 | 0.081184 |
| X208308_s_at | -0.1576 | 0.0562 | -2.806 | 0.008595463 | 0.081355 |
| X203984_s_at | -0.0803 | 0.0286 | -2.805 | 0.008617559 | 0.081497 |
| X218195_at | -0.1062 | 0.0379 | -2.805 | 0.008617852 | 0.081497 |
| X217565_at | 0.3962 | 0.1413 | 2.804 | 0.008629248 | 0.08157 |
| X209281_s_at | -0.2926 | 0.1043 | -2.804 | 0.008636215 | 0.081601 |
| X204355_at | -0.0925 | 0.033 | -2.803 | 0.00864991 | 0.081696 |
| X214749_s_at | 0.1149 | 0.041 | 2.803 | 0.008655875 | 0.081718 |
| X205758_at | -0.2041 | 0.0728 | -2.802 | 0.008667026 | 0.08178 |
| X202180_s_at | 0.23 | 0.0821 | 2.802 | 0.008670307 | 0.08178 |
| X201544_x_at | 0.0569 | 0.0203 | 2.802 | 0.008673577 | 0.08178 |
| X202061_s_at | -0.0843 | 0.0301 | -2.801 | 0.008686252 | 0.081865 |
| X212242_at | 0.196 | 0.07 | 2.799 | 0.008734779 | 0.08227 |
| X205961_s_at | -0.1105 | 0.0395 | -2.799 | 0.008736609 | 0.08227 |
| X201129_at | -0.1166 | 0.0417 | -2.797 | 0.008782199 | 0.082664 |
| X201161_s_at | 0.0232 | 0.0083 | 2.797 | 0.008786762 | 0.082672 |
| X219398_at | -0.0116 | 0.0041 | -2.796 | 0.008798673 | 0.082749 |
| X219587_at | -0.3907 | 0.1398 | -2.795 | 0.008820458 | 0.08291 |
| X205265_s_at | -0.1045 | 0.0374 | -2.795 | 0.008823564 | 0.08291 |
| X203854_at | 0.0337 | 0.0121 | 2.795 | 0.008833534 | 0.08291 |
| X208750_s_at | -0.2995 | 0.1072 | -2.795 | 0.008834659 | 0.08291 |
| X212010_s_at | 0.077 | 0.0275 | 2.794 | 0.008843984 | 0.08291 |
| X214895_s_at | -0.1258 | 0.045 | -2.794 | 0.008844423 | 0.08291 |
| X219866_at | 0.0303 | 0.0108 | 2.794 | 0.008848348 | 0.08291 |
| X219736_at | 0.3831 | 0.1371 | 2.794 | 0.008852415 | 0.08291 |
| X201833_at | -0.0691 | 0.0247 | -2.794 | 0.008855354 | 0.08291 |
| X204984_at | 0.0905 | 0.0324 | 2.794 | 0.008856 | 0.08291 |
| X36830_at | -0.1164 | 0.0417 | -2.794 | 0.008856852 | 0.08291 |
| X207769_s_at | -0.0863 | 0.0309 | -2.793 | 0.00886958 | 0.082983 |
| X214936_at | 0.0213 | 0.0076 | 2.793 | 0.008872139 | 0.082983 |
| X219507_at | 0.1862 | 0.0667 | 2.793 | 0.008880089 | 0.083023 |
| X204560_at | 0.0192 | 0.0069 | 2.792 | 0.008884165 | 0.083026 |
| X217197_x_at | -0.1376 | 0.0493 | -2.792 | 0.008888961 | 0.083036 |
| X221300_at | 0.0074 | 0.0026 | 2.792 | 0.00889889 | 0.08306 |
| X218128_at | -0.0803 | 0.0287 | -2.792 | 0.008899007 | 0.08306 |
| X209376_x_at | 0.2041 | 0.0731 | 2.791 | 0.008911948 | 0.083142 |
| X212418_at | 0.1406 | 0.0504 | 2.791 | 0.008915271 | 0.083142 |
| X221473_x_at | -0.1637 | 0.0587 | -2.791 | 0.008922425 | 0.083174 |
| X212715_s_at | 0.1617 | 0.058 | 2.79 | 0.008929585 | 0.083177 |
| X203000_at | 0.0971 | 0.0348 | 2.79 | 0.008930293 | 0.083177 |
| X217550_at | 0.2783 | 0.0998 | 2.79 | 0.00894345 | 0.083265 |
| X211458_s_at | -0.1262 | 0.0453 | -2.789 | 0.008958069 | 0.083334 |
| X204104_at | -0.0087 | 0.0031 | -2.789 | 0.008958434 | 0.083334 |
| X203614_at | -0.1062 | 0.0381 | -2.789 | 0.008965068 | 0.083361 |
| X208877_at | 0.1409 | 0.0505 | 2.788 | 0.008971456 | 0.083386 |
| X201037_at | 0.1734 | 0.0622 | 2.788 | 0.008983949 | 0.083467 |
| X206590_x_at | -0.2304 | 0.0827 | -2.787 | 0.008992226 | 0.083509 |
| X204566_at | -0.2074 | 0.0744 | -2.787 | 0.009007733 | 0.083618 |
| X213948_x_at | -0.2722 | 0.0977 | -2.785 | 0.009034994 | 0.083836 |
| X37986_at | -0.0291 | 0.0104 | -2.785 | 0.009045963 | 0.083903 |
| X212012_at | 0.1319 | 0.0474 | 2.784 | 0.009068294 | 0.084075 |
| X207266_x_at | -0.1401 | 0.0503 | -2.783 | 0.009084639 | 0.084191 |
| X210183_x_at | 0.0721 | 0.0259 | 2.783 | 0.009091137 | 0.084216 |
| X205689_at | 0.0133 | 0.0048 | 2.782 | 0.009105788 | 0.084317 |
| X205094_at | -0.107 | 0.0385 | -2.782 | 0.009110125 | 0.084322 |
| X219225_at | -0.0994 | 0.0357 | -2.781 | 0.00912537 | 0.084428 |
| X211021_s_at | -0.4404 | 0.1584 | -2.78 | 0.009149429 | 0.084615 |
| X201574_at | -0.0622 | 0.0224 | -2.778 | 0.009191605 | 0.08497 |
| X206101_at | 0.6953 | 0.2503 | 2.778 | 0.009202237 | 0.085033 |
| X215514_at | -0.0884 | 0.0318 | -2.778 | 0.009207372 | 0.085042 |
| X217896_s_at | -0.1066 | 0.0384 | -2.777 | 0.009215705 | 0.085042 |
| X210150_s_at | 0.0148 | 0.0053 | 2.777 | 0.009218088 | 0.085042 |
| X200599_s_at | -0.0851 | 0.0306 | -2.777 | 0.009218574 | 0.085042 |
| X222196_at | -0.0866 | 0.0312 | -2.777 | 0.009224197 | 0.085059 |
| X209044_x_at | 0.013 | 0.0047 | 2.775 | 0.009262331 | 0.085375 |
| X219914_at | 0.0102 | 0.0037 | 2.775 | 0.009274717 | 0.085454 |
| X203118_at | 0.0452 | 0.0163 | 2.774 | 0.009285931 | 0.085512 |
| X205193_at | 0.0419 | 0.0151 | 2.774 | 0.009288759 | 0.085512 |
| X214198_s_at | 0.0818 | 0.0295 | 2.773 | 0.00931689 | 0.085627 |
| X200097_s_at | -0.1102 | 0.0397 | -2.773 | 0.00932019 | 0.085627 |
| X32541_at | 0.1031 | 0.0372 | 2.773 | 0.009320496 | 0.085627 |
| X65472_at | -0.0332 | 0.012 | -2.773 | 0.009323623 | 0.085627 |
| X204727_at | -0.2097 | 0.0756 | -2.773 | 0.009325385 | 0.085627 |
| X216845_x_at | -0.0068 | 0.0025 | -2.772 | 0.009328052 | 0.085627 |
| X202545_at | -0.021 | 0.0076 | -2.772 | 0.009328555 | 0.085627 |
| X215177_s_at | 0.561 | 0.2024 | 2.772 | 0.009332092 | 0.085627 |
| X210501_x_at | -0.0811 | 0.0293 | -2.771 | 0.009353541 | 0.085788 |
| X222015_at | 0.0106 | 0.0038 | 2.771 | 0.009365764 | 0.085865 |
| X220334_at | -0.2022 | 0.073 | -2.77 | 0.009384742 | 0.086004 |
| X218121_at | -0.0382 | 0.0138 | -2.77 | 0.009393519 | 0.086049 |
| X219953_s_at | -0.0771 | 0.0278 | -2.769 | 0.009397412 | 0.086049 |
| X220917_s_at | 0.1479 | 0.0534 | 2.769 | 0.009405401 | 0.086086 |
| X222266_at | 0.1054 | 0.0381 | 2.769 | 0.009418861 | 0.086151 |
| X202181_at | 0.1709 | 0.0617 | 2.768 | 0.00942023 | 0.086151 |
| X214271_x_at | 0.0917 | 0.0331 | 2.768 | 0.009432445 | 0.086196 |
| X213997_at | 0.3902 | 0.141 | 2.768 | 0.009436029 | 0.086196 |
| X213532_at | 0.116 | 0.0419 | 2.768 | 0.009436805 | 0.086196 |
| X210946_at | 0.1643 | 0.0594 | 2.767 | 0.009443247 | 0.08622 |
| X214875_x_at | -0.1929 | 0.0697 | -2.767 | 0.009455904 | 0.0863 |
| X204679_at | -0.1152 | 0.0416 | -2.767 | 0.009464628 | 0.086342 |
| X205850_s_at | -0.0751 | 0.0271 | -2.766 | 0.009468635 | 0.086342 |
| X211112_at | -0.0074 | 0.0027 | -2.766 | 0.009472194 | 0.086342 |
| X213040_s_at | 0.2026 | 0.0733 | 2.766 | 0.009483173 | 0.086368 |
| X209215_at | 0.0296 | 0.0107 | 2.766 | 0.009484727 | 0.086368 |
| X212137_at | 0.0726 | 0.0263 | 2.766 | 0.009486763 | 0.086368 |
| X204058_at | -0.1282 | 0.0464 | -2.765 | 0.009499596 | 0.08645 |
| X221031_s_at | 0.6044 | 0.2186 | 2.765 | 0.009505116 | 0.086465 |
| X202137_s_at | -0.1099 | 0.0397 | -2.764 | 0.009518693 | 0.086553 |
| X37152_at | 0.0877 | 0.0317 | 2.764 | 0.009530679 | 0.086589 |
| X202540_s_at | -0.2281 | 0.0825 | -2.764 | 0.009530839 | 0.086589 |
| X209328_x_at | -0.0334 | 0.0121 | -2.764 | 0.009534327 | 0.086589 |
| X203974_at | -0.1171 | 0.0424 | -2.762 | 0.009567551 | 0.086855 |
| X209093_s_at | -0.1641 | 0.0594 | -2.762 | 0.009581702 | 0.086938 |
| X202802_at | -0.145 | 0.0525 | -2.761 | 0.009586632 | 0.086938 |
| X210822_at | 0.1142 | 0.0414 | 2.761 | 0.009588446 | 0.086938 |
| X213894_at | -0.2144 | 0.0777 | -2.759 | 0.009641749 | 0.087385 |
| X200835_s_at | 0.4349 | 0.1577 | 2.758 | 0.009655709 | 0.087451 |
| X212783_at | 0.1498 | 0.0543 | 2.758 | 0.009656805 | 0.087451 |
| X203226_s_at | -0.0556 | 0.0202 | -2.758 | 0.009667086 | 0.087508 |
| X205003_at | -0.1663 | 0.0603 | -2.757 | 0.00968115 | 0.087566 |
| X203981_s_at | 0.038 | 0.0138 | 2.757 | 0.009681359 | 0.087566 |
| X219767_s_at | -0.1027 | 0.0373 | -2.756 | 0.009708358 | 0.087774 |
| X209539_at | 0.1845 | 0.067 | 2.755 | 0.009725934 | 0.087888 |
| X200736_s_at | 0.2171 | 0.0788 | 2.755 | 0.009728862 | 0.087888 |
| X207198_s_at | 0.1961 | 0.0712 | 2.755 | 0.00973421 | 0.087893 |
| X209268_at | -0.1005 | 0.0365 | -2.755 | 0.009737915 | 0.087893 |
| X204726_at | 0.5167 | 0.1876 | 2.755 | 0.009742443 | 0.087893 |
| X219241_x_at | 0.0126 | 0.0046 | 2.755 | 0.00974522 | 0.087893 |
| X204009_s_at | -0.0991 | 0.036 | -2.754 | 0.009760679 | 0.087965 |
| X204200_s_at | -0.0457 | 0.0166 | -2.754 | 0.009761094 | 0.087965 |
| X200957_s_at | -0.0695 | 0.0252 | -2.754 | 0.009767672 | 0.087988 |
| X212487_at | 0.3403 | 0.1236 | 2.753 | 0.009778229 | 0.088028 |
| X211115_x_at | -0.2013 | 0.0731 | -2.753 | 0.009779972 | 0.088028 |
| X204324_s_at | 0.4072 | 0.1479 | 2.753 | 0.009789858 | 0.088081 |
| X218285_s_at | 0.2034 | 0.0739 | 2.752 | 0.009798526 | 0.088123 |
| X218439_s_at | -0.1814 | 0.0659 | -2.752 | 0.009803239 | 0.088129 |
| X206425_s_at | -0.2834 | 0.103 | -2.752 | 0.009810266 | 0.088129 |
| X214508_x_at | -0.1354 | 0.0492 | -2.752 | 0.009811322 | 0.088129 |
| X221736_at | -0.0981 | 0.0356 | -2.752 | 0.009815058 | 0.088129 |
| X205480_s_at | -0.1383 | 0.0503 | -2.749 | 0.009885125 | 0.0887 |
| X202223_at | -0.1175 | 0.0427 | -2.749 | 0.009886644 | 0.0887 |
| X222250_s_at | -0.2745 | 0.0999 | -2.748 | 0.009893945 | 0.08873 |
| X213198_at | 0.1281 | 0.0466 | 2.748 | 0.009914826 | 0.088876 |
| X213263_s_at | 0.0937 | 0.0341 | 2.747 | 0.009918188 | 0.088876 |
| X217942_at | -0.1351 | 0.0492 | -2.747 | 0.009932566 | 0.088945 |
| X208120_x_at | 0.1106 | 0.0402 | 2.747 | 0.009933891 | 0.088945 |
| X201951_at | -0.1017 | 0.037 | -2.746 | 0.009953751 | 0.08907 |
| X202365_at | 0.1252 | 0.0456 | 2.746 | 0.009955888 | 0.08907 |
| X210147_at | -0.265 | 0.0965 | -2.745 | 0.00997247 | 0.089149 |
| X205186_at | 0.0171 | 0.0062 | 2.745 | 0.00997276 | 0.089149 |
| X214217_at | -0.1816 | 0.0662 | -2.744 | 0.009996642 | 0.089326 |
| X204554_at | 0.2059 | 0.0751 | 2.743 | 0.01002734 | 0.089565 |
| X214277_at | -0.211 | 0.0769 | -2.742 | 0.01004058 | 0.089647 |
| X208490_x_at | -0.023 | 0.0084 | -2.742 | 0.01005137 | 0.089707 |
| X212743_at | -0.071 | 0.0259 | -2.741 | 0.01008581 | 0.089978 |
| X202655_at | -0.1497 | 0.0546 | -2.74 | 0.01010191 | 0.090082 |
| X217918_at | -0.062 | 0.0226 | -2.74 | 0.01010826 | 0.090082 |
| X204745_x_at | 0.2883 | 0.1052 | 2.74 | 0.01010959 | 0.090082 |
| X215143_at | -0.1443 | 0.0527 | -2.739 | 0.01011774 | 0.090118 |
| X217135_x_at | -0.0277 | 0.0101 | -2.739 | 0.01012192 | 0.09012 |
| X206123_at | -0.0201 | 0.0073 | -2.738 | 0.01013907 | 0.090236 |
| X221362_at | -0.0173 | 0.0063 | -2.738 | 0.01014873 | 0.090273 |
| X200804_at | 0.0705 | 0.0258 | 2.738 | 0.01015145 | 0.090273 |
| X217044_s_at | 0.0054 | 0.002 | 2.738 | 0.01015542 | 0.090273 |
| X214878_at | -0.0564 | 0.0206 | -2.737 | 0.01017596 | 0.090371 |
| X200965_s_at | -0.0924 | 0.0338 | -2.737 | 0.01017683 | 0.090371 |
| X221933_at | -0.1002 | 0.0366 | -2.737 | 0.0101786 | 0.090371 |
| X202372_at | -0.0918 | 0.0335 | -2.736 | 0.01018693 | 0.090409 |
| X214870_x_at | 0.1653 | 0.0604 | 2.736 | 0.01020241 | 0.090474 |
| X215296_at | 0.0315 | 0.0115 | 2.736 | 0.01020242 | 0.090474 |
| X203620_s_at | 0.1566 | 0.0572 | 2.735 | 0.01021225 | 0.090525 |
| X218655_s_at | -0.1569 | 0.0574 | -2.735 | 0.01023306 | 0.090673 |
| X217996_at | -0.2423 | 0.0886 | -2.734 | 0.01025034 | 0.09079 |
| X214314_s_at | 0.1451 | 0.0531 | 2.733 | 0.01028216 | 0.091006 |
| X202387_at | -0.1158 | 0.0424 | -2.733 | 0.01028289 | 0.091006 |
| X208017_s_at | -0.1193 | 0.0437 | -2.732 | 0.01030632 | 0.091177 |
| X202427_s_at | -0.0951 | 0.0348 | -2.731 | 0.01031833 | 0.091247 |
| X221918_at | -0.109 | 0.0399 | -2.73 | 0.01034594 | 0.091454 |
| X207707_s_at | -0.0912 | 0.0334 | -2.73 | 0.01035077 | 0.091461 |
| X219491_at | 0.0573 | 0.021 | 2.73 | 0.01035515 | 0.091463 |
| X203548_s_at | -0.3352 | 0.1229 | -2.729 | 0.01038605 | 0.091653 |
| X203586_s_at | -0.1673 | 0.0613 | -2.728 | 0.01038644 | 0.091653 |
| X218304_s_at | 0.2272 | 0.0833 | 2.728 | 0.01038908 | 0.091653 |
| X218138_at | -0.0999 | 0.0367 | -2.727 | 0.01042931 | 0.091941 |
| X35617_at | 0.0095 | 0.0035 | 2.727 | 0.01042996 | 0.091941 |
| X216910_at | -0.0129 | 0.0047 | -2.726 | 0.01045388 | 0.092115 |
| X205168_at | 0.0128 | 0.0047 | 2.724 | 0.01048996 | 0.092397 |
| X212395_s_at | -0.0595 | 0.0218 | -2.724 | 0.01051153 | 0.09255 |
| X206308_at | -0.1229 | 0.0451 | -2.723 | 0.01052265 | 0.092611 |
| X209437_s_at | -0.3296 | 0.1211 | -2.723 | 0.01053549 | 0.092687 |
| X218351_at | -0.1062 | 0.039 | -2.722 | 0.01055136 | 0.092767 |
| X57703_at | -0.0107 | 0.0039 | -2.722 | 0.01055288 | 0.092767 |
| X201151_s_at | -0.148 | 0.0544 | -2.722 | 0.01056049 | 0.092797 |
| X212089_at | 0.0139 | 0.0051 | 2.721 | 0.01057316 | 0.092872 |
| X218627_at | 0.0372 | 0.0137 | 2.72 | 0.01059443 | 0.093022 |
| X202613_at | -0.2471 | 0.0908 | -2.72 | 0.01060671 | 0.093093 |
| X211936_at | -0.1095 | 0.0403 | -2.72 | 0.01061366 | 0.093117 |
| X39650_s_at | 0.1773 | 0.0652 | 2.719 | 0.01062013 | 0.093137 |
| X200995_at | 0.1115 | 0.041 | 2.719 | 0.01063771 | 0.093254 |
| X210315_at | -0.4257 | 0.1566 | -2.718 | 0.01065467 | 0.09333 |
| X208833_s_at | -0.1161 | 0.0427 | -2.718 | 0.01065472 | 0.09333 |
| X217936_at | 0.1319 | 0.0485 | 2.717 | 0.01067515 | 0.093472 |
| X214893_x_at | -0.0142 | 0.0052 | -2.717 | 0.0106905 | 0.093569 |
| X216607_s_at | -0.216 | 0.0795 | -2.716 | 0.01069798 | 0.093598 |
| X218771_at | -0.1286 | 0.0474 | -2.716 | 0.01070944 | 0.093661 |
| X202602_s_at | -0.0744 | 0.0274 | -2.716 | 0.01071823 | 0.093701 |
| X219770_at | 0.1258 | 0.0463 | 2.715 | 0.01072585 | 0.093731 |
| X204552_at | -0.181 | 0.0667 | -2.715 | 0.0107303 | 0.093733 |
| X47069_at | 0.0547 | 0.0202 | 2.714 | 0.01076347 | 0.093986 |
| X214295_at | 0.1407 | 0.0519 | 2.713 | 0.01079681 | 0.094174 |
| X221573_at | -0.0742 | 0.0274 | -2.712 | 0.01079924 | 0.094174 |
| X212460_at | 0.1913 | 0.0705 | 2.712 | 0.01080107 | 0.094174 |
| X209289_at | 0.1643 | 0.0606 | 2.712 | 0.01080199 | 0.094174 |
| X219564_at | 0.5114 | 0.1886 | 2.712 | 0.01080932 | 0.094182 |
| X205631_at | 0.0567 | 0.0209 | 2.712 | 0.01081142 | 0.094182 |
| X201871_s_at | -0.0732 | 0.027 | -2.711 | 0.01083647 | 0.094364 |
| X213922_at | 0.1427 | 0.0526 | 2.711 | 0.01084351 | 0.094382 |
| X200851_s_at | 0.074 | 0.0273 | 2.711 | 0.01084753 | 0.094382 |
| X218100_s_at | 0.1388 | 0.0512 | 2.71 | 0.01085375 | 0.094382 |
| X204478_s_at | -0.1484 | 0.0548 | -2.71 | 0.01085621 | 0.094382 |
| X203194_s_at | -0.0062 | 0.0023 | -2.71 | 0.01086004 | 0.094382 |
| X46947_at | -0.0595 | 0.022 | -2.71 | 0.01086405 | 0.094382 |
| X216112_at | 0.1008 | 0.0372 | 2.71 | 0.01087438 | 0.094435 |
| X201678_s_at | -0.0902 | 0.0333 | -2.709 | 0.01088661 | 0.094504 |
| X213352_at | -0.1429 | 0.0528 | -2.708 | 0.01090399 | 0.094618 |
| X218597_s_at | -0.0705 | 0.026 | -2.708 | 0.01091938 | 0.094714 |
| X200008_s_at | -0.1506 | 0.0556 | -2.708 | 0.0109263 | 0.094737 |
| X218408_at | -0.1464 | 0.0541 | -2.707 | 0.01094982 | 0.094904 |
| X202132_at | 0.1748 | 0.0646 | 2.706 | 0.01096907 | 0.095034 |
| X216080_s_at | 0.133 | 0.0492 | 2.706 | 0.01097708 | 0.095065 |
| X208009_s_at | -0.0117 | 0.0043 | -2.706 | 0.01098121 | 0.095065 |
| X210046_s_at | -0.0803 | 0.0297 | -2.705 | 0.01100867 | 0.095266 |
| X201671_x_at | -0.163 | 0.0603 | -2.704 | 0.01101796 | 0.095308 |
| X222212_s_at | 0.2175 | 0.0804 | 2.704 | 0.01102219 | 0.095308 |
| X204979_s_at | -0.1224 | 0.0453 | -2.703 | 0.01103871 | 0.095414 |
| X635_s_at | 0.1488 | 0.0551 | 2.703 | 0.01105519 | 0.095483 |
| X217608_at | -0.1698 | 0.0628 | -2.703 | 0.01105535 | 0.095483 |
| X214116_at | -0.0204 | 0.0076 | -2.702 | 0.01107439 | 0.095575 |
| X210974_s_at | -0.0402 | 0.0149 | -2.702 | 0.0110768 | 0.095575 |
| X212055_at | 0.1352 | 0.05 | 2.702 | 0.01107957 | 0.095575 |
| X204389_at | 0.158 | 0.0585 | 2.702 | 0.01108449 | 0.095575 |
| X179_at | 0.0053 | 0.002 | 2.702 | 0.01108742 | 0.095575 |
| X211067_s_at | -0.244 | 0.0903 | -2.701 | 0.01109881 | 0.095636 |
| X222243_s_at | 0.1881 | 0.0696 | 2.701 | 0.01111443 | 0.095733 |
| X218557_at | -0.2707 | 0.1003 | -2.7 | 0.01113415 | 0.095866 |
| X202416_at | -0.1145 | 0.0424 | -2.7 | 0.011141 | 0.095868 |
| X219631_at | -0.1309 | 0.0485 | -2.7 | 0.011143 | 0.095868 |
| X203360_s_at | 0.0142 | 0.0053 | 2.698 | 0.01118346 | 0.096171 |
| X203997_at | 0.0133 | 0.0049 | 2.698 | 0.01118686 | 0.096171 |
| X209155_s_at | 0.095 | 0.0352 | 2.698 | 0.01119403 | 0.096195 |
| X213305_s_at | 0.1934 | 0.0717 | 2.697 | 0.01119987 | 0.096208 |
| X215248_at | 0.3098 | 0.1149 | 2.697 | 0.01120491 | 0.096211 |
| X212057_at | 0.3321 | 0.1231 | 2.697 | 0.01120893 | 0.096211 |
| X213595_s_at | 0.0323 | 0.012 | 2.697 | 0.01121405 | 0.096218 |
| X219771_at | -0.1258 | 0.0466 | -2.696 | 0.01122646 | 0.096258 |
| X218259_at | -0.1228 | 0.0456 | -2.696 | 0.0112273 | 0.096258 |
| X208670_s_at | -0.1103 | 0.0409 | -2.696 | 0.0112464 | 0.096382 |
| X213549_at | -0.0845 | 0.0313 | -2.696 | 0.01125051 | 0.096382 |
| X217835_x_at | -0.0704 | 0.0261 | -2.695 | 0.0112556 | 0.096389 |
| X200786_at | -0.0673 | 0.025 | -2.695 | 0.01126936 | 0.096469 |
| X205705_at | -0.1993 | 0.0739 | -2.695 | 0.01127659 | 0.096494 |
| X207627_s_at | -0.2426 | 0.0901 | -2.693 | 0.01130716 | 0.096718 |
| X206989_s_at | 0.1133 | 0.0421 | 2.693 | 0.01131158 | 0.096719 |
| X217862_at | -0.1196 | 0.0444 | -2.693 | 0.01131777 | 0.096734 |
| X212917_x_at | 0.1066 | 0.0396 | 2.692 | 0.0113471 | 0.096948 |
| X205378_s_at | -0.0109 | 0.0041 | -2.691 | 0.01137833 | 0.097177 |
| X211251_x_at | -0.1361 | 0.0506 | -2.689 | 0.01142824 | 0.097566 |
| X209497_s_at | 0.0306 | 0.0114 | 2.689 | 0.01143326 | 0.097571 |
| X220335_x_at | 0.0164 | 0.0061 | 2.688 | 0.01145558 | 0.097724 |
| X217906_at | -0.0756 | 0.0281 | -2.688 | 0.01146906 | 0.097773 |
| X221263_s_at | -0.1531 | 0.057 | -2.687 | 0.01147384 | 0.097773 |
| X210519_s_at | -0.1841 | 0.0685 | -2.687 | 0.01147443 | 0.097773 |
| X214459_x_at | 0.19 | 0.0707 | 2.686 | 0.01150385 | 0.097986 |
| X204194_at | 0.1292 | 0.0481 | 2.684 | 0.01155881 | 0.098353 |
| X202055_at | -0.068 | 0.0253 | -2.684 | 0.01155995 | 0.098353 |
| X201560_at | 0.255 | 0.095 | 2.684 | 0.01156029 | 0.098353 |
| X202863_at | 0.2109 | 0.0786 | 2.684 | 0.01158057 | 0.098488 |
| X212125_at | -0.2226 | 0.083 | -2.682 | 0.01161707 | 0.098745 |
| X200979_at | -0.1102 | 0.0411 | -2.682 | 0.0116211 | 0.098745 |
| X214784_x_at | 0.0175 | 0.0065 | 2.682 | 0.01162413 | 0.098745 |
| X209409_at | -0.1616 | 0.0603 | -2.681 | 0.01164352 | 0.098872 |
| X217408_at | -0.08 | 0.0299 | -2.681 | 0.01165653 | 0.098945 |
| X207483_s_at | -0.1143 | 0.0426 | -2.68 | 0.01167161 | 0.099035 |
| X221961_at | 0.0172 | 0.0064 | 2.68 | 0.0116955 | 0.0992 |
| X213850_s_at | 0.1949 | 0.0727 | 2.679 | 0.01170081 | 0.099207 |
| X213411_at | -0.09 | 0.0336 | -2.679 | 0.01171308 | 0.099273 |
| X202587_s_at | 0.2139 | 0.0799 | 2.679 | 0.01172004 | 0.099294 |
| X210364_at | -0.1997 | 0.0746 | -2.677 | 0.01175387 | 0.099543 |
| X219116_s_at | 0.0259 | 0.0097 | 2.677 | 0.01176475 | 0.099597 |
| X201529_s_at | -0.1354 | 0.0506 | -2.676 | 0.01178834 | 0.099759 |
| X203391_at | -0.126 | 0.0471 | -2.675 | 0.01181044 | 0.099908 |
| X202543_s_at | 0.2508 | 0.0938 | 2.675 | 0.01181862 | 0.099939 |
| X217734_s_at | 0.179 | 0.0669 | 2.675 | 0.01182489 | 0.099954 |
| X212428_at | -0.145 | 0.0542 | -2.675 | 0.01183345 | 0.099988 |
| X209339_at | -0.1965 | 0.0735 | -2.674 | 0.01185002 | 0.10009 |
| X209487_at | 0.2826 | 0.1057 | 2.673 | 0.01186806 | 0.100204 |
| X218079_s_at | -0.0714 | 0.0267 | -2.671 | 0.01194145 | 0.100747 |
| X204137_at | -0.1251 | 0.0469 | -2.671 | 0.01194147 | 0.100747 |
| X219548_at | -0.0375 | 0.014 | -2.671 | 0.01195106 | 0.100757 |
| X219370_at | 0.4772 | 0.1787 | 2.671 | 0.0119517 | 0.100757 |
| X213803_at | -0.1021 | 0.0383 | -2.67 | 0.01197668 | 0.10086 |
| X203597_s_at | 0.104 | 0.039 | 2.67 | 0.01197695 | 0.10086 |
| X213843_x_at | 0.2573 | 0.0964 | 2.67 | 0.01198069 | 0.10086 |
| X212154_at | -0.2112 | 0.0791 | -2.669 | 0.01198442 | 0.10086 |
| X201891_s_at | 0.12 | 0.0449 | 2.669 | 0.01198662 | 0.10086 |
| X214101_s_at | -0.1488 | 0.0558 | -2.669 | 0.01199181 | 0.100866 |
| X218466_at | -0.0191 | 0.0072 | -2.668 | 0.01201384 | 0.101008 |
| X210306_at | 0.1844 | 0.0691 | 2.668 | 0.01202112 | 0.101008 |
| X219042_at | -0.2559 | 0.0959 | -2.668 | 0.01202325 | 0.101008 |
| X207467_x_at | 0.1603 | 0.0601 | 2.668 | 0.01202695 | 0.101008 |
| X206662_at | 0.0618 | 0.0232 | 2.668 | 0.01203714 | 0.101042 |
| X212196_at | 0.1787 | 0.067 | 2.667 | 0.01204563 | 0.101042 |
| X209053_s_at | 0.0304 | 0.0114 | 2.667 | 0.01204788 | 0.101042 |
| X209794_at | 0.1607 | 0.0602 | 2.667 | 0.01204922 | 0.101042 |
| X203092_at | -0.0439 | 0.0165 | -2.666 | 0.01206889 | 0.101169 |
| X202267_at | -0.3054 | 0.1145 | -2.666 | 0.01208417 | 0.101259 |
| X203050_at | -0.1364 | 0.0512 | -2.666 | 0.01209277 | 0.101293 |
| X221886_at | 0.2048 | 0.0768 | 2.665 | 0.01210398 | 0.101349 |
| X220254_at | -0.2755 | 0.1034 | -2.665 | 0.01212441 | 0.101481 |
| X200046_at | -0.1057 | 0.0397 | -2.664 | 0.01213865 | 0.101562 |
| X40569_at | 0.0931 | 0.035 | 2.664 | 0.01214441 | 0.101572 |
| X222010_at | -0.1103 | 0.0414 | -2.663 | 0.01216716 | 0.101724 |
| X215023_s_at | -0.1855 | 0.0697 | -2.662 | 0.0121962 | 0.10188 |
| X217867_x_at | 0.0107 | 0.004 | 2.662 | 0.01220416 | 0.10188 |
| X215290_at | 0.2901 | 0.109 | 2.662 | 0.01220577 | 0.10188 |
| X209455_at | -0.0365 | 0.0137 | -2.662 | 0.01221058 | 0.10188 |
| X203141_s_at | 0.2851 | 0.1071 | 2.662 | 0.01221239 | 0.10188 |
| X202560_s_at | -0.0827 | 0.0311 | -2.662 | 0.01221336 | 0.10188 |
| X208998_at | 0.3808 | 0.1431 | 2.661 | 0.01222895 | 0.101949 |
| X201637_s_at | 0.1259 | 0.0473 | 2.661 | 0.01223072 | 0.101949 |
| X208065_at | -0.3809 | 0.1432 | -2.661 | 0.0122405 | 0.101992 |
| X203197_s_at | -0.1154 | 0.0434 | -2.66 | 0.01225891 | 0.102107 |
| X209196_at | -0.081 | 0.0304 | -2.66 | 0.01226741 | 0.10214 |
| X220007_at | -0.1519 | 0.0571 | -2.659 | 0.01228436 | 0.102242 |
| X209290_s_at | 0.1256 | 0.0472 | 2.659 | 0.01229457 | 0.102289 |
| X214048_at | 0.1592 | 0.0599 | 2.659 | 0.0123024 | 0.102316 |
| X211170_s_at | -0.1083 | 0.0408 | -2.658 | 0.01231546 | 0.102386 |
| X200798_x_at | 0.3435 | 0.1292 | 2.658 | 0.01233139 | 0.10248 |
| X208809_s_at | 0.0864 | 0.0325 | 2.657 | 0.01235677 | 0.102641 |
| X209391_at | -0.0944 | 0.0355 | -2.657 | 0.01235995 | 0.102641 |
| X210762_s_at | -0.2168 | 0.0816 | -2.656 | 0.01238693 | 0.102815 |
| X218125_s_at | -0.1008 | 0.0379 | -2.656 | 0.01239108 | 0.102815 |
| X206860_s_at | -0.1357 | 0.0511 | -2.655 | 0.01239484 | 0.102815 |
| X209859_at | 0.1213 | 0.0457 | 2.655 | 0.01240552 | 0.102865 |
| X91703_at | -0.0061 | 0.0023 | -2.654 | 0.01243523 | 0.103028 |
| X207629_s_at | -0.2052 | 0.0773 | -2.654 | 0.01243833 | 0.103028 |
| X204070_at | 0.2831 | 0.1067 | 2.654 | 0.01244073 | 0.103028 |
| X212175_s_at | 0.0593 | 0.0224 | 2.654 | 0.01244374 | 0.103028 |
| X212353_at | 0.486 | 0.1832 | 2.653 | 0.01248068 | 0.103269 |
| X200626_s_at | -0.0762 | 0.0287 | -2.653 | 0.01248211 | 0.103269 |
| X220025_at | 0.0128 | 0.0048 | 2.652 | 0.0125054 | 0.103423 |
| X206573_at | -0.027 | 0.0102 | -2.651 | 0.01251762 | 0.103486 |
| X222155_s_at | 0.0059 | 0.0022 | 2.651 | 0.01253289 | 0.103566 |
| X217574_at | -0.1946 | 0.0734 | -2.651 | 0.01253662 | 0.103566 |
| X208660_at | -0.0745 | 0.0281 | -2.65 | 0.01254955 | 0.103634 |
| X219505_at | 0.054 | 0.0204 | 2.65 | 0.0125713 | 0.103775 |
| X204848_x_at | -0.0123 | 0.0046 | -2.649 | 0.01258269 | 0.103823 |
| X218496_at | 0.1053 | 0.0398 | 2.649 | 0.01259171 | 0.103823 |
| X205662_at | -0.1658 | 0.0626 | -2.649 | 0.01259554 | 0.103823 |
| X200946_x_at | -0.1634 | 0.0617 | -2.648 | 0.01260739 | 0.103823 |
| X209413_at | 0.0492 | 0.0186 | 2.648 | 0.01260817 | 0.103823 |
| X211993_at | 0.3368 | 0.1272 | 2.648 | 0.01261044 | 0.103823 |
| X200906_s_at | 0.162 | 0.0612 | 2.648 | 0.01261771 | 0.103823 |
| X214394_x_at | 0.1332 | 0.0503 | 2.648 | 0.0126184 | 0.103823 |
| X216264_s_at | 0.0048 | 0.0018 | 2.648 | 0.01261917 | 0.103823 |
| X213710_s_at | -0.369 | 0.1394 | -2.647 | 0.01263972 | 0.103954 |
| X209820_s_at | -0.0721 | 0.0273 | -2.647 | 0.01266287 | 0.104106 |
| X201254_x_at | 0.0593 | 0.0224 | 2.646 | 0.01268803 | 0.104235 |
| X201857_at | -0.0626 | 0.0237 | -2.646 | 0.01268806 | 0.104235 |
| X222122_s_at | 0.081 | 0.0306 | 2.644 | 0.01273347 | 0.10457 |
| X207782_s_at | -0.1301 | 0.0492 | -2.643 | 0.01277864 | 0.104902 |
| X201862_s_at | 0.1579 | 0.0598 | 2.642 | 0.01279559 | 0.105002 |
| X206611_at | 0.0238 | 0.009 | 2.642 | 0.01281008 | 0.105069 |
| X203686_at | -0.1162 | 0.044 | -2.642 | 0.01281316 | 0.105069 |
| X218526_s_at | -0.1494 | 0.0566 | -2.641 | 0.01282243 | 0.105106 |
| X210154_at | -0.2401 | 0.0909 | -2.641 | 0.01282757 | 0.105109 |
| X210317_s_at | -0.1041 | 0.0394 | -2.641 | 0.01283942 | 0.105168 |
| X213074_at | 0.0916 | 0.0347 | 2.641 | 0.01284685 | 0.10519 |
| X37586_at | 0.0154 | 0.0059 | 2.64 | 0.01286331 | 0.105277 |
| X212086_x_at | 0.1293 | 0.049 | 2.64 | 0.01287059 | 0.105277 |
| X207370_at | -0.0207 | 0.0079 | -2.64 | 0.01287172 | 0.105277 |
| X212566_at | 0.1805 | 0.0684 | 2.638 | 0.01291043 | 0.105555 |
| X213794_s_at | -0.0612 | 0.0232 | -2.638 | 0.01293019 | 0.105649 |
| X219607_s_at | 0.2837 | 0.1076 | 2.638 | 0.0129315 | 0.105649 |
| X213506_at | 0.3919 | 0.1486 | 2.637 | 0.01294612 | 0.10573 |
| X202245_at | 0.1578 | 0.0598 | 2.637 | 0.01296078 | 0.105811 |
| X214940_s_at | 0.0239 | 0.0091 | 2.636 | 0.01297524 | 0.10589 |
| X203744_at | 0.1 | 0.0379 | 2.635 | 0.01301278 | 0.106157 |
| X218141_at | 0.0902 | 0.0343 | 2.634 | 0.01303887 | 0.106331 |
| X212620_at | 0.1275 | 0.0484 | 2.634 | 0.01304944 | 0.106378 |
| X221491_x_at | 0.1699 | 0.0645 | 2.633 | 0.01307854 | 0.106576 |
| X201500_s_at | -0.0872 | 0.0331 | -2.633 | 0.01308994 | 0.106588 |
| X205545_x_at | -0.0681 | 0.0259 | -2.633 | 0.01309182 | 0.106588 |
| X212387_at | 0.1795 | 0.0682 | 2.633 | 0.0130944 | 0.106588 |
| X219251_s_at | 0.0798 | 0.0303 | 2.632 | 0.01311708 | 0.106734 |
| X211136_s_at | -0.1293 | 0.0491 | -2.632 | 0.01312663 | 0.106772 |
| X221598_s_at | -0.1858 | 0.0706 | -2.631 | 0.01314403 | 0.106874 |
| X208425_s_at | -0.1456 | 0.0554 | -2.631 | 0.01314871 | 0.106874 |
| X203485_at | 0.0682 | 0.0259 | 2.63 | 0.01317737 | 0.107067 |
| X222037_at | -0.012 | 0.0046 | -2.629 | 0.0132016 | 0.107225 |
| X210016_at | -0.1294 | 0.0492 | -2.629 | 0.01320828 | 0.10724 |
| X201390_s_at | -0.0825 | 0.0314 | -2.628 | 0.01322773 | 0.107359 |
| X214273_x_at | 0.0647 | 0.0246 | 2.627 | 0.01327968 | 0.107741 |
| X211159_s_at | -0.1245 | 0.0474 | -2.626 | 0.01329222 | 0.107803 |
| X208828_at | -0.1367 | 0.0521 | -2.625 | 0.01333314 | 0.108096 |
| X204426_at | -0.2161 | 0.0823 | -2.625 | 0.01334722 | 0.108166 |
| X202085_at | 0.311 | 0.1185 | 2.624 | 0.01335156 | 0.108166 |
| X203045_at | 0.0488 | 0.0186 | 2.624 | 0.01336114 | 0.108204 |
| X208286_x_at | 0.0084 | 0.0032 | 2.624 | 0.01336969 | 0.108234 |
| X213302_at | -0.1085 | 0.0414 | -2.623 | 0.01339983 | 0.108439 |
| X220768_s_at | -0.2311 | 0.0881 | -2.622 | 0.0134245 | 0.108599 |
| X205209_at | 0.1941 | 0.074 | 2.622 | 0.01343238 | 0.108623 |
| X211498_s_at | -0.0077 | 0.0029 | -2.621 | 0.01344755 | 0.108706 |
| X218624_s_at | 0.1329 | 0.0507 | 2.621 | 0.01345916 | 0.10876 |
| X201887_at | 0.3323 | 0.1268 | 2.62 | 0.01350295 | 0.109074 |
| X203397_s_at | 0.0916 | 0.035 | 2.619 | 0.0135197 | 0.109163 |
| X214666_x_at | -0.0619 | 0.0236 | -2.619 | 0.01352369 | 0.109163 |
| X211985_s_at | -0.0966 | 0.0369 | -2.619 | 0.01354011 | 0.109243 |
| X221677_s_at | -0.1042 | 0.0398 | -2.618 | 0.01354343 | 0.109243 |
| X201274_at | -0.1032 | 0.0394 | -2.618 | 0.01356706 | 0.109376 |
| X210552_s_at | -0.0474 | 0.0181 | -2.618 | 0.01357388 | 0.109376 |
| X219738_s_at | 0.1714 | 0.0655 | 2.617 | 0.01357481 | 0.109376 |
| X211658_at | -0.0414 | 0.0158 | -2.617 | 0.01358528 | 0.109421 |
| X214538_x_at | -0.0099 | 0.0038 | -2.617 | 0.0135959 | 0.109467 |
| X221515_s_at | -0.0996 | 0.0381 | -2.616 | 0.01362139 | 0.109621 |
| X217853_at | -0.2215 | 0.0847 | -2.616 | 0.01362489 | 0.109621 |
| X221500_s_at | 0.0577 | 0.022 | 2.616 | 0.01363891 | 0.109694 |
| X201822_at | -0.1076 | 0.0412 | -2.615 | 0.01365198 | 0.109759 |
| X216308_x_at | -0.0549 | 0.021 | -2.614 | 0.0136783 | 0.109908 |
| X212930_at | -0.2512 | 0.0961 | -2.614 | 0.0136803 | 0.109908 |
| X209499_x_at | -0.257 | 0.0983 | -2.614 | 0.0136884 | 0.109919 |
| X207058_s_at | -0.1347 | 0.0515 | -2.614 | 0.01369159 | 0.109919 |
| X205475_at | 0.2225 | 0.0851 | 2.613 | 0.01370484 | 0.10993 |
| X212675_s_at | -0.0591 | 0.0226 | -2.613 | 0.0137057 | 0.10993 |
| X207455_at | -0.2993 | 0.1145 | -2.613 | 0.0137101 | 0.10993 |
| X220677_s_at | -0.4195 | 0.1605 | -2.613 | 0.01371277 | 0.10993 |
| X208030_s_at | 0.0526 | 0.0201 | 2.613 | 0.01372647 | 0.109936 |
| X208963_x_at | 0.1672 | 0.064 | 2.613 | 0.01372766 | 0.109936 |
| X204125_at | -0.0975 | 0.0373 | -2.613 | 0.01372835 | 0.109936 |
| X218980_at | 0.1728 | 0.0661 | 2.613 | 0.01373726 | 0.109964 |
| X209316_s_at | -0.0982 | 0.0376 | -2.612 | 0.0137418 | 0.109964 |
| X202513_s_at | -0.1146 | 0.0439 | -2.612 | 0.01376312 | 0.110095 |
| X204943_at | -0.0103 | 0.0039 | -2.612 | 0.0137688 | 0.110101 |
| X220450_at | -0.3243 | 0.1242 | -2.611 | 0.01379341 | 0.110258 |
| X201319_at | 0.1501 | 0.0575 | 2.61 | 0.01381546 | 0.110395 |
| X204372_s_at | 0.0837 | 0.0321 | 2.61 | 0.01382618 | 0.11044 |
| X201996_s_at | 0.2386 | 0.0915 | 2.608 | 0.01388006 | 0.110831 |
| X208883_at | -0.1184 | 0.0454 | -2.607 | 0.01391747 | 0.11109 |
| X216682_s_at | 0.0575 | 0.0221 | 2.607 | 0.01393293 | 0.111173 |
| X218530_at | -0.0167 | 0.0064 | -2.606 | 0.01394446 | 0.111214 |
| X220253_s_at | -0.325 | 0.1247 | -2.606 | 0.01394801 | 0.111214 |
| X219331_s_at | -0.0108 | 0.0041 | -2.605 | 0.01398034 | 0.111431 |
| X221847_at | -0.0772 | 0.0296 | -2.604 | 0.014007 | 0.111581 |
| X202506_at | 0.147 | 0.0564 | 2.604 | 0.01400916 | 0.111581 |
| X202241_at | -0.2977 | 0.1143 | -2.604 | 0.01401467 | 0.111585 |
| X201635_s_at | 0.209 | 0.0803 | 2.604 | 0.01403529 | 0.111709 |
| X202766_s_at | 0.0433 | 0.0166 | 2.603 | 0.01404146 | 0.111718 |
| X208779_x_at | 0.1812 | 0.0696 | 2.603 | 0.01405563 | 0.111791 |
| X222043_at | 0.1787 | 0.0687 | 2.602 | 0.01406989 | 0.111864 |
| X202078_at | -0.0659 | 0.0253 | -2.602 | 0.01408237 | 0.111887 |
| X203023_at | -0.1864 | 0.0716 | -2.602 | 0.01408288 | 0.111887 |
| X216167_at | -0.0285 | 0.011 | -2.601 | 0.01410582 | 0.111975 |
| X202462_s_at | -0.0965 | 0.0371 | -2.601 | 0.01411101 | 0.111975 |
| X201443_s_at | -0.0811 | 0.0312 | -2.601 | 0.01411271 | 0.111975 |
| X220746_s_at | 0.0662 | 0.0255 | 2.601 | 0.01411411 | 0.111975 |
| X1861_at | -0.0563 | 0.0216 | -2.601 | 0.01413051 | 0.112048 |
| X217127_at | 0.2475 | 0.0952 | 2.6 | 0.01413805 | 0.112048 |
| X214782_at | 0.1898 | 0.073 | 2.6 | 0.01413839 | 0.112048 |
| X1053_at | -0.0813 | 0.0313 | -2.6 | 0.01414566 | 0.112049 |
| X204055_s_at | -0.1549 | 0.0596 | -2.6 | 0.01414856 | 0.112049 |
| X218053_at | 0.1195 | 0.046 | 2.6 | 0.01415729 | 0.112074 |
| X213089_at | 0.304 | 0.1169 | 2.599 | 0.01417006 | 0.112074 |
| X208424_s_at | -0.1631 | 0.0627 | -2.599 | 0.01417167 | 0.112074 |
| X218394_at | -0.1659 | 0.0638 | -2.599 | 0.01417198 | 0.112074 |
| X219132_at | 0.1468 | 0.0565 | 2.599 | 0.01419795 | 0.11224 |
| X204311_at | 0.142 | 0.0546 | 2.598 | 0.01421328 | 0.112321 |
| X208868_s_at | -0.1744 | 0.0672 | -2.597 | 0.01426331 | 0.112634 |
| X214481_at | -0.0205 | 0.0079 | -2.597 | 0.01426596 | 0.112634 |
| X209705_at | -0.0601 | 0.0232 | -2.597 | 0.01426811 | 0.112634 |
| X212281_s_at | -0.2019 | 0.0778 | -2.596 | 0.01427653 | 0.11266 |
| X221514_at | 0.0283 | 0.0109 | 2.596 | 0.01428977 | 0.112683 |
| X213737_x_at | 0.0852 | 0.0328 | 2.596 | 0.01429316 | 0.112683 |
| X218164_at | 0.1503 | 0.0579 | 2.596 | 0.01429461 | 0.112683 |
| X206142_at | -0.0368 | 0.0142 | -2.595 | 0.01433466 | 0.112959 |
| X208895_s_at | 0.1326 | 0.0511 | 2.594 | 0.01434755 | 0.113008 |
| X204023_at | -0.1514 | 0.0584 | -2.594 | 0.01435113 | 0.113008 |
| X201455_s_at | -0.126 | 0.0486 | -2.594 | 0.01437184 | 0.113131 |
| X213729_at | 0.1177 | 0.0454 | 2.593 | 0.01438349 | 0.113183 |
| X212517_at | -0.0798 | 0.0308 | -2.593 | 0.01439363 | 0.113222 |
| X219155_at | 0.1885 | 0.0727 | 2.592 | 0.01440979 | 0.113288 |
| X212983_at | -0.1186 | 0.0458 | -2.592 | 0.01441218 | 0.113288 |
| X202213_s_at | -0.2883 | 0.1112 | -2.592 | 0.01443228 | 0.113406 |
| X213528_at | -0.1219 | 0.0471 | -2.591 | 0.01445877 | 0.113574 |
| X34689_at | 0.0208 | 0.008 | 2.591 | 0.01446382 | 0.113574 |
| X201922_at | -0.0676 | 0.0261 | -2.59 | 0.01449575 | 0.113784 |
| X215424_s_at | -0.1007 | 0.0389 | -2.589 | 0.01451334 | 0.113882 |
| X212605_s_at | -0.0679 | 0.0262 | -2.589 | 0.0145213 | 0.113904 |
| X202442_at | -0.0744 | 0.0287 | -2.589 | 0.01452658 | 0.113905 |
| X212520_s_at | 0.1641 | 0.0634 | 2.589 | 0.01454498 | 0.114009 |
| X201541_s_at | -0.0549 | 0.0212 | -2.588 | 0.01455954 | 0.114083 |
| X211271_x_at | 0.1214 | 0.0469 | 2.588 | 0.01456945 | 0.114121 |
| X221816_s_at | 0.1219 | 0.0471 | 2.587 | 0.0145833 | 0.114176 |
| X220419_s_at | -0.1617 | 0.0625 | -2.587 | 0.0145868 | 0.114176 |
| X204034_at | -0.1114 | 0.0431 | -2.587 | 0.01459598 | 0.114208 |
| X218123_at | 0.0813 | 0.0314 | 2.587 | 0.01461127 | 0.114269 |
| X210079_x_at | -0.5437 | 0.2102 | -2.587 | 0.01461414 | 0.114269 |
| X201718_s_at | -0.213 | 0.0824 | -2.586 | 0.01464395 | 0.114462 |
| X219933_at | -0.1409 | 0.0545 | -2.585 | 0.01465862 | 0.114536 |
| X212653_s_at | 0.0786 | 0.0304 | 2.584 | 0.01469836 | 0.114805 |
| X219079_at | -0.2059 | 0.0797 | -2.584 | 0.01470335 | 0.114805 |
| X203305_at | 0.2031 | 0.0786 | 2.583 | 0.01475408 | 0.115093 |
| X205566_at | 0.0083 | 0.0032 | 2.582 | 0.01475669 | 0.115093 |
| X205074_at | 0.1726 | 0.0668 | 2.582 | 0.01475895 | 0.115093 |
| X208624_s_at | 0.1591 | 0.0616 | 2.582 | 0.01476094 | 0.115093 |
| X208639_x_at | -0.0854 | 0.0331 | -2.582 | 0.01478337 | 0.115227 |
| X200076_s_at | 0.0429 | 0.0166 | 2.581 | 0.01479819 | 0.115302 |
| X201271_s_at | 0.0945 | 0.0366 | 2.581 | 0.01480869 | 0.115344 |
| X40837_at | 0.0935 | 0.0363 | 2.58 | 0.01483305 | 0.115488 |
| X220952_s_at | 0.1446 | 0.056 | 2.58 | 0.01483765 | 0.115488 |
| X207922_s_at | -0.1631 | 0.0632 | -2.58 | 0.01484508 | 0.115495 |
| X213726_x_at | 0.103 | 0.0399 | 2.58 | 0.0148489 | 0.115495 |
| X214848_at | -0.2191 | 0.0849 | -2.579 | 0.01486927 | 0.115613 |
| X207718_x_at | 0.0123 | 0.0048 | 2.579 | 0.01488991 | 0.115713 |
| X206373_at | 0.1298 | 0.0503 | 2.578 | 0.01489524 | 0.115713 |
| X213366_x_at | -0.0399 | 0.0155 | -2.578 | 0.01489771 | 0.115713 |
| X218517_at | 0.0754 | 0.0292 | 2.578 | 0.01492768 | 0.115905 |
| X219023_at | -0.1503 | 0.0583 | -2.577 | 0.0149457 | 0.116004 |
| X204253_s_at | -0.0073 | 0.0028 | -2.577 | 0.01495404 | 0.116024 |
| X205501_at | -0.2508 | 0.0973 | -2.577 | 0.01495868 | 0.116024 |
| X203820_s_at | 0.0172 | 0.0067 | 2.577 | 0.01496553 | 0.116036 |
| X200627_at | -0.094 | 0.0365 | -2.576 | 0.01497765 | 0.11609 |
| X218145_at | 0.0082 | 0.0032 | 2.575 | 0.01501979 | 0.116376 |
| X203280_at | 0.0165 | 0.0064 | 2.575 | 0.01502956 | 0.116411 |
| X214156_at | 0.2218 | 0.0862 | 2.574 | 0.01503926 | 0.116424 |
| X203663_s_at | -0.0618 | 0.024 | -2.574 | 0.01504166 | 0.116424 |
| X200855_at | -0.042 | 0.0163 | -2.574 | 0.01504764 | 0.116429 |
| X220221_at | 0.1711 | 0.0665 | 2.574 | 0.01506773 | 0.116519 |
| X216834_at | 1.2556 | 0.4879 | 2.573 | 0.01507329 | 0.116519 |
| X218291_at | -0.0689 | 0.0268 | -2.573 | 0.01507749 | 0.116519 |
| X220360_at | -0.0171 | 0.0066 | -2.573 | 0.0150802 | 0.116519 |
| X221798_x_at | 0.0522 | 0.0203 | 2.573 | 0.01509653 | 0.116604 |
| X41387_r_at | 0.0281 | 0.0109 | 2.572 | 0.01514275 | 0.116885 |
| X220574_at | 0.0704 | 0.0274 | 2.572 | 0.01514337 | 0.116885 |
| X201531_at | 0.5257 | 0.2046 | 2.569 | 0.01523627 | 0.117561 |
| X45687_at | 0.0754 | 0.0294 | 2.568 | 0.01525708 | 0.117681 |
| X219418_at | -0.0141 | 0.0055 | -2.568 | 0.01528647 | 0.117867 |
| X200056_s_at | -0.1499 | 0.0584 | -2.567 | 0.0153034 | 0.117956 |
| X217562_at | -0.2665 | 0.1038 | -2.567 | 0.01531706 | 0.118021 |
| X205463_s_at | 0.243 | 0.0947 | 2.566 | 0.01532595 | 0.118048 |
| X214322_at | 0.0088 | 0.0034 | 2.566 | 0.01534384 | 0.118125 |
| X221527_s_at | 0.0719 | 0.028 | 2.566 | 0.01534651 | 0.118125 |
| X219728_at | 0.3981 | 0.1552 | 2.566 | 0.01535526 | 0.118151 |
| X214484_s_at | -0.0212 | 0.0082 | -2.565 | 0.0153867 | 0.118352 |
| X209460_at | -0.0833 | 0.0325 | -2.564 | 0.01540157 | 0.118425 |
| X201817_at | -0.0875 | 0.0341 | -2.564 | 0.01542424 | 0.118559 |
| X219636_s_at | -0.177 | 0.069 | -2.563 | 0.01543402 | 0.118575 |
| X200085_s_at | -0.0697 | 0.0272 | -2.563 | 0.015437 | 0.118575 |
| X214170_x_at | -0.0666 | 0.026 | -2.563 | 0.01544553 | 0.118599 |
| X213359_at | 0.1327 | 0.0518 | 2.563 | 0.01546859 | 0.118708 |
| X205119_s_at | 0.0241 | 0.0094 | 2.562 | 0.0154751 | 0.118708 |
| X210672_s_at | -0.0212 | 0.0083 | -2.562 | 0.01547572 | 0.118708 |
| X211142_x_at | -0.0126 | 0.0049 | -2.562 | 0.01549344 | 0.118803 |
| X217142_at | -0.0124 | 0.0048 | -2.562 | 0.01550071 | 0.118817 |
| X214593_at | 0.0542 | 0.0212 | 2.562 | 0.01550652 | 0.118821 |
| X221654_s_at | 0.2452 | 0.0957 | 2.561 | 0.0155242 | 0.118881 |
| X212422_at | -0.0413 | 0.0161 | -2.561 | 0.01552511 | 0.118881 |
| X214308_s_at | -0.0114 | 0.0045 | -2.561 | 0.01553071 | 0.118883 |
| X210995_s_at | -0.3671 | 0.1434 | -2.56 | 0.015555 | 0.119015 |
| X201040_at | 0.176 | 0.0688 | 2.56 | 0.01555866 | 0.119015 |
| X214185_at | 0.0132 | 0.0052 | 2.559 | 0.01559035 | 0.119217 |
| X202741_at | -0.0918 | 0.0359 | -2.559 | 0.01559897 | 0.119241 |
| X204195_s_at | 0.0131 | 0.0051 | 2.559 | 0.01560976 | 0.119283 |
| X207808_s_at | 0.1396 | 0.0546 | 2.558 | 0.01562987 | 0.119395 |
| X211971_s_at | -0.1383 | 0.0541 | -2.557 | 0.01565946 | 0.119529 |
| X211404_s_at | -0.0858 | 0.0336 | -2.557 | 0.01566725 | 0.119529 |
| X203920_at | -0.0514 | 0.0201 | -2.557 | 0.01566823 | 0.119529 |
| X211433_x_at | 0.0997 | 0.039 | 2.557 | 0.01566885 | 0.119529 |
| X202822_at | 0.1424 | 0.0557 | 2.556 | 0.01570986 | 0.119801 |
| X213034_at | -0.1234 | 0.0483 | -2.556 | 0.01571965 | 0.119834 |
| X213368_x_at | 0.0812 | 0.0318 | 2.555 | 0.01573568 | 0.119909 |
| X215054_at | -0.0842 | 0.033 | -2.555 | 0.01574648 | 0.119909 |
| X219870_at | -0.1536 | 0.0601 | -2.555 | 0.01574844 | 0.119909 |
| X221584_s_at | 0.0878 | 0.0344 | 2.555 | 0.01575101 | 0.119909 |
| X200662_s_at | -0.1129 | 0.0442 | -2.554 | 0.01577218 | 0.120029 |
| X200722_s_at | -0.0912 | 0.0357 | -2.554 | 0.01579121 | 0.120132 |
| X203279_at | -0.0845 | 0.0331 | -2.554 | 0.0157992 | 0.120141 |
| X221041_s_at | -0.0745 | 0.0292 | -2.553 | 0.01580319 | 0.120141 |
| X202481_at | 0.1716 | 0.0672 | 2.553 | 0.0158229 | 0.12025 |
| X207985_at | -0.0065 | 0.0025 | -2.552 | 0.01584058 | 0.120343 |
| X202217_at | -0.0973 | 0.0381 | -2.552 | 0.015852 | 0.120389 |
| X218897_at | -0.1211 | 0.0475 | -2.552 | 0.01586449 | 0.120415 |
| X201940_at | -0.1452 | 0.0569 | -2.552 | 0.01586637 | 0.120415 |
| X217855_x_at | 0.0701 | 0.0275 | 2.551 | 0.015897 | 0.120607 |
| X200737_at | -0.1807 | 0.0708 | -2.551 | 0.01590612 | 0.120627 |
| X220494_s_at | -0.1069 | 0.0419 | -2.551 | 0.01591054 | 0.120627 |
| X208789_at | 0.2259 | 0.0886 | 2.55 | 0.01594277 | 0.12083 |
| X221965_at | -0.1337 | 0.0525 | -2.549 | 0.01595719 | 0.120854 |
| X208863_s_at | -0.0632 | 0.0248 | -2.549 | 0.01596075 | 0.120854 |
| X204184_s_at | 0.0802 | 0.0315 | 2.549 | 0.01596227 | 0.120854 |
| X213498_at | 0.0051 | 0.002 | 2.549 | 0.0159791 | 0.12094 |
| X214860_at | -0.3109 | 0.1221 | -2.547 | 0.01605956 | 0.121452 |
| X200041_s_at | -0.0688 | 0.027 | -2.547 | 0.01606059 | 0.121452 |
| X219919_s_at | 0.0103 | 0.004 | 2.546 | 0.01606696 | 0.121452 |
| X210250_x_at | -0.0882 | 0.0346 | -2.546 | 0.0160686 | 0.121452 |
| X219537_x_at | -0.2543 | 0.0999 | -2.546 | 0.01608858 | 0.121562 |
| X218884_s_at | -0.1494 | 0.0587 | -2.546 | 0.01610111 | 0.121615 |
| X201948_at | 0.1488 | 0.0585 | 2.545 | 0.01611524 | 0.121681 |
| X201841_s_at | 0.2975 | 0.1169 | 2.545 | 0.01612411 | 0.121706 |
| X207141_s_at | 0.0085 | 0.0033 | 2.543 | 0.01618057 | 0.122067 |
| X217647_at | -0.0093 | 0.0036 | -2.543 | 0.01618288 | 0.122067 |
| X208648_at | 0.0478 | 0.0188 | 2.542 | 0.01622265 | 0.122291 |
| X214668_at | -0.2898 | 0.114 | -2.542 | 0.01622362 | 0.122291 |
| X216866_s_at | -0.01 | 0.0039 | -2.542 | 0.01623334 | 0.122323 |
| X204590_x_at | -0.0147 | 0.0058 | -2.542 | 0.01625669 | 0.122451 |
| X219992_at | 0.1753 | 0.069 | 2.541 | 0.01626137 | 0.122451 |
| X213149_at | -0.0928 | 0.0365 | -2.541 | 0.01628046 | 0.122518 |
| X217930_s_at | -0.0738 | 0.0291 | -2.541 | 0.01628126 | 0.122518 |
| X212145_at | -0.0727 | 0.0286 | -2.54 | 0.01629636 | 0.12259 |
| X212169_at | 0.2068 | 0.0814 | 2.54 | 0.01630229 | 0.122593 |
| X221899_at | 0.1703 | 0.067 | 2.54 | 0.01631478 | 0.122645 |
| X218502_s_at | -0.1158 | 0.0456 | -2.539 | 0.01633981 | 0.122792 |
| X214328_s_at | -0.0407 | 0.016 | -2.538 | 0.01638778 | 0.123111 |
| X203727_at | -0.0879 | 0.0347 | -2.538 | 0.01639829 | 0.123136 |
| X201826_s_at | -0.1196 | 0.0471 | -2.537 | 0.01641122 | 0.123136 |
| X218674_at | -0.1022 | 0.0403 | -2.537 | 0.01641461 | 0.123136 |
| X219805_at | -0.0357 | 0.0141 | -2.537 | 0.016415 | 0.123136 |
| X204688_at | -0.0805 | 0.0317 | -2.537 | 0.01641921 | 0.123136 |
| X213005_s_at | 0.1509 | 0.0595 | 2.537 | 0.01642435 | 0.123136 |
| X212467_at | -0.0434 | 0.0171 | -2.537 | 0.0164383 | 0.123163 |
| X213533_at | -0.2916 | 0.1149 | -2.537 | 0.01643911 | 0.123163 |
| X203031_s_at | -0.2081 | 0.0821 | -2.536 | 0.01645253 | 0.123222 |
| X200943_at | 0.1115 | 0.044 | 2.536 | 0.01646942 | 0.123256 |
| X217857_s_at | -0.2215 | 0.0873 | -2.536 | 0.01647002 | 0.123256 |
| X219008_at | -0.1838 | 0.0725 | -2.536 | 0.0164737 | 0.123256 |
| X209067_s_at | -0.0873 | 0.0344 | -2.534 | 0.01653657 | 0.123685 |
| X217658_at | -0.0173 | 0.0068 | -2.534 | 0.01654861 | 0.123733 |
| X201253_s_at | -0.0541 | 0.0214 | -2.533 | 0.01658048 | 0.12393 |
| X203641_s_at | 0.0051 | 0.002 | 2.532 | 0.01662351 | 0.124209 |
| X202283_at | 0.0304 | 0.012 | 2.531 | 0.01665257 | 0.124382 |
| X217430_x_at | -0.0124 | 0.0049 | -2.531 | 0.01665781 | 0.124382 |
| X211783_s_at | 0.1102 | 0.0435 | 2.531 | 0.01666846 | 0.12442 |
| X218927_s_at | -0.0287 | 0.0113 | -2.531 | 0.01667929 | 0.124459 |
| X205137_x_at | -0.009 | 0.0035 | -2.529 | 0.01672669 | 0.124771 |
| X205573_s_at | 0.1625 | 0.0642 | 2.529 | 0.01675531 | 0.124911 |
| X213427_at | -0.1624 | 0.0642 | -2.529 | 0.01675682 | 0.124911 |
| X204857_at | -0.3696 | 0.1462 | -2.528 | 0.01677612 | 0.125013 |
| X201091_s_at | -0.106 | 0.0419 | -2.527 | 0.01682265 | 0.125318 |
| X218105_s_at | -0.0939 | 0.0372 | -2.526 | 0.0168628 | 0.125575 |
| X208166_at | -0.0332 | 0.0132 | -2.525 | 0.01689617 | 0.125755 |
| X203942_s_at | -0.0415 | 0.0164 | -2.525 | 0.01690454 | 0.125755 |
| X221232_s_at | -0.0056 | 0.0022 | -2.525 | 0.0169131 | 0.125755 |
| X202620_s_at | 0.2516 | 0.0997 | 2.525 | 0.01692 | 0.125755 |
| X218736_s_at | 0.1359 | 0.0539 | 2.524 | 0.01692077 | 0.125755 |
| X211825_s_at | -0.0085 | 0.0034 | -2.524 | 0.01692091 | 0.125755 |
| X219596_at | -0.1135 | 0.045 | -2.524 | 0.01692672 | 0.125756 |
| X222140_s_at | 0.0882 | 0.0349 | 2.524 | 0.01694552 | 0.125849 |
| X210448_s_at | -0.2479 | 0.0982 | -2.524 | 0.01695055 | 0.125849 |
| X204992_s_at | -0.0747 | 0.0296 | -2.524 | 0.01695835 | 0.125853 |
| X221731_x_at | 0.2452 | 0.0972 | 2.523 | 0.01697119 | 0.125853 |
| X214474_at | 0.1604 | 0.0636 | 2.523 | 0.01697184 | 0.125853 |
| X204342_at | 0.1247 | 0.0494 | 2.523 | 0.01697381 | 0.125853 |
| X200701_at | 0.1658 | 0.0657 | 2.523 | 0.01698904 | 0.125924 |
| X202205_at | 0.1913 | 0.0758 | 2.522 | 0.01701329 | 0.126062 |
| X221783_at | 0.0873 | 0.0347 | 2.521 | 0.01707311 | 0.126463 |
| X218886_at | 0.1186 | 0.0471 | 2.52 | 0.01708875 | 0.126537 |
| X208313_s_at | -0.099 | 0.0393 | -2.52 | 0.01710635 | 0.126584 |
| X205376_at | -0.1633 | 0.0648 | -2.52 | 0.01710649 | 0.126584 |
| X203521_s_at | 0.0636 | 0.0253 | 2.519 | 0.01713813 | 0.126775 |
| X209503_s_at | -0.0417 | 0.0165 | -2.518 | 0.01718987 | 0.127116 |
| X213496_at | -0.1021 | 0.0406 | -2.517 | 0.01720601 | 0.127193 |
| X204007_at | 0.0151 | 0.006 | 2.517 | 0.01722062 | 0.127258 |
| X208078_s_at | 0.4228 | 0.1681 | 2.516 | 0.01726495 | 0.127505 |
| X222270_at | 0.1998 | 0.0794 | 2.516 | 0.01726549 | 0.127505 |
| X210054_at | 0.1538 | 0.0611 | 2.516 | 0.01727471 | 0.127531 |
| X218391_at | -0.1286 | 0.0511 | -2.515 | 0.01730664 | 0.127724 |
| X205905_s_at | 0.0121 | 0.0048 | 2.514 | 0.01733282 | 0.127871 |
| X35671_at | 0.1035 | 0.0412 | 2.514 | 0.01733807 | 0.127871 |
| X209579_s_at | 0.0875 | 0.0348 | 2.514 | 0.01735471 | 0.127951 |
| X201346_at | 0.3059 | 0.1217 | 2.514 | 0.01736108 | 0.127956 |
| X208101_s_at | 0.0269 | 0.0107 | 2.512 | 0.01742195 | 0.128362 |
| X37170_at | 0.1107 | 0.0441 | 2.511 | 0.01745252 | 0.128545 |
| X219142_at | 0.1636 | 0.0652 | 2.51 | 0.01750052 | 0.128855 |
| X205660_at | 0.0063 | 0.0025 | 2.51 | 0.01751646 | 0.12893 |
| X206870_at | -0.0113 | 0.0045 | -2.509 | 0.01753394 | 0.129016 |
| X218176_at | -0.0438 | 0.0175 | -2.508 | 0.01758713 | 0.129364 |
| X201618_x_at | -0.1688 | 0.0673 | -2.507 | 0.01763242 | 0.129655 |
| X33768_at | 0.0247 | 0.0098 | 2.507 | 0.0176405 | 0.129671 |
| X221726_at | -0.0658 | 0.0263 | -2.506 | 0.01767422 | 0.129876 |
| X201504_s_at | -0.2397 | 0.0957 | -2.506 | 0.01768533 | 0.129915 |
| X221214_s_at | 0.1253 | 0.05 | 2.505 | 0.01770127 | 0.129966 |
| X218915_at | 0.1155 | 0.0461 | 2.505 | 0.01770627 | 0.129966 |
| X220331_at | -0.1976 | 0.0789 | -2.505 | 0.01770992 | 0.129966 |
| X201553_s_at | 0.1213 | 0.0484 | 2.505 | 0.01772011 | 0.129998 |
| X208745_at | -0.0692 | 0.0276 | -2.505 | 0.01773074 | 0.130033 |
| X38964_r_at | 0.043 | 0.0172 | 2.504 | 0.01776642 | 0.130252 |
| X209627_s_at | 0.1174 | 0.0469 | 2.503 | 0.0177783 | 0.130296 |
| X219444_at | 0.0073 | 0.0029 | 2.503 | 0.01778559 | 0.130306 |
| X206849_at | -0.4911 | 0.1963 | -2.502 | 0.01781763 | 0.130498 |
| X209689_at | -0.0544 | 0.0217 | -2.502 | 0.01784618 | 0.130634 |
| X221843_s_at | 0.0696 | 0.0278 | 2.502 | 0.01785193 | 0.130634 |
| X201881_s_at | -0.1863 | 0.0745 | -2.502 | 0.01785376 | 0.130634 |
| X209683_at | -0.2088 | 0.0835 | -2.501 | 0.01787375 | 0.130731 |
| X208697_s_at | -0.1 | 0.04 | -2.501 | 0.0178788 | 0.130731 |
| X207128_s_at | -0.1178 | 0.0471 | -2.5 | 0.01790475 | 0.130855 |
| X217242_at | -0.0126 | 0.005 | -2.5 | 0.01790754 | 0.130855 |
| X212019_at | -0.2291 | 0.0917 | -2.499 | 0.01795124 | 0.131113 |
| X1431_at | -0.0412 | 0.0165 | -2.499 | 0.0179547 | 0.131113 |
| X205056_s_at | 0.1208 | 0.0484 | 2.499 | 0.0179641 | 0.131129 |
| X212810_s_at | -0.1737 | 0.0695 | -2.499 | 0.01796864 | 0.131129 |
| X217438_at | -0.0142 | 0.0057 | -2.499 | 0.01798281 | 0.131189 |
| X203928_x_at | 0.1448 | 0.058 | 2.498 | 0.01802487 | 0.131438 |
| X221766_s_at | 0.0866 | 0.0347 | 2.497 | 0.0180288 | 0.131438 |
| X201691_s_at | -0.126 | 0.0505 | -2.497 | 0.01804453 | 0.13151 |
| X203956_at | 0.0898 | 0.036 | 2.496 | 0.01808749 | 0.131779 |
| X213364_s_at | -0.0645 | 0.0259 | -2.496 | 0.01809491 | 0.13179 |
| X209819_at | -0.0323 | 0.0129 | -2.496 | 0.01810553 | 0.131824 |
| X212755_at | -0.0682 | 0.0274 | -2.494 | 0.01815392 | 0.132121 |
| X205212_s_at | -0.0094 | 0.0038 | -2.494 | 0.0181587 | 0.132121 |
| X214743_at | 0.1172 | 0.047 | 2.494 | 0.01816792 | 0.132121 |
| X209965_s_at | -0.0158 | 0.0063 | -2.494 | 0.01817011 | 0.132121 |
| X202770_s_at | -0.1332 | 0.0534 | -2.494 | 0.01819404 | 0.132208 |
| X219279_at | 0.1987 | 0.0797 | 2.493 | 0.01819515 | 0.132208 |
| X208772_at | 0.0944 | 0.0379 | 2.493 | 0.01820745 | 0.132208 |
| X201157_s_at | 0.1236 | 0.0496 | 2.493 | 0.01820883 | 0.132208 |
| X209065_at | -0.0928 | 0.0372 | -2.493 | 0.01821286 | 0.132208 |
| X209846_s_at | 0.2842 | 0.114 | 2.493 | 0.01821766 | 0.132208 |
| X209699_x_at | 0.281 | 0.1127 | 2.492 | 0.01824703 | 0.132377 |
| X200866_s_at | -0.2067 | 0.083 | -2.492 | 0.01826461 | 0.132462 |
| X206486_at | 0.0058 | 0.0023 | 2.491 | 0.01830351 | 0.1327 |
| X213941_x_at | 0.049 | 0.0197 | 2.491 | 0.01831398 | 0.132733 |
| X204245_s_at | -0.1516 | 0.0609 | -2.49 | 0.01835244 | 0.132968 |
| X221605_s_at | -0.2057 | 0.0826 | -2.489 | 0.01837282 | 0.133064 |
| X217379_at | -0.043 | 0.0173 | -2.489 | 0.01837761 | 0.133064 |
| X200742_s_at | 0.1999 | 0.0803 | 2.489 | 0.01839297 | 0.133103 |
| X206906_at | -0.119 | 0.0478 | -2.489 | 0.01839807 | 0.133103 |
| X204026_s_at | -0.2312 | 0.0929 | -2.489 | 0.01840099 | 0.133103 |
| X217871_s_at | -0.0934 | 0.0375 | -2.488 | 0.0184215 | 0.133208 |
| X203966_s_at | 0.1153 | 0.0464 | 2.487 | 0.01845299 | 0.133392 |
| X201787_at | 0.008 | 0.0032 | 2.487 | 0.01846837 | 0.13346 |
| X205931_s_at | 0.3444 | 0.1385 | 2.487 | 0.01848898 | 0.133526 |
| X201704_at | -0.182 | 0.0732 | -2.487 | 0.01848945 | 0.133526 |
| X200659_s_at | -0.1107 | 0.0445 | -2.486 | 0.01850883 | 0.1336 |
| X203853_s_at | 0.1922 | 0.0773 | 2.486 | 0.01851178 | 0.1336 |
| X204155_s_at | -0.1605 | 0.0646 | -2.485 | 0.01856245 | 0.13389 |
| X203619_s_at | -0.1794 | 0.0722 | -2.485 | 0.01856404 | 0.13389 |
| X216867_s_at | 0.3445 | 0.1387 | 2.484 | 0.01860919 | 0.134172 |
| X211488_s_at | 0.0102 | 0.0041 | 2.484 | 0.01861607 | 0.134178 |
| X222111_at | -0.1378 | 0.0555 | -2.482 | 0.01867881 | 0.134587 |
| X204129_at | 0.0265 | 0.0107 | 2.482 | 0.01869498 | 0.13466 |
| X208766_s_at | -0.0491 | 0.0198 | -2.481 | 0.01874912 | 0.135006 |
| X219894_at | -0.4151 | 0.1674 | -2.48 | 0.01876581 | 0.135082 |
| X202004_x_at | -0.068 | 0.0274 | -2.48 | 0.01877324 | 0.135092 |
| X202629_at | -0.0782 | 0.0315 | -2.48 | 0.01879603 | 0.135204 |
| X201133_s_at | -0.1341 | 0.0541 | -2.479 | 0.01880093 | 0.135204 |
| X201908_at | 0.1533 | 0.0618 | 2.479 | 0.01880796 | 0.13521 |
| X204548_at | -0.0502 | 0.0203 | -2.478 | 0.01885632 | 0.135514 |
| X203333_at | -0.1789 | 0.0722 | -2.478 | 0.01886491 | 0.135532 |
| X222335_at | 0.0419 | 0.0169 | 2.477 | 0.01890525 | 0.135778 |
| X200768_s_at | 0.0658 | 0.0266 | 2.475 | 0.01897557 | 0.136239 |
| X209303_at | -0.0832 | 0.0336 | -2.475 | 0.01899839 | 0.136359 |
| X203613_s_at | -0.0794 | 0.0321 | -2.474 | 0.01901877 | 0.136461 |
| X202946_s_at | 0.1893 | 0.0765 | 2.474 | 0.01905506 | 0.136647 |
| X209700_x_at | -0.2526 | 0.1021 | -2.474 | 0.01906123 | 0.136647 |
| X206540_at | 0.0213 | 0.0086 | 2.473 | 0.01906309 | 0.136647 |
| X212660_at | -0.0976 | 0.0395 | -2.473 | 0.01907201 | 0.136666 |
| X213469_at | -0.2384 | 0.0964 | -2.473 | 0.01908068 | 0.136684 |
| X208840_s_at | -0.2759 | 0.1116 | -2.473 | 0.01908781 | 0.136691 |
| X202335_s_at | -0.0453 | 0.0183 | -2.473 | 0.0191007 | 0.136697 |
| X209007_s_at | 0.1169 | 0.0473 | 2.473 | 0.01910188 | 0.136697 |
| X213248_at | 0.0413 | 0.0167 | 2.472 | 0.01910709 | 0.136697 |
| X212110_at | 0.0807 | 0.0326 | 2.472 | 0.01913233 | 0.136834 |
| X52164_at | 0.1587 | 0.0642 | 2.471 | 0.0191797 | 0.137128 |
| X214409_at | -0.0263 | 0.0107 | -2.47 | 0.01920844 | 0.13729 |
| X205849_s_at | -0.0535 | 0.0216 | -2.469 | 0.01924463 | 0.137504 |
| X204556_s_at | 0.1321 | 0.0535 | 2.469 | 0.0192746 | 0.137554 |
| X49051_g_at | -0.0051 | 0.0021 | -2.469 | 0.01927697 | 0.137554 |
| X204033_at | -0.0794 | 0.0321 | -2.469 | 0.01927915 | 0.137554 |
| X208802_at | -0.1487 | 0.0602 | -2.468 | 0.01928767 | 0.137554 |
| X204791_at | 0.1122 | 0.0455 | 2.468 | 0.01929089 | 0.137554 |
| X201028_s_at | 0.2183 | 0.0885 | 2.468 | 0.01929446 | 0.137554 |
| X52731_at | -0.0582 | 0.0236 | -2.468 | 0.0192949 | 0.137554 |
| X218230_at | -0.0913 | 0.037 | -2.468 | 0.01932209 | 0.137677 |
| X210213_s_at | -0.1076 | 0.0436 | -2.468 | 0.01932462 | 0.137677 |
| X201896_s_at | 0.4403 | 0.1784 | 2.467 | 0.01933895 | 0.137735 |
| X212928_at | -0.0815 | 0.0331 | -2.466 | 0.01939639 | 0.138068 |
| X213044_at | 0.0929 | 0.0377 | 2.466 | 0.01939817 | 0.138068 |
| X213588_x_at | 0.0515 | 0.0209 | 2.465 | 0.01942834 | 0.138203 |
| X200873_s_at | -0.0845 | 0.0343 | -2.465 | 0.01942949 | 0.138203 |
| X203542_s_at | 0.2902 | 0.1178 | 2.464 | 0.01946647 | 0.138421 |
| X201203_s_at | -0.009 | 0.0036 | -2.464 | 0.01947426 | 0.138432 |
| X213061_s_at | -0.0886 | 0.036 | -2.464 | 0.01948148 | 0.138439 |
| X201526_at | -0.1053 | 0.0427 | -2.463 | 0.01953166 | 0.138752 |
| X201313_at | -0.1343 | 0.0545 | -2.463 | 0.01953933 | 0.138762 |
| X218847_at | 0.1576 | 0.064 | 2.463 | 0.01954725 | 0.138774 |
| X205709_s_at | 0.1166 | 0.0474 | 2.462 | 0.01956603 | 0.138817 |
| X202929_s_at | -0.191 | 0.0776 | -2.462 | 0.01957775 | 0.138817 |
| X209959_at | -0.6449 | 0.2619 | -2.462 | 0.01957793 | 0.138817 |
| X218205_s_at | 0.1288 | 0.0523 | 2.462 | 0.019583 | 0.138817 |
| X205787_x_at | 0.0322 | 0.0131 | 2.462 | 0.01958465 | 0.138817 |
| X212785_s_at | 0.0992 | 0.0403 | 2.461 | 0.01960487 | 0.138916 |
| X220606_s_at | -0.0523 | 0.0213 | -2.461 | 0.01964389 | 0.139106 |
| X215411_s_at | 0.1365 | 0.0555 | 2.461 | 0.01964422 | 0.139106 |
| X214623_at | -0.0269 | 0.0109 | -2.46 | 0.01965392 | 0.139131 |
| X200617_at | -0.0655 | 0.0266 | -2.46 | 0.01968704 | 0.139249 |
| X210640_s_at | -0.033 | 0.0134 | -2.46 | 0.019689 | 0.139249 |
| X208947_s_at | 0.0133 | 0.0054 | 2.46 | 0.01968947 | 0.139249 |
| X218059_at | 0.064 | 0.026 | 2.458 | 0.01974073 | 0.139512 |
| X202177_at | 0.0729 | 0.0296 | 2.458 | 0.01974399 | 0.139512 |
| X204225_at | 0.1171 | 0.0476 | 2.458 | 0.0197455 | 0.139512 |
| X210975_x_at | 0.011 | 0.0045 | 2.457 | 0.0197871 | 0.139762 |
| X220589_s_at | -0.006 | 0.0024 | -2.457 | 0.01981339 | 0.139875 |
| X206767_at | -0.0103 | 0.0042 | -2.457 | 0.01981575 | 0.139875 |
| X210736_x_at | 0.2887 | 0.1175 | 2.456 | 0.01983202 | 0.139937 |
| X202810_at | -0.0458 | 0.0187 | -2.456 | 0.01983711 | 0.139937 |
| X204933_s_at | 0.0089 | 0.0036 | 2.456 | 0.01985046 | 0.139987 |
| X205101_at | -0.0119 | 0.0049 | -2.456 | 0.01987277 | 0.1401 |
| X211945_s_at | 0.2177 | 0.0887 | 2.455 | 0.01989051 | 0.14018 |
| X221268_s_at | -0.152 | 0.0619 | -2.454 | 0.01992298 | 0.140318 |
| X219165_at | 0.1846 | 0.0752 | 2.454 | 0.01992704 | 0.140318 |
| X213594_x_at | -0.127 | 0.0517 | -2.454 | 0.01992904 | 0.140318 |
| X202019_s_at | -0.1152 | 0.0469 | -2.453 | 0.0199855 | 0.140656 |
| X218389_s_at | 0.1585 | 0.0646 | 2.453 | 0.01998965 | 0.140656 |
| X200634_at | -0.046 | 0.0188 | -2.453 | 0.02000103 | 0.140691 |
| X212727_at | -0.1847 | 0.0753 | -2.452 | 0.0200238 | 0.140766 |
| X200086_s_at | -0.0654 | 0.0267 | -2.452 | 0.02002426 | 0.140766 |
| X220547_s_at | 0.0895 | 0.0365 | 2.452 | 0.02005771 | 0.140944 |
| X213834_at | 0.0389 | 0.0159 | 2.451 | 0.02006236 | 0.140944 |
| X208630_at | 0.0415 | 0.0169 | 2.451 | 0.02008087 | 0.141021 |
| X221356_x_at | 0.0056 | 0.0023 | 2.451 | 0.02008595 | 0.141021 |
| X204958_at | 0.0104 | 0.0043 | 2.451 | 0.02009644 | 0.14105 |
| X201383_s_at | -0.0388 | 0.0158 | -2.45 | 0.02012238 | 0.141175 |
| X219091_s_at | 0.1453 | 0.0593 | 2.45 | 0.02012695 | 0.141175 |
| X202144_s_at | -0.0665 | 0.0272 | -2.45 | 0.0201538 | 0.141318 |
| X205814_at | -0.1851 | 0.0756 | -2.449 | 0.02016914 | 0.141381 |
| X205761_s_at | -0.0829 | 0.0338 | -2.449 | 0.02018323 | 0.14141 |
| X211364_at | 0.0136 | 0.0056 | 2.449 | 0.0201975 | 0.14141 |
| X220588_at | -0.0321 | 0.0131 | -2.449 | 0.02019779 | 0.14141 |
| X210819_x_at | 0.0148 | 0.006 | 2.449 | 0.02020115 | 0.14141 |
| X202455_at | 0.0305 | 0.0124 | 2.448 | 0.02020501 | 0.14141 |
| X209817_at | 0.1623 | 0.0663 | 2.448 | 0.02024752 | 0.141663 |
| X221869_at | -0.0853 | 0.0348 | -2.447 | 0.02025469 | 0.141668 |
| X209505_at | 0.2118 | 0.0865 | 2.447 | 0.0202646 | 0.141693 |
| X203810_at | -0.1334 | 0.0546 | -2.446 | 0.02033631 | 0.142149 |
| X217858_s_at | -0.1153 | 0.0472 | -2.445 | 0.02034644 | 0.142176 |
| X217733_s_at | 0.0538 | 0.022 | 2.445 | 0.02035408 | 0.142184 |
| X219553_at | -0.1692 | 0.0692 | -2.445 | 0.02036308 | 0.142202 |
| X209338_at | -0.1673 | 0.0684 | -2.445 | 0.02037512 | 0.142207 |
| X215778_x_at | -0.0512 | 0.0209 | -2.445 | 0.02037652 | 0.142207 |
| X221217_s_at | -0.1266 | 0.0518 | -2.444 | 0.02040711 | 0.142348 |
| X201598_s_at | 0.0102 | 0.0042 | 2.444 | 0.02040956 | 0.142348 |
| X202135_s_at | -0.0951 | 0.0389 | -2.443 | 0.02044355 | 0.14254 |
| X201885_s_at | 0.0671 | 0.0275 | 2.443 | 0.0204607 | 0.142615 |
| X219440_at | -0.1446 | 0.0592 | -2.443 | 0.02047675 | 0.142682 |
| X200783_s_at | 0.2588 | 0.106 | 2.443 | 0.0204835 | 0.142684 |
| X205371_s_at | -0.1926 | 0.0789 | -2.442 | 0.02049412 | 0.142714 |
| X200937_s_at | -0.0842 | 0.0345 | -2.441 | 0.02053197 | 0.142932 |
| X201967_at | 0.1084 | 0.0444 | 2.441 | 0.02056114 | 0.143091 |
| X218917_s_at | -0.0705 | 0.0289 | -2.441 | 0.02056777 | 0.143092 |
| X216602_s_at | -0.2199 | 0.0901 | -2.44 | 0.02058802 | 0.143188 |
| X211967_at | 0.2121 | 0.087 | 2.44 | 0.02062028 | 0.143367 |
| X208869_s_at | -0.135 | 0.0553 | -2.439 | 0.0206372 | 0.143416 |
| X203113_s_at | 0.152 | 0.0623 | 2.439 | 0.0206402 | 0.143416 |
| X201812_s_at | -0.0811 | 0.0333 | -2.439 | 0.0206585 | 0.143498 |
| X215341_at | -0.0823 | 0.0338 | -2.438 | 0.02067939 | 0.143599 |
| X202233_s_at | -0.0689 | 0.0283 | -2.438 | 0.02069568 | 0.143667 |
| X205547_s_at | 0.1655 | 0.0679 | 2.438 | 0.02070884 | 0.143679 |
| X221069_s_at | -0.1611 | 0.0661 | -2.438 | 0.02071181 | 0.143679 |
| X44669_at | -0.0942 | 0.0386 | -2.438 | 0.02071681 | 0.143679 |
| X207702_s_at | 0.2675 | 0.1098 | 2.437 | 0.02073124 | 0.143734 |
| X202433_at | -0.096 | 0.0394 | -2.437 | 0.02074808 | 0.143792 |
| X206339_at | 0.5645 | 0.2317 | 2.437 | 0.02075555 | 0.143792 |
| X205136_s_at | -0.0448 | 0.0184 | -2.437 | 0.02075907 | 0.143792 |
| X203476_at | -0.47 | 0.1929 | -2.436 | 0.02078986 | 0.143949 |
| X212009_s_at | -0.2246 | 0.0922 | -2.436 | 0.02079469 | 0.143949 |
| X211995_x_at | -0.0856 | 0.0352 | -2.436 | 0.02080373 | 0.143967 |
| X204344_s_at | -0.1182 | 0.0486 | -2.433 | 0.02091399 | 0.144672 |
| X209175_at | -0.1048 | 0.0431 | -2.433 | 0.02091862 | 0.144672 |
| X211593_s_at | 0.0799 | 0.0329 | 2.433 | 0.02093985 | 0.144774 |
| X220246_at | -0.0642 | 0.0264 | -2.433 | 0.02094886 | 0.144791 |
| X213131_at | 0.1799 | 0.074 | 2.43 | 0.02109164 | 0.145732 |
| X214746_s_at | -0.0663 | 0.0273 | -2.429 | 0.02110942 | 0.14581 |
| X219181_at | -0.0102 | 0.0042 | -2.429 | 0.02111624 | 0.145812 |
| X205013_s_at | -0.3144 | 0.1294 | -2.429 | 0.02114004 | 0.145887 |
| X205218_at | -0.0703 | 0.0289 | -2.429 | 0.0211403 | 0.145887 |
| X215707_s_at | -0.2416 | 0.0995 | -2.428 | 0.02120381 | 0.14628 |
| X217832_at | 0.1222 | 0.0504 | 2.426 | 0.0212946 | 0.146861 |
| X200072_s_at | -0.0792 | 0.0327 | -2.424 | 0.02137602 | 0.147376 |
| X209621_s_at | -0.2301 | 0.095 | -2.423 | 0.02142338 | 0.147657 |
| X208815_x_at | 0.0664 | 0.0274 | 2.423 | 0.02143104 | 0.147664 |
| X203340_s_at | 0.1261 | 0.0521 | 2.423 | 0.02144079 | 0.147686 |
| X204927_at | -0.0273 | 0.0113 | -2.422 | 0.02145907 | 0.147766 |
| X203298_s_at | 0.0985 | 0.0407 | 2.422 | 0.02147116 | 0.147798 |
| X201204_s_at | 0.1851 | 0.0764 | 2.422 | 0.02147701 | 0.147798 |
| X208384_s_at | -0.0408 | 0.0168 | -2.422 | 0.02149013 | 0.147842 |
| X211056_s_at | -0.0252 | 0.0104 | -2.421 | 0.02150741 | 0.147902 |
| X217346_at | -0.11 | 0.0454 | -2.421 | 0.02151216 | 0.147902 |
| X203931_s_at | -0.0817 | 0.0337 | -2.421 | 0.02152353 | 0.147925 |
| X221467_at | 0.2703 | 0.1117 | 2.421 | 0.02152885 | 0.147925 |
| X210271_at | 0.0062 | 0.0026 | 2.421 | 0.02154821 | 0.148012 |
| X214691_x_at | -0.127 | 0.0525 | -2.42 | 0.02157469 | 0.1481 |
| X208797_s_at | 0.3573 | 0.1476 | 2.42 | 0.02157992 | 0.1481 |
| X221480_at | -0.1071 | 0.0443 | -2.42 | 0.02158091 | 0.1481 |
| X204512_at | 0.0989 | 0.0409 | 2.42 | 0.02159143 | 0.148115 |
| X219243_at | 0.1979 | 0.0818 | 2.42 | 0.02159648 | 0.148115 |
| X202139_at | -0.1142 | 0.0472 | -2.419 | 0.02160506 | 0.148128 |
| X205196_s_at | -0.2062 | 0.0853 | -2.419 | 0.02162982 | 0.148247 |
| X206144_at | 0.2085 | 0.0862 | 2.419 | 0.02163571 | 0.148247 |
| X217737_x_at | -0.0643 | 0.0266 | -2.418 | 0.02165351 | 0.14827 |
| X209630_s_at | -0.0998 | 0.0413 | -2.418 | 0.02165912 | 0.14827 |
| X217838_s_at | 0.1259 | 0.0521 | 2.418 | 0.0216592 | 0.14827 |
| X201453_x_at | -0.0683 | 0.0282 | -2.418 | 0.02167869 | 0.148326 |
| X209068_at | -0.104 | 0.043 | -2.418 | 0.02168064 | 0.148326 |
| X209274_s_at | -0.1078 | 0.0446 | -2.418 | 0.02169168 | 0.148356 |
| X217250_s_at | 0.1795 | 0.0743 | 2.417 | 0.0217101 | 0.148405 |
| X209047_at | 0.4138 | 0.1712 | 2.417 | 0.02171744 | 0.148405 |
| X218097_s_at | -0.0776 | 0.0321 | -2.417 | 0.02172447 | 0.148405 |
| X218802_at | 0.0282 | 0.0117 | 2.417 | 0.02172566 | 0.148405 |
| X206685_at | -0.4393 | 0.1818 | -2.416 | 0.02175462 | 0.148557 |
| X212348_s_at | -0.0774 | 0.032 | -2.416 | 0.02178003 | 0.148685 |
| X211676_s_at | 0.187 | 0.0774 | 2.416 | 0.02179616 | 0.14875 |
| X220358_at | -0.2322 | 0.0962 | -2.415 | 0.02183272 | 0.148888 |
| X218470_at | -0.113 | 0.0468 | -2.415 | 0.02183693 | 0.148888 |
| X46142_at | 0.1098 | 0.0455 | 2.415 | 0.02183849 | 0.148888 |
| X203060_s_at | 0.4415 | 0.1828 | 2.415 | 0.02184322 | 0.148888 |
| X201377_at | 0.0582 | 0.0241 | 2.413 | 0.02193333 | 0.149415 |
| X202691_at | -0.0827 | 0.0343 | -2.413 | 0.02193406 | 0.149415 |
| X212375_at | -0.0891 | 0.0369 | -2.413 | 0.02194472 | 0.149442 |
| X221992_at | 0.2579 | 0.1069 | 2.412 | 0.02199223 | 0.149679 |
| X203136_at | -0.1256 | 0.0521 | -2.412 | 0.02199293 | 0.149679 |
| X214369_s_at | -0.1752 | 0.0727 | -2.411 | 0.02200619 | 0.149723 |
| X213128_s_at | -0.1235 | 0.0512 | -2.411 | 0.02202167 | 0.149741 |
| X210028_s_at | -0.0827 | 0.0343 | -2.411 | 0.02202987 | 0.149741 |
| X214728_x_at | -0.0833 | 0.0345 | -2.411 | 0.0220434 | 0.149741 |
| X218494_s_at | 0.0212 | 0.0088 | 2.411 | 0.02204767 | 0.149741 |
| X202432_at | -0.1072 | 0.0445 | -2.41 | 0.02205377 | 0.149741 |
| X216266_s_at | -0.1818 | 0.0754 | -2.41 | 0.02207035 | 0.149741 |
| X217730_at | 0.1733 | 0.0719 | 2.41 | 0.0220717 | 0.149741 |
| X213380_x_at | 0.0115 | 0.0048 | 2.41 | 0.02207598 | 0.149741 |
| X209181_s_at | 0.0541 | 0.0225 | 2.41 | 0.02207991 | 0.149741 |
| X209570_s_at | -0.1962 | 0.0814 | -2.41 | 0.02208017 | 0.149741 |
| X203602_s_at | -0.0766 | 0.0318 | -2.41 | 0.02208292 | 0.149741 |
| X202920_at | -0.0968 | 0.0402 | -2.409 | 0.02211578 | 0.149876 |
| X206212_at | -0.0067 | 0.0028 | -2.409 | 0.02212081 | 0.149876 |
| X218144_s_at | -0.1532 | 0.0636 | -2.409 | 0.02212392 | 0.149876 |
| X214260_at | -0.1518 | 0.063 | -2.409 | 0.02212987 | 0.149876 |
| X214162_at | 0.0692 | 0.0287 | 2.408 | 0.02216512 | 0.150001 |
| X202586_at | -0.0073 | 0.003 | -2.408 | 0.02217102 | 0.150001 |
| X207122_x_at | -0.0663 | 0.0275 | -2.408 | 0.0221809 | 0.150001 |
| X222134_at | -0.3462 | 0.1438 | -2.408 | 0.02218221 | 0.150001 |
| X209233_at | -0.0752 | 0.0312 | -2.408 | 0.02218791 | 0.150001 |
| X209695_at | 0.085 | 0.0353 | 2.408 | 0.02218889 | 0.150001 |
| X205666_at | 0.0142 | 0.0059 | 2.407 | 0.02222351 | 0.15019 |
| X203219_s_at | -0.0114 | 0.0047 | -2.407 | 0.02223197 | 0.150201 |
| X213916_at | 0.0946 | 0.0393 | 2.406 | 0.02226763 | 0.150316 |
| X218858_at | 0.4275 | 0.1777 | 2.406 | 0.02227051 | 0.150316 |
| X208647_at | 0.1108 | 0.046 | 2.406 | 0.02227158 | 0.150316 |
| X202862_at | 0.1066 | 0.0443 | 2.406 | 0.02227601 | 0.150316 |
| X218760_at | -0.0102 | 0.0042 | -2.406 | 0.02229455 | 0.150395 |
| X210145_at | 0.1747 | 0.0726 | 2.405 | 0.02232367 | 0.150538 |
| X202142_at | -0.0943 | 0.0392 | -2.405 | 0.02232928 | 0.150538 |
| X202717_s_at | -0.0719 | 0.0299 | -2.404 | 0.02239412 | 0.150929 |
| X211938_at | -0.1023 | 0.0426 | -2.403 | 0.02242925 | 0.151119 |
| X209982_s_at | 0.1251 | 0.0521 | 2.403 | 0.02243584 | 0.151119 |
| X208906_at | -0.1331 | 0.0554 | -2.403 | 0.02245817 | 0.151223 |
| X217999_s_at | -0.0923 | 0.0384 | -2.402 | 0.02247026 | 0.151259 |
| X201148_s_at | 0.0936 | 0.039 | 2.402 | 0.02248833 | 0.151335 |
| X215918_s_at | 0.3671 | 0.1529 | 2.401 | 0.02251329 | 0.151457 |
| X218056_at | -0.1165 | 0.0485 | -2.401 | 0.0225272 | 0.151505 |
| X204500_s_at | 0.1192 | 0.0497 | 2.401 | 0.02253935 | 0.15154 |
| X207002_s_at | 0.1224 | 0.051 | 2.401 | 0.02255754 | 0.151617 |
| X200060_s_at | 0.0471 | 0.0196 | 2.4 | 0.02256446 | 0.151617 |
| X206149_at | -0.0088 | 0.0037 | -2.4 | 0.02258822 | 0.151731 |
| X216017_s_at | -0.0088 | 0.0037 | -2.4 | 0.02261186 | 0.151841 |
| X202381_at | 0.1148 | 0.0478 | 2.399 | 0.02261822 | 0.151841 |
| X212627_s_at | -0.077 | 0.0321 | -2.399 | 0.02263873 | 0.151898 |
| X205889_s_at | 0.12 | 0.05 | 2.399 | 0.02264044 | 0.151898 |
| X200793_s_at | -0.1249 | 0.0521 | -2.398 | 0.02268278 | 0.152135 |
| X213094_at | 0.0508 | 0.0212 | 2.398 | 0.02269224 | 0.152135 |
| X210340_s_at | -0.0099 | 0.0041 | -2.398 | 0.02269632 | 0.152135 |
| X220788_s_at | -0.0667 | 0.0278 | -2.397 | 0.02272764 | 0.152299 |
| X212819_at | -0.1386 | 0.0578 | -2.397 | 0.02275703 | 0.15245 |
| X62212_at | -0.1108 | 0.0462 | -2.396 | 0.02277644 | 0.152534 |
| X201739_at | 0.2514 | 0.1049 | 2.396 | 0.02279682 | 0.152612 |
| X212493_s_at | 0.0773 | 0.0322 | 2.396 | 0.0228042 | 0.152612 |
| X208643_s_at | -0.0523 | 0.0218 | -2.396 | 0.02280872 | 0.152612 |
| X210131_x_at | -0.062 | 0.0259 | -2.395 | 0.02282678 | 0.152687 |
| X212927_at | 0.1456 | 0.0608 | 2.395 | 0.02285112 | 0.152804 |
| X220079_s_at | -0.1166 | 0.0487 | -2.395 | 0.02285823 | 0.152806 |
| X215936_s_at | -0.1569 | 0.0655 | -2.394 | 0.02288582 | 0.152944 |
| X205160_at | 0.0925 | 0.0387 | 2.394 | 0.0229015 | 0.153003 |
| X203462_x_at | -0.0832 | 0.0348 | -2.393 | 0.02294391 | 0.15324 |
| X214925_s_at | 0.2055 | 0.0859 | 2.393 | 0.02295535 | 0.15327 |
| X208044_s_at | 0.0101 | 0.0042 | 2.393 | 0.02297375 | 0.153313 |
| X212871_at | -0.0487 | 0.0204 | -2.393 | 0.02297557 | 0.153313 |
| X221082_s_at | -0.1777 | 0.0743 | -2.392 | 0.0229831 | 0.153317 |
| X221795_at | 0.345 | 0.1443 | 2.392 | 0.02301811 | 0.153474 |
| X210211_s_at | -0.0884 | 0.037 | -2.392 | 0.02302035 | 0.153474 |
| X207081_s_at | -0.158 | 0.0661 | -2.391 | 0.02305083 | 0.153623 |
| X210357_s_at | 0.1803 | 0.0754 | 2.391 | 0.02305895 | 0.153623 |
| X206731_at | -0.1412 | 0.0591 | -2.391 | 0.02306352 | 0.153623 |
| X203225_s_at | -0.1104 | 0.0462 | -2.391 | 0.02307169 | 0.153632 |
| X208427_s_at | 0.1867 | 0.0781 | 2.391 | 0.02308436 | 0.15367 |
| X202569_s_at | 0.0523 | 0.0219 | 2.39 | 0.02310617 | 0.153766 |
| X221649_s_at | -0.058 | 0.0243 | -2.39 | 0.02311268 | 0.153766 |
| X213201_s_at | -0.0108 | 0.0045 | -2.39 | 0.02312152 | 0.153779 |
| X214327_x_at | -0.0316 | 0.0132 | -2.389 | 0.0231397 | 0.153833 |
| X218405_at | -0.0447 | 0.0187 | -2.389 | 0.02314517 | 0.153833 |
| X209494_s_at | 0.0158 | 0.0066 | 2.389 | 0.02315045 | 0.153833 |
| X218514_at | -0.117 | 0.049 | -2.389 | 0.02315805 | 0.153838 |
| X221746_at | -0.0526 | 0.022 | -2.387 | 0.02326088 | 0.154475 |
| X205714_s_at | -0.0173 | 0.0073 | -2.387 | 0.0232702 | 0.15449 |
| X200098_s_at | -0.0762 | 0.0319 | -2.387 | 0.02327773 | 0.154494 |
| X220448_at | 0.095 | 0.0398 | 2.386 | 0.02330762 | 0.154581 |
| X217827_s_at | -0.0848 | 0.0356 | -2.386 | 0.02330846 | 0.154581 |
| X217117_x_at | 0.0113 | 0.0048 | 2.386 | 0.02331174 | 0.154581 |
| X220274_at | 0.1973 | 0.0827 | 2.386 | 0.023329 | 0.154644 |
| X218095_s_at | 0.1729 | 0.0725 | 2.386 | 0.02333511 | 0.154644 |
| X204529_s_at | 0.1428 | 0.0599 | 2.385 | 0.02335216 | 0.154679 |
| X214817_at | -0.1395 | 0.0585 | -2.385 | 0.02336293 | 0.154679 |
| X210770_s_at | 0.184 | 0.0772 | 2.385 | 0.02336567 | 0.154679 |
| X205081_at | 0.0501 | 0.021 | 2.385 | 0.02336856 | 0.154679 |
| X214006_s_at | -0.0278 | 0.0117 | -2.385 | 0.02337522 | 0.154679 |
| X214757_at | -0.0144 | 0.006 | -2.384 | 0.02344001 | 0.155027 |
| X202110_at | -0.0502 | 0.0211 | -2.384 | 0.02344172 | 0.155027 |
| X201997_s_at | 0.0671 | 0.0282 | 2.382 | 0.02351703 | 0.155478 |
| X204892_x_at | -0.0434 | 0.0182 | -2.382 | 0.02354257 | 0.155601 |
| X200960_x_at | -0.0529 | 0.0222 | -2.382 | 0.02355582 | 0.155619 |
| X208642_s_at | -0.0843 | 0.0354 | -2.382 | 0.0235593 | 0.155619 |
| X213370_s_at | 0.0269 | 0.0113 | 2.381 | 0.02359094 | 0.155782 |
| X201752_s_at | 0.1154 | 0.0485 | 2.381 | 0.02360803 | 0.155848 |
| X208905_at | -0.0699 | 0.0294 | -2.38 | 0.02363295 | 0.155966 |
| X205554_s_at | -0.0048 | 0.002 | -2.38 | 0.02364378 | 0.155991 |
| X206059_at | -0.1471 | 0.0618 | -2.38 | 0.02366439 | 0.156081 |
| X203914_x_at | 0.027 | 0.0113 | 2.379 | 0.02369293 | 0.156223 |
| X218448_at | -0.0616 | 0.0259 | -2.379 | 0.02371779 | 0.15634 |
| X203332_s_at | -0.0237 | 0.01 | -2.378 | 0.02373017 | 0.156376 |
| X202579_x_at | -0.1008 | 0.0424 | -2.377 | 0.02378268 | 0.156675 |
| X213336_at | -0.0113 | 0.0048 | -2.377 | 0.02379561 | 0.156714 |
| X218074_at | -0.0525 | 0.0221 | -2.377 | 0.02380398 | 0.156723 |
| X220090_at | 0.0054 | 0.0023 | 2.377 | 0.02381538 | 0.156751 |
| X212596_s_at | 0.1233 | 0.0519 | 2.377 | 0.0238294 | 0.156797 |
| X213666_at | 0.1553 | 0.0654 | 2.376 | 0.0238483 | 0.156875 |
| X221794_at | 0.0102 | 0.0043 | 2.376 | 0.02387019 | 0.156972 |
| X209899_s_at | -0.0402 | 0.0169 | -2.375 | 0.02390176 | 0.157133 |
| X215506_s_at | -0.6662 | 0.2805 | -2.375 | 0.02390982 | 0.15714 |
| X209023_s_at | 0.1249 | 0.0526 | 2.375 | 0.0239253 | 0.157195 |
| X213424_at | 0.1275 | 0.0537 | 2.375 | 0.02393816 | 0.157217 |
| X37005_at | 0.1037 | 0.0437 | 2.375 | 0.02394272 | 0.157217 |
| X214596_at | 0.5364 | 0.2259 | 2.374 | 0.02395239 | 0.157234 |
| X204314_s_at | -0.0931 | 0.0392 | -2.374 | 0.02397084 | 0.157308 |
| X210638_s_at | -0.1274 | 0.0537 | -2.374 | 0.02398853 | 0.157378 |
| X209211_at | -0.1616 | 0.0681 | -2.373 | 0.02403711 | 0.15764 |
| X220650_s_at | -0.0256 | 0.0108 | -2.373 | 0.02404264 | 0.15764 |
| X220892_s_at | -0.1476 | 0.0622 | -2.372 | 0.02405734 | 0.157665 |
| X205633_s_at | -0.1876 | 0.0791 | -2.372 | 0.02406409 | 0.157665 |
| X209018_s_at | -0.1395 | 0.0588 | -2.372 | 0.02406782 | 0.157665 |
| X201910_at | 0.1036 | 0.0437 | 2.372 | 0.02408738 | 0.157712 |
| X200828_s_at | -0.0909 | 0.0383 | -2.372 | 0.02408915 | 0.157712 |
| X201220_x_at | 0.1008 | 0.0425 | 2.371 | 0.02411893 | 0.15786 |
| X219493_at | -0.1964 | 0.0828 | -2.371 | 0.02414219 | 0.157966 |
| X202680_at | -0.1049 | 0.0442 | -2.371 | 0.02415045 | 0.157974 |
| X214500_at | -0.1876 | 0.0792 | -2.37 | 0.02421648 | 0.158359 |
| X202447_at | -0.0786 | 0.0332 | -2.369 | 0.02424593 | 0.158462 |
| X209144_s_at | 0.0067 | 0.0028 | 2.369 | 0.02424655 | 0.158462 |
| X218005_at | 0.1209 | 0.051 | 2.368 | 0.02429819 | 0.158753 |
| X205548_s_at | -0.2334 | 0.0986 | -2.368 | 0.02432917 | 0.158909 |
| X200665_s_at | 0.2086 | 0.0881 | 2.367 | 0.02434762 | 0.158982 |
| X220078_at | -0.1529 | 0.0646 | -2.367 | 0.02435717 | 0.158987 |
| X204097_s_at | 0.0864 | 0.0365 | 2.367 | 0.02436258 | 0.158987 |
| X213959_s_at | -0.1613 | 0.0682 | -2.366 | 0.0243939 | 0.159099 |
| X37512_at | -0.2573 | 0.1087 | -2.366 | 0.02440196 | 0.159099 |
| X200782_at | 0.1852 | 0.0783 | 2.366 | 0.02440538 | 0.159099 |
| X203300_x_at | -0.2078 | 0.0878 | -2.366 | 0.02440837 | 0.159099 |
| X203288_at | 0.1966 | 0.0831 | 2.366 | 0.0244204 | 0.15913 |
| X200001_at | -0.1096 | 0.0463 | -2.366 | 0.02443803 | 0.159198 |
| X219958_at | -0.0528 | 0.0223 | -2.365 | 0.02446388 | 0.159316 |
| X205090_s_at | -0.134 | 0.0566 | -2.365 | 0.02447044 | 0.159316 |
| X208707_at | -0.0575 | 0.0243 | -2.365 | 0.02448238 | 0.159347 |
| X208663_s_at | 0.1001 | 0.0424 | 2.364 | 0.02450486 | 0.159405 |
| X45653_at | -0.1037 | 0.0439 | -2.364 | 0.02450569 | 0.159405 |
| X218657_at | -0.05 | 0.0212 | -2.364 | 0.02452986 | 0.159487 |
| X207842_s_at | 0.1409 | 0.0596 | 2.364 | 0.02453702 | 0.159487 |
| X218874_s_at | -0.1148 | 0.0486 | -2.364 | 0.02453982 | 0.159487 |
| X205539_at | 0.0311 | 0.0131 | 2.364 | 0.02454824 | 0.159495 |
| X209073_s_at | -0.026 | 0.011 | -2.363 | 0.02455904 | 0.159514 |
| X201141_at | 0.4254 | 0.18 | 2.363 | 0.02456541 | 0.159514 |
| X200837_at | -0.0975 | 0.0413 | -2.363 | 0.02459712 | 0.159673 |
| X202343_x_at | -0.0497 | 0.021 | -2.363 | 0.02460902 | 0.159678 |
| X201194_at | -0.0902 | 0.0382 | -2.362 | 0.02461246 | 0.159678 |
| X213276_at | -0.1889 | 0.08 | -2.362 | 0.02461943 | 0.159678 |
| X218909_at | 0.06 | 0.0254 | 2.362 | 0.02464923 | 0.159819 |
| X210006_at | -0.0676 | 0.0286 | -2.362 | 0.02465565 | 0.159819 |
| X207792_at | -0.0066 | 0.0028 | -2.361 | 0.02467495 | 0.159898 |
| X209718_at | -0.0162 | 0.0069 | -2.361 | 0.02468961 | 0.159946 |
| X201728_s_at | 0.183 | 0.0775 | 2.361 | 0.02470576 | 0.159966 |
| X219303_at | -0.0778 | 0.0329 | -2.361 | 0.02470716 | 0.159966 |
| X209513_s_at | 0.1509 | 0.064 | 2.36 | 0.02475391 | 0.160222 |
| X219760_at | -0.1142 | 0.0484 | -2.36 | 0.02476724 | 0.160262 |
| X205000_at | 0.0578 | 0.0245 | 2.359 | 0.02479165 | 0.160373 |
| X205436_s_at | 0.0848 | 0.036 | 2.359 | 0.02483377 | 0.16058 |
| X200080_s_at | 0.0758 | 0.0322 | 2.358 | 0.02484329 | 0.16058 |
| X219214_s_at | -0.0268 | 0.0114 | -2.358 | 0.02484695 | 0.16058 |
| X213455_at | 0.1939 | 0.0822 | 2.358 | 0.02485256 | 0.16058 |
| X213614_x_at | -0.0527 | 0.0224 | -2.358 | 0.0248661 | 0.160621 |
| X210408_s_at | -0.3841 | 0.1629 | -2.358 | 0.0248788 | 0.16064 |
| X205269_at | 0.2232 | 0.0947 | 2.358 | 0.02488349 | 0.16064 |
| X222018_at | 0.1564 | 0.0663 | 2.357 | 0.02490793 | 0.160751 |
| X205794_s_at | 0.0799 | 0.0339 | 2.357 | 0.02494441 | 0.160928 |
| X201128_s_at | 0.0732 | 0.0311 | 2.356 | 0.0249498 | 0.160928 |
| X203488_at | 0.0938 | 0.0398 | 2.356 | 0.02499427 | 0.161069 |
| X207724_s_at | -0.1617 | 0.0687 | -2.356 | 0.0249979 | 0.161069 |
| X221002_s_at | 0.1068 | 0.0453 | 2.356 | 0.02500243 | 0.161069 |
| X39817_s_at | -0.1452 | 0.0616 | -2.355 | 0.02500551 | 0.161069 |
| X208723_at | -0.1172 | 0.0497 | -2.355 | 0.02501139 | 0.161069 |
| X221842_s_at | 0.0725 | 0.0308 | 2.355 | 0.02501517 | 0.161069 |
| X222003_s_at | -0.0052 | 0.0022 | -2.355 | 0.02505176 | 0.161258 |
| X219542_at | 0.1679 | 0.0713 | 2.354 | 0.02506902 | 0.161322 |
| X215504_x_at | 0.0498 | 0.0212 | 2.354 | 0.02509633 | 0.161428 |
| X205369_x_at | -0.0514 | 0.0218 | -2.354 | 0.02510003 | 0.161428 |
| X202776_at | 0.0781 | 0.0332 | 2.354 | 0.02511563 | 0.161436 |
| X221645_s_at | -0.2434 | 0.1034 | -2.354 | 0.0251158 | 0.161436 |
| X214801_at | 0.1069 | 0.0454 | 2.353 | 0.02512788 | 0.161467 |
| X202695_s_at | -0.141 | 0.0599 | -2.352 | 0.02517974 | 0.161753 |
| X208178_x_at | 0.1147 | 0.0487 | 2.352 | 0.025201 | 0.161843 |
| X218692_at | 0.1355 | 0.0576 | 2.352 | 0.02522412 | 0.161945 |
| X202065_s_at | 0.1234 | 0.0525 | 2.351 | 0.02528077 | 0.162262 |
| X204973_at | -0.0919 | 0.0391 | -2.351 | 0.02528865 | 0.162265 |
| X219089_s_at | -0.0303 | 0.0129 | -2.35 | 0.0252987 | 0.162283 |
| X204131_s_at | 0.113 | 0.0481 | 2.35 | 0.02530651 | 0.162286 |
| X222298_at | 0.1732 | 0.0737 | 2.35 | 0.02532151 | 0.162327 |
| X219718_at | -0.1093 | 0.0465 | -2.35 | 0.02532753 | 0.162327 |
| X206320_s_at | 0.0072 | 0.0031 | 2.35 | 0.02534218 | 0.162332 |
| X211758_x_at | -0.082 | 0.0349 | -2.35 | 0.02534289 | 0.162332 |
| X219296_at | 0.1078 | 0.0459 | 2.349 | 0.02535052 | 0.162334 |
| X205577_at | -0.0139 | 0.0059 | -2.349 | 0.02538221 | 0.16249 |
| X207884_at | -0.0036 | 0.0016 | -2.349 | 0.0253972 | 0.162539 |
| X202955_s_at | -0.1849 | 0.0787 | -2.348 | 0.02542178 | 0.16265 |
| X216532_x_at | -0.0188 | 0.008 | -2.347 | 0.02547791 | 0.162962 |
| X214338_at | 0.0229 | 0.0098 | 2.347 | 0.02551014 | 0.163089 |
| X213617_s_at | -0.1364 | 0.0581 | -2.347 | 0.02551755 | 0.163089 |
| X217935_s_at | -0.0972 | 0.0414 | -2.347 | 0.02551979 | 0.163089 |
| X207791_s_at | -0.1532 | 0.0653 | -2.346 | 0.02555228 | 0.16325 |
| X214972_at | -0.0167 | 0.0071 | -2.345 | 0.02558206 | 0.163315 |
| X201946_s_at | -0.159 | 0.0678 | -2.345 | 0.02558401 | 0.163315 |
| X217618_x_at | -0.0873 | 0.0372 | -2.345 | 0.02558457 | 0.163315 |
| X202507_s_at | -0.1508 | 0.0643 | -2.345 | 0.02559362 | 0.163326 |
| X208673_s_at | -0.0582 | 0.0248 | -2.345 | 0.02562354 | 0.163429 |
| X212920_at | 0.1451 | 0.0619 | 2.345 | 0.02562448 | 0.163429 |
| X217370_x_at | -0.1437 | 0.0613 | -2.344 | 0.02565801 | 0.163559 |
| X205392_s_at | -0.0125 | 0.0053 | -2.344 | 0.02565968 | 0.163559 |
| X31837_at | 0.0138 | 0.0059 | 2.344 | 0.02566874 | 0.16357 |
| X203734_at | -0.0915 | 0.039 | -2.344 | 0.02567687 | 0.163575 |
| X243_g_at | 0.1976 | 0.0843 | 2.343 | 0.02570357 | 0.163698 |
| X212673_at | -0.0873 | 0.0373 | -2.342 | 0.02576487 | 0.164042 |
| X218652_s_at | 0.0647 | 0.0276 | 2.341 | 0.02581795 | 0.164332 |
| X204064_at | 0.0635 | 0.0271 | 2.341 | 0.02584102 | 0.164403 |
| X210426_x_at | 0.1998 | 0.0853 | 2.341 | 0.02584377 | 0.164403 |
| X222315_at | -0.0094 | 0.004 | -2.341 | 0.02586187 | 0.164471 |
| X200934_at | 0.0915 | 0.0391 | 2.34 | 0.02588051 | 0.164542 |
| X204308_s_at | 0.0695 | 0.0297 | 2.339 | 0.02598131 | 0.165136 |
| X211958_at | 0.0201 | 0.0086 | 2.338 | 0.02604476 | 0.165492 |
| X213213_at | 0.109 | 0.0467 | 2.337 | 0.02607059 | 0.165572 |
| X212741_at | 0.195 | 0.0835 | 2.337 | 0.0260723 | 0.165572 |
| X215690_x_at | -0.1204 | 0.0515 | -2.337 | 0.02608296 | 0.165592 |
| X202931_x_at | 0.1355 | 0.058 | 2.337 | 0.02609235 | 0.165604 |
| X202500_at | 0.1714 | 0.0734 | 2.336 | 0.02610966 | 0.165667 |
| X217540_at | -0.1438 | 0.0616 | -2.336 | 0.02612287 | 0.165703 |
| X202438_x_at | -0.2541 | 0.1088 | -2.336 | 0.02613596 | 0.165739 |
| X201653_at | -0.0626 | 0.0268 | -2.336 | 0.02615635 | 0.165811 |
| X214182_at | 0.1171 | 0.0502 | 2.336 | 0.02616221 | 0.165811 |
| X206960_at | 0.1878 | 0.0805 | 2.334 | 0.02625171 | 0.166255 |
| X219716_at | 0.014 | 0.006 | 2.334 | 0.02625797 | 0.166255 |
| X207537_at | -0.0049 | 0.0021 | -2.334 | 0.02626059 | 0.166255 |
| X208651_x_at | 0.3099 | 0.1328 | 2.334 | 0.02626926 | 0.166255 |
| X209326_at | -0.1118 | 0.0479 | -2.334 | 0.02627653 | 0.166255 |
| X204890_s_at | -0.005 | 0.0021 | -2.334 | 0.02628149 | 0.166255 |
| X214115_at | 0.0048 | 0.002 | 2.333 | 0.02628458 | 0.166255 |
| X209501_at | -0.1225 | 0.0525 | -2.333 | 0.02630555 | 0.166299 |
| X203743_s_at | -0.0787 | 0.0337 | -2.333 | 0.02630651 | 0.166299 |
| X212818_s_at | -0.1014 | 0.0435 | -2.333 | 0.02632626 | 0.166376 |
| X218400_at | -0.0081 | 0.0035 | -2.333 | 0.02634097 | 0.166379 |
| X215982_s_at | -0.0195 | 0.0084 | -2.332 | 0.02634175 | 0.166379 |
| X218834_s_at | 0.1598 | 0.0685 | 2.332 | 0.026381 | 0.166504 |
| X214671_s_at | -0.1834 | 0.0786 | -2.332 | 0.02638321 | 0.166504 |
| X217512_at | -0.0168 | 0.0072 | -2.332 | 0.02638819 | 0.166504 |
| X202499_s_at | -0.1328 | 0.0569 | -2.332 | 0.02639152 | 0.166504 |
| X205104_at | 0.0979 | 0.042 | 2.331 | 0.02640714 | 0.166555 |
| X215812_s_at | 0.0328 | 0.0141 | 2.331 | 0.02643066 | 0.166657 |
| X213227_at | -0.0765 | 0.0328 | -2.331 | 0.02645775 | 0.16672 |
| X213970_at | -0.0963 | 0.0413 | -2.331 | 0.02645843 | 0.16672 |
| X210263_at | -0.2163 | 0.0928 | -2.33 | 0.02646326 | 0.16672 |
| X215132_at | -0.1745 | 0.0749 | -2.33 | 0.026473 | 0.166734 |
| X205481_at | -0.0975 | 0.0418 | -2.33 | 0.02649128 | 0.166758 |
| X203374_s_at | -0.0249 | 0.0107 | -2.33 | 0.02649187 | 0.166758 |
| X201790_s_at | 0.057 | 0.0245 | 2.329 | 0.02653845 | 0.167004 |
| X212678_at | -0.0877 | 0.0377 | -2.329 | 0.02656711 | 0.167137 |
| X201047_x_at | -0.0611 | 0.0263 | -2.328 | 0.02659351 | 0.167256 |
| X206200_s_at | -0.0688 | 0.0296 | -2.328 | 0.02661942 | 0.167334 |
| X200868_s_at | -0.0216 | 0.0093 | -2.328 | 0.02662098 | 0.167334 |
| X221419_s_at | 0.0675 | 0.029 | 2.327 | 0.02665952 | 0.167518 |
| X205243_at | 0.0245 | 0.0105 | 2.327 | 0.02666532 | 0.167518 |
| X219988_s_at | -0.0957 | 0.0411 | -2.327 | 0.02669995 | 0.167688 |
| X203159_at | -0.1251 | 0.0538 | -2.326 | 0.02672891 | 0.167823 |
| X213892_s_at | -0.0868 | 0.0373 | -2.325 | 0.02679894 | 0.168215 |
| X212399_s_at | 0.1099 | 0.0473 | 2.323 | 0.02688316 | 0.168696 |
| X220925_at | 0.0792 | 0.0341 | 2.323 | 0.02690094 | 0.168697 |
| X213671_s_at | 0.0731 | 0.0315 | 2.323 | 0.0269058 | 0.168697 |
| X204444_at | -0.0163 | 0.007 | -2.323 | 0.02690611 | 0.168697 |
| X206941_x_at | -0.6123 | 0.2636 | -2.323 | 0.02692708 | 0.168734 |
| X203058_s_at | 0.2895 | 0.1246 | 2.323 | 0.02693478 | 0.168734 |
| X209359_x_at | -0.0084 | 0.0036 | -2.323 | 0.02693491 | 0.168734 |
| X201192_s_at | -0.2246 | 0.0967 | -2.321 | 0.02700507 | 0.169126 |
| X200991_s_at | -0.1279 | 0.0551 | -2.321 | 0.0270173 | 0.169155 |
| X202423_at | 0.0787 | 0.0339 | 2.321 | 0.02705281 | 0.169289 |
| X217789_at | 0.228 | 0.0982 | 2.321 | 0.02705387 | 0.169289 |
| X207347_at | 0.0088 | 0.0038 | 2.32 | 0.02709114 | 0.169474 |
| X212504_at | -0.1041 | 0.0449 | -2.319 | 0.02714772 | 0.16978 |
| X202646_s_at | -0.076 | 0.0328 | -2.319 | 0.02716164 | 0.1698 |
| X212602_at | 0.1056 | 0.0456 | 2.319 | 0.02716622 | 0.1698 |
| X203795_s_at | 0.0557 | 0.024 | 2.319 | 0.02718467 | 0.169868 |
| X212553_at | 0.1256 | 0.0542 | 2.318 | 0.02722285 | 0.170059 |
| X212361_s_at | -0.1192 | 0.0514 | -2.317 | 0.02727591 | 0.170342 |
| X219664_s_at | -0.0428 | 0.0185 | -2.317 | 0.0272835 | 0.170342 |
| X218022_at | -0.1477 | 0.0638 | -2.316 | 0.02731323 | 0.170479 |
| X215587_x_at | 0.1138 | 0.0491 | 2.316 | 0.02733218 | 0.17055 |
| X212045_at | -0.0608 | 0.0263 | -2.315 | 0.02737603 | 0.170739 |
| X220602_s_at | 0.0387 | 0.0167 | 2.315 | 0.02737781 | 0.170739 |
| X205443_at | 0.1651 | 0.0713 | 2.315 | 0.02740484 | 0.17082 |
| X209651_at | -0.0199 | 0.0086 | -2.315 | 0.02740624 | 0.17082 |
| X202835_at | 0.0107 | 0.0046 | 2.315 | 0.0274174 | 0.17084 |
| X202886_s_at | -0.1033 | 0.0446 | -2.315 | 0.0274249 | 0.17084 |
| X214997_at | 0.0967 | 0.0418 | 2.314 | 0.02745388 | 0.170937 |
| X218226_s_at | -0.0477 | 0.0206 | -2.314 | 0.02745789 | 0.170937 |
| X212726_at | 0.0321 | 0.0139 | 2.314 | 0.02746752 | 0.170937 |
| X222008_at | 0.0186 | 0.008 | 2.314 | 0.02747287 | 0.170937 |
| X1598_g_at | 0.1553 | 0.0671 | 2.314 | 0.02747884 | 0.170937 |
| X213639_s_at | 0.0549 | 0.0237 | 2.313 | 0.02749553 | 0.17097 |
| X212990_at | -0.0829 | 0.0358 | -2.313 | 0.02749959 | 0.17097 |
| X200720_s_at | -0.1033 | 0.0447 | -2.313 | 0.02754643 | 0.171162 |
| X220085_at | -0.0156 | 0.0068 | -2.313 | 0.02754783 | 0.171162 |
| X201046_s_at | 0.0642 | 0.0278 | 2.313 | 0.02755351 | 0.171162 |
| X213957_s_at | 0.2572 | 0.1112 | 2.312 | 0.02758297 | 0.171297 |
| X215526_at | 0.0418 | 0.0181 | 2.312 | 0.02759114 | 0.1713 |
| X210624_s_at | -0.1366 | 0.0591 | -2.311 | 0.02762408 | 0.171456 |
| X202900_s_at | -0.1064 | 0.046 | -2.311 | 0.02763358 | 0.171458 |
| X213314_at | -0.119 | 0.0515 | -2.311 | 0.02763982 | 0.171458 |
| X204838_s_at | 0.0917 | 0.0397 | 2.311 | 0.02765202 | 0.171486 |
| X214061_at | -0.1607 | 0.0695 | -2.311 | 0.02767847 | 0.171555 |
| X217165_x_at | 0.2099 | 0.0909 | 2.311 | 0.02767866 | 0.171555 |
| X208812_x_at | 0.1551 | 0.0671 | 2.31 | 0.02773669 | 0.171867 |
| X203066_at | -0.1399 | 0.0606 | -2.308 | 0.02781161 | 0.172283 |
| X213333_at | -0.1381 | 0.0598 | -2.308 | 0.02783925 | 0.172406 |
| X40093_at | -0.0051 | 0.0022 | -2.307 | 0.02787578 | 0.172567 |
| X201489_at | 0.092 | 0.0399 | 2.307 | 0.02788302 | 0.172567 |
| X218923_at | 0.1978 | 0.0857 | 2.307 | 0.02789305 | 0.172567 |
| X200741_s_at | -0.0339 | 0.0147 | -2.307 | 0.02789621 | 0.172567 |
| X206204_at | 0.2639 | 0.1144 | 2.307 | 0.02791066 | 0.172594 |
| X205641_s_at | 0.0098 | 0.0043 | 2.307 | 0.02791612 | 0.172594 |
| X204681_s_at | -0.1279 | 0.0555 | -2.307 | 0.02792887 | 0.172624 |
| X216375_s_at | -0.0137 | 0.0059 | -2.305 | 0.02802962 | 0.173199 |
| X210854_x_at | 0.1331 | 0.0578 | 2.304 | 0.02811226 | 0.173661 |
| X221739_at | -0.0895 | 0.0389 | -2.303 | 0.02816836 | 0.173959 |
| X218837_s_at | 0.0428 | 0.0186 | 2.303 | 0.02817687 | 0.173961 |
| X220116_at | 0.1791 | 0.0778 | 2.302 | 0.0281843 | 0.173961 |
| X211184_s_at | 0.0205 | 0.0089 | 2.302 | 0.02819957 | 0.174007 |
| X213780_at | -0.0279 | 0.0121 | -2.302 | 0.02822076 | 0.174089 |
| X214858_at | -0.3637 | 0.158 | -2.301 | 0.0282531 | 0.174241 |
| X203222_s_at | -0.2376 | 0.1033 | -2.301 | 0.02827375 | 0.174296 |
| X222230_s_at | -0.0477 | 0.0207 | -2.301 | 0.02827993 | 0.174296 |
| X207545_s_at | -0.0781 | 0.0339 | -2.301 | 0.02828561 | 0.174296 |
| X200757_s_at | -0.0585 | 0.0254 | -2.301 | 0.02830204 | 0.174335 |
| X219269_at | 0.1873 | 0.0814 | 2.3 | 0.02831893 | 0.174335 |
| X206567_s_at | 0.0707 | 0.0307 | 2.3 | 0.02832136 | 0.174335 |
| X203697_at | 0.239 | 0.1039 | 2.3 | 0.02832939 | 0.174335 |
| X216521_s_at | -0.2158 | 0.0938 | -2.3 | 0.02833115 | 0.174335 |
| X201557_at | -0.119 | 0.0518 | -2.3 | 0.02836922 | 0.174521 |
| X204454_at | -0.0746 | 0.0325 | -2.299 | 0.02839952 | 0.174659 |
| X215626_at | 0.0391 | 0.017 | 2.299 | 0.02842275 | 0.174689 |
| X206441_s_at | -0.0308 | 0.0134 | -2.299 | 0.02842992 | 0.174689 |
| X205485_at | -0.0098 | 0.0043 | -2.299 | 0.02843529 | 0.174689 |
| X209610_s_at | -0.1773 | 0.0771 | -2.299 | 0.0284359 | 0.174689 |
| X204874_x_at | 0.0686 | 0.0298 | 2.298 | 0.02845705 | 0.174771 |
| X209803_s_at | 0.0299 | 0.013 | 2.298 | 0.02849082 | 0.17493 |
| X217834_s_at | 0.1712 | 0.0745 | 2.297 | 0.02850628 | 0.174976 |
| X203015_s_at | -0.299 | 0.1302 | -2.297 | 0.02851782 | 0.174999 |
| X203415_at | -0.1066 | 0.0464 | -2.297 | 0.02855229 | 0.175139 |
| X210999_s_at | -0.0689 | 0.03 | -2.297 | 0.02855872 | 0.175139 |
| X54632_at | -0.0834 | 0.0363 | -2.296 | 0.02856716 | 0.175139 |
| X205852_at | -0.1807 | 0.0787 | -2.296 | 0.02857601 | 0.175139 |
| X204102_s_at | 0.0625 | 0.0272 | 2.296 | 0.02858007 | 0.175139 |
| X213577_at | 0.0519 | 0.0226 | 2.296 | 0.02860787 | 0.175261 |
| X33850_at | 0.188 | 0.0819 | 2.296 | 0.02862404 | 0.175297 |
| X212060_at | -0.084 | 0.0366 | -2.295 | 0.0286499 | 0.175297 |
| X212904_at | -0.0546 | 0.0238 | -2.295 | 0.02865436 | 0.175297 |
| X203033_x_at | -0.0617 | 0.0269 | -2.295 | 0.02865794 | 0.175297 |
| X218371_s_at | -0.0914 | 0.0398 | -2.295 | 0.02866018 | 0.175297 |
| X32723_at | -0.0859 | 0.0374 | -2.295 | 0.02866108 | 0.175297 |
| X202710_at | -0.1285 | 0.056 | -2.294 | 0.0287268 | 0.175623 |
| X209708_at | 0.3479 | 0.1517 | 2.294 | 0.02873436 | 0.175623 |
| X201172_x_at | 0.1216 | 0.053 | 2.294 | 0.0287382 | 0.175623 |
| X200841_s_at | 0.0797 | 0.0348 | 2.293 | 0.02878694 | 0.175873 |
| X207874_s_at | 0.015 | 0.0065 | 2.293 | 0.0287986 | 0.175896 |
| X210368_at | 0.0799 | 0.0349 | 2.292 | 0.02884859 | 0.176152 |
| X206732_at | -0.1644 | 0.0718 | -2.291 | 0.0289227 | 0.176556 |
| X200640_at | -0.1051 | 0.0459 | -2.291 | 0.02893697 | 0.176595 |
| X217911_s_at | 0.2714 | 0.1185 | 2.29 | 0.02895622 | 0.176626 |
| X201909_at | -0.0849 | 0.0371 | -2.29 | 0.02895791 | 0.176626 |
| X216305_s_at | 0.1102 | 0.0481 | 2.29 | 0.02897481 | 0.17668 |
| X204732_s_at | -0.1031 | 0.045 | -2.29 | 0.02899455 | 0.176752 |
| X212811_x_at | -0.1815 | 0.0793 | -2.29 | 0.02900419 | 0.176762 |
| X202472_at | -0.0505 | 0.0221 | -2.289 | 0.02905789 | 0.177008 |
| X200967_at | 0.0887 | 0.0388 | 2.289 | 0.02906036 | 0.177008 |
| X215429_s_at | -0.0739 | 0.0323 | -2.289 | 0.02906952 | 0.177015 |
| X200792_at | -0.0871 | 0.0381 | -2.289 | 0.02908025 | 0.177032 |
| X212905_at | -0.0907 | 0.0396 | -2.288 | 0.02911244 | 0.177179 |
| X222312_s_at | 0.0281 | 0.0123 | 2.288 | 0.02912269 | 0.177193 |
| X215962_at | 0.1078 | 0.0471 | 2.288 | 0.02913699 | 0.177231 |
| X212669_at | 0.0489 | 0.0214 | 2.287 | 0.02916213 | 0.177336 |
| X203615_x_at | -0.1222 | 0.0534 | -2.287 | 0.02919278 | 0.177474 |
| X211628_x_at | 0.0415 | 0.0182 | 2.287 | 0.02920527 | 0.177501 |
| X214931_s_at | -0.2415 | 0.1056 | -2.286 | 0.02922227 | 0.177538 |
| X205078_at | -0.1217 | 0.0532 | -2.286 | 0.02923175 | 0.177538 |
| X212320_at | 0.1241 | 0.0543 | 2.286 | 0.02923534 | 0.177538 |
| X201230_s_at | 0.0574 | 0.0251 | 2.286 | 0.0292601 | 0.177561 |
| X209066_x_at | -0.0517 | 0.0226 | -2.286 | 0.02926888 | 0.177561 |
| X209580_s_at | -0.1043 | 0.0456 | -2.286 | 0.0292704 | 0.177561 |
| X222130_s_at | -0.058 | 0.0254 | -2.286 | 0.02927345 | 0.177561 |
| X211389_x_at | 0.0043 | 0.0019 | 2.285 | 0.02927911 | 0.177561 |
| X222327_x_at | -0.0143 | 0.0063 | -2.285 | 0.02930423 | 0.177665 |
| X212014_x_at | 0.0249 | 0.0109 | 2.285 | 0.02933291 | 0.177691 |
| X214193_s_at | -0.1069 | 0.0468 | -2.285 | 0.02933293 | 0.177691 |
| X221748_s_at | 0.2228 | 0.0975 | 2.285 | 0.02934372 | 0.177691 |
| X205187_at | 0.2149 | 0.0941 | 2.284 | 0.02934772 | 0.177691 |
| X212001_at | 0.0706 | 0.0309 | 2.284 | 0.02934851 | 0.177691 |
| X206789_s_at | 0.0702 | 0.0307 | 2.284 | 0.02936241 | 0.177727 |
| X218799_at | -0.0929 | 0.0407 | -2.284 | 0.02937232 | 0.177738 |
| X207481_at | -0.0122 | 0.0053 | -2.283 | 0.02941308 | 0.177936 |
| X209895_at | -0.1248 | 0.0547 | -2.283 | 0.02942908 | 0.177985 |
| X201767_s_at | -0.0551 | 0.0241 | -2.282 | 0.02948278 | 0.178261 |
| X202522_at | -0.058 | 0.0254 | -2.282 | 0.02949271 | 0.178272 |
| X202555_s_at | 0.2214 | 0.0971 | 2.282 | 0.02953854 | 0.178501 |
| X213123_at | 0.036 | 0.0158 | 2.281 | 0.02958471 | 0.17872 |
| X205981_s_at | -0.1017 | 0.0446 | -2.281 | 0.02959084 | 0.17872 |
| X205283_at | -0.0856 | 0.0376 | -2.28 | 0.02962405 | 0.178841 |
| X206469_x_at | -0.0524 | 0.023 | -2.28 | 0.02962696 | 0.178841 |
| X201795_at | 0.1299 | 0.057 | 2.28 | 0.02963688 | 0.178852 |
| X202779_s_at | -0.1523 | 0.0668 | -2.28 | 0.02966487 | 0.178942 |
| X210232_at | 0.3534 | 0.155 | 2.28 | 0.02966792 | 0.178942 |
| X217969_at | -0.0911 | 0.0399 | -2.279 | 0.02967816 | 0.178955 |
| X202305_s_at | 0.0744 | 0.0326 | 2.279 | 0.02971095 | 0.179104 |
| X203685_at | 0.14 | 0.0615 | 2.279 | 0.02973385 | 0.179194 |
| X218801_at | 0.1128 | 0.0495 | 2.278 | 0.02975188 | 0.179254 |
| X203583_at | -0.0673 | 0.0295 | -2.278 | 0.02978201 | 0.179387 |
| X208093_s_at | -0.0844 | 0.037 | -2.277 | 0.02981956 | 0.179512 |
| X204087_s_at | -0.1137 | 0.0499 | -2.277 | 0.02982615 | 0.179512 |
| X213288_at | 0.1623 | 0.0713 | 2.277 | 0.02982715 | 0.179512 |
| X212528_at | -0.0781 | 0.0343 | -2.277 | 0.02984428 | 0.179521 |
| X215991_s_at | -0.1118 | 0.0491 | -2.277 | 0.02985176 | 0.179521 |
| X205061_s_at | -0.0558 | 0.0245 | -2.277 | 0.02985279 | 0.179521 |
| X200673_at | 0.1075 | 0.0472 | 2.276 | 0.0298783 | 0.179626 |
| X211385_x_at | -0.0329 | 0.0145 | -2.276 | 0.02989391 | 0.179671 |
| X202868_s_at | -0.0893 | 0.0392 | -2.276 | 0.0299126 | 0.179735 |
| X212728_at | -0.1957 | 0.086 | -2.276 | 0.02993453 | 0.179738 |
| X203577_at | -0.1928 | 0.0847 | -2.276 | 0.02993556 | 0.179738 |
| X209598_at | -0.0797 | 0.035 | -2.276 | 0.02993749 | 0.179738 |
| X201280_s_at | 0.1961 | 0.0862 | 2.275 | 0.0299738 | 0.179894 |
| X220287_at | 0.0074 | 0.0032 | 2.275 | 0.02997966 | 0.179894 |
| X209682_at | 0.1069 | 0.047 | 2.274 | 0.03004138 | 0.180216 |
| X209077_at | 0.0448 | 0.0197 | 2.274 | 0.03006045 | 0.180282 |
| X212776_s_at | -0.0033 | 0.0015 | -2.274 | 0.03006991 | 0.18029 |
| X207100_s_at | 0.0074 | 0.0033 | 2.273 | 0.03008249 | 0.180316 |
| X203611_at | -0.0605 | 0.0266 | -2.273 | 0.03009861 | 0.180364 |
| X213179_at | 0.0628 | 0.0276 | 2.273 | 0.03011553 | 0.180417 |
| X208646_at | 0.0179 | 0.0079 | 2.272 | 0.03015054 | 0.180577 |
| X201136_at | 0.0183 | 0.008 | 2.272 | 0.03015855 | 0.180577 |
| X201176_s_at | -0.0557 | 0.0245 | -2.271 | 0.03021863 | 0.180888 |
| X219662_at | 0.0794 | 0.035 | 2.271 | 0.03024613 | 0.181004 |
| X204617_s_at | -0.0645 | 0.0284 | -2.271 | 0.03026273 | 0.181055 |
| X209019_s_at | -0.0977 | 0.043 | -2.27 | 0.03029996 | 0.181229 |
| X201482_at | -0.0156 | 0.0069 | -2.27 | 0.03034245 | 0.181434 |
| X209589_s_at | -0.0149 | 0.0066 | -2.269 | 0.03040242 | 0.181744 |
| X218572_at | -0.0949 | 0.0418 | -2.268 | 0.03042843 | 0.18185 |
| X215155_at | -0.0134 | 0.0059 | -2.268 | 0.03044615 | 0.181907 |
| X218107_at | -0.0762 | 0.0336 | -2.268 | 0.03046756 | 0.181949 |
| X218501_at | 0.0807 | 0.0356 | 2.268 | 0.0304696 | 0.181949 |
| X209605_at | 0.2302 | 0.1015 | 2.267 | 0.03048365 | 0.181984 |
| X221860_at | 0.1812 | 0.0799 | 2.267 | 0.03054099 | 0.182278 |
| X219737_s_at | 0.1998 | 0.0882 | 2.266 | 0.03057429 | 0.182379 |
| X218825_at | 0.0092 | 0.0041 | 2.266 | 0.03057439 | 0.182379 |
| X201897_s_at | -0.2338 | 0.1032 | -2.266 | 0.03058379 | 0.182386 |
| X213560_at | 0.0162 | 0.0071 | 2.265 | 0.03063012 | 0.182613 |
| X217751_at | 0.0815 | 0.036 | 2.264 | 0.03071691 | 0.183082 |
| X216405_at | -0.0118 | 0.0052 | -2.264 | 0.03072792 | 0.183098 |
| X208186_s_at | -0.0368 | 0.0163 | -2.263 | 0.03076383 | 0.183263 |
| X218872_at | -0.1674 | 0.074 | -2.263 | 0.03077763 | 0.183296 |
| X214319_at | -0.0393 | 0.0174 | -2.263 | 0.03079826 | 0.183352 |
| X214255_at | -0.083 | 0.0367 | -2.263 | 0.0308035 | 0.183352 |
| X209056_s_at | 0.1077 | 0.0476 | 2.263 | 0.03081254 | 0.183357 |
| X206034_at | 0.0061 | 0.0027 | 2.262 | 0.03082223 | 0.183365 |
| X211534_x_at | -0.1791 | 0.0792 | -2.262 | 0.03087624 | 0.183637 |
| X219387_at | 0.1829 | 0.0809 | 2.262 | 0.030889 | 0.183664 |
| X202678_at | -0.0733 | 0.0324 | -2.261 | 0.0309027 | 0.183696 |
| X201146_at | 0.1558 | 0.0689 | 2.261 | 0.03091748 | 0.183735 |
| X220486_x_at | -0.1868 | 0.0826 | -2.261 | 0.03093348 | 0.183735 |
| X202179_at | -0.07 | 0.031 | -2.261 | 0.03093403 | 0.183735 |
| X213505_s_at | -0.0661 | 0.0292 | -2.26 | 0.0309704 | 0.183902 |
| X218474_s_at | 0.0961 | 0.0425 | 2.26 | 0.03098132 | 0.183917 |
| X207207_at | 0.0047 | 0.0021 | 2.26 | 0.03100713 | 0.183977 |
| X217649_at | 0.0582 | 0.0258 | 2.26 | 0.03100783 | 0.183977 |
| X211623_s_at | -0.0963 | 0.0426 | -2.259 | 0.03105902 | 0.184231 |
| X219439_at | 0.1452 | 0.0643 | 2.259 | 0.03107361 | 0.184268 |
| X218806_s_at | -0.0183 | 0.0081 | -2.258 | 0.03111193 | 0.184405 |
| X221867_at | 0.011 | 0.0049 | 2.258 | 0.03111326 | 0.184405 |
| X214125_s_at | -0.0043 | 0.0019 | -2.257 | 0.03117141 | 0.1847 |
| X203567_s_at | 0.0173 | 0.0077 | 2.257 | 0.03118071 | 0.184706 |
| X205993_s_at | -0.0124 | 0.0055 | -2.257 | 0.03119854 | 0.184763 |
| X214812_s_at | 0.1701 | 0.0754 | 2.257 | 0.03122281 | 0.184818 |
| X214274_s_at | 0.0691 | 0.0306 | 2.257 | 0.03122641 | 0.184818 |
| X219485_s_at | -0.072 | 0.0319 | -2.257 | 0.03123284 | 0.184818 |
| X211679_x_at | 0.3128 | 0.1386 | 2.256 | 0.03125804 | 0.184904 |
| X209378_s_at | 0.0721 | 0.0319 | 2.256 | 0.03126407 | 0.184904 |
| X204430_s_at | 0.3131 | 0.1388 | 2.256 | 0.03127405 | 0.184914 |
| X33197_at | 0.0102 | 0.0045 | 2.255 | 0.03131265 | 0.185093 |
| X214723_x_at | 0.1688 | 0.0749 | 2.255 | 0.03133627 | 0.185145 |
| X218519_at | -0.0744 | 0.033 | -2.255 | 0.03133809 | 0.185145 |
| X208650_s_at | 0.42 | 0.1863 | 2.255 | 0.03135037 | 0.185168 |
| X201694_s_at | -0.2098 | 0.0931 | -2.255 | 0.03137415 | 0.185259 |
| X204106_at | 0.053 | 0.0235 | 2.254 | 0.03139305 | 0.185284 |
| X213718_at | 0.1229 | 0.0545 | 2.254 | 0.03139495 | 0.185284 |
| X209691_s_at | 0.0819 | 0.0363 | 2.254 | 0.03141046 | 0.1853 |
| X201603_at | -0.1317 | 0.0584 | -2.254 | 0.03141443 | 0.1853 |
| X201243_s_at | -0.0276 | 0.0123 | -2.253 | 0.03144644 | 0.18544 |
| X216383_at | -0.0168 | 0.0075 | -2.253 | 0.03146742 | 0.185514 |
| X210008_s_at | -0.0091 | 0.004 | -2.253 | 0.03147665 | 0.185519 |
| X218936_s_at | 0.0581 | 0.0258 | 2.253 | 0.03149713 | 0.185591 |
| X204459_at | 0.0376 | 0.0167 | 2.252 | 0.03151812 | 0.18563 |
| X218732_at | 0.0477 | 0.0212 | 2.252 | 0.03152594 | 0.18563 |
| X201026_at | 0.0996 | 0.0442 | 2.252 | 0.03153317 | 0.18563 |
| X213977_s_at | 0.0073 | 0.0032 | 2.252 | 0.03154519 | 0.18563 |
| X207277_at | 0.0108 | 0.0048 | 2.252 | 0.03154558 | 0.18563 |
| X212015_x_at | 0.0707 | 0.0314 | 2.252 | 0.03156732 | 0.185709 |
| X212800_at | 0.0837 | 0.0372 | 2.252 | 0.03157818 | 0.185724 |
| X218251_at | 0.1932 | 0.0858 | 2.251 | 0.03163075 | 0.185984 |
| X32837_at | 0.0114 | 0.005 | 2.251 | 0.0316434 | 0.186009 |
| X204488_at | -0.0734 | 0.0326 | -2.25 | 0.03169136 | 0.186241 |
| X200781_s_at | 0.0579 | 0.0257 | 2.25 | 0.03170117 | 0.18625 |
| X204511_at | -0.0082 | 0.0037 | -2.249 | 0.03172663 | 0.18633 |
| X208117_s_at | 0.0483 | 0.0215 | 2.249 | 0.0317328 | 0.18633 |
| X201002_s_at | -0.0599 | 0.0266 | -2.249 | 0.03173994 | 0.18633 |
| X213762_x_at | -0.0574 | 0.0255 | -2.249 | 0.03176736 | 0.186422 |
| X214843_s_at | 0.2855 | 0.1269 | 2.249 | 0.03177245 | 0.186422 |
| X212693_at | -0.0992 | 0.0441 | -2.248 | 0.03185238 | 0.186842 |
| X219495_s_at | -0.1609 | 0.0716 | -2.248 | 0.03186239 | 0.186851 |
| X217720_at | -0.0834 | 0.0371 | -2.247 | 0.03187155 | 0.186855 |
| X213815_x_at | 0.0092 | 0.0041 | 2.247 | 0.03188583 | 0.18689 |
| X219878_s_at | 0.1802 | 0.0802 | 2.247 | 0.03190688 | 0.186964 |
| X219017_at | 0.3441 | 0.1532 | 2.246 | 0.03194076 | 0.187031 |
| X216381_x_at | -0.0225 | 0.01 | -2.246 | 0.03195623 | 0.187031 |
| X205632_s_at | 0.1563 | 0.0696 | 2.246 | 0.03195671 | 0.187031 |
| X201918_at | 0.1161 | 0.0517 | 2.246 | 0.0319569 | 0.187031 |
| X212882_at | -0.0703 | 0.0313 | -2.246 | 0.03196527 | 0.187031 |
| X216109_at | 0.0455 | 0.0202 | 2.246 | 0.03196891 | 0.187031 |
| X201875_s_at | 0.02 | 0.0089 | 2.246 | 0.03200383 | 0.187186 |
| X209025_s_at | -0.0968 | 0.0431 | -2.245 | 0.03204128 | 0.187331 |
| X211875_x_at | 0.0057 | 0.0025 | 2.245 | 0.0320524 | 0.187331 |
| X212594_at | 0.1084 | 0.0483 | 2.245 | 0.03205422 | 0.187331 |
| X214546_s_at | 0.0053 | 0.0024 | 2.245 | 0.03206225 | 0.187331 |
| X209897_s_at | -0.1438 | 0.064 | -2.245 | 0.03207151 | 0.187335 |
| X201611_s_at | -0.1782 | 0.0794 | -2.244 | 0.03208085 | 0.187341 |
| X214414_x_at | 0.3686 | 0.1643 | 2.244 | 0.03209591 | 0.187379 |
| X212405_s_at | -0.0163 | 0.0072 | -2.244 | 0.03211973 | 0.187469 |
| X218034_at | -0.091 | 0.0406 | -2.243 | 0.03215877 | 0.187648 |
| X201077_s_at | -0.0986 | 0.044 | -2.242 | 0.03222648 | 0.187963 |
| X201915_at | -0.0993 | 0.0443 | -2.242 | 0.03223073 | 0.187963 |
| X209902_at | -0.0908 | 0.0405 | -2.242 | 0.03224044 | 0.187963 |
| X208391_s_at | -0.0053 | 0.0024 | -2.242 | 0.03225616 | 0.187963 |
| X219819_s_at | -0.0763 | 0.034 | -2.242 | 0.03225676 | 0.187963 |
| X209037_s_at | -0.062 | 0.0276 | -2.242 | 0.03226356 | 0.187963 |
| X202443_x_at | 0.1266 | 0.0565 | 2.241 | 0.03229355 | 0.188088 |
| X217423_at | 0.0091 | 0.004 | 2.241 | 0.03232639 | 0.188179 |
| X217118_s_at | 0.1534 | 0.0684 | 2.241 | 0.03234001 | 0.188179 |
| X222231_s_at | -0.0497 | 0.0222 | -2.241 | 0.03234334 | 0.188179 |
| X204020_at | 0.0375 | 0.0167 | 2.241 | 0.03234823 | 0.188179 |
| X212807_s_at | 0.2162 | 0.0965 | 2.241 | 0.0323515 | 0.188179 |
| X210402_at | -0.4692 | 0.2094 | -2.241 | 0.03236333 | 0.188199 |
| X209275_s_at | -0.015 | 0.0067 | -2.24 | 0.03237383 | 0.188211 |
| X203756_at | -0.1958 | 0.0874 | -2.24 | 0.03242212 | 0.188407 |
| X218347_at | -0.0997 | 0.0445 | -2.24 | 0.0324299 | 0.188407 |
| X218334_at | -0.0656 | 0.0293 | -2.24 | 0.03243421 | 0.188407 |
| X216894_x_at | 0.1355 | 0.0605 | 2.239 | 0.03244149 | 0.188407 |
| X221683_s_at | 0.1852 | 0.0827 | 2.239 | 0.03245648 | 0.188421 |
| X216120_s_at | -0.1851 | 0.0827 | -2.239 | 0.03246087 | 0.188421 |
| X212557_at | 0.0759 | 0.0339 | 2.238 | 0.03255251 | 0.188875 |
| X201936_s_at | 0.1316 | 0.0588 | 2.238 | 0.03256471 | 0.188875 |
| X57082_at | -0.0228 | 0.0102 | -2.238 | 0.03256696 | 0.188875 |
| X218091_at | -0.0626 | 0.028 | -2.238 | 0.0325731 | 0.188875 |
| X222294_s_at | 0.1503 | 0.0672 | 2.237 | 0.0325891 | 0.188918 |
| X212439_at | -0.1021 | 0.0457 | -2.237 | 0.03260855 | 0.188974 |
| X207451_at | -0.0072 | 0.0032 | -2.237 | 0.03261581 | 0.188974 |
| X220081_x_at | 0.1192 | 0.0533 | 2.237 | 0.03262807 | 0.188991 |
| X221623_at | -0.032 | 0.0143 | -2.237 | 0.03263564 | 0.188991 |
| X213517_at | 0.3058 | 0.1367 | 2.236 | 0.03266491 | 0.189111 |
| X204604_at | 0.1517 | 0.0678 | 2.236 | 0.03269502 | 0.189236 |
| X213004_at | -0.0558 | 0.025 | -2.236 | 0.03270428 | 0.18924 |
| X221719_s_at | -0.0129 | 0.0058 | -2.235 | 0.03275891 | 0.189507 |
| X222116_s_at | -0.1063 | 0.0476 | -2.234 | 0.03281068 | 0.189757 |
| X214954_at | 0.2194 | 0.0982 | 2.234 | 0.03284235 | 0.189891 |
| X217606_at | -0.0447 | 0.02 | -2.233 | 0.03290471 | 0.190202 |
| X210609_s_at | 0.0083 | 0.0037 | 2.233 | 0.03293407 | 0.190322 |
| X207381_at | -0.0056 | 0.0025 | -2.232 | 0.03294945 | 0.190361 |
| X207291_at | -0.0218 | 0.0098 | -2.232 | 0.03296911 | 0.190425 |
| X218489_s_at | -0.0081 | 0.0036 | -2.232 | 0.0329984 | 0.190545 |
| X204235_s_at | -0.1334 | 0.0598 | -2.231 | 0.03301624 | 0.190598 |
| X220914_at | -0.0092 | 0.0041 | -2.231 | 0.03302641 | 0.190608 |
| X209760_at | -0.0573 | 0.0257 | -2.231 | 0.03303514 | 0.190609 |
| X214816_x_at | -0.0149 | 0.0067 | -2.231 | 0.03305553 | 0.19065 |
| X212888_at | 0.0649 | 0.0291 | 2.231 | 0.0330595 | 0.19065 |
| X201260_s_at | 0.1701 | 0.0763 | 2.231 | 0.03308753 | 0.190762 |
| X215236_s_at | -0.2129 | 0.0955 | -2.23 | 0.03310811 | 0.190831 |
| X200675_at | 0.0702 | 0.0315 | 2.23 | 0.03312274 | 0.190865 |
| X217635_s_at | -0.0723 | 0.0324 | -2.23 | 0.03313123 | 0.190865 |
| X212586_at | 0.1122 | 0.0503 | 2.229 | 0.03316266 | 0.190966 |
| X218549_s_at | 0.2239 | 0.1004 | 2.229 | 0.03316583 | 0.190966 |
| X201467_s_at | -0.1558 | 0.0699 | -2.229 | 0.03318781 | 0.191043 |
| X204421_s_at | 0.1036 | 0.0465 | 2.229 | 0.03320222 | 0.191076 |
| X205103_at | -0.0851 | 0.0382 | -2.229 | 0.03322757 | 0.191173 |
| X218163_at | -0.1161 | 0.0521 | -2.228 | 0.03324804 | 0.191241 |
| X218836_at | -0.0189 | 0.0085 | -2.228 | 0.03326568 | 0.191256 |
| X205512_s_at | -0.1567 | 0.0703 | -2.228 | 0.03328004 | 0.191256 |
| X200886_s_at | -0.0607 | 0.0272 | -2.228 | 0.0332814 | 0.191256 |
| X207722_s_at | 0.0096 | 0.0043 | 2.228 | 0.03328503 | 0.191256 |
| X212533_at | 0.1516 | 0.0681 | 2.228 | 0.0332965 | 0.191272 |
| X217432_s_at | -0.1032 | 0.0463 | -2.227 | 0.03331506 | 0.191283 |
| X208121_s_at | -0.3394 | 0.1524 | -2.227 | 0.03332155 | 0.191283 |
| X208598_s_at | -0.1617 | 0.0726 | -2.227 | 0.0333243 | 0.191283 |
| X222366_at | 0.0552 | 0.0248 | 2.227 | 0.03333768 | 0.191311 |
| X205991_s_at | 0.0147 | 0.0066 | 2.226 | 0.0333993 | 0.191615 |
| X205712_at | 0.1081 | 0.0486 | 2.226 | 0.03341807 | 0.191673 |
| X201835_s_at | -0.0075 | 0.0034 | -2.226 | 0.03344235 | 0.191763 |
| X214670_at | -0.0986 | 0.0443 | -2.224 | 0.03355233 | 0.192344 |
| X202363_at | 0.268 | 0.1205 | 2.224 | 0.03357447 | 0.192366 |
| X217955_at | 0.1239 | 0.0557 | 2.224 | 0.0335791 | 0.192366 |
| X212530_at | 0.2175 | 0.0978 | 2.224 | 0.03358452 | 0.192366 |
| X211953_s_at | 0.0679 | 0.0305 | 2.224 | 0.0335908 | 0.192366 |
| X205752_s_at | 0.2355 | 0.1059 | 2.223 | 0.03362259 | 0.192453 |
| X210222_s_at | 0.1299 | 0.0584 | 2.223 | 0.03362346 | 0.192453 |
| X206772_at | 0.0219 | 0.0099 | 2.223 | 0.03364785 | 0.192524 |
| X206280_at | 0.2477 | 0.1114 | 2.223 | 0.03365311 | 0.192524 |
| X204054_at | 0.093 | 0.0418 | 2.222 | 0.03367956 | 0.192626 |
| X203917_at | -0.1369 | 0.0616 | -2.222 | 0.03369202 | 0.192647 |
| X220598_at | 0.0071 | 0.0032 | 2.222 | 0.03370351 | 0.192663 |
| X211799_x_at | 0.145 | 0.0652 | 2.222 | 0.03371562 | 0.192683 |
| X212318_at | 0.1024 | 0.0461 | 2.221 | 0.03376999 | 0.192872 |
| X204149_s_at | -0.0082 | 0.0037 | -2.221 | 0.03377199 | 0.192872 |
| X209898_x_at | -0.0488 | 0.022 | -2.221 | 0.03377477 | 0.192872 |
| X221832_s_at | -0.0983 | 0.0442 | -2.221 | 0.0337919 | 0.192919 |
| X203712_at | 0.0673 | 0.0303 | 2.221 | 0.03380033 | 0.192919 |
| X216252_x_at | 0.0929 | 0.0418 | 2.221 | 0.03381113 | 0.192931 |
| X218859_s_at | 0.0709 | 0.0319 | 2.22 | 0.03389915 | 0.193384 |
| X222301_at | -0.2221 | 0.1001 | -2.219 | 0.03391687 | 0.193425 |
| X218735_s_at | -0.0801 | 0.0361 | -2.219 | 0.03392375 | 0.193425 |
| X201344_at | -0.0601 | 0.0271 | -2.219 | 0.0339365 | 0.193448 |
| X219174_at | 0.1507 | 0.0679 | 2.219 | 0.03397045 | 0.193551 |
| X200638_s_at | -0.1144 | 0.0516 | -2.219 | 0.03397211 | 0.193551 |
| X203650_at | 0.0626 | 0.0282 | 2.218 | 0.03399557 | 0.193605 |
| X208372_s_at | -0.0065 | 0.0029 | -2.218 | 0.03399901 | 0.193605 |
| X212735_at | -0.0665 | 0.03 | -2.218 | 0.03404154 | 0.193798 |
| X201717_at | -0.0548 | 0.0247 | -2.217 | 0.03405977 | 0.193852 |
| X211566_x_at | -0.047 | 0.0212 | -2.217 | 0.0340831 | 0.193913 |
| X219311_at | -0.0272 | 0.0123 | -2.217 | 0.03408803 | 0.193913 |
| X219693_at | 0.1092 | 0.0492 | 2.217 | 0.03411839 | 0.194036 |
| X209003_at | -0.1334 | 0.0602 | -2.216 | 0.03418349 | 0.194357 |
| X206900_x_at | -0.034 | 0.0153 | -2.215 | 0.0342269 | 0.194554 |
| X206777_s_at | -0.0229 | 0.0103 | -2.215 | 0.03425352 | 0.194655 |
| X212638_s_at | -0.1503 | 0.0679 | -2.214 | 0.03430452 | 0.194895 |
| X219581_at | -0.151 | 0.0682 | -2.214 | 0.03431972 | 0.194932 |
| X202288_at | -0.0258 | 0.0117 | -2.214 | 0.03434324 | 0.195016 |
| X200777_s_at | 0.0626 | 0.0283 | 2.213 | 0.03436255 | 0.195075 |
| X203837_at | -0.0746 | 0.0337 | -2.213 | 0.03441033 | 0.195297 |
| X218031_s_at | 0.1834 | 0.0829 | 2.213 | 0.03442886 | 0.195308 |
| X217301_x_at | -0.1325 | 0.0599 | -2.212 | 0.03442993 | 0.195308 |
| X204135_at | 0.0209 | 0.0094 | 2.212 | 0.03445191 | 0.195383 |
| X201333_s_at | 0.0094 | 0.0043 | 2.212 | 0.03450333 | 0.195625 |
| X213045_at | -0.1071 | 0.0484 | -2.211 | 0.03457349 | 0.195972 |
| X221937_at | -0.0503 | 0.0228 | -2.21 | 0.03460231 | 0.196086 |
| X212194_s_at | 0.0059 | 0.0027 | 2.21 | 0.03462383 | 0.196158 |
| X206491_s_at | -0.0326 | 0.0148 | -2.209 | 0.03466869 | 0.196362 |
| X215913_s_at | -0.2152 | 0.0974 | -2.209 | 0.03469919 | 0.196484 |
| X208328_s_at | 0.188 | 0.0851 | 2.209 | 0.03472166 | 0.196561 |
| X204281_at | 0.0061 | 0.0028 | 2.208 | 0.03477604 | 0.196798 |
| X205870_at | 0.0098 | 0.0044 | 2.208 | 0.03478108 | 0.196798 |
| X221210_s_at | 0.0334 | 0.0151 | 2.208 | 0.03480264 | 0.196822 |
| X210109_at | 0.0079 | 0.0036 | 2.208 | 0.03480304 | 0.196822 |
| X203026_at | 0.1086 | 0.0492 | 2.207 | 0.03482456 | 0.196884 |
| X207856_s_at | 0.0936 | 0.0424 | 2.207 | 0.03483216 | 0.196884 |
| X209221_s_at | -0.0124 | 0.0056 | -2.207 | 0.03484367 | 0.196884 |
| X221932_s_at | -0.049 | 0.0222 | -2.207 | 0.03484948 | 0.196884 |
| X220610_s_at | 0.0294 | 0.0133 | 2.207 | 0.03486295 | 0.196908 |
| X206318_at | -0.0211 | 0.0095 | -2.207 | 0.03487159 | 0.196908 |
| X209829_at | -0.13 | 0.0589 | -2.207 | 0.03488338 | 0.196925 |
| X204278_s_at | 0.0891 | 0.0404 | 2.206 | 0.03490682 | 0.196988 |
| X203858_s_at | -0.0375 | 0.017 | -2.206 | 0.03491464 | 0.196988 |
| X221185_s_at | 0.2044 | 0.0926 | 2.206 | 0.03492121 | 0.196988 |
| X218952_at | -0.1522 | 0.069 | -2.206 | 0.03494273 | 0.197015 |
| X212816_s_at | 0.0689 | 0.0313 | 2.206 | 0.0349495 | 0.197015 |
| X203411_s_at | 0.2371 | 0.1075 | 2.206 | 0.0349525 | 0.197015 |
| X217534_at | -0.0921 | 0.0418 | -2.205 | 0.03497296 | 0.197034 |
| X218480_at | -0.0148 | 0.0067 | -2.205 | 0.03497367 | 0.197034 |
| X36829_at | 0.1838 | 0.0834 | 2.205 | 0.03498935 | 0.197072 |
| X202856_s_at | -0.0108 | 0.0049 | -2.204 | 0.03504749 | 0.19735 |
| X221498_at | -0.0639 | 0.029 | -2.204 | 0.03509597 | 0.197535 |
| X209029_at | -0.1018 | 0.0462 | -2.204 | 0.03509809 | 0.197535 |
| X221610_s_at | -0.0113 | 0.0051 | -2.203 | 0.03513154 | 0.197673 |
| X201970_s_at | 0.1154 | 0.0524 | 2.203 | 0.03515537 | 0.197757 |
| X206986_at | -0.009 | 0.0041 | -2.203 | 0.03517565 | 0.197821 |
| X216967_at | -0.5136 | 0.2332 | -2.202 | 0.03523042 | 0.198079 |
| X218708_at | -0.0421 | 0.0191 | -2.202 | 0.03525421 | 0.198162 |
| X205807_s_at | 0.1983 | 0.0901 | 2.201 | 0.03527948 | 0.198254 |
| X207989_at | -0.0093 | 0.0042 | -2.201 | 0.03529866 | 0.198304 |
| X209113_s_at | -0.0146 | 0.0066 | -2.201 | 0.03530617 | 0.198304 |
| X207850_at | 0.0179 | 0.0081 | 2.201 | 0.03531783 | 0.198319 |
| X213141_at | 0.016 | 0.0073 | 2.2 | 0.0353502 | 0.198451 |
| X200696_s_at | 0.1842 | 0.0837 | 2.2 | 0.03537907 | 0.198535 |
| X214786_at | -0.0173 | 0.0079 | -2.2 | 0.03538316 | 0.198535 |
| X208213_s_at | -0.5387 | 0.245 | -2.199 | 0.0354646 | 0.198942 |
| X208762_at | -0.078 | 0.0355 | -2.199 | 0.03550151 | 0.199099 |
| X212363_x_at | -0.0782 | 0.0356 | -2.198 | 0.0355389 | 0.19919 |
| X204909_at | 0.1661 | 0.0756 | 2.198 | 0.03554347 | 0.19919 |
| X219654_at | -0.208 | 0.0946 | -2.198 | 0.03554459 | 0.19919 |
| X221741_s_at | 0.0528 | 0.024 | 2.198 | 0.0355706 | 0.199285 |
| X219114_at | -0.071 | 0.0323 | -2.197 | 0.03558873 | 0.199336 |
| X221964_at | 0.024 | 0.0109 | 2.197 | 0.03563562 | 0.199543 |
| X205173_x_at | 0.1617 | 0.0736 | 2.197 | 0.035656 | 0.199543 |
| X210432_s_at | -0.1483 | 0.0675 | -2.197 | 0.03565623 | 0.199543 |
| X208826_x_at | -0.0785 | 0.0357 | -2.196 | 0.03566163 | 0.199543 |
| X200620_at | -0.0596 | 0.0271 | -2.196 | 0.0356991 | 0.199634 |
| X215605_at | 0.0086 | 0.0039 | 2.196 | 0.03570255 | 0.199634 |
| X204555_s_at | 0.0112 | 0.0051 | 2.196 | 0.03570484 | 0.199634 |
| X203062_s_at | 0.069 | 0.0314 | 2.196 | 0.03572014 | 0.19967 |
| X219002_at | 0.0901 | 0.0411 | 2.196 | 0.03573447 | 0.1997 |
| X201207_at | 0.0589 | 0.0268 | 2.195 | 0.03577341 | 0.199867 |
| X209011_at | 0.0617 | 0.0281 | 2.194 | 0.03582305 | 0.20009 |
| X215303_at | 0.109 | 0.0497 | 2.194 | 0.03583476 | 0.20009 |
| X221016_s_at | 0.0126 | 0.0057 | 2.194 | 0.03584035 | 0.20009 |
| X209617_s_at | 0.0761 | 0.0347 | 2.194 | 0.03586117 | 0.200156 |
| X216609_at | 0.1992 | 0.0908 | 2.194 | 0.03589209 | 0.200247 |
| X219974_x_at | -0.1119 | 0.051 | -2.193 | 0.03589987 | 0.200247 |
| X211952_at | -0.0783 | 0.0357 | -2.193 | 0.03590691 | 0.200247 |
| X221850_x_at | 0.0858 | 0.0391 | 2.193 | 0.03591364 | 0.200247 |
| X201081_s_at | -0.0292 | 0.0133 | -2.193 | 0.03592573 | 0.20026 |
| X214663_at | -0.0656 | 0.0299 | -2.193 | 0.03593386 | 0.20026 |
| X202313_at | -0.142 | 0.0647 | -2.193 | 0.035957 | 0.200317 |
| X218911_at | -0.159 | 0.0725 | -2.193 | 0.03596217 | 0.200317 |
| X217786_at | -0.1678 | 0.0766 | -2.192 | 0.03601245 | 0.200547 |
| X202279_at | -0.0776 | 0.0354 | -2.192 | 0.03602333 | 0.200557 |
| X210473_s_at | 0.166 | 0.0757 | 2.192 | 0.03603629 | 0.200579 |
| X204427_s_at | -0.1648 | 0.0752 | -2.191 | 0.03607469 | 0.200742 |
| X207671_s_at | -0.0404 | 0.0184 | -2.19 | 0.03614802 | 0.201048 |
| X208024_s_at | -0.0881 | 0.0402 | -2.19 | 0.0361546 | 0.201048 |
| X202688_at | -0.4231 | 0.1932 | -2.19 | 0.0361567 | 0.201048 |
| X208964_s_at | 0.1606 | 0.0733 | 2.189 | 0.03622987 | 0.201315 |
| X218615_s_at | 0.029 | 0.0133 | 2.189 | 0.03624319 | 0.201315 |
| X205328_at | -0.2828 | 0.1292 | -2.189 | 0.03625364 | 0.201315 |
| X212688_at | -0.061 | 0.0279 | -2.189 | 0.03625374 | 0.201315 |
| X219560_at | -0.0078 | 0.0035 | -2.189 | 0.03626264 | 0.201315 |
| X205762_s_at | -0.1075 | 0.0491 | -2.189 | 0.03626411 | 0.201315 |
| X214035_x_at | 0.1599 | 0.073 | 2.189 | 0.03627836 | 0.201315 |
| X202122_s_at | 0.1327 | 0.0606 | 2.189 | 0.03628197 | 0.201315 |
| X209429_x_at | -0.1012 | 0.0463 | -2.189 | 0.03628834 | 0.201315 |
| X40189_at | -0.0828 | 0.0378 | -2.188 | 0.03630376 | 0.201315 |
| X209522_s_at | 0.0559 | 0.0255 | 2.188 | 0.03630441 | 0.201315 |
| X212498_at | 0.0612 | 0.028 | 2.188 | 0.03633574 | 0.201393 |
| X208811_s_at | -0.137 | 0.0626 | -2.188 | 0.03633669 | 0.201393 |
| X214755_at | -0.0077 | 0.0035 | -2.188 | 0.03634711 | 0.201401 |
| X202593_s_at | -0.1285 | 0.0587 | -2.188 | 0.03635911 | 0.201417 |
| X212516_at | 0.0092 | 0.0042 | 2.187 | 0.03639906 | 0.201544 |
| X202537_s_at | -0.062 | 0.0283 | -2.187 | 0.03640015 | 0.201544 |
| X218102_at | 0.1397 | 0.0639 | 2.187 | 0.0364166 | 0.201553 |
| X217907_at | -0.0471 | 0.0215 | -2.187 | 0.03642376 | 0.201553 |
| X209163_at | -0.1792 | 0.0819 | -2.187 | 0.03643436 | 0.201553 |
| X202257_s_at | -0.0514 | 0.0235 | -2.187 | 0.03643901 | 0.201553 |
| X200641_s_at | -0.1582 | 0.0723 | -2.186 | 0.03645154 | 0.201553 |
| X213408_s_at | -0.0981 | 0.0449 | -2.186 | 0.03646051 | 0.201553 |
| X218082_s_at | -0.0699 | 0.032 | -2.186 | 0.03649182 | 0.201553 |
| X208706_s_at | -0.0469 | 0.0214 | -2.186 | 0.03649275 | 0.201553 |
| X218495_at | -0.0582 | 0.0266 | -2.186 | 0.03649459 | 0.201553 |
| X217788_s_at | 0.0477 | 0.0218 | 2.186 | 0.036501 | 0.201553 |
| X213656_s_at | 0.077 | 0.0352 | 2.186 | 0.03651573 | 0.201553 |
| X204229_at | 0.7086 | 0.3242 | 2.186 | 0.03651926 | 0.201553 |
| X204757_s_at | -0.1506 | 0.0689 | -2.186 | 0.03651985 | 0.201553 |
| X203887_s_at | 0.0232 | 0.0106 | 2.185 | 0.03656109 | 0.201731 |
| X207494_s_at | -0.0285 | 0.0131 | -2.185 | 0.03657667 | 0.201767 |
| X217223_s_at | -0.1488 | 0.0681 | -2.185 | 0.03659707 | 0.201803 |
| X200604_s_at | -0.2202 | 0.1008 | -2.185 | 0.03660136 | 0.201803 |
| X208107_s_at | -0.0404 | 0.0185 | -2.184 | 0.03661786 | 0.201844 |
| X211240_x_at | 0.0694 | 0.0318 | 2.184 | 0.03667468 | 0.20205 |
| X208800_at | -0.1865 | 0.0854 | -2.184 | 0.03668494 | 0.20205 |
| X216088_s_at | -0.0613 | 0.0281 | -2.183 | 0.03669449 | 0.20205 |
| X214460_at | 0.1493 | 0.0684 | 2.183 | 0.03669563 | 0.20205 |
| X218354_at | -0.142 | 0.065 | -2.183 | 0.03670077 | 0.20205 |
| X202183_s_at | 0.0197 | 0.009 | 2.183 | 0.03674789 | 0.20222 |
| X218330_s_at | 0.1103 | 0.0505 | 2.183 | 0.0367654 | 0.20222 |
| X212623_at | -0.1074 | 0.0492 | -2.182 | 0.03677753 | 0.20222 |
| X201209_at | 0.1144 | 0.0524 | 2.182 | 0.03677948 | 0.20222 |
| X218237_s_at | 0.0656 | 0.0301 | 2.182 | 0.03680148 | 0.20222 |
| X202420_s_at | -0.032 | 0.0147 | -2.182 | 0.03681163 | 0.20222 |
| X211930_at | -0.0595 | 0.0273 | -2.182 | 0.03683074 | 0.20222 |
| X201000_at | -0.087 | 0.0399 | -2.182 | 0.03683233 | 0.20222 |
| X221704_s_at | 0.0791 | 0.0362 | 2.182 | 0.03683453 | 0.20222 |
| X202235_at | 0.0775 | 0.0355 | 2.182 | 0.03683892 | 0.20222 |
| X203353_s_at | -0.0618 | 0.0283 | -2.182 | 0.03684011 | 0.20222 |
| X217762_s_at | 0.1164 | 0.0533 | 2.182 | 0.03684096 | 0.20222 |
| X202622_s_at | 0.0625 | 0.0286 | 2.181 | 0.03685696 | 0.202258 |
| X219877_at | -0.2944 | 0.135 | -2.181 | 0.03688207 | 0.202346 |
| X217977_at | -0.0787 | 0.0361 | -2.181 | 0.0369288 | 0.202476 |
| X218660_at | 0.1336 | 0.0613 | 2.18 | 0.03693288 | 0.202476 |
| X222138_s_at | -0.1001 | 0.0459 | -2.18 | 0.03693311 | 0.202476 |
| X219506_at | 0.189 | 0.0867 | 2.18 | 0.03698204 | 0.202694 |
| X218951_s_at | -0.2187 | 0.1003 | -2.179 | 0.03703336 | 0.202887 |
| X219062_s_at | 0.0735 | 0.0337 | 2.179 | 0.03703541 | 0.202887 |
| X211450_s_at | -0.078 | 0.0358 | -2.179 | 0.03705388 | 0.20291 |
| X212894_at | 0.0472 | 0.0217 | 2.179 | 0.03705803 | 0.20291 |
| X38487_at | 0.0147 | 0.0068 | 2.178 | 0.03713755 | 0.203296 |
| X209273_s_at | -0.0913 | 0.0419 | -2.177 | 0.0372143 | 0.203666 |
| X202141_s_at | -0.0678 | 0.0312 | -2.177 | 0.03723892 | 0.203685 |
| X201233_at | -0.0939 | 0.0431 | -2.177 | 0.03724143 | 0.203685 |
| X203480_s_at | -0.0654 | 0.03 | -2.177 | 0.03724542 | 0.203685 |
| X200947_s_at | -0.1272 | 0.0585 | -2.176 | 0.03727946 | 0.203821 |
| X212036_s_at | 0.0891 | 0.0409 | 2.176 | 0.03730711 | 0.203922 |
| X214310_s_at | -0.0139 | 0.0064 | -2.176 | 0.03732378 | 0.20393 |
| X204847_at | -0.0846 | 0.0389 | -2.176 | 0.03732694 | 0.20393 |
| X65588_at | -0.0146 | 0.0067 | -2.175 | 0.03740732 | 0.204257 |
| X219286_s_at | 0.0844 | 0.0388 | 2.175 | 0.03741304 | 0.204257 |
| X201677_at | -0.064 | 0.0294 | -2.175 | 0.03741728 | 0.204257 |
| X216807_at | 0.0079 | 0.0036 | 2.174 | 0.03742354 | 0.204257 |
| X214280_x_at | 0.0784 | 0.0361 | 2.174 | 0.03747448 | 0.204448 |
| X213214_x_at | -0.0578 | 0.0266 | -2.174 | 0.03747688 | 0.204448 |
| X201634_s_at | 0.0682 | 0.0314 | 2.173 | 0.03751913 | 0.204628 |
| X202554_s_at | -0.1572 | 0.0724 | -2.172 | 0.03765288 | 0.205307 |
| X218791_s_at | -0.042 | 0.0194 | -2.171 | 0.03767803 | 0.205394 |
| X219088_s_at | -0.0731 | 0.0337 | -2.171 | 0.03772105 | 0.205549 |
| X217612_at | -0.0621 | 0.0286 | -2.171 | 0.03772627 | 0.205549 |
| X215215_s_at | -0.0097 | 0.0045 | -2.171 | 0.03773427 | 0.205549 |
| X212290_at | 0.0527 | 0.0243 | 2.17 | 0.03778329 | 0.205766 |
| X218695_at | -0.1345 | 0.062 | -2.17 | 0.03782287 | 0.20591 |
| X202961_s_at | -0.0517 | 0.0239 | -2.17 | 0.03782832 | 0.20591 |
| X202292_x_at | -0.0841 | 0.0388 | -2.169 | 0.03786539 | 0.206061 |
| X219377_at | 0.1277 | 0.0589 | 2.169 | 0.03788552 | 0.20612 |
| X218058_at | -0.0664 | 0.0306 | -2.169 | 0.03789863 | 0.206141 |
| X219078_at | 0.0192 | 0.0089 | 2.168 | 0.03793269 | 0.206276 |
| X205238_at | -0.0293 | 0.0135 | -2.168 | 0.03795368 | 0.20634 |
| X207735_at | -0.1503 | 0.0693 | -2.168 | 0.03798717 | 0.206347 |
| X203755_at | -0.0172 | 0.0079 | -2.167 | 0.03801069 | 0.206347 |
| X207974_s_at | -0.1012 | 0.0467 | -2.167 | 0.0380128 | 0.206347 |
| X201225_s_at | 0.0531 | 0.0245 | 2.167 | 0.03802252 | 0.206347 |
| X207637_at | 0.0058 | 0.0027 | 2.167 | 0.03802297 | 0.206347 |
| X200636_s_at | -0.0634 | 0.0292 | -2.167 | 0.03802516 | 0.206347 |
| X206118_at | -0.2363 | 0.1091 | -2.167 | 0.03803282 | 0.206347 |
| X219263_at | -0.1849 | 0.0853 | -2.167 | 0.03804027 | 0.206347 |
| X209770_at | 0.1638 | 0.0756 | 2.167 | 0.03804668 | 0.206347 |
| X214233_at | -0.009 | 0.0042 | -2.167 | 0.03805375 | 0.206347 |
| X216192_at | -0.0083 | 0.0038 | -2.167 | 0.03805725 | 0.206347 |
| X212219_at | 0.1691 | 0.078 | 2.166 | 0.03808575 | 0.206451 |
| X58696_at | -0.0826 | 0.0381 | -2.166 | 0.03811036 | 0.206534 |
| X208653_s_at | 0.1161 | 0.0537 | 2.165 | 0.03823874 | 0.207165 |
| X206673_at | -0.0056 | 0.0026 | -2.164 | 0.03825215 | 0.207165 |
| X202426_s_at | -0.0092 | 0.0043 | -2.164 | 0.03825468 | 0.207165 |
| X211761_s_at | -0.0719 | 0.0332 | -2.164 | 0.03828194 | 0.207234 |
| X213878_at | -0.0886 | 0.0409 | -2.164 | 0.03828955 | 0.207234 |
| X201229_s_at | 0.088 | 0.0407 | 2.164 | 0.03829541 | 0.207234 |
| X219441_s_at | -0.012 | 0.0055 | -2.164 | 0.03831201 | 0.207273 |
| X204527_at | -0.1454 | 0.0672 | -2.163 | 0.03838259 | 0.207604 |
| X211999_at | 0.1551 | 0.0717 | 2.162 | 0.03843102 | 0.207816 |
| X209075_s_at | -0.1082 | 0.05 | -2.162 | 0.03844342 | 0.207832 |
| X218895_at | -0.0078 | 0.0036 | -2.162 | 0.03846164 | 0.20788 |
| X200825_s_at | -0.0797 | 0.0369 | -2.162 | 0.03849327 | 0.208 |
| X222035_s_at | -0.0829 | 0.0383 | -2.161 | 0.03852869 | 0.208105 |
| X215869_at | -0.01 | 0.0046 | -2.161 | 0.03853143 | 0.208105 |
| X212287_at | -0.0515 | 0.0238 | -2.16 | 0.03860391 | 0.208446 |
| X219574_at | 0.1868 | 0.0865 | 2.16 | 0.03863701 | 0.208524 |
| X206928_at | 0.1535 | 0.0711 | 2.159 | 0.03866387 | 0.208524 |
| X205902_at | 0.1087 | 0.0503 | 2.159 | 0.03866404 | 0.208524 |
| X208092_s_at | -0.2011 | 0.0931 | -2.159 | 0.03866436 | 0.208524 |
| X203605_at | -0.0858 | 0.0397 | -2.159 | 0.03866865 | 0.208524 |
| X204417_at | 0.1 | 0.0463 | 2.159 | 0.03868154 | 0.208524 |
| X222047_s_at | -0.0838 | 0.0388 | -2.159 | 0.03868408 | 0.208524 |
| X210666_at | 0.011 | 0.0051 | 2.159 | 0.03869814 | 0.208549 |
| X216083_s_at | 0.0061 | 0.0028 | 2.159 | 0.03872452 | 0.20862 |
| X206769_at | -0.0305 | 0.0141 | -2.159 | 0.03873836 | 0.20862 |
| X211795_s_at | 0.1304 | 0.0604 | 2.159 | 0.03873939 | 0.20862 |
| X209309_at | 0.0479 | 0.0222 | 2.158 | 0.03874991 | 0.208626 |
| X217139_at | -0.0089 | 0.0041 | -2.158 | 0.03878847 | 0.208783 |
| X220558_x_at | -0.0063 | 0.0029 | -2.158 | 0.0388289 | 0.20895 |
| X218532_s_at | 0.1166 | 0.054 | 2.157 | 0.0388681 | 0.209065 |
| X211036_x_at | -0.0692 | 0.0321 | -2.157 | 0.0388691 | 0.209065 |
| X201554_x_at | -0.0529 | 0.0245 | -2.157 | 0.03890806 | 0.209142 |
| X218653_at | -0.1264 | 0.0586 | -2.157 | 0.0389089 | 0.209142 |
| X207738_s_at | -0.0682 | 0.0316 | -2.157 | 0.03891159 | 0.209142 |
| X209457_at | -0.2828 | 0.1312 | -2.156 | 0.03895831 | 0.209342 |
| X200023_s_at | -0.0623 | 0.0289 | -2.156 | 0.03897339 | 0.209372 |
| X212702_s_at | 0.0703 | 0.0326 | 2.155 | 0.03902153 | 0.20958 |
| X209641_s_at | 0.0063 | 0.0029 | 2.155 | 0.03906775 | 0.209778 |
| X219487_at | -0.0882 | 0.0409 | -2.154 | 0.03910029 | 0.209876 |
| X221459_at | 0.0044 | 0.0021 | 2.154 | 0.0391049 | 0.209876 |
| X209082_s_at | 0.1197 | 0.0556 | 2.154 | 0.03911836 | 0.209897 |
| X220587_s_at | -0.07 | 0.0325 | -2.154 | 0.03913962 | 0.209961 |
| X208671_at | -0.0479 | 0.0222 | -2.154 | 0.03916445 | 0.210043 |
| X206410_at | 0.0047 | 0.0022 | 2.153 | 0.03918284 | 0.210091 |
| X212208_at | 0.1699 | 0.0789 | 2.153 | 0.03924854 | 0.210284 |
| X208907_s_at | -0.087 | 0.0404 | -2.152 | 0.03926819 | 0.210284 |
| X201013_s_at | 0.1263 | 0.0587 | 2.152 | 0.03927261 | 0.210284 |
| X216092_s_at | 0.1172 | 0.0545 | 2.152 | 0.03928191 | 0.210284 |
| X213969_x_at | 0.0503 | 0.0234 | 2.152 | 0.03928402 | 0.210284 |
| X208003_s_at | 0.188 | 0.0874 | 2.152 | 0.03928619 | 0.210284 |
| X212056_at | 0.1003 | 0.0466 | 2.152 | 0.03928737 | 0.210284 |
| X203687_at | -0.1435 | 0.0667 | -2.152 | 0.03929457 | 0.210284 |
| X222154_s_at | 0.1061 | 0.0493 | 2.152 | 0.03931564 | 0.210346 |
| X203157_s_at | -0.1636 | 0.076 | -2.152 | 0.03934031 | 0.210428 |
| X213690_s_at | -0.0087 | 0.0041 | -2.151 | 0.03936261 | 0.210496 |
| X202192_s_at | -0.0991 | 0.0461 | -2.151 | 0.03938182 | 0.210548 |
| X211143_x_at | -0.0103 | 0.0048 | -2.151 | 0.03939133 | 0.210548 |
| X209377_s_at | 0.0555 | 0.0258 | 2.151 | 0.03940592 | 0.210576 |
| X1316_at | 0.2056 | 0.0956 | 2.15 | 0.0394387 | 0.2107 |
| X202239_at | 0.1409 | 0.0655 | 2.15 | 0.03946317 | 0.21073 |
| X209824_s_at | -0.1419 | 0.066 | -2.15 | 0.03947171 | 0.21073 |
| X204977_at | -0.1498 | 0.0697 | -2.15 | 0.03947277 | 0.21073 |
| X212724_at | -0.1775 | 0.0826 | -2.15 | 0.03949509 | 0.210778 |
| X208897_s_at | -0.0358 | 0.0166 | -2.15 | 0.0395034 | 0.210778 |
| X210651_s_at | -0.0122 | 0.0057 | -2.15 | 0.03951016 | 0.210778 |
| X210582_s_at | 0.0637 | 0.0296 | 2.149 | 0.03959107 | 0.211159 |
| X206451_at | 0.1105 | 0.0514 | 2.148 | 0.03962072 | 0.211266 |
| X200833_s_at | 0.0963 | 0.0448 | 2.148 | 0.03964475 | 0.211344 |
| X221425_s_at | -0.1049 | 0.0488 | -2.147 | 0.03971275 | 0.211617 |
| X201888_s_at | 0.1052 | 0.049 | 2.147 | 0.03971504 | 0.211617 |
| X201672_s_at | -0.1084 | 0.0505 | -2.146 | 0.03978046 | 0.211865 |
| X206852_at | -0.0427 | 0.0199 | -2.146 | 0.03978063 | 0.211865 |
| X216515_x_at | 0.071 | 0.0331 | 2.145 | 0.03988495 | 0.212369 |
| X202847_at | 0.0099 | 0.0046 | 2.145 | 0.03990717 | 0.212378 |
| X221064_s_at | 0.014 | 0.0065 | 2.145 | 0.03991514 | 0.212378 |
| X35147_at | 0.0723 | 0.0337 | 2.145 | 0.03991862 | 0.212378 |
| X201625_s_at | 0.056 | 0.0261 | 2.145 | 0.0399249 | 0.212378 |
| X211227_s_at | -0.1035 | 0.0483 | -2.144 | 0.03996044 | 0.212517 |
| X220047_at | -0.0137 | 0.0064 | -2.144 | 0.03997679 | 0.212518 |
| X201117_s_at | -0.0751 | 0.035 | -2.144 | 0.03998739 | 0.212518 |
| X218582_at | 0.1201 | 0.056 | 2.144 | 0.03998949 | 0.212518 |
| X221960_s_at | 0.1043 | 0.0486 | 2.144 | 0.04000383 | 0.212544 |
| X213797_at | -0.0134 | 0.0062 | -2.142 | 0.04013298 | 0.213179 |
| X215146_s_at | -0.0756 | 0.0353 | -2.142 | 0.04014368 | 0.213185 |
| X222325_at | 0.0227 | 0.0106 | 2.142 | 0.04017202 | 0.213284 |
| X203865_s_at | -0.0819 | 0.0382 | -2.141 | 0.0402348 | 0.213567 |
| X210102_at | 0.117 | 0.0547 | 2.141 | 0.04027745 | 0.213709 |
| X212131_at | 0.0707 | 0.033 | 2.141 | 0.04028085 | 0.213709 |
| X200610_s_at | -0.0581 | 0.0271 | -2.14 | 0.0402967 | 0.213742 |
| X65585_at | -0.0319 | 0.0149 | -2.14 | 0.04031813 | 0.213805 |
| X203005_at | 0.0102 | 0.0048 | 2.14 | 0.04034785 | 0.213911 |
| X213194_at | -0.0892 | 0.0417 | -2.139 | 0.04038396 | 0.214051 |
| X203479_s_at | 0.0309 | 0.0144 | 2.139 | 0.0404165 | 0.214173 |
| X218854_at | 0.1372 | 0.0642 | 2.138 | 0.04046546 | 0.214375 |
| X214922_at | -0.0105 | 0.0049 | -2.138 | 0.04048024 | 0.214375 |
| X205109_s_at | -0.1026 | 0.048 | -2.138 | 0.04048468 | 0.214375 |
| X1729_at | 0.0168 | 0.0078 | 2.138 | 0.04049325 | 0.214375 |
| X218903_s_at | -0.0911 | 0.0426 | -2.137 | 0.04055336 | 0.214642 |
| X220154_at | -0.0089 | 0.0042 | -2.137 | 0.04059053 | 0.214739 |
| X210078_s_at | -0.1486 | 0.0695 | -2.137 | 0.04059107 | 0.214739 |
| X209165_at | -0.0714 | 0.0334 | -2.137 | 0.04062734 | 0.21488 |
| X201586_s_at | -0.122 | 0.0571 | -2.136 | 0.04069227 | 0.215125 |
| X220491_at | 0.2839 | 0.1329 | 2.136 | 0.04069419 | 0.215125 |
| X219896_at | -0.2392 | 0.112 | -2.136 | 0.04070345 | 0.215125 |
| X211734_s_at | -0.0186 | 0.0087 | -2.136 | 0.04071235 | 0.215125 |
| X205895_s_at | -0.0315 | 0.0147 | -2.136 | 0.04072437 | 0.215137 |
| X202988_s_at | 0.711 | 0.3331 | 2.135 | 0.04080138 | 0.215493 |
| X207387_s_at | -0.0192 | 0.009 | -2.134 | 0.04081738 | 0.215526 |
| X204059_s_at | -0.1068 | 0.05 | -2.134 | 0.04082725 | 0.215527 |
| X220798_x_at | -0.0132 | 0.0062 | -2.134 | 0.04086893 | 0.215684 |
| X219016_at | -0.098 | 0.0459 | -2.134 | 0.04087637 | 0.215684 |
| X208804_s_at | -0.0775 | 0.0363 | -2.134 | 0.04089292 | 0.21572 |
| X200068_s_at | 0.0642 | 0.0301 | 2.133 | 0.04091931 | 0.215808 |
| X205645_at | -0.0256 | 0.012 | -2.133 | 0.04098917 | 0.216103 |
| X209210_s_at | 0.0898 | 0.0421 | 2.132 | 0.0409947 | 0.216103 |
| X212452_x_at | -0.0451 | 0.0211 | -2.132 | 0.0410729 | 0.21641 |
| X221979_at | -0.0463 | 0.0217 | -2.132 | 0.04108043 | 0.21641 |
| X213185_at | 0.0159 | 0.0075 | 2.131 | 0.04108582 | 0.21641 |
| X215598_at | -0.0117 | 0.0055 | -2.131 | 0.04109203 | 0.21641 |
| X216126_at | 0.0085 | 0.004 | 2.131 | 0.04111795 | 0.216481 |
| X203252_at | -0.1455 | 0.0683 | -2.131 | 0.04113177 | 0.216481 |
| X212703_at | 0.0379 | 0.0178 | 2.131 | 0.04113461 | 0.216481 |
| X203120_at | 0.1293 | 0.0607 | 2.131 | 0.04114677 | 0.216493 |
| X202176_at | -0.0999 | 0.0469 | -2.13 | 0.0411854 | 0.216622 |
| X214405_at | 0.1457 | 0.0684 | 2.13 | 0.04119078 | 0.216622 |
| X213991_s_at | -0.0039 | 0.0018 | -2.13 | 0.04123176 | 0.216786 |
| X213664_at | -0.108 | 0.0507 | -2.13 | 0.04124543 | 0.216807 |
| X204222_s_at | -0.1309 | 0.0615 | -2.129 | 0.04128126 | 0.216944 |
| X218954_s_at | -0.0165 | 0.0077 | -2.129 | 0.04130161 | 0.216988 |
| X207007_at | -0.0114 | 0.0054 | -2.129 | 0.04131529 | 0.216988 |
| X213405_at | -0.0894 | 0.042 | -2.129 | 0.04132182 | 0.216988 |
| X208353_x_at | 0.0076 | 0.0036 | 2.129 | 0.04133378 | 0.216988 |
| X202282_at | 0.0596 | 0.028 | 2.129 | 0.04133844 | 0.216988 |
| X212166_at | -0.1141 | 0.0536 | -2.128 | 0.04135266 | 0.217011 |
| X206958_s_at | -0.1082 | 0.0508 | -2.128 | 0.0413736 | 0.21707 |
| X208727_s_at | 0.2376 | 0.1117 | 2.128 | 0.04140459 | 0.217181 |
| X205388_at | -0.0154 | 0.0073 | -2.128 | 0.04141841 | 0.217202 |
| X204971_at | 0.0238 | 0.0112 | 2.127 | 0.04152471 | 0.217708 |
| X210674_s_at | -0.1062 | 0.0499 | -2.126 | 0.04154347 | 0.217755 |
| X220361_at | 0.0252 | 0.0119 | 2.126 | 0.041579 | 0.217789 |
| X217966_s_at | 0.0188 | 0.0088 | 2.126 | 0.04157949 | 0.217789 |
| X202321_at | -0.1119 | 0.0527 | -2.126 | 0.04158832 | 0.217789 |
| X206770_s_at | -0.126 | 0.0593 | -2.126 | 0.04158921 | 0.217789 |
| X201765_s_at | -0.0659 | 0.031 | -2.126 | 0.04159889 | 0.217789 |
| X206055_s_at | 0.0493 | 0.0232 | 2.126 | 0.04161286 | 0.217811 |
| X206865_at | 0.0052 | 0.0025 | 2.125 | 0.04162998 | 0.217849 |
| X214890_s_at | -0.2255 | 0.1061 | -2.125 | 0.04167224 | 0.21797 |
| X217943_s_at | -0.1112 | 0.0524 | -2.125 | 0.04168771 | 0.21797 |
| X218716_x_at | -0.0708 | 0.0333 | -2.125 | 0.04168799 | 0.21797 |
| X206868_at | 0.0216 | 0.0102 | 2.125 | 0.04169556 | 0.21797 |
| X210105_s_at | 0.0682 | 0.0321 | 2.125 | 0.0417022 | 0.21797 |
| X212961_x_at | -0.1235 | 0.0581 | -2.124 | 0.04171304 | 0.217975 |
| X221440_s_at | -0.0074 | 0.0035 | -2.124 | 0.041767 | 0.218198 |
| X201571_s_at | -0.082 | 0.0386 | -2.124 | 0.04177802 | 0.218198 |
| X204616_at | -0.1753 | 0.0826 | -2.124 | 0.04178514 | 0.218198 |
| X201055_s_at | 0.1144 | 0.0539 | 2.123 | 0.04181743 | 0.218316 |
| X213839_at | 0.2933 | 0.1382 | 2.123 | 0.04185704 | 0.218433 |
| X210050_at | -0.0093 | 0.0044 | -2.123 | 0.04185962 | 0.218433 |
| X213730_x_at | 0.058 | 0.0273 | 2.122 | 0.04193338 | 0.218767 |
| X202640_s_at | 0.0517 | 0.0244 | 2.121 | 0.04197883 | 0.218952 |
| X212560_at | -0.0829 | 0.0391 | -2.121 | 0.04202034 | 0.219077 |
| X202362_at | 0.1262 | 0.0595 | 2.121 | 0.04202244 | 0.219077 |
| X220520_s_at | 0.0741 | 0.035 | 2.121 | 0.04203593 | 0.219092 |
| X205344_at | -0.2119 | 0.0999 | -2.121 | 0.04204503 | 0.219092 |
| X219389_at | -0.1353 | 0.0638 | -2.12 | 0.04207978 | 0.219221 |
| X202681_at | -0.087 | 0.0411 | -2.12 | 0.04213648 | 0.219465 |
| X219147_s_at | 0.1759 | 0.083 | 2.12 | 0.04214733 | 0.21947 |
| X201919_at | 0.0577 | 0.0272 | 2.12 | 0.04215752 | 0.219472 |
| X213262_at | -0.0975 | 0.046 | -2.119 | 0.04221479 | 0.219719 |
| X215280_s_at | 0.0789 | 0.0373 | 2.119 | 0.04223531 | 0.219772 |
| X211569_s_at | -0.115 | 0.0543 | -2.119 | 0.04224611 | 0.219772 |
| X208814_at | -0.1518 | 0.0716 | -2.118 | 0.04225462 | 0.219772 |
| X208775_at | 0.0636 | 0.03 | 2.117 | 0.04236012 | 0.22025 |
| X214094_at | -0.01 | 0.0047 | -2.117 | 0.04236649 | 0.22025 |
| X203604_at | -0.1723 | 0.0814 | -2.117 | 0.0423971 | 0.220358 |
| X203989_x_at | 0.1267 | 0.0599 | 2.116 | 0.04244516 | 0.220556 |
| X222263_at | -0.0205 | 0.0097 | -2.116 | 0.04251822 | 0.220806 |
| X206518_s_at | -0.3316 | 0.1567 | -2.116 | 0.04252095 | 0.220806 |
| X218224_at | -0.088 | 0.0416 | -2.115 | 0.04252759 | 0.220806 |
| X221802_s_at | 0.1843 | 0.0871 | 2.115 | 0.04253302 | 0.220806 |
| X202491_s_at | -0.1178 | 0.0557 | -2.115 | 0.04255032 | 0.220831 |
| X56256_at | -0.0647 | 0.0306 | -2.115 | 0.04256516 | 0.220831 |
| X201843_s_at | -0.2755 | 0.1303 | -2.115 | 0.0425677 | 0.220831 |
| X205322_s_at | 0.0429 | 0.0203 | 2.115 | 0.04260449 | 0.220915 |
| X218071_s_at | 0.0401 | 0.019 | 2.115 | 0.04260549 | 0.220915 |
| X221164_x_at | 0.008 | 0.0038 | 2.115 | 0.04261375 | 0.220915 |
| X203297_s_at | 0.0916 | 0.0433 | 2.114 | 0.0426337 | 0.220967 |
| X221693_s_at | -0.1741 | 0.0824 | -2.114 | 0.0426642 | 0.22105 |
| X201247_at | 0.0666 | 0.0315 | 2.114 | 0.0426696 | 0.22105 |
| X211934_x_at | -0.0585 | 0.0277 | -2.114 | 0.04268828 | 0.221095 |
| X201198_s_at | -0.0872 | 0.0413 | -2.113 | 0.04272028 | 0.221205 |
| X205030_at | 0.2167 | 0.1026 | 2.113 | 0.04273131 | 0.221205 |
| X209130_at | 0.1724 | 0.0816 | 2.113 | 0.04273935 | 0.221205 |
| X203762_s_at | -0.0977 | 0.0462 | -2.113 | 0.04276893 | 0.221304 |
| X210737_at | -0.0064 | 0.003 | -2.113 | 0.04277835 | 0.221304 |
| X212962_at | -0.007 | 0.0033 | -2.112 | 0.0428042 | 0.221386 |
| X209380_s_at | -0.094 | 0.0445 | -2.112 | 0.04282068 | 0.22142 |
| X209715_at | 0.1487 | 0.0704 | 2.112 | 0.04287895 | 0.221669 |
| X206728_at | -0.0578 | 0.0274 | -2.111 | 0.04290059 | 0.221712 |
| X211373_s_at | 0.0404 | 0.0192 | 2.111 | 0.04291549 | 0.221712 |
| X203825_at | 0.0846 | 0.0401 | 2.111 | 0.04291716 | 0.221712 |
| X208250_s_at | -0.0153 | 0.0072 | -2.111 | 0.04297022 | 0.22188 |
| X207855_s_at | -0.0904 | 0.0428 | -2.111 | 0.04297857 | 0.22188 |
| X205195_at | -0.1567 | 0.0742 | -2.111 | 0.0429797 | 0.22188 |
| X215336_at | -0.0194 | 0.0092 | -2.11 | 0.04299998 | 0.221933 |
| X209115_at | -0.0586 | 0.0278 | -2.11 | 0.04303911 | 0.222084 |
| X218202_x_at | -0.0466 | 0.0221 | -2.11 | 0.04305726 | 0.222126 |
| X208692_at | -0.0864 | 0.041 | -2.11 | 0.04307379 | 0.222148 |
| X217457_s_at | -0.1117 | 0.0529 | -2.109 | 0.04308163 | 0.222148 |
| X202889_x_at | -0.0691 | 0.0328 | -2.109 | 0.04309804 | 0.222181 |
| X213038_at | -0.009 | 0.0043 | -2.109 | 0.04315215 | 0.222409 |
| X204659_s_at | -0.0058 | 0.0028 | -2.108 | 0.04317413 | 0.222471 |
| X218592_s_at | -0.0443 | 0.021 | -2.108 | 0.0432415 | 0.222766 |
| X216457_s_at | -0.0677 | 0.0321 | -2.108 | 0.04325454 | 0.222781 |
| X207172_s_at | -0.121 | 0.0574 | -2.107 | 0.04327016 | 0.22281 |
| X207404_s_at | -0.1148 | 0.0545 | -2.107 | 0.04329144 | 0.222868 |
| X208918_s_at | 0.1254 | 0.0595 | 2.107 | 0.04333744 | 0.22302 |
| X211812_s_at | -0.2383 | 0.1131 | -2.107 | 0.04334098 | 0.22302 |
| X208803_s_at | 0.2364 | 0.1122 | 2.106 | 0.0433748 | 0.223076 |
| X216640_s_at | -0.096 | 0.0456 | -2.106 | 0.04338595 | 0.223076 |
| X206970_at | 0.0133 | 0.0063 | 2.106 | 0.04338627 | 0.223076 |
| X218740_s_at | -0.0733 | 0.0348 | -2.106 | 0.04339211 | 0.223076 |
| X203801_at | -0.075 | 0.0356 | -2.106 | 0.0434077 | 0.223105 |
| X221251_x_at | -0.0084 | 0.004 | -2.106 | 0.04341927 | 0.223113 |
| X203266_s_at | -0.0647 | 0.0307 | -2.106 | 0.04343833 | 0.223159 |
| X209147_s_at | 0.1565 | 0.0744 | 2.105 | 0.04351882 | 0.223521 |
| X218402_s_at | -0.0554 | 0.0263 | -2.105 | 0.04353239 | 0.223539 |
| X218958_at | -0.0591 | 0.0281 | -2.104 | 0.04355663 | 0.223612 |
| X214482_at | -0.0061 | 0.0029 | -2.104 | 0.04359039 | 0.223733 |
| X212948_at | 0.0573 | 0.0273 | 2.104 | 0.04363248 | 0.223882 |
| X200012_x_at | -0.059 | 0.0281 | -2.103 | 0.04363959 | 0.223882 |
| X212598_at | 0.0562 | 0.0267 | 2.103 | 0.04367103 | 0.223992 |
| X219123_at | 0.0861 | 0.041 | 2.103 | 0.04368755 | 0.224025 |
| X203366_at | 0.0406 | 0.0193 | 2.103 | 0.04371616 | 0.22412 |
| X218853_s_at | 0.0953 | 0.0454 | 2.102 | 0.0437578 | 0.224281 |
| X204990_s_at | 0.2097 | 0.0998 | 2.102 | 0.04380206 | 0.224368 |
| X64438_at | 0.0359 | 0.0171 | 2.102 | 0.04380499 | 0.224368 |
| X211495_x_at | -0.1404 | 0.0668 | -2.102 | 0.0438076 | 0.224368 |
| X213841_at | 0.1157 | 0.055 | 2.102 | 0.04381502 | 0.224368 |
| X205882_x_at | 0.09 | 0.0428 | 2.101 | 0.04391497 | 0.224828 |
| X200927_s_at | -0.1065 | 0.0507 | -2.1 | 0.04394222 | 0.224915 |
| X215467_x_at | 0.1314 | 0.0626 | 2.1 | 0.04397055 | 0.225009 |
| X203979_at | 0.0246 | 0.0117 | 2.1 | 0.04398809 | 0.225019 |
| X217926_at | -0.0655 | 0.0312 | -2.1 | 0.04399288 | 0.225019 |
| X203804_s_at | 0.0657 | 0.0313 | 2.099 | 0.044016 | 0.225086 |
| X212889_x_at | 0.0038 | 0.0018 | 2.099 | 0.04402991 | 0.225105 |
| X205737_at | 0.1286 | 0.0613 | 2.099 | 0.04406321 | 0.225223 |
| X213398_s_at | -0.0348 | 0.0166 | -2.099 | 0.04409737 | 0.225346 |
| X213836_s_at | 0.0863 | 0.0411 | 2.098 | 0.04412793 | 0.225415 |
| X209556_at | -0.1526 | 0.0727 | -2.098 | 0.04413109 | 0.225415 |
| X214290_s_at | 0.1894 | 0.0903 | 2.098 | 0.04416301 | 0.225526 |
| X220711_at | 0.1057 | 0.0504 | 2.098 | 0.04417618 | 0.225541 |
| X212841_s_at | 0.0996 | 0.0475 | 2.098 | 0.04419259 | 0.22555 |
| X217714_x_at | 0.0556 | 0.0265 | 2.097 | 0.04419906 | 0.22555 |
| X206264_at | 0.014 | 0.0067 | 2.097 | 0.04420841 | 0.22555 |
| X212334_at | 0.0677 | 0.0323 | 2.097 | 0.04424326 | 0.225641 |
| X218196_at | -0.0974 | 0.0464 | -2.097 | 0.04424642 | 0.225641 |
| X214845_s_at | -0.0153 | 0.0073 | -2.097 | 0.04428717 | 0.225797 |
| X217778_at | 0.0264 | 0.0126 | 2.096 | 0.04430189 | 0.22582 |
| X209531_at | -0.0825 | 0.0394 | -2.096 | 0.0443395 | 0.22596 |
| X211586_s_at | -0.0103 | 0.0049 | -2.096 | 0.04437905 | 0.226084 |
| X208850_s_at | -0.1719 | 0.082 | -2.096 | 0.04438436 | 0.226084 |
| X202603_at | -0.0655 | 0.0312 | -2.095 | 0.0444028 | 0.226121 |
| X205924_at | 0.0584 | 0.0278 | 2.095 | 0.04441189 | 0.226121 |
| X203147_s_at | 0.1234 | 0.0589 | 2.095 | 0.04442815 | 0.226152 |
| X218857_s_at | 0.1372 | 0.0655 | 2.095 | 0.04444151 | 0.226168 |
| X201402_at | 0.013 | 0.0062 | 2.094 | 0.04453456 | 0.226544 |
| X210438_x_at | 0.1181 | 0.0564 | 2.094 | 0.04453573 | 0.226544 |
| X215014_at | -0.1108 | 0.0529 | -2.094 | 0.04456061 | 0.226605 |
| X215089_s_at | 0.0662 | 0.0316 | 2.094 | 0.04456876 | 0.226605 |
| X204546_at | -0.1327 | 0.0634 | -2.094 | 0.0445783 | 0.226605 |
| X205143_at | -0.0992 | 0.0474 | -2.093 | 0.04461304 | 0.226729 |
| X219689_at | -0.2791 | 0.1333 | -2.093 | 0.04462634 | 0.226745 |
| X212707_s_at | -0.0456 | 0.0218 | -2.093 | 0.04464199 | 0.226773 |
| X213457_at | 0.1115 | 0.0533 | 2.093 | 0.04466208 | 0.226823 |
| X203522_at | -0.1058 | 0.0506 | -2.093 | 0.0446743 | 0.226833 |
| X221725_at | 0.0997 | 0.0477 | 2.091 | 0.04478344 | 0.227335 |
| X204029_at | -0.1241 | 0.0593 | -2.091 | 0.04479399 | 0.227337 |
| X218172_s_at | -0.137 | 0.0655 | -2.091 | 0.04482225 | 0.227418 |
| X201604_s_at | -0.1801 | 0.0862 | -2.091 | 0.04483047 | 0.227418 |
| X212392_s_at | 0.2927 | 0.14 | 2.091 | 0.0448481 | 0.227456 |
| X208614_s_at | 0.2476 | 0.1184 | 2.09 | 0.04487495 | 0.227498 |
| X210318_at | -0.0058 | 0.0028 | -2.09 | 0.04487687 | 0.227498 |
| X214881_s_at | -0.0181 | 0.0087 | -2.09 | 0.04492927 | 0.227711 |
| X218113_at | 0.0614 | 0.0294 | 2.09 | 0.0449438 | 0.227733 |
| X204834_at | 0.0735 | 0.0352 | 2.089 | 0.04498994 | 0.227875 |
| X212642_s_at | -0.1345 | 0.0644 | -2.089 | 0.04499237 | 0.227875 |
| X202544_at | 0.0655 | 0.0314 | 2.089 | 0.04502471 | 0.227987 |
| X209287_s_at | -0.0042 | 0.002 | -2.089 | 0.04504496 | 0.228038 |
| X211727_s_at | -0.1334 | 0.0639 | -2.088 | 0.04506925 | 0.228109 |
| X216139_s_at | 0.0084 | 0.004 | 2.088 | 0.04510377 | 0.228231 |
| X210205_at | -0.0186 | 0.0089 | -2.088 | 0.04511565 | 0.228239 |
| X200077_s_at | -0.0643 | 0.0308 | -2.087 | 0.04517043 | 0.228424 |
| X202921_s_at | -0.2265 | 0.1085 | -2.087 | 0.04517279 | 0.228424 |
| X219786_at | -0.0078 | 0.0037 | -2.087 | 0.04519808 | 0.228459 |
| X218122_s_at | -0.1083 | 0.0519 | -2.087 | 0.04520025 | 0.228459 |
| X202084_s_at | -0.0914 | 0.0438 | -2.087 | 0.04523921 | 0.228561 |
| X200695_at | -0.1941 | 0.093 | -2.086 | 0.04525916 | 0.228561 |
| X206248_at | 0.0051 | 0.0024 | 2.086 | 0.04526154 | 0.228561 |
| X221606_s_at | 0.0783 | 0.0375 | 2.086 | 0.04526154 | 0.228561 |
| X213552_at | -0.3097 | 0.1485 | -2.086 | 0.04532336 | 0.228821 |
| X218383_at | -0.1177 | 0.0564 | -2.086 | 0.045341 | 0.228858 |
| X208933_s_at | -0.1148 | 0.0551 | -2.085 | 0.04536039 | 0.228904 |
| X212308_at | -0.0616 | 0.0296 | -2.085 | 0.04538402 | 0.228971 |
| X210716_s_at | -0.1753 | 0.0841 | -2.085 | 0.04543374 | 0.229147 |
| X217777_s_at | -0.1078 | 0.0517 | -2.085 | 0.04543943 | 0.229147 |
| X217927_at | -0.051 | 0.0245 | -2.084 | 0.04544972 | 0.229147 |
| X206667_s_at | -0.4653 | 0.2233 | -2.084 | 0.04549466 | 0.229258 |
| X216456_at | 0.1648 | 0.0791 | 2.084 | 0.04552794 | 0.229258 |
| X209883_at | 0.2125 | 0.102 | 2.084 | 0.04553315 | 0.229258 |
| X43977_at | 0.0407 | 0.0195 | 2.084 | 0.04553653 | 0.229258 |
| X212593_s_at | 0.0653 | 0.0313 | 2.084 | 0.04554167 | 0.229258 |
| X219259_at | -0.102 | 0.049 | -2.083 | 0.04554908 | 0.229258 |
| X214794_at | -0.0556 | 0.0267 | -2.083 | 0.04555232 | 0.229258 |
| X202919_at | -0.0992 | 0.0476 | -2.083 | 0.04555446 | 0.229258 |
| X202994_s_at | 0.1845 | 0.0886 | 2.083 | 0.04558066 | 0.229338 |
| X204715_at | -0.1111 | 0.0533 | -2.083 | 0.04561834 | 0.229476 |
| X55065_at | -0.0937 | 0.045 | -2.083 | 0.04562903 | 0.229478 |
| X211275_s_at | 0.0879 | 0.0422 | 2.082 | 0.04565404 | 0.22953 |
| X46256_at | 0.0264 | 0.0127 | 2.082 | 0.0456601 | 0.22953 |
| X219659_at | 0.0484 | 0.0233 | 2.082 | 0.04568222 | 0.229589 |
| X208944_at | 0.2608 | 0.1253 | 2.082 | 0.04569329 | 0.229593 |
| X220979_s_at | -0.3006 | 0.1445 | -2.08 | 0.04584753 | 0.230316 |
| X203754_s_at | 0.0066 | 0.0032 | 2.08 | 0.04586847 | 0.230369 |
| X203073_at | 0.0622 | 0.0299 | 2.08 | 0.04589047 | 0.230427 |
| X202358_s_at | -0.1623 | 0.078 | -2.08 | 0.04591098 | 0.230478 |
| X213716_s_at | 0.0117 | 0.0056 | 2.08 | 0.04592746 | 0.230509 |
| X203123_s_at | 0.1129 | 0.0543 | 2.079 | 0.04597837 | 0.230643 |
| X200700_s_at | 0.1265 | 0.0608 | 2.079 | 0.04598481 | 0.230643 |
| X219706_at | -0.1298 | 0.0624 | -2.079 | 0.04598537 | 0.230643 |
| X205511_at | -0.1408 | 0.0677 | -2.079 | 0.04601937 | 0.230762 |
| X203125_x_at | 0.0078 | 0.0038 | 2.079 | 0.04603014 | 0.230764 |
| X200956_s_at | 0.1002 | 0.0482 | 2.078 | 0.04610218 | 0.231073 |
| X203349_s_at | -0.1613 | 0.0776 | -2.077 | 0.04613552 | 0.231182 |
| X212569_at | 0.0703 | 0.0338 | 2.077 | 0.04614473 | 0.231182 |
| X205019_s_at | -0.0939 | 0.0452 | -2.077 | 0.04615794 | 0.231196 |
| X208380_at | -0.0094 | 0.0045 | -2.077 | 0.0461745 | 0.231226 |
| X209764_at | -0.0369 | 0.0178 | -2.076 | 0.04624413 | 0.231523 |
| X201222_s_at | -0.1167 | 0.0562 | -2.076 | 0.04627706 | 0.231566 |
| X215672_s_at | 0.0062 | 0.003 | 2.076 | 0.04630713 | 0.231566 |
| X203456_at | 0.04 | 0.0192 | 2.076 | 0.0463072 | 0.231566 |
| X203303_at | -0.0692 | 0.0333 | -2.075 | 0.04632968 | 0.231566 |
| X336_at | 0.0103 | 0.005 | 2.075 | 0.04633181 | 0.231566 |
| X201314_at | -0.1208 | 0.0582 | -2.075 | 0.04633809 | 0.231566 |
| X203534_at | 0.0455 | 0.0219 | 2.075 | 0.04634914 | 0.231566 |
| X210577_at | -0.0113 | 0.0054 | -2.075 | 0.04634998 | 0.231566 |
| X217383_at | -0.017 | 0.0082 | -2.075 | 0.04635518 | 0.231566 |
| X201363_s_at | -0.1609 | 0.0775 | -2.075 | 0.04635705 | 0.231566 |
| X208744_x_at | -0.2017 | 0.0972 | -2.075 | 0.04641769 | 0.231817 |
| X200932_s_at | -0.0815 | 0.0393 | -2.074 | 0.0464683 | 0.232018 |
| X220522_at | 0.022 | 0.0106 | 2.074 | 0.04649542 | 0.232066 |
| X202721_s_at | 0.005 | 0.0024 | 2.074 | 0.04649882 | 0.232066 |
| X266_s_at | 0.2913 | 0.1405 | 2.073 | 0.04655528 | 0.232273 |
| X200605_s_at | -0.0779 | 0.0376 | -2.073 | 0.0465612 | 0.232273 |
| X212840_at | 0.1639 | 0.0791 | 2.073 | 0.0465941 | 0.232385 |
| X208949_s_at | 0.1927 | 0.093 | 2.073 | 0.0466214 | 0.232429 |
| X217801_at | -0.0403 | 0.0195 | -2.072 | 0.04663237 | 0.232429 |
| X215297_at | -0.059 | 0.0285 | -2.072 | 0.04664062 | 0.232429 |
| X203689_s_at | -0.0409 | 0.0198 | -2.072 | 0.04664474 | 0.232429 |
| X203104_at | 0.0268 | 0.0129 | 2.072 | 0.04666169 | 0.232461 |
| X204490_s_at | 0.0151 | 0.0073 | 2.072 | 0.04671602 | 0.232679 |
| X212472_at | 0.0132 | 0.0064 | 2.071 | 0.046742 | 0.232757 |
| X216547_at | -0.0259 | 0.0125 | -2.071 | 0.04678547 | 0.232921 |
| X215499_at | 0.0196 | 0.0095 | 2.071 | 0.0468007 | 0.232944 |
| X217811_at | -0.0931 | 0.045 | -2.07 | 0.04684623 | 0.233101 |
| X206357_at | -0.1022 | 0.0493 | -2.07 | 0.04685324 | 0.233101 |
| X215791_at | -0.0291 | 0.014 | -2.07 | 0.04687284 | 0.233147 |
| X203524_s_at | 0.0353 | 0.0171 | 2.069 | 0.0469369 | 0.233413 |
| X209214_s_at | -0.0465 | 0.0225 | -2.069 | 0.04700162 | 0.233683 |
| X203851_at | -0.1176 | 0.0568 | -2.068 | 0.04706269 | 0.23391 |
| X217980_s_at | -0.0462 | 0.0223 | -2.068 | 0.04706844 | 0.23391 |
| X201577_at | -0.1043 | 0.0505 | -2.067 | 0.04713301 | 0.234151 |
| X202026_at | -0.0658 | 0.0318 | -2.067 | 0.04713805 | 0.234151 |
| X202130_at | 0.1004 | 0.0486 | 2.067 | 0.04714962 | 0.234156 |
| X221664_s_at | 0.0331 | 0.016 | 2.066 | 0.04725419 | 0.234534 |
| X208674_x_at | -0.0541 | 0.0262 | -2.066 | 0.0472553 | 0.234534 |
| X203955_at | 0.1028 | 0.0498 | 2.066 | 0.04725729 | 0.234534 |
| X209637_s_at | -0.0929 | 0.045 | -2.066 | 0.04728181 | 0.234575 |
| X213519_s_at | 0.1848 | 0.0895 | 2.066 | 0.04728922 | 0.234575 |
| X210304_at | -0.0226 | 0.0109 | -2.066 | 0.04729725 | 0.234575 |
| X212587_s_at | 0.3021 | 0.1463 | 2.065 | 0.04733791 | 0.234656 |
| X200694_s_at | -0.0778 | 0.0377 | -2.065 | 0.04734498 | 0.234656 |
| X205339_at | -0.0205 | 0.0099 | -2.065 | 0.04736462 | 0.234656 |
| X217029_at | 0.0062 | 0.003 | 2.065 | 0.0473667 | 0.234656 |
| X205048_s_at | 0.5304 | 0.2568 | 2.065 | 0.04737282 | 0.234656 |
| X222310_at | 0.1594 | 0.0772 | 2.065 | 0.04737706 | 0.234656 |
| X216253_s_at | -0.0082 | 0.004 | -2.065 | 0.04740203 | 0.234694 |
| X217756_x_at | 0.0692 | 0.0335 | 2.065 | 0.04740576 | 0.234694 |
| X201359_at | -0.0497 | 0.0241 | -2.064 | 0.04743796 | 0.234801 |
| X222108_at | 0.1455 | 0.0705 | 2.064 | 0.04745135 | 0.234815 |
| X200631_s_at | -0.063 | 0.0305 | -2.064 | 0.0474677 | 0.234844 |
| X221501_x_at | 0.1385 | 0.0671 | 2.063 | 0.04754367 | 0.235167 |
| X50376_at | 0.0282 | 0.0137 | 2.063 | 0.0476124 | 0.235455 |
| X208983_s_at | 0.0115 | 0.0056 | 2.062 | 0.04766894 | 0.235639 |
| X206483_at | 0.0848 | 0.0411 | 2.062 | 0.04767087 | 0.235639 |
| X218409_s_at | 0.0174 | 0.0084 | 2.062 | 0.04768561 | 0.235656 |
| X217947_at | 0.1842 | 0.0893 | 2.062 | 0.04769553 | 0.235656 |
| X202465_at | -0.0048 | 0.0023 | -2.062 | 0.04771942 | 0.235721 |
| X207263_x_at | -0.04 | 0.0194 | -2.061 | 0.04776495 | 0.235894 |
| X205233_s_at | 0.0093 | 0.0045 | 2.061 | 0.04780325 | 0.235952 |
| X204215_at | 0.0668 | 0.0324 | 2.061 | 0.04780747 | 0.235952 |
| X209060_x_at | 0.0205 | 0.01 | 2.061 | 0.04780849 | 0.235952 |
| X205719_s_at | 0.0864 | 0.0419 | 2.061 | 0.04782725 | 0.235992 |
| X203527_s_at | -0.1421 | 0.069 | -2.06 | 0.04787895 | 0.236194 |
| X205152_at | -0.0575 | 0.0279 | -2.06 | 0.04791039 | 0.236297 |
| X206621_s_at | -0.0579 | 0.0281 | -2.059 | 0.04794109 | 0.236368 |
| X214938_x_at | 0.0314 | 0.0153 | 2.059 | 0.04795597 | 0.236368 |
| X200689_x_at | -0.0584 | 0.0284 | -2.059 | 0.04796481 | 0.236368 |
| X202182_at | 0.0546 | 0.0265 | 2.059 | 0.04796745 | 0.236368 |
| X207598_x_at | 0.1594 | 0.0774 | 2.059 | 0.04799235 | 0.236439 |
| X207688_s_at | 0.2413 | 0.1172 | 2.059 | 0.04801134 | 0.23648 |
| X211034_s_at | 0.0459 | 0.0223 | 2.059 | 0.04802527 | 0.236496 |
| X209984_at | -0.0215 | 0.0105 | -2.058 | 0.0480466 | 0.236549 |
| X35160_at | -0.0539 | 0.0262 | -2.058 | 0.04807437 | 0.236633 |
| X204244_s_at | -0.1224 | 0.0595 | -2.058 | 0.04811632 | 0.23675 |
| X214039_s_at | 0.0873 | 0.0424 | 2.058 | 0.04811947 | 0.23675 |
| X221791_s_at | 0.0417 | 0.0202 | 2.057 | 0.04814395 | 0.236818 |
| X203487_s_at | -0.063 | 0.0306 | -2.057 | 0.04818062 | 0.236942 |
| X211318_s_at | -0.1534 | 0.0746 | -2.057 | 0.04819054 | 0.236942 |
| X209436_at | -0.1791 | 0.0871 | -2.057 | 0.04820801 | 0.236975 |
| X219831_at | 0.058 | 0.0282 | 2.057 | 0.0482315 | 0.236975 |
| X209034_at | 0.0548 | 0.0266 | 2.056 | 0.04823786 | 0.236975 |
| X206487_at | 0.0675 | 0.0328 | 2.056 | 0.04823981 | 0.236975 |
| X212563_at | -0.0258 | 0.0126 | -2.056 | 0.04828695 | 0.237154 |
| X205668_at | 0.1956 | 0.0952 | 2.055 | 0.04834785 | 0.2374 |
| X212921_at | 0.0145 | 0.0071 | 2.055 | 0.0483634 | 0.237424 |
| X203894_at | -0.0664 | 0.0323 | -2.054 | 0.04849389 | 0.238012 |
| X201486_at | -0.0918 | 0.0447 | -2.054 | 0.04851977 | 0.238087 |
| X37278_at | 0.0092 | 0.0045 | 2.053 | 0.04859521 | 0.238375 |
| X36564_at | -0.0867 | 0.0422 | -2.053 | 0.04859991 | 0.238375 |
| X204964_s_at | 0.155 | 0.0755 | 2.053 | 0.04863467 | 0.238492 |
| X203940_s_at | 0.0507 | 0.0247 | 2.052 | 0.04869511 | 0.238736 |
| X201336_at | 0.1492 | 0.0727 | 2.051 | 0.04877291 | 0.238972 |
| X202514_at | -0.1435 | 0.07 | -2.051 | 0.04877483 | 0.238972 |
| X204601_at | 0.0174 | 0.0085 | 2.051 | 0.0487755 | 0.238972 |
| X203343_at | -0.0969 | 0.0472 | -2.051 | 0.04882393 | 0.239157 |
| X212831_at | 0.1081 | 0.0527 | 2.051 | 0.04884566 | 0.23921 |
| X220534_at | -0.0283 | 0.0138 | -2.05 | 0.04886539 | 0.239254 |
| X201485_s_at | -0.1325 | 0.0647 | -2.05 | 0.04889777 | 0.23936 |
| X222099_s_at | 0.0572 | 0.0279 | 2.05 | 0.04891235 | 0.239379 |
| X210784_x_at | 0.0149 | 0.0073 | 2.05 | 0.04894714 | 0.239437 |
| X221781_s_at | 0.0126 | 0.0061 | 2.05 | 0.04895189 | 0.239437 |
| X213325_at | 0.1731 | 0.0845 | 2.049 | 0.04895663 | 0.239437 |
| X220947_s_at | 0.0516 | 0.0252 | 2.049 | 0.04899146 | 0.239555 |
| X200756_x_at | -0.0114 | 0.0056 | -2.049 | 0.04901606 | 0.239622 |
| X200043_at | 0.0611 | 0.0298 | 2.048 | 0.04906403 | 0.239804 |
| X213471_at | -0.0502 | 0.0245 | -2.048 | 0.0490831 | 0.239844 |
| X210594_x_at | 0.0217 | 0.0106 | 2.048 | 0.04909566 | 0.239853 |
| X219438_at | -0.012 | 0.0058 | -2.048 | 0.04912008 | 0.239919 |
| X206871_at | -0.0039 | 0.0019 | -2.048 | 0.04915286 | 0.240027 |
| X209550_at | -0.1991 | 0.0972 | -2.047 | 0.04918944 | 0.240106 |
| X209476_at | 0.1173 | 0.0573 | 2.047 | 0.04919074 | 0.240106 |
| X205391_x_at | 0.0042 | 0.002 | 2.046 | 0.04927536 | 0.240466 |
| X218235_s_at | -0.0899 | 0.044 | -2.046 | 0.04932649 | 0.24062 |
| X208096_s_at | 0.2965 | 0.1449 | 2.046 | 0.04932851 | 0.24062 |
| X203271_s_at | 0.0828 | 0.0405 | 2.046 | 0.04934663 | 0.240656 |
| X203525_s_at | -0.0884 | 0.0432 | -2.046 | 0.04936814 | 0.240708 |
| X201801_s_at | 0.0077 | 0.0038 | 2.045 | 0.04946605 | 0.241132 |
| X206178_at | 0.1116 | 0.0546 | 2.044 | 0.04949744 | 0.241232 |
| X203696_s_at | -0.0997 | 0.0488 | -2.044 | 0.04952165 | 0.241286 |
| X211584_s_at | 0.1063 | 0.052 | 2.044 | 0.04953021 | 0.241286 |
| X213017_at | 0.1239 | 0.0606 | 2.044 | 0.04957794 | 0.241466 |
| X202396_at | -0.0801 | 0.0392 | -2.043 | 0.04963652 | 0.241697 |
| X201542_at | 0.0467 | 0.0229 | 2.043 | 0.04964724 | 0.241697 |
| X215378_at | 0.0802 | 0.0393 | 2.043 | 0.04968409 | 0.241824 |
| X211382_s_at | -0.1022 | 0.0501 | -2.042 | 0.04970241 | 0.24186 |
| X205722_s_at | 0.0042 | 0.0021 | 2.042 | 0.04979036 | 0.242228 |
| X218550_s_at | -0.052 | 0.0255 | -2.041 | 0.04980806 | 0.242228 |
| X53071_s_at | 0.1553 | 0.0761 | 2.041 | 0.04981082 | 0.242228 |
| X218170_at | -0.068 | 0.0333 | -2.041 | 0.04988096 | 0.242516 |
| X205561_at | -0.1639 | 0.0803 | -2.04 | 0.04990248 | 0.242533 |
| X217416_x_at | -0.0307 | 0.015 | -2.04 | 0.04990618 | 0.242533 |
| X213933_at | 0.0241 | 0.0118 | 2.039 | 0.05002199 | 0.243042 |
| X201783_s_at | 0.0495 | 0.0243 | 2.039 | 0.05005277 | 0.243139 |
| X208936_x_at | -0.0645 | 0.0316 | -2.039 | 0.0500944 | 0.243288 |
| X204148_s_at | -0.2021 | 0.0991 | -2.038 | 0.05010919 | 0.243306 |
| X204107_at | -0.0065 | 0.0032 | -2.038 | 0.05013721 | 0.243389 |
| X218510_x_at | 0.1375 | 0.0674 | 2.038 | 0.05016194 | 0.243405 |
| X201545_s_at | 0.0718 | 0.0352 | 2.038 | 0.05016243 | 0.243405 |
| X211765_x_at | -0.0267 | 0.0131 | -2.038 | 0.05020975 | 0.243581 |
| X210778_s_at | 0.0212 | 0.0104 | 2.037 | 0.05027529 | 0.243822 |
| X208746_x_at | -0.0338 | 0.0166 | -2.037 | 0.05028138 | 0.243822 |
| X204619_s_at | 0.2006 | 0.0985 | 2.036 | 0.05032008 | 0.243915 |
| X220942_x_at | -0.0528 | 0.0259 | -2.036 | 0.05032239 | 0.243915 |
| X201667_at | 0.1285 | 0.0631 | 2.036 | 0.0503607 | 0.244035 |
| X206645_s_at | -0.011 | 0.0054 | -2.036 | 0.05037291 | 0.244035 |
| X211406_at | -0.0896 | 0.044 | -2.036 | 0.05038012 | 0.244035 |
| X219448_at | -0.0498 | 0.0245 | -2.035 | 0.05044427 | 0.244292 |
| X205470_s_at | 0.0064 | 0.0031 | 2.035 | 0.05046213 | 0.244326 |
| X221523_s_at | -0.1455 | 0.0715 | -2.035 | 0.05052005 | 0.244553 |
| X208377_s_at | 0.0046 | 0.0023 | 2.034 | 0.05061825 | 0.244975 |
| X218477_at | -0.0864 | 0.0425 | -2.034 | 0.0506365 | 0.24501 |
| X200070_at | -0.0281 | 0.0138 | -2.033 | 0.05065603 | 0.245034 |
| X202991_at | 0.0371 | 0.0183 | 2.033 | 0.05067786 | 0.245034 |
| X201737_s_at | 0.1242 | 0.0611 | 2.033 | 0.05067842 | 0.245034 |
| X203927_at | 0.0052 | 0.0026 | 2.033 | 0.05068566 | 0.245034 |
| X212696_s_at | -0.0524 | 0.0258 | -2.032 | 0.05076948 | 0.245321 |
| X221607_x_at | -0.0492 | 0.0242 | -2.032 | 0.05076952 | 0.245321 |
| X220024_s_at | 0.013 | 0.0064 | 2.032 | 0.0507781 | 0.245321 |
| X216149_at | -0.0742 | 0.0365 | -2.032 | 0.05079184 | 0.245334 |
| X205717_x_at | -0.0476 | 0.0234 | -2.032 | 0.05084694 | 0.245547 |
| X201828_x_at | -0.0632 | 0.0311 | -2.031 | 0.05086022 | 0.245557 |
| X205967_at | -0.0739 | 0.0364 | -2.031 | 0.05088075 | 0.245603 |
| X220368_s_at | 0.1414 | 0.0696 | 2.031 | 0.05094962 | 0.245882 |
| X215009_s_at | -0.1207 | 0.0594 | -2.03 | 0.05097768 | 0.245964 |
| X203494_s_at | -0.0703 | 0.0346 | -2.03 | 0.05103125 | 0.246112 |
| X202902_s_at | 0.0499 | 0.0246 | 2.03 | 0.05103626 | 0.246112 |
| X206074_s_at | -0.073 | 0.0359 | -2.03 | 0.05104151 | 0.246112 |
| X202270_at | 0.2369 | 0.1167 | 2.029 | 0.05113706 | 0.246519 |
| X37953_s_at | -0.0246 | 0.0121 | -2.028 | 0.05117511 | 0.246643 |
| X212220_at | 0.1782 | 0.0879 | 2.028 | 0.05119347 | 0.246643 |
| X214968_at | 0.0046 | 0.0022 | 2.028 | 0.05119615 | 0.246643 |
| X206162_x_at | -0.0145 | 0.0072 | -2.028 | 0.0512545 | 0.246871 |
| X209244_s_at | 0.195 | 0.0962 | 2.027 | 0.05129242 | 0.247 |
| X216358_at | -0.0162 | 0.008 | -2.027 | 0.0513125 | 0.247043 |
| X207585_s_at | -0.0878 | 0.0433 | -2.026 | 0.05142973 | 0.247554 |
| X209822_s_at | -0.1608 | 0.0794 | -2.026 | 0.05144088 | 0.247554 |
| X201657_at | -0.1181 | 0.0583 | -2.025 | 0.05151613 | 0.247862 |
| X202538_s_at | -0.0679 | 0.0335 | -2.025 | 0.05155498 | 0.247979 |
| X203409_at | 0.0042 | 0.0021 | 2.025 | 0.05156304 | 0.247979 |
| X204768_s_at | -0.0545 | 0.0269 | -2.025 | 0.05157393 | 0.247979 |
| X218288_s_at | -0.0957 | 0.0473 | -2.024 | 0.05162514 | 0.248072 |
| X200955_at | -0.0923 | 0.0456 | -2.024 | 0.05162626 | 0.248072 |
| X202355_s_at | -0.068 | 0.0336 | -2.024 | 0.05162677 | 0.248072 |
| X220193_at | -0.011 | 0.0054 | -2.024 | 0.05166746 | 0.248214 |
| X214841_at | -0.0929 | 0.0459 | -2.024 | 0.05170603 | 0.248346 |
| X218953_s_at | -0.1412 | 0.0698 | -2.023 | 0.05173072 | 0.248348 |
| X221609_s_at | -0.0264 | 0.013 | -2.023 | 0.05175266 | 0.248348 |
| X203067_at | -0.0611 | 0.0302 | -2.023 | 0.05175924 | 0.248348 |
| X218455_at | -0.1005 | 0.0497 | -2.023 | 0.05176094 | 0.248348 |
| X215400_x_at | 0.0104 | 0.0051 | 2.023 | 0.05176237 | 0.248348 |
| X212468_at | 0.0979 | 0.0484 | 2.023 | 0.05178293 | 0.248393 |
| X207431_s_at | -0.0819 | 0.0405 | -2.022 | 0.05189461 | 0.248875 |
| X206613_s_at | -0.0851 | 0.0421 | -2.022 | 0.05192656 | 0.248949 |
| X208711_s_at | -0.0865 | 0.0428 | -2.022 | 0.05193248 | 0.248949 |
| X209386_at | 0.2141 | 0.1059 | 2.021 | 0.05198481 | 0.249146 |
| X200988_s_at | -0.0355 | 0.0176 | -2.021 | 0.05200041 | 0.249167 |
| X208717_at | -0.0822 | 0.0407 | -2.02 | 0.05206329 | 0.249398 |
| X212425_at | -0.0876 | 0.0434 | -2.02 | 0.05207121 | 0.249398 |
| X210454_s_at | 0.1224 | 0.0606 | 2.02 | 0.05208605 | 0.249416 |
| X201746_at | -0.0076 | 0.0037 | -2.02 | 0.05211752 | 0.249513 |
| X221059_s_at | 0.1299 | 0.0643 | 2.019 | 0.05220918 | 0.249898 |
| X219157_at | -0.0662 | 0.0328 | -2.019 | 0.05225542 | 0.250005 |
| X203124_s_at | 0.115 | 0.057 | 2.018 | 0.05226615 | 0.250005 |
| X211493_x_at | 0.0926 | 0.0459 | 2.018 | 0.05227181 | 0.250005 |
| X210880_s_at | 0.0562 | 0.0278 | 2.018 | 0.05227668 | 0.250005 |
| X213610_s_at | -0.0678 | 0.0336 | -2.018 | 0.05231913 | 0.250125 |
| X212588_at | 0.3564 | 0.1766 | 2.018 | 0.05232415 | 0.250125 |
| X209710_at | -0.0337 | 0.0167 | -2.017 | 0.05238776 | 0.250351 |
| X202604_x_at | -0.0346 | 0.0171 | -2.017 | 0.05239416 | 0.250351 |
| X201413_at | 0.0896 | 0.0444 | 2.017 | 0.05242265 | 0.250414 |
| X202237_at | 0.0338 | 0.0168 | 2.017 | 0.05244232 | 0.250414 |
| X208432_s_at | 0.0116 | 0.0057 | 2.017 | 0.05244747 | 0.250414 |
| X216306_x_at | 0.1406 | 0.0697 | 2.017 | 0.05246341 | 0.250414 |
| X220423_at | 0.0042 | 0.0021 | 2.017 | 0.05247223 | 0.250414 |
| X208778_s_at | -0.063 | 0.0312 | -2.017 | 0.05247493 | 0.250414 |
| X220216_at | -0.0799 | 0.0396 | -2.016 | 0.05249232 | 0.250443 |
| X213609_s_at | 0.5072 | 0.2515 | 2.016 | 0.05250381 | 0.250444 |
| X209195_s_at | 0.0123 | 0.0061 | 2.016 | 0.05252912 | 0.250511 |
| X210267_at | -0.0126 | 0.0062 | -2.016 | 0.05255033 | 0.250559 |
| X205740_s_at | -0.0118 | 0.0058 | -2.016 | 0.0525712 | 0.250559 |
| X205027_s_at | 0.0641 | 0.0318 | 2.016 | 0.05257297 | 0.250559 |
| X222222_s_at | -0.0072 | 0.0036 | -2.015 | 0.0525966 | 0.250616 |
| X204813_at | -0.0574 | 0.0285 | -2.015 | 0.05260744 | 0.250616 |
| X202904_s_at | -0.1552 | 0.077 | -2.015 | 0.05261904 | 0.250617 |
| X212335_at | -0.1297 | 0.0644 | -2.015 | 0.05264812 | 0.250658 |
| X215942_s_at | -0.0104 | 0.0051 | -2.015 | 0.0526535 | 0.250658 |
| X218003_s_at | -0.042 | 0.0208 | -2.015 | 0.05266147 | 0.250658 |
| X215659_at | -0.0109 | 0.0054 | -2.015 | 0.05268371 | 0.25071 |
| X219402_s_at | 0.1287 | 0.0639 | 2.013 | 0.0528338 | 0.251371 |
| X220525_s_at | 0.0556 | 0.0276 | 2.013 | 0.05289735 | 0.25149 |
| X210383_at | 0.151 | 0.075 | 2.013 | 0.05289947 | 0.25149 |
| X210993_s_at | -0.1319 | 0.0656 | -2.013 | 0.05290301 | 0.25149 |
| X213486_at | 0.0962 | 0.0478 | 2.013 | 0.05290405 | 0.25149 |
| X213320_at | -0.0841 | 0.0418 | -2.012 | 0.05300876 | 0.251933 |
| X217137_x_at | 0.2147 | 0.1067 | 2.011 | 0.0530384 | 0.25202 |
| X211630_s_at | -0.1054 | 0.0524 | -2.011 | 0.0531045 | 0.25228 |
| X207336_at | 0.0136 | 0.0068 | 2.011 | 0.05314617 | 0.252424 |
| X201947_s_at | -0.0783 | 0.039 | -2.01 | 0.05317332 | 0.252499 |
| X204252_at | 0.0358 | 0.0178 | 2.01 | 0.0531876 | 0.252513 |
| X209896_s_at | -0.1002 | 0.0499 | -2.01 | 0.05319914 | 0.252514 |
| X213758_at | -0.0901 | 0.0448 | -2.01 | 0.05322874 | 0.252549 |
| X212244_at | 0.1176 | 0.0585 | 2.01 | 0.05322922 | 0.252549 |
| X213994_s_at | -0.1632 | 0.0812 | -2.009 | 0.05326263 | 0.252603 |
| X215820_x_at | -0.0157 | 0.0078 | -2.009 | 0.05326336 | 0.252603 |
| X209192_x_at | -0.0147 | 0.0073 | -2.009 | 0.05327719 | 0.252615 |
| X212938_at | -0.0094 | 0.0047 | -2.009 | 0.05330296 | 0.25268 |
| X200952_s_at | 0.0137 | 0.0068 | 2.009 | 0.05332443 | 0.25268 |
| X216349_at | -0.0233 | 0.0116 | -2.009 | 0.05332844 | 0.25268 |
| X201724_s_at | -0.0946 | 0.0471 | -2.009 | 0.05333642 | 0.25268 |
| X221533_at | -0.009 | 0.0045 | -2.009 | 0.05336317 | 0.252753 |
| X218179_s_at | -0.0738 | 0.0367 | -2.008 | 0.05338875 | 0.25282 |
| X203735_x_at | 0.129 | 0.0642 | 2.008 | 0.05342817 | 0.252953 |
| X204956_at | -0.0283 | 0.0141 | -2.008 | 0.05345113 | 0.25299 |
| X213415_at | 0.0866 | 0.0431 | 2.008 | 0.0534588 | 0.25299 |
| X207305_s_at | -0.0671 | 0.0334 | -2.007 | 0.05350157 | 0.253138 |
| X202307_s_at | 0.0642 | 0.032 | 2.007 | 0.05353223 | 0.253229 |
| X206003_at | 0.0715 | 0.0356 | 2.007 | 0.05358989 | 0.253448 |
| X202457_s_at | -0.1264 | 0.063 | -2.006 | 0.05361253 | 0.253501 |
| X204765_at | -0.0041 | 0.0021 | -2.006 | 0.05363934 | 0.253549 |
| X201185_at | 0.1127 | 0.0562 | 2.006 | 0.05365035 | 0.253549 |
| X211983_x_at | -0.0514 | 0.0256 | -2.006 | 0.05365676 | 0.253549 |
| X35626_at | 0.1231 | 0.0614 | 2.006 | 0.0536815 | 0.253611 |
| X209336_at | -0.0518 | 0.0258 | -2.005 | 0.05373758 | 0.253795 |
| X211061_s_at | -0.0978 | 0.0488 | -2.005 | 0.05374315 | 0.253795 |
| X205026_at | -0.0068 | 0.0034 | -2.005 | 0.05377692 | 0.2539 |
| X214361_s_at | -0.0063 | 0.0032 | -2.005 | 0.05379086 | 0.253909 |
| X220690_s_at | -0.0525 | 0.0262 | -2.005 | 0.05380407 | 0.253909 |
| X222125_s_at | -0.0991 | 0.0494 | -2.004 | 0.05382286 | 0.253909 |
| X219861_at | -0.007 | 0.0035 | -2.004 | 0.05382453 | 0.253909 |
| X202949_s_at | 0.1072 | 0.0535 | 2.004 | 0.05385546 | 0.254001 |
| X211996_s_at | 0.1833 | 0.0915 | 2.004 | 0.05388984 | 0.254063 |
| X216229_x_at | 0.0875 | 0.0437 | 2.004 | 0.05390123 | 0.254063 |
| X211678_s_at | 0.0642 | 0.032 | 2.004 | 0.05391779 | 0.254063 |
| X213655_at | -0.1103 | 0.0551 | -2.004 | 0.0539228 | 0.254063 |
| X209422_at | -0.0678 | 0.0339 | -2.004 | 0.05393103 | 0.254063 |
| X212223_at | -0.1232 | 0.0615 | -2.003 | 0.05393717 | 0.254063 |
| X218739_at | 0.0111 | 0.0055 | 2.003 | 0.05395119 | 0.254075 |
| X205648_at | -0.008 | 0.004 | -2.003 | 0.05396665 | 0.254075 |
| X201660_at | -0.077 | 0.0385 | -2.003 | 0.05397404 | 0.254075 |
| X211792_s_at | 0.0107 | 0.0054 | 2.003 | 0.0540338 | 0.254302 |
| X202780_at | -0.1547 | 0.0772 | -2.002 | 0.05406535 | 0.254397 |
| X218812_s_at | -0.0945 | 0.0472 | -2.002 | 0.05409937 | 0.254503 |
| X217729_s_at | 0.101 | 0.0504 | 2.002 | 0.05413979 | 0.254639 |
| X201006_at | -0.0067 | 0.0034 | -2.001 | 0.05419338 | 0.254732 |
| X208920_at | -0.1042 | 0.0521 | -2.001 | 0.05420973 | 0.254732 |
| X219806_s_at | -0.5523 | 0.276 | -2.001 | 0.05421323 | 0.254732 |
| X212788_x_at | 0.0504 | 0.0252 | 2.001 | 0.05421413 | 0.254732 |
| X202967_at | -0.0791 | 0.0396 | -2.001 | 0.05421674 | 0.254732 |
| X202944_at | 0.016 | 0.008 | 2.001 | 0.05425858 | 0.254874 |
| X212512_s_at | 0.0671 | 0.0335 | 2 | 0.05427722 | 0.254908 |
| X38671_at | 0.123 | 0.0615 | 2 | 0.05437438 | 0.255258 |
| X212732_at | 0.183 | 0.0915 | 1.999 | 0.05438981 | 0.255258 |
| X218249_at | 0.0399 | 0.02 | 1.999 | 0.05439379 | 0.255258 |
| X201615_x_at | 0.2641 | 0.1321 | 1.999 | 0.05441473 | 0.255258 |
| X213701_at | -0.0532 | 0.0266 | -1.999 | 0.05442065 | 0.255258 |
| X203264_s_at | -0.109 | 0.0545 | -1.999 | 0.05442078 | 0.255258 |
| X203610_s_at | 0.0082 | 0.0041 | 1.999 | 0.05443926 | 0.255291 |
| X215481_s_at | -0.0145 | 0.0072 | -1.999 | 0.05446546 | 0.255352 |
| X217890_s_at | 0.156 | 0.0781 | 1.999 | 0.05448715 | 0.255352 |
| X201170_s_at | -0.1307 | 0.0654 | -1.999 | 0.05449746 | 0.255352 |
| X218295_s_at | -0.0224 | 0.0112 | -1.998 | 0.05449821 | 0.255352 |
| X207435_s_at | 0.0814 | 0.0407 | 1.998 | 0.05451809 | 0.255369 |
| X208312_s_at | 0.0074 | 0.0037 | 1.998 | 0.05453596 | 0.255369 |
| X209688_s_at | -0.0615 | 0.0308 | -1.998 | 0.05453636 | 0.255369 |
| X220777_at | 0.0099 | 0.005 | 1.997 | 0.05468249 | 0.255981 |
| X218341_at | -0.0462 | 0.0231 | -1.997 | 0.05469006 | 0.255981 |
| X210115_at | -0.0182 | 0.0091 | -1.997 | 0.0547183 | 0.256059 |
| X204505_s_at | -0.0904 | 0.0453 | -1.996 | 0.05479757 | 0.25622 |
| X212538_at | 0.1174 | 0.0588 | 1.996 | 0.05480792 | 0.25622 |
| X213184_at | 0.0587 | 0.0294 | 1.996 | 0.05481298 | 0.25622 |
| X211370_s_at | -0.1359 | 0.0681 | -1.996 | 0.0548167 | 0.25622 |
| X200859_x_at | 0.0161 | 0.0081 | 1.996 | 0.05482157 | 0.25622 |
| X222164_at | 0.0322 | 0.0161 | 1.996 | 0.05482656 | 0.25622 |
| X218207_s_at | 0.0085 | 0.0043 | 1.996 | 0.05483349 | 0.25622 |
| X201454_s_at | -0.0705 | 0.0353 | -1.995 | 0.0548633 | 0.256306 |
| X221604_s_at | -0.089 | 0.0446 | -1.995 | 0.05491223 | 0.25647 |
| X212061_at | -0.0722 | 0.0362 | -1.995 | 0.05492165 | 0.25647 |
| X210625_s_at | -0.1664 | 0.0834 | -1.994 | 0.05497852 | 0.256682 |
| X208641_s_at | 0.0388 | 0.0194 | 1.994 | 0.05502149 | 0.256829 |
| X208613_s_at | 0.0062 | 0.0031 | 1.994 | 0.05505454 | 0.256929 |
| X219004_s_at | -0.0251 | 0.0126 | -1.993 | 0.05508177 | 0.257002 |
| X211596_s_at | 0.2031 | 0.1019 | 1.993 | 0.05510987 | 0.257079 |
| X217840_at | -0.1006 | 0.0505 | -1.993 | 0.05513445 | 0.25709 |
| X203082_at | -0.1053 | 0.0528 | -1.993 | 0.05513526 | 0.25709 |
| X202266_at | -0.0375 | 0.0188 | -1.993 | 0.05517114 | 0.257165 |
| X205248_at | -0.1519 | 0.0762 | -1.993 | 0.05517463 | 0.257165 |
| X220370_s_at | 0.0911 | 0.0457 | 1.992 | 0.05520527 | 0.257254 |
| X222142_at | 0.0258 | 0.0129 | 1.992 | 0.05522646 | 0.257299 |
| X204810_s_at | -0.0079 | 0.004 | -1.992 | 0.05527995 | 0.257449 |
| X212632_at | -0.0687 | 0.0345 | -1.992 | 0.05528185 | 0.257449 |
| X205340_at | -0.0853 | 0.0429 | -1.991 | 0.05530727 | 0.257475 |
| X203763_at | -0.0751 | 0.0377 | -1.991 | 0.05531057 | 0.257475 |
| X204583_x_at | -0.006 | 0.003 | -1.991 | 0.05533088 | 0.257497 |
| X219796_s_at | -0.0046 | 0.0023 | -1.991 | 0.0553471 | 0.257497 |
| X212911_at | -0.0657 | 0.033 | -1.991 | 0.05535016 | 0.257497 |
| X209049_s_at | 0.0683 | 0.0343 | 1.991 | 0.0553785 | 0.257575 |
| X215554_at | 0.2315 | 0.1163 | 1.99 | 0.05542869 | 0.257754 |
| X212420_at | 0.3001 | 0.1508 | 1.99 | 0.05545692 | 0.257809 |
| X212705_x_at | -0.0063 | 0.0031 | -1.99 | 0.05546368 | 0.257809 |
| X214027_x_at | -0.0106 | 0.0053 | -1.99 | 0.05547926 | 0.257828 |
| X219426_at | 0.1441 | 0.0724 | 1.99 | 0.05550837 | 0.257909 |
| X218531_at | -0.06 | 0.0302 | -1.989 | 0.05553558 | 0.257982 |
| X205877_s_at | 0.1311 | 0.0659 | 1.989 | 0.05555098 | 0.257999 |
| X214687_x_at | -0.0739 | 0.0372 | -1.989 | 0.05557343 | 0.258049 |
| X218682_s_at | -0.049 | 0.0247 | -1.989 | 0.05559289 | 0.258086 |
| X218883_s_at | 0.026 | 0.0131 | 1.988 | 0.05566888 | 0.258385 |
| X214241_at | 0.0415 | 0.0209 | 1.988 | 0.05569721 | 0.258435 |
| X221495_s_at | -0.0815 | 0.041 | -1.988 | 0.05570295 | 0.258435 |
| X222157_s_at | -0.1631 | 0.0821 | -1.988 | 0.0557319 | 0.258515 |
| X209823_x_at | 0.0063 | 0.0032 | 1.987 | 0.05582278 | 0.258883 |
| X217556_at | -0.1308 | 0.0658 | -1.987 | 0.05587241 | 0.259059 |
| X205006_s_at | -0.0932 | 0.0469 | -1.986 | 0.05589883 | 0.259105 |
| X217455_s_at | -0.1068 | 0.0538 | -1.986 | 0.05590566 | 0.259105 |
| X201837_s_at | -0.0805 | 0.0405 | -1.985 | 0.0560064 | 0.259394 |
| X211667_x_at | -0.0058 | 0.0029 | -1.985 | 0.05601852 | 0.259394 |
| X204712_at | -0.1813 | 0.0913 | -1.985 | 0.05603047 | 0.259394 |
| X209332_s_at | 0.0663 | 0.0334 | 1.985 | 0.05603608 | 0.259394 |
| X216759_at | -0.0134 | 0.0067 | -1.985 | 0.05604009 | 0.259394 |
| X206197_at | -0.108 | 0.0544 | -1.985 | 0.05604331 | 0.259394 |
| X32069_at | 0.112 | 0.0564 | 1.985 | 0.05604992 | 0.259394 |
| X44654_at | 0.0514 | 0.0259 | 1.985 | 0.05609771 | 0.259507 |
| X204829_s_at | -0.0218 | 0.011 | -1.985 | 0.05610481 | 0.259507 |
| X210616_s_at | 0.0891 | 0.0449 | 1.984 | 0.05611641 | 0.259507 |
| X213512_at | 0.0179 | 0.009 | 1.984 | 0.05612105 | 0.259507 |
| X205123_s_at | -0.1024 | 0.0516 | -1.984 | 0.05615673 | 0.259577 |
| X823_at | -0.1318 | 0.0664 | -1.984 | 0.05615954 | 0.259577 |
| X206628_at | 0.0038 | 0.0019 | 1.984 | 0.05619227 | 0.259675 |
| X206167_s_at | 0.2464 | 0.1242 | 1.984 | 0.05621927 | 0.259745 |
| X213430_at | 0.1231 | 0.0621 | 1.983 | 0.05626474 | 0.259901 |
| X218013_x_at | -0.1431 | 0.0722 | -1.983 | 0.05628413 | 0.259937 |
| X206529_x_at | 0.1019 | 0.0514 | 1.983 | 0.05630701 | 0.259988 |
| X210992_x_at | 0.0055 | 0.0028 | 1.982 | 0.05638704 | 0.260304 |
| X221010_s_at | -0.0139 | 0.007 | -1.982 | 0.05645381 | 0.260558 |
| X204187_at | 0.1528 | 0.0771 | 1.981 | 0.05657976 | 0.261042 |
| X218680_x_at | 0.0405 | 0.0205 | 1.98 | 0.0565823 | 0.261042 |
| X200966_x_at | -0.0839 | 0.0424 | -1.98 | 0.05665269 | 0.261313 |
| X221776_s_at | -0.0758 | 0.0383 | -1.98 | 0.05666691 | 0.261324 |
| X218664_at | -0.081 | 0.0409 | -1.979 | 0.05673605 | 0.261589 |
| X217564_s_at | -0.0242 | 0.0122 | -1.979 | 0.05675394 | 0.261617 |
| X212974_at | 0.0077 | 0.0039 | 1.979 | 0.05680946 | 0.261692 |
| X220924_s_at | 0.139 | 0.0702 | 1.979 | 0.05680978 | 0.261692 |
| X203908_at | -0.1735 | 0.0877 | -1.978 | 0.05682214 | 0.261692 |
| X218437_s_at | -0.0611 | 0.0309 | -1.978 | 0.05683448 | 0.261692 |
| X207311_at | -0.0068 | 0.0034 | -1.978 | 0.05685434 | 0.261692 |
| X203993_x_at | 0.0035 | 0.0017 | 1.978 | 0.05685579 | 0.261692 |
| X201771_at | -0.0154 | 0.0078 | -1.978 | 0.05685757 | 0.261692 |
| X202401_s_at | -0.0734 | 0.0371 | -1.978 | 0.05686448 | 0.261692 |
| X49077_at | -0.0884 | 0.0447 | -1.978 | 0.05687708 | 0.261696 |
| X213315_x_at | -0.114 | 0.0576 | -1.978 | 0.0568916 | 0.261708 |
| X217529_at | -0.1028 | 0.052 | -1.978 | 0.05691765 | 0.261774 |
| X215905_s_at | -0.1286 | 0.065 | -1.977 | 0.05694368 | 0.261839 |
| X210879_s_at | -0.0327 | 0.0165 | -1.977 | 0.05696539 | 0.261839 |
| X212265_at | 0.1037 | 0.0525 | 1.977 | 0.05697654 | 0.261839 |
| X209334_s_at | 0.0067 | 0.0034 | 1.977 | 0.05697886 | 0.261839 |
| X210074_at | 0.0274 | 0.0139 | 1.977 | 0.05700478 | 0.261904 |
| X218651_s_at | -0.0976 | 0.0494 | -1.977 | 0.0570358 | 0.261992 |
| X217764_s_at | 0.1161 | 0.0588 | 1.976 | 0.05712082 | 0.262303 |
| X215899_at | -0.0111 | 0.0056 | -1.976 | 0.05712715 | 0.262303 |
| X210612_s_at | 0.2987 | 0.1512 | 1.975 | 0.05717273 | 0.262434 |
| X219750_at | 0.3513 | 0.1778 | 1.975 | 0.05717925 | 0.262434 |
| X202050_s_at | -0.0751 | 0.038 | -1.975 | 0.0571981 | 0.262466 |
| X221056_x_at | 0.0493 | 0.025 | 1.975 | 0.05721254 | 0.262478 |
| X206869_at | -0.0118 | 0.006 | -1.974 | 0.05733478 | 0.262985 |
| X212132_at | 0.0581 | 0.0294 | 1.974 | 0.05736491 | 0.263069 |
| X212094_at | -0.2785 | 0.1411 | -1.974 | 0.05738112 | 0.263089 |
| X204502_at | 0.1097 | 0.0556 | 1.973 | 0.05744813 | 0.263342 |
| X201550_x_at | -0.0541 | 0.0274 | -1.972 | 0.05753296 | 0.263676 |
| X203151_at | 0.1009 | 0.0511 | 1.972 | 0.05754988 | 0.263699 |
| X213327_s_at | -0.0703 | 0.0357 | -1.972 | 0.05758603 | 0.26381 |
| X212347_x_at | 0.0059 | 0.003 | 1.972 | 0.05760315 | 0.263834 |
| X219246_s_at | 0.0254 | 0.0129 | 1.972 | 0.05762746 | 0.263891 |
| X204134_at | 0.088 | 0.0447 | 1.971 | 0.05766572 | 0.263982 |
| X205001_s_at | 0.1224 | 0.0621 | 1.971 | 0.05767397 | 0.263982 |
| X216385_at | -0.0107 | 0.0054 | -1.971 | 0.05768296 | 0.263982 |
| X218781_at | 0.0659 | 0.0334 | 1.97 | 0.05784601 | 0.264674 |
| X204217_s_at | -0.0124 | 0.0063 | -1.969 | 0.05790073 | 0.264821 |
| X221381_s_at | -0.1149 | 0.0583 | -1.969 | 0.05790198 | 0.264821 |
| X218536_at | -0.0997 | 0.0506 | -1.969 | 0.05793374 | 0.264824 |
| X201720_s_at | -0.0107 | 0.0054 | -1.969 | 0.05793427 | 0.264824 |
| X215440_s_at | -0.0791 | 0.0402 | -1.969 | 0.05793854 | 0.264824 |
| X220318_at | 0.0046 | 0.0024 | 1.969 | 0.05796567 | 0.264894 |
| X212718_at | 0.0453 | 0.023 | 1.968 | 0.05804032 | 0.265173 |
| X207521_s_at | 0.0054 | 0.0028 | 1.968 | 0.0580505 | 0.265173 |
| X215299_x_at | -0.1015 | 0.0516 | -1.968 | 0.05806879 | 0.265202 |
| X214684_at | 0.0814 | 0.0414 | 1.968 | 0.05809627 | 0.265261 |
| X211578_s_at | 0.0265 | 0.0135 | 1.968 | 0.05810579 | 0.265261 |
| X210445_at | -0.1023 | 0.052 | -1.967 | 0.05816526 | 0.265377 |
| X220371_s_at | -0.0102 | 0.0052 | -1.967 | 0.05818007 | 0.265377 |
| X204783_at | -0.1639 | 0.0833 | -1.967 | 0.05818262 | 0.265377 |
| X221045_s_at | -0.0817 | 0.0416 | -1.967 | 0.05818571 | 0.265377 |
| X218159_at | -0.1048 | 0.0533 | -1.967 | 0.05819074 | 0.265377 |
| X200635_s_at | 0.1251 | 0.0636 | 1.967 | 0.05823617 | 0.265511 |
| X212803_at | -0.023 | 0.0117 | -1.967 | 0.05824419 | 0.265511 |
| X208629_s_at | 0.1505 | 0.0766 | 1.965 | 0.05841944 | 0.26623 |
| X202163_s_at | 0.072 | 0.0366 | 1.965 | 0.05843513 | 0.26623 |
| X218620_s_at | -0.0181 | 0.0092 | -1.965 | 0.0584377 | 0.26623 |
| X220526_s_at | -0.043 | 0.0219 | -1.965 | 0.05846457 | 0.266289 |
| X204367_at | -0.0838 | 0.0426 | -1.965 | 0.05847617 | 0.266289 |
| X205500_at | -0.124 | 0.0631 | -1.964 | 0.05849525 | 0.266289 |
| X204279_at | 0.1005 | 0.0512 | 1.964 | 0.05849875 | 0.266289 |
| X210479_s_at | 0.2134 | 0.1086 | 1.964 | 0.05852161 | 0.266339 |
| X212430_at | 0.0601 | 0.0306 | 1.963 | 0.05869098 | 0.267055 |
| X209960_at | 0.0496 | 0.0253 | 1.962 | 0.05875154 | 0.267276 |
| X219308_s_at | -0.1265 | 0.0645 | -1.962 | 0.05878164 | 0.267358 |
| X218189_s_at | 0.0702 | 0.0358 | 1.962 | 0.05884248 | 0.267537 |
| X214375_at | 0.1886 | 0.0962 | 1.962 | 0.0588452 | 0.267537 |
| X207836_s_at | 0.011 | 0.0056 | 1.961 | 0.05888708 | 0.267673 |
| X214205_x_at | -0.0599 | 0.0306 | -1.961 | 0.05891612 | 0.26775 |
| X218659_at | -0.0583 | 0.0297 | -1.961 | 0.05894481 | 0.267826 |
| X208430_s_at | 0.3366 | 0.1717 | 1.96 | 0.05904454 | 0.268224 |
| X221668_s_at | 0.0074 | 0.0038 | 1.959 | 0.05918184 | 0.268793 |
| X210686_x_at | 0.1204 | 0.0615 | 1.959 | 0.05919755 | 0.268809 |
| X205823_at | -0.2373 | 0.1212 | -1.958 | 0.05923649 | 0.268862 |
| X215243_s_at | 0.0069 | 0.0035 | 1.958 | 0.05923808 | 0.268862 |
| X219584_at | 0.0147 | 0.0075 | 1.958 | 0.05924558 | 0.268862 |
| X219109_at | 0.0505 | 0.0258 | 1.958 | 0.05927283 | 0.268913 |
| X37892_at | 0.1152 | 0.0588 | 1.958 | 0.05928101 | 0.268913 |
| X207235_s_at | -0.0139 | 0.0071 | -1.958 | 0.05932031 | 0.269037 |
| X202231_at | -0.0447 | 0.0228 | -1.957 | 0.05936766 | 0.269196 |
| X201437_s_at | -0.0763 | 0.039 | -1.957 | 0.05944654 | 0.269408 |
| X212510_at | -0.0786 | 0.0402 | -1.956 | 0.05947206 | 0.269408 |
| X218335_x_at | 0.0119 | 0.0061 | 1.956 | 0.05947518 | 0.269408 |
| X212062_at | -0.0575 | 0.0294 | -1.956 | 0.0594758 | 0.269408 |
| X219093_at | -0.0833 | 0.0426 | -1.956 | 0.05948644 | 0.269408 |
| X205337_at | -0.1571 | 0.0803 | -1.956 | 0.05948702 | 0.269408 |
| X204538_x_at | 0.1423 | 0.0728 | 1.956 | 0.05950834 | 0.269449 |
| X214058_at | -0.0185 | 0.0095 | -1.955 | 0.05960058 | 0.269812 |
| X209459_s_at | -0.0497 | 0.0254 | -1.955 | 0.05963379 | 0.269907 |
| X204772_s_at | -0.0946 | 0.0484 | -1.955 | 0.0596736 | 0.270033 |
| X209280_at | -0.0125 | 0.0064 | -1.955 | 0.05970513 | 0.270066 |
| X201072_s_at | 0.2055 | 0.1051 | 1.954 | 0.05971721 | 0.270066 |
| X202651_at | -0.0714 | 0.0365 | -1.954 | 0.05973972 | 0.270066 |
| X209344_at | -0.15 | 0.0767 | -1.954 | 0.05974143 | 0.270066 |
| X56821_at | 0.1269 | 0.0649 | 1.954 | 0.05974175 | 0.270066 |
| X213031_s_at | -0.0542 | 0.0278 | -1.953 | 0.05984508 | 0.270461 |
| X213896_x_at | -0.0902 | 0.0462 | -1.953 | 0.05985354 | 0.270461 |
| X205568_at | 0.0111 | 0.0057 | 1.953 | 0.05989074 | 0.270574 |
| X210680_s_at | -0.0109 | 0.0056 | -1.953 | 0.05992268 | 0.270628 |
| X219071_x_at | -0.0624 | 0.032 | -1.953 | 0.05992705 | 0.270628 |
| X200784_s_at | -0.0112 | 0.0057 | -1.952 | 0.05997062 | 0.27077 |
| X221007_s_at | 0.0882 | 0.0452 | 1.952 | 0.0600169 | 0.270924 |
| X202686_s_at | -0.2192 | 0.1123 | -1.952 | 0.06003122 | 0.270933 |
| X220183_s_at | -0.2373 | 0.1216 | -1.952 | 0.06007438 | 0.271073 |
| X212249_at | 0.248 | 0.1272 | 1.95 | 0.06023349 | 0.271693 |
| X221223_x_at | 0.0051 | 0.0026 | 1.95 | 0.06024359 | 0.271693 |
| X201827_at | 0.026 | 0.0134 | 1.95 | 0.06024852 | 0.271693 |
| X209121_x_at | 0.0745 | 0.0382 | 1.95 | 0.06029293 | 0.271838 |
| X220948_s_at | -0.0976 | 0.05 | -1.95 | 0.06031864 | 0.271886 |
| X205704_s_at | 0.0088 | 0.0045 | 1.95 | 0.06032806 | 0.271886 |
| X219622_at | 0.0183 | 0.0094 | 1.949 | 0.06034325 | 0.2719 |
| X41160_at | 0.1304 | 0.0669 | 1.949 | 0.06037284 | 0.271906 |
| X202262_x_at | 0.0403 | 0.0207 | 1.949 | 0.060373 | 0.271906 |
| X201734_at | -0.0607 | 0.0311 | -1.949 | 0.06038351 | 0.271906 |
| X212259_s_at | 0.0958 | 0.0491 | 1.949 | 0.0603937 | 0.271906 |
| X209469_at | -0.0484 | 0.0249 | -1.949 | 0.06040873 | 0.271919 |
| X206089_at | 0.2058 | 0.1056 | 1.948 | 0.06049609 | 0.272257 |
| X221718_s_at | 0.1251 | 0.0642 | 1.948 | 0.06057801 | 0.27257 |
| X203903_s_at | 0.1434 | 0.0736 | 1.947 | 0.06059312 | 0.272583 |
| X220182_at | -0.4139 | 0.2126 | -1.947 | 0.060616 | 0.272631 |
| X210191_s_at | -0.1389 | 0.0713 | -1.947 | 0.06064791 | 0.2727 |
| X214354_x_at | 0.025 | 0.0128 | 1.947 | 0.06065583 | 0.2727 |
| X215346_at | -0.0119 | 0.0061 | -1.947 | 0.06069545 | 0.272823 |
| X206466_at | -0.135 | 0.0693 | -1.946 | 0.06071992 | 0.272877 |
| X205417_s_at | 0.0687 | 0.0353 | 1.946 | 0.06081917 | 0.273248 |
| X207604_s_at | 0.1335 | 0.0686 | 1.946 | 0.06082702 | 0.273248 |
| X205608_s_at | 0.0062 | 0.0032 | 1.945 | 0.06089912 | 0.273517 |
| X206327_s_at | -0.0056 | 0.0029 | -1.945 | 0.06093899 | 0.273584 |
| X202299_s_at | -0.0487 | 0.025 | -1.944 | 0.06096986 | 0.273584 |
| X212635_at | 0.075 | 0.0386 | 1.944 | 0.0609713 | 0.273584 |
| X221320_at | -0.0061 | 0.0031 | -1.944 | 0.06097961 | 0.273584 |
| X218996_at | -0.1 | 0.0514 | -1.944 | 0.06098863 | 0.273584 |
| X204669_s_at | 0.061 | 0.0314 | 1.944 | 0.06100151 | 0.273584 |
| X218233_s_at | -0.0395 | 0.0203 | -1.944 | 0.06100479 | 0.273584 |
| X210004_at | -0.2646 | 0.1361 | -1.944 | 0.06101267 | 0.273584 |
| X222119_s_at | -0.033 | 0.017 | -1.944 | 0.06106075 | 0.273744 |
| X217629_at | 0.007 | 0.0036 | 1.943 | 0.0611634 | 0.274064 |
| X213073_at | 0.0714 | 0.0368 | 1.943 | 0.06116374 | 0.274064 |
| X48031_r_at | -0.1016 | 0.0523 | -1.943 | 0.06118784 | 0.274064 |
| X208180_s_at | -0.0063 | 0.0032 | -1.943 | 0.06119793 | 0.274064 |
| X204273_at | -0.2403 | 0.1237 | -1.942 | 0.06121394 | 0.274064 |
| X204996_s_at | 0.0037 | 0.0019 | 1.942 | 0.06121647 | 0.274064 |
| X203309_s_at | -0.0095 | 0.0049 | -1.942 | 0.06121832 | 0.274064 |
| X212880_at | -0.0585 | 0.0301 | -1.942 | 0.06128792 | 0.27432 |
| X202308_at | -0.1373 | 0.0707 | -1.941 | 0.06133751 | 0.274487 |
| X213890_x_at | 0.0342 | 0.0176 | 1.941 | 0.06145094 | 0.274939 |
| X216579_at | 0.0063 | 0.0033 | 1.94 | 0.06148697 | 0.275009 |
| X219842_at | 0.0316 | 0.0163 | 1.94 | 0.06149141 | 0.275009 |
| X213645_at | -0.2261 | 0.1166 | -1.94 | 0.06153585 | 0.275153 |
| X200660_at | 0.1384 | 0.0714 | 1.94 | 0.06156537 | 0.275229 |
| X219219_at | -0.1144 | 0.059 | -1.938 | 0.0617348 | 0.275931 |
| X202040_s_at | 0.1479 | 0.0763 | 1.938 | 0.06176217 | 0.275998 |
| X213520_at | -0.0079 | 0.0041 | -1.937 | 0.0618617 | 0.276343 |
| X201179_s_at | 0.0777 | 0.0401 | 1.937 | 0.06186697 | 0.276343 |
| X210011_s_at | -0.0364 | 0.0188 | -1.937 | 0.06187675 | 0.276343 |
| X221619_s_at | -0.0591 | 0.0305 | -1.936 | 0.06204207 | 0.277026 |
| X220761_s_at | 0.0297 | 0.0153 | 1.936 | 0.06206597 | 0.277077 |
| X220990_s_at | -0.0497 | 0.0257 | -1.935 | 0.06210487 | 0.277153 |
| X221814_at | 0.0126 | 0.0065 | 1.935 | 0.06210791 | 0.277153 |
| X218945_at | -0.0234 | 0.0121 | -1.935 | 0.06214305 | 0.277254 |
| X211864_s_at | 0.008 | 0.0042 | 1.935 | 0.0621857 | 0.277376 |
| X213706_at | -0.0141 | 0.0073 | -1.935 | 0.0621987 | 0.277376 |
| X213682_at | -0.0979 | 0.0506 | -1.935 | 0.062208 | 0.277376 |
| X221234_s_at | -0.1385 | 0.0716 | -1.934 | 0.06222687 | 0.277405 |
| X214889_at | -0.2187 | 0.1131 | -1.934 | 0.06225654 | 0.277481 |
| X212912_at | 0.0495 | 0.0256 | 1.934 | 0.06232275 | 0.277721 |
| X219027_s_at | -0.084 | 0.0434 | -1.934 | 0.06233811 | 0.277733 |
| X219203_at | -0.0601 | 0.0311 | -1.933 | 0.06238838 | 0.277852 |
| X203310_at | 0.0901 | 0.0466 | 1.933 | 0.06238963 | 0.277852 |
| X209774_x_at | 0.3306 | 0.171 | 1.933 | 0.06241653 | 0.277916 |
| X221035_s_at | 0.0068 | 0.0035 | 1.933 | 0.0624432 | 0.277979 |
| X209418_s_at | -0.0525 | 0.0272 | -1.932 | 0.06251202 | 0.278202 |
| X218924_s_at | 0.1051 | 0.0544 | 1.932 | 0.0625184 | 0.278202 |
| X219381_at | -0.0858 | 0.0444 | -1.932 | 0.06254072 | 0.278246 |
| X219177_at | -0.0407 | 0.0211 | -1.932 | 0.06256592 | 0.278301 |
| X212502_at | -0.0738 | 0.0382 | -1.932 | 0.06257822 | 0.278301 |
| X214499_s_at | 0.1898 | 0.0983 | 1.931 | 0.06261913 | 0.278427 |
| X216199_s_at | 0.0478 | 0.0248 | 1.931 | 0.06263705 | 0.278451 |
| X203882_at | 0.1642 | 0.085 | 1.931 | 0.0626538 | 0.278451 |
| X220350_at | -0.0133 | 0.0069 | -1.931 | 0.06266223 | 0.278451 |
| X210838_s_at | -0.0065 | 0.0034 | -1.931 | 0.06270041 | 0.278565 |
| X217839_at | -0.0647 | 0.0335 | -1.931 | 0.06273345 | 0.278656 |
| X203247_s_at | 0.1201 | 0.0622 | 1.93 | 0.06278631 | 0.278836 |
| X222201_s_at | 0.0932 | 0.0483 | 1.93 | 0.06281379 | 0.278899 |
| X219443_at | -0.1138 | 0.059 | -1.93 | 0.06283705 | 0.278899 |
| X211219_s_at | 0.1989 | 0.1031 | 1.93 | 0.06283833 | 0.278899 |
| X215995_x_at | 0.0337 | 0.0175 | 1.93 | 0.06285936 | 0.278901 |
| X208662_s_at | 0.0747 | 0.0387 | 1.929 | 0.06287058 | 0.278901 |
| X203598_s_at | -0.0912 | 0.0472 | -1.929 | 0.06287638 | 0.278901 |
| X213341_at | -0.0916 | 0.0475 | -1.929 | 0.06289058 | 0.278908 |
| X221471_at | -0.0722 | 0.0374 | -1.928 | 0.06302337 | 0.279415 |
| X204209_at | -0.0139 | 0.0072 | -1.928 | 0.06302995 | 0.279415 |
| X218965_s_at | -0.0181 | 0.0094 | -1.928 | 0.06306532 | 0.279516 |
| X221848_at | -0.0242 | 0.0125 | -1.928 | 0.06308143 | 0.279531 |
| X219699_at | 0.0093 | 0.0048 | 1.928 | 0.06311201 | 0.279611 |
| X219368_at | -0.0801 | 0.0416 | -1.927 | 0.06316263 | 0.27978 |
| X219709_x_at | 0.0605 | 0.0314 | 1.927 | 0.06319214 | 0.279834 |
| X217818_s_at | -0.0557 | 0.0289 | -1.927 | 0.06320008 | 0.279834 |
| X205937_at | -0.0944 | 0.049 | -1.927 | 0.06323595 | 0.279937 |
| X203496_s_at | 0.0225 | 0.0117 | 1.926 | 0.06326657 | 0.280017 |
| X212658_at | -0.0516 | 0.0268 | -1.926 | 0.06330871 | 0.280147 |
| X202998_s_at | 0.0105 | 0.0054 | 1.926 | 0.06337705 | 0.280394 |
| X220394_at | -0.015 | 0.0078 | -1.925 | 0.06343404 | 0.28059 |
| X211287_x_at | 0.0058 | 0.003 | 1.925 | 0.06347995 | 0.280637 |
| X211454_x_at | 0.0958 | 0.0498 | 1.925 | 0.06348677 | 0.280637 |
| X213010_at | -0.0145 | 0.0076 | -1.925 | 0.06349041 | 0.280637 |
| X218348_s_at | -0.0584 | 0.0304 | -1.925 | 0.06349587 | 0.280637 |
| X201277_s_at | 0.0568 | 0.0295 | 1.925 | 0.06350786 | 0.280637 |
| X209253_at | 0.0278 | 0.0144 | 1.924 | 0.06353375 | 0.280696 |
| X201103_x_at | 0.0622 | 0.0323 | 1.924 | 0.06357037 | 0.280702 |
| X208351_s_at | -0.3016 | 0.1568 | -1.924 | 0.06357045 | 0.280702 |
| X205493_s_at | -0.089 | 0.0463 | -1.924 | 0.06357305 | 0.280702 |
| X203014_x_at | 0.0135 | 0.007 | 1.924 | 0.06359627 | 0.280749 |
| X210800_at | 0.129 | 0.0671 | 1.923 | 0.06364904 | 0.280881 |
| X221691_x_at | -0.0584 | 0.0304 | -1.923 | 0.06365146 | 0.280881 |
| X208234_x_at | 0.0068 | 0.0035 | 1.923 | 0.06372295 | 0.28114 |
| X204037_at | 0.248 | 0.129 | 1.923 | 0.0637392 | 0.281156 |
| X205227_at | -0.0528 | 0.0275 | -1.923 | 0.06376787 | 0.281227 |
| X210336_x_at | 0.0846 | 0.044 | 1.922 | 0.06380918 | 0.281338 |
| X201384_s_at | -0.0485 | 0.0252 | -1.922 | 0.0638299 | 0.281338 |
| X200691_s_at | -0.0241 | 0.0125 | -1.922 | 0.06383282 | 0.281338 |
| X213763_at | 0.2742 | 0.1427 | 1.922 | 0.06384367 | 0.281338 |
| X200951_s_at | -0.2193 | 0.1141 | -1.922 | 0.06388768 | 0.281456 |
| X217781_s_at | -0.0776 | 0.0404 | -1.922 | 0.0638958 | 0.281456 |
| X218709_s_at | -0.0338 | 0.0176 | -1.921 | 0.06396342 | 0.281698 |
| X219915_s_at | -0.3079 | 0.1603 | -1.921 | 0.06397992 | 0.281715 |
| X221231_s_at | -0.0081 | 0.0042 | -1.921 | 0.06401418 | 0.28181 |
| X211248_s_at | 0.0075 | 0.0039 | 1.921 | 0.06403468 | 0.281844 |
| X218414_s_at | 0.2102 | 0.1095 | 1.92 | 0.06409525 | 0.281959 |
| X212975_at | 0.0766 | 0.0399 | 1.92 | 0.06409666 | 0.281959 |
| X215620_at | -0.015 | 0.0078 | -1.92 | 0.064099 | 0.281959 |
| X206171_at | 0.0249 | 0.013 | 1.918 | 0.06442108 | 0.283298 |
| X206911_at | 0.0056 | 0.0029 | 1.918 | 0.06442872 | 0.283298 |
| X205702_at | -0.048 | 0.0251 | -1.917 | 0.06447011 | 0.283424 |
| X208759_at | -0.0152 | 0.0079 | -1.916 | 0.06458552 | 0.283875 |
| X222056_s_at | -0.0679 | 0.0354 | -1.916 | 0.0646062 | 0.283909 |
| X215754_at | 0.0612 | 0.0319 | 1.916 | 0.06464148 | 0.284008 |
| X212615_at | -0.041 | 0.0214 | -1.916 | 0.06468144 | 0.284128 |
| X219522_at | -0.1636 | 0.0855 | -1.915 | 0.06476869 | 0.284288 |
| X203119_at | -0.1006 | 0.0525 | -1.915 | 0.06477784 | 0.284288 |
| X212576_at | -0.0153 | 0.008 | -1.915 | 0.06477913 | 0.284288 |
| X396_f_at | -0.0252 | 0.0132 | -1.915 | 0.06478193 | 0.284288 |
| X208517_x_at | -0.0339 | 0.0177 | -1.915 | 0.06479149 | 0.284288 |
| X44120_at | -0.0337 | 0.0176 | -1.915 | 0.06479461 | 0.284288 |
| X208306_x_at | 0.182 | 0.0951 | 1.914 | 0.06482951 | 0.284338 |
| X205985_x_at | 0.0041 | 0.0021 | 1.914 | 0.06483168 | 0.284338 |
| X218671_s_at | -0.0766 | 0.04 | -1.914 | 0.06486446 | 0.284404 |
| X218340_s_at | -0.1774 | 0.0927 | -1.914 | 0.06487489 | 0.284404 |
| X221510_s_at | -0.1288 | 0.0673 | -1.914 | 0.06488515 | 0.284404 |
| X219982_s_at | -0.1328 | 0.0694 | -1.913 | 0.06497566 | 0.284744 |
| X209517_s_at | -0.0977 | 0.0511 | -1.913 | 0.06505433 | 0.284979 |
| X221882_s_at | -0.0858 | 0.0449 | -1.913 | 0.0650593 | 0.284979 |
| X202092_s_at | -0.0593 | 0.031 | -1.913 | 0.06506775 | 0.284979 |
| X219420_s_at | -0.0839 | 0.0439 | -1.912 | 0.06513117 | 0.285201 |
| X212415_at | 0.0036 | 0.0019 | 1.912 | 0.06518715 | 0.285371 |
| X208249_s_at | 0.1413 | 0.0739 | 1.912 | 0.06519576 | 0.285371 |
| X220336_s_at | 0.0199 | 0.0104 | 1.912 | 0.06521795 | 0.285412 |
| X204496_at | -0.0566 | 0.0296 | -1.911 | 0.06523135 | 0.285415 |
| X217844_at | 0.0426 | 0.0223 | 1.911 | 0.06527738 | 0.285546 |
| X217971_at | -0.0525 | 0.0275 | -1.911 | 0.06528708 | 0.285546 |
| X219352_at | -0.1257 | 0.0658 | -1.911 | 0.0653194 | 0.285631 |
| X209678_s_at | -0.1642 | 0.086 | -1.911 | 0.06535058 | 0.285711 |
| X209618_at | 0.089 | 0.0466 | 1.91 | 0.06539743 | 0.28586 |
| X219694_at | -0.0094 | 0.0049 | -1.91 | 0.06544508 | 0.286012 |
| X221991_at | 0.0112 | 0.0059 | 1.91 | 0.06545817 | 0.286013 |
| X212044_s_at | 0.1488 | 0.0779 | 1.91 | 0.06548237 | 0.286062 |
| X209415_at | -0.0217 | 0.0114 | -1.909 | 0.06555665 | 0.28633 |
| X205848_at | -0.0862 | 0.0452 | -1.908 | 0.06562599 | 0.286577 |
| X213988_s_at | 0.1473 | 0.0772 | 1.908 | 0.06564114 | 0.286587 |
| X205154_at | -0.0552 | 0.0289 | -1.908 | 0.06567153 | 0.286663 |
| X211666_x_at | -0.0405 | 0.0212 | -1.908 | 0.06569289 | 0.2867 |
| X200802_at | -0.0966 | 0.0506 | -1.908 | 0.06572331 | 0.286721 |
| X200680_x_at | 0.0405 | 0.0212 | 1.908 | 0.0657402 | 0.286721 |
| X218258_at | 0.1369 | 0.0717 | 1.907 | 0.06575863 | 0.286721 |
| X207655_s_at | -0.2824 | 0.1481 | -1.907 | 0.06575888 | 0.286721 |
| X201566_x_at | 0.1657 | 0.0869 | 1.907 | 0.06576758 | 0.286721 |
| X205616_at | 0.0121 | 0.0064 | 1.907 | 0.06577513 | 0.286721 |
| X219046_s_at | 0.0289 | 0.0151 | 1.907 | 0.06580472 | 0.286726 |
| X209789_at | -0.1464 | 0.0768 | -1.907 | 0.06581795 | 0.286726 |
| X213561_at | 0.0053 | 0.0028 | 1.907 | 0.06581845 | 0.286726 |
| X218019_s_at | 0.0791 | 0.0415 | 1.907 | 0.06582788 | 0.286726 |
| X205498_at | 0.1837 | 0.0964 | 1.906 | 0.06595355 | 0.287217 |
| X216892_at | 0.0078 | 0.0041 | 1.906 | 0.06602494 | 0.287471 |
| X213170_at | -0.0056 | 0.0029 | -1.905 | 0.06611355 | 0.287801 |
| X212829_at | 0.2216 | 0.1164 | 1.905 | 0.06613873 | 0.287844 |
| X202861_at | 0.1893 | 0.0994 | 1.904 | 0.0661905 | 0.287844 |
| X205700_at | -0.2295 | 0.1205 | -1.904 | 0.06619862 | 0.287844 |
| X213116_at | 0.1187 | 0.0623 | 1.904 | 0.06620273 | 0.287844 |
| X219822_at | -0.0162 | 0.0085 | -1.904 | 0.06620493 | 0.287844 |
| X203628_at | 0.2117 | 0.1112 | 1.904 | 0.06620804 | 0.287844 |
| X38269_at | 0.0739 | 0.0388 | 1.904 | 0.06621426 | 0.287844 |
| X218458_at | 0.0972 | 0.051 | 1.904 | 0.06626236 | 0.287997 |
| X213132_s_at | -0.0993 | 0.0522 | -1.903 | 0.06632144 | 0.288197 |
| X222171_s_at | 0.1384 | 0.0727 | 1.903 | 0.0663868 | 0.288359 |
| X204532_x_at | 0.0107 | 0.0056 | 1.903 | 0.06638923 | 0.288359 |
| X217952_x_at | -0.0433 | 0.0227 | -1.903 | 0.06639755 | 0.288359 |
| X203833_s_at | 0.1818 | 0.0956 | 1.903 | 0.06641217 | 0.288359 |
| X208898_at | -0.0665 | 0.0349 | -1.903 | 0.06642365 | 0.288359 |
| X212529_at | 0.0721 | 0.0379 | 1.902 | 0.06645881 | 0.288363 |
| X220608_s_at | -0.0271 | 0.0143 | -1.902 | 0.06647269 | 0.288363 |
| X204663_at | -0.1238 | 0.0651 | -1.902 | 0.06647479 | 0.288363 |
| X202606_s_at | -0.0502 | 0.0264 | -1.902 | 0.06647645 | 0.288363 |
| X219207_at | -0.0314 | 0.0165 | -1.902 | 0.06651484 | 0.288473 |
| X206512_at | 0.12 | 0.0631 | 1.902 | 0.0665523 | 0.288492 |
| X217758_s_at | 0.044 | 0.0231 | 1.902 | 0.06655369 | 0.288492 |
| X221049_s_at | -0.0143 | 0.0075 | -1.901 | 0.06656994 | 0.288492 |
| X213883_s_at | -0.0631 | 0.0332 | -1.901 | 0.06657097 | 0.288492 |
| X202771_at | 0.0148 | 0.0078 | 1.901 | 0.0666062 | 0.288555 |
| X221535_at | -0.1315 | 0.0692 | -1.901 | 0.06661166 | 0.288555 |
| X212494_at | 0.1136 | 0.0598 | 1.901 | 0.06668606 | 0.288778 |
| X208374_s_at | 0.0596 | 0.0314 | 1.901 | 0.06668902 | 0.288778 |
| X203112_s_at | 0.0368 | 0.0194 | 1.899 | 0.06683831 | 0.289364 |
| X201407_s_at | -0.0773 | 0.0407 | -1.899 | 0.06685045 | 0.289364 |
| X210790_s_at | 0.0046 | 0.0024 | 1.899 | 0.06688206 | 0.289444 |
| X202937_x_at | -0.0834 | 0.0439 | -1.899 | 0.0669322 | 0.289537 |
| X219945_at | -0.0769 | 0.0405 | -1.899 | 0.06694789 | 0.289537 |
| X206343_s_at | 0.2308 | 0.1216 | 1.899 | 0.06695413 | 0.289537 |
| X219260_s_at | -0.1594 | 0.084 | -1.899 | 0.0669555 | 0.289537 |
| X214355_x_at | 0.0079 | 0.0042 | 1.898 | 0.06697427 | 0.289539 |
| X204792_s_at | 0.0188 | 0.0099 | 1.898 | 0.06698313 | 0.289539 |
| X213746_s_at | 0.098 | 0.0516 | 1.898 | 0.06699517 | 0.289539 |
| X208667_s_at | 0.0819 | 0.0431 | 1.898 | 0.06705429 | 0.289707 |
| X214266_s_at | -0.0121 | 0.0064 | -1.898 | 0.06706 | 0.289707 |
| X203379_at | 0.0127 | 0.0067 | 1.898 | 0.06708561 | 0.289732 |
| X221165_s_at | -0.0052 | 0.0027 | -1.898 | 0.06709189 | 0.289732 |
| X202737_s_at | -0.0502 | 0.0265 | -1.897 | 0.06717733 | 0.28999 |
| X208395_s_at | -0.0057 | 0.003 | -1.897 | 0.06717781 | 0.28999 |
| X204136_at | 0.091 | 0.048 | 1.897 | 0.06721453 | 0.290092 |
| X211040_x_at | 0.0691 | 0.0364 | 1.896 | 0.06727792 | 0.290269 |
| X213157_s_at | -0.0051 | 0.0027 | -1.896 | 0.0672816 | 0.290269 |
| X203883_s_at | -0.0557 | 0.0294 | -1.896 | 0.06730968 | 0.290333 |
| X203223_at | -0.2277 | 0.1201 | -1.896 | 0.06734117 | 0.290413 |
| X202301_s_at | 0.0874 | 0.0461 | 1.896 | 0.06735849 | 0.290431 |
| X209998_at | -0.0308 | 0.0163 | -1.895 | 0.06740831 | 0.290536 |
| X202451_at | -0.0875 | 0.0462 | -1.895 | 0.06740896 | 0.290536 |
| X202160_at | 0.0425 | 0.0224 | 1.895 | 0.06744139 | 0.290619 |
| X207563_s_at | 0.0134 | 0.0071 | 1.895 | 0.06746452 | 0.290663 |
| X209874_x_at | -0.0201 | 0.0106 | -1.895 | 0.06751337 | 0.29076 |
| X33579_i_at | 0.0074 | 0.0039 | 1.895 | 0.06751736 | 0.29076 |
| X203021_at | 0.0087 | 0.0046 | 1.894 | 0.06752642 | 0.29076 |
| X200684_s_at | -0.0764 | 0.0403 | -1.894 | 0.06758466 | 0.290954 |
| X202407_s_at | -0.0106 | 0.0056 | -1.894 | 0.0675977 | 0.290954 |
| X211053_at | 0.0063 | 0.0033 | 1.893 | 0.06768993 | 0.291229 |
| X218398_at | -0.1617 | 0.0854 | -1.893 | 0.06769428 | 0.291229 |
| X202762_at | -0.1319 | 0.0697 | -1.893 | 0.06770087 | 0.291229 |
| X203008_x_at | -0.1025 | 0.0542 | -1.892 | 0.06780209 | 0.291608 |
| X208145_at | -0.0068 | 0.0036 | -1.892 | 0.06781831 | 0.291621 |
| X218375_at | -0.0719 | 0.038 | -1.892 | 0.06784301 | 0.291671 |
| X216221_s_at | -0.0344 | 0.0182 | -1.891 | 0.06794952 | 0.292073 |
| X207246_at | -0.0158 | 0.0084 | -1.891 | 0.06796339 | 0.292076 |
| X201458_s_at | 0.0583 | 0.0308 | 1.891 | 0.06798967 | 0.292132 |
| X220129_at | -0.2219 | 0.1173 | -1.891 | 0.06802365 | 0.292222 |
| X213396_s_at | 0.1332 | 0.0705 | 1.89 | 0.06807769 | 0.292353 |
| X212595_s_at | -0.1104 | 0.0584 | -1.89 | 0.06808047 | 0.292353 |
| X215487_x_at | 0.0135 | 0.0072 | 1.89 | 0.06811878 | 0.292406 |
| X206281_at | 0.0192 | 0.0102 | 1.89 | 0.06811917 | 0.292406 |
| X206853_s_at | -0.0557 | 0.0294 | -1.89 | 0.06817667 | 0.292596 |
| X206703_at | -0.0068 | 0.0036 | -1.889 | 0.06824122 | 0.292809 |
| X210378_s_at | -0.0902 | 0.0478 | -1.889 | 0.06826258 | 0.292809 |
| X208930_s_at | 0.1423 | 0.0753 | 1.889 | 0.06826581 | 0.292809 |
| X217792_at | -0.0806 | 0.0427 | -1.889 | 0.06828824 | 0.292848 |
| X204710_s_at | -0.0583 | 0.0309 | -1.888 | 0.06836088 | 0.293103 |
| X202281_at | 0.0159 | 0.0084 | 1.888 | 0.06838372 | 0.293145 |
| X39763_at | 0.0054 | 0.0029 | 1.888 | 0.06846823 | 0.29345 |
| X203985_at | -0.0267 | 0.0141 | -1.887 | 0.06856683 | 0.293816 |
| X201886_at | -0.0899 | 0.0476 | -1.887 | 0.06858692 | 0.293846 |
| X200856_x_at | -0.0654 | 0.0347 | -1.886 | 0.0686527 | 0.294071 |
| X211295_x_at | 0.0041 | 0.0022 | 1.886 | 0.06866812 | 0.29408 |
| X209584_x_at | 0.0162 | 0.0086 | 1.886 | 0.06868352 | 0.294089 |
| X203201_at | -0.0117 | 0.0062 | -1.886 | 0.06873345 | 0.294228 |
| X214080_x_at | 0.0836 | 0.0443 | 1.886 | 0.06874233 | 0.294228 |
| X221264_s_at | -0.0762 | 0.0404 | -1.885 | 0.0687704 | 0.294291 |
| X216925_s_at | 0.0058 | 0.0031 | 1.885 | 0.06887306 | 0.294601 |
| X210532_s_at | -0.0407 | 0.0216 | -1.885 | 0.0688746 | 0.294601 |
| X218149_s_at | 0.1151 | 0.0611 | 1.885 | 0.06888556 | 0.294601 |
| X221887_s_at | 0.0171 | 0.0091 | 1.884 | 0.06890605 | 0.294601 |
| X204986_s_at | -0.0518 | 0.0275 | -1.884 | 0.068909 | 0.294601 |
| X209010_s_at | -0.004 | 0.0021 | -1.884 | 0.0689499 | 0.294719 |
| X207914_x_at | -0.0127 | 0.0067 | -1.884 | 0.06897199 | 0.294726 |
| X220411_x_at | 0.0052 | 0.0028 | 1.884 | 0.06897808 | 0.294726 |
| X217831_s_at | -0.0654 | 0.0347 | -1.884 | 0.06901864 | 0.294792 |
| X203040_s_at | 0.0341 | 0.0181 | 1.884 | 0.06902013 | 0.294792 |
| X207153_s_at | -0.0945 | 0.0502 | -1.884 | 0.06904149 | 0.294827 |
| X215083_at | 0.0921 | 0.0489 | 1.883 | 0.06905501 | 0.294828 |
| X216715_at | -0.0115 | 0.0061 | -1.882 | 0.06922533 | 0.295485 |
| X209631_s_at | 0.269 | 0.1429 | 1.882 | 0.06923551 | 0.295485 |
| X209158_s_at | -0.0848 | 0.0451 | -1.882 | 0.06928685 | 0.295609 |
| X36552_at | 0.0434 | 0.0231 | 1.882 | 0.06929238 | 0.295609 |
| X201342_at | -0.0815 | 0.0433 | -1.882 | 0.06930469 | 0.295609 |
| X205288_at | 0.0789 | 0.0419 | 1.882 | 0.06931781 | 0.295609 |
| X202166_s_at | -0.1371 | 0.0729 | -1.881 | 0.06938449 | 0.295836 |
| X203698_s_at | 0.2424 | 0.1288 | 1.881 | 0.06939794 | 0.295837 |
| X214855_s_at | -0.0732 | 0.0389 | -1.881 | 0.06943303 | 0.295873 |
| X218920_at | 0.0849 | 0.0451 | 1.881 | 0.06943306 | 0.295873 |
| X213414_s_at | 0.0394 | 0.021 | 1.88 | 0.06953967 | 0.29621 |
| X208514_at | 0.0056 | 0.003 | 1.88 | 0.06954727 | 0.29621 |
| X218537_at | -0.1143 | 0.0608 | -1.88 | 0.06955649 | 0.29621 |
| X216086_at | -0.148 | 0.0787 | -1.88 | 0.06957234 | 0.29621 |
| X201652_at | -0.0536 | 0.0285 | -1.88 | 0.0695814 | 0.29621 |
| X221884_at | -0.0093 | 0.005 | -1.88 | 0.06960171 | 0.29621 |
| X200971_s_at | 0.0963 | 0.0513 | 1.879 | 0.0696054 | 0.29621 |
| X202045_s_at | 0.0261 | 0.0139 | 1.879 | 0.06969361 | 0.296467 |
| X215300_s_at | -0.0114 | 0.0061 | -1.879 | 0.0696952 | 0.296467 |
| X219204_s_at | -0.1428 | 0.076 | -1.879 | 0.06970595 | 0.296467 |
| X212004_at | 0.0106 | 0.0056 | 1.878 | 0.06974631 | 0.296487 |
| X208900_s_at | 0.1478 | 0.0787 | 1.878 | 0.0697483 | 0.296487 |
| X211474_s_at | 0.1154 | 0.0614 | 1.878 | 0.06975069 | 0.296487 |
| X221506_s_at | 0.0535 | 0.0285 | 1.878 | 0.06979214 | 0.296607 |
| X200871_s_at | -0.0557 | 0.0297 | -1.878 | 0.06981602 | 0.296651 |
| X218906_x_at | 0.028 | 0.0149 | 1.878 | 0.06986514 | 0.296803 |
| X211984_at | -0.0557 | 0.0297 | -1.877 | 0.06989816 | 0.296886 |
| X218433_at | -0.011 | 0.0058 | -1.877 | 0.06994294 | 0.296886 |
| X207163_s_at | 0.0286 | 0.0152 | 1.877 | 0.06994372 | 0.296886 |
| X208925_at | 0.228 | 0.1215 | 1.877 | 0.06994782 | 0.296886 |
| X213996_at | -0.1807 | 0.0963 | -1.877 | 0.06995583 | 0.296886 |
| X211378_x_at | -0.0232 | 0.0124 | -1.877 | 0.06996474 | 0.296886 |
| X207554_x_at | 0.0133 | 0.0071 | 1.877 | 0.07001161 | 0.297028 |
| X220509_at | -0.012 | 0.0064 | -1.876 | 0.0700301 | 0.29705 |
| X212042_x_at | -0.0596 | 0.0318 | -1.876 | 0.07005114 | 0.297067 |
| X219853_at | 0.0197 | 0.0105 | 1.876 | 0.07006539 | 0.297067 |
| X210415_s_at | -0.0062 | 0.0033 | -1.876 | 0.07008787 | 0.297067 |
| X213391_at | 0.0873 | 0.0465 | 1.876 | 0.07009439 | 0.297067 |
| X219520_s_at | 0.1554 | 0.0828 | 1.876 | 0.07010096 | 0.297067 |
| X204105_s_at | 0.0456 | 0.0243 | 1.876 | 0.07014532 | 0.297169 |
| X200632_s_at | 0.2079 | 0.1108 | 1.876 | 0.07015648 | 0.297169 |
| X201968_s_at | -0.0849 | 0.0453 | -1.876 | 0.07016528 | 0.297169 |
| X208549_x_at | 0.1078 | 0.0575 | 1.875 | 0.07021023 | 0.297303 |
| X208497_x_at | -0.007 | 0.0037 | -1.875 | 0.07023415 | 0.297347 |
| X219952_s_at | -0.0428 | 0.0228 | -1.875 | 0.07027097 | 0.297447 |
| X205098_at | -0.0974 | 0.052 | -1.874 | 0.07039519 | 0.297855 |
| X202818_s_at | 0.227 | 0.1212 | 1.874 | 0.07040817 | 0.297855 |
| X216565_x_at | 0.0151 | 0.0081 | 1.874 | 0.07041726 | 0.297855 |
| X212567_s_at | 0.1589 | 0.0848 | 1.874 | 0.07042116 | 0.297855 |
| X202842_s_at | -0.0935 | 0.0499 | -1.874 | 0.07044869 | 0.297915 |
| X207219_at | 0.2 | 0.1068 | 1.873 | 0.07048079 | 0.297994 |
| X204535_s_at | 0.0091 | 0.0048 | 1.873 | 0.07050673 | 0.298026 |
| X206179_s_at | -0.0776 | 0.0415 | -1.873 | 0.07053562 | 0.298026 |
| X203436_at | -0.0529 | 0.0282 | -1.873 | 0.07054006 | 0.298026 |
| X209468_at | -0.0051 | 0.0027 | -1.873 | 0.07054199 | 0.298026 |
| X215714_s_at | -0.1665 | 0.0889 | -1.873 | 0.07056898 | 0.298048 |
| X207186_s_at | 0.0761 | 0.0406 | 1.873 | 0.07058704 | 0.298048 |
| X220840_s_at | -0.0063 | 0.0034 | -1.873 | 0.07058754 | 0.298048 |
| X222352_at | 0.1109 | 0.0593 | 1.872 | 0.07064869 | 0.298206 |
| X213983_s_at | -0.0168 | 0.009 | -1.872 | 0.07065186 | 0.298206 |
| X202322_s_at | -0.0502 | 0.0268 | -1.872 | 0.07071796 | 0.298378 |
| X210367_s_at | 0.0148 | 0.0079 | 1.872 | 0.07071929 | 0.298378 |
| X205126_at | 0.1524 | 0.0814 | 1.871 | 0.07074509 | 0.29843 |
| X213583_x_at | -0.0193 | 0.0103 | -1.871 | 0.07077773 | 0.29846 |
| X216101_at | 0.2297 | 0.1228 | 1.871 | 0.07077916 | 0.29846 |
| X200705_s_at | -0.0862 | 0.0461 | -1.871 | 0.07084706 | 0.29869 |
| X31799_at | -0.0931 | 0.0498 | -1.87 | 0.07094842 | 0.29906 |
| X202824_s_at | -0.0339 | 0.0181 | -1.87 | 0.07096631 | 0.299079 |
| X202546_at | 0.2441 | 0.1305 | 1.87 | 0.07098966 | 0.299102 |
| X209867_s_at | -0.0616 | 0.033 | -1.87 | 0.07101027 | 0.299102 |
| X221219_s_at | -0.0113 | 0.0061 | -1.87 | 0.0710152 | 0.299102 |
| X221236_s_at | 0.1774 | 0.0949 | 1.869 | 0.07103629 | 0.299102 |
| X208050_s_at | 0.0066 | 0.0035 | 1.869 | 0.07103972 | 0.299102 |
| X208755_x_at | 0.047 | 0.0251 | 1.869 | 0.07105259 | 0.299102 |
| X212988_x_at | -0.0508 | 0.0272 | -1.869 | 0.07109776 | 0.299236 |
| X208817_at | 0.1703 | 0.0911 | 1.869 | 0.07113742 | 0.299346 |
| X213316_at | -0.1925 | 0.103 | -1.868 | 0.07119899 | 0.299548 |
| X221585_at | -0.0058 | 0.0031 | -1.868 | 0.07122979 | 0.299621 |
| X203599_s_at | -0.0514 | 0.0275 | -1.868 | 0.0712596 | 0.299642 |
| X208140_s_at | 0.2525 | 0.1352 | 1.868 | 0.07126174 | 0.299642 |
| X210399_x_at | 0.005 | 0.0027 | 1.868 | 0.0713001 | 0.299747 |
| X215716_s_at | -0.1316 | 0.0705 | -1.867 | 0.07136017 | 0.299942 |
| X202993_at | -0.0837 | 0.0448 | -1.867 | 0.07137775 | 0.299959 |
| X218131_s_at | 0.0934 | 0.0501 | 1.866 | 0.0714955 | 0.300397 |
| X212097_at | 0.2353 | 0.1261 | 1.866 | 0.07152361 | 0.300405 |
| X204537_s_at | 0.0042 | 0.0022 | 1.866 | 0.07152445 | 0.300405 |
| X212388_at | -0.0572 | 0.0306 | -1.866 | 0.07154176 | 0.300421 |
| X209297_at | -0.0936 | 0.0502 | -1.866 | 0.07156605 | 0.300467 |
| X202392_s_at | -0.084 | 0.045 | -1.865 | 0.07160483 | 0.300573 |
| X211090_s_at | -0.2404 | 0.1289 | -1.865 | 0.07167457 | 0.300758 |
| X202029_x_at | 0.0466 | 0.025 | 1.865 | 0.07167599 | 0.300758 |
| X205583_s_at | -0.0615 | 0.033 | -1.865 | 0.071718 | 0.300864 |
| X221864_at | 0.0573 | 0.0307 | 1.865 | 0.07173353 | 0.300864 |
| X215531_s_at | -0.1414 | 0.0759 | -1.865 | 0.0717419 | 0.300864 |
| X207426_s_at | -0.0274 | 0.0147 | -1.864 | 0.07180056 | 0.301053 |
| X215972_at | 0.026 | 0.014 | 1.863 | 0.07192376 | 0.301422 |
| X201528_at | -0.0482 | 0.0259 | -1.863 | 0.07192417 | 0.301422 |
| X201708_s_at | -0.2184 | 0.1172 | -1.863 | 0.07193407 | 0.301422 |
| X212043_at | 0.0404 | 0.0217 | 1.863 | 0.0719435 | 0.301422 |
| X215697_at | 0.0067 | 0.0036 | 1.863 | 0.07195652 | 0.301422 |
| X215417_at | -0.0366 | 0.0196 | -1.863 | 0.07197622 | 0.301448 |
| X201050_at | -0.1347 | 0.0723 | -1.863 | 0.072012 | 0.301541 |
| X214174_s_at | -0.0776 | 0.0416 | -1.862 | 0.07204595 | 0.301626 |
| X204814_at | 0.0445 | 0.0239 | 1.861 | 0.07221024 | 0.302257 |
| X210628_x_at | -0.0086 | 0.0046 | -1.861 | 0.07222678 | 0.302269 |
| X215314_at | 0.1151 | 0.0618 | 1.861 | 0.07224563 | 0.302291 |
| X203450_at | 0.0678 | 0.0364 | 1.86 | 0.07233096 | 0.302518 |
| X203794_at | 0.0866 | 0.0465 | 1.86 | 0.07233495 | 0.302518 |
| X222199_s_at | 0.0692 | 0.0372 | 1.86 | 0.07234074 | 0.302518 |
| X202669_s_at | 0.2582 | 0.1388 | 1.86 | 0.07237077 | 0.302552 |
| X209446_s_at | 0.1752 | 0.0942 | 1.86 | 0.07237592 | 0.302552 |
| X205441_at | 0.0068 | 0.0036 | 1.86 | 0.07239738 | 0.302584 |
| X206457_s_at | -0.0067 | 0.0036 | -1.86 | 0.07244597 | 0.302651 |
| X213390_at | 0.0892 | 0.048 | 1.86 | 0.07246819 | 0.302651 |
| X201347_x_at | -0.0664 | 0.0357 | -1.859 | 0.07247785 | 0.302651 |
| X209786_at | -0.0959 | 0.0516 | -1.859 | 0.07248097 | 0.302651 |
| X215241_at | -0.1505 | 0.0809 | -1.859 | 0.07248153 | 0.302651 |
| X215438_x_at | 0.0613 | 0.033 | 1.859 | 0.07257878 | 0.303 |
| X201125_s_at | 0.1401 | 0.0754 | 1.859 | 0.07261582 | 0.303091 |
| X200714_x_at | 0.0681 | 0.0367 | 1.858 | 0.07262769 | 0.303091 |
| X214802_at | -0.0081 | 0.0043 | -1.858 | 0.072643 | 0.303098 |
| X208453_s_at | -0.1493 | 0.0804 | -1.858 | 0.07272331 | 0.303361 |
| X200884_at | -0.0783 | 0.0421 | -1.858 | 0.07273345 | 0.303361 |
| X212470_at | 0.0768 | 0.0413 | 1.857 | 0.07277475 | 0.303476 |
| X217975_at | 0.1 | 0.0538 | 1.857 | 0.07279272 | 0.303494 |
| X205629_s_at | -0.0167 | 0.009 | -1.857 | 0.07281578 | 0.303528 |
| X200809_x_at | 0.0765 | 0.0412 | 1.857 | 0.07282801 | 0.303528 |
| X211733_x_at | 0.0784 | 0.0422 | 1.857 | 0.07287741 | 0.303676 |
| X212691_at | 0.007 | 0.0038 | 1.856 | 0.07294786 | 0.303913 |
| X203526_s_at | -0.117 | 0.063 | -1.856 | 0.0729831 | 0.303999 |
| X201955_at | -0.0752 | 0.0405 | -1.856 | 0.07299586 | 0.303999 |
| X218628_at | -0.0658 | 0.0355 | -1.855 | 0.07306433 | 0.304208 |
| X220688_s_at | -0.0831 | 0.0448 | -1.855 | 0.07307336 | 0.304208 |
| X220966_x_at | -0.0607 | 0.0327 | -1.855 | 0.07309931 | 0.304259 |
| X215198_s_at | 0.0115 | 0.0062 | 1.855 | 0.07315423 | 0.3044 |
| X211144_x_at | 0.0434 | 0.0234 | 1.855 | 0.07316072 | 0.3044 |
| X202036_s_at | 0.2198 | 0.1185 | 1.855 | 0.07317683 | 0.30441 |
| X212121_at | -0.1152 | 0.0621 | -1.854 | 0.07321192 | 0.304499 |
| X206744_s_at | 0.0506 | 0.0273 | 1.854 | 0.07325218 | 0.30461 |
| X201316_at | -0.0571 | 0.0308 | -1.854 | 0.07330941 | 0.30479 |
| X221548_s_at | -0.0727 | 0.0392 | -1.854 | 0.07334803 | 0.304894 |
| X220828_s_at | 0.0841 | 0.0454 | 1.853 | 0.07336282 | 0.304898 |
| X207087_x_at | 0.0049 | 0.0026 | 1.853 | 0.07338027 | 0.304914 |
| X207600_at | 0.0051 | 0.0027 | 1.853 | 0.07342433 | 0.30504 |
| X215518_at | 0.1935 | 0.1044 | 1.853 | 0.07347769 | 0.305205 |
| X207765_s_at | 0.0401 | 0.0216 | 1.852 | 0.07351143 | 0.30527 |
| X222082_at | 0.0022 | 0.0012 | 1.852 | 0.07352106 | 0.30527 |
| X203316_s_at | -0.0733 | 0.0396 | -1.852 | 0.07357587 | 0.305401 |
| X211609_x_at | -0.0659 | 0.0356 | -1.852 | 0.07357991 | 0.305401 |
| X202643_s_at | -0.01 | 0.0054 | -1.852 | 0.07360651 | 0.305426 |
| X211901_s_at | -0.0083 | 0.0045 | -1.852 | 0.07361449 | 0.305426 |
| X204446_s_at | 0.4106 | 0.2218 | 1.852 | 0.07362729 | 0.305426 |
| X201426_s_at | 0.169 | 0.0913 | 1.851 | 0.07371455 | 0.305687 |
| X211352_s_at | 0.0145 | 0.0078 | 1.851 | 0.07371759 | 0.305687 |
| X205630_at | -0.3309 | 0.1788 | -1.851 | 0.07375436 | 0.305782 |
| X215338_s_at | 0.1304 | 0.0705 | 1.85 | 0.07379313 | 0.305864 |
| X205707_at | -0.2402 | 0.1298 | -1.85 | 0.07380161 | 0.305864 |
| X212577_at | -0.0585 | 0.0316 | -1.85 | 0.07392052 | 0.306203 |
| X218609_s_at | -0.0975 | 0.0527 | -1.85 | 0.0739239 | 0.306203 |
| X209835_x_at | 0.0122 | 0.0066 | 1.85 | 0.07392494 | 0.306203 |
| X214908_s_at | -0.0049 | 0.0027 | -1.849 | 0.07395807 | 0.30628 |
| X201754_at | -0.034 | 0.0184 | -1.849 | 0.07397096 | 0.30628 |
| X220692_at | 0.0472 | 0.0255 | 1.849 | 0.0740155 | 0.306407 |
| X220369_at | 0.0135 | 0.0073 | 1.848 | 0.0742247 | 0.307216 |
| X218869_at | -0.0125 | 0.0068 | -1.847 | 0.0742611 | 0.307259 |
| X216596_at | -0.0057 | 0.0031 | -1.847 | 0.07426271 | 0.307259 |
| X219222_at | -0.0364 | 0.0197 | -1.847 | 0.07432174 | 0.307446 |
| X215179_x_at | 0.0989 | 0.0536 | 1.847 | 0.0743601 | 0.30745 |
| X215926_x_at | -0.0197 | 0.0107 | -1.847 | 0.07436901 | 0.30745 |
| X208447_s_at | -0.2547 | 0.1379 | -1.847 | 0.07437344 | 0.30745 |
| X215825_at | 0.0354 | 0.0192 | 1.847 | 0.07437808 | 0.30745 |
| X201565_s_at | 0.1741 | 0.0943 | 1.846 | 0.07441632 | 0.307551 |
| X202212_at | -0.0102 | 0.0055 | -1.846 | 0.07443569 | 0.307551 |
| X215147_at | 0.1756 | 0.0951 | 1.846 | 0.07444417 | 0.307551 |
| X204878_s_at | 0.011 | 0.006 | 1.846 | 0.07446019 | 0.30756 |
| X204936_at | 0.0094 | 0.0051 | 1.846 | 0.07452577 | 0.307774 |
| X220707_s_at | 0.004 | 0.0022 | 1.845 | 0.07459416 | 0.307999 |
| X221317_x_at | 0.0055 | 0.003 | 1.845 | 0.07463818 | 0.308087 |
| X215136_s_at | 0.067 | 0.0363 | 1.845 | 0.07464314 | 0.308087 |
| X212935_at | 0.057 | 0.0309 | 1.844 | 0.07473654 | 0.308415 |
| X202349_at | -0.0669 | 0.0363 | -1.843 | 0.07484268 | 0.308796 |
| X203054_s_at | -0.0518 | 0.0281 | -1.843 | 0.07487099 | 0.308827 |
| X220135_s_at | 0.0081 | 0.0044 | 1.843 | 0.07487813 | 0.308827 |
| X218901_at | 0.1826 | 0.0991 | 1.843 | 0.07494853 | 0.309003 |
| X214377_s_at | -0.0071 | 0.0039 | -1.843 | 0.07495897 | 0.309003 |
| X204331_s_at | -0.1323 | 0.0718 | -1.843 | 0.07496258 | 0.309003 |
| X213686_at | 0.2212 | 0.1201 | 1.842 | 0.07499253 | 0.309069 |
| X202009_at | -0.0227 | 0.0123 | -1.842 | 0.07513402 | 0.309595 |
| X203096_s_at | -0.3837 | 0.2084 | -1.841 | 0.07518125 | 0.309697 |
| X201644_at | -0.0241 | 0.0131 | -1.841 | 0.07518658 | 0.309697 |
| X201293_x_at | -0.0254 | 0.0138 | -1.841 | 0.07526026 | 0.309943 |
| X213400_s_at | -0.0913 | 0.0496 | -1.84 | 0.07533179 | 0.31018 |
| X215096_s_at | 0.0392 | 0.0213 | 1.84 | 0.07534932 | 0.310195 |
| X219449_s_at | -0.0801 | 0.0435 | -1.84 | 0.07540071 | 0.310349 |
| X37012_at | 0.0297 | 0.0161 | 1.839 | 0.07546621 | 0.310561 |
| X221493_at | -0.0691 | 0.0376 | -1.839 | 0.07554639 | 0.310792 |
| X222282_at | 0.1395 | 0.0759 | 1.839 | 0.07556062 | 0.310792 |
| X220305_at | 0.1599 | 0.087 | 1.839 | 0.07556441 | 0.310792 |
| X201199_s_at | -0.0504 | 0.0274 | -1.838 | 0.07564865 | 0.311066 |
| X205819_at | -0.0057 | 0.0031 | -1.838 | 0.07567647 | 0.311066 |
| X212966_at | 0.2381 | 0.1296 | 1.838 | 0.07568604 | 0.311066 |
| X200950_at | -0.0962 | 0.0523 | -1.838 | 0.07570761 | 0.311066 |
| X201950_x_at | -0.0898 | 0.0489 | -1.838 | 0.07571364 | 0.311066 |
| X210823_s_at | -0.0052 | 0.0028 | -1.838 | 0.07571832 | 0.311066 |
| X213537_at | 0.028 | 0.0152 | 1.837 | 0.07574055 | 0.311066 |
| X218272_at | 0.0055 | 0.003 | 1.837 | 0.07574297 | 0.311066 |
| X213699_s_at | 0.0631 | 0.0343 | 1.837 | 0.07578168 | 0.311167 |
| X204370_at | -0.072 | 0.0392 | -1.837 | 0.0758008 | 0.311186 |
| X203312_x_at | 0.0395 | 0.0215 | 1.837 | 0.07581417 | 0.311186 |
| X219446_at | -0.223 | 0.1214 | -1.837 | 0.07585806 | 0.311225 |
| X218030_at | 0.1496 | 0.0814 | 1.837 | 0.07586175 | 0.311225 |
| X212689_s_at | -0.0496 | 0.027 | -1.837 | 0.07586569 | 0.311225 |
| X217924_at | 0.0065 | 0.0036 | 1.836 | 0.07596269 | 0.311509 |
| X200940_s_at | 0.0512 | 0.0279 | 1.836 | 0.07596302 | 0.311509 |
| X210161_at | -0.0048 | 0.0026 | -1.836 | 0.07598645 | 0.311547 |
| X214808_at | 0.1609 | 0.0877 | 1.836 | 0.07603168 | 0.311675 |
| X207968_s_at | 0.0123 | 0.0067 | 1.835 | 0.07608353 | 0.31183 |
| X201809_s_at | 0.011 | 0.006 | 1.835 | 0.07610361 | 0.311855 |
| X209813_x_at | -0.0138 | 0.0075 | -1.835 | 0.07612345 | 0.311879 |
| X206316_s_at | -0.0155 | 0.0085 | -1.835 | 0.07617246 | 0.312022 |
| X201849_at | 0.072 | 0.0393 | 1.834 | 0.07623857 | 0.312162 |
| X212041_at | -0.1441 | 0.0786 | -1.834 | 0.07624244 | 0.312162 |
| X200728_at | -0.0775 | 0.0423 | -1.834 | 0.07624868 | 0.312162 |
| X214730_s_at | -0.0573 | 0.0313 | -1.834 | 0.07632599 | 0.31237 |
| X218658_s_at | -0.1091 | 0.0595 | -1.834 | 0.07632784 | 0.31237 |
| X221065_s_at | 0.011 | 0.006 | 1.833 | 0.07635412 | 0.31242 |
| X218012_at | -0.1042 | 0.0568 | -1.833 | 0.07640516 | 0.312569 |
| X212993_at | 0.1068 | 0.0583 | 1.833 | 0.07641849 | 0.312569 |
| X211622_s_at | -0.2153 | 0.1175 | -1.832 | 0.0765098 | 0.312854 |
| X215030_at | 0.0605 | 0.033 | 1.832 | 0.07651641 | 0.312854 |
| X213243_at | -0.034 | 0.0186 | -1.832 | 0.07654149 | 0.312899 |
| X209871_s_at | 0.0825 | 0.0451 | 1.832 | 0.07657078 | 0.312944 |
| X218912_at | -0.0192 | 0.0105 | -1.832 | 0.07658071 | 0.312944 |
| X215654_at | -0.0052 | 0.0029 | -1.831 | 0.07669333 | 0.313264 |
| X218136_s_at | -0.162 | 0.0885 | -1.831 | 0.07671188 | 0.313264 |
| X212278_x_at | -0.0075 | 0.0041 | -1.831 | 0.07671236 | 0.313264 |
| X213275_x_at | 0.0733 | 0.04 | 1.831 | 0.07671652 | 0.313264 |
| X217491_x_at | -0.0347 | 0.0189 | -1.831 | 0.07673397 | 0.313264 |
| X215091_s_at | -0.0441 | 0.0241 | -1.831 | 0.07674355 | 0.313264 |
| X208953_at | -0.1051 | 0.0574 | -1.83 | 0.07685576 | 0.313614 |
| X214211_at | 0.0971 | 0.0531 | 1.83 | 0.07685748 | 0.313614 |
| X215732_s_at | -0.0061 | 0.0034 | -1.83 | 0.07689002 | 0.31368 |
| X222288_at | 0.1236 | 0.0675 | 1.83 | 0.07690206 | 0.31368 |
| X39729_at | -0.0535 | 0.0293 | -1.829 | 0.07698572 | 0.313964 |
| X208277_at | -0.0045 | 0.0025 | -1.829 | 0.0770186 | 0.313966 |
| X46167_at | -0.0588 | 0.0321 | -1.829 | 0.07702105 | 0.313966 |
| X214735_at | -0.0896 | 0.049 | -1.829 | 0.07702871 | 0.313966 |
| X209325_s_at | 0.0145 | 0.0079 | 1.829 | 0.07707155 | 0.314006 |
| X215517_at | 0.0123 | 0.0067 | 1.829 | 0.07707733 | 0.314006 |
| X222143_s_at | 0.0311 | 0.017 | 1.829 | 0.07708281 | 0.314006 |
| X218393_s_at | -0.0972 | 0.0531 | -1.829 | 0.07710358 | 0.314006 |
| X209128_s_at | -0.0203 | 0.0111 | -1.829 | 0.07710905 | 0.314006 |
| X201522_x_at | -0.0606 | 0.0332 | -1.828 | 0.07720265 | 0.314329 |
| X210201_x_at | 0.1121 | 0.0613 | 1.827 | 0.07728922 | 0.314624 |
| X200702_s_at | 0.1116 | 0.0611 | 1.827 | 0.07732505 | 0.314712 |
| X209267_s_at | 0.2188 | 0.1198 | 1.827 | 0.07735604 | 0.314756 |
| X206818_s_at | 0.0253 | 0.0138 | 1.827 | 0.07736412 | 0.314756 |
| X212243_at | -0.0468 | 0.0256 | -1.827 | 0.07738496 | 0.314783 |
| X215636_at | -0.0114 | 0.0062 | -1.826 | 0.07742516 | 0.314889 |
| X81811_at | 0.067 | 0.0367 | 1.826 | 0.07744125 | 0.314897 |
| X209379_s_at | -0.0522 | 0.0286 | -1.826 | 0.07747485 | 0.314976 |
| X209246_at | -0.0188 | 0.0103 | -1.826 | 0.07753601 | 0.315167 |
| X218552_at | 0.0974 | 0.0533 | 1.826 | 0.07757144 | 0.315253 |
| X205945_at | 0.0406 | 0.0222 | 1.825 | 0.07761481 | 0.315371 |
| X202783_at | -0.0853 | 0.0468 | -1.825 | 0.07768203 | 0.315587 |
| X218236_s_at | 0.1629 | 0.0893 | 1.825 | 0.0777057 | 0.315625 |
| X220694_at | 0.0125 | 0.0068 | 1.825 | 0.07772563 | 0.315649 |
| X203855_at | -0.0582 | 0.0319 | -1.824 | 0.07784501 | 0.316076 |
| X217368_at | -0.0314 | 0.0172 | -1.823 | 0.07791516 | 0.316286 |
| X202647_s_at | 0.0843 | 0.0463 | 1.823 | 0.07792521 | 0.316286 |
| X207147_at | 0.0211 | 0.0115 | 1.823 | 0.07794426 | 0.316305 |
| X209450_at | -0.025 | 0.0137 | -1.823 | 0.07800827 | 0.316429 |
| X213259_s_at | -0.0533 | 0.0292 | -1.823 | 0.07801733 | 0.316429 |
| X39582_at | -0.0677 | 0.0372 | -1.823 | 0.07802384 | 0.316429 |
| X217917_s_at | -0.0573 | 0.0315 | -1.822 | 0.07805307 | 0.316429 |
| X209843_s_at | 0.0099 | 0.0054 | 1.822 | 0.07805507 | 0.316429 |
| X212793_at | 0.2727 | 0.1497 | 1.822 | 0.07806028 | 0.316429 |
| X207484_s_at | -0.0066 | 0.0036 | -1.822 | 0.0781564 | 0.316761 |
| X201223_s_at | -0.0559 | 0.0307 | -1.822 | 0.07818032 | 0.3168 |
| X213369_at | -0.183 | 0.1005 | -1.821 | 0.07822571 | 0.316926 |
| X208114_s_at | -0.1106 | 0.0607 | -1.821 | 0.07831768 | 0.317198 |
| X217101_at | -0.0135 | 0.0074 | -1.821 | 0.07832522 | 0.317198 |
| X219364_at | 0.0034 | 0.0019 | 1.821 | 0.07833567 | 0.317198 |
| X205271_s_at | -0.125 | 0.0687 | -1.82 | 0.07837546 | 0.317301 |
| X209012_at | 0.0712 | 0.0391 | 1.82 | 0.07840599 | 0.317315 |
| X212359_s_at | 0.0766 | 0.0421 | 1.82 | 0.07840742 | 0.317315 |
| X204465_s_at | -0.0768 | 0.0422 | -1.82 | 0.07843036 | 0.317342 |
| X203713_s_at | -0.0099 | 0.0055 | -1.82 | 0.07844257 | 0.317342 |
| X203038_at | 0.1574 | 0.0865 | 1.82 | 0.07849612 | 0.317501 |
| X210461_s_at | -0.0892 | 0.049 | -1.819 | 0.07852095 | 0.317543 |
| X222011_s_at | -0.074 | 0.0407 | -1.819 | 0.07856619 | 0.317618 |
| X222005_s_at | -0.1033 | 0.0568 | -1.819 | 0.0785727 | 0.317618 |
| X208359_s_at | -0.0969 | 0.0533 | -1.819 | 0.07859195 | 0.317618 |
| X202576_s_at | -0.009 | 0.0049 | -1.819 | 0.0786095 | 0.317618 |
| X206558_at | -0.0059 | 0.0032 | -1.819 | 0.07861091 | 0.317618 |
| X56829_at | -0.034 | 0.0187 | -1.818 | 0.07867695 | 0.317827 |
| X202903_at | -0.0637 | 0.0351 | -1.818 | 0.07869143 | 0.317828 |
| X202215_s_at | -0.0637 | 0.0351 | -1.818 | 0.07877108 | 0.318033 |
| X201596_x_at | 0.0034 | 0.0018 | 1.818 | 0.07877227 | 0.318033 |
| X219039_at | -0.0083 | 0.0046 | -1.818 | 0.07879133 | 0.318033 |
| X202467_s_at | -0.0442 | 0.0243 | -1.818 | 0.07879947 | 0.318033 |
| X219593_at | 0.0055 | 0.003 | 1.817 | 0.07883357 | 0.318112 |
| X219066_at | -0.0095 | 0.0052 | -1.817 | 0.07885393 | 0.318112 |
| X201114_x_at | 0.027 | 0.0149 | 1.817 | 0.07887333 | 0.318112 |
| X213717_at | -0.0074 | 0.0041 | -1.817 | 0.07889002 | 0.318112 |
| X212583_at | -0.0642 | 0.0353 | -1.817 | 0.07889502 | 0.318112 |
| X206824_at | -0.0048 | 0.0027 | -1.817 | 0.07891998 | 0.318112 |
| X217806_s_at | 0.0178 | 0.0098 | 1.817 | 0.0789452 | 0.318112 |
| X212994_at | 0.1275 | 0.0702 | 1.817 | 0.07894658 | 0.318112 |
| X212749_s_at | -0.1196 | 0.0658 | -1.817 | 0.07895987 | 0.318112 |
| X206238_s_at | 0.1287 | 0.0708 | 1.817 | 0.07897118 | 0.318112 |
| X210460_s_at | -0.0783 | 0.0431 | -1.816 | 0.0789836 | 0.318112 |
| X218266_s_at | 0.35 | 0.1927 | 1.816 | 0.07899087 | 0.318112 |
| X211457_at | -0.0197 | 0.0108 | -1.816 | 0.07902464 | 0.318179 |
| X219307_at | 0.0175 | 0.0097 | 1.816 | 0.07904648 | 0.318179 |
| X216039_at | -0.1107 | 0.0609 | -1.816 | 0.07905064 | 0.318179 |
| X220372_at | -0.0289 | 0.0159 | -1.815 | 0.07915717 | 0.3185 |
| X206748_s_at | 0.0164 | 0.009 | 1.815 | 0.07915895 | 0.3185 |
| X201534_s_at | -0.0754 | 0.0415 | -1.815 | 0.07920326 | 0.31862 |
| X214633_at | -0.0121 | 0.0067 | -1.815 | 0.07927556 | 0.318853 |
| X221749_at | -0.0471 | 0.026 | -1.814 | 0.07930547 | 0.318916 |
| X209028_s_at | -0.0747 | 0.0412 | -1.814 | 0.07935571 | 0.31906 |
| X218476_at | -0.053 | 0.0292 | -1.814 | 0.07942881 | 0.319296 |
| X201680_x_at | -0.054 | 0.0298 | -1.813 | 0.07949933 | 0.319522 |
| X205366_s_at | -0.0081 | 0.0045 | -1.813 | 0.07953213 | 0.319596 |
| X219787_s_at | -0.1155 | 0.0637 | -1.813 | 0.07957487 | 0.31971 |
| X219496_at | 0.0414 | 0.0228 | 1.812 | 0.07964049 | 0.319916 |
| X202943_s_at | 0.0143 | 0.0079 | 1.812 | 0.07970835 | 0.32013 |
| X204722_at | 0.0933 | 0.0515 | 1.812 | 0.07973631 | 0.320155 |
| X38521_at | 0.0666 | 0.0367 | 1.812 | 0.07975602 | 0.320155 |
| X213574_s_at | -0.0593 | 0.0327 | -1.812 | 0.07975772 | 0.320155 |
| X211949_s_at | 0.0099 | 0.0055 | 1.811 | 0.07977541 | 0.320168 |
| X201730_s_at | 0.0979 | 0.0541 | 1.811 | 0.07981096 | 0.320253 |
| X211940_x_at | 0.0628 | 0.0347 | 1.811 | 0.07984507 | 0.320332 |
| X203171_s_at | 0.0433 | 0.0239 | 1.811 | 0.07986657 | 0.320333 |
| X206274_s_at | 0.0103 | 0.0057 | 1.811 | 0.07987408 | 0.320333 |
| X210759_s_at | -0.0556 | 0.0307 | -1.811 | 0.07990639 | 0.320404 |
| X220985_s_at | -0.0825 | 0.0456 | -1.81 | 0.07994762 | 0.320455 |
| X201452_at | -0.0083 | 0.0046 | -1.81 | 0.07994794 | 0.320455 |
| X220944_at | 0.0086 | 0.0047 | 1.81 | 0.08000574 | 0.320629 |
| X219317_at | 0.1522 | 0.0841 | 1.81 | 0.08005111 | 0.320701 |
| X208316_s_at | -0.0915 | 0.0506 | -1.81 | 0.08005259 | 0.320701 |
| X40420_at | -0.0148 | 0.0082 | -1.809 | 0.08010948 | 0.320726 |
| X217593_at | -0.0991 | 0.0548 | -1.809 | 0.0801105 | 0.320726 |
| X200762_at | 0.0713 | 0.0394 | 1.809 | 0.08011996 | 0.320726 |
| X214359_s_at | -0.1498 | 0.0828 | -1.809 | 0.08014196 | 0.320726 |
| X214374_s_at | 0.124 | 0.0685 | 1.809 | 0.08015089 | 0.320726 |
| X208669_s_at | -0.0896 | 0.0495 | -1.809 | 0.08016583 | 0.320726 |
| X214447_at | 0.0051 | 0.0028 | 1.809 | 0.08018391 | 0.320726 |
| X202411_at | 0.2302 | 0.1273 | 1.809 | 0.08021909 | 0.320726 |
| X218522_s_at | -0.1097 | 0.0607 | -1.809 | 0.08022334 | 0.320726 |
| X202573_at | 0.0347 | 0.0192 | 1.809 | 0.08023016 | 0.320726 |
| X218675_at | -0.1067 | 0.059 | -1.809 | 0.08023197 | 0.320726 |
| X202460_s_at | -0.0691 | 0.0382 | -1.809 | 0.08023211 | 0.320726 |
| X208786_s_at | 0.0603 | 0.0334 | 1.808 | 0.0803678 | 0.321154 |
| X221903_s_at | -0.0769 | 0.0425 | -1.808 | 0.08036795 | 0.321154 |
| X205541_s_at | -0.1364 | 0.0755 | -1.807 | 0.08042175 | 0.321182 |
| X211514_at | -0.0098 | 0.0054 | -1.807 | 0.08042364 | 0.321182 |
| X209544_at | 0.0875 | 0.0484 | 1.807 | 0.08042455 | 0.321182 |
| X220705_s_at | -0.0055 | 0.003 | -1.807 | 0.08043274 | 0.321182 |
| X214885_at | 0.0045 | 0.0025 | 1.807 | 0.080454 | 0.321209 |
| X205456_at | 0.0078 | 0.0043 | 1.807 | 0.08048773 | 0.321258 |
| X206851_at | 0.0037 | 0.002 | 1.807 | 0.08049538 | 0.321258 |
| X213773_x_at | 0.078 | 0.0432 | 1.807 | 0.08052255 | 0.321309 |
| X203176_s_at | 0.1049 | 0.058 | 1.806 | 0.08056754 | 0.321431 |
| X208872_s_at | -0.0973 | 0.0539 | -1.806 | 0.08062251 | 0.321593 |
| X207055_at | -0.0082 | 0.0046 | -1.806 | 0.08068287 | 0.321721 |
| X211432_s_at | 0.0827 | 0.0458 | 1.806 | 0.08068357 | 0.321721 |
| X213417_at | -0.0067 | 0.0037 | -1.806 | 0.08071321 | 0.321781 |
| X204313_s_at | -0.0757 | 0.0419 | -1.805 | 0.08077806 | 0.321939 |
| X206435_at | -0.0336 | 0.0186 | -1.805 | 0.0807925 | 0.321939 |
| X205746_s_at | -0.0142 | 0.0079 | -1.805 | 0.08079645 | 0.321939 |
| X215725_at | 0.023 | 0.0128 | 1.805 | 0.08084342 | 0.322018 |
| X220392_at | -0.0054 | 0.003 | -1.805 | 0.08084512 | 0.322018 |
| X220064_at | 0.0102 | 0.0057 | 1.804 | 0.08088809 | 0.322131 |
| X44822_s_at | 0.0675 | 0.0374 | 1.804 | 0.080958 | 0.322348 |
| X201889_at | -0.0734 | 0.0407 | -1.804 | 0.08097145 | 0.322348 |
| X207142_at | 0.0283 | 0.0157 | 1.803 | 0.08110611 | 0.322826 |
| X212508_at | -0.0587 | 0.0326 | -1.803 | 0.08113153 | 0.322869 |
| X206729_at | -0.0306 | 0.017 | -1.803 | 0.08119185 | 0.323051 |
| X217961_at | 0.0197 | 0.011 | 1.802 | 0.08123618 | 0.323159 |
| X215740_at | 0.0101 | 0.0056 | 1.802 | 0.08124792 | 0.323159 |
| X206967_at | 0.0311 | 0.0173 | 1.802 | 0.08131811 | 0.32338 |
| X213630_at | -0.0979 | 0.0544 | -1.801 | 0.08136623 | 0.323513 |
| X201954_at | 0.1684 | 0.0935 | 1.801 | 0.08151458 | 0.324045 |
| X205427_at | 0.0665 | 0.037 | 1.8 | 0.08165565 | 0.32453 |
| X208020_s_at | 0.0101 | 0.0056 | 1.8 | 0.08166568 | 0.32453 |
| X201816_s_at | -0.0768 | 0.0427 | -1.799 | 0.08168643 | 0.324554 |
| X38710_at | -0.0753 | 0.0418 | -1.799 | 0.08173518 | 0.324617 |
| X220269_at | 0.1447 | 0.0804 | 1.799 | 0.08173642 | 0.324617 |
| X210233_at | 0.0366 | 0.0203 | 1.799 | 0.08174602 | 0.324617 |
| X203878_s_at | -0.0065 | 0.0036 | -1.799 | 0.08179361 | 0.324748 |
| X203860_at | -0.0503 | 0.028 | -1.799 | 0.08181541 | 0.324776 |
| X37802_r_at | -0.0514 | 0.0286 | -1.798 | 0.08184246 | 0.324826 |
| X218265_at | -0.0701 | 0.039 | -1.798 | 0.08187056 | 0.324879 |
| X217205_at | 0.0096 | 0.0053 | 1.798 | 0.08190806 | 0.32497 |
| X202781_s_at | -0.0074 | 0.0041 | -1.797 | 0.08206137 | 0.32552 |
| X202878_s_at | 0.094 | 0.0523 | 1.797 | 0.08210082 | 0.325613 |
| X208102_s_at | -0.082 | 0.0457 | -1.797 | 0.08212325 | 0.325613 |
| X204647_at | 0.0787 | 0.0438 | 1.797 | 0.08213929 | 0.325613 |
| X204867_at | -0.1521 | 0.0847 | -1.797 | 0.08214357 | 0.325613 |
| X209647_s_at | -0.1081 | 0.0602 | -1.796 | 0.08225476 | 0.325994 |
| X212174_at | 0.1234 | 0.0687 | 1.796 | 0.08226901 | 0.325994 |
| X207391_s_at | 0.0484 | 0.0269 | 1.795 | 0.08233169 | 0.326148 |
| X215258_at | -0.0067 | 0.0038 | -1.795 | 0.08233716 | 0.326148 |
| X210037_s_at | -0.0047 | 0.0026 | -1.795 | 0.08235548 | 0.326162 |
| X209241_x_at | -0.0159 | 0.0089 | -1.795 | 0.08239711 | 0.326269 |
| X202477_s_at | -0.0337 | 0.0188 | -1.795 | 0.08242045 | 0.326303 |
| X202539_s_at | -0.1819 | 0.1014 | -1.795 | 0.08246814 | 0.326434 |
| X221966_at | 0.0112 | 0.0062 | 1.794 | 0.08249918 | 0.326499 |
| X206959_s_at | -0.0585 | 0.0326 | -1.794 | 0.08252763 | 0.326553 |
| X213285_at | -0.1791 | 0.0999 | -1.794 | 0.08257115 | 0.326624 |
| X210871_x_at | -0.0904 | 0.0504 | -1.794 | 0.08257601 | 0.326624 |
| X205257_s_at | 0.1543 | 0.086 | 1.794 | 0.08258957 | 0.326624 |
| X202302_s_at | -0.0578 | 0.0322 | -1.794 | 0.08261236 | 0.326656 |
| X217769_s_at | -0.026 | 0.0145 | -1.794 | 0.08264044 | 0.326708 |
| X204690_at | -0.0412 | 0.023 | -1.793 | 0.0826731 | 0.326779 |
| X203453_at | 0.0044 | 0.0025 | 1.793 | 0.08271346 | 0.326881 |
| X217299_s_at | 0.114 | 0.0636 | 1.793 | 0.08274691 | 0.326955 |
| X204883_s_at | 0.0122 | 0.0068 | 1.792 | 0.08294807 | 0.327442 |
| X202933_s_at | 0.1168 | 0.0652 | 1.792 | 0.08294902 | 0.327442 |
| X217970_s_at | -0.0842 | 0.047 | -1.792 | 0.08296841 | 0.327442 |
| X202377_at | 0.1435 | 0.0801 | 1.791 | 0.08298223 | 0.327442 |
| X221226_s_at | 0.0095 | 0.0053 | 1.791 | 0.08299967 | 0.327442 |
| X50221_at | 0.1633 | 0.0912 | 1.791 | 0.08299967 | 0.327442 |
| X213290_at | -0.0083 | 0.0046 | -1.791 | 0.08301666 | 0.327442 |
| X203319_s_at | -0.0971 | 0.0542 | -1.791 | 0.08301858 | 0.327442 |
| X219278_at | 0.0083 | 0.0046 | 1.791 | 0.08302658 | 0.327442 |
| X203484_at | -0.1296 | 0.0724 | -1.791 | 0.08303131 | 0.327442 |
| X212385_at | 0.1395 | 0.0779 | 1.791 | 0.08303224 | 0.327442 |
| X218024_at | -0.0485 | 0.0271 | -1.791 | 0.08307456 | 0.32755 |
| X219546_at | 0.1384 | 0.0773 | 1.791 | 0.08311115 | 0.327636 |
| X210253_at | 0.1204 | 0.0672 | 1.791 | 0.08313652 | 0.327678 |
| X201135_at | -0.0419 | 0.0234 | -1.79 | 0.08318542 | 0.327758 |
| X203102_s_at | -0.0453 | 0.0253 | -1.79 | 0.08318625 | 0.327758 |
| X200037_s_at | -0.0538 | 0.0301 | -1.79 | 0.08321378 | 0.327808 |
| X219143_s_at | 0.0597 | 0.0334 | 1.79 | 0.08324289 | 0.327864 |
| X219150_s_at | 0.0949 | 0.053 | 1.79 | 0.0832574 | 0.327864 |
| X210515_at | 0.0163 | 0.0091 | 1.79 | 0.08327901 | 0.327891 |
| X221982_x_at | -0.009 | 0.005 | -1.789 | 0.08331268 | 0.327965 |
| X214324_at | -0.0114 | 0.0064 | -1.789 | 0.08335011 | 0.328023 |
| X214313_s_at | 0.3181 | 0.1778 | 1.789 | 0.08335828 | 0.328023 |
| X200715_x_at | -0.0528 | 0.0295 | -1.789 | 0.08337169 | 0.328023 |
| X200975_at | -0.0293 | 0.0164 | -1.789 | 0.08339924 | 0.328039 |
| X201120_s_at | -0.0983 | 0.055 | -1.789 | 0.08340532 | 0.328039 |
| X217518_at | -0.0026 | 0.0015 | -1.789 | 0.08343286 | 0.328063 |
| X213479_at | 0.1931 | 0.108 | 1.789 | 0.08345008 | 0.328063 |
| X200834_s_at | -0.0576 | 0.0322 | -1.788 | 0.08347459 | 0.328063 |
| X210010_s_at | 0.0245 | 0.0137 | 1.788 | 0.08347853 | 0.328063 |
| X205439_at | 0.0108 | 0.006 | 1.788 | 0.08348976 | 0.328063 |
| X214780_s_at | -0.0109 | 0.0061 | -1.788 | 0.0835 | 0.328063 |
| X208138_at | 0.0114 | 0.0064 | 1.788 | 0.0835335 | 0.328137 |
| X211927_x_at | -0.0449 | 0.0251 | -1.788 | 0.08355361 | 0.328158 |
| X203606_at | -0.0819 | 0.0458 | -1.788 | 0.08362824 | 0.328393 |
| X218614_at | 0.0861 | 0.0482 | 1.787 | 0.08371706 | 0.328683 |
| X203007_x_at | 0.1036 | 0.058 | 1.787 | 0.08377988 | 0.328872 |
| X213828_x_at | 0.0592 | 0.0331 | 1.786 | 0.08383213 | 0.329019 |
| X202605_at | 0.1211 | 0.0678 | 1.786 | 0.08389481 | 0.329207 |
| X208270_s_at | -0.0235 | 0.0131 | -1.786 | 0.08392049 | 0.329208 |
| X205448_s_at | 0.0301 | 0.0169 | 1.786 | 0.08392488 | 0.329208 |
| X204180_s_at | 0.1254 | 0.0702 | 1.785 | 0.08403878 | 0.329552 |
| X209193_at | 0.012 | 0.0067 | 1.785 | 0.08404214 | 0.329552 |
| X217922_at | -0.0525 | 0.0294 | -1.785 | 0.08407352 | 0.329617 |
| X212677_s_at | -0.0648 | 0.0363 | -1.785 | 0.08409952 | 0.329661 |
| X214222_at | 0.1842 | 0.1032 | 1.785 | 0.08413304 | 0.329666 |
| X202062_s_at | 0.0444 | 0.0249 | 1.784 | 0.08414143 | 0.329666 |
| X208336_s_at | -0.0814 | 0.0456 | -1.784 | 0.08415893 | 0.329666 |
| X216885_s_at | -0.0504 | 0.0282 | -1.784 | 0.08416031 | 0.329666 |
| X209110_s_at | 0.0603 | 0.0338 | 1.784 | 0.0842187 | 0.329837 |
| X213190_at | -0.0624 | 0.035 | -1.784 | 0.0842564 | 0.329876 |
| X222139_at | 0.089 | 0.0499 | 1.784 | 0.08425843 | 0.329876 |
| X219124_at | -0.025 | 0.014 | -1.782 | 0.08446765 | 0.330637 |
| X209662_at | 0.0953 | 0.0535 | 1.782 | 0.08450457 | 0.33066 |
| X212949_at | 0.0033 | 0.0019 | 1.782 | 0.08451317 | 0.33066 |
| X202168_at | -0.0505 | 0.0284 | -1.782 | 0.08451831 | 0.33066 |
| X221652_s_at | -0.0781 | 0.0438 | -1.782 | 0.0845568 | 0.330729 |
| X212373_at | -0.0935 | 0.0525 | -1.782 | 0.08457691 | 0.330729 |
| X202310_s_at | 0.005 | 0.0028 | 1.782 | 0.08458043 | 0.330729 |
| X201394_s_at | -0.137 | 0.0769 | -1.782 | 0.08462901 | 0.33086 |
| X201941_at | 0.088 | 0.0494 | 1.781 | 0.08467232 | 0.330928 |
| X212996_s_at | -0.0523 | 0.0294 | -1.781 | 0.08467604 | 0.330928 |
| X202173_s_at | 0.1417 | 0.0795 | 1.781 | 0.08469815 | 0.330956 |
| X213558_at | -0.0835 | 0.0469 | -1.781 | 0.08473177 | 0.331029 |
| X202769_at | -0.0619 | 0.0348 | -1.781 | 0.08477539 | 0.331141 |
| X214203_s_at | 0.0472 | 0.0265 | 1.78 | 0.08487313 | 0.331407 |
| X204476_s_at | -0.0498 | 0.028 | -1.78 | 0.08488739 | 0.331407 |
| X215571_at | 0.2395 | 0.1345 | 1.78 | 0.08488806 | 0.331407 |
| X208793_x_at | -0.0126 | 0.0071 | -1.78 | 0.0849077 | 0.331425 |
| X212542_s_at | 0.051 | 0.0287 | 1.779 | 0.08496918 | 0.331559 |
| X219094_at | -0.1227 | 0.069 | -1.779 | 0.08497191 | 0.331559 |
| X208405_s_at | 0.076 | 0.0427 | 1.779 | 0.08501473 | 0.331668 |
| X201004_at | -0.0591 | 0.0332 | -1.779 | 0.08505094 | 0.331751 |
| X216842_x_at | -0.0341 | 0.0192 | -1.779 | 0.08512615 | 0.331941 |
| X205857_at | -0.0099 | 0.0056 | -1.779 | 0.08512957 | 0.331941 |
| X206117_at | -0.0858 | 0.0482 | -1.778 | 0.08516701 | 0.332029 |
| X208232_x_at | -0.0299 | 0.0168 | -1.778 | 0.08520953 | 0.332114 |
| X206026_s_at | 0.1678 | 0.0944 | 1.778 | 0.08522069 | 0.332114 |
| X218864_at | 0.005 | 0.0028 | 1.778 | 0.08523441 | 0.332114 |
| X45297_at | 0.0081 | 0.0046 | 1.778 | 0.08524874 | 0.332114 |
| X205039_s_at | 0.0601 | 0.0338 | 1.777 | 0.08530497 | 0.332275 |
| X204680_s_at | 0.1808 | 0.1017 | 1.777 | 0.08532078 | 0.332278 |
| X204685_s_at | -0.1132 | 0.0637 | -1.777 | 0.08539368 | 0.332437 |
| X205730_s_at | -0.1259 | 0.0709 | -1.777 | 0.08540207 | 0.332437 |
| X209862_s_at | -0.0939 | 0.0528 | -1.777 | 0.08540649 | 0.332437 |
| X218583_s_at | -0.0472 | 0.0266 | -1.777 | 0.08543894 | 0.332488 |
| X218484_at | -0.0038 | 0.0021 | -1.777 | 0.08544953 | 0.332488 |
| X203203_s_at | 0.0931 | 0.0524 | 1.776 | 0.08548959 | 0.332561 |
| X218302_at | 0.0711 | 0.04 | 1.776 | 0.08549815 | 0.332561 |
| X202650_s_at | -0.1145 | 0.0645 | -1.775 | 0.08566976 | 0.333135 |
| X217543_s_at | -0.111 | 0.0625 | -1.775 | 0.08567565 | 0.333135 |
| X212572_at | 0.0668 | 0.0376 | 1.775 | 0.08571107 | 0.333207 |
| X215149_at | -0.181 | 0.102 | -1.775 | 0.08572416 | 0.333207 |
| X208268_at | -0.0213 | 0.012 | -1.774 | 0.08581711 | 0.333498 |
| X218041_x_at | 0.1214 | 0.0684 | 1.774 | 0.08582906 | 0.333498 |
| X212432_at | -0.2011 | 0.1134 | -1.774 | 0.08589169 | 0.333683 |
| X205148_s_at | -0.0696 | 0.0393 | -1.774 | 0.08592729 | 0.333763 |
| X216371_at | -0.004 | 0.0022 | -1.773 | 0.08599386 | 0.333933 |
| X210855_at | -0.0072 | 0.004 | -1.773 | 0.08600131 | 0.333933 |
| X201244_s_at | 0.048 | 0.0271 | 1.773 | 0.08607373 | 0.334156 |
| X208473_s_at | 0.0066 | 0.0037 | 1.773 | 0.08611418 | 0.334255 |
| X220581_at | 0.019 | 0.0107 | 1.772 | 0.08615156 | 0.334342 |
| X209510_at | -0.0595 | 0.0336 | -1.772 | 0.08622448 | 0.334566 |
| X202046_s_at | 0.0191 | 0.0108 | 1.772 | 0.08628958 | 0.33476 |
| X219468_s_at | 0.1199 | 0.0677 | 1.771 | 0.08634534 | 0.334918 |
| X206910_x_at | 0.0164 | 0.0093 | 1.771 | 0.08642399 | 0.33511 |
| X204513_s_at | -0.0829 | 0.0468 | -1.771 | 0.08642507 | 0.33511 |
| X218737_at | -0.1095 | 0.0618 | -1.771 | 0.08645211 | 0.335157 |
| X213993_at | -0.1576 | 0.089 | -1.771 | 0.08647219 | 0.335176 |
| X208149_x_at | 0.0112 | 0.0063 | 1.77 | 0.0865334 | 0.335355 |
| X220679_s_at | -0.0101 | 0.0057 | -1.77 | 0.08656292 | 0.335408 |
| X204636_at | -0.0043 | 0.0025 | -1.77 | 0.08658721 | 0.335408 |
| X220282_at | -0.0091 | 0.0052 | -1.77 | 0.08659238 | 0.335408 |
| X213049_at | 0.0476 | 0.0269 | 1.769 | 0.08668812 | 0.335668 |
| X202957_at | 0.126 | 0.0712 | 1.769 | 0.08668974 | 0.335668 |
| X203275_at | 0.0411 | 0.0232 | 1.769 | 0.08671625 | 0.335694 |
| X221196_x_at | -0.0757 | 0.0428 | -1.769 | 0.08672671 | 0.335694 |
| X209869_at | 0.0108 | 0.0061 | 1.769 | 0.08676631 | 0.335736 |
| X218043_s_at | -0.061 | 0.0345 | -1.769 | 0.08676784 | 0.335736 |
| X205658_s_at | -0.0993 | 0.0562 | -1.768 | 0.08689171 | 0.336142 |
| X204181_s_at | 0.0748 | 0.0423 | 1.768 | 0.08691302 | 0.336142 |
| X213434_at | 0.0831 | 0.047 | 1.768 | 0.08692016 | 0.336142 |
| X203738_at | -0.0829 | 0.0469 | -1.768 | 0.08693317 | 0.336142 |
| X209314_s_at | -0.0789 | 0.0446 | -1.768 | 0.0869623 | 0.336189 |
| X210910_s_at | -0.2599 | 0.1471 | -1.768 | 0.08697564 | 0.336189 |
| X205855_at | -0.0185 | 0.0104 | -1.767 | 0.08703464 | 0.336358 |
| X216938_x_at | -0.0968 | 0.0548 | -1.767 | 0.0871518 | 0.336753 |
| X219660_s_at | -0.1538 | 0.0871 | -1.766 | 0.087294 | 0.337243 |
| X211451_s_at | -0.1127 | 0.0638 | -1.766 | 0.0873191 | 0.337282 |
| X211939_x_at | -0.0346 | 0.0196 | -1.765 | 0.08737333 | 0.337407 |
| X210216_x_at | -0.0568 | 0.0322 | -1.765 | 0.08738192 | 0.337407 |
| X215584_at | 0.0074 | 0.0042 | 1.765 | 0.08740522 | 0.337438 |
| X57715_at | 0.0191 | 0.0108 | 1.765 | 0.08748975 | 0.337671 |
| X201345_s_at | -0.0748 | 0.0424 | -1.765 | 0.08749603 | 0.337671 |
| X220821_at | -0.0113 | 0.0064 | -1.764 | 0.08752828 | 0.337737 |
| X216698_x_at | 0.0103 | 0.0058 | 1.764 | 0.08754664 | 0.337749 |
| X203765_at | 0.1244 | 0.0705 | 1.764 | 0.08757643 | 0.337806 |
| X202244_at | 0.0287 | 0.0163 | 1.763 | 0.08770189 | 0.338231 |
| X201709_s_at | -0.0492 | 0.0279 | -1.763 | 0.08777522 | 0.338455 |
| X31861_at | 0.0135 | 0.0076 | 1.763 | 0.08779279 | 0.338464 |
| X218638_s_at | -0.0676 | 0.0383 | -1.762 | 0.08786198 | 0.338672 |
| X221998_s_at | -0.1693 | 0.0961 | -1.762 | 0.08792865 | 0.33887 |
| X218703_at | -0.0354 | 0.0201 | -1.762 | 0.08797482 | 0.338971 |
| X210422_x_at | 0.0176 | 0.01 | 1.762 | 0.08798537 | 0.338971 |
| X209909_s_at | 0.0448 | 0.0254 | 1.762 | 0.08800343 | 0.338982 |
| X206975_at | 0.0062 | 0.0035 | 1.761 | 0.08806805 | 0.339172 |
| X214081_at | -0.0197 | 0.0112 | -1.761 | 0.08811068 | 0.339277 |
| X216231_s_at | 0.1108 | 0.063 | 1.761 | 0.08817003 | 0.339447 |
| X219970_at | -0.0417 | 0.0237 | -1.76 | 0.08822765 | 0.339551 |
| X219029_at | 0.0723 | 0.0411 | 1.76 | 0.08822768 | 0.339551 |
| X218612_s_at | 0.0225 | 0.0128 | 1.76 | 0.08827697 | 0.339628 |
| X216313_at | -0.0115 | 0.0065 | -1.76 | 0.08827811 | 0.339628 |
| X202408_s_at | 0.052 | 0.0295 | 1.76 | 0.08830892 | 0.339654 |
| X201956_s_at | 0.0692 | 0.0393 | 1.76 | 0.08831565 | 0.339654 |
| X213761_at | 0.0979 | 0.0557 | 1.759 | 0.08838077 | 0.339846 |
| X208966_x_at | 0.2031 | 0.1155 | 1.759 | 0.08843584 | 0.339999 |
| X213251_at | -0.0508 | 0.0289 | -1.759 | 0.08845408 | 0.34001 |
| X209582_s_at | -0.1764 | 0.1003 | -1.759 | 0.08847374 | 0.340027 |
| X211357_s_at | -0.0199 | 0.0113 | -1.759 | 0.08852953 | 0.34016 |
| X209235_at | 0.0159 | 0.0091 | 1.758 | 0.08853903 | 0.34016 |
| X216943_at | 0.0054 | 0.0031 | 1.758 | 0.08855668 | 0.340169 |
| X215976_at | 0.2051 | 0.1167 | 1.758 | 0.08860756 | 0.340272 |
| X220603_s_at | -0.0828 | 0.0471 | -1.758 | 0.08861415 | 0.340272 |
| X215003_at | -0.1195 | 0.068 | -1.758 | 0.08869942 | 0.340539 |
| X208612_at | -0.141 | 0.0802 | -1.757 | 0.08871432 | 0.340539 |
| X218819_at | 0.0963 | 0.0548 | 1.757 | 0.0887608 | 0.340659 |
| X200823_x_at | 0.0447 | 0.0254 | 1.757 | 0.08879048 | 0.340714 |
| X221928_at | 0.0185 | 0.0105 | 1.757 | 0.08882473 | 0.340786 |
| X220018_at | 0.0213 | 0.0121 | 1.757 | 0.08887495 | 0.340919 |
| X203995_at | 0.0092 | 0.0053 | 1.756 | 0.08888991 | 0.340919 |
| X218148_at | 0.0033 | 0.0019 | 1.756 | 0.08891018 | 0.340938 |
| X203797_at | 0.3089 | 0.1759 | 1.756 | 0.08895233 | 0.341026 |
| X60471_at | 0.0057 | 0.0033 | 1.756 | 0.08896567 | 0.341026 |
| X202446_s_at | 0.1442 | 0.0821 | 1.756 | 0.08897922 | 0.341026 |
| X219549_s_at | 0.0444 | 0.0253 | 1.756 | 0.08902118 | 0.341128 |
| X208534_s_at | -0.005 | 0.0028 | -1.755 | 0.08906523 | 0.341238 |
| X217984_at | 0.1178 | 0.0671 | 1.755 | 0.08910632 | 0.341336 |
| X218593_at | 0.0946 | 0.0539 | 1.755 | 0.08915786 | 0.341475 |
| X214045_at | -0.1296 | 0.0739 | -1.754 | 0.08926319 | 0.341819 |
| X214152_at | 0.061 | 0.0348 | 1.754 | 0.08928484 | 0.341843 |
| X207144_s_at | -0.0183 | 0.0104 | -1.754 | 0.08937029 | 0.34209 |
| X204158_s_at | 0.0055 | 0.0031 | 1.754 | 0.08938003 | 0.34209 |
| X206412_at | -0.0568 | 0.0324 | -1.753 | 0.0894291 | 0.342194 |
| X212976_at | -0.1093 | 0.0623 | -1.753 | 0.08943819 | 0.342194 |
| X207527_at | -0.0471 | 0.0269 | -1.753 | 0.0894824 | 0.342305 |
| X219346_at | -0.0055 | 0.0031 | -1.752 | 0.08960452 | 0.342713 |
| X213907_at | 0.0104 | 0.0059 | 1.752 | 0.08971537 | 0.343078 |
| X209845_at | -0.0682 | 0.039 | -1.751 | 0.08976255 | 0.343199 |
| X203870_at | 0.0565 | 0.0323 | 1.751 | 0.0898126 | 0.343331 |
| X47773_at | -0.0772 | 0.0441 | -1.751 | 0.08988622 | 0.34352 |
| X201196_s_at | -0.0912 | 0.0521 | -1.751 | 0.08989286 | 0.34352 |
| X220185_at | 0.0159 | 0.0091 | 1.75 | 0.08994208 | 0.343649 |
| X204912_at | 0.0142 | 0.0081 | 1.75 | 0.09000387 | 0.343826 |
| X213249_at | 0.1456 | 0.0832 | 1.75 | 0.09003437 | 0.343883 |
| X203004_s_at | -0.0071 | 0.0041 | -1.75 | 0.09005202 | 0.343891 |
| X221744_at | -0.0602 | 0.0344 | -1.75 | 0.09007546 | 0.343922 |
| X91617_at | 0.0263 | 0.015 | 1.749 | 0.09011469 | 0.344012 |
| X201350_at | 0.0132 | 0.0075 | 1.749 | 0.09016009 | 0.344081 |
| X215227_x_at | -0.0769 | 0.0439 | -1.749 | 0.09016363 | 0.344081 |
| X220295_x_at | 0.0103 | 0.0059 | 1.749 | 0.0902003 | 0.344127 |
| X212426_s_at | 0.0658 | 0.0376 | 1.749 | 0.09020668 | 0.344127 |
| X214479_at | 0.0043 | 0.0024 | 1.749 | 0.09024167 | 0.344156 |
| X41856_at | 0.0134 | 0.0077 | 1.749 | 0.09024534 | 0.344156 |
| X216295_s_at | -0.0481 | 0.0275 | -1.748 | 0.0903313 | 0.344423 |
| X213151_s_at | 0.0615 | 0.0352 | 1.748 | 0.09036028 | 0.344423 |
| X209836_x_at | -0.0267 | 0.0153 | -1.748 | 0.0903619 | 0.344423 |
| X202808_at | 0.0454 | 0.026 | 1.748 | 0.0903822 | 0.344442 |
| X207622_s_at | -0.0763 | 0.0437 | -1.747 | 0.09046982 | 0.344651 |
| X202171_at | 0.1408 | 0.0806 | 1.747 | 0.09051602 | 0.344651 |
| X218152_at | 0.0501 | 0.0287 | 1.747 | 0.0905161 | 0.344651 |
| X213551_x_at | -0.0075 | 0.0043 | -1.747 | 0.09052902 | 0.344651 |
| X218773_s_at | -0.0436 | 0.025 | -1.747 | 0.09053257 | 0.344651 |
| X215235_at | 0.0718 | 0.0411 | 1.747 | 0.09054742 | 0.344651 |
| X206011_at | 0.0202 | 0.0116 | 1.747 | 0.09054795 | 0.344651 |
| X221855_at | -0.0123 | 0.007 | -1.747 | 0.0905612 | 0.344651 |
| X215287_at | -0.2175 | 0.1245 | -1.746 | 0.09066847 | 0.345 |
| X213188_s_at | 0.0361 | 0.0207 | 1.746 | 0.09072059 | 0.345139 |
| X218679_s_at | -0.0898 | 0.0514 | -1.746 | 0.09074175 | 0.345161 |
| X218696_at | -0.0599 | 0.0343 | -1.746 | 0.09077077 | 0.345212 |
| X210671_x_at | 0.0084 | 0.0048 | 1.745 | 0.09082401 | 0.345345 |
| X202021_x_at | -0.0559 | 0.032 | -1.745 | 0.09085168 | 0.345345 |
| X217805_at | -0.0622 | 0.0356 | -1.745 | 0.09085246 | 0.345345 |
| X200772_x_at | 0.103 | 0.059 | 1.744 | 0.09102844 | 0.345654 |
| X215473_at | 0.0142 | 0.0082 | 1.744 | 0.09103784 | 0.345654 |
| X218424_s_at | 0.0148 | 0.0085 | 1.744 | 0.09105039 | 0.345654 |
| X202985_s_at | -0.0597 | 0.0342 | -1.744 | 0.09105153 | 0.345654 |
| X218504_at | -0.0949 | 0.0544 | -1.744 | 0.09105974 | 0.345654 |
| X205729_at | 0.0112 | 0.0064 | 1.744 | 0.09106202 | 0.345654 |
| X210404_x_at | -0.1494 | 0.0857 | -1.744 | 0.09106283 | 0.345654 |
| X201150_s_at | 0.1031 | 0.0591 | 1.744 | 0.09109534 | 0.345654 |
| X203384_s_at | -0.0291 | 0.0167 | -1.744 | 0.09109686 | 0.345654 |
| X213672_at | 0.0891 | 0.0511 | 1.744 | 0.09110695 | 0.345654 |
| X203562_at | -0.0614 | 0.0352 | -1.744 | 0.091109 | 0.345654 |
| X203934_at | 0.1029 | 0.059 | 1.744 | 0.09112042 | 0.345654 |
| X220139_at | -0.0048 | 0.0027 | -1.744 | 0.09114628 | 0.345657 |
| X202941_at | -0.0385 | 0.0221 | -1.744 | 0.09115241 | 0.345657 |
| X209036_s_at | -0.0986 | 0.0566 | -1.743 | 0.09117151 | 0.345671 |
| X210627_s_at | -0.0048 | 0.0028 | -1.743 | 0.09121908 | 0.345757 |
| X217865_at | 0.1566 | 0.0899 | 1.743 | 0.09122538 | 0.345757 |
| X206103_at | 0.0057 | 0.0033 | 1.743 | 0.0913128 | 0.346029 |
| X206468_s_at | -0.0255 | 0.0147 | -1.743 | 0.09132951 | 0.346033 |
| X215046_at | 0.0934 | 0.0536 | 1.742 | 0.09136155 | 0.346096 |
| X33323_r_at | -0.1575 | 0.0904 | -1.742 | 0.09148604 | 0.346499 |
| X215728_s_at | -0.2096 | 0.1204 | -1.741 | 0.09153221 | 0.346499 |
| X219460_s_at | 0.0476 | 0.0273 | 1.741 | 0.09154543 | 0.346499 |
| X218984_at | 0.066 | 0.0379 | 1.741 | 0.0915509 | 0.346499 |
| X217974_at | -0.0544 | 0.0313 | -1.741 | 0.09155183 | 0.346499 |
| X203741_s_at | 0.1242 | 0.0713 | 1.741 | 0.09156151 | 0.346499 |
| X214297_at | 0.0101 | 0.0058 | 1.741 | 0.09160381 | 0.3466 |
| X221892_at | 0.0641 | 0.0368 | 1.741 | 0.09168086 | 0.346775 |
| X212744_at | 0.042 | 0.0241 | 1.741 | 0.09168141 | 0.346775 |
| X201481_s_at | 0.0684 | 0.0393 | 1.74 | 0.0918014 | 0.34717 |
| X208861_s_at | -0.0478 | 0.0275 | -1.74 | 0.09183013 | 0.347191 |
| X216753_at | -0.0073 | 0.0042 | -1.74 | 0.09183814 | 0.347191 |
| X203471_s_at | 0.0372 | 0.0214 | 1.739 | 0.09190231 | 0.347331 |
| X213470_s_at | -0.0762 | 0.0438 | -1.739 | 0.09191432 | 0.347331 |
| X216580_at | -0.0242 | 0.0139 | -1.739 | 0.09192225 | 0.347331 |
| X207501_s_at | -0.2116 | 0.1216 | -1.739 | 0.09195368 | 0.347365 |
| X200836_s_at | 0.1028 | 0.0591 | 1.739 | 0.0919623 | 0.347365 |
| X213705_at | 0.1685 | 0.0969 | 1.739 | 0.09200989 | 0.347485 |
| X52837_at | 0.1117 | 0.0642 | 1.739 | 0.09204701 | 0.347566 |
| X212084_at | -0.0081 | 0.0047 | -1.738 | 0.09213059 | 0.347742 |
| X212201_at | -0.0279 | 0.0161 | -1.738 | 0.09213444 | 0.347742 |
| X200055_at | -0.0444 | 0.0255 | -1.738 | 0.09214042 | 0.347742 |
| X210323_at | 0.0063 | 0.0036 | 1.738 | 0.09217376 | 0.347749 |
| X213233_s_at | 0.0614 | 0.0353 | 1.738 | 0.09217412 | 0.347749 |
| X201823_s_at | -0.1265 | 0.0728 | -1.738 | 0.09219377 | 0.347749 |
| X222320_at | 0.0478 | 0.0275 | 1.738 | 0.09220501 | 0.347749 |
| X214917_at | 0.034 | 0.0196 | 1.738 | 0.09222403 | 0.347762 |
| X209187_at | -0.0652 | 0.0375 | -1.737 | 0.09224575 | 0.347785 |
| X212133_at | -0.1087 | 0.0626 | -1.737 | 0.09230198 | 0.347917 |
| X204771_s_at | 0.0746 | 0.043 | 1.737 | 0.09231216 | 0.347917 |
| X200018_at | -0.0354 | 0.0204 | -1.736 | 0.09245095 | 0.348381 |
| X210032_s_at | 0.0103 | 0.006 | 1.736 | 0.09252415 | 0.348598 |
| X213825_at | 0.1513 | 0.0872 | 1.736 | 0.09256636 | 0.348698 |
| X205440_s_at | -0.0412 | 0.0238 | -1.735 | 0.0926049 | 0.348784 |
| X206170_at | 0.0126 | 0.0073 | 1.735 | 0.09270652 | 0.349055 |
| X217340_at | -0.0353 | 0.0203 | -1.735 | 0.09270826 | 0.349055 |
| X208820_at | 0.0816 | 0.047 | 1.735 | 0.09276139 | 0.349155 |
| X218786_at | 0.119 | 0.0686 | 1.734 | 0.09276628 | 0.349155 |
| X210329_s_at | -0.0134 | 0.0077 | -1.734 | 0.09282363 | 0.349241 |
| X201845_s_at | 0.0557 | 0.0321 | 1.734 | 0.09282702 | 0.349241 |
| X203039_s_at | -0.06 | 0.0346 | -1.734 | 0.0928414 | 0.349241 |
| X221954_at | -0.0443 | 0.0255 | -1.734 | 0.09285209 | 0.349241 |
| X219538_at | -0.0322 | 0.0186 | -1.733 | 0.0929468 | 0.349538 |
| X221198_at | -0.0119 | 0.0069 | -1.733 | 0.09298751 | 0.349561 |
| X204398_s_at | 0.0693 | 0.04 | 1.733 | 0.0929886 | 0.349561 |
| X204852_s_at | -0.0641 | 0.037 | -1.733 | 0.09300006 | 0.349561 |
| X206903_at | -0.0074 | 0.0043 | -1.732 | 0.09320193 | 0.35026 |
| X211626_x_at | 0.0075 | 0.0043 | 1.731 | 0.0933146 | 0.350584 |
| X203196_at | 0.0576 | 0.0333 | 1.731 | 0.09331972 | 0.350584 |
| X212922_s_at | 0.0919 | 0.0531 | 1.731 | 0.09335758 | 0.350655 |
| X217663_at | -0.0167 | 0.0097 | -1.731 | 0.09337014 | 0.350655 |
| X208611_s_at | -0.061 | 0.0352 | -1.731 | 0.0934136 | 0.350759 |
| X205582_s_at | 0.0055 | 0.0032 | 1.731 | 0.09345435 | 0.350853 |
| X202025_x_at | -0.0176 | 0.0102 | -1.73 | 0.09349142 | 0.350933 |
| X207283_at | 0.1069 | 0.0618 | 1.73 | 0.09354363 | 0.351046 |
| X220367_s_at | -0.0587 | 0.0339 | -1.73 | 0.09355327 | 0.351046 |
| X200911_s_at | 0.0442 | 0.0255 | 1.73 | 0.09358808 | 0.351117 |
| X207353_s_at | -0.0078 | 0.0045 | -1.73 | 0.09361375 | 0.351154 |
| X211819_s_at | 0.0097 | 0.0056 | 1.729 | 0.09369058 | 0.351383 |
| X217172_at | -0.0057 | 0.0033 | -1.729 | 0.0937756 | 0.351643 |
| X207542_s_at | 0.3318 | 0.1919 | 1.728 | 0.09386243 | 0.351909 |
| X216526_x_at | 0.1227 | 0.071 | 1.728 | 0.09389745 | 0.351981 |
| X201215_at | 0.1438 | 0.0832 | 1.728 | 0.09391955 | 0.352004 |
| X201082_s_at | -0.1329 | 0.0769 | -1.728 | 0.09396665 | 0.352078 |
| X201295_s_at | -0.1901 | 0.11 | -1.728 | 0.09397604 | 0.352078 |
| X212130_x_at | -0.0543 | 0.0314 | -1.728 | 0.09398668 | 0.352078 |
| X200821_at | 0.1764 | 0.1021 | 1.727 | 0.09406281 | 0.352303 |
| X201901_s_at | 0.0568 | 0.0329 | 1.727 | 0.09408589 | 0.35233 |
| X209478_at | -0.0665 | 0.0385 | -1.727 | 0.09413591 | 0.352436 |
| X215008_at | -0.1391 | 0.0806 | -1.727 | 0.09414581 | 0.352436 |
| X209162_s_at | -0.1616 | 0.0936 | -1.727 | 0.09419852 | 0.352574 |
| X38290_at | -0.1368 | 0.0793 | -1.726 | 0.09428745 | 0.352834 |
| X202704_at | 0.0798 | 0.0463 | 1.726 | 0.0943102 | 0.352834 |
| X211598_x_at | 0.0079 | 0.0046 | 1.726 | 0.09431569 | 0.352834 |
| X211978_x_at | -0.0181 | 0.0105 | -1.726 | 0.09437124 | 0.352898 |
| X32088_at | -0.0826 | 0.0479 | -1.726 | 0.09437373 | 0.352898 |
| X204145_at | -0.0575 | 0.0333 | -1.726 | 0.09438045 | 0.352898 |
| X208548_at | -0.0234 | 0.0135 | -1.725 | 0.09441935 | 0.352984 |
| X206595_at | -0.0049 | 0.0028 | -1.725 | 0.09450535 | 0.353246 |
| X214945_at | 0.1321 | 0.0766 | 1.725 | 0.09452829 | 0.353272 |
| X201711_x_at | 0.1361 | 0.0789 | 1.724 | 0.09459239 | 0.353453 |
| X215306_at | 0.0179 | 0.0104 | 1.724 | 0.09470683 | 0.353776 |
| X207136_at | 0.0044 | 0.0025 | 1.724 | 0.09471081 | 0.353776 |
| X213624_at | 0.0992 | 0.0576 | 1.724 | 0.09475754 | 0.353891 |
| X213297_at | -0.0093 | 0.0054 | -1.723 | 0.09485204 | 0.354055 |
| X203500_at | -0.0071 | 0.0041 | -1.723 | 0.09486267 | 0.354055 |
| X201649_at | 0.0372 | 0.0216 | 1.723 | 0.09486403 | 0.354055 |
| X217766_s_at | -0.105 | 0.0609 | -1.723 | 0.09486524 | 0.354055 |
| X210980_s_at | -0.0577 | 0.0335 | -1.722 | 0.09496236 | 0.354358 |
| X213490_s_at | -0.0058 | 0.0034 | -1.722 | 0.09500197 | 0.354403 |
| X214765_s_at | -0.1492 | 0.0866 | -1.722 | 0.09500623 | 0.354403 |
| X218045_x_at | -0.0109 | 0.0064 | -1.721 | 0.09514133 | 0.354847 |
| X213787_s_at | -0.1636 | 0.095 | -1.721 | 0.09519126 | 0.354974 |
| X212503_s_at | -0.059 | 0.0343 | -1.721 | 0.09521086 | 0.354987 |
| X209554_at | -0.0095 | 0.0055 | -1.721 | 0.09527753 | 0.355084 |
| X206332_s_at | 0.1705 | 0.0991 | 1.721 | 0.09527838 | 0.355084 |
| X203286_at | 0.0605 | 0.0351 | 1.721 | 0.09528472 | 0.355084 |
| X213508_at | 0.0945 | 0.0549 | 1.72 | 0.09531302 | 0.35513 |
| X216559_x_at | 0.0323 | 0.0188 | 1.72 | 0.09540887 | 0.355427 |
| X218972_at | 0.0345 | 0.02 | 1.72 | 0.09545711 | 0.355493 |
| X217601_at | -0.0061 | 0.0036 | -1.72 | 0.09545852 | 0.355493 |
| X211970_x_at | -0.0401 | 0.0233 | -1.719 | 0.09549765 | 0.355579 |
| X216256_at | -0.0267 | 0.0155 | -1.719 | 0.09556273 | 0.355717 |
| X211351_at | -0.013 | 0.0075 | -1.719 | 0.09556668 | 0.355717 |
| X207068_at | -0.0619 | 0.036 | -1.719 | 0.09562729 | 0.355883 |
| X213153_at | -0.0321 | 0.0187 | -1.718 | 0.09569791 | 0.356086 |
| X214362_at | -0.0072 | 0.0042 | -1.718 | 0.09579756 | 0.356397 |
| X206782_s_at | 0.0063 | 0.0037 | 1.718 | 0.09584758 | 0.356498 |
| X211697_x_at | -0.1379 | 0.0803 | -1.717 | 0.09586398 | 0.356498 |
| X208212_s_at | -0.1537 | 0.0895 | -1.717 | 0.09587282 | 0.356498 |
| X203857_s_at | 0.0254 | 0.0148 | 1.717 | 0.09596543 | 0.356749 |
| X59644_at | 0.1481 | 0.0862 | 1.717 | 0.09597235 | 0.356749 |
| X203206_at | 0.0872 | 0.0508 | 1.717 | 0.09601128 | 0.356834 |
| X215232_at | 0.1443 | 0.084 | 1.717 | 0.09603523 | 0.356863 |
| X209153_s_at | 0.0731 | 0.0426 | 1.716 | 0.0961234 | 0.357131 |
| X206147_x_at | 0.0492 | 0.0286 | 1.716 | 0.09615406 | 0.357151 |
| X207350_s_at | -0.0839 | 0.0489 | -1.716 | 0.09616097 | 0.357151 |
| X207536_s_at | -0.0128 | 0.0074 | -1.716 | 0.09618187 | 0.357169 |
| X217721_at | -0.1737 | 0.1012 | -1.716 | 0.09620545 | 0.357197 |
| X222040_at | 0.0993 | 0.0579 | 1.715 | 0.09625511 | 0.357321 |
| X203265_s_at | -0.2784 | 0.1623 | -1.715 | 0.09628259 | 0.357357 |
| X213927_at | 0.0751 | 0.0438 | 1.715 | 0.09632158 | 0.357357 |
| X205971_s_at | -0.0091 | 0.0053 | -1.715 | 0.09632383 | 0.357357 |
| X214974_x_at | -0.2365 | 0.1379 | -1.715 | 0.09632914 | 0.357357 |
| X219293_s_at | -0.0451 | 0.0263 | -1.715 | 0.09635123 | 0.35738 |
| X208910_s_at | -0.0855 | 0.0499 | -1.714 | 0.09643342 | 0.357625 |
| X217784_at | 0.0582 | 0.034 | 1.714 | 0.09646605 | 0.357686 |
| X218150_at | -0.0449 | 0.0262 | -1.714 | 0.09654459 | 0.357918 |
| X206108_s_at | -0.2293 | 0.1338 | -1.714 | 0.09657754 | 0.35798 |
| X205598_at | 0.0064 | 0.0037 | 1.713 | 0.09663438 | 0.358049 |
| X213908_at | 0.088 | 0.0513 | 1.713 | 0.09663673 | 0.358049 |
| X203011_at | 0.082 | 0.0479 | 1.713 | 0.09665318 | 0.358049 |
| X217973_at | 0.067 | 0.0391 | 1.713 | 0.09666063 | 0.358049 |
| X213654_at | 0.0422 | 0.0246 | 1.713 | 0.09675602 | 0.358307 |
| X220483_s_at | 0.0182 | 0.0106 | 1.713 | 0.09676243 | 0.358307 |
| X204780_s_at | 0.2564 | 0.1498 | 1.712 | 0.09681997 | 0.358408 |
| X211747_s_at | -0.0596 | 0.0348 | -1.712 | 0.09682205 | 0.358408 |
| X209099_x_at | 0.0646 | 0.0377 | 1.712 | 0.09683974 | 0.358414 |
| X213035_at | -0.1001 | 0.0585 | -1.712 | 0.09686045 | 0.358431 |
| X220841_s_at | 0.0558 | 0.0326 | 1.712 | 0.09690206 | 0.358525 |
| X220227_at | -0.1598 | 0.0934 | -1.712 | 0.09694703 | 0.358631 |
| X219335_at | -0.1108 | 0.0648 | -1.711 | 0.09702589 | 0.358863 |
| X203204_s_at | -0.0973 | 0.0569 | -1.711 | 0.0970672 | 0.358956 |
| X218813_s_at | -0.0079 | 0.0046 | -1.711 | 0.09710471 | 0.359035 |
| X216684_s_at | -0.0216 | 0.0126 | -1.71 | 0.09718187 | 0.359261 |
| X213544_at | 0.0064 | 0.0038 | 1.71 | 0.097231 | 0.359383 |
| X208694_at | -0.0434 | 0.0254 | -1.71 | 0.09733032 | 0.35969 |
| X200885_at | 0.0567 | 0.0332 | 1.709 | 0.09738176 | 0.359761 |
| X203564_at | -0.0342 | 0.02 | -1.709 | 0.0973819 | 0.359761 |
| X207405_s_at | -0.0667 | 0.0391 | -1.708 | 0.09754314 | 0.360297 |
| X38398_at | 0.0567 | 0.0332 | 1.708 | 0.09757018 | 0.360337 |
| X215423_at | -0.0788 | 0.0462 | -1.708 | 0.09761431 | 0.36044 |
| X54051_at | -0.0281 | 0.0165 | -1.708 | 0.09766769 | 0.360518 |
| X213879_at | 0.1183 | 0.0693 | 1.708 | 0.09766798 | 0.360518 |
| X218438_s_at | -0.0601 | 0.0352 | -1.708 | 0.09769724 | 0.36052 |
| X205108_s_at | -0.0054 | 0.0032 | -1.708 | 0.09770848 | 0.36052 |
| X203238_s_at | 0.0069 | 0.004 | 1.708 | 0.09771711 | 0.36052 |
| X220882_at | 0.0094 | 0.0055 | 1.707 | 0.09777848 | 0.360686 |
| X213389_at | -0.0307 | 0.018 | -1.707 | 0.09781063 | 0.360745 |
| X201558_at | 0.0294 | 0.0173 | 1.706 | 0.09794715 | 0.361188 |
| X203595_s_at | 0.081 | 0.0475 | 1.706 | 0.09797142 | 0.361218 |
| X207553_at | -0.3646 | 0.2138 | -1.705 | 0.09814835 | 0.36181 |
| X202923_s_at | -0.0953 | 0.0559 | -1.705 | 0.09821462 | 0.361994 |
| X205452_at | -0.0542 | 0.0318 | -1.704 | 0.09832045 | 0.362292 |
| X218492_s_at | 0.0307 | 0.018 | 1.704 | 0.09832789 | 0.362292 |
| X205310_at | -0.0689 | 0.0404 | -1.704 | 0.09836025 | 0.362351 |
| X208968_s_at | -0.075 | 0.044 | -1.704 | 0.09841269 | 0.362484 |
| X211316_x_at | 0.0356 | 0.0209 | 1.704 | 0.09845807 | 0.362583 |
| X213328_at | 0.1939 | 0.1138 | 1.704 | 0.09847222 | 0.362583 |
| X203699_s_at | 0.4479 | 0.263 | 1.703 | 0.09849721 | 0.362615 |
| X219961_s_at | -0.0633 | 0.0372 | -1.703 | 0.09863385 | 0.363058 |
| X217573_at | -0.0084 | 0.0049 | -1.702 | 0.09873164 | 0.363357 |
| X204202_at | 0.0684 | 0.0402 | 1.702 | 0.09876537 | 0.363421 |
| X213374_x_at | 0.0612 | 0.036 | 1.702 | 0.09883662 | 0.363567 |
| X212561_at | 0.0909 | 0.0534 | 1.702 | 0.09883783 | 0.363567 |
| X204923_at | 0.0087 | 0.0051 | 1.701 | 0.09889153 | 0.3636 |
| X211466_at | 0.0281 | 0.0165 | 1.701 | 0.0988929 | 0.3636 |
| X220160_s_at | -0.0079 | 0.0046 | -1.701 | 0.09889589 | 0.3636 |
| X217278_x_at | -0.0108 | 0.0063 | -1.701 | 0.09892904 | 0.363608 |
| X211709_s_at | -0.0395 | 0.0232 | -1.701 | 0.09893063 | 0.363608 |
| X206326_at | 0.0192 | 0.0113 | 1.701 | 0.09898166 | 0.363735 |
| X207089_at | -0.0063 | 0.0037 | -1.701 | 0.09903331 | 0.363865 |
| X221131_at | 0.0061 | 0.0036 | 1.7 | 0.09911811 | 0.363924 |
| X201878_at | -0.0679 | 0.0399 | -1.7 | 0.09911874 | 0.363924 |
| X204921_at | -0.0871 | 0.0513 | -1.7 | 0.09911941 | 0.363924 |
| X221740_x_at | 0.0819 | 0.0482 | 1.7 | 0.09913564 | 0.363924 |
| X221620_s_at | -0.0754 | 0.0444 | -1.7 | 0.09913793 | 0.363924 |
| X218863_s_at | -0.0129 | 0.0076 | -1.7 | 0.09914786 | 0.363924 |
| X206556_at | -0.0078 | 0.0046 | -1.7 | 0.09922937 | 0.364163 |
| X208369_s_at | -0.015 | 0.0088 | -1.699 | 0.09926397 | 0.36423 |
| X222210_at | -0.0047 | 0.0027 | -1.699 | 0.09935208 | 0.364493 |
| X215219_at | -0.0186 | 0.0109 | -1.699 | 0.09937758 | 0.364527 |
| X203566_s_at | -0.0627 | 0.0369 | -1.699 | 0.0994014 | 0.364554 |
| X219313_at | 0.1233 | 0.0726 | 1.698 | 0.09947506 | 0.364764 |
| X213954_at | 0.1077 | 0.0635 | 1.698 | 0.09953175 | 0.364911 |
| X210187_at | -0.1212 | 0.0714 | -1.697 | 0.09964497 | 0.365266 |
| X218147_s_at | -0.0352 | 0.0207 | -1.697 | 0.09977628 | 0.365687 |
| X209050_s_at | 0.0842 | 0.0496 | 1.696 | 0.0998389 | 0.365856 |
| X217848_s_at | -0.0602 | 0.0355 | -1.696 | 0.09986047 | 0.365875 |
| X218939_at | 0.0092 | 0.0054 | 1.696 | 0.09990771 | 0.365988 |
| X220299_at | 0.0109 | 0.0065 | 1.695 | 0.1000068 | 0.366242 |
| X204061_at | 0.0336 | 0.0198 | 1.695 | 0.1000226 | 0.366242 |
| X215028_at | 0.2111 | 0.1245 | 1.695 | 0.1000393 | 0.366242 |
| X201949_x_at | 0.0467 | 0.0276 | 1.695 | 0.100043 | 0.366242 |
| X212421_at | -0.0698 | 0.0412 | -1.695 | 0.1000844 | 0.366333 |
| X215748_at | 0.0038 | 0.0022 | 1.695 | 0.1001037 | 0.366343 |
| X211921_x_at | 0.1049 | 0.0619 | 1.695 | 0.1001406 | 0.366418 |
| X209311_at | 0.0694 | 0.041 | 1.694 | 0.1001964 | 0.366562 |
| X207035_at | 0.0074 | 0.0044 | 1.694 | 0.1002503 | 0.366699 |
| X218961_s_at | -0.0988 | 0.0584 | -1.694 | 0.1002927 | 0.366735 |
| X208275_x_at | -0.0105 | 0.0062 | -1.694 | 0.1002933 | 0.366735 |
| X209894_at | 0.1626 | 0.096 | 1.694 | 0.1003102 | 0.366737 |
| X208199_s_at | -0.0123 | 0.0072 | -1.694 | 0.1003545 | 0.366838 |
| X209967_s_at | -0.0756 | 0.0447 | -1.693 | 0.1004043 | 0.366938 |
| X212881_at | -0.0594 | 0.0351 | -1.693 | 0.1004149 | 0.366938 |
| X210182_at | -0.159 | 0.0939 | -1.693 | 0.1004684 | 0.367073 |
| X212112_s_at | 0.0355 | 0.021 | 1.692 | 0.100598 | 0.367464 |
| X213186_at | -0.0839 | 0.0496 | -1.692 | 0.1006146 | 0.367464 |
| X214345_at | 0.0074 | 0.0043 | 1.692 | 0.1006249 | 0.367464 |
| X206448_at | -0.0892 | 0.0527 | -1.692 | 0.1006648 | 0.367549 |
| X218977_s_at | 0.0083 | 0.0049 | 1.692 | 0.10071 | 0.367615 |
| X212455_at | 0.0316 | 0.0187 | 1.692 | 0.1007159 | 0.367615 |
| X215342_s_at | 0.2104 | 0.1244 | 1.692 | 0.1007517 | 0.367685 |
| X48580_at | 0.0401 | 0.0237 | 1.691 | 0.1008312 | 0.367915 |
| X221889_at | -0.0598 | 0.0354 | -1.691 | 0.1008918 | 0.368006 |
| X204612_at | -0.0609 | 0.036 | -1.691 | 0.1009015 | 0.368006 |
| X160020_at | -0.0197 | 0.0116 | -1.691 | 0.1009059 | 0.368006 |
| X217202_s_at | -0.1062 | 0.0628 | -1.691 | 0.1009534 | 0.368059 |
| X201856_s_at | 0.17 | 0.1006 | 1.691 | 0.1009536 | 0.368059 |
| X213032_at | 0.1328 | 0.0785 | 1.69 | 0.100971 | 0.368062 |
| X201533_at | 0.0581 | 0.0344 | 1.69 | 0.1010219 | 0.368187 |
| X219000_s_at | -0.0124 | 0.0074 | -1.69 | 0.1010734 | 0.3683 |
| X214600_at | 0.0685 | 0.0405 | 1.69 | 0.1010859 | 0.3683 |
| X220991_s_at | 0.0441 | 0.0261 | 1.69 | 0.1011424 | 0.368445 |
| X203224_at | -0.0602 | 0.0356 | -1.689 | 0.1011965 | 0.368582 |
| X202858_at | 0.0623 | 0.0369 | 1.689 | 0.1012473 | 0.368678 |
| X209904_at | -0.0071 | 0.0042 | -1.689 | 0.1012561 | 0.368678 |
| X219393_s_at | 0.0326 | 0.0193 | 1.689 | 0.1012916 | 0.368747 |
| X212729_at | -0.0665 | 0.0394 | -1.689 | 0.1013271 | 0.368815 |
| X200948_at | -0.0651 | 0.0385 | -1.688 | 0.1013656 | 0.368862 |
| X214681_at | -0.0863 | 0.0511 | -1.688 | 0.1013732 | 0.368862 |
| X202436_s_at | 0.1182 | 0.07 | 1.688 | 0.1014063 | 0.368922 |
| X212410_at | 0.0324 | 0.0192 | 1.688 | 0.1014277 | 0.36894 |
| X52285_f_at | -0.1402 | 0.083 | -1.688 | 0.1014556 | 0.368981 |
| X217690_at | -0.0052 | 0.0031 | -1.688 | 0.1014829 | 0.36902 |
| X203719_at | -0.0528 | 0.0313 | -1.687 | 0.1015892 | 0.369297 |
| X212937_s_at | -0.0085 | 0.005 | -1.687 | 0.1015923 | 0.369297 |
| X200621_at | 0.1204 | 0.0713 | 1.687 | 0.1016316 | 0.369379 |
| X212558_at | -0.1745 | 0.1035 | -1.687 | 0.1016736 | 0.369471 |
| X215767_at | -0.146 | 0.0866 | -1.687 | 0.1017104 | 0.369545 |
| X205811_at | -0.03 | 0.0178 | -1.686 | 0.1017615 | 0.36967 |
| X209259_s_at | -0.0562 | 0.0333 | -1.686 | 0.1017917 | 0.369719 |
| X65770_at | 0.0163 | 0.0097 | 1.686 | 0.1018239 | 0.369729 |
| X210477_x_at | 0.0078 | 0.0046 | 1.686 | 0.1018335 | 0.369729 |
| X220936_s_at | -0.1994 | 0.1183 | -1.686 | 0.1018444 | 0.369729 |
| X212830_at | 0.0576 | 0.0342 | 1.685 | 0.102061 | 0.370386 |
| X212093_s_at | 0.0927 | 0.055 | 1.685 | 0.102074 | 0.370386 |
| X213623_at | -0.1651 | 0.098 | -1.685 | 0.1020815 | 0.370386 |
| X211797_s_at | -0.0153 | 0.0091 | -1.685 | 0.1020921 | 0.370386 |
| X207014_at | -0.125 | 0.0742 | -1.684 | 0.102161 | 0.370574 |
| X217669_s_at | -0.0065 | 0.0039 | -1.684 | 0.1021922 | 0.370574 |
| X202418_at | -0.0167 | 0.0099 | -1.684 | 0.1021939 | 0.370574 |
| X204507_s_at | -0.2982 | 0.1771 | -1.684 | 0.1022396 | 0.370679 |
| X212113_at | 0.3836 | 0.2278 | 1.684 | 0.1022575 | 0.370683 |
| X200767_s_at | -0.0354 | 0.021 | -1.684 | 0.1022845 | 0.370721 |
| X201773_at | -0.0522 | 0.031 | -1.684 | 0.1023125 | 0.370722 |
| X214368_at | -0.011 | 0.0065 | -1.684 | 0.1023182 | 0.370722 |
| X220142_at | 0.0132 | 0.0078 | 1.683 | 0.1023678 | 0.370841 |
| X200902_at | -0.0743 | 0.0441 | -1.683 | 0.1024675 | 0.371142 |
| X214072_x_at | 0.0056 | 0.0033 | 1.682 | 0.1025598 | 0.371376 |
| X213880_at | 0.2735 | 0.1626 | 1.682 | 0.1025817 | 0.371376 |
| X202017_at | -0.0137 | 0.0081 | -1.682 | 0.1026072 | 0.371376 |
| X203232_s_at | -0.0973 | 0.0578 | -1.682 | 0.1026118 | 0.371376 |
| X210482_x_at | 0.0334 | 0.0199 | 1.682 | 0.1026157 | 0.371376 |
| X207991_x_at | -0.0142 | 0.0084 | -1.682 | 0.1026768 | 0.371477 |
| X206153_at | -0.0051 | 0.003 | -1.682 | 0.1026772 | 0.371477 |
| X220961_s_at | -0.014 | 0.0083 | -1.681 | 0.10272 | 0.371572 |
| X200717_x_at | -0.0427 | 0.0254 | -1.681 | 0.1027614 | 0.371611 |
| X205281_s_at | 0.1005 | 0.0597 | 1.681 | 0.1027644 | 0.371611 |
| X209164_s_at | -0.1483 | 0.0882 | -1.681 | 0.1027981 | 0.371673 |
| X210108_at | 0.1076 | 0.064 | 1.681 | 0.1028856 | 0.371929 |
| X203348_s_at | -0.1071 | 0.0637 | -1.68 | 0.1029344 | 0.372044 |
| X212767_at | -0.0099 | 0.0059 | -1.68 | 0.1029931 | 0.372069 |
| X210067_at | 0.2057 | 0.1224 | 1.68 | 0.1030067 | 0.372069 |
| X208939_at | 0.0771 | 0.0459 | 1.68 | 0.1030185 | 0.372069 |
| X215216_at | 0.0068 | 0.0041 | 1.68 | 0.1030258 | 0.372069 |
| X221845_s_at | -0.0077 | 0.0046 | -1.68 | 0.1030287 | 0.372069 |
| X216766_at | 0.0154 | 0.0092 | 1.68 | 0.1030418 | 0.372069 |
| X204030_s_at | 0.0728 | 0.0433 | 1.68 | 0.103092 | 0.372176 |
| X209176_at | -0.0472 | 0.0281 | -1.679 | 0.1031106 | 0.372176 |
| X207482_at | -0.009 | 0.0054 | -1.679 | 0.1031344 | 0.372176 |
| X209509_s_at | -0.0925 | 0.0551 | -1.679 | 0.1031467 | 0.372176 |
| X202056_at | -0.0449 | 0.0267 | -1.679 | 0.1031551 | 0.372176 |
| X211047_x_at | -0.0623 | 0.0371 | -1.679 | 0.1032251 | 0.372345 |
| X218250_s_at | -0.038 | 0.0226 | -1.679 | 0.1032356 | 0.372345 |
| X210977_s_at | -0.0056 | 0.0033 | -1.678 | 0.1033354 | 0.372645 |
| X214402_s_at | -0.0145 | 0.0086 | -1.678 | 0.1033844 | 0.372761 |
| X214704_at | 0.0078 | 0.0047 | 1.678 | 0.1034233 | 0.372811 |
| X210776_x_at | 0.0541 | 0.0322 | 1.678 | 0.1034319 | 0.372811 |
| X212411_at | -0.0453 | 0.027 | -1.678 | 0.1034643 | 0.372868 |
| X213973_at | 0.0098 | 0.0058 | 1.678 | 0.1034957 | 0.37292 |
| X214589_at | -0.1647 | 0.0982 | -1.677 | 0.1035224 | 0.372956 |
| X209607_x_at | -0.0919 | 0.0548 | -1.677 | 0.1036422 | 0.373292 |
| X208037_s_at | 0.0341 | 0.0204 | 1.677 | 0.1036493 | 0.373292 |
| X217951_s_at | 0.117 | 0.0698 | 1.677 | 0.1036854 | 0.373362 |
| X205763_s_at | -0.088 | 0.0525 | -1.676 | 0.1038026 | 0.373723 |
| X203776_at | -0.0807 | 0.0482 | -1.675 | 0.1039203 | 0.374086 |
| X202042_at | -0.0404 | 0.0241 | -1.675 | 0.1040007 | 0.374315 |
| X205745_x_at | 0.0234 | 0.0139 | 1.675 | 0.1040208 | 0.374327 |
| X201188_s_at | -0.0053 | 0.0031 | -1.675 | 0.1040501 | 0.374371 |
| X203608_at | -0.0631 | 0.0377 | -1.675 | 0.1040681 | 0.374376 |
| X209337_at | -0.0494 | 0.0295 | -1.674 | 0.1042622 | 0.375013 |
| X208822_s_at | -0.0473 | 0.0283 | -1.673 | 0.1044 | 0.375366 |
| X213273_at | -0.1016 | 0.0607 | -1.673 | 0.1044045 | 0.375366 |
| X209140_x_at | 0.1268 | 0.0758 | 1.673 | 0.1044109 | 0.375366 |
| X211555_s_at | -0.0928 | 0.0555 | -1.672 | 0.1045818 | 0.375888 |
| X206176_at | -0.157 | 0.0939 | -1.672 | 0.10459 | 0.375888 |
| X208636_at | -0.0693 | 0.0414 | -1.672 | 0.1046098 | 0.375898 |
| X213375_s_at | -0.0971 | 0.0581 | -1.672 | 0.1046849 | 0.376107 |
| X209599_s_at | 0.0767 | 0.0459 | 1.671 | 0.1047612 | 0.37632 |
| X214075_at | -0.1125 | 0.0674 | -1.671 | 0.1048118 | 0.37635 |
| X213152_s_at | -0.0753 | 0.0451 | -1.671 | 0.104822 | 0.37635 |
| X204188_s_at | 0.0093 | 0.0056 | 1.671 | 0.1048366 | 0.37635 |
| X211238_at | -0.0089 | 0.0053 | -1.671 | 0.1048694 | 0.37635 |
| X209374_s_at | -0.0044 | 0.0026 | -1.671 | 0.1048708 | 0.37635 |
| X211778_s_at | 0.0046 | 0.0027 | 1.671 | 0.104871 | 0.37635 |
| X219497_s_at | 0.0964 | 0.0577 | 1.67 | 0.1049148 | 0.376442 |
| X203992_s_at | -0.0366 | 0.0219 | -1.67 | 0.1049308 | 0.376442 |
| X220604_x_at | 0.006 | 0.0036 | 1.67 | 0.1049475 | 0.376442 |
| X202713_s_at | -0.0884 | 0.0529 | -1.67 | 0.1050832 | 0.376868 |
| X210681_s_at | 0.0786 | 0.0471 | 1.669 | 0.1051148 | 0.37692 |
| X209217_s_at | -0.1012 | 0.0606 | -1.669 | 0.1052252 | 0.377255 |
| X214933_at | 0.1099 | 0.0659 | 1.668 | 0.105321 | 0.377538 |
| X64486_at | -0.0496 | 0.0298 | -1.668 | 0.1054025 | 0.377769 |
| X212834_at | 0.054 | 0.0324 | 1.667 | 0.1055564 | 0.378259 |
| X205022_s_at | 0.0767 | 0.046 | 1.667 | 0.1055884 | 0.378269 |
| X203002_at | 0.1665 | 0.0999 | 1.667 | 0.1055931 | 0.378269 |
| X208921_s_at | -0.0871 | 0.0523 | -1.667 | 0.1056344 | 0.378356 |
| X205833_s_at | 0.0038 | 0.0023 | 1.667 | 0.105681 | 0.378462 |
| X215583_at | 0.014 | 0.0084 | 1.666 | 0.1056986 | 0.378464 |
| X212752_at | 0.0566 | 0.034 | 1.666 | 0.1057789 | 0.37869 |
| X210424_s_at | -0.0072 | 0.0043 | -1.666 | 0.1058443 | 0.37885 |
| X215888_at | 0.0388 | 0.0233 | 1.666 | 0.1058588 | 0.37885 |
| X215987_at | -0.0182 | 0.0109 | -1.666 | 0.1058748 | 0.37885 |
| X209771_x_at | 0.2604 | 0.1564 | 1.665 | 0.1059463 | 0.379027 |
| X214230_at | 0.1736 | 0.1043 | 1.665 | 0.1059583 | 0.379027 |
| X212936_at | -0.0474 | 0.0285 | -1.665 | 0.1060603 | 0.379331 |
| X206250_x_at | 0.0055 | 0.0033 | 1.664 | 0.1061069 | 0.379436 |
| X201626_at | 0.0805 | 0.0483 | 1.664 | 0.1061287 | 0.379453 |
| X208169_s_at | -0.0116 | 0.007 | -1.664 | 0.1062003 | 0.379608 |
| X209907_s_at | 0.044 | 0.0265 | 1.664 | 0.1062061 | 0.379608 |
| X203421_at | 0.0343 | 0.0206 | 1.664 | 0.1062316 | 0.379638 |
| X214799_at | 0.1162 | 0.0698 | 1.664 | 0.106261 | 0.379682 |
| X219784_at | -0.0045 | 0.0027 | -1.663 | 0.1062974 | 0.379751 |
| X221771_s_at | -0.1052 | 0.0633 | -1.663 | 0.1063542 | 0.379893 |
| X214067_at | 0.0061 | 0.0037 | 1.663 | 0.1063971 | 0.379985 |
| X200039_s_at | -0.0767 | 0.0461 | -1.663 | 0.1064385 | 0.380018 |
| X210348_at | 0.0135 | 0.0081 | 1.663 | 0.1064406 | 0.380018 |
| X218779_x_at | -0.0034 | 0.002 | -1.663 | 0.1064646 | 0.380038 |
| X202265_at | 0.0384 | 0.0231 | 1.663 | 0.1064805 | 0.380038 |
| X220905_at | 0.0456 | 0.0275 | 1.662 | 0.1065523 | 0.380216 |
| X220572_at | 0.0613 | 0.0369 | 1.662 | 0.1065646 | 0.380216 |
| X212025_s_at | 0.033 | 0.0199 | 1.662 | 0.106677 | 0.380556 |
| X204499_at | 0.0255 | 0.0154 | 1.661 | 0.1066952 | 0.38056 |
| X222200_s_at | 0.0306 | 0.0184 | 1.661 | 0.1067854 | 0.380755 |
| X203097_s_at | -0.0408 | 0.0246 | -1.661 | 0.1068138 | 0.380755 |
| X213394_at | 0.0283 | 0.017 | 1.661 | 0.1068293 | 0.380755 |
| X219743_at | -0.1774 | 0.1068 | -1.661 | 0.1068453 | 0.380755 |
| X212808_at | 0.1085 | 0.0653 | 1.661 | 0.1068517 | 0.380755 |
| X209438_at | 0.0085 | 0.0051 | 1.661 | 0.1068541 | 0.380755 |
| X221976_s_at | 0.0115 | 0.0069 | 1.661 | 0.1068927 | 0.380755 |
| X203612_at | -0.0063 | 0.0038 | -1.66 | 0.1069113 | 0.380755 |
| X209167_at | 0.0812 | 0.0489 | 1.66 | 0.1069326 | 0.380755 |
| X213404_s_at | -0.0432 | 0.026 | -1.66 | 0.1069572 | 0.380755 |
| X205140_at | 0.0802 | 0.0483 | 1.66 | 0.1069582 | 0.380755 |
| X203281_s_at | -0.0073 | 0.0044 | -1.66 | 0.1069726 | 0.380755 |
| X214036_at | 0.0947 | 0.057 | 1.66 | 0.1069726 | 0.380755 |
| X221295_at | -0.0069 | 0.0042 | -1.66 | 0.1070095 | 0.380825 |
| X203911_at | -0.0954 | 0.0575 | -1.66 | 0.1070733 | 0.380923 |
| X205576_at | -0.0376 | 0.0227 | -1.66 | 0.1070931 | 0.380923 |
| X201218_at | 0.1062 | 0.064 | 1.66 | 0.1070978 | 0.380923 |
| X207625_s_at | 0.0719 | 0.0433 | 1.659 | 0.1071229 | 0.380923 |
| X219984_s_at | -0.115 | 0.0693 | -1.659 | 0.1071229 | 0.380923 |
| X203901_at | 0.0079 | 0.0048 | 1.659 | 0.1072696 | 0.381287 |
| X216438_s_at | -0.0436 | 0.0263 | -1.659 | 0.1072872 | 0.381287 |
| X204201_s_at | 0.0947 | 0.0571 | 1.659 | 0.1072919 | 0.381287 |
| X210416_s_at | 0.005 | 0.003 | 1.659 | 0.1072938 | 0.381287 |
| X205542_at | -0.0094 | 0.0057 | -1.658 | 0.1073306 | 0.381357 |
| X204754_at | 0.0717 | 0.0432 | 1.658 | 0.1073644 | 0.38137 |
| X205132_at | 0.0156 | 0.0094 | 1.658 | 0.1073686 | 0.38137 |
| X220935_s_at | 0.0339 | 0.0205 | 1.658 | 0.10743 | 0.381527 |
| X205959_at | -0.0087 | 0.0052 | -1.658 | 0.1074556 | 0.381557 |
| X217796_s_at | 0.0508 | 0.0307 | 1.658 | 0.1075026 | 0.381622 |
| X214318_s_at | -0.081 | 0.0489 | -1.657 | 0.1075305 | 0.381622 |
| X212774_at | 0.1064 | 0.0642 | 1.657 | 0.1075371 | 0.381622 |
| X209815_at | -0.1232 | 0.0744 | -1.657 | 0.1075429 | 0.381622 |
| X207618_s_at | -0.0656 | 0.0396 | -1.657 | 0.107577 | 0.381622 |
| X213523_at | 0.009 | 0.0054 | 1.657 | 0.1075802 | 0.381622 |
| X204050_s_at | -0.0526 | 0.0317 | -1.657 | 0.1076149 | 0.381622 |
| X213978_at | 0.0061 | 0.0037 | 1.657 | 0.1076314 | 0.381622 |
| X209400_at | 0.0065 | 0.0039 | 1.657 | 0.1076382 | 0.381622 |
| X221562_s_at | -0.1031 | 0.0622 | -1.657 | 0.1076458 | 0.381622 |
| X213899_at | 0.1083 | 0.0654 | 1.657 | 0.1076655 | 0.381631 |
| X219670_at | 0.0414 | 0.025 | 1.657 | 0.1076909 | 0.38166 |
| X206396_at | 0.0043 | 0.0026 | 1.656 | 0.1077163 | 0.381689 |
| X202131_s_at | 0.0993 | 0.06 | 1.656 | 0.107734 | 0.381691 |
| X205379_at | 0.0722 | 0.0436 | 1.656 | 0.1077941 | 0.381843 |
| X211730_s_at | -0.0448 | 0.0271 | -1.656 | 0.1078276 | 0.381887 |
| X206688_s_at | 0.0604 | 0.0365 | 1.656 | 0.1078408 | 0.381887 |
| X210912_x_at | 0.0815 | 0.0492 | 1.656 | 0.1079099 | 0.382035 |
| X203821_at | 0.0272 | 0.0164 | 1.655 | 0.1079388 | 0.382035 |
| X200744_s_at | -0.1999 | 0.1208 | -1.655 | 0.1079665 | 0.382035 |
| X209473_at | 0.0491 | 0.0297 | 1.655 | 0.10798 | 0.382035 |
| X213477_x_at | -0.0157 | 0.0095 | -1.655 | 0.107994 | 0.382035 |
| X212396_s_at | -0.0168 | 0.0101 | -1.655 | 0.1080114 | 0.382035 |
| X203143_s_at | 0.006 | 0.0036 | 1.655 | 0.1080201 | 0.382035 |
| X218065_s_at | -0.0615 | 0.0372 | -1.655 | 0.1080203 | 0.382035 |
| X201296_s_at | 0.0833 | 0.0504 | 1.654 | 0.1081531 | 0.382444 |
| X206997_s_at | 0.0069 | 0.0042 | 1.654 | 0.1081747 | 0.38246 |
| X217887_s_at | 0.065 | 0.0393 | 1.654 | 0.1082073 | 0.38247 |
| X215269_at | 0.1913 | 0.1156 | 1.654 | 0.108212 | 0.38247 |
| X208352_x_at | 0.0069 | 0.0041 | 1.654 | 0.1083105 | 0.382757 |
| X218889_at | 0.0971 | 0.0587 | 1.653 | 0.1083675 | 0.382848 |
| X207986_x_at | -0.0307 | 0.0186 | -1.653 | 0.1083792 | 0.382848 |
| X204508_s_at | -0.1656 | 0.1002 | -1.653 | 0.1083977 | 0.382848 |
| X212282_at | -0.1154 | 0.0698 | -1.653 | 0.1084052 | 0.382848 |
| X206417_at | -0.0075 | 0.0045 | -1.653 | 0.1084249 | 0.382857 |
| X210870_s_at | -0.0048 | 0.0029 | -1.652 | 0.1086462 | 0.383516 |
| X214148_at | 0.0045 | 0.0027 | 1.652 | 0.1086462 | 0.383516 |
| X209975_at | -0.1921 | 0.1163 | -1.652 | 0.1086937 | 0.383623 |
| X211941_s_at | -0.0376 | 0.0227 | -1.651 | 0.1087639 | 0.383792 |
| X200718_s_at | -0.0568 | 0.0344 | -1.651 | 0.1087842 | 0.383792 |
| X205354_at | -0.0187 | 0.0113 | -1.651 | 0.1088165 | 0.383792 |
| X201433_s_at | -0.0501 | 0.0304 | -1.651 | 0.1088181 | 0.383792 |
| X205898_at | -0.2923 | 0.1771 | -1.651 | 0.1088468 | 0.383792 |
| X219929_s_at | -0.1043 | 0.0632 | -1.651 | 0.1088514 | 0.383792 |
| X216542_x_at | -0.0095 | 0.0058 | -1.651 | 0.1088626 | 0.383792 |
| X212609_s_at | 0.0555 | 0.0336 | 1.651 | 0.1088956 | 0.383848 |
| X216047_x_at | 0.0104 | 0.0063 | 1.65 | 0.1089749 | 0.384029 |
| X221597_s_at | -0.0522 | 0.0316 | -1.65 | 0.108992 | 0.384029 |
| X200601_at | -0.0485 | 0.0294 | -1.65 | 0.1089989 | 0.384029 |
| X208965_s_at | 0.2319 | 0.1406 | 1.65 | 0.109048 | 0.384117 |
| X219762_s_at | 0.066 | 0.04 | 1.65 | 0.1090707 | 0.384117 |
| X57532_at | 0.036 | 0.0218 | 1.65 | 0.1091066 | 0.384117 |
| X206465_at | -0.1981 | 0.1201 | -1.65 | 0.1091201 | 0.384117 |
| X208052_x_at | 0.0077 | 0.0047 | 1.65 | 0.1091337 | 0.384117 |
| X205492_s_at | 0.0049 | 0.003 | 1.65 | 0.1091348 | 0.384117 |
| X221942_s_at | -0.1465 | 0.0888 | -1.649 | 0.1091449 | 0.384117 |
| X215776_at | -0.0037 | 0.0023 | -1.649 | 0.1091653 | 0.384128 |
| X219742_at | -0.0786 | 0.0477 | -1.649 | 0.1092049 | 0.384206 |
| X214448_x_at | 0.005 | 0.003 | 1.649 | 0.1092326 | 0.384243 |
| X222258_s_at | 0.1507 | 0.0914 | 1.649 | 0.1093344 | 0.384463 |
| X214614_at | -0.0059 | 0.0035 | -1.649 | 0.1093423 | 0.384463 |
| X217874_at | -0.043 | 0.0261 | -1.649 | 0.1093472 | 0.384463 |
| X205327_s_at | -0.0281 | 0.0171 | -1.648 | 0.1094355 | 0.384674 |
| X202378_s_at | 0.1063 | 0.0645 | 1.648 | 0.1094437 | 0.384674 |
| X202119_s_at | 0.1013 | 0.0615 | 1.648 | 0.1094635 | 0.384674 |
| X215464_s_at | -0.0318 | 0.0193 | -1.648 | 0.1094763 | 0.384674 |
| X209479_at | -0.0923 | 0.056 | -1.648 | 0.1095115 | 0.384736 |
| X202298_at | -0.042 | 0.0255 | -1.647 | 0.1096446 | 0.385143 |
| X219882_at | 0.1592 | 0.0967 | 1.647 | 0.1097112 | 0.385316 |
| X219825_at | -0.0931 | 0.0566 | -1.646 | 0.1098236 | 0.385632 |
| X206687_s_at | -0.0083 | 0.005 | -1.646 | 0.1098357 | 0.385632 |
| X215022_x_at | -0.0122 | 0.0074 | -1.646 | 0.109899 | 0.385793 |
| X203115_at | -0.0498 | 0.0302 | -1.646 | 0.1099165 | 0.385793 |
| X201404_x_at | -0.0859 | 0.0522 | -1.645 | 0.1099728 | 0.38588 |
| X204076_at | 0.0583 | 0.0354 | 1.645 | 0.1099784 | 0.38588 |
| X219248_at | 0.0455 | 0.0277 | 1.645 | 0.1099932 | 0.38588 |
| X203657_s_at | -0.0832 | 0.0506 | -1.645 | 0.1100202 | 0.385913 |
| X212227_x_at | -0.0544 | 0.0331 | -1.645 | 0.1100776 | 0.386054 |
| X216211_at | 0.0442 | 0.0269 | 1.645 | 0.1101264 | 0.386164 |
| X202558_s_at | -0.1255 | 0.0763 | -1.644 | 0.1101914 | 0.386331 |
| X219774_at | 0.0065 | 0.0039 | 1.644 | 0.1102097 | 0.386334 |
| X218796_at | 0.0727 | 0.0442 | 1.643 | 0.1103915 | 0.38691 |
| X212481_s_at | -0.0322 | 0.0196 | -1.643 | 0.1104409 | 0.387022 |
| X218727_at | 0.0122 | 0.0074 | 1.643 | 0.1104817 | 0.387058 |
| X205091_x_at | -0.0397 | 0.0242 | -1.643 | 0.1105096 | 0.387058 |
| X216913_s_at | -0.0127 | 0.0077 | -1.643 | 0.1105174 | 0.387058 |
| X211082_x_at | -0.0084 | 0.0051 | -1.643 | 0.1105209 | 0.387058 |
| X215493_x_at | -0.0679 | 0.0413 | -1.643 | 0.110591 | 0.387243 |
| X201425_at | -0.0648 | 0.0395 | -1.642 | 0.1106611 | 0.38734 |
| X217011_at | -0.0042 | 0.0026 | -1.642 | 0.1106614 | 0.38734 |
| X205304_s_at | -0.0419 | 0.0255 | -1.642 | 0.110684 | 0.38734 |
| X219175_s_at | -0.0599 | 0.0365 | -1.642 | 0.1106886 | 0.38734 |
| X38069_at | 0.0608 | 0.037 | 1.642 | 0.1107092 | 0.387351 |
| X221868_at | 0.2406 | 0.1465 | 1.642 | 0.1107473 | 0.387424 |
| X218606_at | 0.0406 | 0.0247 | 1.642 | 0.1107755 | 0.387461 |
| X211114_x_at | -0.0521 | 0.0317 | -1.641 | 0.1108097 | 0.38752 |
| X201171_at | 0.0242 | 0.0147 | 1.641 | 0.110844 | 0.38753 |
| X203831_at | 0.0508 | 0.0309 | 1.641 | 0.1108474 | 0.38753 |
| X212423_at | 0.1541 | 0.0939 | 1.641 | 0.1108652 | 0.387531 |
| X205597_at | -0.0062 | 0.0038 | -1.64 | 0.1110572 | 0.388141 |
| X203065_s_at | 0.1497 | 0.0913 | 1.64 | 0.1111173 | 0.388238 |
| X211950_at | -0.0459 | 0.028 | -1.64 | 0.11112 | 0.388238 |
| X213242_x_at | -0.0507 | 0.0309 | -1.64 | 0.1111704 | 0.388353 |
| X202091_at | 0.0065 | 0.004 | 1.64 | 0.1112159 | 0.388392 |
| X215148_s_at | 0.0138 | 0.0084 | 1.639 | 0.1112266 | 0.388392 |
| X214711_at | -0.2075 | 0.1266 | -1.639 | 0.111234 | 0.388392 |
| X205021_s_at | -0.0071 | 0.0043 | -1.639 | 0.1113164 | 0.388615 |
| X211923_s_at | -0.0077 | 0.0047 | -1.639 | 0.1113327 | 0.388615 |
| X219224_x_at | -0.0054 | 0.0033 | -1.638 | 0.1114723 | 0.389041 |
| X221487_s_at | 0.1476 | 0.0901 | 1.638 | 0.1114976 | 0.389068 |
| X217019_at | -0.042 | 0.0257 | -1.638 | 0.1115208 | 0.389088 |
| X222370_x_at | -0.0153 | 0.0093 | -1.638 | 0.1115831 | 0.389225 |
| X207204_at | -0.0062 | 0.0038 | -1.638 | 0.1115952 | 0.389225 |
| X218718_at | -0.0763 | 0.0466 | -1.637 | 0.1117386 | 0.389664 |
| X214543_x_at | 0.0823 | 0.0503 | 1.637 | 0.1117634 | 0.389689 |
| X212842_x_at | -0.0434 | 0.0265 | -1.637 | 0.1117808 | 0.389689 |
| X210212_x_at | 0.0619 | 0.0378 | 1.636 | 0.1118655 | 0.389793 |
| X201616_s_at | 0.135 | 0.0825 | 1.636 | 0.1118687 | 0.389793 |
| X206069_s_at | 0.0091 | 0.0056 | 1.636 | 0.1118798 | 0.389793 |
| X204900_x_at | 0.121 | 0.074 | 1.636 | 0.1118896 | 0.389793 |
| X213747_at | -0.0081 | 0.0049 | -1.636 | 0.1118984 | 0.389793 |
| X218969_at | -0.0335 | 0.0205 | -1.636 | 0.1119818 | 0.389983 |
| X209238_at | 0.0845 | 0.0516 | 1.636 | 0.1119881 | 0.389983 |
| X210231_x_at | 0.0555 | 0.0339 | 1.636 | 0.1120437 | 0.390111 |
| X220817_at | 0.0213 | 0.013 | 1.635 | 0.1120598 | 0.390111 |
| X206161_s_at | -0.0255 | 0.0156 | -1.635 | 0.1121085 | 0.390161 |
| X215606_s_at | -0.1236 | 0.0756 | -1.635 | 0.1121125 | 0.390161 |
| X208894_at | 0.4212 | 0.2576 | 1.635 | 0.112127 | 0.390161 |
| X208993_s_at | 0.0473 | 0.0289 | 1.635 | 0.112195 | 0.390195 |
| X214007_s_at | 0.0714 | 0.0436 | 1.635 | 0.112198 | 0.390195 |
| X208527_x_at | -0.0214 | 0.0131 | -1.635 | 0.1122187 | 0.390195 |
| X206424_at | 0.0061 | 0.0038 | 1.635 | 0.1122289 | 0.390195 |
| X204412_s_at | 0.0779 | 0.0477 | 1.635 | 0.1122325 | 0.390195 |
| X213365_at | -0.0766 | 0.0468 | -1.635 | 0.1122554 | 0.390195 |
| X212838_at | -0.0892 | 0.0546 | -1.635 | 0.1122597 | 0.390195 |
| X214299_at | -0.0065 | 0.004 | -1.634 | 0.1122905 | 0.390241 |
| X216578_at | -0.0066 | 0.004 | -1.634 | 0.1123264 | 0.390305 |
| X206506_s_at | 0.007 | 0.0043 | 1.634 | 0.1124119 | 0.390541 |
| X203466_at | -0.0665 | 0.0407 | -1.634 | 0.1124675 | 0.390673 |
| X200973_s_at | -0.0874 | 0.0535 | -1.633 | 0.1125117 | 0.390742 |
| X206882_at | -0.0597 | 0.0366 | -1.633 | 0.1125226 | 0.390742 |
| X205655_at | 0.0286 | 0.0175 | 1.633 | 0.1126257 | 0.391032 |
| X203633_at | 0.0668 | 0.0409 | 1.633 | 0.1126413 | 0.391032 |
| X221647_s_at | -0.0264 | 0.0162 | -1.632 | 0.1127464 | 0.391336 |
| X217799_x_at | 0.0276 | 0.0169 | 1.632 | 0.1128945 | 0.391749 |
| X205361_s_at | -0.0679 | 0.0416 | -1.632 | 0.1129024 | 0.391749 |
| X201376_s_at | 0.0788 | 0.0483 | 1.631 | 0.1129417 | 0.391749 |
| X201276_at | 0.0328 | 0.0201 | 1.631 | 0.1129536 | 0.391749 |
| X202636_at | -0.0442 | 0.0271 | -1.631 | 0.112963 | 0.391749 |
| X35201_at | 0.0803 | 0.0492 | 1.631 | 0.1129936 | 0.391749 |
| X208563_x_at | -0.0036 | 0.0022 | -1.631 | 0.113005 | 0.391749 |
| X222095_s_at | -0.0318 | 0.0195 | -1.631 | 0.1130065 | 0.391749 |
| X38766_at | 0.0043 | 0.0027 | 1.631 | 0.1130322 | 0.391777 |
| X216527_at | 0.114 | 0.0699 | 1.631 | 0.1130916 | 0.391922 |
| X212017_at | -0.0765 | 0.0469 | -1.63 | 0.1131187 | 0.391954 |
| X218080_x_at | 0.0436 | 0.0268 | 1.63 | 0.1131503 | 0.39196 |
| X201568_at | -0.0362 | 0.0222 | -1.63 | 0.1131555 | 0.39196 |
| X217989_at | 0.1372 | 0.0842 | 1.63 | 0.1131859 | 0.392004 |
| X216379_x_at | 0.2467 | 0.1515 | 1.629 | 0.1134838 | 0.392974 |
| X206374_at | 0.1128 | 0.0693 | 1.628 | 0.1136159 | 0.39337 |
| X217735_s_at | -0.1301 | 0.08 | -1.628 | 0.1137116 | 0.39358 |
| X212539_at | 0.0742 | 0.0456 | 1.628 | 0.113721 | 0.39358 |
| X209717_at | 0.1091 | 0.0671 | 1.628 | 0.1137297 | 0.39358 |
| X210273_at | 0.0108 | 0.0066 | 1.627 | 0.1137986 | 0.393757 |
| X208691_at | 0.1777 | 0.1092 | 1.627 | 0.113817 | 0.39376 |
| X200857_s_at | -0.0509 | 0.0313 | -1.627 | 0.1138602 | 0.393826 |
| X206797_at | -0.0115 | 0.0071 | -1.627 | 0.1138716 | 0.393826 |
| X212837_at | -0.0687 | 0.0422 | -1.627 | 0.1139102 | 0.393898 |
| X204280_at | -0.2055 | 0.1264 | -1.627 | 0.1139469 | 0.393961 |
| X210785_s_at | 0.012 | 0.0074 | 1.626 | 0.113994 | 0.393961 |
| X211881_x_at | 0.0051 | 0.0032 | 1.626 | 0.1139952 | 0.393961 |
| X213397_x_at | 0.1265 | 0.0778 | 1.626 | 0.1139994 | 0.393961 |
| X221042_s_at | 0.0205 | 0.0126 | 1.626 | 0.1140725 | 0.394153 |
| X216283_s_at | 0.0041 | 0.0025 | 1.626 | 0.1140945 | 0.394167 |
| X201285_at | -0.0505 | 0.0311 | -1.626 | 0.1141393 | 0.394258 |
| X221799_at | -0.0215 | 0.0132 | -1.626 | 0.1141564 | 0.394258 |
| X218879_s_at | 0.0069 | 0.0043 | 1.625 | 0.1141862 | 0.3943 |
| X212797_at | 0.0172 | 0.0106 | 1.625 | 0.1142053 | 0.394305 |
| X212929_s_at | 0.056 | 0.0345 | 1.625 | 0.114269 | 0.394446 |
| X202057_at | -0.0396 | 0.0244 | -1.625 | 0.1142816 | 0.394446 |
| X202720_at | -0.0202 | 0.0124 | -1.625 | 0.1143227 | 0.394526 |
| X209399_at | -0.0144 | 0.0089 | -1.625 | 0.1143918 | 0.394703 |
| X208609_s_at | 0.0034 | 0.0021 | 1.624 | 0.114433 | 0.394784 |
| X218186_at | 0.0041 | 0.0025 | 1.624 | 0.1145037 | 0.394854 |
| X219166_at | 0.0484 | 0.0298 | 1.624 | 0.1145342 | 0.394854 |
| X216347_s_at | 0.0638 | 0.0393 | 1.624 | 0.1145388 | 0.394854 |
| X219905_at | -0.0608 | 0.0374 | -1.624 | 0.1145639 | 0.394854 |
| X210691_s_at | -0.1077 | 0.0663 | -1.624 | 0.1145686 | 0.394854 |
| X211512_s_at | 0.0273 | 0.0168 | 1.624 | 0.1145699 | 0.394854 |
| X210007_s_at | -0.1296 | 0.0798 | -1.624 | 0.1145778 | 0.394854 |
| X221221_s_at | -0.1397 | 0.086 | -1.624 | 0.1146024 | 0.394878 |
| X208721_s_at | 0.0033 | 0.002 | 1.623 | 0.1146459 | 0.394967 |
| X206236_at | 0.0299 | 0.0184 | 1.623 | 0.1147595 | 0.395297 |
| X203220_s_at | -0.008 | 0.0049 | -1.623 | 0.1147864 | 0.395328 |
| X207786_at | 0.0053 | 0.0033 | 1.622 | 0.1148501 | 0.395486 |
| X216512_s_at | -0.0063 | 0.0039 | -1.622 | 0.1149462 | 0.395662 |
| X207063_at | 0.0378 | 0.0233 | 1.622 | 0.1149541 | 0.395662 |
| X216194_s_at | -0.0576 | 0.0355 | -1.622 | 0.1149545 | 0.395662 |
| X213344_s_at | 0.0129 | 0.008 | 1.621 | 0.1150589 | 0.39596 |
| X204358_s_at | 0.0312 | 0.0192 | 1.621 | 0.1151311 | 0.396139 |
| X215229_at | -0.0166 | 0.0103 | -1.621 | 0.1151467 | 0.396139 |
| X205346_at | -0.0056 | 0.0035 | -1.62 | 0.1153108 | 0.396642 |
| X221497_x_at | -0.0257 | 0.0159 | -1.62 | 0.115374 | 0.396798 |
| X215196_at | 0.008 | 0.005 | 1.62 | 0.1154561 | 0.397019 |
| X219213_at | 0.1587 | 0.098 | 1.619 | 0.1154855 | 0.397059 |
| X222097_at | -0.0091 | 0.0056 | -1.619 | 0.1155404 | 0.397165 |
| X217722_s_at | -0.0383 | 0.0236 | -1.619 | 0.1155521 | 0.397165 |
| X201351_s_at | -0.0826 | 0.051 | -1.619 | 0.1155909 | 0.397236 |
| X221088_s_at | -0.1318 | 0.0814 | -1.619 | 0.1156356 | 0.397314 |
| X206499_s_at | -0.0114 | 0.0071 | -1.619 | 0.1156747 | 0.397314 |
| X206872_at | 0.005 | 0.0031 | 1.619 | 0.1156752 | 0.397314 |
| X212330_at | -0.0562 | 0.0347 | -1.618 | 0.1157186 | 0.397314 |
| X214244_s_at | 0.0099 | 0.0061 | 1.618 | 0.1157279 | 0.397314 |
| X220141_at | 0.02 | 0.0124 | 1.618 | 0.1157316 | 0.397314 |
| X220738_s_at | 0.0044 | 0.0027 | 1.618 | 0.1157387 | 0.397314 |
| X215224_at | 0.021 | 0.013 | 1.618 | 0.1157986 | 0.397458 |
| X213742_at | 0.146 | 0.0902 | 1.618 | 0.1158226 | 0.397472 |
| X210073_at | 0.1453 | 0.0898 | 1.618 | 0.1158383 | 0.397472 |
| X205529_s_at | 0.1377 | 0.0852 | 1.617 | 0.1159442 | 0.397713 |
| X209523_at | 0.0565 | 0.0349 | 1.617 | 0.1159443 | 0.397713 |
| X201288_at | 0.1515 | 0.0937 | 1.617 | 0.1159983 | 0.397786 |
| X201266_at | -0.0849 | 0.0525 | -1.617 | 0.1160017 | 0.397786 |
| X218029_at | -0.0569 | 0.0352 | -1.617 | 0.1160333 | 0.397786 |
| X209435_s_at | -0.1441 | 0.0891 | -1.617 | 0.1160372 | 0.397786 |
| X212138_at | 0.0484 | 0.0299 | 1.617 | 0.1160909 | 0.397876 |
| X219319_at | 0.0126 | 0.0078 | 1.617 | 0.1160995 | 0.397876 |
| X209935_at | 0.0762 | 0.0471 | 1.616 | 0.1161428 | 0.39788 |
| X209396_s_at | 0.3054 | 0.189 | 1.616 | 0.1161501 | 0.39788 |
| X213760_s_at | -0.0067 | 0.0042 | -1.616 | 0.1161644 | 0.39788 |
| X37966_at | 0.0061 | 0.0038 | 1.616 | 0.1161909 | 0.39788 |
| X206284_x_at | -0.097 | 0.06 | -1.616 | 0.1162039 | 0.39788 |
| X219063_at | -0.0345 | 0.0214 | -1.616 | 0.1162299 | 0.39788 |
| X209088_s_at | 0.0783 | 0.0484 | 1.616 | 0.1162328 | 0.39788 |
| X208111_at | -0.0089 | 0.0055 | -1.616 | 0.1162438 | 0.39788 |
| X214336_s_at | 0.0068 | 0.0042 | 1.616 | 0.1162748 | 0.397925 |
| X210013_at | -0.0044 | 0.0027 | -1.616 | 0.1162968 | 0.397939 |
| X213488_at | 0.0077 | 0.0048 | 1.616 | 0.1163341 | 0.397969 |
| X217682_at | 0.0194 | 0.012 | 1.615 | 0.1163416 | 0.397969 |
| X203937_s_at | -0.0053 | 0.0033 | -1.615 | 0.1164436 | 0.398232 |
| X204544_at | 0.125 | 0.0774 | 1.615 | 0.1164543 | 0.398232 |
| X217615_at | -0.0082 | 0.0051 | -1.615 | 0.1165013 | 0.398293 |
| X210051_at | 0.0329 | 0.0204 | 1.615 | 0.1165079 | 0.398293 |
| X209784_s_at | -0.1234 | 0.0764 | -1.614 | 0.1166066 | 0.398569 |
| X216505_x_at | -0.0461 | 0.0285 | -1.614 | 0.1166371 | 0.398612 |
| X218758_s_at | -0.0182 | 0.0113 | -1.614 | 0.1166743 | 0.398678 |
| X57588_at | -0.0787 | 0.0487 | -1.614 | 0.1167325 | 0.398815 |
| X207631_at | -0.0044 | 0.0028 | -1.614 | 0.1167535 | 0.398826 |
| X204289_at | -0.0081 | 0.005 | -1.613 | 0.1168469 | 0.399083 |
| X206701_x_at | -0.3753 | 0.2327 | -1.613 | 0.1168934 | 0.399137 |
| X202529_at | -0.0593 | 0.0368 | -1.613 | 0.1168985 | 0.399137 |
| X208066_s_at | -0.0519 | 0.0322 | -1.613 | 0.1169265 | 0.399137 |
| X219629_at | -0.0067 | 0.0041 | -1.613 | 0.1169557 | 0.399137 |
| X200099_s_at | -0.0605 | 0.0375 | -1.613 | 0.1169593 | 0.399137 |
| X203796_s_at | 0.1012 | 0.0628 | 1.613 | 0.1169755 | 0.399137 |
| X219475_at | -0.0073 | 0.0045 | -1.613 | 0.1169885 | 0.399137 |
| X213446_s_at | 0.085 | 0.0527 | 1.612 | 0.1170158 | 0.399169 |
| X200065_s_at | -0.0629 | 0.039 | -1.612 | 0.1170473 | 0.399215 |
| X201302_at | 0.1029 | 0.0639 | 1.612 | 0.1170904 | 0.399229 |
| X219924_s_at | 0.0596 | 0.037 | 1.612 | 0.1170997 | 0.399229 |
| X203445_s_at | 0.1061 | 0.0658 | 1.612 | 0.1171053 | 0.399229 |
| X214176_s_at | 0.0404 | 0.0251 | 1.612 | 0.1171524 | 0.399245 |
| X207006_s_at | -0.0513 | 0.0319 | -1.612 | 0.1171612 | 0.399245 |
| X207969_x_at | -0.0195 | 0.0121 | -1.612 | 0.1171739 | 0.399245 |
| X222021_x_at | 0.0422 | 0.0262 | 1.612 | 0.1171818 | 0.399245 |
| X212483_at | 0.0718 | 0.0445 | 1.611 | 0.117221 | 0.399254 |
| X213432_at | -0.0051 | 0.0031 | -1.611 | 0.1172566 | 0.399254 |
| X214246_x_at | -0.0763 | 0.0473 | -1.611 | 0.1172581 | 0.399254 |
| X208776_at | -0.0777 | 0.0482 | -1.611 | 0.1172705 | 0.399254 |
| X202740_at | -0.103 | 0.0639 | -1.611 | 0.1172897 | 0.399254 |
| X214123_s_at | -0.0039 | 0.0024 | -1.611 | 0.1172923 | 0.399254 |
| X206196_s_at | -0.0849 | 0.0527 | -1.611 | 0.1173608 | 0.399426 |
| X207873_x_at | 0.0058 | 0.0036 | 1.61 | 0.1174262 | 0.399587 |
| X220208_at | -0.0065 | 0.004 | -1.61 | 0.1174545 | 0.399598 |
| X202777_at | 0.0626 | 0.0389 | 1.61 | 0.1174652 | 0.399598 |
| X210792_x_at | -0.007 | 0.0044 | -1.61 | 0.1175278 | 0.399749 |
| X65630_at | 0.0317 | 0.0197 | 1.61 | 0.1175714 | 0.399836 |
| X211421_s_at | 0.0231 | 0.0144 | 1.61 | 0.1176048 | 0.399889 |
| X206100_at | 0.2314 | 0.1438 | 1.609 | 0.1176924 | 0.40005 |
| X221908_at | -0.0665 | 0.0413 | -1.609 | 0.1177019 | 0.40005 |
| X212665_at | 0.1313 | 0.0816 | 1.609 | 0.1177063 | 0.40005 |
| X203246_s_at | -0.0552 | 0.0343 | -1.609 | 0.1177271 | 0.40006 |
| X212300_at | 0.041 | 0.0255 | 1.609 | 0.1177835 | 0.400073 |
| X214188_at | 0.0079 | 0.0049 | 1.609 | 0.1177853 | 0.400073 |
| X52169_at | -0.0187 | 0.0116 | -1.609 | 0.1177949 | 0.400073 |
| X220123_at | 0.0259 | 0.0161 | 1.609 | 0.1178169 | 0.400073 |
| X207800_at | -0.0593 | 0.0369 | -1.609 | 0.1178317 | 0.400073 |
| X217367_s_at | 0.0822 | 0.0511 | 1.609 | 0.1178557 | 0.400073 |
| X221187_s_at | -0.0221 | 0.0137 | -1.609 | 0.1178571 | 0.400073 |
| X216125_s_at | -0.0448 | 0.0278 | -1.608 | 0.1179174 | 0.400174 |
| X201391_at | -0.0627 | 0.039 | -1.608 | 0.1179264 | 0.400174 |
| X218753_at | 0.0195 | 0.0121 | 1.608 | 0.117941 | 0.400174 |
| X210542_s_at | 0.1378 | 0.0857 | 1.608 | 0.1179697 | 0.400211 |
| X202259_s_at | -0.1173 | 0.073 | -1.608 | 0.1180595 | 0.400441 |
| X217885_at | 0.0577 | 0.0359 | 1.608 | 0.1180736 | 0.400441 |
| X207348_s_at | -0.0057 | 0.0035 | -1.607 | 0.1180955 | 0.400454 |
| X202582_s_at | 0.0503 | 0.0313 | 1.607 | 0.1181356 | 0.40051 |
| X201414_s_at | -0.0653 | 0.0406 | -1.607 | 0.1181481 | 0.40051 |
| X211410_x_at | 0.0054 | 0.0034 | 1.607 | 0.1181766 | 0.400546 |
| X212391_x_at | -0.0407 | 0.0253 | -1.607 | 0.118262 | 0.400774 |
| X213236_at | 0.0597 | 0.0372 | 1.606 | 0.1183438 | 0.40099 |
| X213935_at | 0.0691 | 0.043 | 1.606 | 0.1184166 | 0.401175 |
| X212311_at | 0.0121 | 0.0075 | 1.606 | 0.1184673 | 0.401264 |
| X204634_at | 0.0767 | 0.0478 | 1.606 | 0.118498 | 0.401264 |
| X208185_x_at | 0.0235 | 0.0147 | 1.606 | 0.1185072 | 0.401264 |
| X206174_s_at | 0.0752 | 0.0468 | 1.605 | 0.1185285 | 0.401264 |
| X205230_at | -0.1414 | 0.0881 | -1.605 | 0.1185331 | 0.401264 |
| X219322_s_at | -0.0148 | 0.0092 | -1.605 | 0.1186352 | 0.401548 |
| X215138_s_at | 0.0053 | 0.0033 | 1.605 | 0.1187338 | 0.401821 |
| X206652_at | 0.2647 | 0.165 | 1.604 | 0.1187638 | 0.401861 |
| X210355_at | -0.0543 | 0.0339 | -1.604 | 0.1188324 | 0.402032 |
| X209146_at | -0.1029 | 0.0642 | -1.604 | 0.1189474 | 0.40236 |
| X201632_at | -0.04 | 0.0249 | -1.603 | 0.1189985 | 0.402461 |
| X215979_s_at | -0.0072 | 0.0045 | -1.603 | 0.1190134 | 0.402461 |
| X205363_at | 0.1484 | 0.0926 | 1.603 | 0.1191002 | 0.402693 |
| X202687_s_at | -0.2411 | 0.1504 | -1.603 | 0.1191705 | 0.402869 |
| X202648_at | 0.0105 | 0.0066 | 1.602 | 0.1192148 | 0.402932 |
| X210510_s_at | 0.0043 | 0.0027 | 1.602 | 0.1192254 | 0.402932 |
| X215452_x_at | 0.0403 | 0.0251 | 1.602 | 0.1192923 | 0.403073 |
| X37254_at | 0.0423 | 0.0264 | 1.602 | 0.1193522 | 0.403073 |
| X221559_s_at | 0.0732 | 0.0457 | 1.602 | 0.1193574 | 0.403073 |
| X220749_at | -0.0055 | 0.0034 | -1.602 | 0.1193617 | 0.403073 |
| X212914_at | -0.058 | 0.0362 | -1.602 | 0.1193764 | 0.403073 |
| X203390_s_at | -0.0631 | 0.0394 | -1.602 | 0.1193906 | 0.403073 |
| X218498_s_at | -0.0301 | 0.0188 | -1.602 | 0.1193945 | 0.403073 |
| X200972_at | 0.0371 | 0.0232 | 1.601 | 0.1194121 | 0.403073 |
| X208846_s_at | -0.07 | 0.0437 | -1.601 | 0.1194722 | 0.403214 |
| X203368_at | -0.0433 | 0.0271 | -1.601 | 0.1195677 | 0.403427 |
| X220939_s_at | 0.0377 | 0.0236 | 1.601 | 0.1195715 | 0.403427 |
| X203517_at | -0.0658 | 0.0411 | -1.6 | 0.1197595 | 0.403999 |
| X203545_at | -0.1311 | 0.0819 | -1.6 | 0.1197774 | 0.403999 |
| X221858_at | -0.1041 | 0.0651 | -1.6 | 0.1198035 | 0.404025 |
| X213593_s_at | 0.2224 | 0.139 | 1.6 | 0.1198374 | 0.404068 |
| X201299_s_at | 0.0981 | 0.0614 | 1.6 | 0.1198526 | 0.404068 |
| X219682_s_at | 0.1047 | 0.0654 | 1.599 | 0.1199253 | 0.404241 |
| X216041_x_at | -0.0565 | 0.0353 | -1.599 | 0.1199577 | 0.404241 |
| X204045_at | -0.079 | 0.0494 | -1.599 | 0.1199584 | 0.404241 |
| X214748_at | 0.0632 | 0.0396 | 1.598 | 0.1200791 | 0.404586 |
| X220219_s_at | 0.1634 | 0.1022 | 1.598 | 0.1201323 | 0.404673 |
| X211345_x_at | -0.0429 | 0.0268 | -1.598 | 0.1201413 | 0.404673 |
| X207696_at | -0.0191 | 0.0119 | -1.598 | 0.1202832 | 0.404884 |
| X219022_at | -0.0977 | 0.0612 | -1.598 | 0.1202837 | 0.404884 |
| X203812_at | -0.0795 | 0.0497 | -1.598 | 0.1202847 | 0.404884 |
| X214789_x_at | -0.0431 | 0.027 | -1.598 | 0.1202913 | 0.404884 |
| X219586_at | 0.0044 | 0.0028 | 1.597 | 0.1203128 | 0.404884 |
| X205747_at | 0.0111 | 0.0069 | 1.597 | 0.1203132 | 0.404884 |
| X212978_at | 0.0718 | 0.0449 | 1.597 | 0.120374 | 0.405027 |
| X208485_x_at | 0.0124 | 0.0077 | 1.597 | 0.1204649 | 0.405271 |
| X213550_s_at | 0.0062 | 0.0039 | 1.597 | 0.1205014 | 0.405301 |
| X206267_s_at | -0.0655 | 0.0411 | -1.597 | 0.1205103 | 0.405301 |
| X217774_s_at | -0.0475 | 0.0297 | -1.596 | 0.1206226 | 0.40554 |
| X200650_s_at | -0.0544 | 0.0341 | -1.596 | 0.1206307 | 0.40554 |
| X208862_s_at | 0.0199 | 0.0125 | 1.596 | 0.1206501 | 0.40554 |
| X212080_at | 0.0892 | 0.0559 | 1.596 | 0.1206543 | 0.40554 |
| X222152_at | 0.0139 | 0.0087 | 1.596 | 0.1207283 | 0.405728 |
| X212757_s_at | 0.0969 | 0.0607 | 1.595 | 0.120848 | 0.40605 |
| X204844_at | -0.0155 | 0.0097 | -1.595 | 0.1208609 | 0.40605 |
| X212336_at | 0.0993 | 0.0623 | 1.595 | 0.12093 | 0.406221 |
| X211780_x_at | -0.0375 | 0.0235 | -1.594 | 0.1210923 | 0.406705 |
| X221947_at | -0.0089 | 0.0056 | -1.593 | 0.1212289 | 0.407058 |
| X204717_s_at | -0.0238 | 0.0149 | -1.593 | 0.1212341 | 0.407058 |
| X209449_at | -0.0737 | 0.0462 | -1.593 | 0.121302 | 0.407224 |
| X212236_x_at | 0.0066 | 0.0042 | 1.593 | 0.1213907 | 0.40746 |
| X218561_s_at | -0.066 | 0.0414 | -1.592 | 0.1214383 | 0.407472 |
| X216927_at | -0.0059 | 0.0037 | -1.592 | 0.1214545 | 0.407472 |
| X217856_at | 0.0828 | 0.052 | 1.592 | 0.1214755 | 0.407472 |
| X221862_at | 0.0082 | 0.0051 | 1.592 | 0.1214854 | 0.407472 |
| X208562_s_at | 0.0083 | 0.0052 | 1.592 | 0.1214948 | 0.407472 |
| X207939_x_at | -0.0048 | 0.003 | -1.592 | 0.1215074 | 0.407472 |
| X202156_s_at | 0.0684 | 0.0429 | 1.592 | 0.1215224 | 0.407472 |
| X208203_x_at | 0.0055 | 0.0034 | 1.592 | 0.1215563 | 0.407524 |
| X209762_x_at | 0.0267 | 0.0168 | 1.591 | 0.1216721 | 0.407822 |
| X205246_at | -0.0243 | 0.0153 | -1.591 | 0.1216819 | 0.407822 |
| X208742_s_at | -0.03 | 0.0189 | -1.591 | 0.1217273 | 0.407912 |
| X209117_at | 0.0719 | 0.0452 | 1.591 | 0.1217656 | 0.407953 |
| X219404_at | 0.0057 | 0.0036 | 1.591 | 0.1217812 | 0.407953 |
| X204040_at | -0.0708 | 0.0445 | -1.591 | 0.121804 | 0.407953 |
| X203590_at | 0.1342 | 0.0844 | 1.591 | 0.1218129 | 0.407953 |
| X218349_s_at | -0.0278 | 0.0175 | -1.59 | 0.1218818 | 0.408106 |
| X200669_s_at | 0.0756 | 0.0475 | 1.59 | 0.1218953 | 0.408106 |
| X218934_s_at | 0.0044 | 0.0028 | 1.59 | 0.1219321 | 0.408168 |
| X212775_at | 0.1079 | 0.0679 | 1.59 | 0.1219677 | 0.408225 |
| X201068_s_at | -0.0348 | 0.0219 | -1.59 | 0.1220064 | 0.408293 |
| X203745_at | -0.0451 | 0.0283 | -1.59 | 0.122055 | 0.408374 |
| X203093_s_at | -0.0089 | 0.0056 | -1.59 | 0.1220799 | 0.408374 |
| X207011_s_at | 0.0065 | 0.0041 | 1.589 | 0.1221019 | 0.408374 |
| X206992_s_at | -0.0206 | 0.013 | -1.589 | 0.122104 | 0.408374 |
| X206154_at | -0.0053 | 0.0033 | -1.589 | 0.1221297 | 0.408398 |
| X221472_at | -0.0367 | 0.0231 | -1.589 | 0.1222302 | 0.408673 |
| X211167_s_at | 0.0079 | 0.005 | 1.588 | 0.1223358 | 0.408964 |
| X220591_s_at | 0.2055 | 0.1294 | 1.588 | 0.1223759 | 0.409005 |
| X201051_at | -0.0783 | 0.0493 | -1.588 | 0.1223943 | 0.409005 |
| X200003_s_at | 0.0482 | 0.0303 | 1.588 | 0.1224051 | 0.409005 |
| X216073_at | -0.0215 | 0.0135 | -1.588 | 0.1224246 | 0.409005 |
| X219867_at | -0.1682 | 0.1059 | -1.588 | 0.1224552 | 0.409005 |
| X218826_at | 0.1083 | 0.0682 | 1.588 | 0.1224585 | 0.409005 |
| X209395_at | 0.3003 | 0.1891 | 1.588 | 0.1224822 | 0.409023 |
| X218204_s_at | 0.1539 | 0.097 | 1.587 | 0.1225981 | 0.409336 |
| X34031_i_at | -0.0842 | 0.0531 | -1.587 | 0.1226127 | 0.409336 |
| X211374_x_at | 0.071 | 0.0447 | 1.587 | 0.1226447 | 0.409381 |
| X213724_s_at | -0.0328 | 0.0207 | -1.587 | 0.1226751 | 0.409421 |
| X214952_at | -0.0233 | 0.0147 | -1.587 | 0.1227324 | 0.409551 |
| X217866_at | 0.0513 | 0.0323 | 1.586 | 0.1227907 | 0.409672 |
| X208400_at | 0.0151 | 0.0095 | 1.586 | 0.1228194 | 0.409672 |
| X201264_at | -0.0229 | 0.0145 | -1.586 | 0.1228242 | 0.409672 |
| X208436_s_at | 0.0142 | 0.009 | 1.586 | 0.1229464 | 0.410018 |
| X204199_at | -0.0786 | 0.0496 | -1.585 | 0.1230033 | 0.41006 |
| X214644_at | 0.0073 | 0.0046 | 1.585 | 0.1230103 | 0.41006 |
| X216144_at | -0.0181 | 0.0114 | -1.585 | 0.1230142 | 0.41006 |
| X210994_x_at | -0.1502 | 0.0948 | -1.585 | 0.1230604 | 0.410152 |
| X213218_at | -0.0508 | 0.032 | -1.585 | 0.1231302 | 0.410292 |
| X219325_s_at | -0.0118 | 0.0075 | -1.585 | 0.1231407 | 0.410292 |
| X210720_s_at | 0.0946 | 0.0597 | 1.585 | 0.1231578 | 0.410292 |
| X219747_at | -0.0043 | 0.0027 | -1.584 | 0.1232431 | 0.410439 |
| X204288_s_at | -0.1083 | 0.0684 | -1.584 | 0.1232533 | 0.410439 |
| X202227_s_at | -0.036 | 0.0227 | -1.584 | 0.1232574 | 0.410439 |
| X206799_at | 0.0073 | 0.0046 | 1.584 | 0.1233124 | 0.410528 |
| X38340_at | 0.07 | 0.0442 | 1.584 | 0.1233211 | 0.410528 |
| X213709_at | 0.1061 | 0.067 | 1.584 | 0.123354 | 0.410571 |
| X201257_x_at | -0.0407 | 0.0257 | -1.584 | 0.1233708 | 0.410571 |
| X213527_s_at | 0.0458 | 0.0289 | 1.584 | 0.123399 | 0.410603 |
| X211139_s_at | 0.0069 | 0.0043 | 1.584 | 0.123431 | 0.410648 |
| X221427_s_at | 0.007 | 0.0044 | 1.583 | 0.1235251 | 0.410877 |
| X207251_at | -0.0083 | 0.0053 | -1.583 | 0.1235369 | 0.410877 |
| X208865_at | 0.0496 | 0.0313 | 1.583 | 0.1235568 | 0.410882 |
| X202158_s_at | 0.0696 | 0.044 | 1.583 | 0.1235872 | 0.41092 |
| X205899_at | -0.1317 | 0.0832 | -1.583 | 0.1236053 | 0.41092 |
| X58900_at | 0.0558 | 0.0352 | 1.583 | 0.1236852 | 0.411124 |
| X221872_at | 0.0096 | 0.0061 | 1.582 | 0.1237131 | 0.411155 |
| X209206_at | -0.0529 | 0.0335 | -1.582 | 0.1238103 | 0.411417 |
| X202049_s_at | 0.0746 | 0.0472 | 1.582 | 0.1238833 | 0.411598 |
| X206036_s_at | -0.0616 | 0.0389 | -1.581 | 0.1239763 | 0.411845 |
| X208593_x_at | -0.0038 | 0.0024 | -1.581 | 0.1241049 | 0.412151 |
| X203728_at | 0.0078 | 0.0049 | 1.581 | 0.1241236 | 0.412151 |
| X201493_s_at | -0.0499 | 0.0315 | -1.581 | 0.1241386 | 0.412151 |
| X216278_at | -0.02 | 0.0126 | -1.581 | 0.1241424 | 0.412151 |
| X208990_s_at | -0.0379 | 0.024 | -1.58 | 0.1242063 | 0.412244 |
| X203912_s_at | -0.0789 | 0.0499 | -1.58 | 0.1242343 | 0.412244 |
| X209083_at | -0.1308 | 0.0828 | -1.58 | 0.1242451 | 0.412244 |
| X212216_at | -0.1734 | 0.1097 | -1.58 | 0.1242487 | 0.412244 |
| X213936_x_at | 0.0308 | 0.0195 | 1.58 | 0.1242729 | 0.412244 |
| X219909_at | 0.1239 | 0.0784 | 1.58 | 0.1242818 | 0.412244 |
| X213300_at | -0.0632 | 0.04 | -1.579 | 0.1244067 | 0.412596 |
| X200815_s_at | 0.0539 | 0.0341 | 1.579 | 0.1244481 | 0.412672 |
| X212326_at | -0.0792 | 0.0501 | -1.579 | 0.1244705 | 0.412685 |
| X214876_s_at | -0.0814 | 0.0515 | -1.579 | 0.1245025 | 0.412729 |
| X216384_x_at | 0.092 | 0.0583 | 1.579 | 0.1245357 | 0.412749 |
| X202792_s_at | 0.0431 | 0.0273 | 1.579 | 0.124557 | 0.412749 |
| X220633_s_at | 0.0149 | 0.0095 | 1.579 | 0.1245642 | 0.412749 |
| X220387_s_at | -0.03 | 0.019 | -1.579 | 0.1245946 | 0.412773 |
| X209285_s_at | 0.0642 | 0.0407 | 1.578 | 0.1246212 | 0.412773 |
| X213901_x_at | -0.0868 | 0.055 | -1.578 | 0.1246435 | 0.412773 |
| X218386_x_at | -0.0573 | 0.0363 | -1.578 | 0.1246458 | 0.412773 |
| X201060_x_at | 0.1633 | 0.1035 | 1.578 | 0.1246817 | 0.41283 |
| X205362_s_at | -0.0523 | 0.0331 | -1.578 | 0.1247258 | 0.412878 |
| X219367_s_at | 0.0044 | 0.0028 | 1.578 | 0.124741 | 0.412878 |
| X205349_at | 0.0097 | 0.0062 | 1.578 | 0.1247517 | 0.412878 |
| X206169_x_at | 0.1168 | 0.074 | 1.578 | 0.1247884 | 0.412938 |
| X210528_at | 0.0238 | 0.0151 | 1.577 | 0.1248363 | 0.413024 |
| X35846_at | -0.0985 | 0.0625 | -1.577 | 0.1248516 | 0.413024 |
| X201459_at | -0.0683 | 0.0433 | -1.577 | 0.1248885 | 0.413084 |
| X206867_at | 0.0034 | 0.0022 | 1.577 | 0.1249173 | 0.413118 |
| X209086_x_at | 0.0786 | 0.0498 | 1.577 | 0.1249612 | 0.413195 |
| X211222_s_at | 0.009 | 0.0057 | 1.577 | 0.1249778 | 0.413195 |
| X210235_s_at | 0.013 | 0.0082 | 1.576 | 0.1251114 | 0.413575 |
| X55662_at | -0.0408 | 0.0259 | -1.576 | 0.1251541 | 0.413655 |
| X212274_at | -0.0578 | 0.0367 | -1.576 | 0.1252154 | 0.413786 |
| X219337_at | -0.0057 | 0.0036 | -1.576 | 0.125231 | 0.413786 |
| X214491_at | 0.0085 | 0.0054 | 1.576 | 0.1252785 | 0.41383 |
| X203678_at | 0.048 | 0.0305 | 1.576 | 0.1252816 | 0.41383 |
| X78383_at | -0.0668 | 0.0424 | -1.575 | 0.125361 | 0.414031 |
| X53720_at | 0.0443 | 0.0281 | 1.575 | 0.1253887 | 0.41406 |
| X217881_s_at | -0.0796 | 0.0506 | -1.575 | 0.1254345 | 0.41415 |
| X34225_at | 0.0407 | 0.0258 | 1.575 | 0.1254731 | 0.414216 |
| X209301_at | -0.1612 | 0.1024 | -1.575 | 0.1255041 | 0.414257 |
| X202679_at | 0.1944 | 0.1235 | 1.574 | 0.1255535 | 0.414358 |
| X211756_at | -0.0712 | 0.0453 | -1.574 | 0.1256107 | 0.414486 |
| X209519_at | -0.0378 | 0.024 | -1.574 | 0.125701 | 0.4147 |
| X37796_at | 0.0054 | 0.0035 | 1.574 | 0.125713 | 0.4147 |
| X201658_at | -0.0544 | 0.0346 | -1.573 | 0.1257703 | 0.414815 |
| X209296_at | 0.0595 | 0.0378 | 1.573 | 0.1257852 | 0.414815 |
| X221572_s_at | 0.0048 | 0.003 | 1.573 | 0.125956 | 0.415316 |
| X209860_s_at | -0.0501 | 0.0318 | -1.572 | 0.1260181 | 0.415422 |
| X219014_at | 0.0044 | 0.0028 | 1.572 | 0.1260254 | 0.415422 |
| X32811_at | 0.0426 | 0.0271 | 1.572 | 0.1260476 | 0.415433 |
| X222169_x_at | -0.022 | 0.014 | -1.572 | 0.1261174 | 0.415602 |
| X203510_at | 0.0424 | 0.027 | 1.572 | 0.1262058 | 0.415832 |
| X202996_at | -0.0056 | 0.0036 | -1.571 | 0.1262943 | 0.416061 |
| X222107_x_at | -0.0076 | 0.0048 | -1.571 | 0.1263445 | 0.416165 |
| X201957_at | -0.0513 | 0.0327 | -1.57 | 0.1265728 | 0.416838 |
| X214511_x_at | 0.2634 | 0.1678 | 1.57 | 0.1265862 | 0.416838 |
| X216048_s_at | 0.1837 | 0.117 | 1.57 | 0.126652 | 0.416992 |
| X221643_s_at | 0.0065 | 0.0041 | 1.569 | 0.1267279 | 0.41718 |
| X205825_at | -0.2655 | 0.1692 | -1.569 | 0.1267831 | 0.4173 |
| X215849_x_at | -0.0209 | 0.0133 | -1.569 | 0.126858 | 0.417485 |
| X207559_s_at | -0.0791 | 0.0504 | -1.569 | 0.1268923 | 0.417536 |
| X201449_at | -0.0634 | 0.0404 | -1.568 | 0.126918 | 0.417542 |
| X221707_s_at | 0.0113 | 0.0072 | 1.568 | 0.1269316 | 0.417542 |
| X204577_s_at | -0.1106 | 0.0705 | -1.568 | 0.1269647 | 0.417589 |
| X204860_s_at | 0.238 | 0.1518 | 1.568 | 0.1269994 | 0.417641 |
| X205171_at | 0.1241 | 0.0791 | 1.568 | 0.1270391 | 0.417655 |
| X212064_x_at | 0.0527 | 0.0336 | 1.568 | 0.1270414 | 0.417655 |
| X204071_s_at | -0.0357 | 0.0228 | -1.568 | 0.1270823 | 0.417728 |
| X211372_s_at | 0.0077 | 0.0049 | 1.567 | 0.1271581 | 0.41788 |
| X213982_s_at | 0.0936 | 0.0597 | 1.567 | 0.127166 | 0.41788 |
| X201880_at | -0.0586 | 0.0374 | -1.567 | 0.1273117 | 0.418174 |
| X201115_at | -0.0615 | 0.0392 | -1.567 | 0.1273118 | 0.418174 |
| X205029_s_at | 0.1855 | 0.1184 | 1.567 | 0.1273348 | 0.418174 |
| X212077_at | 0.0871 | 0.0556 | 1.567 | 0.1273402 | 0.418174 |
| X206602_s_at | 0.0043 | 0.0027 | 1.567 | 0.1273497 | 0.418174 |
| X213542_at | -0.0132 | 0.0084 | -1.567 | 0.1273768 | 0.418201 |
| X215024_at | -0.0451 | 0.0288 | -1.566 | 0.1274873 | 0.418454 |
| X208880_s_at | -0.0621 | 0.0397 | -1.566 | 0.1274915 | 0.418454 |
| X212915_at | 0.1234 | 0.0788 | 1.566 | 0.1275281 | 0.418512 |
| X216997_x_at | -0.1716 | 0.1096 | -1.566 | 0.1276025 | 0.418656 |
| X217337_at | -0.0208 | 0.0133 | -1.566 | 0.1276096 | 0.418656 |
| X208486_at | 0.0056 | 0.0036 | 1.565 | 0.127689 | 0.418737 |
| X205414_s_at | 0.0495 | 0.0317 | 1.565 | 0.1276896 | 0.418737 |
| X213215_at | 0.0452 | 0.0289 | 1.565 | 0.1276908 | 0.418737 |
| X208246_x_at | 0.0744 | 0.0475 | 1.565 | 0.127762 | 0.4189 |
| X204952_at | 0.0051 | 0.0033 | 1.565 | 0.1277781 | 0.4189 |
| X221250_s_at | 0.0099 | 0.0063 | 1.564 | 0.1278613 | 0.41911 |
| X213889_at | -0.1386 | 0.0886 | -1.564 | 0.1278894 | 0.419141 |
| X221123_x_at | 0.0586 | 0.0374 | 1.564 | 0.1279459 | 0.419157 |
| X208884_s_at | -0.06 | 0.0384 | -1.564 | 0.1279574 | 0.419157 |
| X204637_at | 0.0068 | 0.0044 | 1.564 | 0.1279624 | 0.419157 |
| X201381_x_at | -0.0438 | 0.028 | -1.564 | 0.1279801 | 0.419157 |
| X204522_at | -0.0214 | 0.0137 | -1.564 | 0.1279888 | 0.419157 |
| X221233_s_at | -0.0199 | 0.0127 | -1.563 | 0.1281096 | 0.419472 |
| X217695_x_at | 0.0444 | 0.0284 | 1.563 | 0.1281226 | 0.419472 |
| X220021_at | -0.0526 | 0.0337 | -1.563 | 0.1281963 | 0.419651 |
| X207673_at | -0.0084 | 0.0054 | -1.563 | 0.1282354 | 0.419691 |
| X206247_at | 0.0172 | 0.011 | 1.563 | 0.1282462 | 0.419691 |
| X214595_at | 0.0572 | 0.0366 | 1.562 | 0.1283593 | 0.419955 |
| X212165_at | -0.0868 | 0.0556 | -1.562 | 0.1283647 | 0.419955 |
| X212812_at | 0.1076 | 0.0689 | 1.562 | 0.1284231 | 0.420084 |
| X203489_at | -0.0479 | 0.0306 | -1.562 | 0.1284892 | 0.420238 |
| X215204_at | 0.1315 | 0.0842 | 1.562 | 0.1285208 | 0.420253 |
| X202816_s_at | 0.1207 | 0.0773 | 1.562 | 0.1285315 | 0.420253 |
| X200864_s_at | -0.0726 | 0.0465 | -1.561 | 0.12863 | 0.420417 |
| X200953_s_at | -0.0945 | 0.0605 | -1.561 | 0.1286376 | 0.420417 |
| X213813_x_at | 0.2227 | 0.1427 | 1.561 | 0.1286384 | 0.420417 |
| X205614_x_at | 0.007 | 0.0045 | 1.561 | 0.1287584 | 0.420691 |
| X205259_at | -0.0596 | 0.0382 | -1.561 | 0.1287725 | 0.420691 |
| X200796_s_at | -0.0063 | 0.004 | -1.561 | 0.1287924 | 0.420691 |
| X210048_at | 0.0868 | 0.0556 | 1.56 | 0.128798 | 0.420691 |
| X214033_at | 0.0095 | 0.0061 | 1.56 | 0.128842 | 0.420773 |
| X217528_at | -0.0179 | 0.0115 | -1.56 | 0.1288731 | 0.420812 |
| X206909_at | -0.0094 | 0.006 | -1.56 | 0.1288999 | 0.420838 |
| X217702_at | -0.0077 | 0.0049 | -1.56 | 0.1289325 | 0.420882 |
| X214512_s_at | -0.0678 | 0.0435 | -1.56 | 0.1289511 | 0.420882 |
| X207576_x_at | -0.0046 | 0.0029 | -1.56 | 0.1290028 | 0.420942 |
| X217771_at | 0.1101 | 0.0706 | 1.56 | 0.1290075 | 0.420942 |
| X217284_x_at | -0.0038 | 0.0024 | -1.559 | 0.129225 | 0.421473 |
| X217498_at | -0.0155 | 0.01 | -1.559 | 0.1292251 | 0.421473 |
| X213283_s_at | -0.0668 | 0.0428 | -1.559 | 0.1292273 | 0.421473 |
| X212314_at | -0.17 | 0.1091 | -1.558 | 0.129318 | 0.421663 |
| X220128_s_at | 0.0073 | 0.0047 | 1.558 | 0.1293266 | 0.421663 |
| X201236_s_at | 0.2424 | 0.1556 | 1.558 | 0.1293425 | 0.421663 |
| X200593_s_at | -0.0584 | 0.0375 | -1.558 | 0.1293782 | 0.421718 |
| X217978_s_at | -0.0481 | 0.0309 | -1.558 | 0.1294324 | 0.421833 |
| X219690_at | 0.0069 | 0.0044 | 1.558 | 0.1294734 | 0.421904 |
| X208383_s_at | 0.0176 | 0.0113 | 1.558 | 0.1294979 | 0.421922 |
| X219559_at | 0.0083 | 0.0053 | 1.557 | 0.129553 | 0.42204 |
| X220900_at | -0.0253 | 0.0162 | -1.557 | 0.1296754 | 0.422377 |
| X212721_at | 0.0535 | 0.0344 | 1.557 | 0.1297163 | 0.422402 |
| X207180_s_at | 0.0119 | 0.0077 | 1.557 | 0.1297213 | 0.422402 |
| X210411_s_at | -0.0069 | 0.0044 | -1.556 | 0.1297726 | 0.422508 |
| X213229_at | 0.1784 | 0.1146 | 1.556 | 0.1298373 | 0.422526 |
| X222088_s_at | -0.1546 | 0.0994 | -1.556 | 0.1298546 | 0.422526 |
| X217352_at | -0.0163 | 0.0105 | -1.556 | 0.1298735 | 0.422526 |
| X212971_at | 0.0427 | 0.0274 | 1.556 | 0.1298868 | 0.422526 |
| X218033_s_at | -0.1661 | 0.1068 | -1.556 | 0.1298905 | 0.422526 |
| X204150_at | 0.0073 | 0.0047 | 1.556 | 0.1299116 | 0.422526 |
| X218143_s_at | -0.0905 | 0.0582 | -1.556 | 0.129918 | 0.422526 |
| X203231_s_at | -0.1812 | 0.1165 | -1.556 | 0.1299401 | 0.422526 |
| X216222_s_at | 0.1639 | 0.1053 | 1.556 | 0.1299495 | 0.422526 |
| X217723_x_at | -0.0038 | 0.0024 | -1.556 | 0.129972 | 0.422537 |
| X202986_at | -0.0521 | 0.0335 | -1.555 | 0.1300478 | 0.422692 |
| X202671_s_at | -0.0937 | 0.0602 | -1.555 | 0.1300611 | 0.422692 |
| X201864_at | 0.0421 | 0.027 | 1.555 | 0.1300765 | 0.422692 |
| X203075_at | 0.0746 | 0.048 | 1.555 | 0.1301192 | 0.422769 |
| X213556_at | -0.0383 | 0.0246 | -1.555 | 0.1301892 | 0.422934 |
| X204699_s_at | -0.0437 | 0.0281 | -1.555 | 0.1302119 | 0.422946 |
| X201371_s_at | 0.0456 | 0.0293 | 1.554 | 0.1302828 | 0.423114 |
| X214332_s_at | -0.0704 | 0.0453 | -1.554 | 0.1303071 | 0.423131 |
| X207093_s_at | -0.0681 | 0.0438 | -1.554 | 0.1303731 | 0.423284 |
| X201698_s_at | -0.0371 | 0.0239 | -1.553 | 0.1304896 | 0.423551 |
| X220455_at | -0.0872 | 0.0561 | -1.553 | 0.1304934 | 0.423551 |
| X219632_s_at | 0.0196 | 0.0127 | 1.553 | 0.1305499 | 0.42364 |
| X210365_at | -0.0051 | 0.0033 | -1.553 | 0.1305647 | 0.42364 |
| X209092_s_at | -0.0649 | 0.0418 | -1.553 | 0.130578 | 0.42364 |
| X200982_s_at | -0.1415 | 0.0911 | -1.553 | 0.1306143 | 0.423644 |
| X206140_at | 0.1288 | 0.0829 | 1.553 | 0.1306175 | 0.423644 |
| X218277_s_at | 0.0776 | 0.05 | 1.553 | 0.1306378 | 0.423648 |
| X209956_s_at | -0.1243 | 0.0801 | -1.553 | 0.1306852 | 0.42374 |
| X206530_at | 0.0035 | 0.0023 | 1.552 | 0.1307052 | 0.423743 |
| X201659_s_at | -0.0822 | 0.0529 | -1.552 | 0.1307393 | 0.423781 |
| X212035_s_at | -0.0197 | 0.0127 | -1.552 | 0.1307736 | 0.423781 |
| X204335_at | -0.0225 | 0.0145 | -1.552 | 0.1307743 | 0.423781 |
| X221824_s_at | 0.0532 | 0.0343 | 1.552 | 0.1308863 | 0.424059 |
| X221257_x_at | -0.0379 | 0.0244 | -1.552 | 0.1309052 | 0.424059 |
| X220113_x_at | 0.1163 | 0.075 | 1.552 | 0.1309172 | 0.424059 |
| X207680_x_at | -0.0037 | 0.0024 | -1.551 | 0.1310069 | 0.424287 |
| X203262_s_at | 0.0636 | 0.041 | 1.551 | 0.1310263 | 0.424288 |
| X219834_at | -0.0839 | 0.0541 | -1.551 | 0.1310894 | 0.424431 |
| X208541_x_at | 0.0725 | 0.0467 | 1.55 | 0.1311736 | 0.424637 |
| X201906_s_at | 0.0644 | 0.0415 | 1.55 | 0.1311913 | 0.424637 |
| X221455_s_at | -0.0045 | 0.0029 | -1.55 | 0.131215 | 0.424652 |
| X211876_x_at | 0.004 | 0.0026 | 1.55 | 0.1313205 | 0.424931 |
| X201582_at | -0.0695 | 0.0449 | -1.548 | 0.1316615 | 0.425973 |
| X211538_s_at | 0.1913 | 0.1236 | 1.548 | 0.1317024 | 0.426008 |
| X219280_at | -0.0163 | 0.0105 | -1.548 | 0.1317106 | 0.426008 |
| X204124_at | -0.0191 | 0.0123 | -1.548 | 0.131762 | 0.4261 |
| X214250_at | 0.023 | 0.0149 | 1.548 | 0.1317919 | 0.4261 |
| X214220_s_at | 0.0749 | 0.0484 | 1.548 | 0.1317966 | 0.4261 |
| X66053_at | -0.0135 | 0.0087 | -1.548 | 0.1318537 | 0.426212 |
| X207267_s_at | -0.0318 | 0.0206 | -1.548 | 0.1318843 | 0.426212 |
| X213441_x_at | 0.0056 | 0.0036 | 1.548 | 0.131889 | 0.426212 |
| X208240_s_at | 0.1695 | 0.1096 | 1.547 | 0.1319741 | 0.426425 |
| X219405_at | -0.0336 | 0.0217 | -1.547 | 0.1320464 | 0.426597 |
| X200954_at | -0.0498 | 0.0322 | -1.547 | 0.1321014 | 0.426633 |
| X218800_at | -0.0411 | 0.0266 | -1.547 | 0.1321079 | 0.426633 |
| X221616_s_at | 0.0042 | 0.0027 | 1.547 | 0.1321152 | 0.426633 |
| X214534_at | -0.0079 | 0.0051 | -1.546 | 0.1321414 | 0.426655 |
| X210305_at | -0.2632 | 0.1702 | -1.546 | 0.1322135 | 0.426826 |
| X203199_s_at | -0.139 | 0.0899 | -1.546 | 0.1323523 | 0.427212 |
| X216508_x_at | 0.042 | 0.0272 | 1.545 | 0.1325243 | 0.427705 |
| X203457_at | -0.0135 | 0.0088 | -1.545 | 0.1325795 | 0.427821 |
| X220818_s_at | 0.0082 | 0.0053 | 1.545 | 0.132603 | 0.427835 |
| X201688_s_at | -0.1304 | 0.0845 | -1.544 | 0.1327327 | 0.428191 |
| X221005_s_at | 0.005 | 0.0033 | 1.544 | 0.1327711 | 0.428217 |
| X222126_at | -0.0614 | 0.0398 | -1.544 | 0.1327792 | 0.428217 |
| X210685_s_at | 0.0312 | 0.0202 | 1.544 | 0.1328112 | 0.428258 |
| X215259_s_at | 0.0077 | 0.005 | 1.544 | 0.1328457 | 0.428307 |
| X218940_at | -0.0854 | 0.0553 | -1.543 | 0.132868 | 0.428317 |
| X201269_s_at | -0.0086 | 0.0056 | -1.543 | 0.1329545 | 0.428498 |
| X202341_s_at | 0.0551 | 0.0357 | 1.543 | 0.1329875 | 0.428498 |
| X215600_x_at | 0.0815 | 0.0528 | 1.543 | 0.132996 | 0.428498 |
| X208728_s_at | 0.064 | 0.0415 | 1.543 | 0.1330013 | 0.428498 |
| X202577_s_at | -0.0404 | 0.0262 | -1.543 | 0.133062 | 0.428631 |
| X218064_s_at | 0.0706 | 0.0458 | 1.542 | 0.1332025 | 0.42899 |
| X210791_s_at | -0.0857 | 0.0556 | -1.542 | 0.1332158 | 0.42899 |
| X202169_s_at | -0.0431 | 0.0279 | -1.542 | 0.1332461 | 0.42899 |
| X217700_at | -0.0213 | 0.0138 | -1.542 | 0.1332669 | 0.42899 |
| X202290_at | -0.0906 | 0.0588 | -1.542 | 0.13327 | 0.42899 |
| X209097_s_at | -0.0087 | 0.0057 | -1.541 | 0.1333503 | 0.429101 |
| X203603_s_at | 0.1674 | 0.1086 | 1.541 | 0.1333834 | 0.429101 |
| X212158_at | -0.0887 | 0.0576 | -1.541 | 0.133395 | 0.429101 |
| X203174_s_at | -0.0186 | 0.0121 | -1.541 | 0.1333971 | 0.429101 |
| X205599_at | 0.0034 | 0.0022 | 1.541 | 0.1334148 | 0.429101 |
| X217661_x_at | -0.0048 | 0.0031 | -1.541 | 0.1334203 | 0.429101 |
| X218069_at | -0.0538 | 0.0349 | -1.541 | 0.1334459 | 0.429121 |
| X210193_at | 0.2095 | 0.136 | 1.541 | 0.1335071 | 0.429239 |
| X212305_s_at | -0.0393 | 0.0255 | -1.541 | 0.1335211 | 0.429239 |
| X221048_x_at | 0.0059 | 0.0038 | 1.541 | 0.1335498 | 0.429269 |
| X205647_at | -0.0186 | 0.0121 | -1.54 | 0.1335888 | 0.429332 |
| X200984_s_at | -0.0622 | 0.0404 | -1.54 | 0.1336312 | 0.429406 |
| X201375_s_at | 0.0367 | 0.0238 | 1.54 | 0.1336726 | 0.42947 |
| X218154_at | 0.0057 | 0.0037 | 1.54 | 0.1336898 | 0.42947 |
| X201745_at | 0.048 | 0.0312 | 1.54 | 0.1337396 | 0.429568 |
| X201517_at | -0.0422 | 0.0274 | -1.54 | 0.1337627 | 0.42958 |
| X219772_s_at | -0.0063 | 0.0041 | -1.54 | 0.133807 | 0.42963 |
| X208381_s_at | 0.0029 | 0.0019 | 1.54 | 0.133817 | 0.42963 |
| X216570_x_at | 0.0291 | 0.0189 | 1.539 | 0.1338455 | 0.42966 |
| X203960_s_at | -0.0649 | 0.0422 | -1.538 | 0.1340772 | 0.430341 |
| X211617_at | 0.026 | 0.0169 | 1.538 | 0.1341031 | 0.430362 |
| X218702_at | -0.0047 | 0.003 | -1.538 | 0.1341337 | 0.430388 |
| X204338_s_at | -0.3996 | 0.2598 | -1.538 | 0.13415 | 0.430388 |
| X204564_at | -0.0154 | 0.01 | -1.538 | 0.1342395 | 0.430613 |
| X216406_at | -0.0125 | 0.0081 | -1.538 | 0.1342617 | 0.430622 |
| X218175_at | 0.0901 | 0.0586 | 1.538 | 0.134283 | 0.430628 |
| X210779_x_at | -0.0781 | 0.0508 | -1.537 | 0.1344679 | 0.431159 |
| X212200_at | -0.0461 | 0.03 | -1.537 | 0.1344969 | 0.43119 |
| X206489_s_at | 0.1033 | 0.0672 | 1.537 | 0.1345328 | 0.431243 |
| X203802_x_at | 0.0584 | 0.038 | 1.536 | 0.1346473 | 0.431547 |
| X214300_s_at | -0.0121 | 0.0079 | -1.536 | 0.1347379 | 0.431776 |
| X208866_at | 0.0924 | 0.0602 | 1.536 | 0.1347709 | 0.431819 |
| X211910_at | -0.0056 | 0.0036 | -1.536 | 0.1347934 | 0.431829 |
| X207663_x_at | 0.0066 | 0.0043 | 1.535 | 0.1348581 | 0.431974 |
| X201189_s_at | 0.0039 | 0.0025 | 1.535 | 0.1349145 | 0.432092 |
| X208901_s_at | 0.0408 | 0.0266 | 1.535 | 0.134949 | 0.43214 |
| X204737_s_at | -0.0109 | 0.0071 | -1.535 | 0.13498 | 0.432177 |
| X203284_s_at | -0.0638 | 0.0416 | -1.534 | 0.1350586 | 0.432367 |
| X213945_s_at | -0.0121 | 0.0079 | -1.534 | 0.1350898 | 0.432404 |
| X218743_at | 0.006 | 0.0039 | 1.534 | 0.1351164 | 0.432427 |
| X206184_at | 0.0056 | 0.0037 | 1.534 | 0.1351785 | 0.432527 |
| X200603_at | -0.0531 | 0.0346 | -1.534 | 0.1351865 | 0.432527 |
| X211536_x_at | -0.0998 | 0.0651 | -1.534 | 0.1352397 | 0.432635 |
| X203029_s_at | -0.0811 | 0.0529 | -1.533 | 0.1352974 | 0.432757 |
| X209085_x_at | -0.072 | 0.0469 | -1.533 | 0.1353451 | 0.432847 |
| X205992_s_at | 0.0379 | 0.0247 | 1.533 | 0.1353736 | 0.432876 |
| X216750_at | -0.0063 | 0.0041 | -1.533 | 0.1354149 | 0.432908 |
| X205341_at | -0.0041 | 0.0027 | -1.533 | 0.13544 | 0.432908 |
| X220595_at | 0.0644 | 0.042 | 1.533 | 0.135442 | 0.432908 |
| X219817_at | -0.0499 | 0.0326 | -1.533 | 0.1355146 | 0.432969 |
| X218717_s_at | 0.0058 | 0.0038 | 1.533 | 0.1355168 | 0.432969 |
| X221700_s_at | 0.0285 | 0.0186 | 1.533 | 0.1355205 | 0.432969 |
| X212636_at | 0.0791 | 0.0516 | 1.533 | 0.135539 | 0.432969 |
| X217124_at | 0.0065 | 0.0042 | 1.532 | 0.1356262 | 0.433185 |
| X217719_at | -0.023 | 0.015 | -1.532 | 0.1356922 | 0.43328 |
| X204914_s_at | 0.1334 | 0.0871 | 1.532 | 0.135695 | 0.43328 |
| X214096_s_at | 0.0559 | 0.0365 | 1.532 | 0.1357518 | 0.433399 |
| X214330_at | 0.0136 | 0.0089 | 1.531 | 0.1358608 | 0.433685 |
| X206668_s_at | -0.2086 | 0.1362 | -1.531 | 0.1359445 | 0.433879 |
| X207428_x_at | 0.0092 | 0.006 | 1.531 | 0.1359608 | 0.433879 |
| X212661_x_at | -0.0201 | 0.0131 | -1.531 | 0.1360325 | 0.433951 |
| X217490_at | -0.0153 | 0.01 | -1.53 | 0.1360401 | 0.433951 |
| X216207_x_at | -0.0049 | 0.0032 | -1.53 | 0.1360522 | 0.433951 |
| X209451_at | -0.0409 | 0.0267 | -1.53 | 0.1360614 | 0.433951 |
| X216427_at | -0.0073 | 0.0048 | -1.53 | 0.1361498 | 0.434144 |
| X220546_at | 0.0088 | 0.0057 | 1.53 | 0.1361692 | 0.434144 |
| X218988_at | -0.0584 | 0.0382 | -1.53 | 0.13619 | 0.434144 |
| X218242_s_at | 0.055 | 0.036 | 1.53 | 0.1362041 | 0.434144 |
| X221640_s_at | 0.0032 | 0.0021 | 1.53 | 0.1362195 | 0.434144 |
| X221551_x_at | 0.0047 | 0.003 | 1.53 | 0.1362562 | 0.434198 |
| X215994_x_at | 0.0211 | 0.0138 | 1.529 | 0.1362954 | 0.434261 |
| X219752_at | -0.0917 | 0.0599 | -1.529 | 0.136328 | 0.434302 |
| X201986_at | 0.0386 | 0.0252 | 1.529 | 0.1363919 | 0.4344 |
| X218814_s_at | -0.0068 | 0.0045 | -1.529 | 0.1363977 | 0.4344 |
| X204159_at | 0.138 | 0.0902 | 1.529 | 0.1364512 | 0.434508 |
| X213801_x_at | 0.0418 | 0.0274 | 1.529 | 0.1364745 | 0.43452 |
| X202425_x_at | -0.0746 | 0.0488 | -1.528 | 0.1365885 | 0.43482 |
| X220611_at | 0.0031 | 0.002 | 1.528 | 0.1366486 | 0.434904 |
| X220586_at | -0.0983 | 0.0643 | -1.528 | 0.1366538 | 0.434904 |
| X218225_at | -0.055 | 0.036 | -1.528 | 0.1367518 | 0.435109 |
| X220459_at | 0.1099 | 0.0719 | 1.528 | 0.1367575 | 0.435109 |
| X221801_x_at | -0.0888 | 0.0581 | -1.527 | 0.1367998 | 0.435181 |
| X203978_at | -0.0266 | 0.0174 | -1.527 | 0.1368842 | 0.435387 |
| X211320_s_at | 0.0058 | 0.0038 | 1.527 | 0.1369403 | 0.435504 |
| X217958_at | -0.0748 | 0.049 | -1.527 | 0.1369778 | 0.43556 |
| X221600_s_at | 0.0334 | 0.0219 | 1.526 | 0.1370388 | 0.435692 |
| X201132_at | -0.0452 | 0.0296 | -1.526 | 0.1370628 | 0.435706 |
| X203707_at | -0.0669 | 0.0438 | -1.526 | 0.1371247 | 0.43584 |
| X215919_s_at | 0.0443 | 0.029 | 1.526 | 0.1371507 | 0.435857 |
| X215588_x_at | 0.079 | 0.0518 | 1.526 | 0.1371779 | 0.435857 |
| X221220_s_at | 0.0062 | 0.0041 | 1.526 | 0.1371889 | 0.435857 |
| X220323_at | -0.0038 | 0.0025 | -1.525 | 0.1373543 | 0.436296 |
| X207703_at | -0.09 | 0.059 | -1.525 | 0.1373742 | 0.436296 |
| X215544_s_at | -0.023 | 0.0151 | -1.525 | 0.137386 | 0.436296 |
| X213205_s_at | 0.0065 | 0.0043 | 1.525 | 0.1374251 | 0.436358 |
| X208119_s_at | 0.0647 | 0.0425 | 1.524 | 0.137689 | 0.437134 |
| X220050_at | -0.1046 | 0.0687 | -1.524 | 0.1377151 | 0.437147 |
| X221521_s_at | -0.0088 | 0.0058 | -1.524 | 0.1377325 | 0.437147 |
| X206750_at | 0.0051 | 0.0034 | 1.523 | 0.1378017 | 0.437304 |
| X211483_x_at | -0.1205 | 0.0791 | -1.523 | 0.1378382 | 0.437357 |
| X209927_s_at | -0.0095 | 0.0063 | -1.523 | 0.1378766 | 0.437407 |
| X219556_at | 0.0052 | 0.0034 | 1.523 | 0.1378932 | 0.437407 |
| X210393_at | 0.0066 | 0.0043 | 1.523 | 0.1379716 | 0.437593 |
| X200078_s_at | -0.0712 | 0.0468 | -1.522 | 0.1381198 | 0.438001 |
| X207533_at | -0.0024 | 0.0015 | -1.522 | 0.1381566 | 0.438055 |
| X218076_s_at | 0.0136 | 0.0089 | 1.522 | 0.1381882 | 0.438092 |
| X203325_s_at | 0.0137 | 0.009 | 1.521 | 0.1383352 | 0.438496 |
| X221519_at | 0.0804 | 0.0528 | 1.521 | 0.1384349 | 0.438672 |
| X208540_x_at | 0.0097 | 0.0064 | 1.521 | 0.1384491 | 0.438672 |
| X203274_at | 0.0484 | 0.0319 | 1.521 | 0.1384524 | 0.438672 |
| X221753_at | 0.147 | 0.0967 | 1.521 | 0.1384791 | 0.438672 |
| X204346_s_at | -0.0052 | 0.0034 | -1.521 | 0.1384915 | 0.438672 |
| X203449_s_at | 0.0339 | 0.0223 | 1.521 | 0.1385094 | 0.438672 |
| X204762_s_at | -0.1341 | 0.0882 | -1.52 | 0.1385537 | 0.43875 |
| X220712_at | 0.0056 | 0.0037 | 1.52 | 0.1385838 | 0.438783 |
| X220397_at | 0.0206 | 0.0135 | 1.52 | 0.138636 | 0.438886 |
| X218887_at | -0.0047 | 0.0031 | -1.52 | 0.1387174 | 0.439081 |
| X211550_at | -0.0064 | 0.0042 | -1.519 | 0.1388368 | 0.439306 |
| X211728_s_at | 0.0035 | 0.0023 | 1.519 | 0.1388478 | 0.439306 |
| X209769_s_at | -0.0087 | 0.0058 | -1.519 | 0.1388479 | 0.439306 |
| X215700_x_at | -0.0192 | 0.0126 | -1.519 | 0.13888 | 0.439345 |
| X208819_at | 0.0602 | 0.0396 | 1.519 | 0.1389314 | 0.439445 |
| X204994_at | 0.0053 | 0.0035 | 1.519 | 0.1389619 | 0.439479 |
| X213473_at | -0.031 | 0.0204 | -1.518 | 0.1390387 | 0.439659 |
| X212828_at | 0.2452 | 0.1615 | 1.518 | 0.1391297 | 0.439884 |
| X210631_at | 0.0588 | 0.0387 | 1.518 | 0.1392363 | 0.440159 |
| X215330_at | 0.1008 | 0.0664 | 1.517 | 0.1393275 | 0.440368 |
| X207372_s_at | -0.0045 | 0.003 | -1.517 | 0.1393421 | 0.440368 |
| X210845_s_at | 0.0102 | 0.0067 | 1.517 | 0.1394922 | 0.440695 |
| X211582_x_at | 0.0077 | 0.0051 | 1.517 | 0.1394932 | 0.440695 |
| X212146_at | 0.0642 | 0.0423 | 1.517 | 0.1395094 | 0.440695 |
| X219076_s_at | -0.0563 | 0.0372 | -1.517 | 0.1395249 | 0.440695 |
| X206534_at | 0.0065 | 0.0043 | 1.516 | 0.1395838 | 0.440709 |
| X210706_s_at | -0.0294 | 0.0194 | -1.516 | 0.1395885 | 0.440709 |
| X202896_s_at | 0.0244 | 0.0161 | 1.516 | 0.139589 | 0.440709 |
| X212458_at | -0.0835 | 0.0551 | -1.516 | 0.1396366 | 0.440794 |
| X203205_at | -0.0348 | 0.0229 | -1.516 | 0.1396554 | 0.440794 |
| X219555_s_at | 0.0079 | 0.0052 | 1.516 | 0.1396964 | 0.44086 |
| X219912_s_at | -0.0076 | 0.005 | -1.515 | 0.1397919 | 0.441099 |
| X221772_s_at | -0.0397 | 0.0262 | -1.515 | 0.1398626 | 0.44126 |
| X214991_s_at | 0.0046 | 0.0031 | 1.515 | 0.1399595 | 0.441503 |
| X212709_at | 0.0677 | 0.0447 | 1.515 | 0.1400073 | 0.441591 |
| X213611_at | -0.0042 | 0.0028 | -1.514 | 0.1400481 | 0.441657 |
| X212263_at | 0.0978 | 0.0646 | 1.514 | 0.1400844 | 0.441664 |
| X204192_at | 0.0037 | 0.0024 | 1.514 | 0.1400981 | 0.441664 |
| X205047_s_at | 0.082 | 0.0542 | 1.514 | 0.1401172 | 0.441664 |
| X205591_at | 0.1047 | 0.0692 | 1.514 | 0.1401489 | 0.441664 |
| X204141_at | 0.0681 | 0.045 | 1.514 | 0.1401498 | 0.441664 |
| X37433_at | 0.0337 | 0.0222 | 1.513 | 0.1403039 | 0.442087 |
| X214960_at | -0.0611 | 0.0404 | -1.513 | 0.1403268 | 0.442096 |
| X200760_s_at | -0.043 | 0.0284 | -1.513 | 0.1404633 | 0.442463 |
| X204330_s_at | -0.0092 | 0.0061 | -1.513 | 0.1405268 | 0.442541 |
| X207072_at | 0.0051 | 0.0034 | 1.513 | 0.1405303 | 0.442541 |
| X208082_x_at | 0.0519 | 0.0343 | 1.512 | 0.140557 | 0.442541 |
| X65086_at | -0.0032 | 0.0021 | -1.512 | 0.1405675 | 0.442541 |
| X212451_at | 0.1589 | 0.1051 | 1.512 | 0.1406457 | 0.442724 |
| X217665_at | -0.0088 | 0.0058 | -1.511 | 0.1408006 | 0.443149 |
| X210172_at | 0.3034 | 0.2008 | 1.511 | 0.1409108 | 0.443384 |
| X221702_s_at | 0.052 | 0.0344 | 1.511 | 0.1409153 | 0.443384 |
| X201998_at | 0.2124 | 0.1406 | 1.511 | 0.1409961 | 0.443576 |
| X203885_at | -0.0689 | 0.0456 | -1.511 | 0.1410252 | 0.443604 |
| X220127_s_at | 0.0489 | 0.0323 | 1.51 | 0.1411331 | 0.443881 |
| X211068_x_at | -0.0506 | 0.0335 | -1.51 | 0.1411848 | 0.443981 |
| X204812_at | -0.0085 | 0.0056 | -1.51 | 0.1412777 | 0.444168 |
| X219001_s_at | -0.0794 | 0.0526 | -1.51 | 0.1412842 | 0.444168 |
| X209465_x_at | -0.0852 | 0.0564 | -1.509 | 0.1414463 | 0.44457 |
| X216848_at | 0.0059 | 0.0039 | 1.509 | 0.1414521 | 0.44457 |
| X200094_s_at | 0.0565 | 0.0375 | 1.508 | 0.1415652 | 0.444826 |
| X201747_s_at | -0.0106 | 0.0071 | -1.508 | 0.1415736 | 0.444826 |
| X212945_s_at | -0.0831 | 0.0551 | -1.508 | 0.1415984 | 0.444841 |
| X204379_s_at | -0.0947 | 0.0628 | -1.508 | 0.1416776 | 0.445022 |
| X221786_at | -0.0534 | 0.0354 | -1.508 | 0.1417015 | 0.445022 |
| X205687_at | -0.1314 | 0.0871 | -1.508 | 0.1417162 | 0.445022 |
| X214956_at | -0.0481 | 0.0319 | -1.508 | 0.1417683 | 0.445122 |
| X201343_at | -0.0355 | 0.0235 | -1.508 | 0.1418053 | 0.445176 |
| X204186_s_at | -0.0956 | 0.0634 | -1.507 | 0.1418271 | 0.445181 |
| X35156_at | 0.0454 | 0.0301 | 1.507 | 0.1419118 | 0.445349 |
| X214581_x_at | -0.0827 | 0.0549 | -1.507 | 0.1419303 | 0.445349 |
| X214504_at | -0.004 | 0.0027 | -1.507 | 0.1419515 | 0.445349 |
| X210787_s_at | -0.012 | 0.008 | -1.507 | 0.1419606 | 0.445349 |
| X212210_at | -0.0059 | 0.0039 | -1.507 | 0.1420007 | 0.445411 |
| X214937_x_at | 0.0438 | 0.0291 | 1.507 | 0.1420569 | 0.445525 |
| X208502_s_at | 0.0027 | 0.0018 | 1.506 | 0.1420876 | 0.445558 |
| X213349_at | 0.075 | 0.0498 | 1.506 | 0.1421521 | 0.445698 |
| X203336_s_at | -0.1328 | 0.0882 | -1.506 | 0.1422013 | 0.445727 |
| X207025_at | 0.0055 | 0.0037 | 1.506 | 0.1422075 | 0.445727 |
| X213748_at | -0.1319 | 0.0876 | -1.506 | 0.1422217 | 0.445727 |
| X211391_s_at | -0.004 | 0.0026 | -1.506 | 0.1422429 | 0.445731 |
| X208975_s_at | 0.0844 | 0.056 | 1.506 | 0.1422765 | 0.445773 |
| X202653_s_at | -0.056 | 0.0372 | -1.505 | 0.142553 | 0.446576 |
| X203328_x_at | 0.0078 | 0.0052 | 1.504 | 0.1427816 | 0.447229 |
| X202616_s_at | 0.0586 | 0.039 | 1.503 | 0.1429264 | 0.447579 |
| X208551_at | -0.0048 | 0.0032 | -1.503 | 0.1429373 | 0.447579 |
| X220599_s_at | -0.0032 | 0.0021 | -1.503 | 0.1429538 | 0.447579 |
| X202140_s_at | -0.0147 | 0.0098 | -1.503 | 0.1430106 | 0.447685 |
| X214571_at | -0.0032 | 0.0021 | -1.503 | 0.1430376 | 0.447685 |
| X202823_at | -0.066 | 0.0439 | -1.503 | 0.143048 | 0.447685 |
| X212866_at | 0.0589 | 0.0392 | 1.502 | 0.1431171 | 0.44778 |
| X201683_x_at | 0.1352 | 0.09 | 1.502 | 0.1431187 | 0.44778 |
| X219072_at | -0.027 | 0.018 | -1.502 | 0.1431505 | 0.447788 |
| X219160_s_at | 0.0057 | 0.0038 | 1.502 | 0.1431615 | 0.447788 |
| X220623_s_at | 0.0083 | 0.0055 | 1.502 | 0.1432185 | 0.447903 |
| X202105_at | -0.0355 | 0.0236 | -1.501 | 0.1433929 | 0.448324 |
| X204036_at | 0.1872 | 0.1247 | 1.501 | 0.1434347 | 0.448324 |
| X219245_s_at | -0.0209 | 0.0139 | -1.501 | 0.1434356 | 0.448324 |
| X220482_s_at | -0.0632 | 0.0421 | -1.501 | 0.1434672 | 0.448324 |
| X209335_at | 0.1815 | 0.121 | 1.501 | 0.143473 | 0.448324 |
| X217208_s_at | 0.1135 | 0.0757 | 1.501 | 0.1434893 | 0.448324 |
| X212412_at | 0.0545 | 0.0363 | 1.501 | 0.1434944 | 0.448324 |
| X218420_s_at | 0.0071 | 0.0047 | 1.501 | 0.1435463 | 0.448423 |
| X214722_at | 0.064 | 0.0427 | 1.501 | 0.1435831 | 0.448475 |
| X219880_at | -0.0205 | 0.0136 | -1.5 | 0.1437579 | 0.448958 |
| X215082_at | -0.0043 | 0.0029 | -1.5 | 0.1437933 | 0.449005 |
| X206899_at | -0.166 | 0.1107 | -1.5 | 0.1438169 | 0.449016 |
| X206047_at | -0.0085 | 0.0056 | -1.5 | 0.1438415 | 0.44903 |
| X213445_at | 0.0052 | 0.0035 | 1.499 | 0.1438972 | 0.449109 |
| X202496_at | -0.0525 | 0.035 | -1.499 | 0.143926 | 0.449109 |
| X212551_at | -0.0738 | 0.0492 | -1.499 | 0.1439277 | 0.449109 |
| X221426_s_at | 0.0059 | 0.0039 | 1.499 | 0.1439883 | 0.449235 |
| X218742_at | 0.006 | 0.004 | 1.498 | 0.1443169 | 0.450197 |
| X201478_s_at | -0.0335 | 0.0224 | -1.497 | 0.1443959 | 0.45033 |
| X209425_at | -0.1371 | 0.0915 | -1.497 | 0.1443998 | 0.45033 |
| X220131_at | -0.1293 | 0.0864 | -1.497 | 0.1444304 | 0.450362 |
| X216674_at | -0.0047 | 0.0032 | -1.497 | 0.1445103 | 0.450548 |
| X210971_s_at | -0.0217 | 0.0145 | -1.497 | 0.1445798 | 0.450701 |
| X215676_at | -0.006 | 0.004 | -1.496 | 0.1446363 | 0.450754 |
| X216351_x_at | 0.0064 | 0.0043 | 1.496 | 0.1446687 | 0.450754 |
| X217594_at | -0.027 | 0.0181 | -1.496 | 0.1446732 | 0.450754 |
| X206471_s_at | 0.018 | 0.012 | 1.496 | 0.1446949 | 0.450754 |
| X209466_x_at | -0.0681 | 0.0455 | -1.496 | 0.1446982 | 0.450754 |
| X201016_at | 0.0798 | 0.0533 | 1.496 | 0.1447553 | 0.450816 |
| X216568_x_at | -0.0108 | 0.0072 | -1.496 | 0.1447587 | 0.450816 |
| X215268_at | 0.1678 | 0.1122 | 1.496 | 0.1447993 | 0.450879 |
| X206245_s_at | -0.1728 | 0.1156 | -1.496 | 0.1448877 | 0.451091 |
| X217646_at | 0.0077 | 0.0052 | 1.495 | 0.1449518 | 0.451227 |
| X211141_s_at | -0.0043 | 0.0029 | -1.495 | 0.1449786 | 0.451227 |
| X203469_s_at | -0.0258 | 0.0173 | -1.495 | 0.1449924 | 0.451227 |
| X208656_s_at | 0.0591 | 0.0396 | 1.494 | 0.1451718 | 0.451652 |
| X211438_at | -0.0053 | 0.0036 | -1.494 | 0.1451895 | 0.451652 |
| X212250_at | -0.0545 | 0.0365 | -1.494 | 0.1451899 | 0.451652 |
| X218231_at | -0.1149 | 0.0769 | -1.494 | 0.1453587 | 0.45208 |
| X208151_x_at | 0.0098 | 0.0065 | 1.494 | 0.145368 | 0.45208 |
| X219005_at | -0.126 | 0.0844 | -1.493 | 0.145419 | 0.452123 |
| X200845_s_at | 0.0848 | 0.0568 | 1.493 | 0.1454226 | 0.452123 |
| X201597_at | -0.0361 | 0.0241 | -1.493 | 0.1454532 | 0.452146 |
| X204516_at | 0.0459 | 0.0307 | 1.493 | 0.1454706 | 0.452146 |
| X213361_at | -0.0645 | 0.0432 | -1.493 | 0.145568 | 0.452385 |
| X201095_at | 0.0302 | 0.0202 | 1.493 | 0.1455981 | 0.452415 |
| X203511_s_at | -0.0487 | 0.0327 | -1.492 | 0.1457182 | 0.452725 |
| X206677_at | -0.0073 | 0.0049 | -1.492 | 0.1458472 | 0.453063 |
| X218851_s_at | 0.0402 | 0.027 | 1.492 | 0.1458796 | 0.4531 |
| X221156_x_at | 0.0397 | 0.0266 | 1.491 | 0.1460368 | 0.453525 |
| X217585_at | 0.0544 | 0.0365 | 1.491 | 0.1461909 | 0.453906 |
| X219533_at | -0.0037 | 0.0025 | -1.49 | 0.1462166 | 0.453906 |
| X220202_s_at | -0.1632 | 0.1095 | -1.49 | 0.1462208 | 0.453906 |
| X205018_s_at | -0.0911 | 0.0612 | -1.49 | 0.1463573 | 0.454266 |
| X212754_s_at | 0.0548 | 0.0368 | 1.49 | 0.1463862 | 0.454292 |
| X220827_at | 0.006 | 0.004 | 1.489 | 0.1464885 | 0.454546 |
| X205158_at | 0.017 | 0.0114 | 1.489 | 0.1465643 | 0.454718 |
| X210732_s_at | -0.2024 | 0.136 | -1.489 | 0.1466164 | 0.454816 |
| X218173_s_at | 0.0218 | 0.0146 | 1.489 | 0.1466525 | 0.454864 |
| X209178_at | -0.0073 | 0.0049 | -1.488 | 0.1467528 | 0.455112 |
| X201759_at | -0.0296 | 0.0199 | -1.488 | 0.1468638 | 0.455381 |
| X220910_at | -0.0176 | 0.0118 | -1.488 | 0.146883 | 0.455381 |
| X213628_at | 0.0988 | 0.0664 | 1.488 | 0.1469011 | 0.455381 |
| X214477_at | -0.0068 | 0.0046 | -1.487 | 0.1469966 | 0.455576 |
| X203037_s_at | -0.0374 | 0.0252 | -1.487 | 0.1470049 | 0.455576 |
| X211744_s_at | 0.1064 | 0.0715 | 1.487 | 0.147028 | 0.455584 |
| X210127_at | -0.0116 | 0.0078 | -1.487 | 0.1470609 | 0.455622 |
| X210334_x_at | 0.0044 | 0.003 | 1.487 | 0.1470863 | 0.455637 |
| X201930_at | 0.0768 | 0.0516 | 1.487 | 0.1471228 | 0.455687 |
| X204777_s_at | 0.1684 | 0.1133 | 1.486 | 0.1473275 | 0.456152 |
| X221505_at | -0.0486 | 0.0327 | -1.486 | 0.1473306 | 0.456152 |
| X207920_x_at | -0.006 | 0.004 | -1.486 | 0.147348 | 0.456152 |
| X207502_at | -0.0057 | 0.0039 | -1.486 | 0.1473737 | 0.456152 |
| X218418_s_at | 0.1636 | 0.1101 | 1.486 | 0.1473922 | 0.456152 |
| X211464_x_at | 0.0204 | 0.0137 | 1.486 | 0.1474138 | 0.456152 |
| X222127_s_at | -0.0462 | 0.0311 | -1.486 | 0.1474168 | 0.456152 |
| X209950_s_at | -0.0145 | 0.0097 | -1.486 | 0.1474731 | 0.456255 |
| X201842_s_at | -0.1854 | 0.1248 | -1.486 | 0.1474911 | 0.456255 |
| X206382_s_at | 0.0092 | 0.0062 | 1.485 | 0.1475392 | 0.456334 |
| X200901_s_at | -0.0419 | 0.0282 | -1.485 | 0.147575 | 0.456334 |
| X208441_at | 0.0053 | 0.0036 | 1.485 | 0.1475783 | 0.456334 |
| X206571_s_at | 0.1727 | 0.1163 | 1.485 | 0.1476039 | 0.45635 |
| X201869_s_at | -0.0361 | 0.0243 | -1.485 | 0.1476555 | 0.456446 |
| X202152_x_at | -0.0502 | 0.0338 | -1.485 | 0.1477012 | 0.456524 |
| X220753_s_at | -0.0573 | 0.0386 | -1.484 | 0.1478121 | 0.456773 |
| X214458_at | 0.0089 | 0.006 | 1.484 | 0.1478231 | 0.456773 |
| X215029_at | -0.0588 | 0.0396 | -1.484 | 0.1478448 | 0.456777 |
| X204925_at | 0.0061 | 0.0041 | 1.484 | 0.1478997 | 0.456883 |
| X217901_at | 0.0125 | 0.0085 | 1.484 | 0.1479913 | 0.457005 |
| X201629_s_at | -0.1118 | 0.0753 | -1.484 | 0.1479935 | 0.457005 |
| X215978_x_at | 0.0947 | 0.0638 | 1.484 | 0.148017 | 0.457005 |
| X221873_at | 0.023 | 0.0155 | 1.483 | 0.1480499 | 0.457005 |
| X37424_at | -0.0598 | 0.0403 | -1.483 | 0.1480557 | 0.457005 |
| X210875_s_at | -0.1187 | 0.08 | -1.483 | 0.1480657 | 0.457005 |
| X216373_at | -0.0192 | 0.0129 | -1.483 | 0.1480832 | 0.457005 |
| X201169_s_at | -0.0033 | 0.0022 | -1.483 | 0.1481988 | 0.457271 |
| X217502_at | 0.0098 | 0.0066 | 1.483 | 0.1482105 | 0.457271 |
| X214871_x_at | -0.0156 | 0.0105 | -1.483 | 0.1482991 | 0.457379 |
| X209250_at | 0.0478 | 0.0322 | 1.482 | 0.1483428 | 0.457379 |
| X221880_s_at | 0.0131 | 0.0088 | 1.482 | 0.1483462 | 0.457379 |
| X202837_at | 0.0078 | 0.0053 | 1.482 | 0.1483576 | 0.457379 |
| X205913_at | 0.0095 | 0.0064 | 1.482 | 0.1483701 | 0.457379 |
| X220719_at | -0.0596 | 0.0402 | -1.482 | 0.1483754 | 0.457379 |
| X216847_at | -0.006 | 0.004 | -1.482 | 0.1483898 | 0.457379 |
| X209343_at | 0.1747 | 0.1179 | 1.482 | 0.1485647 | 0.457855 |
| X217094_s_at | -0.1148 | 0.0775 | -1.481 | 0.148692 | 0.458183 |
| X205421_at | -0.0221 | 0.0149 | -1.481 | 0.1487141 | 0.458188 |
| X202424_at | 0.0761 | 0.0514 | 1.481 | 0.1487833 | 0.458338 |
| X215115_x_at | 0.083 | 0.0561 | 1.48 | 0.1489243 | 0.458708 |
| X201245_s_at | -0.0988 | 0.0668 | -1.48 | 0.1489933 | 0.458857 |
| X215255_at | -0.0112 | 0.0076 | -1.479 | 0.1491561 | 0.459295 |
| X202875_s_at | 0.0467 | 0.0316 | 1.479 | 0.1492706 | 0.459523 |
| X217739_s_at | 0.0611 | 0.0413 | 1.479 | 0.1492715 | 0.459523 |
| X209759_s_at | 0.0696 | 0.0471 | 1.479 | 0.1493339 | 0.459574 |
| X220470_at | -0.0032 | 0.0022 | -1.479 | 0.1493564 | 0.459574 |
| X203725_at | 0.0923 | 0.0624 | 1.478 | 0.1493731 | 0.459574 |
| X209433_s_at | 0.061 | 0.0413 | 1.478 | 0.1493901 | 0.459574 |
| X211942_x_at | -0.0469 | 0.0318 | -1.478 | 0.1493914 | 0.459574 |
| X216859_x_at | 0.0977 | 0.0661 | 1.478 | 0.1494398 | 0.459659 |
| X207116_s_at | 0.005 | 0.0034 | 1.478 | 0.149474 | 0.4597 |
| X208821_at | 0.0585 | 0.0396 | 1.478 | 0.1495825 | 0.459962 |
| X201713_s_at | -0.0467 | 0.0316 | -1.478 | 0.149609 | 0.459962 |
| X221340_at | -0.0036 | 0.0024 | -1.478 | 0.1496212 | 0.459962 |
| X203731_s_at | -0.0064 | 0.0044 | -1.477 | 0.1496645 | 0.460031 |
| X34260_at | 0.0135 | 0.0091 | 1.477 | 0.1497249 | 0.460153 |
| X207543_s_at | 0.1463 | 0.099 | 1.477 | 0.149781 | 0.460202 |
| X207639_at | 0.0057 | 0.0039 | 1.477 | 0.1498125 | 0.460202 |
| X203684_s_at | -0.0068 | 0.0046 | -1.477 | 0.1498363 | 0.460202 |
| X202876_s_at | 0.1253 | 0.0848 | 1.477 | 0.1498588 | 0.460202 |
| X211751_at | -0.2961 | 0.2005 | -1.477 | 0.1498613 | 0.460202 |
| X212437_at | 0.0609 | 0.0412 | 1.477 | 0.1498649 | 0.460202 |
| X208445_s_at | 0.0411 | 0.0278 | 1.476 | 0.1499999 | 0.46049 |
| X215221_at | 0.1196 | 0.081 | 1.476 | 0.1500003 | 0.46049 |
| X215483_at | 0.1125 | 0.0762 | 1.476 | 0.150027 | 0.460509 |
| X216458_at | 0.0055 | 0.0037 | 1.476 | 0.1500603 | 0.460547 |
| X203655_at | -0.0937 | 0.0635 | -1.476 | 0.1501026 | 0.460613 |
| X213846_at | -0.0542 | 0.0367 | -1.475 | 0.1501762 | 0.460736 |
| X203637_s_at | 0.0992 | 0.0672 | 1.475 | 0.150184 | 0.460736 |
| X205141_at | 0.0061 | 0.0041 | 1.475 | 0.1502213 | 0.460787 |
| X215718_s_at | 0.042 | 0.0284 | 1.475 | 0.1502609 | 0.460797 |
| X200729_s_at | -0.0907 | 0.0615 | -1.475 | 0.1502701 | 0.460797 |
| X202840_at | 0.1314 | 0.0891 | 1.475 | 0.1502869 | 0.460797 |
| X214258_x_at | -0.0417 | 0.0283 | -1.475 | 0.1503813 | 0.461023 |
| X215592_at | 0.0849 | 0.0576 | 1.475 | 0.1504109 | 0.46105 |
| X212338_at | -0.0478 | 0.0324 | -1.474 | 0.1504803 | 0.461199 |
| X216908_x_at | -0.0734 | 0.0498 | -1.474 | 0.1505261 | 0.461276 |
| X201056_at | 0.0429 | 0.0291 | 1.474 | 0.1505525 | 0.461293 |
| X210065_s_at | -0.0802 | 0.0544 | -1.474 | 0.1505857 | 0.461331 |
| X211094_s_at | 0.0074 | 0.005 | 1.474 | 0.150635 | 0.461418 |
| X205117_at | 0.1511 | 0.1025 | 1.474 | 0.1506571 | 0.461422 |
| X213589_s_at | -0.0239 | 0.0162 | -1.473 | 0.1507014 | 0.461459 |
| X218072_at | -0.0392 | 0.0266 | -1.473 | 0.1507105 | 0.461459 |
| X221805_at | -0.1146 | 0.0778 | -1.473 | 0.1507497 | 0.461515 |
| X201062_at | -0.0097 | 0.0066 | -1.473 | 0.1507909 | 0.461578 |
| X216130_at | 0.0047 | 0.0032 | 1.473 | 0.1509226 | 0.461917 |
| X206722_s_at | 0.0044 | 0.003 | 1.472 | 0.1509888 | 0.462005 |
| X210227_at | -0.0894 | 0.0607 | -1.472 | 0.1509962 | 0.462005 |
| X201193_at | 0.0728 | 0.0494 | 1.472 | 0.1510138 | 0.462005 |
| X221587_s_at | -0.0151 | 0.0102 | -1.472 | 0.1510869 | 0.462111 |
| X208074_s_at | -0.0597 | 0.0406 | -1.472 | 0.1510898 | 0.462111 |
| X217821_s_at | -0.0859 | 0.0584 | -1.472 | 0.1511204 | 0.462141 |
| X209424_s_at | -0.052 | 0.0353 | -1.472 | 0.1511858 | 0.462252 |
| X204267_x_at | -0.0071 | 0.0049 | -1.472 | 0.1511983 | 0.462252 |
| X220889_s_at | 0.101 | 0.0687 | 1.471 | 0.1513588 | 0.462679 |
| X209313_at | -0.0452 | 0.0307 | -1.471 | 0.1513814 | 0.462679 |
| X207439_s_at | -0.0058 | 0.0039 | -1.471 | 0.1514006 | 0.462679 |
| X209431_s_at | 0.0703 | 0.0478 | 1.471 | 0.1514423 | 0.462706 |
| X217446_x_at | 0.0268 | 0.0182 | 1.471 | 0.1514577 | 0.462706 |
| X209349_at | -0.1262 | 0.0858 | -1.471 | 0.1514721 | 0.462706 |
| X203191_at | -0.0083 | 0.0056 | -1.47 | 0.1515892 | 0.462963 |
| X215786_at | -0.0512 | 0.0348 | -1.47 | 0.1516083 | 0.462963 |
| X218946_at | -0.0568 | 0.0386 | -1.47 | 0.1516187 | 0.462963 |
| X210646_x_at | -0.0313 | 0.0213 | -1.47 | 0.1517423 | 0.463277 |
| X211974_x_at | 0.0753 | 0.0512 | 1.469 | 0.1517822 | 0.463335 |
| X214910_s_at | 0.0035 | 0.0024 | 1.469 | 0.151854 | 0.463491 |
| X203766_s_at | 0.0051 | 0.0034 | 1.469 | 0.1519298 | 0.463658 |
| X213460_x_at | 0.0243 | 0.0165 | 1.469 | 0.1520028 | 0.463798 |
| X203679_at | -0.0175 | 0.0119 | -1.469 | 0.1520172 | 0.463798 |
| X212791_at | 0.0377 | 0.0257 | 1.468 | 0.1520804 | 0.463927 |
| X214124_x_at | 0.0719 | 0.049 | 1.468 | 0.1521272 | 0.464006 |
| X213395_at | 0.0803 | 0.0547 | 1.468 | 0.1522175 | 0.464099 |
| X222006_at | 0.0202 | 0.0137 | 1.468 | 0.1522298 | 0.464099 |
| X210040_at | -0.0497 | 0.0338 | -1.468 | 0.1522554 | 0.464099 |
| X216574_s_at | -0.0154 | 0.0105 | -1.468 | 0.1522936 | 0.464099 |
| X218830_at | -0.0526 | 0.0358 | -1.468 | 0.1523 | 0.464099 |
| X215230_x_at | -0.0874 | 0.0595 | -1.468 | 0.1523062 | 0.464099 |
| X217636_at | -0.0058 | 0.0039 | -1.467 | 0.1523393 | 0.464099 |
| X201877_s_at | -0.05 | 0.0341 | -1.467 | 0.1523491 | 0.464099 |
| X216185_at | -0.006 | 0.0041 | -1.467 | 0.152383 | 0.464099 |
| X207276_at | -0.0204 | 0.0139 | -1.467 | 0.1524009 | 0.464099 |
| X212738_at | 0.0667 | 0.0455 | 1.467 | 0.1524067 | 0.464099 |
| X212701_at | 0.0851 | 0.058 | 1.467 | 0.1524244 | 0.464099 |
| X219096_at | -0.0262 | 0.0179 | -1.467 | 0.1524364 | 0.464099 |
| X200017_at | -0.0326 | 0.0222 | -1.467 | 0.152462 | 0.464099 |
| X211992_at | 0.1613 | 0.1099 | 1.467 | 0.1524844 | 0.464099 |
| X209979_at | -0.0055 | 0.0037 | -1.467 | 0.1524921 | 0.464099 |
| X214389_at | -0.0232 | 0.0158 | -1.467 | 0.1525813 | 0.464135 |
| X219848_s_at | 0.0768 | 0.0524 | 1.467 | 0.1525829 | 0.464135 |
| X205277_at | -0.0814 | 0.0555 | -1.467 | 0.1525833 | 0.464135 |
| X204840_s_at | 0.1787 | 0.1218 | 1.466 | 0.1525875 | 0.464135 |
| X205750_at | -0.0947 | 0.0646 | -1.466 | 0.1526575 | 0.464257 |
| X201564_s_at | -0.0564 | 0.0385 | -1.466 | 0.1526859 | 0.464257 |
| X65591_at | -0.0599 | 0.0408 | -1.466 | 0.1526904 | 0.464257 |
| X214726_x_at | 0.0404 | 0.0275 | 1.466 | 0.1527247 | 0.464276 |
| X210358_x_at | 0.0035 | 0.0024 | 1.466 | 0.1527382 | 0.464276 |
| X213903_s_at | 0.0052 | 0.0036 | 1.466 | 0.1528107 | 0.464393 |
| X204518_s_at | 0.0082 | 0.0056 | 1.466 | 0.1528271 | 0.464393 |
| X209447_at | -0.0795 | 0.0543 | -1.466 | 0.1528394 | 0.464393 |
| X209917_s_at | -0.0798 | 0.0545 | -1.465 | 0.1528744 | 0.464403 |
| X219611_s_at | -0.01 | 0.0068 | -1.465 | 0.1528847 | 0.464403 |
| X219435_at | -0.0075 | 0.0051 | -1.465 | 0.1529635 | 0.464579 |
| X221642_at | 0.0051 | 0.0035 | 1.465 | 0.1531096 | 0.464869 |
| X220053_at | -0.0102 | 0.007 | -1.465 | 0.1531135 | 0.464869 |
| X200819_s_at | 0.0305 | 0.0208 | 1.464 | 0.1531418 | 0.464869 |
| X217330_at | 0.0051 | 0.0035 | 1.464 | 0.1531426 | 0.464869 |
| X222314_x_at | 0.004 | 0.0027 | 1.464 | 0.1531813 | 0.464923 |
| X205525_at | 0.0571 | 0.039 | 1.464 | 0.1532078 | 0.46494 |
| X221985_at | 0.1519 | 0.1038 | 1.464 | 0.1533562 | 0.465318 |
| X210939_s_at | -0.0048 | 0.0033 | -1.464 | 0.1533869 | 0.465318 |
| X218904_s_at | -0.0226 | 0.0155 | -1.463 | 0.153408 | 0.465318 |
| X208699_x_at | -0.0561 | 0.0383 | -1.463 | 0.1534196 | 0.465318 |
| X202127_at | -0.072 | 0.0492 | -1.463 | 0.1534447 | 0.465318 |
| X219431_at | 0.0092 | 0.0063 | 1.463 | 0.1534621 | 0.465318 |
| X220773_s_at | -0.1284 | 0.0877 | -1.463 | 0.1534791 | 0.465318 |
| X217660_at | -0.0065 | 0.0044 | -1.463 | 0.1535549 | 0.465484 |
| X204307_at | 0.0887 | 0.0607 | 1.463 | 0.1536306 | 0.46565 |
| X216702_x_at | -0.0339 | 0.0232 | -1.463 | 0.1536531 | 0.465655 |
| X206850_at | -0.1551 | 0.1061 | -1.462 | 0.1537256 | 0.465811 |
| X40446_at | 0.0534 | 0.0365 | 1.462 | 0.1537783 | 0.465826 |
| X219401_at | -0.0037 | 0.0025 | -1.462 | 0.1537798 | 0.465826 |
| X202261_at | -0.0438 | 0.03 | -1.462 | 0.1538054 | 0.465826 |
| X203413_at | -0.0515 | 0.0352 | -1.462 | 0.153828 | 0.465826 |
| X203836_s_at | -0.0519 | 0.0355 | -1.462 | 0.1538531 | 0.465826 |
| X218626_at | -0.0611 | 0.0418 | -1.462 | 0.1538789 | 0.465826 |
| X219600_s_at | -0.14 | 0.0958 | -1.462 | 0.1538964 | 0.465826 |
| X218947_s_at | 0.0874 | 0.0598 | 1.462 | 0.1538984 | 0.465826 |
| X221457_s_at | 0.0049 | 0.0034 | 1.461 | 0.1539603 | 0.46595 |
| X215061_at | -0.0048 | 0.0033 | -1.461 | 0.1541357 | 0.466337 |
| X209216_at | -0.0563 | 0.0386 | -1.461 | 0.1541472 | 0.466337 |
| X201840_at | -0.0334 | 0.0229 | -1.461 | 0.154151 | 0.466337 |
| X211954_s_at | 0.0452 | 0.031 | 1.461 | 0.1541965 | 0.466411 |
| X208466_at | 0.0061 | 0.0042 | 1.46 | 0.1542569 | 0.46653 |
| X213372_at | 0.08 | 0.0548 | 1.46 | 0.1542971 | 0.466588 |
| X217472_at | -0.0055 | 0.0038 | -1.46 | 0.1543345 | 0.466638 |
| X205373_at | -0.0679 | 0.0465 | -1.46 | 0.1543877 | 0.466735 |
| X210169_at | -0.144 | 0.0986 | -1.46 | 0.1544107 | 0.466741 |
| X209132_s_at | -0.1132 | 0.0775 | -1.46 | 0.1544443 | 0.466779 |
| X200863_s_at | -0.0401 | 0.0274 | -1.46 | 0.1544758 | 0.466782 |
| X200698_at | 0.0746 | 0.0511 | 1.46 | 0.1544873 | 0.466782 |
| X218057_x_at | -0.0574 | 0.0393 | -1.459 | 0.1545104 | 0.466788 |
| X218844_at | -0.0193 | 0.0132 | -1.459 | 0.1545402 | 0.466815 |
| X212148_at | -0.054 | 0.037 | -1.459 | 0.1546249 | 0.467007 |
| X201083_s_at | 0.1223 | 0.0838 | 1.459 | 0.1546634 | 0.46706 |
| X208360_s_at | 0.0225 | 0.0155 | 1.459 | 0.154704 | 0.467097 |
| X206800_at | 0.0067 | 0.0046 | 1.459 | 0.1547176 | 0.467097 |
| X219081_at | -0.0109 | 0.0074 | -1.458 | 0.1549017 | 0.467589 |
| X221382_at | 0.008 | 0.0055 | 1.457 | 0.1551536 | 0.468265 |
| X206773_at | 0.151 | 0.1036 | 1.457 | 0.155168 | 0.468265 |
| X216067_at | -0.0046 | 0.0031 | -1.456 | 0.1553154 | 0.468577 |
| X218823_s_at | 0.0863 | 0.0593 | 1.456 | 0.1553317 | 0.468577 |
| X221269_s_at | 0.085 | 0.0584 | 1.456 | 0.1553493 | 0.468577 |
| X207468_s_at | 0.0058 | 0.004 | 1.456 | 0.1553556 | 0.468577 |
| X208561_at | 0.0079 | 0.0054 | 1.456 | 0.1554243 | 0.468721 |
| X220533_at | -0.0234 | 0.0161 | -1.456 | 0.1555092 | 0.468761 |
| X218588_s_at | 0.0799 | 0.0549 | 1.456 | 0.1555101 | 0.468761 |
| X215666_at | -0.0107 | 0.0074 | -1.456 | 0.1555395 | 0.468761 |
| X202592_at | 0.0309 | 0.0212 | 1.456 | 0.1555499 | 0.468761 |
| X208888_s_at | 0.0067 | 0.0046 | 1.456 | 0.1555517 | 0.468761 |
| X202784_s_at | -0.0562 | 0.0386 | -1.456 | 0.1555642 | 0.468761 |
| X60474_at | -0.0553 | 0.038 | -1.455 | 0.1556832 | 0.469056 |
| X207099_s_at | -0.0074 | 0.0051 | -1.455 | 0.1557571 | 0.469215 |
| X202690_s_at | -0.037 | 0.0254 | -1.455 | 0.1557962 | 0.469269 |
| X214092_x_at | -0.0445 | 0.0306 | -1.454 | 0.1558638 | 0.469399 |
| X202461_at | -0.0843 | 0.058 | -1.454 | 0.1558817 | 0.469399 |
| X220741_s_at | -0.0442 | 0.0304 | -1.454 | 0.155922 | 0.469457 |
| X216829_at | -0.0091 | 0.0063 | -1.454 | 0.1559659 | 0.469474 |
| X205092_x_at | 0.116 | 0.0798 | 1.454 | 0.1559701 | 0.469474 |
| X45526_g_at | -0.041 | 0.0282 | -1.454 | 0.1560957 | 0.469745 |
| X217466_x_at | 0.0107 | 0.0074 | 1.454 | 0.1561275 | 0.469745 |
| X210729_at | 0.3654 | 0.2514 | 1.453 | 0.1561344 | 0.469745 |
| X204146_at | -0.0584 | 0.0402 | -1.453 | 0.1561445 | 0.469745 |
| X213913_s_at | -0.0758 | 0.0522 | -1.453 | 0.1561695 | 0.469756 |
| X33304_at | 0.0041 | 0.0028 | 1.453 | 0.15625 | 0.469935 |
| X208152_s_at | -0.04 | 0.0275 | -1.453 | 0.1562836 | 0.469972 |
| X211515_s_at | -0.0089 | 0.0061 | -1.453 | 0.1563505 | 0.47011 |
| X210565_at | 0.0026 | 0.0018 | 1.452 | 0.1564656 | 0.470392 |
| X218135_at | -0.0346 | 0.0238 | -1.452 | 0.1565367 | 0.470542 |
| X212297_at | -0.0876 | 0.0603 | -1.452 | 0.1565651 | 0.470564 |
| X217069_at | -0.0077 | 0.0053 | -1.452 | 0.1565929 | 0.470584 |
| X211499_s_at | 0.0492 | 0.0339 | 1.452 | 0.1566341 | 0.470644 |
| X210136_at | 0.1895 | 0.1306 | 1.451 | 0.1567026 | 0.470786 |
| X221025_x_at | -0.0604 | 0.0416 | -1.451 | 0.1567656 | 0.470852 |
| X221923_s_at | -0.0606 | 0.0418 | -1.451 | 0.1567687 | 0.470852 |
| X205843_x_at | 0.0112 | 0.0077 | 1.451 | 0.1567883 | 0.470852 |
| X219803_at | -0.0125 | 0.0086 | -1.451 | 0.1568762 | 0.471053 |
| X203163_at | -0.0232 | 0.016 | -1.451 | 0.1569086 | 0.471086 |
| X203042_at | 0.1902 | 0.1312 | 1.45 | 0.1570538 | 0.471459 |
| X201140_s_at | 0.0651 | 0.0449 | 1.45 | 0.1571146 | 0.471577 |
| X38447_at | 0.006 | 0.0041 | 1.45 | 0.1571446 | 0.471604 |
| X219211_at | 0.0073 | 0.005 | 1.45 | 0.157191 | 0.471626 |
| X202964_s_at | 0.0178 | 0.0123 | 1.45 | 0.1571944 | 0.471626 |
| X204212_at | -0.0694 | 0.0479 | -1.449 | 0.1572519 | 0.471735 |
| X201041_s_at | 0.2504 | 0.1728 | 1.449 | 0.1573021 | 0.47178 |
| X204820_s_at | 0.1273 | 0.0878 | 1.449 | 0.1573095 | 0.47178 |
| X209040_s_at | 0.0082 | 0.0057 | 1.449 | 0.1573471 | 0.471829 |
| X203750_s_at | -0.0069 | 0.0047 | -1.449 | 0.1574042 | 0.471897 |
| X217527_s_at | 0.0397 | 0.0274 | 1.449 | 0.1574124 | 0.471897 |
| X202987_at | -0.03 | 0.0207 | -1.449 | 0.1574596 | 0.471975 |
| X207459_x_at | 0.0052 | 0.0036 | 1.449 | 0.1574892 | 0.47198 |
| X45749_at | -0.0502 | 0.0347 | -1.449 | 0.1575037 | 0.47198 |
| X204887_s_at | -0.0106 | 0.0073 | -1.448 | 0.1575565 | 0.472053 |
| X201074_at | 0.0918 | 0.0634 | 1.448 | 0.1575706 | 0.472053 |
| X203537_at | -0.0658 | 0.0454 | -1.448 | 0.1576053 | 0.472087 |
| X207010_at | -0.0699 | 0.0483 | -1.448 | 0.1576401 | 0.472087 |
| X203090_at | -0.0533 | 0.0368 | -1.448 | 0.157653 | 0.472087 |
| X215076_s_at | -0.0158 | 0.0109 | -1.448 | 0.157667 | 0.472087 |
| X201070_x_at | 0.1147 | 0.0793 | 1.448 | 0.1577706 | 0.472334 |
| X216421_at | 0.0081 | 0.0056 | 1.447 | 0.1578064 | 0.472377 |
| X210202_s_at | 0.1266 | 0.0875 | 1.447 | 0.1579133 | 0.472634 |
| X214426_x_at | 0.016 | 0.0111 | 1.447 | 0.1580449 | 0.472964 |
| X218426_s_at | 0.0126 | 0.0087 | 1.446 | 0.1580888 | 0.473031 |
| X209912_s_at | 0.0128 | 0.0089 | 1.446 | 0.1581853 | 0.473213 |
| X200817_x_at | -0.0343 | 0.0237 | -1.446 | 0.1581922 | 0.473213 |
| X216772_at | -0.007 | 0.0048 | -1.446 | 0.1582842 | 0.47337 |
| X212814_at | -0.0902 | 0.0624 | -1.446 | 0.1582873 | 0.47337 |
| X215447_at | 0.0145 | 0.01 | 1.445 | 0.158402 | 0.47365 |
| X201476_s_at | -0.0123 | 0.0085 | -1.445 | 0.1584657 | 0.473776 |
| X219833_s_at | 0.1275 | 0.0882 | 1.445 | 0.1585412 | 0.473869 |
| X203925_at | 0.0991 | 0.0686 | 1.445 | 0.1585546 | 0.473869 |
| X213615_at | 0.0071 | 0.0049 | 1.445 | 0.1585607 | 0.473869 |
| X221579_s_at | 0.0658 | 0.0455 | 1.445 | 0.1585961 | 0.473911 |
| X207470_at | -0.0072 | 0.005 | -1.445 | 0.158629 | 0.473946 |
| X202778_s_at | 0.0371 | 0.0257 | 1.444 | 0.1586739 | 0.473958 |
| X214024_s_at | -0.0046 | 0.0032 | -1.444 | 0.1586855 | 0.473958 |
| X201334_s_at | 0.0376 | 0.026 | 1.444 | 0.1587078 | 0.473958 |
| X212737_at | 0.1168 | 0.0809 | 1.444 | 0.1587184 | 0.473958 |
| X221672_s_at | 0.0047 | 0.0033 | 1.444 | 0.1587472 | 0.473959 |
| X206087_x_at | 0.034 | 0.0236 | 1.444 | 0.1587615 | 0.473959 |
| X221566_s_at | 0.0117 | 0.0081 | 1.444 | 0.1588993 | 0.474287 |
| X217776_at | 0.0535 | 0.037 | 1.443 | 0.158914 | 0.474287 |
| X218137_s_at | -0.0418 | 0.029 | -1.443 | 0.158957 | 0.474351 |
| X215199_at | -0.0087 | 0.006 | -1.443 | 0.159062 | 0.474601 |
| X215946_x_at | -0.0054 | 0.0037 | -1.443 | 0.1591008 | 0.474653 |
| X208616_s_at | 0.0869 | 0.0602 | 1.443 | 0.1591709 | 0.474757 |
| X220807_at | -0.0084 | 0.0059 | -1.443 | 0.1591786 | 0.474757 |
| X218621_at | -0.0072 | 0.005 | -1.442 | 0.1592216 | 0.474781 |
| X204004_at | -0.1166 | 0.0809 | -1.442 | 0.1592471 | 0.474781 |
| X221032_s_at | -0.0062 | 0.0043 | -1.442 | 0.1592506 | 0.474781 |
| X201818_at | 0.0417 | 0.0289 | 1.442 | 0.159288 | 0.474829 |
| X218127_at | -0.0517 | 0.0358 | -1.442 | 0.1593654 | 0.474996 |
| X209199_s_at | 0.1237 | 0.0858 | 1.442 | 0.1594442 | 0.475167 |
| X219532_at | -0.0753 | 0.0522 | -1.441 | 0.1596968 | 0.475856 |
| X210563_x_at | -0.0894 | 0.0621 | -1.44 | 0.1598731 | 0.476314 |
| X216140_at | 0.0055 | 0.0038 | 1.44 | 0.1598935 | 0.476314 |
| X221949_at | 0.0127 | 0.0088 | 1.44 | 0.1599186 | 0.476325 |
| X41660_at | 0.0247 | 0.0172 | 1.44 | 0.1599461 | 0.476343 |
| X207318_s_at | 0.06 | 0.0417 | 1.44 | 0.1599791 | 0.476377 |
| X207945_s_at | 0.0062 | 0.0043 | 1.439 | 0.1600539 | 0.476536 |
| X208298_at | -0.0058 | 0.004 | -1.439 | 0.1601201 | 0.47667 |
| X206404_at | -0.1384 | 0.0962 | -1.439 | 0.1601853 | 0.4768 |
| X200907_s_at | 0.1025 | 0.0712 | 1.439 | 0.1602539 | 0.47694 |
| X216685_s_at | -0.0099 | 0.0069 | -1.439 | 0.1602838 | 0.47696 |
| X222308_x_at | 0.0117 | 0.0081 | 1.439 | 0.1603037 | 0.47696 |
| X201808_s_at | 0.0053 | 0.0037 | 1.438 | 0.1603777 | 0.477117 |
| X213866_at | -0.0489 | 0.034 | -1.437 | 0.1605989 | 0.477711 |
| X202295_s_at | 0.1236 | 0.086 | 1.437 | 0.1606608 | 0.477831 |
| X203322_at | -0.0336 | 0.0234 | -1.437 | 0.1607694 | 0.478059 |
| X204258_at | 0.082 | 0.0571 | 1.437 | 0.1607804 | 0.478059 |
| X217597_x_at | -0.1358 | 0.0945 | -1.437 | 0.1608501 | 0.478202 |
| X218374_s_at | 0.0866 | 0.0603 | 1.436 | 0.160887 | 0.478248 |
| X221149_at | -0.0069 | 0.0048 | -1.436 | 0.1610308 | 0.478554 |
| X203739_at | 0.0947 | 0.0659 | 1.436 | 0.1610333 | 0.478554 |
| X218461_at | -0.0825 | 0.0575 | -1.435 | 0.1611616 | 0.478872 |
| X210403_s_at | -0.0071 | 0.0049 | -1.435 | 0.1612197 | 0.478931 |
| X206001_at | -0.1235 | 0.086 | -1.435 | 0.1612426 | 0.478931 |
| X207781_s_at | -0.0551 | 0.0384 | -1.435 | 0.1612721 | 0.478931 |
| X214762_at | -0.068 | 0.0474 | -1.435 | 0.1612794 | 0.478931 |
| X221553_at | 0.1226 | 0.0855 | 1.435 | 0.1612933 | 0.478931 |
| X212985_at | -0.0635 | 0.0442 | -1.435 | 0.161311 | 0.478931 |
| X211480_s_at | -0.2017 | 0.1406 | -1.434 | 0.1615156 | 0.479475 |
| X216879_at | 0.0049 | 0.0034 | 1.434 | 0.1615423 | 0.47949 |
| X218392_x_at | 0.0616 | 0.043 | 1.434 | 0.1616978 | 0.479852 |
| X201789_at | 0.0115 | 0.008 | 1.434 | 0.1617074 | 0.479852 |
| X202474_s_at | -0.0222 | 0.0155 | -1.433 | 0.1617917 | 0.480038 |
| X201403_s_at | -0.059 | 0.0412 | -1.433 | 0.1618535 | 0.480041 |
| X212051_at | -0.0601 | 0.042 | -1.433 | 0.1618538 | 0.480041 |
| X209428_s_at | -0.0571 | 0.0398 | -1.433 | 0.1618576 | 0.480041 |
| X212870_at | -0.0614 | 0.0428 | -1.433 | 0.1619087 | 0.480128 |
| X213859_x_at | 0.0279 | 0.0195 | 1.433 | 0.161958 | 0.480199 |
| X201327_s_at | -0.0583 | 0.0407 | -1.433 | 0.1619757 | 0.480199 |
| X202041_s_at | -0.0731 | 0.051 | -1.432 | 0.162018 | 0.48026 |
| X208510_s_at | 0.0025 | 0.0018 | 1.432 | 0.1620414 | 0.480265 |
| X202744_at | -0.0146 | 0.0102 | -1.432 | 0.1620889 | 0.480342 |
| X202002_at | -0.0107 | 0.0075 | -1.432 | 0.162142 | 0.480435 |
| X221870_at | 0.0057 | 0.004 | 1.432 | 0.1621724 | 0.480461 |
| X217336_at | 0.0681 | 0.0476 | 1.432 | 0.1621957 | 0.480466 |
| X202326_at | -0.0886 | 0.0619 | -1.432 | 0.1622339 | 0.480515 |
| X215062_at | -0.065 | 0.0454 | -1.431 | 0.1623159 | 0.480592 |
| X221730_at | 0.0095 | 0.0067 | 1.431 | 0.1623184 | 0.480592 |
| X207016_s_at | 0.0061 | 0.0042 | 1.431 | 0.1623399 | 0.480592 |
| X33307_at | -0.0459 | 0.032 | -1.431 | 0.1623465 | 0.480592 |
| X216530_at | 0.0083 | 0.0058 | 1.431 | 0.1623687 | 0.480593 |
| X207973_x_at | -0.0143 | 0.01 | -1.431 | 0.1623898 | 0.480593 |
| X220308_at | 0.007 | 0.0049 | 1.431 | 0.1624223 | 0.480625 |
| X214536_at | 0.0029 | 0.002 | 1.431 | 0.1624786 | 0.480727 |
| X206815_at | 0.129 | 0.0902 | 1.431 | 0.162512 | 0.480762 |
| X203195_s_at | -0.0094 | 0.0066 | -1.43 | 0.1626099 | 0.480988 |
| X220639_at | -0.0093 | 0.0065 | -1.43 | 0.162649 | 0.481039 |
| X215814_at | -0.0158 | 0.0111 | -1.43 | 0.1627258 | 0.481129 |
| X204558_at | 0.0062 | 0.0043 | 1.43 | 0.162739 | 0.481129 |
| X206025_s_at | -0.0054 | 0.0038 | -1.43 | 0.1627443 | 0.481129 |
| X203236_s_at | -0.0055 | 0.0039 | -1.43 | 0.1627915 | 0.481204 |
| X211719_x_at | 0.119 | 0.0832 | 1.43 | 0.1628372 | 0.481275 |
| X201854_s_at | 0.0517 | 0.0361 | 1.429 | 0.1628934 | 0.481377 |
| X209663_s_at | 0.0092 | 0.0065 | 1.429 | 0.1629399 | 0.481451 |
| X218299_at | 0.0084 | 0.0059 | 1.429 | 0.1629745 | 0.481489 |
| X202628_s_at | 0.0072 | 0.005 | 1.429 | 0.1631174 | 0.481827 |
| X208596_s_at | -0.0044 | 0.0031 | -1.429 | 0.1631323 | 0.481827 |
| X206866_at | -0.0876 | 0.0614 | -1.428 | 0.1631804 | 0.481905 |
| X204265_s_at | 0.0096 | 0.0067 | 1.428 | 0.1632328 | 0.481996 |
| X214395_x_at | 0.03 | 0.021 | 1.428 | 0.1633036 | 0.482083 |
| X204066_s_at | 0.1076 | 0.0753 | 1.428 | 0.1633101 | 0.482083 |
| X217643_x_at | 0.0644 | 0.0451 | 1.428 | 0.1633275 | 0.482083 |
| X220998_s_at | 0.0042 | 0.003 | 1.428 | 0.1633507 | 0.482088 |
| X211220_s_at | 0.0338 | 0.0237 | 1.428 | 0.1633781 | 0.482104 |
| X203575_at | 0.0516 | 0.0361 | 1.428 | 0.1634116 | 0.482139 |
| X202468_s_at | 0.1151 | 0.0806 | 1.427 | 0.1634494 | 0.482187 |
| X222024_s_at | 0.0754 | 0.0528 | 1.427 | 0.1636608 | 0.482746 |
| X214000_s_at | 0.0068 | 0.0048 | 1.426 | 0.163722 | 0.482863 |
| X221536_s_at | -0.042 | 0.0295 | -1.426 | 0.1637855 | 0.482986 |
| X216267_s_at | -0.0093 | 0.0065 | -1.426 | 0.163992 | 0.48353 |
| X217186_at | -0.0061 | 0.0043 | -1.425 | 0.1640722 | 0.483703 |
| X202154_x_at | 0.0735 | 0.0516 | 1.425 | 0.164115 | 0.483765 |
| X205957_at | 0.0071 | 0.005 | 1.425 | 0.164156 | 0.483821 |
| X206966_s_at | 0.0076 | 0.0053 | 1.425 | 0.1641868 | 0.483848 |
| X201786_s_at | -0.0376 | 0.0264 | -1.425 | 0.1642429 | 0.483854 |
| X214139_at | 0.005 | 0.0035 | 1.425 | 0.1642573 | 0.483854 |
| X210175_at | -0.05 | 0.0351 | -1.425 | 0.1642603 | 0.483854 |
| X210072_at | -0.0062 | 0.0043 | -1.425 | 0.164276 | 0.483854 |
| X205276_s_at | -0.0089 | 0.0063 | -1.424 | 0.1643673 | 0.483989 |
| X218605_at | -0.0622 | 0.0437 | -1.424 | 0.1643959 | 0.483989 |
| X201174_s_at | -0.0492 | 0.0346 | -1.424 | 0.1644204 | 0.483989 |
| X202637_s_at | 0.0085 | 0.006 | 1.424 | 0.1644281 | 0.483989 |
| X217219_at | 0.0062 | 0.0043 | 1.424 | 0.1644309 | 0.483989 |
| X202367_at | -0.0083 | 0.0058 | -1.424 | 0.1644666 | 0.48403 |
| X218412_s_at | 0.0089 | 0.0062 | 1.424 | 0.1645501 | 0.484165 |
| X210933_s_at | -0.0153 | 0.0108 | -1.424 | 0.1645559 | 0.484165 |
| X200642_at | -0.0331 | 0.0233 | -1.423 | 0.1645943 | 0.484198 |
| X207823_s_at | 0.0068 | 0.0048 | 1.423 | 0.1646276 | 0.484198 |
| X211947_s_at | 0.2086 | 0.1465 | 1.423 | 0.164645 | 0.484198 |
| X218099_at | 0.0427 | 0.03 | 1.423 | 0.1646544 | 0.484198 |
| X220166_at | -0.0718 | 0.0505 | -1.423 | 0.1647492 | 0.48441 |
| X203834_s_at | -0.0098 | 0.0069 | -1.423 | 0.1647918 | 0.48441 |
| X206555_s_at | -0.0613 | 0.0431 | -1.423 | 0.1648125 | 0.48441 |
| X209947_at | -0.0316 | 0.0222 | -1.423 | 0.1648138 | 0.48441 |
| X203890_s_at | -0.0055 | 0.0039 | -1.422 | 0.1648786 | 0.484537 |
| X214251_s_at | -0.0088 | 0.0062 | -1.422 | 0.1649102 | 0.484566 |
| X218843_at | -0.0649 | 0.0456 | -1.422 | 0.1649496 | 0.484617 |
| X219010_at | 0.0079 | 0.0056 | 1.422 | 0.1650101 | 0.484731 |
| X221081_s_at | 0.0062 | 0.0044 | 1.422 | 0.1650573 | 0.484805 |
| X220276_at | -0.2056 | 0.1446 | -1.421 | 0.1651828 | 0.48511 |
| X219901_at | -0.1325 | 0.0932 | -1.421 | 0.1652175 | 0.485131 |
| X201873_s_at | -0.051 | 0.0359 | -1.421 | 0.1652711 | 0.485131 |
| X214383_x_at | -0.0642 | 0.0451 | -1.421 | 0.1652753 | 0.485131 |
| X215739_s_at | -0.0103 | 0.0073 | -1.421 | 0.1652774 | 0.485131 |
| X220071_x_at | 0.0726 | 0.0511 | 1.421 | 0.1653896 | 0.485396 |
| X203733_at | 0.0444 | 0.0313 | 1.421 | 0.1654385 | 0.485476 |
| X215774_s_at | 0.0039 | 0.0028 | 1.42 | 0.1654763 | 0.485523 |
| X215469_at | -0.1084 | 0.0763 | -1.42 | 0.165518 | 0.485581 |
| X218015_s_at | 0.0068 | 0.0048 | 1.419 | 0.1657567 | 0.486217 |
| X206075_s_at | -0.0682 | 0.0481 | -1.419 | 0.1658424 | 0.486395 |
| X213538_at | -0.0283 | 0.02 | -1.419 | 0.1658612 | 0.486395 |
| X201362_at | -0.1143 | 0.0805 | -1.419 | 0.1658975 | 0.486403 |
| X200773_x_at | 0.0586 | 0.0413 | 1.419 | 0.1659079 | 0.486403 |
| X202709_at | 0.0063 | 0.0044 | 1.419 | 0.165943 | 0.486442 |
| X213819_s_at | 0.0061 | 0.0043 | 1.419 | 0.166004 | 0.48645 |
| X214064_at | -0.187 | 0.1318 | -1.418 | 0.1660275 | 0.48645 |
| X221051_s_at | -0.0058 | 0.0041 | -1.418 | 0.1660362 | 0.48645 |
| X212746_s_at | 0.0569 | 0.0401 | 1.418 | 0.1660528 | 0.48645 |
| X208031_s_at | -0.004 | 0.0028 | -1.418 | 0.1660552 | 0.48645 |
| X209574_s_at | -0.1719 | 0.1212 | -1.418 | 0.1660886 | 0.486484 |
| X202787_s_at | -0.0064 | 0.0045 | -1.418 | 0.1661205 | 0.486513 |
| X201397_at | 0.0909 | 0.0641 | 1.418 | 0.1662426 | 0.4868 |
| X214175_x_at | 0.0165 | 0.0117 | 1.418 | 0.1662622 | 0.4868 |
| X203433_at | -0.0293 | 0.0207 | -1.418 | 0.1662926 | 0.486824 |
| X209535_s_at | 0.0228 | 0.0161 | 1.417 | 0.1663326 | 0.48684 |
| X40148_at | -0.0873 | 0.0616 | -1.417 | 0.1663416 | 0.48684 |
| X222133_s_at | 0.0786 | 0.0555 | 1.417 | 0.1664225 | 0.487012 |
| X212226_s_at | 0.1072 | 0.0757 | 1.417 | 0.1664691 | 0.487084 |
| X202814_s_at | 0.0705 | 0.0497 | 1.417 | 0.1665065 | 0.48713 |
| X216105_x_at | -0.0086 | 0.0061 | -1.416 | 0.1667344 | 0.487695 |
| X216320_x_at | 0.0086 | 0.0061 | 1.416 | 0.1667438 | 0.487695 |
| X209293_x_at | 0.1344 | 0.0949 | 1.416 | 0.1668155 | 0.487841 |
| X205524_s_at | -0.006 | 0.0043 | -1.416 | 0.1668552 | 0.487893 |
| X209901_x_at | -0.0254 | 0.0179 | -1.415 | 0.1668978 | 0.487914 |
| X218998_at | 0.0573 | 0.0405 | 1.415 | 0.1669063 | 0.487914 |
| X220545_s_at | -0.0055 | 0.0039 | -1.415 | 0.1669798 | 0.488064 |
| X212790_x_at | -0.0349 | 0.0247 | -1.415 | 0.167018 | 0.488112 |
| X210412_at | 0.2034 | 0.1438 | 1.415 | 0.1670897 | 0.488228 |
| X214680_at | -0.009 | 0.0064 | -1.415 | 0.1671016 | 0.488228 |
| X219712_s_at | -0.0059 | 0.0042 | -1.414 | 0.1673631 | 0.488927 |
| X212002_at | 0.0542 | 0.0384 | 1.414 | 0.1674438 | 0.489099 |
| X201451_x_at | -0.0128 | 0.0091 | -1.413 | 0.1675474 | 0.489337 |
| X214172_x_at | -0.0212 | 0.015 | -1.413 | 0.1675913 | 0.489401 |
| X41657_at | 0.0076 | 0.0054 | 1.412 | 0.1678045 | 0.489959 |
| X205699_at | 0.0083 | 0.0059 | 1.412 | 0.1678265 | 0.489959 |
| X205228_at | 0.004 | 0.0028 | 1.412 | 0.1679076 | 0.490131 |
| X216450_x_at | 0.1381 | 0.0978 | 1.412 | 0.1679465 | 0.490181 |
| X202318_s_at | 0.041 | 0.0291 | 1.412 | 0.1680309 | 0.490302 |
| X211313_s_at | -0.0115 | 0.0081 | -1.412 | 0.168039 | 0.490302 |
| X208560_at | -0.0068 | 0.0048 | -1.412 | 0.1680545 | 0.490302 |
| X205215_at | -0.0152 | 0.0108 | -1.411 | 0.1681723 | 0.490542 |
| X208523_x_at | -0.0049 | 0.0035 | -1.411 | 0.1681826 | 0.490542 |
| X211301_at | 0.0059 | 0.0041 | 1.411 | 0.168219 | 0.490542 |
| X218119_at | -0.0523 | 0.0371 | -1.411 | 0.1682248 | 0.490542 |
| X214995_s_at | -0.0051 | 0.0036 | -1.411 | 0.1682705 | 0.49061 |
| X213456_at | 0.008 | 0.0057 | 1.411 | 0.1683422 | 0.490705 |
| X209551_at | -0.0592 | 0.0419 | -1.41 | 0.168368 | 0.490705 |
| X202901_x_at | 0.1015 | 0.072 | 1.41 | 0.1683692 | 0.490705 |
| X213085_s_at | -0.0759 | 0.0539 | -1.41 | 0.1684347 | 0.490832 |
| X217280_x_at | -0.0107 | 0.0076 | -1.41 | 0.1685558 | 0.490971 |
| X212954_at | 0.0222 | 0.0157 | 1.41 | 0.1685683 | 0.490971 |
| X220477_s_at | -0.0991 | 0.0703 | -1.41 | 0.1685731 | 0.490971 |
| X221924_at | 0.0058 | 0.0041 | 1.41 | 0.1685785 | 0.490971 |
| X202893_at | -0.1192 | 0.0845 | -1.41 | 0.1685929 | 0.490971 |
| X214305_s_at | 0.0897 | 0.0636 | 1.409 | 0.1686497 | 0.490976 |
| X217713_x_at | 0.0561 | 0.0398 | 1.409 | 0.168655 | 0.490976 |
| X204139_x_at | 0.0815 | 0.0578 | 1.409 | 0.1686632 | 0.490976 |
| X201685_s_at | -0.0327 | 0.0232 | -1.409 | 0.1686857 | 0.490976 |
| X221565_s_at | -0.0049 | 0.0035 | -1.409 | 0.1687053 | 0.490976 |
| X216470_x_at | -0.0065 | 0.0046 | -1.409 | 0.1687927 | 0.491166 |
| X210859_x_at | -0.0149 | 0.0106 | -1.409 | 0.1689165 | 0.491462 |
| X205872_x_at | -0.2024 | 0.1437 | -1.408 | 0.1690309 | 0.49173 |
| X204486_at | -0.0077 | 0.0054 | -1.408 | 0.1691212 | 0.491822 |
| X214169_at | 0.0144 | 0.0102 | 1.408 | 0.1691266 | 0.491822 |
| X207677_s_at | -0.0049 | 0.0035 | -1.408 | 0.1691288 | 0.491822 |
| X211156_at | -0.0064 | 0.0045 | -1.407 | 0.1692411 | 0.492006 |
| X213946_s_at | 0.0514 | 0.0365 | 1.407 | 0.1692507 | 0.492006 |
| X201520_s_at | -0.0581 | 0.0413 | -1.407 | 0.1692653 | 0.492006 |
| X203845_at | 0.0653 | 0.0464 | 1.407 | 0.169294 | 0.492006 |
| X214439_x_at | 0.1017 | 0.0723 | 1.407 | 0.1693028 | 0.492006 |
| X210524_x_at | 0.0884 | 0.0628 | 1.407 | 0.1694354 | 0.492327 |
| X208903_at | -0.0413 | 0.0294 | -1.407 | 0.1694638 | 0.492335 |
| X218252_at | -0.0877 | 0.0623 | -1.407 | 0.1694824 | 0.492335 |
| X214891_at | -0.0058 | 0.0041 | -1.407 | 0.1695118 | 0.492356 |
| X210114_at | 0.0068 | 0.0048 | 1.406 | 0.1695651 | 0.492446 |
| X212739_s_at | 0.085 | 0.0604 | 1.406 | 0.1696178 | 0.492535 |
| X221135_s_at | 0.0542 | 0.0386 | 1.406 | 0.1697186 | 0.492763 |
| X212624_s_at | -0.0418 | 0.0297 | -1.406 | 0.169757 | 0.49281 |
| X204234_s_at | 0.1116 | 0.0794 | 1.406 | 0.1698158 | 0.492916 |
| X216186_at | 0.003 | 0.0022 | 1.405 | 0.1698905 | 0.493069 |
| X208893_s_at | -0.1608 | 0.1144 | -1.405 | 0.1699155 | 0.493077 |
| X210497_x_at | -0.0049 | 0.0035 | -1.405 | 0.1699623 | 0.493148 |
| X206488_s_at | 0.0153 | 0.0109 | 1.405 | 0.1700214 | 0.493255 |
| X211955_at | 0.0468 | 0.0333 | 1.405 | 0.1700581 | 0.493297 |
| X204365_s_at | -0.0724 | 0.0516 | -1.404 | 0.1701142 | 0.493396 |
| X221859_at | 0.0137 | 0.0097 | 1.404 | 0.1701652 | 0.493479 |
| X217519_at | -0.0138 | 0.0098 | -1.404 | 0.1703135 | 0.49376 |
| X220345_at | -0.1014 | 0.0723 | -1.404 | 0.1703486 | 0.49376 |
| X221909_at | -0.0597 | 0.0425 | -1.404 | 0.1703494 | 0.49376 |
| X206401_s_at | 0.0962 | 0.0686 | 1.404 | 0.1703947 | 0.49376 |
| X222032_s_at | -0.051 | 0.0363 | -1.404 | 0.1703993 | 0.49376 |
| X211395_x_at | 0.0045 | 0.0032 | 1.403 | 0.1704097 | 0.49376 |
| X215582_x_at | 0.0141 | 0.01 | 1.403 | 0.1704518 | 0.49376 |
| X55872_at | -0.0553 | 0.0394 | -1.403 | 0.1704779 | 0.49376 |
| X213874_at | 0.0045 | 0.0032 | 1.403 | 0.1704968 | 0.49376 |
| X202872_at | -0.0684 | 0.0488 | -1.403 | 0.1705012 | 0.49376 |
| X216964_at | 0.0081 | 0.0058 | 1.403 | 0.1705065 | 0.49376 |
| X207583_at | -0.0313 | 0.0223 | -1.403 | 0.1706235 | 0.494008 |
| X211691_x_at | -0.0048 | 0.0034 | -1.403 | 0.1706365 | 0.494008 |
| X209572_s_at | 0.0447 | 0.0319 | 1.402 | 0.1707182 | 0.49418 |
| X203077_s_at | 0.0508 | 0.0363 | 1.402 | 0.1708638 | 0.494537 |
| X209685_s_at | -0.1242 | 0.0886 | -1.402 | 0.1709272 | 0.494656 |
| X218324_s_at | -0.0931 | 0.0665 | -1.402 | 0.1709643 | 0.494677 |
| X215947_s_at | -0.0553 | 0.0394 | -1.402 | 0.1709791 | 0.494677 |
| X202832_at | -0.054 | 0.0385 | -1.401 | 0.1711009 | 0.494965 |
| X220583_at | -0.0067 | 0.0048 | -1.401 | 0.1711768 | 0.49512 |
| X209149_s_at | -0.0331 | 0.0237 | -1.401 | 0.1712754 | 0.495235 |
| X213793_s_at | -0.0695 | 0.0496 | -1.401 | 0.1712862 | 0.495235 |
| X200608_s_at | 0.0262 | 0.0187 | 1.4 | 0.1712951 | 0.495235 |
| X221716_s_at | 0.0083 | 0.006 | 1.4 | 0.1713164 | 0.495235 |
| X202981_x_at | -0.0309 | 0.0221 | -1.4 | 0.171328 | 0.495235 |
| X212748_at | 0.0092 | 0.0066 | 1.4 | 0.1713687 | 0.495288 |
| X203508_at | 0.1057 | 0.0755 | 1.4 | 0.1714021 | 0.49532 |
| X203706_s_at | -0.1074 | 0.0767 | -1.4 | 0.17146 | 0.49538 |
| X215461_at | -0.005 | 0.0036 | -1.4 | 0.1714789 | 0.49538 |
| X202570_s_at | -0.0783 | 0.056 | -1.4 | 0.1714896 | 0.49538 |
| X222147_s_at | -0.0129 | 0.0092 | -1.4 | 0.1715395 | 0.495442 |
| X205150_s_at | -0.1304 | 0.0931 | -1.4 | 0.1715577 | 0.495442 |
| X200658_s_at | -0.0667 | 0.0476 | -1.4 | 0.1715782 | 0.495442 |
| X200064_at | -0.086 | 0.0615 | -1.399 | 0.1716402 | 0.495492 |
| X221272_s_at | 0.0986 | 0.0704 | 1.399 | 0.1716453 | 0.495492 |
| X202497_x_at | -0.1162 | 0.0831 | -1.399 | 0.1716624 | 0.495492 |
| X217028_at | -0.1747 | 0.1249 | -1.399 | 0.1717045 | 0.495502 |
| X201263_at | -0.0428 | 0.0306 | -1.399 | 0.1717102 | 0.495502 |
| X209849_s_at | -0.1017 | 0.0727 | -1.399 | 0.1717441 | 0.495535 |
| X205182_s_at | 0.019 | 0.0136 | 1.399 | 0.1718328 | 0.495667 |
| X213207_s_at | -0.0168 | 0.012 | -1.399 | 0.1718345 | 0.495667 |
| X213723_s_at | -0.0044 | 0.0032 | -1.398 | 0.1719138 | 0.495831 |
| X203420_at | -0.0315 | 0.0225 | -1.398 | 0.1719496 | 0.49587 |
| X206093_x_at | 0.0417 | 0.0298 | 1.398 | 0.1720879 | 0.496188 |
| X219163_at | 0.0278 | 0.0199 | 1.398 | 0.1721384 | 0.496188 |
| X201850_at | 0.0212 | 0.0152 | 1.398 | 0.1721396 | 0.496188 |
| X210153_s_at | -0.0604 | 0.0432 | -1.398 | 0.1721492 | 0.496188 |
| X213322_at | -0.0526 | 0.0377 | -1.397 | 0.1723294 | 0.496573 |
| X208999_at | 0.1047 | 0.0749 | 1.397 | 0.1723454 | 0.496573 |
| X213355_at | 0.12 | 0.0859 | 1.397 | 0.1723497 | 0.496573 |
| X209577_at | -0.0911 | 0.0652 | -1.396 | 0.1725194 | 0.496986 |
| X209949_at | 0.0062 | 0.0045 | 1.396 | 0.172538 | 0.496986 |
| X200824_at | 0.053 | 0.0379 | 1.396 | 0.1727084 | 0.497274 |
| X205693_at | 0.0047 | 0.0034 | 1.396 | 0.1727146 | 0.497274 |
| X209323_at | -0.0388 | 0.0278 | -1.396 | 0.1727249 | 0.497274 |
| X206313_at | -0.0037 | 0.0026 | -1.396 | 0.1727272 | 0.497274 |
| X208012_x_at | 0.1319 | 0.0945 | 1.395 | 0.1727971 | 0.49741 |
| X206431_x_at | 0.009 | 0.0065 | 1.395 | 0.172833 | 0.497449 |
| X217718_s_at | -0.0284 | 0.0204 | -1.395 | 0.1728739 | 0.497502 |
| X202672_s_at | 0.2613 | 0.1873 | 1.395 | 0.1729513 | 0.497661 |
| X208058_s_at | -0.0067 | 0.0048 | -1.395 | 0.1729925 | 0.497715 |
| X206790_s_at | -0.0331 | 0.0237 | -1.395 | 0.1730716 | 0.497878 |
| X213826_s_at | -0.036 | 0.0258 | -1.394 | 0.1731938 | 0.498165 |
| X203229_s_at | 0.0165 | 0.0119 | 1.394 | 0.1732844 | 0.498361 |
| X206607_at | 0.0042 | 0.003 | 1.394 | 0.1733326 | 0.498435 |
| X205255_x_at | 0.0891 | 0.0639 | 1.393 | 0.1734231 | 0.498631 |
| X221502_at | -0.044 | 0.0316 | -1.393 | 0.173497 | 0.498779 |
| X221452_s_at | -0.0501 | 0.036 | -1.393 | 0.173536 | 0.498803 |
| X218700_s_at | 0.0877 | 0.0629 | 1.393 | 0.1735631 | 0.498803 |
| X204243_at | -0.1048 | 0.0752 | -1.393 | 0.1735728 | 0.498803 |
| X205684_s_at | -0.0647 | 0.0465 | -1.393 | 0.1736035 | 0.498827 |
| X211234_x_at | 0.006 | 0.0043 | 1.393 | 0.1736634 | 0.498934 |
| X218310_at | 0.0299 | 0.0215 | 1.392 | 0.1736939 | 0.498957 |
| X209807_s_at | -0.0097 | 0.0069 | -1.392 | 0.1738806 | 0.499311 |
| X213295_at | -0.0695 | 0.0499 | -1.392 | 0.1738854 | 0.499311 |
| X209448_at | 0.0875 | 0.0629 | 1.392 | 0.1739184 | 0.499311 |
| X211119_at | -0.0102 | 0.0073 | -1.392 | 0.173919 | 0.499311 |
| X201320_at | 0.0941 | 0.0676 | 1.392 | 0.1739293 | 0.499311 |
| X205768_s_at | 0.171 | 0.1229 | 1.391 | 0.1741234 | 0.499759 |
| X217452_s_at | -0.1073 | 0.0772 | -1.391 | 0.1741306 | 0.499759 |
| X213260_at | 0.0114 | 0.0082 | 1.391 | 0.1741575 | 0.499772 |
| X207243_s_at | -0.0152 | 0.0109 | -1.391 | 0.1741988 | 0.499826 |
| X212413_at | 0.0224 | 0.0161 | 1.391 | 0.1742613 | 0.499941 |
| X221017_s_at | 0.0042 | 0.0031 | 1.39 | 0.1743603 | 0.500122 |
| X214989_x_at | 0.0685 | 0.0493 | 1.39 | 0.1743695 | 0.500122 |
| X217990_at | -0.0419 | 0.0301 | -1.39 | 0.1745182 | 0.500484 |
| X204264_at | 0.0867 | 0.0624 | 1.389 | 0.1745912 | 0.500629 |
| X205611_at | 0.0091 | 0.0066 | 1.389 | 0.1746206 | 0.500648 |
| X216110_x_at | 0.0325 | 0.0234 | 1.389 | 0.174753 | 0.500963 |
| X200015_s_at | 0.0475 | 0.0342 | 1.389 | 0.1748267 | 0.50111 |
| X202369_s_at | 0.0071 | 0.0051 | 1.388 | 0.1749211 | 0.501307 |
| X214669_x_at | -0.0049 | 0.0035 | -1.388 | 0.1749406 | 0.501307 |
| X209258_s_at | 0.0821 | 0.0591 | 1.388 | 0.1749721 | 0.501332 |
| X202999_s_at | -0.0048 | 0.0034 | -1.388 | 0.1750672 | 0.501509 |
| X205348_s_at | -0.0642 | 0.0462 | -1.388 | 0.1750789 | 0.501509 |
| X217196_s_at | -0.0475 | 0.0342 | -1.387 | 0.175184 | 0.501745 |
| X209159_s_at | -0.0681 | 0.0491 | -1.387 | 0.1752355 | 0.501774 |
| X202899_s_at | -0.0491 | 0.0354 | -1.387 | 0.1752393 | 0.501774 |
| X203660_s_at | -0.1095 | 0.079 | -1.387 | 0.1754058 | 0.502102 |
| X214862_x_at | -0.0959 | 0.0692 | -1.387 | 0.1754152 | 0.502102 |
| X207057_at | 0.1415 | 0.1021 | 1.387 | 0.1754214 | 0.502102 |
| X212245_at | 0.0439 | 0.0316 | 1.386 | 0.175496 | 0.502251 |
| X206392_s_at | 0.0052 | 0.0037 | 1.386 | 0.1755202 | 0.502255 |
| X222228_s_at | -0.0201 | 0.0145 | -1.386 | 0.1755502 | 0.502276 |
| X210793_s_at | 0.0437 | 0.0315 | 1.386 | 0.1756709 | 0.502498 |
| X220519_s_at | 0.0063 | 0.0045 | 1.386 | 0.1756964 | 0.502498 |
| X221230_s_at | -0.0329 | 0.0238 | -1.386 | 0.1757024 | 0.502498 |
| X35820_at | 0.0896 | 0.0647 | 1.386 | 0.1757183 | 0.502498 |
| X214634_at | -0.0157 | 0.0114 | -1.386 | 0.1757628 | 0.502561 |
| X203327_at | 0.0152 | 0.0109 | 1.385 | 0.1757902 | 0.502575 |
| X202516_s_at | -0.1195 | 0.0862 | -1.385 | 0.1758349 | 0.502638 |
| X202509_s_at | 0.0023 | 0.0016 | 1.385 | 0.175905 | 0.502759 |
| X201321_s_at | 0.0434 | 0.0314 | 1.385 | 0.1759248 | 0.502759 |
| X209981_at | -0.0784 | 0.0566 | -1.385 | 0.1759451 | 0.502759 |
| X208063_s_at | 0.0072 | 0.0052 | 1.384 | 0.1761187 | 0.50319 |
| X203963_at | -0.1339 | 0.0967 | -1.384 | 0.1762003 | 0.503307 |
| X221212_x_at | -0.0391 | 0.0282 | -1.384 | 0.1762277 | 0.503307 |
| X221403_s_at | 0.0047 | 0.0034 | 1.384 | 0.1762336 | 0.503307 |
| X219484_at | 0.0814 | 0.0588 | 1.384 | 0.1762505 | 0.503307 |
| X209635_at | -0.116 | 0.0838 | -1.384 | 0.1763459 | 0.503515 |
| X202312_s_at | -0.0061 | 0.0044 | -1.383 | 0.1764935 | 0.503872 |
| X214492_at | 0.0039 | 0.0028 | 1.383 | 0.1765231 | 0.503892 |
| X200915_x_at | 0.0377 | 0.0272 | 1.383 | 0.1765581 | 0.503927 |
| X214775_at | -0.0152 | 0.011 | -1.383 | 0.1765932 | 0.503947 |
| X210564_x_at | 0.0603 | 0.0436 | 1.383 | 0.1766104 | 0.503947 |
| X215071_s_at | 0.1601 | 0.1158 | 1.383 | 0.1766516 | 0.503987 |
| X210746_s_at | 0.0049 | 0.0035 | 1.382 | 0.1767054 | 0.503987 |
| X221238_at | -0.0177 | 0.0128 | -1.382 | 0.176752 | 0.503987 |
| X203573_s_at | -0.055 | 0.0398 | -1.382 | 0.1767772 | 0.503987 |
| X202063_s_at | -0.0139 | 0.0101 | -1.382 | 0.1767806 | 0.503987 |
| X222016_s_at | -0.0974 | 0.0705 | -1.382 | 0.1767965 | 0.503987 |
| X217164_at | 0.1135 | 0.0821 | 1.382 | 0.1768221 | 0.503987 |
| X216644_at | 0.0206 | 0.0149 | 1.382 | 0.1768366 | 0.503987 |
| X213669_at | -0.0045 | 0.0033 | -1.382 | 0.1768379 | 0.503987 |
| X220751_s_at | -0.0983 | 0.0712 | -1.382 | 0.1768688 | 0.503987 |
| X216397_s_at | -0.006 | 0.0044 | -1.382 | 0.1768742 | 0.503987 |
| X201814_at | 0.041 | 0.0297 | 1.381 | 0.1770087 | 0.504306 |
| X210541_s_at | 0.0057 | 0.0041 | 1.381 | 0.1771025 | 0.504508 |
| X219450_at | -0.0077 | 0.0056 | -1.381 | 0.1772203 | 0.504754 |
| X201005_at | 0.2034 | 0.1473 | 1.381 | 0.1772341 | 0.504754 |
| X206698_at | -0.1071 | 0.0776 | -1.381 | 0.1772699 | 0.504791 |
| X212753_at | 0.0478 | 0.0346 | 1.38 | 0.1773364 | 0.504884 |
| X218098_at | 0.0691 | 0.05 | 1.38 | 0.1773479 | 0.504884 |
| X209781_s_at | 0.1067 | 0.0773 | 1.38 | 0.177382 | 0.504916 |
| X207132_x_at | -0.0414 | 0.03 | -1.38 | 0.1775387 | 0.505265 |
| X209064_x_at | -0.0568 | 0.0411 | -1.38 | 0.1775501 | 0.505265 |
| X201019_s_at | 0.0296 | 0.0214 | 1.38 | 0.1775891 | 0.505311 |
| X201358_s_at | 0.0512 | 0.0371 | 1.379 | 0.1776311 | 0.505366 |
| X213526_s_at | -0.0587 | 0.0426 | -1.379 | 0.1776685 | 0.505371 |
| X217767_at | 0.2463 | 0.1785 | 1.379 | 0.1776785 | 0.505371 |
| X200612_s_at | 0.1041 | 0.0755 | 1.379 | 0.177818 | 0.505634 |
| X208798_x_at | 0.0774 | 0.0562 | 1.379 | 0.1778297 | 0.505634 |
| X217748_at | -0.0503 | 0.0364 | -1.379 | 0.1778392 | 0.505634 |
| X210056_at | -0.041 | 0.0298 | -1.379 | 0.1779027 | 0.505636 |
| X214659_x_at | -0.0867 | 0.0629 | -1.379 | 0.1779079 | 0.505636 |
| X205822_s_at | 0.0117 | 0.0085 | 1.379 | 0.177908 | 0.505636 |
| X218075_at | -0.0046 | 0.0034 | -1.378 | 0.177986 | 0.505793 |
| X211360_s_at | 0.0045 | 0.0032 | 1.378 | 0.1781113 | 0.506036 |
| X207237_at | 0.0288 | 0.0209 | 1.378 | 0.1781199 | 0.506036 |
| X206136_at | 0.0039 | 0.0029 | 1.378 | 0.1781401 | 0.506036 |
| X212951_at | 0.1397 | 0.1014 | 1.378 | 0.1782067 | 0.506161 |
| X215210_s_at | 0.1083 | 0.0786 | 1.377 | 0.1782344 | 0.506175 |
| X219077_s_at | 0.0739 | 0.0537 | 1.377 | 0.1783757 | 0.506511 |
| X219101_x_at | 0.0528 | 0.0383 | 1.377 | 0.1784398 | 0.506515 |
| X217080_s_at | 0.0062 | 0.0045 | 1.377 | 0.1784416 | 0.506515 |
| X219469_at | 0.0654 | 0.0475 | 1.377 | 0.1784455 | 0.506515 |
| X211022_s_at | -0.0116 | 0.0085 | -1.376 | 0.178543 | 0.506667 |
| X204121_at | 0.008 | 0.0058 | 1.376 | 0.1785446 | 0.506667 |
| X210970_s_at | -0.04 | 0.029 | -1.376 | 0.1785739 | 0.506686 |
| X201061_s_at | 0.1296 | 0.0942 | 1.376 | 0.1786069 | 0.506714 |
| X214763_at | 0.0041 | 0.003 | 1.376 | 0.1787222 | 0.506977 |
| X208873_s_at | -0.0324 | 0.0235 | -1.376 | 0.1788321 | 0.507224 |
| X205424_at | 0.0119 | 0.0086 | 1.375 | 0.1789457 | 0.507478 |
| X217570_x_at | 0.0222 | 0.0161 | 1.375 | 0.1789922 | 0.507478 |
| X212262_at | 0.067 | 0.0487 | 1.375 | 0.1789998 | 0.507478 |
| X1294_at | 0.0079 | 0.0057 | 1.375 | 0.179013 | 0.507478 |
| X208442_s_at | -0.0274 | 0.0199 | -1.375 | 0.1790861 | 0.50762 |
| X201498_at | 0.0215 | 0.0157 | 1.374 | 0.1792208 | 0.507761 |
| X215819_s_at | 0.0036 | 0.0026 | 1.374 | 0.1792243 | 0.507761 |
| X211919_s_at | -0.0349 | 0.0254 | -1.374 | 0.1792247 | 0.507761 |
| X218520_at | -0.0438 | 0.0319 | -1.374 | 0.1792272 | 0.507761 |
| X210570_x_at | -0.0425 | 0.0309 | -1.374 | 0.1792653 | 0.507804 |
| X205004_at | -0.0522 | 0.038 | -1.374 | 0.179333 | 0.507829 |
| X206794_at | 0.0287 | 0.0209 | 1.374 | 0.179364 | 0.507829 |
| X202649_x_at | 0.0321 | 0.0233 | 1.374 | 0.1793736 | 0.507829 |
| X202517_at | -0.0978 | 0.0712 | -1.374 | 0.1793763 | 0.507829 |
| X204018_x_at | 0.2721 | 0.1981 | 1.374 | 0.1794147 | 0.507829 |
| X219233_s_at | 0.0137 | 0.0099 | 1.374 | 0.1794398 | 0.507829 |
| X214107_x_at | -0.0673 | 0.049 | -1.374 | 0.1794471 | 0.507829 |
| X218482_at | 0.0242 | 0.0176 | 1.373 | 0.1794569 | 0.507829 |
| X212648_at | -0.0378 | 0.0275 | -1.373 | 0.1795251 | 0.507957 |
| X201838_s_at | -0.0802 | 0.0584 | -1.373 | 0.1796237 | 0.508171 |
| X208915_s_at | -0.0058 | 0.0042 | -1.373 | 0.179675 | 0.508252 |
| X208830_s_at | -0.0094 | 0.0068 | -1.373 | 0.1797216 | 0.508319 |
| X210966_x_at | 0.0074 | 0.0054 | 1.372 | 0.17976 | 0.508342 |
| X209383_at | -0.0728 | 0.0531 | -1.372 | 0.1797757 | 0.508342 |
| X219415_at | 0.0686 | 0.05 | 1.372 | 0.1798236 | 0.508413 |
| X213289_at | -0.0895 | 0.0652 | -1.372 | 0.1798792 | 0.508506 |
| X217750_s_at | 0.0629 | 0.0459 | 1.372 | 0.179945 | 0.508627 |
| X217895_at | -0.0503 | 0.0367 | -1.372 | 0.1799877 | 0.508683 |
| X202197_at | 0.0345 | 0.0252 | 1.372 | 0.1800587 | 0.508819 |
| X212959_s_at | -0.0331 | 0.0241 | -1.371 | 0.1801074 | 0.508892 |
| X220033_at | -0.0629 | 0.0459 | -1.371 | 0.1801534 | 0.508957 |
| X211651_s_at | -0.1178 | 0.0859 | -1.371 | 0.1801928 | 0.509003 |
| X218355_at | 0.0061 | 0.0044 | 1.371 | 0.1802562 | 0.509118 |
| X212861_at | -0.0491 | 0.0358 | -1.371 | 0.1802911 | 0.509152 |
| X205718_at | 0.0044 | 0.0032 | 1.37 | 0.1803757 | 0.509326 |
| X221580_s_at | -0.0492 | 0.0359 | -1.37 | 0.1806287 | 0.509886 |
| X206268_at | 0.0047 | 0.0035 | 1.37 | 0.1806345 | 0.509886 |
| X211513_s_at | -0.0073 | 0.0053 | -1.37 | 0.180643 | 0.509886 |
| X218344_s_at | -0.0256 | 0.0187 | -1.369 | 0.1807876 | 0.510204 |
| X218456_at | -0.0732 | 0.0535 | -1.369 | 0.1808015 | 0.510204 |
| X201551_s_at | 0.0311 | 0.0227 | 1.369 | 0.1808592 | 0.51025 |
| X216534_at | 0.0024 | 0.0017 | 1.369 | 0.1808823 | 0.51025 |
| X212464_s_at | 0.1117 | 0.0816 | 1.369 | 0.1808868 | 0.51025 |
| X202015_x_at | 0.0688 | 0.0503 | 1.368 | 0.1811134 | 0.510824 |
| X211725_s_at | -0.1127 | 0.0824 | -1.368 | 0.1811502 | 0.510843 |
| X220969_s_at | 0.0151 | 0.011 | 1.368 | 0.1811659 | 0.510843 |
| X214291_at | 0.0476 | 0.0348 | 1.368 | 0.1812029 | 0.510882 |
| X215357_s_at | -0.0128 | 0.0093 | -1.368 | 0.1812442 | 0.510934 |
| X204320_at | 0.0484 | 0.0354 | 1.368 | 0.1812761 | 0.510959 |
| X217928_s_at | 0.0263 | 0.0192 | 1.367 | 0.1813592 | 0.511128 |
| X206776_x_at | -0.0097 | 0.0071 | -1.367 | 0.181536 | 0.511562 |
| X218088_s_at | -0.0415 | 0.0304 | -1.366 | 0.1816411 | 0.511793 |
| X202568_s_at | 0.0592 | 0.0433 | 1.366 | 0.1816685 | 0.511805 |
| X209089_at | 0.0408 | 0.0298 | 1.366 | 0.1817384 | 0.51182 |
| X206753_at | 0.0043 | 0.0031 | 1.366 | 0.1817413 | 0.51182 |
| X206637_at | 0.2375 | 0.1738 | 1.366 | 0.1817427 | 0.51182 |
| X200895_s_at | -0.0498 | 0.0365 | -1.366 | 0.1817874 | 0.511881 |
| X206554_x_at | -0.0541 | 0.0396 | -1.366 | 0.1818438 | 0.511974 |
| X204275_at | -0.0062 | 0.0045 | -1.365 | 0.1819621 | 0.512196 |
| X219195_at | 0.0424 | 0.031 | 1.365 | 0.1819871 | 0.512196 |
| X212303_x_at | 0.0083 | 0.0061 | 1.365 | 0.1819916 | 0.512196 |
| X218239_s_at | -0.0446 | 0.0327 | -1.365 | 0.1820887 | 0.512387 |
| X216985_s_at | -0.0055 | 0.0041 | -1.365 | 0.1821058 | 0.512387 |
| X207634_at | -0.0037 | 0.0027 | -1.365 | 0.1821677 | 0.512497 |
| X209983_s_at | 0.0899 | 0.0659 | 1.364 | 0.1822448 | 0.512649 |
| X44146_at | -0.0144 | 0.0105 | -1.364 | 0.1822821 | 0.512689 |
| X220239_at | -0.0285 | 0.0209 | -1.364 | 0.1824886 | 0.51308 |
| X218364_at | 0.0536 | 0.0393 | 1.364 | 0.1824977 | 0.51308 |
| X203048_s_at | -0.0351 | 0.0258 | -1.364 | 0.1825095 | 0.51308 |
| X204474_at | 0.0149 | 0.0109 | 1.364 | 0.1825138 | 0.51308 |
| X213008_at | 0.0043 | 0.0031 | 1.363 | 0.1827246 | 0.513604 |
| X216311_at | 0.0031 | 0.0022 | 1.363 | 0.1827461 | 0.513604 |
| X219500_at | 0.0065 | 0.0048 | 1.363 | 0.1828026 | 0.513655 |
| X201599_at | -0.0357 | 0.0262 | -1.363 | 0.1828105 | 0.513655 |
| X207799_x_at | -0.0095 | 0.007 | -1.362 | 0.1828706 | 0.513697 |
| X202820_at | -0.0548 | 0.0402 | -1.362 | 0.1828719 | 0.513697 |
| X215225_s_at | -0.0053 | 0.0039 | -1.362 | 0.182973 | 0.513825 |
| X214082_at | -0.0106 | 0.0078 | -1.362 | 0.1829979 | 0.513825 |
| X208333_at | -0.0033 | 0.0024 | -1.362 | 0.1830302 | 0.513825 |
| X207605_x_at | -0.0144 | 0.0106 | -1.362 | 0.1830493 | 0.513825 |
| X216426_at | -0.0079 | 0.0058 | -1.362 | 0.1830578 | 0.513825 |
| X201122_x_at | -0.0455 | 0.0334 | -1.362 | 0.1830745 | 0.513825 |
| X209445_x_at | -0.0555 | 0.0408 | -1.362 | 0.1831129 | 0.513825 |
| X201992_s_at | 0.0854 | 0.0627 | 1.362 | 0.1831186 | 0.513825 |
| X209493_at | -0.0796 | 0.0585 | -1.362 | 0.1831257 | 0.513825 |
| X216958_s_at | 0.0598 | 0.0439 | 1.361 | 0.1831844 | 0.513923 |
| X216235_s_at | 0.0079 | 0.0058 | 1.361 | 0.1832069 | 0.513923 |
| X218262_at | -0.0276 | 0.0203 | -1.361 | 0.183246 | 0.513968 |
| X209027_s_at | -0.0772 | 0.0567 | -1.361 | 0.1832963 | 0.513986 |
| X213093_at | 0.0585 | 0.043 | 1.361 | 0.1832985 | 0.513986 |
| X210908_s_at | -0.0398 | 0.0292 | -1.361 | 0.1833604 | 0.514094 |
| X217125_at | -0.0081 | 0.0059 | -1.361 | 0.1833969 | 0.514105 |
| X206213_at | 0.1169 | 0.0859 | 1.361 | 0.1834107 | 0.514105 |
| X201935_s_at | 0.0531 | 0.039 | 1.361 | 0.183475 | 0.514117 |
| X205176_s_at | -0.1676 | 0.1232 | -1.361 | 0.1834796 | 0.514117 |
| X213584_s_at | 0.0096 | 0.007 | 1.36 | 0.1834888 | 0.514117 |
| X201421_s_at | -0.0284 | 0.0209 | -1.36 | 0.1835266 | 0.514117 |
| X212378_at | 0.0205 | 0.0151 | 1.36 | 0.1835304 | 0.514117 |
| X215233_at | 0.0593 | 0.0436 | 1.36 | 0.1836325 | 0.514338 |
| X210060_at | 0.0063 | 0.0046 | 1.36 | 0.1837738 | 0.514603 |
| X212745_s_at | -0.0302 | 0.0222 | -1.359 | 0.1838212 | 0.514603 |
| X221989_at | -0.1125 | 0.0828 | -1.359 | 0.1838304 | 0.514603 |
| X206636_at | 0.0095 | 0.007 | 1.359 | 0.1838602 | 0.514603 |
| X1494_f_at | -0.0065 | 0.0048 | -1.359 | 0.1838618 | 0.514603 |
| X219765_at | -0.0583 | 0.0429 | -1.359 | 0.1838662 | 0.514603 |
| X201021_s_at | -0.0776 | 0.0571 | -1.359 | 0.1839135 | 0.514671 |
| X221116_at | 0.0029 | 0.0021 | 1.359 | 0.1839657 | 0.514752 |
| X217256_x_at | 0.0291 | 0.0214 | 1.359 | 0.1840463 | 0.514833 |
| X220616_at | 0.005 | 0.0037 | 1.359 | 0.1840501 | 0.514833 |
| X219579_at | 0.007 | 0.0052 | 1.359 | 0.1840641 | 0.514833 |
| X212847_at | -0.0559 | 0.0412 | -1.358 | 0.1841246 | 0.514937 |
| X203431_s_at | -0.0853 | 0.0628 | -1.358 | 0.1842267 | 0.515107 |
| X214661_s_at | 0.0061 | 0.0045 | 1.358 | 0.1842319 | 0.515107 |
| X52159_at | -0.0093 | 0.0068 | -1.358 | 0.1842618 | 0.515126 |
| X205389_s_at | -0.0068 | 0.005 | -1.358 | 0.1843461 | 0.515232 |
| X211570_s_at | -0.0079 | 0.0058 | -1.358 | 0.1843475 | 0.515232 |
| X203832_at | -0.0537 | 0.0396 | -1.358 | 0.1843693 | 0.515232 |
| X202794_at | -0.0585 | 0.0431 | -1.358 | 0.1844046 | 0.515255 |
| X220408_x_at | 0.0363 | 0.0267 | 1.357 | 0.184451 | 0.515255 |
| X221592_at | 0.0042 | 0.0031 | 1.357 | 0.1844597 | 0.515255 |
| X215965_at | -0.0057 | 0.0042 | -1.357 | 0.1844727 | 0.515255 |
| X212759_s_at | 0.0113 | 0.0083 | 1.357 | 0.1844934 | 0.515255 |
| X207170_s_at | -0.0561 | 0.0414 | -1.357 | 0.1845731 | 0.515371 |
| X221114_at | -0.011 | 0.0081 | -1.357 | 0.1845814 | 0.515371 |
| X200034_s_at | -0.0347 | 0.0256 | -1.356 | 0.1847613 | 0.515806 |
| X205462_s_at | 0.0756 | 0.0557 | 1.356 | 0.1847848 | 0.515806 |
| X202189_x_at | 0.0511 | 0.0377 | 1.356 | 0.1848117 | 0.515806 |
| X216022_at | 0.111 | 0.0819 | 1.356 | 0.1848299 | 0.515806 |
| X216673_at | -0.0077 | 0.0057 | -1.356 | 0.1848773 | 0.515873 |
| X218001_at | -0.0611 | 0.0451 | -1.356 | 0.1849624 | 0.516014 |
| X216903_s_at | -0.0563 | 0.0415 | -1.356 | 0.1849743 | 0.516014 |
| X206620_at | 0.0035 | 0.0026 | 1.355 | 0.1850592 | 0.516123 |
| X204376_at | 0.0049 | 0.0036 | 1.355 | 0.1850608 | 0.516123 |
| X207511_s_at | -0.0065 | 0.0048 | -1.355 | 0.1850831 | 0.516123 |
| X208133_at | -0.004 | 0.0029 | -1.355 | 0.1851166 | 0.516152 |
| X206721_at | 0.0736 | 0.0544 | 1.354 | 0.1854725 | 0.51701 |
| X221013_s_at | -0.0239 | 0.0176 | -1.354 | 0.1854923 | 0.51701 |
| X201289_at | 0.24 | 0.1773 | 1.354 | 0.1854944 | 0.51701 |
| X207528_s_at | 0.0048 | 0.0036 | 1.354 | 0.1856122 | 0.51721 |
| X204701_s_at | -0.0606 | 0.0448 | -1.354 | 0.1856212 | 0.51721 |
| X220049_s_at | 0.0067 | 0.005 | 1.354 | 0.1856358 | 0.51721 |
| X203177_x_at | 0.0552 | 0.0408 | 1.353 | 0.1857404 | 0.517436 |
| X203951_at | -0.0026 | 0.0019 | -1.353 | 0.1857824 | 0.517488 |
| X209232_s_at | 0.0206 | 0.0152 | 1.353 | 0.1858271 | 0.517548 |
| X202764_at | -0.0087 | 0.0065 | -1.353 | 0.1858945 | 0.517671 |
| X201408_at | -0.0707 | 0.0523 | -1.353 | 0.1859452 | 0.517747 |
| X201434_at | -0.0463 | 0.0342 | -1.353 | 0.1859872 | 0.517799 |
| X205756_s_at | -0.0674 | 0.0499 | -1.352 | 0.1860624 | 0.517917 |
| X213101_s_at | -0.0279 | 0.0206 | -1.352 | 0.1860763 | 0.517917 |
| X215826_x_at | -0.0097 | 0.0072 | -1.352 | 0.1861543 | 0.51807 |
| X41553_at | -0.007 | 0.0052 | -1.352 | 0.1862874 | 0.51834 |
| X215611_at | -0.0329 | 0.0243 | -1.352 | 0.1863033 | 0.51834 |
| X207316_at | -0.005 | 0.0037 | -1.351 | 0.1863216 | 0.51834 |
| X205604_at | 0.0047 | 0.0034 | 1.351 | 0.1863953 | 0.518459 |
| X212656_at | -0.0513 | 0.0379 | -1.351 | 0.1864193 | 0.518459 |
| X212890_at | 0.0122 | 0.009 | 1.351 | 0.1864344 | 0.518459 |
| X212416_at | -0.0413 | 0.0306 | -1.351 | 0.1865946 | 0.51884 |
| X203666_at | -0.0171 | 0.0127 | -1.35 | 0.1866619 | 0.518962 |
| X208686_s_at | 0.0401 | 0.0297 | 1.35 | 0.1867084 | 0.519027 |
| X206991_s_at | 0.005 | 0.0037 | 1.35 | 0.1867789 | 0.519158 |
| X220980_s_at | 0.0055 | 0.0041 | 1.35 | 0.1868514 | 0.519294 |
| X200651_at | 0.0336 | 0.0249 | 1.349 | 0.1869912 | 0.519551 |
| X221705_s_at | 0.0667 | 0.0494 | 1.349 | 0.1869963 | 0.519551 |
| X222286_at | -0.0794 | 0.0589 | -1.349 | 0.1870319 | 0.519551 |
| X220524_at | -0.0054 | 0.004 | -1.349 | 0.1870373 | 0.519551 |
| X207086_x_at | 0.0059 | 0.0044 | 1.349 | 0.1870877 | 0.519626 |
| X204849_at | 0.0924 | 0.0685 | 1.349 | 0.1871175 | 0.519644 |
| X219341_at | -0.0962 | 0.0713 | -1.349 | 0.1872044 | 0.51982 |
| X217626_at | 0.007 | 0.0052 | 1.349 | 0.1872601 | 0.51991 |
| X202765_s_at | 0.0121 | 0.009 | 1.348 | 0.1873408 | 0.520069 |
| X221973_at | 0.1193 | 0.0885 | 1.348 | 0.1874113 | 0.520199 |
| X207380_x_at | 0.0042 | 0.0032 | 1.347 | 0.1876324 | 0.520748 |
| X200093_s_at | -0.0511 | 0.0379 | -1.347 | 0.1877079 | 0.520892 |
| X213181_s_at | 0.0121 | 0.009 | 1.347 | 0.1878535 | 0.521127 |
| X210794_s_at | 0.1005 | 0.0746 | 1.347 | 0.1878586 | 0.521127 |
| X208801_at | -0.0404 | 0.03 | -1.347 | 0.1878977 | 0.521127 |
| X218701_at | -0.0973 | 0.0723 | -1.346 | 0.1879127 | 0.521127 |
| X203578_s_at | -0.0109 | 0.0081 | -1.346 | 0.187925 | 0.521127 |
| X215832_x_at | 0.0138 | 0.0102 | 1.346 | 0.1879332 | 0.521127 |
| X219557_s_at | -0.0728 | 0.0541 | -1.346 | 0.187993 | 0.521154 |
| X213225_at | 0.0577 | 0.0429 | 1.346 | 0.1879986 | 0.521154 |
| X202391_at | 0.0802 | 0.0596 | 1.346 | 0.1880202 | 0.521154 |
| X209443_at | 0.0067 | 0.005 | 1.346 | 0.1880366 | 0.521154 |
| X212218_s_at | -0.0698 | 0.0519 | -1.346 | 0.1881598 | 0.52143 |
| X215593_at | 0.0065 | 0.0048 | 1.346 | 0.1882204 | 0.521442 |
| X208345_s_at | -0.0082 | 0.0061 | -1.345 | 0.1882378 | 0.521442 |
| X57516_at | -0.0307 | 0.0228 | -1.345 | 0.1882542 | 0.521442 |
| X208412_s_at | -0.0065 | 0.0048 | -1.345 | 0.1882686 | 0.521442 |
| X210066_s_at | 0.1935 | 0.1438 | 1.345 | 0.1882814 | 0.521442 |
| X213858_at | -0.011 | 0.0082 | -1.345 | 0.1884594 | 0.52187 |
| X218301_at | 0.0134 | 0.01 | 1.345 | 0.1885257 | 0.521946 |
| X207155_at | -0.0031 | 0.0023 | -1.344 | 0.1885777 | 0.521946 |
| X204621_s_at | 0.1619 | 0.1204 | 1.344 | 0.1885789 | 0.521946 |
| X216036_x_at | 0.0077 | 0.0057 | 1.344 | 0.1885895 | 0.521946 |
| X216942_s_at | 0.0614 | 0.0457 | 1.344 | 0.1886221 | 0.521946 |
| X207492_at | -0.0086 | 0.0064 | -1.344 | 0.1886482 | 0.521946 |
| X218268_at | 0.0467 | 0.0348 | 1.344 | 0.1886599 | 0.521946 |
| X42361_g_at | -0.015 | 0.0111 | -1.344 | 0.1886749 | 0.521946 |
| X218668_s_at | 0.0403 | 0.03 | 1.344 | 0.1887253 | 0.52197 |
| X202117_at | 0.0461 | 0.0343 | 1.344 | 0.1887428 | 0.52197 |
| X204408_at | -0.0092 | 0.0068 | -1.344 | 0.1887541 | 0.52197 |
| X220052_s_at | -0.0455 | 0.0338 | -1.344 | 0.1888529 | 0.522178 |
| X221336_at | 0.0041 | 0.003 | 1.343 | 0.1889074 | 0.522224 |
| X206548_at | 0.1008 | 0.075 | 1.343 | 0.1889167 | 0.522224 |
| X211032_at | 0.0154 | 0.0114 | 1.343 | 0.1889682 | 0.522302 |
| X215253_s_at | -0.2192 | 0.1633 | -1.343 | 0.1891458 | 0.522728 |
| X206061_s_at | 0.1504 | 0.112 | 1.342 | 0.1892919 | 0.523012 |
| X204172_at | 0.0869 | 0.0647 | 1.342 | 0.1892959 | 0.523012 |
| X217841_s_at | -0.0452 | 0.0337 | -1.342 | 0.1893883 | 0.52317 |
| X202047_s_at | 0.0568 | 0.0423 | 1.342 | 0.1894066 | 0.52317 |
| X200808_s_at | 0.0103 | 0.0077 | 1.342 | 0.1894236 | 0.52317 |
| X200968_s_at | 0.064 | 0.0477 | 1.342 | 0.1894622 | 0.523212 |
| X202730_s_at | -0.0816 | 0.0608 | -1.342 | 0.1894904 | 0.523224 |
| X205194_at | -0.064 | 0.0477 | -1.341 | 0.1895661 | 0.523338 |
| X207227_x_at | 0.1968 | 0.1467 | 1.341 | 0.1895787 | 0.523338 |
| X206273_at | 0.0038 | 0.0028 | 1.341 | 0.1896214 | 0.523355 |
| X206592_s_at | -0.0269 | 0.0201 | -1.341 | 0.1896319 | 0.523355 |
| X208710_s_at | 0.0689 | 0.0514 | 1.341 | 0.1897281 | 0.523555 |
| X207704_s_at | -0.0037 | 0.0028 | -1.341 | 0.1898012 | 0.523692 |
| X220530_at | 0.0088 | 0.0066 | 1.34 | 0.1898526 | 0.523769 |
| X211808_s_at | 0.0036 | 0.0027 | 1.34 | 0.1898835 | 0.523789 |
| X213076_at | 0.005 | 0.0037 | 1.34 | 0.1900305 | 0.524129 |
| X215580_at | -0.007 | 0.0052 | -1.34 | 0.1900848 | 0.524177 |
| X213387_at | 0.0658 | 0.0491 | 1.34 | 0.1901123 | 0.524177 |
| X207732_s_at | 0.0068 | 0.005 | 1.34 | 0.1901187 | 0.524177 |
| X205483_s_at | 0.1131 | 0.0845 | 1.339 | 0.1902244 | 0.524404 |
| X211175_at | -0.0094 | 0.007 | -1.339 | 0.1902841 | 0.524503 |
| X217988_at | 0.0385 | 0.0288 | 1.338 | 0.1905134 | 0.525035 |
| X219343_at | -0.0741 | 0.0554 | -1.338 | 0.1905242 | 0.525035 |
| X201578_at | 0.0793 | 0.0593 | 1.338 | 0.1905772 | 0.525057 |
| X218900_at | -0.0118 | 0.0088 | -1.338 | 0.1905955 | 0.525057 |
| X205263_at | 0.0643 | 0.048 | 1.338 | 0.1906548 | 0.525057 |
| X212758_s_at | 0.1077 | 0.0805 | 1.338 | 0.1906561 | 0.525057 |
| X210687_at | -0.0083 | 0.0062 | -1.338 | 0.1906806 | 0.525057 |
| X205540_s_at | -0.1831 | 0.1369 | -1.338 | 0.1906989 | 0.525057 |
| X211256_x_at | -0.0556 | 0.0415 | -1.338 | 0.1907519 | 0.525057 |
| X204983_s_at | 0.0046 | 0.0034 | 1.338 | 0.1907522 | 0.525057 |
| X212956_at | 0.0549 | 0.0411 | 1.338 | 0.1907577 | 0.525057 |
| X214471_x_at | 0.0036 | 0.0027 | 1.338 | 0.190789 | 0.525057 |
| X215545_at | 0.1183 | 0.0885 | 1.337 | 0.1908001 | 0.525057 |
| X209220_at | 0.0038 | 0.0028 | 1.337 | 0.190816 | 0.525057 |
| X31826_at | 0.0232 | 0.0174 | 1.337 | 0.1908659 | 0.52512 |
| X207930_at | -0.0047 | 0.0035 | -1.337 | 0.1908872 | 0.52512 |
| X221245_s_at | 0.0985 | 0.0736 | 1.337 | 0.1909098 | 0.52512 |
| X206572_x_at | -0.0385 | 0.0288 | -1.337 | 0.190956 | 0.525133 |
| X214400_at | -0.0036 | 0.0027 | -1.337 | 0.1909919 | 0.525133 |
| X209256_s_at | 0.0898 | 0.0672 | 1.337 | 0.19103 | 0.525133 |
| X219208_at | 0.0271 | 0.0203 | 1.337 | 0.1910313 | 0.525133 |
| X209985_s_at | -0.005 | 0.0038 | -1.337 | 0.1910325 | 0.525133 |
| X209388_at | -0.0469 | 0.0351 | -1.336 | 0.1911153 | 0.525175 |
| X208014_x_at | 0.0125 | 0.0093 | 1.336 | 0.1911428 | 0.525175 |
| X200063_s_at | -0.0266 | 0.0199 | -1.336 | 0.1911448 | 0.525175 |
| X216145_at | 0.0046 | 0.0034 | 1.336 | 0.1911891 | 0.525175 |
| X221203_s_at | 0.0159 | 0.0119 | 1.336 | 0.1911902 | 0.525175 |
| X219447_s_at | 0.0124 | 0.0093 | 1.336 | 0.1912105 | 0.525175 |
| X220488_s_at | 0.0083 | 0.0062 | 1.336 | 0.1912135 | 0.525175 |
| X219336_s_at | 0.0681 | 0.051 | 1.336 | 0.1913685 | 0.525437 |
| X205488_at | 0.0073 | 0.0055 | 1.336 | 0.1913774 | 0.525437 |
| X204742_s_at | -0.0515 | 0.0385 | -1.336 | 0.1913796 | 0.525437 |
| X221575_at | 0.0065 | 0.0049 | 1.336 | 0.1914241 | 0.525494 |
| X219697_at | 0.1841 | 0.1378 | 1.335 | 0.1914479 | 0.525494 |
| X201536_at | 0.052 | 0.0389 | 1.335 | 0.1914896 | 0.525502 |
| X213989_x_at | -0.0197 | 0.0148 | -1.335 | 0.1914979 | 0.525502 |
| X200769_s_at | 0.0064 | 0.0048 | 1.335 | 0.1915428 | 0.52556 |
| X209797_at | 0.0525 | 0.0393 | 1.335 | 0.1916428 | 0.525769 |
| X221808_at | -0.0736 | 0.0551 | -1.335 | 0.1917124 | 0.525891 |
| X201183_s_at | 0.0922 | 0.0691 | 1.335 | 0.1917514 | 0.525891 |
| X204826_at | 0.0059 | 0.0044 | 1.335 | 0.1917582 | 0.525891 |
| X219818_s_at | 0.0293 | 0.0219 | 1.334 | 0.1918018 | 0.525946 |
| X212731_at | -0.0492 | 0.0369 | -1.334 | 0.1918354 | 0.525973 |
| X218451_at | 0.0042 | 0.0031 | 1.334 | 0.1918927 | 0.526065 |
| X201308_s_at | -0.0687 | 0.0515 | -1.334 | 0.192026 | 0.526365 |
| X207971_s_at | -0.0986 | 0.074 | -1.333 | 0.1921236 | 0.526524 |
| X215758_x_at | -0.0089 | 0.0067 | -1.333 | 0.1921312 | 0.526524 |
| X203027_s_at | -0.0048 | 0.0036 | -1.333 | 0.1922073 | 0.526667 |
| X200024_at | -0.0439 | 0.0329 | -1.333 | 0.1922854 | 0.526756 |
| X209611_s_at | 0.0057 | 0.0043 | 1.333 | 0.1922871 | 0.526756 |
| X217312_s_at | 0.0074 | 0.0055 | 1.333 | 0.1923663 | 0.526908 |
| X219729_at | -0.0047 | 0.0035 | -1.333 | 0.1924075 | 0.526928 |
| X205901_at | -0.0205 | 0.0154 | -1.332 | 0.1924371 | 0.526928 |
| X205177_at | -0.0062 | 0.0046 | -1.332 | 0.1924446 | 0.526928 |
| X210225_x_at | 0.0071 | 0.0054 | 1.332 | 0.1924777 | 0.526937 |
| X203468_at | 0.0328 | 0.0246 | 1.332 | 0.192496 | 0.526937 |
| X210064_s_at | -0.0098 | 0.0073 | -1.332 | 0.1925388 | 0.526937 |
| X202719_s_at | -0.0037 | 0.0028 | -1.332 | 0.1925429 | 0.526937 |
| X217598_at | 0.0441 | 0.0331 | 1.332 | 0.1925994 | 0.526981 |
| X41329_at | -0.0256 | 0.0192 | -1.332 | 0.1926065 | 0.526981 |
| X208783_s_at | 0.0467 | 0.035 | 1.332 | 0.1926305 | 0.526982 |
| X209486_at | -0.0522 | 0.0392 | -1.332 | 0.1926974 | 0.5271 |
| X218216_x_at | -0.0553 | 0.0416 | -1.332 | 0.1927298 | 0.527114 |
| X201743_at | -0.0112 | 0.0084 | -1.331 | 0.1927504 | 0.527114 |
| X214060_at | 0.0717 | 0.0538 | 1.331 | 0.1927738 | 0.527114 |
| X204961_s_at | 0.0022 | 0.0016 | 1.331 | 0.1928663 | 0.527302 |
| X208689_s_at | -0.0639 | 0.048 | -1.33 | 0.193064 | 0.527608 |
| X203291_at | -0.046 | 0.0346 | -1.33 | 0.1930666 | 0.527608 |
| X216413_at | -0.0122 | 0.0092 | -1.33 | 0.1930697 | 0.527608 |
| X213707_s_at | -0.0068 | 0.0051 | -1.33 | 0.1930732 | 0.527608 |
| X58308_at | 0.0107 | 0.0081 | 1.33 | 0.1931188 | 0.527668 |
| X210842_at | -0.0052 | 0.0039 | -1.33 | 0.1931459 | 0.527677 |
| X206080_at | -0.0042 | 0.0031 | -1.33 | 0.1931863 | 0.52772 |
| X211745_x_at | 0.2589 | 0.1946 | 1.33 | 0.193209 | 0.52772 |
| X211208_s_at | 0.0519 | 0.039 | 1.33 | 0.1932449 | 0.527742 |
| X212700_x_at | 0.0075 | 0.0056 | 1.33 | 0.1932648 | 0.527742 |
| X222045_s_at | -0.0077 | 0.0058 | -1.33 | 0.1933243 | 0.52784 |
| X214432_at | 0.0236 | 0.0177 | 1.33 | 0.193377 | 0.527919 |
| X219782_s_at | 0.0049 | 0.0037 | 1.329 | 0.1934099 | 0.527944 |
| X200929_at | 0.0701 | 0.0527 | 1.329 | 0.19347 | 0.528016 |
| X33778_at | -0.0224 | 0.0169 | -1.329 | 0.1934838 | 0.528016 |
| X215350_at | 0.0129 | 0.0097 | 1.329 | 0.193542 | 0.52811 |
| X211225_at | -0.0062 | 0.0046 | -1.329 | 0.1936699 | 0.528394 |
| X208996_s_at | -0.0527 | 0.0397 | -1.328 | 0.193783 | 0.528638 |
| X212987_at | -0.0618 | 0.0466 | -1.328 | 0.1938537 | 0.528694 |
| X206384_at | -0.1064 | 0.0801 | -1.328 | 0.1938682 | 0.528694 |
| X206564_at | -0.0059 | 0.0044 | -1.328 | 0.1938791 | 0.528694 |
| X216019_x_at | 0.0088 | 0.0067 | 1.328 | 0.1939205 | 0.528694 |
| X203683_s_at | 0.0364 | 0.0274 | 1.328 | 0.1939225 | 0.528694 |
| X204523_at | -0.035 | 0.0264 | -1.327 | 0.1940739 | 0.528967 |
| X205315_s_at | -0.1698 | 0.128 | -1.327 | 0.1941234 | 0.528967 |
| X202285_s_at | 0.0071 | 0.0054 | 1.327 | 0.1941403 | 0.528967 |
| X210061_at | -0.0082 | 0.0062 | -1.327 | 0.1941518 | 0.528967 |
| X200874_s_at | -0.0636 | 0.0479 | -1.327 | 0.1941666 | 0.528967 |
| X217293_at | -0.0085 | 0.0064 | -1.327 | 0.1941909 | 0.528967 |
| X207517_at | -0.0121 | 0.0091 | -1.327 | 0.1941951 | 0.528967 |
| X204114_at | -0.0068 | 0.0052 | -1.327 | 0.1942134 | 0.528967 |
| X220975_s_at | 0.0031 | 0.0023 | 1.327 | 0.1942967 | 0.529129 |
| X219565_at | 0.0072 | 0.0054 | 1.326 | 0.1944489 | 0.529442 |
| X204227_s_at | 0.045 | 0.0339 | 1.326 | 0.1944592 | 0.529442 |
| X219889_at | -0.0124 | 0.0093 | -1.326 | 0.1945941 | 0.529744 |
| X206409_at | 0.0146 | 0.011 | 1.325 | 0.1947015 | 0.529946 |
| X202340_x_at | -0.0923 | 0.0696 | -1.325 | 0.194716 | 0.529946 |
| X209636_at | -0.0047 | 0.0035 | -1.325 | 0.1949206 | 0.530438 |
| X202052_s_at | -0.0284 | 0.0214 | -1.325 | 0.1949631 | 0.530464 |
| X213029_at | 0.0771 | 0.0582 | 1.325 | 0.1949884 | 0.530464 |
| X219679_s_at | 0.1101 | 0.0831 | 1.325 | 0.1950019 | 0.530464 |
| X219255_x_at | 0.1997 | 0.1508 | 1.324 | 0.1950363 | 0.53049 |
| X202153_s_at | -0.0139 | 0.0105 | -1.324 | 0.1950947 | 0.53049 |
| X207049_at | 0.0073 | 0.0055 | 1.324 | 0.1951316 | 0.53049 |
| X212874_at | 0.0048 | 0.0036 | 1.324 | 0.1951361 | 0.53049 |
| X219455_at | -0.0062 | 0.0047 | -1.324 | 0.1951572 | 0.53049 |
| X210789_x_at | 0.0033 | 0.0025 | 1.324 | 0.1951754 | 0.53049 |
| X219677_at | -0.0048 | 0.0036 | -1.324 | 0.1951795 | 0.53049 |
| X204363_at | 0.1496 | 0.113 | 1.324 | 0.1952023 | 0.53049 |
| X220973_s_at | -0.0182 | 0.0137 | -1.324 | 0.1952448 | 0.530513 |
| X216477_at | -0.007 | 0.0053 | -1.324 | 0.1952663 | 0.530513 |
| X218101_s_at | -0.0269 | 0.0203 | -1.324 | 0.1952999 | 0.530513 |
| X222074_at | 0.0139 | 0.0105 | 1.324 | 0.1953063 | 0.530513 |
| X210529_s_at | -0.1531 | 0.1157 | -1.323 | 0.1953658 | 0.53061 |
| X209839_at | -0.0553 | 0.0418 | -1.323 | 0.195492 | 0.530704 |
| X208354_s_at | -0.0043 | 0.0032 | -1.323 | 0.1955008 | 0.530704 |
| X212934_at | -0.0412 | 0.0311 | -1.323 | 0.1955095 | 0.530704 |
| X210774_s_at | 0.0353 | 0.0267 | 1.323 | 0.1955328 | 0.530704 |
| X213774_s_at | 0.0046 | 0.0035 | 1.323 | 0.1955408 | 0.530704 |
| X216382_s_at | 0.0053 | 0.004 | 1.323 | 0.1955438 | 0.530704 |
| X221936_x_at | 0.0055 | 0.0041 | 1.323 | 0.1955973 | 0.530784 |
| X214768_x_at | 0.0044 | 0.0033 | 1.322 | 0.1956879 | 0.530909 |
| X216013_at | -0.0052 | 0.0039 | -1.322 | 0.1956913 | 0.530909 |
| X213529_at | -0.014 | 0.0106 | -1.322 | 0.1957513 | 0.531007 |
| X209330_s_at | -0.06 | 0.0454 | -1.322 | 0.195896 | 0.531335 |
| X221968_s_at | -0.0054 | 0.0041 | -1.322 | 0.1959553 | 0.531431 |
| X206474_at | -0.0662 | 0.0501 | -1.321 | 0.1960552 | 0.531637 |
| X219715_s_at | -0.0086 | 0.0065 | -1.321 | 0.1961677 | 0.531875 |
| X214452_at | -0.0164 | 0.0124 | -1.321 | 0.1962083 | 0.531875 |
| X207490_at | 0.0061 | 0.0046 | 1.321 | 0.1962148 | 0.531875 |
| X221883_at | -0.0982 | 0.0743 | -1.321 | 0.1962439 | 0.531889 |
| X210111_s_at | 0.0371 | 0.0281 | 1.32 | 0.1963862 | 0.53221 |
| X216442_x_at | 0.1095 | 0.0829 | 1.32 | 0.1964237 | 0.532245 |
| X222316_at | 0.0548 | 0.0415 | 1.32 | 0.1964877 | 0.532245 |
| X205482_x_at | -0.024 | 0.0182 | -1.32 | 0.1964901 | 0.532245 |
| X208469_s_at | 0.0077 | 0.0058 | 1.32 | 0.1964952 | 0.532245 |
| X207425_s_at | 0.005 | 0.0038 | 1.32 | 0.196561 | 0.532359 |
| X220171_x_at | -0.0433 | 0.0328 | -1.32 | 0.1965991 | 0.532397 |
| X207474_at | -0.0125 | 0.0095 | -1.319 | 0.1966933 | 0.532587 |
| X211249_at | -0.0056 | 0.0043 | -1.319 | 0.1969075 | 0.533042 |
| X220348_at | 0.02 | 0.0152 | 1.319 | 0.1969098 | 0.533042 |
| X212857_x_at | -0.0483 | 0.0367 | -1.319 | 0.1969332 | 0.533042 |
| X218486_at | 0.0362 | 0.0274 | 1.318 | 0.1970227 | 0.533219 |
| X206496_at | -0.0048 | 0.0037 | -1.318 | 0.1971103 | 0.533372 |
| X222284_at | -0.0092 | 0.0069 | -1.318 | 0.1971284 | 0.533372 |
| X205198_s_at | -0.0442 | 0.0335 | -1.318 | 0.1971512 | 0.533372 |
| X217034_at | -0.0111 | 0.0084 | -1.318 | 0.197289 | 0.533642 |
| X211739_x_at | 0.004 | 0.003 | 1.318 | 0.1972989 | 0.533642 |
| X208123_at | 0.0074 | 0.0056 | 1.317 | 0.1973746 | 0.533767 |
| X34764_at | -0.0719 | 0.0546 | -1.317 | 0.1973932 | 0.533767 |
| X214583_at | 0.0579 | 0.044 | 1.317 | 0.1975767 | 0.534116 |
| X210639_s_at | -0.1089 | 0.0827 | -1.317 | 0.1975829 | 0.534116 |
| X222209_s_at | -0.0622 | 0.0473 | -1.317 | 0.1975945 | 0.534116 |
| X210018_x_at | 0.0056 | 0.0043 | 1.317 | 0.197638 | 0.53414 |
| X211699_x_at | 0.2574 | 0.1955 | 1.316 | 0.1976702 | 0.53414 |
| X219379_x_at | 0.0117 | 0.0089 | 1.316 | 0.1976793 | 0.53414 |
| X204658_at | -0.0498 | 0.0378 | -1.316 | 0.1977305 | 0.53414 |
| X203394_s_at | 0.1642 | 0.1247 | 1.316 | 0.1977698 | 0.53414 |
| X212698_s_at | 0.1448 | 0.11 | 1.316 | 0.1977803 | 0.53414 |
| X209745_at | 0.0059 | 0.0045 | 1.316 | 0.1977832 | 0.53414 |
| X214166_at | -0.0083 | 0.0063 | -1.316 | 0.1977958 | 0.53414 |
| X214522_x_at | 0.0062 | 0.0047 | 1.316 | 0.1978711 | 0.534278 |
| X205134_s_at | 0.053 | 0.0402 | 1.316 | 0.1979408 | 0.534402 |
| X218509_at | 0.0355 | 0.027 | 1.315 | 0.1980261 | 0.534567 |
| X220267_at | -0.0064 | 0.0049 | -1.315 | 0.1980783 | 0.534643 |
| X208489_at | -0.0031 | 0.0024 | -1.315 | 0.198128 | 0.534712 |
| X220063_at | -0.0129 | 0.0098 | -1.315 | 0.1982943 | 0.535081 |
| X213844_at | 0.0036 | 0.0027 | 1.314 | 0.1983273 | 0.535081 |
| X202072_at | 0.0669 | 0.0509 | 1.314 | 0.1983371 | 0.535081 |
| X215370_at | -0.0399 | 0.0304 | -1.314 | 0.198408 | 0.535208 |
| X221067_s_at | 0.006 | 0.0046 | 1.314 | 0.1984447 | 0.535242 |
| X211773_s_at | -0.0123 | 0.0094 | -1.314 | 0.1986012 | 0.535507 |
| X221590_s_at | -0.0869 | 0.0662 | -1.314 | 0.1986094 | 0.535507 |
| X215413_at | -0.0048 | 0.0037 | -1.314 | 0.1986154 | 0.535507 |
| X218320_s_at | -0.0447 | 0.034 | -1.313 | 0.1986779 | 0.535611 |
| X205926_at | -0.0103 | 0.0078 | -1.313 | 0.1988252 | 0.535929 |
| X202331_at | -0.0191 | 0.0145 | -1.313 | 0.1988444 | 0.535929 |
| X201341_at | -0.1384 | 0.1055 | -1.313 | 0.1989602 | 0.536067 |
| X214329_x_at | -0.1426 | 0.1087 | -1.313 | 0.1989626 | 0.536067 |
| X212301_at | -0.0335 | 0.0255 | -1.313 | 0.1989677 | 0.536067 |
| X213003_s_at | 0.0118 | 0.009 | 1.312 | 0.1990025 | 0.536067 |
| X202598_at | -0.0626 | 0.0477 | -1.312 | 0.1990473 | 0.536067 |
| X219135_s_at | 0.0293 | 0.0223 | 1.312 | 0.1990635 | 0.536067 |
| X217775_s_at | 0.0457 | 0.0348 | 1.312 | 0.1990703 | 0.536067 |
| X219423_x_at | 0.0078 | 0.0059 | 1.312 | 0.1990887 | 0.536067 |
| X209463_s_at | -0.0656 | 0.05 | -1.312 | 0.1991461 | 0.536121 |
| X220117_at | 0.139 | 0.106 | 1.312 | 0.199157 | 0.536121 |
| X212761_at | 0.1187 | 0.0905 | 1.312 | 0.1992736 | 0.536298 |
| X218580_x_at | -0.0279 | 0.0212 | -1.312 | 0.1992816 | 0.536298 |
| X216114_at | -0.0033 | 0.0025 | -1.312 | 0.1993146 | 0.536298 |
| X204707_s_at | -0.0026 | 0.002 | -1.312 | 0.1993193 | 0.536298 |
| X219969_at | -0.0062 | 0.0047 | -1.311 | 0.1994335 | 0.536541 |
| X209427_at | -0.0058 | 0.0044 | -1.311 | 0.1994632 | 0.536556 |
| X218943_s_at | 0.0108 | 0.0082 | 1.31 | 0.1997319 | 0.537206 |
| X204455_at | 0.0135 | 0.0103 | 1.31 | 0.1997535 | 0.537206 |
| X205663_at | 0.004 | 0.0031 | 1.31 | 0.1998062 | 0.537283 |
| X209059_s_at | -0.0375 | 0.0286 | -1.31 | 0.1998751 | 0.537402 |
| X213096_at | 0.0762 | 0.0581 | 1.31 | 0.1998988 | 0.537402 |
| X44696_at | 0.0246 | 0.0188 | 1.31 | 0.1999475 | 0.537468 |
| X213530_at | -0.0652 | 0.0498 | -1.309 | 0.1999926 | 0.537524 |
| X216491_x_at | -0.0035 | 0.0027 | -1.309 | 0.2000196 | 0.537532 |
| X201446_s_at | 0.1191 | 0.0909 | 1.309 | 0.2000998 | 0.537616 |
| X207742_s_at | 0.0027 | 0.0021 | 1.309 | 0.2001133 | 0.537616 |
| X201518_at | -0.0228 | 0.0174 | -1.309 | 0.2001237 | 0.537616 |
| X213811_x_at | 0.0455 | 0.0348 | 1.309 | 0.2001644 | 0.537661 |
| X221479_s_at | 0.0776 | 0.0593 | 1.309 | 0.2001932 | 0.537673 |
| X36004_at | -0.006 | 0.0046 | -1.308 | 0.2003556 | 0.537966 |
| X203345_s_at | -0.0767 | 0.0587 | -1.308 | 0.2003722 | 0.537966 |
| X220302_at | 0.0087 | 0.0067 | 1.308 | 0.200375 | 0.537966 |
| X201851_at | 0.0079 | 0.0061 | 1.308 | 0.2004323 | 0.538055 |
| X208482_at | -0.003 | 0.0023 | -1.308 | 0.2005208 | 0.538227 |
| X219113_x_at | -0.0126 | 0.0097 | -1.308 | 0.2005681 | 0.538289 |
| X200628_s_at | -0.0141 | 0.0108 | -1.307 | 0.2006853 | 0.538379 |
| X203364_s_at | 0.0659 | 0.0504 | 1.307 | 0.2006989 | 0.538379 |
| X216697_at | -0.0225 | 0.0172 | -1.307 | 0.2007024 | 0.538379 |
| X221735_at | -0.0607 | 0.0465 | -1.307 | 0.2007143 | 0.538379 |
| X205950_s_at | -0.0127 | 0.0097 | -1.307 | 0.2007423 | 0.538379 |
| X217804_s_at | -0.0532 | 0.0407 | -1.307 | 0.2007471 | 0.538379 |
| X209218_at | -0.084 | 0.0643 | -1.307 | 0.2008247 | 0.538522 |
| X208615_s_at | 0.0902 | 0.0691 | 1.307 | 0.2008669 | 0.538571 |
| X201495_x_at | -0.0061 | 0.0046 | -1.307 | 0.200895 | 0.538581 |
| X203043_at | 0.0222 | 0.017 | 1.307 | 0.2009477 | 0.538584 |
| X213001_at | -0.0234 | 0.0179 | -1.307 | 0.2009477 | 0.538584 |
| X211296_x_at | -0.0281 | 0.0215 | -1.307 | 0.2009872 | 0.538584 |
| X217043_s_at | 0.052 | 0.0398 | 1.306 | 0.2010098 | 0.538584 |
| X209017_s_at | 0.0561 | 0.043 | 1.306 | 0.2010173 | 0.538584 |
| X214023_x_at | 0.0712 | 0.0545 | 1.306 | 0.2010488 | 0.538603 |
| X212323_s_at | 0.0444 | 0.034 | 1.306 | 0.2011983 | 0.538939 |
| X212291_at | 0.1164 | 0.0891 | 1.306 | 0.2012302 | 0.538959 |
| X204352_at | -0.1717 | 0.1315 | -1.305 | 0.201336 | 0.539178 |
| X220731_s_at | 0.0077 | 0.0059 | 1.305 | 0.2013737 | 0.539214 |
| X208879_x_at | 0.1676 | 0.1284 | 1.305 | 0.2014257 | 0.539263 |
| X212292_at | 0.0097 | 0.0074 | 1.305 | 0.2014547 | 0.539263 |
| X211896_s_at | 0.1872 | 0.1434 | 1.305 | 0.2014727 | 0.539263 |
| X220659_s_at | 0.0377 | 0.0289 | 1.305 | 0.2014893 | 0.539263 |
| X210148_at | 0.0109 | 0.0084 | 1.305 | 0.2015271 | 0.539299 |
| X217664_at | -0.0066 | 0.005 | -1.305 | 0.201553 | 0.539304 |
| X211441_x_at | -0.0059 | 0.0045 | -1.305 | 0.2015876 | 0.539331 |
| X204948_s_at | 0.0058 | 0.0044 | 1.305 | 0.2016174 | 0.539346 |
| X220811_at | 0.0043 | 0.0033 | 1.304 | 0.2017258 | 0.539445 |
| X204460_s_at | -0.0828 | 0.0635 | -1.304 | 0.2017271 | 0.539445 |
| X221039_s_at | -0.09 | 0.069 | -1.304 | 0.2017272 | 0.539445 |
| X219531_at | -0.0928 | 0.0712 | -1.304 | 0.2018854 | 0.539803 |
| X200925_at | -0.0351 | 0.0269 | -1.304 | 0.2019304 | 0.539858 |
| X218050_at | 0.0446 | 0.0342 | 1.304 | 0.2019681 | 0.539894 |
| X203664_s_at | 0.0121 | 0.0093 | 1.303 | 0.2021408 | 0.540256 |
| X217678_at | -0.0734 | 0.0563 | -1.303 | 0.2021582 | 0.540256 |
| X203375_s_at | 0.0534 | 0.041 | 1.303 | 0.2021857 | 0.540256 |
| X211155_s_at | 0.0079 | 0.0061 | 1.303 | 0.2022236 | 0.540256 |
| X210167_s_at | -0.0083 | 0.0063 | -1.303 | 0.2022252 | 0.540256 |
| X210832_x_at | 0.0067 | 0.0051 | 1.303 | 0.2023003 | 0.540392 |
| X204380_s_at | 0.0057 | 0.0044 | 1.302 | 0.2023748 | 0.540481 |
| X213165_at | 0.0673 | 0.0517 | 1.302 | 0.2023824 | 0.540481 |
| X216974_at | -0.0148 | 0.0114 | -1.302 | 0.2024085 | 0.540486 |
| X216111_x_at | -0.0672 | 0.0516 | -1.302 | 0.2024703 | 0.540578 |
| X209041_s_at | -0.0086 | 0.0066 | -1.302 | 0.202535 | 0.540578 |
| X213515_x_at | 0.0074 | 0.0057 | 1.302 | 0.202536 | 0.540578 |
| X216745_x_at | -0.028 | 0.0215 | -1.302 | 0.2025404 | 0.540578 |
| X32029_at | 0.0382 | 0.0294 | 1.302 | 0.2025834 | 0.540628 |
| X212822_at | 0.0566 | 0.0435 | 1.301 | 0.2027765 | 0.541078 |
| X214164_x_at | -0.1212 | 0.0932 | -1.301 | 0.2028566 | 0.541181 |
| X216136_at | -0.0102 | 0.0078 | -1.301 | 0.2028637 | 0.541181 |
| X217257_at | -0.021 | 0.0162 | -1.301 | 0.2029633 | 0.541382 |
| X212919_at | -0.0426 | 0.0328 | -1.3 | 0.2030707 | 0.54159 |
| X220223_at | -0.0066 | 0.005 | -1.3 | 0.2030901 | 0.54159 |
| X209832_s_at | -0.0023 | 0.0018 | -1.3 | 0.203183 | 0.541773 |
| X214714_at | -0.012 | 0.0092 | -1.3 | 0.2032225 | 0.541802 |
| X221162_at | 0.0052 | 0.004 | 1.3 | 0.2032738 | 0.541802 |
| X213493_at | 0.0054 | 0.0041 | 1.3 | 0.2032979 | 0.541802 |
| X212849_at | 0.0088 | 0.0068 | 1.3 | 0.2033027 | 0.541802 |
| X201934_at | 0.0405 | 0.0312 | 1.3 | 0.2033336 | 0.541802 |
| X211379_x_at | -0.1088 | 0.0837 | -1.3 | 0.2033405 | 0.541802 |
| X205672_at | -0.0451 | 0.0347 | -1.299 | 0.2034154 | 0.541872 |
| X212147_at | 0.0297 | 0.0229 | 1.299 | 0.2034265 | 0.541872 |
| X207304_at | -0.0074 | 0.0057 | -1.299 | 0.2034398 | 0.541872 |
| X211720_x_at | 0.0436 | 0.0336 | 1.299 | 0.2035808 | 0.542183 |
| X212409_s_at | -0.071 | 0.0547 | -1.299 | 0.2036439 | 0.542184 |
| X212499_s_at | 0.0503 | 0.0388 | 1.299 | 0.2036708 | 0.542184 |
| X221315_s_at | -0.005 | 0.0039 | -1.298 | 0.2037001 | 0.542184 |
| X217812_at | -0.041 | 0.0316 | -1.298 | 0.2037119 | 0.542184 |
| X209453_at | -0.0494 | 0.0381 | -1.298 | 0.2037146 | 0.542184 |
| X219524_s_at | -0.01 | 0.0077 | -1.298 | 0.2037462 | 0.542184 |
| X214898_x_at | 0.0028 | 0.0021 | 1.298 | 0.2037522 | 0.542184 |
| X202315_s_at | -0.0826 | 0.0637 | -1.298 | 0.2037941 | 0.542231 |
| X213776_at | -0.0828 | 0.0638 | -1.298 | 0.2038693 | 0.542366 |
| X206589_at | 0.0102 | 0.0079 | 1.298 | 0.2039443 | 0.5425 |
| X211110_s_at | 0.0052 | 0.004 | 1.297 | 0.204035 | 0.542676 |
| X221444_at | 0.0068 | 0.0052 | 1.297 | 0.2040652 | 0.542692 |
| X211491_at | -0.0081 | 0.0062 | -1.297 | 0.2041142 | 0.542757 |
| X207376_at | 0.0027 | 0.0021 | 1.297 | 0.2042293 | 0.542998 |
| X214851_at | 0.0037 | 0.0029 | 1.297 | 0.2042582 | 0.54301 |
| X213777_s_at | -0.0029 | 0.0022 | -1.296 | 0.2044138 | 0.543359 |
| X220020_at | 0.0046 | 0.0035 | 1.296 | 0.2044451 | 0.543377 |
| X217592_at | 0.0093 | 0.0072 | 1.296 | 0.2045744 | 0.543655 |
| X209706_at | -0.0304 | 0.0235 | -1.296 | 0.2046466 | 0.543782 |
| X207213_s_at | -0.138 | 0.1065 | -1.296 | 0.2047113 | 0.543861 |
| X201755_at | 0.005 | 0.0039 | 1.295 | 0.2047253 | 0.543861 |
| X222237_s_at | -0.0802 | 0.0619 | -1.295 | 0.2047734 | 0.543924 |
| X211289_x_at | 0.0481 | 0.0371 | 1.295 | 0.2048837 | 0.544118 |
| X212202_s_at | 0.0559 | 0.0432 | 1.295 | 0.2049067 | 0.544118 |
| X211138_s_at | -0.0055 | 0.0042 | -1.295 | 0.204966 | 0.544118 |
| X201971_s_at | -0.1913 | 0.1477 | -1.295 | 0.2049906 | 0.544118 |
| X201033_x_at | 0.0459 | 0.0354 | 1.295 | 0.2049932 | 0.544118 |
| X218263_s_at | -0.0369 | 0.0285 | -1.295 | 0.2049935 | 0.544118 |
| X218681_s_at | -0.0266 | 0.0205 | -1.294 | 0.2051055 | 0.544312 |
| X217039_x_at | 0.0617 | 0.0477 | 1.294 | 0.2051389 | 0.544312 |
| X211960_s_at | -0.0615 | 0.0475 | -1.294 | 0.20514 | 0.544312 |
| X210949_s_at | -0.0725 | 0.056 | -1.294 | 0.2053059 | 0.544687 |
| X207328_at | -0.0094 | 0.0072 | -1.293 | 0.2055032 | 0.545016 |
| X212429_s_at | -0.0385 | 0.0297 | -1.293 | 0.2055153 | 0.545016 |
| X220122_at | -0.3682 | 0.2847 | -1.293 | 0.2055477 | 0.545016 |
| X208684_at | -0.0367 | 0.0284 | -1.293 | 0.2055616 | 0.545016 |
| X222175_s_at | -0.0091 | 0.007 | -1.293 | 0.2055705 | 0.545016 |
| X210180_s_at | -0.0104 | 0.008 | -1.293 | 0.2055772 | 0.545016 |
| X205642_at | 0.0263 | 0.0203 | 1.293 | 0.2056291 | 0.545089 |
| X219408_at | -0.0059 | 0.0046 | -1.293 | 0.205672 | 0.545138 |
| X202829_s_at | 0.0598 | 0.0463 | 1.293 | 0.2057253 | 0.545214 |
| X206678_at | 0.1056 | 0.0817 | 1.292 | 0.2057783 | 0.545289 |
| X212876_at | -0.0211 | 0.0163 | -1.292 | 0.2058462 | 0.545404 |
| X219131_at | -0.0094 | 0.0073 | -1.292 | 0.2060436 | 0.545784 |
| X217143_s_at | 0.008 | 0.0062 | 1.292 | 0.20606 | 0.545784 |
| X213080_x_at | -0.0227 | 0.0176 | -1.292 | 0.2060633 | 0.545784 |
| X210988_s_at | 0.0089 | 0.0069 | 1.291 | 0.2063596 | 0.546406 |
| X210507_s_at | -0.0057 | 0.0044 | -1.291 | 0.2063688 | 0.546406 |
| X216302_at | -0.0049 | 0.0038 | -1.291 | 0.2063721 | 0.546406 |
| X207096_at | -0.0044 | 0.0034 | -1.291 | 0.2064019 | 0.546411 |
| X205874_at | -0.1861 | 0.1442 | -1.29 | 0.2064231 | 0.546411 |
| X207443_at | -0.108 | 0.0837 | -1.29 | 0.2064648 | 0.546425 |
| X206735_at | -0.0057 | 0.0044 | -1.29 | 0.206494 | 0.546425 |
| X200749_at | -0.0516 | 0.04 | -1.29 | 0.2065313 | 0.546425 |
| X208465_at | 0.0046 | 0.0036 | 1.29 | 0.2065509 | 0.546425 |
| X40225_at | 0.0304 | 0.0236 | 1.29 | 0.2065514 | 0.546425 |
| X210598_at | 0.0713 | 0.0553 | 1.29 | 0.2065959 | 0.546478 |
| X203486_s_at | 0.0446 | 0.0346 | 1.29 | 0.2066559 | 0.546572 |
| X203100_s_at | -0.0489 | 0.0379 | -1.289 | 0.2068185 | 0.546817 |
| X206501_x_at | -0.0065 | 0.005 | -1.289 | 0.2068268 | 0.546817 |
| X201690_s_at | 0.0315 | 0.0244 | 1.289 | 0.2068639 | 0.546817 |
| X217915_s_at | -0.0804 | 0.0624 | -1.289 | 0.2068662 | 0.546817 |
| X216352_x_at | 0.0024 | 0.0019 | 1.289 | 0.2068716 | 0.546817 |
| X213112_s_at | -0.0046 | 0.0036 | -1.289 | 0.2069003 | 0.546827 |
| X210248_at | -0.0036 | 0.0028 | -1.289 | 0.2070419 | 0.547076 |
| X213036_x_at | 0.0031 | 0.0024 | 1.289 | 0.2070438 | 0.547076 |
| X216822_x_at | -0.0261 | 0.0203 | -1.289 | 0.2070993 | 0.547158 |
| X221957_at | 0.0197 | 0.0153 | 1.288 | 0.207287 | 0.547578 |
| X205046_at | -0.0071 | 0.0055 | -1.288 | 0.2073296 | 0.547578 |
| X203110_at | -0.0376 | 0.0292 | -1.288 | 0.2073323 | 0.547578 |
| X212960_at | -0.0087 | 0.0068 | -1.288 | 0.2073641 | 0.547597 |
| X222058_at | 0.0452 | 0.0351 | 1.287 | 0.2074763 | 0.547828 |
| X201409_s_at | -0.0412 | 0.032 | -1.287 | 0.207506 | 0.547842 |
| X210495_x_at | 0.1089 | 0.0846 | 1.287 | 0.2075507 | 0.547894 |
| X216466_at | -0.0349 | 0.0271 | -1.287 | 0.2075823 | 0.547913 |
| X210041_s_at | -0.0285 | 0.0222 | -1.287 | 0.2076096 | 0.54792 |
| X214505_s_at | -0.1307 | 0.1015 | -1.287 | 0.2076713 | 0.548017 |
| X200674_s_at | 0.0264 | 0.0205 | 1.286 | 0.2078644 | 0.548462 |
| X218534_s_at | -0.0461 | 0.0359 | -1.286 | 0.2080277 | 0.548808 |
| X215373_x_at | 0.0761 | 0.0592 | 1.286 | 0.2080494 | 0.548808 |
| X206575_at | -0.004 | 0.0031 | -1.286 | 0.2080699 | 0.548808 |
| X213771_at | -0.0061 | 0.0047 | -1.285 | 0.2082485 | 0.549135 |
| X204464_s_at | 0.0188 | 0.0146 | 1.285 | 0.2082593 | 0.549135 |
| X214707_x_at | 0.0955 | 0.0743 | 1.285 | 0.2082679 | 0.549135 |
| X206704_at | 0.0141 | 0.0109 | 1.285 | 0.2083329 | 0.549138 |
| X222254_at | -0.006 | 0.0047 | -1.285 | 0.2083349 | 0.549138 |
| X220034_at | 0.0103 | 0.008 | 1.285 | 0.2083432 | 0.549138 |
| X204873_at | -0.0538 | 0.0419 | -1.285 | 0.2083727 | 0.549151 |
| X219471_at | 0.0374 | 0.0291 | 1.285 | 0.2084578 | 0.549252 |
| X215734_at | 0.1037 | 0.0808 | 1.285 | 0.2084707 | 0.549252 |
| X202932_at | 0.0709 | 0.0552 | 1.284 | 0.2084922 | 0.549252 |
| X201803_at | -0.0373 | 0.029 | -1.284 | 0.2085102 | 0.549252 |
| X203381_s_at | -0.1393 | 0.1085 | -1.284 | 0.2086194 | 0.549475 |
| X220539_at | 0.0077 | 0.006 | 1.284 | 0.2086799 | 0.549569 |
| X201622_at | -0.0176 | 0.0137 | -1.284 | 0.2087602 | 0.549677 |
| X204945_at | -0.0231 | 0.018 | -1.284 | 0.2087955 | 0.549677 |
| X218446_s_at | 0.0511 | 0.0398 | 1.284 | 0.2087969 | 0.549677 |
| X218807_at | -0.0901 | 0.0702 | -1.284 | 0.20882 | 0.549677 |
| X217374_x_at | 0.0046 | 0.0036 | 1.283 | 0.2088455 | 0.549679 |
| X215893_x_at | -0.011 | 0.0085 | -1.283 | 0.2089296 | 0.549836 |
| X202498_s_at | -0.1137 | 0.0887 | -1.283 | 0.2089718 | 0.549875 |
| X217230_at | 0.004 | 0.0031 | 1.283 | 0.2089979 | 0.549875 |
| X208260_at | -0.0057 | 0.0044 | -1.283 | 0.2090188 | 0.549875 |
| X203250_at | -0.0417 | 0.0325 | -1.283 | 0.2091153 | 0.549969 |
| X210573_s_at | -0.0126 | 0.0098 | -1.283 | 0.2091333 | 0.549969 |
| X208588_at | -0.0157 | 0.0122 | -1.282 | 0.2092118 | 0.549969 |
| X204784_s_at | -0.0899 | 0.0701 | -1.282 | 0.2092144 | 0.549969 |
| X215586_at | 0.0262 | 0.0204 | 1.282 | 0.2092163 | 0.549969 |
| X218768_at | -0.0674 | 0.0525 | -1.282 | 0.2092236 | 0.549969 |
| X207070_at | 0.0077 | 0.006 | 1.282 | 0.2092279 | 0.549969 |
| X217112_at | 0.0023 | 0.0018 | 1.282 | 0.2092988 | 0.550045 |
| X213083_at | 0.117 | 0.0913 | 1.282 | 0.2093062 | 0.550045 |
| X215873_x_at | 0.0106 | 0.0083 | 1.282 | 0.2094292 | 0.550259 |
| X220875_at | -0.0041 | 0.0032 | -1.282 | 0.2094372 | 0.550259 |
| X203576_at | 0.0282 | 0.022 | 1.281 | 0.2095265 | 0.550421 |
| X216038_x_at | -0.014 | 0.0109 | -1.281 | 0.2095486 | 0.550421 |
| X202018_s_at | 0.0054 | 0.0042 | 1.281 | 0.2095949 | 0.550478 |
| X209490_s_at | 0.0044 | 0.0034 | 1.281 | 0.2096496 | 0.5505 |
| X208725_at | 0.0045 | 0.0035 | 1.281 | 0.2096529 | 0.5505 |
| X204414_at | -0.0303 | 0.0237 | -1.281 | 0.209751 | 0.550692 |
| X205805_s_at | 0.004 | 0.0031 | 1.28 | 0.2099105 | 0.550998 |
| X200860_s_at | -0.047 | 0.0367 | -1.28 | 0.2099341 | 0.550998 |
| X209629_s_at | -0.0076 | 0.006 | -1.28 | 0.209942 | 0.550998 |
| X38918_at | 0.0056 | 0.0044 | 1.28 | 0.2099667 | 0.550998 |
| X213640_s_at | 0.0042 | 0.0033 | 1.28 | 0.2100507 | 0.551134 |
| X213677_s_at | -0.0329 | 0.0257 | -1.28 | 0.2100898 | 0.551134 |
| X213638_at | -0.239 | 0.1868 | -1.28 | 0.2100927 | 0.551134 |
| X202521_at | -0.0451 | 0.0352 | -1.279 | 0.2102392 | 0.551353 |
| X202716_at | 0.0334 | 0.0261 | 1.279 | 0.2102471 | 0.551353 |
| X201262_s_at | 0.0042 | 0.0033 | 1.279 | 0.2102653 | 0.551353 |
| X201914_s_at | 0.0608 | 0.0475 | 1.279 | 0.2102757 | 0.551353 |
| X33760_at | -0.0282 | 0.022 | -1.279 | 0.2103139 | 0.551388 |
| X220399_at | -0.0093 | 0.0072 | -1.279 | 0.2103805 | 0.551453 |
| X206427_s_at | 0.0053 | 0.0041 | 1.279 | 0.2103884 | 0.551453 |
| X203582_s_at | 0.085 | 0.0664 | 1.279 | 0.2104242 | 0.551482 |
| X219761_at | 0.0158 | 0.0124 | 1.279 | 0.2104654 | 0.551525 |
| X214880_x_at | -0.0371 | 0.0291 | -1.278 | 0.2106856 | 0.551999 |
| X59999_at | 0.0587 | 0.0459 | 1.278 | 0.2107472 | 0.551999 |
| X209738_x_at | 0.0045 | 0.0035 | 1.278 | 0.2107518 | 0.551999 |
| X209185_s_at | -0.0663 | 0.0519 | -1.278 | 0.2107638 | 0.551999 |
| X200645_at | -0.0242 | 0.019 | -1.278 | 0.2107704 | 0.551999 |
| X208068_x_at | 0.0046 | 0.0036 | 1.278 | 0.2108387 | 0.552113 |
| X221599_at | 0.0409 | 0.032 | 1.278 | 0.2108705 | 0.552131 |
| X202982_s_at | -0.1116 | 0.0873 | -1.277 | 0.2109673 | 0.552319 |
| X218450_at | -0.0874 | 0.0684 | -1.277 | 0.2110262 | 0.552408 |
| X218465_at | -0.102 | 0.0798 | -1.277 | 0.2110698 | 0.552457 |
| X38892_at | 0.0287 | 0.0225 | 1.277 | 0.2111195 | 0.552504 |
| X222182_s_at | 0.0452 | 0.0354 | 1.277 | 0.2111373 | 0.552504 |
| X219369_s_at | -0.0095 | 0.0075 | -1.277 | 0.2111688 | 0.552521 |
| X214824_at | 0.0039 | 0.0031 | 1.277 | 0.2112496 | 0.552667 |
| X214961_at | -0.0671 | 0.0525 | -1.276 | 0.2113606 | 0.55286 |
| X210168_at | 0.0054 | 0.0043 | 1.276 | 0.2113896 | 0.55286 |
| X213253_at | -0.0603 | 0.0473 | -1.276 | 0.2113977 | 0.55286 |
| X213665_at | -0.0027 | 0.0022 | -1.275 | 0.2116313 | 0.553405 |
| X218359_at | 0.007 | 0.0055 | 1.275 | 0.2116781 | 0.553439 |
| X205291_at | 0.0058 | 0.0046 | 1.275 | 0.2116966 | 0.553439 |
| X219535_at | 0.0059 | 0.0047 | 1.275 | 0.2117191 | 0.553439 |
| X222007_s_at | -0.004 | 0.0031 | -1.275 | 0.2119263 | 0.553916 |
| X218767_at | 0.008 | 0.0063 | 1.274 | 0.2120855 | 0.554163 |
| X210567_s_at | 0.0112 | 0.0088 | 1.274 | 0.2121102 | 0.554163 |
| X222380_s_at | 0.1833 | 0.1439 | 1.274 | 0.2121193 | 0.554163 |
| X205244_s_at | 0.0089 | 0.007 | 1.274 | 0.2121326 | 0.554163 |
| X211077_s_at | -0.014 | 0.011 | -1.274 | 0.2121456 | 0.554163 |
| X205773_at | 0.0584 | 0.0459 | 1.274 | 0.2122292 | 0.55426 |
| X215069_at | -0.1098 | 0.0862 | -1.274 | 0.2122459 | 0.55426 |
| X216840_s_at | -0.0252 | 0.0198 | -1.274 | 0.2122577 | 0.55426 |
| X219206_x_at | -0.0422 | 0.0331 | -1.273 | 0.2123335 | 0.554366 |
| X216882_s_at | -0.0069 | 0.0054 | -1.273 | 0.2123479 | 0.554366 |
| X216563_at | 0.1599 | 0.1256 | 1.273 | 0.2124237 | 0.554498 |
| X210423_s_at | 0.005 | 0.004 | 1.273 | 0.2125202 | 0.554685 |
| X202853_s_at | 0.0476 | 0.0374 | 1.273 | 0.2126098 | 0.554854 |
| X212433_x_at | 0.0441 | 0.0346 | 1.272 | 0.2126812 | 0.554923 |
| X202534_x_at | 0.0679 | 0.0533 | 1.272 | 0.2126947 | 0.554923 |
| X213139_at | 0.0538 | 0.0423 | 1.272 | 0.2127315 | 0.554923 |
| X203672_x_at | 0.0492 | 0.0387 | 1.272 | 0.212742 | 0.554923 |
| X216416_at | 0.0044 | 0.0034 | 1.272 | 0.2127611 | 0.554923 |
| X203793_x_at | 0.012 | 0.0095 | 1.272 | 0.2128383 | 0.555059 |
| X214896_at | -0.077 | 0.0606 | -1.272 | 0.2129449 | 0.555174 |
| X209272_at | 0.0691 | 0.0543 | 1.272 | 0.212955 | 0.555174 |
| X214709_s_at | 0.034 | 0.0267 | 1.272 | 0.2129574 | 0.555174 |
| X203553_s_at | -0.0704 | 0.0554 | -1.271 | 0.2131954 | 0.555727 |
| X222038_s_at | 0.0076 | 0.006 | 1.271 | 0.2132197 | 0.555727 |
| X203173_s_at | -0.0259 | 0.0204 | -1.27 | 0.2133617 | 0.555929 |
| X214754_at | 0.0061 | 0.0048 | 1.27 | 0.2133793 | 0.555929 |
| X218686_s_at | 0.0148 | 0.0116 | 1.27 | 0.2133941 | 0.555929 |
| X217036_at | -0.0075 | 0.0059 | -1.27 | 0.213444 | 0.555929 |
| X210862_s_at | -0.0033 | 0.0026 | -1.27 | 0.2134467 | 0.555929 |
| X214705_at | -0.0489 | 0.0385 | -1.27 | 0.2134594 | 0.555929 |
| X220427_at | -0.0044 | 0.0035 | -1.27 | 0.2134724 | 0.555929 |
| X220757_s_at | -0.0639 | 0.0503 | -1.27 | 0.2136041 | 0.556145 |
| X215909_x_at | -0.0163 | 0.0128 | -1.27 | 0.2136055 | 0.556145 |
| X220691_at | 0.061 | 0.048 | 1.27 | 0.2136896 | 0.556299 |
| X216364_s_at | -0.0032 | 0.0025 | -1.269 | 0.213752 | 0.556356 |
| X221282_x_at | 0.0031 | 0.0024 | 1.269 | 0.2137616 | 0.556356 |
| X219647_at | -0.0058 | 0.0046 | -1.269 | 0.2139094 | 0.556676 |
| X213910_at | 0.0159 | 0.0125 | 1.269 | 0.2139674 | 0.556721 |
| X220815_at | 0.0064 | 0.0051 | 1.269 | 0.213977 | 0.556721 |
| X216323_x_at | 0.0457 | 0.036 | 1.269 | 0.2140138 | 0.556745 |
| X200900_s_at | -0.0847 | 0.0668 | -1.269 | 0.2140485 | 0.556745 |
| X212445_s_at | -0.0781 | 0.0616 | -1.269 | 0.2140612 | 0.556745 |
| X205147_x_at | 0.0047 | 0.0037 | 1.268 | 0.2141519 | 0.556915 |
| X213313_at | 0.0375 | 0.0295 | 1.268 | 0.2142102 | 0.556949 |
| X202483_s_at | -0.0672 | 0.053 | -1.268 | 0.2142334 | 0.556949 |
| X220035_at | 0.0042 | 0.0033 | 1.268 | 0.2142401 | 0.556949 |
| X205682_x_at | 0.0082 | 0.0065 | 1.268 | 0.2142792 | 0.556966 |
| X220083_x_at | 0.0142 | 0.0112 | 1.268 | 0.2142967 | 0.556966 |
| X209406_at | -0.0515 | 0.0406 | -1.267 | 0.2144712 | 0.557354 |
| X202728_s_at | 0.0092 | 0.0073 | 1.267 | 0.2145157 | 0.557405 |
| X209878_s_at | 0.003 | 0.0023 | 1.267 | 0.2146656 | 0.557729 |
| X206527_at | 0.0052 | 0.0041 | 1.267 | 0.214758 | 0.557856 |
| X221432_s_at | 0.0263 | 0.0207 | 1.267 | 0.2147648 | 0.557856 |
| X212402_at | 0.0638 | 0.0503 | 1.266 | 0.2148017 | 0.557887 |
| X216452_at | 0.1406 | 0.111 | 1.266 | 0.2149007 | 0.558079 |
| X216645_at | 0.0079 | 0.0062 | 1.266 | 0.2149361 | 0.558101 |
| X206821_x_at | 0.003 | 0.0024 | 1.266 | 0.214973 | 0.558101 |
| X214186_s_at | 0.0078 | 0.0061 | 1.266 | 0.2149847 | 0.558101 |
| X212649_at | -0.0087 | 0.0069 | -1.266 | 0.2150147 | 0.558114 |
| X57540_at | -0.068 | 0.0537 | -1.266 | 0.2151038 | 0.55828 |
| X203209_at | -0.1003 | 0.0792 | -1.265 | 0.2151412 | 0.558312 |
| X202939_at | -0.0397 | 0.0314 | -1.265 | 0.2153502 | 0.558779 |
| X204877_s_at | -0.0071 | 0.0056 | -1.265 | 0.2153961 | 0.558779 |
| X201883_s_at | 0.0082 | 0.0065 | 1.265 | 0.2154054 | 0.558779 |
| X206661_at | -0.0059 | 0.0047 | -1.265 | 0.2154218 | 0.558779 |
| X207937_x_at | 0.0142 | 0.0113 | 1.265 | 0.2154691 | 0.558836 |
| X202511_s_at | 0.0535 | 0.0423 | 1.264 | 0.2155004 | 0.558852 |
| X214095_at | -0.0057 | 0.0045 | -1.263 | 0.2159692 | 0.559955 |
| X217817_at | -0.0329 | 0.0261 | -1.263 | 0.2159763 | 0.559955 |
| X216828_at | -0.0168 | 0.0133 | -1.263 | 0.2161031 | 0.560219 |
| X219674_s_at | -0.0055 | 0.0044 | -1.262 | 0.2162457 | 0.560434 |
| X209578_s_at | 0.0237 | 0.0187 | 1.262 | 0.2162559 | 0.560434 |
| X212469_at | -0.043 | 0.0341 | -1.262 | 0.216262 | 0.560434 |
| X219625_s_at | -0.0776 | 0.0615 | -1.262 | 0.2163447 | 0.560583 |
| X207522_s_at | -0.0042 | 0.0033 | -1.262 | 0.2164002 | 0.560662 |
| X212479_s_at | -0.0223 | 0.0176 | -1.262 | 0.2164588 | 0.560738 |
| X208960_s_at | -0.0056 | 0.0044 | -1.262 | 0.21648 | 0.560738 |
| X219043_s_at | -0.0467 | 0.0371 | -1.261 | 0.2165986 | 0.56098 |
| X212192_at | -0.089 | 0.0706 | -1.261 | 0.2166446 | 0.561033 |
| X202159_at | -0.0709 | 0.0562 | -1.261 | 0.2166772 | 0.561052 |
| X218181_s_at | 0.114 | 0.0904 | 1.261 | 0.2167198 | 0.561097 |
| X201490_s_at | -0.0655 | 0.0519 | -1.261 | 0.216748 | 0.561105 |
| X48659_at | -0.0074 | 0.0058 | -1.261 | 0.2168191 | 0.561223 |
| X208967_s_at | 0.0723 | 0.0574 | 1.261 | 0.2168489 | 0.561235 |
| X210185_at | -0.0072 | 0.0057 | -1.26 | 0.2169018 | 0.561307 |
| X201589_at | 0.0853 | 0.0677 | 1.26 | 0.2169367 | 0.561332 |
| X220640_at | -0.0061 | 0.0048 | -1.26 | 0.2170741 | 0.561622 |
| X211564_s_at | 0.005 | 0.0039 | 1.26 | 0.2171532 | 0.561703 |
| X207046_at | -0.0049 | 0.0039 | -1.26 | 0.2171776 | 0.561703 |
| X217055_x_at | -0.0075 | 0.0059 | -1.26 | 0.2171812 | 0.561703 |
| X218711_s_at | -0.1008 | 0.08 | -1.26 | 0.2172191 | 0.561723 |
| X206954_at | -0.0051 | 0.004 | -1.26 | 0.2172398 | 0.561723 |
| X212779_at | -0.0391 | 0.031 | -1.259 | 0.217302 | 0.561819 |
| X211791_s_at | -0.0035 | 0.0028 | -1.259 | 0.2173309 | 0.561828 |
| X201525_at | 0.1436 | 0.1141 | 1.259 | 0.2173771 | 0.561852 |
| X219906_at | 0.0101 | 0.0081 | 1.259 | 0.217415 | 0.561852 |
| X218312_s_at | -0.0291 | 0.0231 | -1.259 | 0.2174162 | 0.561852 |
| X207433_at | -0.0091 | 0.0072 | -1.259 | 0.2174712 | 0.561903 |
| X217755_at | -0.0835 | 0.0664 | -1.259 | 0.2175086 | 0.561903 |
| X217604_at | -0.0557 | 0.0442 | -1.259 | 0.2175115 | 0.561903 |
| X203574_at | -0.1092 | 0.0867 | -1.259 | 0.2175524 | 0.561943 |
| X221044_s_at | -0.0072 | 0.0057 | -1.258 | 0.2177583 | 0.562409 |
| X206088_at | -0.0239 | 0.019 | -1.258 | 0.2178241 | 0.562462 |
| X215537_x_at | 0.0545 | 0.0433 | 1.258 | 0.2178294 | 0.562462 |
| X201079_at | 0.1153 | 0.0917 | 1.258 | 0.2178917 | 0.562558 |
| X204010_s_at | -0.051 | 0.0406 | -1.257 | 0.2179888 | 0.562743 |
| X202732_at | -0.0718 | 0.0571 | -1.257 | 0.2180314 | 0.562757 |
| X203347_s_at | -0.0601 | 0.0478 | -1.257 | 0.218045 | 0.562757 |
| X220340_at | 0.0071 | 0.0056 | 1.257 | 0.2181095 | 0.562858 |
| X215557_at | 0.0228 | 0.0181 | 1.257 | 0.2181846 | 0.562987 |
| X219261_at | 0.0045 | 0.0036 | 1.256 | 0.2183877 | 0.563381 |
| X209826_at | 0.0573 | 0.0456 | 1.256 | 0.2184298 | 0.563381 |
| X219884_at | 0.0641 | 0.051 | 1.256 | 0.2184376 | 0.563381 |
| X206560_s_at | 0.0038 | 0.003 | 1.256 | 0.2184748 | 0.563381 |
| X217427_s_at | 0.0312 | 0.0248 | 1.256 | 0.2184792 | 0.563381 |
| X218078_s_at | 0.0474 | 0.0378 | 1.256 | 0.2184896 | 0.563381 |
| X221061_at | 0.0085 | 0.0068 | 1.256 | 0.2185947 | 0.563587 |
| X219763_at | 0.005 | 0.0039 | 1.256 | 0.2186653 | 0.563703 |
| X212532_s_at | 0.0494 | 0.0394 | 1.255 | 0.2187999 | 0.563985 |
| X211066_x_at | -0.0531 | 0.0423 | -1.255 | 0.2188638 | 0.564084 |
| X217533_x_at | 0.0143 | 0.0114 | 1.255 | 0.2188991 | 0.56411 |
| X215129_at | -0.0076 | 0.0061 | -1.255 | 0.2189513 | 0.564179 |
| X215081_at | 0.016 | 0.0128 | 1.255 | 0.2190195 | 0.564223 |
| X202745_at | 0.0433 | 0.0345 | 1.255 | 0.2190316 | 0.564223 |
| X202746_at | 0.149 | 0.1188 | 1.254 | 0.2190498 | 0.564223 |
| X202799_at | -0.0355 | 0.0283 | -1.254 | 0.2190701 | 0.564223 |
| X203069_at | -0.0624 | 0.0498 | -1.254 | 0.2191429 | 0.564272 |
| X205940_at | -0.0079 | 0.0063 | -1.254 | 0.219178 | 0.564272 |
| X208959_s_at | 0.057 | 0.0455 | 1.254 | 0.2192021 | 0.564272 |
| X201419_at | 0.075 | 0.0598 | 1.254 | 0.2192025 | 0.564272 |
| X204748_at | 0.0755 | 0.0602 | 1.254 | 0.2192207 | 0.564272 |
| X210373_at | 0.006 | 0.0048 | 1.254 | 0.2192415 | 0.564272 |
| X217725_x_at | -0.0472 | 0.0377 | -1.254 | 0.2193266 | 0.564292 |
| X208703_s_at | -0.0703 | 0.0561 | -1.254 | 0.2193524 | 0.564292 |
| X204480_s_at | -0.0871 | 0.0695 | -1.254 | 0.2193745 | 0.564292 |
| X206018_at | -0.034 | 0.0271 | -1.254 | 0.2193842 | 0.564292 |
| X213378_s_at | 0.0078 | 0.0062 | 1.254 | 0.2193863 | 0.564292 |
| X203482_at | -0.04 | 0.0319 | -1.253 | 0.2194017 | 0.564292 |
| X117_at | 0.0081 | 0.0064 | 1.253 | 0.2194421 | 0.564331 |
| X202556_s_at | -0.0055 | 0.0044 | -1.253 | 0.2195746 | 0.564548 |
| X208002_s_at | -0.0827 | 0.066 | -1.253 | 0.2195774 | 0.564548 |
| X220893_at | -0.012 | 0.0096 | -1.253 | 0.2196649 | 0.564707 |
| X212942_s_at | 0.1022 | 0.0816 | 1.253 | 0.2196963 | 0.564723 |
| X221752_at | 0.078 | 0.0623 | 1.252 | 0.219818 | 0.564926 |
| X219619_at | 0.0786 | 0.0627 | 1.252 | 0.2198467 | 0.564926 |
| X214187_x_at | 0.0043 | 0.0035 | 1.252 | 0.2198758 | 0.564926 |
| X217894_at | 0.0588 | 0.047 | 1.252 | 0.219877 | 0.564926 |
| X220769_s_at | 0.0127 | 0.0102 | 1.252 | 0.219922 | 0.564958 |
| X207447_s_at | 0.0934 | 0.0746 | 1.252 | 0.2199403 | 0.564958 |
| X208399_s_at | -0.0093 | 0.0074 | -1.252 | 0.2199916 | 0.565024 |
| X221001_at | -0.0054 | 0.0043 | -1.252 | 0.2200695 | 0.565159 |
| X203422_at | -0.0062 | 0.005 | -1.251 | 0.2202082 | 0.565449 |
| X202924_s_at | 0.0041 | 0.0033 | 1.251 | 0.2202333 | 0.565449 |
| X203146_s_at | -0.0533 | 0.0426 | -1.251 | 0.2203164 | 0.565464 |
| X203654_s_at | -0.0255 | 0.0204 | -1.251 | 0.2203199 | 0.565464 |
| X221861_at | -0.0571 | 0.0456 | -1.251 | 0.2203232 | 0.565464 |
| X214601_at | 0.0089 | 0.0071 | 1.251 | 0.2203409 | 0.565464 |
| X208343_s_at | -0.0096 | 0.0077 | -1.251 | 0.2204345 | 0.565595 |
| X202303_x_at | 0.0674 | 0.0539 | 1.251 | 0.2204607 | 0.565595 |
| X207785_s_at | 0.0498 | 0.0398 | 1.251 | 0.2204684 | 0.565595 |
| X216226_at | -0.0069 | 0.0055 | -1.25 | 0.2204997 | 0.56561 |
| X218775_s_at | 0.0878 | 0.0702 | 1.25 | 0.2205385 | 0.565615 |
| X222156_x_at | -0.0574 | 0.0459 | -1.25 | 0.2205527 | 0.565615 |
| X202094_at | 0.0053 | 0.0042 | 1.25 | 0.2206105 | 0.565698 |
| X217062_at | -0.0062 | 0.0049 | -1.25 | 0.220732 | 0.565834 |
| X200724_at | 0.004 | 0.0032 | 1.25 | 0.2207345 | 0.565834 |
| X205308_at | 0.0471 | 0.0377 | 1.25 | 0.2207399 | 0.565834 |
| X212864_at | 0.0718 | 0.0575 | 1.25 | 0.220804 | 0.565933 |
| X206825_at | 0.2125 | 0.1701 | 1.249 | 0.2208823 | 0.566068 |
| X207987_s_at | -0.0568 | 0.0454 | -1.249 | 0.2209513 | 0.56618 |
| X207230_at | 0.0034 | 0.0028 | 1.249 | 0.2210455 | 0.566356 |
| X214281_s_at | -0.0714 | 0.0572 | -1.249 | 0.2211037 | 0.566418 |
| X213127_s_at | 0.068 | 0.0545 | 1.249 | 0.2211291 | 0.566418 |
| X218475_at | -0.0026 | 0.002 | -1.249 | 0.2211464 | 0.566418 |
| X91816_f_at | 0.1237 | 0.0991 | 1.249 | 0.2211926 | 0.566471 |
| X207152_at | -0.0148 | 0.0119 | -1.248 | 0.2212296 | 0.566486 |
| X212255_s_at | 0.0378 | 0.0303 | 1.248 | 0.2212647 | 0.566486 |
| X210837_s_at | 0.0213 | 0.017 | 1.248 | 0.2212748 | 0.566486 |
| X221189_s_at | -0.0076 | 0.0061 | -1.248 | 0.2213558 | 0.566628 |
| X201076_at | -0.0616 | 0.0493 | -1.248 | 0.2214597 | 0.566829 |
| X220858_at | -0.0803 | 0.0644 | -1.248 | 0.2215495 | 0.566944 |
| X213806_at | 0.0051 | 0.0041 | 1.248 | 0.2215557 | 0.566944 |
| X221415_s_at | -0.0058 | 0.0046 | -1.247 | 0.221659 | 0.567143 |
| X218478_s_at | 0.0617 | 0.0495 | 1.247 | 0.2216985 | 0.567147 |
| X209021_x_at | -0.0451 | 0.0361 | -1.247 | 0.2217119 | 0.567147 |
| X201905_s_at | 0.0055 | 0.0044 | 1.247 | 0.2217659 | 0.567207 |
| X206126_at | -0.0029 | 0.0023 | -1.247 | 0.2218506 | 0.567207 |
| X221216_s_at | 0.0283 | 0.0227 | 1.247 | 0.2218814 | 0.567207 |
| X215867_x_at | -0.1184 | 0.095 | -1.247 | 0.2218927 | 0.567207 |
| X201612_at | 0.0518 | 0.0415 | 1.247 | 0.221903 | 0.567207 |
| X218919_at | -0.0641 | 0.0514 | -1.246 | 0.2219225 | 0.567207 |
| X214798_at | -0.0049 | 0.004 | -1.246 | 0.221935 | 0.567207 |
| X208765_s_at | -0.0586 | 0.047 | -1.246 | 0.2219395 | 0.567207 |
| X213687_s_at | -0.0292 | 0.0234 | -1.246 | 0.2220547 | 0.567436 |
| X213021_at | -0.0541 | 0.0434 | -1.246 | 0.2220836 | 0.567445 |
| X206988_at | 0.0047 | 0.0038 | 1.246 | 0.2221212 | 0.567476 |
| X207098_s_at | 0.0849 | 0.0681 | 1.246 | 0.2221804 | 0.567558 |
| X220959_s_at | -0.0071 | 0.0057 | -1.246 | 0.2222047 | 0.567558 |
| X209624_s_at | 0.0054 | 0.0044 | 1.246 | 0.2222635 | 0.567597 |
| X213082_s_at | 0.0396 | 0.0318 | 1.246 | 0.2222708 | 0.567597 |
| X203747_at | -0.0041 | 0.0033 | -1.245 | 0.2223122 | 0.567611 |
| X203880_at | -0.0303 | 0.0243 | -1.245 | 0.2223274 | 0.567611 |
| X203647_s_at | -0.0727 | 0.0584 | -1.245 | 0.2225817 | 0.568059 |
| X220124_at | 0.0104 | 0.0084 | 1.245 | 0.2225831 | 0.568059 |
| X210878_s_at | -0.0448 | 0.036 | -1.245 | 0.2226032 | 0.568059 |
| X51158_at | 0.0189 | 0.0152 | 1.245 | 0.2226052 | 0.568059 |
| X218093_s_at | 0.0434 | 0.0348 | 1.244 | 0.2226944 | 0.568166 |
| X217358_at | 0.0081 | 0.0065 | 1.244 | 0.2226983 | 0.568166 |
| X206346_at | -0.0226 | 0.0182 | -1.244 | 0.2228108 | 0.568351 |
| X203269_at | -0.0542 | 0.0436 | -1.244 | 0.2228222 | 0.568351 |
| X216783_at | -0.0313 | 0.0252 | -1.244 | 0.2229692 | 0.568496 |
| X219656_at | -0.0066 | 0.0053 | -1.243 | 0.2230122 | 0.568496 |
| X201687_s_at | -0.0307 | 0.0247 | -1.243 | 0.2230147 | 0.568496 |
| X211554_s_at | 0.0045 | 0.0036 | 1.243 | 0.2230844 | 0.568496 |
| X201158_at | 0.0547 | 0.044 | 1.243 | 0.2230878 | 0.568496 |
| X217297_s_at | -0.0034 | 0.0028 | -1.243 | 0.2230989 | 0.568496 |
| X208622_s_at | -0.0874 | 0.0703 | -1.243 | 0.2231709 | 0.568496 |
| X219666_at | 0.0118 | 0.0095 | 1.243 | 0.2231797 | 0.568496 |
| X202098_s_at | 0.0655 | 0.0527 | 1.243 | 0.2231845 | 0.568496 |
| X204578_at | -0.0604 | 0.0486 | -1.243 | 0.2231869 | 0.568496 |
| X215516_at | -0.0088 | 0.007 | -1.243 | 0.2231938 | 0.568496 |
| X203434_s_at | -0.2966 | 0.2386 | -1.243 | 0.2232323 | 0.568496 |
| X205294_at | -0.0785 | 0.0631 | -1.243 | 0.2232371 | 0.568496 |
| X31845_at | 0.0051 | 0.0041 | 1.243 | 0.2232614 | 0.568496 |
| X214103_s_at | 0.0042 | 0.0034 | 1.243 | 0.2232736 | 0.568496 |
| X220005_at | -0.0103 | 0.0083 | -1.243 | 0.2232882 | 0.568496 |
| X215519_x_at | 0.0055 | 0.0044 | 1.242 | 0.2234312 | 0.568755 |
| X215220_s_at | 0.2099 | 0.169 | 1.242 | 0.2234493 | 0.568755 |
| X220774_at | 0.0349 | 0.0281 | 1.242 | 0.2234667 | 0.568755 |
| X208477_at | 0.0617 | 0.0497 | 1.242 | 0.2235206 | 0.568827 |
| X216620_s_at | 0.103 | 0.0829 | 1.242 | 0.2236429 | 0.569023 |
| X209398_at | 0.1296 | 0.1044 | 1.242 | 0.2236612 | 0.569023 |
| X209777_s_at | -0.0039 | 0.0032 | -1.242 | 0.2236871 | 0.569023 |
| X215866_at | -0.0053 | 0.0043 | -1.242 | 0.2237002 | 0.569023 |
| X202346_at | -0.038 | 0.0306 | -1.241 | 0.223833 | 0.569216 |
| X202697_at | 0.0243 | 0.0195 | 1.241 | 0.2238384 | 0.569216 |
| X216141_at | 0.0053 | 0.0042 | 1.241 | 0.2238612 | 0.569216 |
| X218388_at | -0.0375 | 0.0302 | -1.241 | 0.2238788 | 0.569216 |
| X212071_s_at | 0.0258 | 0.0208 | 1.241 | 0.2240783 | 0.569658 |
| X215397_x_at | -0.0157 | 0.0127 | -1.24 | 0.2242547 | 0.570004 |
| X202218_s_at | 0.0512 | 0.0413 | 1.24 | 0.2242788 | 0.570004 |
| X201475_x_at | 0.0262 | 0.0211 | 1.24 | 0.2243064 | 0.570004 |
| X216935_at | -0.0058 | 0.0047 | -1.24 | 0.2243342 | 0.570004 |
| X221270_s_at | -0.0356 | 0.0287 | -1.24 | 0.2243426 | 0.570004 |
| X222121_at | -0.1006 | 0.0812 | -1.239 | 0.2245255 | 0.570404 |
| X210849_s_at | -0.0665 | 0.0536 | -1.239 | 0.2245863 | 0.570423 |
| X202033_s_at | 0.0546 | 0.0441 | 1.239 | 0.2246368 | 0.570423 |
| X202772_at | 0.0445 | 0.0359 | 1.239 | 0.2246795 | 0.570423 |
| X217497_at | 0.0129 | 0.0104 | 1.239 | 0.2246835 | 0.570423 |
| X217504_at | 0.0055 | 0.0045 | 1.239 | 0.2246837 | 0.570423 |
| X214692_s_at | 0.0115 | 0.0093 | 1.239 | 0.224692 | 0.570423 |
| X221492_s_at | -0.0453 | 0.0366 | -1.239 | 0.2247127 | 0.570423 |
| X207260_at | 0.0053 | 0.0042 | 1.239 | 0.2247658 | 0.570492 |
| X205334_at | -0.0594 | 0.0479 | -1.239 | 0.2248208 | 0.570567 |
| X200997_at | -0.0471 | 0.038 | -1.238 | 0.2248894 | 0.570676 |
| X222351_at | 0.0175 | 0.0141 | 1.238 | 0.2249673 | 0.57076 |
| X202859_x_at | 0.0529 | 0.0428 | 1.238 | 0.2249917 | 0.57076 |
| X217965_s_at | 0.0579 | 0.0468 | 1.238 | 0.2250133 | 0.57076 |
| X208637_x_at | -0.1174 | 0.0949 | -1.238 | 0.2250254 | 0.57076 |
| X208732_at | -0.0408 | 0.033 | -1.238 | 0.2250676 | 0.570778 |
| X207101_at | -0.0047 | 0.0038 | -1.238 | 0.225084 | 0.570778 |
| X210549_s_at | -0.0118 | 0.0095 | -1.238 | 0.2251279 | 0.570795 |
| X205161_s_at | 0.0042 | 0.0034 | 1.238 | 0.225142 | 0.570795 |
| X204946_s_at | 0.0085 | 0.0068 | 1.238 | 0.225175 | 0.570813 |
| X216594_x_at | 0.0503 | 0.0407 | 1.237 | 0.2252372 | 0.570889 |
| X206388_at | -0.0025 | 0.0021 | -1.237 | 0.2252682 | 0.570889 |
| X204526_s_at | 0.0317 | 0.0257 | 1.237 | 0.2252818 | 0.570889 |
| X220220_at | -0.0137 | 0.011 | -1.237 | 0.2254937 | 0.57136 |
| X215528_at | 0.0097 | 0.0078 | 1.237 | 0.2255272 | 0.57138 |
| X220945_x_at | -0.0422 | 0.0342 | -1.236 | 0.2256008 | 0.571501 |
| X211375_s_at | -0.0484 | 0.0391 | -1.236 | 0.2256333 | 0.571515 |
| X212152_x_at | -0.0122 | 0.0099 | -1.236 | 0.2256577 | 0.571515 |
| X220796_x_at | 0.0707 | 0.0572 | 1.236 | 0.2257676 | 0.571635 |
| X208422_at | -0.0057 | 0.0046 | -1.236 | 0.2258186 | 0.571635 |
| X219869_s_at | -0.0058 | 0.0047 | -1.236 | 0.2258365 | 0.571635 |
| X214834_at | -0.2532 | 0.2049 | -1.236 | 0.2258372 | 0.571635 |
| X213221_s_at | -0.0446 | 0.0361 | -1.236 | 0.2258419 | 0.571635 |
| X211813_x_at | 0.1834 | 0.1484 | 1.236 | 0.2258595 | 0.571635 |
| X220233_at | -0.0274 | 0.0222 | -1.236 | 0.2259081 | 0.571693 |
| X206073_at | 0.0086 | 0.007 | 1.235 | 0.2259855 | 0.571824 |
| X205842_s_at | 0.0123 | 0.01 | 1.235 | 0.2260534 | 0.571931 |
| X203648_at | -0.0401 | 0.0325 | -1.235 | 0.2261365 | 0.572076 |
| X203388_at | 0.0029 | 0.0024 | 1.235 | 0.2262076 | 0.57219 |
| X213893_x_at | -0.0528 | 0.0428 | -1.235 | 0.2262716 | 0.572251 |
| X207919_at | -0.0028 | 0.0023 | -1.235 | 0.226283 | 0.572251 |
| X212839_s_at | 0.0112 | 0.0091 | 1.234 | 0.226339 | 0.572281 |
| X206670_s_at | -0.0046 | 0.0037 | -1.234 | 0.2263511 | 0.572281 |
| X205073_at | -0.1207 | 0.0978 | -1.234 | 0.2263991 | 0.572281 |
| X205151_s_at | -0.1289 | 0.1044 | -1.234 | 0.2264076 | 0.572281 |
| X209865_at | -0.0851 | 0.069 | -1.234 | 0.2264237 | 0.572281 |
| X205469_s_at | -0.0087 | 0.0071 | -1.234 | 0.2264948 | 0.572395 |
| X210539_at | 0.0049 | 0.0039 | 1.234 | 0.2265404 | 0.572418 |
| X205403_at | 0.0119 | 0.0096 | 1.234 | 0.2265706 | 0.572418 |
| X222025_s_at | 0.0187 | 0.0151 | 1.234 | 0.226593 | 0.572418 |
| X203933_at | 0.0573 | 0.0464 | 1.234 | 0.2266357 | 0.572418 |
| X209474_s_at | -0.0038 | 0.0031 | -1.233 | 0.2266743 | 0.572418 |
| X221023_s_at | -0.0462 | 0.0375 | -1.233 | 0.2266792 | 0.572418 |
| X218821_at | 0.0488 | 0.0396 | 1.233 | 0.226684 | 0.572418 |
| X207250_at | -0.0036 | 0.0029 | -1.233 | 0.2269336 | 0.572928 |
| X204204_at | 0.1902 | 0.1543 | 1.233 | 0.2269377 | 0.572928 |
| X218973_at | -0.0131 | 0.0106 | -1.233 | 0.2269895 | 0.572994 |
| X201872_s_at | -0.0297 | 0.0241 | -1.232 | 0.2270419 | 0.573061 |
| X210936_at | 0.0092 | 0.0075 | 1.232 | 0.227089 | 0.573115 |
| X201591_s_at | -0.0704 | 0.0571 | -1.232 | 0.2271715 | 0.573258 |
| X200770_s_at | -0.0154 | 0.0125 | -1.232 | 0.2272704 | 0.573442 |
| X212825_at | -0.0453 | 0.0368 | -1.232 | 0.227355 | 0.573591 |
| X211852_s_at | 0.004 | 0.0033 | 1.232 | 0.2273895 | 0.573612 |
| X207723_s_at | -0.0445 | 0.0361 | -1.231 | 0.2275372 | 0.57392 |
| X214950_at | -0.0053 | 0.0043 | -1.231 | 0.2276046 | 0.574025 |
| X220642_x_at | 0.0374 | 0.0304 | 1.231 | 0.2277209 | 0.574253 |
| X201853_s_at | -0.0827 | 0.0672 | -1.23 | 0.2278861 | 0.574604 |
| X204261_s_at | -0.0081 | 0.0066 | -1.23 | 0.2279374 | 0.574668 |
| X211759_x_at | -0.0442 | 0.0359 | -1.23 | 0.2280723 | 0.574846 |
| X219999_at | -0.0457 | 0.0372 | -1.23 | 0.2280894 | 0.574846 |
| X215354_s_at | -0.0378 | 0.0307 | -1.23 | 0.2280925 | 0.574846 |
| X214731_at | -0.08 | 0.0651 | -1.23 | 0.228126 | 0.574846 |
| X219338_s_at | -0.0572 | 0.0465 | -1.229 | 0.2281374 | 0.574846 |
| X202805_s_at | 0.0049 | 0.004 | 1.229 | 0.2282101 | 0.574964 |
| X208885_at | -0.0063 | 0.0051 | -1.229 | 0.2282894 | 0.575052 |
| X204889_s_at | -0.1003 | 0.0816 | -1.229 | 0.2282967 | 0.575052 |
| X209458_x_at | 0.251 | 0.2043 | 1.229 | 0.2284174 | 0.575228 |
| X217203_at | -0.0127 | 0.0103 | -1.229 | 0.2284183 | 0.575228 |
| X209112_at | 0.0484 | 0.0394 | 1.228 | 0.228507 | 0.575293 |
| X213735_s_at | -0.0155 | 0.0126 | -1.228 | 0.2285173 | 0.575293 |
| X202788_at | 0.006 | 0.0049 | 1.228 | 0.2285219 | 0.575293 |
| X220238_s_at | -0.0415 | 0.0338 | -1.228 | 0.228565 | 0.575335 |
| X219687_at | -0.0071 | 0.0058 | -1.228 | 0.2285977 | 0.575335 |
| X219811_at | 0.0044 | 0.0036 | 1.228 | 0.228624 | 0.575335 |
| X217108_at | 0.0036 | 0.0029 | 1.228 | 0.2286855 | 0.575335 |
| X219371_s_at | 0.0953 | 0.0776 | 1.228 | 0.2286901 | 0.575335 |
| X206965_at | -0.0098 | 0.008 | -1.228 | 0.228694 | 0.575335 |
| X202410_x_at | -0.0035 | 0.0029 | -1.228 | 0.2287711 | 0.575464 |
| X218750_at | 0.0457 | 0.0373 | 1.228 | 0.2288455 | 0.575574 |
| X207956_x_at | 0.0736 | 0.0599 | 1.228 | 0.2288667 | 0.575574 |
| X213598_at | -0.0523 | 0.0427 | -1.227 | 0.2289582 | 0.575739 |
| X212570_at | -0.2062 | 0.168 | -1.227 | 0.2289934 | 0.575762 |
| X214436_at | -0.0596 | 0.0485 | -1.227 | 0.2290416 | 0.575818 |
| X204047_s_at | 0.004 | 0.0033 | 1.227 | 0.229143 | 0.576008 |
| X212901_s_at | -0.0476 | 0.0388 | -1.227 | 0.2291864 | 0.576052 |
| X219205_at | -0.0822 | 0.067 | -1.226 | 0.2293054 | 0.576286 |
| X201893_x_at | 0.1571 | 0.1281 | 1.226 | 0.2293931 | 0.576411 |
| X206158_s_at | -0.035 | 0.0286 | -1.226 | 0.2294071 | 0.576411 |
| X220734_s_at | 0.0291 | 0.0238 | 1.226 | 0.2294578 | 0.576473 |
| X221517_s_at | -0.0407 | 0.0332 | -1.225 | 0.2297221 | 0.577046 |
| X213795_s_at | 0.0474 | 0.0387 | 1.225 | 0.2297798 | 0.577046 |
| X208720_s_at | 0.0415 | 0.0338 | 1.225 | 0.2297886 | 0.577046 |
| X217814_at | -0.0944 | 0.0771 | -1.225 | 0.2298277 | 0.577046 |
| X206370_at | 0.0231 | 0.0189 | 1.225 | 0.2298617 | 0.577046 |
| X213660_s_at | -0.0034 | 0.0028 | -1.225 | 0.2298635 | 0.577046 |
| X210661_at | -0.0263 | 0.0214 | -1.225 | 0.2298678 | 0.577046 |
| X207935_s_at | 0.0037 | 0.0031 | 1.225 | 0.2299856 | 0.577277 |
| X209811_at | -0.0227 | 0.0185 | -1.224 | 0.230065 | 0.577359 |
| X220279_at | -0.0044 | 0.0036 | -1.224 | 0.2301229 | 0.577359 |
| X206650_at | -0.0352 | 0.0288 | -1.224 | 0.2301383 | 0.577359 |
| X207752_x_at | -0.0051 | 0.0042 | -1.224 | 0.2301575 | 0.577359 |
| X202940_at | -0.0106 | 0.0087 | -1.224 | 0.2301718 | 0.577359 |
| X219616_at | -0.0044 | 0.0036 | -1.224 | 0.2301905 | 0.577359 |
| X211748_x_at | -0.0618 | 0.0505 | -1.224 | 0.2302003 | 0.577359 |
| X219527_at | 0.075 | 0.0613 | 1.223 | 0.2303827 | 0.577693 |
| X216693_x_at | 0.0525 | 0.0429 | 1.223 | 0.2303855 | 0.577693 |
| X214111_at | 0.1427 | 0.1166 | 1.223 | 0.2304727 | 0.577847 |
| X213928_s_at | -0.0036 | 0.003 | -1.223 | 0.2305626 | 0.577885 |
| X206908_s_at | -0.0046 | 0.0037 | -1.223 | 0.2305657 | 0.577885 |
| X200894_s_at | 0.0549 | 0.0449 | 1.223 | 0.2305661 | 0.577885 |
| X209288_s_at | -0.0906 | 0.0741 | -1.223 | 0.2306887 | 0.578103 |
| X215811_at | 0.0594 | 0.0486 | 1.223 | 0.230722 | 0.578103 |
| X219855_at | -0.1156 | 0.0946 | -1.223 | 0.2307311 | 0.578103 |
| X206723_s_at | 0.0066 | 0.0054 | 1.222 | 0.2308322 | 0.578291 |
| X219151_s_at | -0.0657 | 0.0538 | -1.222 | 0.230988 | 0.578604 |
| X221895_at | -0.1204 | 0.0985 | -1.222 | 0.2310092 | 0.578604 |
| X202170_s_at | 0.0648 | 0.0531 | 1.221 | 0.2311983 | 0.579003 |
| X204767_s_at | -0.0475 | 0.0389 | -1.221 | 0.2312306 | 0.579003 |
| X206734_at | -0.0552 | 0.0452 | -1.221 | 0.2312465 | 0.579003 |
| X205928_at | 0.0531 | 0.0435 | 1.221 | 0.2312905 | 0.579047 |
| X37652_at | 0.0552 | 0.0452 | 1.221 | 0.2313643 | 0.579136 |
| X205862_at | -0.1014 | 0.0831 | -1.221 | 0.2314116 | 0.579136 |
| X221485_at | -0.0394 | 0.0323 | -1.221 | 0.2314239 | 0.579136 |
| X212526_at | -0.0516 | 0.0423 | -1.221 | 0.2314743 | 0.579136 |
| X203260_at | -0.0519 | 0.0425 | -1.22 | 0.2314941 | 0.579136 |
| X212668_at | -0.0064 | 0.0053 | -1.22 | 0.2314955 | 0.579136 |
| X208297_s_at | 0.2046 | 0.1677 | 1.22 | 0.2315295 | 0.579136 |
| X218650_at | 0.0084 | 0.0069 | 1.22 | 0.2315626 | 0.579136 |
| X206348_s_at | -0.006 | 0.0049 | -1.22 | 0.2315677 | 0.579136 |
| X221468_at | 0.0041 | 0.0034 | 1.22 | 0.23161 | 0.579136 |
| X206105_at | 0.0064 | 0.0052 | 1.22 | 0.2316331 | 0.579136 |
| X218428_s_at | -0.0291 | 0.0238 | -1.22 | 0.2316387 | 0.579136 |
| X202198_s_at | 0.0046 | 0.0037 | 1.22 | 0.2316938 | 0.579208 |
| X221761_at | -0.0592 | 0.0486 | -1.22 | 0.2317712 | 0.579337 |
| X217096_at | 0.0043 | 0.0035 | 1.219 | 0.2318739 | 0.579528 |
| X213025_at | -0.039 | 0.032 | -1.219 | 0.2319218 | 0.579583 |
| X214086_s_at | -0.0304 | 0.0249 | -1.219 | 0.2320762 | 0.579811 |
| X216689_x_at | 0.005 | 0.0041 | 1.219 | 0.2320834 | 0.579811 |
| X219107_at | 0.0449 | 0.0368 | 1.219 | 0.2321065 | 0.579811 |
| X209716_at | -0.0141 | 0.0116 | -1.219 | 0.2321246 | 0.579811 |
| X214985_at | 0.0092 | 0.0075 | 1.219 | 0.2321915 | 0.579811 |
| X216983_s_at | -0.0084 | 0.0069 | -1.219 | 0.2322091 | 0.579811 |
| X205468_s_at | 0.0055 | 0.0045 | 1.219 | 0.2322131 | 0.579811 |
| X215449_at | 0.0043 | 0.0035 | 1.219 | 0.2322219 | 0.579811 |
| X207085_x_at | 0.0045 | 0.0037 | 1.218 | 0.2323107 | 0.579967 |
| X212419_at | 0.1049 | 0.0861 | 1.218 | 0.2323964 | 0.580116 |
| X221568_s_at | 0.045 | 0.0369 | 1.218 | 0.2324449 | 0.580172 |
| X203061_s_at | 0.0048 | 0.004 | 1.218 | 0.2325874 | 0.580398 |
| X217099_s_at | 0.0034 | 0.0028 | 1.218 | 0.2325875 | 0.580398 |
| X210574_s_at | -0.0323 | 0.0265 | -1.217 | 0.2327143 | 0.580595 |
| X218926_at | -0.0447 | 0.0367 | -1.217 | 0.2327188 | 0.580595 |
| X217954_s_at | -0.0358 | 0.0295 | -1.217 | 0.2328596 | 0.580872 |
| X204183_s_at | 0.0047 | 0.0038 | 1.217 | 0.2328821 | 0.580872 |
| X201064_s_at | 0.0357 | 0.0293 | 1.217 | 0.2329181 | 0.580896 |
| X203170_at | -0.0151 | 0.0124 | -1.216 | 0.2329879 | 0.581005 |
| X214509_at | -0.0045 | 0.0037 | -1.216 | 0.2331907 | 0.58141 |
| X219807_x_at | -0.0061 | 0.005 | -1.216 | 0.2332028 | 0.58141 |
| X206455_s_at | -0.007 | 0.0057 | -1.216 | 0.2333232 | 0.581645 |
| X220173_at | 0.0576 | 0.0474 | 1.215 | 0.2334294 | 0.58181 |
| X220349_s_at | 0.0432 | 0.0355 | 1.215 | 0.2334418 | 0.58181 |
| X209654_at | 0.0451 | 0.0371 | 1.215 | 0.2334916 | 0.581869 |
| X219122_s_at | 0.0056 | 0.0046 | 1.215 | 0.2335457 | 0.581939 |
| X203700_s_at | 0.2188 | 0.1801 | 1.215 | 0.2335994 | 0.582007 |
| X214549_x_at | -0.0105 | 0.0086 | -1.215 | 0.2336476 | 0.582062 |
| X213961_s_at | -0.0045 | 0.0037 | -1.215 | 0.2337167 | 0.582169 |
| X201249_at | 0.006 | 0.005 | 1.214 | 0.2338224 | 0.582367 |
| X217035_at | -0.0061 | 0.005 | -1.214 | 0.2338657 | 0.58241 |
| X203216_s_at | 0.0622 | 0.0513 | 1.214 | 0.2339061 | 0.582445 |
| X210179_at | -0.008 | 0.0066 | -1.214 | 0.2339875 | 0.582546 |
| X218193_s_at | 0.0253 | 0.0209 | 1.214 | 0.2340008 | 0.582546 |
| X217652_at | -0.0048 | 0.004 | -1.214 | 0.2340255 | 0.582546 |
| X214844_s_at | -0.0922 | 0.0759 | -1.214 | 0.2340871 | 0.582634 |
| X208705_s_at | -0.0313 | 0.0258 | -1.213 | 0.2341312 | 0.582679 |
| X213853_at | -0.0477 | 0.0393 | -1.213 | 0.2341916 | 0.582764 |
| X207495_at | -0.0653 | 0.0539 | -1.212 | 0.2345102 | 0.583491 |
| X221528_s_at | 0.0682 | 0.0563 | 1.212 | 0.2345639 | 0.583509 |
| X215167_at | -0.0344 | 0.0284 | -1.212 | 0.2346029 | 0.583509 |
| X213064_at | 0.0294 | 0.0243 | 1.212 | 0.2346051 | 0.583509 |
| X204799_at | 0.0128 | 0.0106 | 1.212 | 0.2346352 | 0.583509 |
| X214764_at | -0.0964 | 0.0795 | -1.212 | 0.2346486 | 0.583509 |
| X214387_x_at | 0.0042 | 0.0035 | 1.212 | 0.234708 | 0.583591 |
| X218444_at | 0.0054 | 0.0045 | 1.212 | 0.2347462 | 0.583621 |
| X208856_x_at | 0.0393 | 0.0324 | 1.212 | 0.2347811 | 0.583642 |
| X203959_s_at | -0.0446 | 0.0368 | -1.211 | 0.2349251 | 0.583935 |
| X204533_at | 0.0073 | 0.006 | 1.211 | 0.2349699 | 0.583981 |
| X219474_at | -0.0063 | 0.0052 | -1.211 | 0.2350326 | 0.584024 |
| X201699_at | -0.047 | 0.0388 | -1.211 | 0.2350399 | 0.584024 |
| X206569_at | 0.0055 | 0.0045 | 1.211 | 0.2350835 | 0.584025 |
| X206979_at | -0.0033 | 0.0027 | -1.211 | 0.2351197 | 0.584025 |
| X218979_at | 0.0809 | 0.0668 | 1.211 | 0.2351214 | 0.584025 |
| X208118_x_at | -0.0049 | 0.004 | -1.211 | 0.2351455 | 0.584025 |
| X206775_at | 0.0072 | 0.006 | 1.211 | 0.2352107 | 0.58408 |
| X217559_at | -0.0045 | 0.0037 | -1.211 | 0.2352308 | 0.58408 |
| X219838_at | 0.0048 | 0.004 | 1.21 | 0.2352573 | 0.58408 |
| X216027_at | -0.007 | 0.0058 | -1.21 | 0.23529 | 0.58408 |
| X217613_at | 0.2063 | 0.1705 | 1.21 | 0.2353116 | 0.58408 |
| X208980_s_at | -0.0505 | 0.0417 | -1.21 | 0.2353254 | 0.58408 |
| X217934_x_at | -0.0408 | 0.0337 | -1.21 | 0.2354418 | 0.584304 |
| X208332_at | 0.0048 | 0.0039 | 1.21 | 0.2354692 | 0.584307 |
| X203998_s_at | 0.0698 | 0.0577 | 1.21 | 0.2355125 | 0.584349 |
| X220496_at | -0.0037 | 0.0031 | -1.209 | 0.2356434 | 0.584547 |
| X202330_s_at | 0.0419 | 0.0347 | 1.209 | 0.2356451 | 0.584547 |
| X219695_at | -0.0205 | 0.0169 | -1.209 | 0.2358007 | 0.584824 |
| X209333_at | -0.0633 | 0.0524 | -1.209 | 0.2358092 | 0.584824 |
| X219734_at | 0.0184 | 0.0152 | 1.209 | 0.2358586 | 0.584881 |
| X206492_at | -0.0041 | 0.0034 | -1.209 | 0.2359357 | 0.584984 |
| X217255_at | -0.005 | 0.0041 | -1.209 | 0.2359618 | 0.584984 |
| X205579_at | -0.0288 | 0.0238 | -1.209 | 0.2359799 | 0.584984 |
| X211455_at | -0.0132 | 0.011 | -1.208 | 0.2360055 | 0.584984 |
| X210714_at | 0.0098 | 0.0081 | 1.208 | 0.2360458 | 0.585016 |
| X210375_at | -0.0125 | 0.0104 | -1.208 | 0.2360709 | 0.585016 |
| X219567_s_at | -0.0445 | 0.0368 | -1.208 | 0.2361267 | 0.585089 |
| X206253_at | -0.123 | 0.1018 | -1.208 | 0.2361821 | 0.58511 |
| X206743_s_at | -0.0061 | 0.0051 | -1.208 | 0.2361879 | 0.58511 |
| X203186_s_at | 0.0235 | 0.0194 | 1.208 | 0.2362442 | 0.585121 |
| X201987_at | 0.0376 | 0.0311 | 1.208 | 0.2362453 | 0.585121 |
| X211810_s_at | 0.0044 | 0.0036 | 1.208 | 0.2363238 | 0.585188 |
| X204831_at | -0.056 | 0.0464 | -1.208 | 0.2363247 | 0.585188 |
| X219940_s_at | -0.0411 | 0.034 | -1.207 | 0.2364091 | 0.585331 |
| X206052_s_at | -0.0518 | 0.0429 | -1.207 | 0.236503 | 0.585499 |
| X219591_at | -0.0611 | 0.0506 | -1.207 | 0.2366148 | 0.585571 |
| X214141_x_at | -0.0565 | 0.0468 | -1.207 | 0.2366526 | 0.585571 |
| X214309_s_at | 0.004 | 0.0033 | 1.207 | 0.2366682 | 0.585571 |
| X210486_at | -0.0051 | 0.0042 | -1.207 | 0.2366757 | 0.585571 |
| X209583_s_at | -0.0666 | 0.0552 | -1.207 | 0.2366852 | 0.585571 |
| X202542_s_at | -0.0659 | 0.0546 | -1.207 | 0.2367029 | 0.585571 |
| X200896_x_at | 0.0367 | 0.0305 | 1.206 | 0.2367553 | 0.585571 |
| X216844_at | -0.0122 | 0.0101 | -1.206 | 0.2367629 | 0.585571 |
| X208021_s_at | -0.0361 | 0.03 | -1.206 | 0.2367851 | 0.585571 |
| X202115_s_at | -0.0063 | 0.0052 | -1.206 | 0.236796 | 0.585571 |
| X218267_at | 0.0057 | 0.0047 | 1.206 | 0.2368873 | 0.585732 |
| X213413_at | 0.048 | 0.0398 | 1.206 | 0.236921 | 0.58575 |
| X207659_s_at | 0.1748 | 0.1449 | 1.206 | 0.2369585 | 0.585777 |
| X212151_at | 0.0326 | 0.0271 | 1.206 | 0.2370089 | 0.585777 |
| X206363_at | 0.1005 | 0.0833 | 1.206 | 0.2370387 | 0.585777 |
| X211177_s_at | 0.0103 | 0.0085 | 1.206 | 0.2370499 | 0.585777 |
| X210858_x_at | -0.1514 | 0.1256 | -1.206 | 0.237085 | 0.585777 |
| X212547_at | 0.0398 | 0.033 | 1.206 | 0.237093 | 0.585777 |
| X217747_s_at | -0.0435 | 0.0361 | -1.206 | 0.2371239 | 0.585777 |
| X209974_s_at | 0.0413 | 0.0343 | 1.205 | 0.2371562 | 0.585777 |
| X218506_x_at | -0.0273 | 0.0226 | -1.205 | 0.2371692 | 0.585777 |
| X206691_s_at | 0.0076 | 0.0063 | 1.205 | 0.2372376 | 0.585881 |
| X215854_at | 0.008 | 0.0066 | 1.205 | 0.2373159 | 0.586009 |
| X205236_x_at | 0.0044 | 0.0037 | 1.205 | 0.2373825 | 0.586072 |
| X220627_at | -0.0023 | 0.0019 | -1.205 | 0.2373942 | 0.586072 |
| X204752_x_at | -0.0278 | 0.0231 | -1.205 | 0.2374672 | 0.586187 |
| X212221_x_at | -0.0272 | 0.0226 | -1.204 | 0.2375937 | 0.586434 |
| X218085_at | -0.0453 | 0.0376 | -1.204 | 0.2377251 | 0.586693 |
| X203736_s_at | -0.0083 | 0.0069 | -1.204 | 0.2377785 | 0.586724 |
| X208698_s_at | -0.0482 | 0.04 | -1.204 | 0.2377902 | 0.586724 |
| X208403_x_at | -0.0043 | 0.0035 | -1.203 | 0.2379493 | 0.587018 |
| X217157_x_at | 0.0075 | 0.0063 | 1.203 | 0.2379625 | 0.587018 |
| X219482_at | 0.0127 | 0.0106 | 1.203 | 0.2379911 | 0.587024 |
| X218672_at | -0.0242 | 0.0202 | -1.203 | 0.2381237 | 0.587285 |
| X211359_s_at | -0.0043 | 0.0035 | -1.203 | 0.2382025 | 0.587415 |
| X220046_s_at | 0.0515 | 0.0429 | 1.203 | 0.238254 | 0.587476 |
| X208537_at | 0.0021 | 0.0018 | 1.202 | 0.2383001 | 0.587516 |
| X211543_s_at | -0.0281 | 0.0234 | -1.202 | 0.2383231 | 0.587516 |
| X220386_s_at | 0.0057 | 0.0048 | 1.202 | 0.2383539 | 0.587527 |
| X209726_at | -0.0622 | 0.0517 | -1.202 | 0.2384539 | 0.587708 |
| X201432_at | 0.0987 | 0.0822 | 1.202 | 0.2385173 | 0.587775 |
| X221617_at | 0.03 | 0.0249 | 1.202 | 0.2385502 | 0.587775 |
| X204006_s_at | 0.0059 | 0.0049 | 1.202 | 0.2385788 | 0.587775 |
| X219318_x_at | -0.0066 | 0.0055 | -1.202 | 0.2385866 | 0.587775 |
| X219414_at | 0.0131 | 0.0109 | 1.202 | 0.2386302 | 0.587817 |
| X211918_x_at | 0.0063 | 0.0052 | 1.201 | 0.2387287 | 0.587994 |
| X208144_s_at | -0.0177 | 0.0148 | -1.201 | 0.2388133 | 0.588137 |
| X219938_s_at | 0.0132 | 0.011 | 1.201 | 0.2389131 | 0.588307 |
| X64883_at | -0.0761 | 0.0634 | -1.201 | 0.238935 | 0.588307 |
| X216899_s_at | 0.1069 | 0.089 | 1.201 | 0.238979 | 0.58835 |
| X209452_s_at | -0.0554 | 0.0461 | -1.2 | 0.2391637 | 0.588723 |
| X219290_x_at | 0.0455 | 0.0379 | 1.2 | 0.2391838 | 0.588723 |
| X215185_at | -0.0051 | 0.0043 | -1.2 | 0.2392909 | 0.588922 |
| X217216_x_at | -0.0432 | 0.036 | -1.199 | 0.2394478 | 0.589243 |
| X222293_at | -0.0091 | 0.0076 | -1.199 | 0.239711 | 0.589825 |
| X220075_s_at | -0.0061 | 0.0051 | -1.198 | 0.2398271 | 0.590045 |
| X215954_s_at | 0.0044 | 0.0037 | 1.198 | 0.2399063 | 0.590127 |
| X216945_x_at | 0.1018 | 0.085 | 1.198 | 0.2399133 | 0.590127 |
| X206648_at | 0.029 | 0.0242 | 1.198 | 0.2400041 | 0.590285 |
| X211072_x_at | 0.0433 | 0.0362 | 1.198 | 0.2400424 | 0.590314 |
| X203200_s_at | -0.0445 | 0.0371 | -1.198 | 0.2401066 | 0.590406 |
| X220576_at | 0.1395 | 0.1165 | 1.197 | 0.2401917 | 0.590517 |
| X201642_at | 0.022 | 0.0184 | 1.197 | 0.24021 | 0.590517 |
| X221931_s_at | -0.0303 | 0.0253 | -1.197 | 0.2402314 | 0.590517 |
| X217177_s_at | -0.0122 | 0.0102 | -1.197 | 0.2403633 | 0.590776 |
| X221186_at | -0.0019 | 0.0016 | -1.197 | 0.2404252 | 0.590862 |
| X203926_x_at | -0.0198 | 0.0165 | -1.197 | 0.2405155 | 0.591019 |
| X209889_at | 0.0325 | 0.0272 | 1.196 | 0.2405779 | 0.591107 |
| X209652_s_at | -0.007 | 0.0058 | -1.196 | 0.2406763 | 0.591226 |
| X219055_at | -0.0193 | 0.0161 | -1.196 | 0.2406794 | 0.591226 |
| X212040_at | -0.0746 | 0.0624 | -1.196 | 0.2407769 | 0.591377 |
| X206649_s_at | 0.0397 | 0.0332 | 1.196 | 0.2407944 | 0.591377 |
| X209736_at | 0.0061 | 0.0051 | 1.196 | 0.2408334 | 0.5914 |
| X221773_at | 0.0765 | 0.0639 | 1.196 | 0.2408571 | 0.5914 |
| X216994_s_at | 0.0067 | 0.0056 | 1.196 | 0.2409048 | 0.591452 |
| X202881_x_at | -0.0057 | 0.0047 | -1.196 | 0.2409541 | 0.591452 |
| X208177_at | -0.0042 | 0.0035 | -1.195 | 0.2409581 | 0.591452 |
| X209798_at | -0.041 | 0.0343 | -1.195 | 0.2410673 | 0.591655 |
| X201378_s_at | -0.0249 | 0.0209 | -1.195 | 0.2411343 | 0.591754 |
| X221060_s_at | 0.0594 | 0.0497 | 1.195 | 0.2411728 | 0.591783 |
| X216081_at | -0.0057 | 0.0047 | -1.195 | 0.2412162 | 0.591824 |
| X209666_s_at | -0.0488 | 0.0409 | -1.195 | 0.2413012 | 0.591905 |
| X207145_at | -0.0292 | 0.0244 | -1.195 | 0.2413214 | 0.591905 |
| X221818_at | -0.0038 | 0.0031 | -1.195 | 0.2413291 | 0.591905 |
| X220750_s_at | 0.008 | 0.0067 | 1.194 | 0.2413728 | 0.591947 |
| X206350_at | -0.0047 | 0.0039 | -1.194 | 0.2414136 | 0.591982 |
| X214049_x_at | -0.0056 | 0.0047 | -1.194 | 0.2414649 | 0.592042 |
| X211048_s_at | 0.0682 | 0.0571 | 1.194 | 0.2415064 | 0.592079 |
| X213612_x_at | 0.0364 | 0.0305 | 1.194 | 0.2415964 | 0.592234 |
| X222252_x_at | 0.0441 | 0.0369 | 1.194 | 0.2416847 | 0.592376 |
| X217503_at | 0.0067 | 0.0056 | 1.193 | 0.2417292 | 0.592376 |
| X221146_at | -0.0121 | 0.0101 | -1.193 | 0.2417343 | 0.592376 |
| X212766_s_at | 0.0639 | 0.0536 | 1.193 | 0.2418823 | 0.592673 |
| X209923_s_at | -0.0042 | 0.0035 | -1.193 | 0.2419243 | 0.592711 |
| X220577_at | -0.0273 | 0.0229 | -1.193 | 0.241954 | 0.592718 |
| X203192_at | -0.062 | 0.0519 | -1.193 | 0.2419943 | 0.592726 |
| X205020_s_at | 0.0582 | 0.0488 | 1.193 | 0.2420108 | 0.592726 |
| X212026_s_at | -0.0404 | 0.0339 | -1.193 | 0.2420674 | 0.592777 |
| X205377_s_at | -0.0043 | 0.0036 | -1.193 | 0.2420999 | 0.592777 |
| X217685_at | -0.0049 | 0.0041 | -1.193 | 0.2421115 | 0.592777 |
| X206787_at | -0.0074 | 0.0062 | -1.192 | 0.2421394 | 0.59278 |
| X201255_x_at | -0.0442 | 0.037 | -1.192 | 0.2422207 | 0.592789 |
| X202518_at | -0.0552 | 0.0463 | -1.192 | 0.2422325 | 0.592789 |
| X203051_at | -0.014 | 0.0117 | -1.192 | 0.2422367 | 0.592789 |
| X209189_at | 0.4103 | 0.3442 | 1.192 | 0.2422497 | 0.592789 |
| X218370_s_at | -0.0708 | 0.0594 | -1.192 | 0.2424207 | 0.593115 |
| X222197_s_at | 0.0056 | 0.0047 | 1.192 | 0.2424423 | 0.593115 |
| X219821_s_at | 0.0659 | 0.0553 | 1.192 | 0.2424632 | 0.593115 |
| X206043_s_at | -0.0083 | 0.007 | -1.191 | 0.2425173 | 0.593182 |
| X212488_at | -0.0019 | 0.0016 | -1.191 | 0.2426248 | 0.593344 |
| X209922_at | -0.0151 | 0.0126 | -1.191 | 0.2426369 | 0.593344 |
| X214017_s_at | 0.0048 | 0.0041 | 1.191 | 0.2426712 | 0.593363 |
| X209081_s_at | 0.0071 | 0.006 | 1.191 | 0.242748 | 0.593485 |
| X217782_s_at | 0.0387 | 0.0325 | 1.191 | 0.2427889 | 0.593504 |
| X202111_at | 0.0043 | 0.0036 | 1.191 | 0.2428092 | 0.593504 |
| X213379_at | -0.0589 | 0.0495 | -1.191 | 0.2428754 | 0.593582 |
| X204730_at | 0.0771 | 0.0648 | 1.19 | 0.2428946 | 0.593582 |
| X219425_at | -0.1041 | 0.0875 | -1.19 | 0.2430285 | 0.593844 |
| X216173_at | 0.0418 | 0.0351 | 1.19 | 0.2431074 | 0.593971 |
| X208409_at | -0.0071 | 0.0059 | -1.19 | 0.2431618 | 0.594038 |
| X221621_at | -0.0239 | 0.0201 | -1.19 | 0.2432274 | 0.594038 |
| X207705_s_at | -0.0634 | 0.0533 | -1.19 | 0.2432295 | 0.594038 |
| X215222_x_at | 0.0586 | 0.0492 | 1.19 | 0.2432522 | 0.594038 |
| X221087_s_at | 0.0318 | 0.0268 | 1.19 | 0.2432684 | 0.594038 |
| X211961_s_at | 0.0226 | 0.019 | 1.189 | 0.2434136 | 0.594327 |
| X218376_s_at | 0.0267 | 0.0224 | 1.189 | 0.2435098 | 0.594497 |
| X217473_x_at | 0.0121 | 0.0102 | 1.189 | 0.2435512 | 0.594533 |
| X210337_s_at | -0.1494 | 0.1257 | -1.189 | 0.2436258 | 0.594649 |
| X221325_at | -0.0035 | 0.0029 | -1.188 | 0.243671 | 0.594694 |
| X218573_at | -0.0435 | 0.0366 | -1.188 | 0.2437344 | 0.594777 |
| X214877_at | -0.0153 | 0.0129 | -1.188 | 0.243809 | 0.594777 |
| X216589_at | -0.0059 | 0.005 | -1.188 | 0.2438181 | 0.594777 |
| X213191_at | -0.0079 | 0.0066 | -1.188 | 0.2438275 | 0.594777 |
| X222365_at | 0.0046 | 0.0039 | 1.188 | 0.2438395 | 0.594777 |
| X205880_at | -0.1004 | 0.0845 | -1.188 | 0.2438708 | 0.594777 |
| X212694_s_at | -0.0496 | 0.0418 | -1.188 | 0.2438921 | 0.594777 |
| X214025_at | -0.0042 | 0.0036 | -1.188 | 0.2439375 | 0.594786 |
| X31846_at | 0.0043 | 0.0036 | 1.188 | 0.2439782 | 0.594786 |
| X205911_at | 0.0125 | 0.0105 | 1.188 | 0.2440149 | 0.594786 |
| X209993_at | 0.1808 | 0.1522 | 1.188 | 0.2440305 | 0.594786 |
| X218793_s_at | 0.0914 | 0.0769 | 1.188 | 0.2440477 | 0.594786 |
| X213454_at | -0.0861 | 0.0725 | -1.187 | 0.2440566 | 0.594786 |
| X217933_s_at | -0.0443 | 0.0373 | -1.187 | 0.2442326 | 0.59515 |
| X218473_s_at | -0.0088 | 0.0074 | -1.187 | 0.244352 | 0.595356 |
| X203709_at | 0.0113 | 0.0095 | 1.187 | 0.2443825 | 0.595356 |
| X221596_s_at | 0.051 | 0.043 | 1.187 | 0.2443978 | 0.595356 |
| X207220_at | 0.0089 | 0.0075 | 1.186 | 0.244451 | 0.595421 |
| X205751_at | -0.0611 | 0.0515 | -1.186 | 0.244494 | 0.59546 |
| X204553_x_at | -0.0169 | 0.0143 | -1.186 | 0.2446538 | 0.595712 |
| X214352_s_at | -0.0909 | 0.0767 | -1.186 | 0.2446728 | 0.595712 |
| X207882_at | -0.003 | 0.0025 | -1.186 | 0.2446781 | 0.595712 |
| X202535_at | -0.019 | 0.016 | -1.186 | 0.2448016 | 0.595948 |
| X205657_at | 0.0059 | 0.005 | 1.185 | 0.2448468 | 0.595983 |
| X221916_at | -0.0676 | 0.0571 | -1.185 | 0.2448917 | 0.595983 |
| X37860_at | 0.0419 | 0.0353 | 1.185 | 0.2448964 | 0.595983 |
| X219302_s_at | -0.0087 | 0.0073 | -1.185 | 0.2450126 | 0.596139 |
| X206500_s_at | 0.1173 | 0.099 | 1.185 | 0.2450143 | 0.596139 |
| X219026_s_at | -0.0095 | 0.008 | -1.185 | 0.2450762 | 0.596224 |
| X213618_at | -0.0475 | 0.0401 | -1.185 | 0.2451655 | 0.596376 |
| X201585_s_at | -0.0591 | 0.0499 | -1.185 | 0.2452037 | 0.596404 |
| X219392_x_at | 0.0792 | 0.0669 | 1.184 | 0.2452329 | 0.596409 |
| X204631_at | -0.0095 | 0.008 | -1.184 | 0.2452893 | 0.596481 |
| X37950_at | -0.0645 | 0.0545 | -1.184 | 0.2454793 | 0.596878 |
| X218381_s_at | -0.0431 | 0.0364 | -1.184 | 0.2455524 | 0.596976 |
| X204448_s_at | 0.0059 | 0.005 | 1.183 | 0.2456183 | 0.596976 |
| X207252_at | 0.0039 | 0.0033 | 1.183 | 0.2456692 | 0.596976 |
| X201824_at | -0.0405 | 0.0342 | -1.183 | 0.2456774 | 0.596976 |
| X214237_x_at | -0.0037 | 0.0032 | -1.183 | 0.2457043 | 0.596976 |
| X205301_s_at | -0.036 | 0.0304 | -1.183 | 0.2457061 | 0.596976 |
| X221953_s_at | 0.0243 | 0.0205 | 1.183 | 0.245731 | 0.596976 |
| X218722_s_at | -0.009 | 0.0076 | -1.183 | 0.2457344 | 0.596976 |
| X209462_at | -0.0484 | 0.0409 | -1.183 | 0.2458178 | 0.597113 |
| X210708_x_at | 0.0032 | 0.0027 | 1.183 | 0.2458464 | 0.597115 |
| X203957_at | -0.0221 | 0.0187 | -1.183 | 0.2458735 | 0.597115 |
| X220535_at | 0.0027 | 0.0022 | 1.183 | 0.2459115 | 0.597115 |
| X214160_at | 0.0039 | 0.0033 | 1.183 | 0.245926 | 0.597115 |
| X202209_at | -0.0241 | 0.0204 | -1.182 | 0.2460009 | 0.597231 |
| X217538_at | -0.0594 | 0.0502 | -1.182 | 0.2461065 | 0.597422 |
| X214453_s_at | 0.07 | 0.0592 | 1.182 | 0.2461392 | 0.597436 |
| X217623_at | -0.0208 | 0.0176 | -1.182 | 0.2462228 | 0.597574 |
| X216274_s_at | 0.0555 | 0.0469 | 1.182 | 0.24628 | 0.597599 |
| X208633_s_at | 0.0498 | 0.0422 | 1.182 | 0.2462992 | 0.597599 |
| X216100_s_at | 0.0501 | 0.0424 | 1.182 | 0.2463138 | 0.597599 |
| X219824_at | 0.0044 | 0.0037 | 1.181 | 0.2465626 | 0.598137 |
| X213224_s_at | -0.0482 | 0.0408 | -1.181 | 0.2466391 | 0.598233 |
| X217545_at | -0.0046 | 0.0039 | -1.181 | 0.2466558 | 0.598233 |
| X219832_s_at | -0.0077 | 0.0065 | -1.181 | 0.246695 | 0.598262 |
| X219523_s_at | 0.0809 | 0.0685 | 1.181 | 0.2467227 | 0.598264 |
| X215428_at | 0.0078 | 0.0066 | 1.18 | 0.2468399 | 0.598483 |
| X219396_s_at | 0.0509 | 0.0431 | 1.18 | 0.2468763 | 0.598506 |
| X217950_at | -0.0416 | 0.0352 | -1.18 | 0.2469578 | 0.598638 |
| X207427_at | 0.0049 | 0.0042 | 1.18 | 0.2470615 | 0.598824 |
| X221763_at | 0.0389 | 0.033 | 1.179 | 0.2472951 | 0.599325 |
| X201355_s_at | 0.0081 | 0.0069 | 1.179 | 0.2473387 | 0.599365 |
| X220341_s_at | 0.0087 | 0.0074 | 1.179 | 0.2473902 | 0.599414 |
| X214628_at | -0.0172 | 0.0146 | -1.179 | 0.2474254 | 0.599414 |
| X212058_at | 0.0381 | 0.0323 | 1.179 | 0.2474397 | 0.599414 |
| X213161_at | 0.0564 | 0.0478 | 1.179 | 0.2475265 | 0.599559 |
| X209987_s_at | 0.0692 | 0.0587 | 1.179 | 0.2475604 | 0.599576 |
| X219295_s_at | 0.0279 | 0.0237 | 1.178 | 0.2476174 | 0.599648 |
| X204968_at | 0.0099 | 0.0084 | 1.178 | 0.2477186 | 0.599723 |
| X208748_s_at | -0.0056 | 0.0048 | -1.178 | 0.2477203 | 0.599723 |
| X212367_at | 0.0404 | 0.0343 | 1.178 | 0.2477307 | 0.599723 |
| X208265_at | -0.0083 | 0.007 | -1.178 | 0.2477657 | 0.599723 |
| X217227_x_at | -0.0079 | 0.0067 | -1.178 | 0.2477831 | 0.599723 |
| X205396_at | 0.0041 | 0.0035 | 1.178 | 0.247849 | 0.599765 |
| X203774_at | 0.043 | 0.0365 | 1.178 | 0.2478548 | 0.599765 |
| X204189_at | 0.0053 | 0.0045 | 1.178 | 0.2478949 | 0.599797 |
| X218860_at | -0.0023 | 0.002 | -1.178 | 0.2479345 | 0.599828 |
| X214178_s_at | 0.0045 | 0.0038 | 1.177 | 0.2480203 | 0.59997 |
| X216922_x_at | 0.0045 | 0.0038 | 1.177 | 0.2480628 | 0.600007 |
| X201423_s_at | -0.0624 | 0.053 | -1.177 | 0.248277 | 0.60046 |
| X203148_s_at | 0.0737 | 0.0626 | 1.177 | 0.2483359 | 0.600537 |
| X211423_s_at | 0.0514 | 0.0437 | 1.176 | 0.2484262 | 0.600651 |
| X208805_at | -0.042 | 0.0357 | -1.176 | 0.2484418 | 0.600651 |
| X210483_at | 0.0096 | 0.0081 | 1.176 | 0.2484639 | 0.600651 |
| X222309_at | 0.0823 | 0.07 | 1.176 | 0.2485383 | 0.600765 |
| X206090_s_at | 0.0265 | 0.0225 | 1.175 | 0.2487547 | 0.601189 |
| X204081_at | -0.1515 | 0.1289 | -1.175 | 0.2487762 | 0.601189 |
| X202677_at | 0.0386 | 0.0329 | 1.175 | 0.248795 | 0.601189 |
| X204431_at | 0.0044 | 0.0037 | 1.175 | 0.2489638 | 0.601532 |
| X220923_s_at | -0.0056 | 0.0048 | -1.175 | 0.2490259 | 0.601564 |
| X210096_at | -0.0088 | 0.0075 | -1.175 | 0.2490513 | 0.601564 |
| X212150_at | -0.0321 | 0.0273 | -1.175 | 0.249079 | 0.601564 |
| X220316_at | 0.091 | 0.0775 | 1.175 | 0.2490854 | 0.601564 |
| X204041_at | 0.0448 | 0.0381 | 1.175 | 0.2491297 | 0.601605 |
| X203329_at | -0.039 | 0.0332 | -1.174 | 0.2492302 | 0.601776 |
| X211084_x_at | 0.0164 | 0.014 | 1.174 | 0.2492546 | 0.601776 |
| X200854_at | -0.0416 | 0.0354 | -1.174 | 0.2492989 | 0.601818 |
| X209484_s_at | 0.0569 | 0.0485 | 1.174 | 0.2493342 | 0.601837 |
| X52651_at | 0.0078 | 0.0066 | 1.174 | 0.2493883 | 0.601903 |
| X218885_s_at | 0.0063 | 0.0054 | 1.173 | 0.2495941 | 0.602273 |
| X219610_at | 0.0038 | 0.0032 | 1.173 | 0.2495959 | 0.602273 |
| X213752_at | -0.0486 | 0.0414 | -1.173 | 0.2496256 | 0.602279 |
| X216511_s_at | 0.1007 | 0.0859 | 1.173 | 0.2497225 | 0.602447 |
| X215468_at | 0.0313 | 0.0267 | 1.173 | 0.2498149 | 0.602605 |
| X215456_at | -0.011 | 0.0094 | -1.172 | 0.2500287 | 0.603055 |
| X215500_at | 0.003 | 0.0026 | 1.172 | 0.2501074 | 0.603151 |
| X216445_at | 0.0062 | 0.0053 | 1.172 | 0.2501226 | 0.603151 |
| X205336_at | -0.0048 | 0.0041 | -1.172 | 0.250202 | 0.6032 |
| X203341_at | 0.0572 | 0.0488 | 1.172 | 0.2502122 | 0.6032 |
| X212695_at | -0.036 | 0.0307 | -1.172 | 0.2502358 | 0.6032 |
| X220206_at | 0.0551 | 0.047 | 1.172 | 0.2502515 | 0.6032 |
| X209555_s_at | 0.0115 | 0.0098 | 1.171 | 0.2503678 | 0.603328 |
| X205703_at | 0.0054 | 0.0046 | 1.171 | 0.2503835 | 0.603328 |
| X206256_at | -0.0038 | 0.0033 | -1.171 | 0.2503864 | 0.603328 |
| X219526_at | 0.0443 | 0.0378 | 1.171 | 0.250424 | 0.603353 |
| X200918_s_at | -0.0119 | 0.0102 | -1.171 | 0.2504555 | 0.603364 |
| X211328_x_at | -0.0163 | 0.0139 | -1.171 | 0.2505168 | 0.603412 |
| X203059_s_at | 0.003 | 0.0025 | 1.171 | 0.2505296 | 0.603412 |
| X216262_s_at | -0.0051 | 0.0043 | -1.171 | 0.2505615 | 0.603423 |
| X203259_s_at | 0.0316 | 0.027 | 1.171 | 0.2505949 | 0.603428 |
| X212144_at | 0.1334 | 0.1139 | 1.171 | 0.2506181 | 0.603428 |
| X204123_at | 0.012 | 0.0102 | 1.171 | 0.250659 | 0.603432 |
| X203787_at | 0.0678 | 0.0579 | 1.171 | 0.2506739 | 0.603432 |
| X215152_at | -0.0117 | 0.01 | -1.17 | 0.2507765 | 0.603531 |
| X207900_at | 0.0071 | 0.0061 | 1.17 | 0.2507893 | 0.603531 |
| X219964_at | 0.0036 | 0.0031 | 1.17 | 0.2507967 | 0.603531 |
| X219644_at | -0.0898 | 0.0767 | -1.17 | 0.2509354 | 0.603787 |
| X214781_at | 0.2146 | 0.1834 | 1.17 | 0.2509572 | 0.603787 |
| X205083_at | 0.0075 | 0.0064 | 1.17 | 0.2509857 | 0.60379 |
| X203943_at | -0.0408 | 0.0349 | -1.17 | 0.2510136 | 0.603792 |
| X219015_s_at | -0.0768 | 0.0657 | -1.17 | 0.2511049 | 0.603798 |
| X212449_s_at | 0.0441 | 0.0377 | 1.169 | 0.2511263 | 0.603798 |
| X214483_s_at | 0.0595 | 0.0509 | 1.169 | 0.2511405 | 0.603798 |
| X208331_at | 0.0069 | 0.0059 | 1.169 | 0.251145 | 0.603798 |
| X207191_s_at | 0.0056 | 0.0048 | 1.169 | 0.2511602 | 0.603798 |
| X215250_at | -0.0079 | 0.0068 | -1.169 | 0.2511968 | 0.603798 |
| X213419_at | -0.0848 | 0.0725 | -1.169 | 0.2512065 | 0.603798 |
| X218356_at | -0.0367 | 0.0314 | -1.169 | 0.2513511 | 0.603979 |
| X202768_at | 0.0066 | 0.0056 | 1.169 | 0.251363 | 0.603979 |
| X208682_s_at | 0.0799 | 0.0684 | 1.169 | 0.2513633 | 0.603979 |
| X207334_s_at | -0.0055 | 0.0047 | -1.169 | 0.2514014 | 0.604005 |
| X211871_x_at | -0.0503 | 0.043 | -1.168 | 0.2515351 | 0.604261 |
| X218248_at | 0.0236 | 0.0202 | 1.168 | 0.2516559 | 0.604406 |
| X214347_s_at | -0.0076 | 0.0065 | -1.168 | 0.2516568 | 0.604406 |
| X44702_at | 0.0354 | 0.0303 | 1.168 | 0.2516769 | 0.604406 |
| X51200_at | -0.0638 | 0.0546 | -1.168 | 0.2518353 | 0.604721 |
| X219766_at | 0.0046 | 0.004 | 1.168 | 0.2518653 | 0.604727 |
| X219981_x_at | 0.0484 | 0.0414 | 1.167 | 0.2519581 | 0.604885 |
| X211982_x_at | 0.021 | 0.018 | 1.167 | 0.2520503 | 0.605041 |
| X204592_at | 0.1269 | 0.1087 | 1.167 | 0.2521021 | 0.605045 |
| X213856_at | -0.0114 | 0.0097 | -1.167 | 0.2521068 | 0.605045 |
| X214949_at | -0.0354 | 0.0304 | -1.167 | 0.2521767 | 0.605148 |
| X214732_at | -0.0037 | 0.0032 | -1.166 | 0.2523402 | 0.605475 |
| X218308_at | -0.005 | 0.0043 | -1.166 | 0.2523692 | 0.605479 |
| X219117_s_at | 0.0619 | 0.0531 | 1.166 | 0.252519 | 0.605764 |
| X205165_at | 0.0558 | 0.0478 | 1.166 | 0.2525427 | 0.605764 |
| X210926_at | -0.045 | 0.0386 | -1.166 | 0.2526164 | 0.605876 |
| X200622_x_at | -0.0706 | 0.0606 | -1.165 | 0.2527847 | 0.606213 |
| X208914_at | 0.0421 | 0.0362 | 1.165 | 0.2528307 | 0.606213 |
| X202846_s_at | -0.0367 | 0.0315 | -1.165 | 0.252839 | 0.606213 |
| X206610_s_at | -0.0041 | 0.0035 | -1.165 | 0.2529614 | 0.606441 |
| X215863_at | -0.0076 | 0.0065 | -1.165 | 0.2529947 | 0.606441 |
| X219413_at | -0.0036 | 0.0031 | -1.165 | 0.2530212 | 0.606441 |
| X215992_s_at | 0.1182 | 0.1015 | 1.165 | 0.2530431 | 0.606441 |
| X206523_at | -0.0053 | 0.0046 | -1.164 | 0.2531094 | 0.606452 |
| X221595_at | -0.0654 | 0.0561 | -1.164 | 0.2531478 | 0.606452 |
| X213037_x_at | 0.0236 | 0.0203 | 1.164 | 0.2531725 | 0.606452 |
| X39891_at | -0.0234 | 0.0201 | -1.164 | 0.2531826 | 0.606452 |
| X201084_s_at | -0.0278 | 0.0239 | -1.164 | 0.2532029 | 0.606452 |
| X219249_s_at | 0.0065 | 0.0056 | 1.164 | 0.2532116 | 0.606452 |
| X213337_s_at | -0.0161 | 0.0138 | -1.164 | 0.2532529 | 0.606486 |
| X217783_s_at | -0.0232 | 0.02 | -1.163 | 0.2535107 | 0.607038 |
| X205675_at | -0.0104 | 0.009 | -1.163 | 0.2535711 | 0.607109 |
| X209625_at | -0.075 | 0.0645 | -1.163 | 0.253595 | 0.607109 |
| X220500_s_at | -0.0627 | 0.0539 | -1.163 | 0.2537406 | 0.60734 |
| X205153_s_at | 0.0035 | 0.003 | 1.163 | 0.2537464 | 0.60734 |
| X208195_at | -0.0043 | 0.0037 | -1.162 | 0.2539564 | 0.607754 |
| X214837_at | -0.0083 | 0.0071 | -1.162 | 0.253974 | 0.607754 |
| X214819_at | 0.0364 | 0.0313 | 1.162 | 0.2540506 | 0.607872 |
| X209692_at | -0.0201 | 0.0173 | -1.162 | 0.2541643 | 0.608019 |
| X209536_s_at | 0.0196 | 0.0169 | 1.162 | 0.2541671 | 0.608019 |
| X210184_at | 0.005 | 0.0043 | 1.162 | 0.2542914 | 0.608216 |
| X216103_at | 0.0705 | 0.0607 | 1.162 | 0.2543039 | 0.608216 |
| X56919_at | -0.0802 | 0.069 | -1.161 | 0.2544748 | 0.608559 |
| X218054_s_at | 0.0032 | 0.0028 | 1.161 | 0.2545333 | 0.60861 |
| X205727_at | -0.0087 | 0.0075 | -1.161 | 0.2545511 | 0.60861 |
| X214084_x_at | -0.0042 | 0.0036 | -1.161 | 0.254639 | 0.608755 |
| X207822_at | -0.0064 | 0.0055 | -1.161 | 0.2546871 | 0.608804 |
| X200766_at | 0.0281 | 0.0242 | 1.16 | 0.2547507 | 0.608891 |
| X217485_x_at | -0.0419 | 0.0362 | -1.16 | 0.2548107 | 0.608891 |
| X210401_at | 0.0058 | 0.005 | 1.16 | 0.2548248 | 0.608891 |
| X204312_x_at | -0.0061 | 0.0053 | -1.16 | 0.2548359 | 0.608891 |
| X203947_at | -0.0526 | 0.0453 | -1.16 | 0.2548602 | 0.608891 |
| X210094_s_at | 0.07 | 0.0604 | 1.16 | 0.2548951 | 0.608899 |
| X214438_at | -0.0035 | 0.003 | -1.16 | 0.2549184 | 0.608899 |
| X207292_s_at | 0.0028 | 0.0024 | 1.16 | 0.255054 | 0.609046 |
| X206437_at | 0.0054 | 0.0047 | 1.16 | 0.2550678 | 0.609046 |
| X213860_x_at | 0.03 | 0.0259 | 1.159 | 0.2551074 | 0.609046 |
| X201027_s_at | -0.0242 | 0.0209 | -1.159 | 0.255109 | 0.609046 |
| X210130_s_at | -0.0234 | 0.0202 | -1.159 | 0.2551173 | 0.609046 |
| X213449_at | 0.0067 | 0.0058 | 1.159 | 0.2551569 | 0.609075 |
| X222184_at | -0.1193 | 0.1029 | -1.159 | 0.2552837 | 0.609255 |
| X206657_s_at | -0.0022 | 0.0019 | -1.159 | 0.2552964 | 0.609255 |
| X202412_s_at | 0.1322 | 0.1141 | 1.159 | 0.2553143 | 0.609255 |
| X208264_s_at | 0.0316 | 0.0273 | 1.159 | 0.2553564 | 0.609263 |
| X206619_at | -0.0031 | 0.0026 | -1.159 | 0.2553726 | 0.609263 |
| X217591_at | -0.0213 | 0.0184 | -1.159 | 0.2554311 | 0.609337 |
| X205659_at | 0.0746 | 0.0644 | 1.159 | 0.2554913 | 0.609415 |
| X216465_at | 0.0057 | 0.0049 | 1.158 | 0.255563 | 0.609479 |
| X202838_at | -0.0702 | 0.0606 | -1.158 | 0.255573 | 0.609479 |
| X215050_x_at | 0.003 | 0.0026 | 1.158 | 0.255688 | 0.609627 |
| X216486_x_at | -0.007 | 0.006 | -1.158 | 0.2556897 | 0.609627 |
| X216172_at | 0.0077 | 0.0067 | 1.158 | 0.2557616 | 0.609733 |
| X221015_s_at | 0.0057 | 0.0049 | 1.157 | 0.2559173 | 0.609965 |
| X206580_s_at | 0.0434 | 0.0375 | 1.157 | 0.2559283 | 0.609965 |
| X222272_x_at | -0.011 | 0.0095 | -1.157 | 0.2559413 | 0.609965 |
| X221097_s_at | -0.0101 | 0.0087 | -1.157 | 0.2560029 | 0.610046 |
| X46270_at | 0.0318 | 0.0275 | 1.157 | 0.2560389 | 0.610066 |
| X211064_at | 0.0159 | 0.0137 | 1.157 | 0.2562826 | 0.610521 |
| X203824_at | 0.0146 | 0.0126 | 1.157 | 0.2562846 | 0.610521 |
| X218855_at | -0.0032 | 0.0028 | -1.156 | 0.2564195 | 0.610777 |
| X209008_x_at | 0.0047 | 0.0041 | 1.156 | 0.2566872 | 0.611349 |
| X202915_s_at | -0.0502 | 0.0435 | -1.155 | 0.2567939 | 0.611537 |
| X213575_at | -0.003 | 0.0026 | -1.155 | 0.2568512 | 0.611608 |
| X221733_s_at | 0.0027 | 0.0023 | 1.155 | 0.2569325 | 0.611722 |
| X40465_at | 0.0307 | 0.0266 | 1.155 | 0.2569542 | 0.611722 |
| X211431_s_at | -0.0069 | 0.006 | -1.154 | 0.257188 | 0.612165 |
| X214840_at | 0.0026 | 0.0023 | 1.154 | 0.2571953 | 0.612165 |
| X209837_at | -0.0034 | 0.003 | -1.154 | 0.2573452 | 0.612456 |
| X209612_s_at | -0.0093 | 0.0081 | -1.154 | 0.2574192 | 0.612565 |
| X215362_at | -0.0038 | 0.0033 | -1.154 | 0.2574462 | 0.612565 |
| X212046_x_at | 0.0272 | 0.0236 | 1.153 | 0.2576281 | 0.612933 |
| X219120_at | -0.0046 | 0.004 | -1.153 | 0.2577016 | 0.613042 |
| X207069_s_at | -0.0373 | 0.0324 | -1.153 | 0.2577752 | 0.613145 |
| X214586_at | 0.1929 | 0.1673 | 1.153 | 0.2578265 | 0.613145 |
| X204728_s_at | -0.0147 | 0.0128 | -1.153 | 0.2578306 | 0.613145 |
| X211917_s_at | -0.016 | 0.0139 | -1.153 | 0.2578555 | 0.613145 |
| X217414_x_at | 0.2579 | 0.2238 | 1.152 | 0.2579963 | 0.613388 |
| X215798_at | -0.0047 | 0.0041 | -1.152 | 0.258013 | 0.613388 |
| X207257_at | -0.0059 | 0.0051 | -1.152 | 0.258064 | 0.613412 |
| X221951_at | 0.0412 | 0.0358 | 1.152 | 0.2580904 | 0.613412 |
| X209672_s_at | 0.0476 | 0.0413 | 1.152 | 0.2581059 | 0.613412 |
| X215828_at | -0.0081 | 0.0071 | -1.152 | 0.2582309 | 0.613644 |
| X208503_s_at | 0.043 | 0.0373 | 1.151 | 0.2583387 | 0.613794 |
| X220626_at | -0.0113 | 0.0098 | -1.151 | 0.2583496 | 0.613794 |
| X209055_s_at | 0.111 | 0.0965 | 1.151 | 0.2584404 | 0.613945 |
| X202328_s_at | 0.0654 | 0.0568 | 1.151 | 0.2585073 | 0.613979 |
| X207614_s_at | -0.0476 | 0.0413 | -1.151 | 0.2585413 | 0.613979 |
| X205370_x_at | 0.0646 | 0.0561 | 1.151 | 0.2585598 | 0.613979 |
| X210463_x_at | -0.0113 | 0.0098 | -1.151 | 0.2585928 | 0.613979 |
| X200711_s_at | -0.02 | 0.0174 | -1.151 | 0.2585931 | 0.613979 |
| X208027_s_at | -0.0117 | 0.0102 | -1.15 | 0.2588448 | 0.614511 |
| X210942_s_at | 0.0485 | 0.0422 | 1.15 | 0.2589417 | 0.614675 |
| X218406_x_at | 0.004 | 0.0035 | 1.15 | 0.2590496 | 0.614866 |
| X202225_at | 0.0481 | 0.0419 | 1.15 | 0.2591315 | 0.614961 |
| X218845_at | 0.0286 | 0.0249 | 1.149 | 0.2591631 | 0.614961 |
| X212109_at | 0.0257 | 0.0223 | 1.149 | 0.2591726 | 0.614961 |
| X211005_at | -0.0071 | 0.0062 | -1.149 | 0.2592537 | 0.615087 |
| X214563_at | -0.009 | 0.0078 | -1.149 | 0.2593067 | 0.615147 |
| X205359_at | -0.071 | 0.0618 | -1.149 | 0.2593815 | 0.615158 |
| X216481_at | -0.0067 | 0.0058 | -1.149 | 0.2593863 | 0.615158 |
| X204349_at | -0.0322 | 0.028 | -1.149 | 0.2593941 | 0.615158 |
| X221826_at | -0.0235 | 0.0204 | -1.149 | 0.2594403 | 0.615191 |
| X209937_at | 0.0044 | 0.0038 | 1.149 | 0.2594747 | 0.615191 |
| X219489_s_at | 0.0198 | 0.0172 | 1.149 | 0.2594911 | 0.615191 |
| X207550_at | 0.0166 | 0.0144 | 1.149 | 0.2595381 | 0.615233 |
| X213882_at | -0.0669 | 0.0583 | -1.148 | 0.2595645 | 0.615233 |
| X211105_s_at | -0.0072 | 0.0063 | -1.148 | 0.2596173 | 0.615244 |
| X206325_at | 0.0035 | 0.003 | 1.148 | 0.2596245 | 0.615244 |
| X204828_at | 0.0082 | 0.0071 | 1.148 | 0.259769 | 0.615521 |
| X219075_at | -0.0039 | 0.0034 | -1.148 | 0.2598092 | 0.615551 |
| X201477_s_at | -0.0628 | 0.0547 | -1.148 | 0.2598517 | 0.615556 |
| X209581_at | 0.1133 | 0.0987 | 1.148 | 0.2598671 | 0.615556 |
| X217025_s_at | -0.0755 | 0.0658 | -1.148 | 0.2599021 | 0.615574 |
| X214269_at | -0.0039 | 0.0034 | -1.147 | 0.260056 | 0.615803 |
| X214591_at | -0.1054 | 0.0918 | -1.147 | 0.2600611 | 0.615803 |
| X219937_at | 0.1454 | 0.1267 | 1.147 | 0.2600822 | 0.615803 |
| X204733_at | -0.2141 | 0.1867 | -1.147 | 0.2601908 | 0.615995 |
| X50374_at | -0.0201 | 0.0175 | -1.147 | 0.2602246 | 0.616009 |
| X206522_at | -0.0091 | 0.0079 | -1.147 | 0.2603186 | 0.616101 |
| X202738_s_at | -0.0534 | 0.0466 | -1.147 | 0.2603211 | 0.616101 |
| X205128_x_at | 0.004 | 0.0035 | 1.147 | 0.2603466 | 0.616101 |
| X216035_x_at | 0.1014 | 0.0885 | 1.146 | 0.2604558 | 0.616294 |
| X208683_at | 0.0493 | 0.0431 | 1.146 | 0.2605509 | 0.616453 |
| X200881_s_at | -0.0681 | 0.0594 | -1.146 | 0.2606045 | 0.616514 |
| X212294_at | 0.0584 | 0.051 | 1.145 | 0.2608623 | 0.616967 |
| X205673_s_at | 0.0118 | 0.0103 | 1.145 | 0.2608876 | 0.616967 |
| X204898_at | 0.0029 | 0.0025 | 1.145 | 0.260907 | 0.616967 |
| X221656_s_at | 0.0469 | 0.041 | 1.145 | 0.2609313 | 0.616967 |
| X210265_x_at | 0.0027 | 0.0023 | 1.145 | 0.2609499 | 0.616967 |
| X219857_at | -0.0082 | 0.0071 | -1.145 | 0.2609977 | 0.616967 |
| X204856_at | 0.0054 | 0.0047 | 1.145 | 0.2610101 | 0.616967 |
| X203022_at | -0.0085 | 0.0075 | -1.145 | 0.2610179 | 0.616967 |
| X220107_s_at | -0.0054 | 0.0047 | -1.145 | 0.2610665 | 0.617016 |
| X217170_at | 0.0066 | 0.0058 | 1.145 | 0.261108 | 0.617048 |
| X204031_s_at | 0.028 | 0.0245 | 1.145 | 0.2611507 | 0.617084 |
| X214953_s_at | 0.0717 | 0.0626 | 1.144 | 0.2611906 | 0.617112 |
| X220689_at | 0.0039 | 0.0034 | 1.144 | 0.2612504 | 0.617188 |
| X208417_at | -0.0067 | 0.0059 | -1.144 | 0.2612866 | 0.617196 |
| X209903_s_at | -0.0365 | 0.0319 | -1.144 | 0.2613093 | 0.617196 |
| X215175_at | -0.1875 | 0.1639 | -1.144 | 0.2614127 | 0.617315 |
| X206781_at | -0.0108 | 0.0095 | -1.144 | 0.2614156 | 0.617315 |
| X211517_s_at | -0.0085 | 0.0074 | -1.144 | 0.2614838 | 0.617411 |
| X201116_s_at | -0.0832 | 0.0727 | -1.144 | 0.2615556 | 0.617515 |
| X209488_s_at | 0.0083 | 0.0072 | 1.143 | 0.2615968 | 0.617531 |
| X219342_at | -0.0575 | 0.0503 | -1.143 | 0.2616179 | 0.617531 |
| X210112_at | -0.0056 | 0.0049 | -1.143 | 0.2616591 | 0.617562 |
| X201227_s_at | 0.0422 | 0.0369 | 1.143 | 0.2617306 | 0.617586 |
| X214879_x_at | -0.0389 | 0.034 | -1.143 | 0.2617388 | 0.617586 |
| X212270_x_at | -0.0277 | 0.0242 | -1.143 | 0.2618125 | 0.617586 |
| X204632_at | -0.04 | 0.035 | -1.143 | 0.2618207 | 0.617586 |
| X213951_s_at | 0.0611 | 0.0535 | 1.143 | 0.2618276 | 0.617586 |
| X213266_at | -0.0583 | 0.051 | -1.143 | 0.261837 | 0.617586 |
| X219916_s_at | 0.0046 | 0.004 | 1.143 | 0.2618639 | 0.617586 |
| X209633_at | -0.0505 | 0.0442 | -1.143 | 0.26193 | 0.617635 |
| X205903_s_at | 0.0953 | 0.0834 | 1.143 | 0.2619496 | 0.617635 |
| X210052_s_at | 0.0041 | 0.0035 | 1.143 | 0.2619681 | 0.617635 |
| X221043_at | -0.0068 | 0.006 | -1.142 | 0.261997 | 0.617638 |
| X208310_s_at | -0.0498 | 0.0436 | -1.142 | 0.2620712 | 0.617747 |
| X201511_at | -0.0681 | 0.0597 | -1.142 | 0.262223 | 0.617963 |
| X215033_at | 0.0065 | 0.0057 | 1.142 | 0.2622407 | 0.617963 |
| X205983_at | -0.0028 | 0.0024 | -1.142 | 0.2622836 | 0.617963 |
| X204788_s_at | -0.0452 | 0.0396 | -1.142 | 0.2623021 | 0.617963 |
| X201693_s_at | -0.147 | 0.1287 | -1.142 | 0.262318 | 0.617963 |
| X213055_at | 0.1387 | 0.1215 | 1.142 | 0.2623298 | 0.617963 |
| X208479_at | -0.1803 | 0.158 | -1.142 | 0.2623768 | 0.617979 |
| X213068_at | -0.0064 | 0.0056 | -1.142 | 0.2623919 | 0.617979 |
| X221070_s_at | -0.0063 | 0.0055 | -1.141 | 0.2625442 | 0.618242 |
| X204974_at | 0.029 | 0.0254 | 1.141 | 0.2625595 | 0.618242 |
| X203245_s_at | 0.0393 | 0.0345 | 1.141 | 0.2626321 | 0.618348 |
| X219871_at | -0.0997 | 0.0874 | -1.141 | 0.2627677 | 0.61845 |
| X206228_at | 0.0053 | 0.0047 | 1.141 | 0.2627837 | 0.61845 |
| X203909_at | -0.0601 | 0.0527 | -1.141 | 0.2627853 | 0.61845 |
| X203921_at | -0.0794 | 0.0696 | -1.141 | 0.2628042 | 0.61845 |
| X203047_at | 0.0035 | 0.003 | 1.14 | 0.2628367 | 0.61845 |
| X214171_s_at | 0.0039 | 0.0034 | 1.14 | 0.2628672 | 0.61845 |
| X214289_at | 0.0054 | 0.0047 | 1.14 | 0.2628703 | 0.61845 |
| X205296_at | 0.061 | 0.0535 | 1.14 | 0.2629723 | 0.618624 |
| X205326_at | 0.004 | 0.0035 | 1.14 | 0.2630966 | 0.618851 |
| X206347_at | -0.0097 | 0.0085 | -1.139 | 0.2632716 | 0.61907 |
| X217453_at | -0.0036 | 0.0031 | -1.139 | 0.2632751 | 0.61907 |
| X208934_s_at | -0.0943 | 0.0828 | -1.139 | 0.2632835 | 0.61907 |
| X215771_x_at | -0.0038 | 0.0033 | -1.139 | 0.2633339 | 0.61907 |
| X219298_at | 0.0044 | 0.0039 | 1.139 | 0.2633381 | 0.61907 |
| X205816_at | 0.0055 | 0.0048 | 1.139 | 0.2633641 | 0.61907 |
| X214331_at | -0.0483 | 0.0424 | -1.139 | 0.2633846 | 0.61907 |
| X221115_s_at | -0.0042 | 0.0037 | -1.139 | 0.2634452 | 0.619082 |
| X204462_s_at | -0.0238 | 0.0209 | -1.139 | 0.263455 | 0.619082 |
| X219394_at | 0.0186 | 0.0163 | 1.139 | 0.2634737 | 0.619082 |
| X203441_s_at | 0.0498 | 0.0438 | 1.139 | 0.2635888 | 0.61924 |
| X212065_s_at | 0.0935 | 0.0821 | 1.139 | 0.2635967 | 0.61924 |
| X216214_at | -0.0123 | 0.0108 | -1.138 | 0.2637329 | 0.619495 |
| X205683_x_at | 0.0043 | 0.0037 | 1.138 | 0.2637802 | 0.61952 |
| X212377_s_at | 0.0764 | 0.0671 | 1.138 | 0.2637995 | 0.61952 |
| X202437_s_at | 0.1263 | 0.111 | 1.138 | 0.2639247 | 0.619749 |
| X220060_s_at | -0.0122 | 0.0108 | -1.137 | 0.2641326 | 0.620142 |
| X210561_s_at | 0.0589 | 0.0518 | 1.137 | 0.264148 | 0.620142 |
| X218741_at | 0.0031 | 0.0027 | 1.137 | 0.2642329 | 0.620276 |
| X203138_at | -0.0538 | 0.0474 | -1.137 | 0.2642744 | 0.620308 |
| X217836_s_at | -0.0292 | 0.0257 | -1.137 | 0.2643324 | 0.620378 |
| X213437_at | 0.0531 | 0.0468 | 1.137 | 0.2644022 | 0.620477 |
| X220236_at | -0.0037 | 0.0033 | -1.136 | 0.2644905 | 0.620576 |
| X220016_at | 0.0046 | 0.0041 | 1.136 | 0.2645681 | 0.620576 |
| X203134_at | -0.0159 | 0.014 | -1.136 | 0.264572 | 0.620576 |
| X211137_s_at | -0.0295 | 0.026 | -1.136 | 0.2645771 | 0.620576 |
| X202664_at | 0.1333 | 0.1173 | 1.136 | 0.2645841 | 0.620576 |
| X201462_at | -0.0225 | 0.0198 | -1.136 | 0.2646557 | 0.620673 |
| X211069_s_at | -0.0317 | 0.0279 | -1.136 | 0.2646814 | 0.620673 |
| X219321_at | 0.0486 | 0.0428 | 1.136 | 0.2647838 | 0.620847 |
| X219797_at | -0.0271 | 0.0238 | -1.136 | 0.2648615 | 0.620934 |
| X217676_at | -0.0044 | 0.0039 | -1.135 | 0.2649225 | 0.620934 |
| X213211_s_at | -0.0081 | 0.0071 | -1.135 | 0.2649246 | 0.620934 |
| X32625_at | -0.0083 | 0.0073 | -1.135 | 0.2649475 | 0.620934 |
| X213861_s_at | 0.0081 | 0.0072 | 1.135 | 0.2650419 | 0.620934 |
| X206385_s_at | 0.0369 | 0.0325 | 1.135 | 0.2650697 | 0.620934 |
| X209545_s_at | 0.004 | 0.0035 | 1.135 | 0.2650733 | 0.620934 |
| X205245_at | -0.0283 | 0.025 | -1.135 | 0.2650787 | 0.620934 |
| X221803_s_at | -0.0274 | 0.0241 | -1.135 | 0.2651294 | 0.620934 |
| X210991_s_at | -0.0031 | 0.0027 | -1.135 | 0.265131 | 0.620934 |
| X208511_at | -0.0071 | 0.0063 | -1.135 | 0.2651384 | 0.620934 |
| X208247_at | -0.0095 | 0.0084 | -1.135 | 0.2651801 | 0.620934 |
| X218036_x_at | -0.0533 | 0.047 | -1.135 | 0.2651841 | 0.620934 |
| X215651_at | -0.0028 | 0.0025 | -1.134 | 0.2653471 | 0.62125 |
| X202524_s_at | -0.0462 | 0.0407 | -1.134 | 0.2655878 | 0.621748 |
| X215119_at | -0.0186 | 0.0164 | -1.133 | 0.2657349 | 0.622027 |
| X216917_s_at | -0.0088 | 0.0077 | -1.133 | 0.2657973 | 0.622043 |
| X205451_at | -0.0448 | 0.0395 | -1.133 | 0.2657977 | 0.622043 |
| X219324_at | 0.0387 | 0.0341 | 1.133 | 0.2658748 | 0.622158 |
| X204092_s_at | -0.0111 | 0.0098 | -1.133 | 0.2659492 | 0.622242 |
| X221778_at | -0.0307 | 0.0271 | -1.133 | 0.2659703 | 0.622242 |
| X206594_at | 0.0066 | 0.0058 | 1.133 | 0.2659948 | 0.622242 |
| X215124_at | -0.0043 | 0.0038 | -1.133 | 0.2660232 | 0.622243 |
| X200655_s_at | 0.0291 | 0.0257 | 1.133 | 0.2660869 | 0.622326 |
| X200852_x_at | -0.0344 | 0.0304 | -1.132 | 0.2661787 | 0.622438 |
| X207592_s_at | -0.0077 | 0.0068 | -1.132 | 0.2661908 | 0.622438 |
| X34697_at | 0.0507 | 0.0448 | 1.132 | 0.2662869 | 0.622597 |
| X217151_at | -0.0069 | 0.0061 | -1.132 | 0.2663811 | 0.622661 |
| X206945_at | 0.0046 | 0.0041 | 1.132 | 0.2663833 | 0.622661 |
| X208437_at | -0.0031 | 0.0028 | -1.132 | 0.2664194 | 0.622661 |
| X208258_s_at | -0.003 | 0.0027 | -1.132 | 0.2664263 | 0.622661 |
| X217998_at | -0.0249 | 0.022 | -1.131 | 0.2665246 | 0.622825 |
| X202843_at | -0.0615 | 0.0543 | -1.131 | 0.2665673 | 0.622832 |
| X203939_at | -0.1176 | 0.1039 | -1.131 | 0.2665835 | 0.622832 |
| X204991_s_at | -0.0114 | 0.0101 | -1.131 | 0.266634 | 0.622884 |
| X219247_s_at | 0.0766 | 0.0678 | 1.131 | 0.2666816 | 0.622926 |
| X203423_at | 0.0588 | 0.052 | 1.131 | 0.2667162 | 0.622926 |
| X215477_at | 0.0059 | 0.0052 | 1.131 | 0.2667359 | 0.622926 |
| X212397_at | 0.0801 | 0.0708 | 1.131 | 0.2667718 | 0.622944 |
| X221208_s_at | 0.0579 | 0.0512 | 1.131 | 0.2668355 | 0.623015 |
| X216409_at | 0.0047 | 0.0041 | 1.131 | 0.2668826 | 0.623015 |
| X60815_at | -0.1872 | 0.1656 | -1.131 | 0.2668862 | 0.623015 |
| X220715_at | -0.0024 | 0.0021 | -1.13 | 0.2670052 | 0.623095 |
| X204652_s_at | 0.0073 | 0.0065 | 1.13 | 0.2670642 | 0.623095 |
| X207020_at | -0.0051 | 0.0045 | -1.13 | 0.2670702 | 0.623095 |
| X212376_s_at | 0.0102 | 0.009 | 1.13 | 0.2670836 | 0.623095 |
| X212331_at | 0.0424 | 0.0375 | 1.13 | 0.2670901 | 0.623095 |
| X213591_at | 0.0506 | 0.0447 | 1.13 | 0.2672465 | 0.623095 |
| X214098_at | -0.0488 | 0.0432 | -1.13 | 0.2672887 | 0.623095 |
| X203724_s_at | 0.0313 | 0.0278 | 1.13 | 0.2673086 | 0.623095 |
| X220357_s_at | 0.0047 | 0.0042 | 1.13 | 0.2673203 | 0.623095 |
| X202172_at | 0.075 | 0.0664 | 1.129 | 0.2674151 | 0.623095 |
| X209278_s_at | 0.0052 | 0.0046 | 1.129 | 0.2674401 | 0.623095 |
| X214982_at | 0.0088 | 0.0078 | 1.129 | 0.2674645 | 0.623095 |
| X206504_at | -0.0297 | 0.0263 | -1.129 | 0.2674824 | 0.623095 |
| X206244_at | -0.0071 | 0.0063 | -1.129 | 0.2675062 | 0.623095 |
| X219430_at | -0.0037 | 0.0033 | -1.129 | 0.2675185 | 0.623095 |
| X213166_x_at | -0.0575 | 0.051 | -1.129 | 0.2675329 | 0.623095 |
| X202101_s_at | -0.0222 | 0.0197 | -1.129 | 0.2675374 | 0.623095 |
| X216621_at | -0.0166 | 0.0147 | -1.129 | 0.2675509 | 0.623095 |
| X214906_x_at | -0.0681 | 0.0603 | -1.129 | 0.2675841 | 0.623095 |
| X210284_s_at | -0.0361 | 0.0319 | -1.129 | 0.2675914 | 0.623095 |
| X209105_at | -0.0708 | 0.0627 | -1.129 | 0.2676003 | 0.623095 |
| X203990_s_at | 0.0054 | 0.0048 | 1.129 | 0.2676046 | 0.623095 |
| X201089_at | -0.0942 | 0.0835 | -1.129 | 0.2676248 | 0.623095 |
| X39549_at | -0.0413 | 0.0366 | -1.129 | 0.2676358 | 0.623095 |
| X217607_x_at | -0.0078 | 0.0069 | -1.129 | 0.2676414 | 0.623095 |
| X220949_s_at | 0.0153 | 0.0136 | 1.129 | 0.2676498 | 0.623095 |
| X213384_x_at | 0.0048 | 0.0042 | 1.129 | 0.2677011 | 0.623125 |
| X216875_x_at | -0.0061 | 0.0054 | -1.129 | 0.2677186 | 0.623125 |
| X208498_s_at | -0.0714 | 0.0633 | -1.128 | 0.2677706 | 0.623143 |
| X219755_at | -0.003 | 0.0026 | -1.128 | 0.2677828 | 0.623143 |
| X201608_s_at | -0.0458 | 0.0406 | -1.128 | 0.2678489 | 0.623232 |
| X207408_at | -0.0127 | 0.0112 | -1.128 | 0.2679379 | 0.623374 |
| X218077_s_at | 0.0182 | 0.0161 | 1.128 | 0.268011 | 0.623479 |
| X206405_x_at | 0.0421 | 0.0373 | 1.128 | 0.2681628 | 0.623766 |
| X221347_at | -0.0044 | 0.0039 | -1.127 | 0.2683272 | 0.624083 |
| X203454_s_at | -0.0569 | 0.0505 | -1.127 | 0.2683634 | 0.624102 |
| X201298_s_at | 0.1046 | 0.0928 | 1.127 | 0.2684417 | 0.624219 |
| X209783_at | -0.0055 | 0.0049 | -1.127 | 0.2684769 | 0.624236 |
| X217727_x_at | -0.0386 | 0.0342 | -1.127 | 0.2685307 | 0.624241 |
| X221310_at | -0.0782 | 0.0694 | -1.127 | 0.2685356 | 0.624241 |
| X210268_at | -0.0266 | 0.0236 | -1.127 | 0.2685789 | 0.624242 |
| X220152_at | 0.0063 | 0.0056 | 1.127 | 0.268592 | 0.624242 |
| X217963_s_at | -0.0209 | 0.0185 | -1.126 | 0.2686533 | 0.624263 |
| X209051_s_at | 0.0667 | 0.0592 | 1.126 | 0.2686573 | 0.624263 |
| X203994_s_at | -0.007 | 0.0062 | -1.126 | 0.2687665 | 0.624378 |
| X220140_s_at | 0.0332 | 0.0295 | 1.126 | 0.2687962 | 0.624378 |
| X218463_s_at | 0.0505 | 0.0449 | 1.126 | 0.2688262 | 0.624378 |
| X215327_at | 0.0037 | 0.0033 | 1.126 | 0.2688303 | 0.624378 |
| X218211_s_at | 0.0131 | 0.0116 | 1.126 | 0.2688588 | 0.624378 |
| X208552_at | -0.0025 | 0.0022 | -1.126 | 0.2688754 | 0.624378 |
| X204859_s_at | 0.0466 | 0.0414 | 1.125 | 0.2690862 | 0.624688 |
| X221330_at | 0.0039 | 0.0035 | 1.125 | 0.269132 | 0.624688 |
| X211868_x_at | -0.0034 | 0.0031 | -1.125 | 0.269135 | 0.624688 |
| X220794_at | -0.0596 | 0.053 | -1.125 | 0.2691609 | 0.624688 |
| X216398_at | -0.005 | 0.0045 | -1.125 | 0.2691618 | 0.624688 |
| X216333_x_at | 0.0196 | 0.0174 | 1.125 | 0.2692215 | 0.624688 |
| X204602_at | 0.0099 | 0.0088 | 1.125 | 0.2692294 | 0.624688 |
| X222274_at | 0.0155 | 0.0138 | 1.125 | 0.2692339 | 0.624688 |
| X218387_s_at | 0.0462 | 0.041 | 1.125 | 0.269336 | 0.62474 |
| X204565_at | -0.0467 | 0.0415 | -1.125 | 0.2693371 | 0.62474 |
| X222030_at | -0.006 | 0.0053 | -1.125 | 0.2693406 | 0.62474 |
| X211685_s_at | -0.0417 | 0.0371 | -1.124 | 0.2694497 | 0.624865 |
| X211518_s_at | 0.0033 | 0.0029 | 1.124 | 0.2694635 | 0.624865 |
| X206811_at | 0.0606 | 0.0539 | 1.124 | 0.2694908 | 0.624865 |
| X222240_s_at | 0.0048 | 0.0042 | 1.124 | 0.2695071 | 0.624865 |
| X215689_s_at | -0.0022 | 0.0019 | -1.124 | 0.2695811 | 0.624937 |
| X218670_at | -0.0115 | 0.0102 | -1.124 | 0.2695946 | 0.624937 |
| X204524_at | -0.0396 | 0.0352 | -1.124 | 0.2696494 | 0.624999 |
| X218300_at | 0.0455 | 0.0405 | 1.124 | 0.2697773 | 0.62523 |
| X221629_x_at | 0.0047 | 0.0042 | 1.124 | 0.2698299 | 0.625287 |
| X208396_s_at | 0.2924 | 0.2603 | 1.123 | 0.2698878 | 0.625356 |
| X211501_s_at | -0.037 | 0.033 | -1.123 | 0.2699517 | 0.625376 |
| X201212_at | -0.0785 | 0.0699 | -1.123 | 0.2699528 | 0.625376 |
| X202694_at | -0.0182 | 0.0162 | -1.123 | 0.2701651 | 0.625763 |
| X218554_s_at | -0.1029 | 0.0916 | -1.123 | 0.2701763 | 0.625763 |
| X202403_s_at | 0.1622 | 0.1445 | 1.123 | 0.2702675 | 0.625909 |
| X203498_at | -0.0492 | 0.0438 | -1.122 | 0.2703999 | 0.626057 |
| X202533_s_at | 0.0125 | 0.0111 | 1.122 | 0.2704116 | 0.626057 |
| X215041_s_at | 0.0041 | 0.0037 | 1.122 | 0.2704159 | 0.626057 |
| X203107_x_at | 0.0348 | 0.031 | 1.122 | 0.270615 | 0.626453 |
| X203410_at | -0.0557 | 0.0497 | -1.122 | 0.2706461 | 0.62646 |
| X215524_x_at | -0.0097 | 0.0087 | -1.121 | 0.2707246 | 0.626576 |
| X216865_at | 0.0086 | 0.0077 | 1.121 | 0.2708548 | 0.626778 |
| X206071_s_at | -0.004 | 0.0036 | -1.121 | 0.2708799 | 0.626778 |
| X221767_x_at | -0.0116 | 0.0103 | -1.121 | 0.2708966 | 0.626778 |
| X207972_at | -0.0033 | 0.0029 | -1.121 | 0.2710004 | 0.626911 |
| X209472_at | -0.0858 | 0.0766 | -1.121 | 0.2710105 | 0.626911 |
| X220531_at | 0.0051 | 0.0046 | 1.12 | 0.2711153 | 0.627079 |
| X220286_at | -0.0024 | 0.0021 | -1.12 | 0.2711469 | 0.627079 |
| X221863_at | 0.0035 | 0.0031 | 1.12 | 0.2711678 | 0.627079 |
| X205241_at | -0.0683 | 0.061 | -1.12 | 0.2715011 | 0.627771 |
| X217787_s_at | 0.0123 | 0.011 | 1.12 | 0.2715237 | 0.627771 |
| X206689_x_at | -0.0133 | 0.0119 | -1.119 | 0.271602 | 0.627887 |
| X212379_at | -0.0031 | 0.0028 | -1.119 | 0.2717331 | 0.628125 |
| X207896_s_at | 0.0053 | 0.0048 | 1.119 | 0.2717697 | 0.628144 |
| X201507_at | -0.0152 | 0.0136 | -1.119 | 0.2718046 | 0.628146 |
| X218165_at | -0.0617 | 0.0551 | -1.119 | 0.2718438 | 0.628146 |
| X208878_s_at | 0.0506 | 0.0452 | 1.119 | 0.2718641 | 0.628146 |
| X202339_at | -0.0074 | 0.0066 | -1.119 | 0.271909 | 0.628146 |
| X211000_s_at | 0.1632 | 0.1459 | 1.119 | 0.2719373 | 0.628146 |
| X201173_x_at | -0.0343 | 0.0307 | -1.119 | 0.2719404 | 0.628146 |
| X218617_at | 0.0382 | 0.0341 | 1.118 | 0.2721877 | 0.628652 |
| X215945_s_at | -0.0605 | 0.0541 | -1.118 | 0.2722219 | 0.628666 |
| X211009_s_at | -0.0457 | 0.0409 | -1.118 | 0.2723439 | 0.628882 |
| X217364_x_at | -0.0178 | 0.0159 | -1.117 | 0.2723891 | 0.628905 |
| X203975_s_at | 0.0123 | 0.011 | 1.117 | 0.2724104 | 0.628905 |
| X206894_at | 0.0053 | 0.0047 | 1.117 | 0.2724503 | 0.628932 |
| X212118_at | -0.0351 | 0.0314 | -1.117 | 0.2724878 | 0.628953 |
| X209534_x_at | 0.0855 | 0.0765 | 1.117 | 0.2725829 | 0.629107 |
| X212545_s_at | -0.0037 | 0.0033 | -1.117 | 0.2727133 | 0.629285 |
| X219986_s_at | -0.01 | 0.0089 | -1.117 | 0.2727252 | 0.629285 |
| X210129_s_at | 0.005 | 0.0045 | 1.117 | 0.2727451 | 0.629285 |
| X210982_s_at | 0.3481 | 0.3118 | 1.116 | 0.2728133 | 0.629377 |
| X214814_at | 0.0468 | 0.0419 | 1.116 | 0.2728493 | 0.629395 |
| X203561_at | -0.0127 | 0.0113 | -1.116 | 0.2730336 | 0.629705 |
| X219645_at | 0.007 | 0.0063 | 1.116 | 0.2730508 | 0.629705 |
| X207217_s_at | -0.0041 | 0.0037 | -1.116 | 0.2730689 | 0.629705 |
| X210270_at | -0.0418 | 0.0374 | -1.116 | 0.273158 | 0.629783 |
| X218021_at | -0.0376 | 0.0337 | -1.115 | 0.2732156 | 0.629783 |
| X220111_s_at | -0.0131 | 0.0118 | -1.115 | 0.2732178 | 0.629783 |
| X213984_at | -0.0614 | 0.0551 | -1.115 | 0.2732621 | 0.629783 |
| X215968_at | 0.0217 | 0.0195 | 1.115 | 0.2732688 | 0.629783 |
| X204383_at | 0.0045 | 0.004 | 1.115 | 0.2732727 | 0.629783 |
| X201810_s_at | -0.0707 | 0.0634 | -1.115 | 0.2733823 | 0.62997 |
| X209877_at | -0.0035 | 0.0031 | -1.115 | 0.2734815 | 0.630134 |
| X218333_at | 0.0412 | 0.0369 | 1.115 | 0.2735253 | 0.630169 |
| X203547_at | -0.0038 | 0.0034 | -1.115 | 0.2735766 | 0.630207 |
| X206792_x_at | 0.0715 | 0.0642 | 1.115 | 0.2735985 | 0.630207 |
| X218052_s_at | -0.0243 | 0.0218 | -1.114 | 0.2737389 | 0.630465 |
| X203818_s_at | -0.0315 | 0.0283 | -1.114 | 0.2738931 | 0.630755 |
| X216690_at | 0.0065 | 0.0058 | 1.114 | 0.273932 | 0.630779 |
| X211946_s_at | 0.0249 | 0.0223 | 1.113 | 0.2740553 | 0.630981 |
| X209026_x_at | 0.0593 | 0.0533 | 1.113 | 0.2740763 | 0.630981 |
| X220109_at | 0.0576 | 0.0518 | 1.113 | 0.274107 | 0.630986 |
| X54037_at | -0.0423 | 0.038 | -1.113 | 0.2741377 | 0.630991 |
| X202880_s_at | -0.0372 | 0.0334 | -1.113 | 0.274194 | 0.631015 |
| X221974_at | 0.0615 | 0.0553 | 1.113 | 0.2742068 | 0.631015 |
| X217419_x_at | -0.0058 | 0.0052 | -1.113 | 0.2742335 | 0.631015 |
| X216303_s_at | -0.0071 | 0.0064 | -1.113 | 0.2743155 | 0.631139 |
| X206430_at | -0.0026 | 0.0023 | -1.113 | 0.2743775 | 0.631216 |
| X213129_s_at | 0.0406 | 0.0365 | 1.113 | 0.2744351 | 0.631219 |
| X213740_s_at | -0.0034 | 0.003 | -1.113 | 0.2744358 | 0.631219 |
| X215973_at | 0.0066 | 0.0059 | 1.113 | 0.2744668 | 0.631225 |
| X215348_at | -0.0043 | 0.0039 | -1.112 | 0.2745512 | 0.631298 |
| X206880_at | 0.0042 | 0.0038 | 1.112 | 0.2745553 | 0.631298 |
| X200945_s_at | 0.0381 | 0.0343 | 1.112 | 0.2747486 | 0.631643 |
| X49878_at | -0.0253 | 0.0228 | -1.112 | 0.2747879 | 0.631643 |
| X215479_at | 0.0058 | 0.0052 | 1.112 | 0.2747907 | 0.631643 |
| X210656_at | -0.0138 | 0.0124 | -1.111 | 0.2750016 | 0.632063 |
| X204850_s_at | -0.0662 | 0.0596 | -1.111 | 0.2751083 | 0.632145 |
| X210569_s_at | -0.0029 | 0.0027 | -1.111 | 0.2751464 | 0.632145 |
| X201415_at | -0.0429 | 0.0386 | -1.111 | 0.2751504 | 0.632145 |
| X204032_at | 0.0747 | 0.0672 | 1.111 | 0.2751514 | 0.632145 |
| X206889_at | -0.0046 | 0.0041 | -1.111 | 0.2752065 | 0.632207 |
| X211956_s_at | -0.021 | 0.0189 | -1.11 | 0.2753428 | 0.632413 |
| X201548_s_at | -0.0638 | 0.0575 | -1.11 | 0.2753534 | 0.632413 |
| X221817_at | -0.0078 | 0.0071 | -1.11 | 0.2754771 | 0.632581 |
| X212108_at | 0.0289 | 0.026 | 1.11 | 0.2754832 | 0.632581 |
| X211776_s_at | 0.0762 | 0.0687 | 1.11 | 0.2755154 | 0.632589 |
| X207090_x_at | 0.0255 | 0.0229 | 1.11 | 0.2755882 | 0.632679 |
| X219098_at | -0.0208 | 0.0187 | -1.11 | 0.2756125 | 0.632679 |
| X219178_at | -0.0264 | 0.0238 | -1.11 | 0.2756399 | 0.632679 |
| X214588_s_at | -0.0147 | 0.0133 | -1.11 | 0.2757246 | 0.632773 |
| X220999_s_at | -0.0697 | 0.0628 | -1.11 | 0.2757379 | 0.632773 |
| X209071_s_at | 0.1115 | 0.1005 | 1.109 | 0.2757956 | 0.63284 |
| X212685_s_at | 0.0237 | 0.0213 | 1.109 | 0.2759387 | 0.633103 |
| X209680_s_at | -0.0042 | 0.0038 | -1.109 | 0.2759937 | 0.633117 |
| X214298_x_at | 0.0064 | 0.0058 | 1.109 | 0.2760019 | 0.633117 |
| X208627_s_at | 0.0492 | 0.0444 | 1.109 | 0.2760594 | 0.633184 |
| X213039_at | 0.0413 | 0.0373 | 1.109 | 0.2761178 | 0.633252 |
| X201471_s_at | 0.0369 | 0.0332 | 1.109 | 0.2761593 | 0.633261 |
| X201093_x_at | 0.0432 | 0.039 | 1.108 | 0.2761787 | 0.633261 |
| X213000_at | -0.0319 | 0.0288 | -1.108 | 0.2763463 | 0.633482 |
| X201389_at | 0.0168 | 0.0152 | 1.108 | 0.276373 | 0.633482 |
| X218960_at | -0.0041 | 0.0037 | -1.108 | 0.2763759 | 0.633482 |
| X211638_at | -0.0045 | 0.0041 | -1.108 | 0.2763889 | 0.633482 |
| X219210_s_at | -0.012 | 0.0108 | -1.108 | 0.2765047 | 0.633643 |
| X212485_at | 0.0532 | 0.048 | 1.108 | 0.2765385 | 0.633643 |
| X211742_s_at | -0.103 | 0.093 | -1.108 | 0.276565 | 0.633643 |
| X205297_s_at | -0.0073 | 0.0066 | -1.108 | 0.2765736 | 0.633643 |
| X202359_s_at | 0.0368 | 0.0332 | 1.107 | 0.2766277 | 0.633702 |
| X217500_at | 0.0032 | 0.0029 | 1.107 | 0.276721 | 0.63385 |
| X215502_at | -0.0031 | 0.0028 | -1.107 | 0.2768206 | 0.633958 |
| X211037_s_at | -0.0035 | 0.0031 | -1.107 | 0.2768298 | 0.633958 |
| X212374_at | 0.0043 | 0.0039 | 1.107 | 0.276873 | 0.633958 |
| X206749_at | -0.0059 | 0.0053 | -1.107 | 0.2769032 | 0.633958 |
| X204378_at | 0.1282 | 0.1159 | 1.107 | 0.2769107 | 0.633958 |
| X215737_x_at | 0.0491 | 0.0443 | 1.107 | 0.2770122 | 0.634125 |
| X204549_at | 0.0059 | 0.0053 | 1.106 | 0.277098 | 0.634256 |
| X37566_at | 0.0559 | 0.0506 | 1.106 | 0.2772168 | 0.634463 |
| X219215_s_at | 0.0051 | 0.0046 | 1.106 | 0.2772626 | 0.634483 |
| X202354_s_at | 0.0729 | 0.0659 | 1.106 | 0.2772933 | 0.634483 |
| X210652_s_at | -0.0454 | 0.041 | -1.106 | 0.2773281 | 0.634483 |
| X220645_at | 0.0043 | 0.0039 | 1.106 | 0.2773401 | 0.634483 |
| X208945_s_at | -0.069 | 0.0624 | -1.106 | 0.2774254 | 0.634613 |
| X208769_at | 0.0044 | 0.0039 | 1.105 | 0.2774638 | 0.634636 |
| X209658_at | -0.0316 | 0.0286 | -1.105 | 0.2775353 | 0.634705 |
| X222072_at | 0.0117 | 0.0106 | 1.105 | 0.2775514 | 0.634705 |
| X208567_s_at | 0.0083 | 0.0075 | 1.105 | 0.2776499 | 0.634865 |
| X218712_at | -0.0417 | 0.0377 | -1.105 | 0.2778073 | 0.63516 |
| X204694_at | 0.0039 | 0.0035 | 1.104 | 0.2779392 | 0.635382 |
| X218975_at | -0.005 | 0.0045 | -1.104 | 0.2779616 | 0.635382 |
| X211276_at | 0.0222 | 0.0201 | 1.104 | 0.2780039 | 0.635413 |
| X214797_s_at | -0.0046 | 0.0042 | -1.104 | 0.2781684 | 0.635516 |
| X222185_at | 0.003 | 0.0027 | 1.104 | 0.2781798 | 0.635516 |
| X214678_x_at | 0.0409 | 0.0371 | 1.104 | 0.2781838 | 0.635516 |
| X203970_s_at | -0.0377 | 0.0341 | -1.104 | 0.2781867 | 0.635516 |
| X222381_at | -0.0086 | 0.0078 | -1.104 | 0.2782099 | 0.635516 |
| X211125_x_at | 0.0034 | 0.0031 | 1.104 | 0.2782251 | 0.635516 |
| X212153_at | 0.0271 | 0.0246 | 1.104 | 0.2782634 | 0.635516 |
| X221663_x_at | 0.0089 | 0.008 | 1.104 | 0.2782824 | 0.635516 |
| X203028_s_at | 0.136 | 0.1233 | 1.103 | 0.2783063 | 0.635516 |
| X215334_at | -0.0076 | 0.0069 | -1.103 | 0.2783503 | 0.635526 |
| X202757_at | 0.0468 | 0.0424 | 1.103 | 0.2783679 | 0.635526 |
| X215547_at | 0.0046 | 0.0041 | 1.103 | 0.2784936 | 0.635747 |
| X202064_s_at | 0.0098 | 0.0089 | 1.103 | 0.2786616 | 0.636065 |
| X32836_at | 0.028 | 0.0254 | 1.103 | 0.2786985 | 0.636084 |
| X204089_x_at | 0.0369 | 0.0334 | 1.102 | 0.2787819 | 0.636124 |
| X215193_x_at | 0.2066 | 0.1874 | 1.102 | 0.2787929 | 0.636124 |
| X201221_s_at | 0.0432 | 0.0392 | 1.102 | 0.2788191 | 0.636124 |
| X214323_s_at | -0.0403 | 0.0366 | -1.102 | 0.2788306 | 0.636124 |
| X217061_s_at | -0.0051 | 0.0046 | -1.102 | 0.2788723 | 0.636154 |
| X212608_s_at | -0.0209 | 0.0189 | -1.102 | 0.2790051 | 0.636276 |
| X212120_at | -0.0562 | 0.051 | -1.102 | 0.2790111 | 0.636276 |
| X218875_s_at | -0.0344 | 0.0312 | -1.102 | 0.2790333 | 0.636276 |
| X206864_s_at | 0.0074 | 0.0067 | 1.102 | 0.2790577 | 0.636276 |
| X211653_x_at | 0.0535 | 0.0485 | 1.102 | 0.279069 | 0.636276 |
| X209653_at | 0.0119 | 0.0108 | 1.102 | 0.279135 | 0.636361 |
| X209533_s_at | -0.0578 | 0.0525 | -1.101 | 0.2793644 | 0.636819 |
| X208737_at | 0.0452 | 0.0411 | 1.101 | 0.2794159 | 0.636871 |
| X216977_x_at | -0.0194 | 0.0176 | -1.101 | 0.2794574 | 0.6369 |
| X202419_at | 0.039 | 0.0354 | 1.1 | 0.2796165 | 0.637198 |
| X217067_s_at | -0.017 | 0.0155 | -1.1 | 0.2796666 | 0.637246 |
| X203748_x_at | -0.0627 | 0.057 | -1.1 | 0.2797049 | 0.637268 |
| X206133_at | 0.0815 | 0.074 | 1.1 | 0.2797835 | 0.637382 |
| X211526_s_at | -0.0052 | 0.0047 | -1.1 | 0.2798775 | 0.637531 |
| X207581_s_at | -0.0074 | 0.0067 | -1.1 | 0.2799278 | 0.637567 |
| X201923_at | -0.0656 | 0.0596 | -1.1 | 0.279951 | 0.637567 |
| X203034_s_at | -0.0227 | 0.0206 | -1.099 | 0.2800237 | 0.637606 |
| X203846_at | -0.0918 | 0.0835 | -1.099 | 0.2800254 | 0.637606 |
| X207500_at | 0.0076 | 0.007 | 1.099 | 0.2800978 | 0.637706 |
| X212134_at | 0.0438 | 0.0399 | 1.099 | 0.2803189 | 0.638144 |
| X221003_s_at | 0.0034 | 0.0031 | 1.099 | 0.2804151 | 0.638297 |
| X219643_at | -0.0653 | 0.0594 | -1.099 | 0.280445 | 0.6383 |
| X206181_at | 0.0029 | 0.0027 | 1.098 | 0.2804832 | 0.6383 |
| X219573_at | -0.07 | 0.0638 | -1.098 | 0.2805024 | 0.6383 |
| X201440_at | 0.0076 | 0.0069 | 1.098 | 0.2805391 | 0.638318 |
| X200009_at | -0.0429 | 0.0391 | -1.098 | 0.2806111 | 0.638366 |
| X211429_s_at | 0.0155 | 0.0141 | 1.098 | 0.2806269 | 0.638366 |
| X204271_s_at | -0.1735 | 0.158 | -1.098 | 0.2806497 | 0.638366 |
| X1773_at | -0.0028 | 0.0025 | -1.098 | 0.2806753 | 0.638366 |
| X208891_at | -0.1269 | 0.1156 | -1.098 | 0.2807861 | 0.638483 |
| X213002_at | -0.054 | 0.0492 | -1.098 | 0.2808079 | 0.638483 |
| X203372_s_at | 0.0073 | 0.0066 | 1.098 | 0.2808282 | 0.638483 |
| X202211_at | 0.0677 | 0.0616 | 1.098 | 0.2808607 | 0.638483 |
| X205114_s_at | -0.0092 | 0.0084 | -1.098 | 0.2808706 | 0.638483 |
| X206826_at | 0.0655 | 0.0597 | 1.097 | 0.2809763 | 0.638658 |
| X209980_s_at | 0.0036 | 0.0033 | 1.097 | 0.2810616 | 0.638787 |
| X222180_at | 0.0073 | 0.0066 | 1.097 | 0.2811093 | 0.638788 |
| X214493_s_at | -0.0122 | 0.0111 | -1.097 | 0.2811248 | 0.638788 |
| X202833_s_at | 0.0084 | 0.0076 | 1.097 | 0.2812006 | 0.638788 |
| X201605_x_at | 0.0068 | 0.0062 | 1.097 | 0.2812261 | 0.638788 |
| X210772_at | 0.0074 | 0.0067 | 1.097 | 0.2812648 | 0.638788 |
| X221076_at | -0.011 | 0.01 | -1.097 | 0.2812779 | 0.638788 |
| X203900_at | -0.0053 | 0.0048 | -1.097 | 0.28128 | 0.638788 |
| X205536_at | -0.007 | 0.0064 | -1.097 | 0.2812923 | 0.638788 |
| X208533_at | -0.0031 | 0.0028 | -1.096 | 0.2813219 | 0.63879 |
| X215340_at | 0.004 | 0.0036 | 1.096 | 0.2814059 | 0.638916 |
| X202028_s_at | 0.1028 | 0.0938 | 1.096 | 0.281525 | 0.639087 |
| X204419_x_at | 0.0073 | 0.0066 | 1.096 | 0.2815387 | 0.639087 |
| X208385_at | -0.0023 | 0.0021 | -1.096 | 0.281593 | 0.639107 |
| X222194_at | -0.007 | 0.0064 | -1.096 | 0.2816053 | 0.639107 |
| X201777_s_at | 0.0447 | 0.0408 | 1.096 | 0.2816817 | 0.639215 |
| X208935_s_at | -0.0545 | 0.0498 | -1.095 | 0.2817398 | 0.639282 |
| X207993_s_at | 0.0029 | 0.0026 | 1.095 | 0.2820034 | 0.639815 |
| X208210_at | 0.0039 | 0.0036 | 1.095 | 0.2820596 | 0.639836 |
| X213016_at | -0.057 | 0.0521 | -1.095 | 0.2820703 | 0.639836 |
| X213877_x_at | -0.0288 | 0.0263 | -1.095 | 0.2821178 | 0.639878 |
| X219102_at | -0.0055 | 0.005 | -1.094 | 0.282207 | 0.640015 |
| X219092_s_at | 0.0136 | 0.0124 | 1.094 | 0.2825637 | 0.640759 |
| X207556_s_at | 0.0384 | 0.0351 | 1.093 | 0.2826753 | 0.640915 |
| X202125_s_at | 0.0751 | 0.0687 | 1.093 | 0.2826905 | 0.640915 |
| X212879_x_at | 0.0045 | 0.0041 | 1.093 | 0.2827369 | 0.640955 |
| X212550_at | -0.0044 | 0.0041 | -1.093 | 0.2828291 | 0.641041 |
| X200648_s_at | -0.0736 | 0.0674 | -1.093 | 0.2828676 | 0.641041 |
| X208955_at | -0.0659 | 0.0603 | -1.093 | 0.2828952 | 0.641041 |
| X217357_at | -0.0048 | 0.0044 | -1.093 | 0.2829188 | 0.641041 |
| X208594_x_at | -0.0024 | 0.0022 | -1.093 | 0.2829192 | 0.641041 |
| X212256_at | 0.0614 | 0.0562 | 1.093 | 0.2829737 | 0.641089 |
| X203592_s_at | 0.0051 | 0.0047 | 1.093 | 0.282998 | 0.641089 |
| X203849_s_at | 0.1221 | 0.1118 | 1.092 | 0.2831706 | 0.641415 |
| X208022_s_at | 0.0676 | 0.0619 | 1.092 | 0.2832188 | 0.641438 |
| X217511_at | 0.0059 | 0.0054 | 1.092 | 0.2832515 | 0.641438 |
| X210300_at | -0.0072 | 0.0066 | -1.092 | 0.2832773 | 0.641438 |
| X207162_s_at | -0.0028 | 0.0025 | -1.092 | 0.2833086 | 0.641438 |
| X203474_at | -0.0078 | 0.0072 | -1.092 | 0.2833252 | 0.641438 |
| X217659_at | 0.0146 | 0.0134 | 1.092 | 0.2834091 | 0.641562 |
| X219513_s_at | -0.0038 | 0.0035 | -1.091 | 0.2834764 | 0.641617 |
| X216581_at | 0.0041 | 0.0038 | 1.091 | 0.2834911 | 0.641617 |
| X203149_at | 0.0092 | 0.0085 | 1.091 | 0.2835573 | 0.641702 |
| X215538_at | -0.0039 | 0.0036 | -1.091 | 0.283657 | 0.641767 |
| X217945_at | -0.0182 | 0.0167 | -1.091 | 0.2836663 | 0.641767 |
| X202108_at | -0.019 | 0.0174 | -1.091 | 0.2836727 | 0.641767 |
| X219018_s_at | -0.0081 | 0.0074 | -1.091 | 0.2837138 | 0.641794 |
| X210755_at | 0.0093 | 0.0085 | 1.091 | 0.2838537 | 0.642045 |
| X219804_at | -0.0071 | 0.0066 | -1.09 | 0.2839428 | 0.642157 |
| X213043_s_at | 0.0299 | 0.0274 | 1.09 | 0.283961 | 0.642157 |
| X209312_x_at | 0.1409 | 0.1292 | 1.09 | 0.2840147 | 0.642159 |
| X220720_x_at | 0.0889 | 0.0815 | 1.09 | 0.2840258 | 0.642159 |
| X219799_s_at | -0.0296 | 0.0272 | -1.09 | 0.2840487 | 0.642159 |
| X201983_s_at | 0.0925 | 0.0849 | 1.09 | 0.2843156 | 0.642686 |
| X205252_at | -0.0095 | 0.0087 | -1.089 | 0.2843394 | 0.642686 |
| X207130_at | -0.0574 | 0.0527 | -1.089 | 0.2844059 | 0.642707 |
| X202855_s_at | 0.0028 | 0.0026 | 1.089 | 0.2844068 | 0.642707 |
| X220544_at | -0.0038 | 0.0035 | -1.089 | 0.284517 | 0.642891 |
| X216823_at | -0.0094 | 0.0087 | -1.089 | 0.2846157 | 0.642936 |
| X217888_s_at | 0.0309 | 0.0284 | 1.089 | 0.2846185 | 0.642936 |
| X220934_s_at | -0.0373 | 0.0343 | -1.089 | 0.2846503 | 0.642936 |
| X218772_x_at | -0.0609 | 0.0559 | -1.089 | 0.2846525 | 0.642936 |
| X215782_at | -0.0047 | 0.0043 | -1.089 | 0.284733 | 0.643009 |
| X201758_at | -0.0325 | 0.0299 | -1.089 | 0.2847431 | 0.643009 |
| X201100_s_at | -0.0174 | 0.016 | -1.088 | 0.284828 | 0.643136 |
| X210492_at | -0.02 | 0.0184 | -1.088 | 0.2848739 | 0.643174 |
| X219739_at | 0.0051 | 0.0047 | 1.088 | 0.2850814 | 0.643577 |
| X210400_at | 0.0573 | 0.0527 | 1.088 | 0.285135 | 0.643633 |
| X215974_at | 0.0034 | 0.0031 | 1.088 | 0.285171 | 0.643649 |
| X204928_s_at | 0.0055 | 0.005 | 1.087 | 0.2852506 | 0.643714 |
| X203538_at | 0.0496 | 0.0456 | 1.087 | 0.285258 | 0.643714 |
| X203593_at | 0.0405 | 0.0373 | 1.087 | 0.2853239 | 0.643797 |
| X215876_at | -0.0025 | 0.0023 | -1.087 | 0.2853657 | 0.643826 |
| X217377_x_at | 0.0748 | 0.0688 | 1.087 | 0.2855699 | 0.644069 |
| X222186_at | 0.0061 | 0.0056 | 1.087 | 0.2855795 | 0.644069 |
| X207091_at | -0.0415 | 0.0382 | -1.087 | 0.2855957 | 0.644069 |
| X207916_at | -0.0072 | 0.0067 | -1.087 | 0.2856239 | 0.644069 |
| X202222_s_at | -0.0077 | 0.0071 | -1.086 | 0.28564 | 0.644069 |
| X218413_s_at | -0.0173 | 0.0159 | -1.086 | 0.2856471 | 0.644069 |
| X202800_at | 0.0946 | 0.0871 | 1.086 | 0.2857431 | 0.644157 |
| X208969_at | -0.0267 | 0.0246 | -1.086 | 0.2857441 | 0.644157 |
| X214759_at | 0.0041 | 0.0038 | 1.086 | 0.2858196 | 0.644232 |
| X213810_s_at | -0.0056 | 0.0051 | -1.086 | 0.2858355 | 0.644232 |
| X211977_at | -0.0048 | 0.0045 | -1.086 | 0.2858905 | 0.644291 |
| X217415_at | -0.0031 | 0.0028 | -1.086 | 0.2859195 | 0.644291 |
| X219639_x_at | -0.0305 | 0.0281 | -1.086 | 0.2860132 | 0.644435 |
| X212111_at | 0.0396 | 0.0365 | 1.086 | 0.2860602 | 0.644435 |
| X201593_s_at | 0.0481 | 0.0443 | 1.085 | 0.2860707 | 0.644435 |
| X208768_x_at | 0.031 | 0.0286 | 1.085 | 0.286156 | 0.644562 |
| X220565_at | -0.0028 | 0.0026 | -1.085 | 0.2863671 | 0.644972 |
| X212457_at | 0.0358 | 0.033 | 1.085 | 0.2864796 | 0.645089 |
| X201044_x_at | 0.0126 | 0.0116 | 1.085 | 0.2864821 | 0.645089 |
| X210953_at | -0.0108 | 0.01 | -1.084 | 0.2865061 | 0.645089 |
| X202452_at | 0.0721 | 0.0665 | 1.084 | 0.2865967 | 0.645227 |
| X208777_s_at | 0.0531 | 0.049 | 1.084 | 0.2866969 | 0.64531 |
| X221822_at | -0.006 | 0.0055 | -1.084 | 0.2867375 | 0.64531 |
| X205164_at | -0.0355 | 0.0327 | -1.084 | 0.2867386 | 0.64531 |
| X207595_s_at | 0.0028 | 0.0026 | 1.084 | 0.2867656 | 0.64531 |
| X201904_s_at | 0.0351 | 0.0324 | 1.084 | 0.2867788 | 0.64531 |
| X215886_x_at | -0.0074 | 0.0068 | -1.084 | 0.2868796 | 0.645472 |
| X220856_x_at | 0.0353 | 0.0326 | 1.083 | 0.2869967 | 0.645652 |
| X221339_at | -0.0019 | 0.0017 | -1.083 | 0.2870399 | 0.645652 |
| X216909_at | 0.0032 | 0.003 | 1.083 | 0.2870467 | 0.645652 |
| X218527_at | -0.0358 | 0.033 | -1.083 | 0.2871875 | 0.645903 |
| X220938_s_at | 0.0027 | 0.0025 | 1.083 | 0.2872721 | 0.645973 |
| X219863_at | 0.0572 | 0.0528 | 1.083 | 0.287277 | 0.645973 |
| X202821_s_at | 0.0089 | 0.0083 | 1.083 | 0.2873122 | 0.645987 |
| X208322_s_at | 0.0062 | 0.0058 | 1.082 | 0.287494 | 0.646265 |
| X201778_s_at | 0.0397 | 0.0367 | 1.082 | 0.2875292 | 0.646265 |
| X212684_at | -0.0273 | 0.0252 | -1.082 | 0.287544 | 0.646265 |
| X205093_at | 0.0032 | 0.0029 | 1.082 | 0.2875727 | 0.646265 |
| X211542_x_at | -0.0245 | 0.0226 | -1.082 | 0.2875814 | 0.646265 |
| X215004_s_at | -0.0203 | 0.0188 | -1.082 | 0.2876205 | 0.646288 |
| X209565_at | -0.0272 | 0.0251 | -1.082 | 0.287681 | 0.646358 |
| X216177_at | -0.0092 | 0.0085 | -1.081 | 0.2878252 | 0.646528 |
| X213688_at | 0.1251 | 0.1156 | 1.081 | 0.2878277 | 0.646528 |
| X221054_s_at | -0.0068 | 0.0063 | -1.081 | 0.2878613 | 0.646528 |
| X202597_at | 0.0061 | 0.0056 | 1.081 | 0.2878728 | 0.646528 |
| X213373_s_at | 0.0203 | 0.0188 | 1.081 | 0.2881593 | 0.647106 |
| X210100_s_at | -0.0026 | 0.0024 | -1.08 | 0.2882679 | 0.647223 |
| X209204_at | -0.0586 | 0.0543 | -1.08 | 0.2882721 | 0.647223 |
| X221552_at | 0.0637 | 0.0589 | 1.08 | 0.2882987 | 0.647223 |
| X207414_s_at | -0.1928 | 0.1785 | -1.08 | 0.2883441 | 0.647259 |
| X209161_at | -0.0413 | 0.0383 | -1.08 | 0.2884517 | 0.647435 |
| X206125_s_at | 0.0036 | 0.0033 | 1.08 | 0.2885394 | 0.647523 |
| X206180_x_at | -0.0458 | 0.0424 | -1.08 | 0.2885489 | 0.647523 |
| X206177_s_at | 0.0069 | 0.0064 | 1.08 | 0.2886853 | 0.647763 |
| X211894_x_at | 0.0049 | 0.0045 | 1.079 | 0.2887662 | 0.647815 |
| X213450_s_at | -0.0054 | 0.005 | -1.079 | 0.2887668 | 0.647815 |
| X205088_at | -0.0863 | 0.0799 | -1.079 | 0.288817 | 0.647862 |
| X215661_at | -0.0063 | 0.0058 | -1.079 | 0.2890865 | 0.648401 |
| X207878_at | 0.0043 | 0.004 | 1.078 | 0.2892541 | 0.648712 |
| X213108_at | 0.0921 | 0.0854 | 1.078 | 0.289295 | 0.648738 |
| X203478_at | -0.0346 | 0.0321 | -1.078 | 0.2894103 | 0.648821 |
| X221253_s_at | 0.0206 | 0.0191 | 1.078 | 0.2894635 | 0.648821 |
| X209203_s_at | 0.0169 | 0.0157 | 1.078 | 0.2894875 | 0.648821 |
| X210277_at | -0.0481 | 0.0446 | -1.078 | 0.2894984 | 0.648821 |
| X52255_s_at | -0.0607 | 0.0563 | -1.078 | 0.2895152 | 0.648821 |
| X208836_at | -0.0299 | 0.0277 | -1.078 | 0.2895308 | 0.648821 |
| X213057_at | -0.0052 | 0.0048 | -1.078 | 0.2895364 | 0.648821 |
| X206232_s_at | 0.0069 | 0.0064 | 1.077 | 0.2897316 | 0.648964 |
| X219451_at | -0.0363 | 0.0337 | -1.077 | 0.2897456 | 0.648964 |
| X222349_x_at | 0.0043 | 0.0039 | 1.077 | 0.2897688 | 0.648964 |
| X217509_x_at | 0.022 | 0.0205 | 1.077 | 0.2898115 | 0.648964 |
| X219192_at | 0.062 | 0.0576 | 1.077 | 0.2898157 | 0.648964 |
| X222229_x_at | -0.027 | 0.0251 | -1.077 | 0.2898413 | 0.648964 |
| X219152_at | 0.0066 | 0.0062 | 1.077 | 0.2898433 | 0.648964 |
| X217324_at | 0.0059 | 0.0055 | 1.077 | 0.2899036 | 0.648964 |
| X219358_s_at | 0.0248 | 0.0231 | 1.077 | 0.2899041 | 0.648964 |
| X215324_at | -0.0063 | 0.0059 | -1.077 | 0.2899145 | 0.648964 |
| X214566_at | -0.0094 | 0.0087 | -1.077 | 0.2899217 | 0.648964 |
| X207660_at | 0.0103 | 0.0095 | 1.076 | 0.2900885 | 0.649272 |
| X209470_s_at | -0.0453 | 0.0421 | -1.076 | 0.2901185 | 0.649274 |
| X210535_at | -0.0076 | 0.0071 | -1.076 | 0.2901851 | 0.649306 |
| X216370_s_at | 0.0033 | 0.003 | 1.076 | 0.2901913 | 0.649306 |
| X211377_x_at | -0.0035 | 0.0033 | -1.076 | 0.2902265 | 0.649319 |
| X210092_at | -0.0851 | 0.0791 | -1.076 | 0.290317 | 0.649456 |
| X202918_s_at | -0.0439 | 0.0408 | -1.076 | 0.2904133 | 0.649512 |
| X209810_at | 0.0055 | 0.0052 | 1.076 | 0.2904278 | 0.649512 |
| X215180_at | -0.0076 | 0.0071 | -1.076 | 0.2904294 | 0.649512 |
| X210705_s_at | -0.0205 | 0.0191 | -1.075 | 0.2904822 | 0.649564 |
| X212537_x_at | -0.0256 | 0.0238 | -1.075 | 0.2905526 | 0.649602 |
| X217166_at | 0.0088 | 0.0081 | 1.075 | 0.2905614 | 0.649602 |
| X219291_at | 0.0455 | 0.0423 | 1.075 | 0.2905869 | 0.649602 |
| X215045_at | -0.0562 | 0.0523 | -1.075 | 0.2906354 | 0.649645 |
| X218637_at | -0.0645 | 0.06 | -1.075 | 0.2906845 | 0.64969 |
| X219228_at | -0.0684 | 0.0637 | -1.075 | 0.2907843 | 0.649847 |
| X215436_at | -0.0371 | 0.0346 | -1.075 | 0.2908161 | 0.649853 |
| X200880_at | -0.0634 | 0.059 | -1.074 | 0.2910008 | 0.650056 |
| X221110_x_at | -0.0063 | 0.0058 | -1.074 | 0.2910151 | 0.650056 |
| X207890_s_at | -0.0074 | 0.0069 | -1.074 | 0.2910224 | 0.650056 |
| X217688_at | -0.0047 | 0.0044 | -1.074 | 0.2910259 | 0.650056 |
| X219275_at | -0.0463 | 0.0431 | -1.074 | 0.2910533 | 0.650056 |
| X209357_at | 0.1603 | 0.1493 | 1.074 | 0.2911369 | 0.650163 |
| X207603_at | -0.0088 | 0.0082 | -1.074 | 0.2911597 | 0.650163 |
| X209870_s_at | 0.0692 | 0.0644 | 1.074 | 0.2912379 | 0.650221 |
| X203881_s_at | 0.0579 | 0.0539 | 1.074 | 0.2912443 | 0.650221 |
| X210517_s_at | -0.0657 | 0.0612 | -1.074 | 0.2912954 | 0.650235 |
| X200613_at | -0.0474 | 0.0441 | -1.073 | 0.2913378 | 0.650235 |
| X212139_at | 0.0481 | 0.0448 | 1.073 | 0.2913425 | 0.650235 |
| X211696_x_at | 0.2133 | 0.1987 | 1.073 | 0.2913675 | 0.650235 |
| X203799_at | 0.1162 | 0.1083 | 1.073 | 0.2915365 | 0.650547 |
| X200935_at | -0.0033 | 0.0031 | -1.073 | 0.2916058 | 0.650636 |
| X202561_at | -0.0322 | 0.03 | -1.073 | 0.2916434 | 0.650655 |
| X218111_s_at | 0.062 | 0.0578 | 1.073 | 0.291676 | 0.650662 |
| X218464_s_at | 0.0499 | 0.0465 | 1.072 | 0.2918006 | 0.650874 |
| X220456_at | 0.0747 | 0.0697 | 1.072 | 0.2919036 | 0.651039 |
| X204263_s_at | -0.0052 | 0.0049 | -1.072 | 0.2919787 | 0.651136 |
| X200630_x_at | 0.0352 | 0.0328 | 1.072 | 0.2920058 | 0.651136 |
| X209868_s_at | -0.0682 | 0.0636 | -1.072 | 0.2920403 | 0.651148 |
| X203783_x_at | 0.0023 | 0.0021 | 1.072 | 0.2921046 | 0.651226 |
| X206035_at | 0.0095 | 0.0089 | 1.072 | 0.2921464 | 0.651253 |
| X204101_at | 0.0356 | 0.0332 | 1.071 | 0.2923177 | 0.65157 |
| X211263_s_at | 0.0031 | 0.0029 | 1.071 | 0.2925564 | 0.652037 |
| X206669_at | -0.0084 | 0.0079 | -1.071 | 0.2926424 | 0.652084 |
| X206139_at | 0.0119 | 0.0111 | 1.07 | 0.2926649 | 0.652084 |
| X205034_at | -0.1269 | 0.1186 | -1.07 | 0.2926878 | 0.652084 |
| X208163_s_at | -0.0031 | 0.0029 | -1.07 | 0.2926951 | 0.652084 |
| X210891_s_at | 0.0211 | 0.0197 | 1.07 | 0.2927561 | 0.652144 |
| X212358_at | 0.0461 | 0.0431 | 1.07 | 0.2927895 | 0.652144 |
| X218546_at | -0.0773 | 0.0722 | -1.07 | 0.2928103 | 0.652144 |
| X214165_s_at | -0.0027 | 0.0025 | -1.07 | 0.2928518 | 0.652171 |
| X211717_at | 0.0626 | 0.0585 | 1.07 | 0.2929766 | 0.652384 |
| X206955_at | -0.003 | 0.0029 | -1.07 | 0.2930201 | 0.652415 |
| X212398_at | 0.1148 | 0.1074 | 1.069 | 0.2931373 | 0.652611 |
| X216543_at | -0.0038 | 0.0035 | -1.069 | 0.2932666 | 0.652833 |
| X218276_s_at | 0.0407 | 0.038 | 1.069 | 0.2933751 | 0.65294 |
| X206615_s_at | -0.0085 | 0.0079 | -1.069 | 0.2934343 | 0.65294 |
| X214279_s_at | -0.1203 | 0.1125 | -1.069 | 0.2934455 | 0.65294 |
| X213543_at | -0.0327 | 0.0306 | -1.069 | 0.2934549 | 0.65294 |
| X217992_s_at | 0.0497 | 0.0465 | 1.069 | 0.2935021 | 0.65294 |
| X207845_s_at | -0.0388 | 0.0363 | -1.069 | 0.2935373 | 0.65294 |
| X207913_at | 0.0045 | 0.0042 | 1.068 | 0.2935472 | 0.65294 |
| X216310_at | -0.0115 | 0.0108 | -1.068 | 0.293593 | 0.65294 |
| X202702_at | 0.0155 | 0.0145 | 1.068 | 0.2936223 | 0.65294 |
| X216918_s_at | -0.0047 | 0.0044 | -1.068 | 0.2936421 | 0.65294 |
| X208222_at | -0.006 | 0.0056 | -1.068 | 0.2936508 | 0.65294 |
| X218066_at | 0.0444 | 0.0416 | 1.068 | 0.2936993 | 0.65294 |
| X204130_at | 0.0031 | 0.0029 | 1.068 | 0.2937172 | 0.65294 |
| X219363_s_at | -0.0292 | 0.0273 | -1.068 | 0.2937595 | 0.65294 |
| X213514_s_at | -0.0122 | 0.0114 | -1.068 | 0.2937822 | 0.65294 |
| X204949_at | -0.0059 | 0.0055 | -1.068 | 0.2937849 | 0.65294 |
| X211366_x_at | 0.0073 | 0.0068 | 1.068 | 0.2939123 | 0.653158 |
| X204325_s_at | 0.0036 | 0.0034 | 1.067 | 0.2940767 | 0.653458 |
| X203483_at | -0.0034 | 0.0031 | -1.067 | 0.2943005 | 0.65389 |
| X213120_at | -0.0401 | 0.0376 | -1.067 | 0.2943451 | 0.653924 |
| X211426_x_at | 0.0041 | 0.0038 | 1.067 | 0.2943999 | 0.65398 |
| X207813_s_at | 0.0047 | 0.0044 | 1.066 | 0.2944543 | 0.654035 |
| X216209_at | -0.0046 | 0.0043 | -1.066 | 0.2945818 | 0.654122 |
| X215908_at | 0.1009 | 0.0946 | 1.066 | 0.2945931 | 0.654122 |
| X217825_s_at | -0.0565 | 0.053 | -1.066 | 0.2946092 | 0.654122 |
| X209504_s_at | 0.0781 | 0.0732 | 1.066 | 0.2946598 | 0.654122 |
| X220091_at | 0.0413 | 0.0388 | 1.066 | 0.2946675 | 0.654122 |
| X208235_x_at | 0.0034 | 0.0032 | 1.066 | 0.2946985 | 0.654122 |
| X212267_at | 0.0351 | 0.0329 | 1.066 | 0.2946993 | 0.654122 |
| X219748_at | 0.0039 | 0.0037 | 1.066 | 0.2947567 | 0.654184 |
| X217902_s_at | -0.0207 | 0.0195 | -1.066 | 0.2947923 | 0.654197 |
| X217271_at | -0.0029 | 0.0027 | -1.065 | 0.2949738 | 0.654458 |
| X204394_at | 0.0041 | 0.0038 | 1.065 | 0.2949779 | 0.654458 |
| X200047_s_at | -0.0245 | 0.023 | -1.065 | 0.2950278 | 0.654458 |
| X202600_s_at | 0.0686 | 0.0644 | 1.065 | 0.295047 | 0.654458 |
| X208126_s_at | 0.007 | 0.0066 | 1.065 | 0.2950568 | 0.654458 |
| X220268_at | -0.0103 | 0.0097 | -1.065 | 0.29521 | 0.654732 |
| X215997_s_at | 0.0105 | 0.0099 | 1.065 | 0.2952786 | 0.654819 |
| X211197_s_at | 0.0052 | 0.0048 | 1.064 | 0.2953337 | 0.654876 |
| X203829_at | -0.055 | 0.0517 | -1.064 | 0.2957113 | 0.655557 |
| X202894_at | 0.0053 | 0.005 | 1.064 | 0.2957161 | 0.655557 |
| X222305_at | -0.0054 | 0.0051 | -1.064 | 0.2957352 | 0.655557 |
| X211080_s_at | -0.0085 | 0.008 | -1.064 | 0.2957591 | 0.655557 |
| X213632_at | -0.0035 | 0.0033 | -1.063 | 0.2958094 | 0.655603 |
| X220218_at | -0.006 | 0.0057 | -1.063 | 0.2960435 | 0.656002 |
| X207684_at | 0.005 | 0.0047 | 1.063 | 0.2960485 | 0.656002 |
| X218132_s_at | 0.0453 | 0.0426 | 1.063 | 0.29611 | 0.656037 |
| X221628_s_at | 0.0025 | 0.0024 | 1.063 | 0.2961585 | 0.656037 |
| X221906_at | 0.0031 | 0.0029 | 1.063 | 0.2961659 | 0.656037 |
| X220721_at | -0.0062 | 0.0058 | -1.063 | 0.2961824 | 0.656037 |
| X213136_at | 0.0641 | 0.0603 | 1.062 | 0.2962428 | 0.656105 |
| X215244_at | -0.0268 | 0.0252 | -1.062 | 0.2962815 | 0.656126 |
| X220121_at | -0.0205 | 0.0193 | -1.062 | 0.2963335 | 0.65613 |
| X218747_s_at | 0.0074 | 0.0069 | 1.062 | 0.2963425 | 0.65613 |
| X212114_at | 0.0299 | 0.0282 | 1.062 | 0.2964206 | 0.656238 |
| X208292_at | -0.0023 | 0.0022 | -1.062 | 0.2965226 | 0.656333 |
| X208743_s_at | -0.0374 | 0.0353 | -1.062 | 0.2965526 | 0.656333 |
| X220898_at | 0.0202 | 0.019 | 1.062 | 0.2965681 | 0.656333 |
| X222149_x_at | 0.0396 | 0.0373 | 1.062 | 0.2965821 | 0.656333 |
| X203472_s_at | 0.0023 | 0.0022 | 1.062 | 0.29666 | 0.65644 |
| X203535_at | -0.0044 | 0.0042 | -1.061 | 0.2967335 | 0.656538 |
| X202831_at | 0.0041 | 0.0038 | 1.061 | 0.2968648 | 0.656763 |
| X217566_s_at | -0.0116 | 0.0109 | -1.061 | 0.2969554 | 0.656898 |
| X217179_x_at | 0.0028 | 0.0026 | 1.061 | 0.2971083 | 0.657122 |
| X221929_at | 0.0043 | 0.0041 | 1.06 | 0.297116 | 0.657122 |
| X219667_s_at | -0.0428 | 0.0404 | -1.06 | 0.2974564 | 0.657753 |
| X205846_at | 0.0062 | 0.0058 | 1.06 | 0.2974735 | 0.657753 |
| X215797_at | -0.0045 | 0.0043 | -1.06 | 0.2975162 | 0.657753 |
| X221105_at | 0.0036 | 0.0034 | 1.06 | 0.2975489 | 0.657753 |
| X204083_s_at | 0.0712 | 0.0672 | 1.06 | 0.2975492 | 0.657753 |
| X216747_at | 0.0107 | 0.0101 | 1.059 | 0.297636 | 0.65776 |
| X215750_at | -0.0196 | 0.0185 | -1.059 | 0.2976361 | 0.65776 |
| X213855_s_at | 0.0032 | 0.0031 | 1.059 | 0.2976411 | 0.65776 |
| X219284_at | 0.0806 | 0.0761 | 1.059 | 0.2977142 | 0.657763 |
| X216389_s_at | -0.0037 | 0.0035 | -1.059 | 0.2977395 | 0.657763 |
| X205450_at | 0.005 | 0.0047 | 1.059 | 0.2977816 | 0.657763 |
| X216205_s_at | 0.0045 | 0.0043 | 1.059 | 0.2978021 | 0.657763 |
| X208580_x_at | -0.0044 | 0.0041 | -1.059 | 0.2978776 | 0.657763 |
| X202665_s_at | 0.0121 | 0.0114 | 1.059 | 0.2978785 | 0.657763 |
| X203563_at | -0.0252 | 0.0238 | -1.059 | 0.2978828 | 0.657763 |
| X217759_at | -0.0383 | 0.0361 | -1.059 | 0.2979019 | 0.657763 |
| X217877_s_at | -0.0406 | 0.0384 | -1.059 | 0.2979092 | 0.657763 |
| X64064_at | 0.0096 | 0.0091 | 1.059 | 0.297951 | 0.65779 |
| X220592_at | 0.0024 | 0.0023 | 1.058 | 0.2980851 | 0.658021 |
| X204157_s_at | -0.052 | 0.0492 | -1.058 | 0.2981376 | 0.658027 |
| X207232_s_at | -0.0927 | 0.0876 | -1.058 | 0.2981472 | 0.658027 |
| X203314_at | -0.0401 | 0.0379 | -1.058 | 0.2982997 | 0.658279 |
| X205734_s_at | -0.0056 | 0.0053 | -1.058 | 0.2983207 | 0.658279 |
| X211837_s_at | -0.0087 | 0.0082 | -1.058 | 0.2984103 | 0.658314 |
| X38043_at | 0.0136 | 0.0129 | 1.058 | 0.2984191 | 0.658314 |
| X216934_at | 0.0065 | 0.0061 | 1.058 | 0.2984252 | 0.658314 |
| X218816_at | 0.0782 | 0.074 | 1.057 | 0.2985057 | 0.658373 |
| X212548_s_at | 0.0861 | 0.0814 | 1.057 | 0.298513 | 0.658373 |
| X214219_x_at | -0.0013 | 0.0012 | -1.057 | 0.2985412 | 0.658373 |
| X217087_at | 0.0053 | 0.005 | 1.057 | 0.2985862 | 0.658407 |
| X215116_s_at | 0.0443 | 0.0419 | 1.057 | 0.2986643 | 0.658466 |
| X220250_at | -0.0133 | 0.0126 | -1.057 | 0.2986762 | 0.658466 |
| X207827_x_at | -0.0961 | 0.0909 | -1.057 | 0.298706 | 0.658466 |
| X204905_s_at | -0.0698 | 0.066 | -1.057 | 0.2987312 | 0.658466 |
| X218404_at | 0.049 | 0.0464 | 1.056 | 0.29902 | 0.659022 |
| X203131_at | -0.0863 | 0.0817 | -1.056 | 0.299043 | 0.659022 |
| X216271_x_at | 0.0034 | 0.0032 | 1.056 | 0.2991016 | 0.659086 |
| X212964_at | 0.0716 | 0.0678 | 1.056 | 0.2991852 | 0.659145 |
| X221905_at | -0.0379 | 0.0359 | -1.056 | 0.299188 | 0.659145 |
| X211462_s_at | 0.004 | 0.0038 | 1.056 | 0.2992345 | 0.659149 |
| X207119_at | 0.0073 | 0.0069 | 1.056 | 0.2992488 | 0.659149 |
| X220516_at | -0.0038 | 0.0036 | -1.055 | 0.2994137 | 0.659446 |
| X212657_s_at | 0.0049 | 0.0046 | 1.055 | 0.2994964 | 0.659504 |
| X213160_at | -0.0079 | 0.0075 | -1.055 | 0.2994994 | 0.659504 |
| X218567_x_at | 0.0067 | 0.0063 | 1.055 | 0.299545 | 0.659526 |
| X212817_at | -0.011 | 0.0104 | -1.055 | 0.2995684 | 0.659526 |
| X220056_at | -0.0033 | 0.0031 | -1.055 | 0.2996756 | 0.659696 |
| X207151_at | 0.0041 | 0.0039 | 1.054 | 0.2999238 | 0.660004 |
| X211553_x_at | -0.005 | 0.0048 | -1.054 | 0.2999569 | 0.660004 |
| X204100_at | -0.088 | 0.0835 | -1.054 | 0.2999654 | 0.660004 |
| X221292_at | 0.0041 | 0.0039 | 1.054 | 0.2999656 | 0.660004 |
| X208034_s_at | 0.003 | 0.0028 | 1.054 | 0.299986 | 0.660004 |
| X203512_at | 0.0277 | 0.0262 | 1.054 | 0.2999938 | 0.660004 |
| X213440_at | -0.022 | 0.0208 | -1.054 | 0.3000744 | 0.660116 |
| X219025_at | 0.0035 | 0.0033 | 1.054 | 0.3001422 | 0.660159 |
| X207726_at | -0.0027 | 0.0026 | -1.054 | 0.3001531 | 0.660159 |
| X203509_at | -0.0484 | 0.0459 | -1.053 | 0.3002628 | 0.660334 |
| X203311_s_at | -0.0047 | 0.0044 | -1.053 | 0.3003528 | 0.660467 |
| X213125_at | 0.0053 | 0.005 | 1.053 | 0.3004378 | 0.660543 |
| X221053_s_at | -0.0104 | 0.0099 | -1.053 | 0.300447 | 0.660543 |
| X203424_s_at | 0.1296 | 0.1231 | 1.053 | 0.3004809 | 0.660545 |
| X202486_at | -0.0615 | 0.0584 | -1.053 | 0.3005254 | 0.660545 |
| X217403_s_at | -0.0202 | 0.0191 | -1.053 | 0.3005371 | 0.660545 |
| X221685_s_at | -0.0271 | 0.0257 | -1.053 | 0.3006212 | 0.660665 |
| X218930_s_at | 0.0425 | 0.0404 | 1.053 | 0.3006867 | 0.660743 |
| X212848_s_at | 0.0572 | 0.0543 | 1.052 | 0.3007204 | 0.660752 |
| X205587_at | 0.0032 | 0.003 | 1.052 | 0.3008673 | 0.661009 |
| X213864_s_at | -0.035 | 0.0333 | -1.052 | 0.3009895 | 0.661213 |
| X215335_at | -0.0038 | 0.0036 | -1.052 | 0.3010722 | 0.661329 |
| X215319_at | 0.0039 | 0.0038 | 1.051 | 0.3011722 | 0.661483 |
| X209116_x_at | 0.2257 | 0.2147 | 1.051 | 0.3012652 | 0.661573 |
| X218957_s_at | -0.0486 | 0.0462 | -1.051 | 0.3012924 | 0.661573 |
| X203308_x_at | 0.0114 | 0.0108 | 1.051 | 0.3013241 | 0.661573 |
| X221303_at | 0.0049 | 0.0047 | 1.051 | 0.3013321 | 0.661573 |
| X207526_s_at | 0.0071 | 0.0068 | 1.051 | 0.3014223 | 0.661705 |
| X203639_s_at | 0.1505 | 0.1433 | 1.051 | 0.3014923 | 0.661794 |
| X214391_x_at | 0.004 | 0.0039 | 1.051 | 0.3015234 | 0.661796 |
| X202383_at | 0.0086 | 0.0082 | 1.051 | 0.3015939 | 0.661886 |
| X215934_at | -0.0068 | 0.0064 | -1.05 | 0.3017365 | 0.662119 |
| X221191_at | 0.0046 | 0.0044 | 1.05 | 0.3017732 | 0.662119 |
| X213183_s_at | 0.117 | 0.1115 | 1.05 | 0.3017897 | 0.662119 |
| X206124_s_at | 0.0056 | 0.0053 | 1.05 | 0.3018402 | 0.662151 |
| X204882_at | 0.0028 | 0.0026 | 1.05 | 0.3018783 | 0.662151 |
| X220714_at | -0.004 | 0.0038 | -1.05 | 0.3018934 | 0.662151 |
| X211059_s_at | -0.0057 | 0.0054 | -1.05 | 0.302016 | 0.662354 |
| X205720_at | -0.0031 | 0.0029 | -1.049 | 0.302264 | 0.662832 |
| X212867_at | -0.0248 | 0.0236 | -1.049 | 0.3024743 | 0.66312 |
| X208159_x_at | 0.0048 | 0.0046 | 1.049 | 0.302478 | 0.66312 |
| X213704_at | 0.0313 | 0.0299 | 1.048 | 0.3025349 | 0.66312 |
| X209741_x_at | -0.0351 | 0.0335 | -1.048 | 0.302563 | 0.66312 |
| X205116_at | -0.0093 | 0.0088 | -1.048 | 0.3025681 | 0.66312 |
| X215079_at | -0.0075 | 0.0071 | -1.048 | 0.3025826 | 0.66312 |
| X209724_s_at | 0.0144 | 0.0137 | 1.048 | 0.3026043 | 0.66312 |
| X207183_at | -0.0686 | 0.0654 | -1.048 | 0.302653 | 0.663162 |
| X217956_s_at | -0.0216 | 0.0207 | -1.048 | 0.3027051 | 0.663211 |
| X211563_s_at | -0.0728 | 0.0695 | -1.048 | 0.3027727 | 0.663293 |
| X213925_at | 0.0919 | 0.0877 | 1.048 | 0.3028843 | 0.663438 |
| X39313_at | -0.0916 | 0.0875 | -1.048 | 0.3028984 | 0.663438 |
| X217879_at | -0.0467 | 0.0446 | -1.047 | 0.3030025 | 0.6636 |
| X221409_at | 0.0032 | 0.0031 | 1.047 | 0.3031023 | 0.663753 |
| X216208_s_at | -0.0032 | 0.003 | -1.047 | 0.3031431 | 0.663777 |
| X205595_at | 0.0034 | 0.0033 | 1.047 | 0.3032075 | 0.663812 |
| X218849_s_at | 0.0048 | 0.0046 | 1.047 | 0.3032186 | 0.663812 |
| X201445_at | 0.0676 | 0.0646 | 1.047 | 0.3033873 | 0.664105 |
| X209734_at | 0.0034 | 0.0033 | 1.047 | 0.3034125 | 0.664105 |
| X207929_at | 0.0045 | 0.0043 | 1.046 | 0.3035142 | 0.664205 |
| X220844_at | -0.007 | 0.0067 | -1.046 | 0.3035178 | 0.664205 |
| X206724_at | 0.0027 | 0.0026 | 1.046 | 0.303567 | 0.664247 |
| X214787_at | -0.0302 | 0.0289 | -1.046 | 0.3036335 | 0.664327 |
| X207224_s_at | 0.0067 | 0.0064 | 1.046 | 0.3036964 | 0.664399 |
| X206381_at | -0.0657 | 0.0629 | -1.046 | 0.3037695 | 0.66441 |
| X202634_at | -0.0331 | 0.0317 | -1.046 | 0.303786 | 0.66441 |
| X215599_at | 0.1153 | 0.1102 | 1.046 | 0.3037908 | 0.66441 |
| X216953_s_at | -0.0041 | 0.004 | -1.045 | 0.3039476 | 0.664636 |
| X210952_at | 0.0057 | 0.0055 | 1.045 | 0.3039711 | 0.664636 |
| X219417_s_at | 0.0053 | 0.0051 | 1.045 | 0.3039839 | 0.664636 |
| X211529_x_at | 0.0467 | 0.0447 | 1.045 | 0.3040233 | 0.664656 |
| X208069_x_at | 0.0037 | 0.0036 | 1.045 | 0.3041129 | 0.664676 |
| X201206_s_at | 0.0208 | 0.0199 | 1.045 | 0.3041145 | 0.664676 |
| X221728_x_at | 0.0055 | 0.0053 | 1.045 | 0.3041283 | 0.664676 |
| X202150_s_at | 0.0065 | 0.0062 | 1.045 | 0.3041905 | 0.664676 |
| X206083_at | -0.0094 | 0.009 | -1.045 | 0.3042085 | 0.664676 |
| X216265_x_at | 0.0032 | 0.0031 | 1.045 | 0.3042189 | 0.664676 |
| X219310_at | 0.115 | 0.1101 | 1.045 | 0.304281 | 0.664676 |
| X208072_s_at | -0.0435 | 0.0416 | -1.044 | 0.3043518 | 0.664676 |
| X219199_at | 0.006 | 0.0058 | 1.044 | 0.3043761 | 0.664676 |
| X204315_s_at | 0.0048 | 0.0046 | 1.044 | 0.3043803 | 0.664676 |
| X207489_at | 0.003 | 0.0029 | 1.044 | 0.30443 | 0.664676 |
| X206056_x_at | 0.0301 | 0.0288 | 1.044 | 0.3044376 | 0.664676 |
| X211440_x_at | -0.0047 | 0.0045 | -1.044 | 0.3044403 | 0.664676 |
| X212652_s_at | 0.0378 | 0.0362 | 1.044 | 0.3044513 | 0.664676 |
| X201311_s_at | -0.0265 | 0.0254 | -1.044 | 0.3045495 | 0.664762 |
| X200671_s_at | 0.1481 | 0.1419 | 1.044 | 0.3045505 | 0.664762 |
| X206379_at | 0.0058 | 0.0056 | 1.044 | 0.304583 | 0.664768 |
| X217515_s_at | -0.0055 | 0.0053 | -1.044 | 0.3046471 | 0.664809 |
| X208148_at | -0.0123 | 0.0118 | -1.044 | 0.3046618 | 0.664809 |
| X212939_at | -0.002 | 0.0019 | -1.044 | 0.3047275 | 0.664841 |
| X213613_s_at | -0.0028 | 0.0026 | -1.044 | 0.3047368 | 0.664841 |
| X219608_s_at | 0.0073 | 0.007 | 1.044 | 0.3047662 | 0.664841 |
| X206286_s_at | -0.052 | 0.0498 | -1.043 | 0.3048337 | 0.664923 |
| X208847_s_at | -0.0358 | 0.0343 | -1.043 | 0.3049481 | 0.665107 |
| X218321_x_at | 0.0384 | 0.0368 | 1.043 | 0.304991 | 0.665107 |
| X216473_x_at | 0.0032 | 0.0031 | 1.043 | 0.305008 | 0.665107 |
| X214209_s_at | -0.0088 | 0.0085 | -1.043 | 0.3050764 | 0.665191 |
| X212711_at | -0.0337 | 0.0323 | -1.043 | 0.3051806 | 0.665353 |
| X212193_s_at | -0.0111 | 0.0107 | -1.043 | 0.3052253 | 0.665377 |
| X209911_x_at | -0.1204 | 0.1155 | -1.042 | 0.3052517 | 0.665377 |
| X215687_x_at | 0.008 | 0.0077 | 1.042 | 0.3053081 | 0.665397 |
| X203532_x_at | 0.0585 | 0.0561 | 1.042 | 0.3053207 | 0.665397 |
| X203722_at | 0.0364 | 0.0349 | 1.042 | 0.3053908 | 0.665485 |
| X220087_at | 0.0051 | 0.0049 | 1.042 | 0.3054225 | 0.665489 |
| X206460_at | -0.0096 | 0.0093 | -1.042 | 0.3055279 | 0.665653 |
| X206076_at | 0.0699 | 0.0671 | 1.042 | 0.3056744 | 0.665848 |
| X221027_s_at | 0.0514 | 0.0493 | 1.042 | 0.3056925 | 0.665848 |
| X215897_at | -0.0061 | 0.0058 | -1.041 | 0.3057075 | 0.665848 |
| X219041_s_at | 0.0334 | 0.0321 | 1.041 | 0.3059075 | 0.666219 |
| X215027_at | -0.0145 | 0.0139 | -1.041 | 0.3060307 | 0.666324 |
| X212802_s_at | 0.0237 | 0.0228 | 1.041 | 0.3060476 | 0.666324 |
| X207369_at | 0.0189 | 0.0181 | 1.041 | 0.3060758 | 0.666324 |
| X209838_at | -0.0292 | 0.0281 | -1.041 | 0.306092 | 0.666324 |
| X202136_at | -0.0282 | 0.0271 | -1.041 | 0.3061057 | 0.666324 |
| X51774_s_at | 0.0424 | 0.0407 | 1.04 | 0.3061699 | 0.666335 |
| X217418_x_at | 0.006 | 0.0057 | 1.04 | 0.3061988 | 0.666335 |
| X209752_at | -0.0039 | 0.0037 | -1.04 | 0.306234 | 0.666335 |
| X209152_s_at | 0.0122 | 0.0118 | 1.04 | 0.306248 | 0.666335 |
| X202895_s_at | 0.0032 | 0.0031 | 1.04 | 0.3062874 | 0.666335 |
| X218918_at | 0.0847 | 0.0814 | 1.04 | 0.3062907 | 0.666335 |
| X202703_at | -0.0405 | 0.039 | -1.04 | 0.3063636 | 0.666428 |
| X216339_s_at | 0.0021 | 0.002 | 1.04 | 0.3064207 | 0.666459 |
| X218027_at | -0.0468 | 0.045 | -1.04 | 0.306464 | 0.666459 |
| X214615_at | -0.0111 | 0.0107 | -1.04 | 0.3064844 | 0.666459 |
| X211592_s_at | 0.0086 | 0.0082 | 1.04 | 0.306498 | 0.666459 |
| X212103_at | -0.0456 | 0.0439 | -1.039 | 0.3066855 | 0.666802 |
| X202747_s_at | 0.1332 | 0.1282 | 1.039 | 0.306823 | 0.667035 |
| X218899_s_at | 0.0853 | 0.0821 | 1.039 | 0.3068532 | 0.667036 |
| X215861_at | -0.0149 | 0.0143 | -1.039 | 0.3069806 | 0.667247 |
| X202240_at | 0.002 | 0.0019 | 1.038 | 0.3072622 | 0.667696 |
| X49111_at | -0.0464 | 0.0447 | -1.038 | 0.3072687 | 0.667696 |
| X205982_x_at | 0.003 | 0.0029 | 1.038 | 0.3072771 | 0.667696 |
| X211265_at | -0.0062 | 0.0059 | -1.038 | 0.3073097 | 0.667701 |
| X209022_at | 0.0516 | 0.0497 | 1.038 | 0.3073896 | 0.66781 |
| X220178_at | 0.0033 | 0.0032 | 1.037 | 0.3075816 | 0.668161 |
| X203307_at | -0.0394 | 0.038 | -1.037 | 0.3076339 | 0.66821 |
| X209039_x_at | -0.0038 | 0.0037 | -1.037 | 0.307744 | 0.668383 |
| X202012_s_at | 0.0286 | 0.0275 | 1.037 | 0.3077832 | 0.668403 |
| X204390_at | -0.0052 | 0.005 | -1.036 | 0.3080013 | 0.66877 |
| X213972_at | 0.0047 | 0.0046 | 1.036 | 0.3080125 | 0.66877 |
| X221442_at | -0.0026 | 0.0025 | -1.036 | 0.3080462 | 0.668778 |
| X206737_at | -0.0022 | 0.0022 | -1.036 | 0.3081398 | 0.668916 |
| X203145_at | 0.0108 | 0.0104 | 1.036 | 0.3082703 | 0.669114 |
| X219927_at | 0.0047 | 0.0046 | 1.036 | 0.3082911 | 0.669114 |
| X216324_at | 0.0036 | 0.0035 | 1.036 | 0.3083878 | 0.669161 |
| X220097_s_at | 0.0035 | 0.0034 | 1.036 | 0.3083976 | 0.669161 |
| X211496_s_at | -0.0068 | 0.0066 | -1.036 | 0.3084033 | 0.669161 |
| X209560_s_at | 0.0076 | 0.0074 | 1.035 | 0.3085499 | 0.669345 |
| X211888_x_at | 0.0032 | 0.0031 | 1.035 | 0.3085676 | 0.669345 |
| X216755_at | 0.005 | 0.0048 | 1.035 | 0.3085785 | 0.669345 |
| X221380_at | 0.0048 | 0.0046 | 1.035 | 0.308671 | 0.669473 |
| X200753_x_at | -0.0392 | 0.0378 | -1.035 | 0.3086978 | 0.669473 |
| X204530_s_at | 0.0129 | 0.0124 | 1.035 | 0.3088512 | 0.66973 |
| X204301_at | -0.0338 | 0.0327 | -1.035 | 0.3088764 | 0.66973 |
| X218601_at | -0.0505 | 0.0488 | -1.034 | 0.3090464 | 0.669979 |
| X208184_s_at | -0.0177 | 0.0171 | -1.034 | 0.3090515 | 0.669979 |
| X215596_s_at | -0.0388 | 0.0375 | -1.034 | 0.3091725 | 0.670175 |
| X216304_x_at | -0.0365 | 0.0353 | -1.034 | 0.3092023 | 0.670175 |
| X222162_s_at | 0.1407 | 0.1362 | 1.033 | 0.3094672 | 0.670683 |
| X209416_s_at | 0.0052 | 0.005 | 1.033 | 0.3095465 | 0.67076 |
| X206068_s_at | 0.0045 | 0.0043 | 1.033 | 0.3095631 | 0.67076 |
| X210292_s_at | -0.0562 | 0.0544 | -1.033 | 0.3095949 | 0.670764 |
| X210208_x_at | -0.0333 | 0.0322 | -1.033 | 0.3096255 | 0.670765 |
| X210452_x_at | -0.0041 | 0.004 | -1.033 | 0.3097152 | 0.670847 |
| X203876_s_at | 0.0044 | 0.0043 | 1.033 | 0.3097425 | 0.670847 |
| X221738_at | 0.0059 | 0.0057 | 1.033 | 0.309754 | 0.670847 |
| X31835_at | -0.0136 | 0.0132 | -1.032 | 0.3098451 | 0.670979 |
| X219923_at | 0.0049 | 0.0048 | 1.032 | 0.3099561 | 0.671122 |
| X208902_s_at | -0.0031 | 0.003 | -1.032 | 0.3099719 | 0.671122 |
| X206535_at | -0.005 | 0.0049 | -1.032 | 0.3100505 | 0.671157 |
| X220563_s_at | 0.0039 | 0.0037 | 1.032 | 0.3100805 | 0.671157 |
| X207980_s_at | 0.0058 | 0.0056 | 1.032 | 0.310113 | 0.671157 |
| X215267_s_at | -0.0581 | 0.0563 | -1.032 | 0.3101218 | 0.671157 |
| X201138_s_at | 0.0241 | 0.0234 | 1.032 | 0.3101389 | 0.671157 |
| X207571_x_at | 0.0189 | 0.0184 | 1.032 | 0.3102612 | 0.67132 |
| X205637_s_at | 0.058 | 0.0563 | 1.032 | 0.3102748 | 0.67132 |
| X212212_s_at | 0.0074 | 0.0072 | 1.031 | 0.3103262 | 0.671366 |
| X204653_at | -0.0072 | 0.0069 | -1.031 | 0.310405 | 0.671471 |
| X209269_s_at | -0.008 | 0.0077 | -1.031 | 0.310443 | 0.671488 |
| X213785_at | 0.0348 | 0.0338 | 1.031 | 0.3105138 | 0.671576 |
| X203644_s_at | 0.0076 | 0.0073 | 1.031 | 0.3106553 | 0.671816 |
| X219490_s_at | 0.0079 | 0.0077 | 1.03 | 0.3107688 | 0.671904 |
| X200082_s_at | 0.0259 | 0.0251 | 1.03 | 0.3108293 | 0.671904 |
| X211705_s_at | -0.0046 | 0.0045 | -1.03 | 0.3108357 | 0.671904 |
| X204405_x_at | -0.0272 | 0.0264 | -1.03 | 0.3108569 | 0.671904 |
| X201960_s_at | -0.0354 | 0.0343 | -1.03 | 0.3108901 | 0.671904 |
| X217580_x_at | -0.0635 | 0.0616 | -1.03 | 0.3109042 | 0.671904 |
| X205028_at | -0.0245 | 0.0238 | -1.03 | 0.3109074 | 0.671904 |
| X219462_at | 0.0123 | 0.0119 | 1.03 | 0.3110581 | 0.672164 |
| X210657_s_at | 0.1265 | 0.1228 | 1.03 | 0.3111746 | 0.672342 |
| X220447_at | -0.035 | 0.034 | -1.029 | 0.3112249 | 0.672342 |
| X209597_s_at | -0.0048 | 0.0047 | -1.029 | 0.3112313 | 0.672342 |
| X206419_at | -0.0025 | 0.0024 | -1.029 | 0.311262 | 0.672343 |
| X216916_s_at | 0.004 | 0.0039 | 1.029 | 0.3113074 | 0.672376 |
| X205505_at | -0.0035 | 0.0034 | -1.029 | 0.3114151 | 0.672543 |
| X217900_at | -0.0377 | 0.0366 | -1.029 | 0.3114508 | 0.672555 |
| X207131_x_at | 0.0039 | 0.0038 | 1.029 | 0.3115352 | 0.672671 |
| X209004_s_at | 0.0402 | 0.0391 | 1.029 | 0.3116241 | 0.672798 |
| X203184_at | 0.0168 | 0.0163 | 1.028 | 0.3116984 | 0.672893 |
| X220687_at | -0.0028 | 0.0027 | -1.028 | 0.3117326 | 0.672901 |
| X206191_at | -0.1897 | 0.1845 | -1.028 | 0.3117649 | 0.672906 |
| X215320_at | -0.0133 | 0.0129 | -1.028 | 0.3118516 | 0.672963 |
| X220262_s_at | -0.0067 | 0.0065 | -1.028 | 0.311852 | 0.672963 |
| X204897_at | 0.068 | 0.0661 | 1.028 | 0.3119105 | 0.673024 |
| X206095_s_at | 0.0307 | 0.0298 | 1.028 | 0.3119879 | 0.673125 |
| X201552_at | 0.0654 | 0.0636 | 1.028 | 0.312073 | 0.673146 |
| X221160_s_at | 0.0038 | 0.0037 | 1.028 | 0.312125 | 0.673146 |
| X222285_at | 0.0067 | 0.0065 | 1.027 | 0.3121374 | 0.673146 |
| X207864_at | -0.0067 | 0.0065 | -1.027 | 0.3121573 | 0.673146 |
| X216461_at | -0.0046 | 0.0045 | -1.027 | 0.3121744 | 0.673146 |
| X200920_s_at | -0.0845 | 0.0822 | -1.027 | 0.3121791 | 0.673146 |
| X215298_at | -0.009 | 0.0088 | -1.027 | 0.3122456 | 0.673224 |
| X204096_s_at | -0.0041 | 0.004 | -1.027 | 0.3123151 | 0.673308 |
| X221029_s_at | 0.0032 | 0.0031 | 1.027 | 0.3123577 | 0.673335 |
| X212683_at | -0.0127 | 0.0124 | -1.027 | 0.3124111 | 0.673385 |
| X206600_s_at | 0.0052 | 0.005 | 1.027 | 0.3124641 | 0.673409 |
| X217313_at | -0.0058 | 0.0057 | -1.027 | 0.3124829 | 0.673409 |
| X214315_x_at | -0.0476 | 0.0464 | -1.026 | 0.3126463 | 0.673695 |
| X202945_at | 0.0065 | 0.0063 | 1.026 | 0.3126911 | 0.673727 |
| X211121_s_at | 0.0043 | 0.0042 | 1.026 | 0.3127244 | 0.673733 |
| X209286_at | -0.0657 | 0.0641 | -1.026 | 0.3128302 | 0.673896 |
| X206568_at | -0.0033 | 0.0032 | -1.026 | 0.312885 | 0.673948 |
| X214201_x_at | 0.0509 | 0.0496 | 1.026 | 0.3129894 | 0.67406 |
| X211367_s_at | -0.0079 | 0.0077 | -1.026 | 0.3129975 | 0.67406 |
| X205409_at | -0.0029 | 0.0028 | -1.025 | 0.3131737 | 0.674374 |
| X213967_at | -0.0596 | 0.0582 | -1.025 | 0.3132592 | 0.6744 |
| X220089_at | -0.0189 | 0.0185 | -1.025 | 0.31332 | 0.6744 |
| X206742_at | 0.0046 | 0.0044 | 1.025 | 0.3133548 | 0.6744 |
| X208487_at | 0.0024 | 0.0023 | 1.025 | 0.3133692 | 0.6744 |
| X214883_at | 0.0032 | 0.0032 | 1.025 | 0.3133833 | 0.6744 |
| X204919_at | -0.0341 | 0.0332 | -1.025 | 0.3133843 | 0.6744 |
| X213291_s_at | -0.053 | 0.0517 | -1.025 | 0.3134112 | 0.6744 |
| X214410_at | -0.0044 | 0.0043 | -1.025 | 0.3134285 | 0.6744 |
| X203382_s_at | -0.0795 | 0.0776 | -1.025 | 0.3135225 | 0.674536 |
| X210020_x_at | 0.0047 | 0.0045 | 1.024 | 0.3136036 | 0.674638 |
| X204441_s_at | 0.0063 | 0.0061 | 1.024 | 0.313662 | 0.674638 |
| X64432_at | -0.033 | 0.0322 | -1.024 | 0.3137009 | 0.674638 |
| X222311_s_at | 0.0078 | 0.0076 | 1.024 | 0.3137493 | 0.674638 |
| X207746_at | 0.0051 | 0.005 | 1.024 | 0.3137529 | 0.674638 |
| X209231_s_at | 0.007 | 0.0068 | 1.024 | 0.3137548 | 0.674638 |
| X209879_at | -0.0376 | 0.0367 | -1.024 | 0.3137824 | 0.674638 |
| X206570_s_at | -0.0101 | 0.0099 | -1.023 | 0.3141008 | 0.675216 |
| X210414_at | -0.0954 | 0.0933 | -1.023 | 0.3141118 | 0.675216 |
| X217366_at | -0.0068 | 0.0067 | -1.023 | 0.3143401 | 0.675416 |
| X220134_x_at | -0.004 | 0.0039 | -1.023 | 0.3143582 | 0.675416 |
| X220472_at | 0.004 | 0.0039 | 1.023 | 0.3143674 | 0.675416 |
| X209351_at | -0.0057 | 0.0055 | -1.023 | 0.3143745 | 0.675416 |
| X207893_at | 0.0048 | 0.0046 | 1.023 | 0.3144045 | 0.675416 |
| X202319_at | -0.0676 | 0.0661 | -1.023 | 0.3144075 | 0.675416 |
| X218852_at | 0.0366 | 0.0358 | 1.023 | 0.314418 | 0.675416 |
| X217348_x_at | -0.0059 | 0.0058 | -1.022 | 0.3147046 | 0.675967 |
| X216601_at | -0.0067 | 0.0065 | -1.022 | 0.3148005 | 0.676107 |
| X200726_at | -0.0273 | 0.0267 | -1.022 | 0.3148357 | 0.676118 |
| X213798_s_at | -0.0421 | 0.0412 | -1.021 | 0.3149737 | 0.676349 |
| X203555_at | -0.0051 | 0.0049 | -1.021 | 0.3150693 | 0.67643 |
| X201450_s_at | -0.0488 | 0.0478 | -1.021 | 0.3150724 | 0.67643 |
| X204963_at | 0.0707 | 0.0692 | 1.021 | 0.3151426 | 0.676469 |
| X214820_at | -0.0359 | 0.0352 | -1.021 | 0.3151515 | 0.676469 |
| X212288_at | 0.0463 | 0.0454 | 1.021 | 0.3152156 | 0.676523 |
| X216525_x_at | 0.0142 | 0.0139 | 1.021 | 0.3152378 | 0.676523 |
| X204179_at | 0.0038 | 0.0038 | 1.021 | 0.3153567 | 0.676713 |
| X214902_x_at | 0.0596 | 0.0584 | 1.02 | 0.3155658 | 0.677096 |
| X217875_s_at | 0.0527 | 0.0517 | 1.02 | 0.3156144 | 0.677135 |
| X216837_at | -0.004 | 0.0039 | -1.02 | 0.3157141 | 0.677246 |
| X221940_at | -0.0093 | 0.0091 | -1.02 | 0.315741 | 0.677246 |
| X216071_x_at | 0.0053 | 0.0052 | 1.02 | 0.3157856 | 0.677246 |
| X204479_at | -0.0801 | 0.0785 | -1.02 | 0.3157878 | 0.677246 |
| X213358_at | 0.0492 | 0.0483 | 1.019 | 0.3160814 | 0.677708 |
| X213633_at | 0.0035 | 0.0035 | 1.019 | 0.3160958 | 0.677708 |
| X208276_at | -0.0105 | 0.0103 | -1.019 | 0.3161041 | 0.677708 |
| X212781_at | -0.0366 | 0.0359 | -1.019 | 0.3161253 | 0.677708 |
| X216603_at | -0.0037 | 0.0036 | -1.018 | 0.3163862 | 0.678202 |
| X213140_s_at | 0.0257 | 0.0252 | 1.018 | 0.3164219 | 0.678213 |
| X210456_at | -0.0063 | 0.0062 | -1.018 | 0.3164866 | 0.678286 |
| X201524_x_at | -0.0327 | 0.0321 | -1.017 | 0.3168529 | 0.679005 |
| X219339_s_at | -0.0035 | 0.0034 | -1.017 | 0.3169336 | 0.679098 |
| X213695_at | -0.0044 | 0.0044 | -1.017 | 0.3169699 | 0.679098 |
| X204432_at | 0.0221 | 0.0217 | 1.017 | 0.316995 | 0.679098 |
| X204749_at | 0.0572 | 0.0562 | 1.017 | 0.3170184 | 0.679098 |
| X202752_x_at | 0.0858 | 0.0844 | 1.017 | 0.3170549 | 0.679111 |
| X204291_at | -0.0421 | 0.0414 | -1.017 | 0.317125 | 0.679195 |
| X205089_at | -0.0541 | 0.0532 | -1.017 | 0.3171686 | 0.679223 |
| X209102_s_at | 0.0554 | 0.0545 | 1.017 | 0.3172572 | 0.679348 |
| X220671_at | -0.0042 | 0.0042 | -1.016 | 0.3174335 | 0.67966 |
| X213778_x_at | -0.0031 | 0.003 | -1.016 | 0.3175602 | 0.679782 |
| X201331_s_at | -0.0582 | 0.0572 | -1.016 | 0.3175763 | 0.679782 |
| X211987_at | -0.0232 | 0.0229 | -1.016 | 0.3175867 | 0.679782 |
| X218720_x_at | -0.0474 | 0.0466 | -1.016 | 0.3176129 | 0.679782 |
| X200013_at | -0.0185 | 0.0182 | -1.016 | 0.31769 | 0.679861 |
| X205338_s_at | 0.0674 | 0.0664 | 1.016 | 0.3177111 | 0.679861 |
| X206084_at | 0.0325 | 0.032 | 1.015 | 0.3178538 | 0.680053 |
| X39249_at | -0.0025 | 0.0024 | -1.015 | 0.3178621 | 0.680053 |
| X218607_s_at | -0.0614 | 0.0605 | -1.015 | 0.317913 | 0.680096 |
| X221681_s_at | -0.0319 | 0.0315 | -1.015 | 0.3179727 | 0.680137 |
| X213050_at | 0.0728 | 0.0718 | 1.015 | 0.3179933 | 0.680137 |
| X221358_at | 0.0035 | 0.0035 | 1.015 | 0.3180326 | 0.680156 |
| X210757_x_at | 0.012 | 0.0118 | 1.015 | 0.3180948 | 0.680223 |
| X214076_at | -0.0392 | 0.0386 | -1.015 | 0.3181763 | 0.680262 |
| X212895_s_at | 0.0428 | 0.0422 | 1.015 | 0.3181812 | 0.680262 |
| X217310_s_at | -0.0048 | 0.0048 | -1.014 | 0.3182048 | 0.680262 |
| X218850_s_at | 0.0037 | 0.0036 | 1.014 | 0.3182541 | 0.680302 |
| X44783_s_at | -0.0547 | 0.0539 | -1.014 | 0.3183421 | 0.68041 |
| X213876_x_at | 0.058 | 0.0572 | 1.014 | 0.3183658 | 0.68041 |
| X214897_at | 0.0059 | 0.0058 | 1.014 | 0.318507 | 0.680646 |
| X220797_at | -0.0061 | 0.006 | -1.014 | 0.3185869 | 0.680751 |
| X215404_x_at | 0.0516 | 0.0509 | 1.014 | 0.3186265 | 0.68077 |
| X211891_s_at | -0.0324 | 0.0319 | -1.013 | 0.3187134 | 0.680891 |
| X209404_s_at | 0.039 | 0.0385 | 1.013 | 0.3187877 | 0.680984 |
| X205237_at | 0.0022 | 0.0021 | 1.013 | 0.3188874 | 0.681131 |
| X210807_s_at | -0.0083 | 0.0082 | -1.013 | 0.318944 | 0.681187 |
| X49485_at | -0.0415 | 0.041 | -1.013 | 0.3189913 | 0.681222 |
| X213952_s_at | 0.0036 | 0.0035 | 1.013 | 0.3190354 | 0.681251 |
| X220579_at | -0.0038 | 0.0038 | -1.013 | 0.319123 | 0.681372 |
| X208474_at | -0.0029 | 0.0029 | -1.012 | 0.3193232 | 0.681716 |
| X210788_s_at | 0.059 | 0.0583 | 1.012 | 0.3193454 | 0.681716 |
| X220575_at | -0.0237 | 0.0234 | -1.012 | 0.3195234 | 0.682031 |
| X204377_s_at | 0.0061 | 0.006 | 1.012 | 0.3195897 | 0.682107 |
| X214451_at | 0.0038 | 0.0037 | 1.011 | 0.3196428 | 0.682108 |
| X205256_at | 0.0087 | 0.0086 | 1.011 | 0.3196518 | 0.682108 |
| X200615_s_at | -0.0594 | 0.0587 | -1.011 | 0.3198571 | 0.682481 |
| X208218_s_at | 0.0075 | 0.0075 | 1.01 | 0.3201355 | 0.682955 |
| X205504_at | 0.0069 | 0.0068 | 1.01 | 0.3201411 | 0.682955 |
| X213197_at | -0.045 | 0.0446 | -1.01 | 0.3202362 | 0.683015 |
| X219890_at | 0.0069 | 0.0069 | 1.01 | 0.3202481 | 0.683015 |
| X214462_at | -0.0051 | 0.0051 | -1.01 | 0.3202613 | 0.683015 |
| X218797_s_at | 0.0347 | 0.0344 | 1.01 | 0.3202978 | 0.683027 |
| X216926_s_at | 0.0024 | 0.0024 | 1.01 | 0.3203724 | 0.683109 |
| X202601_s_at | -0.0997 | 0.0988 | -1.01 | 0.3204204 | 0.683109 |
| X218454_at | -0.0054 | 0.0054 | -1.01 | 0.3204282 | 0.683109 |
| X217287_s_at | 0.0035 | 0.0035 | 1.01 | 0.3205496 | 0.683285 |
| X206509_at | 0.0064 | 0.0063 | 1.009 | 0.3205726 | 0.683285 |
| X215194_at | -0.0036 | 0.0036 | -1.009 | 0.3206055 | 0.68329 |
| X64899_at | 0.0523 | 0.0518 | 1.009 | 0.3207663 | 0.683567 |
| X217001_x_at | 0.0029 | 0.0029 | 1.009 | 0.3209216 | 0.683832 |
| X208954_s_at | -0.013 | 0.0129 | -1.009 | 0.3209791 | 0.683889 |
| X221004_s_at | -0.0529 | 0.0525 | -1.009 | 0.3210213 | 0.683906 |
| X209573_s_at | -0.0168 | 0.0167 | -1.008 | 0.3210754 | 0.683906 |
| X201181_at | 0.0297 | 0.0295 | 1.008 | 0.3210792 | 0.683906 |
| X208824_x_at | -0.0078 | 0.0077 | -1.008 | 0.3211618 | 0.683929 |
| X214003_x_at | 0.0179 | 0.0177 | 1.008 | 0.3211879 | 0.683929 |
| X218641_at | -0.0434 | 0.043 | -1.008 | 0.3212008 | 0.683929 |
| X209516_at | 0.0101 | 0.01 | 1.008 | 0.3212554 | 0.683929 |
| X219700_at | -0.0827 | 0.0821 | -1.008 | 0.3212589 | 0.683929 |
| X204351_at | 0.0034 | 0.0034 | 1.008 | 0.3212746 | 0.683929 |
| X219461_at | -0.0747 | 0.0741 | -1.008 | 0.3213173 | 0.683944 |
| X218629_at | -0.0099 | 0.0098 | -1.008 | 0.3213434 | 0.683944 |
| X207649_at | 0.0035 | 0.0034 | 1.008 | 0.3214157 | 0.683949 |
| X205783_at | 0.0041 | 0.004 | 1.008 | 0.3214357 | 0.683949 |
| X207154_at | -0.0055 | 0.0054 | -1.008 | 0.3214383 | 0.683949 |
| X209194_at | 0.0572 | 0.0568 | 1.008 | 0.3214898 | 0.683993 |
| X221104_s_at | -0.0855 | 0.0849 | -1.007 | 0.3216055 | 0.68411 |
| X220198_s_at | -0.008 | 0.008 | -1.007 | 0.3216146 | 0.68411 |
| X205748_s_at | -0.0053 | 0.0053 | -1.007 | 0.32169 | 0.68411 |
| X208099_x_at | 0.0039 | 0.0038 | 1.007 | 0.3217275 | 0.68411 |
| X213940_s_at | 0.0763 | 0.0757 | 1.007 | 0.3217484 | 0.68411 |
| X211830_s_at | 0.0025 | 0.0025 | 1.007 | 0.3217559 | 0.68411 |
| X214699_x_at | -0.0062 | 0.0062 | -1.007 | 0.32176 | 0.68411 |
| X215384_s_at | 0.0208 | 0.0207 | 1.007 | 0.3218079 | 0.684122 |
| X221508_at | 0.0187 | 0.0186 | 1.007 | 0.3218766 | 0.684122 |
| X219873_at | -0.0078 | 0.0078 | -1.007 | 0.3219045 | 0.684122 |
| X216501_at | 0.0028 | 0.0028 | 1.007 | 0.3219286 | 0.684122 |
| X201472_at | -0.0424 | 0.0421 | -1.007 | 0.3219612 | 0.684122 |
| X203976_s_at | -0.0048 | 0.0048 | -1.006 | 0.3219739 | 0.684122 |
| X215766_at | 0.0304 | 0.0302 | 1.006 | 0.3219945 | 0.684122 |
| X219351_at | -0.0331 | 0.0328 | -1.006 | 0.3220123 | 0.684122 |
| X216301_at | -0.0149 | 0.0148 | -1.006 | 0.322182 | 0.684324 |
| X207364_at | -0.0031 | 0.0031 | -1.006 | 0.32219 | 0.684324 |
| X219615_s_at | -0.0074 | 0.0074 | -1.006 | 0.3221995 | 0.684324 |
| X217399_s_at | -0.0042 | 0.0041 | -1.006 | 0.322287 | 0.684444 |
| X219494_at | -0.0111 | 0.011 | -1.006 | 0.3224185 | 0.684527 |
| X201259_s_at | 0.1327 | 0.132 | 1.006 | 0.3224257 | 0.684527 |
| X215746_at | 0.0091 | 0.0091 | 1.006 | 0.3224328 | 0.684527 |
| X207697_x_at | 0.0047 | 0.0047 | 1.005 | 0.3224495 | 0.684527 |
| X205507_at | 0.004 | 0.0039 | 1.005 | 0.3225121 | 0.684595 |
| X206964_at | -0.0043 | 0.0043 | -1.005 | 0.3225563 | 0.684623 |
| X205317_s_at | 0.0745 | 0.0741 | 1.005 | 0.3226697 | 0.684798 |
| X215988_s_at | -0.0027 | 0.0027 | -1.005 | 0.3227023 | 0.684802 |
| X218832_x_at | -0.0023 | 0.0023 | -1.005 | 0.3227964 | 0.684936 |
| X202953_at | 0.1652 | 0.1645 | 1.004 | 0.3229267 | 0.685147 |
| X214847_s_at | 0.0028 | 0.0027 | 1.004 | 0.3229716 | 0.685177 |
| X220819_at | 0.0035 | 0.0035 | 1.004 | 0.3230575 | 0.685181 |
| X206241_at | -0.0132 | 0.0131 | -1.004 | 0.3230583 | 0.685181 |
| X220946_s_at | 0.1414 | 0.1408 | 1.004 | 0.3230659 | 0.685181 |
| X221179_at | 0.0044 | 0.0043 | 1.004 | 0.3231259 | 0.685243 |
| X209182_s_at | 0.004 | 0.0039 | 1.004 | 0.3232072 | 0.685345 |
| X43511_s_at | -0.0387 | 0.0386 | -1.004 | 0.3232359 | 0.685345 |
| X206730_at | 0.0879 | 0.0876 | 1.004 | 0.3233007 | 0.685407 |
| X212033_at | -0.022 | 0.0219 | -1.004 | 0.3233384 | 0.685407 |
| X208572_at | -0.0018 | 0.0018 | -1.004 | 0.323386 | 0.685407 |
| X214753_at | -0.127 | 0.1266 | -1.003 | 0.3234088 | 0.685407 |
| X208713_at | -0.021 | 0.0209 | -1.003 | 0.3234195 | 0.685407 |
| X214376_at | -0.0446 | 0.0444 | -1.003 | 0.3234525 | 0.685412 |
| X207401_at | 0.0164 | 0.0164 | 1.003 | 0.3234842 | 0.685414 |
| X210259_s_at | -0.0039 | 0.0039 | -1.003 | 0.3236284 | 0.68555 |
| X220871_at | 0.0527 | 0.0525 | 1.003 | 0.3236891 | 0.68555 |
| X201579_at | -0.0601 | 0.0599 | -1.003 | 0.3236986 | 0.68555 |
| X207024_at | -0.0044 | 0.0044 | -1.003 | 0.3237022 | 0.68555 |
| X216769_x_at | -0.0145 | 0.0145 | -1.003 | 0.3237026 | 0.68555 |
| X213852_at | -0.0297 | 0.0296 | -1.003 | 0.3238222 | 0.685688 |
| X209674_at | 0.0538 | 0.0537 | 1.003 | 0.3238374 | 0.685688 |
| X204769_s_at | 0.0025 | 0.0025 | 1.002 | 0.3238841 | 0.685688 |
| X220403_s_at | -0.0052 | 0.0052 | -1.002 | 0.3238914 | 0.685688 |
| X216292_at | 0.004 | 0.004 | 1.002 | 0.3239352 | 0.685715 |
| X209441_at | -0.1217 | 0.1214 | -1.002 | 0.3239998 | 0.685739 |
| X207829_s_at | -0.0324 | 0.0324 | -1.002 | 0.3240448 | 0.685739 |
| X206617_s_at | -0.0035 | 0.0035 | -1.002 | 0.3240614 | 0.685739 |
| X212944_at | 0.0809 | 0.0807 | 1.002 | 0.3240972 | 0.685739 |
| X204221_x_at | -0.0865 | 0.0863 | -1.002 | 0.3241101 | 0.685739 |
| X201184_s_at | -0.026 | 0.026 | -1.002 | 0.3241514 | 0.685739 |
| X210465_s_at | -0.0459 | 0.0458 | -1.002 | 0.3241623 | 0.685739 |
| X207262_at | -0.006 | 0.006 | -1.001 | 0.3243417 | 0.686052 |
| X206932_at | -0.1838 | 0.1836 | -1.001 | 0.3243721 | 0.686052 |
| X205854_at | 0.0573 | 0.0573 | 1.001 | 0.3247193 | 0.686721 |
| X221365_at | -0.0024 | 0.0024 | -1 | 0.3248512 | 0.686823 |
| X202804_at | -0.0507 | 0.0507 | -1 | 0.324854 | 0.686823 |
| X203665_at | 0.007 | 0.007 | 1 | 0.3248602 | 0.686823 |
| X213779_at | -0.0078 | 0.0078 | -1 | 0.3249121 | 0.686867 |
| X205303_at | 0.0042 | 0.0042 | 1 | 0.3249978 | 0.686942 |
| X222269_at | 0.0074 | 0.0074 | 1 | 0.3250262 | 0.686942 |
| X211602_s_at | -0.0158 | 0.0158 | -1 | 0.3250443 | 0.686942 |
| X213857_s_at | 0.0368 | 0.0368 | 1 | 0.3251075 | 0.686942 |
| X201015_s_at | 0.0114 | 0.0114 | 1 | 0.3251296 | 0.686942 |
| X214987_at | -0.0117 | 0.0117 | -1 | 0.3251883 | 0.686942 |
| X208323_s_at | 0.0029 | 0.0029 | 1 | 0.3251908 | 0.686942 |
| X218037_at | -0.0476 | 0.0477 | -1 | 0.325195 | 0.686942 |
| X215289_at | -0.0089 | 0.0089 | -0.999 | 0.3253553 | 0.687194 |
| X216247_at | -0.061 | 0.0611 | -0.999 | 0.325376 | 0.687194 |
| X204531_s_at | -0.0171 | 0.0171 | -0.999 | 0.3254315 | 0.687217 |
| X221397_at | -0.0081 | 0.0081 | -0.999 | 0.3254491 | 0.687217 |
| X207396_s_at | 0.0029 | 0.0029 | 0.999 | 0.325608 | 0.687362 |
| X207303_at | 0.0352 | 0.0353 | 0.999 | 0.3256132 | 0.687362 |
| X211342_x_at | -0.0059 | 0.0059 | -0.999 | 0.3256256 | 0.687362 |
| X210107_at | -0.0044 | 0.0044 | -0.999 | 0.3256731 | 0.687362 |
| X204310_s_at | -0.0196 | 0.0196 | -0.999 | 0.325678 | 0.687362 |
| X201499_s_at | -0.0449 | 0.045 | -0.999 | 0.325717 | 0.687362 |
| X204650_s_at | -0.0088 | 0.0088 | -0.999 | 0.3257574 | 0.687362 |
| X208259_x_at | -0.005 | 0.005 | -0.999 | 0.3257652 | 0.687362 |
| X208851_s_at | -0.0636 | 0.0638 | -0.998 | 0.3261654 | 0.688141 |
| X207667_s_at | 0.0092 | 0.0092 | 0.998 | 0.3261986 | 0.688146 |
| X207065_at | 0.0043 | 0.0043 | 0.997 | 0.32629 | 0.68819 |
| X214440_at | 0.008 | 0.008 | 0.997 | 0.3262978 | 0.68819 |
| X204386_s_at | 0.0254 | 0.0255 | 0.997 | 0.3263123 | 0.68819 |
| X207938_at | 0.007 | 0.007 | 0.997 | 0.3263767 | 0.688231 |
| X202493_x_at | 0.003 | 0.003 | 0.997 | 0.3264211 | 0.688231 |
| X221655_x_at | -0.0024 | 0.0025 | -0.997 | 0.3264247 | 0.688231 |
| X212461_at | -0.0205 | 0.0206 | -0.997 | 0.3265055 | 0.688336 |
| X206207_at | 0.0031 | 0.0031 | 0.997 | 0.3265394 | 0.688342 |
| X216787_at | 0.0037 | 0.0038 | 0.997 | 0.3266133 | 0.688402 |
| X215627_at | -0.0077 | 0.0077 | -0.997 | 0.3266482 | 0.688402 |
| X219185_at | -0.0448 | 0.0449 | -0.997 | 0.3266607 | 0.688402 |
| X211663_x_at | -0.0575 | 0.0577 | -0.996 | 0.3267809 | 0.68859 |
| X209500_x_at | 0.0433 | 0.0435 | 0.996 | 0.3268424 | 0.688591 |
| X214813_at | -0.0034 | 0.0034 | -0.996 | 0.3268435 | 0.688591 |
| X217779_s_at | 0.0232 | 0.0233 | 0.996 | 0.3270839 | 0.689032 |
| X218721_s_at | -0.0308 | 0.031 | -0.996 | 0.3271565 | 0.68912 |
| X208566_at | -0.0057 | 0.0058 | -0.995 | 0.3272261 | 0.689201 |
| X220289_s_at | -0.0023 | 0.0023 | -0.995 | 0.3272686 | 0.689225 |
| X210451_at | 0.0039 | 0.0039 | 0.995 | 0.3273042 | 0.689235 |
| X208970_s_at | -0.0199 | 0.02 | -0.995 | 0.3273526 | 0.689271 |
| X205692_s_at | 0.0038 | 0.0038 | 0.995 | 0.3273847 | 0.689273 |
| X221036_s_at | 0.0058 | 0.0059 | 0.995 | 0.3275337 | 0.689343 |
| X219730_at | -0.0078 | 0.0079 | -0.995 | 0.327549 | 0.689343 |
| X213033_s_at | 0.0779 | 0.0783 | 0.995 | 0.3275505 | 0.689343 |
| X205904_at | 0.0847 | 0.0852 | 0.995 | 0.3275629 | 0.689343 |
| X220076_at | -0.042 | 0.0422 | -0.995 | 0.3275833 | 0.689343 |
| X213579_s_at | 0.0164 | 0.0164 | 0.995 | 0.3276038 | 0.689343 |
| X202128_at | -0.0591 | 0.0594 | -0.994 | 0.3278763 | 0.689715 |
| X201121_s_at | 0.0375 | 0.0377 | 0.994 | 0.3279041 | 0.689715 |
| X207050_at | -0.0018 | 0.0018 | -0.994 | 0.3279161 | 0.689715 |
| X210467_x_at | 0.0062 | 0.0062 | 0.994 | 0.3279302 | 0.689715 |
| X203965_at | -0.0672 | 0.0677 | -0.994 | 0.3279466 | 0.689715 |
| X200000_s_at | -0.0262 | 0.0264 | -0.994 | 0.327967 | 0.689715 |
| X216921_s_at | -0.0043 | 0.0043 | -0.994 | 0.328102 | 0.689833 |
| X209482_at | -0.0429 | 0.0432 | -0.994 | 0.3281356 | 0.689833 |
| X207491_at | 0.0052 | 0.0052 | 0.993 | 0.3281938 | 0.689833 |
| X207897_at | 0.0021 | 0.0022 | 0.993 | 0.3282035 | 0.689833 |
| X220763_at | -0.0036 | 0.0036 | -0.993 | 0.3282042 | 0.689833 |
| X214643_x_at | 0.0211 | 0.0213 | 0.993 | 0.3282095 | 0.689833 |
| X210012_s_at | -0.005 | 0.0051 | -0.993 | 0.3282616 | 0.689877 |
| X217063_x_at | -0.0048 | 0.0048 | -0.993 | 0.32834 | 0.689936 |
| X210166_at | 0.0059 | 0.0059 | 0.993 | 0.3283516 | 0.689936 |
| X218642_s_at | -0.0287 | 0.0289 | -0.993 | 0.3284429 | 0.690063 |
| X214114_x_at | 0.0385 | 0.0387 | 0.993 | 0.3285395 | 0.6902 |
| X208904_s_at | 0.0145 | 0.0146 | 0.993 | 0.3285759 | 0.690211 |
| X205383_s_at | 0.0516 | 0.052 | 0.992 | 0.3286386 | 0.690278 |
| X211076_x_at | 0.0237 | 0.0239 | 0.992 | 0.3287405 | 0.690341 |
| X41469_at | -0.0041 | 0.0041 | -0.992 | 0.3287542 | 0.690341 |
| X216376_x_at | 0.0027 | 0.0028 | 0.992 | 0.328766 | 0.690341 |
| X222195_s_at | -0.0074 | 0.0075 | -0.992 | 0.3288266 | 0.690341 |
| X221790_s_at | 0.0197 | 0.0198 | 0.992 | 0.3288743 | 0.690341 |
| X221994_at | -0.0084 | 0.0085 | -0.992 | 0.3288916 | 0.690341 |
| X216005_at | 0.0062 | 0.0063 | 0.992 | 0.3288958 | 0.690341 |
| X211537_x_at | -0.0804 | 0.0811 | -0.992 | 0.3290232 | 0.690341 |
| X201073_s_at | 0.0099 | 0.01 | 0.992 | 0.3290717 | 0.690341 |
| X207859_s_at | 0.0043 | 0.0043 | 0.992 | 0.3290805 | 0.690341 |
| X216432_at | 0.0041 | 0.0042 | 0.992 | 0.3290877 | 0.690341 |
| X220683_at | 0.0036 | 0.0036 | 0.992 | 0.3290927 | 0.690341 |
| X214046_at | -0.051 | 0.0514 | -0.992 | 0.329116 | 0.690341 |
| X204457_s_at | 0.0512 | 0.0517 | 0.991 | 0.3291292 | 0.690341 |
| X219613_s_at | -0.009 | 0.0091 | -0.991 | 0.3291346 | 0.690341 |
| X221732_at | 0.0238 | 0.024 | 0.991 | 0.3292818 | 0.690575 |
| X208320_at | 0.0046 | 0.0046 | 0.991 | 0.3293084 | 0.690575 |
| X218061_at | -0.0219 | 0.0221 | -0.991 | 0.3293851 | 0.69058 |
| X206079_at | 0.0061 | 0.0062 | 0.991 | 0.3293908 | 0.69058 |
| X212252_at | -0.1196 | 0.1207 | -0.991 | 0.3294041 | 0.69058 |
| X211829_s_at | -0.0033 | 0.0033 | -0.991 | 0.3294775 | 0.690668 |
| X214062_x_at | 0.0044 | 0.0044 | 0.991 | 0.3295399 | 0.69071 |
| X214248_s_at | -0.0029 | 0.0029 | -0.991 | 0.3295819 | 0.69071 |
| X204447_at | 0.0669 | 0.0676 | 0.991 | 0.3295906 | 0.69071 |
| X210428_s_at | 0.0714 | 0.0721 | 0.99 | 0.3296386 | 0.690745 |
| X205190_at | 0.013 | 0.0131 | 0.99 | 0.3296792 | 0.690765 |
| X217884_at | 0.0483 | 0.0488 | 0.99 | 0.3297296 | 0.69078 |
| X202656_s_at | -0.0253 | 0.0255 | -0.99 | 0.3297639 | 0.69078 |
| X205914_s_at | 0.0064 | 0.0065 | 0.99 | 0.3297797 | 0.69078 |
| X212497_at | 0.0022 | 0.0022 | 0.99 | 0.3298246 | 0.690786 |
| X206963_s_at | -0.0059 | 0.0059 | -0.99 | 0.3298443 | 0.690786 |
| X215741_x_at | -0.0062 | 0.0063 | -0.99 | 0.3299468 | 0.690935 |
| X217389_s_at | -0.0036 | 0.0036 | -0.99 | 0.3299982 | 0.690978 |
| X205420_at | -0.048 | 0.0485 | -0.989 | 0.3301221 | 0.691158 |
| X211148_s_at | -0.0074 | 0.0075 | -0.989 | 0.3301467 | 0.691158 |
| X36129_at | -0.0435 | 0.0439 | -0.989 | 0.3302155 | 0.691184 |
| X201282_at | 0.0114 | 0.0115 | 0.989 | 0.3302481 | 0.691184 |
| X220532_s_at | 0.1052 | 0.1064 | 0.989 | 0.3302523 | 0.691184 |
| X201890_at | 0.0098 | 0.0099 | 0.989 | 0.3303445 | 0.691236 |
| X213868_s_at | 0.0057 | 0.0058 | 0.989 | 0.330362 | 0.691236 |
| X206840_at | -0.0047 | 0.0048 | -0.989 | 0.3303708 | 0.691236 |
| X210496_at | 0.012 | 0.0122 | 0.989 | 0.3304745 | 0.691388 |
| X215643_at | -0.0142 | 0.0143 | -0.989 | 0.3305537 | 0.691478 |
| X200897_s_at | 0.05 | 0.0506 | 0.988 | 0.3305794 | 0.691478 |
| X218130_at | 0.0365 | 0.0369 | 0.988 | 0.3306468 | 0.691492 |
| X52741_at | 0.0349 | 0.0353 | 0.988 | 0.3306486 | 0.691492 |
| X209251_x_at | 0.0414 | 0.0419 | 0.988 | 0.3307139 | 0.691527 |
| X219086_at | -0.0062 | 0.0063 | -0.988 | 0.3307276 | 0.691527 |
| X205509_at | 0.0051 | 0.0052 | 0.988 | 0.3307804 | 0.691572 |
| X211520_s_at | 0.0512 | 0.0518 | 0.988 | 0.3308961 | 0.69166 |
| X210965_x_at | -0.04 | 0.0405 | -0.988 | 0.3309136 | 0.69166 |
| X213392_at | -0.0474 | 0.048 | -0.988 | 0.3309282 | 0.69166 |
| X203773_x_at | 0.0413 | 0.0418 | 0.988 | 0.3309468 | 0.69166 |
| X202323_s_at | 0.107 | 0.1083 | 0.987 | 0.3310853 | 0.691792 |
| X200637_s_at | 0.0631 | 0.0639 | 0.987 | 0.3311006 | 0.691792 |
| X221353_at | 0.0045 | 0.0046 | 0.987 | 0.3311301 | 0.691792 |
| X220412_x_at | 0.0024 | 0.0025 | 0.987 | 0.3311347 | 0.691792 |
| X210866_s_at | -0.0045 | 0.0046 | -0.987 | 0.3312818 | 0.692011 |
| X222260_at | 0.0038 | 0.0039 | 0.987 | 0.3313019 | 0.692011 |
| X208586_s_at | -0.012 | 0.0122 | -0.987 | 0.3313943 | 0.692139 |
| X205037_at | 0.0443 | 0.0449 | 0.987 | 0.3314949 | 0.692282 |
| X221354_s_at | 0.0017 | 0.0017 | 0.986 | 0.3315303 | 0.692282 |
| X211109_at | -0.0053 | 0.0054 | -0.986 | 0.3315849 | 0.692282 |
| X204395_s_at | 0.0033 | 0.0033 | 0.986 | 0.3316075 | 0.692282 |
| X208237_x_at | 0.0046 | 0.0046 | 0.986 | 0.3316386 | 0.692282 |
| X211735_x_at | 0.0042 | 0.0043 | 0.986 | 0.3316506 | 0.692282 |
| X206072_at | 0.0052 | 0.0053 | 0.986 | 0.3316933 | 0.692282 |
| X217281_x_at | -0.0049 | 0.005 | -0.986 | 0.3317118 | 0.692282 |
| X202482_x_at | -0.0062 | 0.0062 | -0.986 | 0.331798 | 0.692397 |
| X200666_s_at | 0.0825 | 0.0837 | 0.986 | 0.3318625 | 0.692466 |
| X216873_s_at | -0.0046 | 0.0047 | -0.986 | 0.3319395 | 0.692548 |
| X209858_x_at | -0.0227 | 0.023 | -0.986 | 0.3319922 | 0.692548 |
| X220960_x_at | 0.0137 | 0.0139 | 0.986 | 0.3319955 | 0.692548 |
| X209200_at | 0.1001 | 0.1015 | 0.985 | 0.3320679 | 0.692634 |
| X205300_s_at | -0.035 | 0.0356 | -0.985 | 0.3324782 | 0.693425 |
| X204521_at | -0.0401 | 0.0407 | -0.984 | 0.3325462 | 0.693464 |
| X208864_s_at | 0.0306 | 0.0311 | 0.984 | 0.3325595 | 0.693464 |
| X211704_s_at | 0.0321 | 0.0326 | 0.984 | 0.3326163 | 0.693518 |
| X207668_x_at | -0.0328 | 0.0334 | -0.984 | 0.3327726 | 0.693601 |
| X209346_s_at | 0.0126 | 0.0129 | 0.984 | 0.3327736 | 0.693601 |
| X221246_x_at | 0.0099 | 0.0101 | 0.984 | 0.3327802 | 0.693601 |
| X203463_s_at | 0.0681 | 0.0693 | 0.984 | 0.3327811 | 0.693601 |
| X216299_s_at | -0.0036 | 0.0037 | -0.984 | 0.3328584 | 0.693663 |
| X208654_s_at | 0.0517 | 0.0525 | 0.984 | 0.3328765 | 0.693663 |
| X207417_s_at | -0.084 | 0.0854 | -0.984 | 0.3329045 | 0.693663 |
| X200697_at | -0.0427 | 0.0434 | -0.984 | 0.3329565 | 0.693706 |
| X221841_s_at | 0.133 | 0.1352 | 0.983 | 0.3330731 | 0.693884 |
| X202202_s_at | -0.0807 | 0.0821 | -0.983 | 0.3332192 | 0.69412 |
| X212127_at | -0.0189 | 0.0192 | -0.983 | 0.3332807 | 0.69412 |
| X209780_at | -0.0279 | 0.0283 | -0.983 | 0.3332901 | 0.69412 |
| X216688_at | 0.0046 | 0.0047 | 0.983 | 0.3333115 | 0.69412 |
| X222218_s_at | 0.0058 | 0.0059 | 0.982 | 0.3335467 | 0.694545 |
| X206706_at | -0.0032 | 0.0033 | -0.982 | 0.3336653 | 0.694727 |
| X203446_s_at | 0.032 | 0.0326 | 0.982 | 0.3337928 | 0.694833 |
| X200840_at | -0.0206 | 0.021 | -0.982 | 0.333859 | 0.694833 |
| X208948_s_at | 0.018 | 0.0183 | 0.982 | 0.3339241 | 0.694833 |
| X211903_s_at | -0.0048 | 0.0049 | -0.982 | 0.333935 | 0.694833 |
| X206406_at | -0.003 | 0.0031 | -0.981 | 0.333964 | 0.694833 |
| X218046_s_at | -0.0154 | 0.0156 | -0.981 | 0.333987 | 0.694833 |
| X206449_s_at | -0.0041 | 0.0041 | -0.981 | 0.3340015 | 0.694833 |
| X219288_at | 0.0322 | 0.0328 | 0.981 | 0.3340334 | 0.694833 |
| X207179_at | -0.0035 | 0.0036 | -0.981 | 0.3340549 | 0.694833 |
| X214535_s_at | -0.0031 | 0.0032 | -0.981 | 0.3340576 | 0.694833 |
| X218153_at | 0.0213 | 0.0218 | 0.981 | 0.3340602 | 0.694833 |
| X219895_at | 0.1858 | 0.1894 | 0.981 | 0.334124 | 0.6949 |
| X211665_s_at | -0.0251 | 0.0256 | -0.981 | 0.3341706 | 0.694932 |
| X212206_s_at | -0.0317 | 0.0323 | -0.981 | 0.3342597 | 0.695052 |
| X203769_s_at | -0.0433 | 0.0442 | -0.98 | 0.3345314 | 0.695552 |
| X208211_s_at | 0.0024 | 0.0024 | 0.98 | 0.3345731 | 0.695574 |
| X202913_at | -0.0124 | 0.0126 | -0.98 | 0.3346118 | 0.695589 |
| X217311_at | 0.003 | 0.0031 | 0.98 | 0.3346577 | 0.69562 |
| X51192_at | 0.0095 | 0.0097 | 0.98 | 0.3347342 | 0.695714 |
| X219257_s_at | -0.0028 | 0.0029 | -0.98 | 0.3348748 | 0.695941 |
| X220437_at | -0.0035 | 0.0036 | -0.979 | 0.3349846 | 0.696104 |
| X201655_s_at | -0.0049 | 0.005 | -0.979 | 0.3350607 | 0.696132 |
| X214463_x_at | -0.005 | 0.0051 | -0.979 | 0.3350607 | 0.696132 |
| X220730_at | -0.0057 | 0.0058 | -0.979 | 0.3351243 | 0.69618 |
| X212574_x_at | -0.0034 | 0.0035 | -0.979 | 0.3351465 | 0.69618 |
| X218289_s_at | -0.0423 | 0.0433 | -0.979 | 0.3352348 | 0.696211 |
| X217986_s_at | 0.0878 | 0.0898 | 0.979 | 0.3353136 | 0.696211 |
| X217412_at | 0.0037 | 0.0038 | 0.979 | 0.3353257 | 0.696211 |
| X201993_x_at | -0.0277 | 0.0283 | -0.979 | 0.3353291 | 0.696211 |
| X219149_x_at | -0.0392 | 0.0401 | -0.979 | 0.3353429 | 0.696211 |
| X212679_at | 0.0042 | 0.0043 | 0.979 | 0.3353498 | 0.696211 |
| X217018_at | -0.0029 | 0.003 | -0.978 | 0.335441 | 0.696336 |
| X212123_at | -0.0276 | 0.0282 | -0.978 | 0.3355132 | 0.69642 |
| X208251_at | -0.0038 | 0.0039 | -0.978 | 0.3355476 | 0.696427 |
| X205858_at | 0.005 | 0.0052 | 0.978 | 0.335691 | 0.696542 |
| X217041_at | -0.0024 | 0.0024 | -0.978 | 0.3356927 | 0.696542 |
| X207421_at | 0.0044 | 0.0045 | 0.978 | 0.3356973 | 0.696542 |
| X219360_s_at | -0.0037 | 0.0038 | -0.978 | 0.3357361 | 0.696558 |
| X216670_at | 0.0023 | 0.0024 | 0.978 | 0.3358833 | 0.696798 |
| X220669_at | -0.0209 | 0.0214 | -0.977 | 0.3360271 | 0.697019 |
| X215245_x_at | 0.02 | 0.0205 | 0.977 | 0.3360525 | 0.697019 |
| X201182_s_at | 0.0766 | 0.0784 | 0.977 | 0.3361271 | 0.697083 |
| X215859_at | -0.0075 | 0.0077 | -0.977 | 0.3361463 | 0.697083 |
| X222317_at | -0.0697 | 0.0714 | -0.976 | 0.3364007 | 0.697546 |
| X207071_s_at | -0.0504 | 0.0516 | -0.976 | 0.3364656 | 0.697575 |
| X210151_s_at | 0.0039 | 0.004 | 0.976 | 0.3365099 | 0.697575 |
| X207562_at | -0.0022 | 0.0023 | -0.976 | 0.3365594 | 0.697575 |
| X222248_s_at | 0.0022 | 0.0023 | 0.976 | 0.3365625 | 0.697575 |
| X203554_x_at | -0.176 | 0.1803 | -0.976 | 0.3365912 | 0.697575 |
| X211436_at | -0.0097 | 0.01 | -0.976 | 0.3366035 | 0.697575 |
| X213759_at | -0.0041 | 0.0042 | -0.976 | 0.3367386 | 0.69779 |
| X214944_at | 0.0045 | 0.0047 | 0.976 | 0.3368084 | 0.697812 |
| X203835_at | 0.0033 | 0.0034 | 0.976 | 0.3368166 | 0.697812 |
| X221827_at | 0.055 | 0.0564 | 0.976 | 0.3368433 | 0.697812 |
| X203473_at | 0.0981 | 0.1005 | 0.975 | 0.3369257 | 0.697918 |
| X206048_at | -0.0157 | 0.0161 | -0.975 | 0.3369943 | 0.69795 |
| X211205_x_at | 0.0028 | 0.0028 | 0.975 | 0.3370235 | 0.69795 |
| X206887_at | 0.003 | 0.0031 | 0.975 | 0.3370355 | 0.69795 |
| X220000_at | 0.0024 | 0.0025 | 0.975 | 0.3371233 | 0.698066 |
| X206271_at | 0.005 | 0.0051 | 0.975 | 0.3372465 | 0.698066 |
| X211834_s_at | -0.0033 | 0.0034 | -0.975 | 0.3372498 | 0.698066 |
| X221416_at | -0.0038 | 0.0039 | -0.975 | 0.3372666 | 0.698066 |
| X212578_x_at | 0.0189 | 0.0194 | 0.975 | 0.337276 | 0.698066 |
| X218809_at | -0.0472 | 0.0484 | -0.975 | 0.3372802 | 0.698066 |
| X205460_at | 0.0031 | 0.0032 | 0.974 | 0.3373584 | 0.698099 |
| X205860_x_at | 0.1671 | 0.1715 | 0.974 | 0.3373776 | 0.698099 |
| X202553_s_at | -0.0153 | 0.0157 | -0.974 | 0.3373954 | 0.698099 |
| X205715_at | -0.0028 | 0.0028 | -0.974 | 0.3374219 | 0.698099 |
| X204918_s_at | -0.0517 | 0.0531 | -0.974 | 0.3374804 | 0.698155 |
| X214999_s_at | 0.0057 | 0.0059 | 0.974 | 0.3375264 | 0.698185 |
| X208282_x_at | -0.0071 | 0.0073 | -0.974 | 0.3375596 | 0.698189 |
| X204980_at | 0.0272 | 0.0279 | 0.974 | 0.3376004 | 0.698208 |
| X219781_s_at | -0.0038 | 0.0039 | -0.974 | 0.3377174 | 0.698347 |
| X216662_at | -0.0028 | 0.0029 | -0.974 | 0.3377455 | 0.698347 |
| X212544_at | -0.0398 | 0.0409 | -0.974 | 0.3377616 | 0.698347 |
| X211004_s_at | -0.0094 | 0.0097 | -0.973 | 0.3378842 | 0.698454 |
| X215363_x_at | 0.1548 | 0.159 | 0.973 | 0.3379101 | 0.698454 |
| X215530_at | -0.0056 | 0.0057 | -0.973 | 0.3379674 | 0.698454 |
| X214168_s_at | 0.0416 | 0.0428 | 0.973 | 0.3379706 | 0.698454 |
| X210059_s_at | 0.006 | 0.0062 | 0.973 | 0.3379707 | 0.698454 |
| X205897_at | 0.0024 | 0.0025 | 0.973 | 0.3381017 | 0.69866 |
| X215491_at | -0.0046 | 0.0047 | -0.973 | 0.338184 | 0.698765 |
| X216280_s_at | -0.0094 | 0.0096 | -0.973 | 0.33825 | 0.698836 |
| X209070_s_at | -0.0607 | 0.0624 | -0.972 | 0.3383602 | 0.698999 |
| X216215_s_at | -0.031 | 0.0319 | -0.972 | 0.3385793 | 0.699341 |
| X221800_s_at | 0.0026 | 0.0027 | 0.972 | 0.3385974 | 0.699341 |
| X217488_x_at | 0.0029 | 0.003 | 0.972 | 0.3386291 | 0.699341 |
| X217793_at | 0.0034 | 0.0035 | 0.972 | 0.3386516 | 0.699341 |
| X206132_at | 0.0055 | 0.0057 | 0.972 | 0.3387105 | 0.699397 |
| X221557_s_at | 0.0089 | 0.0092 | 0.972 | 0.3387831 | 0.699482 |
| X215984_s_at | -0.0126 | 0.013 | -0.971 | 0.3388688 | 0.699572 |
| X216232_s_at | 0.0467 | 0.0481 | 0.971 | 0.3389577 | 0.699572 |
| X217461_x_at | -0.0076 | 0.0078 | -0.971 | 0.3389779 | 0.699572 |
| X215931_s_at | 0.0021 | 0.0021 | 0.971 | 0.3389812 | 0.699572 |
| X221086_s_at | -0.0651 | 0.067 | -0.971 | 0.3389842 | 0.699572 |
| X209481_at | -0.0233 | 0.024 | -0.971 | 0.3391636 | 0.699859 |
| X213881_x_at | 0.0109 | 0.0113 | 0.971 | 0.3391996 | 0.699859 |
| X220162_s_at | -0.0045 | 0.0046 | -0.971 | 0.3392178 | 0.699859 |
| X211091_s_at | 0.0095 | 0.0098 | 0.97 | 0.3393098 | 0.699984 |
| X205643_s_at | -0.0081 | 0.0084 | -0.97 | 0.3396147 | 0.700548 |
| X221113_s_at | 0.0029 | 0.003 | 0.97 | 0.3397081 | 0.700663 |
| X214935_at | -0.0071 | 0.0073 | -0.97 | 0.3397342 | 0.700663 |
| X202531_at | -0.0036 | 0.0037 | -0.97 | 0.339765 | 0.700663 |
| X209964_s_at | 0.0473 | 0.0487 | 0.969 | 0.3398319 | 0.700693 |
| X211269_s_at | 0.0053 | 0.0055 | 0.969 | 0.3398429 | 0.700693 |
| X206741_at | 0.002 | 0.0021 | 0.969 | 0.3399917 | 0.700883 |
| X210218_s_at | 0.0063 | 0.0065 | 0.969 | 0.339998 | 0.700883 |
| X206898_at | 0.1613 | 0.1665 | 0.969 | 0.3402054 | 0.701187 |
| X203083_at | 0.1624 | 0.1676 | 0.969 | 0.3402086 | 0.701187 |
| X212893_at | -0.0388 | 0.0401 | -0.969 | 0.3402533 | 0.701214 |
| X215474_at | -0.0047 | 0.0049 | -0.968 | 0.3404221 | 0.701488 |
| X211445_x_at | 0.0107 | 0.0111 | 0.968 | 0.3404493 | 0.701488 |
| X211176_s_at | 0.0034 | 0.0035 | 0.968 | 0.3406403 | 0.701737 |
| X221901_at | 0.0724 | 0.0748 | 0.968 | 0.3406707 | 0.701737 |
| X221020_s_at | -0.0492 | 0.0509 | -0.968 | 0.3406717 | 0.701737 |
| X222017_x_at | -0.0032 | 0.0033 | -0.968 | 0.3406965 | 0.701737 |
| X211285_s_at | -0.027 | 0.0279 | -0.968 | 0.340736 | 0.701753 |
| X206338_at | 0.03 | 0.031 | 0.967 | 0.3408171 | 0.701809 |
| X79005_at | -0.0324 | 0.0335 | -0.967 | 0.3408268 | 0.701809 |
| X40149_at | 0.033 | 0.0341 | 0.967 | 0.3408581 | 0.701809 |
| X216204_at | 0.003 | 0.0031 | 0.967 | 0.3409239 | 0.70188 |
| X201508_at | 0.0039 | 0.004 | 0.967 | 0.3409785 | 0.701927 |
| X213350_at | 0.0882 | 0.0913 | 0.967 | 0.3410928 | 0.702097 |
| X202960_s_at | 0.0333 | 0.0345 | 0.967 | 0.3411679 | 0.702187 |
| X204657_s_at | -0.0021 | 0.0022 | -0.967 | 0.3412085 | 0.702205 |
| X220986_s_at | 0.0572 | 0.0592 | 0.967 | 0.3412625 | 0.702252 |
| X214473_x_at | -0.0113 | 0.0117 | -0.966 | 0.3413705 | 0.702282 |
| X204295_at | 0.0495 | 0.0513 | 0.966 | 0.3413986 | 0.702282 |
| X202104_s_at | -0.0523 | 0.0541 | -0.966 | 0.341438 | 0.702282 |
| X205617_at | 0.0026 | 0.0027 | 0.966 | 0.3414555 | 0.702282 |
| X215749_s_at | -0.0425 | 0.044 | -0.966 | 0.3414732 | 0.702282 |
| X217251_x_at | -0.0032 | 0.0033 | -0.966 | 0.3414802 | 0.702282 |
| X214021_x_at | 0.0093 | 0.0097 | 0.966 | 0.3415127 | 0.702282 |
| X201147_s_at | 0.0621 | 0.0643 | 0.966 | 0.34153 | 0.702282 |
| X211911_x_at | 0.0922 | 0.0955 | 0.966 | 0.341645 | 0.70236 |
| X210821_x_at | 0.0038 | 0.0039 | 0.966 | 0.3416898 | 0.70236 |
| X219888_at | 0.0024 | 0.0024 | 0.966 | 0.3416941 | 0.70236 |
| X214793_at | 0.002 | 0.0021 | 0.966 | 0.3416954 | 0.70236 |
| X215763_at | -0.0086 | 0.0089 | -0.966 | 0.3417264 | 0.70236 |
| X202983_at | -0.0316 | 0.0328 | -0.965 | 0.3417944 | 0.702412 |
| X213323_s_at | 0.0068 | 0.0071 | 0.965 | 0.3418671 | 0.702412 |
| X220328_at | -0.0081 | 0.0084 | -0.965 | 0.3418872 | 0.702412 |
| X207869_s_at | 0.0135 | 0.014 | 0.965 | 0.3419116 | 0.702412 |
| X209614_at | -0.0073 | 0.0076 | -0.965 | 0.3419254 | 0.702412 |
| X216720_at | -0.0066 | 0.0068 | -0.965 | 0.3419505 | 0.702412 |
| X212380_at | 0.036 | 0.0373 | 0.965 | 0.341973 | 0.702412 |
| X204466_s_at | -0.0747 | 0.0775 | -0.965 | 0.3420333 | 0.702471 |
| X218571_s_at | -0.0301 | 0.0312 | -0.965 | 0.3420784 | 0.702499 |
| X211990_at | 0.1685 | 0.1747 | 0.965 | 0.3421232 | 0.70251 |
| X204643_s_at | -0.0374 | 0.0387 | -0.965 | 0.3421541 | 0.70251 |
| X216325_x_at | 0.0041 | 0.0043 | 0.965 | 0.3421789 | 0.70251 |
| X216793_x_at | -0.0126 | 0.013 | -0.964 | 0.3422682 | 0.702629 |
| X204075_s_at | 0.0605 | 0.0627 | 0.964 | 0.3423888 | 0.702737 |
| X204861_s_at | 0.0671 | 0.0696 | 0.964 | 0.3424672 | 0.702737 |
| X221090_s_at | 0.0351 | 0.0364 | 0.964 | 0.3424718 | 0.702737 |
| X220926_s_at | -0.0499 | 0.0517 | -0.964 | 0.3424929 | 0.702737 |
| X211897_s_at | -0.0025 | 0.0026 | -0.964 | 0.3424956 | 0.702737 |
| X219373_at | -0.0311 | 0.0323 | -0.964 | 0.3425353 | 0.702737 |
| X213603_s_at | 0.004 | 0.0042 | 0.964 | 0.3425425 | 0.702737 |
| X221729_at | 0.0545 | 0.0566 | 0.964 | 0.3427091 | 0.703014 |
| X212690_at | -0.0338 | 0.0351 | -0.963 | 0.3428572 | 0.703253 |
| X203180_at | -0.0062 | 0.0065 | -0.963 | 0.3429008 | 0.70327 |
| X219242_at | 0.0691 | 0.0718 | 0.963 | 0.3429497 | 0.70327 |
| X213462_at | -0.0222 | 0.0231 | -0.963 | 0.3429692 | 0.70327 |
| X207392_x_at | 0.0078 | 0.0081 | 0.963 | 0.3430158 | 0.70327 |
| X216407_at | -0.0048 | 0.005 | -0.963 | 0.343024 | 0.70327 |
| X217305_s_at | 0.0068 | 0.007 | 0.963 | 0.3432304 | 0.703629 |
| X209713_s_at | -0.0055 | 0.0057 | -0.962 | 0.3433217 | 0.703751 |
| X205404_at | -0.1077 | 0.1119 | -0.962 | 0.3433995 | 0.703782 |
| X219403_s_at | 0.0047 | 0.0049 | 0.962 | 0.3434036 | 0.703782 |
| X205716_at | -0.0084 | 0.0087 | -0.962 | 0.3434317 | 0.703782 |
| X207572_at | 0.0097 | 0.01 | 0.962 | 0.3436336 | 0.70413 |
| X213847_at | 0.0029 | 0.003 | 0.962 | 0.3437105 | 0.704223 |
| X215648_at | -0.0097 | 0.0101 | -0.961 | 0.3437651 | 0.70427 |
| X215745_at | 0.0082 | 0.0086 | 0.961 | 0.3439495 | 0.70448 |
| X213673_x_at | -0.0028 | 0.0029 | -0.961 | 0.3439573 | 0.70448 |
| X207871_s_at | 0.0533 | 0.0555 | 0.961 | 0.3439627 | 0.70448 |
| X216178_x_at | -0.0069 | 0.0072 | -0.961 | 0.3440498 | 0.704534 |
| X201539_s_at | -0.0931 | 0.0969 | -0.961 | 0.3440525 | 0.704534 |
| X215037_s_at | -0.0047 | 0.0049 | -0.961 | 0.3441425 | 0.704653 |
| X204913_s_at | 0.0972 | 0.1012 | 0.96 | 0.3442831 | 0.704876 |
| X203164_at | 0.0422 | 0.044 | 0.96 | 0.3443639 | 0.704977 |
| X213174_at | -0.0052 | 0.0054 | -0.96 | 0.3444021 | 0.704987 |
| X217420_s_at | -0.0058 | 0.006 | -0.96 | 0.3444323 | 0.704987 |
| X201757_at | -0.0215 | 0.0224 | -0.96 | 0.3445159 | 0.705093 |
| X205723_at | -0.0038 | 0.004 | -0.96 | 0.3446095 | 0.705124 |
| X218035_s_at | 0.0066 | 0.0069 | 0.96 | 0.3446526 | 0.705124 |
| X215727_x_at | 0.0053 | 0.0055 | 0.96 | 0.3446565 | 0.705124 |
| X206403_at | 0.1333 | 0.1389 | 0.96 | 0.3446582 | 0.705124 |
| X210761_s_at | 0.0026 | 0.0027 | 0.959 | 0.3447677 | 0.705256 |
| X215390_at | -0.0084 | 0.0088 | -0.959 | 0.3447861 | 0.705256 |
| X219808_at | 0.0022 | 0.0023 | 0.959 | 0.3448958 | 0.705331 |
| X55705_at | 0.0254 | 0.0265 | 0.959 | 0.3449241 | 0.705331 |
| X205471_s_at | -0.1201 | 0.1252 | -0.959 | 0.3449244 | 0.705331 |
| X212000_at | 0.0257 | 0.0268 | 0.959 | 0.3449815 | 0.705331 |
| X218215_s_at | -0.0038 | 0.004 | -0.959 | 0.3450033 | 0.705331 |
| X213148_at | -0.0377 | 0.0393 | -0.959 | 0.3450305 | 0.705331 |
| X218415_at | -0.0351 | 0.0366 | -0.959 | 0.3450806 | 0.705331 |
| X212621_at | -0.0147 | 0.0153 | -0.959 | 0.3450981 | 0.705331 |
| X212543_at | 0.0042 | 0.0043 | 0.959 | 0.3451118 | 0.705331 |
| X221834_at | -0.0336 | 0.0351 | -0.959 | 0.3451405 | 0.705331 |
| X219395_at | -0.0053 | 0.0055 | -0.958 | 0.3454426 | 0.705884 |
| X213582_at | -0.0082 | 0.0086 | -0.958 | 0.3456434 | 0.706229 |
| X207395_at | 0.0036 | 0.0037 | 0.958 | 0.3457032 | 0.706286 |
| X221000_s_at | 0.0021 | 0.0022 | 0.957 | 0.34576 | 0.706325 |
| X201287_s_at | 0.0042 | 0.0044 | 0.957 | 0.3458015 | 0.706325 |
| X217610_at | -0.1419 | 0.1482 | -0.957 | 0.345851 | 0.706325 |
| X212924_s_at | 0.0044 | 0.0046 | 0.957 | 0.3458951 | 0.706325 |
| X206791_s_at | -0.0073 | 0.0076 | -0.957 | 0.3459078 | 0.706325 |
| X206608_s_at | -0.0042 | 0.0044 | -0.957 | 0.3459129 | 0.706325 |
| X205995_x_at | 0.0422 | 0.0441 | 0.957 | 0.3459619 | 0.70636 |
| X204567_s_at | -0.063 | 0.0659 | -0.957 | 0.3459963 | 0.706365 |
| X217807_s_at | 0.0339 | 0.0354 | 0.957 | 0.3461226 | 0.706367 |
| X201620_at | -0.0337 | 0.0353 | -0.957 | 0.346135 | 0.706367 |
| X218229_s_at | 0.0509 | 0.0532 | 0.957 | 0.346146 | 0.706367 |
| X201964_at | -0.0295 | 0.0309 | -0.957 | 0.3461845 | 0.706367 |
| X207487_at | 0.0316 | 0.033 | 0.957 | 0.3461865 | 0.706367 |
| X205917_at | -0.0946 | 0.0989 | -0.957 | 0.3461877 | 0.706367 |
| X207398_at | -0.0017 | 0.0018 | -0.956 | 0.3463203 | 0.706572 |
| X208337_s_at | -0.005 | 0.0052 | -0.956 | 0.3463725 | 0.706614 |
| X217523_at | 0.0135 | 0.0141 | 0.956 | 0.3464267 | 0.70666 |
| X60794_f_at | -0.0705 | 0.0737 | -0.956 | 0.3465358 | 0.706817 |
| X204216_s_at | -0.0193 | 0.0201 | -0.955 | 0.3467201 | 0.707079 |
| X213232_at | 0.003 | 0.0031 | 0.955 | 0.3467277 | 0.707079 |
| X59631_at | 0.006 | 0.0063 | 0.955 | 0.3468432 | 0.707249 |
| X211824_x_at | 0.0025 | 0.0026 | 0.955 | 0.3468968 | 0.707294 |
| X212607_at | 0.0479 | 0.0502 | 0.955 | 0.3470951 | 0.707633 |
| X221601_s_at | 0.0046 | 0.0048 | 0.954 | 0.3473693 | 0.708072 |
| X214480_at | 0.003 | 0.0032 | 0.954 | 0.3473741 | 0.708072 |
| X210139_s_at | 0.1029 | 0.1079 | 0.954 | 0.3475453 | 0.708356 |
| X215246_at | 0.0091 | 0.0096 | 0.953 | 0.3477364 | 0.70868 |
| X214809_at | -0.0149 | 0.0156 | -0.953 | 0.3479513 | 0.709053 |
| X218777_at | -0.0054 | 0.0057 | -0.953 | 0.3481437 | 0.70938 |
| X206788_s_at | -0.0034 | 0.0035 | -0.952 | 0.3482213 | 0.709465 |
| X204359_at | -0.0592 | 0.0622 | -0.952 | 0.3482492 | 0.709465 |
| X213590_at | 0.002 | 0.0021 | 0.952 | 0.3483435 | 0.709504 |
| X219519_s_at | 0.0037 | 0.0039 | 0.952 | 0.3483621 | 0.709504 |
| X215618_at | 0.0044 | 0.0047 | 0.952 | 0.3483641 | 0.709504 |
| X201491_at | -0.0507 | 0.0533 | -0.952 | 0.3486142 | 0.709934 |
| X207796_x_at | 0.0055 | 0.0057 | 0.952 | 0.348639 | 0.709934 |
| X221499_s_at | -0.0302 | 0.0318 | -0.952 | 0.3487 | 0.709993 |
| X221648_s_at | 0.0832 | 0.0874 | 0.951 | 0.3488367 | 0.710206 |
| X206587_at | -0.0069 | 0.0073 | -0.951 | 0.3489784 | 0.71043 |
| X220506_at | 0.0028 | 0.0029 | 0.951 | 0.3490715 | 0.710526 |
| X216132_at | -0.0061 | 0.0064 | -0.951 | 0.3491164 | 0.710526 |
| X201137_s_at | 0.1938 | 0.2039 | 0.951 | 0.3491683 | 0.710526 |
| X205292_s_at | -0.0259 | 0.0273 | -0.951 | 0.3491776 | 0.710526 |
| X220428_at | 0.0041 | 0.0044 | 0.95 | 0.349217 | 0.710526 |
| X215375_x_at | -0.0067 | 0.0071 | -0.95 | 0.3492317 | 0.710526 |
| X47553_at | 0.0457 | 0.0481 | 0.95 | 0.3492498 | 0.710526 |
| X209315_at | -0.1066 | 0.1122 | -0.95 | 0.3493259 | 0.710606 |
| X216830_at | 0.0053 | 0.0056 | 0.95 | 0.3493838 | 0.710606 |
| X205670_at | 0.0042 | 0.0044 | 0.95 | 0.3493849 | 0.710606 |
| X202830_s_at | -0.0043 | 0.0045 | -0.95 | 0.3494282 | 0.710624 |
| X206998_x_at | 0.0084 | 0.0088 | 0.95 | 0.349458 | 0.710624 |
| X211822_s_at | 0.0017 | 0.0018 | 0.95 | 0.3495617 | 0.71077 |
| X213809_x_at | 0.0025 | 0.0027 | 0.95 | 0.3495971 | 0.710777 |
| X214715_x_at | 0.0629 | 0.0662 | 0.949 | 0.3497196 | 0.710961 |
| X206148_at | -0.0033 | 0.0035 | -0.949 | 0.3498269 | 0.711114 |
| X207946_at | 0.0047 | 0.005 | 0.949 | 0.3499392 | 0.711277 |
| X204091_at | -0.0306 | 0.0322 | -0.949 | 0.3499835 | 0.711277 |
| X220762_s_at | -0.0064 | 0.0067 | -0.949 | 0.3500032 | 0.711277 |
| X215669_at | 0.0028 | 0.003 | 0.949 | 0.3500396 | 0.711286 |
| X201804_x_at | -0.034 | 0.0358 | -0.949 | 0.350087 | 0.711317 |
| X219624_at | 0.0069 | 0.0073 | 0.949 | 0.3501529 | 0.711386 |
| X216617_s_at | 0.0109 | 0.0115 | 0.949 | 0.3502042 | 0.711425 |
| X201700_at | 0.0263 | 0.0277 | 0.948 | 0.3502687 | 0.711491 |
| X210223_s_at | -0.0058 | 0.0061 | -0.948 | 0.3503603 | 0.711612 |
| X206478_at | 0.0044 | 0.0047 | 0.948 | 0.3505161 | 0.711864 |
| X221150_at | 0.0042 | 0.0044 | 0.948 | 0.3506126 | 0.711995 |
| X204807_at | -0.0591 | 0.0624 | -0.948 | 0.3506911 | 0.712088 |
| X220043_s_at | -0.0033 | 0.0035 | -0.947 | 0.3507656 | 0.712088 |
| X204138_s_at | 0.0053 | 0.0056 | 0.947 | 0.3507979 | 0.712088 |
| X218942_at | -0.0206 | 0.0217 | -0.947 | 0.3508368 | 0.712088 |
| X213692_s_at | -0.0061 | 0.0064 | -0.947 | 0.3509239 | 0.712088 |
| X203068_at | 0.0161 | 0.017 | 0.947 | 0.3509771 | 0.712088 |
| X214070_s_at | -0.1044 | 0.1103 | -0.947 | 0.3509829 | 0.712088 |
| X208378_x_at | 0.0031 | 0.0033 | 0.947 | 0.3509917 | 0.712088 |
| X209002_s_at | -0.007 | 0.0074 | -0.947 | 0.3509937 | 0.712088 |
| X205968_at | 0.0092 | 0.0097 | 0.947 | 0.350995 | 0.712088 |
| X203041_s_at | 0.1395 | 0.1473 | 0.947 | 0.3510206 | 0.712088 |
| X204739_at | -0.0448 | 0.0473 | -0.947 | 0.3510483 | 0.712088 |
| X218309_at | 0.0573 | 0.0605 | 0.947 | 0.3510754 | 0.712088 |
| X215930_s_at | 0.0309 | 0.0326 | 0.947 | 0.3511606 | 0.712189 |
| X212184_s_at | 0.0285 | 0.0301 | 0.947 | 0.3511893 | 0.712189 |
| X209763_at | 0.1067 | 0.1127 | 0.946 | 0.3512237 | 0.712194 |
| X208420_x_at | 0.0307 | 0.0324 | 0.946 | 0.3512568 | 0.712196 |
| X209219_at | -0.0419 | 0.0443 | -0.946 | 0.3513129 | 0.712245 |
| X205454_at | -0.1023 | 0.1081 | -0.946 | 0.3515077 | 0.712531 |
| X218209_s_at | -0.0616 | 0.0651 | -0.946 | 0.3515183 | 0.712531 |
| X217306_at | -0.0082 | 0.0087 | -0.946 | 0.3516091 | 0.71265 |
| X218533_s_at | -0.0502 | 0.0531 | -0.946 | 0.3516436 | 0.712655 |
| X213220_at | 0.0238 | 0.0252 | 0.945 | 0.3517516 | 0.712768 |
| X219856_at | -0.0023 | 0.0024 | -0.945 | 0.3517941 | 0.712768 |
| X214442_s_at | 0.0468 | 0.0495 | 0.945 | 0.3517958 | 0.712768 |
| X219902_at | -0.0342 | 0.0361 | -0.945 | 0.3520869 | 0.713293 |
| X214455_at | -0.0211 | 0.0224 | -0.945 | 0.3521921 | 0.713381 |
| X221106_at | -0.0034 | 0.0036 | -0.944 | 0.3522384 | 0.713381 |
| X45288_at | 0.0781 | 0.0827 | 0.944 | 0.3522548 | 0.713381 |
| X206605_at | -0.0061 | 0.0065 | -0.944 | 0.3522871 | 0.713381 |
| X222333_at | -0.0172 | 0.0183 | -0.944 | 0.3522945 | 0.713381 |
| X221360_s_at | -0.0025 | 0.0026 | -0.944 | 0.3523231 | 0.713381 |
| X204915_s_at | -0.0785 | 0.0831 | -0.944 | 0.3525014 | 0.713617 |
| X209864_at | -0.0252 | 0.0267 | -0.944 | 0.352504 | 0.713617 |
| X40612_at | -0.0129 | 0.0137 | -0.944 | 0.3526489 | 0.713846 |
| X207679_at | -0.0068 | 0.0072 | -0.943 | 0.352752 | 0.713989 |
| X217732_s_at | -0.0283 | 0.03 | -0.943 | 0.35283 | 0.714069 |
| X220675_s_at | -0.0031 | 0.0032 | -0.943 | 0.352882 | 0.714069 |
| X220010_at | 0.0039 | 0.0041 | 0.943 | 0.3528879 | 0.714069 |
| X213875_x_at | 0.1067 | 0.1132 | 0.943 | 0.3529667 | 0.714107 |
| X209150_s_at | 0.0378 | 0.0401 | 0.943 | 0.353024 | 0.714107 |
| X208573_s_at | 0.0032 | 0.0033 | 0.943 | 0.3530459 | 0.714107 |
| X217976_s_at | -0.0479 | 0.0508 | -0.943 | 0.3530751 | 0.714107 |
| X203982_s_at | 0.0073 | 0.0078 | 0.943 | 0.3530954 | 0.714107 |
| X201049_s_at | 0.0198 | 0.021 | 0.943 | 0.3530996 | 0.714107 |
| X206663_at | 0.006 | 0.0064 | 0.943 | 0.353205 | 0.71423 |
| X206300_s_at | 0.0045 | 0.0048 | 0.942 | 0.3532277 | 0.71423 |
| X210848_at | -0.0109 | 0.0116 | -0.942 | 0.3532567 | 0.71423 |
| X210588_x_at | -0.0349 | 0.037 | -0.942 | 0.3533205 | 0.714294 |
| X208512_s_at | -0.0042 | 0.0044 | -0.942 | 0.353353 | 0.714295 |
| X213347_x_at | 0.0214 | 0.0228 | 0.942 | 0.3534329 | 0.714391 |
| X205299_s_at | -0.0088 | 0.0093 | -0.942 | 0.3534989 | 0.71446 |
| X204638_at | 0.0048 | 0.0051 | 0.942 | 0.3536634 | 0.714683 |
| X208309_s_at | 0.0039 | 0.0041 | 0.942 | 0.3536942 | 0.714683 |
| X204074_s_at | 0.0699 | 0.0743 | 0.942 | 0.3537062 | 0.714683 |
| X208194_s_at | -0.0068 | 0.0072 | -0.941 | 0.3537726 | 0.71474 |
| X218228_s_at | 0.0393 | 0.0417 | 0.941 | 0.3537986 | 0.71474 |
| X214199_at | -0.0463 | 0.0492 | -0.941 | 0.3539915 | 0.715021 |
| X212129_at | -0.0374 | 0.0398 | -0.941 | 0.3540018 | 0.715021 |
| X206710_s_at | 0.0676 | 0.0718 | 0.941 | 0.3540465 | 0.71503 |
| X213046_at | 0.056 | 0.0595 | 0.941 | 0.3540877 | 0.71503 |
| X220402_at | 0.017 | 0.0181 | 0.941 | 0.3541111 | 0.71503 |
| X203273_s_at | 0.0236 | 0.0251 | 0.941 | 0.3541354 | 0.71503 |
| X222075_s_at | -0.004 | 0.0043 | -0.941 | 0.3542072 | 0.71511 |
| X207386_at | -0.0133 | 0.0141 | -0.94 | 0.3543025 | 0.715238 |
| X209842_at | 0.0872 | 0.0927 | 0.94 | 0.3544254 | 0.715421 |
| X218752_at | -0.004 | 0.0043 | -0.94 | 0.3544872 | 0.715474 |
| X221561_at | 0.0495 | 0.0527 | 0.94 | 0.3545316 | 0.715474 |
| X216887_s_at | -0.114 | 0.1213 | -0.94 | 0.3545639 | 0.715474 |
| X221638_s_at | -0.0059 | 0.0062 | -0.94 | 0.3545805 | 0.715474 |
| X34406_at | 0.069 | 0.0734 | 0.94 | 0.3546528 | 0.715506 |
| X204908_s_at | -0.0061 | 0.0065 | -0.94 | 0.3546609 | 0.715506 |
| X201707_at | -0.0287 | 0.0306 | -0.939 | 0.3549327 | 0.715989 |
| X219534_x_at | 0.1053 | 0.1122 | 0.939 | 0.3551196 | 0.716146 |
| X47608_at | 0.0596 | 0.0635 | 0.939 | 0.3551335 | 0.716146 |
| X205180_s_at | -0.0042 | 0.0045 | -0.939 | 0.355151 | 0.716146 |
| X210155_at | 0.0049 | 0.0053 | 0.939 | 0.3551609 | 0.716146 |
| X219416_at | 0.0291 | 0.031 | 0.939 | 0.3551778 | 0.716146 |
| X204117_at | 0.0093 | 0.0099 | 0.939 | 0.355213 | 0.716146 |
| X202199_s_at | 0.0079 | 0.0084 | 0.938 | 0.3552499 | 0.716146 |
| X203558_at | 0.0039 | 0.0042 | 0.938 | 0.3552924 | 0.716146 |
| X202523_s_at | 0.0061 | 0.0065 | 0.938 | 0.3553005 | 0.716146 |
| X221130_s_at | 0.0041 | 0.0044 | 0.938 | 0.3553628 | 0.716207 |
| X220461_at | 0.0052 | 0.0055 | 0.938 | 0.3555004 | 0.716419 |
| X209263_x_at | 0.0448 | 0.0478 | 0.938 | 0.3555823 | 0.716519 |
| X215501_s_at | 0.0778 | 0.083 | 0.938 | 0.3556383 | 0.716567 |
| X213693_s_at | 0.0094 | 0.01 | 0.938 | 0.3557041 | 0.716634 |
| X210470_x_at | -0.0194 | 0.0207 | -0.937 | 0.35578 | 0.716722 |
| X210948_s_at | -0.0069 | 0.0073 | -0.937 | 0.3558579 | 0.716814 |
| X207023_x_at | 0.0194 | 0.0207 | 0.937 | 0.3559466 | 0.716834 |
| X211393_at | -0.0052 | 0.0056 | -0.937 | 0.3560199 | 0.716834 |
| X214565_s_at | -0.005 | 0.0053 | -0.937 | 0.3560326 | 0.716834 |
| X213981_at | 0.0101 | 0.0108 | 0.937 | 0.3560727 | 0.716834 |
| X213443_at | -0.0021 | 0.0022 | -0.937 | 0.3561099 | 0.716834 |
| X208876_s_at | -0.0067 | 0.0072 | -0.937 | 0.3561955 | 0.716834 |
| X214276_at | -0.0089 | 0.0095 | -0.937 | 0.3562164 | 0.716834 |
| X202916_s_at | -0.0324 | 0.0346 | -0.936 | 0.3562757 | 0.716834 |
| X208556_at | 0.0024 | 0.0025 | 0.936 | 0.3562819 | 0.716834 |
| X217246_s_at | -0.0085 | 0.0091 | -0.936 | 0.3563257 | 0.716834 |
| X220404_at | 0.0029 | 0.0031 | 0.936 | 0.356341 | 0.716834 |
| X203711_s_at | 0.0438 | 0.0468 | 0.936 | 0.3563558 | 0.716834 |
| X205329_s_at | -0.0681 | 0.0727 | -0.936 | 0.3563972 | 0.716834 |
| X210000_s_at | -0.0047 | 0.0051 | -0.936 | 0.3564716 | 0.716834 |
| X221925_s_at | 0.0611 | 0.0653 | 0.936 | 0.3564819 | 0.716834 |
| X201686_x_at | -0.0243 | 0.026 | -0.936 | 0.3565267 | 0.716834 |
| X210345_s_at | 0.0034 | 0.0036 | 0.936 | 0.3565426 | 0.716834 |
| X202368_s_at | 0.0042 | 0.0045 | 0.936 | 0.3565704 | 0.716834 |
| X212950_at | 0.1179 | 0.126 | 0.936 | 0.3565909 | 0.716834 |
| X215387_x_at | -0.0394 | 0.0421 | -0.936 | 0.3566024 | 0.716834 |
| X218810_at | 0.0041 | 0.0044 | 0.936 | 0.35661 | 0.716834 |
| X211991_s_at | 0.172 | 0.1838 | 0.936 | 0.3566341 | 0.716834 |
| X208204_s_at | -0.003 | 0.0032 | -0.936 | 0.3566653 | 0.716834 |
| X215441_at | -0.034 | 0.0363 | -0.936 | 0.3566758 | 0.716834 |
| X216474_x_at | 0.0031 | 0.0033 | 0.936 | 0.3567035 | 0.716834 |
| X205062_x_at | -0.0501 | 0.0536 | -0.936 | 0.3567065 | 0.716834 |
| X201676_x_at | 0.0192 | 0.0205 | 0.935 | 0.3567708 | 0.716898 |
| X218436_at | -0.009 | 0.0096 | -0.935 | 0.3569723 | 0.717238 |
| X207647_at | -0.0129 | 0.0138 | -0.935 | 0.3570562 | 0.717342 |
| X211348_s_at | -0.0961 | 0.1028 | -0.935 | 0.3571663 | 0.717365 |
| X213172_at | -0.0412 | 0.0441 | -0.935 | 0.3571844 | 0.717365 |
| X207041_at | 0.0038 | 0.0041 | 0.935 | 0.3572013 | 0.717365 |
| X207984_s_at | 0.0075 | 0.008 | 0.934 | 0.3572842 | 0.717365 |
| X202718_at | 0.003 | 0.0032 | 0.934 | 0.3572888 | 0.717365 |
| X221651_x_at | 0.0045 | 0.0048 | 0.934 | 0.3573029 | 0.717365 |
| X207783_x_at | -0.0091 | 0.0098 | -0.934 | 0.3573286 | 0.717365 |
| X214915_at | 0.0085 | 0.0091 | 0.934 | 0.3573485 | 0.717365 |
| X203401_at | -0.0453 | 0.0485 | -0.934 | 0.3573761 | 0.717365 |
| X216557_x_at | -0.004 | 0.0043 | -0.934 | 0.3573907 | 0.717365 |
| X212079_s_at | 0.1176 | 0.1259 | 0.934 | 0.3574556 | 0.71743 |
| X210101_x_at | 0.0573 | 0.0614 | 0.934 | 0.3574921 | 0.717439 |
| X216434_at | -0.0027 | 0.0029 | -0.934 | 0.3575465 | 0.717483 |
| X204475_at | 0.0059 | 0.0063 | 0.934 | 0.3576958 | 0.717661 |
| X209308_s_at | -0.0223 | 0.0239 | -0.934 | 0.3576995 | 0.717661 |
| X220234_at | -0.0062 | 0.0066 | -0.933 | 0.3579014 | 0.717806 |
| X216736_at | 0.0038 | 0.0041 | 0.933 | 0.3579046 | 0.717806 |
| X222167_at | 0.0041 | 0.0044 | 0.933 | 0.3579198 | 0.717806 |
| X214524_at | -0.0044 | 0.0047 | -0.933 | 0.3579468 | 0.717806 |
| X221768_at | 0.0462 | 0.0495 | 0.933 | 0.3579583 | 0.717806 |
| X210655_s_at | -0.0085 | 0.0091 | -0.933 | 0.3579658 | 0.717806 |
| X210019_at | -0.005 | 0.0053 | -0.933 | 0.3580969 | 0.717974 |
| X202580_x_at | 0.0029 | 0.0031 | 0.933 | 0.3581143 | 0.717974 |
| X209247_s_at | -0.014 | 0.0151 | -0.933 | 0.3581775 | 0.718036 |
| X201053_s_at | 0.0357 | 0.0383 | 0.932 | 0.3583853 | 0.718205 |
| X202431_s_at | 0.0101 | 0.0109 | 0.932 | 0.3583921 | 0.718205 |
| X220009_at | -0.0074 | 0.0079 | -0.932 | 0.3584081 | 0.718205 |
| X216778_s_at | -0.0053 | 0.0057 | -0.932 | 0.3584121 | 0.718205 |
| X204774_at | 0.1502 | 0.1611 | 0.932 | 0.3584237 | 0.718205 |
| X208600_s_at | 0.0025 | 0.0027 | 0.932 | 0.3585275 | 0.718349 |
| X207557_s_at | -0.0953 | 0.1022 | -0.932 | 0.3585701 | 0.718369 |
| X205129_at | 0.0044 | 0.0047 | 0.932 | 0.3586955 | 0.718554 |
| X217104_at | -0.0075 | 0.0081 | -0.932 | 0.3587314 | 0.718554 |
| X205732_s_at | -0.0065 | 0.0069 | -0.932 | 0.3587595 | 0.718554 |
| X206027_at | 0.0049 | 0.0053 | 0.931 | 0.3589191 | 0.718782 |
| X216538_at | 0.0068 | 0.0073 | 0.931 | 0.3589542 | 0.718782 |
| X207805_s_at | -0.0347 | 0.0373 | -0.931 | 0.35898 | 0.718782 |
| X219609_at | -0.0082 | 0.0088 | -0.931 | 0.3590258 | 0.718782 |
| X215410_at | -0.0228 | 0.0245 | -0.931 | 0.3590352 | 0.718782 |
| X218331_s_at | -0.0332 | 0.0357 | -0.931 | 0.3591495 | 0.718946 |
| X220540_at | -0.0046 | 0.005 | -0.931 | 0.3592062 | 0.718995 |
| X221309_at | -0.0055 | 0.0059 | -0.93 | 0.3593737 | 0.719169 |
| X215613_at | -0.0092 | 0.0099 | -0.93 | 0.359375 | 0.719169 |
| X207564_x_at | -0.0829 | 0.0891 | -0.93 | 0.3593901 | 0.719169 |
| X209170_s_at | 0.0392 | 0.0422 | 0.93 | 0.3596271 | 0.719578 |
| X201595_s_at | -0.041 | 0.0441 | -0.93 | 0.3596868 | 0.719595 |
| X214998_at | -0.0359 | 0.0387 | -0.93 | 0.3597003 | 0.719595 |
| X215047_at | -0.0154 | 0.0166 | -0.929 | 0.3598876 | 0.719905 |
| X207360_s_at | 0.0025 | 0.0026 | 0.929 | 0.3599563 | 0.719977 |
| X216378_at | -0.0071 | 0.0077 | -0.929 | 0.3600161 | 0.720032 |
| X208299_at | 0.0023 | 0.0025 | 0.929 | 0.3600752 | 0.720086 |
| X208238_x_at | 0.0176 | 0.019 | 0.929 | 0.3601443 | 0.720113 |
| X211591_s_at | 0.0047 | 0.0051 | 0.929 | 0.3602196 | 0.720113 |
| X213992_at | 0.0056 | 0.006 | 0.929 | 0.3602392 | 0.720113 |
| X209850_s_at | 0.0037 | 0.004 | 0.929 | 0.3602473 | 0.720113 |
| X209148_at | -0.0247 | 0.0266 | -0.929 | 0.3602785 | 0.720113 |
| X220709_at | 0.0079 | 0.0085 | 0.929 | 0.3602836 | 0.720113 |
| X218206_x_at | -0.0355 | 0.0383 | -0.928 | 0.3603283 | 0.720138 |
| X211147_s_at | 0.0022 | 0.0024 | 0.928 | 0.3604684 | 0.720322 |
| X208875_s_at | 0.0422 | 0.0455 | 0.928 | 0.3604854 | 0.720322 |
| X219250_s_at | -0.0992 | 0.1068 | -0.928 | 0.360555 | 0.720354 |
| X206234_s_at | -0.0029 | 0.0032 | -0.928 | 0.3605659 | 0.720354 |
| X209043_at | -0.0211 | 0.0227 | -0.928 | 0.3607012 | 0.720559 |
| X218468_s_at | -0.2216 | 0.2388 | -0.928 | 0.3607771 | 0.720646 |
| X222091_at | -0.1223 | 0.1319 | -0.927 | 0.360998 | 0.720876 |
| X216550_x_at | 0.0453 | 0.0489 | 0.927 | 0.3610145 | 0.720876 |
| X220933_s_at | 0.0454 | 0.0489 | 0.927 | 0.3610501 | 0.720876 |
| X211057_at | 0.0037 | 0.004 | 0.927 | 0.3611316 | 0.720876 |
| X211737_x_at | -0.045 | 0.0486 | -0.927 | 0.3611744 | 0.720876 |
| X211801_x_at | -0.0044 | 0.0048 | -0.927 | 0.3612016 | 0.720876 |
| X212617_at | -0.0041 | 0.0044 | -0.927 | 0.3612075 | 0.720876 |
| X200031_s_at | -0.0174 | 0.0188 | -0.927 | 0.3612623 | 0.720876 |
| X212913_at | 0.08 | 0.0863 | 0.927 | 0.361265 | 0.720876 |
| X217238_s_at | -0.0037 | 0.004 | -0.927 | 0.3612663 | 0.720876 |
| X207887_s_at | 0.0018 | 0.0019 | 0.927 | 0.3612794 | 0.720876 |
| X47560_at | 0.0304 | 0.0328 | 0.927 | 0.3612843 | 0.720876 |
| X201639_s_at | 0.032 | 0.0346 | 0.927 | 0.3613141 | 0.720876 |
| X220201_at | 0.0294 | 0.0318 | 0.926 | 0.3614876 | 0.721157 |
| X208716_s_at | 0.0494 | 0.0534 | 0.926 | 0.3615501 | 0.721181 |
| X219815_at | -0.0096 | 0.0104 | -0.926 | 0.3615932 | 0.721181 |
| X219097_x_at | -0.0306 | 0.0331 | -0.926 | 0.3615996 | 0.721181 |
| X217530_at | -0.005 | 0.0054 | -0.926 | 0.3616295 | 0.721181 |
| X207389_at | 0.0077 | 0.0083 | 0.926 | 0.3617246 | 0.721306 |
| X218221_at | -0.0301 | 0.0325 | -0.925 | 0.3619086 | 0.721564 |
| X209527_at | -0.0385 | 0.0416 | -0.925 | 0.361919 | 0.721564 |
| X202958_at | -0.0037 | 0.004 | -0.925 | 0.3620144 | 0.721591 |
| X206283_s_at | -0.0062 | 0.0067 | -0.925 | 0.362023 | 0.721591 |
| X207001_x_at | 0.0179 | 0.0194 | 0.925 | 0.3620301 | 0.721591 |
| X216016_at | 0.0056 | 0.0061 | 0.925 | 0.3621568 | 0.72172 |
| X216449_x_at | 0.0705 | 0.0763 | 0.925 | 0.3621596 | 0.72172 |
| X217232_x_at | 0.1952 | 0.2111 | 0.925 | 0.362291 | 0.721917 |
| X219963_at | 0.0024 | 0.0026 | 0.924 | 0.3623672 | 0.722004 |
| X217211_at | -0.0102 | 0.011 | -0.924 | 0.3624347 | 0.722015 |
| X206413_s_at | 0.0041 | 0.0045 | 0.924 | 0.3624498 | 0.722015 |
| X202884_s_at | -0.0233 | 0.0252 | -0.924 | 0.3624701 | 0.722015 |
| X212835_at | 0.0522 | 0.0565 | 0.924 | 0.3625635 | 0.722086 |
| X201155_s_at | -0.0195 | 0.0211 | -0.924 | 0.3625724 | 0.722086 |
| X214252_s_at | 0.0494 | 0.0535 | 0.924 | 0.3626145 | 0.722086 |
| X201011_at | 0.0296 | 0.0321 | 0.924 | 0.3626359 | 0.722086 |
| X211452_x_at | 0.0713 | 0.0772 | 0.924 | 0.3627457 | 0.722229 |
| X212393_at | -0.0307 | 0.0332 | -0.924 | 0.3627724 | 0.722229 |
| X219312_s_at | 0.0527 | 0.0571 | 0.923 | 0.3630367 | 0.722644 |
| X212531_at | 0.0034 | 0.0037 | 0.923 | 0.3630462 | 0.722644 |
| X215709_at | -0.0063 | 0.0068 | -0.923 | 0.363151 | 0.722751 |
| X215349_at | 0.0055 | 0.0059 | 0.923 | 0.3631648 | 0.722751 |
| X221978_at | -0.0069 | 0.0074 | -0.923 | 0.3632339 | 0.722778 |
| X221192_x_at | 0.0523 | 0.0567 | 0.923 | 0.3632435 | 0.722778 |
| X201907_x_at | 0.0039 | 0.0043 | 0.923 | 0.3632925 | 0.722811 |
| X210469_at | 0.0032 | 0.0035 | 0.923 | 0.3633544 | 0.722869 |
| X206539_s_at | -0.0074 | 0.008 | -0.922 | 0.3634207 | 0.722936 |
| X218726_at | 0.0033 | 0.0036 | 0.922 | 0.3635054 | 0.72304 |
| X210718_s_at | -0.1378 | 0.1494 | -0.922 | 0.3636059 | 0.723175 |
| X211279_at | -0.0098 | 0.0107 | -0.922 | 0.3637837 | 0.723363 |
| X219941_at | 0.0056 | 0.0061 | 0.921 | 0.3639157 | 0.723363 |
| X221376_at | -0.0025 | 0.0027 | -0.921 | 0.3639161 | 0.723363 |
| X216710_x_at | -0.0259 | 0.0281 | -0.921 | 0.3639463 | 0.723363 |
| X218358_at | -0.0275 | 0.0298 | -0.921 | 0.3639692 | 0.723363 |
| X215591_at | 0.0156 | 0.017 | 0.921 | 0.3640152 | 0.723363 |
| X57739_at | 0.0477 | 0.0518 | 0.921 | 0.3640358 | 0.723363 |
| X200908_s_at | -0.007 | 0.0076 | -0.921 | 0.3640381 | 0.723363 |
| X211892_s_at | -0.0026 | 0.0028 | -0.921 | 0.3640991 | 0.723363 |
| X210381_s_at | -0.0845 | 0.0918 | -0.921 | 0.364118 | 0.723363 |
| X213832_at | -0.0433 | 0.047 | -0.921 | 0.3641261 | 0.723363 |
| X215790_at | 0.0022 | 0.0024 | 0.921 | 0.3641494 | 0.723363 |
| X200090_at | 0.0386 | 0.0419 | 0.921 | 0.3641518 | 0.723363 |
| X220887_at | 0.026 | 0.0283 | 0.921 | 0.3641914 | 0.723363 |
| X220743_at | 0.0031 | 0.0034 | 0.921 | 0.3642206 | 0.723363 |
| X207255_at | -0.0038 | 0.0041 | -0.921 | 0.3642212 | 0.723363 |
| X211909_x_at | -0.0707 | 0.0769 | -0.921 | 0.3644077 | 0.723668 |
| X206259_at | 0.0028 | 0.003 | 0.92 | 0.3645201 | 0.723787 |
| X218770_s_at | -0.0399 | 0.0433 | -0.92 | 0.3645625 | 0.723787 |
| X203514_at | 0.0474 | 0.0515 | 0.92 | 0.3645846 | 0.723787 |
| X216446_at | -0.0026 | 0.0029 | -0.92 | 0.364598 | 0.723787 |
| X207753_at | -0.0323 | 0.0351 | -0.92 | 0.364704 | 0.723814 |
| X207915_at | 0.0033 | 0.0036 | 0.92 | 0.3647041 | 0.723814 |
| X212521_s_at | 0.0819 | 0.089 | 0.92 | 0.3647094 | 0.723814 |
| X213238_at | 0.081 | 0.088 | 0.919 | 0.3649507 | 0.724229 |
| X210764_s_at | 0.158 | 0.1719 | 0.919 | 0.3650822 | 0.724304 |
| X213192_at | -0.0042 | 0.0045 | -0.919 | 0.3650967 | 0.724304 |
| X217467_at | -0.0038 | 0.0041 | -0.919 | 0.3651479 | 0.724304 |
| X221721_s_at | -0.0045 | 0.0049 | -0.919 | 0.3652059 | 0.724304 |
| X222362_at | -0.0041 | 0.0044 | -0.919 | 0.365214 | 0.724304 |
| X209107_x_at | -0.0376 | 0.0409 | -0.919 | 0.3652493 | 0.724304 |
| X207450_s_at | -0.0039 | 0.0042 | -0.919 | 0.3652774 | 0.724304 |
| X212465_at | -0.0173 | 0.0188 | -0.919 | 0.3653312 | 0.724304 |
| X217233_at | 0.0045 | 0.0049 | 0.919 | 0.3653492 | 0.724304 |
| X211749_s_at | 0.0673 | 0.0733 | 0.919 | 0.3653667 | 0.724304 |
| X204382_at | 0.0496 | 0.054 | 0.919 | 0.3654031 | 0.724304 |
| X210371_s_at | 0.0169 | 0.0184 | 0.918 | 0.365456 | 0.724304 |
| X214894_x_at | 0.0324 | 0.0353 | 0.918 | 0.3655706 | 0.724304 |
| X215430_at | 0.0034 | 0.0037 | 0.918 | 0.3655759 | 0.724304 |
| X217463_s_at | -0.003 | 0.0033 | -0.918 | 0.3655764 | 0.724304 |
| X213282_at | -0.0454 | 0.0494 | -0.918 | 0.3656488 | 0.724304 |
| X217344_at | -0.0061 | 0.0067 | -0.918 | 0.3656508 | 0.724304 |
| X216179_x_at | 0.027 | 0.0294 | 0.918 | 0.3656588 | 0.724304 |
| X213960_at | -0.0362 | 0.0395 | -0.918 | 0.3656667 | 0.724304 |
| X37079_at | -0.0079 | 0.0086 | -0.918 | 0.3656724 | 0.724304 |
| X64900_at | 0.0379 | 0.0413 | 0.918 | 0.3656735 | 0.724304 |
| X200928_s_at | -0.0766 | 0.0835 | -0.918 | 0.3657394 | 0.72437 |
| X207221_at | -0.0031 | 0.0034 | -0.918 | 0.3658473 | 0.724519 |
| X202501_at | -0.0371 | 0.0405 | -0.918 | 0.3658898 | 0.724539 |
| X202615_at | -0.0215 | 0.0235 | -0.918 | 0.365949 | 0.724569 |
| X200725_x_at | -0.0251 | 0.0273 | -0.917 | 0.3659704 | 0.724569 |
| X218126_at | -0.0244 | 0.0266 | -0.917 | 0.3660405 | 0.724617 |
| X205538_at | -0.0042 | 0.0046 | -0.917 | 0.3661175 | 0.724617 |
| X204654_s_at | -0.01 | 0.0109 | -0.917 | 0.3661677 | 0.724617 |
| X212006_at | 0.028 | 0.0305 | 0.917 | 0.3661941 | 0.724617 |
| X220745_at | 0.0035 | 0.0039 | 0.917 | 0.3662131 | 0.724617 |
| X220346_at | 0.0569 | 0.062 | 0.917 | 0.3662568 | 0.724617 |
| X221542_s_at | -0.0303 | 0.033 | -0.917 | 0.3663346 | 0.724617 |
| X215489_x_at | 0.005 | 0.0055 | 0.917 | 0.3663646 | 0.724617 |
| X205223_at | 0.0171 | 0.0187 | 0.917 | 0.36637 | 0.724617 |
| X208413_at | -0.0048 | 0.0052 | -0.917 | 0.3663957 | 0.724617 |
| X203523_at | -0.0035 | 0.0038 | -0.917 | 0.3664137 | 0.724617 |
| X217708_x_at | 0.0112 | 0.0122 | 0.917 | 0.3664161 | 0.724617 |
| X209876_at | -0.0119 | 0.013 | -0.917 | 0.3664186 | 0.724617 |
| X203357_s_at | -0.0352 | 0.0384 | -0.916 | 0.3666349 | 0.72498 |
| X205933_at | -0.0237 | 0.0258 | -0.916 | 0.3667095 | 0.725063 |
| X218447_at | -0.0242 | 0.0264 | -0.916 | 0.3667727 | 0.725124 |
| X218199_s_at | -0.0373 | 0.0407 | -0.916 | 0.3669768 | 0.725463 |
| X202997_s_at | -0.0028 | 0.0031 | -0.915 | 0.3671767 | 0.725793 |
| X208986_at | 0.0454 | 0.0496 | 0.915 | 0.3672734 | 0.72592 |
| X216003_at | 0.0031 | 0.0034 | 0.915 | 0.3673164 | 0.72594 |
| X203859_s_at | 0.0592 | 0.0647 | 0.915 | 0.3673671 | 0.725976 |
| X36865_at | 0.0317 | 0.0347 | 0.915 | 0.3674749 | 0.726124 |
| X214078_at | -0.0861 | 0.0941 | -0.914 | 0.3675219 | 0.726152 |
| X209045_at | -0.0318 | 0.0348 | -0.914 | 0.3676015 | 0.726174 |
| X212028_at | 0.0238 | 0.0261 | 0.914 | 0.3676329 | 0.726174 |
| X219671_at | -0.0681 | 0.0745 | -0.914 | 0.3677357 | 0.726174 |
| X207793_s_at | -0.0127 | 0.0139 | -0.914 | 0.3677407 | 0.726174 |
| X214120_at | -0.0357 | 0.0391 | -0.914 | 0.3677467 | 0.726174 |
| X211083_s_at | -0.0062 | 0.0068 | -0.914 | 0.3678463 | 0.726174 |
| X216243_s_at | -0.0034 | 0.0037 | -0.914 | 0.3678499 | 0.726174 |
| X216510_x_at | -0.0046 | 0.005 | -0.914 | 0.3678719 | 0.726174 |
| X208190_s_at | 0.0048 | 0.0052 | 0.914 | 0.367884 | 0.726174 |
| X215536_at | 0.0021 | 0.0023 | 0.914 | 0.3678907 | 0.726174 |
| X216575_at | -0.01 | 0.0109 | -0.914 | 0.3679265 | 0.726174 |
| X202286_s_at | -0.003 | 0.0033 | -0.913 | 0.3680559 | 0.726174 |
| X216345_at | -0.0058 | 0.0064 | -0.913 | 0.3680739 | 0.726174 |
| X218429_s_at | 0.0177 | 0.0194 | 0.913 | 0.3680783 | 0.726174 |
| X212306_at | -0.0508 | 0.0556 | -0.913 | 0.3681297 | 0.726174 |
| X200807_s_at | -0.0228 | 0.025 | -0.913 | 0.3681365 | 0.726174 |
| X212095_s_at | 0.1557 | 0.1705 | 0.913 | 0.3681495 | 0.726174 |
| X210678_s_at | 0.014 | 0.0153 | 0.913 | 0.3681658 | 0.726174 |
| X221408_x_at | 0.0044 | 0.0048 | 0.913 | 0.3681736 | 0.726174 |
| X212973_at | 0.0371 | 0.0406 | 0.913 | 0.3681867 | 0.726174 |
| X202905_x_at | 0.0734 | 0.0804 | 0.913 | 0.3682983 | 0.726282 |
| X221014_s_at | 0.0563 | 0.0617 | 0.913 | 0.3683069 | 0.726282 |
| X219129_s_at | 0.01 | 0.0109 | 0.913 | 0.368493 | 0.726481 |
| X202947_s_at | 0.0812 | 0.089 | 0.913 | 0.3685005 | 0.726481 |
| X205157_s_at | 0.0092 | 0.0101 | 0.913 | 0.3685301 | 0.726481 |
| X206057_x_at | 0.0038 | 0.0041 | 0.913 | 0.3685385 | 0.726481 |
| X216236_s_at | -0.0587 | 0.0644 | -0.912 | 0.3687081 | 0.726751 |
| X209508_x_at | 0.0071 | 0.0078 | 0.912 | 0.3687434 | 0.726756 |
| X207222_at | 0.0036 | 0.0039 | 0.912 | 0.3687806 | 0.726765 |
| X216821_at | -0.0044 | 0.0048 | -0.912 | 0.3688439 | 0.726825 |
| X214263_x_at | -0.0257 | 0.0282 | -0.912 | 0.3689782 | 0.726999 |
| X218353_at | 0.1046 | 0.1147 | 0.912 | 0.3689978 | 0.726999 |
| X44065_at | -0.0347 | 0.0381 | -0.911 | 0.3692214 | 0.727349 |
| X210374_x_at | 0.0034 | 0.0037 | 0.911 | 0.3692854 | 0.727349 |
| X206612_at | -0.0062 | 0.0068 | -0.911 | 0.3692943 | 0.727349 |
| X218218_at | -0.1681 | 0.1846 | -0.911 | 0.3693061 | 0.727349 |
| X215093_at | 0.0188 | 0.0206 | 0.911 | 0.3693457 | 0.727355 |
| X211087_x_at | -0.0039 | 0.0043 | -0.911 | 0.3693805 | 0.727355 |
| X222332_at | -0.0106 | 0.0117 | -0.911 | 0.3694456 | 0.727355 |
| X215759_at | -0.0044 | 0.0049 | -0.911 | 0.3694659 | 0.727355 |
| X217795_s_at | 0.0299 | 0.0328 | 0.911 | 0.369473 | 0.727355 |
| X203977_at | 0.0035 | 0.0038 | 0.91 | 0.3697098 | 0.727724 |
| X205809_s_at | 0.0536 | 0.0589 | 0.91 | 0.3697262 | 0.727724 |
| X206726_at | -0.0124 | 0.0137 | -0.91 | 0.3700137 | 0.728226 |
| X222151_s_at | -0.028 | 0.0307 | -0.91 | 0.3700554 | 0.728234 |
| X213587_s_at | -0.0416 | 0.0458 | -0.91 | 0.3700835 | 0.728234 |
| X220002_at | -0.0064 | 0.007 | -0.909 | 0.370162 | 0.728263 |
| X214189_s_at | 0.0063 | 0.0069 | 0.909 | 0.3701635 | 0.728263 |
| X206533_at | 0.0054 | 0.0059 | 0.909 | 0.3702077 | 0.728285 |
| X217057_s_at | -0.0031 | 0.0034 | -0.909 | 0.370285 | 0.728373 |
| X201696_at | -0.0237 | 0.0261 | -0.909 | 0.3704526 | 0.728594 |
| X212341_at | -0.0168 | 0.0185 | -0.909 | 0.3704877 | 0.728594 |
| X217899_at | 0.0112 | 0.0123 | 0.909 | 0.3705273 | 0.728594 |
| X200879_s_at | -0.005 | 0.0055 | -0.909 | 0.370529 | 0.728594 |
| X214774_x_at | -0.0574 | 0.0632 | -0.909 | 0.3705897 | 0.728635 |
| X212650_at | 0.1164 | 0.1281 | 0.908 | 0.3706694 | 0.728635 |
| X218576_s_at | -0.0751 | 0.0827 | -0.908 | 0.3706821 | 0.728635 |
| X218639_s_at | 0.0125 | 0.0137 | 0.908 | 0.3706998 | 0.728635 |
| X202258_s_at | 0.043 | 0.0474 | 0.908 | 0.3707135 | 0.728635 |
| X206627_s_at | -0.0147 | 0.0162 | -0.908 | 0.3708208 | 0.72874 |
| X218765_at | -0.0082 | 0.009 | -0.908 | 0.3708324 | 0.72874 |
| X221914_at | -0.0719 | 0.0792 | -0.908 | 0.370895 | 0.728748 |
| X220816_at | 0.0032 | 0.0035 | 0.908 | 0.3709024 | 0.728748 |
| X204982_at | -0.0044 | 0.0049 | -0.908 | 0.3709913 | 0.728826 |
| X203627_at | 0.0428 | 0.0472 | 0.908 | 0.3710225 | 0.728826 |
| X204572_s_at | -0.0188 | 0.0207 | -0.908 | 0.371108 | 0.728826 |
| X209477_at | 0.0324 | 0.0357 | 0.908 | 0.3711173 | 0.728826 |
| X203659_s_at | 0.0523 | 0.0576 | 0.908 | 0.3711388 | 0.728826 |
| X220373_at | -0.0047 | 0.0052 | -0.908 | 0.3711389 | 0.728826 |
| X218873_at | -0.0262 | 0.0289 | -0.907 | 0.3713006 | 0.729017 |
| X217869_at | 0.0385 | 0.0424 | 0.907 | 0.3713016 | 0.729017 |
| X220042_x_at | 0.0023 | 0.0026 | 0.907 | 0.3714368 | 0.7292 |
| X218403_at | 0.0322 | 0.0355 | 0.907 | 0.3714608 | 0.7292 |
| X205585_at | 0.0039 | 0.0043 | 0.907 | 0.3715755 | 0.729308 |
| X220418_at | 0.0023 | 0.0025 | 0.907 | 0.3716389 | 0.729308 |
| X207620_s_at | -0.0051 | 0.0056 | -0.907 | 0.3716423 | 0.729308 |
| X206416_at | 0.0129 | 0.0142 | 0.907 | 0.3716529 | 0.729308 |
| X206447_at | -0.004 | 0.0044 | -0.906 | 0.3716796 | 0.729308 |
| X210022_at | -0.007 | 0.0077 | -0.906 | 0.3717886 | 0.729393 |
| X206121_at | 0.0037 | 0.0041 | 0.906 | 0.371821 | 0.729393 |
| X213436_at | -0.0936 | 0.1033 | -0.906 | 0.3718215 | 0.729393 |
| X214562_at | -0.0024 | 0.0027 | -0.906 | 0.3719569 | 0.729521 |
| X210721_s_at | -0.042 | 0.0464 | -0.906 | 0.3719619 | 0.729521 |
| X216951_at | 0.0033 | 0.0037 | 0.906 | 0.3719853 | 0.729521 |
| X201584_s_at | 0.0387 | 0.0428 | 0.906 | 0.3720554 | 0.729594 |
| X201602_s_at | -0.0596 | 0.0658 | -0.906 | 0.3721125 | 0.729595 |
| X201304_at | -0.0286 | 0.0315 | -0.906 | 0.3721297 | 0.729595 |
| X210892_s_at | -0.0088 | 0.0097 | -0.906 | 0.3721544 | 0.729595 |
| X210867_at | 0.0402 | 0.0444 | 0.905 | 0.3723813 | 0.729975 |
| X210684_s_at | -0.0064 | 0.0071 | -0.905 | 0.3724438 | 0.730016 |
| X207868_at | 0.0035 | 0.0039 | 0.905 | 0.3724679 | 0.730016 |
| X208499_s_at | 0.0062 | 0.0069 | 0.905 | 0.3725616 | 0.73003 |
| X207290_at | 0.0931 | 0.1028 | 0.905 | 0.372572 | 0.73003 |
| X214760_at | 0.0534 | 0.0591 | 0.905 | 0.3725738 | 0.73003 |
| X211652_s_at | 0.0038 | 0.0042 | 0.904 | 0.3727106 | 0.730168 |
| X204240_s_at | 0.0042 | 0.0046 | 0.904 | 0.3727252 | 0.730168 |
| X203594_at | -0.0488 | 0.0539 | -0.904 | 0.3727426 | 0.730168 |
| X212801_at | 0.0774 | 0.0856 | 0.904 | 0.3728151 | 0.730198 |
| X44111_at | -0.0301 | 0.0333 | -0.904 | 0.3729031 | 0.730198 |
| X216615_s_at | -0.0083 | 0.0092 | -0.904 | 0.3729458 | 0.730198 |
| X221225_at | -0.0087 | 0.0097 | -0.904 | 0.3729617 | 0.730198 |
| X218937_at | -0.0326 | 0.036 | -0.904 | 0.3729669 | 0.730198 |
| X215201_at | 0.0232 | 0.0257 | 0.904 | 0.3729773 | 0.730198 |
| X216711_s_at | 0.0044 | 0.0048 | 0.904 | 0.3729881 | 0.730198 |
| X220866_at | 0.0042 | 0.0046 | 0.904 | 0.3731205 | 0.730362 |
| X203601_s_at | -0.0034 | 0.0038 | -0.904 | 0.3731425 | 0.730362 |
| X215704_at | -0.0136 | 0.015 | -0.904 | 0.3731704 | 0.730362 |
| X207301_at | 0.0034 | 0.0038 | 0.904 | 0.3732166 | 0.730388 |
| X213926_s_at | 0.0128 | 0.0141 | 0.903 | 0.3733562 | 0.730507 |
| X203848_at | 0.0558 | 0.0618 | 0.903 | 0.3733743 | 0.730507 |
| X215910_s_at | 0.0048 | 0.0054 | 0.903 | 0.3734011 | 0.730507 |
| X210200_at | -0.0058 | 0.0064 | -0.903 | 0.3734087 | 0.730507 |
| X210391_at | 0.0024 | 0.0026 | 0.903 | 0.3735164 | 0.730616 |
| X204093_at | -0.0365 | 0.0405 | -0.903 | 0.3735302 | 0.730616 |
| X214449_s_at | -0.0374 | 0.0415 | -0.903 | 0.3736029 | 0.730693 |
| X222090_at | -0.0061 | 0.0067 | -0.903 | 0.3737416 | 0.730891 |
| X205821_at | 0.0044 | 0.0049 | 0.902 | 0.3737698 | 0.730891 |
| X219759_at | 0.0106 | 0.0117 | 0.902 | 0.3738168 | 0.730919 |
| X209831_x_at | -0.0129 | 0.0143 | -0.902 | 0.3740198 | 0.731251 |
| X221759_at | 0.0028 | 0.0031 | 0.902 | 0.3742083 | 0.731555 |
| X222367_at | -0.0041 | 0.0046 | -0.901 | 0.3743122 | 0.731558 |
| X212664_at | -0.0476 | 0.0528 | -0.901 | 0.3743282 | 0.731558 |
| X207422_at | 0.0049 | 0.0055 | 0.901 | 0.3743347 | 0.731558 |
| X205893_at | -0.0301 | 0.0334 | -0.901 | 0.3743566 | 0.731558 |
| X211473_s_at | 0.0053 | 0.0058 | 0.901 | 0.3743804 | 0.731558 |
| X211782_at | -0.0071 | 0.0079 | -0.901 | 0.3744216 | 0.731558 |
| X212345_s_at | 0.0632 | 0.0701 | 0.901 | 0.3744414 | 0.731558 |
| X210144_at | 0.03 | 0.0333 | 0.901 | 0.3744729 | 0.731558 |
| X216157_at | -0.0022 | 0.0025 | -0.901 | 0.3746376 | 0.731815 |
| X203072_at | 0.0032 | 0.0035 | 0.901 | 0.3747017 | 0.731876 |
| X206166_s_at | -0.0055 | 0.0061 | -0.901 | 0.374769 | 0.731943 |
| X214832_at | -0.0062 | 0.0069 | -0.9 | 0.3749014 | 0.732137 |
| X213138_at | 0.0056 | 0.0062 | 0.9 | 0.374971 | 0.732171 |
| X213143_at | 0.0289 | 0.0321 | 0.9 | 0.3749845 | 0.732171 |
| X218925_s_at | -0.01 | 0.0112 | -0.9 | 0.3750176 | 0.732171 |
| X215358_x_at | -0.0695 | 0.0773 | -0.9 | 0.3750701 | 0.732209 |
| X220775_s_at | 0.0427 | 0.0474 | 0.9 | 0.3751583 | 0.732242 |
| X221789_x_at | -0.0089 | 0.0099 | -0.9 | 0.3751767 | 0.732242 |
| X202276_at | -0.0154 | 0.0171 | -0.9 | 0.3751884 | 0.732242 |
| X210370_s_at | -0.0029 | 0.0033 | -0.9 | 0.3752189 | 0.732242 |
| X202631_s_at | 0.1247 | 0.1386 | 0.9 | 0.3752835 | 0.732304 |
| X204241_at | 0.0067 | 0.0074 | 0.899 | 0.3753732 | 0.732341 |
| X213772_s_at | -0.0053 | 0.0059 | -0.899 | 0.3754104 | 0.732341 |
| X206808_at | -0.0106 | 0.0118 | -0.899 | 0.3754247 | 0.732341 |
| X208950_s_at | 0.059 | 0.0656 | 0.899 | 0.3754344 | 0.732341 |
| X221290_s_at | 0.0485 | 0.0539 | 0.899 | 0.3756854 | 0.732733 |
| X212030_at | -0.0493 | 0.0548 | -0.899 | 0.3757015 | 0.732733 |
| X208208_at | -0.0035 | 0.0039 | -0.898 | 0.3758488 | 0.732944 |
| X209371_s_at | 0.0062 | 0.0069 | 0.898 | 0.3758753 | 0.732944 |
| X208224_at | -0.0032 | 0.0036 | -0.898 | 0.3760056 | 0.733102 |
| X203295_s_at | 0.0465 | 0.0517 | 0.898 | 0.3760718 | 0.733102 |
| X220040_x_at | -0.0099 | 0.011 | -0.898 | 0.3761058 | 0.733102 |
| X206798_x_at | 0.0047 | 0.0052 | 0.898 | 0.3761338 | 0.733102 |
| X215756_at | 0.0038 | 0.0042 | 0.898 | 0.3761511 | 0.733102 |
| X202952_s_at | -0.009 | 0.0101 | -0.898 | 0.3761544 | 0.733102 |
| X214155_s_at | -0.056 | 0.0623 | -0.898 | 0.3762323 | 0.733189 |
| X213827_at | -0.0132 | 0.0147 | -0.897 | 0.3764089 | 0.733469 |
| X200002_at | 0.0289 | 0.0322 | 0.897 | 0.3764529 | 0.733491 |
| X221098_x_at | -0.0088 | 0.0098 | -0.897 | 0.3768134 | 0.734099 |
| X202851_at | -0.0072 | 0.008 | -0.897 | 0.3768792 | 0.734099 |
| X221503_s_at | -0.0538 | 0.06 | -0.896 | 0.3769156 | 0.734099 |
| X203198_at | 0.0484 | 0.054 | 0.896 | 0.3769227 | 0.734099 |
| X211051_s_at | 0.0058 | 0.0064 | 0.896 | 0.3769304 | 0.734099 |
| X205086_s_at | 0.0034 | 0.0038 | 0.896 | 0.3769861 | 0.734104 |
| X201505_at | -0.0528 | 0.0589 | -0.896 | 0.3770263 | 0.734104 |
| X212369_at | -0.0351 | 0.0392 | -0.896 | 0.3770377 | 0.734104 |
| X211274_at | -0.0039 | 0.0043 | -0.896 | 0.377065 | 0.734104 |
| X209518_at | 0.0178 | 0.0198 | 0.896 | 0.3771121 | 0.734122 |
| X201034_at | 0.0358 | 0.04 | 0.896 | 0.37716 | 0.734122 |
| X207524_at | 0.0052 | 0.0058 | 0.896 | 0.3771868 | 0.734122 |
| X212633_at | 0.0378 | 0.0422 | 0.896 | 0.3772096 | 0.734122 |
| X217048_at | -0.0028 | 0.0031 | -0.896 | 0.3772398 | 0.734122 |
| X206878_at | 0.0037 | 0.0041 | 0.896 | 0.3772868 | 0.734149 |
| X206890_at | 0.0025 | 0.0028 | 0.896 | 0.3773413 | 0.73415 |
| X219865_at | -0.0511 | 0.0571 | -0.896 | 0.3773533 | 0.73415 |
| X220921_at | -0.0078 | 0.0088 | -0.895 | 0.3774493 | 0.73425 |
| X216090_x_at | 0.0054 | 0.006 | 0.895 | 0.3774905 | 0.73425 |
| X206930_at | -0.0058 | 0.0065 | -0.895 | 0.3775286 | 0.73425 |
| X200752_s_at | -0.0031 | 0.0035 | -0.895 | 0.3775367 | 0.73425 |
| X202380_s_at | 0.0496 | 0.0554 | 0.895 | 0.3776727 | 0.73445 |
| X218905_at | 0.0358 | 0.04 | 0.895 | 0.3777658 | 0.734567 |
| X216794_at | 0.004 | 0.0045 | 0.895 | 0.3778576 | 0.734591 |
| X201965_s_at | 0.0705 | 0.0788 | 0.895 | 0.377888 | 0.734591 |
| X220101_x_at | -0.0032 | 0.0035 | -0.895 | 0.3779027 | 0.734591 |
| X214235_at | -0.0061 | 0.0068 | -0.894 | 0.3779621 | 0.734591 |
| X209361_s_at | 0.0729 | 0.0815 | 0.894 | 0.3779764 | 0.734591 |
| X212622_at | -0.0296 | 0.0331 | -0.894 | 0.378024 | 0.734591 |
| X202869_at | 0.0093 | 0.0104 | 0.894 | 0.378033 | 0.734591 |
| X207027_at | -0.0027 | 0.003 | -0.894 | 0.3780562 | 0.734591 |
| X215710_at | -0.0031 | 0.0035 | -0.894 | 0.3780761 | 0.734591 |
| X212846_at | -0.0286 | 0.032 | -0.894 | 0.3782919 | 0.734946 |
| X201090_x_at | 0.0331 | 0.0371 | 0.893 | 0.3786612 | 0.7356 |
| X217880_at | -0.0293 | 0.0328 | -0.893 | 0.3787165 | 0.735621 |
| X212187_x_at | -0.0506 | 0.0566 | -0.893 | 0.3787607 | 0.735621 |
| X216614_at | -0.0073 | 0.0082 | -0.893 | 0.3787718 | 0.735621 |
| X207591_s_at | 0.0035 | 0.0039 | 0.893 | 0.3788444 | 0.735648 |
| X207300_s_at | 0.0032 | 0.0036 | 0.893 | 0.3788518 | 0.735648 |
| X210269_s_at | -0.0068 | 0.0076 | -0.893 | 0.3789485 | 0.735749 |
| X220291_at | -0.0059 | 0.0067 | -0.893 | 0.3789821 | 0.735749 |
| X200716_x_at | -0.0197 | 0.0221 | -0.892 | 0.3790033 | 0.735749 |
| X217468_at | -0.0036 | 0.0041 | -0.892 | 0.379069 | 0.735805 |
| X217303_s_at | -0.0029 | 0.0032 | -0.892 | 0.3790982 | 0.735805 |
| X210922_at | 0.0057 | 0.0063 | 0.892 | 0.379201 | 0.73594 |
| X211380_s_at | 0.0039 | 0.0043 | 0.892 | 0.3792602 | 0.735973 |
| X209648_x_at | -0.0332 | 0.0372 | -0.892 | 0.3792841 | 0.735973 |
| X216552_x_at | -0.0027 | 0.003 | -0.892 | 0.3794176 | 0.736158 |
| X216902_s_at | -0.0618 | 0.0694 | -0.892 | 0.3794615 | 0.736158 |
| X202645_s_at | -0.077 | 0.0863 | -0.892 | 0.3795178 | 0.736158 |
| X213092_x_at | -0.0425 | 0.0476 | -0.891 | 0.3795373 | 0.736158 |
| X221422_s_at | 0.0031 | 0.0034 | 0.891 | 0.3795452 | 0.736158 |
| X36499_at | -0.0494 | 0.0554 | -0.891 | 0.3796329 | 0.736264 |
| X219954_s_at | -0.0057 | 0.0064 | -0.891 | 0.3796834 | 0.736297 |
| X222105_s_at | -0.0065 | 0.0073 | -0.891 | 0.3797856 | 0.736431 |
| X217363_x_at | -0.0088 | 0.0099 | -0.891 | 0.3798243 | 0.736442 |
| X204504_s_at | -0.0107 | 0.0121 | -0.891 | 0.3799719 | 0.736644 |
| X221555_x_at | 0.057 | 0.064 | 0.891 | 0.3800074 | 0.736644 |
| X203396_at | 0.0346 | 0.0389 | 0.891 | 0.3800277 | 0.736644 |
| X219191_s_at | -0.0031 | 0.0034 | -0.89 | 0.3801631 | 0.736842 |
| X63305_at | 0.0832 | 0.0934 | 0.89 | 0.3802773 | 0.736999 |
| X208488_s_at | 0.0032 | 0.0036 | 0.89 | 0.3804366 | 0.737192 |
| X210247_at | -0.098 | 0.1101 | -0.89 | 0.3804432 | 0.737192 |
| X219862_s_at | -0.0282 | 0.0317 | -0.889 | 0.3806024 | 0.737428 |
| X203292_s_at | -0.0356 | 0.0401 | -0.889 | 0.3806481 | 0.737428 |
| X220922_s_at | -0.0031 | 0.0035 | -0.889 | 0.3806891 | 0.737428 |
| X219775_s_at | -0.0078 | 0.0087 | -0.889 | 0.380698 | 0.737428 |
| X219648_at | -0.0544 | 0.0612 | -0.889 | 0.3807904 | 0.737466 |
| X214574_x_at | 0.0135 | 0.0152 | 0.889 | 0.3808085 | 0.737466 |
| X216350_s_at | 0.0034 | 0.0038 | 0.889 | 0.3808171 | 0.737466 |
| X201776_s_at | -0.0421 | 0.0474 | -0.889 | 0.3809871 | 0.737731 |
| X221351_at | 0.0023 | 0.0026 | 0.889 | 0.3810827 | 0.7378 |
| X206695_x_at | -0.0529 | 0.0595 | -0.889 | 0.3810929 | 0.7378 |
| X208098_at | 0.0076 | 0.0086 | 0.888 | 0.3811274 | 0.7378 |
| X213196_at | 0.0099 | 0.0111 | 0.888 | 0.3811559 | 0.7378 |
| X210602_s_at | -0.0028 | 0.0032 | -0.888 | 0.3813071 | 0.73796 |
| X209001_s_at | -0.0215 | 0.0242 | -0.888 | 0.3813156 | 0.73796 |
| X216387_x_at | -0.0074 | 0.0083 | -0.888 | 0.3813378 | 0.73796 |
| X211580_s_at | 0.0021 | 0.0023 | 0.888 | 0.3814079 | 0.738031 |
| X205836_s_at | 0.0355 | 0.04 | 0.888 | 0.3814795 | 0.738105 |
| X217842_at | 0.0233 | 0.0263 | 0.888 | 0.3815591 | 0.738176 |
| X216241_s_at | 0.0219 | 0.0247 | 0.888 | 0.3815889 | 0.738176 |
| X210087_s_at | 0.0037 | 0.0042 | 0.888 | 0.3816403 | 0.738176 |
| X204805_s_at | -0.0471 | 0.0531 | -0.887 | 0.3816578 | 0.738176 |
| X209538_at | -0.027 | 0.0304 | -0.887 | 0.3817013 | 0.738176 |
| X207907_at | -0.0028 | 0.0032 | -0.887 | 0.3817155 | 0.738176 |
| X214138_at | -0.0032 | 0.0036 | -0.887 | 0.3818623 | 0.73837 |
| X220981_x_at | 0.0044 | 0.005 | 0.887 | 0.3818823 | 0.73837 |
| X210282_at | 0.0909 | 0.1024 | 0.887 | 0.3819554 | 0.738416 |
| X218967_s_at | -0.0399 | 0.045 | -0.887 | 0.3820003 | 0.738416 |
| X204563_at | 0.0162 | 0.0183 | 0.887 | 0.3820059 | 0.738416 |
| X205930_at | -0.0312 | 0.0352 | -0.887 | 0.3821296 | 0.738429 |
| X222000_at | -0.0284 | 0.032 | -0.887 | 0.3821444 | 0.738429 |
| X204674_at | 0.0037 | 0.0041 | 0.887 | 0.3821653 | 0.738429 |
| X218890_x_at | 0.0649 | 0.0732 | 0.886 | 0.3821806 | 0.738429 |
| X220249_at | -0.004 | 0.0045 | -0.886 | 0.3821869 | 0.738429 |
| X205169_at | -0.0795 | 0.0897 | -0.886 | 0.3822916 | 0.738429 |
| X217749_at | 0.029 | 0.0327 | 0.886 | 0.3823231 | 0.738429 |
| X214406_s_at | 0.0015 | 0.0017 | 0.886 | 0.3823383 | 0.738429 |
| X216940_x_at | -0.0064 | 0.0072 | -0.886 | 0.3823418 | 0.738429 |
| X209872_s_at | -0.003 | 0.0033 | -0.886 | 0.3823766 | 0.738429 |
| X206485_at | 0.0031 | 0.0035 | 0.886 | 0.3824226 | 0.738429 |
| X201488_x_at | -0.0403 | 0.0455 | -0.886 | 0.3824839 | 0.738429 |
| X207739_s_at | -0.0045 | 0.0051 | -0.886 | 0.3826133 | 0.738429 |
| X207139_at | 0.002 | 0.0023 | 0.886 | 0.3826217 | 0.738429 |
| X219829_at | 0.0029 | 0.0032 | 0.886 | 0.3826606 | 0.738429 |
| X203952_at | -0.0044 | 0.005 | -0.886 | 0.3826828 | 0.738429 |
| X221274_s_at | -0.0208 | 0.0235 | -0.885 | 0.3827076 | 0.738429 |
| X218568_at | -0.048 | 0.0543 | -0.885 | 0.3827137 | 0.738429 |
| X216802_at | -0.0055 | 0.0062 | -0.885 | 0.3827229 | 0.738429 |
| X214149_s_at | 0.2405 | 0.2716 | 0.885 | 0.3827338 | 0.738429 |
| X220915_s_at | 0.0242 | 0.0273 | 0.885 | 0.3827435 | 0.738429 |
| X207952_at | -0.0042 | 0.0047 | -0.885 | 0.3827449 | 0.738429 |
| X211865_s_at | -0.0023 | 0.0026 | -0.885 | 0.3827769 | 0.738429 |
| X212484_at | 0.013 | 0.0147 | 0.885 | 0.3828661 | 0.738527 |
| X200707_at | -0.0031 | 0.0035 | -0.885 | 0.3828943 | 0.738527 |
| X206219_s_at | -0.0036 | 0.0041 | -0.885 | 0.3829343 | 0.73854 |
| X219746_at | -0.0043 | 0.0048 | -0.885 | 0.3829997 | 0.738602 |
| X218578_at | 0.0223 | 0.0252 | 0.885 | 0.3830513 | 0.738637 |
| X206255_at | -0.0027 | 0.0031 | -0.885 | 0.3831588 | 0.738781 |
| X212355_at | 0.0494 | 0.0559 | 0.884 | 0.3832895 | 0.738907 |
| X216639_at | 0.0062 | 0.007 | 0.884 | 0.3833111 | 0.738907 |
| X216002_at | -0.0072 | 0.0082 | -0.884 | 0.3833747 | 0.738907 |
| X216636_at | 0.0047 | 0.0053 | 0.884 | 0.3834023 | 0.738907 |
| X206917_at | 0.004 | 0.0045 | 0.884 | 0.3834123 | 0.738907 |
| X212431_at | -0.0511 | 0.0578 | -0.884 | 0.3834241 | 0.738907 |
| X205282_at | 0.0565 | 0.064 | 0.884 | 0.3835101 | 0.739009 |
| X213072_at | -0.0044 | 0.005 | -0.884 | 0.3835963 | 0.739067 |
| X208040_s_at | 0.004 | 0.0045 | 0.884 | 0.3836246 | 0.739067 |
| X213351_s_at | 0.0208 | 0.0236 | 0.884 | 0.3836401 | 0.739067 |
| X204623_at | 0.0028 | 0.0032 | 0.884 | 0.3837161 | 0.739149 |
| X209013_x_at | 0.0122 | 0.0138 | 0.883 | 0.3837972 | 0.739185 |
| X220528_at | 0.003 | 0.0034 | 0.883 | 0.383814 | 0.739185 |
| X207963_at | -0.0076 | 0.0086 | -0.883 | 0.3838523 | 0.739185 |
| X205312_at | -0.0036 | 0.0041 | -0.883 | 0.3839411 | 0.739185 |
| X209091_s_at | 0.0497 | 0.0563 | 0.883 | 0.3839451 | 0.739185 |
| X214761_at | 0.0557 | 0.0631 | 0.883 | 0.3839501 | 0.739185 |
| X219158_s_at | 0.0521 | 0.059 | 0.883 | 0.3839674 | 0.739185 |
| X221774_x_at | 0.0252 | 0.0285 | 0.883 | 0.3840162 | 0.739215 |
| X215892_at | 0.0208 | 0.0236 | 0.883 | 0.3841157 | 0.739342 |
| X215809_at | 0.0048 | 0.0054 | 0.882 | 0.3843437 | 0.739643 |
| X215631_s_at | 0.0062 | 0.007 | 0.882 | 0.3843525 | 0.739643 |
| X220673_s_at | -0.0047 | 0.0053 | -0.882 | 0.3843718 | 0.739643 |
| X218379_at | 0.0467 | 0.0529 | 0.882 | 0.3844109 | 0.739651 |
| X210198_s_at | 0.0595 | 0.0675 | 0.882 | 0.3844805 | 0.739651 |
| X206705_at | 0.0025 | 0.0028 | 0.882 | 0.3845169 | 0.739651 |
| X216701_at | -0.0031 | 0.0035 | -0.882 | 0.3845303 | 0.739651 |
| X38703_at | 0.0263 | 0.0298 | 0.882 | 0.3845426 | 0.739651 |
| X213831_at | -0.0158 | 0.018 | -0.882 | 0.3846703 | 0.739833 |
| X211647_x_at | -0.0029 | 0.0033 | -0.881 | 0.3849036 | 0.740217 |
| X203114_at | -0.0052 | 0.0059 | -0.881 | 0.3850316 | 0.740399 |
| X221807_s_at | 0.0018 | 0.0021 | 0.881 | 0.3850802 | 0.740429 |
| X208386_x_at | 0.0265 | 0.03 | 0.881 | 0.3851462 | 0.740491 |
| X209933_s_at | 0.0044 | 0.005 | 0.881 | 0.3852344 | 0.740597 |
| X204922_at | -0.0138 | 0.0157 | -0.881 | 0.3853106 | 0.740655 |
| X214005_at | -0.0602 | 0.0684 | -0.881 | 0.3853312 | 0.740655 |
| X218689_at | -0.0319 | 0.0363 | -0.88 | 0.3853763 | 0.740677 |
| X204626_s_at | 0.0033 | 0.0038 | 0.88 | 0.3856096 | 0.741062 |
| X214771_x_at | -0.0389 | 0.0442 | -0.88 | 0.3857021 | 0.741175 |
| X200810_s_at | -0.0251 | 0.0285 | -0.88 | 0.3858131 | 0.741322 |
| X221197_s_at | 0.0026 | 0.0029 | 0.88 | 0.385845 | 0.741322 |
| X219388_at | -0.0049 | 0.0056 | -0.879 | 0.3859501 | 0.741417 |
| X205231_s_at | 0.0416 | 0.0473 | 0.879 | 0.3859611 | 0.741417 |
| X216436_at | -0.007 | 0.0079 | -0.879 | 0.386242 | 0.741892 |
| X201984_s_at | 0.0232 | 0.0265 | 0.879 | 0.3863035 | 0.741944 |
| X201109_s_at | 0.0051 | 0.0058 | 0.879 | 0.3863358 | 0.741944 |
| X205368_at | -0.0508 | 0.0578 | -0.878 | 0.3864395 | 0.742079 |
| X214341_at | -0.0043 | 0.0049 | -0.878 | 0.3865419 | 0.742151 |
| X215645_at | 0.089 | 0.1013 | 0.878 | 0.3865599 | 0.742151 |
| X204384_at | -0.0043 | 0.0049 | -0.878 | 0.3865772 | 0.742151 |
| X207206_s_at | 0.0045 | 0.0051 | 0.878 | 0.3866285 | 0.742179 |
| X204098_at | 0.0308 | 0.0351 | 0.878 | 0.386685 | 0.742179 |
| X221675_s_at | 0.0631 | 0.0718 | 0.878 | 0.3866922 | 0.742179 |
| X214050_at | -0.005 | 0.0057 | -0.878 | 0.3868007 | 0.742323 |
| X218311_at | 0.0268 | 0.0305 | 0.878 | 0.3868529 | 0.742359 |
| X220426_at | -0.0023 | 0.0026 | -0.878 | 0.3869078 | 0.7424 |
| X206848_at | 0.0778 | 0.0887 | 0.878 | 0.3869539 | 0.742425 |
| X219976_at | 0.0595 | 0.0678 | 0.877 | 0.3869961 | 0.742442 |
| X207719_x_at | 0.0261 | 0.0297 | 0.877 | 0.3870881 | 0.742478 |
| X212257_s_at | -0.0949 | 0.1082 | -0.877 | 0.3871118 | 0.742478 |
| X221181_at | -0.0028 | 0.0032 | -0.877 | 0.3871465 | 0.742478 |
| X216496_s_at | -0.0035 | 0.004 | -0.877 | 0.3872047 | 0.742478 |
| X203111_s_at | -0.002 | 0.0023 | -0.877 | 0.3872158 | 0.742478 |
| X205415_s_at | 0.0372 | 0.0424 | 0.877 | 0.3872318 | 0.742478 |
| X205239_at | -0.0115 | 0.0131 | -0.877 | 0.3872814 | 0.742478 |
| X205472_s_at | -0.1266 | 0.1444 | -0.877 | 0.3872823 | 0.742478 |
| X210537_s_at | 0.0032 | 0.0036 | 0.877 | 0.3874307 | 0.742677 |
| X210281_s_at | 0.0658 | 0.075 | 0.877 | 0.3874578 | 0.742677 |
| X213842_x_at | 0.0222 | 0.0254 | 0.877 | 0.3874868 | 0.742677 |
| X220644_at | -0.0026 | 0.003 | -0.876 | 0.3875354 | 0.742677 |
| X215706_x_at | 0.0045 | 0.0051 | 0.876 | 0.3875543 | 0.742677 |
| X215266_at | 0.0038 | 0.0044 | 0.876 | 0.3875867 | 0.742677 |
| X206292_s_at | -0.0031 | 0.0035 | -0.876 | 0.3877794 | 0.742907 |
| X208788_at | 0.0556 | 0.0635 | 0.876 | 0.3878146 | 0.742907 |
| X65493_at | -0.0144 | 0.0165 | -0.876 | 0.3878309 | 0.742907 |
| X207549_x_at | -0.0221 | 0.0252 | -0.876 | 0.3878808 | 0.742907 |
| X214472_at | 0.0044 | 0.005 | 0.876 | 0.3879062 | 0.742907 |
| X202798_at | -0.0211 | 0.0241 | -0.876 | 0.3879208 | 0.742907 |
| X201374_x_at | -0.004 | 0.0045 | -0.876 | 0.387941 | 0.742907 |
| X212590_at | 0.0387 | 0.0442 | 0.876 | 0.3879857 | 0.742913 |
| X210343_s_at | -0.0036 | 0.0041 | -0.876 | 0.3880109 | 0.742913 |
| X216098_s_at | -0.0039 | 0.0044 | -0.875 | 0.3880617 | 0.742944 |
| X203080_s_at | 0.0434 | 0.0496 | 0.875 | 0.3881078 | 0.742944 |
| X219239_s_at | -0.015 | 0.0171 | -0.875 | 0.3881274 | 0.742944 |
| X211714_x_at | 0.0462 | 0.0528 | 0.875 | 0.3881952 | 0.742995 |
| X211654_x_at | 0.0119 | 0.0136 | 0.875 | 0.3882286 | 0.742995 |
| X49327_at | 0.0431 | 0.0492 | 0.875 | 0.3882549 | 0.742995 |
| X201567_s_at | -0.0209 | 0.0239 | -0.875 | 0.3883153 | 0.742995 |
| X32209_at | 0.0317 | 0.0362 | 0.875 | 0.3883376 | 0.742995 |
| X211836_s_at | -0.1514 | 0.1731 | -0.875 | 0.3883547 | 0.742995 |
| X215434_x_at | -0.0116 | 0.0132 | -0.875 | 0.3884136 | 0.743043 |
| X211695_x_at | 0.0041 | 0.0047 | 0.874 | 0.3885814 | 0.743238 |
| X220045_at | 0.0028 | 0.0032 | 0.874 | 0.3886693 | 0.743238 |
| X215080_s_at | -0.0028 | 0.0032 | -0.874 | 0.3886882 | 0.743238 |
| X210210_at | -0.0055 | 0.0063 | -0.874 | 0.3886911 | 0.743238 |
| X220074_at | -0.0026 | 0.0029 | -0.874 | 0.3887058 | 0.743238 |
| X202260_s_at | 0.0303 | 0.0346 | 0.874 | 0.3887162 | 0.743238 |
| X216667_at | -0.0033 | 0.0038 | -0.874 | 0.3888568 | 0.743443 |
| X214640_at | -0.0051 | 0.0058 | -0.874 | 0.3889177 | 0.743496 |
| X219920_s_at | -0.0046 | 0.0052 | -0.874 | 0.388955 | 0.743503 |
| X219638_at | -0.0049 | 0.0056 | -0.874 | 0.3890623 | 0.743644 |
| X217259_at | -0.0047 | 0.0054 | -0.873 | 0.3891213 | 0.743693 |
| X216380_x_at | -0.0221 | 0.0253 | -0.873 | 0.3892154 | 0.743809 |
| X218746_at | -0.0047 | 0.0054 | -0.873 | 0.3893043 | 0.743915 |
| X204348_s_at | 0.0488 | 0.0559 | 0.873 | 0.3893488 | 0.743936 |
| X35254_at | 0.0343 | 0.0393 | 0.873 | 0.3893823 | 0.743936 |
| X220446_s_at | -0.0038 | 0.0043 | -0.873 | 0.3894809 | 0.744028 |
| X204470_at | 0.011 | 0.0126 | 0.873 | 0.3894975 | 0.744028 |
| X204293_at | 0.0039 | 0.0044 | 0.873 | 0.3895407 | 0.744046 |
| X210937_s_at | -0.0053 | 0.0061 | -0.872 | 0.3896538 | 0.744058 |
| X201876_at | 0.0654 | 0.0749 | 0.872 | 0.389661 | 0.744058 |
| X202345_s_at | 0.0506 | 0.058 | 0.872 | 0.3896783 | 0.744058 |
| X221813_at | -0.018 | 0.0206 | -0.872 | 0.3896809 | 0.744058 |
| X203587_at | -0.0058 | 0.0066 | -0.872 | 0.389748 | 0.744088 |
| X206438_x_at | 0.0054 | 0.0062 | 0.872 | 0.3898438 | 0.744088 |
| X220855_at | -0.0137 | 0.0157 | -0.872 | 0.3898463 | 0.744088 |
| X206421_s_at | 0.0047 | 0.0054 | 0.872 | 0.3898493 | 0.744088 |
| X211764_s_at | -0.0275 | 0.0315 | -0.872 | 0.3898803 | 0.744088 |
| X211160_x_at | -0.0328 | 0.0376 | -0.872 | 0.3899304 | 0.744088 |
| X210923_at | 0.0041 | 0.0048 | 0.872 | 0.3899386 | 0.744088 |
| X218981_at | -0.0358 | 0.0411 | -0.872 | 0.3899643 | 0.744088 |
| X213629_x_at | 0.0817 | 0.0937 | 0.872 | 0.3900039 | 0.744099 |
| X215140_at | 0.0065 | 0.0074 | 0.872 | 0.3900826 | 0.744131 |
| X207176_s_at | 0.0044 | 0.0051 | 0.872 | 0.3900874 | 0.744131 |
| X200618_at | -0.0392 | 0.045 | -0.872 | 0.3901411 | 0.744169 |
| X212549_at | 0.0295 | 0.0338 | 0.871 | 0.3902481 | 0.744278 |
| X219550_at | 0.015 | 0.0172 | 0.871 | 0.3903102 | 0.744278 |
| X207419_s_at | 0.0051 | 0.0059 | 0.871 | 0.3903266 | 0.744278 |
| X216808_at | -0.004 | 0.0046 | -0.871 | 0.390332 | 0.744278 |
| X219060_at | 0.0381 | 0.0438 | 0.871 | 0.3903903 | 0.744309 |
| X218704_at | 0.0022 | 0.0026 | 0.871 | 0.3904156 | 0.744309 |
| X217516_x_at | -0.0045 | 0.0052 | -0.871 | 0.3905249 | 0.744454 |
| X210884_s_at | -0.0025 | 0.0029 | -0.871 | 0.3905666 | 0.744469 |
| X213015_at | 0.0666 | 0.0765 | 0.87 | 0.3907681 | 0.74479 |
| X214990_at | 0.0094 | 0.0107 | 0.87 | 0.3908916 | 0.744961 |
| X220735_s_at | -0.0093 | 0.0107 | -0.87 | 0.3910649 | 0.745227 |
| X214229_at | -0.0041 | 0.0047 | -0.87 | 0.3911475 | 0.745321 |
| X203715_at | 0.0363 | 0.0417 | 0.87 | 0.3912208 | 0.745397 |
| X212261_at | -0.0331 | 0.0381 | -0.87 | 0.3912558 | 0.745399 |
| X206836_at | 0.0024 | 0.0027 | 0.869 | 0.391344 | 0.74542 |
| X206714_at | 0.0023 | 0.0026 | 0.869 | 0.391358 | 0.74542 |
| X213971_s_at | 0.0396 | 0.0455 | 0.869 | 0.3913777 | 0.74542 |
| X215795_at | -0.0028 | 0.0032 | -0.869 | 0.3914009 | 0.74542 |
| X214831_at | 0.0037 | 0.0042 | 0.869 | 0.3914613 | 0.745471 |
| X219085_s_at | -0.0025 | 0.0029 | -0.869 | 0.3915549 | 0.745586 |
| X208719_s_at | -0.0027 | 0.0031 | -0.869 | 0.3916162 | 0.745638 |
| X216555_at | -0.0281 | 0.0324 | -0.869 | 0.3916673 | 0.745672 |
| X220987_s_at | -0.0058 | 0.0067 | -0.869 | 0.3917579 | 0.74578 |
| X205405_at | 0.0178 | 0.0205 | 0.868 | 0.3918127 | 0.745803 |
| X202246_s_at | -0.0441 | 0.0507 | -0.868 | 0.3918373 | 0.745803 |
| X204645_at | -0.0456 | 0.0525 | -0.868 | 0.3918703 | 0.745803 |
| X211794_at | 0.0357 | 0.0411 | 0.868 | 0.3920178 | 0.74594 |
| X212342_at | 0.0377 | 0.0434 | 0.868 | 0.3920313 | 0.74594 |
| X211880_x_at | 0.0039 | 0.0045 | 0.868 | 0.3920432 | 0.74594 |
| X204836_at | 0.0471 | 0.0542 | 0.868 | 0.3921382 | 0.746057 |
| X205174_s_at | 0.0644 | 0.0742 | 0.868 | 0.3922527 | 0.746066 |
| X222357_at | -0.0435 | 0.0501 | -0.868 | 0.3923294 | 0.746066 |
| X221578_at | 0.0037 | 0.0043 | 0.867 | 0.3923377 | 0.746066 |
| X208513_at | 0.003 | 0.0035 | 0.867 | 0.3923384 | 0.746066 |
| X214087_s_at | 0.0967 | 0.1115 | 0.867 | 0.3923639 | 0.746066 |
| X217050_at | -0.0039 | 0.0045 | -0.867 | 0.3923974 | 0.746066 |
| X208404_x_at | 0.0069 | 0.0079 | 0.867 | 0.3924192 | 0.746066 |
| X208603_s_at | -0.0039 | 0.0045 | -0.867 | 0.3924538 | 0.746066 |
| X202883_s_at | -0.0418 | 0.0482 | -0.867 | 0.3924875 | 0.746066 |
| X208168_s_at | 0.0024 | 0.0028 | 0.867 | 0.3925134 | 0.746066 |
| X202618_s_at | 0.0056 | 0.0064 | 0.867 | 0.3925607 | 0.746066 |
| X215871_at | -0.0039 | 0.0045 | -0.867 | 0.3925862 | 0.746066 |
| X210298_x_at | -0.1237 | 0.1427 | -0.867 | 0.3926291 | 0.746066 |
| X209444_at | -0.0411 | 0.0474 | -0.867 | 0.3926394 | 0.746066 |
| X208127_s_at | -0.0387 | 0.0446 | -0.867 | 0.3926469 | 0.746066 |
| X219599_at | -0.0104 | 0.012 | -0.867 | 0.3926859 | 0.746076 |
| X219119_at | -0.029 | 0.0335 | -0.867 | 0.3927875 | 0.746204 |
| X217195_at | 0.0043 | 0.005 | 0.867 | 0.3928311 | 0.746204 |
| X55583_at | -0.0034 | 0.0039 | -0.867 | 0.3928536 | 0.746204 |
| X210245_at | -0.0046 | 0.0054 | -0.866 | 0.3928906 | 0.74621 |
| X222265_at | 0.0045 | 0.0052 | 0.866 | 0.3929885 | 0.746332 |
| X219064_at | -0.0674 | 0.0778 | -0.866 | 0.3930432 | 0.746363 |
| X211052_s_at | -0.0366 | 0.0423 | -0.866 | 0.3931126 | 0.746363 |
| X216516_at | -0.0095 | 0.011 | -0.866 | 0.3931192 | 0.746363 |
| X221019_s_at | 0.118 | 0.1362 | 0.866 | 0.3931389 | 0.746363 |
| X202306_at | -0.0383 | 0.0442 | -0.866 | 0.3932003 | 0.746415 |
| X206038_s_at | 0.0185 | 0.0213 | 0.866 | 0.3933518 | 0.746515 |
| X222303_at | 0.0135 | 0.0156 | 0.865 | 0.3934238 | 0.746515 |
| X212643_at | 0.041 | 0.0474 | 0.865 | 0.3934321 | 0.746515 |
| X211633_x_at | -0.0025 | 0.0028 | -0.865 | 0.3934396 | 0.746515 |
| X219510_at | 0.0049 | 0.0057 | 0.865 | 0.3934503 | 0.746515 |
| X204085_s_at | 0.0588 | 0.0679 | 0.865 | 0.3934544 | 0.746515 |
| X211599_x_at | 0.0044 | 0.0051 | 0.865 | 0.3936149 | 0.746756 |
| X220759_at | -0.005 | 0.0058 | -0.865 | 0.3936739 | 0.746804 |
| X212655_at | 0.0222 | 0.0257 | 0.865 | 0.3937839 | 0.746949 |
| X211844_s_at | 0.0033 | 0.0038 | 0.864 | 0.3939979 | 0.747177 |
| X206974_at | -0.0065 | 0.0076 | -0.864 | 0.3939993 | 0.747177 |
| X203771_s_at | -0.0148 | 0.0171 | -0.864 | 0.3940053 | 0.747177 |
| X214788_x_at | -0.0446 | 0.0516 | -0.864 | 0.3941332 | 0.747183 |
| X204639_at | -0.0625 | 0.0723 | -0.864 | 0.3941797 | 0.747183 |
| X206303_s_at | 0.004 | 0.0047 | 0.864 | 0.3941885 | 0.747183 |
| X214254_at | -0.0047 | 0.0054 | -0.864 | 0.3941927 | 0.747183 |
| X214009_at | -0.0128 | 0.0148 | -0.864 | 0.3942233 | 0.747183 |
| X215512_at | -0.0052 | 0.006 | -0.864 | 0.3942278 | 0.747183 |
| X219517_at | 0.0238 | 0.0276 | 0.864 | 0.3942727 | 0.747183 |
| X206390_x_at | 0.0026 | 0.003 | 0.864 | 0.3942776 | 0.747183 |
| X221281_at | 0.0042 | 0.0049 | 0.864 | 0.3943224 | 0.747203 |
| X208529_at | -0.0081 | 0.0094 | -0.864 | 0.394357 | 0.747203 |
| X216871_at | 0.0035 | 0.004 | 0.864 | 0.3943888 | 0.747203 |
| X220421_at | 0.0042 | 0.0048 | 0.864 | 0.3944701 | 0.747293 |
| X214261_s_at | 0.0034 | 0.0039 | 0.863 | 0.3945077 | 0.747301 |
| X210330_at | 0.006 | 0.0069 | 0.863 | 0.3946333 | 0.747475 |
| X221724_s_at | -0.0032 | 0.0037 | -0.863 | 0.3949073 | 0.74793 |
| X217622_at | -0.0279 | 0.0324 | -0.863 | 0.3950243 | 0.748061 |
| X220242_x_at | 0.0148 | 0.0171 | 0.862 | 0.3950454 | 0.748061 |
| X201847_at | 0.0814 | 0.0944 | 0.862 | 0.3950776 | 0.748061 |
| X216681_at | -0.0034 | 0.0039 | -0.862 | 0.3951478 | 0.748104 |
| X214253_s_at | -0.0047 | 0.0055 | -0.862 | 0.3951977 | 0.748104 |
| X201937_s_at | 0.0045 | 0.0052 | 0.862 | 0.3952014 | 0.748104 |
| X209854_s_at | -0.0041 | 0.0047 | -0.862 | 0.3952773 | 0.748184 |
| X207203_s_at | 0.0017 | 0.002 | 0.862 | 0.3954744 | 0.748431 |
| X209945_s_at | 0.0899 | 0.1043 | 0.862 | 0.395475 | 0.748431 |
| X217627_at | -0.0365 | 0.0424 | -0.861 | 0.3956059 | 0.748562 |
| X204825_at | -0.0065 | 0.0075 | -0.861 | 0.3956115 | 0.748562 |
| X221368_at | 0.0022 | 0.0025 | 0.861 | 0.3957912 | 0.748838 |
| X215764_x_at | -0.0424 | 0.0492 | -0.861 | 0.3958896 | 0.74896 |
| X219424_at | 0.0042 | 0.0048 | 0.861 | 0.3959954 | 0.748966 |
| X214833_at | -0.0275 | 0.032 | -0.861 | 0.3960291 | 0.748966 |
| X218766_s_at | 0.0094 | 0.011 | 0.861 | 0.3960593 | 0.748966 |
| X210941_at | -0.0084 | 0.0097 | -0.86 | 0.3961446 | 0.748966 |
| X207111_at | 0.0013 | 0.0015 | 0.86 | 0.396145 | 0.748966 |
| X204084_s_at | 0.0492 | 0.0572 | 0.86 | 0.3961537 | 0.748966 |
| X214144_at | 0.0162 | 0.0189 | 0.86 | 0.3961549 | 0.748966 |
| X215683_at | 0.005 | 0.0058 | 0.86 | 0.3961624 | 0.748966 |
| X222244_s_at | 0.0311 | 0.0362 | 0.86 | 0.3962533 | 0.749074 |
| X208176_at | 0.0054 | 0.0063 | 0.86 | 0.3963144 | 0.749126 |
| X214028_x_at | 0.0366 | 0.0425 | 0.86 | 0.3963584 | 0.749134 |
| X203671_at | 0.0364 | 0.0423 | 0.86 | 0.3963886 | 0.749134 |
| X212868_x_at | 0.002 | 0.0024 | 0.86 | 0.39642 | 0.749134 |
| X204190_at | -0.028 | 0.0325 | -0.86 | 0.396626 | 0.749414 |
| X209976_s_at | -0.0053 | 0.0062 | -0.859 | 0.3966944 | 0.749414 |
| X216093_at | -0.0035 | 0.0041 | -0.859 | 0.3967042 | 0.749414 |
| X221308_at | -0.0025 | 0.0029 | -0.859 | 0.396733 | 0.749414 |
| X210607_at | -0.0059 | 0.0069 | -0.859 | 0.3967431 | 0.749414 |
| X207498_s_at | -0.0314 | 0.0366 | -0.859 | 0.3968175 | 0.749414 |
| X200644_at | 0.053 | 0.0617 | 0.859 | 0.3968295 | 0.749414 |
| X210545_at | -0.0022 | 0.0026 | -0.859 | 0.3968394 | 0.749414 |
| X218690_at | 0.002 | 0.0024 | 0.859 | 0.3968834 | 0.749414 |
| X221481_x_at | 0.0264 | 0.0308 | 0.859 | 0.3969056 | 0.749414 |
| X221399_at | -0.0034 | 0.0039 | -0.859 | 0.3969489 | 0.749433 |
| X208626_s_at | 0.0273 | 0.0318 | 0.859 | 0.3970239 | 0.74951 |
| X215391_at | 0.0037 | 0.0043 | 0.859 | 0.3970589 | 0.749513 |
| X216328_at | -0.009 | 0.0105 | -0.858 | 0.3972093 | 0.749733 |
| X215839_at | 0.0034 | 0.004 | 0.858 | 0.397328 | 0.749893 |
| X210715_s_at | 0.0435 | 0.0507 | 0.858 | 0.3973803 | 0.749928 |
| X206128_at | -0.0459 | 0.0535 | -0.858 | 0.397552 | 0.750189 |
| X210447_at | -0.0086 | 0.01 | -0.858 | 0.3976309 | 0.750203 |
| X213643_s_at | 0.0036 | 0.0042 | 0.858 | 0.3976374 | 0.750203 |
| X204845_s_at | 0.0086 | 0.01 | 0.858 | 0.3976722 | 0.750203 |
| X215427_s_at | 0.0032 | 0.0037 | 0.858 | 0.3977166 | 0.750203 |
| X222280_at | 0.0152 | 0.0177 | 0.857 | 0.3977538 | 0.750203 |
| X202519_at | -0.0432 | 0.0504 | -0.857 | 0.3977661 | 0.750203 |
| X201286_at | 0.0026 | 0.003 | 0.857 | 0.3978539 | 0.750203 |
| X218646_at | -0.024 | 0.028 | -0.857 | 0.397862 | 0.750203 |
| X203165_s_at | -0.0431 | 0.0503 | -0.857 | 0.3978637 | 0.750203 |
| X212862_at | 0.0558 | 0.0652 | 0.857 | 0.3980103 | 0.750416 |
| X221770_at | -0.0753 | 0.0879 | -0.857 | 0.3980518 | 0.750426 |
| X220119_at | -0.0025 | 0.0029 | -0.857 | 0.3980832 | 0.750426 |
| X214868_at | -0.0124 | 0.0145 | -0.857 | 0.3981606 | 0.750508 |
| X215844_at | -0.0197 | 0.023 | -0.857 | 0.3982692 | 0.750561 |
| X202785_at | -0.0249 | 0.0291 | -0.857 | 0.3982715 | 0.750561 |
| X222326_at | 0.0674 | 0.0787 | 0.857 | 0.3982897 | 0.750561 |
| X201152_s_at | 0.0247 | 0.0289 | 0.856 | 0.398338 | 0.750588 |
| X216468_s_at | -0.0187 | 0.0219 | -0.856 | 0.3984824 | 0.750796 |
| X201479_at | -0.0248 | 0.029 | -0.856 | 0.3985205 | 0.750804 |
| X207664_at | -0.0051 | 0.006 | -0.856 | 0.3985856 | 0.75086 |
| X206293_at | -0.0077 | 0.0091 | -0.856 | 0.3986333 | 0.75086 |
| X216729_at | -0.0089 | 0.0104 | -0.856 | 0.3986513 | 0.75086 |
| X218734_at | 0.0067 | 0.0078 | 0.856 | 0.3987231 | 0.750931 |
| X214963_at | 0.006 | 0.007 | 0.856 | 0.3988045 | 0.751021 |
| X219745_at | -0.0437 | 0.0512 | -0.855 | 0.3991261 | 0.75152 |
| X200667_at | -0.0209 | 0.0244 | -0.855 | 0.3991652 | 0.75152 |
| X203800_s_at | -0.0276 | 0.0323 | -0.855 | 0.3991836 | 0.75152 |
| X209079_x_at | -0.0347 | 0.0406 | -0.855 | 0.3992046 | 0.75152 |
| X221096_s_at | -0.0024 | 0.0028 | -0.855 | 0.3992632 | 0.751524 |
| X200949_x_at | 0.0221 | 0.0258 | 0.855 | 0.3993047 | 0.751524 |
| X215239_x_at | 0.0516 | 0.0603 | 0.855 | 0.3993737 | 0.751524 |
| X210255_at | -0.0064 | 0.0075 | -0.854 | 0.3994053 | 0.751524 |
| X214478_at | 0.0031 | 0.0037 | 0.854 | 0.3994479 | 0.751524 |
| X214791_at | 0.0036 | 0.0042 | 0.854 | 0.3994564 | 0.751524 |
| X213559_s_at | -0.0023 | 0.0027 | -0.854 | 0.3994585 | 0.751524 |
| X211024_s_at | 0.0636 | 0.0744 | 0.854 | 0.3995078 | 0.751524 |
| X208215_x_at | -0.0013 | 0.0015 | -0.854 | 0.3995115 | 0.751524 |
| X208450_at | -0.0024 | 0.0028 | -0.854 | 0.3995659 | 0.751563 |
| X216009_at | -0.0049 | 0.0058 | -0.854 | 0.3996748 | 0.751588 |
| X208546_x_at | -0.005 | 0.0058 | -0.854 | 0.3996902 | 0.751588 |
| X206258_at | 0.0031 | 0.0036 | 0.854 | 0.3997138 | 0.751588 |
| X216714_at | 0.0031 | 0.0036 | 0.854 | 0.3997381 | 0.751588 |
| X207745_at | 0.0038 | 0.0044 | 0.854 | 0.3997485 | 0.751588 |
| X211012_s_at | 0.003 | 0.0035 | 0.854 | 0.3998114 | 0.751629 |
| X201540_at | -0.0379 | 0.0444 | -0.854 | 0.3998379 | 0.751629 |
| X207795_s_at | 0.0025 | 0.003 | 0.853 | 0.4000958 | 0.75205 |
| X211182_x_at | -0.0022 | 0.0026 | -0.853 | 0.4002086 | 0.752154 |
| X214190_x_at | -0.0026 | 0.003 | -0.853 | 0.4002221 | 0.752154 |
| X208341_x_at | 0.0028 | 0.0033 | 0.853 | 0.4002526 | 0.752154 |
| X221311_x_at | 0.0232 | 0.0272 | 0.853 | 0.4003176 | 0.752212 |
| X210475_at | 0.0042 | 0.0049 | 0.853 | 0.4003777 | 0.752247 |
| X200595_s_at | -0.0227 | 0.0266 | -0.853 | 0.4004578 | 0.752247 |
| X202596_at | -0.0358 | 0.042 | -0.852 | 0.4004856 | 0.752247 |
| X214083_at | -0.0059 | 0.007 | -0.852 | 0.4004954 | 0.752247 |
| X214516_at | -0.0032 | 0.0038 | -0.852 | 0.4005062 | 0.752247 |
| X206938_at | -0.0056 | 0.0066 | -0.852 | 0.4005389 | 0.752247 |
| X216820_at | -0.0042 | 0.0049 | -0.852 | 0.4006178 | 0.752284 |
| X218838_s_at | 0.0158 | 0.0185 | 0.852 | 0.4006793 | 0.752284 |
| X202763_at | -0.0223 | 0.0262 | -0.852 | 0.4006943 | 0.752284 |
| X208410_x_at | 0.0071 | 0.0083 | 0.852 | 0.4007421 | 0.752284 |
| X205210_at | 0.0034 | 0.004 | 0.852 | 0.4007476 | 0.752284 |
| X207692_s_at | 0.0043 | 0.005 | 0.852 | 0.4007619 | 0.752284 |
| X220505_at | 0.0029 | 0.0034 | 0.852 | 0.4008482 | 0.752382 |
| X202874_s_at | -0.0436 | 0.0512 | -0.852 | 0.4008999 | 0.752416 |
| X202668_at | 0.0282 | 0.0331 | 0.852 | 0.4009362 | 0.75242 |
| X203949_at | 0.0066 | 0.0077 | 0.851 | 0.4010758 | 0.752504 |
| X205764_at | -0.0048 | 0.0056 | -0.851 | 0.401076 | 0.752504 |
| X217141_at | 0.0424 | 0.0498 | 0.851 | 0.4010826 | 0.752504 |
| X208507_at | 0.0027 | 0.0032 | 0.851 | 0.4011714 | 0.752588 |
| X208470_s_at | 0.0269 | 0.0316 | 0.851 | 0.4011953 | 0.752588 |
| X218103_at | 0.006 | 0.007 | 0.851 | 0.4012497 | 0.752627 |
| X208774_at | -0.0243 | 0.0285 | -0.851 | 0.401315 | 0.752686 |
| X33132_at | 0.0089 | 0.0105 | 0.851 | 0.4013567 | 0.752701 |
| X215090_x_at | -0.0347 | 0.0407 | -0.851 | 0.4014375 | 0.75273 |
| X202892_at | 0.0199 | 0.0234 | 0.851 | 0.4014401 | 0.75273 |
| X210005_at | -0.032 | 0.0376 | -0.851 | 0.4015097 | 0.752797 |
| X202968_s_at | 0.0133 | 0.0157 | 0.85 | 0.4015784 | 0.752862 |
| X212875_s_at | 0.0373 | 0.0438 | 0.85 | 0.4016253 | 0.752886 |
| X218025_s_at | -0.0422 | 0.0496 | -0.85 | 0.401697 | 0.752893 |
| X220838_at | 0.0074 | 0.0087 | 0.85 | 0.4016975 | 0.752893 |
| X205695_at | -0.0853 | 0.1004 | -0.85 | 0.4017306 | 0.752893 |
| X208054_at | 0.0065 | 0.0076 | 0.85 | 0.4017765 | 0.752916 |
| X219503_s_at | 0.0034 | 0.004 | 0.85 | 0.4018945 | 0.752999 |
| X208831_x_at | -0.039 | 0.0459 | -0.85 | 0.401907 | 0.752999 |
| X217106_x_at | -0.024 | 0.0282 | -0.85 | 0.4019226 | 0.752999 |
| X218166_s_at | -0.0449 | 0.0528 | -0.85 | 0.4020194 | 0.753117 |
| X212020_s_at | 0.0034 | 0.004 | 0.849 | 0.4022948 | 0.753569 |
| X220668_s_at | 0.0048 | 0.0056 | 0.849 | 0.4023838 | 0.753608 |
| X212100_s_at | -0.0327 | 0.0385 | -0.849 | 0.4024032 | 0.753608 |
| X212117_at | -0.0272 | 0.0321 | -0.849 | 0.4024175 | 0.753608 |
| X212940_at | -0.0033 | 0.0039 | -0.849 | 0.4026498 | 0.7539 |
| X219947_at | -0.028 | 0.033 | -0.848 | 0.4027199 | 0.7539 |
| X205053_at | -0.0195 | 0.023 | -0.848 | 0.4028237 | 0.7539 |
| X203617_x_at | 0.0484 | 0.0571 | 0.848 | 0.4028568 | 0.7539 |
| X218180_s_at | 0.0052 | 0.0062 | 0.848 | 0.4028717 | 0.7539 |
| X213580_at | -0.0128 | 0.015 | -0.848 | 0.4028794 | 0.7539 |
| X209628_at | 0.0738 | 0.0871 | 0.848 | 0.4028811 | 0.7539 |
| X219921_s_at | 0.0041 | 0.0049 | 0.848 | 0.4028977 | 0.7539 |
| X214421_x_at | 0.0052 | 0.0061 | 0.848 | 0.4029073 | 0.7539 |
| X217054_at | -0.0106 | 0.0125 | -0.848 | 0.4029128 | 0.7539 |
| X214689_at | -0.0105 | 0.0124 | -0.848 | 0.4029867 | 0.753975 |
| X221885_at | 0.0047 | 0.0056 | 0.848 | 0.4030289 | 0.753982 |
| X202008_s_at | -0.0086 | 0.0101 | -0.848 | 0.403058 | 0.753982 |
| X208255_s_at | 0.0027 | 0.0032 | 0.848 | 0.4030983 | 0.753993 |
| X221091_at | -0.0038 | 0.0044 | -0.847 | 0.4033086 | 0.754271 |
| X201429_s_at | -0.0166 | 0.0195 | -0.847 | 0.4033148 | 0.754271 |
| X220962_s_at | -0.0072 | 0.0085 | -0.847 | 0.4034123 | 0.75439 |
| X211350_s_at | -0.0048 | 0.0056 | -0.847 | 0.4034714 | 0.754423 |
| X218290_at | -0.024 | 0.0283 | -0.847 | 0.4035002 | 0.754423 |
| X220728_at | 0.0707 | 0.0835 | 0.847 | 0.4035319 | 0.754423 |
| X217979_at | -0.0301 | 0.0356 | -0.847 | 0.403568 | 0.754427 |
| X215677_s_at | -0.0025 | 0.003 | -0.847 | 0.4036371 | 0.754475 |
| X208538_at | 0.0058 | 0.0069 | 0.847 | 0.4037058 | 0.754475 |
| X214720_x_at | 0.0073 | 0.0087 | 0.847 | 0.4037139 | 0.754475 |
| X222014_x_at | -0.0379 | 0.0447 | -0.847 | 0.4037291 | 0.754475 |
| X216026_s_at | 0.0061 | 0.0072 | 0.846 | 0.403843 | 0.754624 |
| X220765_s_at | 0.0084 | 0.0099 | 0.846 | 0.4040733 | 0.754988 |
| X213604_at | -0.023 | 0.0272 | -0.846 | 0.4041056 | 0.754988 |
| X204775_at | -0.0036 | 0.0043 | -0.846 | 0.4042399 | 0.755175 |
| X202294_at | 0.02 | 0.0237 | 0.845 | 0.4043684 | 0.755352 |
| X218818_at | 0.0029 | 0.0034 | 0.845 | 0.4044611 | 0.755452 |
| X205435_s_at | 0.0078 | 0.0092 | 0.845 | 0.4044902 | 0.755452 |
| X200092_s_at | -0.0179 | 0.0211 | -0.845 | 0.404526 | 0.755455 |
| X202611_s_at | -0.0488 | 0.0577 | -0.845 | 0.4046732 | 0.755611 |
| X208172_s_at | 0.003 | 0.0036 | 0.845 | 0.4047224 | 0.755611 |
| X215647_at | -0.0167 | 0.0198 | -0.845 | 0.4047552 | 0.755611 |
| X217586_x_at | -0.0727 | 0.0861 | -0.845 | 0.4047641 | 0.755611 |
| X200066_at | -0.0249 | 0.0295 | -0.845 | 0.4047796 | 0.755611 |
| X216275_at | 0.0026 | 0.0031 | 0.845 | 0.4048168 | 0.755617 |
| X206859_s_at | 0.0023 | 0.0027 | 0.844 | 0.4049606 | 0.755822 |
| X36920_at | 0.0727 | 0.0861 | 0.844 | 0.4050553 | 0.755874 |
| X210560_at | -0.0038 | 0.0045 | -0.844 | 0.4050562 | 0.755874 |
| X221211_s_at | 0.0147 | 0.0174 | 0.844 | 0.4051487 | 0.755882 |
| X217307_at | -0.0023 | 0.0028 | -0.844 | 0.4051519 | 0.755882 |
| X221273_s_at | 0.0024 | 0.0028 | 0.844 | 0.4052543 | 0.755882 |
| X213056_at | -0.0798 | 0.0946 | -0.844 | 0.4052577 | 0.755882 |
| X221896_s_at | -0.0271 | 0.0321 | -0.844 | 0.4052649 | 0.755882 |
| X214381_at | 0.0039 | 0.0047 | 0.844 | 0.4053106 | 0.755882 |
| X215279_at | 0.0032 | 0.0038 | 0.844 | 0.4053247 | 0.755882 |
| X208729_x_at | 0.0656 | 0.0778 | 0.844 | 0.4053331 | 0.755882 |
| X213921_at | -0.049 | 0.058 | -0.844 | 0.4053758 | 0.755898 |
| X205221_at | 0.0029 | 0.0035 | 0.843 | 0.4055465 | 0.756153 |
| X217085_at | -0.0097 | 0.0115 | -0.843 | 0.4057031 | 0.756382 |
| X206044_s_at | 0.0066 | 0.0078 | 0.843 | 0.4058548 | 0.756601 |
| X210508_s_at | 0.0032 | 0.0038 | 0.842 | 0.406026 | 0.756857 |
| X213829_x_at | 0.0023 | 0.0028 | 0.842 | 0.4062441 | 0.757182 |
| X207505_at | 0.0054 | 0.0064 | 0.842 | 0.4062689 | 0.757182 |
| X203962_s_at | -0.0354 | 0.0421 | -0.842 | 0.4063169 | 0.757203 |
| X214364_at | -0.0069 | 0.0082 | -0.842 | 0.4064268 | 0.757203 |
| X220916_at | 0.0029 | 0.0034 | 0.842 | 0.4064527 | 0.757203 |
| X215077_at | -0.0047 | 0.0056 | -0.842 | 0.406469 | 0.757203 |
| X211390_at | -0.0033 | 0.0039 | -0.842 | 0.4064933 | 0.757203 |
| X210045_at | -0.0467 | 0.0556 | -0.842 | 0.406501 | 0.757203 |
| X220742_s_at | 0.0321 | 0.0381 | 0.841 | 0.4065451 | 0.757203 |
| X216641_s_at | -0.0035 | 0.0042 | -0.841 | 0.4066506 | 0.757203 |
| X219783_at | 0.0049 | 0.0058 | 0.841 | 0.406745 | 0.757203 |
| X216014_s_at | 0.0028 | 0.0034 | 0.841 | 0.4067716 | 0.757203 |
| X220826_at | 0.0042 | 0.005 | 0.841 | 0.4067946 | 0.757203 |
| X48808_at | 0.042 | 0.05 | 0.841 | 0.4067963 | 0.757203 |
| X207832_at | -0.0045 | 0.0054 | -0.841 | 0.4067995 | 0.757203 |
| X211545_at | -0.0018 | 0.0022 | -0.841 | 0.406812 | 0.757203 |
| X212475_at | 0.0563 | 0.0669 | 0.841 | 0.4068474 | 0.757203 |
| X208273_at | 0.0091 | 0.0108 | 0.841 | 0.4068881 | 0.757203 |
| X211063_s_at | -0.0286 | 0.0341 | -0.841 | 0.4069236 | 0.757203 |
| X204724_s_at | -0.0547 | 0.0651 | -0.841 | 0.4070427 | 0.757203 |
| X207757_at | -0.0074 | 0.0088 | -0.841 | 0.4070604 | 0.757203 |
| X219721_at | 0.0028 | 0.0033 | 0.84 | 0.4071144 | 0.757203 |
| X203618_at | 0.0394 | 0.0468 | 0.84 | 0.4071223 | 0.757203 |
| X201673_s_at | -0.0057 | 0.0068 | -0.84 | 0.4071259 | 0.757203 |
| X205944_s_at | -0.0027 | 0.0032 | -0.84 | 0.4071346 | 0.757203 |
| X218213_s_at | 0.0294 | 0.035 | 0.84 | 0.407135 | 0.757203 |
| X219058_x_at | 0.0039 | 0.0046 | 0.84 | 0.4071655 | 0.757203 |
| X201641_at | 0.0265 | 0.0316 | 0.84 | 0.4071664 | 0.757203 |
| X215723_s_at | -0.0046 | 0.0055 | -0.84 | 0.4072864 | 0.757302 |
| X211861_x_at | -0.0042 | 0.005 | -0.84 | 0.4072878 | 0.757302 |
| X211186_s_at | 0.0032 | 0.0038 | 0.84 | 0.4074354 | 0.757513 |
| X207810_at | -0.0061 | 0.0072 | -0.84 | 0.4075118 | 0.757592 |
| X216633_s_at | -0.0048 | 0.0057 | -0.84 | 0.4075611 | 0.75762 |
| X214785_at | -0.044 | 0.0524 | -0.839 | 0.4076704 | 0.75776 |
| X206725_x_at | -0.0025 | 0.003 | -0.839 | 0.407832 | 0.757915 |
| X200643_at | -0.0387 | 0.0462 | -0.839 | 0.4078552 | 0.757915 |
| X213578_at | 0.043 | 0.0513 | 0.839 | 0.4078746 | 0.757915 |
| X208886_at | 0.0422 | 0.0503 | 0.839 | 0.4079257 | 0.757915 |
| X210540_s_at | 0.0487 | 0.0581 | 0.839 | 0.4079672 | 0.757915 |
| X211650_x_at | -0.0025 | 0.003 | -0.839 | 0.4079921 | 0.757915 |
| X217178_at | -0.0029 | 0.0034 | -0.839 | 0.4079926 | 0.757915 |
| X220839_at | -0.0182 | 0.0217 | -0.839 | 0.4081299 | 0.758107 |
| X205908_s_at | -0.0122 | 0.0145 | -0.838 | 0.4081945 | 0.758137 |
| X212364_at | 0.0506 | 0.0604 | 0.838 | 0.4082243 | 0.758137 |
| X219727_at | 0.0054 | 0.0065 | 0.838 | 0.4083826 | 0.758137 |
| X203638_s_at | 0.0783 | 0.0935 | 0.838 | 0.4083946 | 0.758137 |
| X221438_s_at | -0.0071 | 0.0085 | -0.838 | 0.4084313 | 0.758137 |
| X217931_at | 0.032 | 0.0382 | 0.838 | 0.408468 | 0.758137 |
| X207607_at | -0.0033 | 0.004 | -0.838 | 0.4085017 | 0.758137 |
| X203642_s_at | 0.0886 | 0.1057 | 0.838 | 0.4085064 | 0.758137 |
| X216911_s_at | -0.0025 | 0.003 | -0.838 | 0.4085294 | 0.758137 |
| X218261_at | 0.0026 | 0.0031 | 0.838 | 0.4085414 | 0.758137 |
| X214270_s_at | -0.0034 | 0.0041 | -0.838 | 0.4085528 | 0.758137 |
| X213494_s_at | 0.0035 | 0.0042 | 0.838 | 0.4085556 | 0.758137 |
| X214054_at | 0.0041 | 0.0049 | 0.838 | 0.4086135 | 0.758175 |
| X216743_at | -0.0049 | 0.0058 | -0.838 | 0.4086979 | 0.758175 |
| X215753_at | -0.0035 | 0.0042 | -0.838 | 0.4087172 | 0.758175 |
| X207713_s_at | 0.0054 | 0.0065 | 0.837 | 0.4087292 | 0.758175 |
| X210396_s_at | -0.0733 | 0.0875 | -0.837 | 0.4087469 | 0.758175 |
| X211168_s_at | 0.0272 | 0.0325 | 0.837 | 0.4089234 | 0.758378 |
| X208754_s_at | 0.0317 | 0.0378 | 0.837 | 0.4089545 | 0.758378 |
| X201300_s_at | -0.021 | 0.0251 | -0.837 | 0.4090055 | 0.758378 |
| X207314_x_at | 0.0037 | 0.0045 | 0.837 | 0.4090297 | 0.758378 |
| X206159_at | 0.0152 | 0.0182 | 0.837 | 0.4090668 | 0.758378 |
| X213644_at | 0.0694 | 0.0829 | 0.837 | 0.4090804 | 0.758378 |
| X202174_s_at | -0.0246 | 0.0294 | -0.837 | 0.4090954 | 0.758378 |
| X202635_s_at | -0.0355 | 0.0424 | -0.837 | 0.4091344 | 0.758387 |
| X213631_x_at | -0.0054 | 0.0064 | -0.837 | 0.4091683 | 0.758387 |
| X205918_at | -0.0454 | 0.0542 | -0.836 | 0.4093124 | 0.758591 |
| X212855_at | -0.0265 | 0.0316 | -0.836 | 0.4094161 | 0.758719 |
| X215860_at | -0.0037 | 0.0044 | -0.836 | 0.4094954 | 0.758803 |
| X207743_at | 0.0034 | 0.0041 | 0.836 | 0.4096221 | 0.758975 |
| X214379_at | 0.0094 | 0.0113 | 0.836 | 0.4096765 | 0.75901 |
| X215773_x_at | -0.0191 | 0.0229 | -0.836 | 0.4097627 | 0.75901 |
| X218778_x_at | -0.0035 | 0.0042 | -0.836 | 0.4097743 | 0.75901 |
| X216771_at | -0.0064 | 0.0076 | -0.836 | 0.4097779 | 0.75901 |
| X219733_s_at | -0.0604 | 0.0723 | -0.835 | 0.409871 | 0.759119 |
| X212298_at | 0.1056 | 0.1264 | 0.835 | 0.4100763 | 0.759436 |
| X205943_at | 0.0041 | 0.0049 | 0.835 | 0.4101148 | 0.759444 |
| X202456_s_at | -0.0072 | 0.0086 | -0.835 | 0.4102111 | 0.759559 |
| X213357_at | -0.0308 | 0.0369 | -0.834 | 0.4103846 | 0.759817 |
| X217634_at | -0.0034 | 0.0041 | -0.834 | 0.4105114 | 0.759892 |
| X37590_g_at | 0.025 | 0.03 | 0.834 | 0.4105155 | 0.759892 |
| X209541_at | -0.0231 | 0.0277 | -0.834 | 0.4105547 | 0.759892 |
| X218004_at | 0.0318 | 0.0381 | 0.834 | 0.4105622 | 0.759892 |
| X220181_x_at | 0.0051 | 0.0061 | 0.834 | 0.4105966 | 0.759893 |
| X201201_at | -0.0248 | 0.0298 | -0.834 | 0.4106695 | 0.759964 |
| X221680_s_at | 0.0055 | 0.0066 | 0.834 | 0.4107736 | 0.760078 |
| X215075_s_at | 0.0315 | 0.0378 | 0.834 | 0.4107993 | 0.760078 |
| X213712_at | -0.0579 | 0.0694 | -0.834 | 0.4108498 | 0.760108 |
| X210157_at | 0.0352 | 0.0422 | 0.834 | 0.4108994 | 0.760137 |
| X205058_at | -0.0043 | 0.0052 | -0.833 | 0.4109599 | 0.760154 |
| X215898_at | 0.02 | 0.024 | 0.833 | 0.4110148 | 0.760154 |
| X215777_at | -0.0026 | 0.0031 | -0.833 | 0.4110734 | 0.760154 |
| X215853_at | -0.0061 | 0.0074 | -0.833 | 0.4110818 | 0.760154 |
| X208015_at | 0.0441 | 0.0529 | 0.833 | 0.4111023 | 0.760154 |
| X217506_at | -0.0462 | 0.0555 | -0.833 | 0.4111142 | 0.760154 |
| X215546_at | -0.006 | 0.0072 | -0.833 | 0.4112502 | 0.760342 |
| X201547_at | -0.003 | 0.0036 | -0.833 | 0.4113252 | 0.760418 |
| X213818_x_at | 0.0032 | 0.0038 | 0.833 | 0.4113827 | 0.760461 |
| X214578_s_at | 0.0274 | 0.0329 | 0.833 | 0.4114555 | 0.760511 |
| X205790_at | 0.002 | 0.0024 | 0.833 | 0.4114785 | 0.760511 |
| X201022_s_at | -0.0262 | 0.0314 | -0.832 | 0.4115583 | 0.760533 |
| X221636_s_at | 0.0634 | 0.0762 | 0.832 | 0.411559 | 0.760533 |
| X201436_at | -0.0435 | 0.0522 | -0.832 | 0.4116142 | 0.760572 |
| X206774_at | 0.0052 | 0.0063 | 0.832 | 0.4117213 | 0.760647 |
| X212307_s_at | 0.0596 | 0.0716 | 0.832 | 0.411723 | 0.760647 |
| X210795_s_at | -0.0023 | 0.0027 | -0.832 | 0.4117864 | 0.760701 |
| X202409_at | -0.0375 | 0.045 | -0.832 | 0.4118344 | 0.760726 |
| X208595_s_at | -0.004 | 0.0048 | -0.831 | 0.4120814 | 0.761119 |
| X209719_x_at | 0.0035 | 0.0042 | 0.831 | 0.4121751 | 0.761201 |
| X207590_s_at | 0.0075 | 0.009 | 0.831 | 0.4121942 | 0.761201 |
| X210915_x_at | 0.0034 | 0.0041 | 0.831 | 0.4123984 | 0.761487 |
| X203814_s_at | 0.0837 | 0.1008 | 0.831 | 0.4124421 | 0.761487 |
| X213979_s_at | 0.0556 | 0.0669 | 0.831 | 0.4124582 | 0.761487 |
| X209119_x_at | 0.0061 | 0.0074 | 0.831 | 0.4124976 | 0.761487 |
| X205530_at | 0.0251 | 0.0302 | 0.831 | 0.4125207 | 0.761487 |
| X217938_s_at | -0.0234 | 0.0282 | -0.83 | 0.4127258 | 0.761697 |
| X209223_at | 0.0024 | 0.0029 | 0.83 | 0.4127348 | 0.761697 |
| X214690_at | 0.0701 | 0.0844 | 0.83 | 0.4127374 | 0.761697 |
| X222290_at | -0.0118 | 0.0142 | -0.83 | 0.4128866 | 0.761909 |
| X222356_at | -0.0077 | 0.0093 | -0.83 | 0.4129294 | 0.761925 |
| X210152_at | -0.0026 | 0.0031 | -0.83 | 0.4129879 | 0.761963 |
| X220416_at | -0.0088 | 0.0107 | -0.83 | 0.4130191 | 0.761963 |
| X218294_s_at | -0.0212 | 0.0256 | -0.83 | 0.4130527 | 0.761963 |
| X213768_s_at | -0.0121 | 0.0146 | -0.83 | 0.4131347 | 0.762051 |
| X215038_s_at | -0.0331 | 0.0399 | -0.829 | 0.4132497 | 0.762161 |
| X216028_at | 0.0498 | 0.0601 | 0.829 | 0.4132651 | 0.762161 |
| X218598_at | 0.0327 | 0.0394 | 0.829 | 0.4132973 | 0.762161 |
| X212796_s_at | 0.0478 | 0.0577 | 0.829 | 0.4134049 | 0.76226 |
| X211546_x_at | -0.0642 | 0.0774 | -0.829 | 0.4134807 | 0.76226 |
| X214200_s_at | 0.0049 | 0.0059 | 0.829 | 0.4134892 | 0.76226 |
| X211133_x_at | 0.0094 | 0.0114 | 0.829 | 0.4134934 | 0.76226 |
| X203673_at | 0.0034 | 0.0041 | 0.829 | 0.4135228 | 0.76226 |
| X208077_at | -0.006 | 0.0072 | -0.829 | 0.4136121 | 0.762361 |
| X216471_x_at | 0.0029 | 0.0035 | 0.829 | 0.413701 | 0.762431 |
| X201831_s_at | -0.0991 | 0.1196 | -0.828 | 0.4137699 | 0.762431 |
| X209594_x_at | 0.0031 | 0.0038 | 0.828 | 0.4137922 | 0.762431 |
| X209255_at | -0.1143 | 0.138 | -0.828 | 0.4138128 | 0.762431 |
| X213175_s_at | 0.0305 | 0.0368 | 0.828 | 0.4138216 | 0.762431 |
| X208889_s_at | -0.0033 | 0.004 | -0.828 | 0.4139442 | 0.762594 |
| X221782_at | 0.0432 | 0.0522 | 0.828 | 0.4141261 | 0.762786 |
| X221021_s_at | 0.007 | 0.0085 | 0.828 | 0.4141814 | 0.762786 |
| X214197_s_at | -0.0035 | 0.0043 | -0.828 | 0.4141858 | 0.762786 |
| X220521_s_at | -0.01 | 0.0121 | -0.828 | 0.4141858 | 0.762786 |
| X200653_s_at | -0.0275 | 0.0332 | -0.828 | 0.4142528 | 0.762846 |
| X213086_s_at | 0.0242 | 0.0292 | 0.827 | 0.4145119 | 0.763235 |
| X204854_at | 0.0027 | 0.0032 | 0.827 | 0.4145325 | 0.763235 |
| X212680_x_at | 0.0133 | 0.0161 | 0.827 | 0.4145769 | 0.763253 |
| X215190_at | 0.0454 | 0.0549 | 0.827 | 0.4146473 | 0.763312 |
| X206462_s_at | 0.0637 | 0.077 | 0.827 | 0.4146775 | 0.763312 |
| X213187_x_at | 0.0342 | 0.0414 | 0.827 | 0.4147624 | 0.763405 |
| X204167_at | -0.0097 | 0.0117 | -0.826 | 0.4148791 | 0.763556 |
| X202076_at | 0.0224 | 0.0272 | 0.826 | 0.4150148 | 0.763743 |
| X221959_at | -0.0273 | 0.033 | -0.826 | 0.4150769 | 0.763794 |
| X210181_s_at | -0.0052 | 0.0062 | -0.826 | 0.415114 | 0.763799 |
| X213258_at | 0.0165 | 0.02 | 0.826 | 0.4152021 | 0.763861 |
| X208416_s_at | -0.0041 | 0.005 | -0.826 | 0.4152509 | 0.763861 |
| X201715_s_at | 0.0426 | 0.0516 | 0.826 | 0.415251 | 0.763861 |
| X202836_s_at | 0.0215 | 0.0261 | 0.826 | 0.4152895 | 0.763869 |
| X209492_x_at | -0.0229 | 0.0277 | -0.825 | 0.4154016 | 0.763882 |
| X206968_s_at | -0.0022 | 0.0027 | -0.825 | 0.4154361 | 0.763882 |
| X204595_s_at | 0.0028 | 0.0033 | 0.825 | 0.4154641 | 0.763882 |
| X204888_s_at | 0.0026 | 0.0032 | 0.825 | 0.4154813 | 0.763882 |
| X210093_s_at | -0.0859 | 0.1041 | -0.825 | 0.415483 | 0.763882 |
| X220393_at | -0.0073 | 0.0089 | -0.825 | 0.4155051 | 0.763882 |
| X204816_s_at | -0.0098 | 0.0119 | -0.825 | 0.4155591 | 0.763882 |
| X210763_x_at | 0.0021 | 0.0025 | 0.825 | 0.4155977 | 0.763882 |
| X210733_at | -0.0148 | 0.0179 | -0.825 | 0.4156064 | 0.763882 |
| X204323_x_at | 0.0081 | 0.0099 | 0.825 | 0.4156876 | 0.763956 |
| X219011_at | -0.0026 | 0.0031 | -0.825 | 0.4157156 | 0.763956 |
| X203052_at | -0.0027 | 0.0033 | -0.825 | 0.4157498 | 0.763956 |
| X207586_at | -0.0021 | 0.0025 | -0.825 | 0.415786 | 0.76396 |
| X221373_x_at | -0.003 | 0.0037 | -0.825 | 0.4158557 | 0.764024 |
| X217283_at | 0.0043 | 0.0053 | 0.824 | 0.4160208 | 0.764178 |
| X207388_s_at | 0.0018 | 0.0022 | 0.824 | 0.4160257 | 0.764178 |
| X211937_at | -0.0439 | 0.0532 | -0.824 | 0.4160427 | 0.764178 |
| X221218_s_at | -0.0569 | 0.0691 | -0.824 | 0.4161344 | 0.76426 |
| X219705_at | -0.0042 | 0.0051 | -0.824 | 0.4161643 | 0.76426 |
| X210026_s_at | -0.004 | 0.0049 | -0.824 | 0.4161904 | 0.76426 |
| X218395_at | -0.0251 | 0.0304 | -0.824 | 0.4163024 | 0.764403 |
| X222337_at | 0.0074 | 0.009 | 0.824 | 0.4163934 | 0.764505 |
| X37547_at | -0.0401 | 0.0487 | -0.824 | 0.4164269 | 0.764505 |
| X200939_s_at | 0.0039 | 0.0048 | 0.824 | 0.4164954 | 0.764567 |
| X207685_at | -0.0024 | 0.003 | -0.823 | 0.4168403 | 0.765137 |
| X202089_s_at | -0.0487 | 0.0592 | -0.823 | 0.4169594 | 0.765242 |
| X212279_at | 0.0376 | 0.0458 | 0.823 | 0.4169866 | 0.765242 |
| X205781_at | 0.0042 | 0.0051 | 0.823 | 0.4170576 | 0.765242 |
| X217008_s_at | -0.0081 | 0.0098 | -0.822 | 0.4170805 | 0.765242 |
| X206551_x_at | 0.0308 | 0.0375 | 0.822 | 0.4170961 | 0.765242 |
| X214631_at | -0.0077 | 0.0093 | -0.822 | 0.4171041 | 0.765242 |
| X213353_at | 0.0371 | 0.0451 | 0.822 | 0.4171482 | 0.76526 |
| X208644_at | -0.0287 | 0.035 | -0.822 | 0.4173467 | 0.765438 |
| X206953_s_at | -0.0506 | 0.0615 | -0.822 | 0.4173707 | 0.765438 |
| X220377_at | 0.003 | 0.0036 | 0.822 | 0.417389 | 0.765438 |
| X207773_x_at | -0.0031 | 0.0038 | -0.822 | 0.4173897 | 0.765438 |
| X205784_x_at | 0.0056 | 0.0068 | 0.822 | 0.4174776 | 0.765438 |
| X216952_s_at | -0.0033 | 0.0041 | -0.822 | 0.4174856 | 0.765438 |
| X219598_s_at | 0.027 | 0.0328 | 0.822 | 0.4174864 | 0.765438 |
| X221328_at | 0.0022 | 0.0026 | 0.822 | 0.4175679 | 0.765524 |
| X220779_at | -0.003 | 0.0036 | -0.822 | 0.4176187 | 0.765554 |
| X217666_at | -0.0062 | 0.0076 | -0.821 | 0.4176742 | 0.765592 |
| X206472_s_at | 0.0047 | 0.0057 | 0.821 | 0.4177177 | 0.765609 |
| X215173_at | -0.0037 | 0.0045 | -0.821 | 0.4179043 | 0.76586 |
| X215752_at | 0.0039 | 0.0047 | 0.821 | 0.4179237 | 0.76586 |
| X204689_at | -0.0037 | 0.0045 | -0.821 | 0.4180249 | 0.765982 |
| X216932_at | 0.0043 | 0.0052 | 0.821 | 0.418141 | 0.766132 |
| X213696_s_at | 0.0048 | 0.0058 | 0.82 | 0.4182522 | 0.766248 |
| X212128_s_at | -0.0053 | 0.0065 | -0.82 | 0.4182927 | 0.766248 |
| X217642_at | 0.0032 | 0.0039 | 0.82 | 0.4183076 | 0.766248 |
| X218562_s_at | -0.0327 | 0.0398 | -0.82 | 0.4184376 | 0.766423 |
| X211257_x_at | -0.0174 | 0.0212 | -0.82 | 0.4185448 | 0.76653 |
| X221560_at | -0.0293 | 0.0358 | -0.82 | 0.4185747 | 0.76653 |
| X216708_x_at | -0.0037 | 0.0045 | -0.82 | 0.4185996 | 0.76653 |
| X215285_s_at | 0.0207 | 0.0252 | 0.82 | 0.4187497 | 0.766741 |
| X213164_at | 0.0611 | 0.0746 | 0.819 | 0.4189334 | 0.766822 |
| X215924_at | -0.0091 | 0.0111 | -0.819 | 0.4189426 | 0.766822 |
| X203071_at | 0.1317 | 0.1607 | 0.819 | 0.4190295 | 0.766822 |
| X221119_at | -0.0035 | 0.0043 | -0.819 | 0.4190485 | 0.766822 |
| X205603_s_at | -0.0766 | 0.0935 | -0.819 | 0.4190515 | 0.766822 |
| X210363_s_at | -0.0038 | 0.0047 | -0.819 | 0.4190612 | 0.766822 |
| X203351_s_at | -0.0331 | 0.0404 | -0.819 | 0.4190673 | 0.766822 |
| X210585_s_at | -0.0024 | 0.003 | -0.819 | 0.4190962 | 0.766822 |
| X204014_at | -0.0859 | 0.1049 | -0.819 | 0.4191272 | 0.766822 |
| X220666_at | -0.0535 | 0.0654 | -0.819 | 0.4191387 | 0.766822 |
| X217469_at | -0.0032 | 0.0039 | -0.819 | 0.419193 | 0.766824 |
| X218521_s_at | 0.0504 | 0.0616 | 0.819 | 0.4192093 | 0.766824 |
| X208103_s_at | -0.0193 | 0.0236 | -0.819 | 0.4192676 | 0.766868 |
| X212280_x_at | 0.0065 | 0.0079 | 0.818 | 0.4193341 | 0.766886 |
| X210503_at | -0.0075 | 0.0091 | -0.818 | 0.4193464 | 0.766886 |
| X205307_s_at | -0.0041 | 0.005 | -0.818 | 0.4193889 | 0.7669 |
| X214969_at | 0.0023 | 0.0029 | 0.818 | 0.4194286 | 0.766902 |
| X218246_at | -0.0127 | 0.0155 | -0.818 | 0.4194591 | 0.766902 |
| X218556_at | -0.0704 | 0.086 | -0.818 | 0.4195541 | 0.766999 |
| X204103_at | 0.0024 | 0.0029 | 0.818 | 0.419581 | 0.766999 |
| X215408_at | 0.0039 | 0.0048 | 0.818 | 0.4196816 | 0.767093 |
| X202551_s_at | -0.0421 | 0.0515 | -0.818 | 0.4197016 | 0.767093 |
| X216765_at | 0.0084 | 0.0102 | 0.818 | 0.4197393 | 0.767099 |
| X219858_s_at | 0.1 | 0.1223 | 0.818 | 0.4198739 | 0.767282 |
| X202103_at | -0.0048 | 0.0059 | -0.817 | 0.4199195 | 0.767302 |
| X216006_at | 0.0631 | 0.0772 | 0.817 | 0.4200101 | 0.767364 |
| X207513_s_at | -0.0419 | 0.0513 | -0.817 | 0.4200943 | 0.767364 |
| X212264_s_at | 0.026 | 0.0318 | 0.817 | 0.4201078 | 0.767364 |
| X214532_x_at | 0.0109 | 0.0134 | 0.817 | 0.4201106 | 0.767364 |
| X216524_x_at | 0.025 | 0.0305 | 0.817 | 0.4201261 | 0.767364 |
| X201275_at | -0.052 | 0.0637 | -0.817 | 0.4202213 | 0.767475 |
| X206454_s_at | 0.003 | 0.0037 | 0.817 | 0.4202807 | 0.767483 |
| X217583_at | -0.0079 | 0.0096 | -0.817 | 0.4203176 | 0.767483 |
| X207256_at | 0.0052 | 0.0064 | 0.817 | 0.4203484 | 0.767483 |
| X212190_at | -0.0458 | 0.056 | -0.817 | 0.4203638 | 0.767483 |
| X210420_at | 0.0074 | 0.009 | 0.816 | 0.4204777 | 0.767628 |
| X204613_at | -0.0358 | 0.0439 | -0.816 | 0.4205397 | 0.767676 |
| X220301_at | 0.0196 | 0.024 | 0.816 | 0.4205732 | 0.767676 |
| X215002_at | 0.013 | 0.016 | 0.816 | 0.4206111 | 0.767682 |
| X203417_at | -0.0038 | 0.0047 | -0.816 | 0.4206743 | 0.767734 |
| X206508_at | -0.0026 | 0.0032 | -0.816 | 0.420889 | 0.767964 |
| X220255_at | 0.0028 | 0.0034 | 0.816 | 0.4209142 | 0.767964 |
| X211878_at | -0.0052 | 0.0064 | -0.816 | 0.4209494 | 0.767964 |
| X204113_at | -0.0061 | 0.0075 | -0.816 | 0.420981 | 0.767964 |
| X219710_at | -0.0042 | 0.0052 | -0.816 | 0.4209818 | 0.767964 |
| X205286_at | 0.0049 | 0.006 | 0.815 | 0.4210165 | 0.767964 |
| X208492_at | -0.0042 | 0.0052 | -0.815 | 0.4211095 | 0.767964 |
| X207135_at | 0.1242 | 0.1524 | 0.815 | 0.4211263 | 0.767964 |
| X217850_at | -0.0293 | 0.036 | -0.815 | 0.4211552 | 0.767964 |
| X202812_at | -0.0099 | 0.0122 | -0.815 | 0.4211751 | 0.767964 |
| X213775_x_at | -0.0171 | 0.021 | -0.815 | 0.421197 | 0.767964 |
| X211649_x_at | -0.0022 | 0.0027 | -0.815 | 0.4212532 | 0.767964 |
| X217059_at | -0.006 | 0.0073 | -0.815 | 0.4212811 | 0.767964 |
| X208109_s_at | 0.072 | 0.0883 | 0.815 | 0.4212843 | 0.767964 |
| X211487_x_at | 0.0175 | 0.0215 | 0.815 | 0.4213314 | 0.767987 |
| X216841_s_at | 0.0463 | 0.0568 | 0.815 | 0.421382 | 0.768008 |
| X214636_at | -0.0066 | 0.0081 | -0.815 | 0.4214119 | 0.768008 |
| X214109_at | -0.0764 | 0.0937 | -0.815 | 0.4215328 | 0.76811 |
| X209559_at | 0.0035 | 0.0043 | 0.815 | 0.4215598 | 0.76811 |
| X201235_s_at | 0.0043 | 0.0053 | 0.814 | 0.4215814 | 0.76811 |
| X216827_at | 0.0047 | 0.0057 | 0.814 | 0.4216065 | 0.76811 |
| X213491_x_at | -0.0232 | 0.0285 | -0.814 | 0.4217119 | 0.768239 |
| X208988_at | 0.026 | 0.0319 | 0.814 | 0.4218284 | 0.768382 |
| X211928_at | 0.0303 | 0.0372 | 0.814 | 0.421877 | 0.768382 |
| X210320_s_at | -0.0073 | 0.009 | -0.814 | 0.4219784 | 0.768382 |
| X202639_s_at | -0.0044 | 0.0054 | -0.814 | 0.4219787 | 0.768382 |
| X204839_at | 0.0322 | 0.0396 | 0.814 | 0.4220155 | 0.768382 |
| X209020_at | -0.0436 | 0.0535 | -0.814 | 0.4220562 | 0.768382 |
| X212436_at | -0.0248 | 0.0304 | -0.814 | 0.4220963 | 0.768382 |
| X217051_s_at | 0.0037 | 0.0045 | 0.814 | 0.4221078 | 0.768382 |
| X215443_at | -0.0036 | 0.0044 | -0.814 | 0.4221244 | 0.768382 |
| X221591_s_at | 0.0035 | 0.0043 | 0.813 | 0.4221518 | 0.768382 |
| X218399_s_at | 0.0046 | 0.0056 | 0.813 | 0.422171 | 0.768382 |
| X201335_s_at | 0.0251 | 0.0308 | 0.813 | 0.422299 | 0.76854 |
| X215569_at | -0.004 | 0.005 | -0.813 | 0.4223607 | 0.76854 |
| X213702_x_at | -0.0283 | 0.0347 | -0.813 | 0.4223796 | 0.76854 |
| X207238_s_at | 0.0047 | 0.0058 | 0.813 | 0.4223959 | 0.76854 |
| X214901_at | -0.0092 | 0.0113 | -0.813 | 0.422468 | 0.768591 |
| X201741_x_at | -0.0092 | 0.0113 | -0.813 | 0.4224934 | 0.768591 |
| X211507_s_at | 0.0025 | 0.003 | 0.812 | 0.4227326 | 0.768927 |
| X211994_at | 0.055 | 0.0677 | 0.812 | 0.4227469 | 0.768927 |
| X210047_at | 0.0144 | 0.0177 | 0.812 | 0.4228373 | 0.769028 |
| X220099_s_at | 0.0209 | 0.0257 | 0.812 | 0.42297 | 0.769206 |
| X208874_x_at | 0.0236 | 0.0291 | 0.812 | 0.423218 | 0.769594 |
| X203503_s_at | 0.0033 | 0.0041 | 0.812 | 0.4232635 | 0.769614 |
| X206672_at | 0.0026 | 0.0033 | 0.811 | 0.4233471 | 0.76968 |
| X205543_at | -0.0637 | 0.0786 | -0.811 | 0.423375 | 0.76968 |
| X204573_at | 0.0386 | 0.0476 | 0.811 | 0.4234063 | 0.76968 |
| X206395_at | 0.0158 | 0.0194 | 0.811 | 0.42345 | 0.76968 |
| X207706_at | -0.0052 | 0.0064 | -0.811 | 0.4235099 | 0.76968 |
| X202178_at | -0.0369 | 0.0455 | -0.811 | 0.4235535 | 0.76968 |
| X217296_at | -0.0038 | 0.0047 | -0.811 | 0.4236061 | 0.76968 |
| X212126_at | -0.0321 | 0.0396 | -0.811 | 0.4236365 | 0.76968 |
| X210599_at | -0.0061 | 0.0075 | -0.811 | 0.4236723 | 0.76968 |
| X209015_s_at | 0.0523 | 0.0645 | 0.811 | 0.42368 | 0.76968 |
| X217921_at | 0.0194 | 0.0239 | 0.811 | 0.4237151 | 0.76968 |
| X212663_at | 0.0043 | 0.0053 | 0.811 | 0.4237213 | 0.76968 |
| X217920_at | 0.0191 | 0.0236 | 0.811 | 0.4237709 | 0.76968 |
| X201369_s_at | 0.0411 | 0.0507 | 0.811 | 0.4238243 | 0.76968 |
| X201614_s_at | -0.0271 | 0.0335 | -0.81 | 0.4238406 | 0.76968 |
| X206810_at | -0.0102 | 0.0125 | -0.81 | 0.4238543 | 0.76968 |
| X204651_at | 0.0329 | 0.0406 | 0.81 | 0.4239912 | 0.769762 |
| X205479_s_at | -0.0023 | 0.0029 | -0.81 | 0.4240339 | 0.769762 |
| X221917_s_at | 0.0048 | 0.006 | 0.81 | 0.4240652 | 0.769762 |
| X204246_s_at | -0.0197 | 0.0244 | -0.81 | 0.4240797 | 0.769762 |
| X216744_at | -0.0047 | 0.0058 | -0.81 | 0.424183 | 0.769762 |
| X216377_x_at | 0.0041 | 0.005 | 0.81 | 0.4241895 | 0.769762 |
| X217373_x_at | -0.0033 | 0.0041 | -0.81 | 0.4241974 | 0.769762 |
| X216040_x_at | 0.0036 | 0.0044 | 0.81 | 0.4242635 | 0.769762 |
| X202356_s_at | -0.0731 | 0.0903 | -0.81 | 0.4242644 | 0.769762 |
| X202879_s_at | -0.0044 | 0.0055 | -0.81 | 0.4242959 | 0.769762 |
| X204236_at | 0.0518 | 0.0639 | 0.81 | 0.4243082 | 0.769762 |
| X218479_s_at | 0.0031 | 0.0039 | 0.81 | 0.4243416 | 0.769762 |
| X221057_at | 0.0798 | 0.0985 | 0.81 | 0.4243497 | 0.769762 |
| X202673_at | 0.0235 | 0.029 | 0.81 | 0.4243844 | 0.769762 |
| X220068_at | 0.0022 | 0.0027 | 0.809 | 0.4244299 | 0.769782 |
| X215505_s_at | -0.0873 | 0.1078 | -0.809 | 0.4244977 | 0.769836 |
| X206622_at | 0.0029 | 0.0035 | 0.809 | 0.4245294 | 0.769836 |
| X219939_s_at | 0.0189 | 0.0234 | 0.809 | 0.4245803 | 0.769866 |
| X208957_at | -0.0237 | 0.0293 | -0.809 | 0.4247328 | 0.77008 |
| X212740_at | -0.035 | 0.0433 | -0.809 | 0.424856 | 0.77024 |
| X217599_s_at | -0.0125 | 0.0155 | -0.809 | 0.424925 | 0.770246 |
| X202123_s_at | 0.0332 | 0.0411 | 0.809 | 0.4249382 | 0.770246 |
| X209411_s_at | 0.0217 | 0.0268 | 0.808 | 0.4250046 | 0.770246 |
| X200596_s_at | -0.0378 | 0.0467 | -0.808 | 0.4250115 | 0.770246 |
| X206624_at | -0.0683 | 0.0844 | -0.808 | 0.4250917 | 0.770246 |
| X211530_x_at | -0.0181 | 0.0223 | -0.808 | 0.4250942 | 0.770246 |
| X217740_x_at | -0.0181 | 0.0224 | -0.808 | 0.4251019 | 0.770246 |
| X209143_s_at | 0.0206 | 0.0255 | 0.808 | 0.425172 | 0.77031 |
| X205025_at | -0.0032 | 0.004 | -0.808 | 0.4252494 | 0.770357 |
| X207270_x_at | 0.0019 | 0.0023 | 0.808 | 0.4253289 | 0.770357 |
| X209622_at | -0.0343 | 0.0425 | -0.808 | 0.4253694 | 0.770357 |
| X206861_s_at | 0.0363 | 0.045 | 0.808 | 0.4253921 | 0.770357 |
| X204316_at | 0.0031 | 0.0038 | 0.808 | 0.4253955 | 0.770357 |
| X219021_at | 0.0539 | 0.0667 | 0.808 | 0.4254059 | 0.770357 |
| X219570_at | -0.0236 | 0.0292 | -0.808 | 0.4254766 | 0.770422 |
| X220164_s_at | -0.0035 | 0.0043 | -0.807 | 0.4255799 | 0.770546 |
| X206763_at | -0.0034 | 0.0042 | -0.807 | 0.4256398 | 0.770592 |
| X200746_s_at | -0.0238 | 0.0295 | -0.807 | 0.4257114 | 0.770659 |
| X210124_x_at | -0.0155 | 0.0192 | -0.806 | 0.4261418 | 0.771375 |
| X221666_s_at | -0.0051 | 0.0063 | -0.806 | 0.4263649 | 0.771716 |
| X207879_at | -0.0081 | 0.0101 | -0.806 | 0.4265614 | 0.771922 |
| X214344_at | -0.0055 | 0.0068 | -0.806 | 0.4265761 | 0.771922 |
| X215395_x_at | 0.0053 | 0.0066 | 0.806 | 0.426583 | 0.771922 |
| X207254_at | -0.0057 | 0.0071 | -0.806 | 0.4266635 | 0.77195 |
| X217575_s_at | -0.005 | 0.0062 | -0.805 | 0.4267028 | 0.77195 |
| X219299_at | 0.0186 | 0.0231 | 0.805 | 0.426716 | 0.77195 |
| X205321_at | -0.0538 | 0.0668 | -0.805 | 0.4267373 | 0.77195 |
| X220015_at | 0.014 | 0.0174 | 0.805 | 0.4267743 | 0.771954 |
| X204665_at | 0.0042 | 0.0052 | 0.805 | 0.4268527 | 0.772002 |
| X201080_at | 0.03 | 0.0373 | 0.805 | 0.4268704 | 0.772002 |
| X215660_s_at | -0.0036 | 0.0045 | -0.805 | 0.4270635 | 0.772288 |
| X222117_s_at | 0.0036 | 0.0045 | 0.805 | 0.4271038 | 0.772298 |
| X206854_s_at | -0.0312 | 0.0388 | -0.805 | 0.4271864 | 0.772346 |
| X201920_at | -0.03 | 0.0373 | -0.805 | 0.4271999 | 0.772346 |
| X213212_x_at | 0.0426 | 0.053 | 0.804 | 0.4273697 | 0.772583 |
| X204024_at | 0.0412 | 0.0512 | 0.804 | 0.4274188 | 0.772583 |
| X216779_at | -0.0137 | 0.0171 | -0.804 | 0.4274351 | 0.772583 |
| X217326_x_at | -0.0024 | 0.003 | -0.804 | 0.4274776 | 0.772597 |
| X219566_at | 0.003 | 0.0037 | 0.804 | 0.4275379 | 0.772624 |
| X201339_s_at | -0.0263 | 0.0328 | -0.804 | 0.4275841 | 0.772624 |
| X213566_at | 0.0836 | 0.104 | 0.804 | 0.427597 | 0.772624 |
| X204231_s_at | 0.0072 | 0.0089 | 0.804 | 0.427658 | 0.772671 |
| X220658_s_at | 0.0032 | 0.004 | 0.803 | 0.4279944 | 0.773216 |
| X205390_s_at | 0.0035 | 0.0043 | 0.803 | 0.428078 | 0.773304 |
| X214498_at | 0.0022 | 0.0027 | 0.803 | 0.4281331 | 0.77331 |
| X205588_s_at | 0.0061 | 0.0076 | 0.803 | 0.4281509 | 0.77331 |
| X219465_at | 0.003 | 0.0037 | 0.803 | 0.4282107 | 0.773349 |
| X217653_x_at | -0.076 | 0.0947 | -0.803 | 0.4282706 | 0.773349 |
| X219719_at | 0.0764 | 0.0952 | 0.803 | 0.4282767 | 0.773349 |
| X200019_s_at | 0.0224 | 0.0279 | 0.802 | 0.4283748 | 0.773385 |
| X213497_at | -0.0044 | 0.0055 | -0.802 | 0.4283845 | 0.773385 |
| X209528_s_at | -0.0098 | 0.0122 | -0.802 | 0.4284011 | 0.773385 |
| X219032_x_at | 0.0622 | 0.0775 | 0.802 | 0.4287008 | 0.773863 |
| X206393_at | -0.0032 | 0.004 | -0.802 | 0.4289209 | 0.774162 |
| X211001_at | -0.0021 | 0.0026 | -0.802 | 0.4289361 | 0.774162 |
| X212789_at | -0.005 | 0.0062 | -0.801 | 0.4290269 | 0.77418 |
| X215590_x_at | 0.0073 | 0.0092 | 0.801 | 0.4290385 | 0.77418 |
| X206814_at | 0.0032 | 0.0039 | 0.801 | 0.4290507 | 0.77418 |
| X208266_at | 0.0028 | 0.0035 | 0.801 | 0.4291814 | 0.774321 |
| X221462_x_at | 0.0049 | 0.0061 | 0.801 | 0.4291984 | 0.774321 |
| X217210_at | -0.0042 | 0.0052 | -0.801 | 0.429359 | 0.774548 |
| X204223_at | 0.0106 | 0.0132 | 0.8 | 0.4295192 | 0.774659 |
| X202035_s_at | 0.0671 | 0.0838 | 0.8 | 0.4295413 | 0.774659 |
| X201664_at | -0.048 | 0.06 | -0.8 | 0.4295591 | 0.774659 |
| X220374_at | 0.0042 | 0.0052 | 0.8 | 0.4295604 | 0.774659 |
| X200054_at | 0.0153 | 0.0191 | 0.8 | 0.4296663 | 0.774682 |
| X200745_s_at | -0.0677 | 0.0846 | -0.8 | 0.4297222 | 0.774682 |
| X217677_at | -0.0095 | 0.0118 | -0.8 | 0.4297257 | 0.774682 |
| X205305_at | -0.0071 | 0.0089 | -0.8 | 0.4297266 | 0.774682 |
| X217024_x_at | 0.0027 | 0.0034 | 0.8 | 0.4297613 | 0.774682 |
| X208269_s_at | 0.0048 | 0.006 | 0.8 | 0.4298255 | 0.774682 |
| X213510_x_at | 0.0327 | 0.0409 | 0.8 | 0.4298427 | 0.774682 |
| X217742_s_at | 0.0191 | 0.0239 | 0.8 | 0.4298517 | 0.774682 |
| X201422_at | -0.0061 | 0.0076 | -0.8 | 0.429952 | 0.7748 |
| X208281_x_at | 0.0028 | 0.0035 | 0.8 | 0.4300469 | 0.774908 |
| X40850_at | -0.0045 | 0.0057 | -0.799 | 0.4301019 | 0.774944 |
| X217891_at | 0.0047 | 0.0058 | 0.799 | 0.4301469 | 0.774962 |
| X212024_x_at | 0.0119 | 0.0148 | 0.799 | 0.430194 | 0.774984 |
| X212853_at | 0.0082 | 0.0103 | 0.799 | 0.4303104 | 0.775083 |
| X209114_at | 0.0019 | 0.0024 | 0.799 | 0.4303294 | 0.775083 |
| X213845_at | 0.0548 | 0.0686 | 0.799 | 0.4304068 | 0.775083 |
| X200029_at | 0.0132 | 0.0165 | 0.799 | 0.4304788 | 0.775083 |
| X219776_s_at | -0.0018 | 0.0023 | -0.799 | 0.4304868 | 0.775083 |
| X212751_at | -0.0464 | 0.0581 | -0.799 | 0.4305229 | 0.775083 |
| X212647_at | -0.0086 | 0.0107 | -0.799 | 0.4305554 | 0.775083 |
| X212272_at | 0.0046 | 0.0058 | 0.799 | 0.4305634 | 0.775083 |
| X205960_at | 0.0105 | 0.0132 | 0.799 | 0.4306494 | 0.775083 |
| X216122_at | -0.0047 | 0.0059 | -0.798 | 0.4307316 | 0.775083 |
| X205064_at | 0.0036 | 0.0045 | 0.798 | 0.4307745 | 0.775083 |
| X217156_at | -0.0067 | 0.0084 | -0.798 | 0.4307866 | 0.775083 |
| X207327_at | 0.0059 | 0.0074 | 0.798 | 0.4308055 | 0.775083 |
| X208196_x_at | -0.0047 | 0.0059 | -0.798 | 0.4308236 | 0.775083 |
| X208590_x_at | -0.0033 | 0.0042 | -0.798 | 0.4308318 | 0.775083 |
| X202248_at | 0.0022 | 0.0028 | 0.798 | 0.4308434 | 0.775083 |
| X212579_at | -0.0323 | 0.0405 | -0.798 | 0.4309175 | 0.775083 |
| X207234_at | 0.0061 | 0.0077 | 0.798 | 0.4309436 | 0.775083 |
| X218197_s_at | 0.0528 | 0.0662 | 0.798 | 0.4309659 | 0.775083 |
| X221904_at | -0.038 | 0.0477 | -0.798 | 0.4309704 | 0.775083 |
| X213830_at | -0.004 | 0.005 | -0.798 | 0.4310163 | 0.775083 |
| X210176_at | 0.0048 | 0.006 | 0.798 | 0.4310615 | 0.775083 |
| X211303_x_at | 0.1059 | 0.1327 | 0.798 | 0.4310766 | 0.775083 |
| X214339_s_at | 0.0036 | 0.0046 | 0.798 | 0.4311873 | 0.775083 |
| X218794_s_at | 0.0385 | 0.0483 | 0.798 | 0.4311908 | 0.775083 |
| X209212_s_at | -0.0793 | 0.0994 | -0.798 | 0.4311923 | 0.775083 |
| X210829_s_at | -0.0402 | 0.0504 | -0.798 | 0.4312039 | 0.775083 |
| X216095_x_at | 0.0331 | 0.0416 | 0.798 | 0.4312257 | 0.775083 |
| X214624_at | 0.0022 | 0.0028 | 0.797 | 0.431411 | 0.775138 |
| X205771_s_at | 0.0416 | 0.0522 | 0.797 | 0.43146 | 0.775138 |
| X207575_at | 0.0051 | 0.0063 | 0.797 | 0.4314665 | 0.775138 |
| X215056_at | -0.047 | 0.059 | -0.797 | 0.4314702 | 0.775138 |
| X208688_x_at | -0.0244 | 0.0307 | -0.797 | 0.4314817 | 0.775138 |
| X221757_at | -0.0155 | 0.0195 | -0.797 | 0.4315147 | 0.775138 |
| X208617_s_at | 0.0405 | 0.0508 | 0.797 | 0.4315231 | 0.775138 |
| X213662_at | -0.0062 | 0.0078 | -0.797 | 0.4315524 | 0.775138 |
| X205812_s_at | 0.0157 | 0.0197 | 0.797 | 0.4316404 | 0.775138 |
| X219483_s_at | -0.0169 | 0.0212 | -0.797 | 0.431699 | 0.775138 |
| X202099_s_at | 0.0021 | 0.0026 | 0.797 | 0.4317048 | 0.775138 |
| X64942_at | 0.0201 | 0.0252 | 0.797 | 0.4317054 | 0.775138 |
| X207406_at | -0.0077 | 0.0097 | -0.797 | 0.4317098 | 0.775138 |
| X48612_at | -0.0356 | 0.0447 | -0.797 | 0.4317558 | 0.775158 |
| X220172_at | 0.0067 | 0.0084 | 0.796 | 0.4318172 | 0.775173 |
| X216795_at | -0.0065 | 0.0082 | -0.796 | 0.4318446 | 0.775173 |
| X221200_at | -0.0035 | 0.0044 | -0.796 | 0.4318691 | 0.775173 |
| X209542_x_at | -0.0029 | 0.0037 | -0.796 | 0.4319301 | 0.775219 |
| X210113_s_at | 0.0023 | 0.0029 | 0.796 | 0.4319647 | 0.775219 |
| X219252_s_at | -0.0278 | 0.035 | -0.796 | 0.4321261 | 0.775437 |
| X214304_x_at | -0.0039 | 0.0049 | -0.796 | 0.4321783 | 0.775437 |
| X210038_at | 0.0702 | 0.0882 | 0.796 | 0.4322229 | 0.775437 |
| X202255_s_at | 0.0737 | 0.0926 | 0.796 | 0.4322255 | 0.775437 |
| X212902_at | 0.0077 | 0.0097 | 0.796 | 0.4323103 | 0.775487 |
| X205332_at | -0.0025 | 0.0031 | -0.796 | 0.4323232 | 0.775487 |
| X206881_s_at | -0.0033 | 0.0041 | -0.795 | 0.4323865 | 0.775538 |
| X207430_s_at | -0.006 | 0.0075 | -0.795 | 0.4325434 | 0.775756 |
| X201744_s_at | 0.0233 | 0.0293 | 0.795 | 0.4328211 | 0.776137 |
| X214010_s_at | 0.0046 | 0.0058 | 0.795 | 0.4328256 | 0.776137 |
| X205956_x_at | -0.011 | 0.0139 | -0.794 | 0.4330485 | 0.776474 |
| X208794_s_at | -0.0176 | 0.0222 | -0.794 | 0.4331349 | 0.776567 |
| X216159_s_at | 0.0037 | 0.0047 | 0.794 | 0.4332599 | 0.776728 |
| X221392_at | -0.0035 | 0.0044 | -0.794 | 0.4333333 | 0.77676 |
| X52975_at | -0.034 | 0.0429 | -0.794 | 0.4333746 | 0.77676 |
| X218500_at | -0.0044 | 0.0055 | -0.794 | 0.4333825 | 0.77676 |
| X221206_at | -0.037 | 0.0466 | -0.794 | 0.4334646 | 0.776816 |
| X222249_at | 0.012 | 0.0151 | 0.794 | 0.4334902 | 0.776816 |
| X222328_x_at | -0.0084 | 0.0106 | -0.793 | 0.433527 | 0.776816 |
| X211277_x_at | 0.0373 | 0.047 | 0.793 | 0.4335665 | 0.776816 |
| X49679_s_at | 0.0245 | 0.0309 | 0.793 | 0.4335887 | 0.776816 |
| X212546_s_at | 0.0673 | 0.0848 | 0.793 | 0.4336313 | 0.77683 |
| X221385_s_at | 0.0016 | 0.0021 | 0.793 | 0.4337136 | 0.776862 |
| X221318_at | 0.0048 | 0.0061 | 0.793 | 0.4337194 | 0.776862 |
| X209753_s_at | 0.0034 | 0.0043 | 0.793 | 0.4337631 | 0.776878 |
| X204327_s_at | -0.0073 | 0.0092 | -0.793 | 0.4338187 | 0.776915 |
| X213585_s_at | 0.0035 | 0.0044 | 0.793 | 0.4338886 | 0.776926 |
| X202767_at | 0.0368 | 0.0465 | 0.793 | 0.433911 | 0.776926 |
| X216773_at | -0.0066 | 0.0083 | -0.793 | 0.43393 | 0.776926 |
| X209966_x_at | -0.0703 | 0.0887 | -0.793 | 0.4340014 | 0.776983 |
| X206681_x_at | 0.0027 | 0.0035 | 0.793 | 0.4340449 | 0.776983 |
| X204491_at | 0.0768 | 0.0968 | 0.793 | 0.4340666 | 0.776983 |
| X213095_x_at | -0.0488 | 0.0616 | -0.792 | 0.4342828 | 0.777307 |
| X218274_s_at | -0.004 | 0.005 | -0.792 | 0.4344241 | 0.777432 |
| X211716_x_at | -0.033 | 0.0417 | -0.792 | 0.434443 | 0.777432 |
| X210739_x_at | 0.0887 | 0.112 | 0.792 | 0.4344706 | 0.777432 |
| X213636_at | 0.0634 | 0.0801 | 0.792 | 0.4344925 | 0.777432 |
| X215699_x_at | -0.0023 | 0.0029 | -0.792 | 0.4345488 | 0.77747 |
| X214576_at | -0.002 | 0.0026 | -0.791 | 0.434725 | 0.777723 |
| X206282_at | 0.0053 | 0.0067 | 0.791 | 0.4348035 | 0.777773 |
| X205922_at | 0.0048 | 0.0061 | 0.791 | 0.434823 | 0.777773 |
| X207015_s_at | -0.0042 | 0.0054 | -0.791 | 0.4348619 | 0.77778 |
| X220740_s_at | -0.0032 | 0.0041 | -0.791 | 0.4349025 | 0.77779 |
| X207821_s_at | 0.0435 | 0.055 | 0.791 | 0.4349749 | 0.777842 |
| X207694_at | -0.0045 | 0.0056 | -0.791 | 0.4350172 | 0.777842 |
| X222004_s_at | -0.0014 | 0.0018 | -0.791 | 0.435037 | 0.777842 |
| X220347_at | 0.0031 | 0.0039 | 0.791 | 0.4350732 | 0.777844 |
| X220784_s_at | -0.0052 | 0.0066 | -0.79 | 0.4356091 | 0.77874 |
| X203941_at | 0.0082 | 0.0104 | 0.79 | 0.4357269 | 0.778856 |
| X209277_at | -0.0083 | 0.0105 | -0.79 | 0.4357444 | 0.778856 |
| X214756_x_at | -0.0306 | 0.0388 | -0.789 | 0.4358715 | 0.779021 |
| X221475_s_at | 0.0158 | 0.02 | 0.789 | 0.435933 | 0.779023 |
| X215182_x_at | 0.0567 | 0.0719 | 0.789 | 0.4359426 | 0.779023 |
| X202980_s_at | -0.0274 | 0.0348 | -0.789 | 0.4360821 | 0.779209 |
| X219899_x_at | -0.003 | 0.0038 | -0.789 | 0.4362927 | 0.779391 |
| X211866_x_at | -0.0021 | 0.0026 | -0.789 | 0.4363109 | 0.779391 |
| X204028_s_at | 0.0196 | 0.0249 | 0.789 | 0.4363132 | 0.779391 |
| X211118_x_at | -0.0019 | 0.0024 | -0.789 | 0.4363281 | 0.779391 |
| X213459_at | 0.0614 | 0.0779 | 0.788 | 0.4364088 | 0.779391 |
| X213679_at | -0.0269 | 0.0342 | -0.788 | 0.4364503 | 0.779391 |
| X213135_at | -0.0338 | 0.0428 | -0.788 | 0.4364687 | 0.779391 |
| X204193_at | 0.0334 | 0.0423 | 0.788 | 0.4365152 | 0.779391 |
| X215585_at | -0.0096 | 0.0122 | -0.788 | 0.4365684 | 0.779391 |
| X216987_at | -0.0058 | 0.0074 | -0.788 | 0.4365843 | 0.779391 |
| X208272_at | -0.0068 | 0.0087 | -0.788 | 0.4366348 | 0.779391 |
| X207167_at | -0.0035 | 0.0044 | -0.788 | 0.4366493 | 0.779391 |
| X202667_s_at | 0.007 | 0.0089 | 0.788 | 0.4366665 | 0.779391 |
| X211128_at | 0.0026 | 0.0034 | 0.788 | 0.4367304 | 0.779391 |
| X214089_at | 0.0027 | 0.0034 | 0.788 | 0.4367629 | 0.779391 |
| X214856_at | -0.0105 | 0.0133 | -0.788 | 0.4367796 | 0.779391 |
| X216963_s_at | -0.0497 | 0.063 | -0.788 | 0.4368245 | 0.779391 |
| X215781_s_at | 0.0023 | 0.0029 | 0.788 | 0.4368494 | 0.779391 |
| X200646_s_at | -0.0044 | 0.0056 | -0.788 | 0.4368505 | 0.779391 |
| X215439_x_at | 0.0291 | 0.0369 | 0.788 | 0.4369663 | 0.779453 |
| X202030_at | 0.0048 | 0.0061 | 0.787 | 0.4369749 | 0.779453 |
| X204628_s_at | 0.0048 | 0.0061 | 0.787 | 0.4370131 | 0.779453 |
| X215633_x_at | 0.0027 | 0.0035 | 0.787 | 0.4370376 | 0.779453 |
| X213360_s_at | -0.019 | 0.0242 | -0.787 | 0.4370712 | 0.779453 |
| X211173_at | 0.0037 | 0.0047 | 0.787 | 0.4371569 | 0.779453 |
| X211788_s_at | -0.0018 | 0.0023 | -0.787 | 0.4371936 | 0.779453 |
| X218515_at | -0.0398 | 0.0506 | -0.787 | 0.4372269 | 0.779453 |
| X34471_at | -0.0052 | 0.0066 | -0.787 | 0.4372414 | 0.779453 |
| X220739_s_at | -0.0732 | 0.0931 | -0.787 | 0.4372615 | 0.779453 |
| X209596_at | 0.0052 | 0.0066 | 0.787 | 0.4372712 | 0.779453 |
| X216489_at | 0.0019 | 0.0024 | 0.787 | 0.4374808 | 0.779736 |
| X214965_at | -0.0354 | 0.045 | -0.787 | 0.4375001 | 0.779736 |
| X221897_at | -0.0368 | 0.0468 | -0.786 | 0.4376559 | 0.779923 |
| X215925_s_at | -0.0041 | 0.0053 | -0.786 | 0.4376751 | 0.779923 |
| X204598_at | -0.004 | 0.0051 | -0.786 | 0.4377843 | 0.780055 |
| X211049_at | -0.0032 | 0.004 | -0.786 | 0.4379444 | 0.780277 |
| X211092_s_at | -0.0062 | 0.008 | -0.786 | 0.4379949 | 0.780288 |
| X207320_x_at | 0.0104 | 0.0133 | 0.786 | 0.4380209 | 0.780288 |
| X215597_x_at | 0.0307 | 0.0391 | 0.785 | 0.438134 | 0.780427 |
| X202333_s_at | -0.0349 | 0.0444 | -0.785 | 0.4383709 | 0.780745 |
| X203971_at | -0.0319 | 0.0407 | -0.785 | 0.4383826 | 0.780745 |
| X219516_at | -0.0041 | 0.0052 | -0.785 | 0.4384196 | 0.780748 |
| X219445_at | 0.0054 | 0.0069 | 0.785 | 0.4385572 | 0.780913 |
| X213461_at | -0.0201 | 0.0256 | -0.785 | 0.4385823 | 0.780913 |
| X206915_at | -0.1055 | 0.1345 | -0.784 | 0.4388388 | 0.781246 |
| X218060_s_at | 0.0049 | 0.0062 | 0.784 | 0.4388545 | 0.781246 |
| X211884_s_at | -0.003 | 0.0039 | -0.784 | 0.4388917 | 0.781246 |
| X206066_s_at | -0.0022 | 0.0028 | -0.784 | 0.4389261 | 0.781246 |
| X201882_x_at | -0.0032 | 0.004 | -0.784 | 0.4389454 | 0.781246 |
| X206450_at | 0.0019 | 0.0024 | 0.784 | 0.4389803 | 0.781246 |
| X200909_s_at | -0.0201 | 0.0256 | -0.784 | 0.4390292 | 0.78127 |
| X206324_s_at | -0.0046 | 0.0058 | -0.784 | 0.4392671 | 0.781568 |
| X203025_at | 0.0322 | 0.0411 | 0.784 | 0.4392756 | 0.781568 |
| X214425_at | 0.003 | 0.0038 | 0.783 | 0.4393021 | 0.781568 |
| X215274_at | 0.0031 | 0.004 | 0.783 | 0.4396157 | 0.782063 |
| X217600_at | 0.0529 | 0.0676 | 0.783 | 0.439747 | 0.782085 |
| X220339_s_at | 0.0029 | 0.0037 | 0.783 | 0.4397549 | 0.782085 |
| X204967_at | -0.0654 | 0.0836 | -0.783 | 0.4397569 | 0.782085 |
| X215459_at | 0.0043 | 0.0055 | 0.783 | 0.4398254 | 0.782085 |
| X204277_s_at | 0.0273 | 0.0349 | 0.783 | 0.4398272 | 0.782085 |
| X221684_s_at | 0.0039 | 0.005 | 0.783 | 0.4398392 | 0.782085 |
| X206521_s_at | -0.004 | 0.0052 | -0.782 | 0.4399619 | 0.782239 |
| X219972_s_at | 0.0412 | 0.0527 | 0.782 | 0.44012 | 0.782239 |
| X211882_x_at | 0.002 | 0.0025 | 0.782 | 0.4401303 | 0.782239 |
| X201399_s_at | 0.0445 | 0.057 | 0.782 | 0.4401919 | 0.782239 |
| X221701_s_at | -0.0019 | 0.0024 | -0.782 | 0.4402094 | 0.782239 |
| X216117_at | 0.0035 | 0.0045 | 0.782 | 0.4402268 | 0.782239 |
| X203782_s_at | 0.0276 | 0.0353 | 0.782 | 0.4402282 | 0.782239 |
| X221461_at | 0.0038 | 0.0048 | 0.782 | 0.4403138 | 0.782239 |
| X207853_s_at | 0.0234 | 0.03 | 0.782 | 0.4403157 | 0.782239 |
| X202256_at | -0.0315 | 0.0403 | -0.782 | 0.4403284 | 0.782239 |
| X222353_at | 0.0084 | 0.0107 | 0.782 | 0.4403733 | 0.782239 |
| X51228_at | 0.0552 | 0.0707 | 0.782 | 0.4403754 | 0.782239 |
| X204582_s_at | -0.0055 | 0.007 | -0.782 | 0.4403834 | 0.782239 |
| X222166_at | -0.0023 | 0.0029 | -0.781 | 0.4404452 | 0.782286 |
| X202755_s_at | -0.0028 | 0.0035 | -0.781 | 0.4407581 | 0.782779 |
| X206902_s_at | -0.0071 | 0.0092 | -0.781 | 0.4409547 | 0.783066 |
| X220436_at | -0.0881 | 0.113 | -0.78 | 0.4412306 | 0.783493 |
| X214195_at | -0.006 | 0.0077 | -0.78 | 0.4414011 | 0.783694 |
| X215011_at | -0.0151 | 0.0193 | -0.78 | 0.4414279 | 0.783694 |
| X205206_at | 0.0504 | 0.0646 | 0.78 | 0.4414577 | 0.783694 |
| X201573_s_at | 0.0219 | 0.028 | 0.78 | 0.4415137 | 0.783694 |
| X214032_at | -0.003 | 0.0039 | -0.78 | 0.4415811 | 0.783694 |
| X208073_x_at | 0.0211 | 0.0271 | 0.779 | 0.4416209 | 0.783694 |
| X214506_at | -0.0022 | 0.0028 | -0.779 | 0.4416588 | 0.783694 |
| X212613_at | 0.0161 | 0.0206 | 0.779 | 0.4417014 | 0.783694 |
| X219618_at | 0.0045 | 0.0057 | 0.779 | 0.441713 | 0.783694 |
| X217514_at | 0.0076 | 0.0098 | 0.779 | 0.4417238 | 0.783694 |
| X217410_at | 0.0016 | 0.002 | 0.779 | 0.4417318 | 0.783694 |
| X211116_at | -0.0028 | 0.0036 | -0.779 | 0.4417828 | 0.783722 |
| X203425_s_at | 0.0074 | 0.0095 | 0.779 | 0.4418706 | 0.783815 |
| X201631_s_at | 0.0673 | 0.0865 | 0.779 | 0.4419728 | 0.783887 |
| X205486_at | 0.0104 | 0.0133 | 0.779 | 0.4419921 | 0.783887 |
| X202263_at | -0.0165 | 0.0212 | -0.779 | 0.4420171 | 0.783887 |
| X210275_s_at | 0.0284 | 0.0365 | 0.779 | 0.4420868 | 0.783948 |
| X219885_at | 0.0049 | 0.0063 | 0.779 | 0.4421353 | 0.783971 |
| X205063_at | -0.0296 | 0.038 | -0.778 | 0.4421932 | 0.784012 |
| X216417_x_at | -0.0054 | 0.0069 | -0.778 | 0.4423561 | 0.784209 |
| X218124_at | 0.0353 | 0.0453 | 0.778 | 0.4423754 | 0.784209 |
| X206299_at | 0.0074 | 0.0095 | 0.778 | 0.442509 | 0.784341 |
| X207601_at | 0.005 | 0.0065 | 0.778 | 0.4425204 | 0.784341 |
| X221024_s_at | 0.0793 | 0.102 | 0.778 | 0.4427136 | 0.784621 |
| X34221_at | -0.0334 | 0.0429 | -0.777 | 0.4427607 | 0.784642 |
| X91580_at | -0.0052 | 0.0067 | -0.777 | 0.4428997 | 0.784696 |
| X217153_at | -0.0026 | 0.0033 | -0.777 | 0.4429065 | 0.784696 |
| X212254_s_at | -0.0194 | 0.025 | -0.777 | 0.4429539 | 0.784696 |
| X216360_x_at | -0.0024 | 0.0031 | -0.777 | 0.4429605 | 0.784696 |
| X222373_at | -0.0063 | 0.0082 | -0.777 | 0.4430174 | 0.784696 |
| X203805_s_at | -0.0025 | 0.0032 | -0.777 | 0.4430595 | 0.784696 |
| X210722_at | -0.0036 | 0.0047 | -0.777 | 0.4430621 | 0.784696 |
| X203652_at | 0.058 | 0.0746 | 0.777 | 0.4430744 | 0.784696 |
| X221963_x_at | -0.0489 | 0.0629 | -0.777 | 0.4431389 | 0.784696 |
| X205612_at | 0.0127 | 0.0163 | 0.777 | 0.4432004 | 0.784696 |
| X204953_at | -0.0337 | 0.0433 | -0.777 | 0.4432241 | 0.784696 |
| X206386_at | 0.0035 | 0.0045 | 0.777 | 0.4432451 | 0.784696 |
| X38149_at | 0.0022 | 0.0028 | 0.777 | 0.4433222 | 0.784696 |
| X210525_x_at | 0.0237 | 0.0305 | 0.776 | 0.4433432 | 0.784696 |
| X202421_at | -0.0574 | 0.0739 | -0.776 | 0.4433473 | 0.784696 |
| X220108_at | -0.0831 | 0.107 | -0.776 | 0.4433562 | 0.784696 |
| X213476_x_at | 0.0523 | 0.0674 | 0.776 | 0.4434478 | 0.784726 |
| X205082_s_at | 0.0027 | 0.0034 | 0.776 | 0.4434717 | 0.784726 |
| X205916_at | 0.0048 | 0.0062 | 0.776 | 0.4434796 | 0.784726 |
| X220082_at | 0.0033 | 0.0043 | 0.776 | 0.4437903 | 0.785214 |
| X205145_s_at | -0.0262 | 0.0337 | -0.776 | 0.4438633 | 0.78528 |
| X33494_at | 0.0593 | 0.0765 | 0.775 | 0.4439491 | 0.785311 |
| X202623_at | -0.0304 | 0.0391 | -0.775 | 0.4439511 | 0.785311 |
| X213507_s_at | 0.0217 | 0.028 | 0.775 | 0.4440125 | 0.785314 |
| X213863_s_at | -0.002 | 0.0026 | -0.775 | 0.4440238 | 0.785314 |
| X213622_at | -0.0568 | 0.0733 | -0.775 | 0.444059 | 0.785314 |
| X221142_s_at | -0.0056 | 0.0072 | -0.775 | 0.4442652 | 0.785605 |
| X214708_at | 0.0062 | 0.008 | 0.775 | 0.4442944 | 0.785605 |
| X220066_at | -0.0029 | 0.0037 | -0.775 | 0.4443865 | 0.785705 |
| X216611_s_at | 0.0053 | 0.0069 | 0.775 | 0.4444972 | 0.785723 |
| X206631_at | 0.0066 | 0.0086 | 0.774 | 0.4445413 | 0.785723 |
| X207253_s_at | -0.0087 | 0.0112 | -0.774 | 0.4445475 | 0.785723 |
| X203950_s_at | -0.0181 | 0.0234 | -0.774 | 0.4445686 | 0.785723 |
| X203556_at | -0.0316 | 0.0409 | -0.774 | 0.444608 | 0.785723 |
| X208730_x_at | -0.0288 | 0.0372 | -0.774 | 0.4446493 | 0.785723 |
| X222028_at | -0.0344 | 0.0444 | -0.774 | 0.4446719 | 0.785723 |
| X211979_at | -0.0185 | 0.0239 | -0.774 | 0.4447103 | 0.785723 |
| X211258_s_at | -0.0039 | 0.0051 | -0.774 | 0.4447474 | 0.785723 |
| X202815_s_at | 0.0373 | 0.0482 | 0.774 | 0.4447501 | 0.785723 |
| X214068_at | 0.0057 | 0.0074 | 0.774 | 0.4447937 | 0.785737 |
| X209005_at | 0.033 | 0.0426 | 0.774 | 0.4448642 | 0.785799 |
| X205686_s_at | 0.0032 | 0.0041 | 0.774 | 0.4450332 | 0.786035 |
| X204375_at | -0.0711 | 0.0919 | -0.773 | 0.4450959 | 0.786084 |
| X216042_at | -0.0028 | 0.0036 | -0.773 | 0.4451577 | 0.78613 |
| X218547_at | -0.0057 | 0.0073 | -0.773 | 0.4451999 | 0.786142 |
| X209563_x_at | 0.0565 | 0.0731 | 0.773 | 0.445302 | 0.78618 |
| X208468_at | 0.0017 | 0.0022 | 0.773 | 0.4453959 | 0.78618 |
| X206817_x_at | -0.003 | 0.0039 | -0.773 | 0.4454105 | 0.78618 |
| X213745_at | -0.0506 | 0.0654 | -0.773 | 0.4454394 | 0.78618 |
| X222276_at | -0.038 | 0.0492 | -0.773 | 0.4454716 | 0.78618 |
| X216627_s_at | -0.0048 | 0.0063 | -0.773 | 0.4454865 | 0.78618 |
| X216992_s_at | 0.0682 | 0.0882 | 0.773 | 0.4454949 | 0.78618 |
| X53202_at | -0.0243 | 0.0314 | -0.773 | 0.4455046 | 0.78618 |
| X201036_s_at | -0.0173 | 0.0224 | -0.773 | 0.445645 | 0.786343 |
| X213193_x_at | 0.0036 | 0.0047 | 0.773 | 0.4456675 | 0.786343 |
| X208156_x_at | 0.0033 | 0.0043 | 0.772 | 0.445724 | 0.78638 |
| X210709_at | -0.0059 | 0.0077 | -0.772 | 0.4459102 | 0.786646 |
| X216930_at | -0.0026 | 0.0034 | -0.772 | 0.4459753 | 0.786666 |
| X221296_at | -0.0024 | 0.0032 | -0.772 | 0.4460128 | 0.786666 |
| X202088_at | 0.0234 | 0.0303 | 0.772 | 0.4460432 | 0.786666 |
| X209127_s_at | -0.0223 | 0.0289 | -0.772 | 0.4460628 | 0.786666 |
| X218553_s_at | 0.0037 | 0.0048 | 0.772 | 0.4462427 | 0.78692 |
| X200051_at | 0.0361 | 0.0468 | 0.771 | 0.4463871 | 0.786925 |
| X215543_s_at | -0.0749 | 0.0971 | -0.771 | 0.4464174 | 0.786925 |
| X219798_s_at | -0.0287 | 0.0372 | -0.771 | 0.4464175 | 0.786925 |
| X218232_at | 0.0052 | 0.0067 | 0.771 | 0.4465245 | 0.786925 |
| X204498_s_at | 0.0029 | 0.0037 | 0.771 | 0.4465575 | 0.786925 |
| X208187_s_at | -0.0029 | 0.0037 | -0.771 | 0.4465737 | 0.786925 |
| X209103_s_at | 0.0165 | 0.0214 | 0.771 | 0.4465912 | 0.786925 |
| X220362_at | -0.0044 | 0.0057 | -0.771 | 0.4466001 | 0.786925 |
| X203254_s_at | -0.0044 | 0.0057 | -0.771 | 0.4466797 | 0.786925 |
| X210140_at | -0.0051 | 0.0066 | -0.771 | 0.4467186 | 0.786925 |
| X218317_x_at | 0.0029 | 0.0038 | 0.771 | 0.4467192 | 0.786925 |
| X210950_s_at | 0.0463 | 0.06 | 0.771 | 0.44672 | 0.786925 |
| X203506_s_at | 0.003 | 0.0039 | 0.771 | 0.446782 | 0.786925 |
| X200921_s_at | 0.0544 | 0.0705 | 0.771 | 0.4468259 | 0.786925 |
| X207378_at | -0.0022 | 0.0028 | -0.77 | 0.4468589 | 0.786925 |
| X204975_at | 0.0177 | 0.023 | 0.77 | 0.4469519 | 0.786925 |
| X211677_x_at | -0.0062 | 0.0081 | -0.77 | 0.4469543 | 0.786925 |
| X209934_s_at | 0.0314 | 0.0407 | 0.77 | 0.4470218 | 0.786925 |
| X210359_at | -0.003 | 0.0038 | -0.77 | 0.4470347 | 0.786925 |
| X202109_at | -0.0156 | 0.0203 | -0.77 | 0.4470648 | 0.786925 |
| X201844_s_at | 0.0295 | 0.0383 | 0.77 | 0.4470825 | 0.786925 |
| X213133_s_at | -0.0278 | 0.0361 | -0.77 | 0.4470927 | 0.786925 |
| X213206_at | -0.0271 | 0.0352 | -0.77 | 0.4471065 | 0.786925 |
| X215775_at | 0.0055 | 0.0072 | 0.77 | 0.4471213 | 0.786925 |
| X221124_s_at | -0.0032 | 0.0041 | -0.77 | 0.4471312 | 0.786925 |
| X212625_at | 0.0552 | 0.0717 | 0.77 | 0.4472853 | 0.787121 |
| X214724_at | -0.0488 | 0.0634 | -0.77 | 0.4473132 | 0.787121 |
| X213555_at | 0.0436 | 0.0566 | 0.77 | 0.447363 | 0.787146 |
| X210042_s_at | 0.0097 | 0.0126 | 0.77 | 0.4474004 | 0.78715 |
| X214580_x_at | -0.0035 | 0.0045 | -0.769 | 0.4475162 | 0.787264 |
| X204171_at | 0.0085 | 0.011 | 0.769 | 0.4475364 | 0.787264 |
| X221715_at | 0.0094 | 0.0123 | 0.769 | 0.4476314 | 0.787369 |
| X212878_s_at | -0.0636 | 0.0827 | -0.769 | 0.447704 | 0.787417 |
| X222076_at | 0.0031 | 0.0041 | 0.769 | 0.4477297 | 0.787417 |
| X210261_at | -0.0073 | 0.0095 | -0.769 | 0.4478141 | 0.787428 |
| X206151_x_at | -0.0023 | 0.003 | -0.769 | 0.4478331 | 0.787428 |
| X216453_at | -0.0051 | 0.0066 | -0.769 | 0.447842 | 0.787428 |
| X202962_at | 0.0498 | 0.0648 | 0.769 | 0.4479518 | 0.787511 |
| X203897_at | -0.0331 | 0.043 | -0.769 | 0.44796 | 0.787511 |
| X89476_r_at | -0.0151 | 0.0197 | -0.769 | 0.4479979 | 0.787515 |
| X220852_at | -0.0022 | 0.0029 | -0.768 | 0.4480696 | 0.787579 |
| X203346_s_at | 0.0404 | 0.0526 | 0.768 | 0.4482165 | 0.787737 |
| X219897_at | 0.0107 | 0.0139 | 0.768 | 0.4482548 | 0.787737 |
| X221530_s_at | -0.0347 | 0.0452 | -0.768 | 0.4482877 | 0.787737 |
| X213835_x_at | 0.0431 | 0.0561 | 0.768 | 0.4483292 | 0.787737 |
| X219376_at | -0.0403 | 0.0524 | -0.768 | 0.448337 | 0.787737 |
| X222242_s_at | -0.002 | 0.0025 | -0.768 | 0.4484268 | 0.787764 |
| X220006_at | -0.0022 | 0.0029 | -0.768 | 0.448451 | 0.787764 |
| X209936_at | -0.0582 | 0.0758 | -0.768 | 0.4484585 | 0.787764 |
| X202003_s_at | 0.0326 | 0.0425 | 0.768 | 0.4485417 | 0.787847 |
| X221611_s_at | -0.0061 | 0.0079 | -0.767 | 0.4486194 | 0.787887 |
| X220641_at | -0.0052 | 0.0068 | -0.767 | 0.4486643 | 0.787887 |
| X211044_at | -0.0035 | 0.0046 | -0.767 | 0.4488238 | 0.787887 |
| X221316_at | 0.002 | 0.0026 | 0.767 | 0.4488496 | 0.787887 |
| X206982_at | -0.0029 | 0.0038 | -0.767 | 0.4488786 | 0.787887 |
| X202934_at | 0.0341 | 0.0444 | 0.767 | 0.4489384 | 0.787887 |
| X205777_at | 0.0045 | 0.0059 | 0.767 | 0.4489514 | 0.787887 |
| X218748_s_at | -0.0771 | 0.1005 | -0.767 | 0.448963 | 0.787887 |
| X207293_s_at | 0.0028 | 0.0036 | 0.767 | 0.4490004 | 0.787887 |
| X214613_at | 0.0063 | 0.0083 | 0.767 | 0.449001 | 0.787887 |
| X221946_at | -0.0433 | 0.0564 | -0.767 | 0.4490023 | 0.787887 |
| X216154_at | -0.0043 | 0.0056 | -0.767 | 0.4490071 | 0.787887 |
| X211796_s_at | -0.0032 | 0.0042 | -0.767 | 0.4490604 | 0.787887 |
| X211601_at | 0.0068 | 0.0089 | 0.767 | 0.4490608 | 0.787887 |
| X213732_at | 0.0031 | 0.004 | 0.767 | 0.4491397 | 0.787963 |
| X201701_s_at | -0.0364 | 0.0475 | -0.766 | 0.4492112 | 0.788026 |
| X208376_at | 0.0029 | 0.0038 | 0.766 | 0.4493237 | 0.788107 |
| X215160_x_at | 0.0451 | 0.0589 | 0.766 | 0.4493633 | 0.788107 |
| X222363_at | 0.0029 | 0.0037 | 0.766 | 0.4493963 | 0.788107 |
| X206141_at | -0.004 | 0.0053 | -0.766 | 0.449405 | 0.788107 |
| X204339_s_at | -0.1476 | 0.1926 | -0.766 | 0.4494346 | 0.788107 |
| X206634_at | -0.0555 | 0.0724 | -0.766 | 0.4495337 | 0.788219 |
| X204440_at | 0.0395 | 0.0516 | 0.766 | 0.4496539 | 0.788367 |
| X220396_at | -0.0039 | 0.005 | -0.766 | 0.449699 | 0.788384 |
| X206819_at | 0.0041 | 0.0053 | 0.766 | 0.4497571 | 0.788386 |
| X210804_x_at | 0.0027 | 0.0035 | 0.765 | 0.4497712 | 0.788386 |
| X201848_s_at | 0.0353 | 0.0461 | 0.765 | 0.4498113 | 0.788394 |
| X208632_at | 0.0173 | 0.0226 | 0.765 | 0.4499226 | 0.788527 |
| X201981_at | -0.0049 | 0.0064 | -0.765 | 0.4499604 | 0.788531 |
| X214604_at | -0.0052 | 0.0068 | -0.765 | 0.4500094 | 0.788555 |
| X222070_at | -0.0096 | 0.0125 | -0.765 | 0.4501405 | 0.788665 |
| X218992_at | -0.0426 | 0.0558 | -0.765 | 0.4501518 | 0.788665 |
| X206420_at | -0.0322 | 0.0421 | -0.765 | 0.4501789 | 0.788665 |
| X208130_s_at | -0.003 | 0.0039 | -0.765 | 0.4502445 | 0.788689 |
| X218833_at | 0.0057 | 0.0074 | 0.765 | 0.4502704 | 0.788689 |
| X214590_s_at | -0.0455 | 0.0595 | -0.765 | 0.4502989 | 0.788689 |
| X218707_at | -0.0105 | 0.0137 | -0.764 | 0.4503709 | 0.788753 |
| X220213_at | -0.0028 | 0.0037 | -0.764 | 0.4504181 | 0.788763 |
| X211299_s_at | -0.0032 | 0.0042 | -0.764 | 0.4504475 | 0.788763 |
| X214830_at | 0.0382 | 0.05 | 0.764 | 0.4505177 | 0.78879 |
| X220950_s_at | -0.0019 | 0.0025 | -0.764 | 0.4505344 | 0.78879 |
| X205770_at | -0.0069 | 0.009 | -0.764 | 0.4508208 | 0.78923 |
| X215902_at | -0.0067 | 0.0087 | -0.763 | 0.450941 | 0.789378 |
| X200890_s_at | 0.0527 | 0.069 | 0.763 | 0.4510088 | 0.789434 |
| X207202_s_at | -0.0033 | 0.0043 | -0.763 | 0.4510808 | 0.789498 |
| X219707_at | -0.0077 | 0.0101 | -0.763 | 0.4511246 | 0.789506 |
| X205131_x_at | 0.0022 | 0.0029 | 0.763 | 0.4511566 | 0.789506 |
| X214307_at | 0.0053 | 0.0069 | 0.763 | 0.4512478 | 0.789537 |
| X44563_at | 0.0118 | 0.0155 | 0.763 | 0.4512728 | 0.789537 |
| X205596_s_at | -0.039 | 0.0512 | -0.763 | 0.4513085 | 0.789537 |
| X209756_s_at | -0.0031 | 0.004 | -0.763 | 0.4513161 | 0.789537 |
| X210551_s_at | -0.0021 | 0.0027 | -0.763 | 0.4513811 | 0.789564 |
| X204629_at | -0.0019 | 0.0024 | -0.763 | 0.4514029 | 0.789564 |
| X212289_at | -0.0237 | 0.0311 | -0.763 | 0.4514531 | 0.78959 |
| X200962_at | 0.117 | 0.1534 | 0.762 | 0.4515383 | 0.789641 |
| X210608_s_at | 0.0051 | 0.0067 | 0.762 | 0.4515565 | 0.789641 |
| X205537_s_at | -0.0035 | 0.0046 | -0.762 | 0.4516905 | 0.789641 |
| X221341_s_at | 0.0023 | 0.003 | 0.762 | 0.4516933 | 0.789641 |
| X209393_s_at | -0.0029 | 0.0037 | -0.762 | 0.451721 | 0.789641 |
| X208752_x_at | -0.025 | 0.0329 | -0.762 | 0.4517575 | 0.789641 |
| X216761_at | 0.0035 | 0.0046 | 0.762 | 0.4517802 | 0.789641 |
| X204345_at | 0.0348 | 0.0457 | 0.762 | 0.4517935 | 0.789641 |
| X210954_s_at | 0.0036 | 0.0048 | 0.762 | 0.4518023 | 0.789641 |
| X214408_s_at | -0.0119 | 0.0156 | -0.762 | 0.4518833 | 0.78972 |
| X215743_at | -0.0511 | 0.0671 | -0.762 | 0.4519423 | 0.789761 |
| X204714_s_at | 0.003 | 0.004 | 0.762 | 0.4520439 | 0.789877 |
| X216300_x_at | 0.0073 | 0.0096 | 0.761 | 0.452221 | 0.789953 |
| X202494_at | 0.0343 | 0.045 | 0.761 | 0.4522252 | 0.789953 |
| X206152_at | -0.0497 | 0.0653 | -0.761 | 0.4522313 | 0.789953 |
| X202642_s_at | 0.0252 | 0.0331 | 0.761 | 0.4522558 | 0.789953 |
| X218861_at | -0.0153 | 0.0201 | -0.761 | 0.4522932 | 0.789953 |
| X210024_s_at | -0.0315 | 0.0414 | -0.761 | 0.4523079 | 0.789953 |
| X213237_at | -0.0209 | 0.0275 | -0.761 | 0.4523363 | 0.789953 |
| X201456_s_at | 0.0405 | 0.0532 | 0.761 | 0.4523802 | 0.789963 |
| X213247_at | 0.0035 | 0.0046 | 0.761 | 0.4524391 | 0.789963 |
| X213757_at | 0.0537 | 0.0705 | 0.761 | 0.4524633 | 0.789963 |
| X214071_at | -0.0525 | 0.069 | -0.761 | 0.4525068 | 0.789963 |
| X207342_at | 0.0078 | 0.0103 | 0.761 | 0.4525197 | 0.789963 |
| X221209_s_at | 0.0034 | 0.0045 | 0.761 | 0.4526183 | 0.790073 |
| X211417_x_at | 0.0026 | 0.0035 | 0.761 | 0.4526974 | 0.790149 |
| X202203_s_at | 0.0344 | 0.0452 | 0.76 | 0.4527992 | 0.790234 |
| X217925_s_at | 0.052 | 0.0684 | 0.76 | 0.452843 | 0.790234 |
| X207644_at | 0.0038 | 0.005 | 0.76 | 0.4528736 | 0.790234 |
| X219720_s_at | 0.0032 | 0.0043 | 0.76 | 0.4529066 | 0.790234 |
| X211886_s_at | 0.0027 | 0.0036 | 0.76 | 0.4529299 | 0.790234 |
| X220846_s_at | 0.0021 | 0.0028 | 0.76 | 0.4529631 | 0.790234 |
| X216796_s_at | -0.0032 | 0.0041 | -0.76 | 0.4529953 | 0.790234 |
| X201742_x_at | -0.0777 | 0.1022 | -0.76 | 0.4530624 | 0.790275 |
| X58367_s_at | -0.0221 | 0.0291 | -0.76 | 0.45309 | 0.790275 |
| X218410_s_at | -0.0016 | 0.0022 | -0.76 | 0.4531882 | 0.790384 |
| X202070_s_at | -0.0314 | 0.0414 | -0.759 | 0.4533283 | 0.790481 |
| X206940_s_at | -0.0055 | 0.0073 | -0.759 | 0.4533322 | 0.790481 |
| X203447_at | -0.0476 | 0.0627 | -0.759 | 0.4533701 | 0.790481 |
| X216418_at | -0.0046 | 0.006 | -0.759 | 0.4534105 | 0.790481 |
| X204930_s_at | 0.0087 | 0.0114 | 0.759 | 0.4534216 | 0.790481 |
| X32137_at | -0.0362 | 0.0477 | -0.759 | 0.4535084 | 0.79056 |
| X216588_at | -0.0085 | 0.0112 | -0.759 | 0.4535704 | 0.79056 |
| X220512_at | 0.1022 | 0.1346 | 0.759 | 0.4535739 | 0.79056 |
| X216233_at | 0.0056 | 0.0074 | 0.759 | 0.4538189 | 0.790925 |
| X214437_s_at | 0.0098 | 0.0129 | 0.758 | 0.4538949 | 0.79096 |
| X212997_s_at | 0.0271 | 0.0358 | 0.758 | 0.45391 | 0.79096 |
| X214285_at | -0.1039 | 0.137 | -0.758 | 0.4539713 | 0.791005 |
| X219007_at | 0.0347 | 0.0457 | 0.758 | 0.4540183 | 0.791016 |
| X208291_s_at | -0.0023 | 0.0031 | -0.758 | 0.4540685 | 0.791016 |
| X214296_x_at | 0.0029 | 0.0038 | 0.758 | 0.4540845 | 0.791016 |
| X214694_at | -0.0165 | 0.0218 | -0.758 | 0.4541951 | 0.791079 |
| X214350_at | 0.0032 | 0.0042 | 0.758 | 0.45426 | 0.791079 |
| X206834_at | 0.0049 | 0.0065 | 0.758 | 0.4543121 | 0.791079 |
| X213557_at | 0.0181 | 0.0239 | 0.758 | 0.4543191 | 0.791079 |
| X211506_s_at | -0.0064 | 0.0085 | -0.758 | 0.4543408 | 0.791079 |
| X206996_x_at | -0.0031 | 0.0041 | -0.758 | 0.4543668 | 0.791079 |
| X211646_at | -0.0068 | 0.009 | -0.758 | 0.45437 | 0.791079 |
| X206187_at | 0.0029 | 0.0038 | 0.758 | 0.4544091 | 0.791085 |
| X209735_at | -0.1289 | 0.1702 | -0.757 | 0.4544745 | 0.791137 |
| X217277_at | -0.0033 | 0.0044 | -0.757 | 0.4546286 | 0.791343 |
| X204487_s_at | -0.0036 | 0.0048 | -0.757 | 0.4547059 | 0.791411 |
| X221844_x_at | -0.0196 | 0.0258 | -0.757 | 0.4547389 | 0.791411 |
| X211998_at | 0.0648 | 0.0856 | 0.757 | 0.4548394 | 0.791507 |
| X204966_at | 0.037 | 0.0489 | 0.757 | 0.4548674 | 0.791507 |
| X213509_x_at | 0.007 | 0.0092 | 0.757 | 0.454901 | 0.791507 |
| X213998_s_at | 0.0825 | 0.1091 | 0.756 | 0.4551116 | 0.791812 |
| X215681_at | -0.004 | 0.0053 | -0.756 | 0.4551782 | 0.791865 |
| X201774_s_at | 0.028 | 0.0371 | 0.756 | 0.4552708 | 0.791964 |
| X205522_at | 0.0028 | 0.0037 | 0.756 | 0.4553726 | 0.79208 |
| X210389_x_at | -0.0375 | 0.0496 | -0.756 | 0.4555341 | 0.792267 |
| X212441_at | 0.0219 | 0.029 | 0.756 | 0.455552 | 0.792267 |
| X205197_s_at | 0.0091 | 0.0121 | 0.756 | 0.4555951 | 0.79228 |
| X209773_s_at | -0.0023 | 0.003 | -0.755 | 0.4558451 | 0.792581 |
| X202435_s_at | 0.0499 | 0.0661 | 0.755 | 0.4558682 | 0.792581 |
| X202552_s_at | 0.0301 | 0.0399 | 0.755 | 0.4558749 | 0.792581 |
| X215770_at | -0.0052 | 0.0069 | -0.755 | 0.455957 | 0.792656 |
| X216598_s_at | 0.0088 | 0.0117 | 0.755 | 0.4559896 | 0.792656 |
| X211006_s_at | 0.0478 | 0.0633 | 0.755 | 0.4560266 | 0.792658 |
| X214572_s_at | -0.0024 | 0.0031 | -0.755 | 0.4561705 | 0.79277 |
| X214946_x_at | -0.0211 | 0.0279 | -0.755 | 0.4562204 | 0.79277 |
| X211368_s_at | 0.0058 | 0.0078 | 0.755 | 0.4562265 | 0.79277 |
| X208004_at | -0.0037 | 0.0049 | -0.755 | 0.4562333 | 0.79277 |
| X217235_x_at | 0.0021 | 0.0027 | 0.754 | 0.4562996 | 0.792823 |
| X219048_at | -0.0235 | 0.0311 | -0.754 | 0.4564262 | 0.792981 |
| X216812_at | 0.002 | 0.0027 | 0.754 | 0.4565014 | 0.793049 |
| X204785_x_at | 0.0054 | 0.0071 | 0.754 | 0.4565678 | 0.79309 |
| X217079_at | -0.0083 | 0.011 | -0.754 | 0.4566493 | 0.79309 |
| X219640_at | 0.0103 | 0.0136 | 0.754 | 0.4566527 | 0.79309 |
| X220553_s_at | 0.0359 | 0.0476 | 0.754 | 0.4566678 | 0.79309 |
| X207031_at | 0.0028 | 0.0037 | 0.754 | 0.4567272 | 0.793132 |
| X221371_at | 0.0029 | 0.0039 | 0.754 | 0.4567723 | 0.793148 |
| X214485_at | 0.0047 | 0.0062 | 0.753 | 0.4568596 | 0.79318 |
| X209456_s_at | 0.0042 | 0.0056 | 0.753 | 0.4568725 | 0.79318 |
| X206641_at | -0.0029 | 0.0039 | -0.753 | 0.4569457 | 0.79318 |
| X209257_s_at | -0.041 | 0.0544 | -0.753 | 0.4569479 | 0.79318 |
| X211233_x_at | 0.0033 | 0.0044 | 0.753 | 0.4569993 | 0.79318 |
| X205049_s_at | 0.0022 | 0.0029 | 0.753 | 0.4570049 | 0.79318 |
| X217946_s_at | -0.0237 | 0.0314 | -0.753 | 0.4570988 | 0.793183 |
| X217401_at | -0.0081 | 0.0108 | -0.753 | 0.4571185 | 0.793183 |
| X222324_at | 0.0048 | 0.0064 | 0.753 | 0.4571288 | 0.793183 |
| X204420_at | 0.0036 | 0.0048 | 0.753 | 0.4571495 | 0.793183 |
| X209791_at | 0.0706 | 0.0938 | 0.753 | 0.4572187 | 0.793241 |
| X221261_x_at | -0.0562 | 0.0746 | -0.753 | 0.4573354 | 0.793318 |
| X215085_x_at | 0.0037 | 0.0049 | 0.753 | 0.4573784 | 0.793318 |
| X213930_at | 0.0335 | 0.0445 | 0.753 | 0.4574095 | 0.793318 |
| X211079_s_at | 0.0039 | 0.0051 | 0.753 | 0.457419 | 0.793318 |
| X211877_s_at | -0.0045 | 0.006 | -0.752 | 0.4574419 | 0.793318 |
| X200913_at | 0.0177 | 0.0235 | 0.752 | 0.4576246 | 0.793508 |
| X212316_at | 0.0038 | 0.005 | 0.752 | 0.4576489 | 0.793508 |
| X218840_s_at | -0.0049 | 0.0065 | -0.752 | 0.4577527 | 0.793508 |
| X212275_s_at | -0.0048 | 0.0064 | -0.752 | 0.4577638 | 0.793508 |
| X218728_s_at | 0.0395 | 0.0525 | 0.752 | 0.4577659 | 0.793508 |
| X220652_at | 0.0066 | 0.0088 | 0.752 | 0.4577847 | 0.793508 |
| X211540_s_at | 0.0045 | 0.006 | 0.752 | 0.4578461 | 0.793508 |
| X217130_at | -0.0061 | 0.0081 | -0.752 | 0.4578564 | 0.793508 |
| X213312_at | -0.0388 | 0.0517 | -0.752 | 0.4579064 | 0.793508 |
| X205954_at | -0.0032 | 0.0043 | -0.752 | 0.457921 | 0.793508 |
| X205272_s_at | -0.0031 | 0.0042 | -0.752 | 0.4579889 | 0.793508 |
| X202014_at | 0.0026 | 0.0035 | 0.752 | 0.4579991 | 0.793508 |
| X209237_s_at | 0.0047 | 0.0062 | 0.752 | 0.4580155 | 0.793508 |
| X218361_at | 0.0306 | 0.0407 | 0.751 | 0.458168 | 0.79371 |
| X218430_s_at | 0.0599 | 0.0798 | 0.751 | 0.458276 | 0.793835 |
| X219256_s_at | 0.0019 | 0.0026 | 0.751 | 0.458345 | 0.793864 |
| X217589_at | -0.0055 | 0.0073 | -0.751 | 0.4583867 | 0.793864 |
| X221369_at | -0.0024 | 0.0033 | -0.751 | 0.4584272 | 0.793864 |
| X218560_s_at | 0.0015 | 0.002 | 0.751 | 0.4584572 | 0.793864 |
| X214228_x_at | 0.0028 | 0.0037 | 0.751 | 0.4584713 | 0.793864 |
| X216738_at | 0.0042 | 0.0056 | 0.751 | 0.4585664 | 0.793967 |
| X217744_s_at | 0.0293 | 0.0391 | 0.75 | 0.4586146 | 0.793988 |
| X222339_x_at | 0.0359 | 0.0478 | 0.75 | 0.4586518 | 0.793991 |
| X213728_at | -0.0023 | 0.0031 | -0.75 | 0.4588102 | 0.794203 |
| X210459_at | -0.0053 | 0.007 | -0.75 | 0.4589369 | 0.794303 |
| X214955_at | -0.0245 | 0.0327 | -0.75 | 0.4589396 | 0.794303 |
| X217073_x_at | 0.0021 | 0.0028 | 0.75 | 0.4589851 | 0.79432 |
| X205502_at | 0.0032 | 0.0043 | 0.75 | 0.459056 | 0.794377 |
| X210523_at | 0.0032 | 0.0043 | 0.75 | 0.4590893 | 0.794377 |
| X221660_at | -0.0264 | 0.0352 | -0.75 | 0.4591807 | 0.794473 |
| X201360_at | 0.0651 | 0.0869 | 0.749 | 0.4592221 | 0.794483 |
| X217319_x_at | 0.0038 | 0.0051 | 0.749 | 0.4593023 | 0.79456 |
| X208680_at | 0.0205 | 0.0274 | 0.749 | 0.4593609 | 0.79457 |
| X210983_s_at | 0.0711 | 0.0949 | 0.749 | 0.4594374 | 0.79457 |
| X220232_at | 0.1186 | 0.1583 | 0.749 | 0.4594766 | 0.79457 |
| X210186_s_at | -0.1796 | 0.2398 | -0.749 | 0.459493 | 0.79457 |
| X209063_x_at | 0.023 | 0.0307 | 0.749 | 0.4595345 | 0.79457 |
| X218118_s_at | -0.0209 | 0.028 | -0.749 | 0.4595848 | 0.79457 |
| X212969_x_at | 0.004 | 0.0053 | 0.749 | 0.4595872 | 0.79457 |
| X215510_at | -0.0033 | 0.0043 | -0.749 | 0.4595942 | 0.79457 |
| X217204_at | 0.0106 | 0.0141 | 0.749 | 0.4596782 | 0.794624 |
| X207233_s_at | 0.0672 | 0.0898 | 0.749 | 0.4596971 | 0.794624 |
| X216652_s_at | -0.0505 | 0.0675 | -0.749 | 0.4597511 | 0.794655 |
| X200963_x_at | -0.0129 | 0.0172 | -0.748 | 0.4598483 | 0.794728 |
| X204606_at | 0.0025 | 0.0034 | 0.748 | 0.4599076 | 0.794728 |
| X215845_x_at | 0.0495 | 0.0661 | 0.748 | 0.4599277 | 0.794728 |
| X216544_at | -0.0028 | 0.0038 | -0.748 | 0.4599442 | 0.794728 |
| X215207_x_at | -0.0109 | 0.0146 | -0.748 | 0.4599718 | 0.794728 |
| X211201_at | 0.0026 | 0.0035 | 0.748 | 0.4600436 | 0.79479 |
| X220956_s_at | -0.0276 | 0.0369 | -0.748 | 0.4601901 | 0.794942 |
| X219605_at | 0.0038 | 0.005 | 0.748 | 0.4602168 | 0.794942 |
| X214119_s_at | -0.1753 | 0.2344 | -0.748 | 0.4602849 | 0.794942 |
| X210484_s_at | 0.0042 | 0.0056 | 0.748 | 0.4603106 | 0.794942 |
| X212383_at | -0.0429 | 0.0573 | -0.748 | 0.4603106 | 0.794942 |
| X215183_at | -0.003 | 0.004 | -0.747 | 0.4604687 | 0.795153 |
| X214682_at | 0.0508 | 0.068 | 0.747 | 0.4605755 | 0.795239 |
| X208227_x_at | 0.003 | 0.004 | 0.747 | 0.4605898 | 0.795239 |
| X215401_at | -0.0033 | 0.0044 | -0.747 | 0.460634 | 0.795248 |
| X214646_at | 0.0023 | 0.0031 | 0.747 | 0.46069 | 0.795248 |
| X36742_at | 0.0019 | 0.0025 | 0.747 | 0.4607024 | 0.795248 |
| X202044_at | 0.0029 | 0.0039 | 0.747 | 0.4609196 | 0.79554 |
| X211814_s_at | 0.0048 | 0.0065 | 0.747 | 0.4609434 | 0.79554 |
| X220814_at | -0.0046 | 0.0061 | -0.746 | 0.4610223 | 0.795594 |
| X216480_x_at | 0.0052 | 0.007 | 0.746 | 0.4610465 | 0.795594 |
| X31874_at | 0.0424 | 0.0569 | 0.746 | 0.4611382 | 0.795649 |
| X216425_at | 0.003 | 0.004 | 0.746 | 0.46117 | 0.795649 |
| X215072_x_at | 0.0058 | 0.0078 | 0.746 | 0.461277 | 0.795649 |
| X211310_at | -0.0109 | 0.0146 | -0.746 | 0.4612908 | 0.795649 |
| X203871_at | -0.0502 | 0.0673 | -0.746 | 0.4613168 | 0.795649 |
| X200730_s_at | -0.1054 | 0.1413 | -0.746 | 0.4613226 | 0.795649 |
| X222101_s_at | 0.0357 | 0.0478 | 0.746 | 0.4613807 | 0.795649 |
| X220578_at | -0.0048 | 0.0064 | -0.746 | 0.4613993 | 0.795649 |
| X207818_s_at | -0.0049 | 0.0066 | -0.746 | 0.4614251 | 0.795649 |
| X221634_at | -0.0413 | 0.0554 | -0.746 | 0.461464 | 0.795649 |
| X214047_s_at | -0.029 | 0.0389 | -0.746 | 0.4614722 | 0.795649 |
| X212167_s_at | -0.0063 | 0.0084 | -0.746 | 0.4615394 | 0.795703 |
| X207721_x_at | -0.015 | 0.0201 | -0.745 | 0.4617761 | 0.795995 |
| X211113_s_at | -0.0323 | 0.0433 | -0.745 | 0.46178 | 0.795995 |
| X81737_at | -0.0188 | 0.0253 | -0.745 | 0.4618239 | 0.796008 |
| X211831_s_at | -0.0044 | 0.0059 | -0.745 | 0.461877 | 0.796038 |
| X218803_at | -0.0236 | 0.0316 | -0.745 | 0.4619729 | 0.7961 |
| X207209_at | 0.0056 | 0.0076 | 0.745 | 0.4619848 | 0.7961 |
| X214134_at | -0.0036 | 0.0048 | -0.745 | 0.4620873 | 0.796163 |
| X214828_s_at | -0.035 | 0.047 | -0.745 | 0.4620927 | 0.796163 |
| X217706_at | -0.0121 | 0.0163 | -0.745 | 0.4621688 | 0.796185 |
| X211870_s_at | 0.0019 | 0.0026 | 0.744 | 0.4621856 | 0.796185 |
| X218318_s_at | -0.0305 | 0.041 | -0.744 | 0.4622594 | 0.796185 |
| X213870_at | 0.003 | 0.0041 | 0.744 | 0.4622847 | 0.796185 |
| X221614_s_at | -0.0037 | 0.0049 | -0.744 | 0.4622848 | 0.796185 |
| X217576_x_at | -0.0048 | 0.0065 | -0.744 | 0.4624059 | 0.796302 |
| X220317_at | -0.0262 | 0.0353 | -0.744 | 0.4624246 | 0.796302 |
| X214301_s_at | -0.0033 | 0.0045 | -0.744 | 0.4625167 | 0.796362 |
| X212506_at | 0.031 | 0.0417 | 0.744 | 0.4625884 | 0.796362 |
| X208141_s_at | -0.0416 | 0.0559 | -0.744 | 0.4626002 | 0.796362 |
| X221174_at | -0.0049 | 0.0066 | -0.744 | 0.4626029 | 0.796362 |
| X216284_at | -0.0048 | 0.0064 | -0.744 | 0.4626791 | 0.796432 |
| X207366_at | 0.0158 | 0.0213 | 0.743 | 0.4628166 | 0.796607 |
| X220582_at | -0.0033 | 0.0044 | -0.743 | 0.462866 | 0.79663 |
| X211273_s_at | -0.0034 | 0.0045 | -0.743 | 0.4629739 | 0.796754 |
| X210058_at | 0.0033 | 0.0045 | 0.742 | 0.4633837 | 0.797398 |
| X207192_at | 0.0019 | 0.0025 | 0.742 | 0.4634552 | 0.797459 |
| X201963_at | 0.0532 | 0.0717 | 0.742 | 0.4634962 | 0.797468 |
| X218140_x_at | -0.03 | 0.0404 | -0.742 | 0.4636565 | 0.797682 |
| X204127_at | -0.0542 | 0.073 | -0.742 | 0.4637049 | 0.797703 |
| X209995_s_at | 0.0012 | 0.0016 | 0.742 | 0.4637582 | 0.797733 |
| X220179_at | -0.0023 | 0.003 | -0.742 | 0.4638335 | 0.797801 |
| X210242_x_at | 0.0053 | 0.0071 | 0.742 | 0.4639638 | 0.797963 |
| X204302_s_at | -0.0039 | 0.0052 | -0.741 | 0.4640689 | 0.798065 |
| X211103_at | -0.0041 | 0.0055 | -0.741 | 0.4640948 | 0.798065 |
| X215394_at | -0.0031 | 0.0042 | -0.741 | 0.4642498 | 0.79827 |
| X204725_s_at | -0.0242 | 0.0326 | -0.741 | 0.4642994 | 0.798293 |
| X210713_at | 0.0075 | 0.0101 | 0.741 | 0.4643417 | 0.798304 |
| X215944_at | -0.0061 | 0.0082 | -0.741 | 0.4644326 | 0.798399 |
| X209640_at | 0.0022 | 0.0029 | 0.741 | 0.4644899 | 0.798435 |
| X211843_x_at | 0.0031 | 0.0042 | 0.74 | 0.4645917 | 0.798548 |
| X208913_at | -0.0199 | 0.0269 | -0.74 | 0.4646459 | 0.79858 |
| X207588_at | -0.007 | 0.0094 | -0.74 | 0.4647652 | 0.798685 |
| X203109_at | -0.0413 | 0.0558 | -0.74 | 0.4647791 | 0.798685 |
| X217184_s_at | 0.0021 | 0.0028 | 0.74 | 0.4649227 | 0.798732 |
| X216180_s_at | -0.0029 | 0.004 | -0.74 | 0.4649599 | 0.798732 |
| X210476_s_at | -0.0022 | 0.003 | -0.74 | 0.4649688 | 0.798732 |
| X210579_s_at | -0.006 | 0.0081 | -0.74 | 0.4649833 | 0.798732 |
| X212313_at | -0.0193 | 0.026 | -0.74 | 0.4649862 | 0.798732 |
| X205397_x_at | 0.0028 | 0.0037 | 0.74 | 0.4650666 | 0.798735 |
| X217275_at | 0.0027 | 0.0036 | 0.74 | 0.4650926 | 0.798735 |
| X215558_at | -0.0077 | 0.0105 | -0.74 | 0.4651299 | 0.798735 |
| X219353_at | -0.0203 | 0.0274 | -0.74 | 0.4651314 | 0.798735 |
| X209650_s_at | -0.0023 | 0.0031 | -0.739 | 0.4652996 | 0.798937 |
| X222306_at | 0.0023 | 0.0031 | 0.739 | 0.4653701 | 0.798937 |
| X203089_s_at | -0.0474 | 0.0641 | -0.739 | 0.4653823 | 0.798937 |
| X214854_at | 0.0028 | 0.0038 | 0.739 | 0.4653933 | 0.798937 |
| X220237_at | -0.0183 | 0.0247 | -0.739 | 0.4655444 | 0.799027 |
| X205410_s_at | -0.0027 | 0.0037 | -0.739 | 0.4655488 | 0.799027 |
| X222223_s_at | -0.0092 | 0.0124 | -0.739 | 0.4655914 | 0.799027 |
| X218714_at | 0.0073 | 0.0099 | 0.739 | 0.4656294 | 0.799027 |
| X212651_at | 0.0588 | 0.0796 | 0.739 | 0.4656456 | 0.799027 |
| X210550_s_at | -0.0032 | 0.0043 | -0.739 | 0.4656613 | 0.799027 |
| X219651_at | -0.0066 | 0.009 | -0.739 | 0.4657647 | 0.799079 |
| X201546_at | -0.0195 | 0.0264 | -0.738 | 0.4657716 | 0.799079 |
| X201748_s_at | 0.0128 | 0.0174 | 0.738 | 0.4657996 | 0.799079 |
| X205868_s_at | 0.006 | 0.0081 | 0.738 | 0.4658569 | 0.799116 |
| X220656_at | -0.0079 | 0.0107 | -0.738 | 0.4659731 | 0.799253 |
| X211640_x_at | -0.0023 | 0.0031 | -0.738 | 0.4661332 | 0.799425 |
| X210969_at | 0.0094 | 0.0128 | 0.738 | 0.4661451 | 0.799425 |
| X208494_at | 0.0016 | 0.0022 | 0.738 | 0.4662003 | 0.799444 |
| X207966_s_at | 0.0299 | 0.0405 | 0.738 | 0.4662281 | 0.799444 |
| X203127_s_at | 0.0474 | 0.0642 | 0.737 | 0.4664433 | 0.799704 |
| X219967_at | -0.0041 | 0.0056 | -0.737 | 0.466452 | 0.799704 |
| X206765_at | -0.0729 | 0.0989 | -0.737 | 0.4665253 | 0.799768 |
| X205423_at | -0.0139 | 0.0188 | -0.737 | 0.4666191 | 0.79983 |
| X205393_s_at | -0.0067 | 0.0091 | -0.737 | 0.4666333 | 0.79983 |
| X220899_at | -0.0032 | 0.0043 | -0.737 | 0.4667502 | 0.799948 |
| X215907_at | 0.0129 | 0.0175 | 0.737 | 0.4667743 | 0.799948 |
| X207436_x_at | 0.0457 | 0.0621 | 0.737 | 0.4668182 | 0.799961 |
| X207040_s_at | -0.02 | 0.0272 | -0.736 | 0.4672346 | 0.800505 |
| X204022_at | -0.0198 | 0.0269 | -0.736 | 0.4672369 | 0.800505 |
| X220868_s_at | 0.0274 | 0.0373 | 0.736 | 0.4672434 | 0.800505 |
| X201933_at | 0.0189 | 0.0257 | 0.736 | 0.4673447 | 0.800617 |
| X205214_at | 0.0047 | 0.0064 | 0.736 | 0.4674813 | 0.800722 |
| X216979_at | 0.0037 | 0.005 | 0.736 | 0.4674942 | 0.800722 |
| X220272_at | 0.0031 | 0.0042 | 0.736 | 0.4675146 | 0.800722 |
| X216647_at | -0.0046 | 0.0062 | -0.735 | 0.4676917 | 0.800948 |
| X219013_at | -0.021 | 0.0285 | -0.735 | 0.4677461 | 0.800948 |
| X204729_s_at | -0.0171 | 0.0233 | -0.735 | 0.4677643 | 0.800948 |
| X216595_at | 0.0097 | 0.0132 | 0.735 | 0.4677905 | 0.800948 |
| X207383_s_at | 0.0028 | 0.0038 | 0.735 | 0.4678846 | 0.801047 |
| X201669_s_at | -0.0438 | 0.0595 | -0.735 | 0.4680127 | 0.801121 |
| X212799_at | -0.0189 | 0.0257 | -0.735 | 0.4680218 | 0.801121 |
| X201621_at | -0.0308 | 0.0419 | -0.735 | 0.4680359 | 0.801121 |
| X216982_x_at | -0.0074 | 0.0101 | -0.735 | 0.4680845 | 0.801143 |
| X206185_at | 0.0011 | 0.0015 | 0.734 | 0.4683003 | 0.801289 |
| X212048_s_at | 0.0302 | 0.0411 | 0.734 | 0.4683055 | 0.801289 |
| X208892_s_at | -0.0915 | 0.1246 | -0.734 | 0.4683272 | 0.801289 |
| X217395_at | 0.0028 | 0.0038 | 0.734 | 0.4683567 | 0.801289 |
| X200838_at | -0.0356 | 0.0485 | -0.734 | 0.4684005 | 0.801289 |
| X204306_s_at | -0.0175 | 0.0238 | -0.734 | 0.4684098 | 0.801289 |
| X211621_at | 0.0048 | 0.0065 | 0.734 | 0.4684317 | 0.801289 |
| X219304_s_at | -0.0562 | 0.0765 | -0.734 | 0.4684823 | 0.801289 |
| X212854_x_at | 0.0352 | 0.0479 | 0.734 | 0.4684948 | 0.801289 |
| X215881_x_at | 0.0015 | 0.002 | 0.734 | 0.468715 | 0.801588 |
| X220701_at | 0.0048 | 0.0065 | 0.734 | 0.4687416 | 0.801588 |
| X220485_s_at | -0.0026 | 0.0035 | -0.733 | 0.4688269 | 0.801643 |
| X215552_s_at | -0.0031 | 0.0043 | -0.733 | 0.4688458 | 0.801643 |
| X213586_at | 0.0022 | 0.003 | 0.733 | 0.4688839 | 0.801646 |
| X205222_at | 0.0417 | 0.0569 | 0.733 | 0.4689716 | 0.801697 |
| X204978_at | 0.0235 | 0.0321 | 0.733 | 0.4689856 | 0.801697 |
| X210070_s_at | -0.0146 | 0.0199 | -0.733 | 0.4690218 | 0.801697 |
| X211497_x_at | 0.0028 | 0.0039 | 0.733 | 0.469131 | 0.801791 |
| X201143_s_at | 0.0301 | 0.0411 | 0.733 | 0.4691491 | 0.801791 |
| X203714_s_at | 0.0347 | 0.0474 | 0.733 | 0.469236 | 0.801844 |
| X210825_s_at | 0.0146 | 0.02 | 0.733 | 0.4692521 | 0.801844 |
| X201357_s_at | 0.0033 | 0.0045 | 0.733 | 0.4693473 | 0.801893 |
| X218219_s_at | -0.0044 | 0.0059 | -0.733 | 0.469353 | 0.801893 |
| X207777_s_at | 0.0049 | 0.0066 | 0.732 | 0.4694295 | 0.801962 |
| X220304_s_at | -0.0091 | 0.0124 | -0.732 | 0.4695034 | 0.801962 |
| X217493_x_at | -0.0017 | 0.0023 | -0.732 | 0.4695147 | 0.801962 |
| X212078_s_at | 0.0673 | 0.092 | 0.732 | 0.469566 | 0.801962 |
| X215663_at | -0.0055 | 0.0075 | -0.732 | 0.4695911 | 0.801962 |
| X201779_s_at | 0.0441 | 0.0603 | 0.732 | 0.4696293 | 0.801962 |
| X221658_s_at | 0.0023 | 0.0032 | 0.732 | 0.4696705 | 0.801962 |
| X205431_s_at | 0.0029 | 0.0039 | 0.732 | 0.4696997 | 0.801962 |
| X206892_at | 0.0023 | 0.0032 | 0.732 | 0.4697185 | 0.801962 |
| X220443_s_at | 0.0032 | 0.0043 | 0.732 | 0.4698628 | 0.802147 |
| X216137_s_at | -0.0019 | 0.0026 | -0.732 | 0.4699079 | 0.802162 |
| X206453_s_at | 0.0303 | 0.0414 | 0.731 | 0.4700789 | 0.802363 |
| X210407_at | 0.0261 | 0.0357 | 0.731 | 0.470098 | 0.802363 |
| X201085_s_at | 0.0563 | 0.077 | 0.731 | 0.470147 | 0.802376 |
| X216076_at | 0.0046 | 0.0062 | 0.731 | 0.4701775 | 0.802376 |
| X211741_x_at | -0.0051 | 0.007 | -0.731 | 0.4702275 | 0.802399 |
| X210123_s_at | 0.0038 | 0.0052 | 0.731 | 0.4703108 | 0.802445 |
| X208346_at | -0.003 | 0.0041 | -0.731 | 0.4703266 | 0.802445 |
| X209941_at | 0.0023 | 0.0032 | 0.731 | 0.4704703 | 0.802581 |
| X203442_x_at | 0.0046 | 0.0063 | 0.731 | 0.4704785 | 0.802581 |
| X206317_s_at | -0.0012 | 0.0017 | -0.73 | 0.4707558 | 0.802992 |
| X216584_at | -0.0155 | 0.0213 | -0.73 | 0.470872 | 0.803121 |
| X35179_at | -0.0261 | 0.0357 | -0.73 | 0.4709096 | 0.803121 |
| X220434_at | -0.0037 | 0.0051 | -0.73 | 0.4709773 | 0.803121 |
| X211442_x_at | -0.0031 | 0.0043 | -0.73 | 0.4709859 | 0.803121 |
| X209608_s_at | 0.0279 | 0.0382 | 0.73 | 0.4710119 | 0.803121 |
| X208340_at | -0.0044 | 0.006 | -0.729 | 0.471199 | 0.803357 |
| X220156_at | 0.0074 | 0.0102 | 0.729 | 0.4712459 | 0.803357 |
| X218902_at | -0.0204 | 0.028 | -0.729 | 0.4712588 | 0.803357 |
| X220306_at | -0.0036 | 0.005 | -0.729 | 0.471549 | 0.80379 |
| X204485_s_at | 0.0539 | 0.074 | 0.729 | 0.4716533 | 0.803844 |
| X204611_s_at | 0.0072 | 0.0099 | 0.729 | 0.4716659 | 0.803844 |
| X219314_s_at | 0.0052 | 0.0071 | 0.729 | 0.4717256 | 0.803844 |
| X219828_at | -0.0048 | 0.0066 | -0.729 | 0.471732 | 0.803844 |
| X34063_at | -0.0025 | 0.0034 | -0.729 | 0.4717617 | 0.803844 |
| X204773_at | 0.022 | 0.0303 | 0.728 | 0.4718527 | 0.803902 |
| X218782_s_at | -0.0279 | 0.0383 | -0.728 | 0.4718925 | 0.803902 |
| X205888_s_at | 0.0255 | 0.035 | 0.728 | 0.471949 | 0.803902 |
| X217321_x_at | -0.0083 | 0.0114 | -0.728 | 0.4719643 | 0.803902 |
| X205988_at | 0.0027 | 0.0037 | 0.728 | 0.4719768 | 0.803902 |
| X218738_s_at | 0.0263 | 0.0362 | 0.728 | 0.4721828 | 0.804191 |
| X221670_s_at | 0.0032 | 0.0044 | 0.728 | 0.472336 | 0.804363 |
| X205828_at | 0.0054 | 0.0074 | 0.728 | 0.4723562 | 0.804363 |
| X209136_s_at | 0.0321 | 0.0441 | 0.727 | 0.4725156 | 0.804573 |
| X208780_x_at | 0.0196 | 0.0269 | 0.727 | 0.4726417 | 0.804595 |
| X210568_s_at | 0.0169 | 0.0232 | 0.727 | 0.4727 | 0.804595 |
| X206330_s_at | -0.0724 | 0.0997 | -0.727 | 0.4727038 | 0.804595 |
| X217279_x_at | 0.0034 | 0.0047 | 0.727 | 0.4727167 | 0.804595 |
| X202275_at | -0.0022 | 0.0031 | -0.727 | 0.4727722 | 0.804595 |
| X201852_x_at | 0.0044 | 0.0061 | 0.727 | 0.4727959 | 0.804595 |
| X216586_at | -0.005 | 0.0068 | -0.727 | 0.4728013 | 0.804595 |
| X216357_at | 0.0033 | 0.0045 | 0.727 | 0.4728727 | 0.804595 |
| X207211_at | 0.0026 | 0.0036 | 0.727 | 0.4728771 | 0.804595 |
| X211972_x_at | 0.0237 | 0.0326 | 0.727 | 0.4729165 | 0.804595 |
| X211504_x_at | -0.0169 | 0.0233 | -0.727 | 0.4729604 | 0.804595 |
| X202906_s_at | 0.0425 | 0.0585 | 0.726 | 0.4729953 | 0.804595 |
| X217010_s_at | 0.0044 | 0.0061 | 0.726 | 0.4729993 | 0.804595 |
| X202662_s_at | -0.0354 | 0.0488 | -0.726 | 0.4731356 | 0.804765 |
| X217435_x_at | -0.0019 | 0.0026 | -0.726 | 0.4732777 | 0.804881 |
| X218452_at | 0.0274 | 0.0378 | 0.726 | 0.4732981 | 0.804881 |
| X218555_at | 0.0028 | 0.0038 | 0.726 | 0.4733564 | 0.804881 |
| X203938_s_at | -0.0156 | 0.0215 | -0.726 | 0.473381 | 0.804881 |
| X206944_at | -0.0488 | 0.0673 | -0.726 | 0.4734421 | 0.804881 |
| X201829_at | 0.0499 | 0.0688 | 0.726 | 0.4734659 | 0.804881 |
| X213482_at | -0.0473 | 0.0652 | -0.726 | 0.4734871 | 0.804881 |
| X204095_s_at | -0.0033 | 0.0046 | -0.726 | 0.4734932 | 0.804881 |
| X203354_s_at | -0.0899 | 0.1239 | -0.726 | 0.4735544 | 0.804923 |
| X208853_s_at | -0.0368 | 0.0507 | -0.725 | 0.4736303 | 0.80499 |
| X216843_x_at | -0.0223 | 0.0307 | -0.725 | 0.4736672 | 0.804991 |
| X220013_at | 0.0021 | 0.0029 | 0.725 | 0.4738552 | 0.805239 |
| X217953_at | -0.0289 | 0.0399 | -0.725 | 0.4739403 | 0.805239 |
| X220635_at | 0.0032 | 0.0044 | 0.725 | 0.4739712 | 0.805239 |
| X208666_s_at | 0.0272 | 0.0376 | 0.725 | 0.4740097 | 0.805239 |
| X217937_s_at | -0.0069 | 0.0095 | -0.725 | 0.4740118 | 0.805239 |
| X217765_at | -0.0035 | 0.0049 | -0.725 | 0.4740661 | 0.805239 |
| X209166_s_at | -0.0046 | 0.0063 | -0.725 | 0.4740665 | 0.805239 |
| X207457_s_at | -0.0017 | 0.0023 | -0.724 | 0.4741957 | 0.805318 |
| X221022_s_at | 0.0031 | 0.0043 | 0.724 | 0.4742077 | 0.805318 |
| X214519_s_at | 0.0061 | 0.0085 | 0.724 | 0.4742221 | 0.805318 |
| X219330_at | -0.0057 | 0.0079 | -0.724 | 0.4743556 | 0.805393 |
| X209100_at | 0.0077 | 0.0107 | 0.724 | 0.4743758 | 0.805393 |
| X201075_s_at | 0.0571 | 0.0789 | 0.724 | 0.4743972 | 0.805393 |
| X209698_at | 0.0041 | 0.0056 | 0.724 | 0.4744328 | 0.805393 |
| X220497_at | 0.0095 | 0.0132 | 0.724 | 0.474447 | 0.805393 |
| X212984_at | 0.0306 | 0.0422 | 0.724 | 0.4745107 | 0.805439 |
| X213499_at | 0.0117 | 0.0161 | 0.724 | 0.4745733 | 0.805465 |
| X204950_at | -0.0433 | 0.0598 | -0.724 | 0.4745986 | 0.805465 |
| X218000_s_at | 0.006 | 0.0083 | 0.724 | 0.474721 | 0.805611 |
| X220475_at | 0.0016 | 0.0022 | 0.723 | 0.4749097 | 0.805721 |
| X219685_at | -0.092 | 0.1271 | -0.723 | 0.4749235 | 0.805721 |
| X209381_x_at | 0.0035 | 0.0049 | 0.723 | 0.4749268 | 0.805721 |
| X204811_s_at | -0.0497 | 0.0688 | -0.723 | 0.4749956 | 0.805721 |
| X214435_x_at | 0.0717 | 0.0991 | 0.723 | 0.475003 | 0.805721 |
| X217517_x_at | 0.0049 | 0.0068 | 0.723 | 0.475047 | 0.805721 |
| X205644_s_at | 0.0229 | 0.0317 | 0.723 | 0.4750689 | 0.805721 |
| X210644_s_at | 0.0025 | 0.0034 | 0.723 | 0.4751006 | 0.805721 |
| X205618_at | 0.0711 | 0.0984 | 0.723 | 0.4751118 | 0.805721 |
| X203815_at | -0.1424 | 0.1969 | -0.723 | 0.4751884 | 0.805789 |
| X208984_x_at | 0.0289 | 0.04 | 0.723 | 0.4752505 | 0.805827 |
| X215964_at | 0.0037 | 0.0052 | 0.723 | 0.4752834 | 0.805827 |
| X202585_s_at | -0.0046 | 0.0063 | -0.723 | 0.4753254 | 0.805835 |
| X222331_at | 0.002 | 0.0028 | 0.723 | 0.4753607 | 0.805835 |
| X215052_at | -0.0366 | 0.0507 | -0.722 | 0.4755499 | 0.806094 |
| X216046_at | -0.0033 | 0.0046 | -0.722 | 0.4756026 | 0.806122 |
| X37577_at | -0.0381 | 0.0527 | -0.722 | 0.4757232 | 0.806251 |
| X204735_at | 0.0267 | 0.037 | 0.722 | 0.4757515 | 0.806251 |
| X217944_at | -0.0164 | 0.0227 | -0.722 | 0.4758686 | 0.806344 |
| X217919_s_at | 0.0496 | 0.0687 | 0.722 | 0.4758785 | 0.806344 |
| X213569_at | -0.0229 | 0.0318 | -0.722 | 0.4759282 | 0.806366 |
| X203244_at | -0.0215 | 0.0297 | -0.721 | 0.4760166 | 0.806455 |
| X202683_s_at | 0.0173 | 0.0239 | 0.721 | 0.4762344 | 0.806731 |
| X211405_x_at | 0.0041 | 0.0057 | 0.721 | 0.4762869 | 0.806731 |
| X215455_at | 0.003 | 0.0042 | 0.721 | 0.4763191 | 0.806731 |
| X218142_s_at | -0.0225 | 0.0312 | -0.721 | 0.4763249 | 0.806731 |
| X220114_s_at | -0.0028 | 0.0038 | -0.721 | 0.4764142 | 0.806821 |
| X210940_s_at | -0.0029 | 0.004 | -0.721 | 0.4764563 | 0.806825 |
| X219180_s_at | -0.0339 | 0.047 | -0.721 | 0.4764892 | 0.806825 |
| X204609_at | 0.0034 | 0.0047 | 0.721 | 0.4765765 | 0.806911 |
| X215366_at | -0.0049 | 0.0068 | -0.72 | 0.4766279 | 0.806936 |
| X201884_at | -0.009 | 0.0126 | -0.72 | 0.4767 | 0.806956 |
| X204260_at | -0.0552 | 0.0766 | -0.72 | 0.476746 | 0.806956 |
| X204495_s_at | 0.005 | 0.0069 | 0.72 | 0.4767484 | 0.806956 |
| X206287_s_at | 0.0021 | 0.0029 | 0.72 | 0.4768802 | 0.807081 |
| X209169_at | 0.0264 | 0.0367 | 0.72 | 0.4768948 | 0.807081 |
| X217114_at | -0.0025 | 0.0034 | -0.72 | 0.476999 | 0.807124 |
| X207749_s_at | -0.0037 | 0.0051 | -0.72 | 0.4770285 | 0.807124 |
| X201328_at | 0.0692 | 0.0962 | 0.72 | 0.4770556 | 0.807124 |
| X214145_s_at | -0.0245 | 0.0341 | -0.72 | 0.4770657 | 0.807124 |
| X208845_at | 0.0142 | 0.0198 | 0.72 | 0.4771526 | 0.80721 |
| X212343_at | 0.0216 | 0.03 | 0.719 | 0.4774562 | 0.807513 |
| X203729_at | -0.0028 | 0.0039 | -0.719 | 0.4775239 | 0.807513 |
| X214118_x_at | 0.0187 | 0.0261 | 0.719 | 0.477534 | 0.807513 |
| X214674_at | -0.0041 | 0.0058 | -0.719 | 0.4775415 | 0.807513 |
| X222374_at | 0.0025 | 0.0035 | 0.719 | 0.4775459 | 0.807513 |
| X211802_x_at | 0.0024 | 0.0033 | 0.719 | 0.4775502 | 0.807513 |
| X221324_at | -0.0031 | 0.0043 | -0.719 | 0.4776409 | 0.807605 |
| X211821_x_at | 0.0046 | 0.0064 | 0.719 | 0.4776972 | 0.807639 |
| X211572_s_at | 0.002 | 0.0028 | 0.718 | 0.4779043 | 0.807919 |
| X215879_at | -0.0076 | 0.0106 | -0.718 | 0.4780031 | 0.807919 |
| X210347_s_at | -0.0433 | 0.0603 | -0.718 | 0.4780143 | 0.807919 |
| X219633_at | 0.0045 | 0.0063 | 0.718 | 0.4780608 | 0.807919 |
| X216622_at | -0.0042 | 0.0059 | -0.718 | 0.4780654 | 0.807919 |
| X211879_x_at | 0.0019 | 0.0026 | 0.718 | 0.4780966 | 0.807919 |
| X215602_at | -0.0045 | 0.0062 | -0.718 | 0.4782389 | 0.807919 |
| X213524_s_at | -0.0192 | 0.0267 | -0.718 | 0.4782452 | 0.807919 |
| X202106_at | 0.0367 | 0.0511 | 0.718 | 0.4782534 | 0.807919 |
| X36030_at | -0.0127 | 0.0177 | -0.718 | 0.4782569 | 0.807919 |
| X47105_at | -0.0056 | 0.0078 | -0.718 | 0.4782632 | 0.807919 |
| X217982_s_at | 0.0153 | 0.0213 | 0.718 | 0.4783044 | 0.807928 |
| X202731_at | -0.0424 | 0.0591 | -0.718 | 0.4783668 | 0.807971 |
| X220248_x_at | -0.0205 | 0.0286 | -0.717 | 0.4784459 | 0.808044 |
| X221920_s_at | 0.0569 | 0.0793 | 0.717 | 0.478492 | 0.80806 |
| X202129_s_at | 0.0413 | 0.0576 | 0.717 | 0.478615 | 0.808106 |
| X220363_s_at | -0.0253 | 0.0353 | -0.717 | 0.4786366 | 0.808106 |
| X205938_at | 0.0029 | 0.004 | 0.717 | 0.4786516 | 0.808106 |
| X207610_s_at | 0.0032 | 0.0044 | 0.717 | 0.4787137 | 0.808106 |
| X218599_at | -0.0036 | 0.005 | -0.717 | 0.4787249 | 0.808106 |
| X213171_s_at | 0.0032 | 0.0045 | 0.717 | 0.4787377 | 0.808106 |
| X207438_s_at | 0.0155 | 0.0216 | 0.717 | 0.4788175 | 0.808137 |
| X217020_at | 0.0033 | 0.0046 | 0.717 | 0.4788286 | 0.808137 |
| X209279_s_at | -0.003 | 0.0041 | -0.717 | 0.4789042 | 0.808203 |
| X215747_s_at | -0.0045 | 0.0063 | -0.717 | 0.478965 | 0.808244 |
| X205780_at | 0.0022 | 0.0031 | 0.716 | 0.4790464 | 0.808292 |
| X200656_s_at | 0.0403 | 0.0563 | 0.716 | 0.4790659 | 0.808292 |
| X217886_at | 0.0296 | 0.0413 | 0.716 | 0.4791392 | 0.808304 |
| X201606_s_at | 0.0235 | 0.0328 | 0.716 | 0.4791456 | 0.808304 |
| X203306_s_at | 0.0248 | 0.0347 | 0.716 | 0.4792172 | 0.808363 |
| X219904_at | -0.0339 | 0.0473 | -0.716 | 0.4793923 | 0.808457 |
| X221986_s_at | 0.0671 | 0.0938 | 0.716 | 0.4794053 | 0.808457 |
| X210035_s_at | -0.0061 | 0.0085 | -0.716 | 0.4794543 | 0.808457 |
| X211089_s_at | -0.024 | 0.0336 | -0.716 | 0.4794683 | 0.808457 |
| X203175_at | 0.0586 | 0.0819 | 0.716 | 0.4794909 | 0.808457 |
| X219676_at | 0.0083 | 0.0116 | 0.716 | 0.4794923 | 0.808457 |
| X203208_s_at | 0.0489 | 0.0683 | 0.716 | 0.479541 | 0.808457 |
| X201045_s_at | -0.0028 | 0.0039 | -0.716 | 0.4795641 | 0.808457 |
| X219724_s_at | -0.0131 | 0.0183 | -0.716 | 0.4796435 | 0.808529 |
| X62987_r_at | -0.0272 | 0.0381 | -0.715 | 0.47977 | 0.808681 |
| X216189_at | -0.0073 | 0.0102 | -0.715 | 0.4798237 | 0.80871 |
| X208919_s_at | 0.0193 | 0.027 | 0.715 | 0.4800185 | 0.808923 |
| X222262_s_at | 0.0087 | 0.0121 | 0.715 | 0.4800275 | 0.808923 |
| X35685_at | -0.0221 | 0.0309 | -0.715 | 0.4800592 | 0.808923 |
| X204162_at | -0.0066 | 0.0092 | -0.714 | 0.4803365 | 0.809275 |
| X205802_at | 0.033 | 0.0462 | 0.714 | 0.4803409 | 0.809275 |
| X219816_s_at | -0.0258 | 0.0362 | -0.714 | 0.4804079 | 0.809327 |
| X215883_at | 0.0027 | 0.0037 | 0.714 | 0.4805251 | 0.809463 |
| X222247_at | 0.0029 | 0.0041 | 0.714 | 0.4805675 | 0.809473 |
| X213335_s_at | 0.0074 | 0.0104 | 0.714 | 0.480728 | 0.809655 |
| X205050_s_at | -0.0303 | 0.0425 | -0.714 | 0.4807487 | 0.809655 |
| X216420_at | -0.0035 | 0.005 | -0.713 | 0.4809087 | 0.809863 |
| X200882_s_at | -0.0185 | 0.026 | -0.713 | 0.4810113 | 0.809975 |
| X218271_s_at | -0.0216 | 0.0303 | -0.713 | 0.4810803 | 0.810026 |
| X205360_at | 0.0008 | 0.0011 | 0.713 | 0.4811403 | 0.810026 |
| X207779_at | 0.0038 | 0.0053 | 0.713 | 0.4811812 | 0.810026 |
| X90610_at | -0.0082 | 0.0115 | -0.713 | 0.4811878 | 0.810026 |
| X203126_at | 0.0042 | 0.0058 | 0.713 | 0.4812704 | 0.810104 |
| X207979_s_at | 0.0034 | 0.0048 | 0.713 | 0.4813897 | 0.810243 |
| X209880_s_at | 0.0035 | 0.0049 | 0.713 | 0.4814272 | 0.810245 |
| X203212_s_at | -0.0492 | 0.0691 | -0.712 | 0.481515 | 0.810331 |
| X215621_s_at | -0.0033 | 0.0046 | -0.712 | 0.4816108 | 0.810392 |
| X203103_s_at | -0.0403 | 0.0566 | -0.712 | 0.4816241 | 0.810392 |
| X207476_at | -0.006 | 0.0085 | -0.712 | 0.4816936 | 0.810425 |
| X220958_at | 0.0029 | 0.0041 | 0.712 | 0.4817168 | 0.810425 |
| X208350_at | -0.0045 | 0.0063 | -0.712 | 0.4818323 | 0.810558 |
| X212733_at | -0.0273 | 0.0383 | -0.712 | 0.4819756 | 0.810612 |
| X220994_s_at | 0.1059 | 0.1489 | 0.712 | 0.4820023 | 0.810612 |
| X203211_s_at | -0.0329 | 0.0463 | -0.712 | 0.482037 | 0.810612 |
| X215231_at | 0.0934 | 0.1313 | 0.712 | 0.4820518 | 0.810612 |
| X203588_s_at | -0.0106 | 0.015 | -0.711 | 0.4821235 | 0.810612 |
| X200987_x_at | -0.0324 | 0.0456 | -0.711 | 0.4821273 | 0.810612 |
| X220188_at | 0.0057 | 0.0081 | 0.711 | 0.4821582 | 0.810612 |
| X214905_at | 0.0025 | 0.0035 | 0.711 | 0.482183 | 0.810612 |
| X216314_at | -0.0092 | 0.0129 | -0.711 | 0.4821926 | 0.810612 |
| X217247_at | -0.0079 | 0.0112 | -0.711 | 0.4823045 | 0.810739 |
| X221977_at | -0.0024 | 0.0034 | -0.711 | 0.4823968 | 0.810833 |
| X220200_s_at | -0.0051 | 0.0071 | -0.711 | 0.4825198 | 0.810958 |
| X207789_s_at | -0.0489 | 0.0688 | -0.711 | 0.4825873 | 0.810958 |
| X211648_at | -0.0021 | 0.003 | -0.711 | 0.4825972 | 0.810958 |
| X207734_at | -0.0085 | 0.012 | -0.711 | 0.4826172 | 0.810958 |
| X209526_s_at | 0.0329 | 0.0464 | 0.71 | 0.4827377 | 0.811075 |
| X205077_s_at | -0.0217 | 0.0305 | -0.71 | 0.4827924 | 0.811075 |
| X208460_at | -0.0019 | 0.0027 | -0.71 | 0.4828011 | 0.811075 |
| X207338_s_at | -0.022 | 0.031 | -0.71 | 0.4828332 | 0.811075 |
| X217236_x_at | -0.0018 | 0.0025 | -0.71 | 0.4829323 | 0.81118 |
| X217175_at | 0.0045 | 0.0064 | 0.71 | 0.4829717 | 0.811185 |
| X217218_at | -0.0032 | 0.0045 | -0.71 | 0.483032 | 0.811202 |
| X217434_at | -0.0038 | 0.0053 | -0.71 | 0.483055 | 0.811202 |
| X222348_at | 0.002 | 0.0028 | 0.71 | 0.4832067 | 0.811389 |
| X200936_at | 0.0197 | 0.0278 | 0.71 | 0.4832578 | 0.811389 |
| X205251_at | -0.0525 | 0.074 | -0.71 | 0.4832757 | 0.811389 |
| X203740_at | -0.0259 | 0.0365 | -0.709 | 0.4834189 | 0.811567 |
| X201860_s_at | 0.0917 | 0.1293 | 0.709 | 0.4834881 | 0.811567 |
| X214136_at | -0.0062 | 0.0088 | -0.709 | 0.4834911 | 0.811567 |
| X202488_s_at | -0.0048 | 0.0068 | -0.709 | 0.4835757 | 0.811647 |
| X210296_s_at | -0.0248 | 0.0349 | -0.709 | 0.4837121 | 0.811722 |
| X200069_at | -0.0142 | 0.0201 | -0.709 | 0.4837224 | 0.811722 |
| X214029_at | -0.0016 | 0.0023 | -0.709 | 0.4837299 | 0.811722 |
| X89948_at | -0.0185 | 0.0261 | -0.709 | 0.4837898 | 0.811761 |
| X220556_at | -0.0061 | 0.0086 | -0.708 | 0.483967 | 0.811997 |
| X217602_at | -0.0053 | 0.0075 | -0.708 | 0.4840137 | 0.812013 |
| X212873_at | 0.0031 | 0.0044 | 0.708 | 0.4840543 | 0.812013 |
| X207589_at | 0.0021 | 0.0029 | 0.708 | 0.4841097 | 0.812013 |
| X222347_at | -0.0179 | 0.0253 | -0.708 | 0.4841316 | 0.812013 |
| X210943_s_at | 0.1121 | 0.1583 | 0.708 | 0.484231 | 0.812013 |
| X218535_s_at | -0.032 | 0.0453 | -0.708 | 0.4842878 | 0.812013 |
| X204038_s_at | 0.0728 | 0.1028 | 0.708 | 0.4842998 | 0.812013 |
| X205806_at | -0.0027 | 0.0039 | -0.708 | 0.4843169 | 0.812013 |
| X201018_at | 0.0152 | 0.0214 | 0.708 | 0.4843402 | 0.812013 |
| X205636_at | -0.0033 | 0.0047 | -0.708 | 0.4843419 | 0.812013 |
| X211447_s_at | -0.002 | 0.0028 | -0.708 | 0.4843996 | 0.812048 |
| X214431_at | 0.0244 | 0.0345 | 0.708 | 0.4844858 | 0.812116 |
| X216147_at | 0.0303 | 0.0428 | 0.708 | 0.484513 | 0.812116 |
| X219329_s_at | 0.0263 | 0.0372 | 0.707 | 0.4846258 | 0.812244 |
| X201396_s_at | 0.0035 | 0.005 | 0.707 | 0.4847288 | 0.812355 |
| X206006_s_at | 0.021 | 0.0297 | 0.707 | 0.4849319 | 0.812472 |
| X207343_at | 0.0028 | 0.0039 | 0.707 | 0.4850261 | 0.812472 |
| X202287_s_at | -0.0051 | 0.0072 | -0.707 | 0.4850309 | 0.812472 |
| X210847_x_at | 0.0025 | 0.0035 | 0.707 | 0.4850562 | 0.812472 |
| X219578_s_at | 0.0531 | 0.0752 | 0.707 | 0.4850825 | 0.812472 |
| X216246_at | 0.0701 | 0.0992 | 0.707 | 0.4850885 | 0.812472 |
| X221556_at | -0.0354 | 0.0501 | -0.707 | 0.4851376 | 0.812472 |
| X213091_at | 0.0407 | 0.0576 | 0.707 | 0.4851464 | 0.812472 |
| X208289_s_at | -0.0222 | 0.0314 | -0.707 | 0.4851526 | 0.812472 |
| X210584_s_at | 0.0061 | 0.0087 | 0.706 | 0.4851645 | 0.812472 |
| X210938_at | 0.0032 | 0.0046 | 0.706 | 0.4852272 | 0.812473 |
| X211151_x_at | 0.0036 | 0.0052 | 0.706 | 0.4852378 | 0.812473 |
| X210195_s_at | 0.0057 | 0.008 | 0.706 | 0.4853583 | 0.812613 |
| X214570_x_at | -0.0026 | 0.0037 | -0.706 | 0.4855686 | 0.812904 |
| X210316_at | -0.0043 | 0.0061 | -0.706 | 0.4856945 | 0.812987 |
| X218586_at | -0.0162 | 0.023 | -0.706 | 0.4857441 | 0.812987 |
| X222135_at | 0.0032 | 0.0046 | 0.706 | 0.4857555 | 0.812987 |
| X218829_s_at | 0.0422 | 0.0598 | 0.706 | 0.4857645 | 0.812987 |
| X208823_s_at | 0.0083 | 0.0118 | 0.705 | 0.4858184 | 0.813016 |
| X213975_s_at | 0.0182 | 0.0258 | 0.705 | 0.4858637 | 0.81303 |
| X219991_at | 0.0035 | 0.005 | 0.705 | 0.486034 | 0.813114 |
| X221241_s_at | 0.0027 | 0.0039 | 0.705 | 0.4860535 | 0.813114 |
| X213919_at | -0.0034 | 0.0049 | -0.705 | 0.4860572 | 0.813114 |
| X208519_x_at | -0.0035 | 0.005 | -0.705 | 0.4860751 | 0.813114 |
| X213619_at | -0.0332 | 0.0471 | -0.705 | 0.486108 | 0.813114 |
| X209687_at | 0.0745 | 0.1056 | 0.705 | 0.4861333 | 0.813114 |
| X203268_s_at | 0.0042 | 0.006 | 0.705 | 0.4863703 | 0.813321 |
| X216560_x_at | -0.0018 | 0.0025 | -0.704 | 0.4863855 | 0.813321 |
| X205384_at | -0.0398 | 0.0565 | -0.704 | 0.4863955 | 0.813321 |
| X202638_s_at | 0.0029 | 0.0042 | 0.704 | 0.4864113 | 0.813321 |
| X201588_at | -0.0222 | 0.0315 | -0.704 | 0.4864404 | 0.813321 |
| X221543_s_at | 0.0304 | 0.0432 | 0.704 | 0.4865378 | 0.813339 |
| X213647_at | -0.0047 | 0.0066 | -0.704 | 0.4865408 | 0.813339 |
| X209262_s_at | 0.0171 | 0.0243 | 0.704 | 0.4865607 | 0.813339 |
| X221999_at | -0.005 | 0.0071 | -0.704 | 0.4866779 | 0.813443 |
| X221388_at | 0.0033 | 0.0047 | 0.704 | 0.486735 | 0.813443 |
| X211307_s_at | -0.0025 | 0.0035 | -0.704 | 0.4867814 | 0.813443 |
| X215126_at | -0.0623 | 0.0885 | -0.704 | 0.4867859 | 0.813443 |
| X205534_at | -0.0046 | 0.0066 | -0.704 | 0.4868334 | 0.813443 |
| X212580_at | 0.0056 | 0.008 | 0.704 | 0.4868427 | 0.813443 |
| X202086_at | -0.025 | 0.0355 | -0.704 | 0.4869328 | 0.813532 |
| X216251_s_at | -0.0217 | 0.0308 | -0.703 | 0.487013 | 0.813561 |
| X216288_at | -0.0067 | 0.0095 | -0.703 | 0.4870234 | 0.813561 |
| X201766_at | 0.0036 | 0.0051 | 0.703 | 0.4870737 | 0.813584 |
| X203749_s_at | -0.0033 | 0.0047 | -0.703 | 0.4872504 | 0.813818 |
| X206684_s_at | 0.0049 | 0.007 | 0.703 | 0.487297 | 0.813835 |
| X208046_at | -0.0027 | 0.0039 | -0.703 | 0.4873578 | 0.813875 |
| X221943_x_at | 0.0969 | 0.1379 | 0.703 | 0.487423 | 0.813876 |
| X204214_s_at | 0.0066 | 0.0094 | 0.703 | 0.4874319 | 0.813876 |
| X208280_at | -0.0039 | 0.0056 | -0.703 | 0.487535 | 0.813987 |
| X207709_at | 0.0211 | 0.0301 | 0.702 | 0.4876596 | 0.814134 |
| X219837_s_at | 0.0023 | 0.0033 | 0.702 | 0.4877165 | 0.814168 |
| X207672_at | 0.0123 | 0.0175 | 0.702 | 0.4877541 | 0.81417 |
| X202739_s_at | 0.0259 | 0.037 | 0.702 | 0.4878077 | 0.814198 |
| X202293_at | 0.0536 | 0.0763 | 0.702 | 0.4878705 | 0.814242 |
| X217193_x_at | -0.0027 | 0.0039 | -0.702 | 0.4879632 | 0.814335 |
| X214892_x_at | -0.0034 | 0.0048 | -0.702 | 0.4881032 | 0.814451 |
| X221686_s_at | -0.0041 | 0.0058 | -0.702 | 0.4881058 | 0.814451 |
| X219390_at | -0.0266 | 0.038 | -0.701 | 0.488286 | 0.81469 |
| X216188_at | 0.003 | 0.0042 | 0.701 | 0.4884176 | 0.814849 |
| X220580_at | 0.0038 | 0.0054 | 0.701 | 0.4884853 | 0.814855 |
| X202317_s_at | 0.0215 | 0.0307 | 0.701 | 0.4884947 | 0.814855 |
| X206736_x_at | -0.0027 | 0.0039 | -0.701 | 0.4887108 | 0.815154 |
| X204869_at | 0.0391 | 0.0558 | 0.701 | 0.4888169 | 0.815197 |
| X204822_at | 0.0055 | 0.0079 | 0.7 | 0.4888613 | 0.815197 |
| X222224_at | -0.006 | 0.0085 | -0.7 | 0.4888672 | 0.815197 |
| X205810_s_at | -0.0035 | 0.0049 | -0.7 | 0.4889262 | 0.815197 |
| X203301_s_at | 0.0198 | 0.0282 | 0.7 | 0.4889742 | 0.815197 |
| X215604_x_at | 0.0457 | 0.0653 | 0.7 | 0.4889789 | 0.815197 |
| X208435_s_at | -0.0025 | 0.0036 | -0.7 | 0.4890077 | 0.815197 |
| X215933_s_at | 0.0075 | 0.0107 | 0.7 | 0.4890777 | 0.815197 |
| X204132_s_at | 0.0531 | 0.0758 | 0.7 | 0.4891449 | 0.815197 |
| X215368_at | 0.0119 | 0.017 | 0.7 | 0.489156 | 0.815197 |
| X215284_at | 0.0046 | 0.0066 | 0.7 | 0.4891635 | 0.815197 |
| X213521_at | 0.0044 | 0.0063 | 0.7 | 0.4891766 | 0.815197 |
| X215095_at | 0.0096 | 0.0138 | 0.7 | 0.4892505 | 0.815228 |
| X215912_at | 0.0015 | 0.0022 | 0.7 | 0.4892687 | 0.815228 |
| X208363_s_at | -0.1046 | 0.1495 | -0.7 | 0.4893541 | 0.815309 |
| X208520_at | 0.0032 | 0.0045 | 0.7 | 0.4893955 | 0.815317 |
| X221454_at | -0.0016 | 0.0023 | -0.699 | 0.4894603 | 0.815363 |
| X215841_at | -0.0031 | 0.0044 | -0.699 | 0.4896398 | 0.815565 |
| X222163_s_at | -0.0253 | 0.0362 | -0.699 | 0.4896547 | 0.815565 |
| X213280_at | 0.0403 | 0.0577 | 0.699 | 0.4897074 | 0.815581 |
| X200038_s_at | 0.0123 | 0.0176 | 0.699 | 0.489738 | 0.815581 |
| X207865_s_at | -0.0021 | 0.003 | -0.699 | 0.4898315 | 0.815676 |
| X201675_at | -0.0288 | 0.0413 | -0.699 | 0.48993 | 0.815757 |
| X215535_s_at | 0.0267 | 0.0382 | 0.699 | 0.4899667 | 0.815757 |
| X208471_at | -0.0691 | 0.0988 | -0.699 | 0.4899903 | 0.815757 |
| X214367_at | 0.0036 | 0.0052 | 0.699 | 0.4900402 | 0.815779 |
| X203986_at | 0.0026 | 0.0037 | 0.698 | 0.490077 | 0.815779 |
| X216965_x_at | 0.005 | 0.0071 | 0.698 | 0.4901505 | 0.81584 |
| X219375_at | -0.0275 | 0.0394 | -0.698 | 0.4901943 | 0.815852 |
| X206987_x_at | 0.0024 | 0.0034 | 0.698 | 0.4902911 | 0.815952 |
| X221835_at | -0.0012 | 0.0018 | -0.698 | 0.4904393 | 0.816137 |
| X200994_at | 0.0166 | 0.0239 | 0.698 | 0.4905393 | 0.816181 |
| X206000_at | 0.0035 | 0.0051 | 0.698 | 0.4905636 | 0.816181 |
| X214079_at | 0.0044 | 0.0064 | 0.698 | 0.4905755 | 0.816181 |
| X214135_at | 0.004 | 0.0057 | 0.698 | 0.4906156 | 0.816186 |
| X216833_x_at | 0.0025 | 0.0036 | 0.697 | 0.490726 | 0.816309 |
| X214826_at | -0.0147 | 0.0211 | -0.697 | 0.4908753 | 0.816465 |
| X217322_x_at | -0.0114 | 0.0163 | -0.697 | 0.4908935 | 0.816465 |
| X210382_at | -0.0023 | 0.0033 | -0.697 | 0.4909859 | 0.816558 |
| X214521_at | -0.0044 | 0.0063 | -0.697 | 0.4910732 | 0.816642 |
| X212253_x_at | -0.0329 | 0.0472 | -0.697 | 0.4911691 | 0.816672 |
| X200832_s_at | 0.0556 | 0.0798 | 0.697 | 0.4911742 | 0.816672 |
| X207834_at | 0.003 | 0.0043 | 0.697 | 0.4912018 | 0.816672 |
| X210745_at | -0.0026 | 0.0037 | -0.696 | 0.4913583 | 0.816766 |
| X213547_at | -0.0183 | 0.0263 | -0.696 | 0.4913895 | 0.816766 |
| X211460_at | -0.0061 | 0.0087 | -0.696 | 0.4914056 | 0.816766 |
| X221280_s_at | -0.0103 | 0.0148 | -0.696 | 0.4914165 | 0.816766 |
| X219788_at | -0.0047 | 0.0067 | -0.696 | 0.4914429 | 0.816766 |
| X206653_at | -0.0062 | 0.0089 | -0.696 | 0.4914802 | 0.816766 |
| X218293_x_at | -0.0014 | 0.002 | -0.696 | 0.4915158 | 0.816766 |
| X40687_at | 0.0042 | 0.006 | 0.696 | 0.4915761 | 0.816805 |
| X207424_at | -0.0061 | 0.0087 | -0.696 | 0.491683 | 0.816922 |
| X211803_at | 0.0027 | 0.0039 | 0.696 | 0.4917651 | 0.816997 |
| X216705_s_at | -0.0017 | 0.0024 | -0.696 | 0.4918734 | 0.817116 |
| X205487_s_at | -0.0022 | 0.0032 | -0.695 | 0.4919703 | 0.817186 |
| X218524_at | -0.0299 | 0.0431 | -0.695 | 0.4919893 | 0.817186 |
| X216597_at | -0.0013 | 0.0019 | -0.695 | 0.4920615 | 0.817245 |
| X206104_at | -0.0577 | 0.0831 | -0.695 | 0.4921552 | 0.817296 |
| X214403_x_at | -0.0091 | 0.0131 | -0.695 | 0.4921654 | 0.817296 |
| X215664_s_at | 0.0027 | 0.0038 | 0.695 | 0.4922387 | 0.817315 |
| X208295_x_at | 0.0019 | 0.0027 | 0.695 | 0.4923095 | 0.817315 |
| X221797_at | -0.0045 | 0.0064 | -0.695 | 0.4923108 | 0.817315 |
| X201874_at | 0.0341 | 0.0491 | 0.695 | 0.4923243 | 0.817315 |
| X219327_s_at | 0.0083 | 0.0119 | 0.695 | 0.4923977 | 0.817315 |
| X221662_s_at | -0.002 | 0.0028 | -0.695 | 0.492398 | 0.817315 |
| X216123_x_at | -0.012 | 0.0173 | -0.695 | 0.4924676 | 0.81732 |
| X222052_at | 0.019 | 0.0274 | 0.695 | 0.4924742 | 0.81732 |
| X213914_s_at | 0.0039 | 0.0056 | 0.695 | 0.4925258 | 0.817344 |
| X213421_x_at | -0.0931 | 0.134 | -0.694 | 0.4925684 | 0.817354 |
| X209660_at | 0.0074 | 0.0106 | 0.694 | 0.4927165 | 0.817529 |
| X213657_s_at | 0.0261 | 0.0375 | 0.694 | 0.4927531 | 0.817529 |
| X214396_s_at | 0.0017 | 0.0024 | 0.694 | 0.4927842 | 0.817529 |
| X220037_s_at | 0.0057 | 0.0082 | 0.694 | 0.4928479 | 0.817549 |
| X208174_x_at | 0.0329 | 0.0475 | 0.694 | 0.4928826 | 0.817549 |
| X208300_at | 0.0048 | 0.0069 | 0.694 | 0.4929069 | 0.817549 |
| X205523_at | -0.0521 | 0.0751 | -0.694 | 0.49299 | 0.817626 |
| X215386_at | -0.0027 | 0.004 | -0.694 | 0.4930321 | 0.817635 |
| X217169_at | 0.0032 | 0.0046 | 0.694 | 0.4931173 | 0.817692 |
| X203435_s_at | -0.1239 | 0.1786 | -0.694 | 0.4931404 | 0.817692 |
| X206067_s_at | 0.0636 | 0.0917 | 0.693 | 0.4931935 | 0.817692 |
| X216342_x_at | 0.0244 | 0.0352 | 0.693 | 0.4932716 | 0.817692 |
| X213210_at | -0.0033 | 0.0047 | -0.693 | 0.4932759 | 0.817692 |
| X212708_at | -0.0257 | 0.0371 | -0.693 | 0.4933116 | 0.817692 |
| X204851_s_at | -0.0542 | 0.0782 | -0.693 | 0.4933847 | 0.817692 |
| X217009_at | -0.0033 | 0.0047 | -0.693 | 0.4934434 | 0.817692 |
| X220215_at | -0.0267 | 0.0385 | -0.693 | 0.4934597 | 0.817692 |
| X220570_at | -0.0036 | 0.0051 | -0.693 | 0.493464 | 0.817692 |
| X217149_x_at | -0.0019 | 0.0027 | -0.693 | 0.4934822 | 0.817692 |
| X214031_s_at | 0.0049 | 0.0071 | 0.693 | 0.4935083 | 0.817692 |
| X214558_at | -0.0041 | 0.0059 | -0.693 | 0.4935833 | 0.817695 |
| X202473_x_at | 0.0044 | 0.0063 | 0.693 | 0.4935999 | 0.817695 |
| X218581_at | 0.0049 | 0.007 | 0.693 | 0.4936205 | 0.817695 |
| X204696_s_at | -0.0024 | 0.0035 | -0.693 | 0.4937696 | 0.817723 |
| X204747_at | 0.044 | 0.0635 | 0.692 | 0.4937822 | 0.817723 |
| X211019_s_at | -0.0023 | 0.0033 | -0.692 | 0.4937914 | 0.817723 |
| X210798_x_at | 0.002 | 0.0029 | 0.692 | 0.4937928 | 0.817723 |
| X203730_s_at | 0.0348 | 0.0502 | 0.692 | 0.4938219 | 0.817723 |
| X202484_s_at | 0.0323 | 0.0466 | 0.692 | 0.4938829 | 0.817764 |
| X203784_s_at | -0.003 | 0.0043 | -0.692 | 0.4942216 | 0.818095 |
| X213156_at | 0.0334 | 0.0483 | 0.692 | 0.4942621 | 0.818095 |
| X211437_at | -0.0082 | 0.0118 | -0.692 | 0.4942633 | 0.818095 |
| X205979_at | -0.0038 | 0.0054 | -0.692 | 0.4942673 | 0.818095 |
| X214384_s_at | -0.0044 | 0.0063 | -0.692 | 0.4942675 | 0.818095 |
| X201723_s_at | 0.0266 | 0.0384 | 0.692 | 0.4943399 | 0.81811 |
| X215807_s_at | -0.0732 | 0.1058 | -0.692 | 0.4943498 | 0.81811 |
| X210544_s_at | -0.1064 | 0.1539 | -0.691 | 0.4944147 | 0.818123 |
| X221277_s_at | 0.0176 | 0.0254 | 0.691 | 0.4944316 | 0.818123 |
| X220263_at | -0.0015 | 0.0022 | -0.691 | 0.4945513 | 0.818183 |
| X32062_at | -0.005 | 0.0072 | -0.691 | 0.494577 | 0.818183 |
| X210260_s_at | -0.0089 | 0.0129 | -0.691 | 0.4946012 | 0.818183 |
| X211674_x_at | 0.0015 | 0.0022 | 0.691 | 0.4946395 | 0.818183 |
| X217833_at | 0.024 | 0.0347 | 0.691 | 0.4946516 | 0.818183 |
| X209795_at | 0.0053 | 0.0076 | 0.691 | 0.4947866 | 0.818345 |
| X201703_s_at | -0.0559 | 0.0809 | -0.691 | 0.4948538 | 0.818395 |
| X215520_at | -0.0033 | 0.0048 | -0.691 | 0.4948952 | 0.818403 |
| X214943_s_at | -0.0411 | 0.0595 | -0.691 | 0.4950048 | 0.818523 |
| X203579_s_at | 0.0057 | 0.0082 | 0.69 | 0.4950626 | 0.818558 |
| X203550_s_at | 0.0231 | 0.0334 | 0.69 | 0.4951184 | 0.818589 |
| X205786_s_at | -0.0289 | 0.0419 | -0.69 | 0.4951711 | 0.818615 |
| X210650_s_at | -0.0863 | 0.1251 | -0.69 | 0.4953074 | 0.818779 |
| X203827_at | 0.0545 | 0.0789 | 0.69 | 0.4953441 | 0.818779 |
| X211015_s_at | 0.0313 | 0.0454 | 0.69 | 0.4955143 | 0.818876 |
| X221151_at | 0.0044 | 0.0064 | 0.69 | 0.4955304 | 0.818876 |
| X213100_at | 0.0097 | 0.014 | 0.69 | 0.495579 | 0.818876 |
| X205287_s_at | 0.0029 | 0.0042 | 0.69 | 0.4956004 | 0.818876 |
| X208852_s_at | 0.0388 | 0.0563 | 0.69 | 0.4956092 | 0.818876 |
| X214173_x_at | 0.0219 | 0.0317 | 0.69 | 0.4956306 | 0.818876 |
| X206827_s_at | -0.0125 | 0.0182 | -0.689 | 0.4956605 | 0.818876 |
| X213684_s_at | 0.0296 | 0.0429 | 0.689 | 0.4958144 | 0.818959 |
| X215086_at | -0.01 | 0.0145 | -0.689 | 0.4958442 | 0.818959 |
| X207288_at | 0.0013 | 0.0019 | 0.689 | 0.4958555 | 0.818959 |
| X219112_at | 0.0196 | 0.0285 | 0.689 | 0.4958582 | 0.818959 |
| X210392_x_at | 0.0031 | 0.0046 | 0.689 | 0.4959635 | 0.819072 |
| X221811_at | 0.0158 | 0.0229 | 0.689 | 0.4960295 | 0.81912 |
| X204481_at | 0.0154 | 0.0224 | 0.689 | 0.4961081 | 0.819159 |
| X208307_at | -0.0038 | 0.0055 | -0.689 | 0.4961271 | 0.819159 |
| X216531_at | -0.0022 | 0.0032 | -0.688 | 0.4962871 | 0.819311 |
| X35148_at | -0.0043 | 0.0062 | -0.688 | 0.496293 | 0.819311 |
| X205422_s_at | -0.005 | 0.0073 | -0.688 | 0.4964387 | 0.819491 |
| X207578_s_at | -0.0021 | 0.0031 | -0.688 | 0.4964948 | 0.819521 |
| X210384_at | -0.0038 | 0.0055 | -0.688 | 0.4965304 | 0.819521 |
| X204562_at | 0.0035 | 0.005 | 0.688 | 0.4966419 | 0.819641 |
| X201768_s_at | -0.0258 | 0.0375 | -0.688 | 0.496677 | 0.819641 |
| X204001_at | -0.0213 | 0.031 | -0.688 | 0.4967362 | 0.819662 |
| X201811_x_at | -0.0187 | 0.0271 | -0.688 | 0.4967915 | 0.819662 |
| X220908_at | 0.0024 | 0.0034 | 0.688 | 0.4968071 | 0.819662 |
| X209209_s_at | -0.054 | 0.0786 | -0.687 | 0.4968963 | 0.819662 |
| X208532_x_at | 0.0018 | 0.0026 | 0.687 | 0.4969186 | 0.819662 |
| X209828_s_at | 0.0039 | 0.0057 | 0.687 | 0.4969235 | 0.819662 |
| X216176_at | -0.0114 | 0.0166 | -0.687 | 0.4969484 | 0.819662 |
| X208090_s_at | 0.0028 | 0.0041 | 0.687 | 0.4970807 | 0.819748 |
| X221400_at | 0.0045 | 0.0066 | 0.687 | 0.4970978 | 0.819748 |
| X211784_s_at | 0.0188 | 0.0274 | 0.687 | 0.4971244 | 0.819748 |
| X221120_at | -0.0088 | 0.0128 | -0.687 | 0.4971481 | 0.819748 |
| X206323_x_at | 0.0788 | 0.1148 | 0.687 | 0.4972008 | 0.819774 |
| X210388_at | -0.003 | 0.0043 | -0.686 | 0.4975387 | 0.820257 |
| X210728_s_at | -0.0035 | 0.005 | -0.686 | 0.4975964 | 0.820257 |
| X209812_x_at | -0.0015 | 0.0022 | -0.686 | 0.4976093 | 0.820257 |
| X201721_s_at | 0.1225 | 0.1785 | 0.686 | 0.4976564 | 0.820257 |
| X211926_s_at | 0.0138 | 0.0202 | 0.686 | 0.4976781 | 0.820257 |
| X218828_at | -0.002 | 0.0029 | -0.686 | 0.4978333 | 0.820452 |
| X208000_at | 0.0029 | 0.0043 | 0.686 | 0.4979693 | 0.820615 |
| X217362_x_at | 0.006 | 0.0088 | 0.686 | 0.4980963 | 0.820763 |
| X214019_at | 0.0093 | 0.0136 | 0.685 | 0.4982002 | 0.820874 |
| X205840_x_at | 0.0025 | 0.0036 | 0.685 | 0.498264 | 0.820905 |
| X216460_at | -0.0026 | 0.0038 | -0.685 | 0.4983118 | 0.820905 |
| X211096_at | -0.0043 | 0.0063 | -0.685 | 0.4983302 | 0.820905 |
| X201623_s_at | -0.0218 | 0.0318 | -0.685 | 0.4984818 | 0.821094 |
| X207110_at | -0.0038 | 0.0056 | -0.685 | 0.4986457 | 0.821303 |
| X219588_s_at | -0.015 | 0.0219 | -0.685 | 0.4987028 | 0.821336 |
| X212370_x_at | -0.0237 | 0.0346 | -0.684 | 0.4988636 | 0.821508 |
| X201242_s_at | -0.0211 | 0.0308 | -0.684 | 0.4989117 | 0.821508 |
| X202565_s_at | 0.0149 | 0.0218 | 0.684 | 0.4989181 | 0.821508 |
| X213331_s_at | -0.0259 | 0.0378 | -0.684 | 0.4990157 | 0.821608 |
| X200647_x_at | -0.0566 | 0.0827 | -0.684 | 0.49906 | 0.82162 |
| X221463_at | -0.002 | 0.0029 | -0.684 | 0.4991176 | 0.821654 |
| X215100_at | -0.0037 | 0.0055 | -0.684 | 0.4991793 | 0.821666 |
| X220771_at | -0.0117 | 0.0172 | -0.684 | 0.49924 | 0.821666 |
| X207239_s_at | 0.0025 | 0.0036 | 0.684 | 0.4992507 | 0.821666 |
| X208207_at | -0.003 | 0.0044 | -0.684 | 0.4992726 | 0.821666 |
| X208461_at | -0.0031 | 0.0045 | -0.684 | 0.4993359 | 0.821676 |
| X220931_at | -0.0078 | 0.0114 | -0.684 | 0.4993526 | 0.821676 |
| X205619_s_at | -0.0042 | 0.0061 | -0.683 | 0.4994857 | 0.821772 |
| X217577_at | -0.0051 | 0.0074 | -0.683 | 0.4994983 | 0.821772 |
| X216396_s_at | 0.0152 | 0.0223 | 0.683 | 0.499584 | 0.821772 |
| X36994_at | -0.0222 | 0.0325 | -0.683 | 0.4995996 | 0.821772 |
| X204118_at | 0.0018 | 0.0027 | 0.683 | 0.4996037 | 0.821772 |
| X221657_s_at | -0.0091 | 0.0133 | -0.683 | 0.499633 | 0.821772 |
| X207847_s_at | -0.0029 | 0.0043 | -0.683 | 0.4997116 | 0.821808 |
| X220870_at | -0.0032 | 0.0048 | -0.683 | 0.4997292 | 0.821808 |
| X212923_s_at | 0.0229 | 0.0336 | 0.683 | 0.4998332 | 0.821883 |
| X206260_at | 0.0032 | 0.0046 | 0.683 | 0.4998647 | 0.821883 |
| X210174_at | -0.0033 | 0.0049 | -0.683 | 0.4999076 | 0.821883 |
| X213319_s_at | -0.0029 | 0.0042 | -0.683 | 0.4999229 | 0.821883 |
| X213009_s_at | -0.0193 | 0.0284 | -0.682 | 0.5001354 | 0.822116 |
| X205113_at | 0.0374 | 0.0548 | 0.682 | 0.5001516 | 0.822116 |
| X209324_s_at | 0.0036 | 0.0052 | 0.682 | 0.5001757 | 0.822116 |
| X210753_s_at | 0.0248 | 0.0364 | 0.682 | 0.5003287 | 0.822255 |
| X207651_at | 0.0024 | 0.0035 | 0.682 | 0.5003339 | 0.822255 |
| X220191_at | -0.0032 | 0.0047 | -0.682 | 0.5005378 | 0.822529 |
| X219504_s_at | 0.0202 | 0.0297 | 0.681 | 0.5007771 | 0.822861 |
| X211552_s_at | 0.0057 | 0.0083 | 0.681 | 0.5009648 | 0.823057 |
| X215272_at | -0.0028 | 0.0041 | -0.681 | 0.5009704 | 0.823057 |
| X212081_x_at | -0.0095 | 0.014 | -0.681 | 0.5010476 | 0.823091 |
| X218346_s_at | 0.015 | 0.022 | 0.681 | 0.5010774 | 0.823091 |
| X214719_at | -0.0309 | 0.0455 | -0.681 | 0.5011501 | 0.823091 |
| X204347_at | -0.0328 | 0.0483 | -0.681 | 0.5011749 | 0.823091 |
| X219652_s_at | 0.0025 | 0.0037 | 0.681 | 0.5011764 | 0.823091 |
| X221313_at | -0.0022 | 0.0033 | -0.68 | 0.5012781 | 0.823197 |
| X219990_at | -0.008 | 0.0118 | -0.68 | 0.5013468 | 0.823224 |
| X219382_at | 0.0089 | 0.0131 | 0.68 | 0.5013846 | 0.823224 |
| X211889_x_at | 0.0028 | 0.0041 | 0.68 | 0.5014114 | 0.823224 |
| X207125_at | 0.0071 | 0.0105 | 0.68 | 0.5014423 | 0.823224 |
| X217475_s_at | -0.0031 | 0.0046 | -0.68 | 0.5015276 | 0.823303 |
| X203694_s_at | -0.0233 | 0.0343 | -0.68 | 0.5016216 | 0.823377 |
| X215078_at | 0.0042 | 0.0062 | 0.68 | 0.5016682 | 0.823377 |
| X218587_s_at | -0.0297 | 0.0437 | -0.68 | 0.5016842 | 0.823377 |
| X221205_at | -0.0555 | 0.0817 | -0.679 | 0.501865 | 0.823613 |
| X207488_at | -0.002 | 0.0029 | -0.679 | 0.501922 | 0.823646 |
| X220614_s_at | -0.0062 | 0.0091 | -0.679 | 0.5020831 | 0.823689 |
| X215057_at | 0.0486 | 0.0715 | 0.679 | 0.502091 | 0.823689 |
| X211489_at | 0.0103 | 0.0151 | 0.679 | 0.502127 | 0.823689 |
| X200941_at | -0.0262 | 0.0386 | -0.679 | 0.5021288 | 0.823689 |
| X209126_x_at | -0.0019 | 0.0028 | -0.679 | 0.5021333 | 0.823689 |
| X213126_at | -0.0232 | 0.0342 | -0.679 | 0.5022723 | 0.823856 |
| X206279_at | 0.0047 | 0.0069 | 0.679 | 0.5024556 | 0.824095 |
| X216988_s_at | 0.0469 | 0.0691 | 0.678 | 0.5025589 | 0.82416 |
| X219137_s_at | -0.0189 | 0.0279 | -0.678 | 0.5025694 | 0.82416 |
| X218525_s_at | -0.0164 | 0.0242 | -0.678 | 0.5028843 | 0.824594 |
| X219362_at | -0.0126 | 0.0186 | -0.678 | 0.5029081 | 0.824594 |
| X218516_s_at | -0.0596 | 0.088 | -0.678 | 0.5030674 | 0.824794 |
| X221525_at | 0.0126 | 0.0186 | 0.678 | 0.5031099 | 0.824803 |
| X203202_at | 0.0391 | 0.0577 | 0.677 | 0.5032528 | 0.824977 |
| X205444_at | 0.0019 | 0.0028 | 0.677 | 0.5033125 | 0.825014 |
| X214867_at | 0.0018 | 0.0027 | 0.677 | 0.5033865 | 0.825074 |
| X221304_at | -0.0037 | 0.0054 | -0.677 | 0.5035101 | 0.825182 |
| X204353_s_at | -0.0152 | 0.0224 | -0.677 | 0.5035532 | 0.825182 |
| X203214_x_at | 0.0026 | 0.0038 | 0.677 | 0.5035637 | 0.825182 |
| X212884_x_at | -0.0378 | 0.0558 | -0.677 | 0.5036308 | 0.825231 |
| X205863_at | 0.003 | 0.0044 | 0.677 | 0.5036756 | 0.825243 |
| X217056_at | 0.0056 | 0.0083 | 0.677 | 0.5037262 | 0.825265 |
| X208478_s_at | 0.005 | 0.0073 | 0.676 | 0.5038548 | 0.825408 |
| X209963_s_at | -0.0048 | 0.007 | -0.676 | 0.5039729 | 0.825408 |
| X220881_at | -0.0032 | 0.0048 | -0.676 | 0.5041788 | 0.825408 |
| X213535_s_at | -0.0223 | 0.033 | -0.676 | 0.504182 | 0.825408 |
| X206877_at | -0.0018 | 0.0026 | -0.676 | 0.5042291 | 0.825408 |
| X220136_s_at | -0.0014 | 0.002 | -0.676 | 0.5042616 | 0.825408 |
| X204442_x_at | 0.0043 | 0.0064 | 0.676 | 0.5042713 | 0.825408 |
| X211398_at | -0.0028 | 0.0042 | -0.676 | 0.5042773 | 0.825408 |
| X208440_at | -0.0024 | 0.0035 | -0.676 | 0.5042962 | 0.825408 |
| X209180_at | -0.0199 | 0.0294 | -0.676 | 0.5042974 | 0.825408 |
| X215422_at | -0.0044 | 0.0066 | -0.676 | 0.5043479 | 0.825408 |
| X214392_at | -0.002 | 0.003 | -0.675 | 0.5043854 | 0.825408 |
| X208361_s_at | -0.0017 | 0.0026 | -0.675 | 0.5044312 | 0.825408 |
| X216051_x_at | 0.0411 | 0.0609 | 0.675 | 0.5044457 | 0.825408 |
| X207223_s_at | -0.0034 | 0.005 | -0.675 | 0.5044549 | 0.825408 |
| X218151_x_at | -0.0057 | 0.0084 | -0.675 | 0.5045237 | 0.825408 |
| X204793_at | -0.0395 | 0.0586 | -0.675 | 0.5045544 | 0.825408 |
| X216492_at | -0.0023 | 0.0033 | -0.675 | 0.5045608 | 0.825408 |
| X217396_at | 0.0031 | 0.0046 | 0.675 | 0.5045659 | 0.825408 |
| X213652_at | 0.0243 | 0.0359 | 0.675 | 0.5045705 | 0.825408 |
| X206783_at | -0.0042 | 0.0062 | -0.675 | 0.5045936 | 0.825408 |
| X221117_at | -0.0054 | 0.008 | -0.675 | 0.5048683 | 0.825797 |
| X200616_s_at | 0.0179 | 0.0266 | 0.675 | 0.504989 | 0.825933 |
| X202589_at | -0.1029 | 0.1526 | -0.674 | 0.5050271 | 0.825935 |
| X212604_at | -0.0288 | 0.0427 | -0.674 | 0.5052007 | 0.826075 |
| X216196_at | 0.0038 | 0.0057 | 0.674 | 0.5052448 | 0.826075 |
| X211767_at | 0.0025 | 0.0037 | 0.674 | 0.5052493 | 0.826075 |
| X203695_s_at | 0.046 | 0.0682 | 0.674 | 0.5052618 | 0.826075 |
| X221581_s_at | -0.0155 | 0.0229 | -0.674 | 0.5053794 | 0.826142 |
| X204152_s_at | 0.0018 | 0.0026 | 0.674 | 0.5053962 | 0.826142 |
| X202608_s_at | 0.0025 | 0.0036 | 0.674 | 0.5054577 | 0.826142 |
| X211807_x_at | 0.0051 | 0.0075 | 0.674 | 0.5055232 | 0.826142 |
| X215783_s_at | 0.0025 | 0.0038 | 0.674 | 0.5056012 | 0.826142 |
| X207848_at | 0.0015 | 0.0022 | 0.674 | 0.5056185 | 0.826142 |
| X204896_s_at | 0.0012 | 0.0018 | 0.673 | 0.5056366 | 0.826142 |
| X216663_s_at | -0.0133 | 0.0197 | -0.673 | 0.5056531 | 0.826142 |
| X214926_at | -0.0022 | 0.0033 | -0.673 | 0.5056715 | 0.826142 |
| X210002_at | -0.0061 | 0.009 | -0.673 | 0.5057451 | 0.826142 |
| X221066_at | -0.0027 | 0.004 | -0.673 | 0.505779 | 0.826142 |
| X219708_at | 0.0025 | 0.0037 | 0.673 | 0.5057876 | 0.826142 |
| X216966_at | -0.0079 | 0.0117 | -0.673 | 0.5058172 | 0.826142 |
| X218725_at | 0.026 | 0.0386 | 0.673 | 0.5058231 | 0.826142 |
| X215632_at | -0.0038 | 0.0056 | -0.673 | 0.5058701 | 0.826158 |
| X219154_at | -0.0411 | 0.061 | -0.673 | 0.5061182 | 0.826399 |
| X220176_at | -0.0419 | 0.0624 | -0.673 | 0.5061292 | 0.826399 |
| X214407_x_at | 0.0021 | 0.0031 | 0.673 | 0.5061679 | 0.826399 |
| X213401_s_at | -0.0039 | 0.0059 | -0.673 | 0.5061865 | 0.826399 |
| X206119_at | -0.0047 | 0.007 | -0.673 | 0.5062037 | 0.826399 |
| X205972_at | -0.0026 | 0.0039 | -0.672 | 0.5062628 | 0.826418 |
| X209775_x_at | -0.0014 | 0.0021 | -0.672 | 0.5063824 | 0.826418 |
| X210893_at | -0.0106 | 0.0158 | -0.672 | 0.5064099 | 0.826418 |
| X201722_s_at | 0.023 | 0.0342 | 0.672 | 0.5064206 | 0.826418 |
| X220169_at | 0.0073 | 0.0109 | 0.672 | 0.5064266 | 0.826418 |
| X219601_s_at | -0.0045 | 0.0068 | -0.672 | 0.5064654 | 0.826418 |
| X208726_s_at | -0.0228 | 0.0339 | -0.672 | 0.5064912 | 0.826418 |
| X208173_at | -0.0036 | 0.0053 | -0.672 | 0.5065386 | 0.826418 |
| X632_at | 0.0081 | 0.012 | 0.672 | 0.5065501 | 0.826418 |
| X211325_x_at | 0.0265 | 0.0395 | 0.672 | 0.5066997 | 0.826601 |
| X217638_at | -0.0028 | 0.0041 | -0.672 | 0.5068113 | 0.826722 |
| X221287_at | 0.0029 | 0.0043 | 0.671 | 0.506888 | 0.826787 |
| X218254_s_at | 0.04 | 0.0596 | 0.671 | 0.5071049 | 0.827063 |
| X221926_s_at | 0.0017 | 0.0025 | 0.671 | 0.5071319 | 0.827063 |
| X209260_at | 0.0025 | 0.0037 | 0.671 | 0.5072237 | 0.827152 |
| X218710_at | 0.0117 | 0.0174 | 0.671 | 0.5073202 | 0.827249 |
| X215418_at | 0.0045 | 0.0067 | 0.671 | 0.5074574 | 0.827342 |
| X216806_at | -0.0034 | 0.0051 | -0.671 | 0.5074701 | 0.827342 |
| X213393_at | -0.0047 | 0.007 | -0.67 | 0.5075059 | 0.827342 |
| X213943_at | 0.0078 | 0.0116 | 0.67 | 0.5075265 | 0.827342 |
| X33767_at | 0.0375 | 0.0559 | 0.67 | 0.5075695 | 0.827352 |
| X218959_at | 0.0031 | 0.0046 | 0.67 | 0.5077261 | 0.827465 |
| X220093_at | -0.0033 | 0.0049 | -0.67 | 0.5077464 | 0.827465 |
| X201834_at | -0.0165 | 0.0246 | -0.67 | 0.5077892 | 0.827465 |
| X204594_s_at | -0.0311 | 0.0464 | -0.67 | 0.5078025 | 0.827465 |
| X206755_at | -0.0056 | 0.0083 | -0.67 | 0.5078253 | 0.827465 |
| X56748_at | -0.0051 | 0.0077 | -0.67 | 0.5078886 | 0.827497 |
| X221846_s_at | 0.0253 | 0.0378 | 0.67 | 0.5079509 | 0.827497 |
| X207817_at | -0.003 | 0.0044 | -0.67 | 0.507967 | 0.827497 |
| X216855_s_at | -0.0025 | 0.0038 | -0.67 | 0.5079938 | 0.827497 |
| X206423_at | 0.0016 | 0.0024 | 0.669 | 0.5082063 | 0.8277 |
| X214718_at | 0.0315 | 0.0471 | 0.669 | 0.5082309 | 0.8277 |
| X218729_at | 0.0669 | 0.1 | 0.669 | 0.5082672 | 0.8277 |
| X210996_s_at | 0.0057 | 0.0086 | 0.669 | 0.5082674 | 0.8277 |
| X218366_x_at | 0.0096 | 0.0143 | 0.669 | 0.5085061 | 0.827897 |
| X213939_s_at | 0.0286 | 0.0428 | 0.669 | 0.5085445 | 0.827897 |
| X220244_at | 0.0038 | 0.0057 | 0.669 | 0.508628 | 0.827897 |
| X34726_at | 0.0273 | 0.0408 | 0.669 | 0.5086593 | 0.827897 |
| X217333_at | -0.0041 | 0.0062 | -0.669 | 0.5086663 | 0.827897 |
| X208220_x_at | 0.0048 | 0.0071 | 0.669 | 0.5086843 | 0.827897 |
| X217459_at | 0.0034 | 0.005 | 0.669 | 0.5086845 | 0.827897 |
| X39318_at | 0.002 | 0.003 | 0.669 | 0.5086866 | 0.827897 |
| X202657_s_at | -0.0198 | 0.0296 | -0.668 | 0.5088122 | 0.828041 |
| X202850_at | 0.0289 | 0.0432 | 0.668 | 0.5088525 | 0.828046 |
| X203658_at | 0.0364 | 0.0545 | 0.668 | 0.5089315 | 0.828075 |
| X204580_at | -0.0046 | 0.0069 | -0.668 | 0.508974 | 0.828075 |
| X218139_s_at | 0.0214 | 0.032 | 0.668 | 0.5090427 | 0.828075 |
| X215484_at | 0.0029 | 0.0044 | 0.668 | 0.509043 | 0.828075 |
| X207458_at | 0.0018 | 0.0027 | 0.668 | 0.5090988 | 0.828075 |
| X201796_s_at | -0.0045 | 0.0067 | -0.668 | 0.5091186 | 0.828075 |
| X220011_at | 0.0041 | 0.0062 | 0.668 | 0.5091313 | 0.828075 |
| X206531_at | -0.0053 | 0.008 | -0.668 | 0.5092646 | 0.828142 |
| X207275_s_at | -0.0541 | 0.081 | -0.668 | 0.5092974 | 0.828142 |
| X212168_at | -0.0371 | 0.0555 | -0.668 | 0.5093541 | 0.828142 |
| X208051_s_at | -0.0223 | 0.0334 | -0.668 | 0.5093766 | 0.828142 |
| X217309_s_at | -0.0024 | 0.0036 | -0.668 | 0.5093793 | 0.828142 |
| X207503_at | -0.0044 | 0.0065 | -0.667 | 0.5095093 | 0.828142 |
| X216290_x_at | 0.0591 | 0.0886 | 0.667 | 0.5095829 | 0.828142 |
| X218106_s_at | -0.0164 | 0.0246 | -0.667 | 0.5095953 | 0.828142 |
| X215339_at | 0.0067 | 0.01 | 0.667 | 0.5096017 | 0.828142 |
| X205268_s_at | -0.111 | 0.1664 | -0.667 | 0.509627 | 0.828142 |
| X205987_at | 0.0029 | 0.0043 | 0.667 | 0.5096341 | 0.828142 |
| X218622_at | 0.0329 | 0.0493 | 0.667 | 0.5096344 | 0.828142 |
| X216422_at | -0.0047 | 0.007 | -0.667 | 0.5096573 | 0.828142 |
| X219200_at | -0.0317 | 0.0475 | -0.667 | 0.5097019 | 0.828154 |
| X221967_at | 0.0031 | 0.0047 | 0.667 | 0.509746 | 0.828165 |
| X216975_x_at | 0.0056 | 0.0084 | 0.667 | 0.5099348 | 0.828411 |
| X214552_s_at | -0.0359 | 0.0539 | -0.666 | 0.5100338 | 0.828511 |
| X218669_at | -0.0237 | 0.0356 | -0.666 | 0.5103732 | 0.829002 |
| X204711_at | 0.0165 | 0.0248 | 0.666 | 0.5104374 | 0.829037 |
| X207615_s_at | 0.0023 | 0.0034 | 0.666 | 0.510588 | 0.829037 |
| X205331_s_at | -0.015 | 0.0226 | -0.666 | 0.5106151 | 0.829037 |
| X203158_s_at | -0.1569 | 0.2358 | -0.666 | 0.510648 | 0.829037 |
| X203918_at | 0.004 | 0.0061 | 0.665 | 0.5106719 | 0.829037 |
| X221537_at | 0.0247 | 0.0371 | 0.665 | 0.5106871 | 0.829037 |
| X216043_x_at | 0.002 | 0.003 | 0.665 | 0.5106899 | 0.829037 |
| X211132_at | 0.0021 | 0.0031 | 0.665 | 0.5106933 | 0.829037 |
| X215877_at | -0.0024 | 0.0036 | -0.665 | 0.5108107 | 0.829135 |
| X215286_s_at | -0.0041 | 0.0061 | -0.665 | 0.5108281 | 0.829135 |
| X204981_at | 0.0043 | 0.0065 | 0.665 | 0.5109061 | 0.829201 |
| X214942_at | 0.0049 | 0.0074 | 0.665 | 0.5109466 | 0.829206 |
| X208658_at | 0.0116 | 0.0174 | 0.665 | 0.5110016 | 0.829206 |
| X220722_s_at | -0.0057 | 0.0086 | -0.665 | 0.5110216 | 0.829206 |
| X206477_s_at | -0.009 | 0.0136 | -0.665 | 0.5110613 | 0.82921 |
| X210811_s_at | -0.0142 | 0.0214 | -0.665 | 0.5111144 | 0.829229 |
| X212704_at | -0.0212 | 0.0319 | -0.665 | 0.5111474 | 0.829229 |
| X219442_at | -0.0024 | 0.0036 | -0.665 | 0.5112265 | 0.829285 |
| X35265_at | 0.0229 | 0.0345 | 0.665 | 0.5112606 | 0.829285 |
| X212156_at | -0.036 | 0.0542 | -0.664 | 0.5112943 | 0.829285 |
| X209087_x_at | 0.0537 | 0.0808 | 0.664 | 0.5114058 | 0.829356 |
| X208740_at | 0.0341 | 0.0514 | 0.664 | 0.5114304 | 0.829356 |
| X218749_s_at | 0.006 | 0.009 | 0.664 | 0.5114529 | 0.829356 |
| X213115_at | -0.0371 | 0.0558 | -0.664 | 0.5115068 | 0.829356 |
| X220210_at | 0.0029 | 0.0044 | 0.664 | 0.5115246 | 0.829356 |
| X211416_x_at | 0.0034 | 0.0051 | 0.664 | 0.5117708 | 0.82942 |
| X203070_at | -0.0031 | 0.0047 | -0.664 | 0.5118278 | 0.82942 |
| X220462_at | -0.0923 | 0.1391 | -0.664 | 0.5118349 | 0.82942 |
| X207469_s_at | 0.0763 | 0.115 | 0.664 | 0.511872 | 0.82942 |
| X208005_at | -0.0017 | 0.0026 | -0.664 | 0.5118743 | 0.82942 |
| X221247_s_at | 0.0053 | 0.0079 | 0.663 | 0.511931 | 0.82942 |
| X211319_at | -0.0039 | 0.0058 | -0.663 | 0.5119314 | 0.82942 |
| X211171_s_at | -0.0038 | 0.0058 | -0.663 | 0.5119351 | 0.82942 |
| X210812_at | -0.0043 | 0.0065 | -0.663 | 0.5119578 | 0.82942 |
| X208724_s_at | -0.0258 | 0.0389 | -0.663 | 0.5119685 | 0.82942 |
| X205935_at | 0.009 | 0.0136 | 0.663 | 0.5120851 | 0.82942 |
| X209537_at | -0.0621 | 0.0936 | -0.663 | 0.5120956 | 0.82942 |
| X202272_s_at | -0.0282 | 0.0425 | -0.663 | 0.5121173 | 0.82942 |
| X211218_at | -0.004 | 0.0061 | -0.663 | 0.5121484 | 0.82942 |
| X217555_at | -0.007 | 0.0106 | -0.663 | 0.512165 | 0.82942 |
| X205066_s_at | 0.004 | 0.006 | 0.663 | 0.5121951 | 0.82942 |
| X201202_at | 0.0281 | 0.0424 | 0.663 | 0.5121985 | 0.82942 |
| X211210_x_at | -0.0026 | 0.0039 | -0.663 | 0.5122462 | 0.829436 |
| X203438_at | 0.0021 | 0.0032 | 0.663 | 0.5123091 | 0.829478 |
| X221331_x_at | 0.0012 | 0.0019 | 0.663 | 0.5123585 | 0.829497 |
| X217325_at | -0.0031 | 0.0046 | -0.663 | 0.5125272 | 0.82971 |
| X211762_s_at | -0.0413 | 0.0623 | -0.662 | 0.5128466 | 0.830167 |
| X219673_at | -0.0456 | 0.0689 | -0.662 | 0.5129399 | 0.830211 |
| X209135_at | 0.0402 | 0.0608 | 0.662 | 0.5129488 | 0.830211 |
| X221722_x_at | -0.0015 | 0.0023 | -0.662 | 0.5130167 | 0.830212 |
| X220766_at | 0.0022 | 0.0033 | 0.662 | 0.513024 | 0.830212 |
| X209758_s_at | 0.0038 | 0.0058 | 0.662 | 0.5131353 | 0.830331 |
| X211702_s_at | -0.0216 | 0.0327 | -0.661 | 0.513194 | 0.830366 |
| X212618_at | 0.0071 | 0.0107 | 0.661 | 0.5132511 | 0.830398 |
| X204941_s_at | -0.0031 | 0.0048 | -0.661 | 0.5133294 | 0.830464 |
| X211907_s_at | 0.0036 | 0.0054 | 0.661 | 0.5134556 | 0.830583 |
| X213239_at | -0.0313 | 0.0474 | -0.661 | 0.5134965 | 0.830583 |
| X221176_x_at | 0.021 | 0.0318 | 0.661 | 0.5135688 | 0.830583 |
| X210932_s_at | 0.0058 | 0.0088 | 0.661 | 0.5136535 | 0.830583 |
| X220594_at | -0.0031 | 0.0047 | -0.661 | 0.5136625 | 0.830583 |
| X221938_x_at | -0.0052 | 0.0078 | -0.661 | 0.5136663 | 0.830583 |
| X216161_at | -0.008 | 0.0122 | -0.661 | 0.513701 | 0.830583 |
| X210780_at | -0.003 | 0.0045 | -0.661 | 0.5137191 | 0.830583 |
| X212159_x_at | -0.0354 | 0.0535 | -0.661 | 0.513754 | 0.830583 |
| X209728_at | 0.0034 | 0.0051 | 0.661 | 0.5137913 | 0.830583 |
| X218362_s_at | -0.0376 | 0.0569 | -0.66 | 0.5138143 | 0.830583 |
| X213020_at | 0.0241 | 0.0364 | 0.66 | 0.5138806 | 0.83063 |
| X221322_at | -0.0026 | 0.004 | -0.66 | 0.513974 | 0.83072 |
| X213098_at | 0.0021 | 0.0032 | 0.66 | 0.5141147 | 0.830874 |
| X205820_s_at | 0.0022 | 0.0033 | 0.66 | 0.5142045 | 0.830874 |
| X213270_at | -0.0173 | 0.0263 | -0.66 | 0.5142107 | 0.830874 |
| X200775_s_at | 0.0192 | 0.0292 | 0.66 | 0.5142185 | 0.830874 |
| X215103_at | -0.0041 | 0.0062 | -0.66 | 0.5142766 | 0.830875 |
| X218544_s_at | 0.012 | 0.0181 | 0.66 | 0.514294 | 0.830875 |
| X211039_at | -0.0039 | 0.0058 | -0.66 | 0.5144138 | 0.830931 |
| X200977_s_at | -0.0122 | 0.0185 | -0.659 | 0.5144887 | 0.830931 |
| X208261_x_at | -0.004 | 0.006 | -0.659 | 0.5145573 | 0.830931 |
| X212072_s_at | -0.017 | 0.0258 | -0.659 | 0.5145594 | 0.830931 |
| X204452_s_at | 0.0016 | 0.0024 | 0.659 | 0.5145693 | 0.830931 |
| X211343_s_at | 0.0072 | 0.011 | 0.659 | 0.5146567 | 0.830931 |
| X217625_x_at | 0.0022 | 0.0033 | 0.659 | 0.5147037 | 0.830931 |
| X214302_x_at | 0.0015 | 0.0023 | 0.659 | 0.5147766 | 0.830931 |
| X201164_s_at | -0.0298 | 0.0452 | -0.659 | 0.5147953 | 0.830931 |
| X210596_at | 0.0809 | 0.1228 | 0.659 | 0.5148236 | 0.830931 |
| X215921_at | -0.0035 | 0.0053 | -0.659 | 0.5148548 | 0.830931 |
| X217268_at | -0.0058 | 0.0087 | -0.659 | 0.5148664 | 0.830931 |
| X214042_s_at | -0.0265 | 0.0403 | -0.659 | 0.5148794 | 0.830931 |
| X208228_s_at | 0.0629 | 0.0955 | 0.659 | 0.5149168 | 0.830931 |
| X206759_at | -0.0019 | 0.003 | -0.659 | 0.5149668 | 0.830931 |
| X212116_at | 0.0073 | 0.0111 | 0.659 | 0.5149726 | 0.830931 |
| X207177_at | 0.0075 | 0.0115 | 0.659 | 0.5149815 | 0.830931 |
| X206756_at | -0.0835 | 0.1268 | -0.659 | 0.5150019 | 0.830931 |
| X216904_at | -0.002 | 0.0031 | -0.658 | 0.5150798 | 0.830971 |
| X215549_x_at | 0.0022 | 0.0033 | 0.658 | 0.5151016 | 0.830971 |
| X221075_s_at | 0.0026 | 0.0039 | 0.658 | 0.5151956 | 0.831062 |
| X201130_s_at | -0.0017 | 0.0026 | -0.658 | 0.515302 | 0.831157 |
| X207906_at | -0.0028 | 0.0043 | -0.658 | 0.5153294 | 0.831157 |
| X213816_s_at | -0.0021 | 0.0032 | -0.658 | 0.5153799 | 0.831178 |
| X207852_at | -0.0044 | 0.0067 | -0.658 | 0.5154495 | 0.83123 |
| X205076_s_at | -0.0184 | 0.0279 | -0.658 | 0.5155436 | 0.831322 |
| X206252_s_at | 0.0023 | 0.0036 | 0.658 | 0.51569 | 0.831497 |
| X207650_x_at | -0.0018 | 0.0027 | -0.657 | 0.5157296 | 0.831501 |
| X213782_s_at | 0.0031 | 0.0048 | 0.657 | 0.5157794 | 0.831521 |
| X209957_s_at | -0.0174 | 0.0264 | -0.657 | 0.5159134 | 0.831656 |
| X219221_at | 0.0333 | 0.0506 | 0.657 | 0.5159383 | 0.831656 |
| X217579_x_at | 0.0158 | 0.0241 | 0.657 | 0.516144 | 0.831904 |
| X204865_at | 0.0132 | 0.02 | 0.657 | 0.5161669 | 0.831904 |
| X211753_s_at | 0.0039 | 0.0059 | 0.657 | 0.5162964 | 0.832041 |
| X203507_at | 0.0046 | 0.007 | 0.657 | 0.5163271 | 0.832041 |
| X219809_at | 0.0027 | 0.0042 | 0.656 | 0.5164363 | 0.83212 |
| X210409_at | -0.0111 | 0.017 | -0.656 | 0.5164798 | 0.83212 |
| X214783_s_at | -0.0194 | 0.0296 | -0.656 | 0.5165702 | 0.83212 |
| X217359_s_at | -0.0542 | 0.0826 | -0.656 | 0.516617 | 0.83212 |
| X209419_at | -0.0089 | 0.0136 | -0.656 | 0.5166294 | 0.83212 |
| X210446_at | 0.0015 | 0.0023 | 0.656 | 0.5166447 | 0.83212 |
| X215579_at | 0.0026 | 0.004 | 0.656 | 0.5166447 | 0.83212 |
| X212232_at | 0.0217 | 0.0331 | 0.656 | 0.5167053 | 0.83212 |
| X209664_x_at | -0.0013 | 0.002 | -0.656 | 0.5167127 | 0.83212 |
| X216642_at | 0.0046 | 0.007 | 0.656 | 0.5168253 | 0.832241 |
| X204208_at | -0.0263 | 0.0401 | -0.656 | 0.5168831 | 0.832273 |
| X217098_s_at | -0.0054 | 0.0083 | -0.655 | 0.5170111 | 0.832419 |
| X219216_at | 0.0171 | 0.0262 | 0.655 | 0.5170763 | 0.832425 |
| X207333_at | 0.0029 | 0.0045 | 0.655 | 0.5170897 | 0.832425 |
| X204706_at | 0.0406 | 0.0619 | 0.655 | 0.5171667 | 0.832489 |
| X209229_s_at | -0.0488 | 0.0746 | -0.655 | 0.5174049 | 0.832717 |
| X216613_at | -0.0091 | 0.0139 | -0.655 | 0.5174185 | 0.832717 |
| X211809_x_at | -0.0027 | 0.0042 | -0.655 | 0.517421 | 0.832717 |
| X201113_at | -0.0172 | 0.0263 | -0.655 | 0.5174734 | 0.832741 |
| X222080_s_at | -0.0025 | 0.0038 | -0.655 | 0.5175449 | 0.832747 |
| X208749_x_at | 0.0237 | 0.0362 | 0.655 | 0.517552 | 0.832747 |
| X221132_at | -0.0021 | 0.0031 | -0.654 | 0.5177552 | 0.833013 |
| X216792_at | -0.0035 | 0.0053 | -0.654 | 0.5178818 | 0.833157 |
| X213697_at | 0.0028 | 0.0043 | 0.654 | 0.5181226 | 0.833484 |
| X200985_s_at | 0.0238 | 0.0364 | 0.654 | 0.5182235 | 0.833586 |
| X203718_at | -0.0381 | 0.0583 | -0.653 | 0.5183455 | 0.833687 |
| X215164_at | 0.0027 | 0.0041 | 0.653 | 0.5183616 | 0.833687 |
| X206884_s_at | -0.0042 | 0.0064 | -0.653 | 0.5185187 | 0.833879 |
| X209681_at | 0.0456 | 0.0699 | 0.653 | 0.5186732 | 0.834067 |
| X205008_s_at | 0.0402 | 0.0615 | 0.653 | 0.5187436 | 0.83412 |
| X205894_at | 0.002 | 0.0031 | 0.653 | 0.5188717 | 0.834219 |
| X219552_at | -0.0031 | 0.0048 | -0.653 | 0.518888 | 0.834219 |
| X202914_s_at | 0.0023 | 0.0036 | 0.652 | 0.5190219 | 0.834219 |
| X205803_s_at | -0.0263 | 0.0404 | -0.652 | 0.5190595 | 0.834219 |
| X201043_s_at | -0.0433 | 0.0663 | -0.652 | 0.5190601 | 0.834219 |
| X213340_s_at | -0.0304 | 0.0466 | -0.652 | 0.519097 | 0.834219 |
| X216556_x_at | -0.0065 | 0.01 | -0.652 | 0.5191178 | 0.834219 |
| X212277_at | -0.0238 | 0.0365 | -0.652 | 0.5191227 | 0.834219 |
| X215787_at | 0.0065 | 0.01 | 0.652 | 0.5191432 | 0.834219 |
| X203536_s_at | -0.0047 | 0.0072 | -0.652 | 0.519307 | 0.834359 |
| X215372_x_at | 0.0029 | 0.0044 | 0.652 | 0.5193158 | 0.834359 |
| X200633_at | -0.0135 | 0.0208 | -0.652 | 0.5193428 | 0.834359 |
| X214132_at | 0.0322 | 0.0495 | 0.652 | 0.5194474 | 0.834435 |
| X203135_at | -0.0102 | 0.0156 | -0.652 | 0.5194961 | 0.834435 |
| X221171_at | -0.0057 | 0.0087 | -0.652 | 0.5195141 | 0.834435 |
| X221554_at | 0.0092 | 0.0142 | 0.651 | 0.5195646 | 0.834435 |
| X220453_at | -0.0041 | 0.0063 | -0.651 | 0.5195817 | 0.834435 |
| X202525_at | 0.0025 | 0.0038 | 0.651 | 0.5196157 | 0.834435 |
| X205253_at | 0.0029 | 0.0045 | 0.651 | 0.519796 | 0.834665 |
| X204954_s_at | 0.0043 | 0.0066 | 0.651 | 0.5198979 | 0.834768 |
| X217032_at | -0.0022 | 0.0034 | -0.651 | 0.5200164 | 0.834898 |
| X210029_at | -0.0019 | 0.0029 | -0.65 | 0.5202286 | 0.835173 |
| X203361_s_at | -0.0073 | 0.0113 | -0.65 | 0.520382 | 0.835173 |
| X219349_s_at | -0.0226 | 0.0348 | -0.65 | 0.5203858 | 0.835173 |
| X216757_at | 0.005 | 0.0078 | 0.65 | 0.5203929 | 0.835173 |
| X221737_at | 0.0331 | 0.0509 | 0.65 | 0.5204068 | 0.835173 |
| X215665_at | -0.0056 | 0.0086 | -0.65 | 0.5204712 | 0.835173 |
| X218062_x_at | -0.0464 | 0.0714 | -0.65 | 0.520586 | 0.835173 |
| X209885_at | 0.0019 | 0.0029 | 0.65 | 0.5206643 | 0.835173 |
| X222059_at | -0.0026 | 0.004 | -0.65 | 0.5206765 | 0.835173 |
| X211811_s_at | 0.0029 | 0.0044 | 0.65 | 0.5206807 | 0.835173 |
| X213805_at | 0.0323 | 0.0498 | 0.65 | 0.5207589 | 0.835173 |
| X210419_at | 0.003 | 0.0047 | 0.65 | 0.5207953 | 0.835173 |
| X216163_at | -0.0048 | 0.0075 | -0.649 | 0.5208262 | 0.835173 |
| X216831_s_at | -0.0064 | 0.0099 | -0.649 | 0.5208327 | 0.835173 |
| X202039_at | -0.0164 | 0.0253 | -0.649 | 0.5208872 | 0.835173 |
| X219044_at | 0.0194 | 0.0299 | 0.649 | 0.5209067 | 0.835173 |
| X205560_at | 0.0043 | 0.0066 | 0.649 | 0.5210556 | 0.835173 |
| X207080_s_at | 0.0023 | 0.0035 | 0.649 | 0.5210837 | 0.835173 |
| X219680_at | 0.0035 | 0.0054 | 0.649 | 0.5210989 | 0.835173 |
| X212486_s_at | 0.04 | 0.0617 | 0.649 | 0.5211067 | 0.835173 |
| X202735_at | 0.0247 | 0.0381 | 0.649 | 0.5211081 | 0.835173 |
| X207264_at | -0.0024 | 0.0036 | -0.649 | 0.5211201 | 0.835173 |
| X209754_s_at | -0.0112 | 0.0172 | -0.649 | 0.5211442 | 0.835173 |
| X203844_at | -0.0106 | 0.0164 | -0.649 | 0.5212069 | 0.835173 |
| X206983_at | -0.0072 | 0.0112 | -0.649 | 0.5212246 | 0.835173 |
| X211461_at | 0.0024 | 0.0037 | 0.649 | 0.5212274 | 0.835173 |
| X201156_s_at | -0.0235 | 0.0362 | -0.649 | 0.5212371 | 0.835173 |
| X202660_at | 0.0479 | 0.0738 | 0.649 | 0.521242 | 0.835173 |
| X216478_at | -0.0036 | 0.0055 | -0.649 | 0.521278 | 0.835173 |
| X208807_s_at | 0.0036 | 0.0056 | 0.649 | 0.5213751 | 0.835268 |
| X216030_s_at | -0.0055 | 0.0085 | -0.648 | 0.5214793 | 0.835361 |
| X219159_s_at | -0.0138 | 0.0213 | -0.648 | 0.5215214 | 0.835361 |
| X218944_at | -0.0016 | 0.0025 | -0.648 | 0.5215461 | 0.835361 |
| X204290_s_at | -0.0407 | 0.0628 | -0.648 | 0.52162 | 0.835419 |
| X213872_at | -0.0776 | 0.1197 | -0.648 | 0.5217097 | 0.835503 |
| X203661_s_at | -0.0481 | 0.0742 | -0.648 | 0.5218673 | 0.835695 |
| X201689_s_at | -0.0298 | 0.046 | -0.647 | 0.5221582 | 0.836101 |
| X215247_at | -0.0032 | 0.0049 | -0.647 | 0.5221966 | 0.836102 |
| X218647_s_at | -0.0255 | 0.0395 | -0.647 | 0.5222371 | 0.836106 |
| X212536_at | 0.0258 | 0.0399 | 0.647 | 0.5224292 | 0.836323 |
| X208388_at | 0.0063 | 0.0098 | 0.647 | 0.5224634 | 0.836323 |
| X217630_at | 0.0035 | 0.0054 | 0.647 | 0.5224854 | 0.836323 |
| X207958_at | -0.0037 | 0.0058 | -0.647 | 0.5226088 | 0.836423 |
| X216494_at | 0.0022 | 0.0035 | 0.647 | 0.522623 | 0.836423 |
| X208294_x_at | 0.002 | 0.0031 | 0.646 | 0.5229688 | 0.836875 |
| X219935_at | -0.0159 | 0.0246 | -0.646 | 0.5229812 | 0.836875 |
| X222207_x_at | 0.0196 | 0.0303 | 0.646 | 0.5231366 | 0.837019 |
| X217109_at | 0.0055 | 0.0085 | 0.646 | 0.5231463 | 0.837019 |
| X215684_s_at | 0.0155 | 0.024 | 0.646 | 0.5232047 | 0.837052 |
| X207593_at | 0.0031 | 0.0047 | 0.646 | 0.5233432 | 0.837213 |
| X208393_s_at | -0.0487 | 0.0755 | -0.645 | 0.5234791 | 0.837371 |
| X214888_at | 0.0092 | 0.0143 | 0.645 | 0.5236362 | 0.837536 |
| X215668_s_at | 0.0014 | 0.0022 | 0.645 | 0.5236614 | 0.837536 |
| X210748_at | 0.0058 | 0.009 | 0.645 | 0.5237997 | 0.837536 |
| X219931_s_at | 0.0059 | 0.0091 | 0.645 | 0.5238208 | 0.837536 |
| X219926_at | -0.0995 | 0.1544 | -0.645 | 0.5238659 | 0.837536 |
| X221127_s_at | 0.0787 | 0.1221 | 0.645 | 0.5238818 | 0.837536 |
| X221383_at | -0.0019 | 0.003 | -0.645 | 0.523902 | 0.837536 |
| X212349_at | -0.004 | 0.0062 | -0.645 | 0.5239025 | 0.837536 |
| X220995_at | 0.0019 | 0.0029 | 0.645 | 0.523922 | 0.837536 |
| X202037_s_at | 0.0679 | 0.1054 | 0.645 | 0.5239701 | 0.837553 |
| X219875_s_at | -0.0092 | 0.0143 | -0.644 | 0.5240315 | 0.837591 |
| X206922_at | -0.0035 | 0.0054 | -0.644 | 0.5240738 | 0.837598 |
| X217760_at | -0.0174 | 0.027 | -0.644 | 0.5242088 | 0.837713 |
| X211468_s_at | 0.0018 | 0.0028 | 0.644 | 0.5242211 | 0.837713 |
| X214317_x_at | -0.0073 | 0.0114 | -0.644 | 0.5243157 | 0.837772 |
| X210206_s_at | -0.0033 | 0.0051 | -0.644 | 0.5243597 | 0.837772 |
| X207442_at | -0.0019 | 0.003 | -0.644 | 0.5243854 | 0.837772 |
| X202489_s_at | -0.0025 | 0.0039 | -0.644 | 0.5244257 | 0.837772 |
| X219223_at | 0.0038 | 0.0059 | 0.644 | 0.5244465 | 0.837772 |
| X221615_at | 0.0052 | 0.0081 | 0.644 | 0.5244863 | 0.837775 |
| X217274_x_at | 0.0021 | 0.0033 | 0.644 | 0.5245752 | 0.837857 |
| X206679_at | 0.002 | 0.003 | 0.643 | 0.5246899 | 0.837948 |
| X215351_at | -0.008 | 0.0124 | -0.643 | 0.5247248 | 0.837948 |
| X200803_s_at | 0.0631 | 0.0981 | 0.643 | 0.5247456 | 0.837948 |
| X205099_s_at | -0.0208 | 0.0323 | -0.643 | 0.5248533 | 0.838024 |
| X216656_at | -0.0023 | 0.0036 | -0.643 | 0.5249074 | 0.838024 |
| X213222_at | -0.0589 | 0.0916 | -0.643 | 0.5249423 | 0.838024 |
| X210219_at | 0.0036 | 0.0056 | 0.643 | 0.524944 | 0.838024 |
| X210380_s_at | 0.0353 | 0.0549 | 0.643 | 0.5250906 | 0.838172 |
| X214411_x_at | -0.0038 | 0.0059 | -0.643 | 0.525112 | 0.838172 |
| X210959_s_at | -0.0514 | 0.0799 | -0.643 | 0.5252429 | 0.838321 |
| X204789_at | -0.0035 | 0.0054 | -0.642 | 0.5253485 | 0.838429 |
| X215630_at | -0.0027 | 0.0042 | -0.642 | 0.5253896 | 0.838434 |
| X218548_x_at | 0.0038 | 0.0059 | 0.642 | 0.5254689 | 0.838441 |
| X210645_s_at | 0.0185 | 0.0287 | 0.642 | 0.5254696 | 0.838441 |
| X211356_x_at | 0.0034 | 0.0053 | 0.642 | 0.5255396 | 0.838478 |
| X216219_at | -0.0037 | 0.0058 | -0.642 | 0.5255702 | 0.838478 |
| X208192_at | -0.0077 | 0.0121 | -0.642 | 0.5256622 | 0.838478 |
| X215485_s_at | -0.0064 | 0.0099 | -0.642 | 0.5256804 | 0.838478 |
| X221334_s_at | -0.0024 | 0.0037 | -0.642 | 0.5257159 | 0.838478 |
| X203387_s_at | 0.0276 | 0.0431 | 0.642 | 0.5257865 | 0.838478 |
| X204025_s_at | -0.0232 | 0.0362 | -0.642 | 0.5257975 | 0.838478 |
| X207359_at | -0.0034 | 0.0052 | -0.641 | 0.5259411 | 0.838478 |
| X209502_s_at | -0.0061 | 0.0094 | -0.641 | 0.5259435 | 0.838478 |
| X215237_at | -0.004 | 0.0062 | -0.641 | 0.5259556 | 0.838478 |
| X200829_x_at | 0.0207 | 0.0323 | 0.641 | 0.5259678 | 0.838478 |
| X207019_s_at | -0.0043 | 0.0067 | -0.641 | 0.5260031 | 0.838478 |
| X213483_at | -0.032 | 0.0499 | -0.641 | 0.5260055 | 0.838478 |
| X207368_at | -0.0022 | 0.0034 | -0.641 | 0.5260504 | 0.838478 |
| X206459_s_at | -0.0028 | 0.0043 | -0.641 | 0.5261065 | 0.838478 |
| X208518_s_at | 0.0024 | 0.0037 | 0.641 | 0.5261117 | 0.838478 |
| X219543_at | -0.0406 | 0.0633 | -0.641 | 0.5261341 | 0.838478 |
| X211690_at | -0.0033 | 0.0052 | -0.641 | 0.5261866 | 0.838501 |
| X221821_s_at | -0.0361 | 0.0563 | -0.641 | 0.5262249 | 0.838502 |
| X220593_s_at | -0.0038 | 0.006 | -0.641 | 0.5263451 | 0.83858 |
| X207877_s_at | -0.0024 | 0.0038 | -0.641 | 0.5263706 | 0.83858 |
| X207278_s_at | 0.0028 | 0.0043 | 0.641 | 0.5264426 | 0.83858 |
| X219717_at | 0.0533 | 0.0832 | 0.641 | 0.5264428 | 0.83858 |
| X204443_at | 0.0015 | 0.0023 | 0.641 | 0.5264624 | 0.83858 |
| X221984_s_at | -0.0184 | 0.0288 | -0.64 | 0.5266244 | 0.838778 |
| X213720_s_at | -0.0195 | 0.0304 | -0.64 | 0.526663 | 0.838779 |
| X209529_at | -0.0413 | 0.0644 | -0.64 | 0.5267363 | 0.838794 |
| X208243_s_at | -0.002 | 0.0031 | -0.64 | 0.5267643 | 0.838794 |
| X219236_at | 0.0567 | 0.0886 | 0.64 | 0.5267908 | 0.838794 |
| X214420_s_at | -0.0051 | 0.008 | -0.64 | 0.52684 | 0.838794 |
| X205830_at | -0.028 | 0.0437 | -0.64 | 0.5268612 | 0.838794 |
| X217611_at | 0.0021 | 0.0033 | 0.64 | 0.5270922 | 0.839023 |
| X201559_s_at | 0.0422 | 0.066 | 0.64 | 0.5271504 | 0.839023 |
| X204648_at | 0.0052 | 0.0081 | 0.64 | 0.5271764 | 0.839023 |
| X205127_at | 0.0021 | 0.0033 | 0.639 | 0.5272027 | 0.839023 |
| X214074_s_at | 0.0053 | 0.0083 | 0.639 | 0.5272307 | 0.839023 |
| X213137_s_at | 0.0131 | 0.0205 | 0.639 | 0.5272462 | 0.839023 |
| X209725_at | 0.0379 | 0.0592 | 0.639 | 0.5272692 | 0.839023 |
| X215353_at | -0.0032 | 0.005 | -0.639 | 0.5273376 | 0.839062 |
| X216733_s_at | 0.0326 | 0.051 | 0.639 | 0.5273695 | 0.839062 |
| X215508_at | 0.0044 | 0.0068 | 0.639 | 0.5275891 | 0.839325 |
| X206904_at | -0.0039 | 0.0061 | -0.639 | 0.5276162 | 0.839325 |
| X222307_at | -0.0049 | 0.0076 | -0.639 | 0.5276951 | 0.839325 |
| X208241_at | 0.0046 | 0.0072 | 0.639 | 0.5277388 | 0.839325 |
| X208221_s_at | 0.0018 | 0.0028 | 0.639 | 0.527804 | 0.839325 |
| X218916_at | -0.0268 | 0.042 | -0.639 | 0.5278119 | 0.839325 |
| X213734_at | -0.0281 | 0.044 | -0.639 | 0.5278334 | 0.839325 |
| X214388_at | -0.0047 | 0.0074 | -0.639 | 0.5278372 | 0.839325 |
| X220800_s_at | -0.0066 | 0.0104 | -0.638 | 0.5278764 | 0.839328 |
| X202865_at | -0.009 | 0.0141 | -0.638 | 0.5279505 | 0.839356 |
| X205155_s_at | -0.0016 | 0.0026 | -0.638 | 0.5279697 | 0.839356 |
| X200778_s_at | 0.063 | 0.0987 | 0.638 | 0.528051 | 0.839425 |
| X217239_x_at | -0.0038 | 0.006 | -0.638 | 0.5282323 | 0.839494 |
| X215234_at | -0.003 | 0.0048 | -0.638 | 0.528234 | 0.839494 |
| X209690_s_at | -0.0014 | 0.0023 | -0.638 | 0.5282685 | 0.839494 |
| X216948_at | -0.0091 | 0.0143 | -0.638 | 0.5282756 | 0.839494 |
| X215189_at | -0.003 | 0.0047 | -0.638 | 0.5282831 | 0.839494 |
| X213113_s_at | 0.0048 | 0.0076 | 0.638 | 0.5283221 | 0.839496 |
| X220285_at | 0.0051 | 0.008 | 0.638 | 0.5283969 | 0.839498 |
| X222359_x_at | -0.0017 | 0.0026 | -0.638 | 0.5283992 | 0.839498 |
| X203788_s_at | 0.0049 | 0.0076 | 0.637 | 0.5286064 | 0.839648 |
| X210711_at | -0.0078 | 0.0122 | -0.637 | 0.5286171 | 0.839648 |
| X213824_at | 0.0006 | 0.001 | 0.637 | 0.528645 | 0.839648 |
| X221646_s_at | 0.0692 | 0.1086 | 0.637 | 0.528645 | 0.839648 |
| X218369_s_at | -0.004 | 0.0062 | -0.637 | 0.5287491 | 0.839681 |
| X216804_s_at | -0.0731 | 0.1147 | -0.637 | 0.5287606 | 0.839681 |
| X215607_x_at | 0.0028 | 0.0044 | 0.637 | 0.5288007 | 0.839681 |
| X214105_at | -0.0021 | 0.0033 | -0.637 | 0.5288427 | 0.839681 |
| X220186_s_at | 0.0025 | 0.004 | 0.637 | 0.5288548 | 0.839681 |
| X203758_at | -0.0221 | 0.0348 | -0.637 | 0.5288957 | 0.839686 |
| X204608_at | -0.0056 | 0.0087 | -0.637 | 0.5290105 | 0.839808 |
| X204823_at | -0.0469 | 0.0737 | -0.636 | 0.5291806 | 0.839986 |
| X202320_at | 0.0324 | 0.051 | 0.636 | 0.5291982 | 0.839986 |
| X216334_s_at | -0.0042 | 0.0066 | -0.636 | 0.5292642 | 0.840029 |
| X221627_at | 0.0044 | 0.0069 | 0.636 | 0.5293007 | 0.840029 |
| X204959_at | 0.0667 | 0.1048 | 0.636 | 0.5295105 | 0.840244 |
| X217439_at | -0.0017 | 0.0027 | -0.636 | 0.5295467 | 0.840244 |
| X213330_s_at | -0.0417 | 0.0655 | -0.636 | 0.5295494 | 0.840244 |
| X213736_at | 0.0525 | 0.0825 | 0.636 | 0.5296015 | 0.840266 |
| X212266_s_at | -0.035 | 0.0552 | -0.635 | 0.5299007 | 0.840588 |
| X218691_s_at | 0.0029 | 0.0046 | 0.635 | 0.5299864 | 0.840588 |
| X201662_s_at | -0.0157 | 0.0248 | -0.635 | 0.5300141 | 0.840588 |
| X207075_at | 0.0039 | 0.0061 | 0.635 | 0.5300322 | 0.840588 |
| X202928_s_at | -0.0191 | 0.0301 | -0.635 | 0.5300666 | 0.840588 |
| X221690_s_at | 0.0034 | 0.0054 | 0.635 | 0.5301331 | 0.840588 |
| X221084_at | 0.0017 | 0.0027 | 0.635 | 0.5301712 | 0.840588 |
| X211365_s_at | -0.0026 | 0.0042 | -0.635 | 0.5302354 | 0.840588 |
| X215644_at | 0.0027 | 0.0042 | 0.635 | 0.5302404 | 0.840588 |
| X219868_s_at | -0.0033 | 0.0053 | -0.635 | 0.5303252 | 0.840588 |
| X218275_at | 0.0028 | 0.0044 | 0.635 | 0.5303259 | 0.840588 |
| X208854_s_at | -0.0353 | 0.0557 | -0.635 | 0.5303432 | 0.840588 |
| X204400_at | 0.0539 | 0.085 | 0.635 | 0.5303459 | 0.840588 |
| X203808_at | -0.0023 | 0.0036 | -0.635 | 0.5303933 | 0.840588 |
| X200057_s_at | 0.0147 | 0.0232 | 0.635 | 0.5303985 | 0.840588 |
| X210196_s_at | -0.0083 | 0.0131 | -0.634 | 0.5304098 | 0.840588 |
| X216012_at | 0.003 | 0.0048 | 0.634 | 0.5305588 | 0.840655 |
| X221129_at | 0.0014 | 0.0021 | 0.634 | 0.5305958 | 0.840655 |
| X202990_at | 0.018 | 0.0284 | 0.634 | 0.5306011 | 0.840655 |
| X218744_s_at | -0.0026 | 0.004 | -0.634 | 0.5306044 | 0.840655 |
| X208574_at | -0.0024 | 0.0037 | -0.634 | 0.5306665 | 0.840655 |
| X201802_at | 0.0033 | 0.0052 | 0.634 | 0.5307045 | 0.840655 |
| X211223_at | -0.0038 | 0.0059 | -0.634 | 0.5307578 | 0.840655 |
| X212513_s_at | -0.014 | 0.0221 | -0.634 | 0.5308135 | 0.840655 |
| X210049_at | 0.0018 | 0.0028 | 0.634 | 0.5308236 | 0.840655 |
| X216941_s_at | 0.0367 | 0.0579 | 0.634 | 0.5308626 | 0.840655 |
| X217273_at | -0.0017 | 0.0027 | -0.634 | 0.5308684 | 0.840655 |
| X206341_at | -0.003 | 0.0047 | -0.634 | 0.531046 | 0.840877 |
| X214776_x_at | 0.0129 | 0.0204 | 0.633 | 0.5311458 | 0.840927 |
| X210076_x_at | -0.0169 | 0.0267 | -0.633 | 0.5311533 | 0.840927 |
| X217445_s_at | -0.0063 | 0.01 | -0.633 | 0.5313241 | 0.841137 |
| X203217_s_at | -0.0157 | 0.0248 | -0.633 | 0.5313941 | 0.841168 |
| X221849_s_at | 0.003 | 0.0047 | 0.633 | 0.5314494 | 0.841168 |
| X220442_at | -0.0031 | 0.0049 | -0.633 | 0.5314575 | 0.841168 |
| X220927_s_at | 0.0033 | 0.0052 | 0.633 | 0.5316169 | 0.841329 |
| X220756_s_at | 0.003 | 0.0048 | 0.633 | 0.5316457 | 0.841329 |
| X36084_at | -0.0067 | 0.0106 | -0.633 | 0.5316747 | 0.841329 |
| X216024_at | 0.0024 | 0.0039 | 0.632 | 0.5317103 | 0.841329 |
| X218630_at | -0.0024 | 0.0038 | -0.632 | 0.531773 | 0.841368 |
| X206876_at | -0.006 | 0.0095 | -0.632 | 0.5318598 | 0.841445 |
| X222112_at | -0.0021 | 0.0034 | -0.632 | 0.5319606 | 0.841545 |
| X215311_at | 0.045 | 0.0711 | 0.632 | 0.5320705 | 0.841588 |
| X207506_at | -0.0023 | 0.0037 | -0.632 | 0.532144 | 0.841588 |
| X213481_at | 0.0023 | 0.0037 | 0.632 | 0.5321819 | 0.841588 |
| X212054_x_at | 0.0024 | 0.0038 | 0.632 | 0.5321894 | 0.841588 |
| X210118_s_at | -0.0036 | 0.0056 | -0.632 | 0.5321957 | 0.841588 |
| X203439_s_at | 0.0019 | 0.0031 | 0.632 | 0.5322152 | 0.841588 |
| X207236_at | 0.0361 | 0.0572 | 0.632 | 0.5322722 | 0.841618 |
| X220760_x_at | -0.0173 | 0.0274 | -0.631 | 0.5323749 | 0.841679 |
| X207990_x_at | -0.0063 | 0.0099 | -0.631 | 0.5323862 | 0.841679 |
| X206240_s_at | 0.0455 | 0.072 | 0.631 | 0.5324295 | 0.841687 |
| X207863_at | 0.0027 | 0.0043 | 0.631 | 0.5325414 | 0.841804 |
| X213277_at | 0.0029 | 0.0046 | 0.631 | 0.5325999 | 0.841837 |
| X202134_s_at | 0.0026 | 0.0041 | 0.631 | 0.5326577 | 0.841868 |
| X63825_at | 0.0255 | 0.0405 | 0.631 | 0.5327622 | 0.841974 |
| X221052_at | -0.0038 | 0.006 | -0.631 | 0.5328115 | 0.841989 |
| X221930_at | -0.0027 | 0.0043 | -0.631 | 0.5328477 | 0.841989 |
| X205532_s_at | -0.0635 | 0.1007 | -0.631 | 0.5328912 | 0.841998 |
| X210586_x_at | 0.0021 | 0.0034 | 0.631 | 0.5329459 | 0.842003 |
| X217023_x_at | -0.0034 | 0.0054 | -0.631 | 0.5329703 | 0.842003 |
| X208806_at | 0.0521 | 0.0826 | 0.63 | 0.5332408 | 0.842167 |
| X211046_at | -0.0055 | 0.0088 | -0.63 | 0.5332648 | 0.842167 |
| X217904_s_at | 0.032 | 0.0508 | 0.63 | 0.5332841 | 0.842167 |
| X201928_at | 0.0616 | 0.0978 | 0.63 | 0.5332919 | 0.842167 |
| X210514_x_at | 0.0049 | 0.0078 | 0.63 | 0.5332973 | 0.842167 |
| X209930_s_at | -0.0016 | 0.0026 | -0.63 | 0.5333052 | 0.842167 |
| X207875_at | -0.0069 | 0.0109 | -0.63 | 0.5333709 | 0.842167 |
| X205622_at | -0.0028 | 0.0044 | -0.63 | 0.5333893 | 0.842167 |
| X217328_at | 0.0027 | 0.0042 | 0.63 | 0.5334156 | 0.842167 |
| X202749_at | -0.0239 | 0.0379 | -0.63 | 0.5334551 | 0.84217 |
| X209977_at | 0.0029 | 0.0046 | 0.63 | 0.5335479 | 0.842232 |
| X222181_at | -0.0027 | 0.0043 | -0.63 | 0.5335701 | 0.842232 |
| X209072_at | 0.0477 | 0.0758 | 0.629 | 0.5336456 | 0.842291 |
| X214140_at | 0.0227 | 0.0361 | 0.629 | 0.5336941 | 0.842308 |
| X217160_at | 0.004 | 0.0064 | 0.629 | 0.5338584 | 0.842507 |
| X41386_i_at | 0.002 | 0.0031 | 0.629 | 0.5339152 | 0.842537 |
| X220332_at | 0.0023 | 0.0036 | 0.629 | 0.5340515 | 0.842589 |
| X215031_x_at | -0.0024 | 0.0038 | -0.629 | 0.5340614 | 0.842589 |
| X216286_at | -0.0119 | 0.0189 | -0.629 | 0.534062 | 0.842589 |
| X209423_s_at | -0.0023 | 0.0036 | -0.629 | 0.534162 | 0.842687 |
| X202563_at | -0.0112 | 0.0178 | -0.629 | 0.534216 | 0.842712 |
| X52005_at | 0.0042 | 0.0068 | 0.628 | 0.5342975 | 0.842781 |
| X38158_at | -0.002 | 0.0032 | -0.628 | 0.5344539 | 0.842968 |
| X206218_at | -0.0038 | 0.0061 | -0.628 | 0.5346379 | 0.843198 |
| X205293_x_at | -0.0019 | 0.0031 | -0.628 | 0.5346999 | 0.843236 |
| X216355_at | 0.0018 | 0.0028 | 0.628 | 0.5347936 | 0.843324 |
| X216939_s_at | 0.003 | 0.0048 | 0.628 | 0.5349018 | 0.843408 |
| X204969_s_at | -0.0413 | 0.0658 | -0.627 | 0.5349382 | 0.843408 |
| X218367_x_at | -0.0322 | 0.0514 | -0.627 | 0.5349606 | 0.843408 |
| X215134_at | 0.0125 | 0.02 | 0.627 | 0.5350443 | 0.84348 |
| X208200_at | 0.0037 | 0.0059 | 0.627 | 0.5353827 | 0.843916 |
| X206456_at | -0.0454 | 0.0724 | -0.627 | 0.5353973 | 0.843916 |
| X216153_x_at | 0.0427 | 0.0681 | 0.627 | 0.5354351 | 0.843916 |
| X207911_s_at | 0.0027 | 0.0044 | 0.627 | 0.5355148 | 0.843982 |
| X222344_at | 0.0173 | 0.0276 | 0.626 | 0.5358571 | 0.844438 |
| X215431_at | 0.0022 | 0.0034 | 0.626 | 0.5359505 | 0.844438 |
| X206822_s_at | 0.0018 | 0.0029 | 0.626 | 0.5359585 | 0.844438 |
| X213799_s_at | 0.0805 | 0.1286 | 0.626 | 0.5359757 | 0.844438 |
| X211750_x_at | 0.0251 | 0.0401 | 0.626 | 0.5360034 | 0.844438 |
| X220132_s_at | -0.0053 | 0.0084 | -0.626 | 0.536032 | 0.844438 |
| X208319_s_at | -0.031 | 0.0496 | -0.626 | 0.5361757 | 0.844499 |
| X202448_s_at | 0.0015 | 0.0024 | 0.626 | 0.5361787 | 0.844499 |
| X209498_at | 0.0018 | 0.0028 | 0.626 | 0.5361849 | 0.844499 |
| X206225_at | 0.0026 | 0.0042 | 0.625 | 0.5362594 | 0.844541 |
| X209620_s_at | 0.0324 | 0.0519 | 0.625 | 0.5362874 | 0.844541 |
| X211414_at | 0.0042 | 0.0067 | 0.625 | 0.5363536 | 0.844575 |
| X201329_s_at | -0.0247 | 0.0396 | -0.625 | 0.5363958 | 0.844575 |
| X207712_at | 0.0059 | 0.0095 | 0.625 | 0.5364735 | 0.844575 |
| X212823_s_at | 0.0221 | 0.0354 | 0.625 | 0.5365371 | 0.844575 |
| X207335_x_at | -0.014 | 0.0224 | -0.625 | 0.5365378 | 0.844575 |
| X214647_s_at | -0.0031 | 0.0049 | -0.625 | 0.5365738 | 0.844575 |
| X205760_s_at | 0.0259 | 0.0415 | 0.625 | 0.5365756 | 0.844575 |
| X204593_s_at | -0.0096 | 0.0154 | -0.625 | 0.5368324 | 0.84487 |
| X211968_s_at | 0.0403 | 0.0645 | 0.625 | 0.5368392 | 0.84487 |
| X220537_at | 0.0036 | 0.0057 | 0.624 | 0.5369668 | 0.844902 |
| X207923_x_at | -0.0025 | 0.004 | -0.624 | 0.5369835 | 0.844902 |
| X210636_at | -0.004 | 0.0065 | -0.624 | 0.5370061 | 0.844902 |
| X212142_at | -0.0028 | 0.0045 | -0.624 | 0.5370408 | 0.844902 |
| X219202_at | 0.0031 | 0.005 | 0.624 | 0.537082 | 0.844902 |
| X205757_at | 0.0024 | 0.0039 | 0.624 | 0.5370877 | 0.844902 |
| X216400_at | 0.0017 | 0.0028 | 0.624 | 0.5373037 | 0.845182 |
| X215922_at | -0.0048 | 0.0077 | -0.624 | 0.5374007 | 0.845206 |
| X204614_at | -0.0057 | 0.0092 | -0.624 | 0.5374232 | 0.845206 |
| X219768_at | -0.003 | 0.0048 | -0.624 | 0.5374326 | 0.845206 |
| X213209_at | 0.0022 | 0.0035 | 0.623 | 0.5375193 | 0.845255 |
| X217121_at | -0.0029 | 0.0046 | -0.623 | 0.5375404 | 0.845255 |
| X204284_at | -0.0665 | 0.1067 | -0.623 | 0.537592 | 0.845277 |
| X220538_at | 0.002 | 0.0032 | 0.623 | 0.5376365 | 0.845287 |
| X213158_at | -0.0248 | 0.0398 | -0.623 | 0.5378587 | 0.84555 |
| X206246_at | 0.0021 | 0.0033 | 0.623 | 0.5378799 | 0.84555 |
| X221079_s_at | -0.0194 | 0.0312 | -0.623 | 0.5380505 | 0.845758 |
| X214948_s_at | 0.0247 | 0.0397 | 0.623 | 0.5381364 | 0.845833 |
| X217228_s_at | -0.0079 | 0.0127 | -0.622 | 0.5381871 | 0.845848 |
| X208411_x_at | -0.0058 | 0.0093 | -0.622 | 0.5382325 | 0.845848 |
| X37028_at | -0.0098 | 0.0157 | -0.622 | 0.53826 | 0.845848 |
| X207532_at | 0.0029 | 0.0047 | 0.622 | 0.5383936 | 0.84596 |
| X216972_at | -0.0018 | 0.0029 | -0.622 | 0.5384076 | 0.84596 |
| X208733_at | -0.0748 | 0.1203 | -0.622 | 0.5385517 | 0.846071 |
| X216849_at | 0.0053 | 0.0085 | 0.622 | 0.5385543 | 0.846071 |
| X220785_at | -0.0048 | 0.0078 | -0.622 | 0.5388079 | 0.84641 |
| X211846_s_at | -0.0019 | 0.0031 | -0.621 | 0.53887 | 0.846447 |
| X217413_s_at | -0.0034 | 0.0054 | -0.621 | 0.5389341 | 0.846484 |
| X210301_at | 0.0032 | 0.0052 | 0.621 | 0.5389697 | 0.846484 |
| X204660_at | 0.0034 | 0.0054 | 0.621 | 0.5390399 | 0.846535 |
| X215211_at | 0.0053 | 0.0085 | 0.621 | 0.5391777 | 0.846691 |
| X214568_at | -0.0035 | 0.0057 | -0.621 | 0.5392381 | 0.846726 |
| X200679_x_at | 0.0175 | 0.0281 | 0.621 | 0.5392948 | 0.846752 |
| X212096_s_at | 0.039 | 0.0629 | 0.621 | 0.5393646 | 0.846752 |
| X203732_at | -0.0229 | 0.0369 | -0.621 | 0.5393687 | 0.846752 |
| X210143_at | 0.0035 | 0.0057 | 0.621 | 0.5394251 | 0.84678 |
| X217132_at | -0.0179 | 0.0288 | -0.62 | 0.5394903 | 0.846803 |
| X218505_at | -0.0082 | 0.0132 | -0.62 | 0.5395154 | 0.846803 |
| X210165_at | 0.0009 | 0.0015 | 0.62 | 0.5395877 | 0.846856 |
| X220809_at | -0.007 | 0.0112 | -0.62 | 0.5397057 | 0.846935 |
| X210386_s_at | -0.0111 | 0.0179 | -0.62 | 0.5397142 | 0.846935 |
| X206275_s_at | -0.0465 | 0.075 | -0.62 | 0.5397643 | 0.846954 |
| X220144_s_at | -0.0079 | 0.0127 | -0.62 | 0.539897 | 0.847017 |
| X211634_x_at | 0.0016 | 0.0025 | 0.62 | 0.539938 | 0.847017 |
| X203055_s_at | -0.0037 | 0.0059 | -0.62 | 0.5399624 | 0.847017 |
| X207168_s_at | -0.0168 | 0.0271 | -0.62 | 0.5399857 | 0.847017 |
| X216991_at | -0.0024 | 0.0038 | -0.62 | 0.5399951 | 0.847017 |
| X209694_at | 0.0234 | 0.0377 | 0.62 | 0.540076 | 0.847035 |
| X221194_s_at | 0.0026 | 0.0041 | 0.619 | 0.5401352 | 0.847035 |
| X216174_at | -0.0032 | 0.0052 | -0.619 | 0.5401378 | 0.847035 |
| X219634_at | -0.0105 | 0.017 | -0.619 | 0.5401918 | 0.847035 |
| X204016_at | -0.0331 | 0.0534 | -0.619 | 0.5401974 | 0.847035 |
| X221338_at | 0.0024 | 0.0039 | 0.619 | 0.5402854 | 0.847066 |
| X202911_at | 0.0252 | 0.0407 | 0.619 | 0.5402936 | 0.847066 |
| X212597_s_at | -0.015 | 0.0243 | -0.619 | 0.5403974 | 0.847127 |
| X222019_at | 0.0028 | 0.0045 | 0.619 | 0.5404086 | 0.847127 |
| X201469_s_at | -0.0018 | 0.0029 | -0.619 | 0.5406411 | 0.847393 |
| X216803_at | -0.0084 | 0.0136 | -0.619 | 0.5406545 | 0.847393 |
| X208582_s_at | -0.0029 | 0.0046 | -0.619 | 0.5406997 | 0.847404 |
| X205067_at | 0.003 | 0.0048 | 0.618 | 0.5408034 | 0.847507 |
| X204266_s_at | -0.0077 | 0.0124 | -0.618 | 0.5409901 | 0.84766 |
| X220277_at | 0.0028 | 0.0045 | 0.618 | 0.5409964 | 0.84766 |
| X217447_at | 0.0011 | 0.0018 | 0.618 | 0.5410574 | 0.84766 |
| X214635_at | -0.0012 | 0.0019 | -0.618 | 0.5411192 | 0.84766 |
| X203886_s_at | -0.0035 | 0.0057 | -0.618 | 0.541135 | 0.84766 |
| X221669_s_at | -0.0208 | 0.0337 | -0.618 | 0.5411564 | 0.84766 |
| X208132_x_at | 0.0061 | 0.0099 | 0.618 | 0.541205 | 0.84766 |
| X215035_at | 0.0026 | 0.0042 | 0.618 | 0.5412279 | 0.84766 |
| X205429_s_at | -0.0195 | 0.0316 | -0.618 | 0.5412446 | 0.84766 |
| X204270_at | -0.0526 | 0.0852 | -0.618 | 0.5413659 | 0.84778 |
| X217889_s_at | 0.0145 | 0.0235 | 0.617 | 0.5414345 | 0.84778 |
| X215555_at | 0.1012 | 0.1638 | 0.617 | 0.5414358 | 0.84778 |
| X221080_s_at | 0.0044 | 0.0072 | 0.617 | 0.5415874 | 0.847958 |
| X206333_at | 0.0063 | 0.0102 | 0.617 | 0.5416314 | 0.847967 |
| X221972_s_at | 0.0174 | 0.0283 | 0.617 | 0.5417356 | 0.848018 |
| X212998_x_at | 0.0028 | 0.0046 | 0.617 | 0.5417899 | 0.848018 |
| X213066_at | 0.0135 | 0.0219 | 0.617 | 0.5418288 | 0.848018 |
| X219539_at | 0.0126 | 0.0204 | 0.617 | 0.541862 | 0.848018 |
| X210310_s_at | -0.0016 | 0.0026 | -0.617 | 0.5418782 | 0.848018 |
| X213012_at | 0.0042 | 0.0068 | 0.617 | 0.5419171 | 0.848018 |
| X203760_s_at | -0.0883 | 0.1432 | -0.617 | 0.5419453 | 0.848018 |
| X203677_s_at | 0.0046 | 0.0075 | 0.617 | 0.5419695 | 0.848018 |
| X201668_x_at | -0.0198 | 0.0322 | -0.616 | 0.5421763 | 0.848229 |
| X212573_at | -0.0353 | 0.0572 | -0.616 | 0.5421807 | 0.848229 |
| X220878_at | 0.0051 | 0.0083 | 0.616 | 0.5422261 | 0.84824 |
| X215181_at | -0.0046 | 0.0075 | -0.616 | 0.5425198 | 0.848627 |
| X220767_at | -0.0017 | 0.0028 | -0.616 | 0.542566 | 0.848627 |
| X203726_s_at | -0.002 | 0.0033 | -0.616 | 0.5425881 | 0.848627 |
| X204708_at | -0.0047 | 0.0077 | -0.615 | 0.5427215 | 0.848674 |
| X207034_s_at | -0.0026 | 0.0041 | -0.615 | 0.5427318 | 0.848674 |
| X217320_at | 0.0041 | 0.0067 | 0.615 | 0.5427946 | 0.848674 |
| X206319_s_at | 0.0033 | 0.0054 | 0.615 | 0.542812 | 0.848674 |
| X210333_at | -0.0019 | 0.0032 | -0.615 | 0.5428958 | 0.848674 |
| X220441_at | -0.0012 | 0.002 | -0.615 | 0.5428992 | 0.848674 |
| X32502_at | -0.0341 | 0.0554 | -0.615 | 0.5429065 | 0.848674 |
| X221346_at | 0.0019 | 0.0031 | 0.615 | 0.5429236 | 0.848674 |
| X210626_at | -0.0032 | 0.0051 | -0.615 | 0.5430448 | 0.848775 |
| X214770_at | 0.0106 | 0.0173 | 0.615 | 0.5430648 | 0.848775 |
| X217893_s_at | 0.0231 | 0.0376 | 0.615 | 0.5431172 | 0.848797 |
| X222202_at | -0.0057 | 0.0093 | -0.615 | 0.543257 | 0.848892 |
| X221291_at | 0.0024 | 0.0039 | 0.615 | 0.5433658 | 0.848892 |
| X219080_s_at | 0.0026 | 0.0042 | 0.614 | 0.5433972 | 0.848892 |
| X205372_at | -0.0085 | 0.0139 | -0.614 | 0.5434057 | 0.848892 |
| X201916_s_at | 0.0143 | 0.0233 | 0.614 | 0.5434262 | 0.848892 |
| X210055_at | -0.0055 | 0.0089 | -0.614 | 0.5434719 | 0.848892 |
| X212329_at | 0.0173 | 0.0282 | 0.614 | 0.5435044 | 0.848892 |
| X214430_at | -0.0076 | 0.0123 | -0.614 | 0.5435179 | 0.848892 |
| X201610_at | -0.0031 | 0.005 | -0.614 | 0.5435217 | 0.848892 |
| X206467_x_at | -0.0031 | 0.0051 | -0.614 | 0.5438304 | 0.849308 |
| X206510_at | 0.0026 | 0.0043 | 0.614 | 0.5438848 | 0.849308 |
| X216590_at | -0.0034 | 0.0056 | -0.614 | 0.54391 | 0.849308 |
| X211317_s_at | 0.0072 | 0.0117 | 0.613 | 0.5440372 | 0.849308 |
| X210869_s_at | 0.0534 | 0.0871 | 0.613 | 0.5440492 | 0.849308 |
| X216816_at | 0.0037 | 0.006 | 0.613 | 0.5440642 | 0.849308 |
| X208060_at | -0.0017 | 0.0028 | -0.613 | 0.5440935 | 0.849308 |
| X222206_s_at | 0.0016 | 0.0026 | 0.613 | 0.5441663 | 0.849308 |
| X217522_at | -0.0034 | 0.0055 | -0.613 | 0.5441928 | 0.849308 |
| X216401_x_at | 0.0018 | 0.0029 | 0.613 | 0.5442168 | 0.849308 |
| X200748_s_at | 0.0217 | 0.0354 | 0.613 | 0.5442482 | 0.849308 |
| X222346_at | 0.0018 | 0.003 | 0.613 | 0.5442657 | 0.849308 |
| X201388_at | -0.0242 | 0.0394 | -0.613 | 0.5442852 | 0.849308 |
| X218073_s_at | -0.0028 | 0.0046 | -0.613 | 0.544331 | 0.84932 |
| X210229_s_at | 0.0027 | 0.0044 | 0.613 | 0.5445522 | 0.849535 |
| X203703_s_at | -0.0023 | 0.0037 | -0.613 | 0.5445758 | 0.849535 |
| X201463_s_at | -0.0257 | 0.0419 | -0.613 | 0.5445837 | 0.849535 |
| X208288_at | -0.0034 | 0.0055 | -0.613 | 0.5446421 | 0.849567 |
| X204551_s_at | -0.0026 | 0.0043 | -0.612 | 0.544755 | 0.849582 |
| X202826_at | -0.0026 | 0.0043 | -0.612 | 0.5447675 | 0.849582 |
| X210230_at | -0.1271 | 0.2075 | -0.612 | 0.5447919 | 0.849582 |
| X218490_s_at | 0.0155 | 0.0253 | 0.612 | 0.5448051 | 0.849582 |
| X201682_at | -0.0224 | 0.0365 | -0.612 | 0.5448478 | 0.849589 |
| X217285_at | 0.0042 | 0.0068 | 0.612 | 0.5449473 | 0.849685 |
| X203813_s_at | -0.004 | 0.0065 | -0.612 | 0.5450134 | 0.849709 |
| X219563_at | 0.029 | 0.0474 | 0.612 | 0.5450596 | 0.849709 |
| X212931_at | 0.0217 | 0.0355 | 0.612 | 0.5450778 | 0.849709 |
| X209804_at | -0.0144 | 0.0235 | -0.612 | 0.5451528 | 0.849725 |
| X213873_at | 0.0043 | 0.007 | 0.612 | 0.5451645 | 0.849725 |
| X216724_at | -0.0068 | 0.0111 | -0.612 | 0.5452197 | 0.849752 |
| X211191_at | 0.0023 | 0.0038 | 0.612 | 0.5453197 | 0.849848 |
| X220867_s_at | 0.0028 | 0.0046 | 0.611 | 0.5453769 | 0.84987 |
| X209062_x_at | 0.0018 | 0.0029 | 0.611 | 0.5454106 | 0.84987 |
| X204753_s_at | 0.0425 | 0.0695 | 0.611 | 0.5454602 | 0.849888 |
| X216813_at | -0.077 | 0.1261 | -0.611 | 0.5456054 | 0.849997 |
| X216960_s_at | 0.0228 | 0.0373 | 0.611 | 0.5456194 | 0.849997 |
| X212522_at | 0.0542 | 0.0887 | 0.611 | 0.5456452 | 0.849997 |
| X208245_at | -0.0043 | 0.007 | -0.611 | 0.545712 | 0.850042 |
| X210266_s_at | -0.0243 | 0.0398 | -0.611 | 0.5457647 | 0.850064 |
| X208652_at | -0.0202 | 0.0331 | -0.611 | 0.5458645 | 0.850122 |
| X216672_s_at | 0.0057 | 0.0093 | 0.611 | 0.5458884 | 0.850122 |
| X205739_x_at | 0.033 | 0.054 | 0.611 | 0.5459164 | 0.850122 |
| X205551_at | 0.0465 | 0.0762 | 0.61 | 0.5460284 | 0.850221 |
| X202247_s_at | 0.0021 | 0.0035 | 0.61 | 0.5460796 | 0.850221 |
| X204299_at | 0.0269 | 0.044 | 0.61 | 0.5460947 | 0.850221 |
| X204198_s_at | 0.0039 | 0.0063 | 0.61 | 0.5461746 | 0.850228 |
| X212686_at | -0.0305 | 0.05 | -0.61 | 0.546176 | 0.850228 |
| X222012_at | -0.0019 | 0.003 | -0.61 | 0.5462795 | 0.85033 |
| X202575_at | -0.0022 | 0.0036 | -0.61 | 0.5463356 | 0.850357 |
| X219568_x_at | 0.0009 | 0.0015 | 0.61 | 0.5464119 | 0.850416 |
| X210410_s_at | 0.0031 | 0.005 | 0.61 | 0.5464986 | 0.850492 |
| X201665_x_at | 0.0137 | 0.0224 | 0.61 | 0.5465727 | 0.850548 |
| X216412_x_at | -0.0037 | 0.0061 | -0.609 | 0.5466487 | 0.850606 |
| X221962_s_at | -0.0591 | 0.097 | -0.609 | 0.5467718 | 0.850704 |
| X208188_at | 0.0022 | 0.0036 | 0.609 | 0.546788 | 0.850704 |
| X217200_x_at | -0.0241 | 0.0395 | -0.609 | 0.5470318 | 0.850819 |
| X214133_at | -0.0028 | 0.0046 | -0.609 | 0.5470424 | 0.850819 |
| X214259_s_at | 0.0227 | 0.0372 | 0.609 | 0.5470915 | 0.850819 |
| X216213_at | 0.0037 | 0.0061 | 0.609 | 0.5470976 | 0.850819 |
| X208701_at | 0.0026 | 0.0043 | 0.609 | 0.5471138 | 0.850819 |
| X207851_s_at | -0.0043 | 0.0071 | -0.609 | 0.547116 | 0.850819 |
| X210589_s_at | -0.0117 | 0.0193 | -0.609 | 0.54713 | 0.850819 |
| X212456_at | 0.0102 | 0.0167 | 0.609 | 0.5471713 | 0.850822 |
| X206224_at | -0.0035 | 0.0057 | -0.609 | 0.5472084 | 0.850822 |
| X202828_s_at | -0.0032 | 0.0053 | -0.608 | 0.5473762 | 0.850886 |
| X203443_at | 0.0016 | 0.0027 | 0.608 | 0.5474499 | 0.850886 |
| X209172_s_at | 0.0072 | 0.0119 | 0.608 | 0.5475051 | 0.850886 |
| X91952_at | 0.0012 | 0.0021 | 0.608 | 0.5475059 | 0.850886 |
| X218327_s_at | -0.0254 | 0.0418 | -0.608 | 0.5475222 | 0.850886 |
| X200050_at | 0.0195 | 0.0321 | 0.608 | 0.5475249 | 0.850886 |
| X218610_s_at | -0.0582 | 0.0957 | -0.608 | 0.5475421 | 0.850886 |
| X214612_x_at | 0.0033 | 0.0054 | 0.608 | 0.5475664 | 0.850886 |
| X219860_at | 0.0027 | 0.0044 | 0.608 | 0.5476582 | 0.850886 |
| X203501_at | 0.035 | 0.0577 | 0.608 | 0.5476714 | 0.850886 |
| X219655_at | -0.0034 | 0.0056 | -0.608 | 0.5477018 | 0.850886 |
| X210214_s_at | 0.0501 | 0.0825 | 0.608 | 0.5477094 | 0.850886 |
| X218401_s_at | 0.0254 | 0.0418 | 0.608 | 0.5478441 | 0.851036 |
| X205313_at | 0.0074 | 0.0122 | 0.607 | 0.548014 | 0.85124 |
| X203464_s_at | -0.0365 | 0.0601 | -0.607 | 0.5481988 | 0.851396 |
| X207638_at | -0.0061 | 0.0101 | -0.607 | 0.5482249 | 0.851396 |
| X212473_s_at | 0.0378 | 0.0623 | 0.607 | 0.5482296 | 0.851396 |
| X217158_at | -0.0016 | 0.0026 | -0.607 | 0.5484111 | 0.851416 |
| X204411_at | -0.0483 | 0.0797 | -0.607 | 0.5484306 | 0.851416 |
| X206359_at | 0.0052 | 0.0086 | 0.607 | 0.5484394 | 0.851416 |
| X205653_at | 0.0038 | 0.0062 | 0.607 | 0.5484535 | 0.851416 |
| X213884_s_at | 0.0023 | 0.0039 | 0.607 | 0.5484731 | 0.851416 |
| X215463_at | -0.0037 | 0.0062 | -0.607 | 0.5485144 | 0.851416 |
| X216133_at | -0.0021 | 0.0035 | -0.607 | 0.548555 | 0.851416 |
| X208305_at | 0.0031 | 0.0051 | 0.606 | 0.5486075 | 0.851416 |
| X38691_s_at | -0.0104 | 0.0171 | -0.606 | 0.5486742 | 0.851416 |
| X211397_x_at | 0.0023 | 0.0038 | 0.606 | 0.5486799 | 0.851416 |
| X220383_at | -0.0037 | 0.0061 | -0.606 | 0.548725 | 0.851416 |
| X204939_s_at | -0.0109 | 0.018 | -0.606 | 0.5487595 | 0.851416 |
| X202682_s_at | -0.0172 | 0.0284 | -0.606 | 0.5487726 | 0.851416 |
| X217065_at | 0.0029 | 0.0048 | 0.606 | 0.5488048 | 0.851416 |
| X214677_x_at | -0.0024 | 0.004 | -0.606 | 0.5488174 | 0.851416 |
| X204670_x_at | 0.0665 | 0.1097 | 0.606 | 0.5488766 | 0.851433 |
| X217392_at | -0.0027 | 0.0044 | -0.606 | 0.5489046 | 0.851433 |
| X217637_at | -0.037 | 0.0611 | -0.606 | 0.5490432 | 0.851545 |
| X211490_at | -0.0026 | 0.0044 | -0.606 | 0.549079 | 0.851545 |
| X203099_s_at | 0.0029 | 0.0048 | 0.606 | 0.5491437 | 0.851545 |
| X210435_at | -0.0074 | 0.0123 | -0.606 | 0.5491503 | 0.851545 |
| X207331_at | -0.0054 | 0.0089 | -0.606 | 0.5491863 | 0.851545 |
| X215533_s_at | -0.0141 | 0.0232 | -0.605 | 0.549284 | 0.851545 |
| X217987_at | 0.0228 | 0.0377 | 0.605 | 0.5492848 | 0.851545 |
| X208704_x_at | -0.029 | 0.048 | -0.605 | 0.5493343 | 0.851545 |
| X206380_s_at | -0.0013 | 0.0021 | -0.605 | 0.5493488 | 0.851545 |
| X205628_at | 0.0287 | 0.0474 | 0.605 | 0.5493604 | 0.851545 |
| X203520_s_at | -0.0031 | 0.0051 | -0.605 | 0.5494596 | 0.851615 |
| X220710_at | -0.0066 | 0.0109 | -0.605 | 0.5494822 | 0.851615 |
| X215458_s_at | 0.0015 | 0.0025 | 0.605 | 0.549571 | 0.851638 |
| X204641_at | -0.0092 | 0.0152 | -0.605 | 0.5496021 | 0.851638 |
| X207158_at | -0.0026 | 0.0043 | -0.605 | 0.5496119 | 0.851638 |
| X217632_at | 0.0045 | 0.0074 | 0.605 | 0.5498977 | 0.851761 |
| X222120_at | 0.0045 | 0.0075 | 0.604 | 0.5499391 | 0.851761 |
| X217448_s_at | 0.0062 | 0.0103 | 0.604 | 0.5499496 | 0.851761 |
| X219985_at | 0.0094 | 0.0156 | 0.604 | 0.549966 | 0.851761 |
| X204406_at | 0.0049 | 0.0081 | 0.604 | 0.5499816 | 0.851761 |
| X204600_at | -0.003 | 0.005 | -0.604 | 0.5499828 | 0.851761 |
| X215937_at | 0.0032 | 0.0053 | 0.604 | 0.5500305 | 0.851761 |
| X201969_at | 0.0446 | 0.0737 | 0.604 | 0.5500338 | 0.851761 |
| X219179_at | 0.0644 | 0.1065 | 0.604 | 0.5500368 | 0.851761 |
| X208661_s_at | 0.0287 | 0.0476 | 0.604 | 0.5503608 | 0.852112 |
| X217650_x_at | 0.011 | 0.0182 | 0.604 | 0.550375 | 0.852112 |
| X220791_x_at | 0.0063 | 0.0104 | 0.604 | 0.5503849 | 0.852112 |
| X208790_s_at | 0.0033 | 0.0054 | 0.604 | 0.5504166 | 0.852112 |
| X206266_at | -0.0021 | 0.0034 | -0.604 | 0.5505222 | 0.852216 |
| X205216_s_at | -0.0036 | 0.006 | -0.603 | 0.5505751 | 0.852236 |
| X206841_at | 0.0027 | 0.0045 | 0.603 | 0.5506116 | 0.852236 |
| X200692_s_at | -0.0276 | 0.0458 | -0.603 | 0.5507104 | 0.852255 |
| X206640_x_at | 0.0019 | 0.0031 | 0.603 | 0.5507346 | 0.852255 |
| X219561_at | 0.0032 | 0.0053 | 0.603 | 0.5507393 | 0.852255 |
| X216671_x_at | 0.0021 | 0.0035 | 0.603 | 0.550868 | 0.852287 |
| X213766_x_at | 0.0142 | 0.0236 | 0.603 | 0.5508779 | 0.852287 |
| X211444_at | -0.0061 | 0.0101 | -0.603 | 0.550905 | 0.852287 |
| X221913_at | 0.0023 | 0.0038 | 0.603 | 0.5509133 | 0.852287 |
| X204569_at | 0.0358 | 0.0594 | 0.603 | 0.551038 | 0.852367 |
| X207566_at | 0.0022 | 0.0036 | 0.603 | 0.5510644 | 0.852367 |
| X217563_at | -0.002 | 0.0033 | -0.603 | 0.5510803 | 0.852367 |
| X215827_x_at | -0.0017 | 0.0029 | -0.602 | 0.5513068 | 0.852658 |
| X208373_s_at | -0.0021 | 0.0035 | -0.602 | 0.5513716 | 0.852685 |
| X202031_s_at | -0.0129 | 0.0214 | -0.602 | 0.5514081 | 0.852685 |
| X210436_at | 0.0059 | 0.0097 | 0.602 | 0.5514393 | 0.852685 |
| X214065_s_at | 0.002 | 0.0033 | 0.602 | 0.5515105 | 0.852707 |
| X214275_at | 0.0027 | 0.0044 | 0.602 | 0.55153 | 0.852707 |
| X32032_at | 0.0089 | 0.0148 | 0.602 | 0.5516564 | 0.852759 |
| X206745_at | 0.0029 | 0.0049 | 0.602 | 0.551665 | 0.852759 |
| X217133_x_at | 0.0072 | 0.012 | 0.602 | 0.5516788 | 0.852759 |
| X221126_at | -0.0054 | 0.0089 | -0.602 | 0.5518123 | 0.852906 |
| X212765_at | -0.0147 | 0.0244 | -0.601 | 0.5519404 | 0.853011 |
| X220670_at | 0.0041 | 0.0067 | 0.601 | 0.5519788 | 0.853011 |
| X201187_s_at | -0.0018 | 0.003 | -0.601 | 0.5519957 | 0.853011 |
| X211779_x_at | -0.0313 | 0.052 | -0.601 | 0.5521397 | 0.853174 |
| X209649_at | 0.0364 | 0.0606 | 0.601 | 0.5521954 | 0.853191 |
| X210979_at | -0.0046 | 0.0076 | -0.601 | 0.5522273 | 0.853191 |
| X203296_s_at | 0.0315 | 0.0524 | 0.601 | 0.5523272 | 0.853286 |
| X219190_s_at | 0.0022 | 0.0036 | 0.601 | 0.5524349 | 0.85337 |
| X220829_s_at | 0.0022 | 0.0037 | 0.601 | 0.5524593 | 0.85337 |
| X217639_at | 0.0025 | 0.0041 | 0.601 | 0.552497 | 0.85337 |
| X211192_s_at | 0.0023 | 0.0038 | 0.6 | 0.5525878 | 0.853451 |
| X204770_at | 0.0033 | 0.0054 | 0.6 | 0.5526797 | 0.853534 |
| X221175_at | -0.0031 | 0.0052 | -0.6 | 0.5527449 | 0.853575 |
| X215068_s_at | -0.0275 | 0.0459 | -0.6 | 0.5529235 | 0.853686 |
| X220180_at | 0.0102 | 0.017 | 0.6 | 0.5529492 | 0.853686 |
| X216369_at | 0.0088 | 0.0147 | 0.6 | 0.5530273 | 0.853686 |
| X206263_at | 0.0031 | 0.0052 | 0.6 | 0.5530401 | 0.853686 |
| X220424_at | 0.0023 | 0.0039 | 0.6 | 0.5530857 | 0.853686 |
| X210935_s_at | -0.0038 | 0.0064 | -0.6 | 0.5530953 | 0.853686 |
| X209703_x_at | 0.0446 | 0.0744 | 0.6 | 0.5530956 | 0.853686 |
| X214242_at | 0.0022 | 0.0037 | 0.6 | 0.5531641 | 0.853686 |
| X215498_s_at | -0.0176 | 0.0293 | -0.599 | 0.5532788 | 0.853686 |
| X214804_at | -0.0053 | 0.0089 | -0.599 | 0.5532839 | 0.853686 |
| X211528_x_at | 0.023 | 0.0384 | 0.599 | 0.5532937 | 0.853686 |
| X220429_at | -0.0386 | 0.0644 | -0.599 | 0.5532979 | 0.853686 |
| X217033_x_at | 0.0411 | 0.0686 | 0.599 | 0.5533161 | 0.853686 |
| X211029_x_at | 0.002 | 0.0033 | 0.599 | 0.5534118 | 0.853774 |
| X205284_at | 0.0049 | 0.0081 | 0.599 | 0.5534708 | 0.853806 |
| X216392_s_at | 0.0468 | 0.0781 | 0.599 | 0.5535529 | 0.853873 |
| X213906_at | 0.0686 | 0.1146 | 0.599 | 0.5535989 | 0.853885 |
| X215915_at | 0.0029 | 0.0049 | 0.599 | 0.5536926 | 0.85397 |
| X209954_x_at | 0.0028 | 0.0047 | 0.599 | 0.5537694 | 0.854029 |
| X211396_at | 0.0017 | 0.0028 | 0.598 | 0.5539525 | 0.854142 |
| X217458_at | 0.0025 | 0.0042 | 0.598 | 0.5539603 | 0.854142 |
| X210226_at | 0.004 | 0.0067 | 0.598 | 0.5539683 | 0.854142 |
| X205601_s_at | -0.0025 | 0.0042 | -0.598 | 0.5540037 | 0.854142 |
| X209938_at | -0.0038 | 0.0063 | -0.598 | 0.554038 | 0.854142 |
| X215928_at | 0.0292 | 0.0489 | 0.598 | 0.5540734 | 0.854142 |
| X202043_s_at | 0.0157 | 0.0263 | 0.598 | 0.5541537 | 0.854207 |
| X221981_s_at | -0.0016 | 0.0027 | -0.598 | 0.554266 | 0.8543 |
| X217388_s_at | 0.0056 | 0.0093 | 0.598 | 0.5543638 | 0.8543 |
| X214758_at | -0.002 | 0.0034 | -0.598 | 0.5543683 | 0.8543 |
| X211981_at | 0.0019 | 0.0031 | 0.598 | 0.5543978 | 0.8543 |
| X214413_at | -0.0034 | 0.0057 | -0.598 | 0.5544407 | 0.8543 |
| X219333_s_at | -0.0022 | 0.0037 | -0.598 | 0.5544451 | 0.8543 |
| X201863_at | 0.0169 | 0.0282 | 0.598 | 0.5544851 | 0.854302 |
| X221608_at | -0.0017 | 0.0029 | -0.597 | 0.5545606 | 0.85436 |
| X208108_s_at | -0.0015 | 0.0025 | -0.597 | 0.5546063 | 0.854371 |
| X218018_at | -0.0237 | 0.0396 | -0.597 | 0.5546928 | 0.854445 |
| X208314_at | 0.0038 | 0.0064 | 0.597 | 0.5547444 | 0.854465 |
| X215679_at | -0.0052 | 0.0087 | -0.597 | 0.5548872 | 0.854614 |
| X211403_x_at | -0.0024 | 0.004 | -0.597 | 0.5550739 | 0.854614 |
| X218997_at | 0.0027 | 0.0046 | 0.597 | 0.5551077 | 0.854614 |
| X206045_s_at | -0.0388 | 0.065 | -0.596 | 0.5552106 | 0.854614 |
| X209856_x_at | -0.0036 | 0.0061 | -0.596 | 0.5553228 | 0.854614 |
| X59433_at | -0.0061 | 0.0102 | -0.596 | 0.555328 | 0.854614 |
| X204144_s_at | 0.0019 | 0.0031 | 0.596 | 0.5553468 | 0.854614 |
| X220886_at | 0.0011 | 0.0018 | 0.596 | 0.5553492 | 0.854614 |
| X204920_at | -0.0236 | 0.0396 | -0.596 | 0.5553637 | 0.854614 |
| X220473_s_at | -0.0064 | 0.0107 | -0.596 | 0.5553695 | 0.854614 |
| X219301_s_at | -0.0133 | 0.0223 | -0.596 | 0.5553811 | 0.854614 |
| X208036_at | 0.0015 | 0.0024 | 0.596 | 0.5553886 | 0.854614 |
| X218649_x_at | -0.0189 | 0.0318 | -0.596 | 0.5553957 | 0.854614 |
| X204758_s_at | -0.008 | 0.0135 | -0.596 | 0.5554079 | 0.854614 |
| X215135_at | 0.0035 | 0.0059 | 0.596 | 0.5554185 | 0.854614 |
| X218795_at | 0.0247 | 0.0414 | 0.596 | 0.5555871 | 0.854815 |
| X209910_at | -0.0029 | 0.0049 | -0.596 | 0.5556536 | 0.854858 |
| X208715_at | 0.015 | 0.0252 | 0.596 | 0.5557455 | 0.854918 |
| X213422_s_at | 0.0037 | 0.0061 | 0.596 | 0.5557695 | 0.854918 |
| X203626_s_at | 0.003 | 0.005 | 0.595 | 0.5558813 | 0.85503 |
| X205442_at | 0.0414 | 0.0696 | 0.595 | 0.5559523 | 0.85508 |
| X221265_s_at | 0.0059 | 0.0099 | 0.595 | 0.5560605 | 0.855188 |
| X206479_at | -0.0038 | 0.0064 | -0.595 | 0.5561427 | 0.855255 |
| X219849_at | 0.0032 | 0.0054 | 0.595 | 0.5562539 | 0.855367 |
| X202992_at | 0.0173 | 0.0292 | 0.595 | 0.5563489 | 0.855453 |
| X211242_x_at | -0.002 | 0.0033 | -0.595 | 0.5564309 | 0.85552 |
| X218822_s_at | 0.0014 | 0.0024 | 0.594 | 0.5565652 | 0.85557 |
| X201167_x_at | -0.0034 | 0.0057 | -0.594 | 0.5565691 | 0.85557 |
| X215980_s_at | -0.0033 | 0.0056 | -0.594 | 0.5565789 | 0.85557 |
| X203972_s_at | -0.0321 | 0.054 | -0.594 | 0.5566204 | 0.855575 |
| X208164_s_at | 0.0017 | 0.0028 | 0.594 | 0.5567946 | 0.855751 |
| X214983_at | 0.0147 | 0.0248 | 0.594 | 0.5568447 | 0.855751 |
| X212021_s_at | 0.0019 | 0.0032 | 0.594 | 0.5568509 | 0.855751 |
| X201543_s_at | 0.0122 | 0.0206 | 0.594 | 0.5569499 | 0.855842 |
| X206886_x_at | 0.0027 | 0.0046 | 0.594 | 0.5569867 | 0.855842 |
| X217691_x_at | 0.0067 | 0.0113 | 0.594 | 0.5570439 | 0.85587 |
| X213159_at | 0.004 | 0.0067 | 0.593 | 0.5571924 | 0.856017 |
| X218595_s_at | -0.0261 | 0.0439 | -0.593 | 0.5572328 | 0.856017 |
| X208327_at | -0.0037 | 0.0062 | -0.593 | 0.5573316 | 0.856017 |
| X209042_s_at | -0.0254 | 0.0428 | -0.593 | 0.5573913 | 0.856017 |
| X219642_s_at | 0.144 | 0.2428 | 0.593 | 0.5574149 | 0.856017 |
| X220197_at | -0.0028 | 0.0048 | -0.593 | 0.5574203 | 0.856017 |
| X210251_s_at | -0.0362 | 0.061 | -0.593 | 0.5574601 | 0.856017 |
| X207107_at | -0.0083 | 0.014 | -0.593 | 0.5574632 | 0.856017 |
| X215662_at | -0.0074 | 0.0124 | -0.593 | 0.5575576 | 0.856017 |
| X204736_s_at | 0.0032 | 0.0054 | 0.593 | 0.5575578 | 0.856017 |
| X210335_at | -0.0043 | 0.0072 | -0.593 | 0.557583 | 0.856017 |
| X217343_at | -0.0045 | 0.0076 | -0.593 | 0.5576574 | 0.856017 |
| X207584_at | -0.0043 | 0.0073 | -0.593 | 0.5577364 | 0.856017 |
| X217386_at | -0.0031 | 0.0053 | -0.593 | 0.557768 | 0.856017 |
| X201927_s_at | 0.1028 | 0.1735 | 0.592 | 0.5578618 | 0.856017 |
| X220196_at | -0.0015 | 0.0026 | -0.592 | 0.5579146 | 0.856017 |
| X203967_at | 0.0048 | 0.008 | 0.592 | 0.5579185 | 0.856017 |
| X216296_at | -0.0052 | 0.0087 | -0.592 | 0.5579225 | 0.856017 |
| X218161_s_at | -0.0031 | 0.0052 | -0.592 | 0.5579438 | 0.856017 |
| X219106_s_at | 0.0476 | 0.0804 | 0.592 | 0.5579548 | 0.856017 |
| X220830_at | -0.0028 | 0.0048 | -0.592 | 0.5580014 | 0.856017 |
| X201576_s_at | -0.0157 | 0.0265 | -0.592 | 0.5580201 | 0.856017 |
| X206480_at | -0.0027 | 0.0045 | -0.592 | 0.5580259 | 0.856017 |
| X210442_at | 0.0022 | 0.0037 | 0.592 | 0.5583023 | 0.856382 |
| X207271_x_at | 0.0013 | 0.0021 | 0.592 | 0.5584165 | 0.856475 |
| X211856_x_at | 0.0036 | 0.0061 | 0.592 | 0.5584396 | 0.856475 |
| X209913_x_at | 0.0287 | 0.0486 | 0.591 | 0.5585561 | 0.856594 |
| X216608_at | 0.0013 | 0.0022 | 0.591 | 0.5588166 | 0.856934 |
| X219069_at | 0.0301 | 0.051 | 0.591 | 0.5589579 | 0.85705 |
| X218543_s_at | 0.0073 | 0.0124 | 0.591 | 0.5589693 | 0.85705 |
| X216864_at | 0.0018 | 0.003 | 0.591 | 0.5590855 | 0.857143 |
| X210512_s_at | -0.0522 | 0.0884 | -0.59 | 0.5591506 | 0.857143 |
| X211340_s_at | 0.0376 | 0.0636 | 0.59 | 0.5591978 | 0.857143 |
| X211054_at | -0.0172 | 0.0292 | -0.59 | 0.5592761 | 0.857143 |
| X205947_s_at | 0.0014 | 0.0023 | 0.59 | 0.5592808 | 0.857143 |
| X215805_at | 0.0047 | 0.008 | 0.59 | 0.5593035 | 0.857143 |
| X215154_at | -0.0043 | 0.0072 | -0.59 | 0.5593128 | 0.857143 |
| X210750_s_at | 0.0043 | 0.0072 | 0.59 | 0.5593891 | 0.857143 |
| X211657_at | -0.0052 | 0.0088 | -0.59 | 0.5593897 | 0.857143 |
| X211422_at | -0.0023 | 0.0039 | -0.59 | 0.5594159 | 0.857143 |
| X215432_at | -0.0031 | 0.0052 | -0.59 | 0.5594978 | 0.857156 |
| X207635_s_at | -0.0019 | 0.0032 | -0.59 | 0.5595015 | 0.857156 |
| X208500_x_at | -0.002 | 0.0034 | -0.59 | 0.5595565 | 0.857181 |
| X215257_at | -0.0044 | 0.0075 | -0.59 | 0.5597324 | 0.857392 |
| X220655_at | -0.0061 | 0.0104 | -0.589 | 0.5598241 | 0.857407 |
| X206883_x_at | 0.0015 | 0.0026 | 0.589 | 0.5598593 | 0.857407 |
| X215377_at | -0.004 | 0.0068 | -0.589 | 0.5598962 | 0.857407 |
| X205499_at | -0.0018 | 0.003 | -0.589 | 0.5598968 | 0.857407 |
| X211597_s_at | 0.0463 | 0.0786 | 0.589 | 0.5600541 | 0.857545 |
| X209292_at | 0.0667 | 0.1132 | 0.589 | 0.5600643 | 0.857545 |
| X215829_at | 0.0022 | 0.0038 | 0.589 | 0.5602457 | 0.857764 |
| X215896_at | 0.0032 | 0.0055 | 0.589 | 0.5603839 | 0.857917 |
| X216863_s_at | 0.0063 | 0.0107 | 0.588 | 0.5604728 | 0.857993 |
| X218677_at | -0.0027 | 0.0046 | -0.588 | 0.5605966 | 0.858124 |
| X206262_at | -0.004 | 0.0068 | -0.588 | 0.5607414 | 0.858205 |
| X205146_x_at | 0.0013 | 0.0022 | 0.588 | 0.5607584 | 0.858205 |
| X210264_at | 0.0015 | 0.0026 | 0.588 | 0.5607744 | 0.858205 |
| X215855_s_at | -0.0052 | 0.0089 | -0.588 | 0.5608042 | 0.858205 |
| X214466_at | -0.002 | 0.0034 | -0.588 | 0.5608596 | 0.858231 |
| X205838_at | -0.0027 | 0.0047 | -0.588 | 0.5609903 | 0.858372 |
| X206114_at | 0.0617 | 0.105 | 0.587 | 0.5612063 | 0.858643 |
| X213155_at | 0.0386 | 0.0657 | 0.587 | 0.5614504 | 0.858914 |
| X217338_at | 0.0019 | 0.0032 | 0.587 | 0.561461 | 0.858914 |
| X210985_s_at | 0.009 | 0.0153 | 0.587 | 0.561518 | 0.858942 |
| X211106_at | -0.0679 | 0.1157 | -0.587 | 0.5616113 | 0.858984 |
| X201913_s_at | 0.0267 | 0.0455 | 0.587 | 0.5616953 | 0.858984 |
| X218187_s_at | -0.0234 | 0.04 | -0.587 | 0.5617282 | 0.858984 |
| X219843_at | -0.0366 | 0.0625 | -0.587 | 0.5617326 | 0.858984 |
| X220647_s_at | 0.0162 | 0.0277 | 0.587 | 0.5617543 | 0.858984 |
| X222302_at | -0.0062 | 0.0106 | -0.586 | 0.5617924 | 0.858984 |
| X201751_at | -0.0303 | 0.0517 | -0.586 | 0.5618545 | 0.858984 |
| X201380_at | 0.043 | 0.0734 | 0.586 | 0.5618842 | 0.858984 |
| X222041_at | 0.0082 | 0.014 | 0.586 | 0.5619259 | 0.858984 |
| X212965_at | -0.0047 | 0.008 | -0.586 | 0.5619317 | 0.858984 |
| X216127_at | -0.0039 | 0.0066 | -0.586 | 0.5619743 | 0.85899 |
| X203872_at | 0.004 | 0.0069 | 0.586 | 0.5621494 | 0.859088 |
| X219826_at | -0.011 | 0.0187 | -0.586 | 0.5621986 | 0.859088 |
| X206993_at | -0.0143 | 0.0244 | -0.586 | 0.5622063 | 0.859088 |
| X208254_at | -0.0019 | 0.0032 | -0.586 | 0.5622132 | 0.859088 |
| X204858_s_at | 0.0009 | 0.0016 | 0.586 | 0.5622317 | 0.859088 |
| X221312_at | -0.0022 | 0.0037 | -0.586 | 0.5623124 | 0.859149 |
| X207676_at | -0.0105 | 0.0179 | -0.585 | 0.562457 | 0.859149 |
| X221469_at | 0.002 | 0.0035 | 0.585 | 0.562461 | 0.859149 |
| X211976_at | 0.0191 | 0.0327 | 0.585 | 0.5624838 | 0.859149 |
| X36475_at | -0.0189 | 0.0323 | -0.585 | 0.562575 | 0.859149 |
| X214337_at | -0.0076 | 0.0129 | -0.585 | 0.5625988 | 0.859149 |
| X215818_at | 0.0022 | 0.0037 | 0.585 | 0.5626041 | 0.859149 |
| X208338_at | -0.0016 | 0.0027 | -0.585 | 0.5626754 | 0.859149 |
| X208161_s_at | 0.0023 | 0.0038 | 0.585 | 0.5627553 | 0.859149 |
| X216008_s_at | 0.0061 | 0.0105 | 0.585 | 0.5627592 | 0.859149 |
| X213568_at | 0.0047 | 0.008 | 0.585 | 0.5628117 | 0.859149 |
| X200878_at | 0.0382 | 0.0654 | 0.585 | 0.5628147 | 0.859149 |
| X204242_s_at | 0.002 | 0.0035 | 0.585 | 0.5628312 | 0.859149 |
| X220764_at | 0.0051 | 0.0087 | 0.585 | 0.5629361 | 0.859149 |
| X206156_at | 0.0018 | 0.0031 | 0.585 | 0.5629382 | 0.859149 |
| X201428_at | 0.0021 | 0.0036 | 0.585 | 0.5629479 | 0.859149 |
| X207308_at | -0.1075 | 0.1838 | -0.585 | 0.5629603 | 0.859149 |
| X214192_at | 0.0021 | 0.0035 | 0.585 | 0.562968 | 0.859149 |
| X219951_s_at | 0.0047 | 0.008 | 0.585 | 0.5630463 | 0.859209 |
| X219432_at | -0.0019 | 0.0032 | -0.584 | 0.5631459 | 0.859302 |
| X212955_s_at | -0.0172 | 0.0295 | -0.584 | 0.5632432 | 0.859323 |
| X207296_at | 0.0035 | 0.006 | 0.584 | 0.5632542 | 0.859323 |
| X221448_s_at | 0.0021 | 0.0036 | 0.584 | 0.5632756 | 0.859323 |
| X220869_at | 0.0063 | 0.0108 | 0.584 | 0.5633911 | 0.859414 |
| X217163_at | -0.0018 | 0.0031 | -0.584 | 0.5634122 | 0.859414 |
| X202251_at | -0.0235 | 0.0403 | -0.584 | 0.5636269 | 0.859667 |
| X216394_x_at | -0.0022 | 0.0037 | -0.584 | 0.5636558 | 0.859667 |
| X201246_s_at | -0.0139 | 0.0238 | -0.584 | 0.5637495 | 0.859691 |
| X202100_at | -0.0271 | 0.0464 | -0.583 | 0.5637848 | 0.859691 |
| X212527_at | 0.0024 | 0.0042 | 0.583 | 0.5637876 | 0.859691 |
| X201332_s_at | -0.0016 | 0.0028 | -0.583 | 0.5638336 | 0.8597 |
| X211505_s_at | 0.0167 | 0.0287 | 0.583 | 0.5638709 | 0.8597 |
| X207351_s_at | 0.0029 | 0.0049 | 0.583 | 0.5639637 | 0.859782 |
| X220001_at | -0.0026 | 0.0044 | -0.583 | 0.5640339 | 0.85983 |
| X210188_at | -0.0016 | 0.0028 | -0.583 | 0.5641202 | 0.859903 |
| X216248_s_at | 0.0935 | 0.1605 | 0.583 | 0.5642046 | 0.859973 |
| X206173_x_at | 0.0026 | 0.0044 | 0.583 | 0.5643765 | 0.860176 |
| X222384_at | -0.0033 | 0.0057 | -0.582 | 0.56452 | 0.860217 |
| X91826_at | 0.0018 | 0.003 | 0.582 | 0.5645457 | 0.860217 |
| X212918_at | 0.0323 | 0.0554 | 0.582 | 0.5645515 | 0.860217 |
| X209802_at | -0.003 | 0.0052 | -0.582 | 0.5645687 | 0.860217 |
| X215205_x_at | -0.0023 | 0.0039 | -0.582 | 0.564622 | 0.860217 |
| X204438_at | -0.0347 | 0.0596 | -0.582 | 0.5646649 | 0.860217 |
| X216294_s_at | -0.0022 | 0.0037 | -0.582 | 0.5646756 | 0.860217 |
| X209367_at | -0.0022 | 0.0037 | -0.582 | 0.5647136 | 0.860217 |
| X200751_s_at | -0.0278 | 0.0478 | -0.582 | 0.5649593 | 0.860448 |
| X215026_x_at | -0.0018 | 0.0032 | -0.582 | 0.5649614 | 0.860448 |
| X215550_at | -0.0021 | 0.0037 | -0.582 | 0.564985 | 0.860448 |
| X214697_s_at | -0.0063 | 0.0108 | -0.582 | 0.5650201 | 0.860448 |
| X204509_at | 0.0087 | 0.015 | 0.581 | 0.565191 | 0.860605 |
| X215698_at | 0.0719 | 0.1236 | 0.581 | 0.5652008 | 0.860605 |
| X203502_at | 0.0335 | 0.0575 | 0.581 | 0.5652607 | 0.860622 |
| X211775_x_at | -0.0072 | 0.0124 | -0.581 | 0.5652962 | 0.860622 |
| X213014_at | -0.0015 | 0.0025 | -0.581 | 0.565401 | 0.860622 |
| X218423_x_at | 0.0144 | 0.0248 | 0.581 | 0.565404 | 0.860622 |
| X209697_at | -0.0026 | 0.0044 | -0.581 | 0.565439 | 0.860622 |
| X202404_s_at | -0.01 | 0.0172 | -0.581 | 0.5654528 | 0.860622 |
| X220638_s_at | -0.0021 | 0.0036 | -0.581 | 0.5654966 | 0.860622 |
| X211922_s_at | 0.0456 | 0.0786 | 0.581 | 0.5655215 | 0.860622 |
| X214401_at | 0.0017 | 0.0029 | 0.581 | 0.5656705 | 0.860709 |
| X205400_at | 0.0037 | 0.0063 | 0.581 | 0.5656951 | 0.860709 |
| X217013_at | -0.003 | 0.0052 | -0.581 | 0.5656951 | 0.860709 |
| X211028_s_at | -0.0122 | 0.021 | -0.58 | 0.565779 | 0.860765 |
| X222055_at | 0.0034 | 0.0059 | 0.58 | 0.5658678 | 0.860765 |
| X217687_at | -0.0042 | 0.0072 | -0.58 | 0.5658848 | 0.860765 |
| X211190_x_at | 0.0024 | 0.0041 | 0.58 | 0.5659453 | 0.860765 |
| X212606_at | 0.0142 | 0.0245 | 0.58 | 0.5659468 | 0.860765 |
| X215313_x_at | 0.0395 | 0.0681 | 0.58 | 0.566018 | 0.860765 |
| X201480_s_at | -0.0195 | 0.0335 | -0.58 | 0.5660207 | 0.860765 |
| X207754_at | 0.0091 | 0.0157 | 0.58 | 0.5660772 | 0.860765 |
| X218303_x_at | 0.0272 | 0.0469 | 0.58 | 0.5661142 | 0.860765 |
| X216852_x_at | 0.002 | 0.0035 | 0.58 | 0.5663367 | 0.860765 |
| X218243_at | -0.0115 | 0.0198 | -0.58 | 0.5663533 | 0.860765 |
| X203030_s_at | -0.0327 | 0.0565 | -0.58 | 0.5663645 | 0.860765 |
| X213642_at | 0.0627 | 0.1082 | 0.58 | 0.5663735 | 0.860765 |
| X208181_at | -0.0064 | 0.011 | -0.58 | 0.5664253 | 0.860765 |
| X209294_x_at | 0.0015 | 0.0025 | 0.579 | 0.5664743 | 0.860765 |
| X203389_at | -0.0213 | 0.0368 | -0.579 | 0.5664748 | 0.860765 |
| X213114_at | -0.0018 | 0.0031 | -0.579 | 0.5664749 | 0.860765 |
| X204719_at | 0.0852 | 0.147 | 0.579 | 0.5665325 | 0.860765 |
| X206561_s_at | 0.0027 | 0.0047 | 0.579 | 0.5665497 | 0.860765 |
| X222044_at | -0.0417 | 0.072 | -0.579 | 0.5665833 | 0.860765 |
| X219136_s_at | 0.012 | 0.0207 | 0.579 | 0.5666037 | 0.860765 |
| X207837_at | -0.0072 | 0.0124 | -0.579 | 0.5666233 | 0.860765 |
| X201496_x_at | -0.006 | 0.0104 | -0.579 | 0.5666321 | 0.860765 |
| X221484_at | 0.0424 | 0.0733 | 0.579 | 0.5666965 | 0.860765 |
| X219262_at | -0.0086 | 0.0148 | -0.579 | 0.5667006 | 0.860765 |
| X221215_s_at | -0.0022 | 0.0038 | -0.579 | 0.5667454 | 0.860774 |
| X221326_s_at | -0.005 | 0.0087 | -0.579 | 0.5667892 | 0.860782 |
| X214225_at | 0.0031 | 0.0054 | 0.579 | 0.5670028 | 0.861047 |
| X201617_x_at | 0.0438 | 0.0757 | 0.578 | 0.5672514 | 0.861236 |
| X216897_s_at | 0.0023 | 0.004 | 0.578 | 0.5673192 | 0.861236 |
| X219589_s_at | -0.0027 | 0.0046 | -0.578 | 0.5673358 | 0.861236 |
| X216467_s_at | -0.0077 | 0.0133 | -0.578 | 0.5674096 | 0.861236 |
| X206630_at | -0.0027 | 0.0047 | -0.578 | 0.5674296 | 0.861236 |
| X203168_at | 0.0056 | 0.0097 | 0.578 | 0.567467 | 0.861236 |
| X220481_at | 0.0039 | 0.0067 | 0.578 | 0.5675451 | 0.861236 |
| X203477_at | -0.0069 | 0.0119 | -0.578 | 0.5675539 | 0.861236 |
| X215476_at | -0.0018 | 0.003 | -0.578 | 0.5675865 | 0.861236 |
| X220425_x_at | -0.0015 | 0.0026 | -0.578 | 0.5676891 | 0.861236 |
| X216554_s_at | -0.0037 | 0.0065 | -0.578 | 0.5677217 | 0.861236 |
| X206629_at | -0.0017 | 0.0029 | -0.578 | 0.5677685 | 0.861236 |
| X208321_s_at | 0.0222 | 0.0384 | 0.577 | 0.5678247 | 0.861236 |
| X209657_s_at | -0.0304 | 0.0526 | -0.577 | 0.5678248 | 0.861236 |
| X214978_s_at | 0.0296 | 0.0512 | 0.577 | 0.567827 | 0.861236 |
| X213042_s_at | 0.0033 | 0.0057 | 0.577 | 0.5678779 | 0.861236 |
| X209397_at | -0.0246 | 0.0427 | -0.577 | 0.5679923 | 0.861236 |
| X207164_s_at | -0.0049 | 0.0085 | -0.577 | 0.5680755 | 0.861236 |
| X204962_s_at | -0.0027 | 0.0047 | -0.577 | 0.5680935 | 0.861236 |
| X218146_at | -0.0162 | 0.028 | -0.577 | 0.5681326 | 0.861236 |
| X220507_s_at | -0.0029 | 0.005 | -0.577 | 0.568148 | 0.861236 |
| X207319_s_at | -0.0031 | 0.0054 | -0.577 | 0.5681817 | 0.861236 |
| X219316_s_at | -0.0026 | 0.0046 | -0.577 | 0.5681841 | 0.861236 |
| X216846_at | 0.0024 | 0.0041 | 0.577 | 0.568192 | 0.861236 |
| X210536_s_at | -0.0036 | 0.0063 | -0.577 | 0.5682945 | 0.861236 |
| X219472_at | -0.0093 | 0.0161 | -0.577 | 0.5683851 | 0.861236 |
| X200712_s_at | 0.0355 | 0.0616 | 0.577 | 0.568395 | 0.861236 |
| X206127_at | -0.0018 | 0.0031 | -0.577 | 0.5684025 | 0.861236 |
| X207835_at | 0.0039 | 0.0068 | 0.576 | 0.5684547 | 0.861236 |
| X206375_s_at | 0.0021 | 0.0037 | 0.576 | 0.5684627 | 0.861236 |
| X206024_at | -0.0016 | 0.0029 | -0.576 | 0.5685247 | 0.861236 |
| X218999_at | 0.0047 | 0.0082 | 0.576 | 0.5685375 | 0.861236 |
| X222291_at | -0.0301 | 0.0522 | -0.576 | 0.5685395 | 0.861236 |
| X44040_at | 0.0285 | 0.0494 | 0.576 | 0.5685658 | 0.861236 |
| X220891_at | 0.0052 | 0.009 | 0.576 | 0.568617 | 0.861236 |
| X207953_at | -0.0073 | 0.0127 | -0.576 | 0.5686369 | 0.861236 |
| X221335_x_at | -0.0017 | 0.0029 | -0.576 | 0.5686662 | 0.861236 |
| X218323_at | 0.0195 | 0.0338 | 0.576 | 0.5686798 | 0.861236 |
| X220651_s_at | 0.0036 | 0.0062 | 0.576 | 0.5687122 | 0.861236 |
| X208160_at | 0.0015 | 0.0026 | 0.576 | 0.5687152 | 0.861236 |
| X212356_at | 0.0041 | 0.0071 | 0.576 | 0.5687349 | 0.861236 |
| X203416_at | 0.0941 | 0.1633 | 0.576 | 0.5687553 | 0.861236 |
| X215271_at | 0.003 | 0.0052 | 0.576 | 0.5689304 | 0.861442 |
| X205011_at | -0.0412 | 0.0716 | -0.576 | 0.5690076 | 0.861469 |
| X219987_at | 0.0036 | 0.0062 | 0.576 | 0.5690311 | 0.861469 |
| X213741_s_at | -0.0443 | 0.0769 | -0.576 | 0.5690643 | 0.861469 |
| X213431_x_at | -0.002 | 0.0035 | -0.575 | 0.5692998 | 0.861767 |
| X208795_s_at | 0.0429 | 0.0745 | 0.575 | 0.5694414 | 0.861922 |
| X216493_s_at | 0.0053 | 0.0092 | 0.575 | 0.5695673 | 0.862054 |
| X201648_at | 0.0184 | 0.0321 | 0.575 | 0.5696494 | 0.862062 |
| X214517_at | 0.0022 | 0.0039 | 0.575 | 0.5696694 | 0.862062 |
| X219891_at | 0.0034 | 0.006 | 0.575 | 0.5696892 | 0.862062 |
| X219229_at | 0.0593 | 0.1032 | 0.574 | 0.5698033 | 0.862131 |
| X206543_at | -0.0279 | 0.0486 | -0.574 | 0.5698118 | 0.862131 |
| X32402_s_at | 0.0067 | 0.0116 | 0.574 | 0.5698517 | 0.862132 |
| X201902_s_at | -0.0015 | 0.0026 | -0.574 | 0.5702938 | 0.862739 |
| X219182_at | 0.043 | 0.075 | 0.574 | 0.5703308 | 0.862739 |
| X215018_at | -0.0094 | 0.0164 | -0.574 | 0.5704287 | 0.862829 |
| X210437_at | -0.0038 | 0.0067 | -0.573 | 0.5708814 | 0.86331 |
| X206816_s_at | -0.0064 | 0.0111 | -0.573 | 0.5708816 | 0.86331 |
| X47571_at | 0.0263 | 0.0459 | 0.573 | 0.5708833 | 0.86331 |
| X221745_at | 0.0238 | 0.0416 | 0.573 | 0.5709466 | 0.86331 |
| X216348_at | 0.0244 | 0.0425 | 0.573 | 0.5709717 | 0.86331 |
| X215015_at | 0.0019 | 0.0033 | 0.573 | 0.5710024 | 0.86331 |
| X211565_at | -0.0861 | 0.1504 | -0.573 | 0.5710191 | 0.86331 |
| X206063_x_at | 0.0025 | 0.0044 | 0.573 | 0.5710951 | 0.863325 |
| X212031_at | 0.0208 | 0.0364 | 0.573 | 0.5711068 | 0.863325 |
| X216495_x_at | -0.0029 | 0.005 | -0.572 | 0.5711566 | 0.863342 |
| X208859_s_at | 0.0623 | 0.1088 | 0.572 | 0.5712113 | 0.863366 |
| X221576_at | -0.0017 | 0.003 | -0.572 | 0.571317 | 0.863401 |
| X219105_x_at | 0.0313 | 0.0547 | 0.572 | 0.5713174 | 0.863401 |
| X221240_s_at | -0.0044 | 0.0076 | -0.572 | 0.5713511 | 0.863401 |
| X207265_s_at | 0.0037 | 0.0065 | 0.572 | 0.571676 | 0.863833 |
| X215629_s_at | -0.0432 | 0.0756 | -0.572 | 0.5717395 | 0.86387 |
| X220464_at | 0.0095 | 0.0167 | 0.571 | 0.5717947 | 0.863895 |
| X213234_at | 0.0229 | 0.0401 | 0.571 | 0.5719526 | 0.864075 |
| X204881_s_at | -0.0621 | 0.1087 | -0.571 | 0.5719934 | 0.864078 |
| X222049_s_at | -0.0015 | 0.0027 | -0.571 | 0.5721619 | 0.864147 |
| X220557_s_at | -0.0049 | 0.0085 | -0.571 | 0.5721818 | 0.864147 |
| X215721_at | -0.0051 | 0.009 | -0.571 | 0.5722126 | 0.864147 |
| X216634_at | 0.0033 | 0.0059 | 0.571 | 0.5722928 | 0.864147 |
| X221507_at | -0.0232 | 0.0406 | -0.571 | 0.5723089 | 0.864147 |
| X220527_at | -0.0034 | 0.0059 | -0.571 | 0.5723671 | 0.864147 |
| X220897_at | -0.0082 | 0.0144 | -0.571 | 0.572378 | 0.864147 |
| X201385_at | 0.0153 | 0.0268 | 0.571 | 0.5724107 | 0.864147 |
| X211560_s_at | -0.0006 | 0.001 | -0.571 | 0.5724171 | 0.864147 |
| X210853_at | 0.0041 | 0.0073 | 0.571 | 0.5724494 | 0.864147 |
| X207740_s_at | -0.0022 | 0.0038 | -0.57 | 0.5724763 | 0.864147 |
| X212247_at | 0.0107 | 0.0188 | 0.57 | 0.5725064 | 0.864147 |
| X206378_at | -0.0042 | 0.0074 | -0.57 | 0.572573 | 0.864189 |
| X205766_at | -0.0056 | 0.0099 | -0.57 | 0.5726727 | 0.864213 |
| X213324_at | 0.0057 | 0.01 | 0.57 | 0.572697 | 0.864213 |
| X215833_s_at | 0.0024 | 0.0042 | 0.57 | 0.5727797 | 0.864213 |
| X212946_at | -0.0358 | 0.0629 | -0.57 | 0.5728472 | 0.864213 |
| X216762_at | 0.0048 | 0.0085 | 0.57 | 0.5728713 | 0.864213 |
| X217347_at | 0.0108 | 0.0189 | 0.57 | 0.5728779 | 0.864213 |
| X207201_s_at | -0.0039 | 0.0069 | -0.57 | 0.5729195 | 0.864213 |
| X212478_at | 0.002 | 0.0034 | 0.57 | 0.5729253 | 0.864213 |
| X206229_x_at | 0.0031 | 0.0055 | 0.57 | 0.5729391 | 0.864213 |
| X220968_s_at | 0.0097 | 0.0169 | 0.57 | 0.5729992 | 0.864245 |
| X204937_s_at | -0.0262 | 0.046 | -0.57 | 0.5730419 | 0.864251 |
| X218198_at | 0.0202 | 0.0355 | 0.569 | 0.573258 | 0.864496 |
| X218171_at | 0.0175 | 0.0307 | 0.569 | 0.5732819 | 0.864496 |
| X204109_s_at | 0.0025 | 0.0043 | 0.569 | 0.5733777 | 0.864581 |
| X206030_at | 0.0768 | 0.135 | 0.569 | 0.5734614 | 0.864649 |
| X221893_s_at | -0.04 | 0.0704 | -0.569 | 0.5736261 | 0.864839 |
| X222245_s_at | -0.0036 | 0.0064 | -0.569 | 0.5737417 | 0.864954 |
| X200732_s_at | -0.0289 | 0.0508 | -0.568 | 0.5737947 | 0.864975 |
| X207849_at | -0.0065 | 0.0115 | -0.568 | 0.5738637 | 0.865021 |
| X212233_at | 0.0177 | 0.0311 | 0.568 | 0.5739242 | 0.86503 |
| X214531_s_at | -0.0273 | 0.0481 | -0.568 | 0.5739477 | 0.86503 |
| X205124_at | -0.0026 | 0.0046 | -0.568 | 0.5742653 | 0.865327 |
| X215612_at | 0.0026 | 0.0045 | 0.568 | 0.5743597 | 0.865327 |
| X201638_s_at | 0.002 | 0.0035 | 0.568 | 0.5743611 | 0.865327 |
| X220054_at | 0.0023 | 0.0041 | 0.568 | 0.574367 | 0.865327 |
| X217431_x_at | -0.0044 | 0.0078 | -0.568 | 0.5743769 | 0.865327 |
| X219090_at | -0.0274 | 0.0483 | -0.568 | 0.5743784 | 0.865327 |
| X217483_at | -0.0025 | 0.0044 | -0.567 | 0.5745341 | 0.865417 |
| X215074_at | -0.0056 | 0.0098 | -0.567 | 0.5745501 | 0.865417 |
| X202157_s_at | 0.0223 | 0.0393 | 0.567 | 0.5745751 | 0.865417 |
| X206977_at | -0.0047 | 0.0083 | -0.567 | 0.5745941 | 0.865417 |
| X219612_s_at | -0.0036 | 0.0063 | -0.567 | 0.5747684 | 0.865621 |
| X207992_s_at | 0.0378 | 0.0667 | 0.567 | 0.5749017 | 0.865759 |
| X205302_at | 0.0079 | 0.0139 | 0.567 | 0.5749549 | 0.865759 |
| X208271_at | 0.0034 | 0.0061 | 0.567 | 0.575061 | 0.865759 |
| X206760_s_at | -0.0022 | 0.0039 | -0.567 | 0.5751098 | 0.865759 |
| X206329_at | 0.0029 | 0.0051 | 0.566 | 0.575211 | 0.865759 |
| X214161_at | -0.0046 | 0.0081 | -0.566 | 0.575231 | 0.865759 |
| X213241_at | 0.0388 | 0.0685 | 0.566 | 0.5752401 | 0.865759 |
| X205002_at | 0.0026 | 0.0047 | 0.566 | 0.5752661 | 0.865759 |
| X219594_at | 0.1204 | 0.2127 | 0.566 | 0.5753704 | 0.865759 |
| X216577_at | 0.0023 | 0.0041 | 0.566 | 0.5753788 | 0.865759 |
| X204756_at | -0.0207 | 0.0366 | -0.566 | 0.5753808 | 0.865759 |
| X210576_at | -0.003 | 0.0053 | -0.566 | 0.5754201 | 0.865759 |
| X204402_at | -0.0165 | 0.0292 | -0.566 | 0.5754212 | 0.865759 |
| X214444_s_at | -0.0043 | 0.0075 | -0.566 | 0.5754437 | 0.865759 |
| X214283_at | 0.0076 | 0.0134 | 0.566 | 0.5754547 | 0.865759 |
| X208047_s_at | -0.0314 | 0.0554 | -0.566 | 0.5754835 | 0.865759 |
| X217263_x_at | -0.0029 | 0.0052 | -0.566 | 0.5757576 | 0.86608 |
| X201025_at | 0.0202 | 0.0358 | 0.566 | 0.5757751 | 0.86608 |
| X215457_at | -0.0057 | 0.01 | -0.565 | 0.5758739 | 0.86617 |
| X217240_at | 0.0057 | 0.0101 | 0.565 | 0.5759979 | 0.866298 |
| X203568_s_at | -0.0083 | 0.0146 | -0.565 | 0.5761082 | 0.866405 |
| X219306_at | 0.0026 | 0.0046 | 0.565 | 0.5761722 | 0.866443 |
| X216735_x_at | -0.0071 | 0.0125 | -0.565 | 0.5762626 | 0.86652 |
| X219562_at | 0.0282 | 0.0499 | 0.565 | 0.5763207 | 0.866532 |
| X210326_at | 0.0017 | 0.003 | 0.565 | 0.576349 | 0.866532 |
| X215785_s_at | 0.0141 | 0.0249 | 0.565 | 0.5763962 | 0.866545 |
| X217220_at | 0.0081 | 0.0143 | 0.564 | 0.5764925 | 0.866631 |
| X220885_s_at | 0.0042 | 0.0075 | 0.564 | 0.5767348 | 0.866887 |
| X220632_s_at | -0.0022 | 0.004 | -0.564 | 0.5767412 | 0.866887 |
| X211989_at | 0.0154 | 0.0274 | 0.564 | 0.5770108 | 0.867234 |
| X215768_at | 0.057 | 0.1011 | 0.564 | 0.5770584 | 0.867247 |
| X36566_at | -0.009 | 0.0159 | -0.563 | 0.5772084 | 0.867386 |
| X219357_at | 0.0148 | 0.0263 | 0.563 | 0.577229 | 0.867386 |
| X201035_s_at | -0.0048 | 0.0086 | -0.563 | 0.5772989 | 0.867432 |
| X204750_s_at | 0.0041 | 0.0072 | 0.563 | 0.5773631 | 0.867452 |
| X219300_s_at | 0.052 | 0.0924 | 0.563 | 0.5773902 | 0.867452 |
| X204368_at | 0.0022 | 0.004 | 0.563 | 0.5775512 | 0.867607 |
| X214683_s_at | 0.0268 | 0.0476 | 0.563 | 0.5775864 | 0.867607 |
| X216414_at | -0.0038 | 0.0068 | -0.563 | 0.5776106 | 0.867607 |
| X217103_at | -0.0038 | 0.0067 | -0.562 | 0.5779162 | 0.867953 |
| X205395_s_at | 0.009 | 0.0161 | 0.562 | 0.5780472 | 0.867953 |
| X210356_x_at | 0.0033 | 0.0058 | 0.562 | 0.57805 | 0.867953 |
| X206473_at | 0.0025 | 0.0044 | 0.562 | 0.5780536 | 0.867953 |
| X213683_at | -0.0259 | 0.0461 | -0.562 | 0.5780693 | 0.867953 |
| X202226_s_at | -0.0449 | 0.0799 | -0.562 | 0.578119 | 0.867953 |
| X212717_at | -0.0203 | 0.0361 | -0.562 | 0.5781415 | 0.867953 |
| X221512_at | -0.0034 | 0.0061 | -0.562 | 0.5782125 | 0.867953 |
| X208952_s_at | -0.0142 | 0.0252 | -0.562 | 0.5782166 | 0.867953 |
| X203098_at | -0.0366 | 0.0651 | -0.562 | 0.578259 | 0.867953 |
| X210398_x_at | 0.0015 | 0.0028 | 0.562 | 0.5782831 | 0.867953 |
| X217115_at | -0.0032 | 0.0056 | -0.562 | 0.57831 | 0.867953 |
| X221139_s_at | 0.0239 | 0.0425 | 0.562 | 0.5784089 | 0.867969 |
| X220008_at | 0.002 | 0.0036 | 0.562 | 0.57844 | 0.867969 |
| X64474_g_at | 0.0046 | 0.0082 | 0.562 | 0.5784703 | 0.867969 |
| X214887_at | -0.004 | 0.0071 | -0.561 | 0.5785395 | 0.867969 |
| X211329_x_at | 0.0027 | 0.0047 | 0.561 | 0.5785489 | 0.867969 |
| X216954_x_at | -0.0106 | 0.0189 | -0.561 | 0.5785547 | 0.867969 |
| X213363_at | -0.0017 | 0.0031 | -0.561 | 0.5786188 | 0.868006 |
| X34858_at | -0.0197 | 0.0351 | -0.561 | 0.5786841 | 0.868024 |
| X212101_at | -0.0087 | 0.0156 | -0.561 | 0.5787473 | 0.868024 |
| X216541_x_at | -0.0023 | 0.0041 | -0.561 | 0.578768 | 0.868024 |
| X216124_at | 0.0035 | 0.0062 | 0.561 | 0.5787866 | 0.868024 |
| X215070_x_at | -0.0108 | 0.0193 | -0.561 | 0.5788752 | 0.868065 |
| X217668_at | -0.0034 | 0.0061 | -0.561 | 0.5788931 | 0.868065 |
| X205785_at | -0.0017 | 0.003 | -0.561 | 0.5789313 | 0.868065 |
| X213650_at | 0.0382 | 0.0681 | 0.561 | 0.5790069 | 0.86812 |
| X206397_x_at | 0.0228 | 0.0407 | 0.561 | 0.5790504 | 0.868126 |
| X216270_at | -0.0019 | 0.0034 | -0.56 | 0.5792196 | 0.868321 |
| X220320_at | 0.0015 | 0.0027 | 0.56 | 0.5793509 | 0.868396 |
| X204343_at | 0.0153 | 0.0273 | 0.56 | 0.5793997 | 0.868396 |
| X222053_at | 0.0041 | 0.0074 | 0.56 | 0.5794318 | 0.868396 |
| X216001_at | 0.0031 | 0.0054 | 0.56 | 0.5794395 | 0.868396 |
| X205513_at | 0.0026 | 0.0046 | 0.56 | 0.5794713 | 0.868396 |
| X221490_at | 0.0205 | 0.0366 | 0.56 | 0.5795377 | 0.868396 |
| X208086_s_at | 0.0035 | 0.0062 | 0.56 | 0.5795757 | 0.868396 |
| X220584_at | -0.0024 | 0.0042 | -0.56 | 0.5795824 | 0.868396 |
| X61297_at | 0.0233 | 0.0416 | 0.56 | 0.5797179 | 0.868445 |
| X221632_s_at | 0.002 | 0.0036 | 0.56 | 0.5797374 | 0.868445 |
| X201514_s_at | -0.0103 | 0.0183 | -0.56 | 0.5797481 | 0.868445 |
| X211839_s_at | -0.0024 | 0.0042 | -0.559 | 0.5798647 | 0.868445 |
| X212614_at | -0.0248 | 0.0443 | -0.559 | 0.5798751 | 0.868445 |
| X219270_at | 0.0022 | 0.0039 | 0.559 | 0.5798998 | 0.868445 |
| X221910_at | -0.0035 | 0.0062 | -0.559 | 0.5799209 | 0.868445 |
| X208638_at | -0.0187 | 0.0334 | -0.559 | 0.5799273 | 0.868445 |
| X202910_s_at | 0.0023 | 0.0041 | 0.559 | 0.5801003 | 0.868595 |
| X205385_at | 0.0028 | 0.005 | 0.559 | 0.5801057 | 0.868595 |
| X203840_at | -0.0175 | 0.0313 | -0.559 | 0.5802655 | 0.868764 |
| X218790_s_at | -0.0025 | 0.0046 | -0.559 | 0.5802969 | 0.868764 |
| X207641_at | 0.0019 | 0.0034 | 0.559 | 0.5804313 | 0.868819 |
| X216564_at | -0.0026 | 0.0046 | -0.559 | 0.5804829 | 0.868819 |
| X215623_x_at | -0.0065 | 0.0116 | -0.559 | 0.5804924 | 0.868819 |
| X205514_at | -0.0231 | 0.0414 | -0.558 | 0.5805579 | 0.868819 |
| X206733_at | 0.0015 | 0.0027 | 0.558 | 0.5805641 | 0.868819 |
| X207083_s_at | -0.0021 | 0.0038 | -0.558 | 0.5805684 | 0.868819 |
| X207140_at | -0.0018 | 0.0033 | -0.558 | 0.5806609 | 0.868887 |
| X215191_at | 0.0511 | 0.0916 | 0.558 | 0.5807322 | 0.868887 |
| X222073_at | 0.0353 | 0.0632 | 0.558 | 0.5807456 | 0.868887 |
| X213548_s_at | 0.0028 | 0.0051 | 0.558 | 0.5807703 | 0.868887 |
| X208032_s_at | -0.0544 | 0.0974 | -0.558 | 0.5809193 | 0.86901 |
| X206795_at | -0.0051 | 0.0091 | -0.558 | 0.5809311 | 0.86901 |
| X209955_s_at | -0.0027 | 0.0049 | -0.558 | 0.5810277 | 0.869017 |
| X204233_s_at | -0.0358 | 0.0642 | -0.558 | 0.5810345 | 0.869017 |
| X206758_at | 0.0021 | 0.0038 | 0.558 | 0.581053 | 0.869017 |
| X203899_s_at | -0.0816 | 0.1463 | -0.558 | 0.5811276 | 0.86907 |
| X218042_at | -0.0202 | 0.0362 | -0.557 | 0.5812315 | 0.869167 |
| X218983_at | 0.0464 | 0.0833 | 0.557 | 0.5813621 | 0.869194 |
| X219283_at | 0.0282 | 0.0507 | 0.557 | 0.5813625 | 0.869194 |
| X211604_x_at | -0.0022 | 0.004 | -0.557 | 0.5813671 | 0.869194 |
| X213306_at | 0.0213 | 0.0383 | 0.557 | 0.5816082 | 0.869267 |
| X205017_s_at | -0.0024 | 0.0044 | -0.557 | 0.5816634 | 0.869267 |
| X216045_at | 0.0053 | 0.0095 | 0.557 | 0.5816814 | 0.869267 |
| X212773_s_at | -0.024 | 0.0432 | -0.557 | 0.5817019 | 0.869267 |
| X215122_at | -0.0042 | 0.0075 | -0.557 | 0.5817274 | 0.869267 |
| X218469_at | -0.1274 | 0.2289 | -0.557 | 0.5817302 | 0.869267 |
| X208539_x_at | -0.0038 | 0.0069 | -0.557 | 0.5817907 | 0.869267 |
| X202793_at | 0.0047 | 0.0085 | 0.557 | 0.5817925 | 0.869267 |
| X215521_at | -0.009 | 0.0162 | -0.557 | 0.5818125 | 0.869267 |
| X204891_s_at | 0.0051 | 0.0092 | 0.557 | 0.5818312 | 0.869267 |
| X212858_at | 0.0465 | 0.0836 | 0.556 | 0.5818806 | 0.869267 |
| X207018_s_at | -0.0074 | 0.0133 | -0.556 | 0.5818856 | 0.869267 |
| X216087_at | -0.0053 | 0.0095 | -0.556 | 0.5820723 | 0.869445 |
| X208049_s_at | -0.0028 | 0.0051 | -0.556 | 0.5820826 | 0.869445 |
| X177_at | -0.0034 | 0.0061 | -0.556 | 0.582148 | 0.869484 |
| X217605_at | 0.0294 | 0.0528 | 0.556 | 0.5822406 | 0.869564 |
| X212591_at | -0.0203 | 0.0366 | -0.556 | 0.5824088 | 0.869706 |
| X220321_s_at | 0.0202 | 0.0363 | 0.556 | 0.5824553 | 0.869706 |
| X221267_s_at | 0.0293 | 0.0527 | 0.556 | 0.5824746 | 0.869706 |
| X210290_at | -0.0053 | 0.0095 | -0.556 | 0.5825148 | 0.869706 |
| X209552_at | -0.0027 | 0.0048 | -0.556 | 0.5825318 | 0.869706 |
| X220338_at | -0.0305 | 0.0549 | -0.555 | 0.5825747 | 0.869712 |
| X213079_at | -0.0168 | 0.0303 | -0.555 | 0.5826336 | 0.869733 |
| X214811_at | 0.0392 | 0.0706 | 0.555 | 0.5826954 | 0.869733 |
| X207184_at | -0.0015 | 0.0027 | -0.555 | 0.5827257 | 0.869733 |
| X221323_at | -0.0019 | 0.0034 | -0.555 | 0.5827544 | 0.869733 |
| X215113_s_at | -0.0023 | 0.0041 | -0.555 | 0.5827868 | 0.869733 |
| X209639_s_at | 0.0174 | 0.0313 | 0.555 | 0.5828236 | 0.869733 |
| X208849_at | 0.0052 | 0.0094 | 0.555 | 0.5830614 | 0.869957 |
| X210698_at | -0.0052 | 0.0094 | -0.555 | 0.5831015 | 0.869957 |
| X206009_at | 0.003 | 0.0054 | 0.555 | 0.5831352 | 0.869957 |
| X2028_s_at | -0.001 | 0.0017 | -0.555 | 0.5831484 | 0.869957 |
| X215344_at | 0.0228 | 0.041 | 0.555 | 0.5831696 | 0.869957 |
| X210873_x_at | -0.007 | 0.0125 | -0.554 | 0.5833644 | 0.870153 |
| X210039_s_at | -0.0051 | 0.0091 | -0.554 | 0.5833791 | 0.870153 |
| X217537_x_at | -0.0035 | 0.0062 | -0.554 | 0.5834475 | 0.870162 |
| X215486_at | 0.0033 | 0.006 | 0.554 | 0.5834755 | 0.870162 |
| X206832_s_at | 0.0015 | 0.0027 | 0.554 | 0.5835042 | 0.870162 |
| X208214_at | -0.0065 | 0.0117 | -0.554 | 0.5835423 | 0.870162 |
| X219453_at | 0.0023 | 0.0041 | 0.554 | 0.5836935 | 0.870319 |
| X215609_at | -0.0056 | 0.0102 | -0.554 | 0.5837255 | 0.870319 |
| X210491_at | -0.0051 | 0.0092 | -0.554 | 0.5838172 | 0.870378 |
| X208129_x_at | -0.0022 | 0.0039 | -0.554 | 0.5838439 | 0.870378 |
| X209675_s_at | 0.0029 | 0.0052 | 0.553 | 0.5839809 | 0.870482 |
| X220681_at | 0.0019 | 0.0035 | 0.553 | 0.5839915 | 0.870482 |
| X214609_at | 0.0027 | 0.0049 | 0.553 | 0.5840539 | 0.870516 |
| X216082_at | -0.0014 | 0.0026 | -0.553 | 0.5841348 | 0.870524 |
| X215044_s_at | -0.0025 | 0.0045 | -0.553 | 0.5841651 | 0.870524 |
| X204542_at | -0.0034 | 0.0062 | -0.553 | 0.5841765 | 0.870524 |
| X39248_at | -0.0024 | 0.0043 | -0.553 | 0.5843617 | 0.870741 |
| X216318_at | -0.0029 | 0.0053 | -0.553 | 0.5844659 | 0.870838 |
| X208855_s_at | 0.0139 | 0.0251 | 0.553 | 0.5845455 | 0.870858 |
| X214038_at | -0.0036 | 0.0065 | -0.553 | 0.5845575 | 0.870858 |
| X216600_x_at | -0.0025 | 0.0046 | -0.552 | 0.5847051 | 0.870923 |
| X204995_at | 0.0211 | 0.0383 | 0.552 | 0.5847165 | 0.870923 |
| X210086_at | 0.0019 | 0.0034 | 0.552 | 0.5847576 | 0.870923 |
| X206847_s_at | -0.0012 | 0.0022 | -0.552 | 0.5847635 | 0.870923 |
| X222213_x_at | 0.0041 | 0.0075 | 0.552 | 0.5848104 | 0.870923 |
| X213084_x_at | -0.0084 | 0.0151 | -0.552 | 0.584861 | 0.870923 |
| X217021_at | -0.0038 | 0.0069 | -0.552 | 0.5848755 | 0.870923 |
| X211485_s_at | -0.0015 | 0.0027 | -0.552 | 0.5849798 | 0.87102 |
| X206004_at | -0.0017 | 0.0031 | -0.552 | 0.5850685 | 0.871093 |
| X209994_s_at | 0.059 | 0.1069 | 0.552 | 0.5851871 | 0.871212 |
| X206716_at | 0.0019 | 0.0034 | 0.551 | 0.5852922 | 0.871268 |
| X209712_at | 0.0149 | 0.027 | 0.551 | 0.5853034 | 0.871268 |
| X215263_at | -0.0033 | 0.006 | -0.551 | 0.5853473 | 0.871271 |
| X222033_s_at | -0.0582 | 0.1056 | -0.551 | 0.5853841 | 0.871271 |
| X216956_s_at | -0.0021 | 0.0037 | -0.551 | 0.5854236 | 0.871272 |
| X216551_x_at | 0.0072 | 0.0131 | 0.551 | 0.5855593 | 0.871312 |
| X207609_s_at | -0.0017 | 0.0031 | -0.551 | 0.5855875 | 0.871312 |
| X201178_at | 0.0223 | 0.0405 | 0.551 | 0.5856059 | 0.871312 |
| X213182_x_at | 0.0728 | 0.1322 | 0.551 | 0.5856168 | 0.871312 |
| X204624_at | 0.013 | 0.0236 | 0.551 | 0.5856784 | 0.871312 |
| X220326_s_at | 0.0028 | 0.0051 | 0.551 | 0.5856999 | 0.871312 |
| X204916_at | -0.028 | 0.0508 | -0.551 | 0.5857348 | 0.871312 |
| X218240_at | 0.0038 | 0.0068 | 0.551 | 0.5858288 | 0.871312 |
| X222060_at | 0.005 | 0.0091 | 0.551 | 0.5858623 | 0.871312 |
| X207286_at | -0.0251 | 0.0457 | -0.551 | 0.5858717 | 0.871312 |
| X219266_at | -0.0049 | 0.009 | -0.551 | 0.5858826 | 0.871312 |
| X216217_at | -0.0053 | 0.0096 | -0.551 | 0.5859215 | 0.871312 |
| X209863_s_at | -0.0024 | 0.0044 | -0.55 | 0.5860158 | 0.871315 |
| X215454_x_at | -0.0024 | 0.0044 | -0.55 | 0.5861183 | 0.871315 |
| X216978_x_at | 0.0033 | 0.0059 | 0.55 | 0.5861187 | 0.871315 |
| X221288_at | 0.0574 | 0.1043 | 0.55 | 0.5861481 | 0.871315 |
| X206683_at | -0.0061 | 0.011 | -0.55 | 0.5861628 | 0.871315 |
| X204067_at | -0.0289 | 0.0525 | -0.55 | 0.5861863 | 0.871315 |
| X215801_at | 0.0064 | 0.0116 | 0.55 | 0.5861978 | 0.871315 |
| X222382_x_at | -0.0047 | 0.0085 | -0.55 | 0.5863461 | 0.871477 |
| X210081_at | -0.0024 | 0.0044 | -0.55 | 0.5865039 | 0.871653 |
| X212471_at | -0.0016 | 0.0029 | -0.55 | 0.5865599 | 0.871678 |
| X214056_at | 0.0046 | 0.0083 | 0.549 | 0.5866075 | 0.87169 |
| X44617_at | -0.0032 | 0.0058 | -0.549 | 0.5867159 | 0.871793 |
| X207157_s_at | 0.0203 | 0.037 | 0.549 | 0.5867898 | 0.871845 |
| X220631_at | -0.003 | 0.0055 | -0.549 | 0.5869038 | 0.871956 |
| X210629_x_at | -0.0018 | 0.0033 | -0.549 | 0.5869783 | 0.872008 |
| X209920_at | -0.0468 | 0.0852 | -0.549 | 0.587146 | 0.872199 |
| X222085_at | -0.0021 | 0.0038 | -0.549 | 0.587211 | 0.872237 |
| X220252_x_at | 0.0102 | 0.0186 | 0.549 | 0.5872617 | 0.872254 |
| X215347_at | -0.0037 | 0.0068 | -0.548 | 0.5873125 | 0.872271 |
| X218870_at | 0.0018 | 0.0032 | 0.548 | 0.587356 | 0.872278 |
| X219630_at | 0.0025 | 0.0045 | 0.548 | 0.5874277 | 0.872319 |
| X221133_s_at | 0.0019 | 0.0035 | 0.548 | 0.5874863 | 0.872319 |
| X211145_x_at | -0.0024 | 0.0043 | -0.548 | 0.5875017 | 0.872319 |
| X218068_s_at | 0.0352 | 0.0642 | 0.548 | 0.5877321 | 0.872547 |
| X209198_s_at | -0.03 | 0.0548 | -0.548 | 0.5877419 | 0.872547 |
| X219962_at | -0.004 | 0.0072 | -0.548 | 0.5877734 | 0.872547 |
| X217007_s_at | 0.0016 | 0.003 | 0.547 | 0.5880264 | 0.872865 |
| X209750_at | 0.0576 | 0.1052 | 0.547 | 0.5880688 | 0.872869 |
| X216889_s_at | 0.0032 | 0.0058 | 0.547 | 0.5882429 | 0.872955 |
| X213599_at | -0.003 | 0.0055 | -0.547 | 0.5883403 | 0.872955 |
| X215043_s_at | 0.0768 | 0.1404 | 0.547 | 0.5883954 | 0.872955 |
| X215218_s_at | -0.0026 | 0.0047 | -0.547 | 0.5884019 | 0.872955 |
| X204951_at | 0.0024 | 0.0044 | 0.547 | 0.5884263 | 0.872955 |
| X212554_at | -0.0227 | 0.0415 | -0.547 | 0.5884434 | 0.872955 |
| X203935_at | -0.0273 | 0.05 | -0.547 | 0.5884572 | 0.872955 |
| X215762_at | 0.0029 | 0.0052 | 0.547 | 0.5884775 | 0.872955 |
| X207082_at | 0.002 | 0.0037 | 0.547 | 0.5884974 | 0.872955 |
| X207881_at | -0.0048 | 0.0089 | -0.547 | 0.5885521 | 0.872955 |
| X205753_at | 0.0016 | 0.003 | 0.547 | 0.5885587 | 0.872955 |
| X206436_at | -0.0011 | 0.0021 | -0.546 | 0.5888473 | 0.87316 |
| X202626_s_at | -0.0132 | 0.0242 | -0.546 | 0.5888494 | 0.87316 |
| X204997_at | -0.0011 | 0.002 | -0.546 | 0.5888523 | 0.87316 |
| X214356_s_at | -0.0187 | 0.0343 | -0.546 | 0.5888544 | 0.87316 |
| X207060_at | -0.0033 | 0.006 | -0.546 | 0.5889416 | 0.873175 |
| X207244_x_at | -0.0032 | 0.0058 | -0.546 | 0.588966 | 0.873175 |
| X204429_s_at | 0.0016 | 0.003 | 0.546 | 0.5890195 | 0.873175 |
| X214018_at | -0.0016 | 0.0029 | -0.546 | 0.5890214 | 0.873175 |
| X211790_s_at | -0.002 | 0.0037 | -0.546 | 0.5891235 | 0.873254 |
| X210099_at | -0.0015 | 0.0027 | -0.546 | 0.5892046 | 0.873254 |
| X219603_s_at | -0.03 | 0.055 | -0.546 | 0.5892126 | 0.873254 |
| X217224_at | 0.0013 | 0.0025 | 0.546 | 0.5892585 | 0.873254 |
| X215927_at | 0.0018 | 0.0033 | 0.545 | 0.5893269 | 0.873254 |
| X211407_at | -0.0041 | 0.0075 | -0.545 | 0.5893278 | 0.873254 |
| X213661_at | 0.0558 | 0.1024 | 0.545 | 0.5893503 | 0.873254 |
| X221254_s_at | 0.0016 | 0.003 | 0.545 | 0.5894656 | 0.873367 |
| X208565_at | -0.0023 | 0.0043 | -0.545 | 0.5895261 | 0.873384 |
| X219810_at | 0.002 | 0.0037 | 0.545 | 0.5895628 | 0.873384 |
| X221969_at | -0.0018 | 0.0033 | -0.545 | 0.5896553 | 0.873384 |
| X208922_s_at | -0.0296 | 0.0543 | -0.545 | 0.5896631 | 0.873384 |
| X211656_x_at | 0.0034 | 0.0062 | 0.545 | 0.5896738 | 0.873384 |
| X222061_at | 0.0035 | 0.0064 | 0.545 | 0.5897721 | 0.873422 |
| X220515_at | 0.0015 | 0.0028 | 0.545 | 0.5899103 | 0.873422 |
| X201118_at | 0.0273 | 0.0501 | 0.545 | 0.5899298 | 0.873422 |
| X214742_at | -0.0023 | 0.0043 | -0.545 | 0.5899662 | 0.873422 |
| X215610_at | -0.0023 | 0.0043 | -0.545 | 0.5899817 | 0.873422 |
| X212628_at | 0.024 | 0.0441 | 0.545 | 0.5899847 | 0.873422 |
| X204536_s_at | -0.0021 | 0.0038 | -0.544 | 0.5899936 | 0.873422 |
| X210001_s_at | 0.0161 | 0.0296 | 0.544 | 0.590014 | 0.873422 |
| X216839_at | -0.0073 | 0.0135 | -0.544 | 0.5901389 | 0.873549 |
| X221173_at | 0.0021 | 0.0038 | 0.544 | 0.5902008 | 0.873582 |
| X206995_x_at | 0.0181 | 0.0332 | 0.544 | 0.5902795 | 0.87364 |
| X206470_at | -0.0025 | 0.0046 | -0.544 | 0.5903455 | 0.87368 |
| X206897_at | -0.0027 | 0.0049 | -0.544 | 0.5904496 | 0.873776 |
| X211224_s_at | -0.0015 | 0.0027 | -0.544 | 0.5905648 | 0.873855 |
| X211124_s_at | -0.0029 | 0.0053 | -0.544 | 0.5905819 | 0.873855 |
| X216691_at | 0.0028 | 0.0051 | 0.543 | 0.5907683 | 0.874073 |
| X213990_s_at | -0.0282 | 0.0518 | -0.543 | 0.5908559 | 0.874144 |
| X204461_x_at | -0.0249 | 0.0458 | -0.543 | 0.5909842 | 0.874195 |
| X212943_at | -0.0232 | 0.0428 | -0.543 | 0.5910079 | 0.874195 |
| X200930_s_at | 0.0022 | 0.004 | 0.543 | 0.5910082 | 0.874195 |
| X219047_s_at | -0.0015 | 0.0028 | -0.543 | 0.5911546 | 0.874261 |
| X212285_s_at | -0.0037 | 0.0067 | -0.543 | 0.5912119 | 0.874261 |
| X200848_at | -0.0348 | 0.0641 | -0.543 | 0.591214 | 0.874261 |
| X220110_s_at | 0.0023 | 0.0042 | 0.543 | 0.5912498 | 0.874261 |
| X204543_at | -0.0022 | 0.0041 | -0.543 | 0.5912501 | 0.874261 |
| X201382_at | -0.0148 | 0.0272 | -0.542 | 0.5914909 | 0.874501 |
| X222023_at | -0.0025 | 0.0046 | -0.542 | 0.5914912 | 0.874501 |
| X206519_x_at | -0.0076 | 0.0141 | -0.542 | 0.5915393 | 0.874501 |
| X200875_s_at | -0.0236 | 0.0435 | -0.542 | 0.5915697 | 0.874501 |
| X207051_at | -0.003 | 0.0056 | -0.542 | 0.5917156 | 0.87461 |
| X221319_at | -0.0035 | 0.0065 | -0.542 | 0.5917441 | 0.87461 |
| X215217_at | 0.0023 | 0.0042 | 0.542 | 0.5917615 | 0.87461 |
| X214093_s_at | -0.0254 | 0.0469 | -0.542 | 0.5920092 | 0.874745 |
| X219780_at | -0.0016 | 0.0029 | -0.542 | 0.5920095 | 0.874745 |
| X217128_s_at | -0.0535 | 0.0987 | -0.542 | 0.592011 | 0.874745 |
| X221711_s_at | 0.0189 | 0.035 | 0.542 | 0.5920259 | 0.874745 |
| X207382_at | -0.0028 | 0.0051 | -0.541 | 0.5921873 | 0.874745 |
| X206364_at | -0.0052 | 0.0097 | -0.541 | 0.5922718 | 0.874745 |
| X215559_at | -0.0014 | 0.0025 | -0.541 | 0.5923039 | 0.874745 |
| X208110_x_at | -0.002 | 0.0036 | -0.541 | 0.5923214 | 0.874745 |
| X209743_s_at | -0.0041 | 0.0075 | -0.541 | 0.5923362 | 0.874745 |
| X210513_s_at | 0.0022 | 0.0041 | 0.541 | 0.5923385 | 0.874745 |
| X210558_at | -0.003 | 0.0055 | -0.541 | 0.5925184 | 0.874745 |
| X204318_s_at | -0.0044 | 0.0081 | -0.541 | 0.5926135 | 0.874745 |
| X210194_at | -0.0073 | 0.0135 | -0.541 | 0.5926816 | 0.874745 |
| X210864_x_at | -0.0017 | 0.0032 | -0.541 | 0.5926826 | 0.874745 |
| X203363_s_at | -0.0037 | 0.0069 | -0.541 | 0.5927119 | 0.874745 |
| X201892_s_at | -0.014 | 0.0259 | -0.54 | 0.5927282 | 0.874745 |
| X215426_at | -0.0022 | 0.0041 | -0.54 | 0.5927518 | 0.874745 |
| X222246_at | -0.0039 | 0.0072 | -0.54 | 0.5928277 | 0.874745 |
| X206719_at | 0.002 | 0.0037 | 0.54 | 0.5928345 | 0.874745 |
| X204453_at | 0.0175 | 0.0323 | 0.54 | 0.5928423 | 0.874745 |
| X209873_s_at | 0.0019 | 0.0035 | 0.54 | 0.5928571 | 0.874745 |
| X211198_s_at | 0.001 | 0.0018 | 0.54 | 0.592928 | 0.874745 |
| X207097_s_at | -0.0024 | 0.0044 | -0.54 | 0.5929734 | 0.874745 |
| X200870_at | -0.0165 | 0.0306 | -0.54 | 0.5930463 | 0.874745 |
| X220029_at | 0.0027 | 0.0051 | 0.54 | 0.5930626 | 0.874745 |
| X209525_at | 0.027 | 0.0499 | 0.54 | 0.5930629 | 0.874745 |
| X205130_at | -0.0027 | 0.005 | -0.54 | 0.5930887 | 0.874745 |
| X215715_at | -0.0036 | 0.0067 | -0.54 | 0.5931734 | 0.874745 |
| X213007_at | -0.0047 | 0.0087 | -0.54 | 0.5932357 | 0.874745 |
| X216764_at | -0.0032 | 0.0059 | -0.54 | 0.5932605 | 0.874745 |
| X209426_s_at | -0.0101 | 0.0188 | -0.54 | 0.5932784 | 0.874745 |
| X205778_at | 0.0021 | 0.0038 | 0.54 | 0.5933841 | 0.874745 |
| X214790_at | -0.0298 | 0.0552 | -0.539 | 0.5934138 | 0.874745 |
| X216789_at | -0.003 | 0.0056 | -0.539 | 0.5934231 | 0.874745 |
| X217909_s_at | -0.0076 | 0.014 | -0.539 | 0.5934552 | 0.874745 |
| X215656_at | -0.0019 | 0.0035 | -0.539 | 0.593465 | 0.874745 |
| X204449_at | 0.0231 | 0.0428 | 0.539 | 0.5935451 | 0.874745 |
| X220672_at | -0.0043 | 0.008 | -0.539 | 0.5935642 | 0.874745 |
| X212450_at | 0.0285 | 0.0529 | 0.539 | 0.5935935 | 0.874745 |
| X202164_s_at | 0.0286 | 0.053 | 0.539 | 0.5935973 | 0.874745 |
| X216170_at | -0.0188 | 0.0349 | -0.539 | 0.5936165 | 0.874745 |
| X221836_s_at | 0.0125 | 0.0232 | 0.539 | 0.5936373 | 0.874745 |
| X218910_at | 0.0115 | 0.0213 | 0.539 | 0.5936375 | 0.874745 |
| X200676_s_at | 0.0324 | 0.0601 | 0.539 | 0.5936389 | 0.874745 |
| X222009_at | -0.0026 | 0.0049 | -0.539 | 0.5936608 | 0.874745 |
| X214986_x_at | 0.0025 | 0.0046 | 0.539 | 0.5936644 | 0.874745 |
| X211494_s_at | -0.0818 | 0.1518 | -0.539 | 0.5937067 | 0.87475 |
| X215595_x_at | 0.0094 | 0.0174 | 0.539 | 0.5938822 | 0.87495 |
| X222271_at | 0.0031 | 0.0057 | 0.539 | 0.5939423 | 0.874981 |
| X209962_at | -0.0014 | 0.0026 | -0.539 | 0.5940328 | 0.875056 |
| X211625_s_at | -0.0025 | 0.0046 | -0.538 | 0.5940972 | 0.875093 |
| X213764_s_at | -0.008 | 0.0149 | -0.538 | 0.5941821 | 0.87512 |
| X207349_s_at | -0.0023 | 0.0043 | -0.538 | 0.5942462 | 0.87512 |
| X222233_s_at | 0.0211 | 0.0391 | 0.538 | 0.5942695 | 0.87512 |
| X216223_at | -0.005 | 0.0093 | -0.538 | 0.5942734 | 0.87512 |
| X210376_x_at | -0.0448 | 0.0834 | -0.538 | 0.5943771 | 0.875131 |
| X208767_s_at | -0.0428 | 0.0795 | -0.538 | 0.5944929 | 0.875131 |
| X211330_s_at | -0.0066 | 0.0122 | -0.538 | 0.594603 | 0.875131 |
| X219272_at | 0.0029 | 0.0053 | 0.538 | 0.5946329 | 0.875131 |
| X207445_s_at | 0.0023 | 0.0044 | 0.538 | 0.5946342 | 0.875131 |
| X221222_s_at | 0.0063 | 0.0118 | 0.538 | 0.5946375 | 0.875131 |
| X205411_at | 0.0022 | 0.0041 | 0.538 | 0.594678 | 0.875131 |
| X208042_at | 0.0281 | 0.0523 | 0.538 | 0.5947588 | 0.875131 |
| X208262_x_at | -0.0049 | 0.0091 | -0.537 | 0.594792 | 0.875131 |
| X204354_at | -0.0216 | 0.0402 | -0.537 | 0.5948229 | 0.875131 |
| X203239_s_at | -0.0045 | 0.0083 | -0.537 | 0.5948313 | 0.875131 |
| X202480_s_at | -0.0023 | 0.0043 | -0.537 | 0.5948544 | 0.875131 |
| X214292_at | -0.0022 | 0.004 | -0.537 | 0.59493 | 0.875131 |
| X220439_at | 0.0018 | 0.0034 | 0.537 | 0.5950027 | 0.875131 |
| X211146_at | -0.0031 | 0.0057 | -0.537 | 0.5950049 | 0.875131 |
| X208231_at | 0.003 | 0.0055 | 0.537 | 0.5950582 | 0.875131 |
| X206019_at | -0.0079 | 0.0148 | -0.537 | 0.5950768 | 0.875131 |
| X216618_at | -0.0057 | 0.0107 | -0.537 | 0.5951488 | 0.875131 |
| X210394_x_at | 0.0033 | 0.0061 | 0.537 | 0.5951622 | 0.875131 |
| X217090_at | -0.0031 | 0.0058 | -0.537 | 0.5951672 | 0.875131 |
| X221377_s_at | 0.001 | 0.0019 | 0.537 | 0.5951719 | 0.875131 |
| X216000_at | -0.0396 | 0.0737 | -0.537 | 0.5951983 | 0.875131 |
| X210710_at | 0.0013 | 0.0024 | 0.537 | 0.5952371 | 0.875131 |
| X217657_at | 0.0041 | 0.0077 | 0.537 | 0.5952753 | 0.875131 |
| X208564_at | -0.0022 | 0.004 | -0.537 | 0.5952812 | 0.875131 |
| X205996_s_at | -0.0038 | 0.0072 | -0.537 | 0.595305 | 0.875131 |
| X219545_at | 0.0024 | 0.0046 | 0.537 | 0.5953713 | 0.875171 |
| X209961_s_at | 0.0022 | 0.004 | 0.536 | 0.5954553 | 0.875236 |
| X215935_at | 0.0026 | 0.0049 | 0.536 | 0.5955687 | 0.875279 |
| X41113_at | -0.0144 | 0.0268 | -0.536 | 0.5955718 | 0.875279 |
| X220964_s_at | -0.0271 | 0.0506 | -0.536 | 0.5956148 | 0.875279 |
| X210889_s_at | 0.0066 | 0.0123 | 0.536 | 0.5956421 | 0.875279 |
| X200607_s_at | -0.0395 | 0.0737 | -0.536 | 0.5957491 | 0.875378 |
| X216629_at | -0.0028 | 0.0052 | -0.536 | 0.5958272 | 0.875386 |
| X55093_at | -0.0026 | 0.0049 | -0.536 | 0.5958331 | 0.875386 |
| X206873_at | -0.003 | 0.0056 | -0.536 | 0.5958856 | 0.875405 |
| X209434_s_at | -0.0216 | 0.0404 | -0.536 | 0.5959451 | 0.875432 |
| X209375_at | -0.0158 | 0.0296 | -0.536 | 0.5959828 | 0.875432 |
| X215403_at | -0.0024 | 0.0045 | -0.536 | 0.5960316 | 0.875446 |
| X216893_s_at | -0.0036 | 0.0066 | -0.535 | 0.5962465 | 0.875704 |
| X211899_s_at | 0.0011 | 0.002 | 0.535 | 0.5963879 | 0.875854 |
| X201977_s_at | 0.0368 | 0.0687 | 0.535 | 0.5965803 | 0.876078 |
| X208048_at | -0.0029 | 0.0055 | -0.535 | 0.5967102 | 0.8762 |
| X207683_at | 0.0021 | 0.0039 | 0.535 | 0.5967593 | 0.8762 |
| X202386_s_at | 0.0223 | 0.0416 | 0.535 | 0.5967815 | 0.8762 |
| X220536_at | 0.0029 | 0.0054 | 0.534 | 0.5968715 | 0.876274 |
| X208348_s_at | -0.0038 | 0.0071 | -0.534 | 0.5970244 | 0.876405 |
| X211619_s_at | -0.0017 | 0.0031 | -0.534 | 0.5970393 | 0.876405 |
| X221902_at | -0.0057 | 0.0107 | -0.534 | 0.5971516 | 0.876512 |
| X203676_at | 0.0017 | 0.0032 | 0.534 | 0.5971933 | 0.876515 |
| X207925_at | -0.0017 | 0.0032 | -0.534 | 0.5972759 | 0.876578 |
| X219372_at | 0.0211 | 0.0395 | 0.534 | 0.5973643 | 0.876601 |
| X219328_at | -0.001 | 0.0018 | -0.534 | 0.597492 | 0.876601 |
| X222256_s_at | -0.0024 | 0.0044 | -0.533 | 0.5975058 | 0.876601 |
| X222148_s_at | -0.0177 | 0.0331 | -0.533 | 0.5975414 | 0.876601 |
| X214267_s_at | 0.0026 | 0.0049 | 0.533 | 0.5975459 | 0.876601 |
| X212408_at | 0.0111 | 0.0207 | 0.533 | 0.5976097 | 0.876601 |
| X203515_s_at | -0.0274 | 0.0514 | -0.533 | 0.597615 | 0.876601 |
| X221040_at | -0.0041 | 0.0077 | -0.533 | 0.5976252 | 0.876601 |
| X208201_at | -0.0025 | 0.0047 | -0.533 | 0.5976467 | 0.876601 |
| X200858_s_at | 0.0136 | 0.0255 | 0.533 | 0.597859 | 0.87679 |
| X208302_at | -0.0033 | 0.0061 | -0.533 | 0.5978863 | 0.87679 |
| X212322_at | 0.0276 | 0.0517 | 0.533 | 0.5979574 | 0.87679 |
| X219019_at | 0.0031 | 0.0059 | 0.533 | 0.5979656 | 0.87679 |
| X220792_at | -0.0036 | 0.0068 | -0.533 | 0.5979728 | 0.87679 |
| X203283_s_at | -0.0319 | 0.0598 | -0.533 | 0.5980496 | 0.876845 |
| X202663_at | 0.05 | 0.0938 | 0.533 | 0.5981029 | 0.876865 |
| X214702_at | 0.005 | 0.0094 | 0.532 | 0.5982426 | 0.876945 |
| X214928_at | 0.0027 | 0.005 | 0.532 | 0.5982824 | 0.876945 |
| X204422_s_at | 0.0474 | 0.0891 | 0.532 | 0.5982865 | 0.876945 |
| X208304_at | -0.0015 | 0.0028 | -0.532 | 0.5983154 | 0.876945 |
| X221047_s_at | -0.0505 | 0.095 | -0.532 | 0.598399 | 0.876979 |
| X211772_x_at | -0.0023 | 0.0044 | -0.532 | 0.5984283 | 0.876979 |
| X216319_at | 0.0075 | 0.0141 | 0.532 | 0.5984572 | 0.876979 |
| X219735_s_at | 0.0023 | 0.0043 | 0.532 | 0.5985348 | 0.877035 |
| X218994_s_at | -0.0134 | 0.0253 | -0.532 | 0.5986369 | 0.877086 |
| X203481_at | -0.0352 | 0.0663 | -0.532 | 0.5986488 | 0.877086 |
| X219264_s_at | 0.0028 | 0.0053 | 0.532 | 0.5987594 | 0.877181 |
| X203852_s_at | -0.0459 | 0.0863 | -0.532 | 0.5987921 | 0.877181 |
| X211262_at | 0.0028 | 0.0053 | 0.531 | 0.599142 | 0.877635 |
| X214510_at | -0.0019 | 0.0036 | -0.531 | 0.5991822 | 0.877636 |
| X210025_s_at | -0.0012 | 0.0024 | -0.531 | 0.5992378 | 0.87766 |
| X206715_at | 0.0041 | 0.0077 | 0.531 | 0.5993045 | 0.8777 |
| X211828_s_at | -0.0383 | 0.0722 | -0.531 | 0.5994103 | 0.87773 |
| X203053_at | -0.0132 | 0.0248 | -0.531 | 0.5994267 | 0.87773 |
| X204297_at | -0.0134 | 0.0252 | -0.531 | 0.5994439 | 0.87773 |
| X215943_at | -0.0022 | 0.0042 | -0.53 | 0.599593 | 0.877891 |
| X220069_at | -0.0017 | 0.0032 | -0.53 | 0.5996391 | 0.8779 |
| X207193_at | -0.0012 | 0.0024 | -0.53 | 0.5997483 | 0.878002 |
| X219514_at | 0.0017 | 0.0032 | 0.53 | 0.5998236 | 0.878034 |
| X218191_s_at | -0.0131 | 0.0247 | -0.53 | 0.5998489 | 0.878034 |
| X217614_at | 0.0019 | 0.0035 | 0.53 | 0.5998979 | 0.878048 |
| X217354_s_at | -0.0028 | 0.0054 | -0.53 | 0.6000134 | 0.878159 |
| X203826_s_at | 0.0061 | 0.0115 | 0.53 | 0.6000808 | 0.8782 |
| X204013_s_at | -0.0046 | 0.0086 | -0.53 | 0.6001568 | 0.87821 |
| X213822_s_at | 0.0046 | 0.0086 | 0.53 | 0.6001666 | 0.87821 |
| X202984_s_at | 0.0231 | 0.0437 | 0.529 | 0.6002523 | 0.878221 |
| X202699_s_at | -0.0016 | 0.0029 | -0.529 | 0.6002536 | 0.878221 |
| X210955_at | 0.0032 | 0.006 | 0.529 | 0.600332 | 0.87825 |
| X212172_at | 0.0091 | 0.0171 | 0.529 | 0.600352 | 0.87825 |
| X219188_s_at | -0.0035 | 0.0066 | -0.529 | 0.6004276 | 0.878302 |
| X210280_at | -0.0052 | 0.0098 | -0.529 | 0.6004868 | 0.878331 |
| X34210_at | -0.0043 | 0.0081 | -0.529 | 0.6005797 | 0.878409 |
| X207326_at | -0.0062 | 0.0117 | -0.529 | 0.6007304 | 0.878485 |
| X207365_x_at | 0.0415 | 0.0786 | 0.529 | 0.6007389 | 0.878485 |
| X205602_x_at | -0.002 | 0.0039 | -0.529 | 0.600803 | 0.878485 |
| X210103_s_at | -0.0016 | 0.003 | -0.528 | 0.6010102 | 0.878485 |
| X216010_x_at | 0.0022 | 0.0041 | 0.528 | 0.6010115 | 0.878485 |
| X210827_s_at | 0.0034 | 0.0064 | 0.528 | 0.6010146 | 0.878485 |
| X209914_s_at | -0.0243 | 0.046 | -0.528 | 0.6010353 | 0.878485 |
| X214143_x_at | 0.011 | 0.0209 | 0.528 | 0.6010643 | 0.878485 |
| X207594_s_at | 0.0018 | 0.0033 | 0.528 | 0.6010742 | 0.878485 |
| X220417_s_at | -0.0149 | 0.0283 | -0.528 | 0.6011307 | 0.878485 |
| X213412_at | -0.0019 | 0.0035 | -0.528 | 0.6011425 | 0.878485 |
| X205656_at | -0.0312 | 0.059 | -0.528 | 0.6011453 | 0.878485 |
| X216134_at | 0.003 | 0.0057 | 0.528 | 0.601146 | 0.878485 |
| X220471_s_at | -0.0031 | 0.006 | -0.528 | 0.6012345 | 0.878526 |
| X207009_at | 0.0023 | 0.0043 | 0.528 | 0.6012526 | 0.878526 |
| X210581_x_at | 0.0023 | 0.0043 | 0.528 | 0.60134 | 0.878596 |
| X206924_at | -0.0025 | 0.0047 | -0.528 | 0.6013818 | 0.878599 |
| X207461_at | -0.0075 | 0.0142 | -0.528 | 0.6014616 | 0.878658 |
| X200806_s_at | 0.063 | 0.1195 | 0.527 | 0.6016059 | 0.878811 |
| X217049_x_at | -0.0192 | 0.0364 | -0.527 | 0.6017085 | 0.878903 |
| X212760_at | 0.0129 | 0.0244 | 0.527 | 0.6018547 | 0.879059 |
| X220438_at | 0.0025 | 0.0048 | 0.527 | 0.6020016 | 0.879186 |
| X222100_at | -0.0018 | 0.0034 | -0.527 | 0.6020212 | 0.879186 |
| X220804_s_at | -0.0016 | 0.003 | -0.527 | 0.6021072 | 0.879254 |
| X37201_at | -0.0024 | 0.0045 | -0.527 | 0.6021715 | 0.87929 |
| X202813_at | -0.0371 | 0.0705 | -0.526 | 0.6022897 | 0.879341 |
| X217198_x_at | -0.0012 | 0.0023 | -0.526 | 0.6022909 | 0.879341 |
| X210369_at | 0.002 | 0.0039 | 0.526 | 0.6023253 | 0.879341 |
| X221912_s_at | -0.007 | 0.0133 | -0.526 | 0.6023675 | 0.879345 |
| X207409_at | 0.0029 | 0.0056 | 0.526 | 0.6024196 | 0.879352 |
| X213659_at | -0.0075 | 0.0143 | -0.526 | 0.6024671 | 0.879352 |
| X217567_at | -0.0029 | 0.0056 | -0.526 | 0.6024914 | 0.879352 |
| X219874_at | -0.017 | 0.0324 | -0.526 | 0.6025397 | 0.879365 |
| X217351_at | -0.0026 | 0.005 | -0.526 | 0.6026545 | 0.879475 |
| X207959_s_at | -0.0042 | 0.008 | -0.526 | 0.6027778 | 0.879485 |
| X221034_s_at | 0.0022 | 0.0042 | 0.526 | 0.6028486 | 0.879485 |
| X215856_at | 0.0115 | 0.0219 | 0.526 | 0.6028695 | 0.879485 |
| X215318_at | 0.0571 | 0.1087 | 0.526 | 0.6029223 | 0.879485 |
| X202397_at | -0.0147 | 0.028 | -0.526 | 0.6029464 | 0.879485 |
| X217409_at | 0.0011 | 0.002 | 0.526 | 0.6029548 | 0.879485 |
| X213099_at | -0.0115 | 0.0218 | -0.525 | 0.603016 | 0.879485 |
| X213917_at | -0.0017 | 0.0033 | -0.525 | 0.6030213 | 0.879485 |
| X217443_at | 0.0078 | 0.0149 | 0.525 | 0.6030905 | 0.879485 |
| X207790_at | 0.0263 | 0.05 | 0.525 | 0.6031577 | 0.879485 |
| X203499_at | 0.0022 | 0.0042 | 0.525 | 0.6032245 | 0.879485 |
| X219237_s_at | 0.0381 | 0.0725 | 0.525 | 0.6032511 | 0.879485 |
| X214751_at | 0.0176 | 0.0336 | 0.525 | 0.6032869 | 0.879485 |
| X210920_x_at | -0.0075 | 0.0143 | -0.525 | 0.6032932 | 0.879485 |
| X207279_s_at | -0.0027 | 0.0052 | -0.525 | 0.603329 | 0.879485 |
| X218618_s_at | 0.0456 | 0.0869 | 0.525 | 0.6033822 | 0.879485 |
| X220566_at | 0.0029 | 0.0056 | 0.525 | 0.60343 | 0.879485 |
| X211008_s_at | 0.0012 | 0.0023 | 0.525 | 0.6034435 | 0.879485 |
| X203227_s_at | 0.0195 | 0.0371 | 0.525 | 0.603458 | 0.879485 |
| X219995_s_at | -0.0082 | 0.0156 | -0.525 | 0.6034986 | 0.879485 |
| X205697_at | 0.0013 | 0.0025 | 0.525 | 0.6035287 | 0.879485 |
| X210332_at | -0.0019 | 0.0036 | -0.525 | 0.6035321 | 0.879485 |
| X206916_x_at | 0.0054 | 0.0104 | 0.525 | 0.6035864 | 0.879506 |
| X205183_at | -0.0037 | 0.007 | -0.524 | 0.6036713 | 0.879515 |
| X211500_at | 0.0019 | 0.0037 | 0.524 | 0.6036721 | 0.879515 |
| X205015_s_at | -0.0017 | 0.0033 | -0.524 | 0.6037582 | 0.879557 |
| X209921_at | -0.026 | 0.0497 | -0.524 | 0.6038318 | 0.879557 |
| X216018_at | 0.002 | 0.0038 | 0.524 | 0.6038577 | 0.879557 |
| X48117_at | -0.0058 | 0.011 | -0.524 | 0.603859 | 0.879557 |
| X202885_s_at | 0.0023 | 0.0044 | 0.524 | 0.6040008 | 0.879662 |
| X206524_at | -0.0084 | 0.0161 | -0.524 | 0.6040546 | 0.879662 |
| X206135_at | -0.0626 | 0.1194 | -0.524 | 0.6041032 | 0.879662 |
| X216187_x_at | 0.0313 | 0.0598 | 0.524 | 0.6041074 | 0.879662 |
| X216817_s_at | -0.0034 | 0.0065 | -0.524 | 0.6041452 | 0.879662 |
| X213403_at | -0.0037 | 0.007 | -0.524 | 0.6041727 | 0.879662 |
| X222054_at | 0.0017 | 0.0033 | 0.524 | 0.6042081 | 0.879662 |
| X219234_x_at | -0.0073 | 0.014 | -0.524 | 0.6043233 | 0.879772 |
| X202512_s_at | -0.0243 | 0.0463 | -0.523 | 0.6043705 | 0.879783 |
| X204557_s_at | 0.032 | 0.0612 | 0.523 | 0.6045794 | 0.879999 |
| X205356_at | 0.0356 | 0.0681 | 0.523 | 0.6046118 | 0.879999 |
| X216519_s_at | 0.0023 | 0.0044 | 0.523 | 0.6046376 | 0.879999 |
| X204164_at | -0.0016 | 0.0031 | -0.523 | 0.6047593 | 0.880118 |
| X221812_at | -0.0037 | 0.007 | -0.523 | 0.6047986 | 0.880118 |
| X212515_s_at | -0.0199 | 0.038 | -0.523 | 0.6048646 | 0.880156 |
| X220147_s_at | 0.0481 | 0.0921 | 0.523 | 0.6049253 | 0.880187 |
| X215162_at | -0.0291 | 0.0557 | -0.523 | 0.6049982 | 0.880235 |
| X201127_s_at | 0.0141 | 0.027 | 0.522 | 0.6050814 | 0.880299 |
| X202693_s_at | -0.0483 | 0.0925 | -0.522 | 0.6051394 | 0.880325 |
| X214914_at | -0.0056 | 0.0106 | -0.522 | 0.6051853 | 0.880334 |
| X210126_at | -0.0029 | 0.0055 | -0.522 | 0.6053229 | 0.880477 |
| X221122_at | -0.0026 | 0.005 | -0.522 | 0.6054493 | 0.880543 |
| X216411_s_at | -0.0041 | 0.0079 | -0.522 | 0.605477 | 0.880543 |
| X206712_at | 0.0022 | 0.0043 | 0.522 | 0.6054873 | 0.880543 |
| X215999_at | 0.003 | 0.0057 | 0.522 | 0.6055341 | 0.880553 |
| X206990_at | 0.0016 | 0.0031 | 0.521 | 0.6057594 | 0.880813 |
| X205167_s_at | -0.0021 | 0.0039 | -0.521 | 0.605792 | 0.880813 |
| X215407_s_at | 0.0309 | 0.0594 | 0.521 | 0.6060203 | 0.880979 |
| X200813_s_at | -0.0321 | 0.0617 | -0.521 | 0.6060617 | 0.880979 |
| X206150_at | -0.0016 | 0.003 | -0.521 | 0.606088 | 0.880979 |
| X215446_s_at | 0.0056 | 0.0108 | 0.521 | 0.6060999 | 0.880979 |
| X210439_at | -0.0025 | 0.0049 | -0.521 | 0.6061221 | 0.880979 |
| X213124_at | -0.0045 | 0.0087 | -0.521 | 0.6061734 | 0.880979 |
| X205594_at | 0.0786 | 0.151 | 0.521 | 0.6061838 | 0.880979 |
| X219419_at | -0.004 | 0.0076 | -0.521 | 0.6063886 | 0.881212 |
| X216165_at | -0.0024 | 0.0047 | -0.52 | 0.6064231 | 0.881212 |
| X203991_s_at | 0.0254 | 0.0489 | 0.52 | 0.6067531 | 0.881551 |
| X200938_s_at | -0.003 | 0.0057 | -0.52 | 0.606766 | 0.881551 |
| X216739_at | -0.0016 | 0.003 | -0.52 | 0.6068261 | 0.881551 |
| X216653_at | -0.006 | 0.0116 | -0.52 | 0.6068329 | 0.881551 |
| X204830_x_at | -0.0023 | 0.0045 | -0.52 | 0.6068699 | 0.881551 |
| X216203_at | 0.0037 | 0.0071 | 0.52 | 0.6069293 | 0.881551 |
| X216959_x_at | 0.0062 | 0.0119 | 0.52 | 0.6069624 | 0.881551 |
| X214372_x_at | 0.0078 | 0.015 | 0.52 | 0.6070113 | 0.881551 |
| X210043_at | 0.0014 | 0.0028 | 0.52 | 0.6070257 | 0.881551 |
| X216388_s_at | 0.002 | 0.0039 | 0.52 | 0.6070531 | 0.881551 |
| X213458_at | 0.0071 | 0.0137 | 0.519 | 0.6071144 | 0.881579 |
| X206049_at | 0.002 | 0.0038 | 0.519 | 0.6072277 | 0.881579 |
| X219936_s_at | 0.0031 | 0.0059 | 0.519 | 0.6072306 | 0.881579 |
| X221363_x_at | -0.0016 | 0.003 | -0.519 | 0.6072609 | 0.881579 |
| X211453_s_at | 0.002 | 0.0038 | 0.519 | 0.6072714 | 0.881579 |
| X205664_at | 0.024 | 0.0463 | 0.519 | 0.6073388 | 0.88162 |
| X217167_x_at | 0.0019 | 0.0036 | 0.519 | 0.6073841 | 0.881628 |
| X205437_at | -0.0152 | 0.0293 | -0.519 | 0.6077285 | 0.88207 |
| X216245_at | 0.002 | 0.0038 | 0.518 | 0.6078185 | 0.882122 |
| X213995_at | -0.0271 | 0.0523 | -0.518 | 0.6078437 | 0.882122 |
| X214112_s_at | -0.0194 | 0.0373 | -0.518 | 0.6079614 | 0.882144 |
| X210921_at | -0.0029 | 0.0055 | -0.518 | 0.6079754 | 0.882144 |
| X208283_at | -0.002 | 0.0039 | -0.518 | 0.608021 | 0.882144 |
| X213130_at | 0.003 | 0.0058 | 0.518 | 0.6080286 | 0.882144 |
| X221671_x_at | 0.0017 | 0.0032 | 0.518 | 0.6080599 | 0.882144 |
| X215051_x_at | 0.0231 | 0.0446 | 0.518 | 0.6080974 | 0.882144 |
| X202097_at | 0.0168 | 0.0324 | 0.518 | 0.6082375 | 0.88229 |
| X205357_s_at | -0.003 | 0.0058 | -0.518 | 0.608327 | 0.882331 |
| X205172_x_at | -0.0282 | 0.0544 | -0.518 | 0.608345 | 0.882331 |
| X218422_s_at | -0.0103 | 0.0198 | -0.518 | 0.6084044 | 0.882359 |
| X206553_at | 0.0023 | 0.0044 | 0.517 | 0.6085048 | 0.882404 |
| X203003_at | 0.0037 | 0.0071 | 0.517 | 0.6085667 | 0.882404 |
| X221062_at | -0.0035 | 0.0067 | -0.517 | 0.6085899 | 0.882404 |
| X215101_s_at | -0.0046 | 0.0088 | -0.517 | 0.6085945 | 0.882404 |
| X211842_s_at | 0.0038 | 0.0073 | 0.517 | 0.6086899 | 0.882485 |
| X204808_s_at | 0.0235 | 0.0455 | 0.517 | 0.6087881 | 0.882526 |
| X214282_at | 0.0121 | 0.0235 | 0.517 | 0.6087976 | 0.882526 |
| X212639_x_at | 0.0234 | 0.0453 | 0.517 | 0.6088606 | 0.882546 |
| X207123_s_at | -0.0017 | 0.0033 | -0.517 | 0.608891 | 0.882546 |
| X201261_x_at | 0.0394 | 0.0763 | 0.517 | 0.6089429 | 0.882564 |
| X216054_x_at | 0.0021 | 0.004 | 0.517 | 0.6090757 | 0.882688 |
| X203392_s_at | -0.0234 | 0.0452 | -0.517 | 0.6091158 | 0.882688 |
| X37117_at | 0.0021 | 0.0041 | 0.517 | 0.6091476 | 0.882688 |
| X218602_s_at | -0.0329 | 0.0638 | -0.516 | 0.6092478 | 0.882691 |
| X209643_s_at | 0.016 | 0.0311 | 0.516 | 0.6092504 | 0.882691 |
| X215686_x_at | -0.0038 | 0.0074 | -0.516 | 0.6093813 | 0.882691 |
| X213195_at | -0.0404 | 0.0782 | -0.516 | 0.6094871 | 0.882691 |
| X220787_at | -0.0059 | 0.0115 | -0.516 | 0.6095058 | 0.882691 |
| X207516_at | -0.0023 | 0.0045 | -0.516 | 0.6095185 | 0.882691 |
| X204108_at | 0.0157 | 0.0305 | 0.516 | 0.6095688 | 0.882691 |
| X206222_at | -0.0025 | 0.0048 | -0.516 | 0.6095933 | 0.882691 |
| X211157_at | -0.0034 | 0.0065 | -0.516 | 0.6096104 | 0.882691 |
| X212584_at | -0.0232 | 0.045 | -0.516 | 0.6096817 | 0.882691 |
| X201310_s_at | -0.0332 | 0.0645 | -0.516 | 0.6097066 | 0.882691 |
| X200764_s_at | -0.0237 | 0.0459 | -0.516 | 0.6097344 | 0.882691 |
| X211793_s_at | -0.0071 | 0.0137 | -0.516 | 0.6097534 | 0.882691 |
| X205891_at | -0.074 | 0.1435 | -0.516 | 0.6097795 | 0.882691 |
| X203768_s_at | 0.0029 | 0.0056 | 0.516 | 0.609795 | 0.882691 |
| X207804_s_at | -0.0034 | 0.0066 | -0.516 | 0.6098311 | 0.882691 |
| X216850_at | -0.0421 | 0.0816 | -0.516 | 0.60985 | 0.882691 |
| X221618_s_at | -0.0413 | 0.0802 | -0.515 | 0.609865 | 0.882691 |
| X216402_at | -0.0017 | 0.0032 | -0.515 | 0.6099395 | 0.882741 |
| X220260_at | 0.0034 | 0.0066 | 0.515 | 0.610079 | 0.882886 |
| X219845_at | -0.0019 | 0.0038 | -0.515 | 0.6102617 | 0.883093 |
| X219722_s_at | -0.0019 | 0.0036 | -0.515 | 0.6103691 | 0.883135 |
| X205815_at | -0.0013 | 0.0025 | -0.515 | 0.6103842 | 0.883135 |
| X206660_at | -0.0028 | 0.0054 | -0.515 | 0.6104878 | 0.883135 |
| X216742_at | -0.0023 | 0.0045 | -0.515 | 0.610521 | 0.883135 |
| X207258_at | 0.002 | 0.0039 | 0.514 | 0.6105829 | 0.883135 |
| X205564_at | 0.0024 | 0.0046 | 0.514 | 0.6105914 | 0.883135 |
| X209145_s_at | 0.0013 | 0.0026 | 0.514 | 0.6106251 | 0.883135 |
| X217402_at | -0.005 | 0.0097 | -0.514 | 0.6106394 | 0.883135 |
| X217226_s_at | 0.0219 | 0.0425 | 0.514 | 0.6106761 | 0.883135 |
| X216566_at | -0.0036 | 0.007 | -0.514 | 0.610732 | 0.883135 |
| X206037_at | 0.0287 | 0.0559 | 0.514 | 0.6107382 | 0.883135 |
| X34408_at | -0.0267 | 0.0519 | -0.514 | 0.6107683 | 0.883135 |
| X41397_at | -0.0017 | 0.0033 | -0.514 | 0.6108422 | 0.883185 |
| X203680_at | 0.0201 | 0.039 | 0.514 | 0.6109192 | 0.883195 |
| X201695_s_at | -0.0552 | 0.1074 | -0.514 | 0.6109312 | 0.883195 |
| X216315_x_at | 0.0044 | 0.0086 | 0.514 | 0.6109688 | 0.883195 |
| X211463_at | 0.0028 | 0.0054 | 0.514 | 0.6110449 | 0.883226 |
| X210868_s_at | -0.03 | 0.0583 | -0.514 | 0.6110695 | 0.883226 |
| X208329_at | -0.0031 | 0.0061 | -0.514 | 0.6112368 | 0.883332 |
| X215851_at | 0.0032 | 0.0063 | 0.513 | 0.6112819 | 0.883332 |
| X202016_at | -0.0366 | 0.0712 | -0.513 | 0.6112849 | 0.883332 |
| X37996_s_at | -0.002 | 0.0038 | -0.513 | 0.6113021 | 0.883332 |
| X216785_at | 0.0032 | 0.0063 | 0.513 | 0.6114416 | 0.88342 |
| X207902_at | -0.0025 | 0.0048 | -0.513 | 0.6114474 | 0.88342 |
| X214294_at | -0.0039 | 0.0076 | -0.513 | 0.611482 | 0.88342 |
| X204256_at | -0.015 | 0.0293 | -0.513 | 0.6115476 | 0.883457 |
| X218663_at | -0.005 | 0.0098 | -0.513 | 0.6116475 | 0.883544 |
| X220977_x_at | -0.004 | 0.0078 | -0.513 | 0.6117406 | 0.883597 |
| X213376_at | 0.0136 | 0.0265 | 0.513 | 0.6118065 | 0.883597 |
| X216156_at | -0.003 | 0.0058 | -0.513 | 0.6118244 | 0.883597 |
| X220978_at | 0.0039 | 0.0076 | 0.513 | 0.6118432 | 0.883597 |
| X215981_at | -0.0036 | 0.0071 | -0.512 | 0.6120496 | 0.883806 |
| X215904_at | 0.0043 | 0.0084 | 0.512 | 0.6121241 | 0.883806 |
| X209800_at | 0.0016 | 0.003 | 0.512 | 0.6121483 | 0.883806 |
| X211400_at | 0.0015 | 0.003 | 0.512 | 0.6121505 | 0.883806 |
| X220452_x_at | 0.0193 | 0.0377 | 0.512 | 0.612187 | 0.883806 |
| X202389_s_at | -0.0015 | 0.0029 | -0.512 | 0.6123526 | 0.883949 |
| X209222_s_at | -0.0203 | 0.0396 | -0.512 | 0.6123654 | 0.883949 |
| X218397_at | -0.0169 | 0.033 | -0.512 | 0.6124823 | 0.88406 |
| X207711_at | 0.0061 | 0.0119 | 0.512 | 0.6125501 | 0.8841 |
| X205477_s_at | -0.0019 | 0.0037 | -0.511 | 0.6126725 | 0.88422 |
| X215701_at | 0.0021 | 0.0042 | 0.511 | 0.6128371 | 0.88436 |
| X206738_at | -0.003 | 0.0058 | -0.511 | 0.6128496 | 0.88436 |
| X210106_at | 0.0016 | 0.0031 | 0.511 | 0.6128967 | 0.884371 |
| X35974_at | 0.0044 | 0.0085 | 0.511 | 0.6129606 | 0.884406 |
| X220017_x_at | 0.0032 | 0.0062 | 0.511 | 0.6130611 | 0.884483 |
| X214846_s_at | -0.0014 | 0.0027 | -0.511 | 0.6131324 | 0.884483 |
| X208070_s_at | 0.0267 | 0.0523 | 0.511 | 0.6131403 | 0.884483 |
| X202504_at | 0.0018 | 0.0036 | 0.511 | 0.6132472 | 0.884483 |
| X220698_at | -0.0014 | 0.0027 | -0.511 | 0.6132694 | 0.884483 |
| X218307_at | 0.0157 | 0.0307 | 0.511 | 0.6132842 | 0.884483 |
| X200926_at | 0.01 | 0.0195 | 0.511 | 0.6132931 | 0.884483 |
| X203625_x_at | 0.0276 | 0.054 | 0.51 | 0.6133485 | 0.884506 |
| X209714_s_at | -0.0136 | 0.0266 | -0.51 | 0.6134585 | 0.884607 |
| X215451_s_at | 0.0015 | 0.0029 | 0.51 | 0.6134982 | 0.884607 |
| X210360_s_at | 0.0019 | 0.0038 | 0.51 | 0.6135553 | 0.884632 |
| X220288_at | 0.002 | 0.004 | 0.51 | 0.6136386 | 0.884694 |
| X212424_at | -0.0034 | 0.0066 | -0.51 | 0.6138178 | 0.884876 |
| X217736_s_at | 0.0151 | 0.0295 | 0.51 | 0.6138442 | 0.884876 |
| X220802_at | 0.0015 | 0.003 | 0.509 | 0.6140313 | 0.884994 |
| X209644_x_at | 0.0029 | 0.0056 | 0.509 | 0.6140434 | 0.884994 |
| X205994_at | -0.004 | 0.0079 | -0.509 | 0.6140456 | 0.884994 |
| X214224_s_at | -0.0149 | 0.0293 | -0.509 | 0.6142965 | 0.885298 |
| X202400_s_at | 0.0032 | 0.0064 | 0.509 | 0.6143956 | 0.885383 |
| X220264_s_at | -0.0036 | 0.0071 | -0.509 | 0.6144521 | 0.885407 |
| X221252_s_at | 0.0016 | 0.0032 | 0.509 | 0.614574 | 0.885438 |
| X220621_at | 0.0018 | 0.0035 | 0.509 | 0.6146387 | 0.885438 |
| X201994_at | -0.0139 | 0.0272 | -0.509 | 0.6146441 | 0.885438 |
| X220529_at | -0.0059 | 0.0116 | -0.508 | 0.6147041 | 0.885438 |
| X213406_at | -0.002 | 0.004 | -0.508 | 0.614727 | 0.885438 |
| X203585_at | -0.0036 | 0.0072 | -0.508 | 0.6147537 | 0.885438 |
| X215541_s_at | 0.0036 | 0.0071 | 0.508 | 0.6147905 | 0.885438 |
| X217863_at | -0.0166 | 0.0327 | -0.508 | 0.6147923 | 0.885438 |
| X206164_at | -0.0037 | 0.0073 | -0.508 | 0.6148827 | 0.885475 |
| X207037_at | -0.003 | 0.0058 | -0.508 | 0.61494 | 0.885475 |
| X214445_at | 0.0023 | 0.0045 | 0.508 | 0.6149498 | 0.885475 |
| X211165_x_at | -0.0011 | 0.0022 | -0.508 | 0.6149794 | 0.885475 |
| X214964_at | -0.0057 | 0.0111 | -0.508 | 0.6150541 | 0.885475 |
| X212850_s_at | -0.0523 | 0.103 | -0.508 | 0.6150908 | 0.885475 |
| X201368_at | 0.0464 | 0.0913 | 0.508 | 0.615097 | 0.885475 |
| X208974_x_at | 0.0125 | 0.0246 | 0.508 | 0.6151613 | 0.88551 |
| X202938_x_at | -0.0042 | 0.0082 | -0.508 | 0.6152629 | 0.885599 |
| X202502_at | -0.023 | 0.0452 | -0.508 | 0.615326 | 0.885633 |
| X205671_s_at | 0.0017 | 0.0033 | 0.507 | 0.6155137 | 0.885845 |
| X40359_at | -0.0039 | 0.0077 | -0.507 | 0.6156524 | 0.885988 |
| X217712_at | -0.0054 | 0.0106 | -0.507 | 0.6157063 | 0.886008 |
| X220543_at | 0.0017 | 0.0033 | 0.507 | 0.6158305 | 0.886129 |
| X207736_s_at | -0.0035 | 0.0068 | -0.507 | 0.6159154 | 0.886183 |
| X211284_s_at | -0.023 | 0.0454 | -0.507 | 0.615948 | 0.886183 |
| X214818_at | 0.0018 | 0.0035 | 0.506 | 0.6164736 | 0.886466 |
| X209160_at | -0.0702 | 0.1388 | -0.506 | 0.6164871 | 0.886466 |
| X200678_x_at | -0.0194 | 0.0383 | -0.506 | 0.6164905 | 0.886466 |
| X212813_at | 0.0368 | 0.0727 | 0.506 | 0.6165128 | 0.886466 |
| X216118_at | -0.0037 | 0.0074 | -0.506 | 0.6165248 | 0.886466 |
| X203057_s_at | -0.0223 | 0.0442 | -0.506 | 0.6165776 | 0.886466 |
| X217551_at | 0.0044 | 0.0088 | 0.506 | 0.6165786 | 0.886466 |
| X207788_s_at | -0.0012 | 0.0023 | -0.506 | 0.6166145 | 0.886466 |
| X220918_at | -0.0029 | 0.0057 | -0.506 | 0.6166165 | 0.886466 |
| X215799_at | 0.0016 | 0.0031 | 0.506 | 0.616689 | 0.886466 |
| X211771_s_at | -0.0028 | 0.0056 | -0.506 | 0.6167135 | 0.886466 |
| X217560_at | -0.0026 | 0.0051 | -0.506 | 0.6167346 | 0.886466 |
| X217675_at | -0.0016 | 0.0031 | -0.506 | 0.6167768 | 0.886466 |
| X204581_at | 0.0027 | 0.0054 | 0.505 | 0.6167899 | 0.886466 |
| X213944_x_at | -0.0124 | 0.0245 | -0.505 | 0.6168478 | 0.886466 |
| X219488_at | -0.0023 | 0.0045 | -0.505 | 0.6169215 | 0.886466 |
| X218577_at | 0.0243 | 0.0481 | 0.505 | 0.6170052 | 0.886466 |
| X215673_at | -0.0112 | 0.0222 | -0.505 | 0.6170242 | 0.886466 |
| X219872_at | 0.0064 | 0.0127 | 0.505 | 0.6170971 | 0.886466 |
| X221955_at | -0.0015 | 0.003 | -0.505 | 0.6171247 | 0.886466 |
| X207129_at | 0.0023 | 0.0046 | 0.505 | 0.6171262 | 0.886466 |
| X206007_at | 0.0053 | 0.0105 | 0.505 | 0.6171576 | 0.886466 |
| X219231_at | -0.0049 | 0.0097 | -0.505 | 0.6171721 | 0.886466 |
| X211131_s_at | -0.0061 | 0.0122 | -0.505 | 0.6172621 | 0.886466 |
| X218755_at | -0.0034 | 0.0067 | -0.505 | 0.6172788 | 0.886466 |
| X205958_x_at | -0.0016 | 0.0031 | -0.505 | 0.6172928 | 0.886466 |
| X213685_at | -0.0031 | 0.0062 | -0.505 | 0.6173123 | 0.886466 |
| X201395_at | -0.0249 | 0.0493 | -0.505 | 0.6173341 | 0.886466 |
| X43934_at | 0.0015 | 0.003 | 0.505 | 0.6173476 | 0.886466 |
| X207478_at | 0.0032 | 0.0063 | 0.505 | 0.617357 | 0.886466 |
| X209908_s_at | 0.0021 | 0.0042 | 0.505 | 0.6173818 | 0.886466 |
| X215670_s_at | -0.0015 | 0.0029 | -0.504 | 0.6176697 | 0.886822 |
| X222189_at | 0.0013 | 0.0026 | 0.504 | 0.6178001 | 0.886903 |
| X221201_s_at | -0.0039 | 0.0077 | -0.504 | 0.6178434 | 0.886903 |
| X220597_s_at | -0.0194 | 0.0385 | -0.504 | 0.6178457 | 0.886903 |
| X206690_at | 0.0156 | 0.0309 | 0.504 | 0.6179872 | 0.886995 |
| X213658_at | -0.0232 | 0.046 | -0.504 | 0.6179895 | 0.886995 |
| X221299_at | 0.0016 | 0.0032 | 0.504 | 0.6180849 | 0.887074 |
| X205065_at | -0.0033 | 0.0066 | -0.504 | 0.6181711 | 0.887133 |
| X201805_at | -0.0214 | 0.0425 | -0.503 | 0.6182147 | 0.887133 |
| X214514_at | 0.0021 | 0.0042 | 0.503 | 0.6182458 | 0.887133 |
| X211264_at | 0.0099 | 0.0197 | 0.503 | 0.6184172 | 0.887144 |
| X216482_x_at | -0.0021 | 0.0042 | -0.503 | 0.6184179 | 0.887144 |
| X209430_at | -0.0202 | 0.0402 | -0.503 | 0.6184327 | 0.887144 |
| X204276_at | -0.0152 | 0.0302 | -0.503 | 0.6184521 | 0.887144 |
| X216037_x_at | 0.0297 | 0.0591 | 0.503 | 0.6184525 | 0.887144 |
| X215742_at | -0.0033 | 0.0066 | -0.503 | 0.6186653 | 0.887391 |
| X206182_at | -0.0056 | 0.0112 | -0.503 | 0.6187769 | 0.887453 |
| X205494_at | 0.0021 | 0.0041 | 0.503 | 0.618826 | 0.887453 |
| X202562_s_at | -0.0177 | 0.0352 | -0.503 | 0.6188283 | 0.887453 |
| X203930_s_at | -0.0105 | 0.0209 | -0.502 | 0.6189135 | 0.887518 |
| X220137_at | 0.004 | 0.008 | 0.502 | 0.6190151 | 0.887548 |
| X219635_at | -0.0198 | 0.0394 | -0.502 | 0.6190175 | 0.887548 |
| X204340_at | -0.0034 | 0.0067 | -0.502 | 0.6190611 | 0.887548 |
| X218329_at | 0.0169 | 0.0336 | 0.502 | 0.6190941 | 0.887548 |
| X200727_s_at | 0.0254 | 0.0507 | 0.502 | 0.6191478 | 0.887568 |
| X209298_s_at | 0.033 | 0.0658 | 0.502 | 0.6192503 | 0.887587 |
| X214857_at | -0.0382 | 0.0761 | -0.502 | 0.6192759 | 0.887587 |
| X201492_s_at | -0.0098 | 0.0195 | -0.502 | 0.6193008 | 0.887587 |
| X218298_s_at | -0.0174 | 0.0346 | -0.502 | 0.6193562 | 0.887587 |
| X214919_s_at | 0.0135 | 0.027 | 0.502 | 0.619361 | 0.887587 |
| X37226_at | -0.0059 | 0.0118 | -0.502 | 0.6194466 | 0.887599 |
| X220490_at | -0.0025 | 0.0051 | -0.502 | 0.6194591 | 0.887599 |
| X212047_s_at | 0.004 | 0.008 | 0.502 | 0.6194894 | 0.887599 |
| X209345_s_at | 0.0133 | 0.0265 | 0.502 | 0.6195318 | 0.887603 |
| X221366_at | -0.0012 | 0.0023 | -0.501 | 0.6196105 | 0.887658 |
| X214973_x_at | -0.0019 | 0.0037 | -0.501 | 0.6197899 | 0.887826 |
| X218028_at | 0.0594 | 0.1186 | 0.501 | 0.6198549 | 0.887826 |
| X208284_x_at | 0.0016 | 0.0031 | 0.501 | 0.6198798 | 0.887826 |
| X210882_s_at | 0.0515 | 0.1027 | 0.501 | 0.6199299 | 0.887826 |
| X205191_at | 0.0266 | 0.0531 | 0.501 | 0.6199684 | 0.887826 |
| X213472_at | 0.0089 | 0.0178 | 0.501 | 0.6200433 | 0.887826 |
| X207652_s_at | -0.0017 | 0.0034 | -0.501 | 0.6200484 | 0.887826 |
| X216469_at | 0.0054 | 0.0108 | 0.501 | 0.6200727 | 0.887826 |
| X209254_at | 0.0013 | 0.0025 | 0.501 | 0.6201243 | 0.887826 |
| X217100_s_at | -0.0197 | 0.0393 | -0.501 | 0.6201286 | 0.887826 |
| X222109_at | -0.0022 | 0.0044 | -0.501 | 0.6201674 | 0.887826 |
| X202027_at | 0.0114 | 0.0228 | 0.5 | 0.6202602 | 0.887902 |
| X219836_at | 0.0025 | 0.0051 | 0.5 | 0.6207321 | 0.888423 |
| X213386_at | 0.0303 | 0.0606 | 0.5 | 0.6207407 | 0.888423 |
| X40560_at | 0.0027 | 0.0054 | 0.5 | 0.6207653 | 0.888423 |
| X207756_at | -0.0033 | 0.0067 | -0.5 | 0.620838 | 0.888423 |
| X214903_at | -0.0887 | 0.1776 | -0.499 | 0.6209696 | 0.888423 |
| X216272_x_at | -0.0019 | 0.0038 | -0.499 | 0.6210126 | 0.888423 |
| X206186_at | -0.0034 | 0.0069 | -0.499 | 0.6211185 | 0.888423 |
| X219212_at | 0.0216 | 0.0432 | 0.499 | 0.6211596 | 0.888423 |
| X214419_s_at | -0.003 | 0.006 | -0.499 | 0.6211747 | 0.888423 |
| X220585_at | -0.002 | 0.004 | -0.499 | 0.6211903 | 0.888423 |
| X219883_at | -0.0023 | 0.0045 | -0.499 | 0.6212288 | 0.888423 |
| X214607_at | -0.0294 | 0.0589 | -0.499 | 0.6212516 | 0.888423 |
| X203692_s_at | -0.0175 | 0.035 | -0.499 | 0.6212652 | 0.888423 |
| X211164_at | 0.0052 | 0.0105 | 0.499 | 0.6213205 | 0.888423 |
| X209642_at | -0.0025 | 0.005 | -0.499 | 0.6213233 | 0.888423 |
| X219778_at | -0.0468 | 0.0937 | -0.499 | 0.6213281 | 0.888423 |
| X218087_s_at | 0.0245 | 0.049 | 0.499 | 0.6213416 | 0.888423 |
| X203318_s_at | 0.0152 | 0.0304 | 0.499 | 0.6213444 | 0.888423 |
| X216061_x_at | -0.0055 | 0.011 | -0.499 | 0.6214169 | 0.88847 |
| X220873_at | -0.0023 | 0.0047 | -0.499 | 0.6215193 | 0.888559 |
| X214320_x_at | -0.0015 | 0.0029 | -0.499 | 0.6216159 | 0.88864 |
| X205262_at | -0.0113 | 0.0227 | -0.498 | 0.6217028 | 0.888707 |
| X214836_x_at | 0.0028 | 0.0056 | 0.498 | 0.6217671 | 0.888742 |
| X221294_at | 0.0031 | 0.0062 | 0.498 | 0.6218687 | 0.888745 |
| X214243_s_at | -0.0019 | 0.0038 | -0.498 | 0.6219108 | 0.888745 |
| X219380_x_at | 0.0298 | 0.0599 | 0.498 | 0.6219631 | 0.888745 |
| X206588_at | -0.0055 | 0.0111 | -0.498 | 0.622005 | 0.888745 |
| X218962_s_at | 0.0181 | 0.0364 | 0.498 | 0.6220298 | 0.888745 |
| X215624_at | -0.0018 | 0.0036 | -0.498 | 0.622032 | 0.888745 |
| X200898_s_at | 0.0319 | 0.0641 | 0.498 | 0.6220766 | 0.888745 |
| X202210_x_at | -0.0031 | 0.0062 | -0.498 | 0.6220896 | 0.888745 |
| X220036_s_at | 0.0271 | 0.0544 | 0.498 | 0.622198 | 0.888801 |
| X204015_s_at | -0.0027 | 0.0055 | -0.498 | 0.622209 | 0.888801 |
| X202751_at | -0.0028 | 0.0057 | -0.498 | 0.6222525 | 0.888806 |
| X220648_at | 0.0415 | 0.0834 | 0.498 | 0.6223184 | 0.888843 |
| X201640_x_at | 0.0107 | 0.0214 | 0.497 | 0.6224166 | 0.88892 |
| X202661_at | -0.0103 | 0.0206 | -0.497 | 0.6224535 | 0.88892 |
| X202644_s_at | 0.0341 | 0.0685 | 0.497 | 0.622492 | 0.88892 |
| X215940_at | 0.0022 | 0.0043 | 0.497 | 0.6226662 | 0.889112 |
| X213294_at | 0.0209 | 0.0421 | 0.497 | 0.6227143 | 0.889123 |
| X218835_at | -0.0011 | 0.0022 | -0.497 | 0.6228986 | 0.889306 |
| X207434_s_at | -0.0028 | 0.0057 | -0.497 | 0.6229225 | 0.889306 |
| X35776_at | 0.0307 | 0.0617 | 0.497 | 0.6230392 | 0.889415 |
| X217012_at | -0.0039 | 0.0079 | -0.496 | 0.623181 | 0.889544 |
| X210121_at | -0.0455 | 0.0916 | -0.496 | 0.6232125 | 0.889544 |
| X218893_at | -0.0021 | 0.0043 | -0.496 | 0.6232497 | 0.889544 |
| X217679_x_at | 0.0434 | 0.0875 | 0.496 | 0.623412 | 0.889719 |
| X206097_at | -0.0025 | 0.005 | -0.496 | 0.6235353 | 0.889838 |
| X215900_at | -0.0027 | 0.0054 | -0.496 | 0.6236169 | 0.889897 |
| X200922_at | 0.0018 | 0.0037 | 0.496 | 0.6236572 | 0.889897 |
| X206112_at | 0.0032 | 0.0066 | 0.496 | 0.6237378 | 0.889927 |
| X209350_s_at | -0.0095 | 0.0191 | -0.495 | 0.6237578 | 0.889927 |
| X209951_s_at | -0.001 | 0.0021 | -0.495 | 0.6238052 | 0.889937 |
| X207857_at | -0.0077 | 0.0155 | -0.495 | 0.6239119 | 0.890032 |
| X215525_at | -0.0022 | 0.0045 | -0.495 | 0.6239776 | 0.890069 |
| X210743_s_at | -0.0028 | 0.0056 | -0.495 | 0.6240494 | 0.890073 |
| X205915_x_at | 0.0026 | 0.0053 | 0.495 | 0.6240605 | 0.890073 |
| X209772_s_at | 0.0058 | 0.0116 | 0.495 | 0.6241875 | 0.89009 |
| X209514_s_at | -0.0019 | 0.0039 | -0.495 | 0.6242082 | 0.89009 |
| X213343_s_at | 0.0029 | 0.0059 | 0.495 | 0.6242678 | 0.89009 |
| X202782_s_at | 0.0182 | 0.0367 | 0.495 | 0.6243292 | 0.89009 |
| X218467_at | 0.015 | 0.0303 | 0.495 | 0.624346 | 0.89009 |
| X219125_s_at | -0.0101 | 0.0204 | -0.495 | 0.6243833 | 0.89009 |
| X211914_x_at | 0.0027 | 0.0054 | 0.495 | 0.6243887 | 0.89009 |
| X210913_at | -0.0014 | 0.0028 | -0.495 | 0.624393 | 0.89009 |
| X36554_at | -0.0551 | 0.1113 | -0.494 | 0.6244411 | 0.890101 |
| X215641_at | 0.0051 | 0.0104 | 0.494 | 0.6245248 | 0.890139 |
| X213854_at | -0.0014 | 0.0028 | -0.494 | 0.6245475 | 0.890139 |
| X206211_at | 0.0042 | 0.0085 | 0.494 | 0.6245999 | 0.890156 |
| X221890_at | 0.0035 | 0.007 | 0.494 | 0.624694 | 0.890233 |
| X218084_x_at | 0.0018 | 0.0036 | 0.494 | 0.6248281 | 0.890318 |
| X211425_x_at | 0.0025 | 0.0051 | 0.494 | 0.6248334 | 0.890318 |
| X203702_s_at | 0.0072 | 0.0146 | 0.494 | 0.6248758 | 0.890321 |
| X216268_s_at | 0.0292 | 0.0593 | 0.494 | 0.6250592 | 0.890438 |
| X206786_at | -0.0025 | 0.005 | -0.494 | 0.6251104 | 0.890438 |
| X213348_at | 0.0825 | 0.1672 | 0.493 | 0.625173 | 0.890438 |
| X213501_at | 0.0184 | 0.0374 | 0.493 | 0.6251858 | 0.890438 |
| X203923_s_at | -0.0636 | 0.1288 | -0.493 | 0.6251952 | 0.890438 |
| X40524_at | -0.0018 | 0.0036 | -0.493 | 0.6252366 | 0.890438 |
| X216888_at | -0.0023 | 0.0046 | -0.493 | 0.6252964 | 0.890438 |
| X214611_at | -0.0033 | 0.0066 | -0.493 | 0.6253403 | 0.890438 |
| X203609_s_at | 0.0469 | 0.0951 | 0.493 | 0.6253418 | 0.890438 |
| X218282_at | 0.0042 | 0.0085 | 0.493 | 0.6253589 | 0.890438 |
| X211680_at | 0.0061 | 0.0124 | 0.493 | 0.6254559 | 0.890464 |
| X217081_at | 0.0053 | 0.0107 | 0.493 | 0.6254856 | 0.890464 |
| X208985_s_at | -0.0073 | 0.0147 | -0.493 | 0.6254973 | 0.890464 |
| X221386_at | 0.0022 | 0.0045 | 0.493 | 0.6256301 | 0.890549 |
| X215025_at | -0.0121 | 0.0245 | -0.493 | 0.6256372 | 0.890549 |
| X206226_at | 0.0024 | 0.005 | 0.493 | 0.6257007 | 0.890582 |
| X220601_at | 0.0015 | 0.0031 | 0.493 | 0.6257806 | 0.8906 |
| X208095_s_at | 0.0324 | 0.0657 | 0.493 | 0.625815 | 0.8906 |
| X218932_at | -0.0164 | 0.0333 | -0.493 | 0.6258333 | 0.8906 |
| X210350_x_at | 0.001 | 0.0021 | 0.492 | 0.6260689 | 0.890816 |
| X213279_at | 0.0188 | 0.0382 | 0.492 | 0.626096 | 0.890816 |
| X217102_at | 0.0023 | 0.0046 | 0.492 | 0.6261497 | 0.890816 |
| X206205_at | 0.0057 | 0.0115 | 0.492 | 0.6261804 | 0.890816 |
| X213102_at | -0.0135 | 0.0275 | -0.492 | 0.6261993 | 0.890816 |
| X202445_s_at | 0.0033 | 0.0066 | 0.492 | 0.626251 | 0.890816 |
| X206307_s_at | 0.0044 | 0.0089 | 0.492 | 0.6262661 | 0.890816 |
| X204274_at | -0.0147 | 0.0298 | -0.492 | 0.62645 | 0.890989 |
| X220351_at | -0.006 | 0.0123 | -0.492 | 0.6265005 | 0.890989 |
| X203669_s_at | -0.0061 | 0.0124 | -0.492 | 0.6265079 | 0.890989 |
| X214213_x_at | -0.006 | 0.0122 | -0.491 | 0.6265716 | 0.891022 |
| X205853_at | 0.0019 | 0.0039 | 0.491 | 0.6266747 | 0.891069 |
| X209792_s_at | 0.0019 | 0.0039 | 0.491 | 0.6267833 | 0.891069 |
| X201447_at | -0.026 | 0.053 | -0.491 | 0.6267854 | 0.891069 |
| X200831_s_at | 0.0471 | 0.0959 | 0.491 | 0.6267921 | 0.891069 |
| X213107_at | 0.0379 | 0.0772 | 0.491 | 0.6268048 | 0.891069 |
| X219121_s_at | -0.0026 | 0.0053 | -0.491 | 0.6269619 | 0.891232 |
| X213087_s_at | 0.02 | 0.0407 | 0.491 | 0.6269997 | 0.891232 |
| X216637_at | 0.0014 | 0.0028 | 0.491 | 0.6271134 | 0.891336 |
| X203130_s_at | 0.0139 | 0.0283 | 0.49 | 0.6273012 | 0.891535 |
| X220112_at | -0.082 | 0.1672 | -0.49 | 0.627338 | 0.891535 |
| X208434_at | 0.0016 | 0.0032 | 0.49 | 0.6273735 | 0.891535 |
| X212699_at | -0.0254 | 0.0519 | -0.49 | 0.6276832 | 0.891918 |
| X202005_at | 0.0029 | 0.006 | 0.49 | 0.6278667 | 0.892002 |
| X216851_at | -0.0025 | 0.005 | -0.49 | 0.6278667 | 0.892002 |
| X207909_x_at | -0.0037 | 0.0075 | -0.49 | 0.6278709 | 0.892002 |
| X200073_s_at | 0.0166 | 0.0339 | 0.49 | 0.6279159 | 0.892002 |
| X216113_at | 0.0362 | 0.074 | 0.489 | 0.6279697 | 0.892002 |
| X217375_at | -0.0028 | 0.0057 | -0.489 | 0.6280221 | 0.892002 |
| X200062_s_at | 0.0121 | 0.0248 | 0.489 | 0.6280387 | 0.892002 |
| X202854_at | -0.024 | 0.049 | -0.489 | 0.6280638 | 0.892002 |
| X209530_at | 0.0216 | 0.0442 | 0.489 | 0.628171 | 0.892098 |
| X220212_s_at | -0.0028 | 0.0057 | -0.489 | 0.6282287 | 0.892123 |
| X215065_at | -0.003 | 0.0061 | -0.489 | 0.6283506 | 0.892239 |
| X219099_at | -0.0359 | 0.0735 | -0.489 | 0.6283985 | 0.89225 |
| X215724_at | -0.0026 | 0.0053 | -0.488 | 0.6287214 | 0.892524 |
| X202803_s_at | 0.0205 | 0.042 | 0.488 | 0.6287363 | 0.892524 |
| X222178_s_at | -0.0033 | 0.0067 | -0.488 | 0.6287383 | 0.892524 |
| X215367_at | -0.001 | 0.0021 | -0.488 | 0.6287526 | 0.892524 |
| X215831_at | 0.0048 | 0.0098 | 0.488 | 0.6288283 | 0.892559 |
| X221613_s_at | 0.0161 | 0.0331 | 0.488 | 0.6288577 | 0.892559 |
| X204686_at | 0.0238 | 0.0487 | 0.488 | 0.6289223 | 0.892594 |
| X200774_at | 0.0198 | 0.0407 | 0.488 | 0.6289633 | 0.892595 |
| X219054_at | 0.0026 | 0.0054 | 0.488 | 0.6290126 | 0.892603 |
| X214441_at | 0.0019 | 0.0038 | 0.488 | 0.6290491 | 0.892603 |
| X219686_at | 0.0036 | 0.0074 | 0.488 | 0.6291289 | 0.892659 |
| X205964_at | 0.0215 | 0.0442 | 0.488 | 0.6292071 | 0.892711 |
| X203653_s_at | 0.0123 | 0.0252 | 0.488 | 0.6292718 | 0.892711 |
| X201647_s_at | 0.0407 | 0.0835 | 0.488 | 0.6293249 | 0.892711 |
| X206314_at | 0.0216 | 0.0444 | 0.487 | 0.6293576 | 0.892711 |
| X208406_s_at | -0.0015 | 0.0031 | -0.487 | 0.6293665 | 0.892711 |
| X216558_x_at | 0.007 | 0.0143 | 0.487 | 0.629622 | 0.892924 |
| X203182_s_at | -0.0127 | 0.0261 | -0.487 | 0.6297214 | 0.892924 |
| X211286_x_at | -0.0023 | 0.0048 | -0.487 | 0.62973 | 0.892924 |
| X221390_s_at | -0.0017 | 0.0035 | -0.487 | 0.6297304 | 0.892924 |
| X211102_s_at | -0.0013 | 0.0027 | -0.487 | 0.6297353 | 0.892924 |
| X209540_at | 0.028 | 0.0576 | 0.487 | 0.6297809 | 0.892924 |
| X210239_at | -0.0016 | 0.0033 | -0.487 | 0.6299247 | 0.892924 |
| X203682_s_at | 0.0195 | 0.0401 | 0.487 | 0.629963 | 0.892924 |
| X211358_s_at | -0.0046 | 0.0094 | -0.487 | 0.629963 | 0.892924 |
| X221534_at | -0.0154 | 0.0316 | -0.487 | 0.6299652 | 0.892924 |
| X204393_s_at | -0.0024 | 0.0049 | -0.487 | 0.6300279 | 0.892924 |
| X201442_s_at | -0.0016 | 0.0032 | -0.486 | 0.6300371 | 0.892924 |
| X205449_at | -0.0125 | 0.0257 | -0.486 | 0.6300434 | 0.892924 |
| X219268_at | 0.0044 | 0.009 | 0.486 | 0.6300796 | 0.892924 |
| X221679_s_at | -0.0443 | 0.0911 | -0.486 | 0.6301576 | 0.89294 |
| X205685_at | -0.0047 | 0.0096 | -0.486 | 0.6301775 | 0.89294 |
| X204615_x_at | -0.0288 | 0.0591 | -0.486 | 0.6302252 | 0.89294 |
| X213719_s_at | 0.0022 | 0.0045 | 0.486 | 0.6302961 | 0.89294 |
| X218579_s_at | 0.0019 | 0.004 | 0.486 | 0.6303253 | 0.89294 |
| X215240_at | -0.0038 | 0.0078 | -0.486 | 0.6303774 | 0.89294 |
| X210809_s_at | -0.0026 | 0.0054 | -0.486 | 0.6304078 | 0.89294 |
| X221213_s_at | -0.0222 | 0.0456 | -0.486 | 0.6304941 | 0.89294 |
| X201630_s_at | -0.0164 | 0.0337 | -0.486 | 0.6305001 | 0.89294 |
| X213119_at | -0.0309 | 0.0635 | -0.486 | 0.6305001 | 0.89294 |
| X206209_s_at | -0.0617 | 0.127 | -0.486 | 0.6305706 | 0.89294 |
| X210660_at | 0.0026 | 0.0054 | 0.486 | 0.6305763 | 0.89294 |
| X217847_s_at | 0.0037 | 0.0077 | 0.486 | 0.6306703 | 0.89294 |
| X212863_x_at | -0.0137 | 0.0283 | -0.485 | 0.6307567 | 0.89294 |
| X200788_s_at | 0.0165 | 0.034 | 0.485 | 0.6307613 | 0.89294 |
| X205769_at | 0.0066 | 0.0136 | 0.485 | 0.6307728 | 0.89294 |
| X203009_at | 0.002 | 0.0042 | 0.485 | 0.630774 | 0.89294 |
| X222136_x_at | 0.0274 | 0.0564 | 0.485 | 0.630878 | 0.89303 |
| X218619_s_at | -0.003 | 0.0061 | -0.485 | 0.6309245 | 0.893039 |
| X206614_at | 0.0015 | 0.0032 | 0.485 | 0.6311901 | 0.893358 |
| X213733_at | 0.0026 | 0.0054 | 0.485 | 0.6312919 | 0.893362 |
| X207872_s_at | -0.0034 | 0.0071 | -0.485 | 0.6313038 | 0.893362 |
| X205254_x_at | 0.0018 | 0.0036 | 0.485 | 0.6313137 | 0.893362 |
| X210385_s_at | -0.0071 | 0.0147 | -0.485 | 0.6314021 | 0.893389 |
| X210801_at | 0.0108 | 0.0223 | 0.484 | 0.6314521 | 0.893389 |
| X212611_at | -0.0288 | 0.0594 | -0.484 | 0.6314532 | 0.893389 |
| X213691_at | 0.0027 | 0.0056 | 0.484 | 0.6315894 | 0.893524 |
| X206060_s_at | 0.0028 | 0.0058 | 0.484 | 0.6317187 | 0.893524 |
| X207453_s_at | -0.0012 | 0.0026 | -0.484 | 0.6317254 | 0.893524 |
| X207344_at | 0.0013 | 0.0026 | 0.484 | 0.631785 | 0.893524 |
| X207397_s_at | -0.004 | 0.0082 | -0.484 | 0.6318703 | 0.893524 |
| X219059_s_at | -0.0027 | 0.0057 | -0.484 | 0.6318954 | 0.893524 |
| X210682_at | -0.0019 | 0.004 | -0.484 | 0.6319315 | 0.893524 |
| X204385_at | -0.0069 | 0.0143 | -0.484 | 0.6319763 | 0.893524 |
| X222081_at | -0.0019 | 0.004 | -0.484 | 0.6319799 | 0.893524 |
| X204428_s_at | 0.0016 | 0.0033 | 0.484 | 0.6319827 | 0.893524 |
| X219148_at | -0.0037 | 0.0077 | -0.484 | 0.6320488 | 0.893524 |
| X202186_x_at | -0.0027 | 0.0057 | -0.484 | 0.6320542 | 0.893524 |
| X204778_x_at | -0.0014 | 0.0029 | -0.484 | 0.6321308 | 0.893524 |
| X206288_at | 0.0045 | 0.0094 | 0.483 | 0.6321719 | 0.893524 |
| X206082_at | 0.0022 | 0.0045 | 0.483 | 0.6322427 | 0.893524 |
| X207324_s_at | -0.0024 | 0.0051 | -0.483 | 0.6322439 | 0.893524 |
| X211088_s_at | -0.0041 | 0.0085 | -0.483 | 0.6323736 | 0.893524 |
| X207143_at | 0.0019 | 0.004 | 0.483 | 0.6324379 | 0.893524 |
| X203668_at | 0.0224 | 0.0463 | 0.483 | 0.6324977 | 0.893524 |
| X203803_at | -0.0221 | 0.0457 | -0.483 | 0.6325023 | 0.893524 |
| X203248_at | 0.0047 | 0.0097 | 0.483 | 0.6325025 | 0.893524 |
| X212032_s_at | 0.021 | 0.0436 | 0.483 | 0.6325724 | 0.893524 |
| X218055_s_at | -0.047 | 0.0974 | -0.483 | 0.6326166 | 0.893524 |
| X221398_at | 0.0027 | 0.0056 | 0.483 | 0.6326202 | 0.893524 |
| X215693_x_at | 0.0113 | 0.0233 | 0.483 | 0.6326925 | 0.893524 |
| X209356_x_at | 0.0079 | 0.0164 | 0.483 | 0.6327657 | 0.893524 |
| X207472_at | 0.0036 | 0.0074 | 0.483 | 0.6327736 | 0.893524 |
| X214501_s_at | 0.0143 | 0.0297 | 0.483 | 0.6328087 | 0.893524 |
| X203632_s_at | 0.0412 | 0.0853 | 0.482 | 0.6328912 | 0.893524 |
| X207824_s_at | 0.0013 | 0.0028 | 0.482 | 0.6329319 | 0.893524 |
| X204319_s_at | -0.0709 | 0.1469 | -0.482 | 0.6329761 | 0.893524 |
| X201420_s_at | -0.0022 | 0.0045 | -0.482 | 0.6329777 | 0.893524 |
| X212447_at | -0.0146 | 0.0303 | -0.482 | 0.6330075 | 0.893524 |
| X221888_at | -0.0023 | 0.0047 | -0.482 | 0.6330118 | 0.893524 |
| X211862_x_at | 0.0149 | 0.0309 | 0.482 | 0.6330294 | 0.893524 |
| X205865_at | 0.0016 | 0.0032 | 0.482 | 0.6330338 | 0.893524 |
| X220957_at | -0.0033 | 0.0068 | -0.482 | 0.6330366 | 0.893524 |
| X204437_s_at | 0.0032 | 0.0066 | 0.482 | 0.6330933 | 0.893547 |
| X205467_at | -0.0023 | 0.0047 | -0.482 | 0.6332172 | 0.893624 |
| X203207_s_at | 0.002 | 0.0042 | 0.482 | 0.6332446 | 0.893624 |
| X222042_x_at | -0.0018 | 0.0038 | -0.482 | 0.6332825 | 0.893624 |
| X217237_at | -0.0053 | 0.0109 | -0.482 | 0.6333086 | 0.893624 |
| X205261_at | 0.0017 | 0.0035 | 0.482 | 0.6333556 | 0.893633 |
| X207475_at | -0.005 | 0.0103 | -0.482 | 0.6334263 | 0.893676 |
| X210734_x_at | -0.0025 | 0.0052 | -0.481 | 0.633563 | 0.893759 |
| X213296_at | 0.0091 | 0.0188 | 0.481 | 0.633581 | 0.893759 |
| X211694_at | -0.0014 | 0.0028 | -0.481 | 0.6336057 | 0.893759 |
| X202995_s_at | -0.0303 | 0.063 | -0.481 | 0.6337483 | 0.893826 |
| X218817_at | -0.0352 | 0.0731 | -0.481 | 0.6338143 | 0.893826 |
| X208134_x_at | 0.0019 | 0.004 | 0.481 | 0.6338421 | 0.893826 |
| X218645_at | 0.023 | 0.0478 | 0.481 | 0.6338671 | 0.893826 |
| X205776_at | 0.0048 | 0.0101 | 0.481 | 0.6339681 | 0.893826 |
| X211906_s_at | -0.0042 | 0.0087 | -0.481 | 0.6340321 | 0.893826 |
| X204761_at | -0.0048 | 0.0099 | -0.481 | 0.6340497 | 0.893826 |
| X217231_s_at | 0.0015 | 0.0031 | 0.481 | 0.6341255 | 0.893826 |
| X211873_s_at | -0.0017 | 0.0035 | -0.481 | 0.6341279 | 0.893826 |
| X61734_at | -0.0018 | 0.0036 | -0.481 | 0.6341588 | 0.893826 |
| X200893_at | 0.013 | 0.0271 | 0.481 | 0.6342357 | 0.893826 |
| X207658_s_at | -0.0166 | 0.0345 | -0.48 | 0.6342796 | 0.893826 |
| X219276_x_at | 0.0206 | 0.0428 | 0.48 | 0.634312 | 0.893826 |
| X201580_s_at | -0.0354 | 0.0738 | -0.48 | 0.6343164 | 0.893826 |
| X201764_at | 0.0206 | 0.0428 | 0.48 | 0.6343217 | 0.893826 |
| X200771_at | 0.0217 | 0.0452 | 0.48 | 0.6343359 | 0.893826 |
| X201581_at | 0.028 | 0.0583 | 0.48 | 0.6343375 | 0.893826 |
| X215425_at | 0.0095 | 0.0197 | 0.48 | 0.6344808 | 0.893972 |
| X221417_x_at | 0.0017 | 0.0035 | 0.48 | 0.6345646 | 0.894033 |
| X207695_s_at | -0.048 | 0.0999 | -0.48 | 0.6346197 | 0.894054 |
| X216459_x_at | 0.0086 | 0.0178 | 0.48 | 0.6347248 | 0.8941 |
| X204011_at | 0.0279 | 0.0582 | 0.48 | 0.6347328 | 0.8941 |
| X221107_at | -0.0033 | 0.0069 | -0.48 | 0.6348972 | 0.894183 |
| X217215_s_at | 0.0025 | 0.0053 | 0.48 | 0.6349243 | 0.894183 |
| X217144_at | -0.0215 | 0.0449 | -0.48 | 0.6349398 | 0.894183 |
| X210634_at | -0.0035 | 0.0072 | -0.479 | 0.6349527 | 0.894183 |
| X220836_at | -0.0054 | 0.0113 | -0.479 | 0.6350606 | 0.894278 |
| X209547_s_at | 0.003 | 0.0062 | 0.479 | 0.6351055 | 0.894285 |
| X208183_at | -0.002 | 0.0042 | -0.479 | 0.6352226 | 0.894393 |
| X202797_at | -0.0176 | 0.0367 | -0.479 | 0.6352658 | 0.894397 |
| X217331_at | -0.0026 | 0.0055 | -0.479 | 0.6354477 | 0.894553 |
| X210726_at | -0.0025 | 0.0052 | -0.479 | 0.635457 | 0.894553 |
| X218255_s_at | 0.0032 | 0.0067 | 0.478 | 0.6356649 | 0.894758 |
| X206120_at | 0.0022 | 0.0047 | 0.478 | 0.6357386 | 0.894758 |
| X209342_s_at | 0.0032 | 0.0066 | 0.478 | 0.635775 | 0.894758 |
| X220837_at | -0.0051 | 0.0106 | -0.478 | 0.6357926 | 0.894758 |
| X213700_s_at | 0.0024 | 0.0049 | 0.478 | 0.6358295 | 0.894758 |
| X214733_s_at | 0.0123 | 0.0257 | 0.478 | 0.6358448 | 0.894758 |
| X213821_s_at | -0.0019 | 0.004 | -0.478 | 0.6359247 | 0.894814 |
| X221788_at | -0.0269 | 0.0564 | -0.478 | 0.6359872 | 0.894845 |
| X208233_at | -0.0039 | 0.0082 | -0.478 | 0.6360431 | 0.894854 |
| X218640_s_at | 0.0273 | 0.0572 | 0.478 | 0.6361009 | 0.894854 |
| X222281_s_at | -0.0018 | 0.0037 | -0.478 | 0.6361884 | 0.894854 |
| X213081_at | 0.0034 | 0.007 | 0.478 | 0.6361951 | 0.894854 |
| X221877_at | 0.0153 | 0.032 | 0.478 | 0.6362877 | 0.894854 |
| X214739_at | 0.0177 | 0.037 | 0.478 | 0.6362914 | 0.894854 |
| X200625_s_at | -0.0207 | 0.0433 | -0.478 | 0.6363503 | 0.894854 |
| X209857_s_at | -0.0012 | 0.0026 | -0.477 | 0.6364092 | 0.894854 |
| X215450_at | 0.072 | 0.1509 | 0.477 | 0.6364312 | 0.894854 |
| X212332_at | 0.0013 | 0.0028 | 0.477 | 0.6364606 | 0.894854 |
| X211924_s_at | 0.004 | 0.0084 | 0.477 | 0.6364927 | 0.894854 |
| X218508_at | -0.0266 | 0.0558 | -0.477 | 0.6365299 | 0.894854 |
| X214020_x_at | 0.0064 | 0.0134 | 0.477 | 0.6365507 | 0.894854 |
| X214399_s_at | 0.0018 | 0.0039 | 0.477 | 0.6365727 | 0.894854 |
| X205014_at | 0.0019 | 0.004 | 0.477 | 0.6365977 | 0.894854 |
| X207577_at | -0.0718 | 0.1506 | -0.477 | 0.6366622 | 0.894854 |
| X219409_at | -0.0055 | 0.0116 | -0.477 | 0.6366847 | 0.894854 |
| X219980_at | -0.0289 | 0.0607 | -0.477 | 0.6367568 | 0.894854 |
| X217474_at | 0.0013 | 0.0028 | 0.477 | 0.6367677 | 0.894854 |
| X203580_s_at | -0.0115 | 0.0241 | -0.477 | 0.6367991 | 0.894854 |
| X201457_x_at | -0.0306 | 0.0642 | -0.477 | 0.6369858 | 0.89506 |
| X218974_at | -0.018 | 0.0378 | -0.476 | 0.6371984 | 0.895302 |
| X205526_s_at | 0.0286 | 0.06 | 0.476 | 0.6372837 | 0.895348 |
| X211806_s_at | -0.0023 | 0.0048 | -0.476 | 0.6373116 | 0.895348 |
| X220737_at | -0.0028 | 0.0059 | -0.476 | 0.6375113 | 0.895475 |
| X214262_at | 0.0029 | 0.0061 | 0.476 | 0.6375183 | 0.895475 |
| X203178_at | 0.0252 | 0.053 | 0.476 | 0.6375554 | 0.895475 |
| X217393_x_at | 0.0059 | 0.0123 | 0.476 | 0.6375635 | 0.895475 |
| X219366_at | -0.0307 | 0.0645 | -0.476 | 0.6376877 | 0.895479 |
| X213602_s_at | -0.0011 | 0.0023 | -0.476 | 0.6377267 | 0.895479 |
| X204080_at | -0.0057 | 0.012 | -0.476 | 0.6377494 | 0.895479 |
| X208167_s_at | 0.0025 | 0.0053 | 0.475 | 0.6377953 | 0.895479 |
| X209365_s_at | 0.0013 | 0.0027 | 0.475 | 0.6378658 | 0.895479 |
| X216980_s_at | -0.0013 | 0.0028 | -0.475 | 0.6378813 | 0.895479 |
| X210430_x_at | 0.0015 | 0.0031 | 0.475 | 0.637948 | 0.895479 |
| X214342_at | -0.0019 | 0.0039 | -0.475 | 0.6379586 | 0.895479 |
| X204418_x_at | -0.0261 | 0.0549 | -0.475 | 0.637979 | 0.895479 |
| X205275_at | 0.0031 | 0.0065 | 0.475 | 0.6379993 | 0.895479 |
| X217568_at | 0.0034 | 0.0072 | 0.475 | 0.6380378 | 0.895479 |
| X205600_x_at | -0.0021 | 0.0045 | -0.475 | 0.638078 | 0.895479 |
| X205105_at | 0.0432 | 0.091 | 0.475 | 0.6381159 | 0.895479 |
| X203452_at | 0.0091 | 0.0191 | 0.475 | 0.6381304 | 0.895479 |
| X202817_s_at | 0.0258 | 0.0543 | 0.475 | 0.6382684 | 0.89558 |
| X209999_x_at | 0.0011 | 0.0024 | 0.475 | 0.6382829 | 0.89558 |
| X209300_s_at | -0.0263 | 0.0555 | -0.475 | 0.6383768 | 0.895609 |
| X205881_at | 0.0016 | 0.0033 | 0.475 | 0.6383848 | 0.895609 |
| X216433_s_at | -0.0021 | 0.0045 | -0.474 | 0.638596 | 0.895784 |
| X221011_s_at | -0.0204 | 0.0431 | -0.474 | 0.6386353 | 0.895784 |
| X200683_s_at | -0.0166 | 0.0349 | -0.474 | 0.6386586 | 0.895784 |
| X203809_s_at | -0.0016 | 0.0033 | -0.474 | 0.6386705 | 0.895784 |
| X220224_at | -0.0026 | 0.0054 | -0.474 | 0.6388011 | 0.89587 |
| X218590_at | -0.0032 | 0.0067 | -0.474 | 0.6388912 | 0.89587 |
| X209748_at | 0.0133 | 0.028 | 0.474 | 0.6388928 | 0.89587 |
| X208257_x_at | 0.0017 | 0.0035 | 0.474 | 0.6389202 | 0.89587 |
| X204468_s_at | 0.0015 | 0.0031 | 0.474 | 0.6389333 | 0.89587 |
| X36888_at | 0.0239 | 0.0505 | 0.474 | 0.6390061 | 0.895915 |
| X217269_s_at | -0.0053 | 0.0111 | -0.474 | 0.6390991 | 0.895956 |
| X216677_at | 0.0023 | 0.0049 | 0.474 | 0.6391155 | 0.895956 |
| X217489_s_at | -0.0034 | 0.0072 | -0.473 | 0.6391948 | 0.89601 |
| X210664_s_at | -0.0034 | 0.0072 | -0.473 | 0.6393175 | 0.896059 |
| X211194_s_at | -0.0025 | 0.0052 | -0.473 | 0.639371 | 0.896059 |
| X205678_at | -0.0467 | 0.0987 | -0.473 | 0.6393802 | 0.896059 |
| X207774_at | 0.0022 | 0.0047 | 0.473 | 0.6393912 | 0.896059 |
| X219554_at | 0.0016 | 0.0033 | 0.473 | 0.6395046 | 0.896152 |
| X222220_s_at | -0.0253 | 0.0536 | -0.473 | 0.6395484 | 0.896152 |
| X205159_at | 0.0057 | 0.0121 | 0.473 | 0.6396036 | 0.896152 |
| X209302_at | 0.0085 | 0.0179 | 0.473 | 0.6396549 | 0.896152 |
| X207862_at | -0.0019 | 0.0041 | -0.473 | 0.6396588 | 0.896152 |
| X209138_x_at | 0.0016 | 0.0033 | 0.473 | 0.6397359 | 0.89619 |
| X201738_at | -0.0255 | 0.0539 | -0.473 | 0.6397948 | 0.89619 |
| X214518_at | -0.0021 | 0.0045 | -0.473 | 0.6398274 | 0.89619 |
| X217332_at | -0.0031 | 0.0065 | -0.473 | 0.6398554 | 0.89619 |
| X203948_s_at | 0.0015 | 0.0032 | 0.472 | 0.6398875 | 0.89619 |
| X216814_at | -0.0037 | 0.0079 | -0.472 | 0.6399953 | 0.896238 |
| X211434_s_at | 0.0017 | 0.0036 | 0.472 | 0.6400025 | 0.896238 |
| X207885_at | 0.0025 | 0.0053 | 0.472 | 0.6400433 | 0.896238 |
| X208841_s_at | -0.0181 | 0.0384 | -0.472 | 0.6401013 | 0.896263 |
| X221095_s_at | -0.0023 | 0.0048 | -0.472 | 0.6401861 | 0.896325 |
| X207485_x_at | 0.0016 | 0.0035 | 0.472 | 0.6403234 | 0.896328 |
| X209919_x_at | -0.0015 | 0.0033 | -0.472 | 0.6403528 | 0.896328 |
| X214962_s_at | -0.0042 | 0.0088 | -0.472 | 0.640361 | 0.896328 |
| X211848_s_at | -0.0022 | 0.0047 | -0.472 | 0.6404045 | 0.896328 |
| X204425_at | 0.0032 | 0.0068 | 0.472 | 0.6404383 | 0.896328 |
| X205096_at | 0.0035 | 0.0075 | 0.472 | 0.6404603 | 0.896328 |
| X210287_s_at | 0.0029 | 0.0061 | 0.472 | 0.6404704 | 0.896328 |
| X53076_at | 0.0089 | 0.0188 | 0.472 | 0.6405125 | 0.89633 |
| X213317_at | 0.0379 | 0.0805 | 0.471 | 0.6405905 | 0.896364 |
| X203705_s_at | -0.0603 | 0.1279 | -0.471 | 0.6406171 | 0.896364 |
| X204397_at | 0.0014 | 0.003 | 0.471 | 0.6407528 | 0.896497 |
| X211199_s_at | -0.0016 | 0.0034 | -0.471 | 0.6409318 | 0.896691 |
| X204255_s_at | -0.0028 | 0.0059 | -0.471 | 0.6409854 | 0.89671 |
| X212877_at | 0.028 | 0.0595 | 0.471 | 0.6410382 | 0.896727 |
| X204894_s_at | -0.0041 | 0.0086 | -0.471 | 0.6411242 | 0.896791 |
| X216353_s_at | -0.0023 | 0.0049 | -0.471 | 0.641167 | 0.896794 |
| X220003_at | -0.0034 | 0.0072 | -0.47 | 0.6413515 | 0.896891 |
| X216678_at | 0.0079 | 0.0167 | 0.47 | 0.6413541 | 0.896891 |
| X220203_at | -0.0017 | 0.0036 | -0.47 | 0.6413571 | 0.896891 |
| X207208_at | -0.0027 | 0.0057 | -0.47 | 0.6415213 | 0.897009 |
| X220379_at | 0.0019 | 0.004 | 0.47 | 0.6415224 | 0.897009 |
| X215414_at | 0.0018 | 0.0039 | 0.47 | 0.6415801 | 0.897033 |
| X219218_at | 0.0028 | 0.0059 | 0.47 | 0.6417896 | 0.897269 |
| X209645_s_at | 0.0015 | 0.0033 | 0.47 | 0.6419503 | 0.897438 |
| X214467_at | 0.0032 | 0.0068 | 0.469 | 0.6420102 | 0.897465 |
| X213487_at | 0.0013 | 0.0028 | 0.469 | 0.6422036 | 0.897643 |
| X207355_at | 0.0016 | 0.0034 | 0.469 | 0.6422185 | 0.897643 |
| X209139_s_at | -0.0181 | 0.0387 | -0.469 | 0.6422695 | 0.897658 |
| X220048_at | -0.0035 | 0.0074 | -0.469 | 0.6423569 | 0.897678 |
| X221033_s_at | 0.0025 | 0.0054 | 0.469 | 0.6423722 | 0.897678 |
| X214861_at | -0.0177 | 0.0377 | -0.469 | 0.6424052 | 0.897678 |
| X216853_x_at | -0.0023 | 0.005 | -0.469 | 0.6424564 | 0.897693 |
| X220983_s_at | -0.0067 | 0.0142 | -0.469 | 0.642503 | 0.897702 |
| X214526_x_at | -0.0152 | 0.0324 | -0.469 | 0.6425665 | 0.897734 |
| X214221_at | 0.0258 | 0.0551 | 0.469 | 0.6426815 | 0.897748 |
| X200761_s_at | -0.0113 | 0.0242 | -0.468 | 0.6427533 | 0.897748 |
| X221297_at | 0.001 | 0.0022 | 0.468 | 0.6427559 | 0.897748 |
| X209875_s_at | 0.064 | 0.1366 | 0.468 | 0.6427572 | 0.897748 |
| X207998_s_at | 0.0013 | 0.0027 | 0.468 | 0.6427785 | 0.897748 |
| X214014_at | -0.0011 | 0.0024 | -0.468 | 0.6428255 | 0.897757 |
| X204649_at | 0.0024 | 0.0051 | 0.468 | 0.6429731 | 0.897907 |
| X201714_at | -0.006 | 0.0129 | -0.468 | 0.643088 | 0.897995 |
| X207828_s_at | 0.0021 | 0.0045 | 0.468 | 0.6431172 | 0.897995 |
| X201191_at | 0.0145 | 0.031 | 0.468 | 0.6431878 | 0.898037 |
| X213958_at | 0.0034 | 0.0072 | 0.468 | 0.6432583 | 0.898079 |
| X221466_at | 0.0017 | 0.0035 | 0.468 | 0.6434012 | 0.89808 |
| X222132_s_at | -0.0186 | 0.0398 | -0.467 | 0.6434661 | 0.89808 |
| X210756_s_at | -0.0057 | 0.0122 | -0.467 | 0.6435686 | 0.89808 |
| X205839_s_at | 0.0275 | 0.0588 | 0.467 | 0.6436049 | 0.89808 |
| X207602_at | -0.0025 | 0.0053 | -0.467 | 0.6436417 | 0.89808 |
| X221852_at | 0.0021 | 0.0045 | 0.467 | 0.6436748 | 0.89808 |
| X215628_x_at | 0.0206 | 0.0442 | 0.467 | 0.6436833 | 0.89808 |
| X202360_at | 0.018 | 0.0385 | 0.467 | 0.643689 | 0.89808 |
| X200763_s_at | 0.0086 | 0.0185 | 0.467 | 0.6436894 | 0.89808 |
| X207899_at | 0.0037 | 0.0079 | 0.467 | 0.6437101 | 0.89808 |
| X206604_at | 0.001 | 0.0021 | 0.467 | 0.6437914 | 0.89808 |
| X221298_s_at | -0.0022 | 0.0046 | -0.467 | 0.6438044 | 0.89808 |
| X214725_at | 0.0072 | 0.0154 | 0.467 | 0.6438365 | 0.89808 |
| X220257_x_at | 0.0019 | 0.004 | 0.467 | 0.6438515 | 0.89808 |
| X207337_at | -0.0044 | 0.0095 | -0.467 | 0.6438653 | 0.89808 |
| X203213_at | 0.0083 | 0.0177 | 0.467 | 0.6439707 | 0.898171 |
| X213634_s_at | -0.0075 | 0.0161 | -0.466 | 0.6442343 | 0.898329 |
| X207955_at | 0.0024 | 0.0051 | 0.466 | 0.6442435 | 0.898329 |
| X222093_s_at | -0.0046 | 0.0098 | -0.466 | 0.6442525 | 0.898329 |
| X202338_at | 0.0017 | 0.0037 | 0.466 | 0.6442716 | 0.898329 |
| X204197_s_at | 0.0015 | 0.0032 | 0.466 | 0.6442948 | 0.898329 |
| X207523_at | 0.0048 | 0.0103 | 0.466 | 0.6443268 | 0.898329 |
| X208577_at | 0.0015 | 0.0032 | 0.466 | 0.6443787 | 0.898345 |
| X220376_at | -0.0061 | 0.0132 | -0.466 | 0.6444967 | 0.898453 |
| X219480_at | 0.003 | 0.0064 | 0.466 | 0.6447512 | 0.898633 |
| X214163_at | 0.0077 | 0.0166 | 0.466 | 0.644758 | 0.898633 |
| X207323_s_at | 0.0701 | 0.1506 | 0.465 | 0.6448797 | 0.898633 |
| X216503_s_at | 0.0025 | 0.0055 | 0.465 | 0.6448929 | 0.898633 |
| X201646_at | 0.032 | 0.0689 | 0.465 | 0.6448978 | 0.898633 |
| X213062_at | -0.0155 | 0.0332 | -0.465 | 0.6449022 | 0.898633 |
| X210319_x_at | 0.0034 | 0.0074 | 0.465 | 0.6449088 | 0.898633 |
| X207413_s_at | 0.0029 | 0.0063 | 0.465 | 0.6450222 | 0.898734 |
| X210244_at | -0.0015 | 0.0032 | -0.465 | 0.6450734 | 0.898741 |
| X207670_at | 0.0017 | 0.0037 | 0.465 | 0.6451228 | 0.898741 |
| X217289_s_at | -0.0017 | 0.0037 | -0.465 | 0.6452208 | 0.898741 |
| X219541_at | 0.0034 | 0.0072 | 0.465 | 0.6452683 | 0.898741 |
| X202398_at | -0.0031 | 0.0068 | -0.465 | 0.6453016 | 0.898741 |
| X220748_s_at | 0.0088 | 0.0189 | 0.465 | 0.6453164 | 0.898741 |
| X220783_at | -0.0021 | 0.0045 | -0.465 | 0.6453989 | 0.898741 |
| X208242_at | 0.0024 | 0.0052 | 0.465 | 0.645416 | 0.898741 |
| X214679_x_at | 0.0149 | 0.032 | 0.465 | 0.6454446 | 0.898741 |
| X216055_at | -0.001 | 0.0021 | -0.465 | 0.6454627 | 0.898741 |
| X215824_at | -0.0027 | 0.0057 | -0.465 | 0.6454719 | 0.898741 |
| X208584_at | -0.0025 | 0.0054 | -0.464 | 0.6455705 | 0.898804 |
| X221161_at | 0.0017 | 0.0037 | 0.464 | 0.6456094 | 0.898804 |
| X209156_s_at | -0.0015 | 0.0033 | -0.464 | 0.6456385 | 0.898804 |
| X205593_s_at | -0.0393 | 0.0847 | -0.464 | 0.6457482 | 0.898885 |
| X216031_x_at | -0.0023 | 0.0049 | -0.464 | 0.6457905 | 0.898885 |
| X217145_at | -0.0031 | 0.0066 | -0.464 | 0.6458181 | 0.898885 |
| X217053_x_at | -0.0017 | 0.0037 | -0.464 | 0.6461036 | 0.899178 |
| X219744_at | 0.0009 | 0.0019 | 0.464 | 0.6461095 | 0.899178 |
| X207182_at | 0.003 | 0.0065 | 0.464 | 0.6461602 | 0.899192 |
| X212073_at | 0.0275 | 0.0593 | 0.463 | 0.6462513 | 0.89922 |
| X203084_at | -0.0026 | 0.0057 | -0.463 | 0.6462796 | 0.89922 |
| X208923_at | -0.0257 | 0.0554 | -0.463 | 0.6463026 | 0.89922 |
| X222173_s_at | 0.0042 | 0.0091 | 0.463 | 0.6463455 | 0.89922 |
| X208079_s_at | -0.0032 | 0.0068 | -0.463 | 0.6463955 | 0.89922 |
| X207471_at | -0.0039 | 0.0085 | -0.463 | 0.6464418 | 0.89922 |
| X211611_s_at | 0.0016 | 0.0034 | 0.463 | 0.6464819 | 0.89922 |
| X207444_at | -0.0014 | 0.0031 | -0.463 | 0.6465039 | 0.89922 |
| X210556_at | 0.0044 | 0.0095 | 0.463 | 0.6466199 | 0.899325 |
| X212907_at | -0.0266 | 0.0575 | -0.463 | 0.6467255 | 0.899415 |
| X211516_at | 0.005 | 0.0109 | 0.463 | 0.6467991 | 0.899461 |
| X205418_at | 0.0013 | 0.0029 | 0.463 | 0.6469325 | 0.899492 |
| X221927_s_at | 0.0158 | 0.0341 | 0.462 | 0.6469709 | 0.899492 |
| X203383_s_at | 0.0031 | 0.0068 | 0.462 | 0.6469762 | 0.899492 |
| X219186_at | -0.0155 | 0.0335 | -0.462 | 0.646983 | 0.899492 |
| X210221_at | -0.0772 | 0.1671 | -0.462 | 0.6471113 | 0.899559 |
| X201497_x_at | -0.0075 | 0.0162 | -0.462 | 0.6471227 | 0.899559 |
| X205138_s_at | -0.0014 | 0.0031 | -0.462 | 0.6471533 | 0.899559 |
| X215804_at | -0.0011 | 0.0024 | -0.462 | 0.6471933 | 0.899559 |
| X203270_at | -0.0018 | 0.0038 | -0.462 | 0.6472729 | 0.899573 |
| X211826_s_at | -0.0012 | 0.0027 | -0.462 | 0.6472842 | 0.899573 |
| X207449_s_at | 0.0024 | 0.0051 | 0.462 | 0.64741 | 0.899691 |
| X215985_at | -0.0355 | 0.077 | -0.462 | 0.6475465 | 0.899825 |
| X203915_at | 0.002 | 0.0044 | 0.462 | 0.6476616 | 0.899928 |
| X212016_s_at | -0.0022 | 0.0048 | -0.461 | 0.6477655 | 0.900016 |
| X207787_at | 0.0018 | 0.0039 | 0.461 | 0.6478065 | 0.900017 |
| X209480_at | -0.0078 | 0.0168 | -0.461 | 0.6478925 | 0.900017 |
| X208676_s_at | 0.007 | 0.0151 | 0.461 | 0.6479605 | 0.900017 |
| X208162_s_at | 0.0024 | 0.0051 | 0.461 | 0.6479793 | 0.900017 |
| X211230_s_at | 0.0013 | 0.0028 | 0.461 | 0.6479822 | 0.900017 |
| X35150_at | -0.0065 | 0.0141 | -0.461 | 0.6480092 | 0.900017 |
| X206973_at | -0.0381 | 0.0827 | -0.461 | 0.648151 | 0.900093 |
| X209939_x_at | 0.0335 | 0.0726 | 0.461 | 0.6482077 | 0.900093 |
| X214334_x_at | 0.0146 | 0.0317 | 0.461 | 0.6482266 | 0.900093 |
| X206301_at | 0.0012 | 0.0027 | 0.461 | 0.6482691 | 0.900093 |
| X203777_s_at | 0.0027 | 0.0059 | 0.461 | 0.6482713 | 0.900093 |
| X208198_x_at | 0.0019 | 0.0041 | 0.461 | 0.6483073 | 0.900093 |
| X208367_x_at | -0.002 | 0.0043 | -0.461 | 0.6483474 | 0.900093 |
| X213540_at | -0.0081 | 0.0177 | -0.46 | 0.6484527 | 0.900183 |
| X220832_at | 0.0013 | 0.0027 | 0.46 | 0.6485699 | 0.900267 |
| X204062_s_at | -0.0181 | 0.0393 | -0.46 | 0.6485943 | 0.900267 |
| X220401_at | -0.0065 | 0.0141 | -0.46 | 0.6486593 | 0.900301 |
| X214015_at | 0.0016 | 0.0034 | 0.46 | 0.6487657 | 0.900392 |
| X209261_s_at | -0.0022 | 0.0047 | -0.459 | 0.6491469 | 0.900865 |
| X208634_s_at | 0.0135 | 0.0294 | 0.459 | 0.6493736 | 0.901071 |
| X205910_s_at | -0.0032 | 0.007 | -0.459 | 0.6495317 | 0.901071 |
| X215371_at | -0.0057 | 0.0124 | -0.459 | 0.6495421 | 0.901071 |
| X216655_s_at | 0.0009 | 0.002 | 0.459 | 0.6495858 | 0.901071 |
| X218922_s_at | -0.0036 | 0.0079 | -0.459 | 0.649638 | 0.901071 |
| X210697_at | -0.0048 | 0.0105 | -0.459 | 0.6496573 | 0.901071 |
| X204885_s_at | -0.002 | 0.0043 | -0.459 | 0.649712 | 0.901071 |
| X214740_at | -0.0026 | 0.0057 | -0.459 | 0.6497694 | 0.901071 |
| X203142_s_at | -0.0289 | 0.0629 | -0.459 | 0.6497751 | 0.901071 |
| X209420_s_at | 0.0044 | 0.0096 | 0.459 | 0.6497819 | 0.901071 |
| X210613_s_at | -0.0025 | 0.0055 | -0.458 | 0.6498098 | 0.901071 |
| X212933_x_at | -0.0108 | 0.0236 | -0.458 | 0.6498359 | 0.901071 |
| X216361_s_at | -0.0025 | 0.0055 | -0.458 | 0.6498767 | 0.901071 |
| X217645_at | 0.0333 | 0.0726 | 0.458 | 0.6499115 | 0.901071 |
| X209495_at | -0.0019 | 0.0041 | -0.458 | 0.6499476 | 0.901071 |
| X207645_s_at | 0.0015 | 0.0032 | 0.458 | 0.6500117 | 0.901071 |
| X200974_at | -0.048 | 0.1048 | -0.458 | 0.6500131 | 0.901071 |
| X207504_at | -0.0009 | 0.002 | -0.458 | 0.6500253 | 0.901071 |
| X204972_at | 0.0042 | 0.0091 | 0.458 | 0.6502921 | 0.901314 |
| X208253_at | 0.0054 | 0.0119 | 0.458 | 0.6502997 | 0.901314 |
| X221140_s_at | 0.0016 | 0.0035 | 0.458 | 0.6503465 | 0.901314 |
| X204806_x_at | 0.0216 | 0.0472 | 0.458 | 0.6503627 | 0.901314 |
| X207462_at | 0.0022 | 0.0048 | 0.458 | 0.6504204 | 0.901337 |
| X210530_s_at | -0.0224 | 0.0489 | -0.457 | 0.6506614 | 0.901587 |
| X211306_s_at | -0.0034 | 0.0075 | -0.457 | 0.6507766 | 0.901587 |
| X44673_at | 0.0025 | 0.0054 | 0.457 | 0.6507766 | 0.901587 |
| X214551_s_at | -0.0027 | 0.0059 | -0.457 | 0.6507817 | 0.901587 |
| X205398_s_at | 0.0021 | 0.0045 | 0.457 | 0.6508624 | 0.901587 |
| X206655_s_at | -0.051 | 0.1116 | -0.457 | 0.6508682 | 0.901587 |
| X205314_x_at | -0.0028 | 0.0061 | -0.457 | 0.6508845 | 0.901587 |
| X212034_s_at | -0.0154 | 0.0338 | -0.457 | 0.6511878 | 0.901951 |
| X206070_s_at | -0.0468 | 0.1027 | -0.456 | 0.6513651 | 0.902102 |
| X210063_at | 0.0027 | 0.006 | 0.456 | 0.651432 | 0.902102 |
| X215779_s_at | -0.0029 | 0.0064 | -0.456 | 0.6514518 | 0.902102 |
| X213597_s_at | 0.0019 | 0.0042 | 0.456 | 0.6514592 | 0.902102 |
| X215184_at | 0.007 | 0.0153 | 0.456 | 0.6515124 | 0.902119 |
| X208591_s_at | 0.0025 | 0.0056 | 0.456 | 0.6515624 | 0.902132 |
| X203892_at | 0.0033 | 0.0073 | 0.456 | 0.6516598 | 0.902211 |
| X207784_at | -0.0024 | 0.0052 | -0.456 | 0.6517079 | 0.902221 |
| X201205_at | 0.0026 | 0.0058 | 0.456 | 0.651801 | 0.902275 |
| X215331_at | 0.0014 | 0.003 | 0.456 | 0.6518523 | 0.902275 |
| X219713_at | 0.0069 | 0.0151 | 0.455 | 0.6519399 | 0.902275 |
| X212505_s_at | -0.0076 | 0.0167 | -0.455 | 0.6519639 | 0.902275 |
| X220407_s_at | 0.0031 | 0.0069 | 0.455 | 0.6519883 | 0.902275 |
| X203866_at | 0.0012 | 0.0025 | 0.455 | 0.6520407 | 0.902275 |
| X206985_at | 0.0021 | 0.0045 | 0.455 | 0.6520864 | 0.902275 |
| X203491_s_at | 0.0324 | 0.0713 | 0.455 | 0.6521043 | 0.902275 |
| X206146_s_at | -0.0022 | 0.0047 | -0.455 | 0.6521522 | 0.902275 |
| X219052_at | -0.0021 | 0.0045 | -0.455 | 0.6521529 | 0.902275 |
| X212970_at | -0.0254 | 0.0559 | -0.455 | 0.6522077 | 0.90229 |
| X215780_s_at | -0.0218 | 0.048 | -0.455 | 0.6522449 | 0.90229 |
| X220298_s_at | 0.0072 | 0.0157 | 0.455 | 0.6523057 | 0.902291 |
| X205831_at | -0.0021 | 0.0046 | -0.455 | 0.6523267 | 0.902291 |
| X217026_at | -0.005 | 0.011 | -0.455 | 0.6523757 | 0.902302 |
| X212778_at | 0.026 | 0.0571 | 0.455 | 0.652442 | 0.902338 |
| X206452_x_at | 0.0037 | 0.0082 | 0.455 | 0.652588 | 0.902369 |
| X220354_at | 0.0015 | 0.0034 | 0.455 | 0.6526223 | 0.902369 |
| X216237_s_at | -0.0012 | 0.0027 | -0.454 | 0.6526468 | 0.902369 |
| X216665_s_at | -0.0024 | 0.0052 | -0.454 | 0.6526637 | 0.902369 |
| X204855_at | 0.0019 | 0.0043 | 0.454 | 0.652712 | 0.902369 |
| X222253_s_at | -0.008 | 0.0177 | -0.454 | 0.652745 | 0.902369 |
| X202567_at | 0.0117 | 0.0256 | 0.454 | 0.6527551 | 0.902369 |
| X211915_s_at | -0.0022 | 0.0049 | -0.454 | 0.652817 | 0.902369 |
| X214434_at | -0.0146 | 0.0321 | -0.454 | 0.65283 | 0.902369 |
| X221155_x_at | 0.0131 | 0.0288 | 0.454 | 0.6529306 | 0.902405 |
| X206134_at | -0.0037 | 0.0082 | -0.454 | 0.6529378 | 0.902405 |
| X205681_at | 0.0043 | 0.0095 | 0.454 | 0.6530958 | 0.902568 |
| X202120_x_at | -0.0141 | 0.031 | -0.453 | 0.6533955 | 0.902926 |
| X214747_at | 0.002 | 0.0043 | 0.453 | 0.6536222 | 0.903092 |
| X216723_at | -0.003 | 0.0066 | -0.453 | 0.6536435 | 0.903092 |
| X222128_at | 0.0017 | 0.0038 | 0.453 | 0.6536633 | 0.903092 |
| X220413_at | 0.0021 | 0.0045 | 0.453 | 0.6538017 | 0.903092 |
| X216900_s_at | -0.0017 | 0.0038 | -0.453 | 0.6538164 | 0.903092 |
| X208390_s_at | 0.0041 | 0.009 | 0.453 | 0.653851 | 0.903092 |
| X216129_at | 0.019 | 0.0419 | 0.453 | 0.6538882 | 0.903092 |
| X214664_at | -0.0028 | 0.0062 | -0.453 | 0.6539038 | 0.903092 |
| X37549_g_at | -0.0156 | 0.0344 | -0.453 | 0.6539617 | 0.903092 |
| X210956_at | -0.0015 | 0.0033 | -0.452 | 0.6540885 | 0.903092 |
| X202264_s_at | 0.0294 | 0.0649 | 0.452 | 0.6541091 | 0.903092 |
| X211338_at | -0.0024 | 0.0054 | -0.452 | 0.6541128 | 0.903092 |
| X203169_at | -0.0137 | 0.0302 | -0.452 | 0.6541182 | 0.903092 |
| X205247_at | 0.0215 | 0.0476 | 0.452 | 0.6541386 | 0.903092 |
| X207716_at | 0.0027 | 0.0059 | 0.452 | 0.6541747 | 0.903092 |
| X216053_x_at | 0.0022 | 0.0049 | 0.452 | 0.6542107 | 0.903092 |
| X214696_at | 0.0177 | 0.0392 | 0.452 | 0.6542973 | 0.903092 |
| X216604_s_at | -0.0031 | 0.0069 | -0.452 | 0.654319 | 0.903092 |
| X202059_s_at | 0.0183 | 0.0404 | 0.452 | 0.6543642 | 0.903092 |
| X200992_at | -0.0168 | 0.0371 | -0.452 | 0.6544566 | 0.903092 |
| X212283_at | 0.0014 | 0.0031 | 0.452 | 0.6544691 | 0.903092 |
| X216148_at | -0.0018 | 0.0041 | -0.452 | 0.6544998 | 0.903092 |
| X210462_at | 0.0038 | 0.0084 | 0.452 | 0.654515 | 0.903092 |
| X216190_x_at | -0.0025 | 0.0055 | -0.452 | 0.654528 | 0.903092 |
| X212659_s_at | 0.0023 | 0.0051 | 0.452 | 0.654532 | 0.903092 |
| X216164_at | 0.0012 | 0.0026 | 0.452 | 0.6546133 | 0.903148 |
| X220189_s_at | 0.0224 | 0.0495 | 0.452 | 0.6547293 | 0.903252 |
| X207299_s_at | 0.0556 | 0.1232 | 0.451 | 0.6548042 | 0.903299 |
| X217711_at | 0.0035 | 0.0078 | 0.451 | 0.6549332 | 0.903352 |
| X201373_at | 0.0058 | 0.0128 | 0.451 | 0.6549498 | 0.903352 |
| X206110_at | 0.0022 | 0.0049 | 0.451 | 0.6549644 | 0.903352 |
| X202487_s_at | 0.0167 | 0.037 | 0.451 | 0.6550701 | 0.903399 |
| X222048_at | -0.0165 | 0.0365 | -0.451 | 0.6550804 | 0.903399 |
| X220974_x_at | 0.0142 | 0.0314 | 0.451 | 0.6551514 | 0.903441 |
| X208550_x_at | -0.0015 | 0.0033 | -0.451 | 0.655273 | 0.903446 |
| X220366_at | -0.0024 | 0.0053 | -0.451 | 0.6553193 | 0.903446 |
| X201461_s_at | -0.0026 | 0.0058 | -0.451 | 0.6553399 | 0.903446 |
| X219830_at | 0.0018 | 0.0041 | 0.451 | 0.655349 | 0.903446 |
| X207229_at | 0.0038 | 0.0084 | 0.451 | 0.6553583 | 0.903446 |
| X220073_s_at | 0.0023 | 0.005 | 0.451 | 0.6554553 | 0.903489 |
| X204718_at | -0.0385 | 0.0854 | -0.451 | 0.6554706 | 0.903489 |
| X207510_at | -0.0018 | 0.004 | -0.45 | 0.6555513 | 0.903494 |
| X214923_at | 0.0032 | 0.0072 | 0.45 | 0.6555557 | 0.903494 |
| X214041_x_at | 0.0745 | 0.1656 | 0.45 | 0.6556785 | 0.903607 |
| X207026_s_at | -0.0199 | 0.0442 | -0.45 | 0.6557353 | 0.903622 |
| X217031_at | 0.0014 | 0.0031 | 0.45 | 0.6557783 | 0.903622 |
| X211589_at | -0.0021 | 0.0047 | -0.45 | 0.6558338 | 0.903622 |
| X205767_at | 0.0074 | 0.0163 | 0.45 | 0.6558638 | 0.903622 |
| X205690_s_at | -0.013 | 0.029 | -0.45 | 0.6558936 | 0.903622 |
| X207866_at | -0.0018 | 0.0041 | -0.45 | 0.6559335 | 0.903622 |
| X205515_at | 0.0035 | 0.0078 | 0.45 | 0.656055 | 0.903678 |
| X202548_s_at | -0.0172 | 0.0382 | -0.45 | 0.6560555 | 0.903678 |
| X212860_at | 0.0033 | 0.0074 | 0.449 | 0.6562274 | 0.903859 |
| X207178_s_at | -0.0036 | 0.0079 | -0.449 | 0.6563501 | 0.903972 |
| X208045_at | -0.004 | 0.0089 | -0.449 | 0.6564891 | 0.904034 |
| X204088_at | 0.0018 | 0.0041 | 0.449 | 0.6565158 | 0.904034 |
| X202971_s_at | 0.036 | 0.0802 | 0.449 | 0.6565353 | 0.904034 |
| X202417_at | -0.022 | 0.049 | -0.449 | 0.6566622 | 0.904034 |
| X213920_at | 0.0052 | 0.0115 | 0.449 | 0.6566811 | 0.904034 |
| X219758_at | 0.0019 | 0.0041 | 0.449 | 0.6567365 | 0.904034 |
| X210986_s_at | 0.017 | 0.038 | 0.449 | 0.6567556 | 0.904034 |
| X202337_at | -0.0032 | 0.0071 | -0.449 | 0.6567988 | 0.904034 |
| X211549_s_at | 0.002 | 0.0044 | 0.449 | 0.6568334 | 0.904034 |
| X216128_at | -0.003 | 0.0067 | -0.449 | 0.6568497 | 0.904034 |
| X211312_s_at | -0.0016 | 0.0035 | -0.449 | 0.6568955 | 0.904034 |
| X208481_at | -0.0032 | 0.0071 | -0.448 | 0.656989 | 0.904034 |
| X213178_s_at | -0.0098 | 0.0218 | -0.448 | 0.657016 | 0.904034 |
| X210707_x_at | -0.0185 | 0.0413 | -0.448 | 0.657021 | 0.904034 |
| X218856_at | 0.0353 | 0.0788 | 0.448 | 0.6570625 | 0.904034 |
| X200847_s_at | -0.0194 | 0.0433 | -0.448 | 0.6571156 | 0.904034 |
| X219350_s_at | -0.0153 | 0.0341 | -0.448 | 0.6571211 | 0.904034 |
| X217603_at | 0.0019 | 0.0043 | 0.448 | 0.6571475 | 0.904034 |
| X204899_s_at | -0.0059 | 0.0132 | -0.448 | 0.6572492 | 0.904034 |
| X210397_at | -0.002 | 0.0045 | -0.448 | 0.6572723 | 0.904034 |
| X212589_at | 0.025 | 0.0558 | 0.448 | 0.65731 | 0.904034 |
| X202093_s_at | 0.0109 | 0.0243 | 0.448 | 0.6573287 | 0.904034 |
| X215816_at | -0.0042 | 0.0093 | -0.448 | 0.6573592 | 0.904034 |
| X213019_at | 0.0143 | 0.032 | 0.448 | 0.6573721 | 0.904034 |
| X216462_at | 0.0028 | 0.0062 | 0.448 | 0.657481 | 0.904128 |
| X217092_x_at | -0.0054 | 0.0122 | -0.448 | 0.6575613 | 0.904182 |
| X220879_at | 0.0024 | 0.0053 | 0.448 | 0.6576082 | 0.904191 |
| X208392_x_at | 0.002 | 0.0044 | 0.447 | 0.657904 | 0.904491 |
| X221180_at | -0.0018 | 0.0041 | -0.447 | 0.6580082 | 0.904491 |
| X215107_s_at | -0.0018 | 0.004 | -0.447 | 0.6581129 | 0.904491 |
| X211893_x_at | 0.002 | 0.0045 | 0.447 | 0.6581404 | 0.904491 |
| X205447_s_at | -0.0102 | 0.0227 | -0.447 | 0.6581678 | 0.904491 |
| X211217_s_at | -0.0018 | 0.004 | -0.447 | 0.6581843 | 0.904491 |
| X208089_s_at | -0.0212 | 0.0475 | -0.447 | 0.6582044 | 0.904491 |
| X214146_s_at | 0.0029 | 0.0065 | 0.447 | 0.6582167 | 0.904491 |
| X203969_at | 0.0165 | 0.0368 | 0.447 | 0.6582515 | 0.904491 |
| X221754_s_at | -0.0019 | 0.0043 | -0.447 | 0.6583098 | 0.904491 |
| X212180_at | -0.0099 | 0.0221 | -0.446 | 0.6583531 | 0.904491 |
| X201124_at | -0.0443 | 0.0992 | -0.446 | 0.6584248 | 0.904491 |
| X216624_s_at | -0.0024 | 0.0053 | -0.446 | 0.6584994 | 0.904491 |
| X207371_at | -0.0026 | 0.0059 | -0.446 | 0.6585135 | 0.904491 |
| X217484_at | -0.0023 | 0.0052 | -0.446 | 0.6585455 | 0.904491 |
| X201039_s_at | -0.0167 | 0.0374 | -0.446 | 0.6585459 | 0.904491 |
| X203877_at | 0.0023 | 0.0052 | 0.446 | 0.658595 | 0.904491 |
| X221275_s_at | 0.002 | 0.0045 | 0.446 | 0.6586211 | 0.904491 |
| X200071_at | -0.013 | 0.0291 | -0.446 | 0.6586621 | 0.904491 |
| X217757_at | 0.0459 | 0.103 | 0.446 | 0.6586925 | 0.904491 |
| X209234_at | -0.0075 | 0.0169 | -0.446 | 0.6587029 | 0.904491 |
| X220012_at | 0.0044 | 0.01 | 0.446 | 0.658731 | 0.904491 |
| X211111_at | -0.0029 | 0.0065 | -0.446 | 0.6587628 | 0.904491 |
| X220902_at | 0.0013 | 0.003 | 0.446 | 0.6588637 | 0.904514 |
| X218881_s_at | 0.0019 | 0.0043 | 0.446 | 0.6589137 | 0.904514 |
| X220803_at | 0.0061 | 0.0137 | 0.446 | 0.6589671 | 0.904514 |
| X202715_at | -0.0016 | 0.0036 | -0.446 | 0.6590003 | 0.904514 |
| X215848_at | 0.0021 | 0.0048 | 0.446 | 0.6590163 | 0.904514 |
| X201562_s_at | -0.0043 | 0.0096 | -0.446 | 0.6590242 | 0.904514 |
| X221393_at | 0.002 | 0.0044 | 0.445 | 0.6591831 | 0.904556 |
| X208597_at | 0.0016 | 0.0035 | 0.445 | 0.6591932 | 0.904556 |
| X210289_at | -0.0026 | 0.0059 | -0.445 | 0.6592519 | 0.904556 |
| X216920_s_at | -0.0143 | 0.0322 | -0.445 | 0.6592702 | 0.904556 |
| X210894_s_at | -0.0027 | 0.006 | -0.445 | 0.6593235 | 0.904556 |
| X205558_at | -0.0047 | 0.0105 | -0.445 | 0.6593345 | 0.904556 |
| X221915_s_at | 0.0021 | 0.0046 | 0.445 | 0.6593394 | 0.904556 |
| X212932_at | -0.0253 | 0.0568 | -0.445 | 0.6594813 | 0.904695 |
| X211738_x_at | -0.0009 | 0.0019 | -0.445 | 0.659655 | 0.904847 |
| X220615_s_at | -0.0399 | 0.0897 | -0.445 | 0.6597106 | 0.904847 |
| X204534_at | 0.0019 | 0.0042 | 0.445 | 0.6597148 | 0.904847 |
| X202705_at | 0.0035 | 0.0079 | 0.444 | 0.6598051 | 0.904915 |
| X211523_at | 0.0038 | 0.0085 | 0.444 | 0.6599418 | 0.905047 |
| X208583_x_at | -0.0023 | 0.0051 | -0.444 | 0.6600311 | 0.905107 |
| X206697_s_at | -0.0073 | 0.0165 | -0.444 | 0.6600673 | 0.905107 |
| X219240_s_at | 0.0207 | 0.0465 | 0.444 | 0.6602011 | 0.905201 |
| X221100_at | -0.0022 | 0.005 | -0.444 | 0.6602613 | 0.905201 |
| X210254_at | 0.0024 | 0.0054 | 0.444 | 0.6603126 | 0.905201 |
| X201197_at | -0.0122 | 0.0274 | -0.444 | 0.6603874 | 0.905201 |
| X212535_at | 0.0143 | 0.0323 | 0.444 | 0.6603901 | 0.905201 |
| X207352_s_at | 0.0023 | 0.0052 | 0.444 | 0.6603933 | 0.905201 |
| X217290_at | -0.0021 | 0.0048 | -0.444 | 0.6604211 | 0.905201 |
| X219454_at | 0.0034 | 0.0076 | 0.443 | 0.6604844 | 0.905214 |
| X207846_at | -0.0055 | 0.0125 | -0.443 | 0.6605504 | 0.905214 |
| X206682_at | 0.002 | 0.0045 | 0.443 | 0.660553 | 0.905214 |
| X220967_s_at | -0.0023 | 0.0053 | -0.443 | 0.6606138 | 0.905223 |
| X219104_at | -0.0106 | 0.0239 | -0.443 | 0.660641 | 0.905223 |
| X221470_s_at | -0.0046 | 0.0105 | -0.443 | 0.660771 | 0.905278 |
| X201149_s_at | 0.037 | 0.0834 | 0.443 | 0.6608902 | 0.905278 |
| X210146_x_at | -0.0029 | 0.0065 | -0.443 | 0.6609334 | 0.905278 |
| X220657_at | 0.0017 | 0.0039 | 0.443 | 0.6609669 | 0.905278 |
| X209207_s_at | -0.0138 | 0.0312 | -0.443 | 0.6609756 | 0.905278 |
| X206463_s_at | -0.0013 | 0.0029 | -0.443 | 0.661024 | 0.905278 |
| X212005_at | -0.0022 | 0.005 | -0.443 | 0.6610397 | 0.905278 |
| X215622_x_at | 0.0011 | 0.0025 | 0.443 | 0.6610414 | 0.905278 |
| X212029_s_at | 0.0021 | 0.0046 | 0.443 | 0.6611155 | 0.905278 |
| X215882_at | -0.005 | 0.0114 | -0.443 | 0.6611593 | 0.905278 |
| X218928_s_at | 0.0018 | 0.0041 | 0.442 | 0.6612042 | 0.905278 |
| X221747_at | 0.0018 | 0.0041 | 0.442 | 0.6612467 | 0.905278 |
| X221136_at | -0.0018 | 0.004 | -0.442 | 0.6612602 | 0.905278 |
| X206223_at | -0.001 | 0.0022 | -0.442 | 0.6612874 | 0.905278 |
| X204122_at | 0.0742 | 0.1678 | 0.442 | 0.6612924 | 0.905278 |
| X211058_x_at | 0.0191 | 0.0431 | 0.442 | 0.6614946 | 0.905499 |
| X216202_s_at | -0.0035 | 0.0078 | -0.442 | 0.6615495 | 0.905519 |
| X213600_at | 0.0057 | 0.0129 | 0.442 | 0.6617414 | 0.905674 |
| X205744_at | -0.0024 | 0.0055 | -0.442 | 0.6617444 | 0.905674 |
| X209202_s_at | -0.0058 | 0.0131 | -0.442 | 0.6618649 | 0.905745 |
| X207737_at | -0.0067 | 0.0153 | -0.442 | 0.6618778 | 0.905745 |
| X218769_s_at | -0.0142 | 0.0321 | -0.441 | 0.6619971 | 0.90581 |
| X217302_at | -0.0016 | 0.0036 | -0.441 | 0.6620074 | 0.90581 |
| X218129_s_at | -0.0221 | 0.0501 | -0.441 | 0.6622205 | 0.905923 |
| X215694_at | 0.0039 | 0.0088 | 0.441 | 0.6622339 | 0.905923 |
| X208810_at | -0.0228 | 0.0518 | -0.441 | 0.662248 | 0.905923 |
| X207332_s_at | 0.0422 | 0.0957 | 0.441 | 0.6622527 | 0.905923 |
| X207608_x_at | -0.0279 | 0.0633 | -0.441 | 0.6623175 | 0.905924 |
| X202701_at | -0.0027 | 0.006 | -0.441 | 0.6623349 | 0.905924 |
| X208263_at | 0.0037 | 0.0083 | 0.441 | 0.6623873 | 0.90594 |
| X215941_at | 0.0026 | 0.006 | 0.441 | 0.6624588 | 0.905958 |
| X218608_at | -0.0311 | 0.0707 | -0.441 | 0.6625385 | 0.905958 |
| X203460_s_at | -0.0333 | 0.0756 | -0.441 | 0.6625531 | 0.905958 |
| X205188_s_at | 0.002 | 0.0045 | 0.441 | 0.6625637 | 0.905958 |
| X220810_at | 0.0101 | 0.023 | 0.44 | 0.6627156 | 0.906069 |
| X217539_at | -0.0043 | 0.0097 | -0.44 | 0.6627263 | 0.906069 |
| X200993_at | -0.02 | 0.0453 | -0.44 | 0.6628252 | 0.906148 |
| X207720_at | -0.001 | 0.0022 | -0.44 | 0.6629061 | 0.906203 |
| X217323_at | 0.0015 | 0.0035 | 0.44 | 0.6629533 | 0.906212 |
| X218110_at | -0.0021 | 0.0047 | -0.44 | 0.6630458 | 0.906282 |
| X204591_at | -0.015 | 0.0341 | -0.44 | 0.6631143 | 0.90632 |
| X202617_s_at | -0.0185 | 0.0422 | -0.44 | 0.6632732 | 0.906465 |
| X211333_s_at | 0.002 | 0.0046 | 0.44 | 0.6633016 | 0.906465 |
| X219753_at | 0.0018 | 0.0041 | 0.439 | 0.6634175 | 0.906503 |
| X200872_at | 0.0315 | 0.0717 | 0.439 | 0.6634226 | 0.906503 |
| X209778_at | 0.0038 | 0.0087 | 0.439 | 0.6635254 | 0.906503 |
| X203395_s_at | 0.0191 | 0.0434 | 0.439 | 0.6635454 | 0.906503 |
| X218661_at | 0.0049 | 0.0112 | 0.439 | 0.6635498 | 0.906503 |
| X207261_at | 0.002 | 0.0045 | 0.439 | 0.6636061 | 0.906503 |
| X205563_at | 0.0014 | 0.0032 | 0.439 | 0.6636155 | 0.906503 |
| X209355_s_at | 0.0414 | 0.0943 | 0.439 | 0.6636762 | 0.90653 |
| X212963_at | -0.0025 | 0.0058 | -0.439 | 0.6638599 | 0.906726 |
| X216826_at | -0.0024 | 0.0055 | -0.439 | 0.6639243 | 0.906758 |
| X205832_at | -0.002 | 0.0046 | -0.438 | 0.6640885 | 0.90689 |
| X211204_at | 0.0015 | 0.0033 | 0.438 | 0.6641027 | 0.90689 |
| X219138_at | 0.0026 | 0.0058 | 0.438 | 0.6641781 | 0.906937 |
| X214191_at | -0.0022 | 0.0049 | -0.438 | 0.6642986 | 0.907046 |
| X206443_at | 0.0013 | 0.003 | 0.438 | 0.6644297 | 0.907117 |
| X210203_at | -0.0177 | 0.0404 | -0.438 | 0.6644856 | 0.907117 |
| X221710_x_at | -0.0029 | 0.0065 | -0.438 | 0.6644965 | 0.907117 |
| X206727_at | -0.0028 | 0.0064 | -0.438 | 0.6645294 | 0.907117 |
| X205490_x_at | -0.0012 | 0.0027 | -0.438 | 0.6645545 | 0.907117 |
| X210621_s_at | -0.0297 | 0.0678 | -0.438 | 0.6646652 | 0.907171 |
| X216507_at | 0.0017 | 0.0039 | 0.438 | 0.6647108 | 0.907171 |
| X217342_x_at | 0.0018 | 0.004 | 0.438 | 0.6647428 | 0.907171 |
| X205527_s_at | -0.0058 | 0.0132 | -0.438 | 0.664758 | 0.907171 |
| X200664_s_at | 0.0343 | 0.0784 | 0.437 | 0.6648053 | 0.90718 |
| X215419_at | 0.0032 | 0.0073 | 0.437 | 0.6648874 | 0.907236 |
| X211527_x_at | -0.0014 | 0.0031 | -0.437 | 0.6650945 | 0.907463 |
| X208452_x_at | -0.0019 | 0.0043 | -0.437 | 0.6652344 | 0.907513 |
| X205925_s_at | -0.0014 | 0.0032 | -0.437 | 0.6652535 | 0.907513 |
| X213635_s_at | -0.0019 | 0.0043 | -0.437 | 0.6652537 | 0.907513 |
| X215553_x_at | 0.0224 | 0.0514 | 0.437 | 0.6653933 | 0.907648 |
| X208394_x_at | -0.0053 | 0.0121 | -0.436 | 0.6655263 | 0.90772 |
| X220174_at | -0.0019 | 0.0042 | -0.436 | 0.6655998 | 0.90772 |
| X206905_s_at | -0.0019 | 0.0043 | -0.436 | 0.6656957 | 0.90772 |
| X209373_at | 0.0016 | 0.0037 | 0.436 | 0.6657331 | 0.90772 |
| X220625_s_at | -0.0029 | 0.0066 | -0.436 | 0.6657563 | 0.90772 |
| X220778_x_at | 0.0022 | 0.005 | 0.436 | 0.6657813 | 0.90772 |
| X212417_at | 0.0409 | 0.0937 | 0.436 | 0.6658286 | 0.90772 |
| X206334_at | -0.0027 | 0.0061 | -0.436 | 0.665864 | 0.90772 |
| X37408_at | 0.0374 | 0.0858 | 0.436 | 0.6659161 | 0.90772 |
| X208443_x_at | -0.0021 | 0.0049 | -0.436 | 0.6659527 | 0.90772 |
| X221266_s_at | 0.0024 | 0.0055 | 0.436 | 0.6660534 | 0.90772 |
| X220051_at | 0.0014 | 0.0031 | 0.436 | 0.6660651 | 0.90772 |
| X220118_at | 0.0013 | 0.0029 | 0.436 | 0.6661562 | 0.90772 |
| X203759_at | 0.0027 | 0.0061 | 0.436 | 0.6661566 | 0.90772 |
| X206195_x_at | 0.002 | 0.0047 | 0.435 | 0.6662208 | 0.90772 |
| X207802_at | -0.005 | 0.0116 | -0.435 | 0.6662593 | 0.90772 |
| X202979_s_at | -0.0274 | 0.063 | -0.435 | 0.6662828 | 0.90772 |
| X222115_x_at | -0.0017 | 0.0039 | -0.435 | 0.6663129 | 0.90772 |
| X218368_s_at | 0.0024 | 0.0054 | 0.435 | 0.6663204 | 0.90772 |
| X213871_s_at | 0.0016 | 0.0037 | 0.435 | 0.6663522 | 0.90772 |
| X204833_at | -0.0178 | 0.0409 | -0.435 | 0.6663676 | 0.90772 |
| X208365_s_at | -0.0021 | 0.0049 | -0.435 | 0.6663753 | 0.90772 |
| X201487_at | 0.057 | 0.131 | 0.435 | 0.6664264 | 0.90772 |
| X207934_at | -0.0062 | 0.0142 | -0.435 | 0.6664267 | 0.90772 |
| X216298_at | -0.0036 | 0.0084 | -0.435 | 0.6665099 | 0.907778 |
| X219681_s_at | 0.0028 | 0.0064 | 0.435 | 0.666648 | 0.90791 |
| X200091_s_at | 0.0078 | 0.018 | 0.435 | 0.6667793 | 0.90801 |
| X213231_at | 0.0015 | 0.0034 | 0.435 | 0.6668031 | 0.90801 |
| X207092_at | -0.0023 | 0.0053 | -0.435 | 0.666934 | 0.908132 |
| X210192_at | -0.0007 | 0.0016 | -0.434 | 0.6671792 | 0.908315 |
| X205569_at | 0.0031 | 0.0072 | 0.434 | 0.6672426 | 0.908315 |
| X222159_at | 0.0106 | 0.0245 | 0.434 | 0.6673046 | 0.908315 |
| X220022_at | 0.0116 | 0.0267 | 0.434 | 0.6673702 | 0.908315 |
| X206739_at | -0.0009 | 0.002 | -0.434 | 0.6674033 | 0.908315 |
| X218222_x_at | 0.0014 | 0.0032 | 0.434 | 0.6674535 | 0.908315 |
| X215792_s_at | 0.0222 | 0.0512 | 0.434 | 0.6674911 | 0.908315 |
| X211100_x_at | -0.0015 | 0.0034 | -0.434 | 0.667552 | 0.908315 |
| X218693_at | 0.0402 | 0.0927 | 0.434 | 0.6675528 | 0.908315 |
| X207240_s_at | 0.0034 | 0.0079 | 0.434 | 0.6675764 | 0.908315 |
| X216094_at | 0.0034 | 0.0079 | 0.434 | 0.6676441 | 0.908315 |
| X214992_s_at | -0.0021 | 0.0048 | -0.433 | 0.6676745 | 0.908315 |
| X222221_x_at | 0.002 | 0.0047 | 0.433 | 0.6677036 | 0.908315 |
| X218756_s_at | -0.0336 | 0.0775 | -0.433 | 0.6677223 | 0.908315 |
| X209512_at | 0.0291 | 0.0671 | 0.433 | 0.667791 | 0.908315 |
| X221108_at | 0.0022 | 0.0051 | 0.433 | 0.6677936 | 0.908315 |
| X214026_s_at | 0.0013 | 0.003 | 0.433 | 0.6677955 | 0.908315 |
| X212519_at | -0.0117 | 0.027 | -0.433 | 0.6678041 | 0.908315 |
| X218950_at | 0.0039 | 0.0091 | 0.433 | 0.6679028 | 0.908394 |
| X207306_at | -0.0024 | 0.0055 | -0.433 | 0.6680108 | 0.908422 |
| X206999_at | 0.002 | 0.0047 | 0.433 | 0.6681989 | 0.908422 |
| X212559_at | 0.0391 | 0.0904 | 0.433 | 0.6682353 | 0.908422 |
| X207632_at | 0.0018 | 0.0041 | 0.433 | 0.6682491 | 0.908422 |
| X213895_at | -0.0029 | 0.0066 | -0.433 | 0.6682662 | 0.908422 |
| X220801_s_at | 0.0016 | 0.0038 | 0.433 | 0.6682937 | 0.908422 |
| X221262_s_at | -0.0067 | 0.0156 | -0.433 | 0.6683412 | 0.908422 |
| X216062_at | -0.0066 | 0.0152 | -0.433 | 0.6683644 | 0.908422 |
| X31637_s_at | 0.0371 | 0.0857 | 0.432 | 0.6683841 | 0.908422 |
| X201098_at | -0.0163 | 0.0376 | -0.432 | 0.6684142 | 0.908422 |
| X213097_s_at | 0.0105 | 0.0243 | 0.432 | 0.6684677 | 0.908422 |
| X219779_at | -0.0223 | 0.0516 | -0.432 | 0.6685112 | 0.908422 |
| X220911_s_at | 0.0013 | 0.0031 | 0.432 | 0.6685287 | 0.908422 |
| X205876_at | 0.0178 | 0.0412 | 0.432 | 0.668543 | 0.908422 |
| X216891_at | 0.0014 | 0.0031 | 0.432 | 0.6686153 | 0.908422 |
| X221734_at | 0.0016 | 0.0037 | 0.432 | 0.668629 | 0.908422 |
| X203828_s_at | 0.0016 | 0.0037 | 0.432 | 0.6686592 | 0.908422 |
| X221006_s_at | 0.0259 | 0.0599 | 0.432 | 0.6686594 | 0.908422 |
| X215603_x_at | 0.001 | 0.0024 | 0.432 | 0.6687598 | 0.908435 |
| X220307_at | 0.001 | 0.0024 | 0.432 | 0.6687636 | 0.908435 |
| X201736_s_at | -0.0237 | 0.0549 | -0.432 | 0.6687918 | 0.908435 |
| X206012_at | 0.0019 | 0.0045 | 0.432 | 0.6688606 | 0.908473 |
| X221784_at | -0.0028 | 0.0065 | -0.432 | 0.6689053 | 0.908478 |
| X210257_x_at | -0.0178 | 0.0411 | -0.431 | 0.6691182 | 0.908711 |
| X213788_s_at | -0.0022 | 0.0051 | -0.431 | 0.6693613 | 0.908986 |
| X217217_at | 0.0018 | 0.0042 | 0.431 | 0.6695002 | 0.909051 |
| X207499_x_at | -0.0032 | 0.0075 | -0.431 | 0.6695165 | 0.909051 |
| X215389_s_at | -0.0037 | 0.0087 | -0.431 | 0.6695322 | 0.909051 |
| X204035_at | -0.0178 | 0.0413 | -0.431 | 0.6696758 | 0.909183 |
| X217619_x_at | 0.0257 | 0.0598 | 0.431 | 0.6697503 | 0.909183 |
| X217173_s_at | -0.0013 | 0.003 | -0.431 | 0.6697767 | 0.909183 |
| X210816_s_at | 0.0016 | 0.0037 | 0.431 | 0.6697932 | 0.909183 |
| X213264_at | 0.0196 | 0.0455 | 0.43 | 0.669857 | 0.909209 |
| X206122_at | 0.0061 | 0.0142 | 0.43 | 0.6699242 | 0.909209 |
| X216322_at | -0.0056 | 0.0129 | -0.43 | 0.6699661 | 0.909209 |
| X220310_at | 0.0027 | 0.0063 | 0.43 | 0.6699857 | 0.909209 |
| X208011_at | -0.0037 | 0.0085 | -0.43 | 0.6700169 | 0.909209 |
| X206696_at | -0.0076 | 0.0176 | -0.43 | 0.6700717 | 0.909227 |
| X210603_at | -0.0024 | 0.0056 | -0.43 | 0.6701116 | 0.909227 |
| X212671_s_at | -0.0018 | 0.0042 | -0.43 | 0.6701885 | 0.909276 |
| X204832_s_at | 0.0339 | 0.0789 | 0.43 | 0.6702527 | 0.909307 |
| X210276_s_at | -0.0042 | 0.0098 | -0.43 | 0.6703386 | 0.909368 |
| X204012_s_at | -0.015 | 0.0349 | -0.43 | 0.6704625 | 0.90942 |
| X208631_s_at | -0.0166 | 0.0386 | -0.429 | 0.6705761 | 0.90942 |
| X218787_x_at | 0.0019 | 0.0045 | 0.429 | 0.6705828 | 0.90942 |
| X205920_at | -0.0017 | 0.004 | -0.429 | 0.6706545 | 0.90942 |
| X201278_at | 0.0334 | 0.0779 | 0.429 | 0.6706686 | 0.90942 |
| X60084_at | 0.003 | 0.0069 | 0.429 | 0.6706951 | 0.90942 |
| X214792_x_at | -0.0268 | 0.0625 | -0.429 | 0.6707036 | 0.90942 |
| X216168_at | -0.0023 | 0.0053 | -0.429 | 0.6707156 | 0.90942 |
| X203544_s_at | -0.0165 | 0.0385 | -0.429 | 0.6707456 | 0.90942 |
| X201258_at | 0.0138 | 0.0323 | 0.429 | 0.6708257 | 0.909473 |
| X206394_at | -0.0011 | 0.0026 | -0.429 | 0.6708928 | 0.909509 |
| X213169_at | -0.0215 | 0.0502 | -0.429 | 0.6711116 | 0.909514 |
| X215385_at | 0.0377 | 0.088 | 0.429 | 0.6711172 | 0.909514 |
| X206574_s_at | -0.0017 | 0.0039 | -0.429 | 0.6711211 | 0.909514 |
| X215589_at | -0.0043 | 0.0101 | -0.429 | 0.6711414 | 0.909514 |
| X211724_x_at | 0.0087 | 0.0203 | 0.429 | 0.6711736 | 0.909514 |
| X205731_s_at | 0.0032 | 0.0076 | 0.429 | 0.6712103 | 0.909514 |
| X208179_x_at | 0.0017 | 0.0039 | 0.429 | 0.6712166 | 0.909514 |
| X201763_s_at | -0.0038 | 0.0089 | -0.429 | 0.6712241 | 0.909514 |
| X215685_s_at | -0.0011 | 0.0026 | -0.428 | 0.671374 | 0.909565 |
| X212662_at | 0.0095 | 0.0222 | 0.428 | 0.6713809 | 0.909565 |
| X205676_at | 0.0024 | 0.0056 | 0.428 | 0.6714422 | 0.909565 |
| X212897_at | 0.0401 | 0.0937 | 0.428 | 0.6714656 | 0.909565 |
| X219476_at | 0.0014 | 0.0032 | 0.428 | 0.6714664 | 0.909565 |
| X215073_s_at | -0.0106 | 0.0248 | -0.428 | 0.6716856 | 0.909795 |
| X212524_x_at | -0.0012 | 0.0028 | -0.428 | 0.6717182 | 0.909795 |
| X214750_at | -0.0015 | 0.0035 | -0.428 | 0.6718767 | 0.909924 |
| X217994_x_at | 0.0055 | 0.0129 | 0.428 | 0.6718953 | 0.909924 |
| X216107_at | 0.0016 | 0.0036 | 0.427 | 0.6720163 | 0.910032 |
| X206934_at | 0.0019 | 0.0044 | 0.427 | 0.6721485 | 0.910103 |
| X210498_at | 0.0059 | 0.0139 | 0.427 | 0.6721506 | 0.910103 |
| X216057_at | 0.0065 | 0.0152 | 0.427 | 0.6723713 | 0.910347 |
| X217631_at | -0.0191 | 0.0447 | -0.427 | 0.6725789 | 0.910546 |
| X221697_at | -0.0033 | 0.0076 | -0.427 | 0.6726007 | 0.910546 |
| X204781_s_at | -0.0025 | 0.0059 | -0.426 | 0.6727397 | 0.910606 |
| X214256_at | 0.0022 | 0.0051 | 0.426 | 0.6727607 | 0.910606 |
| X204550_x_at | -0.0143 | 0.0335 | -0.426 | 0.6727788 | 0.910606 |
| X222172_at | -0.0027 | 0.0063 | -0.426 | 0.6728087 | 0.910606 |
| X204603_at | -0.0027 | 0.0064 | -0.426 | 0.6728565 | 0.910615 |
| X203429_s_at | -0.0195 | 0.0458 | -0.426 | 0.6729476 | 0.910643 |
| X215433_at | -0.002 | 0.0048 | -0.426 | 0.6729993 | 0.910643 |
| X207678_s_at | -0.0049 | 0.0115 | -0.426 | 0.6730711 | 0.910643 |
| X212562_s_at | 0.0021 | 0.0048 | 0.426 | 0.6730912 | 0.910643 |
| X214734_at | 0.0074 | 0.0175 | 0.426 | 0.6732058 | 0.910643 |
| X201366_at | 0.0137 | 0.0322 | 0.426 | 0.6732402 | 0.910643 |
| X219051_x_at | -0.0187 | 0.044 | -0.426 | 0.6732463 | 0.910643 |
| X221987_s_at | 0.0071 | 0.0168 | 0.426 | 0.6732733 | 0.910643 |
| X211701_s_at | 0.0236 | 0.0554 | 0.426 | 0.6732827 | 0.910643 |
| X207582_at | -0.0135 | 0.0318 | -0.426 | 0.6733657 | 0.910643 |
| X208311_at | 0.002 | 0.0048 | 0.426 | 0.6733993 | 0.910643 |
| X209943_at | -0.0142 | 0.0335 | -0.425 | 0.6734462 | 0.910643 |
| X222341_x_at | 0.0035 | 0.0082 | 0.425 | 0.6734876 | 0.910643 |
| X206269_at | 0.0025 | 0.0059 | 0.425 | 0.6734882 | 0.910643 |
| X204827_s_at | -0.0011 | 0.0027 | -0.425 | 0.6735238 | 0.910643 |
| X205192_at | -0.0025 | 0.0058 | -0.425 | 0.6735334 | 0.910643 |
| X216746_at | -0.0028 | 0.0066 | -0.425 | 0.6737061 | 0.910793 |
| X217084_at | -0.0023 | 0.0053 | -0.425 | 0.6737474 | 0.910793 |
| X220561_at | -0.0016 | 0.0038 | -0.425 | 0.6737938 | 0.910793 |
| X220993_s_at | 0.0019 | 0.0044 | 0.425 | 0.6738077 | 0.910793 |
| X219932_at | -0.0036 | 0.0084 | -0.425 | 0.6740128 | 0.910978 |
| X219130_at | 0.0241 | 0.0567 | 0.425 | 0.6740929 | 0.910978 |
| X212794_s_at | -0.0267 | 0.0629 | -0.425 | 0.6741022 | 0.910978 |
| X206058_at | 0.0023 | 0.0054 | 0.425 | 0.6741086 | 0.910978 |
| X216354_at | -0.0034 | 0.0081 | -0.424 | 0.674237 | 0.911037 |
| X208239_at | 0.0013 | 0.003 | 0.424 | 0.6742598 | 0.911037 |
| X202909_at | 0.0174 | 0.041 | 0.424 | 0.6743004 | 0.911037 |
| X220102_at | 0.0044 | 0.0103 | 0.424 | 0.6743194 | 0.911037 |
| X214630_at | 0.0037 | 0.0088 | 0.424 | 0.6743799 | 0.911037 |
| X201048_x_at | -0.0043 | 0.0102 | -0.424 | 0.6743984 | 0.911037 |
| X208504_x_at | -0.001 | 0.0023 | -0.424 | 0.6745176 | 0.911142 |
| X220912_at | 0.0069 | 0.0164 | 0.424 | 0.6745704 | 0.911158 |
| X202711_at | -0.0024 | 0.0057 | -0.424 | 0.6746972 | 0.911274 |
| X215049_x_at | -0.0018 | 0.0043 | -0.423 | 0.6749278 | 0.911395 |
| X207095_at | 0.003 | 0.0071 | 0.423 | 0.6749281 | 0.911395 |
| X209133_s_at | -0.0011 | 0.0025 | -0.423 | 0.6749689 | 0.911395 |
| X208456_s_at | 0.006 | 0.0142 | 0.423 | 0.674987 | 0.911395 |
| X211632_at | -0.0036 | 0.0084 | -0.423 | 0.6750605 | 0.911395 |
| X204027_s_at | -0.0023 | 0.0054 | -0.423 | 0.6750672 | 0.911395 |
| X219785_s_at | -0.0131 | 0.0309 | -0.423 | 0.6750954 | 0.911395 |
| X220517_at | 0.0045 | 0.0106 | 0.423 | 0.6751151 | 0.911395 |
| X220270_at | 0.0023 | 0.0055 | 0.423 | 0.6752395 | 0.911482 |
| X217616_at | -0.0031 | 0.0073 | -0.423 | 0.6752612 | 0.911482 |
| X201961_s_at | -0.0298 | 0.0705 | -0.423 | 0.6753272 | 0.911515 |
| X202253_s_at | 0.0021 | 0.0049 | 0.423 | 0.6754231 | 0.911517 |
| X215932_at | -0.002 | 0.0047 | -0.423 | 0.6754955 | 0.911517 |
| X209761_s_at | -0.0291 | 0.0689 | -0.422 | 0.6755838 | 0.911517 |
| X204232_at | 0.0303 | 0.0716 | 0.422 | 0.6756014 | 0.911517 |
| X206298_at | 0.0015 | 0.0036 | 0.422 | 0.6756454 | 0.911517 |
| X212183_at | -0.0365 | 0.0865 | -0.422 | 0.6756462 | 0.911517 |
| X214673_s_at | 0.0023 | 0.0054 | 0.422 | 0.6756476 | 0.911517 |
| X209665_at | -0.0025 | 0.0059 | -0.422 | 0.6756568 | 0.911517 |
| X37943_at | 0.0155 | 0.0368 | 0.422 | 0.675745 | 0.911581 |
| X209722_s_at | 0.0014 | 0.0034 | 0.422 | 0.6759062 | 0.911647 |
| X210782_x_at | 0.0023 | 0.0055 | 0.422 | 0.6759136 | 0.911647 |
| X222153_at | 0.0032 | 0.0075 | 0.422 | 0.6759169 | 0.911647 |
| X210342_s_at | -0.0013 | 0.0032 | -0.422 | 0.6761504 | 0.911896 |
| X202607_at | 0.0183 | 0.0435 | 0.422 | 0.6762108 | 0.911896 |
| X207423_s_at | 0.0033 | 0.0077 | 0.421 | 0.6763002 | 0.911896 |
| X211401_s_at | 0.0035 | 0.0082 | 0.421 | 0.6763726 | 0.911896 |
| X206017_at | -0.0205 | 0.0486 | -0.421 | 0.6763962 | 0.911896 |
| X207249_s_at | 0.0022 | 0.0053 | 0.421 | 0.6764793 | 0.911896 |
| X221935_s_at | -0.0241 | 0.0573 | -0.421 | 0.6764801 | 0.911896 |
| X220303_at | -0.0012 | 0.0029 | -0.421 | 0.6764886 | 0.911896 |
| X208619_at | 0.0068 | 0.016 | 0.421 | 0.6764932 | 0.911896 |
| X210758_at | 0.0095 | 0.0224 | 0.421 | 0.6765419 | 0.911896 |
| X211743_s_at | 0.0013 | 0.003 | 0.421 | 0.6765736 | 0.911896 |
| X221152_at | -0.0041 | 0.0096 | -0.421 | 0.6766119 | 0.911896 |
| X215929_at | -0.0041 | 0.0097 | -0.421 | 0.6767042 | 0.911896 |
| X218339_at | -0.0156 | 0.037 | -0.421 | 0.6767603 | 0.911896 |
| X217424_at | -0.0022 | 0.0052 | -0.421 | 0.6768643 | 0.911896 |
| X220460_at | -0.0454 | 0.1079 | -0.421 | 0.6768684 | 0.911896 |
| X205669_at | 0.0038 | 0.009 | 0.421 | 0.6769385 | 0.911896 |
| X207188_at | 0.0011 | 0.0025 | 0.421 | 0.6770087 | 0.911896 |
| X219501_at | -0.0107 | 0.0254 | -0.42 | 0.6770427 | 0.911896 |
| X220309_at | 0.0014 | 0.0032 | 0.42 | 0.6770918 | 0.911896 |
| X209038_s_at | 0.0014 | 0.0034 | 0.42 | 0.677092 | 0.911896 |
| X203399_x_at | -0.0022 | 0.0051 | -0.42 | 0.6771039 | 0.911896 |
| X212197_x_at | 0.0179 | 0.0425 | 0.42 | 0.6771415 | 0.911896 |
| X212736_at | -0.02 | 0.0476 | -0.42 | 0.6771502 | 0.911896 |
| X219265_at | -0.002 | 0.0048 | -0.42 | 0.6772188 | 0.911896 |
| X207538_at | -0.0017 | 0.0041 | -0.42 | 0.6772823 | 0.911896 |
| X210533_at | -0.0026 | 0.0062 | -0.42 | 0.6772847 | 0.911896 |
| X221775_x_at | 0.0136 | 0.0323 | 0.42 | 0.6772853 | 0.911896 |
| X219769_at | -0.001 | 0.0024 | -0.42 | 0.6773491 | 0.911896 |
| X215017_s_at | -0.0229 | 0.0545 | -0.42 | 0.6774611 | 0.911896 |
| X200735_x_at | 0.0064 | 0.0153 | 0.42 | 0.6774696 | 0.911896 |
| X212083_at | -0.0226 | 0.0539 | -0.42 | 0.6775904 | 0.911896 |
| X215688_at | -0.002 | 0.0048 | -0.42 | 0.6775993 | 0.911896 |
| X216258_s_at | -0.0041 | 0.0097 | -0.42 | 0.677618 | 0.911896 |
| X221418_s_at | -0.0025 | 0.0059 | -0.42 | 0.6776587 | 0.911896 |
| X210662_at | -0.0046 | 0.0109 | -0.42 | 0.677704 | 0.911896 |
| X207748_at | 0.0019 | 0.0045 | 0.42 | 0.6777048 | 0.911896 |
| X207464_at | -0.0021 | 0.005 | -0.42 | 0.6777092 | 0.911896 |
| X221673_s_at | -0.0012 | 0.0028 | -0.419 | 0.6777537 | 0.911896 |
| X213329_at | 0.0034 | 0.008 | 0.419 | 0.6777712 | 0.911896 |
| X210288_at | 0.0011 | 0.0026 | 0.419 | 0.677785 | 0.911896 |
| X221235_s_at | -0.0061 | 0.0146 | -0.419 | 0.6778567 | 0.911899 |
| X217066_s_at | -0.0016 | 0.0039 | -0.419 | 0.6778695 | 0.911899 |
| X219354_at | -0.0247 | 0.059 | -0.419 | 0.6779504 | 0.911944 |
| X220795_s_at | 0.0111 | 0.0264 | 0.419 | 0.6780087 | 0.911944 |
| X221727_at | -0.0155 | 0.0371 | -0.419 | 0.6780257 | 0.911944 |
| X220375_s_at | 0.0026 | 0.0062 | 0.419 | 0.6780879 | 0.911972 |
| X215380_s_at | -0.0194 | 0.0462 | -0.419 | 0.6781881 | 0.91204 |
| X204142_at | 0.0327 | 0.0781 | 0.419 | 0.6782205 | 0.91204 |
| X205295_at | 0.013 | 0.0311 | 0.419 | 0.6782898 | 0.912078 |
| X207045_at | 0.0033 | 0.0078 | 0.419 | 0.6784319 | 0.912157 |
| X215308_at | -0.0035 | 0.0084 | -0.419 | 0.6784563 | 0.912157 |
| X218227_at | -0.0055 | 0.0132 | -0.418 | 0.6784713 | 0.912157 |
| X220004_at | -0.0028 | 0.0067 | -0.418 | 0.6785477 | 0.912204 |
| X207241_at | 0.001 | 0.0025 | 0.418 | 0.6786156 | 0.91224 |
| X65438_at | 0.0054 | 0.013 | 0.418 | 0.6786677 | 0.91225 |
| X208524_at | 0.0013 | 0.003 | 0.418 | 0.6787051 | 0.91225 |
| X210346_s_at | -0.0189 | 0.0453 | -0.418 | 0.6788894 | 0.912373 |
| X216084_at | 0.0041 | 0.0098 | 0.418 | 0.6789033 | 0.912373 |
| X212389_at | 0.0016 | 0.0037 | 0.418 | 0.6789197 | 0.912373 |
| X206277_at | 0.0013 | 0.0031 | 0.417 | 0.6792043 | 0.912674 |
| X216799_at | -0.003 | 0.0071 | -0.417 | 0.6792259 | 0.912674 |
| X213485_s_at | 0.0259 | 0.062 | 0.417 | 0.6793122 | 0.912735 |
| X213833_x_at | -0.0013 | 0.0032 | -0.417 | 0.6793947 | 0.91279 |
| X208599_at | -0.0024 | 0.0058 | -0.417 | 0.6795921 | 0.913 |
| X215957_at | -0.0047 | 0.0114 | -0.417 | 0.6796681 | 0.913047 |
| X201058_s_at | 0.0017 | 0.004 | 0.417 | 0.6797711 | 0.913054 |
| X216905_s_at | -0.0023 | 0.0056 | -0.417 | 0.6797801 | 0.913054 |
| X207888_at | -0.0016 | 0.0038 | -0.417 | 0.6798078 | 0.913054 |
| X222096_x_at | 0.0015 | 0.0036 | 0.417 | 0.6798376 | 0.913054 |
| X208018_s_at | -0.0031 | 0.0074 | -0.416 | 0.6799594 | 0.913056 |
| X208112_x_at | 0.0039 | 0.0094 | 0.416 | 0.6800409 | 0.913056 |
| X209668_x_at | 0.0199 | 0.0478 | 0.416 | 0.6800589 | 0.913056 |
| X215808_at | -0.002 | 0.0047 | -0.416 | 0.6800696 | 0.913056 |
| X210620_s_at | 0.0013 | 0.0031 | 0.416 | 0.6800755 | 0.913056 |
| X217738_at | -0.0196 | 0.0472 | -0.416 | 0.6800855 | 0.913056 |
| X219989_s_at | -0.0031 | 0.0075 | -0.416 | 0.6801296 | 0.91306 |
| X57163_at | 0.0711 | 0.1709 | 0.416 | 0.6802317 | 0.913142 |
| X220055_at | -0.0223 | 0.0536 | -0.416 | 0.6802898 | 0.913148 |
| X215108_x_at | 0.0176 | 0.0423 | 0.416 | 0.680335 | 0.913148 |
| X208088_s_at | -0.0024 | 0.0058 | -0.416 | 0.68036 | 0.913148 |
| X217254_s_at | -0.001 | 0.0024 | -0.416 | 0.680401 | 0.913148 |
| X221828_s_at | -0.0081 | 0.0195 | -0.416 | 0.680468 | 0.91316 |
| X204666_s_at | 0.0065 | 0.0157 | 0.416 | 0.6805111 | 0.91316 |
| X222204_s_at | -0.0156 | 0.0376 | -0.416 | 0.6805333 | 0.91316 |
| X217014_s_at | -0.0039 | 0.0095 | -0.416 | 0.6805998 | 0.913194 |
| X203907_s_at | 0.0241 | 0.058 | 0.415 | 0.6806803 | 0.913212 |
| X203426_s_at | 0.0039 | 0.0093 | 0.415 | 0.680695 | 0.913212 |
| X203016_s_at | -0.0165 | 0.0398 | -0.415 | 0.6808127 | 0.913273 |
| X207205_at | -0.0027 | 0.0066 | -0.415 | 0.6808226 | 0.913273 |
| X211123_at | 0.0021 | 0.005 | 0.415 | 0.6809048 | 0.913276 |
| X220695_at | 0.002 | 0.0048 | 0.415 | 0.6809238 | 0.913276 |
| X221378_at | -0.0014 | 0.0034 | -0.415 | 0.6809706 | 0.913276 |
| X210981_s_at | 0.0126 | 0.0303 | 0.415 | 0.6809899 | 0.913276 |
| X220080_at | 0.0013 | 0.0032 | 0.415 | 0.6811824 | 0.913479 |
| X211272_s_at | -0.0013 | 0.0032 | -0.415 | 0.6812941 | 0.913574 |
| X211149_at | -0.0151 | 0.0364 | -0.414 | 0.681462 | 0.913656 |
| X207313_x_at | -0.0013 | 0.0031 | -0.414 | 0.6815041 | 0.913656 |
| X213743_at | -0.0095 | 0.0229 | -0.414 | 0.6815535 | 0.913656 |
| X218194_at | 0.0174 | 0.0421 | 0.414 | 0.6815636 | 0.913656 |
| X217794_at | 0.0107 | 0.0257 | 0.414 | 0.6815759 | 0.913656 |
| X215730_at | -0.0043 | 0.0104 | -0.414 | 0.6816144 | 0.913656 |
| X216950_s_at | 0.0153 | 0.0369 | 0.414 | 0.68176 | 0.913656 |
| X216569_at | -0.0029 | 0.007 | -0.414 | 0.6817867 | 0.913656 |
| X207317_s_at | -0.0018 | 0.0044 | -0.414 | 0.6818042 | 0.913656 |
| X202161_at | -0.001 | 0.0025 | -0.414 | 0.6818284 | 0.913656 |
| X205069_s_at | 0.0481 | 0.1162 | 0.414 | 0.6818683 | 0.913656 |
| X219864_s_at | -0.0031 | 0.0075 | -0.414 | 0.6818703 | 0.913656 |
| X216857_at | 0.0016 | 0.0038 | 0.414 | 0.6818898 | 0.913656 |
| X202714_s_at | -0.0021 | 0.0051 | -0.414 | 0.6820338 | 0.913794 |
| X216089_at | -0.0044 | 0.0106 | -0.413 | 0.6820941 | 0.913819 |
| X208576_s_at | 0.0024 | 0.0058 | 0.413 | 0.682141 | 0.913827 |
| X211003_x_at | -0.0013 | 0.0031 | -0.413 | 0.6822712 | 0.913946 |
| X219038_at | 0.0066 | 0.016 | 0.413 | 0.6823552 | 0.914004 |
| X210425_x_at | -0.0386 | 0.0934 | -0.413 | 0.6824921 | 0.91404 |
| X214560_at | 0.002 | 0.0048 | 0.413 | 0.6825314 | 0.91404 |
| X216654_at | -0.0017 | 0.004 | -0.413 | 0.6825408 | 0.91404 |
| X219910_at | 0.0042 | 0.0102 | 0.413 | 0.682556 | 0.91404 |
| X217683_at | 0.0019 | 0.0047 | 0.413 | 0.6826056 | 0.91404 |
| X208219_at | 0.002 | 0.0048 | 0.413 | 0.6826703 | 0.91404 |
| X213468_at | 0.0014 | 0.0035 | 0.413 | 0.682676 | 0.91404 |
| X202583_s_at | 0.0193 | 0.0467 | 0.413 | 0.682761 | 0.91404 |
| X208008_at | -0.0013 | 0.0032 | -0.412 | 0.6828835 | 0.91404 |
| X221154_at | -0.0052 | 0.0125 | -0.412 | 0.6829438 | 0.91404 |
| X213274_s_at | -0.0406 | 0.0984 | -0.412 | 0.6830484 | 0.91404 |
| X214183_s_at | -0.0023 | 0.0056 | -0.412 | 0.6830812 | 0.91404 |
| X208951_at | 0.0359 | 0.087 | 0.412 | 0.6831378 | 0.91404 |
| X221489_s_at | 0.0043 | 0.0105 | 0.412 | 0.6831865 | 0.91404 |
| X214602_at | -0.0032 | 0.0078 | -0.412 | 0.6832768 | 0.91404 |
| X217353_at | -0.0087 | 0.0211 | -0.412 | 0.6832777 | 0.91404 |
| X218908_at | -0.0025 | 0.0061 | -0.412 | 0.6834004 | 0.91404 |
| X203490_at | 0.0018 | 0.0044 | 0.412 | 0.6834261 | 0.91404 |
| X218964_at | -0.0026 | 0.0063 | -0.412 | 0.6834943 | 0.91404 |
| X207540_s_at | 0.0016 | 0.0039 | 0.412 | 0.6835041 | 0.91404 |
| X203277_at | 0.0147 | 0.0358 | 0.411 | 0.683558 | 0.91404 |
| X211890_x_at | -0.0579 | 0.1408 | -0.411 | 0.6835794 | 0.91404 |
| X210760_x_at | -0.0014 | 0.0034 | -0.411 | 0.6836228 | 0.91404 |
| X215252_at | -0.0032 | 0.0078 | -0.411 | 0.6836495 | 0.91404 |
| X205517_at | -0.0025 | 0.0061 | -0.411 | 0.6837066 | 0.91404 |
| X206939_at | -0.0019 | 0.0046 | -0.411 | 0.683712 | 0.91404 |
| X203988_s_at | 0.0289 | 0.0702 | 0.411 | 0.6837704 | 0.91404 |
| X211413_s_at | -0.0028 | 0.0069 | -0.411 | 0.6838037 | 0.91404 |
| X200036_s_at | -0.0172 | 0.0418 | -0.411 | 0.6838073 | 0.91404 |
| X216390_at | 0.0007 | 0.0018 | 0.411 | 0.6838077 | 0.91404 |
| X210299_s_at | -0.0227 | 0.0552 | -0.411 | 0.6838102 | 0.91404 |
| X208557_at | -0.0013 | 0.0032 | -0.411 | 0.6840157 | 0.91404 |
| X215295_at | 0.0023 | 0.0057 | 0.411 | 0.6840234 | 0.91404 |
| X205738_s_at | -0.0418 | 0.1017 | -0.411 | 0.6840236 | 0.91404 |
| X216962_at | -0.0026 | 0.0063 | -0.411 | 0.6840311 | 0.91404 |
| X210887_s_at | -0.0016 | 0.0038 | -0.411 | 0.6840379 | 0.91404 |
| X216686_at | 0.0067 | 0.0162 | 0.411 | 0.6840544 | 0.91404 |
| X221820_s_at | 0.0116 | 0.0282 | 0.411 | 0.684063 | 0.91404 |
| X212171_x_at | 0.008 | 0.0194 | 0.411 | 0.684066 | 0.91404 |
| X215067_x_at | 0.0273 | 0.0664 | 0.411 | 0.6840804 | 0.91404 |
| X219996_at | 0.0025 | 0.0061 | 0.411 | 0.6840979 | 0.91404 |
| X204947_at | -0.0037 | 0.0091 | -0.411 | 0.6841105 | 0.91404 |
| X203155_at | -0.0036 | 0.0088 | -0.411 | 0.6842204 | 0.914094 |
| X208136_s_at | 0.002 | 0.0049 | 0.411 | 0.6842328 | 0.914094 |
| X219530_at | 0.0124 | 0.0302 | 0.41 | 0.6842812 | 0.914103 |
| X218871_x_at | 0.0175 | 0.0426 | 0.41 | 0.6843634 | 0.914121 |
| X206768_at | 0.0012 | 0.003 | 0.41 | 0.6843852 | 0.914121 |
| X209866_s_at | 0.021 | 0.0512 | 0.41 | 0.6844182 | 0.914121 |
| X214236_at | -0.0029 | 0.0072 | -0.41 | 0.6844903 | 0.914163 |
| X217680_x_at | 0.0043 | 0.0104 | 0.41 | 0.6845558 | 0.914164 |
| X220860_at | -0.0039 | 0.0094 | -0.41 | 0.6845735 | 0.914164 |
| X219614_s_at | -0.002 | 0.0049 | -0.41 | 0.68462 | 0.914169 |
| X218014_at | -0.0315 | 0.0769 | -0.41 | 0.6846599 | 0.914169 |
| X220629_at | 0.0013 | 0.0032 | 0.41 | 0.6847133 | 0.914186 |
| X202621_at | -0.003 | 0.0074 | -0.41 | 0.6847713 | 0.914208 |
| X200598_s_at | 0.0381 | 0.0931 | 0.41 | 0.6849296 | 0.914365 |
| X221327_s_at | 0.0008 | 0.002 | 0.409 | 0.6851092 | 0.914535 |
| X205665_at | -0.003 | 0.0073 | -0.409 | 0.6852082 | 0.914535 |
| X218827_s_at | 0.0217 | 0.0531 | 0.409 | 0.6852188 | 0.914535 |
| X215444_s_at | 0.0014 | 0.0033 | 0.409 | 0.6852251 | 0.914535 |
| X216281_at | 0.0017 | 0.0042 | 0.409 | 0.6852628 | 0.914535 |
| X206368_at | 0.0025 | 0.006 | 0.409 | 0.6854813 | 0.914739 |
| X216150_at | 0.0023 | 0.0056 | 0.409 | 0.6854986 | 0.914739 |
| X211706_s_at | -0.0012 | 0.0029 | -0.409 | 0.6855594 | 0.914766 |
| X221102_s_at | 0.0028 | 0.0069 | 0.408 | 0.6857298 | 0.914925 |
| X213807_x_at | 0.002 | 0.0049 | 0.408 | 0.6857771 | 0.914925 |
| X211610_at | -0.0011 | 0.0027 | -0.408 | 0.6858027 | 0.914925 |
| X207733_x_at | 0.0017 | 0.0043 | 0.408 | 0.6858686 | 0.914928 |
| X220314_at | 0.0019 | 0.0047 | 0.408 | 0.685887 | 0.914928 |
| X212444_at | 0.0028 | 0.0069 | 0.408 | 0.6859932 | 0.914972 |
| X210689_at | -0.0026 | 0.0065 | -0.408 | 0.6860275 | 0.914972 |
| X221157_s_at | -0.0014 | 0.0033 | -0.408 | 0.6860435 | 0.914972 |
| X203528_at | 0.0511 | 0.1253 | 0.408 | 0.6861058 | 0.915 |
| X206296_x_at | 0.0008 | 0.0019 | 0.408 | 0.6862093 | 0.915083 |
| X209410_s_at | 0.002 | 0.0049 | 0.408 | 0.6862649 | 0.915102 |
| X200743_s_at | 0.0279 | 0.0685 | 0.408 | 0.6863343 | 0.915115 |
| X219928_s_at | 0.0015 | 0.0038 | 0.408 | 0.686357 | 0.915115 |
| X201895_at | -0.0034 | 0.0084 | -0.407 | 0.6865318 | 0.915185 |
| X210633_x_at | 0.0082 | 0.0201 | 0.407 | 0.6865572 | 0.915185 |
| X215322_at | 0.0469 | 0.1152 | 0.407 | 0.6865698 | 0.915185 |
| X220058_at | -0.0019 | 0.0048 | -0.407 | 0.6865817 | 0.915185 |
| X201154_x_at | -0.0077 | 0.0189 | -0.407 | 0.6866637 | 0.915185 |
| X221540_x_at | 0.0301 | 0.0739 | 0.407 | 0.6867074 | 0.915185 |
| X221359_at | -0.0013 | 0.0033 | -0.407 | 0.6867304 | 0.915185 |
| X221717_at | -0.0031 | 0.0077 | -0.407 | 0.6867467 | 0.915185 |
| X207961_x_at | -0.0032 | 0.008 | -0.407 | 0.6867803 | 0.915185 |
| X203287_at | 0.0017 | 0.0042 | 0.407 | 0.6869058 | 0.915295 |
| X219040_at | -0.0067 | 0.0164 | -0.407 | 0.6869954 | 0.915295 |
| X219649_at | -0.0183 | 0.0451 | -0.407 | 0.6870243 | 0.915295 |
| X219678_x_at | 0.0197 | 0.0483 | 0.407 | 0.6870276 | 0.915295 |
| X205696_s_at | 0.0033 | 0.0082 | 0.406 | 0.6872135 | 0.915469 |
| X208165_s_at | -0.0021 | 0.0051 | -0.406 | 0.6873226 | 0.915469 |
| X220498_at | 0.0019 | 0.0047 | 0.406 | 0.6873874 | 0.915469 |
| X217206_at | 0.0047 | 0.0116 | 0.406 | 0.6874207 | 0.915469 |
| X216395_at | 0.0012 | 0.0029 | 0.406 | 0.6874298 | 0.915469 |
| X215977_x_at | 0.0011 | 0.0026 | 0.406 | 0.6875069 | 0.915469 |
| X206476_s_at | -0.0011 | 0.0027 | -0.406 | 0.6875159 | 0.915469 |
| X219509_at | 0.0015 | 0.0038 | 0.406 | 0.6875424 | 0.915469 |
| X202478_at | 0.0229 | 0.0565 | 0.406 | 0.6875551 | 0.915469 |
| X212037_at | 0.0062 | 0.0153 | 0.406 | 0.6876289 | 0.915469 |
| X206835_at | 0.0028 | 0.0069 | 0.406 | 0.6876397 | 0.915469 |
| X205075_at | -0.0011 | 0.0028 | -0.406 | 0.6876523 | 0.915469 |
| X206545_at | -0.0029 | 0.0072 | -0.406 | 0.6877292 | 0.915516 |
| X222029_x_at | 0.0122 | 0.0302 | 0.406 | 0.6878597 | 0.915547 |
| X216694_at | -0.0016 | 0.0039 | -0.405 | 0.6879571 | 0.915547 |
| X221787_at | -0.0267 | 0.0658 | -0.405 | 0.6880025 | 0.915547 |
| X214085_x_at | -0.0838 | 0.2067 | -0.405 | 0.6880379 | 0.915547 |
| X208175_s_at | -0.0045 | 0.011 | -0.405 | 0.6880638 | 0.915547 |
| X87100_at | -0.0032 | 0.008 | -0.405 | 0.6880813 | 0.915547 |
| X215437_x_at | 0.0015 | 0.0038 | 0.405 | 0.6882203 | 0.915547 |
| X201538_s_at | 0.0008 | 0.0019 | 0.405 | 0.688237 | 0.915547 |
| X216782_at | 0.0023 | 0.0057 | 0.405 | 0.6882873 | 0.915547 |
| X219930_at | 0.0038 | 0.0093 | 0.405 | 0.6882924 | 0.915547 |
| X219359_at | 0.0022 | 0.0055 | 0.405 | 0.6883146 | 0.915547 |
| X220480_at | 0.0019 | 0.0048 | 0.405 | 0.6883827 | 0.915547 |
| X210450_at | 0.0058 | 0.0142 | 0.405 | 0.6883919 | 0.915547 |
| X221833_at | 0.0233 | 0.0575 | 0.405 | 0.6884865 | 0.915547 |
| X206192_at | 0.0014 | 0.0035 | 0.405 | 0.6884933 | 0.915547 |
| X216984_x_at | 0.0021 | 0.0051 | 0.405 | 0.6885111 | 0.915547 |
| X219544_at | 0.002 | 0.0049 | 0.404 | 0.6886246 | 0.915547 |
| X216052_x_at | -0.0015 | 0.0037 | -0.404 | 0.688657 | 0.915547 |
| X213784_at | 0.012 | 0.0297 | 0.404 | 0.6886637 | 0.915547 |
| X202571_s_at | 0.0073 | 0.0181 | 0.404 | 0.6886785 | 0.915547 |
| X217201_at | -0.0056 | 0.0138 | -0.404 | 0.6886852 | 0.915547 |
| X220726_at | -0.0056 | 0.0138 | -0.404 | 0.6887205 | 0.915547 |
| X202841_x_at | -0.0021 | 0.0052 | -0.404 | 0.6887376 | 0.915547 |
| X205557_at | -0.0014 | 0.0036 | -0.404 | 0.6887419 | 0.915547 |
| X206943_at | 0.0023 | 0.0056 | 0.404 | 0.6888061 | 0.915578 |
| X217549_at | 0.003 | 0.0075 | 0.404 | 0.6888789 | 0.91562 |
| X202470_s_at | 0.0115 | 0.0284 | 0.404 | 0.6890014 | 0.915661 |
| X214700_x_at | -0.0136 | 0.0338 | -0.404 | 0.689017 | 0.915661 |
| X211603_s_at | -0.0017 | 0.0041 | -0.404 | 0.6890643 | 0.915661 |
| X214533_at | 0.0017 | 0.0043 | 0.404 | 0.6891567 | 0.915661 |
| X208508_s_at | -0.0038 | 0.0093 | -0.404 | 0.6891677 | 0.915661 |
| X205355_at | -0.0121 | 0.0301 | -0.404 | 0.6892031 | 0.915661 |
| X216327_s_at | -0.0015 | 0.0038 | -0.404 | 0.6892511 | 0.915661 |
| X207017_at | -0.0038 | 0.0094 | -0.404 | 0.6892527 | 0.915661 |
| X220620_at | -0.0012 | 0.003 | -0.404 | 0.6892807 | 0.915661 |
| X217361_at | 0.002 | 0.005 | 0.404 | 0.6893371 | 0.915681 |
| X206503_x_at | -0.0012 | 0.003 | -0.403 | 0.6895425 | 0.915816 |
| X219585_at | -0.0034 | 0.0084 | -0.403 | 0.6895454 | 0.915816 |
| X206389_s_at | -0.003 | 0.0073 | -0.403 | 0.6895621 | 0.915816 |
| X210918_at | 0.0016 | 0.0041 | 0.403 | 0.6896409 | 0.915866 |
| X205679_x_at | -0.0011 | 0.0028 | -0.403 | 0.6898113 | 0.916037 |
| X201820_at | 0.0008 | 0.0019 | 0.403 | 0.6898969 | 0.916096 |
| X212440_at | 0.0149 | 0.0371 | 0.403 | 0.6899863 | 0.916146 |
| X214603_at | -0.0035 | 0.0087 | -0.403 | 0.6900172 | 0.916146 |
| X202402_s_at | 0.0247 | 0.0615 | 0.403 | 0.6900653 | 0.916153 |
| X210555_s_at | -0.0275 | 0.0684 | -0.402 | 0.6901422 | 0.916153 |
| X208509_s_at | 0.0021 | 0.0051 | 0.402 | 0.6901457 | 0.916153 |
| X214404_x_at | 0.0015 | 0.0036 | 0.402 | 0.6902102 | 0.916183 |
| X208407_s_at | 0.0279 | 0.0692 | 0.402 | 0.6902881 | 0.916184 |
| X214979_at | -0.0044 | 0.011 | -0.402 | 0.6902975 | 0.916184 |
| X218914_at | 0.0018 | 0.0045 | 0.402 | 0.6903341 | 0.916184 |
| X216626_at | 0.0049 | 0.0121 | 0.402 | 0.6904107 | 0.916228 |
| X204584_at | -0.0283 | 0.0704 | -0.402 | 0.6904676 | 0.916228 |
| X201059_at | -0.0086 | 0.0214 | -0.402 | 0.6904914 | 0.916228 |
| X219942_at | -0.0013 | 0.0031 | -0.402 | 0.6905835 | 0.916267 |
| X201232_s_at | -0.0152 | 0.0377 | -0.402 | 0.6906132 | 0.916267 |
| X202977_s_at | -0.0021 | 0.0052 | -0.402 | 0.6906445 | 0.916267 |
| X215719_x_at | 0.0608 | 0.1515 | 0.402 | 0.6906983 | 0.91628 |
| X205789_at | 0.0027 | 0.0067 | 0.402 | 0.6907369 | 0.91628 |
| X206858_s_at | 0.0027 | 0.0068 | 0.401 | 0.6908278 | 0.916319 |
| X220381_at | 0.0014 | 0.0034 | 0.401 | 0.6908742 | 0.916319 |
| X37020_at | -0.0023 | 0.0058 | -0.401 | 0.6908897 | 0.916319 |
| X210874_s_at | -0.0015 | 0.0037 | -0.401 | 0.6909788 | 0.916382 |
| X210249_s_at | -0.0129 | 0.0321 | -0.401 | 0.6911783 | 0.916563 |
| X217476_at | 0.0015 | 0.0036 | 0.401 | 0.6912383 | 0.916563 |
| X209881_s_at | 0.0016 | 0.0039 | 0.401 | 0.6912387 | 0.916563 |
| X216344_at | -0.0007 | 0.0018 | -0.401 | 0.6914213 | 0.916659 |
| X213261_at | 0.0022 | 0.0056 | 0.401 | 0.6914271 | 0.916659 |
| X213796_at | 0.0012 | 0.0031 | 0.401 | 0.6914518 | 0.916659 |
| X208837_at | -0.0267 | 0.0668 | -0.401 | 0.6914766 | 0.916659 |
| X207114_at | -0.0026 | 0.0064 | -0.401 | 0.6915199 | 0.916662 |
| X203334_at | 0.0022 | 0.0055 | 0.4 | 0.691606 | 0.916722 |
| X205367_at | 0.0106 | 0.0264 | 0.4 | 0.6918595 | 0.916945 |
| X202566_s_at | 0.002 | 0.0049 | 0.4 | 0.6918716 | 0.916945 |
| X215402_at | -0.0067 | 0.0168 | -0.4 | 0.6918982 | 0.916945 |
| X211187_at | -0.005 | 0.0126 | -0.4 | 0.6920116 | 0.91704 |
| X207138_at | -0.0016 | 0.0041 | -0.4 | 0.6921805 | 0.917209 |
| X212980_at | -0.0571 | 0.143 | -0.399 | 0.6922975 | 0.917259 |
| X219139_s_at | -0.0016 | 0.0041 | -0.399 | 0.692317 | 0.917259 |
| X216797_at | -0.0016 | 0.0039 | -0.399 | 0.6923679 | 0.917259 |
| X207105_s_at | -0.0019 | 0.0049 | -0.399 | 0.692394 | 0.917259 |
| X214934_at | -0.0067 | 0.0169 | -0.399 | 0.692459 | 0.917259 |
| X212443_at | -0.003 | 0.0076 | -0.399 | 0.6924933 | 0.917259 |
| X215713_at | -0.0029 | 0.0074 | -0.399 | 0.6925364 | 0.917259 |
| X211766_s_at | -0.0017 | 0.0041 | -0.399 | 0.692698 | 0.917259 |
| X203085_s_at | 0.0015 | 0.0039 | 0.399 | 0.6927078 | 0.917259 |
| X217345_at | -0.0028 | 0.0071 | -0.399 | 0.6927233 | 0.917259 |
| X220158_at | 0.0018 | 0.0046 | 0.399 | 0.6928024 | 0.917259 |
| X222161_at | 0.0293 | 0.0734 | 0.399 | 0.6928102 | 0.917259 |
| X205951_at | -0.0039 | 0.0098 | -0.399 | 0.6928607 | 0.917259 |
| X219950_s_at | 0.0018 | 0.0044 | 0.399 | 0.6928827 | 0.917259 |
| X210641_at | 0.002 | 0.0051 | 0.399 | 0.6928878 | 0.917259 |
| X216592_at | -0.0019 | 0.0047 | -0.399 | 0.6929089 | 0.917259 |
| X204366_s_at | -0.0137 | 0.0344 | -0.399 | 0.6929199 | 0.917259 |
| X218245_at | 0.0015 | 0.0036 | 0.398 | 0.6930729 | 0.917334 |
| X218907_s_at | 0.0024 | 0.006 | 0.398 | 0.6931101 | 0.917334 |
| X205135_s_at | 0.0021 | 0.0053 | 0.398 | 0.6931232 | 0.917334 |
| X204341_at | -0.007 | 0.0176 | -0.398 | 0.6931552 | 0.917334 |
| X206537_at | -0.0032 | 0.008 | -0.398 | 0.6931829 | 0.917334 |
| X61732_r_at | 0.0213 | 0.0535 | 0.398 | 0.693253 | 0.917339 |
| X215482_s_at | 0.0085 | 0.0213 | 0.398 | 0.6933046 | 0.917339 |
| X210562_at | -0.0016 | 0.004 | -0.398 | 0.6933106 | 0.917339 |
| X210701_at | -0.012 | 0.0302 | -0.398 | 0.6935194 | 0.91756 |
| X217495_x_at | 0.0013 | 0.0034 | 0.398 | 0.6935697 | 0.917572 |
| X200010_at | 0.01 | 0.0253 | 0.398 | 0.693643 | 0.917615 |
| X211636_at | 0.0019 | 0.0047 | 0.397 | 0.693845 | 0.917782 |
| X216435_at | -0.0038 | 0.0095 | -0.397 | 0.6938522 | 0.917782 |
| X218896_s_at | -0.0032 | 0.008 | -0.397 | 0.6939404 | 0.917844 |
| X217006_x_at | -0.0016 | 0.0041 | -0.397 | 0.6939966 | 0.917859 |
| X204286_s_at | 0.0097 | 0.0245 | 0.397 | 0.6940504 | 0.917859 |
| X214729_at | 0.0046 | 0.0116 | 0.397 | 0.6941084 | 0.917859 |
| X206693_at | -0.0049 | 0.0123 | -0.397 | 0.6942204 | 0.917859 |
| X221549_at | 0.0236 | 0.0595 | 0.397 | 0.6942372 | 0.917859 |
| X206377_at | 0.0303 | 0.0764 | 0.397 | 0.6942516 | 0.917859 |
| X211339_s_at | 0.0035 | 0.0089 | 0.397 | 0.6942647 | 0.917859 |
| X218538_s_at | -0.0236 | 0.0594 | -0.397 | 0.6942953 | 0.917859 |
| X214605_x_at | -0.0165 | 0.0415 | -0.397 | 0.6943233 | 0.917859 |
| X202229_s_at | -0.001 | 0.0025 | -0.396 | 0.6945322 | 0.918039 |
| X204907_s_at | -0.001 | 0.0024 | -0.396 | 0.6945426 | 0.918039 |
| X219956_at | -0.0501 | 0.1263 | -0.396 | 0.6946076 | 0.918071 |
| X207194_s_at | -0.0017 | 0.0043 | -0.396 | 0.6946641 | 0.918091 |
| X219846_at | -0.0066 | 0.0166 | -0.396 | 0.6948293 | 0.918217 |
| X220125_at | -0.002 | 0.0051 | -0.396 | 0.6948423 | 0.918217 |
| X213381_at | 0.0012 | 0.0029 | 0.396 | 0.6948998 | 0.918238 |
| X220266_s_at | 0.0018 | 0.0046 | 0.396 | 0.6949596 | 0.918263 |
| X201418_s_at | 0.0027 | 0.0067 | 0.396 | 0.6950629 | 0.918345 |
| X205225_at | -0.0018 | 0.0046 | -0.396 | 0.69516 | 0.918345 |
| X207843_x_at | 0.0167 | 0.0421 | 0.396 | 0.6951681 | 0.918345 |
| X41858_at | 0.0196 | 0.0495 | 0.395 | 0.6951874 | 0.918345 |
| X220445_s_at | -0.0013 | 0.0033 | -0.395 | 0.6953272 | 0.918434 |
| X206751_s_at | -0.0016 | 0.004 | -0.395 | 0.6953371 | 0.918434 |
| X202547_s_at | 0.0051 | 0.013 | 0.395 | 0.6953783 | 0.918434 |
| X203461_at | 0.0024 | 0.0061 | 0.395 | 0.695518 | 0.918497 |
| X216476_at | -0.0047 | 0.0118 | -0.395 | 0.6955353 | 0.918497 |
| X206065_s_at | -0.0033 | 0.0084 | -0.395 | 0.6955503 | 0.918497 |
| X206923_at | -0.0014 | 0.0036 | -0.395 | 0.6958179 | 0.918631 |
| X201470_at | -0.0108 | 0.0274 | -0.394 | 0.6960042 | 0.918631 |
| X204911_s_at | 0.0024 | 0.0061 | 0.394 | 0.696011 | 0.918631 |
| X221723_s_at | 0.0014 | 0.0036 | 0.394 | 0.6960139 | 0.918631 |
| X215691_x_at | 0.0083 | 0.0209 | 0.394 | 0.69603 | 0.918631 |
| X220229_s_at | -0.0039 | 0.0099 | -0.394 | 0.6960601 | 0.918631 |
| X215466_at | -0.002 | 0.0052 | -0.394 | 0.6960618 | 0.918631 |
| X216490_x_at | -0.0042 | 0.0107 | -0.394 | 0.6960753 | 0.918631 |
| X206680_at | -0.002 | 0.005 | -0.394 | 0.6960773 | 0.918631 |
| X207950_s_at | 0.0263 | 0.0667 | 0.394 | 0.6960918 | 0.918631 |
| X211169_s_at | -0.0038 | 0.0097 | -0.394 | 0.6961101 | 0.918631 |
| X207073_at | -0.0019 | 0.0049 | -0.394 | 0.6961916 | 0.918631 |
| X205289_at | -0.006 | 0.0153 | -0.394 | 0.6962638 | 0.918631 |
| X221229_s_at | -0.0177 | 0.0448 | -0.394 | 0.6963351 | 0.918631 |
| X219547_at | -0.0143 | 0.0362 | -0.394 | 0.6963425 | 0.918631 |
| X219993_at | -0.0016 | 0.0041 | -0.394 | 0.6963472 | 0.918631 |
| X208344_x_at | 0.0019 | 0.0049 | 0.394 | 0.6963547 | 0.918631 |
| X203635_at | -0.007 | 0.0177 | -0.394 | 0.696402 | 0.918639 |
| X219922_s_at | 0.0345 | 0.0877 | 0.394 | 0.6965609 | 0.918767 |
| X209799_at | 0.0048 | 0.0123 | 0.394 | 0.6966333 | 0.918767 |
| X206626_x_at | 0.0025 | 0.0063 | 0.394 | 0.696641 | 0.918767 |
| X214351_x_at | 0.0138 | 0.0351 | 0.393 | 0.6966646 | 0.918767 |
| X206632_s_at | -0.0039 | 0.0098 | -0.393 | 0.6968489 | 0.918909 |
| X216023_at | 0.0013 | 0.0033 | 0.393 | 0.6968555 | 0.918909 |
| X214907_at | -0.0012 | 0.0031 | -0.393 | 0.6969512 | 0.918924 |
| X222203_s_at | 0.0128 | 0.0327 | 0.393 | 0.6970306 | 0.918924 |
| X208010_s_at | -0.0031 | 0.0079 | -0.393 | 0.6970643 | 0.918924 |
| X205934_at | -0.0304 | 0.0773 | -0.393 | 0.6971787 | 0.918924 |
| X212495_at | 0.0139 | 0.0353 | 0.393 | 0.6972216 | 0.918924 |
| X220153_at | 0.0025 | 0.0065 | 0.393 | 0.6972257 | 0.918924 |
| X221838_at | 0.0015 | 0.0037 | 0.393 | 0.6972529 | 0.918924 |
| X203193_at | 0.003 | 0.0076 | 0.393 | 0.6972553 | 0.918924 |
| X221260_s_at | -0.0139 | 0.0354 | -0.393 | 0.6973157 | 0.918924 |
| X207294_at | -0.0061 | 0.0155 | -0.393 | 0.6973659 | 0.918924 |
| X207036_x_at | 0.0009 | 0.0022 | 0.392 | 0.6973988 | 0.918924 |
| X211117_x_at | 0.0009 | 0.0024 | 0.392 | 0.697569 | 0.918924 |
| X204998_s_at | 0.013 | 0.0332 | 0.392 | 0.6976116 | 0.918924 |
| X41037_at | -0.0017 | 0.0045 | -0.392 | 0.6976774 | 0.918924 |
| X221871_s_at | 0.0027 | 0.0068 | 0.392 | 0.697726 | 0.918924 |
| X215761_at | -0.0018 | 0.0045 | -0.392 | 0.697734 | 0.918924 |
| X219658_at | 0.0127 | 0.0325 | 0.392 | 0.6977526 | 0.918924 |
| X210197_at | 0.0012 | 0.0032 | 0.392 | 0.6977571 | 0.918924 |
| X212088_at | 0.0135 | 0.0344 | 0.392 | 0.697805 | 0.918924 |
| X204682_at | 0.0011 | 0.0029 | 0.392 | 0.6978892 | 0.918924 |
| X214494_s_at | -0.0169 | 0.0431 | -0.392 | 0.6979931 | 0.918924 |
| X220410_s_at | 0.0049 | 0.0124 | 0.392 | 0.6980202 | 0.918924 |
| X204458_at | 0.0015 | 0.0039 | 0.392 | 0.6980639 | 0.918924 |
| X212365_at | -0.0228 | 0.0583 | -0.392 | 0.6980773 | 0.918924 |
| X211643_x_at | 0.001 | 0.0027 | 0.392 | 0.6980935 | 0.918924 |
| X214378_at | -0.0045 | 0.0116 | -0.391 | 0.6981076 | 0.918924 |
| X200747_s_at | -0.0192 | 0.0491 | -0.391 | 0.6981694 | 0.918924 |
| X221166_at | -0.0017 | 0.0042 | -0.391 | 0.6982207 | 0.918924 |
| X221441_at | -0.0014 | 0.0035 | -0.391 | 0.6982236 | 0.918924 |
| X207535_s_at | -0.0023 | 0.0058 | -0.391 | 0.6982324 | 0.918924 |
| X202464_s_at | 0.0201 | 0.0515 | 0.391 | 0.6983219 | 0.918924 |
| X205946_at | -0.0016 | 0.0041 | -0.391 | 0.6983269 | 0.918924 |
| X209033_s_at | 0.0104 | 0.0266 | 0.391 | 0.6983317 | 0.918924 |
| X215895_x_at | 0.0116 | 0.0298 | 0.391 | 0.6983345 | 0.918924 |
| X203898_at | -0.0107 | 0.0274 | -0.391 | 0.6983469 | 0.918924 |
| X201139_s_at | 0.0177 | 0.0451 | 0.391 | 0.6983739 | 0.918924 |
| X211260_at | -0.0042 | 0.0107 | -0.391 | 0.6983972 | 0.918924 |
| X200629_at | 0.0151 | 0.0385 | 0.391 | 0.6984408 | 0.918927 |
| X220630_s_at | 0.0012 | 0.0032 | 0.391 | 0.6985642 | 0.919002 |
| X206102_at | -0.0192 | 0.049 | -0.391 | 0.6985808 | 0.919002 |
| X204404_at | -0.0341 | 0.0874 | -0.391 | 0.6987683 | 0.919195 |
| X215852_x_at | -0.0066 | 0.0169 | -0.391 | 0.6988349 | 0.919195 |
| X221145_at | -0.0027 | 0.0069 | -0.39 | 0.6988513 | 0.919195 |
| X208358_s_at | -0.0908 | 0.2327 | -0.39 | 0.6990508 | 0.919375 |
| X213023_at | 0.001 | 0.0024 | 0.39 | 0.6990709 | 0.919375 |
| X222078_at | -0.0039 | 0.0101 | -0.39 | 0.6992264 | 0.919412 |
| X211673_s_at | -0.0099 | 0.0253 | -0.39 | 0.6992432 | 0.919412 |
| X208356_x_at | 0.0013 | 0.0033 | 0.39 | 0.6992642 | 0.919412 |
| X215894_at | 0.0024 | 0.0061 | 0.39 | 0.6992645 | 0.919412 |
| X216175_at | 0.0037 | 0.0094 | 0.39 | 0.6994097 | 0.919482 |
| X217816_s_at | 0.0112 | 0.0288 | 0.39 | 0.6994476 | 0.919482 |
| X205506_at | 0.0016 | 0.0042 | 0.39 | 0.6994777 | 0.919482 |
| X212780_at | -0.0131 | 0.0335 | -0.39 | 0.6994836 | 0.919482 |
| X217308_at | 0.0026 | 0.0067 | 0.389 | 0.699587 | 0.919564 |
| X201208_s_at | 0.0029 | 0.0074 | 0.389 | 0.6996422 | 0.919582 |
| X204853_at | 0.0062 | 0.0158 | 0.389 | 0.6998349 | 0.919606 |
| X217557_s_at | -0.0049 | 0.0126 | -0.389 | 0.6998522 | 0.919606 |
| X211465_x_at | 0.0042 | 0.0107 | 0.389 | 0.6999134 | 0.919606 |
| X222304_x_at | 0.0018 | 0.0047 | 0.389 | 0.6999431 | 0.919606 |
| X216649_at | -0.0013 | 0.0034 | -0.389 | 0.6999861 | 0.919606 |
| X207519_at | 0.0012 | 0.0032 | 0.389 | 0.6999964 | 0.919606 |
| X221793_at | 0.0017 | 0.0043 | 0.389 | 0.7000916 | 0.919606 |
| X217222_at | 0.001 | 0.0026 | 0.389 | 0.7001599 | 0.919606 |
| X201806_s_at | -0.0029 | 0.0073 | -0.389 | 0.7002269 | 0.919606 |
| X221659_s_at | 0.0014 | 0.0037 | 0.389 | 0.7002295 | 0.919606 |
| X222322_at | -0.006 | 0.0154 | -0.389 | 0.7002673 | 0.919606 |
| X200891_s_at | 0.0163 | 0.042 | 0.388 | 0.7003233 | 0.919606 |
| X201361_at | 0.0136 | 0.035 | 0.388 | 0.7003466 | 0.919606 |
| X204675_at | 0.0176 | 0.0454 | 0.388 | 0.7003999 | 0.919606 |
| X207854_at | 0.0034 | 0.0088 | 0.388 | 0.7004533 | 0.919606 |
| X219452_at | 0.0009 | 0.0022 | 0.388 | 0.7004584 | 0.919606 |
| X219068_x_at | -0.0034 | 0.0087 | -0.388 | 0.7004595 | 0.919606 |
| X202469_s_at | 0.012 | 0.0309 | 0.388 | 0.7005093 | 0.919606 |
| X200020_at | -0.0096 | 0.0248 | -0.388 | 0.7006026 | 0.919606 |
| X215712_s_at | -0.0017 | 0.0045 | -0.388 | 0.7006224 | 0.919606 |
| X207412_x_at | 0.0011 | 0.003 | 0.388 | 0.7006707 | 0.919606 |
| X215260_s_at | 0.0026 | 0.0068 | 0.388 | 0.7007502 | 0.919606 |
| X205610_at | -0.0014 | 0.0035 | -0.388 | 0.7007983 | 0.919606 |
| X217161_x_at | -0.0014 | 0.0037 | -0.388 | 0.700829 | 0.919606 |
| X203552_at | 0.0183 | 0.0471 | 0.388 | 0.7008751 | 0.919606 |
| X209988_s_at | 0.0418 | 0.1077 | 0.388 | 0.7008852 | 0.919606 |
| X219569_s_at | -0.0178 | 0.046 | -0.388 | 0.7009394 | 0.919606 |
| X208501_at | 0.0007 | 0.0019 | 0.388 | 0.7009472 | 0.919606 |
| X214610_at | -0.0026 | 0.0068 | -0.388 | 0.7009583 | 0.919606 |
| X205900_at | 0.0013 | 0.0034 | 0.388 | 0.7009643 | 0.919606 |
| X212952_at | 0.0256 | 0.0662 | 0.388 | 0.7010135 | 0.919606 |
| X210303_at | -0.0059 | 0.0153 | -0.387 | 0.7010899 | 0.919606 |
| X207623_at | -0.0018 | 0.0047 | -0.387 | 0.7011209 | 0.919606 |
| X207067_s_at | 0.0024 | 0.0062 | 0.387 | 0.7011971 | 0.919606 |
| X215830_at | -0.0015 | 0.0038 | -0.387 | 0.7013035 | 0.919606 |
| X212228_s_at | -0.008 | 0.0208 | -0.387 | 0.7013606 | 0.919606 |
| X213117_at | 0.0175 | 0.0451 | 0.387 | 0.7013845 | 0.919606 |
| X218933_at | -0.0119 | 0.0307 | -0.387 | 0.7014157 | 0.919606 |
| X215735_s_at | -0.0088 | 0.0228 | -0.387 | 0.7014953 | 0.919606 |
| X207420_at | 0.0018 | 0.0047 | 0.387 | 0.7015078 | 0.919606 |
| X204374_s_at | -0.001 | 0.0025 | -0.387 | 0.7015112 | 0.919606 |
| X222057_at | 0.0018 | 0.0046 | 0.387 | 0.701516 | 0.919606 |
| X207912_s_at | -0.0051 | 0.0133 | -0.387 | 0.7015339 | 0.919606 |
| X219793_at | -0.0293 | 0.0757 | -0.387 | 0.7015614 | 0.919606 |
| X216660_at | -0.0028 | 0.0073 | -0.387 | 0.7016085 | 0.919606 |
| X220508_at | -0.0012 | 0.0032 | -0.387 | 0.7016342 | 0.919606 |
| X216343_at | 0.0012 | 0.0032 | 0.387 | 0.7017425 | 0.919606 |
| X217400_at | -0.005 | 0.0128 | -0.386 | 0.7017783 | 0.919606 |
| X213399_x_at | -0.0109 | 0.0283 | -0.386 | 0.7017801 | 0.919606 |
| X211027_s_at | 0.0016 | 0.0042 | 0.386 | 0.7017972 | 0.919606 |
| X216183_at | -0.0015 | 0.0038 | -0.386 | 0.701845 | 0.919606 |
| X219477_s_at | -0.0132 | 0.0341 | -0.386 | 0.7018685 | 0.919606 |
| X204174_at | -0.0534 | 0.1383 | -0.386 | 0.7018927 | 0.919606 |
| X215105_at | 0.0019 | 0.0048 | 0.386 | 0.7018963 | 0.919606 |
| X210843_s_at | 0.0019 | 0.005 | 0.386 | 0.7019657 | 0.919643 |
| X221851_at | 0.001 | 0.0025 | 0.386 | 0.7020886 | 0.91975 |
| X213250_at | -0.0009 | 0.0024 | -0.386 | 0.7021316 | 0.919752 |
| X204469_at | -0.0235 | 0.0608 | -0.386 | 0.7021979 | 0.919784 |
| X206830_at | -0.0034 | 0.0087 | -0.386 | 0.702254 | 0.919804 |
| X220541_at | -0.0014 | 0.0038 | -0.386 | 0.7023718 | 0.919863 |
| X206839_at | -0.0019 | 0.0048 | -0.386 | 0.7023929 | 0.919863 |
| X201503_at | 0.0123 | 0.0319 | 0.385 | 0.7025563 | 0.919863 |
| X200867_at | 0.018 | 0.0468 | 0.385 | 0.7026633 | 0.919863 |
| X217482_at | -0.0083 | 0.0216 | -0.385 | 0.702703 | 0.919863 |
| X219294_at | -0.0164 | 0.0425 | -0.385 | 0.7027498 | 0.919863 |
| X213271_s_at | -0.0149 | 0.0387 | -0.385 | 0.7028372 | 0.919863 |
| X217464_at | -0.0029 | 0.0075 | -0.385 | 0.702883 | 0.919863 |
| X213075_at | -0.0016 | 0.0041 | -0.385 | 0.7029001 | 0.919863 |
| X201234_at | -0.0016 | 0.0042 | -0.385 | 0.7029125 | 0.919863 |
| X203968_s_at | 0.0028 | 0.0073 | 0.385 | 0.7029151 | 0.919863 |
| X216732_at | 0.001 | 0.0027 | 0.385 | 0.7029896 | 0.919863 |
| X205163_at | -0.001 | 0.0025 | -0.385 | 0.7030635 | 0.919863 |
| X204902_s_at | -0.0103 | 0.0269 | -0.385 | 0.7030759 | 0.919863 |
| X202873_at | -0.002 | 0.0051 | -0.385 | 0.7031062 | 0.919863 |
| X221279_at | -0.0074 | 0.0192 | -0.385 | 0.7031475 | 0.919863 |
| X213974_at | 0.0021 | 0.0054 | 0.385 | 0.7031816 | 0.919863 |
| X222124_at | 0.0036 | 0.0094 | 0.385 | 0.7031842 | 0.919863 |
| X204656_at | -0.0048 | 0.0125 | -0.385 | 0.703186 | 0.919863 |
| X213339_at | -0.0014 | 0.0036 | -0.384 | 0.7032374 | 0.919863 |
| X221395_at | 0.0021 | 0.0054 | 0.384 | 0.7032743 | 0.919863 |
| X200089_s_at | 0.0071 | 0.0185 | 0.384 | 0.7032843 | 0.919863 |
| X216091_s_at | 0.0013 | 0.0035 | 0.384 | 0.7032966 | 0.919863 |
| X216703_at | -0.0029 | 0.0076 | -0.384 | 0.7033185 | 0.919863 |
| X206383_s_at | -0.0058 | 0.0151 | -0.384 | 0.7033342 | 0.919863 |
| X212404_s_at | -0.0013 | 0.0033 | -0.384 | 0.7034761 | 0.919953 |
| X205544_s_at | 0.0022 | 0.0057 | 0.384 | 0.7034886 | 0.919953 |
| X217394_at | -0.004 | 0.0105 | -0.384 | 0.7035596 | 0.919953 |
| X210673_x_at | -0.0016 | 0.004 | -0.384 | 0.7035692 | 0.919953 |
| X212260_at | -0.0035 | 0.0092 | -0.384 | 0.7036497 | 0.920001 |
| X212565_at | -0.018 | 0.0469 | -0.384 | 0.7036883 | 0.920001 |
| X219502_at | 0.0027 | 0.007 | 0.384 | 0.7038591 | 0.920057 |
| X211567_at | -0.0038 | 0.01 | -0.384 | 0.7038766 | 0.920057 |
| X203444_s_at | -0.0011 | 0.0028 | -0.384 | 0.7039365 | 0.920057 |
| X213303_x_at | -0.0125 | 0.0325 | -0.384 | 0.7039527 | 0.920057 |
| X205170_at | -0.0015 | 0.0038 | -0.383 | 0.703979 | 0.920057 |
| X211261_at | -0.0037 | 0.0097 | -0.383 | 0.7040458 | 0.920057 |
| X208472_at | -0.0012 | 0.0032 | -0.383 | 0.7041494 | 0.920057 |
| X220315_at | -0.0037 | 0.0096 | -0.383 | 0.7041643 | 0.920057 |
| X207531_at | -0.0017 | 0.0043 | -0.383 | 0.7041791 | 0.920057 |
| X202434_s_at | -0.0007 | 0.0019 | -0.383 | 0.7041866 | 0.920057 |
| X204734_at | 0.0017 | 0.0045 | 0.383 | 0.7041867 | 0.920057 |
| X201556_s_at | -0.0121 | 0.0315 | -0.383 | 0.7042556 | 0.920093 |
| X202966_at | 0.0044 | 0.0115 | 0.383 | 0.7043152 | 0.920116 |
| X218332_at | -0.0106 | 0.0277 | -0.383 | 0.7045328 | 0.920243 |
| X203662_s_at | -0.0321 | 0.084 | -0.383 | 0.7045672 | 0.920243 |
| X221037_s_at | -0.0065 | 0.0169 | -0.383 | 0.7046307 | 0.920243 |
| X206251_s_at | -0.0025 | 0.0064 | -0.383 | 0.7046494 | 0.920243 |
| X205949_at | 0.002 | 0.0053 | 0.383 | 0.7046866 | 0.920243 |
| X202013_s_at | -0.0133 | 0.0349 | -0.382 | 0.7047122 | 0.920243 |
| X204561_x_at | -0.0049 | 0.0129 | -0.382 | 0.7047538 | 0.920243 |
| X216151_at | -0.0047 | 0.0122 | -0.382 | 0.7047904 | 0.920243 |
| X213026_at | -0.0181 | 0.0474 | -0.382 | 0.7048008 | 0.920243 |
| X203701_s_at | 0.0035 | 0.0091 | 0.382 | 0.7048386 | 0.920243 |
| X203954_x_at | -0.0027 | 0.007 | -0.382 | 0.7048801 | 0.920243 |
| X205635_at | 0.0256 | 0.0671 | 0.382 | 0.7049096 | 0.920243 |
| X211486_s_at | -0.0008 | 0.0022 | -0.382 | 0.7049892 | 0.920293 |
| X204493_at | 0.0472 | 0.1235 | 0.382 | 0.7050878 | 0.920354 |
| X210699_at | 0.002 | 0.0052 | 0.382 | 0.7051575 | 0.920354 |
| X219050_s_at | 0.0011 | 0.0029 | 0.382 | 0.7051601 | 0.920354 |
| X208191_x_at | -0.0021 | 0.0056 | -0.382 | 0.7052701 | 0.920444 |
| X215575_at | 0.0045 | 0.0118 | 0.382 | 0.7053784 | 0.9205 |
| X211713_x_at | -0.0074 | 0.0195 | -0.382 | 0.7053965 | 0.9205 |
| X216391_s_at | -0.0008 | 0.0021 | -0.381 | 0.7055366 | 0.920629 |
| X211449_at | -0.0025 | 0.0065 | -0.381 | 0.7056583 | 0.92066 |
| X203841_x_at | -0.0014 | 0.0038 | -0.381 | 0.7056749 | 0.92066 |
| X208317_at | -0.0035 | 0.0092 | -0.381 | 0.7057025 | 0.92066 |
| X213077_at | 0.0141 | 0.0371 | 0.381 | 0.705742 | 0.92066 |
| X209151_x_at | -0.0018 | 0.0046 | -0.381 | 0.7057675 | 0.92066 |
| X203596_s_at | -0.0187 | 0.0492 | -0.381 | 0.7059194 | 0.920748 |
| X202707_at | -0.0014 | 0.0038 | -0.381 | 0.7059417 | 0.920748 |
| X206361_at | -0.0008 | 0.0022 | -0.381 | 0.7059596 | 0.920748 |
| X212769_at | 0.0019 | 0.005 | 0.381 | 0.7060269 | 0.920769 |
| X205792_at | 0.0018 | 0.0048 | 0.381 | 0.7060582 | 0.920769 |
| X210844_x_at | 0.0171 | 0.0449 | 0.381 | 0.706107 | 0.920779 |
| X214312_at | -0.0013 | 0.0033 | -0.381 | 0.7061532 | 0.920785 |
| X220736_at | 0.0021 | 0.0055 | 0.38 | 0.7062207 | 0.920819 |
| X213703_at | -0.0441 | 0.1161 | -0.38 | 0.7064446 | 0.920971 |
| X216488_s_at | -0.001 | 0.0026 | -0.38 | 0.7064549 | 0.920971 |
| X205185_at | 0.0017 | 0.0046 | 0.38 | 0.7064858 | 0.920971 |
| X200083_at | 0.017 | 0.0447 | 0.38 | 0.7065142 | 0.920971 |
| X222318_at | 0.0025 | 0.0065 | 0.38 | 0.7065445 | 0.920971 |
| X210228_at | 0.0016 | 0.0041 | 0.38 | 0.7067938 | 0.921164 |
| X200850_s_at | 0.0244 | 0.0642 | 0.38 | 0.7068213 | 0.921164 |
| X202316_x_at | -0.0011 | 0.003 | -0.38 | 0.7068577 | 0.921164 |
| X205921_s_at | -0.0024 | 0.0064 | -0.38 | 0.7068585 | 0.921164 |
| X204917_s_at | -0.0298 | 0.0786 | -0.379 | 0.7069082 | 0.921169 |
| X207047_s_at | -0.0032 | 0.0084 | -0.379 | 0.7069454 | 0.921169 |
| X221177_at | 0.0042 | 0.0111 | 0.379 | 0.7070884 | 0.921301 |
| X205203_at | 0.0029 | 0.0077 | 0.379 | 0.7071389 | 0.921313 |
| X211095_at | -0.0076 | 0.0201 | -0.379 | 0.7072597 | 0.921416 |
| X217378_x_at | 0.0011 | 0.003 | 0.379 | 0.7073534 | 0.921484 |
| X203591_s_at | 0.0007 | 0.0018 | 0.379 | 0.7075257 | 0.921596 |
| X206926_s_at | 0.0018 | 0.0048 | 0.379 | 0.7075312 | 0.921596 |
| X209179_s_at | -0.0292 | 0.0771 | -0.379 | 0.7076306 | 0.921596 |
| X217168_s_at | 0.0197 | 0.0522 | 0.378 | 0.7076865 | 0.921596 |
| X215608_at | -0.0151 | 0.0399 | -0.378 | 0.7077004 | 0.921596 |
| X214069_at | -0.0017 | 0.0044 | -0.378 | 0.7077649 | 0.921596 |
| X219627_at | 0.0193 | 0.0512 | 0.378 | 0.7078221 | 0.921596 |
| X204099_at | -0.0167 | 0.0441 | -0.378 | 0.707832 | 0.921596 |
| X204703_at | -0.0115 | 0.0303 | -0.378 | 0.7078514 | 0.921596 |
| X214947_at | 0.0025 | 0.0065 | 0.378 | 0.7078542 | 0.921596 |
| X208973_at | 0.0014 | 0.0036 | 0.378 | 0.7080188 | 0.921751 |
| X221583_s_at | 0.0396 | 0.1048 | 0.378 | 0.708056 | 0.921751 |
| X221567_at | 0.0012 | 0.0032 | 0.378 | 0.7081821 | 0.921854 |
| X208458_at | -0.0009 | 0.0023 | -0.378 | 0.708219 | 0.921854 |
| X206130_s_at | -0.0019 | 0.0049 | -0.378 | 0.7082596 | 0.921854 |
| X212258_s_at | 0.0019 | 0.005 | 0.378 | 0.7083492 | 0.921872 |
| X205250_s_at | 0.0133 | 0.0354 | 0.377 | 0.7084001 | 0.921872 |
| X203868_s_at | -0.06 | 0.1589 | -0.377 | 0.7084467 | 0.921872 |
| X221558_s_at | -0.0398 | 0.1054 | -0.377 | 0.7084749 | 0.921872 |
| X216696_s_at | 0.0017 | 0.0044 | 0.377 | 0.7085309 | 0.921872 |
| X204292_x_at | -0.0018 | 0.0048 | -0.377 | 0.708542 | 0.921872 |
| X212571_at | 0.0203 | 0.0539 | 0.377 | 0.7085637 | 0.921872 |
| X217234_s_at | 0.0401 | 0.1064 | 0.377 | 0.7086686 | 0.921913 |
| X203419_at | -0.014 | 0.0372 | -0.377 | 0.7087036 | 0.921913 |
| X212327_at | 0.0204 | 0.054 | 0.377 | 0.7087198 | 0.921913 |
| X221357_at | -0.0009 | 0.0025 | -0.377 | 0.7088276 | 0.92195 |
| X207509_s_at | 0.0014 | 0.0037 | 0.377 | 0.7088313 | 0.92195 |
| X210170_at | -0.0155 | 0.0411 | -0.377 | 0.708881 | 0.92196 |
| X218678_at | 0.0186 | 0.0494 | 0.377 | 0.7089428 | 0.921972 |
| X220789_s_at | -0.0014 | 0.0037 | -0.377 | 0.708986 | 0.921972 |
| X215567_at | -0.0199 | 0.0528 | -0.377 | 0.7090313 | 0.921972 |
| X217411_s_at | -0.0019 | 0.0051 | -0.377 | 0.7090558 | 0.921972 |
| X221089_at | 0.0019 | 0.0049 | 0.377 | 0.7091056 | 0.921983 |
| X216561_x_at | -0.0015 | 0.0041 | -0.376 | 0.7091514 | 0.921988 |
| X215692_s_at | 0.0226 | 0.06 | 0.376 | 0.7092369 | 0.922045 |
| X210030_at | -0.0013 | 0.0034 | -0.376 | 0.7093174 | 0.922061 |
| X204720_s_at | -0.0125 | 0.0331 | -0.376 | 0.7093317 | 0.922061 |
| X212805_at | -0.0107 | 0.0284 | -0.376 | 0.7094047 | 0.922102 |
| X216700_at | -0.0009 | 0.0023 | -0.376 | 0.7094713 | 0.922134 |
| X219795_at | -0.0039 | 0.0104 | -0.376 | 0.709735 | 0.922335 |
| X217709_at | -0.0031 | 0.0083 | -0.376 | 0.7097465 | 0.922335 |
| X218372_at | 0.0013 | 0.0034 | 0.376 | 0.7097504 | 0.922335 |
| X207698_at | 0.0013 | 0.0035 | 0.375 | 0.7098873 | 0.922459 |
| X201702_s_at | -0.0031 | 0.0083 | -0.375 | 0.7099882 | 0.922475 |
| X214645_at | 0.0022 | 0.006 | 0.375 | 0.7100433 | 0.922475 |
| X212827_at | -0.0025 | 0.0067 | -0.375 | 0.7100601 | 0.922475 |
| X222174_at | -0.0012 | 0.0031 | -0.375 | 0.710066 | 0.922475 |
| X215304_at | -0.0016 | 0.0041 | -0.375 | 0.7103429 | 0.922701 |
| X215283_at | -0.0173 | 0.0461 | -0.375 | 0.7103435 | 0.922701 |
| X202413_s_at | 0.0119 | 0.0318 | 0.375 | 0.7103807 | 0.922701 |
| X207189_s_at | 0.0032 | 0.0086 | 0.375 | 0.7104613 | 0.922701 |
| X205374_at | -0.0108 | 0.0287 | -0.375 | 0.7104824 | 0.922701 |
| X212898_at | 0.0115 | 0.0306 | 0.375 | 0.7105025 | 0.922701 |
| X219172_at | 0.0021 | 0.0055 | 0.375 | 0.7105489 | 0.922701 |
| X211347_at | -0.0469 | 0.1252 | -0.374 | 0.7106103 | 0.922701 |
| X220953_s_at | 0.0017 | 0.0045 | 0.374 | 0.7106134 | 0.922701 |
| X208484_at | 0.0021 | 0.0057 | 0.374 | 0.7106737 | 0.922701 |
| X213751_at | -0.0018 | 0.0048 | -0.374 | 0.7106967 | 0.922701 |
| X204309_at | 0.0015 | 0.0039 | 0.374 | 0.7107773 | 0.922752 |
| X214970_s_at | -0.0033 | 0.0088 | -0.374 | 0.7108569 | 0.922801 |
| X214779_s_at | -0.0188 | 0.0502 | -0.374 | 0.7109393 | 0.922805 |
| X219665_at | 0.0061 | 0.0164 | 0.374 | 0.7109428 | 0.922805 |
| X214465_at | 0.0015 | 0.004 | 0.374 | 0.711013 | 0.922837 |
| X208542_x_at | 0.0021 | 0.0057 | 0.374 | 0.7110509 | 0.922837 |
| X204545_at | 0.0045 | 0.0121 | 0.374 | 0.7111483 | 0.92291 |
| X217813_s_at | -0.0244 | 0.0652 | -0.374 | 0.7112534 | 0.922992 |
| X218632_at | -0.0034 | 0.009 | -0.373 | 0.7114122 | 0.923043 |
| X211334_at | -0.0048 | 0.0129 | -0.373 | 0.7114429 | 0.923043 |
| X205728_at | -0.0409 | 0.1095 | -0.373 | 0.711492 | 0.923043 |
| X210559_s_at | 0.0015 | 0.0041 | 0.373 | 0.7115116 | 0.923043 |
| X211135_x_at | -0.003 | 0.0079 | -0.373 | 0.7115214 | 0.923043 |
| X213800_at | 0.0034 | 0.0091 | 0.373 | 0.7115641 | 0.923043 |
| X204191_at | -0.0011 | 0.003 | -0.373 | 0.7115834 | 0.923043 |
| X205495_s_at | 0.0015 | 0.004 | 0.373 | 0.7117167 | 0.923149 |
| X210967_x_at | -0.0014 | 0.0039 | -0.373 | 0.7117551 | 0.923149 |
| X203161_s_at | -0.0016 | 0.0042 | -0.373 | 0.7117895 | 0.923149 |
| X37232_at | -0.0072 | 0.0193 | -0.373 | 0.7119552 | 0.923273 |
| X206231_at | 0.0066 | 0.0178 | 0.373 | 0.7119686 | 0.923273 |
| X204219_s_at | 0.0081 | 0.0218 | 0.372 | 0.7120632 | 0.923314 |
| X207759_s_at | 0.0029 | 0.0079 | 0.372 | 0.7120829 | 0.923314 |
| X210619_s_at | 0.0011 | 0.0031 | 0.372 | 0.7122115 | 0.923427 |
| X212720_at | -0.0543 | 0.146 | -0.372 | 0.7123179 | 0.923511 |
| X210321_at | -0.0011 | 0.0031 | -0.372 | 0.7123724 | 0.923527 |
| X216976_s_at | 0.0011 | 0.0029 | 0.372 | 0.7124345 | 0.923554 |
| X208007_at | 0.0013 | 0.0036 | 0.372 | 0.7125846 | 0.923662 |
| X214245_at | -0.0018 | 0.0048 | -0.372 | 0.7126007 | 0.923662 |
| X211827_s_at | -0.0017 | 0.0045 | -0.372 | 0.7126686 | 0.923662 |
| X214040_s_at | 0.0019 | 0.005 | 0.372 | 0.7126845 | 0.923662 |
| X218284_at | 0.0265 | 0.0713 | 0.372 | 0.7127275 | 0.923664 |
| X216687_x_at | -0.0036 | 0.0098 | -0.371 | 0.7131362 | 0.92414 |
| X205386_s_at | -0.0061 | 0.0164 | -0.371 | 0.7132336 | 0.924212 |
| X213563_s_at | 0.0017 | 0.0045 | 0.371 | 0.7133356 | 0.924219 |
| X216316_x_at | 0.0024 | 0.0065 | 0.371 | 0.7133956 | 0.924219 |
| X205133_s_at | -0.0133 | 0.036 | -0.371 | 0.7134246 | 0.924219 |
| X200074_s_at | 0.009 | 0.0244 | 0.371 | 0.7134874 | 0.924219 |
| X216883_x_at | -0.0043 | 0.0117 | -0.37 | 0.7135739 | 0.924219 |
| X208916_at | -0.0034 | 0.0092 | -0.37 | 0.7136747 | 0.924219 |
| X204664_at | -0.0011 | 0.0031 | -0.37 | 0.7136939 | 0.924219 |
| X217681_at | -0.0012 | 0.0032 | -0.37 | 0.7137454 | 0.924219 |
| X211557_x_at | 0.0019 | 0.0052 | 0.37 | 0.7137932 | 0.924219 |
| X205980_s_at | 0.0011 | 0.0031 | 0.37 | 0.7138433 | 0.924219 |
| X217174_s_at | 0.001 | 0.0026 | 0.37 | 0.7138855 | 0.924219 |
| X221379_at | 0.0014 | 0.0037 | 0.37 | 0.7140009 | 0.924219 |
| X202394_s_at | -0.0061 | 0.0166 | -0.37 | 0.7140258 | 0.924219 |
| X201633_s_at | -0.0162 | 0.0438 | -0.37 | 0.7140282 | 0.924219 |
| X206828_at | -0.0018 | 0.005 | -0.37 | 0.7140636 | 0.924219 |
| X212182_at | -0.0057 | 0.0155 | -0.37 | 0.7140744 | 0.924219 |
| X204886_at | -0.0092 | 0.025 | -0.37 | 0.7141268 | 0.924219 |
| X203888_at | 0.0021 | 0.0058 | 0.37 | 0.7141372 | 0.924219 |
| X221414_s_at | -0.0016 | 0.0043 | -0.37 | 0.7141984 | 0.924219 |
| X210487_at | -0.002 | 0.0054 | -0.37 | 0.7142028 | 0.924219 |
| X211644_x_at | -0.001 | 0.0026 | -0.37 | 0.7142146 | 0.924219 |
| X200706_s_at | 0.035 | 0.0948 | 0.37 | 0.7142648 | 0.924219 |
| X216706_x_at | -0.002 | 0.0053 | -0.369 | 0.7143123 | 0.924219 |
| X202124_s_at | -0.034 | 0.092 | -0.369 | 0.7143381 | 0.924219 |
| X222227_at | 0.0054 | 0.0147 | 0.369 | 0.7143903 | 0.924219 |
| X220682_s_at | -0.005 | 0.0136 | -0.369 | 0.7144052 | 0.924219 |
| X217571_at | 0.0025 | 0.0069 | 0.369 | 0.7144249 | 0.924219 |
| X203874_s_at | 0.0139 | 0.0378 | 0.369 | 0.7144644 | 0.924219 |
| X205724_at | -0.0008 | 0.0021 | -0.369 | 0.7145146 | 0.924219 |
| X210286_s_at | 0.0042 | 0.0114 | 0.369 | 0.7145256 | 0.924219 |
| X211174_s_at | -0.002 | 0.0054 | -0.369 | 0.7145353 | 0.924219 |
| X215392_at | 0.006 | 0.0163 | 0.369 | 0.7145984 | 0.924219 |
| X220854_at | 0.006 | 0.0161 | 0.369 | 0.7146118 | 0.924219 |
| X221875_x_at | 0.0221 | 0.0599 | 0.369 | 0.7147596 | 0.924356 |
| X204163_at | -0.0022 | 0.0059 | -0.368 | 0.7150396 | 0.924568 |
| X211547_s_at | -0.0262 | 0.0712 | -0.368 | 0.7150434 | 0.924568 |
| X214468_at | -0.002 | 0.0053 | -0.368 | 0.7150484 | 0.924568 |
| X207281_x_at | 0.0025 | 0.0067 | 0.368 | 0.7152534 | 0.92474 |
| X221520_s_at | -0.0018 | 0.005 | -0.368 | 0.7153236 | 0.92474 |
| X217790_s_at | -0.0413 | 0.1122 | -0.368 | 0.7153476 | 0.92474 |
| X210771_at | -0.0078 | 0.0211 | -0.368 | 0.7153835 | 0.92474 |
| X214196_s_at | 0.0022 | 0.0061 | 0.368 | 0.7154146 | 0.92474 |
| X214617_at | -0.0033 | 0.0088 | -0.368 | 0.7154648 | 0.92474 |
| X208252_s_at | 0.0013 | 0.0037 | 0.368 | 0.7155881 | 0.92474 |
| X216155_at | 0.0038 | 0.0102 | 0.368 | 0.7155986 | 0.92474 |
| X220226_at | -0.0007 | 0.002 | -0.368 | 0.7156148 | 0.92474 |
| X213938_at | -0.0318 | 0.0866 | -0.368 | 0.7157167 | 0.92474 |
| X213770_at | 0.0019 | 0.0051 | 0.368 | 0.7157292 | 0.92474 |
| X216033_s_at | 0.0136 | 0.0369 | 0.367 | 0.7158732 | 0.92474 |
| X207770_x_at | 0.0015 | 0.0041 | 0.367 | 0.7158833 | 0.92474 |
| X200961_at | -0.0105 | 0.0287 | -0.367 | 0.7158962 | 0.92474 |
| X211226_at | -0.0012 | 0.0033 | -0.367 | 0.7159303 | 0.92474 |
| X215254_at | -0.0018 | 0.0049 | -0.367 | 0.7159502 | 0.92474 |
| X221278_at | 0.001 | 0.0027 | 0.367 | 0.7159722 | 0.92474 |
| X221431_s_at | 0.0018 | 0.0049 | 0.367 | 0.7160681 | 0.92474 |
| X214502_at | -0.0019 | 0.0051 | -0.367 | 0.716116 | 0.92474 |
| X208535_x_at | 0.0016 | 0.0043 | 0.367 | 0.7161858 | 0.92474 |
| X216861_at | -0.001 | 0.0029 | -0.367 | 0.7162589 | 0.92474 |
| X214239_x_at | 0.0177 | 0.0482 | 0.367 | 0.716276 | 0.92474 |
| X215726_s_at | 0.018 | 0.0491 | 0.367 | 0.7162841 | 0.92474 |
| X213953_at | -0.0026 | 0.0072 | -0.367 | 0.7163134 | 0.92474 |
| X220225_at | 0.0013 | 0.0037 | 0.367 | 0.7163324 | 0.92474 |
| X217846_at | -0.0125 | 0.034 | -0.367 | 0.7163604 | 0.92474 |
| X222083_at | -0.0012 | 0.0033 | -0.367 | 0.7163748 | 0.92474 |
| X215561_s_at | -0.0029 | 0.008 | -0.367 | 0.7164487 | 0.92474 |
| X222264_at | -0.011 | 0.0299 | -0.367 | 0.7164603 | 0.92474 |
| X212832_s_at | -0.0075 | 0.0204 | -0.367 | 0.7164655 | 0.92474 |
| X213367_at | 0.005 | 0.0138 | 0.366 | 0.7165082 | 0.92474 |
| X209408_at | 0.0008 | 0.0023 | 0.366 | 0.7165486 | 0.92474 |
| X36019_at | 0.0062 | 0.0169 | 0.366 | 0.7165681 | 0.92474 |
| X218338_at | 0.0263 | 0.0717 | 0.366 | 0.7165961 | 0.92474 |
| X222226_at | 0.0021 | 0.0058 | 0.366 | 0.7166422 | 0.924745 |
| X208368_s_at | 0.0041 | 0.0112 | 0.366 | 0.7167119 | 0.924779 |
| X210082_at | -0.001 | 0.0027 | -0.366 | 0.7167879 | 0.924779 |
| X207473_at | 0.0012 | 0.0032 | 0.366 | 0.716804 | 0.924779 |
| X205861_at | 0.0016 | 0.0045 | 0.366 | 0.7168892 | 0.924779 |
| X206094_x_at | -0.0023 | 0.0063 | -0.366 | 0.7169381 | 0.924779 |
| X221073_s_at | 0.0015 | 0.0041 | 0.366 | 0.7169391 | 0.924779 |
| X207357_s_at | 0.0051 | 0.014 | 0.366 | 0.717019 | 0.924779 |
| X210957_s_at | -0.002 | 0.0054 | -0.366 | 0.7170379 | 0.924779 |
| X208555_x_at | -0.0021 | 0.0057 | -0.366 | 0.717084 | 0.924779 |
| X207747_s_at | 0.0034 | 0.0093 | 0.366 | 0.7171056 | 0.924779 |
| X209322_s_at | 0.0038 | 0.0103 | 0.366 | 0.7171264 | 0.924779 |
| X207448_at | -0.0025 | 0.0069 | -0.366 | 0.7171965 | 0.924807 |
| X203867_s_at | 0.0011 | 0.0031 | 0.365 | 0.7172928 | 0.924807 |
| X209827_s_at | -0.0017 | 0.0046 | -0.365 | 0.7172967 | 0.924807 |
| X216070_at | -0.0162 | 0.0443 | -0.365 | 0.7173608 | 0.924807 |
| X214216_s_at | -0.0024 | 0.0065 | -0.365 | 0.7173827 | 0.924807 |
| X220167_s_at | -0.0061 | 0.0166 | -0.365 | 0.7174506 | 0.924807 |
| X217479_at | 0.0031 | 0.0084 | 0.365 | 0.7174544 | 0.924807 |
| X216712_at | -0.0031 | 0.0085 | -0.365 | 0.7174808 | 0.924807 |
| X215751_at | -0.004 | 0.0111 | -0.365 | 0.7175939 | 0.924899 |
| X210630_s_at | -0.002 | 0.0055 | -0.365 | 0.7177509 | 0.925036 |
| X216676_x_at | 0.0011 | 0.0031 | 0.365 | 0.7177839 | 0.925036 |
| X206442_at | -0.0015 | 0.0041 | -0.365 | 0.7178687 | 0.92506 |
| X207000_s_at | 0.0039 | 0.0108 | 0.365 | 0.7178857 | 0.92506 |
| X222089_s_at | -0.0017 | 0.0047 | -0.364 | 0.7180297 | 0.925153 |
| X220150_s_at | 0.014 | 0.0383 | 0.364 | 0.718049 | 0.925153 |
| X207816_at | 0.0015 | 0.004 | 0.364 | 0.7180918 | 0.925153 |
| X215722_s_at | -0.0082 | 0.0226 | -0.364 | 0.7181242 | 0.925153 |
| X222330_at | 0.0041 | 0.0112 | 0.364 | 0.7183322 | 0.925194 |
| X221242_at | 0.0024 | 0.0067 | 0.364 | 0.718519 | 0.925194 |
| X207497_s_at | -0.0011 | 0.0031 | -0.364 | 0.7186138 | 0.925194 |
| X217878_s_at | 0.0885 | 0.2433 | 0.364 | 0.718626 | 0.925194 |
| X210828_s_at | -0.0009 | 0.0024 | -0.364 | 0.7186592 | 0.925194 |
| X204870_s_at | -0.0025 | 0.0068 | -0.364 | 0.7186863 | 0.925194 |
| X212023_s_at | -0.0021 | 0.0058 | -0.363 | 0.7187127 | 0.925194 |
| X206270_at | -0.0013 | 0.0035 | -0.363 | 0.7187351 | 0.925194 |
| X209327_s_at | -0.0015 | 0.0042 | -0.363 | 0.7187362 | 0.925194 |
| X209372_x_at | -0.0381 | 0.1047 | -0.363 | 0.7187585 | 0.925194 |
| X211681_s_at | 0.001 | 0.0027 | 0.363 | 0.7187836 | 0.925194 |
| X206371_at | 0.0023 | 0.0062 | 0.363 | 0.7187999 | 0.925194 |
| X211708_s_at | 0.001 | 0.0027 | 0.363 | 0.7188169 | 0.925194 |
| X201270_x_at | -0.0123 | 0.0338 | -0.363 | 0.7188206 | 0.925194 |
| X216368_s_at | -0.0014 | 0.0038 | -0.363 | 0.7188906 | 0.925194 |
| X205907_s_at | -0.0461 | 0.127 | -0.363 | 0.7188987 | 0.925194 |
| X216599_x_at | -0.0006 | 0.0018 | -0.363 | 0.7189045 | 0.925194 |
| X214701_s_at | -0.0013 | 0.0036 | -0.363 | 0.7189177 | 0.925194 |
| X212224_at | 0.1124 | 0.3095 | 0.363 | 0.7189472 | 0.925194 |
| X203132_at | 0.017 | 0.0468 | 0.363 | 0.7190565 | 0.925281 |
| X216612_x_at | 0.0062 | 0.0171 | 0.363 | 0.7192416 | 0.925465 |
| X202079_s_at | 0.0094 | 0.0259 | 0.363 | 0.719329 | 0.925488 |
| X202476_s_at | -0.0038 | 0.0105 | -0.363 | 0.7193424 | 0.925488 |
| X206360_s_at | 0.0041 | 0.0112 | 0.363 | 0.7194053 | 0.925491 |
| X38241_at | -0.0366 | 0.101 | -0.363 | 0.719428 | 0.925491 |
| X216143_at | 0.0006 | 0.0015 | 0.362 | 0.7195134 | 0.925547 |
| X214515_at | 0.0072 | 0.0198 | 0.362 | 0.7196993 | 0.925691 |
| X211215_x_at | 0.0015 | 0.0042 | 0.362 | 0.7197424 | 0.925691 |
| X211252_x_at | 0.0029 | 0.0081 | 0.362 | 0.71975 | 0.925691 |
| X222160_at | -0.0014 | 0.0038 | -0.362 | 0.7197984 | 0.925699 |
| X216269_s_at | 0.0021 | 0.0057 | 0.362 | 0.719901 | 0.925778 |
| X217451_at | -0.0018 | 0.0051 | -0.362 | 0.7201707 | 0.925956 |
| X209392_at | 0.0399 | 0.1103 | 0.361 | 0.7202366 | 0.925956 |
| X214218_s_at | 0.0039 | 0.0109 | 0.361 | 0.7202507 | 0.925956 |
| X211341_at | 0.003 | 0.0082 | 0.361 | 0.7203608 | 0.925956 |
| X204661_at | 0.0022 | 0.006 | 0.361 | 0.720395 | 0.925956 |
| X215256_x_at | 0.0014 | 0.0039 | 0.361 | 0.7204283 | 0.925956 |
| X218763_at | -0.0091 | 0.0253 | -0.361 | 0.7204419 | 0.925956 |
| X215186_at | 0.0194 | 0.0538 | 0.361 | 0.7204481 | 0.925956 |
| X212883_at | 0.0018 | 0.0051 | 0.361 | 0.7204565 | 0.925956 |
| X222067_x_at | -0.0318 | 0.0881 | -0.361 | 0.7204565 | 0.925956 |
| X201338_x_at | -0.0095 | 0.0265 | -0.361 | 0.7206541 | 0.926061 |
| X217265_at | 0.0014 | 0.0038 | 0.361 | 0.7206692 | 0.926061 |
| X219907_at | -0.0014 | 0.0039 | -0.361 | 0.720746 | 0.926061 |
| X51146_at | 0.0123 | 0.034 | 0.361 | 0.7207749 | 0.926061 |
| X203108_at | 0.0016 | 0.0045 | 0.361 | 0.7207971 | 0.926061 |
| X207440_at | 0.0019 | 0.0052 | 0.361 | 0.7208027 | 0.926061 |
| X202773_s_at | 0.0025 | 0.007 | 0.361 | 0.7208492 | 0.926061 |
| X208825_x_at | -0.0047 | 0.0131 | -0.361 | 0.7209114 | 0.926061 |
| X211221_at | -0.0009 | 0.0025 | -0.36 | 0.7209629 | 0.926061 |
| X215803_at | -0.0042 | 0.0116 | -0.36 | 0.7209953 | 0.926061 |
| X202492_at | -0.0132 | 0.0366 | -0.36 | 0.7210213 | 0.926061 |
| X221143_at | 0.0019 | 0.0053 | 0.36 | 0.7210384 | 0.926061 |
| X215310_at | 0.0029 | 0.008 | 0.36 | 0.7212881 | 0.926093 |
| X221083_at | -0.0013 | 0.0037 | -0.36 | 0.7212959 | 0.926093 |
| X210159_s_at | -0.0021 | 0.0058 | -0.36 | 0.7213294 | 0.926093 |
| X221071_at | -0.0029 | 0.0082 | -0.36 | 0.7213668 | 0.926093 |
| X205201_at | 0.0398 | 0.1106 | 0.36 | 0.7213747 | 0.926093 |
| X212186_at | 0.0101 | 0.0281 | 0.36 | 0.7214035 | 0.926093 |
| X209591_s_at | 0.025 | 0.0695 | 0.36 | 0.7214196 | 0.926093 |
| X206931_at | -0.0029 | 0.0081 | -0.36 | 0.721427 | 0.926093 |
| X214637_at | -0.0011 | 0.0032 | -0.36 | 0.7214383 | 0.926093 |
| X216455_at | -0.0012 | 0.0033 | -0.36 | 0.7215421 | 0.926111 |
| X217843_s_at | 0.0091 | 0.0254 | 0.36 | 0.7215698 | 0.926111 |
| X221093_at | -0.0019 | 0.0054 | -0.36 | 0.7216146 | 0.926111 |
| X204625_s_at | -0.001 | 0.0028 | -0.359 | 0.7216625 | 0.926111 |
| X210366_at | -0.0064 | 0.0177 | -0.359 | 0.721677 | 0.926111 |
| X203869_at | 0.0134 | 0.0374 | 0.359 | 0.7217026 | 0.926111 |
| X209996_x_at | -0.0049 | 0.0135 | -0.359 | 0.7218908 | 0.926299 |
| X217655_at | 0.002 | 0.0055 | 0.359 | 0.7220404 | 0.926437 |
| X217819_at | 0.0222 | 0.062 | 0.359 | 0.7221449 | 0.926466 |
| X206085_s_at | 0.0025 | 0.0071 | 0.359 | 0.7221464 | 0.926466 |
| X203255_at | -0.0138 | 0.0384 | -0.359 | 0.7222053 | 0.926488 |
| X207982_at | 0.0038 | 0.0107 | 0.359 | 0.7223256 | 0.926575 |
| X210580_x_at | -0.0224 | 0.0624 | -0.359 | 0.7223562 | 0.926575 |
| X211731_x_at | -0.0011 | 0.003 | -0.358 | 0.7224194 | 0.926586 |
| X216191_s_at | 0.0024 | 0.0068 | 0.358 | 0.7224836 | 0.926586 |
| X216234_s_at | 0.0011 | 0.0031 | 0.358 | 0.7225125 | 0.926586 |
| X214632_at | 0.0024 | 0.0067 | 0.358 | 0.7225321 | 0.926586 |
| X213554_s_at | 0.0038 | 0.0105 | 0.358 | 0.7225823 | 0.926597 |
| X211086_x_at | -0.0216 | 0.0604 | -0.358 | 0.722689 | 0.926638 |
| X220327_at | -0.0085 | 0.0237 | -0.358 | 0.7226977 | 0.926638 |
| X204407_at | 0.0109 | 0.0305 | 0.358 | 0.7227475 | 0.926649 |
| X213516_at | -0.0022 | 0.0061 | -0.358 | 0.7230401 | 0.92697 |
| X215680_at | 0.0011 | 0.0032 | 0.358 | 0.7231237 | 0.926996 |
| X206243_at | 0.002 | 0.0055 | 0.357 | 0.7232451 | 0.926996 |
| X209844_at | 0.0011 | 0.0031 | 0.357 | 0.7232679 | 0.926996 |
| X206276_at | 0.0015 | 0.0042 | 0.357 | 0.7232882 | 0.926996 |
| X38707_r_at | 0.0043 | 0.012 | 0.357 | 0.7233688 | 0.926996 |
| X200619_at | 0.0067 | 0.0187 | 0.357 | 0.7234316 | 0.926996 |
| X203838_s_at | 0.0018 | 0.005 | 0.357 | 0.7234717 | 0.926996 |
| X210142_x_at | 0.0127 | 0.0356 | 0.357 | 0.7234924 | 0.926996 |
| X217288_at | -0.0031 | 0.0087 | -0.357 | 0.7235098 | 0.926996 |
| X213522_s_at | 0.0015 | 0.0041 | 0.357 | 0.7235333 | 0.926996 |
| X200095_x_at | 0.0064 | 0.0179 | 0.357 | 0.723534 | 0.926996 |
| X208067_x_at | -0.0054 | 0.0152 | -0.357 | 0.7235656 | 0.926996 |
| X205520_at | -0.002 | 0.0057 | -0.357 | 0.7236028 | 0.926996 |
| X216517_at | 0.0013 | 0.0037 | 0.357 | 0.7237189 | 0.927045 |
| X207927_at | -0.0022 | 0.0062 | -0.357 | 0.7237245 | 0.927045 |
| X214741_at | -0.0147 | 0.0412 | -0.357 | 0.7238109 | 0.927102 |
| X202754_at | 0.0147 | 0.0412 | 0.356 | 0.723896 | 0.927134 |
| X211200_s_at | -0.0138 | 0.0388 | -0.356 | 0.7239193 | 0.927134 |
| X216989_at | -0.0021 | 0.0058 | -0.356 | 0.7240075 | 0.927147 |
| X204704_s_at | -0.0061 | 0.0171 | -0.356 | 0.7240363 | 0.927147 |
| X220920_at | -0.0084 | 0.0235 | -0.356 | 0.7240543 | 0.927147 |
| X209590_at | 0.0293 | 0.0823 | 0.356 | 0.7241231 | 0.927181 |
| X222279_at | 0.0191 | 0.0537 | 0.356 | 0.724196 | 0.927191 |
| X208402_at | 0.002 | 0.0055 | 0.356 | 0.7242137 | 0.927191 |
| X221199_at | 0.0009 | 0.0026 | 0.356 | 0.7243379 | 0.927275 |
| X215949_x_at | -0.002 | 0.0058 | -0.356 | 0.7243898 | 0.927275 |
| X203842_s_at | 0.0031 | 0.0087 | 0.356 | 0.7244048 | 0.927275 |
| X220685_at | 0.0014 | 0.0039 | 0.356 | 0.7245117 | 0.927339 |
| X206289_at | 0.002 | 0.0057 | 0.356 | 0.7245502 | 0.927339 |
| X219305_x_at | -0.0196 | 0.055 | -0.356 | 0.7246111 | 0.927339 |
| X215325_x_at | -0.0016 | 0.0046 | -0.355 | 0.7246423 | 0.927339 |
| X219012_s_at | -0.0052 | 0.0146 | -0.355 | 0.7246887 | 0.927339 |
| X206345_s_at | 0.0015 | 0.0041 | 0.355 | 0.7247916 | 0.927339 |
| X211017_s_at | -0.0019 | 0.0054 | -0.355 | 0.724818 | 0.927339 |
| X212309_at | 0.0102 | 0.0288 | 0.355 | 0.7248229 | 0.927339 |
| X210162_s_at | -0.0011 | 0.0032 | -0.355 | 0.7248981 | 0.927339 |
| X205416_s_at | 0.0128 | 0.0361 | 0.355 | 0.724933 | 0.927339 |
| X215478_at | -0.0029 | 0.0081 | -0.355 | 0.7249492 | 0.927339 |
| X220619_at | -0.0022 | 0.0062 | -0.355 | 0.7249556 | 0.927339 |
| X204570_at | -0.0303 | 0.0854 | -0.355 | 0.7250408 | 0.927394 |
| X218253_s_at | -0.019 | 0.0537 | -0.355 | 0.725125 | 0.927424 |
| X205565_s_at | -0.016 | 0.0452 | -0.355 | 0.7251889 | 0.927424 |
| X217910_x_at | -0.0047 | 0.0131 | -0.355 | 0.7251889 | 0.927424 |
| X220716_at | 0.0022 | 0.0061 | 0.355 | 0.7252636 | 0.927446 |
| X203651_at | -0.0206 | 0.0581 | -0.355 | 0.7252923 | 0.927446 |
| X216835_s_at | -0.0024 | 0.0068 | -0.355 | 0.7253317 | 0.927446 |
| X214194_at | 0.0033 | 0.0093 | 0.354 | 0.7255018 | 0.92761 |
| X204147_s_at | -0.0012 | 0.0034 | -0.354 | 0.7255784 | 0.927655 |
| X207666_x_at | -0.0021 | 0.0058 | -0.354 | 0.7256436 | 0.927685 |
| X217171_at | -0.0016 | 0.0045 | -0.354 | 0.7258773 | 0.92793 |
| X203398_s_at | -0.0067 | 0.0188 | -0.353 | 0.7261441 | 0.928155 |
| X214167_s_at | 0.0139 | 0.0392 | 0.353 | 0.7261595 | 0.928155 |
| X210341_at | 0.0041 | 0.0117 | 0.353 | 0.7262114 | 0.928155 |
| X207216_at | 0.0013 | 0.0036 | 0.353 | 0.7263778 | 0.928155 |
| X205095_s_at | 0.0011 | 0.0032 | 0.353 | 0.7264029 | 0.928155 |
| X216007_at | 0.0027 | 0.0075 | 0.353 | 0.7264134 | 0.928155 |
| X216463_at | -0.0041 | 0.0116 | -0.353 | 0.7264189 | 0.928155 |
| X216230_x_at | -0.0009 | 0.0026 | -0.353 | 0.7264288 | 0.928155 |
| X65718_at | 0.0026 | 0.0073 | 0.353 | 0.7264458 | 0.928155 |
| X204835_at | 0.0216 | 0.0612 | 0.353 | 0.7264879 | 0.928155 |
| X216730_at | -0.001 | 0.0028 | -0.353 | 0.7265126 | 0.928155 |
| X204525_at | -0.0237 | 0.0673 | -0.353 | 0.7266473 | 0.928273 |
| X205974_at | -0.0036 | 0.0103 | -0.353 | 0.7267358 | 0.928296 |
| X217107_at | -0.0044 | 0.0124 | -0.353 | 0.7267486 | 0.928296 |
| X215059_at | -0.0039 | 0.0109 | -0.352 | 0.7268685 | 0.928356 |
| X211435_at | -0.002 | 0.0057 | -0.352 | 0.7269078 | 0.928356 |
| X210034_s_at | -0.008 | 0.0228 | -0.352 | 0.726921 | 0.928356 |
| X214806_at | 0.0223 | 0.0632 | 0.352 | 0.7270334 | 0.928446 |
| X204369_at | 0.0238 | 0.0675 | 0.352 | 0.7270797 | 0.928452 |
| X209615_s_at | -0.0031 | 0.0089 | -0.352 | 0.7272368 | 0.928547 |
| X220613_s_at | 0.0018 | 0.0052 | 0.352 | 0.7273751 | 0.928547 |
| X206230_at | -0.0012 | 0.0035 | -0.352 | 0.727383 | 0.928547 |
| X215961_at | 0.0009 | 0.0027 | 0.352 | 0.7274061 | 0.928547 |
| X215106_at | -0.0007 | 0.0021 | -0.352 | 0.7274536 | 0.928547 |
| X210815_s_at | -0.0013 | 0.0037 | -0.352 | 0.7275854 | 0.928547 |
| X206493_at | 0.0026 | 0.0074 | 0.351 | 0.7276011 | 0.928547 |
| X214823_at | -0.022 | 0.0625 | -0.351 | 0.7276073 | 0.928547 |
| X220564_at | -0.008 | 0.0226 | -0.351 | 0.7276328 | 0.928547 |
| X210069_at | 0.0019 | 0.0053 | 0.351 | 0.7276589 | 0.928547 |
| X207771_at | 0.0019 | 0.0055 | 0.351 | 0.7277102 | 0.928547 |
| X202599_s_at | -0.0156 | 0.0443 | -0.351 | 0.7277991 | 0.928547 |
| X202011_at | -0.0181 | 0.0516 | -0.351 | 0.7278513 | 0.928547 |
| X219235_s_at | -0.0101 | 0.0287 | -0.351 | 0.7278988 | 0.928547 |
| X220518_at | 0.0026 | 0.0074 | 0.351 | 0.7279359 | 0.928547 |
| X211712_s_at | 0.0027 | 0.0076 | 0.351 | 0.727967 | 0.928547 |
| X206143_at | -0.0028 | 0.0081 | -0.351 | 0.7280074 | 0.928547 |
| X204942_s_at | -0.002 | 0.0057 | -0.351 | 0.7280657 | 0.928547 |
| X215955_x_at | 0.0022 | 0.0063 | 0.351 | 0.7280685 | 0.928547 |
| X210642_at | 0.0009 | 0.0024 | 0.351 | 0.7280775 | 0.928547 |
| X205309_at | 0.0009 | 0.0026 | 0.351 | 0.7280952 | 0.928547 |
| X215282_at | -0.0037 | 0.0105 | -0.351 | 0.7281033 | 0.928547 |
| X207361_at | -0.0029 | 0.0084 | -0.351 | 0.7281618 | 0.928547 |
| X203330_s_at | 0.0014 | 0.004 | 0.351 | 0.7281675 | 0.928547 |
| X213292_s_at | -0.0188 | 0.0535 | -0.351 | 0.7282265 | 0.928547 |
| X207860_at | 0.0017 | 0.0049 | 0.351 | 0.7282406 | 0.928547 |
| X207798_s_at | 0.0023 | 0.0065 | 0.351 | 0.7282932 | 0.92856 |
| X220843_s_at | -0.0014 | 0.0039 | -0.35 | 0.7283818 | 0.928591 |
| X211085_s_at | 0.0296 | 0.0846 | 0.35 | 0.7284012 | 0.928591 |
| X219433_at | 0.0157 | 0.0448 | 0.35 | 0.7284992 | 0.928645 |
| X205343_at | -0.0022 | 0.0062 | -0.35 | 0.7285575 | 0.928645 |
| X207245_at | -0.0032 | 0.009 | -0.35 | 0.7285683 | 0.928645 |
| X217187_at | -0.0092 | 0.0262 | -0.35 | 0.7287073 | 0.928685 |
| X209776_s_at | -0.0008 | 0.0023 | -0.35 | 0.7287131 | 0.928685 |
| X208267_at | 0.0014 | 0.004 | 0.35 | 0.7287254 | 0.928685 |
| X210294_at | 0.0015 | 0.0043 | 0.35 | 0.7288078 | 0.928728 |
| X213189_at | -0.0139 | 0.0397 | -0.35 | 0.7288428 | 0.928728 |
| X214424_s_at | -0.0017 | 0.0047 | -0.35 | 0.7289809 | 0.928795 |
| X221412_at | -0.0042 | 0.0121 | -0.35 | 0.7290229 | 0.928795 |
| X222110_at | -0.0016 | 0.0045 | -0.35 | 0.729077 | 0.928795 |
| X205365_at | 0.0027 | 0.0078 | 0.349 | 0.7291241 | 0.928795 |
| X52940_at | -0.025 | 0.0716 | -0.349 | 0.7291385 | 0.928795 |
| X216169_at | -0.0026 | 0.0074 | -0.349 | 0.7292614 | 0.928795 |
| X217060_at | -0.0022 | 0.0062 | -0.349 | 0.7293057 | 0.928795 |
| X214110_s_at | -0.0208 | 0.0596 | -0.349 | 0.7293091 | 0.928795 |
| X221854_at | -0.0011 | 0.0033 | -0.349 | 0.7293621 | 0.928795 |
| X213054_at | -0.0052 | 0.0148 | -0.349 | 0.7293837 | 0.928795 |
| X204003_s_at | -0.0026 | 0.0074 | -0.349 | 0.7293856 | 0.928795 |
| X213621_s_at | 0.0014 | 0.004 | 0.349 | 0.7294276 | 0.928795 |
| X204817_at | 0.002 | 0.0059 | 0.349 | 0.7294726 | 0.928795 |
| X221698_s_at | 0.0079 | 0.0228 | 0.349 | 0.7294809 | 0.928795 |
| X208071_s_at | 0.0016 | 0.0045 | 0.349 | 0.7297062 | 0.92895 |
| X222383_s_at | -0.0021 | 0.0061 | -0.349 | 0.7297227 | 0.92895 |
| X221511_x_at | 0.0129 | 0.037 | 0.349 | 0.729728 | 0.92895 |
| X209168_at | 0.0171 | 0.049 | 0.348 | 0.729846 | 0.92899 |
| X222375_at | -0.0323 | 0.0927 | -0.348 | 0.7298896 | 0.92899 |
| X205578_at | -0.0029 | 0.0084 | -0.348 | 0.7300193 | 0.92899 |
| X206969_at | 0.0017 | 0.0048 | 0.348 | 0.7300583 | 0.92899 |
| X216216_at | 0.0023 | 0.0066 | 0.348 | 0.7300751 | 0.92899 |
| X207310_s_at | 0.0008 | 0.0022 | 0.348 | 0.7300938 | 0.92899 |
| X221305_s_at | 0.002 | 0.0058 | 0.348 | 0.730101 | 0.92899 |
| X219073_s_at | -0.0129 | 0.0372 | -0.348 | 0.7301169 | 0.92899 |
| X46665_at | 0.0153 | 0.0441 | 0.348 | 0.7301558 | 0.92899 |
| X211384_s_at | -0.0019 | 0.0056 | -0.348 | 0.7302096 | 0.92899 |
| X218115_at | 0.0013 | 0.0036 | 0.348 | 0.7302606 | 0.92899 |
| X208987_s_at | 0.0031 | 0.009 | 0.348 | 0.7302868 | 0.92899 |
| X214994_at | -0.002 | 0.0058 | -0.348 | 0.7303432 | 0.92899 |
| X220502_s_at | -0.0059 | 0.0168 | -0.348 | 0.7303446 | 0.92899 |
| X220653_at | -0.003 | 0.0088 | -0.348 | 0.7305403 | 0.929138 |
| X216628_at | 0.0023 | 0.0065 | 0.347 | 0.7305982 | 0.929138 |
| X220062_s_at | 0.0021 | 0.0062 | 0.347 | 0.7306192 | 0.929138 |
| X216439_at | 0.0019 | 0.0054 | 0.347 | 0.7307 | 0.929138 |
| X205640_at | 0.0018 | 0.0052 | 0.347 | 0.7307116 | 0.929138 |
| X218314_s_at | 0.0167 | 0.0483 | 0.347 | 0.7308317 | 0.929138 |
| X207330_at | 0.0013 | 0.0038 | 0.347 | 0.7308472 | 0.929138 |
| X207599_at | -0.0022 | 0.0064 | -0.347 | 0.7309329 | 0.929138 |
| X209058_at | -0.0083 | 0.0238 | -0.347 | 0.7309442 | 0.929138 |
| X207587_at | -0.0006 | 0.0016 | -0.347 | 0.7309735 | 0.929138 |
| X211551_at | -0.001 | 0.0029 | -0.347 | 0.7310065 | 0.929138 |
| X210617_at | 0.0013 | 0.0038 | 0.347 | 0.7310864 | 0.929138 |
| X207212_at | -0.0013 | 0.0038 | -0.347 | 0.7311524 | 0.929138 |
| X215513_at | 0.0044 | 0.0126 | 0.347 | 0.7311532 | 0.929138 |
| X215789_s_at | 0.0232 | 0.067 | 0.347 | 0.7311634 | 0.929138 |
| X201177_s_at | -0.0087 | 0.0252 | -0.347 | 0.7311771 | 0.929138 |
| X201474_s_at | -0.0015 | 0.0044 | -0.347 | 0.7311824 | 0.929138 |
| X211239_s_at | -0.0028 | 0.008 | -0.347 | 0.7312143 | 0.929138 |
| X220634_at | -0.0014 | 0.0041 | -0.346 | 0.7313175 | 0.929188 |
| X211038_s_at | -0.0284 | 0.0821 | -0.346 | 0.7314018 | 0.929188 |
| X211107_s_at | -0.0011 | 0.0033 | -0.346 | 0.7314464 | 0.929188 |
| X216337_at | 0.0023 | 0.0066 | 0.346 | 0.7314647 | 0.929188 |
| X210767_at | 0.0031 | 0.0091 | 0.346 | 0.7315186 | 0.929188 |
| X204328_at | 0.0287 | 0.0829 | 0.346 | 0.7315373 | 0.929188 |
| X220876_at | -0.002 | 0.0058 | -0.346 | 0.7316751 | 0.929188 |
| X222071_s_at | 0.0058 | 0.0167 | 0.346 | 0.7316887 | 0.929188 |
| X206919_at | 0.0029 | 0.0084 | 0.346 | 0.7317837 | 0.929188 |
| X218063_s_at | -0.0009 | 0.0026 | -0.346 | 0.7318451 | 0.929188 |
| X220913_at | -0.0018 | 0.0052 | -0.346 | 0.7318561 | 0.929188 |
| X203267_s_at | -0.0023 | 0.0067 | -0.346 | 0.7318708 | 0.929188 |
| X220548_at | 0.0016 | 0.0046 | 0.346 | 0.7319164 | 0.929188 |
| X221588_x_at | 0.0145 | 0.0419 | 0.346 | 0.7319469 | 0.929188 |
| X205743_at | -0.0153 | 0.0441 | -0.346 | 0.73198 | 0.929188 |
| X205620_at | 0.0009 | 0.0025 | 0.345 | 0.7321004 | 0.929188 |
| X213651_at | -0.0019 | 0.0055 | -0.345 | 0.7321507 | 0.929188 |
| X222092_at | 0.0008 | 0.0023 | 0.345 | 0.7321876 | 0.929188 |
| X219232_s_at | 0.0426 | 0.1233 | 0.345 | 0.7322257 | 0.929188 |
| X205345_at | 0.0024 | 0.0068 | 0.345 | 0.7322332 | 0.929188 |
| X215959_at | 0.0015 | 0.0045 | 0.345 | 0.7322589 | 0.929188 |
| X205909_at | 0.0012 | 0.0035 | 0.345 | 0.732282 | 0.929188 |
| X219691_at | 0.0035 | 0.01 | 0.345 | 0.7323058 | 0.929188 |
| X220100_at | 0.0013 | 0.0039 | 0.345 | 0.7323596 | 0.929188 |
| X219814_at | -0.002 | 0.0058 | -0.345 | 0.7323812 | 0.929188 |
| X217590_s_at | -0.0018 | 0.0052 | -0.345 | 0.7323841 | 0.929188 |
| X218257_s_at | 0.0023 | 0.0067 | 0.345 | 0.732404 | 0.929188 |
| X219823_at | -0.0025 | 0.0072 | -0.345 | 0.7324547 | 0.929188 |
| X204692_at | 0.001 | 0.0029 | 0.345 | 0.7324661 | 0.929188 |
| X221603_at | -0.0007 | 0.002 | -0.345 | 0.7325262 | 0.929211 |
| X209709_s_at | 0.0023 | 0.0066 | 0.345 | 0.7326299 | 0.92925 |
| X214346_at | -0.0011 | 0.0032 | -0.345 | 0.7326591 | 0.92925 |
| X207568_at | 0.0013 | 0.0038 | 0.345 | 0.7327575 | 0.92925 |
| X215702_s_at | -0.0029 | 0.0083 | -0.345 | 0.7327585 | 0.92925 |
| X215640_at | 0.0033 | 0.0095 | 0.345 | 0.7327836 | 0.92925 |
| X215619_at | -0.0065 | 0.0189 | -0.344 | 0.7329054 | 0.92925 |
| X212722_s_at | -0.0019 | 0.0055 | -0.344 | 0.7329159 | 0.92925 |
| X202007_at | -0.0448 | 0.1301 | -0.344 | 0.7329653 | 0.92925 |
| X207159_x_at | 0.0017 | 0.0049 | 0.344 | 0.7330195 | 0.92925 |
| X209317_at | -0.0119 | 0.0345 | -0.344 | 0.7330199 | 0.92925 |
| X221271_at | -0.0018 | 0.0051 | -0.344 | 0.7330545 | 0.92925 |
| X213371_at | 0.0404 | 0.1173 | 0.344 | 0.7330812 | 0.92925 |
| X208578_at | -0.0023 | 0.0068 | -0.344 | 0.7331294 | 0.92925 |
| X215212_at | -0.0162 | 0.0472 | -0.344 | 0.7331426 | 0.92925 |
| X206785_s_at | 0.0875 | 0.2544 | 0.344 | 0.733242 | 0.929323 |
| X209052_s_at | 0.0009 | 0.0025 | 0.344 | 0.7334291 | 0.929359 |
| X215060_at | -0.0011 | 0.0033 | -0.344 | 0.7334983 | 0.929359 |
| X216906_at | -0.001 | 0.0029 | -0.344 | 0.7334992 | 0.929359 |
| X203850_s_at | -0.004 | 0.0117 | -0.344 | 0.7335266 | 0.929359 |
| X212106_at | 0.0234 | 0.068 | 0.343 | 0.7335843 | 0.929359 |
| X202311_s_at | 0.0045 | 0.013 | 0.343 | 0.7336591 | 0.929359 |
| X216731_s_at | -0.0028 | 0.0083 | -0.343 | 0.7337072 | 0.929359 |
| X218986_s_at | 0.02 | 0.0584 | 0.343 | 0.7337512 | 0.929359 |
| X206717_at | -0.0039 | 0.0114 | -0.343 | 0.7338125 | 0.929359 |
| X209942_x_at | 0.0015 | 0.0043 | 0.343 | 0.7338709 | 0.929359 |
| X205827_at | -0.0802 | 0.2338 | -0.343 | 0.7339056 | 0.929359 |
| X205986_at | 0.0171 | 0.0497 | 0.343 | 0.7339734 | 0.929359 |
| X212809_at | 0.0015 | 0.0044 | 0.343 | 0.7339796 | 0.929359 |
| X217526_at | 0.0138 | 0.0403 | 0.343 | 0.734 | 0.929359 |
| X208881_x_at | -0.0239 | 0.0698 | -0.343 | 0.7340228 | 0.929359 |
| X217508_s_at | 0.0018 | 0.0051 | 0.343 | 0.7341061 | 0.929359 |
| X205976_at | -0.0068 | 0.0197 | -0.343 | 0.7341884 | 0.929359 |
| X215291_at | -0.002 | 0.0059 | -0.343 | 0.734189 | 0.929359 |
| X203393_at | -0.0023 | 0.0066 | -0.343 | 0.7341989 | 0.929359 |
| X208605_s_at | 0.0014 | 0.004 | 0.343 | 0.7342659 | 0.929359 |
| X212119_at | -0.0172 | 0.0503 | -0.342 | 0.7343238 | 0.929359 |
| X213980_s_at | -0.0111 | 0.0323 | -0.342 | 0.7343454 | 0.929359 |
| X215975_x_at | 0.0014 | 0.004 | 0.342 | 0.734351 | 0.929359 |
| X207322_at | 0.002 | 0.0059 | 0.342 | 0.7343824 | 0.929359 |
| X208528_x_at | -0.0007 | 0.0022 | -0.342 | 0.7343881 | 0.929359 |
| X216362_at | -0.0016 | 0.0047 | -0.342 | 0.7344139 | 0.929359 |
| X221411_at | -0.0017 | 0.0048 | -0.342 | 0.7344345 | 0.929359 |
| X204473_s_at | 0.0124 | 0.0364 | 0.342 | 0.7344795 | 0.929359 |
| X202080_s_at | -0.0036 | 0.0106 | -0.342 | 0.7344836 | 0.929359 |
| X216257_at | 0.0017 | 0.005 | 0.342 | 0.7346505 | 0.929517 |
| X215412_x_at | 0.0155 | 0.0453 | 0.342 | 0.7347307 | 0.929536 |
| X215572_at | 0.0043 | 0.0125 | 0.342 | 0.7347494 | 0.929536 |
| X215382_x_at | -0.0012 | 0.0034 | -0.342 | 0.7348428 | 0.929573 |
| X209454_s_at | -0.0024 | 0.0069 | -0.342 | 0.7349437 | 0.929573 |
| X204043_at | 0.0008 | 0.0023 | 0.342 | 0.7349499 | 0.929573 |
| X203792_x_at | -0.0016 | 0.0046 | -0.342 | 0.7349598 | 0.929573 |
| X203172_at | 0.0127 | 0.0372 | 0.342 | 0.7349878 | 0.929573 |
| X217040_x_at | 0.001 | 0.0029 | 0.341 | 0.735091 | 0.929651 |
| X204044_at | 0.0066 | 0.0194 | 0.341 | 0.7351465 | 0.929668 |
| X218805_at | 0.0033 | 0.0096 | 0.341 | 0.7353073 | 0.929811 |
| X203717_at | 0.0025 | 0.0073 | 0.341 | 0.7353589 | 0.929811 |
| X207228_at | -0.0019 | 0.0055 | -0.341 | 0.7354319 | 0.929811 |
| X205461_at | -0.0015 | 0.0043 | -0.341 | 0.7355093 | 0.929811 |
| X206562_s_at | -0.0275 | 0.0808 | -0.341 | 0.7355098 | 0.929811 |
| X222355_at | -0.0048 | 0.014 | -0.341 | 0.7355107 | 0.929811 |
| X209031_at | -0.0178 | 0.0522 | -0.341 | 0.7357064 | 0.929941 |
| X215010_s_at | -0.0013 | 0.0038 | -0.341 | 0.7357149 | 0.929941 |
| X47530_at | 0.0077 | 0.0227 | 0.341 | 0.7357392 | 0.929941 |
| X206656_s_at | 0.0102 | 0.0299 | 0.34 | 0.7358596 | 0.930034 |
| X213223_at | 0.0032 | 0.0095 | 0.34 | 0.7358964 | 0.930034 |
| X204484_at | 0.0147 | 0.0431 | 0.34 | 0.7360038 | 0.930049 |
| X211817_s_at | -0.0015 | 0.0044 | -0.34 | 0.7360209 | 0.930049 |
| X201867_s_at | 0.0554 | 0.163 | 0.34 | 0.7360342 | 0.930049 |
| X214433_s_at | -0.0335 | 0.0985 | -0.34 | 0.7360846 | 0.93006 |
| X205892_s_at | 0.0015 | 0.0045 | 0.34 | 0.736192 | 0.930103 |
| X203629_s_at | 0.0186 | 0.0546 | 0.34 | 0.7362188 | 0.930103 |
| X221058_s_at | -0.004 | 0.0119 | -0.34 | 0.7362439 | 0.930103 |
| X210987_x_at | -0.0156 | 0.0458 | -0.34 | 0.736448 | 0.930307 |
| X206391_at | -0.0015 | 0.0043 | -0.34 | 0.736493 | 0.930311 |
| X205199_at | -0.001 | 0.0028 | -0.339 | 0.7365519 | 0.930328 |
| X201726_at | -0.0192 | 0.0567 | -0.339 | 0.7366017 | 0.930328 |
| X217180_at | 0.0015 | 0.0043 | 0.339 | 0.7366318 | 0.930328 |
| X216500_at | 0.0014 | 0.004 | 0.339 | 0.7367867 | 0.930448 |
| X214204_at | 0.0257 | 0.0757 | 0.339 | 0.7368106 | 0.930448 |
| X214497_s_at | -0.0017 | 0.0051 | -0.339 | 0.7368959 | 0.930503 |
| X217212_s_at | 0.002 | 0.0058 | 0.339 | 0.7369768 | 0.930552 |
| X208677_s_at | 0.0309 | 0.0912 | 0.339 | 0.737102 | 0.930657 |
| X218096_at | 0.012 | 0.0354 | 0.339 | 0.7371753 | 0.930673 |
| X215109_at | -0.0085 | 0.025 | -0.339 | 0.7372493 | 0.930673 |
| X208912_s_at | 0.034 | 0.1005 | 0.338 | 0.7372687 | 0.930673 |
| X54970_at | 0.0169 | 0.05 | 0.338 | 0.7373113 | 0.930673 |
| X221349_at | -0.0017 | 0.0051 | -0.338 | 0.7373292 | 0.930673 |
| X216786_at | -0.0052 | 0.0155 | -0.338 | 0.7374313 | 0.930673 |
| X210572_at | -0.0028 | 0.0083 | -0.338 | 0.7374585 | 0.930673 |
| X208571_at | -0.0036 | 0.0107 | -0.338 | 0.7375233 | 0.930673 |
| X206165_s_at | -0.0025 | 0.0074 | -0.338 | 0.7375323 | 0.930673 |
| X213865_at | 0.0022 | 0.0065 | 0.338 | 0.7375438 | 0.930673 |
| X215125_s_at | 0.0018 | 0.0052 | 0.338 | 0.7375756 | 0.930673 |
| X211241_at | 0.0025 | 0.0075 | 0.338 | 0.7378007 | 0.930905 |
| X209603_at | -0.0013 | 0.0038 | -0.338 | 0.7379535 | 0.930955 |
| X206464_at | -0.0014 | 0.004 | -0.338 | 0.7379649 | 0.930955 |
| X221612_at | -0.0009 | 0.0028 | -0.338 | 0.7379664 | 0.930955 |
| X206008_at | 0.0013 | 0.0037 | 0.337 | 0.7380351 | 0.930967 |
| X216448_at | 0.0013 | 0.0039 | 0.337 | 0.7380599 | 0.930967 |
| X206644_at | -0.0028 | 0.0083 | -0.337 | 0.7381207 | 0.930991 |
| X212768_s_at | -0.0083 | 0.0245 | -0.337 | 0.7382129 | 0.931054 |
| X216995_x_at | 0.0012 | 0.0034 | 0.337 | 0.7382687 | 0.931072 |
| X216996_s_at | -0.0068 | 0.0202 | -0.337 | 0.7383603 | 0.93108 |
| X220667_at | 0.003 | 0.0089 | 0.337 | 0.7383665 | 0.93108 |
| X217245_at | -0.0022 | 0.0064 | -0.337 | 0.7384008 | 0.93108 |
| X215496_at | 0.0013 | 0.0037 | 0.337 | 0.7385255 | 0.931155 |
| X221779_at | -0.002 | 0.0059 | -0.337 | 0.7385439 | 0.931155 |
| X206823_at | 0.002 | 0.0058 | 0.337 | 0.7386929 | 0.931227 |
| X210444_at | 0.0016 | 0.0048 | 0.337 | 0.7387273 | 0.931227 |
| X209366_x_at | 0.0157 | 0.0466 | 0.337 | 0.7387509 | 0.931227 |
| X217710_x_at | -0.0013 | 0.0038 | -0.336 | 0.7388204 | 0.931227 |
| X220663_at | -0.0151 | 0.0449 | -0.336 | 0.7388679 | 0.931227 |
| X218831_s_at | 0.0045 | 0.0135 | 0.336 | 0.738901 | 0.931227 |
| X221101_at | 0.0016 | 0.0048 | 0.336 | 0.7389172 | 0.931227 |
| X205624_at | 0.002 | 0.0059 | 0.336 | 0.7389863 | 0.931227 |
| X220344_at | -0.0016 | 0.0047 | -0.336 | 0.7390175 | 0.931227 |
| X217407_x_at | -0.001 | 0.0031 | -0.336 | 0.7390208 | 0.931227 |
| X207931_s_at | 0.0028 | 0.0084 | 0.336 | 0.7390779 | 0.931247 |
| X215471_s_at | -0.0125 | 0.0372 | -0.336 | 0.7391876 | 0.931253 |
| X206265_s_at | -0.0018 | 0.0052 | -0.336 | 0.7391929 | 0.931253 |
| X215021_s_at | 0.0162 | 0.0481 | 0.336 | 0.7392086 | 0.931253 |
| X213646_x_at | 0.0151 | 0.045 | 0.336 | 0.7393337 | 0.931358 |
| X210723_x_at | -0.0035 | 0.0105 | -0.336 | 0.7394432 | 0.931443 |
| X210610_at | -0.0013 | 0.0039 | -0.336 | 0.7394869 | 0.931445 |
| X204112_s_at | 0.0193 | 0.0576 | 0.335 | 0.7396206 | 0.931466 |
| X201643_x_at | 0.0097 | 0.0289 | 0.335 | 0.7398038 | 0.931466 |
| X212833_at | 0.0176 | 0.0526 | 0.335 | 0.7398431 | 0.931466 |
| X208607_s_at | -0.0019 | 0.0056 | -0.335 | 0.7398499 | 0.931466 |
| X205458_at | -0.0016 | 0.0049 | -0.335 | 0.7398508 | 0.931466 |
| X216504_s_at | -0.0024 | 0.0071 | -0.335 | 0.7398629 | 0.931466 |
| X211641_x_at | 0.0016 | 0.0048 | 0.335 | 0.7398825 | 0.931466 |
| X200007_at | -0.0078 | 0.0232 | -0.335 | 0.7399495 | 0.931466 |
| X205106_at | -0.0023 | 0.0067 | -0.335 | 0.7399722 | 0.931466 |
| X216479_at | -0.0037 | 0.011 | -0.335 | 0.7399845 | 0.931466 |
| X207967_at | -0.001 | 0.0031 | -0.335 | 0.7399958 | 0.931466 |
| X215560_x_at | 0.009 | 0.0269 | 0.335 | 0.7400066 | 0.931466 |
| X211988_at | -0.0107 | 0.0319 | -0.335 | 0.7400568 | 0.931466 |
| X216704_at | 0.0026 | 0.0077 | 0.335 | 0.740119 | 0.931466 |
| X221586_s_at | -0.0172 | 0.0513 | -0.335 | 0.7401547 | 0.931466 |
| X214456_x_at | 0.0017 | 0.005 | 0.335 | 0.7401747 | 0.931466 |
| X212892_at | -0.0019 | 0.0056 | -0.334 | 0.7402617 | 0.931523 |
| X218673_s_at | 0.0044 | 0.0131 | 0.334 | 0.7403714 | 0.931557 |
| X214214_s_at | 0.0125 | 0.0373 | 0.334 | 0.7403727 | 0.931557 |
| X222106_at | 0.0032 | 0.0095 | 0.334 | 0.7405266 | 0.931655 |
| X208041_at | 0.0013 | 0.004 | 0.334 | 0.740567 | 0.931655 |
| X206606_at | 0.0011 | 0.0033 | 0.334 | 0.7405961 | 0.931655 |
| X203290_at | -0.0028 | 0.0083 | -0.334 | 0.7406184 | 0.931655 |
| X216049_at | 0.0022 | 0.0067 | 0.334 | 0.7408083 | 0.931796 |
| X207005_s_at | -0.0019 | 0.0057 | -0.334 | 0.7408599 | 0.931796 |
| X205240_at | 0.0306 | 0.0917 | 0.334 | 0.7409117 | 0.931796 |
| X201108_s_at | -0.0012 | 0.0035 | -0.334 | 0.7409549 | 0.931796 |
| X216206_x_at | -0.0054 | 0.016 | -0.333 | 0.7410052 | 0.931796 |
| X214688_at | -0.0042 | 0.0126 | -0.333 | 0.7410418 | 0.931796 |
| X207029_at | -0.003 | 0.0089 | -0.333 | 0.7410591 | 0.931796 |
| X219757_s_at | 0.0269 | 0.0806 | 0.333 | 0.7411302 | 0.931796 |
| X205010_at | -0.0129 | 0.0387 | -0.333 | 0.741163 | 0.931796 |
| X219661_at | 0.0012 | 0.0037 | 0.333 | 0.7411729 | 0.931796 |
| X204746_s_at | -0.0013 | 0.0038 | -0.333 | 0.7412302 | 0.931796 |
| X217300_at | 0.0029 | 0.0086 | 0.333 | 0.7412334 | 0.931796 |
| X207983_s_at | 0.0206 | 0.0619 | 0.333 | 0.7413093 | 0.931838 |
| X214777_at | 0.0018 | 0.0053 | 0.333 | 0.7414781 | 0.931857 |
| X207648_at | 0.0014 | 0.0042 | 0.333 | 0.7414951 | 0.931857 |
| X220467_at | 0.0101 | 0.0304 | 0.333 | 0.7415163 | 0.931857 |
| X206994_at | -0.0028 | 0.0083 | -0.333 | 0.741523 | 0.931857 |
| X220300_at | -0.0011 | 0.0034 | -0.333 | 0.7415573 | 0.931857 |
| X214393_at | 0.0254 | 0.0763 | 0.333 | 0.741576 | 0.931857 |
| X206666_at | 0.0012 | 0.0037 | 0.333 | 0.7417343 | 0.932003 |
| X207778_at | 0.0008 | 0.0023 | 0.332 | 0.7419778 | 0.932257 |
| X219702_at | -0.0008 | 0.0025 | -0.332 | 0.7420462 | 0.93229 |
| X206581_at | -0.003 | 0.0092 | -0.332 | 0.7422103 | 0.932443 |
| X215772_x_at | -0.0226 | 0.0681 | -0.332 | 0.7422559 | 0.932448 |
| X216201_at | 0.0017 | 0.0051 | 0.332 | 0.7424008 | 0.932577 |
| X217964_at | 0.0153 | 0.0461 | 0.331 | 0.7425384 | 0.932659 |
| X205491_s_at | -0.0011 | 0.0032 | -0.331 | 0.7425839 | 0.932659 |
| X220130_x_at | -0.0016 | 0.0049 | -0.331 | 0.7425918 | 0.932659 |
| X200685_at | 0.0113 | 0.0341 | 0.331 | 0.7426661 | 0.932699 |
| X214752_x_at | 0.0014 | 0.0043 | 0.331 | 0.7428332 | 0.932855 |
| X222238_s_at | -0.0012 | 0.0037 | -0.331 | 0.7428739 | 0.932855 |
| X204716_at | -0.0226 | 0.0684 | -0.331 | 0.7430648 | 0.932963 |
| X217015_at | -0.0014 | 0.0043 | -0.331 | 0.7431124 | 0.932963 |
| X204607_at | 0.0015 | 0.0044 | 0.331 | 0.7431448 | 0.932963 |
| X210077_s_at | -0.0021 | 0.0062 | -0.331 | 0.743168 | 0.932963 |
| X220562_at | -0.0018 | 0.0053 | -0.331 | 0.7431719 | 0.932963 |
| X206757_at | 0.0048 | 0.0147 | 0.33 | 0.74326 | 0.932963 |
| X204695_at | -0.0012 | 0.0036 | -0.33 | 0.7432635 | 0.932963 |
| X216536_at | 0.0016 | 0.0049 | 0.33 | 0.74331 | 0.932963 |
| X211786_at | -0.0024 | 0.0073 | -0.33 | 0.7433379 | 0.932963 |
| X215880_at | -0.0019 | 0.0056 | -0.33 | 0.7434061 | 0.932996 |
| X204843_s_at | 0.0013 | 0.0039 | 0.33 | 0.7435775 | 0.93311 |
| X218360_at | -0.0139 | 0.0421 | -0.33 | 0.7436628 | 0.93311 |
| X53968_at | 0.0032 | 0.0097 | 0.33 | 0.7436665 | 0.93311 |
| X202352_s_at | -0.0136 | 0.0413 | -0.33 | 0.7437171 | 0.93311 |
| X220190_s_at | -0.0035 | 0.0107 | -0.33 | 0.7437353 | 0.93311 |
| X216171_at | -0.0037 | 0.0112 | -0.33 | 0.7437491 | 0.93311 |
| X216064_s_at | -0.0107 | 0.0323 | -0.33 | 0.7438186 | 0.933144 |
| X222267_at | 0.0028 | 0.0085 | 0.33 | 0.7438958 | 0.933189 |
| X215445_x_at | 0.0125 | 0.0378 | 0.33 | 0.7439644 | 0.933222 |
| X220776_at | -0.0023 | 0.007 | -0.329 | 0.7440185 | 0.933237 |
| X220717_at | 0.0035 | 0.0107 | 0.329 | 0.7440869 | 0.933248 |
| X209970_x_at | -0.0029 | 0.0088 | -0.329 | 0.7441143 | 0.933248 |
| X217494_s_at | 0.0028 | 0.0086 | 0.329 | 0.744153 | 0.933248 |
| X208387_s_at | 0.0013 | 0.0038 | 0.329 | 0.7442264 | 0.933287 |
| X211078_s_at | 0.0056 | 0.0172 | 0.329 | 0.7443776 | 0.933424 |
| X213753_x_at | -0.0076 | 0.0232 | -0.329 | 0.7444416 | 0.933451 |
| X207840_at | -0.002 | 0.0061 | -0.329 | 0.7445274 | 0.933463 |
| X212654_at | -0.0025 | 0.0076 | -0.329 | 0.7445346 | 0.933463 |
| X208871_at | 0.0012 | 0.0038 | 0.329 | 0.7446316 | 0.933512 |
| X210339_s_at | -0.0021 | 0.0064 | -0.329 | 0.744658 | 0.933512 |
| X205772_s_at | 0.002 | 0.006 | 0.329 | 0.7447222 | 0.93354 |
| X220184_at | 0.0669 | 0.2037 | 0.328 | 0.7448408 | 0.933601 |
| X205264_at | -0.0132 | 0.0402 | -0.328 | 0.7449179 | 0.933601 |
| X201448_at | -0.0093 | 0.0283 | -0.328 | 0.7449239 | 0.933601 |
| X206010_at | 0.0012 | 0.0037 | 0.328 | 0.744939 | 0.933601 |
| X216886_at | 0.0014 | 0.0043 | 0.328 | 0.7451287 | 0.933786 |
| X218643_s_at | -0.0196 | 0.0599 | -0.328 | 0.7451997 | 0.933822 |
| X220842_at | 0.0022 | 0.0068 | 0.328 | 0.7452475 | 0.933829 |
| X201279_s_at | 0.0031 | 0.0094 | 0.328 | 0.7452939 | 0.933835 |
| X213006_at | -0.0029 | 0.0089 | -0.328 | 0.7453914 | 0.933904 |
| X220665_at | 0.0015 | 0.0047 | 0.328 | 0.7454453 | 0.933919 |
| X205351_at | -0.0168 | 0.0513 | -0.327 | 0.7455405 | 0.933944 |
| X205791_x_at | -0.002 | 0.006 | -0.327 | 0.7455767 | 0.933944 |
| X218425_at | 0.0137 | 0.0417 | 0.327 | 0.745598 | 0.933944 |
| X207214_at | 0.0013 | 0.0041 | 0.327 | 0.7456548 | 0.933944 |
| X207161_at | -0.0023 | 0.0071 | -0.327 | 0.7456751 | 0.933944 |
| X212714_at | 0.0094 | 0.0286 | 0.327 | 0.7457654 | 0.934003 |
| X207815_at | 0.0053 | 0.0161 | 0.327 | 0.745864 | 0.934003 |
| X201387_s_at | -0.0129 | 0.0396 | -0.327 | 0.7458699 | 0.934003 |
| X207127_s_at | -0.014 | 0.0427 | -0.327 | 0.7458903 | 0.934003 |
| X202610_s_at | 0.0143 | 0.0437 | 0.327 | 0.7459322 | 0.934003 |
| X216878_x_at | -0.0017 | 0.0053 | -0.326 | 0.7463573 | 0.934364 |
| X206807_s_at | 0.0021 | 0.0065 | 0.326 | 0.7463701 | 0.934364 |
| X220469_at | -0.0012 | 0.0036 | -0.326 | 0.7464154 | 0.934364 |
| X204471_at | 0.0166 | 0.0509 | 0.326 | 0.7464288 | 0.934364 |
| X210084_x_at | 0.0013 | 0.004 | 0.326 | 0.7464467 | 0.934364 |
| X210719_s_at | 0.0251 | 0.077 | 0.326 | 0.7464735 | 0.934364 |
| X217859_s_at | -0.0013 | 0.0039 | -0.326 | 0.7465712 | 0.934382 |
| X215470_at | -0.0499 | 0.1531 | -0.326 | 0.7465839 | 0.934382 |
| X220454_s_at | 0.0017 | 0.0051 | 0.326 | 0.7466315 | 0.934382 |
| X221389_at | -0.0008 | 0.0025 | -0.326 | 0.746671 | 0.934382 |
| X206804_at | -0.0011 | 0.0033 | -0.326 | 0.7467278 | 0.934382 |
| X217422_s_at | -0.0011 | 0.0033 | -0.326 | 0.7467397 | 0.934382 |
| X204893_s_at | -0.0137 | 0.042 | -0.326 | 0.7468764 | 0.934477 |
| X212786_at | 0.0012 | 0.0038 | 0.325 | 0.7469911 | 0.934477 |
| X210272_at | -0.0035 | 0.0106 | -0.325 | 0.7470354 | 0.934477 |
| X208193_at | -0.0015 | 0.0047 | -0.325 | 0.7470483 | 0.934477 |
| X210659_at | 0.0013 | 0.004 | 0.325 | 0.7471015 | 0.934477 |
| X205535_s_at | -0.0018 | 0.0057 | -0.325 | 0.7471693 | 0.934477 |
| X216365_x_at | -0.0018 | 0.0056 | -0.325 | 0.7471782 | 0.934477 |
| X204467_s_at | 0.0199 | 0.0612 | 0.325 | 0.7472025 | 0.934477 |
| X206720_at | -0.0011 | 0.0033 | -0.325 | 0.7472209 | 0.934477 |
| X220550_at | -0.0026 | 0.0081 | -0.325 | 0.7472857 | 0.934477 |
| X218051_s_at | 0.0009 | 0.0027 | 0.325 | 0.7472894 | 0.934477 |
| X210500_at | -0.0024 | 0.0074 | -0.325 | 0.7473309 | 0.934477 |
| X200044_at | 0.0057 | 0.0175 | 0.325 | 0.7473625 | 0.934477 |
| X203806_s_at | 0.0022 | 0.0066 | 0.325 | 0.7474621 | 0.934549 |
| X205845_at | 0.0015 | 0.0047 | 0.325 | 0.7475925 | 0.934659 |
| X207833_s_at | -0.0009 | 0.0029 | -0.324 | 0.7478145 | 0.934878 |
| X210637_at | 0.0015 | 0.0047 | 0.324 | 0.7478517 | 0.934878 |
| X208496_x_at | -0.0011 | 0.0034 | -0.324 | 0.7480008 | 0.934979 |
| X209616_s_at | 0.0026 | 0.0079 | 0.324 | 0.7480167 | 0.934979 |
| X221244_s_at | 0.0013 | 0.0039 | 0.324 | 0.7480966 | 0.934995 |
| X221695_s_at | -0.0015 | 0.0047 | -0.324 | 0.7481536 | 0.934995 |
| X211684_s_at | 0.0138 | 0.0426 | 0.324 | 0.7481558 | 0.934995 |
| X208029_s_at | 0.0212 | 0.0656 | 0.324 | 0.7482131 | 0.935014 |
| X216901_s_at | -0.0014 | 0.0042 | -0.324 | 0.7482716 | 0.935034 |
| X213268_at | 0.0159 | 0.0492 | 0.324 | 0.748441 | 0.935193 |
| X211575_s_at | -0.0188 | 0.0582 | -0.323 | 0.7485324 | 0.935255 |
| X217667_at | -0.0042 | 0.0131 | -0.323 | 0.7486297 | 0.935265 |
| X215594_at | 0.0014 | 0.0043 | 0.323 | 0.7486379 | 0.935265 |
| X222257_s_at | 0.0018 | 0.0055 | 0.323 | 0.7486665 | 0.935265 |
| X204800_s_at | -0.0058 | 0.0179 | -0.323 | 0.7487737 | 0.935304 |
| X205042_at | -0.0135 | 0.0417 | -0.323 | 0.7487823 | 0.935304 |
| X218811_at | -0.001 | 0.0032 | -0.323 | 0.7488464 | 0.935332 |
| X209173_at | 0.0012 | 0.0036 | 0.323 | 0.7489073 | 0.935344 |
| X221170_at | -0.0023 | 0.0073 | -0.323 | 0.7489621 | 0.935344 |
| X212446_s_at | -0.0152 | 0.0471 | -0.323 | 0.7489827 | 0.935344 |
| X202809_s_at | -0.0143 | 0.0443 | -0.323 | 0.7490804 | 0.935409 |
| X211820_x_at | -0.0021 | 0.0064 | -0.323 | 0.7491187 | 0.935409 |
| X221477_s_at | 0.0257 | 0.0797 | 0.322 | 0.7493428 | 0.935596 |
| X205306_x_at | 0.0015 | 0.0046 | 0.322 | 0.7493526 | 0.935596 |
| X209656_s_at | -0.0149 | 0.0463 | -0.322 | 0.7494678 | 0.935684 |
| X213180_s_at | -0.0193 | 0.06 | -0.322 | 0.7495165 | 0.935684 |
| X210951_x_at | 0.0018 | 0.0057 | 0.322 | 0.74955 | 0.935684 |
| X203263_s_at | -0.0269 | 0.0837 | -0.322 | 0.7498215 | 0.935962 |
| X216725_at | 0.0016 | 0.005 | 0.322 | 0.7498569 | 0.935962 |
| X213987_s_at | 0.0014 | 0.0043 | 0.321 | 0.7500285 | 0.936012 |
| X207570_at | 0.0019 | 0.0059 | 0.321 | 0.7500411 | 0.936012 |
| X219518_s_at | -0.0013 | 0.0041 | -0.321 | 0.7500753 | 0.936012 |
| X215705_at | 0.0016 | 0.005 | 0.321 | 0.7500945 | 0.936012 |
| X210134_x_at | -0.0011 | 0.0033 | -0.321 | 0.7501217 | 0.936012 |
| X207341_at | 0.0012 | 0.0037 | 0.321 | 0.7501498 | 0.936012 |
| X200888_s_at | 0.0103 | 0.0322 | 0.321 | 0.7503934 | 0.936222 |
| X221256_s_at | -0.0022 | 0.007 | -0.321 | 0.7504017 | 0.936222 |
| X218611_at | -0.0162 | 0.0506 | -0.321 | 0.7504863 | 0.936234 |
| X207525_s_at | 0.0047 | 0.0147 | 0.321 | 0.750496 | 0.936234 |
| X204693_at | 0.0015 | 0.0046 | 0.321 | 0.7505406 | 0.936237 |
| X205977_s_at | 0.0006 | 0.002 | 0.321 | 0.7506474 | 0.936261 |
| X210703_at | -0.0055 | 0.0172 | -0.321 | 0.7506841 | 0.936261 |
| X213480_at | 0.0088 | 0.0274 | 0.321 | 0.7507374 | 0.936261 |
| X215200_x_at | 0.0025 | 0.0079 | 0.32 | 0.750837 | 0.936261 |
| X204304_s_at | -0.0284 | 0.0886 | -0.32 | 0.7508888 | 0.936261 |
| X220799_at | 0.0027 | 0.0084 | 0.32 | 0.750905 | 0.936261 |
| X220468_at | -0.0033 | 0.0103 | -0.32 | 0.7509314 | 0.936261 |
| X208848_at | -0.0174 | 0.0543 | -0.32 | 0.750965 | 0.936261 |
| X212173_at | -0.0012 | 0.0038 | -0.32 | 0.7509653 | 0.936261 |
| X216630_at | 0.0017 | 0.0053 | 0.32 | 0.7509809 | 0.936261 |
| X209483_s_at | 0.0055 | 0.0171 | 0.32 | 0.7510662 | 0.936265 |
| X209693_at | 0.0113 | 0.0353 | 0.32 | 0.7511058 | 0.936265 |
| X217531_at | 0.0015 | 0.0047 | 0.32 | 0.7512023 | 0.936265 |
| X200740_s_at | 0.0088 | 0.0274 | 0.32 | 0.7512024 | 0.936265 |
| X201294_s_at | 0.0675 | 0.211 | 0.32 | 0.7512333 | 0.936265 |
| X205879_x_at | -0.0013 | 0.0041 | -0.32 | 0.7512377 | 0.936265 |
| X219944_at | -0.0018 | 0.0056 | -0.32 | 0.7513303 | 0.936328 |
| X220240_s_at | -0.0011 | 0.0034 | -0.32 | 0.7514435 | 0.93637 |
| X203764_at | -0.0026 | 0.0081 | -0.32 | 0.7514483 | 0.93637 |
| X215460_x_at | 0.0045 | 0.0142 | 0.319 | 0.751556 | 0.936425 |
| X206137_at | 0.0136 | 0.0424 | 0.319 | 0.7516153 | 0.936425 |
| X217276_x_at | -0.0008 | 0.0026 | -0.319 | 0.7516385 | 0.936425 |
| X222297_x_at | 0.0086 | 0.0271 | 0.319 | 0.7516608 | 0.936425 |
| X220861_at | -0.0013 | 0.004 | -0.319 | 0.7519056 | 0.936649 |
| X215042_at | 0.0006 | 0.0019 | 0.319 | 0.7519248 | 0.936649 |
| X215503_at | 0.0014 | 0.0043 | 0.319 | 0.7521135 | 0.936831 |
| X215834_x_at | -0.0011 | 0.0035 | -0.319 | 0.7521906 | 0.936853 |
| X215064_at | -0.0012 | 0.0037 | -0.319 | 0.7522152 | 0.936853 |
| X220554_at | 0.0034 | 0.0108 | 0.318 | 0.7524124 | 0.937046 |
| X220955_x_at | 0.0016 | 0.005 | 0.318 | 0.7524796 | 0.937077 |
| X221450_x_at | 0.0018 | 0.0057 | 0.318 | 0.7525262 | 0.937082 |
| X211259_s_at | -0.001 | 0.003 | -0.318 | 0.7525681 | 0.937082 |
| X214537_at | 0.0013 | 0.0042 | 0.318 | 0.7527903 | 0.937206 |
| X218419_s_at | -0.0036 | 0.0114 | -0.318 | 0.7527936 | 0.937206 |
| X37793_r_at | -0.0061 | 0.0191 | -0.318 | 0.7528117 | 0.937206 |
| X202791_s_at | -0.0062 | 0.0194 | -0.318 | 0.7528359 | 0.937206 |
| X212059_s_at | -0.0019 | 0.006 | -0.318 | 0.7529366 | 0.937278 |
| X214866_at | 0.0019 | 0.0059 | 0.318 | 0.7529883 | 0.93729 |
| X208209_s_at | 0.0014 | 0.0046 | 0.317 | 0.7530788 | 0.93735 |
| X220297_at | 0.0022 | 0.0068 | 0.317 | 0.7532061 | 0.937451 |
| X216876_s_at | -0.0033 | 0.0103 | -0.317 | 0.7532442 | 0.937451 |
| X208459_s_at | -0.0039 | 0.0122 | -0.317 | 0.7534001 | 0.937593 |
| X208053_at | 0.0012 | 0.0039 | 0.317 | 0.7535341 | 0.937665 |
| X221526_x_at | 0.0202 | 0.0637 | 0.317 | 0.7535482 | 0.937665 |
| X219385_at | -0.0009 | 0.0028 | -0.317 | 0.7535849 | 0.937665 |
| X214625_s_at | 0.0016 | 0.0049 | 0.317 | 0.7536332 | 0.937673 |
| X216811_at | -0.0016 | 0.0051 | -0.316 | 0.7538871 | 0.937936 |
| X206702_at | 0.0109 | 0.0346 | 0.316 | 0.7540283 | 0.938008 |
| X220406_at | 0.0011 | 0.0035 | 0.316 | 0.7540295 | 0.938008 |
| X221184_at | -0.0037 | 0.0117 | -0.316 | 0.754154 | 0.9381 |
| X213731_s_at | -0.0008 | 0.0026 | -0.316 | 0.7542244 | 0.9381 |
| X217939_s_at | -0.0083 | 0.0263 | -0.316 | 0.7542906 | 0.9381 |
| X218067_s_at | 0.0138 | 0.0436 | 0.316 | 0.7543018 | 0.9381 |
| X220228_at | -0.0015 | 0.0048 | -0.316 | 0.7543564 | 0.9381 |
| X221995_s_at | 0.0233 | 0.0739 | 0.316 | 0.7543931 | 0.9381 |
| X219168_s_at | 0.0008 | 0.0026 | 0.316 | 0.7543988 | 0.9381 |
| X203117_s_at | -0.0207 | 0.0655 | -0.315 | 0.7545382 | 0.93814 |
| X210905_x_at | 0.0012 | 0.004 | 0.315 | 0.7545769 | 0.93814 |
| X220451_s_at | 0.002 | 0.0063 | 0.315 | 0.7545924 | 0.93814 |
| X214721_x_at | -0.0172 | 0.0546 | -0.315 | 0.7545999 | 0.93814 |
| X210924_at | 0.0013 | 0.0043 | 0.315 | 0.7546542 | 0.938155 |
| X218676_s_at | 0.0177 | 0.0562 | 0.315 | 0.7547194 | 0.938183 |
| X204713_s_at | -0.0016 | 0.0049 | -0.315 | 0.7548703 | 0.938318 |
| X213949_s_at | 0.0012 | 0.0039 | 0.315 | 0.7549945 | 0.93842 |
| X202220_at | -0.0103 | 0.0328 | -0.315 | 0.7550716 | 0.938464 |
| X208408_at | 0.0014 | 0.0044 | 0.315 | 0.7552211 | 0.938597 |
| X221374_at | -0.0016 | 0.005 | -0.314 | 0.7552904 | 0.938631 |
| X216947_at | -0.0049 | 0.0155 | -0.314 | 0.755385 | 0.938685 |
| X214212_x_at | 0.0234 | 0.0745 | 0.314 | 0.7554186 | 0.938685 |
| X206408_at | -0.0123 | 0.0391 | -0.314 | 0.7557118 | 0.938997 |
| X213345_at | -0.0024 | 0.0076 | -0.314 | 0.7558645 | 0.939061 |
| X219917_at | -0.0028 | 0.009 | -0.314 | 0.7559416 | 0.939061 |
| X220194_at | -0.0016 | 0.0051 | -0.313 | 0.7560164 | 0.939061 |
| X210917_at | -0.0023 | 0.0075 | -0.313 | 0.7560186 | 0.939061 |
| X211746_x_at | -0.0079 | 0.0253 | -0.313 | 0.7560247 | 0.939061 |
| X36936_at | -0.0068 | 0.0216 | -0.313 | 0.7561183 | 0.939061 |
| X221306_at | -0.0148 | 0.0473 | -0.313 | 0.7561456 | 0.939061 |
| X220865_s_at | -0.015 | 0.0479 | -0.313 | 0.7561511 | 0.939061 |
| X64488_at | 0.0151 | 0.0481 | 0.313 | 0.7562573 | 0.939061 |
| X209805_at | 0.0081 | 0.0258 | 0.313 | 0.7562608 | 0.939061 |
| X215019_x_at | 0.01 | 0.032 | 0.313 | 0.7562743 | 0.939061 |
| X208033_s_at | 0.0012 | 0.0039 | 0.313 | 0.7562801 | 0.939061 |
| X215875_at | 0.0012 | 0.004 | 0.313 | 0.7563354 | 0.939061 |
| X207242_s_at | -0.0153 | 0.049 | -0.313 | 0.7563744 | 0.939061 |
| X211166_at | -0.0015 | 0.0047 | -0.313 | 0.7563975 | 0.939061 |
| X205742_at | 0.0021 | 0.0069 | 0.313 | 0.7565525 | 0.93919 |
| X210173_at | -0.0019 | 0.0062 | -0.313 | 0.7566814 | 0.93919 |
| X206358_at | -0.001 | 0.0034 | -0.313 | 0.7567512 | 0.93919 |
| X200983_x_at | -0.0227 | 0.0725 | -0.312 | 0.7567873 | 0.93919 |
| X218574_s_at | 0.0045 | 0.0145 | 0.312 | 0.756855 | 0.93919 |
| X206297_at | 0.0019 | 0.0061 | 0.312 | 0.756859 | 0.93919 |
| X211097_s_at | 0.0063 | 0.0201 | 0.312 | 0.7568705 | 0.93919 |
| X215036_at | 0.0015 | 0.0048 | 0.312 | 0.7569079 | 0.93919 |
| X209586_s_at | -0.0129 | 0.0412 | -0.312 | 0.756955 | 0.93919 |
| X220790_s_at | 0.0015 | 0.0047 | 0.312 | 0.756965 | 0.93919 |
| X203128_at | 0.027 | 0.0864 | 0.312 | 0.7569685 | 0.93919 |
| X201980_s_at | -0.0136 | 0.0436 | -0.312 | 0.7570688 | 0.93919 |
| X219258_at | -0.028 | 0.0897 | -0.312 | 0.7570965 | 0.93919 |
| X201057_s_at | -0.0178 | 0.0572 | -0.312 | 0.7571109 | 0.93919 |
| X212328_at | 0.0149 | 0.0477 | 0.312 | 0.7572116 | 0.93919 |
| X205847_at | -0.0014 | 0.0045 | -0.312 | 0.7573308 | 0.93919 |
| X220155_s_at | -0.0126 | 0.0405 | -0.312 | 0.7573765 | 0.93919 |
| X215865_at | 0.0009 | 0.003 | 0.312 | 0.7573889 | 0.93919 |
| X215639_at | 0.0014 | 0.0044 | 0.312 | 0.7574487 | 0.93919 |
| X219173_at | 0.0017 | 0.0053 | 0.312 | 0.757487 | 0.93919 |
| X211556_at | -0.0023 | 0.0075 | -0.311 | 0.7575755 | 0.93919 |
| X209604_s_at | -0.0015 | 0.0049 | -0.311 | 0.7576224 | 0.93919 |
| X205032_at | 0.0019 | 0.0062 | 0.311 | 0.7576236 | 0.93919 |
| X214941_s_at | -0.0017 | 0.0055 | -0.311 | 0.7576768 | 0.93919 |
| X35666_at | 0.0129 | 0.0414 | 0.311 | 0.7577009 | 0.93919 |
| X206163_at | 0.0015 | 0.0047 | 0.311 | 0.7577256 | 0.93919 |
| X204938_s_at | -0.0014 | 0.0046 | -0.311 | 0.7577266 | 0.93919 |
| X200964_at | -0.015 | 0.0484 | -0.311 | 0.7577786 | 0.93919 |
| X205989_s_at | -0.0495 | 0.1591 | -0.311 | 0.7577874 | 0.93919 |
| X220283_at | 0.001 | 0.0033 | 0.311 | 0.7578335 | 0.93919 |
| X205878_at | 0.0128 | 0.0412 | 0.311 | 0.757845 | 0.93919 |
| X204568_at | -0.0129 | 0.0414 | -0.311 | 0.7578549 | 0.93919 |
| X206813_at | 0.0011 | 0.0035 | 0.311 | 0.7579738 | 0.939243 |
| X216545_at | -0.0014 | 0.0045 | -0.311 | 0.7579816 | 0.939243 |
| X206194_at | 0.0029 | 0.0093 | 0.311 | 0.7582135 | 0.939314 |
| X215956_at | -0.0083 | 0.0267 | -0.31 | 0.7583056 | 0.939314 |
| X215264_at | 0.0009 | 0.0029 | 0.31 | 0.7583084 | 0.939314 |
| X202230_s_at | 0.0068 | 0.0219 | 0.31 | 0.7584329 | 0.939314 |
| X216249_at | 0.0018 | 0.0057 | 0.31 | 0.7585273 | 0.939314 |
| X205347_s_at | 0.0021 | 0.0068 | 0.31 | 0.7585477 | 0.939314 |
| X216679_at | 0.0022 | 0.0071 | 0.31 | 0.7585725 | 0.939314 |
| X207520_at | 0.0029 | 0.0094 | 0.31 | 0.7586455 | 0.939314 |
| X212248_at | 0.0119 | 0.0382 | 0.31 | 0.758657 | 0.939314 |
| X207477_at | 0.0023 | 0.0073 | 0.31 | 0.7588063 | 0.939314 |
| X213377_x_at | -0.0112 | 0.0361 | -0.31 | 0.758813 | 0.939314 |
| X218891_at | 0.0014 | 0.0046 | 0.31 | 0.7588322 | 0.939314 |
| X213739_at | -0.0051 | 0.0164 | -0.31 | 0.758862 | 0.939314 |
| X211613_s_at | 0.0011 | 0.0036 | 0.31 | 0.7588658 | 0.939314 |
| X215817_at | 0.0017 | 0.0053 | 0.31 | 0.7589303 | 0.939314 |
| X41512_at | 0.0104 | 0.0338 | 0.31 | 0.7589707 | 0.939314 |
| X211388_s_at | 0.0011 | 0.0035 | 0.31 | 0.7589722 | 0.939314 |
| X210405_x_at | 0.0008 | 0.0026 | 0.309 | 0.7590654 | 0.939314 |
| X208256_at | 0.0023 | 0.0073 | 0.309 | 0.7591369 | 0.939314 |
| X217828_at | 0.0065 | 0.0209 | 0.309 | 0.7591457 | 0.939314 |
| X218090_s_at | -0.0115 | 0.0371 | -0.309 | 0.759165 | 0.939314 |
| X210440_s_at | 0.0014 | 0.0044 | 0.309 | 0.7591793 | 0.939314 |
| X213525_at | -0.0014 | 0.0045 | -0.309 | 0.7592923 | 0.939314 |
| X215843_s_at | 0.0013 | 0.0043 | 0.309 | 0.7593025 | 0.939314 |
| X211726_s_at | -0.0023 | 0.0073 | -0.309 | 0.7593661 | 0.939314 |
| X211579_at | -0.0026 | 0.0083 | -0.309 | 0.7595549 | 0.939314 |
| X213235_at | 0.0441 | 0.143 | 0.309 | 0.7596359 | 0.939314 |
| X214207_s_at | 0.0014 | 0.0044 | 0.309 | 0.7596387 | 0.939314 |
| X215887_at | -0.0047 | 0.0153 | -0.309 | 0.7597385 | 0.939314 |
| X217070_at | -0.0022 | 0.0072 | -0.309 | 0.7597485 | 0.939314 |
| X209188_x_at | -0.0139 | 0.0449 | -0.309 | 0.7597519 | 0.939314 |
| X218566_s_at | 0.0145 | 0.047 | 0.308 | 0.7597752 | 0.939314 |
| X215507_x_at | 0.0072 | 0.0232 | 0.308 | 0.7598194 | 0.939314 |
| X210669_at | -0.0017 | 0.0056 | -0.308 | 0.7598874 | 0.939314 |
| X91920_at | 0.0013 | 0.0043 | 0.308 | 0.7599076 | 0.939314 |
| X219965_s_at | 0.0033 | 0.0106 | 0.308 | 0.7600421 | 0.939314 |
| X219792_at | 0.0024 | 0.0078 | 0.308 | 0.7600638 | 0.939314 |
| X211120_x_at | -0.0012 | 0.004 | -0.308 | 0.7600892 | 0.939314 |
| X204409_s_at | 0.0182 | 0.0591 | 0.308 | 0.76012 | 0.939314 |
| X212205_at | 0.0073 | 0.0238 | 0.308 | 0.7601534 | 0.939314 |
| X212104_s_at | -0.0172 | 0.0559 | -0.308 | 0.7601905 | 0.939314 |
| X222131_x_at | 0.0013 | 0.0043 | 0.308 | 0.7602646 | 0.939314 |
| X218715_at | -0.009 | 0.0294 | -0.308 | 0.7604958 | 0.939314 |
| X216713_at | -0.0084 | 0.0274 | -0.308 | 0.7605059 | 0.939314 |
| X206616_s_at | 0.0023 | 0.0074 | 0.307 | 0.7605222 | 0.939314 |
| X217698_at | 0.0037 | 0.0121 | 0.307 | 0.7605504 | 0.939314 |
| X210480_s_at | -0.0022 | 0.0072 | -0.307 | 0.7605742 | 0.939314 |
| X211966_at | 0.0008 | 0.0026 | 0.307 | 0.7605996 | 0.939314 |
| X210571_s_at | 0.0017 | 0.0054 | 0.307 | 0.7606481 | 0.939314 |
| X215098_at | 0.0015 | 0.0049 | 0.307 | 0.7607803 | 0.939314 |
| X211101_x_at | -0.0012 | 0.0039 | -0.307 | 0.7607946 | 0.939314 |
| X205079_s_at | -0.0136 | 0.0441 | -0.307 | 0.7608476 | 0.939314 |
| X216464_x_at | 0.001 | 0.0033 | 0.307 | 0.760952 | 0.939314 |
| X221624_at | -0.0014 | 0.0045 | -0.307 | 0.7610302 | 0.939314 |
| X202444_s_at | -0.0011 | 0.0035 | -0.307 | 0.7610478 | 0.939314 |
| X205966_at | 0.0297 | 0.0968 | 0.307 | 0.761062 | 0.939314 |
| X220754_at | -0.0032 | 0.0105 | -0.307 | 0.7610628 | 0.939314 |
| X206549_at | 0.0012 | 0.0038 | 0.307 | 0.7611217 | 0.939314 |
| X213426_s_at | 0.0011 | 0.0035 | 0.307 | 0.7611294 | 0.939314 |
| X221476_s_at | -0.0066 | 0.0216 | -0.307 | 0.7611566 | 0.939314 |
| X219529_at | -0.001 | 0.0032 | -0.307 | 0.7611743 | 0.939314 |
| X212667_at | 0.0211 | 0.0688 | 0.307 | 0.7611761 | 0.939314 |
| X216506_x_at | -0.0009 | 0.0029 | -0.307 | 0.7611802 | 0.939314 |
| X206190_at | 0.0334 | 0.1091 | 0.307 | 0.7611858 | 0.939314 |
| X203076_s_at | 0.0102 | 0.0333 | 0.307 | 0.7612145 | 0.939314 |
| X37462_i_at | 0.001 | 0.0032 | 0.307 | 0.7612398 | 0.939314 |
| X204597_x_at | 0.002 | 0.0064 | 0.307 | 0.7612559 | 0.939314 |
| X201273_s_at | 0.0125 | 0.0407 | 0.306 | 0.761275 | 0.939314 |
| X201819_at | -0.0037 | 0.0122 | -0.306 | 0.7613246 | 0.939314 |
| X211698_at | -0.0118 | 0.0386 | -0.306 | 0.7613609 | 0.939314 |
| X211642_at | -0.0019 | 0.0063 | -0.306 | 0.7614799 | 0.939314 |
| X202908_at | 0.0375 | 0.1224 | 0.306 | 0.7615097 | 0.939314 |
| X214053_at | 0.0153 | 0.0499 | 0.306 | 0.7615261 | 0.939314 |
| X216986_s_at | -0.0011 | 0.0036 | -0.306 | 0.7615273 | 0.939314 |
| X205552_s_at | 0.002 | 0.0066 | 0.306 | 0.7615418 | 0.939314 |
| X211908_x_at | 0.0016 | 0.0053 | 0.306 | 0.7615421 | 0.939314 |
| X202194_at | 0.0203 | 0.0663 | 0.306 | 0.7615712 | 0.939314 |
| X59375_at | -0.0015 | 0.005 | -0.306 | 0.7616606 | 0.939314 |
| X221204_s_at | -0.0021 | 0.007 | -0.306 | 0.7616616 | 0.939314 |
| X201674_s_at | 0.0149 | 0.0488 | 0.306 | 0.7616886 | 0.939314 |
| X217477_at | -0.0014 | 0.0047 | -0.306 | 0.7617278 | 0.939314 |
| X217385_at | -0.0025 | 0.008 | -0.306 | 0.7617631 | 0.939314 |
| X216716_at | 0.0023 | 0.0077 | 0.306 | 0.7617838 | 0.939314 |
| X218762_at | 0.0021 | 0.0067 | 0.306 | 0.761805 | 0.939314 |
| X205394_at | -0.0034 | 0.0112 | -0.306 | 0.7618558 | 0.939314 |
| X206845_s_at | -0.0028 | 0.0093 | -0.306 | 0.7619243 | 0.939314 |
| X213168_at | 0.0106 | 0.0346 | 0.306 | 0.7619911 | 0.939314 |
| X216279_at | -0.0017 | 0.0054 | -0.305 | 0.7620355 | 0.939314 |
| X220095_at | -0.0018 | 0.0057 | -0.305 | 0.7620955 | 0.939314 |
| X218416_s_at | 0.0016 | 0.0053 | 0.305 | 0.7621006 | 0.939314 |
| X218698_at | -0.0247 | 0.0808 | -0.305 | 0.7621766 | 0.939314 |
| X213711_at | -0.0023 | 0.0074 | -0.305 | 0.7622598 | 0.939314 |
| X217030_at | -0.0029 | 0.0094 | -0.305 | 0.7622855 | 0.939314 |
| X213769_at | 0.0014 | 0.0047 | 0.305 | 0.7622872 | 0.939314 |
| X214240_at | -0.0011 | 0.0035 | -0.305 | 0.7623512 | 0.939314 |
| X204698_at | 0.0009 | 0.0031 | 0.305 | 0.7624145 | 0.939314 |
| X201958_s_at | 0.0012 | 0.004 | 0.305 | 0.7624533 | 0.939314 |
| X206344_at | 0.0024 | 0.008 | 0.305 | 0.7624638 | 0.939314 |
| X219606_at | -0.0028 | 0.0092 | -0.305 | 0.7624772 | 0.939314 |
| X217189_s_at | 0.0016 | 0.0053 | 0.305 | 0.7625049 | 0.939314 |
| X210658_s_at | -0.002 | 0.0066 | -0.305 | 0.7625319 | 0.939314 |
| X210313_at | 0.0208 | 0.0681 | 0.305 | 0.7625436 | 0.939314 |
| X202083_s_at | 0.0217 | 0.0712 | 0.305 | 0.7625746 | 0.939314 |
| X204116_at | -0.0024 | 0.0077 | -0.305 | 0.7626036 | 0.939314 |
| X221168_at | -0.0024 | 0.0079 | -0.305 | 0.7626547 | 0.939314 |
| X216451_at | -0.0017 | 0.0057 | -0.305 | 0.7626769 | 0.939314 |
| X209587_at | -0.0007 | 0.0024 | -0.305 | 0.7627522 | 0.939314 |
| X207309_at | -0.0019 | 0.0063 | -0.304 | 0.7628185 | 0.939314 |
| X213299_at | -0.0016 | 0.0052 | -0.304 | 0.7628445 | 0.939314 |
| X216763_at | -0.0019 | 0.0063 | -0.304 | 0.7628695 | 0.939314 |
| X214967_at | -0.0019 | 0.0063 | -0.304 | 0.7628714 | 0.939314 |
| X219943_s_at | 0.0016 | 0.0054 | 0.304 | 0.7628896 | 0.939314 |
| X208335_s_at | -0.0021 | 0.007 | -0.304 | 0.762924 | 0.939314 |
| X211818_s_at | 0.0009 | 0.0029 | 0.304 | 0.7629561 | 0.939314 |
| X215534_at | -0.001 | 0.0033 | -0.304 | 0.7629787 | 0.939314 |
| X218417_s_at | 0.0137 | 0.0449 | 0.304 | 0.762986 | 0.939314 |
| X218842_at | 0.0141 | 0.0464 | 0.304 | 0.7630232 | 0.939314 |
| X207043_s_at | 0.0011 | 0.0035 | 0.304 | 0.7630824 | 0.939314 |
| X40489_at | 0.001 | 0.0031 | 0.304 | 0.7630829 | 0.939314 |
| X206933_s_at | 0.0008 | 0.0027 | 0.304 | 0.7631395 | 0.939314 |
| X205556_at | -0.003 | 0.0099 | -0.304 | 0.7631553 | 0.939314 |
| X206766_at | -0.0033 | 0.0108 | -0.304 | 0.7634255 | 0.939594 |
| X210283_x_at | -0.0088 | 0.0289 | -0.303 | 0.7635475 | 0.939667 |
| X216669_at | -0.0023 | 0.0076 | -0.303 | 0.7636709 | 0.939667 |
| X213013_at | 0.0069 | 0.0228 | 0.303 | 0.763731 | 0.939667 |
| X214932_at | -0.002 | 0.0067 | -0.303 | 0.7637338 | 0.939667 |
| X207977_s_at | -0.0013 | 0.0044 | -0.303 | 0.7637959 | 0.939667 |
| X207954_at | 0.0016 | 0.0054 | 0.303 | 0.7638096 | 0.939667 |
| X40829_at | -0.006 | 0.0198 | -0.303 | 0.763846 | 0.939667 |
| X211428_at | -0.0018 | 0.006 | -0.303 | 0.7638593 | 0.939667 |
| X211832_s_at | -0.0027 | 0.0089 | -0.303 | 0.7638978 | 0.939667 |
| X214539_at | -0.0019 | 0.0063 | -0.303 | 0.763908 | 0.939667 |
| X204086_at | 0.0012 | 0.0038 | 0.303 | 0.7640192 | 0.939712 |
| X213269_at | 0.017 | 0.0561 | 0.303 | 0.7641098 | 0.939712 |
| X218483_s_at | 0.0088 | 0.0292 | 0.303 | 0.7641194 | 0.939712 |
| X43544_at | -0.019 | 0.0629 | -0.303 | 0.7642165 | 0.939712 |
| X218070_s_at | -0.0016 | 0.0052 | -0.303 | 0.7642266 | 0.939712 |
| X211371_at | 0.0017 | 0.0055 | 0.303 | 0.764269 | 0.939712 |
| X202541_at | 0.0135 | 0.0448 | 0.302 | 0.764299 | 0.939712 |
| X205844_at | -0.002 | 0.0065 | -0.302 | 0.7643239 | 0.939712 |
| X214001_x_at | 0.0575 | 0.1903 | 0.302 | 0.7643771 | 0.939712 |
| X212706_at | 0.0208 | 0.0688 | 0.302 | 0.7644057 | 0.939712 |
| X221314_at | 0.0012 | 0.0041 | 0.302 | 0.7644093 | 0.939712 |
| X220032_at | -0.0012 | 0.0041 | -0.302 | 0.7644998 | 0.93972 |
| X204903_x_at | 0.0185 | 0.0613 | 0.302 | 0.7645008 | 0.93972 |
| X208516_at | 0.0013 | 0.0042 | 0.302 | 0.7645743 | 0.939752 |
| X203154_s_at | 0.0017 | 0.0057 | 0.302 | 0.7646113 | 0.939752 |
| X216096_s_at | -0.0195 | 0.0644 | -0.302 | 0.7647362 | 0.939839 |
| X217542_at | 0.0045 | 0.0148 | 0.302 | 0.7647992 | 0.939839 |
| X207273_at | -0.0025 | 0.0081 | -0.302 | 0.7648344 | 0.939839 |
| X220385_at | -0.0004 | 0.0014 | -0.302 | 0.7648576 | 0.939839 |
| X205285_s_at | 0.0019 | 0.0064 | 0.302 | 0.7648934 | 0.939839 |
| X215094_at | 0.0027 | 0.0089 | 0.302 | 0.7649951 | 0.939887 |
| X216121_at | 0.0021 | 0.0068 | 0.301 | 0.7652242 | 0.939887 |
| X210527_x_at | -0.0048 | 0.0158 | -0.301 | 0.7652804 | 0.939887 |
| X216075_at | 0.0018 | 0.0058 | 0.301 | 0.7652839 | 0.939887 |
| X201340_s_at | 0.044 | 0.1462 | 0.301 | 0.7653413 | 0.939887 |
| X214550_s_at | 0.0176 | 0.0586 | 0.301 | 0.7653932 | 0.939887 |
| X204627_s_at | 0.0017 | 0.0057 | 0.301 | 0.7654108 | 0.939887 |
| X216969_s_at | -0.0029 | 0.0095 | -0.301 | 0.7654255 | 0.939887 |
| X220449_at | -0.0059 | 0.0197 | -0.301 | 0.7654451 | 0.939887 |
| X220607_x_at | 0.015 | 0.0499 | 0.301 | 0.7654533 | 0.939887 |
| X201292_at | -0.0035 | 0.0118 | -0.301 | 0.7654753 | 0.939887 |
| X32259_at | -0.009 | 0.03 | -0.301 | 0.765485 | 0.939887 |
| X204207_s_at | -0.003 | 0.0101 | -0.301 | 0.7654981 | 0.939887 |
| X216293_at | 0.0037 | 0.0122 | 0.301 | 0.7655748 | 0.939887 |
| X205826_at | -0.0068 | 0.0226 | -0.301 | 0.7655819 | 0.939887 |
| X203589_s_at | 0.0035 | 0.0118 | 0.301 | 0.76561 | 0.939887 |
| X200990_at | -0.0044 | 0.0145 | -0.301 | 0.7656775 | 0.939918 |
| X201200_at | -0.0082 | 0.0272 | -0.301 | 0.7657376 | 0.93994 |
| X215581_s_at | 0.0016 | 0.0052 | 0.3 | 0.7658815 | 0.940028 |
| X209928_s_at | -0.001 | 0.0032 | -0.3 | 0.7659321 | 0.940028 |
| X216774_at | -0.0012 | 0.0039 | -0.3 | 0.765984 | 0.940028 |
| X209348_s_at | 0.0272 | 0.0905 | 0.3 | 0.7660254 | 0.940028 |
| X214541_s_at | 0.0032 | 0.0107 | 0.3 | 0.766043 | 0.940028 |
| X220605_s_at | 0.0151 | 0.0503 | 0.3 | 0.766063 | 0.940028 |
| X215637_at | -0.0035 | 0.0117 | -0.3 | 0.7661141 | 0.940039 |
| X210164_at | -0.0016 | 0.0052 | -0.3 | 0.7662839 | 0.940177 |
| X214996_at | 0.0015 | 0.0049 | 0.3 | 0.7663115 | 0.940177 |
| X221975_s_at | 0.0017 | 0.0058 | 0.3 | 0.7665001 | 0.940306 |
| X220120_s_at | -0.001 | 0.0034 | -0.3 | 0.7665011 | 0.940306 |
| X208834_x_at | 0.0035 | 0.0116 | 0.299 | 0.7665599 | 0.940326 |
| X211670_x_at | 0.001 | 0.0032 | 0.299 | 0.7666872 | 0.940352 |
| X33148_at | -0.0463 | 0.1548 | -0.299 | 0.7666941 | 0.940352 |
| X220395_at | 0.0015 | 0.0049 | 0.299 | 0.7668047 | 0.940352 |
| X216239_at | 0.0018 | 0.0059 | 0.299 | 0.7668555 | 0.940352 |
| X219646_at | 0.0101 | 0.0337 | 0.299 | 0.7668703 | 0.940352 |
| X217138_x_at | 0.001 | 0.0035 | 0.299 | 0.766907 | 0.940352 |
| X220463_at | -0.0015 | 0.005 | -0.299 | 0.7669585 | 0.940352 |
| X217587_at | -0.0021 | 0.0069 | -0.299 | 0.7669848 | 0.940352 |
| X216635_at | -0.0022 | 0.0073 | -0.299 | 0.7670754 | 0.940352 |
| X209186_at | -0.0153 | 0.0514 | -0.299 | 0.7671191 | 0.940352 |
| X211588_s_at | 0.0016 | 0.0055 | 0.299 | 0.7671255 | 0.940352 |
| X220105_at | 0.001 | 0.0034 | 0.299 | 0.7672011 | 0.940352 |
| X213090_s_at | 0.014 | 0.0469 | 0.299 | 0.7672131 | 0.940352 |
| X215649_s_at | 0.004 | 0.0134 | 0.299 | 0.7672222 | 0.940352 |
| X216181_at | -0.0017 | 0.0057 | -0.299 | 0.7672378 | 0.940352 |
| X213409_s_at | -0.0173 | 0.0579 | -0.299 | 0.7672587 | 0.940352 |
| X202789_at | 0.0123 | 0.0413 | 0.299 | 0.7673073 | 0.940357 |
| X220086_at | -0.0017 | 0.0057 | -0.298 | 0.767347 | 0.940357 |
| X221028_s_at | -0.0045 | 0.015 | -0.298 | 0.7674118 | 0.940384 |
| X214980_at | -0.003 | 0.0099 | -0.298 | 0.7674853 | 0.940403 |
| X212630_at | 0.0202 | 0.0676 | 0.298 | 0.7675479 | 0.940403 |
| X206599_at | 0.0011 | 0.0036 | 0.298 | 0.767554 | 0.940403 |
| X205574_x_at | 0.001 | 0.0035 | 0.298 | 0.7676558 | 0.940476 |
| X216363_at | -0.0026 | 0.0087 | -0.298 | 0.7677047 | 0.940484 |
| X210587_at | -0.002 | 0.0067 | -0.298 | 0.7677897 | 0.940527 |
| X215755_at | -0.0019 | 0.0064 | -0.298 | 0.7678245 | 0.940527 |
| X216430_x_at | 0.0008 | 0.0027 | 0.298 | 0.7679183 | 0.940559 |
| X215345_x_at | 0.0028 | 0.0093 | 0.298 | 0.7680086 | 0.940559 |
| X207346_at | 0.0021 | 0.0071 | 0.298 | 0.7680239 | 0.940559 |
| X217272_s_at | -0.002 | 0.0068 | -0.297 | 0.7681977 | 0.940559 |
| X40665_at | 0.0028 | 0.0094 | 0.297 | 0.7682904 | 0.940559 |
| X209751_s_at | -0.0125 | 0.042 | -0.297 | 0.7683407 | 0.940559 |
| X207839_s_at | -0.0033 | 0.011 | -0.297 | 0.7683607 | 0.940559 |
| X210805_x_at | -0.0009 | 0.0029 | -0.297 | 0.7683707 | 0.940559 |
| X211729_x_at | 0.0154 | 0.052 | 0.297 | 0.7683719 | 0.940559 |
| X208013_s_at | -0.0017 | 0.0057 | -0.297 | 0.7684503 | 0.940559 |
| X215197_at | 0.0022 | 0.0074 | 0.297 | 0.7684726 | 0.940559 |
| X221792_at | -0.0129 | 0.0433 | -0.297 | 0.7684981 | 0.940559 |
| X65635_at | -0.008 | 0.0268 | -0.297 | 0.7685016 | 0.940559 |
| X212972_x_at | 0.0017 | 0.0059 | 0.297 | 0.7686088 | 0.940559 |
| X206978_at | 0.0014 | 0.0046 | 0.297 | 0.7686687 | 0.940559 |
| X211002_s_at | -0.0015 | 0.0049 | -0.297 | 0.7686862 | 0.940559 |
| X215822_x_at | 0.0014 | 0.0046 | 0.297 | 0.7687487 | 0.940559 |
| X205054_at | -0.0022 | 0.0075 | -0.297 | 0.7687769 | 0.940559 |
| X211042_x_at | 0.0089 | 0.03 | 0.297 | 0.7688128 | 0.940559 |
| X218808_at | -0.0044 | 0.015 | -0.296 | 0.768826 | 0.940559 |
| X211872_s_at | -0.0011 | 0.0039 | -0.296 | 0.7688407 | 0.940559 |
| X215360_at | -0.0027 | 0.0089 | -0.296 | 0.7688752 | 0.940559 |
| X201168_x_at | -0.0034 | 0.0115 | -0.296 | 0.768876 | 0.940559 |
| X210062_s_at | 0.0017 | 0.0058 | 0.296 | 0.7688853 | 0.940559 |
| X221509_at | 0.006 | 0.0204 | 0.296 | 0.7689089 | 0.940559 |
| X216529_at | 0.0028 | 0.0094 | 0.296 | 0.7689665 | 0.940566 |
| X204166_at | 0.0013 | 0.0045 | 0.296 | 0.7689999 | 0.940566 |
| X206295_at | 0.0094 | 0.0317 | 0.296 | 0.7690926 | 0.940574 |
| X207690_at | -0.0007 | 0.0023 | -0.296 | 0.7691082 | 0.940574 |
| X222295_x_at | 0.0055 | 0.0185 | 0.296 | 0.7691858 | 0.940574 |
| X217861_s_at | 0.0128 | 0.0434 | 0.296 | 0.7692057 | 0.940574 |
| X216661_x_at | 0.0016 | 0.0054 | 0.296 | 0.7692243 | 0.940574 |
| X207190_at | 0.0014 | 0.0047 | 0.296 | 0.7692987 | 0.940574 |
| X206208_at | -0.002 | 0.0068 | -0.296 | 0.7693028 | 0.940574 |
| X212843_at | -0.0146 | 0.0495 | -0.296 | 0.7694673 | 0.940724 |
| X213755_s_at | -0.0019 | 0.0066 | -0.295 | 0.7696458 | 0.94089 |
| X214358_at | 0.0015 | 0.005 | 0.295 | 0.7697822 | 0.941005 |
| X208958_at | -0.0127 | 0.043 | -0.295 | 0.7698743 | 0.941066 |
| X216741_at | 0.0012 | 0.0042 | 0.295 | 0.7700075 | 0.941153 |
| X210945_at | -0.0012 | 0.0042 | -0.295 | 0.7700716 | 0.941153 |
| X210861_s_at | -0.0029 | 0.0098 | -0.295 | 0.7700726 | 0.941153 |
| X208449_s_at | -0.0009 | 0.0031 | -0.295 | 0.7701197 | 0.941159 |
| X218804_at | -0.0024 | 0.0083 | -0.295 | 0.7701853 | 0.941187 |
| X208536_s_at | -0.0013 | 0.0044 | -0.295 | 0.7702594 | 0.941226 |
| X209390_at | -0.0059 | 0.0199 | -0.295 | 0.7703279 | 0.941253 |
| X205936_s_at | -0.0008 | 0.0029 | -0.294 | 0.7703667 | 0.941253 |
| X212454_x_at | 0.0094 | 0.032 | 0.294 | 0.7704263 | 0.941268 |
| X216758_at | -0.0043 | 0.0146 | -0.294 | 0.7704822 | 0.941268 |
| X212640_at | -0.0176 | 0.0598 | -0.294 | 0.7705458 | 0.941268 |
| X217582_at | 0.0048 | 0.0164 | 0.294 | 0.7705771 | 0.941268 |
| X217176_s_at | 0.0027 | 0.0093 | 0.294 | 0.7705907 | 0.941268 |
| X211255_x_at | 0.001 | 0.0034 | 0.294 | 0.7709458 | 0.941551 |
| X205759_s_at | 0.0016 | 0.0055 | 0.294 | 0.7709802 | 0.941551 |
| X219908_at | -0.0027 | 0.0093 | -0.293 | 0.7711728 | 0.941551 |
| X217148_x_at | 0.0008 | 0.0026 | 0.293 | 0.7711969 | 0.941551 |
| X213607_x_at | 0.0016 | 0.0056 | 0.293 | 0.7712354 | 0.941551 |
| X208741_at | 0.0157 | 0.0535 | 0.293 | 0.7712983 | 0.941551 |
| X211920_at | -0.0026 | 0.0087 | -0.293 | 0.7713345 | 0.941551 |
| X207766_at | 0.002 | 0.0067 | 0.293 | 0.7713377 | 0.941551 |
| X217441_at | 0.0009 | 0.0032 | 0.293 | 0.7713715 | 0.941551 |
| X206583_at | 0.013 | 0.0442 | 0.293 | 0.7714247 | 0.941551 |
| X207416_s_at | -0.004 | 0.0137 | -0.293 | 0.7714462 | 0.941551 |
| X205121_at | 0.0035 | 0.0119 | 0.293 | 0.7717088 | 0.941551 |
| X204433_s_at | 0.004 | 0.0136 | 0.293 | 0.7717196 | 0.941551 |
| X204751_x_at | 0.0025 | 0.0087 | 0.293 | 0.7717229 | 0.941551 |
| X222102_at | 0.0026 | 0.0088 | 0.293 | 0.771752 | 0.941551 |
| X208570_at | 0.0006 | 0.002 | 0.293 | 0.7717627 | 0.941551 |
| X207044_at | 0.0007 | 0.0023 | 0.293 | 0.7717746 | 0.941551 |
| X213694_at | -0.011 | 0.0376 | -0.293 | 0.7717767 | 0.941551 |
| X206771_at | 0.0009 | 0.003 | 0.293 | 0.7717795 | 0.941551 |
| X205572_at | -0.0039 | 0.0133 | -0.293 | 0.7717966 | 0.941551 |
| X203902_at | -0.0021 | 0.0073 | -0.293 | 0.7718256 | 0.941551 |
| X205782_at | -0.0118 | 0.0405 | -0.293 | 0.7718386 | 0.941551 |
| X212555_at | 0.0032 | 0.0111 | 0.292 | 0.7718706 | 0.941551 |
| X216068_at | -0.0044 | 0.015 | -0.292 | 0.7718723 | 0.941551 |
| X208929_x_at | 0.0107 | 0.0365 | 0.292 | 0.7718818 | 0.941551 |
| X218636_s_at | -0.0168 | 0.0576 | -0.292 | 0.7719736 | 0.941592 |
| X212434_at | 0.0064 | 0.0219 | 0.292 | 0.7720001 | 0.941592 |
| X202758_s_at | 0.0039 | 0.0133 | 0.292 | 0.7721301 | 0.941699 |
| X204317_at | -0.0016 | 0.0054 | -0.292 | 0.7722399 | 0.941708 |
| X216115_at | 0.0069 | 0.0236 | 0.292 | 0.772279 | 0.941708 |
| X217136_at | -0.0019 | 0.0065 | -0.292 | 0.7722952 | 0.941708 |
| X206111_at | 0.0025 | 0.0086 | 0.292 | 0.7723171 | 0.941708 |
| X215383_x_at | 0.007 | 0.024 | 0.292 | 0.7723547 | 0.941708 |
| X207362_at | -0.0017 | 0.0059 | -0.292 | 0.7724089 | 0.941708 |
| X216646_at | -0.0013 | 0.0043 | -0.292 | 0.7724349 | 0.941708 |
| X204779_s_at | 0.0026 | 0.009 | 0.292 | 0.7725491 | 0.941796 |
| X210663_s_at | -0.0016 | 0.0056 | -0.291 | 0.7727397 | 0.941941 |
| X219704_at | 0.0017 | 0.0059 | 0.291 | 0.7727815 | 0.941941 |
| X216770_at | -0.0052 | 0.0179 | -0.291 | 0.7728525 | 0.941941 |
| X204801_s_at | -0.0012 | 0.0042 | -0.291 | 0.772855 | 0.941941 |
| X218223_s_at | 0.0079 | 0.0272 | 0.291 | 0.7728803 | 0.941941 |
| X220278_at | -0.0014 | 0.0049 | -0.291 | 0.7729428 | 0.941954 |
| X222287_at | -0.0025 | 0.0086 | -0.291 | 0.7729938 | 0.941954 |
| X211912_at | 0.0013 | 0.0044 | 0.291 | 0.7730182 | 0.941954 |
| X210632_s_at | -0.0009 | 0.0032 | -0.291 | 0.7731091 | 0.942013 |
| X208543_at | 0.0014 | 0.0047 | 0.291 | 0.7731971 | 0.942014 |
| X211510_s_at | 0.0011 | 0.0037 | 0.291 | 0.7732381 | 0.942014 |
| X211362_s_at | 0.0008 | 0.0027 | 0.291 | 0.7732632 | 0.942014 |
| X218460_at | -0.0083 | 0.0286 | -0.29 | 0.7733786 | 0.942014 |
| X214767_s_at | 0.0019 | 0.0066 | 0.29 | 0.7733987 | 0.942014 |
| X218644_at | 0.0009 | 0.0032 | 0.29 | 0.7734187 | 0.942014 |
| X212477_at | 0.0027 | 0.0094 | 0.29 | 0.7734341 | 0.942014 |
| X215658_at | -0.0024 | 0.0082 | -0.29 | 0.7734488 | 0.942014 |
| X219227_at | 0.0011 | 0.0038 | 0.29 | 0.7735981 | 0.942144 |
| X211655_at | 0.0014 | 0.0049 | 0.29 | 0.7737675 | 0.942299 |
| X215145_s_at | 0.0378 | 0.1303 | 0.29 | 0.7738318 | 0.942325 |
| X208230_s_at | 0.0011 | 0.0038 | 0.29 | 0.773996 | 0.942366 |
| X206157_at | 0.0055 | 0.0191 | 0.29 | 0.7740404 | 0.942366 |
| X215494_at | -0.0024 | 0.0082 | -0.29 | 0.7740647 | 0.942366 |
| X209958_s_at | 0.0024 | 0.0084 | 0.29 | 0.7741154 | 0.942366 |
| X203183_s_at | 0.0013 | 0.0044 | 0.289 | 0.7741448 | 0.942366 |
| X219082_at | -0.0008 | 0.0028 | -0.289 | 0.7741507 | 0.942366 |
| X221900_at | 0.0012 | 0.0041 | 0.289 | 0.7741865 | 0.942366 |
| X204115_at | 0.0191 | 0.066 | 0.289 | 0.7742385 | 0.942366 |
| X213442_x_at | -0.0016 | 0.0055 | -0.289 | 0.774287 | 0.942366 |
| X218008_at | 0.0161 | 0.0556 | 0.289 | 0.7744102 | 0.942366 |
| X207033_at | -0.0014 | 0.0049 | -0.289 | 0.7744576 | 0.942366 |
| X217372_at | -0.0015 | 0.0051 | -0.289 | 0.7744967 | 0.942366 |
| X205654_at | -0.0009 | 0.0031 | -0.289 | 0.7745162 | 0.942366 |
| X37022_at | 0.0014 | 0.0049 | 0.289 | 0.7745289 | 0.942366 |
| X221078_s_at | 0.0015 | 0.005 | 0.289 | 0.774554 | 0.942366 |
| X216097_at | -0.0042 | 0.0147 | -0.289 | 0.7746165 | 0.942366 |
| X212534_at | 0.0141 | 0.0489 | 0.289 | 0.7746327 | 0.942366 |
| X208525_s_at | 0.0016 | 0.0054 | 0.289 | 0.7746829 | 0.942366 |
| X207452_s_at | -0.0015 | 0.0052 | -0.289 | 0.7746897 | 0.942366 |
| X215717_s_at | 0.0021 | 0.0074 | 0.289 | 0.7747135 | 0.942366 |
| X209299_x_at | 0.0008 | 0.0029 | 0.289 | 0.7748539 | 0.942485 |
| X220628_s_at | 0.0018 | 0.0061 | 0.288 | 0.7749424 | 0.942541 |
| X214357_at | 0.0012 | 0.004 | 0.288 | 0.7749994 | 0.942559 |
| X212394_at | -0.012 | 0.0415 | -0.288 | 0.775065 | 0.942587 |
| X209030_s_at | 0.017 | 0.059 | 0.288 | 0.7751963 | 0.942695 |
| X212384_at | 0.0259 | 0.0899 | 0.288 | 0.7754031 | 0.942895 |
| X211363_s_at | -0.001 | 0.0033 | -0.288 | 0.7755759 | 0.94297 |
| X214525_x_at | 0.0024 | 0.0083 | 0.288 | 0.7755768 | 0.94297 |
| X213244_at | 0.0013 | 0.0044 | 0.288 | 0.7755923 | 0.94297 |
| X219230_at | 0.0362 | 0.1261 | 0.287 | 0.7758241 | 0.943129 |
| X222002_at | 0.0008 | 0.0029 | 0.287 | 0.7758744 | 0.943129 |
| X220894_x_at | -0.0012 | 0.0042 | -0.287 | 0.775875 | 0.943129 |
| X204126_s_at | -0.0008 | 0.0027 | -0.287 | 0.7758927 | 0.943129 |
| X205100_at | -0.0186 | 0.0649 | -0.287 | 0.7760463 | 0.943212 |
| X216999_at | -0.0025 | 0.0087 | -0.287 | 0.7760745 | 0.943212 |
| X216195_at | 0.0378 | 0.1317 | 0.287 | 0.7760887 | 0.943212 |
| X206764_x_at | -0.0021 | 0.0073 | -0.287 | 0.7763108 | 0.943431 |
| X215012_at | 0.0311 | 0.1084 | 0.287 | 0.7763944 | 0.943481 |
| X218824_at | -0.0146 | 0.0509 | -0.286 | 0.7764416 | 0.943485 |
| X215006_at | 0.0033 | 0.0115 | 0.286 | 0.776483 | 0.943485 |
| X208075_s_at | -0.0023 | 0.0081 | -0.286 | 0.7765935 | 0.943534 |
| X201650_at | 0.0011 | 0.0039 | 0.286 | 0.7766085 | 0.943534 |
| X221435_x_at | -0.0054 | 0.0187 | -0.286 | 0.7766816 | 0.943572 |
| X201367_s_at | 0.0012 | 0.0043 | 0.286 | 0.7767411 | 0.943592 |
| X209787_s_at | -0.01 | 0.0352 | -0.286 | 0.7768655 | 0.943608 |
| X201868_s_at | -0.0014 | 0.0049 | -0.286 | 0.7768743 | 0.943608 |
| X206188_at | -0.0138 | 0.0484 | -0.286 | 0.7769135 | 0.943608 |
| X209932_s_at | -0.0049 | 0.0171 | -0.286 | 0.7770417 | 0.943608 |
| X212612_at | -0.0142 | 0.0497 | -0.286 | 0.7770518 | 0.943608 |
| X205407_at | -0.0141 | 0.0494 | -0.286 | 0.777142 | 0.943608 |
| X204506_at | -0.0333 | 0.1168 | -0.285 | 0.7771679 | 0.943608 |
| X212670_at | 0.0007 | 0.0023 | 0.285 | 0.7771969 | 0.943608 |
| X221594_at | -0.0021 | 0.0074 | -0.285 | 0.7772048 | 0.943608 |
| X209414_at | -0.0021 | 0.0075 | -0.285 | 0.7772111 | 0.943608 |
| X209154_at | -0.0222 | 0.0776 | -0.285 | 0.7772214 | 0.943608 |
| X213840_s_at | 0.0011 | 0.004 | 0.285 | 0.7773008 | 0.943618 |
| X218589_at | 0.0259 | 0.0907 | 0.285 | 0.7774387 | 0.943618 |
| X205175_s_at | 0.0013 | 0.0047 | 0.285 | 0.7774536 | 0.943618 |
| X205166_at | 0.002 | 0.0069 | 0.285 | 0.7775289 | 0.943618 |
| X221364_at | -0.0008 | 0.0029 | -0.285 | 0.7775731 | 0.943618 |
| X208522_s_at | -0.0012 | 0.0042 | -0.285 | 0.7776059 | 0.943618 |
| X217129_at | -0.0007 | 0.0024 | -0.285 | 0.7776191 | 0.943618 |
| X205605_at | 0.0007 | 0.0025 | 0.285 | 0.7776695 | 0.943618 |
| X216915_s_at | -0.0034 | 0.012 | -0.285 | 0.7777163 | 0.943618 |
| X207751_at | 0.0013 | 0.0046 | 0.285 | 0.7777217 | 0.943618 |
| X206091_at | 0.0096 | 0.0337 | 0.285 | 0.7777236 | 0.943618 |
| X55616_at | -0.0082 | 0.0287 | -0.285 | 0.777739 | 0.943618 |
| X208602_x_at | 0.0011 | 0.004 | 0.285 | 0.7779132 | 0.94368 |
| X220126_at | 0.0013 | 0.0046 | 0.285 | 0.7779136 | 0.94368 |
| X216760_at | -0.0008 | 0.0028 | -0.285 | 0.7779179 | 0.94368 |
| X218044_x_at | 0.0021 | 0.0073 | 0.284 | 0.777998 | 0.943726 |
| X220848_x_at | -0.0007 | 0.0026 | -0.284 | 0.7781211 | 0.943761 |
| X200844_s_at | -0.0094 | 0.0332 | -0.284 | 0.7781705 | 0.943761 |
| X204824_at | -0.0159 | 0.0561 | -0.284 | 0.7782024 | 0.943761 |
| X203624_at | -0.0054 | 0.0191 | -0.284 | 0.7782685 | 0.943761 |
| X203873_at | 0.0012 | 0.0043 | 0.284 | 0.7782785 | 0.943761 |
| X201312_s_at | -0.0057 | 0.02 | -0.284 | 0.778283 | 0.943761 |
| X209721_s_at | 0.0017 | 0.0058 | 0.284 | 0.7783396 | 0.943761 |
| X211736_at | 0.0022 | 0.0077 | 0.284 | 0.7783666 | 0.943761 |
| X207870_at | 0.0017 | 0.0061 | 0.284 | 0.7784455 | 0.943801 |
| X207496_at | -0.0024 | 0.0085 | -0.284 | 0.7785515 | 0.943801 |
| X213789_at | -0.0005 | 0.0019 | -0.284 | 0.7785632 | 0.943801 |
| X220772_at | -0.0025 | 0.0087 | -0.284 | 0.7785802 | 0.943801 |
| X205867_at | -0.0037 | 0.0132 | -0.284 | 0.7786642 | 0.943801 |
| X212161_at | 0.0053 | 0.0185 | 0.283 | 0.7786893 | 0.943801 |
| X217155_at | -0.0019 | 0.0065 | -0.283 | 0.7788453 | 0.943801 |
| X204944_at | 0.0173 | 0.061 | 0.283 | 0.7788559 | 0.943801 |
| X204683_at | -0.0032 | 0.0113 | -0.283 | 0.7789065 | 0.943801 |
| X222013_x_at | 0.0014 | 0.0051 | 0.283 | 0.7789409 | 0.943801 |
| X216648_s_at | 0.001 | 0.0034 | 0.283 | 0.7789643 | 0.943801 |
| X217341_at | 0.0017 | 0.006 | 0.283 | 0.778976 | 0.943801 |
| X221602_s_at | 0.001 | 0.0035 | 0.283 | 0.7790408 | 0.943801 |
| X211448_s_at | -0.0011 | 0.0039 | -0.283 | 0.7790614 | 0.943801 |
| X207084_at | 0.0343 | 0.1212 | 0.283 | 0.7790766 | 0.943801 |
| X213208_at | -0.006 | 0.0211 | -0.283 | 0.7790801 | 0.943801 |
| X217692_at | 0.0026 | 0.009 | 0.283 | 0.7791602 | 0.943812 |
| X201692_at | -0.0052 | 0.0185 | -0.283 | 0.7792254 | 0.943812 |
| X210773_s_at | 0.0012 | 0.0044 | 0.283 | 0.7792485 | 0.943812 |
| X209057_x_at | 0.0101 | 0.0356 | 0.283 | 0.7792585 | 0.943812 |
| X221751_at | -0.0101 | 0.0356 | -0.283 | 0.7794304 | 0.943968 |
| X205813_s_at | 0.0012 | 0.0042 | 0.282 | 0.779605 | 0.944128 |
| X210752_s_at | 0.0022 | 0.0078 | 0.282 | 0.7797664 | 0.944245 |
| X210930_s_at | 0.0008 | 0.0028 | 0.282 | 0.7798066 | 0.944245 |
| X213668_s_at | -0.0012 | 0.0042 | -0.282 | 0.7798287 | 0.944245 |
| X214088_s_at | 0.0013 | 0.0047 | 0.282 | 0.7798987 | 0.944278 |
| X214648_at | 0.0015 | 0.0054 | 0.282 | 0.7800167 | 0.94437 |
| X220907_at | 0.0019 | 0.0067 | 0.282 | 0.7800606 | 0.944371 |
| X207284_s_at | 0.0016 | 0.0058 | 0.281 | 0.7802011 | 0.944413 |
| X216993_s_at | -0.0015 | 0.0055 | -0.281 | 0.7802123 | 0.944413 |
| X219764_at | -0.0021 | 0.0073 | -0.281 | 0.7802225 | 0.944413 |
| X217091_at | -0.0022 | 0.0077 | -0.281 | 0.7803203 | 0.94448 |
| X211768_at | -0.003 | 0.0105 | -0.281 | 0.7804123 | 0.94454 |
| X215102_at | -0.0019 | 0.0066 | -0.281 | 0.7805535 | 0.944659 |
| X201624_at | 0.0142 | 0.0506 | 0.281 | 0.7806698 | 0.944748 |
| X218363_at | 0.0148 | 0.0528 | 0.281 | 0.7807383 | 0.944755 |
| X216971_s_at | 0.0018 | 0.0063 | 0.281 | 0.780825 | 0.944755 |
| X215168_at | -0.0067 | 0.024 | -0.281 | 0.7808918 | 0.944755 |
| X218038_at | -0.0021 | 0.0074 | -0.28 | 0.7809669 | 0.944755 |
| X217267_s_at | -0.0018 | 0.0064 | -0.28 | 0.781013 | 0.944755 |
| X216261_at | 0.0016 | 0.0057 | 0.28 | 0.7810186 | 0.944755 |
| X203737_s_at | -0.002 | 0.0073 | -0.28 | 0.7810329 | 0.944755 |
| X210694_s_at | 0.0095 | 0.0338 | 0.28 | 0.781066 | 0.944755 |
| X215361_at | 0.0022 | 0.0078 | 0.28 | 0.7811165 | 0.944755 |
| X213063_at | -0.0073 | 0.0259 | -0.28 | 0.7811404 | 0.944755 |
| X215635_at | 0.0285 | 0.1017 | 0.28 | 0.7811815 | 0.944755 |
| X202361_at | 0.0109 | 0.039 | 0.28 | 0.7811856 | 0.944755 |
| X204078_at | 0.0072 | 0.0257 | 0.28 | 0.7813753 | 0.944933 |
| X217074_at | -0.0013 | 0.0048 | -0.28 | 0.7814606 | 0.944985 |
| X222098_s_at | -0.0015 | 0.0054 | -0.28 | 0.7815241 | 0.94501 |
| X206513_at | -0.0015 | 0.0055 | -0.279 | 0.7818065 | 0.945181 |
| X217501_at | -0.0078 | 0.0279 | -0.279 | 0.7818204 | 0.945181 |
| X214366_s_at | 0.001 | 0.0036 | 0.279 | 0.7818329 | 0.945181 |
| X219620_x_at | -0.0017 | 0.0061 | -0.279 | 0.7818727 | 0.945181 |
| X211010_s_at | 0.0008 | 0.0027 | 0.279 | 0.7818781 | 0.945181 |
| X221956_at | -0.0015 | 0.0053 | -0.279 | 0.7820326 | 0.945316 |
| X204269_at | 0.0018 | 0.0065 | 0.279 | 0.7820844 | 0.945327 |
| X220256_s_at | -0.0008 | 0.0028 | -0.279 | 0.782171 | 0.94538 |
| X205323_s_at | -0.0044 | 0.0157 | -0.279 | 0.7823275 | 0.9455 |
| X202347_s_at | 0.0111 | 0.0399 | 0.279 | 0.7823787 | 0.9455 |
| X215261_at | -0.0009 | 0.0032 | -0.279 | 0.7823974 | 0.9455 |
| X215114_at | 0.0022 | 0.0081 | 0.278 | 0.7825712 | 0.945658 |
| X219434_at | 0.0015 | 0.0052 | 0.278 | 0.7826774 | 0.945681 |
| X222334_at | 0.0022 | 0.0081 | 0.278 | 0.7826961 | 0.945681 |
| X209310_s_at | 0.0014 | 0.0051 | 0.278 | 0.7827179 | 0.945681 |
| X220884_at | -0.0035 | 0.0126 | -0.278 | 0.7828979 | 0.945847 |
| X215277_at | -0.0009 | 0.0033 | -0.278 | 0.783009 | 0.94593 |
| X218867_s_at | 0.0017 | 0.0061 | 0.278 | 0.7830895 | 0.945976 |
| X204381_at | -0.0116 | 0.0418 | -0.278 | 0.7831932 | 0.94605 |
| X201439_at | -0.011 | 0.0396 | -0.277 | 0.7833149 | 0.946145 |
| X211689_s_at | 0.0006 | 0.0023 | 0.277 | 0.7834306 | 0.946234 |
| X215667_x_at | 0.0132 | 0.0478 | 0.277 | 0.7834951 | 0.94626 |
| X212352_s_at | 0.0072 | 0.026 | 0.277 | 0.7835609 | 0.946288 |
| X215337_at | 0.0005 | 0.0018 | 0.277 | 0.7836357 | 0.946327 |
| X218114_at | -0.0023 | 0.0084 | -0.277 | 0.7837675 | 0.946385 |
| X205184_at | -0.036 | 0.1302 | -0.277 | 0.7838317 | 0.946385 |
| X216329_at | -0.0009 | 0.0031 | -0.277 | 0.7838678 | 0.946385 |
| X205634_x_at | 0.0018 | 0.0066 | 0.276 | 0.784002 | 0.946385 |
| X209952_s_at | 0.0011 | 0.004 | 0.276 | 0.7840089 | 0.946385 |
| X217349_s_at | 0.0013 | 0.0046 | 0.276 | 0.7840213 | 0.946385 |
| X215601_at | -0.0022 | 0.0079 | -0.276 | 0.7840466 | 0.946385 |
| X217192_s_at | -0.0014 | 0.0052 | -0.276 | 0.7841039 | 0.946385 |
| X210057_at | -0.0127 | 0.0458 | -0.276 | 0.7841918 | 0.946385 |
| X64440_at | 0.0013 | 0.0045 | 0.276 | 0.7842058 | 0.946385 |
| X219187_at | 0.0014 | 0.005 | 0.276 | 0.7842299 | 0.946385 |
| X213565_s_at | 0.0013 | 0.0049 | 0.276 | 0.7842705 | 0.946385 |
| X220503_at | 0.0042 | 0.0153 | 0.276 | 0.7842864 | 0.946385 |
| X202756_s_at | 0.0146 | 0.053 | 0.276 | 0.7843627 | 0.946385 |
| X219126_at | 0.002 | 0.0074 | 0.276 | 0.7843722 | 0.946385 |
| X211900_x_at | -0.0017 | 0.0062 | -0.276 | 0.7844001 | 0.946385 |
| X208439_s_at | 0.0014 | 0.005 | 0.276 | 0.7844501 | 0.946385 |
| X220319_s_at | 0.0221 | 0.0802 | 0.276 | 0.784501 | 0.946385 |
| X212299_at | -0.0033 | 0.0119 | -0.276 | 0.7845756 | 0.946385 |
| X205459_s_at | 0.0014 | 0.0052 | 0.276 | 0.7846684 | 0.946385 |
| X211532_x_at | -0.0013 | 0.0047 | -0.276 | 0.7846831 | 0.946385 |
| X212885_at | 0.0144 | 0.0523 | 0.276 | 0.784699 | 0.946385 |
| X200061_s_at | 0.0071 | 0.0259 | 0.275 | 0.7847937 | 0.946385 |
| X214142_at | 0.0009 | 0.0034 | 0.275 | 0.7848029 | 0.946385 |
| X217365_at | -0.0011 | 0.004 | -0.275 | 0.784838 | 0.946385 |
| X207534_at | -0.0012 | 0.0044 | -0.275 | 0.7848563 | 0.946385 |
| X202385_s_at | 0.0014 | 0.0051 | 0.275 | 0.7848961 | 0.946385 |
| X219881_s_at | -0.0028 | 0.0102 | -0.275 | 0.7849188 | 0.946385 |
| X210325_at | 0.0018 | 0.0065 | 0.275 | 0.7849196 | 0.946385 |
| X216276_s_at | 0.0044 | 0.0158 | 0.275 | 0.7850476 | 0.946458 |
| X210833_at | 0.0028 | 0.01 | 0.275 | 0.7851757 | 0.946458 |
| X209853_s_at | 0.0124 | 0.0449 | 0.275 | 0.7852697 | 0.946458 |
| X221948_s_at | 0.0004 | 0.0016 | 0.275 | 0.7853085 | 0.946458 |
| X211018_at | -0.0043 | 0.0158 | -0.275 | 0.7853175 | 0.946458 |
| X214827_at | 0.0014 | 0.005 | 0.275 | 0.7853713 | 0.946458 |
| X207924_x_at | 0.0012 | 0.0045 | 0.275 | 0.7853714 | 0.946458 |
| X202959_at | -0.0114 | 0.0416 | -0.275 | 0.7853796 | 0.946458 |
| X209884_s_at | -0.0121 | 0.0441 | -0.275 | 0.7854212 | 0.946458 |
| X204776_at | -0.0215 | 0.0782 | -0.275 | 0.7854479 | 0.946458 |
| X205710_at | -0.0323 | 0.1175 | -0.275 | 0.785491 | 0.946458 |
| X210929_s_at | 0.0023 | 0.0084 | 0.274 | 0.7855297 | 0.946458 |
| X205798_at | 0.0009 | 0.0033 | 0.274 | 0.7855338 | 0.946458 |
| X210931_at | -0.0026 | 0.0095 | -0.274 | 0.7856247 | 0.946516 |
| X214268_s_at | -0.0111 | 0.0406 | -0.274 | 0.7858087 | 0.946687 |
| X211969_at | 0.0054 | 0.0196 | 0.274 | 0.7859063 | 0.946708 |
| X210116_at | 0.0014 | 0.005 | 0.274 | 0.7859119 | 0.946708 |
| X37145_at | 0.0008 | 0.003 | 0.274 | 0.7861071 | 0.946815 |
| X217981_s_at | -0.0015 | 0.0056 | -0.274 | 0.786123 | 0.946815 |
| X212619_at | 0.0015 | 0.0055 | 0.274 | 0.7861929 | 0.946815 |
| X203822_s_at | -0.004 | 0.0148 | -0.274 | 0.7862097 | 0.946815 |
| X211885_x_at | -0.001 | 0.0035 | -0.274 | 0.786238 | 0.946815 |
| X219966_x_at | -0.0142 | 0.0519 | -0.274 | 0.7862738 | 0.946815 |
| X206322_at | -0.0025 | 0.009 | -0.273 | 0.7863148 | 0.946815 |
| X208026_at | 0.0021 | 0.0079 | 0.273 | 0.7863415 | 0.946815 |
| X203356_at | -0.0102 | 0.0374 | -0.273 | 0.786419 | 0.946857 |
| X211682_x_at | 0.0019 | 0.0068 | 0.273 | 0.7866113 | 0.947037 |
| X201427_s_at | 0.0278 | 0.102 | 0.273 | 0.7867093 | 0.94705 |
| X216727_at | -0.0014 | 0.005 | -0.273 | 0.7867268 | 0.94705 |
| X202965_s_at | 0.0019 | 0.0071 | 0.273 | 0.7867948 | 0.94705 |
| X216880_at | -0.0014 | 0.0052 | -0.273 | 0.7867969 | 0.94705 |
| X202405_at | -0.007 | 0.0258 | -0.273 | 0.7868413 | 0.94705 |
| X209264_s_at | -0.0016 | 0.0059 | -0.273 | 0.7869028 | 0.94705 |
| X212235_at | 0.0013 | 0.0047 | 0.273 | 0.7869202 | 0.94705 |
| X210934_at | 0.0013 | 0.0047 | 0.273 | 0.7869668 | 0.947055 |
| X221433_at | -0.001 | 0.0036 | -0.272 | 0.7870975 | 0.947161 |
| X219751_at | -0.019 | 0.0697 | -0.272 | 0.7872062 | 0.947222 |
| X221074_at | 0.0008 | 0.003 | 0.272 | 0.787234 | 0.947222 |
| X216297_at | -0.0021 | 0.0077 | -0.272 | 0.7873008 | 0.947251 |
| X210443_x_at | 0.0015 | 0.0054 | 0.272 | 0.7874832 | 0.947364 |
| X218963_s_at | -0.0012 | 0.0043 | -0.272 | 0.7875134 | 0.947364 |
| X205701_at | -0.001 | 0.0035 | -0.272 | 0.7875364 | 0.947364 |
| X208649_s_at | -0.0103 | 0.0377 | -0.272 | 0.7875648 | 0.947364 |
| X212191_x_at | -0.0111 | 0.041 | -0.272 | 0.7876849 | 0.947397 |
| X214470_at | 0.0013 | 0.0049 | 0.272 | 0.7877025 | 0.947397 |
| X203160_s_at | 0.006 | 0.0219 | 0.272 | 0.7877263 | 0.947397 |
| X206972_s_at | -0.0014 | 0.0051 | -0.272 | 0.7877627 | 0.947397 |
| X210704_at | 0.0016 | 0.006 | 0.271 | 0.7878324 | 0.947401 |
| X211502_s_at | -0.0239 | 0.0879 | -0.271 | 0.7878518 | 0.947401 |
| X211062_s_at | -0.0014 | 0.0051 | -0.271 | 0.7879274 | 0.947401 |
| X210677_at | 0.0009 | 0.0034 | 0.271 | 0.7880335 | 0.947401 |
| X207674_at | 0.0032 | 0.0117 | 0.271 | 0.7880397 | 0.947401 |
| X216225_at | 0.0025 | 0.009 | 0.271 | 0.7880453 | 0.947401 |
| X205430_at | -0.0027 | 0.0101 | -0.271 | 0.7880649 | 0.947401 |
| X211411_at | -0.0024 | 0.0088 | -0.271 | 0.7881557 | 0.947459 |
| X205120_s_at | 0.0164 | 0.0604 | 0.271 | 0.7882271 | 0.947461 |
| X218600_at | -0.0013 | 0.0048 | -0.271 | 0.7882923 | 0.947461 |
| X218846_at | 0.0118 | 0.0434 | 0.271 | 0.7883086 | 0.947461 |
| X207841_at | -0.001 | 0.0038 | -0.271 | 0.7883284 | 0.947461 |
| X205698_s_at | -0.004 | 0.0149 | -0.271 | 0.7884825 | 0.947557 |
| X215738_at | 0.001 | 0.0038 | 0.27 | 0.7885701 | 0.947557 |
| X215312_at | -0.0024 | 0.0088 | -0.27 | 0.7886012 | 0.947557 |
| X215515_at | -0.001 | 0.0039 | -0.27 | 0.7886529 | 0.947557 |
| X221661_at | -0.0017 | 0.0062 | -0.27 | 0.7886645 | 0.947557 |
| X209767_s_at | -0.0101 | 0.0373 | -0.27 | 0.7886657 | 0.947557 |
| X204463_s_at | -0.004 | 0.0149 | -0.27 | 0.7887525 | 0.947557 |
| X205035_at | 0.0023 | 0.0084 | 0.27 | 0.7887533 | 0.947557 |
| X206635_at | 0.0119 | 0.0441 | 0.27 | 0.7889208 | 0.947557 |
| X218092_s_at | 0.013 | 0.048 | 0.27 | 0.7889211 | 0.947557 |
| X205260_s_at | 0.0149 | 0.0551 | 0.27 | 0.7889317 | 0.947557 |
| X217548_at | -0.0024 | 0.0089 | -0.27 | 0.7889878 | 0.947557 |
| X212237_at | -0.0058 | 0.0216 | -0.27 | 0.7889884 | 0.947557 |
| X221239_s_at | -0.001 | 0.0037 | -0.27 | 0.7890324 | 0.947557 |
| X216659_at | -0.0028 | 0.0104 | -0.27 | 0.7890476 | 0.947557 |
| X53912_at | -0.0046 | 0.0171 | -0.27 | 0.7891134 | 0.947585 |
| X220909_at | 0.0008 | 0.003 | 0.27 | 0.7892477 | 0.947695 |
| X209588_at | 0.0008 | 0.0031 | 0.269 | 0.7893546 | 0.947744 |
| X213420_at | -0.0097 | 0.036 | -0.269 | 0.7893741 | 0.947744 |
| X203791_at | -0.0094 | 0.035 | -0.269 | 0.7896071 | 0.947909 |
| X210683_at | -0.0011 | 0.0043 | -0.269 | 0.7896271 | 0.947909 |
| X206306_at | -0.0237 | 0.0881 | -0.269 | 0.7896399 | 0.947909 |
| X213862_at | -0.0011 | 0.004 | -0.269 | 0.7897186 | 0.947949 |
| X205818_at | 0.0095 | 0.0353 | 0.269 | 0.7897598 | 0.947949 |
| X222323_at | 0.0012 | 0.0044 | 0.269 | 0.7898085 | 0.947949 |
| X200865_at | -0.002 | 0.0075 | -0.269 | 0.7898499 | 0.947949 |
| X215376_at | -0.0009 | 0.0034 | -0.269 | 0.7898862 | 0.947949 |
| X220971_at | 0.001 | 0.0037 | 0.269 | 0.7899768 | 0.947992 |
| X203049_s_at | -0.0088 | 0.0328 | -0.269 | 0.7900196 | 0.947992 |
| X220065_at | 0.0012 | 0.0045 | 0.268 | 0.7901365 | 0.947992 |
| X216740_at | 0.0015 | 0.0057 | 0.268 | 0.7901468 | 0.947992 |
| X222144_at | -0.0009 | 0.0033 | -0.268 | 0.7901537 | 0.947992 |
| X220664_at | -0.0018 | 0.0067 | -0.268 | 0.7902176 | 0.947992 |
| X217791_s_at | 0.0038 | 0.0141 | 0.268 | 0.7902513 | 0.947992 |
| X205402_x_at | 0.0015 | 0.0057 | 0.268 | 0.7902637 | 0.947992 |
| X204063_s_at | -0.0104 | 0.039 | -0.268 | 0.7904429 | 0.948099 |
| X222345_at | -0.0012 | 0.0045 | -0.268 | 0.7905145 | 0.948099 |
| X211196_at | -0.0021 | 0.008 | -0.268 | 0.7905148 | 0.948099 |
| X220104_at | 0.0034 | 0.0126 | 0.268 | 0.7905231 | 0.948099 |
| X214872_at | 0.0013 | 0.0047 | 0.268 | 0.7906504 | 0.948181 |
| X205419_at | 0.0274 | 0.1023 | 0.268 | 0.7906771 | 0.948181 |
| X205516_x_at | 0.002 | 0.0074 | 0.268 | 0.7907704 | 0.948194 |
| X209766_at | 0.0019 | 0.0071 | 0.267 | 0.790862 | 0.948194 |
| X203541_s_at | 0.0012 | 0.0044 | 0.267 | 0.7908964 | 0.948194 |
| X217183_at | 0.0006 | 0.0022 | 0.267 | 0.7909791 | 0.948194 |
| X215480_at | 0.0008 | 0.0031 | 0.267 | 0.7909817 | 0.948194 |
| X208467_at | 0.0007 | 0.0026 | 0.267 | 0.7910019 | 0.948194 |
| X215989_at | -0.001 | 0.0039 | -0.267 | 0.7910333 | 0.948194 |
| X205667_at | -0.0066 | 0.0248 | -0.267 | 0.7910422 | 0.948194 |
| X209765_at | -0.0034 | 0.0128 | -0.267 | 0.7911133 | 0.948194 |
| X211153_s_at | 0.0012 | 0.0044 | 0.267 | 0.7911307 | 0.948194 |
| X220780_at | 0.0011 | 0.0042 | 0.267 | 0.7911576 | 0.948194 |
| X214530_x_at | 0.0008 | 0.0029 | 0.267 | 0.7912188 | 0.948216 |
| X215760_s_at | -0.0014 | 0.0053 | -0.267 | 0.7914426 | 0.948371 |
| X217387_at | -0.0012 | 0.0045 | -0.267 | 0.7914735 | 0.948371 |
| X217674_at | 0.0011 | 0.0041 | 0.267 | 0.7915011 | 0.948371 |
| X210676_x_at | 0.0249 | 0.0932 | 0.267 | 0.7915189 | 0.948371 |
| X208019_at | -0.0026 | 0.0098 | -0.267 | 0.7915848 | 0.948399 |
| X219711_at | 0.0018 | 0.0068 | 0.266 | 0.7916579 | 0.9484 |
| X200789_at | 0.0056 | 0.0212 | 0.266 | 0.7917053 | 0.9484 |
| X201240_s_at | -0.0083 | 0.0313 | -0.266 | 0.7917544 | 0.9484 |
| X221259_s_at | -0.0018 | 0.0066 | -0.266 | 0.7917567 | 0.9484 |
| X206311_s_at | -0.0019 | 0.007 | -0.266 | 0.7918913 | 0.94851 |
| X213321_at | 0.007 | 0.0263 | 0.266 | 0.7920053 | 0.948522 |
| X205267_at | -0.0025 | 0.0093 | -0.266 | 0.7920347 | 0.948522 |
| X204815_s_at | 0.0007 | 0.0026 | 0.266 | 0.7920529 | 0.948522 |
| X207665_at | 0.002 | 0.0074 | 0.266 | 0.792072 | 0.948522 |
| X203770_s_at | -0.0009 | 0.0032 | -0.266 | 0.7921192 | 0.948528 |
| X219323_s_at | -0.0014 | 0.0052 | -0.266 | 0.7922052 | 0.948567 |
| X206925_at | 0.0013 | 0.0048 | 0.266 | 0.7922378 | 0.948567 |
| X220649_at | -0.0055 | 0.0207 | -0.266 | 0.792309 | 0.948602 |
| X218529_at | -0.0027 | 0.0101 | -0.265 | 0.7924181 | 0.94865 |
| X204910_s_at | -0.0015 | 0.0055 | -0.265 | 0.792435 | 0.94865 |
| X215137_at | -0.0026 | 0.0098 | -0.265 | 0.7925556 | 0.948743 |
| X206913_at | 0.0013 | 0.0048 | 0.265 | 0.7929186 | 0.949127 |
| X211508_s_at | -0.001 | 0.0039 | -0.265 | 0.792973 | 0.949141 |
| X217436_x_at | 0.015 | 0.0569 | 0.264 | 0.7932584 | 0.949359 |
| X204337_at | 0.0446 | 0.1686 | 0.264 | 0.7933041 | 0.949359 |
| X218731_s_at | -0.0012 | 0.0046 | -0.264 | 0.7933343 | 0.949359 |
| X214977_at | -0.0013 | 0.0049 | -0.264 | 0.7933872 | 0.949359 |
| X202581_at | 0.0306 | 0.1161 | 0.264 | 0.7936051 | 0.949359 |
| X205274_at | -0.0006 | 0.0024 | -0.264 | 0.7936965 | 0.949359 |
| X219802_at | 0.0153 | 0.0579 | 0.264 | 0.7936991 | 0.949359 |
| X206372_at | 0.001 | 0.0038 | 0.264 | 0.793727 | 0.949359 |
| X205694_at | -0.0025 | 0.0096 | -0.264 | 0.7937353 | 0.949359 |
| X201972_at | 0.0136 | 0.0516 | 0.264 | 0.7937614 | 0.949359 |
| X204926_at | -0.0034 | 0.0128 | -0.264 | 0.793797 | 0.949359 |
| X217823_s_at | 0.0128 | 0.0487 | 0.264 | 0.7938304 | 0.949359 |
| X208318_s_at | 0.0007 | 0.0026 | 0.264 | 0.7938573 | 0.949359 |
| X215916_at | 0.001 | 0.004 | 0.263 | 0.7939123 | 0.949359 |
| X203616_at | -0.0103 | 0.0391 | -0.263 | 0.7939236 | 0.949359 |
| X220573_at | 0.0009 | 0.0035 | 0.263 | 0.7939272 | 0.949359 |
| X210583_at | -0.0008 | 0.0029 | -0.263 | 0.7940087 | 0.949359 |
| X217068_at | 0.002 | 0.0075 | 0.263 | 0.7940228 | 0.949359 |
| X200602_at | -0.0067 | 0.0254 | -0.263 | 0.7940287 | 0.949359 |
| X216784_at | 0.0021 | 0.0081 | 0.263 | 0.7940371 | 0.949359 |
| X218987_at | -0.0235 | 0.0892 | -0.263 | 0.7940528 | 0.949359 |
| X219045_at | 0.0015 | 0.0057 | 0.263 | 0.7941311 | 0.949401 |
| X208553_at | 0.0012 | 0.0044 | 0.263 | 0.7941988 | 0.949431 |
| X220243_at | 0.0019 | 0.0071 | 0.263 | 0.7943781 | 0.949476 |
| X214864_s_at | -0.0092 | 0.0352 | -0.263 | 0.7944339 | 0.949476 |
| X202048_s_at | -0.0119 | 0.0453 | -0.263 | 0.7944368 | 0.949476 |
| X220872_at | -0.0014 | 0.0052 | -0.263 | 0.7944503 | 0.949476 |
| X221401_at | 0.001 | 0.0037 | 0.263 | 0.7944843 | 0.949476 |
| X207048_at | 0.0008 | 0.0032 | 0.263 | 0.7944924 | 0.949476 |
| X216718_at | -0.0013 | 0.005 | -0.263 | 0.7945731 | 0.949516 |
| X203742_s_at | -0.0132 | 0.0503 | -0.263 | 0.7946112 | 0.949516 |
| X219886_at | 0.0018 | 0.0068 | 0.262 | 0.7947053 | 0.949577 |
| X205890_s_at | -0.0012 | 0.0044 | -0.262 | 0.7948123 | 0.949632 |
| X220806_x_at | 0.0022 | 0.0082 | 0.262 | 0.7948771 | 0.949632 |
| X217510_at | 0.0008 | 0.0031 | 0.262 | 0.7948799 | 0.949632 |
| X209978_s_at | 0.0011 | 0.0042 | 0.262 | 0.7949708 | 0.949632 |
| X206597_at | 0.0009 | 0.0035 | 0.262 | 0.794996 | 0.949632 |
| X217697_at | 0.0026 | 0.0098 | 0.262 | 0.7950084 | 0.949632 |
| X208559_at | -0.0011 | 0.0044 | -0.262 | 0.7950519 | 0.949632 |
| X220758_s_at | 0.0011 | 0.0041 | 0.262 | 0.7951421 | 0.949632 |
| X206598_at | -0.0009 | 0.0033 | -0.262 | 0.7951654 | 0.949632 |
| X206981_at | 0.0013 | 0.0048 | 0.262 | 0.7952014 | 0.949632 |
| X205885_s_at | 0.0015 | 0.0057 | 0.262 | 0.7952218 | 0.949632 |
| X219238_at | 0.0141 | 0.054 | 0.261 | 0.7954474 | 0.949808 |
| X200889_s_at | -0.0181 | 0.0693 | -0.261 | 0.795458 | 0.949808 |
| X212230_at | -0.0184 | 0.0703 | -0.261 | 0.7955027 | 0.949808 |
| X201679_at | -0.0012 | 0.0048 | -0.261 | 0.7955559 | 0.949808 |
| X202819_s_at | 0.0009 | 0.0036 | 0.261 | 0.7955832 | 0.949808 |
| X221990_at | 0.001 | 0.0038 | 0.261 | 0.7956603 | 0.949849 |
| X213985_s_at | -0.0015 | 0.0058 | -0.261 | 0.7957228 | 0.949873 |
| X204960_at | 0.0013 | 0.0049 | 0.261 | 0.7958156 | 0.949933 |
| X214371_at | -0.0009 | 0.0035 | -0.261 | 0.7959753 | 0.95003 |
| X211849_s_at | -0.0015 | 0.0058 | -0.261 | 0.7960449 | 0.95003 |
| X202297_s_at | -0.007 | 0.0269 | -0.261 | 0.796046 | 0.95003 |
| X210353_s_at | 0.0009 | 0.0036 | 0.261 | 0.7960683 | 0.95003 |
| X211687_x_at | 0.0011 | 0.0044 | 0.26 | 0.7962441 | 0.950189 |
| X203338_at | -0.0111 | 0.0426 | -0.26 | 0.7963148 | 0.950222 |
| X214698_at | -0.0076 | 0.0291 | -0.26 | 0.7964424 | 0.950286 |
| X208023_at | 0.0011 | 0.0041 | 0.26 | 0.7964541 | 0.950286 |
| X217633_at | 0.0009 | 0.0033 | 0.26 | 0.7965428 | 0.950292 |
| X200682_s_at | 0.007 | 0.027 | 0.26 | 0.796566 | 0.950292 |
| X218345_at | 0.0115 | 0.0441 | 0.26 | 0.7966256 | 0.950292 |
| X201942_s_at | 0.0008 | 0.0031 | 0.26 | 0.7966905 | 0.950292 |
| X204702_s_at | 0.0026 | 0.01 | 0.26 | 0.7967999 | 0.950292 |
| X213511_s_at | 0.0118 | 0.0456 | 0.26 | 0.7968062 | 0.950292 |
| X217003_s_at | 0.0014 | 0.0053 | 0.26 | 0.7968436 | 0.950292 |
| X210295_at | -0.0007 | 0.0027 | -0.26 | 0.7968646 | 0.950292 |
| X219458_s_at | 0.0138 | 0.0532 | 0.26 | 0.7968822 | 0.950292 |
| X209600_s_at | -0.0163 | 0.0629 | -0.26 | 0.7969242 | 0.950292 |
| X214863_at | -0.0009 | 0.0033 | -0.259 | 0.796998 | 0.950292 |
| X205024_s_at | 0.0021 | 0.0083 | 0.259 | 0.7970139 | 0.950292 |
| X32699_s_at | -0.0011 | 0.0041 | -0.259 | 0.7970378 | 0.950292 |
| X207682_s_at | -0.0007 | 0.0028 | -0.259 | 0.7970813 | 0.950292 |
| X204684_at | -0.0247 | 0.0955 | -0.259 | 0.7971879 | 0.950292 |
| X211099_s_at | 0.0008 | 0.0033 | 0.259 | 0.7972797 | 0.950292 |
| X210890_x_at | 0.0015 | 0.006 | 0.259 | 0.7972941 | 0.950292 |
| X208043_at | 0.001 | 0.0038 | 0.259 | 0.7972957 | 0.950292 |
| X220559_at | -0.0014 | 0.0054 | -0.259 | 0.7973718 | 0.950292 |
| X220415_at | 0.0013 | 0.0049 | 0.259 | 0.7973844 | 0.950292 |
| X201716_at | -0.0199 | 0.0768 | -0.259 | 0.7973912 | 0.950292 |
| X207185_at | 0.0013 | 0.0048 | 0.259 | 0.7974003 | 0.950292 |
| X221182_at | -0.0024 | 0.0093 | -0.259 | 0.7976528 | 0.950532 |
| X208139_s_at | 0.0019 | 0.0074 | 0.259 | 0.7976868 | 0.950532 |
| X204298_s_at | 0.001 | 0.0037 | 0.258 | 0.7979793 | 0.950812 |
| X212105_s_at | -0.0029 | 0.0112 | -0.258 | 0.7980148 | 0.950812 |
| X217561_at | 0.0014 | 0.0056 | 0.258 | 0.7980503 | 0.950812 |
| X215874_at | -0.001 | 0.0037 | -0.258 | 0.7982258 | 0.950917 |
| X208796_s_at | -0.0076 | 0.0296 | -0.258 | 0.798253 | 0.950917 |
| X211081_s_at | 0.0475 | 0.1844 | 0.258 | 0.798301 | 0.950917 |
| X200668_s_at | -0.0088 | 0.0343 | -0.258 | 0.7983363 | 0.950917 |
| X206952_at | -0.0032 | 0.0123 | -0.258 | 0.7983528 | 0.950917 |
| X208083_s_at | -0.0012 | 0.0047 | -0.258 | 0.798419 | 0.95092 |
| X221682_s_at | 0.0017 | 0.0067 | 0.258 | 0.7984407 | 0.95092 |
| X216025_x_at | 0.0017 | 0.0066 | 0.258 | 0.798491 | 0.950929 |
| X205464_at | 0.0009 | 0.0035 | 0.257 | 0.7985809 | 0.950985 |
| X206497_at | -0.0154 | 0.0599 | -0.257 | 0.7986892 | 0.950994 |
| X216522_at | -0.0018 | 0.0069 | -0.257 | 0.7987106 | 0.950994 |
| X221713_s_at | -0.0207 | 0.0806 | -0.257 | 0.7987569 | 0.950994 |
| X219282_s_at | 0.0009 | 0.0037 | 0.257 | 0.7987635 | 0.950994 |
| X214567_s_at | 0.0014 | 0.0053 | 0.257 | 0.7988168 | 0.950994 |
| X217470_at | 0.0019 | 0.0073 | 0.257 | 0.7988452 | 0.950994 |
| X206033_s_at | -0.0016 | 0.0061 | -0.257 | 0.7989232 | 0.951029 |
| X208347_at | 0.002 | 0.0078 | 0.257 | 0.7989959 | 0.951029 |
| X203649_s_at | -0.0017 | 0.0066 | -0.257 | 0.7990032 | 0.951029 |
| X208693_s_at | 0.0081 | 0.0317 | 0.256 | 0.7993032 | 0.951335 |
| X206593_s_at | -0.002 | 0.0079 | -0.256 | 0.7996727 | 0.951639 |
| X220863_at | -0.0013 | 0.005 | -0.256 | 0.7996751 | 0.951639 |
| X206415_at | 0.0038 | 0.0148 | 0.256 | 0.7996868 | 0.951639 |
| X214249_at | -0.0017 | 0.0065 | -0.256 | 0.7998281 | 0.951676 |
| X219946_x_at | 0.0014 | 0.0054 | 0.256 | 0.7998545 | 0.951676 |
| X215765_at | -0.0011 | 0.0044 | -0.256 | 0.7999399 | 0.951676 |
| X213162_at | 0.0008 | 0.0032 | 0.256 | 0.7999745 | 0.951676 |
| X222234_s_at | 0.0064 | 0.0249 | 0.255 | 0.8000491 | 0.951676 |
| X208464_at | 0.0013 | 0.0051 | 0.255 | 0.8000546 | 0.951676 |
| X217588_at | -0.0158 | 0.062 | -0.255 | 0.8001943 | 0.951676 |
| X213429_at | 0.0077 | 0.0302 | 0.255 | 0.8002083 | 0.951676 |
| X220702_at | 0.0027 | 0.0106 | 0.255 | 0.8002136 | 0.951676 |
| X222022_at | -0.0007 | 0.0027 | -0.255 | 0.8002184 | 0.951676 |
| X206642_at | -0.0018 | 0.0071 | -0.255 | 0.8002207 | 0.951676 |
| X217442_at | 0.0009 | 0.0035 | 0.255 | 0.8002532 | 0.951676 |
| X213802_at | 0.0032 | 0.0124 | 0.255 | 0.8003141 | 0.951676 |
| X207104_x_at | 0.0007 | 0.0027 | 0.255 | 0.8003181 | 0.951676 |
| X211467_s_at | 0.0022 | 0.0087 | 0.255 | 0.8005805 | 0.951801 |
| X204841_s_at | -0.002 | 0.0077 | -0.255 | 0.8006394 | 0.951801 |
| X216424_at | -0.0017 | 0.0065 | -0.254 | 0.8008066 | 0.951801 |
| X215302_at | 0.0022 | 0.0088 | 0.254 | 0.8008429 | 0.951801 |
| X205242_at | -0.0019 | 0.0075 | -0.254 | 0.8008448 | 0.951801 |
| X210546_x_at | -0.0006 | 0.0024 | -0.254 | 0.8008837 | 0.951801 |
| X208229_at | 0.0015 | 0.006 | 0.254 | 0.8009207 | 0.951801 |
| X206802_at | 0.0009 | 0.0036 | 0.254 | 0.8009475 | 0.951801 |
| X206651_s_at | 0.0011 | 0.0042 | 0.254 | 0.8010128 | 0.951801 |
| X210741_at | -0.0019 | 0.0073 | -0.254 | 0.8011556 | 0.951801 |
| X204077_x_at | -0.0053 | 0.0208 | -0.254 | 0.8011707 | 0.951801 |
| X217671_at | 0.0272 | 0.1071 | 0.254 | 0.8011768 | 0.951801 |
| X211973_at | 0.037 | 0.1458 | 0.254 | 0.8011897 | 0.951801 |
| X205829_at | 0.0008 | 0.0033 | 0.254 | 0.8012251 | 0.951801 |
| X221384_at | -0.0006 | 0.0025 | -0.254 | 0.8012294 | 0.951801 |
| X215396_at | -0.0014 | 0.0055 | -0.254 | 0.8012675 | 0.951801 |
| X206239_s_at | -0.0024 | 0.0093 | -0.254 | 0.8013535 | 0.951801 |
| X215802_at | 0.0028 | 0.0112 | 0.254 | 0.801371 | 0.951801 |
| X206643_at | -0.0024 | 0.0093 | -0.254 | 0.8013773 | 0.951801 |
| X219975_x_at | 0.0023 | 0.0089 | 0.254 | 0.8014206 | 0.951801 |
| X208285_at | 0.0013 | 0.0049 | 0.254 | 0.8014277 | 0.951801 |
| X207530_s_at | -0.0011 | 0.0042 | -0.254 | 0.8015076 | 0.951801 |
| X216854_at | 0.0011 | 0.0045 | 0.254 | 0.8015313 | 0.951801 |
| X213653_at | -0.0116 | 0.0456 | -0.253 | 0.8015611 | 0.951801 |
| X222211_x_at | -0.0012 | 0.0047 | -0.253 | 0.8015863 | 0.951801 |
| X214685_at | 0.0021 | 0.0083 | 0.253 | 0.8015928 | 0.951801 |
| X39854_r_at | 0.0015 | 0.0059 | 0.253 | 0.8016706 | 0.951801 |
| X205211_s_at | -0.0056 | 0.022 | -0.253 | 0.8016909 | 0.951801 |
| X201727_s_at | 0.001 | 0.0041 | 0.253 | 0.8017175 | 0.951801 |
| X220624_s_at | -0.0005 | 0.0021 | -0.253 | 0.8017229 | 0.951801 |
| X206312_at | -0.001 | 0.0038 | -0.253 | 0.8017513 | 0.951801 |
| X206633_at | 0.001 | 0.0039 | 0.253 | 0.8018755 | 0.951898 |
| X221068_at | 0.0015 | 0.0058 | 0.253 | 0.8019688 | 0.951917 |
| X208558_at | 0.0013 | 0.0052 | 0.253 | 0.8020489 | 0.951917 |
| X216263_s_at | 0.002 | 0.0081 | 0.253 | 0.8020664 | 0.951917 |
| X202273_at | 0.0086 | 0.0339 | 0.253 | 0.8021273 | 0.951917 |
| X222050_at | -0.0011 | 0.0045 | -0.253 | 0.8021361 | 0.951917 |
| X213626_at | -0.0099 | 0.0392 | -0.253 | 0.8021492 | 0.951917 |
| X222278_at | 0.0011 | 0.0043 | 0.253 | 0.8022603 | 0.951998 |
| X218575_at | -0.0045 | 0.018 | -0.252 | 0.8024676 | 0.952194 |
| X207375_s_at | 0.0009 | 0.0037 | 0.252 | 0.8026556 | 0.952266 |
| X214226_at | 0.0019 | 0.0074 | 0.252 | 0.802657 | 0.952266 |
| X207744_at | 0.0047 | 0.0188 | 0.252 | 0.8026576 | 0.952266 |
| X217609_at | 0.002 | 0.0079 | 0.252 | 0.8027984 | 0.952366 |
| X215381_at | -0.0013 | 0.005 | -0.252 | 0.802827 | 0.952366 |
| X202054_s_at | 0.0155 | 0.0615 | 0.252 | 0.8028715 | 0.952368 |
| X213909_at | 0.0014 | 0.0056 | 0.252 | 0.802968 | 0.952431 |
| X207926_at | -0.0021 | 0.0084 | -0.252 | 0.8030161 | 0.952437 |
| X201921_at | -0.0049 | 0.0196 | -0.251 | 0.8031092 | 0.952453 |
| X205364_at | -0.0015 | 0.0058 | -0.251 | 0.8031148 | 0.952453 |
| X38157_at | -0.0124 | 0.0495 | -0.251 | 0.8031958 | 0.952498 |
| X212676_at | 0.006 | 0.0239 | 0.251 | 0.8033698 | 0.952653 |
| X219998_at | -0.0217 | 0.0864 | -0.251 | 0.803424 | 0.952667 |
| X221545_x_at | 0.0011 | 0.0045 | 0.251 | 0.8034865 | 0.95269 |
| X212682_s_at | 0.0018 | 0.0073 | 0.251 | 0.8035563 | 0.952706 |
| X203056_s_at | -0.0239 | 0.0951 | -0.251 | 0.8035856 | 0.952706 |
| X205179_s_at | 0.0014 | 0.0056 | 0.251 | 0.8036537 | 0.952713 |
| X222094_at | -0.0017 | 0.0068 | -0.251 | 0.8036904 | 0.952713 |
| X210328_at | 0.0007 | 0.0027 | 0.251 | 0.8037795 | 0.952713 |
| X217569_x_at | 0.0014 | 0.0056 | 0.251 | 0.8037969 | 0.952713 |
| X203916_at | -0.0068 | 0.0273 | -0.251 | 0.8038056 | 0.952713 |
| X214529_at | -0.0015 | 0.0059 | -0.25 | 0.8039057 | 0.952778 |
| X208645_s_at | -0.0044 | 0.0175 | -0.25 | 0.8039781 | 0.952778 |
| X206511_s_at | 0.0017 | 0.0066 | 0.25 | 0.8039896 | 0.952778 |
| X217397_at | -0.0013 | 0.0052 | -0.25 | 0.804097 | 0.952855 |
| X208940_at | -0.0037 | 0.0148 | -0.25 | 0.8041993 | 0.952925 |
| X222118_at | -0.0019 | 0.0078 | -0.25 | 0.8044215 | 0.953137 |
| X216106_at | 0.0013 | 0.0054 | 0.25 | 0.8046121 | 0.953269 |
| X219968_at | -0.0008 | 0.0033 | -0.25 | 0.8046185 | 0.953269 |
| X220569_at | 0.0009 | 0.0038 | 0.249 | 0.8046658 | 0.953274 |
| X215195_at | 0.0016 | 0.0064 | 0.249 | 0.8047818 | 0.953304 |
| X220057_at | -0.0011 | 0.0045 | -0.249 | 0.8048425 | 0.953304 |
| X200917_s_at | -0.0009 | 0.0035 | -0.249 | 0.8048514 | 0.953304 |
| X215332_s_at | -0.0027 | 0.0107 | -0.249 | 0.8048733 | 0.953304 |
| X201735_s_at | 0.0094 | 0.0378 | 0.249 | 0.8049271 | 0.953304 |
| X207156_at | -0.003 | 0.0119 | -0.249 | 0.8050039 | 0.953304 |
| X222191_s_at | 0.0011 | 0.0045 | 0.249 | 0.8050334 | 0.953304 |
| X222168_at | -0.0027 | 0.0108 | -0.249 | 0.805046 | 0.953304 |
| X204575_s_at | -0.0009 | 0.0037 | -0.249 | 0.8050774 | 0.953304 |
| X207741_x_at | 0.0009 | 0.0035 | 0.249 | 0.805244 | 0.953308 |
| X204483_at | -0.0009 | 0.0035 | -0.249 | 0.8052454 | 0.953308 |
| X213110_s_at | 0.0283 | 0.1139 | 0.249 | 0.8052796 | 0.953308 |
| X215316_at | 0.0018 | 0.0074 | 0.249 | 0.8053185 | 0.953308 |
| X216780_at | 0.0011 | 0.0045 | 0.249 | 0.805335 | 0.953308 |
| X205627_at | 0.0007 | 0.0028 | 0.249 | 0.805338 | 0.953308 |
| X201379_s_at | 0.0108 | 0.0436 | 0.248 | 0.8055443 | 0.953448 |
| X210220_at | -0.0013 | 0.0052 | -0.248 | 0.8055505 | 0.953448 |
| X221243_s_at | -0.0035 | 0.014 | -0.248 | 0.8056524 | 0.953448 |
| X213444_at | 0.0036 | 0.0146 | 0.248 | 0.8057337 | 0.953448 |
| X215858_at | 0.0008 | 0.0032 | 0.248 | 0.8057522 | 0.953448 |
| X209619_at | 0.0137 | 0.0552 | 0.248 | 0.8057556 | 0.953448 |
| X222062_at | 0.0013 | 0.0052 | 0.248 | 0.8057569 | 0.953448 |
| X210083_at | 0.0008 | 0.0031 | 0.248 | 0.8058075 | 0.953457 |
| X201846_s_at | -0.0166 | 0.067 | -0.248 | 0.8059417 | 0.953565 |
| X209439_s_at | -0.0121 | 0.049 | -0.248 | 0.8059961 | 0.953579 |
| X217421_at | 0.0007 | 0.0026 | 0.248 | 0.8060492 | 0.953583 |
| X202898_at | 0.0128 | 0.0516 | 0.248 | 0.8060863 | 0.953583 |
| X214503_x_at | -0.001 | 0.0041 | -0.247 | 0.8062035 | 0.953583 |
| X220996_s_at | -0.0027 | 0.0111 | -0.247 | 0.8062536 | 0.953583 |
| X209553_at | 0.0077 | 0.031 | 0.247 | 0.8062837 | 0.953583 |
| X210377_at | 0.0015 | 0.0063 | 0.247 | 0.8062844 | 0.953583 |
| X210457_x_at | -0.0009 | 0.0038 | -0.247 | 0.8063004 | 0.953583 |
| X217830_s_at | -0.0176 | 0.0711 | -0.247 | 0.8064096 | 0.953662 |
| X222214_at | -0.0132 | 0.0533 | -0.247 | 0.8065091 | 0.953729 |
| X202082_s_at | -0.0223 | 0.0904 | -0.247 | 0.8066803 | 0.95383 |
| X213334_x_at | 0.0115 | 0.0467 | 0.247 | 0.8067143 | 0.95383 |
| X204073_s_at | 0.0362 | 0.1465 | 0.247 | 0.8067232 | 0.95383 |
| X206077_at | 0.0008 | 0.003 | 0.247 | 0.8067814 | 0.953848 |
| X220571_at | 0.0006 | 0.0024 | 0.247 | 0.80685 | 0.953878 |
| X218898_at | -0.0055 | 0.0223 | -0.247 | 0.8068979 | 0.953884 |
| X207715_at | 0.0009 | 0.0037 | 0.246 | 0.8070308 | 0.953929 |
| X207775_at | -0.0016 | 0.0067 | -0.246 | 0.8070381 | 0.953929 |
| X210989_at | 0.0013 | 0.0052 | 0.246 | 0.8070951 | 0.953929 |
| X205258_at | -0.0009 | 0.0038 | -0.246 | 0.8071079 | 0.953929 |
| X213681_at | -0.0008 | 0.0034 | -0.246 | 0.8071523 | 0.953931 |
| X220365_at | -0.0013 | 0.0053 | -0.246 | 0.807215 | 0.953954 |
| X206532_at | -0.0023 | 0.0095 | -0.246 | 0.807326 | 0.954035 |
| X217390_x_at | -0.0021 | 0.0085 | -0.246 | 0.8074118 | 0.954065 |
| X211093_at | -0.0037 | 0.0148 | -0.246 | 0.8074375 | 0.954065 |
| X214582_at | 0.0052 | 0.0212 | 0.246 | 0.8074892 | 0.954075 |
| X204755_x_at | 0.0135 | 0.0549 | 0.246 | 0.8075963 | 0.954119 |
| X61874_at | 0.0033 | 0.0133 | 0.246 | 0.8076121 | 0.954119 |
| X206028_s_at | 0.0188 | 0.0766 | 0.245 | 0.8077449 | 0.95418 |
| X201780_s_at | 0.0173 | 0.0706 | 0.245 | 0.8077495 | 0.95418 |
| X212824_at | 0.0068 | 0.0278 | 0.245 | 0.8078585 | 0.954213 |
| X219973_at | -0.0071 | 0.029 | -0.245 | 0.8078638 | 0.954213 |
| X203497_at | -0.0061 | 0.025 | -0.245 | 0.807938 | 0.954226 |
| X217433_at | 0.0009 | 0.0038 | 0.245 | 0.8080006 | 0.954226 |
| X221077_at | 0.0012 | 0.0051 | 0.245 | 0.8080037 | 0.954226 |
| X217162_at | 0.0009 | 0.0036 | 0.245 | 0.8080613 | 0.954244 |
| X205496_at | 0.0077 | 0.0313 | 0.245 | 0.8082632 | 0.954279 |
| X210234_at | -0.001 | 0.0042 | -0.245 | 0.8082961 | 0.954279 |
| X215163_at | -0.0019 | 0.0076 | -0.245 | 0.8083093 | 0.954279 |
| X221593_s_at | -0.0146 | 0.0599 | -0.245 | 0.808327 | 0.954279 |
| X203688_at | 0.0095 | 0.0387 | 0.245 | 0.8083431 | 0.954279 |
| X211130_x_at | -0.0013 | 0.0053 | -0.245 | 0.8083488 | 0.954279 |
| X212222_at | 0.0139 | 0.057 | 0.245 | 0.8084007 | 0.954289 |
| X217083_at | -0.0013 | 0.0052 | -0.244 | 0.8084925 | 0.954347 |
| X200849_s_at | 0.0152 | 0.0623 | 0.244 | 0.8087477 | 0.95457 |
| X210431_at | -0.0012 | 0.0047 | -0.244 | 0.8087674 | 0.95457 |
| X200754_x_at | -0.0054 | 0.022 | -0.244 | 0.808962 | 0.954712 |
| X200842_s_at | 0.0103 | 0.0421 | 0.244 | 0.8089741 | 0.954712 |
| X205886_at | -0.0007 | 0.003 | -0.244 | 0.8091632 | 0.954844 |
| X205320_at | 0.005 | 0.0205 | 0.244 | 0.8091714 | 0.954844 |
| X216896_at | -0.001 | 0.0042 | -0.243 | 0.8092471 | 0.954882 |
| X206646_at | -0.0009 | 0.0039 | -0.243 | 0.8093538 | 0.954958 |
| X210818_s_at | -0.0011 | 0.0045 | -0.243 | 0.8094471 | 0.954984 |
| X215326_at | -0.0009 | 0.0037 | -0.243 | 0.8094693 | 0.954984 |
| X206310_at | 0.0009 | 0.0036 | 0.243 | 0.8095439 | 0.954984 |
| X214971_s_at | -0.0019 | 0.008 | -0.243 | 0.8095744 | 0.954984 |
| X213111_at | -0.0085 | 0.0349 | -0.243 | 0.809591 | 0.954984 |
| X215187_at | 0.005 | 0.0207 | 0.242 | 0.8101055 | 0.955448 |
| X221367_at | 0.0006 | 0.0025 | 0.242 | 0.8101717 | 0.955448 |
| X200800_s_at | -0.0287 | 0.1185 | -0.242 | 0.8101745 | 0.955448 |
| X220230_s_at | 0.0299 | 0.1236 | 0.242 | 0.810207 | 0.955448 |
| X213284_at | 0.0011 | 0.0045 | 0.242 | 0.8102292 | 0.955448 |
| X220895_at | -0.001 | 0.0043 | -0.242 | 0.8102429 | 0.955448 |
| X204782_at | 0.0014 | 0.0057 | 0.242 | 0.8104163 | 0.95557 |
| X204182_s_at | -0.0053 | 0.022 | -0.242 | 0.8104761 | 0.95557 |
| X201110_s_at | 0.0284 | 0.1176 | 0.242 | 0.8105111 | 0.95557 |
| X221137_at | -0.0014 | 0.0059 | -0.242 | 0.8105528 | 0.95557 |
| X220479_at | -0.0022 | 0.0091 | -0.242 | 0.8105609 | 0.95557 |
| X217058_at | 0.0016 | 0.0066 | 0.242 | 0.8106729 | 0.955645 |
| X209638_x_at | -0.0051 | 0.021 | -0.242 | 0.8107107 | 0.955645 |
| X214686_at | -0.0157 | 0.0649 | -0.241 | 0.8107889 | 0.955664 |
| X50277_at | -0.0062 | 0.0259 | -0.241 | 0.8108128 | 0.955664 |
| X205651_x_at | -0.0159 | 0.0657 | -0.241 | 0.8108853 | 0.955699 |
| X208278_s_at | -0.0013 | 0.0056 | -0.241 | 0.8109834 | 0.955763 |
| X206565_x_at | -0.027 | 0.1121 | -0.241 | 0.8110929 | 0.955842 |
| X208274_at | -0.0018 | 0.0076 | -0.241 | 0.8111427 | 0.95585 |
| X207390_s_at | -0.0004 | 0.0017 | -0.241 | 0.8112411 | 0.955888 |
| X49329_at | -0.0131 | 0.0543 | -0.241 | 0.8112615 | 0.955888 |
| X215857_at | 0.0008 | 0.0034 | 0.241 | 0.8113165 | 0.955898 |
| X214446_at | 0.0009 | 0.0037 | 0.241 | 0.8113658 | 0.955898 |
| X220192_x_at | 0.001 | 0.004 | 0.241 | 0.8114291 | 0.955898 |
| X204196_x_at | -0.0019 | 0.0078 | -0.241 | 0.811442 | 0.955898 |
| X214744_s_at | 0.0076 | 0.0316 | 0.241 | 0.8114955 | 0.955911 |
| X220414_at | -0.0007 | 0.0028 | -0.24 | 0.8116053 | 0.955969 |
| X220259_at | 0.0005 | 0.0021 | 0.24 | 0.8117285 | 0.955969 |
| X217123_x_at | -0.0025 | 0.0103 | -0.24 | 0.8117495 | 0.955969 |
| X211387_x_at | -0.0049 | 0.0204 | -0.24 | 0.8117882 | 0.955969 |
| X213885_at | 0.0016 | 0.0068 | 0.24 | 0.8118228 | 0.955969 |
| X220906_at | 0.0091 | 0.0378 | 0.24 | 0.8118253 | 0.955969 |
| X208182_x_at | 0.0011 | 0.0047 | 0.24 | 0.8118465 | 0.955969 |
| X209855_s_at | -0.001 | 0.0041 | -0.24 | 0.8119779 | 0.956012 |
| X206888_s_at | -0.0015 | 0.0064 | -0.24 | 0.812042 | 0.956012 |
| X218325_s_at | -0.0058 | 0.024 | -0.24 | 0.812069 | 0.956012 |
| X221148_at | -0.0011 | 0.0046 | -0.24 | 0.8121073 | 0.956012 |
| X204177_s_at | -0.0068 | 0.0283 | -0.24 | 0.8121599 | 0.956012 |
| X208751_at | -0.0085 | 0.0356 | -0.24 | 0.8121837 | 0.956012 |
| X216260_at | 0.0016 | 0.0069 | 0.24 | 0.812184 | 0.956012 |
| X207463_x_at | -0.0289 | 0.1208 | -0.24 | 0.812293 | 0.956046 |
| X215315_at | -0.0013 | 0.0054 | -0.239 | 0.8123031 | 0.956046 |
| X219777_at | 0.0181 | 0.0757 | 0.239 | 0.8123425 | 0.956046 |
| X202165_at | -0.0104 | 0.0435 | -0.239 | 0.8124123 | 0.956078 |
| X216709_at | -0.0025 | 0.0104 | -0.239 | 0.8124782 | 0.956105 |
| X210493_s_at | 0.0172 | 0.072 | 0.239 | 0.8126928 | 0.956162 |
| X215161_at | -0.0202 | 0.0846 | -0.239 | 0.8126993 | 0.956162 |
| X210504_at | -0.001 | 0.004 | -0.239 | 0.8127373 | 0.956162 |
| X200687_s_at | 0.0104 | 0.0437 | 0.239 | 0.8127829 | 0.956162 |
| X215682_at | 0.0008 | 0.0034 | 0.239 | 0.8127887 | 0.956162 |
| X217005_at | -0.001 | 0.0043 | -0.239 | 0.8127904 | 0.956162 |
| X210814_at | -0.0347 | 0.1453 | -0.239 | 0.8128685 | 0.956162 |
| X214918_at | -0.0038 | 0.016 | -0.239 | 0.8128818 | 0.956162 |
| X214873_at | -0.0014 | 0.006 | -0.239 | 0.8129272 | 0.956162 |
| X213536_s_at | 0.0013 | 0.0056 | 0.239 | 0.8129573 | 0.956162 |
| X1438_at | -0.0007 | 0.003 | -0.238 | 0.8131242 | 0.95626 |
| X206579_at | 0.0019 | 0.008 | 0.238 | 0.8131609 | 0.95626 |
| X221769_at | -0.0021 | 0.0087 | -0.238 | 0.81317 | 0.95626 |
| X219422_at | -0.0011 | 0.0045 | -0.238 | 0.8132588 | 0.956299 |
| X219197_s_at | -0.001 | 0.0041 | -0.238 | 0.8132885 | 0.956299 |
| X221513_s_at | 0.0092 | 0.0386 | 0.238 | 0.8133548 | 0.956304 |
| X214939_x_at | -0.0008 | 0.0035 | -0.238 | 0.8134561 | 0.956304 |
| X207429_at | -0.0009 | 0.004 | -0.238 | 0.8134974 | 0.956304 |
| X201283_s_at | 0.0009 | 0.0038 | 0.238 | 0.8135476 | 0.956304 |
| X203237_s_at | -0.0008 | 0.0035 | -0.238 | 0.8135914 | 0.956304 |
| X214325_at | -0.0007 | 0.0029 | -0.238 | 0.8136054 | 0.956304 |
| X205181_at | 0.0007 | 0.0031 | 0.238 | 0.8136159 | 0.956304 |
| X220311_at | 0.0015 | 0.0062 | 0.238 | 0.8136886 | 0.956304 |
| X200839_s_at | 0.0087 | 0.0368 | 0.238 | 0.8137559 | 0.956304 |
| X221570_s_at | 0.0066 | 0.0279 | 0.238 | 0.8137832 | 0.956304 |
| X213932_x_at | 0.0032 | 0.0137 | 0.237 | 0.8138666 | 0.956304 |
| X211823_s_at | -0.0006 | 0.0027 | -0.237 | 0.81387 | 0.956304 |
| X39548_at | 0.0092 | 0.0387 | 0.237 | 0.8139042 | 0.956304 |
| X208426_x_at | 0.001 | 0.0041 | 0.237 | 0.8139129 | 0.956304 |
| X207730_x_at | -0.0201 | 0.0847 | -0.237 | 0.8139608 | 0.956304 |
| X222219_s_at | -0.0009 | 0.004 | -0.237 | 0.8140284 | 0.956304 |
| X210839_s_at | 0.0301 | 0.127 | 0.237 | 0.8140591 | 0.956304 |
| X221128_at | 0.0012 | 0.005 | 0.237 | 0.8141206 | 0.956304 |
| X205333_s_at | 0.0013 | 0.0053 | 0.237 | 0.8141437 | 0.956304 |
| X216981_x_at | 0.001 | 0.0044 | 0.237 | 0.8142277 | 0.956304 |
| X212844_at | 0.0015 | 0.0063 | 0.237 | 0.8142321 | 0.956304 |
| X214154_s_at | -0.0007 | 0.0029 | -0.237 | 0.8142406 | 0.956304 |
| X217097_s_at | 0.0018 | 0.0078 | 0.237 | 0.8144339 | 0.956434 |
| X204042_at | -0.0091 | 0.0386 | -0.237 | 0.8144447 | 0.956434 |
| X215292_s_at | 0.0009 | 0.0036 | 0.237 | 0.8144805 | 0.956434 |
| X210505_at | -0.0022 | 0.0093 | -0.236 | 0.8146261 | 0.956493 |
| X219399_at | -0.0105 | 0.0442 | -0.236 | 0.8147201 | 0.956493 |
| X215847_at | -0.0019 | 0.0081 | -0.236 | 0.8147242 | 0.956493 |
| X214727_at | -0.0021 | 0.009 | -0.236 | 0.8148056 | 0.956493 |
| X49049_at | 0.0007 | 0.003 | 0.236 | 0.8148145 | 0.956493 |
| X217404_s_at | -0.001 | 0.0043 | -0.236 | 0.8148662 | 0.956493 |
| X216224_s_at | 0.0007 | 0.0029 | 0.236 | 0.8148686 | 0.956493 |
| X215960_at | 0.0011 | 0.0045 | 0.236 | 0.8148752 | 0.956493 |
| X217689_at | -0.001 | 0.0042 | -0.236 | 0.815088 | 0.956661 |
| X212476_at | -0.0116 | 0.0492 | -0.236 | 0.8151234 | 0.956661 |
| X204056_s_at | -0.0008 | 0.0036 | -0.236 | 0.8152221 | 0.956661 |
| X216625_at | 0.0059 | 0.0251 | 0.236 | 0.8152422 | 0.956661 |
| X220026_at | -0.0371 | 0.1574 | -0.236 | 0.8153049 | 0.956661 |
| X206160_at | 0.0009 | 0.0038 | 0.235 | 0.815377 | 0.956661 |
| X220352_x_at | 0.0148 | 0.0629 | 0.235 | 0.815537 | 0.956661 |
| X45714_at | -0.0088 | 0.0376 | -0.235 | 0.8155783 | 0.956661 |
| X201406_at | 0.007 | 0.03 | 0.235 | 0.815613 | 0.956661 |
| X208157_at | -0.0007 | 0.0031 | -0.235 | 0.8156238 | 0.956661 |
| X203630_s_at | 0.0126 | 0.0535 | 0.235 | 0.8156493 | 0.956661 |
| X219273_at | -0.0007 | 0.0029 | -0.235 | 0.8156607 | 0.956661 |
| X220972_s_at | 0.0018 | 0.0078 | 0.235 | 0.8156706 | 0.956661 |
| X205873_at | 0.0013 | 0.0054 | 0.235 | 0.8156767 | 0.956661 |
| X220514_at | -0.0008 | 0.0034 | -0.235 | 0.815728 | 0.956661 |
| X208421_at | 0.0011 | 0.0046 | 0.235 | 0.8157371 | 0.956661 |
| X210506_at | -0.0005 | 0.0023 | -0.235 | 0.8157499 | 0.956661 |
| X210125_s_at | -0.0008 | 0.0036 | -0.235 | 0.815884 | 0.956767 |
| X208480_s_at | -0.0005 | 0.0022 | -0.235 | 0.8160398 | 0.956767 |
| X209098_s_at | -0.0098 | 0.042 | -0.235 | 0.8160692 | 0.956767 |
| X200016_x_at | -0.0031 | 0.0131 | -0.235 | 0.8161041 | 0.956767 |
| X215087_at | 0.0011 | 0.0045 | 0.235 | 0.8161167 | 0.956767 |
| X212489_at | -0.002 | 0.0086 | -0.234 | 0.8162051 | 0.956767 |
| X207886_s_at | -0.0041 | 0.0176 | -0.234 | 0.8162203 | 0.956767 |
| X215176_x_at | -0.0008 | 0.0035 | -0.234 | 0.8162443 | 0.956767 |
| X217380_s_at | 0.0035 | 0.015 | 0.234 | 0.8162608 | 0.956767 |
| X212238_at | 0.0012 | 0.0051 | 0.234 | 0.8162952 | 0.956767 |
| X214476_at | 0.001 | 0.0041 | 0.234 | 0.8163138 | 0.956767 |
| X216484_x_at | 0.0054 | 0.0231 | 0.234 | 0.816465 | 0.956837 |
| X221258_s_at | 0.0026 | 0.0109 | 0.234 | 0.8165203 | 0.956837 |
| X202466_at | -0.0063 | 0.027 | -0.234 | 0.8165573 | 0.956837 |
| X215657_at | -0.0026 | 0.011 | -0.234 | 0.8165634 | 0.956837 |
| X208462_s_at | 0.0012 | 0.0051 | 0.234 | 0.8166256 | 0.956837 |
| X214667_s_at | 0.0015 | 0.0064 | 0.234 | 0.816632 | 0.956837 |
| X215121_x_at | -0.0008 | 0.0034 | -0.234 | 0.8167414 | 0.956914 |
| X204515_at | -0.0014 | 0.0061 | -0.234 | 0.8168504 | 0.956991 |
| X213065_at | 0.0078 | 0.0333 | 0.233 | 0.8171293 | 0.957268 |
| X220409_at | -0.0009 | 0.004 | -0.233 | 0.8173678 | 0.957484 |
| X65884_at | -0.0135 | 0.058 | -0.233 | 0.8173998 | 0.957484 |
| X214450_at | 0.0006 | 0.0025 | 0.233 | 0.8175515 | 0.957551 |
| X222283_at | -0.0033 | 0.0144 | -0.233 | 0.8175727 | 0.957551 |
| X220247_at | -0.0026 | 0.0113 | -0.233 | 0.8176331 | 0.957551 |
| X219592_at | 0.0008 | 0.0036 | 0.233 | 0.8176508 | 0.957551 |
| X210516_at | -0.0018 | 0.0077 | -0.232 | 0.8177086 | 0.957551 |
| X218328_at | 0.0018 | 0.0079 | 0.232 | 0.8178093 | 0.957551 |
| X220231_at | 0.0021 | 0.009 | 0.232 | 0.8178121 | 0.957551 |
| X205837_s_at | 0.004 | 0.0171 | 0.232 | 0.8178251 | 0.957551 |
| X209825_s_at | 0.0012 | 0.005 | 0.232 | 0.8178654 | 0.957551 |
| X210909_x_at | 0.0009 | 0.0039 | 0.232 | 0.8178886 | 0.957551 |
| X216029_at | -0.0011 | 0.0047 | -0.232 | 0.817946 | 0.957566 |
| X219934_s_at | -0.0014 | 0.006 | -0.232 | 0.8180026 | 0.957566 |
| X214321_at | 0.0176 | 0.0757 | 0.232 | 0.8180694 | 0.957566 |
| X207446_at | 0.0008 | 0.0033 | 0.232 | 0.8180746 | 0.957566 |
| X215151_at | -0.0028 | 0.0123 | -0.232 | 0.818117 | 0.957566 |
| X219835_at | -0.0012 | 0.0053 | -0.232 | 0.8181944 | 0.957606 |
| X215951_at | 0.0005 | 0.0021 | 0.232 | 0.8183912 | 0.957786 |
| X221402_at | -0.0011 | 0.0046 | -0.231 | 0.8185143 | 0.95788 |
| X213888_s_at | -0.0013 | 0.0057 | -0.231 | 0.8186246 | 0.957891 |
| X208784_s_at | -0.0131 | 0.0567 | -0.231 | 0.8186395 | 0.957891 |
| X211576_s_at | 0.0012 | 0.0051 | 0.231 | 0.8186534 | 0.957891 |
| X209989_at | -0.0137 | 0.0593 | -0.231 | 0.8188773 | 0.957976 |
| X212462_at | 0.0076 | 0.0328 | 0.231 | 0.8188928 | 0.957976 |
| X206980_s_at | -0.0009 | 0.0038 | -0.231 | 0.8189086 | 0.957976 |
| X212968_at | 0.0033 | 0.0142 | 0.231 | 0.8189332 | 0.957976 |
| X204387_x_at | -0.0079 | 0.0344 | -0.231 | 0.8189766 | 0.957976 |
| X208226_x_at | 0.0011 | 0.0048 | 0.231 | 0.8189847 | 0.957976 |
| X215836_s_at | -0.0065 | 0.0281 | -0.231 | 0.819122 | 0.958086 |
| X210635_s_at | -0.0012 | 0.0052 | -0.23 | 0.8192772 | 0.95817 |
| X208764_s_at | -0.0047 | 0.0205 | -0.23 | 0.8193294 | 0.95817 |
| X204111_at | -0.0022 | 0.0095 | -0.23 | 0.8193462 | 0.95817 |
| X219309_at | -0.0021 | 0.0091 | -0.23 | 0.8193658 | 0.95817 |
| X209701_at | -0.001 | 0.0044 | -0.23 | 0.81956 | 0.958346 |
| X218648_at | -0.0084 | 0.0364 | -0.23 | 0.8197275 | 0.958438 |
| X90265_at | 0.0126 | 0.0548 | 0.23 | 0.8197328 | 0.958438 |
| X205043_at | 0.0008 | 0.0035 | 0.23 | 0.819827 | 0.958438 |
| X202890_at | -0.0085 | 0.037 | -0.23 | 0.8198618 | 0.958438 |
| X206820_at | 0.0011 | 0.0049 | 0.23 | 0.8198842 | 0.958438 |
| X203761_at | 0.0145 | 0.0631 | 0.23 | 0.8199394 | 0.958438 |
| X205589_at | -0.0012 | 0.0051 | -0.23 | 0.8199686 | 0.958438 |
| X221703_at | -0.0026 | 0.0111 | -0.229 | 0.8199939 | 0.958438 |
| X219084_at | -0.0007 | 0.003 | -0.229 | 0.8200699 | 0.958438 |
| X219108_x_at | 0.0087 | 0.0379 | 0.229 | 0.8201109 | 0.958438 |
| X211521_s_at | 0.0007 | 0.0028 | 0.229 | 0.8201128 | 0.958438 |
| X221125_s_at | 0.001 | 0.0045 | 0.229 | 0.8202624 | 0.958478 |
| X221008_s_at | 0.0226 | 0.0986 | 0.229 | 0.8202722 | 0.958478 |
| X218921_at | -0.0188 | 0.0823 | -0.229 | 0.8203182 | 0.958478 |
| X220175_s_at | -0.0089 | 0.039 | -0.229 | 0.82032 | 0.958478 |
| X206700_s_at | 0.0058 | 0.0253 | 0.229 | 0.8205224 | 0.958603 |
| X215097_at | -0.0015 | 0.0064 | -0.229 | 0.8205428 | 0.958603 |
| X221072_at | 0.001 | 0.0044 | 0.229 | 0.820597 | 0.958603 |
| X216737_at | 0.0287 | 0.1256 | 0.229 | 0.8206205 | 0.958603 |
| X216346_at | 0.0008 | 0.0035 | 0.229 | 0.8206426 | 0.958603 |
| X215539_at | 0.0044 | 0.0193 | 0.229 | 0.8207145 | 0.958634 |
| X219840_s_at | 0.0014 | 0.0061 | 0.228 | 0.8207629 | 0.958634 |
| X205057_s_at | 0.0008 | 0.0033 | 0.228 | 0.8207986 | 0.958634 |
| X201929_s_at | 0.0257 | 0.1125 | 0.228 | 0.820884 | 0.958683 |
| X221158_at | 0.0027 | 0.012 | 0.228 | 0.8209343 | 0.958692 |
| X216158_at | -0.0016 | 0.0072 | -0.228 | 0.8210828 | 0.95877 |
| X207731_at | -0.0034 | 0.0149 | -0.228 | 0.8210876 | 0.95877 |
| X207108_s_at | -0.0111 | 0.0485 | -0.228 | 0.8211576 | 0.958801 |
| X205884_at | 0.0024 | 0.0103 | 0.228 | 0.8212285 | 0.958834 |
| X214108_at | -0.0008 | 0.0037 | -0.228 | 0.8213396 | 0.958896 |
| X220281_at | 0.0092 | 0.0405 | 0.228 | 0.8213679 | 0.958896 |
| X209601_at | -0.0012 | 0.0055 | -0.228 | 0.8214693 | 0.958929 |
| X220568_at | 0.0014 | 0.0062 | 0.228 | 0.8214835 | 0.958929 |
| X205571_at | -0.016 | 0.0703 | -0.227 | 0.821556 | 0.958929 |
| X220937_s_at | -0.0028 | 0.0122 | -0.227 | 0.8215691 | 0.958929 |
| X220290_at | 0.0045 | 0.0197 | 0.227 | 0.8216372 | 0.958958 |
| X216914_at | -0.0011 | 0.0048 | -0.227 | 0.8217747 | 0.959053 |
| X50965_at | 0.0168 | 0.0739 | 0.227 | 0.8218047 | 0.959053 |
| X221633_at | 0.0009 | 0.0041 | 0.227 | 0.8219218 | 0.959078 |
| X221458_at | 0.0014 | 0.0062 | 0.227 | 0.8219246 | 0.959078 |
| X202463_s_at | 0.0008 | 0.0035 | 0.227 | 0.8219559 | 0.959078 |
| X217382_at | -0.0016 | 0.0072 | -0.227 | 0.8220318 | 0.959116 |
| X213744_at | -0.0147 | 0.0648 | -0.227 | 0.8221167 | 0.959165 |
| X220680_at | -0.0014 | 0.0064 | -0.227 | 0.8222318 | 0.95922 |
| X220245_at | 0.0009 | 0.0041 | 0.227 | 0.8222502 | 0.95922 |
| X37004_at | -0.0012 | 0.0053 | -0.226 | 0.8224033 | 0.959335 |
| X220143_x_at | -0.0104 | 0.046 | -0.226 | 0.8224692 | 0.959335 |
| X207814_at | 0.001 | 0.0044 | 0.226 | 0.8224784 | 0.959335 |
| X220954_s_at | 0.0198 | 0.0874 | 0.226 | 0.8225349 | 0.959351 |
| X204760_s_at | 0.0006 | 0.0029 | 0.226 | 0.8226255 | 0.959406 |
| X221577_x_at | 0.0009 | 0.0039 | 0.226 | 0.8226932 | 0.959434 |
| X215462_at | -0.0028 | 0.0123 | -0.226 | 0.8228529 | 0.959563 |
| X206198_s_at | -0.0015 | 0.0068 | -0.226 | 0.8228897 | 0.959563 |
| X216990_at | 0.0022 | 0.01 | 0.225 | 0.823126 | 0.959771 |
| X210314_x_at | -0.0135 | 0.0599 | -0.225 | 0.8231779 | 0.959771 |
| X216444_at | -0.002 | 0.0089 | -0.225 | 0.8231978 | 0.959771 |
| X215203_at | -0.026 | 0.1155 | -0.225 | 0.8232494 | 0.959781 |
| X210023_s_at | 0.0007 | 0.0033 | 0.225 | 0.8233445 | 0.959781 |
| X211267_at | 0.0009 | 0.0041 | 0.225 | 0.8233793 | 0.959781 |
| X33322_i_at | -0.0152 | 0.0677 | -0.225 | 0.8233986 | 0.959781 |
| X215251_at | 0.0016 | 0.0069 | 0.225 | 0.8234226 | 0.959781 |
| X209749_s_at | -0.0008 | 0.0034 | -0.225 | 0.8236019 | 0.95994 |
| X221026_s_at | 0.001 | 0.0045 | 0.225 | 0.823756 | 0.96002 |
| X220433_at | -0.0013 | 0.0057 | -0.225 | 0.8237714 | 0.96002 |
| X205650_s_at | -0.0008 | 0.0035 | -0.225 | 0.8238354 | 0.96002 |
| X219169_s_at | -0.0092 | 0.0412 | -0.224 | 0.8238593 | 0.96002 |
| X216623_x_at | 0.0413 | 0.1839 | 0.224 | 0.823887 | 0.96002 |
| X207544_s_at | -0.0012 | 0.0056 | -0.224 | 0.8240467 | 0.960133 |
| X215016_x_at | 0.0051 | 0.0228 | 0.224 | 0.8240699 | 0.960133 |
| X203904_x_at | -0.0145 | 0.0649 | -0.224 | 0.8241425 | 0.960148 |
| X40284_at | 0.0013 | 0.006 | 0.224 | 0.8241691 | 0.960148 |
| X207160_at | -0.001 | 0.0043 | -0.224 | 0.8242522 | 0.960194 |
| X207454_at | -0.0007 | 0.0032 | -0.224 | 0.824307 | 0.960194 |
| X211585_at | -0.002 | 0.009 | -0.224 | 0.824339 | 0.960194 |
| X205927_s_at | -0.0013 | 0.0059 | -0.224 | 0.8244255 | 0.960204 |
| X201353_s_at | 0.001 | 0.0047 | 0.224 | 0.8244439 | 0.960204 |
| X213931_at | 0.0363 | 0.1625 | 0.224 | 0.8244773 | 0.960204 |
| X210830_s_at | 0.0217 | 0.0971 | 0.223 | 0.8246172 | 0.960233 |
| X213502_x_at | 0.0056 | 0.0251 | 0.223 | 0.8246633 | 0.960233 |
| X221439_at | -0.0012 | 0.0056 | -0.223 | 0.8247585 | 0.960233 |
| X216356_x_at | -0.0097 | 0.0435 | -0.223 | 0.8247931 | 0.960233 |
| X211254_x_at | 0.0007 | 0.0031 | 0.223 | 0.8248227 | 0.960233 |
| X217449_at | -0.0018 | 0.0081 | -0.223 | 0.8248702 | 0.960233 |
| X218683_at | 0.007 | 0.0313 | 0.223 | 0.8249151 | 0.960233 |
| X212995_x_at | 0.0074 | 0.0331 | 0.223 | 0.8249217 | 0.960233 |
| X213942_at | -0.0006 | 0.0026 | -0.223 | 0.8249248 | 0.960233 |
| X205009_at | 0.0016 | 0.007 | 0.223 | 0.8249428 | 0.960233 |
| X200059_s_at | 0.0096 | 0.0431 | 0.223 | 0.8249775 | 0.960233 |
| X206665_s_at | -0.0016 | 0.0071 | -0.223 | 0.8251131 | 0.960341 |
| X201697_s_at | -0.0066 | 0.0297 | -0.223 | 0.8253304 | 0.960494 |
| X207642_at | -0.0006 | 0.0029 | -0.223 | 0.825379 | 0.960494 |
| X204250_s_at | -0.0009 | 0.004 | -0.222 | 0.8253843 | 0.960494 |
| X220637_at | -0.0009 | 0.0042 | -0.222 | 0.8254298 | 0.960494 |
| X220273_at | -0.0008 | 0.0037 | -0.222 | 0.8254915 | 0.960494 |
| X210796_x_at | -0.0012 | 0.0053 | -0.222 | 0.8255556 | 0.960494 |
| X208575_at | -0.0007 | 0.0032 | -0.222 | 0.8256331 | 0.960494 |
| X216895_at | -0.0012 | 0.0056 | -0.222 | 0.8256915 | 0.960494 |
| X201349_at | -0.0181 | 0.0814 | -0.222 | 0.8257053 | 0.960494 |
| X220877_at | -0.0013 | 0.0059 | -0.222 | 0.8257203 | 0.960494 |
| X210015_s_at | 0.0318 | 0.1435 | 0.222 | 0.8257825 | 0.960494 |
| X215309_at | -0.0035 | 0.0156 | -0.222 | 0.8258333 | 0.960494 |
| X206331_at | 0.0024 | 0.0107 | 0.222 | 0.8258476 | 0.960494 |
| X216287_at | -0.0012 | 0.0053 | -0.222 | 0.8258498 | 0.960494 |
| X219650_at | -0.0016 | 0.0074 | -0.222 | 0.8258965 | 0.960498 |
| X216726_at | 0.0012 | 0.0056 | 0.222 | 0.8260866 | 0.96054 |
| X220380_at | 0.0009 | 0.0041 | 0.222 | 0.826141 | 0.96054 |
| X206021_at | -0.0015 | 0.007 | -0.221 | 0.8261558 | 0.96054 |
| X220510_at | -0.0005 | 0.0024 | -0.221 | 0.8261849 | 0.96054 |
| X201166_s_at | 0.0058 | 0.0264 | 0.221 | 0.8261969 | 0.96054 |
| X206145_at | -0.0015 | 0.007 | -0.221 | 0.8262991 | 0.96054 |
| X203235_at | 0.0034 | 0.0154 | 0.221 | 0.8263583 | 0.96054 |
| X218016_s_at | -0.0061 | 0.0274 | -0.221 | 0.8263584 | 0.96054 |
| X206002_at | -0.0009 | 0.0041 | -0.221 | 0.826363 | 0.96054 |
| X216085_at | 0.0013 | 0.0061 | 0.221 | 0.8263652 | 0.96054 |
| X215092_s_at | 0.057 | 0.2578 | 0.221 | 0.8265184 | 0.960599 |
| X215953_at | -0.0007 | 0.0031 | -0.221 | 0.8265289 | 0.960599 |
| X204510_at | 0.0114 | 0.0515 | 0.221 | 0.8265455 | 0.960599 |
| X203405_at | -0.0135 | 0.061 | -0.221 | 0.8266335 | 0.960603 |
| X208429_x_at | 0.0006 | 0.0027 | 0.221 | 0.8266648 | 0.960603 |
| X222343_at | 0.0022 | 0.0099 | 0.221 | 0.8266991 | 0.960603 |
| X211232_x_at | 0.0007 | 0.0031 | 0.221 | 0.8267357 | 0.960603 |
| X206064_s_at | 0.0012 | 0.0056 | 0.221 | 0.8267742 | 0.960603 |
| X217648_at | -0.0012 | 0.0056 | -0.221 | 0.826849 | 0.960603 |
| X207225_at | 0.0009 | 0.004 | 0.221 | 0.8268817 | 0.960603 |
| X209861_s_at | 0.0075 | 0.0341 | 0.221 | 0.8268954 | 0.960603 |
| X207062_at | -0.0017 | 0.0076 | -0.22 | 0.8270016 | 0.96062 |
| X217318_x_at | 0.0008 | 0.0037 | 0.22 | 0.8270438 | 0.96062 |
| X205045_at | -0.0011 | 0.0051 | -0.22 | 0.8271754 | 0.96062 |
| X214013_s_at | -0.0008 | 0.0038 | -0.22 | 0.8271848 | 0.96062 |
| X217282_at | 0.0045 | 0.0203 | 0.22 | 0.8272535 | 0.96062 |
| X215084_s_at | 0.0012 | 0.0055 | 0.22 | 0.8273062 | 0.96062 |
| X211481_at | -0.0121 | 0.0552 | -0.22 | 0.8273373 | 0.96062 |
| X220941_s_at | -0.0008 | 0.0035 | -0.22 | 0.8273627 | 0.96062 |
| X203187_at | 0.0196 | 0.089 | 0.22 | 0.8273844 | 0.96062 |
| X213354_s_at | 0.0012 | 0.0053 | 0.22 | 0.8274487 | 0.96062 |
| X205473_at | -0.0008 | 0.0038 | -0.22 | 0.827516 | 0.96062 |
| X217715_x_at | -0.0045 | 0.0207 | -0.22 | 0.8275397 | 0.96062 |
| X203046_s_at | 0.0009 | 0.0041 | 0.22 | 0.8275924 | 0.96062 |
| X208105_at | 0.0023 | 0.0103 | 0.22 | 0.8276615 | 0.96062 |
| X221111_at | -0.0027 | 0.0123 | -0.22 | 0.8276777 | 0.96062 |
| X220342_x_at | -0.008 | 0.0365 | -0.219 | 0.8277041 | 0.96062 |
| X208924_at | 0.0074 | 0.0335 | 0.219 | 0.8278321 | 0.96062 |
| X211605_s_at | -0.0011 | 0.005 | -0.219 | 0.8278908 | 0.96062 |
| X204864_s_at | -0.0007 | 0.0031 | -0.219 | 0.8279451 | 0.96062 |
| X37872_at | -0.0013 | 0.006 | -0.219 | 0.8279766 | 0.96062 |
| X211193_at | 0.0013 | 0.0061 | 0.219 | 0.8279932 | 0.96062 |
| X217088_s_at | 0.0006 | 0.0028 | 0.219 | 0.8280248 | 0.96062 |
| X217929_s_at | -0.0073 | 0.0334 | -0.219 | 0.828045 | 0.96062 |
| X210177_at | -0.0019 | 0.0089 | -0.219 | 0.8280454 | 0.96062 |
| X211353_at | -0.0006 | 0.0027 | -0.219 | 0.8280633 | 0.96062 |
| X220148_at | 0.0294 | 0.1344 | 0.219 | 0.8280751 | 0.96062 |
| X221193_s_at | -0.0085 | 0.0387 | -0.219 | 0.8280776 | 0.96062 |
| X217716_s_at | 0.0093 | 0.0425 | 0.219 | 0.8282011 | 0.960636 |
| X215615_x_at | 0.0054 | 0.0245 | 0.219 | 0.8282869 | 0.960636 |
| X218182_s_at | -0.0014 | 0.0066 | -0.219 | 0.8282923 | 0.960636 |
| X210738_s_at | 0.0038 | 0.0172 | 0.219 | 0.8283086 | 0.960636 |
| X203670_at | -0.0012 | 0.0057 | -0.219 | 0.8283186 | 0.960636 |
| X222188_at | 0.0013 | 0.0061 | 0.219 | 0.8283509 | 0.960636 |
| X205220_at | 0.001 | 0.0048 | 0.219 | 0.8284528 | 0.960647 |
| X216919_at | 0.0007 | 0.0034 | 0.219 | 0.8284568 | 0.960647 |
| X214606_at | 0.0042 | 0.0194 | 0.218 | 0.82849 | 0.960647 |
| X206694_at | 0.0008 | 0.0035 | 0.218 | 0.8286304 | 0.960709 |
| X206440_at | 0.0005 | 0.0022 | 0.218 | 0.828648 | 0.960709 |
| X211122_s_at | 0.002 | 0.0091 | 0.218 | 0.8286921 | 0.960709 |
| X220149_at | -0.0011 | 0.0052 | -0.218 | 0.8287424 | 0.960709 |
| X212003_at | 0.0006 | 0.0028 | 0.218 | 0.8287791 | 0.960709 |
| X203530_s_at | 0.0089 | 0.0406 | 0.218 | 0.8288334 | 0.960709 |
| X203024_s_at | 0.0094 | 0.0431 | 0.218 | 0.8288873 | 0.960709 |
| X209521_s_at | -0.0016 | 0.0074 | -0.218 | 0.8289003 | 0.960709 |
| X206490_at | 0.0185 | 0.0851 | 0.218 | 0.8289605 | 0.960709 |
| X216528_at | -0.0016 | 0.0075 | -0.218 | 0.8289757 | 0.960709 |
| X217717_s_at | -0.0108 | 0.0496 | -0.218 | 0.8290461 | 0.96074 |
| X208357_x_at | 0.0007 | 0.0032 | 0.218 | 0.8291047 | 0.960752 |
| X219672_at | 0.0009 | 0.004 | 0.218 | 0.8291805 | 0.960752 |
| X207809_s_at | -0.0121 | 0.0556 | -0.218 | 0.8292071 | 0.960752 |
| X216923_at | -0.0006 | 0.0028 | -0.218 | 0.8292288 | 0.960752 |
| X208475_at | -0.0021 | 0.0096 | -0.217 | 0.8292889 | 0.960771 |
| X204999_s_at | -0.0008 | 0.0038 | -0.217 | 0.8293931 | 0.960842 |
| X209905_at | -0.0013 | 0.0061 | -0.217 | 0.8296155 | 0.961049 |
| X216419_at | -0.0009 | 0.0043 | -0.217 | 0.8297058 | 0.961104 |
| X210846_x_at | -0.0009 | 0.0041 | -0.217 | 0.8297639 | 0.961113 |
| X204571_x_at | 0.0057 | 0.0262 | 0.217 | 0.8298007 | 0.961113 |
| X208493_at | -0.0005 | 0.0023 | -0.217 | 0.8299249 | 0.961207 |
| X221589_s_at | 0.0141 | 0.065 | 0.217 | 0.829979 | 0.96122 |
| X201537_s_at | -0.001 | 0.0047 | -0.216 | 0.8300856 | 0.961264 |
| X217291_at | 0.0011 | 0.0051 | 0.216 | 0.830148 | 0.961264 |
| X65517_at | -0.0008 | 0.0036 | -0.216 | 0.8301487 | 0.961264 |
| X220187_at | -0.0022 | 0.0103 | -0.216 | 0.8301908 | 0.961264 |
| X210474_s_at | 0.0014 | 0.0064 | 0.216 | 0.8302742 | 0.961311 |
| X221342_at | 0.0007 | 0.0033 | 0.216 | 0.8304672 | 0.961398 |
| X220493_at | -0.0016 | 0.0075 | -0.216 | 0.8305027 | 0.961398 |
| X204294_at | 0.015 | 0.0696 | 0.216 | 0.8305251 | 0.961398 |
| X219971_at | 0.0011 | 0.005 | 0.216 | 0.8305314 | 0.961398 |
| X1320_at | 0.001 | 0.0046 | 0.216 | 0.8305676 | 0.961398 |
| X211336_x_at | 0.0007 | 0.0033 | 0.216 | 0.8306463 | 0.961398 |
| X219003_s_at | -0.0084 | 0.0391 | -0.216 | 0.8306526 | 0.961398 |
| X222179_at | -0.0005 | 0.0025 | -0.215 | 0.8309664 | 0.96166 |
| X220023_at | -0.0005 | 0.0025 | -0.215 | 0.8310282 | 0.96166 |
| X220207_at | -0.0011 | 0.0049 | -0.215 | 0.8310484 | 0.96166 |
| X211718_at | -0.001 | 0.0045 | -0.215 | 0.8310521 | 0.96166 |
| X205375_at | -0.0014 | 0.0063 | -0.215 | 0.8312031 | 0.961785 |
| X204528_s_at | 0.0055 | 0.0256 | 0.215 | 0.8313175 | 0.961792 |
| X203980_at | 0.001 | 0.0049 | 0.215 | 0.8313704 | 0.961792 |
| X207187_at | -0.001 | 0.0046 | -0.215 | 0.8313777 | 0.961792 |
| X211660_at | -0.0014 | 0.0064 | -0.215 | 0.8313827 | 0.961792 |
| X206155_at | -0.0009 | 0.0042 | -0.215 | 0.8314366 | 0.961805 |
| X221894_at | 0.0057 | 0.0265 | 0.214 | 0.831639 | 0.961868 |
| X219115_s_at | -0.0129 | 0.0601 | -0.214 | 0.831699 | 0.961868 |
| X217547_x_at | -0.0045 | 0.0212 | -0.214 | 0.8317011 | 0.961868 |
| X221138_s_at | -0.0024 | 0.0111 | -0.214 | 0.831712 | 0.961868 |
| X1487_at | 0.0016 | 0.0075 | 0.214 | 0.8317949 | 0.961868 |
| X219723_x_at | -0.001 | 0.0045 | -0.214 | 0.8318018 | 0.961868 |
| X215958_at | -0.0011 | 0.0052 | -0.214 | 0.8318025 | 0.961868 |
| X201510_at | -0.0017 | 0.0081 | -0.214 | 0.8318528 | 0.961868 |
| X209402_s_at | -0.0008 | 0.0039 | -0.214 | 0.8318809 | 0.961868 |
| X216754_at | -0.0008 | 0.0038 | -0.214 | 0.831978 | 0.96193 |
| X211127_x_at | -0.0016 | 0.0076 | -0.214 | 0.8321623 | 0.962064 |
| X207867_at | 0.0023 | 0.0105 | 0.214 | 0.8321806 | 0.962064 |
| X221372_s_at | -0.0008 | 0.0036 | -0.214 | 0.8322678 | 0.962069 |
| X210089_s_at | -0.0014 | 0.0068 | -0.214 | 0.8322814 | 0.962069 |
| X212634_at | 0.0051 | 0.0237 | 0.213 | 0.8323621 | 0.962069 |
| X215523_at | 0.001 | 0.0047 | 0.213 | 0.8324001 | 0.962069 |
| X220965_s_at | 0.0007 | 0.0033 | 0.213 | 0.832401 | 0.962069 |
| X206515_at | 0.0025 | 0.0116 | 0.213 | 0.8327392 | 0.962284 |
| X212022_s_at | -0.0012 | 0.0056 | -0.213 | 0.8327441 | 0.962284 |
| X222198_at | 0.001 | 0.0047 | 0.213 | 0.8327597 | 0.962284 |
| X220567_at | -0.0016 | 0.0073 | -0.213 | 0.8327605 | 0.962284 |
| X208454_s_at | -0.0018 | 0.0083 | -0.213 | 0.8328232 | 0.962306 |
| X213463_s_at | 0.0124 | 0.0583 | 0.213 | 0.8329655 | 0.962391 |
| X214716_at | -0.0051 | 0.0241 | -0.213 | 0.8330323 | 0.962391 |
| X217111_at | -0.0015 | 0.0072 | -0.213 | 0.8330396 | 0.962391 |
| X203122_at | -0.0094 | 0.044 | -0.212 | 0.8331128 | 0.962391 |
| X218435_at | 0.0155 | 0.0728 | 0.212 | 0.8332091 | 0.962391 |
| X207437_at | 0.0129 | 0.0609 | 0.212 | 0.8332677 | 0.962391 |
| X209354_at | -0.0011 | 0.0052 | -0.212 | 0.8333175 | 0.962391 |
| X208844_at | 0.001 | 0.0047 | 0.212 | 0.8333738 | 0.962391 |
| X213869_x_at | -0.0088 | 0.0413 | -0.212 | 0.8333783 | 0.962391 |
| X221687_s_at | -0.0018 | 0.0083 | -0.212 | 0.8333932 | 0.962391 |
| X217316_at | -0.0022 | 0.0102 | -0.212 | 0.8334323 | 0.962391 |
| X215850_s_at | 0.003 | 0.0141 | 0.212 | 0.8334963 | 0.962391 |
| X214043_at | 0.0136 | 0.0641 | 0.212 | 0.8335729 | 0.962391 |
| X216870_x_at | -0.0044 | 0.0209 | -0.212 | 0.8335984 | 0.962391 |
| X221443_x_at | -0.0008 | 0.0036 | -0.212 | 0.8336779 | 0.962391 |
| X205189_s_at | 0.0023 | 0.0108 | 0.212 | 0.833725 | 0.962391 |
| X218877_s_at | -0.0097 | 0.0457 | -0.212 | 0.8337527 | 0.962391 |
| X211845_at | -0.0014 | 0.0067 | -0.212 | 0.8337673 | 0.962391 |
| X201830_s_at | 0.0206 | 0.0975 | 0.212 | 0.833778 | 0.962391 |
| X206407_s_at | 0.0012 | 0.0058 | 0.212 | 0.8337896 | 0.962391 |
| X217496_s_at | -0.0012 | 0.0055 | -0.212 | 0.8338313 | 0.962391 |
| X206893_at | 0.022 | 0.1039 | 0.212 | 0.8338497 | 0.962391 |
| X211760_s_at | 0.013 | 0.0617 | 0.211 | 0.8339276 | 0.962398 |
| X217126_at | 0.0025 | 0.0118 | 0.211 | 0.8339648 | 0.962398 |
| X215784_at | 0.0013 | 0.0062 | 0.211 | 0.8340172 | 0.962398 |
| X207210_at | 0.0564 | 0.2667 | 0.211 | 0.8340417 | 0.962398 |
| X201088_at | 0.0062 | 0.0292 | 0.211 | 0.8340726 | 0.962398 |
| X207259_at | 0.0017 | 0.008 | 0.211 | 0.8342629 | 0.962559 |
| X217905_at | 0.0027 | 0.0126 | 0.211 | 0.8343336 | 0.962559 |
| X207567_at | -0.0014 | 0.0065 | -0.211 | 0.8343424 | 0.962559 |
| X204642_at | 0.011 | 0.0521 | 0.211 | 0.8343989 | 0.962575 |
| X205102_at | 0.0009 | 0.0042 | 0.211 | 0.8345293 | 0.962598 |
| X219254_at | 0.0009 | 0.0041 | 0.211 | 0.8345817 | 0.962598 |
| X210160_at | -0.0015 | 0.0073 | -0.211 | 0.8346046 | 0.962598 |
| X209887_at | -0.0006 | 0.0029 | -0.211 | 0.8346221 | 0.962598 |
| X210692_s_at | 0.0014 | 0.0066 | 0.21 | 0.8346965 | 0.962598 |
| X217829_s_at | -0.0104 | 0.0496 | -0.21 | 0.8347042 | 0.962598 |
| X211635_x_at | 0.0007 | 0.0033 | 0.21 | 0.8347279 | 0.962598 |
| X205353_s_at | 0.0048 | 0.0229 | 0.21 | 0.8347657 | 0.962598 |
| X208100_x_at | -0.0005 | 0.0023 | -0.21 | 0.8348316 | 0.962617 |
| X218780_at | -0.0007 | 0.0032 | -0.21 | 0.834869 | 0.962617 |
| X202274_at | -0.0014 | 0.0065 | -0.21 | 0.8349556 | 0.962637 |
| X220027_s_at | 0.0009 | 0.0041 | 0.21 | 0.8350795 | 0.962637 |
| X217701_x_at | -0.0034 | 0.0162 | -0.21 | 0.8351039 | 0.962637 |
| X206369_s_at | 0.0012 | 0.0056 | 0.21 | 0.8351098 | 0.962637 |
| X207943_x_at | 0.0067 | 0.032 | 0.21 | 0.8351647 | 0.962637 |
| X221163_s_at | 0.001 | 0.0049 | 0.21 | 0.8351829 | 0.962637 |
| X222255_at | -0.0005 | 0.0025 | -0.21 | 0.8351892 | 0.962637 |
| X221396_at | 0.0011 | 0.0053 | 0.21 | 0.8352832 | 0.962678 |
| X201104_x_at | -0.0113 | 0.0538 | -0.21 | 0.8353122 | 0.962678 |
| X205033_s_at | 0.0014 | 0.0066 | 0.209 | 0.8355112 | 0.96277 |
| X219626_at | 0.0011 | 0.0054 | 0.209 | 0.83552 | 0.96277 |
| X205997_at | -0.0042 | 0.0201 | -0.209 | 0.8355528 | 0.96277 |
| X221375_at | 0.0008 | 0.004 | 0.209 | 0.8355982 | 0.96277 |
| X55692_at | 0.0046 | 0.022 | 0.209 | 0.8356084 | 0.96277 |
| X200795_at | -0.0115 | 0.0549 | -0.209 | 0.8356943 | 0.962802 |
| X220732_at | 0.0016 | 0.0074 | 0.209 | 0.8357676 | 0.962802 |
| X206891_at | -0.0006 | 0.0029 | -0.209 | 0.835788 | 0.962802 |
| X209006_s_at | -0.0237 | 0.1136 | -0.209 | 0.8358096 | 0.962802 |
| X204790_at | 0.0143 | 0.0687 | 0.209 | 0.8359703 | 0.962932 |
| X221183_at | 0.0009 | 0.0043 | 0.209 | 0.8360291 | 0.962932 |
| X207908_at | 0.0009 | 0.0042 | 0.209 | 0.8360526 | 0.962932 |
| X207280_at | 0.0008 | 0.0038 | 0.208 | 0.8362351 | 0.962968 |
| X209096_at | -0.009 | 0.0429 | -0.208 | 0.8362384 | 0.962968 |
| X205754_at | -0.0005 | 0.0023 | -0.208 | 0.8362484 | 0.962968 |
| X209679_s_at | 0.0008 | 0.0039 | 0.208 | 0.8362986 | 0.962968 |
| X219127_at | 0.0012 | 0.0057 | 0.208 | 0.8363308 | 0.962968 |
| X221625_at | -0.0022 | 0.0107 | -0.208 | 0.8363811 | 0.962968 |
| X211013_x_at | -0.0005 | 0.0023 | -0.208 | 0.8363929 | 0.962968 |
| X205706_s_at | 0.0085 | 0.041 | 0.208 | 0.8365015 | 0.962968 |
| X206206_at | -0.001 | 0.0049 | -0.208 | 0.8365207 | 0.962968 |
| X212333_at | 0.0087 | 0.0416 | 0.208 | 0.8365233 | 0.962968 |
| X212098_at | 0.0061 | 0.0295 | 0.208 | 0.8365601 | 0.962968 |
| X207546_at | -0.001 | 0.005 | -0.208 | 0.8366439 | 0.963014 |
| X217350_at | -0.0009 | 0.0043 | -0.208 | 0.8367486 | 0.963085 |
| X216341_s_at | 0.0012 | 0.0059 | 0.207 | 0.837029 | 0.963292 |
| X220420_at | -0.0013 | 0.0065 | -0.207 | 0.8370451 | 0.963292 |
| X207880_at | 0.0006 | 0.0028 | 0.207 | 0.8371175 | 0.963292 |
| X208155_x_at | 0.0008 | 0.004 | 0.207 | 0.8371364 | 0.963292 |
| X208635_x_at | -0.0039 | 0.0189 | -0.207 | 0.8371875 | 0.963292 |
| X215104_at | 0.0106 | 0.051 | 0.207 | 0.8371892 | 0.963292 |
| X214486_x_at | 0.0009 | 0.0043 | 0.207 | 0.8374592 | 0.963553 |
| X218158_s_at | 0.007 | 0.034 | 0.207 | 0.837595 | 0.963659 |
| X215574_at | 0.0043 | 0.021 | 0.206 | 0.8377582 | 0.963707 |
| X213966_at | 0.0008 | 0.0038 | 0.206 | 0.8378446 | 0.963707 |
| X215793_at | -0.0018 | 0.0089 | -0.206 | 0.8378631 | 0.963707 |
| X204143_s_at | -0.012 | 0.0584 | -0.206 | 0.8378959 | 0.963707 |
| X218162_at | -0.0235 | 0.1138 | -0.206 | 0.837938 | 0.963707 |
| X214122_at | -0.0008 | 0.0037 | -0.206 | 0.8380198 | 0.963707 |
| X202532_s_at | 0.0125 | 0.0605 | 0.206 | 0.838026 | 0.963707 |
| X209968_s_at | -0.0165 | 0.0803 | -0.206 | 0.8380501 | 0.963707 |
| X209240_at | 0.0085 | 0.0414 | 0.206 | 0.83806 | 0.963707 |
| X214179_s_at | -0.0062 | 0.0303 | -0.206 | 0.8381284 | 0.963707 |
| X207134_x_at | 0.0006 | 0.0029 | 0.206 | 0.8382151 | 0.963707 |
| X220697_at | 0.0023 | 0.0112 | 0.206 | 0.838217 | 0.963707 |
| X207820_at | -0.0015 | 0.0075 | -0.206 | 0.8382825 | 0.963707 |
| X219153_s_at | -0.0007 | 0.0034 | -0.206 | 0.8382931 | 0.963707 |
| X209368_at | -0.0011 | 0.0052 | -0.206 | 0.838345 | 0.963707 |
| X208202_s_at | -0.0011 | 0.0052 | -0.206 | 0.838374 | 0.963707 |
| X34868_at | -0.0031 | 0.0153 | -0.206 | 0.8384032 | 0.963707 |
| X212564_at | 0.0092 | 0.045 | 0.206 | 0.838437 | 0.963707 |
| X206387_at | -0.0005 | 0.0024 | -0.206 | 0.8384604 | 0.963707 |
| X208097_s_at | -0.0057 | 0.0275 | -0.205 | 0.8385456 | 0.963755 |
| X216104_at | -0.0019 | 0.0092 | -0.205 | 0.8387319 | 0.963877 |
| X210593_at | -0.0009 | 0.0042 | -0.205 | 0.838739 | 0.963877 |
| X209779_at | 0.0017 | 0.0084 | 0.205 | 0.8389984 | 0.964081 |
| X206692_at | -0.0009 | 0.0046 | -0.205 | 0.8390031 | 0.964081 |
| X207933_at | -0.0008 | 0.0037 | -0.205 | 0.8391636 | 0.964166 |
| X206844_at | 0.0008 | 0.0037 | 0.205 | 0.8391982 | 0.964166 |
| X214594_x_at | 0.0173 | 0.0847 | 0.205 | 0.8392257 | 0.964166 |
| X208438_s_at | -0.0015 | 0.0072 | -0.205 | 0.8392504 | 0.964166 |
| X207626_s_at | -0.0007 | 0.0034 | -0.204 | 0.8393711 | 0.964255 |
| X211722_s_at | 0.0014 | 0.0071 | 0.204 | 0.8394175 | 0.964258 |
| X220382_s_at | 0.001 | 0.005 | 0.204 | 0.8394688 | 0.964267 |
| X207558_s_at | 0.0023 | 0.0112 | 0.204 | 0.8395471 | 0.964271 |
| X220293_at | -0.0008 | 0.004 | -0.204 | 0.839559 | 0.964271 |
| X214579_at | 0.015 | 0.0737 | 0.204 | 0.8396598 | 0.964273 |
| X213838_at | 0.0063 | 0.0309 | 0.204 | 0.8396876 | 0.964273 |
| X210274_at | -0.0014 | 0.0069 | -0.204 | 0.8396912 | 0.964273 |
| X217624_at | 0.0022 | 0.0108 | 0.204 | 0.8397601 | 0.964278 |
| X216872_at | -0.0014 | 0.007 | -0.204 | 0.8397819 | 0.964278 |
| X204079_at | 0.0134 | 0.0658 | 0.204 | 0.8398938 | 0.964334 |
| X211800_s_at | 0.0071 | 0.0347 | 0.204 | 0.8399527 | 0.964334 |
| X210361_s_at | -0.0109 | 0.0536 | -0.204 | 0.8399788 | 0.964334 |
| X206351_s_at | -0.0022 | 0.0109 | -0.204 | 0.8400685 | 0.964334 |
| X216657_at | -0.0032 | 0.0155 | -0.203 | 0.8401473 | 0.964334 |
| X213570_at | 0.0011 | 0.0054 | 0.203 | 0.8402023 | 0.964334 |
| X201962_s_at | -0.0089 | 0.0438 | -0.203 | 0.8402035 | 0.964334 |
| X206016_at | -0.0048 | 0.0237 | -0.203 | 0.8402223 | 0.964334 |
| X209655_s_at | -0.0113 | 0.0557 | -0.203 | 0.8402259 | 0.964334 |
| X211189_x_at | 0.0008 | 0.004 | 0.203 | 0.840318 | 0.964334 |
| X222235_s_at | 0.0104 | 0.0512 | 0.203 | 0.8403482 | 0.964334 |
| X210895_s_at | 0.001 | 0.0047 | 0.203 | 0.8403767 | 0.964334 |
| X220405_at | 0.0204 | 0.1004 | 0.203 | 0.8404546 | 0.964334 |
| X214660_at | -0.0039 | 0.0192 | -0.203 | 0.8404703 | 0.964334 |
| X212631_at | 0.0068 | 0.0336 | 0.203 | 0.8404819 | 0.964334 |
| X219057_at | -0.0009 | 0.0045 | -0.203 | 0.8406143 | 0.964402 |
| X206475_x_at | 0.0006 | 0.0028 | 0.203 | 0.8406281 | 0.964402 |
| X216200_at | 0.0017 | 0.0086 | 0.203 | 0.84074 | 0.964449 |
| X214800_x_at | 0.0049 | 0.0243 | 0.202 | 0.8408608 | 0.964449 |
| X215159_s_at | -0.001 | 0.0048 | -0.202 | 0.8408725 | 0.964449 |
| X218699_at | 0.0108 | 0.0534 | 0.202 | 0.8410151 | 0.964449 |
| X218039_at | 0.003 | 0.0151 | 0.202 | 0.8410177 | 0.964449 |
| X219597_s_at | -0.0009 | 0.0042 | -0.202 | 0.8410225 | 0.964449 |
| X211011_at | 0.0006 | 0.0029 | 0.202 | 0.8410826 | 0.964449 |
| X221178_at | -0.0012 | 0.0058 | -0.202 | 0.8410838 | 0.964449 |
| X203380_x_at | 0.0096 | 0.0476 | 0.202 | 0.841208 | 0.964449 |
| X215806_x_at | -0.0107 | 0.053 | -0.202 | 0.8412681 | 0.964449 |
| X210675_s_at | -0.0175 | 0.0865 | -0.202 | 0.841301 | 0.964449 |
| X216874_at | -0.0018 | 0.0089 | -0.202 | 0.8413332 | 0.964449 |
| X212666_at | -0.0044 | 0.0216 | -0.202 | 0.841388 | 0.964449 |
| X213332_at | 0.001 | 0.0049 | 0.202 | 0.8414061 | 0.964449 |
| X201211_s_at | 0.002 | 0.0102 | 0.202 | 0.8415128 | 0.964449 |
| X204596_s_at | -0.0007 | 0.0034 | -0.202 | 0.841536 | 0.964449 |
| X220823_at | -0.0018 | 0.0088 | -0.202 | 0.8416114 | 0.964449 |
| X220617_s_at | 0.009 | 0.0447 | 0.201 | 0.8416456 | 0.964449 |
| X212712_at | 0.0046 | 0.023 | 0.201 | 0.8416789 | 0.964449 |
| X210712_at | -0.0015 | 0.0073 | -0.201 | 0.8416937 | 0.964449 |
| X203667_at | -0.0054 | 0.0266 | -0.201 | 0.8416975 | 0.964449 |
| X204795_at | 0.0047 | 0.0232 | 0.201 | 0.8417435 | 0.964449 |
| X216675_at | 0.0008 | 0.0042 | 0.201 | 0.8417851 | 0.964449 |
| X216330_s_at | -0.0007 | 0.0034 | -0.201 | 0.8418344 | 0.964449 |
| X206921_at | 0.001 | 0.0052 | 0.201 | 0.8418646 | 0.964449 |
| X214386_at | 0.0014 | 0.0069 | 0.201 | 0.8418792 | 0.964449 |
| X218757_s_at | -0.0076 | 0.0377 | -0.201 | 0.8419051 | 0.964449 |
| X211710_x_at | -0.004 | 0.0199 | -0.201 | 0.8419243 | 0.964449 |
| X208094_s_at | 0.0013 | 0.0064 | 0.201 | 0.8419281 | 0.964449 |
| X217584_at | -0.0013 | 0.0066 | -0.201 | 0.8420112 | 0.964494 |
| X212525_s_at | 0.0008 | 0.0042 | 0.201 | 0.8421288 | 0.964579 |
| X214882_s_at | -0.0069 | 0.0345 | -0.201 | 0.8422148 | 0.964593 |
| X215278_at | -0.001 | 0.0051 | -0.201 | 0.8422277 | 0.964593 |
| X215333_x_at | 0.0101 | 0.0503 | 0.201 | 0.8423322 | 0.964599 |
| X209851_at | -0.0016 | 0.0078 | -0.201 | 0.8423356 | 0.964599 |
| X216331_at | -0.0088 | 0.0441 | -0.201 | 0.8423634 | 0.964599 |
| X207995_s_at | -0.0014 | 0.0068 | -0.2 | 0.8424276 | 0.964623 |
| X202371_at | 0.0033 | 0.0164 | 0.2 | 0.8425208 | 0.964673 |
| X214822_at | 0.0091 | 0.0456 | 0.2 | 0.8426774 | 0.964673 |
| X203513_at | 0.0089 | 0.0443 | 0.2 | 0.8426837 | 0.964673 |
| X219790_s_at | -0.0006 | 0.0031 | -0.2 | 0.8427582 | 0.964673 |
| X222183_x_at | 0.008 | 0.0402 | 0.2 | 0.8428231 | 0.964673 |
| X209201_x_at | -0.0018 | 0.0089 | -0.2 | 0.8428303 | 0.964673 |
| X203218_at | 0.0093 | 0.0463 | 0.2 | 0.8429238 | 0.964673 |
| X203144_s_at | 0.001 | 0.0049 | 0.2 | 0.8429247 | 0.964673 |
| X216440_at | 0.0013 | 0.0065 | 0.2 | 0.8429628 | 0.964673 |
| X221169_s_at | 0.0009 | 0.0044 | 0.2 | 0.842968 | 0.964673 |
| X215374_at | 0.0047 | 0.0234 | 0.2 | 0.8430262 | 0.964673 |
| X204588_s_at | 0.0008 | 0.0042 | 0.2 | 0.8430341 | 0.964673 |
| X205691_at | 0.0088 | 0.0441 | 0.199 | 0.8431874 | 0.964673 |
| X217327_at | 0.001 | 0.0051 | 0.199 | 0.8432644 | 0.964673 |
| X220061_at | 0.0017 | 0.0087 | 0.199 | 0.8433458 | 0.964673 |
| X205235_s_at | 0.0051 | 0.0255 | 0.199 | 0.84338 | 0.964673 |
| X219391_at | 0.0014 | 0.0072 | 0.199 | 0.8434259 | 0.964673 |
| X209886_s_at | -0.0014 | 0.007 | -0.199 | 0.8434505 | 0.964673 |
| X204296_at | -0.0015 | 0.0077 | -0.199 | 0.8435657 | 0.964673 |
| X220430_at | -0.0009 | 0.0046 | -0.199 | 0.8435721 | 0.964673 |
| X216063_at | 0.0007 | 0.0036 | 0.199 | 0.843573 | 0.964673 |
| X219456_s_at | -0.0005 | 0.0026 | -0.199 | 0.8436229 | 0.964673 |
| X209684_at | 0.0118 | 0.0594 | 0.199 | 0.8436264 | 0.964673 |
| X221121_at | 0.001 | 0.0051 | 0.199 | 0.8437065 | 0.964673 |
| X213267_at | -0.0172 | 0.0864 | -0.199 | 0.8437123 | 0.964673 |
| X221516_s_at | -0.0074 | 0.0372 | -0.199 | 0.8437192 | 0.964673 |
| X215365_at | -0.0016 | 0.0079 | -0.199 | 0.8437256 | 0.964673 |
| X214158_s_at | -0.0009 | 0.0046 | -0.199 | 0.8437426 | 0.964673 |
| X215369_at | -0.0011 | 0.0055 | -0.199 | 0.8437805 | 0.964673 |
| X209992_at | 0.0211 | 0.1061 | 0.199 | 0.8438169 | 0.964673 |
| X217258_x_at | 0.001 | 0.0051 | 0.199 | 0.8438185 | 0.964673 |
| X210413_x_at | 0.0009 | 0.0046 | 0.199 | 0.8439247 | 0.964673 |
| X217621_at | -0.002 | 0.0101 | -0.198 | 0.8439813 | 0.964673 |
| X208753_s_at | 0.0064 | 0.0321 | 0.198 | 0.8439826 | 0.964673 |
| X204743_at | -0.0073 | 0.037 | -0.198 | 0.8439924 | 0.964673 |
| X215242_at | -0.0023 | 0.0114 | -0.198 | 0.8440343 | 0.964673 |
| X211355_x_at | 0.0013 | 0.0067 | 0.198 | 0.8443349 | 0.96492 |
| X202722_s_at | -0.0247 | 0.1249 | -0.198 | 0.844402 | 0.96492 |
| X218305_at | 0.0015 | 0.0076 | 0.198 | 0.8444198 | 0.96492 |
| X210472_at | -0.0012 | 0.0061 | -0.198 | 0.8444241 | 0.96492 |
| X218745_x_at | 0.0008 | 0.0038 | 0.198 | 0.8445161 | 0.964936 |
| X209986_at | -0.0006 | 0.0032 | -0.198 | 0.8445254 | 0.964936 |
| X213573_at | 0.0055 | 0.0276 | 0.198 | 0.8445766 | 0.964945 |
| X34478_at | 0.0009 | 0.0045 | 0.198 | 0.8447003 | 0.964955 |
| X214642_x_at | 0.0012 | 0.0062 | 0.197 | 0.8447461 | 0.964955 |
| X220324_at | -0.0027 | 0.0135 | -0.197 | 0.8447542 | 0.964955 |
| X206189_at | 0.0009 | 0.0046 | 0.197 | 0.8447592 | 0.964955 |
| X210820_x_at | 0.0072 | 0.0364 | 0.197 | 0.8448408 | 0.964966 |
| X203681_at | 0.0006 | 0.0028 | 0.197 | 0.8449196 | 0.964966 |
| X216548_x_at | 0.0012 | 0.0059 | 0.197 | 0.8449673 | 0.964966 |
| X218892_at | 0.0004 | 0.0023 | 0.197 | 0.8449932 | 0.964966 |
| X220205_at | 0.0019 | 0.0097 | 0.197 | 0.8450871 | 0.964966 |
| X220693_at | 0.0007 | 0.0034 | 0.197 | 0.8451012 | 0.964966 |
| X217444_at | 0.0013 | 0.0066 | 0.197 | 0.8451104 | 0.964966 |
| X220555_s_at | -0.0013 | 0.0068 | -0.197 | 0.8453192 | 0.964966 |
| X216756_at | 0.0035 | 0.0176 | 0.197 | 0.8453628 | 0.964966 |
| X206237_s_at | 0.0008 | 0.0043 | 0.197 | 0.8453698 | 0.964966 |
| X216138_at | 0.0007 | 0.0037 | 0.197 | 0.8454574 | 0.964966 |
| X211541_s_at | -0.0006 | 0.0033 | -0.197 | 0.8454809 | 0.964966 |
| X221709_s_at | -0.0019 | 0.0096 | -0.197 | 0.8454829 | 0.964966 |
| X213934_s_at | 0.0078 | 0.0396 | 0.196 | 0.8455114 | 0.964966 |
| X217554_at | -0.0014 | 0.0069 | -0.196 | 0.8455247 | 0.964966 |
| X210984_x_at | 0.0006 | 0.003 | 0.196 | 0.8455376 | 0.964966 |
| X205779_at | -0.0005 | 0.0028 | -0.196 | 0.8456326 | 0.964966 |
| X204362_at | 0.0246 | 0.1254 | 0.196 | 0.8457456 | 0.964966 |
| X208081_s_at | 0.004 | 0.0206 | 0.196 | 0.8457502 | 0.964966 |
| X201094_at | 0.0057 | 0.0288 | 0.196 | 0.8457997 | 0.964966 |
| X215150_at | -0.0017 | 0.0088 | -0.196 | 0.845963 | 0.964966 |
| X207285_x_at | -0.0006 | 0.0032 | -0.196 | 0.8459648 | 0.964966 |
| X207345_at | -0.0014 | 0.0071 | -0.196 | 0.8459873 | 0.964966 |
| X216193_at | 0.0012 | 0.0059 | 0.196 | 0.846065 | 0.964966 |
| X207363_at | 0.0015 | 0.0077 | 0.196 | 0.8460925 | 0.964966 |
| X216907_x_at | -0.0008 | 0.004 | -0.196 | 0.8461174 | 0.964966 |
| X204445_s_at | -0.0008 | 0.0039 | -0.196 | 0.8461647 | 0.964966 |
| X200042_at | -0.0109 | 0.056 | -0.196 | 0.8461853 | 0.964966 |
| X217122_s_at | -0.0092 | 0.0469 | -0.196 | 0.8461939 | 0.964966 |
| X220312_at | -0.0005 | 0.0025 | -0.196 | 0.8462269 | 0.964966 |
| X203691_at | 0.0008 | 0.0043 | 0.195 | 0.8462808 | 0.964966 |
| X211188_at | -0.0006 | 0.0031 | -0.195 | 0.8462898 | 0.964966 |
| X206708_at | 0.0007 | 0.0034 | 0.195 | 0.8463675 | 0.964966 |
| X210973_s_at | 0.0009 | 0.0047 | 0.195 | 0.8463756 | 0.964966 |
| X209014_at | 0.0121 | 0.0621 | 0.195 | 0.8464121 | 0.964966 |
| X212967_x_at | -0.0048 | 0.0245 | -0.195 | 0.8464587 | 0.964966 |
| X211587_x_at | -0.0006 | 0.0032 | -0.195 | 0.8464638 | 0.964966 |
| X207539_s_at | -0.0007 | 0.0035 | -0.195 | 0.8464979 | 0.964966 |
| X203350_at | 0.0095 | 0.0487 | 0.195 | 0.8465207 | 0.964966 |
| X211300_s_at | 0.001 | 0.0051 | 0.195 | 0.8465564 | 0.964966 |
| X211975_at | -0.007 | 0.0359 | -0.195 | 0.8465774 | 0.964966 |
| X210885_s_at | 0.0007 | 0.0034 | 0.195 | 0.8466092 | 0.964966 |
| X218487_at | -0.0082 | 0.0418 | -0.195 | 0.8466365 | 0.964966 |
| X209009_at | 0.0035 | 0.0177 | 0.195 | 0.8466808 | 0.964967 |
| X207844_at | 0.0011 | 0.0054 | 0.195 | 0.8468042 | 0.965002 |
| X208349_at | -0.0017 | 0.0089 | -0.195 | 0.8468349 | 0.965002 |
| X221765_at | -0.0147 | 0.0752 | -0.195 | 0.8468424 | 0.965002 |
| X218192_at | 0.0085 | 0.0436 | 0.195 | 0.8469253 | 0.965047 |
| X210916_s_at | 0.0006 | 0.0029 | 0.195 | 0.8470356 | 0.965096 |
| X210421_s_at | -0.0007 | 0.0034 | -0.194 | 0.8470548 | 0.965096 |
| X208039_at | 0.0011 | 0.0059 | 0.194 | 0.8471018 | 0.9651 |
| X214951_at | 0.0006 | 0.0033 | 0.194 | 0.8473099 | 0.965223 |
| X207858_s_at | -0.0008 | 0.004 | -0.194 | 0.8473365 | 0.965223 |
| X202970_at | 0.0058 | 0.0301 | 0.194 | 0.8473402 | 0.965223 |
| X214852_x_at | 0.0012 | 0.0064 | 0.194 | 0.8475025 | 0.965224 |
| X220504_at | 0.0034 | 0.0177 | 0.194 | 0.8475378 | 0.965224 |
| X221248_s_at | 0.0013 | 0.007 | 0.194 | 0.8475989 | 0.965224 |
| X207662_at | -0.002 | 0.0105 | -0.194 | 0.8476229 | 0.965224 |
| X203924_at | -0.0013 | 0.0066 | -0.194 | 0.8476612 | 0.965224 |
| X210291_s_at | -0.0007 | 0.0038 | -0.194 | 0.8477048 | 0.965224 |
| X219820_at | 0.0012 | 0.0063 | 0.194 | 0.8477159 | 0.965224 |
| X205446_s_at | -0.0013 | 0.0065 | -0.194 | 0.847721 | 0.965224 |
| X210578_at | 0.0007 | 0.0037 | 0.194 | 0.8477445 | 0.965224 |
| X218776_s_at | 0.0016 | 0.0081 | 0.194 | 0.8477753 | 0.965224 |
| X219031_s_at | 0.0087 | 0.0452 | 0.193 | 0.8479613 | 0.965383 |
| X220474_at | 0.0023 | 0.0121 | 0.193 | 0.8480021 | 0.965383 |
| X216078_at | 0.001 | 0.005 | 0.193 | 0.8480642 | 0.965404 |
| X202761_s_at | -0.0104 | 0.054 | -0.193 | 0.8481141 | 0.965412 |
| X212734_x_at | -0.0066 | 0.0343 | -0.193 | 0.8481674 | 0.96542 |
| X205575_at | 0.0013 | 0.0067 | 0.193 | 0.8482085 | 0.96542 |
| X211637_x_at | 0.0006 | 0.003 | 0.193 | 0.8482519 | 0.96542 |
| X210688_s_at | 0.0007 | 0.0036 | 0.193 | 0.8483785 | 0.96542 |
| X205887_x_at | 0.006 | 0.0313 | 0.193 | 0.8484141 | 0.96542 |
| X203470_s_at | 0.0038 | 0.0195 | 0.193 | 0.8484182 | 0.96542 |
| X45828_at | 0.0076 | 0.0395 | 0.193 | 0.8484259 | 0.96542 |
| X215652_at | 0.0024 | 0.0123 | 0.193 | 0.8485708 | 0.965533 |
| X214513_s_at | 0.001 | 0.0052 | 0.192 | 0.8486117 | 0.965533 |
| X215920_s_at | -0.0006 | 0.0032 | -0.192 | 0.8487249 | 0.965551 |
| X217304_at | -0.0012 | 0.0064 | -0.192 | 0.848747 | 0.965551 |
| X211179_at | 0.0014 | 0.0074 | 0.192 | 0.8487585 | 0.965551 |
| X217450_at | 0.0007 | 0.0036 | 0.192 | 0.8489123 | 0.965647 |
| X202116_at | 0.0068 | 0.0354 | 0.192 | 0.8489293 | 0.965647 |
| X216431_at | 0.0006 | 0.0031 | 0.192 | 0.8491665 | 0.965867 |
| X209848_s_at | -0.0007 | 0.0035 | -0.192 | 0.8493266 | 0.965982 |
| X205484_at | 0.0011 | 0.0058 | 0.192 | 0.8493545 | 0.965982 |
| X218565_at | 0.0011 | 0.0057 | 0.191 | 0.8494402 | 0.966016 |
| X214638_s_at | 0.0013 | 0.0066 | 0.191 | 0.8494826 | 0.966016 |
| X221819_at | -0.0018 | 0.0097 | -0.191 | 0.8495524 | 0.966016 |
| X221780_s_at | -0.0068 | 0.0358 | -0.191 | 0.8495581 | 0.966016 |
| X217190_x_at | 0.001 | 0.0054 | 0.191 | 0.8496273 | 0.966045 |
| X213749_at | -0.0007 | 0.0034 | -0.191 | 0.8497452 | 0.966122 |
| X220431_at | 0.001 | 0.0052 | 0.191 | 0.8498248 | 0.966122 |
| X208896_at | -0.0064 | 0.0333 | -0.191 | 0.8498254 | 0.966122 |
| X206520_x_at | 0.0009 | 0.0047 | 0.191 | 0.849879 | 0.966129 |
| X215532_x_at | 0.0041 | 0.0214 | 0.191 | 0.8499621 | 0.966129 |
| X220755_s_at | -0.0048 | 0.0252 | -0.191 | 0.8500636 | 0.966129 |
| X206365_at | -0.0013 | 0.0066 | -0.191 | 0.8500721 | 0.966129 |
| X219397_at | -0.0066 | 0.0346 | -0.191 | 0.8500832 | 0.966129 |
| X212122_at | -0.0122 | 0.064 | -0.191 | 0.8500923 | 0.966129 |
| X221856_s_at | -0.0067 | 0.035 | -0.191 | 0.8501577 | 0.966146 |
| X210433_at | -0.0008 | 0.0042 | -0.19 | 0.8502044 | 0.966146 |
| X211108_s_at | -0.0006 | 0.0029 | -0.19 | 0.8502384 | 0.966146 |
| X217480_x_at | -0.0007 | 0.0037 | -0.19 | 0.8503578 | 0.966183 |
| X202439_s_at | -0.0157 | 0.0824 | -0.19 | 0.850358 | 0.966183 |
| X205859_at | 0.0121 | 0.0635 | 0.19 | 0.8505718 | 0.966306 |
| X204051_s_at | 0.0006 | 0.0033 | 0.19 | 0.8506343 | 0.966306 |
| X204439_at | -0.018 | 0.0949 | -0.19 | 0.8506711 | 0.966306 |
| X212763_at | -0.0142 | 0.0746 | -0.19 | 0.8506844 | 0.966306 |
| X208491_s_at | 0.0007 | 0.0037 | 0.19 | 0.8506976 | 0.966306 |
| X218941_at | -0.0064 | 0.0338 | -0.19 | 0.8507292 | 0.966306 |
| X215305_at | -0.001 | 0.0055 | -0.19 | 0.8507699 | 0.966306 |
| X208035_at | -0.0011 | 0.0057 | -0.189 | 0.8510265 | 0.966548 |
| X208287_at | 0.0015 | 0.008 | 0.189 | 0.8511108 | 0.966594 |
| X210997_at | 0.0014 | 0.0076 | 0.189 | 0.8512926 | 0.966636 |
| X210679_x_at | 0.0152 | 0.0802 | 0.189 | 0.851297 | 0.966636 |
| X204154_at | 0.0088 | 0.0463 | 0.189 | 0.8513619 | 0.966636 |
| X221919_at | -0.0082 | 0.0435 | -0.189 | 0.8514292 | 0.966636 |
| X222376_at | -0.0023 | 0.0121 | -0.189 | 0.8514296 | 0.966636 |
| X220932_at | 0.0003 | 0.0018 | 0.189 | 0.8514669 | 0.966636 |
| X200721_s_at | -0.0098 | 0.0522 | -0.189 | 0.8515097 | 0.966636 |
| X209000_s_at | -0.0237 | 0.1254 | -0.189 | 0.8515122 | 0.966636 |
| X217471_at | 0.0021 | 0.0113 | 0.189 | 0.8515481 | 0.966636 |
| X215415_s_at | -0.0007 | 0.0039 | -0.189 | 0.8515832 | 0.966636 |
| X218542_at | -0.0017 | 0.009 | -0.189 | 0.8516449 | 0.966657 |
| X216877_at | 0.001 | 0.0053 | 0.188 | 0.8517479 | 0.966724 |
| X220499_at | 0.0004 | 0.0024 | 0.188 | 0.8518044 | 0.966739 |
| X206586_at | -0.0008 | 0.0041 | -0.188 | 0.8518664 | 0.96676 |
| X215616_s_at | 0.0005 | 0.0028 | 0.188 | 0.8519142 | 0.966765 |
| X217335_at | -0.0011 | 0.0058 | -0.188 | 0.8520255 | 0.966826 |
| X216255_s_at | 0.0007 | 0.0038 | 0.188 | 0.8520553 | 0.966826 |
| X206831_s_at | -0.0007 | 0.0039 | -0.188 | 0.8521492 | 0.966883 |
| X205408_at | -0.0022 | 0.0115 | -0.188 | 0.8522172 | 0.966911 |
| X212302_at | 0.0045 | 0.0238 | 0.188 | 0.852272 | 0.966924 |
| X203495_at | -0.0009 | 0.0049 | -0.188 | 0.8524609 | 0.96705 |
| X204667_at | -0.001 | 0.0051 | -0.187 | 0.8525583 | 0.96705 |
| X207118_s_at | -0.0003 | 0.0016 | -0.187 | 0.8525986 | 0.96705 |
| X209137_s_at | 0.0032 | 0.0172 | 0.187 | 0.852603 | 0.96705 |
| X210618_at | -0.0004 | 0.0023 | -0.187 | 0.8526343 | 0.96705 |
| X206779_s_at | -0.0011 | 0.006 | -0.187 | 0.852644 | 0.96705 |
| X206658_at | -0.001 | 0.0053 | -0.187 | 0.8526996 | 0.967063 |
| X210538_s_at | 0.0054 | 0.0288 | 0.187 | 0.8528001 | 0.967106 |
| X205555_s_at | -0.0008 | 0.0044 | -0.187 | 0.8528363 | 0.967106 |
| X221944_at | 0.0013 | 0.0069 | 0.187 | 0.8528675 | 0.967106 |
| X210810_s_at | 0.0006 | 0.0034 | 0.187 | 0.8529674 | 0.967157 |
| X204435_at | 0.0099 | 0.0532 | 0.187 | 0.8530354 | 0.967157 |
| X205765_at | -0.002 | 0.0105 | -0.187 | 0.8530785 | 0.967157 |
| X204586_at | -0.0149 | 0.0796 | -0.187 | 0.8531588 | 0.967157 |
| X212315_s_at | -0.0009 | 0.005 | -0.187 | 0.8531615 | 0.967157 |
| X220138_at | 0.001 | 0.0055 | 0.187 | 0.8531821 | 0.967157 |
| X209405_s_at | -0.0016 | 0.0083 | -0.187 | 0.8532588 | 0.967157 |
| X214272_at | 0.0011 | 0.0061 | 0.187 | 0.8532611 | 0.967157 |
| X220833_at | 0.001 | 0.0054 | 0.186 | 0.8533164 | 0.96717 |
| X201661_s_at | -0.0081 | 0.0435 | -0.186 | 0.8535831 | 0.967423 |
| X216423_at | -0.0011 | 0.0058 | -0.186 | 0.8536884 | 0.967493 |
| X220422_at | 0.0005 | 0.0028 | 0.186 | 0.8537653 | 0.967528 |
| X222104_x_at | 0.0158 | 0.0849 | 0.186 | 0.8538316 | 0.967528 |
| X213240_s_at | 0.001 | 0.0055 | 0.186 | 0.8538781 | 0.967528 |
| X217315_s_at | -0.0006 | 0.0033 | -0.186 | 0.8538932 | 0.967528 |
| X206585_at | 0.0015 | 0.0079 | 0.186 | 0.8539755 | 0.967572 |
| X213053_at | -0.0008 | 0.0044 | -0.185 | 0.854136 | 0.967587 |
| X213071_at | 0.0006 | 0.003 | 0.185 | 0.854139 | 0.967587 |
| X204723_at | -0.0127 | 0.0684 | -0.185 | 0.8541533 | 0.967587 |
| X221293_s_at | -0.0006 | 0.0034 | -0.185 | 0.854163 | 0.967587 |
| X211692_s_at | -0.0007 | 0.0039 | -0.185 | 0.8542808 | 0.967671 |
| X214850_at | 0.0161 | 0.0872 | 0.185 | 0.8544082 | 0.967698 |
| X210171_s_at | 0.0015 | 0.0081 | 0.185 | 0.8544881 | 0.967698 |
| X209613_s_at | 0.0048 | 0.0258 | 0.185 | 0.8545447 | 0.967698 |
| X219558_at | 0.0009 | 0.0049 | 0.185 | 0.8545743 | 0.967698 |
| X214390_s_at | 0.0009 | 0.0047 | 0.185 | 0.8546104 | 0.967698 |
| X214051_at | -0.0058 | 0.0316 | -0.185 | 0.8546242 | 0.967698 |
| X206336_at | 0.0022 | 0.0118 | 0.185 | 0.8546524 | 0.967698 |
| X210786_s_at | 0.002 | 0.0108 | 0.185 | 0.8546531 | 0.967698 |
| X214090_at | -0.0013 | 0.0069 | -0.185 | 0.8546982 | 0.9677 |
| X221825_at | -0.0074 | 0.0403 | -0.185 | 0.8547573 | 0.967717 |
| X215172_at | 0.03 | 0.1625 | 0.184 | 0.8548385 | 0.96776 |
| X220163_s_at | 0.0007 | 0.0036 | 0.184 | 0.8550416 | 0.967914 |
| X207514_s_at | -0.0006 | 0.0033 | -0.184 | 0.8550617 | 0.967914 |
| X217252_at | 0.0006 | 0.0035 | 0.184 | 0.8551109 | 0.967919 |
| X216065_at | -0.0017 | 0.009 | -0.184 | 0.8551667 | 0.967919 |
| X210521_s_at | 0.0011 | 0.0059 | 0.184 | 0.855261 | 0.967919 |
| X216108_at | -0.0014 | 0.0076 | -0.184 | 0.8553066 | 0.967919 |
| X204364_s_at | -0.0094 | 0.0509 | -0.184 | 0.8553181 | 0.967919 |
| X206843_at | 0.0005 | 0.0028 | 0.184 | 0.8553832 | 0.967919 |
| X207008_at | 0.0017 | 0.0092 | 0.184 | 0.8554198 | 0.967919 |
| X220824_at | -0.0005 | 0.0027 | -0.184 | 0.8554717 | 0.967919 |
| X208997_s_at | 0.0005 | 0.0026 | 0.184 | 0.8555009 | 0.967919 |
| X207056_s_at | 0.0155 | 0.0846 | 0.184 | 0.8555238 | 0.967919 |
| X50400_at | -0.0011 | 0.006 | -0.184 | 0.8555552 | 0.967919 |
| X207410_s_at | -0.0006 | 0.0033 | -0.183 | 0.8556165 | 0.967919 |
| X207302_at | -0.0011 | 0.0061 | -0.183 | 0.8556939 | 0.967919 |
| X217572_at | -0.0011 | 0.0061 | -0.183 | 0.855811 | 0.967919 |
| X215840_at | 0.0008 | 0.0043 | 0.183 | 0.8558529 | 0.967919 |
| X212575_at | -0.0008 | 0.0043 | -0.183 | 0.8559397 | 0.967919 |
| X206220_s_at | -0.0008 | 0.0044 | -0.183 | 0.855993 | 0.967919 |
| X220943_s_at | 0.0087 | 0.0477 | 0.183 | 0.8560047 | 0.967919 |
| X206131_at | 0.0007 | 0.0036 | 0.183 | 0.8560294 | 0.967919 |
| X214417_s_at | 0.0008 | 0.0043 | 0.183 | 0.8560337 | 0.967919 |
| X217532_x_at | -0.0011 | 0.0063 | -0.183 | 0.8560483 | 0.967919 |
| X217741_s_at | -0.0119 | 0.0651 | -0.183 | 0.8560596 | 0.967919 |
| X217093_at | 0.0006 | 0.0033 | 0.183 | 0.8560684 | 0.967919 |
| X207384_at | 0.0005 | 0.003 | 0.183 | 0.8562765 | 0.968056 |
| X220596_at | -0.0048 | 0.0265 | -0.183 | 0.8562765 | 0.968056 |
| X200004_at | -0.004 | 0.0218 | -0.182 | 0.8566238 | 0.968336 |
| X211916_s_at | -0.0007 | 0.0038 | -0.182 | 0.8566412 | 0.968336 |
| X201126_s_at | 0.0041 | 0.0226 | 0.182 | 0.8566548 | 0.968336 |
| X219436_s_at | 0.0007 | 0.0039 | 0.182 | 0.8567789 | 0.968427 |
| X205546_s_at | 0.0067 | 0.0366 | 0.182 | 0.8568918 | 0.968475 |
| X201945_at | 0.0014 | 0.0078 | 0.182 | 0.8569345 | 0.968475 |
| X211661_x_at | 0.0005 | 0.003 | 0.182 | 0.8569642 | 0.968475 |
| X201190_s_at | 0.0063 | 0.0346 | 0.182 | 0.857074 | 0.968475 |
| X210702_s_at | -0.0008 | 0.0044 | -0.182 | 0.8570805 | 0.968475 |
| X201959_s_at | -0.01 | 0.0549 | -0.182 | 0.8571271 | 0.968475 |
| X213820_s_at | 0.0006 | 0.0032 | 0.182 | 0.8571272 | 0.968475 |
| X215872_at | 0.0008 | 0.0046 | 0.181 | 0.8573253 | 0.96865 |
| X65521_at | 0.0059 | 0.0328 | 0.181 | 0.8573988 | 0.968684 |
| X216160_at | 0.001 | 0.0053 | 0.181 | 0.8574713 | 0.968693 |
| X208296_x_at | 0.002 | 0.0113 | 0.181 | 0.8575293 | 0.968693 |
| X207699_at | 0.0015 | 0.0085 | 0.181 | 0.8575399 | 0.968693 |
| X217581_at | 0.0007 | 0.004 | 0.181 | 0.8575817 | 0.968693 |
| X215174_at | 0.001 | 0.0056 | 0.181 | 0.8577053 | 0.968784 |
| X206428_s_at | -0.0003 | 0.0016 | -0.181 | 0.8577626 | 0.968799 |
| X206305_s_at | 0.001 | 0.0056 | 0.18 | 0.8579553 | 0.968898 |
| X211804_s_at | -0.0006 | 0.0033 | -0.18 | 0.8579801 | 0.968898 |
| X202528_at | 0.0009 | 0.0053 | 0.18 | 0.8579808 | 0.968898 |
| X222079_at | 0.0012 | 0.0066 | 0.18 | 0.8581415 | 0.96899 |
| X220292_at | -0.0008 | 0.0042 | -0.18 | 0.8581501 | 0.96899 |
| X220805_at | 0.0006 | 0.0033 | 0.18 | 0.8582737 | 0.969077 |
| X218380_at | -0.009 | 0.0497 | -0.18 | 0.8583754 | 0.969077 |
| X204017_at | -0.0014 | 0.0076 | -0.18 | 0.8584031 | 0.969077 |
| X214528_s_at | 0.0008 | 0.0044 | 0.18 | 0.8584158 | 0.969077 |
| X214316_x_at | 0.0089 | 0.0493 | 0.18 | 0.8584446 | 0.969077 |
| X216707_at | 0.0007 | 0.0038 | 0.18 | 0.8586829 | 0.969216 |
| X216539_at | -0.001 | 0.0056 | -0.179 | 0.8587728 | 0.969216 |
| X216695_s_at | 0.0051 | 0.0283 | 0.179 | 0.8588078 | 0.969216 |
| X207247_s_at | 0.001 | 0.0057 | 0.179 | 0.8588442 | 0.969216 |
| X200814_at | 0.0073 | 0.041 | 0.179 | 0.8588793 | 0.969216 |
| X222336_at | -0.0041 | 0.023 | -0.179 | 0.8589001 | 0.969216 |
| X206961_s_at | -0.0007 | 0.004 | -0.179 | 0.8589222 | 0.969216 |
| X210441_at | 0.0007 | 0.0038 | 0.179 | 0.8589696 | 0.969216 |
| X208080_at | -0.0013 | 0.0071 | -0.179 | 0.8590062 | 0.969216 |
| X33736_at | 0.005 | 0.0278 | 0.179 | 0.8590314 | 0.969216 |
| X215542_at | -0.0015 | 0.0085 | -0.179 | 0.8590479 | 0.969216 |
| X221478_at | -0.0062 | 0.0346 | -0.179 | 0.8591332 | 0.969263 |
| X207456_at | 0.0011 | 0.0061 | 0.179 | 0.8591961 | 0.969273 |
| X213204_at | -0.006 | 0.0335 | -0.179 | 0.8592345 | 0.969273 |
| X217214_s_at | -0.0014 | 0.0078 | -0.179 | 0.8594353 | 0.969273 |
| X217038_at | -0.0009 | 0.0048 | -0.178 | 0.8595033 | 0.969273 |
| X221447_s_at | -0.0011 | 0.006 | -0.178 | 0.8595282 | 0.969273 |
| X213150_at | -0.0015 | 0.0087 | -0.178 | 0.8595737 | 0.969273 |
| X210654_at | -0.0006 | 0.0032 | -0.178 | 0.8595786 | 0.969273 |
| X219095_at | -0.0006 | 0.0035 | -0.178 | 0.8596171 | 0.969273 |
| X216166_at | -0.0008 | 0.0047 | -0.178 | 0.8596275 | 0.969273 |
| X219876_s_at | -0.0009 | 0.0053 | -0.178 | 0.8596463 | 0.969273 |
| X216099_at | 0.0011 | 0.006 | 0.178 | 0.8596485 | 0.969273 |
| X210053_at | -0.008 | 0.0448 | -0.178 | 0.8596907 | 0.969273 |
| X211471_s_at | 0.001 | 0.0059 | 0.178 | 0.8597091 | 0.969273 |
| X216182_at | 0.0005 | 0.003 | 0.178 | 0.8598208 | 0.969335 |
| X203451_at | 0.0141 | 0.0795 | 0.178 | 0.8598514 | 0.969335 |
| X213915_at | 0.0007 | 0.0037 | 0.178 | 0.8599791 | 0.969429 |
| X221546_at | 0.0013 | 0.0074 | 0.178 | 0.8601971 | 0.969535 |
| X213018_at | -0.0101 | 0.0569 | -0.178 | 0.8602008 | 0.969535 |
| X219133_at | 0.0028 | 0.0157 | 0.178 | 0.8602322 | 0.969535 |
| X218687_s_at | -0.0014 | 0.008 | -0.177 | 0.8602753 | 0.969535 |
| X207078_at | -0.0151 | 0.0849 | -0.177 | 0.8602906 | 0.969535 |
| X211606_at | 0.0009 | 0.0049 | 0.177 | 0.8603867 | 0.969582 |
| X206676_at | -0.0018 | 0.01 | -0.177 | 0.8604204 | 0.969582 |
| X204039_at | 0.0096 | 0.0543 | 0.177 | 0.8607058 | 0.969762 |
| X203807_x_at | 0.0005 | 0.0031 | 0.177 | 0.8609572 | 0.969762 |
| X205553_s_at | -0.0006 | 0.0032 | -0.177 | 0.8610066 | 0.969762 |
| X210098_s_at | 0.0196 | 0.1112 | 0.176 | 0.8610699 | 0.969762 |
| X216722_at | 0.0016 | 0.0092 | 0.176 | 0.8610922 | 0.969762 |
| X208334_at | 0.0005 | 0.0031 | 0.176 | 0.8611178 | 0.969762 |
| X40273_at | -0.002 | 0.0114 | -0.176 | 0.861218 | 0.969762 |
| X216751_at | 0.0013 | 0.0072 | 0.176 | 0.8612615 | 0.969762 |
| X219791_s_at | -0.0007 | 0.0039 | -0.176 | 0.8613503 | 0.969762 |
| X59437_at | 0.0105 | 0.0595 | 0.176 | 0.8613723 | 0.969762 |
| X205793_x_at | -0.0003 | 0.0015 | -0.176 | 0.8613987 | 0.969762 |
| X207003_at | 0.0009 | 0.0051 | 0.176 | 0.8614721 | 0.969762 |
| X218378_s_at | 0.0042 | 0.0236 | 0.176 | 0.8614976 | 0.969762 |
| X221544_s_at | -0.0009 | 0.0048 | -0.176 | 0.8615025 | 0.969762 |
| X210623_at | -0.0023 | 0.0131 | -0.176 | 0.8615215 | 0.969762 |
| X203543_s_at | 0.0154 | 0.0877 | 0.176 | 0.861578 | 0.969762 |
| X213608_s_at | 0.0099 | 0.0565 | 0.176 | 0.8616604 | 0.969762 |
| X202595_s_at | 0.0112 | 0.0636 | 0.176 | 0.8616612 | 0.969762 |
| X207861_at | 0.0006 | 0.0037 | 0.176 | 0.8616975 | 0.969762 |
| X206927_s_at | -0.0004 | 0.0024 | -0.176 | 0.8617213 | 0.969762 |
| X216410_at | 0.0007 | 0.0039 | 0.176 | 0.8617465 | 0.969762 |
| X213722_at | -0.001 | 0.006 | -0.176 | 0.8617894 | 0.969762 |
| X213069_at | 0.0107 | 0.061 | 0.176 | 0.8617967 | 0.969762 |
| X211607_x_at | -0.0006 | 0.0037 | -0.176 | 0.861797 | 0.969762 |
| X202692_s_at | -0.0113 | 0.0643 | -0.176 | 0.861805 | 0.969762 |
| X211065_x_at | 0.0015 | 0.0085 | 0.176 | 0.8618114 | 0.969762 |
| X211459_at | -0.002 | 0.0112 | -0.176 | 0.8618262 | 0.969762 |
| X48106_at | 0.0087 | 0.0496 | 0.175 | 0.861848 | 0.969762 |
| X200032_s_at | 0.002 | 0.0114 | 0.175 | 0.8618734 | 0.969762 |
| X212286_at | -0.0184 | 0.1047 | -0.175 | 0.8619089 | 0.969762 |
| X216815_at | -0.0004 | 0.0025 | -0.175 | 0.8619677 | 0.969762 |
| X205973_at | 0.0057 | 0.0325 | 0.175 | 0.8619882 | 0.969762 |
| X216810_at | 0.0018 | 0.0101 | 0.175 | 0.8620207 | 0.969762 |
| X203129_s_at | 0.01 | 0.0572 | 0.175 | 0.8621843 | 0.969897 |
| X211050_x_at | 0.0007 | 0.004 | 0.175 | 0.8623512 | 0.969916 |
| X221159_at | 0.0011 | 0.006 | 0.175 | 0.8623552 | 0.969916 |
| X208423_s_at | 0.001 | 0.0057 | 0.175 | 0.8623676 | 0.969916 |
| X214159_at | -0.0025 | 0.0143 | -0.175 | 0.8623757 | 0.969916 |
| X201153_s_at | 0.0045 | 0.0255 | 0.175 | 0.8624238 | 0.969921 |
| X222215_at | 0.0007 | 0.0042 | 0.175 | 0.8625527 | 0.969996 |
| X217641_at | -0.0013 | 0.0076 | -0.174 | 0.8626177 | 0.969996 |
| X206699_x_at | -0.001 | 0.0058 | -0.174 | 0.8626294 | 0.969996 |
| X219740_at | 0.0007 | 0.0042 | 0.174 | 0.8626647 | 0.969996 |
| X44790_s_at | -0.0085 | 0.0485 | -0.174 | 0.8628196 | 0.970121 |
| X219827_at | -0.0011 | 0.0062 | -0.174 | 0.8628983 | 0.970134 |
| X220822_at | -0.0013 | 0.0076 | -0.174 | 0.8630307 | 0.970134 |
| X209924_at | 0.0008 | 0.0044 | 0.174 | 0.8630385 | 0.970134 |
| X121_at | -0.0045 | 0.0259 | -0.174 | 0.8630593 | 0.970134 |
| X220476_s_at | -0.0006 | 0.0032 | -0.174 | 0.8630866 | 0.970134 |
| X203996_s_at | -0.0107 | 0.0615 | -0.174 | 0.86317 | 0.970134 |
| X201144_s_at | 0.0042 | 0.0241 | 0.174 | 0.86318 | 0.970134 |
| X215053_at | 0.0013 | 0.0077 | 0.174 | 0.8632163 | 0.970134 |
| X221118_at | -0.0016 | 0.0093 | -0.174 | 0.8633134 | 0.970134 |
| X215678_at | -0.0005 | 0.0029 | -0.174 | 0.8633238 | 0.970134 |
| X219508_at | 0.0007 | 0.0042 | 0.174 | 0.8633845 | 0.970134 |
| X215889_at | 0.001 | 0.0059 | 0.173 | 0.8633993 | 0.970134 |
| X216748_at | -0.0016 | 0.0092 | -0.173 | 0.8635437 | 0.970134 |
| X219812_at | 0.0006 | 0.0037 | 0.173 | 0.8635739 | 0.970134 |
| X221329_at | 0.0009 | 0.0054 | 0.173 | 0.8635834 | 0.970134 |
| X217095_x_at | 0.0003 | 0.002 | 0.173 | 0.8636071 | 0.970134 |
| X207580_at | -0.0019 | 0.0107 | -0.173 | 0.8636466 | 0.970134 |
| X209790_s_at | 0.0017 | 0.01 | 0.173 | 0.8636976 | 0.970134 |
| X206812_at | 0.0009 | 0.0054 | 0.173 | 0.8637285 | 0.970134 |
| X221103_s_at | -0.0019 | 0.0107 | -0.173 | 0.8637491 | 0.970134 |
| X214385_s_at | -0.0011 | 0.0062 | -0.173 | 0.8637618 | 0.970134 |
| X207113_s_at | -0.0006 | 0.0035 | -0.173 | 0.8637918 | 0.970134 |
| X204514_at | -0.001 | 0.0059 | -0.173 | 0.8638611 | 0.970162 |
| X203516_at | -0.0112 | 0.0651 | -0.173 | 0.8639321 | 0.970193 |
| X222241_at | -0.0009 | 0.0053 | -0.172 | 0.8642005 | 0.970417 |
| X203320_at | -0.0082 | 0.0476 | -0.172 | 0.8642427 | 0.970417 |
| X208414_s_at | -0.0016 | 0.0093 | -0.172 | 0.8642622 | 0.970417 |
| X216428_x_at | -0.0026 | 0.015 | -0.172 | 0.8643364 | 0.970451 |
| X214561_at | -0.0011 | 0.0064 | -0.172 | 0.8645995 | 0.970531 |
| X209084_s_at | 0.0061 | 0.0357 | 0.172 | 0.8646495 | 0.970531 |
| X219657_s_at | 0.0023 | 0.0135 | 0.172 | 0.8646551 | 0.970531 |
| X212246_at | -0.0068 | 0.0398 | -0.172 | 0.864738 | 0.970531 |
| X216573_at | -0.0009 | 0.0051 | -0.172 | 0.8647729 | 0.970531 |
| X222205_x_at | -0.0023 | 0.0135 | -0.172 | 0.8648865 | 0.970531 |
| X205592_at | -0.0017 | 0.0102 | -0.172 | 0.8648921 | 0.970531 |
| X214795_at | 0.0009 | 0.005 | 0.172 | 0.8648942 | 0.970531 |
| X200942_s_at | 0.0051 | 0.03 | 0.172 | 0.8649033 | 0.970531 |
| X213976_at | 0.0016 | 0.0091 | 0.172 | 0.8649211 | 0.970531 |
| X218558_s_at | 0.0057 | 0.0335 | 0.171 | 0.8649573 | 0.970531 |
| X203240_at | -0.0009 | 0.005 | -0.171 | 0.8649638 | 0.970531 |
| X215868_x_at | -0.002 | 0.0116 | -0.171 | 0.8650306 | 0.970531 |
| X205453_at | -0.0005 | 0.0031 | -0.171 | 0.8650544 | 0.970531 |
| X209032_s_at | -0.0141 | 0.0824 | -0.171 | 0.8650633 | 0.970531 |
| X202786_at | 0.01 | 0.0587 | 0.171 | 0.8651919 | 0.970626 |
| X203519_s_at | -0.0049 | 0.0288 | -0.171 | 0.8652653 | 0.970633 |
| X208137_x_at | -0.0113 | 0.0659 | -0.171 | 0.8653389 | 0.970633 |
| X216781_at | -0.0004 | 0.0025 | -0.171 | 0.8653819 | 0.970633 |
| X200704_at | 0.0142 | 0.0832 | 0.171 | 0.8654441 | 0.970633 |
| X206918_s_at | -0.0028 | 0.0166 | -0.171 | 0.8654534 | 0.970633 |
| X220151_at | 0.0006 | 0.0034 | 0.171 | 0.8654599 | 0.970633 |
| X205581_s_at | 0.0004 | 0.0026 | 0.171 | 0.8655204 | 0.970636 |
| X216443_at | -0.001 | 0.0059 | -0.171 | 0.8655499 | 0.970636 |
| X201775_s_at | -0.0018 | 0.0103 | -0.171 | 0.8656839 | 0.970714 |
| X202204_s_at | 0.0075 | 0.0439 | 0.17 | 0.8657346 | 0.970714 |
| X221355_at | 0.0009 | 0.0055 | 0.17 | 0.8657502 | 0.970714 |
| X214587_at | -0.0018 | 0.0107 | -0.17 | 0.8658398 | 0.970765 |
| X222378_at | 0.0013 | 0.0077 | 0.17 | 0.8658838 | 0.970765 |
| X203458_at | -0.0045 | 0.0264 | -0.17 | 0.8659632 | 0.970769 |
| X221063_x_at | 0.0012 | 0.0072 | 0.17 | 0.8660118 | 0.970769 |
| X58916_at | -0.0006 | 0.0037 | -0.17 | 0.8660182 | 0.970769 |
| X220333_at | 0.0008 | 0.0047 | 0.17 | 0.8661159 | 0.970819 |
| X201769_at | -0.0049 | 0.029 | -0.17 | 0.8661499 | 0.970819 |
| X222379_at | 0.0051 | 0.03 | 0.17 | 0.8662746 | 0.970905 |
| X205070_at | 0.0044 | 0.0257 | 0.17 | 0.8663144 | 0.970905 |
| X208339_at | -0.0021 | 0.0127 | -0.17 | 0.8664266 | 0.970982 |
| X219315_s_at | 0.0024 | 0.0142 | 0.169 | 0.8665233 | 0.970985 |
| X217628_at | -0.001 | 0.0056 | -0.169 | 0.8665856 | 0.970985 |
| X206507_at | -0.0091 | 0.054 | -0.169 | 0.8666886 | 0.970985 |
| X215209_at | 0.0012 | 0.007 | 0.169 | 0.8666949 | 0.970985 |
| X221429_x_at | 0.0004 | 0.0026 | 0.169 | 0.8667563 | 0.970985 |
| X201107_s_at | 0.0005 | 0.0032 | 0.169 | 0.8667692 | 0.970985 |
| X219074_at | -0.0053 | 0.0311 | -0.169 | 0.8667985 | 0.970985 |
| X217426_at | 0.0008 | 0.0048 | 0.169 | 0.8668042 | 0.970985 |
| X217082_at | 0.0008 | 0.0046 | 0.169 | 0.8668723 | 0.970985 |
| X209412_at | 0.0067 | 0.0398 | 0.169 | 0.8668838 | 0.970985 |
| X217440_at | -0.0024 | 0.014 | -0.169 | 0.8669255 | 0.970985 |
| X219602_s_at | 0.0242 | 0.1431 | 0.169 | 0.8669921 | 0.970985 |
| X215472_at | -0.0074 | 0.0441 | -0.169 | 0.8670506 | 0.970985 |
| X214311_at | 0.0021 | 0.0123 | 0.169 | 0.8670511 | 0.970985 |
| X204371_s_at | 0.0007 | 0.0042 | 0.169 | 0.8671865 | 0.970985 |
| X209205_s_at | 0.0081 | 0.048 | 0.169 | 0.8672395 | 0.970985 |
| X213715_s_at | -0.0012 | 0.0071 | -0.169 | 0.867241 | 0.970985 |
| X200869_at | -0.0066 | 0.0392 | -0.169 | 0.8672425 | 0.970985 |
| X203767_s_at | -0.0105 | 0.0625 | -0.169 | 0.86726 | 0.970985 |
| X220355_s_at | -0.0064 | 0.0377 | -0.168 | 0.8673166 | 0.970999 |
| X207312_at | -0.0005 | 0.0029 | -0.168 | 0.8674375 | 0.971086 |
| X221046_s_at | 0.0057 | 0.034 | 0.168 | 0.8675232 | 0.971121 |
| X219741_x_at | -0.0123 | 0.0733 | -0.168 | 0.8675948 | 0.971121 |
| X219813_at | -0.0034 | 0.0205 | -0.168 | 0.8676004 | 0.971121 |
| X209929_s_at | 0.0018 | 0.0107 | 0.168 | 0.8677837 | 0.971278 |
| X211324_s_at | -0.0021 | 0.0126 | -0.168 | 0.8678463 | 0.971289 |
| X210237_at | -0.0008 | 0.0051 | -0.168 | 0.867893 | 0.971289 |
| X216220_s_at | -0.0011 | 0.0064 | -0.168 | 0.8679956 | 0.971289 |
| X220457_at | -0.0005 | 0.0027 | -0.168 | 0.8680289 | 0.971289 |
| X207764_s_at | 0.0009 | 0.0053 | 0.168 | 0.8680547 | 0.971289 |
| X208709_s_at | 0.0049 | 0.0295 | 0.167 | 0.8681248 | 0.971289 |
| X207269_at | 0.0006 | 0.0036 | 0.167 | 0.8681561 | 0.971289 |
| X208592_s_at | 0.0007 | 0.0043 | 0.167 | 0.8682023 | 0.971289 |
| X220400_at | -0.0009 | 0.0054 | -0.167 | 0.868267 | 0.971289 |
| X210670_at | -0.0004 | 0.0023 | -0.167 | 0.8682861 | 0.971289 |
| X221644_s_at | -0.0007 | 0.004 | -0.167 | 0.8683035 | 0.971289 |
| X205335_s_at | 0.0049 | 0.0293 | 0.167 | 0.8683331 | 0.971289 |
| X211548_s_at | -0.0017 | 0.0101 | -0.167 | 0.8684067 | 0.971289 |
| X212989_at | -0.0078 | 0.0467 | -0.167 | 0.8684159 | 0.971289 |
| X204161_s_at | 0.0089 | 0.0532 | 0.167 | 0.868488 | 0.971289 |
| X206957_at | -0.0011 | 0.0063 | -0.167 | 0.8685505 | 0.971289 |
| X207755_at | 0.0011 | 0.0064 | 0.167 | 0.8685864 | 0.971289 |
| X217119_s_at | 0.0005 | 0.0032 | 0.167 | 0.8686208 | 0.971289 |
| X216198_at | -0.0013 | 0.0077 | -0.167 | 0.8687859 | 0.971289 |
| X219859_at | -0.0015 | 0.009 | -0.166 | 0.8689051 | 0.971289 |
| X200794_x_at | 0.0053 | 0.032 | 0.166 | 0.8689484 | 0.971289 |
| X205942_s_at | 0.0006 | 0.0034 | 0.166 | 0.8690189 | 0.971289 |
| X211645_x_at | -0.0007 | 0.0042 | -0.166 | 0.869025 | 0.971289 |
| X215055_at | 0.0006 | 0.0038 | 0.166 | 0.8690557 | 0.971289 |
| X64371_at | -0.0097 | 0.0585 | -0.166 | 0.8690811 | 0.971289 |
| X207148_x_at | -0.0009 | 0.0053 | -0.166 | 0.8692232 | 0.971289 |
| X209461_x_at | 0.0012 | 0.0072 | 0.166 | 0.8692346 | 0.971289 |
| X204268_at | 0.0004 | 0.0025 | 0.166 | 0.8692445 | 0.971289 |
| X214370_at | 0.0024 | 0.0147 | 0.166 | 0.8693019 | 0.971289 |
| X209069_s_at | 0.0068 | 0.0411 | 0.166 | 0.869303 | 0.971289 |
| X221405_at | 0.0004 | 0.0024 | 0.166 | 0.8693812 | 0.971289 |
| X207681_at | 0.0006 | 0.0034 | 0.166 | 0.8694038 | 0.971289 |
| X216868_s_at | 0.0009 | 0.0053 | 0.166 | 0.8694156 | 0.971289 |
| X208545_x_at | -0.0007 | 0.004 | -0.166 | 0.8694797 | 0.971289 |
| X203465_at | -0.0054 | 0.0324 | -0.166 | 0.8695301 | 0.971289 |
| X205399_at | -0.0059 | 0.0356 | -0.166 | 0.8696125 | 0.971289 |
| X209852_x_at | 0.0022 | 0.0134 | 0.165 | 0.8696367 | 0.971289 |
| X206664_at | 0.0019 | 0.0113 | 0.165 | 0.8696611 | 0.971289 |
| X221370_at | 0.0011 | 0.0069 | 0.165 | 0.8697274 | 0.971289 |
| X211214_s_at | 0.001 | 0.0058 | 0.165 | 0.8697284 | 0.971289 |
| X221980_at | -0.0013 | 0.0078 | -0.165 | 0.8697696 | 0.971289 |
| X207315_at | -0.002 | 0.012 | -0.165 | 0.8698072 | 0.971289 |
| X204254_s_at | 0.0011 | 0.0068 | 0.165 | 0.8698367 | 0.971289 |
| X216498_at | -0.0015 | 0.0088 | -0.165 | 0.8698615 | 0.971289 |
| X208006_at | -0.0007 | 0.0042 | -0.165 | 0.8698729 | 0.971289 |
| X219773_at | 0.001 | 0.006 | 0.165 | 0.8700747 | 0.971289 |
| X217465_at | 0.0013 | 0.008 | 0.165 | 0.8700897 | 0.971289 |
| X217525_at | -0.0011 | 0.0067 | -0.165 | 0.8701023 | 0.971289 |
| X220204_s_at | 0.0005 | 0.0032 | 0.165 | 0.8701031 | 0.971289 |
| X215815_at | 0.0005 | 0.003 | 0.165 | 0.870109 | 0.971289 |
| X216632_at | -0.0013 | 0.0076 | -0.165 | 0.8701639 | 0.971289 |
| X208585_at | 0.0009 | 0.0054 | 0.165 | 0.8701833 | 0.971289 |
| X215355_at | -0.0009 | 0.0056 | -0.165 | 0.8701864 | 0.971289 |
| X211898_s_at | 0.0005 | 0.0028 | 0.165 | 0.8702245 | 0.971289 |
| X220874_at | -0.013 | 0.0791 | -0.165 | 0.8702606 | 0.971289 |
| X216970_at | -0.0008 | 0.005 | -0.165 | 0.8703005 | 0.971289 |
| X216819_at | -0.0008 | 0.0049 | -0.165 | 0.8703451 | 0.971289 |
| X208431_s_at | -0.0006 | 0.0036 | -0.165 | 0.8703884 | 0.971289 |
| X212050_at | 0.0052 | 0.0318 | 0.164 | 0.8704392 | 0.971289 |
| X206578_at | -0.0007 | 0.0045 | -0.164 | 0.8704394 | 0.971289 |
| X211211_x_at | -0.0005 | 0.0031 | -0.164 | 0.8704819 | 0.971289 |
| X210765_at | -0.0009 | 0.0057 | -0.164 | 0.8705044 | 0.971289 |
| X218616_at | -0.0059 | 0.0361 | -0.164 | 0.8706108 | 0.971359 |
| X219701_at | -0.0023 | 0.0142 | -0.164 | 0.8706848 | 0.971392 |
| X212856_at | -0.0037 | 0.0225 | -0.164 | 0.870833 | 0.971509 |
| X214542_x_at | 0.0009 | 0.0056 | 0.164 | 0.8709311 | 0.97157 |
| X216367_at | -0.0009 | 0.0053 | -0.164 | 0.8710569 | 0.971579 |
| X214622_at | 0.0005 | 0.0032 | 0.164 | 0.87106 | 0.971579 |
| X207728_at | -0.0014 | 0.0085 | -0.164 | 0.8710707 | 0.971579 |
| X206321_at | 0.0006 | 0.0038 | 0.164 | 0.8711807 | 0.971653 |
| X208061_at | -0.0018 | 0.0108 | -0.163 | 0.8713549 | 0.971791 |
| X203326_x_at | -0.0006 | 0.0037 | -0.163 | 0.8714368 | 0.971791 |
| X216788_at | 0.0015 | 0.0092 | 0.163 | 0.8714548 | 0.971791 |
| X217808_s_at | 0.0109 | 0.0671 | 0.163 | 0.87148 | 0.971791 |
| X207653_at | -0.0006 | 0.0039 | -0.163 | 0.8715482 | 0.971805 |
| X204931_at | -0.0006 | 0.0039 | -0.163 | 0.8716053 | 0.971805 |
| X207727_s_at | 0.0056 | 0.0342 | 0.163 | 0.871623 | 0.971805 |
| X206948_at | -0.0007 | 0.0042 | -0.163 | 0.871718 | 0.971826 |
| X206971_at | 0.0006 | 0.0039 | 0.163 | 0.8717403 | 0.971826 |
| X206418_at | 0.001 | 0.0062 | 0.163 | 0.8717735 | 0.971826 |
| X217131_at | -0.0026 | 0.0157 | -0.163 | 0.8718977 | 0.971839 |
| X203847_s_at | 0.0014 | 0.0088 | 0.163 | 0.8719059 | 0.971839 |
| X220845_at | -0.002 | 0.0123 | -0.163 | 0.8719298 | 0.971839 |
| X217752_s_at | -0.004 | 0.0244 | -0.163 | 0.8719603 | 0.971839 |
| X210783_x_at | 0.0006 | 0.0036 | 0.162 | 0.872018 | 0.971855 |
| X207226_at | 0.0015 | 0.009 | 0.162 | 0.8721451 | 0.971893 |
| X208025_s_at | 0.0011 | 0.0066 | 0.162 | 0.8721931 | 0.971893 |
| X205606_at | -0.0014 | 0.0084 | -0.162 | 0.8722297 | 0.971893 |
| X216890_at | -0.0005 | 0.0028 | -0.162 | 0.8722802 | 0.971893 |
| X205149_s_at | -0.0007 | 0.0046 | -0.162 | 0.8723236 | 0.971893 |
| X205801_s_at | -0.0203 | 0.1254 | -0.162 | 0.8723271 | 0.971893 |
| X211331_x_at | 0.0006 | 0.0036 | 0.162 | 0.8724087 | 0.971893 |
| X219118_at | -0.0011 | 0.0071 | -0.162 | 0.8724605 | 0.971893 |
| X200690_at | -0.0054 | 0.0336 | -0.162 | 0.8724978 | 0.971893 |
| X211841_s_at | -0.0006 | 0.0035 | -0.162 | 0.8724992 | 0.971893 |
| X218485_s_at | -0.0004 | 0.0025 | -0.162 | 0.8725334 | 0.971893 |
| X206278_at | 0.0016 | 0.0096 | 0.162 | 0.8726488 | 0.971973 |
| X216998_s_at | -0.0007 | 0.0041 | -0.162 | 0.8727376 | 0.972023 |
| X209571_at | 0.0056 | 0.0348 | 0.161 | 0.8727955 | 0.972039 |
| X212241_at | -0.0063 | 0.0389 | -0.161 | 0.8729886 | 0.972191 |
| X207910_at | 0.0006 | 0.0036 | 0.161 | 0.8730324 | 0.972191 |
| X203572_s_at | -0.0051 | 0.0318 | -0.161 | 0.8731751 | 0.972191 |
| X201627_s_at | 0.0075 | 0.0468 | 0.161 | 0.8731848 | 0.972191 |
| X214803_at | -0.0238 | 0.148 | -0.161 | 0.8731918 | 0.972191 |
| X208601_s_at | -0.0008 | 0.0051 | -0.161 | 0.8732372 | 0.972191 |
| X215731_s_at | -0.0109 | 0.0677 | -0.161 | 0.8732665 | 0.972191 |
| X217075_x_at | -0.0012 | 0.0074 | -0.161 | 0.8732848 | 0.972191 |
| X220492_s_at | 0.0008 | 0.005 | 0.161 | 0.8733656 | 0.972191 |
| X216587_s_at | -0.0004 | 0.0024 | -0.161 | 0.8733705 | 0.972191 |
| X210354_at | 0.0008 | 0.0053 | 0.161 | 0.873467 | 0.972248 |
| X206337_at | -0.0007 | 0.0045 | -0.16 | 0.8735873 | 0.972248 |
| X205144_at | 0.001 | 0.0063 | 0.16 | 0.8735963 | 0.972248 |
| X220930_s_at | -0.0008 | 0.0049 | -0.16 | 0.8736397 | 0.972248 |
| X210961_s_at | -0.0011 | 0.0071 | -0.16 | 0.8736638 | 0.972248 |
| X204764_at | 0.0004 | 0.0025 | 0.16 | 0.873684 | 0.972248 |
| X210919_at | 0.0017 | 0.0105 | 0.16 | 0.8738086 | 0.972338 |
| X206907_at | 0.0012 | 0.0074 | 0.16 | 0.8740187 | 0.972421 |
| X211835_at | 0.0005 | 0.0032 | 0.16 | 0.8740271 | 0.972421 |
| X213147_at | -0.0006 | 0.0038 | -0.16 | 0.8741215 | 0.972421 |
| X201031_s_at | -0.0045 | 0.028 | -0.16 | 0.8741273 | 0.972421 |
| X217334_at | 0.0005 | 0.0034 | 0.16 | 0.8741293 | 0.972421 |
| X215497_s_at | -0.0011 | 0.0066 | -0.16 | 0.8741459 | 0.972421 |
| X216487_at | 0.0007 | 0.0044 | 0.16 | 0.8742536 | 0.972427 |
| X201020_at | -0.0123 | 0.0769 | -0.16 | 0.8743036 | 0.972427 |
| X221224_s_at | -0.0009 | 0.0057 | -0.159 | 0.8743256 | 0.972427 |
| X210133_at | -0.001 | 0.0063 | -0.159 | 0.874379 | 0.972427 |
| X221421_s_at | -0.0005 | 0.0034 | -0.159 | 0.8744049 | 0.972427 |
| X221413_at | 0.0006 | 0.0038 | 0.159 | 0.8744186 | 0.972427 |
| X212163_at | 0.003 | 0.0189 | 0.159 | 0.8744575 | 0.972427 |
| X216228_s_at | -0.0011 | 0.0071 | -0.159 | 0.8745377 | 0.972467 |
| X220703_at | 0.0047 | 0.0297 | 0.159 | 0.8746406 | 0.972521 |
| X214916_x_at | 0.001 | 0.006 | 0.159 | 0.8746734 | 0.972521 |
| X216546_s_at | -0.0008 | 0.0049 | -0.159 | 0.8749259 | 0.972683 |
| X221085_at | 0.0022 | 0.0136 | 0.159 | 0.8749341 | 0.972683 |
| X208016_s_at | 0.0014 | 0.0091 | 0.159 | 0.8749509 | 0.972683 |
| X221424_s_at | -0.0006 | 0.004 | -0.159 | 0.8750281 | 0.972712 |
| X219756_s_at | 0.002 | 0.0126 | 0.159 | 0.8750641 | 0.972712 |
| X213781_at | -0.0005 | 0.0032 | -0.158 | 0.8751537 | 0.972732 |
| X216259_at | 0.0008 | 0.0049 | 0.158 | 0.8751949 | 0.972732 |
| X217704_x_at | 0.0085 | 0.054 | 0.158 | 0.8752133 | 0.972732 |
| X204329_s_at | 0.0008 | 0.0051 | 0.158 | 0.8754206 | 0.972878 |
| X208076_at | 0.0008 | 0.0048 | 0.158 | 0.8754872 | 0.972878 |
| X211838_x_at | -0.0007 | 0.0047 | -0.158 | 0.875523 | 0.972878 |
| X200681_at | 0.0056 | 0.0356 | 0.158 | 0.8755496 | 0.972878 |
| X206193_s_at | 0.0007 | 0.0042 | 0.158 | 0.875564 | 0.972878 |
| X205476_at | -0.0008 | 0.005 | -0.158 | 0.875726 | 0.972975 |
| X221996_s_at | -0.0006 | 0.0038 | -0.158 | 0.8757385 | 0.972975 |
| X216102_at | -0.0004 | 0.0022 | -0.157 | 0.8759722 | 0.973019 |
| X210309_at | -0.0008 | 0.0051 | -0.157 | 0.8759986 | 0.973019 |
| X213964_x_at | 0.0022 | 0.0142 | 0.157 | 0.8760131 | 0.973019 |
| X210695_s_at | 0.0065 | 0.0416 | 0.157 | 0.8760182 | 0.973019 |
| X219161_s_at | 0.0087 | 0.0556 | 0.157 | 0.8760459 | 0.973019 |
| X211618_s_at | -0.0004 | 0.0024 | -0.157 | 0.8760499 | 0.973019 |
| X218337_at | 0.001 | 0.0061 | 0.157 | 0.8761587 | 0.973019 |
| X220888_s_at | -0.0007 | 0.0044 | -0.157 | 0.8762709 | 0.973019 |
| X60528_at | 0.0022 | 0.0142 | 0.157 | 0.8763193 | 0.973019 |
| X206022_at | 0.0086 | 0.0552 | 0.157 | 0.8764229 | 0.973019 |
| X207329_at | -0.001 | 0.0063 | -0.157 | 0.8764616 | 0.973019 |
| X216585_at | 0.001 | 0.0066 | 0.157 | 0.8764821 | 0.973019 |
| X211574_s_at | 0.0024 | 0.0152 | 0.157 | 0.8765281 | 0.973019 |
| X215769_at | 0.0008 | 0.005 | 0.157 | 0.8765659 | 0.973019 |
| X210834_s_at | 0.0013 | 0.0083 | 0.157 | 0.8765813 | 0.973019 |
| X217002_s_at | 0.0003 | 0.0017 | 0.156 | 0.8766546 | 0.973019 |
| X212541_at | 0.0022 | 0.0141 | 0.156 | 0.8767252 | 0.973019 |
| X51226_at | -0.0013 | 0.0081 | -0.156 | 0.8767435 | 0.973019 |
| X202734_at | 0.0005 | 0.0033 | 0.156 | 0.8767722 | 0.973019 |
| X221404_at | -0.0007 | 0.0044 | -0.156 | 0.8768139 | 0.973019 |
| X219479_at | -0.0014 | 0.0089 | -0.156 | 0.8768573 | 0.973019 |
| X221352_at | 0.0005 | 0.0033 | 0.156 | 0.8768765 | 0.973019 |
| X215281_x_at | -0.0143 | 0.0916 | -0.156 | 0.8769008 | 0.973019 |
| X215110_at | -0.0006 | 0.0039 | -0.156 | 0.8769858 | 0.973019 |
| X219267_at | -0.0188 | 0.1204 | -0.156 | 0.877012 | 0.973019 |
| X218733_at | 0.0108 | 0.069 | 0.156 | 0.8770134 | 0.973019 |
| X208989_s_at | -0.0042 | 0.0268 | -0.156 | 0.8770607 | 0.973019 |
| X215288_at | 0.0005 | 0.0033 | 0.156 | 0.877068 | 0.973019 |
| X204220_at | 0.001 | 0.0063 | 0.156 | 0.8770895 | 0.973019 |
| X220211_at | -0.0006 | 0.0037 | -0.156 | 0.8771054 | 0.973019 |
| X208142_at | -0.0012 | 0.0079 | -0.156 | 0.8771851 | 0.973019 |
| X215720_s_at | -0.0009 | 0.0058 | -0.156 | 0.8771891 | 0.973019 |
| X203256_at | 0.0005 | 0.0033 | 0.156 | 0.877224 | 0.973019 |
| X222299_x_at | 0.0035 | 0.0222 | 0.156 | 0.8774216 | 0.973122 |
| X206950_at | -0.0029 | 0.0184 | -0.156 | 0.8774245 | 0.973122 |
| X218551_at | 0.0033 | 0.0211 | 0.155 | 0.8774479 | 0.973122 |
| X220793_at | 0.0014 | 0.0093 | 0.155 | 0.877579 | 0.973202 |
| X202848_s_at | 0.0005 | 0.0032 | 0.155 | 0.8777083 | 0.973202 |
| X219925_at | -0.0034 | 0.0219 | -0.155 | 0.8778067 | 0.973202 |
| X201102_s_at | -0.0045 | 0.0291 | -0.155 | 0.877826 | 0.973202 |
| X214326_x_at | -0.0006 | 0.004 | -0.155 | 0.8778548 | 0.973202 |
| X37965_at | 0.0004 | 0.0028 | 0.155 | 0.877863 | 0.973202 |
| X219194_at | 0.0011 | 0.0073 | 0.155 | 0.8778749 | 0.973202 |
| X204988_at | -0.0012 | 0.008 | -0.155 | 0.8779385 | 0.973202 |
| X208062_s_at | 0.0008 | 0.0053 | 0.155 | 0.8779942 | 0.973202 |
| X206354_at | -0.0013 | 0.0087 | -0.155 | 0.87802 | 0.973202 |
| X206031_s_at | 0.0008 | 0.0051 | 0.155 | 0.8781755 | 0.973202 |
| X206647_at | 0.0004 | 0.0029 | 0.155 | 0.8781874 | 0.973202 |
| X214495_at | -0.0007 | 0.0045 | -0.154 | 0.8782346 | 0.973202 |
| X220849_at | 0.0007 | 0.0042 | 0.154 | 0.878284 | 0.973202 |
| X209925_at | -0.0011 | 0.007 | -0.154 | 0.8783824 | 0.973202 |
| X216499_at | -0.0009 | 0.0062 | -0.154 | 0.8784425 | 0.973202 |
| X46323_at | 0.0015 | 0.0097 | 0.154 | 0.8784437 | 0.973202 |
| X208700_s_at | 0.0038 | 0.0244 | 0.154 | 0.8784484 | 0.973202 |
| X220549_at | 0.0018 | 0.0115 | 0.154 | 0.8785453 | 0.973202 |
| X215323_at | 0.0196 | 0.127 | 0.154 | 0.8785584 | 0.973202 |
| X200765_x_at | 0.0063 | 0.0407 | 0.154 | 0.8785769 | 0.973202 |
| X213310_at | 0.0029 | 0.0185 | 0.154 | 0.8786231 | 0.973202 |
| X210925_at | 0.0009 | 0.0058 | 0.154 | 0.8786714 | 0.973202 |
| X221112_at | 0.0007 | 0.0045 | 0.154 | 0.8787226 | 0.973202 |
| X212523_s_at | -0.0041 | 0.0268 | -0.154 | 0.8787289 | 0.973202 |
| X213495_s_at | 0.0005 | 0.0032 | 0.154 | 0.878755 | 0.973202 |
| X204668_at | -0.0012 | 0.0077 | -0.154 | 0.8787552 | 0.973202 |
| X220077_at | 0.001 | 0.0067 | 0.154 | 0.8787988 | 0.973202 |
| X220465_at | -0.0029 | 0.0188 | -0.154 | 0.8788224 | 0.973202 |
| X208931_s_at | -0.0054 | 0.0348 | -0.154 | 0.8788346 | 0.973202 |
| X211250_s_at | -0.0007 | 0.0043 | -0.154 | 0.8788862 | 0.973211 |
| X210119_at | -0.0013 | 0.0082 | -0.154 | 0.8789299 | 0.973211 |
| X220770_s_at | -0.006 | 0.0392 | -0.153 | 0.8790236 | 0.973247 |
| X206053_at | -0.0036 | 0.0234 | -0.153 | 0.8790509 | 0.973247 |
| X217768_at | -0.0037 | 0.0239 | -0.153 | 0.8791021 | 0.973254 |
| X214208_at | 0.0006 | 0.0042 | 0.153 | 0.8791445 | 0.973254 |
| X206304_at | -0.0005 | 0.003 | -0.153 | 0.8792008 | 0.973268 |
| X219164_s_at | -0.0078 | 0.0513 | -0.153 | 0.8794286 | 0.97347 |
| X204210_s_at | 0.0009 | 0.0057 | 0.153 | 0.8795041 | 0.97347 |
| X211314_at | 0.0008 | 0.0049 | 0.153 | 0.8795732 | 0.97347 |
| X210279_at | 0.001 | 0.0064 | 0.153 | 0.8795956 | 0.97347 |
| X216798_at | 0.0007 | 0.0047 | 0.153 | 0.8796025 | 0.97347 |
| X222329_x_at | -0.0015 | 0.0097 | -0.153 | 0.8796645 | 0.97349 |
| X217270_s_at | 0.0008 | 0.0051 | 0.153 | 0.8797245 | 0.973504 |
| X218948_at | 0.015 | 0.0981 | 0.153 | 0.8797782 | 0.973504 |
| X207052_at | 0.0009 | 0.0056 | 0.152 | 0.879816 | 0.973504 |
| X212672_at | -0.0076 | 0.0501 | -0.152 | 0.8799796 | 0.973504 |
| X201210_at | 0.0047 | 0.0307 | 0.152 | 0.8800402 | 0.973504 |
| X207274_at | -0.0005 | 0.0036 | -0.152 | 0.8800415 | 0.973504 |
| X216116_at | 0.0006 | 0.0041 | 0.152 | 0.8800477 | 0.973504 |
| X221945_at | -0.0045 | 0.0298 | -0.152 | 0.8800898 | 0.973504 |
| X217520_x_at | 0.0135 | 0.0887 | 0.152 | 0.8802525 | 0.973504 |
| X218993_at | -0.0027 | 0.0177 | -0.152 | 0.8802939 | 0.973504 |
| X215139_at | -0.0077 | 0.051 | -0.152 | 0.8803202 | 0.973504 |
| X207552_at | 0.0006 | 0.0042 | 0.152 | 0.880368 | 0.973504 |
| X203716_s_at | -0.0008 | 0.0053 | -0.152 | 0.8805225 | 0.973504 |
| X212362_at | 0.0008 | 0.0051 | 0.152 | 0.8805261 | 0.973504 |
| X221464_at | 0.001 | 0.0065 | 0.151 | 0.8805738 | 0.973504 |
| X211161_s_at | -0.0023 | 0.015 | -0.151 | 0.8806148 | 0.973504 |
| X211180_x_at | 0.0007 | 0.0048 | 0.151 | 0.880641 | 0.973504 |
| X211392_s_at | 0.0009 | 0.0061 | 0.151 | 0.8806435 | 0.973504 |
| X220098_at | -0.0005 | 0.0032 | -0.151 | 0.8806511 | 0.973504 |
| X214206_at | -0.0007 | 0.0049 | -0.151 | 0.8806574 | 0.973504 |
| X214398_s_at | -0.0004 | 0.0028 | -0.151 | 0.880667 | 0.973504 |
| X219571_s_at | 0.0052 | 0.0342 | 0.151 | 0.8807256 | 0.973504 |
| X207197_at | -0.0008 | 0.0055 | -0.151 | 0.8807357 | 0.973504 |
| X212414_s_at | -0.003 | 0.0197 | -0.151 | 0.8807421 | 0.973504 |
| X203020_at | 0.0043 | 0.0284 | 0.151 | 0.8807725 | 0.973504 |
| X208364_at | -0.0262 | 0.1738 | -0.151 | 0.8810207 | 0.97373 |
| X217371_s_at | -0.0021 | 0.0139 | -0.151 | 0.8812402 | 0.973764 |
| X209918_at | 0.0004 | 0.0027 | 0.151 | 0.8812899 | 0.973764 |
| X202666_s_at | -0.0061 | 0.0403 | -0.151 | 0.8813166 | 0.973764 |
| X220322_at | 0.0005 | 0.0033 | 0.151 | 0.8813369 | 0.973764 |
| X221407_at | 0.0006 | 0.0039 | 0.151 | 0.8813406 | 0.973764 |
| X59705_at | 0.0015 | 0.0097 | 0.15 | 0.8813549 | 0.973764 |
| X215495_s_at | 0.0006 | 0.0037 | 0.15 | 0.8813584 | 0.973764 |
| X220901_at | 0.0006 | 0.0037 | 0.15 | 0.881493 | 0.973818 |
| X215638_at | -0.0008 | 0.0056 | -0.15 | 0.8814949 | 0.973818 |
| X202625_at | 0.0065 | 0.0435 | 0.15 | 0.8815724 | 0.973855 |
| X218497_s_at | -0.0107 | 0.0716 | -0.15 | 0.8816642 | 0.973861 |
| X211181_x_at | -0.0007 | 0.0045 | -0.15 | 0.8816964 | 0.973861 |
| X216824_at | 0.0007 | 0.0043 | 0.15 | 0.8817956 | 0.973861 |
| X220458_at | -0.0008 | 0.0053 | -0.15 | 0.8818838 | 0.973861 |
| X208028_s_at | -0.0008 | 0.005 | -0.15 | 0.8818847 | 0.973861 |
| X217696_at | 0.0039 | 0.0262 | 0.15 | 0.8819171 | 0.973861 |
| X215890_at | -0.0008 | 0.0053 | -0.15 | 0.8820158 | 0.973861 |
| X206032_at | -0.002 | 0.0137 | -0.15 | 0.8820739 | 0.973861 |
| X215885_at | 0.0006 | 0.0037 | 0.149 | 0.8822167 | 0.973861 |
| X211476_at | 0.0019 | 0.0127 | 0.149 | 0.882228 | 0.973861 |
| X220106_at | 0.0005 | 0.003 | 0.149 | 0.8822587 | 0.973861 |
| X212269_s_at | 0.0084 | 0.0564 | 0.149 | 0.8822688 | 0.973861 |
| X205984_at | -0.0034 | 0.0229 | -0.149 | 0.8822818 | 0.973861 |
| X214547_at | -0.0013 | 0.0087 | -0.149 | 0.8822914 | 0.973861 |
| X210876_at | -0.0007 | 0.0046 | -0.149 | 0.8823043 | 0.973861 |
| X201976_s_at | 0.0136 | 0.0909 | 0.149 | 0.8824347 | 0.973861 |
| X207367_at | 0.0005 | 0.0034 | 0.149 | 0.8824474 | 0.973861 |
| X207325_x_at | -0.0007 | 0.0047 | -0.149 | 0.8824511 | 0.973861 |
| X216928_at | -0.0004 | 0.0028 | -0.149 | 0.8825669 | 0.973861 |
| X222020_s_at | -0.0102 | 0.0688 | -0.149 | 0.8827047 | 0.973861 |
| X217654_at | 0.001 | 0.0065 | 0.149 | 0.8828529 | 0.973861 |
| X221134_at | -0.0006 | 0.004 | -0.149 | 0.8828819 | 0.973861 |
| X204993_at | -0.0081 | 0.0547 | -0.148 | 0.8829685 | 0.973861 |
| X220296_at | 0.0015 | 0.01 | 0.148 | 0.8829997 | 0.973861 |
| X206199_at | -0.0011 | 0.0075 | -0.148 | 0.8830158 | 0.973861 |
| X217194_at | -0.0036 | 0.0243 | -0.148 | 0.8830243 | 0.973861 |
| X208154_at | -0.0016 | 0.0105 | -0.148 | 0.8830373 | 0.973861 |
| X222354_at | 0.0017 | 0.0112 | 0.148 | 0.8830503 | 0.973861 |
| X207059_at | 0.0005 | 0.0036 | 0.148 | 0.8831818 | 0.973861 |
| X210690_at | 0.0021 | 0.0139 | 0.148 | 0.8831888 | 0.973861 |
| X200022_at | 0.0032 | 0.0214 | 0.148 | 0.8832023 | 0.973861 |
| X217876_at | 0.0014 | 0.0092 | 0.148 | 0.8832187 | 0.973861 |
| X220258_s_at | -0.0006 | 0.0041 | -0.148 | 0.8832197 | 0.973861 |
| X216020_at | -0.001 | 0.0067 | -0.148 | 0.8832234 | 0.973861 |
| X216317_x_at | -0.0009 | 0.0061 | -0.148 | 0.8832284 | 0.973861 |
| X207611_at | -0.0004 | 0.0028 | -0.148 | 0.8832917 | 0.973861 |
| X210665_at | -0.001 | 0.0065 | -0.148 | 0.8832965 | 0.973861 |
| X206040_s_at | -0.0005 | 0.0031 | -0.148 | 0.883298 | 0.973861 |
| X206366_x_at | 0.0003 | 0.0021 | 0.148 | 0.8834474 | 0.973861 |
| X221050_s_at | 0.0005 | 0.0034 | 0.148 | 0.8834646 | 0.973861 |
| X222358_x_at | 0.0017 | 0.0112 | 0.148 | 0.8834854 | 0.973861 |
| X215406_at | -0.0006 | 0.0044 | -0.148 | 0.8836061 | 0.973861 |
| X220718_at | 0.001 | 0.0065 | 0.148 | 0.8836105 | 0.973861 |
| X221810_at | 0.0087 | 0.0587 | 0.148 | 0.8836442 | 0.973861 |
| X217462_at | 0.0015 | 0.0102 | 0.148 | 0.8836724 | 0.973861 |
| X217295_at | 0.0008 | 0.0054 | 0.147 | 0.8837366 | 0.973861 |
| X216056_at | 0.0009 | 0.006 | 0.147 | 0.883746 | 0.973861 |
| X201788_at | -0.0043 | 0.0293 | -0.147 | 0.8837506 | 0.973861 |
| X211381_x_at | 0.0004 | 0.0026 | 0.147 | 0.8839155 | 0.973861 |
| X210742_at | -0.001 | 0.0068 | -0.147 | 0.8839498 | 0.973861 |
| X204332_s_at | 0.0162 | 0.1104 | 0.147 | 0.8839623 | 0.973861 |
| X215703_at | 0.0014 | 0.0095 | 0.147 | 0.8839771 | 0.973861 |
| X218185_s_at | -0.0043 | 0.0291 | -0.147 | 0.8840317 | 0.973861 |
| X217146_at | -0.0005 | 0.0037 | -0.147 | 0.8840746 | 0.973861 |
| X220622_at | -0.0009 | 0.0059 | -0.147 | 0.8840814 | 0.973861 |
| X216374_at | -0.0007 | 0.0046 | -0.147 | 0.8841172 | 0.973861 |
| X212438_at | -0.0071 | 0.0484 | -0.147 | 0.8841408 | 0.973861 |
| X206422_at | -0.0007 | 0.005 | -0.147 | 0.8841577 | 0.973861 |
| X205266_at | -0.0007 | 0.0045 | -0.147 | 0.8842338 | 0.973861 |
| X204451_at | -0.0149 | 0.1013 | -0.147 | 0.8842408 | 0.973861 |
| X221109_at | 0.0006 | 0.0039 | 0.147 | 0.8842524 | 0.973861 |
| X207289_at | 0.0009 | 0.0064 | 0.147 | 0.8843345 | 0.973878 |
| X203101_s_at | 0.0008 | 0.0055 | 0.147 | 0.8843592 | 0.973878 |
| X212899_at | -0.0098 | 0.0672 | -0.147 | 0.8843992 | 0.973878 |
| X200087_s_at | 0.0031 | 0.0213 | 0.146 | 0.8845478 | 0.973936 |
| X211178_s_at | -0.0005 | 0.0033 | -0.146 | 0.8845795 | 0.973936 |
| X34187_at | 0.0022 | 0.0153 | 0.146 | 0.8845835 | 0.973936 |
| X216403_at | -0.0007 | 0.0047 | -0.146 | 0.884705 | 0.974006 |
| X214130_s_at | 0.0008 | 0.0055 | 0.146 | 0.8847343 | 0.974006 |
| X218040_at | -0.0061 | 0.0421 | -0.146 | 0.8848072 | 0.974038 |
| X217810_x_at | -0.0073 | 0.0501 | -0.146 | 0.8849407 | 0.974127 |
| X218798_at | -0.0011 | 0.0074 | -0.146 | 0.8850053 | 0.974127 |
| X212340_at | 0.0012 | 0.0085 | 0.146 | 0.8850322 | 0.974127 |
| X31807_at | -0.0038 | 0.0257 | -0.146 | 0.8850963 | 0.974127 |
| X211030_s_at | 0.0006 | 0.0042 | 0.146 | 0.8851073 | 0.974127 |
| X213287_s_at | -0.0052 | 0.0359 | -0.145 | 0.8853187 | 0.974311 |
| X207686_s_at | -0.0005 | 0.0033 | -0.145 | 0.8854277 | 0.974383 |
| X220723_s_at | -0.0016 | 0.011 | -0.145 | 0.8856156 | 0.974421 |
| X220161_s_at | -0.0007 | 0.0047 | -0.145 | 0.8856286 | 0.974421 |
| X205087_at | -0.0064 | 0.0442 | -0.145 | 0.8857095 | 0.974421 |
| X210727_at | -0.0011 | 0.0073 | -0.145 | 0.8857235 | 0.974421 |
| X208956_x_at | 0.0034 | 0.0233 | 0.145 | 0.8857858 | 0.974421 |
| X205713_s_at | -0.0007 | 0.0046 | -0.145 | 0.8857896 | 0.974421 |
| X215891_s_at | 0.0007 | 0.0048 | 0.145 | 0.8858483 | 0.974421 |
| X222225_at | -0.0005 | 0.0035 | -0.145 | 0.8858553 | 0.974421 |
| X221907_at | -0.0011 | 0.0075 | -0.145 | 0.8858571 | 0.974421 |
| X215475_at | -0.0008 | 0.0059 | -0.145 | 0.8859172 | 0.974439 |
| X205939_at | 0.0006 | 0.0044 | 0.145 | 0.8860236 | 0.974474 |
| X206096_at | 0.0036 | 0.0252 | 0.145 | 0.886037 | 0.974474 |
| X207901_at | -0.0018 | 0.0124 | -0.144 | 0.8861056 | 0.974484 |
| X209729_at | 0.0062 | 0.0429 | 0.144 | 0.8862051 | 0.974484 |
| X212372_at | 0.0078 | 0.0539 | 0.144 | 0.8862583 | 0.974484 |
| X219386_s_at | 0.0008 | 0.0058 | 0.144 | 0.8863143 | 0.974484 |
| X207133_x_at | -0.0047 | 0.0323 | -0.144 | 0.886358 | 0.974484 |
| X201017_at | 0.0107 | 0.0744 | 0.144 | 0.8863588 | 0.974484 |
| X210080_x_at | -0.0004 | 0.0028 | -0.144 | 0.8863761 | 0.974484 |
| X215568_x_at | -0.001 | 0.0068 | -0.144 | 0.8864608 | 0.974484 |
| X210730_s_at | -0.0006 | 0.0043 | -0.144 | 0.8865144 | 0.974484 |
| X216285_at | -0.0007 | 0.0049 | -0.144 | 0.8865194 | 0.974484 |
| X213963_s_at | 0.002 | 0.0139 | 0.144 | 0.8865289 | 0.974484 |
| X216366_x_at | -0.0008 | 0.0055 | -0.143 | 0.8868403 | 0.974643 |
| X216576_x_at | -0.0005 | 0.0036 | -0.143 | 0.8868583 | 0.974643 |
| X214929_s_at | 0.0005 | 0.0036 | 0.143 | 0.8868677 | 0.974643 |
| X220820_at | 0.001 | 0.0072 | 0.143 | 0.8868681 | 0.974643 |
| X215614_at | -0.0006 | 0.0043 | -0.143 | 0.8869786 | 0.974643 |
| X204494_s_at | 0.001 | 0.0067 | 0.143 | 0.8869973 | 0.974643 |
| X210499_s_at | 0.0034 | 0.024 | 0.143 | 0.8870556 | 0.974643 |
| X218684_at | 0.0064 | 0.045 | 0.143 | 0.8871309 | 0.974643 |
| X207460_at | 0.0008 | 0.0056 | 0.143 | 0.8871508 | 0.974643 |
| X216011_at | -0.0005 | 0.0035 | -0.143 | 0.8871816 | 0.974643 |
| X212474_at | -0.0049 | 0.0346 | -0.143 | 0.8872535 | 0.974643 |
| X215842_s_at | -0.0009 | 0.006 | -0.143 | 0.8872881 | 0.974643 |
| X202685_s_at | 0.0006 | 0.0045 | 0.143 | 0.8873377 | 0.974643 |
| X214117_s_at | -0.0007 | 0.0048 | -0.143 | 0.8874424 | 0.974643 |
| X220552_at | -0.001 | 0.0071 | -0.143 | 0.8875434 | 0.974643 |
| X205674_x_at | 0.0005 | 0.0034 | 0.143 | 0.8875531 | 0.974643 |
| X219604_s_at | -0.0009 | 0.0063 | -0.142 | 0.8876118 | 0.974643 |
| X221839_s_at | 0.0039 | 0.0273 | 0.142 | 0.8876219 | 0.974643 |
| X211707_s_at | 0.0071 | 0.05 | 0.142 | 0.8876296 | 0.974643 |
| X215364_s_at | 0.0009 | 0.0066 | 0.142 | 0.8876302 | 0.974643 |
| X210262_at | 0.0012 | 0.0084 | 0.142 | 0.8876638 | 0.974643 |
| X211887_x_at | 0.0004 | 0.0031 | 0.142 | 0.8877178 | 0.974643 |
| X215262_at | 0.0016 | 0.0113 | 0.142 | 0.8877465 | 0.974643 |
| X202612_s_at | 0.0007 | 0.0047 | 0.142 | 0.8878525 | 0.974643 |
| X203185_at | 0.0152 | 0.1067 | 0.142 | 0.8878558 | 0.974643 |
| X202034_x_at | 0.0039 | 0.0274 | 0.142 | 0.8879259 | 0.974643 |
| X36553_at | -0.0042 | 0.0298 | -0.142 | 0.8880795 | 0.974643 |
| X220612_at | -0.0008 | 0.0056 | -0.142 | 0.8880927 | 0.974643 |
| X211172_x_at | -0.0008 | 0.006 | -0.142 | 0.8881776 | 0.974643 |
| X221524_s_at | -0.01 | 0.0703 | -0.142 | 0.8881794 | 0.974643 |
| X222371_at | -0.0106 | 0.0746 | -0.142 | 0.888222 | 0.974643 |
| X219983_at | 0.0046 | 0.0326 | 0.142 | 0.888249 | 0.974643 |
| X216805_at | -0.0012 | 0.0087 | -0.142 | 0.8883061 | 0.974643 |
| X207415_at | -0.0012 | 0.0087 | -0.142 | 0.8883093 | 0.974643 |
| X211332_x_at | 0.0006 | 0.0042 | 0.142 | 0.8883105 | 0.974643 |
| X214766_s_at | 0.0054 | 0.0383 | 0.142 | 0.8883363 | 0.974643 |
| X215118_s_at | 0.0004 | 0.0032 | 0.141 | 0.8883941 | 0.974643 |
| X216514_at | 0.0006 | 0.0043 | 0.141 | 0.8884423 | 0.974643 |
| X221099_at | -0.0005 | 0.0038 | -0.141 | 0.8884774 | 0.974643 |
| X205906_at | -0.0013 | 0.0092 | -0.141 | 0.8884855 | 0.974643 |
| X215788_at | 0.0007 | 0.0049 | 0.141 | 0.8885326 | 0.974643 |
| X205834_s_at | 0.0007 | 0.005 | 0.141 | 0.8885466 | 0.974643 |
| X213059_at | -0.001 | 0.0068 | -0.141 | 0.8885603 | 0.974643 |
| X212616_at | 0.0042 | 0.0297 | 0.141 | 0.888719 | 0.974663 |
| X219879_s_at | -0.0009 | 0.0067 | -0.141 | 0.8887274 | 0.974663 |
| X207165_at | 0.0011 | 0.0077 | 0.141 | 0.8887672 | 0.974663 |
| X206242_at | -0.0003 | 0.0024 | -0.141 | 0.8888717 | 0.974663 |
| X32128_at | -0.0015 | 0.011 | -0.141 | 0.8889248 | 0.974663 |
| X206885_x_at | 0.0004 | 0.0029 | 0.141 | 0.8889403 | 0.974663 |
| X206439_at | -0.0014 | 0.0098 | -0.141 | 0.8889946 | 0.974663 |
| X219183_s_at | 0.0008 | 0.0059 | 0.14 | 0.8891877 | 0.974663 |
| X207701_at | 0.0005 | 0.0035 | 0.14 | 0.889221 | 0.974663 |
| X209407_s_at | -0.0099 | 0.0708 | -0.14 | 0.889227 | 0.974663 |
| X214422_at | 0.0021 | 0.0147 | 0.14 | 0.8892454 | 0.974663 |
| X220636_at | -0.0015 | 0.0104 | -0.14 | 0.8892585 | 0.974663 |
| X207803_s_at | -0.0006 | 0.0044 | -0.14 | 0.8892766 | 0.974663 |
| X221202_at | 0.0009 | 0.0066 | 0.14 | 0.8893865 | 0.974663 |
| X212052_s_at | 0.0045 | 0.0324 | 0.14 | 0.8894061 | 0.974663 |
| X211774_s_at | -0.0012 | 0.0088 | -0.14 | 0.8894327 | 0.974663 |
| X210395_x_at | 0.0006 | 0.0042 | 0.14 | 0.889433 | 0.974663 |
| X211519_s_at | -0.0003 | 0.002 | -0.14 | 0.8894482 | 0.974663 |
| X210128_s_at | 0.0005 | 0.0034 | 0.14 | 0.8894592 | 0.974663 |
| X213308_at | 0.0058 | 0.0412 | 0.14 | 0.8895085 | 0.974663 |
| X208587_s_at | 0.0013 | 0.009 | 0.14 | 0.8895206 | 0.974663 |
| X217045_x_at | -0.0005 | 0.0038 | -0.14 | 0.8895434 | 0.974663 |
| X200758_s_at | -0.0058 | 0.0416 | -0.14 | 0.8896503 | 0.974669 |
| X221286_s_at | 0.0008 | 0.0059 | 0.14 | 0.8896589 | 0.974669 |
| X216610_at | -0.0007 | 0.0052 | -0.14 | 0.8896803 | 0.974669 |
| X213121_at | -0.0008 | 0.0055 | -0.14 | 0.8897656 | 0.974694 |
| X217800_s_at | -0.0059 | 0.0425 | -0.14 | 0.8898364 | 0.974694 |
| X221451_s_at | 0.001 | 0.0071 | 0.14 | 0.8898658 | 0.974694 |
| X205325_at | 0.0098 | 0.0703 | 0.14 | 0.8898792 | 0.974694 |
| X212091_s_at | 0.0004 | 0.003 | 0.139 | 0.8900756 | 0.974861 |
| X207708_at | 0.0003 | 0.0022 | 0.139 | 0.8901493 | 0.974867 |
| X216415_at | 0.0014 | 0.01 | 0.139 | 0.8901684 | 0.974867 |
| X214181_x_at | 0.0083 | 0.0596 | 0.139 | 0.8902134 | 0.974868 |
| X33814_at | -0.0022 | 0.016 | -0.139 | 0.890339 | 0.974958 |
| X214557_at | 0.0008 | 0.0054 | 0.139 | 0.8904609 | 0.975043 |
| X209347_s_at | 0.0009 | 0.0062 | 0.139 | 0.8905296 | 0.97507 |
| X32091_at | -0.0034 | 0.0244 | -0.139 | 0.8906121 | 0.975082 |
| X205962_at | 0.001 | 0.0074 | 0.139 | 0.8906323 | 0.975082 |
| X214651_s_at | -0.0015 | 0.0105 | -0.139 | 0.8906859 | 0.975082 |
| X208738_x_at | 0.0021 | 0.0153 | 0.139 | 0.8907155 | 0.975082 |
| X222114_x_at | 0.0004 | 0.0031 | 0.138 | 0.890793 | 0.975118 |
| X218411_s_at | -0.0059 | 0.0429 | -0.138 | 0.8908418 | 0.975124 |
| X205615_at | -0.0006 | 0.0041 | -0.138 | 0.890965 | 0.975211 |
| X220313_at | 0.0141 | 0.1019 | 0.138 | 0.8911358 | 0.975236 |
| X217266_at | 0.0038 | 0.0273 | 0.138 | 0.8911407 | 0.975236 |
| X217037_at | -0.0006 | 0.0044 | -0.138 | 0.8911418 | 0.975236 |
| X215570_s_at | -0.0009 | 0.0062 | -0.138 | 0.8911642 | 0.975236 |
| X214619_at | -0.0008 | 0.0061 | -0.138 | 0.8912579 | 0.975291 |
| X213606_s_at | -0.0012 | 0.0089 | -0.138 | 0.891497 | 0.975386 |
| X210998_s_at | 0.0006 | 0.0044 | 0.137 | 0.8915712 | 0.975386 |
| X203181_x_at | 0.0051 | 0.0374 | 0.137 | 0.891574 | 0.975386 |
| X214469_at | 0.0005 | 0.0037 | 0.137 | 0.8916764 | 0.975386 |
| X218326_s_at | -0.0112 | 0.0816 | -0.137 | 0.8916886 | 0.975386 |
| X203999_at | 0.0058 | 0.0423 | 0.137 | 0.8917125 | 0.975386 |
| X206603_at | -0.0007 | 0.0053 | -0.137 | 0.8917127 | 0.975386 |
| X212225_at | -0.0014 | 0.0103 | -0.137 | 0.8917167 | 0.975386 |
| X209471_s_at | 0.0072 | 0.0526 | 0.137 | 0.8917397 | 0.975386 |
| X201265_at | 0.0005 | 0.0035 | 0.137 | 0.8917844 | 0.975386 |
| X214059_at | 0.0027 | 0.0198 | 0.137 | 0.891837 | 0.975396 |
| X210963_s_at | 0.0006 | 0.0044 | 0.137 | 0.8919573 | 0.97548 |
| X202095_s_at | -0.0004 | 0.0029 | -0.137 | 0.8920137 | 0.975493 |
| X211562_s_at | 0.0008 | 0.0058 | 0.137 | 0.8920808 | 0.975519 |
| X39402_at | -0.0005 | 0.0039 | -0.137 | 0.8922441 | 0.975571 |
| X218017_s_at | -0.0032 | 0.0237 | -0.137 | 0.892253 | 0.975571 |
| X205755_at | -0.0003 | 0.0023 | -0.137 | 0.8922609 | 0.975571 |
| X211478_s_at | 0.0011 | 0.0078 | 0.136 | 0.8923217 | 0.97559 |
| X202684_s_at | 0.0035 | 0.026 | 0.136 | 0.8925224 | 0.975697 |
| X216513_at | -0.0011 | 0.0082 | -0.136 | 0.8925413 | 0.975697 |
| X204410_at | 0.0056 | 0.0409 | 0.136 | 0.892551 | 0.975697 |
| X217948_at | -0.0037 | 0.0274 | -0.136 | 0.8926307 | 0.975706 |
| X206576_s_at | 0.0007 | 0.0052 | 0.136 | 0.892709 | 0.975706 |
| X217940_s_at | 0.0056 | 0.041 | 0.136 | 0.8927164 | 0.975706 |
| X219749_at | -0.0009 | 0.0069 | -0.136 | 0.892735 | 0.975706 |
| X217150_s_at | -0.0028 | 0.0209 | -0.136 | 0.8928162 | 0.975745 |
| X217261_at | -0.0003 | 0.0025 | -0.136 | 0.8928593 | 0.975745 |
| X207838_x_at | 0.0008 | 0.006 | 0.136 | 0.8929427 | 0.975769 |
| X214427_at | -0.004 | 0.0297 | -0.136 | 0.8929686 | 0.975769 |
| X221881_s_at | -0.0011 | 0.0084 | -0.135 | 0.8934442 | 0.976226 |
| X207394_at | 0.0039 | 0.0286 | 0.135 | 0.8935276 | 0.976226 |
| X217798_at | -0.0028 | 0.0209 | -0.135 | 0.8935466 | 0.976226 |
| X215270_at | 0.0003 | 0.0021 | 0.135 | 0.8935628 | 0.976226 |
| X221321_s_at | 0.0006 | 0.0046 | 0.135 | 0.8937539 | 0.976345 |
| X206294_at | -0.001 | 0.0072 | -0.135 | 0.8937671 | 0.976345 |
| X218978_s_at | -0.0011 | 0.0085 | -0.135 | 0.8938267 | 0.976345 |
| X206862_at | 0.0039 | 0.0291 | 0.135 | 0.8938511 | 0.976345 |
| X205387_s_at | -0.0005 | 0.004 | -0.134 | 0.8938916 | 0.976345 |
| X211470_s_at | 0.0014 | 0.0107 | 0.134 | 0.8940898 | 0.976488 |
| X201750_s_at | -0.0004 | 0.0031 | -0.134 | 0.8941532 | 0.976488 |
| X204090_at | 0.0012 | 0.0088 | 0.134 | 0.8941932 | 0.976488 |
| X76897_s_at | -0.001 | 0.0071 | -0.134 | 0.8942469 | 0.976488 |
| X215939_at | 0.0012 | 0.009 | 0.134 | 0.894301 | 0.976488 |
| X204213_at | -0.0004 | 0.0033 | -0.134 | 0.8943357 | 0.976488 |
| X210520_at | 0.0005 | 0.0038 | 0.134 | 0.8943878 | 0.976488 |
| X210888_s_at | -0.0005 | 0.0039 | -0.134 | 0.8944212 | 0.976488 |
| X218188_s_at | 0.0028 | 0.0207 | 0.134 | 0.8944681 | 0.976488 |
| X213176_s_at | 0.0004 | 0.003 | 0.134 | 0.8945023 | 0.976488 |
| X208932_at | -0.0005 | 0.0035 | -0.134 | 0.8945062 | 0.976488 |
| X218155_x_at | 0.008 | 0.0599 | 0.133 | 0.8947209 | 0.976675 |
| X203289_s_at | -0.0007 | 0.0053 | -0.133 | 0.894793 | 0.976705 |
| X214335_at | 0.0004 | 0.003 | 0.133 | 0.8948436 | 0.976713 |
| X214127_s_at | 0.0004 | 0.0033 | 0.133 | 0.8949127 | 0.976728 |
| X209394_at | 0.0047 | 0.0352 | 0.133 | 0.8949459 | 0.976728 |
| X217369_at | -0.0004 | 0.0028 | -0.133 | 0.8950595 | 0.976737 |
| X218287_s_at | 0.004 | 0.03 | 0.133 | 0.8950801 | 0.976737 |
| X207932_at | -0.001 | 0.0077 | -0.133 | 0.8950856 | 0.976737 |
| X208315_x_at | -0.0008 | 0.0059 | -0.133 | 0.8951302 | 0.976738 |
| X204673_at | -0.0004 | 0.0031 | -0.133 | 0.895238 | 0.97676 |
| X222084_s_at | 0.0005 | 0.0038 | 0.133 | 0.8952391 | 0.97676 |
| X215488_at | 0.0006 | 0.0047 | 0.133 | 0.8953534 | 0.976783 |
| X206784_at | 0.0005 | 0.0034 | 0.133 | 0.8953784 | 0.976783 |
| X205044_at | -0.0007 | 0.0051 | -0.133 | 0.8954135 | 0.976783 |
| X215142_at | -0.0003 | 0.0019 | -0.132 | 0.8954738 | 0.976783 |
| X211668_s_at | -0.0003 | 0.0025 | -0.132 | 0.89548 | 0.976783 |
| X222277_at | 0.0006 | 0.0046 | 0.132 | 0.8956894 | 0.976807 |
| X203402_at | 0.007 | 0.0526 | 0.132 | 0.8956982 | 0.976807 |
| X207569_at | 0.0006 | 0.0044 | 0.132 | 0.8957036 | 0.976807 |
| X217541_x_at | -0.0012 | 0.0093 | -0.132 | 0.8957567 | 0.976807 |
| X202073_at | -0.0069 | 0.0523 | -0.132 | 0.895759 | 0.976807 |
| X216898_s_at | -0.0018 | 0.0134 | -0.132 | 0.8957656 | 0.976807 |
| X215966_x_at | -0.0008 | 0.006 | -0.132 | 0.8958104 | 0.976808 |
| X204803_s_at | -0.0005 | 0.0036 | -0.132 | 0.8958912 | 0.976826 |
| X205142_x_at | 0.0007 | 0.0053 | 0.132 | 0.8959579 | 0.976826 |
| X217078_s_at | -0.0004 | 0.0032 | -0.132 | 0.89601 | 0.976826 |
| X217381_s_at | -0.0007 | 0.0053 | -0.132 | 0.8960792 | 0.976826 |
| X206601_s_at | 0.0009 | 0.0068 | 0.132 | 0.8960916 | 0.976826 |
| X205213_at | 0.0006 | 0.0048 | 0.132 | 0.8961071 | 0.976826 |
| X205955_at | -0.0018 | 0.0134 | -0.132 | 0.8961346 | 0.976826 |
| X220275_at | 0.0007 | 0.0054 | 0.131 | 0.8963864 | 0.977003 |
| X208773_s_at | -0.0037 | 0.0279 | -0.131 | 0.8963917 | 0.977003 |
| X200028_s_at | -0.0025 | 0.0191 | -0.131 | 0.8964369 | 0.977003 |
| X214884_at | -0.0017 | 0.0129 | -0.131 | 0.8964778 | 0.977003 |
| X221147_x_at | 0.0004 | 0.0033 | 0.131 | 0.8966603 | 0.977003 |
| X214102_at | -0.0092 | 0.0706 | -0.131 | 0.8966958 | 0.977003 |
| X214569_at | -0.0007 | 0.0055 | -0.131 | 0.8967238 | 0.977003 |
| X209606_at | -0.0024 | 0.018 | -0.131 | 0.8967274 | 0.977003 |
| X221195_at | 0.0023 | 0.0172 | 0.131 | 0.8968112 | 0.977003 |
| X216408_at | 0.0008 | 0.0058 | 0.131 | 0.8968585 | 0.977003 |
| X211026_s_at | 0.0096 | 0.0733 | 0.131 | 0.8968678 | 0.977003 |
| X213539_at | 0.0005 | 0.0041 | 0.131 | 0.896892 | 0.977003 |
| X219193_at | -0.0023 | 0.0176 | -0.131 | 0.8969307 | 0.977003 |
| X221997_s_at | 0.0004 | 0.0032 | 0.131 | 0.8969334 | 0.977003 |
| X214738_s_at | -0.0003 | 0.0025 | -0.13 | 0.8970187 | 0.977003 |
| X211531_x_at | 0.0008 | 0.0064 | 0.13 | 0.8970513 | 0.977003 |
| X200703_at | 0.0038 | 0.0293 | 0.13 | 0.8971984 | 0.977003 |
| X210429_at | -0.0005 | 0.0042 | -0.13 | 0.8972678 | 0.977003 |
| X221228_s_at | -0.001 | 0.0074 | -0.13 | 0.8972906 | 0.977003 |
| X219411_at | -0.0003 | 0.0024 | -0.13 | 0.8973014 | 0.977003 |
| X211154_at | 0.001 | 0.0077 | 0.13 | 0.8973244 | 0.977003 |
| X211874_s_at | -0.001 | 0.0078 | -0.13 | 0.8973345 | 0.977003 |
| X203565_s_at | -0.0006 | 0.0048 | -0.13 | 0.8973397 | 0.977003 |
| X207028_at | 0.0006 | 0.0049 | 0.13 | 0.8973527 | 0.977003 |
| X202696_at | -0.0052 | 0.0399 | -0.13 | 0.8975007 | 0.977063 |
| X217076_s_at | 0.001 | 0.008 | 0.13 | 0.897508 | 0.977063 |
| X207126_x_at | 0.0008 | 0.006 | 0.13 | 0.8976345 | 0.977063 |
| X217209_at | -0.0006 | 0.0045 | -0.13 | 0.8977434 | 0.977063 |
| X207061_at | -0.0004 | 0.0035 | -0.13 | 0.8977466 | 0.977063 |
| X204965_at | -0.0006 | 0.0049 | -0.13 | 0.8977569 | 0.977063 |
| X203153_at | -0.0075 | 0.0579 | -0.129 | 0.8978191 | 0.977063 |
| X213962_s_at | -0.0006 | 0.0047 | -0.129 | 0.8978195 | 0.977063 |
| X213905_x_at | -0.0099 | 0.0764 | -0.129 | 0.8978587 | 0.977063 |
| X207377_at | -0.0006 | 0.005 | -0.129 | 0.8978724 | 0.977063 |
| X212324_s_at | 0.001 | 0.0074 | 0.129 | 0.8979126 | 0.977063 |
| X206210_s_at | 0.0004 | 0.0028 | 0.129 | 0.8979641 | 0.977063 |
| X214343_s_at | 0.0003 | 0.0027 | 0.129 | 0.8979959 | 0.977063 |
| X206563_s_at | -0.0005 | 0.004 | -0.129 | 0.8980492 | 0.977063 |
| X202852_s_at | -0.0108 | 0.0834 | -0.129 | 0.898105 | 0.977063 |
| X210553_x_at | -0.0004 | 0.0029 | -0.129 | 0.8981174 | 0.977063 |
| X219030_at | 0.0048 | 0.0371 | 0.129 | 0.8981552 | 0.977063 |
| X203875_at | -0.0054 | 0.0416 | -0.129 | 0.8982091 | 0.977074 |
| X206623_at | -0.0004 | 0.0033 | -0.129 | 0.8982928 | 0.977117 |
| X206398_s_at | -0.0004 | 0.003 | -0.129 | 0.8983411 | 0.977121 |
| X203091_at | -0.0051 | 0.0395 | -0.129 | 0.8984101 | 0.977149 |
| X214488_at | -0.0008 | 0.0064 | -0.128 | 0.8985845 | 0.977247 |
| X202422_s_at | 0.0089 | 0.0692 | 0.128 | 0.8985889 | 0.977247 |
| X207621_s_at | -0.0042 | 0.0327 | -0.128 | 0.898648 | 0.977264 |
| X218434_s_at | -0.0054 | 0.0424 | -0.128 | 0.8987125 | 0.977278 |
| X214618_at | -0.001 | 0.0077 | -0.128 | 0.8988183 | 0.977278 |
| X219918_s_at | 0.0009 | 0.0069 | 0.128 | 0.8988193 | 0.977278 |
| X219521_at | 0.0073 | 0.0572 | 0.128 | 0.8988893 | 0.977278 |
| X202147_s_at | -0.0064 | 0.0502 | -0.128 | 0.8988996 | 0.977278 |
| X212140_at | 0.0018 | 0.0138 | 0.128 | 0.8989254 | 0.977278 |
| X212211_at | 0.0048 | 0.0374 | 0.128 | 0.8989813 | 0.977287 |
| X203253_s_at | 0.0046 | 0.0363 | 0.128 | 0.8990213 | 0.977287 |
| X206202_at | -0.0012 | 0.0091 | -0.128 | 0.8992684 | 0.977448 |
| X214278_s_at | 0.0007 | 0.0054 | 0.128 | 0.899291 | 0.977448 |
| X217507_at | -0.0009 | 0.007 | -0.128 | 0.8993017 | 0.977448 |
| X217110_s_at | 0.0006 | 0.0048 | 0.127 | 0.8993868 | 0.977458 |
| X203953_s_at | 0.0004 | 0.0031 | 0.127 | 0.8994732 | 0.977458 |
| X216277_at | -0.0009 | 0.0071 | -0.127 | 0.8994816 | 0.977458 |
| X217870_s_at | 0.0037 | 0.029 | 0.127 | 0.8995489 | 0.977458 |
| X211412_at | -0.0009 | 0.0072 | -0.127 | 0.8996089 | 0.977458 |
| X216549_s_at | -0.0007 | 0.0052 | -0.127 | 0.8996335 | 0.977458 |
| X205929_at | -0.0005 | 0.0036 | -0.127 | 0.8996347 | 0.977458 |
| X202195_s_at | -0.0015 | 0.0119 | -0.127 | 0.8997349 | 0.977458 |
| X214657_s_at | -0.0008 | 0.0064 | -0.127 | 0.8997949 | 0.977458 |
| X215276_at | 0.0012 | 0.0098 | 0.127 | 0.8998235 | 0.977458 |
| X208544_at | 0.0006 | 0.0044 | 0.127 | 0.8998365 | 0.977458 |
| X214656_x_at | -0.0056 | 0.044 | -0.127 | 0.8998786 | 0.977458 |
| X219145_at | -0.0034 | 0.0269 | -0.127 | 0.899922 | 0.977458 |
| X214958_s_at | 0.0004 | 0.0035 | 0.127 | 0.8999263 | 0.977458 |
| X211524_at | -0.0008 | 0.006 | -0.127 | 0.9000396 | 0.977533 |
| X220847_x_at | 0.0005 | 0.0043 | 0.127 | 0.9001088 | 0.977537 |
| X219499_at | -0.005 | 0.0394 | -0.127 | 0.9001316 | 0.977537 |
| X206129_s_at | 0.0006 | 0.0047 | 0.126 | 0.9001886 | 0.97755 |
| X214380_at | -0.0006 | 0.0045 | -0.126 | 0.9002622 | 0.97755 |
| X221876_at | 0.0009 | 0.0069 | 0.126 | 0.9002916 | 0.97755 |
| X210449_x_at | -0.0004 | 0.003 | -0.126 | 0.9003527 | 0.97755 |
| X211833_s_at | -0.0007 | 0.0059 | -0.126 | 0.9004043 | 0.97755 |
| X220684_at | -0.0007 | 0.0052 | -0.126 | 0.9004078 | 0.97755 |
| X221394_at | 0.0006 | 0.005 | 0.126 | 0.9004841 | 0.977586 |
| X213439_x_at | 0.0073 | 0.058 | 0.126 | 0.9006883 | 0.977606 |
| X209353_s_at | 0.0006 | 0.0045 | 0.126 | 0.9007509 | 0.977606 |
| X205432_at | 0.0016 | 0.0124 | 0.126 | 0.9008009 | 0.977606 |
| X207597_at | -0.001 | 0.0076 | -0.126 | 0.900836 | 0.977606 |
| X220919_s_at | -0.0016 | 0.0126 | -0.126 | 0.9008435 | 0.977606 |
| X221750_at | -0.0113 | 0.09 | -0.126 | 0.9008988 | 0.977606 |
| X220398_at | -0.0004 | 0.0032 | -0.126 | 0.9009022 | 0.977606 |
| X203249_at | -0.0038 | 0.0306 | -0.125 | 0.9009647 | 0.977606 |
| X216961_s_at | 0.0008 | 0.0067 | 0.125 | 0.900972 | 0.977606 |
| X209893_s_at | 0.0007 | 0.0059 | 0.125 | 0.9009758 | 0.977606 |
| X210097_s_at | -0.0078 | 0.0624 | -0.125 | 0.9009873 | 0.977606 |
| X207074_s_at | 0.0007 | 0.0052 | 0.125 | 0.9012128 | 0.977803 |
| X209245_s_at | -0.0013 | 0.0105 | -0.125 | 0.9012591 | 0.977806 |
| X214693_x_at | 0.0099 | 0.0795 | 0.125 | 0.9013655 | 0.977873 |
| X215813_s_at | 0.0006 | 0.0046 | 0.125 | 0.9014338 | 0.9779 |
| X221815_at | 0.0061 | 0.0487 | 0.125 | 0.901566 | 0.97798 |
| X211816_x_at | -0.0003 | 0.0027 | -0.125 | 0.901612 | 0.97798 |
| X211304_x_at | 0.0009 | 0.0072 | 0.125 | 0.9016398 | 0.97798 |
| X207964_x_at | 0.0006 | 0.0046 | 0.124 | 0.9019057 | 0.978009 |
| X214137_at | 0.0007 | 0.0055 | 0.124 | 0.9019275 | 0.978009 |
| X219621_at | -0.0012 | 0.0095 | -0.124 | 0.9019471 | 0.978009 |
| X217731_s_at | -0.0034 | 0.0276 | -0.124 | 0.9019526 | 0.978009 |
| X211869_at | 0.0003 | 0.0028 | 0.124 | 0.9019709 | 0.978009 |
| X209667_at | -0.0066 | 0.0534 | -0.124 | 0.9019785 | 0.978009 |
| X210135_s_at | -0.0009 | 0.0072 | -0.124 | 0.9020888 | 0.978009 |
| X201932_at | -0.0051 | 0.0412 | -0.124 | 0.9021167 | 0.978009 |
| X222086_s_at | -0.0004 | 0.0032 | -0.124 | 0.9021181 | 0.978009 |
| X220857_at | 0.0004 | 0.0031 | 0.124 | 0.9021182 | 0.978009 |
| X211209_x_at | -0.0004 | 0.0034 | -0.124 | 0.9021763 | 0.978009 |
| X207021_at | 0.0007 | 0.0056 | 0.124 | 0.9021949 | 0.978009 |
| X206863_x_at | -0.0004 | 0.0035 | -0.124 | 0.902342 | 0.978078 |
| X221665_s_at | 0.0005 | 0.0043 | 0.124 | 0.902347 | 0.978078 |
| X213541_s_at | -0.001 | 0.0079 | -0.124 | 0.902409 | 0.978098 |
| X209744_x_at | 0.0148 | 0.1198 | 0.124 | 0.9024845 | 0.978118 |
| X217913_at | 0.0043 | 0.035 | 0.123 | 0.9025641 | 0.978118 |
| X207555_s_at | 0.001 | 0.0083 | 0.123 | 0.9026992 | 0.978118 |
| X206895_at | 0.0007 | 0.0055 | 0.123 | 0.9027416 | 0.978118 |
| X203294_s_at | 0.001 | 0.0077 | 0.123 | 0.902751 | 0.978118 |
| X212076_at | 0.0142 | 0.1153 | 0.123 | 0.9028585 | 0.978118 |
| X206484_s_at | -0.0003 | 0.0027 | -0.123 | 0.9028693 | 0.978118 |
| X214099_s_at | -0.0091 | 0.0739 | -0.123 | 0.9029053 | 0.978118 |
| X217147_s_at | 0.0022 | 0.0178 | 0.123 | 0.9029419 | 0.978118 |
| X203790_s_at | -0.0097 | 0.0786 | -0.123 | 0.9029425 | 0.978118 |
| X210190_at | 0.0006 | 0.0049 | 0.123 | 0.9029612 | 0.978118 |
| X205677_s_at | 0.0008 | 0.0068 | 0.123 | 0.9029959 | 0.978118 |
| X217656_at | 0.0006 | 0.0048 | 0.123 | 0.9030118 | 0.978118 |
| X214287_s_at | 0.0006 | 0.0045 | 0.123 | 0.9030441 | 0.978118 |
| X218279_s_at | 0.0005 | 0.004 | 0.123 | 0.9031759 | 0.978212 |
| X211326_x_at | -0.0005 | 0.0042 | -0.123 | 0.9032533 | 0.978212 |
| X220976_s_at | -0.0006 | 0.0046 | -0.123 | 0.903263 | 0.978212 |
| X213173_at | -0.0016 | 0.013 | -0.122 | 0.9033324 | 0.97824 |
| X201732_s_at | 0.009 | 0.0736 | 0.122 | 0.9034334 | 0.978301 |
| X204333_s_at | -0.0122 | 0.1003 | -0.122 | 0.9036046 | 0.97837 |
| X216066_at | -0.0008 | 0.0069 | -0.122 | 0.9036931 | 0.97837 |
| X202314_at | 0.0044 | 0.036 | 0.122 | 0.903698 | 0.97837 |
| X220862_s_at | 0.0003 | 0.0028 | 0.122 | 0.9037408 | 0.97837 |
| X218991_at | -0.0021 | 0.0172 | -0.122 | 0.9037507 | 0.97837 |
| X215564_at | -0.0012 | 0.0097 | -0.122 | 0.9038298 | 0.97837 |
| X203775_at | 0.0065 | 0.0533 | 0.122 | 0.903831 | 0.97837 |
| X216801_at | 0.0002 | 0.0019 | 0.122 | 0.9038816 | 0.97837 |
| X219198_at | 0.0024 | 0.0201 | 0.122 | 0.9040193 | 0.97837 |
| X219189_at | 0.0004 | 0.003 | 0.122 | 0.9040706 | 0.97837 |
| X217486_s_at | -0.001 | 0.0081 | -0.121 | 0.9040801 | 0.97837 |
| X220674_at | 0.0006 | 0.0051 | 0.121 | 0.9041516 | 0.97837 |
| X210806_at | 0.0005 | 0.0042 | 0.121 | 0.9041641 | 0.97837 |
| X212979_s_at | 0.0112 | 0.0921 | 0.121 | 0.9041647 | 0.97837 |
| X221823_at | 0.0043 | 0.0356 | 0.121 | 0.9042274 | 0.97837 |
| X203321_s_at | 0.0053 | 0.0433 | 0.121 | 0.9042736 | 0.97837 |
| X220103_s_at | -0.0055 | 0.0455 | -0.121 | 0.904315 | 0.97837 |
| X200672_x_at | -0.006 | 0.0496 | -0.121 | 0.9043545 | 0.97837 |
| X208244_at | 0.0005 | 0.0038 | 0.121 | 0.9043552 | 0.97837 |
| X220982_s_at | 0.0008 | 0.0068 | 0.121 | 0.904378 | 0.97837 |
| X205969_at | 0.0007 | 0.0059 | 0.121 | 0.9044837 | 0.978371 |
| X214153_at | 0.0007 | 0.006 | 0.121 | 0.904489 | 0.978371 |
| X218365_s_at | -0.0004 | 0.0032 | -0.121 | 0.9045107 | 0.978371 |
| X221038_at | -0.0009 | 0.0072 | -0.121 | 0.9045734 | 0.97838 |
| X206842_at | -0.0004 | 0.0036 | -0.121 | 0.9046072 | 0.97838 |
| X34449_at | -0.0009 | 0.0073 | -0.121 | 0.9046845 | 0.978409 |
| X217072_at | 0.0004 | 0.003 | 0.121 | 0.9047223 | 0.978409 |
| X221141_x_at | -0.0009 | 0.0077 | -0.12 | 0.9048907 | 0.978544 |
| X220678_at | 0.0006 | 0.0047 | 0.12 | 0.9049997 | 0.978555 |
| X220390_at | -0.0007 | 0.0057 | -0.12 | 0.9050191 | 0.978555 |
| X213286_at | -0.0202 | 0.1679 | -0.12 | 0.9051351 | 0.978555 |
| X213968_at | -0.0007 | 0.0057 | -0.12 | 0.9051632 | 0.978555 |
| X222340_at | 0.0007 | 0.0059 | 0.12 | 0.9051935 | 0.978555 |
| X209118_s_at | -0.004 | 0.0331 | -0.12 | 0.905228 | 0.978555 |
| X214461_at | 0.0006 | 0.0047 | 0.12 | 0.9052888 | 0.978555 |
| X209677_at | -0.002 | 0.0163 | -0.12 | 0.9052994 | 0.978555 |
| X209742_s_at | -0.0005 | 0.0039 | -0.12 | 0.9053009 | 0.978555 |
| X203634_s_at | 0.0005 | 0.0038 | 0.12 | 0.9053556 | 0.978555 |
| X207385_at | -0.0007 | 0.0058 | -0.12 | 0.9054148 | 0.978555 |
| X202726_at | 0.0004 | 0.0033 | 0.12 | 0.9054562 | 0.978555 |
| X202146_at | 0.0031 | 0.026 | 0.12 | 0.9055389 | 0.978555 |
| X214984_at | -0.0014 | 0.0117 | -0.12 | 0.9055533 | 0.978555 |
| X218089_at | 0.0008 | 0.0068 | 0.12 | 0.9056329 | 0.978555 |
| X205800_at | -0.0006 | 0.005 | -0.12 | 0.9056455 | 0.978555 |
| X202185_at | -0.0064 | 0.0531 | -0.12 | 0.90565 | 0.978555 |
| X203906_at | -0.0069 | 0.0582 | -0.119 | 0.9058654 | 0.97874 |
| X205919_at | -0.0008 | 0.0066 | -0.119 | 0.9059774 | 0.978813 |
| X40640_at | -0.0004 | 0.0033 | -0.119 | 0.9061562 | 0.978959 |
| X212325_at | 0.0091 | 0.0764 | 0.119 | 0.9062197 | 0.978972 |
| X202238_s_at | 0.0006 | 0.005 | 0.119 | 0.9063246 | 0.978972 |
| X209891_at | -0.0013 | 0.0113 | -0.119 | 0.9063497 | 0.978972 |
| X207272_at | -0.002 | 0.0171 | -0.119 | 0.9064263 | 0.978972 |
| X208115_x_at | -0.0005 | 0.004 | -0.118 | 0.9064512 | 0.978972 |
| X205725_at | 0.0004 | 0.0035 | 0.118 | 0.9064777 | 0.978972 |
| X207282_s_at | -0.0006 | 0.0049 | -0.118 | 0.9064896 | 0.978972 |
| X209659_s_at | -0.0034 | 0.0287 | -0.118 | 0.906521 | 0.978972 |
| X214639_s_at | -0.0006 | 0.0047 | -0.118 | 0.9066269 | 0.979028 |
| X207962_at | 0.0006 | 0.0053 | 0.118 | 0.9066859 | 0.979028 |
| X215213_at | 0.0019 | 0.0158 | 0.118 | 0.9067053 | 0.979028 |
| X213756_s_at | -0.0003 | 0.0024 | -0.118 | 0.9068377 | 0.979081 |
| X215393_s_at | 0.0005 | 0.0047 | 0.118 | 0.9068573 | 0.979081 |
| X207039_at | 0.0005 | 0.0043 | 0.118 | 0.9068866 | 0.979081 |
| X203317_at | -0.0005 | 0.004 | -0.118 | 0.9070609 | 0.979153 |
| X202221_s_at | -0.0076 | 0.0643 | -0.118 | 0.907103 | 0.979153 |
| X209737_at | 0.0056 | 0.0479 | 0.118 | 0.9071246 | 0.979153 |
| X222350_at | 0.0015 | 0.0126 | 0.118 | 0.9071513 | 0.979153 |
| X212189_s_at | 0.0005 | 0.0038 | 0.118 | 0.9071849 | 0.979153 |
| X205465_x_at | -0.0005 | 0.0044 | -0.118 | 0.9072177 | 0.979153 |
| X208655_at | 0.0045 | 0.0382 | 0.117 | 0.9074112 | 0.97928 |
| X214662_at | 0.0036 | 0.031 | 0.117 | 0.907459 | 0.97928 |
| X212164_at | -0.0035 | 0.03 | -0.117 | 0.9074814 | 0.97928 |
| X207175_at | 0.0014 | 0.0117 | 0.117 | 0.9075212 | 0.97928 |
| X214382_at | 0.0006 | 0.0048 | 0.117 | 0.9075943 | 0.97928 |
| X214627_at | 0.0005 | 0.0047 | 0.117 | 0.9076687 | 0.97928 |
| X216059_at | -0.0003 | 0.0029 | -0.117 | 0.9076843 | 0.97928 |
| X217558_at | -0.0009 | 0.0076 | -0.117 | 0.9076879 | 0.97928 |
| X207656_s_at | 0.0007 | 0.0058 | 0.117 | 0.9077948 | 0.979348 |
| X206221_at | 0.0005 | 0.0047 | 0.117 | 0.9078706 | 0.979382 |
| X215131_at | -0.0007 | 0.0057 | -0.116 | 0.9081641 | 0.979541 |
| X202074_s_at | -0.006 | 0.052 | -0.116 | 0.9082314 | 0.979541 |
| X206328_at | -0.0003 | 0.0029 | -0.116 | 0.9082618 | 0.979541 |
| X212463_at | 0.0043 | 0.0367 | 0.116 | 0.9082648 | 0.979541 |
| X209746_s_at | 0.0056 | 0.0481 | 0.116 | 0.9083201 | 0.979541 |
| X216616_at | -0.0004 | 0.0033 | -0.116 | 0.9083455 | 0.979541 |
| X207295_at | 0.0005 | 0.0043 | 0.116 | 0.9083729 | 0.979541 |
| X200887_s_at | 0.0042 | 0.0362 | 0.116 | 0.9084235 | 0.979541 |
| X207904_s_at | 0.0008 | 0.0072 | 0.116 | 0.9084944 | 0.979541 |
| X221420_at | 0.0006 | 0.0054 | 0.116 | 0.9085522 | 0.979541 |
| X211840_s_at | 0.0005 | 0.0042 | 0.116 | 0.9086618 | 0.979541 |
| X214423_x_at | 0.0003 | 0.0026 | 0.116 | 0.9086777 | 0.979541 |
| X222145_at | 0.001 | 0.0082 | 0.116 | 0.9086912 | 0.979541 |
| X220984_s_at | -0.0006 | 0.0051 | -0.116 | 0.9087069 | 0.979541 |
| X204519_s_at | 0.015 | 0.13 | 0.116 | 0.9087726 | 0.979541 |
| X216562_at | 0.0002 | 0.002 | 0.116 | 0.908787 | 0.979541 |
| X220706_at | -0.0004 | 0.0036 | -0.115 | 0.9088086 | 0.979541 |
| X214147_at | 0.0005 | 0.0046 | 0.115 | 0.9088844 | 0.979541 |
| X200025_s_at | -0.0028 | 0.0245 | -0.115 | 0.9089419 | 0.979541 |
| X215671_at | 0.0018 | 0.0152 | 0.115 | 0.9089997 | 0.979541 |
| X209340_at | -0.007 | 0.0611 | -0.115 | 0.9090176 | 0.979541 |
| X216968_at | -0.0005 | 0.0042 | -0.115 | 0.9090265 | 0.979541 |
| X204740_at | -0.0013 | 0.0112 | -0.115 | 0.9090319 | 0.979541 |
| X216472_at | -0.0007 | 0.0057 | -0.115 | 0.9091468 | 0.979617 |
| X216884_at | -0.0007 | 0.0058 | -0.115 | 0.9092548 | 0.979666 |
| X212229_s_at | 0.0036 | 0.0313 | 0.115 | 0.9093064 | 0.979666 |
| X203335_at | 0.0057 | 0.0495 | 0.115 | 0.9094226 | 0.979666 |
| X206536_s_at | 0.001 | 0.0085 | 0.115 | 0.9094322 | 0.979666 |
| X211014_s_at | -0.0003 | 0.0023 | -0.115 | 0.9094437 | 0.979666 |
| X203546_at | 0.0069 | 0.0603 | 0.115 | 0.9094756 | 0.979666 |
| X78495_at | 0.001 | 0.0091 | 0.115 | 0.9095798 | 0.979666 |
| X210297_s_at | -0.001 | 0.0088 | -0.114 | 0.9095958 | 0.979666 |
| X204540_at | -0.0051 | 0.0449 | -0.114 | 0.9096254 | 0.979666 |
| X222296_at | 0.0007 | 0.0057 | 0.114 | 0.9096335 | 0.979666 |
| X208589_at | 0.0005 | 0.0042 | 0.114 | 0.9096897 | 0.979679 |
| X214927_at | -0.0007 | 0.0058 | -0.114 | 0.9099934 | 0.979897 |
| X215986_at | -0.0018 | 0.0155 | -0.114 | 0.910034 | 0.979897 |
| X205232_s_at | 0.0005 | 0.0048 | 0.114 | 0.9101784 | 0.979897 |
| X220209_at | 0.0009 | 0.0078 | 0.114 | 0.9102499 | 0.979897 |
| X216572_at | -0.0005 | 0.0047 | -0.114 | 0.9103227 | 0.979897 |
| X213088_s_at | 0.0056 | 0.0494 | 0.114 | 0.9103288 | 0.979897 |
| X214584_x_at | 0.0007 | 0.0064 | 0.114 | 0.9103335 | 0.979897 |
| X212075_s_at | 0.0102 | 0.0899 | 0.114 | 0.9103439 | 0.979897 |
| X219184_x_at | 0.0062 | 0.0544 | 0.113 | 0.9103938 | 0.979897 |
| X220851_at | -0.001 | 0.0085 | -0.113 | 0.9105108 | 0.979897 |
| X213986_s_at | 0.0004 | 0.0031 | 0.113 | 0.9105828 | 0.979897 |
| X202200_s_at | 0.0063 | 0.056 | 0.113 | 0.9105948 | 0.979897 |
| X220752_at | -0.0009 | 0.0076 | -0.113 | 0.9106011 | 0.979897 |
| X207561_s_at | 0.0004 | 0.0039 | 0.113 | 0.9106041 | 0.979897 |
| X208505_s_at | -0.0004 | 0.0037 | -0.113 | 0.9106947 | 0.979897 |
| X211043_s_at | -0.0047 | 0.0416 | -0.113 | 0.9107761 | 0.979897 |
| X205380_at | 0.0004 | 0.0037 | 0.113 | 0.9108266 | 0.979897 |
| X213492_at | 0.0005 | 0.0044 | 0.113 | 0.9108281 | 0.979897 |
| X202058_s_at | 0.0113 | 0.1005 | 0.113 | 0.9108408 | 0.979897 |
| X209661_at | 0.0009 | 0.008 | 0.113 | 0.9108499 | 0.979897 |
| X211913_s_at | -0.0005 | 0.0042 | -0.113 | 0.9108504 | 0.979897 |
| X221030_s_at | -0.0007 | 0.0062 | -0.113 | 0.910892 | 0.979897 |
| X222137_at | 0.001 | 0.0087 | 0.113 | 0.9109065 | 0.979897 |
| X32540_at | 0.0015 | 0.0137 | 0.113 | 0.9110557 | 0.979968 |
| X210575_at | -0.0004 | 0.0039 | -0.113 | 0.9110606 | 0.979968 |
| X222031_at | -0.0011 | 0.0098 | -0.112 | 0.911182 | 0.979988 |
| X214838_at | 0.0003 | 0.0029 | 0.112 | 0.9112005 | 0.979988 |
| X207750_at | -0.0024 | 0.0213 | -0.112 | 0.9112201 | 0.979988 |
| X206482_at | 0.0009 | 0.008 | 0.112 | 0.9112555 | 0.979988 |
| X202032_s_at | -0.0047 | 0.0419 | -0.112 | 0.9113203 | 0.98001 |
| X206546_at | -0.0021 | 0.0189 | -0.112 | 0.9114318 | 0.980052 |
| X211162_x_at | 0.0004 | 0.0033 | 0.112 | 0.9114861 | 0.980052 |
| X221650_s_at | 0.0007 | 0.0059 | 0.112 | 0.9114912 | 0.980052 |
| X220733_at | 0.0005 | 0.0044 | 0.112 | 0.9117019 | 0.980231 |
| X320_at | -0.0005 | 0.004 | -0.112 | 0.9117576 | 0.980235 |
| X222208_s_at | 0.0009 | 0.008 | 0.112 | 0.9117938 | 0.980235 |
| X211104_s_at | -0.0007 | 0.0059 | -0.111 | 0.911954 | 0.980265 |
| X214365_at | -0.0011 | 0.0096 | -0.111 | 0.9119554 | 0.980265 |
| X201501_s_at | 0.0034 | 0.0302 | 0.111 | 0.9120483 | 0.980265 |
| X207298_at | 0.0005 | 0.0041 | 0.111 | 0.9121045 | 0.980265 |
| X208917_x_at | -0.0003 | 0.0024 | -0.111 | 0.9121398 | 0.980265 |
| X203418_at | -0.0004 | 0.0036 | -0.111 | 0.9121426 | 0.980265 |
| X215757_at | -0.0004 | 0.0037 | -0.111 | 0.9121755 | 0.980265 |
| X213202_at | 0.0005 | 0.0045 | 0.111 | 0.9121958 | 0.980265 |
| X216244_at | -0.0012 | 0.0105 | -0.111 | 0.9122193 | 0.980265 |
| X205533_s_at | 0.0011 | 0.0095 | 0.111 | 0.912346 | 0.980354 |
| X206914_at | 0.0007 | 0.006 | 0.111 | 0.9124131 | 0.980356 |
| X204259_at | -0.0004 | 0.004 | -0.111 | 0.9124362 | 0.980356 |
| X221639_x_at | -0.0007 | 0.0067 | -0.111 | 0.912574 | 0.980357 |
| X205425_at | 0.0117 | 0.1058 | 0.111 | 0.9126697 | 0.980357 |
| X215967_s_at | -0.0004 | 0.0039 | -0.111 | 0.9126711 | 0.980357 |
| X213790_at | -0.0136 | 0.1232 | -0.11 | 0.9127386 | 0.980357 |
| X209282_at | -0.0003 | 0.0027 | -0.11 | 0.9128296 | 0.980357 |
| X214564_s_at | -0.0002 | 0.0021 | -0.11 | 0.9128332 | 0.980357 |
| X215158_s_at | -0.0036 | 0.0326 | -0.11 | 0.9128463 | 0.980357 |
| X205406_s_at | -0.0063 | 0.0576 | -0.11 | 0.9128621 | 0.980357 |
| X200801_x_at | 0.0033 | 0.0297 | 0.11 | 0.9129099 | 0.980357 |
| X210614_at | -0.0005 | 0.005 | -0.11 | 0.9129488 | 0.980357 |
| X210883_x_at | -0.0008 | 0.0076 | -0.11 | 0.9129963 | 0.980357 |
| X205200_at | -0.0153 | 0.1388 | -0.11 | 0.9130059 | 0.980357 |
| X209892_at | -0.0012 | 0.0106 | -0.11 | 0.9130861 | 0.980357 |
| X213257_at | 0.0027 | 0.0242 | 0.11 | 0.9131132 | 0.980357 |
| X220214_at | 0.0003 | 0.0026 | 0.11 | 0.9132009 | 0.980357 |
| X220146_at | -0.001 | 0.0087 | -0.11 | 0.9132362 | 0.980357 |
| X214886_s_at | 0.0005 | 0.0042 | 0.11 | 0.9132614 | 0.980357 |
| X216749_at | -0.0007 | 0.0062 | -0.11 | 0.9132657 | 0.980357 |
| X215208_x_at | 0.0052 | 0.0478 | 0.11 | 0.9133063 | 0.980357 |
| X213814_s_at | 0.0004 | 0.0038 | 0.11 | 0.9133431 | 0.980357 |
| X220708_at | -0.0005 | 0.0044 | -0.11 | 0.9133643 | 0.980357 |
| X210464_at | -0.0006 | 0.0055 | -0.11 | 0.9134569 | 0.980379 |
| X205817_at | -0.0004 | 0.0037 | -0.11 | 0.913473 | 0.980379 |
| X215020_at | 0.0077 | 0.0707 | 0.109 | 0.9135267 | 0.98039 |
| X213466_at | -0.0003 | 0.0031 | -0.109 | 0.9136002 | 0.980421 |
| X206747_at | 0.0006 | 0.0056 | 0.109 | 0.9136836 | 0.980463 |
| X208293_x_at | 0.0004 | 0.0034 | 0.109 | 0.9139224 | 0.980672 |
| X220388_at | 0.0005 | 0.0044 | 0.109 | 0.9141777 | 0.98083 |
| X208606_s_at | 0.0002 | 0.0016 | 0.109 | 0.9141833 | 0.98083 |
| X219955_at | 0.0006 | 0.0053 | 0.108 | 0.9142998 | 0.98083 |
| X204895_x_at | 0.0003 | 0.0031 | 0.108 | 0.9143028 | 0.98083 |
| X217684_at | 0.0005 | 0.0048 | 0.108 | 0.9143786 | 0.98083 |
| X209793_at | -0.0059 | 0.0541 | -0.108 | 0.9143814 | 0.98083 |
| X221785_at | -0.0004 | 0.0032 | -0.108 | 0.9144241 | 0.98083 |
| X221922_at | -0.0094 | 0.087 | -0.108 | 0.9144657 | 0.98083 |
| X210896_s_at | -0.0101 | 0.0936 | -0.108 | 0.9144667 | 0.98083 |
| X213167_s_at | 0.0009 | 0.0082 | 0.108 | 0.9145231 | 0.980834 |
| X210036_s_at | -0.0005 | 0.0043 | -0.108 | 0.9145808 | 0.980834 |
| X203830_at | 0.0049 | 0.045 | 0.108 | 0.9146137 | 0.980834 |
| X207466_at | 0.0005 | 0.0044 | 0.108 | 0.9146476 | 0.980834 |
| X220157_x_at | 0.0004 | 0.0033 | 0.108 | 0.9146933 | 0.980836 |
| X205503_at | 0.0004 | 0.0035 | 0.108 | 0.9148916 | 0.98096 |
| X222039_at | -0.0007 | 0.0066 | -0.108 | 0.9148976 | 0.98096 |
| X201052_s_at | -0.0051 | 0.0474 | -0.108 | 0.91496 | 0.98098 |
| X216485_s_at | 0.0002 | 0.002 | 0.108 | 0.9150136 | 0.98099 |
| X207617_at | 0.0006 | 0.0054 | 0.108 | 0.9150795 | 0.981013 |
| X59697_at | -0.0085 | 0.0794 | -0.107 | 0.9152101 | 0.981048 |
| X206023_at | 0.0007 | 0.0063 | 0.107 | 0.9152139 | 0.981048 |
| X220133_at | 0.0006 | 0.0058 | 0.107 | 0.9152443 | 0.981048 |
| X206951_at | -0.0003 | 0.0027 | -0.107 | 0.9153106 | 0.981072 |
| X210417_s_at | 0.0042 | 0.0396 | 0.107 | 0.9154696 | 0.981084 |
| X217221_x_at | -0.0043 | 0.04 | -0.107 | 0.9154969 | 0.981084 |
| X220356_at | -0.001 | 0.0095 | -0.107 | 0.9156251 | 0.981084 |
| X213382_at | -0.0009 | 0.0083 | -0.107 | 0.9156256 | 0.981084 |
| X221348_at | -0.0002 | 0.0021 | -0.107 | 0.915692 | 0.981084 |
| X220330_s_at | 0.0213 | 0.1997 | 0.107 | 0.9157325 | 0.981084 |
| X205841_at | -0.0026 | 0.024 | -0.107 | 0.9157406 | 0.981084 |
| X89977_at | 0.0017 | 0.0161 | 0.107 | 0.9157622 | 0.981084 |
| X210252_s_at | 0.0043 | 0.0404 | 0.107 | 0.9157844 | 0.981084 |
| X216650_at | -0.0005 | 0.0048 | -0.107 | 0.915805 | 0.981084 |
| X208569_at | -0.0005 | 0.0042 | -0.107 | 0.9158635 | 0.981084 |
| X222261_at | 0.0002 | 0.0022 | 0.106 | 0.9159639 | 0.981084 |
| X205519_at | -0.0007 | 0.007 | -0.106 | 0.9159682 | 0.981084 |
| X218528_s_at | -0.004 | 0.0375 | -0.106 | 0.9160631 | 0.981084 |
| X212821_at | 0.005 | 0.0473 | 0.106 | 0.9160752 | 0.981084 |
| X211631_x_at | 0.0002 | 0.0016 | 0.106 | 0.9160902 | 0.981084 |
| X206175_x_at | -0.0019 | 0.0175 | -0.106 | 0.9161403 | 0.981084 |
| X221361_at | -0.0002 | 0.0021 | -0.106 | 0.9161687 | 0.981084 |
| X202053_s_at | -0.0161 | 0.1516 | -0.106 | 0.9162323 | 0.981084 |
| X214859_at | 0.0003 | 0.0024 | 0.106 | 0.9162463 | 0.981084 |
| X218705_s_at | 0.0073 | 0.0685 | 0.106 | 0.9162496 | 0.981084 |
| X212357_at | 0.0032 | 0.03 | 0.106 | 0.9163386 | 0.98111 |
| X202652_at | -0.003 | 0.0279 | -0.106 | 0.9163801 | 0.98111 |
| X219978_s_at | -0.0004 | 0.0034 | -0.106 | 0.9164219 | 0.98111 |
| X204520_x_at | -0.0034 | 0.0325 | -0.106 | 0.9164708 | 0.98111 |
| X204262_s_at | -0.0019 | 0.0184 | -0.106 | 0.9165288 | 0.98111 |
| X215998_at | -0.0004 | 0.0039 | -0.106 | 0.9166023 | 0.98111 |
| X216775_at | -0.0018 | 0.0167 | -0.106 | 0.9166402 | 0.98111 |
| X217120_s_at | 0.0003 | 0.0026 | 0.105 | 0.9166955 | 0.98111 |
| X213667_at | 0.0005 | 0.0043 | 0.105 | 0.9167138 | 0.98111 |
| X221762_s_at | -0.0004 | 0.0039 | -0.105 | 0.9167152 | 0.98111 |
| X215048_at | 0.0004 | 0.0042 | 0.105 | 0.9167811 | 0.981133 |
| X207032_s_at | -0.0005 | 0.0044 | -0.105 | 0.9169567 | 0.981205 |
| X202454_s_at | 0.0177 | 0.1688 | 0.105 | 0.9169611 | 0.981205 |
| X207689_at | -0.0003 | 0.003 | -0.105 | 0.9169809 | 0.981205 |
| X217578_at | -0.0009 | 0.0087 | -0.105 | 0.9170903 | 0.981238 |
| X217405_x_at | 0.0004 | 0.004 | 0.105 | 0.9171318 | 0.981238 |
| X211282_x_at | 0.0004 | 0.0034 | 0.105 | 0.9171553 | 0.981238 |
| X215917_at | 0.0011 | 0.0105 | 0.105 | 0.9171969 | 0.981238 |
| X208757_at | -0.0036 | 0.0346 | -0.105 | 0.9172406 | 0.981238 |
| X216643_at | -0.0007 | 0.0071 | -0.105 | 0.9172766 | 0.981238 |
| X214900_at | -0.0003 | 0.0031 | -0.104 | 0.9175012 | 0.981415 |
| X214621_at | -0.0008 | 0.0077 | -0.104 | 0.917531 | 0.981415 |
| X215625_at | 0.0006 | 0.0057 | 0.104 | 0.9176328 | 0.981477 |
| X204940_at | -0.0021 | 0.0203 | -0.104 | 0.9177541 | 0.981508 |
| X216582_at | -0.0004 | 0.0034 | -0.104 | 0.9177644 | 0.981508 |
| X217873_at | -0.0029 | 0.0277 | -0.104 | 0.9177945 | 0.981508 |
| X218584_at | 0.0028 | 0.0266 | 0.104 | 0.9178945 | 0.981512 |
| X215969_at | -0.0005 | 0.0046 | -0.104 | 0.917915 | 0.981512 |
| X203779_s_at | 0.0008 | 0.0076 | 0.104 | 0.9179307 | 0.981512 |
| X213318_s_at | -0.0056 | 0.054 | -0.104 | 0.9180433 | 0.981544 |
| X211439_at | 0.0004 | 0.0038 | 0.104 | 0.9180491 | 0.981544 |
| X216957_at | 0.0009 | 0.0089 | 0.104 | 0.9181537 | 0.98157 |
| X201165_s_at | -0.0034 | 0.0327 | -0.104 | 0.9181612 | 0.98157 |
| X211544_s_at | -0.0007 | 0.0069 | -0.103 | 0.9183112 | 0.981613 |
| X211798_x_at | 0.0004 | 0.0038 | 0.103 | 0.9183742 | 0.981613 |
| X219688_at | 0.0083 | 0.0807 | 0.103 | 0.9183773 | 0.981613 |
| X219893_at | 0.0084 | 0.0817 | 0.103 | 0.9183784 | 0.981613 |
| X210799_at | 0.0004 | 0.0039 | 0.103 | 0.9185216 | 0.981719 |
| X220115_s_at | -0.0108 | 0.1051 | -0.103 | 0.9186047 | 0.981744 |
| X214592_s_at | -0.007 | 0.0678 | -0.103 | 0.9186341 | 0.981744 |
| X201879_at | 0.0049 | 0.0477 | 0.103 | 0.9187123 | 0.981781 |
| X213727_x_at | 0.0032 | 0.0315 | 0.103 | 0.9188885 | 0.981866 |
| X206217_at | -0.0004 | 0.004 | -0.103 | 0.9189208 | 0.981866 |
| X215112_x_at | -0.0038 | 0.0374 | -0.102 | 0.9190245 | 0.981866 |
| X215099_s_at | -0.0005 | 0.0045 | -0.102 | 0.9190452 | 0.981866 |
| X213311_s_at | 0.0047 | 0.046 | 0.102 | 0.9190786 | 0.981866 |
| X213030_s_at | -0.0054 | 0.0525 | -0.102 | 0.9191314 | 0.981866 |
| X220951_s_at | -0.0004 | 0.004 | -0.102 | 0.9191721 | 0.981866 |
| X210831_s_at | -0.0004 | 0.0042 | -0.102 | 0.9192355 | 0.981866 |
| X204876_at | -0.0006 | 0.006 | -0.102 | 0.9192539 | 0.981866 |
| X217406_at | 0.0007 | 0.007 | 0.102 | 0.9192715 | 0.981866 |
| X220542_s_at | -0.0003 | 0.0026 | -0.102 | 0.9193444 | 0.981866 |
| X207714_s_at | -0.0001 | 0.0014 | -0.102 | 0.9193905 | 0.981866 |
| X219577_s_at | -0.0004 | 0.0043 | -0.102 | 0.9194066 | 0.981866 |
| X214454_at | -0.0006 | 0.0056 | -0.102 | 0.9194269 | 0.981866 |
| X213200_at | -0.0008 | 0.0079 | -0.102 | 0.9195119 | 0.981866 |
| X202708_s_at | -0.0097 | 0.0951 | -0.102 | 0.9195144 | 0.981866 |
| X201982_s_at | 0.0002 | 0.0023 | 0.102 | 0.9195821 | 0.981866 |
| X200759_x_at | 0.0025 | 0.0247 | 0.102 | 0.9195963 | 0.981866 |
| X214286_at | 0.0004 | 0.0038 | 0.102 | 0.9196834 | 0.981866 |
| X207640_x_at | 0.0005 | 0.0052 | 0.102 | 0.9197084 | 0.981866 |
| X209816_at | -0.0013 | 0.0126 | -0.102 | 0.9197204 | 0.981866 |
| X210960_at | 0.0005 | 0.0053 | 0.101 | 0.9199008 | 0.981901 |
| X212251_at | -0.0034 | 0.0332 | -0.101 | 0.9199163 | 0.981901 |
| X202609_at | -0.0048 | 0.0476 | -0.101 | 0.9199252 | 0.981901 |
| X211163_s_at | 0.0008 | 0.008 | 0.101 | 0.9199302 | 0.981901 |
| X200933_x_at | 0.0022 | 0.0219 | 0.101 | 0.9199792 | 0.981906 |
| X215214_at | 0.0008 | 0.0082 | 0.101 | 0.9200274 | 0.981911 |
| X207940_x_at | -0.0004 | 0.0037 | -0.101 | 0.9201977 | 0.982035 |
| X215352_at | -0.0007 | 0.0065 | -0.101 | 0.9202834 | 0.982035 |
| X210768_x_at | 0.0037 | 0.0366 | 0.101 | 0.9203083 | 0.982035 |
| X219253_at | 0.0072 | 0.0715 | 0.101 | 0.9203208 | 0.982035 |
| X207403_at | 0.0005 | 0.0052 | 0.101 | 0.9204177 | 0.982091 |
| X216015_s_at | 0.0004 | 0.0042 | 0.1 | 0.9206169 | 0.982194 |
| X216929_x_at | -0.0008 | 0.0083 | -0.1 | 0.9206342 | 0.982194 |
| X214772_at | 0.011 | 0.1095 | 0.1 | 0.9206465 | 0.982194 |
| X217703_x_at | 0.0038 | 0.0383 | 0.1 | 0.9207082 | 0.982213 |
| X221018_s_at | 0.0006 | 0.0061 | 0.1 | 0.920817 | 0.982225 |
| X216973_s_at | -0.0009 | 0.0093 | -0.1 | 0.9208236 | 0.982225 |
| X209686_at | 0.0059 | 0.0586 | 0.1 | 0.9208521 | 0.982225 |
| X203358_s_at | -0.0005 | 0.0052 | -0.1 | 0.9209214 | 0.982251 |
| X209808_x_at | -0.0004 | 0.0035 | -0.1 | 0.921034 | 0.982324 |
| X208398_s_at | 0.0053 | 0.0529 | 0.1 | 0.9211702 | 0.982422 |
| X210606_x_at | 0.0004 | 0.0036 | 0.1 | 0.9212709 | 0.982437 |
| X220883_at | 0.0008 | 0.0083 | 0.1 | 0.9212721 | 0.982437 |
| X217967_s_at | -0.0039 | 0.0388 | -0.1 | 0.9213806 | 0.982472 |
| X222051_s_at | 0.0005 | 0.0048 | 0.099 | 0.9213934 | 0.982472 |
| X205016_at | 0.0102 | 0.1029 | 0.099 | 0.9214792 | 0.982487 |
| X214626_s_at | -0.0038 | 0.0379 | -0.099 | 0.9215059 | 0.982487 |
| X205808_at | -0.0003 | 0.0026 | -0.099 | 0.9215415 | 0.982487 |
| X206203_at | -0.0004 | 0.0039 | -0.099 | 0.9215848 | 0.982487 |
| X203600_s_at | 0.0031 | 0.0317 | 0.099 | 0.921648 | 0.982507 |
| X205528_s_at | -0.0048 | 0.0488 | -0.099 | 0.9217809 | 0.982602 |
| X207811_at | 0.0008 | 0.0079 | 0.099 | 0.9219031 | 0.982636 |
| X207354_at | -0.0005 | 0.0053 | -0.099 | 0.9219661 | 0.982636 |
| X210751_s_at | 0.0075 | 0.076 | 0.099 | 0.9220034 | 0.982636 |
| X214034_at | 0.0014 | 0.0144 | 0.099 | 0.9220842 | 0.982636 |
| X205059_s_at | -0.0003 | 0.0029 | -0.098 | 0.9221958 | 0.982636 |
| X212319_at | -0.0032 | 0.0323 | -0.098 | 0.92223 | 0.982636 |
| X205401_at | 0.0044 | 0.0443 | 0.098 | 0.9222794 | 0.982636 |
| X208382_s_at | 0.0008 | 0.0077 | 0.098 | 0.9223058 | 0.982636 |
| X205952_at | -0.0111 | 0.1128 | -0.098 | 0.9223353 | 0.982636 |
| X219340_s_at | -0.004 | 0.0404 | -0.098 | 0.9223355 | 0.982636 |
| X211492_s_at | 0.0007 | 0.0072 | 0.098 | 0.9223548 | 0.982636 |
| X200021_at | -0.0011 | 0.0114 | -0.098 | 0.9224007 | 0.982636 |
| X210744_s_at | -0.0003 | 0.0033 | -0.098 | 0.9224123 | 0.982636 |
| X219851_at | -0.0009 | 0.009 | -0.098 | 0.9225816 | 0.982636 |
| X215729_s_at | 0.0005 | 0.0051 | 0.098 | 0.9226034 | 0.982636 |
| X201228_s_at | -0.0003 | 0.0036 | -0.098 | 0.9226109 | 0.982636 |
| X216072_at | -0.0005 | 0.0051 | -0.098 | 0.9227003 | 0.982636 |
| X213475_s_at | -0.0012 | 0.0119 | -0.098 | 0.9227219 | 0.982636 |
| X219070_s_at | -0.0025 | 0.026 | -0.098 | 0.9227525 | 0.982636 |
| X202925_s_at | 0.0009 | 0.009 | 0.098 | 0.9227875 | 0.982636 |
| X206432_at | 0.0009 | 0.0096 | 0.098 | 0.9228426 | 0.982636 |
| X218518_at | -0.0041 | 0.0416 | -0.098 | 0.9228548 | 0.982636 |
| X206949_s_at | 0.0047 | 0.048 | 0.097 | 0.9229614 | 0.982636 |
| X214231_s_at | -0.0016 | 0.0163 | -0.097 | 0.9230186 | 0.982636 |
| X200719_at | -0.0024 | 0.0248 | -0.097 | 0.9230477 | 0.982636 |
| X216437_at | 0.0005 | 0.0047 | 0.097 | 0.9230514 | 0.982636 |
| X217536_x_at | -0.0074 | 0.0758 | -0.097 | 0.9231531 | 0.982636 |
| X208059_at | -0.0006 | 0.006 | -0.097 | 0.9231538 | 0.982636 |
| X206718_at | -0.0005 | 0.005 | -0.097 | 0.9231728 | 0.982636 |
| X214675_at | -0.0006 | 0.0059 | -0.097 | 0.9232059 | 0.982636 |
| X205567_at | 0.0036 | 0.0366 | 0.097 | 0.9232312 | 0.982636 |
| X218457_s_at | -0.0039 | 0.0403 | -0.097 | 0.9232479 | 0.982636 |
| X207758_at | 0.0007 | 0.0074 | 0.097 | 0.9233107 | 0.982636 |
| X204052_s_at | -0.0009 | 0.009 | -0.097 | 0.9233487 | 0.982636 |
| X204169_at | 0.0011 | 0.0113 | 0.097 | 0.9234131 | 0.982636 |
| X204361_s_at | -0.0163 | 0.1687 | -0.097 | 0.9234329 | 0.982636 |
| X204356_at | -0.0003 | 0.0029 | -0.097 | 0.9235144 | 0.982636 |
| X211315_s_at | -0.0006 | 0.0059 | -0.097 | 0.9235466 | 0.982636 |
| X215421_at | 0.0006 | 0.0061 | 0.097 | 0.9235812 | 0.982636 |
| X219334_s_at | 0.001 | 0.0108 | 0.097 | 0.9235827 | 0.982636 |
| X209890_at | 0.0046 | 0.0476 | 0.097 | 0.9236739 | 0.982656 |
| X220662_s_at | -0.0003 | 0.0032 | -0.097 | 0.9237015 | 0.982656 |
| X220903_at | 0.0008 | 0.0087 | 0.097 | 0.923734 | 0.982656 |
| X220284_at | -0.0004 | 0.0037 | -0.096 | 0.9238188 | 0.982678 |
| X211608_at | 0.0004 | 0.0038 | 0.096 | 0.9238433 | 0.982678 |
| X213067_at | 0.0069 | 0.0715 | 0.096 | 0.9239256 | 0.982719 |
| X209123_at | -0.0055 | 0.0569 | -0.096 | 0.9239777 | 0.982721 |
| X207892_at | -0.0002 | 0.0026 | -0.096 | 0.9240156 | 0.982721 |
| X207358_x_at | 0.0027 | 0.028 | 0.096 | 0.924221 | 0.982812 |
| X214957_at | 0.0004 | 0.0038 | 0.096 | 0.9242517 | 0.982812 |
| X214599_at | 0.0005 | 0.0049 | 0.096 | 0.9242605 | 0.982812 |
| X209906_at | 0.0103 | 0.1074 | 0.096 | 0.924279 | 0.982812 |
| X212916_at | 0.0007 | 0.0076 | 0.096 | 0.9243944 | 0.982879 |
| X220989_s_at | -0.0008 | 0.0082 | -0.096 | 0.9244305 | 0.982879 |
| X205298_s_at | 0.0013 | 0.0136 | 0.095 | 0.924549 | 0.982942 |
| X217454_at | 0.0012 | 0.0131 | 0.095 | 0.9246596 | 0.982942 |
| X207876_s_at | 0.0011 | 0.0116 | 0.095 | 0.9246938 | 0.982942 |
| X212804_s_at | 0.011 | 0.116 | 0.095 | 0.9247496 | 0.982942 |
| X211045_s_at | -0.0006 | 0.0059 | -0.095 | 0.9247752 | 0.982942 |
| X201003_x_at | -0.0021 | 0.0218 | -0.095 | 0.9248184 | 0.982942 |
| X215864_at | -0.0004 | 0.0037 | -0.095 | 0.9248223 | 0.982942 |
| X216475_at | -0.0003 | 0.0031 | -0.095 | 0.9248559 | 0.982942 |
| X215000_s_at | -0.0027 | 0.0282 | -0.095 | 0.9248872 | 0.982942 |
| X208397_x_at | -0.0006 | 0.0063 | -0.095 | 0.924942 | 0.98295 |
| X211430_s_at | -0.0002 | 0.002 | -0.095 | 0.9249833 | 0.98295 |
| X216289_at | -0.0007 | 0.0074 | -0.095 | 0.9250747 | 0.982956 |
| X215970_at | 0.0006 | 0.0063 | 0.095 | 0.9250775 | 0.982956 |
| X213265_at | 0.0004 | 0.0037 | 0.095 | 0.9251523 | 0.982959 |
| X215398_at | 0.0013 | 0.0136 | 0.095 | 0.9251689 | 0.982959 |
| X217851_s_at | 0.0046 | 0.0484 | 0.095 | 0.92523 | 0.982977 |
| X207465_at | 0.0009 | 0.0096 | 0.094 | 0.9253535 | 0.983061 |
| X202324_s_at | 0.0022 | 0.023 | 0.094 | 0.9254597 | 0.983127 |
| X214063_s_at | 0.012 | 0.1279 | 0.094 | 0.9255767 | 0.983172 |
| X202510_s_at | 0.001 | 0.0111 | 0.094 | 0.9255909 | 0.983172 |
| X221410_x_at | 0.0004 | 0.0042 | 0.094 | 0.9257865 | 0.983247 |
| X201438_at | -0.0004 | 0.0043 | -0.094 | 0.9257936 | 0.983247 |
| X207693_at | 0.0104 | 0.1109 | 0.094 | 0.9257944 | 0.983247 |
| X203946_s_at | 0.009 | 0.0957 | 0.094 | 0.9258806 | 0.983267 |
| X203961_at | 0.0042 | 0.0447 | 0.094 | 0.9259013 | 0.983267 |
| X217042_at | 0.0004 | 0.0044 | 0.094 | 0.925983 | 0.983306 |
| X214842_s_at | -0.0005 | 0.0054 | -0.093 | 0.9261687 | 0.983457 |
| X204655_at | -0.0003 | 0.0036 | -0.093 | 0.9262174 | 0.983461 |
| X208124_s_at | 0.0008 | 0.0082 | 0.093 | 0.9262842 | 0.983485 |
| X210362_x_at | -0.0004 | 0.0039 | -0.093 | 0.9263319 | 0.983489 |
| X219320_at | -0.0007 | 0.007 | -0.093 | 0.9264026 | 0.983517 |
| X207780_at | 0.0006 | 0.006 | 0.093 | 0.9264938 | 0.983567 |
| X213765_at | 0.0009 | 0.0102 | 0.093 | 0.9265492 | 0.983579 |
| X216692_at | -0.0005 | 0.0052 | -0.093 | 0.9266869 | 0.983678 |
| X211305_x_at | -0.0008 | 0.0085 | -0.093 | 0.9267322 | 0.983679 |
| X213024_at | -0.0049 | 0.0525 | -0.093 | 0.9268547 | 0.983762 |
| X221809_at | -0.0013 | 0.0143 | -0.092 | 0.926916 | 0.983774 |
| X206272_at | 0.0032 | 0.0349 | 0.092 | 0.9269546 | 0.983774 |
| X207894_s_at | 0.0003 | 0.0031 | 0.092 | 0.9271423 | 0.983926 |
| X202827_s_at | 0.0002 | 0.0025 | 0.092 | 0.9272355 | 0.983934 |
| X214303_x_at | 0.0005 | 0.0051 | 0.092 | 0.9272383 | 0.983934 |
| X218086_at | -0.0046 | 0.0497 | -0.092 | 0.9273443 | 0.983999 |
| X220859_at | -0.0012 | 0.0127 | -0.092 | 0.927533 | 0.984128 |
| X217022_s_at | -0.0003 | 0.0032 | -0.092 | 0.9275708 | 0.984128 |
| X208941_s_at | -0.0036 | 0.0397 | -0.092 | 0.9275982 | 0.984128 |
| X216273_at | 0.0004 | 0.0039 | 0.091 | 0.9278441 | 0.984151 |
| X210595_at | 0.0006 | 0.0065 | 0.091 | 0.9278782 | 0.984151 |
| X220660_at | -0.0004 | 0.0043 | -0.091 | 0.9278952 | 0.984151 |
| X215800_at | 0.0009 | 0.01 | 0.091 | 0.9279439 | 0.984151 |
| X206516_at | 0.0004 | 0.0043 | 0.091 | 0.9279971 | 0.984151 |
| X217248_s_at | 0.0003 | 0.0036 | 0.091 | 0.9280473 | 0.984151 |
| X210331_at | -0.0039 | 0.0429 | -0.091 | 0.9281384 | 0.984151 |
| X215565_at | -0.0009 | 0.0104 | -0.091 | 0.9281632 | 0.984151 |
| X209623_at | -0.0039 | 0.0434 | -0.091 | 0.9282352 | 0.984151 |
| X214066_x_at | 0.0013 | 0.0141 | 0.091 | 0.928242 | 0.984151 |
| X217686_at | -0.0002 | 0.002 | -0.091 | 0.9282466 | 0.984151 |
| X208882_s_at | 0.0042 | 0.0468 | 0.091 | 0.9282485 | 0.984151 |
| X216447_at | -0.0004 | 0.0048 | -0.091 | 0.928271 | 0.984151 |
| X220478_at | 0.0003 | 0.0029 | 0.091 | 0.9283203 | 0.984151 |
| X206838_at | -0.0004 | 0.0043 | -0.091 | 0.9283699 | 0.984151 |
| X45572_s_at | -0.0027 | 0.0298 | -0.091 | 0.9283949 | 0.984151 |
| X219087_at | 0.0035 | 0.0382 | 0.091 | 0.9284025 | 0.984151 |
| X210327_s_at | -0.0003 | 0.0031 | -0.091 | 0.9284673 | 0.984151 |
| X208495_at | -0.0004 | 0.0049 | -0.09 | 0.9284819 | 0.984151 |
| X200058_s_at | -0.002 | 0.0217 | -0.09 | 0.9285062 | 0.984151 |
| X202353_s_at | 0.0034 | 0.0376 | 0.09 | 0.9285693 | 0.984171 |
| X215711_s_at | 0.0004 | 0.0047 | 0.09 | 0.9287733 | 0.984248 |
| X218212_s_at | -0.0028 | 0.0315 | -0.09 | 0.9288855 | 0.984248 |
| X221714_s_at | -0.0009 | 0.0103 | -0.09 | 0.9288895 | 0.984248 |
| X211020_at | -0.0005 | 0.0052 | -0.09 | 0.9289258 | 0.984248 |
| X212068_s_at | -0.0036 | 0.0397 | -0.09 | 0.9289312 | 0.984248 |
| X212293_at | 0.003 | 0.0329 | 0.09 | 0.9289562 | 0.984248 |
| X215178_x_at | 0.0026 | 0.0294 | 0.09 | 0.9290196 | 0.984248 |
| X205273_s_at | 0.0065 | 0.0728 | 0.09 | 0.929026 | 0.984248 |
| X204763_s_at | 0.0048 | 0.0534 | 0.09 | 0.9290618 | 0.984248 |
| X222026_at | -0.0076 | 0.0848 | -0.09 | 0.9290855 | 0.984248 |
| X216946_at | 0.0005 | 0.0061 | 0.09 | 0.9291577 | 0.984278 |
| X207402_at | 0.0008 | 0.009 | 0.09 | 0.9292403 | 0.984315 |
| X77508_r_at | 0.0006 | 0.0063 | 0.089 | 0.9292809 | 0.984315 |
| X217262_s_at | -0.0003 | 0.0036 | -0.089 | 0.929388 | 0.984363 |
| X213678_at | 0.0005 | 0.0058 | 0.089 | 0.9294778 | 0.984363 |
| X203459_s_at | -0.0028 | 0.0315 | -0.089 | 0.9294797 | 0.984363 |
| X213177_at | -0.0005 | 0.0052 | -0.089 | 0.9295042 | 0.984363 |
| X213362_at | 0.0077 | 0.0866 | 0.089 | 0.929565 | 0.984379 |
| X206639_x_at | 0.0007 | 0.0081 | 0.089 | 0.929634 | 0.984379 |
| X211427_s_at | 0.001 | 0.0111 | 0.089 | 0.9296743 | 0.984379 |
| X208734_x_at | 0.0035 | 0.0389 | 0.089 | 0.9296957 | 0.984379 |
| X208038_at | 0.0002 | 0.0026 | 0.089 | 0.9298478 | 0.984415 |
| X209830_s_at | 0.0004 | 0.0047 | 0.089 | 0.9298688 | 0.984415 |
| X217027_x_at | -0.0027 | 0.0307 | -0.089 | 0.9298843 | 0.984415 |
| X216238_s_at | -0.0009 | 0.0097 | -0.089 | 0.929907 | 0.984415 |
| X216832_at | 0.0003 | 0.0034 | 0.088 | 0.9302192 | 0.984698 |
| X204069_at | -0.0045 | 0.051 | -0.088 | 0.9304185 | 0.984789 |
| X204175_at | -0.0044 | 0.0501 | -0.088 | 0.9304504 | 0.984789 |
| X210141_s_at | -0.0004 | 0.0048 | -0.088 | 0.9305248 | 0.984789 |
| X219037_at | -0.0056 | 0.064 | -0.088 | 0.9306424 | 0.984789 |
| X221460_at | -0.0002 | 0.0025 | -0.088 | 0.9307135 | 0.984789 |
| X208547_at | -0.0006 | 0.0067 | -0.088 | 0.9307509 | 0.984789 |
| X213950_s_at | 0.0007 | 0.0079 | 0.088 | 0.9307974 | 0.984789 |
| X204336_s_at | 0.0032 | 0.0364 | 0.088 | 0.9308008 | 0.984789 |
| X204574_s_at | -0.0003 | 0.0039 | -0.087 | 0.930864 | 0.984789 |
| X208325_s_at | -0.0095 | 0.1082 | -0.087 | 0.9309205 | 0.984789 |
| X36545_s_at | -0.0069 | 0.0788 | -0.087 | 0.9309603 | 0.984789 |
| X217071_s_at | 0.0007 | 0.0086 | 0.087 | 0.9309843 | 0.984789 |
| X214555_at | -0.0002 | 0.0019 | -0.087 | 0.9309971 | 0.984789 |
| X209035_at | -0.0006 | 0.0065 | -0.087 | 0.9310194 | 0.984789 |
| X212339_at | 0.0049 | 0.0556 | 0.087 | 0.9310544 | 0.984789 |
| X214234_s_at | -0.001 | 0.012 | -0.087 | 0.9310791 | 0.984789 |
| X204008_at | 0.0056 | 0.0639 | 0.087 | 0.9311556 | 0.984789 |
| X205508_at | 0.0057 | 0.0653 | 0.087 | 0.9311962 | 0.984789 |
| X214008_at | -0.0007 | 0.0075 | -0.087 | 0.9312177 | 0.984789 |
| X218815_s_at | -0.0006 | 0.007 | -0.087 | 0.931291 | 0.984789 |
| X202526_at | 0.0023 | 0.0264 | 0.087 | 0.9313067 | 0.984789 |
| X216079_at | -0.0001 | 0.0017 | -0.087 | 0.9313174 | 0.984789 |
| X203293_s_at | -0.001 | 0.0117 | -0.087 | 0.9313248 | 0.984789 |
| X202022_at | 0.0045 | 0.0514 | 0.087 | 0.931491 | 0.984828 |
| X220145_at | 0.0031 | 0.0354 | 0.087 | 0.9315902 | 0.984828 |
| X221406_s_at | 0.0003 | 0.0036 | 0.087 | 0.9316106 | 0.984828 |
| X213767_at | -0.0003 | 0.0032 | -0.087 | 0.9316128 | 0.984828 |
| X204322_at | 0.0004 | 0.0046 | 0.087 | 0.9316159 | 0.984828 |
| X205797_s_at | 0.0004 | 0.0051 | 0.086 | 0.9316272 | 0.984828 |
| X203584_at | 0.0029 | 0.0331 | 0.086 | 0.9317009 | 0.984842 |
| X216717_at | 0.0005 | 0.0057 | 0.086 | 0.9317678 | 0.984842 |
| X208928_at | -0.0014 | 0.0168 | -0.086 | 0.931774 | 0.984842 |
| X209566_at | -0.0036 | 0.0418 | -0.086 | 0.9320417 | 0.985019 |
| X201337_s_at | 0.0095 | 0.111 | 0.086 | 0.9320605 | 0.985019 |
| X210747_at | 0.0005 | 0.0058 | 0.086 | 0.9320838 | 0.985019 |
| X213754_s_at | 0.0033 | 0.0381 | 0.086 | 0.9321191 | 0.985019 |
| X220813_at | -0.0007 | 0.0079 | -0.086 | 0.9322521 | 0.985113 |
| X215576_at | 0.0018 | 0.0216 | 0.086 | 0.9323188 | 0.985137 |
| X216912_at | 0.0002 | 0.0023 | 0.085 | 0.9325593 | 0.985261 |
| X208301_at | 0.0006 | 0.0069 | 0.085 | 0.9325873 | 0.985261 |
| X201307_at | -0.0029 | 0.0337 | -0.085 | 0.9326709 | 0.985261 |
| X202978_s_at | -0.0005 | 0.0062 | -0.085 | 0.9327423 | 0.985261 |
| X220825_s_at | -0.0003 | 0.0038 | -0.085 | 0.9327725 | 0.985261 |
| X214284_s_at | -0.0003 | 0.0037 | -0.085 | 0.9327896 | 0.985261 |
| X211503_s_at | -0.0041 | 0.0485 | -0.085 | 0.9329774 | 0.985261 |
| X218985_at | -0.0004 | 0.0043 | -0.085 | 0.9330539 | 0.985261 |
| X215674_at | 0.0012 | 0.014 | 0.085 | 0.9330789 | 0.985261 |
| X207146_at | -0.0004 | 0.005 | -0.085 | 0.9330953 | 0.985261 |
| X213534_s_at | 0.0085 | 0.1006 | 0.084 | 0.9332293 | 0.985261 |
| X221764_at | 0.0017 | 0.0207 | 0.084 | 0.9332342 | 0.985261 |
| X217429_at | -0.0003 | 0.0038 | -0.084 | 0.9332407 | 0.985261 |
| X207825_s_at | 0.0002 | 0.002 | 0.084 | 0.9332953 | 0.985261 |
| X204802_at | -0.0003 | 0.003 | -0.084 | 0.9333525 | 0.985261 |
| X216291_at | 0.0005 | 0.0065 | 0.084 | 0.9334141 | 0.985261 |
| X206541_at | -0.001 | 0.0118 | -0.084 | 0.9334566 | 0.985261 |
| X215708_s_at | -0.0006 | 0.007 | -0.084 | 0.933461 | 0.985261 |
| X207976_at | -0.0014 | 0.0163 | -0.084 | 0.9336179 | 0.985261 |
| X207999_s_at | 0.0003 | 0.0038 | 0.084 | 0.9336604 | 0.985261 |
| X212442_s_at | 0.0059 | 0.0709 | 0.084 | 0.933701 | 0.985261 |
| X204305_at | 0.0006 | 0.0068 | 0.084 | 0.9337148 | 0.985261 |
| X205965_at | 0.0005 | 0.0056 | 0.084 | 0.9337293 | 0.985261 |
| X210769_at | 0.0003 | 0.0042 | 0.084 | 0.9337569 | 0.985261 |
| X220170_at | -0.0007 | 0.0086 | -0.084 | 0.9337767 | 0.985261 |
| X215188_at | 0.0006 | 0.0075 | 0.084 | 0.9337873 | 0.985261 |
| X206654_s_at | -0.0016 | 0.0186 | -0.084 | 0.9337885 | 0.985261 |
| X208303_s_at | -0.0003 | 0.0041 | -0.084 | 0.9338221 | 0.985261 |
| X211071_s_at | 0.0037 | 0.0441 | 0.084 | 0.9338269 | 0.985261 |
| X210236_at | 0.0033 | 0.0394 | 0.084 | 0.9338306 | 0.985261 |
| X201473_at | -0.0045 | 0.0538 | -0.084 | 0.9338362 | 0.985261 |
| X207399_at | -0.0004 | 0.0044 | -0.084 | 0.9338554 | 0.985261 |
| X204989_s_at | -0.0002 | 0.0028 | -0.084 | 0.9339622 | 0.985327 |
| X214921_at | -0.0004 | 0.0049 | -0.083 | 0.9341953 | 0.985526 |
| X220271_x_at | 0.0004 | 0.0043 | 0.083 | 0.9343739 | 0.985667 |
| X209946_at | 0.0006 | 0.0071 | 0.083 | 0.9344898 | 0.985715 |
| X206591_at | -0.0007 | 0.008 | -0.083 | 0.934546 | 0.985715 |
| X213416_at | 0.0005 | 0.006 | 0.083 | 0.9345526 | 0.985715 |
| X216502_at | 0.0005 | 0.0056 | 0.083 | 0.9347063 | 0.985831 |
| X214544_s_at | 0.0008 | 0.0102 | 0.083 | 0.9347736 | 0.985855 |
| X205735_s_at | -0.0004 | 0.0054 | -0.082 | 0.9348283 | 0.985857 |
| X217260_x_at | 0.0004 | 0.0047 | 0.082 | 0.9348639 | 0.985857 |
| X201483_s_at | 0.0061 | 0.0743 | 0.082 | 0.9350342 | 0.985878 |
| X204503_at | -0.0004 | 0.0043 | -0.082 | 0.9351514 | 0.985878 |
| X220831_at | 0.0005 | 0.006 | 0.082 | 0.9351915 | 0.985878 |
| X221465_at | -0.0004 | 0.0044 | -0.082 | 0.9352177 | 0.985878 |
| X211583_x_at | -0.0002 | 0.0029 | -0.082 | 0.9353201 | 0.985878 |
| X203505_at | 0.0068 | 0.0827 | 0.082 | 0.9353938 | 0.985878 |
| X208135_at | 0.0003 | 0.0039 | 0.082 | 0.9354784 | 0.985878 |
| X219977_at | -0.0006 | 0.0078 | -0.082 | 0.9354798 | 0.985878 |
| X212592_at | 0.0013 | 0.0161 | 0.082 | 0.9355398 | 0.985878 |
| X215359_x_at | -0.0049 | 0.0606 | -0.081 | 0.9356344 | 0.985878 |
| X208672_s_at | 0.0028 | 0.0349 | 0.081 | 0.9356756 | 0.985878 |
| X214523_at | -0.0004 | 0.0046 | -0.081 | 0.9356798 | 0.985878 |
| X206362_x_at | 0.0005 | 0.0067 | 0.081 | 0.9357225 | 0.985878 |
| X211424_x_at | 0.0027 | 0.0329 | 0.081 | 0.935729 | 0.985878 |
| X210085_s_at | -0.0003 | 0.0033 | -0.081 | 0.9357352 | 0.985878 |
| X217016_x_at | 0.0011 | 0.0138 | 0.081 | 0.9357511 | 0.985878 |
| X220654_at | -0.0002 | 0.0029 | -0.081 | 0.9357524 | 0.985878 |
| X206081_at | 0.0019 | 0.023 | 0.081 | 0.9357949 | 0.985878 |
| X220195_at | 0.0022 | 0.0267 | 0.081 | 0.9358085 | 0.985878 |
| X211322_s_at | 0.0005 | 0.0061 | 0.081 | 0.9358175 | 0.985878 |
| X201815_s_at | -0.0002 | 0.0026 | -0.081 | 0.9358271 | 0.985878 |
| X214904_at | -0.0004 | 0.005 | -0.081 | 0.9358609 | 0.985878 |
| X207137_at | -0.0003 | 0.0038 | -0.081 | 0.9359483 | 0.985903 |
| X208515_at | 0.0006 | 0.0073 | 0.081 | 0.9359916 | 0.985903 |
| X219383_at | -0.0116 | 0.1437 | -0.081 | 0.9360171 | 0.985903 |
| X207951_at | -0.0018 | 0.0225 | -0.081 | 0.9360892 | 0.985903 |
| X215733_x_at | 0.0002 | 0.0021 | 0.081 | 0.9361538 | 0.985903 |
| X217916_s_at | 0.0026 | 0.032 | 0.081 | 0.9361763 | 0.985903 |
| X214073_at | -0.001 | 0.0127 | -0.081 | 0.9362305 | 0.985903 |
| X203623_at | 0.0005 | 0.0059 | 0.081 | 0.9362399 | 0.985903 |
| X220487_at | -0.0003 | 0.0042 | -0.08 | 0.9364117 | 0.986038 |
| X216605_s_at | -0.0002 | 0.0028 | -0.08 | 0.9365509 | 0.986132 |
| X221337_s_at | -0.0005 | 0.0062 | -0.08 | 0.9365897 | 0.986132 |
| X206352_s_at | 0.0009 | 0.0117 | 0.08 | 0.9367867 | 0.986211 |
| X215465_at | -0.0006 | 0.0072 | -0.08 | 0.9367915 | 0.986211 |
| X218956_s_at | 0.001 | 0.013 | 0.08 | 0.9367982 | 0.986211 |
| X206901_at | -0.0002 | 0.0019 | -0.08 | 0.93692 | 0.986262 |
| X213851_at | -0.0005 | 0.0064 | -0.08 | 0.9369354 | 0.986262 |
| X213680_at | 0.0003 | 0.0041 | 0.08 | 0.9371005 | 0.986389 |
| X212179_at | 0.0028 | 0.0353 | 0.079 | 0.9371955 | 0.986399 |
| X214745_at | -0.0029 | 0.0368 | -0.079 | 0.9373158 | 0.986399 |
| X205835_s_at | 0.0005 | 0.0062 | 0.079 | 0.9373194 | 0.986399 |
| X212906_at | 0.0027 | 0.034 | 0.079 | 0.9373307 | 0.986399 |
| X217213_at | -0.0004 | 0.005 | -0.079 | 0.9373567 | 0.986399 |
| X218047_at | -0.0019 | 0.0245 | -0.079 | 0.9373768 | 0.986399 |
| X216372_at | 0.0002 | 0.0027 | 0.079 | 0.9374762 | 0.98642 |
| X216533_at | 0.0012 | 0.0146 | 0.079 | 0.9374849 | 0.98642 |
| X215562_at | 0.0002 | 0.0029 | 0.079 | 0.9375484 | 0.986433 |
| X202954_at | -0.0004 | 0.0053 | -0.079 | 0.9375939 | 0.986433 |
| X219285_s_at | 0.0012 | 0.0148 | 0.079 | 0.937649 | 0.986433 |
| X206638_at | -0.0004 | 0.0045 | -0.079 | 0.937719 | 0.986433 |
| X207654_x_at | 0.0015 | 0.0185 | 0.079 | 0.9377199 | 0.986433 |
| X211614_at | -0.0005 | 0.0061 | -0.079 | 0.9378222 | 0.986445 |
| X206674_at | 0.0003 | 0.0033 | 0.078 | 0.9379354 | 0.986445 |
| X201975_at | 0.0029 | 0.0375 | 0.078 | 0.9379696 | 0.986445 |
| X219850_s_at | -0.0006 | 0.0073 | -0.078 | 0.9380022 | 0.986445 |
| X221153_s_at | -0.0005 | 0.0061 | -0.078 | 0.9380353 | 0.986445 |
| X219576_at | 0.0013 | 0.0166 | 0.078 | 0.9380659 | 0.986445 |
| X216838_at | -0.0004 | 0.0046 | -0.078 | 0.938085 | 0.986445 |
| X215837_x_at | -0.0003 | 0.0042 | -0.078 | 0.9381321 | 0.986445 |
| X201401_s_at | -0.0004 | 0.0048 | -0.078 | 0.9383568 | 0.986445 |
| X217154_s_at | -0.0004 | 0.0052 | -0.078 | 0.9383824 | 0.986445 |
| X217552_x_at | 0.0009 | 0.012 | 0.078 | 0.9384388 | 0.986445 |
| X220880_at | -0.0006 | 0.0074 | -0.078 | 0.9385186 | 0.986445 |
| X201793_x_at | 0.0009 | 0.0116 | 0.078 | 0.9385233 | 0.986445 |
| X203932_at | 0.0066 | 0.0848 | 0.078 | 0.9385522 | 0.986445 |
| X201663_s_at | -0.0006 | 0.0082 | -0.078 | 0.9385584 | 0.986445 |
| X209331_s_at | 0.0024 | 0.0311 | 0.078 | 0.9386517 | 0.986445 |
| X217903_at | -0.0007 | 0.0086 | -0.078 | 0.9387028 | 0.986445 |
| X207393_at | -0.0004 | 0.0053 | -0.077 | 0.9387394 | 0.986445 |
| X212697_at | -0.0031 | 0.0404 | -0.077 | 0.9388138 | 0.986445 |
| X204206_at | -0.0043 | 0.056 | -0.077 | 0.9388365 | 0.986445 |
| X207117_at | 0.0007 | 0.0085 | 0.077 | 0.9388515 | 0.986445 |
| X221430_s_at | 0.0055 | 0.0718 | 0.077 | 0.9388844 | 0.986445 |
| X222123_s_at | -0.0004 | 0.0048 | -0.077 | 0.9388936 | 0.986445 |
| X206547_s_at | 0.0003 | 0.0045 | 0.077 | 0.9390154 | 0.986445 |
| X207606_s_at | 0.0047 | 0.0615 | 0.077 | 0.9390332 | 0.986445 |
| X213199_at | 0.0009 | 0.012 | 0.077 | 0.9390424 | 0.986445 |
| X214676_x_at | -0.0004 | 0.0047 | -0.077 | 0.9390868 | 0.986445 |
| X207004_at | 0.0005 | 0.007 | 0.077 | 0.9391673 | 0.986445 |
| X222321_at | -0.0007 | 0.0088 | -0.077 | 0.9391971 | 0.986445 |
| X219427_at | 0.0047 | 0.0614 | 0.077 | 0.9392087 | 0.986445 |
| X202989_at | -0.0007 | 0.0091 | -0.077 | 0.9392343 | 0.986445 |
| X220834_at | -0.0004 | 0.0047 | -0.077 | 0.9392349 | 0.986445 |
| X216441_at | -0.0007 | 0.0091 | -0.077 | 0.9392578 | 0.986445 |
| X214736_s_at | 0.002 | 0.0257 | 0.077 | 0.9393643 | 0.986445 |
| X205162_at | 0.0051 | 0.0659 | 0.077 | 0.9393871 | 0.986445 |
| X218878_s_at | 0.0033 | 0.0437 | 0.077 | 0.939449 | 0.986445 |
| X210836_x_at | 0.0006 | 0.0076 | 0.076 | 0.9395528 | 0.986445 |
| X213418_at | 0.0005 | 0.0072 | 0.076 | 0.9395572 | 0.986445 |
| X216606_x_at | 0.0005 | 0.0062 | 0.076 | 0.9396496 | 0.986445 |
| X220378_at | 0.0002 | 0.0026 | 0.076 | 0.9396608 | 0.986445 |
| X206400_at | -0.0003 | 0.0036 | -0.076 | 0.939669 | 0.986445 |
| X208087_s_at | -0.0006 | 0.008 | -0.076 | 0.9397006 | 0.986445 |
| X205999_x_at | 0.0002 | 0.0031 | 0.076 | 0.9397191 | 0.986445 |
| X204046_at | 0.0004 | 0.0047 | 0.076 | 0.9397297 | 0.986445 |
| X207022_s_at | 0.0003 | 0.004 | 0.076 | 0.9397898 | 0.986445 |
| X204057_at | -0.0023 | 0.0308 | -0.076 | 0.9397981 | 0.986445 |
| X219844_at | 0.0004 | 0.0054 | 0.076 | 0.9398186 | 0.986445 |
| X219789_at | -0.0005 | 0.0066 | -0.076 | 0.9399371 | 0.986468 |
| X216768_x_at | 0.0003 | 0.0044 | 0.076 | 0.9399701 | 0.986468 |
| X211693_at | -0.0005 | 0.0069 | -0.076 | 0.9401551 | 0.986468 |
| X203504_s_at | 0.0108 | 0.1428 | 0.076 | 0.9401909 | 0.986468 |
| X204285_s_at | 0.0048 | 0.0636 | 0.076 | 0.9401986 | 0.986468 |
| X218208_at | 0.0032 | 0.043 | 0.076 | 0.9402296 | 0.986468 |
| X200970_s_at | 0.0039 | 0.0521 | 0.076 | 0.9402962 | 0.986468 |
| X214865_at | -0.0002 | 0.0026 | -0.075 | 0.940352 | 0.986468 |
| X220904_at | 0.0006 | 0.0079 | 0.075 | 0.94037 | 0.986468 |
| X207432_at | -0.0003 | 0.0033 | -0.075 | 0.9404421 | 0.986468 |
| X212188_at | 0.0055 | 0.0733 | 0.075 | 0.9404565 | 0.986468 |
| X206201_s_at | 0.0012 | 0.0154 | 0.075 | 0.9405053 | 0.986468 |
| X56197_at | 0.002 | 0.0267 | 0.075 | 0.9405312 | 0.986468 |
| X218662_s_at | -0.0017 | 0.0229 | -0.075 | 0.9405532 | 0.986468 |
| X215265_at | 0.0002 | 0.0029 | 0.075 | 0.9405797 | 0.986468 |
| X211213_at | 0.0012 | 0.0161 | 0.075 | 0.9405839 | 0.986468 |
| X221453_at | -0.0002 | 0.0033 | -0.075 | 0.9406145 | 0.986468 |
| X217428_s_at | -0.0006 | 0.0085 | -0.075 | 0.9406615 | 0.986468 |
| X220384_at | -0.0004 | 0.0051 | -0.075 | 0.9407125 | 0.986468 |
| X205052_at | -0.0034 | 0.0449 | -0.075 | 0.9407843 | 0.986468 |
| X212540_at | 0.0023 | 0.0304 | 0.075 | 0.9408303 | 0.986468 |
| X201706_s_at | -0.0008 | 0.0102 | -0.075 | 0.9408596 | 0.986468 |
| X216721_at | -0.0003 | 0.0045 | -0.075 | 0.9408734 | 0.986468 |
| X215551_at | 0.0005 | 0.0069 | 0.075 | 0.9409059 | 0.986468 |
| X216924_s_at | -0.0011 | 0.0144 | -0.075 | 0.9410465 | 0.986569 |
| X220808_at | 0.0002 | 0.0025 | 0.074 | 0.9411159 | 0.986595 |
| X207518_at | -0.0007 | 0.0092 | -0.074 | 0.941164 | 0.986599 |
| X204128_s_at | 0.0005 | 0.0073 | 0.074 | 0.9412732 | 0.986667 |
| X218862_at | -0.0043 | 0.0575 | -0.074 | 0.9415134 | 0.986861 |
| X205851_at | 0.0022 | 0.0302 | 0.074 | 0.9415476 | 0.986861 |
| X203936_s_at | -0.0005 | 0.0071 | -0.074 | 0.9416265 | 0.986897 |
| X204492_at | -0.0006 | 0.0076 | -0.074 | 0.941865 | 0.987025 |
| X203365_s_at | 0.0003 | 0.0038 | 0.074 | 0.9418748 | 0.987025 |
| X217417_at | -0.0006 | 0.0081 | -0.073 | 0.941896 | 0.987025 |
| X207944_at | 0.0002 | 0.0028 | 0.073 | 0.9419368 | 0.987025 |
| X210120_s_at | -0.0004 | 0.0053 | -0.073 | 0.9419724 | 0.987025 |
| X207112_s_at | -0.0009 | 0.0121 | -0.073 | 0.9420352 | 0.987025 |
| X216250_s_at | -0.0025 | 0.0335 | -0.073 | 0.942059 | 0.987025 |
| X204005_s_at | 0.0026 | 0.0349 | 0.073 | 0.9421831 | 0.987091 |
| X219536_s_at | -0.0002 | 0.0027 | -0.073 | 0.9422511 | 0.987091 |
| X221172_at | 0.0005 | 0.0067 | 0.073 | 0.9422721 | 0.987091 |
| X206172_at | 0.0059 | 0.0813 | 0.073 | 0.9423459 | 0.987091 |
| X222187_x_at | -0.0031 | 0.0426 | -0.073 | 0.9424715 | 0.987091 |
| X214412_at | -0.0002 | 0.003 | -0.073 | 0.9424972 | 0.987091 |
| X207094_at | 0.0002 | 0.0031 | 0.073 | 0.9425068 | 0.987091 |
| X212231_at | -0.0022 | 0.03 | -0.073 | 0.9425075 | 0.987091 |
| X214184_at | 0.0003 | 0.0042 | 0.073 | 0.9425626 | 0.987091 |
| X212459_x_at | 0.0053 | 0.0731 | 0.073 | 0.9425664 | 0.987091 |
| X218591_s_at | 0.0005 | 0.0064 | 0.073 | 0.9426476 | 0.987124 |
| X216658_at | -0.0006 | 0.0084 | -0.072 | 0.9427257 | 0.987124 |
| X208604_s_at | 0.0004 | 0.0053 | 0.072 | 0.942731 | 0.987124 |
| X221857_s_at | -0.0005 | 0.0062 | -0.072 | 0.9428622 | 0.987204 |
| X33646_g_at | -0.0004 | 0.0053 | -0.072 | 0.9428962 | 0.987204 |
| X200731_s_at | 0.003 | 0.0418 | 0.072 | 0.9431134 | 0.98721 |
| X219209_at | 0.0074 | 0.1023 | 0.072 | 0.943118 | 0.98721 |
| X208170_s_at | 0.0004 | 0.0058 | 0.072 | 0.9431331 | 0.98721 |
| X206686_at | -0.0002 | 0.0033 | -0.072 | 0.9431443 | 0.98721 |
| X201654_s_at | -0.0002 | 0.0025 | -0.072 | 0.9432026 | 0.98721 |
| X212603_at | 0.002 | 0.0278 | 0.072 | 0.9432111 | 0.98721 |
| X217460_at | -0.0006 | 0.008 | -0.072 | 0.9432134 | 0.98721 |
| X215993_at | 0.0007 | 0.0095 | 0.072 | 0.9433334 | 0.987257 |
| X217914_at | -0.001 | 0.014 | -0.072 | 0.9433849 | 0.987257 |
| X202342_s_at | 0.0013 | 0.018 | 0.072 | 0.9434158 | 0.987257 |
| X221144_at | 0.0008 | 0.0114 | 0.071 | 0.9434864 | 0.987257 |
| X214976_at | -0.0005 | 0.0069 | -0.071 | 0.9435214 | 0.987257 |
| X217845_x_at | -0.002 | 0.0287 | -0.071 | 0.9435425 | 0.987257 |
| X216518_at | -0.0014 | 0.0194 | -0.071 | 0.9435693 | 0.987257 |
| X213474_at | 0.0035 | 0.0496 | 0.071 | 0.9437309 | 0.987366 |
| X210754_s_at | -0.001 | 0.0145 | -0.071 | 0.9438492 | 0.987366 |
| X220970_s_at | 0.0004 | 0.0057 | 0.071 | 0.9438984 | 0.987366 |
| X216058_s_at | -0.0004 | 0.0053 | -0.071 | 0.9439127 | 0.987366 |
| X212381_at | -0.0026 | 0.0371 | -0.071 | 0.9439238 | 0.987366 |
| X209972_s_at | -0.0012 | 0.017 | -0.071 | 0.9439809 | 0.987366 |
| X211349_at | -0.0009 | 0.0129 | -0.071 | 0.9439844 | 0.987366 |
| X214975_s_at | 0.0005 | 0.0074 | 0.071 | 0.9440996 | 0.987397 |
| X222268_x_at | -0.0004 | 0.0051 | -0.071 | 0.9441035 | 0.987397 |
| X205426_s_at | -0.0003 | 0.0046 | -0.071 | 0.9442149 | 0.987413 |
| X205680_at | -0.0004 | 0.0058 | -0.071 | 0.9442335 | 0.987413 |
| X202790_at | 0.0004 | 0.0053 | 0.07 | 0.9442523 | 0.987413 |
| X209370_s_at | 0.0005 | 0.0072 | 0.07 | 0.9443763 | 0.987497 |
| X200776_s_at | -0.0033 | 0.0467 | -0.07 | 0.9444851 | 0.987513 |
| X216666_at | -0.0004 | 0.0057 | -0.07 | 0.9445 | 0.987513 |
| X216152_at | 0.0002 | 0.0035 | 0.07 | 0.9445252 | 0.987513 |
| X209997_x_at | -0.0012 | 0.0177 | -0.07 | 0.9445969 | 0.987513 |
| X209442_x_at | 0.0032 | 0.045 | 0.07 | 0.9446144 | 0.987513 |
| X218880_at | 0.0003 | 0.0049 | 0.07 | 0.9447049 | 0.987561 |
| X210597_x_at | -0.0009 | 0.0131 | -0.07 | 0.9447676 | 0.98758 |
| X220786_s_at | -0.0008 | 0.0113 | -0.07 | 0.9448923 | 0.987636 |
| X47083_at | -0.0011 | 0.016 | -0.07 | 0.9449521 | 0.987636 |
| X211055_s_at | -0.0004 | 0.0064 | -0.07 | 0.9449622 | 0.987636 |
| X209464_at | 0.0004 | 0.0058 | 0.07 | 0.9449991 | 0.987636 |
| X207195_at | 0.0007 | 0.0106 | 0.069 | 0.9450634 | 0.987657 |
| X221446_at | -0.0003 | 0.0042 | -0.069 | 0.9452186 | 0.987726 |
| X209696_at | -0.0002 | 0.003 | -0.069 | 0.9452739 | 0.987726 |
| X213867_x_at | 0.0017 | 0.0253 | 0.069 | 0.9453743 | 0.987726 |
| X201555_at | 0.0033 | 0.0483 | 0.069 | 0.9453873 | 0.987726 |
| X206216_at | 0.0006 | 0.0081 | 0.069 | 0.9453952 | 0.987726 |
| X206092_x_at | -0.0002 | 0.0024 | -0.069 | 0.9454004 | 0.987726 |
| X208401_s_at | -0.0005 | 0.0068 | -0.069 | 0.9454462 | 0.987726 |
| X218865_at | 0.0007 | 0.0108 | 0.069 | 0.9454846 | 0.987726 |
| X204396_s_at | 0.0024 | 0.0347 | 0.069 | 0.9455357 | 0.987732 |
| X216312_at | 0.0004 | 0.0054 | 0.069 | 0.9457146 | 0.987862 |
| X204160_s_at | -0.0038 | 0.0551 | -0.069 | 0.9457489 | 0.987862 |
| X201101_s_at | -0.0099 | 0.1453 | -0.068 | 0.9459512 | 0.988012 |
| X209266_s_at | -0.0005 | 0.0067 | -0.068 | 0.9459814 | 0.988012 |
| X216571_at | -0.0003 | 0.0044 | -0.068 | 0.9460804 | 0.988035 |
| X214128_at | -0.0003 | 0.0042 | -0.068 | 0.9460925 | 0.988035 |
| X221630_s_at | 0.0004 | 0.0064 | 0.068 | 0.9462445 | 0.988083 |
| X214616_at | -0.0003 | 0.0051 | -0.068 | 0.9463139 | 0.988083 |
| X220560_at | -0.0003 | 0.0051 | -0.068 | 0.9463258 | 0.988083 |
| X213567_at | -0.0025 | 0.0365 | -0.068 | 0.9463984 | 0.988083 |
| X216619_at | -0.0006 | 0.0087 | -0.068 | 0.9464007 | 0.988083 |
| X208415_x_at | 0.0005 | 0.0073 | 0.068 | 0.9464391 | 0.988083 |
| X211231_x_at | 0.0004 | 0.006 | 0.068 | 0.9464492 | 0.988083 |
| X203475_at | -0.0005 | 0.0068 | -0.067 | 0.9467813 | 0.988307 |
| X214238_at | 0.0003 | 0.004 | 0.067 | 0.9468225 | 0.988307 |
| X214807_at | 0.0029 | 0.0432 | 0.067 | 0.9468569 | 0.988307 |
| X210088_x_at | -0.0002 | 0.0031 | -0.067 | 0.9468588 | 0.988307 |
| X207166_at | 0.0011 | 0.0168 | 0.067 | 0.9469214 | 0.988307 |
| X205084_at | -0.0035 | 0.0523 | -0.067 | 0.9469308 | 0.988307 |
| X215317_at | -0.0005 | 0.0078 | -0.067 | 0.9470252 | 0.988341 |
| X220781_at | -0.0003 | 0.0048 | -0.067 | 0.9470528 | 0.988341 |
| X216254_at | 0.0009 | 0.0131 | 0.066 | 0.9475601 | 0.988805 |
| X204282_s_at | -0.0004 | 0.0065 | -0.066 | 0.9475858 | 0.988805 |
| X204640_s_at | -0.0019 | 0.0284 | -0.066 | 0.9476416 | 0.988816 |
| X214002_at | -0.0008 | 0.0114 | -0.066 | 0.9477875 | 0.988915 |
| X209364_at | 0.0009 | 0.0131 | 0.066 | 0.947825 | 0.988915 |
| X216184_s_at | -0.0018 | 0.0267 | -0.066 | 0.9480342 | 0.989048 |
| X219277_s_at | -0.0028 | 0.0433 | -0.066 | 0.9480877 | 0.989048 |
| X220059_at | -0.0004 | 0.0062 | -0.066 | 0.9481618 | 0.989048 |
| X218929_at | -0.0021 | 0.0317 | -0.066 | 0.9481698 | 0.989048 |
| X216680_s_at | -0.0007 | 0.0103 | -0.066 | 0.9481751 | 0.989048 |
| X203751_x_at | 0.0003 | 0.0048 | 0.065 | 0.9482283 | 0.989057 |
| X207883_s_at | -0.0003 | 0.0046 | -0.065 | 0.9483248 | 0.9891 |
| X217513_at | -0.0006 | 0.0087 | -0.065 | 0.9483778 | 0.9891 |
| X221092_at | -0.0004 | 0.0055 | -0.065 | 0.9484641 | 0.9891 |
| X201974_s_at | 0.0008 | 0.012 | 0.065 | 0.9484687 | 0.9891 |
| X220199_s_at | 0.0027 | 0.0411 | 0.065 | 0.9484924 | 0.9891 |
| X213144_at | -0.001 | 0.0152 | -0.065 | 0.9487448 | 0.989229 |
| X210199_at | -0.0001 | 0.002 | -0.065 | 0.9487635 | 0.989229 |
| X210803_at | 0.0007 | 0.011 | 0.065 | 0.9487641 | 0.989229 |
| X202107_s_at | 0.0002 | 0.0037 | 0.065 | 0.9489275 | 0.989229 |
| X219841_at | -0.0003 | 0.0047 | -0.065 | 0.9489351 | 0.989229 |
| X214993_at | -0.0057 | 0.0886 | -0.065 | 0.9489614 | 0.989229 |
| X212427_at | -0.0038 | 0.0588 | -0.064 | 0.949089 | 0.989229 |
| X222063_s_at | -0.0005 | 0.0078 | -0.064 | 0.9491107 | 0.989229 |
| X208829_at | -0.0041 | 0.064 | -0.064 | 0.9491518 | 0.989229 |
| X214496_x_at | -0.002 | 0.0305 | -0.064 | 0.9491737 | 0.989229 |
| X206937_at | -0.0004 | 0.0055 | -0.064 | 0.9492405 | 0.989229 |
| X218264_at | 0.0032 | 0.0499 | 0.064 | 0.9492415 | 0.989229 |
| X216567_at | 0.0003 | 0.0039 | 0.064 | 0.9492713 | 0.989229 |
| X216767_at | -0.0004 | 0.0061 | -0.064 | 0.9493126 | 0.989229 |
| X207794_at | 0.0003 | 0.0047 | 0.064 | 0.9494535 | 0.989229 |
| X206505_at | 0.0007 | 0.0106 | 0.064 | 0.9494648 | 0.989229 |
| X217481_x_at | -0.0009 | 0.0134 | -0.064 | 0.9495473 | 0.989229 |
| X216664_at | 0.0003 | 0.0044 | 0.064 | 0.9495616 | 0.989229 |
| X222103_at | -0.0026 | 0.0409 | -0.064 | 0.9496073 | 0.989229 |
| X207710_at | -0.0003 | 0.0053 | -0.064 | 0.9496143 | 0.989229 |
| X219024_at | 0.0062 | 0.0978 | 0.064 | 0.9496296 | 0.989229 |
| X218759_at | -0.0004 | 0.0069 | -0.064 | 0.9496399 | 0.989229 |
| X207918_s_at | -0.0004 | 0.0063 | -0.064 | 0.9496401 | 0.989229 |
| X205085_at | 0.0002 | 0.003 | 0.064 | 0.9497009 | 0.989246 |
| X216404_at | 0.0004 | 0.0064 | 0.063 | 0.9498292 | 0.989313 |
| X219994_at | 0.0004 | 0.0058 | 0.063 | 0.9498546 | 0.989313 |
| X207936_x_at | -0.0021 | 0.0335 | -0.063 | 0.9499272 | 0.989342 |
| X205224_at | -0.0005 | 0.0075 | -0.063 | 0.9500055 | 0.989357 |
| X206796_at | -0.0003 | 0.0045 | -0.063 | 0.95003 | 0.989357 |
| X203557_s_at | 0.0006 | 0.0103 | 0.063 | 0.9501595 | 0.989385 |
| X214849_at | 0.0002 | 0.0038 | 0.063 | 0.9501752 | 0.989385 |
| X217199_s_at | -0.0002 | 0.0031 | -0.063 | 0.9502077 | 0.989385 |
| X201007_at | 0.0029 | 0.0465 | 0.063 | 0.9503319 | 0.989385 |
| X205941_s_at | 0.0003 | 0.0049 | 0.063 | 0.9503688 | 0.989385 |
| X217640_x_at | -0.0006 | 0.0094 | -0.063 | 0.9503823 | 0.989385 |
| X215275_at | -0.0003 | 0.0047 | -0.063 | 0.9504075 | 0.989385 |
| X219361_s_at | 0.0003 | 0.0042 | 0.063 | 0.9504137 | 0.989385 |
| X207579_at | 0.0006 | 0.0092 | 0.062 | 0.9506043 | 0.989456 |
| X215166_at | 0.0002 | 0.0036 | 0.062 | 0.95061 | 0.989456 |
| X208568_at | -0.0003 | 0.004 | -0.062 | 0.9506209 | 0.989456 |
| X205455_at | 0.0002 | 0.0032 | 0.062 | 0.9506599 | 0.989456 |
| X203036_s_at | -0.0003 | 0.0053 | -0.062 | 0.9507889 | 0.989504 |
| X210137_s_at | -0.0031 | 0.0496 | -0.062 | 0.9507954 | 0.989504 |
| X222065_s_at | -0.0018 | 0.029 | -0.062 | 0.9509289 | 0.989523 |
| X205023_at | 0.0006 | 0.0092 | 0.062 | 0.9510334 | 0.989523 |
| X215328_at | 0.0003 | 0.0043 | 0.062 | 0.9510861 | 0.989523 |
| X204837_at | -0.0023 | 0.0372 | -0.062 | 0.9512119 | 0.989523 |
| X207268_x_at | 0.0012 | 0.0188 | 0.062 | 0.951243 | 0.989523 |
| X217159_x_at | -0.0002 | 0.0028 | -0.062 | 0.9513517 | 0.989523 |
| X215573_at | -0.0009 | 0.0148 | -0.061 | 0.9514037 | 0.989523 |
| X211805_s_at | -0.0002 | 0.0032 | -0.061 | 0.951414 | 0.989523 |
| X204804_at | 0.0002 | 0.0029 | 0.061 | 0.9514547 | 0.989523 |
| X217292_at | 0.0005 | 0.0083 | 0.061 | 0.9514827 | 0.989523 |
| X216728_at | -0.0004 | 0.0073 | -0.061 | 0.9514862 | 0.989523 |
| X214712_at | -0.0007 | 0.0108 | -0.061 | 0.9514912 | 0.989523 |
| X213060_s_at | -0.0003 | 0.0051 | -0.061 | 0.9515375 | 0.989523 |
| X206947_at | -0.0002 | 0.0031 | -0.061 | 0.9515377 | 0.989523 |
| X204970_s_at | -0.0017 | 0.0281 | -0.061 | 0.951573 | 0.989523 |
| X214215_s_at | 0.0002 | 0.0033 | 0.061 | 0.9516019 | 0.989523 |
| X202479_s_at | -0.0053 | 0.0872 | -0.061 | 0.9516538 | 0.989523 |
| X217264_s_at | -0.0007 | 0.011 | -0.061 | 0.9516545 | 0.989523 |
| X204456_s_at | 0.0002 | 0.0039 | 0.061 | 0.9516609 | 0.989523 |
| X204251_s_at | 0.0007 | 0.011 | 0.061 | 0.9517428 | 0.989523 |
| X212198_s_at | 0.0006 | 0.0092 | 0.061 | 0.9517483 | 0.989523 |
| X215971_at | -0.0003 | 0.0042 | -0.061 | 0.951845 | 0.989558 |
| X206086_x_at | 0.0002 | 0.004 | 0.061 | 0.9519096 | 0.989558 |
| X216004_s_at | -0.0004 | 0.0059 | -0.061 | 0.951916 | 0.989558 |
| X220096_at | -0.0003 | 0.0043 | -0.061 | 0.9520781 | 0.989635 |
| X211195_s_at | -0.0003 | 0.0042 | -0.061 | 0.9520795 | 0.989635 |
| X217046_s_at | 0.0003 | 0.0057 | 0.061 | 0.9521416 | 0.989637 |
| X212925_at | 0.0003 | 0.0051 | 0.06 | 0.9521704 | 0.989637 |
| X201912_s_at | -0.0031 | 0.0519 | -0.06 | 0.9522903 | 0.989715 |
| X212999_x_at | 0.0017 | 0.0287 | 0.06 | 0.9523641 | 0.989746 |
| X48030_i_at | 0.0034 | 0.0562 | 0.06 | 0.952418 | 0.989756 |
| X206517_at | 0.0002 | 0.0034 | 0.06 | 0.9524837 | 0.989778 |
| X210387_at | 0.0005 | 0.0077 | 0.06 | 0.9526099 | 0.989823 |
| X205869_at | 0.0004 | 0.0072 | 0.06 | 0.9526166 | 0.989823 |
| X211245_x_at | -0.0002 | 0.0033 | -0.059 | 0.9529623 | 0.990028 |
| X205736_at | 0.0002 | 0.0034 | 0.059 | 0.9529712 | 0.990028 |
| X206054_at | -0.0003 | 0.0043 | -0.059 | 0.9530467 | 0.990028 |
| X219400_at | -0.0042 | 0.0702 | -0.059 | 0.9530808 | 0.990028 |
| X202366_at | -0.0003 | 0.0051 | -0.059 | 0.9530956 | 0.990028 |
| X207613_s_at | -0.0003 | 0.0052 | -0.059 | 0.9531202 | 0.990028 |
| X216429_at | -0.0003 | 0.0042 | -0.059 | 0.9531441 | 0.990028 |
| X220646_s_at | 0.0005 | 0.0081 | 0.059 | 0.9531709 | 0.990028 |
| X219274_at | 0.0056 | 0.095 | 0.059 | 0.9532665 | 0.990081 |
| X221564_at | -0.0016 | 0.0271 | -0.059 | 0.9533401 | 0.990107 |
| X206029_at | 0.0004 | 0.0075 | 0.059 | 0.9533805 | 0.990107 |
| X39835_at | -0.0037 | 0.0629 | -0.059 | 0.9535362 | 0.990189 |
| X204862_s_at | 0.0029 | 0.0494 | 0.059 | 0.9535484 | 0.990189 |
| X208248_x_at | -0.0018 | 0.0304 | -0.059 | 0.9537085 | 0.990265 |
| X201979_s_at | -0.0002 | 0.0028 | -0.059 | 0.9537105 | 0.990265 |
| X201719_s_at | 0.0028 | 0.0487 | 0.058 | 0.9540143 | 0.990534 |
| X203437_at | -0.0004 | 0.0072 | -0.058 | 0.9541965 | 0.990677 |
| X203210_s_at | 0.0001 | 0.0025 | 0.058 | 0.9542671 | 0.990704 |
| X202750_s_at | 0.0011 | 0.0182 | 0.058 | 0.9543557 | 0.99075 |
| X211402_x_at | 0.0002 | 0.0043 | 0.058 | 0.9545101 | 0.990784 |
| X213467_at | 0.0015 | 0.0256 | 0.057 | 0.9545386 | 0.990784 |
| X207150_at | 0.0003 | 0.0047 | 0.057 | 0.9545538 | 0.990784 |
| X204360_s_at | 0.0013 | 0.0225 | 0.057 | 0.9546279 | 0.990784 |
| X219156_at | 0.0023 | 0.0393 | 0.057 | 0.9546309 | 0.990784 |
| X221720_s_at | 0.0002 | 0.0043 | 0.057 | 0.9546567 | 0.990784 |
| X215563_s_at | 0.0004 | 0.0073 | 0.057 | 0.9547477 | 0.990815 |
| X206039_at | 0.0074 | 0.1303 | 0.057 | 0.954829 | 0.990815 |
| X215301_at | -0.0007 | 0.0115 | -0.057 | 0.9548574 | 0.990815 |
| X209491_s_at | -0.0002 | 0.0032 | -0.057 | 0.9548647 | 0.990815 |
| X203188_at | 0.0023 | 0.0399 | 0.057 | 0.954942 | 0.990849 |
| X213027_at | -0.0024 | 0.0417 | -0.057 | 0.955129 | 0.990959 |
| X202399_s_at | -0.0021 | 0.0363 | -0.057 | 0.9551376 | 0.990959 |
| X215578_at | -0.005 | 0.0896 | -0.056 | 0.9554211 | 0.991115 |
| X215013_s_at | -0.0014 | 0.0245 | -0.056 | 0.9554417 | 0.991115 |
| X212851_at | -0.0019 | 0.0333 | -0.056 | 0.9554858 | 0.991115 |
| X213898_at | -0.0002 | 0.0044 | -0.056 | 0.9554994 | 0.991115 |
| X207379_at | 0.0005 | 0.0086 | 0.056 | 0.9555485 | 0.991115 |
| X210911_at | -0.0004 | 0.0064 | -0.056 | 0.9555551 | 0.991115 |
| X218931_at | 0.0002 | 0.0041 | 0.056 | 0.9557651 | 0.991239 |
| X215923_s_at | -0.0003 | 0.0052 | -0.056 | 0.9558221 | 0.991239 |
| X205382_s_at | 0.0002 | 0.0035 | 0.056 | 0.9558247 | 0.991239 |
| X207960_at | -0.0002 | 0.0037 | -0.056 | 0.9558694 | 0.991239 |
| X208366_at | 0.0005 | 0.0097 | 0.056 | 0.9559176 | 0.991239 |
| X220241_at | 0.0006 | 0.0112 | 0.056 | 0.9559423 | 0.991239 |
| X207612_at | -0.0002 | 0.0043 | -0.055 | 0.9562182 | 0.991373 |
| X216021_s_at | -0.0008 | 0.0139 | -0.055 | 0.9562271 | 0.991373 |
| X203913_s_at | -0.0007 | 0.0134 | -0.055 | 0.9563091 | 0.991373 |
| X216818_s_at | -0.0002 | 0.0038 | -0.055 | 0.9564335 | 0.991373 |
| X215202_at | -0.0001 | 0.0021 | -0.055 | 0.9565158 | 0.991373 |
| X208621_s_at | -0.0054 | 0.0979 | -0.055 | 0.9565465 | 0.991373 |
| X208057_s_at | -0.0002 | 0.004 | -0.055 | 0.9565585 | 0.991373 |
| X216131_at | 0.0007 | 0.0128 | 0.055 | 0.9565597 | 0.991373 |
| X222176_at | -0.0005 | 0.0082 | -0.055 | 0.9565641 | 0.991373 |
| X216776_at | 0.0004 | 0.0065 | 0.055 | 0.9565686 | 0.991373 |
| X206429_at | -0.0002 | 0.0044 | -0.055 | 0.9566692 | 0.991373 |
| X205948_at | 0.0099 | 0.1805 | 0.055 | 0.9567136 | 0.991373 |
| X205342_s_at | -0.0003 | 0.0052 | -0.055 | 0.9567735 | 0.991373 |
| X209131_s_at | -0.0003 | 0.006 | -0.055 | 0.9567965 | 0.991373 |
| X210781_x_at | -0.0002 | 0.0029 | -0.055 | 0.9568287 | 0.991373 |
| X209236_at | 0.0013 | 0.0246 | 0.055 | 0.9568297 | 0.991373 |
| X204000_at | 0.0024 | 0.0446 | 0.055 | 0.956836 | 0.991373 |
| X214227_at | 0.0002 | 0.0031 | 0.055 | 0.9568751 | 0.991373 |
| X219473_at | -0.0003 | 0.006 | -0.054 | 0.9569287 | 0.991382 |
| X211688_x_at | -0.0002 | 0.0044 | -0.054 | 0.9570893 | 0.991464 |
| X220484_at | -0.0005 | 0.0084 | -0.054 | 0.9570974 | 0.991464 |
| X208428_at | 0.0002 | 0.0045 | 0.054 | 0.9571735 | 0.991494 |
| X209568_s_at | 0.0016 | 0.0299 | 0.054 | 0.9572155 | 0.991494 |
| X207356_at | -0.0003 | 0.0055 | -0.054 | 0.9573318 | 0.991507 |
| X203757_s_at | -0.0002 | 0.0038 | -0.054 | 0.9574137 | 0.991507 |
| X214457_at | -0.0003 | 0.0062 | -0.054 | 0.9574692 | 0.991507 |
| X203879_at | 0.0004 | 0.008 | 0.054 | 0.9574791 | 0.991507 |
| X216809_at | -0.0004 | 0.0077 | -0.054 | 0.9575 | 0.991507 |
| X216227_at | -0.0003 | 0.0049 | -0.054 | 0.9575206 | 0.991507 |
| X212337_at | -0.0017 | 0.0311 | -0.054 | 0.9575398 | 0.991507 |
| X203539_s_at | -0.0002 | 0.003 | -0.053 | 0.9577811 | 0.991678 |
| X216077_s_at | 0.0002 | 0.0035 | 0.053 | 0.9578317 | 0.991678 |
| X201760_s_at | -0.0019 | 0.035 | -0.053 | 0.9578832 | 0.991678 |
| X221674_s_at | 0.0001 | 0.0026 | 0.053 | 0.9578962 | 0.991678 |
| X210352_at | -0.0002 | 0.0041 | -0.053 | 0.957949 | 0.991678 |
| X206538_at | 0.0007 | 0.0128 | 0.053 | 0.9579732 | 0.991678 |
| X218540_at | -0.0028 | 0.0524 | -0.053 | 0.9580532 | 0.991715 |
| X215001_s_at | -0.0023 | 0.044 | -0.053 | 0.9580984 | 0.991715 |
| X220440_at | 0.0002 | 0.0039 | 0.053 | 0.9582808 | 0.991858 |
| X212107_s_at | 0.0141 | 0.2688 | 0.053 | 0.9583827 | 0.991858 |
| X209054_s_at | 0.0015 | 0.0291 | 0.053 | 0.9584261 | 0.991858 |
| X200787_s_at | 0.0034 | 0.0654 | 0.053 | 0.9584269 | 0.991858 |
| X216497_at | -0.0012 | 0.0226 | -0.052 | 0.9585246 | 0.991858 |
| X215420_at | 0.0002 | 0.0039 | 0.052 | 0.9585592 | 0.991858 |
| X207687_at | 0.0004 | 0.007 | 0.052 | 0.9585633 | 0.991858 |
| X219794_at | -0.0007 | 0.0125 | -0.052 | 0.9585939 | 0.991858 |
| X205433_at | -0.005 | 0.0951 | -0.052 | 0.9587495 | 0.991909 |
| X215870_s_at | -0.0009 | 0.0174 | -0.052 | 0.9587603 | 0.991909 |
| X221436_s_at | 0.0002 | 0.0044 | 0.052 | 0.9587772 | 0.991909 |
| X204413_at | 0.0001 | 0.0023 | 0.052 | 0.9588976 | 0.991988 |
| X221866_at | -0.0006 | 0.0114 | -0.052 | 0.9589579 | 0.992004 |
| X220165_at | 0.0002 | 0.0036 | 0.052 | 0.9590893 | 0.992004 |
| X219887_at | -0.0002 | 0.0032 | -0.052 | 0.959137 | 0.992004 |
| X210824_at | -0.0002 | 0.0031 | -0.052 | 0.9592015 | 0.992004 |
| X218697_at | 0.0023 | 0.0438 | 0.052 | 0.9592044 | 0.992004 |
| X216359_at | 0.0002 | 0.0037 | 0.052 | 0.9592424 | 0.992004 |
| X222300_at | 0.0005 | 0.0093 | 0.051 | 0.9592928 | 0.992004 |
| X202870_s_at | -0.0001 | 0.0025 | -0.051 | 0.9592954 | 0.992004 |
| X209543_s_at | 0.0002 | 0.0045 | 0.051 | 0.9593874 | 0.992004 |
| X213103_at | -0.0042 | 0.0827 | -0.051 | 0.9594443 | 0.992004 |
| X214397_at | 0.0003 | 0.0057 | 0.051 | 0.9594854 | 0.992004 |
| X213663_s_at | 0.0007 | 0.0141 | 0.051 | 0.9595668 | 0.992004 |
| X209585_s_at | 0.0025 | 0.0481 | 0.051 | 0.9595975 | 0.992004 |
| X200799_at | 0.005 | 0.0983 | 0.051 | 0.9596652 | 0.992004 |
| X220039_s_at | 0.0009 | 0.0172 | 0.051 | 0.9597425 | 0.992004 |
| X220177_s_at | -0.0019 | 0.0372 | -0.051 | 0.959766 | 0.992004 |
| X213230_at | 0.0032 | 0.0635 | 0.051 | 0.9598248 | 0.992004 |
| X219997_s_at | 0.0004 | 0.007 | 0.051 | 0.9598722 | 0.992004 |
| X209125_at | 0.0003 | 0.0067 | 0.051 | 0.9598726 | 0.992004 |
| X215238_s_at | 0.0002 | 0.0047 | 0.051 | 0.9599018 | 0.992004 |
| X213625_at | -0.0022 | 0.0428 | -0.051 | 0.9599584 | 0.992004 |
| X205795_at | -0.0023 | 0.0454 | -0.051 | 0.9600015 | 0.992004 |
| X215878_at | -0.0005 | 0.01 | -0.051 | 0.9600357 | 0.992004 |
| X214598_at | 0.0004 | 0.0072 | 0.05 | 0.9600688 | 0.992004 |
| X213402_at | 0.0005 | 0.0107 | 0.05 | 0.9601074 | 0.992004 |
| X206528_at | 0.0005 | 0.0108 | 0.05 | 0.9601184 | 0.992004 |
| X217207_s_at | 0.0003 | 0.006 | 0.05 | 0.9601189 | 0.992004 |
| X214348_at | -0.0002 | 0.0043 | -0.05 | 0.9601876 | 0.992017 |
| X202151_s_at | -0.0026 | 0.0512 | -0.05 | 0.9602227 | 0.992017 |
| X208217_at | -0.0002 | 0.0033 | -0.05 | 0.9605218 | 0.992017 |
| X211904_x_at | 0.0002 | 0.0046 | 0.05 | 0.9605429 | 0.992017 |
| X215435_at | 0.0005 | 0.0108 | 0.05 | 0.9606146 | 0.992017 |
| X220890_s_at | 0.0013 | 0.026 | 0.05 | 0.9606392 | 0.992017 |
| X220835_s_at | -0.0004 | 0.0079 | -0.05 | 0.9606405 | 0.992017 |
| X213823_at | -0.0002 | 0.0036 | -0.05 | 0.9607129 | 0.992017 |
| X204709_s_at | 0.0006 | 0.0121 | 0.049 | 0.9608678 | 0.992017 |
| X209948_at | 0.0002 | 0.004 | 0.049 | 0.9609078 | 0.992017 |
| X215810_x_at | 0.0005 | 0.0094 | 0.049 | 0.9609099 | 0.992017 |
| X214912_at | -0.0003 | 0.0061 | -0.049 | 0.9609111 | 0.992017 |
| X212087_s_at | 0.0002 | 0.0037 | 0.049 | 0.9609222 | 0.992017 |
| X215223_s_at | 0.0021 | 0.0426 | 0.049 | 0.9609433 | 0.992017 |
| X219470_x_at | -0.0007 | 0.0145 | -0.049 | 0.9609585 | 0.992017 |
| X215117_at | 0.001 | 0.0197 | 0.049 | 0.9609758 | 0.992017 |
| X214416_at | -0.0003 | 0.0058 | -0.049 | 0.961043 | 0.992017 |
| X217000_at | 0.0002 | 0.0039 | 0.049 | 0.9611357 | 0.992017 |
| X207066_at | 0.0001 | 0.0018 | 0.049 | 0.96114 | 0.992017 |
| X221283_at | 0.0001 | 0.0026 | 0.049 | 0.9611636 | 0.992017 |
| X215540_at | -0.0002 | 0.0035 | -0.049 | 0.9611732 | 0.992017 |
| X211361_s_at | -0.0007 | 0.0148 | -0.049 | 0.9611825 | 0.992017 |
| X208455_at | -0.0003 | 0.0053 | -0.049 | 0.9612304 | 0.992017 |
| X211522_s_at | 0.0005 | 0.0104 | 0.049 | 0.9612712 | 0.992017 |
| X204415_at | -0.0032 | 0.066 | -0.049 | 0.9612724 | 0.992017 |
| X207565_s_at | -0.0002 | 0.0041 | -0.049 | 0.9613675 | 0.992017 |
| X202627_s_at | -0.0003 | 0.0057 | -0.049 | 0.9614222 | 0.992017 |
| X201710_at | -0.0003 | 0.0062 | -0.049 | 0.9614543 | 0.992017 |
| X206502_s_at | 0.0034 | 0.069 | 0.049 | 0.9614617 | 0.992017 |
| X218596_at | -0.0002 | 0.0042 | -0.049 | 0.9614715 | 0.992017 |
| X201670_s_at | 0.003 | 0.0625 | 0.049 | 0.9615842 | 0.992033 |
| X206446_s_at | -0.0003 | 0.0054 | -0.048 | 0.9616416 | 0.992033 |
| X218390_s_at | -0.0011 | 0.0234 | -0.048 | 0.9616925 | 0.992033 |
| X220590_at | -0.0007 | 0.015 | -0.048 | 0.9617535 | 0.992033 |
| X215617_at | 0.0004 | 0.0084 | 0.048 | 0.9617885 | 0.992033 |
| X203095_at | 0.0015 | 0.0316 | 0.048 | 0.9618089 | 0.992033 |
| X222292_at | 0.0004 | 0.0078 | 0.048 | 0.9618281 | 0.992033 |
| X206609_at | -0.0003 | 0.0061 | -0.048 | 0.9618443 | 0.992033 |
| X214899_at | -0.0008 | 0.0161 | -0.048 | 0.9619244 | 0.992045 |
| X216069_at | 0.003 | 0.0622 | 0.048 | 0.9619449 | 0.992045 |
| X206582_s_at | 0.0002 | 0.0032 | 0.048 | 0.9619989 | 0.992054 |
| X215675_at | -0.0001 | 0.0027 | -0.048 | 0.9620516 | 0.992063 |
| X203690_at | -0.0018 | 0.0385 | -0.048 | 0.962107 | 0.992074 |
| X208084_at | -0.0002 | 0.0039 | -0.048 | 0.9622312 | 0.992156 |
| X202888_s_at | -0.0003 | 0.0066 | -0.048 | 0.962402 | 0.992286 |
| X207109_at | 0.0005 | 0.0107 | 0.047 | 0.9624893 | 0.992288 |
| X216454_at | -0.0003 | 0.0056 | -0.047 | 0.962539 | 0.992288 |
| X210693_at | -0.0001 | 0.0021 | -0.047 | 0.9625606 | 0.992288 |
| X217086_at | -0.0002 | 0.0036 | -0.047 | 0.962625 | 0.992288 |
| X218094_s_at | -0.0049 | 0.1039 | -0.047 | 0.9626704 | 0.992288 |
| X216734_s_at | 0.0003 | 0.0058 | 0.047 | 0.96272 | 0.992288 |
| X204399_s_at | 0.0001 | 0.0027 | 0.047 | 0.9627427 | 0.992288 |
| X213999_at | -0.0003 | 0.0063 | -0.047 | 0.9627612 | 0.992288 |
| X34846_at | 0.001 | 0.0204 | 0.047 | 0.9629932 | 0.992481 |
| X214815_at | -0.0044 | 0.0943 | -0.047 | 0.9630455 | 0.992489 |
| X215655_at | 0.0006 | 0.0127 | 0.047 | 0.9631099 | 0.992497 |
| X211883_x_at | -0.0003 | 0.0062 | -0.047 | 0.9631425 | 0.992497 |
| X202067_s_at | -0.0002 | 0.0035 | -0.046 | 0.9633262 | 0.992632 |
| X221167_s_at | -0.0003 | 0.0069 | -0.046 | 0.9633631 | 0.992632 |
| X205912_at | -0.0003 | 0.0057 | -0.046 | 0.9636384 | 0.992679 |
| X215120_s_at | -0.0004 | 0.0083 | -0.046 | 0.9636477 | 0.992679 |
| X221539_at | 0.0002 | 0.0047 | 0.046 | 0.9636477 | 0.992679 |
| X218631_at | 0.0016 | 0.0351 | 0.046 | 0.9636509 | 0.992679 |
| X208938_at | 0.0016 | 0.0355 | 0.046 | 0.9637352 | 0.992679 |
| X217339_x_at | -0.0002 | 0.0037 | -0.046 | 0.9637352 | 0.992679 |
| X211581_x_at | 0.0003 | 0.0063 | 0.046 | 0.963792 | 0.992679 |
| X219684_at | -0.0004 | 0.0089 | -0.046 | 0.9638402 | 0.992679 |
| X204110_at | -0.0002 | 0.0053 | -0.046 | 0.9638517 | 0.992679 |
| X207218_at | -0.0003 | 0.0073 | -0.046 | 0.9638693 | 0.992679 |
| X217524_x_at | -0.0008 | 0.0181 | -0.046 | 0.963925 | 0.992679 |
| X202527_s_at | -0.0023 | 0.0502 | -0.046 | 0.9639453 | 0.992679 |
| X206856_at | 0.0002 | 0.0039 | 0.045 | 0.964013 | 0.992703 |
| X201572_x_at | -0.0013 | 0.0278 | -0.045 | 0.9641472 | 0.992742 |
| X203242_s_at | -0.0036 | 0.0802 | -0.045 | 0.9641835 | 0.992742 |
| X215509_s_at | -0.0003 | 0.0066 | -0.045 | 0.9641851 | 0.992742 |
| X217244_at | 0.0003 | 0.0059 | 0.045 | 0.9642456 | 0.992749 |
| X211620_x_at | 0.0001 | 0.0032 | 0.045 | 0.9642809 | 0.992749 |
| X208448_x_at | -0.0003 | 0.0057 | -0.045 | 0.9643723 | 0.992797 |
| X200026_at | -0.0012 | 0.0274 | -0.045 | 0.9644276 | 0.992808 |
| X203778_at | 0.0008 | 0.0188 | 0.045 | 0.9644887 | 0.992825 |
| X206709_x_at | -0.0002 | 0.0053 | -0.045 | 0.9646574 | 0.992916 |
| X209646_x_at | -0.0005 | 0.011 | -0.045 | 0.9647078 | 0.992916 |
| X205749_at | -0.0001 | 0.003 | -0.044 | 0.9648042 | 0.992916 |
| X216309_x_at | 0.0005 | 0.0101 | 0.044 | 0.9648332 | 0.992916 |
| X216777_at | -0.0003 | 0.0058 | -0.044 | 0.964838 | 0.992916 |
| X215911_x_at | 0.0004 | 0.0094 | 0.044 | 0.9648512 | 0.992916 |
| X213433_at | -0.0003 | 0.0075 | -0.044 | 0.9648901 | 0.992916 |
| X207321_s_at | 0.0003 | 0.0077 | 0.044 | 0.9649687 | 0.992951 |
| X203012_x_at | -0.0006 | 0.0135 | -0.044 | 0.9650432 | 0.992981 |
| X220501_at | -0.0001 | 0.0021 | -0.044 | 0.9651431 | 0.993038 |
| X207994_s_at | -0.0004 | 0.0094 | -0.044 | 0.9652637 | 0.993054 |
| X214012_at | 0.0052 | 0.1184 | 0.044 | 0.965282 | 0.993054 |
| X207199_at | -0.0004 | 0.01 | -0.044 | 0.9653856 | 0.993054 |
| X220019_s_at | -0.0008 | 0.0177 | -0.044 | 0.9653961 | 0.993054 |
| X221756_at | -0.0035 | 0.0794 | -0.044 | 0.9654014 | 0.993054 |
| X217912_at | -0.0014 | 0.0323 | -0.044 | 0.9654268 | 0.993054 |
| X208143_s_at | 0.0005 | 0.0122 | 0.043 | 0.9656242 | 0.99315 |
| X214475_x_at | -0.007 | 0.1617 | -0.043 | 0.9656883 | 0.99315 |
| X220084_at | -0.0005 | 0.0124 | -0.043 | 0.9657288 | 0.99315 |
| X217596_at | 0.0003 | 0.0075 | 0.043 | 0.9657291 | 0.99315 |
| X202051_s_at | 0.0019 | 0.0447 | 0.043 | 0.9657434 | 0.99315 |
| X218313_s_at | 0.0021 | 0.0479 | 0.043 | 0.9657897 | 0.993151 |
| X206837_at | -0.0001 | 0.0031 | -0.043 | 0.965836 | 0.993153 |
| X202485_s_at | 0.0002 | 0.0037 | 0.043 | 0.965898 | 0.993171 |
| X216683_at | 0.0002 | 0.0055 | 0.043 | 0.9659935 | 0.993223 |
| X200693_at | -0.0022 | 0.0507 | -0.043 | 0.9660625 | 0.993248 |
| X215796_at | -0.0003 | 0.0073 | -0.043 | 0.9661613 | 0.993279 |
| X216699_s_at | -0.0002 | 0.0053 | -0.043 | 0.9662164 | 0.993279 |
| X207646_s_at | -0.0002 | 0.0055 | -0.043 | 0.9662274 | 0.993279 |
| X210601_at | -0.0004 | 0.0105 | -0.043 | 0.9663643 | 0.99328 |
| X221237_s_at | -0.0003 | 0.0065 | -0.042 | 0.9663849 | 0.99328 |
| X209847_at | -0.0007 | 0.0161 | -0.042 | 0.9664159 | 0.99328 |
| X215032_at | 0.0004 | 0.0104 | 0.042 | 0.9664161 | 0.99328 |
| X218445_at | 0.0002 | 0.0056 | 0.042 | 0.9665175 | 0.99328 |
| X207928_s_at | -0.0007 | 0.0177 | -0.042 | 0.9665611 | 0.99328 |
| X221445_at | -0.0002 | 0.0052 | -0.042 | 0.9667576 | 0.99328 |
| X204819_at | 0.0002 | 0.0049 | 0.042 | 0.9667658 | 0.99328 |
| X214981_at | 0.0006 | 0.0148 | 0.042 | 0.9667847 | 0.99328 |
| X216240_at | -0.0002 | 0.0042 | -0.042 | 0.9667882 | 0.99328 |
| X222361_at | 0.0002 | 0.0055 | 0.042 | 0.9668052 | 0.99328 |
| X212782_x_at | -0.0013 | 0.0311 | -0.042 | 0.9668317 | 0.99328 |
| X214418_at | -0.0003 | 0.007 | -0.042 | 0.9668402 | 0.99328 |
| X210163_at | 0.0006 | 0.0137 | 0.042 | 0.9668538 | 0.99328 |
| X215846_at | -0.0004 | 0.0094 | -0.042 | 0.9669287 | 0.993303 |
| X220686_s_at | -0.0001 | 0.0036 | -0.042 | 0.9669961 | 0.993303 |
| X207763_at | 0.0002 | 0.005 | 0.042 | 0.9670346 | 0.993303 |
| X217391_x_at | -0.0002 | 0.0046 | -0.042 | 0.9671016 | 0.993303 |
| X205623_at | 0.0002 | 0.0048 | 0.042 | 0.9671121 | 0.993303 |
| X210481_s_at | 0.0002 | 0.0037 | 0.041 | 0.9671849 | 0.993303 |
| X208483_x_at | -0.0001 | 0.0035 | -0.041 | 0.9671893 | 0.993303 |
| X215634_at | -0.0042 | 0.1027 | -0.041 | 0.967283 | 0.99333 |
| X207895_at | 0.0001 | 0.0026 | 0.041 | 0.9673051 | 0.99333 |
| X210643_at | -0.0004 | 0.0086 | -0.041 | 0.9673808 | 0.993343 |
| X221343_at | -0.0002 | 0.0042 | -0.041 | 0.9674073 | 0.993343 |
| X220724_at | 0.0005 | 0.0119 | 0.041 | 0.9674883 | 0.993356 |
| X218453_s_at | 0.0016 | 0.0389 | 0.041 | 0.9675344 | 0.993356 |
| X207548_at | -0.0028 | 0.068 | -0.041 | 0.9675544 | 0.993356 |
| X212155_at | 0.0013 | 0.0321 | 0.041 | 0.9676179 | 0.993376 |
| X212049_at | -0.0014 | 0.0336 | -0.041 | 0.9676836 | 0.993379 |
| X210344_at | 0.0002 | 0.0051 | 0.041 | 0.9677105 | 0.993379 |
| X205350_at | -0.0034 | 0.0838 | -0.041 | 0.9678413 | 0.993467 |
| X209602_s_at | 0.0003 | 0.0064 | 0.041 | 0.9678885 | 0.99347 |
| X207013_s_at | 0.0002 | 0.0047 | 0.04 | 0.9679793 | 0.993517 |
| X218913_s_at | 0.0002 | 0.0059 | 0.04 | 0.9681889 | 0.993686 |
| X203241_at | -0.0013 | 0.0329 | -0.04 | 0.9682493 | 0.993703 |
| X200785_s_at | -0.0021 | 0.052 | -0.04 | 0.9683536 | 0.993734 |
| X217753_s_at | -0.0038 | 0.0946 | -0.04 | 0.9683699 | 0.993734 |
| X207106_s_at | 0.0002 | 0.0042 | 0.04 | 0.9684669 | 0.993788 |
| X211577_s_at | -0.0002 | 0.0049 | -0.04 | 0.968552 | 0.993804 |
| X215066_at | 0.0002 | 0.006 | 0.04 | 0.9686676 | 0.993804 |
| X207633_s_at | 0.0002 | 0.0061 | 0.04 | 0.9686924 | 0.993804 |
| X214559_at | 0.0001 | 0.0028 | 0.04 | 0.9687111 | 0.993804 |
| X217182_at | 0.0009 | 0.0236 | 0.039 | 0.96877 | 0.993804 |
| X222342_at | 0.0005 | 0.0125 | 0.039 | 0.9688653 | 0.993804 |
| X221344_at | -0.0001 | 0.0038 | -0.039 | 0.9689296 | 0.993804 |
| X206740_x_at | -0.0003 | 0.0067 | -0.039 | 0.9689409 | 0.993804 |
| X221941_at | -0.0001 | 0.0023 | -0.039 | 0.9689516 | 0.993804 |
| X213278_at | -0.0011 | 0.0279 | -0.039 | 0.9689779 | 0.993804 |
| X219668_at | -0.0017 | 0.0441 | -0.039 | 0.9689832 | 0.993804 |
| X220929_at | 0.0001 | 0.0025 | 0.039 | 0.9690947 | 0.993804 |
| X208147_s_at | 0.001 | 0.0246 | 0.039 | 0.9691679 | 0.993804 |
| X207691_x_at | -0.0002 | 0.0041 | -0.039 | 0.9691921 | 0.993804 |
| X221993_s_at | 0.0001 | 0.0037 | 0.039 | 0.9692282 | 0.993804 |
| X213848_at | 0.003 | 0.0768 | 0.039 | 0.9692397 | 0.993804 |
| X201731_s_at | -0.0011 | 0.0281 | -0.039 | 0.9693362 | 0.993804 |
| X204904_at | -0.0001 | 0.0029 | -0.039 | 0.9693441 | 0.993804 |
| X212986_s_at | -0.001 | 0.0263 | -0.039 | 0.9693477 | 0.993804 |
| X212909_at | -0.0025 | 0.0654 | -0.039 | 0.9694517 | 0.993804 |
| X202148_s_at | 0.0013 | 0.0346 | 0.039 | 0.9695029 | 0.993804 |
| X210944_s_at | -0.0049 | 0.1266 | -0.038 | 0.9695538 | 0.993804 |
| X210311_at | 0.0002 | 0.0042 | 0.038 | 0.9695655 | 0.993804 |
| X217229_at | 0.0005 | 0.012 | 0.038 | 0.9696122 | 0.993804 |
| X205445_at | 0.0002 | 0.0044 | 0.038 | 0.9696312 | 0.993804 |
| X215577_at | 0.0003 | 0.0085 | 0.038 | 0.969718 | 0.993804 |
| X218784_s_at | -0.0001 | 0.0039 | -0.038 | 0.9698101 | 0.993804 |
| X220782_x_at | 0.0001 | 0.0028 | 0.038 | 0.9699299 | 0.993804 |
| X220444_at | -0.0002 | 0.0048 | -0.038 | 0.9699864 | 0.993804 |
| X203331_s_at | 0.0001 | 0.0023 | 0.038 | 0.97 | 0.993804 |
| X207675_x_at | 0.0002 | 0.0042 | 0.038 | 0.9700928 | 0.993804 |
| X214773_x_at | -0.0034 | 0.089 | -0.038 | 0.9700943 | 0.993804 |
| X214097_at | -0.0001 | 0.0038 | -0.038 | 0.9701337 | 0.993804 |
| X207965_at | -0.0002 | 0.0043 | -0.038 | 0.9701571 | 0.993804 |
| X222259_s_at | -0.0004 | 0.0115 | -0.038 | 0.9701927 | 0.993804 |
| X217595_at | -0.0006 | 0.0172 | -0.038 | 0.9702032 | 0.993804 |
| X212713_at | 0.0002 | 0.0057 | 0.038 | 0.9702207 | 0.993804 |
| X211851_x_at | -0.0001 | 0.0033 | -0.038 | 0.9702268 | 0.993804 |
| X203370_s_at | 0.0002 | 0.0042 | 0.038 | 0.9702275 | 0.993804 |
| X222141_at | -0.0002 | 0.0056 | -0.037 | 0.9703879 | 0.993847 |
| X221631_at | -0.0042 | 0.112 | -0.037 | 0.9704678 | 0.993847 |
| X219731_at | -0.0005 | 0.0146 | -0.037 | 0.9705075 | 0.993847 |
| X222364_at | 0.0044 | 0.1191 | 0.037 | 0.9705908 | 0.993847 |
| X216949_s_at | -0.0002 | 0.0048 | -0.037 | 0.9706143 | 0.993847 |
| X211624_s_at | -0.0003 | 0.0071 | -0.037 | 0.9706412 | 0.993847 |
| X211905_s_at | 0.0002 | 0.0044 | 0.037 | 0.9706435 | 0.993847 |
| X217181_at | -0.0001 | 0.0026 | -0.037 | 0.9707269 | 0.993847 |
| X204622_x_at | 0.0066 | 0.1778 | 0.037 | 0.9707512 | 0.993847 |
| X219903_s_at | -0.0004 | 0.0103 | -0.037 | 0.9707605 | 0.993847 |
| X216146_at | -0.0003 | 0.0076 | -0.037 | 0.9707813 | 0.993847 |
| X214650_x_at | -0.0052 | 0.1415 | -0.037 | 0.9708064 | 0.993847 |
| X207978_s_at | 0.0002 | 0.0061 | 0.037 | 0.9710248 | 0.994025 |
| X215294_s_at | 0.0031 | 0.0841 | 0.037 | 0.9710988 | 0.994055 |
| X220280_s_at | -0.0001 | 0.0032 | -0.036 | 0.9712679 | 0.994182 |
| X204450_x_at | 0.0002 | 0.0045 | 0.036 | 0.9713578 | 0.994185 |
| X215914_at | -0.0003 | 0.0073 | -0.036 | 0.9713934 | 0.994185 |
| X210904_s_at | 0.0003 | 0.0074 | 0.036 | 0.9714046 | 0.994185 |
| X212725_s_at | 0.001 | 0.0291 | 0.036 | 0.97153 | 0.994249 |
| X203819_s_at | 0.0002 | 0.005 | 0.036 | 0.9715564 | 0.994249 |
| X208531_at | -0.0002 | 0.0065 | -0.036 | 0.9716788 | 0.994282 |
| X219498_s_at | 0.0034 | 0.0956 | 0.036 | 0.9717083 | 0.994282 |
| X201733_at | -0.0035 | 0.0981 | -0.036 | 0.9717234 | 0.994282 |
| X217478_s_at | 0.0008 | 0.0235 | 0.036 | 0.9718814 | 0.994359 |
| X209090_s_at | 0.0021 | 0.0603 | 0.036 | 0.9718885 | 0.994359 |
| X202249_s_at | 0.0005 | 0.0134 | 0.035 | 0.9720894 | 0.994439 |
| X208867_s_at | 0.0019 | 0.0534 | 0.035 | 0.9721144 | 0.994439 |
| X215835_at | 0.0003 | 0.0097 | 0.035 | 0.972172 | 0.994439 |
| X215249_at | -0.0003 | 0.008 | -0.035 | 0.9721988 | 0.994439 |
| X205107_s_at | -0.0001 | 0.0036 | -0.035 | 0.9722376 | 0.994439 |
| X202807_s_at | 0.0001 | 0.0038 | 0.035 | 0.9723708 | 0.994439 |
| X215821_x_at | -0.0001 | 0.0022 | -0.035 | 0.9723775 | 0.994439 |
| X211129_x_at | 0.0001 | 0.0039 | 0.035 | 0.9723954 | 0.994439 |
| X206353_at | 0.0001 | 0.0036 | 0.035 | 0.9724417 | 0.994439 |
| X211659_at | 0.0001 | 0.002 | 0.035 | 0.9724458 | 0.994439 |
| X215399_s_at | 0.0005 | 0.0143 | 0.035 | 0.9724698 | 0.994439 |
| X204605_at | 0.0021 | 0.0594 | 0.035 | 0.9725031 | 0.994439 |
| X208128_x_at | -0.0002 | 0.0048 | -0.035 | 0.9725947 | 0.994487 |
| X218585_s_at | 0.0003 | 0.0079 | 0.035 | 0.9726515 | 0.994499 |
| X221474_at | 0.0009 | 0.0248 | 0.034 | 0.9727154 | 0.994518 |
| X215127_s_at | 0.0022 | 0.0631 | 0.034 | 0.9727747 | 0.994533 |
| X207921_x_at | 0.0001 | 0.0039 | 0.034 | 0.9728464 | 0.994561 |
| X201069_at | 0.0003 | 0.009 | 0.034 | 0.9728947 | 0.994564 |
| X219583_s_at | 0.0022 | 0.0642 | 0.034 | 0.9729533 | 0.994579 |
| X217693_x_at | 0.0003 | 0.0097 | 0.034 | 0.9730002 | 0.994581 |
| X204901_at | 0.0002 | 0.0058 | 0.034 | 0.9730905 | 0.994606 |
| X216936_at | -0.0001 | 0.0029 | -0.034 | 0.9731209 | 0.994606 |
| X211203_s_at | -0.0002 | 0.0045 | -0.034 | 0.9731591 | 0.994606 |
| X222177_s_at | 0.0001 | 0.0042 | 0.034 | 0.9732377 | 0.99464 |
| X210547_x_at | 0.0005 | 0.0139 | 0.034 | 0.9734569 | 0.994774 |
| X219580_s_at | 0.0003 | 0.0098 | 0.033 | 0.9735783 | 0.994774 |
| X215996_at | -0.0001 | 0.002 | -0.033 | 0.9735901 | 0.994774 |
| X208446_s_at | -0.0001 | 0.0036 | -0.033 | 0.9735957 | 0.994774 |
| X215642_at | 0.0003 | 0.0079 | 0.033 | 0.9736512 | 0.994774 |
| X205118_at | 0.0004 | 0.0126 | 0.033 | 0.973667 | 0.994774 |
| X220850_at | -0.0005 | 0.0154 | -0.033 | 0.9736821 | 0.994774 |
| X209360_s_at | 0.0002 | 0.0076 | 0.033 | 0.9739177 | 0.994947 |
| X218688_at | -0.0004 | 0.0107 | -0.033 | 0.973989 | 0.994947 |
| X212836_at | 0.0016 | 0.0486 | 0.033 | 0.9740053 | 0.994947 |
| X219412_at | 0.0001 | 0.0044 | 0.033 | 0.9740307 | 0.994947 |
| X216791_at | 0.0006 | 0.0182 | 0.033 | 0.9741054 | 0.994978 |
| X215206_at | -0.0001 | 0.0045 | -0.033 | 0.9741811 | 0.994995 |
| X220031_at | 0.0002 | 0.0052 | 0.033 | 0.9742616 | 0.994995 |
| X206566_at | -0.0001 | 0.0043 | -0.033 | 0.9742774 | 0.994995 |
| X211399_at | -0.0002 | 0.0069 | -0.032 | 0.9743016 | 0.994995 |
| X206168_at | -0.0001 | 0.0033 | -0.032 | 0.9744743 | 0.995103 |
| X205207_at | -0.0001 | 0.0044 | -0.032 | 0.9744966 | 0.995103 |
| X207053_at | -0.0001 | 0.0046 | -0.032 | 0.9745418 | 0.995103 |
| X220092_s_at | -0.0002 | 0.0065 | -0.032 | 0.9746325 | 0.995123 |
| X206713_at | 0.0001 | 0.0043 | 0.032 | 0.9746506 | 0.995123 |
| X220997_s_at | -0.0002 | 0.0056 | -0.032 | 0.9747993 | 0.995221 |
| X220963_s_at | -0.0001 | 0.0033 | -0.032 | 0.9748661 | 0.995221 |
| X217662_x_at | -0.0007 | 0.0226 | -0.032 | 0.9748814 | 0.995221 |
| X214966_at | 0.0002 | 0.005 | 0.032 | 0.9749661 | 0.995251 |
| X207102_at | 0.0004 | 0.0124 | 0.032 | 0.9750001 | 0.995251 |
| X216631_s_at | 0.0001 | 0.0043 | 0.031 | 0.9751575 | 0.995366 |
| X58780_s_at | -0.0011 | 0.035 | -0.031 | 0.9752325 | 0.995397 |
| X216869_at | 0.0005 | 0.0173 | 0.031 | 0.9753342 | 0.995424 |
| X218730_s_at | 0.0005 | 0.0151 | 0.031 | 0.9754279 | 0.995424 |
| X204153_s_at | 0.0003 | 0.0094 | 0.031 | 0.9754364 | 0.995424 |
| X211740_at | -0.0004 | 0.0114 | -0.031 | 0.9754384 | 0.995424 |
| X211590_x_at | 0.0001 | 0.0017 | 0.031 | 0.9755026 | 0.995444 |
| X214641_at | 0.0002 | 0.006 | 0.031 | 0.975578 | 0.995475 |
| X35436_at | -0.0012 | 0.0402 | -0.031 | 0.9756384 | 0.995491 |
| X208375_at | -0.0003 | 0.0104 | -0.031 | 0.9757846 | 0.995573 |
| X206962_x_at | -0.0002 | 0.0069 | -0.03 | 0.9759703 | 0.995573 |
| X216535_at | -0.0001 | 0.0041 | -0.03 | 0.9761344 | 0.995573 |
| X208330_at | 0.0001 | 0.0031 | 0.03 | 0.976196 | 0.995573 |
| X213596_at | -0.0002 | 0.0056 | -0.03 | 0.9762689 | 0.995573 |
| X210324_at | 0.0001 | 0.0024 | 0.03 | 0.9762799 | 0.995573 |
| X222289_at | 0.0001 | 0.0037 | 0.03 | 0.9762862 | 0.995573 |
| X220812_s_at | -0.0004 | 0.0125 | -0.03 | 0.9764178 | 0.995573 |
| X201911_s_at | -0.0016 | 0.0549 | -0.03 | 0.9764774 | 0.995573 |
| X220699_s_at | -0.0002 | 0.0067 | -0.03 | 0.9765031 | 0.995573 |
| X216933_x_at | 0.0021 | 0.0725 | 0.03 | 0.9765629 | 0.995573 |
| X217052_x_at | 0.0007 | 0.0222 | 0.03 | 0.976585 | 0.995573 |
| X216034_at | -0.0001 | 0.0036 | -0.03 | 0.9765992 | 0.995573 |
| X209888_s_at | -0.0001 | 0.0028 | -0.029 | 0.976772 | 0.995573 |
| X204287_at | 0.0019 | 0.0645 | 0.029 | 0.9768007 | 0.995573 |
| X209213_at | -0.0026 | 0.0884 | -0.029 | 0.9768234 | 0.995573 |
| X217425_at | 0.0001 | 0.0041 | 0.029 | 0.9768248 | 0.995573 |
| X211075_s_at | 0.0024 | 0.0803 | 0.029 | 0.9768307 | 0.995573 |
| X216937_s_at | 0.0001 | 0.0024 | 0.029 | 0.9768572 | 0.995573 |
| X216856_s_at | 0.0002 | 0.008 | 0.029 | 0.9768577 | 0.995573 |
| X211266_s_at | -0.0001 | 0.0038 | -0.029 | 0.9768589 | 0.995573 |
| X202882_x_at | 0.0019 | 0.0638 | 0.029 | 0.9770504 | 0.995573 |
| X219020_at | 0.0003 | 0.0116 | 0.029 | 0.9770617 | 0.995573 |
| X217376_at | -0.0001 | 0.0047 | -0.029 | 0.9771351 | 0.995573 |
| X210928_at | -0.0001 | 0.0046 | -0.029 | 0.9771493 | 0.995573 |
| X202023_at | -0.0015 | 0.0515 | -0.029 | 0.9772214 | 0.995573 |
| X216790_at | -0.0001 | 0.0045 | -0.029 | 0.9772325 | 0.995573 |
| X214575_s_at | 0.0001 | 0.0029 | 0.029 | 0.977279 | 0.995573 |
| X207339_s_at | -0.0002 | 0.0073 | -0.029 | 0.9773071 | 0.995573 |
| X219801_at | -0.0002 | 0.0053 | -0.029 | 0.9773507 | 0.995573 |
| X215490_at | -0.0002 | 0.0053 | -0.029 | 0.9773809 | 0.995573 |
| X214695_at | -0.0002 | 0.0086 | -0.029 | 0.9774255 | 0.995573 |
| X216050_at | -0.0002 | 0.0068 | -0.028 | 0.9775609 | 0.995573 |
| X204539_s_at | 0.0001 | 0.0046 | 0.028 | 0.9775677 | 0.995573 |
| X208216_at | 0.0002 | 0.0064 | 0.028 | 0.9776088 | 0.995573 |
| X214540_at | -0.0001 | 0.0031 | -0.028 | 0.9776516 | 0.995573 |
| X216393_at | -0.0001 | 0.0047 | -0.028 | 0.9776626 | 0.995573 |
| X221878_at | 0.0002 | 0.0062 | 0.028 | 0.9776955 | 0.995573 |
| X207407_x_at | -0.0002 | 0.008 | -0.028 | 0.9776997 | 0.995573 |
| X204272_at | 0.0001 | 0.003 | 0.028 | 0.9777467 | 0.995573 |
| X218990_s_at | 0.0001 | 0.0041 | 0.028 | 0.9777949 | 0.995573 |
| X203064_s_at | 0.0015 | 0.0519 | 0.028 | 0.9778077 | 0.995573 |
| X201291_s_at | -0.0002 | 0.0085 | -0.028 | 0.9778299 | 0.995573 |
| X206367_at | 0.0001 | 0.0026 | 0.028 | 0.9778597 | 0.995573 |
| X210204_s_at | -0.0002 | 0.0054 | -0.028 | 0.9778609 | 0.995573 |
| X209417_s_at | 0.0001 | 0.003 | 0.028 | 0.9778773 | 0.995573 |
| X1405_i_at | -0.0004 | 0.014 | -0.028 | 0.9780024 | 0.995573 |
| X205041_s_at | 0.0002 | 0.0057 | 0.028 | 0.9780102 | 0.995573 |
| X211863_x_at | -0.0001 | 0.0031 | -0.028 | 0.9780137 | 0.995573 |
| X210808_s_at | 0.0002 | 0.0088 | 0.028 | 0.9780145 | 0.995573 |
| X211867_s_at | 0.0001 | 0.0043 | 0.028 | 0.9780251 | 0.995573 |
| X212953_x_at | -0.0005 | 0.0164 | -0.028 | 0.9780489 | 0.995573 |
| X220700_at | 0.0004 | 0.016 | 0.028 | 0.9781942 | 0.995578 |
| X210775_x_at | -0.0007 | 0.0246 | -0.027 | 0.9782579 | 0.995578 |
| X203559_s_at | 0.0001 | 0.0054 | 0.027 | 0.9782594 | 0.995578 |
| X210302_s_at | 0.0002 | 0.0081 | 0.027 | 0.9784055 | 0.995578 |
| X219669_at | 0.0002 | 0.0072 | 0.027 | 0.9784234 | 0.995578 |
| X212102_s_at | -0.0002 | 0.0076 | -0.027 | 0.9786369 | 0.995578 |
| X212719_at | -0.002 | 0.0727 | -0.027 | 0.9786828 | 0.995578 |
| X217864_s_at | -0.0006 | 0.023 | -0.027 | 0.9787359 | 0.995578 |
| X212777_at | 0.0002 | 0.0066 | 0.027 | 0.9788689 | 0.995578 |
| X214649_s_at | 0.001 | 0.0364 | 0.027 | 0.9789298 | 0.995578 |
| X204818_at | -0.0001 | 0.0028 | -0.027 | 0.9789354 | 0.995578 |
| X210224_at | 0.0002 | 0.007 | 0.027 | 0.9789411 | 0.995578 |
| X216752_at | -0.0005 | 0.0191 | -0.026 | 0.979054 | 0.995578 |
| X216955_at | -0.0003 | 0.0112 | -0.026 | 0.9791259 | 0.995578 |
| X206227_at | 0.0002 | 0.006 | 0.026 | 0.979167 | 0.995578 |
| X216340_s_at | -0.0002 | 0.0064 | -0.026 | 0.979192 | 0.995578 |
| X219486_at | -0.0002 | 0.0071 | -0.026 | 0.9791926 | 0.995578 |
| X219466_s_at | -0.0001 | 0.0036 | -0.026 | 0.9791943 | 0.995578 |
| X210886_x_at | -0.0007 | 0.0271 | -0.026 | 0.9791975 | 0.995578 |
| X201226_at | 0.0007 | 0.0254 | 0.026 | 0.9792246 | 0.995578 |
| X210850_s_at | 0.0001 | 0.0042 | 0.026 | 0.9792455 | 0.995578 |
| X203243_s_at | -0.0013 | 0.0489 | -0.026 | 0.9793036 | 0.995578 |
| X207762_at | 0.0001 | 0.0033 | 0.026 | 0.9793355 | 0.995578 |
| X216553_x_at | 0.0003 | 0.0119 | 0.026 | 0.9793371 | 0.995578 |
| X215405_at | 0.0002 | 0.0078 | 0.026 | 0.9794129 | 0.995578 |
| X212204_at | 0.0013 | 0.0496 | 0.026 | 0.9794736 | 0.995578 |
| X209401_s_at | 0.0001 | 0.0046 | 0.026 | 0.9794742 | 0.995578 |
| X214805_at | -0.0001 | 0.0057 | -0.026 | 0.9794852 | 0.995578 |
| X216825_s_at | 0.0001 | 0.0046 | 0.026 | 0.9795459 | 0.995578 |
| X207669_at | -0.0001 | 0.0057 | -0.026 | 0.9795846 | 0.995578 |
| X200699_at | -0.0007 | 0.0253 | -0.026 | 0.9796016 | 0.995578 |
| X207529_at | 0.0001 | 0.0036 | 0.026 | 0.9796553 | 0.995578 |
| X207889_at | 0.0001 | 0.0057 | 0.026 | 0.9797022 | 0.995578 |
| X202268_s_at | -0.0006 | 0.0246 | -0.026 | 0.9797214 | 0.995578 |
| X212981_s_at | 0.0011 | 0.0441 | 0.026 | 0.9797248 | 0.995578 |
| X215226_at | -0.0002 | 0.0092 | -0.026 | 0.9797562 | 0.995578 |
| X216399_s_at | -0.0016 | 0.0629 | -0.025 | 0.9798537 | 0.995578 |
| X202280_at | 0.0001 | 0.0047 | 0.025 | 0.9798552 | 0.995578 |
| X216719_s_at | 0.0002 | 0.0085 | 0.025 | 0.9798769 | 0.995578 |
| X206340_at | 0.0001 | 0.0042 | 0.025 | 0.9798903 | 0.995578 |
| X222087_at | 0.0001 | 0.0026 | 0.025 | 0.9799502 | 0.995578 |
| X213674_x_at | -0.0001 | 0.0052 | -0.025 | 0.9800033 | 0.995578 |
| X213902_at | 0.0006 | 0.0238 | 0.025 | 0.9800125 | 0.995578 |
| X38037_at | 0.0002 | 0.0091 | 0.025 | 0.9800266 | 0.995578 |
| X207819_s_at | -0.0001 | 0.0035 | -0.025 | 0.9801329 | 0.995641 |
| X202175_at | 0.0001 | 0.0039 | 0.025 | 0.9803781 | 0.995782 |
| X220353_at | -0.0001 | 0.0033 | -0.025 | 0.980467 | 0.995782 |
| X206761_at | 0.0002 | 0.0076 | 0.025 | 0.9805849 | 0.995782 |
| X209940_at | -0.0001 | 0.0033 | -0.024 | 0.9806765 | 0.995782 |
| X208608_s_at | -0.0001 | 0.0033 | -0.024 | 0.9806919 | 0.995782 |
| X216074_x_at | -0.0009 | 0.038 | -0.024 | 0.9807336 | 0.995782 |
| X204618_s_at | -0.0005 | 0.0217 | -0.024 | 0.9807689 | 0.995782 |
| X221391_at | -0.0001 | 0.0043 | -0.024 | 0.9807926 | 0.995782 |
| X214126_at | 0.0007 | 0.0269 | 0.024 | 0.9808082 | 0.995782 |
| X215379_x_at | -0.0001 | 0.0031 | -0.024 | 0.9809299 | 0.995782 |
| X205122_at | 0.0013 | 0.0531 | 0.024 | 0.9809567 | 0.995782 |
| X211237_s_at | -0.0001 | 0.0044 | -0.024 | 0.9809785 | 0.995782 |
| X214796_at | -0.0002 | 0.0091 | -0.024 | 0.9810103 | 0.995782 |
| X220600_at | -0.0001 | 0.0045 | -0.024 | 0.9810117 | 0.995782 |
| X205998_x_at | 0.0009 | 0.0372 | 0.024 | 0.9810894 | 0.995782 |
| X215492_x_at | 0.0001 | 0.0053 | 0.024 | 0.9812398 | 0.995782 |
| X201785_at | 0.0036 | 0.1503 | 0.024 | 0.9812919 | 0.995782 |
| X202632_at | -0.0005 | 0.023 | -0.024 | 0.9812951 | 0.995782 |
| X211925_s_at | -0.0018 | 0.0748 | -0.024 | 0.9813004 | 0.995782 |
| X210914_at | -0.0001 | 0.0035 | -0.023 | 0.9814267 | 0.995782 |
| X204585_s_at | 0.0001 | 0.0024 | 0.023 | 0.9814363 | 0.995782 |
| X206778_at | -0.0001 | 0.0034 | -0.023 | 0.9815032 | 0.995782 |
| X207441_at | -0.0002 | 0.0074 | -0.023 | 0.9815255 | 0.995782 |
| X214874_at | 0.0016 | 0.0685 | 0.023 | 0.981554 | 0.995782 |
| X208623_s_at | -0.0015 | 0.0648 | -0.023 | 0.9817249 | 0.995782 |
| X204401_at | 0.0001 | 0.0052 | 0.023 | 0.981758 | 0.995782 |
| X206402_s_at | 0.0001 | 0.0034 | 0.023 | 0.9817895 | 0.995782 |
| X212115_at | -0.0021 | 0.0921 | -0.023 | 0.9817985 | 0.995782 |
| X211997_x_at | -0.0012 | 0.0504 | -0.023 | 0.9818 | 0.995782 |
| X205708_s_at | 0.0011 | 0.0487 | 0.023 | 0.9818044 | 0.995782 |
| X216800_at | 0.0001 | 0.0044 | 0.023 | 0.9818295 | 0.995782 |
| X206946_at | 0.0001 | 0.0025 | 0.023 | 0.98185 | 0.995782 |
| X210604_at | 0.0001 | 0.0045 | 0.023 | 0.9819373 | 0.995782 |
| X216881_x_at | 0.0002 | 0.007 | 0.023 | 0.9819533 | 0.995782 |
| X218594_at | -0.0005 | 0.0224 | -0.023 | 0.9820387 | 0.995782 |
| X209788_s_at | 0.0028 | 0.1243 | 0.023 | 0.9820941 | 0.995782 |
| X219653_at | -0.0014 | 0.0632 | -0.023 | 0.9820975 | 0.995782 |
| X210557_x_at | 0.0001 | 0.0032 | 0.023 | 0.9821373 | 0.995782 |
| X219725_at | 0.0008 | 0.0374 | 0.023 | 0.9821383 | 0.995782 |
| X201352_at | -0.0007 | 0.0302 | -0.023 | 0.9821425 | 0.995782 |
| X221387_at | -0.0001 | 0.0046 | -0.022 | 0.9822438 | 0.995782 |
| X201613_s_at | 0.001 | 0.0466 | 0.022 | 0.9822524 | 0.995782 |
| X211016_x_at | 0.0002 | 0.0067 | 0.022 | 0.9823538 | 0.995782 |
| X208279_s_at | 0.0001 | 0.0034 | 0.022 | 0.982388 | 0.995782 |
| X218296_x_at | -0.0001 | 0.0032 | -0.022 | 0.9824005 | 0.995782 |
| X207374_at | 0.0001 | 0.0056 | 0.022 | 0.982407 | 0.995782 |
| X210797_s_at | -0.0002 | 0.0085 | -0.022 | 0.982462 | 0.995782 |
| X209730_at | -0.0002 | 0.0091 | -0.022 | 0.9825547 | 0.995782 |
| X210531_at | 0.0004 | 0.0204 | 0.022 | 0.9826174 | 0.995782 |
| X218966_at | 0.0018 | 0.082 | 0.022 | 0.9827086 | 0.995782 |
| X210717_at | 0.0002 | 0.0101 | 0.022 | 0.9827854 | 0.995782 |
| X208205_at | -0.0002 | 0.0109 | -0.022 | 0.9827964 | 0.995782 |
| X205474_at | -0.0009 | 0.0412 | -0.022 | 0.9828771 | 0.995782 |
| X214869_x_at | 0.0035 | 0.1633 | 0.022 | 0.9828879 | 0.995782 |
| X220265_at | 0.0001 | 0.0049 | 0.022 | 0.9828985 | 0.995782 |
| X215736_at | 0.0006 | 0.0268 | 0.022 | 0.9829085 | 0.995782 |
| X205040_at | -0.0001 | 0.0032 | -0.022 | 0.982916 | 0.995782 |
| X208718_at | -0.0006 | 0.0298 | -0.022 | 0.9829726 | 0.995782 |
| X207012_at | 0.0002 | 0.0076 | 0.022 | 0.9829758 | 0.995782 |
| X205804_s_at | 0.0001 | 0.0034 | 0.021 | 0.9829903 | 0.995782 |
| X212346_s_at | -0.0006 | 0.0296 | -0.021 | 0.9830619 | 0.995782 |
| X211600_at | -0.0013 | 0.0621 | -0.021 | 0.9830781 | 0.995782 |
| X211469_s_at | 0.0001 | 0.0037 | 0.021 | 0.9831498 | 0.995782 |
| X222319_at | -0.0002 | 0.0094 | -0.021 | 0.9831803 | 0.995782 |
| X211446_at | 0.0001 | 0.0051 | 0.021 | 0.9832302 | 0.995782 |
| X201065_s_at | 0.0008 | 0.0392 | 0.021 | 0.9833035 | 0.995782 |
| X206285_at | -0.0002 | 0.0092 | -0.021 | 0.9833075 | 0.995782 |
| X218117_at | -0.0004 | 0.0212 | -0.021 | 0.9833645 | 0.995782 |
| X219429_at | -0.0024 | 0.1144 | -0.021 | 0.9834051 | 0.995782 |
| X217487_x_at | -0.0032 | 0.1538 | -0.021 | 0.9834095 | 0.995782 |
| X206257_at | 0.0001 | 0.0059 | 0.021 | 0.9835342 | 0.99584 |
| X214835_s_at | 0.0012 | 0.0565 | 0.021 | 0.9835568 | 0.99584 |
| X212764_at | 0.0012 | 0.0568 | 0.021 | 0.9837507 | 0.995876 |
| X215511_at | -0.0001 | 0.0071 | -0.02 | 0.983777 | 0.995876 |
| X216282_x_at | -0.0001 | 0.0044 | -0.02 | 0.9838827 | 0.995876 |
| X219948_x_at | 0.0004 | 0.0199 | 0.02 | 0.9839033 | 0.995876 |
| X205038_at | -0.0001 | 0.0052 | -0.02 | 0.9839621 | 0.995876 |
| X201855_s_at | 0.0007 | 0.035 | 0.02 | 0.9839634 | 0.995876 |
| X210090_at | -0.0001 | 0.0045 | -0.02 | 0.9839872 | 0.995876 |
| X210322_x_at | 0.0001 | 0.0046 | 0.02 | 0.9840949 | 0.995876 |
| X214489_at | 0.0001 | 0.0045 | 0.02 | 0.9840985 | 0.995876 |
| X209557_s_at | -0.0001 | 0.0038 | -0.02 | 0.9841231 | 0.995876 |
| X213649_at | 0.0012 | 0.0599 | 0.02 | 0.9841261 | 0.995876 |
| X221332_at | 0.0001 | 0.0029 | 0.02 | 0.9841306 | 0.995876 |
| X208122_x_at | -0.0001 | 0.0029 | -0.02 | 0.9842834 | 0.995889 |
| X214443_at | -0.0001 | 0.0045 | -0.02 | 0.9843063 | 0.995889 |
| X215039_at | -0.0001 | 0.0049 | -0.02 | 0.9843865 | 0.995889 |
| X219457_s_at | 0.0001 | 0.0037 | 0.02 | 0.9843913 | 0.995889 |
| X208379_x_at | -0.0001 | 0.0042 | -0.02 | 0.9844054 | 0.995889 |
| X206514_s_at | 0.0001 | 0.0061 | 0.02 | 0.9844119 | 0.995889 |
| X205438_at | -0.0001 | 0.0037 | -0.019 | 0.9846393 | 0.996023 |
| X217754_at | 0.0008 | 0.0432 | 0.019 | 0.9846394 | 0.996023 |
| X208389_s_at | -0.0026 | 0.1323 | -0.019 | 0.9846792 | 0.996023 |
| X206525_at | -0.0001 | 0.0059 | -0.019 | 0.984752 | 0.996051 |
| X213109_at | -0.002 | 0.1057 | -0.019 | 0.9849907 | 0.996248 |
| X203074_at | -0.0001 | 0.0048 | -0.019 | 0.9852074 | 0.996288 |
| X220337_at | 0.0001 | 0.0048 | 0.018 | 0.9853625 | 0.996288 |
| X217521_at | -0.0001 | 0.0035 | -0.018 | 0.9853929 | 0.996288 |
| X218564_at | 0.0013 | 0.0683 | 0.018 | 0.9854932 | 0.996288 |
| X217505_at | -0.0002 | 0.013 | -0.018 | 0.9855011 | 0.996288 |
| X200969_at | 0.0008 | 0.0442 | 0.018 | 0.985519 | 0.996288 |
| X200033_at | -0.0007 | 0.0383 | -0.018 | 0.9855651 | 0.996288 |
| X210044_s_at | 0.0001 | 0.0043 | 0.018 | 0.9856275 | 0.996288 |
| X215556_at | 0.0001 | 0.0066 | 0.018 | 0.9857034 | 0.996288 |
| X210881_s_at | 0.0001 | 0.0042 | 0.018 | 0.9857102 | 0.996288 |
| X215938_s_at | 0.0001 | 0.0067 | 0.018 | 0.9857315 | 0.996288 |
| X209632_at | -0.0009 | 0.0507 | -0.018 | 0.9857339 | 0.996288 |
| X201159_s_at | 0.0001 | 0.005 | 0.018 | 0.9857467 | 0.996288 |
| X207725_at | 0.0001 | 0.0051 | 0.018 | 0.9858348 | 0.996288 |
| X217535_at | -0.0002 | 0.0116 | -0.018 | 0.9858876 | 0.996288 |
| X202162_s_at | 0.001 | 0.0563 | 0.018 | 0.9858921 | 0.996288 |
| X205580_s_at | -0.0001 | 0.0034 | -0.018 | 0.9859168 | 0.996288 |
| X214223_at | -0.0002 | 0.0098 | -0.018 | 0.9859569 | 0.996288 |
| X36612_at | -0.0008 | 0.0426 | -0.018 | 0.9859635 | 0.996288 |
| X212074_at | 0.0004 | 0.0249 | 0.018 | 0.9860323 | 0.996288 |
| X211935_at | 0.0004 | 0.024 | 0.018 | 0.9860403 | 0.996288 |
| X203378_at | -0.0008 | 0.0431 | -0.018 | 0.9861096 | 0.996288 |
| X219344_at | 0.0001 | 0.008 | 0.017 | 0.9861526 | 0.996288 |
| X217892_s_at | 0.0009 | 0.0509 | 0.017 | 0.9861962 | 0.996288 |
| X217134_at | -0.0001 | 0.0043 | -0.017 | 0.9862388 | 0.996288 |
| X221755_at | 0.0001 | 0.0041 | 0.017 | 0.9863049 | 0.996288 |
| X210455_at | -0.0001 | 0.0046 | -0.017 | 0.9863247 | 0.996288 |
| X210841_s_at | 0 | 0.0026 | -0.017 | 0.9863631 | 0.996288 |
| X205205_at | 0.0001 | 0.003 | 0.017 | 0.9863911 | 0.996288 |
| X217105_at | -0.0001 | 0.0078 | -0.017 | 0.9864144 | 0.996288 |
| X217360_x_at | -0.0001 | 0.0065 | -0.017 | 0.9864211 | 0.996288 |
| X220523_at | 0.0002 | 0.0129 | 0.017 | 0.9866424 | 0.996342 |
| X217983_s_at | 0.0016 | 0.0966 | 0.017 | 0.9866603 | 0.996342 |
| X204821_at | -0.0016 | 0.0966 | -0.017 | 0.98669 | 0.996342 |
| X215156_at | 0.0005 | 0.028 | 0.017 | 0.9866988 | 0.996342 |
| X209440_at | 0.0007 | 0.0422 | 0.017 | 0.9867719 | 0.996342 |
| X218971_s_at | -0.0001 | 0.0046 | -0.017 | 0.9867843 | 0.996342 |
| X203400_s_at | 0.0023 | 0.1402 | 0.017 | 0.9868026 | 0.996342 |
| X207215_at | -0.0001 | 0.0061 | -0.017 | 0.986865 | 0.996342 |
| X220747_at | -0.0001 | 0.006 | -0.017 | 0.9869133 | 0.996342 |
| X209252_at | 0.0005 | 0.0274 | 0.017 | 0.9869308 | 0.996342 |
| X205649_s_at | -0.0002 | 0.0115 | -0.016 | 0.9869677 | 0.996342 |
| X207077_at | 0.0001 | 0.005 | 0.016 | 0.9870769 | 0.996407 |
| X214520_at | -0.0001 | 0.0038 | -0.016 | 0.9872056 | 0.99649 |
| X208526_at | 0.0001 | 0.0052 | 0.016 | 0.9872496 | 0.99649 |
| X221302_at | 0.0001 | 0.0037 | 0.016 | 0.9872941 | 0.99649 |
| X201825_s_at | 0.0005 | 0.0315 | 0.016 | 0.9873586 | 0.996496 |
| X216540_at | 0.0001 | 0.0057 | 0.016 | 0.9873896 | 0.996496 |
| X211561_x_at | -0.0001 | 0.0039 | -0.016 | 0.987437 | 0.996499 |
| X78330_at | -0.0001 | 0.0039 | -0.016 | 0.9874976 | 0.996514 |
| X208554_at | -0.0001 | 0.0051 | -0.016 | 0.9876277 | 0.996543 |
| X215144_at | 0 | 0.0023 | 0.016 | 0.9877198 | 0.996543 |
| X217355_at | -0.0001 | 0.0038 | -0.016 | 0.9877267 | 0.996543 |
| X210724_at | 0.0001 | 0.0042 | 0.015 | 0.9877481 | 0.996543 |
| X202118_s_at | -0.0028 | 0.1822 | -0.015 | 0.9877498 | 0.996543 |
| X206746_at | 0.0002 | 0.0111 | 0.015 | 0.987945 | 0.996594 |
| X217705_at | -0.0002 | 0.0144 | -0.015 | 0.9879574 | 0.996594 |
| X207479_at | 0.0001 | 0.0067 | 0.015 | 0.9879586 | 0.996594 |
| X215906_at | 0 | 0.0029 | 0.015 | 0.9879804 | 0.996594 |
| X205007_s_at | 0 | 0.0027 | 0.015 | 0.9882762 | 0.996749 |
| X204934_s_at | 0 | 0.0028 | 0.015 | 0.9883013 | 0.996749 |
| X214554_at | 0.0001 | 0.007 | 0.015 | 0.9883707 | 0.996749 |
| X222165_x_at | -0.0005 | 0.0313 | -0.015 | 0.9883752 | 0.996749 |
| X57539_at | 0.0004 | 0.0306 | 0.015 | 0.9883916 | 0.996749 |
| X203276_at | -0.0004 | 0.0248 | -0.015 | 0.9885234 | 0.996749 |
| X206596_s_at | 0 | 0.0032 | -0.014 | 0.9885506 | 0.996749 |
| X207493_x_at | 0.0001 | 0.0045 | 0.014 | 0.988573 | 0.996749 |
| X206550_s_at | -0.0001 | 0.0071 | -0.014 | 0.988575 | 0.996749 |
| X218724_s_at | 0.0001 | 0.0037 | 0.014 | 0.9886246 | 0.996749 |
| X211140_s_at | 0 | 0.003 | 0.014 | 0.988681 | 0.996749 |
| X209094_at | -0.0007 | 0.0492 | -0.014 | 0.9886998 | 0.996749 |
| X215088_s_at | 0.0002 | 0.0171 | 0.014 | 0.9887176 | 0.996749 |
| X210307_s_at | 0.0001 | 0.0059 | 0.014 | 0.9888477 | 0.996788 |
| X221529_s_at | 0 | 0.0035 | -0.014 | 0.9889352 | 0.996788 |
| X201894_s_at | 0.0005 | 0.0383 | 0.014 | 0.9889497 | 0.996788 |
| X210122_at | 0.0001 | 0.0052 | 0.014 | 0.9889621 | 0.996788 |
| X215123_at | -0.0021 | 0.1524 | -0.014 | 0.9890162 | 0.996788 |
| X215862_at | 0 | 0.0031 | -0.014 | 0.9890264 | 0.996788 |
| X221350_at | -0.0001 | 0.0038 | -0.014 | 0.9890862 | 0.996788 |
| X220511_s_at | 0.0001 | 0.0061 | 0.014 | 0.9891651 | 0.996788 |
| X207917_at | 0.0001 | 0.0048 | 0.014 | 0.9891801 | 0.996788 |
| X215901_at | 0.0002 | 0.0145 | 0.014 | 0.9892043 | 0.996788 |
| X208342_x_at | 0 | 0.0024 | -0.014 | 0.9892738 | 0.996813 |
| X206526_at | 0 | 0.0028 | 0.013 | 0.9893763 | 0.996833 |
| X212806_at | 0.0005 | 0.0409 | 0.013 | 0.9893842 | 0.996833 |
| X204809_at | -0.0005 | 0.0362 | -0.013 | 0.9894641 | 0.996853 |
| X211235_s_at | 0 | 0.0027 | -0.013 | 0.9894931 | 0.996853 |
| X213783_at | -0.0001 | 0.004 | -0.013 | 0.989593 | 0.996853 |
| X218848_at | 0.0001 | 0.0041 | 0.013 | 0.9897444 | 0.996853 |
| X220928_s_at | 0 | 0.0023 | 0.013 | 0.9897521 | 0.996853 |
| X220489_s_at | -0.0001 | 0.0049 | -0.013 | 0.9898045 | 0.996853 |
| X207596_at | -0.0001 | 0.0065 | -0.013 | 0.989813 | 0.996853 |
| X211525_s_at | -0.0001 | 0.0081 | -0.013 | 0.9898286 | 0.996853 |
| X219407_s_at | 0 | 0.0025 | 0.013 | 0.9898346 | 0.996853 |
| X215529_x_at | 0.0008 | 0.0637 | 0.013 | 0.9898649 | 0.996853 |
| X213708_s_at | -0.0004 | 0.0342 | -0.013 | 0.9899341 | 0.996853 |
| X201248_s_at | 0 | 0.0033 | -0.013 | 0.9899665 | 0.996853 |
| X217699_at | 0.0001 | 0.007 | 0.013 | 0.990037 | 0.996853 |
| X217253_at | 0.0005 | 0.0407 | 0.013 | 0.9900761 | 0.996853 |
| X200649_at | 0 | 0.0029 | 0.012 | 0.9901158 | 0.996853 |
| X211354_s_at | -0.0001 | 0.0049 | -0.012 | 0.9901885 | 0.996853 |
| X220704_at | 0.0002 | 0.0161 | 0.012 | 0.9901923 | 0.996853 |
| X213356_x_at | -0.0001 | 0.0094 | -0.012 | 0.990229 | 0.996853 |
| X221678_at | -0.0001 | 0.006 | -0.012 | 0.9902566 | 0.996853 |
| X208476_s_at | -0.0001 | 0.0045 | -0.012 | 0.9903687 | 0.996893 |
| X206936_x_at | 0.0006 | 0.0489 | 0.012 | 0.9903859 | 0.996893 |
| X211700_s_at | 0.0013 | 0.1148 | 0.012 | 0.9907341 | 0.997198 |
| X203088_at | 0.0006 | 0.0539 | 0.012 | 0.9908417 | 0.997211 |
| X219959_at | 0 | 0.004 | 0.012 | 0.9908776 | 0.997211 |
| X222372_at | 0.0005 | 0.041 | 0.011 | 0.990904 | 0.997211 |
| X206020_at | -0.0005 | 0.0453 | -0.011 | 0.9909426 | 0.997211 |
| X216142_at | -0.0001 | 0.0059 | -0.011 | 0.9909709 | 0.997211 |
| X219083_at | -0.0003 | 0.0264 | -0.011 | 0.9910485 | 0.997244 |
| X216931_at | 0 | 0.0041 | -0.011 | 0.9912034 | 0.997271 |
| X207200_at | 0.0001 | 0.0052 | 0.011 | 0.9912091 | 0.997271 |
| X206912_at | -0.0001 | 0.0096 | -0.011 | 0.9912105 | 0.997271 |
| X211639_x_at | 0 | 0.0045 | 0.011 | 0.9914156 | 0.997432 |
| X217314_at | -0.0001 | 0.0047 | -0.011 | 0.991506 | 0.997457 |
| X211207_s_at | -0.0008 | 0.077 | -0.011 | 0.9915298 | 0.997457 |
| X216332_at | 0 | 0.0025 | 0.011 | 0.9915974 | 0.99748 |
| X212784_at | 0.0003 | 0.0286 | 0.01 | 0.9916938 | 0.997508 |
| X214202_at | -0.0007 | 0.0623 | -0.01 | 0.9917188 | 0.997508 |
| X208055_s_at | -0.0004 | 0.0427 | -0.01 | 0.9917811 | 0.997508 |
| X211098_x_at | 0.0002 | 0.0161 | 0.01 | 0.9918312 | 0.997508 |
| X209016_s_at | 0 | 0.0027 | -0.01 | 0.9918498 | 0.997508 |
| X217113_at | 0 | 0.0026 | -0.01 | 0.9920124 | 0.997626 |
| X221345_at | 0 | 0.0027 | -0.01 | 0.9921668 | 0.997714 |
| X220713_at | -0.0001 | 0.0055 | -0.01 | 0.9922225 | 0.997714 |
| X222069_s_at | 0 | 0.0041 | -0.01 | 0.9922346 | 0.997714 |
| X205036_at | 0.0002 | 0.0176 | 0.01 | 0.9923553 | 0.99779 |
| X216638_s_at | -0.0001 | 0.0073 | -0.01 | 0.9924181 | 0.997797 |
| X203958_s_at | 0 | 0.0032 | 0.01 | 0.9924516 | 0.997797 |
| X211472_at | 0 | 0.005 | -0.009 | 0.992614 | 0.997878 |
| X210390_s_at | 0 | 0.0052 | 0.009 | 0.992632 | 0.997878 |
| X220725_x_at | 0.0006 | 0.0618 | 0.009 | 0.9926675 | 0.997878 |
| X217644_s_at | 0.0001 | 0.0061 | 0.009 | 0.9927503 | 0.997916 |
| X220853_at | 0.0001 | 0.0057 | 0.009 | 0.9928797 | 0.998001 |
| X201281_at | -0.0002 | 0.0206 | -0.009 | 0.9929847 | 0.998062 |
| X221012_s_at | -0.0003 | 0.0285 | -0.009 | 0.9930359 | 0.998068 |
| X222066_at | -0.0001 | 0.0062 | -0.009 | 0.9931131 | 0.998101 |
| X208085_s_at | 0 | 0.0037 | -0.009 | 0.9931808 | 0.998112 |
| X214340_at | 0 | 0.0032 | -0.009 | 0.9932147 | 0.998112 |
| X211280_s_at | 0 | 0.0058 | -0.008 | 0.9933504 | 0.998204 |
| X214349_at | 0 | 0.0041 | 0.008 | 0.9934004 | 0.998209 |
| X214706_at | 0.0003 | 0.0316 | 0.008 | 0.9934485 | 0.998212 |
| X202081_at | -0.0006 | 0.0719 | -0.008 | 0.9935159 | 0.998234 |
| X205864_at | 0 | 0.0032 | 0.008 | 0.9935765 | 0.99825 |
| X207373_at | 0 | 0.0049 | 0.008 | 0.9936765 | 0.998305 |
| X220041_at | 0.0005 | 0.069 | 0.008 | 0.9937446 | 0.998306 |
| X220618_s_at | 0 | 0.0056 | 0.008 | 0.9938326 | 0.998306 |
| X219847_at | 0 | 0.0049 | -0.008 | 0.9939395 | 0.998306 |
| X221285_at | 0 | 0.0035 | 0.008 | 0.9940203 | 0.998306 |
| X205688_at | -0.0001 | 0.0075 | -0.007 | 0.9941001 | 0.998306 |
| X212610_at | -0.0002 | 0.0275 | -0.007 | 0.9941078 | 0.998306 |
| X204866_at | -0.0004 | 0.0616 | -0.007 | 0.9942346 | 0.998306 |
| X218523_at | 0.0006 | 0.0785 | 0.007 | 0.9942404 | 0.998306 |
| X204579_at | 0 | 0.0034 | -0.007 | 0.9942439 | 0.998306 |
| X209341_s_at | -0.0004 | 0.0492 | -0.007 | 0.9942632 | 0.998306 |
| X204880_at | -0.0002 | 0.0294 | -0.007 | 0.9942765 | 0.998306 |
| X222193_at | -0.0001 | 0.0072 | -0.007 | 0.994348 | 0.998306 |
| X41047_at | 0.0003 | 0.0475 | 0.007 | 0.9944625 | 0.998306 |
| X211533_at | 0 | 0.0067 | 0.007 | 0.9944635 | 0.998306 |
| X215128_at | 0.0005 | 0.0658 | 0.007 | 0.9944645 | 0.998306 |
| X219852_s_at | 0 | 0.0043 | 0.007 | 0.9945155 | 0.998306 |
| X209720_s_at | -0.0001 | 0.013 | -0.007 | 0.9945452 | 0.998306 |
| X215192_at | -0.0001 | 0.0073 | -0.007 | 0.9945726 | 0.998306 |
| X211789_s_at | 0 | 0.0032 | -0.007 | 0.9945908 | 0.998306 |
| X216537_s_at | 0 | 0.0053 | -0.007 | 0.994649 | 0.998306 |
| X217437_s_at | -0.0002 | 0.036 | -0.007 | 0.9946907 | 0.998306 |
| X213146_at | 0 | 0.0066 | -0.007 | 0.9947068 | 0.998306 |
| X205497_at | -0.0002 | 0.0281 | -0.007 | 0.9947105 | 0.998306 |
| X203789_s_at | -0.0012 | 0.1782 | -0.007 | 0.9947999 | 0.99835 |
| X210246_s_at | 0.0004 | 0.0573 | 0.006 | 0.9949433 | 0.998449 |
| X208225_at | 0 | 0.0045 | 0.006 | 0.995037 | 0.998498 |
| X218247_s_at | -0.0003 | 0.0509 | -0.006 | 0.995083 | 0.998499 |
| X214360_at | 0 | 0.0035 | -0.006 | 0.9952349 | 0.998606 |
| X220696_at | 0.0001 | 0.009 | 0.006 | 0.995321 | 0.998648 |
| X210622_x_at | 0 | 0.0029 | -0.006 | 0.9954187 | 0.99868 |
| X218373_at | -0.0001 | 0.0193 | -0.006 | 0.9954691 | 0.99868 |
| X210258_at | -0.0001 | 0.0159 | -0.006 | 0.9954881 | 0.99868 |
| X212390_at | -0.0002 | 0.0429 | -0.005 | 0.9956945 | 0.998842 |
| X221830_at | 0.0004 | 0.0768 | 0.005 | 0.9957781 | 0.998881 |
| X221456_at | 0 | 0.0029 | 0.005 | 0.9959204 | 0.998943 |
| X210615_at | 0 | 0.007 | -0.005 | 0.9959488 | 0.998943 |
| X209676_at | -0.0001 | 0.0118 | -0.005 | 0.9959824 | 0.998943 |
| X205319_at | 0 | 0.0055 | -0.005 | 0.9961863 | 0.998943 |
| X210110_x_at | -0.0001 | 0.0286 | -0.005 | 0.9962354 | 0.998943 |
| X216162_at | 0 | 0.0073 | 0.005 | 0.9962626 | 0.998943 |
| X212466_at | 0 | 0.0036 | 0.005 | 0.9963146 | 0.998943 |
| X208506_at | 0 | 0.0046 | 0.005 | 0.9964041 | 0.998943 |
| X214658_at | 0.0003 | 0.0595 | 0.005 | 0.996414 | 0.998943 |
| X212514_x_at | 0.0002 | 0.0529 | 0.005 | 0.9964293 | 0.998943 |
| X204884_s_at | 0.0001 | 0.0136 | 0.005 | 0.9964328 | 0.998943 |
| X208324_at | 0 | 0.0089 | -0.004 | 0.9964481 | 0.998943 |
| X208979_at | -0.0001 | 0.0281 | -0.004 | 0.9964611 | 0.998943 |
| X211369_at | 0 | 0.0091 | 0.004 | 0.9964698 | 0.998943 |
| X203636_at | 0.0003 | 0.0804 | 0.004 | 0.9965856 | 0.999014 |
| X216386_at | 0 | 0.0043 | -0.004 | 0.9966878 | 0.999058 |
| X221188_s_at | 0 | 0.0051 | -0.004 | 0.9967188 | 0.999058 |
| X220222_at | 0 | 0.0035 | 0.004 | 0.996801 | 0.999095 |
| X205733_at | 0 | 0.0056 | -0.004 | 0.9969053 | 0.99911 |
| X208223_s_at | 0 | 0.0101 | -0.004 | 0.9969064 | 0.99911 |
| X220088_at | -0.0001 | 0.0157 | -0.004 | 0.9969757 | 0.999135 |
| X210865_at | 0 | 0.0039 | -0.004 | 0.997076 | 0.99919 |
| X217694_at | 0 | 0.0086 | 0.004 | 0.997181 | 0.999205 |
| X216509_x_at | 0 | 0.0082 | -0.004 | 0.9972147 | 0.999205 |
| X206426_at | 0 | 0.0075 | -0.003 | 0.9972544 | 0.999205 |
| X201326_at | -0.0001 | 0.03 | -0.003 | 0.9972703 | 0.999205 |
| X207287_at | 0 | 0.0037 | 0.003 | 0.9973703 | 0.99926 |
| X215650_at | 0 | 0.0042 | -0.003 | 0.9976178 | 0.999398 |
| X220391_at | -0.0002 | 0.0518 | -0.003 | 0.9976488 | 0.999398 |
| X216860_s_at | 0 | 0.0022 | 0.003 | 0.9976798 | 0.999398 |
| X218713_at | -0.0001 | 0.0359 | -0.003 | 0.9976879 | 0.999398 |
| X219692_at | 0 | 0.0035 | 0.003 | 0.9978606 | 0.999525 |
| X212865_s_at | 0 | 0.0136 | 0.003 | 0.997986 | 0.999533 |
| X202503_s_at | 0 | 0.0068 | -0.002 | 0.9980416 | 0.999533 |
| X221301_at | 0 | 0.0033 | -0.002 | 0.9981081 | 0.999533 |
| X53987_at | 0.0001 | 0.0415 | 0.002 | 0.9982978 | 0.999533 |
| X215838_at | 0 | 0.0039 | -0.002 | 0.9983003 | 0.999533 |
| X211573_x_at | 0 | 0.0029 | -0.002 | 0.998331 | 0.999533 |
| X220067_at | 0 | 0.0041 | -0.002 | 0.9983314 | 0.999533 |
| X217544_at | 0 | 0.0081 | 0.002 | 0.9983579 | 0.999533 |
| X207115_x_at | 0.0001 | 0.0499 | 0.002 | 0.9983611 | 0.999533 |
| X211627_x_at | 0.0001 | 0.0274 | 0.002 | 0.9983957 | 0.999533 |
| X221284_s_at | 0 | 0.0044 | 0.002 | 0.9984103 | 0.999533 |
| X217017_at | 0 | 0.0048 | 0.002 | 0.9984624 | 0.999533 |
| X222377_at | 0 | 0.003 | 0.002 | 0.9984952 | 0.999533 |
| X217298_at | 0 | 0.0035 | -0.002 | 0.9984978 | 0.999533 |
| X213048_s_at | -0.0001 | 0.046 | -0.002 | 0.9985707 | 0.999561 |
| X220896_at | 0 | 0.0038 | 0.002 | 0.9986387 | 0.999584 |
| X207042_at | 0 | 0.0033 | -0.002 | 0.9987239 | 0.999615 |
| X211253_x_at | 0 | 0.0027 | -0.002 | 0.9987604 | 0.999615 |
| X217670_at | 0 | 0.0062 | 0.001 | 0.9988301 | 0.999639 |
| X205115_s_at | 0 | 0.0062 | -0.001 | 0.998874 | 0.999639 |
| X220729_at | 0 | 0.0093 | 0.001 | 0.9989198 | 0.99964 |
| X220343_at | 0 | 0.0053 | -0.001 | 0.9989953 | 0.999646 |
| X212772_s_at | 0.0001 | 0.0457 | 0.001 | 0.9990162 | 0.999646 |
| X212066_s_at | 0 | 0.025 | 0.001 | 0.9990671 | 0.999652 |
| X210178_x_at | 0 | 0.0469 | 0.001 | 0.999162 | 0.999684 |
| X221837_at | 0 | 0.004 | -0.001 | 0.9991888 | 0.999684 |
| X220389_at | 0 | 0.0045 | 0.001 | 0.9992908 | 0.999741 |
| X209358_at | 0 | 0.0293 | -0.001 | 0.9994151 | 0.999743 |
| X210158_at | 0 | 0.0022 | -0.001 | 0.9994847 | 0.999743 |
| X203772_at | 0 | 0.0056 | 0.001 | 0.9994948 | 0.999743 |
| X210548_at | 0 | 0.0036 | -0.001 | 0.9995006 | 0.999743 |
| X218169_at | 0 | 0.0058 | -0.001 | 0.9996039 | 0.999743 |
| X206342_x_at | 0 | 0.0497 | 0 | 0.9996382 | 0.999743 |
| X215442_s_at | 0 | 0.0107 | 0 | 0.9996529 | 0.999743 |
| X219839_x_at | 0 | 0.0037 | 0 | 0.9996533 | 0.999743 |
| X208521_at | 0 | 0.0055 | 0 | 0.9997434 | 0.999788 |
| X217384_x_at | 0 | 0.0102 | 0 | 0.999887 | 0.999887 |

(B)

| Probe-ID | b(i) | SE | T-Value | P-Values | q-value |
| --- | --- | --- | --- | --- | --- |
| hsa-miR-1538-st | 0.2693 | 0.0571 | 4.719 | 0 | 0 |
| hsa-miR-371-5p-st | 0.3292 | 0.0525 | 6.267 | 0 | 0 |
| hsa-miR-4763-5p-st | 0.378 | 0.0797 | 4.745 | 0 | 0 |
| hsa-miR-129-star-st | -0.2791 | 0.0644 | -4.336 | 0.0001 | 0.02418659 |
| hsa-miR-378c-st | -0.148 | 0.0342 | -4.328 | 0.0001 | 0.02418659 |
| hsa-miR-448-st | -0.3092 | 0.071 | -4.355 | 0.0001 | 0.02418659 |
| hsa-miR-154-st | -0.1668 | 0.0399 | -4.182 | 0.0002 | 0.02418659 |
| hsa-miR-1972-st | 0.4924 | 0.1146 | 4.295 | 0.0002 | 0.02418659 |
| hsa-miR-4295-st | 0.0595 | 0.0141 | 4.222 | 0.0002 | 0.02418659 |
| hsa-miR-4682-st | 0.2041 | 0.0488 | 4.182 | 0.0002 | 0.02418659 |
| hsa-miR-516a-5p-st | -0.2182 | 0.0519 | -4.207 | 0.0002 | 0.02418659 |
| hsa-miR-543-st | -0.2267 | 0.0537 | -4.219 | 0.0002 | 0.02418659 |
| hsa-miR-193a-3p-st | 0.4244 | 0.1044 | 4.063 | 0.0003 | 0.03348912 |
| hsa-miR-3689c-st | -0.1384 | 0.0352 | -3.937 | 0.0004 | 0.04146272 |
| hsa-miR-378f-st | -0.1284 | 0.0331 | -3.874 | 0.0005 | 0.04837318 |
| hsa-miR-4724-5p-st | 0.5134 | 0.1344 | 3.82 | 0.0006 | 0.05121866 |
| hsa-miR-523-st | -0.0737 | 0.0193 | -3.816 | 0.0006 | 0.05121866 |
| hsa-miR-614-st | -0.0831 | 0.0221 | -3.75 | 0.0007 | 0.05643537 |
| hsa-miR-1247-st | -0.5419 | 0.1477 | -3.67 | 0.0009 | 0.06219408 |
| hsa-miR-1273d-st | 0.2345 | 0.0637 | 3.683 | 0.0009 | 0.06219408 |
| hsa-miR-516b-st | -0.3971 | 0.1081 | -3.672 | 0.0009 | 0.06219408 |
| hsa-miR-4436b-3p-st | 0.187 | 0.0522 | 3.582 | 0.0011 | 0.06940499 |
| hsa-miR-4720-3p-st | -0.0892 | 0.0248 | -3.592 | 0.0011 | 0.06940499 |
| hsa-miR-3119-st | -0.0836 | 0.0234 | -3.581 | 0.0012 | 0.07255976 |
| hsa-miR-1283-st | -0.0505 | 0.0143 | -3.539 | 0.0013 | 0.07546215 |
| hsa-miR-330-5p-st | -0.1398 | 0.0404 | -3.456 | 0.0016 | 0.08507007 |
| hsa-miR-487a-st | -0.1725 | 0.0499 | -3.455 | 0.0016 | 0.08507007 |
| hsa-miR-335-star-st | -0.1968 | 0.0574 | -3.426 | 0.0017 | 0.08507007 |
| hsa-miR-34c-3p-st | 0.64 | 0.1859 | 3.442 | 0.0017 | 0.08507007 |
| hsa-miR-3160-5p-st | 0.0989 | 0.0296 | 3.347 | 0.0022 | 0.1052117 |
| hsa-miR-421-st | -0.1291 | 0.039 | -3.31 | 0.0024 | 0.1052117 |
| hsa-miR-3653-st | -0.0635 | 0.0193 | -3.283 | 0.0025 | 0.1052117 |
| hsa-miR-802-st | 0.0742 | 0.0225 | 3.296 | 0.0025 | 0.1052117 |
| hsa-miR-1291-st | -0.1667 | 0.0509 | -3.279 | 0.0026 | 0.1052117 |
| hsa-miR-498-st | 0.3646 | 0.1112 | 3.278 | 0.0026 | 0.1052117 |
| hsa-miR-558-st | 0.094 | 0.0289 | 3.254 | 0.0027 | 0.1052117 |
| hsa-miR-378-st | -0.162 | 0.0499 | -3.247 | 0.0028 | 0.1052117 |
| hsa-miR-3169-st | -0.0916 | 0.0284 | -3.229 | 0.0029 | 0.1052117 |
| hsa-miR-3183-st | 0.0858 | 0.0265 | 3.24 | 0.0029 | 0.1052117 |
| hsa-miR-4539-st | 0.2921 | 0.0903 | 3.234 | 0.0029 | 0.1052117 |
| hsa-miR-4270-st | 0.2895 | 0.0904 | 3.202 | 0.0031 | 0.1096459 |
| hsa-miR-4438-st | -0.1104 | 0.0347 | -3.182 | 0.0033 | 0.1096459 |
| hsa-miR-575-st | 0.3611 | 0.1136 | 3.179 | 0.0033 | 0.1096459 |
| hsa-miR-3619-3p-st | -0.198 | 0.0624 | -3.175 | 0.0034 | 0.1096459 |
| hsa-miR-4281-st | 0.2818 | 0.0889 | 3.17 | 0.0034 | 0.1096459 |
| hsa-miR-4788-st | 0.1952 | 0.0621 | 3.144 | 0.0037 | 0.1167266 |
| hsa-miR-10a-star-st | 0.0872 | 0.0279 | 3.12 | 0.0039 | 0.1204183 |
| hsa-miR-4278-st | 0.0589 | 0.0192 | 3.072 | 0.0044 | 0.1330262 |
| hsa-miR-4784-st | 0.171 | 0.0558 | 3.064 | 0.0045 | 0.133273 |
| hsa-miR-34c-5p-st | 0.8333 | 0.2738 | 3.043 | 0.0047 | 0.1364124 |
| hsa-miR-4748-st | 0.1649 | 0.0543 | 3.034 | 0.0049 | 0.1369052 |
| hsa-miR-1258-st | 0.2283 | 0.0756 | 3.021 | 0.005 | 0.1369052 |
| hsa-miR-1912-st | -0.2176 | 0.072 | -3.022 | 0.005 | 0.1369052 |
| hsa-miR-34b-star-st | 0.8309 | 0.2776 | 2.993 | 0.0054 | 0.1426599 |
| hsa-miR-424-st | -0.2492 | 0.0836 | -2.979 | 0.0056 | 0.1426599 |
| hsa-miR-431-st | -0.129 | 0.0433 | -2.978 | 0.0056 | 0.1426599 |
| hsa-miR-3200-3p-st | -0.1364 | 0.046 | -2.965 | 0.0058 | 0.1426599 |
| hsa-miR-33b-st | -0.0612 | 0.0206 | -2.965 | 0.0058 | 0.1426599 |
| hsa-miR-616-star-st | -0.1183 | 0.0399 | -2.965 | 0.0058 | 0.1426599 |
| hsa-miR-517a-st | -0.3381 | 0.1143 | -2.958 | 0.0059 | 0.1427009 |
| hsa-miR-129-3p-st | -0.1854 | 0.0628 | -2.953 | 0.006 | 0.1427405 |
| hsa-miR-941-st | 0.1631 | 0.0556 | 2.933 | 0.0063 | 0.1474602 |
| hsa-miR-4760-3p-st | -0.0829 | 0.0287 | -2.886 | 0.007 | 0.1573831 |
| hsa-miR-1273e-st | 0.3226 | 0.1118 | 2.885 | 0.0071 | 0.1573831 |
| hsa-miR-3934-st | 0.2228 | 0.0773 | 2.881 | 0.0071 | 0.1573831 |
| hsa-miR-4786-3p-st | 0.1838 | 0.064 | 2.872 | 0.0073 | 0.1573831 |
| hsa-miR-3074-5p-st | 0.2158 | 0.0754 | 2.861 | 0.0075 | 0.1573831 |
| hsa-miR-3123-st | -0.0521 | 0.0182 | -2.863 | 0.0075 | 0.1573831 |
| hsa-miR-623-st | 0.2269 | 0.0795 | 2.855 | 0.0076 | 0.1573831 |
| hsa-miR-15a-star-st | 0.1755 | 0.0616 | 2.85 | 0.0077 | 0.1573831 |
| hsa-miR-518e-st | -0.0949 | 0.0333 | -2.85 | 0.0077 | 0.1573831 |
| hsa-miR-525-5p-st | 0.1101 | 0.039 | 2.824 | 0.0082 | 0.165275 |
| hsa-miR-373-star-st | 0.4281 | 0.1523 | 2.81 | 0.0085 | 0.1664037 |
| hsa-miR-601-st | -0.1356 | 0.0482 | -2.812 | 0.0085 | 0.1664037 |
| hsa-miR-720-st | 0.4314 | 0.1538 | 2.805 | 0.0086 | 0.1664037 |
| hsa-miR-381-st | -0.087 | 0.0311 | -2.794 | 0.0089 | 0.1699426 |
| hsa-miR-3189-5p-st | -0.123 | 0.0442 | -2.781 | 0.0091 | 0.1715049 |
| hsa-miR-631-st | 0.1702 | 0.0615 | 2.766 | 0.0095 | 0.1767481 |
| hsa-miR-378e-st | -0.2157 | 0.0788 | -2.738 | 0.0102 | 0.1873695 |
| hsa-miR-2355-5p-st | -0.0804 | 0.0295 | -2.723 | 0.0105 | 0.1899095 |
| hsa-let-7a-star-st | -0.0517 | 0.019 | -2.721 | 0.0106 | 0.1899095 |
| hsa-miR-377-star-st | -0.1441 | 0.0532 | -2.71 | 0.0109 | 0.1929028 |
| hsa-miR-4307-st | -0.0658 | 0.0244 | -2.698 | 0.0112 | 0.1958239 |
| hsa-miR-4713-5p-st | 0.1633 | 0.061 | 2.677 | 0.0118 | 0.2008049 |
| hsa-miR-4726-3p-st | 0.1998 | 0.0746 | 2.678 | 0.0118 | 0.2008049 |
| hsa-miR-4656-st | 0.2696 | 0.1009 | 2.671 | 0.0119 | 0.2008049 |
| hsa-miR-4636-st | -0.0778 | 0.0293 | -2.658 | 0.0123 | 0.205169 |
| hsa-miR-34b-st | 0.527 | 0.2003 | 2.63 | 0.0132 | 0.2144544 |
| hsa-miR-4693-5p-st | 0.0476 | 0.0181 | 2.628 | 0.0132 | 0.2144544 |
| hsa-miR-376a-star-st | -0.0847 | 0.0323 | -2.626 | 0.0133 | 0.2144544 |
| hsa-miR-3679-5p-st | 0.1824 | 0.0698 | 2.614 | 0.0137 | 0.2169155 |
| hsa-miR-212-st | -0.1855 | 0.0711 | -2.608 | 0.0139 | 0.2169155 |
| hsa-miR-4273-st | -0.1307 | 0.0502 | -2.604 | 0.014 | 0.2169155 |
| hsa-miR-4762-5p-st | -0.0764 | 0.0294 | -2.601 | 0.0141 | 0.2169155 |
| hsa-miR-224-star-st | 0.2752 | 0.1059 | 2.599 | 0.0142 | 0.2169155 |
| hsa-miR-32-st | -0.0954 | 0.037 | -2.58 | 0.0148 | 0.2212016 |
| hsa-miR-523-star-st | -0.1904 | 0.0739 | -2.575 | 0.015 | 0.2212016 |
| hsa-miR-132-st | -0.095 | 0.0369 | -2.572 | 0.0151 | 0.2212016 |
| hsa-miR-3648-st | 0.2417 | 0.0939 | 2.574 | 0.0151 | 0.2212016 |
| hsa-miR-4771-st | -0.0678 | 0.0264 | -2.566 | 0.0154 | 0.2212016 |
| hsa-miR-494-st | -0.0795 | 0.031 | -2.562 | 0.0155 | 0.2212016 |
| hsa-miR-339-5p-st | 0.1638 | 0.0641 | 2.555 | 0.0157 | 0.2212016 |
| hsa-miR-3618-st | -0.0526 | 0.0206 | -2.556 | 0.0157 | 0.2212016 |
| hsa-miR-3166-st | -0.0581 | 0.0228 | -2.551 | 0.0159 | 0.2218654 |
| hsa-miR-1282-st | -0.0527 | 0.0208 | -2.535 | 0.0165 | 0.2254536 |
| hsa-miR-21-star-st | 0.1427 | 0.0563 | 2.534 | 0.0165 | 0.2254536 |
| hsa-miR-495-st | -0.1579 | 0.0625 | -2.526 | 0.0169 | 0.2254536 |
| hsa-miR-501-5p-st | -0.1175 | 0.0466 | -2.524 | 0.0169 | 0.2254536 |
| hsa-miR-483-3p-st | -0.1684 | 0.0669 | -2.518 | 0.0172 | 0.2254536 |
| hsa-miR-3657-st | -0.0474 | 0.0189 | -2.513 | 0.0174 | 0.2254536 |
| hsa-miR-514-st | 0.0652 | 0.0259 | 2.513 | 0.0174 | 0.2254536 |
| hsa-miR-639-st | 0.1521 | 0.0605 | 2.512 | 0.0174 | 0.2254536 |
| hsa-miR-99a-st | 0.0634 | 0.0253 | 2.508 | 0.0176 | 0.2260269 |
| hsa-miR-4266-st | -0.0642 | 0.0258 | -2.493 | 0.0182 | 0.2316821 |
| hsa-miR-3664-5p-st | 0.0538 | 0.0217 | 2.483 | 0.0187 | 0.2317736 |
| hsa-miR-323-3p-st | -0.1107 | 0.0446 | -2.479 | 0.0188 | 0.2317736 |
| hsa-miR-380-st | -0.1134 | 0.0459 | -2.469 | 0.0192 | 0.2317736 |
| hsa-miR-1286-st | 0.1313 | 0.0532 | 2.467 | 0.0193 | 0.2317736 |
| hsa-miR-374b-star-st | -0.0588 | 0.0239 | -2.466 | 0.0194 | 0.2317736 |
| hsa-miR-4532-st | 0.3169 | 0.1286 | 2.465 | 0.0194 | 0.2317736 |
| hsa-miR-1264-st | -0.2177 | 0.0885 | -2.46 | 0.0197 | 0.2317736 |
| hsa-miR-4745-3p-st | -0.0745 | 0.0304 | -2.451 | 0.0201 | 0.2317736 |
| hsa-miR-4502-st | 0.08 | 0.0327 | 2.448 | 0.0202 | 0.2317736 |
| hsa-miR-628-3p-st | -0.086 | 0.0351 | -2.449 | 0.0202 | 0.2317736 |
| hsa-miR-145-star-st | 0.1641 | 0.0673 | 2.437 | 0.0207 | 0.2317736 |
| hsa-miR-95-st | -0.2344 | 0.0961 | -2.438 | 0.0207 | 0.2317736 |
| hsa-miR-4516-st | 0.1834 | 0.0753 | 2.436 | 0.0208 | 0.2317736 |
| hsa-miR-449c-st | -0.2853 | 0.1176 | -2.426 | 0.0213 | 0.2317736 |
| hsa-miR-485-3p-st | -0.145 | 0.0598 | -2.426 | 0.0213 | 0.2317736 |
| hsa-miR-4311-st | -0.0759 | 0.0314 | -2.419 | 0.0216 | 0.2317736 |
| hsa-miR-4758-5p-st | 0.2336 | 0.0965 | 2.42 | 0.0216 | 0.2317736 |
| hsa-miR-487b-st | -0.0923 | 0.0382 | -2.415 | 0.0218 | 0.2317736 |
| hsa-miR-378-star-st | -0.1351 | 0.056 | -2.414 | 0.0219 | 0.2317736 |
| hsa-miR-4703-3p-st | 0.0653 | 0.0271 | 2.414 | 0.0219 | 0.2317736 |
| hsa-miR-3607-5p-st | -0.1482 | 0.0615 | -2.41 | 0.0221 | 0.2317736 |
| hsa-miR-373-st | 0.2039 | 0.0846 | 2.409 | 0.0221 | 0.2317736 |
| hsa-miR-4315-st | 0.0597 | 0.0248 | 2.409 | 0.0221 | 0.2317736 |
| hsa-miR-4446-5p-st | -0.114 | 0.0473 | -2.41 | 0.0221 | 0.2317736 |
| hsa-miR-4793-3p-st | 0.3976 | 0.1651 | 2.408 | 0.0222 | 0.2317736 |
| hsa-miR-620-st | -0.059 | 0.0246 | -2.397 | 0.0227 | 0.2353009 |
| hsa-miR-3132-st | 0.1087 | 0.0456 | 2.384 | 0.0234 | 0.2384831 |
| hsa-miR-555-st | -0.0651 | 0.0273 | -2.385 | 0.0234 | 0.2384831 |
| hsa-miR-4689-st | 0.2712 | 0.1138 | 2.383 | 0.0235 | 0.2384831 |
| hsa-miR-378g-st | -0.1563 | 0.0658 | -2.377 | 0.0238 | 0.2398503 |
| hsa-miR-302f-st | 0.0554 | 0.0233 | 2.374 | 0.024 | 0.2401978 |
| hsa-miR-1289-st | -0.0688 | 0.0291 | -2.367 | 0.0243 | 0.2415346 |
| hsa-miR-646-st | -0.0661 | 0.028 | -2.358 | 0.0248 | 0.2425152 |
| hsa-miR-939-st | 0.2278 | 0.0965 | 2.36 | 0.0248 | 0.2425152 |
| hsa-miR-4716-3p-st | 0.144 | 0.0611 | 2.358 | 0.0249 | 0.2425152 |
| hsa-miR-375-st | 0.504 | 0.2143 | 2.352 | 0.0252 | 0.2438008 |
| hsa-miR-1180-st | -0.1861 | 0.0793 | -2.348 | 0.0255 | 0.2450694 |
| hsa-miR-4436b-5p-st | 0.212 | 0.0906 | 2.34 | 0.0259 | 0.247276 |
| hsa-miR-3192-st | 0.1423 | 0.061 | 2.332 | 0.0264 | 0.2482548 |
| hsa-miR-3176-st | 0.1528 | 0.0656 | 2.328 | 0.0266 | 0.2482548 |
| hsa-miR-4454-st | 0.192 | 0.0825 | 2.326 | 0.0267 | 0.2482548 |
| hsa-miR-4497-st | 0.15 | 0.0645 | 2.325 | 0.0268 | 0.2482548 |
| hsa-miR-222-star-st | 0.0769 | 0.0331 | 2.32 | 0.0271 | 0.2482548 |
| hsa-miR-383-st | -0.1584 | 0.0683 | -2.318 | 0.0272 | 0.2482548 |
| hsa-miR-665-st | 0.2662 | 0.1148 | 2.318 | 0.0272 | 0.2482548 |
| hsa-miR-4701-3p-st | 0.1935 | 0.0837 | 2.313 | 0.0275 | 0.2490323 |
| hsa-miR-105-st | 0.1544 | 0.0669 | 2.309 | 0.0278 | 0.2490323 |
| hsa-miR-4419a-st | 0.1925 | 0.0834 | 2.308 | 0.0278 | 0.2490323 |
| hsa-miR-184-st | 0.1463 | 0.0636 | 2.3 | 0.0283 | 0.249151 |
| hsa-miR-4652-3p-st | 0.0829 | 0.036 | 2.301 | 0.0283 | 0.249151 |
| hsa-miR-1253-st | 0.0914 | 0.0397 | 2.299 | 0.0284 | 0.249151 |
| hsa-miR-30b-star-st | -0.1448 | 0.063 | -2.298 | 0.0285 | 0.249151 |
| hsa-miR-4633-5p-st | -0.0654 | 0.0285 | -2.294 | 0.0287 | 0.249397 |
| hsa-miR-4666-3p-st | 0.0675 | 0.0295 | 2.291 | 0.0289 | 0.2496401 |
| hsa-miR-4707-3p-st | 0.1041 | 0.0458 | 2.272 | 0.0302 | 0.2556466 |
| hsa-miR-571-st | 0.059 | 0.026 | 2.271 | 0.0302 | 0.2556466 |
| hsa-miR-608-st | -0.2033 | 0.0895 | -2.272 | 0.0302 | 0.2556466 |
| hsa-miR-4651-st | 0.2282 | 0.1005 | 2.271 | 0.0303 | 0.2556466 |
| hsa-miR-455-5p-st | 0.2618 | 0.1156 | 2.263 | 0.0308 | 0.2568693 |
| hsa-miR-518f-star-st | -0.1013 | 0.0449 | -2.256 | 0.0312 | 0.2568693 |
| hsa-miR-4734-st | 0.2079 | 0.0922 | 2.255 | 0.0313 | 0.2568693 |
| hsa-miR-629-star-st | 0.164 | 0.0729 | 2.249 | 0.0318 | 0.2568693 |
| hsa-miR-148a-st | -0.1402 | 0.0624 | -2.247 | 0.0319 | 0.2568693 |
| hsa-miR-141-st | -0.0966 | 0.0431 | -2.244 | 0.0321 | 0.2568693 |
| hsa-miR-3193-st | -0.1945 | 0.0866 | -2.245 | 0.0321 | 0.2568693 |
| hsa-miR-577-st | 0.0507 | 0.0226 | 2.244 | 0.0321 | 0.2568693 |
| hsa-miR-711-st | 0.2068 | 0.0923 | 2.239 | 0.0325 | 0.2568693 |
| hsa-miR-301b-st | 0.077 | 0.0344 | 2.237 | 0.0326 | 0.2568693 |
| hsa-miR-1273-st | 0.0737 | 0.033 | 2.236 | 0.0327 | 0.2568693 |
| hsa-miR-3606-st | -0.0534 | 0.0239 | -2.233 | 0.0329 | 0.2568693 |
| hsa-miR-4783-3p-st | 0.2839 | 0.1271 | 2.234 | 0.0329 | 0.2568693 |
| hsa-miR-4639-5p-st | -0.0814 | 0.0365 | -2.231 | 0.033 | 0.2568693 |
| hsa-miR-103b-st | 0.3302 | 0.148 | 2.231 | 0.0331 | 0.2568693 |
| hsa-miR-624-star-st | -0.0664 | 0.0298 | -2.225 | 0.0335 | 0.2579903 |
| hsa-miR-3916-st | -0.1378 | 0.062 | -2.223 | 0.0336 | 0.2579903 |
| hsa-miR-449b-st | -0.2995 | 0.1349 | -2.221 | 0.0338 | 0.25816 |
| hsa-miR-452-st | 0.2317 | 0.1046 | 2.215 | 0.0343 | 0.2606073 |
| hsa-miR-96-star-st | -0.0591 | 0.0268 | -2.209 | 0.0347 | 0.2622733 |
| hsa-miR-1292-st | -0.1969 | 0.0896 | -2.198 | 0.0355 | 0.2655538 |
| hsa-miR-4655-5p-st | 0.2032 | 0.0924 | 2.199 | 0.0355 | 0.2655538 |
| hsa-miR-3065-3p-st | 0.1633 | 0.0745 | 2.192 | 0.036 | 0.2665461 |
| hsa-miR-4481-st | 0.2089 | 0.0953 | 2.192 | 0.036 | 0.2665461 |
| hsa-miR-2861-st | 0.1715 | 0.0783 | 2.189 | 0.0362 | 0.2666663 |
| hsa-miR-5095-st | 0.301 | 0.1378 | 2.184 | 0.0367 | 0.2689842 |
| hsa-miR-3164-st | 0.0705 | 0.0323 | 2.181 | 0.0369 | 0.269091 |
| hsa-miR-4756-5p-st | -0.0689 | 0.0317 | -2.175 | 0.0373 | 0.2706479 |
| hsa-miR-3605-5p-st | 0.141 | 0.065 | 2.167 | 0.038 | 0.2723672 |
| hsa-miR-4423-3p-st | 0.1693 | 0.0781 | 2.167 | 0.038 | 0.2723672 |
| hsa-miR-1207-5p-st | 0.1857 | 0.0857 | 2.166 | 0.0381 | 0.2723672 |
| hsa-miR-658-st | -0.0624 | 0.0289 | -2.155 | 0.039 | 0.2774344 |
| hsa-miR-148a-star-st | -0.1574 | 0.0732 | -2.15 | 0.0395 | 0.2796206 |
| hsa-miR-3195-st | 0.3579 | 0.1667 | 2.147 | 0.0397 | 0.2796721 |
| hsa-miR-370-st | -0.1534 | 0.0717 | -2.139 | 0.0404 | 0.2801613 |
| hsa-miR-449a-st | -0.2429 | 0.1136 | -2.139 | 0.0404 | 0.2801613 |
| hsa-miR-195-star-st | 0.1705 | 0.0798 | 2.137 | 0.0406 | 0.2801613 |
| hsa-miR-26a-1-star-st | 0.0495 | 0.0233 | 2.13 | 0.0412 | 0.2801613 |
| hsa-miR-920-st | 0.0662 | 0.0311 | 2.129 | 0.0413 | 0.2801613 |
| hsa-miR-936-st | 0.1927 | 0.0905 | 2.128 | 0.0413 | 0.2801613 |
| hsa-miR-551b-star-st | 0.1535 | 0.0722 | 2.127 | 0.0415 | 0.2801613 |
| hsa-let-7g-star-st | 0.1569 | 0.0739 | 2.125 | 0.0417 | 0.2801613 |
| hsa-miR-422a-st | -0.1409 | 0.0663 | -2.125 | 0.0417 | 0.2801613 |
| hsa-miR-4468-st | -0.0561 | 0.0264 | -2.125 | 0.0417 | 0.2801613 |
| hsa-miR-3139-st | -0.0704 | 0.0332 | -2.122 | 0.0419 | 0.2802078 |
| hsa-miR-1279-st | -0.0615 | 0.0291 | -2.116 | 0.0425 | 0.2803445 |
| hsa-miR-3683-st | -0.0485 | 0.0229 | -2.116 | 0.0425 | 0.2803445 |
| hsa-miR-652-st | -0.1513 | 0.0715 | -2.116 | 0.0425 | 0.2803445 |
| hsa-miR-548b-3p-st | 0.0477 | 0.0226 | 2.111 | 0.0429 | 0.2804337 |
| hsa-miR-548b-5p-st | -0.0437 | 0.0207 | -2.111 | 0.0429 | 0.2804337 |
| hsa-miR-361-5p-st | -0.0751 | 0.0356 | -2.108 | 0.0432 | 0.2811284 |
| hsa-miR-4763-3p-st | 0.2062 | 0.0979 | 2.106 | 0.0434 | 0.2811691 |
| hsa-miR-4742-3p-st | -0.0862 | 0.041 | -2.103 | 0.0437 | 0.281289 |
| hsa-miR-4540-st | -0.0561 | 0.0267 | -2.1 | 0.044 | 0.281289 |
| hsa-miR-518e-star-st | -0.1576 | 0.0751 | -2.099 | 0.044 | 0.281289 |
| hsa-miR-4728-3p-st | -0.1596 | 0.0761 | -2.097 | 0.0442 | 0.2813282 |
| hsa-miR-3136-5p-st | -0.1659 | 0.0792 | -2.095 | 0.0445 | 0.2820008 |
| hsa-miR-214-star-st | 0.0656 | 0.0314 | 2.088 | 0.0451 | 0.2845605 |
| hsa-miR-3667-3p-st | -0.0695 | 0.0333 | -2.086 | 0.0453 | 0.284585 |
| hsa-miR-134-st | -0.1652 | 0.0793 | -2.084 | 0.0455 | 0.2846094 |
| hsa-miR-4721-st | 0.1354 | 0.0652 | 2.076 | 0.0463 | 0.2883706 |
| hsa-miR-3187-3p-st | 0.2828 | 0.1366 | 2.071 | 0.0468 | 0.289004 |
| hsa-miR-3676-st | -0.0946 | 0.0457 | -2.07 | 0.0468 | 0.289004 |
| hsa-miR-382-st | -0.0725 | 0.0352 | -2.061 | 0.0477 | 0.2932878 |
| hsa-miR-4673-st | 0.235 | 0.1142 | 2.058 | 0.048 | 0.2932878 |
| hsa-miR-1294-st | 0.0975 | 0.0474 | 2.058 | 0.0481 | 0.2932878 |
| hsa-miR-154-star-st | -0.1728 | 0.0844 | -2.048 | 0.0491 | 0.2968904 |
| hsa-miR-554-st | 0.0865 | 0.0422 | 2.048 | 0.0491 | 0.2968904 |
| hsa-miR-217-st | -0.3782 | 0.1857 | -2.037 | 0.0503 | 0.3028843 |
| hsa-miR-520d-3p-st | -0.0489 | 0.0241 | -2.03 | 0.051 | 0.3058304 |
| hsa-miR-1229-st | -0.184 | 0.0908 | -2.027 | 0.0514 | 0.3069606 |
| hsa-miR-4459-st | 0.1333 | 0.0659 | 2.023 | 0.0517 | 0.3074869 |
| hsa-miR-650-st | 0.139 | 0.0688 | 2.021 | 0.052 | 0.3080088 |
| hsa-miR-218-1-star-st | -0.0532 | 0.0264 | -2.016 | 0.0526 | 0.3102962 |
| hsa-miR-3913-5p-st | 0.0426 | 0.0212 | 2.011 | 0.0531 | 0.3113048 |
| hsa-miR-3135b-st | -0.1632 | 0.0812 | -2.01 | 0.0532 | 0.3113048 |
| hsa-miR-548j-st | -0.0593 | 0.0296 | -2.003 | 0.0539 | 0.3141342 |
| hsa-miR-548d-5p-st | 0.0637 | 0.0319 | 1.999 | 0.0545 | 0.3163606 |
| hsa-miR-181c-st | 0.2114 | 0.106 | 1.993 | 0.0551 | 0.3185692 |
| hsa-miR-3168-st | -0.0537 | 0.027 | -1.99 | 0.0554 | 0.3189188 |
| hsa-miR-3671-st | -0.0457 | 0.023 | -1.989 | 0.0556 | 0.3189188 |
| hsa-miR-4766-3p-st | -0.0646 | 0.0326 | -1.979 | 0.0568 | 0.3245193 |
| hsa-miR-329-st | -0.1084 | 0.055 | -1.972 | 0.0575 | 0.3272303 |
| hsa-miR-3612-st | 0.2914 | 0.148 | 1.969 | 0.0579 | 0.3273627 |
| hsa-miR-4664-3p-st | 0.137 | 0.0696 | 1.968 | 0.058 | 0.3273627 |
| hsa-miR-409-3p-st | -0.1204 | 0.0612 | -1.967 | 0.0582 | 0.3273627 |
| hsa-miR-518a-3p-st | 0.0567 | 0.0289 | 1.963 | 0.0586 | 0.3283399 |
| hsa-miR-3678-3p-st | -0.071 | 0.0362 | -1.961 | 0.0589 | 0.3287515 |
| hsa-miR-3928-st | -0.1565 | 0.0803 | -1.951 | 0.0602 | 0.3343819 |
| hsa-miR-30d-st | -0.0552 | 0.0283 | -1.949 | 0.0604 | 0.3343819 |
| hsa-miR-4776-5p-st | 0.3178 | 0.1632 | 1.948 | 0.0606 | 0.3343819 |
| hsa-miR-4495-st | 0.0832 | 0.043 | 1.936 | 0.0621 | 0.3400726 |
| hsa-miR-574-5p-st | 0.1202 | 0.0621 | 1.936 | 0.0621 | 0.3400726 |
| hsa-miR-4477a-st | -0.0454 | 0.0235 | -1.932 | 0.0626 | 0.3412701 |
| hsa-miR-429-st | -0.0535 | 0.0277 | -1.928 | 0.063 | 0.3412701 |
| hsa-miR-1280-st | 0.1481 | 0.0768 | 1.927 | 0.0632 | 0.3412701 |
| hsa-miR-1245-st | 0.0524 | 0.0272 | 1.925 | 0.0634 | 0.3412701 |
| hsa-miR-520d-5p-st | -0.0523 | 0.0272 | -1.923 | 0.0637 | 0.3412701 |
| hsa-miR-522-star-st | -0.0987 | 0.0514 | -1.922 | 0.0638 | 0.3412701 |
| hsa-miR-300-st | -0.0682 | 0.0355 | -1.921 | 0.064 | 0.3412701 |
| hsa-miR-4755-5p-st | 0.0575 | 0.03 | 1.919 | 0.0642 | 0.3412701 |
| hsa-miR-1236-st | 0.095 | 0.0496 | 1.917 | 0.0645 | 0.3414267 |
| hsa-miR-520c-3p-st | 0.0474 | 0.0248 | 1.915 | 0.0647 | 0.3414267 |
| hsa-miR-19b-2-star-st | -0.0353 | 0.0185 | -1.912 | 0.0652 | 0.3418485 |
| hsa-miR-150-star-st | 0.1866 | 0.0978 | 1.908 | 0.0657 | 0.3418485 |
| hsa-miR-1224-3p-st | -0.11 | 0.0577 | -1.907 | 0.0658 | 0.3418485 |
| hsa-miR-1183-st | 0.0677 | 0.0355 | 1.907 | 0.0659 | 0.3418485 |
| hsa-miR-642a-st | -0.0491 | 0.0258 | -1.905 | 0.066 | 0.3418485 |
| hsa-miR-1208-st | 0.3135 | 0.1647 | 1.904 | 0.0663 | 0.3418485 |
| hsa-miR-4522-st | -0.06 | 0.0316 | -1.9 | 0.0668 | 0.3418485 |
| hsa-miR-497-st | 0.06 | 0.0316 | 1.9 | 0.0668 | 0.3418485 |
| hsa-miR-624-st | -0.0504 | 0.0265 | -1.899 | 0.0669 | 0.3418485 |
| hsa-miR-133b-st | -0.164 | 0.0865 | -1.897 | 0.0672 | 0.3421766 |
| hsa-miR-4530-st | 0.2019 | 0.1068 | 1.89 | 0.0681 | 0.3455468 |
| hsa-miR-4325-st | -0.0391 | 0.0207 | -1.887 | 0.0685 | 0.3456667 |
| hsa-miR-3188-st | 0.2445 | 0.1296 | 1.886 | 0.0686 | 0.3456667 |
| hsa-miR-432-st | -0.1258 | 0.067 | -1.879 | 0.0696 | 0.349492 |
| hsa-miR-193a-5p-st | 0.1269 | 0.0677 | 1.874 | 0.0703 | 0.3515437 |
| hsa-miR-3133-st | -0.0488 | 0.0261 | -1.87 | 0.071 | 0.3515437 |
| hsa-miR-4718-st | 0.0525 | 0.0281 | 1.87 | 0.071 | 0.3515437 |
| hsa-miR-143-star-st | 0.1576 | 0.0845 | 1.864 | 0.0718 | 0.3515437 |
| hsa-miR-3610-st | 0.1778 | 0.0954 | 1.864 | 0.0718 | 0.3515437 |
| hsa-miR-564-st | 0.1517 | 0.0814 | 1.863 | 0.072 | 0.3515437 |
| hsa-miR-4797-3p-st | 0.058 | 0.0311 | 1.861 | 0.0722 | 0.3515437 |
| hsa-miR-5096-st | 0.3006 | 0.1615 | 1.861 | 0.0723 | 0.3515437 |
| hsa-miR-4511-st | 0.0659 | 0.0354 | 1.859 | 0.0725 | 0.3515437 |
| hsa-miR-4687-5p-st | 0.0726 | 0.039 | 1.859 | 0.0726 | 0.3515437 |
| hsa-miR-4717-3p-st | 0.2093 | 0.1128 | 1.855 | 0.0731 | 0.3515437 |
| hsa-miR-3654-st | 0.0743 | 0.04 | 1.855 | 0.0732 | 0.3515437 |
| hsa-miR-4463-st | 0.1959 | 0.1057 | 1.853 | 0.0734 | 0.3515437 |
| hsa-miR-762-st | 0.1928 | 0.104 | 1.853 | 0.0734 | 0.3515437 |
| hsa-miR-770-5p-st | -0.1509 | 0.0823 | -1.833 | 0.0765 | 0.3646958 |
| hsa-miR-7-st | -0.2233 | 0.122 | -1.831 | 0.0768 | 0.3646958 |
| hsa-miR-141-star-st | 0.0369 | 0.0202 | 1.83 | 0.0769 | 0.3646958 |
| hsa-miR-3153-st | 0.0682 | 0.0374 | 1.824 | 0.0778 | 0.3677622 |
| hsa-miR-27a-star-st | -0.0742 | 0.041 | -1.811 | 0.0798 | 0.374502 |
| hsa-miR-378h-st | -0.1454 | 0.0802 | -1.812 | 0.0798 | 0.374502 |
| hsa-miR-3545-3p-st | -0.0504 | 0.0278 | -1.81 | 0.08 | 0.374502 |
| hsa-miR-4683-st | 0.1051 | 0.0581 | 1.807 | 0.0804 | 0.3751643 |
| hsa-miR-942-st | -0.061 | 0.0338 | -1.804 | 0.081 | 0.3757394 |
| hsa-miR-1304-st | 0.0621 | 0.0345 | 1.802 | 0.0812 | 0.3757394 |
| hsa-miR-4300-st | 0.0565 | 0.0314 | 1.802 | 0.0813 | 0.3757394 |
| hsa-miR-1243-st | -0.0583 | 0.0324 | -1.799 | 0.0818 | 0.3768501 |
| hsa-miR-33b-star-st | -0.0942 | 0.0525 | -1.794 | 0.0825 | 0.3788722 |
| hsa-miR-4466-st | 0.1411 | 0.0787 | 1.793 | 0.0828 | 0.3790504 |
| hsa-miR-3147-st | 0.1278 | 0.0714 | 1.79 | 0.0833 | 0.3801401 |
| hsa-miR-3673-st | -0.0469 | 0.0262 | -1.786 | 0.0839 | 0.381678 |
| hsa-miR-4687-3p-st | 0.1728 | 0.0969 | 1.783 | 0.0843 | 0.381776 |
| hsa-miR-569-st | 0.0529 | 0.0298 | 1.779 | 0.085 | 0.381776 |
| hsa-miR-615-3p-st | 0.1413 | 0.0794 | 1.779 | 0.085 | 0.381776 |
| hsa-miR-1298-st | -0.2066 | 0.1162 | -1.778 | 0.0853 | 0.381776 |
| hsa-miR-378i-st | -0.0901 | 0.0507 | -1.777 | 0.0854 | 0.381776 |
| hsa-miR-4429-st | 0.0732 | 0.0412 | 1.777 | 0.0855 | 0.381776 |
| hsa-miR-29b-2-star-st | -0.0233 | 0.0132 | -1.773 | 0.0861 | 0.3818236 |
| hsa-miR-663b-st | 0.206 | 0.1162 | 1.772 | 0.0862 | 0.3818236 |
| hsa-miR-4741-st | 0.2031 | 0.1146 | 1.772 | 0.0863 | 0.3818236 |
| hsa-miR-16-1-star-st | 0.0415 | 0.0235 | 1.769 | 0.0868 | 0.3825878 |
| hsa-miR-3646-st | 0.2262 | 0.128 | 1.767 | 0.087 | 0.3825878 |
| hsa-miR-378d-st | -0.1283 | 0.0728 | -1.762 | 0.0878 | 0.3837417 |
| hsa-miR-4524-star-st | -0.0669 | 0.0379 | -1.763 | 0.0878 | 0.3837417 |
| hsa-miR-337-5p-st | -0.0601 | 0.0342 | -1.759 | 0.0885 | 0.3837417 |
| hsa-miR-195-st | 0.0811 | 0.0461 | 1.758 | 0.0886 | 0.3837417 |
| hsa-miR-765-st | 0.0982 | 0.0559 | 1.758 | 0.0886 | 0.3837417 |
| hsa-miR-4274-st | 0.1111 | 0.0634 | 1.753 | 0.0896 | 0.3837417 |
| hsa-miR-4426-st | 0.0547 | 0.0312 | 1.753 | 0.0896 | 0.3837417 |
| hsa-miR-3126-3p-st | -0.0736 | 0.0421 | -1.75 | 0.0901 | 0.3837417 |
| hsa-miR-342-5p-st | -0.0667 | 0.0381 | -1.749 | 0.0901 | 0.3837417 |
| hsa-miR-1273f-st | 0.2796 | 0.16 | 1.748 | 0.0904 | 0.3837417 |
| hsa-miR-4643-st | 0.0366 | 0.021 | 1.747 | 0.0905 | 0.3837417 |
| hsa-miR-655-st | -0.0461 | 0.0264 | -1.747 | 0.0906 | 0.3837417 |
| hsa-miR-4680-3p-st | 0.036 | 0.0206 | 1.746 | 0.0907 | 0.3837417 |
| hsa-miR-3922-5p-st | 0.0927 | 0.0532 | 1.744 | 0.0911 | 0.3843136 |
| hsa-miR-1203-st | 0.0856 | 0.0492 | 1.74 | 0.0918 | 0.3861441 |
| hsa-miR-4743-st | 0.1322 | 0.0764 | 1.73 | 0.0935 | 0.3921583 |
| hsa-miR-140-3p-st | 0.0506 | 0.0293 | 1.727 | 0.0942 | 0.3939556 |
| hsa-miR-3140-3p-st | 0.0547 | 0.0319 | 1.717 | 0.096 | 0.3997006 |
| hsa-miR-1249-st | -0.0877 | 0.0511 | -1.716 | 0.0962 | 0.3997006 |
| hsa-miR-3689b-star-st | 0.0454 | 0.0265 | 1.715 | 0.0964 | 0.3997006 |
| hsa-miR-155-st | 0.0887 | 0.0519 | 1.71 | 0.0973 | 0.4022829 |
| hsa-miR-4507-st | 0.1832 | 0.1073 | 1.708 | 0.0977 | 0.4027891 |
| hsa-miR-1184-st | -0.2437 | 0.1432 | -1.702 | 0.0988 | 0.4061702 |
| hsa-miR-4494-st | 0.0448 | 0.0264 | 1.696 | 0.0998 | 0.4087874 |
| hsa-miR-3167-st | -0.0422 | 0.0249 | -1.696 | 0.1 | 0.4087874 |
| hsa-miR-3179-st | 0.0455 | 0.0269 | 1.691 | 0.1009 | 0.4113079 |
| hsa-miR-3591-5p-st | -0.0453 | 0.0268 | -1.688 | 0.1014 | 0.4114422 |
| hsa-miR-323b-5p-st | 0.1336 | 0.0792 | 1.688 | 0.1015 | 0.4114422 |
| hsa-miR-384-st | -0.0392 | 0.0233 | -1.686 | 0.1018 | 0.4115089 |
| hsa-miR-4449-st | 0.2249 | 0.1337 | 1.682 | 0.1026 | 0.4135907 |
| hsa-let-7b-star-st | 0.0885 | 0.0526 | 1.68 | 0.1029 | 0.413651 |
| hsa-miR-3158-5p-st | 0.1098 | 0.0655 | 1.677 | 0.1037 | 0.4154246 |
| hsa-miR-3908-st | -0.0314 | 0.0187 | -1.675 | 0.104 | 0.4154246 |
| hsa-miR-1193-st | -0.109 | 0.0651 | -1.674 | 0.1042 | 0.4154246 |
| hsa-miR-890-st | -0.0641 | 0.0385 | -1.666 | 0.1057 | 0.4202502 |
| hsa-miR-1268-st | 0.1499 | 0.0901 | 1.663 | 0.1063 | 0.421481 |
| hsa-miR-2117-st | 0.0243 | 0.0147 | 1.652 | 0.1086 | 0.4281594 |
| hsa-miR-20b-st | 0.0594 | 0.036 | 1.648 | 0.1094 | 0.4281594 |
| hsa-miR-3117-3p-st | 0.1417 | 0.086 | 1.648 | 0.1095 | 0.4281594 |
| hsa-miR-519b-5p-st | -0.1122 | 0.0682 | -1.647 | 0.1097 | 0.4281594 |
| hsa-miR-1469-st | 0.1634 | 0.0994 | 1.644 | 0.1104 | 0.4281594 |
| hsa-miR-424-star-st | -0.1298 | 0.0791 | -1.641 | 0.1108 | 0.4281594 |
| hsa-miR-4764-5p-st | 0.0438 | 0.0267 | 1.642 | 0.1108 | 0.4281594 |
| hsa-miR-215-st | -0.0642 | 0.0391 | -1.64 | 0.1111 | 0.4281594 |
| hsa-miR-3158-3p-st | 0.0434 | 0.0264 | 1.64 | 0.1111 | 0.4281594 |
| hsa-miR-345-st | 0.1001 | 0.0611 | 1.638 | 0.1116 | 0.4281594 |
| hsa-miR-106b-st | -0.0414 | 0.0253 | -1.635 | 0.1122 | 0.4281594 |
| hsa-miR-671-5p-st | 0.1303 | 0.0797 | 1.635 | 0.1122 | 0.4281594 |
| hsa-miR-615-5p-st | -0.0847 | 0.0518 | -1.634 | 0.1123 | 0.4281594 |
| hsa-miR-152-st | 0.0906 | 0.0555 | 1.633 | 0.1125 | 0.4281594 |
| hsa-miR-548s-st | 0.0393 | 0.024 | 1.632 | 0.1127 | 0.4281594 |
| hsa-miR-132-star-st | -0.1199 | 0.0735 | -1.632 | 0.1128 | 0.4281594 |
| hsa-miR-455-3p-st | 0.0945 | 0.0579 | 1.631 | 0.113 | 0.4281594 |
| hsa-miR-4768-3p-st | -0.0541 | 0.0332 | -1.628 | 0.1137 | 0.4289673 |
| hsa-miR-4706-st | -0.0787 | 0.0484 | -1.627 | 0.1139 | 0.4289673 |
| hsa-miR-30a-st | 0.0353 | 0.0217 | 1.626 | 0.1141 | 0.4289673 |
| hsa-miR-4255-st | -0.0406 | 0.0251 | -1.621 | 0.1152 | 0.4316184 |
| hsa-miR-512-5p-st | -0.0416 | 0.0257 | -1.62 | 0.1154 | 0.4316184 |
| hsa-miR-4505-st | 0.1882 | 0.1164 | 1.617 | 0.1159 | 0.4323741 |
| hsa-miR-4472-st | -0.0899 | 0.0557 | -1.615 | 0.1165 | 0.4327605 |
| hsa-miR-767-5p-st | -0.1753 | 0.1086 | -1.614 | 0.1166 | 0.4327605 |
| hsa-miR-4640-3p-st | 0.1839 | 0.1142 | 1.61 | 0.1175 | 0.4349884 |
| hsa-miR-1321-st | 0.0471 | 0.0293 | 1.609 | 0.1178 | 0.4349893 |
| hsa-miR-504-st | -0.131 | 0.0816 | -1.606 | 0.1185 | 0.435726 |
| hsa-miR-224-st | 0.0657 | 0.0409 | 1.605 | 0.1186 | 0.435726 |
| hsa-miR-873-st | -0.1418 | 0.0884 | -1.603 | 0.119 | 0.4360915 |
| hsa-miR-376a-st | -0.1367 | 0.0854 | -1.602 | 0.1194 | 0.4364552 |
| hsa-miR-196a-st | -0.0423 | 0.0264 | -1.599 | 0.12 | 0.4375463 |
| hsa-miR-4320-st | -0.0453 | 0.0284 | -1.594 | 0.1211 | 0.4404505 |
| hsa-miR-1228-st | 0.1317 | 0.0828 | 1.59 | 0.122 | 0.4418565 |
| hsa-miR-4727-5p-st | -0.0457 | 0.0288 | -1.589 | 0.1222 | 0.4418565 |
| hsa-miR-222-st | -0.0546 | 0.0344 | -1.588 | 0.1224 | 0.4418565 |
| hsa-miR-4798-3p-st | -0.0441 | 0.0278 | -1.586 | 0.1229 | 0.4425427 |
| hsa-miR-3173-3p-st | -0.1425 | 0.0899 | -1.585 | 0.1232 | 0.4425427 |
| hsa-let-7d-st | -0.023 | 0.0145 | -1.582 | 0.1238 | 0.4435999 |
| hsa-miR-1278-st | -0.0422 | 0.0267 | -1.58 | 0.1244 | 0.444652 |
| hsa-miR-3622b-3p-st | 0.1825 | 0.1158 | 1.577 | 0.125 | 0.4452451 |
| hsa-miR-4456-st | 0.0435 | 0.0276 | 1.575 | 0.1253 | 0.4452451 |
| hsa-miR-4751-st | 0.048 | 0.0305 | 1.573 | 0.126 | 0.4452451 |
| hsa-miR-191-st | -0.0164 | 0.0104 | -1.572 | 0.1261 | 0.4452451 |
| hsa-miR-27a-st | 0.067 | 0.0426 | 1.572 | 0.1261 | 0.4452451 |
| hsa-miR-330-3p-st | -0.0649 | 0.0414 | -1.567 | 0.1272 | 0.4464949 |
| hsa-miR-1301-st | -0.1208 | 0.0772 | -1.566 | 0.1275 | 0.4464949 |
| hsa-miR-4267-st | -0.1188 | 0.0759 | -1.566 | 0.1275 | 0.4464949 |
| hsa-miR-576-5p-st | -0.045 | 0.0288 | -1.565 | 0.1278 | 0.4464949 |
| hsa-miR-3191-st | -0.1095 | 0.0701 | -1.564 | 0.128 | 0.4464949 |
| hsa-miR-1255b-st | -0.065 | 0.0416 | -1.563 | 0.1283 | 0.4464949 |
| hsa-miR-92b-st | 0.0874 | 0.056 | 1.56 | 0.129 | 0.4478569 |
| hsa-miR-4538-st | 0.1269 | 0.0816 | 1.556 | 0.1297 | 0.4492125 |
| hsa-miR-379-st | -0.0683 | 0.044 | -1.554 | 0.1304 | 0.4493914 |
| hsa-miR-875-3p-st | 0.0534 | 0.0344 | 1.553 | 0.1305 | 0.4493914 |
| hsa-miR-4773-st | -0.0734 | 0.0473 | -1.552 | 0.1307 | 0.4493914 |
| hsa-miR-659-st | 0.1385 | 0.0893 | 1.55 | 0.1312 | 0.4493914 |
| hsa-miR-149-st | -0.0886 | 0.0571 | -1.55 | 0.1313 | 0.4493914 |
| hsa-miR-411-star-st | -0.1136 | 0.0734 | -1.548 | 0.1317 | 0.4496998 |
| hsa-miR-4649-3p-st | 0.1124 | 0.0727 | 1.547 | 0.1321 | 0.4500068 |
| hsa-miR-518a-5p-st | -0.0708 | 0.0459 | -1.542 | 0.1333 | 0.4514465 |
| hsa-miR-3689e-st | -0.0512 | 0.0332 | -1.541 | 0.1335 | 0.4514465 |
| hsa-miR-744-star-st | 0.1263 | 0.0821 | 1.538 | 0.1341 | 0.4514465 |
| hsa-miR-640-st | 0.1957 | 0.1274 | 1.536 | 0.1347 | 0.4514465 |
| hsa-miR-3917-st | 0.1925 | 0.1256 | 1.533 | 0.1355 | 0.4514465 |
| hsa-miR-561-st | 0.0341 | 0.0223 | 1.531 | 0.136 | 0.4514465 |
| hsa-miR-519c-3p-st | -0.0326 | 0.0213 | -1.53 | 0.1361 | 0.4514465 |
| hsa-miR-3666-st | -0.0529 | 0.0346 | -1.528 | 0.1366 | 0.4514465 |
| hsa-miR-3915-st | -0.0473 | 0.031 | -1.528 | 0.1366 | 0.4514465 |
| hsa-miR-4753-3p-st | -0.047 | 0.0308 | -1.528 | 0.1367 | 0.4514465 |
| hsa-miR-3622b-5p-st | 0.1287 | 0.0843 | 1.527 | 0.137 | 0.4514465 |
| hsa-miR-889-st | -0.0883 | 0.0578 | -1.527 | 0.137 | 0.4514465 |
| hsa-miR-4688-st | 0.148 | 0.097 | 1.526 | 0.1372 | 0.4514465 |
| hsa-miR-582-3p-st | 0.1014 | 0.0665 | 1.526 | 0.1372 | 0.4514465 |
| hsa-miR-1539-st | -0.0607 | 0.0398 | -1.525 | 0.1375 | 0.4514465 |
| hsa-miR-3150b-5p-st | -0.058 | 0.0381 | -1.525 | 0.1375 | 0.4514465 |
| hsa-miR-1285-st | 0.0969 | 0.0638 | 1.519 | 0.1388 | 0.4544277 |
| hsa-miR-3129-3p-st | 0.1369 | 0.0903 | 1.516 | 0.1396 | 0.4544277 |
| hsa-miR-4761-3p-st | -0.0505 | 0.0333 | -1.516 | 0.1396 | 0.4544277 |
| hsa-miR-182-st | 0.1901 | 0.1254 | 1.516 | 0.1397 | 0.4544277 |
| hsa-miR-609-st | -0.0236 | 0.0156 | -1.514 | 0.1402 | 0.4544277 |
| hsa-miR-3144-3p-st | -0.0338 | 0.0224 | -1.512 | 0.1406 | 0.4544277 |
| hsa-miR-520b-st | -0.0379 | 0.0251 | -1.512 | 0.1406 | 0.4544277 |
| hsa-miR-4746-3p-st | -0.1245 | 0.0825 | -1.509 | 0.1415 | 0.4563203 |
| hsa-miR-363-star-st | -0.1342 | 0.0891 | -1.506 | 0.1423 | 0.4578827 |
| hsa-miR-766-st | 0.1217 | 0.0809 | 1.503 | 0.1428 | 0.458475 |
| hsa-miR-541-st | -0.1788 | 0.1191 | -1.501 | 0.1434 | 0.4589604 |
| hsa-miR-149-star-st | 0.1589 | 0.1059 | 1.501 | 0.1436 | 0.4589604 |
| hsa-miR-3924-st | 0.0498 | 0.0332 | 1.499 | 0.1439 | 0.4589604 |
| hsa-miR-4695-5p-st | 0.1549 | 0.1035 | 1.496 | 0.1448 | 0.4601273 |
| hsa-miR-4428-st | 0.1224 | 0.0818 | 1.495 | 0.1449 | 0.4601273 |
| hsa-miR-4787-3p-st | -0.0789 | 0.0529 | -1.493 | 0.1455 | 0.4610238 |
| hsa-miR-4711-5p-st | 0.0498 | 0.0334 | 1.49 | 0.1462 | 0.4622326 |
| hsa-miR-611-st | 0.0625 | 0.0421 | 1.487 | 0.1471 | 0.464067 |
| hsa-miR-181d-st | -0.0602 | 0.0405 | -1.485 | 0.1477 | 0.4648642 |
| hsa-miR-1297-st | -0.0429 | 0.0289 | -1.483 | 0.1481 | 0.4648642 |
| hsa-miR-3960-st | 0.0969 | 0.0654 | 1.481 | 0.1486 | 0.4648642 |
| hsa-miR-325-st | 0.0455 | 0.0307 | 1.48 | 0.149 | 0.4648642 |
| hsa-miR-3925-3p-st | -0.0364 | 0.0246 | -1.478 | 0.1496 | 0.4648642 |
| hsa-miR-885-5p-st | -0.0566 | 0.0383 | -1.476 | 0.15 | 0.4648642 |
| hsa-miR-365-star-st | 0.1357 | 0.092 | 1.474 | 0.1505 | 0.4648642 |
| hsa-miR-4277-st | -0.0576 | 0.0392 | -1.471 | 0.1515 | 0.4648642 |
| hsa-miR-526b-star-st | 0.0284 | 0.0193 | 1.469 | 0.1518 | 0.4648642 |
| hsa-miR-147b-st | -0.058 | 0.0395 | -1.469 | 0.1519 | 0.4648642 |
| hsa-miR-3670-st | 0.035 | 0.0238 | 1.468 | 0.1521 | 0.4648642 |
| hsa-miR-1287-st | -0.0715 | 0.0487 | -1.467 | 0.1524 | 0.4648642 |
| hsa-miR-3944-3p-st | 0.1845 | 0.1259 | 1.466 | 0.1528 | 0.4648642 |
| hsa-miR-4324-st | -0.1715 | 0.1171 | -1.465 | 0.153 | 0.4648642 |
| hsa-miR-3686-st | -0.0484 | 0.0331 | -1.464 | 0.1532 | 0.4648642 |
| hsa-miR-3074-3p-st | -0.0614 | 0.042 | -1.464 | 0.1534 | 0.4648642 |
| hsa-miR-603-st | -0.0581 | 0.0397 | -1.464 | 0.1534 | 0.4648642 |
| hsa-miR-4499-st | 0.1144 | 0.0782 | 1.463 | 0.1535 | 0.4648642 |
| hsa-miR-4660-st | 0.0567 | 0.0388 | 1.462 | 0.1537 | 0.4648642 |
| hsa-miR-3668-st | -0.03 | 0.0205 | -1.462 | 0.1539 | 0.4648642 |
| hsa-miR-4458-st | 0.067 | 0.0459 | 1.461 | 0.1541 | 0.4648642 |
| hsa-miR-146a-star-st | -0.0536 | 0.0367 | -1.46 | 0.1544 | 0.4648642 |
| hsa-miR-4796-5p-st | -0.0354 | 0.0243 | -1.458 | 0.155 | 0.4657045 |
| hsa-miR-4753-5p-st | -0.0573 | 0.0394 | -1.453 | 0.1562 | 0.4676074 |
| hsa-miR-1976-st | 0.1275 | 0.0878 | 1.453 | 0.1563 | 0.4676074 |
| hsa-miR-2115-st | 0.0355 | 0.0244 | 1.452 | 0.1566 | 0.4676074 |
| hsa-miR-101-st | -0.1711 | 0.1181 | -1.448 | 0.1575 | 0.4689621 |
| hsa-miR-4735-5p-st | 0.0353 | 0.0244 | 1.448 | 0.1577 | 0.4689621 |
| hsa-miR-1227-st | -0.0607 | 0.042 | -1.445 | 0.1585 | 0.4703772 |
| hsa-miR-604-st | -0.0494 | 0.0342 | -1.442 | 0.1592 | 0.471058 |
| hsa-miR-3665-st | 0.0855 | 0.0594 | 1.441 | 0.1597 | 0.471058 |
| hsa-miR-3140-5p-st | 0.0344 | 0.0239 | 1.44 | 0.1599 | 0.471058 |
| hsa-miR-4441-st | 0.0519 | 0.0361 | 1.439 | 0.1603 | 0.471058 |
| hsa-miR-548w-st | 0.0738 | 0.0514 | 1.435 | 0.1613 | 0.471058 |
| hsa-miR-4779-st | 0.0422 | 0.0294 | 1.435 | 0.1614 | 0.471058 |
| hsa-miR-4797-5p-st | -0.0869 | 0.0605 | -1.435 | 0.1614 | 0.471058 |
| hsa-miR-4766-5p-st | -0.0542 | 0.0378 | -1.434 | 0.1615 | 0.471058 |
| hsa-miR-3681-star-st | -0.0776 | 0.0542 | -1.433 | 0.162 | 0.471058 |
| hsa-miR-126-star-st | 0.0509 | 0.0355 | 1.432 | 0.1622 | 0.471058 |
| hsa-miR-155-star-st | 0.0524 | 0.0366 | 1.431 | 0.1623 | 0.471058 |
| hsa-miR-4304-st | -0.1432 | 0.1003 | -1.428 | 0.1634 | 0.4712821 |
| hsa-miR-4513-st | 0.1517 | 0.1063 | 1.427 | 0.1635 | 0.4712821 |
| hsa-miR-4443-st | -0.1178 | 0.0826 | -1.427 | 0.1636 | 0.4712821 |
| hsa-miR-4776-3p-st | -0.068 | 0.0477 | -1.426 | 0.1637 | 0.4712821 |
| hsa-miR-4328-st | 0.0393 | 0.0276 | 1.424 | 0.1643 | 0.4712821 |
| hsa-miR-4670-5p-st | -0.0328 | 0.023 | -1.423 | 0.1646 | 0.4712821 |
| hsa-miR-4526-st | 0.1387 | 0.0975 | 1.422 | 0.1649 | 0.4712821 |
| hsa-miR-1276-st | 0.087 | 0.0612 | 1.422 | 0.165 | 0.4712821 |
| hsa-miR-758-st | -0.1107 | 0.0779 | -1.421 | 0.1653 | 0.4712821 |
| hsa-miR-4442-st | 0.1319 | 0.0933 | 1.414 | 0.1672 | 0.4741137 |
| hsa-miR-1288-st | -0.0487 | 0.0345 | -1.414 | 0.1674 | 0.4741137 |
| hsa-miR-4462-st | 0.1258 | 0.089 | 1.414 | 0.1675 | 0.4741137 |
| hsa-miR-4634-st | 0.1646 | 0.1165 | 1.413 | 0.1676 | 0.4741137 |
| hsa-miR-3647-5p-st | 0.3157 | 0.2239 | 1.41 | 0.1685 | 0.4757323 |
| hsa-miR-1915-st | 0.1214 | 0.0862 | 1.408 | 0.169 | 0.4761383 |
| hsa-miR-4425-st | -0.045 | 0.032 | -1.407 | 0.1693 | 0.4761383 |
| hsa-miR-3185-st | 0.1507 | 0.1073 | 1.405 | 0.1699 | 0.4767414 |
| hsa-miR-4474-3p-st | 0.0675 | 0.0481 | 1.404 | 0.1703 | 0.4767414 |
| hsa-miR-628-5p-st | 0.1171 | 0.0835 | 1.403 | 0.1705 | 0.4767414 |
| hsa-miR-4257-st | 0.1353 | 0.0966 | 1.401 | 0.1711 | 0.4771396 |
| hsa-miR-1225-5p-st | 0.0827 | 0.0591 | 1.4 | 0.1713 | 0.4771396 |
| hsa-miR-4670-3p-st | 0.0493 | 0.0353 | 1.398 | 0.172 | 0.4776506 |
| hsa-miR-3134-st | 0.0321 | 0.023 | 1.396 | 0.1725 | 0.4776506 |
| hsa-miR-519a-star-st | -0.086 | 0.0616 | -1.396 | 0.1726 | 0.4776506 |
| hsa-miR-4520b-5p-st | -0.0376 | 0.0269 | -1.395 | 0.1728 | 0.4776506 |
| hsa-miR-1269b-st | -0.0442 | 0.0318 | -1.391 | 0.1742 | 0.480605 |
| hsa-miR-526a-st | -0.0969 | 0.0699 | -1.387 | 0.1753 | 0.4827221 |
| hsa-miR-4657-st | 0.1458 | 0.1055 | 1.381 | 0.177 | 0.4850755 |
| hsa-miR-200c-st | -0.0566 | 0.041 | -1.381 | 0.1772 | 0.4850755 |
| hsa-miR-4313-st | 0.0789 | 0.0571 | 1.381 | 0.1772 | 0.4850755 |
| hsa-miR-302c-star-st | 0.0334 | 0.0242 | 1.379 | 0.1779 | 0.4850755 |
| hsa-miR-183-st | 0.1233 | 0.0894 | 1.378 | 0.178 | 0.4850755 |
| hsa-miR-3177-5p-st | -0.0569 | 0.0414 | -1.376 | 0.1785 | 0.4850755 |
| hsa-miR-181b-st | -0.0594 | 0.0432 | -1.376 | 0.1786 | 0.4850755 |
| hsa-miR-1914-star-st | -0.1351 | 0.0983 | -1.375 | 0.1789 | 0.4850755 |
| hsa-miR-3142-st | -0.0419 | 0.0305 | -1.373 | 0.1796 | 0.4850755 |
| hsa-miR-4790-5p-st | -0.0332 | 0.0242 | -1.372 | 0.18 | 0.4850755 |
| hsa-miR-4786-5p-st | 0.1644 | 0.1199 | 1.371 | 0.1801 | 0.4850755 |
| hsa-miR-1-st | -0.0756 | 0.0551 | -1.371 | 0.1803 | 0.4850755 |
| hsa-miR-147-st | 0.0467 | 0.0341 | 1.37 | 0.1805 | 0.4850755 |
| hsa-miR-200a-st | 0.1988 | 0.1452 | 1.369 | 0.1809 | 0.4852518 |
| hsa-miR-1271-st | -0.064 | 0.0469 | -1.364 | 0.1825 | 0.4867005 |
| hsa-miR-4286-st | 0.1483 | 0.1089 | 1.363 | 0.1828 | 0.4867005 |
| hsa-miR-18b-st | 0.0964 | 0.0708 | 1.361 | 0.1834 | 0.4867005 |
| hsa-miR-1273g-st | 0.0965 | 0.071 | 1.359 | 0.1838 | 0.4867005 |
| hsa-miR-3611-st | -0.0378 | 0.0278 | -1.359 | 0.184 | 0.4867005 |
| hsa-miR-369-5p-st | -0.1257 | 0.0927 | -1.356 | 0.185 | 0.4867005 |
| hsa-miR-4436a-st | -0.1164 | 0.0859 | -1.356 | 0.185 | 0.4867005 |
| hsa-miR-4804-5p-st | -0.0275 | 0.0203 | -1.356 | 0.185 | 0.4867005 |
| hsa-miR-1256-st | 0.0495 | 0.0365 | 1.355 | 0.1851 | 0.4867005 |
| hsa-miR-127-5p-st | -0.0851 | 0.0628 | -1.355 | 0.1852 | 0.4867005 |
| hsa-miR-539-st | -0.0908 | 0.067 | -1.355 | 0.1852 | 0.4867005 |
| hsa-miR-23a-st | 0.0446 | 0.033 | 1.353 | 0.1858 | 0.4867005 |
| hsa-miR-708-star-st | 0.1245 | 0.092 | 1.353 | 0.1858 | 0.4867005 |
| hsa-miR-542-5p-st | -0.1074 | 0.0798 | -1.346 | 0.1882 | 0.492099 |
| hsa-miR-3977-st | -0.0401 | 0.0298 | -1.344 | 0.1886 | 0.492258 |
| hsa-miR-3156-5p-st | -0.1223 | 0.0911 | -1.341 | 0.1895 | 0.493719 |
| hsa-miR-367-st | 0.0366 | 0.0275 | 1.332 | 0.1926 | 0.50026 |
| hsa-miR-4789-3p-st | 0.0446 | 0.0335 | 1.332 | 0.1927 | 0.50026 |
| hsa-miR-93-star-st | -0.0906 | 0.0681 | -1.33 | 0.1932 | 0.500624 |
| hsa-miR-1268b-st | 0.1088 | 0.082 | 1.328 | 0.194 | 0.500624 |
| hsa-miR-764-st | 0.1374 | 0.1035 | 1.327 | 0.194 | 0.500624 |
| hsa-miR-379-star-st | -0.152 | 0.1148 | -1.324 | 0.1951 | 0.500624 |
| hsa-miR-199b-5p-st | -0.0485 | 0.0366 | -1.324 | 0.1952 | 0.500624 |
| hsa-miR-3128-st | 0.0512 | 0.0387 | 1.323 | 0.1955 | 0.500624 |
| hsa-miR-25-star-st | -0.1 | 0.0756 | -1.323 | 0.1956 | 0.500624 |
| hsa-miR-630-st | 0.0356 | 0.0269 | 1.323 | 0.1956 | 0.500624 |
| hsa-miR-486-3p-st | 0.171 | 0.1298 | 1.318 | 0.1972 | 0.5038305 |
| hsa-miR-4647-st | 0.0775 | 0.0589 | 1.316 | 0.1979 | 0.5040994 |
| hsa-miR-503-st | -0.1054 | 0.0801 | -1.315 | 0.198 | 0.5040994 |
| hsa-miR-33a-star-st | -0.0496 | 0.0378 | -1.31 | 0.1999 | 0.5075398 |
| hsa-let-7f-1-star-st | 0.1231 | 0.094 | 1.309 | 0.2002 | 0.5075398 |
| hsa-miR-185-st | -0.0321 | 0.0246 | -1.307 | 0.2008 | 0.5075398 |
| hsa-miR-125a-3p-st | -0.0781 | 0.0597 | -1.307 | 0.2009 | 0.5075398 |
| hsa-miR-520g-st | -0.058 | 0.0444 | -1.306 | 0.2011 | 0.5075398 |
| hsa-miR-3675-5p-st | 0.0317 | 0.0244 | 1.302 | 0.2026 | 0.5089226 |
| hsa-miR-520a-5p-st | -0.0425 | 0.0327 | -1.302 | 0.2026 | 0.5089226 |
| hsa-miR-548h-st | -0.077 | 0.0592 | -1.302 | 0.2027 | 0.5089226 |
| hsa-miR-18b-star-st | 0.0572 | 0.0441 | 1.299 | 0.2035 | 0.50992 |
| hsa-miR-4487-st | 0.083 | 0.0639 | 1.298 | 0.2038 | 0.50992 |
| hsa-miR-4778-5p-st | -0.1172 | 0.0903 | -1.297 | 0.2042 | 0.5100414 |
| hsa-miR-221-st | -0.0327 | 0.0253 | -1.293 | 0.2056 | 0.5122744 |
| hsa-miR-4769-5p-st | -0.0658 | 0.0509 | -1.292 | 0.2058 | 0.5122744 |
| hsa-miR-1225-3p-st | 0.0516 | 0.04 | 1.291 | 0.2063 | 0.5126397 |
| hsa-miR-552-st | -0.2289 | 0.1779 | -1.287 | 0.2077 | 0.5151 |
| hsa-miR-4661-3p-st | -0.0486 | 0.0378 | -1.286 | 0.208 | 0.5151 |
| hsa-miR-874-st | -0.0701 | 0.0547 | -1.282 | 0.2092 | 0.5171892 |
| hsa-miR-1910-st | 0.1302 | 0.102 | 1.277 | 0.2109 | 0.5205052 |
| hsa-miR-3118-st | 0.0261 | 0.0204 | 1.276 | 0.2116 | 0.5207085 |
| hsa-miR-129-5p-st | -0.0543 | 0.0426 | -1.275 | 0.2117 | 0.5207085 |
| hsa-miR-1911-star-st | -0.0688 | 0.054 | -1.273 | 0.2123 | 0.5210109 |
| hsa-miR-3667-5p-st | 0.0577 | 0.0453 | 1.273 | 0.2126 | 0.5210109 |
| hsa-miR-27b-st | -0.0655 | 0.0515 | -1.272 | 0.2129 | 0.5210109 |
| hsa-miR-3154-st | -0.1214 | 0.0956 | -1.27 | 0.2137 | 0.5220407 |
| hsa-miR-4457-st | -0.0378 | 0.0298 | -1.268 | 0.2143 | 0.5220407 |
| hsa-miR-135a-st | -0.0244 | 0.0193 | -1.268 | 0.2144 | 0.5220407 |
| hsa-miR-4790-3p-st | 0.0267 | 0.0211 | 1.267 | 0.2148 | 0.5221386 |
| hsa-miR-4510-st | -0.0846 | 0.0668 | -1.265 | 0.2152 | 0.5222362 |
| hsa-miR-4695-3p-st | 0.1268 | 0.1005 | 1.262 | 0.2165 | 0.5241488 |
| hsa-miR-548l-st | 0.0326 | 0.0259 | 1.258 | 0.2178 | 0.5241488 |
| hsa-miR-3714-st | 0.1217 | 0.0968 | 1.258 | 0.2179 | 0.5241488 |
| hsa-miR-605-st | 0.0943 | 0.0751 | 1.257 | 0.2183 | 0.5241488 |
| hsa-miR-499a-3p-st | -0.0353 | 0.0281 | -1.255 | 0.219 | 0.5241488 |
| hsa-miR-4781-3p-st | 0.036 | 0.0287 | 1.254 | 0.2191 | 0.5241488 |
| hsa-miR-18a-star-st | 0.1009 | 0.0805 | 1.254 | 0.2193 | 0.5241488 |
| hsa-miR-3679-3p-st | -0.0463 | 0.0369 | -1.254 | 0.2193 | 0.5241488 |
| hsa-miR-4704-5p-st | 0.0559 | 0.0446 | 1.253 | 0.2194 | 0.5241488 |
| hsa-miR-1228-star-st | 0.1319 | 0.1053 | 1.253 | 0.2196 | 0.5241488 |
| hsa-miR-4684-3p-st | 0.1497 | 0.1201 | 1.247 | 0.2217 | 0.526322 |
| hsa-miR-4272-st | 0.0336 | 0.027 | 1.243 | 0.2232 | 0.526322 |
| hsa-miR-433-st | -0.0952 | 0.0767 | -1.242 | 0.2234 | 0.526322 |
| hsa-miR-4440-st | 0.1519 | 0.1223 | 1.242 | 0.2237 | 0.526322 |
| hsa-miR-4745-5p-st | 0.1389 | 0.112 | 1.24 | 0.2242 | 0.526322 |
| hsa-miR-3131-st | 0.154 | 0.1242 | 1.24 | 0.2243 | 0.526322 |
| hsa-miR-625-st | -0.1018 | 0.0821 | -1.24 | 0.2243 | 0.526322 |
| hsa-miR-4666-5p-st | 0.0313 | 0.0253 | 1.24 | 0.2244 | 0.526322 |
| hsa-miR-4707-5p-st | 0.1413 | 0.114 | 1.24 | 0.2244 | 0.526322 |
| hsa-miR-888-star-st | -0.0378 | 0.0305 | -1.24 | 0.2244 | 0.526322 |
| hsa-miR-2681-st | -0.0821 | 0.0662 | -1.24 | 0.2245 | 0.526322 |
| hsa-miR-548ab-st | -0.0334 | 0.027 | -1.237 | 0.2253 | 0.5273456 |
| hsa-miR-3148-st | 0.1558 | 0.1262 | 1.235 | 0.2263 | 0.5284497 |
| hsa-miR-3909-st | -0.077 | 0.0624 | -1.234 | 0.2265 | 0.5284497 |
| hsa-miR-208b-st | -0.0475 | 0.0386 | -1.231 | 0.2276 | 0.5301638 |
| hsa-miR-3677-3p-st | 0.1043 | 0.0851 | 1.227 | 0.2292 | 0.5324895 |
| hsa-miR-1909-star-st | 0.103 | 0.084 | 1.226 | 0.2294 | 0.5324895 |
| hsa-miR-4310-st | 0.0953 | 0.0778 | 1.224 | 0.2303 | 0.5324895 |
| hsa-miR-412-st | -0.0483 | 0.0395 | -1.223 | 0.2305 | 0.5324895 |
| hsa-miR-3940-5p-st | 0.1247 | 0.102 | 1.223 | 0.2306 | 0.5324895 |
| hsa-miR-3200-5p-st | -0.0681 | 0.0557 | -1.222 | 0.2308 | 0.5324895 |
| hsa-miR-3678-5p-st | 0.023 | 0.0188 | 1.22 | 0.2316 | 0.5328715 |
| hsa-miR-4427-st | 0.0292 | 0.024 | 1.22 | 0.2317 | 0.5328715 |
| hsa-miR-3174-st | -0.0619 | 0.0511 | -1.211 | 0.235 | 0.5385598 |
| hsa-miR-3137-st | -0.104 | 0.086 | -1.21 | 0.2356 | 0.5385598 |
| hsa-miR-548v-st | 0.0329 | 0.0272 | 1.21 | 0.2356 | 0.5385598 |
| hsa-miR-1915-star-st | 0.1425 | 0.118 | 1.208 | 0.2362 | 0.5385598 |
| hsa-miR-885-3p-st | -0.0985 | 0.0815 | -1.208 | 0.2363 | 0.5385598 |
| hsa-miR-3690-st | 0.0561 | 0.0464 | 1.207 | 0.2364 | 0.5385598 |
| hsa-miR-573-st | 0.025 | 0.0207 | 1.206 | 0.2371 | 0.538691 |
| hsa-miR-4432-st | -0.0455 | 0.0378 | -1.205 | 0.2372 | 0.538691 |
| hsa-miR-3615-st | 0.1052 | 0.0875 | 1.203 | 0.2381 | 0.53989 |
| hsa-miR-340-st | -0.1324 | 0.1102 | -1.202 | 0.2386 | 0.5401797 |
| hsa-miR-891b-st | -0.0399 | 0.0334 | -1.196 | 0.2407 | 0.5425134 |
| hsa-miR-556-5p-st | -0.0287 | 0.024 | -1.195 | 0.2413 | 0.5425134 |
| hsa-miR-1471-st | 0.1053 | 0.0882 | 1.194 | 0.2414 | 0.5425134 |
| hsa-miR-508-3p-st | -0.0297 | 0.0248 | -1.194 | 0.2414 | 0.5425134 |
| hsa-miR-4791-st | -0.0537 | 0.045 | -1.194 | 0.2415 | 0.5425134 |
| hsa-miR-1251-st | -0.1114 | 0.0938 | -1.188 | 0.2437 | 0.5440316 |
| hsa-miR-516a-3p-st | 0.0327 | 0.0275 | 1.188 | 0.2437 | 0.5440316 |
| hsa-miR-181c-star-st | 0.0721 | 0.0607 | 1.188 | 0.2439 | 0.5440316 |
| hsa-miR-3940-3p-st | 0.0761 | 0.0641 | 1.187 | 0.2441 | 0.5440316 |
| hsa-miR-511-st | -0.0281 | 0.0237 | -1.186 | 0.2447 | 0.5440316 |
| hsa-miR-105-star-st | -0.0463 | 0.0391 | -1.186 | 0.2448 | 0.5440316 |
| hsa-miR-450b-3p-st | 0.0286 | 0.0241 | 1.186 | 0.2448 | 0.5440316 |
| hsa-miR-130a-st | 0.0618 | 0.0523 | 1.182 | 0.246 | 0.5458624 |
| hsa-miR-4712-5p-st | 0.0316 | 0.0268 | 1.181 | 0.2467 | 0.54658 |
| hsa-miR-3177-3p-st | -0.0722 | 0.0614 | -1.176 | 0.2486 | 0.5499499 |
| hsa-miR-4637-st | -0.031 | 0.0264 | -1.175 | 0.249 | 0.5499964 |
| hsa-miR-548ad-st | -0.033 | 0.0283 | -1.168 | 0.2518 | 0.5545325 |
| hsa-miR-124-star-st | 0.0563 | 0.0483 | 1.167 | 0.2522 | 0.5545325 |
| hsa-miR-664-st | 0.0548 | 0.047 | 1.167 | 0.2522 | 0.5545325 |
| hsa-miR-675-st | 0.1402 | 0.1208 | 1.161 | 0.2546 | 0.55753 |
| hsa-let-7e-star-st | 0.0868 | 0.0748 | 1.16 | 0.2549 | 0.55753 |
| hsa-miR-361-3p-st | -0.0804 | 0.0693 | -1.16 | 0.255 | 0.55753 |
| hsa-miR-509-3p-st | -0.1246 | 0.1074 | -1.159 | 0.2551 | 0.55753 |
| hsa-miR-340-star-st | -0.0537 | 0.0467 | -1.15 | 0.2589 | 0.56329 |
| hsa-miR-4293-st | 0.028 | 0.0243 | 1.15 | 0.2589 | 0.56329 |
| hsa-miR-431-star-st | -0.0807 | 0.0702 | -1.15 | 0.2589 | 0.56329 |
| hsa-miR-662-st | 0.0446 | 0.039 | 1.144 | 0.2616 | 0.5676798 |
| hsa-miR-570-st | 0.0564 | 0.0494 | 1.143 | 0.2617 | 0.5676798 |
| hsa-miR-4724-3p-st | -0.0365 | 0.032 | -1.14 | 0.263 | 0.5696483 |
| hsa-miR-4327-st | 0.1916 | 0.1683 | 1.139 | 0.2636 | 0.570097 |
| hsa-miR-4329-st | -0.045 | 0.0397 | -1.134 | 0.2654 | 0.5731358 |
| hsa-miR-1237-st | 0.1059 | 0.0937 | 1.13 | 0.2672 | 0.5738721 |
| hsa-miR-23c-st | -0.0863 | 0.0764 | -1.13 | 0.2673 | 0.5738721 |
| hsa-miR-4431-st | -0.0409 | 0.0363 | -1.129 | 0.2677 | 0.5738721 |
| hsa-miR-1537-st | 0.0292 | 0.0259 | 1.128 | 0.268 | 0.5738721 |
| hsa-miR-3151-st | -0.1226 | 0.1088 | -1.127 | 0.2685 | 0.5738721 |
| hsa-miR-4646-3p-st | 0.0569 | 0.0505 | 1.126 | 0.2687 | 0.5738721 |
| hsa-miR-302c-st | -0.0224 | 0.0199 | -1.125 | 0.2691 | 0.5738721 |
| hsa-miR-3196-st | 0.1183 | 0.1051 | 1.125 | 0.2691 | 0.5738721 |
| hsa-miR-581-st | -0.0232 | 0.0206 | -1.125 | 0.2693 | 0.5738721 |
| hsa-miR-4679-st | -0.0338 | 0.0302 | -1.122 | 0.2706 | 0.5751818 |
| hsa-miR-3912-st | -0.0353 | 0.0315 | -1.121 | 0.2711 | 0.5751818 |
| hsa-miR-4652-5p-st | 0.0334 | 0.0298 | 1.119 | 0.2715 | 0.5751818 |
| hsa-miR-626-st | -0.0308 | 0.0275 | -1.12 | 0.2715 | 0.5751818 |
| hsa-miR-4665-3p-st | -0.0505 | 0.0452 | -1.118 | 0.2724 | 0.5756197 |
| hsa-miR-1293-st | -0.0432 | 0.0387 | -1.117 | 0.2725 | 0.5756197 |
| hsa-miR-196b-star-st | -0.055 | 0.0493 | -1.115 | 0.2733 | 0.5756408 |
| hsa-miR-876-5p-st | -0.035 | 0.0314 | -1.115 | 0.2734 | 0.5756408 |
| hsa-miR-29a-star-st | -0.0294 | 0.0264 | -1.114 | 0.2737 | 0.5756408 |
| hsa-miR-4640-5p-st | 0.1266 | 0.1138 | 1.113 | 0.2742 | 0.5758578 |
| hsa-miR-125b-1-star-st | -0.0608 | 0.0547 | -1.111 | 0.2749 | 0.5764936 |
| hsa-miR-135a-star-st | 0.1139 | 0.1027 | 1.108 | 0.2762 | 0.5779868 |
| hsa-miR-371b-5p-st | 0.1985 | 0.1797 | 1.105 | 0.2777 | 0.5779868 |
| hsa-miR-3180-5p-st | -0.0362 | 0.0327 | -1.105 | 0.2778 | 0.5779868 |
| hsa-miR-4709-3p-st | -0.0804 | 0.0728 | -1.105 | 0.2778 | 0.5779868 |
| hsa-miR-4258-st | 0.0788 | 0.0713 | 1.105 | 0.2779 | 0.5779868 |
| hsa-miR-4527-st | 0.0258 | 0.0233 | 1.104 | 0.2782 | 0.5779868 |
| hsa-miR-16-st | 0.0283 | 0.0257 | 1.103 | 0.2784 | 0.5779868 |
| hsa-miR-3605-3p-st | -0.0953 | 0.0865 | -1.101 | 0.2792 | 0.5784513 |
| hsa-miR-524-3p-st | -0.0392 | 0.0356 | -1.1 | 0.2796 | 0.5784513 |
| hsa-miR-3186-3p-st | 0.0698 | 0.0636 | 1.098 | 0.2807 | 0.5784513 |
| hsa-miR-559-st | -0.0482 | 0.0439 | -1.098 | 0.2808 | 0.5784513 |
| hsa-miR-3129-5p-st | 0.0341 | 0.0311 | 1.095 | 0.2819 | 0.5784513 |
| hsa-miR-200b-st | 0.0974 | 0.089 | 1.095 | 0.2821 | 0.5784513 |
| hsa-miR-3652-st | 0.1021 | 0.0933 | 1.094 | 0.2822 | 0.5784513 |
| hsa-miR-23a-star-st | 0.0781 | 0.0715 | 1.092 | 0.2831 | 0.5784513 |
| hsa-miR-4728-5p-st | 0.0706 | 0.0647 | 1.091 | 0.2835 | 0.5784513 |
| hsa-miR-4524-st | 0.0302 | 0.0277 | 1.091 | 0.2838 | 0.5784513 |
| hsa-miR-2276-st | -0.1014 | 0.093 | -1.09 | 0.284 | 0.5784513 |
| hsa-miR-449c-star-st | -0.0422 | 0.0387 | -1.09 | 0.2842 | 0.5784513 |
| hsa-miR-572-st | 0.0973 | 0.0893 | 1.089 | 0.2844 | 0.5784513 |
| hsa-miR-3689b-st | 0.0356 | 0.0327 | 1.088 | 0.2849 | 0.5784513 |
| hsa-miR-505-st | -0.0822 | 0.0755 | -1.088 | 0.285 | 0.5784513 |
| hsa-miR-20a-st | 0.0353 | 0.0325 | 1.087 | 0.2854 | 0.5784513 |
| hsa-miR-20b-star-st | 0.0684 | 0.0629 | 1.087 | 0.2854 | 0.5784513 |
| hsa-miR-3935-st | 0.0745 | 0.0687 | 1.084 | 0.2869 | 0.5806805 |
| hsa-miR-4291-st | -0.0702 | 0.0649 | -1.082 | 0.2877 | 0.5808818 |
| hsa-miR-29c-st | -0.0739 | 0.0683 | -1.081 | 0.2878 | 0.5808818 |
| hsa-miR-598-st | -0.0689 | 0.0638 | -1.079 | 0.289 | 0.5824937 |
| hsa-miR-103a-st | -0.0176 | 0.0164 | -1.075 | 0.2905 | 0.584096 |
| hsa-miR-4672-st | 0.0421 | 0.0392 | 1.075 | 0.2906 | 0.584096 |
| hsa-miR-188-3p-st | -0.0433 | 0.0403 | -1.074 | 0.2913 | 0.5846932 |
| hsa-miR-496-st | -0.0656 | 0.0612 | -1.072 | 0.292 | 0.5852887 |
| hsa-miR-613-st | 0.0294 | 0.0275 | 1.069 | 0.2932 | 0.5868834 |
| hsa-miR-3065-5p-st | 0.1437 | 0.1345 | 1.068 | 0.2938 | 0.5872743 |
| hsa-miR-4676-3p-st | -0.0339 | 0.0318 | -1.066 | 0.2948 | 0.5881993 |
| hsa-miR-376b-st | -0.0726 | 0.0682 | -1.065 | 0.2952 | 0.5881993 |
| hsa-miR-4716-5p-st | -0.0617 | 0.0581 | -1.062 | 0.2963 | 0.5881993 |
| hsa-miR-629-st | 0.0474 | 0.0446 | 1.062 | 0.2963 | 0.5881993 |
| hsa-miR-4671-3p-st | -0.0407 | 0.0383 | -1.062 | 0.2966 | 0.5881993 |
| hsa-let-7a-2-star-st | -0.058 | 0.0547 | -1.061 | 0.2968 | 0.5881993 |
| hsa-miR-4492-st | 0.1797 | 0.1694 | 1.061 | 0.2971 | 0.5881993 |
| hsa-miR-4508-st | 0.1493 | 0.1412 | 1.058 | 0.2984 | 0.5895113 |
| hsa-miR-206-st | 0.0935 | 0.0885 | 1.057 | 0.2987 | 0.5895113 |
| hsa-miR-3663-5p-st | 0.1292 | 0.1224 | 1.056 | 0.2993 | 0.5895113 |
| hsa-miR-588-st | -0.0251 | 0.0238 | -1.055 | 0.2995 | 0.5895113 |
| hsa-miR-3156-3p-st | 0.0425 | 0.0403 | 1.054 | 0.3001 | 0.5895113 |
| hsa-miR-4736-st | 0.0269 | 0.0256 | 1.054 | 0.3002 | 0.5895113 |
| hsa-miR-3621-st | 0.1337 | 0.1278 | 1.046 | 0.3036 | 0.5953421 |
| hsa-miR-452-star-st | -0.0287 | 0.0275 | -1.045 | 0.304 | 0.5953421 |
| hsa-miR-3674-st | -0.0266 | 0.0255 | -1.044 | 0.3044 | 0.5953421 |
| hsa-miR-3187-5p-st | 0.1047 | 0.1006 | 1.041 | 0.306 | 0.5966458 |
| hsa-miR-4283-st | 0.0426 | 0.0409 | 1.041 | 0.306 | 0.5966458 |
| hsa-miR-4787-5p-st | 0.0718 | 0.069 | 1.04 | 0.3063 | 0.5966458 |
| hsa-miR-3150b-3p-st | -0.0428 | 0.0413 | -1.038 | 0.3073 | 0.5977913 |
| hsa-miR-4322-st | 0.1349 | 0.1303 | 1.036 | 0.3082 | 0.5982321 |
| hsa-miR-100-star-st | 0.0368 | 0.0356 | 1.033 | 0.3094 | 0.5982321 |
| hsa-miR-3146-st | -0.0352 | 0.0341 | -1.033 | 0.3097 | 0.5982321 |
| hsa-miR-548f-st | -0.0323 | 0.0313 | -1.032 | 0.3099 | 0.5982321 |
| hsa-miR-1260-st | 0.1112 | 0.1077 | 1.032 | 0.31 | 0.5982321 |
| hsa-miR-891a-st | -0.0793 | 0.0769 | -1.032 | 0.31 | 0.5982321 |
| hsa-miR-2116-st | -0.0996 | 0.0966 | -1.031 | 0.3106 | 0.598594 |
| hsa-miR-708-st | -0.0604 | 0.0587 | -1.03 | 0.3111 | 0.5987624 |
| hsa-miR-3178-st | 0.1297 | 0.1262 | 1.028 | 0.312 | 0.598906 |
| hsa-miR-3691-5p-st | -0.0473 | 0.046 | -1.028 | 0.312 | 0.598906 |
| hsa-miR-483-5p-st | 0.076 | 0.0741 | 1.025 | 0.3135 | 0.600389 |
| hsa-miR-590-5p-st | 0.0993 | 0.0969 | 1.024 | 0.3136 | 0.600389 |
| hsa-miR-1295-st | -0.0592 | 0.0579 | -1.021 | 0.3151 | 0.6007229 |
| hsa-miR-663-st | 0.119 | 0.1168 | 1.019 | 0.3161 | 0.6007229 |
| hsa-miR-4732-5p-st | 0.0489 | 0.048 | 1.019 | 0.3163 | 0.6007229 |
| hsa-miR-576-3p-st | -0.0271 | 0.0266 | -1.018 | 0.3164 | 0.6007229 |
| hsa-miR-10b-star-st | 0.0285 | 0.028 | 1.017 | 0.3169 | 0.6007229 |
| hsa-miR-4423-5p-st | 0.0306 | 0.0301 | 1.017 | 0.317 | 0.6007229 |
| hsa-miR-23b-st | -0.0193 | 0.019 | -1.017 | 0.3171 | 0.6007229 |
| hsa-miR-1204-st | 0.0585 | 0.0576 | 1.017 | 0.3172 | 0.6007229 |
| hsa-miR-4764-3p-st | -0.0542 | 0.0534 | -1.016 | 0.3175 | 0.6007229 |
| hsa-miR-4275-st | -0.0252 | 0.025 | -1.01 | 0.3202 | 0.6050426 |
| hsa-miR-192-star-st | 0.0789 | 0.0784 | 1.006 | 0.3222 | 0.6061315 |
| hsa-miR-1178-st | -0.0315 | 0.0313 | -1.006 | 0.3224 | 0.6061315 |
| hsa-miR-4696-st | -0.0364 | 0.0363 | -1.005 | 0.3226 | 0.6061315 |
| hsa-miR-3616-3p-st | -0.0495 | 0.0492 | -1.005 | 0.3227 | 0.6061315 |
| hsa-miR-4646-5p-st | 0.1081 | 0.1077 | 1.004 | 0.3231 | 0.6061315 |
| hsa-miR-28-3p-st | 0.0355 | 0.0353 | 1.004 | 0.3233 | 0.6061315 |
| hsa-miR-139-3p-st | -0.074 | 0.0738 | -1.003 | 0.3237 | 0.6061315 |
| hsa-miR-4520a-3p-st | -0.1111 | 0.1109 | -1.002 | 0.3242 | 0.6062855 |
| hsa-miR-657-st | -0.0343 | 0.0344 | -0.998 | 0.3261 | 0.6075374 |
| hsa-miR-30e-st | 0.0528 | 0.0531 | 0.995 | 0.3273 | 0.6075374 |
| hsa-miR-636-st | -0.0697 | 0.07 | -0.995 | 0.3275 | 0.6075374 |
| hsa-miR-3199-st | -0.0341 | 0.0342 | -0.995 | 0.3276 | 0.6075374 |
| hsa-miR-2682-st | -0.0777 | 0.0781 | -0.994 | 0.3277 | 0.6075374 |
| hsa-miR-190-st | -0.0277 | 0.0278 | -0.994 | 0.3278 | 0.6075374 |
| hsa-miR-2278-st | 0.0655 | 0.0659 | 0.994 | 0.3278 | 0.6075374 |
| hsa-miR-489-st | 0.1857 | 0.1875 | 0.99 | 0.3297 | 0.6078142 |
| hsa-miR-4452-st | 0.0229 | 0.0231 | 0.99 | 0.3299 | 0.6078142 |
| hsa-miR-3614-5p-st | 0.0582 | 0.0588 | 0.989 | 0.3301 | 0.6078142 |
| hsa-miR-3942-3p-st | -0.0279 | 0.0282 | -0.99 | 0.3301 | 0.6078142 |
| hsa-miR-1911-st | -0.089 | 0.09 | -0.988 | 0.3306 | 0.6078142 |
| hsa-miR-4799-5p-st | -0.0399 | 0.0404 | -0.988 | 0.331 | 0.6078142 |
| hsa-miR-451b-st | 0.0282 | 0.0285 | 0.987 | 0.3312 | 0.6078142 |
| hsa-miR-372-st | 0.0288 | 0.0292 | 0.987 | 0.3313 | 0.6078142 |
| hsa-miR-2392-st | 0.0795 | 0.0808 | 0.985 | 0.3323 | 0.6084772 |
| hsa-miR-4321-st | 0.0992 | 0.1008 | 0.985 | 0.3325 | 0.6084772 |
| hsa-miR-769-3p-st | -0.0782 | 0.0795 | -0.983 | 0.3331 | 0.6088075 |
| hsa-miR-4635-st | 0.0344 | 0.0351 | 0.981 | 0.3342 | 0.6100496 |
| hsa-miR-4713-3p-st | 0.0353 | 0.0362 | 0.976 | 0.3365 | 0.6120103 |
| hsa-miR-759-st | 0.0322 | 0.033 | 0.975 | 0.3371 | 0.6120103 |
| hsa-miR-548g-st | -0.0198 | 0.0203 | -0.975 | 0.3372 | 0.6120103 |
| hsa-miR-4749-5p-st | 0.1064 | 0.1093 | 0.973 | 0.3379 | 0.6120103 |
| hsa-miR-1324-st | -0.0456 | 0.0469 | -0.973 | 0.3381 | 0.6120103 |
| hsa-miR-1185-st | 0.1116 | 0.1148 | 0.972 | 0.3386 | 0.6120103 |
| hsa-miR-24-2-star-st | 0.0696 | 0.0716 | 0.972 | 0.3387 | 0.6120103 |
| hsa-miR-107-st | -0.0105 | 0.0108 | -0.971 | 0.3392 | 0.6120103 |
| hsa-miR-519e-star-st | -0.1487 | 0.1533 | -0.97 | 0.3394 | 0.6120103 |
| hsa-miR-550a-st | 0.0476 | 0.049 | 0.97 | 0.3398 | 0.6120103 |
| hsa-miR-4655-3p-st | -0.049 | 0.0506 | -0.969 | 0.3402 | 0.6120103 |
| hsa-miR-4534-st | 0.0631 | 0.0654 | 0.966 | 0.3414 | 0.6120103 |
| hsa-let-7e-st | -0.0228 | 0.0236 | -0.966 | 0.3416 | 0.6120103 |
| hsa-miR-29b-1-star-st | -0.0647 | 0.067 | -0.966 | 0.3416 | 0.6120103 |
| hsa-miR-548i-st | -0.0344 | 0.0356 | -0.966 | 0.3416 | 0.6120103 |
| hsa-miR-515-3p-st | 0.0236 | 0.0245 | 0.964 | 0.3424 | 0.6120724 |
| hsa-miR-423-3p-st | -0.0448 | 0.0465 | -0.963 | 0.3428 | 0.6120724 |
| hsa-miR-23b-star-st | -0.0422 | 0.0438 | -0.963 | 0.3429 | 0.6120724 |
| hsa-miR-3157-3p-st | 0.0793 | 0.0824 | 0.961 | 0.3438 | 0.612925 |
| hsa-miR-551a-st | -0.046 | 0.048 | -0.959 | 0.345 | 0.6139125 |
| hsa-miR-106a-st | 0.0238 | 0.0248 | 0.958 | 0.3452 | 0.6139125 |
| hsa-miR-1281-st | 0.0785 | 0.0821 | 0.956 | 0.3464 | 0.6143335 |
| hsa-miR-3975-st | -0.0171 | 0.0178 | -0.956 | 0.3464 | 0.6143335 |
| hsa-miR-181a-2-star-st | -0.059 | 0.0619 | -0.954 | 0.3476 | 0.6143335 |
| hsa-miR-19a-star-st | -0.0238 | 0.0249 | -0.954 | 0.3476 | 0.6143335 |
| hsa-miR-4448-st | 0.0475 | 0.0499 | 0.953 | 0.3479 | 0.6143335 |
| hsa-miR-4735-3p-st | 0.028 | 0.0294 | 0.953 | 0.3482 | 0.6143335 |
| hsa-miR-622-st | -0.0448 | 0.0471 | -0.952 | 0.3484 | 0.6143335 |
| hsa-miR-324-3p-st | -0.0357 | 0.0375 | -0.951 | 0.3489 | 0.6144685 |
| hsa-miR-200a-star-st | 0.0319 | 0.0336 | 0.947 | 0.3509 | 0.6172417 |
| hsa-miR-211-st | 0.0875 | 0.0929 | 0.942 | 0.3533 | 0.620711 |
| hsa-miR-610-st | -0.029 | 0.0308 | -0.941 | 0.3539 | 0.6210133 |
| hsa-miR-4475-st | -0.0428 | 0.0455 | -0.94 | 0.3546 | 0.6213421 |
| hsa-miR-4319-st | -0.0291 | 0.031 | -0.939 | 0.355 | 0.6213421 |
| hsa-miR-146b-5p-st | 0.0894 | 0.0953 | 0.938 | 0.3557 | 0.6213421 |
| hsa-miR-4671-5p-st | 0.0386 | 0.0412 | 0.937 | 0.3558 | 0.6213421 |
| hsa-miR-3616-5p-st | -0.0263 | 0.0283 | -0.931 | 0.3591 | 0.6263512 |
| hsa-miR-567-st | 0.0389 | 0.0421 | 0.924 | 0.3627 | 0.631871 |
| hsa-miR-4419b-st | 0.0362 | 0.0394 | 0.92 | 0.3646 | 0.6323673 |
| hsa-miR-4537-st | 0.0398 | 0.0432 | 0.92 | 0.3649 | 0.6323673 |
| hsa-miR-301a-st | 0.0768 | 0.0835 | 0.919 | 0.365 | 0.6323673 |
| hsa-miR-4692-st | -0.0443 | 0.0482 | -0.919 | 0.3651 | 0.6323673 |
| hsa-miR-3688-5p-st | -0.0181 | 0.0197 | -0.917 | 0.3663 | 0.6323673 |
| hsa-miR-190b-st | 0.0282 | 0.0309 | 0.915 | 0.3671 | 0.6323673 |
| hsa-miR-3136-3p-st | 0.0236 | 0.0258 | 0.915 | 0.3671 | 0.6323673 |
| hsa-miR-4418-st | 0.035 | 0.0383 | 0.912 | 0.369 | 0.6323673 |
| hsa-miR-4777-5p-st | 0.0298 | 0.0327 | 0.912 | 0.369 | 0.6323673 |
| hsa-miR-3677-5p-st | -0.0232 | 0.0255 | -0.911 | 0.3692 | 0.6323673 |
| hsa-miR-4282-st | -0.0182 | 0.02 | -0.911 | 0.3695 | 0.6323673 |
| hsa-miR-4444-st | 0.0763 | 0.0838 | 0.91 | 0.3698 | 0.6323673 |
| hsa-let-7d-star-st | 0.0564 | 0.0622 | 0.908 | 0.371 | 0.6323673 |
| hsa-miR-1825-st | -0.0307 | 0.0338 | -0.907 | 0.3712 | 0.6323673 |
| hsa-miR-4684-5p-st | 0.0261 | 0.0288 | 0.907 | 0.3712 | 0.6323673 |
| hsa-miR-499-3p-st | -0.0298 | 0.0329 | -0.907 | 0.3712 | 0.6323673 |
| hsa-miR-377-st | -0.1318 | 0.1453 | -0.907 | 0.3713 | 0.6323673 |
| hsa-miR-488-star-st | -0.0968 | 0.1067 | -0.907 | 0.3713 | 0.6323673 |
| hsa-miR-320c-st | 0.0346 | 0.0381 | 0.907 | 0.3716 | 0.6323673 |
| hsa-miR-145-st | 0.0393 | 0.0433 | 0.906 | 0.3717 | 0.6323673 |
| hsa-miR-4326-st | 0.0486 | 0.0538 | 0.904 | 0.373 | 0.6335706 |
| hsa-miR-1257-st | -0.0254 | 0.0281 | -0.903 | 0.3735 | 0.6335706 |
| hsa-miR-4794-st | 0.0246 | 0.0273 | 0.902 | 0.3742 | 0.6335706 |
| hsa-miR-551b-st | -0.047 | 0.0522 | -0.9 | 0.3751 | 0.6335706 |
| hsa-miR-297-st | 0.1463 | 0.1627 | 0.9 | 0.3753 | 0.6335706 |
| hsa-miR-146a-st | -0.0698 | 0.0777 | -0.899 | 0.3757 | 0.6335706 |
| hsa-miR-203-st | 0.1208 | 0.1344 | 0.899 | 0.3757 | 0.6335706 |
| hsa-miR-3149-st | 0.064 | 0.0712 | 0.898 | 0.3759 | 0.6335706 |
| hsa-miR-374b-st | -0.0853 | 0.0953 | -0.895 | 0.3775 | 0.6343128 |
| hsa-miR-1245b-5p-st | -0.0227 | 0.0254 | -0.894 | 0.3781 | 0.6343128 |
| hsa-miR-223-st | 0.0853 | 0.0954 | 0.894 | 0.3784 | 0.6343128 |
| hsa-miR-96-st | 0.0323 | 0.0361 | 0.893 | 0.3785 | 0.6343128 |
| hsa-miR-4446-3p-st | 0.095 | 0.1065 | 0.893 | 0.3789 | 0.6343128 |
| hsa-miR-4782-3p-st | -0.0202 | 0.0227 | -0.892 | 0.3791 | 0.6343128 |
| hsa-miR-4720-5p-st | 0.0991 | 0.1111 | 0.892 | 0.3794 | 0.6343128 |
| hsa-miR-202-st | 0.0685 | 0.077 | 0.89 | 0.3805 | 0.6348562 |
| hsa-miR-198-st | 0.081 | 0.0911 | 0.89 | 0.3806 | 0.6348562 |
| hsa-miR-492-st | -0.0436 | 0.0492 | -0.885 | 0.3829 | 0.6372352 |
| hsa-miR-30c-1-star-st | -0.0521 | 0.0589 | -0.885 | 0.3831 | 0.6372352 |
| hsa-miR-648-st | -0.0358 | 0.0406 | -0.883 | 0.384 | 0.6372352 |
| hsa-miR-519e-st | 0.0238 | 0.027 | 0.882 | 0.3845 | 0.6372352 |
| hsa-miR-3121-5p-st | -0.0256 | 0.0291 | -0.881 | 0.3849 | 0.6372352 |
| hsa-miR-410-st | -0.0539 | 0.0611 | -0.881 | 0.385 | 0.6372352 |
| hsa-miR-4795-5p-st | -0.0323 | 0.0366 | -0.881 | 0.3851 | 0.6372352 |
| hsa-miR-647-st | 0.0768 | 0.0875 | 0.878 | 0.3867 | 0.639154 |
| hsa-miR-4453-st | -0.0285 | 0.0325 | -0.876 | 0.3875 | 0.6396803 |
| hsa-miR-3656-st | 0.0902 | 0.103 | 0.876 | 0.3879 | 0.6396803 |
| hsa-miR-4480-st | -0.0307 | 0.0352 | -0.873 | 0.3895 | 0.6415897 |
| hsa-miR-4491-st | 0.0283 | 0.0327 | 0.866 | 0.3932 | 0.6462635 |
| hsa-miR-205-st | -0.0394 | 0.0456 | -0.865 | 0.3939 | 0.6462635 |
| hsa-miR-513c-st | 0.0362 | 0.0418 | 0.864 | 0.394 | 0.6462635 |
| hsa-miR-199a-5p-st | 0.0617 | 0.0715 | 0.863 | 0.3946 | 0.6462635 |
| hsa-miR-146b-3p-st | 0.1133 | 0.1314 | 0.862 | 0.3951 | 0.6462635 |
| hsa-miR-1914-st | 0.04 | 0.0464 | 0.862 | 0.3953 | 0.6462635 |
| hsa-miR-517b-st | -0.0529 | 0.0614 | -0.861 | 0.3957 | 0.6462635 |
| hsa-miR-4727-3p-st | -0.0698 | 0.081 | -0.861 | 0.3959 | 0.6462635 |
| hsa-miR-532-3p-st | 0.0372 | 0.0433 | 0.859 | 0.397 | 0.6470982 |
| hsa-miR-606-st | -0.019 | 0.0221 | -0.858 | 0.3974 | 0.6470982 |
| hsa-miR-4793-5p-st | 0.0738 | 0.0862 | 0.856 | 0.3987 | 0.6470982 |
| hsa-miR-3545-5p-st | -0.0289 | 0.0338 | -0.855 | 0.399 | 0.6470982 |
| hsa-miR-3650-st | -0.0263 | 0.0308 | -0.854 | 0.3994 | 0.6470982 |
| hsa-miR-3649-st | -0.0203 | 0.0237 | -0.854 | 0.3998 | 0.6470982 |
| hsa-miR-4747-5p-st | 0.0289 | 0.0338 | 0.853 | 0.4 | 0.6470982 |
| hsa-miR-3675-3p-st | 0.0398 | 0.0467 | 0.853 | 0.4004 | 0.6470982 |
| hsa-miR-4290-st | 0.0807 | 0.0949 | 0.85 | 0.4017 | 0.6470982 |
| hsa-miR-4783-5p-st | 0.0457 | 0.0539 | 0.849 | 0.4022 | 0.6470982 |
| hsa-miR-335-st | -0.0495 | 0.0583 | -0.849 | 0.4023 | 0.6470982 |
| hsa-miR-4795-3p-st | 0.0179 | 0.021 | 0.849 | 0.4024 | 0.6470982 |
| hsa-miR-4697-3p-st | 0.0613 | 0.0722 | 0.849 | 0.4025 | 0.6470982 |
| hsa-miR-4693-3p-st | -0.0245 | 0.0289 | -0.848 | 0.4027 | 0.6470982 |
| hsa-miR-4509-st | 0.0214 | 0.0252 | 0.848 | 0.4031 | 0.6470982 |
| hsa-miR-3918-st | 0.0784 | 0.0927 | 0.846 | 0.4041 | 0.6472842 |
| hsa-miR-25-st | 0.0619 | 0.0733 | 0.845 | 0.4043 | 0.6472842 |
| hsa-miR-4529-5p-st | 0.0205 | 0.0243 | 0.845 | 0.4048 | 0.6472842 |
| hsa-miR-1226-star-st | -0.0969 | 0.1148 | -0.844 | 0.405 | 0.6472842 |
| hsa-miR-4731-5p-st | 0.0745 | 0.0884 | 0.842 | 0.406 | 0.6474564 |
| hsa-miR-549-st | -0.0303 | 0.036 | -0.842 | 0.406 | 0.6474564 |
| hsa-miR-128-st | -0.0341 | 0.0407 | -0.838 | 0.4084 | 0.6483795 |
| hsa-miR-3155-st | -0.0414 | 0.0494 | -0.837 | 0.4088 | 0.6483795 |
| hsa-miR-4669-st | -0.0567 | 0.0677 | -0.837 | 0.4091 | 0.6483795 |
| hsa-miR-3145-5p-st | 0.0296 | 0.0354 | 0.836 | 0.4094 | 0.6483795 |
| hsa-miR-143-st | 0.0369 | 0.0442 | 0.835 | 0.4099 | 0.6483795 |
| hsa-miR-1238-st | 0.0622 | 0.0745 | 0.835 | 0.4102 | 0.6483795 |
| hsa-let-7b-st | 0.0032 | 0.0038 | 0.834 | 0.4104 | 0.6483795 |
| hsa-miR-194-st | -0.024 | 0.0288 | -0.834 | 0.4105 | 0.6483795 |
| hsa-miR-4473-st | 0.0288 | 0.0345 | 0.834 | 0.4106 | 0.6483795 |
| hsa-miR-4755-3p-st | -0.0321 | 0.0386 | -0.833 | 0.4112 | 0.6486212 |
| hsa-miR-92a-st | -0.0325 | 0.0391 | -0.832 | 0.4117 | 0.6487048 |
| hsa-miR-548ae-st | -0.0539 | 0.0648 | -0.831 | 0.4126 | 0.6494178 |
| hsa-miR-374c-st | -0.1226 | 0.1481 | -0.828 | 0.4138 | 0.6503737 |
| hsa-miR-2682-star-st | -0.0282 | 0.0341 | -0.828 | 0.4143 | 0.6503737 |
| hsa-miR-4740-3p-st | 0.1102 | 0.1333 | 0.827 | 0.4147 | 0.6503737 |
| hsa-miR-3152-5p-st | -0.0228 | 0.0277 | -0.826 | 0.415 | 0.6503737 |
| hsa-miR-4758-3p-st | -0.082 | 0.0996 | -0.823 | 0.417 | 0.6528031 |
| hsa-miR-133a-st | -0.11 | 0.1339 | -0.821 | 0.4177 | 0.6529598 |
| hsa-miR-4299-st | 0.0974 | 0.1187 | 0.821 | 0.418 | 0.6529598 |
| hsa-miR-760-st | 0.0579 | 0.0708 | 0.817 | 0.4199 | 0.6552225 |
| hsa-miR-485-5p-st | 0.0419 | 0.0515 | 0.814 | 0.422 | 0.6562128 |
| hsa-miR-4645-3p-st | -0.0236 | 0.029 | -0.813 | 0.4223 | 0.6562128 |
| hsa-miR-4725-3p-st | 0.136 | 0.1673 | 0.813 | 0.4224 | 0.6562128 |
| hsa-miR-3184-st | 0.0464 | 0.0571 | 0.813 | 0.4227 | 0.6562128 |
| hsa-miR-4424-st | -0.0233 | 0.0287 | -0.812 | 0.4231 | 0.6562128 |
| hsa-miR-4691-5p-st | 0.0362 | 0.0446 | 0.812 | 0.4233 | 0.6562128 |
| hsa-miR-4437-st | -0.0194 | 0.0239 | -0.811 | 0.4237 | 0.6562128 |
| hsa-miR-208a-st | -0.0225 | 0.0277 | -0.81 | 0.4244 | 0.6565962 |
| hsa-miR-138-1-star-st | -0.0569 | 0.0705 | -0.807 | 0.4257 | 0.6573606 |
| hsa-miR-4678-st | -0.0195 | 0.0242 | -0.807 | 0.4258 | 0.6573606 |
| hsa-miR-4711-3p-st | -0.0167 | 0.0208 | -0.806 | 0.4266 | 0.6578958 |
| hsa-miR-548ai-st | 0.0803 | 0.0999 | 0.804 | 0.4275 | 0.6582426 |
| hsa-miR-3647-3p-st | -0.0297 | 0.037 | -0.802 | 0.4289 | 0.6582426 |
| hsa-miR-2467-3p-st | 0.1031 | 0.1287 | 0.801 | 0.429 | 0.6582426 |
| hsa-miR-3684-st | -0.0241 | 0.0301 | -0.801 | 0.4294 | 0.6582426 |
| hsa-miR-4445-star-st | 0.0312 | 0.0389 | 0.8 | 0.4295 | 0.6582426 |
| hsa-miR-4800-5p-st | 0.0664 | 0.0829 | 0.8 | 0.4296 | 0.6582426 |
| hsa-miR-634-st | -0.0365 | 0.0456 | -0.8 | 0.43 | 0.6582426 |
| hsa-miR-661-st | 0.0803 | 0.1007 | 0.798 | 0.4311 | 0.659218 |
| hsa-miR-4484-st | 0.049 | 0.0615 | 0.796 | 0.432 | 0.659218 |
| hsa-miR-545-star-st | 0.02 | 0.0252 | 0.796 | 0.432 | 0.659218 |
| hsa-miR-1266-st | 0.0439 | 0.0553 | 0.794 | 0.433 | 0.66005 |
| hsa-miR-4512-st | 0.0206 | 0.0262 | 0.786 | 0.4381 | 0.6671235 |
| hsa-miR-4489-st | 0.0392 | 0.05 | 0.784 | 0.439 | 0.6673391 |
| hsa-miR-1261-st | 0.0343 | 0.0438 | 0.782 | 0.4399 | 0.6673391 |
| hsa-miR-4471-st | -0.0217 | 0.0278 | -0.781 | 0.4409 | 0.6673391 |
| hsa-miR-493-star-st | -0.022 | 0.0282 | -0.781 | 0.4409 | 0.6673391 |
| hsa-let-7f-2-star-st | -0.036 | 0.0461 | -0.78 | 0.4414 | 0.6673391 |
| hsa-miR-586-st | 0.0252 | 0.0323 | 0.78 | 0.4416 | 0.6673391 |
| hsa-miR-643-st | 0.0301 | 0.0387 | 0.778 | 0.4423 | 0.6673391 |
| hsa-miR-92b-star-st | 0.0811 | 0.1042 | 0.778 | 0.4423 | 0.6673391 |
| hsa-miR-520c-5p-st | -0.0411 | 0.0529 | -0.777 | 0.4432 | 0.6673391 |
| hsa-miR-656-st | -0.0227 | 0.0292 | -0.777 | 0.4432 | 0.6673391 |
| hsa-miR-924-st | -0.0219 | 0.0282 | -0.776 | 0.4433 | 0.6673391 |
| hsa-miR-933-st | 0.0835 | 0.1077 | 0.775 | 0.444 | 0.6677002 |
| hsa-miR-1299-st | -0.2029 | 0.2624 | -0.773 | 0.4453 | 0.6680296 |
| hsa-miR-323b-3p-st | 0.0765 | 0.099 | 0.773 | 0.4456 | 0.6680296 |
| hsa-miR-4447-st | 0.0887 | 0.1149 | 0.773 | 0.4456 | 0.6680296 |
| hsa-miR-4661-5p-st | -0.0199 | 0.0258 | -0.77 | 0.4471 | 0.6693451 |
| hsa-miR-4422-st | -0.0336 | 0.0437 | -0.77 | 0.4474 | 0.6693451 |
| hsa-miR-4528-st | -0.0226 | 0.0295 | -0.768 | 0.4482 | 0.6696102 |
| hsa-miR-1224-5p-st | 0.048 | 0.0625 | 0.768 | 0.4485 | 0.6696102 |
| hsa-miR-670-st | 0.0616 | 0.0805 | 0.765 | 0.4501 | 0.6704552 |
| hsa-miR-513a-3p-st | -0.0228 | 0.0298 | -0.763 | 0.4513 | 0.6704552 |
| hsa-miR-1973-st | -0.0506 | 0.0665 | -0.761 | 0.4521 | 0.6704552 |
| hsa-miR-2277-3p-st | 0.0587 | 0.0771 | 0.761 | 0.4522 | 0.6704552 |
| hsa-miR-3161-st | 0.0258 | 0.0339 | 0.761 | 0.4522 | 0.6704552 |
| hsa-miR-124-st | 0.0062 | 0.0082 | 0.761 | 0.4523 | 0.6704552 |
| hsa-miR-524-5p-st | 0.0264 | 0.0347 | 0.761 | 0.4523 | 0.6704552 |
| hsa-miR-3622a-5p-st | 0.0547 | 0.072 | 0.759 | 0.4533 | 0.6712519 |
| hsa-miR-4268-st | -0.0135 | 0.0178 | -0.758 | 0.4541 | 0.671751 |
| hsa-miR-2115-star-st | -0.0746 | 0.0987 | -0.756 | 0.4551 | 0.6721326 |
| hsa-miR-4738-5p-st | -0.0179 | 0.0237 | -0.754 | 0.4566 | 0.6721326 |
| hsa-miR-3691-3p-st | 0.0537 | 0.0713 | 0.753 | 0.4571 | 0.6721326 |
| hsa-miR-1246-st | 0.1141 | 0.1515 | 0.753 | 0.4572 | 0.6721326 |
| hsa-miR-4701-5p-st | 0.0492 | 0.0654 | 0.753 | 0.4573 | 0.6721326 |
| hsa-miR-4752-st | 0.023 | 0.0306 | 0.753 | 0.4573 | 0.6721326 |
| hsa-miR-4767-st | 0.0564 | 0.075 | 0.752 | 0.4576 | 0.6721326 |
| hsa-miR-216b-st | -0.0865 | 0.1154 | -0.749 | 0.4593 | 0.6735953 |
| hsa-miR-153-st | -0.0565 | 0.0755 | -0.748 | 0.4598 | 0.6735953 |
| hsa-miR-653-st | -0.0287 | 0.0384 | -0.747 | 0.4606 | 0.6735953 |
| hsa-miR-4461-st | -0.0619 | 0.083 | -0.745 | 0.4619 | 0.6735953 |
| hsa-miR-197-st | -0.0336 | 0.0451 | -0.744 | 0.4626 | 0.6735953 |
| hsa-miR-2355-3p-st | 0.03 | 0.0404 | 0.744 | 0.4627 | 0.6735953 |
| hsa-miR-3942-5p-st | -0.0307 | 0.0412 | -0.743 | 0.4628 | 0.6735953 |
| hsa-miR-187-st | -0.1031 | 0.1387 | -0.743 | 0.4629 | 0.6735953 |
| hsa-miR-122-star-st | -0.0216 | 0.0292 | -0.74 | 0.4648 | 0.6735953 |
| hsa-miR-15b-st | -0.0571 | 0.0771 | -0.74 | 0.4649 | 0.6735953 |
| hsa-miR-675-star-st | 0.0567 | 0.0767 | 0.739 | 0.4658 | 0.6735953 |
| hsa-miR-3938-st | 0.0253 | 0.0342 | 0.738 | 0.4659 | 0.6735953 |
| hsa-miR-4260-st | 0.0425 | 0.0576 | 0.738 | 0.4664 | 0.6735953 |
| hsa-miR-4279-st | -0.0342 | 0.0464 | -0.737 | 0.4667 | 0.6735953 |
| hsa-miR-3175-st | 0.0855 | 0.1162 | 0.736 | 0.4673 | 0.6735953 |
| hsa-miR-1265-st | -0.0182 | 0.0247 | -0.735 | 0.4678 | 0.6735953 |
| hsa-miR-4285-st | 0.0329 | 0.0448 | 0.735 | 0.4679 | 0.6735953 |
| hsa-miR-3064-5p-st | 0.0435 | 0.0592 | 0.735 | 0.4681 | 0.6735953 |
| hsa-miR-130a-star-st | -0.0231 | 0.0314 | -0.734 | 0.4683 | 0.6735953 |
| hsa-miR-521-st | 0.0165 | 0.0225 | 0.734 | 0.4686 | 0.6735953 |
| hsa-miR-585-st | -0.0178 | 0.0244 | -0.733 | 0.4692 | 0.6735953 |
| hsa-miR-182-star-st | 0.0196 | 0.0268 | 0.732 | 0.4697 | 0.6735953 |
| hsa-miR-4777-3p-st | 0.0227 | 0.031 | 0.732 | 0.4697 | 0.6735953 |
| hsa-miR-127-3p-st | -0.0175 | 0.0239 | -0.732 | 0.4698 | 0.6735953 |
| hsa-miR-490-3p-st | 0.0696 | 0.0952 | 0.731 | 0.4702 | 0.6735953 |
| hsa-miR-510-st | -0.0359 | 0.0493 | -0.729 | 0.4715 | 0.6747776 |
| hsa-miR-4259-st | 0.1625 | 0.2241 | 0.725 | 0.4737 | 0.6747776 |
| hsa-miR-4733-5p-st | 0.0337 | 0.0464 | 0.725 | 0.4738 | 0.6747776 |
| hsa-miR-4506-st | 0.0534 | 0.0736 | 0.725 | 0.4739 | 0.6747776 |
| hsa-miR-3941-st | 0.0269 | 0.0371 | 0.725 | 0.4741 | 0.6747776 |
| hsa-miR-548an-st | -0.0292 | 0.0403 | -0.725 | 0.4741 | 0.6747776 |
| hsa-miR-3937-st | 0.0766 | 0.1059 | 0.724 | 0.4748 | 0.6747776 |
| hsa-miR-320a-st | 0.0356 | 0.0492 | 0.723 | 0.4753 | 0.6747776 |
| hsa-miR-3663-3p-st | 0.0858 | 0.1188 | 0.722 | 0.4758 | 0.6747776 |
| hsa-miR-4533-st | 0.0647 | 0.0897 | 0.721 | 0.4762 | 0.6747776 |
| hsa-miR-541-star-st | 0.0289 | 0.0401 | 0.719 | 0.4774 | 0.6747776 |
| hsa-miR-3138-st | 0.0541 | 0.0753 | 0.719 | 0.4775 | 0.6747776 |
| hsa-miR-627-st | 0.0275 | 0.0383 | 0.719 | 0.4776 | 0.6747776 |
| hsa-miR-4531-st | 0.0164 | 0.0229 | 0.719 | 0.4778 | 0.6747776 |
| hsa-miR-4769-3p-st | -0.0315 | 0.0439 | -0.718 | 0.478 | 0.6747776 |
| hsa-miR-1250-st | -0.0737 | 0.1029 | -0.716 | 0.4792 | 0.6758142 |
| hsa-miR-16-2-star-st | -0.0396 | 0.0556 | -0.712 | 0.482 | 0.6779737 |
| hsa-miR-519d-st | -0.0209 | 0.0294 | -0.711 | 0.4823 | 0.6779737 |
| hsa-miR-4417-st | 0.138 | 0.1942 | 0.711 | 0.4825 | 0.6779737 |
| hsa-miR-99b-star-st | -0.0396 | 0.0557 | -0.711 | 0.4826 | 0.6779737 |
| hsa-miR-4514-st | 0.0318 | 0.0447 | 0.71 | 0.4832 | 0.6781601 |
| hsa-miR-365-st | -0.0329 | 0.0464 | -0.708 | 0.4841 | 0.6787668 |
| hsa-miR-544-st | -0.0194 | 0.0275 | -0.706 | 0.4854 | 0.679741 |
| hsa-miR-3622a-3p-st | 0.0826 | 0.1171 | 0.705 | 0.4859 | 0.679741 |
| hsa-miR-3135-st | 0.0259 | 0.0367 | 0.705 | 0.4862 | 0.679741 |
| hsa-miR-302b-star-st | -0.0216 | 0.0307 | -0.702 | 0.4879 | 0.6808919 |
| hsa-miR-4665-5p-st | 0.0738 | 0.1053 | 0.701 | 0.4884 | 0.6808919 |
| hsa-miR-644-st | 0.0193 | 0.0276 | 0.701 | 0.4886 | 0.6808919 |
| hsa-miR-4503-st | -0.0148 | 0.0211 | -0.7 | 0.4889 | 0.6808919 |
| hsa-miR-31-st | 0.0455 | 0.0652 | 0.698 | 0.4902 | 0.6816726 |
| hsa-miR-4729-st | -0.0227 | 0.0325 | -0.698 | 0.4904 | 0.6816726 |
| hsa-miR-26a-st | -0.0129 | 0.0187 | -0.691 | 0.4947 | 0.6859197 |
| hsa-miR-635-st | -0.0309 | 0.0447 | -0.69 | 0.4952 | 0.6859197 |
| hsa-miR-938-st | -0.0199 | 0.0289 | -0.689 | 0.4956 | 0.6859197 |
| hsa-miR-616-st | 0.038 | 0.0552 | 0.689 | 0.4959 | 0.6859197 |
| hsa-miR-3655-st | -0.0323 | 0.0469 | -0.688 | 0.4966 | 0.6859197 |
| hsa-miR-99a-star-st | 0.0461 | 0.0671 | 0.687 | 0.497 | 0.6859197 |
| hsa-miR-602-st | -0.0696 | 0.1014 | -0.687 | 0.4975 | 0.6859197 |
| hsa-miR-4708-3p-st | 0.0633 | 0.0923 | 0.686 | 0.4978 | 0.6859197 |
| hsa-miR-4306-st | -0.0391 | 0.0571 | -0.685 | 0.4984 | 0.6859197 |
| hsa-miR-302d-st | 0.0278 | 0.0407 | 0.684 | 0.4988 | 0.6859197 |
| hsa-miR-3687-st | 0.1138 | 0.1663 | 0.684 | 0.4989 | 0.6859197 |
| hsa-miR-3162-5p-st | 0.1202 | 0.1758 | 0.684 | 0.4993 | 0.6859197 |
| hsa-miR-4292-st | -0.0765 | 0.112 | -0.683 | 0.4996 | 0.6859197 |
| hsa-miR-4715-5p-st | 0.0193 | 0.0283 | 0.681 | 0.5012 | 0.687466 |
| hsa-miR-1908-st | 0.0753 | 0.1112 | 0.677 | 0.5032 | 0.6895576 |
| hsa-miR-4433-st | 0.0715 | 0.1057 | 0.676 | 0.5039 | 0.6898654 |
| hsa-miR-3939-st | -0.0335 | 0.0498 | -0.673 | 0.5056 | 0.6906488 |
| hsa-miR-4709-5p-st | -0.0281 | 0.0418 | -0.673 | 0.5057 | 0.6906488 |
| hsa-miR-3907-st | 0.0775 | 0.1152 | 0.673 | 0.5059 | 0.6906488 |
| hsa-miR-4488-st | 0.0641 | 0.0959 | 0.669 | 0.5085 | 0.6935459 |
| hsa-miR-2114-star-st | 0.0273 | 0.0409 | 0.667 | 0.5096 | 0.6942867 |
| hsa-miR-4680-5p-st | -0.0181 | 0.0272 | -0.667 | 0.51 | 0.6942867 |
| hsa-miR-3115-st | -0.0165 | 0.0248 | -0.665 | 0.5109 | 0.69486 |
| hsa-miR-3689d-st | 0.0169 | 0.0255 | 0.662 | 0.5128 | 0.6961669 |
| hsa-miR-520f-st | -0.0187 | 0.0282 | -0.662 | 0.5132 | 0.6961669 |
| hsa-miR-1909-st | 0.0846 | 0.1279 | 0.661 | 0.5133 | 0.6961669 |
| hsa-miR-3614-3p-st | -0.0229 | 0.0348 | -0.659 | 0.5147 | 0.6968158 |
| hsa-miR-943-st | 0.0579 | 0.0882 | 0.657 | 0.516 | 0.6968158 |
| hsa-miR-520h-st | -0.0195 | 0.0298 | -0.657 | 0.5161 | 0.6968158 |
| hsa-miR-654-5p-st | -0.0411 | 0.0627 | -0.656 | 0.5166 | 0.6968158 |
| hsa-miR-1182-st | 0.0583 | 0.089 | 0.656 | 0.5169 | 0.6968158 |
| hsa-miR-4725-5p-st | 0.0483 | 0.0737 | 0.655 | 0.5175 | 0.6968158 |
| hsa-miR-3181-st | -0.0866 | 0.1324 | -0.654 | 0.5176 | 0.6968158 |
| hsa-miR-4754-st | 0.0384 | 0.0586 | 0.654 | 0.5177 | 0.6968158 |
| hsa-miR-4723-3p-st | 0.039 | 0.0596 | 0.654 | 0.5181 | 0.6968158 |
| hsa-miR-618-st | -0.0133 | 0.0204 | -0.651 | 0.5198 | 0.697943 |
| hsa-miR-548aj-st | -0.04 | 0.0614 | -0.651 | 0.5199 | 0.697943 |
| hsa-miR-32-star-st | 0.0279 | 0.0431 | 0.648 | 0.5218 | 0.6992001 |
| hsa-miR-3913-3p-st | 0.0201 | 0.0311 | 0.648 | 0.5218 | 0.6992001 |
| hsa-miR-4803-st | -0.0159 | 0.0247 | -0.645 | 0.5237 | 0.7003137 |
| hsa-miR-150-st | 0.0421 | 0.0653 | 0.644 | 0.5241 | 0.7003137 |
| hsa-miR-3189-3p-st | -0.021 | 0.0326 | -0.643 | 0.5247 | 0.7003137 |
| hsa-miR-4650-3p-st | -0.0197 | 0.0307 | -0.641 | 0.5265 | 0.7003137 |
| hsa-miR-380-star-st | 0.073 | 0.114 | 0.64 | 0.5267 | 0.7003137 |
| hsa-miR-4726-5p-st | 0.0736 | 0.115 | 0.64 | 0.5269 | 0.7003137 |
| hsa-miR-3976-st | 0.0254 | 0.0398 | 0.64 | 0.527 | 0.7003137 |
| hsa-miR-509-5p-st | -0.0299 | 0.0468 | -0.639 | 0.5273 | 0.7003137 |
| hsa-miR-3682-3p-st | 0.079 | 0.1238 | 0.638 | 0.5282 | 0.7003137 |
| hsa-miR-591-st | 0.0194 | 0.0304 | 0.638 | 0.5283 | 0.7003137 |
| hsa-miR-1277-st | -0.019 | 0.0299 | -0.637 | 0.5287 | 0.7003137 |
| hsa-miR-3163-st | -0.0408 | 0.0641 | -0.637 | 0.5288 | 0.7003137 |
| hsa-miR-4677-3p-st | 0.0317 | 0.0498 | 0.636 | 0.5294 | 0.7003137 |
| hsa-miR-450a-st | -0.016 | 0.0252 | -0.635 | 0.5298 | 0.7003137 |
| hsa-miR-186-st | -0.0579 | 0.0913 | -0.634 | 0.5306 | 0.7003137 |
| hsa-miR-877-st | -0.0494 | 0.078 | -0.634 | 0.5309 | 0.7003137 |
| hsa-miR-1284-st | 0.0354 | 0.0559 | 0.633 | 0.5312 | 0.7003137 |
| hsa-miR-183-star-st | -0.0243 | 0.0385 | -0.633 | 0.5314 | 0.7003137 |
| hsa-let-7g-st | 0.0525 | 0.083 | 0.632 | 0.5318 | 0.7003137 |
| hsa-miR-22-star-st | -0.0471 | 0.0748 | -0.63 | 0.5334 | 0.7008435 |
| hsa-miR-519c-5p-st | -0.0338 | 0.0537 | -0.629 | 0.5338 | 0.7008435 |
| hsa-miR-302a-star-st | -0.0169 | 0.0269 | -0.629 | 0.5341 | 0.7008435 |
| hsa-miR-205-star-st | 0.0218 | 0.0347 | 0.628 | 0.5344 | 0.7008435 |
| hsa-miR-2053-st | 0.0229 | 0.0365 | 0.627 | 0.5351 | 0.7008435 |
| hsa-miR-3122-st | -0.0616 | 0.0982 | -0.627 | 0.5351 | 0.7008435 |
| hsa-miR-525-3p-st | -0.0168 | 0.0269 | -0.625 | 0.5364 | 0.7015418 |
| hsa-miR-3661-st | -0.0244 | 0.0391 | -0.625 | 0.5366 | 0.7015418 |
| hsa-miR-4493-st | 0.027 | 0.0433 | 0.624 | 0.5374 | 0.7019553 |
| hsa-miR-4714-3p-st | -0.0106 | 0.0171 | -0.622 | 0.5386 | 0.7027801 |
| hsa-miR-4700-5p-st | 0.0357 | 0.0575 | 0.621 | 0.539 | 0.7027801 |
| hsa-miR-515-5p-st | -0.0191 | 0.0309 | -0.62 | 0.5399 | 0.7030813 |
| hsa-miR-3920-st | 0.0142 | 0.0229 | 0.619 | 0.5402 | 0.7030813 |
| hsa-miR-769-5p-st | -0.0245 | 0.0397 | -0.617 | 0.5415 | 0.7035312 |
| hsa-miR-9-st | -0.0458 | 0.0742 | -0.617 | 0.5419 | 0.7035312 |
| hsa-miR-550a-star-st | 0.0403 | 0.0654 | 0.617 | 0.542 | 0.7035312 |
| hsa-miR-4271-st | 0.0991 | 0.162 | 0.612 | 0.5451 | 0.7069227 |
| hsa-miR-3973-st | -0.0169 | 0.0277 | -0.611 | 0.546 | 0.7074577 |
| hsa-miR-346-st | -0.0502 | 0.0825 | -0.609 | 0.547 | 0.7081212 |
| hsa-miR-651-st | -0.0171 | 0.0282 | -0.607 | 0.5482 | 0.7084413 |
| hsa-miR-4474-5p-st | 0.0185 | 0.0305 | 0.606 | 0.5489 | 0.7084413 |
| hsa-miR-3144-5p-st | -0.0126 | 0.0208 | -0.606 | 0.549 | 0.7084413 |
| hsa-miR-4722-5p-st | 0.1077 | 0.1778 | 0.606 | 0.5492 | 0.7084413 |
| hsa-miR-4768-5p-st | 0.0172 | 0.0286 | 0.603 | 0.5508 | 0.70875 |
| hsa-miR-518d-3p-st | -0.0159 | 0.0264 | -0.602 | 0.5515 | 0.70875 |
| hsa-miR-548z-st | 0.0384 | 0.0637 | 0.602 | 0.5515 | 0.70875 |
| hsa-miR-3194-5p-st | 0.0475 | 0.0789 | 0.602 | 0.5517 | 0.70875 |
| hsa-miR-4465-st | -0.0257 | 0.0428 | -0.6 | 0.5529 | 0.70875 |
| hsa-miR-505-star-st | -0.0504 | 0.0841 | -0.599 | 0.5534 | 0.70875 |
| hsa-miR-219-1-3p-st | 0.0248 | 0.0414 | 0.598 | 0.554 | 0.70875 |
| hsa-miR-3165-st | 0.0208 | 0.0347 | 0.598 | 0.5541 | 0.70875 |
| hsa-miR-3689f-st | 0.0181 | 0.0304 | 0.597 | 0.5549 | 0.70875 |
| hsa-miR-4664-5p-st | -0.0255 | 0.0427 | -0.596 | 0.5552 | 0.70875 |
| hsa-miR-592-st | 0.0799 | 0.134 | 0.597 | 0.5552 | 0.70875 |
| hsa-miR-15b-star-st | -0.017 | 0.0285 | -0.596 | 0.5553 | 0.70875 |
| hsa-miR-4638-3p-st | 0.0592 | 0.0994 | 0.596 | 0.5558 | 0.7087648 |
| hsa-miR-3619-5p-st | -0.0646 | 0.1087 | -0.594 | 0.5569 | 0.709544 |
| hsa-miR-4694-3p-st | 0.0145 | 0.0244 | 0.592 | 0.5579 | 0.7101946 |
| hsa-miR-4749-3p-st | 0.0282 | 0.0477 | 0.591 | 0.5589 | 0.710844 |
| hsa-miR-1269-st | -0.0287 | 0.0486 | -0.59 | 0.5597 | 0.7112382 |
| hsa-miR-4703-5p-st | -0.0196 | 0.0334 | -0.589 | 0.5604 | 0.7115047 |
| hsa-miR-3182-st | 0.0172 | 0.0293 | 0.587 | 0.5617 | 0.7119095 |
| hsa-miR-625-star-st | -0.0286 | 0.0487 | -0.587 | 0.5617 | 0.7119095 |
| hsa-miR-320b-st | 0.0296 | 0.0506 | 0.585 | 0.5626 | 0.7120717 |
| hsa-miR-4700-3p-st | -0.0092 | 0.0158 | -0.585 | 0.5629 | 0.7120717 |
| hsa-miR-4804-3p-st | 0.0234 | 0.0401 | 0.584 | 0.5633 | 0.7120717 |
| hsa-miR-454-star-st | 0.0149 | 0.0257 | 0.582 | 0.5648 | 0.7133465 |
| hsa-miR-337-3p-st | -0.0225 | 0.0389 | -0.579 | 0.567 | 0.7139185 |
| hsa-miR-1179-st | -0.0221 | 0.0384 | -0.576 | 0.5688 | 0.7139185 |
| hsa-miR-4523-st | 0.1159 | 0.2015 | 0.575 | 0.5694 | 0.7139185 |
| hsa-miR-19b-st | 0.0375 | 0.0653 | 0.575 | 0.5697 | 0.7139185 |
| hsa-miR-4252-st | -0.028 | 0.0487 | -0.575 | 0.5698 | 0.7139185 |
| hsa-miR-518c-star-st | -0.0354 | 0.0617 | -0.574 | 0.5698 | 0.7139185 |
| hsa-miR-484-st | -0.0439 | 0.0765 | -0.573 | 0.5705 | 0.7139185 |
| hsa-miR-4460-st | 0.0165 | 0.0288 | 0.573 | 0.571 | 0.7139185 |
| hsa-miR-3911-st | -0.0368 | 0.0643 | -0.572 | 0.5715 | 0.7139185 |
| hsa-miR-3943-st | 0.0595 | 0.1041 | 0.571 | 0.5719 | 0.7139185 |
| hsa-miR-98-st | -0.0481 | 0.0842 | -0.571 | 0.5719 | 0.7139185 |
| hsa-miR-4690-5p-st | 0.0729 | 0.1278 | 0.571 | 0.5725 | 0.7139185 |
| hsa-miR-3974-st | 0.0172 | 0.0301 | 0.57 | 0.5726 | 0.7139185 |
| hsa-miR-24-st | 0.0118 | 0.0208 | 0.57 | 0.5731 | 0.7139185 |
| hsa-miR-4639-3p-st | -0.0121 | 0.0212 | -0.569 | 0.5737 | 0.7139185 |
| hsa-miR-92a-1-star-st | -0.0439 | 0.0773 | -0.568 | 0.5738 | 0.7139185 |
| hsa-miR-1262-st | 0.0246 | 0.0433 | 0.568 | 0.5739 | 0.7139185 |
| hsa-miR-362-3p-st | 0.047 | 0.0828 | 0.568 | 0.5743 | 0.7139185 |
| hsa-miR-17-star-st | 0.032 | 0.0563 | 0.567 | 0.5746 | 0.7139185 |
| hsa-miR-33a-st | -0.0276 | 0.0487 | -0.566 | 0.5752 | 0.7140526 |
| hsa-miR-15a-st | 0.036 | 0.0639 | 0.563 | 0.5773 | 0.716047 |
| hsa-miR-4731-3p-st | -0.024 | 0.0428 | -0.561 | 0.5785 | 0.7169227 |
| hsa-miR-518b-st | -0.0109 | 0.0195 | -0.56 | 0.5795 | 0.7175492 |
| hsa-miR-200c-star-st | 0.0278 | 0.0498 | 0.559 | 0.58 | 0.7175561 |
| hsa-miR-3617-st | -0.0644 | 0.1154 | -0.558 | 0.5808 | 0.7179337 |
| hsa-miR-4309-st | 0.015 | 0.027 | 0.556 | 0.5819 | 0.7186813 |
| hsa-miR-934-st | -0.0142 | 0.0257 | -0.553 | 0.5841 | 0.7205425 |
| hsa-miR-3127-5p-st | -0.0439 | 0.0794 | -0.553 | 0.5844 | 0.7205425 |
| hsa-miR-4760-5p-st | 0.0162 | 0.0293 | 0.552 | 0.5849 | 0.7205468 |
| hsa-miR-4289-st | -0.0656 | 0.119 | -0.551 | 0.5855 | 0.7206742 |
| hsa-miR-3923-st | -0.0124 | 0.0227 | -0.547 | 0.5886 | 0.7228966 |
| hsa-miR-508-5p-st | 0.0355 | 0.065 | 0.546 | 0.5886 | 0.7228966 |
| hsa-miR-4318-st | 0.0192 | 0.0351 | 0.546 | 0.5888 | 0.7228966 |
| hsa-miR-1307-st | -0.0369 | 0.0679 | -0.544 | 0.5902 | 0.7240029 |
| hsa-miR-4482-st | -0.0166 | 0.0308 | -0.541 | 0.5927 | 0.7255976 |
| hsa-miR-4653-5p-st | -0.0159 | 0.0294 | -0.54 | 0.5932 | 0.7255976 |
| hsa-miR-937-st | -0.0304 | 0.0563 | -0.54 | 0.5932 | 0.7255976 |
| hsa-miR-4782-5p-st | 0.0145 | 0.0269 | 0.539 | 0.5937 | 0.7255976 |
| hsa-miR-545-st | -0.0146 | 0.0272 | -0.539 | 0.594 | 0.7255976 |
| hsa-miR-4744-st | 0.0144 | 0.0268 | 0.537 | 0.5954 | 0.7266961 |
| hsa-miR-3651-st | 0.0415 | 0.0778 | 0.534 | 0.5974 | 0.7282738 |
| hsa-miR-4792-st | -0.0291 | 0.0546 | -0.533 | 0.5981 | 0.7282738 |
| hsa-miR-409-5p-st | -0.0355 | 0.0668 | -0.532 | 0.5984 | 0.7282738 |
| hsa-miR-34a-star-st | 0.0441 | 0.0829 | 0.532 | 0.5987 | 0.7282738 |
| hsa-miR-4469-st | -0.035 | 0.0661 | -0.53 | 0.6 | 0.7292439 |
| hsa-miR-4694-5p-st | -0.0154 | 0.0292 | -0.529 | 0.6006 | 0.7293622 |
| hsa-miR-3159-st | -0.019 | 0.036 | -0.527 | 0.6017 | 0.7297197 |
| hsa-miR-4801-st | 0.0193 | 0.0366 | 0.527 | 0.6019 | 0.7297197 |
| hsa-miR-122-st | 0.1048 | 0.1995 | 0.525 | 0.6032 | 0.7306853 |
| hsa-miR-3669-st | -0.0172 | 0.033 | -0.521 | 0.6059 | 0.7324679 |
| hsa-miR-3680-star-st | -0.0201 | 0.0386 | -0.521 | 0.6059 | 0.7324679 |
| hsa-miR-3680-st | 0.0142 | 0.0273 | 0.519 | 0.6071 | 0.7324679 |
| hsa-miR-3198-st | 0.0305 | 0.0588 | 0.519 | 0.6073 | 0.7324679 |
| hsa-miR-518f-st | 0.0107 | 0.0207 | 0.519 | 0.6076 | 0.7324679 |
| hsa-let-7a-st | 0.0208 | 0.0402 | 0.519 | 0.6077 | 0.7324679 |
| hsa-miR-4464-st | 0.0134 | 0.0261 | 0.514 | 0.611 | 0.7358343 |
| hsa-miR-302d-star-st | 0.0222 | 0.0435 | 0.51 | 0.614 | 0.7388341 |
| hsa-miR-193b-star-st | -0.0474 | 0.0933 | -0.508 | 0.6153 | 0.739785 |
| hsa-miR-432-star-st | -0.0362 | 0.0717 | -0.505 | 0.6172 | 0.7408041 |
| hsa-miR-4730-st | 0.1186 | 0.2351 | 0.504 | 0.6176 | 0.7408041 |
| hsa-miR-3613-3p-st | -0.0899 | 0.1787 | -0.503 | 0.6184 | 0.7408041 |
| hsa-miR-151b-st | 0.0357 | 0.071 | 0.503 | 0.6186 | 0.7408041 |
| hsa-miR-4770-st | 0.012 | 0.0239 | 0.503 | 0.6187 | 0.7408041 |
| hsa-miR-4434-st | 0.0117 | 0.0235 | 0.498 | 0.6217 | 0.7432893 |
| hsa-miR-548m-st | 0.0165 | 0.0332 | 0.498 | 0.6218 | 0.7432893 |
| hsa-miR-574-3p-st | 0.0354 | 0.0714 | 0.496 | 0.6231 | 0.7441691 |
| hsa-miR-3945-st | 0.0231 | 0.0466 | 0.495 | 0.6242 | 0.7441691 |
| hsa-miR-4654-st | 0.0434 | 0.0878 | 0.494 | 0.6245 | 0.7441691 |
| hsa-miR-593-st | 0.0145 | 0.0293 | 0.494 | 0.6247 | 0.7441691 |
| hsa-miR-1197-st | -0.0214 | 0.0433 | -0.494 | 0.6251 | 0.7441691 |
| hsa-miR-744-st | 0.0407 | 0.0829 | 0.491 | 0.6268 | 0.7455813 |
| hsa-miR-4297-st | 0.014 | 0.0289 | 0.485 | 0.6308 | 0.7487838 |
| hsa-miR-1587-st | 0.0475 | 0.0982 | 0.484 | 0.6318 | 0.7487838 |
| hsa-miR-1270-st | -0.0578 | 0.1195 | -0.484 | 0.6321 | 0.7487838 |
| hsa-miR-193b-st | -0.0285 | 0.059 | -0.483 | 0.6322 | 0.7487838 |
| hsa-miR-4699-3p-st | 0.0125 | 0.0258 | 0.483 | 0.6322 | 0.7487838 |
| hsa-miR-761-st | -0.011 | 0.0229 | -0.482 | 0.6332 | 0.7487838 |
| hsa-miR-3150a-5p-st | 0.0215 | 0.0448 | 0.481 | 0.634 | 0.7487838 |
| hsa-miR-3972-st | 0.02 | 0.0416 | 0.48 | 0.6343 | 0.7487838 |
| hsa-miR-7-2-star-st | -0.0537 | 0.1119 | -0.48 | 0.6347 | 0.7487838 |
| hsa-miR-22-st | 0.0087 | 0.0181 | 0.48 | 0.6348 | 0.7487838 |
| hsa-miR-1305-st | -0.0136 | 0.0283 | -0.479 | 0.6355 | 0.7487838 |
| hsa-miR-4450-st | 0.0217 | 0.0454 | 0.478 | 0.636 | 0.7487838 |
| hsa-miR-633-st | -0.0129 | 0.027 | -0.478 | 0.6362 | 0.7487838 |
| hsa-miR-4719-st | 0.0126 | 0.0265 | 0.477 | 0.6368 | 0.7488826 |
| hsa-miR-4302-st | -0.0268 | 0.0565 | -0.474 | 0.6389 | 0.7507439 |
| hsa-miR-26b-st | -0.042 | 0.089 | -0.472 | 0.6403 | 0.7512825 |
| hsa-miR-493-st | -0.0559 | 0.1188 | -0.471 | 0.6411 | 0.7512825 |
| hsa-miR-139-5p-st | -0.0134 | 0.0284 | -0.471 | 0.6412 | 0.7512825 |
| hsa-miR-4525-st | -0.0587 | 0.125 | -0.47 | 0.642 | 0.7512825 |
| hsa-miR-1205-st | -0.0103 | 0.0219 | -0.469 | 0.6426 | 0.7512825 |
| hsa-miR-3124-3p-st | 0.0153 | 0.0327 | 0.468 | 0.6432 | 0.7512825 |
| hsa-miR-4276-st | -0.0157 | 0.0335 | -0.468 | 0.6433 | 0.7512825 |
| hsa-miR-940-st | 0.0304 | 0.0651 | 0.467 | 0.6435 | 0.7512825 |
| hsa-miR-500a-star-st | 0.022 | 0.0472 | 0.466 | 0.6443 | 0.7516118 |
| hsa-miR-3162-3p-st | -0.0402 | 0.0867 | -0.463 | 0.6466 | 0.7536891 |
| hsa-miR-4774-3p-st | -0.0157 | 0.0343 | -0.458 | 0.6503 | 0.7564471 |
| hsa-miR-3126-5p-st | -0.0213 | 0.0467 | -0.457 | 0.6508 | 0.7564471 |
| hsa-miR-548k-st | 0.0168 | 0.037 | 0.455 | 0.6522 | 0.7564471 |
| hsa-miR-302a-st | 0.0103 | 0.0227 | 0.455 | 0.6524 | 0.7564471 |
| hsa-miR-500b-st | 0.014 | 0.0308 | 0.455 | 0.6524 | 0.7564471 |
| hsa-miR-506-st | -0.0227 | 0.05 | -0.454 | 0.6529 | 0.7564471 |
| hsa-miR-4714-5p-st | 0.018 | 0.0397 | 0.454 | 0.6531 | 0.7564471 |
| hsa-miR-4667-3p-st | 0.0233 | 0.0515 | 0.453 | 0.6535 | 0.7564471 |
| hsa-miR-612-st | 0.0241 | 0.0533 | 0.452 | 0.6543 | 0.7564471 |
| hsa-miR-520a-3p-st | -0.0159 | 0.0352 | -0.452 | 0.6544 | 0.7564471 |
| hsa-miR-548am-st | 0.0103 | 0.0227 | 0.452 | 0.6547 | 0.7564471 |
| hsa-miR-3921-st | 0.0249 | 0.0552 | 0.45 | 0.6557 | 0.7569998 |
| hsa-miR-1827-st | -0.0348 | 0.0781 | -0.446 | 0.6589 | 0.7600895 |
| hsa-miR-9-star-st | -0.0267 | 0.0603 | -0.444 | 0.6603 | 0.7609281 |
| hsa-miR-1234-st | -0.0249 | 0.0563 | -0.443 | 0.6609 | 0.7609281 |
| hsa-miR-1302-st | -0.0134 | 0.0303 | -0.442 | 0.6612 | 0.7609281 |
| hsa-miR-362-5p-st | -0.0194 | 0.044 | -0.441 | 0.6625 | 0.7617627 |
| hsa-miR-599-st | -0.0112 | 0.0255 | -0.44 | 0.6632 | 0.7617627 |
| hsa-miR-499a-5p-st | -0.0106 | 0.0241 | -0.439 | 0.6635 | 0.7617627 |
| hsa-miR-30c-st | -0.0183 | 0.0418 | -0.438 | 0.6647 | 0.7622787 |
| hsa-miR-142-5p-st | 0.0207 | 0.0474 | 0.437 | 0.665 | 0.7622787 |
| hsa-miR-642b-st | 0.0283 | 0.0648 | 0.436 | 0.6656 | 0.7623643 |
| hsa-miR-2052-st | -0.0098 | 0.0226 | -0.435 | 0.6667 | 0.7624207 |
| hsa-miR-3145-3p-st | 0.0121 | 0.0278 | 0.435 | 0.6667 | 0.7624207 |
| hsa-miR-500a-st | 0.019 | 0.0441 | 0.432 | 0.6691 | 0.7628853 |
| hsa-miR-4785-st | 0.0403 | 0.0934 | 0.431 | 0.6693 | 0.7628853 |
| hsa-miR-548ag-st | -0.011 | 0.0257 | -0.429 | 0.671 | 0.7628853 |
| hsa-miR-320e-st | 0.0223 | 0.052 | 0.429 | 0.6712 | 0.7628853 |
| hsa-miR-563-st | 0.0379 | 0.0885 | 0.428 | 0.6714 | 0.7628853 |
| hsa-miR-597-st | -0.0145 | 0.034 | -0.428 | 0.6717 | 0.7628853 |
| hsa-miR-876-3p-st | -0.0133 | 0.0311 | -0.426 | 0.6732 | 0.7628853 |
| hsa-miR-3689a-3p-st | -0.0099 | 0.0233 | -0.426 | 0.6733 | 0.7628853 |
| hsa-miR-671-3p-st | 0.0268 | 0.0629 | 0.425 | 0.6737 | 0.7628853 |
| hsa-miR-4517-st | 0.0161 | 0.038 | 0.424 | 0.6744 | 0.7628853 |
| hsa-miR-595-st | -0.0434 | 0.1024 | -0.424 | 0.6748 | 0.7628853 |
| hsa-miR-4478-st | 0.0369 | 0.0873 | 0.423 | 0.6749 | 0.7628853 |
| hsa-miR-210-st | -0.0344 | 0.082 | -0.419 | 0.678 | 0.7628853 |
| hsa-miR-4802-3p-st | -0.0144 | 0.0345 | -0.419 | 0.6784 | 0.7628853 |
| hsa-miR-1267-st | -0.0162 | 0.0387 | -0.418 | 0.6785 | 0.7628853 |
| hsa-miR-3171-st | 0.0133 | 0.0319 | 0.418 | 0.6791 | 0.7628853 |
| hsa-miR-323-5p-st | 0.0261 | 0.0624 | 0.418 | 0.6791 | 0.7628853 |
| hsa-miR-548n-st | 0.0118 | 0.0283 | 0.418 | 0.6792 | 0.7628853 |
| hsa-miR-4659a-5p-st | -0.0159 | 0.038 | -0.417 | 0.6793 | 0.7628853 |
| hsa-miR-4746-5p-st | -0.0209 | 0.05 | -0.417 | 0.6794 | 0.7628853 |
| hsa-miR-4479-st | 0.0779 | 0.1868 | 0.417 | 0.6795 | 0.7628853 |
| hsa-miR-921-st | -0.0128 | 0.0307 | -0.417 | 0.6798 | 0.7628853 |
| hsa-miR-3117-5p-st | 0.0122 | 0.0293 | 0.416 | 0.6799 | 0.7628853 |
| hsa-miR-4750-st | 0.0497 | 0.1196 | 0.416 | 0.6804 | 0.7628853 |
| hsa-miR-423-5p-st | -0.0336 | 0.0807 | -0.416 | 0.6805 | 0.7628853 |
| hsa-miR-371b-3p-st | -0.02 | 0.0482 | -0.415 | 0.6811 | 0.7628853 |
| hsa-miR-519b-3p-st | 0.0091 | 0.022 | 0.415 | 0.6813 | 0.7628853 |
| hsa-miR-4298-st | -0.0311 | 0.0753 | -0.413 | 0.6823 | 0.763416 |
| hsa-miR-4667-5p-st | 0.0322 | 0.0787 | 0.409 | 0.6851 | 0.7659583 |
| hsa-miR-2054-st | 0.0115 | 0.0281 | 0.407 | 0.687 | 0.7674913 |
| hsa-miR-5047-st | -0.0069 | 0.0171 | -0.404 | 0.689 | 0.7687563 |
| hsa-miR-186-star-st | 0.0161 | 0.04 | 0.402 | 0.6905 | 0.7687563 |
| hsa-miR-19b-1-star-st | -0.0126 | 0.0314 | -0.401 | 0.6911 | 0.7687563 |
| hsa-miR-4294-st | -0.0155 | 0.0388 | -0.401 | 0.6914 | 0.7687563 |
| hsa-miR-466-st | 0.0226 | 0.0567 | 0.399 | 0.6924 | 0.7687563 |
| hsa-miR-4520b-3p-st | 0.0268 | 0.067 | 0.399 | 0.6925 | 0.7687563 |
| hsa-miR-875-5p-st | 0.0107 | 0.0268 | 0.399 | 0.6925 | 0.7687563 |
| hsa-miR-3910-st | 0.0247 | 0.062 | 0.399 | 0.6929 | 0.7687563 |
| hsa-miR-4518-st | -0.0179 | 0.0449 | -0.399 | 0.6929 | 0.7687563 |
| hsa-miR-3613-5p-st | 0.0616 | 0.1554 | 0.396 | 0.6945 | 0.7692551 |
| hsa-let-7c-st | 0.0066 | 0.0166 | 0.396 | 0.695 | 0.7692551 |
| hsa-miR-4704-3p-st | 0.0094 | 0.0238 | 0.395 | 0.6957 | 0.7692551 |
| hsa-miR-4662b-st | 0.0092 | 0.0234 | 0.394 | 0.6959 | 0.7692551 |
| hsa-miR-30d-star-st | -0.0137 | 0.0347 | -0.394 | 0.696 | 0.7692551 |
| hsa-miR-568-st | -0.01 | 0.0255 | -0.392 | 0.6977 | 0.7705471 |
| hsa-miR-4504-st | 0.0112 | 0.0287 | 0.391 | 0.6984 | 0.7707337 |
| hsa-miR-185-star-st | 0.0215 | 0.0555 | 0.388 | 0.7007 | 0.7726843 |
| hsa-miR-450b-5p-st | 0.0231 | 0.0598 | 0.386 | 0.702 | 0.7735301 |
| hsa-miR-17-st | 0.0094 | 0.0244 | 0.384 | 0.7034 | 0.7741175 |
| hsa-miR-544b-st | -0.0119 | 0.031 | -0.384 | 0.7036 | 0.7741175 |
| hsa-miR-378b-st | 0.0253 | 0.0665 | 0.38 | 0.7064 | 0.7766094 |
| hsa-miR-1231-st | 0.0463 | 0.1221 | 0.379 | 0.7071 | 0.7767904 |
| hsa-miR-1233-st | -0.0287 | 0.0762 | -0.376 | 0.7093 | 0.7786179 |
| hsa-miR-4796-3p-st | -0.0172 | 0.0462 | -0.373 | 0.7119 | 0.7800312 |
| hsa-miR-4288-st | -0.0223 | 0.06 | -0.373 | 0.712 | 0.7800312 |
| hsa-miR-3660-st | 0.0116 | 0.0313 | 0.372 | 0.7122 | 0.7800312 |
| hsa-miR-93-st | 0.0124 | 0.0335 | 0.371 | 0.7133 | 0.7806468 |
| hsa-miR-514b-3p-st | 0.0146 | 0.0397 | 0.369 | 0.715 | 0.7817659 |
| hsa-miR-4303-st | 0.0167 | 0.0455 | 0.368 | 0.7154 | 0.7817659 |
| hsa-miR-3919-st | 0.0105 | 0.0287 | 0.367 | 0.716 | 0.7818328 |
| hsa-miR-30c-2-star-st | 0.0158 | 0.0432 | 0.365 | 0.7177 | 0.7830999 |
| hsa-miR-3150a-3p-st | 0.0303 | 0.0832 | 0.364 | 0.7183 | 0.7831657 |
| hsa-miR-3064-3p-st | -0.0285 | 0.0786 | -0.362 | 0.7195 | 0.7836238 |
| hsa-miR-328-st | -0.0202 | 0.0559 | -0.361 | 0.7204 | 0.7836238 |
| hsa-miR-4644-st | -0.016 | 0.0446 | -0.359 | 0.7217 | 0.7836238 |
| hsa-miR-324-5p-st | 0.015 | 0.0417 | 0.359 | 0.7218 | 0.7836238 |
| hsa-miR-4265-st | -0.0104 | 0.0289 | -0.359 | 0.7221 | 0.7836238 |
| hsa-miR-10a-st | 0.0369 | 0.1028 | 0.359 | 0.7223 | 0.7836238 |
| hsa-miR-4256-st | -0.0104 | 0.0289 | -0.358 | 0.7225 | 0.7836238 |
| hsa-miR-944-st | -0.0118 | 0.0332 | -0.356 | 0.7239 | 0.7845118 |
| hsa-miR-4521-st | -0.0251 | 0.0707 | -0.356 | 0.7244 | 0.7845118 |
| hsa-miR-4645-5p-st | -0.0172 | 0.0485 | -0.354 | 0.7258 | 0.7846604 |
| hsa-miR-654-3p-st | -0.0208 | 0.0588 | -0.354 | 0.726 | 0.7846604 |
| hsa-miR-4535-st | 0.025 | 0.0708 | 0.353 | 0.7267 | 0.7846604 |
| hsa-miR-4723-5p-st | 0.0283 | 0.0801 | 0.353 | 0.7267 | 0.7846604 |
| hsa-miR-587-st | 0.0061 | 0.0172 | 0.352 | 0.7273 | 0.7847244 |
| hsa-miR-619-st | 0.0134 | 0.0383 | 0.35 | 0.7287 | 0.7847601 |
| hsa-miR-3692-star-st | 0.0105 | 0.03 | 0.349 | 0.7292 | 0.7847601 |
| hsa-miR-877-star-st | -0.0361 | 0.1035 | -0.349 | 0.7293 | 0.7847601 |
| hsa-miR-1255a-st | -0.0239 | 0.0686 | -0.349 | 0.7296 | 0.7847601 |
| hsa-miR-548t-st | 0.0094 | 0.027 | 0.347 | 0.7308 | 0.7847601 |
| hsa-miR-4757-3p-st | -0.0101 | 0.0292 | -0.347 | 0.731 | 0.7847601 |
| hsa-miR-4633-3p-st | -0.0193 | 0.0557 | -0.346 | 0.7316 | 0.7847601 |
| hsa-miR-140-5p-st | 0.0213 | 0.0615 | 0.346 | 0.732 | 0.7847601 |
| hsa-miR-4781-5p-st | 0.0119 | 0.0345 | 0.345 | 0.7322 | 0.7847601 |
| hsa-miR-3190-st | 0.0222 | 0.0644 | 0.344 | 0.7333 | 0.785359 |
| hsa-miR-1202-st | 0.0988 | 0.292 | 0.338 | 0.7373 | 0.7881418 |
| hsa-miR-676-st | 0.0089 | 0.0263 | 0.338 | 0.738 | 0.7881418 |
| hsa-miR-4262-st | -0.0118 | 0.0352 | -0.336 | 0.7389 | 0.7881418 |
| hsa-miR-4659a-3p-st | -0.0103 | 0.0305 | -0.336 | 0.7389 | 0.7881418 |
| hsa-miR-29c-star-st | -0.0136 | 0.0405 | -0.336 | 0.7391 | 0.7881418 |
| hsa-miR-1296-st | 0.0274 | 0.0816 | 0.335 | 0.7397 | 0.7881418 |
| hsa-miR-7-1-star-st | 0.0287 | 0.0855 | 0.335 | 0.7397 | 0.7881418 |
| hsa-miR-486-5p-st | -0.0311 | 0.0933 | -0.334 | 0.741 | 0.7884757 |
| hsa-miR-3658-st | -0.0093 | 0.028 | -0.333 | 0.7411 | 0.7884757 |
| hsa-miR-30a-star-st | 0.0111 | 0.0335 | 0.332 | 0.7418 | 0.7886422 |
| hsa-miR-3922-3p-st | -0.0085 | 0.0257 | -0.332 | 0.7425 | 0.7888086 |
| hsa-miR-1275-st | -0.031 | 0.0941 | -0.33 | 0.7438 | 0.7893703 |
| hsa-miR-4708-5p-st | 0.0263 | 0.08 | 0.329 | 0.7446 | 0.7893703 |
| hsa-miR-517c-st | -0.0097 | 0.0297 | -0.328 | 0.7453 | 0.7893703 |
| hsa-miR-138-st | -0.0118 | 0.0361 | -0.327 | 0.7458 | 0.7893703 |
| hsa-miR-218-2-star-st | -0.0238 | 0.0729 | -0.327 | 0.7462 | 0.7893703 |
| hsa-miR-557-st | 0.0328 | 0.1007 | 0.325 | 0.7471 | 0.7893703 |
| hsa-miR-607-st | 0.0123 | 0.0377 | 0.325 | 0.7471 | 0.7893703 |
| hsa-miR-4308-st | 0.0086 | 0.0266 | 0.325 | 0.7475 | 0.7893703 |
| hsa-miR-4483-st | -0.0156 | 0.0481 | -0.324 | 0.7484 | 0.7893703 |
| hsa-miR-3936-st | -0.0229 | 0.071 | -0.322 | 0.7496 | 0.7893703 |
| hsa-miR-4435-st | 0.0125 | 0.0388 | 0.322 | 0.7497 | 0.7893703 |
| hsa-miR-3157-5p-st | -0.0268 | 0.0832 | -0.322 | 0.75 | 0.7893703 |
| hsa-miR-1322-st | -0.016 | 0.0497 | -0.321 | 0.7501 | 0.7893703 |
| hsa-miR-4638-5p-st | 0.0286 | 0.0894 | 0.32 | 0.7509 | 0.7896395 |
| hsa-miR-2113-st | 0.0085 | 0.0266 | 0.319 | 0.7521 | 0.7903287 |
| hsa-miR-548a-3p-st | -0.033 | 0.1041 | -0.317 | 0.7535 | 0.7912269 |
| hsa-miR-4653-3p-st | 0.0115 | 0.0364 | 0.316 | 0.7544 | 0.7915992 |
| hsa-miR-302b-st | 0.0069 | 0.0219 | 0.314 | 0.7553 | 0.7919709 |
| hsa-miR-520e-st | 0.0106 | 0.0339 | 0.312 | 0.7574 | 0.7931533 |
| hsa-miR-888-st | -0.0104 | 0.0335 | -0.31 | 0.7587 | 0.7931533 |
| hsa-miR-582-5p-st | -0.0366 | 0.1185 | -0.309 | 0.7592 | 0.7931533 |
| hsa-miR-4323-st | 0.0132 | 0.0427 | 0.309 | 0.7594 | 0.7931533 |
| hsa-miR-548u-st | 0.0126 | 0.0409 | 0.309 | 0.7595 | 0.7931533 |
| hsa-miR-3121-3p-st | -0.0128 | 0.0417 | -0.308 | 0.7602 | 0.7931533 |
| hsa-miR-3914-st | -0.0112 | 0.0363 | -0.307 | 0.7606 | 0.7931533 |
| hsa-miR-4642-st | -0.0146 | 0.0476 | -0.307 | 0.7608 | 0.7931533 |
| hsa-miR-188-5p-st | 0.0155 | 0.0506 | 0.306 | 0.7618 | 0.7936257 |
| hsa-miR-18a-st | 0.0168 | 0.0556 | 0.302 | 0.7649 | 0.7948243 |
| hsa-miR-4668-5p-st | -0.0485 | 0.161 | -0.301 | 0.7652 | 0.7948243 |
| hsa-miR-4467-st | -0.035 | 0.1165 | -0.301 | 0.7658 | 0.7948243 |
| hsa-miR-1206-st | -0.0085 | 0.0284 | -0.3 | 0.7659 | 0.7948243 |
| hsa-miR-518d-5p-st | 0.0182 | 0.0607 | 0.3 | 0.7659 | 0.7948243 |
| hsa-miR-3141-st | 0.0308 | 0.103 | 0.299 | 0.767 | 0.7948243 |
| hsa-miR-4699-5p-st | -0.0092 | 0.0309 | -0.299 | 0.7673 | 0.7948243 |
| hsa-miR-331-5p-st | -0.0138 | 0.0462 | -0.298 | 0.7676 | 0.7948243 |
| hsa-miR-2110-st | -0.0217 | 0.073 | -0.298 | 0.768 | 0.7948243 |
| hsa-miR-589-st | -0.0234 | 0.0788 | -0.297 | 0.7687 | 0.7948243 |
| hsa-miR-4280-st | 0.0102 | 0.0345 | 0.296 | 0.7693 | 0.7948243 |
| hsa-miR-526b-st | 0.0123 | 0.0419 | 0.294 | 0.7704 | 0.7948243 |
| hsa-miR-4263-st | -0.0098 | 0.0335 | -0.294 | 0.7708 | 0.7948243 |
| hsa-miR-194-star-st | 0.0144 | 0.049 | 0.294 | 0.7709 | 0.7948243 |
| hsa-miR-2114-st | -0.0089 | 0.0305 | -0.293 | 0.7714 | 0.7948243 |
| hsa-miR-100-st | -0.0101 | 0.0348 | -0.292 | 0.7724 | 0.7948243 |
| hsa-miR-130b-st | 0.0141 | 0.0483 | 0.292 | 0.7724 | 0.7948243 |
| hsa-miR-632-st | 0.0082 | 0.0281 | 0.29 | 0.7735 | 0.7948243 |
| hsa-miR-299-3p-st | -0.025 | 0.0863 | -0.29 | 0.7741 | 0.7948243 |
| hsa-miR-369-3p-st | 0.0095 | 0.0327 | 0.289 | 0.7743 | 0.7948243 |
| hsa-miR-522-st | -0.0066 | 0.0229 | -0.289 | 0.7745 | 0.7948243 |
| hsa-miR-497-star-st | -0.0071 | 0.0247 | -0.288 | 0.775 | 0.7948243 |
| hsa-miR-4715-3p-st | -0.0073 | 0.0254 | -0.286 | 0.7766 | 0.7956483 |
| hsa-miR-28-5p-st | 0.0078 | 0.0273 | 0.286 | 0.7769 | 0.7956483 |
| hsa-miR-491-5p-st | -0.0159 | 0.056 | -0.283 | 0.7787 | 0.7958788 |
| hsa-miR-4762-3p-st | -0.006 | 0.0212 | -0.282 | 0.7795 | 0.7958788 |
| hsa-miR-181a-st | -0.0088 | 0.0314 | -0.282 | 0.7799 | 0.7958788 |
| hsa-miR-3664-3p-st | 0.0087 | 0.0309 | 0.282 | 0.78 | 0.7958788 |
| hsa-miR-3609-st | -0.0169 | 0.0603 | -0.281 | 0.7809 | 0.7958788 |
| hsa-miR-548y-st | -0.0075 | 0.0268 | -0.281 | 0.7809 | 0.7958788 |
| hsa-miR-3927-st | -0.0174 | 0.0623 | -0.28 | 0.7816 | 0.7958788 |
| hsa-miR-137-st | 0.0176 | 0.0632 | 0.278 | 0.7828 | 0.7958788 |
| hsa-miR-4287-st | 0.0117 | 0.0424 | 0.276 | 0.7841 | 0.7958788 |
| hsa-miR-4747-3p-st | 0.0092 | 0.0333 | 0.276 | 0.7841 | 0.7958788 |
| hsa-miR-3197-st | 0.0319 | 0.1158 | 0.275 | 0.7849 | 0.7958788 |
| hsa-miR-4519-st | -0.0159 | 0.0578 | -0.275 | 0.785 | 0.7958788 |
| hsa-miR-1200-st | -0.0101 | 0.0369 | -0.275 | 0.7855 | 0.7958788 |
| hsa-miR-527-st | -0.0171 | 0.0623 | -0.274 | 0.7856 | 0.7958788 |
| hsa-miR-4778-3p-st | -0.0072 | 0.0262 | -0.274 | 0.7859 | 0.7958788 |
| hsa-miR-617-st | 0.0138 | 0.0505 | 0.274 | 0.7859 | 0.7958788 |
| hsa-miR-4800-3p-st | 0.0222 | 0.0826 | 0.269 | 0.7899 | 0.7993718 |
| hsa-miR-4421-st | -0.0113 | 0.0423 | -0.267 | 0.7915 | 0.8002737 |
| hsa-miR-2909-st | -0.0087 | 0.0329 | -0.265 | 0.7926 | 0.8002737 |
| hsa-miR-638-st | -0.0019 | 0.0073 | -0.264 | 0.7936 | 0.8002737 |
| hsa-miR-4668-3p-st | -0.0157 | 0.0595 | -0.263 | 0.7939 | 0.8002737 |
| hsa-miR-374a-st | 0.0154 | 0.0585 | 0.263 | 0.794 | 0.8002737 |
| hsa-miR-767-3p-st | -0.017 | 0.0645 | -0.263 | 0.7941 | 0.8002737 |
| hsa-miR-202-star-st | -0.0107 | 0.0411 | -0.261 | 0.7959 | 0.800923 |
| hsa-miR-221-star-st | -0.0206 | 0.079 | -0.261 | 0.796 | 0.800923 |
| hsa-miR-451-st | -0.0358 | 0.1378 | -0.26 | 0.7964 | 0.800923 |
| hsa-miR-4476-st | -0.0319 | 0.1236 | -0.258 | 0.798 | 0.8009675 |
| hsa-miR-4496-st | 0.0173 | 0.0671 | 0.258 | 0.798 | 0.8009675 |
| hsa-miR-1470-st | -0.0109 | 0.0422 | -0.258 | 0.7981 | 0.8009675 |
| hsa-miR-4733-3p-st | 0.0101 | 0.0394 | 0.256 | 0.7997 | 0.8017653 |
| hsa-miR-30e-star-st | -0.0204 | 0.0799 | -0.256 | 0.8 | 0.8017653 |
| hsa-miR-1207-3p-st | -0.006 | 0.0236 | -0.255 | 0.8006 | 0.8018129 |
| hsa-miR-4445-st | 0.0083 | 0.0329 | 0.252 | 0.8028 | 0.8031922 |
| hsa-miR-548x-st | -0.014 | 0.0558 | -0.251 | 0.8032 | 0.8031922 |
| hsa-miR-3155b-st | -0.0062 | 0.025 | -0.25 | 0.8045 | 0.8031922 |
| hsa-miR-4455-st | -0.0262 | 0.1051 | -0.249 | 0.8052 | 0.8031922 |
| hsa-miR-4485-st | -0.0154 | 0.0619 | -0.248 | 0.8058 | 0.8031922 |
| hsa-miR-513a-5p-st | 0.0194 | 0.0781 | 0.248 | 0.8058 | 0.8031922 |
| hsa-miR-548c-3p-st | 0.0069 | 0.0281 | 0.247 | 0.8068 | 0.8031922 |
| hsa-miR-4658-st | -0.0076 | 0.031 | -0.245 | 0.8078 | 0.8031922 |
| hsa-miR-363-st | -0.0159 | 0.0648 | -0.245 | 0.808 | 0.8031922 |
| hsa-miR-92a-2-star-st | 0.0096 | 0.0394 | 0.245 | 0.8081 | 0.8031922 |
| hsa-let-7i-star-st | -0.0139 | 0.0569 | -0.244 | 0.8091 | 0.8031922 |
| hsa-miR-27b-star-st | -0.0159 | 0.0655 | -0.243 | 0.8093 | 0.8031922 |
| hsa-miR-1245b-3p-st | -0.0082 | 0.0338 | -0.243 | 0.8097 | 0.8031922 |
| hsa-miR-4500-st | -0.0232 | 0.0957 | -0.243 | 0.8098 | 0.8031922 |
| hsa-miR-4712-3p-st | 0.0084 | 0.0347 | 0.241 | 0.8114 | 0.8031922 |
| hsa-miR-3659-st | 0.0158 | 0.066 | 0.239 | 0.8127 | 0.8031922 |
| hsa-miR-26b-star-st | 0.0161 | 0.0676 | 0.239 | 0.8129 | 0.8031922 |
| hsa-miR-3124-5p-st | 0.0175 | 0.0733 | 0.238 | 0.8133 | 0.8031922 |
| hsa-miR-3186-5p-st | -0.0067 | 0.0282 | -0.238 | 0.8135 | 0.8031922 |
| hsa-miR-3681-st | -0.0072 | 0.0301 | -0.238 | 0.8136 | 0.8031922 |
| hsa-miR-4690-3p-st | -0.0105 | 0.0442 | -0.238 | 0.8136 | 0.8031922 |
| hsa-miR-425-st | -0.0051 | 0.0217 | -0.237 | 0.8143 | 0.8033367 |
| hsa-miR-339-3p-st | -0.0154 | 0.0655 | -0.236 | 0.8151 | 0.8035797 |
| hsa-miR-501-3p-st | -0.0073 | 0.0315 | -0.232 | 0.8182 | 0.8060882 |
| hsa-miR-4430-st | -0.0119 | 0.0517 | -0.231 | 0.8189 | 0.8062305 |
| hsa-miR-4515-st | -0.0109 | 0.0476 | -0.229 | 0.8201 | 0.8068646 |
| hsa-miR-214-st | 0.0226 | 0.099 | 0.228 | 0.8209 | 0.8071045 |
| hsa-miR-4662a-3p-st | 0.0057 | 0.0253 | 0.226 | 0.8227 | 0.8083266 |
| hsa-miR-138-2-star-st | -0.0151 | 0.0671 | -0.224 | 0.8239 | 0.8086769 |
| hsa-miR-922-st | 0.0097 | 0.0436 | 0.223 | 0.8247 | 0.8086769 |
| hsa-miR-3926-st | 0.0098 | 0.0441 | 0.223 | 0.8248 | 0.8086769 |
| hsa-miR-3672-st | 0.008 | 0.0361 | 0.222 | 0.8254 | 0.8086769 |
| hsa-miR-502-5p-st | -0.0168 | 0.076 | -0.221 | 0.8262 | 0.8086769 |
| hsa-miR-1263-st | -0.02 | 0.0905 | -0.221 | 0.8264 | 0.8086769 |
| hsa-miR-3944-5p-st | -0.0119 | 0.0544 | -0.218 | 0.8289 | 0.81017 |
| hsa-miR-516b-star-st | 0.0055 | 0.0253 | 0.216 | 0.8304 | 0.81017 |
| hsa-miR-579-st | 0.006 | 0.0281 | 0.215 | 0.8309 | 0.81017 |
| hsa-miR-371-3p-st | -0.0057 | 0.0263 | -0.215 | 0.831 | 0.81017 |
| hsa-miR-3591-3p-st | 0.0401 | 0.1873 | 0.214 | 0.832 | 0.81017 |
| hsa-miR-20a-star-st | 0.0087 | 0.0407 | 0.213 | 0.8324 | 0.81017 |
| hsa-miR-548aa-st | -0.0052 | 0.0243 | -0.212 | 0.8333 | 0.81017 |
| hsa-miR-151-5p-st | -0.0073 | 0.0342 | -0.212 | 0.8335 | 0.81017 |
| hsa-miR-4536-st | 0.0069 | 0.0327 | 0.212 | 0.8336 | 0.81017 |
| hsa-miR-296-3p-st | -0.01 | 0.0475 | -0.211 | 0.834 | 0.81017 |
| hsa-let-7i-st | -0.0143 | 0.0685 | -0.209 | 0.836 | 0.81017 |
| hsa-miR-4649-5p-st | 0.025 | 0.1196 | 0.209 | 0.836 | 0.81017 |
| hsa-miR-151-3p-st | -0.0115 | 0.0551 | -0.209 | 0.8362 | 0.81017 |
| hsa-miR-4676-5p-st | 0.0113 | 0.0543 | 0.208 | 0.8362 | 0.81017 |
| hsa-miR-3929-st | -0.0064 | 0.0307 | -0.208 | 0.8363 | 0.81017 |
| hsa-miR-4662a-5p-st | 0.005 | 0.0243 | 0.206 | 0.838 | 0.8112753 |
| hsa-miR-199a-3p-st | 0.0175 | 0.0857 | 0.205 | 0.8392 | 0.8118954 |
| hsa-miR-3194-3p-st | 0.0071 | 0.0352 | 0.202 | 0.8413 | 0.8132861 |
| hsa-miR-645-st | -0.0057 | 0.0283 | -0.201 | 0.8421 | 0.8132861 |
| hsa-miR-4641-st | -0.0104 | 0.052 | -0.2 | 0.8432 | 0.8132861 |
| hsa-miR-4705-st | -0.0073 | 0.0364 | -0.199 | 0.8432 | 0.8132861 |
| hsa-miR-583-st | -0.0051 | 0.0259 | -0.199 | 0.8439 | 0.8132861 |
| hsa-miR-4659b-3p-st | 0.0037 | 0.0188 | 0.198 | 0.844 | 0.8132861 |
| hsa-miR-4269-st | -0.016 | 0.081 | -0.197 | 0.8451 | 0.8138057 |
| hsa-miR-4780-st | -0.0074 | 0.038 | -0.195 | 0.8468 | 0.8143295 |
| hsa-miR-4498-st | 0.0259 | 0.1337 | 0.194 | 0.8478 | 0.8143295 |
| hsa-miR-331-3p-st | -0.0066 | 0.0342 | -0.193 | 0.8481 | 0.8143295 |
| hsa-miR-4477b-st | 0.0063 | 0.0324 | 0.193 | 0.8481 | 0.8143295 |
| hsa-miR-892a-st | -0.0062 | 0.0326 | -0.192 | 0.8492 | 0.8143295 |
| hsa-miR-1273c-st | 0.0171 | 0.0893 | 0.192 | 0.8493 | 0.8143295 |
| hsa-miR-196a-star-st | -0.0066 | 0.0347 | -0.191 | 0.8497 | 0.8143295 |
| hsa-miR-106b-star-st | 0.0114 | 0.0601 | 0.19 | 0.8503 | 0.8143295 |
| hsa-miR-4737-st | 0.0062 | 0.0329 | 0.189 | 0.8513 | 0.8143295 |
| hsa-miR-4772-5p-st | -0.0051 | 0.0271 | -0.188 | 0.8521 | 0.8143295 |
| hsa-miR-4632-st | 0.0076 | 0.0406 | 0.188 | 0.8523 | 0.8143295 |
| hsa-miR-99b-st | 0.0062 | 0.0331 | 0.187 | 0.8527 | 0.8143295 |
| hsa-miR-641-st | 0.0161 | 0.0865 | 0.186 | 0.8534 | 0.8143295 |
| hsa-miR-3978-st | 0.006 | 0.032 | 0.186 | 0.8535 | 0.8143295 |
| hsa-miR-660-st | -0.0097 | 0.0531 | -0.184 | 0.8555 | 0.8156423 |
| hsa-miR-4681-st | 0.0068 | 0.037 | 0.183 | 0.856 | 0.8156423 |
| hsa-miR-1260b-st | 0.0179 | 0.0998 | 0.18 | 0.8584 | 0.8170467 |
| hsa-miR-4261-st | -0.0089 | 0.0495 | -0.18 | 0.8586 | 0.8170467 |
| hsa-miR-125b-st | -0.0033 | 0.0184 | -0.178 | 0.8595 | 0.8170532 |
| hsa-miR-3127-3p-st | -0.0046 | 0.0257 | -0.178 | 0.8603 | 0.8170532 |
| hsa-miR-4685-3p-st | -0.0154 | 0.0869 | -0.177 | 0.8604 | 0.8170532 |
| hsa-miR-449b-star-st | 0.0115 | 0.0658 | 0.175 | 0.8624 | 0.8170532 |
| hsa-miR-637-st | 0.0246 | 0.141 | 0.174 | 0.8626 | 0.8170532 |
| hsa-miR-548q-st | 0.0135 | 0.0773 | 0.174 | 0.8627 | 0.8170532 |
| hsa-miR-148b-star-st | -0.0072 | 0.0414 | -0.174 | 0.8632 | 0.8170532 |
| hsa-miR-491-3p-st | -0.0092 | 0.0529 | -0.174 | 0.8634 | 0.8170532 |
| hsa-miR-676-star-st | -0.0049 | 0.0285 | -0.173 | 0.8637 | 0.8170532 |
| hsa-miR-3607-3p-st | 0.0047 | 0.0276 | 0.172 | 0.8645 | 0.8170532 |
| hsa-miR-1303-st | 0.0191 | 0.1111 | 0.172 | 0.8648 | 0.8170532 |
| hsa-miR-219-2-3p-st | 0.0127 | 0.0746 | 0.17 | 0.8663 | 0.8173464 |
| hsa-miR-4520a-5p-st | -0.004 | 0.0239 | -0.169 | 0.8666 | 0.8173464 |
| hsa-miR-454-st | 0.0184 | 0.1086 | 0.169 | 0.8668 | 0.8173464 |
| hsa-let-7f-st | -0.0159 | 0.0954 | -0.167 | 0.8688 | 0.8182111 |
| hsa-miR-4253-st | -0.0205 | 0.1241 | -0.165 | 0.8698 | 0.8182111 |
| hsa-miR-4761-5p-st | -0.0076 | 0.0461 | -0.165 | 0.8701 | 0.8182111 |
| hsa-miR-19a-st | 0.0179 | 0.1089 | 0.164 | 0.8705 | 0.8182111 |
| hsa-miR-2964a-3p-st | 0.0062 | 0.0377 | 0.164 | 0.8706 | 0.8182111 |
| hsa-miR-3125-st | -0.0074 | 0.0449 | -0.164 | 0.8711 | 0.8182111 |
| hsa-miR-548d-3p-st | -0.004 | 0.0253 | -0.157 | 0.8763 | 0.8215638 |
| hsa-miR-2467-5p-st | 0.004 | 0.0255 | 0.157 | 0.8764 | 0.8215638 |
| hsa-miR-578-st | -0.0048 | 0.0306 | -0.157 | 0.8764 | 0.8215638 |
| hsa-miR-548c-5p-st | -0.0064 | 0.0411 | -0.156 | 0.877 | 0.8215638 |
| hsa-miR-3201-st | 0.0123 | 0.0794 | 0.155 | 0.8775 | 0.8215638 |
| hsa-miR-4420-st | -0.0057 | 0.0368 | -0.155 | 0.8781 | 0.8215955 |
| hsa-miR-562-st | 0.0036 | 0.0235 | 0.153 | 0.8791 | 0.8216587 |
| hsa-miR-509-3-5p-st | 0.0146 | 0.0954 | 0.153 | 0.8793 | 0.8216587 |
| hsa-miR-29a-st | -0.0079 | 0.0523 | -0.151 | 0.8807 | 0.8221576 |
| hsa-miR-4314-st | -0.0219 | 0.1457 | -0.15 | 0.8815 | 0.8221576 |
| hsa-miR-3682-5p-st | 0.0035 | 0.0232 | 0.15 | 0.8817 | 0.8221576 |
| hsa-miR-556-3p-st | 0.0048 | 0.0318 | 0.149 | 0.8821 | 0.8221576 |
| hsa-miR-299-5p-st | -0.0102 | 0.0691 | -0.147 | 0.884 | 0.8227468 |
| hsa-miR-892b-st | 0.0062 | 0.0427 | 0.146 | 0.8847 | 0.8227468 |
| hsa-miR-1252-st | 0.0029 | 0.0197 | 0.146 | 0.8848 | 0.8227468 |
| hsa-miR-4765-st | -0.0048 | 0.0329 | -0.146 | 0.885 | 0.8227468 |
| hsa-miR-4774-5p-st | 0.0047 | 0.0329 | 0.142 | 0.8878 | 0.8238865 |
| hsa-miR-548ak-st | 0.0062 | 0.0437 | 0.142 | 0.8884 | 0.8238865 |
| hsa-miR-4757-5p-st | 0.0033 | 0.0237 | 0.141 | 0.8887 | 0.8238865 |
| hsa-miR-4742-5p-st | -0.0095 | 0.0679 | -0.14 | 0.8892 | 0.8238865 |
| hsa-miR-125a-5p-st | -0.0043 | 0.031 | -0.14 | 0.8896 | 0.8238865 |
| hsa-miR-192-st | 0.0065 | 0.047 | 0.139 | 0.8901 | 0.8238865 |
| hsa-miR-4301-st | 0.005 | 0.0362 | 0.139 | 0.8902 | 0.8238865 |
| hsa-miR-125b-2-star-st | -0.0045 | 0.033 | -0.137 | 0.8922 | 0.8241469 |
| hsa-miR-4529-3p-st | 0.0172 | 0.1256 | 0.137 | 0.8923 | 0.8241469 |
| hsa-miR-181a-star-st | 0.0109 | 0.0804 | 0.136 | 0.8926 | 0.8241469 |
| hsa-miR-3120-5p-st | -0.0071 | 0.0525 | -0.135 | 0.8938 | 0.8241469 |
| hsa-miR-513b-st | -0.005 | 0.0377 | -0.133 | 0.8947 | 0.8241469 |
| hsa-miR-24-1-star-st | 0.0145 | 0.1092 | 0.133 | 0.8953 | 0.8241469 |
| hsa-miR-4451-st | 0.0061 | 0.0465 | 0.132 | 0.8957 | 0.8241469 |
| hsa-miR-519a-st | 0.0064 | 0.049 | 0.131 | 0.8965 | 0.8241469 |
| hsa-miR-548al-st | -0.0051 | 0.0388 | -0.13 | 0.8971 | 0.8241469 |
| hsa-miR-1306-st | -0.0112 | 0.0861 | -0.13 | 0.8976 | 0.8241469 |
| hsa-miR-4312-st | -0.0074 | 0.0575 | -0.129 | 0.8978 | 0.8241469 |
| hsa-miR-3689a-5p-st | -0.0033 | 0.0259 | -0.129 | 0.8981 | 0.8241469 |
| hsa-miR-4490-st | 0.0064 | 0.0502 | 0.128 | 0.899 | 0.8241469 |
| hsa-miR-4663-st | 0.0044 | 0.0343 | 0.128 | 0.899 | 0.8241469 |
| hsa-miR-550b-st | 0.0036 | 0.0285 | 0.128 | 0.899 | 0.8241469 |
| hsa-miR-30b-st | 0.0079 | 0.0621 | 0.127 | 0.9 | 0.8245428 |
| hsa-miR-367-star-st | -0.0034 | 0.0275 | -0.125 | 0.9012 | 0.8251213 |
| hsa-miR-4674-st | 0.0165 | 0.1327 | 0.124 | 0.902 | 0.825333 |
| hsa-miR-566-st | 0.004 | 0.0326 | 0.121 | 0.9043 | 0.8266696 |
| hsa-miR-4789-5p-st | -0.0042 | 0.0345 | -0.121 | 0.9046 | 0.8266696 |
| hsa-miR-26a-2-star-st | 0.0034 | 0.0294 | 0.117 | 0.9076 | 0.8270906 |
| hsa-miR-4722-3p-st | 0.0145 | 0.1247 | 0.116 | 0.9084 | 0.8270906 |
| hsa-miR-4486-st | 0.0144 | 0.1245 | 0.116 | 0.9085 | 0.8270906 |
| hsa-miR-512-3p-st | -0.0047 | 0.0407 | -0.116 | 0.9087 | 0.8270906 |
| hsa-miR-3160-3p-st | -0.0038 | 0.0336 | -0.114 | 0.9099 | 0.8270906 |
| hsa-miR-4738-3p-st | -0.0106 | 0.0925 | -0.114 | 0.9099 | 0.8270906 |
| hsa-miR-1244-st | 0.0057 | 0.0501 | 0.114 | 0.9101 | 0.8270906 |
| hsa-miR-507-st | -0.0037 | 0.0328 | -0.114 | 0.9103 | 0.8270906 |
| hsa-miR-887-st | 0.007 | 0.0616 | 0.113 | 0.9107 | 0.8270906 |
| hsa-miR-548ac-st | -0.012 | 0.106 | -0.113 | 0.9109 | 0.8270906 |
| hsa-miR-126-st | -0.0046 | 0.0409 | -0.112 | 0.9119 | 0.8270906 |
| hsa-miR-4650-5p-st | -0.003 | 0.0273 | -0.112 | 0.9119 | 0.8270906 |
| hsa-miR-548o-st | -0.0033 | 0.0299 | -0.11 | 0.9131 | 0.8275074 |
| hsa-miR-3202-st | 0.0046 | 0.0422 | 0.109 | 0.9135 | 0.8275074 |
| hsa-miR-600-st | -0.0068 | 0.0629 | -0.108 | 0.915 | 0.8281041 |
| hsa-miR-3152-3p-st | 0.0056 | 0.0522 | 0.107 | 0.9153 | 0.8281041 |
| hsa-miR-3180-3p-st | -0.0141 | 0.1338 | -0.105 | 0.9168 | 0.828357 |
| hsa-miR-223-star-st | 0.0028 | 0.0267 | 0.104 | 0.9177 | 0.828357 |
| hsa-miR-1226-st | -0.0066 | 0.0633 | -0.104 | 0.9179 | 0.828357 |
| hsa-miR-1323-st | -0.0073 | 0.0711 | -0.103 | 0.9186 | 0.828357 |
| hsa-miR-548a-5p-st | -0.0033 | 0.0323 | -0.103 | 0.9188 | 0.828357 |
| hsa-miR-302e-st | 0.0038 | 0.0368 | 0.102 | 0.9195 | 0.828357 |
| hsa-miR-4254-st | 0.0094 | 0.0922 | 0.102 | 0.9198 | 0.828357 |
| hsa-miR-2681-star-st | 0.0047 | 0.0469 | 0.101 | 0.9202 | 0.828357 |
| hsa-miR-548p-st | -0.003 | 0.0301 | -0.099 | 0.9215 | 0.828357 |
| hsa-miR-4264-st | -0.0026 | 0.0265 | -0.099 | 0.922 | 0.828357 |
| hsa-miR-4732-3p-st | 0.0055 | 0.056 | 0.099 | 0.9221 | 0.828357 |
| hsa-miR-542-3p-st | 0.0023 | 0.0234 | 0.098 | 0.9228 | 0.828357 |
| hsa-miR-3713-st | -0.0034 | 0.0351 | -0.097 | 0.923 | 0.828357 |
| hsa-miR-1254-st | -0.0074 | 0.0771 | -0.096 | 0.924 | 0.8287419 |
| hsa-miR-589-star-st | 0.0061 | 0.0638 | 0.095 | 0.9247 | 0.8288575 |
| hsa-miR-4697-5p-st | -0.0126 | 0.1342 | -0.094 | 0.9259 | 0.8294208 |
| hsa-miR-3130-3p-st | -0.0056 | 0.06 | -0.093 | 0.9268 | 0.8297149 |
| hsa-miR-4501-st | -0.0034 | 0.0388 | -0.088 | 0.9302 | 0.8299106 |
| hsa-miR-548ah-st | -0.0027 | 0.0306 | -0.088 | 0.9304 | 0.8299106 |
| hsa-miR-1913-st | 0.0098 | 0.1137 | 0.087 | 0.9315 | 0.8299106 |
| hsa-miR-621-st | 0.0029 | 0.0337 | 0.086 | 0.9316 | 0.8299106 |
| hsa-miR-514b-5p-st | -0.0045 | 0.0523 | -0.086 | 0.9317 | 0.8299106 |
| hsa-miR-296-5p-st | -0.0049 | 0.0571 | -0.086 | 0.9318 | 0.8299106 |
| hsa-miR-590-3p-st | 0.0017 | 0.0204 | 0.085 | 0.9329 | 0.8299106 |
| hsa-miR-4685-5p-st | -0.0035 | 0.0415 | -0.085 | 0.933 | 0.8299106 |
| hsa-miR-376c-st | -0.006 | 0.071 | -0.085 | 0.9332 | 0.8299106 |
| hsa-miR-21-st | -0.0079 | 0.0937 | -0.084 | 0.9335 | 0.8299106 |
| hsa-miR-4251-st | -0.0022 | 0.0272 | -0.082 | 0.9356 | 0.8299106 |
| hsa-miR-338-5p-st | 0.0086 | 0.1061 | 0.081 | 0.9357 | 0.8299106 |
| hsa-miR-502-3p-st | -0.0041 | 0.0504 | -0.081 | 0.9358 | 0.8299106 |
| hsa-miR-34a-st | -0.0046 | 0.0575 | -0.081 | 0.9361 | 0.8299106 |
| hsa-miR-136-st | -0.0021 | 0.026 | -0.08 | 0.9367 | 0.8299106 |
| hsa-miR-196b-st | 0.0024 | 0.0297 | 0.08 | 0.937 | 0.8299106 |
| hsa-miR-1248-st | 0.0021 | 0.0267 | 0.079 | 0.9373 | 0.8299106 |
| hsa-miR-10b-st | -0.003 | 0.0378 | -0.079 | 0.9378 | 0.8299106 |
| hsa-miR-548e-st | 0.0027 | 0.0348 | 0.078 | 0.9386 | 0.8299106 |
| hsa-miR-664-star-st | 0.0034 | 0.0442 | 0.077 | 0.939 | 0.8299106 |
| hsa-miR-3130-5p-st | 0.0039 | 0.0504 | 0.077 | 0.9393 | 0.8299106 |
| hsa-miR-4798-5p-st | 0.0019 | 0.0249 | 0.076 | 0.9396 | 0.8299106 |
| hsa-miR-103a-2-star-st | 0.0054 | 0.0722 | 0.075 | 0.9405 | 0.8301366 |
| hsa-miR-4330-st | 0.0034 | 0.0454 | 0.075 | 0.941 | 0.8301366 |
| hsa-miR-4802-5p-st | -0.0024 | 0.0322 | -0.074 | 0.9419 | 0.8301717 |
| hsa-miR-218-st | -0.0066 | 0.0906 | -0.073 | 0.9422 | 0.8301717 |
| hsa-miR-3685-st | 0.0025 | 0.0354 | 0.071 | 0.9436 | 0.8301717 |
| hsa-miR-4439-st | -0.0042 | 0.0585 | -0.071 | 0.9437 | 0.8301717 |
| hsa-miR-718-st | 0.0045 | 0.0628 | 0.071 | 0.9439 | 0.8301717 |
| hsa-miR-4698-st | -0.0041 | 0.0591 | -0.069 | 0.9456 | 0.8303482 |
| hsa-miR-144-st | -0.0022 | 0.0327 | -0.068 | 0.9461 | 0.8303482 |
| hsa-miR-3120-3p-st | 0.0039 | 0.058 | 0.068 | 0.9462 | 0.8303482 |
| hsa-miR-142-3p-st | -0.002 | 0.0319 | -0.063 | 0.9499 | 0.8303482 |
| hsa-miR-4717-5p-st | -0.004 | 0.0632 | -0.063 | 0.9499 | 0.8303482 |
| hsa-miR-4772-3p-st | 0.0017 | 0.0274 | 0.062 | 0.951 | 0.8303482 |
| hsa-miR-488-st | 0.0057 | 0.0939 | 0.061 | 0.9518 | 0.8303482 |
| hsa-miR-1272-st | -0.0042 | 0.0695 | -0.061 | 0.952 | 0.8303482 |
| hsa-miR-4740-5p-st | 0.0042 | 0.0694 | 0.06 | 0.9524 | 0.8303482 |
| hsa-miR-187-star-st | 0.0057 | 0.0945 | 0.06 | 0.9526 | 0.8303482 |
| hsa-miR-4739-st | 0.0007 | 0.0116 | 0.06 | 0.9526 | 0.8303482 |
| hsa-miR-374a-star-st | -0.0014 | 0.0242 | -0.059 | 0.9532 | 0.8303482 |
| hsa-miR-219-5p-st | -0.0117 | 0.198 | -0.059 | 0.9534 | 0.8303482 |
| hsa-miR-518c-st | 0.0014 | 0.0247 | 0.059 | 0.9536 | 0.8303482 |
| hsa-miR-1343-st | -0.0049 | 0.085 | -0.058 | 0.954 | 0.8303482 |
| hsa-miR-200b-star-st | 0.0037 | 0.0635 | 0.058 | 0.9541 | 0.8303482 |
| hsa-miR-425-star-st | 0.0027 | 0.0468 | 0.058 | 0.9543 | 0.8303482 |
| hsa-miR-320d-st | -0.0022 | 0.039 | -0.058 | 0.9544 | 0.8303482 |
| hsa-miR-2964a-5p-st | -0.004 | 0.0705 | -0.057 | 0.9553 | 0.8306332 |
| hsa-miR-3180-st | -0.0067 | 0.1237 | -0.054 | 0.957 | 0.8312495 |
| hsa-miR-135b-st | 0.0011 | 0.0201 | 0.053 | 0.9578 | 0.8312495 |
| hsa-miR-553-st | 0.0015 | 0.0289 | 0.053 | 0.9583 | 0.8312495 |
| hsa-miR-668-st | -0.0038 | 0.072 | -0.053 | 0.9583 | 0.8312495 |
| hsa-miR-106a-star-st | -0.001 | 0.0192 | -0.052 | 0.9589 | 0.8312731 |
| hsa-miR-135b-star-st | 0.0048 | 0.0943 | 0.05 | 0.9601 | 0.8318165 |
| hsa-miR-3925-5p-st | -0.0021 | 0.0417 | -0.05 | 0.9607 | 0.8318397 |
| hsa-miR-4759-st | -0.0014 | 0.0284 | -0.048 | 0.9616 | 0.8321225 |
| hsa-miR-4305-st | -0.0012 | 0.025 | -0.047 | 0.9629 | 0.832687 |
| hsa-miR-2277-5p-st | 0.0038 | 0.0823 | 0.046 | 0.9634 | 0.832687 |
| hsa-miR-148b-st | 0.003 | 0.0687 | 0.043 | 0.966 | 0.8332144 |
| hsa-miR-2116-star-st | 0.0027 | 0.0648 | 0.042 | 0.9666 | 0.8332144 |
| hsa-miR-3662-st | -0.0015 | 0.0353 | -0.042 | 0.9668 | 0.8332144 |
| hsa-miR-411-st | -0.0018 | 0.0446 | -0.041 | 0.9678 | 0.8332144 |
| hsa-miR-216a-st | -0.0082 | 0.2018 | -0.04 | 0.968 | 0.8332144 |
| hsa-miR-4316-st | -0.0015 | 0.0393 | -0.039 | 0.9692 | 0.8332144 |
| hsa-miR-3620-st | -0.0018 | 0.0461 | -0.038 | 0.9696 | 0.8332144 |
| hsa-miR-4756-3p-st | -0.0018 | 0.0461 | -0.038 | 0.9696 | 0.8332144 |
| hsa-miR-3529-st | -0.0009 | 0.0235 | -0.038 | 0.9698 | 0.8332144 |
| hsa-miR-3116-st | 0.0011 | 0.0293 | 0.038 | 0.9699 | 0.8332144 |
| hsa-miR-298-st | 0.0016 | 0.0419 | 0.037 | 0.9704 | 0.8332144 |
| hsa-miR-4675-st | 0.001 | 0.0276 | 0.037 | 0.9709 | 0.8332144 |
| hsa-miR-1181-st | -0.0034 | 0.0961 | -0.035 | 0.9722 | 0.8332751 |
| hsa-miR-3692-st | -0.0008 | 0.0247 | -0.032 | 0.9745 | 0.8332751 |
| hsa-miR-4470-st | 0.0018 | 0.0576 | 0.03 | 0.9759 | 0.8332751 |
| hsa-miR-101-star-st | -0.0023 | 0.076 | -0.03 | 0.9761 | 0.8332751 |
| hsa-miR-326-st | -0.0012 | 0.0401 | -0.029 | 0.9771 | 0.8332751 |
| hsa-miR-4691-3p-st | 0.0012 | 0.0399 | 0.029 | 0.9772 | 0.8332751 |
| hsa-miR-4659b-5p-st | -0.0007 | 0.0241 | -0.028 | 0.9778 | 0.8332751 |
| hsa-miR-130b-star-st | -0.0016 | 0.0561 | -0.028 | 0.9779 | 0.8332751 |
| hsa-miR-191-star-st | -0.0019 | 0.0695 | -0.028 | 0.9779 | 0.8332751 |
| hsa-miR-4677-5p-st | 0.0006 | 0.0216 | 0.027 | 0.9784 | 0.8332751 |
| hsa-miR-935-st | -0.002 | 0.0748 | -0.027 | 0.9785 | 0.8332751 |
| hsa-miR-204-st | 0.0033 | 0.1261 | 0.026 | 0.9793 | 0.8332751 |
| hsa-miR-342-3p-st | -0.0009 | 0.0358 | -0.026 | 0.9794 | 0.8332751 |
| hsa-miR-517-star-st | 0.0012 | 0.0465 | 0.026 | 0.9797 | 0.8332751 |
| hsa-miR-199b-3p-st | -0.0021 | 0.0852 | -0.025 | 0.9803 | 0.8332751 |
| hsa-miR-4648-st | 0.0042 | 0.1695 | 0.025 | 0.9803 | 0.8332751 |
| hsa-miR-4799-3p-st | -0.0006 | 0.0259 | -0.024 | 0.9812 | 0.8332751 |
| hsa-miR-338-3p-st | 0.0027 | 0.1235 | 0.022 | 0.9825 | 0.8332751 |
| hsa-miR-4775-st | -0.0005 | 0.0225 | -0.022 | 0.9826 | 0.8332751 |
| hsa-miR-584-st | -0.0029 | 0.1352 | -0.022 | 0.9829 | 0.8332751 |
| hsa-miR-144-star-st | 0.001 | 0.0477 | 0.021 | 0.9837 | 0.8332751 |
| hsa-miR-31-star-st | -0.002 | 0.1026 | -0.02 | 0.9843 | 0.8332751 |
| hsa-miR-29b-st | -0.0012 | 0.0615 | -0.02 | 0.9844 | 0.8332751 |
| hsa-miR-580-st | -0.0005 | 0.0252 | -0.019 | 0.9853 | 0.8332751 |
| hsa-miR-3143-st | 0.0007 | 0.0385 | 0.018 | 0.9856 | 0.8332751 |
| hsa-miR-4710-st | -0.0015 | 0.0859 | -0.018 | 0.9859 | 0.8332751 |
| hsa-miR-3170-st | -0.0005 | 0.0314 | -0.017 | 0.9869 | 0.8334031 |
| hsa-miR-4296-st | 0.001 | 0.0585 | 0.016 | 0.9872 | 0.8334031 |
| hsa-miR-3688-3p-st | 0.0004 | 0.0278 | 0.014 | 0.9887 | 0.8339524 |
| hsa-miR-4284-st | -0.0012 | 0.0854 | -0.014 | 0.989 | 0.8339524 |
| hsa-miR-490-5p-st | -0.0012 | 0.092 | -0.013 | 0.99 | 0.8343109 |
| hsa-miR-1468-st | 0.0008 | 0.0792 | 0.01 | 0.9918 | 0.8350265 |
| hsa-miR-593-star-st | -0.0005 | 0.0479 | -0.01 | 0.992 | 0.8350265 |
| hsa-miR-499-5p-st | 0.001 | 0.107 | 0.009 | 0.9929 | 0.8352996 |
| hsa-miR-3173-5p-st | -0.0003 | 0.0459 | -0.008 | 0.994 | 0.8357405 |
| hsa-miR-1290-st | 0.0011 | 0.1769 | 0.006 | 0.9951 | 0.8361809 |
| hsa-miR-4686-st | 0.0003 | 0.0655 | 0.004 | 0.9966 | 0.8367192 |
| hsa-miR-649-st | -0.0001 | 0.0256 | -0.002 | 0.9982 | 0.8367192 |
| hsa-miR-596-st | 0.0002 | 0.1104 | 0.002 | 0.9983 | 0.8367192 |
| hsa-miR-532-5p-st | 0.0001 | 0.0411 | 0.002 | 0.9984 | 0.8367192 |
| hsa-miR-136-star-st | 0.0002 | 0.1198 | 0.001 | 0.9989 | 0.8367192 |
| hsa-miR-4317-st | 0.0001 | 0.0677 | 0.001 | 0.9992 | 0.8367192 |
